# Preferential regulation of miRNA targets by environmental chemicals in the human genome

**DOI:** 10.1186/1471-2164-12-244

**Published:** 2011-05-18

**Authors:** Xudong Wu, Yijiang Song

**Affiliations:** 1 Key laboratory of Photosynthesis and Environmental Molecular Physiology, the Institute of Botany, Chinese Academy of Sciences, Beijing, 100093, China; 2 Institute of Genomic Medicine, Wenzhou Medical College, Wenzhou, 325035, China; 3 State key laboratory of Molecular Reaction Dynamics, Dalian Institute of Chemical Physics, Chinese Academy of Sciences, Dalian, 116023, China

## Abstract

**Background:**

microRNAs (miRNAs) represent a class of small (typically 22 nucleotides in length) non-coding RNAs that can degrade their target mRNAs or block their translation. Recent disease research showed the exposure to some environmental chemicals (ECs) can regulate the expression patterns of miRNAs, which raises the intriguing question of how miRNAs and their targets cope with the exposure to ECs throughout the genome.

**Results:**

In this study, we comprehensively analyzed the properties of genes regulated by ECs (EC-genes) and found miRNA targets were significantly enriched among the EC-genes. Compared with the non-miRNA-targets, miRNA targets were roughly twice as likely to be EC-genes. By investigating the collection methods and other properties of the EC-genes, we demonstrated that the enrichment of miRNA targets was not attributed to either the potential collection bias of EC-genes, the presence of paralogs, longer 3'UTRs or more conserved 3'UTRs. Finally, we identified 1,842 significant concurrent interactions between 407 miRNAs and 497 ECs. This association network of miRNAs-ECs was highly modular and could be separated into 14 interconnected modules. In each module, miRNAs and ECs were closely connected, providing a good method to design accurate miRNA markers for ECs in toxicology research.

**Conclusions:**

Our analyses indicated that miRNAs and their targets played important roles in cellular responses to ECs. Association analyses of miRNAs and ECs will help to broaden the understanding of the pathogenesis of such chemical components.

## Background

miRNAs are a class of small non-coding RNAs, which act through binding in a sequence-specific manner to the 3'UTR of target genes [1]. With a very short recognition sequence (~8bp), each miRNA can potentially regulate hundreds of transcripts. At least one-third of human genes are estimated to be miRNA targets, so the regulation mediated by miRNA at the post-transcriptional level is pervasive in animals [2]. Transcriptomic studies suggest that miRNAs can regulate the expression and stability of targets [3-6]. miRNAs also provide a genetic buffer to constrain the variation of their targets' expression, playing an important role in regulating embryo development and maintaining the identity of mature tissues [7]. In many situations, miRNAs and their targets are co-expressed at intermediate levels; miRNAs serve to buffer the fluctuation of the targets' expression through feed-forward loop architecture [8], such as the relationship between *miR-9a *and *E(spl) *in *Drosophila *[9,10] and *miR-17 *and *E2F1 *in human [11].

Cells change physiologically in response to signals from their external environments. To achieve this, they must activate or repress various genes and tune their products to a proper level under different situations. Many toxicological researchers, adopting RT-PCR, Northern-blotting or microarray technologies to investigate the expression of protein-coding genes, have demonstrated that exposure to ECs often has a negative effect on the normal growth of cells [12,13]. The Comparative Toxicogenomics Database (CTD, http://ctd.mdibl.org/) is a manually curated database, which stores high-quality chemical-gene regulatory data [14]. Its current dataset includes a large number of associations among chemicals and proteins in *Homo sapiens*, *Mus musculus*, *Rattus norvegicus*, *Drosophila melanogaster*, *Caenorhabditis elegans *and other species; therefore, it can be used to determine whether a chemical binds to or regulates the expression of a protein-coding gene.

A two-tiered review system was implemented in the CTD to identify the high-quality chemicals-genes regulatory data [14,15]. First, the curators used text mining to select literature where interactions between chemicals and genes were identifiable, so that data were supported by their source references. The senior curators then proofread the entries from other curators, ensuring that the correct chemical names and gene symbols were chosen. Second, a group of prominent senior immunologists evaluated the curation guidelines and contacted the authors of papers to clarify details of the experimental procedures, to assure that the data were exactly presented as in the reference. Recently, the datasets of the CTD has been used by several independent groups and demonstrated great utility for meta-analyses of ECs [13,16,17].

miRNAs are essential for regulating many cellular processes, such as apoptosis, proliferation and metastasis [18,19]. How miRNAs function in regulating human responses to environmental chemical (EC) stimuli is an unexplored field of compound risk evaluation. In this study, we retrieved the dataset of EC-genes from the CTD and explored their propensities to be miRNA targets. By evaluating the factors that may potentially result in the enrichment, we found that miRNA targets were preferentially regulated by ECs. Through simulations and statistical analyses, we identified significantly occurring miRNA-EC pairs and reconstructed the association network. The identified miRNAs specific to EC-exposure could be used as biomarkers for determining the genotoxicity and carcinogenicity of chemicals [20,21] The following module analysis provides us with an in-depth view of miRNA function in toxicological research.

## Results

### miRNA targets are preferentially regulated by environmental chemicals

We first collected the genes regulated by ECs (EC-genes). According to the expression regulatory information from the CTD [14], such as "chemical x results in increased expression of protein y", "compound x results in decreased expression of protein y" or "compound x affects the expression of protein y", we compiled the dataset of proteins regulated by ECs (see Methods), and then transformed the gene symbols to their Ensembl gene IDs using the BioMart program (http://biomart.org). Based on the reports of 4,162 literatures, we retrieved 42,770 regulatory relationships among compounds and human protein-coding genes (Additional file 1). Specifically, the expression profiles of 9,692 protein-encoding genes were regulated by at least one of the 1,938 ECs, including polycyclic compounds, organic chemicals, heterocyclic compounds, inorganic chemicals, hormones and so on.

We next evaluated the probability of these EC-genes being targeted by miRNAs using TargetScan5.1 [22] and PicTar (four-way) [23], which predict miRNA targets based on sequence complementarities, sequence context information, binding energy, and were regarded by previous surveys having high confidence [24]. As the reliance of TargetScan and PicTar upon cross-species conservation might introduce potential bias, we also included a third set of predicted human miRNA targets derived from PITA [25], which only considered the sequence complementarities and site accessibility; therefore, many more genes were annotated as miRNA targets with the advantage of detecting human-specific miRNA targets.

**Figure 1:**
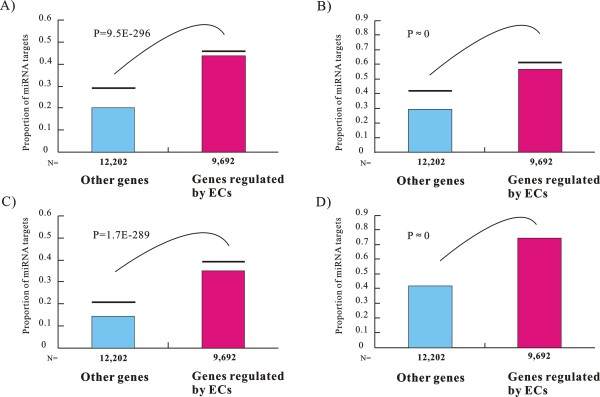
**miRNA targets are enriched among human EC-genes**. This figure shows the proportion of miRNA targets predicted (*A*) by PicTar, (B) by TargetScan5.1, (*C*) by both programs of PicTar and TargetScan5.1 (intersections), and (*D*) by PITA. The horizontal lines above the histogram bars represent the proportion of miRNA targets using genes with mouse orthologs as background.

Using each dataset of miRNA targets (Additional file 2), we observed that miRNA targets were significantly enriched among EC-genes. As shown in Figure 1A, miRNA targets comprise 43% of the EC-genes (4,195 out of 9,692), but only make up 20% of the non-EC-genes (2,473 out of 12,202) as predicted by PicTar. Using the targets detected by TargetScan (Figure 1B), both programs of PicTar and TargetScan (Figure 1C), and PITA (Figure 1D), we obtained similar results. Therefore, miRNA targets were roughly twice as likely to be EC-genes as comparable to the other genes (*Chi-square test*, p-values < 1.7E-289 for the four datasets).

Because the methods of TargetScan and PicTar depend upon alignments of human-mouse orthologs, we only included the human genes with mouse orthologs and repeated the comparative analyses. In Figure 1A-C, the horizontal lines above the histogram bars represent the proportion of miRNA targets using the genes with mouse orthologs as background. Significant enrichment of miRNA targets among EC-genes were observed in this dataset.

In *Mus musculus *and *Rattus norvegicus*, we retrieved 9,552 and 5,064 genes respectively, which were regulated by ECs and found miRNA targets were over-represented among them as compared to the other genes (Additional file 3), indicating that the enrichment of miRNA targets in EC-genes seems to be common in mammal systems. In the following analysis, we only focused on the human genes because similar conclusions were drawn from the analysis results of *Mus musculus *and *Rattus norvegicus*.

### Enrichment of miRNA targets is not dependent on the collection bias of EC-genes

To evaluate whether the above-observed enrichment of miRNA targets among EC-genes was caused by some sample collection bias, we performed the following analysis. First, if the experiments in the literatures from which the EC-genes were extracted, were designed to deal with miRNA-related scientific questions, the enrichment would be intuitively expected but with serious bias. We downloaded all of the abstracts of the 4,261 papers from the NCBI via the PubMed IDs (http://www.ncbi.nlm.nih.gov/sites/batchentrez) and searched keywords such as "miR", "miRNA", "microRNA" or "let-7". As a result, only 5 papers were directly related to the study of miRNAs and about 30 genes appearing in the datasets were included by them (Additional file 4); therefore, there should be no bias in the literature collection results.

Second, some EC-genes may belong to a certain class of genes that are preferentially regulated by miRNAs; therefore, the enrichment may be only contributed by that class of genes. As cancer-related genes were extensively studied [26] and often found to be miRNAs targets [18,19], it is possible that the exposure of the cancer-related genes to ECs were more likely to be investigated and eventually made the miRNA targets over-represented among the EC-genes. To test this, we retrieved a separate set of genes over-expressed in cancer tissues [27]. Specifically, 2,362 proteins corresponding to 2,062 Ensembl genes were at least over-expressed 4-fold in brain (*astrocytoma *and *glioblastoma*), breast, colon, endometrial, kidney, liver, lung, ovary, prostate, skin, and thyroid cancers as compared to healthy tissues of the same type. Significant enrichment of miRNA targets among EC-genes were still observed even after filtering out these cancer-related genes from the datasets (Additional file 5).

Third, for the 12,202 genes not observed to be regulated by ECs (non-EC-genes), some of them may in fact be regulated by ECs but not analyzed or reported thus far. If this is indeed the case, the potential false-negatives from the non-EC-genes may seriously challenge the enrichment conclusion. Because 45% of human genes (calculated by 9,692/(9,692 + 12,202)) were confirmed to be regulated by ECs genome-widely, we arbitrarily sampled genes with the probability of 0.45 from the non-EC-genes and assumed them to be non-annotated EC-genes. To investigate the impact of the potential false-negatives of non-EC-genes, we performed the following procedures: (a) randomly sampled genes from non-EC-genes with the probability of 0.45, *S = 0.45*; (b) constructing the dataset of pseudo-EC-genes with n = 9,692 + 12,202*S and a dataset of pseudo-non-EC-genes with n = 12,202-12,202*S; (c) comparing the proportion of miRNA targets between the pseudo-EC-genes and pseudo-non-EC-genes. We repeated this simulation several times and always obtained significant differences of miRNA targets between pseudo-EC-genes and pseudo-non-EC-genes (see Additional file 6 for the results of eight simulations). Thus, the potential false-negatives of non-EC-genes would not affect the enrichment tendencies of miRNA targets.

Fourth, it is interesting and important to know whether the enrichment of miRNA targets could still be observed in a single experiment. In Perl scripts, the keywords such as "microarray", "array", "affymetrix" and "chip" were used to search the abstracts of 4,162 papers. Many reports did not provide the raw datasets (*.cel *files), but rather only displayed the differentially expressed genes in tables in the main texts or supplemental materials. We read the full-text of ≥50 papers and investigated whether the raw datasets (*.cel *files) were available from GEO (NCBI Gene Expression Omnibus, http://www.ncbi.nlm.nih.gov/geo/) or ArrayExpress (EBI Gene Expression Atlas, http://www.ebi.ac.uk/gxa/). Finally, we manually selected six affymetrix raw datasets and used a uniform pipeline to identify the differentially expressed genes in seven cell lines treated with chemicals (see Methods). The enrichment of miRNA targets among differentially expressed genes was observed in each dataset, respectively (Additional file 7), implying the pervasive roles of miRNAs in responding to the various chemicals in different cells.

### Enrichment of miRNA targets is not caused by other properties of EC-genes

Previous studies have reported significant differences between miRNA-targets and non-miRNA-targets. These differences may be potential sources of bias and contribute to the enrichment of miRNA targets in EC-genes. We adopted a sampling method to control these biases and then examined whether the enrichment of miRNA targets in EC-genes was still observable.

**Figure 2:**
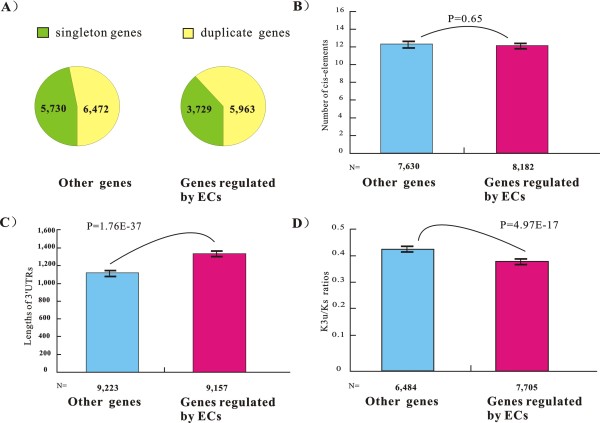
**Comparison of four properties between EC-genes and non-EC-genes in the human genome**. This figure shows the comparison in (*A*) proportion of gene with paralogs, (*B*) the number of transcription factor binding sites, (*C*) the lengths of 3'UTRs and (*D*) evolutionary constraints on the 3'UTRs.

**Figure 3:**
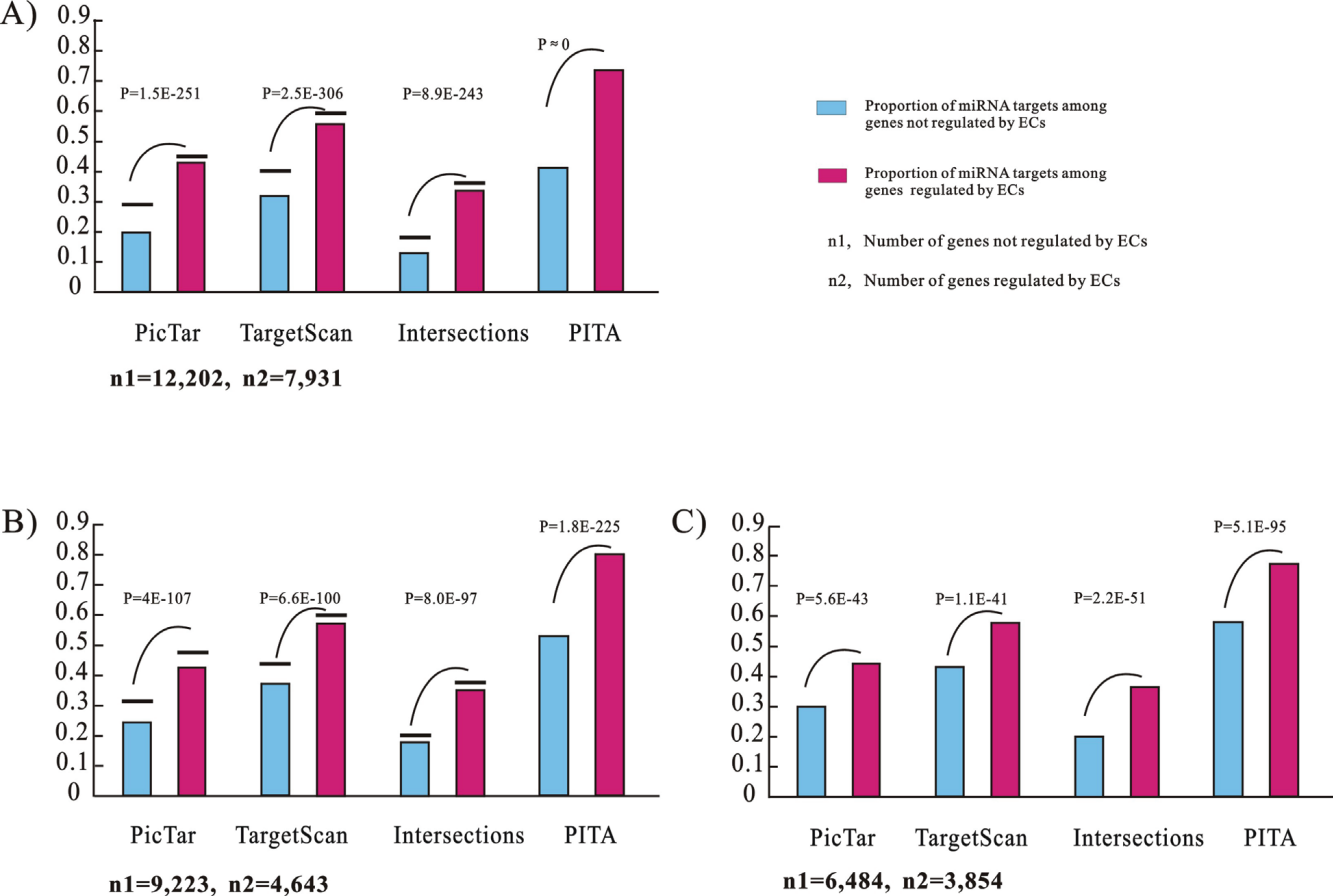
**miRNA targets are enriched among human EC-genes after filtering out the bias caused by three properties**. The EC-genes were sampled to have similar (*A*) proportion of genes with paralogs, (*B*) lengths of 3'UTRs and (*C*) evolutionary constraints on the 3'UTRs as compared to non-EC-genes. The horizontal lines above the histogram bars represent the proportion of miRNA targets using genes with mouse orthologs as background.

The first potential bias stems from the observation showing that genes with paralogs (gene duplication mechanisms) have a high probability of being targeted by miRNAs [28]. In Figure 2A, the EC-genes have a higher tendency of having paralogs compared with non-EC-genes (61% vs. 53%, *p = 2.45E-36, Chi-square test, two tailed*). We sampled genes with a probability of 0.71 (*S = 0.71*, calculated by [3,729*(6,472/5,730)]/5,963) from the 5,963 EC-genes with paralogs. In this way, the dataset of sampled EC-genes were constructed with n = 3,729 + 5,963*S and had the same proportion of genes with paralogs as that of the non-EC-genes. By eliminating potential bias from the higher propensity for gene duplication, we observed similar enrichment of miRNA targets among EC-genes (Figure 3A).

A second potential bias comes from the observation that genes with more TF-binding sites in the 5' upstream regions have a higher probability to become targets of miRNAs [29]; thus, an increased number of transcription factor binding sites may result in an over-representation of miRNA targets among EC-genes. We tested this hypothesis by using cis-elements that were exclusively predicted from conserved motif sequences among a set of vertebrate genome sequences (cisRED database, http://www.cisred.org/) [30]. A total of 94,321 and 100,112 predicted cis-elements were found to be in the proximity of 7,630 non-EC-genes and 8,182 EC-genes, respectively. Thus, EC-genes and non-EC-genes have a similar average number of cis-elements (*p = 0.65*, Manny-Whitney U, two-tail test) (Figure 2B), suggesting that the enrichment of miRNA targets among EC-genes was not likely related to the over-presence of TF-binding sites in 5'-upstream regions.

A third potential bias results from the observation showing that genes with longer 3'UTRs are more likely to be regulated by distinct types of miRNA [31].As shown in Figure 2C, the EC-genes tended to have longer 3'UTRs than non-EC-genes (*p = 1.76E-37*, Manny-Whitney U, two-tailed test), indicating that the enrichment may be attributed to the higher probability of EC-genes of being detected as miRNA targets. To test this, we sampled 4,643 EC-genes whose 3'UTR length falls into the 1^st ^to 3^rd ^quartiles of the 3'UTR lengths among the 9,223 non-EC-genes with available 3'UTR annotation. By eliminating potential bias caused by longer 3'UTRs, we further observed that miRNA targets were significantly enriched among EC-genes (Figure 3B).

Fourth, we inspected whether the 3'UTRs of the EC-genes and non-EC-genes were under the same level of selective pressure. The substitution rates of the 3'UTR (*K3u*) of each gene were normalized against the synonymous substitutions per synonymous site (*Ks*) in the coding region of the same gene (see Methods). Using the ratio of *K3u*/*Ks *to estimate the evolutionary constraints on the 3'UTRs, we found the 3'UTR of the EC-genes tended to evolve more conservatively than non-EC-genes (*p = 4.97E-17*, Manny-Whitney U, two-tailed test) (Figure 2D). To explore the possibility that the elevated level of overall sequence conservation led to an over-representation of miRNA targets among EC-genes, we sampled 3,854 EC-genes whose *K3u/Ks *ratios fall into 1^st ^to 3^rd ^quartiles of the *K3u/Ks *ratios calculated from the 6,484 non-EC-genes. After getting rid of the potential bias caused by the more conserved 3'UTR, the enrichment of miRNA targets was again observable among sampled EC-genes (Figure 3C).

Collectively, these results demonstrate that the enrichment of miRNA targets is not a simple by-product of ancillary features of the analyzed gene set, but is a reflection of the propensity of being targeted by miRNAs increasing the genes' probabilities of being regulated by ECs.

### Target preference of miRNAs on genes regulated by different environmental chemicals

Based on the above statistical analyses, we have confirmed that genome-widely miRNA targets were preferentially regulated by ECs; but, whether miRNAs have different targeting preference for genes regulated by different ECs is still an open question, and vice versa. If the preference exists, we would expect a large number of concurrent miRNA-EC pairs, which tend to co-regulate the same genes.

**Figure 4:**
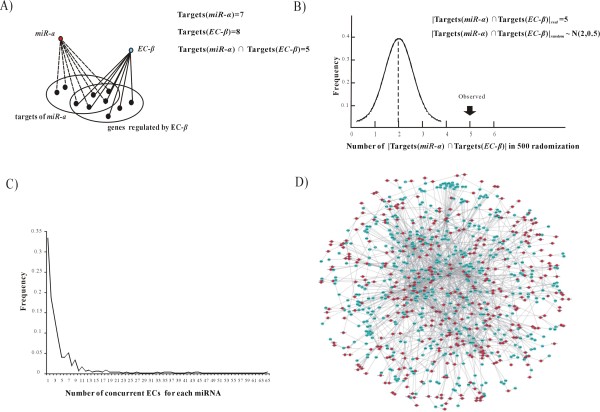
**Associations among the miRNAs and ECs in the human genome**. (*A*) The five targets of miRNA-*a *are regulated by EC-*β*. (*B*) A schematic picture showing a significantly concurrent pair of miRNA-*a *and EC-*β*. (*C*) The frequency distribution of the number of concurrent ECs for each miRNA. (*D*) The association network of ECs and miRNAs. The blue nodes represent ECs, the red nodes represent miRNAs.

We devised a randomization method to identify significant concurrent miRNA-ECs pairs (see a detailed description in Methods). As an example to illustrate the identifying pipelines: In Figure 4A, miRNA-*a *has 7 target genes, 5 of which are regulated by EC-*β*; therefore, the |Targets(miR-*a*)| is assigned to 7, |Targets(EC-*β*)| to 8 and |Targets(miR-*a*) ∩ Targets(EC-*β*)|_*real *_to 5. In each simulated run, the 7 targets for miRNA-*a *were randomly replaced by the targets of other miRNAs by an edge-swapping procedure [32] (the algorithm of edge-swapping can sufficiently randomize the content of targets, while keeping the number of targets for each miRNA), then |Targets(*miR*-*α*) ∩ Targets(*EC-β*)|_*random *_was recorded. Repeating this simulation 500 times, the |Targets(*miR*-*α*) ∩ Targets(*EC-β*)|_*random *_followed a normal distribution as *N(2, 0.5) *(Figure 4B). The *Z*-score was adopted to assess the statistical significance of whether the miRNAs and ECs tend to regulate the same genes. Here, the Z-score was calculated by (5-2)/0.5 = 6 and transformed to a *p-value *as 9.87E-10; thus, the targets of miRNA-*a *were considered to be preferentially regulated by the component-*β*.

Using two lists, EC-Genes (Additional file 1) and miRNA-Target by TargetScan5.1, a miRNA-EC pair was considered to be significantly concurrent if the FDR-corrected *p-value *(the *q-value*) was less than 0.01. Finally, we identified 1,842 concurrent interactions among 407 miRNAs and 497 ECs (Additional file 8), which tend to synergistically regulate the same gene sets. Therefore, distinct miRNAs tend to be "adopted" to regulate genes in response to different ECs.

### Association network of miRNAs and ECs

Graph theory provides paradigms to study biological networks [33]. Here, miRNAs and ECs can be represented respectively by different colored nodes, the concurrent relationship by links. We constructed the association network of miRNAs and ECs to provide a global view of how miRNAs function in concert with ECs. As shown in Figure 4C, the number of the concurrent ECs for each miRNA followed a power-law distribution, where a small proportion of miRNAs connect to many ECs; whereas, a large number of miRNAs only connected to one or two ECs. In this way, it is possible to select a single miRNA or a combination of miRNAs as biological markers in functional studies of their concurrent ECs.

**Figure 5:**
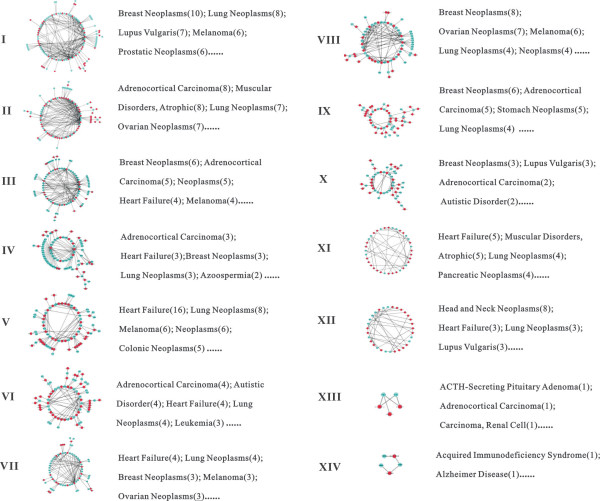
**The topological structure of the 14 separated modules and the miRNA-associated diseases in each module**. The blue nodes represent ECs, the red nodes represent the miRNAs. The number in parentheses indicates the number of miRNAs involved in the corresponding disease categories.

Besides a scale-free structure, the network also demonstrated a modular structure (Figure 4D); that is, a set of miRNAs and ECs were found to be densely concurrent in community-like modules, suggesting that miRNAs in some functional pathway may be co-operatively "adopted" to respond to ECs [34]. We used the algorithm of *Guimera *and *Amaral *[35] to measure the modularity (see Methods), because it performed well in making links within modules much denser than those across modules [36] and has been validated in our previous network analysis [37]. With a final value of modularity being 0.65, 14 topological modules could be separated. In each module, the miRNAs and ECs were closely connected (see Figure 5 for topological structures and Additional file 9 for description of the 14 modules).

We used available disease information of miRNAs to explore the potential function of each module. Three databases have been recently developed, HMDD [38], miR2Disease [39] and PhenomiR [40], which contain a large number of miRNA-disease associations from the literatures (*i.e*., the abnormal regulations of miRNAs correlated with or leading to diseases). Therefore, the correlation of chemicals associated with human diseases could be interpreted by integrating the available disease information on miRNAs with the network modules. As shown in Figure 5, eight out of 19 miRNAs of module XII involved in "Head and Neck neoplasm", indicated that the concurrent ECs of this module had a high probability to be risk factors for head and neck neoplasms. For 16 out of 40 miRNAs of module V involved in"Heart Failure", the information on the concurrent ECs of this module could aid greater understanding of the regulatory mechanisms of heart disease.

## Discussion

In this study, we showed that miRNA targets were preferentially regulated by ECs in *Homo sapiens, Mus musculus and Rattus norvegicus*. The enrichment of miRNA targets in genes associated with ECs was also confirmed by the STITCH database (http://stitch.embl.de/), another well-known free resource of associations between chemicals and proteins [41] (see Additional file 10 for the results of comparative analyses). Therefore, miRNA mediated post-transcriptional regulation may be a pervasive strategy for mammals to cope with irritation caused by ECs. The concurrent analysis revealed that distinct miRNAs regulated genes in response to different ECs. Based on these findings, we proposed that abnormal regulation of miRNAs and protein-coding genes by ECs may eventually disrupt normal signal transduction pathways or destroy the dosage balance of protein complexes. Hence, miRNAs and their targets should be given more attention in studies on environmental health.

**Figure 6:**
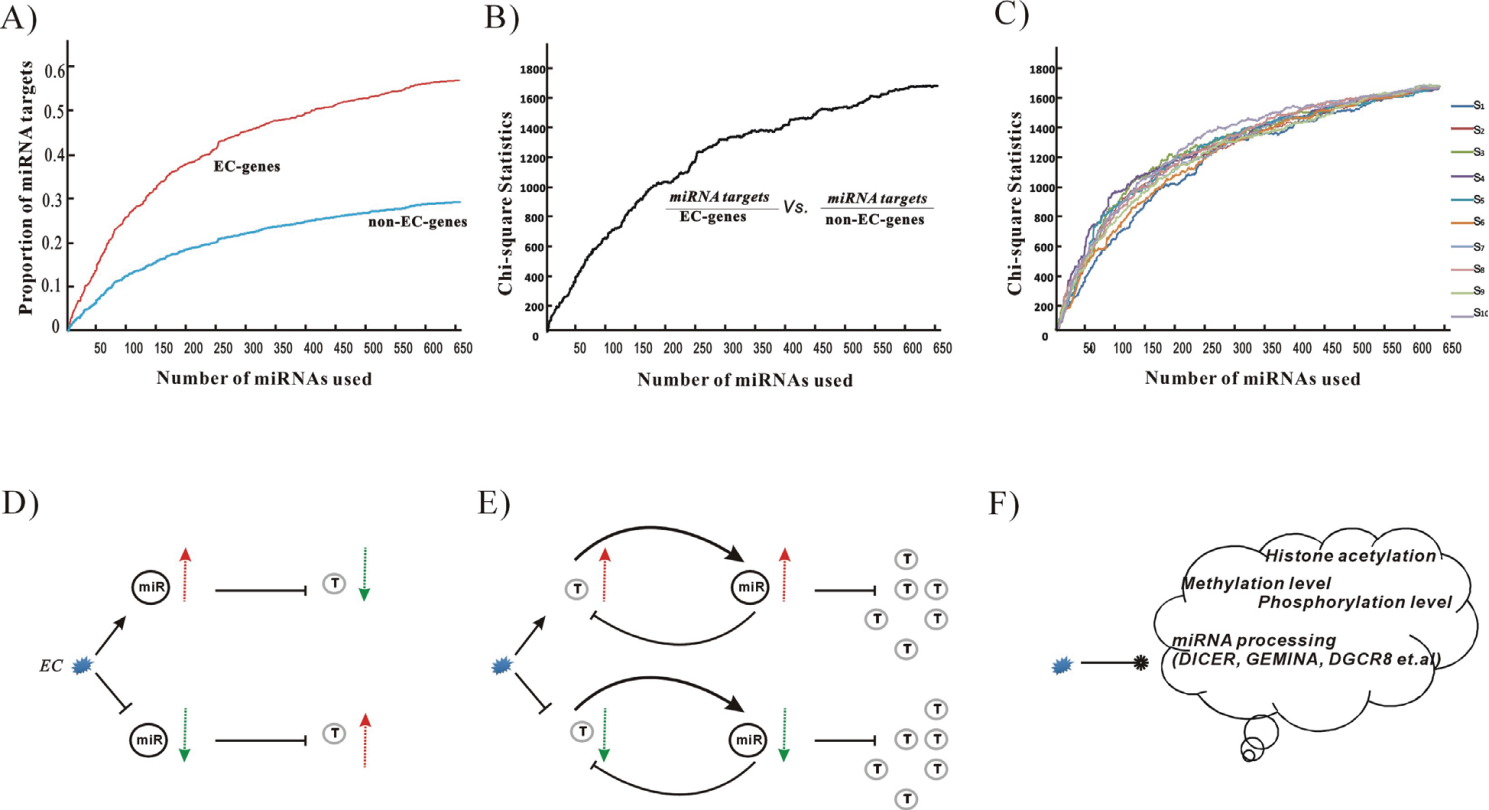
**The impact of incompleteness of miRNAs on the enrichment of miRNA targets among human EC-genes and the biological significance of concurrent miRNA-EC**. (*A*) The proportion of miRNA targets between EC-genes and non-EC-genes in the case of successive addition of miRNAs. (*B*) The plot of *Chi-square *statistics, which measure significant differences in the proportions of miRNA targets between EC-genes and non-EC-genes. A value of *x*^*2 *^≥ *3.84 *(df = 1) corresponds to a *p-*value ≤ *0.05*. (*C*) The plot of *Chi-square *statistics from ten simulations, where each color represents one simulation. (*D*) ECs directly down- or up-regulate the miRNA, then a simple repression motif is involved where miRNA reduces the expression of its target T. (*E*) ECs directly down- or up-regulate the miRNA targets, then a negative feedback loop is involved where miRNA and its target T mutually buffer each other's expression from perturbation. (*F*) ECs lead to the epigenetic changes of the genome.

It is known that the miRNAs identified to date are incomplete. More and more miRNAs have been recently discovered using next-generation sequencing platforms. One may suspect that the enrichment of miRNA targets may be only established using the current version of miRNAs and these tendencies might disappear with the identification of more miRNAs. However, considering the principles of miRNA-Target recognition, we speculate that findings of more miRNAs will not weaken but rather consolidate this enrichment. First, if a new miRNA belongs to a known family, its targets should have been predicted by their homologous miRNAs because of similar mature seed sequences [42]. Second, different miRNAs tend to synergistically target the same genes [43]. Although a new miRNA cannot be classified into a known family, it may not dramatically increase the number of new targets. This expectation has been further confirmed by the following simulation. Of 659 miRNAs contained in TargetScan 5.1, we successively sampled one miRNA into a new miRNA dataset, and then used these miRNAs to compare the proportion of their targets between EC-genes and non-EC-genes. As more miRNAs were placed into the new miRNA dataset (*n *= 1, 2......658, 659), the difference in proportion of miRNA targets between EC-genes and non-EC-genes became more and more pronounced (Figure 6A and 6B). Surprisingly, in the very early stage, when accumulatively transferring ≥5 miRNAs, we observed the enrichment of miRNA targets among EC-genes (see Figure 6C for the plot of *C*hi-square statistics from ten simulations). Therefore, miRNA-target propensity increases the preference of being regulated by ECs and discovering new miRNAs will consolidate the tendency for enrichment.

Using statistical analysis, we found miRNAs had different preferences for targeting genes in response to different ECs, (i.e., a set of miRNAs were often concurrent with a specific EC). From a biological view, three paradigms could be used to explain the strong concurrence between ECs and miRNAs, where some have been verified by recent toxicological studies.

In the first paradigm, the ECs directly down- or up-regulate the miRNAs [44] and subsequently their targets are up- or down-regulated accordingly (Figure 6D). In brain tissue, hexahydro-1,3,5-trinitro-1,3,5-triazine (RDX), a common environmental contaminant, induced the over-expression of *miR-206*, *miR-30 and miR-195*, which then inhibited the expression of the target *BDNF *gene and contributed to neuro-toxicity and CNS disorders. Exposure to RDX also induced aberrant expressions of other onco-miRNAs and tumor-suppressing miRNAs, such as *let-7*, *miR-10b*, *miR-15*, *miR-16*, *miR-26 *and *miR-181*, which regulated tumor pathogenesis or genes related to the cell cycle (e.g., *TNKS*) [45]. In human airway epithelial cells, diesel exhaust particles (DEP), the largest source of emitted airborne particulate matter (PM), induced *miR-513b*, *miR-513c*, *miR-923*, *miR-494 *and *miR-338*, and repressed miR-31*, *miR-26b*, *miR-96*, *miR-27a*, *miR-135 *and *miR-374a*. The subsequent target genes such as *IL-8, IL-6*, *tumor necrosis factor-α, B7-H1 and PRMT5*, were found to be strongly associated with inflammatory-response pathways and tumorigenic disease signatures [46].

The second paradigm is that ECs do not regulate miRNAs but rather their targets directly (Figure 6E). In the expression-buffering motifs, where a miRNA and its target mutually buffer each other's expression from perturbation in a negative feedback loop [47], miRNAs may be induced to be inversely regulated to buffer the expression fluctuation of their targets when exposed to ECs [9,10]. It is difficult to distinguish the second paradigm from the first, but the biological significance is clear. The proposal for drug design follows that, if the drug is directly designed to a miRNA target (*e.g*., down-regulating an onco-gene), whose expression level is maintained by miRNA, the down-regulation of targets may promote the corresponding miRNA to be down-regulated, leading to the unexpected up-regulation of other targets and even deleterious phenotypes.

The third paradigm is that exposure to ECs alters the methylation level of global DNA, histone acetylation [48], methylation [49], and phosphorylation. In this way, epigenetic changes will lead to expression variation of both the miRNAs and their targets (Figure 6F). For example, the association between the chemical black-carbon and blood pressure was mediated by the modification of nucleotide polymorphisms in miRNA processing genes, such as *DICER, GEMIN4, DGCR8,GEMIN3 and GEMIN4 *[50].

No matter which particular paradigm is, the interactions between miRNAs and ECs add to the potential roles of miRNAs in chemical modulation of gene expression throughout the entire genome. The regulatory mechanisms of miRNAs will help us to design more accurate biological markers of ECs or drugs in toxicology research.

## Conclusions

To the best of our knowledge, this is the first genome-wide association analysis among human miRNAs, their targets and ECs. Our analysis will pave the way for future studies for the functional characterization of miRNAs. This network study reveals more clear roles of miRNAs involvement in toxicology and is also valuable for studying the impact of ECs on human health.

## Methods

### The compilation of EC-genes

The protein-coding genes regulated by various ECs were retrieved from the publicly-available Comparative Toxico-genomics Database (CTD, http://ctd.mdibl.org/downloads/) [14]. Gene expression data are presented in the CTD such as "chemical x can increase, decrease, affect or not affect the expression of protein y". Perl scripts were used to remove associations with negation such as ''chemical x does not affect the expression of protein y''.

### The compilation of miRNA targets

The miRNAs and their predicted targets were taken from two previously published studies: TargetScan (http://www.targetscan.org version 5.1, updated April, 2009) [22] and PicTar (USSC genome browser database, http://genome.ucsc.edu four-way) [23]. Targets predicted by TargetScan 5.1 with a total context score of -0.3 or lower were ignored, where the score could quantitatively measure the overall target efficacy [51]. Targets with at least one conserved 7-mer or 8-mer were selected as reliable miRNA targets. The intersection dataset was constructed by the targets predicted both by the TargetScan5.1 and PicTar (four-way). The PITA targets were downloaded from the Weizman Institute website (http://genie.weizmann.ac.il/pubs/mir07/mir07_data.html, updated August, 2008) [25], where a score less than -10 was used as the cutoff to select reliable miRNA targets.

### Analysis of microarray datasets

The Affymetrix raw datasets were downloaded from ftp://ftp.ncbi.nih.gov/pub/geo/data/geo/raw_data/series/ and http://www.ebi.ac.uk/arrayexpress/ GSE6013, the lung adenocarcinoma cells (A549) and SV40-transformed bronchial epithelial cells (Beas-2B) were treated with asbestos; GSE5679, normal dendritic cells were treated with RARa-specific agonists (AM580) and the synthetic PPARg ligand rosiglitazone (RSG), respectively; GSE6907, the HepG2 cells were treated with N-nitrosodimethylamine (NMN) and phenol, respectively; E-MEXP-1327, normal epithelia prostatic cells were treated with selenium and vitamin E, respectively, and the stromal cells were treated with selenium; E-MEXP-390, colorectal carcinoma cells (HCT116) were treated with fluorouracil; E-MEXP-1171, *HCT116 *cells were treated with 7-ethyl-10-hydroxy-20(S)-camptothecin (SN38).

In each test, the raw datasets were normalized using the Affymetrix detection algorithms in the MAS5 library and the background levels and PM/MM ratios were corrected according to the Affymetrix Statistical Algorithms. Based on the estimated expression values of probes (Affy library), the expression values of corresponding Ensembl genes were obtained by BioMart. Finally, each test consisted of one control (no treatment) and a series of treatments (a chemical). Afterwards, the t-test (two-tailed) was used to determine if a gene's expression intensity after treatment (Ga) was significantly different from that before treatment (Gb).

The null hypothesis was *H0 = Ga-Gb = 0*.

A gene was considered to be differentially expressed if *H0 *was rejected (*p-value *≤ 0.01) after treatment.

### The compilation of human genes

The human protein-coding genes annotated as '*known genes*', human paralogs, human-mouse orthologs, the sequences of proteins, coding regions and 3'UTRs were downloaded from Ensembl using BioMart software (http://www.biomart.org/ ) [52]. For genes with multiple splice isoforms, the transcripts with the longest sequences were used for analysis.

### Measurement of the evolutionary constraints on the 3'UTRs of human genes

The Clustalw software [53] was used to globally align the protein sequences of human-mouse orthologs, and the corresponding coding sequences were realigned with the gaps in the alignment trimmed. The *Ks *was estimated from the codon-based nucleotide sequence alignment by using the *Yang-Nielsen *maximum-likelihood method implemented in the *yn00 *program of the PAML package [54]. The Clustalw software was used to globally align the 3'UTRs of orthologs, the substitution rate per site *K3u *with the *Kimura *two-parameter model was calculated by *distmat *program of the EMBOSS package [55]. Finally, the ratios of *K3u*/*Ks *were used to estimate the evolutionary constraints on the 3'UTRs for individual genes.

### Identification of significant concurrence between miRNAs and ECs

This model tests whether a given pair of miRNA-EC co-regulates the same genes at a higher rate, while considering the distribution of the number of different miRNAs regulating each target as a background. Two steps were followed: (a) the assignment matrix of miRNAs to targets were subjected to 100,000 iterations of the edge-swapping procedure, while keeping the number of targets for each miRNA and keeping the number of regulator miRNA for each target [32], (b) for a pair of miRNA, *α*, and EC, *β*, with their set of targets, Targets(*miR-a*) and Targets(*EC-β*), respectively, the number of |Targets(*miR*-*α*) ∩ Targets(*EC-β*)|_*random *_was recorded if it was larger than 0. The steps from (a) to (b) were repeated 500 times to obtain the distribution of |Targets(*miR-α*) ∩ Targets(*EC-β*)|_*random*_.

*Z-*scores and *p-*values were employed to determine whether the value of |Targets(*miR-a*) ∩ Targets(*EC-β*)|_*real *_significantly deviated from the distribution of |Targets(*miR-a*) ∩ Targets(*EC-β*)|_*random *_generated from 500 random simulations.

Where *N*_*real *_was the number of |Targets(*miR-a*) ∩ Targets(*EC-β*)|_*real*_, *N*_*random *_was the number of |Targets(*miR-a*) ∩ Targets(*EC-β*)|_*random*_, *μ *and σ denoted the mean and the standard deviation of the |Targets(*miR-α*) ∩ Targets(*EC-β*)|_*random*_, respectively.

The *Z*-scor*e *was then transformed to the *p*-valu*e * (Calculated by the *NORMDIST *function in *Microsoft Excel*)

Because the above statistical significance analyses involve the simultaneous testing of thousands of hypotheses, multiple hypotheses testing is important to control the overall Type I error rate. The *p-*values in EC-miRNA concurrent analysis were FDR corrected using the *Q-value *program from R package [56].

Finally, the obtained *q-values *were used to assess the statistical significance of the concurrence between a miRNA and an EC.

### The network and modules analysis

The association network of miRNAs and ECs was displayed by the Cytoscape software http://www.cytoscape.org/[57]. The least-squares method was used to estimate power-law exponent of *p(K)∞K*^*-t *^for log-transformed data (*t*, power exponent; *K*, degree). Since the estimated power-law exponent was 2.0, the method for study of scale-free structure was applied in analysis of miRNA-EC network. The algorithm of *Guimera *and *Amaral *[35], with parameter settings as iteration factor = 1.0, cooling factor = 0.95 and number of randomization = 100, was used to measure the extent of modularity of network and separate the network into topological modules.

### The disease information of miRNAs

The disease categories associated with miRNAs were integrated based on the following three published studies: http://202.38.126.151/hmdd/mirna/md/[38], http://www.mir2disease.org/[39] and http://mips.helmholtz-muenchen.de/phenomir/[40].

### Statistical analysis and computational methods

Comparison of proportions from miRNA-targets between EC-genes and non-EC-genes were performed using the *Chi-square *two-tailed test.

Where *O*_*ij *_was the observed number in row *i *of column *j*, *E*_*ij *_was the expected number in row *i *of column *j*. For the condition of *df *= 1, the value of *x*^2 ^≥ 3.84 corresponded to *p *≤ 0.05, indicating a significant difference.

The comparison of the number of cis-elements, *K3u/Ks *and the lengths of 3'UTRs between EC-genes and non-EC-genes were performed using the Manny-Whitney U with two-tailed test.

A preliminary analysis of datasets and computations were performed on a Linux cluster with 16 nodes (Intel 5130, 2.0 GHz CPU, 4G memory, Research Center for Systematic and Evolutionary Botany, Institute of Botany, CAS). The updating of datasets was performed on the Linux clusters provided by the Institute of Genomic Medicine (Wenzhou Medical College) and Dalian Institute of Chemical Physics (CAS), respectively. Perl (http://perl.org) and R (http://www.r-project.org/) scripts were used for analyses, and can be obtained on request.

## Abbreviations

CTD: comparative toxicogenomics database; EC: environmental chemicals; EC-genes: genes observed to be regulated by environmental chemicals; Non-EC-genes: genes not observed to be regulated by the environmental chemicals; UTR: untranslated region; *Ks*: synonymous substitution rates of coding region; *K3u*: substitution rate of 3'UTR;

## Competing interests

The authors declare that they have no competing interests.

## Authors' contributions

XW designed and performed the experiments. XW analyzed the datasets and wrote the paper. YS participated in the analysis of datasets. All authors read and approved the final manuscript.

## Supplementary Material

Additional file 1 **Table S1: The 42,770 regulatory relationships among 1938 chemicals and 9,692 human protein-coding genes**. Click here for file

Additional file 2 **Table S2: The list of miRNA targets predicted by PicTar, TargetScan5.1, both programs of PicTar and TargetScan5.1 (intersections), and by PITA**. Click here for file

Additional file 3 **Figure S1: miRNA targets are enriched among EC-genes in *Mus musculus *and *Rattus norvegicus***. This figure shows the proportion of miRNA targets predicted (*A*) by *Mus musculus *TargetScan5.1, (*B*) by *Mus musculus *PITA and (*C*) by *Rattus norvegicus *TargetScan5.1. As there are no miRNA targets from the PicTar prediction for both species and PITA prediction for *Rattus norvegicus*, this figure shows the proportion of miRNA targets predicted by TargetScan5.1 and the *Rattus norvegicus *PITA. The horizontal lines above histogram bars represent the proportion of miRNA targets using genes with human orthologs as background. Click here for file

Additional file 4 **Table S3: The 4,126 papers investigating the expression profiles of human genes in response to ECs**. Click here for file

Additional file 5 **Figure S2: miRNA targets are enriched among human EC-genes after filtering out the cancer-related genes in simulations**. This figure shows the proportion of miRNA targets predicted by PicTar, TargetScan5.1, both programs of PicTar and TargetScan5.1 (intersections), and PITA. The horizontal lines above the histogram bars represent the proportion of miRNA targets using genes with mouse orthologs as background. Click here for file

Additional file 6 **Figure S3: miRNA targets are enriched among human EC-genes after controlling for potential false-negatives of non-EC-genes**. This figure shows the proportion of miRNA targets predicted by PicTar, TargetScan5.1, both programs of PicTar and TargetScan5.1 (intersections), and PITA. The horizontal lines above the histogram bars represent the proportion of miRNA targets using genes with mouse orthologs as background. Click here for file

Additional file 7 **Figure S4: miRNA targets are enriched among human EC-genes in 11 raw-data-based microarray datasets**. This figure shows the proportion of miRNA targets between differentially vs. non-differentially regulated genes where (*A*) A549 cells treated with asbestos, (*B*) Beas-2B cells treated with asbestos, (*C*) Dendritic cells treated with AM580, (*D*) Dendritic cells treated with rosiglitazone, (*E*) Epithelia cells treated with selenium, (*F*) Epithelia cells treated with Vitamin E, (*G*) Stromal cells treated with selenium, (H) HCT116 cells treated with fluorouracil, (*I*) HCT116 cells treated with SN38, (*J*) HepG2 cells treated with NMN and (*K*) HepG2 cells treated with phenol. The horizontal lines above the histogram bars represent the proportion of miRNA targets using genes with mouse orthologs as background. Click here for file

Additional file 8 **Table S4: The 1,842 pairs of significantly concurrent EC-miRNA based on the TargetScan5.1**. Click here for file

Additional file 9 **Table S5: The 14 topological modules with their miRNAs and ECs**. The number in the bracket indicates the number of miRNAs associated with the corresponding disease categories. Click here for file

Additional file 10 **Figure S5: miRNA targets are enriched among human EC-associated genes by STITCH2.0**. This figure shows the proportion of miRNA targets predicted (*A*) by PicTar, (*B*) by TargetScan5.1, (*C*) by both programs of PicTar and TargetScan5.1 (intersections), and (*D*) by PITA. The horizontal lines above the histogram bars represent the proportion of miRNA targets using genes with mouse orthologs as background. Click here for file
