# Supplementary material for: Preferential regulation of miRNA targets by environmental chemicals in the human genome
Source: BMC Genomics. 2011 May 18;12:244. doi: 10.1186/1471-2164-12-244 (PMC3118786; doi:10.1186/1471-2164-12-244)
Supplement: Additional file 1 — Table S1: The 42,770 regulatory relationships among 1938 chemicals and 9,692 human protein-coding genes. [file 1471-2164-12-244-S1.PDF]

Environmental chemical	Regulatory information	Gene symbol	Ensembl ID	Reference
C000188	increase	BCL2	ENSG00000171791	12730627;10545406;
C000188	increase	CYP3A4	ENSG00000160868	10475613;16565514;
C001277	affect	HSPA5	ENSG00000044574	14576064;
C001277	decrease	AKT1	ENSG00000142208	15781649;17293044;
C001277	decrease	BCL2	ENSG00000171791	15781649;
C001277	decrease	BCL2L1	ENSG00000171552	15781649;
C001277	decrease	BIRC2	ENSG00000110330	15781649;
C001277	decrease	CCND1	ENSG00000110092	15781649;17959518;
C001277	decrease	ESR1	ENSG00000091831	16037132;11448926;
C001277	decrease	HIF1A	ENSG00000100644	11980636;16432534;14726529;15319539;
C001277	decrease	KCNH2	ENSG00000055118	12775586;
C001277	decrease	PGR	ENSG00000082175	11448926;
C001277	decrease	PLAU	ENSG00000122861	12724357;
C001277	increase	CDH1	ENSG00000039068	17024233;
C001277	increase	FGF7	ENSG00000140285	14656992;
C001277	increase	HPGD	ENSG00000164120	15680906;
C001277	increase	HSPA1A	ENSG00000232804	11448926;
C001277	increase	HSPA4	ENSG00000170606	16311509;
C001277	increase	IL24	ENSG00000162892	17024233;
C001277	increase	IL8	ENSG00000169429	11472980;
C001277	increase	MGMT	ENSG00000170430	16950796;
C001277	increase	MMP1	ENSG00000196611	12724357;
C001277	increase	PTHLH	ENSG00000087494	12724357;
C001277	increase	TNF	ENSG00000223952	11472980;
C001329	decrease	NFKBIA	ENSG00000100906	16024638;
C001329	increase	CD40	ENSG00000101017	15894585;
C001329	increase	CD80	ENSG00000121594	15894585;
C001329	increase	CD83	ENSG00000112149	15894585;
C001329	increase	CD86	ENSG00000114013	15894585;
C001329	increase	ICAM1	ENSG00000090339	15894585;
C001329	increase	TNFRSF10A	ENSG00000104689	16024638;
C001390	increase	PTGS2	ENSG00000073756	18480072;
C001478	decrease	GNB5	ENSG00000069966	16129123;
C001478	decrease	PTPN3	ENSG00000070159	16129123;
C001478	decrease	TFDP2	ENSG00000114126	16129123;
C001478	decrease	VIM	ENSG00000026025	16129123;
C001478	increase	ASNS	ENSG00000070669	16129123;
C001478	increase	BTG1	ENSG00000133639	16129123;
C001478	increase	JAG1	ENSG00000101384	16129123;
C001478	increase	NUPL1	ENSG00000139496	16129123;
C001478	increase	PDE4B	ENSG00000184588	16129123;
C001478	increase	PIR	ENSG00000087842	16129123;
C001478	increase	PMAIP1	ENSG00000141682	16129123;
C001478	increase	PPIG	ENSG00000138398	16129123;
C001478	increase	PTPN12	ENSG00000127947	16129123;
C001478	increase	RGS16	ENSG00000143333	16129123;
C001478	increase	RIT1	ENSG00000143622	16129123;
C001478	increase	S100P	ENSG00000163993	16129123;
C001478	increase	TNFAIP2	ENSG00000185215	16129123;
C001570	decrease	MAPT	ENSG00000186868	16930453;

C001654	affect	NR1I2	ENSG00000144852	14600250;15630458;15856409;
C001654	increase	ABCB1	ENSG00000085563	15266218;16005588;
C001654	increase	CYP24A1	ENSG00000019186	15630458;
C001654	increase	CYP2C9	ENSG00000138109	14600250;15100173;
C001654	increase	CYP3A4	ENSG00000160868	15100173;16005588;16442130;17954527;15266218;
C001654	increase	ICAM1	ENSG00000090339	15622447;
C001654	increase	IL8	ENSG00000169429	15622447;
C001758	decrease	BIRC2	ENSG00000110330	15359644;
C001758	decrease	BIRC3	ENSG00000023445	12948851;
C001758	decrease	DAXX	ENSG00000231617	12948851;
C001758	decrease	PAWR	ENSG00000177425	12948851;
C001758	decrease	XIAP	ENSG00000101966	12948851;15359644;
C001758	increase	TP53	ENSG00000141510	12948851;
C001765	increase	CGB	ENSG00000213030	15941851;
C001765	increase	CYP19A1	ENSG00000137869	15941851;
C001765	increase	HSD17B1	ENSG00000108786	16513093;
C001803	increase	CDKN1A	ENSG00000124762	15601469;
C001803	increase	TP53	ENSG00000141510	15601469;
C001870	increase	CYP2B6	ENSG00000197408	15548381;
C001870	increase	CYP3A4	ENSG00000160868	15548381;
C001899	decrease	BIRC3	ENSG00000023445	15972136;
C001899	decrease	CD80	ENSG00000121594	15663903;
C001899	decrease	CD86	ENSG00000114013	15663903;
C001899	decrease	DUSP1	ENSG00000120129	17507666;
C001899	decrease	ICAM1	ENSG00000090339	17931847;
C001899	decrease	IL12A	ENSG00000168811	15663903;
C001899	decrease	IL12B	ENSG00000113302	15663903;
C002010	affect	EGR1	ENSG00000120738	10739673;
C002010	affect	FOS	ENSG00000170345	10739673;
C002010	affect	MYC	ENSG00000136997	10739673;
C002055	decrease	CCNA2	ENSG00000145386	18930000;
C002055	decrease	CCND1	ENSG00000110092	18930000;
C002055	decrease	CCNE1	ENSG00000105173	18930000;
C002055	increase	CDKN1B	ENSG00000111276	18930000;
C002055	increase	TP53	ENSG00000141510	18930000;
C002055	increase	TXNIP	ENSG00000117289	18930000;
C002070	increase	BIRC5	ENSG00000089685	16077934;
C002202	affect	CDH1	ENSG00000039068	17034753;
C002202	decrease	AIP	ENSG00000110711	15982314;
C002202	decrease	CBX3	ENSG00000122565	15982314;
C002202	decrease	CKB	ENSG00000166165	15982314;
C002202	decrease	COL6A1	ENSG00000142156	15982314;
C002202	decrease	COL8A2	ENSG00000171812	15982314;
C002202	decrease	CXCL1	ENSG00000163739	15982314;
C002202	decrease	CYP27A1	ENSG00000135929	15982314;
C002202	decrease	CYP3A7	ENSG00000160870	15982314;
C002202	decrease	CYP4A11	ENSG00000187048	15982314;
C002202	decrease	DSC1	ENSG00000134765	15982314;
C002202	decrease	DUSP11	ENSG00000144048	15982314;
C002202	decrease	E2F1	ENSG00000101412	15982314;
C002202	decrease	EGFR	ENSG00000146648	15982314;
C002202	decrease	EIF4EBP2	ENSG00000148730	15982314;

C002202	decrease	FABP5	ENSG00000164687	15982314;
C002202	decrease	FAS	ENSG00000026103	17034753;
C002202	decrease	FASTK	ENSG00000164896	15982314;
C002202	decrease	FGF1	ENSG00000113578	15982314;
C002202	decrease	FLG	ENSG00000143631	15982314;
C002202	decrease	GBA	ENSG00000177628	15982314;
C002202	decrease	GDF2	ENSG00000128802	15982314;
C002202	decrease	GRB7	ENSG00000141738	15982314;
C002202	decrease	GSTM1	ENSG00000134184	15982314;
C002202	decrease	IGF2	ENSG00000167244	15982314;
C002202	decrease	IL10RA	ENSG00000110324	15982314;
C002202	decrease	IL24	ENSG00000162892	15982314;
C002202	decrease	KRT10	ENSG00000186395	15982314;
C002202	decrease	KRT14	ENSG00000186847	15982314;
C002202	decrease	KRT17	ENSG00000128422	15982314;
C002202	decrease	KRT1	ENSG00000167768	15982314;
C002202	decrease	KRT5	ENSG00000186081	15982314;
C002202	decrease	KRT6A	ENSG00000205420	15982314;
C002202	decrease	KRT75	ENSG00000170454	15982314;
C002202	decrease	LAMA2	ENSG00000196569	15982314;
C002202	decrease	LAMA3	ENSG00000053747	15982314;
C002202	decrease	MCL1	ENSG00000143384	17034753;
C002202	decrease	MFAP2	ENSG00000117122	15982314;
C002202	decrease	MMP10	ENSG00000166670	15982314;
C002202	decrease	NEFL	ENSG00000104725	15982314;
C002202	decrease	ODC1	ENSG00000115758	15982314;
C002202	decrease	PTGS1	ENSG00000095303	15982314;
C002202	decrease	PTGS2	ENSG00000073756	15982314;
C002202	decrease	RHOA	ENSG00000067560	15982314;
C002202	decrease	RHOB	ENSG00000143878	15982314;
C002202	decrease	RNF5	ENSG00000225452	15982314;
C002202	decrease	S100A1	ENSG00000160678	15982314;
C002202	decrease	SERPINB2	ENSG00000197632	15982314;
C002202	decrease	SERPINB9	ENSG00000170542	15982314;
C002202	decrease	SLC16A1	ENSG00000155380	15982314;
C002202	decrease	SOCS1	ENSG00000185338	15982314;
C002202	decrease	SPRR1B	ENSG00000169469	15982314;
C002202	decrease	SULT2A1	ENSG00000105398	15982314;
C002202	decrease	TGM1	ENSG00000092295	15982314;
C002202	decrease	THBS1	ENSG00000137801	15982314;
C002202	decrease	VEGFC	ENSG00000150630	15982314;
C002202	increase	ANXA1	ENSG00000135046	15982314;
C002202	increase	APOD	ENSG00000189058	15982314;
C002202	increase	CDKN1C	ENSG00000129757	15982314;
C002202	increase	COL1A1	ENSG00000108821	17034753;
C002202	increase	COL4A2	ENSG00000134871	17034753;
C002202	increase	CRABP2	ENSG00000143320	17034753;
C002202	increase	DUSP10	ENSG00000143507	15982314;
C002202	increase	EMP1	ENSG00000134531	15982314;
C002202	increase	FAM3C	ENSG00000196937	15982314;
C002202	increase	FASN	ENSG00000169710	15982314;
C002202	increase	FKBP8	ENSG00000105701	15982314;
C002202	increase	FOXA1	ENSG00000129514	17034753;
C002202	increase	G6PD	ENSG00000160211	15982314;

C002202	increase	GATA6	ENSG00000141448	17034753;
C002202	increase	GDF15	ENSG00000130513	15982314;
C002202	increase	GLUL	ENSG00000135821	15982314;
C002202	increase	HBEGF	ENSG00000113070	15982314;
C002202	increase	IGF1	ENSG00000017427	15982314;
C002202	increase	IGFBP3	ENSG00000146674	15982314;
C002202	increase	IL1B	ENSG00000125538	15982314;
C002202	increase	ITGA4	ENSG00000115232	15982314;
C002202	increase	IVL	ENSG00000163207	17034753;
C002202	increase	KRT15	ENSG00000171346	15982314;
C002202	increase	KRT18	ENSG00000111057	17034753;
C002202	increase	KRT19	ENSG00000171345	15982314;17034753;
C002202	increase	KRT7	ENSG00000135480	15982314;17034753;
C002202	increase	KRT8	ENSG00000170421	17034753;
C002202	increase	LAMC3	ENSG00000050555	15982314;
C002202	increase	LCN2	ENSG00000148346	15982314;
C002202	increase	MEIS1	ENSG00000143995	17034753;
C002202	increase	MEIS2	ENSG00000134138	17034753;
C002202	increase	MEOX1	ENSG00000005102	17034753;
C002202	increase	NEDD9	ENSG00000111859	15982314;
C002202	increase	OLR1	ENSG00000173391	17034753;
C002202	increase	PIR	ENSG00000087842	15982314;
C002202	increase	PLAT	ENSG00000104368	15982314;
C002202	increase	RARA	ENSG00000131759	17034753;
C002202	increase	RARB	ENSG00000077092	17034753;
C002202	increase	RARRES1	ENSG00000118849	17034753;
C002202	increase	RARRES3	ENSG00000133321	17034753;
C002202	increase	RPS13	ENSG00000110700	15982314;
C002202	increase	S100A7	ENSG00000143556	15982314;
C002202	increase	S100A8	ENSG00000143546	15982314;
C002202	increase	S100A9	ENSG00000163220	15982314;
C002202	increase	SERPINA3	ENSG00000196136	15982314;
C002202	increase	SOCS2	ENSG00000120833	15982314;
C002202	increase	TGFB1I1	ENSG00000140682	15982314;
C002202	increase	TGM2	ENSG00000198959	17034753;
C002202	increase	TMSB4X	ENSG00000205542	15982314;
C002385	increase	CASP3	ENSG00000164305	12880480;
C002385	increase	HMOX1	ENSG00000100292	8764571;
C002385	increase	ITPR1	ENSG00000150995	17241155;
C002385	increase	PARK2	ENSG00000185345	12880480;
C002385	increase	SNCA	ENSG00000145335	12880480;
C002669	decrease	CFB	ENSG00000243570	16921510;
C002669	decrease	FAS	ENSG00000026103	16024633;
C002669	decrease	FASLG	ENSG00000117560	16024633;
C002669	decrease	IFNA1	ENSG00000197919	16921510;
C002669	decrease	IL12B	ENSG00000113302	16921510;
C002669	decrease	IL1A	ENSG00000115008	16921510;
C002669	decrease	LTC4S	ENSG00000213316	12574384;
C002669	decrease	SAA1	ENSG00000173432	16921510;
C002669	decrease	SELE	ENSG00000007908	16921510;
C002669	decrease	SELP	ENSG00000174175	16921510;
C002669	decrease	TLR5	ENSG00000187554	16921510;
C002669	decrease	TLR6	ENSG00000174130	16921510;
C002669	decrease	VCAM1	ENSG00000162692	16921510;

C002669	increase	BAK1	ENSG00000030110	16024633;
C002669	increase	BAX	ENSG00000087088	16024633;
C002669	increase	DDIT3	ENSG00000175197	11360202;
C002669	increase	FGF2	ENSG00000138685	16921510;
C002669	increase	IL8	ENSG00000169429	18413660;
C002669	increase	MAPK8	ENSG00000107643	16921510;
C002669	increase	MAPK9	ENSG00000050748	16921510;
C002669	increase	TP53	ENSG00000141510	16024633;
C002669	increase	TRADD	ENSG00000102871	16921510;
C002669	increase	TRAF1	ENSG00000056558	16921510;
C002669	increase	TRAF2	ENSG00000127191	16921510;
C002669	increase	VEGFA	ENSG00000112715	16921510;
C002842	increase	CYP3A4	ENSG00000160868	19249324;
C002963	increase	PON1	ENSG00000005421	15690306;14500290;
C002979	decrease	ABCC1	ENSG00000103222	15897249;
C002979	decrease	BCL2	ENSG00000171791	19360359;
C002979	decrease	CCND1	ENSG00000110092	18855134;
C002979	decrease	COL1A1	ENSG00000108821	18514395;
C002979	decrease	COL1A2	ENSG00000164692	18514395;
C002979	decrease	COL7A1	ENSG00000114270	18514395;
C002979	decrease	DRG1	ENSG00000185721	15897249;
C002979	decrease	ESR1	ENSG00000091831	18855134;
C002979	decrease	MKI67	ENSG00000148773	18855134;
C002979	decrease	PGR	ENSG00000082175	18855134;
C002979	decrease	SNAP23	ENSG00000092531	18514395;
C002979	decrease	TNFSF10	ENSG00000121858	19360359;
C002979	increase	BANP	ENSG00000172530	18514395;
C002979	increase	CES1	ENSG00000198848	15897249;
C002979	increase	CHMP1A	ENSG00000131165	18514395;
C002979	increase	COL4A5	ENSG00000188153	18514395;
C002979	increase	COL6A1	ENSG00000142156	18514395;
C002979	increase	CYP3A5	ENSG00000106258	15897249;
C002979	increase	FASLG	ENSG00000117560	15897249;
C002979	increase	GRIK1	ENSG00000171189	18514395;
C002979	increase	GRIK3	ENSG00000163873	18514395;
C002979	increase	IGF1R	ENSG00000140443	18514395;
C002979	increase	P4HA1	ENSG00000122884	18514395;
C002979	increase	RAB3B	ENSG00000169213	18514395;
C002979	increase	RAB4A	ENSG00000168118	18514395;
C002979	increase	SCARB1	ENSG00000073060	18514395;
C002979	increase	SORT1	ENSG00000134243	18514395;
C003001	increase	AHR	ENSG00000106546	16480812;
C003001	increase	NOS2	ENSG00000007171	12085989;
C003001	increase	PON1	ENSG00000005421	15169886;15458977;
C003001	increase	TGM2	ENSG00000198959	16480812;
C003060	decrease	IL6	ENSG00000136244	15319175;
C003135	affect	ARL6IP5	ENSG00000144746	15799245;
C003243	increase	CCL2	ENSG00000108691	17655880;
C003297	decrease	APOA1	ENSG00000118137	16153628;
C003297	increase	APOB	ENSG00000084674	16153628;
C003377	decrease	APOB	ENSG00000084674	11108730;15380446;
C003377	decrease	CCNA1	ENSG00000133101	17541156;
C003377	decrease	CDC25A	ENSG00000164045	17541156;
C003377	decrease	CYP2E1	ENSG00000130649	17541156;

C003377	decrease	EGF	ENSG00000138798	17541156;
C003377	decrease	FGF18	ENSG00000156427	17541156;
C003377	decrease	FGF3	ENSG00000186895	17541156;
C003377	decrease	JUN	ENSG00000177606	17541156;
C003377	decrease	PDGFB	ENSG00000100311	17541156;
C003377	decrease	RASA1	ENSG00000145715	17541156;
C003377	increase	APOA1	ENSG00000118137	11108730;
C003377	increase	DCC	ENSG00000187323	17541156;
C003377	increase	GAS6	ENSG00000248501	17541156;
C003377	increase	GDF15	ENSG00000130513	17541156;
C003377	increase	GSTM1	ENSG00000134184	17541156;
C003377	increase	HMOX1	ENSG00000100292	16799064;
C003377	increase	ICAM1	ENSG00000090339	12044887;19103272;
C003377	increase	IFNG	ENSG00000111537	12044887;19103272;
C003377	increase	NFE2L2	ENSG00000116044	16799064;
C003377	increase	NQO1	ENSG00000181019	17541156;
C003377	increase	TRAF3	ENSG00000131323	17541156;
C003377	increase	XPA	ENSG00000136936	17541156;
C003377	increase	XRCC4	ENSG00000152422	17541156;
C003585	decrease	LDLR	ENSG00000130164	16142410;
C003585	increase	ABCA1	ENSG00000165029	16142410;16730733;
C003585	increase	ABCG1	ENSG00000160179	16730733;
C003585	increase	CETP	ENSG00000087237	16142410;
C003585	increase	LPL	ENSG00000175445	16142410;
C003853	increase	ICAM1	ENSG00000090339	12952965;
C003959	increase	IL8	ENSG00000169429	19162161;
C003959	increase	MSR1	ENSG00000038945	9614211;
C004369	decrease	ESR1	ENSG00000091831	12822488;
C004369	increase	PGR	ENSG00000082175	12822488;
C004479	decrease	CFTR	ENSG00000001626	19061877;
C004479	decrease	PLAT	ENSG00000104368	10856522;
C004479	increase	CYP2B6	ENSG00000197408	18206661;
C004479	increase	LPA	ENSG00000198670	10856522;
C004479	increase	SERPINE1	ENSG00000106366	10856522;
C004532	increase	CCL11	ENSG00000172156	17572062;
C004532	increase	CCL2	ENSG00000108691	17572062;
C004532	increase	CCL3	ENSG00000006075	17572062;
C004532	increase	CCL4	ENSG00000129277	17572062;
C004532	increase	CCL5	ENSG00000161570	17572062;
C004532	increase	CDKN1A	ENSG00000124762	14976336;
C004532	increase	IL10	ENSG00000136634	17572062;
C004532	increase	IL6	ENSG00000136244	17572062;
C004532	increase	IL8	ENSG00000169429	17572062;
C004532	increase	MYB	ENSG00000118513	18778717;
C004532	increase	NQO1	ENSG00000181019	8652659;
C004532	increase	TOP2A	ENSG00000131747	18778717;15935817;16906772;11468185;15833037;
C004541	decrease	AGRN	ENSG00000188157	12111696;
C004541	decrease	ALDH7A1	ENSG00000164904	12111696;
C004541	decrease	C18ORF1	ENSG00000168675	12111696;
C004541	decrease	C9ORF86	ENSG00000196642	12111696;
C004541	decrease	CDH2	ENSG00000170558	12111696;
C004541	decrease	CDKN1A	ENSG00000124762	12111696;
C004541	decrease	CYP19A1	ENSG00000137869	16965912;9435150;160233

C004541	decrease	DERA	ENSG00000023697	12111696;
C004541	decrease	EIF5	ENSG00000100664	12111696;
C004541	decrease	ENPP5	ENSG00000112796	12111696;
C004541	decrease	EPS15	ENSG00000085832	12111696;
C004541	decrease	FOLR1	ENSG00000110195	12111696;
C004541	decrease	GM2A	ENSG00000196743	12111696;
C004541	decrease	GNAI3	ENSG00000065135	12111696;
C004541	decrease	MMP14	ENSG00000157227	16598420;
C004541	decrease	NDUFA5	ENSG00000128609	12111696;
C004541	decrease	NONO	ENSG00000147140	12111696;
C004541	decrease	PFDN5	ENSG00000123349	12111696;
C004541	decrease	PLAUR	ENSG00000011422	16598420;
C004541	decrease	RELA	ENSG00000173039	12111696;
C004541	decrease	RIF1	ENSG00000080345	12111696;
C004541	decrease	RPUSD3	ENSG00000156990	12111696;
C004541	decrease	SFRS1	ENSG00000136450	12111696;
C004541	decrease	SLC25A3	ENSG00000075415	12111696;
C004541	decrease	TNFRSF21	ENSG00000146072	12111696;
C004541	decrease	UBE2D2	ENSG00000131508	12111696;
C004541	decrease	WDR74	ENSG00000133316	12111696;
C004541	decrease	WWC2	ENSG00000151718	12111696;
C004541	increase	ARID5B	ENSG00000150347	12111696;
C004541	increase	BRE	ENSG00000158019	12111696;
C004541	increase	CYP27B1	ENSG00000111012	17659868;15795327;
C004541	increase	FBL	ENSG00000105202	12111696;
C004541	increase	KIAA0247	ENSG00000100647	12111696;
C004541	increase	LXN	ENSG00000079257	12111696;
C004541	increase	MT2A	ENSG00000125148	10656621;
C004541	increase	PGR	ENSG00000082175	14579009;16076101;
C004541	increase	SH3GL1	ENSG00000141985	12111696;
C004541	increase	TUBB	ENSG00000229684	12111696;
C004648	affect	ABCA7	ENSG00000064687	17440010;
C004648	affect	ABHD2	ENSG00000140526	17440010;
C004648	affect	ABLIM3	ENSG00000173210	17440010;
C004648	affect	ACBD3	ENSG00000182827	17440010;
C004648	affect	ACVR2B	ENSG00000114739	17440010;
C004648	affect	ADAM21	ENSG00000139985	17440010;
C004648	affect	ADAMTSL3	ENSG00000156218	17440010;
C004648	affect	ADNP2	ENSG00000101544	17440010;
C004648	affect	AGGF1	ENSG00000164252	17440010;
C004648	affect	AKAP13	ENSG00000170776	17440010;
C004648	affect	AKR1C1	ENSG00000187134	17440010;
C004648	affect	AKR1C2	ENSG00000151632	17440010;
C004648	affect	AKR1C3	ENSG00000196139	17440010;
C004648	affect	ALDH1A3	ENSG00000184254	17440010;
C004648	affect	ALDH1B1	ENSG00000137124	17440010;
C004648	affect	ALKBH4	ENSG00000160993	17440010;
C004648	affect	AMOT	ENSG00000126016	17440010;
C004648	affect	ANAPC5	ENSG00000089053	17440010;
C004648	affect	ANGPT1	ENSG00000154188	17440010;
C004648	affect	ANK1	ENSG00000029534	17440010;
C004648	affect	ANK3	ENSG00000151150	17440010;
C004648	affect	ANKRD2	ENSG00000165887	17440010;
C004648	affect	AP2B1	ENSG00000006125	17440010;

C004648	affect	AP4S1	ENSG00000100478	17440010;
C004648	affect	API5	ENSG00000166181	17440010;
C004648	affect	APOBEC3C	ENSG00000244509	17440010;
C004648	affect	APP	ENSG00000142192	17440010;
C004648	affect	AREG	ENSG00000205595	17440010;
C004648	affect	ARMCX3	ENSG00000102401	17440010;
C004648	affect	ARPC1B	ENSG00000130429	17440010;
C004648	affect	ASB13	ENSG00000196372	17440010;
C004648	affect	ASPN	ENSG00000106819	17440010;
C004648	affect	ASS1	ENSG00000130707	17440010;
C004648	affect	ASTN2	ENSG00000148219	17440010;
C004648	affect	ATHL1	ENSG00000142102	17440010;
C004648	affect	ATP2B1	ENSG00000070961	17440010;
C004648	affect	ATP6V1A	ENSG00000114573	17440010;
C004648	affect	ATP8B1	ENSG00000081923	17440010;
C004648	affect	B4GALT4	ENSG00000121578	17440010;
C004648	affect	BAG5	ENSG00000166170	17440010;
C004648	affect	BAT2L2	ENSG00000117523	17440010;
C004648	affect	BAZ1A	ENSG00000198604	17440010;
C004648	affect	BBX	ENSG00000114439	17440010;
C004648	affect	BCLAF1	ENSG00000029363	17440010;
C004648	affect	BCORL1	ENSG00000085185	17440010;
C004648	affect	BHMT2	ENSG00000132840	17440010;
C004648	affect	BMP2K	ENSG00000138756	17440010;
C004648	affect	BNIP3L	ENSG00000104765	17440010;
C004648	affect	BRD2	ENSG00000204256	17440010;
C004648	affect	BTD	ENSG00000169814	17440010;
C004648	affect	C17ORF39	ENSG00000141034	17440010;
C004648	affect	C17ORF81	ENSG00000170291	17440010;
C004648	affect	C19ORF6	ENSG00000182087	17440010;
C004648	affect	C1ORF54	ENSG00000118292	17440010;
C004648	affect	C1QA	ENSG00000173372	17440010;
C004648	affect	C2ORF34	ENSG00000143919	17440010;
C004648	affect	C4ORF31	ENSG00000173376	17440010;
C004648	affect	C6ORF108	ENSG00000112667	17440010;
C004648	affect	CAMSAP1L	ENSG00000118200	17440010;
C004648	affect	CAPN2	ENSG00000162909	17440010;
C004648	affect	CAPN6	ENSG00000077274	17440010;
C004648	affect	CASP6	ENSG00000138794	17440010;
C004648	affect	CBR1	ENSG00000159228	17440010;
C004648	affect	CBX5	ENSG00000094916	17440010;
C004648	affect	CCDC85B	ENSG00000175602	17440010;
C004648	affect	CCHCR1	ENSG00000204536	17440010;
C004648	affect	CCNE2	ENSG00000175305	17440010;
C004648	affect	CCR1	ENSG00000163823	17440010;
C004648	affect	CCS	ENSG00000173992	17440010;
C004648	affect	CD151	ENSG00000177697	17440010;
C004648	affect	CD53	ENSG00000143119	17440010;
C004648	affect	CDK1	ENSG00000170312	17440010;
C004648	affect	CDKAL1	ENSG00000145996	17440010;
C004648	affect	CEP57	ENSG00000166037	17440010;
C004648	affect	CES2	ENSG00000172831	17440010;
C004648	affect	CFH	ENSG00000000971	17440010;
C004648	affect	CHD4	ENSG00000111642	17440010;

C004648	affect	CHD8	ENSG00000100888	17440010;
C004648	affect	CKAP2	ENSG00000136108	17440010;
C004648	affect	CKS2	ENSG00000123975	17440010;
C004648	affect	CLCN3	ENSG00000109572	17440010;
C004648	affect	CLEC2B	ENSG00000110852	17440010;
C004648	affect	CLIC4	ENSG00000169504	17440010;
C004648	affect	CLUAP1	ENSG00000103351	17440010;
C004648	affect	CNOT3	ENSG00000088038	17440010;
C004648	affect	CNOT4	ENSG00000080802	17440010;
C004648	affect	COL14A1	ENSG00000187955	17440010;
C004648	affect	COL1A1	ENSG00000108821	17440010;
C004648	affect	COL1A2	ENSG00000164692	17440010;
C004648	affect	COL3A1	ENSG00000168542	17440010;
C004648	affect	COL4A2	ENSG00000134871	17440010;
C004648	affect	COL5A1	ENSG00000130635	17440010;
C004648	affect	COL5A2	ENSG00000204262	17440010;
C004648	affect	COL6A1	ENSG00000142156	17440010;
C004648	affect	COL6A2	ENSG00000142173	17440010;
C004648	affect	CPNE3	ENSG00000085719	17440010;
C004648	affect	CRIM1	ENSG00000150938	17440010;
C004648	affect	CTDP1	ENSG00000060069	17440010;
C004648	affect	CTSS	ENSG00000163131	17440010;
C004648	affect	CUL5	ENSG00000166266	17440010;
C004648	affect	CYBA	ENSG00000051523	17440010;
C004648	affect	CYHR1	ENSG00000187954	17440010;
C004648	affect	DAB2	ENSG00000153071	17440010;
C004648	affect	DDX17	ENSG00000100201	17440010;
C004648	affect	DDX24	ENSG00000089737	17440010;
C004648	affect	DDX3Y	ENSG00000067048	17440010;
C004648	affect	DHRS4	ENSG00000157326	17440010;
C004648	affect	DHX29	ENSG00000067248	17440010;
C004648	affect	DHX38	ENSG00000140829	17440010;
C004648	affect	DIAPH2	ENSG00000147202	17440010;
C004648	affect	DIP2A	ENSG00000248658	17440010;
C004648	affect	DKK2	ENSG00000155011	17440010;
C004648	affect	DNAJB14	ENSG00000164031	17440010;
C004648	affect	DNAJC4	ENSG00000110011	17440010;
C004648	affect	DNAJC7	ENSG00000168259	17440010;
C004648	affect	DPT	ENSG00000143196	17440010;
C004648	affect	DSN1	ENSG00000149636	17440010;
C004648	affect	E2F3	ENSG00000112242	17440010;
C004648	affect	ECT2	ENSG00000114346	17440010;
C004648	affect	EDNRA	ENSG00000151617	17440010;
C004648	affect	EDNRB	ENSG00000136160	17440010;
C004648	affect	EFEMP2	ENSG00000172638	17440010;
C004648	affect	EFNB2	ENSG00000125266	17440010;
C004648	affect	EHBP1L1	ENSG00000173442	17440010;
C004648	affect	EIF2AK2	ENSG00000055332	17440010;
C004648	affect	EIF2C3	ENSG00000126070	17440010;
C004648	affect	EIF2S1	ENSG00000134001	17440010;
C004648	affect	EIF4G1	ENSG00000114867	17440010;
C004648	affect	EIF4G3	ENSG00000075151	17440010;
C004648	affect	ELF1	ENSG00000120690	17440010;
C004648	affect	ELK1	ENSG00000126767	17440010;

C004648	affect	EMD	ENSG00000102119	17440010;
C004648	affect	ENG	ENSG00000106991	17440010;
C004648	affect	ENPP1	ENSG00000197594	17440010;
C004648	affect	ENTPD1	ENSG00000138185	17440010;
C004648	affect	EPHB4	ENSG00000196411	17440010;
C004648	affect	EPRS	ENSG00000136628	17440010;
C004648	affect	ERAP2	ENSG00000164308	17440010;
C004648	affect	ERC1	ENSG00000082805	17440010;
C004648	affect	ESF1	ENSG00000089048	17440010;
C004648	affect	ESRRG	ENSG00000196482	17440010;
C004648	affect	EXTL1	ENSG00000158008	17440010;
C004648	affect	EZR	ENSG00000092820	17440010;
C004648	affect	FAF2	ENSG00000113194	17440010;
C004648	affect	FAM115A	ENSG00000198420	17440010;
C004648	affect	FAM134C	ENSG00000141699	17440010;
C004648	affect	FAM149A	ENSG00000109794	17440010;
C004648	affect	FANCC	ENSG00000158169	17440010;
C004648	affect	FASTK	ENSG00000164896	17440010;
C004648	affect	FBXO40	ENSG00000163833	17440010;
C004648	affect	FCER1G	ENSG00000158869	17440010;
C004648	affect	FCGR2A	ENSG00000143226	17440010;
C004648	affect	FEZ1	ENSG00000149557	17440010;
C004648	affect	FHIT	ENSG00000189283	17440010;
C004648	affect	FKBP4	ENSG00000004478	17440010;
C004648	affect	FKBP5	ENSG00000096060	17440010;
C004648	affect	FKBP8	ENSG00000105701	17440010;
C004648	affect	FLRT2	ENSG00000185070	17440010;
C004648	affect	FMO2	ENSG00000094963	17440010;
C004648	affect	FRZB	ENSG00000162998	17440010;
C004648	affect	FXR1	ENSG00000114416	17440010;
C004648	affect	FXR2	ENSG00000129245	17440010;
C004648	affect	GALK2	ENSG00000156958	17440010;
C004648	affect	GBP1	ENSG00000117228	17440010;
C004648	affect	GBP2	ENSG00000162645	17440010;
C004648	affect	GCAT	ENSG00000100116	17440010;
C004648	affect	GLG1	ENSG00000090863	17440010;
C004648	affect	GNB1	ENSG00000078369	17440010;
C004648	affect	GNL3L	ENSG00000130119	17440010;
C004648	affect	GOLGA1	ENSG00000136935	17440010;
C004648	affect	GOLGA2	ENSG00000167110	17440010;
C004648	affect	GOLGA4	ENSG00000144674	17440010;
C004648	affect	GOLIM4	ENSG00000173905	17440010;
C004648	affect	GPATCH8	ENSG00000186566	17440010;
C004648	affect	GPC1	ENSG00000063660	17440010;
C004648	affect	GPM6B	ENSG00000046653	17440010;
C004648	affect	GPSM2	ENSG00000121957	17440010;
C004648	affect	GREM1	ENSG00000166923	17440010;
C004648	affect	GSK3B	ENSG00000082701	17440010;
C004648	affect	GSTM1	ENSG00000134184	17440010;
C004648	affect	GTPBP8	ENSG00000163607	17440010;
C004648	affect	HADHA	ENSG00000084754	17440010;
C004648	affect	HBG1	ENSG00000213934	17440010;
C004648	affect	HBG2	ENSG00000196565	17440010;
C004648	affect	HCK	ENSG00000101336	17440010;

C004648	affect	HCLS1	ENSG00000180353	17440010;
C004648	affect	HEPH	ENSG00000089472	17440010;
C004648	affect	HIBCH	ENSG00000198130	17440010;
C004648	affect	HIPK1	ENSG00000163349	17440010;
C004648	affect	HIVEP2	ENSG00000010818	17440010;
C004648	affect	HLA-DPA1	ENSG00000168384	17440010;
C004648	affect	HLA-DQA1	ENSG00000196735	17440010;
C004648	affect	HLA-DQB1	ENSG00000179344	17440010;
C004648	affect	HOXA7	ENSG00000122592	17440010;
C004648	affect	HOXB6	ENSG00000108511	17440010;
C004648	affect	HOXB7	ENSG00000120087	17440010;
C004648	affect	HPS1	ENSG00000107521	17440010;
C004648	affect	HSPA1B	ENSG00000232804	17440010;
C004648	affect	HSPA4	ENSG00000170606	17440010;
C004648	affect	HTATIP2	ENSG00000109854	17440010;
C004648	affect	HTATSF1	ENSG00000102241	17440010;
C004648	affect	HYAL4	ENSG00000106302	17440010;
C004648	affect	IDS	ENSG00000010404	17440010;
C004648	affect	IFI16	ENSG00000163565	17440010;
C004648	affect	IFI27	ENSG00000165949	17440010;
C004648	affect	IFI44L	ENSG00000137959	17440010;
C004648	affect	IFI6	ENSG00000126709	17440010;
C004648	affect	IFIT3	ENSG00000119917	17440010;
C004648	affect	IL10RB	ENSG00000243646	17440010;
C004648	affect	IL6R	ENSG00000160712	17440010;
C004648	affect	IL6ST	ENSG00000134352	17440010;
C004648	affect	INPPL1	ENSG00000165458	17440010;
C004648	affect	INTS6	ENSG00000249168	17440010;
C004648	affect	ISG15	ENSG00000187608	17440010;
C004648	affect	ITGAV	ENSG00000138448	17440010;
C004648	affect	KCNJ12	ENSG00000184185	17440010;
C004648	affect	KDM5A	ENSG00000073614	17440010;
C004648	affect	KIAA0090	ENSG00000127463	17440010;
C004648	affect	KIAA0100	ENSG00000007202	17440010;
C004648	affect	KIAA0562	ENSG00000116198	17440010;
C004648	affect	KIAA0776	ENSG00000014123	17440010;
C004648	affect	KIAA1033	ENSG00000136051	17440010;
C004648	affect	KLHL24	ENSG00000114796	17440010;
C004648	affect	KPNB1	ENSG00000108424	17440010;
C004648	affect	KRAS	ENSG00000133703	17440010;
C004648	affect	LAMA4	ENSG00000112769	17440010;
C004648	affect	LAPTM5	ENSG00000162511	17440010;
C004648	affect	LIMK2	ENSG00000182541	17440010;
C004648	affect	LIMS1	ENSG00000169756	17440010;
C004648	affect	LOXL2	ENSG00000134013	17440010;
C004648	affect	LRRC8B	ENSG00000197147	17440010;
C004648	affect	LTBP1	ENSG00000049323	17440010;
C004648	affect	LTBP3	ENSG00000168056	17440010;
C004648	affect	LUC7L3	ENSG00000108848	17440010;
C004648	affect	LUM	ENSG00000139329	17440010;
C004648	affect	MACF1	ENSG00000127603	17440010;
C004648	affect	MAN2B2	ENSG00000013288	17440010;
C004648	affect	MAPT	ENSG00000186868	17440010;
C004648	affect	MARCKS	ENSG00000155130	17440010;

C004648	affect	MBD4	ENSG00000129071	17440010;
C004648	affect	MBNL2	ENSG00000139793	17440010;
C004648	affect	MED8	ENSG00000159479	17440010;
C004648	affect	METTL4	ENSG00000101574	17440010;
C004648	affect	MLL	ENSG00000118058	17440010;
C004648	affect	MPDZ	ENSG00000107186	17440010;
C004648	affect	MPHOSPH9	ENSG00000051825	17440010;
C004648	affect	MRPS31	ENSG00000102738	17440010;
C004648	affect	MRPS6	ENSG00000243927	17440010;
C004648	affect	MSN	ENSG00000147065	17440010;
C004648	affect	MTUS1	ENSG00000129422	17440010;
C004648	affect	MUM1	ENSG00000160953	17440010;
C004648	affect	MX1	ENSG00000157601	17440010;
C004648	affect	MX2	ENSG00000183486	17440010;
C004648	affect	MXD4	ENSG00000123933	17440010;
C004648	affect	MXRA5	ENSG00000101825	17440010;
C004648	affect	MYH11	ENSG00000133392	17440010;
C004648	affect	MYH3	ENSG00000109063	17440010;
C004648	affect	MYL5	ENSG00000215375	17440010;
C004648	affect	MYO9A	ENSG00000066933	17440010;
C004648	affect	N4BP2L2	ENSG00000244754	17440010;
C004648	affect	NAA15	ENSG00000164134	17440010;
C004648	affect	NDRG2	ENSG00000165795	17440010;
C004648	affect	NEK1	ENSG00000137601	17440010;
C004648	affect	NES	ENSG00000132688	17440010;
C004648	affect	NEURL	ENSG00000107954	17440010;
C004648	affect	NFATC2IP	ENSG00000176953	17440010;
C004648	affect	NKTR	ENSG00000114857	17440010;
C004648	affect	NLK	ENSG00000087095	17440010;
C004648	affect	NNMT	ENSG00000166741	17440010;
C004648	affect	NPEPPS	ENSG00000141279	17440010;
C004648	affect	OAS2	ENSG00000111335	17440010;
C004648	affect	OAZ2	ENSG00000180304	17440010;
C004648	affect	OGDH	ENSG00000105953	17440010;
C004648	affect	OGFOD2	ENSG00000111325	17440010;
C004648	affect	OGFR	ENSG00000060491	17440010;
C004648	affect	ORC4L	ENSG00000115947	17440010;
C004648	affect	OSBPL8	ENSG00000091039	17440010;
C004648	affect	PALLD	ENSG00000129116	17440010;
C004648	affect	PALM2	ENSG00000243444	17440010;
C004648	affect	PARD3	ENSG00000148498	17440010;
C004648	affect	PCDH17	ENSG00000118946	17440010;
C004648	affect	PCYOX1	ENSG00000116005	17440010;
C004648	affect	PDCD4	ENSG00000150593	17440010;
C004648	affect	PDE4DIP	ENSG00000178104	17440010;
C004648	affect	PDK4	ENSG00000004799	17440010;
C004648	affect	PDXK	ENSG00000160209	17440010;
C004648	affect	PFKL	ENSG00000141959	17440010;
C004648	affect	PHF14	ENSG00000106443	17440010;
C004648	affect	PHF3	ENSG00000118482	17440010;
C004648	affect	PHLDA1	ENSG00000139289	17440010;
C004648	affect	PICALM	ENSG00000073921	17440010;
C004648	affect	PITPNA	ENSG00000174238	17440010;
C004648	affect	PLEKHA1	ENSG00000107679	17440010;

C004648	affect	PLOD2	ENSG00000152952	17440010;
C004648	affect	PMVK	ENSG00000163344	17440010;
C004648	affect	PPAP2B	ENSG00000162407	17440010;
C004648	affect	PPIB	ENSG00000166794	17440010;
C004648	affect	PPIG	ENSG00000138398	17440010;
C004648	affect	PPM1A	ENSG00000100614	17440010;
C004648	affect	PPP1R12A	ENSG00000058272	17440010;
C004648	affect	PPP1R3A	ENSG00000154415	17440010;
C004648	affect	PPP1R7	ENSG00000115685	17440010;
C004648	affect	PPP1R9A	ENSG00000158528	17440010;
C004648	affect	PPP4C	ENSG00000149923	17440010;
C004648	affect	PPPDE1	ENSG00000121644	17440010;
C004648	affect	PRF1	ENSG00000180644	17440010;
C004648	affect	PRKAA2	ENSG00000162409	17440010;
C004648	affect	PRKAB2	ENSG00000131791	17440010;
C004648	affect	PRKACB	ENSG00000142875	17440010;
C004648	affect	PRKAR2A	ENSG00000114302	17440010;
C004648	affect	PRKG1	ENSG00000185532	17440010;
C004648	affect	PROX1	ENSG00000117707	17440010;
C004648	affect	PRSS22	ENSG00000005001	17440010;
C004648	affect	PRUNE2	ENSG00000106772	17440010;
C004648	affect	PSME4	ENSG00000068878	17440010;
C004648	affect	PSPH	ENSG00000146733	17440010;
C004648	affect	PTBP1	ENSG00000011304	17440010;
C004648	affect	PTCD2	ENSG00000049883	17440010;
C004648	affect	PTMA	ENSG00000187514	17440010;
C004648	affect	PTN	ENSG00000105894	17440010;
C004648	affect	PTPN4	ENSG00000088179	17440010;
C004648	affect	PTPRD	ENSG00000153707	17440010;
C004648	affect	PTRF	ENSG00000177469	17440010;
C004648	affect	PVALB	ENSG00000100362	17440010;
C004648	affect	PXDN	ENSG00000130508	17440010;
C004648	affect	RAB14	ENSG00000119396	17440010;
C004648	affect	RAB27A	ENSG00000069974	17440010;
C004648	affect	RAD50	ENSG00000113522	17440010;
C004648	affect	RANBP2	ENSG00000153201	17440010;
C004648	affect	RAP2A	ENSG00000125249	17440010;
C004648	affect	RAPGEF2	ENSG00000109756	17440010;
C004648	affect	RBBP5	ENSG00000117222	17440010;
C004648	affect	RBL2	ENSG00000103479	17440010;
C004648	affect	RBM25	ENSG00000119707	17440010;
C004648	affect	RBM41	ENSG00000089682	17440010;
C004648	affect	RGN	ENSG00000130988	17440010;
C004648	affect	RHOBTB3	ENSG00000164292	17440010;
C004648	affect	RNGTT	ENSG00000111880	17440010;
C004648	affect	RPL30	ENSG00000156482	17440010;
C004648	affect	RRAD	ENSG00000166592	17440010;
C004648	affect	RRBP1	ENSG00000125844	17440010;
C004648	affect	RRP7A	ENSG00000189306	17440010;
C004648	affect	RSAD2	ENSG00000134321	17440010;
C004648	affect	RSL1D1	ENSG00000171490	17440010;
C004648	affect	RSRC1	ENSG00000174891	17440010;
C004648	affect	RUFY3	ENSG00000018189	17440010;
C004648	affect	RXRB	ENSG00000235712	17440010;

C004648	affect	SART3	ENSG00000075856	17440010;
C004648	affect	SC65	ENSG00000141696	17440010;
C004648	affect	SCAMP1	ENSG00000085365	17440010;
C004648	affect	SCD	ENSG00000099194	17440010;
C004648	affect	SCPEP1	ENSG00000121064	17440010;
C004648	affect	SCRG1	ENSG00000164106	17440010;
C004648	affect	SEC14L1	ENSG00000129657	17440010;
C004648	affect	SEC23B	ENSG00000101310	17440010;
C004648	affect	SEC23IP	ENSG00000107651	17440010;
C004648	affect	SEC61A1	ENSG00000058262	17440010;
C004648	affect	SERBP1	ENSG00000142864	17440010;
C004648	affect	SERPINA3	ENSG00000196136	17440010;
C004648	affect	SF3B1	ENSG00000115524	17440010;
C004648	affect	SFRS14	ENSG00000064607	17440010;
C004648	affect	SFRS2IP	ENSG00000139218	17440010;
C004648	affect	SFRS6	ENSG00000124193	17440010;
C004648	affect	SFXN3	ENSG00000107819	17440010;
C004648	affect	SGK3	ENSG00000104205	17440010;
C004648	affect	SLC16A7	ENSG00000118596	17440010;
C004648	affect	SLC25A16	ENSG00000122912	17440010;
C004648	affect	SLC2A14	ENSG00000173262	17440010;
C004648	affect	SLC35D1	ENSG00000116704	17440010;
C004648	affect	SLC38A4	ENSG00000139209	17440010;
C004648	affect	SLC38A6	ENSG00000139974	17440010;
C004648	affect	SLC4A7	ENSG00000033867	17440010;
C004648	affect	SLCO1A2	ENSG00000084453	17440010;
C004648	affect	SMAD4	ENSG00000141646	17440010;
C004648	affect	SMAD5	ENSG00000113658	17440010;
C004648	affect	SMC2	ENSG00000136824	17440010;
C004648	affect	SMC3	ENSG00000108055	17440010;
C004648	affect	SMC5	ENSG00000198887	17440010;
C004648	affect	SMG1	ENSG00000157106	17440010;
C004648	affect	SNAP29	ENSG00000099940	17440010;
C004648	affect	SNAPC1	ENSG00000023608	17440010;
C004648	affect	SNCA	ENSG00000145335	17440010;
C004648	affect	SNRNP35	ENSG00000184209	17440010;
C004648	affect	SNRPE	ENSG00000182004	17440010;
C004648	affect	SNRPN	ENSG00000128739	17440010;
C004648	affect	SORBS1	ENSG00000095637	17440010;
C004648	affect	SOS1	ENSG00000115904	17440010;
C004648	affect	SOS2	ENSG00000100485	17440010;
C004648	affect	SOX4	ENSG00000124766	17440010;
C004648	affect	SP110	ENSG00000135899	17440010;
C004648	affect	SPAG9	ENSG00000008294	17440010;
C004648	affect	SQSTM1	ENSG00000161011	17440010;
C004648	affect	SRPK2	ENSG00000135250	17440010;
C004648	affect	SRPX	ENSG00000101955	17440010;
C004648	affect	SRRM2	ENSG00000167978	17440010;
C004648	affect	SSB	ENSG00000138385	17440010;
C004648	affect	SSBP1	ENSG00000106028	17440010;
C004648	affect	SSPN	ENSG00000123096	17440010;
C004648	affect	ST3GAL6	ENSG00000064225	17440010;
C004648	affect	ST7	ENSG00000004866	17440010;
C004648	affect	STAT1	ENSG00000115415	17440010;

C004648	affect	STK10	ENSG00000072786	17440010;
C004648	affect	STK38	ENSG00000112079	17440010;
C004648	affect	STRN3	ENSG00000196792	17440010;
C004648	affect	SYNCRIP	ENSG00000135316	17440010;
C004648	affect	TBX1	ENSG00000184058	17440010;
C004648	affect	TCAP	ENSG00000173991	17440010;
C004648	affect	TCEB3	ENSG00000011007	17440010;
C004648	affect	TCF25	ENSG00000141002	17440010;
C004648	affect	TCOF1	ENSG00000070814	17440010;
C004648	affect	TGOLN2	ENSG00000152291	17440010;
C004648	affect	THBS4	ENSG00000113296	17440010;
C004648	affect	THNSL1	ENSG00000185875	17440010;
C004648	affect	TIMP3	ENSG00000100234	17440010;
C004648	affect	TIPIN	ENSG00000075131	17440010;
C004648	affect	TIPRL	ENSG00000143155	17440010;
C004648	affect	TLR3	ENSG00000164342	17440010;
C004648	affect	TMBIM1	ENSG00000135926	17440010;
C004648	affect	TMEM11	ENSG00000178307	17440010;
C004648	affect	TMEM159	ENSG00000011638	17440010;
C004648	affect	TMF1	ENSG00000144747	17440010;
C004648	affect	TNFSF10	ENSG00000121858	17440010;
C004648	affect	TNPO3	ENSG00000064419	17440010;
C004648	affect	TOR3A	ENSG00000186283	17440010;
C004648	affect	TOX4	ENSG00000092203	17440010;
C004648	affect	TPD52L1	ENSG00000111907	17440010;
C004648	affect	TPD52L2	ENSG00000101150	17440010;
C004648	affect	TPR	ENSG00000047410	17440010;
C004648	affect	TRA2A	ENSG00000164548	17440010;
C004648	affect	TRAPPC6A	ENSG00000007255	17440010;
C004648	affect	TRIM16	ENSG00000221926	17440010;
C004648	affect	TRIM38	ENSG00000112343	17440010;
C004648	affect	TSHZ1	ENSG00000179981	17440010;
C004648	affect	TTC3	ENSG00000182670	17440010;
C004648	affect	TTC9	ENSG00000133985	17440010;
C004648	affect	TXNIP	ENSG00000117289	17440010;
C004648	affect	TYRP1	ENSG00000107165	17440010;
C004648	affect	UBE2W	ENSG00000104343	17440010;
C004648	affect	UBN1	ENSG00000118900	17440010;
C004648	affect	UGCG	ENSG00000148154	17440010;
C004648	affect	USP1	ENSG00000162607	17440010;
C004648	affect	USP2	ENSG00000036672	17440010;
C004648	affect	USP31	ENSG00000103404	17440010;
C004648	affect	USP33	ENSG00000077254	17440010;
C004648	affect	USP34	ENSG00000115464	17440010;
C004648	affect	USP47	ENSG00000170242	17440010;
C004648	affect	VASP	ENSG00000125753	17440010;
C004648	affect	VCP	ENSG00000165280	17440010;
C004648	affect	WASL	ENSG00000106299	17440010;
C004648	affect	WDR18	ENSG00000065268	17440010;
C004648	affect	WDR46	ENSG00000227057	17440010;
C004648	affect	WDR48	ENSG00000114742	17440010;
C004648	affect	WIPF1	ENSG00000115935	17440010;
C004648	affect	WNK1	ENSG00000060237	17440010;
C004648	affect	WWP2	ENSG00000198373	17440010;

C004648	affect	YEATS2	ENSG00000163872	17440010;
C004648	affect	YKT6	ENSG00000106636	17440010;
C004648	affect	YWHAB	ENSG00000166913	17440010;
C004648	affect	ZEB1	ENSG00000148516	17440010;
C004648	affect	ZFP37	ENSG00000136866	17440010;
C004648	affect	ZGPAT	ENSG00000197114	17440010;
C004648	affect	ZIC1	ENSG00000152977	17440010;
C004648	affect	ZMYM1	ENSG00000197056	17440010;
C004648	affect	ZMYM5	ENSG00000132950	17440010;
C004648	affect	ZNF148	ENSG00000163848	17440010;
C004648	affect	ZNF264	ENSG00000083844	17440010;
C004648	affect	ZNF443	ENSG00000180855	17440010;
C004648	affect	ZNF44	ENSG00000197857	17440010;
C004648	affect	ZNF510	ENSG00000081386	17440010;
C004648	affect	ZNF562	ENSG00000171466	17440010;
C004648	increase	AR	ENSG00000169083	17440010;
C004648	increase	ATRX	ENSG00000085224	17440010;
C004648	increase	NCOA3	ENSG00000124151	17440010;
C004648	increase	NFAT5	ENSG00000102908	17440010;
C004648	increase	RORA	ENSG00000069667	17440010;
C004648	increase	SPEN	ENSG00000065526	17440010;
C004648	increase	TCF4	ENSG00000196628	17440010;
C004742	affect	VCAM1	ENSG00000162692	16332659;
C004742	decrease	ABCA1	ENSG00000165029	16963248;
C004742	decrease	ADD2	ENSG00000075340	16963248;
C004742	decrease	ARHGDIA	ENSG00000141522	15757668;
C004742	decrease	ATP6V0A2	ENSG00000185344	16963248;
C004742	decrease	COMT	ENSG00000093010	18192686;18497090;
C004742	decrease	CRP	ENSG00000132693	16332659;
C004742	decrease	CYP19A1	ENSG00000137869	16965912;16023337;
C004742	decrease	CYP1A1	ENSG00000140465	18497090;
C004742	decrease	DOCK3	ENSG00000088538	16963248;
C004742	decrease	DUSP4	ENSG00000120875	16865672;
C004742	decrease	ENO3	ENSG00000108515	15757668;
C004742	decrease	ESPN	ENSG00000187017	16963248;
C004742	decrease	ESR1	ENSG00000091831	16965913;12650720;14579009;16506809;16773531;9751507;16076101;15876408;17266178;15084758;16118406;16033089;
C004742	decrease	IGF1R	ENSG00000140443	16865672;
C004742	decrease	KLK3	ENSG00000142515	16865672;
C004742	decrease	NKX3-1	ENSG00000167034	16865672;
C004742	decrease	NQO1	ENSG00000181019	18497090;
C004742	decrease	POU4F2	ENSG00000151615	16963248;
C004742	decrease	PRKCD	ENSG00000163932	15757668;
C004742	decrease	SGPP1	ENSG00000126821	16963248;
C004742	decrease	SLC12A2	ENSG00000064651	15757668;
C004742	decrease	STC1	ENSG00000159167	16963248;
C004742	decrease	STK39	ENSG00000198648	16865672;
C004742	decrease	TNFAIP6	ENSG00000123610	16963248;
C004742	decrease	TP53I11	ENSG00000175274	15757668;
C004742	decrease	UCHL1	ENSG00000154277	16963248;
C004742	increase	ALPPL2	ENSG00000163286	15993745;

C004742	increase	BAZ1A	ENSG00000198604	16963248;
C004742	increase	BRCA1	ENSG00000012048	17266178;
C004742	increase	CCND1	ENSG00000110092	17266178;
C004742	increase	COX7A2L	ENSG00000115944	17266178;
C004742	increase	EBAG9	ENSG00000147654	17266178;
C004742	increase	EPHA1	ENSG00000146904	16963248;
C004742	increase	ESR2	ENSG00000140009	16332659;16965913;15993745;16566672;16506809;16773531;9751507;16076101;15084758;16118406;18937077;17266178;16033089;
C004742	increase	FMN2	ENSG00000155816	16963248;
C004742	increase	FOS	ENSG00000170345	17142977;
C004742	increase	IFRD2	ENSG00000214706	16963248;
C004742	increase	IGFBP4	ENSG00000141753	15757668;
C004742	increase	IRF2BP1	ENSG00000170604	16963248;
C004742	increase	JUN	ENSG00000177606	17266178;
C004742	increase	KCNG2	ENSG00000178342	16963248;
C004742	increase	ME1	ENSG00000065833	16865672;
C004742	increase	MT2A	ENSG00000125148	10656621;
C004742	increase	MYC	ENSG00000136997	17266178;
C004742	increase	NRIP1	ENSG00000180530	15757668;
C004742	increase	OLFML3	ENSG00000116774	16963248;
C004742	increase	PDLIM2	ENSG00000120913	16963248;
C004742	increase	PDZK1	ENSG00000174827	15757668;
C004742	increase	PGR	ENSG00000082175	17142977;
C004742	increase	PNN	ENSG00000100941	16963248;
C004742	increase	PTGS2	ENSG00000073756	16870006;
C004742	increase	RBBP8	ENSG00000101773	15757668;
C004742	increase	RPS6KA3	ENSG00000177189	15757668;
C004742	increase	SLC7A5	ENSG00000103257	15757668;
C004742	increase	TACR3	ENSG00000169836	16963248;
C004742	increase	TFF1	ENSG00000160182	16965913;
C004742	increase	TPD52L1	ENSG00000111907	15757668;
C004742	increase	UGT2B17	ENSG00000197888	16865672;
C004808	increase	PON1	ENSG00000005421	15690306;14500290;
C004809	decrease	CCR5	ENSG00000160791	11994442;
C004809	increase	FGF9	ENSG00000102678	14704367;
C004809	increase	IFNG	ENSG00000111537	15879110;
C004809	increase	IL2RA	ENSG00000134460	15879110;
C004809	increase	VDR	ENSG00000111424	15879110;
C004912	increase	HSPA5	ENSG00000044574	12183065;
C004934	decrease	BCL2	ENSG00000171791	15961301;
C004934	increase	GADD45A	ENSG00000116717	15961301;
C004934	increase	PPARG	ENSG00000132170	15961301;
C004934	increase	TP53	ENSG00000141510	15961301;
C004965	increase	ABCB1	ENSG00000085563	15266218;16005588;
C004965	increase	CYP3A4	ENSG00000160868	15266218;16005588;
C004984	affect	KRT7	ENSG00000135480	17034753;
C004984	affect	MCL1	ENSG00000143384	17034753;
C004984	affect	SPTLC2	ENSG00000100596	17034753;
C004984	decrease	ALDH1B1	ENSG00000137124	17034753;
C004984	decrease	ALDH4A1	ENSG00000159423	17034753;
C004984	decrease	BAX	ENSG00000087088	17034753;

C004984	decrease	COL1A1	ENSG00000108821	17034753;
C004984	decrease	COL4A1	ENSG00000187498	17034753;
C004984	decrease	COL4A2	ENSG00000134871	17034753;
C004984	decrease	CRABP2	ENSG00000143320	17034753;
C004984	decrease	CTSB	ENSG00000164733	17034753;
C004984	decrease	ERCC2	ENSG00000104884	17034753;
C004984	decrease	ETS1	ENSG00000134954	17034753;
C004984	decrease	FASN	ENSG00000169710	17034753;
C004984	decrease	GSTK1	ENSG00000250907	17034753;
C004984	decrease	GSTM2	ENSG00000213366	17034753;
C004984	decrease	GSTM4	ENSG00000168765	17034753;
C004984	decrease	GSTZ1	ENSG00000100577	17034753;
C004984	decrease	IRX4	ENSG00000113430	17034753;
C004984	decrease	ITGB4	ENSG00000132470	17034753;
C004984	decrease	KRT17	ENSG00000128422	17034753;
C004984	decrease	RARA	ENSG00000131759	17034753;
C004984	decrease	RARRES3	ENSG00000133321	17034753;
C004984	decrease	SOD1	ENSG00000142168	17034753;
C004984	increase	ACADM	ENSG00000117054	17034753;
C004984	increase	ADH1A	ENSG00000187758	17034753;
C004984	increase	ADH5	ENSG00000197894	17034753;
C004984	increase	ANK2	ENSG00000145362	17034753;
C004984	increase	ANK3	ENSG00000151150	17034753;
C004984	increase	ASAH1	ENSG00000104763	17034753;
C004984	increase	ASB1	ENSG00000065802	17034753;
C004984	increase	ASB9	ENSG00000102048	17034753;
C004984	increase	CASP8AP2	ENSG00000118412	17034753;
C004984	increase	CDH1	ENSG00000039068	17034753;
C004984	increase	CLDN10	ENSG00000134873	17034753;
C004984	increase	CLDN18	ENSG00000066405	17034753;
C004984	increase	CXCL12	ENSG00000107562	17034753;
C004984	increase	CXCL6	ENSG00000124875	17034753;
C004984	increase	CXCL9	ENSG00000138755	17034753;
C004984	increase	CXCR4	ENSG00000121966	17034753;
C004984	increase	CYP2C9	ENSG00000138109	17034753;
C004984	increase	DLX2	ENSG00000115844	17034753;
C004984	increase	DLX4	ENSG00000108813	17034753;
C004984	increase	FAS	ENSG00000026103	17034753;
C004984	increase	GATA6	ENSG00000141448	17034753;
C004984	increase	GBP1	ENSG00000117228	17034753;
C004984	increase	GJA9	ENSG00000131233	17034753;
C004984	increase	GSTM5	ENSG00000134201	17034753;
C004984	increase	HOXA7	ENSG00000122592	17034753;
C004984	increase	HOXB6	ENSG00000108511	17034753;
C004984	increase	HOXC10	ENSG00000180818	17034753;
C004984	increase	IFNA14	ENSG00000228083	17034753;
C004984	increase	IFNA16	ENSG00000147885	17034753;
C004984	increase	IFNA17	ENSG00000234829	17034753;
C004984	increase	IFNA5	ENSG00000147873	17034753;
C004984	increase	IRF8	ENSG00000140968	17034753;
C004984	increase	IVL	ENSG00000163207	17034753;
C004984	increase	KRT2	ENSG00000172867	17034753;
C004984	increase	LHX3	ENSG00000107187	17034753;
C004984	increase	MEIS2	ENSG00000134138	17034753;

C004984	increase	MEOX1	ENSG00000005102	17034753;
C004984	increase	MNX1	ENSG00000130675	17034753;
C004984	increase	MSH5	ENSG00000227314	17034753;
C004984	increase	NOS1	ENSG00000089250	17034753;
C004984	increase	OLR1	ENSG00000173391	17034753;
C004984	increase	PCDHGC3	ENSG00000240184	17034753;
C004984	increase	PLSCR1	ENSG00000188313	17034753;
C004984	increase	PLSCR3	ENSG00000187838	17034753;
C004984	increase	PLSCR4	ENSG00000114698	17034753;
C004984	increase	RAD51AP1	ENSG00000111247	17034753;
C004984	increase	RAD51L3	ENSG00000185379	17034753;
C004984	increase	RARB	ENSG00000077092	17034753;
C004984	increase	RARRES1	ENSG00000118849	17034753;
C004984	increase	SIX6	ENSG00000184302	17034753;
C004984	increase	SREBF1	ENSG00000072310	17034753;
C004984	increase	TGM4	ENSG00000163810	17034753;
C004984	increase	TNFRSF9	ENSG00000049249	17034753;
C004984	increase	TNFSF10	ENSG00000121858	17034753;
C004984	increase	TXNDC9	ENSG00000115514	17034753;
C004984	increase	XRCC3	ENSG00000126215	17034753;
C004999	decrease	CCND1	ENSG00000110092	15486966;
C004999	decrease	PTGS2	ENSG00000073756	15841493;
C004999	increase	BACE1	ENSG00000186318	18583042;
C004999	increase	CDKN1A	ENSG00000124762	15486966;
C005266	increase	AKR1C1	ENSG00000187134	18214805;
C005266	increase	CYP2A13	ENSG00000197838	18214805;
C005273	increase	CYP1A1	ENSG00000140465	11454723;
C005273	increase	PON1	ENSG00000005421	15169886;
C005290	increase	TFF1	ENSG00000160182	16183391;
C005406	decrease	HSP90B1	ENSG00000166598	16884889;
C005406	decrease	HSPA5	ENSG00000044574	16884889;
C005406	decrease	HSPB1	ENSG00000106211	16884889;
C005460	affect	AADAT	ENSG00000109576	15672453;
C005460	affect	AANAT	ENSG00000129673	15672453;
C005460	affect	AASDHPPT	ENSG00000149313	15672453;
C005460	affect	ABCC8	ENSG00000006071	15672453;
C005460	affect	ABCF2	ENSG00000033050	15672453;
C005460	affect	ACAT2	ENSG00000120437	15672453;
C005460	affect	ACTB	ENSG00000075624	15672453;
C005460	affect	ACTR2	ENSG00000138071	15672453;
C005460	affect	ADAR	ENSG00000160710	15672453;
C005460	affect	ADNP	ENSG00000101126	15672453;
C005460	affect	AKAP1	ENSG00000121057	15672453;
C005460	affect	AKR1C3	ENSG00000196139	15672453;
C005460	affect	APBB1	ENSG00000166313	15672453;
C005460	affect	APC	ENSG00000134982	15672453;
C005460	affect	APP	ENSG00000142192	15672453;
C005460	affect	APPL1	ENSG00000157500	15672453;
C005460	affect	ATP6V1A	ENSG00000114573	15672453;
C005460	affect	ATP9B	ENSG00000166377	15672453;
C005460	affect	ATXN10	ENSG00000130638	15672453;
C005460	affect	B2M	ENSG00000166710	15672453;
C005460	affect	BAALC	ENSG00000164929	15672453;
C005460	affect	BGN	ENSG00000182492	15672453;

C005460	affect	BTF3	ENSG00000145741	15672453;
C005460	affect	C11ORF49	ENSG00000149179	15672453;
C005460	affect	C12ORF43	ENSG00000157895	15672453;
C005460	affect	C1ORF43	ENSG00000143612	15672453;
C005460	affect	C20ORF30	ENSG00000089063	15672453;
C005460	affect	C21ORF56	ENSG00000160284	15672453;
C005460	affect	C7ORF44	ENSG00000106603	15672453;
C005460	affect	CALM2	ENSG00000143933	15672453;
C005460	affect	CAMK2D	ENSG00000145349	15672453;
C005460	affect	CANX	ENSG00000127022	15672453;
C005460	affect	CBARA1	ENSG00000107745	15672453;
C005460	affect	CBFA2T2	ENSG00000078699	15672453;
C005460	affect	CGB	ENSG00000213030	15672453;
C005460	affect	CLINT1	ENSG00000113282	15672453;
C005460	affect	CLSTN3	ENSG00000139182	15672453;
C005460	affect	COG7	ENSG00000168434	15672453;
C005460	affect	COPS3	ENSG00000141030	15672453;
C005460	affect	CRLS1	ENSG00000088766	15672453;
C005460	affect	CRYBB2	ENSG00000244752	15672453;
C005460	affect	CSDE1	ENSG00000009307	15672453;
C005460	affect	CTCF	ENSG00000102974	15672453;
C005460	affect	CTRL	ENSG00000141086	15672453;
C005460	affect	CUL2	ENSG00000108094	15672453;
C005460	affect	DGKA	ENSG00000065357	15672453;
C005460	affect	DIXDC1	ENSG00000150764	15672453;
C005460	affect	DNAJA2	ENSG00000069345	15672453;
C005460	affect	DNAJC9	ENSG00000213551	15672453;
C005460	affect	DTYMK	ENSG00000168393	15672453;
C005460	affect	EEF1A1	ENSG00000156508	15672453;
C005460	affect	EEF1G	ENSG00000149016	15672453;
C005460	affect	EPS15	ENSG00000085832	15672453;
C005460	affect	ERBB3	ENSG00000065361	15672453;
C005460	affect	EVI5	ENSG00000067208	15672453;
C005460	affect	EVL	ENSG00000196405	15672453;
C005460	affect	FAM3C	ENSG00000196937	15672453;
C005460	affect	FUT8	ENSG00000033170	15672453;
C005460	affect	GLO1	ENSG00000124767	15672453;
C005460	affect	GLUL	ENSG00000135821	15672453;
C005460	affect	GRB2	ENSG00000177885	15672453;
C005460	affect	GTF2H2	ENSG00000145736	15672453;
C005460	affect	HADH	ENSG00000138796	15672453;
C005460	affect	HBP1	ENSG00000105856	15672453;
C005460	affect	HLA-E	ENSG00000225201	15672453;
C005460	affect	HMGB1	ENSG00000189403	15672453;
C005460	affect	HMP19	ENSG00000170091	15672453;
C005460	affect	IARS2	ENSG00000067704	15672453;
C005460	affect	ID2	ENSG00000115738	15672453;
C005460	affect	IFRD2	ENSG00000214706	15672453;
C005460	affect	IK	ENSG00000113141	15672453;
C005460	affect	INSIG1	ENSG00000186480	15672453;
C005460	affect	IQGAP2	ENSG00000145703	15672453;
C005460	affect	KAZ	ENSG00000189337	15672453;
C005460	affect	KDM2B	ENSG00000089094	15672453;
C005460	affect	KDM5B	ENSG00000117139	15672453;

C005460	affect	KIF3B	ENSG00000101350	15672453;
C005460	affect	KIF3C	ENSG00000084731	15672453;
C005460	affect	KPNA3	ENSG00000102753	15672453;
C005460	affect	LAD1	ENSG00000159166	15672453;
C005460	affect	LAMP2	ENSG00000005893	15672453;
C005460	affect	LDHA	ENSG00000134333	15672453;
C005460	affect	LPIN1	ENSG00000134324	15672453;
C005460	affect	M6PR	ENSG00000003056	15672453;
C005460	affect	MARCKS	ENSG00000155130	15672453;
C005460	affect	MAT2B	ENSG00000038274	15672453;
C005460	affect	MCFD2	ENSG00000180398	15672453;
C005460	affect	MDH2	ENSG00000146701	15672453;
C005460	affect	METAP2	ENSG00000111142	15672453;
C005460	affect	MFSD4	ENSG00000174514	15672453;
C005460	affect	MLLT10	ENSG00000078403	15672453;
C005460	affect	MSH2	ENSG00000095002	15672453;
C005460	affect	NAIP	ENSG00000250628	15672453;
C005460	affect	NAMPT	ENSG00000105835	15672453;
C005460	affect	NAP1L1	ENSG00000187109	15672453;
C005460	affect	NARS	ENSG00000134440	15672453;
C005460	affect	NCOA7	ENSG00000111912	15672453;
C005460	affect	NDFIP1	ENSG00000131507	15672453;
C005460	affect	NDRG2	ENSG00000165795	15672453;
C005460	affect	NEFL	ENSG00000104725	15672453;
C005460	affect	NEIL2	ENSG00000154328	15672453;
C005460	affect	NOTCH2	ENSG00000134250	15672453;
C005460	affect	NPEPL1	ENSG00000215440	15672453;
C005460	affect	NRF1	ENSG00000106459	15672453;
C005460	affect	NT5E	ENSG00000135318	15672453;
C005460	affect	OAT	ENSG00000065154	15672453;
C005460	affect	PAIP2B	ENSG00000124374	15672453;
C005460	affect	PBX3	ENSG00000167081	15672453;
C005460	affect	PDCD6IP	ENSG00000170248	15672453;
C005460	affect	PDCD7	ENSG00000090470	15672453;
C005460	affect	PDE1A	ENSG00000115252	15672453;
C005460	affect	PFKFB4	ENSG00000114268	15672453;
C005460	affect	PGD	ENSG00000142657	15672453;
C005460	affect	PHF10	ENSG00000130024	15672453;
C005460	affect	PI4K2A	ENSG00000155252	15672453;
C005460	affect	PIK3R1	ENSG00000145675	15672453;
C005460	affect	PIP4K2B	ENSG00000141720	15672453;
C005460	affect	PLEKHB2	ENSG00000115762	15672453;
C005460	affect	PPIA	ENSG00000196262	15672453;
C005460	affect	PPP2CA	ENSG00000113575	15672453;
C005460	affect	PRDX2	ENSG00000167815	15672453;
C005460	affect	PRPF4B	ENSG00000112739	15672453;
C005460	affect	PSMA4	ENSG00000041357	15672453;
C005460	affect	PSMG1	ENSG00000183527	15672453;
C005460	affect	PTGES3	ENSG00000110958	15672453;
C005460	affect	PTK2	ENSG00000169398	15672453;
C005460	affect	PTMA	ENSG00000187514	15672453;
C005460	affect	PUM1	ENSG00000134644	15672453;
C005460	affect	RAB11FIP4	ENSG00000131242	15672453;
C005460	affect	RABGGTB	ENSG00000137955	15672453;

C005460	affect	RAC1	ENSG00000136238	15672453;
C005460	affect	RAN	ENSG00000132341	15672453;
C005460	affect	RBBP7	ENSG00000102054	15672453;
C005460	affect	RBM8A	ENSG00000131795	15672453;
C005460	affect	RDX	ENSG00000137710	15672453;
C005460	affect	REEP1	ENSG00000068615	15672453;
C005460	affect	RHOA	ENSG00000067560	15672453;
C005460	affect	RHOT1	ENSG00000126858	15672453;
C005460	affect	RMI1	ENSG00000178966	15672453;
C005460	affect	RNF10	ENSG00000022840	15672453;
C005460	affect	RNF123	ENSG00000164068	15672453;
C005460	affect	RNF144A	ENSG00000151692	15672453;
C005460	affect	RNGTT	ENSG00000111880	15672453;
C005460	affect	RPA1	ENSG00000132383	15672453;
C005460	affect	RPL15	ENSG00000174748	15672453;
C005460	affect	RPL22	ENSG00000116251	15672453;
C005460	affect	RPL24	ENSG00000114391	15672453;
C005460	affect	SCAMP1	ENSG00000085365	15672453;
C005460	affect	SCN2B	ENSG00000149575	15672453;
C005460	affect	SCP2	ENSG00000251612	15672453;
C005460	affect	SDC3	ENSG00000162512	15672453;
C005460	affect	SDCBP	ENSG00000137575	15672453;
C005460	affect	SEC24B	ENSG00000138802	15672453;
C005460	affect	SERBP1	ENSG00000142864	15672453;
C005460	affect	SFXN3	ENSG00000107819	15672453;
C005460	affect	SLC17A2	ENSG00000112337	15672453;
C005460	affect	SLC25A6	ENSG00000169100	15672453;
C005460	affect	SLC2A3	ENSG00000059804	15672453;
C005460	affect	SLC44A5	ENSG00000137968	15672453;
C005460	affect	SMAP1	ENSG00000112305	15672453;
C005460	affect	SMARCC1	ENSG00000173473	15672453;
C005460	affect	SMC5	ENSG00000198887	15672453;
C005460	affect	SNAP91	ENSG00000065609	15672453;
C005460	affect	SNX6	ENSG00000129515	15672453;
C005460	affect	SPP2	ENSG00000072080	15672453;
C005460	affect	SRRM1	ENSG00000133226	15672453;
C005460	affect	SSX2IP	ENSG00000117155	15672453;
C005460	affect	STK36	ENSG00000163482	15672453;
C005460	affect	STMN1	ENSG00000117632	15672453;
C005460	affect	STMN2	ENSG00000104435	15672453;
C005460	affect	TALDO1	ENSG00000177156	15672453;
C005460	affect	TDG	ENSG00000139372	15672453;
C005460	affect	TFAM	ENSG00000108064	15672453;
C005460	affect	TGIF2	ENSG00000118707	15672453;
C005460	affect	TGOLN2	ENSG00000152291	15672453;
C005460	affect	TLK1	ENSG00000198586	15672453;
C005460	affect	TNFRSF1B	ENSG00000028137	15672453;
C005460	affect	TNFRSF9	ENSG00000049249	15672453;
C005460	affect	TNP1	ENSG00000118245	15672453;
C005460	affect	TOMM20	ENSG00000173726	15672453;
C005460	affect	TRAK2	ENSG00000115993	15672453;
C005460	affect	TRIM28	ENSG00000130726	15672453;
C005460	affect	TUBA1A	ENSG00000167552	15672453;
C005460	affect	UBE2D2	ENSG00000131508	15672453;

C005460	affect	ULK2	ENSG00000083290	15672453;
C005460	affect	UNG	ENSG00000076248	15672453;
C005460	affect	VDAC2	ENSG00000165637	15672453;
C005460	affect	VPS13B	ENSG00000132549	15672453;
C005460	affect	VPS13D	ENSG00000048707	15672453;
C005460	affect	VPS26B	ENSG00000151502	15672453;
C005460	affect	VSNL1	ENSG00000163032	15672453;
C005460	affect	VTA1	ENSG00000009844	15672453;
C005460	affect	WASL	ENSG00000106299	15672453;
C005460	affect	WDR59	ENSG00000103091	15672453;
C005460	affect	WFDC2	ENSG00000101443	15672453;
C005460	affect	WNT7B	ENSG00000188064	15672453;
C005460	affect	WSB2	ENSG00000176871	15672453;
C005460	affect	YTHDF3	ENSG00000185728	15672453;
C005460	affect	YWHAB	ENSG00000166913	15672453;
C005460	affect	YWHAQ	ENSG00000134308	15672453;
C005460	affect	ZFP82	ENSG00000249760	15672453;
C005460	increase	AKIRIN1	ENSG00000174574	15672453;
C005460	increase	ARMC9	ENSG00000135931	15672453;
C005460	increase	C20ORF12	ENSG00000089091	15672453;
C005460	increase	C5ORF33	ENSG00000152620	15672453;
C005460	increase	CHCHD5	ENSG00000125611	15672453;
C005460	increase	CHRDL1	ENSG00000101938	15672453;
C005460	increase	CHURC1	ENSG00000125954	15672453;
C005460	increase	DPYSL5	ENSG00000157851	15672453;
C005460	increase	DTNA	ENSG00000134769	15672453;
C005460	increase	GSPT1	ENSG00000103342	15672453;
C005460	increase	KIAA1143	ENSG00000163807	15672453;
C005460	increase	KIF1B	ENSG00000054523	15672453;
C005460	increase	LONP2	ENSG00000102910	15672453;
C005460	increase	NDRG4	ENSG00000103034	15672453;
C005460	increase	PARP4	ENSG00000102699	15672453;
C005460	increase	RBM3	ENSG00000102317	15672453;
C005466	decrease	BCL2	ENSG00000171791	16012738;
C005466	increase	BAX	ENSG00000087088	16012738;
C005499	decrease	MCL1	ENSG00000143384	15802533;
C005499	increase	BAX	ENSG00000087088	15802533;
C005556	increase	PTGIS	ENSG00000124212	17255567;
C005581	increase	AGER	ENSG00000204305	17597607;
C005581	increase	S100B	ENSG00000160307	17597607;
C005581	increase	TGFB1	ENSG00000105329	17597607;
C005705	increase	CYP1A1	ENSG00000140465	15993743;
C005705	increase	CYP1B1	ENSG00000138061	15993743;
C005705	increase	GSTP1	ENSG00000084207	15313406;
C005705	increase	TNF	ENSG00000223952	15313406;
C005926	decrease	BID	ENSG00000015475	16019850;
C005969	increase	ICAM1	ENSG00000090339	11698162;
C005975	decrease	CCL5	ENSG00000161570	16797527;
C005975	decrease	IL5	ENSG00000113525	16797527;
C005975	decrease	IL6	ENSG00000136244	16797527;
C005975	decrease	NFE2L1	ENSG00000082641	17949413;
C005975	decrease	STAT2	ENSG00000170581	16797527;
C005975	decrease	TFAM	ENSG00000108064	17949413;
C005975	increase	RPA3	ENSG00000106399	16797527;

C005975	increase	TNF	ENSG00000223952	17586618;
C005975	increase	VCAM1	ENSG00000162692	17586618;
C005984	increase	PCNA	ENSG00000132646	16870008;
C006012	decrease	ADIPOR1	ENSG00000159346	16023994;
C006012	increase	CYP2C8	ENSG00000138115	15771232;
C006012	increase	CYP3A4	ENSG00000160868	15771232;
C006012	increase	PPARA	ENSG00000186951	16680075;
C006012	increase	UGT1A1	ENSG00000241635	15771232;
C006014	increase	PTGS2	ENSG00000073756	12493747;
C006068	increase	TFF1	ENSG00000160182	16797915;
C006166	decrease	ESR1	ENSG00000091831	10966511;15118252;
C006166	decrease	ESR2	ENSG00000140009	15118252;
C006166	increase	TFF1	ENSG00000160182	10064545;15118252;
C006166	increase	WNT10B	ENSG00000169884	12437293;
C006253	decrease	CES1	ENSG00000198848	11409902;
C006253	decrease	PPARA	ENSG00000186951	16292757;17610302;9013583;15491415;16326050;17185614;10581215;15178457;
C006253	decrease	RXRA	ENSG00000186350	16292757;
C006253	decrease	UGDH	ENSG00000109814	16292757;
C006253	increase	CYP1A1	ENSG00000140465	15521013;
C006253	increase	CYP4X1	ENSG00000186377	15797250;
C006253	increase	SULT2A1	ENSG00000105398	15635043;
C006303	increase	CFTR	ENSG00000001626	14766015;
C006453	increase	FGF2	ENSG00000138685	16351512;
C006453	increase	TP53	ENSG00000141510	16351512;
C006552	decrease	CCL5	ENSG00000161570	16797527;
C006552	decrease	CCNB1	ENSG00000134057	18331776;19397994;
C006552	decrease	CDKN2C	ENSG00000123080	18331776;
C006552	decrease	CRP	ENSG00000132693	17184768;
C006552	decrease	CYP1B1	ENSG00000138061	16271822;19794518;
C006552	decrease	FBP1	ENSG00000165140	18331776;
C006552	decrease	IL6	ENSG00000136244	16797527;
C006552	decrease	NOS2	ENSG00000007171	17184768;
C006552	decrease	PSCA	ENSG00000167653	18331776;
C006552	decrease	STAT2	ENSG00000170581	16797527;
C006552	increase	CDKN1A	ENSG00000124762	19397994;
C006552	increase	CYP1A1	ENSG00000140465	17012224;16271822;11454723;
C006552	increase	CYP1A2	ENSG00000140505	19034627;
C006552	increase	CYP3A4	ENSG00000160868	15266218;16442130;
C006552	increase	ESR1	ENSG00000091831	16118406;14706564;14579009;15182386;9751507;
C006552	increase	GADD45B	ENSG00000099860	18331776;
C006552	increase	GCLC	ENSG00000001084	11864778;
C006552	increase	ICAM1	ENSG00000090339	15322261;
C006552	increase	IL1B	ENSG00000125538	19367675;
C006552	increase	IL4	ENSG00000113520	12789233;
C006552	increase	IL5	ENSG00000113525	16797527;12789233;
C006552	increase	JUN	ENSG00000177606	15322261;
C006552	increase	MT2A	ENSG00000125148	10656621;
C006552	increase	PPARGC1A	ENSG00000109819	17327447;
C006552	increase	PTGS2	ENSG00000073756	10783318;17184768;
C006552	increase	RPA3	ENSG00000106399	16797527;

C006552	increase	SFN	ENSG00000175793	18331776;
C006552	increase	TFAM	ENSG00000108064	17327447;
C006552	increase	TFF1	ENSG00000160182	14706564;
C006552	increase	TGFA	ENSG00000163235	10783318;
C006552	increase	TNF	ENSG00000223952	15322261;
C006552	increase	TP53I3	ENSG00000115129	18331776;19397994;
C006552	increase	TP63	ENSG00000073282	18331776;19397994;
C006552	increase	TP73	ENSG00000078900	18331776;19397994;
C006552	increase	UCP3	ENSG00000175564	17327447;
C006632	affect	EGR1	ENSG00000120738	12749819;
C006632	affect	HSPA1A	ENSG00000232804	12749819;
C006632	affect	IER2	ENSG00000160888	12749819;
C006632	affect	JUN	ENSG00000177606	12749819;
C006632	affect	ZFP36	ENSG00000128016	12749819;
C006632	decrease	ABCB1	ENSG00000085563	15979894;14642128;
C006632	decrease	ABL1	ENSG00000097007	14633726;
C006632	decrease	ADCY9	ENSG00000162104	15761015;
C006632	decrease	AKR1B1	ENSG00000085662	17547211;
C006632	decrease	AKT1	ENSG00000142208	17077332;16882451;
C006632	decrease	ALDH6A1	ENSG00000119711	17547211;
C006632	decrease	ALDOC	ENSG00000109107	15725085;
C006632	decrease	ANPEP	ENSG00000166825	15949261;
C006632	decrease	AP3S1	ENSG00000177879	15761015;
C006632	decrease	ARL4C	ENSG00000188042	15725085;
C006632	decrease	ARMC9	ENSG00000135931	15761015;
C006632	decrease	ASNS	ENSG00000070669	17547211;
C006632	decrease	ASS1	ENSG00000130707	15070760;15761015;
C006632	decrease	ATP1B1	ENSG00000143153	15761015;
C006632	decrease	ATP2B1	ENSG00000070961	12852829;17547211;
C006632	decrease	ATP5A1	ENSG00000152234	15949261;
C006632	decrease	AZGP1	ENSG00000160862	17547211;
C006632	decrease	BACH2	ENSG00000112182	15725085;
C006632	decrease	BAMBI	ENSG00000095739	15725085;
C006632	decrease	BCL2L1	ENSG00000171552	11468182;15622746;15665116;16105982;
C006632	decrease	BCMO1	ENSG00000135697	15761015;
C006632	decrease	BCR	ENSG00000186716	14633726;
C006632	decrease	BTBD2	ENSG00000133243	15725085;
C006632	decrease	BTBD3	ENSG00000132640	15725085;
C006632	decrease	C5ORF13	ENSG00000134986	15725085;
C006632	decrease	C6ORF48	ENSG00000234728	17547211;
C006632	decrease	CARS	ENSG00000110619	12852829;
C006632	decrease	CAV1	ENSG00000105974	15725085;
C006632	decrease	CCNA1	ENSG00000133101	15761015;
C006632	decrease	CD1D	ENSG00000158473	15761015;
C006632	decrease	CD44	ENSG00000026508	15553829;
C006632	decrease	CD70	ENSG00000125726	15761015;
C006632	decrease	CD86	ENSG00000114013	15761015;
C006632	decrease	CFB	ENSG00000243570	17547211;
C006632	decrease	CFLAR	ENSG00000003402	16105982;16174796;
C006632	decrease	CIDEB	ENSG00000136305	17547211;
C006632	decrease	CITED2	ENSG00000164442	15725085;
C006632	decrease	CKAP4	ENSG00000136026	12852829;
C006632	decrease	CLEC7A	ENSG00000172243	15725085;

C006632	decrease	CLGN	ENSG00000153132	17547211;
C006632	decrease	CMTM8	ENSG00000170293	15761015;
C006632	decrease	CNOT2	ENSG00000111596	15761015;
C006632	decrease	COLEC10	ENSG00000184374	15761015;
C006632	decrease	COPS5	ENSG00000121022	17077332;
C006632	decrease	CRH	ENSG00000147571	15761015;
C006632	decrease	CSH1	ENSG00000136488	15761015;
C006632	decrease	CSPP1	ENSG00000104218	15761015;
C006632	decrease	CSRP3	ENSG00000129170	15725085;
C006632	decrease	CST3	ENSG00000101439	15725085;
C006632	decrease	CTNNA1	ENSG00000044115	15761015;
C006632	decrease	CTSH	ENSG00000103811	15761015;
C006632	decrease	CX3CL1	ENSG00000006210	17547211;
C006632	decrease	CYLD	ENSG00000083799	15761015;
C006632	decrease	CYP39A1	ENSG00000146233	15761015;
C006632	decrease	CYTL1	ENSG00000170891	15761015;15070760;
C006632	decrease	DNMT3A	ENSG00000119772	12679007;
C006632	decrease	DNMT3B	ENSG00000088305	12679007;
C006632	decrease	DUSP4	ENSG00000120875	15725085;
C006632	decrease	EPB41L2	ENSG00000079819	15761015;
C006632	decrease	EPHX1	ENSG00000143819	17547211;
C006632	decrease	ESR1	ENSG00000091831	12014631;
C006632	decrease	EVL	ENSG00000196405	15725085;
C006632	decrease	F3	ENSG00000117525	15761015;16206674;
C006632	decrease	FAM46C	ENSG00000183508	15725085;
C006632	decrease	FBXL7	ENSG00000183580	15725085;
C006632	decrease	FGFR2	ENSG00000066468	15761015;
C006632	decrease	FNBP1L	ENSG00000137942	15761015;
C006632	decrease	FRK	ENSG00000111816	15761015;
C006632	decrease	FSCN1	ENSG00000075618	15070760;15761015;
C006632	decrease	GABBR2	ENSG00000136928	15761015;
C006632	decrease	GABPB2	ENSG00000143458	12852829;
C006632	decrease	GJA1	ENSG00000152661	15070760;15761015;
C006632	decrease	GPER	ENSG00000164850	17547211;
C006632	decrease	GPRC5D	ENSG00000111291	15725085;
C006632	decrease	HCRTR1	ENSG00000121764	15761015;
C006632	decrease	HGF	ENSG00000019991	15761015;
C006632	decrease	HIST1H2BM	ENSG00000196374	15761015;
C006632	decrease	HLA-B	ENSG00000234745	15725085;15761015;
C006632	decrease	HLA-C	ENSG00000204525	15725085;15761015;
C006632	decrease	HLA-DRA	ENSG00000204287	15725085;
C006632	decrease	HLA-F	ENSG00000204642	15725085;
C006632	decrease	HLA-G	ENSG00000237216	15725085;15761015;
C006632	decrease	HSD17B2	ENSG00000086696	17547211;
C006632	decrease	HSPB1	ENSG00000106211	15070760;15761015;
C006632	decrease	ID2	ENSG00000115738	15761015;
C006632	decrease	IFI16	ENSG00000163565	15761015;
C006632	decrease	IFIH1	ENSG00000115267	15761015;
C006632	decrease	IFNA2	ENSG00000188379	17077332;12560223;
C006632	decrease	IFRD1	ENSG00000006652	17547211;
C006632	decrease	IGFBP2	ENSG00000115457	15070760;15761015;
C006632	decrease	IGFBP7	ENSG00000163453	15725085;
C006632	decrease	IL6	ENSG00000136244	12560223;
C006632	decrease	ITGB1	ENSG00000150093	12852829;

C006632	decrease	ITM2A	ENSG00000078596	15725085;
C006632	decrease	KCTD12	ENSG00000178695	15761015;
C006632	decrease	KITLG	ENSG00000049130	15761015;
C006632	decrease	KLF11	ENSG00000172059	15761015;
C006632	decrease	LAMC1	ENSG00000135862	12852829;
C006632	decrease	LGMN	ENSG00000100600	15725085;
C006632	decrease	LHFP	ENSG00000183722	15070760;15761015;
C006632	decrease	LHX6	ENSG00000106852	15761015;
C006632	decrease	MCF2L	ENSG00000126217	12852829;
C006632	decrease	MMP1	ENSG00000196611	15761015;
C006632	decrease	MMP28	ENSG00000129270	15761015;
C006632	decrease	MMP2	ENSG00000087245	16624393;15553829;
C006632	decrease	MPL	ENSG00000117400	15761015;
C006632	decrease	MPO	ENSG00000005381	12130515;
C006632	decrease	MSC	ENSG00000178860	15761015;
C006632	decrease	MSRB2	ENSG00000148450	15725085;
C006632	decrease	MTA1	ENSG00000182979	12478894;
C006632	decrease	MYBL1	ENSG00000185697	15725085;
C006632	decrease	MYC	ENSG00000136997	11775218;15622746;15761015;11714746;12903512;12903497;
C006632	decrease	MYCN	ENSG00000134323	12478894;
C006632	decrease	MYO1B	ENSG00000128641	15070760;15761015;
C006632	decrease	MYST4	ENSG00000156650	15761015;
C006632	decrease	N4BP2L1	ENSG00000139597	15761015;
C006632	decrease	NAB2	ENSG00000166886	15761015;
C006632	decrease	NAV3	ENSG00000067798	15761015;
C006632	decrease	NFKBIA	ENSG00000100906	12560223;
C006632	decrease	NUCB2	ENSG00000070081	17547211;
C006632	decrease	ORC4L	ENSG00000115947	15761015;
C006632	decrease	OS9	ENSG00000135506	17547211;
C006632	decrease	P4HA1	ENSG00000122884	15725085;
C006632	decrease	PAQR6	ENSG00000160781	15725085;
C006632	decrease	PDE4A	ENSG00000065989	15070760;
C006632	decrease	PDE4B	ENSG00000184588	15761015;
C006632	decrease	PDZD2	ENSG00000133401	15761015;
C006632	decrease	PGC	ENSG00000096088	17547211;
C006632	decrease	PGF	ENSG00000119630	17547211;
C006632	decrease	PHLDA2	ENSG00000181649	15761015;
C006632	decrease	PI4KA	ENSG00000249793	12852829;
C006632	decrease	PKNOX1	ENSG00000160199	15761015;
C006632	decrease	PLAUR	ENSG00000011422	15761015;
C006632	decrease	PLEKHO1	ENSG00000023902	15761015;
C006632	decrease	PPP1CB	ENSG00000213639	12852829;
C006632	decrease	PRKCB	ENSG00000166501	15725085;
C006632	decrease	PSMB8	ENSG00000226201	15725085;
C006632	decrease	PXDN	ENSG00000130508	15761015;
C006632	decrease	RAB27B	ENSG00000041353	15761015;
C006632	decrease	RBMX	ENSG00000147274	12852829;
C006632	decrease	RGS2	ENSG00000116741	12852829;
C006632	decrease	ROBO1	ENSG00000169855	15725085;
C006632	decrease	RPL17	ENSG00000215472	12852829;
C006632	decrease	RPL18A	ENSG00000105640	15725085;
C006632	decrease	RPL7	ENSG00000147604	12852829;

C006632	decrease	RSL1D1	ENSG00000171490	15761015;
C006632	decrease	RYK	ENSG00000163785	15761015;
C006632	decrease	SATB2	ENSG00000119042	15761015;
C006632	decrease	SCAMP5	ENSG00000198794	15761015;
C006632	decrease	SCT	ENSG00000070031	15761015;
C006632	decrease	SDC2	ENSG00000169439	12852829;15761015;15725085;
C006632	decrease	SDC4	ENSG00000124145	15761015;
C006632	decrease	SELP	ENSG00000174175	16206674;
C006632	decrease	SERTAD2	ENSG00000179833	15725085;
C006632	decrease	SLC15A1	ENSG00000088386	15761015;
C006632	decrease	SLC1A2	ENSG00000110436	17547211;
C006632	decrease	SLC22A18	ENSG00000110628	15725085;
C006632	decrease	SLC44A1	ENSG00000070214	15761015;
C006632	decrease	SMAD1	ENSG00000170365	15761015;
C006632	decrease	SOX18	ENSG00000203883	15761015;
C006632	decrease	SOX4	ENSG00000124766	15725085;
C006632	decrease	ST6GALNAC4	ENSG00000136840	17547211;
C006632	decrease	STAB1	ENSG00000010327	15761015;
C006632	decrease	STATH	ENSG00000126549	15761015;
C006632	decrease	SULF1	ENSG00000137573	15725085;
C006632	decrease	SYN3	ENSG00000185666	15761015;
C006632	decrease	SYNJ2	ENSG00000078269	12852829;
C006632	decrease	TFAP2C	ENSG00000087510	15761015;
C006632	decrease	TGFBR2	ENSG00000163513	12852829;
C006632	decrease	TIMP2	ENSG00000035862	16624393;
C006632	decrease	TMEM158	ENSG00000249992	15761015;
C006632	decrease	TNF	ENSG00000223952	12560223;
C006632	decrease	TNFRSF1B	ENSG00000028137	15761015;
C006632	decrease	TNFRSF25	ENSG00000215788	15761015;
C006632	decrease	TOP2A	ENSG00000131747	16884364;
C006632	decrease	TPT1	ENSG00000133112	15949261;
C006632	decrease	TRA2A	ENSG00000164548	15761015;
C006632	decrease	TXNIP	ENSG00000117289	15725085;
C006632	decrease	VNN1	ENSG00000112299	15761015;
C006632	decrease	WSB2	ENSG00000176871	15725085;
C006632	decrease	WT1	ENSG00000184937	16966277;
C006632	decrease	XIAP	ENSG00000101966	16105982;16174796;
C006632	decrease	XRCC6	ENSG00000196419	15761015;
C006632	decrease	ZFP36L1	ENSG00000185650	15725085;
C006632	decrease	ZNF334	ENSG00000198185	15761015;
C006632	decrease	ZNF771	ENSG00000179965	15761015;
C006632	increase	ABCG2	ENSG00000118777	17547211;
C006632	increase	ACAA2	ENSG00000167315	15761015;
C006632	increase	AFAP1	ENSG00000196526	15761015;
C006632	increase	AKAP12	ENSG00000131016	17547211;
C006632	increase	ALAS1	ENSG00000023330	15725085;
C006632	increase	ALG13	ENSG00000101901	15761015;
C006632	increase	ALOX5AP	ENSG00000132965	15070760;15761015;
C006632	increase	ANK3	ENSG00000151150	15761015;
C006632	increase	ANKRD12	ENSG00000101745	15761015;
C006632	increase	ANXA2	ENSG00000182718	17547211;
C006632	increase	AP1S1	ENSG00000106367	17258074;

C006632	increase	AQP9	ENSG00000103569	16968895;15336539;
C006632	increase	ARL6IP1	ENSG00000170540	14703492;
C006632	increase	ARL6IP5	ENSG00000144746	16468075;16430862;
C006632	increase	ATP6	ENSG00000198899	15949261;14703492;
C006632	increase	ATP8A1	ENSG00000124406	15761015;
C006632	increase	BAX	ENSG00000087088	16867262;16972261;15665116;11775218;16010437;15622746;16882451;16020671;17258074;11135700;
C006632	increase	BCL2A1	ENSG00000140379	12130515;
C006632	increase	BCL2	ENSG00000171791	16867262;11775218;16904648;16007134;16818652;16010437;15979894;16029599;15622746;11135700;11780464;16105982;12845720;11589617;12490120;169662
C006632	increase	BECN1	ENSG00000126581	16882451;
C006632	increase	BIRC5	ENSG00000089685	15587394;16328441;
C006632	increase	BLVRB	ENSG00000090013	15725085;
C006632	increase	BLZF1	ENSG00000117475	15761015;
C006632	increase	BNIP3	ENSG00000176171	15592527;
C006632	increase	BNIP3L	ENSG00000104765	15592527;
C006632	increase	BPI	ENSG00000101425	15761015;
C006632	increase	C14ORF105	ENSG00000100557	15761015;
C006632	increase	C16ORF58	ENSG00000140688	15761015;
C006632	increase	C8ORF4	ENSG00000176907	15761015;
C006632	increase	CACNA2D2	ENSG00000007402	15761015;
C006632	increase	CAPN10	ENSG00000142330	15761015;
C006632	increase	CAPZB	ENSG00000077549	12852829;
C006632	increase	CASP10	ENSG00000003400	12388546;
C006632	increase	CASP3	ENSG00000164305	16867262;14668793;16951922;15665116;15979894;
C006632	increase	CAV2	ENSG00000105971	15725085;15761015;
C006632	increase	CCDC102B	ENSG00000150636	15761015;
C006632	increase	CCDC52	ENSG00000163611	15761015;
C006632	increase	CCL15	ENSG00000161574	15761015;
C006632	increase	CCL23	ENSG00000167236	15761015;
C006632	increase	CCL2	ENSG00000108691	15761015;
C006632	increase	CCNB1	ENSG00000134057	12783709;
C006632	increase	CCNB2	ENSG00000157456	17547211;
C006632	increase	CD52	ENSG00000169442	15761015;
C006632	increase	CDC42BPA	ENSG00000143776	15761015;
C006632	increase	CDC73	ENSG00000134371	15761015;
C006632	increase	CDKN1A	ENSG00000124762	15961274;12749819;
C006632	increase	CDKN2A	ENSG00000147889	15191659;16008847;
C006632	increase	CDKN2B	ENSG00000147883	12679007;11877046;
C006632	increase	CDKN3	ENSG00000100526	17547211;
C006632	increase	CDV3	ENSG00000091527	17258074;
C006632	increase	CEBPE	ENSG00000092067	12130515;
C006632	increase	CEP57	ENSG00000166037	14703492;
C006632	increase	CHGB	ENSG00000089199	15761015;
C006632	increase	CHI3L1	ENSG00000133048	15761015;
C006632	increase	CHST6	ENSG00000183196	15070760;15761015;
C006632	increase	CLC	ENSG00000105205	15070760;15761015;
C006632	increase	CLIC4	ENSG00000169504	17258074;

C006632	increase	CLTB	ENSG00000175416	17258074;
C006632	increase	COBLL1	ENSG00000082438	15761015;
C006632	increase	CREB5	ENSG00000146592	15761015;
C006632	increase	CRIM1	ENSG00000150938	15725085;15761015;
C006632	increase	CSF2RB	ENSG00000100368	15070760;
C006632	increase	CYB5R3	ENSG00000100243	15725085;
C006632	increase	CYBA	ENSG00000051523	15070760;
C006632	increase	CYBRD1	ENSG00000071967	15761015;
C006632	increase	CYP1A1	ENSG00000140465	11678611;12490585;
C006632	increase	CYP3A43	ENSG00000021461	15761015;
C006632	increase	DAZ4	ENSG00000205944	15761015;
C006632	increase	DBC1	ENSG00000078725	15761015;
C006632	increase	DDIT3	ENSG00000175197	11678611;12749819;
C006632	increase	DEFA1	ENSG00000240247	15070760;15761015;
C006632	increase	DEFA4	ENSG00000164821	15070760;15761015;
C006632	increase	DKK1	ENSG00000107984	17547211;
C006632	increase	DNAJB4	ENSG00000162616	15761015;
C006632	increase	DNAJC9	ENSG00000213551	15761015;
C006632	increase	DYNC1H1	ENSG00000197102	15725085;
C006632	increase	DYRK1B	ENSG00000105204	15070760;
C006632	increase	EPX	ENSG00000121053	15070760;15761015;
C006632	increase	ERC2	ENSG00000187672	15761015;
C006632	increase	FAM110B	ENSG00000169122	15761015;
C006632	increase	FANCC	ENSG00000158169	15070760;
C006632	increase	FAS	ENSG00000026103	12478894;15979894;11135700;16029599;15382040;12126518;12452020;
C006632	increase	FBLN5	ENSG00000140092	15761015;
C006632	increase	FGFR1	ENSG00000249195	15761015;17027752;17258074;
C006632	increase	FGR	ENSG00000000938	15761015;
C006632	increase	FHL1	ENSG00000022267	17258074;
C006632	increase	FN1	ENSG00000115414	17258074;
C006632	increase	FOS	ENSG00000170345	11678611;12749819;14682389;
C006632	increase	FOXRED2	ENSG00000100350	15761015;
C006632	increase	FTH1	ENSG00000167996	14703492;15725085;
C006632	increase	FYB	ENSG00000082074	15761015;
C006632	increase	GADD45A	ENSG00000116717	11678611;15761015;
C006632	increase	GBA	ENSG00000177628	15761015;
C006632	increase	GCLM	ENSG00000023909	15725085;
C006632	increase	GIMAP6	ENSG00000133561	15761015;
C006632	increase	GIT2	ENSG00000139436	15761015;
C006632	increase	GLA	ENSG00000102393	17547211;
C006632	increase	GNAL	ENSG00000141404	15761015;
C006632	increase	GPM6A	ENSG00000150625	15761015;
C006632	increase	GPR44	ENSG00000183134	15761015;
C006632	increase	GRAMD3	ENSG00000155324	15070760;
C006632	increase	GRK4	ENSG00000125388	15761015;
C006632	increase	GSR	ENSG00000104687	17258074;
C006632	increase	GSTA1	ENSG00000243955	11678611;
C006632	increase	GSTZ1	ENSG00000100577	15761015;
C006632	increase	HIF1A	ENSG00000100644	16330433;12482858;
C006632	increase	HIST1H4C	ENSG00000182217	17547211;

C006632	increase	HK1	ENSG00000156515	15761015;
C006632	increase	HMGA1	ENSG00000137309	17547211;
C006632	increase	HMGB2	ENSG00000164104	14703492;
C006632	increase	HMGN2	ENSG00000198830	17547211;
C006632	increase	HMOX1	ENSG00000100292	17258074;15725085;17547211;16487037;
C006632	increase	HSPA4	ENSG00000170606	11678611;14682389;15665116;
C006632	increase	HSPA5	ENSG00000044574	11678611;
C006632	increase	HSPA6	ENSG00000173110	15978632;
C006632	increase	HTATIP2	ENSG00000109854	15761015;
C006632	increase	ICAM3	ENSG00000076662	15761015;
C006632	increase	ID1	ENSG00000125968	15761015;
C006632	increase	IDS	ENSG00000010404	15761015;
C006632	increase	IFNG	ENSG00000111537	14668793;16914093;
C006632	increase	IL12RB2	ENSG00000081985	15761015;
C006632	increase	INPP5B	ENSG00000204084	15761015;
C006632	increase	INSM1	ENSG00000173404	15761015;
C006632	increase	IQCH	ENSG00000103599	15761015;
C006632	increase	IRF1	ENSG00000125347	14668793;16914093;
C006632	increase	ITGA2B	ENSG00000005961	15761015;
C006632	increase	ITGAM	ENSG00000169896	16468075;16430862;
C006632	increase	ITGB3BP	ENSG00000142856	17547211;
C006632	increase	ITGB7	ENSG00000139626	15761015;
C006632	increase	JUND	ENSG00000130522	17077332;15761015;
C006632	increase	KCNH2	ENSG00000055118	15213294;15070760;
C006632	increase	KIAA1609	ENSG00000140950	15761015;
C006632	increase	KIF21B	ENSG00000116852	15761015;
C006632	increase	KLF5	ENSG00000102554	15761015;
C006632	increase	KRT6A	ENSG00000205420	15949261;
C006632	increase	KYNU	ENSG00000115919	15761015;
C006632	increase	LAT	ENSG00000213658	15761015;
C006632	increase	LGALS9	ENSG00000168961	15761015;
C006632	increase	LSP1	ENSG00000130592	15761015;
C006632	increase	LTC4S	ENSG00000213316	15761015;
C006632	increase	MAFF	ENSG00000185022	16487037;
C006632	increase	MAN1A1	ENSG00000111885	15761015;
C006632	increase	MAOA	ENSG00000189221	15761015;
C006632	increase	MAP2K4	ENSG00000065559	15978632;
C006632	increase	MAP7	ENSG00000135525	15761015;
C006632	increase	MAPK1	ENSG00000100030	15961274;16328441;15580305;17050201;17258074;
C006632	increase	2-Mar	ENSG00000099785	15761015;
C006632	increase	MCM2	ENSG00000073111	17547211;
C006632	increase	ME1	ENSG00000065833	15725085;
C006632	increase	MKI67IP	ENSG00000155438	14703492;
C006632	increase	MMP9	ENSG00000100985	16624393;15949266;
C006632	increase	MRPS16	ENSG00000182180	14703492;
C006632	increase	MT1E	ENSG00000169715	15725085;
C006632	increase	MT1G	ENSG00000125144	15725085;
C006632	increase	MT1H	ENSG00000205358	15725085;
C006632	increase	MT1X	ENSG00000187193	15725085;
C006632	increase	MT2A	ENSG00000125148	11678611;14682389;15725085;

C006632	increase	MXRA7	ENSG00000182534	17547211;
C006632	increase	MYO6	ENSG00000196586	17258074;
C006632	increase	N4BP1	ENSG00000102921	15761015;
C006632	increase	NCF1	ENSG00000158517	15070760;15761015;
C006632	increase	NCF2	ENSG00000116701	15070760;
C006632	increase	ND4	ENSG00000198886	14703492;
C006632	increase	NEU3	ENSG00000162139	15070760;
C006632	increase	NFE2L1	ENSG00000082641	16487037;
C006632	increase	NME1	ENSG00000239672	12478894;12452020;
C006632	increase	NPAS1	ENSG00000130751	15761015;
C006632	increase	NPTX2	ENSG00000106236	15761015;
C006632	increase	NUDT18	ENSG00000173566	15761015;
C006632	increase	NUP107	ENSG00000111581	17547211;
C006632	increase	OBSL1	ENSG00000124006	15761015;
C006632	increase	PAFAH2	ENSG00000158006	15761015;
C006632	increase	PAOX	ENSG00000148832	15761015;
C006632	increase	PAWR	ENSG00000177425	16966277;
C006632	increase	PCNA	ENSG00000132646	16029599;12783709;
C006632	increase	PCSK5	ENSG00000099139	15761015;
C006632	increase	PDCD4	ENSG00000150593	17259349;
C006632	increase	PDE4DIP	ENSG00000178104	15761015;
C006632	increase	PDLIM7	ENSG00000196923	15761015;
C006632	increase	PEX3	ENSG00000034693	15761015;
C006632	increase	PIGO	ENSG00000165282	15761015;
C006632	increase	PIR	ENSG00000087842	15761015;
C006632	increase	PLXNC1	ENSG00000136040	15761015;
C006632	increase	PPIH	ENSG00000171960	17547211;
C006632	increase	PRG3	ENSG00000156575	15761015;
C006632	increase	PRR16	ENSG00000184838	15761015;
C006632	increase	PRSS1	ENSG00000204983	15761015;
C006632	increase	PSMB6	ENSG00000142507	12852829;
C006632	increase	PTGFR	ENSG00000122420	15761015;
C006632	increase	PTRH2	ENSG00000141378	14703492;
C006632	increase	PTTG1	ENSG00000164611	17547211;
C006632	increase	PURG	ENSG00000172733	15761015;
C006632	increase	RACGAP1	ENSG00000161800	17547211;
C006632	increase	RAPGEF4	ENSG00000091428	15761015;
C006632	increase	RASSF8	ENSG00000123094	15761015;
C006632	increase	RERE	ENSG00000142599	15761015;
C006632	increase	RFC2	ENSG00000049541	17547211;
C006632	increase	RNASEH2A	ENSG00000104889	17547211;
C006632	increase	RNF144A	ENSG00000151692	15761015;
C006632	increase	RNF24	ENSG00000101236	15761015;
C006632	increase	RNF6	ENSG00000127870	15761015;
C006632	increase	ROD1	ENSG00000119314	17258074;
C006632	increase	RPL23	ENSG00000125691	14703492;
C006632	increase	RPS15A	ENSG00000134419	14703492;
C006632	increase	RRBP1	ENSG00000125844	15761015;
C006632	increase	S100A10	ENSG00000197747	15949261;
C006632	increase	S100A8	ENSG00000143546	15070760;15761015;
C006632	increase	S100A9	ENSG00000163220	15761015;
C006632	increase	SCGB2A2	ENSG00000110484	15761015;
C006632	increase	SCPEP1	ENSG00000121064	15725085;
C006632	increase	11-Sep	ENSG00000138758	17258074;

C006632	increase	6-Sep	ENSG00000125354	17258074;
C006632	increase	SERPINB1	ENSG00000021355	15761015;
C006632	increase	SFRS5	ENSG00000100650	14703492;
C006632	increase	SH3GL2	ENSG00000107295	15761015;
C006632	increase	SIGLEC6	ENSG00000105492	15761015;
C006632	increase	SLC14A2	ENSG00000132874	15761015;
C006632	increase	SLC48A1	ENSG00000211584	15761015;15725085;
C006632	increase	SLC5A12	ENSG00000148942	15761015;
C006632	increase	SLC6A5	ENSG00000165970	15761015;
C006632	increase	SLCO3A1	ENSG00000176463	15761015;
C006632	increase	SMC4	ENSG00000113810	17547211;
C006632	increase	SOX30	ENSG00000039600	15761015;
C006632	increase	SP1	ENSG00000185591	11714746;15761015;
C006632	increase	SPTAN1	ENSG00000197694	12852829;
C006632	increase	STAT1	ENSG00000115415	14668793;16914093;
C006632	increase	SUSD5	ENSG00000173705	15761015;
C006632	increase	TCF4	ENSG00000196628	15761015;
C006632	increase	TERT	ENSG00000164362	15761015;11714746;15996315;16129045;16966277;16285558;
C006632	increase	TFF1	ENSG00000160182	12014631;
C006632	increase	TIMP1	ENSG00000102265	16624393;
C006632	increase	TNFAIP3	ENSG00000118503	17547211;
C006632	increase	TNFAIP8	ENSG00000145779	17547211;
C006632	increase	TNFRSF9	ENSG00000049249	16010437;
C006632	increase	TNFSF13	ENSG00000161955	15761015;
C006632	increase	TNFSF8	ENSG00000106952	15761015;
C006632	increase	TP53AIP1	ENSG00000120471	15031205;16467208;
C006632	increase	TP53	ENSG00000141510	11775218;11714746;15622746;14682389;12490120;15979894;16467208;
C006632	increase	TP53I11	ENSG00000175274	15225615;15761015;12883691;
C006632	increase	TP73	ENSG00000078900	16467208;15031205;
C006632	increase	TRAF3IP2	ENSG00000056972	15761015;
C006632	increase	TRIM16	ENSG00000221926	15725085;
C006632	increase	TRIP13	ENSG00000071539	17547211;
C006632	increase	TRMT1	ENSG00000104907	15725085;
C006632	increase	TSC22D3	ENSG00000157514	17081986;
C006632	increase	TTC38	ENSG00000075234	15761015;
C006632	increase	TYMS	ENSG00000176890	17547211;
C006632	increase	UBE2C	ENSG00000175063	17547211;
C006632	increase	UBE2D1	ENSG00000072401	15761015;
C006632	increase	UCHL1	ENSG00000154277	15725085;
C006632	increase	UCP2	ENSG00000175567	15761015;
C006632	increase	VEGFA	ENSG00000112715	16928304;15949266;12482858;
C006632	increase	WDR12	ENSG00000138442	15725085;
C006632	increase	WDR82	ENSG00000164091	15761015;
C006632	increase	ZNF37A	ENSG00000075407	15761015;
C006632	increase	ZNF682	ENSG00000197124	15761015;
C006632	increase	ZWINT	ENSG00000122952	17547211;
C006680	decrease	CSF2	ENSG00000164400	10718847;
C006680	decrease	NAT1	ENSG00000171428	15796204;
C006680	decrease	VEGFA	ENSG00000112715	16638750;

C006680	increase	AHR	ENSG00000106546	14644660;
C006680	increase	CYP1A1	ENSG00000140465	12224597;14644660;
C006680	increase	CYP1B1	ENSG00000138061	12224597;
C006680	increase	PMAIP1	ENSG00000141682	17216584;
C006680	increase	PTGS2	ENSG00000073756	19804834;
C006698	increase	CYP19A1	ENSG00000137869	14691014;
C006703	increase	CYP1A1	ENSG00000140465	15566942;
C006703	increase	CYP1B1	ENSG00000138061	15566942;
C006703	increase	UGT1A6	ENSG00000167165	15566942;
C006703	increase	UGT1A7	ENSG00000244122	15566942;
C006757	decrease	OGG1	ENSG00000114026	16112689;
C006780	decrease	ATF3	ENSG00000162772	16474171;
C006780	decrease	ATP1B1	ENSG00000143153	16474171;
C006780	decrease	BASP1	ENSG00000176788	16474171;
C006780	decrease	BRCA1	ENSG00000012048	16029874;
C006780	decrease	BTG1	ENSG00000133639	16474171;
C006780	decrease	CAMK2N1	ENSG00000162545	16474171;
C006780	decrease	CDK2AP2	ENSG00000167797	16474171;
C006780	decrease	CDKN1A	ENSG00000124762	16474171;
C006780	decrease	CDKN2C	ENSG00000123080	16474171;
C006780	decrease	CDKN3	ENSG00000100526	16474171;
C006780	decrease	CEBPD	ENSG00000221869	16474171;
C006780	decrease	CLDN4	ENSG00000189143	16474171;
C006780	decrease	EFNB2	ENSG00000125266	16474171;
C006780	decrease	ERBB3	ENSG00000065361	16029874;
C006780	decrease	ESR1	ENSG00000091831	16328721;14975756;12650720;12753421;11693576;11162928;11518614;17400923;15521089;14579009;9751507;11530281;15026083;15952375;16029874;11867264;15713566;11485867;
C006780	decrease	ETS2	ENSG00000157557	16474171;
C006780	decrease	FZD5	ENSG00000163251	16029874;
C006780	decrease	GADD45A	ENSG00000116717	16474171;
C006780	decrease	GPRC5A	ENSG00000013588	16474171;
C006780	decrease	H1FX	ENSG00000184897	16029874;
C006780	decrease	HIST1H2AC	ENSG00000180573	16474171;
C006780	decrease	HIST2H2BE	ENSG00000184678	16474171;
C006780	decrease	HOXB7	ENSG00000120087	16029874;
C006780	decrease	HOXC6	ENSG00000197757	16029874;
C006780	decrease	HSPA6	ENSG00000173110	16029874;
C006780	decrease	ID3	ENSG00000117318	16474171;
C006780	decrease	JUP	ENSG00000173801	16474171;
C006780	decrease	KRAS	ENSG00000133703	16474171;
C006780	decrease	L1CAM	ENSG00000198910	16474171;
C006780	decrease	MAP3K7	ENSG00000135341	16029874;
C006780	decrease	MXD4	ENSG00000123933	16474171;
C006780	decrease	MYH6	ENSG00000197616	16474171;
C006780	decrease	NCOA3	ENSG00000124151	16474171;
C006780	decrease	NEDD9	ENSG00000111859	16474171;
C006780	decrease	PIAS3	ENSG00000131788	16029874;
C006780	decrease	PMEPA1	ENSG00000124225	16474171;
C006780	decrease	POLD4	ENSG00000175482	16474171;

C006780	decrease	PPAP2B	ENSG00000162407	16474171;
C006780	decrease	PPAP2C	ENSG00000141934	16474171;
C006780	decrease	SELENBP1	ENSG00000143416	15159206;
C006780	decrease	SLC7A11	ENSG00000151012	16474171;
C006780	decrease	SOCS2	ENSG00000120833	16029874;
C006780	decrease	SOX2	ENSG00000181449	16474171;
C006780	decrease	SOX4	ENSG00000124766	16474171;
C006780	decrease	SQSTM1	ENSG00000161011	16474171;
C006780	decrease	STEAP1	ENSG00000164647	16474171;
C006780	decrease	TCF25	ENSG00000141002	16474171;
C006780	decrease	TGFB2	ENSG00000092969	16029874;16474171;
C006780	decrease	TIMP3	ENSG00000100234	16474171;
C006780	decrease	TRIM16	ENSG00000221926	16474171;
C006780	decrease	TRIM24	ENSG00000122779	16029874;
C006780	decrease	UGT1A3	ENSG00000243135	16474171;
C006780	decrease	WNT5A	ENSG00000114251	16029874;
C006780	decrease	YPEL5	ENSG00000119801	16474171;
C006780	decrease	ZFP36L1	ENSG00000185650	16474171;
C006780	increase	AGR2	ENSG00000106541	16474171;
C006780	increase	AP1S2	ENSG00000182287	16474171;
C006780	increase	APOBEC3B	ENSG00000179750	16474171;
C006780	increase	AREG	ENSG00000205595	16474171;
C006780	increase	AR	ENSG00000169083	16893599;11867264;16169144;15084347;11403899;9846162;15159206;
C006780	increase	ARL3	ENSG00000138175	16474171;
C006780	increase	ASPM	ENSG00000066279	16474171;
C006780	increase	ATAD2	ENSG00000156802	16474171;
C006780	increase	AURKA	ENSG00000087586	16474171;17268063;
C006780	increase	AURKB	ENSG00000178999	16474171;
C006780	increase	BIRC5	ENSG00000089685	16474171;
C006780	increase	BLM	ENSG00000197299	17268063;
C006780	increase	BUB1B	ENSG00000156970	16474171;
C006780	increase	CA12	ENSG00000074410	16474171;
C006780	increase	CCNA2	ENSG00000145386	16474171;
C006780	increase	CCNB2	ENSG00000157456	16474171;
C006780	increase	CDC20	ENSG00000117399	16474171;
C006780	increase	CDC6	ENSG00000094804	16474171;17268063;
C006780	increase	CDK1	ENSG00000170312	16474171;
C006780	increase	CDK2	ENSG00000123374	16474171;
C006780	increase	CDT1	ENSG00000167513	16474171;
C006780	increase	CENPA	ENSG00000115163	16474171;
C006780	increase	CENPF	ENSG00000117724	16474171;
C006780	increase	CENPJ	ENSG00000151849	16474171;
C006780	increase	CENPN	ENSG00000166451	16474171;
C006780	increase	CHEK1	ENSG00000149554	16474171;
C006780	increase	CHSY1	ENSG00000131873	16474171;
C006780	increase	CKS1B	ENSG00000173207	16474171;
C006780	increase	CLIC6	ENSG00000159212	16474171;
C006780	increase	COL12A1	ENSG00000111799	16474171;
C006780	increase	CSRP2BP	ENSG00000149474	16474171;
C006780	increase	CTPS	ENSG00000171793	16474171;
C006780	increase	CYP1A1	ENSG00000140465	16029874;16474171;
C006780	increase	CYP1B1	ENSG00000138061	16474171;

C006780	increase	CYP3A4	ENSG00000160868	15650019;
C006780	increase	DAD1	ENSG00000129562	16474171;
C006780	increase	DEPDC1B	ENSG00000035499	16474171;
C006780	increase	DHRS2	ENSG00000100867	16474171;
C006780	increase	DTL	ENSG00000143476	16474171;
C006780	increase	DTYMK	ENSG00000168393	16474171;
C006780	increase	EBP	ENSG00000147155	16474171;
C006780	increase	ECT2	ENSG00000114346	16474171;
C006780	increase	EGR3	ENSG00000179388	15159206;
C006780	increase	EMP2	ENSG00000213853	16474171;
C006780	increase	FANCI	ENSG00000140525	16474171;
C006780	increase	FBXO32	ENSG00000156804	16474171;
C006780	increase	FBXO5	ENSG00000112029	16474171;
C006780	increase	FEN1	ENSG00000168496	16474171;
C006780	increase	FKBP4	ENSG00000004478	16474171;
C006780	increase	FOXC1	ENSG00000054598	16029874;
C006780	increase	GFRA1	ENSG00000151892	16474171;
C006780	increase	GGH	ENSG00000137563	16474171;
C006780	increase	GINS1	ENSG00000101003	16474171;
C006780	increase	GINS2	ENSG00000131153	16474171;
C006780	increase	GMNN	ENSG00000112312	16474171;
C006780	increase	GREB1	ENSG00000196208	16474171;
C006780	increase	H2AFX	ENSG00000188486	16474171;16462019;
C006780	increase	H2AFZ	ENSG00000164032	16474171;
C006780	increase	HADH	ENSG00000138796	16474171;
C006780	increase	HIST1H2BJ	ENSG00000124635	16029874;
C006780	increase	HMGB2	ENSG00000164104	16474171;
C006780	increase	HOXA10	ENSG00000078399	16632680;17093138;
C006780	increase	IGFBP4	ENSG00000141753	16474171;
C006780	increase	ILF2	ENSG00000143621	16474171;
C006780	increase	IRS1	ENSG00000169047	16029874;
C006780	increase	ITGB2	ENSG00000160255	14555285;
C006780	increase	ITPK1	ENSG00000100605	16474171;
C006780	increase	KIAA0101	ENSG00000166803	16474171;
C006780	increase	KIF11	ENSG00000138160	16474171;
C006780	increase	KIF14	ENSG00000118193	16474171;
C006780	increase	KIF15	ENSG00000163808	16474171;
C006780	increase	KIF20A	ENSG00000112984	16474171;
C006780	increase	KIF23	ENSG00000137807	16474171;
C006780	increase	KIF4A	ENSG00000090889	16474171;
C006780	increase	KPNA2	ENSG00000182481	16474171;
C006780	increase	MAD1L1	ENSG00000002822	16474171;
C006780	increase	MASTL	ENSG00000120539	16474171;
C006780	increase	MCM10	ENSG00000065328	16474171;
C006780	increase	MCM2	ENSG00000073111	16474171;17268063;
C006780	increase	MCM5	ENSG00000100297	17268063;
C006780	increase	MCM6	ENSG00000076003	16474171;
C006780	increase	MCM7	ENSG00000166508	16474171;
C006780	increase	MELK	ENSG00000165304	16474171;
C006780	increase	MLF1	ENSG00000178053	16474171;
C006780	increase	MLF1IP	ENSG00000151725	16474171;
C006780	increase	MPHOSPH9	ENSG00000051825	16474171;
C006780	increase	MYB	ENSG00000118513	16029874;16474171;
C006780	increase	MYBL2	ENSG00000101057	16474171;

C006780	increase	MYC	ENSG00000136997	16474171;
C006780	increase	MYT1	ENSG00000196132	17268063;
C006780	increase	NCAPD2	ENSG00000010292	16474171;
C006780	increase	NCAPG	ENSG00000109805	16474171;
C006780	increase	NCF1	ENSG00000158517	14555285;
C006780	increase	NCF2	ENSG00000116701	14555285;
C006780	increase	NDC80	ENSG00000080986	16474171;
C006780	increase	OLFM1	ENSG00000130558	16474171;
C006780	increase	OLFML3	ENSG00000116774	16474171;
C006780	increase	PBK	ENSG00000168078	16474171;
C006780	increase	PCNA	ENSG00000132646	16474171;17268063;
C006780	increase	PCP4	ENSG00000183036	16474171;
C006780	increase	PGR	ENSG00000082175	14579009;11867264;12753421;11693576;
C006780	increase	PLK4	ENSG00000142731	16474171;
C006780	increase	PLOD1	ENSG00000083444	16474171;
C006780	increase	PPP2R2C	ENSG00000074211	16474171;
C006780	increase	PRC1	ENSG00000198901	16474171;
C006780	increase	PRIM1	ENSG00000198056	16474171;
C006780	increase	PTGER3	ENSG00000050628	16474171;
C006780	increase	RACGAP1	ENSG00000161800	16474171;
C006780	increase	RAD51AP1	ENSG00000111247	16474171;
C006780	increase	RAD51C	ENSG00000108384	16474171;
C006780	increase	RAD51	ENSG00000051180	16474171;
C006780	increase	RNASEH1	ENSG00000171865	16474171;
C006780	increase	RRM2	ENSG00000171848	16474171;
C006780	increase	SAC3D1	ENSG00000168061	16474171;
C006780	increase	SFXN2	ENSG00000156398	16474171;
C006780	increase	SLC39A8	ENSG00000138821	16474171;
C006780	increase	SLC6A14	ENSG00000087916	16474171;
C006780	increase	SMC2	ENSG00000136824	16474171;
C006780	increase	SMC4	ENSG00000113810	16474171;
C006780	increase	STC2	ENSG00000113739	16474171;
C006780	increase	STMN1	ENSG00000117632	16474171;
C006780	increase	TACC3	ENSG00000013810	16474171;
C006780	increase	TERT	ENSG00000164362	15368248;
C006780	increase	TFF1	ENSG00000160182	15525692;16474171;12753421;11693576;
C006780	increase	TFF3	ENSG00000160180	16474171;
C006780	increase	THBS1	ENSG00000137801	16474171;
C006780	increase	TK1	ENSG00000167900	16474171;
C006780	increase	TMEM38B	ENSG00000095209	16474171;
C006780	increase	TMEM97	ENSG00000109084	16474171;
C006780	increase	TMPO	ENSG00000120802	16474171;
C006780	increase	TOP2A	ENSG00000131747	16474171;
C006780	increase	TPM3	ENSG00000143549	16474171;
C006780	increase	TRIP13	ENSG00000071539	16474171;
C006780	increase	TUBB6	ENSG00000176014	16474171;
C006780	increase	TYMS	ENSG00000176890	16474171;
C006780	increase	UBE2C	ENSG00000175063	16474171;
C006780	increase	UBE2T	ENSG00000077152	16474171;
C006780	increase	UHRF1	ENSG00000034063	16474171;
C006780	increase	UNG	ENSG00000076248	16474171;
C006780	increase	ZNF703	ENSG00000183779	16474171;

C006780	increase	ZWINT	ENSG00000122952	16474171;
C006821	decrease	IL4	ENSG00000113520	15454121;
C006821	decrease	IL5	ENSG00000113525	15454121;
C006821	increase	IFNG	ENSG00000111537	15454121;
C006862	decrease	FAS	ENSG00000026103	15816839;
C006862	increase	ADORA2B	ENSG00000170425	10571726;
C006862	increase	LRP1	ENSG00000123384	15816839;
C006862	increase	RPL24	ENSG00000114391	10571726;
C006862	increase	TNFRSF10A	ENSG00000104689	15816839;
C006862	increase	TNFRSF10B	ENSG00000120889	15816839;
C006862	increase	TNFRSF1A	ENSG00000067182	15816839;
C006862	increase	TNFRSF1B	ENSG00000028137	15816839;
C006862	increase	TYRP1	ENSG00000107165	15816839;
C006923	increase	RHOA	ENSG00000067560	12671036;
C006923	increase	RHOB	ENSG00000143878	12671036;
C006963	increase	CDKN1A	ENSG00000124762	16439135;
C007164	increase	GAPDH	ENSG00000111640	11295360;
C007164	increase	HSPB1	ENSG00000106211	11295360;
C007164	increase	PRDX1	ENSG00000117450	11295360;
C007164	increase	PRDX2	ENSG00000167815	11295360;
C007164	increase	PRDX6	ENSG00000117592	11295360;
C007262	affect	IL2	ENSG00000109471	17166736;
C007262	decrease	BCL2	ENSG00000171791	17166419;
C007262	decrease	TAP1	ENSG00000230705	15766417;
C007262	increase	ASCL2	ENSG00000183734	19438726;
C007262	increase	ATF3	ENSG00000162772	19101521;19360757;
C007262	increase	BAX	ENSG00000087088	17166419;
C007262	increase	CASP3	ENSG00000164305	17166419;
C007262	increase	CD14	ENSG00000170458	19549553;
C007262	increase	CDKN1A	ENSG00000124762	18006205;
C007262	increase	EGR1	ENSG00000120738	18485432;17707346;19360757;
C007262	increase	EIF2AK2	ENSG00000055332	18599499;
C007262	increase	ELAVL1	ENSG00000066044	19591856;
C007262	increase	FOS	ENSG00000170345	19360757;
C007262	increase	HOXA7	ENSG00000122592	19438726;
C007262	increase	ICAM1	ENSG00000090339	19549553;
C007262	increase	IFNG	ENSG00000111537	17166736;19549553;
C007262	increase	IFNGR1	ENSG00000027697	19549553;
C007262	increase	IL1B	ENSG00000125538	18308354;16687389;16364386;
C007262	increase	IL6	ENSG00000136244	16364386;
C007262	increase	IL8	ENSG00000169429	18485432;18599499;20181660;18308354;19591856;17636245;16364386;17707346;17166736;
C007262	increase	JUN	ENSG00000177606	19360757;
C007262	increase	MAPK1	ENSG00000100030	18485432;17707346;19360757;18006205;
C007262	increase	MAPK3	ENSG00000102882	18485432;17707346;19360757;18006205;
C007262	increase	MLL	ENSG00000118058	19438726;
C007262	increase	PPARG	ENSG00000132170	18485432;
C007262	increase	RBBP5	ENSG00000117222	19438726;
C007262	increase	RELA	ENSG00000173039	18599499;17636245;

C007262	increase	SP1	ENSG00000185591	19438726;
C007262	increase	WDR5	ENSG00000196363	19438726;
C007350	decrease	BCL2L1	ENSG00000171552	17439724;
C007350	decrease	FOXA2	ENSG00000125798	16490937;
C007350	decrease	NKX2-1	ENSG00000136352	15083329;16490937;10370019;
C007350	decrease	PCNA	ENSG00000132646	17439724;
C007350	decrease	SFTPB	ENSG00000168878	16490937;15083329;
C007366	increase	TP53	ENSG00000141510	14998682;
C007379	increase	CYP1B1	ENSG00000138061	16040568;
C007482	decrease	DNMT1	ENSG00000130816	11912126;
C007517	decrease	ADIPOQ	ENSG00000181092	17668557;
C007517	decrease	LEP	ENSG00000174697	17668557;
C007517	increase	CCL2	ENSG00000108691	17668557;16600694;14576080;
C007517	increase	CDKN1A	ENSG00000124762	15978937;
C007517	increase	IL6	ENSG00000136244	17668557;11950692;
C007517	increase	IL8	ENSG00000169429	12208513;
C007517	increase	ITGAM	ENSG00000169896	16357311;
C007517	increase	PTGS2	ENSG00000073756	19804834;
C007517	increase	TNF	ENSG00000223952	14576080;
C007567	increase	CGB	ENSG00000213030	15941851;
C007567	increase	CYP19A1	ENSG00000137869	15941851;
C007567	increase	HSD17B1	ENSG00000108786	16513093;
C007567	increase	RXRA	ENSG00000186350	15941851;
C007610	decrease	CCNA2	ENSG00000145386	16865672;
C007610	decrease	CCNB2	ENSG00000157456	16865672;
C007610	decrease	CDC25B	ENSG00000101224	16865672;
C007610	decrease	CDC25C	ENSG00000158402	16865672;
C007610	decrease	CHEK1	ENSG00000149554	16865672;
C007610	decrease	DBF4	ENSG00000006634	16865672;
C007610	decrease	DUSP4	ENSG00000120875	16865672;
C007610	decrease	IGF1R	ENSG00000140443	16865672;
C007610	decrease	KLK3	ENSG00000142515	16865672;
C007610	decrease	MCM4	ENSG00000104738	16865672;
C007610	decrease	NKX3-1	ENSG00000167034	16865672;
C007610	decrease	PRIM2	ENSG00000146143	16865672;
C007610	decrease	STK39	ENSG00000198648	16865672;
C007610	decrease	THBS1	ENSG00000137801	16865672;
C007610	increase	ALPPL2	ENSG00000163286	15993745;
C007610	increase	ME1	ENSG00000065833	16865672;
C007610	increase	PTGS2	ENSG00000073756	16870006;
C007610	increase	TFF1	ENSG00000160182	16965913;
C007610	increase	UGT2B15	ENSG00000196620	16865672;
C007610	increase	UGT2B17	ENSG00000197888	16865672;
C007619	increase	HMOX1	ENSG00000100292	16799064;
C007619	increase	NFE2L2	ENSG00000116044	16799064;
C007628	decrease	BCL2L1	ENSG00000171552	16051289;
C007628	decrease	MAPK14	ENSG00000112062	16051289;
C007628	decrease	MAPK1	ENSG00000100030	16051289;
C007628	decrease	MAPK3	ENSG00000102882	16051289;
C007628	increase	BAX	ENSG00000087088	16051289;
C007628	increase	FAS	ENSG00000026103	16051289;
C007628	increase	FASLG	ENSG00000117560	16051289;

C007628	increase	MAPK8	ENSG00000107643	16051289;
C007628	increase	MAPK9	ENSG00000050748	16051289;
C007650	increase	CCL5	ENSG00000161570	11306435;
C007650	increase	CSF2	ENSG00000164400	11306435;
C007789	decrease	BIRC2	ENSG00000110330	16201850;
C007789	decrease	BIRC5	ENSG00000089685	16201850;
C007789	decrease	CCL5	ENSG00000161570	16201850;
C007789	decrease	CCND2	ENSG00000118971	16201850;
C007789	decrease	MYC	ENSG00000136997	16201850;
C007789	decrease	RB1	ENSG00000139687	16201850;
C007836	decrease	TK1	ENSG00000167900	15374632;
C007836	decrease	TYMS	ENSG00000176890	15374632;
C007845	affect	GSK3B	ENSG00000082701	18058799;
C007845	affect	PRKCE	ENSG00000171132	18058799;
C007845	affect	PTGS2	ENSG00000073756	17454143;
C007845	decrease	CCND1	ENSG00000110092	18058799;
C007845	decrease	CDK4	ENSG00000135446	18058799;
C007845	decrease	MMP2	ENSG00000087245	17683926;
C007845	decrease	MMP9	ENSG00000100985	17683926;
C007845	increase	APAF1	ENSG00000120868	19481070;
C007845	increase	BAX	ENSG00000087088	18030663;19481070;
C007845	increase	BCL2	ENSG00000171791	18030663;19481070;
C007845	increase	BIRC5	ENSG00000089685	18030663;
C007845	increase	CASP3	ENSG00000164305	18030663;19481070;
C007845	increase	CASP9	ENSG00000132906	18030663;19481070;
C007845	increase	DUSP10	ENSG00000143507	17151092;
C007845	increase	GDF15	ENSG00000130513	18058799;
C007845	increase	KLKB1	ENSG00000164344	18030663;
C007845	increase	TP53	ENSG00000141510	18030663;
C007997	decrease	ADIPOR1	ENSG00000159346	16023994;
C007997	increase	ABCA1	ENSG00000165029	16023994;
C007997	increase	NOS2	ENSG00000007171	12085989;
C008261	decrease	APBA1	ENSG00000107282	12634122;
C008261	decrease	BAG1	ENSG00000250477	12634122;
C008261	decrease	BMP2	ENSG00000125845	12634122;
C008261	decrease	C1ORF61	ENSG00000125462	12634122;
C008261	decrease	CAT	ENSG00000121691	12634122;
C008261	decrease	CDKN1A	ENSG00000124762	12634122;
C008261	decrease	CKB	ENSG00000166165	12634122;
C008261	decrease	CKM	ENSG00000104879	12634122;
C008261	decrease	CXCR4	ENSG00000121966	12634122;
C008261	decrease	DAB2	ENSG00000153071	12634122;
C008261	decrease	EPB41L3	ENSG00000082397	12634122;
C008261	decrease	EPHA5	ENSG00000145242	12634122;
C008261	decrease	ERBB3	ENSG00000065361	12634122;
C008261	decrease	ERCC2	ENSG00000104884	12634122;
C008261	decrease	ERCC5	ENSG00000134899	12634122;
C008261	decrease	GATA6	ENSG00000141448	12634122;
C008261	decrease	GDF15	ENSG00000130513	12634122;
C008261	decrease	GLI1	ENSG00000111087	12634122;
C008261	decrease	HCK	ENSG00000101336	12634122;
C008261	decrease	ID1	ENSG00000125968	12634122;
C008261	decrease	ID2	ENSG00000115738	12634122;
C008261	decrease	IGFBP7	ENSG00000163453	12634122;

C008261	decrease	MAP2K6	ENSG00000108984	12634122;
C008261	decrease	MAPK4	ENSG00000141639	12634122;
C008261	decrease	MAPK9	ENSG00000050748	12634122;
C008261	decrease	MATK	ENSG00000007264	12634122;
C008261	decrease	MCC	ENSG00000171444	12634122;
C008261	decrease	MCF2	ENSG00000101977	12634122;
C008261	decrease	MSH2	ENSG00000095002	12634122;
C008261	decrease	MYCN	ENSG00000134323	12634122;
C008261	decrease	PIP5K1B	ENSG00000107242	12634122;
C008261	decrease	PTP4A1	ENSG00000112245	12634122;
C008261	decrease	RARRES3	ENSG00000133321	12634122;
C008261	decrease	RET	ENSG00000165731	12634122;
C008261	decrease	SGK1	ENSG00000118515	12634122;
C008261	decrease	SLC2A3	ENSG00000059804	12634122;
C008261	decrease	SOD1	ENSG00000142168	12634122;
C008261	decrease	SPINK1	ENSG00000164266	12634122;
C008261	decrease	SSPN	ENSG00000123096	12634122;
C008261	decrease	STOM	ENSG00000148175	12634122;
C008261	decrease	TGFB2	ENSG00000092969	12634122;
C008261	decrease	TM4SF1	ENSG00000169908	12634122;
C008261	decrease	TNFRSF1B	ENSG00000028137	12634122;
C008261	decrease	TNFSF10	ENSG00000121858	12634122;
C008261	decrease	VIM	ENSG00000026025	12634122;
C008261	decrease	WNT1	ENSG00000125084	12634122;
C008261	decrease	WNT2	ENSG00000105989	12634122;
C008261	increase	BMP4	ENSG00000125378	12634122;
C008261	increase	CASP10	ENSG00000003400	12634122;
C008261	increase	CDKN2A	ENSG00000147889	12634122;
C008261	increase	COL6A2	ENSG00000142173	12634122;
C008261	increase	FOSL1	ENSG00000175592	12634122;
C008261	increase	HIST1H2AC	ENSG00000180573	12634122;
C008261	increase	ITGB4	ENSG00000132470	12634122;
C008261	increase	JUNB	ENSG00000171223	12634122;
C008261	increase	LRPAP1	ENSG00000163956	12634122;
C008261	increase	MT1X	ENSG00000187193	12634122;
C008261	increase	MT3	ENSG00000087250	12634122;
C008261	increase	NFIX	ENSG00000008441	12634122;
C008261	increase	NTHL1	ENSG00000065057	12634122;
C008288	increase	IL1B	ENSG00000125538	16804125;
C008340	decrease	IFNG	ENSG00000111537	10883730;
C008340	decrease	TNF	ENSG00000223952	10883730;
C008461	affect	CCNE1	ENSG00000105173	19513903;
C008461	affect	CDK2	ENSG00000123374	19513903;
C008461	affect	CDKN1A	ENSG00000124762	19513903;
C008461	affect	TP53	ENSG00000141510	19513903;
C008461	increase	IFNG	ENSG00000111537	19295137;
C008461	increase	IL10	ENSG00000136634	19295137;
C008461	increase	IL2	ENSG00000109471	19295137;
C008461	increase	IL4	ENSG00000113520	19295137;
C008480	decrease	MKI67	ENSG00000148773	18418871;
C008480	increase	AKR1C1	ENSG00000187134	19800895;
C008480	increase	AKR1C2	ENSG00000151632	19800895;
C008480	increase	CDKN1A	ENSG00000124762	18418871;
C008480	increase	CYP1A1	ENSG00000140465	19800895;

C008480	increase	GADD45A	ENSG00000116717	18418871;
C008480	increase	MDM2	ENSG00000135679	18418871;
C008480	increase	TP53	ENSG00000141510	18418871;
C008493	affect	ACPP	ENSG00000014257	14617803;
C008493	affect	APOD	ENSG00000189058	14617803;
C008493	affect	ATF3	ENSG00000162772	14617803;
C008493	affect	B4GALT1	ENSG00000086062	14617803;
C008493	affect	BIRC2	ENSG00000110330	14617803;
C008493	affect	BIRC3	ENSG00000023445	14617803;
C008493	affect	BUB1B	ENSG00000156970	14617803;
C008493	affect	CDC14B	ENSG00000081377	14617803;
C008493	affect	CDC20	ENSG00000117399	14617803;
C008493	affect	CENPA	ENSG00000115163	14617803;
C008493	affect	CLU	ENSG00000120885	14617803;
C008493	affect	CREB3L4	ENSG00000143578	14617803;
C008493	affect	DNAJB9	ENSG00000128590	14617803;
C008493	affect	FKBP5	ENSG00000096060	14617803;
C008493	affect	FN1	ENSG00000115414	14617803;
C008493	affect	H1F0	ENSG00000189060	14617803;
C008493	affect	ID2	ENSG00000115738	14617803;
C008493	affect	IGSF3	ENSG00000143061	14617803;
C008493	affect	ING1	ENSG00000153487	14617803;
C008493	affect	INPP4B	ENSG00000109452	14617803;
C008493	affect	IRF1	ENSG00000125347	14617803;
C008493	affect	KLHL2	ENSG00000109466	14617803;
C008493	affect	MCM2	ENSG00000073111	14617803;
C008493	affect	MEN1	ENSG00000133895	14617803;
C008493	affect	MXD1	ENSG00000059728	14617803;
C008493	affect	NDRG1	ENSG00000104419	14617803;
C008493	affect	NFIL3	ENSG00000165030	14617803;
C008493	affect	NKX3-1	ENSG00000167034	14617803;
C008493	affect	NR3C1	ENSG00000113580	16928829;
C008493	affect	PEG3	ENSG00000198300	14617803;
C008493	affect	PGR	ENSG00000082175	16928829;
C008493	affect	PPFIA1	ENSG00000131626	14617803;
C008493	affect	RDH11	ENSG00000072042	14617803;
C008493	affect	RHOB	ENSG00000143878	14617803;
C008493	affect	RRM1	ENSG00000167325	14617803;
C008493	affect	RRM2	ENSG00000171848	14617803;
C008493	affect	SAT1	ENSG00000130066	14617803;
C008493	affect	SCD	ENSG00000099194	14617803;
C008493	affect	SEC24D	ENSG00000150961	14617803;
C008493	affect	SGK1	ENSG00000118515	14617803;
C008493	affect	SH3BP5	ENSG00000131370	14617803;
C008493	affect	STX1A	ENSG00000106089	14617803;
C008493	affect	TMPRSS2	ENSG00000184012	14617803;
C008493	affect	TNFAIP3	ENSG00000118503	14617803;
C008493	affect	TOP2A	ENSG00000131747	14617803;
C008493	affect	UGDH	ENSG00000109814	14617803;
C008493	affect	VDR	ENSG00000111424	16928829;
C008493	affect	WASF1	ENSG00000112290	14617803;
C008493	decrease	ABCC4	ENSG00000125257	16020662;14617803;18548127;
C008493	decrease	ACSL3	ENSG00000123983	14617803;18548127;

C008493	decrease	AKT2	ENSG00000105221	11830524;
C008493	decrease	ALG3	ENSG00000214160	18548127;
C008493	decrease	ALG8	ENSG00000159063	18548127;
C008493	decrease	ANK3	ENSG00000151150	18548127;
C008493	decrease	AR	ENSG00000169083	16020662;16928829;17346688;16648561;14617803;15141018;
C008493	decrease	ATF5	ENSG00000169136	12517777;18548127;
C008493	decrease	AURKA	ENSG00000087586	14617803;12517777;
C008493	decrease	BIRC5	ENSG00000089685	17876054;12517777;18567002;
C008493	decrease	C1QBP	ENSG00000108561	18548127;
C008493	decrease	C21ORF33	ENSG00000160221	18548127;
C008493	decrease	C6ORF108	ENSG00000112667	18548127;
C008493	decrease	CAPZA1	ENSG00000116489	18548127;
C008493	decrease	CCNA2	ENSG00000145386	12517777;
C008493	decrease	CCNB1	ENSG00000134057	11830524;
C008493	decrease	CCND1	ENSG00000110092	18314491;11830524;17847021;
C008493	decrease	CCNE2	ENSG00000175305	12517777;
C008493	decrease	CD14	ENSG00000170458	18548127;
C008493	decrease	CD44	ENSG00000026508	12517777;
C008493	decrease	CDC25A	ENSG00000164045	14617803;11830524;
C008493	decrease	CDC25C	ENSG00000158402	11830524;
C008493	decrease	CDC42	ENSG00000070831	11830524;
C008493	decrease	CDC6	ENSG00000094804	14617803;11830524;
C008493	decrease	CDK10	ENSG00000185324	11830524;
C008493	decrease	CDK1	ENSG00000170312	16205645;12517777;11830524;
C008493	decrease	CDK2	ENSG00000123374	16205645;17847021;12517777;
C008493	decrease	CDK4	ENSG00000135446	17847021;12517777;18548127;11830524;
C008493	decrease	CDKL1	ENSG00000100490	11830524;
C008493	decrease	CENPF	ENSG00000117724	12517777;
C008493	decrease	CHAF1A	ENSG00000167670	12517777;
C008493	decrease	CNPY2	ENSG00000144785	18548127;
C008493	decrease	CREB3L1	ENSG00000157613	18548127;
C008493	decrease	DCI	ENSG00000167969	18548127;
C008493	decrease	DEAF1	ENSG00000177030	18548127;
C008493	decrease	DHCR24	ENSG00000116133	16020662;18548127;
C008493	decrease	DHFR	ENSG00000228716	12517777;
C008493	decrease	DKC1	ENSG00000130826	18548127;
C008493	decrease	DTYMK	ENSG00000168393	12517777;
C008493	decrease	E2F1	ENSG00000101412	14617803;17847021;11830524;
C008493	decrease	E2F5	ENSG00000133740	11830524;
C008493	decrease	EFHA1	ENSG00000165487	18548127;
C008493	decrease	EIF4G2	ENSG00000110321	12517777;
C008493	decrease	ESR1	ENSG00000091831	16093440;15833885;16155795;
C008493	decrease	FGF2	ENSG00000138685	12517777;
C008493	decrease	FKBP4	ENSG00000004478	18548127;
C008493	decrease	FOXA1	ENSG00000129514	12517777;18548127;
C008493	decrease	GALNT1	ENSG00000141429	18548127;

C008493	decrease	GTF3C1	ENSG00000077235	18548127;
C008493	decrease	GUCY1A3	ENSG00000164116	16020662;14617803;18548127;
C008493	decrease	HIST1H2AE	ENSG00000168274	18548127;
C008493	decrease	HIST1H4J	ENSG00000182217	18548127;
C008493	decrease	HIST2H2AA	ENSG00000203812	18548127;
C008493	decrease	HNRNPAB	ENSG00000197451	18548127;
C008493	decrease	HOXA9	ENSG00000078399	18548127;
C008493	decrease	ID1	ENSG00000125968	12517777;
C008493	decrease	IFRD2	ENSG00000214706	18548127;
C008493	decrease	ITGB3BP	ENSG00000142856	12517777;
C008493	decrease	ITGB4	ENSG00000132470	12517777;
C008493	decrease	ITGB5	ENSG00000082781	12517777;
C008493	decrease	JUNB	ENSG00000171223	12517777;
C008493	decrease	JUN	ENSG00000177606	11830524;
C008493	decrease	KDELR2	ENSG00000136240	18548127;
C008493	decrease	KIF11	ENSG00000138160	12517777;
C008493	decrease	KIF20B	ENSG00000138182	12517777;
C008493	decrease	KLK2	ENSG00000167751	16020662;14617803;18548127;
C008493	decrease	LAGE3	ENSG00000196976	18548127;
C008493	decrease	LMNB2	ENSG00000176619	12517777;
C008493	decrease	LYPLA1	ENSG00000120992	18548127;
C008493	decrease	MAEA	ENSG00000090316	18548127;
C008493	decrease	MAPK9	ENSG00000050748	11830524;
C008493	decrease	MAPT	ENSG00000186868	12517777;
C008493	decrease	MAZ	ENSG00000103495	12517777;
C008493	decrease	MCCC2	ENSG00000131844	18548127;
C008493	decrease	MCM3	ENSG00000112118	12517777;
C008493	decrease	MCM5	ENSG00000100297	12517777;
C008493	decrease	MCM6	ENSG00000076003	14617803;12517777;
C008493	decrease	MCM7	ENSG00000166508	12517777;
C008493	decrease	MKI67	ENSG00000148773	12517777;
C008493	decrease	MRPL19	ENSG00000115364	18548127;
C008493	decrease	MYBL2	ENSG00000101057	12517777;
C008493	decrease	MYO10	ENSG00000145555	18548127;
C008493	decrease	MYRIP	ENSG00000170011	18548127;
C008493	decrease	NFKB1	ENSG00000109320	12517777;
C008493	decrease	NME1	ENSG00000239672	18548127;
C008493	decrease	NME4	ENSG00000103202	18548127;
C008493	decrease	NMI	ENSG00000123609	12517777;
C008493	decrease	PATZ1	ENSG00000100105	12517777;
C008493	decrease	PAXIP1	ENSG00000157212	18548127;
C008493	decrease	PCBD1	ENSG00000166228	18548127;
C008493	decrease	PCNA	ENSG00000132646	14617803;12517777;11830524;
C008493	decrease	PFDN5	ENSG00000123349	11830524;
C008493	decrease	PGAP2	ENSG00000249611	18548127;
C008493	decrease	PLK1	ENSG00000166851	14617803;11830524;
C008493	decrease	POLA1	ENSG00000101868	12517777;
C008493	decrease	POLR2H	ENSG00000163882	18548127;
C008493	decrease	PPIL2	ENSG00000100023	18548127;
C008493	decrease	PTGES	ENSG00000148344	11830524;
C008493	decrease	PTPLB	ENSG00000206527	18548127;

C008493	decrease	RABAC1	ENSG00000105404	12517777;
C008493	decrease	RB1	ENSG00000139687	17847021;
C008493	decrease	RCC1	ENSG00000180198	18548127;
C008493	decrease	RFC5	ENSG00000111445	11830524;
C008493	decrease	RPA2	ENSG00000117748	12517777;
C008493	decrease	RPIA	ENSG00000153574	18548127;
C008493	decrease	RPL27A	ENSG00000166441	18548127;
C008493	decrease	SERPINB6	ENSG00000124570	18548127;
C008493	decrease	SLC19A1	ENSG00000173638	18548127;
C008493	decrease	SMARCA4	ENSG00000127616	18548127;
C008493	decrease	SMARCE1	ENSG00000073584	12517777;
C008493	decrease	SND1	ENSG00000197157	18548127;
C008493	decrease	SRP19	ENSG00000153037	18548127;
C008493	decrease	ST7	ENSG00000004866	14617803;18548127;
C008493	decrease	TACSTD2	ENSG00000184292	18548127;
C008493	decrease	TARBP2	ENSG00000139546	18548127;
C008493	decrease	TMEM5	ENSG00000118600	18548127;
C008493	decrease	TRIB1	ENSG00000173334	18548127;
C008493	decrease	TRIM14	ENSG00000106785	18548127;
C008493	decrease	TTK	ENSG00000112742	18548127;
C008493	decrease	TYMS	ENSG00000176890	14617803;12517777;
C008493	decrease	UCK2	ENSG00000143179	18548127;
C008493	decrease	UTRN	ENSG00000152818	18548127;
C008493	decrease	VEGFC	ENSG00000150630	12517777;
C008493	decrease	WNT7A	ENSG00000154764	12517777;
C008493	decrease	WSB2	ENSG00000176871	18548127;
C008493	decrease	YWHAE	ENSG00000108953	18548127;
C008493	decrease	ZNF24	ENSG00000172466	18548127;
C008493	decrease	ZNF43	ENSG00000198521	18548127;
C008493	increase	ACTA2	ENSG00000107796	18548127;
C008493	increase	ACTG2	ENSG00000163017	18548127;
C008493	increase	ADAM22	ENSG00000008277	18548127;
C008493	increase	ADD3	ENSG00000148700	12517777;
C008493	increase	AHR	ENSG00000106546	18548127;
C008493	increase	AKAP12	ENSG00000131016	18548127;
C008493	increase	AKR1B1	ENSG00000085662	18548127;
C008493	increase	ALAD	ENSG00000148218	18548127;
C008493	increase	AMT	ENSG00000145020	18548127;
C008493	increase	ANK2	ENSG00000145362	18548127;
C008493	increase	ARHGEF2	ENSG00000116584	18548127;
C008493	increase	ARL4A	ENSG00000122644	18548127;
C008493	increase	ATP2A2	ENSG00000174437	18548127;
C008493	increase	ATP2B4	ENSG00000058668	18548127;
C008493	increase	BACH1	ENSG00000156273	12517777;
C008493	increase	BCL2A1	ENSG00000140379	11830524;
C008493	increase	BCL2L11	ENSG00000153094	17339365;
C008493	increase	BMP4	ENSG00000125378	12517777;
C008493	increase	BRCA2	ENSG00000139618	12517777;
C008493	increase	BTG3	ENSG00000154640	18548127;
C008493	increase	CALM1	ENSG00000143933	18548127;
C008493	increase	CAMK2G	ENSG00000148660	18548127;
C008493	increase	CAMK4	ENSG00000152495	12517777;
C008493	increase	CASP9	ENSG00000132906	17339365;12517777;
C008493	increase	CAST	ENSG00000153113	18548127;

C008493	increase	CBX7	ENSG00000100307	18548127;
C008493	increase	CCK	ENSG00000187094	18548127;
C008493	increase	CDC42EP3	ENSG00000163171	18548127;
C008493	increase	CDH15	ENSG00000129910	12517777;
C008493	increase	CDH7	ENSG00000081138	12517777;
C008493	increase	CDK5	ENSG00000164885	11830524;
C008493	increase	CDKN1A	ENSG00000124762	16205645;18314491;14617803;17847021;12517777;
C008493	increase	CDKN1B	ENSG00000111276	18314491;17847021;
C008493	increase	CDKN1C	ENSG00000129757	14617803;18548127;
C008493	increase	CDKN2A	ENSG00000147889	17847021;
C008493	increase	CDKN2D	ENSG00000129355	14617803;12517777;
C008493	increase	CHEK2	ENSG00000183765	12517777;
C008493	increase	COL16A1	ENSG00000084636	18548127;
C008493	increase	COL6A1	ENSG00000142156	18548127;
C008493	increase	CORO1A	ENSG00000102879	12517777;
C008493	increase	CRABP2	ENSG00000143320	18548127;
C008493	increase	CREB1	ENSG00000118260	12517777;
C008493	increase	CRYAB	ENSG00000109846	18548127;
C008493	increase	CSRP1	ENSG00000159176	18548127;
C008493	increase	CTSK	ENSG00000143387	18548127;
C008493	increase	CUL1	ENSG00000055130	18548127;
C008493	increase	CYLD	ENSG00000083799	18548127;
C008493	increase	DDIT3	ENSG00000175197	16205645;16204082;19276161;11830524;12517777;
C008493	increase	DIXDC1	ENSG00000150764	18548127;
C008493	increase	DLC1	ENSG00000164741	12517777;
C008493	increase	DNAJB5	ENSG00000137094	18548127;
C008493	increase	DPYD	ENSG00000188641	18548127;
C008493	increase	DPYSL2	ENSG00000092964	18548127;
C008493	increase	DTNA	ENSG00000134769	18548127;
C008493	increase	DUSP10	ENSG00000143507	12517777;
C008493	increase	DVL3	ENSG00000161202	18548127;
C008493	increase	ECM2	ENSG00000106823	18548127;
C008493	increase	EDNRA	ENSG00000151617	18548127;
C008493	increase	EDNRB	ENSG00000136160	18548127;
C008493	increase	EFEMP2	ENSG00000172638	18548127;
C008493	increase	ELF3	ENSG00000163435	14617803;12517777;
C008493	increase	ENO2	ENSG00000111674	18548127;
C008493	increase	EPB41L2	ENSG00000079819	18548127;
C008493	increase	EPS8	ENSG00000151491	18548127;
C008493	increase	ERCC1	ENSG00000012061	12517777;
C008493	increase	ESR2	ENSG00000140009	16928829;16093440;15833885;
C008493	increase	ETV5	ENSG00000244405	18548127;
C008493	increase	ETV6	ENSG00000139083	18548127;
C008493	increase	EXT1	ENSG00000182197	18548127;
C008493	increase	FAM107A	ENSG00000168309	18548127;
C008493	increase	FBXL8	ENSG00000135722	18548127;
C008493	increase	FEZ1	ENSG00000149557	18548127;
C008493	increase	FHL2	ENSG00000115641	18548127;
C008493	increase	FLNC	ENSG00000128591	18548127;
C008493	increase	FNBP1	ENSG00000187239	18548127;
C008493	increase	FYN	ENSG00000010810	18548127;

C008493	increase	GABARAPL	ENSG00000139112	18548127;
C008493	increase	GADD45A	ENSG00000116717	19276161;12517777;
C008493	increase	GAS1	ENSG00000180447	18548127;
C008493	increase	GBP2	ENSG00000162645	18548127;
C008493	increase	GJA1	ENSG00000152661	18548127;
C008493	increase	GPR161	ENSG00000143147	18548127;
C008493	increase	GPR37	ENSG00000170775	12517777;
C008493	increase	GRK5	ENSG00000198873	18548127;
C008493	increase	GSN	ENSG00000148180	18548127;
C008493	increase	HABP4	ENSG00000130956	18548127;
C008493	increase	HEPH	ENSG00000089472	18548127;
C008493	increase	HMGN4	ENSG00000182952	18548127;
C008493	increase	HOXD8	ENSG00000175879	12517777;
C008493	increase	HSP90B1	ENSG00000166598	16204082;
C008493	increase	HSPA4L	ENSG00000164070	18548127;
C008493	increase	HSPA5	ENSG00000044574	16205645;16204082;
C008493	increase	ID3	ENSG00000117318	12517777;18548127;
C008493	increase	IDS	ENSG00000010404	18548127;
C008493	increase	IFI16	ENSG00000163565	18548127;
C008493	increase	IGF1	ENSG00000017427	11830524;
C008493	increase	IPO8	ENSG00000133704	12517777;
C008493	increase	ITGA3	ENSG00000005884	18548127;
C008493	increase	ITPR1	ENSG00000150995	18548127;
C008493	increase	JAK1	ENSG00000162434	14617803;18548127;
C008493	increase	JMJD6	ENSG00000070495	18548127;
C008493	increase	KCNAB1	ENSG00000169282	18548127;
C008493	increase	KCNH2	ENSG00000055118	18548127;
C008493	increase	KCNJ8	ENSG00000121361	18548127;
C008493	increase	KLF4	ENSG00000136826	18314491;14617803;
C008493	increase	KLK3	ENSG00000142515	16020662;16928829;14617803;16648561;15141018;18548127;
C008493	increase	KRT15	ENSG00000171346	18548127;
C008493	increase	KRT20	ENSG00000171431	12517777;
C008493	increase	LAMA4	ENSG00000112769	18548127;
C008493	increase	LAMB3	ENSG00000196878	18548127;
C008493	increase	LPHN3	ENSG00000150471	18548127;
C008493	increase	MAF	ENSG00000178573	18548127;
C008493	increase	MAP1B	ENSG00000131711	14617803;18548127;
C008493	increase	MAP2K1	ENSG00000169032	11830524;12517777;
C008493	increase	MAP2K5	ENSG00000137764	12517777;
C008493	increase	MAPK3	ENSG00000102882	11830524;15845651;
C008493	increase	MAPK8	ENSG00000107643	12517777;
C008493	increase	MAPRE2	ENSG00000166974	18548127;
C008493	increase	MBNL2	ENSG00000139793	18548127;
C008493	increase	ME1	ENSG00000065833	18548127;
C008493	increase	MEF2C	ENSG00000081189	18548127;
C008493	increase	MITF	ENSG00000187098	18548127;
C008493	increase	MXI1	ENSG00000119950	18548127;
C008493	increase	MYC	ENSG00000136997	14617803;16093440;16155795;15833885;
C008493	increase	MYL9	ENSG00000101335	18548127;
C008493	increase	NEBL	ENSG00000078114	18548127;
C008493	increase	OAT	ENSG00000065154	18548127;

C008493	increase	OLFML2A	ENSG00000185585	18548127;
C008493	increase	OPTN	ENSG00000123240	18548127;
C008493	increase	PALM2	ENSG00000243444	18548127;
C008493	increase	PBX1	ENSG00000185630	18548127;
C008493	increase	PGM5	ENSG00000154330	18548127;
C008493	increase	PJA2	ENSG00000198961	18548127;
C008493	increase	PLA2G4A	ENSG00000116711	18548127;
C008493	increase	PLCB4	ENSG00000101333	12517777;
C008493	increase	PMS2	ENSG00000122512	12517777;
C008493	increase	PPM1B	ENSG00000138032	12517777;
C008493	increase	PRKAR2B	ENSG00000005249	18548127;
C008493	increase	PRKCA	ENSG00000154229	12517777;
C008493	increase	PRKCB	ENSG00000166501	18548127;
C008493	increase	PRNP	ENSG00000171867	18548127;
C008493	increase	PTEN	ENSG00000171862	12517777;
C008493	increase	QKI	ENSG00000112531	18548127;
C008493	increase	RAB28	ENSG00000157869	12517777;
C008493	increase	RAB31	ENSG00000168461	14617803;12517777;18548127;
C008493	increase	RAPGEF4	ENSG00000091428	12517777;
C008493	increase	REEP1	ENSG00000068615	18548127;
C008493	increase	RGL1	ENSG00000143344	18548127;
C008493	increase	RGS10	ENSG00000148908	12517777;
C008493	increase	RIMS3	ENSG00000117016	18548127;
C008493	increase	RND3	ENSG00000115963	18548127;
C008493	increase	S100A4	ENSG00000196154	18548127;
C008493	increase	SDC2	ENSG00000169439	18548127;
C008493	increase	SEMA5A	ENSG00000112902	18548127;
C008493	increase	SERPINB5	ENSG00000206075	14617803;18548127;
C008493	increase	SERPINE2	ENSG00000135919	18548127;
C008493	increase	SERPINF1	ENSG00000132386	18548127;
C008493	increase	SH3BGR	ENSG00000185437	18548127;
C008493	increase	SLC14A1	ENSG00000141469	18548127;
C008493	increase	SLC16A7	ENSG00000118596	18548127;
C008493	increase	SMAD7	ENSG00000101665	18548127;
C008493	increase	SMARCD3	ENSG00000082014	18548127;
C008493	increase	SNAI2	ENSG00000019549	18548127;
C008493	increase	SNAP25	ENSG00000132639	18548127;
C008493	increase	SNCG	ENSG00000173267	18548127;
C008493	increase	SNX1	ENSG00000028528	18548127;
C008493	increase	SORBS1	ENSG00000095637	18548127;
C008493	increase	SPDEF	ENSG00000124664	18567002;
C008493	increase	SPTAN1	ENSG00000197694	18548127;
C008493	increase	SSBP2	ENSG00000145687	18548127;
C008493	increase	STOM	ENSG00000148175	18548127;
C008493	increase	STX12	ENSG00000117758	18548127;
C008493	increase	STX2	ENSG00000111450	18548127;
C008493	increase	SVIL	ENSG00000197321	18548127;
C008493	increase	TBL1X	ENSG00000101849	18548127;
C008493	increase	TCN2	ENSG00000185339	18548127;
C008493	increase	TFF1	ENSG00000160182	16093440;16155795;
C008493	increase	TGFB1	ENSG00000105329	12517777;
C008493	increase	TGFB2	ENSG00000092969	18548127;
C008493	increase	TGFBR2	ENSG00000163513	18548127;

C008493	increase	TGFBR3	ENSG00000069702	12517777;18548127;
C008493	increase	TLE2	ENSG00000065717	18548127;
C008493	increase	TLR2	ENSG00000137462	12517777;
C008493	increase	TNFRSF25	ENSG00000215788	11830524;
C008493	increase	TOR1AIP1	ENSG00000143337	18548127;
C008493	increase	TPM1	ENSG00000140416	18548127;
C008493	increase	VAMP3	ENSG00000049245	18548127;
C008493	increase	VIM	ENSG00000026025	18548127;
C008493	increase	ZCCHC24	ENSG00000165424	18548127;
C008493	increase	ZNF165	ENSG00000197279	12517777;
C008493	increase	ZNF217	ENSG00000171940	12517777;
C008493	increase	ZNF267	ENSG00000185947	12517777;
C008493	increase	ZNF274	ENSG00000171606	12517777;
C008493	increase	ZNF345	ENSG00000251247	12517777;
C008493	increase	ZNF75D	ENSG00000186376	12517777;
C008500	decrease	COL1A1	ENSG00000108821	15983038;
C008500	decrease	COL1A2	ENSG00000164692	15983038;
C008542	decrease	BAD	ENSG00000002330	16170020;
C008542	decrease	BIRC5	ENSG00000089685	16170020;
C008542	decrease	GSK3B	ENSG00000082701	16170020;
C008542	increase	BCL2L11	ENSG00000153094	16170020;
C008636	decrease	HSD3B2	ENSG00000203859	18336853;
C008636	increase	HSD17B4	ENSG00000133835	18336853;
C008651	decrease	MGMT	ENSG00000170430	15542103;
C008655	increase	BCL2	ENSG00000171791	16835749;16582591;
C008655	increase	GATA3	ENSG00000107485	16835749;16582591;
C008655	increase	NFKB1	ENSG00000109320	16835749;16582591;
C008655	increase	PCNA	ENSG00000132646	16835749;16582591;
C008655	increase	STAT1	ENSG00000115415	16582591;16835749;
C008661	decrease	CD74	ENSG00000019582	18818744;
C008661	decrease	MMP7	ENSG00000137673	15725655;
C008729	increase	NGFR	ENSG00000064300	17409433;
C008922	decrease	CSF2	ENSG00000164400	15531295;
C008922	decrease	IL1B	ENSG00000125538	15531295;
C008922	decrease	IL6	ENSG00000136244	15531295;
C008922	decrease	MMP2	ENSG00000087245	15531295;
C008922	decrease	MMP9	ENSG00000100985	15531295;
C008922	decrease	TNF	ENSG00000223952	15531295;
C008922	decrease	UGDH	ENSG00000109814	16292757;
C008922	increase	CYP1A1	ENSG00000140465	17012224;
C008954	decrease	CYP21A2	ENSG00000231852	16650473;
C008954	decrease	HSD3B2	ENSG00000203859	16650473;
C008954	increase	AHR	ENSG00000106546	16650473;
C008954	increase	CYP11B2	ENSG00000179142	16650473;
C008958	increase	CRP	ENSG00000132693	15855670;
C008970	increase	COL10A1	ENSG00000123500	16371897;
C008999	affect	FHIT	ENSG00000189283	17019711;
C009131	affect	APAF1	ENSG00000120868	17133271;
C009131	decrease	ANKRD6	ENSG00000135299	19194470;
C009131	decrease	HSP90AA1	ENSG00000080824	18830594;
C009131	decrease	PHLDB1	ENSG00000019144	19194470;
C009131	decrease	PML	ENSG00000140464	19194470;
C009131	decrease	RABGAP1L	ENSG00000250898	19194470;
C009131	decrease	RBM34	ENSG00000188739	19194470;

C009131	decrease	SNIP1	ENSG00000163877	19194470;
C009131	decrease	WDR47	ENSG00000085433	19194470;
C009131	increase	CSGALNACT2	ENSG00000169826	19194470;
C009131	increase	DERL1	ENSG00000136986	19194470;
C009131	increase	H2AFX	ENSG00000188486	17991895;
C009131	increase	HLA-DQB1	ENSG00000179344	19194470;
C009131	increase	LARP7	ENSG00000174720	19194470;
C009131	increase	MAP1A	ENSG00000166963	19194470;
C009131	increase	MAPK8IP3	ENSG00000138834	19194470;
C009131	increase	MTRF1L	ENSG00000112031	19194470;
C009131	increase	NFATC2IP	ENSG00000176953	19194470;
C009131	increase	NFIC	ENSG00000141905	19194470;
C009131	increase	RRM2B	ENSG00000048392	19010910;
C009131	increase	S100P	ENSG00000163993	19194470;
C009131	increase	SLA	ENSG00000155926	19194470;
C009131	increase	ST3GAL6	ENSG00000064225	19194470;
C009131	increase	TFE3	ENSG00000068323	19194470;
C009277	decrease	ABP1	ENSG00000002726	12727804;
C009277	decrease	ALDH1A3	ENSG00000184254	12727804;
C009277	decrease	ARHGDIB	ENSG00000111348	12727804;
C009277	decrease	C10ORF116	ENSG00000148671	12727804;
C009277	decrease	CAMK2G	ENSG00000148660	12727804;
C009277	decrease	CAPNS1	ENSG00000126247	12727804;
C009277	decrease	CDC20	ENSG00000117399	12727804;
C009277	decrease	CDKN2A	ENSG00000147889	11825526;
C009277	decrease	CTSH	ENSG00000103811	12727804;
C009277	decrease	DHRS3	ENSG00000162496	12727804;
C009277	decrease	EMP1	ENSG00000134531	12727804;
C009277	decrease	FABP5	ENSG00000164687	12727804;
C009277	decrease	FLG	ENSG00000143631	12727804;11485391;
C009277	decrease	GJA1	ENSG00000152661	12727804;
C009277	decrease	GLUL	ENSG00000135821	12727804;
C009277	decrease	HSD11B2	ENSG00000176387	12727804;
C009277	decrease	ID1	ENSG00000125968	12727804;
C009277	decrease	ID2	ENSG00000115738	12727804;
C009277	decrease	ID3	ENSG00000117318	12727804;
C009277	decrease	IGFBP2	ENSG00000115457	12727804;
C009277	decrease	IGFBP3	ENSG00000146674	12727804;
C009277	decrease	ITGB6	ENSG00000115221	12727804;
C009277	decrease	IVL	ENSG00000163207	12727804;11485391;
C009277	decrease	KRT10	ENSG00000186395	12727804;
C009277	decrease	LGALS7	ENSG00000178934	12727804;
C009277	decrease	MED21	ENSG00000152944	12727804;
C009277	decrease	NRIP1	ENSG00000180530	12727804;
C009277	decrease	PLA2G16	ENSG00000176485	12727804;
C009277	decrease	POLR2E	ENSG00000099817	12727804;
C009277	decrease	PSCA	ENSG00000167653	12727804;
C009277	decrease	PSMB8	ENSG00000226201	12727804;
C009277	decrease	RARRES3	ENSG00000133321	12727804;
C009277	decrease	SERPINB4	ENSG00000206073	12727804;
C009277	decrease	SPRR1A	ENSG00000169474	11485391;
C009277	decrease	SPRR1B	ENSG00000169469	11485391;
C009277	decrease	TNFAIP2	ENSG00000185215	12727804;

C009277	decrease	TP53I3	ENSG00000115129	12727804;
C009277	decrease	TRIM31	ENSG00000223531	12727804;
C009277	increase	ABCC2	ENSG00000023839	12727804;
C009277	increase	AKR1B1	ENSG00000085662	12727804;
C009277	increase	AKR1C1	ENSG00000187134	12727804;
C009277	increase	ASNS	ENSG00000070669	12727804;
C009277	increase	DDIT3	ENSG00000175197	12727804;
C009277	increase	EEF1A1	ENSG00000156508	12727804;
C009277	increase	EEF1A2	ENSG00000101210	12727804;
C009277	increase	FTH1	ENSG00000167996	12727804;
C009277	increase	FTL	ENSG00000087086	12727804;
C009277	increase	GCLC	ENSG00000001084	12727804;
C009277	increase	HMOX1	ENSG00000100292	12727804;
C009277	increase	MLLT11	ENSG00000213190	12727804;
C009277	increase	NQO1	ENSG00000181019	12727804;
C009277	increase	PDGFA	ENSG00000197461	12727804;
C009277	increase	SPINK1	ENSG00000164266	12727804;
C009277	increase	SPRR2A	ENSG00000241794	12727804;
C009277	increase	TGM1	ENSG00000092295	11485391;
C009277	increase	TKT	ENSG00000163931	12727804;
C009277	increase	TRAP1	ENSG00000126602	12727804;
C009277	increase	TRIM16	ENSG00000221926	12727804;
C009277	increase	UPP1	ENSG00000183696	12727804;
C009424	decrease	AR	ENSG00000169083	16621434;12711008;
C009424	decrease	FOLH1	ENSG00000086205	12711008;
C009424	decrease	NKX3-1	ENSG00000167034	12711008;
C009424	increase	KLK3	ENSG00000142515	12711008;
C009424	increase	TMPRSS2	ENSG00000184012	12711008;
C009438	decrease	BCL2	ENSG00000171791	18495333;
C009438	decrease	BIRC5	ENSG00000089685	18495333;
C009687	affect	ERBB2	ENSG00000141736	16488535;
C009687	affect	FGF2	ENSG00000138685	16289960;
C009687	affect	FN1	ENSG00000115414	11343241;
C009687	affect	IL6	ENSG00000136244	11410519;
C009687	affect	KLK3	ENSG00000142515	11410519;
C009687	affect	NFKBIA	ENSG00000100906	15571967;17935137;
C009687	affect	NRP1	ENSG00000099250	16289960;
C009687	decrease	BIRC5	ENSG00000089685	15065019;
C009687	decrease	C5	ENSG00000106804	16116186;
C009687	decrease	CTNNB1	ENSG00000168036	11739494;
C009687	decrease	IL12A	ENSG00000168811	16116186;
C009687	decrease	IL12B	ENSG00000113302	16116186;
C009687	decrease	IL23A	ENSG00000110944	16116186;
C009687	increase	BAX	ENSG00000087088	15970427;
C009687	increase	BIRC2	ENSG00000110330	19596284;15065019;
C009687	increase	BIRC3	ENSG00000023445	16154993;19596284;
C009687	increase	CDKN1B	ENSG00000111276	17935137;
C009687	increase	COL1A2	ENSG00000164692	11343241;
C009687	increase	DDIT3	ENSG00000175197	17171638;
C009687	increase	FGF7	ENSG00000140285	15803460;
C009687	increase	H2AFX	ENSG00000188486	17991895;
C009687	increase	HIF1A	ENSG00000100644	16682453;12482858;15050414;18927491;11424089;14726529;

C009687	increase	HMOX1	ENSG00000100292	18048804;
C009687	increase	HSF1	ENSG00000185122	11424089;
C009687	increase	HSPA5	ENSG00000044574	19428936;
C009687	increase	IL1B	ENSG00000125538	16359550;
C009687	increase	MCL1	ENSG00000143384	15970427;17935137;
C009687	increase	MMP12	ENSG00000110347	16359550;
C009687	increase	PDCD4	ENSG00000150593	17259349;
C009687	increase	PLAC1	ENSG00000170965	15803460;
C009687	increase	TP53	ENSG00000141510	19111594;12676607;15837074;15489221;16024610;17534123;
C009687	increase	VEGFA	ENSG00000112715	16682453;12482858;15050414;18252963;18790786;
C009828	decrease	HSD17B1	ENSG00000108786	16216300;
C009828	decrease	STAR	ENSG00000147465	16216300;
C009828	increase	CYP11A1	ENSG00000140459	16216300;
C009828	increase	CYP11B1	ENSG00000160882	16216300;
C009828	increase	CYP11B2	ENSG00000179142	16216300;
C009828	increase	CYP19A1	ENSG00000137869	16216300;
C009828	increase	CYP21A2	ENSG00000231852	16216300;
C009828	increase	HSD3B1	ENSG00000203857	16216300;
C009828	increase	HSD3B2	ENSG00000203859	16216300;
C009970	affect	RB1	ENSG00000139687	9586815;
C009970	increase	CDKN1A	ENSG00000124762	8943236;9586815;9343371;
C010160	increase	IL1B	ENSG00000125538	16641320;
C010160	increase	IL6	ENSG00000136244	9344885;
C010160	increase	IL8	ENSG00000169429	9344885;
C010160	increase	TNF	ENSG00000223952	9344885;
C010210	increase	EDN1	ENSG00000078401	16895544;
C010238	affect	ITPR1	ENSG00000150995	17241155;
C010238	decrease	HAMP	ENSG00000105697	16737972;
C010238	decrease	OPRK1	ENSG00000082556	17934066;
C010238	decrease	PDYN	ENSG00000101327	17934066;
C010238	decrease	PENK	ENSG00000181195	17934066;
C010238	decrease	POMC	ENSG00000115138	17934066;
C010238	increase	CYP2E1	ENSG00000130649	8531136;9143349;18845238;8074729;
C010327	decrease	ACOT13	ENSG00000112304	19682730;
C010327	decrease	ACTG1	ENSG00000184009	19682730;
C010327	decrease	ADAM12	ENSG00000148848	19682730;
C010327	decrease	AEBP1	ENSG00000106624	19682730;
C010327	decrease	AGPS	ENSG00000018510	19682730;
C010327	decrease	AIDA	ENSG00000186063	19682730;
C010327	decrease	AIMP2	ENSG00000106305	19682730;
C010327	decrease	ALG2	ENSG00000119523	19682730;
C010327	decrease	ALKBH1	ENSG00000100601	19682730;
C010327	decrease	AMMECR1	ENSG00000101935	19682730;
C010327	decrease	ANKRD13C	ENSG00000118454	19682730;
C010327	decrease	ANXA5	ENSG00000164111	19682730;
C010327	decrease	ARF3	ENSG00000134287	19682730;
C010327	decrease	ARPC5	ENSG00000162704	19682730;
C010327	decrease	ATIC	ENSG00000138363	19682730;
C010327	decrease	ATP13A3	ENSG00000133657	19682730;
C010327	decrease	ATP1B3	ENSG00000069849	19682730;

C010327	decrease	ATXN3	ENSG00000066427	19682730;
C010327	decrease	BAT3	ENSG00000204463	19682730;
C010327	decrease	C17ORF71	ENSG00000167447	19682730;
C010327	decrease	C1ORF163	ENSG00000162377	19682730;
C010327	decrease	C2ORF60	ENSG00000162971	19682730;
C010327	decrease	C7ORF25	ENSG00000136197	19682730;
C010327	decrease	C7ORF36	ENSG00000241127	19682730;
C010327	decrease	C9ORF64	ENSG00000165118	19682730;
C010327	decrease	CALD1	ENSG00000122786	19682730;
C010327	decrease	CASP8	ENSG00000064012	19682730;
C010327	decrease	CCDC43	ENSG00000180329	19682730;
C010327	decrease	CCL2	ENSG00000108691	19682730;
C010327	decrease	CCND2	ENSG00000118971	19682730;
C010327	decrease	CCNY	ENSG00000108100	19682730;
C010327	decrease	CDC123	ENSG00000151465	19682730;
C010327	decrease	CEP350	ENSG00000135837	19682730;
C010327	decrease	CLEC11A	ENSG00000105472	19682730;
C010327	decrease	CLIC1	ENSG00000226417	19682730;
C010327	decrease	CLTC	ENSG00000141367	19682730;
C010327	decrease	CNIH4	ENSG00000143771	19682730;
C010327	decrease	CNOT4	ENSG00000080802	19682730;
C010327	decrease	COPB2	ENSG00000184432	19682730;
C010327	decrease	CSNK2A1	ENSG00000101266	19682730;
C010327	decrease	CSTF1	ENSG00000101138	19682730;
C010327	decrease	CTBS	ENSG00000117151	19682730;
C010327	decrease	DBR1	ENSG00000138231	19682730;
C010327	decrease	DCAF7	ENSG00000136485	19682730;
C010327	decrease	DCBLD1	ENSG00000164465	19682730;
C010327	decrease	DCN	ENSG00000011465	19682730;
C010327	decrease	DCUN1D4	ENSG00000109184	19682730;
C010327	decrease	DNAJC13	ENSG00000138246	19682730;
C010327	decrease	DNMT3A	ENSG00000119772	19682730;
C010327	decrease	DPF2	ENSG00000133884	19682730;
C010327	decrease	DPYSL3	ENSG00000113657	19682730;
C010327	decrease	DVL3	ENSG00000161202	19682730;
C010327	decrease	EIF4E2	ENSG00000135930	19682730;
C010327	decrease	ELP4	ENSG00000109911	19682730;
C010327	decrease	EMP3	ENSG00000142227	19682730;
C010327	decrease	ETAA1	ENSG00000143971	19682730;
C010327	decrease	EXT1	ENSG00000182197	19682730;
C010327	decrease	FAM160B1	ENSG00000151553	19682730;
C010327	decrease	FGFR1	ENSG00000249195	19682730;
C010327	decrease	FSD1L	ENSG00000106701	19682730;
C010327	decrease	FTL	ENSG00000087086	19682730;
C010327	decrease	FYN	ENSG00000010810	19682730;
C010327	decrease	FZD2	ENSG00000180340	19682730;
C010327	decrease	GAP43	ENSG00000172020	19682730;
C010327	decrease	GAPDH	ENSG00000111640	19682730;
C010327	decrease	GAS6	ENSG00000248501	19682730;
C010327	decrease	GHR	ENSG00000112964	19682730;
C010327	decrease	GNB2L1	ENSG00000204628	19682730;
C010327	decrease	GNPDA1	ENSG00000113552	19682730;
C010327	decrease	GORAB	ENSG00000120370	19682730;
C010327	decrease	GPC4	ENSG00000076716	19682730;

C010327	decrease	GPNMB	ENSG00000136235	19682730;
C010327	decrease	GPRC5A	ENSG00000013588	19682730;
C010327	decrease	GSK3B	ENSG00000082701	19682730;
C010327	decrease	GTF3C3	ENSG00000119041	19682730;
C010327	decrease	HIF1A	ENSG00000100644	19682730;
C010327	decrease	HS2ST1	ENSG00000153936	19682730;
C010327	decrease	ICMT	ENSG00000116237	19682730;
C010327	decrease	IGF2R	ENSG00000197081	19682730;
C010327	decrease	IGFBP4	ENSG00000141753	19682730;
C010327	decrease	IGFBP7	ENSG00000163453	19682730;
C010327	decrease	INTS2	ENSG00000108506	19682730;
C010327	decrease	INTS8	ENSG00000164941	19682730;
C010327	decrease	IRF9	ENSG00000213928	19682730;
C010327	decrease	KDELR2	ENSG00000136240	19682730;
C010327	decrease	KDELR3	ENSG00000100196	19682730;
C010327	decrease	KLHL20	ENSG00000076321	19682730;
C010327	decrease	LARP1	ENSG00000155506	19682730;
C010327	decrease	LDHA	ENSG00000134333	19682730;
C010327	decrease	LMBR1	ENSG00000105983	19682730;
C010327	decrease	LPIN2	ENSG00000101577	19682730;
C010327	decrease	MAPK14	ENSG00000112062	19682730;
C010327	decrease	MARS	ENSG00000166986	19682730;
C010327	decrease	MMP10	ENSG00000166670	19682730;
C010327	decrease	MMP14	ENSG00000157227	19682730;
C010327	decrease	MT2A	ENSG00000125148	19682730;
C010327	decrease	MYO10	ENSG00000145555	19682730;
C010327	decrease	NAT10	ENSG00000135372	19682730;
C010327	decrease	NID1	ENSG00000116962	19682730;
C010327	decrease	NID2	ENSG00000087303	19682730;
C010327	decrease	NOL8	ENSG00000198000	19682730;
C010327	decrease	NOTCH3	ENSG00000074181	19682730;
C010327	decrease	NSF	ENSG00000073969	19682730;
C010327	decrease	NUAK2	ENSG00000163545	19682730;
C010327	decrease	NUCKS1	ENSG00000069275	19682730;
C010327	decrease	NUDT5	ENSG00000165609	19682730;
C010327	decrease	NUP37	ENSG00000075188	19682730;
C010327	decrease	NUP62	ENSG00000213024	19682730;
C010327	decrease	PAK2	ENSG00000180370	19682730;
C010327	decrease	PDE8A	ENSG00000073417	19682730;
C010327	decrease	PDGFRB	ENSG00000113721	19682730;
C010327	decrease	PFKFB3	ENSG00000170525	19682730;
C010327	decrease	PGK1	ENSG00000102144	19682730;
C010327	decrease	PKD2	ENSG00000118762	19682730;
C010327	decrease	PLAA	ENSG00000137055	19682730;
C010327	decrease	PLOD2	ENSG00000152952	19682730;
C010327	decrease	PLP2	ENSG00000102007	19682730;
C010327	decrease	PNKD	ENSG00000127838	19682730;
C010327	decrease	PPPDE1	ENSG00000121644	19682730;
C010327	decrease	PREPL	ENSG00000138078	19682730;
C010327	decrease	PSMA5	ENSG00000143106	19682730;
C010327	decrease	PSMB7	ENSG00000136930	19682730;
C010327	decrease	PSMD8	ENSG00000099341	19682730;
C010327	decrease	PTOV1	ENSG00000104960	19682730;
C010327	decrease	RAB23	ENSG00000112210	19682730;

C010327	decrease	RAD23B	ENSG00000119318	19682730;
C010327	decrease	RIN2	ENSG00000132669	19682730;
C010327	decrease	RNF8	ENSG00000112130	19682730;
C010327	decrease	RYBP	ENSG00000163602	19682730;
C010327	decrease	SCNM1	ENSG00000163156	19682730;
C010327	decrease	SDF4	ENSG00000078808	19682730;
C010327	decrease	SETD5	ENSG00000168137	19682730;
C010327	decrease	SKIV2L2	ENSG00000039123	19682730;
C010327	decrease	SLC38A9	ENSG00000177058	19682730;
C010327	decrease	SNRNP25	ENSG00000161981	19682730;
C010327	decrease	SNX6	ENSG00000129515	19682730;
C010327	decrease	SPATS2L	ENSG00000196141	19682730;
C010327	decrease	SPP1	ENSG00000118785	19682730;
C010327	decrease	SRP54	ENSG00000100883	19682730;
C010327	decrease	SSR1	ENSG00000124783	19682730;
C010327	decrease	STAM	ENSG00000136738	19682730;
C010327	decrease	STAU1	ENSG00000124214	19682730;
C010327	decrease	STK39	ENSG00000198648	19682730;
C010327	decrease	THUMPD3	ENSG00000134077	19682730;
C010327	decrease	TICAM2	ENSG00000243414	19682730;
C010327	decrease	TIMP3	ENSG00000100234	19682730;
C010327	decrease	TNC	ENSG00000041982	19682730;
C010327	decrease	TUBB3	ENSG00000198211	19682730;
C010327	decrease	TUBB	ENSG00000229684	19682730;
C010327	decrease	UBE2F	ENSG00000184182	19682730;
C010327	decrease	UBE2S	ENSG00000108106	19682730;
C010327	decrease	UHRF1BP1	ENSG00000065060	19682730;
C010327	decrease	VOPP1	ENSG00000154978	19682730;
C010327	decrease	WIPF1	ENSG00000115935	19682730;
C010327	decrease	XPNPEP1	ENSG00000108039	19682730;
C010327	decrease	XPR1	ENSG00000143324	19682730;
C010327	decrease	ZMAT3	ENSG00000172667	19682730;
C010327	decrease	ZYG11B	ENSG00000162378	19682730;
C010643	decrease	ABCA1	ENSG00000165029	17070507;
C010643	decrease	FLT1	ENSG00000102755	17992120;
C010643	decrease	KDR	ENSG00000128052	17992120;
C010643	increase	ACTA2	ENSG00000107796	16757516;
C010643	increase	FN1	ENSG00000115414	15086456;
C010643	increase	HGF	ENSG00000019991	16757516;
C010643	increase	IL6	ENSG00000136244	16757516;
C010643	increase	MMP1	ENSG00000196611	10836612;
C010643	increase	PGK1	ENSG00000102144	18603805;
C010643	increase	PON2	ENSG00000105854	17916643;
C010643	increase	TGFB1	ENSG00000105329	16757516;
C010680	affect	MUT	ENSG00000146085	19427250;
C010715	increase	SLC5A5	ENSG00000105641	16257484;
C010722	decrease	EDN1	ENSG00000078401	12536650;
C010722	decrease	TNFSF11	ENSG00000120659	16114553;
C010722	increase	CALCA	ENSG00000110680	12536650;
C010722	increase	TNFRSF11B	ENSG00000164761	16114553;
C010809	increase	PER1	ENSG00000179094	17364588;
C010920	decrease	IGF1R	ENSG00000140443	16728583;
C010953	increase	BNIP3	ENSG00000176171	12460908;
C010953	increase	EPAS1	ENSG00000116016	12460908;

C010953	increase	HIF1A	ENSG00000100644	12460908;
C010953	increase	VEGFA	ENSG00000112715	12460908;
C011157	decrease	BIRC2	ENSG00000110330	15498119;
C011157	decrease	BIRC3	ENSG00000023445	15498119;
C011164	decrease	DCT	ENSG00000080166	17473463;
C011164	decrease	NOS2	ENSG00000007171	12144868;
C011164	decrease	PTGS2	ENSG00000073756	12144868;
C011310	increase	IL1B	ENSG00000125538	12177049;
C011310	increase	SERPINB9	ENSG00000170542	10681578;12177049;14617632;
C011310	increase	TFF1	ENSG00000160182	14617632;
C011512	increase	CYP1A1	ENSG00000140465	9169082;15385644;15893423;15276085;15766918;15698582;
C011521	increase	CYP1A2	ENSG00000140505	14709624;
C011559	increase	CGB	ENSG00000213030	15941851;19497422;
C011559	increase	CYP19A1	ENSG00000137869	15941851;11751016;11522339;16996190;
C011559	increase	HSD17B1	ENSG00000108786	16513093;
C011559	increase	RXRA	ENSG00000186350	15941851;
C011585	decrease	MAPT	ENSG00000186868	16930453;
C011585	increase	FKBP5	ENSG00000096060	19255438;
C011890	decrease	ABCA13	ENSG00000179869	16595896;
C011890	decrease	ACMSD	ENSG00000153086	16595896;
C011890	decrease	ACVRL1	ENSG00000139567	16595896;
C011890	decrease	ADAM20	ENSG00000134007	16595896;
C011890	decrease	ADRB3	ENSG00000188778	16595896;
C011890	decrease	AGBL4	ENSG00000186094	16595896;
C011890	decrease	AHNAK	ENSG00000124942	16595896;
C011890	decrease	AKT1	ENSG00000142208	16595896;
C011890	decrease	ALPI	ENSG00000163295	16595896;
C011890	decrease	AMAC1L2	ENSG00000177710	16595896;
C011890	decrease	AMTN	ENSG00000187689	16595896;
C011890	decrease	ANXA1	ENSG00000135046	16595896;
C011890	decrease	AP1S1	ENSG00000106367	16595896;
C011890	decrease	APOBEC1	ENSG00000111701	16595896;
C011890	decrease	APTX	ENSG00000137074	16595896;
C011890	decrease	ARFIP2	ENSG00000132254	16595896;
C011890	decrease	ARHGEF16	ENSG00000130762	16595896;
C011890	decrease	ASAM	ENSG00000166250	16595896;
C011890	decrease	ASNSD1	ENSG00000138381	16595896;
C011890	decrease	ATF6B	ENSG00000228628	16595896;
C011890	decrease	B4GALNT4	ENSG00000182272	16595896;
C011890	decrease	BAG5	ENSG00000166170	16595896;
C011890	decrease	BIRC2	ENSG00000110330	16595896;
C011890	decrease	BMP5	ENSG00000112175	16595896;
C011890	decrease	BRSK1	ENSG00000160469	16595896;
C011890	decrease	BST2	ENSG00000130303	16595896;
C011890	decrease	C12ORF4	ENSG00000047621	16595896;
C011890	decrease	C1ORF131	ENSG00000143633	16595896;
C011890	decrease	C1ORF87	ENSG00000162598	16595896;
C011890	decrease	C20ORF165	ENSG00000149634	16595896;
C011890	decrease	C6ORF162	ENSG00000111850	16595896;
C011890	decrease	C9ORF167	ENSG00000198113	16595896;

C011890	decrease	CCDC144A	ENSG00000170160	16595896;
C011890	decrease	CCPG1	ENSG00000214882	16595896;
C011890	decrease	CCR4	ENSG00000183813	16595896;
C011890	decrease	CD22	ENSG00000012124	16595896;
C011890	decrease	CD3G	ENSG00000160654	16595896;
C011890	decrease	CDK10	ENSG00000185324	16595896;
C011890	decrease	CEACAM1	ENSG00000079385	16595896;
C011890	decrease	CENPQ	ENSG00000031691	16595896;
C011890	decrease	CLDN3	ENSG00000165215	16595896;
C011890	decrease	CLDND2	ENSG00000160318	16595896;
C011890	decrease	CLEC4C	ENSG00000198178	16595896;
C011890	decrease	CNKSR2	ENSG00000149970	16595896;
C011890	decrease	COL17A1	ENSG00000065618	16595896;
C011890	decrease	COL24A1	ENSG00000171502	16595896;
C011890	decrease	COPS2	ENSG00000166200	16595896;
C011890	decrease	CPB1	ENSG00000153002	16595896;
C011890	decrease	CSE1L	ENSG00000124207	16595896;
C011890	decrease	CTNNAL1	ENSG00000119326	16595896;
C011890	decrease	DEK	ENSG00000124795	16595896;
C011890	decrease	DLX1	ENSG00000144355	16595896;
C011890	decrease	DMXL1	ENSG00000172869	16595896;
C011890	decrease	DNTTIP2	ENSG00000067334	16595896;
C011890	decrease	DOK4	ENSG00000125170	16595896;
C011890	decrease	DSPP	ENSG00000152591	16595896;
C011890	decrease	DYNLT3	ENSG00000165169	16595896;
C011890	decrease	EFNA3	ENSG00000143590	16595896;
C011890	decrease	EIF4E	ENSG00000151247	16595896;
C011890	decrease	EPHA10	ENSG00000183317	16595896;
C011890	decrease	FGF5	ENSG00000138675	16595896;
C011890	decrease	FGFR3	ENSG00000068078	16595896;
C011890	decrease	FIBCD1	ENSG00000130720	16595896;
C011890	decrease	FPGT	ENSG00000116783	16595896;
C011890	decrease	FRMPD4	ENSG00000169933	16595896;
C011890	decrease	GALNT1	ENSG00000141429	16595896;
C011890	decrease	GFOD1	ENSG00000145990	16595896;
C011890	decrease	GMPR	ENSG00000137198	16595896;
C011890	decrease	GPR146	ENSG00000164849	16595896;
C011890	decrease	GPR26	ENSG00000154478	16595896;
C011890	decrease	GPR61	ENSG00000156097	16595896;
C011890	decrease	GSDMA	ENSG00000167914	16595896;
C011890	decrease	GUCY1A2	ENSG00000152402	16595896;
C011890	decrease	HIBCH	ENSG00000198130	16595896;
C011890	decrease	HLF	ENSG00000108924	16595896;
C011890	decrease	HMMR	ENSG00000072571	16595896;
C011890	decrease	HPS3	ENSG00000163755	16595896;
C011890	decrease	IL6	ENSG00000136244	12727023;
C011890	decrease	IL6ST	ENSG00000134352	16595896;
C011890	decrease	KARS	ENSG00000065427	16595896;
C011890	decrease	KCNAB3	ENSG00000170049	16595896;
C011890	decrease	KCTD17	ENSG00000100379	16595896;
C011890	decrease	KLKB1	ENSG00000164344	16595896;
C011890	decrease	KTN1	ENSG00000126777	16595896;
C011890	decrease	LARP7	ENSG00000174720	16595896;
C011890	decrease	LILRB5	ENSG00000105609	16595896;

C011890	decrease	LRP6	ENSG00000070018	16595896;
C011890	decrease	LRRC4	ENSG00000128594	16595896;
C011890	decrease	MAFK	ENSG00000198517	16595896;
C011890	decrease	MAP7D2	ENSG00000184368	16595896;
C011890	decrease	MAPK8	ENSG00000107643	16595896;
C011890	decrease	MBNL1	ENSG00000152601	16595896;
C011890	decrease	MDFI	ENSG00000112559	16595896;
C011890	decrease	ME3	ENSG00000151376	16595896;
C011890	decrease	MFAP4	ENSG00000166482	16595896;
C011890	decrease	MIDN	ENSG00000167470	16595896;
C011890	decrease	MRPL30	ENSG00000185414	16595896;
C011890	decrease	NCALD	ENSG00000104490	16595896;
C011890	decrease	NKX6-1	ENSG00000163623	16595896;
C011890	decrease	NLGN2	ENSG00000169992	16595896;
C011890	decrease	NOC3L	ENSG00000173145	16595896;
C011890	decrease	NOL11	ENSG00000130935	16595896;
C011890	decrease	NUDT10	ENSG00000122824	16595896;
C011890	decrease	OLFML1	ENSG00000183801	16595896;
C011890	decrease	OR51B4	ENSG00000183251	16595896;
C011890	decrease	OSBP2	ENSG00000184792	16595896;
C011890	decrease	OSTM1	ENSG00000081087	16595896;
C011890	decrease	PAH	ENSG00000171759	16595896;
C011890	decrease	PAPD5	ENSG00000121274	16595896;
C011890	decrease	PCNXL3	ENSG00000197136	16595896;
C011890	decrease	PCSK4	ENSG00000115257	16595896;
C011890	decrease	PEX5L	ENSG00000114757	16595896;
C011890	decrease	PIGQ	ENSG00000007541	16595896;
C011890	decrease	POU2F3	ENSG00000137709	16595896;
C011890	decrease	PRG2	ENSG00000186652	16595896;
C011890	decrease	PRICKLE3	ENSG00000012211	16595896;
C011890	decrease	PRIM1	ENSG00000198056	16595896;
C011890	decrease	PRMT2	ENSG00000160310	16595896;
C011890	decrease	PRPF40A	ENSG00000196504	16595896;
C011890	decrease	PRR18	ENSG00000176381	16595896;
C011890	decrease	PRRX1	ENSG00000116132	16595896;
C011890	decrease	PSMC6	ENSG00000100519	16595896;
C011890	decrease	PSMD3	ENSG00000108344	16595896;
C011890	decrease	PUS7L	ENSG00000129317	16595896;
C011890	decrease	RAB6B	ENSG00000154917	16595896;
C011890	decrease	RASGEF1B	ENSG00000138670	16595896;
C011890	decrease	RB1CC1	ENSG00000023287	16595896;
C011890	decrease	RBM38	ENSG00000132819	16595896;
C011890	decrease	RECQL	ENSG00000004700	16595896;
C011890	decrease	RG9MTD1	ENSG00000174173	16595896;
C011890	decrease	RIMS2	ENSG00000176406	16595896;
C011890	decrease	RSBN1	ENSG00000081019	16595896;
C011890	decrease	SAA4	ENSG00000148965	16595896;
C011890	decrease	SCN10A	ENSG00000185313	16595896;
C011890	decrease	SCP2	ENSG00000251612	16595896;
C011890	decrease	SDC4	ENSG00000124145	16595896;
C011890	decrease	SECTM1	ENSG00000141574	16595896;
C011890	decrease	7-Sep	ENSG00000122545	16595896;
C011890	decrease	SERPINA5	ENSG00000188488	16595896;
C011890	decrease	SIN3B	ENSG00000127511	16595896;

C011890	decrease	SKIV2L2	ENSG00000039123	16595896;
C011890	decrease	SLC17A2	ENSG00000112337	16595896;
C011890	decrease	SLC25A2	ENSG00000120329	16595896;
C011890	decrease	SLC38A4	ENSG00000139209	16595896;
C011890	decrease	SMC6	ENSG00000163029	16595896;
C011890	decrease	SNX10	ENSG00000086300	16595896;
C011890	decrease	ST18	ENSG00000147488	16595896;
C011890	decrease	STARD8	ENSG00000130052	16595896;
C011890	decrease	SUZ12	ENSG00000178691	16595896;
C011890	decrease	SYF2	ENSG00000117614	16595896;
C011890	decrease	SYMPK	ENSG00000125755	16595896;
C011890	decrease	TAF15	ENSG00000172660	16595896;
C011890	decrease	TAL1	ENSG00000162367	16595896;
C011890	decrease	TCF21	ENSG00000118526	16595896;
C011890	decrease	TCN1	ENSG00000134827	16595896;
C011890	decrease	THRB	ENSG00000151090	16595896;
C011890	decrease	TIRAP	ENSG00000150455	16595896;
C011890	decrease	TMEM59L	ENSG00000105696	16595896;
C011890	decrease	TMX1	ENSG00000139921	16595896;
C011890	decrease	TNFAIP8L1	ENSG00000185361	16595896;
C011890	decrease	TRAM1	ENSG00000067167	16595896;
C011890	decrease	TRAM2	ENSG00000065308	16595896;
C011890	decrease	TREM1	ENSG00000124731	16595896;
C011890	decrease	TTK	ENSG00000112742	16595896;
C011890	decrease	TULP3	ENSG00000078246	16595896;
C011890	decrease	TWF1	ENSG00000151239	16595896;
C011890	decrease	UBE2D4	ENSG00000078967	16595896;
C011890	decrease	UBE2E1	ENSG00000170142	16595896;
C011890	decrease	UBE2M	ENSG00000130725	16595896;
C011890	decrease	UNC5C	ENSG00000182168	16595896;
C011890	decrease	USP1	ENSG00000162607	16595896;
C011890	decrease	USP44	ENSG00000136014	16595896;
C011890	decrease	WNT10A	ENSG00000135925	16595896;
C011890	decrease	ZC3H15	ENSG00000065548	16595896;
C011890	decrease	ZDHHC20	ENSG00000180776	16595896;
C011890	decrease	ZFAND1	ENSG00000104231	16595896;
C011890	decrease	ZMYND15	ENSG00000141497	16595896;
C011890	decrease	ZNF146	ENSG00000167635	16595896;
C011890	decrease	ZNF192	ENSG00000198315	16595896;
C011890	decrease	ZNF414	ENSG00000133250	16595896;
C011890	decrease	ZNF483	ENSG00000173258	16595896;
C011890	decrease	ZNF497	ENSG00000174586	16595896;
C011890	decrease	ZP2	ENSG00000103310	16595896;
C011890	increase	AGAP3	ENSG00000133612	16595896;
C011890	increase	APP	ENSG00000142192	16595896;
C011890	increase	AQP1	ENSG00000240583	16595896;
C011890	increase	ARL6IP5	ENSG00000144746	16595896;
C011890	increase	ARMC4	ENSG00000169126	16595896;
C011890	increase	BAT2L2	ENSG00000117523	16595896;
C011890	increase	BCL2A1	ENSG00000140379	16595896;
C011890	increase	C15ORF41	ENSG00000186073	16595896;
C011890	increase	C16ORF53	ENSG00000185928	16595896;
C011890	increase	C1ORF54	ENSG00000118292	16595896;
C011890	increase	C4ORF32	ENSG00000174749	16595896;

C011890	increase	CA14	ENSG00000118298	16595896;
C011890	increase	CAB39L	ENSG00000102547	16595896;
C011890	increase	CABC1	ENSG00000163050	16595896;
C011890	increase	CCAR1	ENSG00000060339	16595896;
C011890	increase	CCDC50	ENSG00000152492	16595896;
C011890	increase	CD274	ENSG00000120217	16595896;
C011890	increase	CEBPD	ENSG00000221869	16595896;
C011890	increase	CEP192	ENSG00000101639	16595896;
C011890	increase	CEP72	ENSG00000112877	16595896;
C011890	increase	CNIH	ENSG00000100528	16595896;
C011890	increase	COLQ	ENSG00000206561	16595896;
C011890	increase	COQ10A	ENSG00000135469	16595896;
C011890	increase	CPE	ENSG00000109472	16595896;
C011890	increase	CPNE5	ENSG00000124772	16595896;
C011890	increase	CTSZ	ENSG00000101160	16595896;
C011890	increase	CYP26B1	ENSG00000003137	16595896;
C011890	increase	DAGLA	ENSG00000134780	16595896;
C011890	increase	DAZAP2	ENSG00000183283	16595896;
C011890	increase	DLGAP4	ENSG00000080845	16595896;
C011890	increase	DOCK4	ENSG00000128512	16595896;
C011890	increase	DSTN	ENSG00000249789	16595896;
C011890	increase	E2F3	ENSG00000112242	16595896;
C011890	increase	E2F8	ENSG00000129173	16595896;
C011890	increase	EIF5A2	ENSG00000163577	16595896;
C011890	increase	EP300	ENSG00000100393	16595896;
C011890	increase	EPHA4	ENSG00000116106	16595896;
C011890	increase	ESAM	ENSG00000149564	16595896;
C011890	increase	EXOC2	ENSG00000112685	16595896;
C011890	increase	F10	ENSG00000126218	16595896;
C011890	increase	FAM122A	ENSG00000187866	16595896;
C011890	increase	FAM92A1	ENSG00000188343	16595896;
C011890	increase	FBN2	ENSG00000138829	16595896;
C011890	increase	FBXO16	ENSG00000214050	16595896;
C011890	increase	FGF12	ENSG00000114279	16595896;
C011890	increase	FKBP10	ENSG00000141756	16595896;
C011890	increase	FZD8	ENSG00000177283	16595896;
C011890	increase	GNB1	ENSG00000078369	16595896;
C011890	increase	GPR32	ENSG00000142511	16595896;
C011890	increase	HAND1	ENSG00000113196	16595896;
C011890	increase	HINT3	ENSG00000111911	16595896;
C011890	increase	HIP1	ENSG00000127946	16595896;
C011890	increase	HIST1H1A	ENSG00000124610	16595896;
C011890	increase	HIST1H2AC	ENSG00000180573	16595896;
C011890	increase	HIST1H2AJ	ENSG00000182611	16595896;
C011890	increase	IL22RA1	ENSG00000142677	16595896;
C011890	increase	INCENP	ENSG00000149503	16595896;
C011890	increase	KDELC1	ENSG00000134901	16595896;
C011890	increase	KLF5	ENSG00000102554	16595896;
C011890	increase	KLHL13	ENSG00000003096	16595896;
C011890	increase	KRT7	ENSG00000135480	16595896;
C011890	increase	MAEA	ENSG00000090316	16595896;
C011890	increase	MAGEC2	ENSG00000046774	16595896;
C011890	increase	MAGEF1	ENSG00000177383	16595896;
C011890	increase	MED14	ENSG00000180182	16595896;

C011890	increase	MEX3B	ENSG00000183496	16595896;
C011890	increase	NDUFA1	ENSG00000125356	16595896;
C011890	increase	NFIL3	ENSG00000165030	16595896;
C011890	increase	NFKBIE	ENSG00000146232	16595896;
C011890	increase	OTX1	ENSG00000115507	16595896;
C011890	increase	PARG	ENSG00000227345	16595896;
C011890	increase	PDCL	ENSG00000136940	16595896;
C011890	increase	PDHA1	ENSG00000131828	16595896;
C011890	increase	PDLIM2	ENSG00000120913	16595896;
C011890	increase	PGM2	ENSG00000169299	16595896;
C011890	increase	PITPNB	ENSG00000180957	16595896;
C011890	increase	PLAGL1	ENSG00000118495	16595896;
C011890	increase	PPP4R1	ENSG00000154845	16595896;
C011890	increase	PRPS2	ENSG00000101911	16595896;
C011890	increase	PVRL4	ENSG00000143217	16595896;
C011890	increase	RAB3GAP1	ENSG00000115839	16595896;
C011890	increase	RAD1	ENSG00000113456	16595896;
C011890	increase	RASD1	ENSG00000108551	16595896;
C011890	increase	RASSF3	ENSG00000153179	16595896;
C011890	increase	SEC24B	ENSG00000138802	16595896;
C011890	increase	SFRS2	ENSG00000161547	16595896;
C011890	increase	SHCBP1	ENSG00000171241	16595896;
C011890	increase	SOAT2	ENSG00000167780	16595896;
C011890	increase	SPDYE1	ENSG00000136206	16595896;
C011890	increase	SPP1	ENSG00000118785	16595896;
C011890	increase	SPTLC1	ENSG00000090054	16595896;
C011890	increase	SSX1	ENSG00000126752	16595896;
C011890	increase	STX6	ENSG00000135823	16595896;
C011890	increase	SWAP70	ENSG00000133789	16595896;
C011890	increase	SYNCRIP	ENSG00000135316	16595896;
C011890	increase	TMEM217	ENSG00000172738	16595896;
C011890	increase	TP53BP2	ENSG00000143514	16595896;
C011890	increase	TPCN1	ENSG00000186815	16595896;
C011890	increase	TSPAN8	ENSG00000127324	16595896;
C011890	increase	TTC9	ENSG00000133985	16595896;
C011890	increase	UFD1L	ENSG00000070010	16595896;
C011890	increase	UTP11L	ENSG00000183520	16595896;
C011890	increase	YEATS2	ENSG00000163872	16595896;
C011890	increase	ZNF438	ENSG00000183621	16595896;
C011959	decrease	BCL2	ENSG00000171791	16019488;
C011978	affect	TMPRSS2	ENSG00000184012	12711008;
C011978	decrease	KLK3	ENSG00000142515	12711008;
C011978	decrease	NKX3-1	ENSG00000167034	12711008;
C011978	decrease	PMEPA1	ENSG00000124225	12711008;
C011978	increase	AR	ENSG00000169083	12711008;
C011978	increase	FOLH1	ENSG00000086205	12711008;
C011994	decrease	ESR1	ENSG00000091831	15261991;
C011994	decrease	HSPA1A	ENSG00000232804	16271832;
C011994	decrease	HSPA8	ENSG00000109971	16271832;
C011994	decrease	HSPB2	ENSG00000170276	16271832;
C011994	increase	HSPA5	ENSG00000044574	16271832;
C012258	increase	CYP3A4	ENSG00000160868	15466163;
C012466	increase	CYP3A4	ENSG00000160868	16565514;
C012589	affect	ABCB1	ENSG00000085563	18673531;

C012589	affect	AKAP12	ENSG00000131016	18803327;
C012589	affect	APOD	ENSG00000189058	16865689;
C012589	affect	ARHGAP27	ENSG00000159314	18803327;
C012589	affect	ARHGEF16	ENSG00000130762	18803327;
C012589	affect	ATP1B2	ENSG00000129244	16865689;
C012589	affect	BIRC3	ENSG00000023445	15899819;
C012589	affect	CASP8	ENSG00000064012	15781658;18819746;
C012589	affect	CD44	ENSG00000026508	18538736;
C012589	affect	CDH13	ENSG00000140945	18553387;
C012589	affect	CDKN2A	ENSG00000147889	18813790;17019711;18069755;
C012589	affect	CTNNB1	ENSG00000168036	18538736;
C012589	affect	DACT3	ENSG00000197380	18538736;
C012589	affect	DCBLD2	ENSG00000057019	18314483;
C012589	affect	DNMT3B	ENSG00000088305	18544619;
C012589	affect	DVL2	ENSG00000004975	18538736;
C012589	affect	ENC1	ENSG00000171617	18803327;
C012589	affect	FBP1	ENSG00000165140	18938139;
C012589	affect	FBP2	ENSG00000130957	18938139;
C012589	affect	FHIT	ENSG00000189283	17019711;
C012589	affect	GPX7	ENSG00000116157	18664505;
C012589	affect	GSTM2	ENSG00000213366	18664505;
C012589	affect	HHEX	ENSG00000152804	16854221;
C012589	affect	HIST3H3	ENSG00000168148	18538736;18584348;15959780;18069755;18291368;18310117;17698632;17330857;18676679;17502362;19503093;16740727;17431121;
C012589	affect	HIST4H4	ENSG00000182217	18538736;18310117;17698632;17330857;19503093;16740727;16009488;
C012589	affect	IRF8	ENSG00000140968	18922617;
C012589	affect	LEF1	ENSG00000138795	18538736;
C012589	affect	LRRC3B	ENSG00000179796	18757430;
C012589	affect	POU5F1	ENSG00000235068	18440631;
C012589	affect	PPP1R14C	ENSG00000198729	18803327;
C012589	affect	PPP1R3C	ENSG00000119938	18803327;
C012589	affect	PYCARD	ENSG00000103490	17471463;
C012589	affect	RARRES1	ENSG00000118849	18803327;
C012589	affect	RASSF1	ENSG00000068028	18819746;17019711;
C012589	affect	S1PR1	ENSG00000170989	17189669;
C012589	affect	S1PR2	ENSG00000175898	17189669;
C012589	affect	S1PR3	ENSG00000213694	17189669;
C012589	affect	SSTR5	ENSG00000162009	18575731;
C012589	affect	TERT	ENSG00000164362	16051188;
C012589	affect	TFPI2	ENSG00000105825	17464989;
C012589	affect	TP53INP1	ENSG00000164938	18803327;
C012589	affect	UCHL1	ENSG00000154277	18666234;
C012589	affect	WWOX	ENSG00000186153	17019711;
C012589	affect	ZMYND10	ENSG00000004838	18819746;
C012589	decrease	ABCC2	ENSG00000023839	10691972;
C012589	decrease	ABCC3	ENSG00000108846	10691972;
C012589	decrease	AZI2	ENSG00000163512	17891453;
C012589	decrease	BAK1	ENSG00000030110	15781658;16009488;
C012589	decrease	BCL2	ENSG00000171791	16009488;16010430;

C012589	decrease	BCL2L1	ENSG00000171552	15781649;16009488;
C012589	decrease	CCNA1	ENSG00000133101	17698632;
C012589	decrease	CCNA2	ENSG00000145386	15179185;
C012589	decrease	CCNB1	ENSG00000134057	16009488;
C012589	decrease	CCND1	ENSG00000110092	18538736;17431121;15179185;
C012589	decrease	CDH11	ENSG00000140937	18025290;
C012589	decrease	CLIC1	ENSG00000226417	19294695;
C012589	decrease	DCT	ENSG00000080166	15892717;
C012589	decrease	DVL1	ENSG00000107404	17891453;
C012589	decrease	FOXA1	ENSG00000129514	17891453;
C012589	decrease	FOXK1	ENSG00000164916	17891453;
C012589	decrease	GAPDH	ENSG00000111640	19294695;
C012589	decrease	GATA3	ENSG00000107485	17891453;
C012589	decrease	HDAC1	ENSG00000116478	18310117;15103026;15927959;15930892;
C012589	decrease	HDAC3	ENSG00000171720	15103026;15930892;18676679;
C012589	decrease	HNRNPA2B	ENSG00000122566	19294695;
C012589	decrease	HSP90AA1	ENSG00000080824	19294695;
C012589	decrease	HSPA9	ENSG00000113013	19294695;
C012589	decrease	KRT8	ENSG00000170421	19294695;
C012589	decrease	LETM1	ENSG00000168924	17891453;
C012589	decrease	MBD5	ENSG00000204406	17891453;
C012589	decrease	MCL1	ENSG00000143384	16009488;
C012589	decrease	MECP2	ENSG00000169057	17891453;18310117;17698632;
C012589	decrease	NOTCH1	ENSG00000148400	17185465;
C012589	decrease	PPIA	ENSG00000196262	19294695;
C012589	decrease	PSMB4	ENSG00000159377	19294695;
C012589	decrease	PTGS2	ENSG00000073756	16959971;16010430;
C012589	decrease	RB1	ENSG00000139687	18544619;15494029;15179185;
C012589	decrease	RPLP0	ENSG00000089157	19294695;
C012589	decrease	SOD3	ENSG00000109610	18310117;
C012589	decrease	TPM3	ENSG00000143549	19294695;
C012589	decrease	TPM4	ENSG00000167460	19294695;
C012589	decrease	TPT1	ENSG00000133112	19294695;
C012589	decrease	TYMS	ENSG00000176890	17172411;
C012589	increase	ABCC10	ENSG00000124574	12566991;
C012589	increase	ANXA2	ENSG00000182718	19294695;
C012589	increase	AR	ENSG00000169083	18092350;
C012589	increase	ATP5B	ENSG00000110955	19294695;
C012589	increase	AZGP1	ENSG00000160862	18978557;
C012589	increase	BAX	ENSG00000087088	15781658;16009488;16010430;
C012589	increase	BMI1	ENSG00000168283	17185465;
C012589	increase	CAGE1	ENSG00000164304	17891453;
C012589	increase	CASP3	ENSG00000164305	15781658;17019711;16009488;16010430;16865256;
C012589	increase	CASZ1	ENSG00000130940	17940511;
C012589	increase	CCND2	ENSG00000118971	16009488;
C012589	increase	CCND3	ENSG00000112576	15179185;
C012589	increase	CCNE1	ENSG00000105173	16009488;15179185;
C012589	increase	CDH1	ENSG00000039068	18584348;18025290;

C012589	increase	CDKN1A	ENSG00000124762	17230511;17431121;17185465;18544619;16043219;18223691;16865256;15179185;16009488;19234609;1605
C012589	increase	CDKN1B	ENSG00000111276	17431121;17185465;18544619;
C012589	increase	CDX2	ENSG00000165556	16990345;
C012589	increase	CLDN3	ENSG00000165215	17986852;
C012589	increase	CLDN6	ENSG00000184697	18661270;
C012589	increase	CLDN8	ENSG00000156284	18661270;
C012589	increase	CLDN9	ENSG00000213937	18661270;
C012589	increase	CLU	ENSG00000120885	19234609;
C012589	increase	COX5B	ENSG00000135940	19294695;
C012589	increase	CST6	ENSG00000175315	19503093;
C012589	increase	CXCL12	ENSG00000107562	19289100;
C012589	increase	CXCR4	ENSG00000121966	17616702;
C012589	increase	CYP1A1	ENSG00000140465	15713371;
C012589	increase	CYP1B1	ENSG00000138061	15713371;
C012589	increase	DAPK1	ENSG00000196730	16865256;
C012589	increase	DAPK2	ENSG00000035664	16865256;
C012589	increase	DNMT1	ENSG00000130816	18310117;
C012589	increase	DUSP1	ENSG00000120129	11689710;
C012589	increase	EGR1	ENSG00000120738	17330857;18801729;
C012589	increase	EP300	ENSG00000100393	15647279;17330857;
C012589	increase	ERRFI1	ENSG00000116285	17940511;
C012589	increase	ESR1	ENSG00000091831	19458056;18264725;17891453;17533736;18092350;
C012589	increase	ESR2	ENSG00000140009	19331143;18092350;15103026;
C012589	increase	FOLR2	ENSG00000165457	16740727;
C012589	increase	FOXC1	ENSG00000054598	17891453;
C012589	increase	GATA1	ENSG00000102145	17185465;
C012589	increase	GATA2	ENSG00000179348	17185465;
C012589	increase	GDF15	ENSG00000130513	18801729;
C012589	increase	GSTP1	ENSG00000084207	17891453;19294695;
C012589	increase	HMHA1	ENSG00000180448	19096014;
C012589	increase	HOXA5	ENSG00000106004	17891453;
C012589	increase	HOXB4	ENSG00000182742	17185465;
C012589	increase	HPGD	ENSG00000164120	19584167;
C012589	increase	IKZF3	ENSG00000161405	18206652;
C012589	increase	IL12A	ENSG00000168811	12470611;
C012589	increase	IL12B	ENSG00000113302	12470611;
C012589	increase	IL1B	ENSG00000125538	16365456;
C012589	increase	IL6ST	ENSG00000134352	19223499;
C012589	increase	IL8	ENSG00000169429	15899819;12470611;
C012589	increase	KLK3	ENSG00000142515	18092350;
C012589	increase	LEP	ENSG00000174697	17502362;
C012589	increase	LOXL2	ENSG00000134013	19330836;
C012589	increase	LYZ	ENSG00000090382	17000900;
C012589	increase	MGMT	ENSG00000170430	15657354;12807730;
C012589	increase	MMP13	ENSG00000137745	17891453;
C012589	increase	MPO	ENSG00000005381	17185465;
C012589	increase	MUC4	ENSG00000145113	19127263;
C012589	increase	MYC	ENSG00000136997	18538736;17185465;12414619;

C012589	increase	NFKBIA	ENSG00000100906	15899819;
C012589	increase	NR3C1	ENSG00000113580	17498915;
C012589	increase	NRP1	ENSG00000099250	16330548;
C012589	increase	OCLN	ENSG00000197822	18661270;
C012589	increase	PARP1	ENSG00000143799	18801729;17431121;16010430;
C012589	increase	PCDH19	ENSG00000165194	17891453;
C012589	increase	PDK4	ENSG00000004799	16757381;
C012589	increase	PGR	ENSG00000082175	18264725;17533736;16024066;
C012589	increase	PIK3CD	ENSG00000171608	17940511;
C012589	increase	PRDX1	ENSG00000117450	19294695;
C012589	increase	PTEN	ENSG00000171862	17330857;
C012589	increase	RARB	ENSG00000077092	15959780;16740727;
C012589	increase	RBP7	ENSG00000162444	17940511;
C012589	increase	RERG	ENSG00000134533	19458056;
C012589	increase	RHOB	ENSG00000143878	18047684;
C012589	increase	S100A3	ENSG00000188015	17891453;
C012589	increase	SET	ENSG00000119335	19294695;
C012589	increase	SFRP1	ENSG00000104332	17923031;
C012589	increase	SFRP2	ENSG00000145423	17923031;18404682;
C012589	increase	SFRP4	ENSG00000106483	17923031;
C012589	increase	SFRP5	ENSG00000120057	17923031;
C012589	increase	SLC25A1	ENSG00000100075	18706393;
C012589	increase	SLC5A5	ENSG00000105641	16954431;
C012589	increase	SNCA	ENSG00000145335	17891453;
C012589	increase	SOD2	ENSG00000112096	19294695;
C012589	increase	SP1	ENSG00000185591	18801729;18706393;17986852;16757381;16740727;
C012589	increase	TFAP2C	ENSG00000087510	19458056;
C012589	increase	TFF1	ENSG00000160182	18264725;17533736;
C012589	increase	TGFB1	ENSG00000105329	16365456;
C012589	increase	TGFBR2	ENSG00000163513	15647279;
C012589	increase	TJP1	ENSG00000104067	18661270;
C012589	increase	TNFRSF10B	ENSG00000120889	15964798;
C012589	increase	TP53	ENSG00000141510	17230511;16467109;16051188;15179185;15964798;16865256;
C012589	increase	TSHR	ENSG00000165409	17679169;
C012589	increase	VDR	ENSG00000111424	17891453;
C012589	increase	WIF1	ENSG00000156076	17923031;
C012594	increase	CGB	ENSG00000213030	15941851;19497422;
C012594	increase	CYP19A1	ENSG00000137869	15941851;
C012594	increase	HSD17B1	ENSG00000108786	16513093;
C012596	increase	UGT1A1	ENSG00000241635	16819192;
C012596	increase	UGT1A6	ENSG00000167165	16819192;
C012629	decrease	BCL2	ENSG00000171791	14687023;
C012655	decrease	CAT	ENSG00000121691	18848576;
C012655	decrease	CYP19A1	ENSG00000137869	15964185;
C012655	decrease	SOD2	ENSG00000112096	18848576;
C012655	decrease	VEGFA	ENSG00000112715	17431384;
C012655	increase	CAV1	ENSG00000105974	18848576;
C012655	increase	IL1B	ENSG00000125538	12946449;
C012655	increase	IL6	ENSG00000136244	12946449;
C012655	increase	IL8	ENSG00000169429	12946449;

C012655	increase	PAWR	ENSG00000177425	10833474;
C012655	increase	PTGS1	ENSG00000095303	15482327;
C012655	increase	PTGS2	ENSG00000073756	17431384;12946449;18848576;17164136;15155531;15482327;
C012843	decrease	BCL2	ENSG00000171791	15566391;17510524;15964311;
C012843	decrease	BIRC2	ENSG00000110330	15566391;
C012843	decrease	BIRC3	ENSG00000023445	15566391;
C012843	decrease	FOS	ENSG00000170345	17510524;
C012843	decrease	JUN	ENSG00000177606	17510524;
C012843	decrease	MCL1	ENSG00000143384	15964311;
C012843	decrease	XIAP	ENSG00000101966	15566391;
C012843	increase	BAX	ENSG00000087088	15566391;15964311;
C012843	increase	CASP3	ENSG00000164305	15566391;16343908;17510524;
C012843	increase	HMOX1	ENSG00000100292	19033392;
C012843	increase	KEAP1	ENSG00000079999	19033392;
C012843	increase	NFE2L2	ENSG00000116044	19033392;
C012843	increase	NQO1	ENSG00000181019	19033392;
C012906	increase	CAT	ENSG00000121691	18818525;
C012906	increase	MAP1LC3A	ENSG00000101460	18818525;
C012920	decrease	CDKN1A	ENSG00000124762	18418871;
C012920	increase	MKI67	ENSG00000148773	18418871;
C012920	increase	TP53	ENSG00000141510	18418871;
C013015	affect	BAX	ENSG00000087088	15975156;
C013015	affect	BCL2	ENSG00000171791	15975156;
C013031	increase	CYP1A1	ENSG00000140465	15596260;
C013031	increase	CYP1B1	ENSG00000138061	15596260;
C013038	affect	CCL11	ENSG00000172156	17572062;
C013038	affect	CCL2	ENSG00000108691	17572062;
C013038	affect	CCL3	ENSG00000006075	17572062;
C013038	affect	CCL4	ENSG00000129277	17572062;
C013038	affect	CCL5	ENSG00000161570	17572062;
C013038	affect	IL8	ENSG00000169429	17572062;
C013038	decrease	ADSL	ENSG00000239900	17572062;
C013038	decrease	ARL6IP5	ENSG00000144746	17572062;
C013038	decrease	HLA-DPA1	ENSG00000168384	17572062;
C013038	decrease	HLA-DPB1	ENSG00000223865	17572062;
C013038	decrease	KCND1	ENSG00000102057	17572062;
C013038	decrease	RPL15	ENSG00000174748	17572062;
C013038	decrease	RPL5	ENSG00000122406	17572062;
C013038	decrease	SERPINF1	ENSG00000132386	17572062;
C013038	decrease	SFRS9	ENSG00000111786	17572062;
C013038	decrease	SORL1	ENSG00000137642	17572062;
C013038	increase	ABCC1	ENSG00000103222	17572062;
C013038	increase	ACLY	ENSG00000131473	17572062;
C013038	increase	BACH1	ENSG00000156273	17572062;
C013038	increase	CTTN	ENSG00000085733	17572062;
C013038	increase	CYP1B1	ENSG00000138061	17572062;
C013038	increase	CYP27A1	ENSG00000135929	17572062;
C013038	increase	DNAJB1	ENSG00000132002	17572062;
C013038	increase	ERO1L	ENSG00000197930	17572062;
C013038	increase	FDXR	ENSG00000161513	17572062;

C013038	increase	GABPB2	ENSG00000143458	17572062;
C013038	increase	GGA1	ENSG00000248512	17572062;
C013038	increase	GNAZ	ENSG00000128266	17572062;
C013038	increase	GP1BB	ENSG00000203618	17572062;
C013038	increase	HES1	ENSG00000114315	17572062;
C013038	increase	HIST1H2BG	ENSG00000168242	17572062;
C013038	increase	HIST2H2AA	ENSG00000203812	17572062;
C013038	increase	HSP90AA1	ENSG00000080824	17572062;
C013038	increase	HSPA1A	ENSG00000232804	17572062;
C013038	increase	HSPA1B	ENSG00000232804	17572062;
C013038	increase	IL10	ENSG00000136634	17572062;
C013038	increase	IL6	ENSG00000136244	17572062;
C013038	increase	ITGA2B	ENSG00000005961	17572062;
C013038	increase	ITGB3	ENSG00000056345	17572062;
C013038	increase	JARID2	ENSG00000008083	17572062;
C013038	increase	KEAP1	ENSG00000079999	17572062;
C013038	increase	KIAA0317	ENSG00000119682	17572062;
C013038	increase	NCK2	ENSG00000071051	17572062;
C013038	increase	PHLDA1	ENSG00000139289	17572062;
C013038	increase	PPP1R12A	ENSG00000058272	17572062;
C013038	increase	PRUNE	ENSG00000143363	17572062;
C013038	increase	RAB22A	ENSG00000124209	17572062;
C013038	increase	SH3BP2	ENSG00000087266	17572062;
C013038	increase	TMEM158	ENSG00000249992	17572062;
C013038	increase	TNF	ENSG00000223952	17572062;
C013038	increase	TTLL4	ENSG00000135912	17572062;
C013091	decrease	NFE2L2	ENSG00000116044	18258206;
C013099	affect	IL2	ENSG00000109471	9012323;
C013099	increase	TNF	ENSG00000223952	16112155;15893782;
C013185	increase	CCL2	ENSG00000108691	16253226;
C013185	increase	ICAM1	ENSG00000090339	16253226;
C013185	increase	TNF	ENSG00000223952	16253226;
C013185	increase	VCAM1	ENSG00000162692	16253226;
C013221	decrease	CCR2	ENSG00000121807	12462994;
C013221	decrease	CCR3	ENSG00000183625	12462994;
C013221	decrease	CCR5	ENSG00000160791	12462994;
C013229	increase	CYP1A1	ENSG00000140465	12147272;
C013531	decrease	CDKN1A	ENSG00000124762	12807743;
C013531	decrease	TFRC	ENSG00000072274	16760464;
C013550	decrease	SELE	ENSG00000007908	15623601;
C013592	decrease	ABL1	ENSG00000097007	15498116;
C013592	increase	FAS	ENSG00000026103	17634039;
C013592	increase	ICAM1	ENSG00000090339	15161907;
C013592	increase	LPL	ENSG00000175445	15975614;
C013592	increase	PTGS2	ENSG00000073756	15161907;
C013592	increase	TNF	ENSG00000223952	15161907;
C013598	decrease	PMF1	ENSG00000160783	15103026;
C013598	increase	ACSL1	ENSG00000151726	15103026;
C013598	increase	AP1G1	ENSG00000166747	15103026;
C013598	increase	C12ORF5	ENSG00000078237	15103026;
C013598	increase	DLG5	ENSG00000151208	15103026;
C013598	increase	GLUL	ENSG00000135821	15103026;
C013598	increase	GPSM2	ENSG00000121957	15103026;
C013598	increase	MT1B	ENSG00000169688	15103026;

C013598	increase	MT1X	ENSG00000187193	15103026;
C013598	increase	MT2A	ENSG00000125148	15103026;
C013598	increase	MYO10	ENSG00000145555	15103026;
C013598	increase	PIP	ENSG00000159763	15103026;
C013598	increase	PLK2	ENSG00000145632	15103026;
C013598	increase	QSOX1	ENSG00000116260	15103026;
C013598	increase	RBBP7	ENSG00000102054	15103026;
C013598	increase	S100P	ENSG00000163993	15103026;
C013649	increase	ABCB1	ENSG00000085563	15269186;
C013649	increase	CYP3A4	ENSG00000160868	15269186;16008107;15649653;
C013649	increase	UGT1A1	ENSG00000241635	15269186;
C013690	affect	BAX	ENSG00000087088	11230554;
C013690	affect	BCL2	ENSG00000171791	11230554;
C013690	affect	CDKN1A	ENSG00000124762	11230554;
C013690	affect	TGFB2	ENSG00000092969	16705669;
C013690	affect	TGFBR3	ENSG00000069702	16705669;
C013690	affect	TP53	ENSG00000141510	11230554;
C013690	increase	BCL2L1	ENSG00000171552	16705669;
C013690	increase	HMOX1	ENSG00000100292	15582351;
C013690	increase	MYC	ENSG00000136997	16705669;
C013905	increase	CDKN1B	ENSG00000111276	16061222;
C013908	increase	FOS	ENSG00000170345	17142977;
C013908	increase	PGR	ENSG00000082175	17142977;
C014024	affect	CDKN1A	ENSG00000124762	12473173;
C014024	decrease	GJA1	ENSG00000152661	8820588;
C014024	decrease	IFNG	ENSG00000111537	15319175;
C014024	decrease	IL10	ENSG00000136634	15319175;
C014024	decrease	TNF	ENSG00000223952	15319175;
C014024	increase	BRCA1	ENSG00000012048	12473173;
C014026	affect	BAX	ENSG00000087088	15543656;
C014026	affect	BCL2	ENSG00000171791	15543656;
C014026	affect	BCL2L1	ENSG00000171552	15543656;
C014026	decrease	CCNE1	ENSG00000105173	17349212;
C014026	decrease	CD44	ENSG00000026508	17349212;
C014026	decrease	MPO	ENSG00000005381	12130515;
C014026	increase	BCL2A1	ENSG00000140379	12130515;
C014026	increase	CDKN1A	ENSG00000124762	12130515;17349212;
C014026	increase	CDKN1B	ENSG00000111276	17349212;
C014026	increase	CEBPE	ENSG00000092067	12130515;
C014026	increase	HOXA9	ENSG00000078399	16973558;
C014026	increase	IFIT3	ENSG00000119917	12130515;
C014036	increase	HIF1A	ENSG00000100644	15050414;
C014036	increase	PTGS2	ENSG00000073756	18480072;
C014036	increase	VEGFA	ENSG00000112715	15050414;
C014138	increase	GSTM1	ENSG00000134184	18036774;
C014138	increase	HMOX1	ENSG00000100292	18036774;
C014138	increase	NQO1	ENSG00000181019	18036774;
C014290	affect	UGT2B15	ENSG00000196620	16690804;
C014290	increase	ADAMTS1	ENSG00000154734	17018655;
C014290	increase	IL6	ENSG00000136244	14532843;
C014290	increase	PIP	ENSG00000159763	10402478;
C014347	affect	ABCB1	ENSG00000085563	18673531;
C014347	affect	ACP5	ENSG00000102575	17145863;

C014347	affect	ADRB2	ENSG00000169252	17194187;
C014347	affect	AEBP1	ENSG00000106624	17145863;
C014347	affect	AGRN	ENSG00000188157	17330099;
C014347	affect	AKAP12	ENSG00000131016	18803327;17571247;
C014347	affect	ANXA4	ENSG00000196975	17330099;
C014347	affect	ANXA6	ENSG00000197043	17330099;
C014347	affect	APOD	ENSG00000189058	16865689;
C014347	affect	ARHGAP27	ENSG00000159314	18803327;
C014347	affect	ARHGEF16	ENSG00000130762	18803327;
C014347	affect	ASMTL	ENSG00000169093	17330099;
C014347	affect	ATM	ENSG00000149311	17991895;17488656;
C014347	affect	ATP1B1	ENSG00000143153	17330099;
C014347	affect	ATP1B2	ENSG00000129244	16865689;
C014347	affect	ATR	ENSG00000175054	17977830;17991895;
C014347	affect	B3GALNT1	ENSG00000169255	17145863;
C014347	affect	BBC3	ENSG00000105327	18573879;17488656;
C014347	affect	BIRC2	ENSG00000110330	18636160;
C014347	affect	BIRC3	ENSG00000023445	18636160;
C014347	affect	BMP6	ENSG00000153162	18688853;
C014347	affect	BNC1	ENSG00000169594	17194187;
C014347	affect	BST2	ENSG00000130303	17145863;
C014347	affect	CADM1	ENSG00000182985	17260099;
C014347	affect	CAPN2	ENSG00000162909	17330099;
C014347	affect	CASP3	ENSG00000164305	18649362;17019711;17653094;
C014347	affect	CASP7	ENSG00000165806	18649362;17330099;
C014347	affect	CASP9	ENSG00000132906	18649362;18636160;
C014347	affect	CCNA1	ENSG00000133101	17194187;17488656;
C014347	affect	CCR4	ENSG00000183813	17145863;
C014347	affect	CD69	ENSG00000110848	17145863;
C014347	affect	CD9	ENSG00000010278	17330099;
C014347	affect	CDH13	ENSG00000140945	18729198;18553387;18425332;18055864;
C014347	affect	CDH8	ENSG00000150394	17145863;
C014347	affect	CEBPD	ENSG00000221869	17234736;
C014347	affect	CFLAR	ENSG00000003402	18636160;
C014347	affect	CHD6	ENSG00000124177	18577749;
C014347	affect	CNTN2	ENSG00000184144	18791715;
C014347	affect	CPEB1	ENSG00000214575	17145863;
C014347	affect	CRABP1	ENSG00000166426	17438526;
C014347	affect	CST3	ENSG00000101439	17330099;
C014347	affect	CST6	ENSG00000175315	17043665;
C014347	affect	CTCF	ENSG00000102974	17267411;
C014347	affect	CTNNA1	ENSG00000044115	17330099;
C014347	affect	CTSL2	ENSG00000136943	18636205;
C014347	affect	CTSZ	ENSG00000101160	17330099;17194187;
C014347	affect	CYLD	ENSG00000083799	18431742;
C014347	affect	CYP1B1	ENSG00000138061	17145863;17630775;
C014347	affect	DACT1	ENSG00000165617	18538736;
C014347	affect	DACT2	ENSG00000164488	18538736;
C014347	affect	DACT3	ENSG00000197380	18538736;
C014347	affect	DCBLD2	ENSG00000057019	18314483;
C014347	affect	DCLK1	ENSG00000133083	17145863;
C014347	affect	DDX43	ENSG00000080007	17145863;

C014347	affect	DNAJC15	ENSG00000120675	17145863;
C014347	affect	DPP4	ENSG00000197635	17145863;
C014347	affect	DSC3	ENSG00000134762	16799634;
C014347	affect	EHD3	ENSG00000013016	17330099;
C014347	affect	ENC1	ENSG00000171617	18803327;
C014347	affect	ENO2	ENSG00000111674	17330099;
C014347	affect	EPB41	ENSG00000159023	17145863;
C014347	affect	EPB41L3	ENSG00000082397	17260099;
C014347	affect	EPCAM	ENSG00000119888	18398839;16624485;
C014347	affect	F2RL1	ENSG00000164251	17145863;
C014347	affect	FABP5	ENSG00000164687	18636205;
C014347	affect	FBP1	ENSG00000165140	18938139;
C014347	affect	FBP2	ENSG00000130957	18938139;
C014347	affect	FHIT	ENSG00000189283	17019711;
C014347	affect	FHL2	ENSG00000115641	17330099;
C014347	affect	FKBP1B	ENSG00000119782	17145863;
C014347	affect	FOS	ENSG00000170345	17145863;
C014347	affect	FOXO1	ENSG00000150907	17330099;
C014347	affect	FUT3	ENSG00000171124	16427187;
C014347	affect	FXYD6	ENSG00000137726	17330099;
C014347	affect	GAS2L1	ENSG00000185340	17330099;
C014347	affect	GDF15	ENSG00000130513	17145863;18801729;
C014347	affect	GFI1	ENSG00000162676	17330099;
C014347	affect	GJA1	ENSG00000152661	17306607;
C014347	affect	GJB1	ENSG00000169562	18264126;
C014347	affect	GLI1	ENSG00000111087	18559595;
C014347	affect	GPR34	ENSG00000171659	17145863;
C014347	affect	GPRC5B	ENSG00000167191	17145863;
C014347	affect	GPX7	ENSG00000116157	18664505;
C014347	affect	GSTM2	ENSG00000213366	18664505;
C014347	affect	GSTM3	ENSG00000134202	18664505;
C014347	affect	GSTM5	ENSG00000134201	18664505;
C014347	affect	H1F0	ENSG00000189060	17330099;
C014347	affect	HAS2	ENSG00000170961	17145863;
C014347	affect	HDHD2	ENSG00000167220	18559491;
C014347	affect	HHIP	ENSG00000164161	18559595;
C014347	affect	HIST1H1C	ENSG00000187837	17330099;
C014347	affect	HIST3H3	ENSG00000168148	19030781;18584348;18410530;15959780;18069755;18291368;18310117;15657354;18676679;19080631;1908
C014347	affect	HOXB13	ENSG00000159184	17145863;
C014347	affect	HPS1	ENSG00000107521	17145863;
C014347	affect	HPSE	ENSG00000173083	18320071;
C014347	affect	HSPB1	ENSG00000106211	17330099;
C014347	affect	IFI30	ENSG00000216490	17330099;
C014347	affect	IFT74	ENSG00000096872	17145863;
C014347	affect	IL4R	ENSG00000077238	17145863;
C014347	affect	IL7R	ENSG00000168685	17442928;
C014347	affect	IRF8	ENSG00000140968	18922617;
C014347	affect	IRX1	ENSG00000170549	18559491;
C014347	affect	IRX4	ENSG00000113430	17194187;
C014347	affect	ITGAM	ENSG00000169896	18460780;
C014347	affect	KCNJ13	ENSG00000115474	17145863;

C014347	affect	KEAP1	ENSG00000079999	18555005;
C014347	affect	LATS1	ENSG00000131023	17049657;
C014347	affect	LATS2	ENSG00000150457	17049657;
C014347	affect	LRRC2	ENSG00000163827	17145863;
C014347	affect	LXN	ENSG00000079257	17145863;
C014347	affect	MAF	ENSG00000178573	17194187;
C014347	affect	MAGEA10	ENSG00000124260	18791715;
C014347	affect	MAGEA12	ENSG00000213401	18791715;
C014347	affect	MAGEA6	ENSG00000197172	18791715;18240144;
C014347	affect	MAGEA9B	ENSG00000166008	18240144;
C014347	affect	MAGEA9	ENSG00000166008	17290406;
C014347	affect	MAPRE3	ENSG00000084764	18559491;
C014347	affect	MDM2	ENSG00000135679	17145863;
C014347	affect	MFAP2	ENSG00000117122	17145863;
C014347	affect	MMP1	ENSG00000196611	17145863;
C014347	affect	MYLK	ENSG00000251351	17145863;
C014347	affect	NEFH	ENSG00000100285	17145863;
C014347	affect	NELL1	ENSG00000165973	17452981;
C014347	affect	NRCAM	ENSG00000091129	17194187;
C014347	affect	PAX4	ENSG00000106331	16701883;
C014347	affect	PCSK1	ENSG00000175426	17145863;
C014347	affect	PDE4B	ENSG00000184588	17330099;
C014347	affect	PDE4DIP	ENSG00000178104	17145863;
C014347	affect	PEG10	ENSG00000242265	17145863;
C014347	affect	PHLDA1	ENSG00000139289	17194187;
C014347	affect	PLD1	ENSG00000075651	17145863;
C014347	affect	PLEKHF1	ENSG00000166289	17145863;
C014347	affect	PMAIP1	ENSG00000141682	18608210;
C014347	affect	POU5F1	ENSG00000235068	18440631;
C014347	affect	PPM1H	ENSG00000111110	17145863;
C014347	affect	PPP1R14C	ENSG00000198729	18803327;
C014347	affect	PPP1R3C	ENSG00000119938	18803327;
C014347	affect	PRDM2	ENSG00000116731	17103461;
C014347	affect	PROM1	ENSG00000007062	18679414;18829568;
C014347	affect	PSIP1	ENSG00000164985	17330099;
C014347	affect	PTCH1	ENSG00000185920	18559595;
C014347	affect	PTGS2	ENSG00000073756	17145863;
C014347	affect	PXDN	ENSG00000130508	17330099;
C014347	affect	QPCT	ENSG00000115828	17145863;
C014347	affect	RAC2	ENSG00000128340	17145863;
C014347	affect	RALGDS	ENSG00000160271	17145863;
C014347	affect	RARRES1	ENSG00000118849	18803327;
C014347	affect	RASSF2	ENSG00000101265	17013896;
C014347	affect	RB1	ENSG00000139687	18544619;
C014347	affect	RECK	ENSG00000122707	17443689;17233834;
C014347	affect	RRAD	ENSG00000166592	17195088;
C014347	affect	RUVBL1	ENSG00000175792	17145863;
C014347	affect	S100A8	ENSG00000143546	18460780;
C014347	affect	S1PR1	ENSG00000170989	17189669;
C014347	affect	S1PR2	ENSG00000175898	17189669;
C014347	affect	S1PR3	ENSG00000213694	17189669;
C014347	affect	SATB1	ENSG00000182568	17145863;
C014347	affect	SDC2	ENSG00000169439	17145863;
C014347	affect	SEMG1	ENSG00000124233	18468680;

C014347	affect	SGK1	ENSG00000118515	17330099;
C014347	affect	SLC16A4	ENSG00000168679	17145863;
C014347	affect	SLC46A1	ENSG00000076351	18817749;
C014347	affect	SLC5A8	ENSG00000139357	18559491;
C014347	affect	SMARCA1	ENSG00000102038	17145863;
C014347	affect	SNCG	ENSG00000173267	18320071;
C014347	affect	SRC	ENSG00000197122	18264126;
C014347	affect	SSTR5	ENSG00000162009	18575731;
C014347	affect	STAT1	ENSG00000115415	18649362;
C014347	affect	SVIL	ENSG00000197321	17330099;
C014347	affect	SYCP1	ENSG00000198765	18240144;
C014347	affect	SYK	ENSG00000165025	17145863;
C014347	affect	SYNGR1	ENSG00000100321	17330099;
C014347	affect	TCEA2	ENSG00000171703	17330099;
C014347	affect	TFPI2	ENSG00000105825	17464989;
C014347	affect	TNFSF13	ENSG00000161955	17145863;
C014347	affect	TNNT1	ENSG00000105048	17145863;
C014347	affect	TP53AIP1	ENSG00000120471	18608210;
C014347	affect	TP53I3	ENSG00000115129	18608210;
C014347	affect	TP53INP1	ENSG00000164938	18803327;
C014347	affect	TPM2	ENSG00000198467	17145863;
C014347	affect	TTBK2	ENSG00000128881	17145863;
C014347	affect	TUBB2A	ENSG00000137267	17330099;
C014347	affect	TUBB3	ENSG00000198211	18497984;
C014347	affect	VAMP8	ENSG00000118640	17145863;
C014347	affect	WFDC1	ENSG00000103175	17145863;
C014347	affect	WWOX	ENSG00000186153	17019711;17200365;
C014347	affect	ZMYND10	ENSG00000004838	18819746;
C014347	decrease	ALDH6A1	ENSG00000119711	19363521;
C014347	decrease	AMH	ENSG00000104899	19363521;
C014347	decrease	AXL	ENSG00000167601	18522535;
C014347	decrease	AZI2	ENSG00000163512	17891453;
C014347	decrease	BAG2	ENSG00000112208	19363521;
C014347	decrease	BCL2	ENSG00000171791	18636160;
C014347	decrease	BCL2L1	ENSG00000171552	17064661;19372550;
C014347	decrease	BIRC5	ENSG00000089685	19363521;18636160;
C014347	decrease	BSN	ENSG00000164061	19363521;
C014347	decrease	C3ORF62	ENSG00000188315	19363521;
C014347	decrease	C4ORF29	ENSG00000164074	19363521;
C014347	decrease	CAPN3	ENSG00000248897	17785578;
C014347	decrease	CAPN5	ENSG00000149260	19363521;
C014347	decrease	CCDC18	ENSG00000122483	19363521;
C014347	decrease	CCND1	ENSG00000110092	17064661;17417771;19387464;
C014347	decrease	CCNF	ENSG00000162063	19363521;
C014347	decrease	CDC25C	ENSG00000158402	19363521;
C014347	decrease	CDH11	ENSG00000140937	18025290;
C014347	decrease	CENPF	ENSG00000117724	19363521;
C014347	decrease	CEP110	ENSG00000119397	19363521;
C014347	decrease	CEP57	ENSG00000166037	19363521;
C014347	decrease	CLEC2B	ENSG00000110852	19194470;
C014347	decrease	CLIC1	ENSG00000226417	19294695;
C014347	decrease	COL22A1	ENSG00000169436	19559774;
C014347	decrease	DNAJC12	ENSG00000108176	19194470;

C014347	decrease	DNMT3A	ENSG00000119772	16887905;19177197;18676679;19221000;17991895;
C014347	decrease	DNMT3B	ENSG00000088305	17991895;16887905;18223691;18544619;18676679;19221000;
C014347	decrease	DVL1	ENSG00000107404	17891453;
C014347	decrease	E2F6	ENSG00000169016	19194470;
C014347	decrease	ENG	ENSG00000106991	19559774;
C014347	decrease	ENOX1	ENSG00000120658	19363521;
C014347	decrease	FBXO41	ENSG00000163013	19194470;
C014347	decrease	FGFR1	ENSG00000249195	17785578;
C014347	decrease	FOXA1	ENSG00000129514	17891453;
C014347	decrease	FOXK1	ENSG00000164916	17891453;
C014347	decrease	FPR1	ENSG00000171051	19037090;
C014347	decrease	FZD7	ENSG00000155760	19363521;
C014347	decrease	GATA3	ENSG00000107485	17891453;
C014347	decrease	GK	ENSG00000198814	19194470;
C014347	decrease	GLI2	ENSG00000074047	19363521;
C014347	decrease	GMDS	ENSG00000112699	19194470;
C014347	decrease	GPR162	ENSG00000250510	19363521;
C014347	decrease	GYPC	ENSG00000136732	19194470;
C014347	decrease	HDAC1	ENSG00000116478	16954373;17464181;16043219;18310117;
C014347	decrease	HDAC2	ENSG00000196591	17908484;
C014347	decrease	HMGB1	ENSG00000189403	19363521;
C014347	decrease	HMGCS1	ENSG00000112972	19194470;
C014347	decrease	HNRNPA2B	ENSG00000122566	19294695;
C014347	decrease	HSDL2	ENSG00000119471	19194470;
C014347	decrease	HSP90AA1	ENSG00000080824	19294695;
C014347	decrease	IGF2	ENSG00000167244	19559774;
C014347	decrease	IGFBP5	ENSG00000115461	16507463;
C014347	decrease	INSIG1	ENSG00000186480	19194470;
C014347	decrease	KRT8	ENSG00000170421	19294695;
C014347	decrease	LEF1	ENSG00000138795	19387464;
C014347	decrease	LETM1	ENSG00000168924	17891453;
C014347	decrease	LMNB1	ENSG00000113368	19363521;
C014347	decrease	LRRC3B	ENSG00000179796	18757430;18815942;18377727;
C014347	decrease	LZTFL1	ENSG00000163818	19363521;
C014347	decrease	MAP3K8	ENSG00000107968	17621267;
C014347	decrease	MASP1	ENSG00000127241	19194470;
C014347	decrease	MBD5	ENSG00000204406	17891453;
C014347	decrease	MECP2	ENSG00000169057	17891453;18310117;
C014347	decrease	MET	ENSG00000105976	19363521;
C014347	decrease	MICB	ENSG00000231179	18395517;
C014347	decrease	MITF	ENSG00000187098	17785578;
C014347	decrease	MMP2	ENSG00000087245	16507463;
C014347	decrease	MRPS28	ENSG00000147586	19194470;
C014347	decrease	MSI1	ENSG00000135097	19363521;
C014347	decrease	MYEOV	ENSG00000172927	19559774;
C014347	decrease	MYO18A	ENSG00000196535	19363521;
C014347	decrease	NDN	ENSG00000182636	19559774;
C014347	decrease	NDUFA7	ENSG00000167774	19194470;
C014347	decrease	NEDD4L	ENSG00000049759	19363521;
C014347	decrease	NEK2	ENSG00000117650	19363521;

C014347	decrease	NFATC1	ENSG00000131196	17785578;
C014347	decrease	NFIX	ENSG00000008441	19363521;
C014347	decrease	NOTCH1	ENSG00000148400	17185465;
C014347	decrease	NR2F1	ENSG00000175745	19363521;
C014347	decrease	NRP1	ENSG00000099250	17064661;
C014347	decrease	NRTN	ENSG00000171119	19363521;
C014347	decrease	NUMA1	ENSG00000137497	19363521;
C014347	decrease	NXPH4	ENSG00000182379	19363521;
C014347	decrease	OLA1	ENSG00000138430	19363521;
C014347	decrease	PABPC3	ENSG00000151846	19363521;
C014347	decrease	PIP4K2B	ENSG00000141720	19194470;
C014347	decrease	POLR3E	ENSG00000058600	19194470;
C014347	decrease	PPIA	ENSG00000196262	19294695;
C014347	decrease	PSMB4	ENSG00000159377	19294695;
C014347	decrease	PTPN9	ENSG00000169410	19363521;
C014347	decrease	RAD17	ENSG00000152942	19194470;
C014347	decrease	RAD23B	ENSG00000119318	17064661;
C014347	decrease	RAPGEFL1	ENSG00000108352	19363521;
C014347	decrease	RBM34	ENSG00000188739	19194470;
C014347	decrease	RECQL5	ENSG00000108469	19194470;
C014347	decrease	REEP3	ENSG00000165476	19363521;
C014347	decrease	RPLP0	ENSG00000089157	19294695;
C014347	decrease	RPSA	ENSG00000168028	19363521;
C014347	decrease	RTEL1	ENSG00000026036	19194470;
C014347	decrease	RTN3	ENSG00000133318	19363521;
C014347	decrease	SAMD5	ENSG00000203727	19363521;
C014347	decrease	SC4MOL	ENSG00000052802	19194470;
C014347	decrease	SGSM3	ENSG00000100359	19194470;
C014347	decrease	SH3RF2	ENSG00000156463	19363521;
C014347	decrease	SLC29A2	ENSG00000174669	19194470;
C014347	decrease	SNX13	ENSG00000071189	19194470;
C014347	decrease	SOD3	ENSG00000109610	18310117;
C014347	decrease	TBC1D17	ENSG00000104946	19363521;
C014347	decrease	TCF7	ENSG00000081059	19387464;
C014347	decrease	TERT	ENSG00000164362	18021753;18045574;17267411;
C014347	decrease	TIA1	ENSG00000116001	17064661;
C014347	decrease	TIAL1	ENSG00000151923	17064661;
C014347	decrease	TMEM18	ENSG00000151353	19363521;
C014347	decrease	TNKS1BP1	ENSG00000149115	19363521;
C014347	decrease	TPM3	ENSG00000143549	19294695;
C014347	decrease	TPM4	ENSG00000167460	19294695;
C014347	decrease	TPT1	ENSG00000133112	19294695;
C014347	decrease	TRIM10	ENSG00000229381	19194470;
C014347	decrease	TRPM2	ENSG00000142185	19194470;
C014347	decrease	WBSCR22	ENSG00000071462	17621267;
C014347	decrease	ZC3H3	ENSG00000014164	19363521;
C014347	decrease	ZFP36L1	ENSG00000185650	17064661;
C014347	decrease	ZNF22	ENSG00000165512	19559774;
C014347	increase	ABCC5	ENSG00000114770	19151715;
C014347	increase	ABCG2	ENSG00000118777	16954373;
C014347	increase	ACADVL	ENSG00000072778	19151715;
C014347	increase	ADAMTS1	ENSG00000154734	19027488;
C014347	increase	ADRA1D	ENSG00000171873	17384146;

C014347	increase	ADRA2A	ENSG00000150594	17145863;19194470;
C014347	increase	AGPAT2	ENSG00000169692	17908484;
C014347	increase	ALDH1A3	ENSG00000184254	17194187;16367923;
C014347	increase	ALDH2	ENSG00000111275	19194470;
C014347	increase	ALDOC	ENSG00000109107	17908484;
C014347	increase	ANGPTL4	ENSG00000167772	17908484;
C014347	increase	ANPEP	ENSG00000166825	19194470;
C014347	increase	ANXA2	ENSG00000182718	17330099;19294695;
C014347	increase	ANXA5	ENSG00000164111	17330099;16367923;
C014347	increase	AP1S1	ENSG00000106367	17908484;
C014347	increase	AP1S2	ENSG00000182287	19194470;
C014347	increase	APAF1	ENSG00000120868	17133271;15972851;
C014347	increase	APLP2	ENSG00000084234	17630775;17908484;
C014347	increase	APOBEC3F	ENSG00000128394	19194470;
C014347	increase	AREG	ENSG00000205595	16367923;
C014347	increase	AR	ENSG00000169083	18092350;19235587;18174752;
C014347	increase	ARHGEF10	ENSG00000104728	19194470;
C014347	increase	ARL4D	ENSG00000175906	17908484;
C014347	increase	ATP5B	ENSG00000110955	19294695;
C014347	increase	AZGP1	ENSG00000160862	18978557;
C014347	increase	B2M	ENSG00000166710	19294695;
C014347	increase	BAK1	ENSG00000030110	17908484;
C014347	increase	BAX	ENSG00000087088	18608210;18636160;19372550;
C014347	increase	BCL2L11	ENSG00000153094	19403302;
C014347	increase	BDNF	ENSG00000176697	16367923;
C014347	increase	BECN1	ENSG00000126581	19577553;
C014347	increase	BIK	ENSG00000100290	17064661;
C014347	increase	BMF	ENSG00000104081	17064661;
C014347	increase	BMI1	ENSG00000168283	17185465;
C014347	increase	BMP7	ENSG00000101144	19283074;16367923;
C014347	increase	BNIP3	ENSG00000176171	17195906;15942716;16367923;
C014347	increase	BRF2	ENSG00000104221	19194470;
C014347	increase	BTG3	ENSG00000154640	19221000;
C014347	increase	C1ORF106	ENSG00000163362	19194470;
C014347	increase	C1ORF89	ENSG00000132881	19194470;
C014347	increase	C1RL	ENSG00000139178	19194470;
C014347	increase	C6ORF145	ENSG00000168994	19194470;
C014347	increase	CAGE1	ENSG00000164304	17891453;
C014347	increase	CAMTA2	ENSG00000108509	19194470;
C014347	increase	CARHSP1	ENSG00000153048	17908484;
C014347	increase	CASP6	ENSG00000138794	19037991;
C014347	increase	CASP8	ENSG00000064012	18819746;18649362;18636160;19440673;19214542;
C014347	increase	CASZ1	ENSG00000130940	17940511;
C014347	increase	CAV1	ENSG00000105974	17064661;16367923;
C014347	increase	CCK	ENSG00000187094	19151715;
C014347	increase	CD14	ENSG00000170458	19117987;19151715;
C014347	increase	CD40	ENSG00000101017	19401350;
C014347	increase	CD4	ENSG00000010610	19194470;
C014347	increase	CD80	ENSG00000121594	19401350;
C014347	increase	CD86	ENSG00000114013	19401350;

C014347	increase	CDH1	ENSG00000039068	16887905;18584348;17194187;17417771;18055864;18974268;18025290;
C014347	increase	CDH2	ENSG00000170558	16367923;
C014347	increase	CDKN1A	ENSG00000124762	18223691;19422044;19234609;17977830;17210717;18544619;17185465;17389721;17230511;16043219;18845559;18025290;17417771;
C014347	increase	CDKN1B	ENSG00000111276	18544619;18622747;17185465;
C014347	increase	CDKN1C	ENSG00000129757	18454857;17417771;17145863;19194470;
C014347	increase	CDKN2A	ENSG00000147889	17389721;17210717;18813790;18410530;18454857;17019711;18069755;17195906;17194187;17571247;19372550;19417133;18845559;1
C014347	increase	CDKN2B	ENSG00000147883	18704418;16882708;17056112;19194470;17785578;17679729;
C014347	increase	CDKN2D	ENSG00000129355	17785578;16367923;
C014347	increase	CDX1	ENSG00000113722	18845559;
C014347	increase	CDX2	ENSG00000165556	16990345;18845559;
C014347	increase	CEACAM6	ENSG00000086548	18845559;
C014347	increase	CHFR	ENSG00000072609	16367923;
C014347	increase	CIDEA	ENSG00000176194	18033804;
C014347	increase	CIITA	ENSG00000179583	16650406;
C014347	increase	CLCF1	ENSG00000175505	19194470;
C014347	increase	CLDN2	ENSG00000165376	18845559;
C014347	increase	CLDN3	ENSG00000165215	17986852;16367923;
C014347	increase	CLU	ENSG00000120885	19234609;17064661;
C014347	increase	COL14A1	ENSG00000187955	19194470;
C014347	increase	COL1A1	ENSG00000108821	19424577;
C014347	increase	COL1A2	ENSG00000164692	19424577;
C014347	increase	COX5B	ENSG00000135940	19294695;
C014347	increase	CPA3	ENSG00000163751	19194470;
C014347	increase	CRISP2	ENSG00000124490	19401350;
C014347	increase	CROCC	ENSG00000058453	19194470;
C014347	increase	CSNK2A1	ENSG00000101266	19027835;
C014347	increase	CSNK2A2	ENSG00000070770	19027835;
C014347	increase	CST7	ENSG00000077984	19194470;16367923;
C014347	increase	CTAG1B	ENSG00000184033	19030781;18791715;19401350;
C014347	increase	CTAG2	ENSG00000126890	18240144;19401350;
C014347	increase	CTNNB1	ENSG00000168036	19234609;19387464;
C014347	increase	CTSH	ENSG00000103811	17330099;19194470;
C014347	increase	CXCL12	ENSG00000107562	19289100;
C014347	increase	CXCR4	ENSG00000121966	18320071;17616702;17145863;
C014347	increase	DAPK1	ENSG00000196730	17133271;19577553;17690560;19215824;19037991;
C014347	increase	DAPK2	ENSG00000035664	19037991;
C014347	increase	DAPK3	ENSG00000167657	19037991;
C014347	increase	DAZL	ENSG00000092345	19194470;
C014347	increase	DCC	ENSG00000187323	19329758;

C014347	increase	DCHS1	ENSG00000166341	19194470;
C014347	increase	DHRS9	ENSG00000073737	19194470;
C014347	increase	DIP2C	ENSG00000151240	19194470;
C014347	increase	DKK1	ENSG00000107984	19387464;17785578;
C014347	increase	DLK1	ENSG00000185559	19194470;
C014347	increase	DNMT1	ENSG00000130816	17991895;18223691;17621267;18310117;18676679;19221000;19470736;19117987;17653094;17571247;17616700;19417133;
C014347	increase	DOK4	ENSG00000125170	17908484;
C014347	increase	DPYD	ENSG00000188641	17612628;
C014347	increase	DUSP6	ENSG00000139318	17145863;19151715;
C014347	increase	DYNLL1	ENSG00000088986	19151715;
C014347	increase	EBI3	ENSG00000105246	16367923;
C014347	increase	EEF1A2	ENSG00000101210	16367923;
C014347	increase	EGFL7	ENSG00000172889	19116145;
C014347	increase	EGFR	ENSG00000146648	19151715;
C014347	increase	EIF4G1	ENSG00000114867	17908484;
C014347	increase	ENO1	ENSG00000074800	17908484;
C014347	increase	EPHA1	ENSG00000146904	19277044;
C014347	increase	EPHX1	ENSG00000143819	19194470;
C014347	increase	EPHX3	ENSG00000105131	16367923;
C014347	increase	EPO	ENSG00000130427	19194470;
C014347	increase	ERG	ENSG00000157554	19108891;19194470;
C014347	increase	ERRFI1	ENSG00000116285	17785578;
C014347	increase	ESR1	ENSG00000091831	19458056;18264725;17891453;17533736;18092350;18701604;
C014347	increase	ESR2	ENSG00000140009	19331143;18092350;17625110;
C014347	increase	ETS1	ENSG00000134954	19027835;
C014347	increase	F2R	ENSG00000181104	16367923;
C014347	increase	FADS1	ENSG00000149485	17908484;16367923;
C014347	increase	FAM111A	ENSG00000166801	19194470;
C014347	increase	FAM149B1	ENSG00000138286	19194470;
C014347	increase	FAM8A1	ENSG00000137414	19194470;
C014347	increase	FATE1	ENSG00000147378	19401350;
C014347	increase	FCER1G	ENSG00000158869	19194470;
C014347	increase	FCGBP	ENSG00000090920	16367923;
C014347	increase	FILIP1	ENSG00000118407	16367923;
C014347	increase	FMR1NB	ENSG00000176988	19401350;
C014347	increase	FOSL2	ENSG00000075426	17908484;
C014347	increase	FOXC1	ENSG00000054598	17891453;
C014347	increase	FOXP3	ENSG00000049768	18567616;
C014347	increase	FSD1	ENSG00000105255	16367923;
C014347	increase	FST	ENSG00000134363	16367923;
C014347	increase	FSTL1	ENSG00000163430	19194470;
C014347	increase	FTH1	ENSG00000167996	19194470;
C014347	increase	FYN	ENSG00000010810	18337055;19151715;16367923;
C014347	increase	GABARAPL	ENSG00000139112	17145863;19194470;
C014347	increase	GADD45A	ENSG00000116717	18472964;19190346;
C014347	increase	GAGE4	ENSG00000205777	19401350;
C014347	increase	GAGE7	ENSG00000215269	19401350;

C014347	increase	GAPDH	ENSG00000111640	19294695;17908484;
C014347	increase	GATA1	ENSG00000102145	17185465;
C014347	increase	GATA2	ENSG00000179348	17185465;
C014347	increase	GCH1	ENSG00000250845	19151715;19194470;
C014347	increase	GDF10	ENSG00000107623	18949431;
C014347	increase	GGT1	ENSG00000100031	19194470;
C014347	increase	GLDC	ENSG00000178445	19151715;
C014347	increase	GREM1	ENSG00000166923	16367923;
C014347	increase	GSTP1	ENSG00000084207	19396019;17891453;18174752;18676679;
C014347	increase	GTPBP1	ENSG00000100226	19194470;
C014347	increase	H2AFX	ENSG00000188486	17991895;18223691;19215824;
C014347	increase	HCK	ENSG00000101336	17344919;
C014347	increase	HIC1	ENSG00000177374	19440673;
C014347	increase	HIST2H2AA	ENSG00000203812	19194470;
C014347	increase	HIST4H4	ENSG00000182217	18310117;15657354;19221000;
C014347	increase	HLA-G	ENSG00000237216	18823661;18498645;17949227;
C014347	increase	HMHA1	ENSG00000180448	19096014;
C014347	increase	HNRNPF	ENSG00000169813	17908484;
C014347	increase	HOXA5	ENSG00000106004	17891453;
C014347	increase	HOXA9	ENSG00000078399	19194470;
C014347	increase	HOXB4	ENSG00000182742	17185465;
C014347	increase	HOXD3	ENSG00000128652	19283074;
C014347	increase	HSD11B2	ENSG00000176387	15489962;
C014347	increase	HSPA6	ENSG00000173110	19194470;
C014347	increase	HSPA8	ENSG00000109971	17630775;19151715;
C014347	increase	HSPA9	ENSG00000113013	17389721;19294695;
C014347	increase	ICAM1	ENSG00000090339	17630775;17545540;
C014347	increase	ICAM2	ENSG00000108622	16367923;
C014347	increase	ID1	ENSG00000125968	19194470;
C014347	increase	ID2	ENSG00000115738	17330099;19194470;
C014347	increase	ID3	ENSG00000117318	19194470;
C014347	increase	ID4	ENSG00000172201	16367923;
C014347	increase	IER3	ENSG00000137331	19117987;
C014347	increase	IFITM3	ENSG00000142089	19151715;
C014347	increase	IFNG	ENSG00000111537	18953428;18567616;16650406;17545540;
C014347	increase	IGFBP3	ENSG00000146674	19151715;16367923;19215824;
C014347	increase	IGFBP6	ENSG00000167779	17908484;
C014347	increase	IGFBP7	ENSG00000163453	17334979;18981723;16367923;
C014347	increase	IKZF3	ENSG00000161405	18206652;
C014347	increase	IL12RB2	ENSG00000081985	15173892;
C014347	increase	IL17A	ENSG00000112115	18567616;
C014347	increase	IL24	ENSG00000162892	17145863;17785578;
C014347	increase	IL2	ENSG00000109471	18567616;
C014347	increase	IL6	ENSG00000136244	17621267;18025290;
C014347	increase	IL6R	ENSG00000160712	16367923;
C014347	increase	IL6ST	ENSG00000134352	19223499;
C014347	increase	INSIG2	ENSG00000125629	19194470;
C014347	increase	IRF1	ENSG00000125347	17785578;

C014347	increase	IRF7	ENSG00000185507	19117987;17785578;
C014347	increase	IRS2	ENSG00000185950	16367923;
C014347	increase	JUND	ENSG00000130522	17064661;
C014347	increase	JUN	ENSG00000177606	19117987;
C014347	increase	JUP	ENSG00000173801	16887905;18974268;
C014347	increase	KAL1	ENSG00000011201	19151715;
C014347	increase	KCNJ8	ENSG00000121361	19151715;
C014347	increase	KCNN4	ENSG00000104783	17785578;
C014347	increase	KISS1	ENSG00000170498	16367923;
C014347	increase	KISS1R	ENSG00000116014	16367923;
C014347	increase	KLK3	ENSG00000142515	18092350;18174752;
C014347	increase	KRCC1	ENSG00000172086	19194470;
C014347	increase	KRT20	ENSG00000171431	18845559;
C014347	increase	KRT5	ENSG00000186081	17908484;
C014347	increase	LARP7	ENSG00000174720	19194470;
C014347	increase	LCT	ENSG00000115850	18845559;
C014347	increase	LEP	ENSG00000174697	17502362;
C014347	increase	LGALS3	ENSG00000131981	19194470;
C014347	increase	LIF	ENSG00000128342	17785578;
C014347	increase	LIMS1	ENSG00000169756	19194470;
C014347	increase	LOX	ENSG00000113083	17194187;17908484;
C014347	increase	LOXL2	ENSG00000134013	19330836;
C014347	increase	LPXN	ENSG00000110031	19194470;
C014347	increase	LRCH4	ENSG00000077454	19194470;
C014347	increase	LRRC23	ENSG00000010626	17908484;
C014347	increase	LST1	ENSG00000234514	19194470;
C014347	increase	LYZ	ENSG00000090382	17000900;19194470;
C014347	increase	MAGEA1	ENSG00000198681	19259094;18791715;18240144;17957795;19421002;17925555;19531572;1940135
C014347	increase	MAGEA3	ENSG00000221867	18791715;18240144;19401350;
C014347	increase	MAGEA4	ENSG00000147381	18791715;19401350;
C014347	increase	MAGEB2	ENSG00000099399	19401350;
C014347	increase	MAL	ENSG00000172005	19208741;19445022;
C014347	increase	MAN1B1	ENSG00000177239	17908484;
C014347	increase	MAP1LC3B	ENSG00000140941	19577553;
C014347	increase	MAP2K1	ENSG00000169032	17785578;
C014347	increase	MAP3K3	ENSG00000198909	17064661;
C014347	increase	MARK1	ENSG00000116141	16367923;
C014347	increase	MBD1	ENSG00000141644	19329758;
C014347	increase	MBD3	ENSG00000071655	17908484;
C014347	increase	MBD3L2	ENSG00000230522	16367923;
C014347	increase	MECR	ENSG00000116353	17908484;
C014347	increase	MEF2C	ENSG00000081189	19151715;
C014347	increase	METTL7A	ENSG00000185432	19194470;
C014347	increase	MGMT	ENSG00000170430	15657354;18021753;15703815;19215824;
C014347	increase	MIA	ENSG00000213054	16367923;
C014347	increase	MLF1	ENSG00000178053	16367923;
C014347	increase	MLH1	ENSG00000076242	19259094;18069755;19080631;19032668;19417133;
C014347	increase	MMP13	ENSG00000137745	17891453;17502362;
C014347	increase	MORC3	ENSG00000159256	19194470;
C014347	increase	MPO	ENSG00000005381	17185465;

C014347	increase	MRPS18A	ENSG00000096080	17908484;
C014347	increase	MS4A3	ENSG00000149516	19194470;
C014347	increase	MSH2	ENSG00000095002	19032668;
C014347	increase	MSX1	ENSG00000163132	16367923;
C014347	increase	MT1X	ENSG00000187193	19151715;
C014347	increase	MT2A	ENSG00000125148	19151715;
C014347	increase	MTMR6	ENSG00000139505	19151715;
C014347	increase	MTRF1L	ENSG00000112031	19194470;
C014347	increase	MTSS1	ENSG00000170873	16921485;16367923;
C014347	increase	MUC4	ENSG00000145113	19127263;
C014347	increase	MX1	ENSG00000157601	17330099;16367923;
C014347	increase	MYC	ENSG00000136997	17185465;17417771;19363521;18045574;
C014347	increase	MYL7	ENSG00000106631	18989703;
C014347	increase	MYO1F	ENSG00000142347	19194470;
C014347	increase	NDRG1	ENSG00000104419	17908484;
C014347	increase	NFKB1	ENSG00000109320	19117987;19027835;
C014347	increase	NFKBIA	ENSG00000100906	19151715;16763620;
C014347	increase	NIPSNAP1	ENSG00000184117	17145863;17908484;
C014347	increase	NKX2-1	ENSG00000136352	19524134;18852534;19506552;
C014347	increase	NKX2-8	ENSG00000136327	17908484;
C014347	increase	NKX3-1	ENSG00000167034	18174752;
C014347	increase	NNAT	ENSG00000053438	17762496;
C014347	increase	NOS3	ENSG00000164867	16367923;
C014347	increase	NQO1	ENSG00000181019	15763338;19194470;
C014347	increase	NR3C1	ENSG00000113580	17498915;
C014347	increase	NT5E	ENSG00000135318	16367923;
C014347	increase	NTRK2	ENSG00000148053	19151715;
C014347	increase	NUBP2	ENSG00000095906	17908484;
C014347	increase	NXF2	ENSG00000185945	19401350;
C014347	increase	NXT2	ENSG00000101888	19194470;
C014347	increase	P4HA2	ENSG00000072682	19194470;
C014347	increase	PAX6	ENSG00000007372	16367923;
C014347	increase	PAX8	ENSG00000125618	19524134;
C014347	increase	PCDH19	ENSG00000165194	17891453;
C014347	increase	PCYT2	ENSG00000185813	17908484;
C014347	increase	PDK2	ENSG00000005882	17908484;
C014347	increase	PDLIM4	ENSG00000131435	17634552;
C014347	increase	PEX19	ENSG00000250901	17389721;19194470;
C014347	increase	PGR	ENSG00000082175	18264725;17533736;16024066;
C014347	increase	PHOX2B	ENSG00000109132	17690560;
C014347	increase	PIK3CD	ENSG00000171608	17940511;
C014347	increase	PIK3CG	ENSG00000105851	17785578;
C014347	increase	PJA1	ENSG00000181191	19194470;
C014347	increase	PKD2	ENSG00000118762	19194470;
C014347	increase	PLA2G2A	ENSG00000188257	18953428;
C014347	increase	PLA2G5	ENSG00000127472	18953428;
C014347	increase	PLA2R1	ENSG00000153246	18953428;
C014347	increase	PLAGL1	ENSG00000118495	16367923;
C014347	increase	PLAU	ENSG00000122861	18320071;18025290;16367923;
C014347	increase	PLP2	ENSG00000102007	19194470;

C014347	increase	PMEPA1	ENSG00000124225	18174752;
C014347	increase	PMP22	ENSG00000109099	17785578;
C014347	increase	PPAP2C	ENSG00000141934	17908484;
C014347	increase	PPARG	ENSG00000132170	16763620;
C014347	increase	PPIC	ENSG00000168938	16367923;
C014347	increase	PRDX2	ENSG00000167815	19194470;
C014347	increase	PRG2	ENSG00000186652	19194470;
C014347	increase	PRKAG1	ENSG00000181929	17908484;
C014347	increase	PROX1	ENSG00000117707	17415710;
C014347	increase	PSTPIP1	ENSG00000140368	19194470;
C014347	increase	PTEN	ENSG00000171862	18431742;18622747;
C014347	increase	PTPN6	ENSG00000111679	19551406;
C014347	increase	PYCARD	ENSG00000103490	17471463;16367923;
C014347	increase	RAB13	ENSG00000143545	19194470;
C014347	increase	RAB5C	ENSG00000108774	17908484;
C014347	increase	RAB6C	ENSG00000222014	18992151;
C014347	increase	RANGAP1	ENSG00000100401	17908484;
C014347	increase	RAP1GAP	ENSG00000076864	19147557;
C014347	increase	RARA	ENSG00000131759	19524134;17417771;
C014347	increase	RARB	ENSG00000077092	15959780;18852534;19524134;17079450;16832676;
C014347	increase	RARG	ENSG00000172819	19524134;17908484;
C014347	increase	RASSF1	ENSG00000068028	18203293;18819746;17019711;18320071;17671114;17641842;17571247;17526942;19172108;19440673;19442645;19215824;19037991;
C014347	increase	RBP1	ENSG00000248478	17079450;16367923;
C014347	increase	RBP4	ENSG00000138207	16367923;
C014347	increase	RBP7	ENSG00000162444	17940511;
C014347	increase	RDX	ENSG00000137710	19194470;
C014347	increase	RELB	ENSG00000104856	17785578;
C014347	increase	RERG	ENSG00000134533	19458056;
C014347	increase	RETNLB	ENSG00000163515	18845559;
C014347	increase	RGS12	ENSG00000159788	19194470;
C014347	increase	RGS2	ENSG00000116741	17330099;16367923;
C014347	increase	RORA	ENSG00000069667	16367923;
C014347	increase	ROS1	ENSG00000047936	19276365;
C014347	increase	RRM2B	ENSG00000048392	19010910;
C014347	increase	RRP7A	ENSG00000189306	17908484;
C014347	increase	RUNX3	ENSG00000020633	19087683;17488656;17571247;16367923;
C014347	increase	RXRB	ENSG00000235712	17908484;
C014347	increase	S100A10	ENSG00000197747	17330099;17579622;19151715;
C014347	increase	S100A2	ENSG00000196754	17200013;17579622;17785578;
C014347	increase	S100A3	ENSG00000188015	17891453;17579622;17785578;
C014347	increase	S100A4	ENSG00000196154	17579622;19194470;
C014347	increase	S100A5	ENSG00000196420	17785578;
C014347	increase	S100A6	ENSG00000197956	17579622;19194470;
C014347	increase	S100A9	ENSG00000163220	18460780;19194470;
C014347	increase	S100P	ENSG00000163993	19194470;
C014347	increase	SCAND1	ENSG00000171222	17908484;

C014347	increase	SCG5	ENSG00000166922	17334394;
C014347	increase	SCGB3A1	ENSG00000161055	19442645;
C014347	increase	SCRN1	ENSG00000136193	16367923;
C014347	increase	SDC4	ENSG00000124145	19151715;
C014347	increase	SELL	ENSG00000188404	19194470;
C014347	increase	SERPINA1	ENSG00000197249	19194470;
C014347	increase	SERPINB2	ENSG00000197632	16367923;
C014347	increase	SERPINB5	ENSG00000206075	18291368;16799634;
C014347	increase	SERPINE1	ENSG00000106366	16367923;
C014347	increase	SET	ENSG00000119335	19294695;
C014347	increase	SFRP1	ENSG00000104332	17923031;18538736;17882284;19387464;19234609;
C014347	increase	SFRP2	ENSG00000145423	18404682;17923031;17882284;17848950;19387464;
C014347	increase	SFRP4	ENSG00000106483	17923031;
C014347	increase	SFRP5	ENSG00000120057	17923031;17882284;19387464;
C014347	increase	SHANK3	ENSG00000251322	17419801;
C014347	increase	SHC1	ENSG00000160691	19151715;
C014347	increase	SLA	ENSG00000155926	19194470;
C014347	increase	SLC12A4	ENSG00000124067	17908484;
C014347	increase	SLC16A3	ENSG00000141526	19194470;
C014347	increase	SLC25A1	ENSG00000100075	18706393;
C014347	increase	SLC26A3	ENSG00000091138	18845559;
C014347	increase	SLC31A1	ENSG00000136868	19470736;
C014347	increase	SLC43A3	ENSG00000134802	19194470;
C014347	increase	SLC5A5	ENSG00000105641	17967635;19524134;
C014347	increase	SLC6A8	ENSG00000130821	17908484;
C014347	increase	SLC9A2	ENSG00000115616	18845559;
C014347	increase	SLIT2	ENSG00000145147	19100240;
C014347	increase	SMAD7	ENSG00000101665	19194470;
C014347	increase	SNAI1	ENSG00000124216	16367923;
C014347	increase	SNCA	ENSG00000145335	17891453;19151715;
C014347	increase	SNX10	ENSG00000086300	19194470;
C014347	increase	SOCS1	ENSG00000185338	19551406;
C014347	increase	SOD2	ENSG00000112096	19294695;
C014347	increase	SOX9	ENSG00000125398	19151715;
C014347	increase	SP1	ENSG00000185591	18706393;17986852;18033804;19027835;
C014347	increase	SPARC	ENSG00000113140	18458674;19177197;17397030;
C014347	increase	SPHK1	ENSG00000176170	19194470;
C014347	increase	SPRR2A	ENSG00000241794	16367923;
C014347	increase	SPTAN1	ENSG00000197694	17064661;
C014347	increase	SPZ1	ENSG00000164299	16367923;
C014347	increase	SQRDL	ENSG00000137767	19194470;
C014347	increase	SREBF1	ENSG00000072310	17064661;
C014347	increase	SRGN	ENSG00000122862	19194470;
C014347	increase	SSBP2	ENSG00000145687	18559593;19194470;
C014347	increase	SSX1	ENSG00000126752	18240144;19401350;
C014347	increase	SSX2	ENSG00000157950	17145863;18240144;19401350;
C014347	increase	SSX4	ENSG00000198946	18240144;19401350;
C014347	increase	STC2	ENSG00000113739	18394600;
C014347	increase	STK17B	ENSG00000081320	17064661;

C014347	increase	SWAP70	ENSG00000133789	19194470;
C014347	increase	TAF9B	ENSG00000187325	19194470;
C014347	increase	TBX3	ENSG00000135111	16367923;
C014347	increase	TFAP2C	ENSG00000087510	19458056;16367923;
C014347	increase	TFF1	ENSG00000160182	18264725;17533736;
C014347	increase	TG	ENSG00000042832	19524134;
C014347	increase	TGFB1	ENSG00000105329	18567616;18320071;19234609;
C014347	increase	TGFB1I1	ENSG00000140682	19194470;
C014347	increase	THBD	ENSG00000178726	16367923;
C014347	increase	THUMPD1	ENSG00000066654	19194470;
C014347	increase	TIMP3	ENSG00000100234	17671114;19215824;19417133;16367923;
C014347	increase	TKTL1	ENSG00000007350	17194187;
C014347	increase	TMEM212	ENSG00000186329	19194470;
C014347	increase	TNF	ENSG00000223952	19117987;18567616;
C014347	increase	TNFRSF1B	ENSG00000028137	19117987;
C014347	increase	TNFSF9	ENSG00000125657	16367923;
C014347	increase	TP53	ENSG00000141510	17977830;17133271;19363521;18223691;19010910;18608210;18992151;17389721;19037090;17991895;19037991;16025287;17785578;1
C014347	increase	TRIM23	ENSG00000113595	17908484;
C014347	increase	TRIM8	ENSG00000171206	19194470;
C014347	increase	TXNIP	ENSG00000117289	19194470;
C014347	increase	UBAC1	ENSG00000130560	17908484;
C014347	increase	UCHL1	ENSG00000154277	18666234;19194470;
C014347	increase	ULBP2	ENSG00000131015	16367923;
C014347	increase	VDR	ENSG00000111424	17891453;
C014347	increase	VHL	ENSG00000134086	16954373;
C014347	increase	VIM	ENSG00000026025	19194470;18025290;
C014347	increase	WIF1	ENSG00000156076	17384664;17923031;16367923;
C014347	increase	WRB	ENSG00000182093	19151715;
C014347	increase	XAB2	ENSG00000076924	17908484;
C014347	increase	XAF1	ENSG00000132530	17195906;15843754;
C014347	increase	XAGE1A	ENSG00000204375	19531572;
C014347	increase	XAGE1D	ENSG00000204375	19401350;
C014347	increase	YY2	ENSG00000230797	19026728;
C014347	increase	ZNF177	ENSG00000188629	16367923;
C014347	increase	ZNF559	ENSG00000188321	16367923;
C014476	decrease	CCNA1	ENSG00000133101	11875189;
C014476	decrease	CCND1	ENSG00000110092	11875189;
C014476	decrease	CCNK	ENSG00000090061	11875189;
C014476	decrease	PCNA	ENSG00000132646	11875189;
C014476	decrease	TRAP1	ENSG00000126602	17853063;
C014476	increase	APAF1	ENSG00000120868	11875189;
C014476	increase	ATM	ENSG00000149311	11875189;
C014476	increase	CASP2	ENSG00000106144	11875189;
C014476	increase	CASP3	ENSG00000164305	11875189;
C014476	increase	CASP9	ENSG00000132906	11875189;
C014476	increase	CDKN1A	ENSG00000124762	11875189;
C014553	decrease	AMH	ENSG00000104899	17726078;

C014553	increase	FSHB	ENSG00000131808	17981817;14752856;17965791;17515571;17726078;
C014553	increase	LHB	ENSG00000104826	14752856;17981817;17965791;
C014638	increase	GSTP1	ENSG00000084207	15313406;
C014638	increase	TNF	ENSG00000223952	15313406;
C014659	decrease	IL8	ENSG00000169429	15762874;
C014970	decrease	OGG1	ENSG00000114026	16112689;
C015001	decrease	CCL2	ENSG00000108691	16085347;
C015001	decrease	IL18	ENSG00000150782	16085347;
C015001	decrease	IL1A	ENSG00000115008	16085347;
C015001	decrease	IL2	ENSG00000109471	16085347;
C015001	decrease	IL8	ENSG00000169429	16085347;
C015001	decrease	TGFB2	ENSG00000092969	16085347;
C015001	decrease	TNF	ENSG00000223952	16085347;
C015001	decrease	VDR	ENSG00000111424	15037631;
C015001	increase	ABCC2	ENSG00000023839	11836020;
C015001	increase	APEX1	ENSG00000100823	15996700;
C015001	increase	CYBA	ENSG00000051523	10720412;
C015001	increase	CYP1A1	ENSG00000140465	15630080;
C015001	increase	DDIT4	ENSG00000168209	16008523;
C015001	increase	DUSP1	ENSG00000120129	11689710;
C015001	increase	HIF1A	ENSG00000100644	14971644;
C015001	increase	JUN	ENSG00000177606	16085347;15037631;
C015001	increase	LIG1	ENSG00000105486	15996700;
C015001	increase	POLB	ENSG00000070501	15996700;
C015001	increase	SOD1	ENSG00000142168	16008523;
C015001	increase	TP53	ENSG00000141510	12126965;11489357;
C015001	increase	VEGFA	ENSG00000112715	14971644;
C015008	increase	CDKN1A	ENSG00000124762	15993080;
C015008	increase	TP53	ENSG00000141510	15993080;
C015234	decrease	HMGCR	ENSG00000113161	19819230;
C015234	decrease	RAP1A	ENSG00000116473	19819230;
C015262	increase	ICAM1	ENSG00000090339	11698162;
C015329	affect	CDKN1B	ENSG00000111276	16565789;
C015329	decrease	ADD1	ENSG00000087274	16886892;
C015329	decrease	AHR	ENSG00000106546	16886892;
C015329	decrease	AR	ENSG00000169083	17337101;16886892;
C015329	decrease	BRAF	ENSG00000157764	17337101;
C015329	decrease	CASP8	ENSG00000064012	16886892;
C015329	decrease	CDK1	ENSG00000170312	17337101;
C015329	decrease	CDK4	ENSG00000135446	17337101;
C015329	decrease	CDK5	ENSG00000164885	17337101;
C015329	decrease	CDK7	ENSG00000134058	17337101;
C015329	decrease	CDK9	ENSG00000136807	17337101;
C015329	decrease	CHGB	ENSG00000089199	16886892;
C015329	decrease	CRB1	ENSG00000134376	16886892;
C015329	decrease	FANCC	ENSG00000158169	16886892;
C015329	decrease	FANCF	ENSG00000183161	16886892;
C015329	decrease	FASLG	ENSG00000117560	16886892;
C015329	decrease	GJA4	ENSG00000187513	16886892;
C015329	decrease	IGF1R	ENSG00000140443	17337101;
C015329	decrease	IVL	ENSG00000163207	10769631;
C015329	decrease	JUN	ENSG00000177606	16886892;

C015329	decrease	MAP2K2	ENSG00000126934	17337101;
C015329	decrease	MAP2K4	ENSG00000065559	17337101;
C015329	decrease	MAP2K6	ENSG00000108984	17337101;
C015329	decrease	MAPK3	ENSG00000102882	17337101;
C015329	decrease	MAPK8	ENSG00000107643	17337101;
C015329	decrease	NOS1	ENSG00000089250	16886892;
C015329	decrease	NUP214	ENSG00000126883	16886892;
C015329	decrease	PRKDC	ENSG00000121031	16886892;
C015329	decrease	RDX	ENSG00000137710	16886892;
C015329	decrease	RPS6KA1	ENSG00000117676	17337101;
C015329	decrease	RPS6KA3	ENSG00000177189	17337101;
C015329	decrease	SMC1A	ENSG00000072501	16886892;
C015329	decrease	TFF1	ENSG00000160182	16886892;
C015329	decrease	TFPI2	ENSG00000105825	16886892;
C015329	decrease	TP53	ENSG00000141510	16886892;
C015329	decrease	WRB	ENSG00000182093	16886892;
C015329	increase	BACH1	ENSG00000156273	16886892;
C015329	increase	BAP1	ENSG00000163930	16886892;
C015329	increase	CCND1	ENSG00000110092	17051425;16565789;17337101;
C015329	increase	CCNE1	ENSG00000105173	17337101;16886892;
C015329	increase	CDKN1A	ENSG00000124762	17051425;16565789;
C015329	increase	CHGA	ENSG00000100604	16886892;
C015329	increase	DDB2	ENSG00000134574	16886892;
C015329	increase	EIF4G1	ENSG00000114867	16886892;
C015329	increase	ESR1	ENSG00000091831	17051425;16886892;
C015329	increase	FOS	ENSG00000170345	16886892;
C015329	increase	GJA5	ENSG00000143140	16886892;
C015329	increase	IGF1	ENSG00000017427	16565789;16886892;
C015329	increase	IGFBP2	ENSG00000115457	17337101;
C015329	increase	JAK1	ENSG00000162434	16886892;
C015329	increase	KIF2A	ENSG00000068796	16886892;
C015329	increase	KPNA2	ENSG00000182481	16886892;
C015329	increase	LDLR	ENSG00000130164	16886892;
C015329	increase	LIPA	ENSG00000107798	16886892;
C015329	increase	MAP2K1	ENSG00000169032	16886892;
C015329	increase	MAPK14	ENSG00000112062	17337101;16886892;
C015329	increase	MGAT2	ENSG00000168282	16886892;
C015329	increase	MICA	ENSG00000183214	16886892;
C015329	increase	MLH1	ENSG00000076242	16886892;
C015329	increase	MSH2	ENSG00000095002	16886892;
C015329	increase	MSH6	ENSG00000116062	16886892;
C015329	increase	RAD50	ENSG00000113522	16886892;
C015329	increase	RASSF7	ENSG00000099849	16886892;
C015329	increase	RRM2	ENSG00000171848	16886892;
C015329	increase	SMAD3	ENSG00000166949	16886892;
C015329	increase	STAT3	ENSG00000168610	16886892;
C015329	increase	TGFA	ENSG00000163235	16886892;
C015329	increase	VCP	ENSG00000165280	16886892;
C015329	increase	ZNF138	ENSG00000197008	16886892;
C015358	increase	TFF1	ENSG00000160182	16797915;
C015499	decrease	CCND1	ENSG00000110092	18161920;
C015688	increase	TIMP1	ENSG00000102265	16311067;
C015802	decrease	ACO2	ENSG00000100412	11227219;

C015802	decrease	FH	ENSG00000091483	11227219;
C015802	decrease	H2AFX	ENSG00000188486	11227219;
C015802	decrease	IDH3A	ENSG00000166411	11227219;
C015802	decrease	MDH1	ENSG00000014641	11227219;
C015802	decrease	PDHA1	ENSG00000131828	11227219;
C015802	decrease	SDHA	ENSG00000073578	11227219;
C015802	decrease	SDHB	ENSG00000117118	11227219;
C015802	decrease	SDHD	ENSG00000204370	11227219;
C015802	increase	GAPDH	ENSG00000111640	11227219;
C015802	increase	RPL13A	ENSG00000142541	11227219;
C015802	increase	RPS3	ENSG00000149273	11227219;
C015802	increase	TUBA1B	ENSG00000123416	11227219;
C015802	increase	UBC	ENSG00000150991	11227219;
C015854	decrease	FSHB	ENSG00000131808	10843175;
C015854	decrease	IGF1	ENSG00000017427	10843175;
C015854	decrease	IGFALS	ENSG00000099769	10843175;
C015854	decrease	IGFBP3	ENSG00000146674	10843175;
C015854	decrease	LHB	ENSG00000104826	10843175;
C015854	increase	CCND1	ENSG00000110092	15955616;
C015854	increase	CRP	ENSG00000132693	15970291;
C015854	increase	GH1	ENSG00000189162	10843175;
C015959	increase	ABCB1	ENSG00000085563	8632764;
C015959	increase	CYP3A4	ENSG00000160868	8632764;
C016063	increase	TGM2	ENSG00000198959	18295389;
C016104	decrease	AKT1	ENSG00000142208	12760830;
C016104	decrease	ATP1A1	ENSG00000163399	12760830;
C016104	decrease	BMP4	ENSG00000125378	12760830;
C016104	decrease	CCL2	ENSG00000108691	12760830;
C016104	decrease	CCNK	ENSG00000090061	12760830;
C016104	decrease	COL4A2	ENSG00000134871	12760830;
C016104	decrease	CTNNA1	ENSG00000044115	12760830;
C016104	decrease	CYP1B1	ENSG00000138061	12760830;
C016104	decrease	DNAJB1	ENSG00000132002	12760830;
C016104	decrease	EGR1	ENSG00000120738	12760830;
C016104	decrease	EPHA2	ENSG00000142627	12760830;
C016104	decrease	FGFR1	ENSG00000249195	12760830;
C016104	decrease	FOSL1	ENSG00000175592	12760830;
C016104	decrease	HDGF	ENSG00000143321	12760830;
C016104	decrease	HSP90AA1	ENSG00000080824	12760830;
C016104	decrease	HSPD1	ENSG00000144381	12760830;
C016104	decrease	HYAL2	ENSG00000068001	12760830;
C016104	decrease	ID1	ENSG00000125968	12760830;
C016104	decrease	IER2	ENSG00000160888	12760830;
C016104	decrease	IER3	ENSG00000137331	12760830;
C016104	decrease	IL10	ENSG00000136634	12760830;
C016104	decrease	IL6	ENSG00000136244	12760830;
C016104	decrease	ITGB4	ENSG00000132470	12760830;
C016104	decrease	MAPKAPK2	ENSG00000162889	12760830;
C016104	decrease	MARCKSL1	ENSG00000175130	12760830;
C016104	decrease	MYC	ENSG00000136997	12760830;
C016104	decrease	PLAUR	ENSG00000011422	12760830;
C016104	decrease	PPP1CA	ENSG00000172531	12760830;
C016104	decrease	RFC4	ENSG00000163918	12760830;
C016104	decrease	RPS6KA1	ENSG00000117676	12760830;

C016104	decrease	SERPINA1	ENSG00000197249	12760830;
C016104	decrease	SLC2A1	ENSG00000117394	12760830;
C016104	decrease	TNK1	ENSG00000174292	12760830;
C016104	decrease	TRRAP	ENSG00000196367	12760830;
C016104	decrease	VEZF1	ENSG00000136451	12760830;
C016104	decrease	YWHAH	ENSG00000128245	12760830;
C016104	decrease	ZFP36L1	ENSG00000185650	12760830;
C016104	decrease	ZYX	ENSG00000159840	12760830;
C016104	increase	ATF4	ENSG00000128272	12760830;
C016104	increase	RBBP7	ENSG00000102054	12760830;
C016104	increase	RPS3A	ENSG00000145425	12760830;
C016104	increase	UBC	ENSG00000150991	12760830;
C016299	increase	FN1	ENSG00000115414	12388107;
C016299	increase	IL6	ENSG00000136244	19074641;
C016299	increase	IL8	ENSG00000169429	15831558;19074641;
C016300	decrease	SELP	ENSG00000174175	11441983;
C016340	increase	BCL2	ENSG00000171791	10545406;
C016340	increase	CYP2B6	ENSG00000197408	15548381;
C016340	increase	CYP3A4	ENSG00000160868	15795092;17035600;15548381;
C016340	increase	ESR1	ENSG00000091831	11922773;12676605;14579009;9751507;16626760;8930550;11867264;15064155;
C016340	increase	PGR	ENSG00000082175	14579009;18262749;11867264;
C016391	increase	MYC	ENSG00000136997	18045574;
C016391	increase	TERT	ENSG00000164362	18045574;
C016392	decrease	CDH11	ENSG00000140937	18025290;
C016392	increase	ATF3	ENSG00000162772	15670751;
C016392	increase	BCL2L1	ENSG00000171552	18025290;
C016392	increase	CDKN1A	ENSG00000124762	18025290;
C016392	increase	CYP1A1	ENSG00000140465	15672752;
C016392	increase	CYP1A2	ENSG00000140505	15672752;16544949;
C016392	increase	GDF15	ENSG00000130513	15670751;
C016392	increase	IL6	ENSG00000136244	18025290;
C016392	increase	NQO1	ENSG00000181019	15672752;
C016392	increase	PLAU	ENSG00000122861	18025290;
C016403	increase	DDIT3	ENSG00000175197	11409192;16705839;
C016403	increase	FOS	ENSG00000170345	11409192;16705839;
C016403	increase	GADD45A	ENSG00000116717	11409192;16705839;
C016403	increase	HSPA5	ENSG00000044574	11409192;
C016517	decrease	ABCB1	ENSG00000085563	16082211;
C016517	decrease	BCL2L1	ENSG00000171552	16082211;
C016517	decrease	CCNE1	ENSG00000105173	16082211;
C016517	decrease	CDK2	ENSG00000123374	16082211;
C016517	decrease	CDK4	ENSG00000135446	16082211;
C016517	decrease	CDK6	ENSG00000105810	16082211;
C016517	decrease	CTSD	ENSG00000117984	11110848;
C016517	decrease	EGFR	ENSG00000146648	18025290;
C016517	decrease	MUC1	ENSG00000185499	15027113;
C016517	decrease	TFF1	ENSG00000160182	11110848;
C016517	decrease	VIM	ENSG00000026025	18025290;
C016517	increase	ABCG2	ENSG00000118777	17077187;
C016517	increase	AHR	ENSG00000106546	17311112;9407059;

C016517	increase	BAX	ENSG00000087088	16082211;
C016517	increase	BCL2A1	ENSG00000140379	15811958;
C016517	increase	BCL2	ENSG00000171791	15811958;16082211;
C016517	increase	BIRC2	ENSG00000110330	15811958;
C016517	increase	BIRC3	ENSG00000023445	15811958;
C016517	increase	BIRC5	ENSG00000089685	15811958;16082211;
C016517	increase	BRCA1	ENSG00000012048	11110848;16434996;
C016517	increase	BRCA2	ENSG00000139618	16434996;
C016517	increase	CCND1	ENSG00000110092	15811958;16082211;
C016517	increase	CDKN1A	ENSG00000124762	16082211;18025290;17311112;
C016517	increase	CDKN1B	ENSG00000111276	16082211;
C016517	increase	CDKN2B	ENSG00000147883	16082211;
C016517	increase	CFLAR	ENSG00000003402	15811958;16082211;
C016517	increase	CYP1A1	ENSG00000140465	17311112;15890477;
C016517	increase	CYP1B1	ENSG00000138061	17311112;15890477;
C016517	increase	GDF15	ENSG00000130513	15670751;
C016517	increase	IL6	ENSG00000136244	18025290;
C016517	increase	MMP9	ENSG00000100985	15811958;
C016517	increase	PLAU	ENSG00000122861	18025290;
C016517	increase	PTGS2	ENSG00000073756	15811958;
C016517	increase	TNF	ENSG00000223952	15811958;
C016517	increase	TRAF1	ENSG00000056558	15811958;
C016517	increase	XIAP	ENSG00000101966	15811958;16082211;
C016527	decrease	MMP2	ENSG00000087245	18715546;
C016527	decrease	MMP9	ENSG00000100985	18715546;
C016527	increase	ACAT2	ENSG00000120437	18032389;
C016527	increase	CYP51A1	ENSG00000001630	18032389;
C016527	increase	DHCR7	ENSG00000172893	18032389;
C016527	increase	FDFT1	ENSG00000079459	18032389;
C016527	increase	FDPS	ENSG00000160752	18032389;
C016527	increase	HMGCR	ENSG00000113161	18032389;
C016527	increase	HMGCS1	ENSG00000112972	18032389;
C016527	increase	INSIG1	ENSG00000186480	18032389;
C016527	increase	LDLR	ENSG00000130164	18032389;
C016527	increase	SC5DL	ENSG00000109929	18032389;
C016527	increase	SQLE	ENSG00000104549	18032389;
C016527	increase	SREBF2	ENSG00000198911	18032389;
C016527	increase	TIMP1	ENSG00000102265	18715546;
C016583	affect	RELA	ENSG00000173039	18508827;
C016583	decrease	BAD	ENSG00000002330	18508827;
C016583	decrease	CCL2	ENSG00000108691	15762874;
C016583	decrease	IL6	ENSG00000136244	15762874;
C016583	decrease	IL8	ENSG00000169429	15762874;
C016583	increase	BCL2	ENSG00000171791	16835749;16582591;18508827;
C016583	increase	BIRC3	ENSG00000023445	18508827;
C016583	increase	CDKN1A	ENSG00000124762	18508827;
C016583	increase	GATA3	ENSG00000107485	16835749;16582591;
C016583	increase	HMOX1	ENSG00000100292	18508827;
C016583	increase	NFKB1	ENSG00000109320	18508827;16835749;16582591;
C016583	increase	PCNA	ENSG00000132646	16835749;16582591;
C016583	increase	STAT1	ENSG00000115415	16582591;16835749;

C016601	affect	ESR2	ENSG00000140009	15721411;9751507;17203231;15103026;15821116;
C016601	decrease	AHNAK	ENSG00000124942	16514628;
C016601	decrease	AREG	ENSG00000205595	16514628;
C016601	decrease	ASNS	ENSG00000070669	16514628;
C016601	decrease	AURKA	ENSG00000087586	16514628;
C016601	decrease	C6ORF106	ENSG00000196821	16514628;
C016601	decrease	CBX1	ENSG00000108468	16514628;
C016601	decrease	CCND1	ENSG00000110092	15665275;
C016601	decrease	CENPA	ENSG00000115163	16514628;
C016601	decrease	CLSTN1	ENSG00000171603	16514628;
C016601	decrease	COPB2	ENSG00000184432	16514628;
C016601	decrease	DDX21	ENSG00000165732	16514628;
C016601	decrease	DDX3Y	ENSG00000067048	16514628;
C016601	decrease	DHX9	ENSG00000135829	16514628;
C016601	decrease	DNAJA1	ENSG00000086061	16514628;
C016601	decrease	EIF2S1	ENSG00000134001	16514628;
C016601	decrease	EIF5	ENSG00000100664	16514628;
C016601	decrease	ELOVL5	ENSG00000012660	16514628;
C016601	decrease	FLNC	ENSG00000128591	16514628;
C016601	decrease	FYTTD1	ENSG00000122068	16514628;
C016601	decrease	GOLGA5	ENSG00000066455	16514628;
C016601	decrease	HMGB1	ENSG00000189403	16514628;
C016601	decrease	HNRNPA2B	ENSG00000122566	16514628;
C016601	decrease	HNRNPU	ENSG00000153187	16514628;
C016601	decrease	HSP90AB1	ENSG00000096384	16514628;
C016601	decrease	HSP90B1	ENSG00000166598	16514628;
C016601	decrease	HSPA4	ENSG00000170606	16514628;
C016601	decrease	HSPA8	ENSG00000109971	16514628;
C016601	decrease	HSPD1	ENSG00000144381	16514628;
C016601	decrease	IFRD1	ENSG00000006652	16514628;
C016601	decrease	IQGAP1	ENSG00000140575	16514628;
C016601	decrease	KIAA0101	ENSG00000166803	16514628;
C016601	decrease	KIF20A	ENSG00000112984	16514628;
C016601	decrease	KPNA2	ENSG00000182481	16514628;
C016601	decrease	KPNB1	ENSG00000108424	16514628;
C016601	decrease	MATR3	ENSG00000015479	16514628;
C016601	decrease	MGP	ENSG00000111341	16514628;
C016601	decrease	MST4	ENSG00000134602	16514628;
C016601	decrease	NARS	ENSG00000134440	16514628;
C016601	decrease	NCAPH	ENSG00000121152	16514628;
C016601	decrease	NCL	ENSG00000115053	16514628;
C016601	decrease	NPM1	ENSG00000181163	16514628;
C016601	decrease	NUS1	ENSG00000153989	16514628;
C016601	decrease	POLR2B	ENSG00000047315	16514628;
C016601	decrease	PPM1D	ENSG00000170836	16514628;
C016601	decrease	PRKDC	ENSG00000121031	16514628;
C016601	decrease	PSMD14	ENSG00000115233	16514628;
C016601	decrease	PSMD6	ENSG00000163636	16514628;
C016601	decrease	PSME4	ENSG00000068878	16514628;
C016601	decrease	RACGAP1	ENSG00000161800	16514628;
C016601	decrease	RAP1A	ENSG00000116473	16514628;
C016601	decrease	RBBP7	ENSG00000102054	16514628;
C016601	decrease	RPL6	ENSG00000089009	16514628;

C016601	decrease	RRM1	ENSG00000167325	16514628;
C016601	decrease	SFPQ	ENSG00000116560	16514628;
C016601	decrease	SFRS1	ENSG00000136450	16514628;
C016601	decrease	SLC26A2	ENSG00000155850	16514628;
C016601	decrease	SLC39A6	ENSG00000141424	16514628;
C016601	decrease	SON	ENSG00000159140	16514628;
C016601	decrease	STAT1	ENSG00000115415	16514628;
C016601	decrease	STMN1	ENSG00000117632	16514628;
C016601	decrease	TAF9	ENSG00000085231	16514628;
C016601	decrease	TARS	ENSG00000113407	16514628;
C016601	decrease	TCEB2	ENSG00000103363	16514628;
C016601	decrease	THBS2	ENSG00000186340	16514628;
C016601	decrease	TMPO	ENSG00000120802	16514628;
C016601	decrease	TNPO1	ENSG00000083312	16514628;
C016601	decrease	TOP1	ENSG00000198900	16514628;
C016601	decrease	TOP2B	ENSG00000077097	16514628;
C016601	decrease	TRAK2	ENSG00000115993	16514628;
C016601	decrease	UNG	ENSG00000076248	16514628;
C016601	decrease	USP32	ENSG00000170832	16514628;
C016601	decrease	XPO1	ENSG00000082898	16514628;
C016601	decrease	XRCC5	ENSG00000079246	16514628;
C016601	increase	APOD	ENSG00000189058	16514628;
C016601	increase	ARNT2	ENSG00000172379	16514628;
C016601	increase	CDKN1B	ENSG00000111276	15665275;
C016601	increase	CTSH	ENSG00000103811	16514628;
C016601	increase	CYP2A6	ENSG00000198077	17646279;
C016601	increase	ESR1	ENSG00000091831	11162928;15521089;17400923;17203231;16037132;10397250;9751507;17049190;12765246;11530281;15721411;11751013;15885261;9846162;15941852;17237823;14617632;15665275;16093[illegible]
C016601	increase	MYC	ENSG00000136997	16093440;
C016601	increase	NR4A1	ENSG00000123358	16514628;
C016601	increase	SERPINB9	ENSG00000170542	17237823;14617632;
C016601	increase	TFF1	ENSG00000160182	16514628;16093440;14617632;
C016766	decrease	BCL2	ENSG00000171791	16033772;
C016766	decrease	BCL2L1	ENSG00000171552	16033772;
C016766	increase	ABCC2	ENSG00000023839	11264000;15896333;
C016766	increase	BAX	ENSG00000087088	16033772;
C016766	increase	CCNB1	ENSG00000134057	15231455;
C016766	increase	CDKN1A	ENSG00000124762	15231455;
C016766	increase	GSTA1	ENSG00000243955	15090468;15672752;12949046;12756216;12151360;
C016766	increase	HMOX1	ENSG00000100292	19033392;
C016766	increase	KEAP1	ENSG00000079999	19033392;
C016766	increase	MGMT	ENSG00000170430	16950796;
C016766	increase	MT1A	ENSG00000205362	16033772;
C016766	increase	MT2A	ENSG00000125148	16033772;
C016766	increase	NFE2L2	ENSG00000116044	16033772;
C016766	increase	NQO1	ENSG00000181019	19033392;15896333;15672752;
C016766	increase	RELA	ENSG00000173039	15090468;

C016766	increase	UGT1A1	ENSG00000241635	15090468;12949046;12151360;12756216;15896333;
C016837	decrease	ADAM2	ENSG00000104755	16870260;
C016837	decrease	BCL2	ENSG00000171791	16624386;
C016837	decrease	BCL2L1	ENSG00000171552	16624386;
C016837	decrease	C20ORF7	ENSG00000101247	16870260;
C016837	decrease	CCDC52	ENSG00000163611	16870260;
C016837	decrease	CDR1	ENSG00000184258	16870260;
C016837	decrease	F2RL2	ENSG00000164220	16870260;
C016837	decrease	MTA2	ENSG00000149480	16870260;
C016837	decrease	NF2	ENSG00000186575	16870260;
C016837	decrease	PEG3	ENSG00000198300	16870260;
C016837	decrease	SCARA3	ENSG00000168077	16870260;
C016837	decrease	TRA2A	ENSG00000164548	16870260;
C016837	increase	BAG3	ENSG00000151929	16870260;
C016837	increase	BAX	ENSG00000087088	16624386;
C016837	increase	CALD1	ENSG00000122786	16870260;
C016837	increase	CCDC102B	ENSG00000150636	16870260;
C016837	increase	CD9	ENSG00000010278	16870260;
C016837	increase	CDKN1A	ENSG00000124762	16624386;
C016837	increase	CFTR	ENSG00000001626	16870260;
C016837	increase	CPS1	ENSG00000021826	16870260;
C016837	increase	DHRS2	ENSG00000100867	16870260;
C016837	increase	GRIK2	ENSG00000164418	16870260;
C016837	increase	HIF1A	ENSG00000100644	16579968;
C016837	increase	HMOX1	ENSG00000100292	16870260;
C016837	increase	HSPA1A	ENSG00000232804	16870260;
C016837	increase	HSPA6	ENSG00000173110	16870260;
C016837	increase	ITGB6	ENSG00000115221	16870260;
C016837	increase	MAFG	ENSG00000197063	11162468;
C016837	increase	MT1E	ENSG00000169715	16870260;
C016837	increase	MT1F	ENSG00000198417	16870260;
C016837	increase	MT1G	ENSG00000125144	16870260;
C016837	increase	MT1H	ENSG00000205358	16870260;
C016837	increase	MT1M	ENSG00000205364	16870260;
C016837	increase	MT1X	ENSG00000187193	16870260;
C016837	increase	MT2A	ENSG00000125148	16402917;16870260;
C016837	increase	NOX4	ENSG00000086991	16870260;
C016837	increase	PGF	ENSG00000119630	16870260;
C016837	increase	PIK3R4	ENSG00000196455	16870260;
C016837	increase	SBNO1	ENSG00000139697	16870260;
C016837	increase	SLC48A1	ENSG00000211584	16870260;
C016837	increase	ST5	ENSG00000166444	16870260;
C016837	increase	TFPI2	ENSG00000105825	16870260;
C016957	increase	SELE	ENSG00000007908	8780174;
C016957	increase	TNF	ENSG00000223952	8780174;
C017032	increase	VDAC1	ENSG00000213585	18802751;
C017037	increase	FKBP5	ENSG00000096060	19255438;
C017133	affect	APOA1	ENSG00000118137	12679171;
C017133	decrease	APBA1	ENSG00000107282	12634122;
C017133	decrease	BAG1	ENSG00000250477	12634122;
C017133	decrease	BMP2	ENSG00000125845	12634122;
C017133	decrease	C1ORF61	ENSG00000125462	12634122;
C017133	decrease	CAT	ENSG00000121691	12634122;

C017133	decrease	CDKN1A	ENSG00000124762	12634122;
C017133	decrease	CKB	ENSG00000166165	12634122;
C017133	decrease	CKM	ENSG00000104879	12634122;
C017133	decrease	CXCR4	ENSG00000121966	12634122;
C017133	decrease	DAB2	ENSG00000153071	12634122;
C017133	decrease	EPB41L3	ENSG00000082397	12634122;
C017133	decrease	EPHA5	ENSG00000145242	12634122;
C017133	decrease	ERBB3	ENSG00000065361	12634122;
C017133	decrease	ERCC2	ENSG00000104884	12634122;
C017133	decrease	ERCC5	ENSG00000134899	12634122;
C017133	decrease	GATA6	ENSG00000141448	12634122;
C017133	decrease	GDF15	ENSG00000130513	12634122;
C017133	decrease	GLI1	ENSG00000111087	12634122;
C017133	decrease	HCK	ENSG00000101336	12634122;
C017133	decrease	ID1	ENSG00000125968	12634122;
C017133	decrease	ID2	ENSG00000115738	12634122;
C017133	decrease	IGFBP7	ENSG00000163453	12634122;
C017133	decrease	MAP2K6	ENSG00000108984	12634122;
C017133	decrease	MAPK4	ENSG00000141639	12634122;
C017133	decrease	MAPK9	ENSG00000050748	12634122;
C017133	decrease	MATK	ENSG00000007264	12634122;
C017133	decrease	MCC	ENSG00000171444	12634122;
C017133	decrease	MCF2	ENSG00000101977	12634122;
C017133	decrease	MSH2	ENSG00000095002	12634122;
C017133	decrease	MYCN	ENSG00000134323	12634122;
C017133	decrease	PIP5K1B	ENSG00000107242	12634122;
C017133	decrease	PTP4A1	ENSG00000112245	12634122;
C017133	decrease	RARRES3	ENSG00000133321	12634122;
C017133	decrease	RET	ENSG00000165731	12634122;
C017133	decrease	SGK1	ENSG00000118515	12634122;
C017133	decrease	SLC2A3	ENSG00000059804	12634122;
C017133	decrease	SOD1	ENSG00000142168	12634122;
C017133	decrease	SPINK1	ENSG00000164266	12634122;
C017133	decrease	SSPN	ENSG00000123096	12634122;
C017133	decrease	STOM	ENSG00000148175	12634122;
C017133	decrease	TGFB2	ENSG00000092969	12634122;
C017133	decrease	TM4SF1	ENSG00000169908	12634122;
C017133	decrease	TNFRSF1B	ENSG00000028137	12634122;
C017133	decrease	TNFSF10	ENSG00000121858	12634122;
C017133	decrease	VIM	ENSG00000026025	12634122;
C017133	decrease	WNT1	ENSG00000125084	12634122;
C017133	decrease	WNT2	ENSG00000105989	12634122;
C017133	increase	BMP4	ENSG00000125378	12634122;
C017133	increase	CASP10	ENSG00000003400	12634122;
C017133	increase	CDKN2A	ENSG00000147889	12634122;
C017133	increase	COL6A2	ENSG00000142173	12634122;
C017133	increase	FOSL1	ENSG00000175592	12634122;
C017133	increase	HIST1H2AC	ENSG00000180573	12634122;
C017133	increase	ITGB4	ENSG00000132470	12634122;
C017133	increase	JUNB	ENSG00000171223	12634122;
C017133	increase	LRPAP1	ENSG00000163956	12634122;
C017133	increase	MT1X	ENSG00000187193	12634122;
C017133	increase	MT3	ENSG00000087250	12634122;
C017133	increase	NFIX	ENSG00000008441	12634122;

C017133	increase	NTHL1	ENSG00000065057	12634122;
C017154	increase	NOS3	ENSG00000164867	15740983;
C017160	decrease	AR	ENSG00000169083	16857237;17980950;
C017160	increase	CYP2B6	ENSG00000197408	15548381;
C017160	increase	CYP3A4	ENSG00000160868	15548381;
C017160	increase	TFF1	ENSG00000160182	12396874;
C017180	increase	CYP3A4	ENSG00000160868	16790556;19249324;
C017185	decrease	BCL2	ENSG00000171791	15740068;
C017185	increase	ARNT	ENSG00000143437	18603805;
C017185	increase	BAX	ENSG00000087088	15740068;
C017185	increase	EPAS1	ENSG00000116016	18603805;
C017185	increase	HIF1A	ENSG00000100644	18603805;
C017185	increase	HIF3A	ENSG00000124440	18603805;
C017185	increase	JUN	ENSG00000177606	15740068;
C017185	increase	PGK1	ENSG00000102144	18603805;
C017228	decrease	BAD	ENSG00000002330	15698582;
C017228	decrease	BCL2L1	ENSG00000171552	15698582;
C017228	decrease	BID	ENSG00000015475	15698582;
C017228	increase	HRAS	ENSG00000174775	11008122;
C017228	increase	NQO1	ENSG00000181019	11008122;
C017228	increase	TP53	ENSG00000141510	15698582;
C017338	affect	RHOA	ENSG00000067560	15324309;
C017338	decrease	CYR61	ENSG00000142871	19530242;
C017338	decrease	HMGCR	ENSG00000113161	19819230;
C017338	decrease	RAP1A	ENSG00000116473	19819230;16539721;
C017338	increase	IBSP	ENSG00000029559	15324309;
C017461	increase	DDIT3	ENSG00000175197	14971657;
C017461	increase	FOS	ENSG00000170345	14971657;
C017461	increase	GADD45A	ENSG00000116717	14971657;
C017461	increase	HSPA5	ENSG00000044574	14971657;
C017557	decrease	ATN1	ENSG00000111676	12760830;
C017557	decrease	CASP4	ENSG00000196954	12760830;
C017557	decrease	CCL2	ENSG00000108691	12760830;
C017557	decrease	CCNK	ENSG00000090061	12760830;
C017557	decrease	COL4A2	ENSG00000134871	12760830;
C017557	decrease	CTTN	ENSG00000085733	12760830;
C017557	decrease	CYP1B1	ENSG00000138061	12760830;
C017557	decrease	EGR1	ENSG00000120738	12760830;
C017557	decrease	EPHA2	ENSG00000142627	12760830;
C017557	decrease	HDGF	ENSG00000143321	12760830;
C017557	decrease	HSP90AA1	ENSG00000080824	12760830;
C017557	decrease	ITGB4	ENSG00000132470	12760830;
C017557	decrease	JUP	ENSG00000173801	12760830;
C017557	decrease	MAPKAPK2	ENSG00000162889	12760830;
C017557	decrease	MYC	ENSG00000136997	12760830;
C017557	decrease	RFC4	ENSG00000163918	12760830;
C017557	decrease	TFAP2A	ENSG00000137203	12760830;
C017557	decrease	TNFAIP2	ENSG00000185215	12760830;
C017557	decrease	TNK1	ENSG00000174292	12760830;
C017557	decrease	TRRAP	ENSG00000196367	12760830;
C017557	decrease	VEZF1	ENSG00000136451	12760830;
C017557	decrease	ZFP36L1	ENSG00000185650	12760830;
C017557	decrease	ZYX	ENSG00000159840	12760830;
C017557	increase	IGFBP3	ENSG00000146674	12760830;

C017557	increase	PSMA3	ENSG00000100567	12760830;
C017557	increase	RPA3	ENSG00000106399	12760830;
C017557	increase	RPS3A	ENSG00000145425	12760830;
C017557	increase	UBC	ENSG00000150991	12760830;
C017558	decrease	FHIT	ENSG00000189283	17044645;
C017573	increase	PGR	ENSG00000082175	16009159;
C017576	decrease	AR	ENSG00000169083	17505938;
C017576	decrease	PLAT	ENSG00000104368	12618251;
C017576	decrease	PROS1	ENSG00000184500	12618251;
C017576	decrease	SERPINC1	ENSG00000117601	12618251;
C017576	increase	CRP	ENSG00000132693	16982228;
C017576	increase	F7	ENSG00000057593	12618251;
C017576	increase	F8	ENSG00000185010	12618251;
C017576	increase	FGA	ENSG00000171560	12618251;
C017576	increase	FGB	ENSG00000171564	12618251;
C017576	increase	FGG	ENSG00000171557	12618251;
C017576	increase	PLG	ENSG00000122194	12618251;
C017576	increase	PROC	ENSG00000115718	12618251;
C017576	increase	SERPINA6	ENSG00000170099	16982228;
C017576	increase	SERPINA7	ENSG00000123561	16982228;
C017576	increase	SHBG	ENSG00000129214	16982228;12586319;
C017618	decrease	AR	ENSG00000169083	16857237;
C017674	decrease	RB1	ENSG00000139687	15827326;
C017679	increase	CYP1A1	ENSG00000140465	12147272;11849738;
C017690	decrease	AR	ENSG00000169083	15064155;16857237;16125883;
C017690	increase	CYP2B6	ENSG00000197408	15548381;
C017690	increase	CYP3A4	ENSG00000160868	19249324;15548381;
C017690	increase	PGR	ENSG00000082175	16326432;11137321;
C017690	increase	TFF1	ENSG00000160182	10064545;12396874;
C017690	increase	WNT10B	ENSG00000169884	12437293;
C017803	affect	CDKN1A	ENSG00000124762	18508827;
C017803	increase	BIRC3	ENSG00000023445	18508827;
C017803	increase	HMOX1	ENSG00000100292	18508827;16462769;
C017875	increase	AR	ENSG00000169083	18922931;
C017875	increase	CD40	ENSG00000101017	16601352;
C017875	increase	HMOX1	ENSG00000100292	16799064;
C017875	increase	IL13	ENSG00000169194	12789233;
C017875	increase	IL1B	ENSG00000125538	18958421;
C017875	increase	IL4	ENSG00000113520	12789233;16343431;
C017875	increase	IL5	ENSG00000113525	12789233;
C017875	increase	IL6	ENSG00000136244	18958421;
C017875	increase	IL8	ENSG00000169429	17884996;18958421;
C017875	increase	KLK3	ENSG00000142515	18922931;
C017875	increase	NFE2L2	ENSG00000116044	16799064;
C017875	increase	TNF	ENSG00000223952	18958421;
C017947	affect	CEBPB	ENSG00000172216	14523996;
C017947	affect	FZD2	ENSG00000180340	16966095;
C017947	affect	PRKAA1	ENSG00000132356	16678800;
C017947	affect	UBE2C	ENSG00000175063	16966095;
C017947	affect	UBE2K	ENSG00000078140	16966095;
C017947	affect	UBE2N	ENSG00000177889	16966095;
C017947	decrease	ABP1	ENSG00000002726	12727804;
C017947	decrease	ADAMTS1	ENSG00000154734	12679051;

C017947	decrease	AKT1	ENSG00000142208	12760830;12482858;
C017947	decrease	ALDH1A3	ENSG00000184254	12727804;
C017947	decrease	ALOX5AP	ENSG00000132965	16507463;
C017947	decrease	APBA1	ENSG00000107282	12634122;
C017947	decrease	APEX1	ENSG00000100823	12760830;12377979;
C017947	decrease	APOL3	ENSG00000128284	12634122;
C017947	decrease	ARHGAP5	ENSG00000100852	12760830;
C017947	decrease	ARHGDIA	ENSG00000141522	12760830;
C017947	decrease	ARHGDIB	ENSG00000111348	12634122;12727804;
C017947	decrease	AURKB	ENSG00000178999	12760830;
C017947	decrease	BAG1	ENSG00000250477	12634122;12760830;
C017947	decrease	BAX	ENSG00000087088	12760830;
C017947	decrease	BCL2L1	ENSG00000171552	12760830;
C017947	decrease	BIRC3	ENSG00000023445	12676792;
C017947	decrease	BMP2	ENSG00000125845	12634122;
C017947	decrease	BNIP3	ENSG00000176171	12760830;
C017947	decrease	C10ORF116	ENSG00000148671	12727804;
C017947	decrease	C1ORF61	ENSG00000125462	12634122;
C017947	decrease	C1QTNF6	ENSG00000133466	18448484;
C017947	decrease	CALR	ENSG00000179218	15899475;
C017947	decrease	CAMK2G	ENSG00000148660	12727804;
C017947	decrease	CAPN2	ENSG00000162909	12760830;
C017947	decrease	CAPNS1	ENSG00000126247	12727804;
C017947	decrease	CASP2	ENSG00000106144	12760830;
C017947	decrease	CASP7	ENSG00000165806	12760830;
C017947	decrease	CAT	ENSG00000121691	8526746;12634122;166005
C017947	decrease	CCL2	ENSG00000108691	12016162;16507463;12760<br>830;
C017947	decrease	CCND2	ENSG00000118971	12377979;
C017947	decrease	CCR7	ENSG00000126353	12676792;
C017947	decrease	CD40LG	ENSG00000102245	12760830;
C017947	decrease	CD74	ENSG00000019582	12634122;
C017947	decrease	CDC20	ENSG00000117399	12727804;
C017947	decrease	CDC25B	ENSG00000101224	12377979;
C017947	decrease	CDH1	ENSG00000039068	12634122;
C017947	decrease	CENPE	ENSG00000138778	12679051;
C017947	decrease	CKB	ENSG00000166165	12634122;
C017947	decrease	CKM	ENSG00000104879	12634122;
C017947	decrease	CLDN4	ENSG00000189143	12634122;
C017947	decrease	COL1A1	ENSG00000108821	16507463;
C017947	decrease	CRABP2	ENSG00000143320	12016162;
C017947	decrease	CREBBP	ENSG00000005339	12377979;
C017947	decrease	CTSH	ENSG00000103811	12727804;
C017947	decrease	CTTN	ENSG00000085733	12760830;
C017947	decrease	CXCR4	ENSG00000121966	12634122;
C017947	decrease	CYP1B1	ENSG00000138061	12727804;
C017947	decrease	DAB2	ENSG00000153071	12634122;
C017947	decrease	DAD1	ENSG00000129562	12760830;
C017947	decrease	DAPK1	ENSG00000196730	19577553;
C017947	decrease	DARC	ENSG00000213088	12016162;
C017947	decrease	DAXX	ENSG00000231617	12760830;
C017947	decrease	DBC1	ENSG00000078725	18448484;
C017947	decrease	DDB2	ENSG00000134574	12377979;
C017947	decrease	DFFA	ENSG00000160049	12760830;

C017947	decrease	DHRS3	ENSG00000162496	12727804;
C017947	decrease	DSP	ENSG00000096696	12634122;
C017947	decrease	DUSP6	ENSG00000139318	12377979;
C017947	decrease	EFNA4	ENSG00000243364	12760830;
C017947	decrease	EFNB2	ENSG00000125266	12727804;
C017947	decrease	ELF3	ENSG00000163435	12634122;
C017947	decrease	EMP1	ENSG00000134531	12727804;
C017947	decrease	ENDOG	ENSG00000167136	12377979;
C017947	decrease	EPB41L3	ENSG00000082397	12634122;
C017947	decrease	EPCAM	ENSG00000119888	12634122;
C017947	decrease	ERBB3	ENSG00000065361	12634122;
C017947	decrease	ERCC2	ENSG00000104884	12634122;12760830;
C017947	decrease	ERCC5	ENSG00000134899	12634122;
C017947	decrease	FAM83A	ENSG00000147689	18448484;
C017947	decrease	FASTK	ENSG00000164896	12760830;
C017947	decrease	FEN1	ENSG00000168496	12377979;
C017947	decrease	FLG	ENSG00000143631	12727804;11485391;
C017947	decrease	FLT1	ENSG00000102755	12634122;
C017947	decrease	GATA3	ENSG00000107485	12634122;
C017947	decrease	GATA6	ENSG00000141448	12634122;16507463;
C017947	decrease	GBP2	ENSG00000162645	12377979;
C017947	decrease	GDF15	ENSG00000130513	18448484;12634122;
C017947	decrease	GJA1	ENSG00000152661	12727804;16507463;
C017947	decrease	GLI1	ENSG00000111087	12634122;
C017947	decrease	GNB2	ENSG00000172354	12760830;
C017947	decrease	GSS	ENSG00000100983	12634122;
C017947	decrease	GSTO1	ENSG00000148834	12634122;
C017947	decrease	HCK	ENSG00000101336	12634122;
C017947	decrease	HMGCS1	ENSG00000112972	16507463;
C017947	decrease	HOXA10	ENSG00000078399	14989901;
C017947	decrease	HSD11B2	ENSG00000176387	12727804;
C017947	decrease	HSPA5	ENSG00000044574	15788408;15899475;
C017947	decrease	HTRA1	ENSG00000166033	16507463;
C017947	decrease	ICAM1	ENSG00000090339	12634122;
C017947	decrease	IFIT1	ENSG00000185745	12727804;
C017947	decrease	IFITM2	ENSG00000185201	18448484;12377979;
C017947	decrease	IFITM3	ENSG00000142089	12727804;
C017947	decrease	IGFBP2	ENSG00000115457	12727804;
C017947	decrease	IGFBP3	ENSG00000146674	12727804;
C017947	decrease	IGFBP5	ENSG00000115461	16507463;
C017947	decrease	IGFBP7	ENSG00000163453	12634122;
C017947	decrease	IL13	ENSG00000169194	12676792;
C017947	decrease	IL1A	ENSG00000115008	12679051;
C017947	decrease	ISG15	ENSG00000187608	18448484;12377979;
C017947	decrease	ITGB3	ENSG00000056345	16507463;
C017947	decrease	ITGB6	ENSG00000115221	12727804;
C017947	decrease	JAK1	ENSG00000162434	12679051;
C017947	decrease	JUP	ENSG00000173801	12760830;
C017947	decrease	KLF4	ENSG00000136826	12377979;
C017947	decrease	KLK7	ENSG00000169035	12634122;
C017947	decrease	KRT13	ENSG00000171401	12634122;
C017947	decrease	KRT15	ENSG00000171346	12016162;
C017947	decrease	KRT19	ENSG00000171345	12016162;
C017947	decrease	KRT6C	ENSG00000170465	12634122;

C017947	decrease	L1CAM	ENSG00000198910	12016162;
C017947	decrease	LIG1	ENSG00000105486	12760830;
C017947	decrease	LMNB2	ENSG00000176619	12377979;
C017947	decrease	LY6E	ENSG00000160932	12377979;
C017947	decrease	MAD2L1	ENSG00000164109	12377979;
C017947	decrease	MAP2K1	ENSG00000169032	12016162;
C017947	decrease	MAP2K6	ENSG00000108984	12634122;
C017947	decrease	MAPK4	ENSG00000141639	12634122;
C017947	decrease	MAPK9	ENSG00000050748	12634122;12475910;12482858;
C017947	decrease	MAPKAPK3	ENSG00000114738	12547826;12760830;
C017947	decrease	MARCKS	ENSG00000155130	16507463;
C017947	decrease	MATK	ENSG00000007264	12634122;
C017947	decrease	MCC	ENSG00000171444	12634122;
C017947	decrease	MCM2	ENSG00000073111	12760830;
C017947	decrease	MCM7	ENSG00000166508	12760830;
C017947	decrease	MED21	ENSG00000152944	12727804;
C017947	decrease	MGMT	ENSG00000170430	12760830;
C017947	decrease	MMP13	ENSG00000137745	12679051;
C017947	decrease	MMP1	ENSG00000196611	12377979;
C017947	decrease	MSH2	ENSG00000095002	12634122;
C017947	decrease	MSH5	ENSG00000227314	12377979;
C017947	decrease	MYL9	ENSG00000101335	12727804;
C017947	decrease	MYLK	ENSG00000251351	16507463;
C017947	decrease	NDFIP2	ENSG00000102471	16507463;
C017947	decrease	NEFL	ENSG00000104725	18448484;
C017947	decrease	NFKB1	ENSG00000109320	16809336;12377979;
C017947	decrease	NME1	ENSG00000239672	12016162;
C017947	decrease	NRGN	ENSG00000154146	12760830;
C017947	decrease	NRIP1	ENSG00000180530	12727804;
C017947	decrease	NUP88	ENSG00000108559	16507463;
C017947	decrease	OGG1	ENSG00000114026	12359242;
C017947	decrease	P4HB	ENSG00000185624	15899475;
C017947	decrease	PDCD6	ENSG00000249915	12760830;
C017947	decrease	PEX14	ENSG00000142655	16507463;
C017947	decrease	PGF	ENSG00000119630	12016162;
C017947	decrease	PHLDB2	ENSG00000144824	12679051;
C017947	decrease	PI4KA	ENSG00000249793	12760830;
C017947	decrease	PIP5K1B	ENSG00000107242	12634122;
C017947	decrease	PLA2G16	ENSG00000176485	12727804;
C017947	decrease	PLAU	ENSG00000122861	12016162;
C017947	decrease	PLCG1	ENSG00000124181	12760830;
C017947	decrease	PML	ENSG00000140464	12016162;
C017947	decrease	POLD1	ENSG00000062822	12377979;12760830;
C017947	decrease	POLE	ENSG00000177084	12377979;
C017947	decrease	POLG	ENSG00000140521	12760830;
C017947	decrease	POLR2E	ENSG00000099817	12727804;
C017947	decrease	PRKACA	ENSG00000072062	12760830;
C017947	decrease	PRSS8	ENSG00000052344	12634122;
C017947	decrease	PSCA	ENSG00000167653	12727804;
C017947	decrease	PSMB1	ENSG00000008018	16507463;
C017947	decrease	PSMB8	ENSG00000226201	12727804;
C017947	decrease	PTPN11	ENSG00000179295	12679051;
C017947	decrease	PTPRF	ENSG00000142949	12377979;

C017947	decrease	RARG	ENSG00000172819	12377979;
C017947	decrease	RARRES3	ENSG00000133321	12634122;12727804;
C017947	decrease	RELA	ENSG00000173039	12676792;12241537;12377979;12760830;
C017947	decrease	RET	ENSG00000165731	12634122;
C017947	decrease	REV3L	ENSG00000009413	12377979;
C017947	decrease	RFC1	ENSG00000035928	12760830;
C017947	decrease	RFC2	ENSG00000049541	12760830;
C017947	decrease	RHOB	ENSG00000143878	12760830;
C017947	decrease	RPS6KA1	ENSG00000117676	12760830;
C017947	decrease	RXRA	ENSG00000186350	15833926;
C017947	decrease	SAA2	ENSG00000134339	12727804;
C017947	decrease	SERPINB1	ENSG00000021355	12727804;
C017947	decrease	SERPINB4	ENSG00000206073	12727804;
C017947	decrease	SFN	ENSG00000175793	12760830;
C017947	decrease	SGK1	ENSG00000118515	12634122;
C017947	decrease	SLC2A1	ENSG00000117394	12760830;
C017947	decrease	SLC2A3	ENSG00000059804	12634122;
C017947	decrease	SMAD7	ENSG00000101665	16507463;
C017947	decrease	SOX4	ENSG00000124766	12727804;
C017947	decrease	SPINK1	ENSG00000164266	12634122;
C017947	decrease	SPINT2	ENSG00000167642	12634122;
C017947	decrease	SPRR1A	ENSG00000169474	11485391;
C017947	decrease	SPRR1B	ENSG00000169469	11485391;
C017947	decrease	SSPN	ENSG00000123096	12634122;
C017947	decrease	STOM	ENSG00000148175	12634122;
C017947	decrease	TERT	ENSG00000164362	16966277;
C017947	decrease	TFAP4	ENSG00000090447	12679051;
C017947	decrease	TNC	ENSG00000041982	12727804;
C017947	decrease	TNFAIP2	ENSG00000185215	12727804;12760830;
C017947	decrease	TNFAIP6	ENSG00000123610	12016162;
C017947	decrease	TNF	ENSG00000223952	12377979;
C017947	decrease	TNFRSF1B	ENSG00000028137	12634122;
C017947	decrease	TP53I3	ENSG00000115129	12727804;
C017947	decrease	TPR	ENSG00000047410	12679051;
C017947	decrease	TRAF1	ENSG00000056558	12676792;
C017947	decrease	TRAF3	ENSG00000131323	12760830;
C017947	decrease	TRAP1	ENSG00000126602	12377979;
C017947	decrease	TRIM31	ENSG00000223531	12727804;
C017947	decrease	TSC2	ENSG00000103197	12760830;
C017947	decrease	TYK2	ENSG00000105397	12760830;
C017947	decrease	UBE2L6	ENSG00000156587	12727804;
C017947	decrease	UNG	ENSG00000076248	12377979;12760830;
C017947	decrease	VIM	ENSG00000026025	12634122;
C017947	decrease	WNT1	ENSG00000125084	12634122;
C017947	decrease	WNT2	ENSG00000105989	12634122;
C017947	decrease	WNT5A	ENSG00000114251	12679051;
C017947	decrease	XPC	ENSG00000154767	12377979;12760830;
C017947	decrease	YWHAH	ENSG00000128245	12760830;
C017947	decrease	ZSCAN12	ENSG00000158691	18448484;
C017947	increase	ABCC2	ENSG00000023839	11408547;12727804;
C017947	increase	ABL1	ENSG00000097007	12016162;
C017947	increase	ABL2	ENSG00000143322	12760830;
C017947	increase	AKR1C3	ENSG00000196139	12727804;

C017947	increase	ALDOA	ENSG00000149925	15899475;
C017947	increase	ANXA1	ENSG00000135046	15899475;
C017947	increase	APP	ENSG00000142192	12760830;
C017947	increase	ATF2	ENSG00000115966	12475910;12220541;15292961;12016162;
C017947	increase	ATF3	ENSG00000162772	12220541;12760830;
C017947	increase	ATF4	ENSG00000128272	15788408;12760830;
C017947	increase	ATM	ENSG00000149311	11507245;
C017947	increase	ATP1B3	ENSG00000069849	12760830;
C017947	increase	BCL2A1	ENSG00000140379	12016162;
C017947	increase	BCL2	ENSG00000171791	12679051;16966277;
C017947	increase	BCL6	ENSG00000113916	12760830;
C017947	increase	BDNF	ENSG00000176697	12760830;
C017947	increase	BECN1	ENSG00000126581	19577553;
C017947	increase	BIRC2	ENSG00000110330	14970392;
C017947	increase	BIRC5	ENSG00000089685	14988404;
C017947	increase	BLVRA	ENSG00000106605	15741166;
C017947	increase	BMP4	ENSG00000125378	12634122;12760830;
C017947	increase	BRCA1	ENSG00000012048	12760830;
C017947	increase	BRCA2	ENSG00000139618	12760830;
C017947	increase	CAPZA1	ENSG00000116489	12377979;
C017947	increase	CASP10	ENSG00000003400	12634122;12016162;
C017947	increase	CAST	ENSG00000153113	12016162;
C017947	increase	CCNB1	ENSG00000134057	12016162;
C017947	increase	CCND1	ENSG00000110092	19168569;12634122;16283521;17005224;
C017947	increase	CCNF	ENSG00000162063	12016162;
C017947	increase	CCNG1	ENSG00000113328	12016162;12377979;
C017947	increase	CDC25A	ENSG00000164045	12016162;
C017947	increase	CDK1	ENSG00000170312	12016162;
C017947	increase	CDKN1A	ENSG00000124762	11507245;12634122;12760830;12377979;11813266;
C017947	increase	CDKN2A	ENSG00000147889	12634122;11825526;12016162;
C017947	increase	CDKN3	ENSG00000100526	12016162;
C017947	increase	CFLAR	ENSG00000003402	12016162;
C017947	increase	CLK1	ENSG00000013441	12016162;12760830;
C017947	increase	CLU	ENSG00000120885	12760830;12377979;
C017947	increase	COL6A2	ENSG00000142173	12634122;
C017947	increase	CPVL	ENSG00000106066	12727804;
C017947	increase	CRADD	ENSG00000169372	12016162;
C017947	increase	CREM	ENSG00000095794	14523996;
C017947	increase	CSDA	ENSG00000060138	12760830;
C017947	increase	CSDE1	ENSG00000009307	12016162;
C017947	increase	CSNK2A1	ENSG00000101266	18572023;
C017947	increase	CSNK2A2	ENSG00000070770	18572023;
C017947	increase	CSNK2B	ENSG00000224398	18572023;
C017947	increase	CTGF	ENSG00000118523	12377979;
C017947	increase	CTSC	ENSG00000109861	12760830;
C017947	increase	CXCL1	ENSG00000163739	12016162;12727804;12377979;
C017947	increase	CXCL2	ENSG00000081041	12016162;12760830;
C017947	increase	CYCS	ENSG00000172115	15741166;
C017947	increase	CYP1A1	ENSG00000140465	15576448;15630080;16713074;

C017947	increase	CYP3A4	ENSG00000160868	15833926;
C017947	increase	DBP	ENSG00000105516	12760830;
C017947	increase	DDIT3	ENSG00000175197	12016162;12760830;
C017947	increase	DLX2	ENSG00000115844	16966095;
C017947	increase	DNAJB1	ENSG00000132002	12679051;12760830;16729991;
C017947	increase	DNAJB4	ENSG00000162616	12634122;
C017947	increase	DUSP1	ENSG00000120129	16966095;12377979;
C017947	increase	E2F3	ENSG00000112242	12760830;
C017947	increase	EEF1A1	ENSG00000156508	12727804;
C017947	increase	EEF1A2	ENSG00000101210	12727804;
C017947	increase	EGFR	ENSG00000146648	15504454;19168569;12016162;11723127;
C017947	increase	EGR1	ENSG00000120738	15292961;12016162;12760830;12377979;12634122;
C017947	increase	EIF4E	ENSG00000151247	16283521;16951406;12016162;
C017947	increase	ELK1	ENSG00000126767	12660819;15292961;12016162;
C017947	increase	ELK3	ENSG00000111145	12760830;
C017947	increase	EPHA1	ENSG00000146904	12760830;
C017947	increase	EPHA2	ENSG00000142627	12760830;
C017947	increase	EPHA5	ENSG00000145242	12634122;
C017947	increase	ETS1	ENSG00000134954	12760830;
C017947	increase	ETS2	ENSG00000157557	12760830;
C017947	increase	ETV6	ENSG00000139083	12016162;12760830;
C017947	increase	EXT1	ENSG00000182197	12634122;
C017947	increase	F11R	ENSG00000158769	12016162;
C017947	increase	F2R	ENSG00000181104	12760830;
C017947	increase	FADD	ENSG00000168040	12016162;
C017947	increase	FAS	ENSG00000026103	12016162;
C017947	increase	FGF2	ENSG00000138685	12703962;
C017947	increase	FGFR4	ENSG00000160867	12377979;
C017947	increase	FLI1	ENSG00000151702	12634122;
C017947	increase	FOS	ENSG00000170345	12547826;14523996;12475910;12377979;17409708;
C017947	increase	FOSL1	ENSG00000175592	12634122;14523996;12760830;12475910;
C017947	increase	FYN	ENSG00000010810	12016162;
C017947	increase	GABPB2	ENSG00000143458	12760830;
C017947	increase	GADD45A	ENSG00000116717	12016162;12760830;
C017947	increase	GADD45B	ENSG00000099860	12760830;
C017947	increase	GALR1	ENSG00000166573	12760830;
C017947	increase	GAP43	ENSG00000172020	12016162;
C017947	increase	GAPDH	ENSG00000111640	15899475;
C017947	increase	GCLM	ENSG00000023909	12679051;
C017947	increase	GLRX	ENSG00000173221	12016162;12377979;
C017947	increase	GRB2	ENSG00000177885	12016162;
C017947	increase	GRLF1	ENSG00000160007	12760830;
C017947	increase	GTF2H2	ENSG00000145736	12760830;
C017947	increase	HBEGF	ENSG00000113070	19168569;12760830;
C017947	increase	HDAC9	ENSG00000048052	16966095;
C017947	increase	HIF1A	ENSG00000100644	12482858;16678800;12760830;
C017947	increase	HIST1H2AC	ENSG00000180573	12634122;

C017947	increase	HIST2H2BE	ENSG00000184678	12679051;
C017947	increase	HMOX1	ENSG00000100292	16959797;15741166;15618017;16775837;12016162;17409708;16966095;12679051;12220541;12760830;15788408;12727804;
C017947	increase	HOXA5	ENSG00000106004	12760830;
C017947	increase	HRAS	ENSG00000174775	16039940;
C017947	increase	HSF1	ENSG00000185122	15618017;11322385;
C017947	increase	HSF2	ENSG00000025156	12016162;
C017947	increase	HSP90AA1	ENSG00000080824	12760830;
C017947	increase	HSPA1A	ENSG00000232804	16966095;12016162;12760830;16729991;
C017947	increase	HSPA2	ENSG00000126803	12760830;
C017947	increase	HSPA4	ENSG00000170606	11322385;16959797;
C017947	increase	HSPA6	ENSG00000173110	12760830;16966095;
C017947	increase	HSPA8	ENSG00000109971	12760830;
C017947	increase	HSPB1	ENSG00000106211	12760830;15899475;16729991;
C017947	increase	HSPD1	ENSG00000144381	12016162;12760830;
C017947	increase	HTR3A	ENSG00000166736	12760830;
C017947	increase	HYAL1	ENSG00000114378	12377979;
C017947	increase	ID1	ENSG00000125968	16966095;12634122;12727804;
C017947	increase	ID2	ENSG00000115738	12634122;12727804;16966095;
C017947	increase	ID3	ENSG00000117318	12727804;12760830;16966095;
C017947	increase	IER2	ENSG00000160888	12760830;
C017947	increase	IER3	ENSG00000137331	12760830;
C017947	increase	IFNGR1	ENSG00000027697	12016162;
C017947	increase	IGF2	ENSG00000167244	12016162;
C017947	increase	IL11	ENSG00000095752	12760830;
C017947	increase	IL12B	ENSG00000113302	12760830;
C017947	increase	IL1B	ENSG00000125538	12241537;14523996;
C017947	increase	IL1R2	ENSG00000115590	12760830;16507463;
C017947	increase	IL2	ENSG00000109471	12760830;
C017947	increase	IL2RA	ENSG00000134460	12760830;
C017947	increase	IL5RA	ENSG00000091181	12760830;
C017947	increase	IL6	ENSG00000136244	12241537;14523996;12016162;12760830;11893605;
C017947	increase	IL7	ENSG00000104432	12760830;
C017947	increase	IL8	ENSG00000169429	12016162;12760830;
C017947	increase	INSIG1	ENSG00000186480	12679051;16966095;
C017947	increase	ITGA4	ENSG00000115232	12760830;
C017947	increase	ITGAV	ENSG00000138448	12016162;
C017947	increase	ITGB4	ENSG00000132470	12634122;12377979;
C017947	increase	IVL	ENSG00000163207	12727804;11485391;18572023;
C017947	increase	JUNB	ENSG00000171223	12634122;12016162;12220541;12475910;
C017947	increase	JUND	ENSG00000130522	12220541;12760830;12475910;
C017947	increase	JUN	ENSG00000177606	15741166;16775837;12482858;12547826;14523996;16809336;12377979;12760830;12016162;12475910;

C017947	increase	KIT	ENSG00000157404	12016162;
C017947	increase	KLF5	ENSG00000102554	12760830;
C017947	increase	KLF8	ENSG00000102349	12377979;
C017947	increase	KRT10	ENSG00000186395	12727804;18572023;
C017947	increase	KRT1	ENSG00000167768	18572023;
C017947	increase	KRT5	ENSG00000186081	12634122;12016162;
C017947	increase	KRT6A	ENSG00000205420	12016162;
C017947	increase	KRT8	ENSG00000170421	12634122;12016162;
C017947	increase	LIF	ENSG00000128342	12760830;
C017947	increase	LOR	ENSG00000203782	18572023;
C017947	increase	LRPAP1	ENSG00000163956	12634122;
C017947	increase	MAFF	ENSG00000185022	16966095;
C017947	increase	MAFG	ENSG00000197063	12220541;11162468;
C017947	increase	MAP1LC3B	ENSG00000140941	19577553;
C017947	increase	MAP2K3	ENSG00000034152	12760830;
C017947	increase	MAP2K4	ENSG00000065559	12760830;
C017947	increase	MAP3K8	ENSG00000107968	12016162;
C017947	increase	MAPK1	ENSG00000100030	12547826;12660819;12482858;15504454;19577553;16959797;19168569;12475910;11322385;11723127;
C017947	increase	MAPK3	ENSG00000102882	12547826;12482858;15504454;19577553;16959797;19168569;12475910;11322385;11723127;
C017947	increase	MAPK6	ENSG00000069956	12760830;
C017947	increase	MAPRE1	ENSG00000101367	12760830;
C017947	increase	MARK3	ENSG00000075413	12760830;
C017947	increase	MCF2	ENSG00000101977	12634122;12016162;
C017947	increase	MCL1	ENSG00000143384	12016162;12760830;
C017947	increase	MDM2	ENSG00000135679	12016162;11813266;
C017947	increase	MLH1	ENSG00000076242	12634122;
C017947	increase	MMP2	ENSG00000087245	16507463;12377979;
C017947	increase	MNAT1	ENSG00000020426	12016162;
C017947	increase	MT1A	ENSG00000205362	12679051;11693492;
C017947	increase	MT1E	ENSG00000169715	12679051;12377979;14555400;
C017947	increase	MT1G	ENSG00000125144	12679051;11693492;
C017947	increase	MT1H	ENSG00000205358	12679051;12377979;
C017947	increase	MT1M	ENSG00000205364	12679051;
C017947	increase	MT1X	ENSG00000187193	12634122;12679051;11693492;16966095;14555400;
C017947	increase	MT2A	ENSG00000125148	12679051;14555400;16966095;12377979;11693492;
C017947	increase	MT3	ENSG00000087250	12634122;11693492;
C017947	increase	MXD1	ENSG00000059728	12377979;12760830;
C017947	increase	MYBL2	ENSG00000101057	12016162;
C017947	increase	MYC	ENSG00000136997	12679051;12760830;
C017947	increase	MYCL1	ENSG00000116990	12016162;
C017947	increase	MYCN	ENSG00000134323	12634122;12016162;
C017947	increase	NAT1	ENSG00000171428	12016162;
C017947	increase	NCK1	ENSG00000158092	12016162;
C017947	increase	NEK6	ENSG00000119408	12016162;
C017947	increase	NFATC4	ENSG00000100968	16809336;
C017947	increase	NFIX	ENSG00000008441	12634122;

C017947	increase	NFKB2	ENSG00000077150	12760830;
C017947	increase	NFX1	ENSG00000086102	12760830;
C017947	increase	NIT1	ENSG00000158793	12016162;
C017947	increase	NKTR	ENSG00000114857	12634122;
C017947	increase	NMBR	ENSG00000135577	12760830;
C017947	increase	NOS3	ENSG00000164867	12703962;
C017947	increase	NOV	ENSG00000136999	12016162;
C017947	increase	NPAT	ENSG00000149308	12016162;
C017947	increase	NQO1	ENSG00000181019	12377979;17409708;12426128;
C017947	increase	NR2F6	ENSG00000160113	12016162;12760830;
C017947	increase	NR4A1	ENSG00000123358	12760830;
C017947	increase	NTHL1	ENSG00000065057	12634122;
C017947	increase	PAWR	ENSG00000177425	16966277;
C017947	increase	PBX1	ENSG00000185630	12760830;
C017947	increase	PCNA	ENSG00000132646	12760830;12634122;
C017947	increase	PDGFA	ENSG00000197461	12727804;12760830;
C017947	increase	PFDN4	ENSG00000101132	12016162;
C017947	increase	PFDN5	ENSG00000123349	12377979;
C017947	increase	PGAM1	ENSG00000171314	15899475;
C017947	increase	PIK3CA	ENSG00000121879	12016162;
C017947	increase	PIK3R2	ENSG00000105647	12760830;
C017947	increase	PIM1	ENSG00000137193	12016162;
C017947	increase	PLK2	ENSG00000145632	12760830;
C017947	increase	POR	ENSG00000127948	12760830;12016162;
C017947	increase	POU2F2	ENSG00000028277	12377979;
C017947	increase	PPP1CB	ENSG00000213639	12016162;
C017947	increase	PRDX1	ENSG00000117450	15899475;
C017947	increase	PRKCA	ENSG00000154229	16507463;12016162;
C017947	increase	PRKCD	ENSG00000163932	12016162;12377979;
C017947	increase	PRNP	ENSG00000171867	12760830;
C017947	increase	PTGS2	ENSG00000073756	16809336;11835400;12377979;
C017947	increase	PTK2	ENSG00000169398	12016162;
C017947	increase	PTP4A1	ENSG00000112245	12634122;12760830;
C017947	increase	RAB3A	ENSG00000105649	12016162;
C017947	increase	RAB5A	ENSG00000144566	12016162;
C017947	increase	RAD23A	ENSG00000179262	12634122;
C017947	increase	RAP1A	ENSG00000116473	12016162;
C017947	increase	RAP1B	ENSG00000127314	12760830;
C017947	increase	RASA1	ENSG00000145715	12016162;
C017947	increase	RASSF1	ENSG00000068028	12634122;
C017947	increase	RFC5	ENSG00000111445	12016162;
C017947	increase	RNF5	ENSG00000225452	12016162;
C017947	increase	ROBO1	ENSG00000169855	12760830;
C017947	increase	RPL6	ENSG00000089009	12760830;
C017947	increase	S100A10	ENSG00000197747	12377979;
C017947	increase	SEMA3C	ENSG00000075223	12634122;
C017947	increase	SERPINB8	ENSG00000166401	12760830;
C017947	increase	SERPINH1	ENSG00000149257	12377979;
C017947	increase	SHB	ENSG00000107338	12760830;
C017947	increase	SLC16A1	ENSG00000155380	12760830;
C017947	increase	SLC1A4	ENSG00000115902	12760830;
C017947	increase	SLC20A2	ENSG00000168575	16966095;

C017947	increase	SLC30A1	ENSG00000170385	16966095;
C017947	increase	SLC7A5	ENSG00000103257	12760830;
C017947	increase	SLC9A1	ENSG00000090020	12760830;
C017947	increase	SMAD1	ENSG00000170365	12760830;
C017947	increase	SMAD4	ENSG00000141646	12016162;12760830;
C017947	increase	SMAD6	ENSG00000137834	12016162;16966095;
C017947	increase	SMARCA1	ENSG00000102038	12016162;
C017947	increase	SMN1	ENSG00000172062	12760830;
C017947	increase	SOD1	ENSG00000142168	12634122;12377979;
C017947	increase	SP3	ENSG00000172845	12016162;
C017947	increase	STK25	ENSG00000115694	12377979;12426128;
C017947	increase	SYN3	ENSG00000185666	12760830;
C017947	increase	TACSTD2	ENSG00000184292	12377979;
C017947	increase	TAGLN	ENSG00000149591	12634122;
C017947	increase	TANK	ENSG00000136560	12016162;
C017947	increase	TAPBP	ENSG00000206208	12016162;
C017947	increase	TCF12	ENSG00000140262	16507463;12016162;
C017947	increase	TFAM	ENSG00000108064	12016162;
C017947	increase	TFRC	ENSG00000072274	12377979;
C017947	increase	TGFB2	ENSG00000092969	12634122;
C017947	increase	TGFB3	ENSG00000119699	12016162;
C017947	increase	TGM1	ENSG00000092295	11485391;
C017947	increase	TIMP3	ENSG00000100234	12634122;
C017947	increase	TM4SF1	ENSG00000169908	12634122;12377979;
C017947	increase	TMSB4X	ENSG00000205542	12760830;
C017947	increase	TNFRSF10B	ENSG00000120889	15741166;
C017947	increase	TNFSF10	ENSG00000121858	15741166;12634122;12727804;
C017947	increase	TOB1	ENSG00000141232	12760830;
C017947	increase	TOP2A	ENSG00000131747	12760830;12016162;
C017947	increase	TP53	ENSG00000141510	11507245;16966095;12377979;11813266;
C017947	increase	TPI1	ENSG00000111669	15899475;
C017947	increase	TRIM24	ENSG00000122779	12760830;
C017947	increase	TSPAN31	ENSG00000135452	12016162;
C017947	increase	TST	ENSG00000128311	12760830;
C017947	increase	TTR	ENSG00000118271	12760830;
C017947	increase	UAP1	ENSG00000117143	12679051;
C017947	increase	UBC	ENSG00000150991	12760830;
C017947	increase	USP4	ENSG00000114316	12016162;
C017947	increase	USP8	ENSG00000138592	12016162;
C017947	increase	VBP1	ENSG00000155959	12016162;
C017947	increase	VEGFA	ENSG00000112715	12482858;15788408;16678800;12703962;12760830;
C017947	increase	VWF	ENSG00000110799	12703962;
C017947	increase	WT1	ENSG00000184937	16966277;12016162;
C017947	increase	XK	ENSG00000047597	12016162;
C017947	increase	XRCC1	ENSG00000073050	12634122;
C017947	increase	XRCC6	ENSG00000196419	12016162;
C017947	increase	YES1	ENSG00000176105	12377979;
C017947	increase	ZEB1	ENSG00000148516	12760830;
C017947	increase	ZFP36	ENSG00000128016	12016162;
C017947	increase	ZFP36L1	ENSG00000185650	12634122;12760830;
C017953	decrease	APOC3	ENSG00000110245	15520497;

C017953	increase	ACCN3	ENSG00000213199	15520497;
C017953	increase	ATP2A2	ENSG00000174437	15520497;
C017953	increase	CYP11B1	ENSG00000160882	15520497;
C018003	increase	HRAS	ENSG00000174775	11008122;
C018003	increase	NQO1	ENSG00000181019	11008122;
C018021	affect	CAT	ENSG00000121691	16678800;
C018021	affect	IFNG	ENSG00000111537	17034590;
C018021	affect	PRKAA1	ENSG00000132356	16678800;
C018021	decrease	BCAS2	ENSG00000116752	16622835;
C018021	decrease	COPE	ENSG00000105669	16622835;
C018021	decrease	COX5A	ENSG00000178741	16622835;
C018021	decrease	EGLN2	ENSG00000171570	15695372;
C018021	decrease	EIF1AX	ENSG00000173674	16622835;
C018021	decrease	GRPEL1	ENSG00000109519	16622835;
C018021	decrease	HIF1AN	ENSG00000166135	15695372;
C018021	decrease	ITPA	ENSG00000125877	16622835;
C018021	decrease	LDHB	ENSG00000111716	16622835;
C018021	decrease	NFKBIA	ENSG00000100906	17034590;
C018021	decrease	NSFL1C	ENSG00000088833	16622835;
C018021	decrease	NUDT5	ENSG00000165609	16622835;
C018021	decrease	P4HTM	ENSG00000178467	15695372;
C018021	decrease	PCMT1	ENSG00000120265	16622835;
C018021	decrease	SEC13	ENSG00000157020	16622835;
C018021	decrease	SSBP1	ENSG00000106028	16622835;
C018021	decrease	SUGT1	ENSG00000165416	16622835;
C018021	decrease	UCHL3	ENSG00000118939	16622835;
C018021	increase	BCL2	ENSG00000171791	16827160;
C018021	increase	CD44	ENSG00000026508	16798617;
C018021	increase	CLU	ENSG00000120885	16827160;
C018021	increase	CTGF	ENSG00000118523	17034590;
C018021	increase	CYP2A6	ENSG00000198077	11038160;
C018021	increase	EGLN1	ENSG00000135766	15695372;
C018021	increase	EPO	ENSG00000130427	15743794;16827160;
C018021	increase	HIF1A	ENSG00000100644	15695405;16682453;17082639;16678800;16330433;19196431;17034590;16775626;16504308;16859120;16932349;16026872;16827160;1
C018021	increase	HIF3A	ENSG00000124440	16775626;
C018021	increase	HMOX1	ENSG00000100292	16859120;
C018021	increase	ICAM1	ENSG00000090339	16798617;
C018021	increase	IL1B	ENSG00000125538	16504308;
C018021	increase	IL8	ENSG00000169429	17082639;
C018021	increase	ITGA2	ENSG00000164171	16798617;
C018021	increase	ITGA5	ENSG00000161638	16798617;
C018021	increase	MCL1	ENSG00000143384	15611089;
C018021	increase	NDRG1	ENSG00000104419	16622835;
C018021	increase	PLD1	ENSG00000075651	17640750;
C018021	increase	PLD2	ENSG00000129219	17640750;
C018021	increase	PTGS2	ENSG00000073756	17640750;
C018021	increase	RNF7	ENSG00000114125	16874460;
C018021	increase	SLC2A1	ENSG00000117394	7493931;
C018021	increase	TGFB1	ENSG00000105329	17034590;
C018021	increase	TP53	ENSG00000141510	10951577;

C018021	increase	VEGFA	ENSG00000112715	16682453;16678800;15075239;15611089;16026872;15695372;16827160;
C018021	increase	WT1	ENSG00000184937	12738801;
C018145	increase	CCL2	ENSG00000108691	16376386;
C018145	increase	IL8	ENSG00000169429	16376386;
C018421	increase	CYP3A4	ENSG00000160868	16837568;
C018465	increase	CYP3A4	ENSG00000160868	19249324;
C018465	increase	IFNG	ENSG00000111537	10414782;9860237;
C018465	increase	IL4	ENSG00000113520	10414782;9860237;
C018562	increase	CYP3A4	ENSG00000160868	15075359;
C018566	decrease	GJA1	ENSG00000152661	8820588;
C018855	affect	GCLC	ENSG00000001084	15946948;
C018855	increase	ABCC1	ENSG00000103222	11836020;
C018855	increase	ABCC2	ENSG00000023839	11836020;
C018855	increase	ABCG2	ENSG00000118777	17077187;
C018855	increase	AHR	ENSG00000106546	17077187;
C018855	increase	AKR1C1	ENSG00000187134	9973208;
C018855	increase	CD86	ENSG00000114013	19033392;
C018855	increase	CYP1A1	ENSG00000140465	16581943;15608132;99732
C018855	increase	GSTP1	ENSG00000084207	15533597;
C018855	increase	HMOX1	ENSG00000100292	19033392;18227147;
C018855	increase	KEAP1	ENSG00000079999	19033392;
C018855	increase	NFE2L2	ENSG00000116044	12446695;19033392;
C018855	increase	NQO1	ENSG00000181019	9029048;19033392;
C019003	decrease	BCL2A1	ENSG00000140379	19625696;
C019003	decrease	HHIP	ENSG00000164161	19625696;
C019003	decrease	KLK3	ENSG00000142515	15141018;
C019003	decrease	MYC	ENSG00000136997	19625696;
C019003	increase	CDKN1C	ENSG00000129757	19625696;
C019003	increase	EGR1	ENSG00000120738	19625696;
C019003	increase	HMOX1	ENSG00000100292	19625696;
C019003	increase	PPARG	ENSG00000132170	19625696;
C019046	increase	PGR	ENSG00000082175	12753421;
C019046	increase	TFF1	ENSG00000160182	12753421;
C019107	increase	IL8	ENSG00000169429	9628260;9707512;
C019248	decrease	BCL2	ENSG00000171791	10780527;
C019248	decrease	COL1A1	ENSG00000108821	19597852;
C019248	decrease	FGF23	ENSG00000118972	15838626;
C019248	increase	CRP	ENSG00000132693	9351880;
C019248	increase	IL6	ENSG00000136244	9351880;
C019248	increase	TNF	ENSG00000223952	9351880;
C019248	increase	TNFRSF11B	ENSG00000164761	11855844;
C019273	affect	DDX58	ENSG00000107201	16303604;
C019273	increase	NPHS1	ENSG00000161270	16687628;
C019304	increase	PPARA	ENSG00000186951	16680075;
C019304	increase	SULT2A1	ENSG00000105398	15635043;
C020174	decrease	GSTP1	ENSG00000084207	12007958;
C020174	decrease	MYC	ENSG00000136997	12007958;
C020174	increase	CCL5	ENSG00000161570	12007958;
C020174	increase	IL2RA	ENSG00000134460	12007958;
C020174	increase	MYB	ENSG00000118513	12007958;
C020174	increase	SOD2	ENSG00000112096	12007958;
C020243	increase	CXCL10	ENSG00000169245	14600836;

C020243	increase	CXCL12	ENSG00000107562	14600836;
C020326	increase	ABCB1	ENSG00000085563	18385176;
C020326	increase	CYP2E1	ENSG00000130649	17034788;
C020326	increase	KRT18	ENSG00000111057	17034788;
C020326	increase	KRT8	ENSG00000170421	17034788;
C020365	increase	FKBP5	ENSG00000096060	19255438;
C020365	increase	TNF	ENSG00000223952	16318612;
C020365	increase	TSC22D3	ENSG00000157514	19255438;
C020711	increase	ACTA2	ENSG00000107796	16314022;
C020711	increase	ANXA5	ENSG00000164111	16314022;
C020711	increase	CCL5	ENSG00000161570	16314022;
C020711	increase	CD44	ENSG00000026508	16314022;
C020711	increase	CDKN1A	ENSG00000124762	17395767;
C020711	increase	CDKN2A	ENSG00000147889	17395767;
C020711	increase	CTSL2	ENSG00000136943	16314022;
C020711	increase	CXCL3	ENSG00000163734	16314022;
C020711	increase	EGR2	ENSG00000122877	16314022;
C020711	increase	HLA-A	ENSG00000206503	16314022;
C020711	increase	HLA-C	ENSG00000204525	16314022;
C020711	increase	HLA-DRB1	ENSG00000196126	16272364;16314022;
C020711	increase	ICAM1	ENSG00000090339	18539414;
C020711	increase	IL18	ENSG00000150782	16100009;
C020711	increase	ITGB3	ENSG00000056345	16314022;
C020711	increase	NEDD8	ENSG00000129559	16314022;
C020711	increase	NOVA1	ENSG00000248163	16314022;
C020711	increase	RHOA	ENSG00000067560	16314022;
C020711	increase	RPL22	ENSG00000116251	16314022;
C020711	increase	RPL30	ENSG00000156482	16314022;
C020711	increase	RPL37A	ENSG00000197756	16314022;
C020711	increase	RPS7	ENSG00000171863	16314022;
C020711	increase	SERPINB2	ENSG00000197632	16314022;
C020711	increase	SFTPD	ENSG00000133661	16314022;
C020711	increase	TMSB4X	ENSG00000205542	16314022;
C020711	increase	TP53	ENSG00000141510	17395767;
C020711	increase	WNT4	ENSG00000162552	16314022;
C020711	increase	ZNF77	ENSG00000175691	16314022;
C020748	decrease	CRP	ENSG00000132693	15963385;17119383;
C020748	decrease	IL6	ENSG00000136244	17119383;
C020748	increase	EPAS1	ENSG00000116016	17973296;
C020748	increase	HIF1A	ENSG00000100644	16386771;17973296;
C020748	increase	IL4	ENSG00000113520	15963385;
C020748	increase	IL8	ENSG00000169429	15081272;
C020748	increase	TNF	ENSG00000223952	15963385;
C020754	increase	ICAM1	ENSG00000090339	12044887;
C020754	increase	IFNG	ENSG00000111537	12044887;
C020806	increase	CYP21A2	ENSG00000231852	18313098;
C020806	increase	PGR	ENSG00000082175	12753421;11693576;
C020806	increase	TFF1	ENSG00000160182	12753421;11693576;
C020888	decrease	CEACAM7	ENSG00000007306	14668052;
C020888	decrease	CPA3	ENSG00000163751	14668052;
C020888	decrease	CXCL9	ENSG00000138755	14668052;
C020888	decrease	DUSP1	ENSG00000120129	14668052;
C020888	decrease	EDNRB	ENSG00000136160	14668052;
C020888	decrease	GLRX	ENSG00000173221	14668052;

C020888	decrease	RPL6	ENSG00000089009	14668052;
C020888	increase	RPA1	ENSG00000132383	14668052;
C020922	increase	IL2	ENSG00000109471	18423386;
C020924	increase	F2	ENSG00000180210	18597608;
C020924	increase	FOS	ENSG00000170345	18597608;
C020924	increase	MYC	ENSG00000136997	18597608;
C020972	decrease	MST1R	ENSG00000164078	18593918;
C020972	increase	ALB	ENSG00000163631	11961005;
C020972	increase	AR	ENSG00000169083	16139439;
C020972	increase	BCL2	ENSG00000171791	16139439;
C020972	increase	BIRC3	ENSG00000023445	14527959;
C020972	increase	BIRC5	ENSG00000089685	16139439;
C020972	increase	CCL2	ENSG00000108691	11961005;16600694;12016129;
C020972	increase	CP	ENSG00000047457	17032174;
C020972	increase	DDIT3	ENSG00000175197	17049495;16972258;
C020972	increase	EGF	ENSG00000138798	16340751;
C020972	increase	FN1	ENSG00000115414	12388107;
C020972	increase	FOS	ENSG00000170345	17032174;
C020972	increase	GATA1	ENSG00000102145	10656287;
C020972	increase	HMBS	ENSG00000149397	10656287;
C020972	increase	HMOX1	ENSG00000100292	16972258;
C020972	increase	ICAM1	ENSG00000090339	14572618;
C020972	increase	IL17A	ENSG00000112115	12016129;
C020972	increase	IL6	ENSG00000136244	12016129;
C020972	increase	IL8	ENSG00000169429	15622447;11306435;12016129;
C020972	increase	IRF1	ENSG00000125347	11739238;
C020972	increase	JUN	ENSG00000177606	17032174;
C020972	increase	KLK3	ENSG00000142515	16139439;
C020972	increase	MMP9	ENSG00000100985	16340751;
C020972	increase	NFE2	ENSG00000123405	10656287;
C020972	increase	PLAU	ENSG00000122861	16340751;
C020972	increase	PTGS2	ENSG00000073756	11835400;
C020972	increase	TNF	ENSG00000223952	11699074;16112155;15161907;14572618;11675405;
C021277	increase	GREB1	ENSG00000196208	16496412;
C021287	increase	AR	ENSG00000169083	15336702;
C021287	increase	KLK3	ENSG00000142515	15336702;
C021304	decrease	SIRT1	ENSG00000096717	18552516;
C021751	decrease	ALDH1A3	ENSG00000184254	15849732;
C021751	decrease	ALDH3A2	ENSG00000072210	15849732;
C021751	decrease	ASNS	ENSG00000070669	15849732;
C021751	decrease	ASS1	ENSG00000130707	15849732;
C021751	decrease	BCKDHB	ENSG00000083123	15849732;
C021751	decrease	BCL2	ENSG00000171791	16009009;
C021751	decrease	BIRC3	ENSG00000023445	15849732;
C021751	decrease	CD33	ENSG00000105383	16009009;
C021751	decrease	CEACAM6	ENSG00000086548	15849732;
C021751	decrease	COL6A1	ENSG00000142156	15849732;
C021751	decrease	DYRK4	ENSG00000010219	15849732;
C021751	decrease	ETS2	ENSG00000157557	15849732;
C021751	decrease	IL8	ENSG00000169429	15849732;
C021751	decrease	ITGA6	ENSG00000091409	15849732;

C021751	decrease	LRP5	ENSG00000162337	15849732;
C021751	decrease	LRP8	ENSG00000157193	15849732;
C021751	decrease	MAP3K1	ENSG00000095015	15849732;
C021751	decrease	MIPEP	ENSG00000027001	15849732;
C021751	decrease	MMP7	ENSG00000137673	15849732;
C021751	decrease	MYC	ENSG00000136997	16009009;
C021751	decrease	NES	ENSG00000132688	16955220;
C021751	decrease	PLOD2	ENSG00000152952	15849732;
C021751	decrease	PRPF6	ENSG00000101161	15849732;
C021751	decrease	PTPRK	ENSG00000152894	15849732;
C021751	decrease	SP2	ENSG00000167182	15849732;
C021751	decrease	TFAP4	ENSG00000090447	15849732;
C021751	increase	ACADVL	ENSG00000072778	15849732;
C021751	increase	ACSL1	ENSG00000151726	15849732;
C021751	increase	APOC1	ENSG00000130208	15849732;
C021751	increase	APOM	ENSG00000226215	15849732;
C021751	increase	ATP6V0B	ENSG00000117410	15849732;
C021751	increase	ATP6V1E1	ENSG00000131100	15849732;
C021751	increase	CALCOCO2	ENSG00000136436	15849732;
C021751	increase	CASP3	ENSG00000164305	15849732;
C021751	increase	CDKN1A	ENSG00000124762	15849732;
C021751	increase	CLU	ENSG00000120885	15849732;
C021751	increase	CST6	ENSG00000175315	15849732;
C021751	increase	CYP1A1	ENSG00000140465	16955220;15849732;
C021751	increase	DCK	ENSG00000156136	15849732;
C021751	increase	DUSP11	ENSG00000144048	15849732;
C021751	increase	DUSP5	ENSG00000138166	15849732;
C021751	increase	ENC1	ENSG00000171617	15849732;
C021751	increase	F2RL1	ENSG00000164251	15849732;
C021751	increase	FLAD1	ENSG00000160688	15849732;
C021751	increase	FOS	ENSG00000170345	16009009;
C021751	increase	GBA	ENSG00000177628	15849732;
C021751	increase	GFAP	ENSG00000131095	16955220;
C021751	increase	GRB7	ENSG00000141738	15849732;
C021751	increase	HDAC3	ENSG00000171720	15849732;
C021751	increase	ID2	ENSG00000115738	15849732;
C021751	increase	INPPL1	ENSG00000165458	15849732;
C021751	increase	ISG20	ENSG00000172183	15849732;
C021751	increase	ITGAM	ENSG00000169896	16009009;
C021751	increase	MAOB	ENSG00000069535	15849732;
C021751	increase	MYBL2	ENSG00000101057	15849732;
C021751	increase	MYL1	ENSG00000168530	15849732;
C021751	increase	NDUFB2	ENSG00000090266	15849732;
C021751	increase	NDUFV1	ENSG00000167792	15849732;
C021751	increase	NOS3	ENSG00000164867	15849732;
C021751	increase	PARP4	ENSG00000102699	16955220;15849732;
C021751	increase	PCSK7	ENSG00000160613	15849732;
C021751	increase	PSMB5	ENSG00000251114	15849732;
C021751	increase	PSMD2	ENSG00000175166	15849732;
C021751	increase	RHOC	ENSG00000155366	15849732;
C021751	increase	TESK1	ENSG00000107140	15849732;
C021751	increase	TK1	ENSG00000167900	15849732;
C021751	increase	TP53	ENSG00000141510	16009009;
C021751	increase	WFDC2	ENSG00000101443	15849732;

C022189	increase	TH	ENSG00000180176	20196486;
C022776	decrease	FAS	ENSG00000026103	16712844;
C022776	increase	CYP4A11	ENSG00000187048	16712844;
C022777	increase	FOS	ENSG00000170345	10712238;
C022777	increase	IL1B	ENSG00000125538	10712238;
C022777	increase	TNF	ENSG00000223952	10712238;
C022830	decrease	TUBB4	ENSG00000104833	15924336;
C022830	decrease	XIAP	ENSG00000101966	16356831;
C022830	increase	CCNB1	ENSG00000134057	16356831;
C022830	increase	PRDX4	ENSG00000123131	18272409;
C022838	decrease	ACSL1	ENSG00000151726	17547211;
C022838	decrease	AKR1B1	ENSG00000085662	17547211;
C022838	decrease	CLPTM1	ENSG00000104853	17547211;
C022838	decrease	F5	ENSG00000198734	17547211;
C022838	decrease	FBXW2	ENSG00000119402	17547211;
C022838	decrease	FDPS	ENSG00000160752	17547211;
C022838	decrease	FURIN	ENSG00000140564	17547211;
C022838	decrease	GGCX	ENSG00000115486	17547211;
C022838	decrease	GPER	ENSG00000164850	17547211;
C022838	decrease	IGSF1	ENSG00000147255	17547211;
C022838	decrease	LIPA	ENSG00000107798	17547211;
C022838	decrease	LSS	ENSG00000160285	17547211;
C022838	decrease	NFKBIA	ENSG00000100906	16806455;17547211;
C022838	decrease	NR0B2	ENSG00000131910	17547211;
C022838	decrease	NUCB2	ENSG00000070081	17547211;
C022838	decrease	PDIA4	ENSG00000155660	17547211;
C022838	decrease	PGC	ENSG00000096088	17547211;
C022838	decrease	RAB9A	ENSG00000123595	17547211;
C022838	decrease	SERPINB1	ENSG00000021355	17547211;
C022838	decrease	SERPINC1	ENSG00000117601	17547211;
C022838	decrease	SFRS5	ENSG00000100650	17547211;
C022838	decrease	ST6GALNA C4	ENSG00000136840	17547211;
C022838	decrease	TNFSF10	ENSG00000121858	17547211;
C022838	decrease	TST	ENSG00000128311	17547211;
C022838	increase	ADM	ENSG00000148926	17547211;
C022838	increase	ASNS	ENSG00000070669	17547211;
C022838	increase	CCNB2	ENSG00000157456	17547211;
C022838	increase	CCR7	ENSG00000126353	15737199;
C022838	increase	CD86	ENSG00000114013	15737199;
C022838	increase	CDKN1A	ENSG00000124762	17547211;
C022838	increase	CKS2	ENSG00000123975	17547211;
C022838	increase	DKK1	ENSG00000107984	17547211;
C022838	increase	DUSP1	ENSG00000120129	17547211;
C022838	increase	FOSL1	ENSG00000175592	17547211;
C022838	increase	GCLC	ENSG00000001084	17547211;
C022838	increase	GCNT3	ENSG00000140297	17547211;
C022838	increase	GLA	ENSG00000102393	17547211;
C022838	increase	GLRX	ENSG00000173221	17547211;
C022838	increase	HIF1A	ENSG00000100644	16649251;
C022838	increase	HIST1H4C	ENSG00000182217	17547211;
C022838	increase	HMOX1	ENSG00000100292	16806455;17547211;
C022838	increase	ICAM1	ENSG00000090339	15737199;
C022838	increase	IER2	ENSG00000160888	17547211;

C022838	increase	ITGB3BP	ENSG00000142856	17547211;
C022838	increase	KLF10	ENSG00000155090	17547211;
C022838	increase	KLF6	ENSG00000067082	17547211;
C022838	increase	KPNA4	ENSG00000186432	17547211;
C022838	increase	MAFF	ENSG00000185022	17547211;
C022838	increase	NFE2L2	ENSG00000116044	16806455;
C022838	increase	NFYA	ENSG00000001167	17547211;
C022838	increase	PHLDA2	ENSG00000181649	17547211;
C022838	increase	PTTG1	ENSG00000164611	17547211;
C022838	increase	RACGAP1	ENSG00000161800	17547211;
C022838	increase	SFRS2	ENSG00000161547	17547211;
C022838	increase	SIK1	ENSG00000142178	17547211;
C022838	increase	SIRT1	ENSG00000096717	17547211;
C022838	increase	SPP1	ENSG00000118785	17547211;
C022838	increase	TNFAIP8	ENSG00000145779	17547211;
C022838	increase	TRIB1	ENSG00000173334	17547211;
C022838	increase	TUBA1A	ENSG00000167552	17547211;
C022838	increase	TUFT1	ENSG00000143367	17547211;
C022838	increase	UBE2C	ENSG00000175063	17547211;
C022838	increase	VIP	ENSG00000146469	16563610;
C022921	increase	ABCB1	ENSG00000085563	15566942;
C022921	increase	ABCG2	ENSG00000118777	17077187;15917307;
C022921	increase	AHR	ENSG00000106546	17077187;15917307;
C022921	increase	CYP1A1	ENSG00000140465	15576448;15566942;
C022921	increase	CYP1B1	ENSG00000138061	15566942;
C022921	increase	UGT1A6	ENSG00000167165	15566942;
C022921	increase	UGT1A7	ENSG00000244122	15566942;
C022999	decrease	BAX	ENSG00000087088	15863893;
C022999	decrease	XIAP	ENSG00000101966	15863893;
C023035	affect	CTSD	ENSG00000117984	16489053;
C023035	increase	AGTR1	ENSG00000144891	14962509;
C023035	increase	AHR	ENSG00000106546	16705413;16049271;10448124;16489053;
C023035	increase	CYP11B1	ENSG00000160882	16396990;15703266;16216300;
C023035	increase	CYP11B2	ENSG00000179142	16396990;14962509;15703266;16216300;
C023035	increase	CYP17A1	ENSG00000148795	15703266;16216300;
C023035	increase	CYP19A1	ENSG00000137869	16216300;
C023035	increase	CYP1A1	ENSG00000140465	16396990;16489053;15659568;16049271;
C023035	increase	CYP1A2	ENSG00000140505	15659567;16049271;
C023035	increase	CYP21A2	ENSG00000231852	15703266;16216300;
C023035	increase	ESR1	ENSG00000091831	16489053;
C023035	increase	HSD3B2	ENSG00000203859	16216300;
C023386	decrease	CYP21A2	ENSG00000231852	16650473;
C023386	decrease	HSD3B2	ENSG00000203859	16650473;
C023386	increase	AHR	ENSG00000106546	16650473;
C023386	increase	CYP11B2	ENSG00000179142	16650473;
C023514	increase	DDIT3	ENSG00000175197	16705839;
C023514	increase	FOS	ENSG00000170345	16705839;
C023514	increase	GADD45A	ENSG00000116717	16705839;
C023600	decrease	APBA1	ENSG00000107282	12634122;
C023600	decrease	BAG1	ENSG00000250477	12634122;

C023600	decrease	BMP2	ENSG00000125845	12634122;
C023600	decrease	C1ORF61	ENSG00000125462	12634122;
C023600	decrease	CAT	ENSG00000121691	12634122;
C023600	decrease	CDKN1A	ENSG00000124762	12634122;
C023600	decrease	CKB	ENSG00000166165	12634122;
C023600	decrease	CKM	ENSG00000104879	12634122;
C023600	decrease	CXCR4	ENSG00000121966	12634122;
C023600	decrease	DAB2	ENSG00000153071	12634122;
C023600	decrease	EPB41L3	ENSG00000082397	12634122;
C023600	decrease	EPHA5	ENSG00000145242	12634122;
C023600	decrease	ERBB3	ENSG00000065361	12634122;
C023600	decrease	ERCC2	ENSG00000104884	12634122;
C023600	decrease	ERCC5	ENSG00000134899	12634122;
C023600	decrease	GATA6	ENSG00000141448	12634122;
C023600	decrease	GDF15	ENSG00000130513	12634122;
C023600	decrease	GLI1	ENSG00000111087	12634122;
C023600	decrease	HCK	ENSG00000101336	12634122;
C023600	decrease	ID1	ENSG00000125968	12634122;
C023600	decrease	ID2	ENSG00000115738	12634122;
C023600	decrease	IGFBP7	ENSG00000163453	12634122;
C023600	decrease	MAP2K6	ENSG00000108984	12634122;
C023600	decrease	MAPK4	ENSG00000141639	12634122;
C023600	decrease	MAPK9	ENSG00000050748	12634122;
C023600	decrease	MATK	ENSG00000007264	12634122;
C023600	decrease	MCC	ENSG00000171444	12634122;
C023600	decrease	MCF2	ENSG00000101977	12634122;
C023600	decrease	MSH2	ENSG00000095002	12634122;
C023600	decrease	MYCN	ENSG00000134323	12634122;
C023600	decrease	PIP5K1B	ENSG00000107242	12634122;
C023600	decrease	PTP4A1	ENSG00000112245	12634122;
C023600	decrease	RARRES3	ENSG00000133321	12634122;
C023600	decrease	RET	ENSG00000165731	12634122;
C023600	decrease	SGK1	ENSG00000118515	12634122;
C023600	decrease	SLC2A3	ENSG00000059804	12634122;
C023600	decrease	SOD1	ENSG00000142168	12634122;
C023600	decrease	SPINK1	ENSG00000164266	12634122;
C023600	decrease	SSPN	ENSG00000123096	12634122;
C023600	decrease	STOM	ENSG00000148175	12634122;
C023600	decrease	TGFB2	ENSG00000092969	12634122;
C023600	decrease	TM4SF1	ENSG00000169908	12634122;
C023600	decrease	TNFRSF1B	ENSG00000028137	12634122;
C023600	decrease	TNFSF10	ENSG00000121858	12634122;
C023600	decrease	VIM	ENSG00000026025	12634122;
C023600	decrease	WNT1	ENSG00000125084	12634122;
C023600	decrease	WNT2	ENSG00000105989	12634122;
C023600	increase	BMP4	ENSG00000125378	12634122;
C023600	increase	CASP10	ENSG00000003400	12634122;
C023600	increase	CDKN2A	ENSG00000147889	12634122;
C023600	increase	COL6A2	ENSG00000142173	12634122;
C023600	increase	FOSL1	ENSG00000175592	12634122;
C023600	increase	HIST1H2AC	ENSG00000180573	12634122;
C023600	increase	ITGB4	ENSG00000132470	12634122;
C023600	increase	JUNB	ENSG00000171223	12634122;
C023600	increase	LRPAP1	ENSG00000163956	12634122;

C023600	increase	MT1X	ENSG00000187193	12634122;
C023600	increase	MT3	ENSG00000087250	12634122;
C023600	increase	NFIX	ENSG00000008441	12634122;
C023600	increase	NTHL1	ENSG00000065057	12634122;
C023617	increase	ABCB11	ENSG00000073734	12525500;12705905;
C023617	increase	BCL2A1	ENSG00000140379	15322087;
C023617	increase	BCL2	ENSG00000171791	15322087;
C023617	increase	BIRC2	ENSG00000110330	15322087;
C023617	increase	BIRC5	ENSG00000089685	15322087;
C023617	increase	CCND1	ENSG00000110092	15322087;
C023617	increase	CFLAR	ENSG00000003402	15322087;
C023617	increase	CYP3A4	ENSG00000160868	15075359;
C023617	increase	CYP7A1	ENSG00000167910	12705905;
C023617	increase	MMP9	ENSG00000100985	15322087;
C023617	increase	MYC	ENSG00000136997	15322087;
C023617	increase	NR1H4	ENSG00000012504	12705905;
C023617	increase	NR1I2	ENSG00000144852	12705905;15075359;
C023617	increase	PTGS2	ENSG00000073756	15322087;
C023617	increase	TNF	ENSG00000223952	15322087;
C023617	increase	TRAF1	ENSG00000056558	15322087;
C023617	increase	VEGFA	ENSG00000112715	15322087;
C023617	increase	XIAP	ENSG00000101966	15322087;
C023623	decrease	BCL2	ENSG00000171791	17557751;
C023635	decrease	CD4	ENSG00000010610	10813102;
C023635	decrease	IGF1	ENSG00000017427	11172797;
C023635	decrease	PLAT	ENSG00000104368	15288212;
C023635	decrease	PROS1	ENSG00000184500	15288212;
C023635	decrease	SERPINE1	ENSG00000106366	15288212;
C023635	increase	CRP	ENSG00000132693	15970291;
C023635	increase	F2	ENSG00000180210	15288212;
C023635	increase	F7	ENSG00000057593	15288212;
C023635	increase	FGA	ENSG00000171560	15288212;
C023635	increase	FGB	ENSG00000171564	15288212;
C023635	increase	FGG	ENSG00000171557	15288212;
C023635	increase	IL8	ENSG00000169429	15866594;
C023635	increase	TNF	ENSG00000223952	15866594;
C023650	affect	CDKN1A	ENSG00000124762	16039525;
C023650	decrease	CYP19A1	ENSG00000137869	16480277;
C023650	decrease	SKP2	ENSG00000145604	16039525;
C023650	increase	CDKN1B	ENSG00000111276	16039525;
C023663	decrease	IL8	ENSG00000169429	16973179;
C023663	decrease	PTGS2	ENSG00000073756	16973179;
C023663	decrease	TNF	ENSG00000223952	16973179;
C023666	increase	CYP1A1	ENSG00000140465	12147272;11849738;
C023666	increase	CYP2E1	ENSG00000130649	12147272;12464242;80747
C023844	decrease	CCDC120	ENSG00000147144	15878651;
C023844	decrease	CLK1	ENSG00000013441	15878651;
C023844	decrease	7-Sep	ENSG00000122545	15878651;
C023844	decrease	VEGFA	ENSG00000112715	15878651;
C023844	increase	DCLRE1C	ENSG00000152457	15878651;
C023844	increase	FTH1	ENSG00000167996	15878651;
C023844	increase	FTL	ENSG00000087086	15878651;
C023844	increase	HMGB1	ENSG00000189403	15878651;
C023844	increase	HMGB2	ENSG00000164104	15878651;

C023844	increase	PCNA	ENSG00000132646	15878651;
C023844	increase	STMN1	ENSG00000117632	15878651;
C023888	decrease	ESR1	ENSG00000091831	12948864;15064155;
C023888	decrease	MAPK1	ENSG00000100030	12948864;
C023888	decrease	MAPK3	ENSG00000102882	12948864;
C023888	increase	ERBB2	ENSG00000141736	11222871;12948864;
C023888	increase	MMP9	ENSG00000100985	12948864;
C023888	increase	PGR	ENSG00000082175	16242299;
C023888	increase	TFF1	ENSG00000160182	16242299;
C023889	decrease	ESR1	ENSG00000091831	17904202;
C023889	increase	PGR	ENSG00000082175	17904202;
C023889	increase	TFF1	ENSG00000160182	17904202;
C023891	increase	ALB	ENSG00000163631	2015207;
C023990	decrease	CD40	ENSG00000101017	11306145;
C023990	decrease	HRH2	ENSG00000113749	11306145;
C023990	decrease	PTPRC	ENSG00000081237	11306145;
C024262	decrease	CCL5	ENSG00000161570	16842799;
C024262	decrease	VCAM1	ENSG00000162692	17903073;
C024262	increase	CRP	ENSG00000132693	17903073;
C024348	increase	ALOX15	ENSG00000161905	11406566;
C024352	affect	TNFRSF10A	ENSG00000104689	14614459;
C024352	decrease	CCNA2	ENSG00000145386	15059916;
C024352	decrease	CCND1	ENSG00000110092	15059916;
C024352	decrease	CCNE1	ENSG00000105173	15059916;
C024352	decrease	CDKN1B	ENSG00000111276	15059916;
C024352	decrease	HIF1A	ENSG00000100644	15213310;
C024352	decrease	VEGFA	ENSG00000112715	15213310;
C024352	increase	BBC3	ENSG00000105327	16439685;
C024352	increase	CDKN1A	ENSG00000124762	16439685;16439677;
C024352	increase	FAS	ENSG00000026103	11675354;
C024352	increase	GDF15	ENSG00000130513	16439677;
C024352	increase	MCL1	ENSG00000143384	15059916;
C024352	increase	PCNA	ENSG00000132646	16439677;
C024352	increase	TNFRSF10B	ENSG00000120889	14614459;18092340;
C024352	increase	TP53	ENSG00000141510	17226861;18092340;16439677;
C024352	increase	XIAP	ENSG00000101966	15059916;
C024352	increase	ZMAT3	ENSG00000172667	16439685;
C024555	increase	IL1B	ENSG00000125538	9851740;
C024555	increase	IL8	ENSG00000169429	15081272;
C024587	decrease	TNFRSF11B	ENSG00000164761	18264100;
C024587	increase	TNFSF11	ENSG00000120659	18264100;
C024746	decrease	AIM1L	ENSG00000176092	19559774;
C024746	decrease	ANKRD1	ENSG00000148677	19559774;
C024746	decrease	ANXA10	ENSG00000109511	19559774;
C024746	decrease	APOE	ENSG00000130203	19559774;
C024746	decrease	ARID5B	ENSG00000150347	19559774;
C024746	decrease	ARL4D	ENSG00000175906	19559774;
C024746	decrease	ATP2B4	ENSG00000058668	19559774;
C024746	decrease	AUTS2	ENSG00000158321	19559774;
C024746	decrease	C17ORF98	ENSG00000214556	19559774;
C024746	decrease	C18ORF56	ENSG00000176912	19559774;
C024746	decrease	C22ORF43	ENSG00000189269	19559774;
C024746	decrease	C3ORF32	ENSG00000125046	19559774;

C024746	decrease	C9ORF16	ENSG00000171159	19559774;
C024746	decrease	CASZ1	ENSG00000130940	19559774;
C024746	decrease	CBR4	ENSG00000145439	19559774;
C024746	decrease	CD164L2	ENSG00000174950	19559774;
C024746	decrease	CD86	ENSG00000114013	19559774;
C024746	decrease	CEBPA	ENSG00000245848	19559774;
C024746	decrease	CLDN5	ENSG00000184113	19559774;
C024746	decrease	CLK1	ENSG00000013441	19559774;
C024746	decrease	CNTN1	ENSG00000018236	19559774;
C024746	decrease	CNTNAP2	ENSG00000174469	19559774;
C024746	decrease	CNTNAP3B	ENSG00000236029	19559774;
C024746	decrease	COL22A1	ENSG00000169436	19559774;
C024746	decrease	CRBN	ENSG00000113851	19559774;
C024746	decrease	CRLF1	ENSG00000006016	19559774;
C024746	decrease	CSPG4	ENSG00000173546	19559774;
C024746	decrease	CYORF15B	ENSG00000131002	19559774;
C024746	decrease	DDX60	ENSG00000137628	19559774;
C024746	decrease	DHRS9	ENSG00000073737	19559774;
C024746	decrease	DIAPH2	ENSG00000147202	19559774;
C024746	decrease	DMD	ENSG00000198947	19559774;
C024746	decrease	DMRTA1	ENSG00000176399	19559774;
C024746	decrease	DPYSL4	ENSG00000151640	19559774;
C024746	decrease	DSG3	ENSG00000134757	19559774;
C024746	decrease	DTNA	ENSG00000134769	19559774;
C024746	decrease	DUSP10	ENSG00000143507	19559774;
C024746	decrease	EFHD1	ENSG00000115468	19559774;
C024746	decrease	EIF1AY	ENSG00000198692	19559774;
C024746	decrease	ELF3	ENSG00000163435	19559774;
C024746	decrease	ENG	ENSG00000106991	19559774;
C024746	decrease	FAM84B	ENSG00000168672	19559774;
C024746	decrease	FHDC1	ENSG00000137460	19559774;
C024746	decrease	FN1	ENSG00000115414	19559774;
C024746	decrease	FOSB	ENSG00000125740	19559774;
C024746	decrease	FOXF2	ENSG00000137273	19559774;
C024746	decrease	FOXQ1	ENSG00000164379	19559774;
C024746	decrease	FYN	ENSG00000010810	19559774;
C024746	decrease	FZD10	ENSG00000111432	19559774;
C024746	decrease	GALNT5	ENSG00000136542	19559774;
C024746	decrease	GDA	ENSG00000119125	19559774;
C024746	decrease	GLS2	ENSG00000135423	19559774;
C024746	decrease	GMPR	ENSG00000137198	19559774;
C024746	decrease	GPR37	ENSG00000170775	19559774;
C024746	decrease	GRAMD2	ENSG00000175318	19559774;
C024746	decrease	GSN	ENSG00000148180	19559774;
C024746	decrease	HES2	ENSG00000069812	19559774;
C024746	decrease	HOOK1	ENSG00000134709	19559774;
C024746	decrease	HSD17B3	ENSG00000130948	19559774;
C024746	decrease	IER5L	ENSG00000188483	19559774;
C024746	decrease	IFIT2	ENSG00000119922	19559774;
C024746	decrease	IGF2	ENSG00000167244	19559774;
C024746	decrease	IL18R1	ENSG00000115604	19559774;
C024746	decrease	INA	ENSG00000148798	19559774;
C024746	decrease	IRX4	ENSG00000113430	19559774;
C024746	decrease	IRX5	ENSG00000176842	19559774;

C024746	decrease	ITGB2	ENSG00000160255	19559774;
C024746	decrease	KCNS3	ENSG00000170745	19559774;
C024746	decrease	KDM5D	ENSG00000012817	19559774;
C024746	decrease	KIAA1199	ENSG00000103888	19559774;
C024746	decrease	KRT13	ENSG00000171401	19559774;
C024746	decrease	KRT15	ENSG00000171346	19559774;
C024746	decrease	KRT81	ENSG00000205426	19559774;
C024746	decrease	LFNG	ENSG00000106003	19559774;
C024746	decrease	LMCD1	ENSG00000071282	19559774;
C024746	decrease	LY96	ENSG00000154589	19559774;
C024746	decrease	MAP2	ENSG00000078018	19559774;
C024746	decrease	MEX3B	ENSG00000183496	19559774;
C024746	decrease	MKRN2	ENSG00000075975	19559774;
C024746	decrease	MYEOV	ENSG00000172927	19559774;
C024746	decrease	NDN	ENSG00000182636	19559774;
C024746	decrease	NEDD9	ENSG00000111859	19559774;
C024746	decrease	NFIL3	ENSG00000165030	19559774;
C024746	decrease	NIPAL4	ENSG00000172548	19559774;
C024746	decrease	NIPBL	ENSG00000164190	19559774;
C024746	decrease	NLGN4Y	ENSG00000165246	19559774;
C024746	decrease	NPAS1	ENSG00000130751	19559774;
C024746	decrease	NRCAM	ENSG00000091129	19559774;
C024746	decrease	NTNG1	ENSG00000162631	19559774;
C024746	decrease	PALM	ENSG00000099864	19559774;
C024746	decrease	PIM1	ENSG00000137193	19559774;
C024746	decrease	PKN3	ENSG00000160447	19559774;
C024746	decrease	PLVAP	ENSG00000130300	19559774;
C024746	decrease	PPAP2B	ENSG00000162407	19559774;
C024746	decrease	PRSS23	ENSG00000150687	19559774;
C024746	decrease	RBM20	ENSG00000203867	19559774;
C024746	decrease	RCN3	ENSG00000142552	19559774;
C024746	decrease	RFTN1	ENSG00000131378	19559774;
C024746	decrease	RGS2	ENSG00000116741	19559774;
C024746	decrease	RPL32	ENSG00000144713	19559774;
C024746	decrease	S100A4	ENSG00000196154	19559774;
C024746	decrease	SBK1	ENSG00000188322	19559774;
C024746	decrease	SCNN1G	ENSG00000166828	19559774;
C024746	decrease	SERTAD4	ENSG00000082497	19559774;
C024746	decrease	SH3KBP1	ENSG00000147010	19559774;
C024746	decrease	SH3RF1	ENSG00000154447	19559774;
C024746	decrease	SLCO2A1	ENSG00000174640	19559774;
C024746	decrease	SMAD7	ENSG00000101665	19559774;
C024746	decrease	SPANXA1	ENSG00000203926	19559774;
C024746	decrease	SPANXB2	ENSG00000235604	19559774;
C024746	decrease	SPANXC	ENSG00000198573	19559774;
C024746	decrease	SRCRB4D	ENSG00000146700	19559774;
C024746	decrease	ST6GALNAC2	ENSG00000070731	19559774;
C024746	decrease	STMN3	ENSG00000197457	19559774;
C024746	decrease	TFF3	ENSG00000160180	19559774;
C024746	decrease	TGM2	ENSG00000198959	19559774;
C024746	decrease	TMC4	ENSG00000167608	19559774;
C024746	decrease	TRIM29	ENSG00000137699	19559774;
C024746	decrease	TRIM9	ENSG00000100505	19559774;

C024746	decrease	TRNT1	ENSG00000072756	19559774;
C024746	decrease	VANGL2	ENSG00000162738	19559774;
C024746	decrease	VSTM2L	ENSG00000132821	19559774;
C024746	decrease	ZIC2	ENSG00000043355	19559774;
C024746	decrease	ZIK1	ENSG00000171649	19559774;
C024746	decrease	ZNF22	ENSG00000165512	19559774;
C024746	decrease	ZNF585A	ENSG00000196967	19559774;
C024746	decrease	ZNF585B	ENSG00000245680	19559774;
C024746	decrease	ZNF618	ENSG00000157657	19559774;
C024746	decrease	ZNF79	ENSG00000196152	19559774;
C024746	increase	ARPM1	ENSG00000184378	19559774;
C024746	increase	B3GALNT1	ENSG00000169255	19559774;
C024746	increase	C5ORF25	ENSG00000170085	19559774;
C024746	increase	C9ORF103	ENSG00000148057	19559774;
C024746	increase	CSF3	ENSG00000108342	19559774;
C024746	increase	CXCL1	ENSG00000163739	19559774;
C024746	increase	EIF5A2	ENSG00000163577	19559774;
C024746	increase	ESM1	ENSG00000164283	19559774;
C024746	increase	FECH	ENSG00000066926	19559774;
C024746	increase	FRAS1	ENSG00000138759	19559774;
C024746	increase	GLDC	ENSG00000178445	19559774;
C024746	increase	HRAS	ENSG00000174775	10905503;
C024746	increase	IL8	ENSG00000169429	19559774;
C024746	increase	KCNK5	ENSG00000164626	19559774;
C024746	increase	LAMP3	ENSG00000078081	19559774;
C024746	increase	LMLN	ENSG00000185621	19559774;
C024746	increase	MMP3	ENSG00000149968	19559774;
C024746	increase	NEK11	ENSG00000114670	19559774;
C024746	increase	NNT	ENSG00000112992	19559774;
C024746	increase	NTSR1	ENSG00000101188	19559774;
C024746	increase	PI3	ENSG00000124102	19559774;
C024746	increase	PRICKLE1	ENSG00000139174	19559774;
C024746	increase	RNMTL1	ENSG00000171861	19559774;
C024746	increase	RTN1	ENSG00000139970	19559774;
C024746	increase	SCAMP5	ENSG00000198794	19559774;
C024746	increase	SEMA4D	ENSG00000187764	19559774;
C024746	increase	SLC10A7	ENSG00000120519	19559774;
C024746	increase	TFIP11	ENSG00000100109	19559774;
C024746	increase	TNFAIP6	ENSG00000123610	19559774;
C024746	increase	TSPAN18	ENSG00000157570	19559774;
C024746	increase	UCHL1	ENSG00000154277	19559774;
C024808	increase	CYB5A	ENSG00000166347	3689822;
C024989	increase	NDUFB6	ENSG00000165264	19523936;
C024989	increase	NDUFS7	ENSG00000115286	19523936;
C024989	increase	SNCA	ENSG00000145335	15114628;
C025034	affect	DPYD	ENSG00000188641	16646564;16734730;18630517;
C025034	affect	TYMS	ENSG00000176890	16734730;18630517;
C025034	increase	IFNA1	ENSG00000197919	17988526;
C025034	increase	IFNG	ENSG00000111537	15301717;
C025034	increase	TNF	ENSG00000223952	17988526;
C025034	increase	TYMP	ENSG00000025708	15864645;16457948;15301717;18630517;17988526;
C025160	decrease	CCND1	ENSG00000110092	15670817;

C025160	decrease	CTNNB1	ENSG00000168036	15670817;
C025160	decrease	ID2	ENSG00000115738	15670817;
C025160	decrease	JUN	ENSG00000177606	15670817;
C025160	decrease	MYC	ENSG00000136997	15670817;
C025160	increase	BGLAP	ENSG00000242252	15670817;
C025160	increase	CDKN1A	ENSG00000124762	15670817;
C025160	increase	INS	ENSG00000129965	16505238;
C025160	increase	SPP1	ENSG00000118785	15670817;
C025256	decrease	ARHGDIA	ENSG00000141522	15159206;
C025256	decrease	ENO2	ENSG00000111674	15159206;
C025256	decrease	IGFBP5	ENSG00000115461	15159206;
C025256	decrease	SELENBP1	ENSG00000143416	15159206;
C025256	decrease	SLC12A2	ENSG00000064651	15159206;
C025256	increase	AR	ENSG00000169083	15159206;
C025256	increase	EGR3	ENSG00000179388	15159206;
C025256	increase	GSTA2	ENSG00000244067	16054331;
C025256	increase	LGALS3	ENSG00000131981	16054331;
C025256	increase	PGR	ENSG00000082175	14579009;18262749;
C025256	increase	PRDX1	ENSG00000117450	16054331;
C025256	increase	S100P	ENSG00000163993	15159206;
C025256	increase	SLC7A11	ENSG00000151012	15159206;
C025256	increase	WARS	ENSG00000140105	15159206;
C025292	increase	ACAN	ENSG00000157766	18321735;
C025292	increase	COL1A1	ENSG00000108821	18321735;
C025292	increase	COL2A1	ENSG00000139219	18321735;
C025292	increase	IL1B	ENSG00000125538	18321735;
C025299	decrease	ABCC1	ENSG00000103222	15465739;
C025299	decrease	APOB	ENSG00000084674	16046716;
C025299	decrease	APOE	ENSG00000130203	16046716;
C025299	decrease	ARHGAP4	ENSG00000089820	15465739;
C025299	decrease	BCR	ENSG00000186716	15465739;
C025299	decrease	BRCC3	ENSG00000185515	15465739;
C025299	decrease	CD79A	ENSG00000105369	15465739;
C025299	decrease	CEBPG	ENSG00000153879	15465739;
C025299	decrease	CYBA	ENSG00000051523	19450372;
C025299	decrease	CYBB	ENSG00000165168	19450372;
C025299	decrease	FLT3LG	ENSG00000090554	15465739;
C025299	decrease	FTH1	ENSG00000167996	15465739;
C025299	decrease	GSTP1	ENSG00000084207	15465739;
C025299	decrease	HSPD1	ENSG00000144381	15465739;
C025299	decrease	IL6	ENSG00000136244	16702388;
C025299	decrease	IRF4	ENSG00000137265	15465739;
C025299	decrease	IVL	ENSG00000163207	10769631;
C025299	decrease	MAP2K1	ENSG00000169032	15465739;
C025299	decrease	NCF1	ENSG00000158517	19450372;
C025299	decrease	NPM1	ENSG00000181163	15465739;
C025299	decrease	PARP1	ENSG00000143799	15465739;
C025299	decrease	RAC1	ENSG00000136238	15465739;
C025299	decrease	RPL6	ENSG00000089009	15465739;
C025299	decrease	STAT1	ENSG00000115415	15465739;
C025299	decrease	TCF3	ENSG00000071564	15465739;
C025299	decrease	TK1	ENSG00000167900	15465739;
C025299	decrease	TNF	ENSG00000223952	16702388;16046716;
C025299	decrease	TOP1	ENSG00000198900	15465739;

C025299	decrease	TOP2A	ENSG00000131747	15465739;
C025299	decrease	XCL1	ENSG00000143184	15465739;
C025299	decrease	XRCC1	ENSG00000073050	15465739;
C025299	increase	BAT2	ENSG00000204469	15465739;
C025299	increase	BSG	ENSG00000172270	15465739;
C025299	increase	CD81	ENSG00000110651	15465739;
C025299	increase	CSF2RB	ENSG00000100368	15465739;
C025299	increase	CYP1A1	ENSG00000140465	17177569;
C025299	increase	ETS1	ENSG00000134954	15465739;
C025299	increase	GRN	ENSG00000030582	15465739;
C025299	increase	HCFC1	ENSG00000172534	15465739;
C025299	increase	HOXD3	ENSG00000128652	15465739;
C025299	increase	ICAM1	ENSG00000090339	15465739;
C025299	increase	IL10	ENSG00000136634	15465739;
C025299	increase	IL2RA	ENSG00000134460	15465739;
C025299	increase	IL4	ENSG00000113520	15465739;
C025299	increase	IRAK1	ENSG00000184216	15465739;
C025299	increase	IRF2	ENSG00000168310	15465739;
C025299	increase	ITGA2B	ENSG00000005961	15465739;
C025299	increase	ITGAL	ENSG00000005844	15465739;
C025299	increase	MIF	ENSG00000240972	15465739;
C025299	increase	MLF2	ENSG00000089693	15465739;
C025299	increase	NUP214	ENSG00000126883	15465739;
C025299	increase	PBX2	ENSG00000236353	15465739;
C025299	increase	POU3F2	ENSG00000184486	15465739;
C025299	increase	SELL	ENSG00000188404	15465739;
C025299	increase	SLC20A1	ENSG00000144136	15465739;
C025299	increase	STAT6	ENSG00000166888	15465739;
C025299	increase	TCF7	ENSG00000081059	15465739;
C025299	increase	TNK2	ENSG00000061938	15465739;
C025299	increase	XCR1	ENSG00000173578	15465739;
C025299	increase	ZNF593	ENSG00000142684	15465739;
C025314	increase	HIF1A	ENSG00000100644	17364964;
C025314	increase	IL8	ENSG00000169429	17097691;
C025314	increase	TFRC	ENSG00000072274	17364964;
C025340	decrease	ACO2	ENSG00000100412	16625280;
C025340	increase	CD86	ENSG00000114013	15737199;
C025430	increase	PTPN13	ENSG00000163629	15876410;
C025462	affect	PTGS1	ENSG00000095303	11700559;12203115;11245490;
C025462	decrease	ACTN1	ENSG00000072110	16184548;
C025462	decrease	ADAM9	ENSG00000168615	16184548;
C025462	decrease	ADRA2C	ENSG00000184160	12734198;
C025462	decrease	ALDOC	ENSG00000109107	16184548;
C025462	decrease	APOL1	ENSG00000100342	16184548;
C025462	decrease	AR	ENSG00000169083	14581372;
C025462	decrease	ARL6IP1	ENSG00000170540	12734198;
C025462	decrease	ARPC1B	ENSG00000130429	12734198;
C025462	decrease	ASAH1	ENSG00000104763	16184548;
C025462	decrease	AURKA	ENSG00000087586	12734198;
C025462	decrease	BAD	ENSG00000002330	18678619;17409426;
C025462	decrease	BCAM	ENSG00000187244	12734198;
C025462	decrease	BCL2	ENSG00000171791	18678619;
C025462	decrease	BCL2L1	ENSG00000171552	11912124;

C025462	decrease	BUB1B	ENSG00000156970	16184548;
C025462	decrease	CD46	ENSG00000117335	16184548;
C025462	decrease	CDH17	ENSG00000079112	16184548;
C025462	decrease	CFLAR	ENSG00000003402	11912124;
C025462	decrease	CLP1	ENSG00000172409	12734198;
C025462	decrease	CTSC	ENSG00000109861	16184548;
C025462	decrease	CYB5B	ENSG00000103018	12734198;
C025462	decrease	DDX11	ENSG00000013573	16184548;
C025462	decrease	DHCR24	ENSG00000116133	16184548;
C025462	decrease	DHCR7	ENSG00000172893	16184548;
C025462	decrease	DYNLL1	ENSG00000088986	12734198;
C025462	decrease	ESR1	ENSG00000091831	16740773;
C025462	decrease	ETFB	ENSG00000105379	16184548;
C025462	decrease	FCGRT	ENSG00000104870	16184548;
C025462	decrease	FDPS	ENSG00000160752	16184548;
C025462	decrease	FGFR4	ENSG00000160867	16184548;
C025462	decrease	FOXM1	ENSG00000111206	16184548;
C025462	decrease	GAL3ST4	ENSG00000197093	16184548;
C025462	decrease	GRN	ENSG00000030582	16184548;
C025462	decrease	HDAC1	ENSG00000116478	16184548;
C025462	decrease	HGF	ENSG00000019991	10807402;
C025462	decrease	HSD3B1	ENSG00000203857	16184548;
C025462	decrease	ID1	ENSG00000125968	16184548;
C025462	decrease	IFITM1	ENSG00000185885	16184548;
C025462	decrease	IFITM3	ENSG00000142089	16184548;
C025462	decrease	IL13RA1	ENSG00000131724	16184548;
C025462	decrease	ILF3	ENSG00000129351	16184548;
C025462	decrease	INSIG1	ENSG00000186480	12734198;
C025462	decrease	ITPR1	ENSG00000150995	15767558;
C025462	decrease	KALRN	ENSG00000160145	16184548;
C025462	decrease	KLK3	ENSG00000142515	14581372;
C025462	decrease	KRT19	ENSG00000171345	12734198;
C025462	decrease	KRT5	ENSG00000186081	12734198;
C025462	decrease	LAMP1	ENSG00000185896	16184548;
C025462	decrease	LDHA	ENSG00000134333	12734198;
C025462	decrease	LDLR	ENSG00000130164	16184548;
C025462	decrease	MAD2L1	ENSG00000164109	12734198;
C025462	decrease	MAL	ENSG00000172005	16184548;
C025462	decrease	MAP3K1	ENSG00000095015	12734198;
C025462	decrease	MCM2	ENSG00000073111	16184548;
C025462	decrease	MSX1	ENSG00000163132	12734198;
C025462	decrease	MUC1	ENSG00000185499	16184548;
C025462	decrease	MX1	ENSG00000157601	16184548;
C025462	decrease	NCAPD3	ENSG00000151503	16184548;
C025462	decrease	PARP1	ENSG00000143799	16184548;18678619;12663503;
C025462	decrease	PCSK9	ENSG00000169174	16184548;
C025462	decrease	PGAM1	ENSG00000171314	12734198;
C025462	decrease	PGR	ENSG00000082175	16740773;
C025462	decrease	PKM2	ENSG00000067225	16184548;
C025462	decrease	PLAU	ENSG00000122861	15767558;
C025462	decrease	POLD3	ENSG00000077514	16184548;
C025462	decrease	PPP2R1A	ENSG00000105568	12734198;
C025462	decrease	PPT1	ENSG00000131238	16184548;

C025462	decrease	PRKCSH	ENSG00000130175	16184548;
C025462	decrease	PRRX1	ENSG00000116132	16184548;
C025462	decrease	PRSS23	ENSG00000150687	12734198;
C025462	decrease	PSG6	ENSG00000170848	12734198;
C025462	decrease	PSG8	ENSG00000124467	12734198;
C025462	decrease	PSMC2	ENSG00000161057	16184548;
C025462	decrease	PTPRF	ENSG00000142949	16184548;
C025462	decrease	RECQL4	ENSG00000160957	16184548;
C025462	decrease	REG1A	ENSG00000115386	16184548;
C025462	decrease	RERE	ENSG00000142599	12734198;
C025462	decrease	RRM2	ENSG00000171848	12734198;
C025462	decrease	SC4MOL	ENSG00000052802	12734198;
C025462	decrease	SERPINB5	ENSG00000206075	15897233;
C025462	decrease	SKP2	ENSG00000145604	16184548;
C025462	decrease	SLC25A1	ENSG00000100075	16184548;
C025462	decrease	SPTBN1	ENSG00000115306	12734198;
C025462	decrease	SREBF2	ENSG00000198911	16184548;
C025462	decrease	STAT3	ENSG00000168610	17566705;
C025462	decrease	TFF3	ENSG00000160180	16184548;
C025462	decrease	TK1	ENSG00000167900	16184548;
C025462	decrease	TMPO	ENSG00000120802	16184548;
C025462	decrease	TMSB4X	ENSG00000205542	15292456;
C025462	decrease	TSPAN8	ENSG00000127324	16184548;
C025462	decrease	TUBA4A	ENSG00000127824	12734198;
C025462	decrease	UBA7	ENSG00000182179	16184548;
C025462	decrease	YWHAE	ENSG00000108953	17409426;18678619;
C025462	increase	ABCG2	ENSG00000118777	16184548;
C025462	increase	ADM	ENSG00000148926	16184548;
C025462	increase	AGXT2L1	ENSG00000164089	12734198;
C025462	increase	AKAP12	ENSG00000131016	12734198;
C025462	increase	AKR1C1	ENSG00000187134	16184548;
C025462	increase	ALOX15	ENSG00000161905	16166323;
C025462	increase	ANXA5	ENSG00000164111	18678619;
C025462	increase	APOA1	ENSG00000118137	19013290;
C025462	increase	AREG	ENSG00000205595	16184548;
C025462	increase	ATF3	ENSG00000162772	15897233;16079301;16184548;12734198;
C025462	increase	ATP6V0A1	ENSG00000033627	16184548;
C025462	increase	BIRC3	ENSG00000023445	16184548;
C025462	increase	CACNG3	ENSG00000006116	16184548;
C025462	increase	CCAR1	ENSG00000060339	12734198;
C025462	increase	CCND1	ENSG00000110092	14710233;15767558;15188006;
C025462	increase	CD4	ENSG00000010610	12734198;
C025462	increase	CDH1	ENSG00000039068	12391285;
C025462	increase	CDKN1A	ENSG00000124762	16091736;16184548;15767558;
C025462	increase	CEBPB	ENSG00000172216	16184548;12734198;
C025462	increase	CRYAB	ENSG00000109846	16184548;
C025462	increase	CTGF	ENSG00000118523	12734198;16184548;
C025462	increase	CTNNB1	ENSG00000168036	14555707;15188006;14710233;
C025462	increase	CYP1A1	ENSG00000140465	16184548;17985343;12734198;
C025462	increase	CYP1A2	ENSG00000140505	17985343;

C025462	increase	CYP1B1	ENSG00000138061	17985343;12734198;
C025462	increase	DCBLD2	ENSG00000057019	12734198;
C025462	increase	EGR1	ENSG00000120738	16227405;16079301;15509713;17599376;
C025462	increase	ELK1	ENSG00000126767	17599376;
C025462	increase	ESD	ENSG00000139684	12734198;
C025462	increase	FABP1	ENSG00000163586	17602619;18665581;
C025462	increase	FABP4	ENSG00000170323	17602619;18665581;
C025462	increase	FAM129A	ENSG00000135842	12734198;
C025462	increase	GARS	ENSG00000106105	12734198;
C025462	increase	GDF15	ENSG00000130513	15509713;15767558;12734198;11925476;15180942;15555568;
C025462	increase	GK	ENSG00000198814	16184548;
C025462	increase	GLCCI1	ENSG00000106415	16184548;
C025462	increase	HSPA1B	ENSG00000232804	16184548;
C025462	increase	HSPA6	ENSG00000173110	16184548;
C025462	increase	ICAM2	ENSG00000108622	16184548;
C025462	increase	IFRD1	ENSG00000006652	12734198;
C025462	increase	IL1R1	ENSG00000115594	16184548;
C025462	increase	IL8	ENSG00000169429	16184548;
C025462	increase	ISG20	ENSG00000172183	16184548;
C025462	increase	JAG2	ENSG00000184916	16184548;
C025462	increase	JUN	ENSG00000177606	16704987;
C025462	increase	KLHL21	ENSG00000162413	16184548;
C025462	increase	KRT14	ENSG00000186847	16184548;
C025462	increase	KRT16	ENSG00000186832	16184548;
C025462	increase	KRT17	ENSG00000128422	16184548;
C025462	increase	LAMC1	ENSG00000135862	15561105;16184548;
C025462	increase	MAGI1	ENSG00000151276	12734198;
C025462	increase	MAN2B2	ENSG00000013288	12734198;
C025462	increase	MAP2K1	ENSG00000169032	15548677;16079301;
C025462	increase	MAPK10	ENSG00000109339	12734198;
C025462	increase	MAPK1	ENSG00000100030	15548677;17599376;16079301;18261746;
C025462	increase	MAPK3	ENSG00000102882	15548677;17599376;16079301;18261746;
C025462	increase	MARS2	ENSG00000152428	16184548;
C025462	increase	MET	ENSG00000105976	14710233;
C025462	increase	MMP10	ENSG00000166670	16184548;
C025462	increase	MMP1	ENSG00000196611	15767558;16184548;
C025462	increase	MRAS	ENSG00000158186	16184548;
C025462	increase	MT1F	ENSG00000198417	16184548;
C025462	increase	MTFR1	ENSG00000066855	16184548;
C025462	increase	MTHFD2	ENSG00000065911	12734198;
C025462	increase	MYH10	ENSG00000133026	12734198;
C025462	increase	NQO1	ENSG00000181019	17985343;
C025462	increase	NUP133	ENSG00000069248	12734198;
C025462	increase	ODC1	ENSG00000115758	16184548;
C025462	increase	PAWR	ENSG00000177425	10833474;
C025462	increase	PMAIP1	ENSG00000141682	16184548;
C025462	increase	PPARA	ENSG00000186951	11948457;
C025462	increase	PPARD	ENSG00000112033	17409426;16091736;10555149;18678619;11948457;

C025462	increase	PPARG	ENSG00000132170	18665581;16091736;10866999;17602619;11948457;
C025462	increase	PREP	ENSG00000085377	12734198;
C025462	increase	PRKACA	ENSG00000072062	16184548;
C025462	increase	PTGS2	ENSG00000073756	11700559;12203115;12414664;10866999;12208739;11245490;11948457;1048548
C025462	increase	RANBP1	ENSG00000099901	12734198;
C025462	increase	RAP1GAP	ENSG00000076864	12734198;
C025462	increase	RGS16	ENSG00000143333	16184548;
C025462	increase	RHOA	ENSG00000067560	18261746;
C025462	increase	RRS1	ENSG00000179041	12734198;
C025462	increase	S100P	ENSG00000163993	16184548;
C025462	increase	SERPINE1	ENSG00000106366	15897233;
C025462	increase	SLC25A45	ENSG00000162241	12734198;
C025462	increase	SLC3A2	ENSG00000168003	16184548;
C025462	increase	SPRR1B	ENSG00000169469	16184548;
C025462	increase	SQSTM1	ENSG00000161011	16184548;
C025462	increase	STC2	ENSG00000113739	12734198;
C025462	increase	STK17B	ENSG00000081320	16184548;
C025462	increase	SYDE1	ENSG00000105137	12734198;
C025462	increase	TFAP2B	ENSG00000008196	16184548;
C025462	increase	THBS1	ENSG00000137801	16227405;18261746;
C025462	increase	TMEM49	ENSG00000062716	12734198;
C025462	increase	TNF	ENSG00000223952	10866999;
C025462	increase	TNFRSF10A	ENSG00000104689	15180942;
C025462	increase	TNFRSF10B	ENSG00000120889	12203115;12208739;11559570;15180942;
C025462	increase	TRIP4	ENSG00000103671	16184548;
C025462	increase	UBE2D1	ENSG00000072401	16184548;
C025462	increase	UGT1A1	ENSG00000241635	17985343;
C025462	increase	UPP1	ENSG00000183696	16184548;
C025462	increase	XRCC6	ENSG00000196419	12734198;
C025463	decrease	AR	ENSG00000169083	14581372;
C025463	decrease	CCND1	ENSG00000110092	12392815;15188006;
C025463	decrease	ESR1	ENSG00000091831	16740773;
C025463	decrease	KLK3	ENSG00000142515	14581372;11490238;
C025463	decrease	MMP7	ENSG00000137673	15725655;
C025463	decrease	NFKBIA	ENSG00000100906	18059344;
C025463	decrease	PGR	ENSG00000082175	16740773;
C025463	decrease	YWHAE	ENSG00000108953	17409426;
C025463	increase	ALOX15	ENSG00000161905	16166323;11861401;
C025463	increase	CDKN1A	ENSG00000124762	18593937;
C025463	increase	CDKN1B	ENSG00000111276	18593937;
C025463	increase	CTNNB1	ENSG00000168036	14555707;12392815;10910034;11602670;
C025463	increase	GATA6	ENSG00000141448	11861401;
C025463	increase	HINT1	ENSG00000169567	18593937;
C025463	increase	IL24	ENSG00000162892	15713900;
C025463	increase	NGFR	ENSG00000064300	17447067;
C025463	increase	NQO1	ENSG00000181019	17985343;
C025463	increase	PPARA	ENSG00000186951	11948457;
C025463	increase	PPARD	ENSG00000112033	11948457;
C025463	increase	PPARG	ENSG00000132170	14506281;11948457;
C025463	increase	PRKG1	ENSG00000185532	16731751;11602670;

C025463	increase	PTGS2	ENSG00000073756	14506281;11948457;
C025463	increase	SAT1	ENSG00000130066	14506281;16262603;
C025463	increase	TNFRSF10B	ENSG00000120889	11559570;
C025463	increase	UGT1A1	ENSG00000241635	15843492;17985343;
C025473	decrease	BCL2	ENSG00000171791	14612938;
C025473	decrease	BIRC2	ENSG00000110330	14612938;
C025473	increase	BAX	ENSG00000087088	14612938;
C025566	affect	CD14	ENSG00000170458	14499251;
C025566	affect	HRH2	ENSG00000113749	14499251;
C025566	decrease	CCL2	ENSG00000108691	17507084;
C025589	increase	IL1B	ENSG00000125538	18308354;
C025589	increase	IL8	ENSG00000169429	18308354;
C025603	increase	MMP9	ENSG00000100985	19371603;
C025643	increase	CYP19A1	ENSG00000137869	12127262;
C025643	increase	CYP1A1	ENSG00000140465	15979257;
C025643	increase	PGR	ENSG00000082175	14579009;16750840;18262749;
C025946	decrease	HIF1A	ENSG00000100644	18927491;
C026105	decrease	CCND1	ENSG00000110092	16987298;
C026105	decrease	CCNE1	ENSG00000105173	16987298;
C026105	decrease	CDK2	ENSG00000123374	16987298;
C026105	decrease	CDK4	ENSG00000135446	16987298;
C026105	decrease	CDK6	ENSG00000105810	16987298;
C026105	decrease	MKI67	ENSG00000148773	16987298;
C026105	increase	AIFM1	ENSG00000156709	16987298;
C026105	increase	BAX	ENSG00000087088	16987298;
C026105	increase	CDKN1A	ENSG00000124762	16987298;
C026105	increase	CDKN2A	ENSG00000147889	16987298;
C026105	increase	FAS	ENSG00000026103	16987298;
C026105	increase	TP53	ENSG00000141510	16987298;
C026209	decrease	IVL	ENSG00000163207	10769631;
C026209	increase	CYP1A1	ENSG00000140465	15890477;
C026209	increase	CYP1B1	ENSG00000138061	15890477;
C026209	increase	GSTP1	ENSG00000084207	16950796;
C026209	increase	MGMT	ENSG00000170430	16950796;
C026609	decrease	BCL2	ENSG00000171791	17619073;
C026609	decrease	BCL2L1	ENSG00000171552	17619073;
C026609	increase	BAX	ENSG00000087088	17619073;
C026609	increase	FADD	ENSG00000168040	17619073;
C026609	increase	FAS	ENSG00000026103	17619073;
C026609	increase	TNFRSF1A	ENSG00000067182	17619073;
C026609	increase	TRADD	ENSG00000102871	17619073;
C026702	increase	PRDX4	ENSG00000123131	18272409;
C026777	increase	MAPK1	ENSG00000100030	11675405;
C026777	increase	MAPK3	ENSG00000102882	11675405;
C026777	increase	TNF	ENSG00000223952	11675405;
C027005	increase	IL1B	ENSG00000125538	12573452;
C027005	increase	PTGS2	ENSG00000073756	12573452;
C027185	decrease	IFNG	ENSG00000111537	11134660;
C027185	increase	CSF3R	ENSG00000119535	16115123;
C027185	increase	ITGB2	ENSG00000160255	16115123;
C027185	increase	NCF1	ENSG00000158517	16115123;
C027235	increase	BAX	ENSG00000087088	17121930;
C027309	increase	CYP11B1	ENSG00000160882	16396990;

C027309	increase	CYP11B2	ENSG00000179142	16396990;
C027385	decrease	ICAM1	ENSG00000090339	17882670;
C027385	decrease	SELE	ENSG00000007908	17882670;
C027385	decrease	VCAM1	ENSG00000162692	17882670;
C027385	increase	CRP	ENSG00000132693	17882670;
C027385	increase	F3	ENSG00000117525	16011840;
C027385	increase	SERPINE1	ENSG00000106366	16011840;
C027478	decrease	IL12RB2	ENSG00000081985	12047756;12864811;
C027478	increase	CD80	ENSG00000121594	12864811;
C027478	increase	CD86	ENSG00000114013	12864811;
C027478	increase	CTLA4	ENSG00000163599	12864811;
C027561	increase	SLC5A5	ENSG00000105641	16257484;
C027576	decrease	ADORA1	ENSG00000163485	12419474;
C027576	decrease	AMPH	ENSG00000078053	12419474;
C027576	decrease	APC	ENSG00000134982	12419474;
C027576	decrease	APP	ENSG00000142192	12419474;
C027576	decrease	ARHGAP5	ENSG00000100852	12419474;
C027576	decrease	ARHGDIA	ENSG00000141522	12419474;
C027576	decrease	ATP1B3	ENSG00000069849	12419474;
C027576	decrease	ATR	ENSG00000175054	12419474;
C027576	decrease	BAD	ENSG00000002330	12419474;
C027576	decrease	BCL2L1	ENSG00000171552	12419474;
C027576	decrease	BMP1	ENSG00000168487	12419474;
C027576	decrease	BNIP3	ENSG00000176171	12419474;
C027576	decrease	BRAF	ENSG00000157764	12419474;
C027576	decrease	C6ORF108	ENSG00000112667	12419474;
C027576	decrease	CAPN2	ENSG00000162909	12419474;
C027576	decrease	CAPNS1	ENSG00000126247	12419474;
C027576	decrease	CASK	ENSG00000147044	12419474;
C027576	decrease	CASP8	ENSG00000064012	12419474;
C027576	decrease	CBLB	ENSG00000114423	12419474;
C027576	decrease	CCL3	ENSG00000006075	12419474;
C027576	decrease	CCNA2	ENSG00000145386	12419474;
C027576	decrease	CCND2	ENSG00000118971	15607904;
C027576	decrease	CCNK	ENSG00000090061	12419474;
C027576	decrease	CCNT1	ENSG00000129315	12419474;
C027576	decrease	CCR1	ENSG00000163823	12419474;
C027576	decrease	CD47	ENSG00000196776	12419474;
C027576	decrease	CDC20	ENSG00000117399	12419474;
C027576	decrease	CDC25A	ENSG00000164045	12419474;
C027576	decrease	CDH2	ENSG00000170558	12419474;
C027576	decrease	CDH3	ENSG00000062038	12419474;
C027576	decrease	CDK9	ENSG00000136807	12419474;
C027576	decrease	CDKN1C	ENSG00000129757	12419474;
C027576	decrease	CDKN2D	ENSG00000129355	12419474;
C027576	decrease	CLK3	ENSG00000179335	12419474;
C027576	decrease	COMT	ENSG00000093010	12419474;
C027576	decrease	CPE	ENSG00000109472	12419474;
C027576	decrease	CREB1	ENSG00000118260	12419474;16337876;
C027576	decrease	CRYZ	ENSG00000116791	12419474;
C027576	decrease	CSDA	ENSG00000060138	12419474;
C027576	decrease	CSNK1G2	ENSG00000133275	12419474;
C027576	decrease	CTNNB1	ENSG00000168036	12419474;
C027576	decrease	CTSL1	ENSG00000135047	12419474;

C027576	decrease	CTTN	ENSG00000085733	12419474;
C027576	decrease	CUX1	ENSG00000160967	12419474;
C027576	decrease	CXCL5	ENSG00000163735	12419474;
C027576	decrease	CYP1B1	ENSG00000138061	12419474;
C027576	decrease	DAPK1	ENSG00000196730	12419474;
C027576	decrease	DDR2	ENSG00000162733	12419474;
C027576	decrease	DFFA	ENSG00000160049	12419474;
C027576	decrease	DFFB	ENSG00000169598	12419474;
C027576	decrease	DNAJC3	ENSG00000102580	12419474;
C027576	decrease	DST	ENSG00000151914	12419474;
C027576	decrease	E2F1	ENSG00000101412	12419474;
C027576	decrease	EFNA4	ENSG00000243364	12419474;
C027576	decrease	ELK1	ENSG00000126767	12419474;
C027576	decrease	EPS15	ENSG00000085832	12419474;
C027576	decrease	EPS8	ENSG00000151491	12419474;
C027576	decrease	ERBB2	ENSG00000141736	12419474;
C027576	decrease	ERBB4	ENSG00000178568	12419474;
C027576	decrease	ERCC2	ENSG00000104884	12419474;
C027576	decrease	ERCC5	ENSG00000134899	12419474;
C027576	decrease	ETS1	ENSG00000134954	12419474;
C027576	decrease	ETS2	ENSG00000157557	12419474;
C027576	decrease	ETV3	ENSG00000117036	12419474;
C027576	decrease	ETV6	ENSG00000139083	12419474;
C027576	decrease	EZR	ENSG00000092820	12419474;
C027576	decrease	F2R	ENSG00000181104	12419474;
C027576	decrease	FASTK	ENSG00000164896	12419474;
C027576	decrease	FECH	ENSG00000066926	12419474;
C027576	decrease	FGFR1	ENSG00000249195	12419474;
C027576	decrease	FOS	ENSG00000170345	12419474;
C027576	decrease	FUBP1	ENSG00000162613	12419474;
C027576	decrease	GAK	ENSG00000178950	12419474;
C027576	decrease	GATA3	ENSG00000107485	12419474;
C027576	decrease	GNAS	ENSG00000087460	12419474;
C027576	decrease	GNB1	ENSG00000078369	12419474;
C027576	decrease	GNB2	ENSG00000172354	12419474;
C027576	decrease	GSK3B	ENSG00000082701	12419474;
C027576	decrease	GTF2B	ENSG00000137947	12419474;
C027576	decrease	HBEGF	ENSG00000113070	12419474;
C027576	decrease	HGF	ENSG00000019991	12419474;
C027576	decrease	HINT1	ENSG00000169567	12419474;
C027576	decrease	HIVEP2	ENSG00000010818	12419474;
C027576	decrease	HMGA1	ENSG00000137309	12419474;
C027576	decrease	HNF4A	ENSG00000101076	12419474;
C027576	decrease	HSPA2	ENSG00000126803	12419474;
C027576	decrease	ID1	ENSG00000125968	12419474;
C027576	decrease	IER3	ENSG00000137331	12419474;
C027576	decrease	IGF1R	ENSG00000140443	12419474;
C027576	decrease	IKBKAP	ENSG00000070061	12419474;
C027576	decrease	IL12RB1	ENSG00000096996	12419474;
C027576	decrease	IL17A	ENSG00000112115	12419474;
C027576	decrease	IL18	ENSG00000150782	12419474;
C027576	decrease	IL3	ENSG00000164399	12419474;
C027576	decrease	IL6R	ENSG00000160712	12419474;
C027576	decrease	IL6ST	ENSG00000134352	12419474;

C027576	decrease	IL7R	ENSG00000168685	12419474;
C027576	decrease	IL9	ENSG00000145839	12419474;
C027576	decrease	ITGA5	ENSG00000161638	12419474;
C027576	decrease	ITGA6	ENSG00000091409	12419474;
C027576	decrease	ITGA7	ENSG00000135424	12419474;
C027576	decrease	ITGB6	ENSG00000115221	12419474;
C027576	decrease	ITSN1	ENSG00000205726	12419474;
C027576	decrease	JUND	ENSG00000130522	12419474;
C027576	decrease	JUN	ENSG00000177606	12419474;11488593;16337876;
C027576	decrease	LAT	ENSG00000213658	12419474;
C027576	decrease	LCAT	ENSG00000213398	12419474;
C027576	decrease	LCK	ENSG00000182866	12419474;
C027576	decrease	LYN	ENSG00000249529	12419474;
C027576	decrease	MAP2K5	ENSG00000137764	12419474;
C027576	decrease	MARCKSL1	ENSG00000175130	12419474;
C027576	decrease	MARK3	ENSG00000075413	12419474;
C027576	decrease	MCM4	ENSG00000104738	12419474;
C027576	decrease	MLH1	ENSG00000076242	12419474;
C027576	decrease	MNDA	ENSG00000163563	12419474;
C027576	decrease	MYBL2	ENSG00000101057	12419474;
C027576	decrease	MYC	ENSG00000136997	12419474;
C027576	decrease	MYLK	ENSG00000251351	12419474;
C027576	decrease	NBN	ENSG00000104320	12419474;
C027576	decrease	NCK1	ENSG00000158092	12419474;
C027576	decrease	NEO1	ENSG00000067141	12419474;
C027576	decrease	NF2	ENSG00000186575	12419474;
C027576	decrease	NFATC1	ENSG00000131196	12419474;
C027576	decrease	NFE2L1	ENSG00000082641	12419474;
C027576	decrease	NFIX	ENSG00000008441	12419474;
C027576	decrease	NFKB1	ENSG00000109320	12419474;
C027576	decrease	NFRKB	ENSG00000170322	12419474;
C027576	decrease	NFX1	ENSG00000086102	12419474;
C027576	decrease	NMB	ENSG00000197696	12419474;
C027576	decrease	NME2	ENSG00000243678	12419474;
C027576	decrease	NR1I3	ENSG00000143257	12419474;
C027576	decrease	NR2F6	ENSG00000160113	12419474;
C027576	decrease	P2RX5	ENSG00000083454	12419474;
C027576	decrease	PAIP1	ENSG00000172239	12419474;
C027576	decrease	PCSK2	ENSG00000125851	12419474;
C027576	decrease	PDYN	ENSG00000101327	12419474;
C027576	decrease	PHB	ENSG00000167085	12419474;
C027576	decrease	PIK3R2	ENSG00000105647	12419474;
C027576	decrease	POLB	ENSG00000070501	12419474;
C027576	decrease	POLD1	ENSG00000062822	12419474;
C027576	decrease	PPP1CA	ENSG00000172531	12419474;
C027576	decrease	PPP3CA	ENSG00000138814	12419474;
C027576	decrease	PRDM1	ENSG00000057657	12419474;
C027576	decrease	PRKDC	ENSG00000121031	12419474;
C027576	decrease	PTK2	ENSG00000169398	12419474;
C027576	decrease	PTPN1	ENSG00000196396	12419474;
C027576	decrease	PVRL1	ENSG00000110400	12419474;
C027576	decrease	RALGDS	ENSG00000160271	12419474;
C027576	decrease	RARB	ENSG00000077092	12419474;

C027576	decrease	RBL2	ENSG00000103479	12419474;
C027576	decrease	REL	ENSG00000162924	12419474;
C027576	decrease	RPS6KA2	ENSG00000071242	12419474;
C027576	decrease	RXRB	ENSG00000235712	12419474;
C027576	decrease	SATB1	ENSG00000182568	12419474;
C027576	decrease	SELENBP1	ENSG00000143416	12419474;
C027576	decrease	SERPINB5	ENSG00000206075	12419474;
C027576	decrease	SERPINI1	ENSG00000163536	12419474;
C027576	decrease	SHOC2	ENSG00000108061	12419474;
C027576	decrease	SMAD1	ENSG00000170365	12419474;
C027576	decrease	SP2	ENSG00000167182	12419474;
C027576	decrease	SPRY2	ENSG00000136158	12419474;
C027576	decrease	STAT6	ENSG00000166888	12419474;
C027576	decrease	SYP	ENSG00000102003	12419474;
C027576	decrease	TANK	ENSG00000136560	12419474;
C027576	decrease	TCEB1	ENSG00000154582	12419474;
C027576	decrease	TCF3	ENSG00000071564	12419474;
C027576	decrease	TERF1	ENSG00000147601	12419474;
C027576	decrease	TFDP2	ENSG00000114126	12419474;
C027576	decrease	TJP1	ENSG00000104067	12419474;
C027576	decrease	TNFAIP2	ENSG00000185215	12419474;
C027576	decrease	TNFRSF1A	ENSG00000067182	12419474;
C027576	decrease	TOP1	ENSG00000198900	12419474;
C027576	decrease	TOP2A	ENSG00000131747	12419474;
C027576	decrease	TRIM24	ENSG00000122779	12419474;
C027576	decrease	TSC2	ENSG00000103197	12419474;
C027576	decrease	TSG101	ENSG00000074319	12419474;
C027576	decrease	TSPAN31	ENSG00000135452	12419474;
C027576	decrease	UBE2A	ENSG00000077721	12419474;
C027576	decrease	XPC	ENSG00000154767	12419474;
C027576	decrease	YES1	ENSG00000176105	12419474;
C027576	decrease	YWHAH	ENSG00000128245	12419474;
C027576	decrease	ZFP36	ENSG00000128016	12419474;
C027576	decrease	ZNF91	ENSG00000167232	12419474;
C027576	increase	BAX	ENSG00000087088	12419474;15607904;
C027576	increase	CDKN1A	ENSG00000124762	15607904;
C027576	increase	COL1A1	ENSG00000108821	14672615;
C027576	increase	MMP1	ENSG00000196611	14672615;
C027576	increase	MMP2	ENSG00000087245	14672615;
C027576	increase	TIMP1	ENSG00000102265	14672615;
C027576	increase	TP53	ENSG00000141510	15607904;
C027576	increase	TP63	ENSG00000073282	15607904;
C027576	increase	TP73	ENSG00000078900	15607904;
C027579	increase	CD38	ENSG00000004468	15964820;
C027579	increase	CYP27A1	ENSG00000135929	15964820;
C027579	increase	TGM2	ENSG00000198959	15964820;
C027696	decrease	UCP3	ENSG00000175564	12824081;
C027869	decrease	ATP5H	ENSG00000167863	18422750;
C027869	decrease	ECHS1	ENSG00000127884	18422750;
C027869	decrease	EIF5A	ENSG00000132507	18422750;
C027869	decrease	HSP90B1	ENSG00000166598	18422750;
C027869	decrease	KRT19	ENSG00000171345	18422750;
C027869	decrease	LDHB	ENSG00000111716	18422750;
C027869	decrease	PPP2R1A	ENSG00000105568	18422750;

C027869	decrease	PRDX2	ENSG00000167815	18422750;
C027869	decrease	PSMA1	ENSG00000129084	18422750;
C027869	decrease	RANBP1	ENSG00000099901	18422750;
C027869	decrease	SERPINB6	ENSG00000124570	18422750;
C027869	decrease	WARS	ENSG00000140105	18422750;
C027869	decrease	XRCC5	ENSG00000079246	18422750;
C027869	decrease	YWHAB	ENSG00000166913	18422750;
C028009	decrease	CCNB1	ENSG00000134057	16691315;
C028009	decrease	CDK1	ENSG00000170312	16691315;
C028009	increase	ATF3	ENSG00000162772	15897233;
C028009	increase	GDF15	ENSG00000130513	11925476;
C028009	increase	TP53	ENSG00000141510	11925476;
C028358	decrease	CD1A	ENSG00000158477	17475839;
C028358	decrease	CD36	ENSG00000135218	17475839;
C028358	decrease	CD80	ENSG00000121594	17475839;
C028358	increase	CD86	ENSG00000114013	17475839;
C028419	increase	NQO1	ENSG00000181019	9029048;
C028432	decrease	BCL2	ENSG00000171791	15485790;11342237;
C028432	increase	TP53	ENSG00000141510	15485790;11342237;
C028451	increase	CYP11B1	ENSG00000160882	16396990;
C028451	increase	CYP11B2	ENSG00000179142	16396990;
C028474	increase	CYP1A2	ENSG00000140505	15764585;
C028474	increase	NCOA6	ENSG00000198646	15764585;
C028559	increase	CP	ENSG00000047457	17032174;
C028559	increase	FOS	ENSG00000170345	17032174;
C028559	increase	JUN	ENSG00000177606	17032174;
C028577	increase	IL4	ENSG00000113520	12423647;
C029010	increase	NOS3	ENSG00000164867	15740983;
C029108	decrease	MAPT	ENSG00000186868	16930453;
C029108	increase	CDKN1A	ENSG00000124762	9343371;8943236;
C029108	increase	NQO1	ENSG00000181019	9343371;
C029167	increase	AGT	ENSG00000135744	17519148;
C029167	increase	SHBG	ENSG00000129214	17519148;
C029408	decrease	FOS	ENSG00000170345	18003792;
C029415	increase	MAP2K3	ENSG00000034152	15817653;
C029536	increase	GSTP1	ENSG00000084207	16950796;
C029536	increase	MGMT	ENSG00000170430	16950796;
C029569	increase	HMOX1	ENSG00000100292	16959797;
C029728	increase	ABCC3	ENSG00000108846	16314067;
C029728	increase	ANXA1	ENSG00000135046	16314067;
C029728	increase	ATF3	ENSG00000162772	16314067;
C029728	increase	CD86	ENSG00000114013	19033392;
C029728	increase	CDKN1A	ENSG00000124762	16314067;
C029728	increase	DUSP1	ENSG00000120129	16314067;
C029728	increase	FTH1	ENSG00000167996	16314067;
C029728	increase	GCLM	ENSG00000023909	16314067;
C029728	increase	HIF1A	ENSG00000100644	16314067;
C029728	increase	HMOX1	ENSG00000100292	19033392;16314067;
C029728	increase	HTATIP2	ENSG00000109854	16314067;
C029728	increase	ME1	ENSG00000065833	16314067;
C029728	increase	MT1H	ENSG00000205358	16314067;
C029728	increase	MT2A	ENSG00000125148	16314067;
C029728	increase	NFE2L2	ENSG00000116044	19033392;
C029728	increase	NINJ1	ENSG00000131669	16314067;

C029728	increase	NQO1	ENSG00000181019	19033392;16314067;
C029728	increase	PGD	ENSG00000142657	16314067;
C029728	increase	PIR	ENSG00000087842	16314067;
C029728	increase	PMAIP1	ENSG00000141682	16314067;
C029728	increase	PPARG	ENSG00000132170	16314067;
C029728	increase	PTGS1	ENSG00000095303	18571826;
C029728	increase	PTGS2	ENSG00000073756	18571826;
C029728	increase	RIT1	ENSG00000143622	16314067;
C029728	increase	S100P	ENSG00000163993	16314067;
C029728	increase	SI	ENSG00000090402	16314067;
C029728	increase	SPTA1	ENSG00000163554	16314067;
C029790	affect	CDKN1A	ENSG00000124762	12473173;
C029790	increase	BRCA1	ENSG00000012048	12473173;
C029876	increase	TP53	ENSG00000141510	17690521;15748509;
C029892	decrease	AR	ENSG00000169083	19789329;
C029892	decrease	CCS	ENSG00000173992	16531609;
C029892	decrease	MCM2	ENSG00000073111	19789329;
C029892	decrease	MCM5	ENSG00000100297	19789329;
C029892	increase	MT1B	ENSG00000169688	19789329;
C029892	increase	MT1F	ENSG00000198417	19789329;
C029892	increase	MT1G	ENSG00000125144	19789329;
C029892	increase	MT1X	ENSG00000187193	19789329;
C029892	increase	MT2A	ENSG00000125148	19789329;17409708;
C029938	decrease	ABCA6	ENSG00000154262	16780908;
C029938	decrease	ACO1	ENSG00000122729	16780908;
C029938	decrease	ADCY7	ENSG00000121281	16780908;
C029938	decrease	ALOX5AP	ENSG00000132965	16780908;
C029938	decrease	AQP3	ENSG00000165272	16780908;
C029938	decrease	ASAH1	ENSG00000104763	16780908;
C029938	decrease	ASGR1	ENSG00000141505	16780908;
C029938	decrease	ASGR2	ENSG00000161944	16780908;
C029938	decrease	B3GAT1	ENSG00000109956	16780908;
C029938	decrease	C14ORF118	ENSG00000089916	16780908;
C029938	decrease	C1QB	ENSG00000173369	16780908;
C029938	decrease	C1QC	ENSG00000159189	16780908;
C029938	decrease	C5ORF13	ENSG00000134986	16780908;
C029938	decrease	CA2	ENSG00000104267	16780908;
C029938	decrease	CANX	ENSG00000127022	16780908;
C029938	decrease	CCL13	ENSG00000181374	16780908;
C029938	decrease	CD36	ENSG00000135218	16780908;
C029938	decrease	CDH1	ENSG00000039068	16780908;
C029938	decrease	CEPT1	ENSG00000134255	16780908;
C029938	decrease	CLTB	ENSG00000175416	16780908;
C029938	decrease	CTSC	ENSG00000109861	16780908;
C029938	decrease	DKC1	ENSG00000130826	16780908;
C029938	decrease	DPP4	ENSG00000197635	16780908;
C029938	decrease	ENC1	ENSG00000171617	16780908;
C029938	decrease	EXOC6	ENSG00000138190	16780908;
C029938	decrease	F13A1	ENSG00000124491	16780908;
C029938	decrease	FGL2	ENSG00000127951	16780908;
C029938	decrease	FKBP1B	ENSG00000119782	16780908;
C029938	decrease	FUCA1	ENSG00000179163	16780908;
C029938	decrease	GATM	ENSG00000171766	16780908;
C029938	decrease	GFRA2	ENSG00000168546	16780908;

C029938	decrease	HERC4	ENSG00000148634	16780908;
C029938	decrease	HLA-DMA	ENSG00000239463	16780908;
C029938	decrease	IPCEF1	ENSG00000074706	16780908;
C029938	decrease	KCNJ10	ENSG00000177807	16780908;
C029938	decrease	KCNJ2	ENSG00000123700	16780908;
C029938	decrease	KCTD15	ENSG00000153885	16780908;
C029938	decrease	KLF4	ENSG00000136826	16780908;
C029938	decrease	KRR1	ENSG00000111615	16780908;
C029938	decrease	LGALS8	ENSG00000116977	16780908;
C029938	decrease	LINS1	ENSG00000140471	16780908;
C029938	decrease	MAF	ENSG00000178573	16780908;
C029938	decrease	MPZL1	ENSG00000197965	16780908;
C029938	decrease	MS4A6A	ENSG00000110077	16780908;
C029938	decrease	MYCL1	ENSG00000116990	16780908;
C029938	decrease	MYH10	ENSG00000133026	16780908;
C029938	decrease	NCOA4	ENSG00000138293	16780908;
C029938	decrease	NPL	ENSG00000135838	16780908;
C029938	decrease	NT5C3	ENSG00000122643	16780908;
C029938	decrease	OXCT1	ENSG00000083720	16780908;
C029938	decrease	PLAUR	ENSG00000011422	16780908;
C029938	decrease	PTGER3	ENSG00000050628	16780908;
C029938	decrease	RAB32	ENSG00000118508	16780908;
C029938	decrease	RBM17	ENSG00000134453	16780908;
C029938	decrease	RCVRN	ENSG00000109047	16780908;
C029938	decrease	RHPN2	ENSG00000131941	16780908;
C029938	decrease	RNASE6	ENSG00000169413	16780908;
C029938	decrease	SACM1L	ENSG00000211456	16780908;
C029938	decrease	SLAMF8	ENSG00000158714	16780908;
C029938	decrease	TAF1B	ENSG00000115750	16780908;
C029938	decrease	TCF4	ENSG00000196628	16780908;
C029938	decrease	TFE3	ENSG00000068323	16780908;
C029938	decrease	TOX	ENSG00000198846	16780908;
C029938	decrease	TRIM22	ENSG00000132274	16780908;
C029938	decrease	TRIM32	ENSG00000119401	16780908;
C029938	decrease	TSPAN7	ENSG00000156298	16780908;
C029938	decrease	USP13	ENSG00000058056	16780908;
C029938	decrease	ZFP36L1	ENSG00000185650	16780908;
C029938	decrease	ZFYVE26	ENSG00000072121	16780908;
C029938	increase	ABCC1	ENSG00000103222	16780908;
C029938	increase	ABCC3	ENSG00000108846	16314067;
C029938	increase	ABP1	ENSG00000002726	16780908;
C029938	increase	ACOT2	ENSG00000119673	16780908;
C029938	increase	ACOX2	ENSG00000168306	16314067;
C029938	increase	ACTG2	ENSG00000163017	16780908;
C029938	increase	ADORA2A	ENSG00000128271	16780908;
C029938	increase	ADORA2B	ENSG00000170425	16314067;
C029938	increase	ALCAM	ENSG00000170017	16780908;
C029938	increase	ANTXR2	ENSG00000163297	16780908;
C029938	increase	ANXA1	ENSG00000135046	16314067;
C029938	increase	ANXA2	ENSG00000182718	16314067;
C029938	increase	APOE	ENSG00000130203	16780908;
C029938	increase	ARG2	ENSG00000081181	16314067;
C029938	increase	ARPC2	ENSG00000163466	16780908;
C029938	increase	ATF3	ENSG00000162772	16314067;

C029938	increase	ATP1B3	ENSG00000069849	16780908;
C029938	increase	BCL2A1	ENSG00000140379	16780908;
C029938	increase	BID	ENSG00000015475	16780908;
C029938	increase	BIRC2	ENSG00000110330	16780908;
C029938	increase	BIRC3	ENSG00000023445	16780908;
C029938	increase	BMP6	ENSG00000153162	16780908;
C029938	increase	BTG1	ENSG00000133639	16780908;
C029938	increase	C10ORF10	ENSG00000165507	16780908;
C029938	increase	C1ORF198	ENSG00000248579	16780908;
C029938	increase	CALCRL	ENSG00000064989	16780908;
C029938	increase	CAMK1	ENSG00000134072	16780908;
C029938	increase	CCL2	ENSG00000108691	16780908;
C029938	increase	CCL3	ENSG00000006075	16780908;
C029938	increase	CCL3L1	ENSG00000205021	16780908;
C029938	increase	CCL4	ENSG00000129277	16314067;16780908;
C029938	increase	CCR7	ENSG00000126353	16780908;
C029938	increase	CD1C	ENSG00000158481	16780908;
C029938	increase	CD276	ENSG00000103855	16780908;
C029938	increase	CD69	ENSG00000110848	16780908;
C029938	increase	CD83	ENSG00000112149	16780908;
C029938	increase	CD86	ENSG00000114013	16780908;19033392;
C029938	increase	CD8A	ENSG00000153563	16780908;
C029938	increase	CDKN1A	ENSG00000124762	16314067;
C029938	increase	CFLAR	ENSG00000003402	16780908;
C029938	increase	CHKA	ENSG00000110721	16780908;
C029938	increase	CIDEC	ENSG00000187288	16780908;
C029938	increase	CLIC4	ENSG00000169504	16314067;
C029938	increase	CREM	ENSG00000095794	16780908;
C029938	increase	CRIM1	ENSG00000150938	16780908;
C029938	increase	CTPS	ENSG00000171793	16780908;
C029938	increase	CTSB	ENSG00000164733	16314067;
C029938	increase	CTSL1	ENSG00000135047	16780908;
C029938	increase	CXCL1	ENSG00000163739	16780908;
C029938	increase	CXCR4	ENSG00000121966	16780908;
C029938	increase	DAB1	ENSG00000173406	16780908;
C029938	increase	DCT	ENSG00000080166	16780908;
C029938	increase	DEAF1	ENSG00000177030	16780908;
C029938	increase	DSG2	ENSG00000046604	16780908;
C029938	increase	DUSP1	ENSG00000120129	16314067;
C029938	increase	DUSP4	ENSG00000120875	16780908;
C029938	increase	DUSP5	ENSG00000138166	16780908;
C029938	increase	EBI3	ENSG00000105246	16780908;
C029938	increase	ECE1	ENSG00000117298	16780908;
C029938	increase	EGR1	ENSG00000120738	16780908;
C029938	increase	ENTPD1	ENSG00000138185	16780908;
C029938	increase	ETS2	ENSG00000157557	16780908;
C029938	increase	FABP5	ENSG00000164687	16780908;
C029938	increase	FARP1	ENSG00000152767	16780908;
C029938	increase	FASN	ENSG00000169710	16780908;
C029938	increase	FGFR2	ENSG00000066468	12930308;
C029938	increase	FOS	ENSG00000170345	16780908;
C029938	increase	FOSL2	ENSG00000075426	16780908;
C029938	increase	FTH1	ENSG00000167996	16314067;
C029938	increase	FYN	ENSG00000010810	16780908;

C029938	increase	GCLM	ENSG00000023909	16314067;
C029938	increase	GM2A	ENSG00000196743	16780908;
C029938	increase	GP9	ENSG00000169704	16780908;
C029938	increase	GPNMB	ENSG00000136235	16314067;
C029938	increase	HIF1A	ENSG00000100644	16314067;
C029938	increase	HIP1	ENSG00000127946	16780908;
C029938	increase	HIPK2	ENSG00000064393	16314067;
C029938	increase	HIVEP1	ENSG00000095951	16780908;
C029938	increase	HMOX1	ENSG00000100292	19033392;16314067;
C029938	increase	HSPA6	ENSG00000173110	16314067;
C029938	increase	HTATIP2	ENSG00000109854	16314067;
C029938	increase	ID2	ENSG00000115738	16780908;
C029938	increase	IER2	ENSG00000160888	16780908;
C029938	increase	IGFBP6	ENSG00000167779	16780908;
C029938	increase	IL1B	ENSG00000125538	16314067;16780908;
C029938	increase	IL1R2	ENSG00000115590	16780908;
C029938	increase	IL1RN	ENSG00000136689	16780908;
C029938	increase	IL32	ENSG00000008517	16780908;
C029938	increase	IL6	ENSG00000136244	16780908;
C029938	increase	IL7R	ENSG00000168685	16780908;
C029938	increase	IL8	ENSG00000169429	15081272;16780908;
C029938	increase	INHBA	ENSG00000122641	16780908;
C029938	increase	INSM1	ENSG00000173404	16780908;
C029938	increase	IRF5	ENSG00000128604	16780908;
C029938	increase	ITGB3	ENSG00000056345	16780908;
C029938	increase	JUNB	ENSG00000171223	16780908;
C029938	increase	JUN	ENSG00000177606	16314067;
C029938	increase	KIAA0226	ENSG00000145016	16780908;
C029938	increase	KLF6	ENSG00000067082	16314067;
C029938	increase	LAD1	ENSG00000159166	16780908;
C029938	increase	LAMP3	ENSG00000078081	16780908;
C029938	increase	MAOA	ENSG00000189221	16780908;
C029938	increase	MAP3K14	ENSG00000006062	16780908;
C029938	increase	MARCKS	ENSG00000155130	16314067;
C029938	increase	MDFI	ENSG00000112559	16780908;
C029938	increase	ME1	ENSG00000065833	16314067;
C029938	increase	MED22	ENSG00000148297	16780908;
C029938	increase	MFSD9	ENSG00000135953	16780908;
C029938	increase	MGLL	ENSG00000074416	16780908;
C029938	increase	MITF	ENSG00000187098	16780908;
C029938	increase	MMP10	ENSG00000166670	16780908;
C029938	increase	MMP1	ENSG00000196611	16780908;
C029938	increase	MMP3	ENSG00000149968	16780908;
C029938	increase	MS4A7	ENSG00000166927	16780908;
C029938	increase	MST1R	ENSG00000164078	16780908;
C029938	increase	MT1F	ENSG00000198417	16780908;
C029938	increase	MT1G	ENSG00000125144	16780908;
C029938	increase	MT1H	ENSG00000205358	16314067;
C029938	increase	MT2A	ENSG00000125148	16314067;
C029938	increase	MTSS1	ENSG00000170873	16780908;
C029938	increase	MYO1E	ENSG00000157483	16780908;
C029938	increase	NET1	ENSG00000173848	16780908;
C029938	increase	NFE2L2	ENSG00000116044	19033392;
C029938	increase	NFIL3	ENSG00000165030	16780908;

C029938	increase	NFKB1	ENSG00000109320	16780908;
C029938	increase	NFKBIA	ENSG00000100906	16780908;
C029938	increase	NINJ1	ENSG00000131669	16314067;16780908;
C029938	increase	NQO1	ENSG00000181019	19033392;16314067;
C029938	increase	NR2F6	ENSG00000160113	16780908;
C029938	increase	NR4A1	ENSG00000123358	16780908;
C029938	increase	NR4A2	ENSG00000153234	16780908;
C029938	increase	NRP2	ENSG00000118257	16780908;
C029938	increase	NUB1	ENSG00000013374	16780908;
C029938	increase	NUP88	ENSG00000108559	16780908;
C029938	increase	OSTF1	ENSG00000134996	16780908;
C029938	increase	PDE4B	ENSG00000184588	16780908;
C029938	increase	PDGFA	ENSG00000197461	16780908;
C029938	increase	PGD	ENSG00000142657	16314067;
C029938	increase	PHLDA1	ENSG00000139289	16780908;
C029938	increase	PICALM	ENSG00000073921	16780908;
C029938	increase	PIR	ENSG00000087842	16314067;
C029938	increase	PLA2G16	ENSG00000176485	16780908;
C029938	increase	PLAT	ENSG00000104368	16780908;
C029938	increase	PLEK	ENSG00000115956	16780908;
C029938	increase	PMAIP1	ENSG00000141682	16314067;16780908;
C029938	increase	POLR2A	ENSG00000181222	16780908;
C029938	increase	PORCN	ENSG00000102312	16780908;
C029938	increase	PPARG	ENSG00000132170	16314067;
C029938	increase	PPP2R1B	ENSG00000137713	16780908;
C029938	increase	PTEN	ENSG00000171862	16780908;
C029938	increase	PTGS2	ENSG00000073756	16780908;
C029938	increase	RAB8B	ENSG00000166128	16780908;
C029938	increase	RALB	ENSG00000144118	16780908;
C029938	increase	RAMP1	ENSG00000132329	16780908;
C029938	increase	RCAN1	ENSG00000159200	16780908;
C029938	increase	REL	ENSG00000162924	16780908;
C029938	increase	RGS16	ENSG00000143333	16780908;
C029938	increase	RGS1	ENSG00000090104	16780908;
C029938	increase	RHOC	ENSG00000155366	16314067;
C029938	increase	RIT1	ENSG00000143622	16314067;
C029938	increase	S100A10	ENSG00000197747	16314067;
C029938	increase	S100P	ENSG00000163993	16314067;
C029938	increase	SAMSN1	ENSG00000155307	16780908;
C029938	increase	SAT1	ENSG00000130066	16780908;
C029938	increase	SATB1	ENSG00000182568	16780908;
C029938	increase	SDC2	ENSG00000169439	16780908;
C029938	increase	SEMA4C	ENSG00000168758	16314067;
C029938	increase	SEMA5A	ENSG00000112902	16780908;
C029938	increase	SFRS5	ENSG00000100650	16780908;
C029938	increase	SGK1	ENSG00000118515	16780908;
C029938	increase	SLC16A10	ENSG00000112394	16780908;
C029938	increase	SLC2A3	ENSG00000059804	16780908;
C029938	increase	SLC43A3	ENSG00000134802	16780908;
C029938	increase	SLC7A5	ENSG00000103257	16780908;
C029938	increase	SNED1	ENSG00000162804	16780908;
C029938	increase	SNX14	ENSG00000135317	16780908;
C029938	increase	SOCS3	ENSG00000184557	16780908;
C029938	increase	SOD2	ENSG00000112096	16314067;16780908;

C029938	increase	SPTA1	ENSG00000163554	16314067;
C029938	increase	STAT4	ENSG00000138378	16780908;
C029938	increase	STAT5A	ENSG00000126561	16780908;
C029938	increase	STX11	ENSG00000135604	16780908;
C029938	increase	STX1B	ENSG00000099365	16780908;
C029938	increase	SUGT1	ENSG00000165416	16780908;
C029938	increase	TAC3	ENSG00000166863	16780908;
C029938	increase	TBC1D13	ENSG00000107021	16780908;
C029938	increase	TIAM1	ENSG00000156299	16780908;
C029938	increase	TIMP1	ENSG00000102265	16780908;
C029938	increase	TIMP3	ENSG00000100234	16314067;
C029938	increase	TLN2	ENSG00000171914	16780908;
C029938	increase	TMCC3	ENSG00000057704	16780908;
C029938	increase	TNFAIP3	ENSG00000118503	16780908;
C029938	increase	TNFRSF1B	ENSG00000028137	16780908;
C029938	increase	TOB1	ENSG00000141232	16780908;
C029938	increase	TOMM34	ENSG00000025772	16780908;
C029938	increase	TPD52	ENSG00000076554	16780908;
C029938	increase	TRAF1	ENSG00000056558	16780908;
C029938	increase	TRIP10	ENSG00000125733	16780908;
C029938	increase	TTYH3	ENSG00000136295	16780908;
C029938	increase	UBD	ENSG00000213886	16780908;
C029938	increase	VAV2	ENSG00000160293	16780908;
C029938	increase	YWHAH	ENSG00000128245	16780908;
C029938	increase	ZDHHC18	ENSG00000204160	16780908;
C030110	decrease	ADA	ENSG00000196839	17762391;
C030110	decrease	ALDOC	ENSG00000109107	17762391;
C030110	decrease	ASNS	ENSG00000070669	17762391;
C030110	decrease	ATXN7	ENSG00000163635	16510598;
C030110	decrease	AURKA	ENSG00000087586	17762391;
C030110	decrease	BCL2	ENSG00000171791	16024531;
C030110	decrease	BCL2L1	ENSG00000171552	16024531;
C030110	decrease	BNIP3	ENSG00000176171	17762391;
C030110	decrease	CA9	ENSG00000107159	17762391;
C030110	decrease	CFLAR	ENSG00000003402	16373718;
C030110	decrease	COL12A1	ENSG00000111799	16510598;
C030110	decrease	COL13A1	ENSG00000197467	16510598;
C030110	decrease	CXCL12	ENSG00000107562	17762391;
C030110	decrease	DUT	ENSG00000128951	19015155;
C030110	decrease	ETFA	ENSG00000140374	17762391;
C030110	decrease	F3	ENSG00000117525	16510598;
C030110	decrease	FHL1	ENSG00000022267	17762391;
C030110	decrease	GADD45A	ENSG00000116717	16227409;
C030110	decrease	GRK7	ENSG00000114124	17762391;
C030110	decrease	HDGF	ENSG00000143321	17762391;
C030110	decrease	HEY1	ENSG00000164683	17762391;
C030110	decrease	IGFBP3	ENSG00000146674	17762391;
C030110	decrease	INSIG1	ENSG00000186480	16510598;
C030110	decrease	ITGB8	ENSG00000105855	16510598;
C030110	decrease	KCNK15	ENSG00000124249	17762391;
C030110	decrease	MNS1	ENSG00000138587	16510598;
C030110	decrease	MYC	ENSG00000136997	15204521;
C030110	decrease	NCBP1	ENSG00000136937	17762391;
C030110	decrease	NEK2	ENSG00000117650	17762391;

C030110	decrease	P4HA1	ENSG00000122884	17762391;
C030110	decrease	P4HA2	ENSG00000072682	17762391;
C030110	decrease	PAK2	ENSG00000180370	17762391;
C030110	decrease	PCK2	ENSG00000100889	17762391;
C030110	decrease	PFKFB4	ENSG00000114268	17762391;
C030110	decrease	PFKP	ENSG00000067057	17762391;
C030110	decrease	PKD1	ENSG00000008710	17762391;
C030110	decrease	PKIG	ENSG00000168734	17762391;
C030110	decrease	POLR2E	ENSG00000099817	17762391;
C030110	decrease	RAP1B	ENSG00000127314	17762391;
C030110	decrease	RGS4	ENSG00000117152	17762391;
C030110	decrease	S100P	ENSG00000163993	18636193;17762391;
C030110	decrease	SDHB	ENSG00000117118	17762391;
C030110	decrease	SENP1	ENSG00000079387	17762391;
C030110	decrease	SERPINH1	ENSG00000149257	16510598;
C030110	decrease	SH2B3	ENSG00000111252	17762391;
C030110	decrease	SLC2A3	ENSG00000059804	17762391;
C030110	decrease	SLC38A1	ENSG00000111371	16510598;
C030110	decrease	SLC7A5	ENSG00000103257	17762391;
C030110	decrease	SRP72	ENSG00000174780	17762391;
C030110	decrease	STC1	ENSG00000159167	17762391;
C030110	decrease	STK17A	ENSG00000164543	17762391;
C030110	decrease	TGIF1	ENSG00000177426	17762391;
C030110	decrease	TSPO	ENSG00000100300	17762391;
C030110	decrease	TYMS	ENSG00000176890	15213713;18448328;19074750;15057142;
C030110	decrease	ZAK	ENSG00000091436	16510598;
C030110	decrease	ZEB1	ENSG00000148516	17762391;
C030110	increase	AAMP	ENSG00000127837	17762391;
C030110	increase	ADAM19	ENSG00000135074	16510598;
C030110	increase	ANKRD22	ENSG00000152766	16510598;
C030110	increase	ANXA2	ENSG00000182718	15152939;
C030110	increase	ARHGDIA	ENSG00000141522	17762391;
C030110	increase	BAX	ENSG00000087088	16227409;
C030110	increase	BDH2	ENSG00000164039	16510598;
C030110	increase	BIRC5	ENSG00000089685	16004971;16024531;19147571;
C030110	increase	CALB2	ENSG00000172137	16510598;
C030110	increase	CCNA2	ENSG00000145386	15204521;
C030110	increase	CCNB1	ENSG00000134057	15204521;
C030110	increase	CCND1	ENSG00000110092	19147571;
C030110	increase	CCNE1	ENSG00000105173	15204521;
C030110	increase	CDC37	ENSG00000105401	17762391;
C030110	increase	CDC42BPB	ENSG00000198752	17762391;
C030110	increase	CDK1	ENSG00000170312	16024531;15204521;
C030110	increase	CDK2	ENSG00000123374	15204521;
C030110	increase	CDKN1A	ENSG00000124762	16227409;
C030110	increase	CDKN1B	ENSG00000111276	15204521;
C030110	increase	CNOT3	ENSG00000088038	17762391;
C030110	increase	CSTF3	ENSG00000176102	17762391;
C030110	increase	DAG1	ENSG00000173402	17762391;
C030110	increase	DDB1	ENSG00000167986	17762391;
C030110	increase	DVL3	ENSG00000161202	17762391;
C030110	increase	EPN3	ENSG00000049283	16510598;

C030110	increase	FGFR2	ENSG00000066468	17762391;
C030110	increase	FLT1	ENSG00000102755	18790786;
C030110	increase	FXYD3	ENSG00000089356	15152939;
C030110	increase	HES1	ENSG00000114315	19147571;
C030110	increase	HSPE1	ENSG00000115541	15152939;
C030110	increase	MAN1A1	ENSG00000111885	16510598;
C030110	increase	MAP2	ENSG00000078018	16510598;
C030110	increase	MARCKS	ENSG00000155130	17762391;
C030110	increase	NCSTN	ENSG00000162736	19147571;
C030110	increase	NOTCH1	ENSG00000148400	19147571;
C030110	increase	NR2F1	ENSG00000175745	17762391;
C030110	increase	NRP1	ENSG00000099250	18790786;
C030110	increase	NUMA1	ENSG00000137497	17762391;
C030110	increase	PAICS	ENSG00000128050	17762391;
C030110	increase	PDF	ENSG00000213380	16510598;
C030110	increase	PGF	ENSG00000119630	18790786;
C030110	increase	PIK3R2	ENSG00000105647	17762391;
C030110	increase	PPM1G	ENSG00000115241	17762391;
C030110	increase	PSAP	ENSG00000197746	17762391;
C030110	increase	PSEN1	ENSG00000080815	19147571;
C030110	increase	PXDN	ENSG00000130508	16510598;
C030110	increase	RNF43	ENSG00000108375	16510598;
C030110	increase	RPA1	ENSG00000132383	17762391;
C030110	increase	S100A14	ENSG00000189334	16510598;
C030110	increase	S100A4	ENSG00000196154	16510598;
C030110	increase	SAT1	ENSG00000130066	17237273;16510598;15152939;
C030110	increase	SDC1	ENSG00000115884	17762391;
C030110	increase	SMOX	ENSG00000088826	17987291;
C030110	increase	SOX4	ENSG00000124766	16510598;
C030110	increase	SPTBN1	ENSG00000115306	17762391;
C030110	increase	TMSB10	ENSG00000034510	15152939;
C030110	increase	TNIK	ENSG00000154310	16510598;
C030110	increase	TP53	ENSG00000141510	15041737;19015155;16227409;
C030110	increase	VEGFA	ENSG00000112715	18790786;
C030110	increase	VEGFC	ENSG00000150630	18790786;
C030110	increase	ZNF238	ENSG00000179456	16510598;
C030123	decrease	BCL2	ENSG00000171791	15964220;
C030123	increase	BAX	ENSG00000087088	15964220;
C030358	increase	IL8	ENSG00000169429	18413660;
C030371	decrease	IGF1R	ENSG00000140443	16728583;
C030371	decrease	MTR	ENSG00000116984	16598758;
C030419	decrease	MMP7	ENSG00000137673	19426720;
C030517	increase	SLC6A12	ENSG00000111181	16303854;
C030601	increase	COL1A2	ENSG00000164692	11343241;
C030601	increase	FN1	ENSG00000115414	11343241;
C030665	decrease	NOTCH1	ENSG00000148400	12788061;
C030665	decrease	PTPN7	ENSG00000143851	12788061;
C030665	increase	CCL2	ENSG00000108691	12788061;
C030665	increase	CD14	ENSG00000170458	12788061;
C030665	increase	CSF3R	ENSG00000119535	12788061;
C030665	increase	CYBA	ENSG00000051523	12788061;
C030665	increase	HCK	ENSG00000101336	12788061;

C030665	increase	ITGB2	ENSG00000160255	12788061;
C030665	increase	MCL1	ENSG00000143384	12788061;
C030665	increase	NCF1	ENSG00000158517	12788061;
C030665	increase	NCF2	ENSG00000116701	12788061;
C030682	decrease	CCR5	ENSG00000160791	18486366;
C030682	increase	CCL11	ENSG00000172156	18486366;
C030682	increase	CCL20	ENSG00000115009	18486366;
C030682	increase	CCR2	ENSG00000121807	18486366;
C030682	increase	IFNG	ENSG00000111537	18486366;
C030682	increase	IL21	ENSG00000138684	18486366;
C030682	increase	IL9	ENSG00000145839	18486366;
C030682	increase	UGT1A10	ENSG00000242515	18486366;
C030682	increase	XCL2	ENSG00000143185	18486366;
C030682	increase	XRCC4	ENSG00000152422	18486366;
C030852	decrease	CDKN2A	ENSG00000147889	17904787;
C030852	decrease	PTGS2	ENSG00000073756	17904787;
C030935	increase	CYP1A1	ENSG00000140465	11377097;8961944;12224597;11454723;
C030935	increase	CYP1B1	ENSG00000138061	12224597;
C030935	increase	ESR1	ENSG00000091831	11922773;12441364;
C030935	increase	NQO1	ENSG00000181019	9029048;
C030973	increase	HMOX1	ENSG00000100292	19683516;
C030984	increase	CYP1A1	ENSG00000140465	15016593;15566942;
C030984	increase	CYP1B1	ENSG00000138061	15016593;15566942;
C030984	increase	UGT1A6	ENSG00000167165	15566942;
C030984	increase	UGT1A7	ENSG00000244122	15566942;
C031143	decrease	CCNA2	ENSG00000145386	12730239;
C031143	decrease	CCNB1	ENSG00000134057	12730239;
C031180	affect	PGR	ENSG00000082175	9144411;
C031180	increase	ABCB1	ENSG00000085563	15566942;
C031180	increase	CYP1A1	ENSG00000140465	8961944;15566942;
C031180	increase	CYP1B1	ENSG00000138061	15566942;
C031180	increase	ESR1	ENSG00000091831	11922773;
C031180	increase	UGT1A6	ENSG00000167165	15566942;
C031180	increase	UGT1A7	ENSG00000244122	15566942;
C031181	increase	CYP1A1	ENSG00000140465	17035385;15566942;
C031181	increase	CYP1B1	ENSG00000138061	17035385;15566942;
C031181	increase	UGT1A6	ENSG00000167165	15566942;
C031181	increase	UGT1A7	ENSG00000244122	15566942;
C031183	increase	ABCG1	ENSG00000160179	16730733;
C031183	increase	EDN1	ENSG00000078401	16895544;
C031278	decrease	AKT1	ENSG00000142208	12208782;
C031278	decrease	BAK1	ENSG00000030110	12208782;
C031278	decrease	BAX	ENSG00000087088	12208782;
C031278	decrease	BCL2	ENSG00000171791	12208782;
C031278	decrease	BCL2L1	ENSG00000171552	12208782;
C031278	decrease	BIK	ENSG00000100290	12208782;
C031278	decrease	IL1R2	ENSG00000115590	11031321;11428619;
C031278	decrease	IL1RN	ENSG00000136689	11428619;11031321;
C031278	decrease	IL6	ENSG00000136244	11428619;
C031278	decrease	MCL1	ENSG00000143384	12208782;
C031278	decrease	PDK1	ENSG00000152256	12208782;
C031278	increase	CASP3	ENSG00000164305	12208782;
C031278	increase	CASP4	ENSG00000196954	12208782;

C031278	increase	CASP6	ENSG00000138794	12208782;
C031278	increase	CASP8	ENSG00000064012	12208782;
C031278	increase	CASP9	ENSG00000132906	12208782;
C031278	increase	IL1A	ENSG00000115008	11031321;11428619;
C031278	increase	TP53	ENSG00000141510	19111594;
C031280	increase	CYP2A6	ENSG00000198077	11038160;
C031280	increase	CYP2E1	ENSG00000130649	8074729;
C031285	increase	CCL2	ENSG00000108691	17993253;
C031285	increase	IL8	ENSG00000169429	17993253;
C031291	increase	CCL2	ENSG00000108691	15139008;16794257;
C031291	increase	IL8	ENSG00000169429	15139008;16794257;
C031291	increase	TNF	ENSG00000223952	10802230;16794257;
C031294	increase	CCL2	ENSG00000108691	18645721;18514500;17993253;
C031294	increase	HSPA5	ENSG00000044574	12183065;
C031294	increase	IL13	ENSG00000169194	18514500;
C031294	increase	IL8	ENSG00000169429	18514500;17993253;
C031327	increase	ABCB1	ENSG00000085563	15761118;
C031327	increase	CYP2B6	ENSG00000197408	12920490;18332078;15761118;
C031327	increase	CYP3A4	ENSG00000160868	12920490;18332078;15761118;
C031338	increase	CYP3A4	ENSG00000160868	16632523;
C031338	increase	NR1I2	ENSG00000144852	16632523;
C031338	increase	RXRA	ENSG00000186350	16632523;
C031389	increase	PTGS2	ENSG00000073756	10783318;
C031389	increase	TGFA	ENSG00000163235	10783318;
C031390	increase	CYP3A4	ENSG00000160868	16632523;
C031390	increase	NR1I2	ENSG00000144852	16632523;
C031390	increase	RXRA	ENSG00000186350	16632523;
C031403	increase	MMP7	ENSG00000137673	17928719;
C031463	increase	ALPL	ENSG00000162551	18500657;
C031463	increase	ENPP1	ENSG00000197594	18500657;
C031463	increase	FGF23	ENSG00000118972	15542045;
C031463	increase	PHEX	ENSG00000102174	18500657;
C031477	decrease	BCL2	ENSG00000171791	15964220;
C031477	decrease	CCND1	ENSG00000110092	11679177;
C031477	increase	BAX	ENSG00000087088	15964220;
C031556	decrease	FGF23	ENSG00000118972	16076378;
C031655	decrease	IFNA1	ENSG00000197919	15885359;
C031655	decrease	NR3C1	ENSG00000113580	15885359;
C031721	decrease	FUS	ENSG00000089280	14595851;
C031721	increase	BCL2	ENSG00000171791	14595851;
C031721	increase	FOS	ENSG00000170345	14595851;
C031721	increase	IL4	ENSG00000113520	12112628;
C031721	increase	IL8	ENSG00000169429	14595851;
C031721	increase	JUN	ENSG00000177606	14595851;
C031721	increase	RAF1	ENSG00000132155	14595851;
C031721	increase	TCF7	ENSG00000081059	14595851;
C031818	increase	IFNG	ENSG00000111537	12496413;
C031818	increase	IL31	ENSG00000204671	19281909;
C031927	affect	ARHGDIA	ENSG00000141522	17181942;
C031927	affect	CD69	ENSG00000110848	12749906;
C031927	affect	CYR61	ENSG00000142871	15452088;

C031927	affect	FN1	ENSG00000115414	15452088;
C031927	affect	H2AFX	ENSG00000188486	17875398;
C031927	affect	IL2	ENSG00000109471	12749906;
C031927	affect	IL2RA	ENSG00000134460	12749906;
C031927	affect	LMNA	ENSG00000160789	17181942;
C031927	affect	MMP15	ENSG00000102996	15452088;
C031927	affect	MMP3	ENSG00000149968	15452088;
C031927	affect	PA2G4	ENSG00000170515	17181942;
C031927	affect	PGLS	ENSG00000130313	17181942;
C031927	affect	RELA	ENSG00000173039	16928326;
C031927	affect	WRN	ENSG00000165392	17875398;
C031927	decrease	BAD	ENSG00000002330	15452088;
C031927	decrease	BNIP3	ENSG00000176171	15452088;
C031927	decrease	CASP3	ENSG00000164305	15816534;15452088;15777082;
C031927	decrease	CASP6	ENSG00000138794	15452088;
C031927	decrease	CASP9	ENSG00000132906	15452088;15816534;15777082;
C031927	decrease	DAP	ENSG00000112977	15452088;
C031927	decrease	FADD	ENSG00000168040	15452088;
C031927	decrease	FOS	ENSG00000170345	15452088;
C031927	decrease	SOD1	ENSG00000142168	15816534;
C031927	decrease	SOD2	ENSG00000112096	15816534;
C031927	decrease	TNFRSF10B	ENSG00000120889	15452088;
C031927	decrease	TNFRSF25	ENSG00000215788	15452088;
C031927	decrease	TNFSF12	ENSG00000239697	15452088;
C031927	decrease	TRAF4	ENSG00000076604	15452088;
C031927	increase	ALDH2	ENSG00000111275	15452088;
C031927	increase	ATF4	ENSG00000128272	15452088;
C031927	increase	BIRC3	ENSG00000023445	15452088;
C031927	increase	CCL11	ENSG00000172156	17572062;
C031927	increase	CCL2	ENSG00000108691	17572062;
C031927	increase	CCL3	ENSG00000006075	17572062;
C031927	increase	CCL4	ENSG00000129277	17572062;
C031927	increase	CCL5	ENSG00000161570	17572062;
C031927	increase	COL1A1	ENSG00000108821	18055817;
C031927	increase	COL4A1	ENSG00000187498	18055817;
C031927	increase	DNAJA1	ENSG00000086061	15452088;
C031927	increase	DNAJB1	ENSG00000132002	15452088;
C031927	increase	FTH1	ENSG00000167996	15452088;
C031927	increase	HSPA2	ENSG00000126803	15452088;
C031927	increase	IL10	ENSG00000136634	17572062;
C031927	increase	IL8	ENSG00000169429	15356918;17572062;
C031927	increase	LAMA4	ENSG00000112769	18055817;
C031927	increase	MGST1	ENSG00000008394	15452088;
C031927	increase	MMP14	ENSG00000157227	18055817;
C031927	increase	MMP9	ENSG00000100985	17669387;
C031927	increase	MYC	ENSG00000136997	12749906;
C031927	increase	NQO1	ENSG00000181019	8652659;
C031927	increase	PRDX1	ENSG00000117450	15452088;
C031927	increase	RECQL4	ENSG00000160957	15452088;
C031927	increase	TFRC	ENSG00000072274	12749906;
C031927	increase	TIMP2	ENSG00000035862	18055817;
C031927	increase	TNF	ENSG00000223952	17669387;10890505;

C031927	increase	TP53	ENSG00000141510	17875398;
C031927	increase	TRADD	ENSG00000102871	15452088;
C031927	increase	TXN	ENSG00000136810	15452088;
C031938	increase	CYP11B2	ENSG00000179142	12193581;
C032171	decrease	PTGS2	ENSG00000073756	15841493;
C032171	increase	NOS3	ENSG00000164867	15740983;
C032201	increase	CYP2B6	ENSG00000197408	14977870;
C032208	decrease	IVL	ENSG00000163207	10769631;
C032208	increase	RAP1A	ENSG00000116473	12671036;
C032208	increase	RHOA	ENSG00000067560	12671036;
C032208	increase	RHOB	ENSG00000143878	12671036;
C032279	increase	CYP1A1	ENSG00000140465	15521013;
C032279	increase	CYP3A4	ENSG00000160868	15650019;
C032311	decrease	IGFBP4	ENSG00000141753	11996908;
C032311	increase	IGF2	ENSG00000167244	11996908;
C032311	increase	IGFBP5	ENSG00000115461	11996908;
C032628	affect	CCL2	ENSG00000108691	17191021;
C032628	affect	IL1B	ENSG00000125538	17191021;
C032668	increase	IL8	ENSG00000169429	12393170;
C032668	increase	NFKBIA	ENSG00000100906	12393170;
C032727	decrease	IL2	ENSG00000109471	11859000;
C032854	affect	IL1A	ENSG00000115008	11031321;
C032854	affect	IL1R2	ENSG00000115590	11031321;
C032854	affect	IL1RN	ENSG00000136689	11031321;
C032881	increase	HIF1A	ENSG00000100644	17919812;
C032881	increase	LAMA3	ENSG00000053747	15541073;
C032881	increase	LAMB3	ENSG00000196878	15541073;
C032881	increase	LAMC2	ENSG00000248691	15541073;
C032881	increase	VEGFA	ENSG00000112715	17919812;
C032910	increase	ABCB1	ENSG00000085563	19409404;
C033006	decrease	PPARA	ENSG00000186951	15585952;
C033006	decrease	RXRA	ENSG00000186350	15585952;
C033020	increase	HMOX1	ENSG00000100292	18227147;
C033110	affect	TP53	ENSG00000141510	15781256;
C033110	increase	CDKN1A	ENSG00000124762	15781256;
C033146	decrease	PGR	ENSG00000082175	9144411;
C033170	increase	IVL	ENSG00000163207	16621454;
C033170	increase	KRT10	ENSG00000186395	16621454;
C033246	increase	IL1B	ENSG00000125538	12573452;
C033246	increase	PTGS2	ENSG00000073756	12573452;
C033273	decrease	F2	ENSG00000180210	16522526;15504374;
C033273	decrease	PLAT	ENSG00000104368	12618251;
C033273	decrease	SERPINC1	ENSG00000117601	11994571;12618251;
C033273	increase	CP	ENSG00000047457	12616983;
C033273	increase	CRP	ENSG00000132693	12616983;
C033273	increase	F7	ENSG00000057593	12616980;12618251;
C033273	increase	F8	ENSG00000185010	11720199;12618251;
C033273	increase	FGA	ENSG00000171560	12618251;12616980;12616983;
C033273	increase	FGB	ENSG00000171564	12618251;12616983;12616980;
C033273	increase	FGG	ENSG00000171557	12618251;12616980;12616983;
C033273	increase	FN1	ENSG00000115414	15504374;

C033273	increase	PLG	ENSG00000122194	11720199;12618251;
C033273	increase	PROC	ENSG00000115718	11720199;11994571;15669648;12618251;
C033273	increase	PROS1	ENSG00000184500	11720199;11994571;16522526;12618251;15669648;
C033273	increase	SERPIND1	ENSG00000099937	15669648;
C033273	increase	SHBG	ENSG00000129214	10899481;
C033273	increase	VWF	ENSG00000110799	12616983;
C033342	increase	RAP1A	ENSG00000116473	12671036;
C033342	increase	RHOA	ENSG00000067560	12671036;
C033342	increase	RHOB	ENSG00000143878	12671036;
C033363	increase	BCL2	ENSG00000171791	17690738;
C033363	increase	NFKB2	ENSG00000077150	17690738;
C033457	increase	CYP2B6	ENSG00000197408	15382119;15849716;
C033457	increase	CYP2C9	ENSG00000138109	15382119;
C033457	increase	CYP3A4	ENSG00000160868	15382119;
C033457	increase	GSTA1	ENSG00000243955	15382119;
C033457	increase	GSTA2	ENSG00000244067	15382119;
C033457	increase	IL1B	ENSG00000125538	15382119;
C033457	increase	SLCO1B1	ENSG00000134538	15382119;
C033457	increase	UGT1A1	ENSG00000241635	15382119;15849716;
C033607	decrease	BCL2	ENSG00000171791	16055262;
C033607	decrease	MYC	ENSG00000136997	16055262;
C033607	increase	BAX	ENSG00000087088	16055262;
C033607	increase	FOS	ENSG00000170345	17142977;
C033607	increase	PGR	ENSG00000082175	17142977;
C033729	decrease	ERBB2	ENSG00000141736	16393665;
C033729	increase	ESR1	ENSG00000091831	17603182;16393665;
C033729	increase	PDZK1	ENSG00000174827	16393665;
C033729	increase	PGR	ENSG00000082175	16393665;
C033729	increase	TFF1	ENSG00000160182	16393665;
C033789	decrease	ESR1	ENSG00000091831	15253041;10789375;16626760;15261991;15064155;
C033789	increase	ESR2	ENSG00000140009	15261991;15064155;
C034028	decrease	ACPP	ENSG00000014257	16330358;
C034028	decrease	ARNT	ENSG00000143437	16330358;
C034028	decrease	ARRDC3	ENSG00000113369	16330358;
C034028	decrease	ARRDC4	ENSG00000140450	16330358;
C034028	decrease	BAT1	ENSG00000229496	16330358;
C034028	decrease	BCL6	ENSG00000113916	16330358;
C034028	decrease	BCOR	ENSG00000183337	16330358;
C034028	decrease	CALM1	ENSG00000143933	16330358;
C034028	decrease	CCNF	ENSG00000162063	16330358;
C034028	decrease	CINP	ENSG00000100865	16330358;
C034028	decrease	CIRBP	ENSG00000099622	16330358;
C034028	decrease	CRH	ENSG00000147571	16330358;
C034028	decrease	CYBB	ENSG00000165168	16330358;
C034028	decrease	DENR	ENSG00000139726	16330358;
C034028	decrease	FBXO5	ENSG00000112029	16330358;
C034028	decrease	FYB	ENSG00000082074	16330358;
C034028	decrease	GALT	ENSG00000213930	16330358;
C034028	decrease	GPR137B	ENSG00000077585	16330358;
C034028	decrease	HNRNPA2B	ENSG00000122566	16330358;
C034028	decrease	ICA1	ENSG00000003147	16330358;

C034028	decrease	ID3	ENSG00000117318	16330358;
C034028	decrease	KBTBD6	ENSG00000165572	16330358;
C034028	decrease	KCTD6	ENSG00000168301	16330358;
C034028	decrease	LHX9	ENSG00000143355	16330358;
C034028	decrease	LINS1	ENSG00000140471	16330358;
C034028	decrease	MAPK14	ENSG00000112062	16330358;
C034028	decrease	MYO3B	ENSG00000071909	16330358;
C034028	decrease	P2RY2	ENSG00000175591	16330358;
C034028	decrease	PAK6	ENSG00000137843	16330358;
C034028	decrease	PDXP	ENSG00000241360	16330358;
C034028	decrease	PML	ENSG00000140464	16330358;
C034028	decrease	PTH	ENSG00000152266	16330358;
C034028	decrease	RARA	ENSG00000131759	16330358;
C034028	decrease	RCHY1	ENSG00000163743	16330358;
C034028	decrease	RIMS3	ENSG00000117016	16330358;
C034028	decrease	S100A6	ENSG00000197956	16330358;
C034028	decrease	SCARF2	ENSG00000244486	16330358;
C034028	decrease	SDCCAG8	ENSG00000054282	16330358;
C034028	decrease	SIX1	ENSG00000126778	16330358;
C034028	decrease	SIX2	ENSG00000170577	16330358;
C034028	decrease	SLC26A3	ENSG00000091138	16330358;
C034028	decrease	SMAD4	ENSG00000141646	16330358;
C034028	decrease	SOX2	ENSG00000181449	16330358;
C034028	decrease	STAU2	ENSG00000040341	16330358;
C034028	decrease	TAF6L	ENSG00000162227	16330358;
C034028	decrease	TBX1	ENSG00000184058	16330358;
C034028	decrease	TERF2	ENSG00000132604	16330358;
C034028	decrease	TIFA	ENSG00000145365	16330358;
C034028	decrease	TOR2A	ENSG00000250525	16330358;
C034028	decrease	TPM1	ENSG00000140416	16330358;
C034028	decrease	TPP2	ENSG00000134900	16330358;
C034028	decrease	XAF1	ENSG00000132530	16330358;
C034028	decrease	ZBTB1	ENSG00000126804	16330358;
C034028	decrease	ZBTB24	ENSG00000112365	16330358;
C034028	decrease	ZDHHC21	ENSG00000175893	16330358;
C034028	decrease	ZFP30	ENSG00000120784	16330358;
C034028	decrease	ZNF436	ENSG00000125945	16330358;
C034028	decrease	ZNF44	ENSG00000197857	16330358;
C034028	decrease	ZNF503	ENSG00000165655	16330358;
C034028	decrease	ZNF555	ENSG00000186300	16330358;
C034028	increase	ALB	ENSG00000163631	16330358;
C034028	increase	ANGPTL1	ENSG00000116194	16330358;
C034028	increase	CACNG2	ENSG00000166862	16330358;
C034028	increase	CCDC85B	ENSG00000175602	16330358;
C034028	increase	CCL2	ENSG00000108691	16330358;
C034028	increase	CLDN1	ENSG00000163347	16330358;
C034028	increase	CLDN4	ENSG00000189143	16330358;
C034028	increase	CPVL	ENSG00000106066	16330358;
C034028	increase	CSNK1D	ENSG00000141551	16330358;
C034028	increase	CTSG	ENSG00000100448	16330358;
C034028	increase	CXCL1	ENSG00000163739	16330358;
C034028	increase	CXCL3	ENSG00000163734	16330358;
C034028	increase	CYP11A1	ENSG00000140459	16330358;
C034028	increase	CYP3A7	ENSG00000160870	16330358;

C034028	increase	DRP2	ENSG00000102385	16330358;
C034028	increase	DUSP1	ENSG00000120129	16330358;
C034028	increase	DUSP21	ENSG00000189037	16330358;
C034028	increase	DUSP5	ENSG00000138166	16330358;
C034028	increase	EDN1	ENSG00000078401	16330358;
C034028	increase	ERRFI1	ENSG00000116285	16330358;
C034028	increase	FGD6	ENSG00000180263	16330358;
C034028	increase	FGFR2	ENSG00000066468	16330358;
C034028	increase	FOS	ENSG00000170345	16330358;
C034028	increase	GCNT2	ENSG00000111846	16330358;
C034028	increase	HAL	ENSG00000084110	16330358;
C034028	increase	HOXD9	ENSG00000128709	16330358;
C034028	increase	HS3ST1	ENSG00000002587	16330358;
C034028	increase	ICAM2	ENSG00000108622	16330358;
C034028	increase	IFNE	ENSG00000184995	16330358;
C034028	increase	IL1A	ENSG00000115008	16330358;
C034028	increase	IL1F9	ENSG00000136688	16330358;
C034028	increase	IL8	ENSG00000169429	16330358;
C034028	increase	ITPR3	ENSG00000096433	16330358;
C034028	increase	JAM2	ENSG00000154721	16330358;
C034028	increase	KBTBD10	ENSG00000239474	16330358;
C034028	increase	KRT36	ENSG00000126337	16330358;
C034028	increase	KRT86	ENSG00000170442	16330358;
C034028	increase	LIF	ENSG00000128342	16330358;
C034028	increase	MCHR1	ENSG00000128285	16330358;
C034028	increase	MMP25	ENSG00000008516	16330358;
C034028	increase	PDCD6	ENSG00000249915	16330358;
C034028	increase	PDE4DIP	ENSG00000178104	16330358;
C034028	increase	PDK2	ENSG00000005882	16330358;
C034028	increase	PDLIM3	ENSG00000154553	16330358;
C034028	increase	PLA2G12B	ENSG00000138308	16330358;
C034028	increase	PLA2G1B	ENSG00000170890	16330358;
C034028	increase	PLAUR	ENSG00000011422	16330358;
C034028	increase	PLEKHB1	ENSG00000021300	16330358;
C034028	increase	POLR3G	ENSG00000113356	16330358;
C034028	increase	PPEF2	ENSG00000156194	16330358;
C034028	increase	PPP1R10	ENSG00000235291	16330358;
C034028	increase	PRDM1	ENSG00000057657	16330358;
C034028	increase	PTGS2	ENSG00000073756	16330358;
C034028	increase	PTPRE	ENSG00000132334	16330358;
C034028	increase	RARS2	ENSG00000146282	16330358;
C034028	increase	RCE1	ENSG00000173653	16330358;
C034028	increase	RGS2	ENSG00000116741	16330358;
C034028	increase	RHOJ	ENSG00000126785	16330358;
C034028	increase	RPL13	ENSG00000167526	16330358;
C034028	increase	SERPINB2	ENSG00000197632	16330358;
C034028	increase	SERPINI2	ENSG00000114204	16330358;
C034028	increase	SIGLEC8	ENSG00000105366	16330358;
C034028	increase	SIPA1L3	ENSG00000105738	16330358;
C034028	increase	SLC13A3	ENSG00000158296	16330358;
C034028	increase	SLC22A16	ENSG00000004809	16330358;
C034028	increase	STAT3	ENSG00000168610	16330358;
C034028	increase	STX6	ENSG00000135823	16330358;
C034028	increase	TNFAIP3	ENSG00000118503	16330358;

C034028	increase	TREX1	ENSG00000213689	16330358;
C034028	increase	WARS2	ENSG00000116874	16330358;
C034028	increase	ZKSCAN3	ENSG00000189298	16330358;
C034192	decrease	BCL2	ENSG00000171791	16027529;
C034192	decrease	CCNB1	ENSG00000134057	16027529;
C034192	decrease	CDC25C	ENSG00000158402	16027529;
C034192	decrease	CDK1	ENSG00000170312	16027529;
C034192	decrease	XIAP	ENSG00000101966	16027529;
C034192	increase	BAX	ENSG00000087088	16027529;
C034221	affect	CD69	ENSG00000110848	12749906;
C034221	affect	IL2	ENSG00000109471	12749906;
C034221	affect	IL2RA	ENSG00000134460	12749906;
C034221	increase	CCL11	ENSG00000172156	17572062;
C034221	increase	CCL2	ENSG00000108691	17572062;
C034221	increase	CCL3	ENSG00000006075	17572062;
C034221	increase	CCL4	ENSG00000129277	17572062;
C034221	increase	CCL5	ENSG00000161570	17572062;
C034221	increase	IL10	ENSG00000136634	17572062;
C034221	increase	IL8	ENSG00000169429	15356918;17572062;
C034221	increase	MYC	ENSG00000136997	12749906;
C034221	increase	NQO1	ENSG00000181019	8652659;
C034221	increase	TFRC	ENSG00000072274	12749906;
C034240	increase	IL1B	ENSG00000125538	12573452;
C034240	increase	PTGS2	ENSG00000073756	12573452;
C034244	decrease	MYC	ENSG00000136997	11489473;
C034244	increase	NRAS	ENSG00000213281	11489473;
C034244	increase	TP53	ENSG00000141510	11489473;
C034364	decrease	TNFSF11	ENSG00000120659	18264100;
C034364	increase	TNFRSF11B	ENSG00000164761	18264100;
C034528	decrease	F7	ENSG00000057593	14561536;
C034528	decrease	PROC	ENSG00000115718	14561536;
C034534	decrease	CCND1	ENSG00000110092	15475462;16596235;
C034534	increase	CDKN1A	ENSG00000124762	15475462;16596235;
C034534	increase	RARB	ENSG00000077092	15475462;
C034534	increase	TP53	ENSG00000141510	15475462;
C034554	affect	PGR	ENSG00000082175	15806153;
C034587	increase	NPPA	ENSG00000175206	16247790;
C034587	increase	NPPB	ENSG00000120937	16247790;
C034613	decrease	ABCD3	ENSG00000117528	16704987;
C034613	decrease	ADARB1	ENSG00000197381	16704987;
C034613	decrease	AQP3	ENSG00000165272	16704987;
C034613	decrease	BDNF	ENSG00000176697	16704987;
C034613	decrease	C14ORF1	ENSG00000133935	16704987;
C034613	decrease	COL18A1	ENSG00000182871	16704987;
C034613	decrease	CTDSPL	ENSG00000144677	16704987;
C034613	decrease	DDX24	ENSG00000089737	16704987;
C034613	decrease	DHCR7	ENSG00000172893	16704987;
C034613	decrease	EBP	ENSG00000147155	16704987;
C034613	decrease	FGFR3	ENSG00000068078	16704987;
C034613	decrease	GNPDA1	ENSG00000113552	16704987;
C034613	decrease	HMGCR	ENSG00000113161	16704987;
C034613	decrease	HS2ST1	ENSG00000153936	16704987;
C034613	decrease	HSPB11	ENSG00000081870	16704987;
C034613	decrease	ID3	ENSG00000117318	16704987;

C034613	decrease	IDI1	ENSG00000067064	16704987;
C034613	decrease	IGFBP7	ENSG00000163453	16704987;
C034613	decrease	ILVBL	ENSG00000105135	16704987;
C034613	decrease	ISL1	ENSG00000016082	16704987;
C034613	decrease	LSS	ENSG00000160285	16704987;
C034613	decrease	LUC7L3	ENSG00000108848	16704987;
C034613	decrease	NR2F1	ENSG00000175745	16704987;
C034613	decrease	NR4A2	ENSG00000153234	16704987;
C034613	decrease	NRTN	ENSG00000171119	16704987;
C034613	decrease	PDE4DIP	ENSG00000178104	16704987;
C034613	decrease	PDSS2	ENSG00000164494	16704987;
C034613	decrease	SKP2	ENSG00000145604	16704987;
C034613	decrease	SOX9	ENSG00000125398	16704987;
C034613	decrease	SPARC	ENSG00000113140	16704987;
C034613	decrease	SPAST	ENSG00000021574	16704987;
C034613	decrease	SRGAP2	ENSG00000163486	16704987;
C034613	decrease	TNS3	ENSG00000136205	16704987;
C034613	decrease	TOP2A	ENSG00000131747	16704987;
C034613	decrease	ZBED2	ENSG00000177494	16704987;
C034613	increase	ARHGDIA	ENSG00000141522	16704987;
C034613	increase	ATF3	ENSG00000162772	16704987;
C034613	increase	DDIT4	ENSG00000168209	16704987;
C034613	increase	EGR1	ENSG00000120738	16704987;
C034613	increase	FOS	ENSG00000170345	16704987;
C034613	increase	FOSL1	ENSG00000175592	16704987;
C034613	increase	GCLC	ENSG00000001084	16704987;
C034613	increase	GCLM	ENSG00000023909	16704987;
C034613	increase	HMOX1	ENSG00000100292	16704987;
C034613	increase	ITGA6	ENSG00000091409	16704987;
C034613	increase	JUN	ENSG00000177606	16704987;
C034613	increase	KLF5	ENSG00000102554	16704987;
C034613	increase	MAFF	ENSG00000185022	16704987;
C034613	increase	PHLDA1	ENSG00000139289	16704987;
C034613	increase	PMAIP1	ENSG00000141682	16704987;
C034613	increase	PSAT1	ENSG00000135069	16704987;
C034613	increase	PTP4A1	ENSG00000112245	16704987;
C034613	increase	RAPGEF2	ENSG00000109756	16704987;
C034613	increase	SLC7A11	ENSG00000151012	16704987;
C034613	increase	SNAI2	ENSG00000019549	16704987;
C034613	increase	SQSTM1	ENSG00000161011	16704987;
C034613	increase	STC2	ENSG00000113739	16704987;
C034613	increase	TNFRSF12A	ENSG00000006327	16704987;
C034613	increase	YWHAZ	ENSG00000164924	16704987;
C034632	increase	FSHB	ENSG00000131808	14752856;17965791;17515571;
C034632	increase	LHB	ENSG00000104826	14752856;17965791;
C034653	decrease	PGR	ENSG00000082175	11041225;
C034786	decrease	MMP14	ENSG00000157227	18495463;
C034786	decrease	TIMP2	ENSG00000035862	18495463;
C034786	decrease	WT1	ENSG00000184937	18034345;
C034786	increase	GCLM	ENSG00000023909	19188863;
C034786	increase	HMOX1	ENSG00000100292	19188863;
C034786	increase	MGAT3	ENSG00000128268	19090986;
C034786	increase	NQO1	ENSG00000181019	19188863;

C034930	decrease	IL2	ENSG00000109471	11859000;
C035054	affect	CCNE1	ENSG00000105173	15606011;
C035054	affect	CDK2	ENSG00000123374	15606011;
C035054	affect	CDKN1A	ENSG00000124762	15606011;
C035133	increase	HTR2C	ENSG00000147246	18083778;
C035144	increase	SERPINA6	ENSG00000170099	11245553;
C035144	increase	SHBG	ENSG00000129214	11245553;
C035208	increase	TP53	ENSG00000141510	14577614;
C035359	decrease	ESR1	ENSG00000091831	11448926;
C035359	decrease	KCNH2	ENSG00000055118	12775586;
C035359	decrease	PGR	ENSG00000082175	11448926;
C035359	increase	HSPA1A	ENSG00000232804	11448926;
C035605	decrease	BCL2	ENSG00000171791	16019514;
C035965	decrease	TNF	ENSG00000223952	15868479;
C035976	increase	PTGS2	ENSG00000073756	15673847;
C036116	increase	AGT	ENSG00000135744	17214612;
C036116	increase	LDLR	ENSG00000130164	9712728;
C036837	decrease	HSD11B2	ENSG00000176387	18032417;
C036837	increase	TNFRSF10B	ENSG00000120889	17645780;
C036986	increase	FSHB	ENSG00000131808	17515571;
C037032	increase	EPAS1	ENSG00000116016	17973296;
C037032	increase	HIF1A	ENSG00000100644	17973296;
C037112	decrease	CES1	ENSG00000198848	11409902;
C037123	increase	HMOX1	ENSG00000100292	17244614;
C037123	increase	HSPA1A	ENSG00000232804	16460681;17244614;
C037219	affect	E2F1	ENSG00000101412	15450938;
C037219	decrease	ANP32A	ENSG00000140350	18645022;
C037219	decrease	BTF3	ENSG00000145741	18645022;
C037219	decrease	CBX3	ENSG00000122565	18645022;
C037219	decrease	EEF2	ENSG00000167658	18645022;
C037219	decrease	FUBP1	ENSG00000162613	18645022;
C037219	decrease	HNRNPL	ENSG00000104824	18645022;
C037219	decrease	MTHFD2	ENSG00000065911	18645022;
C037219	decrease	NACA	ENSG00000196531	18645022;
C037219	decrease	OLA1	ENSG00000138430	18645022;
C037219	decrease	PDAP1	ENSG00000106244	18645022;
C037219	decrease	PSMA3	ENSG00000100567	18645022;
C037219	decrease	PSMD4	ENSG00000159352	18645022;
C037219	decrease	RANBP1	ENSG00000099901	18645022;
C037219	decrease	RCN2	ENSG00000117906	18645022;
C037219	decrease	RRM1	ENSG00000167325	18645022;
C037219	decrease	TXNDC5	ENSG00000239264	18645022;
C037219	decrease	YBX1	ENSG00000065978	18645022;
C037219	increase	ACOT7	ENSG00000097021	18645022;
C037219	increase	ANXA1	ENSG00000135046	18645022;
C037219	increase	ANXA2	ENSG00000182718	18645022;
C037219	increase	CCNB1	ENSG00000134057	18645022;
C037219	increase	CDKN1A	ENSG00000124762	18645022;
C037219	increase	CDKN2A	ENSG00000147889	19417133;
C037219	increase	CNDP2	ENSG00000133313	18645022;
C037219	increase	CSDE1	ENSG00000009307	18645022;
C037219	increase	EEF1G	ENSG00000149016	18645022;
C037219	increase	FSCN1	ENSG00000075618	18645022;
C037219	increase	LGALS3	ENSG00000131981	18645022;

C037219	increase	LMNA	ENSG00000160789	18645022;
C037219	increase	LTA4H	ENSG00000111144	18645022;
C037219	increase	MLH1	ENSG00000076242	19417133;
C037219	increase	NAMPT	ENSG00000105835	18645022;
C037219	increase	NME2	ENSG00000243678	18645022;
C037219	increase	PAICS	ENSG00000128050	18645022;
C037219	increase	PDHA1	ENSG00000131828	18645022;
C037219	increase	PRKAR1A	ENSG00000108946	18645022;
C037219	increase	PROSC	ENSG00000147471	18645022;
C037219	increase	PSMC1	ENSG00000100764	18645022;
C037219	increase	PSMC5	ENSG00000087191	18645022;
C037219	increase	PSMD7	ENSG00000103035	18645022;
C037219	increase	SERPINB1	ENSG00000021355	18645022;
C037219	increase	SERPINB5	ENSG00000206075	18645022;
C037219	increase	TIMP3	ENSG00000100234	19417133;
C037219	increase	TNF	ENSG00000223952	16973359;
C037219	increase	TP53	ENSG00000141510	18645022;12082016;
C037219	increase	WDR1	ENSG00000071127	18645022;
C037304	decrease	ESR1	ENSG00000091831	18419197;
C037304	decrease	ESR2	ENSG00000140009	18419197;
C037304	increase	CYP3A4	ENSG00000160868	19249324;
C037304	increase	PGR	ENSG00000082175	18419197;
C037304	increase	TFF1	ENSG00000160182	18419197;
C037311	increase	ESR1	ENSG00000091831	15922381;
C037406	decrease	DAP3	ENSG00000132676	16413536;
C037431	increase	TNF	ENSG00000223952	12729831;
C037850	decrease	BCL2	ENSG00000171791	12730627;15519653;
C037850	decrease	BCL2L1	ENSG00000171552	15519653;
C037850	increase	BAX	ENSG00000087088	15519653;
C038018	increase	KEAP1	ENSG00000079999	19033392;
C038091	increase	TFF1	ENSG00000160182	16797915;
C038237	decrease	ABCA1	ENSG00000165029	16118212;
C038293	increase	CD40	ENSG00000101017	16299289;
C038405	affect	IFNG	ENSG00000111537	17166736;
C038405	affect	IL2	ENSG00000109471	17166736;
C038405	increase	ATF3	ENSG00000162772	19101521;
C038405	increase	IL8	ENSG00000169429	17166736;
C038501	decrease	CRP	ENSG00000132693	15990257;
C038753	affect	AHR	ENSG00000106546	15385644;
C038753	increase	TP53	ENSG00000141510	15625077;16467109;
C038864	decrease	BCL2	ENSG00000171791	16909596;16109544;
C038864	decrease	CCND1	ENSG00000110092	16273314;
C038864	decrease	CCND3	ENSG00000112576	16273314;
C038864	increase	BAX	ENSG00000087088	16909596;16109544;
C038864	increase	CASP3	ENSG00000164305	16909596;16157052;16109544;
C038864	increase	CYP19A1	ENSG00000137869	16996190;
C038864	increase	CYP26A1	ENSG00000095596	15589975;
C038864	increase	NRL	ENSG00000129535	16854989;
C038864	increase	TGM2	ENSG00000198959	16158052;
C038867	affect	BIRC5	ENSG00000089685	16061352;
C038867	increase	CDKN1A	ENSG00000124762	16061352;
C038939	increase	TFF1	ENSG00000160182	16183391;
C038983	decrease	EDN1	ENSG00000078401	11603923;

C038983	increase	ALB	ENSG00000163631	11961005;
C038983	increase	CCL2	ENSG00000108691	11961005;17529908;14576080;
C038983	increase	ELANE	ENSG00000197561	11919081;17456367;10843967;
C038983	increase	ICAM1	ENSG00000090339	14572618;
C038983	increase	IL1B	ENSG00000125538	9851740;
C038983	increase	IL6	ENSG00000136244	9344885;
C038983	increase	IL8	ENSG00000169429	16091123;9344885;
C038983	increase	MSR1	ENSG00000038945	9614211;
C038983	increase	TNF	ENSG00000223952	9851740;14576080;14572618;16091123;
C038983	increase	TNFRSF1A	ENSG00000067182	9870925;
C039389	increase	AKR1C1	ENSG00000187134	9973208;
C039537	increase	CD40	ENSG00000101017	16299289;
C039537	increase	CD80	ENSG00000121594	16299289;
C039537	increase	CD83	ENSG00000112149	16299289;
C039537	increase	CD86	ENSG00000114013	16299289;
C039537	increase	IFNG	ENSG00000111537	16299289;
C039537	increase	IL12A	ENSG00000168811	16299289;
C039537	increase	IL12B	ENSG00000113302	16299289;
C039537	increase	IL15	ENSG00000164136	16299289;
C039537	increase	IL8	ENSG00000169429	16299289;
C039671	affect	DDX58	ENSG00000107201	16303604;
C039671	decrease	CES1	ENSG00000198848	11409902;
C039671	increase	CDH1	ENSG00000039068	12391285;
C039671	increase	PTGS2	ENSG00000073756	16417216;
C039961	increase	APAF1	ENSG00000120868	15967204;
C039961	increase	BAX	ENSG00000087088	15967204;
C039961	increase	BCL2	ENSG00000171791	15967204;
C039961	increase	JUN	ENSG00000177606	15967204;
C039961	increase	RELA	ENSG00000173039	15967204;
C039961	increase	TP53	ENSG00000141510	15967204;
C040015	decrease	BAX	ENSG00000087088	15748703;
C040015	decrease	BCL2	ENSG00000171791	15748703;
C040015	decrease	CCNB1	ENSG00000134057	19397994;
C040015	decrease	FBP1	ENSG00000165140	18331776;
C040015	decrease	PSCA	ENSG00000167653	18331776;
C040015	increase	CDKN1A	ENSG00000124762	19397994;
C040015	increase	CDKN2C	ENSG00000123080	18331776;
C040015	increase	HMOX1	ENSG00000100292	16799064;
C040015	increase	NFE2L2	ENSG00000116044	16799064;
C040015	increase	PTGS2	ENSG00000073756	19804834;
C040015	increase	TP53I3	ENSG00000115129	19397994;
C040015	increase	TP63	ENSG00000073282	19397994;
C040015	increase	TP73	ENSG00000078900	19397994;
C040048	affect	TP53	ENSG00000141510	11522280;
C040048	increase	IL8	ENSG00000169429	17636245;
C040048	increase	NOS3	ENSG00000164867	15740983;
C040115	decrease	BCL2	ENSG00000171791	18384088;
C040115	decrease	BCL2L1	ENSG00000171552	18384088;
C040115	increase	BAX	ENSG00000087088	18384088;
C040115	increase	DDIT3	ENSG00000175197	18384088;
C040115	increase	FAS	ENSG00000026103	18384088;

C040115	increase	FASLG	ENSG00000117560	18384088;
C040527	increase	IL1B	ENSG00000125538	10377187;
C040527	increase	TNF	ENSG00000223952	10377187;
C040641	increase	G6PD	ENSG00000160211	16719502;
C040641	increase	MT1A	ENSG00000205362	16719502;
C040641	increase	MT1E	ENSG00000169715	16719502;
C040641	increase	MT1X	ENSG00000187193	16719502;
C040641	increase	MT2A	ENSG00000125148	16719502;
C040641	increase	SLC30A1	ENSG00000170385	16719502;
C040920	decrease	BCL2L1	ENSG00000171552	15729620;
C040920	decrease	BIRC2	ENSG00000110330	15729620;
C040920	decrease	BIRC3	ENSG00000023445	15729620;
C040920	increase	NFKBIA	ENSG00000100906	15729620;
C040937	decrease	CCND1	ENSG00000110092	17332266;
C040937	increase	ATF3	ENSG00000162772	17332266;
C040937	increase	CCNG2	ENSG00000138764	17332266;
C040937	increase	DDIT3	ENSG00000175197	17332266;
C040937	increase	EIF2A	ENSG00000144895	17332266;
C040937	increase	GADD45A	ENSG00000116717	17332266;
C040937	increase	HSPA5	ENSG00000044574	17332266;
C040937	increase	TRPV1	ENSG00000196689	17332266;
C041181	increase	PGR	ENSG00000082175	12753421;
C041181	increase	TFF1	ENSG00000160182	12753421;
C041359	decrease	IL4	ENSG00000113520	15284827;
C041359	decrease	IL5	ENSG00000113525	15284827;
C041359	decrease	MMP2	ENSG00000087245	17172416;
C041359	decrease	MMP9	ENSG00000100985	17172416;
C041359	decrease	RHOB	ENSG00000143878	17172416;
C041517	increase	ABCB1	ENSG00000085563	15566942;
C041517	increase	CDH1	ENSG00000039068	15735009;
C041517	increase	CDKN1A	ENSG00000124762	15735009;
C041517	increase	CTGF	ENSG00000118523	15735009;
C041517	increase	CYP1A1	ENSG00000140465	15566942;15735009;
C041517	increase	CYP1B1	ENSG00000138061	15566942;15735009;
C041517	increase	DDB2	ENSG00000134574	15735009;
C041517	increase	FAS	ENSG00000026103	15735009;
C041517	increase	GADD45A	ENSG00000116717	15735009;
C041517	increase	MTUS1	ENSG00000129422	15735009;
C041517	increase	RAB6A	ENSG00000175582	15735009;
C041517	increase	TFPI2	ENSG00000105825	15735009;
C041517	increase	UGT1A6	ENSG00000167165	15566942;
C041517	increase	UGT1A7	ENSG00000244122	15566942;
C041524	decrease	ADAM9	ENSG00000168615	15961160;
C041524	decrease	GATA2	ENSG00000179348	15961160;
C041524	decrease	GTF2H1	ENSG00000110768	15961160;
C041524	decrease	MTF1	ENSG00000188786	15961160;
C041524	decrease	NEFL	ENSG00000104725	15961160;
C041524	decrease	P2RX1	ENSG00000108405	15961160;
C041524	decrease	ROBO2	ENSG00000185008	15961160;
C041524	decrease	SYN1	ENSG00000008056	15961160;
C041524	decrease	SYP	ENSG00000102003	15961160;
C041524	increase	APP	ENSG00000142192	15961160;
C041524	increase	DAXX	ENSG00000231617	15961160;
C041524	increase	IL18	ENSG00000150782	16100009;

C041524	increase	NFKB1	ENSG00000109320	15961160;
C041524	increase	PLA2G4A	ENSG00000116711	15961160;
C041524	increase	PTGS2	ENSG00000073756	15961160;
C041525	increase	ICAM1	ENSG00000090339	18539414;
C041525	increase	IFNG	ENSG00000111537	15779068;
C041525	increase	IL8	ENSG00000169429	15779068;
C041525	increase	TNF	ENSG00000223952	15779068;
C041594	decrease	ERBB2	ENSG00000141736	16393665;
C041594	decrease	ESR1	ENSG00000091831	11518614;11530281;16393665;17141713;
C041594	increase	PDZK1	ENSG00000174827	16393665;
C041594	increase	PGR	ENSG00000082175	16393665;
C041594	increase	TFF1	ENSG00000160182	16393665;
C041819	affect	CDH1	ENSG00000039068	17034753;
C041819	decrease	FAS	ENSG00000026103	17034753;
C041819	decrease	MCL1	ENSG00000143384	17034753;
C041819	increase	COL1A1	ENSG00000108821	17034753;
C041819	increase	COL4A2	ENSG00000134871	17034753;
C041819	increase	CRABP2	ENSG00000143320	17034753;
C041819	increase	FOXA1	ENSG00000129514	17034753;
C041819	increase	GATA6	ENSG00000141448	17034753;
C041819	increase	IVL	ENSG00000163207	17034753;
C041819	increase	KRT18	ENSG00000111057	17034753;
C041819	increase	KRT19	ENSG00000171345	17034753;
C041819	increase	KRT7	ENSG00000135480	17034753;
C041819	increase	KRT8	ENSG00000170421	17034753;
C041819	increase	MEIS1	ENSG00000143995	17034753;
C041819	increase	MEIS2	ENSG00000134138	17034753;
C041819	increase	MEOX1	ENSG00000005102	17034753;
C041819	increase	OLR1	ENSG00000173391	17034753;
C041819	increase	RARA	ENSG00000131759	17034753;
C041819	increase	RARRES1	ENSG00000118849	17034753;
C041819	increase	RARRES3	ENSG00000133321	17034753;
C041819	increase	TGM2	ENSG00000198959	17034753;
C042080	decrease	CDO1	ENSG00000129596	16223563;
C042080	decrease	PAPSS1	ENSG00000138801	16223563;
C042080	decrease	PAPSS2	ENSG00000198682	16223563;
C042080	decrease	SUOX	ENSG00000139531	16223563;
C042080	increase	SLC5A5	ENSG00000105641	16257484;
C042375	increase	CD40	ENSG00000101017	16879495;
C042431	increase	TP53	ENSG00000141510	17555331;
C042577	decrease	BCL2	ENSG00000171791	17513609;
C042577	decrease	BCL2L1	ENSG00000171552	17513609;
C042577	decrease	FTH1	ENSG00000167996	16707465;
C042577	decrease	FTL	ENSG00000087086	16707465;
C042577	increase	BAK1	ENSG00000030110	17513609;
C042986	increase	ACTA2	ENSG00000107796	16757516;
C042986	increase	ADAM9	ENSG00000168615	17018608;
C042986	increase	IL8	ENSG00000169429	14572605;
C042986	increase	TGFB1	ENSG00000105329	16757516;
C043060	increase	IL12RB1	ENSG00000096996	12932289;
C043060	increase	TNFRSF1B	ENSG00000028137	12932289;
C043062	decrease	CD14	ENSG00000170458	11556524;
C043062	decrease	CD33	ENSG00000105383	11306145;

C043062	decrease	CD40	ENSG00000101017	11306145;
C043062	decrease	CD86	ENSG00000114013	11306145;
C043062	decrease	ITGA4	ENSG00000115232	11556524;
C043062	decrease	ITGAX	ENSG00000140678	11556524;11306145;
C043062	decrease	LRP1	ENSG00000123384	11556524;
C043062	decrease	PTPRC	ENSG00000081237	11306145;
C043561	decrease	BCL2	ENSG00000171791	16215670;
C043561	decrease	CCNB1	ENSG00000134057	18331776;19397994;
C043561	decrease	CDKN2C	ENSG00000123080	18331776;
C043561	decrease	FBP1	ENSG00000165140	18331776;
C043561	decrease	PSCA	ENSG00000167653	18331776;
C043561	decrease	XIAP	ENSG00000101966	16215670;
C043561	increase	ABCG2	ENSG00000118777	17077187;
C043561	increase	CDKN1A	ENSG00000124762	19397994;
C043561	increase	CYP1A1	ENSG00000140465	19766177;17012224;
C043561	increase	EDN1	ENSG00000078401	18457675;
C043561	increase	GADD45B	ENSG00000099860	18331776;
C043561	increase	ICAM1	ENSG00000090339	15322261;
C043561	increase	IL6	ENSG00000136244	18457675;
C043561	increase	IL8	ENSG00000169429	18457675;
C043561	increase	JUN	ENSG00000177606	15322261;
C043561	increase	PTGS2	ENSG00000073756	18457675;
C043561	increase	SFN	ENSG00000175793	18331776;
C043561	increase	TNF	ENSG00000223952	15322261;
C043561	increase	TP53I3	ENSG00000115129	18331776;19397994;
C043561	increase	TP63	ENSG00000073282	18331776;19397994;
C043561	increase	TP73	ENSG00000078900	18331776;19397994;
C043561	increase	UGT1A1	ENSG00000241635	10950852;14557274;11442279;16003560;15104253;
C043561	increase	VCAM1	ENSG00000162692	18457675;
C043562	increase	ABCG2	ENSG00000118777	17077187;
C043562	increase	CYP1A1	ENSG00000140465	15890477;
C043562	increase	CYP1B1	ENSG00000138061	15890477;
C043562	increase	PON1	ENSG00000005421	15169886;
C044387	decrease	G6PD	ENSG00000160211	17018880;
C044387	decrease	GSTA1	ENSG00000243955	17018880;
C044387	increase	HIF1A	ENSG00000100644	16579968;16973622;
C044387	increase	MT1A	ENSG00000205362	17018880;
C044387	increase	MT1E	ENSG00000169715	17018880;
C044387	increase	MT1X	ENSG00000187193	17018880;
C044387	increase	MT2A	ENSG00000125148	17018880;
C044387	increase	MTF1	ENSG00000188786	17018880;
C044387	increase	SLC30A1	ENSG00000170385	17018880;
C044387	increase	SLC30A7	ENSG00000162695	17068819;
C044578	decrease	BCL2	ENSG00000171791	11064002;
C044578	decrease	CSE1L	ENSG00000124207	11064002;
C044815	increase	PGR	ENSG00000082175	11041225;
C044919	increase	CCL2	ENSG00000108691	14576080;
C044919	increase	TNF	ENSG00000223952	14576080;
C045229	decrease	VEGFA	ENSG00000112715	16638750;
C045362	decrease	CYP11A1	ENSG00000140459	19733639;
C045362	decrease	CYP11B1	ENSG00000160882	19733639;
C045362	decrease	CYP17A1	ENSG00000148795	19733639;
C045362	decrease	CYP21A2	ENSG00000231852	19733639;

C045362	decrease	ESR1	ENSG00000091831	15130599;10789375;15261991;
C045362	decrease	HSD3B2	ENSG00000203859	19733639;
C045362	decrease	STAR	ENSG00000147465	19733639;
C045362	increase	ESR2	ENSG00000140009	15130599;15261991;
C045463	increase	CCL26	ENSG00000006606	16045735;
C045463	increase	IL4	ENSG00000113520	16045735;
C045651	decrease	ABCB1	ENSG00000085563	18851785;
C045651	decrease	ABCG1	ENSG00000160179	16084531;
C045651	decrease	ART4	ENSG00000111339	16084531;
C045651	decrease	BAK1	ENSG00000030110	18851785;
C045651	decrease	BCL2	ENSG00000171791	15975156;19557821;12672908;15657356;
C045651	decrease	BPGM	ENSG00000172331	16084531;
C045651	decrease	BTG1	ENSG00000133639	16084531;
C045651	decrease	CCND1	ENSG00000110092	15142168;
C045651	decrease	CDH13	ENSG00000140945	16084531;
C045651	decrease	CDK4	ENSG00000135446	15142168;
C045651	decrease	CDK6	ENSG00000105810	15142168;
C045651	decrease	COL4A1	ENSG00000187498	16084531;
C045651	decrease	CPE	ENSG00000109472	16084531;
C045651	decrease	CRADD	ENSG00000169372	16084531;
C045651	decrease	CTNNB1	ENSG00000168036	16968065;
C045651	decrease	DAPK1	ENSG00000196730	12628509;
C045651	decrease	DTYMK	ENSG00000168393	18851785;
C045651	decrease	EFNB2	ENSG00000125266	16084531;
C045651	decrease	FGF2	ENSG00000138685	16084531;
C045651	decrease	FILIP1L	ENSG00000168386	16084531;
C045651	decrease	GBP1	ENSG00000117228	16084531;
C045651	decrease	GJA4	ENSG00000187513	16084531;
C045651	decrease	GSS	ENSG00000100983	18851785;
C045651	decrease	GSTP1	ENSG00000084207	18851785;
C045651	decrease	GULP1	ENSG00000144366	16084531;
C045651	decrease	HDAC5	ENSG00000108840	18851785;
C045651	decrease	HSPA1B	ENSG00000232804	18851785;
C045651	decrease	HSPA1L	ENSG00000204390	18851785;
C045651	decrease	HSPB7	ENSG00000173641	18851785;
C045651	decrease	HTATIP2	ENSG00000109854	16084531;
C045651	decrease	ID2	ENSG00000115738	16084531;
C045651	decrease	IL10RB	ENSG00000243646	16084531;
C045651	decrease	IL27RA	ENSG00000104998	16084531;
C045651	decrease	IL2	ENSG00000109471	19761891;
C045651	decrease	ITGAV	ENSG00000138448	16084531;
C045651	decrease	ITGB4	ENSG00000132470	12628509;
C045651	decrease	JAG1	ENSG00000101384	16084531;
C045651	decrease	KDR	ENSG00000128052	17992120;
C045651	decrease	MAP3K14	ENSG00000006062	12628509;
C045651	decrease	MGEA5	ENSG00000198408	16084531;
C045651	decrease	MOAP1	ENSG00000165943	16084531;
C045651	decrease	MVP	ENSG00000013364	18851785;
C045651	decrease	NEDD9	ENSG00000111859	16084531;
C045651	decrease	NFKBIA	ENSG00000100906	19557821;
C045651	decrease	NR3C1	ENSG00000113580	16084531;
C045651	decrease	PDLIM5	ENSG00000163110	16084531;

C045651	decrease	PLAT	ENSG00000104368	12628509;
C045651	decrease	PLAU	ENSG00000122861	16084531;
C045651	decrease	RAB11B	ENSG00000185236	18851785;
C045651	decrease	RABEPK	ENSG00000136933	18851785;
C045651	decrease	RHOBTB1	ENSG00000072422	16084531;
C045651	decrease	RHOD	ENSG00000173156	18851785;
C045651	decrease	SELE	ENSG00000007908	16084531;
C045651	decrease	TANK	ENSG00000136560	16084531;
C045651	decrease	TIMP3	ENSG00000100234	12628509;
C045651	decrease	TNFSF10	ENSG00000121858	16084531;
C045651	decrease	TSPAN6	ENSG00000000003	16084531;
C045651	decrease	TXNL1	ENSG00000091164	16084531;
C045651	decrease	TYRO3	ENSG00000092445	12628509;
C045651	decrease	USP5	ENSG00000111667	18851785;
C045651	decrease	XIAP	ENSG00000101966	15705601;
C045651	decrease	ZMYM2	ENSG00000121741	16084531;
C045651	increase	ABCB10	ENSG00000135776	18851785;
C045651	increase	ABCG2	ENSG00000118777	18851785;
C045651	increase	APAF1	ENSG00000120868	15657356;
C045651	increase	ATP2A2	ENSG00000174437	16084531;
C045651	increase	BAX	ENSG00000087088	15764647;15975156;12672908;15705601;18851785;15657356;
C045651	increase	BCL2L1	ENSG00000171552	18348186;
C045651	increase	CCNG1	ENSG00000113328	18851785;
C045651	increase	CCNG2	ENSG00000138764	18851785;
C045651	increase	CDKN1A	ENSG00000124762	15764647;12628509;18851785;
C045651	increase	CDKN2A	ENSG00000147889	16037419;17683969;14633667;
C045651	increase	DDIT3	ENSG00000175197	12628509;16084531;
C045651	increase	DNAJB9	ENSG00000128590	16084531;
C045651	increase	DUSP4	ENSG00000120875	16084531;
C045651	increase	F2	ENSG00000180210	19944065;
C045651	increase	F3	ENSG00000117525	19944065;
C045651	increase	HERPUD1	ENSG00000051108	16084531;
C045651	increase	HIF1A	ENSG00000100644	16005427;
C045651	increase	HK2	ENSG00000159399	16084531;
C045651	increase	HMOX1	ENSG00000100292	16799064;
C045651	increase	IL1B	ENSG00000125538	12628509;19557821;
C045651	increase	IL6	ENSG00000136244	18025290;
C045651	increase	IL8	ENSG00000169429	18413660;
C045651	increase	MCL1	ENSG00000143384	18348186;
C045651	increase	MGMT	ENSG00000170430	14633667;15703815;
C045651	increase	MLH1	ENSG00000076242	14633667;
C045651	increase	MMP7	ENSG00000137673	17928719;
C045651	increase	NFE2L2	ENSG00000116044	16799064;
C045651	increase	PIGA	ENSG00000165195	16084531;
C045651	increase	PIM2	ENSG00000102096	16084531;
C045651	increase	PLD1	ENSG00000075651	15210717;
C045651	increase	PLD2	ENSG00000129219	15210717;
C045651	increase	PPP1CA	ENSG00000172531	18348186;
C045651	increase	PPP1R15A	ENSG00000087074	18851785;
C045651	increase	PTGS2	ENSG00000073756	15210717;19557821;16084531;

C045651	increase	RARA	ENSG00000131759	12628509;
C045651	increase	RARB	ENSG00000077092	16037419;14633667;
C045651	increase	RB1	ENSG00000139687	15142168;18851785;
C045651	increase	RBBP4	ENSG00000162521	18851785;
C045651	increase	SLC39A14	ENSG00000104635	16084531;
C045651	increase	SMN2	ENSG00000172062	17962980;
C045651	increase	TNF	ENSG00000223952	19557821;
C045651	increase	TOP2A	ENSG00000131747	18851785;
C045651	increase	TOP2B	ENSG00000077097	18851785;
C045651	increase	TP53	ENSG00000141510	15764647;15795422;15657356;
C045651	increase	UGDH	ENSG00000109814	16084531;
C045651	increase	ZFP36L2	ENSG00000152518	16084531;
C045816	affect	ABCF1	ENSG00000236342	12015082;
C045816	affect	CCNG2	ENSG00000138764	12015082;
C045816	affect	CHMP5	ENSG00000086065	12015082;
C045816	decrease	PML	ENSG00000140464	12513809;
C045816	decrease	RARA	ENSG00000131759	12513809;
C045856	increase	ABCB1	ENSG00000085563	19034627;
C045856	increase	ABCC2	ENSG00000023839	19034627;
C045856	increase	CYP2B6	ENSG00000197408	19034627;
C045856	increase	CYP3A4	ENSG00000160868	19034627;
C045856	increase	UGT1A1	ENSG00000241635	19034627;
C045950	increase	ABCB1	ENSG00000085563	19070657;19409404;
C045950	increase	CYP1A1	ENSG00000140465	19070657;19409404;
C045950	increase	CYP1A2	ENSG00000140505	19409404;
C046243	decrease	ABCB1	ENSG00000085563	17482571;
C046243	decrease	TP53	ENSG00000141510	17482571;
C046498	increase	APOA1	ENSG00000118137	19013290;
C046498	increase	TNF	ENSG00000223952	10640779;
C046627	increase	ACSL1	ENSG00000151726	19682441;
C046627	increase	CPT1A	ENSG00000110090	19682441;
C046627	increase	PPARA	ENSG00000186951	19682441;
C046760	increase	ATF3	ENSG00000162772	19101521;
C046782	increase	FGF2	ENSG00000138685	16286479;
C046785	decrease	IL10	ENSG00000136634	15614042;
C046785	increase	BIRC3	ENSG00000023445	14527959;
C046785	increase	IL12B	ENSG00000113302	15614042;
C047246	increase	NFKBIA	ENSG00000100906	17638900;
C047246	increase	PMAIP1	ENSG00000141682	17216584;
C047246	increase	PTGS2	ENSG00000073756	17638900;
C047246	increase	SOD2	ENSG00000112096	17638900;
C047246	increase	TNF	ENSG00000223952	17638900;
C047246	increase	TP53	ENSG00000141510	17216584;
C047246	increase	XIAP	ENSG00000101966	17638900;
C047368	decrease	CYP1B1	ENSG00000138061	19794518;
C047368	decrease	NOS3	ENSG00000164867	16891912;
C047368	increase	OLR1	ENSG00000173391	16891912;
C047368	increase	TFF1	ENSG00000160182	14706564;
C047382	increase	BCL2A1	ENSG00000140379	11877450;
C047382	increase	BIRC2	ENSG00000110330	11877450;
C047382	increase	BIRC3	ENSG00000023445	11877450;
C047382	increase	TNF	ENSG00000223952	11877450;
C047426	decrease	ACP5	ENSG00000102575	16341940;

C047426	decrease	KLK10	ENSG00000129451	16341940;
C047426	decrease	LARGE	ENSG00000133424	16341940;
C047426	decrease	LMO7	ENSG00000136153	16341940;
C047426	decrease	MVP	ENSG00000013364	16341940;
C047426	decrease	NSUN2	ENSG00000037474	16341940;
C047426	decrease	TCF12	ENSG00000140262	16341940;
C047426	decrease	TRIM13	ENSG00000204977	16341940;
C047426	decrease	UBE2Q1	ENSG00000160714	16341940;
C047426	increase	ABCB1	ENSG00000085563	17191263;
C047426	increase	ABCC1	ENSG00000103222	17191263;
C047426	increase	ANKRD55	ENSG00000249922	16341940;
C047426	increase	ARHGAP10	ENSG00000071205	16341940;
C047426	increase	C9ORF123	ENSG00000137038	16341940;
C047426	increase	FAM5C	ENSG00000162670	16341940;
C047426	increase	FMNL1	ENSG00000184922	16341940;
C047426	increase	LIPG	ENSG00000101670	16341940;
C047426	increase	NASP	ENSG00000132780	16341940;
C047426	increase	RCC1	ENSG00000180198	16341940;
C047426	increase	RING1	ENSG00000231115	16341940;
C047426	increase	SALL1	ENSG00000103449	16341940;
C047426	increase	SYT4	ENSG00000132872	16341940;
C047637	increase	CRP	ENSG00000132693	17882670;
C047681	decrease	IL5	ENSG00000113525	15650315;
C047686	decrease	TNF	ENSG00000223952	10215738;
C047686	increase	ITGAM	ENSG00000169896	10215738;
C047686	increase	ITGB2	ENSG00000160255	10215738;
C047948	affect	RELA	ENSG00000173039	15905586;
C047948	increase	BCL2A1	ENSG00000140379	15905586;
C047948	increase	BCL2	ENSG00000171791	15905586;
C047948	increase	BCL2L1	ENSG00000171552	15905586;
C047948	increase	BIRC2	ENSG00000110330	15905586;
C047948	increase	BIRC3	ENSG00000023445	15905586;
C047948	increase	BIRC5	ENSG00000089685	15905586;
C047948	increase	CCND1	ENSG00000110092	15905586;
C047948	increase	CFLAR	ENSG00000003402	15905586;
C047948	increase	ICAM1	ENSG00000090339	15905586;
C047948	increase	IKBKB	ENSG00000104365	15905586;
C047948	increase	MAP3K14	ENSG00000006062	15905586;
C047948	increase	MMP9	ENSG00000100985	15905586;
C047948	increase	MYC	ENSG00000136997	15905586;
C047948	increase	PTGS2	ENSG00000073756	15905586;
C047948	increase	TNF	ENSG00000223952	15905586;
C047948	increase	TNFRSF1A	ENSG00000067182	15905586;
C047948	increase	TRADD	ENSG00000102871	15905586;
C047948	increase	TRAF1	ENSG00000056558	15905586;
C047948	increase	TRAF2	ENSG00000127191	15905586;
C047948	increase	VEGFA	ENSG00000112715	15905586;
C047948	increase	XIAP	ENSG00000101966	15905586;
C047981	decrease	TIMP1	ENSG00000102265	15547672;
C047981	increase	MMP1	ENSG00000196611	15547672;
C048336	affect	TERT	ENSG00000164362	18045574;
C048336	increase	MYC	ENSG00000136997	18045574;
C048460	affect	CCND1	ENSG00000110092	18538736;
C048460	affect	CD44	ENSG00000026508	18538736;

C048460	affect	CTNNB1	ENSG00000168036	18538736;
C048460	affect	DACT3	ENSG00000197380	18538736;
C048460	affect	DVL2	ENSG00000004975	18538736;
C048460	affect	HIST3H3	ENSG00000168148	18538736;
C048460	affect	HIST4H4	ENSG00000182217	18538736;
C048460	affect	LEF1	ENSG00000138795	18538736;
C048460	affect	MYC	ENSG00000136997	18538736;
C049051	increase	TF	ENSG00000091513	16772442;
C049051	increase	TFRC	ENSG00000072274	16772442;
C049325	decrease	IVL	ENSG00000163207	10769631;
C049584	increase	CDKN1A	ENSG00000124762	15635149;
C049584	increase	MDM2	ENSG00000135679	15635149;
C049584	increase	TP53	ENSG00000141510	15635149;
C049639	increase	ABCB1	ENSG00000085563	15710601;
C049639	increase	BCL2A1	ENSG00000140379	15710601;
C049639	increase	BCL2	ENSG00000171791	15710601;
C049639	increase	BCL2L1	ENSG00000171552	15710601;
C049639	increase	BIRC2	ENSG00000110330	15710601;
C049639	increase	BIRC3	ENSG00000023445	15710601;
C049639	increase	BIRC5	ENSG00000089685	15710601;
C049639	increase	CCND1	ENSG00000110092	15710601;
C049639	increase	CFLAR	ENSG00000003402	15710601;
C049639	increase	ICAM1	ENSG00000090339	15710601;
C049639	increase	MMP9	ENSG00000100985	15710601;
C049639	increase	MYC	ENSG00000136997	15710601;
C049639	increase	PTGS2	ENSG00000073756	15710601;
C049639	increase	TNF	ENSG00000223952	15710601;
C049639	increase	XIAP	ENSG00000101966	15710601;
C049740	increase	IL1B	ENSG00000125538	12573452;
C049740	increase	PTGS2	ENSG00000073756	12573452;
C050229	affect	KDR	ENSG00000128052	17960570;
C050229	affect	VEGFA	ENSG00000112715	17960570;
C050229	decrease	MMP14	ENSG00000157227	18495463;
C050229	decrease	TIMP2	ENSG00000035862	18495463;
C050229	decrease	WT1	ENSG00000184937	18034345;
C050229	increase	GCLM	ENSG00000023909	19188863;
C050229	increase	HMOX1	ENSG00000100292	19188863;
C050229	increase	NQO1	ENSG00000181019	19188863;
C050414	decrease	AR	ENSG00000169083	17010675;
C050414	decrease	ARID5B	ENSG00000150347	17010675;
C050414	decrease	BIRC5	ENSG00000089685	17010675;
C050414	decrease	BTG1	ENSG00000133639	17010675;
C050414	decrease	C21ORF33	ENSG00000160221	17010675;
C050414	decrease	C5ORF13	ENSG00000134986	17010675;
C050414	decrease	CDC7	ENSG00000097046	17010675;
C050414	decrease	CDK2	ENSG00000123374	17010675;
C050414	decrease	CITED2	ENSG00000164442	17010675;
C050414	decrease	CYP1A1	ENSG00000140465	17010675;
C050414	decrease	EFNB2	ENSG00000125266	17010675;
C050414	decrease	EGFR	ENSG00000146648	17010675;
C050414	decrease	FLT3	ENSG00000122025	17010675;
C050414	decrease	IL1RN	ENSG00000136689	17010675;
C050414	decrease	JAG1	ENSG00000101384	17010675;
C050414	decrease	KCNN2	ENSG00000080709	17010675;

C050414	decrease	MAFB	ENSG00000204103	17010675;
C050414	decrease	MDH2	ENSG00000146701	17010675;
C050414	decrease	MSH6	ENSG00000116062	17010675;
C050414	decrease	MYCBP2	ENSG00000005810	17010675;
C050414	decrease	MYNN	ENSG00000085274	17010675;
C050414	decrease	NBN	ENSG00000104320	17010675;
C050414	decrease	NKX3-1	ENSG00000167034	17010675;
C050414	decrease	OPRK1	ENSG00000082556	17010675;
C050414	decrease	PDE9A	ENSG00000160191	17010675;
C050414	decrease	PIK3CD	ENSG00000171608	17010675;
C050414	decrease	PIK3R1	ENSG00000145675	17010675;
C050414	decrease	PPIF	ENSG00000108179	17010675;
C050414	decrease	PURA	ENSG00000185129	17010675;
C050414	decrease	RRM2	ENSG00000171848	17010675;
C050414	decrease	SESN1	ENSG00000080546	17010675;
C050414	decrease	SLTM	ENSG00000137776	17010675;
C050414	decrease	SOX4	ENSG00000124766	17010675;
C050414	decrease	TAOK3	ENSG00000135090	17010675;
C050414	decrease	TMPRSS2	ENSG00000184012	17010675;
C050414	decrease	TXNIP	ENSG00000117289	17010675;
C050414	decrease	WDR77	ENSG00000116455	17010675;
C050414	decrease	ZBTB43	ENSG00000169155	17010675;
C050414	decrease	ZC3H12A	ENSG00000163874	17010675;
C050414	increase	AHSA1	ENSG00000100591	17010675;
C050414	increase	AKR1C1	ENSG00000187134	17010675;
C050414	increase	AKR1C2	ENSG00000151632	17010675;
C050414	increase	ATF3	ENSG00000162772	17010675;
C050414	increase	BAG3	ENSG00000151929	17010675;
C050414	increase	C20ORF111	ENSG00000132823	17010675;
C050414	increase	CACYBP	ENSG00000116161	17010675;
C050414	increase	CBR3	ENSG00000159231	17010675;
C050414	increase	CHORDC1	ENSG00000110172	17010675;
C050414	increase	DDIT3	ENSG00000175197	17010675;
C050414	increase	DDIT4	ENSG00000168209	17010675;
C050414	increase	DNAJA1	ENSG00000086061	17010675;
C050414	increase	DNAJB4	ENSG00000162616	17010675;
C050414	increase	FICD	ENSG00000198855	17010675;
C050414	increase	GCLM	ENSG00000023909	17010675;
C050414	increase	GDF15	ENSG00000130513	17010675;
C050414	increase	GTPBP2	ENSG00000172432	17010675;
C050414	increase	HMGCS1	ENSG00000112972	17010675;
C050414	increase	HMOX1	ENSG00000100292	17010675;
C050414	increase	HSPA1A	ENSG00000232804	17010675;
C050414	increase	HSPA1B	ENSG00000232804	17010675;
C050414	increase	HSPA5	ENSG00000044574	17010675;
C050414	increase	HSPH1	ENSG00000120694	17010675;
C050414	increase	ICAM1	ENSG00000090339	16769766;
C050414	increase	IFNG	ENSG00000111537	16769766;
C050414	increase	IL1B	ENSG00000125538	16769766;
C050414	increase	INSIG1	ENSG00000186480	17010675;
C050414	increase	LDLR	ENSG00000130164	17010675;
C050414	increase	LPIN1	ENSG00000134324	17010675;
C050414	increase	ME1	ENSG00000065833	17010675;
C050414	increase	MNT	ENSG00000070444	17010675;

C050414	increase	MRPL18	ENSG00000112110	17010675;
C050414	increase	SC4MOL	ENSG00000052802	17010675;
C050414	increase	SELE	ENSG00000007908	16769766;
C050414	increase	SERP1	ENSG00000120742	17010675;
C050414	increase	SLC3A2	ENSG00000168003	17010675;
C050414	increase	SLC7A11	ENSG00000151012	17010675;
C050414	increase	STIP1	ENSG00000168439	17010675;
C050414	increase	TNF	ENSG00000223952	16769766;
C050414	increase	UBE2B	ENSG00000119048	17010675;
C050414	increase	UGDH	ENSG00000109814	17010675;
C050414	increase	VCAM1	ENSG00000162692	16769766;
C050510	increase	GADD45A	ENSG00000116717	16814109;16421274;
C050718	affect	CAT	ENSG00000121691	15576159;
C050718	increase	PTGS2	ENSG00000073756	15576159;
C050950	affect	BAX	ENSG00000087088	16002045;
C050950	affect	BCL2	ENSG00000171791	16002045;
C050950	affect	TP53	ENSG00000141510	16002045;
C051140	decrease	APOA1	ENSG00000118137	10898742;
C051140	decrease	APOA4	ENSG00000110244	10898742;
C051140	decrease	APOB	ENSG00000084674	10898742;
C051140	decrease	NR1H2	ENSG00000131408	17070507;
C051140	decrease	NR1H3	ENSG00000025434	17070507;
C051140	decrease	PPARG	ENSG00000132170	17070507;
C051140	increase	ABCA1	ENSG00000165029	17070507;
C051140	increase	CD36	ENSG00000135218	17070507;
C051140	increase	PON2	ENSG00000105854	17916643;
C051781	increase	PGR	ENSG00000082175	16242299;
C051781	increase	TFF1	ENSG00000160182	10064545;16242299;
C051786	increase	CYP3A4	ENSG00000160868	11133395;16565514;
C051883	increase	CDKN1A	ENSG00000124762	16039115;
C051883	increase	CDKN1B	ENSG00000111276	16039115;
C051890	affect	CDK6	ENSG00000105810	18927307;
C051890	affect	CDKN1A	ENSG00000124762	15132777;
C051890	affect	EIF2AK4	ENSG00000128829	18927307;
C051890	affect	FCF1	ENSG00000119616	18927307;
C051890	affect	GJA1	ENSG00000152661	18927307;
C051890	affect	GLRX3	ENSG00000108010	18927307;
C051890	affect	IMMT	ENSG00000132305	18927307;
C051890	affect	MDN1	ENSG00000112159	18927307;
C051890	affect	RB1	ENSG00000139687	15132777;
C051890	affect	SAMHD1	ENSG00000101347	18927307;
C051890	affect	SMURF1	ENSG00000198742	18927307;
C051890	affect	TRIB2	ENSG00000071575	18927307;
C051890	decrease	ABCC1	ENSG00000103222	16815871;15897249;18927307;
C051890	decrease	ABHD13	ENSG00000139826	18927307;
C051890	decrease	ACACA	ENSG00000132142	18927307;
C051890	decrease	ADD3	ENSG00000148700	15956246;
C051890	decrease	ADI1	ENSG00000182551	18927307;
C051890	decrease	AGT	ENSG00000135744	18927307;
C051890	decrease	AKAP10	ENSG00000108599	15956246;
C051890	decrease	ALB	ENSG00000163631	18927307;
C051890	decrease	ALDH1A1	ENSG00000165092	15956246;
C051890	decrease	ANKRD17	ENSG00000132466	18927307;

C051890	decrease	ANKRD28	ENSG00000206560	18927307;
C051890	decrease	ANXA13	ENSG00000104537	18927307;
C051890	decrease	AP2B1	ENSG00000006125	18927307;
C051890	decrease	AP2S1	ENSG00000042753	16391804;
C051890	decrease	APC	ENSG00000134982	15956246;
C051890	decrease	ARF1	ENSG00000143761	18927307;
C051890	decrease	ARHGEF12	ENSG00000196914	18927307;
C051890	decrease	ARID2	ENSG00000189079	18927307;
C051890	decrease	ARID4A	ENSG00000032219	15956246;
C051890	decrease	ARMC8	ENSG00000114098	18927307;
C051890	decrease	ARSB	ENSG00000113273	18927307;
C051890	decrease	ASS1	ENSG00000130707	16391804;
C051890	decrease	ATG10	ENSG00000152348	15956246;
C051890	decrease	ATP5S	ENSG00000125375	18927307;
C051890	decrease	ATPBD4	ENSG00000134146	18927307;
C051890	decrease	ATRX	ENSG00000085224	18927307;
C051890	decrease	AURKB	ENSG00000178999	15956246;
C051890	decrease	BCL2	ENSG00000171791	18949393;16391804;
C051890	decrease	BCL2L1	ENSG00000171552	18182997;
C051890	decrease	BCR	ENSG00000186716	18927307;
C051890	decrease	BIRC5	ENSG00000089685	16373703;15956246;18182997;
C051890	decrease	BTBD3	ENSG00000132640	18927307;
C051890	decrease	BUB1	ENSG00000169679	15956246;
C051890	decrease	C14ORF118	ENSG00000089916	18927307;
C051890	decrease	C16ORF87	ENSG00000155330	18927307;
C051890	decrease	C1ORF43	ENSG00000143612	18927307;
C051890	decrease	C5ORF28	ENSG00000151881	18927307;
C051890	decrease	C7ORF44	ENSG00000106603	18927307;
C051890	decrease	C9ORF100	ENSG00000137135	18927307;
C051890	decrease	C9ORF25	ENSG00000164970	18927307;
C051890	decrease	CABIN1	ENSG00000099991	18927307;
C051890	decrease	CACNA1I	ENSG00000100346	18927307;
C051890	decrease	CASP4	ENSG00000196954	15956246;
C051890	decrease	CCDC76	ENSG00000122435	18927307;
C051890	decrease	CCNA2	ENSG00000145386	15956246;
C051890	decrease	CCNB2	ENSG00000157456	16734730;15956246;
C051890	decrease	CCNF	ENSG00000162063	15956246;
C051890	decrease	CCR6	ENSG00000112486	15956246;
C051890	decrease	CCT6A	ENSG00000146731	18927307;
C051890	decrease	CDC25B	ENSG00000101224	15956246;
C051890	decrease	CDC42BPA	ENSG00000143776	18927307;
C051890	decrease	CDH4	ENSG00000179242	18927307;
C051890	decrease	CDK1	ENSG00000170312	15956246;
C051890	decrease	CDKN3	ENSG00000100526	15956246;
C051890	decrease	CDON	ENSG00000064309	18927307;
C051890	decrease	CENPE	ENSG00000138778	18927307;15956246;
C051890	decrease	CENPF	ENSG00000117724	15956246;
C051890	decrease	CEP192	ENSG00000101639	18927307;
C051890	decrease	CEP290	ENSG00000198707	18927307;
C051890	decrease	CFLAR	ENSG00000003402	16373718;
C051890	decrease	CHD1	ENSG00000153922	15956246;
C051890	decrease	CLTC	ENSG00000141367	18927307;
C051890	decrease	COL4A4	ENSG00000081052	18927307;

C051890	decrease	CREBBP	ENSG00000005339	18927307;15956246;
C051890	decrease	CRIM1	ENSG00000150938	18927307;
C051890	decrease	CSH2	ENSG00000213218	18927307;
C051890	decrease	CTBP2	ENSG00000175029	18927307;16391804;
C051890	decrease	CUL4A	ENSG00000139842	18927307;
C051890	decrease	CXCL12	ENSG00000107562	18927307;
C051890	decrease	DAGLB	ENSG00000164535	18927307;
C051890	decrease	DCAF8	ENSG00000132716	18927307;
C051890	decrease	DCLRE1A	ENSG00000198924	15956246;
C051890	decrease	DDHD1	ENSG00000100523	18927307;
C051890	decrease	DEDD	ENSG00000158796	18927307;
C051890	decrease	DEFA1	ENSG00000240247	15956246;
C051890	decrease	DENND4A	ENSG00000174485	15956246;
C051890	decrease	DENR	ENSG00000139726	18927307;
C051890	decrease	DEPDC1B	ENSG00000035499	18927307;
C051890	decrease	DGCR8	ENSG00000128191	18927307;
C051890	decrease	DHFRL1	ENSG00000178700	18927307;
C051890	decrease	DIP2A	ENSG00000248658	18927307;
C051890	decrease	DLC1	ENSG00000164741	18927307;
C051890	decrease	DLG1	ENSG00000075711	18927307;
C051890	decrease	DPH2	ENSG00000132768	18927307;
C051890	decrease	DPYD	ENSG00000188641	16734730;15956246;
C051890	decrease	DRG1	ENSG00000185721	15897249;
C051890	decrease	DST	ENSG00000151914	18927307;
C051890	decrease	DYRK1A	ENSG00000157540	15956246;
C051890	decrease	DYRK2	ENSG00000127334	15956246;
C051890	decrease	ENO1	ENSG00000074800	18927307;
C051890	decrease	EP300	ENSG00000100393	15956246;
C051890	decrease	ERC1	ENSG00000082805	18927307;
C051890	decrease	EREG	ENSG00000124882	18927307;
C051890	decrease	ETV6	ENSG00000139083	15956246;
C051890	decrease	EXOC4	ENSG00000131558	18927307;
C051890	decrease	FAM3C	ENSG00000196937	18927307;
C051890	decrease	FARSB	ENSG00000116120	18927307;
C051890	decrease	FGF9	ENSG00000102678	18927307;
C051890	decrease	FRMD5	ENSG00000171877	18927307;
C051890	decrease	FTO	ENSG00000140718	18927307;
C051890	decrease	FYB	ENSG00000082074	18927307;
C051890	decrease	GATA3	ENSG00000107485	15956246;
C051890	decrease	GGT1	ENSG00000100031	18927307;
C051890	decrease	GK	ENSG00000198814	18927307;
C051890	decrease	GPATCH4	ENSG00000160818	18927307;
C051890	decrease	HBG1	ENSG00000213934	18927307;
C051890	decrease	HCFC1	ENSG00000172534	18927307;
C051890	decrease	HECTD2	ENSG00000165338	18927307;
C051890	decrease	HELLS	ENSG00000119969	18927307;
C051890	decrease	HIST1H2AC	ENSG00000180573	15956246;
C051890	decrease	HIST1H4C	ENSG00000182217	18927307;
C051890	decrease	HLA-B	ENSG00000234745	18927307;
C051890	decrease	HMMR	ENSG00000072571	15956246;
C051890	decrease	HN1	ENSG00000189159	18927307;
C051890	decrease	HNRPDL	ENSG00000152795	18927307;
C051890	decrease	HSPA4L	ENSG00000164070	15956246;
C051890	decrease	ID2	ENSG00000115738	15956246;

C051890	decrease	IL18	ENSG00000150782	16391804;
C051890	decrease	IMMP2L	ENSG00000184903	18927307;
C051890	decrease	ING3	ENSG00000071243	18927307;
C051890	decrease	INSIG1	ENSG00000186480	18927307;
C051890	decrease	ITGAV	ENSG00000138448	15956246;
C051890	decrease	ITGB3BP	ENSG00000142856	15956246;
C051890	decrease	KHDRBS1	ENSG00000121774	18927307;
C051890	decrease	KIAA0101	ENSG00000166803	16391804;
C051890	decrease	KIAA1267	ENSG00000120071	18927307;
C051890	decrease	KIAA1731	ENSG00000166004	18927307;
C051890	decrease	KIF14	ENSG00000118193	18927307;
C051890	decrease	KNG1	ENSG00000113889	16391804;
C051890	decrease	KRIT1	ENSG00000001631	18927307;
C051890	decrease	KRT5	ENSG00000186081	15956246;
C051890	decrease	LBH	ENSG00000213626	18927307;
C051890	decrease	LOXL3	ENSG00000115318	18927307;
C051890	decrease	LPCAT1	ENSG00000153395	18927307;
C051890	decrease	LRCH3	ENSG00000186001	18927307;
C051890	decrease	LTF	ENSG00000012223	15956246;
C051890	decrease	LTV1	ENSG00000135521	18927307;
C051890	decrease	MAD2L1	ENSG00000164109	15956246;
C051890	decrease	MAP3K5	ENSG00000197442	15956246;
C051890	decrease	MAP4K3	ENSG00000011566	18927307;
C051890	decrease	MATR3	ENSG00000015479	18927307;
C051890	decrease	MBNL1	ENSG00000152601	18927307;
C051890	decrease	MBP	ENSG00000197971	18927307;
C051890	decrease	MCCC2	ENSG00000131844	18927307;
C051890	decrease	MCF2L	ENSG00000126217	15956246;
C051890	decrease	MCM4	ENSG00000104738	15956246;
C051890	decrease	MED13	ENSG00000108510	18927307;
C051890	decrease	MIP	ENSG00000135517	18927307;
C051890	decrease	MKX	ENSG00000150051	18927307;
C051890	decrease	MPO	ENSG00000005381	15956246;
C051890	decrease	MRPS30	ENSG00000112996	18927307;
C051890	decrease	MSH2	ENSG00000095002	18949393;15956246;
C051890	decrease	MTHFD1L	ENSG00000120254	18927307;
C051890	decrease	MTHFS	ENSG00000136371	15956246;
C051890	decrease	MX2	ENSG00000183486	15956246;
C051890	decrease	MYH10	ENSG00000133026	18927307;
C051890	decrease	MYSM1	ENSG00000162601	18927307;
C051890	decrease	N4BP1	ENSG00000102921	18927307;
C051890	decrease	NCK1	ENSG00000158092	15956246;
C051890	decrease	NCOR1	ENSG00000141027	18927307;
C051890	decrease	NEK2	ENSG00000117650	15956246;
C051890	decrease	NFKBIA	ENSG00000100906	18182997;
C051890	decrease	NQO1	ENSG00000181019	18927307;
C051890	decrease	NSF	ENSG00000073969	18927307;
C051890	decrease	NUP153	ENSG00000124789	18927307;
C051890	decrease	NUSAP1	ENSG00000137804	18927307;
C051890	decrease	PAM	ENSG00000145730	18927307;
C051890	decrease	PAN3	ENSG00000152520	18927307;
C051890	decrease	PAX5	ENSG00000196092	18927307;
C051890	decrease	PAX7	ENSG00000009709	18927307;
C051890	decrease	PCBD2	ENSG00000132570	18927307;

C051890	decrease	PDE4B	ENSG00000184588	15956246;
C051890	decrease	PDLIM5	ENSG00000163110	18927307;
C051890	decrease	PHLDA1	ENSG00000139289	18927307;
C051890	decrease	PIGW	ENSG00000184886	18927307;
C051890	decrease	PLAT	ENSG00000104368	18927307;
C051890	decrease	PLEKHA5	ENSG00000052126	18927307;
C051890	decrease	PLK1	ENSG00000166851	15956246;
C051890	decrease	POGZ	ENSG00000248742	18927307;
C051890	decrease	POLK	ENSG00000122008	18927307;
C051890	decrease	PPP3CB	ENSG00000107758	15956246;
C051890	decrease	PPPDE1	ENSG00000121644	18927307;
C051890	decrease	PRKAA1	ENSG00000132356	18927307;
C051890	decrease	PROM1	ENSG00000007062	15956246;
C051890	decrease	PTK2	ENSG00000169398	18927307;
C051890	decrease	PTP4A1	ENSG00000112245	18927307;
C051890	decrease	PTPN13	ENSG00000163629	15956246;
C051890	decrease	PTPRG	ENSG00000144724	18927307;
C051890	decrease	RAD23B	ENSG00000119318	15956246;
C051890	decrease	RAD51L1	ENSG00000182185	18927307;
C051890	decrease	RASA1	ENSG00000145715	18927307;
C051890	decrease	RBL1	ENSG00000080839	15956246;
C051890	decrease	RBPJ	ENSG00000168214	16391804;
C051890	decrease	REPS1	ENSG00000135597	18927307;
C051890	decrease	RFC3	ENSG00000133119	16734730;15956246;
C051890	decrease	RGS22	ENSG00000132554	18927307;
C051890	decrease	RPL4	ENSG00000174444	18927307;
C051890	decrease	RUNX1	ENSG00000159216	15956246;
C051890	decrease	SCAI	ENSG00000173611	18927307;
C051890	decrease	SCAMP3	ENSG00000116521	16391804;
C051890	decrease	SEC31A	ENSG00000138674	18927307;
C051890	decrease	SENP6	ENSG00000112701	18927307;
C051890	decrease	SERPINB5	ENSG00000206075	16391804;
C051890	decrease	SETD1B	ENSG00000139718	18927307;
C051890	decrease	SETD5	ENSG00000168137	18927307;
C051890	decrease	SFN	ENSG00000175793	16373703;
C051890	decrease	SFRS15	ENSG00000156304	18927307;
C051890	decrease	SGOL2	ENSG00000163535	18927307;
C051890	decrease	SLC16A4	ENSG00000168679	15956246;
C051890	decrease	SLC19A1	ENSG00000173638	18927307;
C051890	decrease	SLC25A21	ENSG00000183032	18927307;
C051890	decrease	SLC30A1	ENSG00000170385	18927307;
C051890	decrease	SMAD3	ENSG00000166949	18927307;
C051890	decrease	SMCHD1	ENSG00000101596	18927307;
C051890	decrease	SMEK2	ENSG00000138041	18927307;
C051890	decrease	SMU1	ENSG00000122692	18927307;
C051890	decrease	SNRPA1	ENSG00000131876	18927307;
C051890	decrease	SNX18	ENSG00000178996	15956246;
C051890	decrease	SP1	ENSG00000185591	18927307;17441964;
C051890	decrease	SRP19	ENSG00000153037	16391804;
C051890	decrease	STK4	ENSG00000101109	18927307;15956246;
C051890	decrease	SYT1	ENSG00000067715	18927307;
C051890	decrease	TAF2	ENSG00000064313	15956246;
C051890	decrease	TAF5L	ENSG00000135801	16391804;
C051890	decrease	TCF12	ENSG00000140262	18927307;

C051890	decrease	TCF7L2	ENSG00000148737	18927307;
C051890	decrease	TFCP2	ENSG00000135457	18927307;
C051890	decrease	TFPI	ENSG00000003436	18927307;
C051890	decrease	TGFBI	ENSG00000120708	18927307;
C051890	decrease	THEM4	ENSG00000159445	18927307;
C051890	decrease	THNSL1	ENSG00000185875	18927307;
C051890	decrease	THRAP3	ENSG00000054118	18927307;
C051890	decrease	TMEM123	ENSG00000152558	18927307;
C051890	decrease	TMEM41B	ENSG00000166471	15956246;
C051890	decrease	TNFAIP6	ENSG00000123610	15956246;
C051890	decrease	TOP2A	ENSG00000131747	15239142;15956246;
C051890	decrease	TOPORS	ENSG00000197579	15956246;
C051890	decrease	TPX2	ENSG00000088325	15956246;
C051890	decrease	TSPAN8	ENSG00000127324	16391804;
C051890	decrease	TTK	ENSG00000112742	15956246;
C051890	decrease	UBE2C	ENSG00000175063	15956246;
C051890	decrease	UBE2E3	ENSG00000170035	18927307;
C051890	decrease	UBE2J2	ENSG00000160087	18927307;
C051890	decrease	UCHL5	ENSG00000116750	15956246;
C051890	decrease	UGGT2	ENSG00000102595	18927307;
C051890	decrease	ULK4	ENSG00000168038	18927307;
C051890	decrease	USO1	ENSG00000138768	18927307;
C051890	decrease	USP24	ENSG00000162402	18927307;
C051890	decrease	VPS4A	ENSG00000132612	18927307;
C051890	decrease	VTI1A	ENSG00000151532	18927307;
C051890	decrease	WAPAL	ENSG00000062650	18927307;
C051890	decrease	WASL	ENSG00000106299	18927307;
C051890	decrease	XPO7	ENSG00000130227	18927307;
C051890	decrease	XRCC4	ENSG00000152422	15956246;
C051890	decrease	XRCC5	ENSG00000079246	18927307;
C051890	decrease	YAF2	ENSG00000015153	18927307;
C051890	decrease	YES1	ENSG00000176105	15956246;
C051890	decrease	ZBTB20	ENSG00000181722	18927307;
C051890	decrease	ZC3HAV1	ENSG00000105939	18927307;
C051890	decrease	ZDHHC21	ENSG00000175893	18927307;
C051890	decrease	ZFP161	ENSG00000198081	18927307;
C051890	decrease	ZFP36L2	ENSG00000152518	15956246;
C051890	decrease	ZFX	ENSG00000005889	15956246;
C051890	decrease	ZNF207	ENSG00000010244	18927307;
C051890	decrease	ZNF323	ENSG00000235109	18927307;
C051890	decrease	ZNF574	ENSG00000105732	18927307;
C051890	decrease	ZNF668	ENSG00000167394	18927307;
C051890	decrease	ZNRF1	ENSG00000186187	18927307;
C051890	increase	ADAM19	ENSG00000135074	18927307;
C051890	increase	ALAS2	ENSG00000158578	15956246;
C051890	increase	AMIGO2	ENSG00000139211	18927307;
C051890	increase	ANXA2	ENSG00000182718	15152939;
C051890	increase	ANXA6	ENSG00000197043	15956246;
C051890	increase	ARAF	ENSG00000078061	15956246;
C051890	increase	AREG	ENSG00000205595	15723263;
C051890	increase	ARHGAP5	ENSG00000100852	15956246;
C051890	increase	ARL4C	ENSG00000188042	18927307;
C051890	increase	ATF3	ENSG00000162772	15956246;
C051890	increase	ATL2	ENSG00000119787	18927307;

C051890	increase	ATP6	ENSG00000198899	16391804;
C051890	increase	BAMBI	ENSG00000095739	18927307;
C051890	increase	BCL7A	ENSG00000110987	15956246;
C051890	increase	BCL9	ENSG00000116128	15956246;
C051890	increase	BMP8A	ENSG00000183682	15956246;
C051890	increase	C11ORF80	ENSG00000173715	18927307;
C051890	increase	CCNG2	ENSG00000138764	18927307;
C051890	increase	CCR1	ENSG00000163823	15956246;
C051890	increase	CD47	ENSG00000196776	18927307;
C051890	increase	CD70	ENSG00000125726	15956246;
C051890	increase	CD80	ENSG00000121594	15956246;
C051890	increase	CDK5R2	ENSG00000171450	15956246;
C051890	increase	CES2	ENSG00000172831	16203781;16033949;16963839;15655543;12171891;11716702;15592324;12171903;10728672;15475733;15100172;14581373;
C051890	increase	CLGN	ENSG00000153132	18927307;
C051890	increase	CNNM2	ENSG00000148842	18927307;
C051890	increase	COL13A1	ENSG00000197467	18927307;
C051890	increase	COL6A1	ENSG00000142156	15956246;
C051890	increase	COMMD6	ENSG00000188243	15956246;
C051890	increase	COPA	ENSG00000122218	18927307;
C051890	increase	CPE	ENSG00000109472	18927307;
C051890	increase	CTH	ENSG00000116761	18927307;
C051890	increase	CYP3A5	ENSG00000106258	15897249;
C051890	increase	DCTN2	ENSG00000175203	18927307;
C051890	increase	DDB2	ENSG00000134574	15956246;
C051890	increase	DLX4	ENSG00000108813	18927307;
C051890	increase	DMWD	ENSG00000185800	15956246;
C051890	increase	DUSP2	ENSG00000158050	15956246;
C051890	increase	EGR1	ENSG00000120738	15956246;
C051890	increase	ERBB4	ENSG00000178568	15956246;
C051890	increase	ETS2	ENSG00000157557	15956246;
C051890	increase	FABP6	ENSG00000170231	18927307;
C051890	increase	FANCG	ENSG00000221829	15956246;
C051890	increase	FAS	ENSG00000026103	16204068;
C051890	increase	FASLG	ENSG00000117560	15897249;
C051890	increase	FGF2	ENSG00000138685	15956246;
C051890	increase	FN1	ENSG00000115414	18927307;15956246;
C051890	increase	FOSB	ENSG00000125740	15956246;
C051890	increase	FOS	ENSG00000170345	15956246;
C051890	increase	FXYD3	ENSG00000089356	15152939;
C051890	increase	GDF15	ENSG00000130513	15956246;
C051890	increase	GNA13	ENSG00000120063	18927307;
C051890	increase	GPR109B	ENSG00000182782	15956246;
C051890	increase	H2AFX	ENSG00000188486	15956246;
C051890	increase	HBEGF	ENSG00000113070	15723263;
C051890	increase	HLA-DRB1	ENSG00000196126	18927307;
C051890	increase	HLA-DRB4	ENSG00000231021	18927307;
C051890	increase	HRG	ENSG00000113905	15956246;
C051890	increase	HS3ST3B1	ENSG00000125430	18927307;
C051890	increase	HTATIP2	ENSG00000109854	16391804;
C051890	increase	IFI27L2	ENSG00000119632	16391804;

C051890	increase	IFI6	ENSG00000126709	18927307;
C051890	increase	IFITM1	ENSG00000185885	18927307;
C051890	increase	IGF1	ENSG00000017427	15956246;
C051890	increase	IL1B	ENSG00000125538	15956246;
C051890	increase	IL8	ENSG00000169429	15723263;
C051890	increase	ITGA2	ENSG00000164171	18927307;
C051890	increase	ITGAX	ENSG00000140678	15956246;
C051890	increase	JUN	ENSG00000177606	15956246;
C051890	increase	KLF4	ENSG00000136826	18927307;
C051890	increase	KLF9	ENSG00000119138	15956246;
C051890	increase	KLHL24	ENSG00000114796	18927307;
C051890	increase	LMF1	ENSG00000103227	16391804;
C051890	increase	MAL	ENSG00000172005	15956246;
C051890	increase	MALL	ENSG00000144063	15956246;
C051890	increase	6-Mar	ENSG00000145495	18927307;
C051890	increase	MATN2	ENSG00000132561	18927307;
C051890	increase	MDM2	ENSG00000135679	15956246;
C051890	increase	MEF2B	ENSG00000213999	15956246;
C051890	increase	MMP9	ENSG00000100985	15956246;
C051890	increase	MPP7	ENSG00000150054	18927307;
C051890	increase	NCOA1	ENSG00000084676	15956246;
C051890	increase	NID1	ENSG00000116962	15956246;
C051890	increase	NRIP1	ENSG00000180530	16391804;
C051890	increase	OPHN1	ENSG00000079482	18927307;
C051890	increase	OSMR	ENSG00000145623	16734730;15956246;
C051890	increase	PAG1	ENSG00000076641	18927307;
C051890	increase	PCDHGC3	ENSG00000240184	15956246;
C051890	increase	PGM2L1	ENSG00000165434	18927307;
C051890	increase	PLCXD2	ENSG00000240891	18927307;
C051890	increase	PLEKHH3	ENSG00000068137	15956246;
C051890	increase	PLK2	ENSG00000145632	15956246;
C051890	increase	PLK3	ENSG00000173846	15956246;
C051890	increase	PLRG1	ENSG00000171566	16391804;
C051890	increase	PMAIP1	ENSG00000141682	15956246;
C051890	increase	PNRC1	ENSG00000146278	18927307;
C051890	increase	PPM1D	ENSG00000170836	18927307;
C051890	increase	PPP3CA	ENSG00000138814	18927307;16391804;
C051890	increase	PRR13	ENSG00000205352	16391804;
C051890	increase	PTCH1	ENSG00000185920	16391804;
C051890	increase	PTGES	ENSG00000148344	15956246;
C051890	increase	PTPN22	ENSG00000248536	15956246;
C051890	increase	PTPRD	ENSG00000153707	15956246;
C051890	increase	PTPRN	ENSG00000054356	15956246;
C051890	increase	RAC3	ENSG00000169750	15956246;
C051890	increase	RBBP5	ENSG00000117222	15956246;
C051890	increase	RBM24	ENSG00000112183	18927307;
C051890	increase	RECK	ENSG00000122707	18927307;
C051890	increase	RELA	ENSG00000173039	15161687;16685529;18182997;
C051890	increase	RGS1	ENSG00000090104	15956246;
C051890	increase	RHOU	ENSG00000116574	18927307;
C051890	increase	SAMD9	ENSG00000205413	18927307;
C051890	increase	SAT1	ENSG00000130066	15152939;
C051890	increase	SDC4	ENSG00000124145	15956246;

C051890	increase	SEMA3C	ENSG00000075223	18927307;
C051890	increase	SESN2	ENSG00000130766	15956246;
C051890	increase	SGK1	ENSG00000118515	18927307;15956246;
C051890	increase	SIGLEC1	ENSG00000088827	16391804;
C051890	increase	SLC9A3R2	ENSG00000065054	15956246;
C051890	increase	SPIB	ENSG00000142539	15956246;
C051890	increase	STK38	ENSG00000112079	15956246;
C051890	increase	STX5	ENSG00000162236	16391804;
C051890	increase	SULT1A4	ENSG00000213599	15956246;
C051890	increase	SYDE2	ENSG00000097096	18927307;
C051890	increase	TBCC	ENSG00000124659	15956246;
C051890	increase	TFRC	ENSG00000072274	18927307;
C051890	increase	THY1	ENSG00000154096	15956246;
C051890	increase	TNF	ENSG00000223952	18182997;
C051890	increase	TOB1	ENSG00000141232	15956246;
C051890	increase	TOB2	ENSG00000183864	15956246;
C051890	increase	TP53	ENSG00000141510	16373703;16963839;16204068;15041737;
C051890	increase	TP53INP1	ENSG00000164938	18927307;
C051890	increase	TPMT	ENSG00000137364	18927307;
C051890	increase	TPPP	ENSG00000171368	18927307;
C051890	increase	TRADD	ENSG00000102871	15956246;
C051890	increase	TRAF1	ENSG00000056558	15956246;
C051890	increase	TRIB1	ENSG00000173334	18927307;
C051890	increase	TRIM4	ENSG00000146833	18927307;
C051890	increase	TRIM9	ENSG00000100505	18927307;
C051890	increase	ULBP2	ENSG00000131015	18927307;
C051890	increase	UTP3	ENSG00000132467	16391804;
C051890	increase	WNT16	ENSG00000002745	18927307;
C051890	increase	WSB1	ENSG00000109046	18927307;
C051890	increase	ZCCHC12	ENSG00000174460	18927307;
C051890	increase	ZNF408	ENSG00000175213	16391804;
C052026	decrease	MAPK14	ENSG00000112062	15753397;
C052026	decrease	TOP2A	ENSG00000131747	15753397;
C052075	increase	IL16	ENSG00000172349	12235264;
C052087	affect	IL1A	ENSG00000115008	11031321;
C052087	affect	IL1R2	ENSG00000115590	11031321;
C052087	affect	IL1RN	ENSG00000136689	11031321;
C052342	increase	CYP3A4	ENSG00000160868	14636322;
C052498	decrease	MKI67	ENSG00000148773	16507397;
C052498	decrease	PTGS2	ENSG00000073756	16507397;
C052498	decrease	TP53	ENSG00000141510	16507397;
C052498	increase	IFNG	ENSG00000111537	18488220;
C052498	increase	IL2	ENSG00000109471	18488220;
C052498	increase	IL4	ENSG00000113520	18488220;
C052498	increase	KRT18	ENSG00000111057	17545523;
C052498	increase	TYMP	ENSG00000025708	15150550;
C052570	decrease	HYDIN	ENSG00000157423	16790487;
C052570	decrease	SLC6A14	ENSG00000087916	16790487;
C052570	increase	BCR	ENSG00000186716	16790487;
C052570	increase	CLIP3	ENSG00000105270	16790487;
C052570	increase	CYP3A4	ENSG00000160868	19249324;
C052570	increase	IL6	ENSG00000136244	16790487;
C052570	increase	MAPK1	ENSG00000100030	16790487;

C052570	increase	MAPK3	ENSG00000102882	16790487;
C052570	increase	PPP2R3B	ENSG00000167393	16790487;
C052659	decrease	BCL2	ENSG00000171791	19084572;15968715;
C052659	decrease	CASP3	ENSG00000164305	19084572;
C052659	decrease	PARP1	ENSG00000143799	19084572;
C052659	increase	BAX	ENSG00000087088	15968715;
C052659	increase	TYMS	ENSG00000176890	19084572;
C052659	increase	UMPS	ENSG00000114491	19084572;
C052753	increase	DDAH2	ENSG00000225635	17977009;
C052785	decrease	AURKB	ENSG00000178999	15993841;
C052785	decrease	BIRC5	ENSG00000089685	15993841;
C052787	increase	CTGF	ENSG00000118523	15703175;
C053001	increase	MUT	ENSG00000146085	19427250;19199343;
C053079	increase	BCL2L1	ENSG00000171552	18348186;
C053079	increase	MCL1	ENSG00000143384	18348186;
C053400	increase	GSTM1	ENSG00000134184	11346484;
C053401	increase	GSTM1	ENSG00000134184	11346484;
C053429	increase	FSHB	ENSG00000131808	17515571;
C053541	affect	GDF15	ENSG00000130513	16523695;
C053541	decrease	ACPP	ENSG00000014257	15638997;
C053541	decrease	AMACR	ENSG00000242110	15638997;
C053541	decrease	BARD1	ENSG00000138376	15638997;
C053541	decrease	BAX	ENSG00000087088	11845989;
C053541	decrease	BCHE	ENSG00000114200	15638997;
C053541	decrease	BIRC5	ENSG00000089685	15638997;
C053541	decrease	BUB1B	ENSG00000156970	15638997;
C053541	decrease	CCNA2	ENSG00000145386	15887248;
C053541	decrease	CCNB1	ENSG00000134057	15887248;
C053541	decrease	CDC20	ENSG00000117399	15638997;
C053541	decrease	CDC6	ENSG00000094804	15887248;
C053541	decrease	CDKN2C	ENSG00000123080	15638997;
C053541	decrease	CKS2	ENSG00000123975	15638997;
C053541	decrease	CREBBP	ENSG00000005339	15378487;
C053541	decrease	DKC1	ENSG00000130826	15638997;
C053541	decrease	EBP	ENSG00000147155	15638997;
C053541	decrease	GCG	ENSG00000115263	15638997;
C053541	decrease	GPC1	ENSG00000063660	15638997;
C053541	decrease	GPRC5A	ENSG00000013588	15638997;
C053541	decrease	IFIT1	ENSG00000185745	15638997;
C053541	decrease	KIAA1217	ENSG00000120549	17624924;
C053541	decrease	KIF20A	ENSG00000112984	15638997;
C053541	decrease	KLK2	ENSG00000167751	15638997;
C053541	decrease	LMNB1	ENSG00000113368	15638997;
C053541	decrease	MAD2L1	ENSG00000164109	15638997;
C053541	decrease	MCM2	ENSG00000073111	15638997;
C053541	decrease	MKI67	ENSG00000148773	15638997;
C053541	decrease	MYC	ENSG00000136997	15638997;
C053541	decrease	NEK2	ENSG00000117650	15638997;
C053541	decrease	NKX3-1	ENSG00000167034	15638997;
C053541	decrease	NRP1	ENSG00000099250	15638997;
C053541	decrease	NUSAP1	ENSG00000137804	15638997;
C053541	decrease	PBK	ENSG00000168078	15638997;
C053541	decrease	PCNA	ENSG00000132646	15638997;
C053541	decrease	PRC1	ENSG00000198901	15638997;

C053541	decrease	PTEN	ENSG00000171862	16637073;
C053541	decrease	RACGAP1	ENSG00000161800	15638997;
C053541	decrease	RANBP1	ENSG00000099901	15638997;
C053541	decrease	RRM2	ENSG00000171848	15638997;
C053541	decrease	RXRB	ENSG00000235712	11845989;
C053541	decrease	SLC4A4	ENSG00000080493	15638997;
C053541	decrease	STIP1	ENSG00000168439	15638997;
C053541	decrease	TERT	ENSG00000164362	15638997;
C053541	decrease	TK1	ENSG00000167900	15638997;
C053541	decrease	TPX2	ENSG00000088325	15638997;
C053541	decrease	TYMS	ENSG00000176890	17599054;
C053541	decrease	UGT2B17	ENSG00000197888	15638997;
C053541	decrease	ZWINT	ENSG00000122952	15638997;
C053541	increase	AAMP	ENSG00000127837	16631469;
C053541	increase	ACSL1	ENSG00000151726	15790403;
C053541	increase	ADAM9	ENSG00000168615	17342749;
C053541	increase	ADRB2	ENSG00000169252	18454446;
C053541	increase	AGR2	ENSG00000106541	15638997;
C053541	increase	ALDH1A3	ENSG00000184254	17526768;
C053541	increase	ALDH3A2	ENSG00000072210	15638997;
C053541	increase	AMD1	ENSG00000123505	15790403;
C053541	increase	ANKIB1	ENSG00000001629	16631469;
C053541	increase	AR	ENSG00000169083	16015592;18487222;17804755;18324645;15308689;12376534;15336702;19359544;11981028;15994348;16266977;15171712;12730620;16877366;11956172;10053169;18922931;12114440;18486176;19131511;11845989;[illegible]
C053541	increase	ATF3	ENSG00000162772	16516039;15638997;
C053541	increase	ATP5I	ENSG00000169020	16631469;
C053541	increase	B4GALT6	ENSG00000118276	16631469;
C053541	increase	BCL2	ENSG00000171791	11845989;
C053541	increase	BNIP3	ENSG00000176171	15790403;
C053541	increase	BTBD3	ENSG00000132640	16631469;
C053541	increase	C3ORF31	ENSG00000144559	16631469;
C053541	increase	C7ORF44	ENSG00000106603	16631469;
C053541	increase	CCL2	ENSG00000108691	16317058;
C053541	increase	CCNB1IP1	ENSG00000100814	15638997;
C053541	increase	CCNG2	ENSG00000138764	15790403;
C053541	increase	CCPG1	ENSG00000214882	15638997;
C053541	increase	CD40	ENSG00000101017	16317058;
C053541	increase	CD9	ENSG00000010278	15790403;
C053541	increase	CDKN1A	ENSG00000124762	11845989;15638997;
C053541	increase	CDKN1B	ENSG00000111276	11845989;
C053541	increase	CLGN	ENSG00000153132	15638997;
C053541	increase	CLU	ENSG00000120885	17148459;17325745;
C053541	increase	COPB2	ENSG00000184432	16631469;
C053541	increase	CXCL3	ENSG00000163734	15638997;
C053541	increase	DNAJB9	ENSG00000128590	15638997;
C053541	increase	EGF	ENSG00000138798	15466214;
C053541	increase	EZR	ENSG00000092820	16873375;
C053541	increase	FAM129A	ENSG00000135842	15638997;

C053541	increase	FGF13	ENSG00000129682	15638997;
C053541	increase	FKBP11	ENSG00000134285	15638997;
C053541	increase	FOS	ENSG00000170345	15466214;
C053541	increase	GADD45A	ENSG00000116717	15638997;
C053541	increase	GPNMB	ENSG00000136235	15638997;
C053541	increase	GREB1	ENSG00000196208	16496412;
C053541	increase	GTPBP2	ENSG00000172432	15638997;
C053541	increase	HERC5	ENSG00000138646	15638997;
C053541	increase	HIST1H2BB	ENSG00000196226	15790403;
C053541	increase	HYOU1	ENSG00000149428	15638997;
C053541	increase	ICAM1	ENSG00000090339	16317058;
C053541	increase	IGF1	ENSG00000017427	16847815;16368782;
C053541	increase	IGFBP1	ENSG00000146678	16847815;
C053541	increase	IGFBP2	ENSG00000115457	16368782;
C053541	increase	IGFBP3	ENSG00000146674	16847815;16368782;
C053541	increase	IL6	ENSG00000136244	19240160;14532843;16317058;11410519;
C053541	increase	KLK3	ENSG00000142515	18487222;15308689;12376534;16753443;15994348;11059779;17503469;17624924;11410519;15336702;15994225;16637073;15638997;
C053541	increase	LAMP2	ENSG00000005893	16631469;
C053541	increase	LAMP3	ENSG00000078081	15638997;
C053541	increase	LCN2	ENSG00000148346	15638997;
C053541	increase	LRBA	ENSG00000198589	15790403;
C053541	increase	MAN1A1	ENSG00000111885	15638997;
C053541	increase	MBD2	ENSG00000134046	16631469;
C053541	increase	MDK	ENSG00000110492	15638997;
C053541	increase	MME	ENSG00000196549	15790403;
C053541	increase	MMP13	ENSG00000137745	15138554;
C053541	increase	MMP2	ENSG00000087245	16888681;
C053541	increase	MYO6	ENSG00000196586	15790403;16631469;
C053541	increase	NCOA2	ENSG00000140396	19240160;
C053541	increase	NEBL	ENSG00000078114	15638997;
C053541	increase	PECR	ENSG00000115425	15638997;
C053541	increase	PHB	ENSG00000167085	18487222;
C053541	increase	PIP	ENSG00000159763	10402478;
C053541	increase	PTGS2	ENSG00000073756	16317058;
C053541	increase	RARB	ENSG00000077092	15790403;
C053541	increase	ROM1	ENSG00000149489	15790403;
C053541	increase	S100P	ENSG00000163993	15638997;
C053541	increase	SCGB2A1	ENSG00000124939	16020486;
C053541	increase	SERPINE1	ENSG00000106366	16317058;
C053541	increase	SHFM1	ENSG00000127922	16631469;
C053541	increase	SLC3A2	ENSG00000168003	15638997;
C053541	increase	SLC7A11	ENSG00000151012	15638997;
C053541	increase	SNAP25	ENSG00000132639	15790403;
C053541	increase	SPINT2	ENSG00000167642	15790403;
C053541	increase	SQSTM1	ENSG00000161011	15638997;
C053541	increase	TLR4	ENSG00000136869	16317058;
C053541	increase	TMSB4X	ENSG00000205542	17916567;
C053541	increase	TNF	ENSG00000223952	16317058;
C053541	increase	TP53	ENSG00000141510	15638997;

C053541	increase	TP53I3	ENSG00000115129	15638997;
C053541	increase	TRAM1	ENSG00000067167	16631469;
C053541	increase	TRIB3	ENSG00000101255	15638997;
C053541	increase	UAP1	ENSG00000117143	15790403;
C053541	increase	UPP1	ENSG00000183696	15638997;
C053541	increase	VCAM1	ENSG00000162692	16317058;
C053541	increase	WARS	ENSG00000140105	15638997;
C053541	increase	YTHDF3	ENSG00000185728	16631469;
C053541	increase	ZMAT3	ENSG00000172667	15638997;
C053603	decrease	GSTA1	ENSG00000243955	7986210;
C053603	decrease	GSTM1	ENSG00000134184	7986210;
C053629	increase	IGF2	ENSG00000167244	11996908;
C053629	increase	IGFBP5	ENSG00000115461	11996908;
C054105	increase	FGF7	ENSG00000140285	14656992;
C054189	decrease	CD1A	ENSG00000158477	17475839;
C054189	decrease	CD36	ENSG00000135218	17475839;
C054189	decrease	CD80	ENSG00000121594	17475839;
C054189	increase	CD86	ENSG00000114013	17475839;
C054189	increase	RARB	ENSG00000077092	7556191;
C054189	increase	RBP1	ENSG00000248478	7556191;
C054189	increase	RXRA	ENSG00000186350	7556191;
C054284	decrease	CD40	ENSG00000101017	11306145;
C054284	decrease	PTPRC	ENSG00000081237	11306145;
C054474	decrease	ENOSF1	ENSG00000132199	18357371;
C054527	decrease	SOD2	ENSG00000112096	14981915;
C054597	decrease	BCL2	ENSG00000171791	16052481;
C054597	decrease	MMP7	ENSG00000137673	16052481;
C054649	increase	ALDOC	ENSG00000109107	10821843;
C054649	increase	BTG2	ENSG00000159388	10821843;
C054649	increase	CCL2	ENSG00000108691	10218491;10821843;
C054649	increase	CCL4	ENSG00000129277	10821843;
C054649	increase	CNR2	ENSG00000188822	10688601;17397826;10821843;10218491;17558435;
C054649	increase	IL8	ENSG00000169429	15115777;17558435;10218491;10821843;
C054649	increase	JUNB	ENSG00000171223	10821843;
C054649	increase	NFKBIA	ENSG00000100906	10821843;
C054649	increase	TNF	ENSG00000223952	17558435;10821843;
C054852	decrease	BIRC5	ENSG00000089685	16012789;
C054852	decrease	TP53	ENSG00000141510	11378265;
C054852	increase	RB1	ENSG00000139687	14653808;
C054852	increase	SP1	ENSG00000185591	14653808;
C054919	increase	ACVR1C	ENSG00000123612	19422813;
C054919	increase	ACVRL1	ENSG00000139567	19422813;
C054919	increase	ALK	ENSG00000171094	19422813;
C054919	increase	ERBB3	ENSG00000065361	19422813;
C054919	increase	KIT	ENSG00000157404	19422813;
C054919	increase	PDGFRA	ENSG00000134853	19422813;
C054919	increase	PDGFRB	ENSG00000113721	19422813;
C054919	increase	PGR	ENSG00000082175	14579009;18262749;
C055162	decrease	ABCB1	ENSG00000085563	19106083;17112805;19934793;20180610;
C055162	increase	CCL4	ENSG00000129277	16856976;
C055162	increase	CCL5	ENSG00000161570	16856976;

C055162	increase	CD40LG	ENSG00000102245	11297035;
C055162	increase	THBS1	ENSG00000137801	11875746;9447704;
C055162	increase	TP53	ENSG00000141510	9447704;
C055494	decrease	AHR	ENSG00000106546	17204746;
C055494	decrease	BIRC2	ENSG00000110330	15291876;
C055494	decrease	BIRC3	ENSG00000023445	15291876;
C055494	decrease	CCL2	ENSG00000108691	16085347;
C055494	decrease	CCNB1	ENSG00000134057	16085347;
C055494	decrease	CDK1	ENSG00000170312	16085347;
C055494	decrease	IL18	ENSG00000150782	16085347;
C055494	decrease	IL1A	ENSG00000115008	16085347;
C055494	decrease	IL2	ENSG00000109471	16085347;
C055494	decrease	IL8	ENSG00000169429	17709599;16085347;
C055494	decrease	TGFB2	ENSG00000092969	16085347;
C055494	decrease	TNF	ENSG00000223952	15291876;16085347;
C055494	decrease	XIAP	ENSG00000101966	15291876;
C055494	increase	CD14	ENSG00000170458	16766008;
C055494	increase	CDKN1A	ENSG00000124762	16766008;
C055494	increase	CEBPE	ENSG00000092067	16766008;
C055494	increase	IFNG	ENSG00000111537	18953428;
C055494	increase	ITGAM	ENSG00000169896	16766008;
C055494	increase	JUN	ENSG00000177606	16085347;
C055494	increase	NFE2L2	ENSG00000116044	17204746;
C055494	increase	PLA2G2A	ENSG00000188257	18953428;
C055494	increase	RARA	ENSG00000131759	16766008;
C055542	increase	GSTA2	ENSG00000244067	16829687;
C055656	increase	ALOX15	ENSG00000161905	16166323;
C055778	decrease	ID1	ENSG00000125968	19450571;
C055778	decrease	ID2	ENSG00000115738	19450571;
C055778	increase	CDKN1A	ENSG00000124762	19450571;
C055778	increase	RB1	ENSG00000139687	19450571;
C056068	decrease	BCL2	ENSG00000171791	17499812;
C056068	increase	BAX	ENSG00000087088	17499812;
C056068	increase	CYCS	ENSG00000172115	17499812;
C056068	increase	TP53	ENSG00000141510	17499812;
C056165	increase	CCL2	ENSG00000108691	19158351;16845898;
C056165	increase	CXCL10	ENSG00000169245	18322232;
C056165	increase	IFNB1	ENSG00000171855	18322232;
C056165	increase	ITGAM	ENSG00000169896	16357311;
C056165	increase	MSR1	ENSG00000038945	9614211;
C056213	increase	APOA1	ENSG00000118137	19013290;
C056493	decrease	FGF2	ENSG00000138685	16368197;
C056498	increase	GDF15	ENSG00000130513	15555568;
C056507	affect	ANTXR1	ENSG00000169604	17039268;
C056507	affect	BCL2L1	ENSG00000171552	16929163;
C056507	affect	BIRC3	ENSG00000023445	17039268;
C056507	affect	DDIT4	ENSG00000168209	17039268;
C056507	affect	FGG	ENSG00000171557	17039268;
C056507	affect	GTF2A1	ENSG00000165417	17039268;
C056507	affect	NFKB2	ENSG00000077150	17039268;
C056507	affect	NFKBIE	ENSG00000146232	17039268;
C056507	affect	PTEN	ENSG00000171862	17039268;
C056507	affect	PTPN11	ENSG00000179295	17039268;
C056507	affect	TNF	ENSG00000223952	17039268;

C056507	affect	TRAF1	ENSG00000056558	17039268;
C056507	affect	TYRP1	ENSG00000107165	17039268;
C056507	decrease	AGPAT2	ENSG00000169692	17039268;
C056507	decrease	ARF1	ENSG00000143761	17039268;
C056507	decrease	BCL2	ENSG00000171791	15770523;16929163;
C056507	decrease	BIRC5	ENSG00000089685	16929163;
C056507	decrease	C1ORF144	ENSG00000055070	17039268;
C056507	decrease	CCND1	ENSG00000110092	17039268;
C056507	decrease	CD40	ENSG00000101017	17039268;
C056507	decrease	CKB	ENSG00000166165	17039268;
C056507	decrease	CYP24A1	ENSG00000019186	17039268;
C056507	decrease	DHX9	ENSG00000135829	17039268;
C056507	decrease	EDN2	ENSG00000127129	17039268;
C056507	decrease	EVI2B	ENSG00000185862	17039268;
C056507	decrease	FOSL2	ENSG00000075426	17039268;
C056507	decrease	GAL3ST1	ENSG00000128242	17039268;
C056507	decrease	GAS1	ENSG00000180447	17039268;
C056507	decrease	HIST1H4B	ENSG00000182217	17039268;
C056507	decrease	HIST1H4C	ENSG00000182217	17039268;
C056507	decrease	HSPA1L	ENSG00000204390	17039268;
C056507	decrease	IRF3	ENSG00000126456	17039268;
C056507	decrease	JAM3	ENSG00000166086	17039268;
C056507	decrease	KCTD12	ENSG00000178695	17039268;
C056507	decrease	KRT8	ENSG00000170421	17039268;
C056507	decrease	LYZ	ENSG00000090382	17039268;
C056507	decrease	MAGEA10	ENSG00000124260	17039268;
C056507	decrease	MGMT	ENSG00000170430	17039268;
C056507	decrease	NPEPPS	ENSG00000141279	17039268;
C056507	decrease	PTGS1	ENSG00000095303	17039268;
C056507	decrease	RAB8A	ENSG00000167461	17039268;
C056507	decrease	SLC43A3	ENSG00000134802	17039268;
C056507	decrease	SYNCRIP	ENSG00000135316	17039268;
C056507	decrease	TCOF1	ENSG00000070814	17039268;
C056507	decrease	UBE2C	ENSG00000175063	17039268;
C056507	decrease	VGF	ENSG00000128564	17039268;
C056507	decrease	WNT5A	ENSG00000114251	17039268;
C056507	decrease	ZNF521	ENSG00000198795	17039268;
C056507	increase	ALDH1A3	ENSG00000184254	17039268;
C056507	increase	ALDH6A1	ENSG00000119711	17039268;
C056507	increase	ANAPC1	ENSG00000153107	17039268;
C056507	increase	ANK3	ENSG00000151150	17039268;
C056507	increase	ANKRD1	ENSG00000148677	17039268;
C056507	increase	ATF3	ENSG00000162772	17039268;
C056507	increase	BAX	ENSG00000087088	15770523;
C056507	increase	BCL2L11	ENSG00000153094	17039268;
C056507	increase	BCL6	ENSG00000113916	17039268;
C056507	increase	BIRC2	ENSG00000110330	17039268;
C056507	increase	BNIP3L	ENSG00000104765	17039268;
C056507	increase	CCL20	ENSG00000115009	17039268;
C056507	increase	CCNG2	ENSG00000138764	17039268;
C056507	increase	CDH1	ENSG00000039068	17039268;
C056507	increase	CDKN1A	ENSG00000124762	15770523;17039268;
C056507	increase	CEBPB	ENSG00000172216	17039268;
C056507	increase	CLIC4	ENSG00000169504	17039268;

C056507	increase	CSRP2	ENSG00000175183	17039268;
C056507	increase	CTHRC1	ENSG00000164932	17039268;
C056507	increase	CXCL1	ENSG00000163739	17039268;
C056507	increase	CXCL2	ENSG00000081041	17039268;
C056507	increase	CXCL3	ENSG00000163734	17039268;
C056507	increase	DLG2	ENSG00000150672	17039268;
C056507	increase	EGFR	ENSG00000146648	17146438;
C056507	increase	EGR1	ENSG00000120738	17039268;
C056507	increase	ETV1	ENSG00000006468	17039268;
C056507	increase	FBXO25	ENSG00000147364	17039268;
C056507	increase	FXYD3	ENSG00000089356	17039268;
C056507	increase	GLRX	ENSG00000173221	17039268;
C056507	increase	GPC3	ENSG00000147257	17039268;
C056507	increase	HDAC9	ENSG00000048052	17039268;
C056507	increase	HOXB2	ENSG00000173917	17039268;
C056507	increase	HSPA5	ENSG00000044574	17039268;
C056507	increase	IFIT1	ENSG00000185745	17039268;
C056507	increase	IL6	ENSG00000136244	17039268;
C056507	increase	INSIG1	ENSG00000186480	17039268;
C056507	increase	IQGAP1	ENSG00000140575	17039268;
C056507	increase	IRAK2	ENSG00000134070	17039268;
C056507	increase	ITSN2	ENSG00000198399	17039268;
C056507	increase	KLF8	ENSG00000102349	17039268;
C056507	increase	LIFR	ENSG00000113594	17039268;
C056507	increase	LRRC28	ENSG00000168904	17039268;
C056507	increase	MAF	ENSG00000178573	17039268;
C056507	increase	MAGEC1	ENSG00000155495	17039268;
C056507	increase	MARCKS	ENSG00000155130	17039268;
C056507	increase	MARS	ENSG00000166986	17039268;
C056507	increase	MDM2	ENSG00000135679	17039268;
C056507	increase	MXI1	ENSG00000119950	17039268;
C056507	increase	MYB	ENSG00000118513	17039268;
C056507	increase	NCBP1	ENSG00000136937	17039268;
C056507	increase	NEDD9	ENSG00000111859	17039268;
C056507	increase	NEU1	ENSG00000227129	17039268;
C056507	increase	NNMT	ENSG00000166741	17039268;
C056507	increase	NR4A3	ENSG00000119508	17039268;
C056507	increase	NRG1	ENSG00000157168	17039268;
C056507	increase	PAQR8	ENSG00000170915	17039268;
C056507	increase	PCSK9	ENSG00000169174	17039268;
C056507	increase	PDZK1IP1	ENSG00000162366	17039268;
C056507	increase	PJA2	ENSG00000198961	17039268;
C056507	increase	PLAU	ENSG00000122861	17039268;
C056507	increase	PPFIA4	ENSG00000143847	17039268;
C056507	increase	PPP1R15A	ENSG00000087074	17039268;
C056507	increase	PSAT1	ENSG00000135069	17039268;
C056507	increase	RELA	ENSG00000173039	17039268;
C056507	increase	RGS2	ENSG00000116741	17039268;
C056507	increase	RNF149	ENSG00000163162	17039268;
C056507	increase	RPL37	ENSG00000145592	17039268;
C056507	increase	RRAGD	ENSG00000025039	17039268;
C056507	increase	SCD	ENSG00000099194	17039268;
C056507	increase	SLC25A29	ENSG00000197119	17039268;

C056507	increase	SLC29A1	ENSG00000112759	17695509;16868479;18490900;18452103;18728667;18992248;18383843;
C056507	increase	SLC2A14	ENSG00000173262	17039268;
C056507	increase	SLC2A3	ENSG00000059804	17039268;
C056507	increase	SLITRK2	ENSG00000185985	17039268;
C056507	increase	SPP1	ENSG00000118785	17039268;
C056507	increase	TGFB2	ENSG00000092969	17039268;
C056507	increase	TNFAIP3	ENSG00000118503	17039268;
C056507	increase	TNFRSF11B	ENSG00000164761	17039268;
C056507	increase	TP53BP1	ENSG00000067369	17039268;
C056507	increase	TP53	ENSG00000141510	12082016;
C056507	increase	TP53INP1	ENSG00000164938	17039268;
C056507	increase	TUBE1	ENSG00000074935	17039268;
C056507	increase	UGGT2	ENSG00000102595	17039268;
C056507	increase	VEGFA	ENSG00000112715	17039268;
C056507	increase	VLDLR	ENSG00000147852	17039268;
C056507	increase	WBP5	ENSG00000185222	17039268;
C056507	increase	ZNF274	ENSG00000171606	17039268;
C056507	increase	ZNF449	ENSG00000173275	17039268;
C056516	decrease	MKI67	ENSG00000148773	16002280;
C056516	decrease	PGR	ENSG00000082175	16002280;15909811;
C056516	increase	ABCG4	ENSG00000172350	17254854;
C056516	increase	ADCYAP1R	ENSG00000078549	17254854;
C056516	increase	C20ORF194	ENSG00000088854	17254854;
C056516	increase	CD83	ENSG00000112149	17254854;
C056516	increase	DAB2	ENSG00000153071	17254854;
C056516	increase	EBF1	ENSG00000164330	17254854;
C056516	increase	GPC2	ENSG00000213420	17254854;
C056516	increase	HOXD11	ENSG00000128713	17254854;
C056516	increase	INTS4	ENSG00000149262	17254854;
C056516	increase	KIF13B	ENSG00000197892	17254854;
C056516	increase	KLK7	ENSG00000169035	17254854;
C056516	increase	METRN	ENSG00000103260	17254854;
C056516	increase	MYBL2	ENSG00000101057	17254854;
C056516	increase	MYO9B	ENSG00000099331	17254854;
C056516	increase	OSTM1	ENSG00000081087	17254854;
C056516	increase	PLCG2	ENSG00000197943	17254854;
C056516	increase	RNF123	ENSG00000164068	17254854;
C056516	increase	RRP12	ENSG00000052749	17254854;
C056516	increase	SLC13A4	ENSG00000164707	17254854;
C056516	increase	TBC1D17	ENSG00000104946	17254854;
C056516	increase	TMEM155	ENSG00000164112	17254854;
C056516	increase	TNFRSF14	ENSG00000157873	17254854;
C056516	increase	TRIOBP	ENSG00000100106	17254854;
C056599	increase	BMP2	ENSG00000125845	17355822;15724410;
C056599	increase	NOS3	ENSG00000164867	17499557;
C056599	increase	PGR	ENSG00000082175	15541416;
C056599	increase	TFF1	ENSG00000160182	15541416;
C056709	affect	PTPRC	ENSG00000081237	17157857;
C056709	decrease	IL2RA	ENSG00000134460	17157857;
C056709	decrease	ITGAM	ENSG00000169896	17157857;
C056709	decrease	NOS3	ENSG00000164867	17157857;
C056709	decrease	SELL	ENSG00000188404	17157857;

C056709	decrease	TNF	ENSG00000223952	17157857;
C056933	increase	UGCG	ENSG00000148154	18245173;
C057224	decrease	AR	ENSG00000169083	17505938;
C057416	increase	IL8	ENSG00000169429	17097691;
C057416	increase	MAPK1	ENSG00000100030	11675405;
C057416	increase	MAPK3	ENSG00000102882	11675405;
C057416	increase	TNF	ENSG00000223952	11675405;
C057693	decrease	ADIPOR1	ENSG00000159346	16023994;
C057693	decrease	CCNA2	ENSG00000145386	15729575;
C057693	decrease	CCNB1	ENSG00000134057	15729575;
C057693	decrease	CCND3	ENSG00000112576	15729575;
C057693	decrease	CCNE1	ENSG00000105173	15729575;
C057693	decrease	CDK2	ENSG00000123374	15729575;
C057693	decrease	CDKN1A	ENSG00000124762	15729575;
C057693	decrease	CYP3A5	ENSG00000106258	15618651;
C057693	decrease	CYP3A7	ENSG00000160870	15618651;
C057693	decrease	FN1	ENSG00000115414	15908479;
C057693	decrease	GLO1	ENSG00000124767	16085563;12572858;
C057693	decrease	PPARG	ENSG00000132170	15345676;15908479;18665581;15641079;12065695;15729575;15618651;
C057693	decrease	RB1	ENSG00000139687	15729575;
C057693	decrease	SEMA6B	ENSG00000167680	16525649;
C057693	decrease	SP1	ENSG00000185591	15908479;
C057693	increase	ADIPOQ	ENSG00000181092	16076985;
C057693	increase	ATF3	ENSG00000162772	16079301;15897233;
C057693	increase	CD36	ENSG00000135218	14977855;
C057693	increase	CYP2B6	ENSG00000197408	17954527;12642470;
C057693	increase	CYP3A4	ENSG00000160868	12642470;15860655;16837568;12505310;17954527;15466163;
C057693	increase	EGR1	ENSG00000120738	16079301;
C057693	increase	MAP2K1	ENSG00000169032	16079301;
C057693	increase	MAPK1	ENSG00000100030	16079301;
C057693	increase	MAPK3	ENSG00000102882	16079301;
C057693	increase	PTGS2	ENSG00000073756	10866999;15641079;
C057693	increase	SLC5A5	ENSG00000105641	16954431;
C057823	decrease	BCL2	ENSG00000171791	14687023;
C057823	increase	ADRB2	ENSG00000169252	12621035;10516654;16980553;14722251;
C057823	increase	CFTR	ENSG00000001626	12621035;
C057823	increase	DUSP1	ENSG00000120129	15862954;
C057823	increase	SLPI	ENSG00000124107	15862954;
C058305	decrease	BCL2	ENSG00000171791	16054126;
C058305	decrease	BCL2L1	ENSG00000171552	16054126;
C058305	decrease	MCL1	ENSG00000143384	16054126;
C058305	decrease	XIAP	ENSG00000101966	16054126;
C058305	increase	APAF1	ENSG00000120868	16054126;
C058305	increase	BAX	ENSG00000087088	16054126;
C058305	increase	BID	ENSG00000015475	16054126;
C058305	increase	CYP1A1	ENSG00000140465	15672752;
C058305	increase	CYP1A2	ENSG00000140505	15672752;
C058305	increase	NQO1	ENSG00000181019	15672752;
C058305	increase	TP53	ENSG00000141510	16054126;

C058317	affect	TXNIP	ENSG00000117289	16562669;
C058317	affect	ZFYVE16	ENSG00000039319	16529693;
C058317	decrease	ABL1	ENSG00000097007	12421474;
C058317	decrease	AQR	ENSG00000021776	12890387;
C058317	decrease	ARHGEF18	ENSG00000104880	12890387;
C058317	decrease	ATG7	ENSG00000197548	12890387;
C058317	decrease	BCR	ENSG00000186716	12421474;
C058317	decrease	BIRC5	ENSG00000089685	15972126;
C058317	decrease	CHMP5	ENSG00000086065	16778962;
C058317	decrease	COG3	ENSG00000136152	12890387;
C058317	decrease	DDHD2	ENSG00000085788	12890387;
C058317	decrease	EVL	ENSG00000196405	12890387;
C058317	decrease	GSN	ENSG00000148180	12890387;
C058317	decrease	HIPK1	ENSG00000163349	12890387;
C058317	decrease	HOMER3	ENSG00000051128	12890387;
C058317	decrease	IFITM1	ENSG00000185885	12890387;
C058317	decrease	IFT57	ENSG00000114446	12890387;
C058317	decrease	ITGB1	ENSG00000150093	12776498;12890387;
C058317	decrease	KIF3A	ENSG00000131437	12890387;
C058317	decrease	MDM4	ENSG00000198625	12890387;
C058317	decrease	MERTK	ENSG00000153208	12890387;
C058317	decrease	OGT	ENSG00000147162	12890387;
C058317	decrease	PEX5	ENSG00000139197	12890387;
C058317	decrease	PKIA	ENSG00000171033	12890387;
C058317	decrease	PRCP	ENSG00000137509	12890387;
C058317	decrease	PRDM2	ENSG00000116731	12890387;
C058317	decrease	PTP4A3	ENSG00000184489	12890387;
C058317	decrease	RBMX	ENSG00000147274	12890387;
C058317	decrease	RGS2	ENSG00000116741	12890387;
C058317	decrease	RPL13A	ENSG00000142541	12890387;
C058317	decrease	RPL17	ENSG00000215472	12890387;
C058317	decrease	RPS27A	ENSG00000143947	12890387;
C058317	decrease	RTN1	ENSG00000139970	12890387;
C058317	decrease	SNX17	ENSG00000115234	12890387;
C058317	decrease	SORL1	ENSG00000137642	12890387;
C058317	decrease	STX2	ENSG00000111450	12890387;
C058317	decrease	SV2B	ENSG00000185518	12890387;
C058317	decrease	TGFBR2	ENSG00000163513	12890387;
C058317	decrease	ZKSCAN5	ENSG00000196652	12890387;
C058317	increase	BTG1	ENSG00000133639	16562669;16918137;
C058317	increase	CASP3	ENSG00000164305	15972126;
C058317	increase	CCL2	ENSG00000108691	16918137;
C058317	increase	CCL3	ENSG00000006075	16918137;
C058317	increase	CCNB1	ENSG00000134057	12890387;
C058317	increase	IGFBP4	ENSG00000141753	16918137;
C058317	increase	PSMC2	ENSG00000161057	12776498;12890387;
C058317	increase	PSMD1	ENSG00000173692	12776498;12890387;
C058317	increase	SLC38A2	ENSG00000134294	16918137;
C058317	increase	TNFAIP3	ENSG00000118503	16918137;
C058317	increase	TNFAIP8	ENSG00000145779	16918137;
C058544	increase	HIF1A	ENSG00000100644	17082639;
C058687	decrease	IL8	ENSG00000169429	15089887;
C058687	increase	CYP1A1	ENSG00000140465	12623754;
C058687	increase	CYP1A2	ENSG00000140505	12623754;

C058687	increase	CYP1B1	ENSG00000138061	12623754;
C058787	increase	TFF1	ENSG00000160182	15946813;
C058787	increase	VEGFA	ENSG00000112715	15946813;
C058815	increase	BIRC2	ENSG00000110330	15520213;
C058815	increase	TNFRSF10A	ENSG00000104689	15520213;
C058815	increase	TNFRSF10B	ENSG00000120889	15520213;
C058815	increase	TNFSF10	ENSG00000121858	15520213;
C058815	increase	XIAP	ENSG00000101966	15520213;
C058819	decrease	ABCB4	ENSG00000005471	11331069;
C058819	increase	ALB	ENSG00000163631	11961005;
C058819	increase	CCL2	ENSG00000108691	11961005;
C058819	increase	COL1A2	ENSG00000164692	11343241;
C058819	increase	FN1	ENSG00000115414	11343241;15086456;
C058819	increase	TNFRSF1A	ENSG00000067182	9870925;
C058858	decrease	BCL2	ENSG00000171791	15949805;
C058858	increase	BAX	ENSG00000087088	15949805;
C059041	affect	CYP1A1	ENSG00000140465	10696073;
C059041	decrease	CCND1	ENSG00000110092	18790744;
C059041	increase	BCL2L1	ENSG00000171552	18348186;
C059041	increase	CYP1B1	ENSG00000138061	10696073;
C059041	increase	FOS	ENSG00000170345	7963561;
C059041	increase	MCL1	ENSG00000143384	18348186;
C059356	increase	MYC	ENSG00000136997	18045574;
C059356	increase	TERT	ENSG00000164362	18045574;
C059514	affect	FADD	ENSG00000168040	15763656;
C059514	affect	HRAS	ENSG00000174775	16369916;
C059514	affect	TIMP1	ENSG00000102265	17651959;
C059514	decrease	ANLN	ENSG00000011426	15767336;
C059514	decrease	API5	ENSG00000166181	12569576;
C059514	decrease	AR	ENSG00000169083	17486135;15767336;
C059514	decrease	ARID4A	ENSG00000032219	12569576;
C059514	decrease	BAP1	ENSG00000163930	12569576;
C059514	decrease	BCL2A1	ENSG00000140379	17164350;
C059514	decrease	BIRC5	ENSG00000089685	15763656;17270149;17261084;17164350;17636462;17007014;
C059514	decrease	BMP6	ENSG00000153162	12002526;
C059514	decrease	BRF1	ENSG00000185024	12569576;12002526;
C059514	decrease	BUB1B	ENSG00000156970	15767336;
C059514	decrease	BUB1	ENSG00000169679	15767336;
C059514	decrease	CALR	ENSG00000179218	12002526;
C059514	decrease	CCNB1	ENSG00000134057	15670636;17050787;
C059514	decrease	CCNB2	ENSG00000157456	15767336;
C059514	decrease	CCNE2	ENSG00000175305	15767336;
C059514	decrease	CCR2	ENSG00000121807	17499741;
C059514	decrease	CDC123	ENSG00000151465	12002526;
C059514	decrease	CDC27	ENSG00000004897	15767336;
C059514	decrease	CDC6	ENSG00000094804	15767336;
C059514	decrease	CDK1	ENSG00000170312	15670636;17050787;15767336;
C059514	decrease	CDK6	ENSG00000105810	16761963;
C059514	decrease	CDKN2A	ENSG00000147889	17804521;
C059514	decrease	CENPE	ENSG00000138778	15767336;
C059514	decrease	CENPF	ENSG00000117724	15767336;

C059514	decrease	CKS2	ENSG00000123975	15767336;
C059514	decrease	COL2A1	ENSG00000139219	17404069;
C059514	decrease	CYP19A1	ENSG00000137869	17910019;16611627;17766065;
C059514	decrease	DAXX	ENSG00000231617	19631782;12002526;
C059514	decrease	DCT	ENSG00000080166	17460731;
C059514	decrease	DEK	ENSG00000124795	12002526;
C059514	decrease	DYRK1A	ENSG00000157540	12569576;
C059514	decrease	EIF4A2	ENSG00000156976	15767336;
C059514	decrease	ENO1	ENSG00000074800	15582268;
C059514	decrease	ENO2	ENSG00000111674	15582268;
C059514	decrease	ENO3	ENSG00000108515	15582268;
C059514	decrease	ESR1	ENSG00000091831	17513867;15615701;16393696;15664452;15084758;15120416;15876408;17486135;16506809;16118406;10854714;17935960;17141713;
C059514	decrease	F3	ENSG00000117525	12871381;
C059514	decrease	FGF2	ENSG00000138685	17935668;
C059514	decrease	FLT1	ENSG00000102755	17992120;
C059514	decrease	FOXN3	ENSG00000053254	12569576;
C059514	decrease	GAP43	ENSG00000172020	12002526;
C059514	decrease	GDF1	ENSG00000130283	12002526;
C059514	decrease	GPLD1	ENSG00000112293	12002526;
C059514	decrease	GRB2	ENSG00000177885	12002526;
C059514	decrease	GSPT1	ENSG00000103342	12002526;
C059514	decrease	GTF3A	ENSG00000122034	12002526;
C059514	decrease	HDGF	ENSG00000143321	12002526;
C059514	decrease	HELLS	ENSG00000119969	15767336;
C059514	decrease	HES1	ENSG00000114315	17868649;
C059514	decrease	HK2	ENSG00000159399	16979140;
C059514	decrease	HMGB2	ENSG00000164104	15767336;
C059514	decrease	IFRD1	ENSG00000006652	12002526;
C059514	decrease	INSM1	ENSG00000173404	12569576;
C059514	decrease	ITGAL	ENSG00000005844	16267019;
C059514	decrease	ITGB1	ENSG00000150093	17404069;
C059514	decrease	KDR	ENSG00000128052	17992120;
C059514	decrease	KIF11	ENSG00000138160	15767336;
C059514	decrease	KIF23	ENSG00000137807	15767336;
C059514	decrease	KLK3	ENSG00000142515	15767336;12569576;
C059514	decrease	KPNA2	ENSG00000182481	15767336;
C059514	decrease	KRAS	ENSG00000133703	12569576;12002526;
C059514	decrease	LPL	ENSG00000175445	16901463;
C059514	decrease	MAD2L1BP	ENSG00000124688	12002526;
C059514	decrease	MAD2L1	ENSG00000164109	15767336;
C059514	decrease	MADD	ENSG00000110514	12002526;
C059514	decrease	MAP1A	ENSG00000166963	12569576;
C059514	decrease	MAP4	ENSG00000047849	12569576;
C059514	decrease	MAPT	ENSG00000186868	12569576;
C059514	decrease	MCM4	ENSG00000104738	15767336;
C059514	decrease	MCM6	ENSG00000076003	15767336;
C059514	decrease	MKI67	ENSG00000148773	15767336;17992120;
C059514	decrease	MMP2	ENSG00000087245	17935668;16317118;16084059;17049120;

C059514	decrease	MYC	ENSG00000136997	16209266;17088997;17521618;
C059514	decrease	MYO18A	ENSG00000196535	12569576;
C059514	decrease	NAIP	ENSG00000250628	12569576;12002526;
C059514	decrease	NDC80	ENSG00000080986	15767336;
C059514	decrease	NEK2	ENSG00000117650	15767336;
C059514	decrease	NOTCH1	ENSG00000148400	17868649;
C059514	decrease	ODC1	ENSG00000115758	17521618;
C059514	decrease	PBK	ENSG00000168078	15767336;
C059514	decrease	PDE2A	ENSG00000186642	12569576;12002526;
C059514	decrease	PDHA1	ENSG00000131828	15582268;
C059514	decrease	PGAM1	ENSG00000171314	15582268;16979140;
C059514	decrease	PLCG1	ENSG00000124181	12569576;
C059514	decrease	PLK1	ENSG00000166851	15767336;
C059514	decrease	POLA1	ENSG00000101868	15767336;
C059514	decrease	PPARD	ENSG00000112033	12569576;12002526;
C059514	decrease	PRDM2	ENSG00000116731	12569576;
C059514	decrease	PRIM1	ENSG00000198056	15767336;
C059514	decrease	PSEN2	ENSG00000143801	11606380;
C059514	decrease	PTPRA	ENSG00000132670	12569576;
C059514	decrease	PTPRG	ENSG00000144724	16624241;
C059514	decrease	RAC3	ENSG00000169750	17804521;
C059514	decrease	RAD51AP1	ENSG00000111247	15767336;
C059514	decrease	RAN	ENSG00000132341	12569576;12002526;
C059514	decrease	RASGRF1	ENSG00000058335	17804521;
C059514	decrease	RELA	ENSG00000173039	17164350;15489888;16614140;16267019;12569576;12002526;16490592;1793818
C059514	decrease	RFC4	ENSG00000163918	15767336;
C059514	decrease	RGS5	ENSG00000143248	12569576;
C059514	decrease	RNF4	ENSG00000063978	12569576;
C059514	decrease	RPA1	ENSG00000132383	12569576;
C059514	decrease	RPS6	ENSG00000137154	17534123;17554206;
C059514	decrease	RPS6KB1	ENSG00000108443	17919812;17534123;16979140;
C059514	decrease	RRM2	ENSG00000171848	15767336;
C059514	decrease	SFRP1	ENSG00000104332	12569576;
C059514	decrease	SLC45A2	ENSG00000164175	17460731;
C059514	decrease	SMC4	ENSG00000113810	15767336;
C059514	decrease	SPARC	ENSG00000113140	16084059;
C059514	decrease	STIL	ENSG00000123473	15767336;
C059514	decrease	STK17B	ENSG00000081320	15767336;
C059514	decrease	TFAM	ENSG00000108064	12002526;
C059514	decrease	TOP2A	ENSG00000131747	16536579;15767336;
C059514	decrease	TOPBP1	ENSG00000163781	15767336;
C059514	decrease	TRAF2	ENSG00000127191	17164350;
C059514	decrease	TSN	ENSG00000211460	15767336;
C059514	decrease	TTK	ENSG00000112742	15767336;
C059514	decrease	TYR	ENSG00000077498	17460731;
C059514	decrease	TYRP1	ENSG00000107165	17460731;
C059514	decrease	UBE2D3	ENSG00000109332	17804521;
C059514	decrease	UGP2	ENSG00000169764	12002526;
C059514	decrease	YWHAE	ENSG00000108953	12569576;
C059514	decrease	YWHAZ	ENSG00000164924	12569576;

C059514	decrease	ZBTB16	ENSG00000109906	12002526;
C059514	decrease	ZFAND5	ENSG00000107372	12569576;
C059514	decrease	ZWINT	ENSG00000122952	15767336;
C059514	increase	ABCA1	ENSG00000165029	16901463;
C059514	increase	ABCG1	ENSG00000160179	16901463;
C059514	increase	ABCG2	ENSG00000118777	17032904;17077187;
C059514	increase	ABLIM1	ENSG00000099204	12569576;
C059514	increase	AHR	ENSG00000106546	17077187;15458977;15634023;16359657;19376845;15142886;
C059514	increase	ALDOC	ENSG00000109107	15582268;
C059514	increase	APAF1	ENSG00000120868	12002526;14749477;12569576;
C059514	increase	ASCL1	ENSG00000139352	12002526;
C059514	increase	ASNS	ENSG00000070669	12569576;12002526;
C059514	increase	ATF3	ENSG00000162772	12002526;15897233;
C059514	increase	ATG5	ENSG00000057663	12569576;12002526;
C059514	increase	BAD	ENSG00000002330	16731767;
C059514	increase	BAK1	ENSG00000030110	16550006;16617056;12569576;12002526;17636462;16731767;
C059514	increase	BAT2	ENSG00000204469	12002526;
C059514	increase	BAX	ENSG00000087088	16518869;16550006;16617056;17116725;16624241;16087638;17007014;16998810;14749477;17868649;16731767;17823925;17164350;17049120;17636462;
C059514	increase	BBC3	ENSG00000105327	17636462;
C059514	increase	BCL2	ENSG00000171791	15763656;19631782;17690738;16087638;17007014;17603292;16209266;17049120;17164350;17636462;16731767;15688415;16490592;17261084;19549761;
C059514	increase	BCL2L11	ENSG00000153094	17636462;
C059514	increase	BCL2L1	ENSG00000171552	17007014;16546976;14749477;17636462;17164350;16490592;17049120;1256957
C059514	increase	BCL6	ENSG00000113916	17088997;12569576;12002526;
C059514	increase	BGLAP	ENSG00000242252	17689939;16267019;
C059514	increase	BID	ENSG00000015475	17007014;16617056;16731767;
C059514	increase	BIRC2	ENSG00000110330	12569576;
C059514	increase	BMP2	ENSG00000125845	17513867;16973825;
C059514	increase	BNIP2	ENSG00000140299	12569576;
C059514	increase	BOK	ENSG00000176720	15767336;
C059514	increase	BSG	ENSG00000172270	17055343;
C059514	increase	BUB3	ENSG00000154473	12002526;
C059514	increase	C10ORF137	ENSG00000107938	17257620;
C059514	increase	C15ORF48	ENSG00000166920	17257620;

C059514	increase	CASP3	ENSG00000164305	19631782;17116725;15763656;15688415;17823925;17603292;17404069;17959154;17929310;12002526;19549761;17007014;17050787;16087638;17164350;17044934;16617056;17636462;125[illegible]
C059514	increase	CCL2	ENSG00000108691	17191021;16212921;
C059514	increase	CCNA2	ENSG00000145386	15767336;16761963;15583814;
C059514	increase	CCND1	ENSG00000110092	16546976;15489888;16317118;17050787;16761963;17804756;15670636;17486135;17164350;
C059514	increase	CCNE1	ENSG00000105173	16317118;17050787;15670636;
C059514	increase	CD14	ENSG00000170458	16267019;
C059514	increase	CD209	ENSG00000090659	17938187;
C059514	increase	CD36	ENSG00000135218	17938187;
C059514	increase	CD69	ENSG00000110848	17088997;
C059514	increase	CDK2	ENSG00000123374	16317118;15583814;
C059514	increase	CDK4	ENSG00000135446	16317118;17050787;12569576;17804756;
C059514	increase	CDKN1A	ENSG00000124762	17174366;15993843;16317118;15583814;17868649;17261084;16761963;16624241;17804756;17050787;
C059514	increase	CDKN1B	ENSG00000111276	16317118;17513867;16761963;17804756;15829497;17050787;17088997;
C059514	increase	CDKN1C	ENSG00000129757	12569576;
C059514	increase	CHRNA4	ENSG00000101204	12569576;
C059514	increase	CLDN1	ENSG00000163347	17257620;
C059514	increase	CLEC7A	ENSG00000172243	17938187;
C059514	increase	COPS3	ENSG00000141030	12002526;
C059514	increase	CRABP2	ENSG00000143320	12002526;
C059514	increase	CRP	ENSG00000132693	17388968;
C059514	increase	CSF1	ENSG00000184371	16267019;
C059514	increase	CSF1R	ENSG00000182578	16267019;
C059514	increase	CST4	ENSG00000101441	17257620;
C059514	increase	CTSD	ENSG00000117984	17116725;16393696;
C059514	increase	CTSK	ENSG00000143387	16267019;
C059514	increase	CX3CL1	ENSG00000006210	16614140;
C059514	increase	CYP1A1	ENSG00000140465	15276085;15893423;16985168;16581943;15162144;15142886;19376845;17012224;17959153;15316568;15182955;11454723;
C059514	increase	CYP1A2	ENSG00000140505	15276085;16544949;19376845;17959153;
C059514	increase	CYP1B1	ENSG00000138061	15276085;15893423;15905203;16530937;15162144;15142886;19376845;15316568;15182955;14990334;
C059514	increase	CYP3A4	ENSG00000160868	17210444;
C059514	increase	DDIT3	ENSG00000175197	17049495;17914584;
C059514	increase	DFFA	ENSG00000160049	12002526;12569576;

C059514	increase	DLD	ENSG00000091140	15582268;
C059514	increase	DUSP10	ENSG00000143507	17151092;
C059514	increase	E4F1	ENSG00000167967	12002526;
C059514	increase	EDN1	ENSG00000078401	18234130;11755377;
C059514	increase	EGF	ENSG00000138798	15542774;16343977;
C059514	increase	EI24	ENSG00000149547	12569576;
C059514	increase	ENC1	ENSG00000171617	12002526;12569576;
C059514	increase	EP300	ENSG00000100393	16928824;12569576;12002526;
C059514	increase	EPHA4	ENSG00000116106	12569576;
C059514	increase	EPS15	ENSG00000085832	12002526;
C059514	increase	ERBB2	ENSG00000141736	16488535;17651959;15688416;
C059514	increase	EZH1	ENSG00000108799	12002526;
C059514	increase	F2	ENSG00000180210	16051184;
C059514	increase	FABP7	ENSG00000164434	12002526;
C059514	increase	FAS	ENSG00000026103	17261084;
C059514	increase	FGFR1	ENSG00000249195	12002526;
C059514	increase	FLOT1	ENSG00000137312	12002526;
C059514	increase	FOS	ENSG00000170345	15542774;17174366;16761963;16928824;
C059514	increase	FOXA1	ENSG00000129514	17513867;
C059514	increase	FST	ENSG00000134363	17257620;
C059514	increase	G0S2	ENSG00000123689	17257620;
C059514	increase	GADD45A	ENSG00000116717	17261084;16624241;
C059514	increase	GCLC	ENSG00000001084	11909699;
C059514	increase	GDF15	ENSG00000130513	17257620;15670751;
C059514	increase	GRINA	ENSG00000178719	17257620;
C059514	increase	GSR	ENSG00000104687	17261084;12569576;12002526;
C059514	increase	GSTA4	ENSG00000170899	12002526;
C059514	increase	GSTP1	ENSG00000084207	17261084;12002526;
C059514	increase	GTF2B	ENSG00000137947	12002526;
C059514	increase	GTF2E2	ENSG00000197265	12569576;12002526;
C059514	increase	GTF2H4	ENSG00000234370	12002526;
C059514	increase	HDAC3	ENSG00000171720	15767336;
C059514	increase	HIF1A	ENSG00000100644	17473185;16227395;17919812;12002526;
C059514	increase	HIST1H2BE	ENSG00000168242	17257620;
C059514	increase	HIST1H4F	ENSG00000182217	17257620;
C059514	increase	HLA-F	ENSG00000204642	17257620;
C059514	increase	HMOX1	ENSG00000100292	17191021;17210444;
C059514	increase	HSPA1B	ENSG00000232804	17956190;
C059514	increase	HSPA5	ENSG00000044574	17956190;17914584;
C059514	increase	ICAM1	ENSG00000090339	16973825;
C059514	increase	IER2	ENSG00000160888	12002526;
C059514	increase	IFNG	ENSG00000111537	15779068;16154495;
C059514	increase	IGF1	ENSG00000017427	17513867;12002526;
C059514	increase	IGF2	ENSG00000167244	16037384;16393696;
C059514	increase	IGF2R	ENSG00000197081	12002526;
C059514	increase	IGFBP2	ENSG00000115457	12569576;12002526;
C059514	increase	IL1A	ENSG00000115008	17929310;
C059514	increase	IL1B	ENSG00000125538	17191021;15180920;17388968;17404069;17959154;12871381;16338976;

C059514	increase	IL2	ENSG00000109471	16154495;
C059514	increase	IL6	ENSG00000136244	17388968;17164350;16973825;16490592;
C059514	increase	IL8	ENSG00000169429	15180920;16212921;15779068;16624241;
C059514	increase	IRF9	ENSG00000213928	12002526;
C059514	increase	JUND	ENSG00000130522	16624241;15767336;
C059514	increase	JUN	ENSG00000177606	15542774;17174366;16761963;16928824;
C059514	increase	KRT20	ENSG00000171431	16849586;
C059514	increase	LHX2	ENSG00000106689	12002526;
C059514	increase	LITAF	ENSG00000189067	12569576;12002526;
C059514	increase	MCL1	ENSG00000143384	16546976;14749477;
C059514	increase	MDM2	ENSG00000135679	15542774;16998810;15767336;
C059514	increase	MEF2C	ENSG00000081189	12002526;
C059514	increase	MGMT	ENSG00000170430	16950796;
C059514	increase	MMD	ENSG00000108960	12002526;
C059514	increase	MMP9	ENSG00000100985	17055343;17651959;16317118;17935668;15688415;16490592;17049120;
C059514	increase	MSR1	ENSG00000038945	17938187;16901463;
C059514	increase	MTF1	ENSG00000188786	12002526;
C059514	increase	NDRG1	ENSG00000104419	17261084;
C059514	increase	NFATC1	ENSG00000131196	16267019;
C059514	increase	NFIX	ENSG00000008441	12002526;
C059514	increase	NFKB2	ENSG00000077150	17690738;12002526;
C059514	increase	NMU	ENSG00000109255	17257620;
C059514	increase	NOS3	ENSG00000164867	16759640;15740983;18234130;17278969;17052855;17499741;
C059514	increase	NPAS2	ENSG00000170485	12569576;12002526;
C059514	increase	NPC2	ENSG00000119655	17257620;
C059514	increase	NQO1	ENSG00000181019	15993843;12632063;16948474;16759640;
C059514	increase	NQO2	ENSG00000124588	15350128;15993843;16759640;
C059514	increase	NR1H3	ENSG00000025434	16901463;
C059514	increase	NRG1	ENSG00000157168	17651959;12002526;
C059514	increase	OBFC2A	ENSG00000173559	17257620;
C059514	increase	PAPPA	ENSG00000182752	16338976;
C059514	increase	PDCD2	ENSG00000071994	12569576;
C059514	increase	PDCD4	ENSG00000150593	15767336;
C059514	increase	PDGFB	ENSG00000100311	16343977;12002526;
C059514	increase	PDZK1IP1	ENSG00000162366	17257620;
C059514	increase	PELP1	ENSG00000141456	17804729;
C059514	increase	PERP	ENSG00000112378	15767336;
C059514	increase	PGK1	ENSG00000102144	15582268;
C059514	increase	PGR	ENSG00000082175	11606380;
C059514	increase	PLA2G6	ENSG00000184381	15767336;
C059514	increase	PLAT	ENSG00000104368	11236827;
C059514	increase	PLAU	ENSG00000122861	11236827;
C059514	increase	PLP2	ENSG00000102007	12002526;
C059514	increase	PMAIP1	ENSG00000141682	17636462;
C059514	increase	PON1	ENSG00000005421	15458977;17879943;
C059514	increase	PPARGC1A	ENSG00000109819	16849586;

C059514	increase	PTEN	ENSG00000171862	15829497;
C059514	increase	PTGES2	ENSG00000148334	15779068;
C059514	increase	PTGS2	ENSG00000073756	16928824;17984113;10783318;15489888;15155531;8985016;17044934;
C059514	increase	PTPRN2	ENSG00000155093	12569576;
C059514	increase	RRM2B	ENSG00000048392	15767336;
C059514	increase	RSAD2	ENSG00000134321	17257620;
C059514	increase	RUNX2	ENSG00000250096	17689939;
C059514	increase	SARS	ENSG00000031698	12569576;
C059514	increase	SC4MOL	ENSG00000052802	12002526;
C059514	increase	SELP	ENSG00000174175	16051184;
C059514	increase	SESN1	ENSG00000080546	15767336;
C059514	increase	SIRT1	ENSG00000096717	16626303;17885668;16628003;15152190;17103016;15749705;17327447;1684958
C059514	increase	SMN2	ENSG00000172062	17962980;
C059514	increase	SOD2	ENSG00000112096	17261084;
C059514	increase	SP7	ENSG00000170374	17689939;
C059514	increase	SPHK1	ENSG00000176170	16624241;
C059514	increase	SPP1	ENSG00000118785	16267019;
C059514	increase	ST13	ENSG00000100380	12569576;
C059514	increase	STAT3	ENSG00000168610	17164350;16546976;
C059514	increase	STK17A	ENSG00000164543	15767336;
C059514	increase	SULT1E1	ENSG00000109193	17293380;16418064;17287390;
C059514	increase	TCF12	ENSG00000140262	12002526;
C059514	increase	TGFA	ENSG00000163235	10783318;15120416;
C059514	increase	TGFB2	ENSG00000092969	12002526;
C059514	increase	TGM1	ENSG00000092295	16985168;
C059514	increase	TGM3	ENSG00000125780	16985168;
C059514	increase	TGM5	ENSG00000104055	16985168;
C059514	increase	TNF	ENSG00000223952	17055343;16317118;17929310;15489888;12871381;16614140;16338976;16973825;17586618;15779068;16154495;17938187;
C059514	increase	TNFRSF10A	ENSG00000104689	17823925;17636462;
C059514	increase	TNFRSF10B	ENSG00000120889	17823925;12569576;17636462;
C059514	increase	TNFRSF11A	ENSG00000141655	16267019;
C059514	increase	TNFSF11	ENSG00000120659	16267019;
C059514	increase	TNIP1	ENSG00000145901	17257620;
C059514	increase	TNK2	ENSG00000061938	12569576;
C059514	increase	TNS1	ENSG00000079308	15735708;
C059514	increase	TP53	ENSG00000141510	17534123;15542774;19631782;16928824;16814113;16518869;17174366;16790523;11522280;16317118;17959154;16209266;17261084;15993843;16759640;16091005;17007014;17088997;170[illegible]
C059514	increase	TP53INP1	ENSG00000164938	15767336;
C059514	increase	TSG101	ENSG00000074319	15767336;
C059514	increase	UGT1A1	ENSG00000241635	17991766;17287390;
C059514	increase	UGT2B7	ENSG00000171234	17287390;

C059514	increase	USP4	ENSG00000114316	12002526;
C059514	increase	VCAM1	ENSG00000162692	17586618;
C059514	increase	VDR	ENSG00000111424	15664452;16267019;
C059514	increase	VEGFA	ENSG00000112715	17473185;16973825;17919812;17935668;16227395;16490592;17992120;
C059514	increase	WARS	ENSG00000140105	12569576;12002526;
C059514	increase	WFDC2	ENSG00000101443	17257620;
C059514	increase	XIAP	ENSG00000101966	17164350;17049120;16490592;17261084;
C059514	increase	ZMYM2	ENSG00000121741	12569576;
C059514	increase	ZNF135	ENSG00000176293	12569576;
C059685	decrease	CCNA2	ENSG00000145386	15546879;
C059685	decrease	CCNB1	ENSG00000134057	15546879;
C059685	decrease	NFKB1	ENSG00000109320	16637064;
C059685	decrease	NFKBIA	ENSG00000100906	16637064;
C059685	decrease	RELA	ENSG00000173039	16637064;
C059685	increase	CDKN1A	ENSG00000124762	15546879;
C059685	increase	FAS	ENSG00000026103	15546879;
C059685	increase	MDM2	ENSG00000135679	15546879;
C059685	increase	SAT1	ENSG00000130066	16637064;19576191;17237273;16510598;
C059685	increase	TP53	ENSG00000141510	15546879;
C060229	increase	APOA1	ENSG00000118137	19013290;
C060229	increase	CD40	ENSG00000101017	16879495;
C060298	increase	CYP1A1	ENSG00000140465	15661813;15905203;
C060298	increase	CYP1B1	ENSG00000138061	15905203;16530937;
C060327	decrease	BCL2	ENSG00000171791	15684474;
C060327	decrease	BCL2L1	ENSG00000171552	15215653;
C060327	increase	BAX	ENSG00000087088	16864444;15215653;15813022;15684474;
C060327	increase	CDKN1A	ENSG00000124762	15684474;
C060327	increase	TP53	ENSG00000141510	15684474;
C060506	decrease	KDR	ENSG00000128052	15741222;
C060506	decrease	NRP1	ENSG00000099250	15741222;
C060506	decrease	NRP2	ENSG00000118257	15741222;
C060506	increase	CYP17A1	ENSG00000148795	15666826;
C060506	increase	IL8	ENSG00000169429	12039947;
C060506	increase	LAMA3	ENSG00000053747	15541073;
C060506	increase	LAMB3	ENSG00000196878	15541073;
C060506	increase	LAMC2	ENSG00000248691	15541073;
C060658	increase	ACTA2	ENSG00000107796	16757516;
C060658	increase	TGFB1	ENSG00000105329	16757516;
C060836	affect	PPARG	ENSG00000132170	17030193;17138841;15729575;16687628;
C060836	decrease	APP	ENSG00000142192	14586007;15817521;
C060836	decrease	CCNA2	ENSG00000145386	15729575;
C060836	decrease	CCNB1	ENSG00000134057	15729575;
C060836	decrease	CCND3	ENSG00000112576	15729575;
C060836	decrease	CCNE1	ENSG00000105173	15729575;
C060836	decrease	CDK2	ENSG00000123374	15729575;
C060836	decrease	CDKN1A	ENSG00000124762	15729575;
C060836	decrease	HSD3B2	ENSG00000203859	17138841;15072549;
C060836	decrease	RB1	ENSG00000139687	15729575;
C060836	increase	BACE1	ENSG00000186318	14586007;

C060836	increase	BSG	ENSG00000172270	17055343;
C060836	increase	CYP17A1	ENSG00000148795	17138841;15072549;
C060836	increase	CYP2B6	ENSG00000197408	12642470;
C060836	increase	CYP3A4	ENSG00000160868	15900286;12642470;15860655;16837568;
C060836	increase	FABP4	ENSG00000170323	16573735;
C060836	increase	IFNG	ENSG00000111537	14586007;
C060836	increase	NPHS1	ENSG00000161270	16687628;
C060836	increase	TNF	ENSG00000223952	17055343;14586007;
C060893	increase	LPL	ENSG00000175445	15975614;
C061133	decrease	CD44	ENSG00000026508	17229644;
C061133	decrease	DUSP10	ENSG00000143507	17229644;
C061133	decrease	IL6	ENSG00000136244	17229644;
C061133	decrease	PTPRC	ENSG00000081237	17229644;
C061133	decrease	RASSF6	ENSG00000169435	17229644;
C061133	decrease	RHOU	ENSG00000116574	17229644;
C061133	decrease	S100A4	ENSG00000196154	17229644;
C061133	decrease	SH2D1A	ENSG00000183918	17229644;
C061133	decrease	TRAF5	ENSG00000082512	17229644;
C061133	decrease	VAV3	ENSG00000134215	17229644;
C061133	increase	ARHGAP8	ENSG00000241484	17229644;
C061133	increase	ASB2	ENSG00000100628	17229644;
C061133	increase	CD36	ENSG00000135218	16484594;
C061133	increase	CD74	ENSG00000019582	17229644;
C061133	increase	CD79A	ENSG00000105369	17229644;
C061133	increase	CDKN1A	ENSG00000124762	17229644;
C061133	increase	CIITA	ENSG00000179583	17229644;
C061133	increase	CTSA	ENSG00000064601	17229644;
C061133	increase	CYP24A1	ENSG00000019186	16289102;
C061133	increase	GRINA	ENSG00000178719	17229644;
C061133	increase	HLA-DMA	ENSG00000239463	17229644;
C061133	increase	HLA-DMB	ENSG00000242092	17229644;
C061133	increase	HLA-DPA1	ENSG00000168384	17229644;
C061133	increase	HLA-DQB1	ENSG00000179344	17229644;
C061133	increase	HLA-DRA	ENSG00000204287	17229644;
C061133	increase	HLA-DRB5	ENSG00000198502	17229644;
C061133	increase	HRASLS2	ENSG00000133328	17229644;
C061133	increase	IRAK2	ENSG00000134070	17229644;
C061133	increase	IRF1	ENSG00000125347	17229644;
C061133	increase	ITGA3	ENSG00000005884	17229644;
C061133	increase	ITGB7	ENSG00000139626	17229644;
C061133	increase	LZTFL1	ENSG00000163818	17229644;
C061133	increase	MAP4K1	ENSG00000104814	17229644;
C061133	increase	MAPKAPK2	ENSG00000162889	17229644;
C061133	increase	MDK	ENSG00000110492	17229644;
C061133	increase	MFGE8	ENSG00000140545	17229644;
C061133	increase	MSR1	ENSG00000038945	16484594;
C061133	increase	NCOR2	ENSG00000196498	17229644;
C061133	increase	OCLN	ENSG00000197822	16982755;
C061133	increase	P2RX5	ENSG00000083454	17229644;
C061133	increase	PLEK	ENSG00000115956	17229644;
C061133	increase	PLXNA1	ENSG00000114554	17229644;
C061133	increase	PPP1R15A	ENSG00000087074	17229644;
C061133	increase	PRKACA	ENSG00000072062	17229644;

C061133	increase	PRTN3	ENSG00000196415	17229644;
C061133	increase	RARB	ENSG00000077092	16289102;
C061133	increase	RELB	ENSG00000104856	17229644;
C061133	increase	SH2B2	ENSG00000160999	17229644;
C061133	increase	SLC2A4RG	ENSG00000125520	17229644;
C061133	increase	SRI	ENSG00000075142	17229644;
C061133	increase	TFEB	ENSG00000112561	17229644;
C061133	increase	TNFRSF17	ENSG00000048462	17229644;
C061133	increase	VAV1	ENSG00000141968	17229644;
C061133	increase	VEGFA	ENSG00000112715	17170094;
C061133	increase	XBP1	ENSG00000100219	17229644;
C061282	decrease	MMP9	ENSG00000100985	16783407;
C061465	increase	F3	ENSG00000117525	15371228;
C061481	increase	ABCG2	ENSG00000118777	17077187;
C061481	increase	BCL2	ENSG00000171791	16051634;
C061481	increase	HRAS	ENSG00000174775	16051634;
C061481	increase	TERT	ENSG00000164362	16051634;
C061636	decrease	BID	ENSG00000015475	19616521;
C061636	increase	BAD	ENSG00000002330	19616521;
C061636	increase	FADD	ENSG00000168040	19616521;
C061636	increase	FAS	ENSG00000026103	19616521;
C062047	decrease	MAPT	ENSG00000186868	16930453;
C062198	decrease	AMIGO2	ENSG00000139211	16951191;
C062198	decrease	BARD1	ENSG00000138376	16951191;
C062198	decrease	BLM	ENSG00000197299	16951191;
C062198	decrease	BMP7	ENSG00000101144	16951191;
C062198	decrease	CAV1	ENSG00000105974	16951191;
C062198	decrease	CAV2	ENSG00000105971	16951191;
C062198	decrease	CCNA2	ENSG00000145386	16951191;
C062198	decrease	CCND1	ENSG00000110092	16951191;
C062198	decrease	CRABP2	ENSG00000143320	16951191;
C062198	decrease	CXCL12	ENSG00000107562	16951191;
C062198	decrease	ECT2	ENSG00000114346	16951191;
C062198	decrease	ENTPD5	ENSG00000187097	16951191;
C062198	decrease	FGFR3	ENSG00000068078	16951191;
C062198	decrease	G6PD	ENSG00000160211	16951191;
C062198	decrease	GPER	ENSG00000164850	16951191;
C062198	decrease	GREB1	ENSG00000196208	16951191;
C062198	decrease	HMMR	ENSG00000072571	16951191;
C062198	decrease	IRS1	ENSG00000169047	16951191;
C062198	decrease	LYPD3	ENSG00000124466	16951191;
C062198	decrease	MAFB	ENSG00000204103	16951191;
C062198	decrease	MSX2	ENSG00000120149	16951191;
C062198	decrease	MYB	ENSG00000118513	16951191;
C062198	decrease	MYBL1	ENSG00000185697	16951191;
C062198	decrease	NAT1	ENSG00000171428	16951191;
C062198	decrease	PBX3	ENSG00000167081	16951191;
C062198	decrease	PDLIM2	ENSG00000120913	16951191;
C062198	decrease	PHLDA2	ENSG00000181649	16951191;
C062198	decrease	PIK3R1	ENSG00000145675	16951191;
C062198	decrease	PTTG1	ENSG00000164611	16951191;
C062198	decrease	RET	ENSG00000165731	16951191;
C062198	decrease	SFN	ENSG00000175793	16951191;
C062198	decrease	SPDEF	ENSG00000124664	16951191;

C062198	decrease	TFF3	ENSG00000160180	16951191;
C062198	decrease	TOB1	ENSG00000141232	16951191;
C062198	decrease	VAV3	ENSG00000134215	16951191;
C062198	increase	ATF3	ENSG00000162772	16951191;
C062198	increase	BATF	ENSG00000156127	16951191;
C062198	increase	BTG1	ENSG00000133639	16951191;
C062198	increase	BTG2	ENSG00000159388	16951191;
C062198	increase	C13ORF15	ENSG00000102760	16951191;
C062198	increase	CDKN2B	ENSG00000147883	16951191;
C062198	increase	CEACAM1	ENSG00000079385	16951191;
C062198	increase	CLU	ENSG00000120885	16951191;
C062198	increase	CXCR4	ENSG00000121966	16951191;
C062198	increase	DDIT3	ENSG00000175197	16951191;
C062198	increase	EDN1	ENSG00000078401	16951191;
C062198	increase	EI24	ENSG00000149547	16951191;
C062198	increase	ELF3	ENSG00000163435	16951191;
C062198	increase	FBLN5	ENSG00000140092	16951191;
C062198	increase	FGF13	ENSG00000129682	16951191;
C062198	increase	FGFR2	ENSG00000066468	16951191;
C062198	increase	FOXO3	ENSG00000118689	16951191;
C062198	increase	GADD45G	ENSG00000130222	16951191;
C062198	increase	GDF15	ENSG00000130513	16951191;
C062198	increase	GSN	ENSG00000148180	16951191;
C062198	increase	HIPK2	ENSG00000064393	16951191;
C062198	increase	HOXA1	ENSG00000105991	16951191;
C062198	increase	HTRA1	ENSG00000166033	16951191;
C062198	increase	IGFBP3	ENSG00000146674	16951191;
C062198	increase	IGFBP6	ENSG00000167779	16951191;
C062198	increase	IL8	ENSG00000169429	16951191;
C062198	increase	IRF1	ENSG00000125347	16951191;
C062198	increase	JAG1	ENSG00000101384	16951191;
C062198	increase	JUN	ENSG00000177606	16951191;
C062198	increase	KLF6	ENSG00000067082	16951191;
C062198	increase	LIMA1	ENSG00000050405	16951191;
C062198	increase	LYN	ENSG00000249529	16951191;
C062198	increase	MARCKS	ENSG00000155130	16951191;
C062198	increase	MDM2	ENSG00000135679	16951191;
C062198	increase	MITF	ENSG00000187098	16951191;
C062198	increase	NBL1	ENSG00000158747	16951191;
C062198	increase	NKX3-1	ENSG00000167034	16951191;
C062198	increase	PDCD4	ENSG00000150593	16951191;
C062198	increase	PDGFC	ENSG00000145431	16951191;
C062198	increase	PHLDA1	ENSG00000139289	16951191;
C062198	increase	PLA2G2A	ENSG00000188257	16951191;
C062198	increase	PPARG	ENSG00000132170	16951191;
C062198	increase	PRSS8	ENSG00000052344	16951191;
C062198	increase	PSAP	ENSG00000197746	16951191;
C062198	increase	S100P	ENSG00000163993	16951191;
C062198	increase	SOD2	ENSG00000112096	16951191;
C062198	increase	SULF1	ENSG00000137573	16951191;
C062198	increase	TGFA	ENSG00000163235	16951191;
C062198	increase	TGFBI	ENSG00000120708	16951191;
C062198	increase	TGM2	ENSG00000198959	16951191;
C062198	increase	TRIM31	ENSG00000223531	16951191;

C062198	increase	UBD	ENSG00000213886	16951191;
C062735	increase	EDN1	ENSG00000078401	11372388;
C062876	decrease	LHB	ENSG00000104826	16914592;
C063002	decrease	ACOT2	ENSG00000119673	17547211;
C063002	decrease	ACP2	ENSG00000134575	17547211;
C063002	decrease	ACSL1	ENSG00000151726	17547211;
C063002	decrease	ADH6	ENSG00000172955	17547211;
C063002	decrease	AKR1B1	ENSG00000085662	17547211;
C063002	decrease	ALDH6A1	ENSG00000119711	17547211;
C063002	decrease	ARSE	ENSG00000157399	17547211;
C063002	decrease	ASNS	ENSG00000070669	17547211;
C063002	decrease	ATP2B1	ENSG00000070961	17547211;
C063002	decrease	AZGP1	ENSG00000160862	17547211;
C063002	decrease	BCAT1	ENSG00000060982	17547211;
C063002	decrease	C6ORF48	ENSG00000234728	17547211;
C063002	decrease	CALR	ENSG00000179218	17547211;
C063002	decrease	CDH2	ENSG00000170558	17547211;
C063002	decrease	CETN2	ENSG00000147400	19031421;
C063002	decrease	CFB	ENSG00000243570	17547211;
C063002	decrease	CHPT1	ENSG00000111666	19031421;
C063002	decrease	CIDEB	ENSG00000136305	17547211;
C063002	decrease	CLGN	ENSG00000153132	17547211;
C063002	decrease	CLPTM1	ENSG00000104853	17547211;
C063002	decrease	CLU	ENSG00000120885	17547211;
C063002	decrease	CRYAA	ENSG00000160202	19031421;
C063002	decrease	CX3CL1	ENSG00000006210	17547211;
C063002	decrease	EPHX1	ENSG00000143819	17547211;
C063002	decrease	F10	ENSG00000126218	17547211;
C063002	decrease	F5	ENSG00000198734	17547211;
C063002	decrease	FURIN	ENSG00000140564	17547211;
C063002	decrease	GGCX	ENSG00000115486	17547211;
C063002	decrease	GHITM	ENSG00000165678	17547211;
C063002	decrease	GPER	ENSG00000164850	17547211;
C063002	decrease	GSN	ENSG00000148180	17547211;
C063002	decrease	IFRD1	ENSG00000006652	17547211;
C063002	decrease	IGSF1	ENSG00000147255	17547211;
C063002	decrease	IL17RB	ENSG00000056736	17547211;
C063002	decrease	ITIH3	ENSG00000162267	17547211;
C063002	decrease	MGAT2	ENSG00000168282	17547211;
C063002	decrease	NEDD9	ENSG00000111859	19031421;
C063002	decrease	NEK4	ENSG00000114904	19031421;
C063002	decrease	NUCB2	ENSG00000070081	17547211;
C063002	decrease	OS9	ENSG00000135506	17547211;
C063002	decrease	PGC	ENSG00000096088	17547211;
C063002	decrease	PGF	ENSG00000119630	17547211;
C063002	decrease	PSPH	ENSG00000146733	17547211;
C063002	decrease	RAD23B	ENSG00000119318	19031421;
C063002	decrease	RECQL	ENSG00000004700	19031421;
C063002	decrease	SCD	ENSG00000099194	17547211;
C063002	decrease	SERPINA5	ENSG00000188488	17547211;
C063002	decrease	SERPINA6	ENSG00000170099	17547211;
C063002	decrease	SERPINB1	ENSG00000021355	17547211;
C063002	decrease	SERPINC1	ENSG00000117601	17547211;
C063002	decrease	SLC1A2	ENSG00000110436	17547211;

C063002	decrease	ST6GALNAC4	ENSG00000136840	17547211;
C063002	decrease	TMED2	ENSG00000086598	17547211;
C063002	decrease	TNFSF10	ENSG00000121858	17547211;
C063002	decrease	TSPAN7	ENSG00000156298	17547211;
C063002	decrease	TST	ENSG00000128311	17547211;
C063002	decrease	VTN	ENSG00000109072	17547211;
C063002	increase	ADM	ENSG00000148926	17547211;
C063002	increase	AKAP12	ENSG00000131016	17547211;
C063002	increase	AKR1C1	ENSG00000187134	9973208;
C063002	increase	ANXA2	ENSG00000182718	17547211;
C063002	increase	AURKA	ENSG00000087586	19031421;
C063002	increase	BIRC5	ENSG00000089685	19031421;
C063002	increase	BUB1B	ENSG00000156970	19031421;
C063002	increase	CCNA2	ENSG00000145386	19031421;
C063002	increase	CCNB2	ENSG00000157456	19031421;17547211;
C063002	increase	CDC20	ENSG00000117399	19031421;
C063002	increase	CDC25B	ENSG00000101224	19031421;
C063002	increase	CDKN3	ENSG00000100526	17547211;
C063002	increase	CKS2	ENSG00000123975	19031421;17547211;
C063002	increase	CYP1A1	ENSG00000140465	9973208;
C063002	increase	DKK1	ENSG00000107984	17547211;
C063002	increase	DUSP13	ENSG00000079393	19031421;
C063002	increase	FOSL1	ENSG00000175592	17547211;
C063002	increase	GLA	ENSG00000102393	17547211;
C063002	increase	GRB10	ENSG00000106070	17547211;
C063002	increase	GTPBP6	ENSG00000178605	17547211;
C063002	increase	H2AFX	ENSG00000188486	19031421;
C063002	increase	HIST1H4C	ENSG00000182217	17547211;
C063002	increase	HMGA1	ENSG00000137309	17547211;
C063002	increase	HMGN2	ENSG00000198830	17547211;
C063002	increase	HMOX1	ENSG00000100292	17547211;
C063002	increase	IER2	ENSG00000160888	17547211;
C063002	increase	ITGB3BP	ENSG00000142856	17547211;
C063002	increase	KIF20B	ENSG00000138182	19031421;
C063002	increase	KIF22	ENSG00000079616	19031421;
C063002	increase	KIF23	ENSG00000137807	19031421;
C063002	increase	KIF2C	ENSG00000142945	19031421;
C063002	increase	KRT19	ENSG00000171345	17547211;
C063002	increase	LGALS1	ENSG00000100097	17547211;
C063002	increase	MAD2L1	ENSG00000164109	19031421;
C063002	increase	MAFF	ENSG00000185022	17547211;
C063002	increase	MAPRE2	ENSG00000166974	19031421;
C063002	increase	MCM2	ENSG00000073111	17547211;
C063002	increase	MXRA7	ENSG00000182534	17547211;
C063002	increase	NCAPG	ENSG00000109805	19031421;
C063002	increase	NEK2	ENSG00000117650	19031421;
C063002	increase	NOLC1	ENSG00000166197	19031421;
C063002	increase	NUP107	ENSG00000111581	17547211;
C063002	increase	NUSAP1	ENSG00000137804	19031421;
C063002	increase	PAK3	ENSG00000077264	19031421;
C063002	increase	PBK	ENSG00000168078	19031421;
C063002	increase	PLK1	ENSG00000166851	19031421;
C063002	increase	POLE2	ENSG00000100479	19031421;

C063002	increase	PPIH	ENSG00000171960	17547211;
C063002	increase	PTTG1	ENSG00000164611	17547211;
C063002	increase	RACGAP1	ENSG00000161800	17547211;
C063002	increase	RAD51C	ENSG00000108384	19031421;
C063002	increase	RBBP8	ENSG00000101773	19031421;
C063002	increase	RBPMS	ENSG00000157110	17547211;
C063002	increase	RFC2	ENSG00000049541	17547211;
C063002	increase	RFC4	ENSG00000163918	19031421;
C063002	increase	RNASEH2A	ENSG00000104889	17547211;
C063002	increase	RUVBL2	ENSG00000183207	19031421;
C063002	increase	SFN	ENSG00000175793	17547211;
C063002	increase	SFRS2	ENSG00000161547	17547211;
C063002	increase	SFRS7	ENSG00000115875	17547211;
C063002	increase	SHC1	ENSG00000160691	19031421;
C063002	increase	SMC4	ENSG00000113810	19031421;17547211;
C063002	increase	SNRPA	ENSG00000077312	17547211;
C063002	increase	SPAG5	ENSG00000076382	19031421;
C063002	increase	SPP1	ENSG00000118785	17547211;
C063002	increase	SQRDL	ENSG00000137767	17547211;
C063002	increase	TIMP1	ENSG00000102265	17547211;
C063002	increase	TNFAIP3	ENSG00000118503	17547211;
C063002	increase	TNFAIP8	ENSG00000145779	17547211;
C063002	increase	TPX2	ENSG00000088325	19031421;
C063002	increase	TRIP13	ENSG00000071539	17547211;
C063002	increase	TUBA1A	ENSG00000167552	17547211;
C063002	increase	TYMS	ENSG00000176890	19031421;17547211;
C063002	increase	UBE2C	ENSG00000175063	17547211;
C063002	increase	UBE2V2	ENSG00000169139	19031421;
C063002	increase	ZW10	ENSG00000086827	19031421;
C063002	increase	ZWINT	ENSG00000122952	19031421;17547211;
C063129	increase	CYP1A1	ENSG00000140465	12623754;
C063129	increase	CYP1A2	ENSG00000140505	12623754;
C063159	increase	BIRC3	ENSG00000023445	16154993;
C063159	increase	TNF	ENSG00000223952	16154993;
C063170	decrease	APP	ENSG00000142192	18001288;19464278;
C063170	decrease	BDNF	ENSG00000176697	18001288;
C063170	increase	ABCB1	ENSG00000085563	19034627;
C063170	increase	ABCC2	ENSG00000023839	19034627;
C063170	increase	CYP1A2	ENSG00000140505	15147983;19034627;
C063170	increase	CYP2B6	ENSG00000197408	19034627;
C063170	increase	CYP3A4	ENSG00000160868	15147983;19034627;
C063170	increase	GCLC	ENSG00000001084	11673643;
C063170	increase	UGT1A1	ENSG00000241635	19034627;
C063261	decrease	CCND1	ENSG00000110092	16024653;
C063261	decrease	CCND3	ENSG00000112576	16024653;
C063261	decrease	NFKBIA	ENSG00000100906	15930313;17291458;
C063261	decrease	SCNN1G	ENSG00000166828	15564131;
C063261	increase	IL8	ENSG00000169429	12208513;
C063302	decrease	BIRC3	ENSG00000023445	14666661;
C063302	increase	ITGAM	ENSG00000169896	17431504;
C063509	affect	VDR	ENSG00000111424	15879110;
C063509	decrease	ABCB4	ENSG00000005471	12926078;
C063509	decrease	BIRC5	ENSG00000089685	18377872;
C063509	increase	ABCB1	ENSG00000085563	12926078;

C063509	increase	AREG	ENSG00000205595	15967414;
C063509	increase	CDKN1A	ENSG00000124762	16361081;
C063509	increase	CDKN2B	ENSG00000147883	16361081;
C063509	increase	FSHB	ENSG00000131808	15967414;
C063509	increase	LHB	ENSG00000104826	15967414;
C063509	increase	MAPK1	ENSG00000100030	16671086;11675405;
C063509	increase	MAPK3	ENSG00000102882	16671086;11675405;
C063509	increase	PLD1	ENSG00000075651	17640750;
C063509	increase	PTGS2	ENSG00000073756	17640750;
C063509	increase	SLC2A5	ENSG00000142583	12820898;
C063509	increase	TGFB1	ENSG00000105329	16361081;
C063509	increase	TNF	ENSG00000223952	11675405;
C063871	decrease	ABCB1	ENSG00000085563	18414057;
C063871	decrease	GSTP1	ENSG00000084207	18414057;
C063871	decrease	TOP2A	ENSG00000131747	18414057;
C064515	increase	CTGF	ENSG00000118523	15703175;
C064515	increase	TGFB1	ENSG00000105329	15703175;
C064545	increase	TGM2	ENSG00000198959	9516142;
C064758	increase	ACTA2	ENSG00000107796	16757516;
C064758	increase	DPP3	ENSG00000174483	17360324;
C064758	increase	GDF15	ENSG00000130513	18058799;
C064758	increase	HMOX1	ENSG00000100292	18048804;18357586;
C064758	increase	IL1B	ENSG00000125538	11854442;10712238;
C064758	increase	IL8	ENSG00000169429	17000667;
C064758	increase	NQO1	ENSG00000181019	17360324;
C064758	increase	PTGS2	ENSG00000073756	11854442;
C064758	increase	SQSTM1	ENSG00000161011	17360324;
C064758	increase	TGFB1	ENSG00000105329	16757516;
C064769	decrease	KDR	ENSG00000128052	15741222;
C064769	decrease	NRP1	ENSG00000099250	15741222;
C064769	decrease	NRP2	ENSG00000118257	15741222;
C064976	increase	CDKN1A	ENSG00000124762	15735757;
C064976	increase	TP53	ENSG00000141510	15735757;
C065179	affect	IL2RA	ENSG00000134460	16387846;
C065179	affect	NOS3	ENSG00000164867	16962929;15178637;
C065179	affect	TFRC	ENSG00000072274	16387846;
C065179	decrease	APOB	ENSG00000084674	12612140;
C065179	decrease	CD40	ENSG00000101017	16387846;
C065179	decrease	CD40LG	ENSG00000102245	16368305;
C065179	decrease	CD83	ENSG00000112149	16387846;
C065179	decrease	CD86	ENSG00000114013	16387846;
C065179	decrease	CRP	ENSG00000132693	16360360;
C065179	decrease	EDN1	ENSG00000078401	16360360;
C065179	decrease	IL12A	ENSG00000168811	12492458;
C065179	decrease	TNFRSF1A	ENSG00000067182	16360360;
C065179	increase	CYP2B6	ENSG00000197408	15802384;
C065179	increase	HMGCR	ENSG00000113161	12612140;
C065179	increase	PON1	ENSG00000005421	16238680;15969877;15324535;15690306;
C065179	increase	PON2	ENSG00000105854	14592851;
C065179	increase	TLR2	ENSG00000137462	16387846;
C065179	increase	TLR4	ENSG00000136869	16387846;
C065180	decrease	MCL1	ENSG00000143384	16996129;
C065180	increase	ARHGAP29	ENSG00000137962	16996129;

C065180	increase	ARHGEF12	ENSG00000196914	16996129;
C065180	increase	BCAP29	ENSG00000075790	16996129;
C065180	increase	BHLHE40	ENSG00000134107	16996129;
C065180	increase	BUB1	ENSG00000169679	16996129;
C065180	increase	CASP1	ENSG00000137752	16996129;
C065180	increase	CDK2AP2	ENSG00000167797	16996129;
C065180	increase	CKS2	ENSG00000123975	16996129;
C065180	increase	CYP2B6	ENSG00000197408	15802384;
C065180	increase	EMP3	ENSG00000142227	16996129;
C065180	increase	ERCC5	ENSG00000134899	16996129;
C065180	increase	ERG	ENSG00000157554	16996129;
C065180	increase	FDFT1	ENSG00000079459	16996129;
C065180	increase	FNTA	ENSG00000168522	16996129;
C065180	increase	GADD45B	ENSG00000099860	16996129;
C065180	increase	IGF1	ENSG00000017427	16996129;
C065180	increase	IGFBP5	ENSG00000115461	16996129;
C065180	increase	IL10RA	ENSG00000110324	16996129;
C065180	increase	IL2RG	ENSG00000147168	16996129;
C065180	increase	KAT2B	ENSG00000114166	16996129;
C065180	increase	LZTS1	ENSG00000061337	16996129;
C065180	increase	MED23	ENSG00000112282	16996129;
C065180	increase	MPHOSPH8	ENSG00000196199	16996129;
C065180	increase	MPP5	ENSG00000072415	16996129;
C065180	increase	NDC80	ENSG00000080986	16996129;
C065180	increase	OPTN	ENSG00000123240	16996129;
C065180	increase	PAPPA2	ENSG00000116183	16996129;
C065180	increase	PPP1R15A	ENSG00000087074	16996129;
C065180	increase	PRDX3	ENSG00000165672	16996129;
C065180	increase	PROCR	ENSG00000101000	16142594;
C065180	increase	RAB27B	ENSG00000041353	16996129;
C065180	increase	RAB5B	ENSG00000111540	16996129;
C065180	increase	RABGAP1L	ENSG00000250898	16996129;
C065180	increase	RGS11	ENSG00000076344	16996129;
C065180	increase	RHOB	ENSG00000143878	16996129;
C065180	increase	RIMS2	ENSG00000176406	16996129;
C065180	increase	RIN2	ENSG00000132669	16996129;
C065180	increase	SOS2	ENSG00000100485	16996129;
C065180	increase	STK17A	ENSG00000164543	16996129;
C065180	increase	TFPI	ENSG00000003436	16142594;
C065180	increase	TNFRSF10A	ENSG00000104689	16996129;
C065180	increase	TP53INP1	ENSG00000164938	16996129;
C065180	increase	WNT11	ENSG00000085741	16996129;
C065250	decrease	ACOX1	ENSG00000161533	15135645;
C065250	decrease	ARG2	ENSG00000081181	15135645;
C065250	decrease	BRCA1	ENSG00000012048	15135645;
C065250	decrease	CHAF1B	ENSG00000159259	15135645;
C065250	decrease	CYP24A1	ENSG00000019186	15135645;
C065250	decrease	ETFB	ENSG00000105379	15135645;
C065250	decrease	GRM5	ENSG00000168959	15135645;
C065250	decrease	GSTM2	ENSG00000213366	15135645;
C065250	decrease	HNMT	ENSG00000150540	15135645;
C065250	decrease	MRPS12	ENSG00000128626	15135645;
C065250	decrease	MT2A	ENSG00000125148	15135645;
C065250	decrease	MVK	ENSG00000110921	15135645;

C065250	decrease	MYC	ENSG00000136997	15135645;
C065250	decrease	NCL	ENSG00000115053	15135645;
C065250	decrease	NNAT	ENSG00000053438	15135645;
C065250	decrease	PFN1	ENSG00000108518	15135645;
C065250	decrease	PLCG2	ENSG00000197943	15135645;
C065250	decrease	PLOD1	ENSG00000083444	15135645;
C065250	decrease	PRDX1	ENSG00000117450	15135645;
C065250	decrease	PSMD4	ENSG00000159352	15135645;
C065250	decrease	RAB4B	ENSG00000167578	15135645;
C065250	decrease	SERPINB6	ENSG00000124570	15135645;
C065250	decrease	SREBF2	ENSG00000198911	15135645;
C065250	decrease	TAF12	ENSG00000120656	15135645;
C065250	decrease	TP53	ENSG00000141510	15135645;
C065250	decrease	VPS72	ENSG00000163159	15135645;
C065250	increase	CD48	ENSG00000117091	15135645;
C065250	increase	CD83	ENSG00000112149	15135645;
C065250	increase	CDH3	ENSG00000062038	15135645;
C065250	increase	FOXA3	ENSG00000170608	15135645;
C065250	increase	FOXO1	ENSG00000150907	15135645;
C065250	increase	FTH1	ENSG00000167996	15135645;
C065250	increase	FXYD3	ENSG00000089356	15135645;
C065250	increase	GAA	ENSG00000171298	15135645;
C065250	increase	PABPC1	ENSG00000070756	15135645;
C065250	increase	RPL21	ENSG00000122026	15135645;
C065250	increase	RPL23A	ENSG00000198242	15135645;
C065250	increase	RPL32	ENSG00000144713	15135645;
C065250	increase	RPL37A	ENSG00000197756	15135645;
C065250	increase	RPS27	ENSG00000177954	15135645;
C065250	increase	RPS4X	ENSG00000198034	15135645;
C065250	increase	S100A11	ENSG00000163191	15135645;
C065250	increase	SULT1E1	ENSG00000109193	15135645;
C065250	increase	TIMP3	ENSG00000100234	15135645;
C065250	increase	TXN	ENSG00000136810	15135645;
C065299	decrease	BCL2	ENSG00000171791	17155983;
C065299	increase	CDH1	ENSG00000039068	17155983;
C065299	increase	JUP	ENSG00000173801	17155983;
C065382	affect	CCL11	ENSG00000172156	15753895;
C065382	affect	ITGAM	ENSG00000169896	15753895;
C065382	decrease	IL5	ENSG00000113525	15113433;15753895;15634547;
C065382	decrease	PRKCA	ENSG00000154229	15634547;
C065382	increase	DUSP1	ENSG00000120129	15862954;
C065382	increase	SLPI	ENSG00000124107	15862954;
C065757	increase	ABCB1	ENSG00000085563	15501994;
C065861	decrease	BCL2	ENSG00000171791	15964118;
C065861	increase	BAX	ENSG00000087088	15964118;
C065861	increase	MAPK14	ENSG00000112062	15964118;
C066075	affect	BMP2	ENSG00000125845	17459161;
C066075	affect	CCNT2	ENSG00000082258	17459161;
C066075	affect	CD44	ENSG00000026508	17459161;
C066075	affect	DUSP6	ENSG00000139318	17459161;
C066075	affect	EGR1	ENSG00000120738	17459161;
C066075	affect	EGR3	ENSG00000179388	17459161;
C066075	affect	GATA6	ENSG00000141448	17459161;

C066075	affect	GBP1	ENSG00000117228	17459161;
C066075	affect	IRF1	ENSG00000125347	17459161;
C066075	affect	LYST	ENSG00000143669	17459161;
C066075	affect	NEDD9	ENSG00000111859	17459161;
C066075	affect	SLC2A3	ENSG00000059804	17459161;
C066075	affect	SPRED2	ENSG00000198369	17459161;
C066075	decrease	BTG1	ENSG00000133639	17459161;
C066075	decrease	BTG2	ENSG00000159388	17459161;
C066075	decrease	CCNG2	ENSG00000138764	17459161;
C066075	decrease	CDK7	ENSG00000134058	17459161;
C066075	decrease	CDK9	ENSG00000136807	17459161;
C066075	decrease	CDKN1B	ENSG00000111276	17459161;
C066075	decrease	CDKN1C	ENSG00000129757	17459161;
C066075	decrease	CDKN2C	ENSG00000123080	17459161;
C066075	decrease	COL1A1	ENSG00000108821	17459161;
C066075	decrease	COL1A2	ENSG00000164692	17459161;
C066075	decrease	COL3A1	ENSG00000168542	17459161;
C066075	decrease	CTGF	ENSG00000118523	17459161;
C066075	decrease	FGF2	ENSG00000138685	17459161;
C066075	decrease	FGF9	ENSG00000102678	17459161;
C066075	decrease	GAS1	ENSG00000180447	17459161;
C066075	decrease	IFNGR1	ENSG00000027697	17459161;
C066075	decrease	IL15	ENSG00000164136	17459161;
C066075	decrease	JAK2	ENSG00000096968	17459161;
C066075	decrease	KEAP1	ENSG00000079999	17459161;
C066075	decrease	MARCKS	ENSG00000155130	17459161;
C066075	decrease	OXR1	ENSG00000164830	17459161;
C066075	decrease	OXSR1	ENSG00000172939	17459161;
C066075	decrease	SMAD1	ENSG00000170365	17459161;
C066075	decrease	SMURF1	ENSG00000198742	17459161;
C066075	decrease	SOCS1	ENSG00000185338	17459161;
C066075	decrease	STAT1	ENSG00000115415	18322232;17459161;
C066075	decrease	TGFB2	ENSG00000092969	17459161;
C066075	decrease	TRAF4	ENSG00000076604	17459161;
C066075	decrease	VCAM1	ENSG00000162692	17459161;
C066075	increase	CCL8	ENSG00000108700	17459161;
C066075	increase	CX3CR1	ENSG00000168329	17459161;
C066075	increase	CXCL10	ENSG00000169245	18322232;17459161;
C066075	increase	CXCL9	ENSG00000138755	17459161;
C066075	increase	GDF15	ENSG00000130513	17459161;
C066075	increase	HBEGF	ENSG00000113070	12676768;11159045;17459161;
C066075	increase	HGF	ENSG00000019991	17459161;
C066075	increase	IFNB1	ENSG00000171855	18322232;
C066075	increase	IL6	ENSG00000136244	17459161;
C066075	increase	IL8	ENSG00000169429	9707512;17459161;
C066075	increase	JUN	ENSG00000177606	17459161;
C066075	increase	KLF7	ENSG00000118263	17459161;
C066075	increase	NAMPT	ENSG00000105835	17459161;
C066075	increase	NQO1	ENSG00000181019	17459161;
C066075	increase	PIPOX	ENSG00000179761	17459161;
C066075	increase	SOCS3	ENSG00000184557	17459161;
C066075	increase	SOD2	ENSG00000112096	17459161;
C066075	increase	STC1	ENSG00000159167	17459161;

C066075	increase	TNF	ENSG00000223952	9707512;
C066075	increase	VEGFA	ENSG00000112715	17459161;
C066229	increase	IL8	ENSG00000169429	18308354;
C066515	increase	CCND1	ENSG00000110092	15276080;
C066515	increase	CDKN1A	ENSG00000124762	15276080;
C066515	increase	TP53	ENSG00000141510	15276080;
C066851	decrease	BIRC5	ENSG00000089685	17124180;
C066851	decrease	HSD17B2	ENSG00000086696	16807381;
C066851	increase	BAK1	ENSG00000030110	15489892;
C066851	increase	BBC3	ENSG00000105327	15489892;
C066851	increase	CDKN1A	ENSG00000124762	15489892;
C066851	increase	SP1	ENSG00000185591	16807381;15489892;
C066851	increase	TNFRSF10B	ENSG00000120889	18980244;
C066851	increase	TP53	ENSG00000141510	15489892;17124180;
C067311	decrease	BCL2	ENSG00000171791	19103299;
C067311	decrease	CASP3	ENSG00000164305	17562444;15970518;15282376;
C067311	decrease	CDKN2A	ENSG00000147889	16797627;
C067311	decrease	CYP19A1	ENSG00000137869	15623590;
C067311	decrease	MCM2	ENSG00000073111	17562444;
C067311	decrease	PGR	ENSG00000082175	15623590;
C067311	decrease	RB1	ENSG00000139687	15297405;
C067311	decrease	TNF	ENSG00000223952	15623590;
C067311	increase	ABCB1	ENSG00000085563	15239142;17284363;15239124;
C067311	increase	ABCC1	ENSG00000103222	15239124;
C067311	increase	BAX	ENSG00000087088	15641988;
C067311	increase	BIRC5	ENSG00000089685	19102932;16224667;17035597;
C067311	increase	DPYD	ENSG00000188641	16557585;
C067311	increase	GADD45A	ENSG00000116717	19190346;
C067311	increase	HRAS	ENSG00000174775	15970518;
C067311	increase	IL6	ENSG00000136244	15623590;
C067311	increase	MDM2	ENSG00000135679	16797627;16080190;
C067311	increase	PTGS2	ENSG00000073756	15623590;
C067311	increase	RAF1	ENSG00000132155	15970518;
C067311	increase	TP53	ENSG00000141510	17369602;16080190;15297405;15970518;16797627;
C067311	increase	TUBB	ENSG00000229684	16080190;15239142;
C067311	increase	TYMP	ENSG00000025708	15150550;
C067311	increase	TYMS	ENSG00000176890	16557585;19074750;
C067311	increase	UMPS	ENSG00000114491	16557585;
C067372	increase	BAX	ENSG00000087088	15447660;
C067372	increase	BCL2L1	ENSG00000171552	15447660;
C067372	increase	BID	ENSG00000015475	15447660;
C067431	decrease	CCND1	ENSG00000110092	16033851;
C067431	decrease	CCND2	ENSG00000118971	16033851;
C067431	decrease	MKI67	ENSG00000148773	16002280;
C067431	decrease	PGR	ENSG00000082175	16002280;
C067431	increase	LHB	ENSG00000104826	16046582;
C067513	increase	DCD	ENSG00000161634	15451026;
C067513	increase	PSMA2	ENSG00000106588	15451026;
C067513	increase	PSMB5	ENSG00000251114	15451026;
C067713	decrease	CTNNB1	ENSG00000168036	11756231;

C067713	increase	CEBPB	ENSG00000172216	11779194;
C067713	increase	CEBPD	ENSG00000221869	11779194;
C067713	increase	COMT	ENSG00000093010	17138778;
C067713	increase	GSTA2	ENSG00000244067	12446695;
C067713	increase	IL6	ENSG00000136244	11893605;
C067713	increase	IL8	ENSG00000169429	12208513;
C067713	increase	NFE2L2	ENSG00000116044	12446695;
C067713	increase	TNF	ENSG00000223952	17138778;
C068040	increase	MT2A	ENSG00000125148	9851254;
C068051	affect	BIRC5	ENSG00000089685	15916722;
C068051	affect	CASP10	ENSG00000003400	15916722;
C068051	affect	CASP6	ENSG00000138794	15916722;
C068051	increase	TP53	ENSG00000141510	15916722;
C068073	decrease	AGRN	ENSG00000188157	16982809;
C068073	decrease	ATP2B1	ENSG00000070961	16982809;
C068073	decrease	C16ORF46	ENSG00000166455	16982809;
C068073	decrease	C4ORF32	ENSG00000174749	16982809;
C068073	decrease	C7ORF45	ENSG00000165120	16982809;
C068073	decrease	CALCRL	ENSG00000064989	16982809;
C068073	decrease	CD1A	ENSG00000158477	16982809;
C068073	decrease	CDR2L	ENSG00000109089	16982809;
C068073	decrease	CTTNBP2	ENSG00000077063	16982809;
C068073	decrease	DMPK	ENSG00000104936	16982809;
C068073	decrease	DMWD	ENSG00000185800	16982809;
C068073	decrease	FAM109A	ENSG00000198324	16982809;
C068073	decrease	FUT7	ENSG00000180549	16982809;
C068073	decrease	GOLGA8A	ENSG00000175265	16982809;
C068073	decrease	GPD1L	ENSG00000152642	16982809;
C068073	decrease	HIST1H2BD	ENSG00000158373	16982809;
C068073	decrease	HLX	ENSG00000136630	16982809;
C068073	decrease	IL1R1	ENSG00000115594	16982809;
C068073	decrease	IL1RAP	ENSG00000196083	16982809;
C068073	decrease	IPCEF1	ENSG00000074706	16982809;
C068073	decrease	KIAA1161	ENSG00000164976	16982809;
C068073	decrease	KIAA1274	ENSG00000107719	16982809;
C068073	decrease	KIFC2	ENSG00000167702	16982809;
C068073	decrease	LELP1	ENSG00000203784	16982809;
C068073	decrease	LRP11	ENSG00000120256	16982809;
C068073	decrease	MAN1C1	ENSG00000117643	16982809;
C068073	decrease	MAP3K6	ENSG00000142733	16982809;
C068073	decrease	MAP4K1	ENSG00000104814	16982809;
C068073	decrease	METRN	ENSG00000103260	16982809;
C068073	decrease	MGAT4C	ENSG00000182050	16982809;
C068073	decrease	NDRG2	ENSG00000165795	16982809;
C068073	decrease	NUDT7	ENSG00000140876	16982809;
C068073	decrease	PDLIM2	ENSG00000120913	16982809;
C068073	decrease	PDLIM4	ENSG00000131435	16982809;
C068073	decrease	PHLDA2	ENSG00000181649	16982809;
C068073	decrease	PON2	ENSG00000105854	16982809;
C068073	decrease	QPRT	ENSG00000103485	16982809;
C068073	decrease	RAB15	ENSG00000139998	16982809;
C068073	decrease	RAP1GAP	ENSG00000076864	16982809;
C068073	decrease	REPS2	ENSG00000169891	16982809;
C068073	decrease	RFXAP	ENSG00000133111	16982809;

C068073	decrease	SELPLG	ENSG00000110876	16982809;
C068073	decrease	TACSTD2	ENSG00000184292	16982809;
C068073	decrease	TBL1X	ENSG00000101849	16982809;
C068073	decrease	TERT	ENSG00000164362	16482212;
C068073	decrease	TMEM14A	ENSG00000096092	16982809;
C068073	decrease	TNFSF12	ENSG00000239697	16982809;
C068073	decrease	TNS1	ENSG00000079308	16982809;
C068073	decrease	ZBTB7B	ENSG00000160685	16982809;
C068073	increase	ADA	ENSG00000196839	16982809;
C068073	increase	AGPAT4	ENSG00000026652	16982809;
C068073	increase	AJAP1	ENSG00000196581	16982809;
C068073	increase	APLP2	ENSG00000084234	16982809;
C068073	increase	BAG2	ENSG00000112208	16982809;
C068073	increase	C6ORF142	ENSG00000146147	16982809;
C068073	increase	CD1D	ENSG00000158473	16982809;17392484;
C068073	increase	CD300A	ENSG00000167851	16982809;
C068073	increase	CD300LB	ENSG00000178789	16982809;
C068073	increase	CD300LF	ENSG00000186074	16982809;
C068073	increase	CD38	ENSG00000004468	16329108;
C068073	increase	CGB	ENSG00000213030	15941851;
C068073	increase	COL5A3	ENSG00000080573	16982809;
C068073	increase	CYP19A1	ENSG00000137869	15941851;
C068073	increase	DHRS3	ENSG00000162496	16982809;
C068073	increase	DNAJC5B	ENSG00000147570	16982809;
C068073	increase	ETS2	ENSG00000157557	16982809;
C068073	increase	FAM101B	ENSG00000183688	16982809;
C068073	increase	FN1	ENSG00000115414	16982809;
C068073	increase	GDF15	ENSG00000130513	16982809;
C068073	increase	GUSB	ENSG00000169919	16982809;
C068073	increase	HIPK2	ENSG00000064393	16982809;
C068073	increase	IFNGR1	ENSG00000027697	16085646;
C068073	increase	INPP5F	ENSG00000198825	16982809;
C068073	increase	IRF1	ENSG00000125347	16636311;16085646;
C068073	increase	ITGAE	ENSG00000083457	16982809;
C068073	increase	LAIR1	ENSG00000167613	16982809;
C068073	increase	LILRA1	ENSG00000104974	16982809;
C068073	increase	NRIP1	ENSG00000180530	16982809;
C068073	increase	NRIP3	ENSG00000175352	16982809;
C068073	increase	OAS2	ENSG00000111335	16636311;
C068073	increase	OCLN	ENSG00000197822	16982755;
C068073	increase	OLR1	ENSG00000173391	16982809;
C068073	increase	OMA1	ENSG00000162600	16982809;
C068073	increase	PAPSS2	ENSG00000198682	16982809;
C068073	increase	PLBD1	ENSG00000121316	16982809;
C068073	increase	PPAP2B	ENSG00000162407	16982809;
C068073	increase	PRAM1	ENSG00000133246	16982809;
C068073	increase	RAB20	ENSG00000139832	16982809;
C068073	increase	RARA	ENSG00000131759	10779427;16178010;16636311;
C068073	increase	RBP1	ENSG00000248478	16982809;
C068073	increase	RHOBTB3	ENSG00000164292	16982809;
C068073	increase	SLC26A11	ENSG00000181045	16982809;
C068073	increase	SLITRK4	ENSG00000179542	16982809;
C068073	increase	SNAI3	ENSG00000185669	16982809;

C068073	increase	TBC1D2	ENSG00000095383	16982809;
C068073	increase	TBL2	ENSG00000106638	16982809;
C068073	increase	TGM2	ENSG00000198959	9516142;16982809;
C068073	increase	THBS3	ENSG00000169231	16982809;
C068073	increase	TM7SF4	ENSG00000164935	16982809;
C068291	decrease	IFNG	ENSG00000111537	14644929;
C068291	increase	CCL11	ENSG00000172156	14644929;
C068291	increase	IL5	ENSG00000113525	14644929;
C068337	decrease	CD74	ENSG00000019582	18818744;
C068430	increase	HMOX1	ENSG00000100292	17225922;
C068870	increase	INS	ENSG00000129965	16505238;
C068874	affect	TP53	ENSG00000141510	17339891;15161716;16204068;
C068874	increase	ANXA2	ENSG00000182718	15152939;
C068874	increase	FAS	ENSG00000026103	16204068;15161716;
C068874	increase	FXYD3	ENSG00000089356	15152939;
C068874	increase	HSPE1	ENSG00000115541	15152939;
C068874	increase	SAT1	ENSG00000130066	15152939;
C068874	increase	TMSB10	ENSG00000034510	15152939;
C068874	increase	TYMS	ENSG00000176890	10482907;17339891;15152939;15161716;
C069357	increase	HRH4	ENSG00000134489	12235264;11181941;
C069357	increase	IL16	ENSG00000172349	12235264;
C069741	decrease	ANGPT2	ENSG00000091879	16681930;
C069741	decrease	PCNA	ENSG00000132646	16681930;
C069741	decrease	VEGFA	ENSG00000112715	16681930;
C069741	increase	THBS1	ENSG00000137801	16681930;
C069741	increase	TIMP2	ENSG00000035862	16681930;
C070081	affect	PON1	ENSG00000005421	15458977;
C070081	affect	THBS1	ENSG00000137801	17010073;
C070081	decrease	ABCC11	ENSG00000121270	18310281;
C070081	decrease	ABCC5	ENSG00000114770	15072547;
C070081	decrease	AHNAK	ENSG00000124942	16514628;
C070081	decrease	AR	ENSG00000169083	15171712;15316697;11867264;
C070081	decrease	ASNS	ENSG00000070669	16514628;
C070081	decrease	AURKA	ENSG00000087586	16514628;
C070081	decrease	BAX	ENSG00000087088	15870704;
C070081	decrease	C10ORF10	ENSG00000165507	16298037;
C070081	decrease	C6ORF106	ENSG00000196821	16514628;
C070081	decrease	CBX1	ENSG00000108468	16514628;
C070081	decrease	CCNA2	ENSG00000145386	17445807;15072547;
C070081	decrease	CDKN1A	ENSG00000124762	12473173;15870704;
C070081	decrease	CENPA	ENSG00000115163	16514628;
C070081	decrease	CLSTN1	ENSG00000171603	16514628;
C070081	decrease	COMT	ENSG00000093010	18497090;18192686;
C070081	decrease	COPB2	ENSG00000184432	16514628;
C070081	decrease	CSF3	ENSG00000108342	16298037;
C070081	decrease	CSTA	ENSG00000121552	15072547;
C070081	decrease	DDX21	ENSG00000165732	16514628;
C070081	decrease	DDX3Y	ENSG00000067048	16514628;
C070081	decrease	DHX9	ENSG00000135829	16514628;
C070081	decrease	DNAJA1	ENSG00000086061	16514628;
C070081	decrease	EIF2S1	ENSG00000134001	16514628;

C070081	decrease	EIF5	ENSG00000100664	16514628;
C070081	decrease	ELOVL5	ENSG00000012660	16514628;
C070081	decrease	ESRRA	ENSG00000173153	15968320;
C070081	decrease	FGF9	ENSG00000102678	14602803;
C070081	decrease	FLNC	ENSG00000128591	16514628;
C070081	decrease	FYTTD1	ENSG00000122068	16514628;
C070081	decrease	GOLGA5	ENSG00000066455	16514628;
C070081	decrease	HMGB1	ENSG00000189403	16514628;
C070081	decrease	HMOX1	ENSG00000100292	16298037;
C070081	decrease	HNRNPA2B	ENSG00000122566	16514628;
C070081	decrease	HNRNPU	ENSG00000153187	16514628;
C070081	decrease	HSP90AB1	ENSG00000096384	16514628;
C070081	decrease	HSP90B1	ENSG00000166598	16514628;
C070081	decrease	HSPA4	ENSG00000170606	16514628;
C070081	decrease	HSPA8	ENSG00000109971	16514628;
C070081	decrease	HSPD1	ENSG00000144381	16514628;
C070081	decrease	IFRD1	ENSG00000006652	16514628;
C070081	decrease	IGFBP4	ENSG00000141753	15072547;
C070081	decrease	IQGAP1	ENSG00000140575	16514628;
C070081	decrease	IRS1	ENSG00000169047	15072547;
C070081	decrease	KIAA0101	ENSG00000166803	16514628;
C070081	decrease	KIF20A	ENSG00000112984	16514628;
C070081	decrease	KPNA2	ENSG00000182481	16514628;
C070081	decrease	KPNB1	ENSG00000108424	16514628;
C070081	decrease	MATR3	ENSG00000015479	16514628;
C070081	decrease	MGP	ENSG00000111341	16514628;
C070081	decrease	MST4	ENSG00000134602	16514628;
C070081	decrease	MYC	ENSG00000136997	15766595;15072547;
C070081	decrease	NARS	ENSG00000134440	16514628;
C070081	decrease	NCAPH	ENSG00000121152	16514628;
C070081	decrease	NCL	ENSG00000115053	16514628;
C070081	decrease	NFKBIA	ENSG00000100906	16298037;
C070081	decrease	NPM1	ENSG00000181163	16514628;
C070081	decrease	NUS1	ENSG00000153989	16514628;
C070081	decrease	PCNA	ENSG00000132646	16202921;15072547;
C070081	decrease	POLR2B	ENSG00000047315	16514628;
C070081	decrease	PPM1D	ENSG00000170836	16514628;
C070081	decrease	PRKDC	ENSG00000121031	16514628;
C070081	decrease	PSMD14	ENSG00000115233	16514628;
C070081	decrease	PSMD6	ENSG00000163636	16514628;
C070081	decrease	PSME4	ENSG00000068878	16514628;
C070081	decrease	RACGAP1	ENSG00000161800	16514628;
C070081	decrease	RAP1A	ENSG00000116473	16514628;
C070081	decrease	RBBP7	ENSG00000102054	16514628;
C070081	decrease	RPL6	ENSG00000089009	16514628;
C070081	decrease	RRM1	ENSG00000167325	16514628;
C070081	decrease	SFPQ	ENSG00000116560	16514628;
C070081	decrease	SFRS1	ENSG00000136450	16514628;
C070081	decrease	SLC26A2	ENSG00000155850	16514628;
C070081	decrease	SLC39A6	ENSG00000141424	16514628;
C070081	decrease	SON	ENSG00000159140	16514628;
C070081	decrease	STAT1	ENSG00000115415	16514628;
C070081	decrease	STMN1	ENSG00000117632	16514628;
C070081	decrease	TAF9	ENSG00000085231	16514628;

C070081	decrease	TARS	ENSG00000113407	16514628;
C070081	decrease	TCEB2	ENSG00000103363	16514628;
C070081	decrease	TFF3	ENSG00000160180	16202921;
C070081	decrease	TFPI	ENSG00000003436	17029634;
C070081	decrease	THBS2	ENSG00000186340	16514628;
C070081	decrease	TMPO	ENSG00000120802	16514628;
C070081	decrease	TNPO1	ENSG00000083312	16514628;
C070081	decrease	TOP1	ENSG00000198900	16514628;
C070081	decrease	TOP2B	ENSG00000077097	16514628;
C070081	decrease	TRAK2	ENSG00000115993	16514628;
C070081	decrease	UNG	ENSG00000076248	16514628;
C070081	decrease	USP32	ENSG00000170832	16514628;
C070081	decrease	WNT2	ENSG00000105989	15072547;
C070081	decrease	WNT7A	ENSG00000154764	16534752;
C070081	decrease	XPO1	ENSG00000082898	16514628;
C070081	decrease	XRCC5	ENSG00000079246	16514628;
C070081	increase	ABCC3	ENSG00000108846	15072547;
C070081	increase	AGER	ENSG00000204305	15878629;
C070081	increase	APOD	ENSG00000189058	16514628;
C070081	increase	AREG	ENSG00000205595	16202921;16514628;15072547;
C070081	increase	ARNT2	ENSG00000172379	16514628;
C070081	increase	BCL2	ENSG00000171791	10545406;15870704;
C070081	increase	BGLAP	ENSG00000242252	17689939;
C070081	increase	BLM	ENSG00000197299	17268063;
C070081	increase	BMP2	ENSG00000125845	17513867;
C070081	increase	BRCA1	ENSG00000012048	12473173;
C070081	increase	CCL20	ENSG00000115009	16298037;
C070081	increase	CCNB1	ENSG00000134057	17445807;
C070081	increase	CTSD	ENSG00000117984	15072547;
C070081	increase	CTSH	ENSG00000103811	16514628;
C070081	increase	CXCL10	ENSG00000169245	15557169;
C070081	increase	CXCL11	ENSG00000169248	15557169;
C070081	increase	CXCL3	ENSG00000163734	16298037;
C070081	increase	CYP2A6	ENSG00000198077	17646279;
C070081	increase	DMBT1	ENSG00000187908	15564322;
C070081	increase	DST	ENSG00000151914	16202921;
C070081	increase	EFNA1	ENSG00000169242	15072547;
C070081	increase	ERBB2	ENSG00000141736	15072547;
C070081	increase	ESR1	ENSG00000091831	15899841;19351862;15950373;17299137;16037132;17203231;11518614;15842231;16489053;17003281;12441364;15130599;16076840;12650720;14742696;17513867;16010691;11867264;162[illegible]
C070081	increase	ESR2	ENSG00000140009	17638070;17203231;15950373;17935960;17003281;15171712;15130599;14742696;15103026;17692514;1620
C070081	increase	FARP1	ENSG00000152767	16849584;
C070081	increase	FOS	ENSG00000170345	17125913;15072547;
C070081	increase	FST	ENSG00000134363	16298037;
C070081	increase	IER3	ENSG00000137331	16849584;
C070081	increase	IFI6	ENSG00000126709	16202921;

C070081	increase	IL1B	ENSG00000125538	16298037;
C070081	increase	IL24	ENSG00000162892	16298037;
C070081	increase	IL6	ENSG00000136244	16298037;
C070081	increase	IL8	ENSG00000169429	16298037;
C070081	increase	JUN	ENSG00000177606	17125913;
C070081	increase	KCNH1	ENSG00000143473	19351862;
C070081	increase	KLK3	ENSG00000142515	17131305;17503469;
C070081	increase	KRT13	ENSG00000171401	16849584;
C070081	increase	MME	ENSG00000196549	16202921;
C070081	increase	MMP15	ENSG00000102996	15072547;
C070081	increase	MYH10	ENSG00000133026	16901965;
C070081	increase	NKX3-1	ENSG00000167034	17131305;
C070081	increase	NOS3	ENSG00000164867	14742696;
C070081	increase	NOV	ENSG00000136999	15072547;
C070081	increase	NR4A1	ENSG00000123358	16514628;
C070081	increase	PDGFA	ENSG00000197461	17125913;
C070081	increase	PGR	ENSG00000082175	15806153;16323292;16892383;15541416;11867264;15072547;
C070081	increase	PIM1	ENSG00000137193	16298037;
C070081	increase	PTPRG	ENSG00000144724	16849584;
C070081	increase	RAB30	ENSG00000137502	16849584;
C070081	increase	RUNX2	ENSG00000250096	17689939;
C070081	increase	SERPINB2	ENSG00000197632	16298037;
C070081	increase	SERPINB9	ENSG00000170542	14617632;17237823;10681578;
C070081	increase	SOCS1	ENSG00000185338	16849584;
C070081	increase	SOCS3	ENSG00000184557	16055089;
C070081	increase	SP7	ENSG00000170374	17689939;
C070081	increase	STK39	ENSG00000198648	17131305;
C070081	increase	TACC1	ENSG00000147526	15072547;
C070081	increase	TFF1	ENSG00000160182	15525692;18310281;12473173;16892383;9276643;15541416;16514628;16202921;15072547;
C070081	increase	TNF	ENSG00000223952	12547825;
C070081	increase	UGT1A1	ENSG00000241635	15072547;
C070081	increase	UGT2B15	ENSG00000196620	16690804;
C070081	increase	VCAM1	ENSG00000162692	12547825;
C070081	increase	VDR	ENSG00000111424	17088408;15930183;
C070081	increase	ZNF217	ENSG00000171940	15072547;
C070272	increase	ABCB1	ENSG00000085563	18673531;15788683;17947497;16221533;16223781;11934808;
C070279	increase	CCNB1	ENSG00000134057	16061671;
C070279	increase	CDKN1A	ENSG00000124762	16061671;
C070379	increase	IL8	ENSG00000169429	15831558;17000667;17129690;
C070441	decrease	JUN	ENSG00000177606	9007151;
C070441	decrease	MYC	ENSG00000136997	9007151;
C070515	decrease	ABCB4	ENSG00000005471	11331069;
C070515	decrease	CNN1	ENSG00000130176	15380616;
C070515	decrease	TAGLN	ENSG00000149591	15380616;
C070515	decrease	TP53	ENSG00000141510	16055726;
C070515	increase	IL8	ENSG00000169429	17000667;

C070515	increase	JUN	ENSG00000177606	18628248;
C070515	increase	RB1	ENSG00000139687	14653808;
C070515	increase	SP1	ENSG00000185591	14653808;
C070840	affect	IL1B	ENSG00000125538	16354411;
C070840	affect	IL6	ENSG00000136244	16354411;
C070840	affect	IL8	ENSG00000169429	16354411;
C070840	affect	PTGS2	ENSG00000073756	16354411;
C071423	increase	AR	ENSG00000169083	19059400;
C071423	increase	CALCA	ENSG00000110680	18584893;
C071423	increase	CALCB	ENSG00000175868	18584893;
C071423	increase	IL6	ENSG00000136244	17306835;
C071423	increase	MMP1	ENSG00000196611	17508023;
C071834	decrease	ALDH3B2	ENSG00000132746	17822822;
C071834	decrease	ALDH4A1	ENSG00000159423	17822822;
C071834	decrease	ANKRD34A	ENSG00000181039	17822822;
C071834	decrease	ANXA1	ENSG00000135046	17822822;
C071834	decrease	CA12	ENSG00000074410	17822822;
C071834	decrease	CAPN13	ENSG00000162949	17822822;
C071834	decrease	CAPN2	ENSG00000162909	17822822;
C071834	decrease	CASP7	ENSG00000165806	17822822;
C071834	decrease	CAV1	ENSG00000105974	17822822;
C071834	decrease	CAV2	ENSG00000105971	17822822;
C071834	decrease	CDH12	ENSG00000154162	17822822;
C071834	decrease	CRABP2	ENSG00000143320	17822822;
C071834	decrease	CTNND2	ENSG00000169862	17822822;
C071834	decrease	CYP1A1	ENSG00000140465	17822822;
C071834	decrease	CYP1B1	ENSG00000138061	17822822;
C071834	decrease	CYP2J2	ENSG00000134716	17822822;
C071834	decrease	CYP4B1	ENSG00000142973	17822822;
C071834	decrease	DOCK8	ENSG00000107099	17822822;
C071834	decrease	FHOD3	ENSG00000134775	17822822;
C071834	decrease	GADD45B	ENSG00000099860	17822822;
C071834	decrease	HS6ST2	ENSG00000171004	17822822;
C071834	decrease	IDH2	ENSG00000182054	17822822;
C071834	decrease	IFITM1	ENSG00000185885	17822822;
C071834	decrease	IGFBP2	ENSG00000115457	17822822;
C071834	decrease	IGFBP4	ENSG00000141753	17822822;
C071834	decrease	IGFBP5	ENSG00000115461	17822822;
C071834	decrease	IL1R1	ENSG00000115594	17822822;
C071834	decrease	ING3	ENSG00000071243	17822822;
C071834	decrease	ITGA6	ENSG00000091409	17822822;
C071834	decrease	ITGB6	ENSG00000115221	17822822;
C071834	decrease	MMP16	ENSG00000156103	17822822;
C071834	decrease	PCSK6	ENSG00000140479	17822822;
C071834	decrease	PGM2L1	ENSG00000165434	17822822;
C071834	decrease	PRLR	ENSG00000113494	17822822;
C071834	decrease	PTPN13	ENSG00000163629	17822822;
C071834	decrease	SASH1	ENSG00000111961	17822822;
C071834	decrease	SELENBP1	ENSG00000143416	17822822;
C071834	decrease	SHANK2	ENSG00000162105	17822822;
C071834	decrease	SIDT1	ENSG00000072858	17822822;
C071834	decrease	ST3GAL1	ENSG00000008513	17822822;
C071834	decrease	ST7	ENSG00000004866	17822822;
C071834	decrease	TIMP2	ENSG00000035862	17822822;

C071834	decrease	TIMP3	ENSG00000100234	17822822;
C071834	decrease	TM4SF1	ENSG00000169908	17822822;
C071834	decrease	TMPRSS2	ENSG00000184012	17822822;
C071834	decrease	TRAF5	ENSG00000082512	17822822;
C071834	decrease	WISP2	ENSG00000064205	17822822;
C071834	decrease	WWOX	ENSG00000186153	17822822;
C071834	increase	ABCB6	ENSG00000115657	17822822;
C071834	increase	AKR1B10	ENSG00000198074	17822822;
C071834	increase	AKR1C1	ENSG00000187134	17822822;
C071834	increase	AKR1C2	ENSG00000151632	17822822;
C071834	increase	AKR1C3	ENSG00000196139	17822822;
C071834	increase	ATG3	ENSG00000144848	17822822;
C071834	increase	CAPN7	ENSG00000131375	17822822;
C071834	increase	CBR1	ENSG00000159228	17822822;
C071834	increase	CBR3	ENSG00000159231	17822822;
C071834	increase	CCNI	ENSG00000118816	17822822;
C071834	increase	CLTC	ENSG00000141367	17822822;
C071834	increase	COMMD10	ENSG00000145781	17822822;
C071834	increase	CYP4F11	ENSG00000171903	17822822;
C071834	increase	DHRS2	ENSG00000100867	17822822;
C071834	increase	EME1	ENSG00000154920	17822822;
C071834	increase	EPHX1	ENSG00000143819	15668107;17822822;
C071834	increase	ESD	ENSG00000139684	17822822;
C071834	increase	FECH	ENSG00000066926	17822822;
C071834	increase	FTH1	ENSG00000167996	17822822;
C071834	increase	G6PD	ENSG00000160211	17822822;
C071834	increase	GCLM	ENSG00000023909	17822822;
C071834	increase	HMOX1	ENSG00000100292	17822822;
C071834	increase	IGF2R	ENSG00000197081	17822822;
C071834	increase	KITLG	ENSG00000049130	17822822;
C071834	increase	LIAS	ENSG00000121897	17822822;
C071834	increase	MAFG	ENSG00000197063	17822822;
C071834	increase	MAPK12	ENSG00000188130	17822822;
C071834	increase	ME1	ENSG00000065833	17822822;
C071834	increase	METAP2	ENSG00000111142	17822822;
C071834	increase	MGST1	ENSG00000008394	17822822;
C071834	increase	NQO1	ENSG00000181019	17822822;
C071834	increase	PRDX3	ENSG00000165672	17822822;
C071834	increase	PRDX6	ENSG00000117592	17822822;
C071834	increase	PTGR1	ENSG00000106853	17822822;
C071834	increase	SERINC2	ENSG00000168528	17822822;
C071834	increase	SLC7A11	ENSG00000151012	17822822;
C071834	increase	SQSTM1	ENSG00000161011	17822822;
C071834	increase	SRXN1	ENSG00000172070	17822822;
C071834	increase	SULT1A1	ENSG00000196502	17822822;
C071834	increase	SULT1A3	ENSG00000213599	17822822;
C071834	increase	SULT1A4	ENSG00000213599	17822822;
C071834	increase	TMED4	ENSG00000158604	17822822;
C071834	increase	TNFRSF11A	ENSG00000141655	17822822;
C071834	increase	TNFRSF11B	ENSG00000164761	17822822;
C071834	increase	TNFRSF25	ENSG00000215788	17822822;
C071834	increase	TNFSF9	ENSG00000125657	17822822;
C071834	increase	TRPM7	ENSG00000092439	17822822;
C071834	increase	TXN	ENSG00000136810	17822822;

C071834	increase	USP40	ENSG00000085982	17822822;
C071924	increase	MIF	ENSG00000240972	16872482;
C071924	increase	MMP2	ENSG00000087245	16872482;
C072019	decrease	BAD	ENSG00000002330	15698582;
C072019	decrease	BCL2L1	ENSG00000171552	15698582;
C072019	decrease	BID	ENSG00000015475	15698582;
C072019	increase	TP53	ENSG00000141510	15698582;
C072105	decrease	CCND1	ENSG00000110092	15958590;
C072105	increase	CYP11B2	ENSG00000179142	12193581;
C072553	affect	CAV1	ENSG00000105974	18848576;
C072553	affect	HAX1	ENSG00000143575	19763263;
C072553	decrease	ACTB	ENSG00000075624	15857753;
C072553	decrease	AHR	ENSG00000106546	15385644;15899923;
C072553	decrease	ARNT	ENSG00000143437	16880289;15899923;
C072553	decrease	BAX	ENSG00000087088	15782132;
C072553	decrease	BCL2L1	ENSG00000171552	16960866;
C072553	decrease	CCND1	ENSG00000110092	15247282;16051428;
C072553	decrease	CFLAR	ENSG00000003402	18980244;
C072553	decrease	CTNNB1	ENSG00000168036	17219422;14555707;17030184;
C072553	decrease	EP300	ENSG00000100393	17927689;
C072553	decrease	FTH1	ENSG00000167996	16707465;
C072553	decrease	FTL	ENSG00000087086	16707465;
C072553	decrease	HDAC1	ENSG00000116478	17927689;
C072553	decrease	HDAC2	ENSG00000196591	18421014;
C072553	decrease	PPIA	ENSG00000196262	15857753;
C072553	decrease	TYMS	ENSG00000176890	17172411;
C072553	increase	ABCG2	ENSG00000118777	17077187;
C072553	increase	AR	ENSG00000169083	12376534;
C072553	increase	BIRC3	ENSG00000023445	14527959;
C072553	increase	BIRC5	ENSG00000089685	16328441;15993841;
C072553	increase	CCL2	ENSG00000108691	11230753;11698246;
C072553	increase	CEBPB	ENSG00000172216	11779194;
C072553	increase	CEBPD	ENSG00000221869	11779194;
C072553	increase	CX3CL1	ENSG00000006210	11698246;
C072553	increase	CXCL1	ENSG00000163739	11698246;
C072553	increase	EDN1	ENSG00000078401	18457675;
C072553	increase	FOSL1	ENSG00000175592	17148446;
C072553	increase	GSTA2	ENSG00000244067	12446695;
C072553	increase	GSTP1	ENSG00000084207	15863507;
C072553	increase	HIF1A	ENSG00000100644	17973296;18682687;17764071;11980636;
C072553	increase	HSD11B2	ENSG00000176387	15857753;
C072553	increase	HTRA2	ENSG00000115317	19763263;
C072553	increase	ICAM1	ENSG00000090339	14572618;11698246;
C072553	increase	IL6	ENSG00000136244	11893605;18457675;
C072553	increase	IL8	ENSG00000169429	12208513;11698246;16392133;18457675;
C072553	increase	JUNB	ENSG00000171223	17148446;
C072553	increase	KLK2	ENSG00000167751	12376534;
C072553	increase	NCOR1	ENSG00000141027	15857753;
C072553	increase	NCOR2	ENSG00000196498	15857753;
C072553	increase	NFE2L2	ENSG00000116044	12446695;
C072553	increase	PGR	ENSG00000082175	15218360;

C072553	increase	PTGS2	ENSG00000073756	11230753;18457675;
C072553	increase	RXRA	ENSG00000186350	17170071;15707588;12970875;
C072553	increase	TNF	ENSG00000223952	12883408;11698246;14572618;11230753;
C072553	increase	TNFRSF10A	ENSG00000104689	16287099;
C072553	increase	TNFRSF10B	ENSG00000120889	16287099;
C072553	increase	VCAM1	ENSG00000162692	11698246;18457675;
C072876	affect	IL2	ENSG00000109471	8719445;
C072876	increase	CCNA2	ENSG00000145386	12181422;
C072876	increase	CCNE1	ENSG00000105173	12181422;
C072876	increase	CDKN1A	ENSG00000124762	12181422;
C072876	increase	GYPA	ENSG00000170180	12181422;
C072876	increase	IL10RA	ENSG00000110324	10076546;
C072876	increase	NFKB1	ENSG00000109320	9973483;
C072876	increase	RELA	ENSG00000173039	9973483;
C072876	increase	TNF	ENSG00000223952	9973483;
C072876	increase	TP53	ENSG00000141510	10076546;
C073539	increase	ABCC2	ENSG00000023839	15896333;
C073539	increase	NQO1	ENSG00000181019	15896333;
C073539	increase	UGT1A1	ENSG00000241635	15896333;
C074153	affect	CFL2	ENSG00000165410	18444160;
C074153	affect	HSPE1	ENSG00000115541	18444160;
C074153	affect	NME6	ENSG00000172113	18444160;
C074153	affect	SSBP1	ENSG00000106028	18444160;
C074153	decrease	AKT1	ENSG00000142208	16399772;
C074153	decrease	CCND1	ENSG00000110092	12844480;16399772;
C074153	decrease	CCND2	ENSG00000118971	12844480;
C074153	decrease	KLK3	ENSG00000142515	17205524;
C074153	decrease	MYC	ENSG00000136997	12844480;
C074153	decrease	NFKB1	ENSG00000109320	16399772;
C074153	decrease	PCNA	ENSG00000132646	12844480;
C074153	decrease	PTGS2	ENSG00000073756	16399772;
C074153	increase	AR	ENSG00000169083	17205524;
C074153	increase	CASP3	ENSG00000164305	12844480;
C074153	increase	CDKN1A	ENSG00000124762	12844480;
C074153	increase	CDKN1B	ENSG00000111276	12844480;
C074153	increase	FAS	ENSG00000026103	12844480;
C074153	increase	GPX5	ENSG00000224586	16399772;
C074153	increase	TP53	ENSG00000141510	17205524;
C074279	decrease	CCNE1	ENSG00000105173	12907249;
C074279	decrease	CDK2	ENSG00000123374	12907249;
C074279	decrease	CDKN1A	ENSG00000124762	12907249;
C074279	increase	BGLAP	ENSG00000242252	12898515;
C074279	increase	CAMP	ENSG00000164047	15985530;
C074279	increase	CDKN1B	ENSG00000111276	12907249;
C074619	decrease	CRP	ENSG00000132693	16118546;
C074619	decrease	MPO	ENSG00000005381	18701766;
C074619	increase	F2	ENSG00000180210	1290488;18449412;17696810;15080313;16084352;19124943;15155122;19468011;16845256;19644995;84564
C074619	increase	SELP	ENSG00000174175	16845256;
C074702	affect	HMOX1	ENSG00000100292	12716893;

C074702	affect	LCK	ENSG00000182866	17078813;
C074702	affect	STAT1	ENSG00000115415	19403854;17078813;
C074702	decrease	SOD1	ENSG00000142168	17098216;
C074702	increase	HIF1A	ENSG00000100644	19403854;
C074702	increase	IL6	ENSG00000136244	17078813;
C074702	increase	MT1A	ENSG00000205362	12716893;
C074702	increase	MT2A	ENSG00000125148	12716893;
C074702	increase	VEGFA	ENSG00000112715	19403854;
C074761	increase	CAT	ENSG00000121691	17473450;
C075750	increase	PTGS2	ENSG00000073756	8621535;
C075773	affect	HDAC1	ENSG00000116478	18497984;
C075773	affect	MAGEA9	ENSG00000166008	17290406;
C075773	affect	MECP2	ENSG00000169057	18497984;
C075773	affect	TUBB3	ENSG00000198211	18497984;
C075773	decrease	C3ORF26	ENSG00000184220	14583596;
C075773	decrease	CIRH1A	ENSG00000141076	14583596;
C075773	decrease	CITED2	ENSG00000164442	14583596;
C075773	decrease	CLDN1	ENSG00000163347	14583596;
C075773	decrease	CNIH4	ENSG00000143771	14583596;
C075773	decrease	CNOT2	ENSG00000111596	14583596;
C075773	decrease	CSDE1	ENSG00000009307	14583596;
C075773	decrease	DDX55	ENSG00000111364	14583596;
C075773	decrease	EEF1E1	ENSG00000124802	14583596;
C075773	decrease	EFTUD2	ENSG00000108883	14583596;
C075773	decrease	FARSB	ENSG00000116120	14583596;
C075773	decrease	FGF2	ENSG00000138685	14583596;
C075773	decrease	GAS6	ENSG00000248501	14583596;
C075773	decrease	HMGA1	ENSG00000137309	14583596;
C075773	decrease	HNRNPA2B	ENSG00000122566	14583596;
C075773	decrease	ID1	ENSG00000125968	14583596;
C075773	decrease	IPO11	ENSG00000086200	14583596;
C075773	decrease	JUB	ENSG00000129474	14583596;
C075773	decrease	MAT2A	ENSG00000168906	14583596;
C075773	decrease	MKI67IP	ENSG00000155438	14583596;
C075773	decrease	MRPS18C	ENSG00000163319	14583596;
C075773	decrease	MRPS22	ENSG00000175110	14583596;
C075773	decrease	NDE1	ENSG00000072864	14583596;
C075773	decrease	NLN	ENSG00000123213	14583596;
C075773	decrease	NOLC1	ENSG00000166197	14583596;
C075773	decrease	NOM1	ENSG00000146909	14583596;
C075773	decrease	PAWR	ENSG00000177425	14583596;
C075773	decrease	PDCD2L	ENSG00000126249	14583596;
C075773	decrease	POLE4	ENSG00000115350	14583596;
C075773	decrease	PPIL1	ENSG00000137168	14583596;
C075773	decrease	RBM8A	ENSG00000131795	14583596;
C075773	decrease	RPL7A	ENSG00000148303	14583596;
C075773	decrease	SLU7	ENSG00000164609	14583596;
C075773	decrease	SMCR7L	ENSG00000100335	14583596;
C075773	decrease	SNX5	ENSG00000089006	14583596;
C075773	decrease	SOX4	ENSG00000124766	14583596;
C075773	decrease	SUV420H1	ENSG00000110066	14583596;
C075773	decrease	SYVN1	ENSG00000162298	14583596;
C075773	decrease	TAX1BP1	ENSG00000106052	14583596;
C075773	decrease	TAX1BP3	ENSG00000213977	14583596;

C075773	decrease	TIMM50	ENSG00000105197	14583596;
C075773	decrease	TMPO	ENSG00000120802	14583596;
C075773	decrease	TRA2A	ENSG00000164548	14583596;
C075773	decrease	TSR1	ENSG00000167721	14583596;
C075773	decrease	UBE2W	ENSG00000104343	14583596;
C075773	decrease	WDR4	ENSG00000160193	14583596;
C075773	decrease	WWTR1	ENSG00000018408	14583596;
C075773	decrease	XPO5	ENSG00000124571	14583596;
C075773	decrease	YTHDF2	ENSG00000198492	14583596;
C075773	decrease	ZBTB2	ENSG00000181472	14583596;
C075773	decrease	ZNRD1	ENSG00000236808	14583596;
C075773	increase	AARS	ENSG00000090861	14583596;
C075773	increase	AHSA1	ENSG00000100591	14583596;
C075773	increase	AKR1C1	ENSG00000187134	14583596;
C075773	increase	BCL6	ENSG00000113916	14583596;
C075773	increase	CARS	ENSG00000110619	14583596;
C075773	increase	CDKN1A	ENSG00000124762	17210717;
C075773	increase	CDKN2A	ENSG00000147889	17210717;
C075773	increase	COL5A1	ENSG00000130635	14583596;
C075773	increase	CSPG5	ENSG00000114646	14583596;
C075773	increase	EGFL7	ENSG00000172889	19116145;
C075773	increase	EHD3	ENSG00000013016	14583596;
C075773	increase	FAAH	ENSG00000117480	14583596;
C075773	increase	FBLN1	ENSG00000077942	14583596;
C075773	increase	GRB10	ENSG00000106070	14583596;
C075773	increase	GSTA4	ENSG00000170899	14583596;
C075773	increase	HLA-E	ENSG00000225201	14583596;
C075773	increase	HSPA1A	ENSG00000232804	14583596;
C075773	increase	HSPA2	ENSG00000126803	14583596;
C075773	increase	HSPA4L	ENSG00000164070	14583596;
C075773	increase	HSPH1	ENSG00000120694	14583596;
C075773	increase	IL1B	ENSG00000125538	17709599;
C075773	increase	IL6	ENSG00000136244	17709599;
C075773	increase	IL8	ENSG00000169429	17709599;
C075773	increase	MT1F	ENSG00000198417	14583596;
C075773	increase	NR1D2	ENSG00000174738	14583596;
C075773	increase	POLR2A	ENSG00000181222	14583596;
C075773	increase	PTGS2	ENSG00000073756	17709599;
C075773	increase	SLC22A18	ENSG00000110628	14583596;
C075773	increase	TMED5	ENSG00000117500	14583596;
C075773	increase	TSC2	ENSG00000103197	14583596;
C075856	increase	RHOB	ENSG00000143878	12671036;
C075954	increase	ABCG2	ENSG00000118777	17077187;15917307;
C076196	increase	ICAM1	ENSG00000090339	15358673;
C076196	increase	NOS3	ENSG00000164867	15358673;
C076196	increase	VCAM1	ENSG00000162692	15358673;
C076207	decrease	IL12A	ENSG00000168811	9819373;
C076207	decrease	IL12B	ENSG00000113302	9819373;
C076588	increase	AKR1A1	ENSG00000117448	15720144;16411658;99732
C076588	increase	AKR1C1	ENSG00000187134	9973208;
C076588	increase	CYP1A1	ENSG00000140465	9973208;
C076588	increase	CYP1B1	ENSG00000138061	16411658;
C076852	decrease	BIRC5	ENSG00000089685	15958646;
C076852	decrease	CDK1	ENSG00000170312	15958646;

C076852	increase	ABCB1	ENSG00000085563	16544145;
C076852	increase	SP1	ENSG00000185591	16544145;
C077990	affect	H2AFX	ENSG00000188486	15078984;
C077990	decrease	BAG1	ENSG00000250477	15972445;
C077990	decrease	BCL2	ENSG00000171791	12170773;12517783;15770523;15972445;
C077990	decrease	BCL2L1	ENSG00000171552	12517783;15634644;
C077990	decrease	BIRC3	ENSG00000023445	12517783;
C077990	decrease	BIRC5	ENSG00000089685	16012789;12517783;
C077990	decrease	CCNB1	ENSG00000134057	12517783;
C077990	decrease	CCND1	ENSG00000110092	15634644;12517783;
C077990	decrease	MCL1	ENSG00000143384	12517783;15634644;15972445;
C077990	decrease	RB1	ENSG00000139687	15297405;15078984;15180955;
C077990	decrease	XIAP	ENSG00000101966	12517783;15972445;15385934;
C077990	increase	BAX	ENSG00000087088	12517783;15634644;15770523;
C077990	increase	CDKN1A	ENSG00000124762	15770523;15180955;
C077990	increase	CDKN1B	ENSG00000111276	15180955;
C077990	increase	TP53	ENSG00000141510	15297405;15180955;
C078131	increase	MMP9	ENSG00000100985	18006877;
C078706	increase	RHOA	ENSG00000067560	12671036;
C078706	increase	RHOB	ENSG00000143878	12671036;
C078814	increase	CD36	ENSG00000135218	17360047;
C078846	decrease	PACRG	ENSG00000112530	18586549;
C078846	decrease	PARK2	ENSG00000185345	18586549;
C078903	decrease	BIRC5	ENSG00000089685	18567002;
C078903	decrease	CCNE1	ENSG00000105173	12907249;
C078903	decrease	CDK2	ENSG00000123374	12907249;
C078903	decrease	PTHLH	ENSG00000087494	16243370;
C078903	increase	BAX	ENSG00000087088	16340750;
C078903	increase	BCL2L11	ENSG00000153094	16340750;
C078903	increase	CA2	ENSG00000104267	16340750;
C078903	increase	CAMP	ENSG00000164047	15985530;
C078903	increase	CDKN1A	ENSG00000124762	12907249;16340750;
C078903	increase	CDKN1B	ENSG00000111276	16340750;12907249;
C078903	increase	SPDEF	ENSG00000124664	18567002;
C079198	decrease	CD34	ENSG00000174059	18607998;
C079198	decrease	FGF2	ENSG00000138685	18607998;
C079198	decrease	TEK	ENSG00000120156	18607998;
C079198	increase	DPYD	ENSG00000188641	18506536;
C079198	increase	SLC29A1	ENSG00000112759	18383843;
C079198	increase	TYMP	ENSG00000025708	18506536;
C079391	increase	CDKN1A	ENSG00000124762	17045927;
C079391	increase	NOS3	ENSG00000164867	17045927;
C079391	increase	TP53	ENSG00000141510	17045927;
C079905	increase	ABCC2	ENSG00000023839	15342794;
C080163	increase	IL1B	ENSG00000125538	10822086;
C080163	increase	SELE	ENSG00000007908	10822086;
C080222	increase	ABCB1	ENSG00000085563	16026610;
C080417	decrease	CDO1	ENSG00000129596	16223563;
C080417	decrease	PAPSS1	ENSG00000138801	16223563;
C080417	decrease	PAPSS2	ENSG00000198682	16223563;

C080417	decrease	SUOX	ENSG00000139531	16223563;
C080418	decrease	HMOX1	ENSG00000100292	17855475;
C080418	increase	CYBB	ENSG00000165168	17855475;
C080418	increase	NOS1	ENSG00000089250	17855475;
C080418	increase	NOS2	ENSG00000007171	17855475;
C080418	increase	PTGS2	ENSG00000073756	17855475;
C080815	increase	FSHB	ENSG00000131808	17515571;
C080955	decrease	CAT	ENSG00000121691	18848576;
C080955	decrease	CAV1	ENSG00000105974	18848576;
C080955	decrease	CCNK	ENSG00000090061	11494140;
C080955	decrease	CYP19A1	ENSG00000137869	16480277;15964185;
C080955	decrease	DDIT3	ENSG00000175197	17709599;
C080955	decrease	HGF	ENSG00000019991	10807402;
C080955	decrease	KIAA0101	ENSG00000166803	11494140;
C080955	decrease	PTGER2	ENSG00000125384	17709599;
C080955	decrease	SOD2	ENSG00000112096	18848576;
C080955	decrease	TPX2	ENSG00000088325	11494140;
C080955	increase	ALOX15	ENSG00000161905	11861401;10904086;11406566;
C080955	increase	DNM2	ENSG00000079805	11494140;
C080955	increase	EGR1	ENSG00000120738	11595463;
C080955	increase	GATA6	ENSG00000141448	11861401;
C080955	increase	GDF15	ENSG00000130513	15555568;15180942;
C080955	increase	IL1B	ENSG00000125538	16354411;12946449;
C080955	increase	IL6	ENSG00000136244	16354411;12946449;15379866;17709599;
C080955	increase	IL8	ENSG00000169429	16354411;12946449;17709599;
C080955	increase	MMP9	ENSG00000100985	19804834;
C080955	increase	PAWR	ENSG00000177425	10833474;
C080955	increase	PCDH7	ENSG00000169851	11494140;
C080955	increase	PDCD4	ENSG00000150593	11494140;
C080955	increase	PDLIM5	ENSG00000163110	11494140;
C080955	increase	PPFIA1	ENSG00000131626	11494140;
C080955	increase	PTGS2	ENSG00000073756	16354411;12946449;12414664;18596194;16928824;18848576;17709599;15155531;14566837;11494140;15843042;15144595;10485483;
C080955	increase	VEGFA	ENSG00000112715	11595463;18596194;
C081020	increase	IL6	ENSG00000136244	11410519;
C081020	increase	KLK3	ENSG00000142515	11410519;
C081021	decrease	PRKCA	ENSG00000154229	16374459;
C081021	increase	BTG2	ENSG00000159388	15302583;
C081021	increase	CCNB1	ENSG00000134057	17320279;
C081021	increase	CRH	ENSG00000147571	17170093;
C081021	increase	CYP11A1	ENSG00000140459	17170093;
C081021	increase	HMOX1	ENSG00000100292	18357586;
C081021	increase	HSD3B1	ENSG00000203857	17170093;
C081021	increase	IL2	ENSG00000109471	16241859;
C081021	increase	PLD2	ENSG00000129219	15210717;
C081021	increase	PTGS2	ENSG00000073756	15210717;19804834;
C081021	increase	VDR	ENSG00000111424	15879110;
C081306	decrease	BCL2	ENSG00000171791	15519653;
C081306	decrease	BCL2L1	ENSG00000171552	15519653;

C081306	increase	BAX	ENSG00000087088	15519653;
C081489	increase	NPPB	ENSG00000120937	16247790;
C081510	decrease	CCL2	ENSG00000108691	15762874;
C081510	decrease	IL6	ENSG00000136244	15762874;
C081510	decrease	IL8	ENSG00000169429	15762874;
C081972	increase	GUSB	ENSG00000169919	11238657;
C081972	increase	HRH1	ENSG00000196639	11238657;
C081972	increase	IL6	ENSG00000136244	11238657;
C082032	increase	ABCB1	ENSG00000085563	15269186;
C082032	increase	CYP3A4	ENSG00000160868	15269186;
C082032	increase	UGT1A1	ENSG00000241635	15269186;
C082089	increase	ABCB1	ENSG00000085563	15269186;
C082089	increase	CYP3A4	ENSG00000160868	15269186;
C082089	increase	UGT1A1	ENSG00000241635	15269186;
C082097	increase	ABCB1	ENSG00000085563	15269186;
C082097	increase	CYP3A4	ENSG00000160868	15269186;
C082097	increase	UGT1A1	ENSG00000241635	15269186;
C082137	increase	CES1	ENSG00000198848	15681896;
C082137	increase	CES2	ENSG00000172831	15681896;
C082137	increase	CHST1	ENSG00000175264	15681896;
C082137	increase	CYP1A1	ENSG00000140465	15681896;
C082137	increase	CYP1A2	ENSG00000140505	15681896;
C082137	increase	CYP3A4	ENSG00000160868	15681896;
C082137	increase	SULT2A1	ENSG00000105398	15681896;
C082161	increase	HMOX1	ENSG00000100292	16806455;
C082161	increase	NFE2L2	ENSG00000116044	16806455;
C082290	increase	NQO1	ENSG00000181019	9029048;
C082359	decrease	HSP90B1	ENSG00000166598	16884889;
C082359	decrease	HSPA5	ENSG00000044574	16884889;
C082359	decrease	HSPB1	ENSG00000106211	16884889;
C083079	decrease	CD74	ENSG00000019582	11046123;
C083079	decrease	ICAM1	ENSG00000090339	11046123;
C083079	decrease	SELE	ENSG00000007908	11046123;
C083781	decrease	FSHB	ENSG00000131808	11228061;
C083781	decrease	LHB	ENSG00000104826	11228061;
C084012	increase	CALCA	ENSG00000110680	16996168;
C084527	increase	PTGS2	ENSG00000073756	11854442;
C084597	decrease	SLC2A3	ENSG00000059804	15342952;
C084597	decrease	TAGLN	ENSG00000149591	15342952;
C084597	increase	ASAH1	ENSG00000104763	15342952;
C084597	increase	C10ORF10	ENSG00000165507	15342952;
C084597	increase	FABP1	ENSG00000163586	15342952;
C084597	increase	FNDC4	ENSG00000115226	15342952;
C084597	increase	GDPD3	ENSG00000102886	15342952;
C084597	increase	HPN	ENSG00000105707	15342952;
C084597	increase	INHBE	ENSG00000139269	15342952;
C084597	increase	LSS	ENSG00000160285	15342952;
C084597	increase	NR0B2	ENSG00000131910	15342952;
C084597	increase	NUPR1	ENSG00000176046	15342952;
C084597	increase	SERPINA3	ENSG00000196136	15342952;
C084597	increase	WIPI1	ENSG00000070540	15342952;
C084656	affect	CPT1A	ENSG00000110090	17715259;
C084656	affect	EDNRB	ENSG00000136160	17715259;
C084656	affect	IL11	ENSG00000095752	17715259;

C084656	affect	IL6	ENSG00000136244	17715259;
C084656	affect	NR4A1	ENSG00000123358	17715259;
C084656	affect	NR4A2	ENSG00000153234	17715259;
C084656	affect	PEX11A	ENSG00000166821	17715259;
C084656	decrease	APOE	ENSG00000130203	17715259;
C084656	decrease	CPN2	ENSG00000178772	17715259;
C084656	decrease	CTDSPL	ENSG00000144677	17715259;
C084656	decrease	CYP1B1	ENSG00000138061	17715259;
C084656	decrease	CYP2B6	ENSG00000197408	17715259;
C084656	decrease	IL1B	ENSG00000125538	17715259;
C084656	decrease	IL1F5	ENSG00000136695	17715259;
C084656	decrease	IL1RN	ENSG00000136689	17715259;
C084656	decrease	IL24	ENSG00000162892	17715259;
C084656	decrease	IL8	ENSG00000169429	17715259;
C084656	decrease	NCOR1	ENSG00000141027	17715259;
C084656	decrease	POSTN	ENSG00000133110	17715259;
C084656	decrease	SERPINE1	ENSG00000106366	17715259;
C084656	decrease	THBS1	ENSG00000137801	17715259;
C084656	decrease	TNFRSF11B	ENSG00000164761	17715259;
C084656	increase	AADAC	ENSG00000114771	17715259;
C084656	increase	ALPL	ENSG00000162551	17715259;
C084656	increase	B4GALT1	ENSG00000086062	17715259;
C084656	increase	BMP4	ENSG00000125378	17715259;
C084656	increase	CD14	ENSG00000170458	17715259;
C084656	increase	CES1	ENSG00000198848	17715259;
C084656	increase	COL15A1	ENSG00000204291	17715259;
C084656	increase	COL1A1	ENSG00000108821	17715259;
C084656	increase	CP	ENSG00000047457	17715259;
C084656	increase	CRIP1	ENSG00000213145	17715259;
C084656	increase	CYP24A1	ENSG00000019186	17715259;
C084656	increase	CYP3A4	ENSG00000160868	11723248;
C084656	increase	FBLN2	ENSG00000163520	17715259;
C084656	increase	ID3	ENSG00000117318	17715259;
C084656	increase	MAPK13	ENSG00000156711	17715259;
C084656	increase	MFI2	ENSG00000163975	17715259;
C084656	increase	MGP	ENSG00000111341	17715259;
C084656	increase	MRPL33	ENSG00000243147	17715259;
C084656	increase	PTGS1	ENSG00000095303	17715259;
C084656	increase	PTGS2	ENSG00000073756	17715259;
C084656	increase	RARRES1	ENSG00000118849	17715259;
C084656	increase	SOD3	ENSG00000109610	17715259;
C084656	increase	SPP1	ENSG00000118785	17715259;
C084656	increase	TGFB3	ENSG00000119699	17715259;
C084656	increase	THBD	ENSG00000178726	17715259;
C084656	increase	TNXB	ENSG00000168477	17715259;
C084656	increase	TREM1	ENSG00000124731	17715259;
C084656	increase	VDR	ENSG00000111424	17715259;15898824;16076355;
C084904	decrease	STAT1	ENSG00000115415	16085646;16007204;
C084904	increase	BCMO1	ENSG00000135697	16186334;
C084904	increase	CD1D	ENSG00000158473	17392484;
C084904	increase	IFNG	ENSG00000111537	16007204;16872382;
C084904	increase	IFNGR1	ENSG00000027697	16085646;
C084904	increase	IRF1	ENSG00000125347	16085646;16636311;

C084904	increase	MUC4	ENSG00000145113	16007204;
C084904	increase	OAS2	ENSG00000111335	16636311;
C084904	increase	PLAT	ENSG00000104368	7556191;
C084904	increase	RARA	ENSG00000131759	10779427;7556191;172165
C084924	increase	SNCA	ENSG00000145335	15114628;
C084956	decrease	BIRC3	ENSG00000023445	16088125;
C084956	decrease	IL12A	ENSG00000168811	16116186;
C084956	decrease	IL12B	ENSG00000113302	16116186;
C084956	decrease	NAIP	ENSG00000250628	16088125;
C085118	increase	FGF7	ENSG00000140285	15678371;
C085514	decrease	NAT1	ENSG00000171428	15816529;
C085586	affect	TP53	ENSG00000141510	17339891;15161716;
C085586	increase	FAS	ENSG00000026103	15161716;
C085586	increase	TYMS	ENSG00000176890	17339891;15161716;
C085746	decrease	ABL1	ENSG00000097007	17030193;
C085746	decrease	PPARG	ENSG00000132170	17030193;
C085746	increase	BIRC3	ENSG00000023445	14527959;
C085746	increase	CDKN1A	ENSG00000124762	15976015;
C085746	increase	COL1A1	ENSG00000108821	17030193;
C085746	increase	DDIT3	ENSG00000175197	17171638;
C085746	increase	HMOX1	ENSG00000100292	18048804;18357586;
C085746	increase	IFNA2	ENSG00000188379	16260419;
C085746	increase	IL2	ENSG00000109471	16241859;
C085746	increase	IL8	ENSG00000169429	17097691;
C085746	increase	JUN	ENSG00000177606	18628248;
C085746	increase	MIF	ENSG00000240972	16872482;
C085746	increase	MMP2	ENSG00000087245	16872482;
C085746	increase	PLD1	ENSG00000075651	17640750;
C085746	increase	PLSCR1	ENSG00000188313	16260419;
C085746	increase	PTGS2	ENSG00000073756	17363696;17640750;
C085911	affect	BCL2	ENSG00000171791	18325115;
C085911	affect	F7	ENSG00000057593	18325115;
C085911	affect	ITGA5	ENSG00000161638	16465378;
C085911	affect	ITGB3	ENSG00000056345	16465378;
C085911	decrease	APP	ENSG00000142192	16648635;
C085911	decrease	BCL2L1	ENSG00000171552	14762343;
C085911	decrease	CCND1	ENSG00000110092	14757120;
C085911	decrease	CCND3	ENSG00000112576	14757120;
C085911	decrease	CCNE1	ENSG00000105173	14757120;
C085911	decrease	CFLAR	ENSG00000003402	12663669;12970779;
C085911	decrease	KLK3	ENSG00000142515	16472761;
C085911	increase	ABCC2	ENSG00000023839	15896333;
C085911	increase	ANGPT1	ENSG00000154188	15763944;
C085911	increase	AQP3	ENSG00000165272	18214481;16848764;
C085911	increase	AR	ENSG00000169083	19059400;16472761;18486176;
C085911	increase	BIRC2	ENSG00000110330	19596284;15065019;14762343;12970779;
C085911	increase	BIRC3	ENSG00000023445	16154993;19596284;15339911;14762343;12970779;
C085911	increase	BIRC5	ENSG00000089685	16222118;15065019;
C085911	increase	CALCA	ENSG00000110680	16222118;
C085911	increase	CCL11	ENSG00000172156	11980903;
C085911	increase	CCL2	ENSG00000108691	16794257;

C085911	increase	CDKN1B	ENSG00000111276	11322924;18622747;
C085911	increase	DDIT3	ENSG00000175197	17049495;17171638;
C085911	increase	DPP3	ENSG00000174483	17360324;
C085911	increase	EGF	ENSG00000138798	16472761;16343977;18214481;16848764;16619521;
C085911	increase	F3	ENSG00000117525	18325115;15371228;
C085911	increase	FAS	ENSG00000026103	16162944;
C085911	increase	FGF2	ENSG00000138685	15631803;
C085911	increase	FGF7	ENSG00000140285	16162944;
C085911	increase	GSTA1	ENSG00000243955	15090468;
C085911	increase	HIF1A	ENSG00000100644	16682453;15050414;15631803;18924134;11424089;17082639;15213310;16227395;14726529;
C085911	increase	HMOX1	ENSG00000100292	18048804;18357586;17420286;
C085911	increase	HSF1	ENSG00000185122	11424089;
C085911	increase	HSPA5	ENSG00000044574	19428936;
C085911	increase	IGF1	ENSG00000017427	15947004;16682453;16465378;
C085911	increase	IL1B	ENSG00000125538	16359550;
C085911	increase	IL8	ENSG00000169429	16611624;17606477;16794257;17082639;
C085911	increase	ITGAM	ENSG00000169896	11980903;16617325;
C085911	increase	KIT	ENSG00000157404	17420286;
C085911	increase	MMP12	ENSG00000110347	16359550;
C085911	increase	NQO1	ENSG00000181019	17360324;15896333;
C085911	increase	PDCD4	ENSG00000150593	17259349;
C085911	increase	PTEN	ENSG00000171862	18622747;
C085911	increase	SCD	ENSG00000099194	16162944;
C085911	increase	SQSTM1	ENSG00000161011	17360324;
C085911	increase	SREBF1	ENSG00000072310	16162944;
C085911	increase	TNF	ENSG00000223952	12663669;12805067;16794257;
C085911	increase	TNFRSF10A	ENSG00000104689	15942663;
C085911	increase	TNFRSF10B	ENSG00000120889	15942663;
C085911	increase	TP53	ENSG00000141510	15763944;16024610;17534123;
C085911	increase	UGT1A1	ENSG00000241635	15896333;
C085911	increase	VEGFA	ENSG00000112715	15050414;18252963;16682453;15930297;16638750;
C085911	increase	XIAP	ENSG00000101966	15339911;18071906;
C085989	increase	ACTA2	ENSG00000107796	16757516;
C085989	increase	TGFB1	ENSG00000105329	16757516;
C086108	decrease	CCL2	ENSG00000108691	17507084;
C086108	increase	HRH2	ENSG00000113749	12235264;
C086108	increase	IL16	ENSG00000172349	12235264;
C086232	increase	CYP2B6	ENSG00000197408	18332078;
C086232	increase	CYP3A4	ENSG00000160868	18332078;
C086232	increase	FN1	ENSG00000115414	12388107;
C086276	decrease	ENTPD1	ENSG00000138185	12067895;
C086276	decrease	F2	ENSG00000180210	12067895;
C086276	increase	CYP2B6	ENSG00000197408	15802384;
C086511	affect	BCL2	ENSG00000171791	15868441;
C086511	affect	IGF1R	ENSG00000140443	15868441;
C086511	affect	IGFBP2	ENSG00000115457	15868441;

C086511	affect	PGR	ENSG00000082175	15868441;
C086511	decrease	ESR1	ENSG00000091831	15868441;
C086539	increase	FN1	ENSG00000115414	12388107;
C086566	decrease	ABCA1	ENSG00000165029	16963248;
C086566	decrease	ADD2	ENSG00000075340	16963248;
C086566	decrease	ARHGDIA	ENSG00000141522	15757668;
C086566	decrease	ATP6V0A2	ENSG00000185344	16963248;
C086566	decrease	DOCK3	ENSG00000088538	16963248;
C086566	decrease	ESPN	ENSG00000187017	16963248;
C086566	decrease	POU4F2	ENSG00000151615	16963248;
C086566	decrease	SGPP1	ENSG00000126821	16963248;
C086566	decrease	SLC12A2	ENSG00000064651	15757668;
C086566	decrease	STC1	ENSG00000159167	16963248;
C086566	decrease	TNFAIP6	ENSG00000123610	16963248;
C086566	decrease	TP53I11	ENSG00000175274	15757668;
C086566	decrease	UCHL1	ENSG00000154277	16963248;
C086566	increase	BAZ1A	ENSG00000198604	16963248;
C086566	increase	EPHA1	ENSG00000146904	16963248;
C086566	increase	FMN2	ENSG00000155816	16963248;
C086566	increase	IFRD2	ENSG00000214706	16963248;
C086566	increase	IRF2BP1	ENSG00000170604	16963248;
C086566	increase	KCNG2	ENSG00000178342	16963248;
C086566	increase	NRIP1	ENSG00000180530	15757668;
C086566	increase	OLFML3	ENSG00000116774	16963248;
C086566	increase	PDLIM2	ENSG00000120913	16963248;
C086566	increase	PNN	ENSG00000100941	16963248;
C086566	increase	RPS6KA3	ENSG00000177189	15757668;
C086566	increase	TACR3	ENSG00000169836	16963248;
C086566	increase	VDR	ENSG00000111424	17088408;
C086664	increase	CGB	ENSG00000213030	15941851;19497422;
C086664	increase	CYP19A1	ENSG00000137869	15941851;
C086827	affect	FSHB	ENSG00000131808	15244497;
C086827	affect	LHB	ENSG00000104826	15244497;
C086827	decrease	BCL2	ENSG00000171791	16196285;16109552;
C086827	increase	BAX	ENSG00000087088	16196285;16109552;
C086827	increase	SLC5A5	ENSG00000105641	16954431;
C087123	affect	TP53	ENSG00000141510	17230511;
C087123	decrease	EWSR1	ENSG00000182944	15849726;
C087123	decrease	FLI1	ENSG00000151702	15849726;
C087123	increase	ABCB1	ENSG00000085563	15634944;16049968;16223781;15833893;
C087123	increase	CDKN1A	ENSG00000124762	17230511;16223781;15849726;
C087123	increase	FOLR2	ENSG00000165457	16740727;
C087123	increase	NR1I3	ENSG00000143257	16006969;
C087203	decrease	PGR	ENSG00000082175	9144411;
C087667	increase	CYP11B1	ENSG00000160882	16396990;
C087667	increase	CYP11B2	ENSG00000179142	16396990;
C087926	increase	GATA1	ENSG00000102145	14757314;
C087926	increase	GATA2	ENSG00000179348	14757314;
C087926	increase	GYPA	ENSG00000170180	14757314;
C087926	increase	HMBS	ENSG00000149397	14757314;
C087926	increase	ITGB3	ENSG00000056345	14757314;
C087926	increase	NFE2	ENSG00000123405	14757314;

C088060	increase	CDKN1A	ENSG00000124762	15976015;
C088453	decrease	CYP2C19	ENSG00000165841	15448115;
C088453	decrease	CYP2C9	ENSG00000138109	15448115;
C088453	decrease	CYP2D6	ENSG00000100197	15448115;
C088453	decrease	CYP2E1	ENSG00000130649	15448115;
C088453	decrease	CYP3A5	ENSG00000106258	15448115;
C088453	increase	CYP1A1	ENSG00000140465	15448115;
C088453	increase	CYP1A2	ENSG00000140505	15448115;
C088453	increase	CYP2A6	ENSG00000198077	15448115;
C088453	increase	CYP2B6	ENSG00000197408	15448115;
C088453	increase	CYP3A4	ENSG00000160868	15448115;
C088658	affect	BCL2L10	ENSG00000137875	19036117;
C088658	affect	CASP2	ENSG00000106144	19036117;
C088658	affect	CASP5	ENSG00000137757	19036117;
C088658	affect	CD27	ENSG00000139193	19036117;
C088658	affect	FASLG	ENSG00000117560	19036117;
C088658	affect	GZMB	ENSG00000100453	19446661;
C088658	affect	IFNG	ENSG00000111537	19400555;
C088658	affect	IL2	ENSG00000109471	19446661;19400555;
C088658	affect	NAIP	ENSG00000250628	19036117;
C088658	decrease	BCL2A1	ENSG00000140379	19036117;
C088658	decrease	BIRC2	ENSG00000110330	20349282;19036117;
C088658	decrease	BIRC5	ENSG00000089685	20349282;19036117;
C088658	decrease	BIRC6	ENSG00000115760	19036117;
C088658	decrease	BIRC8	ENSG00000163098	19036117;
C088658	decrease	BNIP3	ENSG00000176171	19036117;
C088658	decrease	CASP14	ENSG00000105141	19036117;
C088658	decrease	CCL2	ENSG00000108691	19825521;
C088658	decrease	CCND1	ENSG00000110092	16621245;
C088658	decrease	CD40LG	ENSG00000102245	19036117;
C088658	decrease	CDKN1B	ENSG00000111276	16322342;19530242;
C088658	decrease	COL1A1	ENSG00000108821	19597852;
C088658	decrease	CTSB	ENSG00000164733	16475674;
C088658	decrease	CXCL1	ENSG00000163739	19825521;
C088658	decrease	CYR61	ENSG00000142871	19530242;
C088658	decrease	DAPK1	ENSG00000196730	19036117;
C088658	decrease	DIABLO	ENSG00000184047	20349282;
C088658	decrease	EPHB4	ENSG00000196411	19825521;
C088658	decrease	FGF2	ENSG00000138685	19825521;
C088658	decrease	FGFR3	ENSG00000068078	19825521;
C088658	decrease	ID1	ENSG00000125968	19825521;
C088658	decrease	LAMA5	ENSG00000130702	19825521;
C088658	decrease	MCL1	ENSG00000143384	20349282;
C088658	decrease	MCM2	ENSG00000073111	17562444;
C088658	decrease	MDK	ENSG00000110492	19825521;
C088658	decrease	MKI67	ENSG00000148773	18695136;
C088658	decrease	MMP2	ENSG00000087245	16475674;
C088658	decrease	MSH2	ENSG00000095002	17070308;
C088658	decrease	PDGFA	ENSG00000197461	19825521;
C088658	decrease	PTGS2	ENSG00000073756	16322342;
C088658	decrease	PTHLH	ENSG00000087494	16475708;16278781;
C088658	decrease	RAF1	ENSG00000132155	12799645;
C088658	decrease	RHOA	ENSG00000067560	15324309;
C088658	decrease	TNFRSF25	ENSG00000215788	19036117;

C088658	decrease	TNFRSF9	ENSG00000049249	19036117;
C088658	decrease	TNFSF11	ENSG00000120659	14753746;17041912;18980195;
C088658	decrease	TP53	ENSG00000141510	20349282;
C088658	decrease	XIAP	ENSG00000101966	19036117;
C088658	increase	ABCG2	ENSG00000118777	18225567;
C088658	increase	ADAM17	ENSG00000151694	14753746;
C088658	increase	ARHGAP29	ENSG00000137962	16996129;
C088658	increase	ARHGEF12	ENSG00000196914	16996129;
C088658	increase	BAD	ENSG00000002330	20349282;19036117;
C088658	increase	BAG1	ENSG00000250477	19036117;
C088658	increase	BAG3	ENSG00000151929	19036117;
C088658	increase	BAX	ENSG00000087088	20349282;19036117;
C088658	increase	BCAP29	ENSG00000075790	16996129;
C088658	increase	BCL2	ENSG00000171791	11986784;20349282;19103299;19036117;18225567;
C088658	increase	BCL2L1	ENSG00000171552	19036117;
C088658	increase	BCL2L2	ENSG00000129473	19036117;
C088658	increase	BGLAP	ENSG00000242252	14751568;
C088658	increase	BHLHE40	ENSG00000134107	16996129;
C088658	increase	BID	ENSG00000015475	19036117;
C088658	increase	BMP2	ENSG00000125845	14751568;
C088658	increase	BUB1	ENSG00000169679	16996129;
C088658	increase	CASP1	ENSG00000137752	16996129;19036117;
C088658	increase	CASP3	ENSG00000164305	20349282;18695136;19825521;17562444;18025282;12799645;17070308;12796698;11986784;19915386;1903
C088658	increase	CASP8	ENSG00000064012	19036117;
C088658	increase	CD80	ENSG00000121594	19446661;
C088658	increase	CD86	ENSG00000114013	19446661;
C088658	increase	CDK2AP2	ENSG00000167797	16996129;
C088658	increase	CDKN1A	ENSG00000124762	19530242;
C088658	increase	CKS2	ENSG00000123975	16996129;
C088658	increase	EMP3	ENSG00000142227	16996129;
C088658	increase	ERCC5	ENSG00000134899	16996129;
C088658	increase	ERG	ENSG00000157554	16996129;
C088658	increase	FADD	ENSG00000168040	20349282;
C088658	increase	FAS	ENSG00000026103	20349282;19036117;
C088658	increase	FDFT1	ENSG00000079459	16996129;
C088658	increase	FNTA	ENSG00000168522	16996129;
C088658	increase	GADD45B	ENSG00000099860	16996129;
C088658	increase	HMOX2	ENSG00000103415	20349282;
C088658	increase	IBSP	ENSG00000029559	15324309;
C088658	increase	ICAM1	ENSG00000090339	19446661;
C088658	increase	IGF1	ENSG00000017427	16475708;16996129;
C088658	increase	IGFBP5	ENSG00000115461	16996129;
C088658	increase	IL10RA	ENSG00000110324	16996129;
C088658	increase	IL2RG	ENSG00000147168	16996129;
C088658	increase	IL6	ENSG00000136244	16475708;18601766;93518
C088658	increase	KAT2B	ENSG00000114166	16996129;
C088658	increase	LTBR	ENSG00000111321	20349282;19036117;
C088658	increase	LZTS1	ENSG00000061337	16996129;
C088658	increase	MED23	ENSG00000112282	16996129;

C088658	increase	MPHOSPH8	ENSG00000196199	16996129;
C088658	increase	MPP5	ENSG00000072415	16996129;
C088658	increase	MVP	ENSG00000013364	18225567;
C088658	increase	NDC80	ENSG00000080986	16996129;
C088658	increase	OPTN	ENSG00000123240	16996129;
C088658	increase	PAPPA2	ENSG00000116183	16996129;
C088658	increase	PON2	ENSG00000105854	20349282;
C088658	increase	PPP1R15A	ENSG00000087074	16996129;
C088658	increase	PRDX3	ENSG00000165672	16996129;
C088658	increase	RAB27B	ENSG00000041353	16996129;
C088658	increase	RAB5B	ENSG00000111540	16996129;
C088658	increase	RABGAP1L	ENSG00000250898	16996129;
C088658	increase	RAD17	ENSG00000152942	20349282;
C088658	increase	RGS11	ENSG00000076344	16996129;
C088658	increase	RHOB	ENSG00000143878	16996129;
C088658	increase	RIMS2	ENSG00000176406	16996129;
C088658	increase	RIN2	ENSG00000132669	16996129;
C088658	increase	SOS2	ENSG00000100485	16996129;
C088658	increase	STK17A	ENSG00000164543	16996129;
C088658	increase	TNF	ENSG00000223952	17059425;19036117;93518
C088658	increase	TNFRSF10A	ENSG00000104689	16996129;19036117;
C088658	increase	TNFRSF10B	ENSG00000120889	20349282;
C088658	increase	TNFRSF11B	ENSG00000164761	17041912;14753746;11855844;19036117;18980195;
C088658	increase	TNFRSF12A	ENSG00000006327	20349282;
C088658	increase	TNFRSF1A	ENSG00000067182	20349282;19036117;
C088658	increase	TNFRSF21	ENSG00000146072	19036117;
C088658	increase	TP53INP1	ENSG00000164938	16996129;
C088658	increase	TRADD	ENSG00000102871	19036117;
C088658	increase	TRAF2	ENSG00000127191	19036117;
C088658	increase	TRAF3	ENSG00000131323	19036117;
C088658	increase	TYMP	ENSG00000025708	19825521;
C088658	increase	WNT11	ENSG00000085741	16996129;
C088860	decrease	PTHLH	ENSG00000087494	15072578;
C088860	increase	AQP3	ENSG00000165272	18214481;16848764;
C088860	increase	BIRC5	ENSG00000089685	16211241;16077934;
C088860	increase	EGF	ENSG00000138798	15613483;18214481;16848764;
C088860	increase	RARB	ENSG00000077092	17608728;
C089730	decrease	AGRN	ENSG00000188157	16982809;
C089730	decrease	ATP2B1	ENSG00000070961	16982809;
C089730	decrease	BCL2	ENSG00000171791	17461445;
C089730	decrease	C16ORF46	ENSG00000166455	16982809;
C089730	decrease	C4ORF32	ENSG00000174749	16982809;
C089730	decrease	C7ORF45	ENSG00000165120	16982809;
C089730	decrease	CALCRL	ENSG00000064989	16982809;
C089730	decrease	CCNA2	ENSG00000145386	15729575;
C089730	decrease	CCNB1	ENSG00000134057	15729575;
C089730	decrease	CCND3	ENSG00000112576	15729575;
C089730	decrease	CCNE1	ENSG00000105173	15729575;
C089730	decrease	CCR7	ENSG00000126353	17475839;
C089730	decrease	CD1A	ENSG00000158477	17475839;16982809;
C089730	decrease	CD80	ENSG00000121594	17475839;
C089730	decrease	CDK2	ENSG00000123374	15729575;

C089730	decrease	CDKN1A	ENSG00000124762	15254749;15729575;
C089730	decrease	CDR2L	ENSG00000109089	16982809;
C089730	decrease	CTTNBP2	ENSG00000077063	16982809;
C089730	decrease	DMPK	ENSG00000104936	16982809;
C089730	decrease	DMWD	ENSG00000185800	16982809;
C089730	decrease	FAM109A	ENSG00000198324	16982809;
C089730	decrease	FUT7	ENSG00000180549	16982809;
C089730	decrease	GOLGA8A	ENSG00000175265	16982809;
C089730	decrease	GPD1L	ENSG00000152642	16982809;
C089730	decrease	HIST1H2BD	ENSG00000158373	16982809;
C089730	decrease	HLX	ENSG00000136630	16982809;
C089730	decrease	IL1R1	ENSG00000115594	16982809;
C089730	decrease	IL1RAP	ENSG00000196083	16982809;
C089730	decrease	IPCEF1	ENSG00000074706	16982809;
C089730	decrease	KIAA1161	ENSG00000164976	16982809;
C089730	decrease	KIAA1274	ENSG00000107719	16982809;
C089730	decrease	KIFC2	ENSG00000167702	16982809;
C089730	decrease	LELP1	ENSG00000203784	16982809;
C089730	decrease	LEP	ENSG00000174697	15949695;
C089730	decrease	LRP11	ENSG00000120256	16982809;
C089730	decrease	MAN1C1	ENSG00000117643	16982809;
C089730	decrease	MAP3K6	ENSG00000142733	16982809;
C089730	decrease	MAP4K1	ENSG00000104814	16982809;
C089730	decrease	METRN	ENSG00000103260	16982809;
C089730	decrease	MGAT4C	ENSG00000182050	16982809;
C089730	decrease	NDRG2	ENSG00000165795	16982809;
C089730	decrease	NUDT7	ENSG00000140876	16982809;
C089730	decrease	PDLIM2	ENSG00000120913	16982809;
C089730	decrease	PDLIM4	ENSG00000131435	16982809;
C089730	decrease	PHLDA2	ENSG00000181649	16982809;
C089730	decrease	PON2	ENSG00000105854	16982809;
C089730	decrease	QPRT	ENSG00000103485	16982809;
C089730	decrease	RAB15	ENSG00000139998	16982809;
C089730	decrease	RAP1GAP	ENSG00000076864	16982809;
C089730	decrease	RB1	ENSG00000139687	15729575;
C089730	decrease	REPS2	ENSG00000169891	16982809;
C089730	decrease	RFXAP	ENSG00000133111	16982809;
C089730	decrease	SELPLG	ENSG00000110876	16982809;
C089730	decrease	SP1	ENSG00000185591	15908479;
C089730	decrease	TACSTD2	ENSG00000184292	16982809;
C089730	decrease	TBL1X	ENSG00000101849	16982809;
C089730	decrease	TMEM14A	ENSG00000096092	16982809;
C089730	decrease	TNFSF12	ENSG00000239697	16982809;
C089730	decrease	TNS1	ENSG00000079308	16982809;
C089730	decrease	ZBTB7B	ENSG00000160685	16982809;
C089730	increase	ADA	ENSG00000196839	16982809;
C089730	increase	AGPAT4	ENSG00000026652	16982809;
C089730	increase	AJAP1	ENSG00000196581	16982809;
C089730	increase	ALDH1A2	ENSG00000128918	16982809;
C089730	increase	APLP2	ENSG00000084234	16982809;
C089730	increase	B3GNT4	ENSG00000176383	16982809;
C089730	increase	BAG2	ENSG00000112208	16982809;
C089730	increase	BAX	ENSG00000087088	17461445;
C089730	increase	C18ORF1	ENSG00000168675	16982809;

C089730	increase	C6ORF142	ENSG00000146147	16982809;
C089730	increase	CARD14	ENSG00000141527	16982809;
C089730	increase	CD1D	ENSG00000158473	16982809;
C089730	increase	CD300A	ENSG00000167851	16982809;
C089730	increase	CD300LB	ENSG00000178789	16982809;
C089730	increase	CD300LF	ENSG00000186074	16982809;
C089730	increase	CD36	ENSG00000135218	17475839;
C089730	increase	CD86	ENSG00000114013	17475839;
C089730	increase	CGB	ENSG00000213030	15941851;19497422;
C089730	increase	COL5A3	ENSG00000080573	16982809;
C089730	increase	CYP2B6	ENSG00000197408	12642470;
C089730	increase	CYP3A4	ENSG00000160868	12642470;15860655;16837568;
C089730	increase	DHRS3	ENSG00000162496	16982809;
C089730	increase	DHRS9	ENSG00000073737	16982809;
C089730	increase	DNAJC5B	ENSG00000147570	16982809;
C089730	increase	EHF	ENSG00000135373	16982809;
C089730	increase	EML4	ENSG00000143924	16982809;
C089730	increase	ETS2	ENSG00000157557	16982809;
C089730	increase	FABP4	ENSG00000170323	16982809;
C089730	increase	FAM101B	ENSG00000183688	16982809;
C089730	increase	FBXO15	ENSG00000141665	16982809;
C089730	increase	FN1	ENSG00000115414	15908479;16982809;
C089730	increase	GABARAPL	ENSG00000139112	16982809;
C089730	increase	GDF15	ENSG00000130513	16982809;
C089730	increase	GUSB	ENSG00000169919	16982809;
C089730	increase	HIPK2	ENSG00000064393	16982809;
C089730	increase	INPP5F	ENSG00000198825	16982809;
C089730	increase	ITGAE	ENSG00000083457	16982809;
C089730	increase	LAIR1	ENSG00000167613	16982809;
C089730	increase	LILRA1	ENSG00000104974	16982809;
C089730	increase	NRIP1	ENSG00000180530	16982809;
C089730	increase	OLR1	ENSG00000173391	16982809;
C089730	increase	OMA1	ENSG00000162600	16982809;
C089730	increase	PAPSS2	ENSG00000198682	16982809;
C089730	increase	PCK1	ENSG00000124253	14739078;
C089730	increase	PLBD1	ENSG00000121316	16982809;
C089730	increase	PPAP2B	ENSG00000162407	16982809;
C089730	increase	PPARGC1A	ENSG00000109819	16513826;
C089730	increase	PPARG	ENSG00000132170	17030193;9013583;15908479;16527820;16982809;19497422;15254749;15491415;15729575;15866424;17185614;16112107;17461445;
C089730	increase	PRAM1	ENSG00000133246	16982809;
C089730	increase	RAB20	ENSG00000139832	16982809;
C089730	increase	RARB	ENSG00000077092	15254749;
C089730	increase	RBKS	ENSG00000171174	16982809;
C089730	increase	RBP1	ENSG00000248478	16982809;
C089730	increase	RDH10	ENSG00000121039	16982809;
C089730	increase	RHOBTB3	ENSG00000164292	16982809;
C089730	increase	RPIA	ENSG00000153574	16982809;
C089730	increase	RXRA	ENSG00000186350	15254749;
C089730	increase	SIRPB1	ENSG00000101307	16982809;

C089730	increase	SLC26A11	ENSG00000181045	16982809;
C089730	increase	SLITRK4	ENSG00000179542	16982809;
C089730	increase	SNAI3	ENSG00000185669	16982809;
C089730	increase	TBC1D2	ENSG00000095383	16982809;
C089730	increase	TBL2	ENSG00000106638	16982809;
C089730	increase	TGM2	ENSG00000198959	16982809;
C089730	increase	THBS3	ENSG00000169231	16982809;
C089730	increase	TM7SF4	ENSG00000164935	16982809;
C089730	increase	WFS1	ENSG00000109501	16982809;
C089946	decrease	BCL2	ENSG00000171791	15916743;
C089946	decrease	CCND1	ENSG00000110092	15916743;
C089946	decrease	CDK4	ENSG00000135446	15916743;
C089946	decrease	CYP1A1	ENSG00000140465	15521013;
C089946	decrease	PCNA	ENSG00000132646	15916743;
C089946	decrease	RB1	ENSG00000139687	15916743;
C089946	increase	BAX	ENSG00000087088	15916743;
C089946	increase	CDKN1A	ENSG00000124762	15916743;
C089946	increase	CDKN1B	ENSG00000111276	15916743;
C090046	increase	RB1	ENSG00000139687	14653808;
C090046	increase	SP1	ENSG00000185591	14653808;
C090450	decrease	MKI67	ENSG00000148773	15767642;16002280;
C090450	decrease	PGR	ENSG00000082175	16002280;
C090840	affect	FSHB	ENSG00000131808	15387353;
C090840	affect	LHB	ENSG00000104826	15387353;
C090859	decrease	CYP19A1	ENSG00000137869	15964185;
C090859	increase	ATF3	ENSG00000162772	14633654;15897233;
C090859	increase	PAWR	ENSG00000177425	10833474;
C090937	decrease	BCL2	ENSG00000171791	19481069;
C090937	decrease	BID	ENSG00000015475	19481069;
C090937	increase	ALOX15	ENSG00000161905	16166323;
C090937	increase	BAX	ENSG00000087088	19481069;
C090937	increase	PRKG1	ENSG00000185532	11602670;
C090937	increase	TP53	ENSG00000141510	19481069;
C090942	affect	BIRC3	ENSG00000023445	16154993;
C090942	decrease	BIRC5	ENSG00000089685	14988404;
C090942	decrease	CCR2	ENSG00000121807	16712875;
C090942	increase	CCL11	ENSG00000172156	11980903;
C090942	increase	CCL26	ENSG00000006606	16045735;
C090942	increase	CCL2	ENSG00000108691	18645721;
C090942	increase	CD14	ENSG00000170458	14729647;
C090942	increase	CEBPB	ENSG00000172216	14729647;
C090942	increase	CYP1A1	ENSG00000140465	15698582;
C090942	increase	FOS	ENSG00000170345	12547826;
C090942	increase	HIF1A	ENSG00000100644	12482858;
C090942	increase	IL4	ENSG00000113520	16045735;
C090942	increase	IL6	ENSG00000136244	11410519;
C090942	increase	ITGAM	ENSG00000169896	11980903;
C090942	increase	KLK3	ENSG00000142515	11410519;
C090942	increase	NGFR	ENSG00000064300	18056468;
C090942	increase	PLAT	ENSG00000104368	18419748;
C090942	increase	PLAU	ENSG00000122861	15557793;
C090942	increase	RB1	ENSG00000139687	14729647;
C090942	increase	VEGFA	ENSG00000112715	12482858;
C091547	decrease	CYP1B1	ENSG00000138061	17637178;

C091696	increase	NQO1	ENSG00000181019	9029048;
C091768	increase	CYP1A1	ENSG00000140465	15385644;17012224;
C091861	increase	ANXA5	ENSG00000164111	16024610;
C092152	increase	ELANE	ENSG00000197561	17456367;
C092312	decrease	P2RY2	ENSG00000175591	10496880;
C092312	decrease	P2RY4	ENSG00000186912	10496880;
C093216	decrease	BCL2L1	ENSG00000171552	18292947;
C093216	increase	BAX	ENSG00000087088	18292947;
C093230	decrease	ICAM1	ENSG00000090339	16270723;
C093230	increase	GUSB	ENSG00000169919	11238657;
C093230	increase	HRH1	ENSG00000196639	11238657;
C093230	increase	IL6	ENSG00000136244	11238657;
C093285	increase	CYP2B6	ENSG00000197408	18206661;
C093285	increase	CYP2E1	ENSG00000130649	18206661;
C093285	increase	CYP3A4	ENSG00000160868	18206661;
C093323	decrease	BIRC5	ENSG00000089685	16780807;
C093323	decrease	CCND1	ENSG00000110092	16780807;
C093323	decrease	E2F1	ENSG00000101412	16780807;
C093323	decrease	HRAS	ENSG00000174775	16780807;
C093622	affect	SHBG	ENSG00000129214	10709161;
C093642	affect	ABCC1	ENSG00000103222	15585369;
C093642	affect	APOA1	ENSG00000118137	19013290;
C093642	affect	BGLAP	ENSG00000242252	17689939;
C093642	affect	CD40	ENSG00000101017	15894585;
C093642	affect	CD80	ENSG00000121594	15894585;
C093642	affect	ERBB2	ENSG00000141736	16488535;
C093642	affect	FGF2	ENSG00000138685	15631803;15249425;
C093642	affect	HSPA6	ENSG00000173110	15978632;
C093642	affect	ICAM1	ENSG00000090339	15894585;
C093642	affect	RUNX2	ENSG00000250096	17689939;
C093642	affect	SP7	ENSG00000170374	17689939;
C093642	decrease	BCL2	ENSG00000171791	16818652;
C093642	decrease	BIRC5	ENSG00000089685	16328441;
C093642	decrease	MAPK9	ENSG00000050748	16818652;18583539;
C093642	decrease	TP53	ENSG00000141510	15963507;16024610;19020741;18583539;
C093642	increase	ALB	ENSG00000163631	16908450;
C093642	increase	APP	ENSG00000142192	18583042;
C093642	increase	BACE1	ENSG00000186318	18583042;
C093642	increase	CAV1	ENSG00000105974	17108117;
C093642	increase	CCL2	ENSG00000108691	17655880;16143069;12016129;18645721;
C093642	increase	CCL5	ENSG00000161570	16908450;
C093642	increase	CD14	ENSG00000170458	18180316;
C093642	increase	CD83	ENSG00000112149	15894585;17698565;
C093642	increase	CD86	ENSG00000114013	15894585;17698565;
C093642	increase	CDK1	ENSG00000170312	18583539;
C093642	increase	CDK2	ENSG00000123374	18583539;
C093642	increase	CDKN1A	ENSG00000124762	15249425;14647439;18583539;
C093642	increase	CRP	ENSG00000132693	17388968;
C093642	increase	CSF2	ENSG00000164400	16908450;
C093642	increase	DDIT3	ENSG00000175197	17049495;17341418;
C093642	increase	DUSP6	ENSG00000139318	18314537;

C093642	increase	EDN1	ENSG00000078401	18457675;
C093642	increase	EGR1	ENSG00000120738	18757417;
C093642	increase	F3	ENSG00000117525	15371228;
C093642	increase	FOS	ENSG00000170345	18982426;15322261;12547826;
C093642	increase	GABPA	ENSG00000154727	16308312;
C093642	increase	HBEGF	ENSG00000113070	12676768;11159045;
C093642	increase	HIF1A	ENSG00000100644	15631803;17082639;12482858;14695184;
C093642	increase	HMOX1	ENSG00000100292	14647439;18357586;
C093642	increase	HSPA5	ENSG00000044574	17341418;
C093642	increase	IFNG	ENSG00000111537	10952721;
C093642	increase	IL12A	ENSG00000168811	15894585;10952721;
C093642	increase	IL12B	ENSG00000113302	15894585;10952721;
C093642	increase	IL17A	ENSG00000112115	12016129;
C093642	increase	IL1B	ENSG00000125538	16359550;18982426;11854442;15251176;17388968;10712238;
C093642	increase	IL6	ENSG00000136244	18457675;12016129;17698565;15251176;17388968;17196171;
C093642	increase	IL8	ENSG00000169429	15831558;16373669;15622447;19074641;11504702;11306435;18457675;10540334;12016129;11527995;12734376;15251176;16091123;16908450;17082639;163643
C093642	increase	MAPK14	ENSG00000112062	15963507;15631803;15964311;16864444;15585369;15242773;15249425;15254963;15817653;
C093642	increase	MYC	ENSG00000136997	18583539;
C093642	increase	PLD2	ENSG00000129219	15210717;
C093642	increase	PPARD	ENSG00000112033	17341418;
C093642	increase	PTGS2	ENSG00000073756	16928824;18457675;11854442;15210717;18596194;
C093642	increase	SP1	ENSG00000185591	17108117;
C093642	increase	SULT1A1	ENSG00000196502	16308312;
C093642	increase	THBS1	ENSG00000137801	18757417;
C093642	increase	TNF	ENSG00000223952	18314537;15603917;12016129;17698565;15251176;11675405;15322261;16091123;10712238;
C093642	increase	VCAM1	ENSG00000162692	18457675;
C093642	increase	VEGFA	ENSG00000112715	18252963;18596194;12482858;
C093875	decrease	IL5	ENSG00000113525	15061398;14619337;
C093875	decrease	IL8	ENSG00000169429	15061398;
C093973	affect	ALDH1A3	ENSG00000184254	17526768;
C093973	affect	BIRC3	ENSG00000023445	16154993;
C093973	affect	C5	ENSG00000106804	16116186;
C093973	affect	CD40	ENSG00000101017	15894585;
C093973	affect	CD80	ENSG00000121594	15894585;
C093973	affect	CD83	ENSG00000112149	15894585;
C093973	affect	CD86	ENSG00000114013	15894585;
C093973	affect	DDIT3	ENSG00000175197	17049495;
C093973	affect	ERBB2	ENSG00000141736	16488535;

C093973	affect	FAS	ENSG00000026103	16162944;
C093973	affect	FGF2	ENSG00000138685	15631803;
C093973	affect	FN1	ENSG00000115414	11343241;
C093973	affect	HSPA6	ENSG00000173110	15978632;
C093973	affect	ICAM1	ENSG00000090339	15894585;
C093973	affect	IL12A	ENSG00000168811	16116186;15894585;
C093973	affect	IL12B	ENSG00000113302	16116186;15894585;
C093973	affect	LTC4S	ENSG00000213316	12574384;
C093973	affect	MCM6	ENSG00000076003	14516785;
C093973	affect	MCM7	ENSG00000166508	14516785;
C093973	affect	SCD	ENSG00000099194	16162944;
C093973	affect	SREBF1	ENSG00000072310	16162944;
C093973	decrease	ABCG2	ENSG00000118777	15917307;
C093973	decrease	ABL1	ENSG00000097007	17030193;
C093973	decrease	BIRC5	ENSG00000089685	16328441;18377872;
C093973	decrease	CDK1	ENSG00000170312	18583539;
C093973	decrease	CDK2	ENSG00000123374	18583539;
C093973	decrease	CFTR	ENSG00000001626	15304546;15563584;
C093973	decrease	MAPK1	ENSG00000100030	15231676;16149052;11343241;17030193;15531749;12660819;16966607;16111618 6;15608143;19059400;15466214;17526808;12207323;15930183;17689939;18295389;15631803;16407847;16251475;19686714;10640773;12016129;11854442;16373414;16763222;15671028;18377872;16361081;15322261;18628248;16091123;17174366;16648635;16328441;16008523;15795063;16864444;15129224;16814113;17554206;12482858;15563584;15486966;17492661;11322
C093973	decrease	MAPK3	ENSG00000102882	15231676;16149052;11343241;17030193;15531749;12660819;16966607;16111618 6;15608143;19059400;15466214;17526808;12207323;15930183;17689939;18295389;15631803;16407847;18001288;16251475;19686714;10640773;12016129;11854442;16373414;16763222;15671028;18377872;16361081;15322261;14645110;18628248;16091123;17174366;16648635;16328441;16008523;15795063;16864444;15129224;16814113;17554206;12482858;15563584;15486966;17492661;11322385;18
C093973	decrease	MYC	ENSG00000136997	18583539;
C093973	decrease	NFE2L2	ENSG00000116044	12446695;
C093973	decrease	PPARG	ENSG00000132170	17030193;
C093973	decrease	TIMP1	ENSG00000102265	10866818;
C093973	decrease	ZFP42	ENSG00000179059	18583539;
C093973	increase	ABCC2	ENSG00000023839	15896333;

C093973	increase	APP	ENSG00000142192	18001288;18583042;
C093973	increase	AQP3	ENSG00000165272	18214481;
C093973	increase	AR	ENSG00000169083	16472761;19059400;12114440;
C093973	increase	ATF3	ENSG00000162772	16079301;
C093973	increase	BACE1	ENSG00000186318	18583042;
C093973	increase	BAX	ENSG00000087088	15970427;
C093973	increase	BGLAP	ENSG00000242252	17689939;
C093973	increase	CCL2	ENSG00000108691	12016129;
C093973	increase	CCNE1	ENSG00000105173	17273796;
C093973	increase	CD14	ENSG00000170458	14729647;18180316;
C093973	increase	CD38	ENSG00000004468	15494208;16329108;
C093973	increase	CDKN1A	ENSG00000124762	14647439;15978937;
C093973	increase	CEBPB	ENSG00000172216	14729647;
C093973	increase	COL1A1	ENSG00000108821	17030193;
C093973	increase	COL1A2	ENSG00000164692	11343241;
C093973	increase	CRP	ENSG00000132693	17388968;
C093973	increase	CYP11B2	ENSG00000179142	15666830;
C093973	increase	CYP17A1	ENSG00000148795	17138841;
C093973	increase	CYP1A1	ENSG00000140465	15313166;15698582;
C093973	increase	EDN1	ENSG00000078401	17974986;
C093973	increase	EGF	ENSG00000138798	16472761;15947004;15613483;18214481;15531749;15466214;17805209;
C093973	increase	EGR1	ENSG00000120738	15292961;16079301;
C093973	increase	FGF7	ENSG00000140285	16162944;15803460;14656992;
C093973	increase	FOS	ENSG00000170345	15466214;15090535;18982426;15322261;10712238;
C093973	increase	GCLC	ENSG00000001084	11909699;
C093973	increase	GREM1	ENSG00000166923	17975199;
C093973	increase	GSTP1	ENSG00000084207	19396019;
C093973	increase	HBEGF	ENSG00000113070	12676768;11159045;
C093973	increase	HIF1A	ENSG00000100644	15050414;15631803;17082639;12482858;14695184;15169887;16227395;
C093973	increase	HMOX1	ENSG00000100292	14647439;18357586;
C093973	increase	HSD3B2	ENSG00000203859	17138841;
C093973	increase	HSPA4	ENSG00000170606	11322385;
C093973	increase	IFNG	ENSG00000111537	18953428;
C093973	increase	IL17A	ENSG00000112115	12016129;
C093973	increase	IL1B	ENSG00000125538	16359550;18982426;11854442;17388968;10712238;
C093973	increase	IL6	ENSG00000136244	17196171;19074641;12016129;11410519;17388968;
C093973	increase	IL8	ENSG00000169429	17082639;16611624;19074641;15952644;12016129;11110672;15831558;11527995;17000667;16373669;1562
C093973	increase	ITGAM	ENSG00000169896	16617325;
C093973	increase	JUNB	ENSG00000171223	15666830;
C093973	increase	JUN	ENSG00000177606	14645110;15930183;18628248;
C093973	increase	KLK3	ENSG00000142515	16472761;17131305;11410519;
C093973	increase	LCP2	ENSG00000043462	16439309;

C093973	increase	MCL1	ENSG00000143384	15970427;
C093973	increase	MMP12	ENSG00000110347	16359550;
C093973	increase	MMP1	ENSG00000196611	11414687;
C093973	increase	MMP3	ENSG00000149968	16778083;
C093973	increase	MMP9	ENSG00000100985	18006877;16251475;15831558;19804834;18628248;15504454;
C093973	increase	NKX3-1	ENSG00000167034	17131305;
C093973	increase	NQO1	ENSG00000181019	17360324;15896333;
C093973	increase	PCNA	ENSG00000132646	17174366;
C093973	increase	PLA2G2A	ENSG00000188257	18953428;
C093973	increase	PLAC1	ENSG00000170965	15803460;
C093973	increase	PTGS2	ENSG00000073756	19804834;16894348;18596194;16928824;
C093973	increase	RB1	ENSG00000139687	14729647;15138593;17273796;14516785;
C093973	increase	RUNX2	ENSG00000250096	17689939;
C093973	increase	SP7	ENSG00000170374	17689939;
C093973	increase	SQSTM1	ENSG00000161011	17360324;
C093973	increase	STK39	ENSG00000198648	17131305;
C093973	increase	TNF	ENSG00000223952	11675405;17273796;15322261;
C093973	increase	TP53AIP1	ENSG00000120471	15031205;
C093973	increase	TP73	ENSG00000078900	15031205;
C093973	increase	UGT1A1	ENSG00000241635	15896333;15090468;
C093973	increase	VDR	ENSG00000111424	15930183;15879110;
C093973	increase	VEGFA	ENSG00000112715	15050414;12482858;18252963;18596194;15169887;15930297;
C094210	increase	ICAM1	ENSG00000090339	18508964;
C094299	increase	CCNE1	ENSG00000105173	15342418;
C094299	increase	CDC6	ENSG00000094804	15342418;
C094299	increase	CDK1	ENSG00000170312	15342418;
C094299	increase	CDK2	ENSG00000123374	15342418;
C094299	increase	CDKN1A	ENSG00000124762	15342418;
C094503	increase	CCL2	ENSG00000108691	15115777;
C094503	increase	CNR2	ENSG00000188822	15115777;10617657;
C094503	increase	IL8	ENSG00000169429	15115777;
C094645	decrease	PRL	ENSG00000172179	19519261;
C094859	increase	CYP11B1	ENSG00000160882	16396990;
C094859	increase	CYP11B2	ENSG00000179142	16396990;
C094868	increase	HTR2C	ENSG00000147246	18083778;
C095104	decrease	CCNA2	ENSG00000145386	15729575;
C095104	decrease	CCNB1	ENSG00000134057	15729575;
C095104	decrease	CCND1	ENSG00000110092	16273314;
C095104	decrease	CCND3	ENSG00000112576	15729575;
C095104	decrease	CCNE1	ENSG00000105173	15729575;
C095104	decrease	CDK2	ENSG00000123374	15729575;
C095104	decrease	NR5A2	ENSG00000116833	16357189;
C095104	decrease	RB1	ENSG00000139687	16273314;15729575;
C095104	increase	ABCB11	ENSG00000073734	12519787;
C095104	increase	CGB	ENSG00000213030	15941851;
C095104	increase	CYP19A1	ENSG00000137869	15941851;
C095104	increase	HSD17B1	ENSG00000108786	16513093;
C095104	increase	ID1	ENSG00000125968	17178900;

C095104	increase	IGFBP6	ENSG00000167779	17178900;
C095104	increase	RXRA	ENSG00000186350	12519787;15544927;15941851;
C095104	increase	SCD	ENSG00000099194	17178900;
C095105	decrease	ADRB2	ENSG00000169252	17178900;
C095105	decrease	AMOTL2	ENSG00000114019	17178900;
C095105	decrease	ASNS	ENSG00000070669	17178900;
C095105	decrease	BNIP2	ENSG00000140299	17178900;
C095105	decrease	CEBPG	ENSG00000153879	17178900;
C095105	decrease	COX2	ENSG00000198712	17178900;
C095105	decrease	CTH	ENSG00000116761	17178900;
C095105	decrease	CYCS	ENSG00000172115	17178900;
C095105	decrease	CYR61	ENSG00000142871	17178900;
C095105	decrease	DUSP1	ENSG00000120129	17178900;
C095105	decrease	DUSP5	ENSG00000138166	17178900;
C095105	decrease	EGR3	ENSG00000179388	17178900;
C095105	decrease	EIF1	ENSG00000173812	17178900;
C095105	decrease	EPHA2	ENSG00000142627	17178900;
C095105	decrease	GARS	ENSG00000106105	17178900;
C095105	decrease	HERPUD1	ENSG00000051108	17178900;
C095105	decrease	IER2	ENSG00000160888	17178900;
C095105	decrease	KLF10	ENSG00000155090	17178900;
C095105	decrease	KRT17	ENSG00000128422	17178900;
C095105	decrease	MAFF	ENSG00000185022	17178900;
C095105	decrease	MMP1	ENSG00000196611	17178900;
C095105	decrease	MTHFD2	ENSG00000065911	17178900;
C095105	decrease	ODC1	ENSG00000115758	17178900;
C095105	decrease	PER2	ENSG00000132326	17178900;
C095105	decrease	PLAUR	ENSG00000011422	17178900;
C095105	decrease	PNO1	ENSG00000115946	17178900;
C095105	decrease	SFRS2	ENSG00000161547	17178900;
C095105	decrease	SIAH2	ENSG00000181788	17178900;
C095105	decrease	SLC7A1	ENSG00000139514	17178900;
C095105	decrease	SLC7A5	ENSG00000103257	17178900;
C095105	decrease	UAP1	ENSG00000117143	17178900;
C095105	decrease	XBP1	ENSG00000100219	17178900;
C095105	increase	ADD3	ENSG00000148700	17178900;
C095105	increase	AKR1C1	ENSG00000187134	17178900;
C095105	increase	AKR1C3	ENSG00000196139	17178900;
C095105	increase	ARL6IP5	ENSG00000144746	17178900;
C095105	increase	C10ORF10	ENSG00000165507	17178900;
C095105	increase	C10ORF116	ENSG00000148671	17178900;
C095105	increase	CTSH	ENSG00000103811	17178900;
C095105	increase	CYP26A1	ENSG00000095596	17178900;
C095105	increase	DDIT4	ENSG00000168209	17178900;
C095105	increase	DHRS3	ENSG00000162496	17178900;
C095105	increase	DKK1	ENSG00000107984	17178900;
C095105	increase	GPM6B	ENSG00000046653	17178900;
C095105	increase	ID1	ENSG00000125968	17178900;
C095105	increase	IGFBP6	ENSG00000167779	17178900;
C095105	increase	IL15	ENSG00000164136	17178900;
C095105	increase	ITGB6	ENSG00000115221	17178900;
C095105	increase	ITM2A	ENSG00000078596	17178900;
C095105	increase	ITPR1	ENSG00000150995	17178900;

C095105	increase	KRT15	ENSG00000171346	17178900;
C095105	increase	LOXL2	ENSG00000134013	17178900;
C095105	increase	MAF	ENSG00000178573	17178900;
C095105	increase	MLLT11	ENSG00000213190	17178900;
C095105	increase	PLAT	ENSG00000104368	17178900;
C095105	increase	PLOD2	ENSG00000152952	17178900;
C095105	increase	PNRC1	ENSG00000146278	17178900;
C095105	increase	RARB	ENSG00000077092	17178900;
C095105	increase	S100A9	ENSG00000163220	17178900;
C095105	increase	SCD	ENSG00000099194	17178900;
C095105	increase	SMAD5	ENSG00000113658	17178900;
C095105	increase	SPARC	ENSG00000113140	17178900;
C095105	increase	TGM2	ENSG00000198959	17178900;
C095105	increase	TM4SF1	ENSG00000169908	17178900;
C095105	increase	TSC22D3	ENSG00000157514	17178900;
C095105	increase	VAMP8	ENSG00000118640	17178900;
C095108	increase	CCL2	ENSG00000108691	17668557;
C095108	increase	CCL3	ENSG00000006075	17668557;
C095284	affect	HIF1A	ENSG00000100644	15465035;
C095284	increase	BAX	ENSG00000087088	16288207;
C095284	increase	CDKN1A	ENSG00000124762	16288207;
C095284	increase	MDM2	ENSG00000135679	16288207;
C095284	increase	TP53	ENSG00000141510	16288207;
C095512	affect	HSPA6	ENSG00000173110	15978632;
C095512	decrease	CFTR	ENSG00000001626	19061877;
C095512	decrease	CREBBP	ENSG00000005339	15378487;
C095512	decrease	IL6	ENSG00000136244	15378487;
C095512	increase	BIRC3	ENSG00000023445	12393423;15845643;
C095512	increase	CCL2	ENSG00000108691	17666914;
C095512	increase	CCND3	ENSG00000112576	16920920;
C095512	increase	CSF3	ENSG00000108342	12393423;
C095512	increase	ICAM1	ENSG00000090339	17666914;
C095512	increase	IFNA1	ENSG00000197919	15845643;15937643;
C095512	increase	IFNB1	ENSG00000171855	15937643;
C095512	increase	IFNG	ENSG00000111537	15845643;15937643;
C095512	increase	IRF1	ENSG00000125347	11739238;
C095512	increase	TNF	ENSG00000223952	17666914;
C095512	increase	TYMP	ENSG00000025708	15937643;
C095591	decrease	BCL2	ENSG00000171791	16012733;
C095591	decrease	BCL2L1	ENSG00000171552	16012733;
C095591	decrease	PTGS2	ENSG00000073756	16012733;
C095591	increase	BAX	ENSG00000087088	16012733;
C096064	decrease	CCND1	ENSG00000110092	14758092;
C096064	decrease	CDK4	ENSG00000135446	14758092;
C096064	decrease	RB1	ENSG00000139687	14758092;
C096064	increase	CDKN1A	ENSG00000124762	14758092;
C096856	increase	ATP1A1	ENSG00000163399	12804770;
C096856	increase	CDKN1A	ENSG00000124762	12866041;
C096856	increase	FGF10	ENSG00000070193	12804770;
C096910	increase	SLC29A1	ENSG00000112759	16868479;
C096918	decrease	COX2	ENSG00000198712	16940060;
C096920	affect	PTGS2	ENSG00000073756	16894348;
C096920	increase	CYP26A1	ENSG00000095596	15589975;
C096920	increase	DDIT3	ENSG00000175197	16972258;

C096920	increase	HMOX1	ENSG00000100292	16972258;
C096920	increase	RARB	ENSG00000077092	15589975;16894348;
C096920	increase	RARRES1	ENSG00000118849	16575387;
C096920	increase	TGM2	ENSG00000198959	16158052;
C097240	decrease	BIRC2	ENSG00000110330	15498850;
C097240	decrease	BIRC3	ENSG00000023445	15498850;
C097240	decrease	CCNA2	ENSG00000145386	15729575;
C097240	decrease	CCNB1	ENSG00000134057	15729575;
C097240	decrease	CCND3	ENSG00000112576	15729575;
C097240	decrease	CCNE1	ENSG00000105173	15729575;
C097240	decrease	CDK2	ENSG00000123374	15729575;
C097240	decrease	CDKN1B	ENSG00000111276	15729575;
C097240	decrease	CFLAR	ENSG00000003402	15498850;
C097240	decrease	CYP4A11	ENSG00000187048	16712844;
C097240	decrease	FAS	ENSG00000026103	16712844;
C097240	decrease	HSPB1	ENSG00000106211	16299251;
C097240	decrease	RB1	ENSG00000139687	15729575;
C097240	decrease	VIM	ENSG00000026025	15254749;
C097240	decrease	XIAP	ENSG00000101966	15498850;16299251;
C097240	increase	CDKN1A	ENSG00000124762	15729575;15254749;
C097240	increase	CGB	ENSG00000213030	15941851;
C097240	increase	DDX58	ENSG00000107201	16303604;
C097240	increase	FABP4	ENSG00000170323	16573735;
C097240	increase	GFAP	ENSG00000131095	15254749;
C097240	increase	HIF1A	ENSG00000100644	17658243;
C097240	increase	HMOX1	ENSG00000100292	15618017;
C097240	increase	HSF1	ENSG00000185122	15618017;
C097240	increase	PPARD	ENSG00000112033	17341418;
C097240	increase	PPARG	ENSG00000132170	15254749;9013583;12065695;18485432;14979731;15866424;15729575;15491415;17341418;15707588;
C097240	increase	PTGS2	ENSG00000073756	15254749;
C097240	increase	RARB	ENSG00000077092	15254749;
C097240	increase	RXRA	ENSG00000186350	15254749;
C097284	increase	NDUFB6	ENSG00000165264	19523936;
C097284	increase	NDUFS7	ENSG00000115286	19523936;
C097599	increase	CD38	ENSG00000004468	16329108;
C097613	decrease	BCL2L1	ENSG00000171552	16960866;
C097613	decrease	BIRC5	ENSG00000089685	16166298;16254145;
C097613	decrease	CCND3	ENSG00000112576	15100154;
C097613	decrease	FGFR3	ENSG00000068078	14562121;
C097613	decrease	PDGFB	ENSG00000100311	15786421;
C097613	decrease	PDGFRA	ENSG00000134853	17614352;15786421;
C097613	decrease	PDGFRB	ENSG00000113721	15786421;
C097613	decrease	RB1	ENSG00000139687	15100154;
C097613	decrease	WT1	ENSG00000184937	15329907;
C097613	increase	ABCB1	ENSG00000085563	15970668;
C097613	increase	ABCG2	ENSG00000118777	15970668;
C097613	increase	COL1A1	ENSG00000108821	17030193;
C097613	increase	HMOX1	ENSG00000100292	17420286;
C097613	increase	KIT	ENSG00000157404	17420286;
C097949	decrease	NPPA	ENSG00000175206	15837528;
C097949	decrease	RXRA	ENSG00000186350	15837528;15331595;

C097949	increase	COL1A1	ENSG00000108821	15331595;
C097949	increase	CYP24A1	ENSG00000019186	15331595;
C097949	increase	TRPV6	ENSG00000165125	16362534;
C097949	increase	TXNIP	ENSG00000117289	16061374;
C097949	increase	VDR	ENSG00000111424	15837528;16059639;15331595;
C098320	increase	CYP3A4	ENSG00000160868	15980690;16837568;
C098384	increase	TNF	ENSG00000223952	12721113;
C098751	increase	ABCB1	ENSG00000085563	16047355;16051478;
C098869	increase	GSTP1	ENSG00000084207	15863507;
C098869	increase	RBL2	ENSG00000103479	16936753;
C099150	decrease	CCNA2	ENSG00000145386	15961672;
C099150	decrease	CCNB1	ENSG00000134057	15961672;
C099150	decrease	CCNB2	ENSG00000157456	15961672;
C099150	increase	E2F1	ENSG00000101412	15961672;
C099158	increase	SNCA	ENSG00000145335	15114628;
C099410	decrease	TNFSF11	ENSG00000120659	14753746;
C099555	affect	ARL6IP5	ENSG00000144746	16720286;
C099555	affect	TP53	ENSG00000141510	16720286;
C099555	decrease	AK2	ENSG00000004455	12958071;
C099555	decrease	AKR1B1	ENSG00000085662	12958071;
C099555	decrease	ALDOA	ENSG00000149925	12958071;
C099555	decrease	AMH	ENSG00000104899	12958071;
C099555	decrease	AP2M1	ENSG00000161203	12958071;
C099555	decrease	AP2S1	ENSG00000042753	12958071;
C099555	decrease	APC2	ENSG00000115266	12958071;
C099555	decrease	API5	ENSG00000166181	12958071;
C099555	decrease	ARF1	ENSG00000143761	12958071;
C099555	decrease	ARHGDIB	ENSG00000111348	12958071;
C099555	decrease	ARL2	ENSG00000213465	12958071;
C099555	decrease	ARPC2	ENSG00000163466	12958071;
C099555	decrease	ARPC3	ENSG00000111229	12958071;
C099555	decrease	ARSA	ENSG00000100299	12958071;
C099555	decrease	ATP5A1	ENSG00000152234	12958071;
C099555	decrease	ATP5B	ENSG00000110955	12958071;
C099555	decrease	ATP5C1	ENSG00000165629	12958071;
C099555	decrease	ATP5G3	ENSG00000154518	12958071;
C099555	decrease	ATP5J	ENSG00000154723	12958071;
C099555	decrease	ATP5L	ENSG00000167283	12958071;
C099555	decrease	ATP5O	ENSG00000241837	12958071;
C099555	decrease	ATP6V0C	ENSG00000185883	12958071;
C099555	decrease	BANF1	ENSG00000175334	12958071;
C099555	decrease	C11ORF10	ENSG00000134825	12958071;
C099555	decrease	C14ORF156	ENSG00000119705	12958071;
C099555	decrease	CCL5	ENSG00000161570	12958071;
C099555	decrease	CCT2	ENSG00000166226	12958071;
C099555	decrease	CCT3	ENSG00000163468	12958071;
C099555	decrease	CCT6A	ENSG00000146731	12958071;
C099555	decrease	CDK4	ENSG00000135446	12958071;
C099555	decrease	CEBPB	ENSG00000172216	12958071;
C099555	decrease	CLIC1	ENSG00000226417	12958071;
C099555	decrease	COX6C	ENSG00000164919	12958071;
C099555	decrease	COX7A2	ENSG00000112695	12958071;
C099555	decrease	COX7A2L	ENSG00000115944	12958071;

C099555	decrease	COX7B	ENSG00000131174	12958071;
C099555	decrease	COX7C	ENSG00000127184	12958071;
C099555	decrease	CST7	ENSG00000077984	12958071;
C099555	decrease	DNAJA1	ENSG00000086061	12958071;
C099555	decrease	EEF2	ENSG00000167658	12958071;
C099555	decrease	EIF3D	ENSG00000100353	12958071;
C099555	decrease	EIF4A1	ENSG00000161960	12958071;
C099555	decrease	EIF4A2	ENSG00000156976	12958071;
C099555	decrease	EIF4B	ENSG00000063046	12958071;
C099555	decrease	EIF5A	ENSG00000132507	12958071;
C099555	decrease	ENO1	ENSG00000074800	12958071;
C099555	decrease	ERH	ENSG00000100632	12958071;
C099555	decrease	FKBP5	ENSG00000096060	12958071;
C099555	decrease	FLT3LG	ENSG00000090554	12958071;
C099555	decrease	FSCN1	ENSG00000075618	12958071;
C099555	decrease	FTH1	ENSG00000167996	12958071;
C099555	decrease	GHSR	ENSG00000121853	12958071;
C099555	decrease	GPR6	ENSG00000146360	12958071;
C099555	decrease	HADHA	ENSG00000084754	12958071;
C099555	decrease	HIC1	ENSG00000177374	12958071;
C099555	decrease	HLA-C	ENSG00000204525	12958071;
C099555	decrease	HMGB1	ENSG00000189403	12958071;
C099555	decrease	HSP90AA1	ENSG00000080824	12958071;
C099555	decrease	HSPA8	ENSG00000109971	12958071;
C099555	decrease	ILF2	ENSG00000143621	12958071;
C099555	decrease	IMP3	ENSG00000177971	12958071;
C099555	decrease	ITGA11	ENSG00000137809	12958071;
C099555	decrease	ITM2B	ENSG00000136156	12958071;
C099555	decrease	KARS	ENSG00000065427	12958071;
C099555	decrease	KIAA0101	ENSG00000166803	12958071;
C099555	decrease	KIF2C	ENSG00000142945	12958071;
C099555	decrease	KIF3A	ENSG00000131437	12958071;
C099555	decrease	KPNA2	ENSG00000182481	12958071;
C099555	decrease	LGALS1	ENSG00000100097	12958071;
C099555	decrease	LSM3	ENSG00000170860	12958071;
C099555	decrease	MAN1B1	ENSG00000177239	12958071;
C099555	decrease	MEN1	ENSG00000133895	12958071;
C099555	decrease	MGST1	ENSG00000008394	12958071;
C099555	decrease	MOBKL1B	ENSG00000114978	12958071;
C099555	decrease	MRE11A	ENSG00000020922	15059897;
C099555	decrease	MTCH2	ENSG00000109919	12958071;
C099555	decrease	NACA	ENSG00000196531	12958071;
C099555	decrease	NARS	ENSG00000134440	12958071;
C099555	decrease	NDUFB9	ENSG00000147684	12958071;
C099555	decrease	NDUFS5	ENSG00000168653	12958071;
C099555	decrease	NFKBIA	ENSG00000100906	16024638;
C099555	decrease	NKX2-5	ENSG00000183072	12958071;
C099555	decrease	NME1	ENSG00000239672	12958071;
C099555	decrease	NPM1	ENSG00000181163	12958071;
C099555	decrease	ODC1	ENSG00000115758	12958071;
C099555	decrease	PABPC1	ENSG00000070756	12958071;
C099555	decrease	PCBP1	ENSG00000169564	12958071;
C099555	decrease	PCBP2	ENSG00000197111	12958071;
C099555	decrease	PDIA3	ENSG00000167004	12958071;

C099555	decrease	PFN1	ENSG00000108518	12958071;
C099555	decrease	PGAM1	ENSG00000171314	12958071;
C099555	decrease	PHYH	ENSG00000107537	12958071;
C099555	decrease	PIN1	ENSG00000127445	12958071;
C099555	decrease	PLP2	ENSG00000102007	12958071;
C099555	decrease	PNPLA6	ENSG00000032444	12958071;
C099555	decrease	POLR2G	ENSG00000168002	12958071;
C099555	decrease	POU3F1	ENSG00000185668	12958071;
C099555	decrease	PRICKLE4	ENSG00000124593	12958071;
C099555	decrease	PSMA4	ENSG00000041357	12958071;
C099555	decrease	PSMB1	ENSG00000008018	12958071;
C099555	decrease	PSMD3	ENSG00000108344	12958071;
C099555	decrease	PSMF1	ENSG00000125818	12958071;
C099555	decrease	PURB	ENSG00000146676	12958071;
C099555	decrease	RASL10B	ENSG00000141150	12958071;
C099555	decrease	RBMX	ENSG00000147274	12958071;
C099555	decrease	RBX1	ENSG00000100387	12958071;
C099555	decrease	ROBO4	ENSG00000154133	12958071;
C099555	decrease	RPL10	ENSG00000147403	12958071;
C099555	decrease	RPL11	ENSG00000142676	12958071;
C099555	decrease	RPL13A	ENSG00000142541	12958071;
C099555	decrease	RPL13	ENSG00000167526	12958071;
C099555	decrease	RPL19	ENSG00000108298	12958071;
C099555	decrease	RPL24	ENSG00000114391	12958071;
C099555	decrease	RPL36A	ENSG00000241343	12958071;
C099555	decrease	RPL36AL	ENSG00000165502	12958071;
C099555	decrease	RPL39	ENSG00000198918	12958071;
C099555	decrease	RPL8	ENSG00000161016	12958071;
C099555	decrease	RPLP1	ENSG00000137818	12958071;
C099555	decrease	RPLP2	ENSG00000177600	12958071;
C099555	decrease	RPS10	ENSG00000124614	12958071;
C099555	decrease	RPS15	ENSG00000115268	12958071;
C099555	decrease	RPS16	ENSG00000105193	12958071;
C099555	decrease	RPS17	ENSG00000182774	12958071;
C099555	decrease	RPS27	ENSG00000177954	12958071;
C099555	decrease	RPS2	ENSG00000140988	12958071;
C099555	decrease	RYBP	ENSG00000163602	12958071;
C099555	decrease	S100P	ENSG00000163993	12958071;
C099555	decrease	SAP18	ENSG00000150459	12958071;
C099555	decrease	7-Sep	ENSG00000122545	12958071;
C099555	decrease	SET	ENSG00000119335	12958071;
C099555	decrease	SFRS3	ENSG00000112081	12958071;
C099555	decrease	SH3BGRL3	ENSG00000142669	12958071;
C099555	decrease	SHFM1	ENSG00000127922	12958071;
C099555	decrease	SLC25A37	ENSG00000147454	12958071;
C099555	decrease	SLC25A3	ENSG00000075415	12958071;
C099555	decrease	SLC25A5	ENSG00000005022	12958071;
C099555	decrease	SLC25A6	ENSG00000169100	12958071;
C099555	decrease	SLC30A5	ENSG00000145740	12958071;
C099555	decrease	SLC38A7	ENSG00000103042	12958071;
C099555	decrease	SNRPB	ENSG00000125835	12958071;
C099555	decrease	SNRPD2	ENSG00000125743	12958071;
C099555	decrease	SNRPF	ENSG00000139343	12958071;
C099555	decrease	SNRPG	ENSG00000143977	12958071;

C099555	decrease	SOD1	ENSG00000142168	12958071;
C099555	decrease	SOX11	ENSG00000176887	12958071;
C099555	decrease	SOX4	ENSG00000124766	12958071;
C099555	decrease	SSBP1	ENSG00000106028	12958071;
C099555	decrease	SSR2	ENSG00000163479	12958071;
C099555	decrease	ST13	ENSG00000100380	12958071;
C099555	decrease	STOML2	ENSG00000165283	12958071;
C099555	decrease	SUMO1	ENSG00000116030	12958071;
C099555	decrease	TBL1XR1	ENSG00000177565	12958071;
C099555	decrease	TIMM8B	ENSG00000150779	12958071;
C099555	decrease	TMEM14C	ENSG00000111843	12958071;
C099555	decrease	TMSB10	ENSG00000034510	12958071;
C099555	decrease	TOMM7	ENSG00000196683	12958071;
C099555	decrease	TOP2A	ENSG00000131747	15059897;
C099555	decrease	TOPBP1	ENSG00000163781	12958071;
C099555	decrease	TUBA1A	ENSG00000167552	12958071;
C099555	decrease	TXN	ENSG00000136810	12958071;
C099555	decrease	UBA52	ENSG00000221983	12958071;
C099555	decrease	UBE2M	ENSG00000130725	12958071;
C099555	decrease	UBE2N	ENSG00000177889	12958071;
C099555	decrease	UBL5	ENSG00000198258	12958071;
C099555	decrease	UGCG	ENSG00000148154	12958071;
C099555	decrease	UQCRC2	ENSG00000140740	12958071;
C099555	decrease	UQCRH	ENSG00000173660	12958071;
C099555	decrease	UXT	ENSG00000126756	12958071;
C099555	decrease	VDAC2	ENSG00000165637	12958071;
C099555	decrease	WDR45	ENSG00000196998	12958071;
C099555	decrease	XRN2	ENSG00000088930	12958071;
C099555	decrease	YBX1	ENSG00000065978	12958071;
C099555	decrease	YWHAZ	ENSG00000164924	12958071;
C099555	increase	CASP3	ENSG00000164305	16024638;16720286;
C099555	increase	CORO1A	ENSG00000102879	12958071;
C099555	increase	GADD45A	ENSG00000116717	17474084;
C099555	increase	GADD45B	ENSG00000099860	17474084;
C099555	increase	IL8	ENSG00000169429	12958071;
C099555	increase	JUN	ENSG00000177606	15059897;
C099555	increase	POLR3E	ENSG00000058600	15059897;
C099555	increase	PRDX4	ENSG00000123131	12958071;
C099555	increase	RBL2	ENSG00000103479	15059897;
C099555	increase	RPL35	ENSG00000136942	12958071;
C099555	increase	SETD5	ENSG00000168137	12958071;
C099555	increase	SP1	ENSG00000185591	15059897;
C099555	increase	STK3	ENSG00000104375	12958071;
C099555	increase	STXBP5	ENSG00000164506	15059897;
C099555	increase	SUB1	ENSG00000113387	12958071;
C099555	increase	TNFRSF10A	ENSG00000104689	16024638;
C099630	increase	NAIP	ENSG00000250628	15729293;
C099681	decrease	IL4R	ENSG00000077238	12239597;
C099681	increase	IFNG	ENSG00000111537	12239597;
C099734	increase	CGB	ENSG00000213030	15941851;
C099734	increase	CYP19A1	ENSG00000137869	15941851;
C099952	decrease	HSPA1A	ENSG00000232804	16271832;
C099952	decrease	HSPA8	ENSG00000109971	16271832;
C099952	decrease	HSPB2	ENSG00000170276	16271832;

C099952	increase	CYP3A4	ENSG00000160868	19249324;
C099952	increase	HSPA5	ENSG00000044574	16271832;
C100043	increase	CYP19A1	ENSG00000137869	10746939;11675267;
C100075	increase	ADI1	ENSG00000182551	17786183;
C100075	increase	AR	ENSG00000169083	15661808;15509168;12676605;15253041;17075824;15466214;
C100075	increase	KLK3	ENSG00000142515	15141018;
C100163	decrease	HSPA1A	ENSG00000232804	16271832;
C100163	decrease	HSPA8	ENSG00000109971	16271832;
C100163	increase	HSPA5	ENSG00000044574	16271832;
C100355	increase	CYP3A4	ENSG00000160868	16565514;
C100692	decrease	TYMS	ENSG00000176890	17172411;
C101044	decrease	AR	ENSG00000169083	16472761;
C101044	decrease	EGF	ENSG00000138798	16472761;11527950;
C101044	decrease	KLK3	ENSG00000142515	16472761;
C101044	increase	ALB	ENSG00000163631	16908450;
C101044	increase	ANGPT1	ENSG00000154188	16373414;
C101044	increase	ANGPT2	ENSG00000091879	16373414;
C101044	increase	CSF2	ENSG00000164400	16908450;
C101044	increase	EDN1	ENSG00000078401	17974986;
C101044	increase	EGR1	ENSG00000120738	15292961;
C101044	increase	FGF2	ENSG00000138685	16373414;
C101044	increase	FOS	ENSG00000170345	15090535;
C101044	increase	HBEGF	ENSG00000113070	11159045;
C101044	increase	IL8	ENSG00000169429	15247267;16908450;
C101044	increase	MMP9	ENSG00000100985	18006877;15504454;
C101044	increase	NTS	ENSG00000133636	15247267;15177934;
C101670	increase	ACTA2	ENSG00000107796	15703175;
C101670	increase	COL1A1	ENSG00000108821	15703175;
C101670	increase	CTGF	ENSG00000118523	15703175;
C101670	increase	FOS	ENSG00000170345	10712238;
C101670	increase	IL1B	ENSG00000125538	10712238;
C101670	increase	TGFB1	ENSG00000105329	15703175;
C101954	decrease	MYC	ENSG00000136997	17521618;
C101954	decrease	ODC1	ENSG00000115758	17521618;
C101954	increase	ICAM1	ENSG00000090339	12952965;
C101954	increase	UGCG	ENSG00000148154	18245173;
C102210	increase	CDKN1A	ENSG00000124762	12556972;
C102351	affect	MAGEA9	ENSG00000166008	17290406;
C102834	affect	PGR	ENSG00000082175	9144411;
C102881	increase	CGB	ENSG00000213030	15941851;19497422;
C102881	increase	CYP19A1	ENSG00000137869	15941851;
C102881	increase	HSD17B1	ENSG00000108786	16513093;
C103016	decrease	CYP11A1	ENSG00000140459	16014403;
C103016	decrease	CYP17A1	ENSG00000148795	16014403;
C103016	decrease	STAR	ENSG00000147465	16014403;
C103016	decrease	SULT2A1	ENSG00000105398	16014403;
C103112	decrease	CYP1B1	ENSG00000138061	17637178;
C103303	affect	CDH1	ENSG00000039068	17034753;
C103303	affect	S100G	ENSG00000169906	16491319;
C103303	affect	TRPV6	ENSG00000165125	16491319;
C103303	decrease	ADIPOR1	ENSG00000159346	16023994;
C103303	decrease	AIP	ENSG00000110711	15982314;

C103303	decrease	BCL2	ENSG00000171791	12189556;16909596;16109544;17077328;
C103303	decrease	CBX3	ENSG00000122565	15982314;
C103303	decrease	CCND3	ENSG00000112576	16273314;
C103303	decrease	CCR2	ENSG00000121807	16712875;
C103303	decrease	CD1A	ENSG00000158477	17475839;
C103303	decrease	CD80	ENSG00000121594	17475839;
C103303	decrease	CDKN1A	ENSG00000124762	16340750;
C103303	decrease	CDKN1C	ENSG00000129757	15982314;
C103303	decrease	COL17A1	ENSG00000065618	15982314;
C103303	decrease	COL6A1	ENSG00000142156	15982314;
C103303	decrease	CXCL1	ENSG00000163739	15982314;
C103303	decrease	CYP3A7	ENSG00000160870	15982314;
C103303	decrease	DSC1	ENSG00000134765	15982314;
C103303	decrease	DUSP11	ENSG00000144048	15982314;
C103303	decrease	EGFR	ENSG00000146648	15982314;
C103303	decrease	EIF4EBP2	ENSG00000148730	15982314;
C103303	decrease	FABP5	ENSG00000164687	15982314;
C103303	decrease	FASN	ENSG00000169710	15982314;
C103303	decrease	FASTK	ENSG00000164896	15982314;
C103303	decrease	FGF1	ENSG00000113578	15982314;
C103303	decrease	FLG	ENSG00000143631	15982314;
C103303	decrease	FN1	ENSG00000115414	11139446;
C103303	decrease	GBA	ENSG00000177628	15982314;
C103303	decrease	GDF2	ENSG00000128802	15982314;
C103303	decrease	GRB7	ENSG00000141738	15982314;
C103303	decrease	GSTM1	ENSG00000134184	15982314;
C103303	decrease	HSD11B1	ENSG00000117594	15982314;
C103303	decrease	IFNG	ENSG00000111537	17118196;
C103303	decrease	IL10RA	ENSG00000110324	15982314;
C103303	decrease	IL12A	ENSG00000168811	17475839;17118196;
C103303	decrease	IL12B	ENSG00000113302	17475839;17118196;
C103303	decrease	IL24	ENSG00000162892	15982314;
C103303	decrease	IL2	ENSG00000109471	17118196;
C103303	decrease	KRT10	ENSG00000186395	15982314;
C103303	decrease	KRT14	ENSG00000186847	15982314;
C103303	decrease	KRT17	ENSG00000128422	15982314;
C103303	decrease	KRT1	ENSG00000167768	15982314;
C103303	decrease	KRT5	ENSG00000186081	15982314;
C103303	decrease	KRT6A	ENSG00000205420	15982314;
C103303	decrease	KRT75	ENSG00000170454	15982314;
C103303	decrease	LAMA2	ENSG00000196569	15982314;
C103303	decrease	LAMA3	ENSG00000053747	15982314;
C103303	decrease	MFAP2	ENSG00000117122	15982314;
C103303	decrease	MMP10	ENSG00000166670	15982314;
C103303	decrease	NEFL	ENSG00000104725	15982314;
C103303	decrease	ODC1	ENSG00000115758	15982314;
C103303	decrease	PPARA	ENSG00000186951	16292757;
C103303	decrease	PTGS1	ENSG00000095303	15982314;
C103303	decrease	PTGS2	ENSG00000073756	15982314;
C103303	decrease	PTHLH	ENSG00000087494	16243370;
C103303	decrease	RAB27A	ENSG00000069974	16449964;
C103303	decrease	RARG	ENSG00000172819	15615532;15375546;
C103303	decrease	RHOA	ENSG00000067560	15982314;

C103303	decrease	RHOB	ENSG00000143878	15982314;
C103303	decrease	RNF5	ENSG00000225452	15982314;
C103303	decrease	RXRG	ENSG00000249671	15941851;11139446;
C103303	decrease	S100A1	ENSG00000160678	15982314;
C103303	decrease	S100A8	ENSG00000143546	15982314;
C103303	decrease	SEMA6B	ENSG00000167680	16525649;
C103303	decrease	SERPINB2	ENSG00000197632	15982314;
C103303	decrease	SERPINB8	ENSG00000166401	15982314;
C103303	decrease	SERPINB9	ENSG00000170542	15982314;
C103303	decrease	SLC16A1	ENSG00000155380	15982314;
C103303	decrease	SOCS1	ENSG00000185338	15982314;
C103303	decrease	SPP1	ENSG00000118785	11139446;
C103303	decrease	SPRR1B	ENSG00000169469	15982314;
C103303	decrease	SULT2A1	ENSG00000105398	15982314;
C103303	decrease	TGM1	ENSG00000092295	15982314;
C103303	decrease	THBS1	ENSG00000137801	15982314;
C103303	decrease	TNF	ENSG00000223952	17118196;
C103303	decrease	UGDH	ENSG00000109814	16292757;
C103303	decrease	UVRAG	ENSG00000198382	15982314;
C103303	decrease	VEGFC	ENSG00000150630	15982314;
C103303	decrease	WNT5B	ENSG00000111186	15972578;
C103303	increase	ABCA1	ENSG00000165029	16023994;16142410;
C103303	increase	ABCC6	ENSG00000091262	17045963;
C103303	increase	ABCG1	ENSG00000160179	16024918;16730733;
C103303	increase	BAX	ENSG00000087088	16909596;12189556;16340750;16109544;
C103303	increase	BCL2L11	ENSG00000153094	16340750;
C103303	increase	BGLAP	ENSG00000242252	12898515;
C103303	increase	CA2	ENSG00000104267	16340750;
C103303	increase	CASP3	ENSG00000164305	16909596;16109544;
C103303	increase	CCR3	ENSG00000183625	16712875;
C103303	increase	CCR7	ENSG00000126353	17475839;
C103303	increase	CD36	ENSG00000135218	17475839;
C103303	increase	CD38	ENSG00000004468	15964820;
C103303	increase	CD83	ENSG00000112149	17475839;
C103303	increase	CD86	ENSG00000114013	17475839;
C103303	increase	CDKN1B	ENSG00000111276	16340750;
C103303	increase	CETP	ENSG00000087237	16142410;
C103303	increase	CGB	ENSG00000213030	15941851;
C103303	increase	CIITA	ENSG00000179583	17229644;
C103303	increase	COL1A1	ENSG00000108821	17034753;
C103303	increase	COL4A2	ENSG00000134871	17034753;
C103303	increase	COL8A2	ENSG00000171812	15982314;
C103303	increase	CRABP2	ENSG00000143320	17034753;
C103303	increase	CYP19A1	ENSG00000137869	15941851;16357189;
C103303	increase	CYP26A1	ENSG00000095596	16194896;
C103303	increase	CYP27A1	ENSG00000135929	15964820;15982314;
C103303	increase	CYP3A4	ENSG00000160868	16632523;
C103303	increase	DHRS9	ENSG00000073737	11304534;
C103303	increase	DUSP10	ENSG00000143507	15982314;
C103303	increase	EMP1	ENSG00000134531	15982314;
C103303	increase	FAM3C	ENSG00000196937	15982314;
C103303	increase	FAS	ENSG00000026103	17034753;16712844;
C103303	increase	FOXA1	ENSG00000129514	17034753;

C103303	increase	G6PD	ENSG00000160211	15982314;
C103303	increase	GATA6	ENSG00000141448	17034753;
C103303	increase	GDF15	ENSG00000130513	15982314;
C103303	increase	GLUL	ENSG00000135821	15982314;
C103303	increase	HBEGF	ENSG00000113070	15982314;
C103303	increase	HGF	ENSG00000019991	16192631;
C103303	increase	HSD17B1	ENSG00000108786	16513093;
C103303	increase	IFNGR1	ENSG00000027697	16085646;
C103303	increase	IGF1	ENSG00000017427	15994153;15982314;
C103303	increase	IGFBP3	ENSG00000146674	16760641;15994153;
C103303	increase	IL13	ENSG00000169194	17118196;
C103303	increase	IL1B	ENSG00000125538	15982314;
C103303	increase	IL4	ENSG00000113520	17118196;
C103303	increase	IL5	ENSG00000113525	17118196;
C103303	increase	IRF1	ENSG00000125347	16636311;17229644;16085646;
C103303	increase	ITGA4	ENSG00000115232	15982314;
C103303	increase	ITGAM	ENSG00000169896	15964820;
C103303	increase	ITGAX	ENSG00000140678	15964820;
C103303	increase	ITGB2	ENSG00000160255	15964820;
C103303	increase	IVL	ENSG00000163207	17034753;
C103303	increase	KLK10	ENSG00000129451	16800735;
C103303	increase	KRT15	ENSG00000171346	15982314;
C103303	increase	KRT18	ENSG00000111057	17034753;
C103303	increase	KRT19	ENSG00000171345	15982314;17034753;
C103303	increase	KRT7	ENSG00000135480	15982314;17034753;
C103303	increase	KRT8	ENSG00000170421	17034753;
C103303	increase	LAMC3	ENSG00000050555	15982314;
C103303	increase	LCN2	ENSG00000148346	15982314;
C103303	increase	LPL	ENSG00000175445	16142410;
C103303	increase	MEIS1	ENSG00000143995	17034753;
C103303	increase	MEIS2	ENSG00000134138	17034753;
C103303	increase	MEOX1	ENSG00000005102	17034753;
C103303	increase	NEDD9	ENSG00000111859	15982314;
C103303	increase	NKX3-1	ENSG00000167034	16763719;
C103303	increase	NR1I2	ENSG00000144852	16632523;
C103303	increase	NR5A2	ENSG00000116833	16357189;
C103303	increase	NRL	ENSG00000129535	16854989;
C103303	increase	OLR1	ENSG00000173391	17034753;
C103303	increase	PADI4	ENSG00000159339	15964820;
C103303	increase	PCSK2	ENSG00000125851	15585599;
C103303	increase	PIR	ENSG00000087842	15982314;
C103303	increase	PLAT	ENSG00000104368	15982314;
C103303	increase	PPARGC1A	ENSG00000109819	16513826;
C103303	increase	PSG5	ENSG00000204941	17475324;
C103303	increase	RARA	ENSG00000131759	17045963;16179254;15375546;11139446;17034753;
C103303	increase	RARB	ENSG00000077092	16760641;11139446;15375546;
C103303	increase	RARRES1	ENSG00000118849	17034753;
C103303	increase	RARRES3	ENSG00000133321	17034753;

C103303	increase	RXRA	ENSG00000186350	17170071;16460010;17223708;15941851;16204233;17475324;16292757;16946489;15866424;7641808;16632523;15375546;11139446;
C103303	increase	S100A7	ENSG00000143556	15982314;
C103303	increase	S100A9	ENSG00000163220	15982314;
C103303	increase	SCARB1	ENSG00000073060	16142410;
C103303	increase	SERPINA3	ENSG00000196136	15982314;
C103303	increase	SERPINE1	ENSG00000106366	16192631;
C103303	increase	SLC5A5	ENSG00000105641	16954431;
C103303	increase	SOCS2	ENSG00000120833	15982314;
C103303	increase	TACSTD2	ENSG00000184292	15982314;
C103303	increase	TGFB1	ENSG00000105329	16192631;
C103303	increase	TGFB1I1	ENSG00000140682	15982314;
C103303	increase	TGIF1	ENSG00000177426	16192631;
C103303	increase	TGM2	ENSG00000198959	15964820;9516142;17034753;16158052;
C103303	increase	TNFRSF10A	ENSG00000104689	16449964;
C103303	increase	TNFRSF10B	ENSG00000120889	16449964;
C103303	increase	TNFSF10	ENSG00000121858	16449964;
C103303	increase	VEGFA	ENSG00000112715	16386082;
C103303	increase	VIPR1	ENSG00000114812	16037943;
C103436	decrease	CCNE1	ENSG00000105173	12907249;
C103436	decrease	CDK2	ENSG00000123374	12907249;
C103436	decrease	CDKN1A	ENSG00000124762	12907249;
C103436	increase	CDKN1B	ENSG00000111276	12907249;
C103439	increase	CBR1	ENSG00000159228	18755171;
C103481	affect	ITGB3	ENSG00000056345	14769215;
C103481	decrease	ACHE	ENSG00000087085	14769215;
C103481	increase	GATA1	ENSG00000102145	14769215;
C103481	increase	GATA2	ENSG00000179348	14769215;
C103481	increase	GYPA	ENSG00000170180	14769215;
C103481	increase	HMBS	ENSG00000149397	14769215;
C103481	increase	NFE2	ENSG00000123405	14769215;
C103505	decrease	ARPC4	ENSG00000241553	16500647;
C103505	decrease	C6ORF192	ENSG00000146409	16500647;
C103505	decrease	CAP2	ENSG00000112186	16500647;
C103505	decrease	CCDC86	ENSG00000110104	16500647;
C103505	decrease	DHX9	ENSG00000135829	16500647;
C103505	decrease	EFTUD2	ENSG00000108883	16500647;
C103505	decrease	FEN1	ENSG00000168496	16500647;
C103505	decrease	GTF3C5	ENSG00000148308	16500647;
C103505	decrease	HSPA1B	ENSG00000232804	16500647;
C103505	decrease	HSPA8	ENSG00000109971	16500647;
C103505	decrease	LMNB2	ENSG00000176619	16500647;
C103505	decrease	MAP2K3	ENSG00000034152	16500647;
C103505	decrease	MCM3	ENSG00000112118	16500647;
C103505	decrease	MCM5	ENSG00000100297	16500647;
C103505	decrease	MFN2	ENSG00000116688	16500647;
C103505	decrease	NCAPD3	ENSG00000151503	16500647;
C103505	decrease	PAPOLA	ENSG00000090060	16500647;
C103505	decrease	PPIF	ENSG00000108179	16500647;
C103505	decrease	PTBP1	ENSG00000011304	16500647;

C103505	decrease	RACGAP1	ENSG00000161800	16500647;
C103505	decrease	RAD54L	ENSG00000085999	16500647;
C103505	decrease	RCC1	ENSG00000180198	16500647;
C103505	decrease	RFC5	ENSG00000111445	16500647;
C103505	decrease	RPA1	ENSG00000132383	16500647;
C103505	decrease	SDPR	ENSG00000168497	16500647;
C103505	decrease	SFRS14	ENSG00000064607	16500647;
C103505	decrease	SFRS1	ENSG00000136450	16500647;
C103505	decrease	SPTBN1	ENSG00000115306	16500647;
C103505	decrease	STAM	ENSG00000136738	16500647;
C103505	decrease	STAT3	ENSG00000168610	16500647;
C103505	decrease	TACC3	ENSG00000013810	16500647;
C103505	decrease	UGCG	ENSG00000148154	16500647;
C103505	decrease	UHRF1	ENSG00000034063	16500647;
C103505	decrease	WBSCR16	ENSG00000174374	16500647;
C103505	decrease	ZNF142	ENSG00000115568	16500647;
C103505	increase	ASS1	ENSG00000130707	16500647;
C103505	increase	ATF5	ENSG00000169136	16500647;
C103505	increase	C14ORF4	ENSG00000119669	16500647;
C103505	increase	C6ORF48	ENSG00000234728	16500647;
C103505	increase	CARS	ENSG00000110619	16500647;
C103505	increase	CD55	ENSG00000196352	16500647;
C103505	increase	CEBPG	ENSG00000153879	16500647;
C103505	increase	CHRNB3	ENSG00000147432	16500647;
C103505	increase	CKLF	ENSG00000217555	16500647;
C103505	increase	CTH	ENSG00000116761	16500647;
C103505	increase	DDIT3	ENSG00000175197	16500647;
C103505	increase	DDIT4	ENSG00000168209	16500647;
C103505	increase	HAX1	ENSG00000143575	16500647;
C103505	increase	HIST1H2AC	ENSG00000180573	16500647;
C103505	increase	HMOX1	ENSG00000100292	16500647;
C103505	increase	HNMT	ENSG00000150540	16500647;
C103505	increase	IMMP2L	ENSG00000184903	16500647;
C103505	increase	NCOA7	ENSG00000111912	16500647;
C103505	increase	PEA15	ENSG00000162734	16500647;
C103505	increase	RNASEH1	ENSG00000171865	16500647;
C103505	increase	RWDD1	ENSG00000111832	16500647;
C103505	increase	S100A2	ENSG00000196754	16500647;
C103505	increase	SARS	ENSG00000031698	16500647;
C103505	increase	SESN2	ENSG00000130766	16500647;
C103505	increase	SLC1A4	ENSG00000115902	16500647;
C103505	increase	SLC30A1	ENSG00000170385	16500647;
C103505	increase	SLC7A5	ENSG00000103257	16500647;
C103505	increase	TBL1X	ENSG00000101849	16500647;
C103505	increase	TMEM126A	ENSG00000171202	16500647;
C103505	increase	TRIB3	ENSG00000101255	16500647;
C103505	increase	TSPAN10	ENSG00000182612	16500647;
C103505	increase	TXNIP	ENSG00000117289	16500647;
C103505	increase	VLDLR	ENSG00000147852	16500647;
C103505	increase	WARS	ENSG00000140105	16500647;
C103505	increase	XBP1	ENSG00000100219	16500647;
C103505	increase	YARS	ENSG00000134684	16500647;
C103505	increase	ZMYM6	ENSG00000163867	16500647;
C103712	increase	MAP1LC3A	ENSG00000101460	18818525;

C103873	increase	CXCL10	ENSG00000169245	15867097;
C103873	increase	IL12B	ENSG00000113302	15867097;
C104182	affect	CYP24A1	ENSG00000019186	16289102;
C104183	affect	PTGS2	ENSG00000073756	16894348;
C104183	increase	PIK3CB	ENSG00000051382	16043647;
C104416	decrease	CYP11A1	ENSG00000140459	16014403;
C104416	decrease	CYP17A1	ENSG00000148795	16014403;
C104416	decrease	STAR	ENSG00000147465	16014403;
C104416	decrease	SULT2A1	ENSG00000105398	16014403;
C104450	increase	CYP27B1	ENSG00000111012	16362534;
C104450	increase	TRPV6	ENSG00000165125	16362534;
C104536	decrease	BCL2	ENSG00000171791	15995977;
C104536	decrease	IL6	ENSG00000136244	16019205;
C104536	increase	ALPL	ENSG00000162551	16019205;
C104536	increase	TFF1	ENSG00000160182	16019205;
C104536	increase	VWF	ENSG00000110799	16019205;
C104586	decrease	BIRC5	ENSG00000089685	16230394;
C104586	decrease	CDKN1A	ENSG00000124762	16275999;15342418;
C104586	decrease	MCL1	ENSG00000143384	16275999;
C104586	decrease	XIAP	ENSG00000101966	16275999;16230394;16140939;
C104586	increase	BAX	ENSG00000087088	16275999;16230394;
C104586	increase	RB1	ENSG00000139687	15231455;15741232;14653808;
C104586	increase	SP1	ENSG00000185591	14653808;
C104586	increase	TP53	ENSG00000141510	16003486;16140939;
C104727	decrease	IFNG	ENSG00000111537	16006447;
C104727	decrease	IL4	ENSG00000113520	16006447;
C104727	decrease	RELA	ENSG00000173039	16352735;
C104820	increase	CYP2B6	ENSG00000197408	18206661;
C105195	decrease	PGR	ENSG00000082175	9144411;
C105351	increase	CCL3	ENSG00000006075	10540334;
C105351	increase	IL8	ENSG00000169429	10540334;
C105427	increase	CDKN1A	ENSG00000124762	11741290;
C105427	increase	TP53	ENSG00000141510	11741290;
C105671	decrease	BAX	ENSG00000087088	15996779;
C105671	increase	CAT	ENSG00000121691	15904944;
C105671	increase	SOD2	ENSG00000112096	15904944;
C105686	affect	FGFR3	ENSG00000068078	14871245;15029211;16091734;
C105686	decrease	MAPK1	ENSG00000100030	15631803;14871245;15029211;
C105686	decrease	MAPK3	ENSG00000102882	15631803;14871245;15029211;
C105686	decrease	STAT3	ENSG00000168610	14871245;
C105686	increase	FGF2	ENSG00000138685	16188231;16154193;15631803;
C105686	increase	FOS	ENSG00000170345	16188231;
C105686	increase	HIF1A	ENSG00000100644	15631803;
C105686	increase	TACR1	ENSG00000115353	16154193;
C105686	increase	TNF	ENSG00000223952	16154193;
C105905	decrease	BAK1	ENSG00000030110	15781658;
C105934	decrease	AKT1	ENSG00000142208	16123214;14654083;17270149;

C105934	decrease	BIRC5	ENSG00000089685	17270149;16707021;16004971;16123214;
C105934	decrease	CAT	ENSG00000121691	18848576;
C105934	decrease	CYP19A1	ENSG00000137869	15964185;
C105934	decrease	IL6	ENSG00000136244	16702388;
C105934	decrease	MCL1	ENSG00000143384	14654083;
C105934	decrease	MKI67	ENSG00000148773	16507397;
C105934	decrease	SOD2	ENSG00000112096	18848576;
C105934	decrease	TP53	ENSG00000141510	16507397;
C105934	increase	ABCC4	ENSG00000125257	18690847;
C105934	increase	ABCC5	ENSG00000114770	18690847;
C105934	increase	CAV1	ENSG00000105974	18848576;
C105934	increase	CCND1	ENSG00000110092	15489888;17270149;
C105934	increase	DES	ENSG00000175084	18089846;
C105934	increase	GDF15	ENSG00000130513	18089846;
C105934	increase	GSTP1	ENSG00000084207	18089846;
C105934	increase	IL1B	ENSG00000125538	16357062;
C105934	increase	NGFR	ENSG00000064300	17447067;
C105934	increase	NRG1	ENSG00000157168	16357062;
C105934	increase	PTGS2	ENSG00000073756	18089846;16507397;16004971;15489888;18848576;17164136;
C105934	increase	RELA	ENSG00000173039	16685529;18848576;17097285;15489888;
C105934	increase	TNF	ENSG00000223952	16702388;15489888;
C106014	decrease	AR	ENSG00000169083	17010675;
C106014	decrease	ARID5B	ENSG00000150347	17010675;
C106014	decrease	BIRC5	ENSG00000089685	17010675;
C106014	decrease	BTG1	ENSG00000133639	17010675;
C106014	decrease	C21ORF33	ENSG00000160221	17010675;
C106014	decrease	C5ORF13	ENSG00000134986	17010675;
C106014	decrease	CDC7	ENSG00000097046	17010675;
C106014	decrease	CDK2	ENSG00000123374	17010675;
C106014	decrease	CITED2	ENSG00000164442	17010675;
C106014	decrease	CYP1A1	ENSG00000140465	17010675;
C106014	decrease	EFNB2	ENSG00000125266	17010675;
C106014	decrease	EGFR	ENSG00000146648	17010675;
C106014	decrease	FLT3	ENSG00000122025	17010675;
C106014	decrease	IL1RN	ENSG00000136689	17010675;
C106014	decrease	JAG1	ENSG00000101384	17010675;
C106014	decrease	KCNN2	ENSG00000080709	17010675;
C106014	decrease	MAFB	ENSG00000204103	17010675;
C106014	decrease	MDH2	ENSG00000146701	17010675;
C106014	decrease	MSH6	ENSG00000116062	17010675;
C106014	decrease	MYCBP2	ENSG00000005810	17010675;
C106014	decrease	MYNN	ENSG00000085274	17010675;
C106014	decrease	NBN	ENSG00000104320	17010675;
C106014	decrease	NKX3-1	ENSG00000167034	17010675;
C106014	decrease	OPRK1	ENSG00000082556	17010675;
C106014	decrease	PDE9A	ENSG00000160191	17010675;
C106014	decrease	PIK3CD	ENSG00000171608	17010675;
C106014	decrease	PIK3R1	ENSG00000145675	17010675;
C106014	decrease	PPIF	ENSG00000108179	17010675;
C106014	decrease	PURA	ENSG00000185129	17010675;

C106014	decrease	RRM2	ENSG00000171848	17010675;
C106014	decrease	SESN1	ENSG00000080546	17010675;
C106014	decrease	SLTM	ENSG00000137776	17010675;
C106014	decrease	SOX4	ENSG00000124766	17010675;
C106014	decrease	TAOK3	ENSG00000135090	17010675;
C106014	decrease	TMPRSS2	ENSG00000184012	17010675;
C106014	decrease	TXNIP	ENSG00000117289	17010675;
C106014	decrease	WDR77	ENSG00000116455	17010675;
C106014	decrease	ZBTB43	ENSG00000169155	17010675;
C106014	decrease	ZC3H12A	ENSG00000163874	17010675;
C106014	increase	AHSA1	ENSG00000100591	17010675;
C106014	increase	AKR1C1	ENSG00000187134	17010675;
C106014	increase	AKR1C2	ENSG00000151632	17010675;
C106014	increase	ATF3	ENSG00000162772	17010675;
C106014	increase	BAG3	ENSG00000151929	17010675;
C106014	increase	C20ORF111	ENSG00000132823	17010675;
C106014	increase	CACYBP	ENSG00000116161	17010675;
C106014	increase	CBR3	ENSG00000159231	17010675;
C106014	increase	CHORDC1	ENSG00000110172	17010675;
C106014	increase	DDIT3	ENSG00000175197	17010675;
C106014	increase	DDIT4	ENSG00000168209	17010675;
C106014	increase	DNAJA1	ENSG00000086061	17010675;
C106014	increase	DNAJB4	ENSG00000162616	17010675;
C106014	increase	FICD	ENSG00000198855	17010675;
C106014	increase	GCLM	ENSG00000023909	17010675;
C106014	increase	GDF15	ENSG00000130513	17010675;
C106014	increase	GTPBP2	ENSG00000172432	17010675;
C106014	increase	HMGCS1	ENSG00000112972	17010675;
C106014	increase	HMOX1	ENSG00000100292	17010675;
C106014	increase	HSPA1A	ENSG00000232804	17010675;
C106014	increase	HSPA1B	ENSG00000232804	17010675;
C106014	increase	HSPA5	ENSG00000044574	17010675;
C106014	increase	HSPH1	ENSG00000120694	17010675;
C106014	increase	INSIG1	ENSG00000186480	17010675;
C106014	increase	LDLR	ENSG00000130164	17010675;
C106014	increase	LPIN1	ENSG00000134324	17010675;
C106014	increase	ME1	ENSG00000065833	17010675;
C106014	increase	MNT	ENSG00000070444	17010675;
C106014	increase	MRPL18	ENSG00000112110	17010675;
C106014	increase	SC4MOL	ENSG00000052802	17010675;
C106014	increase	SERP1	ENSG00000120742	17010675;
C106014	increase	SLC3A2	ENSG00000168003	17010675;
C106014	increase	SLC7A11	ENSG00000151012	17010675;
C106014	increase	STIP1	ENSG00000168439	17010675;
C106014	increase	UBE2B	ENSG00000119048	17010675;
C106014	increase	UGDH	ENSG00000109814	17010675;
C106266	increase	NPPA	ENSG00000175206	11762555;
C106276	increase	FN1	ENSG00000115414	12388107;
C106448	increase	ICAM1	ENSG00000090339	17517105;
C106448	increase	IL1B	ENSG00000125538	17517105;
C106448	increase	VCAM1	ENSG00000162692	17517105;
C106538	increase	CRP	ENSG00000132693	19891054;
C106538	increase	GFAP	ENSG00000131095	19358275;
C106538	increase	MMP9	ENSG00000100985	19891054;

C106538	increase	MPO	ENSG00000005381	19891054;
C106538	increase	SYP	ENSG00000102003	19358275;
C106538	increase	UCP1	ENSG00000109424	14518702;
C107058	decrease	CRP	ENSG00000132693	16616026;
C107135	decrease	CCND1	ENSG00000110092	16033851;
C107135	decrease	CCND2	ENSG00000118971	16033851;
C107448	decrease	BCL2	ENSG00000171791	16007152;
C107448	decrease	BCL2L1	ENSG00000171552	16007152;
C107594	increase	TNF	ENSG00000223952	17196940;
C107676	decrease	AKT1	ENSG00000142208	17015749;19139008;19336726;18593368;
C107676	decrease	BCL2	ENSG00000171791	19365708;
C107676	decrease	BCL2L1	ENSG00000171552	19365708;
C107676	decrease	CDK4	ENSG00000135446	19139008;19336726;
C107676	decrease	CDKN1A	ENSG00000124762	19139008;
C107676	decrease	EGFR	ENSG00000146648	19139008;
C107676	decrease	EIF4EBP1	ENSG00000187840	19139008;
C107676	decrease	EPAS1	ENSG00000116016	19336726;
C107676	decrease	IGF1R	ENSG00000140443	19139008;
C107676	decrease	KDR	ENSG00000128052	19139008;
C107676	decrease	MAP2K1	ENSG00000169032	19139008;19336726;
C107676	decrease	MAP2K2	ENSG00000126934	19336726;
C107676	decrease	MTOR	ENSG00000198793	19139008;
C107676	decrease	NCOA3	ENSG00000124151	19365708;
C107676	decrease	NOS3	ENSG00000164867	19336726;
C107676	decrease	NUP98	ENSG00000110713	17490514;
C107676	decrease	PDK1	ENSG00000152256	19139008;
C107676	decrease	PIK3CA	ENSG00000121879	19139008;
C107676	decrease	RB1	ENSG00000139687	15960896;
C107676	decrease	RELA	ENSG00000173039	17015749;19365708;
C107676	decrease	RPS6	ENSG00000137154	19139008;
C107676	decrease	RPS6KB1	ENSG00000108443	19139008;
C107676	decrease	XIAP	ENSG00000101966	17035597;
C107676	increase	BIRC5	ENSG00000089685	17035597;18593368;
C107676	increase	CASP7	ENSG00000165806	19139008;
C107676	increase	CCND1	ENSG00000110092	17015749;15960896;
C107676	increase	HIF1A	ENSG00000100644	17764071;19139008;18575727;
C107676	increase	ICAM1	ENSG00000090339	17015749;
C107676	increase	IGF1	ENSG00000017427	17764071;
C107676	increase	MMP9	ENSG00000100985	17015749;
C107676	increase	MYC	ENSG00000136997	17015749;
C107676	increase	PARP1	ENSG00000143799	17015749;19139008;17035597;
C107676	increase	PTGS2	ENSG00000073756	17015749;
C107676	increase	TNF	ENSG00000223952	17015749;
C107676	increase	VEGFA	ENSG00000112715	17764071;18575727;
C107773	affect	RELA	ENSG00000173039	18852136;
C107773	affect	SP1	ENSG00000185591	18852136;
C107773	decrease	BCL2	ENSG00000171791	18852136;
C107773	increase	SOD2	ENSG00000112096	18852136;
C107820	increase	HTR2C	ENSG00000147246	18083778;
C107875	decrease	RBL1	ENSG00000080839	15572027;
C107875	increase	CDKN1B	ENSG00000111276	15572027;

C107875	increase	RBL2	ENSG00000103479	15572027;
C108123	affect	ALDH1A3	ENSG00000184254	17526768;
C108123	decrease	MAPK1	ENSG00000100030	15961274;17492661;
C108123	decrease	MAPK3	ENSG00000102882	15961274;17492661;
C108123	decrease	RAF1	ENSG00000132155	17492661;
C108123	increase	CDKN1A	ENSG00000124762	15961274;
C108181	increase	IFNG	ENSG00000111537	17827067;
C108181	increase	IL2	ENSG00000109471	17827067;
C108373	decrease	DHCR24	ENSG00000116133	17636412;
C108373	decrease	KLK2	ENSG00000167751	17636412;
C108373	decrease	KLK3	ENSG00000142515	17636412;
C108373	decrease	SRD5A1	ENSG00000145545	16806904;
C108373	decrease	SRD5A2	ENSG00000049319	16806904;
C108373	increase	AKR1C1	ENSG00000187134	16806904;
C108373	increase	AKR1C2	ENSG00000151632	16806904;
C108373	increase	AKR1C3	ENSG00000196139	16806904;
C108373	increase	AR	ENSG00000169083	17636412;
C108373	increase	CCND1	ENSG00000110092	17636412;
C108373	increase	CYP11B2	ENSG00000179142	17636412;
C108373	increase	ERBB2	ENSG00000141736	17636412;
C108373	increase	HSD17B1	ENSG00000108786	17636412;
C108373	increase	HSD17B3	ENSG00000130948	17636412;
C108373	increase	SOS1	ENSG00000115904	17636412;
C108373	increase	VCAM1	ENSG00000162692	17636412;
C108475	increase	PON1	ENSG00000005421	15690306;
C108606	decrease	ABCA1	ENSG00000165029	16177187;
C108606	decrease	NPC1L1	ENSG00000015520	15679830;16177187;
C108606	decrease	NR1H2	ENSG00000131408	16177187;
C108606	decrease	RARG	ENSG00000172819	16177187;
C108606	decrease	SCARB1	ENSG00000073060	16177187;
C108606	decrease	SREBF1	ENSG00000072310	16177187;
C108606	decrease	SREBF2	ENSG00000198911	16177187;
C108732	increase	VDR	ENSG00000111424	15930183;
C109238	decrease	ACTA2	ENSG00000107796	17510436;
C109238	decrease	CCL1	ENSG00000108702	17510436;
C109238	decrease	CTGF	ENSG00000118523	17510436;
C109238	decrease	DCN	ENSG00000011465	17510436;
C109238	decrease	EDN3	ENSG00000124205	17510436;
C109238	decrease	EGF	ENSG00000138798	17510436;
C109238	decrease	FKBP5	ENSG00000096060	17510436;
C109238	decrease	GSTM1	ENSG00000134184	17510436;
C109238	decrease	IFNG	ENSG00000111537	16352669;
C109238	decrease	IGFBP5	ENSG00000115461	17510436;
C109238	decrease	KLK3	ENSG00000142515	17510436;
C109238	decrease	LMAN1L	ENSG00000140506	17510436;
C109238	decrease	MAF	ENSG00000178573	17510436;
C109238	decrease	MME	ENSG00000196549	17510436;
C109238	decrease	MT2A	ENSG00000125148	17510436;
C109238	decrease	MYBPC1	ENSG00000196091	17510436;
C109238	decrease	MYLK	ENSG00000251351	17510436;
C109238	decrease	NBL1	ENSG00000158747	17510436;
C109238	decrease	NCOA4	ENSG00000138293	17510436;
C109238	decrease	NDRG1	ENSG00000104419	17510436;
C109238	decrease	ORM2	ENSG00000228278	17510436;

C109238	decrease	PPAP2A	ENSG00000067113	17510436;
C109238	decrease	PSCA	ENSG00000167653	17510436;
C109238	decrease	SPON2	ENSG00000159674	17510436;
C109238	decrease	TBC1D25	ENSG00000068354	17510436;
C109238	decrease	TMPRSS2	ENSG00000184012	17510436;
C109238	increase	C6ORF136	ENSG00000224120	17510436;
C109238	increase	COG4	ENSG00000103051	17510436;
C109238	increase	CRISPLD1	ENSG00000121005	17510436;
C109238	increase	DMXL1	ENSG00000172869	17510436;
C109238	increase	ETS1	ENSG00000134954	17510436;
C109238	increase	FAM60A	ENSG00000139146	17510436;
C109238	increase	FOLH1	ENSG00000086205	17510436;
C109238	increase	HNRNPF	ENSG00000169813	17510436;
C109238	increase	HNRNPR	ENSG00000125944	17510436;
C109238	increase	HSP90AA1	ENSG00000080824	17510436;
C109238	increase	HSPD1	ENSG00000144381	17510436;
C109238	increase	MATR3	ENSG00000015479	17510436;
C109238	increase	PLA2G7	ENSG00000146070	17510436;
C109238	increase	PYGL	ENSG00000100504	17510436;
C109238	increase	RPS15A	ENSG00000134419	17510436;
C109238	increase	SCCPDH	ENSG00000143653	17510436;
C109238	increase	SFN	ENSG00000175793	17510436;
C109238	increase	SON	ENSG00000159140	17510436;
C109238	increase	TBCE	ENSG00000116957	17510436;
C109238	increase	TFF1	ENSG00000160182	17510436;
C109238	increase	TSPAN8	ENSG00000127324	17510436;
C109238	increase	ZFAND5	ENSG00000107372	17510436;
C109238	increase	ZNF292	ENSG00000188994	17510436;
C109628	increase	TGM2	ENSG00000198959	9516142;
C109695	increase	KLK3	ENSG00000142515	11580929;
C109695	increase	PGR	ENSG00000082175	11368622;
C109695	increase	TFF1	ENSG00000160182	11580929;11368622;
C109799	increase	CCL2	ENSG00000108691	12676771;
C109799	increase	CSF1	ENSG00000184371	12676771;
C109799	increase	CSF2	ENSG00000164400	12676771;
C109799	increase	CSF3	ENSG00000108342	12676771;
C109799	increase	IL1B	ENSG00000125538	12676771;
C109799	increase	IL8	ENSG00000169429	12676771;
C109799	increase	TNF	ENSG00000223952	12676771;
C109860	decrease	P2RY2	ENSG00000175591	10496880;
C109860	decrease	P2RY4	ENSG00000186912	10496880;
C109986	affect	BIRC3	ENSG00000023445	16154993;
C109986	affect	TNF	ENSG00000223952	16154993;
C110417	affect	TP53	ENSG00000141510	15781256;
C110417	increase	CDKN1A	ENSG00000124762	15781256;
C110630	increase	CCL2	ENSG00000108691	10218491;
C110630	increase	CNR2	ENSG00000188822	10688601;10617657;10218491;17558435;15115777;
C110630	increase	IL8	ENSG00000169429	10218491;15115777;
C110772	decrease	XIAP	ENSG00000101966	16054126;
C110772	increase	BAX	ENSG00000087088	16054126;
C110845	increase	ABCB1	ENSG00000085563	16047355;
C110881	decrease	BIRC5	ENSG00000089685	14988404;
C110881	decrease	CYP19A1	ENSG00000137869	10338361;

C110904	affect	TYMS	ENSG00000176890	16734730;16438929;
C110904	decrease	CDKN2A	ENSG00000147889	17904787;
C110904	decrease	PTGS2	ENSG00000073756	17904787;
C110904	increase	DPYD	ENSG00000188641	18846242;16734730;17611699;
C111237	affect	AGRN	ENSG00000188157	17330099;
C111237	affect	ANXA2	ENSG00000182718	17330099;
C111237	affect	ANXA4	ENSG00000196975	17330099;
C111237	affect	ANXA5	ENSG00000164111	17330099;
C111237	affect	ANXA6	ENSG00000197043	17330099;
C111237	affect	ASMTL	ENSG00000169093	17330099;
C111237	affect	ATP1B1	ENSG00000143153	17330099;
C111237	affect	CAPN2	ENSG00000162909	17330099;
C111237	affect	CASP7	ENSG00000165806	17330099;15897598;
C111237	affect	CD9	ENSG00000010278	17330099;
C111237	affect	CST3	ENSG00000101439	17330099;
C111237	affect	CTNNA1	ENSG00000044115	17330099;
C111237	affect	CTSH	ENSG00000103811	17330099;
C111237	affect	CTSZ	ENSG00000101160	17330099;
C111237	affect	EHD3	ENSG00000013016	17330099;
C111237	affect	ENO2	ENSG00000111674	17330099;
C111237	affect	FHL2	ENSG00000115641	17330099;
C111237	affect	FOXO1	ENSG00000150907	17330099;
C111237	affect	FXYD6	ENSG00000137726	17330099;
C111237	affect	GAS2L1	ENSG00000185340	17330099;
C111237	affect	GFI1	ENSG00000162676	17330099;
C111237	affect	H1F0	ENSG00000189060	17330099;
C111237	affect	HIST1H1C	ENSG00000187837	17330099;
C111237	affect	HSPB1	ENSG00000106211	17330099;
C111237	affect	ID2	ENSG00000115738	17330099;
C111237	affect	IFI30	ENSG00000216490	17330099;
C111237	affect	MX1	ENSG00000157601	17330099;
C111237	affect	PDE4B	ENSG00000184588	17330099;
C111237	affect	PSIP1	ENSG00000164985	17330099;
C111237	affect	PXDN	ENSG00000130508	17330099;
C111237	affect	RGS2	ENSG00000116741	17330099;
C111237	affect	S100A10	ENSG00000197747	17330099;
C111237	affect	SGK1	ENSG00000118515	17330099;
C111237	affect	SVIL	ENSG00000197321	17330099;
C111237	affect	SYNGR1	ENSG00000100321	17330099;
C111237	affect	TCEA2	ENSG00000171703	17330099;
C111237	affect	TUBB2A	ENSG00000137267	17330099;
C111237	decrease	AKT1	ENSG00000142208	15781658;16144943;
C111237	decrease	AR	ENSG00000169083	17218635;
C111237	decrease	BCL2A1	ENSG00000140379	15897598;
C111237	decrease	BCL2	ENSG00000171791	15897598;19631782;16144943;
C111237	decrease	BCL2L1	ENSG00000171552	12893773;15897598;16144943;
C111237	decrease	BID	ENSG00000015475	17410615;12893773;15897598;
C111237	decrease	BIRC5	ENSG00000089685	17410615;18156316;16144943;
C111237	decrease	CCNB1	ENSG00000134057	17417771;

C111237	decrease	CCND1	ENSG00000110092	17417771;12893773;17218635;17431121;
C111237	decrease	CFLAR	ENSG00000003402	12893773;15897598;
C111237	decrease	ERBB2	ENSG00000141736	17218635;16144943;
C111237	decrease	KLK2	ENSG00000167751	17218635;
C111237	decrease	KLK3	ENSG00000142515	17218635;
C111237	decrease	MAP2K1	ENSG00000169032	15781658;12893773;
C111237	decrease	MAPK1	ENSG00000100030	15781658;12893773;
C111237	decrease	MAPK3	ENSG00000102882	15781658;12893773;
C111237	decrease	MYC	ENSG00000136997	17417771;
C111237	decrease	NEDD4L	ENSG00000049759	17218635;
C111237	decrease	RAF1	ENSG00000132155	12893773;16144943;
C111237	decrease	TYMS	ENSG00000176890	17172411;
C111237	decrease	XIAP	ENSG00000101966	17410615;15964800;12893773;16144943;
C111237	increase	BAK1	ENSG00000030110	15781658;16144943;
C111237	increase	BAX	ENSG00000087088	15781658;15897598;
C111237	increase	BCL2L11	ENSG00000153094	16144943;
C111237	increase	BIRC2	ENSG00000110330	12893773;16377638;
C111237	increase	BIRC3	ENSG00000023445	12893773;16377638;15897598;
C111237	increase	CDH1	ENSG00000039068	17417771;
C111237	increase	CDKN1A	ENSG00000124762	17431121;12893773;17417771;17218635;14707268;15897598;16144943;
C111237	increase	CDKN1B	ENSG00000111276	17431121;16144943;
C111237	increase	CDKN1C	ENSG00000129757	17417771;
C111237	increase	CTNNB1	ENSG00000168036	17417771;
C111237	increase	CYP1B1	ENSG00000138061	15713371;
C111237	increase	RARA	ENSG00000131759	17417771;
C111237	increase	RARB	ENSG00000077092	16832676;
C111237	increase	RB1	ENSG00000139687	12893773;14707268;15897598;
C111237	increase	TNF	ENSG00000223952	16377638;
C111237	increase	TNFSF10	ENSG00000121858	17410615;
C111237	increase	TXNIP	ENSG00000117289	17218635;
C111238	decrease	BCL2A1	ENSG00000140379	15897598;
C111238	decrease	BCL2	ENSG00000171791	15897598;
C111238	decrease	BCL2L1	ENSG00000171552	15897598;
C111238	increase	BAX	ENSG00000087088	15897598;
C111238	increase	CDKN1A	ENSG00000124762	15897598;
C111238	increase	RB1	ENSG00000139687	15897598;
C111654	increase	IVL	ENSG00000163207	10469331;
C111654	increase	TGM1	ENSG00000092295	10469331;
C111914	increase	ITGA2B	ENSG00000005961	18404433;
C111914	increase	ITGB3	ENSG00000056345	18404433;
C111914	increase	SELP	ENSG00000174175	18404433;
C112035	decrease	ADIPOQ	ENSG00000181092	17668557;
C112035	decrease	LEP	ENSG00000174697	17668557;
C112035	increase	CCL2	ENSG00000108691	17668557;
C112035	increase	CCL3	ENSG00000006075	17668557;
C112035	increase	IL1B	ENSG00000125538	17668557;
C112035	increase	IL6	ENSG00000136244	17668557;
C112035	increase	TNF	ENSG00000223952	17668557;
C112106	decrease	TERT	ENSG00000164362	17273765;

C112765	decrease	AKT1	ENSG00000142208	17157164;
C112765	decrease	AR	ENSG00000169083	17010675;
C112765	decrease	CCND1	ENSG00000110092	17157164;
C112765	decrease	CDK4	ENSG00000135446	17157164;
C112765	decrease	CDK8	ENSG00000132964	17157164;
C112765	decrease	EGFR	ENSG00000146648	17010675;16061882;
C112765	decrease	ERBB2	ENSG00000141736	16061882;
C112765	decrease	FGFR3	ENSG00000068078	16061882;
C112765	decrease	FLT3	ENSG00000122025	17010675;
C112765	decrease	HSP90AA1	ENSG00000080824	18830594;
C112765	decrease	KIT	ENSG00000157404	16213582;16061882;
C112765	decrease	RAF1	ENSG00000132155	10564678;
C112765	decrease	TNFSF10	ENSG00000121858	15993848;
C112765	decrease	TP53	ENSG00000141510	10564678;
C112765	decrease	TYMS	ENSG00000176890	18830594;
C112765	decrease	XIAP	ENSG00000101966	15993848;
C112765	increase	HSPA4	ENSG00000170606	16311509;
C113580	affect	NEFM	ENSG00000104722	16149052;
C113580	decrease	ABCB1	ENSG00000085563	15526378;
C113580	decrease	ABCC1	ENSG00000103222	15526378;
C113580	decrease	BAD	ENSG00000002330	18508827;
C113580	decrease	CCND1	ENSG00000110092	15547725;14757120;15930308;
C113580	decrease	CCND3	ENSG00000112576	14757120;
C113580	decrease	CCNE1	ENSG00000105173	15930308;14757120;
C113580	decrease	CDH1	ENSG00000039068	17596522;
C113580	decrease	CDK2	ENSG00000123374	15930308;15547725;
C113580	decrease	CDK4	ENSG00000135446	15930308;
C113580	decrease	DAPK1	ENSG00000196730	19577553;
C113580	decrease	DKK1	ENSG00000107984	17320366;
C113580	decrease	HRH4	ENSG00000134489	15843518;
C113580	decrease	MVP	ENSG00000013364	15526378;
C113580	decrease	NFE2L2	ENSG00000116044	12446695;
C113580	increase	ACTA2	ENSG00000107796	17596522;
C113580	increase	APOA1	ENSG00000118137	19013290;
C113580	increase	AQP3	ENSG00000165272	18214481;16848764;
C113580	increase	ATP1A1	ENSG00000163399	12804770;
C113580	increase	BCL2	ENSG00000171791	18508827;
C113580	increase	BECN1	ENSG00000126581	19577553;
C113580	increase	BIRC3	ENSG00000023445	18508827;
C113580	increase	CCL11	ENSG00000172156	11980903;
C113580	increase	CCL2	ENSG00000108691	12016129;
C113580	increase	CDH2	ENSG00000170558	16149052;
C113580	increase	CDKN1A	ENSG00000124762	18508827;18006205;
C113580	increase	CDKN1B	ENSG00000111276	15930308;
C113580	increase	CDKN2C	ENSG00000123080	15930308;
C113580	increase	COL1A1	ENSG00000108821	15331595;
C113580	increase	CYP1A1	ENSG00000140465	15698582;
C113580	increase	CYP24A1	ENSG00000019186	15331595;
C113580	increase	DDIT3	ENSG00000175197	17341418;
C113580	increase	EGF	ENSG00000138798	15231676;18214481;16848764;16175315;15103026;
C113580	increase	EGR1	ENSG00000120738	18316600;17599376;
C113580	increase	EPHB6	ENSG00000106123	17320366;

C113580	increase	F3	ENSG00000117525	15371228;
C113580	increase	FGF10	ENSG00000070193	12804770;12574212;
C113580	increase	FGF2	ENSG00000138685	16289960;
C113580	increase	FOS	ENSG00000170345	12547826;
C113580	increase	GREM1	ENSG00000166923	17975199;
C113580	increase	HIF1A	ENSG00000100644	11504687;
C113580	increase	HMOX1	ENSG00000100292	18508827;
C113580	increase	HSPA5	ENSG00000044574	17341418;
C113580	increase	IFNG	ENSG00000111537	10952721;
C113580	increase	IL12A	ENSG00000168811	15843518;10952721;
C113580	increase	IL12B	ENSG00000113302	15843518;10952721;
C113580	increase	IL17A	ENSG00000112115	12016129;
C113580	increase	IL2	ENSG00000109471	18423386;
C113580	increase	IL6	ENSG00000136244	12016129;
C113580	increase	IL8	ENSG00000169429	12016129;17000667;15622447;
C113580	increase	ITGAM	ENSG00000169896	11980903;
C113580	increase	JUN	ENSG00000177606	12547826;
C113580	increase	MAP1LC3B	ENSG00000140941	19577553;
C113580	increase	MAPK1	ENSG00000100030	15231676;16149052;18508827;15901486;15955068;15888667;18468633;15557793;12574212;16251475;12016129;15177934;15919668;16211241;16175315;18486176;19577553;12547826;15456914;17254629;15563584;15930308;14757120;17003[illegible]
C113580	increase	MAPK3	ENSG00000102882	15231676;16149052;18508827;15901486;15955068;15888667;18468633;15557793;12574212;16251475;12016129;15177934;15919668;16211241;16175315;18423386;18486176;19577553;12547826;15456914;17254629;15563584;15930308;14757[illegible]
C113580	increase	NRP1	ENSG00000099250	16289960;
C113580	increase	PLAU	ENSG00000122861	15557793;
C113580	increase	PPARG	ENSG00000132170	17030193;18485432;
C113580	increase	SERPINE1	ENSG00000106366	17379280;11504687;
C113580	increase	SNAI1	ENSG00000124216	17596522;
C113580	increase	TAGLN	ENSG00000149591	17320366;
C113580	increase	THBS1	ENSG00000137801	18261746;
C113580	increase	VDR	ENSG00000111424	15331595;17088408;15879110;
C113580	increase	VEGFA	ENSG00000112715	19403854;18790786;
C113580	increase	WISP1	ENSG00000104415	17320366;
C113580	increase	WNT5A	ENSG00000114251	17320366;
C113642	decrease	ESR2	ENSG00000140009	16406510;
C113642	increase	ESR1	ENSG00000091831	16406510;
C115058	affect	AR	ENSG00000169083	12711008;
C115058	decrease	KLK3	ENSG00000142515	12711008;
C115058	decrease	NKX3-1	ENSG00000167034	12711008;
C115058	decrease	PMEPA1	ENSG00000124225	12711008;
C115058	increase	FOLH1	ENSG00000086205	12711008;

C115174	decrease	CCNE1	ENSG00000105173	14977855;
C115174	decrease	KLK3	ENSG00000142515	14977855;
C115174	increase	CD36	ENSG00000135218	14977855;
C115174	increase	CDH1	ENSG00000039068	14977855;
C115174	increase	CDKN1A	ENSG00000124762	14977855;
C115354	increase	BCL2A1	ENSG00000140379	15090542;
C115354	increase	BCL2	ENSG00000171791	15090542;
C115354	increase	BIRC2	ENSG00000110330	15090542;
C115354	increase	BIRC3	ENSG00000023445	15090542;
C115354	increase	BIRC5	ENSG00000089685	15090542;
C115354	increase	CCND1	ENSG00000110092	15090542;
C115354	increase	CFLAR	ENSG00000003402	15090542;
C115354	increase	MMP9	ENSG00000100985	15090542;
C115354	increase	PTGS2	ENSG00000073756	15090542;
C115354	increase	TNF	ENSG00000223952	15090542;
C115354	increase	TRAF1	ENSG00000056558	15090542;
C115354	increase	XIAP	ENSG00000101966	15090542;
C115461	affect	PTGS2	ENSG00000073756	16894348;
C115461	decrease	BIRC5	ENSG00000089685	17270149;16391822;
C115461	decrease	CCND1	ENSG00000110092	17270149;
C115461	decrease	CYP19A1	ENSG00000137869	15964185;
C115461	decrease	INSIG1	ENSG00000186480	14633654;
C115461	decrease	MAD2L1	ENSG00000164109	14633654;
C115461	decrease	MSX1	ENSG00000163132	14633654;
C115461	decrease	NRG1	ENSG00000157168	14633654;
C115461	decrease	XIAP	ENSG00000101966	16391822;
C115461	increase	ATF3	ENSG00000162772	14633654;15897233;
C115461	increase	CEBPB	ENSG00000172216	14633654;
C115461	increase	GDF15	ENSG00000130513	14633654;15509713;
C115461	increase	TMSB4X	ENSG00000205542	15292456;
C115703	decrease	CD74	ENSG00000019582	11046123;
C115703	decrease	ICAM1	ENSG00000090339	11046123;
C115703	decrease	SELE	ENSG00000007908	11046123;
C115703	decrease	VCAM1	ENSG00000162692	11046123;
C115711	decrease	CCND1	ENSG00000110092	15377668;
C116174	increase	HSF1	ENSG00000185122	11424089;
C116890	decrease	HIF1A	ENSG00000100644	15474452;
C116890	decrease	VEGFA	ENSG00000112715	15474452;
C116926	affect	ADAMTS8	ENSG00000134917	17070997;
C116926	affect	ALOX15B	ENSG00000179593	17070997;
C116926	affect	ANXA1	ENSG00000135046	17070997;
C116926	affect	CDH2	ENSG00000170558	17070997;
C116926	affect	CDSN	ENSG00000204539	17070997;
C116926	affect	CEACAM1	ENSG00000079385	17070997;
C116926	affect	CX3CL1	ENSG00000006210	17070997;
C116926	affect	CXCL1	ENSG00000163739	17070997;
C116926	affect	DSC1	ENSG00000134765	17070997;
C116926	affect	FAF1	ENSG00000185104	17070997;
C116926	affect	FRZB	ENSG00000162998	17070997;
C116926	affect	GPR110	ENSG00000153292	17070997;
C116926	affect	GPR126	ENSG00000112414	17070997;
C116926	affect	IL1A	ENSG00000115008	17070997;
C116926	affect	ITGBL1	ENSG00000198542	17070997;
C116926	affect	KLF12	ENSG00000118922	17070997;

C116926	affect	MEF2C	ENSG00000081189	17070997;
C116926	affect	MLLT4	ENSG00000249273	17070997;
C116926	affect	NOS1	ENSG00000089250	17070997;
C116926	affect	NQO1	ENSG00000181019	17070997;
C116926	affect	PLA2G2A	ENSG00000188257	17070997;
C116926	affect	POSTN	ENSG00000133110	17070997;
C116926	affect	PRRX1	ENSG00000116132	17070997;
C116926	affect	RASA1	ENSG00000145715	17070997;
C116926	affect	TFRC	ENSG00000072274	17070997;
C116926	affect	TRIM17	ENSG00000162931	17070997;
C116926	decrease	ANGPT1	ENSG00000154188	17070997;
C116926	decrease	ANGPTL1	ENSG00000116194	17070997;
C116926	decrease	ANXA9	ENSG00000143412	17070997;
C116926	decrease	AOX1	ENSG00000138356	17070997;
C116926	decrease	ARHGAP29	ENSG00000137962	17070997;
C116926	decrease	CACNA2D3	ENSG00000157445	17070997;
C116926	decrease	COL14A1	ENSG00000187955	17070997;
C116926	decrease	COLEC12	ENSG00000158270	17070997;
C116926	decrease	EPHA3	ENSG00000044524	17070997;
C116926	decrease	EPHB6	ENSG00000106123	17070997;
C116926	decrease	ETV5	ENSG00000244405	17070997;
C116926	decrease	FABP4	ENSG00000170323	17070997;
C116926	decrease	FAIM2	ENSG00000135472	17070997;
C116926	decrease	GATA3	ENSG00000107485	17070997;
C116926	decrease	HGF	ENSG00000019991	17070997;
C116926	decrease	HPGD	ENSG00000164120	17070997;
C116926	decrease	INSM1	ENSG00000173404	17070997;
C116926	decrease	LIFR	ENSG00000113594	17070997;
C116926	decrease	MAPRE2	ENSG00000166974	17070997;
C116926	decrease	MBTD1	ENSG00000011258	17070997;
C116926	decrease	MEOX2	ENSG00000106511	17070997;
C116926	decrease	MTA2	ENSG00000149480	17070997;
C116926	decrease	NAALADL2	ENSG00000177694	17070997;
C116926	decrease	NFASC	ENSG00000163531	17070997;
C116926	decrease	NPR2	ENSG00000159899	17070997;
C116926	decrease	PI4KA	ENSG00000249793	17070997;
C116926	decrease	PTGER3	ENSG00000050628	17070997;
C116926	decrease	PTGIS	ENSG00000124212	17175104;
C116926	decrease	PTGS1	ENSG00000095303	16141368;17175104;
C116926	decrease	RORC	ENSG00000143365	17070997;
C116926	decrease	SH2D1A	ENSG00000183918	17070997;
C116926	decrease	TIMP3	ENSG00000100234	16580899;
C116926	decrease	TSPAN2	ENSG00000134198	17070997;
C116926	decrease	VCAN	ENSG00000038427	17070997;
C116926	decrease	ZNF559	ENSG00000188321	17070997;
C116926	increase	ANXA3	ENSG00000138772	17070997;
C116926	increase	C3	ENSG00000125730	17070997;
C116926	increase	CCL2	ENSG00000108691	17070997;
C116926	increase	CDH3	ENSG00000062038	17070997;
C116926	increase	CLU	ENSG00000120885	17070997;
C116926	increase	CRISP3	ENSG00000096006	17070997;
C116926	increase	FLNB	ENSG00000136068	17070997;
C116926	increase	IL1RN	ENSG00000136689	17070997;
C116926	increase	IL6	ENSG00000136244	17070997;

C116926	increase	IL8	ENSG00000169429	16580899;
C116926	increase	LMX1A	ENSG00000162761	17070997;
C116926	increase	MAL	ENSG00000172005	17070997;
C116926	increase	MMP1	ENSG00000196611	16580899;
C116926	increase	MMP3	ENSG00000149968	16580899;
C116926	increase	NPAS3	ENSG00000151322	17070997;
C116926	increase	PLAT	ENSG00000104368	16580899;
C116926	increase	PML	ENSG00000140464	17070997;
C116926	increase	PTGS2	ENSG00000073756	16678543;15705740;17164136;17070997;
C116926	increase	RNF144B	ENSG00000137393	17070997;
C116926	increase	SCD	ENSG00000099194	17070997;
C116926	increase	SERPINA3	ENSG00000196136	17070997;
C116926	increase	SOCS3	ENSG00000184557	17070997;
C116926	increase	SOD2	ENSG00000112096	17070997;
C116926	increase	STAT3	ENSG00000168610	17070997;
C116926	increase	TAC1	ENSG00000006128	16449370;
C116926	increase	TEAD4	ENSG00000197905	17070997;
C116926	increase	TMPRSS4	ENSG00000137648	17070997;
C116926	increase	TNC	ENSG00000041982	17070997;
C117155	decrease	BCL2	ENSG00000171791	16039551;
C117155	increase	BAX	ENSG00000087088	16039551;
C117155	increase	TNF	ENSG00000223952	16134060;
C117342	increase	SFTPA1	ENSG00000122852	14617519;
C117342	increase	SFTPA2	ENSG00000185303	14617519;
C117342	increase	TNF	ENSG00000223952	14617519;
C117446	decrease	F3	ENSG00000117525	20200314;
C117446	increase	ITGA2B	ENSG00000005961	18404433;
C117446	increase	ITGB3	ENSG00000056345	18404433;
C117446	increase	SELP	ENSG00000174175	18404433;
C118050	increase	CYP3A4	ENSG00000160868	16565514;
C118180	decrease	CCNB1	ENSG00000134057	11774253;
C118180	increase	CCNA2	ENSG00000145386	11774253;
C118180	increase	TP53	ENSG00000141510	11774253;
C118258	decrease	BAD	ENSG00000002330	18508827;12970779;
C118258	decrease	BCL2A1	ENSG00000140379	12466137;
C118258	decrease	BIRC2	ENSG00000110330	12970779;
C118258	decrease	CFLAR	ENSG00000003402	12970779;
C118258	increase	BAX	ENSG00000087088	12466137;
C118258	increase	BCL2	ENSG00000171791	18508827;12466137;
C118258	increase	BIRC3	ENSG00000023445	18508827;12466137;12970779;
C118258	increase	CDKN1A	ENSG00000124762	18508827;
C118258	increase	FN1	ENSG00000115414	12388107;
C118258	increase	GSTA1	ENSG00000243955	15090468;
C118258	increase	HMOX1	ENSG00000100292	18508827;
C118258	increase	IL6	ENSG00000136244	19074641;
C118258	increase	IL8	ENSG00000169429	19074641;
C118258	increase	TNF	ENSG00000223952	11753679;
C118258	increase	TNFRSF21	ENSG00000146072	11753679;
C118559	decrease	BCL2L1	ENSG00000171552	16815550;
C118559	increase	BDNF	ENSG00000176697	19189864;
C118580	increase	TFF1	ENSG00000160182	16183391;
C118739	affect	MAGEA9	ENSG00000166008	17290406;

C118739	decrease	CCNA2	ENSG00000145386	15059916;
C118739	decrease	CCND1	ENSG00000110092	15059916;
C118739	decrease	CCNE1	ENSG00000105173	15059916;
C118739	decrease	CDKN1B	ENSG00000111276	15059916;
C118739	increase	CDKN1A	ENSG00000124762	18223691;
C118739	increase	MCL1	ENSG00000143384	15059916;
C118739	increase	XIAP	ENSG00000101966	15964800;15059916;
C118852	increase	NOS2	ENSG00000007171	19497413;
C119130	affect	BAX	ENSG00000087088	12203115;
C119130	decrease	BAD	ENSG00000002330	17409426;
C119130	decrease	BIRC3	ENSG00000023445	12837940;
C119130	decrease	CDKN1B	ENSG00000111276	16322342;
C119130	decrease	PTGS2	ENSG00000073756	17409426;16322342;
C119130	decrease	YWHAE	ENSG00000108953	17409426;
C119130	increase	TNFRSF10B	ENSG00000120889	12203115;
C119141	increase	TNFRSF11B	ENSG00000164761	17420779;
C119222	increase	ESRRA	ENSG00000173153	16024613;
C119222	increase	IL13	ENSG00000169194	12789233;
C119222	increase	IL4	ENSG00000113520	12789233;
C119222	increase	IL5	ENSG00000113525	12789233;
C119222	increase	IL6	ENSG00000136244	12789233;
C119222	increase	IL8	ENSG00000169429	12789233;
C119222	increase	TFF1	ENSG00000160182	16024613;
C119536	decrease	UBE2A	ENSG00000077721	16870173;
C119536	increase	UBE2E1	ENSG00000170142	16870173;
C119543	increase	BACE1	ENSG00000186318	14586007;
C119543	increase	IFNG	ENSG00000111537	14586007;
C119543	increase	TNF	ENSG00000223952	14586007;
C119620	increase	ABCB1	ENSG00000085563	18624906;
C119737	decrease	IL6	ENSG00000136244	16019205;
C119737	increase	ALPL	ENSG00000162551	16019205;12650720;
C119737	increase	CYP1A2	ENSG00000140505	15672752;
C119737	increase	PGR	ENSG00000082175	16076101;
C119737	increase	TFF1	ENSG00000160182	16019205;
C119737	increase	VWF	ENSG00000110799	16019205;
C120029	increase	CASP2	ENSG00000106144	14757846;
C120129	increase	CYP11A1	ENSG00000140459	16216300;
C120129	increase	CYP11B1	ENSG00000160882	16216300;
C120129	increase	CYP11B2	ENSG00000179142	16216300;
C120129	increase	CYP19A1	ENSG00000137869	16216300;
C120129	increase	CYP21A2	ENSG00000231852	16216300;
C120129	increase	HSD17B1	ENSG00000108786	16216300;
C120129	increase	HSD3B1	ENSG00000203857	16216300;
C120129	increase	HSD3B2	ENSG00000203859	16216300;
C120227	increase	CD38	ENSG00000004468	17077328;
C120227	increase	ITGAM	ENSG00000169896	17077328;
C120227	increase	RARB	ENSG00000077092	17077328;
C120227	increase	TP53AIP1	ENSG00000120471	15031205;16467208;
C120227	increase	TP73	ENSG00000078900	16467208;15031205;
C120275	affect	TP53	ENSG00000141510	12869419;12807743;
C120275	decrease	CCNA2	ENSG00000145386	11468187;
C120275	decrease	CCNB1	ENSG00000134057	11468187;
C120275	decrease	CCND1	ENSG00000110092	11468187;
C120275	decrease	CCND2	ENSG00000118971	11468187;

C120275	decrease	CCND3	ENSG00000112576	11468187;
C120275	decrease	CDK2	ENSG00000123374	17631934;11468187;
C120275	increase	CCNE1	ENSG00000105173	11468187;
C120275	increase	CDKN1A	ENSG00000124762	11468187;12869419;12807743;
C120275	increase	GADD45A	ENSG00000116717	11468187;
C120275	increase	TFRC	ENSG00000072274	12807743;
C120508	increase	DDIT3	ENSG00000175197	19040731;
C120508	increase	GADD45A	ENSG00000116717	19040731;
C120508	increase	PPP1R15A	ENSG00000087074	19040731;
C120508	increase	SESN2	ENSG00000130766	19040731;
C120508	increase	TNFRSF10B	ENSG00000120889	19040731;
C120793	decrease	TP53	ENSG00000141510	16055726;
C121153	increase	CD36	ENSG00000135218	17360047;
C121329	decrease	IL12A	ENSG00000168811	12847216;
C121329	decrease	IL12B	ENSG00000113302	12847216;
C121329	decrease	IL23A	ENSG00000110944	12847216;
C121345	decrease	IL5	ENSG00000113525	15061398;
C121345	decrease	IL8	ENSG00000169429	15061398;
C121479	increase	TFF1	ENSG00000160182	16183391;
C121565	decrease	BAX	ENSG00000087088	16357363;16946128;15547111;12893085;
C121565	decrease	BCL2	ENSG00000171791	18516295;15547111;
C121565	decrease	DNMT1	ENSG00000130816	15547111;
C121565	decrease	DNMT3A	ENSG00000119772	15547111;
C121565	decrease	EDN1	ENSG00000078401	19098008;
C121565	decrease	EDNRB	ENSG00000136160	19098008;
C121565	decrease	KIT	ENSG00000157404	19098008;
C121565	decrease	KITLG	ENSG00000049130	19098008;
C121565	decrease	MC1R	ENSG00000198211	19098008;
C121565	decrease	MITF	ENSG00000187098	19098008;
C121565	decrease	MMP1	ENSG00000196611	18557930;
C121565	decrease	TYR	ENSG00000077498	19098008;
C121565	increase	CCNB1	ENSG00000134057	18516295;15547111;16620711;
C121565	increase	CDK1	ENSG00000170312	18516295;16620711;
C121565	increase	CDKN1A	ENSG00000124762	15547111;18516295;18223691;12893085;
C121565	increase	CYP1A1	ENSG00000140465	16258175;15843497;15698582;
C121565	increase	CYP1A2	ENSG00000140505	16258175;
C121565	increase	CYP1B1	ENSG00000138061	16258175;
C121565	increase	FAS	ENSG00000026103	11779855;
C121565	increase	GADD45A	ENSG00000116717	15547111;
C121565	increase	PLK1	ENSG00000166851	18516295;
C121565	increase	TP53	ENSG00000141510	16170029;16357363;15547111;16258175;19098008;16467109;16814113;16777994;12893085;
C121616	increase	DDIT3	ENSG00000175197	16972258;
C121616	increase	HMOX1	ENSG00000100292	16972258;
C122062	increase	CYP1A1	ENSG00000140465	15355884;11752201;17674193;
C122062	increase	CYP1B1	ENSG00000138061	17674193;11752201;
C400082	decrease	AKT1	ENSG00000142208	15781649;

C400082	decrease	BCL2	ENSG00000171791	15781649;16024631;16022909;
C400082	decrease	BCL2L1	ENSG00000171552	15781649;12893773;16024631;15543232;
C400082	decrease	BID	ENSG00000015475	12893773;
C400082	decrease	BIRC2	ENSG00000110330	12893773;15781649;16024631;15543232;
C400082	decrease	BIRC5	ENSG00000089685	16373703;
C400082	decrease	CA9	ENSG00000107159	16061869;
C400082	decrease	CCL3	ENSG00000006075	18089816;
C400082	decrease	CCND1	ENSG00000110092	12893773;15781649;
C400082	decrease	MAP2K1	ENSG00000169032	12893773;
C400082	decrease	MAPK1	ENSG00000100030	12893773;
C400082	decrease	MAPK3	ENSG00000102882	12893773;
C400082	decrease	MKI67	ENSG00000148773	19372569;
C400082	decrease	PTHLH	ENSG00000087494	18089816;
C400082	decrease	RAF1	ENSG00000132155	12893773;
C400082	decrease	SFN	ENSG00000175793	16373703;
C400082	decrease	VEGFA	ENSG00000112715	19372569;
C400082	decrease	XIAP	ENSG00000101966	12893773;16024631;15543232;
C400082	increase	BCL2L11	ENSG00000153094	16024631;
C400082	increase	CDKN1A	ENSG00000124762	12893773;15543232;
C400082	increase	DDIT3	ENSG00000175197	17709599;16024631;
C400082	increase	HIF1A	ENSG00000100644	16061869;
C400082	increase	HSPA5	ENSG00000044574	16024631;
C400082	increase	MCL1	ENSG00000143384	12893773;
C400082	increase	PMAIP1	ENSG00000141682	16024631;
C400184	increase	AGTR1	ENSG00000144891	16386258;
C400184	increase	ALOX5	ENSG00000012779	16386258;
C400184	increase	CCR2	ENSG00000121807	16386258;
C400184	increase	CD68	ENSG00000129226	16386258;
C400184	increase	CRP	ENSG00000132693	16386258;
C400184	increase	IL1A	ENSG00000115008	16386258;
C400184	increase	IL1B	ENSG00000125538	16386258;
C400184	increase	IL8	ENSG00000169429	16386258;
C400184	increase	OLR1	ENSG00000173391	16386258;
C400184	increase	PTGS1	ENSG00000095303	16386258;
C400184	increase	PTGS2	ENSG00000073756	16386258;
C400184	increase	SELE	ENSG00000007908	16386258;
C400184	increase	TIMP4	ENSG00000157150	16386258;
C400184	increase	TNF	ENSG00000223952	16386258;
C400278	increase	CCND1	ENSG00000110092	19168569;
C401858	decrease	ESR1	ENSG00000091831	17033922;
C401859	decrease	CCND1	ENSG00000110092	16954435;
C401859	decrease	RB1	ENSG00000139687	16954435;
C402365	increase	IFNG	ENSG00000111537	18490488;
C402365	increase	IL10	ENSG00000136634	18490488;
C402365	increase	IL12A	ENSG00000168811	18490488;
C402365	increase	IL12B	ENSG00000113302	18490488;
C402365	increase	IL23A	ENSG00000110944	18490488;
C402665	decrease	BIRC3	ENSG00000023445	15155752;
C402665	decrease	PDIA6	ENSG00000143870	15155752;
C402665	decrease	PODXL	ENSG00000128567	15155752;

C402665	decrease	RNASE3	ENSG00000169397	15155752;
C402665	decrease	TAX1BP3	ENSG00000213977	15155752;
C402665	increase	ATF3	ENSG00000162772	15155752;
C402665	increase	CEBPB	ENSG00000172216	15155752;
C402665	increase	COL1A2	ENSG00000164692	15155752;
C402665	increase	CYR61	ENSG00000142871	15155752;
C402665	increase	DUSP6	ENSG00000139318	15155752;
C402665	increase	EGR1	ENSG00000120738	15155752;
C402665	increase	EPHA2	ENSG00000142627	15155752;
C402665	increase	FGFR1OP	ENSG00000213066	15155752;
C402665	increase	FOS	ENSG00000170345	15155752;
C402665	increase	GADD45A	ENSG00000116717	15155752;
C402665	increase	GDF15	ENSG00000130513	15155752;
C402665	increase	GNA11	ENSG00000088256	15155752;
C402665	increase	HAS2	ENSG00000170961	15155752;
C402665	increase	HSP90AA1	ENSG00000080824	15155752;
C402665	increase	HSPA1B	ENSG00000232804	15155752;
C402665	increase	IL2RB	ENSG00000100385	15155752;
C402665	increase	JUNB	ENSG00000171223	15155752;
C402665	increase	JUN	ENSG00000177606	15155752;
C402665	increase	MYC	ENSG00000136997	15155752;
C402665	increase	RASA1	ENSG00000145715	15155752;
C402665	increase	TFPI2	ENSG00000105825	15155752;
C402665	increase	TNFRSF10C	ENSG00000173535	15155752;
C402665	increase	VASP	ENSG00000125753	15155752;
C402665	increase	VEGFC	ENSG00000150630	15155752;
C402665	increase	ZFP36	ENSG00000128016	15155752;
C402892	increase	CYP24A1	ENSG00000019186	16289102;
C402892	increase	RARB	ENSG00000077092	16289102;
C403304	decrease	BCL2A1	ENSG00000140379	16007145;
C403304	decrease	BCL2	ENSG00000171791	16007145;
C403304	decrease	BCL2L1	ENSG00000171552	16007145;
C403304	decrease	BIRC2	ENSG00000110330	16007145;
C403304	decrease	BIRC3	ENSG00000023445	16007145;
C403304	decrease	BIRC5	ENSG00000089685	16007145;
C403304	decrease	CCND1	ENSG00000110092	16007145;
C403304	decrease	CFLAR	ENSG00000003402	16007145;
C403304	decrease	ICAM1	ENSG00000090339	16007145;
C403304	decrease	MMP9	ENSG00000100985	16007145;
C403304	decrease	MYC	ENSG00000136997	16007145;
C403304	decrease	PTGS2	ENSG00000073756	16007145;
C403304	decrease	TRAF1	ENSG00000056558	16007145;
C403304	decrease	XIAP	ENSG00000101966	16007145;
C403899	decrease	CCNA2	ENSG00000145386	15202011;
C403899	decrease	CCND1	ENSG00000110092	15202011;
C403899	decrease	CCNE1	ENSG00000105173	15202011;
C403899	decrease	CDK2	ENSG00000123374	15202011;
C403899	decrease	CDK4	ENSG00000135446	15202011;
C403899	decrease	CDK6	ENSG00000105810	15202011;
C403899	decrease	RB1	ENSG00000139687	15202011;
C403899	increase	CDKN1A	ENSG00000124762	15202011;
C403900	decrease	CCNA2	ENSG00000145386	15202011;
C403900	decrease	CCND1	ENSG00000110092	15202011;
C403900	decrease	CCNE1	ENSG00000105173	12969788;15202011;

C403900	decrease	CDK2	ENSG00000123374	12969788;15202011;
C403900	decrease	CDK4	ENSG00000135446	15202011;
C403900	decrease	CDK6	ENSG00000105810	15202011;
C403900	decrease	RB1	ENSG00000139687	15202011;
C403900	increase	CDKN1A	ENSG00000124762	12969788;15202011;
C404397	decrease	AKT1	ENSG00000142208	14647418;
C404397	decrease	CCND1	ENSG00000110092	14647418;
C404397	decrease	RAF1	ENSG00000132155	14647418;
C404743	decrease	BCL2	ENSG00000171791	16391850;
C404743	decrease	BCL2L1	ENSG00000171552	16391850;
C404743	increase	BAX	ENSG00000087088	16391850;
C405346	decrease	FGF23	ENSG00000118972	16076378;
C405354	decrease	BCL2	ENSG00000171791	16489027;
C405354	decrease	BCL2L1	ENSG00000171552	16489027;
C405354	increase	BAX	ENSG00000087088	16489027;
C405354	increase	TNFSF10	ENSG00000121858	16489027;
C405603	increase	PON1	ENSG00000005421	14767865;
C405904	increase	IL8	ENSG00000169429	17366569;
C406082	decrease	AKAP5	ENSG00000179841	16507463;
C406082	decrease	C1QTNF6	ENSG00000133466	18448484;
C406082	decrease	CCL2	ENSG00000108691	16507463;
C406082	decrease	CLK3	ENSG00000179335	16507463;
C406082	decrease	COL1A1	ENSG00000108821	16507463;
C406082	decrease	FAM83A	ENSG00000147689	18448484;
C406082	decrease	GATA6	ENSG00000141448	16507463;
C406082	decrease	GJA1	ENSG00000152661	16507463;
C406082	decrease	HTRA1	ENSG00000166033	16507463;
C406082	decrease	IGFBP5	ENSG00000115461	16507463;
C406082	decrease	ITGB3	ENSG00000056345	16507463;
C406082	decrease	MMP2	ENSG00000087245	16507463;
C406082	decrease	MYCL1	ENSG00000116990	16507463;
C406082	decrease	MYLK	ENSG00000251351	16507463;
C406082	decrease	NEFL	ENSG00000104725	18448484;
C406082	decrease	NUP88	ENSG00000108559	16507463;
C406082	decrease	PRKCA	ENSG00000154229	16507463;
C406082	decrease	ZSCAN12	ENSG00000158691	18448484;
C406082	increase	IL1R2	ENSG00000115590	16507463;
C406734	decrease	TOP2A	ENSG00000131747	17089011;
C407502	decrease	IVL	ENSG00000163207	10769631;
C408162	decrease	BCL2	ENSG00000171791	15867202;11095261;
C408604	increase	CYP1A1	ENSG00000140465	15698582;
C408604	increase	FOS	ENSG00000170345	12547826;
C408604	increase	PLD1	ENSG00000075651	17640750;
C408604	increase	PLD2	ENSG00000129219	17640750;
C408604	increase	PTGS2	ENSG00000073756	17640750;
C408982	increase	HSPA1B	ENSG00000232804	17046822;
C408982	increase	MYD88	ENSG00000172936	17046822;
C408982	increase	TLR9	ENSG00000239732	17046822;
C410026	decrease	CCND1	ENSG00000110092	15377668;
C410127	affect	CDKN1A	ENSG00000124762	12473173;
C410127	increase	BRCA1	ENSG00000012048	12473173;
C410216	decrease	AKT1	ENSG00000142208	17918158;
C410216	decrease	EGFR	ENSG00000146648	17918158;
C410216	decrease	ERBB2	ENSG00000141736	17918158;

C410216	decrease	ERBB3	ENSG00000065361	17918158;
C410228	increase	CALB1	ENSG00000104327	15922086;
C410228	increase	CD14	ENSG00000170458	14729647;
C410228	increase	CEBPB	ENSG00000172216	14729647;
C410228	increase	RB1	ENSG00000139687	14729647;
C410228	increase	VDR	ENSG00000111424	15922086;
C410666	decrease	IL10	ENSG00000136634	17185614;
C410666	decrease	TNF	ENSG00000223952	17185614;
C410666	increase	ACO1	ENSG00000122729	17185614;
C410666	increase	CPT1A	ENSG00000110090	17185614;
C410666	increase	CPT2	ENSG00000157184	17185614;
C410666	increase	IL2	ENSG00000109471	17185614;
C410666	increase	IL4	ENSG00000113520	17185614;
C410733	affect	ANAPC1	ENSG00000153107	18813790;
C410733	affect	ANLN	ENSG00000011426	18813790;
C410733	affect	CAV1	ENSG00000105974	18813790;
C410733	affect	CCNB2	ENSG00000157456	18813790;
C410733	affect	CCNE2	ENSG00000175305	18813790;
C410733	affect	CCNJ	ENSG00000107443	18813790;
C410733	affect	CD82	ENSG00000085117	18813790;
C410733	affect	CDC6	ENSG00000094804	18813790;
C410733	affect	CDK2AP1	ENSG00000111328	18813790;
C410733	affect	CDK3	ENSG00000250506	18813790;
C410733	affect	CDKN2A	ENSG00000147889	18813790;
C410733	affect	CDKN2D	ENSG00000129355	18813790;
C410733	affect	CETN2	ENSG00000147400	18813790;
C410733	affect	CKS1B	ENSG00000173207	18813790;
C410733	affect	CST3	ENSG00000101439	18813790;
C410733	affect	CTNNB1	ENSG00000168036	18813790;
C410733	affect	CTSD	ENSG00000117984	18813790;
C410733	affect	CTSO	ENSG00000151792	18813790;
C410733	affect	DUSP6	ENSG00000139318	18813790;
C410733	affect	E2F3	ENSG00000112242	18813790;
C410733	affect	EIF4G2	ENSG00000110321	18813790;
C410733	affect	EZR	ENSG00000092820	18813790;
C410733	affect	F2R	ENSG00000181104	18813790;
C410733	affect	FLT4	ENSG00000037280	18813790;
C410733	affect	GAS1	ENSG00000180447	18813790;
C410733	affect	ITGA6	ENSG00000091409	18813790;
C410733	affect	ITGB1	ENSG00000150093	18813790;
C410733	affect	KIF11	ENSG00000138160	18813790;
C410733	affect	MMP13	ENSG00000137745	18813790;
C410733	affect	MOS	ENSG00000172680	18813790;
C410733	affect	MTSS1	ENSG00000170873	18813790;
C410733	affect	NCAPD2	ENSG00000010292	18813790;
C410733	affect	NCAPG	ENSG00000109805	18813790;
C410733	affect	NDC80	ENSG00000080986	18813790;
C410733	affect	NPM1	ENSG00000181163	18813790;
C410733	affect	NR4A3	ENSG00000119508	18813790;
C410733	affect	NRG1	ENSG00000157168	18813790;
C410733	affect	NRP2	ENSG00000118257	18813790;
C410733	affect	RAD51L1	ENSG00000182185	18813790;
C410733	affect	RGS2	ENSG00000116741	18813790;
C410733	affect	3-Sep	ENSG00000100167	18813790;

C410733	affect	SERPINE1	ENSG00000106366	18813790;
C410733	affect	SESN3	ENSG00000149212	18813790;
C410733	affect	STAG2	ENSG00000101972	18813790;
C410733	affect	SYK	ENSG00000165025	18813790;
C410733	affect	TGFA	ENSG00000163235	18813790;
C410733	affect	THBS1	ENSG00000137801	18813790;
C410733	affect	TIMP2	ENSG00000035862	18813790;
C410733	affect	TIMP4	ENSG00000157150	18813790;
C410733	affect	TPTE2	ENSG00000132958	18813790;
C411007	increase	HMOX1	ENSG00000100292	17420286;
C411007	increase	KIT	ENSG00000157404	17420286;
C411652	affect	MDM2	ENSG00000135679	16027726;
C411652	affect	TP53	ENSG00000141510	16027726;
C411652	increase	CSNK1D	ENSG00000141551	16027726;
C411652	increase	CSNK1E	ENSG00000213923	16027726;
C411675	decrease	SPHK1	ENSG00000176170	16281067;
C411675	decrease	XIAP	ENSG00000101966	16281067;
C412373	decrease	TYMS	ENSG00000176890	18794807;
C412373	increase	BIRC3	ENSG00000023445	17671207;
C412373	increase	BMP2	ENSG00000125845	17513867;
C412373	increase	FOS	ENSG00000170345	15090535;
C412373	increase	HOXA7	ENSG00000122592	19438726;
C412373	increase	IL8	ENSG00000169429	15831558;20181660;
C412373	increase	MIF	ENSG00000240972	16872482;
C412373	increase	MLL	ENSG00000118058	19438726;
C412373	increase	MMP2	ENSG00000087245	16872482;
C412373	increase	VDR	ENSG00000111424	15930183;
C412373	increase	VEGFA	ENSG00000112715	19403854;18790786;
C412815	decrease	PON1	ENSG00000005421	16269825;
C412815	decrease	SREBF1	ENSG00000072310	16269825;
C412815	increase	ABCB11	ENSG00000073734	15307955;12519787;12525500;15911693;
C412815	increase	ABCB4	ENSG00000005471	14527955;14623915;
C412815	increase	FGF19	ENSG00000162344	16269825;
C412815	increase	KNG1	ENSG00000113889	15307955;
C412815	increase	NR0B2	ENSG00000131910	16269825;
C412815	increase	NR1H4	ENSG00000012504	12519787;12525500;14684751;14527955;17567710;
C412815	increase	VIPR1	ENSG00000114812	16037943;
C412963	decrease	PTGS2	ENSG00000073756	10920275;
C413408	increase	CCL2	ENSG00000108691	17668557;
C413408	increase	CCL3	ENSG00000006075	17668557;
C414690	decrease	CDH1	ENSG00000039068	17596522;
C414690	increase	ACTA2	ENSG00000107796	17596522;
C414690	increase	SNAI1	ENSG00000124216	17596522;
C415032	decrease	BIRC5	ENSG00000089685	16166596;
C415032	decrease	MCL1	ENSG00000143384	16166596;
C416282	increase	BCL2	ENSG00000171791	16407826;
C416282	increase	BCL2L1	ENSG00000171552	16407826;
C416282	increase	CD14	ENSG00000170458	18180316;
C416282	increase	PTGS2	ENSG00000073756	18480072;
C416282	increase	RNF34	ENSG00000170633	16407826;
C416531	affect	BAX	ENSG00000087088	16003723;
C416531	decrease	BCL2	ENSG00000171791	16003723;

C416531	increase	ABCB1	ENSG00000085563	16051478;
C417207	increase	IL8	ENSG00000169429	16611624;
C417207	increase	MMP3	ENSG00000149968	16778083;
C417207	increase	SRC	ENSG00000197122	16611624;
C417521	affect	CCND3	ENSG00000112576	16024653;
C417521	decrease	CCND1	ENSG00000110092	16024653;
C417521	increase	CDKN1A	ENSG00000124762	15958644;
C417521	increase	CTNNB1	ENSG00000168036	16968065;16968061;
C417521	increase	TP53	ENSG00000141510	15958644;17210701;
C418365	decrease	F10	ENSG00000126218	14561536;
C418365	decrease	F7	ENSG00000057593	14561536;
C418365	decrease	PROC	ENSG00000115718	14561536;
C418365	decrease	SERPINC1	ENSG00000117601	14561536;
C419708	decrease	ADORA1	ENSG00000163485	16685379;
C419708	decrease	CDKN1B	ENSG00000111276	16322342;
C419708	decrease	CYP2F1	ENSG00000197446	16685379;
C419708	decrease	DUSP9	ENSG00000130829	16685379;
C419708	decrease	E2F1	ENSG00000101412	18347146;
C419708	decrease	GADD45G	ENSG00000130222	16685379;
C419708	decrease	GNB2	ENSG00000172354	16685379;
C419708	decrease	GUCY2D	ENSG00000132518	16685379;
C419708	decrease	IFI6	ENSG00000126709	16685379;
C419708	decrease	LEPR	ENSG00000116678	16685379;
C419708	decrease	MLH1	ENSG00000076242	16685379;
C419708	decrease	NFKB1	ENSG00000109320	16685379;
C419708	decrease	NPTX2	ENSG00000106236	16685379;
C419708	decrease	NRL	ENSG00000129535	16685379;
C419708	decrease	PTGS2	ENSG00000073756	16322342;
C419708	decrease	SFN	ENSG00000175793	16685379;
C419708	decrease	TNFRSF1B	ENSG00000028137	16685379;
C419708	decrease	TYMS	ENSG00000176890	18347146;
C419708	increase	AREG	ENSG00000205595	15496427;16230376;15723263;
C419708	increase	CGRRF1	ENSG00000100532	16685379;
C419708	increase	EPOR	ENSG00000187266	16685379;
C419708	increase	EPS15	ENSG00000085832	16685379;
C419708	increase	FGF6	ENSG00000111241	16685379;
C419708	increase	GADD45A	ENSG00000116717	16685379;
C419708	increase	GARS	ENSG00000106105	16685379;
C419708	increase	HBEGF	ENSG00000113070	15723263;
C419708	increase	IL8	ENSG00000169429	15723263;
C419708	increase	QSOX1	ENSG00000116260	16685379;
C419708	increase	RPA1	ENSG00000132383	16685379;
C419708	increase	SKI	ENSG00000157933	16685379;
C420268	decrease	IL8	ENSG00000169429	15956251;
C420268	decrease	VEGFA	ENSG00000112715	15956251;
C421458	decrease	TFRC	ENSG00000072274	17257079;
C422175	decrease	CD1A	ENSG00000158477	17475839;
C422175	decrease	CD36	ENSG00000135218	17475839;
C422175	increase	CD80	ENSG00000121594	17475839;
C422175	increase	CD86	ENSG00000114013	17475839;
C422648	decrease	NRF1	ENSG00000106459	16579640;
C422648	decrease	SDHC	ENSG00000143252	16579640;
C422648	decrease	TFF1	ENSG00000160182	16579640;

C422648	increase	ABCB1	ENSG00000085563	16579640;
C422648	increase	APAF1	ENSG00000120868	15939500;
C422648	increase	ATP6V0E1	ENSG00000113732	16579640;
C422648	increase	BAX	ENSG00000087088	15939500;
C422648	increase	BCL2	ENSG00000171791	15939500;
C422648	increase	BCL2L1	ENSG00000171552	15939500;
C422648	increase	CASP3	ENSG00000164305	15939500;
C422648	increase	CASP9	ENSG00000132906	15939500;
C422648	increase	DNAJA1	ENSG00000086061	16579640;
C422648	increase	HSPA8	ENSG00000109971	16579640;
C422648	increase	MT1G	ENSG00000125144	16579640;
C422648	increase	MT2A	ENSG00000125148	16579640;
C422648	increase	PDCD5	ENSG00000105185	16579640;
C422648	increase	TP53	ENSG00000141510	15939500;
C422648	increase	TUBB	ENSG00000229684	16579640;
C422649	decrease	ICAM1	ENSG00000090339	17931847;
C423012	increase	TNFRSF1A	ENSG00000067182	9870925;
C423185	increase	CYP2B6	ENSG00000197408	15333513;
C423185	increase	CYP2C9	ENSG00000138109	15333513;
C423185	increase	CYP3A4	ENSG00000160868	16837568;
C423222	decrease	ETS2	ENSG00000157557	16129123;
C423222	decrease	PTPN3	ENSG00000070159	16129123;
C423222	decrease	RABEPK	ENSG00000136933	16129123;
C423222	decrease	TFDP2	ENSG00000114126	16129123;
C423222	increase	ALOX5	ENSG00000012779	16129123;
C423222	increase	ANXA1	ENSG00000135046	16129123;
C423222	increase	BST1	ENSG00000109743	16129123;
C423222	increase	BTG1	ENSG00000133639	16129123;
C423222	increase	CCNG2	ENSG00000138764	18754885;
C423222	increase	CD48	ENSG00000117091	16129123;
C423222	increase	DUSP6	ENSG00000139318	16129123;
C423222	increase	IFI6	ENSG00000126709	16129123;
C423222	increase	IL32	ENSG00000008517	16129123;
C423222	increase	LASP1	ENSG00000002834	16129123;
C423222	increase	LGALS3BP	ENSG00000108679	16129123;
C423222	increase	OLIG2	ENSG00000205927	16129123;
C423222	increase	PDE4B	ENSG00000184588	16129123;
C423222	increase	PPIG	ENSG00000138398	16129123;
C423222	increase	PTGS1	ENSG00000095303	16129123;
C423222	increase	PTPRC	ENSG00000081237	16129123;
C423222	increase	RAB31	ENSG00000168461	16129123;
C423222	increase	RAC2	ENSG00000128340	16129123;
C423222	increase	S100P	ENSG00000163993	16129123;
C423222	increase	TIMP2	ENSG00000035862	16129123;
C423222	increase	TNFAIP2	ENSG00000185215	16129123;
C423915	increase	ABCA1	ENSG00000165029	16142410;17449538;
C423915	increase	ABCG1	ENSG00000160179	16024918;17449538;
C423915	increase	APOE	ENSG00000130203	16142410;17449538;
C423915	increase	CETP	ENSG00000087237	16142410;
C423915	increase	LDLR	ENSG00000130164	16142410;
C423915	increase	LIPG	ENSG00000101670	16142410;
C423915	increase	LPL	ENSG00000175445	16142410;
C423915	increase	NR1H3	ENSG00000025434	12932788;12970175;17449538;

C423915	increase	PLTP	ENSG00000100979	16142410;17449538;
C423915	increase	SCARB1	ENSG00000073060	16142410;
C423917	decrease	CTNNB1	ENSG00000168036	17219422;
C423917	increase	IL8	ENSG00000169429	12208513;
C423917	increase	RXRA	ENSG00000186350	15707588;
C425784	increase	CYP1A1	ENSG00000140465	15355884;14707267;
C425784	increase	CYP1B1	ENSG00000138061	15355884;14707267;
C425784	increase	IL6	ENSG00000136244	15655410;
C427824	decrease	PGR	ENSG00000082175	9144411;
C428725	increase	ODC1	ENSG00000115758	9834970;
C430898	decrease	CCNB1	ENSG00000134057	15318936;
C430898	decrease	CCNE1	ENSG00000105173	15318936;
C430898	decrease	CDK1	ENSG00000170312	15318936;
C430898	decrease	CDK4	ENSG00000135446	15318936;
C430898	decrease	CDK6	ENSG00000105810	15318936;
C430898	decrease	EGFR	ENSG00000146648	15318936;
C430898	decrease	FOSL1	ENSG00000175592	15318936;
C430898	decrease	MAPK3	ENSG00000102882	15318936;
C430898	decrease	MAPK4	ENSG00000141639	15318936;
C430898	decrease	MET	ENSG00000105976	15318936;
C432165	affect	BIRC3	ENSG00000023445	16154993;
C432165	affect	CDC25C	ENSG00000158402	16179969;
C432165	affect	NRP1	ENSG00000099250	16289960;
C432165	decrease	APP	ENSG00000142192	16648635;
C432165	decrease	BIRC5	ENSG00000089685	16328441;
C432165	decrease	FGF19	ENSG00000162344	16269825;
C432165	decrease	PON1	ENSG00000005421	16269825;
C432165	decrease	SERPINE1	ENSG00000106366	17379280;
C432165	decrease	TAT	ENSG00000198650	15744361;
C432165	increase	APOA1	ENSG00000118137	19013290;
C432165	increase	BIRC2	ENSG00000110330	12832416;
C432165	increase	CCL8	ENSG00000108700	12832416;
C432165	increase	CD14	ENSG00000170458	14729647;18180316;
C432165	increase	CDKN1A	ENSG00000124762	15961274;16061660;
C432165	increase	CEBPB	ENSG00000172216	14729647;
C432165	increase	CXCL10	ENSG00000169245	12832416;
C432165	increase	CXCL3	ENSG00000163734	12832416;
C432165	increase	CYP17A1	ENSG00000148795	16269825;
C432165	increase	CYP1A1	ENSG00000140465	17959153;
C432165	increase	CYP1A2	ENSG00000140505	17959153;
C432165	increase	CYP3A4	ENSG00000160868	17965521;
C432165	increase	DDIT3	ENSG00000175197	17341418;17049495;
C432165	increase	EGF	ENSG00000138798	15961274;
C432165	increase	EGR1	ENSG00000120738	18316600;
C432165	increase	F2	ENSG00000180210	19944065;
C432165	increase	F3	ENSG00000117525	19944065;
C432165	increase	FAS	ENSG00000026103	16162944;16061660;
C432165	increase	FGF2	ENSG00000138685	16289960;12832416;
C432165	increase	FGF7	ENSG00000140285	16162944;15677771;
C432165	increase	GABPA	ENSG00000154727	16308312;
C432165	increase	GCH1	ENSG00000250845	12832416;
C432165	increase	HSPA5	ENSG00000044574	17341418;
C432165	increase	HSPA6	ENSG00000173110	15978632;
C432165	increase	ICAM1	ENSG00000090339	12832416;

C432165	increase	IL1A	ENSG00000115008	12832416;
C432165	increase	IL1B	ENSG00000125538	12832416;16359550;18982426;16365456;15677771;
C432165	increase	IL6	ENSG00000136244	17196171;19074641;12832416;
C432165	increase	IL8	ENSG00000169429	17082639;12832416;16373669;
C432165	increase	JUN	ENSG00000177606	16041517;15930183;16407847;12832416;18982426;15677771;16872482;16061660;15322261;16932349;
C432165	increase	MAPK8	ENSG00000107643	15831436;16041517;18728404;15322261;16648635;15964311;16864444;1677799
C432165	increase	MMP12	ENSG00000110347	16359550;
C432165	increase	MMP1	ENSG00000196611	12832416;
C432165	increase	MMP3	ENSG00000149968	16778083;12832416;
C432165	increase	PPARD	ENSG00000112033	17341418;
C432165	increase	PTGS2	ENSG00000073756	12832416;18596194;
C432165	increase	PTX3	ENSG00000163661	12832416;
C432165	increase	RB1	ENSG00000139687	14729647;
C432165	increase	SAA1	ENSG00000173432	12832416;
C432165	increase	SCD	ENSG00000099194	16162944;
C432165	increase	SERPINB2	ENSG00000197632	12832416;
C432165	increase	SOD2	ENSG00000112096	12832416;
C432165	increase	SREBF1	ENSG00000072310	16162944;
C432165	increase	ST8SIA3	ENSG00000177511	17069899;
C432165	increase	SULT1A1	ENSG00000196502	16308312;
C432165	increase	TGFB1	ENSG00000105329	16365456;
C432165	increase	TNF	ENSG00000223952	15322261;
C432165	increase	TP53	ENSG00000141510	17292493;16061660;
C432165	increase	VDR	ENSG00000111424	15930183;
C432165	increase	VEGFA	ENSG00000112715	18596194;
C432511	decrease	MMP2	ENSG00000087245	18715546;
C432511	decrease	MMP9	ENSG00000100985	18715546;
C432511	increase	TIMP1	ENSG00000102265	18715546;
C433788	increase	NFKB1	ENSG00000109320	11437639;
C433788	increase	PTGS2	ENSG00000073756	8621535;
C433788	increase	RELA	ENSG00000173039	11437639;
C433985	decrease	TYMS	ENSG00000176890	18425338;
C434003	affect	DDIT3	ENSG00000175197	17049495;
C434003	decrease	BCL2L1	ENSG00000171552	16173963;16960866;
C434003	decrease	BIRC2	ENSG00000110330	16380446;
C434003	decrease	BIRC3	ENSG00000023445	16380446;
C434003	decrease	CFLAR	ENSG00000003402	16380446;16173963;
C434003	decrease	IL6	ENSG00000136244	16380446;
C434003	decrease	XIAP	ENSG00000101966	16173963;15964800;
C434003	increase	CCL2	ENSG00000108691	17666914;
C434003	increase	EFEMP1	ENSG00000115380	19208748;
C434003	increase	ICAM1	ENSG00000090339	14572618;17666914;
C434003	increase	IFNG	ENSG00000111537	19208748;
C434003	increase	TNF	ENSG00000223952	17666914;14572618;
C436083	increase	FGF7	ENSG00000140285	14656992;
C436947	increase	ITGA2	ENSG00000164171	11545736;
C436947	increase	LDLR	ENSG00000130164	11545736;

C436947	increase	ZBTB17	ENSG00000116809	11545736;
C437043	increase	CDKN1A	ENSG00000124762	17045927;
C437043	increase	NOS3	ENSG00000164867	17045927;
C437043	increase	TP53	ENSG00000141510	17045927;
C437683	increase	MT1A	ENSG00000205362	15867382;
C437683	increase	MT1E	ENSG00000169715	15867382;
C437683	increase	MT1F	ENSG00000198417	15867382;
C437683	increase	MT1G	ENSG00000125144	15867382;
C437683	increase	MT1H	ENSG00000205358	15867382;
C437683	increase	MT1X	ENSG00000187193	15867382;
C437683	increase	MT2A	ENSG00000125148	15867382;
C437683	increase	RNF19B	ENSG00000116514	15867382;
C437683	increase	SLC30A1	ENSG00000170385	15867382;
C439285	decrease	CCND1	ENSG00000110092	15475462;
C439285	decrease	CTNNB1	ENSG00000168036	12392815;
C439285	increase	ALOX15	ENSG00000161905	16166323;
C439285	increase	CDKN1A	ENSG00000124762	15475462;
C439285	increase	PRKG1	ENSG00000185532	11602670;
C439285	increase	RARB	ENSG00000077092	15475462;
C439285	increase	TP53	ENSG00000141510	15475462;
C439584	decrease	CCND1	ENSG00000110092	16954435;
C439584	decrease	ESR1	ENSG00000091831	16954435;
C440499	increase	CCL3	ENSG00000006075	12538206;
C440499	increase	F3	ENSG00000117525	7600656;
C440499	increase	IL6	ENSG00000136244	10700573;
C440499	increase	SELE	ENSG00000007908	8780174;
C440499	increase	SPP1	ENSG00000118785	18703563;
C440499	increase	TNF	ENSG00000223952	10700573;
C440614	decrease	BIRC5	ENSG00000089685	14988404;
C440780	increase	F3	ENSG00000117525	18974383;
C440780	increase	TNF	ENSG00000223952	18974383;
C440975	affect	CYP1A2	ENSG00000140505	15977188;
C440975	affect	CYP3A4	ENSG00000160868	15977188;15155549;
C440975	increase	MGMT	ENSG00000170430	16950796;
C440975	increase	TP53	ENSG00000141510	16777994;
C441209	affect	FSHB	ENSG00000131808	16291413;
C441209	affect	LHB	ENSG00000104826	16291413;
C442659	decrease	C4BPB	ENSG00000123843	14551147;
C442659	decrease	F5	ENSG00000198734	14551147;
C442659	decrease	LHB	ENSG00000104826	17105841;
C442659	decrease	PLAT	ENSG00000104368	12618251;
C442659	decrease	PROS1	ENSG00000184500	14551147;11994571;12618251;15318540;10928463;
C442659	decrease	VCAM1	ENSG00000162692	17105841;
C442659	increase	A2M	ENSG00000175899	10928463;
C442659	increase	CD19	ENSG00000177455	10813102;
C442659	increase	CD4	ENSG00000010610	10813102;
C442659	increase	CP	ENSG00000047457	12616983;
C442659	increase	CRP	ENSG00000132693	17105841;16637303;12616983;
C442659	increase	F7	ENSG00000057593	12616980;12618251;
C442659	increase	F8	ENSG00000185010	12618251;
C442659	increase	FCER2	ENSG00000104921	10813102;

C442659	increase	FGA	ENSG00000171560	16637303;14981370;12616980;12618251;12616983;
C442659	increase	FGB	ENSG00000171564	16637303;14981370;12616980;12616983;12618251;
C442659	increase	FGG	ENSG00000171557	16637303;14981370;12616983;12616980;12618251;
C442659	increase	PLG	ENSG00000122194	15318540;12618251;
C442659	increase	PROC	ENSG00000115718	15318540;11994571;14551147;12618251;10928463;
C442659	increase	SERPINA1	ENSG00000197249	10928463;
C442659	increase	SERPINA5	ENSG00000188488	14551147;10928463;
C442659	increase	SERPINC1	ENSG00000117601	11994571;14981370;12618251;10928463;15318540;
C442659	increase	SERPIND1	ENSG00000099937	15318540;
C442659	increase	SHBG	ENSG00000129214	14981370;
C442659	increase	VWF	ENSG00000110799	12616983;
C442781	increase	CCL17	ENSG00000102970	18325701;
C442781	increase	IL4	ENSG00000113520	18325701;
C442781	increase	IL5	ENSG00000113525	18325701;
C442781	increase	TNF	ENSG00000223952	18325701;
C442817	increase	LDLR	ENSG00000130164	12524230;
C443401	affect	CCL2	ENSG00000108691	18489909;
C443401	affect	ICAM1	ENSG00000090339	18489909;
C443401	increase	TNF	ENSG00000223952	18489909;17880982;
C443401	increase	VCAM1	ENSG00000162692	18489909;17880982;
C443641	affect	MET	ENSG00000105976	16308212;
C443641	decrease	ALOX5	ENSG00000012779	16308212;
C443641	decrease	CD27	ENSG00000139193	16308212;
C443641	decrease	CDK1	ENSG00000170312	16308212;
C443641	decrease	DBF4	ENSG00000006634	16308212;
C443641	decrease	DUSP1	ENSG00000120129	16308212;
C443641	decrease	GMPS	ENSG00000163655	16308212;
C443641	decrease	HSPD1	ENSG00000144381	16308212;
C443641	decrease	IK	ENSG00000113141	16308212;
C443641	decrease	PCCB	ENSG00000114054	16308212;
C443641	decrease	PCNA	ENSG00000132646	16308212;
C443641	decrease	PSMB4	ENSG00000159377	16308212;
C443641	decrease	RPA3	ENSG00000106399	16308212;
C443641	decrease	RPL8	ENSG00000161016	16308212;
C443641	decrease	SMARCA4	ENSG00000127616	16308212;
C443641	decrease	YWHAQ	ENSG00000134308	16308212;
C443641	increase	ANXA2	ENSG00000182718	16308212;
C443641	increase	CCNA1	ENSG00000133101	16308212;
C443641	increase	CD14	ENSG00000170458	16308212;
C443641	increase	CDK4	ENSG00000135446	16308212;
C443641	increase	HIST1H2AD	ENSG00000196866	16308212;
C443641	increase	HSP90B1	ENSG00000166598	16308212;
C443641	increase	IL9R	ENSG00000124334	16308212;
C443641	increase	PSMA4	ENSG00000041357	16308212;
C443641	increase	RXRA	ENSG00000186350	16308212;
C443641	increase	TGFA	ENSG00000163235	16308212;
C443641	increase	TPR	ENSG00000047410	16308212;
C443641	increase	TUBB4	ENSG00000104833	16308212;
C444299	affect	CYCS	ENSG00000172115	16051428;
C444299	decrease	BCL2	ENSG00000171791	16051428;

C444299	decrease	CCND1	ENSG00000110092	16051428;
C446520	decrease	BCL2	ENSG00000171791	16077967;
C446520	decrease	BCL2L1	ENSG00000171552	16096431;16077967;
C446520	decrease	CDC25A	ENSG00000164045	16077967;
C446520	decrease	CDC25B	ENSG00000101224	16077967;
C446520	decrease	CDC25C	ENSG00000158402	16077967;
C446520	decrease	XIAP	ENSG00000101966	16096431;
C446520	increase	BAX	ENSG00000087088	16077967;
C446685	increase	CYP1A2	ENSG00000140505	19409404;
C447119	increase	LIPC	ENSG00000166035	15961563;
C448659	decrease	CHEK1	ENSG00000149554	17085670;
C450682	increase	CRABP2	ENSG00000143320	17486130;
C450682	increase	CYP26B1	ENSG00000003137	17486130;
C450682	increase	MYCN	ENSG00000134323	17486130;
C450682	increase	RARB	ENSG00000077092	17486130;
C450832	increase	C3	ENSG00000125730	17060630;
C450832	increase	COX2	ENSG00000198712	16531283;
C450832	increase	COX3	ENSG00000198938	16531283;
C451219	increase	VEGFA	ENSG00000112715	15860260;
C451735	decrease	BCL2L1	ENSG00000171552	15555623;
C451735	decrease	BCL2L2	ENSG00000129473	15555623;
C451735	decrease	CCND1	ENSG00000110092	15555623;
C451735	decrease	NFKBIA	ENSG00000100906	15555623;
C451735	increase	CDKN1A	ENSG00000124762	15555623;
C451735	increase	TP53	ENSG00000141510	15555623;
C452139	increase	CXCL1	ENSG00000163739	14997032;
C452139	increase	IL8	ENSG00000169429	14997032;
C452423	decrease	MSH3	ENSG00000113318	16052530;
C452423	decrease	PTMA	ENSG00000187514	16052530;
C452423	increase	ADM	ENSG00000148926	16052530;
C452423	increase	ANGPTL4	ENSG00000167772	16052530;
C452423	increase	BNIP3L	ENSG00000104765	16052530;
C452423	increase	CALB1	ENSG00000104327	16052530;
C452423	increase	COL4A3	ENSG00000169031	16052530;
C452423	increase	DUSP1	ENSG00000120129	16052530;
C452423	increase	ENO2	ENSG00000111674	16052530;
C452423	increase	HIST1H1C	ENSG00000187837	16052530;
C452423	increase	IGFBP1	ENSG00000146678	16052530;
C452423	increase	IGFBP3	ENSG00000146674	16052530;
C452423	increase	INSIG1	ENSG00000186480	16052530;
C452423	increase	INSIG2	ENSG00000125629	16052530;
C452423	increase	MUC1	ENSG00000185499	16052530;
C452423	increase	NDRG1	ENSG00000104419	16052530;
C452423	increase	PGK1	ENSG00000102144	16052530;
C452423	increase	PLOD2	ENSG00000152952	16052530;
C452423	increase	PRSS2	ENSG00000204983	16052530;
C452423	increase	SCD	ENSG00000099194	16052530;
C452423	increase	SLC2A14	ENSG00000173262	16052530;
C452423	increase	SLC2A3	ENSG00000059804	16052530;
C452423	increase	TSC22D3	ENSG00000157514	16052530;
C454300	decrease	PLAT	ENSG00000104368	15288212;
C454300	decrease	PROS1	ENSG00000184500	15288212;
C454300	decrease	SERPINE1	ENSG00000106366	15288212;
C454300	increase	F2	ENSG00000180210	15288212;

C454300	increase	F7	ENSG00000057593	15288212;
C454300	increase	FGA	ENSG00000171560	15288212;
C454300	increase	FGB	ENSG00000171564	15288212;
C454300	increase	FGG	ENSG00000171557	15288212;
C454323	decrease	IFNG	ENSG00000111537	10883730;
C454323	decrease	TNF	ENSG00000223952	10883730;
C455846	decrease	SLC7A11	ENSG00000151012	16443240;
C455846	increase	MYC	ENSG00000136997	11198217;
C455860	increase	CYP3A4	ENSG00000160868	17965521;
C456099	decrease	AKT1	ENSG00000142208	11800023;
C456099	decrease	ERBB2	ENSG00000141736	11800023;
C456099	decrease	RAF1	ENSG00000132155	11800023;
C456739	affect	IL8	ENSG00000169429	17558435;
C456739	affect	TNF	ENSG00000223952	17558435;
C456800	decrease	BCL2L1	ENSG00000171552	15634644;
C456800	decrease	CCND1	ENSG00000110092	15634644;
C456800	decrease	MCL1	ENSG00000143384	15634644;
C457499	decrease	BCL2	ENSG00000171791	17461445;
C457499	decrease	CD1A	ENSG00000158477	17475839;
C457499	decrease	CD36	ENSG00000135218	17475839;
C457499	decrease	CD80	ENSG00000121594	17475839;
C457499	decrease	FN1	ENSG00000115414	15908479;
C457499	increase	BAX	ENSG00000087088	17461445;
C457499	increase	BSG	ENSG00000172270	17055343;
C457499	increase	CD86	ENSG00000114013	17475839;
C457499	increase	CDKN1A	ENSG00000124762	15345676;
C457499	increase	FABP1	ENSG00000163586	17602619;
C457499	increase	FABP4	ENSG00000170323	17602619;
C457499	increase	PTGS2	ENSG00000073756	16417216;
C459179	increase	GREM1	ENSG00000166923	17975199;
C459179	increase	TGFB1	ENSG00000105329	17975199;
C459559	decrease	MAP2K1	ENSG00000169032	15725661;
C459559	decrease	MAP2K2	ENSG00000126934	15725661;
C459559	decrease	MAPK1	ENSG00000100030	15725661;
C459559	decrease	MAPK3	ENSG00000102882	15725661;
C459604	decrease	ACY1	ENSG00000243989	16309724;
C459604	decrease	AK3L1	ENSG00000162433	16309724;
C459604	decrease	ALDOC	ENSG00000109107	16309724;
C459604	decrease	AQP3	ENSG00000165272	16309724;
C459604	decrease	ASS1	ENSG00000130707	16309724;
C459604	decrease	AUH	ENSG00000148090	16309724;
C459604	decrease	BAG1	ENSG00000250477	16309724;
C459604	decrease	BASP1	ENSG00000176788	16309724;
C459604	decrease	BNIP3	ENSG00000176171	16309724;
C459604	decrease	CA9	ENSG00000107159	16309724;
C459604	decrease	CCNG2	ENSG00000138764	16309724;
C459604	decrease	CD55	ENSG00000196352	16309724;
C459604	decrease	CHRNE	ENSG00000108556	16309724;
C459604	decrease	CKB	ENSG00000166165	16309724;
C459604	decrease	CLNS1A	ENSG00000074201	16309724;
C459604	decrease	COL4A1	ENSG00000187498	16309724;
C459604	decrease	CPS1	ENSG00000021826	16309724;
C459604	decrease	CSAD	ENSG00000139631	16309724;
C459604	decrease	CSRP2	ENSG00000175183	16309724;

C459604	decrease	CUL4A	ENSG00000139842	16309724;
C459604	decrease	DDX17	ENSG00000100201	16309724;
C459604	decrease	DDX21	ENSG00000165732	16309724;
C459604	decrease	DUSP9	ENSG00000130829	16309724;
C459604	decrease	EEF1A2	ENSG00000101210	16309724;
C459604	decrease	EIF5A	ENSG00000132507	16309724;
C459604	decrease	FBN2	ENSG00000138829	16309724;
C459604	decrease	GALNT2	ENSG00000143641	16309724;
C459604	decrease	GLUD1	ENSG00000148672	16309724;
C459604	decrease	GMDS	ENSG00000112699	16309724;
C459604	decrease	GPRC5A	ENSG00000013588	16309724;
C459604	decrease	HTATIP2	ENSG00000109854	16309724;
C459604	decrease	IFITM1	ENSG00000185885	16309724;
C459604	decrease	IFITM2	ENSG00000185201	16309724;
C459604	decrease	IFITM3	ENSG00000142089	16309724;
C459604	decrease	IGF1R	ENSG00000140443	16309724;
C459604	decrease	IGFBP4	ENSG00000141753	16309724;
C459604	decrease	IL1A	ENSG00000115008	16309724;
C459604	decrease	IRS2	ENSG00000185950	16309724;
C459604	decrease	JUNB	ENSG00000171223	16309724;
C459604	decrease	KLF9	ENSG00000119138	16309724;
C459604	decrease	L1CAM	ENSG00000198910	16309724;
C459604	decrease	LPL	ENSG00000175445	16309724;
C459604	decrease	LPPR1	ENSG00000148123	16309724;
C459604	decrease	MAP3K5	ENSG00000197442	16309724;
C459604	decrease	MINK1	ENSG00000141503	16309724;
C459604	decrease	MYC	ENSG00000136997	16309724;
C459604	decrease	NDRG1	ENSG00000104419	16309724;
C459604	decrease	NR4A2	ENSG00000153234	16309724;
C459604	decrease	OLR1	ENSG00000173391	16309724;
C459604	decrease	OSMR	ENSG00000145623	16309724;
C459604	decrease	PDE3B	ENSG00000152270	16309724;
C459604	decrease	PLA2G4A	ENSG00000116711	16309724;
C459604	decrease	PLTP	ENSG00000100979	16309724;
C459604	decrease	PTGES	ENSG00000148344	16309724;
C459604	decrease	PTGS1	ENSG00000095303	16309724;
C459604	decrease	PTGS2	ENSG00000073756	16309724;
C459604	decrease	PTP4A1	ENSG00000112245	16309724;
C459604	decrease	PTPRM	ENSG00000173482	16309724;
C459604	decrease	RPL13A	ENSG00000142541	16309724;
C459604	decrease	RPS21	ENSG00000171858	16309724;
C459604	decrease	SFN	ENSG00000175793	16309724;
C459604	decrease	SLC12A7	ENSG00000113504	16309724;
C459604	decrease	SNCA	ENSG00000145335	16309724;
C459604	decrease	TFAM	ENSG00000108064	16309724;
C459604	decrease	TIMP3	ENSG00000100234	16309724;
C459604	decrease	TPD52	ENSG00000076554	16309724;
C459604	decrease	UCP2	ENSG00000175567	16309724;
C459604	decrease	ZEB1	ENSG00000148516	16309724;
C459604	increase	AUTS2	ENSG00000158321	16309724;
C459604	increase	CASP1	ENSG00000137752	16309724;
C459604	increase	CD44	ENSG00000026508	16309724;
C459604	increase	CDH2	ENSG00000170558	16309724;
C459604	increase	CLIC4	ENSG00000169504	16309724;

C459604	increase	CNN3	ENSG00000117519	16309724;
C459604	increase	DAPK3	ENSG00000167657	16309724;
C459604	increase	DNAJB6	ENSG00000105993	16309724;
C459604	increase	GABRA2	ENSG00000151834	16309724;
C459604	increase	GADD45A	ENSG00000116717	16309724;
C459604	increase	GAS6	ENSG00000248501	16309724;
C459604	increase	GBP1	ENSG00000117228	16309724;
C459604	increase	GPR39	ENSG00000183840	16309724;
C459604	increase	GPR56	ENSG00000205336	16309724;
C459604	increase	HSPA1A	ENSG00000232804	16309724;
C459604	increase	IFI44	ENSG00000137965	16309724;
C459604	increase	IL13RA2	ENSG00000123496	16309724;
C459604	increase	IL7R	ENSG00000168685	16309724;
C459604	increase	NRP1	ENSG00000099250	16309724;
C459604	increase	PEA15	ENSG00000162734	16309724;
C459604	increase	PTX3	ENSG00000163661	16309724;
C459604	increase	RAP1GDS1	ENSG00000138698	16309724;
C459604	increase	SLC7A11	ENSG00000151012	16309724;
C459604	increase	SOCS1	ENSG00000185338	16309724;
C459604	increase	SQSTM1	ENSG00000161011	16309724;
C459604	increase	SRPR	ENSG00000182934	16309724;
C459604	increase	THBS1	ENSG00000137801	16309724;
C459604	increase	TNFRSF12A	ENSG00000006327	16309724;
C459604	increase	TPM1	ENSG00000140416	16309724;
C459604	increase	UBE2D3	ENSG00000109332	16309724;
C459604	increase	UBE2E3	ENSG00000170035	16309724;
C459623	decrease	IL4	ENSG00000113520	16343431;
C460864	increase	CD209	ENSG00000090659	17938187;
C460864	increase	CD36	ENSG00000135218	17938187;
C460864	increase	CLEC7A	ENSG00000172243	17938187;
C460864	increase	MSR1	ENSG00000038945	17938187;
C463260	decrease	BIRC5	ENSG00000089685	16473406;
C463260	decrease	FLT3	ENSG00000122025	16473406;
C463759	decrease	BAX	ENSG00000087088	15863893;
C463759	decrease	XIAP	ENSG00000101966	15863893;
C464444	decrease	BIRC2	ENSG00000110330	15501958;
C464444	decrease	BIRC3	ENSG00000023445	15501958;
C464444	decrease	BIRC5	ENSG00000089685	16211219;
C464444	decrease	CFLAR	ENSG00000003402	16211219;
C464444	decrease	TNF	ENSG00000223952	16211219;
C464444	decrease	XIAP	ENSG00000101966	15501958;
C464660	decrease	CYP7A1	ENSG00000167910	15911693;
C464660	decrease	SLC10A1	ENSG00000100652	15911693;
C464660	increase	ABCB11	ENSG00000073734	15911693;
C464660	increase	NR0B2	ENSG00000131910	15911693;
C465546	increase	CYP1A1	ENSG00000140465	11454723;
C466420	affect	CCL2	ENSG00000108691	14576080;
C466420	affect	TNF	ENSG00000223952	14576080;
C466913	decrease	TYMS	ENSG00000176890	17599054;
C466913	increase	CDKN1B	ENSG00000111276	17599054;
C467001	decrease	TP53	ENSG00000141510	16055726;
C467566	decrease	GATA1	ENSG00000102145	15292067;
C467566	decrease	GATA2	ENSG00000179348	15292067;
C467566	decrease	GFI1B	ENSG00000165702	15292067;

C467566	decrease	JUND	ENSG00000130522	15292067;
C467566	decrease	KLF1	ENSG00000105610	15292067;
C467566	decrease	LMO2	ENSG00000135363	15292067;
C467566	decrease	NFE2	ENSG00000123405	15292067;
C467566	increase	CEBPA	ENSG00000245848	15292067;
C467566	increase	CEBPD	ENSG00000221869	15292067;
C467566	increase	CEBPE	ENSG00000092067	15292067;
C467566	increase	GFI1	ENSG00000162676	15292067;
C467566	increase	ID1	ENSG00000125968	15292067;
C467566	increase	ID2	ENSG00000115738	15292067;
C467566	increase	IL2	ENSG00000109471	16241859;
C467566	increase	SPI1	ENSG00000066336	16373662;15292067;
C467567	decrease	F2R	ENSG00000181104	15618473;
C467567	decrease	IL1R1	ENSG00000115594	15618473;
C467567	decrease	IL6ST	ENSG00000134352	15618473;
C467567	decrease	TEK	ENSG00000120156	15618473;
C467567	decrease	TGFBR1	ENSG00000106799	15618473;
C467567	decrease	TNFRSF1A	ENSG00000067182	15618473;
C467567	decrease	TNFRSF1B	ENSG00000028137	15618473;
C467746	increase	DHCR24	ENSG00000116133	15585566;
C467746	increase	ESR1	ENSG00000091831	12399409;15585566;17935960;
C467746	increase	IGFBP4	ENSG00000141753	12399409;
C467746	increase	UGT2B15	ENSG00000196620	16690804;
C468130	increase	AR	ENSG00000169083	19059400;
C468753	increase	PGR	ENSG00000082175	16892383;
C468753	increase	TFF1	ENSG00000160182	16892383;
C469298	increase	APP	ENSG00000142192	18583042;
C469298	increase	BACE1	ENSG00000186318	18583042;
C469298	increase	CD83	ENSG00000112149	17698565;
C469298	increase	CD86	ENSG00000114013	17698565;
C469852	affect	HSPB1	ENSG00000106211	15583868;
C469852	affect	HSPD1	ENSG00000144381	15583868;
C469937	decrease	XIAP	ENSG00000101966	16140939;
C469937	increase	TP53	ENSG00000141510	16140939;
C470148	decrease	EDN1	ENSG00000078401	12934402;
C470148	decrease	SELP	ENSG00000174175	12934402;
C470148	decrease	TF	ENSG00000091513	12934402;
C470148	decrease	VWF	ENSG00000110799	12934402;
C471405	decrease	MCL1	ENSG00000143384	16007148;
C471843	decrease	BIRC5	ENSG00000089685	14988404;15958644;16170024;
C471843	decrease	MDM2	ENSG00000135679	15958644;
C471843	increase	CDKN1A	ENSG00000124762	15958644;
C471843	increase	CDKN2A	ENSG00000147889	15958644;
C471843	increase	TP53	ENSG00000141510	15958644;
C471992	decrease	CCND1	ENSG00000110092	16489032;
C472511	decrease	ALOX5AP	ENSG00000132965	16507463;
C472511	decrease	CCL2	ENSG00000108691	16507463;
C472511	decrease	CCNA1	ENSG00000133101	16507463;
C472511	decrease	CEBPB	ENSG00000172216	16507463;
C472511	decrease	COL1A1	ENSG00000108821	16507463;
C472511	decrease	DDIT3	ENSG00000175197	16507463;
C472511	decrease	FGFR1	ENSG00000249195	16507463;

C472511	decrease	GATA6	ENSG00000141448	16507463;
C472511	decrease	GJA1	ENSG00000152661	16507463;
C472511	decrease	HBEGF	ENSG00000113070	16507463;
C472511	decrease	HIF1A	ENSG00000100644	16507463;
C472511	decrease	IGFBP5	ENSG00000115461	16507463;
C472511	decrease	ITGB3	ENSG00000056345	16507463;
C472511	decrease	MMP2	ENSG00000087245	16507463;
C472511	decrease	MYLK	ENSG00000251351	16507463;
C472511	decrease	NSF	ENSG00000073969	16507463;
C472511	decrease	NUP88	ENSG00000108559	16507463;
C472511	decrease	ODC1	ENSG00000115758	16507463;
C472511	decrease	PRKCA	ENSG00000154229	16507463;
C472511	decrease	PSMA3	ENSG00000100567	16507463;
C472511	decrease	RPUSD4	ENSG00000165526	16507463;
C472511	decrease	SMAD7	ENSG00000101665	16507463;
C472511	decrease	TCF12	ENSG00000140262	16507463;
C472511	decrease	THBS1	ENSG00000137801	16507463;
C472511	decrease	TMEM50B	ENSG00000142188	16507463;
C472511	decrease	TXNDC17	ENSG00000129235	16507463;
C472511	increase	IL1R2	ENSG00000115590	16507463;
C472777	affect	LAT	ENSG00000213658	12493747;
C472777	affect	LCK	ENSG00000182866	12493747;
C472777	affect	NFATC2	ENSG00000101096	12493747;
C472777	affect	PTPN2	ENSG00000175354	12493747;
C472777	affect	PTPN6	ENSG00000111679	12493747;
C472777	affect	ZAP70	ENSG00000115085	12493747;
C472777	decrease	LTC4S	ENSG00000213316	12574384;
C472777	increase	PTGS2	ENSG00000073756	12493747;
C472791	decrease	ACTB	ENSG00000075624	12958071;
C472791	decrease	AK2	ENSG00000004455	12958071;
C472791	decrease	AKR1B1	ENSG00000085662	12958071;
C472791	decrease	AMH	ENSG00000104899	12958071;
C472791	decrease	AP2M1	ENSG00000161203	12958071;
C472791	decrease	AP2S1	ENSG00000042753	12958071;
C472791	decrease	ARF1	ENSG00000143761	12958071;
C472791	decrease	ARPC2	ENSG00000163466	12958071;
C472791	decrease	ARPC3	ENSG00000111229	12958071;
C472791	decrease	ARSA	ENSG00000100299	12958071;
C472791	decrease	ATP2B2	ENSG00000157087	12958071;
C472791	decrease	ATP5A1	ENSG00000152234	12958071;
C472791	decrease	ATP5B	ENSG00000110955	12958071;
C472791	decrease	ATP5I	ENSG00000169020	12958071;
C472791	decrease	ATP5J	ENSG00000154723	12958071;
C472791	decrease	BANF1	ENSG00000175334	12958071;
C472791	decrease	C11ORF10	ENSG00000134825	12958071;
C472791	decrease	C14ORF156	ENSG00000119705	12958071;
C472791	decrease	C19ORF43	ENSG00000123144	12958071;
C472791	decrease	CAMK2G	ENSG00000148660	12958071;
C472791	decrease	CCT2	ENSG00000166226	12958071;
C472791	decrease	CCT3	ENSG00000163468	12958071;
C472791	decrease	CCT6A	ENSG00000146731	12958071;
C472791	decrease	CDK4	ENSG00000135446	12958071;
C472791	decrease	CEBPB	ENSG00000172216	12958071;
C472791	decrease	CFL1	ENSG00000172757	12958071;

C472791	decrease	CHCHD2	ENSG00000106153	12958071;
C472791	decrease	CLIC1	ENSG00000226417	12958071;
C472791	decrease	COX5B	ENSG00000135940	12958071;
C472791	decrease	COX6C	ENSG00000164919	12958071;
C472791	decrease	COX7A2	ENSG00000112695	12958071;
C472791	decrease	COX7A2L	ENSG00000115944	12958071;
C472791	decrease	COX7B	ENSG00000131174	12958071;
C472791	decrease	COX7C	ENSG00000127184	12958071;
C472791	decrease	DAPK3	ENSG00000167657	12958071;
C472791	decrease	DYNLL1	ENSG00000088986	12958071;
C472791	decrease	EEF2	ENSG00000167658	12958071;
C472791	decrease	EIF3D	ENSG00000100353	12958071;
C472791	decrease	EIF3E	ENSG00000104408	12958071;
C472791	decrease	EIF4A1	ENSG00000161960	12958071;
C472791	decrease	EIF4A2	ENSG00000156976	12958071;
C472791	decrease	EIF4B	ENSG00000063046	12958071;
C472791	decrease	EIF4G2	ENSG00000110321	12958071;
C472791	decrease	EIF5A	ENSG00000132507	12958071;
C472791	decrease	ENO1	ENSG00000074800	12958071;
C472791	decrease	FKBP5	ENSG00000096060	12958071;
C472791	decrease	H2AFZ	ENSG00000164032	12958071;
C472791	decrease	HIC1	ENSG00000177374	12958071;
C472791	decrease	HSP90AA1	ENSG00000080824	12958071;
C472791	decrease	ILF2	ENSG00000143621	12958071;
C472791	decrease	IMP3	ENSG00000177971	12958071;
C472791	decrease	ITGA11	ENSG00000137809	12958071;
C472791	decrease	ITM2B	ENSG00000136156	12958071;
C472791	decrease	KARS	ENSG00000065427	12958071;
C472791	decrease	KIAA0101	ENSG00000166803	12958071;
C472791	decrease	KIF2C	ENSG00000142945	12958071;
C472791	decrease	KIF3A	ENSG00000131437	12958071;
C472791	decrease	KPNA2	ENSG00000182481	12958071;
C472791	decrease	LGALS1	ENSG00000100097	12958071;
C472791	decrease	LSM3	ENSG00000170860	12958071;
C472791	decrease	MAN1B1	ENSG00000177239	12958071;
C472791	decrease	MEN1	ENSG00000133895	12958071;
C472791	decrease	MGST1	ENSG00000008394	12958071;
C472791	decrease	MOBKL1B	ENSG00000114978	12958071;
C472791	decrease	MTCH2	ENSG00000109919	12958071;
C472791	decrease	NACA	ENSG00000196531	12958071;
C472791	decrease	NARS	ENSG00000134440	12958071;
C472791	decrease	NDUFB9	ENSG00000147684	12958071;
C472791	decrease	NDUFS5	ENSG00000168653	12958071;
C472791	decrease	NKX2-5	ENSG00000183072	12958071;
C472791	decrease	NME1	ENSG00000239672	12958071;
C472791	decrease	NPM1	ENSG00000181163	12958071;
C472791	decrease	ODC1	ENSG00000115758	12958071;
C472791	decrease	PABPC1	ENSG00000070756	12958071;
C472791	decrease	PCBP2	ENSG00000197111	12958071;
C472791	decrease	PDIA3	ENSG00000167004	12958071;
C472791	decrease	PFN1	ENSG00000108518	12958071;
C472791	decrease	PIN1	ENSG00000127445	12958071;
C472791	decrease	POLR2G	ENSG00000168002	12958071;
C472791	decrease	POU3F1	ENSG00000185668	12958071;

C472791	decrease	PRICKLE4	ENSG00000124593	12958071;
C472791	decrease	PSMA4	ENSG00000041357	12958071;
C472791	decrease	PSMB1	ENSG00000008018	12958071;
C472791	decrease	PSMD3	ENSG00000108344	12958071;
C472791	decrease	PSMF1	ENSG00000125818	12958071;
C472791	decrease	PURB	ENSG00000146676	12958071;
C472791	decrease	RBX1	ENSG00000100387	12958071;
C472791	decrease	ROBO4	ENSG00000154133	12958071;
C472791	decrease	RPL10	ENSG00000147403	12958071;
C472791	decrease	RPL13	ENSG00000167526	12958071;
C472791	decrease	RPL18A	ENSG00000105640	12958071;
C472791	decrease	RPL19	ENSG00000108298	12958071;
C472791	decrease	RPL24	ENSG00000114391	12958071;
C472791	decrease	RPL29	ENSG00000162244	12958071;
C472791	decrease	RPL32	ENSG00000144713	12958071;
C472791	decrease	RPL36A	ENSG00000241343	12958071;
C472791	decrease	RPL36AL	ENSG00000165502	12958071;
C472791	decrease	RPL39	ENSG00000198918	12958071;
C472791	decrease	RPL41	ENSG00000229117	12958071;
C472791	decrease	RPL6	ENSG00000089009	12958071;
C472791	decrease	RPL8	ENSG00000161016	12958071;
C472791	decrease	RPLP1	ENSG00000137818	12958071;
C472791	decrease	RPS10	ENSG00000124614	12958071;
C472791	decrease	RPS15	ENSG00000115268	12958071;
C472791	decrease	RPS26	ENSG00000197728	12958071;
C472791	decrease	RPS27A	ENSG00000143947	12958071;
C472791	decrease	RPS27	ENSG00000177954	12958071;
C472791	decrease	RPS2	ENSG00000140988	12958071;
C472791	decrease	RYBP	ENSG00000163602	12958071;
C472791	decrease	S100P	ENSG00000163993	12958071;
C472791	decrease	SAP18	ENSG00000150459	12958071;
C472791	decrease	7-Sep	ENSG00000122545	12958071;
C472791	decrease	SFRS3	ENSG00000112081	12958071;
C472791	decrease	SHFM1	ENSG00000127922	12958071;
C472791	decrease	SLC25A37	ENSG00000147454	12958071;
C472791	decrease	SLC25A3	ENSG00000075415	12958071;
C472791	decrease	SLC25A6	ENSG00000169100	12958071;
C472791	decrease	SLC30A5	ENSG00000145740	12958071;
C472791	decrease	SLC38A7	ENSG00000103042	12958071;
C472791	decrease	SNRPB	ENSG00000125835	12958071;
C472791	decrease	SNRPD2	ENSG00000125743	12958071;
C472791	decrease	SNRPF	ENSG00000139343	12958071;
C472791	decrease	SNRPG	ENSG00000143977	12958071;
C472791	decrease	SOD1	ENSG00000142168	12958071;
C472791	decrease	SOX11	ENSG00000176887	12958071;
C472791	decrease	SOX4	ENSG00000124766	12958071;
C472791	decrease	SSBP1	ENSG00000106028	12958071;
C472791	decrease	SSR2	ENSG00000163479	12958071;
C472791	decrease	SSTR3	ENSG00000183473	12958071;
C472791	decrease	ST13	ENSG00000100380	12958071;
C472791	decrease	TBL1XR1	ENSG00000177565	12958071;
C472791	decrease	TIMM8B	ENSG00000150779	12958071;
C472791	decrease	TMEM14C	ENSG00000111843	12958071;
C472791	decrease	TMSB10	ENSG00000034510	12958071;

C472791	decrease	TMSB4X	ENSG00000205542	12958071;
C472791	decrease	TOMM7	ENSG00000196683	12958071;
C472791	decrease	TPT1	ENSG00000133112	12958071;
C472791	decrease	TXN	ENSG00000136810	12958071;
C472791	decrease	UBA52	ENSG00000221983	12958071;
C472791	decrease	UBE2M	ENSG00000130725	12958071;
C472791	decrease	UBL5	ENSG00000198258	12958071;
C472791	decrease	UGCG	ENSG00000148154	12958071;
C472791	decrease	UQCRB	ENSG00000156467	12958071;
C472791	decrease	UQCRC2	ENSG00000140740	12958071;
C472791	decrease	UQCRH	ENSG00000173660	12958071;
C472791	decrease	UXT	ENSG00000126756	12958071;
C472791	decrease	VAMP8	ENSG00000118640	12958071;
C472791	decrease	VDAC2	ENSG00000165637	12958071;
C472791	decrease	YBX1	ENSG00000065978	12958071;
C472791	decrease	YWHAE	ENSG00000108953	12958071;
C472791	decrease	YWHAZ	ENSG00000164924	12958071;
C472791	increase	IL8	ENSG00000169429	12958071;
C472791	increase	MLL4	ENSG00000105663	12958071;
C472791	increase	SETD5	ENSG00000168137	12958071;
C472791	increase	STK3	ENSG00000104375	12958071;
C472791	increase	SUB1	ENSG00000113387	12958071;
C472791	increase	UGT2B15	ENSG00000196620	12958071;
C473027	decrease	CYP11A1	ENSG00000140459	17449538;
C473027	decrease	STAR	ENSG00000147465	17449538;
C473027	increase	ABCA1	ENSG00000165029	16107141;17449538;
C473027	increase	ABCG1	ENSG00000160179	17449538;
C473027	increase	APOE	ENSG00000130203	17449538;
C473027	increase	NR1H3	ENSG00000025434	12932788;17449538;
C473027	increase	PLTP	ENSG00000100979	17449538;
C473027	increase	SREBF1	ENSG00000072310	16107141;
C474928	decrease	CCNE1	ENSG00000105173	12694875;
C474928	increase	CCNB1	ENSG00000134057	12694875;
C475093	increase	CYP2A6	ENSG00000198077	16857725;
C475093	increase	CYP2B6	ENSG00000197408	18332078;
C475093	increase	CYP2C8	ENSG00000138115	15933212;
C475093	increase	CYP3A4	ENSG00000160868	18332078;
C475587	increase	CDKN1A	ENSG00000124762	16003486;
C475587	increase	TP53	ENSG00000141510	16003486;
C475882	decrease	ESR1	ENSG00000091831	17904202;
C475882	increase	PGR	ENSG00000082175	17904202;
C475882	increase	TFF1	ENSG00000160182	17904202;
C475919	decrease	ABAT	ENSG00000183044	16849584;
C475919	decrease	CSTA	ENSG00000121552	15072547;
C475919	decrease	DNAJC12	ENSG00000108176	16849584;
C475919	decrease	EFNA1	ENSG00000169242	15072547;
C475919	decrease	ERBB2	ENSG00000141736	15072547;
C475919	decrease	GALNT6	ENSG00000139629	16849584;
C475919	decrease	MEGF9	ENSG00000106780	16849584;
C475919	decrease	MMP15	ENSG00000102996	15072547;
C475919	decrease	MYLIP	ENSG00000007944	16849584;
C475919	decrease	NAT9	ENSG00000109065	16849584;
C475919	decrease	NOV	ENSG00000136999	15072547;
C475919	decrease	PCNA	ENSG00000132646	15072547;

C475919	decrease	PDCD4	ENSG00000150593	16849584;
C475919	decrease	SOX9	ENSG00000125398	16849584;
C475919	decrease	STS	ENSG00000101846	16849584;
C475919	decrease	TACC1	ENSG00000147526	15072547;
C475919	decrease	UGT1A1	ENSG00000241635	15072547;
C475919	decrease	ZNF217	ENSG00000171940	15072547;
C475919	increase	ABCC3	ENSG00000108846	15072547;
C475919	increase	ABCC5	ENSG00000114770	15072547;
C475919	increase	AREG	ENSG00000205595	15072547;
C475919	increase	ASCL1	ENSG00000139352	16849584;
C475919	increase	B3GNT2	ENSG00000170340	16849584;
C475919	increase	C3ORF63	ENSG00000163946	16849584;
C475919	increase	CCNA2	ENSG00000145386	15072547;
C475919	increase	CD44	ENSG00000026508	16849584;
C475919	increase	CLIC3	ENSG00000169583	16849584;
C475919	increase	CPM	ENSG00000135678	16849584;
C475919	increase	CTSD	ENSG00000117984	15072547;
C475919	increase	DAB2	ENSG00000153071	16849584;
C475919	increase	EFNB2	ENSG00000125266	16849584;
C475919	increase	ELF3	ENSG00000163435	16849584;
C475919	increase	ESR2	ENSG00000140009	16849584;
C475919	increase	ETNK1	ENSG00000139163	16849584;
C475919	increase	FAM129A	ENSG00000135842	16849584;
C475919	increase	FARP1	ENSG00000152767	16849584;
C475919	increase	FOS	ENSG00000170345	15072547;
C475919	increase	GBE1	ENSG00000114480	16849584;
C475919	increase	GGA2	ENSG00000103365	16849584;
C475919	increase	GPR56	ENSG00000205336	16849584;
C475919	increase	IER3	ENSG00000137331	16849584;
C475919	increase	IGFBP4	ENSG00000141753	15072547;
C475919	increase	IRS1	ENSG00000169047	15072547;
C475919	increase	KBTBD2	ENSG00000170852	16849584;
C475919	increase	KLC1	ENSG00000126214	16849584;
C475919	increase	KRT13	ENSG00000171401	16849584;
C475919	increase	LASP1	ENSG00000002834	16849584;
C475919	increase	LSS	ENSG00000160285	16849584;
C475919	increase	LTBP1	ENSG00000049323	16849584;
C475919	increase	MSH2	ENSG00000095002	16849584;
C475919	increase	MYC	ENSG00000136997	15072547;
C475919	increase	OAZ3	ENSG00000143450	16849584;
C475919	increase	PCYOX1	ENSG00000116005	16849584;
C475919	increase	PGR	ENSG00000082175	15072547;
C475919	increase	PGRMC1	ENSG00000101856	16849584;
C475919	increase	PKIA	ENSG00000171033	16849584;
C475919	increase	PRPS1	ENSG00000147224	16849584;
C475919	increase	PTPRG	ENSG00000144724	16849584;
C475919	increase	RAB30	ENSG00000137502	16849584;
C475919	increase	RGS3	ENSG00000249544	16849584;
C475919	increase	SDC1	ENSG00000115884	16849584;
C475919	increase	SERPINA1	ENSG00000197249	16849584;
C475919	increase	SHANK2	ENSG00000162105	16849584;
C475919	increase	SLC25A1	ENSG00000100075	16849584;
C475919	increase	SOCS1	ENSG00000185338	16849584;
C475919	increase	SRD5A1	ENSG00000145545	16849584;

C475919	increase	STAG1	ENSG00000118007	16849584;
C475919	increase	TFF1	ENSG00000160182	15072547;
C475919	increase	TM7SF2	ENSG00000149809	16849584;
C475919	increase	UPF1	ENSG00000005007	16849584;
C475919	increase	WNT2	ENSG00000105989	15072547;
C475919	increase	WNT4	ENSG00000162552	16849584;
C475919	increase	YWHAZ	ENSG00000164924	16849584;
C475919	increase	ZNF185	ENSG00000147394	16849584;
C476036	decrease	MMP14	ENSG00000157227	16783407;
C476200	increase	RXRG	ENSG00000249671	12766334;
C477042	increase	ABCB1	ENSG00000085563	19034627;
C477042	increase	ABCC2	ENSG00000023839	19034627;
C477042	increase	CYP2B6	ENSG00000197408	19034627;
C477042	increase	CYP3A4	ENSG00000160868	19034627;
C477042	increase	UGT1A1	ENSG00000241635	19034627;
C477330	decrease	CCND2	ENSG00000118971	12907618;
C477330	decrease	CCND3	ENSG00000112576	12907618;
C477330	decrease	CCNE1	ENSG00000105173	12907618;
C477330	decrease	MAPK14	ENSG00000112062	12907618;
C477330	increase	CDKN1B	ENSG00000111276	12907618;
C477361	decrease	MCL1	ENSG00000143384	16189296;16275999;
C477361	decrease	XIAP	ENSG00000101966	16189296;16275999;
C477361	increase	CDKN1A	ENSG00000124762	17237267;16189296;16275999;
C477361	increase	RARB	ENSG00000077092	17237267;
C477361	increase	TAGLN	ENSG00000149591	17237267;
C477659	increase	AR	ENSG00000169083	19059400;
C477819	decrease	BIRC2	ENSG00000110330	12623786;
C477819	decrease	FN1	ENSG00000115414	15908479;
C477819	decrease	SP1	ENSG00000185591	15908479;
C477819	increase	IL1A	ENSG00000115008	10866999;
C477819	increase	IL1B	ENSG00000125538	15641079;
C477819	increase	NOS2	ENSG00000007171	10866999;
C477819	increase	PTGS2	ENSG00000073756	15641079;10866999;
C477819	increase	TNF	ENSG00000223952	10866999;
C477931	decrease	BIRC2	ENSG00000110330	12970779;
C477931	decrease	BIRC3	ENSG00000023445	12970779;
C477931	decrease	CFLAR	ENSG00000003402	12970779;
C477941	decrease	CCND1	ENSG00000110092	12844480;
C477941	decrease	CCND2	ENSG00000118971	12844480;
C477941	decrease	MYC	ENSG00000136997	12844480;
C477941	decrease	PCNA	ENSG00000132646	12844480;
C477941	increase	CASP3	ENSG00000164305	12844480;
C477941	increase	CDKN1A	ENSG00000124762	12844480;
C477941	increase	CDKN1B	ENSG00000111276	12844480;
C477941	increase	FAS	ENSG00000026103	12844480;
C478160	decrease	BCL2	ENSG00000171791	15982805;
C478160	decrease	CASP3	ENSG00000164305	15982805;
C478160	increase	BAX	ENSG00000087088	15982805;
C479067	increase	TP53	ENSG00000141510	15724842;
C479072	increase	CRABP2	ENSG00000143320	15225641;
C479128	decrease	CCNA2	ENSG00000145386	15202011;
C479128	decrease	CCND1	ENSG00000110092	12969788;15202011;
C479128	decrease	CCND3	ENSG00000112576	12969788;

C479128	decrease	CCNE1	ENSG00000105173	12969788;15202011;
C479128	decrease	CDK2	ENSG00000123374	12969788;15202011;
C479128	decrease	CDK4	ENSG00000135446	12969788;15202011;
C479128	decrease	CDK6	ENSG00000105810	15202011;
C479128	decrease	RB1	ENSG00000139687	15202011;12969788;
C479128	increase	CDKN1A	ENSG00000124762	12969788;15202011;
C479799	decrease	BIRC5	ENSG00000089685	15528975;
C479799	decrease	CALCOCO2	ENSG00000136436	15528975;
C479799	decrease	CCNG2	ENSG00000138764	15528975;
C479799	decrease	CLGN	ENSG00000153132	15528975;
C479799	decrease	CRKL	ENSG00000099942	15528975;
C479799	decrease	EEF1A1	ENSG00000156508	15528975;
C479799	decrease	EPS15	ENSG00000085832	15528975;
C479799	decrease	FAM48A	ENSG00000102710	15528975;
C479799	decrease	GADD45A	ENSG00000116717	15528975;
C479799	decrease	GRB10	ENSG00000106070	15528975;
C479799	decrease	HGF	ENSG00000019991	15528975;
C479799	decrease	HIST2H4B	ENSG00000182217	15528975;
C479799	decrease	LDHA	ENSG00000134333	15528975;
C479799	decrease	MAPK9	ENSG00000050748	15528975;
C479799	decrease	PCDH1	ENSG00000156453	15528975;
C479799	decrease	POSTN	ENSG00000133110	15528975;
C479799	decrease	POU2F2	ENSG00000028277	15528975;
C479799	decrease	RAP1A	ENSG00000116473	15528975;
C479799	decrease	RHOD	ENSG00000173156	15528975;
C479799	decrease	SPTA1	ENSG00000163554	15528975;
C479799	decrease	TERT	ENSG00000164362	15528975;
C479799	decrease	TOP2A	ENSG00000131747	15528975;
C479799	decrease	VEGFA	ENSG00000112715	15528975;
C479799	increase	BTG2	ENSG00000159388	15528975;
C479799	increase	CDKN1A	ENSG00000124762	15528975;
C479799	increase	CPNE5	ENSG00000124772	15528975;
C479799	increase	CTGF	ENSG00000118523	15528975;
C479799	increase	EGR1	ENSG00000120738	15528975;
C479799	increase	GDF15	ENSG00000130513	15528975;
C479799	increase	TIMP1	ENSG00000102265	15528975;
C479799	increase	TP53I3	ENSG00000115129	15528975;
C480030	decrease	BCL2L1	ENSG00000171552	15930313;
C480030	decrease	BIRC2	ENSG00000110330	15930313;
C480030	decrease	NFKBIA	ENSG00000100906	15930313;
C480030	decrease	XIAP	ENSG00000101966	15930313;
C480030	increase	FAS	ENSG00000026103	15930313;
C480030	increase	HSP90AB1	ENSG00000096384	15930313;
C480030	increase	RALBP1	ENSG00000017797	15930313;
C480030	increase	TNFRSF10A	ENSG00000104689	15930313;
C480030	increase	TNFRSF10B	ENSG00000120889	15930313;
C480546	increase	CD1A	ENSG00000158477	16388738;
C480546	increase	CD80	ENSG00000121594	16388738;
C480546	increase	CD83	ENSG00000112149	16388738;
C480546	increase	CD86	ENSG00000114013	16388738;
C480848	increase	BIRC2	ENSG00000110330	18841906;
C480848	increase	BIRC3	ENSG00000023445	18841906;
C480848	increase	MMP9	ENSG00000100985	18841906;
C480848	increase	NOS2	ENSG00000007171	18841906;

C480848	increase	PTGS2	ENSG00000073756	18841906;
C480848	increase	TNF	ENSG00000223952	18841906;
C482199	affect	PPARG	ENSG00000132170	18485432;
C482199	increase	CCL2	ENSG00000108691	17880982;
C482199	increase	ICAM1	ENSG00000090339	17880982;
C482199	increase	IL8	ENSG00000169429	18485432;
C482199	increase	TNF	ENSG00000223952	17880982;
C482199	increase	VCAM1	ENSG00000162692	17880982;
C482205	increase	BAX	ENSG00000087088	16439685;16014563;
C482205	increase	BBC3	ENSG00000105327	16439685;16014563;
C482205	increase	CDKN1A	ENSG00000124762	16439685;16439677;16014563;19098008;
C482205	increase	DDB1	ENSG00000167986	16439677;
C482205	increase	EDN1	ENSG00000078401	19098008;
C482205	increase	GADD45A	ENSG00000116717	19098008;
C482205	increase	GDF15	ENSG00000130513	16439677;
C482205	increase	KITLG	ENSG00000049130	19098008;
C482205	increase	MDM2	ENSG00000135679	16439685;19098008;16014563;
C482205	increase	PCNA	ENSG00000132646	16439677;
C482205	increase	PMAIP1	ENSG00000141682	16014563;
C482205	increase	TNFRSF10B	ENSG00000120889	16439677;18092340;
C482205	increase	TP53	ENSG00000141510	16439685;16014563;19098008;18092340;16439677;
C482205	increase	TP53I3	ENSG00000115129	16439677;16439685;
C482205	increase	ZMAT3	ENSG00000172667	16439685;
C482267	increase	NOS3	ENSG00000164867	14508237;
C482378	decrease	BCL2	ENSG00000171791	16749867;
C482378	decrease	BCL2L1	ENSG00000171552	16749867;
C482378	increase	BAD	ENSG00000002330	16749867;
C482378	increase	BAX	ENSG00000087088	16749867;
C482402	decrease	FGFR3	ENSG00000068078	12963989;
C482492	affect	BAX	ENSG00000087088	15668717;
C482492	affect	BCL2	ENSG00000171791	15668717;
C482492	affect	TP53	ENSG00000141510	15668717;
C482884	decrease	BCL2	ENSG00000171791	16497706;
C482884	decrease	BID	ENSG00000015475	16497706;
C482884	decrease	CCNB1	ENSG00000134057	16497706;
C482884	decrease	CCND1	ENSG00000110092	16497706;
C482884	decrease	CCNE1	ENSG00000105173	16497706;
C482884	decrease	CDC25C	ENSG00000158402	16497706;
C482884	decrease	CDK1	ENSG00000170312	16497706;
C482884	decrease	CDK2	ENSG00000123374	16497706;
C482884	decrease	CDK4	ENSG00000135446	16497706;
C482884	decrease	CDK6	ENSG00000105810	16497706;
C482884	increase	BAX	ENSG00000087088	16497706;
C482884	increase	CDKN1B	ENSG00000111276	16497706;
C482884	increase	FADD	ENSG00000168040	16497706;
C482884	increase	FAS	ENSG00000026103	16497706;
C482884	increase	TNFSF10	ENSG00000121858	16497706;
C483321	increase	ABCB1	ENSG00000085563	18624906;
C483321	increase	ABCC2	ENSG00000023839	18624906;
C483321	increase	ABCC4	ENSG00000125257	18624906;
C483321	increase	ABCG2	ENSG00000118777	18624906;

C483321	increase	CCND1	ENSG00000110092	18624906;
C483321	increase	CTNNB1	ENSG00000168036	18624906;
C484027	decrease	ANXA2	ENSG00000182718	14668052;
C484027	decrease	LDHA	ENSG00000134333	14668052;
C484027	decrease	RXRA	ENSG00000186350	14668052;
C484027	increase	ALOX5	ENSG00000012779	14668052;
C484027	increase	BCL2A1	ENSG00000140379	14668052;
C484027	increase	CASP8	ENSG00000064012	14668052;
C484027	increase	CDO1	ENSG00000129596	14668052;
C484027	increase	DBF4	ENSG00000006634	14668052;
C484027	increase	DUSP1	ENSG00000120129	14668052;
C484027	increase	EDNRB	ENSG00000136160	14668052;
C484027	increase	EIF2S1	ENSG00000134001	14668052;
C484027	increase	ERCC1	ENSG00000012061	14668052;
C484027	increase	GINS1	ENSG00000101003	14668052;
C484027	increase	GMPS	ENSG00000163655	14668052;
C484027	increase	HSPA4	ENSG00000170606	14668052;
C484027	increase	HSPD1	ENSG00000144381	14668052;
C484027	increase	HYAL2	ENSG00000068001	14668052;
C484027	increase	MET	ENSG00000105976	14668052;
C484027	increase	MMP8	ENSG00000118113	14668052;
C484027	increase	MYC	ENSG00000136997	14668052;
C484027	increase	PFDN4	ENSG00000101132	14668052;
C484027	increase	POU2AF1	ENSG00000110777	14668052;
C484027	increase	POU2F2	ENSG00000028277	14668052;
C484027	increase	PTPRR	ENSG00000153233	14668052;
C484027	increase	RPL32	ENSG00000144713	14668052;
C484027	increase	TCEA2	ENSG00000171703	14668052;
C484327	decrease	BCL2	ENSG00000171791	14660655;
C484327	decrease	BCL2L2	ENSG00000129473	14660655;
C484919	increase	FSHB	ENSG00000131808	17190377;
C484919	increase	LHB	ENSG00000104826	17190377;
C485706	increase	VEGFA	ENSG00000112715	17010073;
C487081	increase	CDKN1A	ENSG00000124762	19234609;
C487081	increase	CLU	ENSG00000120885	19234609;
C487081	increase	MAGEA1	ENSG00000198681	19259094;
C487081	increase	MLH1	ENSG00000076242	19259094;
C487498	decrease	CNN1	ENSG00000130176	15380616;
C487498	decrease	TAGLN	ENSG00000149591	15380616;
C487498	increase	IL8	ENSG00000169429	17000667;
C487689	decrease	BIRC3	ENSG00000023445	16546985;
C487689	decrease	CCND1	ENSG00000110092	16546985;
C487689	decrease	CCND2	ENSG00000118971	16546985;
C487689	decrease	XIAP	ENSG00000101966	16546985;
C487689	increase	NFKBIA	ENSG00000100906	16546985;
C487928	increase	KLK3	ENSG00000142515	11580929;
C487928	increase	TFF1	ENSG00000160182	11580929;
C488176	increase	NGFR	ENSG00000064300	17447067;
C488234	decrease	ESRRA	ENSG00000173153	19464277;
C488234	decrease	PPARGC1A	ENSG00000109819	19464277;
C488623	increase	BAX	ENSG00000087088	16012788;
C488623	increase	TP53	ENSG00000141510	16012788;
C489023	decrease	CCND1	ENSG00000110092	15246556;
C489023	decrease	CCNE1	ENSG00000105173	15246556;

C489023	decrease	RB1	ENSG00000139687	15246556;
C490191	increase	MAPK14	ENSG00000112062	15963507;
C490191	increase	TP53	ENSG00000141510	15963507;
C490707	increase	CEACAM8	ENSG00000124469	15652230;
C490707	increase	IL1B	ENSG00000125538	15652230;
C490707	increase	IL8	ENSG00000169429	15652230;
C490707	increase	ITGAM	ENSG00000169896	15652230;
C490728	decrease	AKT1	ENSG00000142208	15665275;16091755;
C490728	decrease	BIRC5	ENSG00000089685	16091755;
C490728	decrease	CCND1	ENSG00000110092	15665275;
C490728	decrease	ERBB2	ENSG00000141736	15665275;16091755;
C490728	decrease	MAPK1	ENSG00000100030	16091755;
C490728	decrease	MAPK3	ENSG00000102882	16091755;
C490728	increase	CDKN1B	ENSG00000111276	15665275;
C492519	increase	DHCR24	ENSG00000116133	15585566;
C492651	increase	CYP1A1	ENSG00000140465	15377855;15355884;
C492719	increase	FOS	ENSG00000170345	12547826;
C492810	decrease	CDK4	ENSG00000135446	15733867;
C492810	decrease	CDKN1B	ENSG00000111276	15733867;
C492810	increase	CDKN1A	ENSG00000124762	15733867;
C492909	affect	SP1	ENSG00000185591	15345676;
C492909	affect	SP4	ENSG00000105866	15345676;
C492909	increase	CDKN1A	ENSG00000124762	15345676;
C494060	decrease	CYP17A1	ENSG00000148795	18336853;
C494620	decrease	MCL1	ENSG00000143384	15858615;
C494622	decrease	MCL1	ENSG00000143384	15858615;
C494622	decrease	XIAP	ENSG00000101966	15858615;
C494898	decrease	CCNA2	ENSG00000145386	16540676;
C494898	decrease	CDC25C	ENSG00000158402	16540676;
C494898	increase	CDKN1A	ENSG00000124762	16540676;
C494898	increase	TP53	ENSG00000141510	16540676;
C495287	decrease	PLAT	ENSG00000104368	16202731;
C495818	increase	CDKN1A	ENSG00000124762	18347191;18223691;
C496197	decrease	ABCA1	ENSG00000165029	17951470;
C496197	decrease	AIF1	ENSG00000235985	17951470;
C496197	decrease	ANLN	ENSG00000011426	17951470;
C496197	decrease	ASPM	ENSG00000066279	17951470;
C496197	decrease	BCAT1	ENSG00000060982	17951470;
C496197	decrease	BIRC5	ENSG00000089685	17951470;
C496197	decrease	BLM	ENSG00000197299	17951470;
C496197	decrease	BUB1B	ENSG00000156970	17951470;
C496197	decrease	CCNE2	ENSG00000175305	17951470;
C496197	decrease	CDC25B	ENSG00000101224	17951470;
C496197	decrease	CDC7	ENSG00000097046	17951470;
C496197	decrease	CDK1	ENSG00000170312	17951470;
C496197	decrease	CDK2	ENSG00000123374	17951470;
C496197	decrease	CDK4	ENSG00000135446	17951470;
C496197	decrease	CDKN2C	ENSG00000123080	17951470;
C496197	decrease	CDKN3	ENSG00000100526	17951470;
C496197	decrease	CENPA	ENSG00000115163	17951470;
C496197	decrease	CENPE	ENSG00000138778	17951470;
C496197	decrease	CENPF	ENSG00000117724	17951470;
C496197	decrease	CLDN1	ENSG00000163347	16650747;
C496197	decrease	CSRP2	ENSG00000175183	17951470;

C496197	decrease	DDX11	ENSG00000013573	17951470;
C496197	decrease	DNASE1	ENSG00000213918	17951470;
C496197	decrease	E2F7	ENSG00000165891	17951470;
C496197	decrease	E2F8	ENSG00000129173	17951470;
C496197	decrease	EMP2	ENSG00000213853	17951470;
C496197	decrease	EZH2	ENSG00000106462	17951470;
C496197	decrease	FEN1	ENSG00000168496	17951470;
C496197	decrease	FGA	ENSG00000171560	17951470;
C496197	decrease	FGB	ENSG00000171564	17951470;
C496197	decrease	FGG	ENSG00000171557	17951470;
C496197	decrease	G0S2	ENSG00000123689	17951470;
C496197	decrease	GAS2	ENSG00000148935	17951470;
C496197	decrease	GINS1	ENSG00000101003	17951470;
C496197	decrease	GINS2	ENSG00000131153	17951470;
C496197	decrease	GTSE1	ENSG00000075218	17951470;
C496197	decrease	HIRIP3	ENSG00000149929	17951470;
C496197	decrease	HMGB2	ENSG00000164104	17951470;
C496197	decrease	KCNMA1	ENSG00000156113	17951470;
C496197	decrease	KIF11	ENSG00000138160	17951470;
C496197	decrease	KIF15	ENSG00000163808	17951470;
C496197	decrease	KIF2C	ENSG00000142945	17951470;
C496197	decrease	KITLG	ENSG00000049130	17951470;
C496197	decrease	MCM2	ENSG00000073111	17951470;
C496197	decrease	MCM4	ENSG00000104738	17951470;
C496197	decrease	MCM7	ENSG00000166508	17951470;
C496197	decrease	MKI67	ENSG00000148773	17951470;
C496197	decrease	MSH2	ENSG00000095002	17951470;
C496197	decrease	MSH6	ENSG00000116062	17951470;
C496197	decrease	MYBL2	ENSG00000101057	17951470;
C496197	decrease	NASP	ENSG00000132780	17951470;
C496197	decrease	NCAPG	ENSG00000109805	17951470;
C496197	decrease	NCK2	ENSG00000071051	17951470;
C496197	decrease	NEIL3	ENSG00000109674	17951470;
C496197	decrease	NEK2	ENSG00000117650	17951470;
C496197	decrease	PBK	ENSG00000168078	17951470;
C496197	decrease	PDGFRA	ENSG00000134853	17951470;
C496197	decrease	PLK1	ENSG00000166851	17951470;
C496197	decrease	PLK4	ENSG00000142731	17951470;
C496197	decrease	POLA2	ENSG00000014138	17951470;
C496197	decrease	POLD3	ENSG00000077514	17951470;
C496197	decrease	POLE2	ENSG00000100479	17951470;
C496197	decrease	POLQ	ENSG00000051341	17951470;
C496197	decrease	PRC1	ENSG00000198901	17951470;
C496197	decrease	RAD51AP1	ENSG00000111247	17951470;
C496197	decrease	RAD51C	ENSG00000108384	17951470;
C496197	decrease	RARRES1	ENSG00000118849	17951470;
C496197	decrease	RFC3	ENSG00000133119	17951470;
C496197	decrease	RFC5	ENSG00000111445	17951470;
C496197	decrease	RNASEH2A	ENSG00000104889	17951470;
C496197	decrease	RRM2	ENSG00000171848	17951470;
C496197	decrease	SASH1	ENSG00000111961	17951470;
C496197	decrease	SESN1	ENSG00000080546	17951470;
C496197	decrease	SESN3	ENSG00000149212	17951470;
C496197	decrease	SI	ENSG00000090402	17951470;

C496197	decrease	SKP2	ENSG00000145604	17951470;
C496197	decrease	SMC4	ENSG00000113810	17951470;
C496197	decrease	SSTR1	ENSG00000139874	17951470;
C496197	decrease	TCF7L1	ENSG00000152284	17951470;
C496197	decrease	TGFBI	ENSG00000120708	17951470;
C496197	decrease	TK1	ENSG00000167900	17951470;
C496197	decrease	TOP2A	ENSG00000131747	17951470;
C496197	decrease	TPX2	ENSG00000088325	17951470;
C496197	decrease	TTK	ENSG00000112742	17951470;
C496197	decrease	UHRF1	ENSG00000034063	17951470;
C496197	decrease	ZFP36L2	ENSG00000152518	17951470;
C496197	increase	AREG	ENSG00000205595	17951470;
C496197	increase	ARHGEF2	ENSG00000116584	17951470;
C496197	increase	BRF2	ENSG00000104221	17951470;
C496197	increase	CDKN1A	ENSG00000124762	17951470;
C496197	increase	CDKN1C	ENSG00000129757	17951470;
C496197	increase	CDKN2B	ENSG00000147883	17951470;
C496197	increase	CLDN4	ENSG00000189143	17951470;16650747;
C496197	increase	CREB3L3	ENSG00000060566	17951470;
C496197	increase	CRIP1	ENSG00000213145	17951470;
C496197	increase	DUSP1	ENSG00000120129	17951470;
C496197	increase	DUSP6	ENSG00000139318	17951470;
C496197	increase	EGF	ENSG00000138798	17951470;
C496197	increase	EMP1	ENSG00000134531	17951470;
C496197	increase	ENPP7	ENSG00000182156	17951470;
C496197	increase	EREG	ENSG00000124882	17951470;
C496197	increase	FABP6	ENSG00000170231	17951470;
C496197	increase	GPNMB	ENSG00000136235	17951470;
C496197	increase	HBEGF	ENSG00000113070	17951470;
C496197	increase	HK2	ENSG00000159399	17951470;
C496197	increase	HMGA1	ENSG00000137309	17951470;
C496197	increase	HMGA2	ENSG00000149948	17951470;
C496197	increase	IFITM1	ENSG00000185885	17951470;
C496197	increase	INSIG1	ENSG00000186480	17951470;
C496197	increase	ISG20	ENSG00000172183	17951470;
C496197	increase	JAG1	ENSG00000101384	17951470;
C496197	increase	KLF4	ENSG00000136826	17951470;
C496197	increase	MAP2K5	ENSG00000137764	17951470;
C496197	increase	MAPRE3	ENSG00000084764	17951470;
C496197	increase	MXD1	ENSG00000059728	17951470;
C496197	increase	MXI1	ENSG00000119950	17951470;
C496197	increase	MYC	ENSG00000136997	17951470;
C496197	increase	OCLN	ENSG00000197822	16650747;
C496197	increase	POLD4	ENSG00000175482	17951470;
C496197	increase	POLR3D	ENSG00000168495	17951470;
C496197	increase	PPARG	ENSG00000132170	17951470;
C496197	increase	RECQL	ENSG00000004700	17951470;
C496197	increase	RGS2	ENSG00000116741	17951470;
C496197	increase	RHOB	ENSG00000143878	17951470;
C496197	increase	S100G	ENSG00000169906	17951470;
C496197	increase	SFN	ENSG00000175793	17951470;
C496197	increase	SLC22A4	ENSG00000197208	17951470;
C496197	increase	SLC29A2	ENSG00000174669	17951470;
C496197	increase	TJP1	ENSG00000104067	16650747;

C496197	increase	TM4SF4	ENSG00000169903	17951470;
C496197	increase	TP53INP2	ENSG00000078804	17951470;
C496197	increase	UCHL1	ENSG00000154277	17951470;
C496197	increase	VDR	ENSG00000111424	17951470;
C496197	increase	YWHAH	ENSG00000128245	17951470;
C496751	decrease	BCL2	ENSG00000171791	17145897;
C496751	decrease	CCND1	ENSG00000110092	17387344;17145897;
C496751	decrease	CDK4	ENSG00000135446	17145897;
C496751	decrease	ERBB2	ENSG00000141736	17145897;
C496751	decrease	NCOA3	ENSG00000124151	17145897;
C496751	increase	BAD	ENSG00000002330	17387344;17145897;
C496751	increase	BAX	ENSG00000087088	17145897;
C496751	increase	ESR1	ENSG00000091831	17145897;17387344;
C496751	increase	KRT18	ENSG00000111057	17387344;17145897;
C496751	increase	KRT8	ENSG00000170421	17387344;17145897;
C496879	decrease	SLC2A1	ENSG00000117394	15695405;
C496879	decrease	VEGFA	ENSG00000112715	15695405;
C496879	increase	HIF1A	ENSG00000100644	15695405;
C497101	decrease	CCND3	ENSG00000112576	16027227;
C497101	decrease	CDK2	ENSG00000123374	16027227;
C497101	decrease	CDK4	ENSG00000135446	16027227;
C497101	decrease	E2F1	ENSG00000101412	16027227;
C497101	increase	CCND1	ENSG00000110092	16027227;
C497101	increase	CCNE1	ENSG00000105173	16027227;
C497103	decrease	BCL2L1	ENSG00000171552	16009488;
C497103	decrease	CCNB1	ENSG00000134057	16009488;
C497103	decrease	MCL1	ENSG00000143384	16009488;
C497103	increase	CCND2	ENSG00000118971	16009488;
C497103	increase	CDKN1A	ENSG00000124762	16009488;
C498475	decrease	AR	ENSG00000169083	15547720;
C498475	decrease	CCND1	ENSG00000110092	15547720;
C498475	decrease	CCNE1	ENSG00000105173	15547720;
C498475	decrease	KLK3	ENSG00000142515	15547720;
C498475	increase	CDKN1B	ENSG00000111276	15547720;
C499403	increase	PGR	ENSG00000082175	16323292;
C499404	increase	PGR	ENSG00000082175	16323292;15541416;
C499404	increase	TFF1	ENSG00000160182	15541416;
C499814	decrease	CCND1	ENSG00000110092	17516867;
C499814	decrease	CDK1	ENSG00000170312	17516867;
C499814	decrease	KLK3	ENSG00000142515	17516867;
C499827	increase	HMOX1	ENSG00000100292	19683516;
C499970	decrease	BCL2	ENSG00000171791	15547680;
C499970	decrease	BIRC2	ENSG00000110330	15547680;
C499970	increase	BAX	ENSG00000087088	15547680;
C500218	decrease	IL10	ENSG00000136634	15658607;
C500218	decrease	IL1B	ENSG00000125538	15658607;
C500218	decrease	IL6	ENSG00000136244	15658607;
C500218	decrease	TNF	ENSG00000223952	15658607;
C500218	increase	IL4	ENSG00000113520	15658607;
C500344	decrease	KDR	ENSG00000128052	17426705;
C500344	decrease	MKI67	ENSG00000148773	17426705;
C500810	decrease	XIAP	ENSG00000101966	16322690;
C501280	decrease	BCL2L1	ENSG00000171552	15756023;
C501280	decrease	BIRC2	ENSG00000110330	15756023;

C501280	decrease	CCND1	ENSG00000110092	15756023;
C501280	decrease	XIAP	ENSG00000101966	15756023;
C501517	increase	HSPA8	ENSG00000109971	19763263;
C501517	increase	IDH3A	ENSG00000166411	19763263;
C501517	increase	LDHB	ENSG00000111716	19763263;
C501517	increase	PDHB	ENSG00000168291	19763263;
C501517	increase	PDIA3	ENSG00000167004	19763263;
C501520	increase	BIRC2	ENSG00000110330	19596284;
C501520	increase	BIRC3	ENSG00000023445	19596284;
C502230	decrease	KLK3	ENSG00000142515	16928829;
C502471	increase	CDKN1A	ENSG00000124762	16826403;
C503118	decrease	IL5	ENSG00000113525	15821026;
C503119	affect	CDC25C	ENSG00000158402	16179969;
C503119	decrease	CCNA2	ENSG00000145386	16179969;
C503119	increase	CCNB1	ENSG00000134057	16179969;
C503536	decrease	CCNA2	ENSG00000145386	15202011;
C503536	decrease	CCND1	ENSG00000110092	15202011;
C503536	decrease	CCNE1	ENSG00000105173	15202011;
C503536	decrease	CDK2	ENSG00000123374	15202011;
C503536	decrease	CDK4	ENSG00000135446	15202011;
C503536	decrease	CDK6	ENSG00000105810	15202011;
C503536	decrease	RB1	ENSG00000139687	15202011;
C503536	increase	CDKN1A	ENSG00000124762	15202011;
C503643	affect	CDC25C	ENSG00000158402	16179969;
C503643	affect	MAPK8	ENSG00000107643	16179969;
C503643	decrease	CCNA2	ENSG00000145386	16179969;
C503643	increase	CCNB1	ENSG00000134057	16179969;
C504517	increase	ABCA1	ENSG00000165029	16107141;
C504517	increase	SREBF1	ENSG00000072310	16107141;
C506002	decrease	BCL2	ENSG00000171791	16163705;
C506002	decrease	BCL2L1	ENSG00000171552	16163705;
C506002	decrease	BIRC2	ENSG00000110330	16163705;
C506002	decrease	CCND1	ENSG00000110092	16163705;
C506002	decrease	CCND3	ENSG00000112576	16163705;
C506002	decrease	CDK4	ENSG00000135446	16163705;
C506002	decrease	CDK6	ENSG00000105810	16163705;
C506002	decrease	PCNA	ENSG00000132646	16163705;
C506002	increase	BAD	ENSG00000002330	16163705;
C506002	increase	BAX	ENSG00000087088	16163705;
C506002	increase	CDKN1A	ENSG00000124762	16163705;
C506002	increase	CDKN1B	ENSG00000111276	16163705;
C506002	increase	FAS	ENSG00000026103	16163705;
C506002	increase	FASLG	ENSG00000117560	16163705;
C506606	decrease	BIRC5	ENSG00000089685	16170024;
C506698	decrease	BIRC5	ENSG00000089685	16707021;16123214;
C507164	decrease	MMP1	ENSG00000196611	16055107;
C507164	increase	ELN	ENSG00000049540	16055107;
C507164	increase	FBN1	ENSG00000166147	16055107;
C507553	increase	CDKN1A	ENSG00000124762	19091749;
C507553	increase	HIF1A	ENSG00000100644	19091749;
C507553	increase	SLC2A1	ENSG00000117394	19091749;
C507553	increase	VEGFA	ENSG00000112715	19091749;
C508075	increase	IFNG	ENSG00000111537	18490488;
C508075	increase	IL12A	ENSG00000168811	18490488;

C508075	increase	IL12B	ENSG00000113302	18490488;
C508075	increase	IL23A	ENSG00000110944	18490488;
C508178	increase	IL8	ENSG00000169429	12207893;
C508178	increase	TNF	ENSG00000223952	12207893;
C508406	decrease	ERBB2	ENSG00000141736	16393665;
C508406	increase	ESR1	ENSG00000091831	17603182;16393665;
C508406	increase	PDZK1	ENSG00000174827	16393665;
C508406	increase	PGR	ENSG00000082175	16393665;
C508406	increase	TFF1	ENSG00000160182	16393665;
C508470	decrease	NR5A2	ENSG00000116833	16357189;
C508490	increase	BBC3	ENSG00000105327	16432175;
C508490	increase	CDKN1A	ENSG00000124762	16432175;
C508490	increase	MDM2	ENSG00000135679	16432175;
C508490	increase	TP53I3	ENSG00000115129	16432175;
C508967	increase	CYP1A2	ENSG00000140505	19034627;
C509204	increase	BAD	ENSG00000002330	16413505;
C509204	increase	CDKN1A	ENSG00000124762	16413505;
C509204	increase	TP53	ENSG00000141510	16413505;
C509288	increase	CYP11B1	ENSG00000160882	16396990;
C509288	increase	CYP11B2	ENSG00000179142	16396990;
C509288	increase	CYP1A1	ENSG00000140465	16396990;19794518;
C509288	increase	CYP1B1	ENSG00000138061	16530937;19794518;17637178;
C510003	decrease	CFL1	ENSG00000172757	18232056;
C510003	decrease	EIF5A	ENSG00000132507	18232056;
C510003	decrease	PCNA	ENSG00000132646	18232056;
C510003	decrease	PRDX1	ENSG00000117450	18232056;
C510003	decrease	VIM	ENSG00000026025	18232056;
C510003	increase	CXCL12	ENSG00000107562	17415525;
C510003	increase	CXCR4	ENSG00000121966	17415525;
C510003	increase	ENO1	ENSG00000074800	18232056;
C510003	increase	GAPDH	ENSG00000111640	18232056;
C510003	increase	GFAP	ENSG00000131095	18232056;
C510003	increase	GSTP1	ENSG00000084207	18232056;
C510003	increase	IL8	ENSG00000169429	17415525;
C510003	increase	VEGFA	ENSG00000112715	17415525;17377739;
C510621	decrease	BAD	ENSG00000002330	16170020;
C510621	decrease	BIRC5	ENSG00000089685	16170020;
C510621	decrease	GSK3B	ENSG00000082701	16170020;
C511292	increase	CRP	ENSG00000132693	16982228;
C511292	increase	SERPINA6	ENSG00000170099	16982228;
C511292	increase	SERPINA7	ENSG00000123561	16982228;
C511292	increase	SHBG	ENSG00000129214	16982228;
C511402	affect	BCL2	ENSG00000171791	12587719;
C511402	affect	MYC	ENSG00000136997	12587719;
C511402	affect	TP53	ENSG00000141510	12587719;
C511704	affect	ITPR1	ENSG00000150995	17241155;
C511766	affect	PGR	ENSG00000082175	9144411;
C512273	increase	CDKN1A	ENSG00000124762	18347191;
C512910	increase	CYP1A2	ENSG00000140505	15672752;
C513485	decrease	CDKN1A	ENSG00000124762	16039525;
C513485	decrease	CDKN1B	ENSG00000111276	16039525;
C513485	decrease	SKP2	ENSG00000145604	16039525;
C514580	affect	TP53	ENSG00000141510	17341627;

C514580	increase	BCL2	ENSG00000171791	17341627;
C514580	increase	CDKN1A	ENSG00000124762	17341627;
C514835	decrease	ABCB1	ENSG00000085563	15378274;
C516024	decrease	AKT1	ENSG00000142208	17289836;
C516024	decrease	BCL2	ENSG00000171791	17289836;
C516024	decrease	BCL2L1	ENSG00000171552	17289836;
C516024	decrease	RELA	ENSG00000173039	17289836;
C516024	decrease	XIAP	ENSG00000101966	17289836;
C516216	increase	NR0B2	ENSG00000131910	17154495;
C516216	increase	NR5A2	ENSG00000116833	17154495;
C516322	decrease	SRC	ENSG00000197122	16600465;
C516322	increase	HSPA8	ENSG00000109971	16600465;
C516519	decrease	PLAT	ENSG00000104368	15157790;
C516519	decrease	SERPINE1	ENSG00000106366	15157790;
C516519	increase	F7	ENSG00000057593	15157790;
C516519	increase	PLG	ENSG00000122194	15157790;
C517629	affect	APP	ENSG00000142192	17287498;
C517975	increase	CDKN1A	ENSG00000124762	19422044;
C518327	decrease	NAT1	ENSG00000171428	16314733;
C518368	decrease	BCL2	ENSG00000171791	17352252;
C518368	decrease	CCNB1	ENSG00000134057	17352252;
C518368	increase	CDKN1A	ENSG00000124762	17352252;
C519899	decrease	BCL2L1	ENSG00000171552	18182997;
C519899	decrease	BIRC5	ENSG00000089685	18182997;
C519899	decrease	NFKBIA	ENSG00000100906	18182997;
C521787	decrease	KLK3	ENSG00000142515	17499997;
C522803	increase	TP53	ENSG00000141510	17285121;
C524042	decrease	ARNT	ENSG00000143437	18682687;
C524042	decrease	HIF1A	ENSG00000100644	18682687;
C524042	decrease	VHL	ENSG00000134086	18682687;
C524063	increase	BCL2	ENSG00000171791	18325115;
C524063	increase	F3	ENSG00000117525	18325115;
C524063	increase	F7	ENSG00000057593	18325115;
C524947	decrease	BAX	ENSG00000087088	15369734;
C524947	decrease	BCL2	ENSG00000171791	15369734;
C526145	decrease	CFLAR	ENSG00000003402	16868541;
C526145	increase	MAPK8	ENSG00000107643	16868541;
C526219	decrease	CTNNB1	ENSG00000168036	16968061;
C526219	decrease	EPHX1	ENSG00000143819	16369997;
C526219	increase	ABCC1	ENSG00000103222	18351577;
C526219	increase	ABCC2	ENSG00000023839	18351577;
C526219	increase	CASP10	ENSG00000003400	18351577;
C526219	increase	CHST3	ENSG00000122863	18351577;
C526219	increase	CHST5	ENSG00000135702	16369997;
C526219	increase	CHST6	ENSG00000183196	16369997;
C526219	increase	CHST7	ENSG00000147119	16369997;
C526219	increase	CYP24A1	ENSG00000019186	18351577;
C526219	increase	CYP4F3	ENSG00000186529	16369997;
C526219	increase	EPO	ENSG00000130427	18351577;
C526219	increase	GSK3B	ENSG00000082701	16968061;
C526219	increase	GSTA4	ENSG00000170899	18351577;
C526219	increase	GSTP1	ENSG00000084207	16369997;
C526219	increase	GSTT2	ENSG00000133433	18351577;16369997;
C526219	increase	MGST2	ENSG00000085871	16369997;

C526219	increase	NFKB2	ENSG00000077150	18351577;
C526219	increase	NFKBIB	ENSG00000104825	18351577;
C526219	increase	PTPN6	ENSG00000111679	18351577;
C526219	increase	PTPRJ	ENSG00000149177	18351577;
C526219	increase	TGFA	ENSG00000163235	18351577;
C526219	increase	UGT2B7	ENSG00000171234	18351577;
C526437	decrease	BIRC5	ENSG00000089685	19846905;
C526437	decrease	XIAP	ENSG00000101966	19846905;
C526438	decrease	BIRC5	ENSG00000089685	19846905;
C526438	decrease	XIAP	ENSG00000101966	19846905;
C526438	increase	CCNB1	ENSG00000134057	18223236;
C527236	decrease	BCL2L1	ENSG00000171552	19148494;
C527236	increase	CASP3	ENSG00000164305	19148494;
C527236	increase	CDKN1A	ENSG00000124762	19148494;
C527236	increase	CDKN2B	ENSG00000147883	19148494;
C527236	increase	MMP9	ENSG00000100985	19148494;
C527236	increase	NR4A1	ENSG00000123358	19148494;
C528002	decrease	BCL2	ENSG00000171791	18205257;18161298;17498489;
C528002	decrease	PCNA	ENSG00000132646	18205257;18161298;
C528002	decrease	ROD1	ENSG00000119314	18205257;18161298;
C528002	decrease	RUFY3	ENSG00000018189	18205257;18161298;
C528002	decrease	STAT3	ENSG00000168610	18205257;18161298;17498489;
C529061	affect	BBC3	ENSG00000105327	18424558;
C529061	affect	CDKN1A	ENSG00000124762	18424558;
C529061	affect	TP53	ENSG00000141510	18424558;
C529061	affect	TP73	ENSG00000078900	18424558;
C530804	increase	CDKN1A	ENSG00000124762	19497413;
C530804	increase	CDKN1B	ENSG00000111276	19497413;
C530804	increase	NOS2	ENSG00000007171	19497413;
C530804	increase	TP53	ENSG00000141510	19497413;
C531389	affect	CCL2	ENSG00000108691	18489909;
C531389	affect	ICAM1	ENSG00000090339	18489909;
C531389	decrease	PTGS2	ENSG00000073756	14988445;
C531389	increase	TNF	ENSG00000223952	18489909;17880982;
C531389	increase	VCAM1	ENSG00000162692	18489909;17880982;
C531975	increase	TNF	ENSG00000223952	18602074;
C531975	increase	VCAM1	ENSG00000162692	18602074;
C533894	decrease	AMIGO2	ENSG00000139211	16951191;
C533894	decrease	BARD1	ENSG00000138376	16951191;
C533894	decrease	BLM	ENSG00000197299	16951191;
C533894	decrease	BMP7	ENSG00000101144	16951191;
C533894	decrease	CAV1	ENSG00000105974	16951191;
C533894	decrease	CAV2	ENSG00000105971	16951191;
C533894	decrease	CCNA2	ENSG00000145386	16951191;
C533894	decrease	CCND1	ENSG00000110092	16951191;
C533894	decrease	CRABP2	ENSG00000143320	16951191;
C533894	decrease	CXCL12	ENSG00000107562	16951191;
C533894	decrease	ECT2	ENSG00000114346	16951191;
C533894	decrease	ENTPD5	ENSG00000187097	16951191;
C533894	decrease	FGFR3	ENSG00000068078	16951191;
C533894	decrease	G6PD	ENSG00000160211	16951191;
C533894	decrease	GPER	ENSG00000164850	16951191;

C533894	decrease	GREB1	ENSG00000196208	16951191;
C533894	decrease	HMMR	ENSG00000072571	16951191;
C533894	decrease	IRS1	ENSG00000169047	16951191;
C533894	decrease	LYPD3	ENSG00000124466	16951191;
C533894	decrease	MAFB	ENSG00000204103	16951191;
C533894	decrease	MSX2	ENSG00000120149	16951191;
C533894	decrease	MYB	ENSG00000118513	16951191;
C533894	decrease	MYBL1	ENSG00000185697	16951191;
C533894	decrease	NAT1	ENSG00000171428	16951191;
C533894	decrease	PBX3	ENSG00000167081	16951191;
C533894	decrease	PDLIM2	ENSG00000120913	16951191;
C533894	decrease	PHLDA2	ENSG00000181649	16951191;
C533894	decrease	PIK3R1	ENSG00000145675	16951191;
C533894	decrease	PTTG1	ENSG00000164611	16951191;
C533894	decrease	RET	ENSG00000165731	16951191;
C533894	decrease	SFN	ENSG00000175793	16951191;
C533894	decrease	SPDEF	ENSG00000124664	16951191;
C533894	decrease	TFF3	ENSG00000160180	16951191;
C533894	decrease	TOB1	ENSG00000141232	16951191;
C533894	decrease	VAV3	ENSG00000134215	16951191;
C533894	increase	ATF3	ENSG00000162772	16951191;
C533894	increase	BATF	ENSG00000156127	16951191;
C533894	increase	BTG1	ENSG00000133639	16951191;
C533894	increase	BTG2	ENSG00000159388	16951191;
C533894	increase	C13ORF15	ENSG00000102760	16951191;
C533894	increase	CDKN2B	ENSG00000147883	16951191;
C533894	increase	CEACAM1	ENSG00000079385	16951191;
C533894	increase	CLU	ENSG00000120885	16951191;
C533894	increase	CXCR4	ENSG00000121966	16951191;
C533894	increase	DDIT3	ENSG00000175197	16951191;
C533894	increase	EDN1	ENSG00000078401	16951191;
C533894	increase	EI24	ENSG00000149547	16951191;
C533894	increase	ELF3	ENSG00000163435	16951191;
C533894	increase	FBLN5	ENSG00000140092	16951191;
C533894	increase	FGF13	ENSG00000129682	16951191;
C533894	increase	FGFR2	ENSG00000066468	16951191;
C533894	increase	FOXO3	ENSG00000118689	16951191;
C533894	increase	GADD45G	ENSG00000130222	16951191;
C533894	increase	GDF15	ENSG00000130513	16951191;
C533894	increase	GSN	ENSG00000148180	16951191;
C533894	increase	HIPK2	ENSG00000064393	16951191;
C533894	increase	HOXA1	ENSG00000105991	16951191;
C533894	increase	HTRA1	ENSG00000166033	16951191;
C533894	increase	IGFBP3	ENSG00000146674	16951191;
C533894	increase	IGFBP6	ENSG00000167779	16951191;
C533894	increase	IL8	ENSG00000169429	16951191;
C533894	increase	IRF1	ENSG00000125347	16951191;
C533894	increase	JAG1	ENSG00000101384	16951191;
C533894	increase	JUN	ENSG00000177606	16951191;
C533894	increase	KLF6	ENSG00000067082	16951191;
C533894	increase	LIMA1	ENSG00000050405	16951191;
C533894	increase	LYN	ENSG00000249529	16951191;
C533894	increase	MARCKS	ENSG00000155130	16951191;
C533894	increase	MDM2	ENSG00000135679	16951191;

C533894	increase	MITF	ENSG00000187098	16951191;
C533894	increase	NBL1	ENSG00000158747	16951191;
C533894	increase	NKX3-1	ENSG00000167034	16951191;
C533894	increase	PDCD4	ENSG00000150593	16951191;
C533894	increase	PDGFC	ENSG00000145431	16951191;
C533894	increase	PHLDA1	ENSG00000139289	16951191;
C533894	increase	PLA2G2A	ENSG00000188257	16951191;
C533894	increase	PPARG	ENSG00000132170	16951191;
C533894	increase	PRSS8	ENSG00000052344	16951191;
C533894	increase	PSAP	ENSG00000197746	16951191;
C533894	increase	S100P	ENSG00000163993	16951191;
C533894	increase	SOD2	ENSG00000112096	16951191;
C533894	increase	SULF1	ENSG00000137573	16951191;
C533894	increase	TGFA	ENSG00000163235	16951191;
C533894	increase	TGFBI	ENSG00000120708	16951191;
C533894	increase	TGM2	ENSG00000198959	16951191;
C533894	increase	UBD	ENSG00000213886	16951191;
C534043	decrease	HIF1A	ENSG00000100644	18927491;
C534043	decrease	NOS2	ENSG00000007171	18927491;
C534043	decrease	PTGS2	ENSG00000073756	18927491;
C534305	increase	RHOA	ENSG00000067560	12671036;
C534305	increase	RHOB	ENSG00000143878	12671036;
C534317	increase	RAP1A	ENSG00000116473	12671036;
C534317	increase	RHOA	ENSG00000067560	12671036;
C534317	increase	RHOB	ENSG00000143878	12671036;
C539236	increase	IL1B	ENSG00000125538	18854230;
C539236	increase	IL4	ENSG00000113520	18854230;
C540278	decrease	KLK3	ENSG00000142515	19359544;
D000001	decrease	UHRF1	ENSG00000034063	15964557;
D000001	increase	CD40	ENSG00000101017	16601352;
D000001	increase	DDIT3	ENSG00000175197	11360202;19428936;
D000001	increase	HDC	ENSG00000140287	17191106;
D000001	increase	HSPA5	ENSG00000044574	19428936;
D000001	increase	IFNG	ENSG00000111537	14994387;
D000001	increase	IL13	ENSG00000169194	12789233;
D000001	increase	IL18R1	ENSG00000115604	14994387;
D000001	increase	IL1B	ENSG00000125538	18958421;17588137;15705740;
D000001	increase	IL4	ENSG00000113520	12789233;11861792;16601352;18325701;
D000001	increase	IL5	ENSG00000113525	12789233;12797483;18325701;
D000001	increase	IL6	ENSG00000136244	11238657;12789233;17191106;18958421;17588137;
D000001	increase	IL8	ENSG00000169429	17000667;12789233;16134060;18958421;17588137;15286717;
D000001	increase	RELA	ENSG00000173039	17588137;18958421;11360202;
D000001	increase	TNF	ENSG00000223952	16134060;18958421;17588137;
D000079	affect	IL10	ENSG00000136634	15698409;
D000079	affect	ITPR1	ENSG00000150995	17241155;
D000079	decrease	BCL2	ENSG00000171791	16814256;
D000079	decrease	COL4A1	ENSG00000187498	8135774;

D000079	decrease	HAMP	ENSG00000105697	16737972;
D000079	decrease	MMP1	ENSG00000196611	7934638;
D000079	increase	ADIPOQ	ENSG00000181092	16814256;
D000079	increase	BAX	ENSG00000087088	16814256;
D000079	increase	CCL2	ENSG00000108691	18716790;17050345;
D000079	increase	CCR2	ENSG00000121807	19036374;
D000079	increase	COL1A1	ENSG00000108821	17030193;
D000079	increase	COL1A2	ENSG00000164692	11343241;16025520;
D000079	increase	CYBB	ENSG00000165168	18845238;
D000079	increase	DDIT3	ENSG00000175197	12612910;
D000079	increase	FANCD2	ENSG00000144554	18482162;
D000079	increase	FN1	ENSG00000115414	11343241;8135774;
D000079	increase	IL1B	ENSG00000125538	18716790;17050345;11344824;
D000079	increase	IL4	ENSG00000113520	15698409;
D000079	increase	IL6	ENSG00000136244	18716790;17050345;
D000079	increase	IL8	ENSG00000169429	18716790;14619338;11344824;15698409;
D000079	increase	JUN	ENSG00000177606	10799556;11978551;
D000079	increase	MAPK1	ENSG00000100030	11343241;17030193;14722101;
D000079	increase	MAPK3	ENSG00000102882	11343241;17030193;14722101;
D000079	increase	MMP11	ENSG00000099953	11978551;
D000079	increase	MMP2	ENSG00000087245	7934638;
D000079	increase	MMP9	ENSG00000100985	19110045;
D000079	increase	NOS2	ENSG00000007171	18845238;
D000079	increase	PPARG	ENSG00000132170	17030193;
D000079	increase	PRKCD	ENSG00000163932	17030193;
D000079	increase	PTGS1	ENSG00000095303	18716790;17050345;
D000079	increase	PTGS2	ENSG00000073756	18716790;19110045;17050345;
D000079	increase	SELP	ENSG00000174175	19036374;
D000079	increase	SMAD3	ENSG00000166949	16025520;
D000079	increase	SMAD4	ENSG00000141646	16025520;
D000079	increase	SP1	ENSG00000185591	16025520;
D000079	increase	SPP1	ENSG00000118785	18703563;
D000079	increase	SREBF1	ENSG00000072310	12612910;
D000079	increase	TGFB1	ENSG00000105329	16025520;8135774;
D000079	increase	TNF	ENSG00000223952	18716790;11344824;19036374;17050345;
D000079	increase	XDH	ENSG00000158125	18845238;
D000082	affect	BCL2L1	ENSG00000171552	11896290;
D000082	affect	CDKN1A	ENSG00000124762	11896290;
D000082	affect	FOS	ENSG00000170345	11896290;
D000082	affect	MCL1	ENSG00000143384	11896290;
D000082	decrease	ACAT2	ENSG00000120437	11793227;
D000082	decrease	ALDH18A1	ENSG00000059573	11793227;
D000082	decrease	ANG	ENSG00000214274	11793227;
D000082	decrease	ATP5J2	ENSG00000241468	11793227;
D000082	decrease	BNIP3	ENSG00000176171	17093179;
D000082	decrease	CALU	ENSG00000128595	11793227;
D000082	decrease	CD2	ENSG00000116824	17093179;
D000082	decrease	CD69	ENSG00000110848	17093179;
D000082	decrease	CDK4	ENSG00000135446	11793227;

D000082	decrease	CYC1	ENSG00000179091	11793227;
D000082	decrease	DAD1	ENSG00000129562	11793227;
D000082	decrease	GLRX	ENSG00000173221	11793227;
D000082	decrease	GLT8D1	ENSG00000016864	11793227;
D000082	decrease	GLUD1	ENSG00000148672	11793227;
D000082	decrease	GSTO1	ENSG00000148834	11793227;
D000082	decrease	GZMA	ENSG00000145649	17093179;
D000082	decrease	KLRD1	ENSG00000134539	17093179;
D000082	decrease	KLRF1	ENSG00000150045	17093179;
D000082	decrease	LDHA	ENSG00000134333	11793227;
D000082	decrease	LDHB	ENSG00000111716	17093179;
D000082	decrease	LEF1	ENSG00000138795	17093179;
D000082	decrease	MAL	ENSG00000172005	17093179;
D000082	decrease	MGST1	ENSG00000008394	11793227;
D000082	decrease	MOAP1	ENSG00000165943	17093179;
D000082	decrease	NFKBIA	ENSG00000100906	17093179;
D000082	decrease	PDCD4	ENSG00000150593	17093179;
D000082	decrease	POMC	ENSG00000115138	16449370;
D000082	decrease	PRELID1	ENSG00000169230	11793227;
D000082	decrease	PRKCH	ENSG00000027075	17093179;
D000082	decrease	PRKCQ	ENSG00000065675	17093179;
D000082	decrease	PSME3	ENSG00000131467	11793227;
D000082	decrease	PTGS1	ENSG00000095303	16141368;17045584;11113024;15263079;17175104;
D000082	decrease	RASGRP1	ENSG00000172575	17093179;
D000082	decrease	RPS6KA3	ENSG00000177189	11793227;
D000082	decrease	SCFD1	ENSG00000249037	11793227;
D000082	decrease	SCP2	ENSG00000251612	17093179;
D000082	decrease	SEC13	ENSG00000157020	11793227;
D000082	decrease	SEC23A	ENSG00000100934	11793227;
D000082	decrease	SERPINA6	ENSG00000170099	11793227;
D000082	decrease	SFRS1	ENSG00000136450	11793227;
D000082	decrease	SQLE	ENSG00000104549	11793227;
D000082	decrease	STRBP	ENSG00000165209	11793227;
D000082	decrease	TFDP1	ENSG00000198176	11793227;
D000082	decrease	TM9SF1	ENSG00000100926	11793227;
D000082	decrease	TMEM97	ENSG00000109084	11793227;
D000082	decrease	UQCRC2	ENSG00000140740	11793227;
D000082	increase	ABCB1	ENSG00000085563	16930294;17526808;17627974;
D000082	increase	ABCC1	ENSG00000103222	17627974;
D000082	increase	ABCC4	ENSG00000125257	17627974;
D000082	increase	ABCC5	ENSG00000114770	17627974;
D000082	increase	ABCG2	ENSG00000118777	17627974;
D000082	increase	ACIN1	ENSG00000100813	11793227;
D000082	increase	ACSL3	ENSG00000123983	11793227;
D000082	increase	ARL6IP1	ENSG00000170540	11793227;
D000082	increase	CD14	ENSG00000170458	17093179;
D000082	increase	CD93	ENSG00000125810	17093179;
D000082	increase	DAPK1	ENSG00000196730	17093179;
D000082	increase	DDIT4	ENSG00000168209	17093179;
D000082	increase	DHRS9	ENSG00000073737	17093179;
D000082	increase	EIF1	ENSG00000173812	11793227;
D000082	increase	GAA	ENSG00000171298	17093179;

D000082	increase	HAL	ENSG00000084110	17093179;
D000082	increase	HMOX1	ENSG00000100292	18227147;
D000082	increase	ID1	ENSG00000125968	15120964;
D000082	increase	IGFBP1	ENSG00000146678	11793227;
D000082	increase	IMPDH1	ENSG00000106348	17093179;
D000082	increase	MT1B	ENSG00000169688	11793227;
D000082	increase	MT1G	ENSG00000125144	11793227;
D000082	increase	MT1H	ENSG00000205358	11793227;
D000082	increase	MYC	ENSG00000136997	11896290;
D000082	increase	NQO1	ENSG00000181019	16610002;
D000082	increase	OAT	ENSG00000065154	11793227;
D000082	increase	PCNA	ENSG00000132646	17627974;
D000082	increase	PHYH	ENSG00000107537	15342952;
D000082	increase	PLOD2	ENSG00000152952	15997088;
D000082	increase	PSAP	ENSG00000197746	17093179;
D000082	increase	PTGS2	ENSG00000073756	15705740;11113024;17175104;
D000082	increase	PYGL	ENSG00000100504	17093179;
D000082	increase	RRS1	ENSG00000179041	11793227;
D000082	increase	SIGLEC5	ENSG00000105501	17093179;
D000082	increase	SMOX	ENSG00000088826	11793227;
D000082	increase	TAC1	ENSG00000006128	16449370;
D000082	increase	TNFSF13	ENSG00000161955	17093179;
D000096	increase	CCND1	ENSG00000110092	12720008;
D000096	increase	FGFR2	ENSG00000066468	12720008;
D000096	increase	ITGA5	ENSG00000161638	12720008;
D000111	decrease	ABCG1	ENSG00000160179	16084531;
D000111	decrease	AR	ENSG00000169083	16928829;
D000111	decrease	ART4	ENSG00000111339	16084531;
D000111	decrease	BCL2	ENSG00000171791	17542780;
D000111	decrease	BPGM	ENSG00000172331	16084531;
D000111	decrease	BTG1	ENSG00000133639	16084531;
D000111	decrease	CDH13	ENSG00000140945	16084531;
D000111	decrease	CFTR	ENSG00000001626	19061877;
D000111	decrease	COL1A1	ENSG00000108821	11710721;
D000111	decrease	COL1A2	ENSG00000164692	11710721;
D000111	decrease	COL4A1	ENSG00000187498	16084531;
D000111	decrease	CPE	ENSG00000109472	16084531;
D000111	decrease	CRADD	ENSG00000169372	16084531;
D000111	decrease	CXCL1	ENSG00000163739	17395010;
D000111	decrease	EFNB2	ENSG00000125266	16084531;
D000111	decrease	FGF2	ENSG00000138685	16084531;
D000111	decrease	FILIP1L	ENSG00000168386	16084531;
D000111	decrease	GBP1	ENSG00000117228	16084531;
D000111	decrease	GJA4	ENSG00000187513	16084531;
D000111	decrease	GULP1	ENSG00000144366	16084531;
D000111	decrease	HTATIP2	ENSG00000109854	16084531;
D000111	decrease	ID2	ENSG00000115738	16084531;
D000111	decrease	IL10RB	ENSG00000243646	16084531;
D000111	decrease	IL27RA	ENSG00000104998	16084531;
D000111	decrease	ITGAV	ENSG00000138448	16084531;
D000111	decrease	JAG1	ENSG00000101384	16084531;
D000111	decrease	MGEA5	ENSG00000198408	16084531;
D000111	decrease	MOAP1	ENSG00000165943	16084531;

D000111	decrease	NEDD9	ENSG00000111859	16084531;
D000111	decrease	NR3C1	ENSG00000113580	16084531;
D000111	decrease	PDLIM5	ENSG00000163110	16084531;
D000111	decrease	PLAU	ENSG00000122861	16084531;
D000111	decrease	RHOBTB1	ENSG00000072422	16084531;
D000111	decrease	SELE	ENSG00000007908	16084531;
D000111	decrease	SP1	ENSG00000185591	16928829;
D000111	decrease	TANK	ENSG00000136560	16084531;
D000111	decrease	TNFSF10	ENSG00000121858	16084531;
D000111	decrease	TSPAN6	ENSG00000000003	16084531;
D000111	decrease	TXNL1	ENSG00000091164	16084531;
D000111	decrease	ZMYM2	ENSG00000121741	16084531;
D000111	increase	ABCC1	ENSG00000103222	11820781;15473893;
D000111	increase	ABCC3	ENSG00000108846	11820781;
D000111	increase	ACTA2	ENSG00000107796	16757516;
D000111	increase	ATF4	ENSG00000128272	15788408;
D000111	increase	ATP2A2	ENSG00000174437	16084531;
D000111	increase	BAX	ENSG00000087088	17542780;
D000111	increase	CCL2	ENSG00000108691	15139008;19158351;17529908;
D000111	increase	CCND1	ENSG00000110092	15897899;
D000111	increase	CD86	ENSG00000114013	19033392;
D000111	increase	CDKN1A	ENSG00000124762	15978937;9343371;894323
D000111	increase	CXCL2	ENSG00000081041	10718115;
D000111	increase	DDIT3	ENSG00000175197	17171638;18384088;16084531;
D000111	increase	DNAJB9	ENSG00000128590	16084531;
D000111	increase	DUSP4	ENSG00000120875	16084531;
D000111	increase	FLT1	ENSG00000102755	17992120;
D000111	increase	FOS	ENSG00000170345	9525474;
D000111	increase	GATA1	ENSG00000102145	10656287;
D000111	increase	GCLC	ENSG00000001084	11909699;11820781;
D000111	increase	HBEGF	ENSG00000113070	12676768;
D000111	increase	HERPUD1	ENSG00000051108	16084531;
D000111	increase	HGF	ENSG00000019991	16757516;
D000111	increase	HIF1A	ENSG00000100644	15840558;17658243;17066447;
D000111	increase	HK2	ENSG00000159399	16084531;
D000111	increase	HMBS	ENSG00000149397	10656287;
D000111	increase	HMOX1	ENSG00000100292	16959797;18357586;19033392;16806455;
D000111	increase	ICAM1	ENSG00000090339	17666914;
D000111	increase	IL6	ENSG00000136244	16757516;
D000111	increase	IL8	ENSG00000169429	9628260;11306435;9707512;15139008;12208513;
D000111	increase	INS	ENSG00000129965	16505238;
D000111	increase	KDR	ENSG00000128052	17992120;
D000111	increase	KEAP1	ENSG00000079999	19033392;11909699;
D000111	increase	MGMT	ENSG00000170430	16950796;
D000111	increase	MMP7	ENSG00000137673	17928719;
D000111	increase	MMP9	ENSG00000100985	17015178;19371603;16251475;
D000111	increase	NDUFB6	ENSG00000165264	19523936;
D000111	increase	NDUFS7	ENSG00000115286	19523936;
D000111	increase	NFE2	ENSG00000123405	10656287;

D000111	increase	NFE2L2	ENSG00000116044	19033392;11909699;16806455;16959797;
D000111	increase	NQO1	ENSG00000181019	19033392;
D000111	increase	PARK7	ENSG00000116288	18377993;
D000111	increase	PIGA	ENSG00000165195	16084531;
D000111	increase	PIM2	ENSG00000102096	16084531;
D000111	increase	PLD1	ENSG00000075651	17640750;
D000111	increase	PLD2	ENSG00000129219	17640750;
D000111	increase	PON2	ENSG00000105854	17916643;
D000111	increase	PTGS2	ENSG00000073756	18480072;17640750;17255567;15576159;16084531;
D000111	increase	SLC39A14	ENSG00000104635	16084531;
D000111	increase	SNAI1	ENSG00000124216	17596522;
D000111	increase	SNCA	ENSG00000145335	15114628;
D000111	increase	SOD2	ENSG00000112096	8760145;15904944;
D000111	increase	TGFB1	ENSG00000105329	16757516;
D000111	increase	TNF	ENSG00000223952	9721806;15161907;8760145;17666914;15650392;
D000111	increase	TP53	ENSG00000141510	15489221;17555331;10951577;
D000111	increase	UGDH	ENSG00000109814	16084531;
D000111	increase	VEGFA	ENSG00000112715	15788408;17992120;
D000111	increase	ZFP36L2	ENSG00000152518	16084531;
D000119	increase	CD40	ENSG00000101017	16299289;
D000119	increase	CD80	ENSG00000121594	16299289;
D000119	increase	CD83	ENSG00000112149	16299289;
D000119	increase	CD86	ENSG00000114013	16299289;
D000119	increase	IFNG	ENSG00000111537	18490488;16299289;
D000119	increase	IL12A	ENSG00000168811	16299289;18490488;
D000119	increase	IL12B	ENSG00000113302	15905553;16299289;18490488;
D000119	increase	IL15	ENSG00000164136	16299289;
D000119	increase	IL23A	ENSG00000110944	18490488;
D000119	increase	IL8	ENSG00000169429	16299289;18413660;
D000143	decrease	CDX2	ENSG00000165556	16990345;
D000143	increase	IL6	ENSG00000136244	19074641;
D000143	increase	IL8	ENSG00000169429	19074641;
D000165	increase	GSTP1	ENSG00000084207	16814760;
D000171	affect	EGFR	ENSG00000146648	11329622;15531749;
D000171	decrease	NFE2L1	ENSG00000082641	17949413;
D000171	decrease	PTGIS	ENSG00000124212	17255567;
D000171	decrease	TFAM	ENSG00000108064	17949413;
D000171	increase	ADAM17	ENSG00000151694	15531749;
D000171	increase	AKT1	ENSG00000142208	19596284;
D000171	increase	BIRC2	ENSG00000110330	19596284;
D000171	increase	BIRC3	ENSG00000023445	19596284;
D000171	increase	GCLC	ENSG00000001084	12084617;
D000171	increase	HMOX1	ENSG00000100292	18048804;
D000171	increase	IL6	ENSG00000136244	16860297;
D000171	increase	IL8	ENSG00000169429	17491020;15650393;18201443;11504702;16860297;
D000171	increase	MMP9	ENSG00000100985	19371603;18006877;15531749;
D000171	increase	NFE2L2	ENSG00000116044	18048804;12084617;18258206;17197552;

D000171	increase	NFKBIA	ENSG00000100906	15650393;
D000171	increase	NQO1	ENSG00000181019	12084617;
D000171	increase	PTGS2	ENSG00000073756	17363696;
D000171	increase	TIMP3	ENSG00000100234	15531749;
D000171	increase	TNF	ENSG00000223952	15650393;17491020;
D000181	decrease	BCL2	ENSG00000171791	16173056;
D000181	decrease	CCL2	ENSG00000108691	16173056;
D000181	decrease	CXCR4	ENSG00000121966	16173056;
D000181	decrease	FOS	ENSG00000170345	16173056;
D000181	decrease	JUN	ENSG00000177606	16173056;
D000181	decrease	S100A8	ENSG00000143546	16173056;
D000181	increase	BAX	ENSG00000087088	16173056;
D000181	increase	HMOX1	ENSG00000100292	14644359;
D000181	increase	NQO1	ENSG00000181019	9029048;
D000225	increase	IL8	ENSG00000169429	18308354;
D000241	affect	ADORA1	ENSG00000163485	16611738;
D000241	affect	ADORA2A	ENSG00000128271	16611738;
D000241	affect	ADORA2B	ENSG00000170425	16611738;
D000241	affect	ADORA3	ENSG00000121933	16611738;
D000241	decrease	DPP4	ENSG00000197635	16611738;
D000241	decrease	MAPK1	ENSG00000100030	16611738;
D000241	decrease	MAPK3	ENSG00000102882	16611738;
D000242	affect	SLC2A5	ENSG00000142583	8554516;12820898;
D000242	decrease	HRH2	ENSG00000113749	9681472;15843518;918726
D000242	decrease	IL12A	ENSG00000168811	15843518;
D000242	decrease	IL12B	ENSG00000113302	15843518;
D000242	increase	IL8	ENSG00000169429	9258252;
D000244	affect	CYP2C19	ENSG00000165841	18004210;18394438;16772608;
D000244	affect	P2RY12	ENSG00000169313	11196645;18404433;
D000244	increase	CD40LG	ENSG00000102245	11297035;
D000244	increase	ITGA2B	ENSG00000005961	18404433;
D000244	increase	ITGB3	ENSG00000056345	18404433;
D000244	increase	SELP	ENSG00000174175	18394438;18404433;
D000255	increase	ICAM1	ENSG00000090339	18508964;
D000255	increase	SLC5A5	ENSG00000105641	16439463;
D000255	increase	TSHB	ENSG00000134200	16439463;
D000305	decrease	IL12B	ENSG00000113302	11080718;
D000305	increase	IL3	ENSG00000164399	10224351;
D000305	increase	IL5	ENSG00000113525	10224351;
D000305	increase	IL5RA	ENSG00000091181	10224351;
D000420	decrease	ADRB2	ENSG00000169252	15687340;16980553;12920204;10516654;10952688;14722251;
D000420	decrease	UCP3	ENSG00000175564	12824081;
D000420	increase	CCL3	ENSG00000006075	12818968;
D000420	increase	IL8	ENSG00000169429	12818968;15679717;
D000420	increase	PDE4A	ENSG00000065989	10763856;
D000420	increase	PDE4B	ENSG00000184588	10763856;
D000431	decrease	ADAT1	ENSG00000065457	15963989;
D000431	decrease	C6ORF108	ENSG00000112667	11566570;
D000431	decrease	CDC37L1	ENSG00000106993	15963989;
D000431	decrease	CEBPA	ENSG00000245848	16737972;
D000431	decrease	CRYBB3	ENSG00000100053	11566570;

D000431	decrease	HAMP	ENSG00000105697	16737972;
D000431	decrease	MAPK3	ENSG00000102882	18295389;11427526;11566570;
D000431	decrease	MUTYH	ENSG00000132781	11566570;
D000431	decrease	OPRK1	ENSG00000082556	17934066;
D000431	decrease	PDYN	ENSG00000101327	17934066;
D000431	decrease	PENK	ENSG00000181195	17934066;
D000431	decrease	PFDN5	ENSG00000123349	11566570;
D000431	decrease	PMS2	ENSG00000122512	11566570;
D000431	decrease	POMC	ENSG00000115138	17934066;
D000431	decrease	POU2F1	ENSG00000143190	11566570;
D000431	decrease	RRAD	ENSG00000166592	11566570;
D000431	decrease	SLC19A1	ENSG00000173638	19616087;
D000431	increase	CAV1	ENSG00000105974	15963989;
D000431	increase	CCND1	ENSG00000110092	12720008;
D000431	increase	CLU	ENSG00000120885	15963989;
D000431	increase	CYBB	ENSG00000165168	18845238;
D000431	increase	CYP2E1	ENSG00000130649	8798623;12464242;10826103;10573527;18845238;11212141;16235733;15802389;8531136;9143349;8074729;17590308;3782137;170347
D000431	increase	ESR1	ENSG00000091831	15659783;
D000431	increase	FANCD2	ENSG00000144554	18482162;
D000431	increase	FGFR2	ENSG00000066468	12720008;
D000431	increase	FN1	ENSG00000115414	15963989;
D000431	increase	FOLR1	ENSG00000110195	17531458;19616087;
D000431	increase	FTL	ENSG00000087086	15963989;
D000431	increase	HMOX1	ENSG00000100292	17295091;
D000431	increase	IGFBP3	ENSG00000146674	15963989;
D000431	increase	IL11	ENSG00000095752	17295091;
D000431	increase	IL1B	ENSG00000125538	11344824;
D000431	increase	ITGA5	ENSG00000161638	12720008;
D000431	increase	ITPR1	ENSG00000150995	17241155;
D000431	increase	KRT18	ENSG00000111057	17034788;
D000431	increase	KRT8	ENSG00000170421	17034788;
D000431	increase	LOXL2	ENSG00000134013	15963989;
D000431	increase	NOS2	ENSG00000007171	16235733;18845238;
D000431	increase	PLOD2	ENSG00000152952	15997088;
D000431	increase	PPARA	ENSG00000186951	18003597;
D000431	increase	S100A4	ENSG00000196154	15963989;
D000431	increase	SPARC	ENSG00000113140	15963989;
D000431	increase	SPP1	ENSG00000118785	18703563;
D000431	increase	TFRC	ENSG00000072274	16737972;15963989;
D000431	increase	TGFB1	ENSG00000105329	11344824;15963989;15147944;
D000431	increase	TGM2	ENSG00000198959	18295389;
D000431	increase	TNF	ENSG00000223952	11344824;
D000431	increase	XDH	ENSG00000158125	18845238;
D000432	increase	CSF2	ENSG00000164400	11306435;
D000432	increase	ESR1	ENSG00000091831	15659783;
D000432	increase	IL1B	ENSG00000125538	15225597;
D000432	increase	PTGS2	ENSG00000073756	15225597;
D000450	decrease	CDH1	ENSG00000039068	17596522;
D000450	increase	ABCB1	ENSG00000085563	12387747;

D000450	increase	ACTA2	ENSG00000107796	17596522;
D000450	increase	SNAI1	ENSG00000124216	17596522;
D000452	increase	ACVR1C	ENSG00000123612	19422813;
D000452	increase	ACVRL1	ENSG00000139567	19422813;
D000452	increase	ALK	ENSG00000171094	19422813;
D000452	increase	CYP19A1	ENSG00000137869	16996190;
D000452	increase	CYP26A1	ENSG00000095596	15589975;
D000452	increase	CYP2B6	ENSG00000197408	15548381;
D000452	increase	CYP3A4	ENSG00000160868	15548381;
D000452	increase	ERBB3	ENSG00000065361	19422813;
D000452	increase	KIT	ENSG00000157404	19422813;
D000452	increase	PDGFRA	ENSG00000134853	19422813;
D000452	increase	PDGFRB	ENSG00000113721	19422813;
D000452	increase	RARB	ENSG00000077092	15589975;
D000452	increase	RARG	ENSG00000172819	15589975;
D000487	increase	WNT10B	ENSG00000169884	12437293;
D000493	increase	CXCL10	ENSG00000169245	18322232;
D000493	increase	IFNB1	ENSG00000171855	18322232;
D000493	increase	MMP9	ENSG00000100985	19371603;
D000535	affect	EPOR	ENSG00000187266	15777837;
D000535	decrease	ACO2	ENSG00000100412	16906525;
D000535	decrease	FH	ENSG00000091483	16906525;
D000535	decrease	OGDH	ENSG00000105953	16906525;
D000535	decrease	SCGB1A1	ENSG00000149021	16099050;
D000535	increase	ACLY	ENSG00000131473	17762189;
D000535	increase	APOB	ENSG00000084674	17762189;
D000535	increase	GPD1	ENSG00000167588	17762189;
D000535	increase	LITAF	ENSG00000189067	17408380;
D000535	increase	MPO	ENSG00000005381	17402216;
D000535	increase	SLC2A1	ENSG00000117394	16979867;
D000535	increase	TNF	ENSG00000223952	17408380;
D000537	increase	IL10	ENSG00000136634	15183442;
D000537	increase	IL1A	ENSG00000115008	15183442;
D000537	increase	IL1B	ENSG00000125538	15183442;
D000537	increase	IL8	ENSG00000169429	15183442;
D000537	increase	TNF	ENSG00000223952	15183442;
D000622	affect	MMP1	ENSG00000196611	12542540;
D000622	affect	MMP3	ENSG00000149968	12542540;
D000638	decrease	ADRB1	ENSG00000043591	1653643;
D000638	decrease	ADRB2	ENSG00000169252	1653643;
D000638	decrease	AP1S1	ENSG00000106367	17567588;
D000638	decrease	SLC26A4	ENSG00000091137	18020914;
D000638	decrease	SLC2A3	ENSG00000059804	17567588;15342952;
D000638	decrease	SLC5A5	ENSG00000105641	18020914;
D000638	decrease	TAGLN	ENSG00000149591	17567588;
D000638	decrease	TG	ENSG00000042832	18020914;
D000638	decrease	TPO	ENSG00000115705	18020914;
D000638	increase	ABCA2	ENSG00000107331	19774075;
D000638	increase	ABCC11	ENSG00000121270	19774075;
D000638	increase	ABL1	ENSG00000097007	19774075;
D000638	increase	ACACA	ENSG00000132142	19774075;
D000638	increase	ACOT8	ENSG00000101473	19774075;
D000638	increase	ACSF3	ENSG00000176715	19774075;
D000638	increase	ACTR3B	ENSG00000133627	19774075;

D000638	increase	ACVR1B	ENSG00000135503	19774075;
D000638	increase	ACYP1	ENSG00000119640	19774075;
D000638	increase	ADORA3	ENSG00000121933	19774075;
D000638	increase	ADRA2A	ENSG00000150594	19774075;
D000638	increase	AGT	ENSG00000135744	17214612;
D000638	increase	AHNAK	ENSG00000124942	19774075;
D000638	increase	ALAS1	ENSG00000023330	19774075;
D000638	increase	ALB	ENSG00000163631	19774075;
D000638	increase	ALG12	ENSG00000182858	19774075;
D000638	increase	ALG2	ENSG00000119523	19774075;
D000638	increase	ALK	ENSG00000171094	19774075;
D000638	increase	AMIGO1	ENSG00000181754	19774075;
D000638	increase	ANXA4	ENSG00000196975	19774075;
D000638	increase	ARC	ENSG00000198576	19774075;
D000638	increase	ARHGAP15	ENSG00000075884	19774075;
D000638	increase	ARHGAP4	ENSG00000089820	19774075;
D000638	increase	ARHGAP9	ENSG00000123329	19774075;
D000638	increase	ARHGEF10	ENSG00000074964	19774075;
D000638	increase	ARHGEF7	ENSG00000102606	19774075;
D000638	increase	ARHGEF9	ENSG00000131089	19774075;
D000638	increase	ARID5A	ENSG00000196843	19774075;
D000638	increase	ARMC9	ENSG00000135931	19774075;
D000638	increase	ARPC4	ENSG00000241553	19774075;
D000638	increase	ASAH1	ENSG00000104763	17567588;15342952;
D000638	increase	ASB7	ENSG00000183475	19774075;
D000638	increase	ASNS	ENSG00000070669	17567588;
D000638	increase	ATF3	ENSG00000162772	19774075;
D000638	increase	ATF6B	ENSG00000228628	19774075;
D000638	increase	ATOH1	ENSG00000172238	19774075;
D000638	increase	ATP11C	ENSG00000101974	19774075;
D000638	increase	BAG1	ENSG00000250477	19774075;
D000638	increase	BAIAP2L1	ENSG00000006453	19774075;
D000638	increase	BAX	ENSG00000087088	12084619;
D000638	increase	BBOX1	ENSG00000129151	19774075;
D000638	increase	BCL2L11	ENSG00000153094	19774075;
D000638	increase	BMP2K	ENSG00000138756	19774075;
D000638	increase	BOLA2	ENSG00000169627	19774075;
D000638	increase	BOLL	ENSG00000152430	19774075;
D000638	increase	BRCC3	ENSG00000185515	19774075;
D000638	increase	BRPF1	ENSG00000156983	19774075;
D000638	increase	BSG	ENSG00000172270	19774075;
D000638	increase	C10ORF47	ENSG00000148426	19774075;
D000638	increase	C11ORF16	ENSG00000176029	19774075;
D000638	increase	C16ORF74	ENSG00000154102	19774075;
D000638	increase	C17ORF68	ENSG00000178971	19774075;
D000638	increase	C17ORF87	ENSG00000161929	19774075;
D000638	increase	C19ORF51	ENSG00000167646	19774075;
D000638	increase	C1ORF183	ENSG00000197852	19774075;
D000638	increase	C1ORF88	ENSG00000173947	19774075;
D000638	increase	C1ORF95	ENSG00000203685	19774075;
D000638	increase	C20ORF94	ENSG00000149346	19774075;
D000638	increase	C21ORF91	ENSG00000154642	19774075;
D000638	increase	C3ORF18	ENSG00000088543	19774075;
D000638	increase	C3ORF23	ENSG00000179152	19774075;

D000638	increase	C3ORF33	ENSG00000174928	19774075;
D000638	increase	C6ORF114	ENSG00000187461	19774075;
D000638	increase	C7ORF31	ENSG00000153790	19774075;
D000638	increase	CAMK2A	ENSG00000070808	19774075;
D000638	increase	CAPN11	ENSG00000137225	19774075;
D000638	increase	CAPN3	ENSG00000248897	19774075;
D000638	increase	CARD9	ENSG00000187796	19774075;
D000638	increase	CASKIN2	ENSG00000177303	19774075;
D000638	increase	CASP3	ENSG00000164305	12084619;
D000638	increase	CASP9	ENSG00000132906	19774075;
D000638	increase	CAV3	ENSG00000182533	19774075;
D000638	increase	CCDC134	ENSG00000100147	19774075;
D000638	increase	CCDC135	ENSG00000159625	19774075;
D000638	increase	CCDC17	ENSG00000159588	19774075;
D000638	increase	CCDC48	ENSG00000114654	19774075;
D000638	increase	CCDC96	ENSG00000173013	19774075;
D000638	increase	CCDC9	ENSG00000105321	19774075;
D000638	increase	CCL2	ENSG00000108691	19225199;
D000638	increase	CCNT1	ENSG00000129315	19774075;
D000638	increase	CCR9	ENSG00000173585	19774075;
D000638	increase	CD320	ENSG00000167775	19774075;
D000638	increase	CDC25A	ENSG00000164045	19774075;
D000638	increase	CDC42SE1	ENSG00000197622	19774075;
D000638	increase	CDH15	ENSG00000129910	19774075;
D000638	increase	CDH26	ENSG00000124215	19774075;
D000638	increase	CEBPB	ENSG00000172216	19774075;
D000638	increase	CECR1	ENSG00000093072	19774075;
D000638	increase	CHAC1	ENSG00000128965	19774075;
D000638	increase	CHGA	ENSG00000100604	19774075;
D000638	increase	CHRNB3	ENSG00000147432	19774075;
D000638	increase	CHST6	ENSG00000183196	19774075;
D000638	increase	CLCN5	ENSG00000171365	19774075;
D000638	increase	CLCN6	ENSG00000011021	19774075;
D000638	increase	CLTCL1	ENSG00000249108	19774075;
D000638	increase	CMTM7	ENSG00000153551	19774075;
D000638	increase	CMYA5	ENSG00000164309	19774075;
D000638	increase	CNIH3	ENSG00000143786	19774075;
D000638	increase	CNNM2	ENSG00000148842	19774075;
D000638	increase	CNOT7	ENSG00000198791	19774075;
D000638	increase	CNP	ENSG00000173786	19774075;
D000638	increase	COL11A1	ENSG00000060718	19774075;
D000638	increase	COL27A1	ENSG00000196739	19774075;
D000638	increase	COL8A2	ENSG00000171812	19774075;
D000638	increase	CORO2A	ENSG00000106789	19774075;
D000638	increase	CPLX2	ENSG00000145920	19774075;
D000638	increase	CRB3	ENSG00000130545	19774075;
D000638	increase	CRP	ENSG00000132693	19225199;
D000638	increase	CST6	ENSG00000175315	19774075;
D000638	increase	CST7	ENSG00000077984	19774075;
D000638	increase	CTLA4	ENSG00000163599	19774075;
D000638	increase	CYP26A1	ENSG00000095596	19774075;
D000638	increase	CYP2D6	ENSG00000100197	19774075;15541258;
D000638	increase	CYP2J2	ENSG00000134716	19774075;
D000638	increase	CYP2R1	ENSG00000186104	19774075;

D000638	increase	DAPK2	ENSG00000035664	19774075;
D000638	increase	DBN1	ENSG00000113758	19774075;
D000638	increase	DBNDD1	ENSG00000003249	19774075;
D000638	increase	DCST2	ENSG00000163354	19774075;
D000638	increase	DCTN1	ENSG00000204843	19774075;
D000638	increase	DDIT3	ENSG00000175197	19774075;
D000638	increase	DDIT4	ENSG00000168209	19774075;
D000638	increase	DDX17	ENSG00000100201	19774075;
D000638	increase	DDX31	ENSG00000125485	19774075;
D000638	increase	DDX39	ENSG00000123136	19774075;
D000638	increase	DDX49	ENSG00000105671	19774075;
D000638	increase	DEDD	ENSG00000158796	19774075;
D000638	increase	DEPDC6	ENSG00000155792	19774075;
D000638	increase	DHRS2	ENSG00000100867	19774075;
D000638	increase	DHX57	ENSG00000163214	19774075;
D000638	increase	DLG4	ENSG00000132535	19774075;
D000638	increase	DLK2	ENSG00000171462	19774075;
D000638	increase	DNAH7	ENSG00000118997	19774075;
D000638	increase	DNAJB12	ENSG00000148719	19774075;
D000638	increase	DNAJB7	ENSG00000172404	19774075;
D000638	increase	DNAJB9	ENSG00000128590	19774075;
D000638	increase	DNASE1	ENSG00000213918	19774075;
D000638	increase	DOCK5	ENSG00000147459	19774075;
D000638	increase	DPM2	ENSG00000136908	19774075;
D000638	increase	DUSP10	ENSG00000143507	19774075;
D000638	increase	DUSP1	ENSG00000120129	19774075;
D000638	increase	DUT	ENSG00000128951	19774075;
D000638	increase	EAF2	ENSG00000145088	19774075;
D000638	increase	EED	ENSG00000074266	19774075;
D000638	increase	EEPD1	ENSG00000122547	19774075;
D000638	increase	EFCAB5	ENSG00000176927	19774075;
D000638	increase	EGFL6	ENSG00000198759	19774075;
D000638	increase	EGR1	ENSG00000120738	19774075;
D000638	increase	EGR2	ENSG00000122877	19774075;
D000638	increase	EHMT1	ENSG00000181090	19774075;
D000638	increase	EIF2AK3	ENSG00000172071	19774075;
D000638	increase	EIF5	ENSG00000100664	19774075;
D000638	increase	ELMO3	ENSG00000102890	19774075;
D000638	increase	ENPP7	ENSG00000182156	19774075;
D000638	increase	EPC1	ENSG00000120616	19774075;
D000638	increase	EPC2	ENSG00000135999	19774075;
D000638	increase	ERMN	ENSG00000136541	19774075;
D000638	increase	ETV7	ENSG00000010030	19774075;
D000638	increase	EXO1	ENSG00000174371	19774075;
D000638	increase	F2	ENSG00000180210	18974383;
D000638	increase	F3	ENSG00000117525	18974383;
D000638	increase	FABP1	ENSG00000163586	17567588;15342952;
D000638	increase	FAM119B	ENSG00000123427	19774075;
D000638	increase	FAM129A	ENSG00000135842	19774075;
D000638	increase	FAM150A	ENSG00000196711	19774075;
D000638	increase	FAM153A	ENSG00000170074	19774075;
D000638	increase	FAM165B	ENSG00000205670	19774075;
D000638	increase	FAM81A	ENSG00000157470	19774075;
D000638	increase	FAM89A	ENSG00000182118	19774075;

D000638	increase	FANCA	ENSG00000187741	19774075;
D000638	increase	FARP2	ENSG00000006607	19774075;
D000638	increase	FBXO16	ENSG00000214050	19774075;
D000638	increase	FBXO32	ENSG00000156804	19774075;
D000638	increase	FCRL1	ENSG00000163534	19774075;
D000638	increase	FCRL2	ENSG00000132704	19774075;
D000638	increase	FCRL3	ENSG00000160856	19774075;
D000638	increase	FFAR3	ENSG00000185897	19774075;
D000638	increase	FGD4	ENSG00000139132	19774075;
D000638	increase	FGD5	ENSG00000154783	19774075;
D000638	increase	FGFR1OP2	ENSG00000111790	19774075;
D000638	increase	FIBCD1	ENSG00000130720	19774075;
D000638	increase	FKBP10	ENSG00000141756	19774075;
D000638	increase	FLCN	ENSG00000154803	19774075;
D000638	increase	FNDC4	ENSG00000115226	17567588;
D000638	increase	FOSB	ENSG00000125740	19774075;
D000638	increase	FOS	ENSG00000170345	19774075;17214612;
D000638	increase	FOXP3	ENSG00000049768	19774075;
D000638	increase	FRAS1	ENSG00000138759	19774075;
D000638	increase	FRMD3	ENSG00000172159	19774075;
D000638	increase	FRMD5	ENSG00000171877	19774075;
D000638	increase	FUNDC2	ENSG00000165775	19774075;
D000638	increase	FUZ	ENSG00000010361	19774075;
D000638	increase	FXYD1	ENSG00000221857	19774075;
D000638	increase	G6PD	ENSG00000160211	19774075;
D000638	increase	GADD45A	ENSG00000116717	19774075;
D000638	increase	GADD45G	ENSG00000130222	19774075;
D000638	increase	GAL3ST3	ENSG00000175229	19774075;
D000638	increase	GBP2	ENSG00000162645	19774075;
D000638	increase	GDF15	ENSG00000130513	19774075;
D000638	increase	GDF5	ENSG00000125965	19774075;
D000638	increase	GDPD3	ENSG00000102886	17567588;
D000638	increase	GEM	ENSG00000164949	19774075;
D000638	increase	GINS1	ENSG00000101003	19774075;
D000638	increase	GJA5	ENSG00000143140	19774075;
D000638	increase	GK5	ENSG00000175066	19774075;
D000638	increase	GKAP1	ENSG00000165113	19774075;
D000638	increase	GNAS	ENSG00000087460	19774075;
D000638	increase	GON4L	ENSG00000116580	19774075;
D000638	increase	GPR133	ENSG00000111452	19774075;
D000638	increase	GPRC5C	ENSG00000170412	19774075;
D000638	increase	GRLF1	ENSG00000160007	19774075;
D000638	increase	GRM2	ENSG00000164082	19774075;
D000638	increase	GSTO2	ENSG00000065621	19774075;
D000638	increase	GSTZ1	ENSG00000100577	19774075;
D000638	increase	GUCY1A2	ENSG00000152402	19774075;
D000638	increase	GUCY1B3	ENSG00000061918	19774075;
D000638	increase	H2AFJ	ENSG00000111332	19774075;
D000638	increase	HCN1	ENSG00000164588	19774075;
D000638	increase	HDX	ENSG00000165259	19774075;
D000638	increase	HELB	ENSG00000127311	19774075;
D000638	increase	HERPUD1	ENSG00000051108	19774075;
D000638	increase	HEXIM1	ENSG00000186834	19774075;
D000638	increase	HGFAC	ENSG00000109758	19774075;

D000638	increase	HIRA	ENSG00000100084	19774075;
D000638	increase	HIST1H2AM	ENSG00000233224	19774075;
D000638	increase	HIST1H2BE	ENSG00000168242	19774075;
D000638	increase	HIST1H3I	ENSG00000197153	19774075;
D000638	increase	HIST1H3J	ENSG00000197153	19774075;
D000638	increase	HIST1H4H	ENSG00000182217	19774075;
D000638	increase	HLA-DPB1	ENSG00000223865	19774075;
D000638	increase	HMHA1	ENSG00000180448	19774075;
D000638	increase	HMOX1	ENSG00000100292	19774075;18020914;
D000638	increase	HNF4G	ENSG00000164749	19774075;
D000638	increase	HOXB6	ENSG00000108511	19774075;
D000638	increase	HOXC4	ENSG00000198353	19774075;
D000638	increase	HPN	ENSG00000105707	17567588;15342952;
D000638	increase	HRK	ENSG00000135116	19774075;
D000638	increase	HUS1	ENSG00000136273	19774075;
D000638	increase	ICAM1	ENSG00000090339	19774075;
D000638	increase	IGFALS	ENSG00000099769	19774075;
D000638	increase	IGSF8	ENSG00000162729	19774075;
D000638	increase	IKBKAP	ENSG00000070061	19774075;
D000638	increase	IL10RA	ENSG00000110324	19774075;
D000638	increase	IL1A	ENSG00000115008	19774075;
D000638	increase	IL6	ENSG00000136244	11288978;
D000638	increase	INHBE	ENSG00000139269	19774075;17567588;15342952;
D000638	increase	IPPK	ENSG00000127080	19774075;
D000638	increase	IRAK2	ENSG00000134070	19774075;
D000638	increase	IRF1	ENSG00000125347	19774075;
D000638	increase	ISX	ENSG00000175329	19774075;
D000638	increase	ITIH2	ENSG00000151655	19774075;
D000638	increase	JRKL	ENSG00000183340	19774075;
D000638	increase	JUN	ENSG00000177606	17214612;
D000638	increase	KCNE1L	ENSG00000176076	19774075;
D000638	increase	KCNJ2	ENSG00000123700	19774075;
D000638	increase	KCNK12	ENSG00000184261	19774075;
D000638	increase	KCNMA1	ENSG00000156113	19774075;
D000638	increase	KCTD14	ENSG00000151364	19774075;
D000638	increase	KIF16B	ENSG00000089177	19774075;
D000638	increase	KIF17	ENSG00000117245	19774075;
D000638	increase	KIFC1	ENSG00000237649	19774075;
D000638	increase	KIR2DL5B	ENSG00000215764	19774075;
D000638	increase	KITLG	ENSG00000049130	19774075;
D000638	increase	KLF10	ENSG00000155090	19774075;
D000638	increase	KLF15	ENSG00000163884	19774075;
D000638	increase	KLF16	ENSG00000129911	19774075;
D000638	increase	KLF4	ENSG00000136826	19774075;
D000638	increase	KLHL28	ENSG00000179454	19774075;
D000638	increase	KLK14	ENSG00000129437	19774075;
D000638	increase	KRT17	ENSG00000128422	19774075;
D000638	increase	L3MBTL	ENSG00000185513	19774075;
D000638	increase	LACRT	ENSG00000135413	19774075;
D000638	increase	LCTL	ENSG00000188501	19774075;
D000638	increase	LDLR	ENSG00000130164	9712728;
D000638	increase	LELP1	ENSG00000203784	19774075;
D000638	increase	LHCGR	ENSG00000138039	19774075;

D000638	increase	LIF	ENSG00000128342	19774075;
D000638	increase	LIMS3	ENSG00000184115	19774075;
D000638	increase	LIN7A	ENSG00000111052	19774075;
D000638	increase	LRP5	ENSG00000162337	19774075;
D000638	increase	LSS	ENSG00000160285	17567588;15342952;
D000638	increase	LYZ	ENSG00000090382	19774075;
D000638	increase	MACROD2	ENSG00000172264	19774075;
D000638	increase	MAFG	ENSG00000197063	19774075;
D000638	increase	MAGED4B	ENSG00000154545	19774075;
D000638	increase	MASP1	ENSG00000127241	19774075;
D000638	increase	MCAM	ENSG00000076706	19774075;
D000638	increase	MCF2	ENSG00000101977	19774075;
D000638	increase	MED11	ENSG00000161920	19774075;
D000638	increase	MEF2C	ENSG00000081189	19774075;
D000638	increase	MFHAS1	ENSG00000147324	19774075;
D000638	increase	MGAT4A	ENSG00000071073	19774075;
D000638	increase	MGST1	ENSG00000008394	19774075;
D000638	increase	MICALL2	ENSG00000164877	19774075;
D000638	increase	MID2	ENSG00000080561	19774075;
D000638	increase	MITF	ENSG00000187098	19774075;
D000638	increase	MPI	ENSG00000178802	19774075;
D000638	increase	MRPL10	ENSG00000159111	19774075;
D000638	increase	MS4A14	ENSG00000166928	19774075;
D000638	increase	MT1F	ENSG00000198417	19774075;
D000638	increase	MT1G	ENSG00000125144	19774075;
D000638	increase	MT1X	ENSG00000187193	19774075;
D000638	increase	MXD1	ENSG00000059728	19774075;
D000638	increase	MYEF2	ENSG00000104177	19774075;
D000638	increase	MYH11	ENSG00000133392	19774075;
D000638	increase	MYL4	ENSG00000198336	19774075;
D000638	increase	MYLK2	ENSG00000101306	19774075;
D000638	increase	MYO3A	ENSG00000095777	19774075;
D000638	increase	N4BP3	ENSG00000145911	19774075;
D000638	increase	NANOS1	ENSG00000188613	19774075;
D000638	increase	NBPF3	ENSG00000142794	19774075;
D000638	increase	NEK8	ENSG00000160602	19774075;
D000638	increase	NF2	ENSG00000186575	19774075;
D000638	increase	NFIC	ENSG00000141905	19774075;
D000638	increase	NFKBIA	ENSG00000100906	19774075;
D000638	increase	NFKBIZ	ENSG00000144802	19774075;
D000638	increase	NOL6	ENSG00000165271	19774075;
D000638	increase	NOS1AP	ENSG00000251671	19774075;
D000638	increase	NPSR1	ENSG00000187258	19774075;
D000638	increase	NPTXR	ENSG00000221890	19774075;
D000638	increase	NR4A2	ENSG00000153234	19774075;
D000638	increase	NT5M	ENSG00000205309	19774075;
D000638	increase	NTRK3	ENSG00000140538	19774075;
D000638	increase	NUPR1	ENSG00000176046	17567588;15342952;
D000638	increase	OCM	ENSG00000122543	19774075;
D000638	increase	OPHN1	ENSG00000079482	19774075;
D000638	increase	OR14I1	ENSG00000189181	19774075;
D000638	increase	OR2B2	ENSG00000168131	19774075;
D000638	increase	OR2D2	ENSG00000166368	19774075;
D000638	increase	OR4C16	ENSG00000181935	19774075;

D000638	increase	OR4F5	ENSG00000177693	19774075;
D000638	increase	OR52N2	ENSG00000180988	19774075;
D000638	increase	OR6T1	ENSG00000181499	19774075;
D000638	increase	OR9Q2	ENSG00000186513	19774075;
D000638	increase	OSBP2	ENSG00000184792	19774075;
D000638	increase	OXSM	ENSG00000151093	19774075;
D000638	increase	PAX3	ENSG00000135903	19774075;
D000638	increase	PCDH1	ENSG00000156453	19774075;
D000638	increase	PCDH7	ENSG00000169851	19774075;
D000638	increase	PCDH8	ENSG00000136099	19774075;
D000638	increase	PCDHAC2	ENSG00000243232	19774075;
D000638	increase	PCDHGC3	ENSG00000240184	19774075;
D000638	increase	PCSK9	ENSG00000169174	19774075;
D000638	increase	PDCD10	ENSG00000114209	19774075;
D000638	increase	PDE6G	ENSG00000185527	19774075;
D000638	increase	PDE7B	ENSG00000171408	19774075;
D000638	increase	PDF	ENSG00000213380	19774075;
D000638	increase	PDGFRL	ENSG00000104213	19774075;
D000638	increase	PDLIM2	ENSG00000120913	19774075;
D000638	increase	PDYN	ENSG00000101327	19774075;
D000638	increase	PER1	ENSG00000179094	19774075;
D000638	increase	PGK2	ENSG00000170950	19774075;
D000638	increase	PHF20L1	ENSG00000129292	19774075;
D000638	increase	PHF5A	ENSG00000100410	19774075;
D000638	increase	PHKA1	ENSG00000067177	19774075;
D000638	increase	PIGA	ENSG00000165195	19774075;
D000638	increase	PIP5K1A	ENSG00000143398	19774075;
D000638	increase	PLAA	ENSG00000137055	19774075;
D000638	increase	PLAC8L1	ENSG00000173261	19774075;
D000638	increase	PLEKHG4	ENSG00000196155	19774075;
D000638	increase	PLS1	ENSG00000120756	19774075;
D000638	increase	PODN	ENSG00000174348	19774075;
D000638	increase	POLL	ENSG00000166169	19774075;
D000638	increase	POTEH	ENSG00000198062	19774075;
D000638	increase	PPM1B	ENSG00000138032	19774075;
D000638	increase	PPP1R15A	ENSG00000087074	19774075;
D000638	increase	PPP2R5C	ENSG00000078304	19774075;
D000638	increase	PPP3R1	ENSG00000221823	19774075;
D000638	increase	PRDM2	ENSG00000116731	19774075;
D000638	increase	PRDM8	ENSG00000152784	19774075;
D000638	increase	PRIM2	ENSG00000146143	19774075;
D000638	increase	PRMT1	ENSG00000126457	19774075;
D000638	increase	PROX1	ENSG00000117707	19774075;
D000638	increase	PRR19	ENSG00000188368	19774075;
D000638	increase	PSEN1	ENSG00000080815	19774075;
D000638	increase	PSIP1	ENSG00000164985	19774075;
D000638	increase	PSMD11	ENSG00000108671	19774075;
D000638	increase	PTGS2	ENSG00000073756	19774075;
D000638	increase	PTPN3	ENSG00000070159	19774075;
D000638	increase	PTPRF	ENSG00000142949	19774075;
D000638	increase	PTPRN	ENSG00000054356	19774075;
D000638	increase	PYGO1	ENSG00000171016	19774075;
D000638	increase	RAB39B	ENSG00000155961	19774075;
D000638	increase	RAB9B	ENSG00000123570	19774075;

D000638	increase	RAC3	ENSG00000169750	19774075;
D000638	increase	RBBP8	ENSG00000101773	19774075;
D000638	increase	RBKS	ENSG00000171174	19774075;
D000638	increase	RBM33	ENSG00000184863	19774075;
D000638	increase	RBPMS	ENSG00000157110	19774075;
D000638	increase	RCN3	ENSG00000142552	19774075;
D000638	increase	RDH8	ENSG00000080511	19774075;
D000638	increase	RELB	ENSG00000104856	19774075;
D000638	increase	REPIN1	ENSG00000214022	19774075;
D000638	increase	RG9MTD3	ENSG00000165275	19774075;
D000638	increase	RGS6	ENSG00000182732	19774075;
D000638	increase	RHEBL1	ENSG00000167550	19774075;
D000638	increase	RIN3	ENSG00000100599	19774075;
D000638	increase	RNF146	ENSG00000118518	19774075;
D000638	increase	RNF19A	ENSG00000034677	19774075;
D000638	increase	RNF212	ENSG00000178222	19774075;
D000638	increase	RNF6	ENSG00000127870	19774075;
D000638	increase	RNF8	ENSG00000112130	19774075;
D000638	increase	RPL36A	ENSG00000241343	19774075;
D000638	increase	RUNDC3A	ENSG00000108309	19774075;
D000638	increase	SARDH	ENSG00000123453	19774075;
D000638	increase	SEMA3D	ENSG00000153993	19774075;
D000638	increase	SEMA4D	ENSG00000187764	19774075;
D000638	increase	SEMA6B	ENSG00000167680	19774075;
D000638	increase	SEMG1	ENSG00000124233	19774075;
D000638	increase	SERPINA10	ENSG00000140093	19774075;
D000638	increase	SERTAD3	ENSG00000167565	19774075;
D000638	increase	SESN2	ENSG00000130766	19774075;
D000638	increase	SFRS12IP1	ENSG00000153006	19774075;
D000638	increase	SH2D1A	ENSG00000183918	19774075;
D000638	increase	SIPA1L3	ENSG00000105738	19774075;
D000638	increase	SIRT4	ENSG00000089163	19774075;
D000638	increase	SIX1	ENSG00000126778	19774075;
D000638	increase	SLC13A4	ENSG00000164707	19774075;
D000638	increase	SLC14A2	ENSG00000132874	19774075;
D000638	increase	SLC25A18	ENSG00000182902	19774075;
D000638	increase	SLC25A25	ENSG00000148339	19774075;
D000638	increase	SLC25A26	ENSG00000144741	19774075;
D000638	increase	SLC25A30	ENSG00000174032	19774075;
D000638	increase	SLC30A1	ENSG00000170385	19774075;
D000638	increase	SLC35A2	ENSG00000102100	19774075;
D000638	increase	SLC38A11	ENSG00000169507	19774075;
D000638	increase	SLC3A2	ENSG00000168003	19774075;
D000638	increase	SLC5A3	ENSG00000198743	19774075;
D000638	increase	SLCO4C1	ENSG00000173930	19774075;
D000638	increase	SLCO5A1	ENSG00000137571	19774075;
D000638	increase	SLFN5	ENSG00000166750	19774075;
D000638	increase	SLITRK3	ENSG00000121871	19774075;
D000638	increase	SMG6	ENSG00000070366	19774075;
D000638	increase	SMOX	ENSG00000088826	19774075;
D000638	increase	SNRNP35	ENSG00000184209	19774075;
D000638	increase	SNX18	ENSG00000178996	19774075;
D000638	increase	SOCS6	ENSG00000170677	19774075;
D000638	increase	SPAG1	ENSG00000104450	19774075;

D000638	increase	SPATA17	ENSG00000162814	19774075;
D000638	increase	SPATA2L	ENSG00000158792	19774075;
D000638	increase	SPEF2	ENSG00000152582	19774075;
D000638	increase	SPHAR	ENSG00000213029	19774075;
D000638	increase	SPOPL	ENSG00000144228	19774075;
D000638	increase	SPR	ENSG00000116096	19774075;
D000638	increase	SPTBN1	ENSG00000115306	19774075;
D000638	increase	SQSTM1	ENSG00000161011	19774075;
D000638	increase	SS18L1	ENSG00000184402	19774075;
D000638	increase	STARD5	ENSG00000172345	19774075;
D000638	increase	STK16	ENSG00000115661	19774075;
D000638	increase	STX11	ENSG00000135604	19774075;
D000638	increase	STXBP2	ENSG00000076944	19774075;
D000638	increase	SULT1A3	ENSG00000213599	19774075;
D000638	increase	SVIL	ENSG00000197321	19774075;
D000638	increase	TAF5L	ENSG00000135801	19774075;
D000638	increase	TAPBPL	ENSG00000139192	19774075;
D000638	increase	TAS2R10	ENSG00000121318	19774075;
D000638	increase	TAZ	ENSG00000102125	19774075;
D000638	increase	TBC1D3	ENSG00000197681	19774075;
D000638	increase	TBX4	ENSG00000121075	19774075;
D000638	increase	TCF20	ENSG00000100207	19774075;
D000638	increase	TCF7	ENSG00000081059	19774075;
D000638	increase	TCL1B	ENSG00000213231	19774075;
D000638	increase	TDO2	ENSG00000151790	19774075;
D000638	increase	TEF	ENSG00000167074	19774075;
D000638	increase	TERF1	ENSG00000147601	19774075;
D000638	increase	TFAP2C	ENSG00000087510	19774075;
D000638	increase	TFG	ENSG00000114354	19774075;
D000638	increase	THRB	ENSG00000151090	11806713;19774075;
D000638	increase	TIGD6	ENSG00000164296	19774075;
D000638	increase	TIMM13	ENSG00000099800	19774075;
D000638	increase	TIMM17A	ENSG00000134375	19774075;
D000638	increase	TINAG	ENSG00000137251	19774075;
D000638	increase	TKTL1	ENSG00000007350	19774075;
D000638	increase	TMCC2	ENSG00000133069	19774075;
D000638	increase	TMEM161B	ENSG00000164180	19774075;
D000638	increase	TMEM31	ENSG00000179363	19774075;
D000638	increase	TMEM68	ENSG00000167904	19774075;
D000638	increase	TMLHE	ENSG00000185973	19774075;
D000638	increase	TMPRSS9	ENSG00000178297	19774075;
D000638	increase	TMUB2	ENSG00000168591	19774075;
D000638	increase	TNFAIP3	ENSG00000118503	19774075;
D000638	increase	TNF	ENSG00000223952	19225199;18974383;
D000638	increase	TNNT1	ENSG00000105048	19774075;
D000638	increase	TNRC6A	ENSG00000090905	19774075;
D000638	increase	TOM1L1	ENSG00000141198	19774075;
D000638	increase	TREX1	ENSG00000213689	19774075;
D000638	increase	TRIB3	ENSG00000101255	19774075;
D000638	increase	TRIM36	ENSG00000152503	19774075;
D000638	increase	TRIM61	ENSG00000183439	19774075;
D000638	increase	TRNT1	ENSG00000072756	19774075;
D000638	increase	TRPV2	ENSG00000187688	19774075;
D000638	increase	TSGA10	ENSG00000135951	19774075;

D000638	increase	TSLP	ENSG00000251640	19774075;
D000638	increase	TSPYL2	ENSG00000184205	19774075;
D000638	increase	TTBK2	ENSG00000128881	19774075;
D000638	increase	TTLL3	ENSG00000214021	19774075;
D000638	increase	TUSC3	ENSG00000104723	19774075;
D000638	increase	TXNDC3	ENSG00000086288	19774075;
D000638	increase	UBE2D2	ENSG00000131508	19774075;
D000638	increase	UBE2D3	ENSG00000109332	19774075;
D000638	increase	ULK3	ENSG00000140474	19774075;
D000638	increase	USF1	ENSG00000158773	19774075;
D000638	increase	USP25	ENSG00000155313	19774075;
D000638	increase	USP2	ENSG00000036672	19774075;
D000638	increase	UTS2	ENSG00000049247	19774075;
D000638	increase	VCAM1	ENSG00000162692	19774075;
D000638	increase	VEGFA	ENSG00000112715	19774075;
D000638	increase	VMAC	ENSG00000187650	19774075;
D000638	increase	VPS11	ENSG00000160695	19774075;
D000638	increase	VPS13B	ENSG00000132549	19774075;
D000638	increase	VPS16	ENSG00000215305	19774075;
D000638	increase	WBSCR16	ENSG00000174374	19774075;
D000638	increase	WBSCR17	ENSG00000185274	19774075;
D000638	increase	WIPI1	ENSG00000070540	17567588;15342952;
D000638	increase	WNK1	ENSG00000060237	19774075;
D000638	increase	WTAP	ENSG00000146457	19774075;
D000638	increase	XBP1	ENSG00000100219	19774075;
D000638	increase	XK	ENSG00000047597	19774075;
D000638	increase	XPNPEP2	ENSG00000122121	19774075;
D000638	increase	ZBED3	ENSG00000132846	19774075;
D000638	increase	ZC3HAV1	ENSG00000105939	19774075;
D000638	increase	ZDHHC15	ENSG00000102383	19774075;
D000638	increase	ZFP36	ENSG00000128016	19774075;
D000638	increase	ZMYND10	ENSG00000004838	19774075;
D000638	increase	ZNF101	ENSG00000181896	19774075;
D000638	increase	ZNF121	ENSG00000197961	19774075;
D000638	increase	ZNF197	ENSG00000186448	19774075;
D000638	increase	ZNF205	ENSG00000122386	19774075;
D000638	increase	ZNF225	ENSG00000159882	19774075;
D000638	increase	ZNF311	ENSG00000225351	19774075;
D000638	increase	ZNF420	ENSG00000197050	19774075;
D000638	increase	ZNF497	ENSG00000174586	19774075;
D000638	increase	ZNF554	ENSG00000172006	19774075;
D000638	increase	ZNF584	ENSG00000171574	19774075;
D000638	increase	ZNF592	ENSG00000166716	19774075;
D000638	increase	ZNF624	ENSG00000197566	19774075;
D000638	increase	ZNF683	ENSG00000176083	19774075;
D000638	increase	ZNF703	ENSG00000183779	19774075;
D000638	increase	ZNF704	ENSG00000164684	19774075;
D000638	increase	ZNF710	ENSG00000140548	19774075;
D000638	increase	ZNF771	ENSG00000179965	19774075;
D000638	increase	ZNHIT2	ENSG00000174276	19774075;
D000638	increase	ZSCAN20	ENSG00000121903	19774075;
D000638	increase	ZXDA	ENSG00000198205	19774075;
D000639	decrease	AP1S1	ENSG00000106367	17567588;
D000639	decrease	SLC2A3	ENSG00000059804	17567588;15342952;

D000639	decrease	TAGLN	ENSG00000149591	17567588;15342952;
D000639	increase	ASAH1	ENSG00000104763	17567588;15342952;
D000639	increase	ASNS	ENSG00000070669	17567588;15342952;
D000639	increase	C10ORF10	ENSG00000165507	17567588;15342952;
D000639	increase	FABP1	ENSG00000163586	17567588;15342952;
D000639	increase	FNDC4	ENSG00000115226	15342952;
D000639	increase	GDPD3	ENSG00000102886	17567588;15342952;
D000639	increase	HPN	ENSG00000105707	17567588;15342952;
D000639	increase	INHBE	ENSG00000139269	17567588;15342952;
D000639	increase	LSS	ENSG00000160285	17567588;15342952;
D000639	increase	NR0B2	ENSG00000131910	15342952;
D000639	increase	NUPR1	ENSG00000176046	17567588;15342952;
D000639	increase	PHYH	ENSG00000107537	15342952;
D000639	increase	SERPINA3	ENSG00000196136	17567588;15342952;
D000639	increase	WIPI1	ENSG00000070540	15342952;
D000640	decrease	EPO	ENSG00000130427	7980410;
D000640	increase	MAP1LC3A	ENSG00000101460	18818525;
D000667	increase	SLC1A2	ENSG00000110436	17138558;
D000677	decrease	CCNA2	ENSG00000145386	11774253;
D000677	decrease	CCNB1	ENSG00000134057	11774253;
D000677	decrease	EGF	ENSG00000138798	16969495;
D000677	decrease	MYC	ENSG00000136997	11585056;
D000677	decrease	PRC1	ENSG00000198901	17374387;
D000677	decrease	TOP2A	ENSG00000131747	11676865;1651812;12569090;11470519;10727526;16969495;
D000677	decrease	UBE2C	ENSG00000175063	17374387;
D000677	increase	CASP2	ENSG00000106144	14757846;
D000677	increase	CCNE1	ENSG00000105173	11774253;
D000677	increase	RARB	ENSG00000077092	17608728;
D000677	increase	TP53	ENSG00000141510	12082016;11774253;17555331;16177561;
D000677	increase	TP53I3	ENSG00000115129	17374387;
D000728	affect	GRPR	ENSG00000126010	17204703;
D000728	affect	KLK3	ENSG00000142515	17918750;
D000728	affect	PSCA	ENSG00000167653	16015594;
D000728	affect	PTPN1	ENSG00000196396	16652382;
D000728	affect	RALA	ENSG00000006451	16964283;
D000728	affect	RAP2A	ENSG00000125249	17918750;
D000728	affect	VEGFC	ENSG00000150630	16964283;
D000728	decrease	IGFBP5	ENSG00000115461	17823924;
D000728	decrease	SELENBP1	ENSG00000143416	16380993;
D000728	decrease	SLC7A1	ENSG00000139514	11401523;
D000728	increase	B2M	ENSG00000166710	17404077;
D000728	increase	FGF8	ENSG00000107831	12404063;
D000728	increase	FOSL2	ENSG00000075426	15486991;
D000728	increase	JUND	ENSG00000130522	15486991;
D000728	increase	MMP13	ENSG00000137745	15138554;
D000728	increase	NKX3-1	ENSG00000167034	16817226;17202838;
D000728	increase	TP53	ENSG00000141510	17202838;
D000735	decrease	CYP17A1	ENSG00000148795	15625562;16030167;15562394;14522586;
D000735	increase	AR	ENSG00000169083	17804755;12730620;15336702;
D000735	increase	KLK3	ENSG00000142515	15336702;

D000841	decrease	AHR	ENSG00000106546	15385644;
D000841	increase	ATF3	ENSG00000162772	19101521;
D000841	increase	CYP1A1	ENSG00000140465	15385644;
D000841	increase	FOS	ENSG00000170345	12660819;
D000875	increase	CAT	ENSG00000121691	9628260;
D000875	increase	CSF2	ENSG00000164400	9628260;
D000875	increase	IL6	ENSG00000136244	9628260;
D000875	increase	IL8	ENSG00000169429	9628260;
D000875	increase	TNF	ENSG00000223952	9628260;
D000894	decrease	CYP19A1	ENSG00000137869	15964185;
D000965	decrease	A2M	ENSG00000175899	17547211;
D000965	decrease	ACP2	ENSG00000134575	17547211;
D000965	decrease	ACSL1	ENSG00000151726	17547211;
D000965	decrease	AKR1B1	ENSG00000085662	17547211;
D000965	decrease	ALDH6A1	ENSG00000119711	17547211;
D000965	decrease	ATP2B1	ENSG00000070961	17547211;
D000965	decrease	AZGP1	ENSG00000160862	17547211;
D000965	decrease	CFB	ENSG00000243570	17547211;
D000965	decrease	CLGN	ENSG00000153132	17547211;
D000965	decrease	CLPTM1	ENSG00000104853	17547211;
D000965	decrease	F5	ENSG00000198734	17547211;
D000965	decrease	FDFT1	ENSG00000079459	17547211;
D000965	decrease	FDPS	ENSG00000160752	17547211;
D000965	decrease	GHITM	ENSG00000165678	17547211;
D000965	decrease	HSD17B2	ENSG00000086696	17547211;
D000965	decrease	HSD17B7	ENSG00000132196	17547211;
D000965	decrease	IFRD1	ENSG00000006652	17547211;
D000965	decrease	ITIH3	ENSG00000162267	17547211;
D000965	decrease	LIPA	ENSG00000107798	17547211;
D000965	decrease	LSS	ENSG00000160285	17547211;
D000965	decrease	MAN1A2	ENSG00000198162	17547211;
D000965	decrease	MGAT2	ENSG00000168282	17547211;
D000965	decrease	MT1X	ENSG00000187193	17547211;
D000965	decrease	NDRG2	ENSG00000165795	17547211;
D000965	decrease	NFKBIA	ENSG00000100906	17547211;
D000965	decrease	NR0B2	ENSG00000131910	17547211;
D000965	decrease	OS9	ENSG00000135506	17547211;
D000965	decrease	PDIA4	ENSG00000155660	17547211;
D000965	decrease	PGC	ENSG00000096088	17547211;
D000965	decrease	PSPH	ENSG00000146733	17547211;
D000965	decrease	SCD	ENSG00000099194	17547211;
D000965	decrease	SERPINB1	ENSG00000021355	17547211;
D000965	decrease	SERPINC1	ENSG00000117601	17547211;
D000965	decrease	SFRS1	ENSG00000136450	17547211;
D000965	decrease	SFRS5	ENSG00000100650	17547211;
D000965	decrease	SQLE	ENSG00000104549	17547211;
D000965	decrease	ST6GALNAC4	ENSG00000136840	17547211;
D000965	decrease	TARDBP	ENSG00000120948	17547211;
D000965	decrease	TNFSF10	ENSG00000121858	17547211;
D000965	decrease	TOB1	ENSG00000141232	17547211;
D000965	decrease	TSPAN7	ENSG00000156298	17547211;
D000965	increase	ADM	ENSG00000148926	17547211;
D000965	increase	CCNB2	ENSG00000157456	17547211;

D000965	increase	CDKN1A	ENSG00000124762	17547211;
D000965	increase	CDKN3	ENSG00000100526	17547211;
D000965	increase	CKS2	ENSG00000123975	17547211;
D000965	increase	DAZAP1	ENSG00000071626	17547211;
D000965	increase	DCP1A	ENSG00000162290	17547211;
D000965	increase	DUSP1	ENSG00000120129	17547211;
D000965	increase	FOSL1	ENSG00000175592	17547211;
D000965	increase	GCNT3	ENSG00000140297	17547211;
D000965	increase	GLA	ENSG00000102393	17547211;
D000965	increase	GTPBP6	ENSG00000178605	17547211;
D000965	increase	HIST1H4C	ENSG00000182217	17547211;
D000965	increase	HMGA1	ENSG00000137309	17547211;
D000965	increase	HMOX1	ENSG00000100292	17547211;
D000965	increase	IER2	ENSG00000160888	17547211;
D000965	increase	ING3	ENSG00000071243	17547211;
D000965	increase	ITGB3BP	ENSG00000142856	17547211;
D000965	increase	KLF6	ENSG00000067082	17547211;
D000965	increase	MAFF	ENSG00000185022	17547211;
D000965	increase	MCM2	ENSG00000073111	17547211;
D000965	increase	PPIH	ENSG00000171960	17547211;
D000965	increase	PTTG1	ENSG00000164611	17547211;
D000965	increase	RBPMS	ENSG00000157110	17547211;
D000965	increase	RFC2	ENSG00000049541	17547211;
D000965	increase	RNASEH2A	ENSG00000104889	17547211;
D000965	increase	SIK1	ENSG00000142178	17547211;
D000965	increase	SNRPA	ENSG00000077312	17547211;
D000965	increase	TNFAIP3	ENSG00000118503	17547211;
D000965	increase	TUFT1	ENSG00000143367	17547211;
D000965	increase	TYMS	ENSG00000176890	17547211;
D000965	increase	UBE2C	ENSG00000175063	17547211;
D000965	increase	ZWINT	ENSG00000122952	17547211;
D000966	decrease	A2M	ENSG00000175899	17547211;
D000966	decrease	ACP2	ENSG00000134575	17547211;
D000966	decrease	ACSL1	ENSG00000151726	17547211;
D000966	decrease	AKR1B1	ENSG00000085662	17547211;
D000966	decrease	ALDH6A1	ENSG00000119711	17547211;
D000966	decrease	ATP2B1	ENSG00000070961	17547211;
D000966	decrease	AZGP1	ENSG00000160862	17547211;
D000966	decrease	CFB	ENSG00000243570	17547211;
D000966	decrease	CLGN	ENSG00000153132	17547211;
D000966	decrease	CLPTM1	ENSG00000104853	17547211;
D000966	decrease	F5	ENSG00000198734	17547211;
D000966	decrease	FDFT1	ENSG00000079459	17547211;
D000966	decrease	FDPS	ENSG00000160752	17547211;
D000966	decrease	GHITM	ENSG00000165678	17547211;
D000966	decrease	HSD17B2	ENSG00000086696	17547211;
D000966	decrease	HSD17B7	ENSG00000132196	17547211;
D000966	decrease	IFRD1	ENSG00000006652	17547211;
D000966	decrease	ITIH3	ENSG00000162267	17547211;
D000966	decrease	LIPA	ENSG00000107798	17547211;
D000966	decrease	LSS	ENSG00000160285	17547211;
D000966	decrease	MAN1A2	ENSG00000198162	17547211;
D000966	decrease	MGAT2	ENSG00000168282	17547211;
D000966	decrease	MT1X	ENSG00000187193	17547211;

D000966	decrease	NDRG2	ENSG00000165795	17547211;
D000966	decrease	NFKBIA	ENSG00000100906	17547211;
D000966	decrease	NR0B2	ENSG00000131910	17547211;
D000966	decrease	OS9	ENSG00000135506	17547211;
D000966	decrease	PDIA4	ENSG00000155660	17547211;
D000966	decrease	PGC	ENSG00000096088	17547211;
D000966	decrease	PSPH	ENSG00000146733	17547211;
D000966	decrease	SCD	ENSG00000099194	17547211;
D000966	decrease	SERPINB1	ENSG00000021355	17547211;
D000966	decrease	SERPINC1	ENSG00000117601	17547211;
D000966	decrease	SFRS1	ENSG00000136450	17547211;
D000966	decrease	SFRS5	ENSG00000100650	17547211;
D000966	decrease	SQLE	ENSG00000104549	17547211;
D000966	decrease	ST6GALNAC4	ENSG00000136840	17547211;
D000966	decrease	TARDBP	ENSG00000120948	17547211;
D000966	decrease	TNFSF10	ENSG00000121858	17547211;
D000966	decrease	TOB1	ENSG00000141232	17547211;
D000966	decrease	TSPAN7	ENSG00000156298	17547211;
D000966	increase	ADM	ENSG00000148926	17547211;
D000966	increase	CCNB2	ENSG00000157456	17547211;
D000966	increase	CDKN1A	ENSG00000124762	17547211;
D000966	increase	CDKN3	ENSG00000100526	17547211;
D000966	increase	CKS2	ENSG00000123975	17547211;
D000966	increase	DAZAP1	ENSG00000071626	17547211;
D000966	increase	DCP1A	ENSG00000162290	17547211;
D000966	increase	DUSP1	ENSG00000120129	17547211;
D000966	increase	FOSL1	ENSG00000175592	17547211;
D000966	increase	GCNT3	ENSG00000140297	17547211;
D000966	increase	GLA	ENSG00000102393	17547211;
D000966	increase	GTPBP6	ENSG00000178605	17547211;
D000966	increase	HIST1H4C	ENSG00000182217	17547211;
D000966	increase	HMGA1	ENSG00000137309	17547211;
D000966	increase	HMOX1	ENSG00000100292	17547211;
D000966	increase	IER2	ENSG00000160888	17547211;
D000966	increase	ING3	ENSG00000071243	17547211;
D000966	increase	ITGB3BP	ENSG00000142856	17547211;
D000966	increase	KLF6	ENSG00000067082	17547211;
D000966	increase	MAFF	ENSG00000185022	17547211;
D000966	increase	MCM2	ENSG00000073111	17547211;
D000966	increase	PPIH	ENSG00000171960	17547211;
D000966	increase	PTTG1	ENSG00000164611	17547211;
D000966	increase	RBPMS	ENSG00000157110	17547211;
D000966	increase	RFC2	ENSG00000049541	17547211;
D000966	increase	RNASEH2A	ENSG00000104889	17547211;
D000966	increase	SIK1	ENSG00000142178	17547211;
D000966	increase	SNRPA	ENSG00000077312	17547211;
D000966	increase	TNFAIP3	ENSG00000118503	17547211;
D000966	increase	TUFT1	ENSG00000143367	17547211;
D000966	increase	TYMS	ENSG00000176890	17547211;
D000966	increase	UBE2C	ENSG00000175063	17547211;
D000966	increase	ZWINT	ENSG00000122952	17547211;
D000968	decrease	CCNA2	ENSG00000145386	12730239;
D000968	decrease	CCNB1	ENSG00000134057	12730239;

D000968	decrease	CP	ENSG00000047457	19019832;
D000968	decrease	FAS	ENSG00000026103	8902626;
D000968	increase	CD14	ENSG00000170458	10491287;
D000968	increase	CD38	ENSG00000004468	10491287;
D000968	increase	ICAM1	ENSG00000090339	12952965;
D000968	increase	IL8	ENSG00000169429	12208513;
D000968	increase	JUN	ENSG00000177606	16237197;
D000975	decrease	IL6	ENSG00000136244	16702388;
D000975	decrease	TNF	ENSG00000223952	16702388;
D001104	decrease	ABCA10	ENSG00000154263	17103032;
D001104	decrease	ABCA13	ENSG00000179869	17103032;
D001104	decrease	ACMSD	ENSG00000153086	17103032;
D001104	decrease	ACVRL1	ENSG00000139567	17103032;
D001104	decrease	ADAM19	ENSG00000135074	17103032;
D001104	decrease	ADAM20	ENSG00000134007	17103032;
D001104	decrease	ADAMTS3	ENSG00000156140	17103032;
D001104	decrease	ADAMTS9	ENSG00000163638	17103032;
D001104	decrease	ADRB3	ENSG00000188778	17103032;
D001104	decrease	AFAP1	ENSG00000196526	17103032;
D001104	decrease	AGBL4	ENSG00000186094	17103032;
D001104	decrease	AGPAT3	ENSG00000160216	17103032;
D001104	decrease	AHNAK	ENSG00000124942	17103032;
D001104	decrease	AKT1	ENSG00000142208	17103032;
D001104	decrease	AMTN	ENSG00000187689	17103032;
D001104	decrease	AP1S1	ENSG00000106367	17103032;
D001104	decrease	APTX	ENSG00000137074	17103032;
D001104	decrease	ARFIP2	ENSG00000132254	17103032;
D001104	decrease	ARHGEF2	ENSG00000116584	17103032;
D001104	decrease	ARL2	ENSG00000213465	17103032;
D001104	decrease	ARX	ENSG00000004848	17103032;
D001104	decrease	ASAP3	ENSG00000088280	17103032;
D001104	decrease	ASNA1	ENSG00000198356	17103032;
D001104	decrease	ATAD3B	ENSG00000160072	17103032;
D001104	decrease	ATF6B	ENSG00000228628	17103032;
D001104	decrease	B4GALNT4	ENSG00000182272	17103032;
D001104	decrease	BAG5	ENSG00000166170	17103032;
D001104	decrease	BCKDK	ENSG00000103507	17103032;
D001104	decrease	BMPR1B	ENSG00000138696	17103032;
D001104	decrease	BRSK1	ENSG00000160469	17103032;
D001104	decrease	BST2	ENSG00000130303	17103032;
D001104	decrease	BTN2A1	ENSG00000112763	17103032;
D001104	decrease	BZRAP1	ENSG00000005379	17103032;
D001104	decrease	C14ORF148	ENSG00000165555	17103032;
D001104	decrease	C15ORF26	ENSG00000156206	17103032;
D001104	decrease	C19ORF33	ENSG00000167644	17103032;
D001104	decrease	C1ORF61	ENSG00000125462	17103032;
D001104	decrease	C1ORF87	ENSG00000162598	17103032;
D001104	decrease	C1QB	ENSG00000173369	17103032;
D001104	decrease	C6ORF97	ENSG00000120262	17103032;
D001104	decrease	C9ORF167	ENSG00000198113	17103032;
D001104	decrease	CACNG7	ENSG00000105605	17103032;
D001104	decrease	CASP8	ENSG00000064012	17103032;
D001104	decrease	CBX6	ENSG00000183741	17103032;
D001104	decrease	CCDC28B	ENSG00000160050	17103032;

D001104	decrease	CD151	ENSG00000177697	17103032;
D001104	decrease	CD160	ENSG00000117281	17103032;
D001104	decrease	CD163L1	ENSG00000177675	17103032;
D001104	decrease	CD2BP2	ENSG00000169217	17103032;
D001104	decrease	CD3G	ENSG00000160654	17103032;
D001104	decrease	CEACAM1	ENSG00000079385	17103032;
D001104	decrease	CLEC4C	ENSG00000198178	17103032;
D001104	decrease	CNKSR2	ENSG00000149970	17103032;
D001104	decrease	COL17A1	ENSG00000065618	17103032;
D001104	decrease	CPB1	ENSG00000153002	17103032;
D001104	decrease	DIAPH2	ENSG00000147202	17103032;
D001104	decrease	DLX1	ENSG00000144355	17103032;
D001104	decrease	DMXL1	ENSG00000172869	17103032;
D001104	decrease	DNAH7	ENSG00000118997	17103032;
D001104	decrease	DPP7	ENSG00000176978	17103032;
D001104	decrease	DPYSL2	ENSG00000092964	17103032;
D001104	decrease	EFHC2	ENSG00000183690	17103032;
D001104	decrease	EFNA3	ENSG00000143590	17103032;
D001104	decrease	EFS	ENSG00000100842	17103032;
D001104	decrease	EMX2	ENSG00000170370	17103032;
D001104	decrease	ENPP1	ENSG00000197594	17103032;
D001104	decrease	EVI2B	ENSG00000185862	17103032;
D001104	decrease	F13B	ENSG00000143278	17103032;
D001104	decrease	FAM3D	ENSG00000198643	17103032;
D001104	decrease	FASLG	ENSG00000117560	17103032;
D001104	decrease	FGF5	ENSG00000138675	17103032;
D001104	decrease	FGFR3	ENSG00000068078	17103032;
D001104	decrease	FMNL3	ENSG00000161791	17103032;
D001104	decrease	FPGT	ENSG00000116783	17103032;
D001104	decrease	FRAS1	ENSG00000138759	17103032;
D001104	decrease	FRMPD4	ENSG00000169933	17103032;
D001104	decrease	GIPC1	ENSG00000123159	17103032;
D001104	decrease	GLT8D2	ENSG00000120820	17103032;
D001104	decrease	GMPR	ENSG00000137198	17103032;
D001104	decrease	GPC3	ENSG00000147257	17103032;
D001104	decrease	GPR155	ENSG00000163328	17103032;
D001104	decrease	GPR26	ENSG00000154478	17103032;
D001104	decrease	GPR55	ENSG00000135898	17103032;
D001104	decrease	GPRC5D	ENSG00000111291	17103032;
D001104	decrease	GSDMA	ENSG00000167914	17103032;
D001104	decrease	GUCY1A2	ENSG00000152402	17103032;
D001104	decrease	HLF	ENSG00000108924	17103032;
D001104	decrease	HOXB8	ENSG00000120068	17103032;
D001104	decrease	HPS3	ENSG00000163755	17103032;
D001104	decrease	HSD3B1	ENSG00000203857	17103032;
D001104	decrease	IFIT3	ENSG00000119917	17103032;
D001104	decrease	IL23A	ENSG00000110944	17103032;
D001104	decrease	IL6ST	ENSG00000134352	17103032;
D001104	decrease	INA	ENSG00000148798	17103032;
D001104	decrease	JUN	ENSG00000177606	17103032;
D001104	decrease	KCNAB3	ENSG00000170049	17103032;
D001104	decrease	KCTD17	ENSG00000100379	17103032;
D001104	decrease	KIF26B	ENSG00000162849	17103032;
D001104	decrease	LCTL	ENSG00000188501	17103032;

D001104	decrease	LGALS14	ENSG00000006659	17103032;
D001104	decrease	LRP5	ENSG00000162337	17103032;
D001104	decrease	LRP6	ENSG00000070018	17103032;
D001104	decrease	LRRC4	ENSG00000128594	17103032;
D001104	decrease	LRRTM4	ENSG00000176204	17103032;
D001104	decrease	MAFK	ENSG00000198517	17103032;
D001104	decrease	MAP4K5	ENSG00000012983	17103032;
D001104	decrease	MAPK8	ENSG00000107643	17103032;
D001104	decrease	MDFI	ENSG00000112559	17103032;
D001104	decrease	MFAP4	ENSG00000166482	17103032;
D001104	decrease	MIDN	ENSG00000167470	17103032;
D001104	decrease	MS4A6E	ENSG00000166926	17103032;
D001104	decrease	MTHFD1L	ENSG00000120254	17103032;
D001104	decrease	MVD	ENSG00000167508	17103032;
D001104	decrease	MYO3B	ENSG00000071909	17103032;
D001104	decrease	NCALD	ENSG00000104490	17103032;
D001104	decrease	NEGR1	ENSG00000172260	17103032;
D001104	decrease	NKD1	ENSG00000140807	17103032;
D001104	decrease	NTF4	ENSG00000167744	17103032;
D001104	decrease	OLFML1	ENSG00000183801	17103032;
D001104	decrease	OSBP2	ENSG00000184792	17103032;
D001104	decrease	OTX1	ENSG00000115507	17103032;
D001104	decrease	PACSIN2	ENSG00000100266	17103032;
D001104	decrease	PCNXL2	ENSG00000135749	17103032;
D001104	decrease	PDZK1	ENSG00000174827	17103032;
D001104	decrease	PF4	ENSG00000163737	17103032;
D001104	decrease	PIGQ	ENSG00000007541	17103032;
D001104	decrease	PIN1	ENSG00000127445	17103032;
D001104	decrease	PKHD1	ENSG00000170927	17103032;
D001104	decrease	PRG2	ENSG00000186652	17103032;
D001104	decrease	PRKX	ENSG00000183943	17103032;
D001104	decrease	PRMT2	ENSG00000160310	17103032;
D001104	decrease	PRR18	ENSG00000176381	17103032;
D001104	decrease	PRRX1	ENSG00000116132	17103032;
D001104	decrease	PRSS2	ENSG00000204983	17103032;
D001104	decrease	PSMD13	ENSG00000185627	17103032;
D001104	decrease	PSMD3	ENSG00000108344	17103032;
D001104	decrease	RAB6B	ENSG00000154917	17103032;
D001104	decrease	RASGEF1B	ENSG00000138670	17103032;
D001104	decrease	RFPL2	ENSG00000249824	17103032;
D001104	decrease	RFPL3	ENSG00000128276	17103032;
D001104	decrease	RNF208	ENSG00000212864	17103032;
D001104	decrease	RSBN1	ENSG00000081019	17103032;
D001104	decrease	SAA4	ENSG00000148965	17103032;
D001104	decrease	SDC4	ENSG00000124145	17103032;
D001104	decrease	SEMG1	ENSG00000124233	17103032;
D001104	decrease	SERPINA5	ENSG00000188488	17103032;
D001104	decrease	SERPIND1	ENSG00000099937	17103032;
D001104	decrease	SFRP5	ENSG00000120057	17103032;
D001104	decrease	SFXN2	ENSG00000156398	17103032;
D001104	decrease	SH3GL2	ENSG00000107295	17103032;
D001104	decrease	SIN3B	ENSG00000127511	17103032;
D001104	decrease	SLC13A2	ENSG00000007216	17103032;
D001104	decrease	SLC15A3	ENSG00000110446	17103032;

D001104	decrease	SLC18A2	ENSG00000165646	17103032;
D001104	decrease	SLC25A2	ENSG00000120329	17103032;
D001104	decrease	SLC27A4	ENSG00000167114	17103032;
D001104	decrease	SLC7A3	ENSG00000165349	17103032;
D001104	decrease	SLCO5A1	ENSG00000137571	17103032;
D001104	decrease	SNIP1	ENSG00000163877	17103032;
D001104	decrease	SOCS4	ENSG00000180008	17103032;
D001104	decrease	SPATA20	ENSG00000006282	17103032;
D001104	decrease	STARD8	ENSG00000130052	17103032;
D001104	decrease	SYDE1	ENSG00000105137	17103032;
D001104	decrease	SYMPK	ENSG00000125755	17103032;
D001104	decrease	TAL1	ENSG00000162367	17103032;
D001104	decrease	TCN1	ENSG00000134827	17103032;
D001104	decrease	TEX13B	ENSG00000170925	17103032;
D001104	decrease	TGFB3	ENSG00000119699	17103032;
D001104	decrease	TLL1	ENSG00000038295	17103032;
D001104	decrease	TMEM59L	ENSG00000105696	17103032;
D001104	decrease	TRAM2	ENSG00000065308	17103032;
D001104	decrease	TREH	ENSG00000118094	17103032;
D001104	decrease	TSC22D2	ENSG00000196428	17103032;
D001104	decrease	TSHZ2	ENSG00000182463	17103032;
D001104	decrease	TTC18	ENSG00000156042	17103032;
D001104	decrease	TTC22	ENSG00000006555	17103032;
D001104	decrease	TULP3	ENSG00000078246	17103032;
D001104	decrease	UBE2D4	ENSG00000078967	17103032;
D001104	decrease	UBE2M	ENSG00000130725	17103032;
D001104	decrease	USP44	ENSG00000136014	17103032;
D001104	decrease	VWA3A	ENSG00000175267	17103032;
D001104	decrease	WDR63	ENSG00000162643	17103032;
D001104	decrease	WNT10A	ENSG00000135925	17103032;
D001104	decrease	ZBTB7B	ENSG00000160685	17103032;
D001104	decrease	ZDHHC2	ENSG00000104219	17103032;
D001104	decrease	ZNF141	ENSG00000131127	17103032;
D001104	decrease	ZNF167	ENSG00000196345	17103032;
D001104	decrease	ZNF320	ENSG00000182986	17103032;
D001104	decrease	ZNF354B	ENSG00000178338	17103032;
D001104	decrease	ZNF414	ENSG00000133250	17103032;
D001104	decrease	ZNF41	ENSG00000147124	17103032;
D001104	decrease	ZNF483	ENSG00000173258	17103032;
D001104	decrease	ZNF497	ENSG00000174586	17103032;
D001104	decrease	ZNF81	ENSG00000197779	17103032;
D001104	decrease	ZP2	ENSG00000103310	17103032;
D001104	increase	ADAM7	ENSG00000069206	17103032;
D001104	increase	AEBP2	ENSG00000139154	17103032;
D001104	increase	ANKRD17	ENSG00000132466	17103032;
D001104	increase	ANKRD23	ENSG00000163126	17103032;
D001104	increase	APOM	ENSG00000226215	17103032;
D001104	increase	ARL6IP1	ENSG00000170540	17103032;
D001104	increase	BCAS2	ENSG00000116752	17103032;
D001104	increase	BNIP2	ENSG00000140299	17103032;
D001104	increase	C15ORF41	ENSG00000186073	17103032;
D001104	increase	CCAR1	ENSG00000060339	17103032;
D001104	increase	CCNYL1	ENSG00000163249	17103032;
D001104	increase	CCT8	ENSG00000156261	17103032;

D001104	increase	CPE	ENSG00000109472	17103032;
D001104	increase	CPNE5	ENSG00000124772	17103032;
D001104	increase	CYP2U1	ENSG00000155016	17103032;
D001104	increase	DDX46	ENSG00000145833	17103032;
D001104	increase	DLGAP4	ENSG00000080845	17103032;
D001104	increase	DNAJB9	ENSG00000128590	17103032;
D001104	increase	ECE1	ENSG00000117298	17103032;
D001104	increase	EEPD1	ENSG00000122547	17103032;
D001104	increase	EFTUD1	ENSG00000140598	17103032;
D001104	increase	EPS8	ENSG00000151491	17103032;
D001104	increase	EPX	ENSG00000121053	17103032;
D001104	increase	ERC1	ENSG00000082805	17103032;
D001104	increase	FAM49B	ENSG00000153310	17103032;
D001104	increase	FAM59A	ENSG00000141441	17103032;
D001104	increase	FBXO11	ENSG00000250152	17103032;
D001104	increase	FBXO16	ENSG00000214050	17103032;
D001104	increase	GALC	ENSG00000054983	17103032;
D001104	increase	GNL2	ENSG00000134697	17103032;
D001104	increase	GPD2	ENSG00000115159	17103032;
D001104	increase	GPR32	ENSG00000142511	17103032;
D001104	increase	HAND1	ENSG00000113196	17103032;
D001104	increase	HIST1H1A	ENSG00000124610	17103032;
D001104	increase	HIST1H2AC	ENSG00000180573	17103032;
D001104	increase	HTATSF1	ENSG00000102241	17103032;
D001104	increase	IL3RA	ENSG00000185291	17103032;
D001104	increase	INPP4B	ENSG00000109452	17103032;
D001104	increase	KDELC1	ENSG00000134901	17103032;
D001104	increase	KLHL13	ENSG00000003096	17103032;
D001104	increase	KLRC4	ENSG00000183542	17103032;
D001104	increase	MCM8	ENSG00000125885	17103032;
D001104	increase	MKI67	ENSG00000148773	17103032;
D001104	increase	NEU4	ENSG00000204099	17103032;
D001104	increase	NMI	ENSG00000123609	17103032;
D001104	increase	NR2F1	ENSG00000175745	17103032;
D001104	increase	NRP2	ENSG00000118257	17103032;
D001104	increase	OSBPL11	ENSG00000144909	17103032;
D001104	increase	PARG	ENSG00000227345	17103032;
D001104	increase	PCGF6	ENSG00000156374	17103032;
D001104	increase	PCMTD2	ENSG00000203880	17103032;
D001104	increase	PGM2	ENSG00000169299	17103032;
D001104	increase	PITPNB	ENSG00000180957	17103032;
D001104	increase	POLE2	ENSG00000100479	17103032;
D001104	increase	PPHLN1	ENSG00000134283	17103032;
D001104	increase	RAB3GAP1	ENSG00000115839	17103032;
D001104	increase	RPS6KA3	ENSG00000177189	17103032;
D001104	increase	SCCPDH	ENSG00000143653	17103032;
D001104	increase	SCN1B	ENSG00000105711	17103032;
D001104	increase	SF3B1	ENSG00000115524	17103032;
D001104	increase	SLC23A3	ENSG00000213901	17103032;
D001104	increase	SPATA13	ENSG00000182957	17103032;
D001104	increase	SPTLC1	ENSG00000090054	17103032;
D001104	increase	TARDBP	ENSG00000120948	17103032;
D001104	increase	TCF12	ENSG00000140262	17103032;
D001104	increase	TES	ENSG00000135269	17103032;

D001104	increase	TSC1	ENSG00000165699	17103032;
D001104	increase	UBQLN1	ENSG00000135018	17103032;
D001104	increase	UTP11L	ENSG00000183520	17103032;
D001104	increase	VPS39	ENSG00000166887	17103032;
D001104	increase	XAGE2	ENSG00000185751	17103032;
D001104	increase	ZBTB20	ENSG00000181722	17103032;
D001104	increase	ZNF670	ENSG00000135747	17103032;
D001115	increase	MT1A	ENSG00000205362	17418620;
D001115	increase	PTGS2	ENSG00000073756	16311067;
D001115	increase	TIMP1	ENSG00000102265	16311067;
D001149	decrease	DAB2	ENSG00000153071	12773770;
D001149	decrease	GDF15	ENSG00000130513	12773770;
D001149	decrease	MAP2K6	ENSG00000108984	12773770;
D001149	decrease	VIM	ENSG00000026025	12773770;
D001149	increase	BMP4	ENSG00000125378	12773770;
D001149	increase	CDKN2A	ENSG00000147889	12773770;
D001149	increase	MT3	ENSG00000087250	12773770;
D001151	affect	AES	ENSG00000104964	18414638;
D001151	affect	AHCTF1	ENSG00000153207	18414638;
D001151	affect	ALOX5	ENSG00000012779	18414638;
D001151	affect	ANKRD10	ENSG00000088448	18414638;
D001151	affect	ANXA2	ENSG00000182718	18414638;
D001151	affect	AP3D1	ENSG00000065000	18414638;
D001151	affect	APBB1	ENSG00000166313	18414638;
D001151	affect	APC2	ENSG00000115266	18414638;
D001151	affect	ARIH2	ENSG00000177479	18414638;
D001151	affect	ATL3	ENSG00000184743	18414638;
D001151	affect	ATP2B4	ENSG00000058668	18414638;
D001151	affect	ATP5G1	ENSG00000159199	18414638;
D001151	affect	ATXN1	ENSG00000124788	18414638;
D001151	affect	AUTS2	ENSG00000158321	18414638;
D001151	affect	BAG3	ENSG00000151929	18414638;
D001151	affect	BRE	ENSG00000158019	18414638;
D001151	affect	BTN3A3	ENSG00000111801	18414638;
D001151	affect	C10ORF67	ENSG00000179133	18414638;
D001151	affect	C10ORF76	ENSG00000120029	18414638;
D001151	affect	C1ORF55	ENSG00000143751	18414638;
D001151	affect	C2CD3	ENSG00000168014	18414638;
D001151	affect	C2ORF27A	ENSG00000197927	18414638;
D001151	affect	C6ORF62	ENSG00000112308	18414638;
D001151	affect	C7ORF50	ENSG00000146540	18414638;
D001151	affect	CA6	ENSG00000131686	18414638;
D001151	affect	CCDC59	ENSG00000133773	18414638;
D001151	affect	CD247	ENSG00000198821	18414638;
D001151	affect	CD69	ENSG00000110848	18414638;
D001151	affect	CDADC1	ENSG00000102543	18414638;
D001151	affect	CDC42	ENSG00000070831	18414638;
D001151	affect	CDCP1	ENSG00000163814	18414638;
D001151	affect	CDH16	ENSG00000166589	18414638;
D001151	affect	CEL	ENSG00000170835	18414638;
D001151	affect	CENPL	ENSG00000120334	18414638;
D001151	affect	CEP72	ENSG00000112877	18414638;
D001151	affect	CFL1	ENSG00000172757	18414638;
D001151	affect	CHD2	ENSG00000173575	18414638;

D001151	affect	CHML	ENSG00000203668	18414638;
D001151	affect	CMTM8	ENSG00000170293	18414638;
D001151	affect	CPEB4	ENSG00000113742	18414638;
D001151	affect	CYP2E1	ENSG00000130649	18414638;
D001151	affect	DCAF7	ENSG00000136485	18414638;
D001151	affect	DHRS3	ENSG00000162496	18414638;
D001151	affect	DYNLL2	ENSG00000121083	18414638;
D001151	affect	EFCAB2	ENSG00000203666	18414638;
D001151	affect	EIF4EBP2	ENSG00000148730	18414638;
D001151	affect	EPB41L3	ENSG00000082397	18414638;
D001151	affect	ERBB2IP	ENSG00000112851	18414638;
D001151	affect	ERMP1	ENSG00000099219	18414638;
D001151	affect	ERN2	ENSG00000134398	18414638;
D001151	affect	EXOC8	ENSG00000116903	18414638;
D001151	affect	F2R	ENSG00000181104	18414638;
D001151	affect	FAM105B	ENSG00000154124	18414638;
D001151	affect	FAM108A1	ENSG00000129968	18414638;
D001151	affect	FAM53C	ENSG00000120709	18414638;
D001151	affect	FBXL5	ENSG00000118564	18414638;
D001151	affect	FBXO32	ENSG00000156804	18414638;
D001151	affect	FBXO3	ENSG00000110429	18414638;
D001151	affect	FCRL1	ENSG00000163534	18414638;
D001151	affect	FXYD2	ENSG00000137731	18414638;
D001151	affect	GALNT10	ENSG00000164574	18414638;
D001151	affect	GBP5	ENSG00000154451	18414638;
D001151	affect	GCN1L1	ENSG00000089154	18414638;
D001151	affect	GPR56	ENSG00000205336	18414638;
D001151	affect	GRPEL1	ENSG00000109519	18414638;
D001151	affect	GTPBP3	ENSG00000130299	18414638;
D001151	affect	GTPBP5	ENSG00000101181	18414638;
D001151	affect	GUCA1B	ENSG00000112599	18414638;
D001151	affect	HAPLN2	ENSG00000132702	18414638;
D001151	affect	HLA-DPA1	ENSG00000168384	18414638;
D001151	affect	HLA-DQA1	ENSG00000196735	18414638;
D001151	affect	HLA-DQB1	ENSG00000179344	18414638;
D001151	affect	HLA-DRB1	ENSG00000196126	18414638;
D001151	affect	HSPA9	ENSG00000113013	18414638;
D001151	affect	ICAM1	ENSG00000090339	18414638;
D001151	affect	IFRD1	ENSG00000006652	18414638;
D001151	affect	IGSF6	ENSG00000140749	18414638;
D001151	affect	IL2RB	ENSG00000100385	18414638;
D001151	affect	ING3	ENSG00000071243	18414638;
D001151	affect	ISCA1	ENSG00000135070	18414638;
D001151	affect	ITGA7	ENSG00000135424	18414638;
D001151	affect	JMJD6	ENSG00000070495	18414638;
D001151	affect	KBTBD8	ENSG00000163376	18414638;
D001151	affect	KCNH2	ENSG00000055118	18414638;
D001151	affect	KCNK17	ENSG00000124780	18414638;
D001151	affect	KIR2DL1	ENSG00000125498	18414638;
D001151	affect	KIR2DL2	ENSG00000215764	18414638;
D001151	affect	KIR2DL3	ENSG00000243772	18414638;
D001151	affect	KIR2DL4	ENSG00000189013	18414638;
D001151	affect	KIR2DL5A	ENSG00000215764	18414638;
D001151	affect	KIR2DS1	ENSG00000125498	18414638;

D001151	affect	KIR3DL1	ENSG00000167633	18414638;
D001151	affect	KIR3DL2	ENSG00000240403	18414638;
D001151	affect	KIR3DL3	ENSG00000242019	18414638;
D001151	affect	KLHL15	ENSG00000174010	18414638;
D001151	affect	KLRF1	ENSG00000150045	18414638;
D001151	affect	KLRK1	ENSG00000213809	18414638;
D001151	affect	KPNA2	ENSG00000182481	18414638;
D001151	affect	LMF1	ENSG00000103227	18414638;
D001151	affect	LRIG1	ENSG00000144749	18414638;
D001151	affect	MALT1	ENSG00000172175	18414638;
D001151	affect	MATK	ENSG00000007264	18414638;
D001151	affect	MEF2C	ENSG00000081189	18414638;
D001151	affect	MEX3C	ENSG00000176624	18414638;
D001151	affect	MLC1	ENSG00000100427	18414638;
D001151	affect	MRAS	ENSG00000158186	18414638;
D001151	affect	MXD3	ENSG00000213347	18414638;
D001151	affect	NCAM1	ENSG00000149294	18414638;
D001151	affect	NCF1	ENSG00000158517	18414638;
D001151	affect	NFATC3	ENSG00000072736	18414638;
D001151	affect	NHP2L1	ENSG00000100138	18414638;
D001151	affect	NMD3	ENSG00000169251	18414638;
D001151	affect	NR1D2	ENSG00000174738	18414638;
D001151	affect	NRIP3	ENSG00000175352	18414638;
D001151	affect	NUAK2	ENSG00000163545	18414638;
D001151	affect	NUP210	ENSG00000132182	18414638;
D001151	affect	NUPL1	ENSG00000139496	18414638;
D001151	affect	OSBPL5	ENSG00000021762	18414638;
D001151	affect	PARP10	ENSG00000178685	18414638;
D001151	affect	PDE4C	ENSG00000105650	18414638;
D001151	affect	PLA2G5	ENSG00000127472	18414638;
D001151	affect	PLAC9	ENSG00000189129	18414638;
D001151	affect	PLEKHF1	ENSG00000166289	18414638;
D001151	affect	PLEKHG3	ENSG00000126822	18414638;
D001151	affect	PPARA	ENSG00000186951	18414638;
D001151	affect	PPP2R1A	ENSG00000105568	18414638;
D001151	affect	PRF1	ENSG00000180644	18414638;
D001151	affect	PRKD2	ENSG00000105287	18414638;
D001151	affect	PSMD14	ENSG00000115233	18414638;
D001151	affect	RAC2	ENSG00000128340	18414638;
D001151	affect	RANBP6	ENSG00000137040	18414638;
D001151	affect	RASAL2	ENSG00000075391	18414638;
D001151	affect	RBMS1	ENSG00000153250	18414638;
D001151	affect	REEP2	ENSG00000132563	18414638;
D001151	affect	RHEBL1	ENSG00000167550	18414638;
D001151	affect	RIMKLB	ENSG00000166532	18414638;
D001151	affect	RNF13	ENSG00000082996	18414638;
D001151	affect	RNF216	ENSG00000011275	18414638;
D001151	affect	RPP25	ENSG00000178718	18414638;
D001151	affect	SARDH	ENSG00000123453	18414638;
D001151	affect	SBF1	ENSG00000100241	18414638;
D001151	affect	9-Sep	ENSG00000184640	18414638;
D001151	affect	SH2D1B	ENSG00000198574	18414638;
D001151	affect	SH2D3C	ENSG00000095370	18414638;
D001151	affect	SIDT2	ENSG00000149577	18414638;

D001151	affect	SLC11A2	ENSG00000110911	18414638;
D001151	affect	SLC44A2	ENSG00000129353	18414638;
D001151	affect	SLC6A13	ENSG00000010379	18414638;
D001151	affect	SLC6A6	ENSG00000131389	18414638;
D001151	affect	SLC9A3R1	ENSG00000109062	18414638;
D001151	affect	SMARCC1	ENSG00000173473	18414638;
D001151	affect	SORL1	ENSG00000137642	18414638;
D001151	affect	SOS1	ENSG00000115904	18414638;
D001151	affect	SPSB1	ENSG00000171621	18414638;
D001151	affect	SSH1	ENSG00000084112	18414638;
D001151	affect	SSRP1	ENSG00000149136	18414638;
D001151	affect	STAM	ENSG00000136738	18414638;
D001151	affect	STAT2	ENSG00000170581	18414638;
D001151	affect	STK17B	ENSG00000081320	18414638;
D001151	affect	SYNJ2	ENSG00000078269	18414638;
D001151	affect	TAGAP	ENSG00000164691	18414638;
D001151	affect	TAGLN	ENSG00000149591	18414638;
D001151	affect	TAL1	ENSG00000162367	18414638;
D001151	affect	TCTN3	ENSG00000119977	18414638;
D001151	affect	TESK2	ENSG00000070759	18414638;
D001151	affect	THAP11	ENSG00000168286	18414638;
D001151	affect	TIGD1	ENSG00000221944	18414638;
D001151	affect	TMC8	ENSG00000167895	18414638;
D001151	affect	TMEM185A	ENSG00000155984	18414638;
D001151	affect	TOMM40	ENSG00000130204	18414638;
D001151	affect	TP53I11	ENSG00000175274	18414638;
D001151	affect	TPSB2	ENSG00000172236	18414638;
D001151	affect	TTC7A	ENSG00000068724	18414638;
D001151	affect	VAMP5	ENSG00000168899	18414638;
D001151	affect	VASH1	ENSG00000071246	18414638;
D001151	affect	VEPH1	ENSG00000197415	18414638;
D001151	affect	WDR26	ENSG00000162923	18414638;
D001151	affect	WDR34	ENSG00000119333	18414638;
D001151	affect	WNT10A	ENSG00000135925	18414638;
D001151	affect	YPEL1	ENSG00000100027	18414638;
D001151	affect	ZBTB10	ENSG00000205189	18414638;
D001151	affect	ZBTB25	ENSG00000089775	18414638;
D001151	affect	ZEB2	ENSG00000169554	18414638;
D001151	affect	ZNF500	ENSG00000103199	18414638;
D001151	affect	ZNF579	ENSG00000218891	18414638;
D001151	affect	ZNF771	ENSG00000179965	18414638;
D001151	decrease	ADAM17	ENSG00000151694	11134558;
D001151	decrease	AKAP9	ENSG00000127914	16835338;
D001151	decrease	ATXN7	ENSG00000163635	16835338;
D001151	decrease	CASP4	ENSG00000196954	11134558;
D001151	decrease	CASP6	ENSG00000138794	11134558;
D001151	decrease	CCL20	ENSG00000115009	16835338;
D001151	decrease	CCL3L3	ENSG00000205021	16835338;
D001151	decrease	CCL4	ENSG00000129277	16835338;
D001151	decrease	CCRL2	ENSG00000121797	16835338;
D001151	decrease	CD44	ENSG00000026508	16835338;
D001151	decrease	CD83	ENSG00000112149	16835338;
D001151	decrease	CXCL3	ENSG00000163734	16835338;
D001151	decrease	ERCC1	ENSG00000012061	12569548;

D001151	decrease	ERCC3	ENSG00000163161	12569548;
D001151	decrease	ERCC4	ENSG00000175595	12569548;
D001151	decrease	FASLG	ENSG00000117560	11134558;
D001151	decrease	GADD45B	ENSG00000099860	16835338;
D001151	decrease	GDF15	ENSG00000130513	12773770;
D001151	decrease	GOLGA4	ENSG00000144674	16835338;
D001151	decrease	HSPA1B	ENSG00000232804	16835338;
D001151	decrease	ID2	ENSG00000115738	16835338;
D001151	decrease	IER3	ENSG00000137331	16835338;
D001151	decrease	IL1A	ENSG00000115008	16835338;
D001151	decrease	IL1RN	ENSG00000136689	16835338;
D001151	decrease	KCNJ2	ENSG00000123700	16835338;
D001151	decrease	KRT18	ENSG00000111057	11134558;12773770;
D001151	decrease	KRT8	ENSG00000170421	11134558;12773770;
D001151	decrease	MCL1	ENSG00000143384	11134558;
D001151	decrease	MINPP1	ENSG00000107789	16835338;
D001151	decrease	N4BP2L2	ENSG00000244754	16835338;
D001151	decrease	NDUFB8	ENSG00000166136	16835338;
D001151	decrease	NR4A2	ENSG00000153234	16835338;
D001151	decrease	PDE4B	ENSG00000184588	16835338;
D001151	decrease	PELI1	ENSG00000197329	16835338;
D001151	decrease	PFKFB3	ENSG00000170525	16835338;
D001151	decrease	PIAS1	ENSG00000033800	16835338;
D001151	decrease	PTX3	ENSG00000163661	18414638;16835338;
D001151	decrease	RFX3	ENSG00000080298	16835338;
D001151	decrease	RGS1	ENSG00000090104	18414638;16835338;
D001151	decrease	RUFY3	ENSG00000018189	16835338;
D001151	decrease	SERPINE1	ENSG00000106366	11134558;
D001151	decrease	SFPQ	ENSG00000116560	16835338;
D001151	decrease	SKIL	ENSG00000136603	16835338;
D001151	decrease	SOD2	ENSG00000112096	16835338;16076760;
D001151	decrease	SOX18	ENSG00000203883	16835338;
D001151	decrease	SRP68	ENSG00000167881	16835338;
D001151	decrease	SSBP1	ENSG00000106028	16835338;
D001151	decrease	TAF1D	ENSG00000166012	16835338;
D001151	decrease	TNFAIP6	ENSG00000123610	16835338;
D001151	decrease	TNF	ENSG00000223952	16835338;
D001151	decrease	TNFRSF1A	ENSG00000067182	11134558;
D001151	decrease	TNIK	ENSG00000154310	16835338;
D001151	decrease	TRA2A	ENSG00000164548	16835338;
D001151	decrease	TRAPPC10	ENSG00000160218	16835338;
D001151	decrease	UBE2E1	ENSG00000170142	16835338;
D001151	decrease	USO1	ENSG00000138768	16835338;
D001151	decrease	USP13	ENSG00000058056	16835338;
D001151	decrease	ZFAND6	ENSG00000086666	16835338;
D001151	decrease	ZNF267	ENSG00000185947	16835338;
D001151	decrease	ZNF331	ENSG00000130844	16835338;
D001151	increase	BCL2	ENSG00000171791	12899209;
D001151	increase	BMP4	ENSG00000125378	12773770;
D001151	increase	CASP9	ENSG00000132906	11134558;
D001151	increase	CCL2	ENSG00000108691	12928151;
D001151	increase	CCNA2	ENSG00000145386	11134558;
D001151	increase	CCNE1	ENSG00000105173	11134558;
D001151	increase	CCNG1	ENSG00000113328	11134558;

D001151	increase	CD14	ENSG00000170458	12928151;
D001151	increase	CDKN1A	ENSG00000124762	11134558;
D001151	increase	CDKN2A	ENSG00000147889	12773770;16251483;
D001151	increase	CDKN2D	ENSG00000129355	11134558;
D001151	increase	CXCL1	ENSG00000163739	12928151;18039032;
D001151	increase	CXCL2	ENSG00000081041	16835338;12928151;
D001151	increase	DUSP1	ENSG00000120129	18039032;
D001151	increase	E2F3	ENSG00000112242	11134558;
D001151	increase	E2F5	ENSG00000133740	11134558;
D001151	increase	EDN2	ENSG00000127129	11134558;
D001151	increase	EDN3	ENSG00000124205	11134558;
D001151	increase	EGR1	ENSG00000120738	18039032;
D001151	increase	ERCC2	ENSG00000104884	11134558;17050553;12749816;
D001151	increase	ERCC5	ENSG00000134899	11134558;
D001151	increase	HGF	ENSG00000019991	11134558;
D001151	increase	HIF1A	ENSG00000100644	16386771;
D001151	increase	IER2	ENSG00000160888	18039032;
D001151	increase	IL1B	ENSG00000125538	16835338;11134558;12928151;
D001151	increase	IL6	ENSG00000136244	12928151;
D001151	increase	IL6R	ENSG00000160712	11134558;
D001151	increase	JUNB	ENSG00000171223	18039032;
D001151	increase	KRT1	ENSG00000167768	11134558;
D001151	increase	MMP1	ENSG00000196611	12928151;
D001151	increase	MT1A	ENSG00000205362	16984198;17409696;
D001151	increase	MT1X	ENSG00000187193	17409696;
D001151	increase	MT2A	ENSG00000125148	17409696;
D001151	increase	MT3	ENSG00000087250	16854967;
D001151	increase	OGG1	ENSG00000114026	16759981;
D001151	increase	OSM	ENSG00000099985	18039032;
D001151	increase	PCNA	ENSG00000132646	12899209;
D001151	increase	PTGS2	ENSG00000073756	18039032;
D001151	increase	RAD23A	ENSG00000179262	11134558;12773770;
D001151	increase	RFC2	ENSG00000049541	11134558;
D001151	increase	RNF149	ENSG00000163162	18039032;
D001151	increase	SFRS5	ENSG00000100650	18039032;
D001151	increase	SOCS3	ENSG00000184557	18414638;18039032;
D001151	increase	SOD1	ENSG00000142168	11134558;
D001151	increase	TFDP2	ENSG00000114126	11134558;
D001151	increase	TGFA	ENSG00000163235	17056473;
D001151	increase	TNFSF10	ENSG00000121858	11134558;
D001151	increase	TOP2A	ENSG00000131747	11134558;
D001151	increase	TP53	ENSG00000141510	18414638;12899209;16251483;15967209;16930632;
D001151	increase	VEGFB	ENSG00000173511	18414638;11134558;
D001151	increase	XRCC1	ENSG00000073050	12773770;
D001152	increase	APAF1	ENSG00000120868	16778962;
D001194	affect	ADD1	ENSG00000087274	17297452;
D001194	affect	AGAP7	ENSG00000204169	17297452;
D001194	affect	ASB9	ENSG00000102048	17297452;
D001194	affect	ATL2	ENSG00000119787	17297452;
D001194	affect	ATM	ENSG00000149311	17297452;
D001194	affect	ATRN	ENSG00000088812	17297452;

D001194	affect	CCR2	ENSG00000121807	17297452;
D001194	affect	CD86	ENSG00000114013	17297452;
D001194	affect	CTBP2	ENSG00000175029	17297452;
D001194	affect	FBXL12	ENSG00000127452	17297452;
D001194	affect	FGL2	ENSG00000127951	17297452;
D001194	affect	FKBP1A	ENSG00000088832	17297452;
D001194	affect	FNTA	ENSG00000168522	17297452;
D001194	affect	GIMAP6	ENSG00000133561	17297452;
D001194	affect	GLRX	ENSG00000173221	17297452;
D001194	affect	GPR126	ENSG00000112414	17297452;
D001194	affect	GSTM3	ENSG00000134202	7614465;
D001194	affect	HMGXB3	ENSG00000113716	17297452;
D001194	affect	IFT74	ENSG00000096872	17297452;
D001194	affect	ITGA4	ENSG00000115232	17297452;
D001194	affect	LIFR	ENSG00000113594	17297452;
D001194	affect	LRPAP1	ENSG00000163956	17297452;
D001194	affect	LZTR1	ENSG00000099949	17297452;
D001194	affect	MAEA	ENSG00000090316	17297452;
D001194	affect	MAFB	ENSG00000204103	17297452;
D001194	affect	MS4A6A	ENSG00000110077	17297452;
D001194	affect	MUSK	ENSG00000030304	17297452;
D001194	affect	MYO5A	ENSG00000197535	17297452;
D001194	affect	MYO7A	ENSG00000137474	17297452;
D001194	affect	NIPSNAP3B	ENSG00000165028	17297452;
D001194	affect	PATZ1	ENSG00000100105	17297452;
D001194	affect	PPIG	ENSG00000138398	17297452;
D001194	affect	PREPL	ENSG00000138078	17297452;
D001194	affect	PRKACA	ENSG00000072062	17297452;
D001194	affect	PRKCSH	ENSG00000130175	17297452;
D001194	affect	RUNX1	ENSG00000159216	17297452;
D001194	affect	SERINC3	ENSG00000132824	17297452;
D001194	affect	SIRT6	ENSG00000077463	17297452;
D001194	affect	SLC44A4	ENSG00000204385	17297452;
D001194	affect	SLC6A14	ENSG00000087916	17297452;
D001194	affect	SMCR7L	ENSG00000100335	17297452;
D001194	affect	SORBS2	ENSG00000154556	17297452;
D001194	affect	TRAT1	ENSG00000163519	17297452;
D001194	affect	UVRAG	ENSG00000198382	17297452;
D001194	affect	WFDC2	ENSG00000101443	17297452;
D001194	affect	YWHAB	ENSG00000166913	17297452;
D001194	affect	ZNF140	ENSG00000196387	17297452;
D001194	decrease	TGFBI	ENSG00000120708	16920672;
D001194	increase	F3	ENSG00000117525	15636197;
D001194	increase	SP1	ENSG00000185591	15636197;
D001194	increase	TNFRSF1A	ENSG00000067182	18709470;
D001205	affect	NOS3	ENSG00000164867	15719745;
D001205	decrease	ABCA1	ENSG00000165029	17639512;
D001205	decrease	ACE2	ENSG00000130234	17639512;
D001205	decrease	ALPI	ENSG00000163295	17639512;
D001205	decrease	ANPEP	ENSG00000166825	17639512;
D001205	decrease	APOB	ENSG00000084674	17475462;17639512;
D001205	decrease	APOM	ENSG00000226215	17639512;
D001205	decrease	AQP3	ENSG00000165272	17639512;
D001205	decrease	BCL2	ENSG00000171791	16867262;17166419;

D001205	decrease	BCL2L14	ENSG00000121380	17639512;
D001205	decrease	CD34	ENSG00000174059	16443354;
D001205	decrease	CD40	ENSG00000101017	16443354;
D001205	decrease	CFB	ENSG00000243570	17639512;
D001205	decrease	CR2	ENSG00000117322	16443354;
D001205	decrease	CRP	ENSG00000132693	16517955;
D001205	decrease	CTSO	ENSG00000151792	17639512;
D001205	decrease	CYP19A1	ENSG00000137869	16443354;
D001205	decrease	DNASE1	ENSG00000213918	17639512;
D001205	decrease	DPP4	ENSG00000197635	17639512;
D001205	decrease	EDN3	ENSG00000124205	16443354;
D001205	decrease	EPHX1	ENSG00000143819	17639512;
D001205	decrease	EPHX2	ENSG00000120915	17639512;
D001205	decrease	FABP6	ENSG00000170231	17639512;
D001205	decrease	FMO1	ENSG00000010932	17639512;
D001205	decrease	FMO5	ENSG00000131781	17639512;
D001205	decrease	G0S2	ENSG00000123689	17639512;
D001205	decrease	GAK	ENSG00000178950	16443354;
D001205	decrease	GLS	ENSG00000115419	17639512;
D001205	decrease	GSTA1	ENSG00000243955	17639512;
D001205	decrease	GTF2H1	ENSG00000110768	17639512;
D001205	decrease	HEPH	ENSG00000089472	17639512;
D001205	decrease	IGFBP5	ENSG00000115461	16443354;
D001205	decrease	MEP1A	ENSG00000112818	17639512;
D001205	decrease	MST1	ENSG00000173531	17639512;
D001205	decrease	MYO1A	ENSG00000166866	17639512;
D001205	decrease	NFYA	ENSG00000001167	16443354;
D001205	decrease	NINJ1	ENSG00000131669	16443354;
D001205	decrease	NKX2-1	ENSG00000136352	15083329;
D001205	decrease	NME1	ENSG00000239672	16443354;
D001205	decrease	OS9	ENSG00000135506	16443354;
D001205	decrease	PON1	ENSG00000005421	12871208;16052486;15375178;
D001205	decrease	RAB6A	ENSG00000175582	16443354;
D001205	decrease	S100G	ENSG00000169906	17639512;
D001205	decrease	SFTPB	ENSG00000168878	16490937;15083329;
D001205	decrease	SI	ENSG00000090402	17639512;
D001205	decrease	SULT2A1	ENSG00000105398	17639512;
D001205	decrease	TF	ENSG00000091513	17639512;
D001205	decrease	TFF2	ENSG00000160181	17639512;
D001205	decrease	TMPRSS6	ENSG00000187045	17639512;
D001205	decrease	TNFSF10	ENSG00000121858	17639512;
D001205	decrease	TRIP10	ENSG00000125733	16443354;
D001205	decrease	UBE2A	ENSG00000077721	16443354;
D001205	decrease	UGT2B15	ENSG00000196620	17639512;
D001205	decrease	VCAM1	ENSG00000162692	16443354;
D001205	decrease	VRK1	ENSG00000100749	16443354;
D001205	decrease	WWTR1	ENSG00000018408	17639512;
D001205	decrease	XPNPEP2	ENSG00000122121	17639512;
D001205	decrease	XRCC1	ENSG00000073050	16124896;
D001205	increase	ACTA2	ENSG00000107796	17053324;
D001205	increase	ANXA1	ENSG00000135046	17639512;
D001205	increase	ARHGDIB	ENSG00000111348	17639512;
D001205	increase	ATF3	ENSG00000162772	17639512;

D001205	increase	AURKB	ENSG00000178999	17639512;
D001205	increase	BAX	ENSG00000087088	16867262;17166419;
D001205	increase	BIRC3	ENSG00000023445	17639512;
D001205	increase	BUB1	ENSG00000169679	17639512;
D001205	increase	CASP3	ENSG00000164305	16407847;16124896;17166419;16867262;
D001205	increase	CCND2	ENSG00000118971	17639512;
D001205	increase	CDC6	ENSG00000094804	17639512;
D001205	increase	CDK11A	ENSG00000008128	16443354;
D001205	increase	CIT	ENSG00000122966	17639512;
D001205	increase	CXCL9	ENSG00000138755	16443354;
D001205	increase	CXCR5	ENSG00000160683	16443354;
D001205	increase	CYP1A1	ENSG00000140465	17512695;17639512;
D001205	increase	DLGAP5	ENSG00000126787	17639512;
D001205	increase	DNAJC10	ENSG00000077232	17353921;
D001205	increase	EDN1	ENSG00000078401	16443354;
D001205	increase	EGLN3	ENSG00000129521	17639512;
D001205	increase	EMP1	ENSG00000134531	17639512;
D001205	increase	EMP3	ENSG00000142227	17639512;
D001205	increase	FN1	ENSG00000115414	16443354;
D001205	increase	FOXJ1	ENSG00000129654	16443354;
D001205	increase	FZD2	ENSG00000180340	17639512;
D001205	increase	GATA1	ENSG00000102145	16443354;
D001205	increase	GFRA1	ENSG00000151892	16443354;
D001205	increase	HIF1A	ENSG00000100644	17364964;
D001205	increase	HK1	ENSG00000156515	16443354;
D001205	increase	HMOX1	ENSG00000100292	8743975;19683516;
D001205	increase	HSPA1A	ENSG00000232804	17639512;
D001205	increase	HSPA5	ENSG00000044574	17353921;
D001205	increase	HSPB1	ENSG00000106211	17639512;
D001205	increase	IER3	ENSG00000137331	17639512;
D001205	increase	KCNB1	ENSG00000158445	16443354;
D001205	increase	KIF23	ENSG00000137807	17639512;
D001205	increase	LGALS1	ENSG00000100097	17639512;
D001205	increase	MAFF	ENSG00000185022	17639512;
D001205	increase	MALT1	ENSG00000172175	17639512;
D001205	increase	MCM4	ENSG00000104738	17639512;
D001205	increase	MKI67	ENSG00000148773	17639512;
D001205	increase	MMP1	ENSG00000196611	10836612;
D001205	increase	MMP7	ENSG00000137673	17639512;
D001205	increase	MYBL2	ENSG00000101057	17639512;
D001205	increase	NR3C1	ENSG00000113580	16443354;
D001205	increase	NR4A3	ENSG00000119508	16443354;
D001205	increase	OLR1	ENSG00000173391	17639512;
D001205	increase	PDIA3	ENSG00000167004	17353921;
D001205	increase	PHLDA1	ENSG00000139289	17639512;
D001205	increase	PHLDA2	ENSG00000181649	17639512;
D001205	increase	PLA2G16	ENSG00000176485	17639512;
D001205	increase	PLK1	ENSG00000166851	17639512;
D001205	increase	PMAIP1	ENSG00000141682	17216584;
D001205	increase	PRKCQ	ENSG00000065675	16443354;
D001205	increase	PSMC3	ENSG00000165916	16443354;
D001205	increase	RAB3B	ENSG00000169213	17639512;
D001205	increase	RAD51C	ENSG00000108384	16443354;

D001205	increase	RAD54B	ENSG00000197275	17639512;
D001205	increase	RCAN1	ENSG00000159200	16443354;
D001205	increase	SOD1	ENSG00000142168	16867262;16443354;
D001205	increase	STK17B	ENSG00000081320	17639512;
D001205	increase	TGFB1	ENSG00000105329	17639512;
D001205	increase	TM4SF1	ENSG00000169908	17639512;
D001205	increase	TRIB3	ENSG00000101255	17639512;
D001205	increase	UGT1A10	ENSG00000242515	17639512;
D001205	increase	UGT1A9	ENSG00000241119	17639512;
D001205	increase	ZNF165	ENSG00000197279	17639512;
D001218	increase	INS	ENSG00000129965	1805284;
D001218	increase	POMC	ENSG00000115138	1805284;
D001241	decrease	ACTR3	ENSG00000115091	11906190;
D001241	decrease	AP2B1	ENSG00000006125	11906190;
D001241	decrease	BCL2	ENSG00000171791	12490308;
D001241	decrease	CACNG1	ENSG00000108878	11906190;
D001241	decrease	DDX18	ENSG00000088205	11906190;
D001241	decrease	DLST	ENSG00000119689	11906190;
D001241	decrease	EGR4	ENSG00000135625	11906190;
D001241	decrease	ELOVL5	ENSG00000012660	11906190;
D001241	decrease	GTF2I	ENSG00000077809	11906190;
D001241	decrease	IARS	ENSG00000196305	11906190;
D001241	decrease	ICAM1	ENSG00000090339	18059344;
D001241	decrease	KCNMB4	ENSG00000135643	11906190;
D001241	decrease	KIF20B	ENSG00000138182	11906190;
D001241	decrease	MYC	ENSG00000136997	11906190;
D001241	decrease	NPEPPS	ENSG00000141279	11906190;
D001241	decrease	NPR1	ENSG00000169418	11906190;
D001241	decrease	PCSK1	ENSG00000175426	11906190;
D001241	decrease	PFKFB3	ENSG00000170525	11906190;
D001241	decrease	PICALM	ENSG00000073921	11906190;
D001241	decrease	PIK3C2A	ENSG00000011405	11906190;
D001241	decrease	PPARA	ENSG00000186951	16816107;
D001241	decrease	PPARD	ENSG00000112033	18678619;
D001241	decrease	PPP1R12A	ENSG00000058272	11906190;
D001241	decrease	PPP1R2	ENSG00000184203	11906190;
D001241	decrease	PRH2	ENSG00000134551	11906190;
D001241	decrease	PSMC6	ENSG00000100519	11906190;
D001241	decrease	RALB	ENSG00000144118	11906190;
D001241	decrease	SLC16A1	ENSG00000155380	19023563;11906190;
D001241	decrease	SMC4	ENSG00000113810	11906190;
D001241	decrease	STAT3	ENSG00000168610	11906190;
D001241	decrease	SUMO1	ENSG00000116030	11906190;
D001241	decrease	TAF6	ENSG00000106290	11906190;
D001241	decrease	TOB1	ENSG00000141232	11906190;
D001241	decrease	VCL	ENSG00000035403	11906190;
D001241	decrease	WARS	ENSG00000140105	11906190;
D001241	decrease	YWHAE	ENSG00000108953	18678619;
D001241	increase	ACIN1	ENSG00000100813	11906190;
D001241	increase	AKT2	ENSG00000105221	11906190;
D001241	increase	ASTN2	ENSG00000148219	11906190;
D001241	increase	ATP2A1	ENSG00000196296	11906190;
D001241	increase	AURKA	ENSG00000087586	11906190;
D001241	increase	BAX	ENSG00000087088	15986848;

D001241	increase	BIRC5	ENSG00000089685	11906190;
D001241	increase	BRD4	ENSG00000141867	11906190;
D001241	increase	C14ORF45	ENSG00000119636	11906190;
D001241	increase	C21ORF33	ENSG00000160221	11906190;
D001241	increase	C9ORF16	ENSG00000171159	11906190;
D001241	increase	CALM3	ENSG00000143933	11906190;
D001241	increase	CAPSL	ENSG00000152611	11906190;
D001241	increase	CASP3	ENSG00000164305	12490308;
D001241	increase	CCL19	ENSG00000172724	11906190;
D001241	increase	CCL2	ENSG00000108691	16600694;
D001241	increase	CCNB1	ENSG00000134057	11906190;
D001241	increase	CCND1	ENSG00000110092	15489888;11313997;11593435;
D001241	increase	CCNJ	ENSG00000107443	11906190;
D001241	increase	CD274	ENSG00000120217	11906190;
D001241	increase	CDK3	ENSG00000250506	11906190;
D001241	increase	CLIP3	ENSG00000105270	11906190;
D001241	increase	CPNE1	ENSG00000214078	11906190;
D001241	increase	CREBBP	ENSG00000005339	11906190;
D001241	increase	CSNK2A1	ENSG00000101266	11906190;
D001241	increase	DENND5A	ENSG00000184014	11906190;
D001241	increase	EFCAB4A	ENSG00000177685	11906190;
D001241	increase	EHMT2	ENSG00000204371	11906190;
D001241	increase	ERCC5	ENSG00000134899	11906190;
D001241	increase	FAM27E3	ENSG00000237198	11906190;
D001241	increase	FAT3	ENSG00000165323	11906190;
D001241	increase	G6PC3	ENSG00000141349	11906190;
D001241	increase	GDF15	ENSG00000130513	15180942;
D001241	increase	HLCS	ENSG00000159267	11906190;
D001241	increase	HMMR	ENSG00000072571	11906190;
D001241	increase	IL1B	ENSG00000125538	19164858;
D001241	increase	IL1RN	ENSG00000136689	12045164;
D001241	increase	JUN	ENSG00000177606	16600694;
D001241	increase	KIF21A	ENSG00000139116	11906190;
D001241	increase	LY9	ENSG00000122224	11906190;
D001241	increase	NDST2	ENSG00000166507	11906190;
D001241	increase	NGFR	ENSG00000064300	17447067;
D001241	increase	ORC4L	ENSG00000115947	11906190;
D001241	increase	PCDH17	ENSG00000118946	11906190;
D001241	increase	PITX1	ENSG00000069011	11906190;
D001241	increase	PLA2G6	ENSG00000184381	11906190;
D001241	increase	POLR2A	ENSG00000181222	11906190;
D001241	increase	PSD	ENSG00000059915	11906190;
D001241	increase	PTGS2	ENSG00000073756	15489888;15843042;
D001241	increase	RABGGTA	ENSG00000100949	11906190;
D001241	increase	REEP6	ENSG00000115255	11906190;
D001241	increase	RELA	ENSG00000173039	16600694;10328874;15489888;
D001241	increase	RNASE1	ENSG00000129538	11906190;
D001241	increase	RNASE6	ENSG00000169413	11906190;
D001241	increase	RND2	ENSG00000108830	11906190;
D001241	increase	RNF26	ENSG00000173456	11906190;
D001241	increase	SAT1	ENSG00000130066	16262603;
D001241	increase	SCARB1	ENSG00000073060	16816107;

D001241	increase	SCFD2	ENSG00000184178	11906190;
D001241	increase	SCN1B	ENSG00000105711	11906190;
D001241	increase	SDS	ENSG00000135094	11906190;
D001241	increase	SERPINA3	ENSG00000196136	11906190;
D001241	increase	SLC22A8	ENSG00000149452	11906190;
D001241	increase	SPAG6	ENSG00000077327	11906190;
D001241	increase	STRN4	ENSG00000090372	11906190;
D001241	increase	SUMO3	ENSG00000184900	11906190;
D001241	increase	TBC1D10B	ENSG00000169221	11906190;
D001241	increase	TCIRG1	ENSG00000110719	11906190;
D001241	increase	TIMM17B	ENSG00000126768	11906190;
D001241	increase	TMED9	ENSG00000184840	11906190;
D001241	increase	TMOD4	ENSG00000163157	11906190;
D001241	increase	TNF	ENSG00000223952	10328874;15379866;15489888;12045164;
D001241	increase	TNNI3	ENSG00000129991	11906190;
D001241	increase	TNS3	ENSG00000136205	11906190;
D001241	increase	TOP3B	ENSG00000100038	11906190;
D001241	increase	TP53I13	ENSG00000167543	11906190;
D001241	increase	TRAF6	ENSG00000175104	11906190;
D001241	increase	TRAPPC6A	ENSG00000007255	11906190;
D001241	increase	TRIT1	ENSG00000043514	11906190;
D001241	increase	TRPM4	ENSG00000130529	11906190;
D001241	increase	TSNAX	ENSG00000116918	11906190;
D001241	increase	UBE2K	ENSG00000078140	11906190;
D001241	increase	UBTD1	ENSG00000165886	11906190;
D001241	increase	UNG	ENSG00000076248	11906190;
D001241	increase	USP15	ENSG00000135655	11906190;
D001241	increase	WIF1	ENSG00000156076	11906190;
D001241	increase	ZNF37A	ENSG00000075407	11906190;
D001241	increase	ZNF385A	ENSG00000161642	11906190;
D001241	increase	ZSCAN16	ENSG00000196812	11906190;
D001262	decrease	FKBP5	ENSG00000096060	19915711;
D001262	decrease	SLC19A1	ENSG00000173638	19616087;
D001262	decrease	SLC6A8	ENSG00000130821	19915711;
D001262	increase	CLEC2B	ENSG00000110852	19915711;
D001262	increase	SLCO4C1	ENSG00000173930	19915711;
D001262	increase	SNRPN	ENSG00000128739	19915711;
D001262	increase	TXN	ENSG00000136810	19915711;
D001280	affect	CYP1A1	ENSG00000140465	11678611;16248553;
D001280	decrease	IFNG	ENSG00000111537	10883730;15319175;
D001280	decrease	IL5	ENSG00000113525	10883730;15319175;
D001280	decrease	TNF	ENSG00000223952	10883730;
D001280	increase	AVPI1	ENSG00000119986	18461179;
D001280	increase	C15ORF48	ENSG00000166920	18461179;
D001280	increase	CGA	ENSG00000135346	18461179;
D001280	increase	CGB	ENSG00000213030	18461179;
D001280	increase	CYP11A1	ENSG00000140459	18461179;
D001280	increase	CYP19A1	ENSG00000137869	11675267;17520059;12127262;10746939;16996190;18461179;
D001280	increase	DDIT3	ENSG00000175197	11678611;
D001280	increase	FOS	ENSG00000170345	11678611;
D001280	increase	FSTL3	ENSG00000070404	18461179;

D001280	increase	GADD45A	ENSG00000116717	11678611;
D001280	increase	GSTA1	ENSG00000243955	11678611;
D001280	increase	HSD11B2	ENSG00000176387	18461179;
D001280	increase	HSPA4	ENSG00000170606	11678611;
D001280	increase	HSPA5	ENSG00000044574	11678611;
D001280	increase	INHA	ENSG00000123999	18461179;
D001280	increase	INSL4	ENSG00000120211	18461179;
D001280	increase	LHB	ENSG00000104826	18461179;
D001280	increase	MB	ENSG00000198125	1916698;
D001280	increase	MT2A	ENSG00000125148	11678611;
D001280	increase	NR4A1	ENSG00000123358	18461179;
D001280	increase	NR4A3	ENSG00000119508	18461179;
D001280	increase	NR5A1	ENSG00000136931	17520059;18461179;
D001280	increase	PDE4D	ENSG00000113448	18461179;
D001280	increase	PSG8	ENSG00000124467	18461179;
D001280	increase	SGK1	ENSG00000118515	18461179;
D001280	increase	STAR	ENSG00000147465	18461179;
D001285	increase	CD40	ENSG00000101017	16879495;
D001285	increase	PCNA	ENSG00000132646	12762645;
D001285	increase	TP53	ENSG00000141510	12762645;
D001335	increase	CCL5	ENSG00000161570	11306435;
D001335	increase	CSF2	ENSG00000164400	11306435;
D001335	increase	IL8	ENSG00000169429	11306435;16819095;
D001335	increase	SFTPA2	ENSG00000185303	19181744;
D001371	decrease	SLC2A3	ENSG00000059804	15342952;
D001371	decrease	TAGLN	ENSG00000149591	15342952;
D001371	increase	ASAH1	ENSG00000104763	15342952;
D001371	increase	C10ORF10	ENSG00000165507	15342952;
D001371	increase	FABP1	ENSG00000163586	15342952;
D001371	increase	GDPD3	ENSG00000102886	15342952;
D001371	increase	HPN	ENSG00000105707	15342952;
D001371	increase	IL12A	ENSG00000168811	9756745;
D001371	increase	IL12B	ENSG00000113302	9756745;
D001371	increase	IL2RA	ENSG00000134460	9756745;
D001371	increase	LSS	ENSG00000160285	15342952;
D001371	increase	NR0B2	ENSG00000131910	15342952;
D001371	increase	SERPINA3	ENSG00000196136	15342952;
D001371	increase	WIPI1	ENSG00000070540	15342952;
D001374	affect	BUB1B	ENSG00000156970	18720364;
D001374	affect	CCNA2	ENSG00000145386	18720364;
D001374	affect	CCND2	ENSG00000118971	17016690;
D001374	affect	CDC20	ENSG00000117399	18720364;
D001374	affect	CENPA	ENSG00000115163	18720364;
D001374	affect	CENPF	ENSG00000117724	18720364;
D001374	affect	E2F1	ENSG00000101412	19451745;
D001374	affect	FEN1	ENSG00000168496	18720364;
D001374	affect	IL6R	ENSG00000160712	18443271;
D001374	affect	KIF2C	ENSG00000142945	18720364;
D001374	affect	MCM5	ENSG00000100297	18720364;
D001374	affect	MKI67	ENSG00000148773	18720364;
D001374	affect	NUSAP1	ENSG00000137804	18720364;
D001374	affect	RFC3	ENSG00000133119	18720364;
D001374	affect	RRM2	ENSG00000171848	18720364;
D001374	affect	SOCS3	ENSG00000184557	18443271;

D001374	affect	SPAG5	ENSG00000076382	18720364;
D001374	affect	STAT3	ENSG00000168610	18443271;
D001374	affect	TMPO	ENSG00000120802	18720364;
D001374	affect	TSC1	ENSG00000165699	18538015;
D001374	affect	TTK	ENSG00000112742	18720364;
D001374	affect	UBE2C	ENSG00000175063	18720364;
D001374	decrease	ALAD	ENSG00000148218	19194470;
D001374	decrease	BAX	ENSG00000087088	15547111;
D001374	decrease	BCL2	ENSG00000171791	15547111;
D001374	decrease	BCL2L1	ENSG00000171552	18443271;19148494;
D001374	decrease	CCNA1	ENSG00000133101	17698632;
D001374	decrease	CCNB1	ENSG00000134057	15547111;18720364;
D001374	decrease	CDK2	ENSG00000123374	18324645;
D001374	decrease	CDK4	ENSG00000135446	18324645;
D001374	decrease	CLDN10	ENSG00000134873	19194470;
D001374	decrease	CNNM2	ENSG00000148842	19194470;
D001374	decrease	DNMT1	ENSG00000130816	17616700;15547111;19139132;
D001374	decrease	DNMT3A	ENSG00000119772	15547111;
D001374	decrease	DPYD	ENSG00000188641	15501990;
D001374	decrease	ITGB2	ENSG00000160255	19194470;
D001374	decrease	LITAF	ENSG00000189067	19194470;
D001374	decrease	MAD1L1	ENSG00000002822	19194470;
D001374	decrease	MAPT	ENSG00000186868	16930453;
D001374	decrease	MEGF6	ENSG00000162591	19194470;
D001374	decrease	MYC	ENSG00000136997	18045574;19451745;
D001374	decrease	NRN1	ENSG00000124785	19194470;
D001374	decrease	PANK3	ENSG00000120137	19194470;
D001374	decrease	PIP5K1A	ENSG00000143398	19194470;
D001374	decrease	SLC28A1	ENSG00000156222	19139132;
D001374	decrease	SLC29A2	ENSG00000174669	19194470;
D001374	decrease	SRPX	ENSG00000101955	19194470;
D001374	decrease	STC2	ENSG00000113739	19194470;
D001374	decrease	TBC1D4	ENSG00000136111	19194470;
D001374	decrease	TERT	ENSG00000164362	18045574;19451745;
D001374	decrease	TOP2A	ENSG00000131747	18720364;18348204;
D001374	decrease	TRH	ENSG00000170893	19194470;
D001374	decrease	TRIB1	ENSG00000173334	19194470;
D001374	decrease	TSPAN32	ENSG00000064201	19194470;
D001374	decrease	WDR47	ENSG00000085433	19194470;
D001374	increase	AGGF1	ENSG00000164252	19194470;
D001374	increase	APOBEC3F	ENSG00000128394	19194470;
D001374	increase	APOBEC3G	ENSG00000239713	19194470;
D001374	increase	AR	ENSG00000169083	18324645;
D001374	increase	BAK1	ENSG00000030110	15547111;
D001374	increase	BLK	ENSG00000136573	15547111;
D001374	increase	C3AR1	ENSG00000171860	19194470;
D001374	increase	C6ORF62	ENSG00000112308	19194470;
D001374	increase	CASP10	ENSG00000003400	19194470;
D001374	increase	CASP3	ENSG00000164305	18443271;19148494;
D001374	increase	CASP5	ENSG00000137757	15547111;
D001374	increase	CASP6	ENSG00000138794	15547111;
D001374	increase	CASP8	ENSG00000064012	18082045;
D001374	increase	CDK1	ENSG00000170312	18324645;

D001374	increase	CDKN1A	ENSG00000124762	18324645;15547111;19148494;
D001374	increase	CDKN1C	ENSG00000129757	19194470;
D001374	increase	CDKN2A	ENSG00000147889	18443271;17616700;15547111;
D001374	increase	CDKN2B	ENSG00000147883	19194470;17611569;19148494;
D001374	increase	CENPO	ENSG00000138092	19194470;
D001374	increase	CIITA	ENSG00000179583	18829986;
D001374	increase	CLDN6	ENSG00000184697	18661270;
D001374	increase	CLDN8	ENSG00000156284	18661270;
D001374	increase	CLDN9	ENSG00000213937	18661270;
D001374	increase	COL14A1	ENSG00000187955	19194470;
D001374	increase	CYTH1	ENSG00000108669	19194470;
D001374	increase	FAM8A1	ENSG00000137414	19194470;
D001374	increase	GABRB1	ENSG00000163288	19194470;
D001374	increase	GADD45A	ENSG00000116717	19190346;15547111;
D001374	increase	GADD45B	ENSG00000099860	18225533;
D001374	increase	H1F0	ENSG00000189060	16006241;
D001374	increase	H2AFX	ENSG00000188486	17991895;
D001374	increase	HGF	ENSG00000019991	19194470;
D001374	increase	HLA-DRA	ENSG00000204287	18829986;
D001374	increase	HPGD	ENSG00000164120	19584167;
D001374	increase	HRK	ENSG00000135116	15547111;
D001374	increase	HSPA6	ENSG00000173110	19194470;
D001374	increase	ID1	ENSG00000125968	19194470;
D001374	increase	ID3	ENSG00000117318	19194470;
D001374	increase	IFNG	ENSG00000111537	18829986;18945643;
D001374	increase	ISCA1	ENSG00000135070	19194470;
D001374	increase	KIR2DL1	ENSG00000125498	19394699;18945643;
D001374	increase	KIR2DL2	ENSG00000215764	18945643;19394699;
D001374	increase	KIR2DL3	ENSG00000243772	19394699;18945643;
D001374	increase	KIR2DL4	ENSG00000189013	18945643;
D001374	increase	KIR3DL1	ENSG00000167633	19394699;18945643;
D001374	increase	KLK3	ENSG00000142515	18324645;
D001374	increase	LIMS1	ENSG00000169756	19194470;
D001374	increase	LYZ	ENSG00000090382	19194470;
D001374	increase	MGMT	ENSG00000170430	12807730;
D001374	increase	MMP9	ENSG00000100985	19148494;
D001374	increase	MSRB2	ENSG00000148450	19194470;
D001374	increase	NKX2-5	ENSG00000183072	18557828;
D001374	increase	NR4A1	ENSG00000123358	19148494;
D001374	increase	NXT2	ENSG00000101888	19194470;
D001374	increase	OCLN	ENSG00000197822	18661270;
D001374	increase	PAX4	ENSG00000106331	18949370;
D001374	increase	PJA1	ENSG00000181191	19194470;
D001374	increase	PLCB1	ENSG00000182621	19805378;
D001374	increase	PML	ENSG00000140464	19194470;
D001374	increase	PPAP2A	ENSG00000067113	19194470;
D001374	increase	PPP2CA	ENSG00000113575	19155497;
D001374	increase	PTPRC	ENSG00000081237	19194470;
D001374	increase	RANBP2	ENSG00000153201	19194470;
D001374	increase	RHOB	ENSG00000143878	18047684;
D001374	increase	RIPK2	ENSG00000104312	15547111;

D001374	increase	RRM2B	ENSG00000048392	19010910;
D001374	increase	S100A9	ENSG00000163220	19194470;
D001374	increase	S100P	ENSG00000163993	19194470;
D001374	increase	SLC5A5	ENSG00000105641	16954431;17164311;
D001374	increase	ST3GAL6	ENSG00000064225	19194470;
D001374	increase	SULT1C2	ENSG00000198203	19194470;
D001374	increase	TECPR2	ENSG00000196663	19194470;
D001374	increase	TFE3	ENSG00000068323	19194470;
D001374	increase	THUMPD1	ENSG00000066654	19194470;
D001374	increase	TJP1	ENSG00000104067	18661270;
D001374	increase	TNFRSF1B	ENSG00000028137	15547111;
D001374	increase	TP53	ENSG00000141510	15547111;
D001374	increase	TPO	ENSG00000115705	17679169;
D001374	increase	TRIM8	ENSG00000171206	19194470;
D001374	increase	TSC2	ENSG00000103197	19250671;18538015;
D001374	increase	TSHR	ENSG00000165409	17679169;
D001374	increase	XAF1	ENSG00000132530	19077051;
D001375	decrease	MAPT	ENSG00000186868	16930453;
D001388	decrease	BCL2	ENSG00000171791	15174162;
D001388	decrease	IL8	ENSG00000169429	16243974;
D001388	increase	IL6	ENSG00000136244	16243974;
D001388	increase	NR3C1	ENSG00000113580	16243974;
D001388	increase	TNF	ENSG00000223952	16243974;
D001463	increase	CYP3A4	ENSG00000160868	15554232;15955870;
D001466	increase	IL6	ENSG00000136244	11403239;
D001498	increase	CYP11B2	ENSG00000179142	9139807;
D001498	increase	FOS	ENSG00000170345	16188231;
D001500	affect	AR	ENSG00000169083	14532843;
D001500	increase	IL6	ENSG00000136244	14532843;
D001507	increase	ADRB2	ENSG00000169252	15679717;
D001514	decrease	BCL2	ENSG00000171791	15922390;
D001514	increase	BAX	ENSG00000087088	15922390;
D001514	increase	CASP3	ENSG00000164305	15922390;
D001514	increase	CYP19A1	ENSG00000137869	15894134;
D001542	increase	BUB1B	ENSG00000156970	16903866;
D001542	increase	CYP19A1	ENSG00000137869	14691014;
D001554	decrease	BAX	ENSG00000087088	18724895;
D001554	decrease	BCL2	ENSG00000171791	18724895;
D001554	decrease	CALM1	ENSG00000143933	15929907;
D001554	decrease	GP1BB	ENSG00000203618	15929907;
D001554	decrease	HSPA1A	ENSG00000232804	15929907;
D001554	decrease	IFNG	ENSG00000111537	15929907;
D001554	decrease	JUN	ENSG00000177606	17119257;15929907;
D001554	decrease	MAP4	ENSG00000047849	15929907;
D001554	decrease	MYNN	ENSG00000085274	15929907;
D001554	decrease	PDE7B	ENSG00000171408	15929907;
D001554	decrease	PF4	ENSG00000163737	17119257;15929907;16286641;
D001554	decrease	PKIG	ENSG00000168734	15929907;
D001554	decrease	PPBP	ENSG00000163736	16286641;
D001554	decrease	PSRC1	ENSG00000134222	15929907;
D001554	decrease	RAD51	ENSG00000051180	18724895;
D001554	decrease	RNF31	ENSG00000092098	15929907;
D001554	decrease	TFPI	ENSG00000003436	15929907;

D001554	decrease	TGOLN2	ENSG00000152291	15929907;
D001554	decrease	TMEM107	ENSG00000179029	15929907;
D001554	decrease	TMEM40	ENSG00000088726	15929907;
D001554	decrease	TMEM66	ENSG00000133872	15929907;
D001554	decrease	UCP1	ENSG00000109424	15929907;
D001554	decrease	USP16	ENSG00000156256	15929907;
D001554	decrease	XPA	ENSG00000136936	18724895;
D001554	decrease	XPC	ENSG00000154767	18724895;
D001554	increase	ACOT9	ENSG00000123130	15929907;
D001554	increase	CCL5	ENSG00000161570	11306435;
D001554	increase	CCNB1	ENSG00000134057	15826604;
D001554	increase	CCNT2	ENSG00000082258	15929907;
D001554	increase	CD1B	ENSG00000158485	14673790;
D001554	increase	CD44	ENSG00000026508	15929907;
D001554	increase	CISH	ENSG00000114737	15929907;
D001554	increase	CLTC	ENSG00000141367	15929907;
D001554	increase	CREM	ENSG00000095794	15929907;
D001554	increase	CRK	ENSG00000167193	15929907;
D001554	increase	CSF2	ENSG00000164400	11306435;
D001554	increase	CXCL16	ENSG00000161921	17119257;15929907;
D001554	increase	CXORF40A	ENSG00000197620	15929907;
D001554	increase	FKBP1A	ENSG00000088832	14673790;
D001554	increase	FKBP5	ENSG00000096060	15036117;
D001554	increase	HSPA5	ENSG00000044574	12183065;
D001554	increase	IFNGR1	ENSG00000027697	15929907;
D001554	increase	IL10RA	ENSG00000110324	15929907;
D001554	increase	IL11RA	ENSG00000137070	15929907;
D001554	increase	IL16	ENSG00000172349	15929907;
D001554	increase	IL2RB	ENSG00000100385	15929907;
D001554	increase	IL4R	ENSG00000077238	15929907;14673790;
D001554	increase	IL6R	ENSG00000160712	15929907;
D001554	increase	IL8	ENSG00000169429	11306435;15356918;
D001554	increase	KLF13	ENSG00000169926	15929907;
D001554	increase	KPNA1	ENSG00000114030	15929907;
D001554	increase	LGALS8	ENSG00000116977	15929907;
D001554	increase	MMP13	ENSG00000137745	15036117;14673790;
D001554	increase	NAMPT	ENSG00000105835	15929907;
D001554	increase	PDE4D	ENSG00000113448	15929907;
D001554	increase	PIK3R5	ENSG00000141506	15929907;
D001554	increase	PPP1CB	ENSG00000213639	15929907;
D001554	increase	PPPDE1	ENSG00000121644	15929907;
D001554	increase	PTPRE	ENSG00000132334	15929907;
D001554	increase	PURB	ENSG00000146676	15929907;
D001554	increase	SLFN11	ENSG00000172716	15929907;
D001554	increase	SOCS3	ENSG00000184557	15929907;
D001554	increase	TBC1D7	ENSG00000145979	15929907;
D001554	increase	TLR2	ENSG00000137462	15929907;
D001554	increase	USP3	ENSG00000140455	15929907;
D001554	increase	ZNF302	ENSG00000089335	15929907;
D001554	increase	ZNF331	ENSG00000130844	17119257;15929907;
D001555	increase	IL8	ENSG00000169429	15356918;
D001556	decrease	IFNG	ENSG00000111537	15319175;
D001556	decrease	IL5	ENSG00000113525	15319175;
D001556	decrease	PGR	ENSG00000082175	14579009;9144411;

D001556	decrease	SULT2A1	ENSG00000105398	17080404;
D001556	decrease	TNF	ENSG00000223952	15319175;
D001556	increase	BAX	ENSG00000087088	14688026;
D001556	increase	BCL2	ENSG00000171791	14688026;
D001556	increase	CDKN1A	ENSG00000124762	14688026;
D001556	increase	CYP11A1	ENSG00000140459	17080404;
D001556	increase	CYP11B1	ENSG00000160882	17080404;
D001556	increase	CYP11B2	ENSG00000179142	17080404;
D001556	increase	CYP17A1	ENSG00000148795	17080404;
D001556	increase	CYP19A1	ENSG00000137869	12849724;17080404;
D001556	increase	CYP2B6	ENSG00000197408	15548381;
D001556	increase	CYP3A4	ENSG00000160868	15548381;
D001556	increase	CYP3A7	ENSG00000160870	8812189;
D001556	increase	ESR1	ENSG00000091831	15253041;14579009;16329587;
D001556	increase	GNRH1	ENSG00000147437	16329587;
D001556	increase	HSD3B2	ENSG00000203859	17080404;
D001556	increase	POMC	ENSG00000115138	16329587;
D001556	increase	STAR	ENSG00000147465	17080404;
D001564	decrease	AMHR2	ENSG00000135409	18247414;
D001564	decrease	BAD	ENSG00000002330	18247414;15698582;
D001564	decrease	BCL2A1	ENSG00000140379	18247414;
D001564	decrease	BCL2L1	ENSG00000171552	15698582;
D001564	decrease	BRCA1	ENSG00000012048	18247414;
D001564	decrease	BTF3	ENSG00000145741	18247414;
D001564	decrease	C5AR1	ENSG00000197405	18247414;
D001564	decrease	CCL16	ENSG00000161573	18247414;
D001564	decrease	CCR6	ENSG00000112486	18247414;
D001564	decrease	CDK1	ENSG00000170312	15837074;
D001564	decrease	CREB1	ENSG00000118260	18247414;
D001564	decrease	DFFB	ENSG00000169598	18247414;
D001564	decrease	DNASE2	ENSG00000105612	18247414;
D001564	decrease	EDNRA	ENSG00000151617	18247414;
D001564	decrease	EGR1	ENSG00000120738	15808406;
D001564	decrease	EPHA3	ENSG00000044524	18247414;
D001564	decrease	EPHB3	ENSG00000182580	18247414;
D001564	decrease	FLT3	ENSG00000122025	18247414;
D001564	decrease	FN1	ENSG00000115414	18247414;
D001564	decrease	HLTF	ENSG00000071794	18247414;
D001564	decrease	IFI6	ENSG00000126709	18247414;
D001564	decrease	IFNAR1	ENSG00000142166	18247414;
D001564	decrease	IL10	ENSG00000136634	18247414;
D001564	decrease	IL3RA	ENSG00000185291	18247414;
D001564	decrease	IL9	ENSG00000145839	18247414;
D001564	decrease	ITGAL	ENSG00000005844	18247414;
D001564	decrease	KLF11	ENSG00000172059	18247414;
D001564	decrease	LMNB1	ENSG00000113368	17292933;
D001564	decrease	LTB	ENSG00000236237	18247414;
D001564	decrease	MADD	ENSG00000110514	18247414;
D001564	decrease	MST1	ENSG00000173531	18247414;
D001564	decrease	NFKBIE	ENSG00000146232	18247414;
D001564	decrease	NFYB	ENSG00000120837	18247414;
D001564	decrease	NME3	ENSG00000103024	18247414;
D001564	decrease	OPRK1	ENSG00000082556	18247414;

D001564	decrease	POU2AF1	ENSG00000110777	18247414;
D001564	decrease	PRDX2	ENSG00000167815	17292933;
D001564	decrease	PSMB6	ENSG00000142507	18247414;
D001564	decrease	PTAFR	ENSG00000169403	18247414;
D001564	decrease	PTX3	ENSG00000163661	18247414;
D001564	decrease	RARG	ENSG00000172819	18247414;
D001564	decrease	RPL7A	ENSG00000148303	18247414;
D001564	decrease	RUNX1T1	ENSG00000079102	18247414;
D001564	decrease	SLC9A3R2	ENSG00000065054	18247414;
D001564	decrease	SREBF1	ENSG00000072310	18247414;
D001564	decrease	STAT2	ENSG00000170581	18247414;
D001564	decrease	TFDP1	ENSG00000198176	18247414;
D001564	decrease	UBE2I	ENSG00000103275	18247414;
D001564	decrease	VIM	ENSG00000026025	18247414;
D001564	increase	AATK	ENSG00000181409	18247414;
D001564	increase	ABCB1	ENSG00000085563	15566942;
D001564	increase	ABCG2	ENSG00000118777	15917307;
D001564	increase	AKR1C1	ENSG00000187134	19800895;15735009;
D001564	increase	AKR1C2	ENSG00000151632	19800895;15735009;
D001564	increase	ALDOA	ENSG00000149925	17292933;
D001564	increase	ANP32A	ENSG00000140350	17292933;
D001564	increase	ARNT	ENSG00000143437	18247414;
D001564	increase	BAX	ENSG00000087088	15735009;16041517;
D001564	increase	BCL2	ENSG00000171791	16041517;
D001564	increase	BCL2L11	ENSG00000153094	15735009;
D001564	increase	BID	ENSG00000015475	15698582;16041517;
D001564	increase	BTK	ENSG00000010671	18247414;
D001564	increase	CARTPT	ENSG00000164326	18247414;
D001564	increase	CBL	ENSG00000110395	18247414;
D001564	increase	CCL5	ENSG00000161570	18247414;11306435;
D001564	increase	CCR5	ENSG00000160791	18247414;
D001564	increase	CD4	ENSG00000010610	18247414;
D001564	increase	CD59	ENSG00000085063	18247414;17045307;
D001564	increase	CDKN1A	ENSG00000124762	15625077;18418871;15735009;15837074;15808406;
D001564	increase	CSF2	ENSG00000164400	11306435;
D001564	increase	CYP11B2	ENSG00000179142	15808406;
D001564	increase	CYP1A1	ENSG00000140465	15661813;15905203;19070657;15627480;15808406;15566942;15735009;15713371;19800895;16258175;15808407;11849738;18214805;15608128;15298956;2791199;8961944;12490585;15162[illegible]
D001564	increase	CYP1A2	ENSG00000140505	11849738;2791199;896194
D001564	increase	CYP1B1	ENSG00000138061	16530937;15905203;18214805;15297370;15713371;15566942;15808406;15735009;15808407;16381670;16258175;15958554;15162144;
D001564	increase	CYP2B6	ENSG00000197408	15735009;
D001564	increase	DDB2	ENSG00000134574	15735009;
D001564	increase	DEDD	ENSG00000158796	18247414;
D001564	increase	EHHADH	ENSG00000113790	17292933;
D001564	increase	ENO1	ENSG00000074800	17292933;

D001564	increase	EPHX1	ENSG00000143819	15566942;15735009;15298956;8961944;
D001564	increase	EPO	ENSG00000130427	18247414;
D001564	increase	ESR1	ENSG00000091831	12441364;11922773;
D001564	increase	EZR	ENSG00000092820	18247414;
D001564	increase	FAS	ENSG00000026103	16041517;
D001564	increase	FASLG	ENSG00000117560	18247414;16041517;
D001564	increase	FGF9	ENSG00000102678	16002475;
D001564	increase	G0S2	ENSG00000123689	15808406;
D001564	increase	GADD45A	ENSG00000116717	18418871;15735009;
D001564	increase	GAPDH	ENSG00000111640	17292933;
D001564	increase	GDF1	ENSG00000130283	18247414;
D001564	increase	GLUD1	ENSG00000148672	17292933;
D001564	increase	HRAS	ENSG00000174775	11008122;
D001564	increase	IL11	ENSG00000095752	16002475;
D001564	increase	IL1A	ENSG00000115008	16002475;
D001564	increase	IL1B	ENSG00000125538	15808406;15792794;
D001564	increase	IL6	ENSG00000136244	16002475;
D001564	increase	IL8	ENSG00000169429	11306435;12393170;
D001564	increase	JUN	ENSG00000177606	16041517;
D001564	increase	KRT19	ENSG00000171345	17292933;
D001564	increase	KRT8	ENSG00000170421	17292933;
D001564	increase	MAP3K1	ENSG00000095015	16041517;
D001564	increase	MAPK8	ENSG00000107643	16041517;15698582;
D001564	increase	MCL1	ENSG00000143384	16041517;
D001564	increase	MDM2	ENSG00000135679	15625077;18418871;
D001564	increase	MLLT11	ENSG00000213190	15808406;
D001564	increase	MMP1	ENSG00000196611	15808406;
D001564	increase	MT1A	ENSG00000205362	17418620;
D001564	increase	NOS2	ENSG00000007171	18247414;
D001564	increase	NQO1	ENSG00000181019	11008122;15808406;15735009;
D001564	increase	NR5A1	ENSG00000136931	17520059;
D001564	increase	OSGIN1	ENSG00000140961	15735009;
D001564	increase	PLD2	ENSG00000129219	18247414;
D001564	increase	PON1	ENSG00000005421	15169886;
D001564	increase	POU2F1	ENSG00000143190	18247414;
D001564	increase	PPARG	ENSG00000132170	15735009;
D001564	increase	PPIA	ENSG00000196262	17292933;
D001564	increase	PPIB	ENSG00000166794	17292933;
D001564	increase	PRDX1	ENSG00000117450	17292933;
D001564	increase	PRKAR2B	ENSG00000005249	18247414;
D001564	increase	PSMC4	ENSG00000013275	17292933;
D001564	increase	PTGES2	ENSG00000148334	16086235;
D001564	increase	PTGS2	ENSG00000073756	16086235;
D001564	increase	RAN	ENSG00000132341	17292933;
D001564	increase	RGS1	ENSG00000090104	18247414;
D001564	increase	RPS25	ENSG00000118181	18247414;
D001564	increase	S100A10	ENSG00000197747	18247414;
D001564	increase	SMAD9	ENSG00000120693	18247414;
D001564	increase	SNCA	ENSG00000145335	18247414;
D001564	increase	STK25	ENSG00000115694	18247414;
D001564	increase	SUPT4H1	ENSG00000213246	18247414;
D001564	increase	TNF	ENSG00000223952	17092623;15792794;

D001564	increase	TNFRSF1A	ENSG00000067182	18247414;15735009;
D001564	increase	TP53	ENSG00000141510	12807757;15808406;15625077;18247414;16258175;16041517;15698582;1583707
D001564	increase	TSHR	ENSG00000165409	18247414;
D001564	increase	TSKU	ENSG00000182704	15735009;
D001564	increase	TUFM	ENSG00000178952	17292933;
D001564	increase	UGP2	ENSG00000169764	18247414;
D001564	increase	UGT1A10	ENSG00000242515	15735009;14557274;
D001564	increase	UGT1A6	ENSG00000167165	15566942;
D001564	increase	UGT1A7	ENSG00000244122	15566942;14557274;
D001585	increase	IL6	ENSG00000136244	11403239;
D001599	decrease	BCL2	ENSG00000171791	16505103;
D001599	decrease	BCL2L1	ENSG00000171552	16505103;
D001599	decrease	CCND1	ENSG00000110092	16505103;
D001599	decrease	CCND2	ENSG00000118971	16505103;
D001599	decrease	CCNE1	ENSG00000105173	16505103;
D001599	decrease	CDK2	ENSG00000123374	16505103;
D001599	decrease	CDK4	ENSG00000135446	16505103;
D001599	decrease	CDK6	ENSG00000105810	16505103;
D001599	increase	BAX	ENSG00000087088	16505103;
D001599	increase	CCL2	ENSG00000108691	16391493;
D001599	increase	CDKN1A	ENSG00000124762	16505103;
D001599	increase	CDKN1B	ENSG00000111276	16505103;
D001599	increase	CYP1A1	ENSG00000140465	16046213;
D001599	increase	IL1B	ENSG00000125538	16391493;
D001599	increase	IL8	ENSG00000169429	16391493;
D001599	increase	TNF	ENSG00000223952	16391493;
D001608	increase	IL1B	ENSG00000125538	8629860;
D001608	increase	TNF	ENSG00000223952	8629860;12449171;151279
D001623	increase	HPGD	ENSG00000164120	15680906;
D001629	increase	ABCB4	ENSG00000005471	15258199;14685799;15588777;
D001629	increase	ABCG5	ENSG00000138075	14685799;
D001629	increase	ACSL1	ENSG00000151726	19682441;
D001629	increase	CPT1A	ENSG00000110090	19682441;
D001629	increase	CYP1A1	ENSG00000140465	15521013;
D001629	increase	CYP4A11	ENSG00000187048	16712844;
D001629	increase	CYP7A1	ENSG00000167910	14685799;
D001629	increase	FAS	ENSG00000026103	16712844;
D001629	increase	PPARA	ENSG00000186951	15491415;19682441;
D001629	increase	SREBF2	ENSG00000198911	14685799;
D001647	decrease	CYP7A1	ENSG00000167910	15145977;15696852;
D001647	increase	ABCB11	ENSG00000073734	15696852;15841457;12663868;15582136;15145977;12705905;
D001647	increase	NR0B2	ENSG00000131910	15145977;
D001663	increase	CYP2B6	ENSG00000197408	15382119;
D001663	increase	CYP2C9	ENSG00000138109	15382119;
D001663	increase	CYP3A4	ENSG00000160868	15382119;
D001663	increase	GSTA1	ENSG00000243955	15382119;
D001663	increase	GSTA2	ENSG00000244067	15382119;
D001663	increase	IL1B	ENSG00000125538	15382119;
D001663	increase	SLCO1B1	ENSG00000134538	15382119;

D001663	increase	UGT1A1	ENSG00000241635	15560369;15334623;15382119;17898154;15988124;15557560;10950852;
D001710	affect	SLC5A6	ENSG00000138074	15561972;
D001710	decrease	CXCL11	ENSG00000169248	15219929;
D001710	decrease	HIRA	ENSG00000100084	15219929;
D001710	decrease	LAMP1	ENSG00000185896	15219929;
D001710	increase	CYP1B1	ENSG00000138061	15219929;15333708;
D001710	increase	HERC4	ENSG00000148634	15219929;
D001710	increase	TPR	ENSG00000047410	15219929;
D001728	increase	CDKN1A	ENSG00000124762	16061648;9343371;
D001728	increase	IL1B	ENSG00000125538	17959154;
D001728	increase	IL8	ENSG00000169429	19059883;
D001728	increase	MMP9	ENSG00000100985	17669387;
D001728	increase	RB1CC1	ENSG00000023287	16061648;
D001728	increase	TNF	ENSG00000223952	17669387;10531305;
D001728	increase	TP53	ENSG00000141510	14634213;17959154;16061648;
D001761	decrease	SOCS1	ENSG00000185338	17374387;
D001761	increase	IL8	ENSG00000169429	14617519;
D001761	increase	SFTPA1	ENSG00000122852	14617519;
D001761	increase	SFTPA2	ENSG00000185303	14617519;
D001761	increase	TNF	ENSG00000223952	14617519;
D001865	increase	ICAM1	ENSG00000090339	12952965;
D002038	increase	APP	ENSG00000142192	16627626;
D002038	increase	BCL2	ENSG00000171791	16835749;
D002038	increase	GATA3	ENSG00000107485	16835749;
D002038	increase	NFKB1	ENSG00000109320	16835749;
D002038	increase	PCNA	ENSG00000132646	16835749;
D002038	increase	STAT1	ENSG00000115415	16582591;16835749;
D002045	increase	PTGS1	ENSG00000095303	18165598;
D002045	increase	PTGS2	ENSG00000073756	18165598;
D002049	increase	HRH2	ENSG00000113749	9681472;
D002064	increase	BAX	ENSG00000087088	15965100;
D002064	increase	CASP3	ENSG00000164305	15965100;
D002064	increase	CASP9	ENSG00000132906	15965100;
D002083	decrease	ATP6V1A	ENSG00000114573	12470298;
D002083	decrease	COX2	ENSG00000198712	12470298;
D002083	decrease	ESR1	ENSG00000091831	12822488;14579009;
D002083	decrease	PSMC6	ENSG00000100519	12470298;
D002083	decrease	SOD2	ENSG00000112096	14981915;
D002083	decrease	TNF	ENSG00000223952	8740460;12470298;
D002083	increase	ABCC2	ENSG00000023839	16426233;
D002083	increase	CES1	ENSG00000198848	18227147;
D002083	increase	GCLC	ENSG00000001084	16426233;
D002083	increase	HMOX1	ENSG00000100292	18227147;
D002083	increase	NQO1	ENSG00000181019	18227147;
D002083	increase	PGR	ENSG00000082175	14579009;12822488;
D002083	increase	UGT1A1	ENSG00000241635	18227147;
D002084	affect	HMOX1	ENSG00000100292	8743975;
D002084	decrease	ATP6V1A	ENSG00000114573	12470298;
D002084	decrease	COX2	ENSG00000198712	12470298;
D002084	decrease	NR1H2	ENSG00000131408	17070507;
D002084	decrease	NR1H3	ENSG00000025434	17070507;

D002084	decrease	PON1	ENSG00000005421	16052486;
D002084	decrease	PPARG	ENSG00000132170	17070507;
D002084	decrease	PSMC6	ENSG00000100519	12470298;
D002084	decrease	SOD2	ENSG00000112096	14981915;
D002084	increase	ABCA1	ENSG00000165029	17070507;
D002084	increase	ACTA2	ENSG00000107796	16757516;
D002084	increase	CYP2B6	ENSG00000197408	18570159;
D002084	increase	CYP3A4	ENSG00000160868	18570159;
D002084	increase	LPA	ENSG00000198670	10856522;
D002084	increase	PGK1	ENSG00000102144	18603805;
D002084	increase	PLAT	ENSG00000104368	10856522;
D002084	increase	PON2	ENSG00000105854	17916643;
D002084	increase	SERPINE1	ENSG00000106366	10856522;
D002084	increase	TGFB1	ENSG00000105329	16757516;
D002084	increase	TNF	ENSG00000223952	9721806;12470298;
D002087	affect	CFTR	ENSG00000001626	15304546;
D002087	affect	HHEX	ENSG00000152804	16854221;
D002087	decrease	BID	ENSG00000015475	12893773;
D002087	decrease	BIRC5	ENSG00000089685	16042588;16007142;16061681;
D002087	decrease	CCNA1	ENSG00000133101	17698632;
D002087	decrease	CCNA2	ENSG00000145386	16007142;
D002087	decrease	CCNB1	ENSG00000134057	16007142;16061681;
D002087	decrease	CCND1	ENSG00000110092	16042588;12893773;15177505;17431121;
D002087	decrease	CCNE1	ENSG00000105173	15177505;
D002087	decrease	CCT5	ENSG00000150753	16042588;
D002087	decrease	CFLAR	ENSG00000003402	16042588;12893773;
D002087	decrease	CTNNB1	ENSG00000168036	15177505;
D002087	decrease	GTF2A1	ENSG00000165417	16646664;
D002087	decrease	GTF2A1L	ENSG00000242441	16646664;
D002087	decrease	IL12A	ENSG00000168811	11024006;
D002087	decrease	IL12B	ENSG00000113302	11024006;
D002087	decrease	IL12RB1	ENSG00000096996	11024006;
D002087	decrease	IL12RB2	ENSG00000081985	11024006;
D002087	decrease	MAP2K1	ENSG00000169032	12893773;15781658;
D002087	decrease	MAPK1	ENSG00000100030	12893773;15781658;
D002087	decrease	MAPK3	ENSG00000102882	12893773;15781658;
D002087	decrease	MECP2	ENSG00000169057	17698632;16042588;
D002087	decrease	MMP7	ENSG00000137673	16042588;
D002087	decrease	MPDU1	ENSG00000129255	15541394;
D002087	decrease	MPO	ENSG00000005381	12130515;
D002087	decrease	PCNA	ENSG00000132646	16042588;
D002087	decrease	PIK3CG	ENSG00000105851	16042588;
D002087	decrease	PTGS2	ENSG00000073756	16042588;
D002087	decrease	RAF1	ENSG00000132155	12893773;
D002087	decrease	RB1	ENSG00000139687	16042588;15177505;14707268;12893773;
D002087	decrease	SPP1	ENSG00000118785	16042588;
D002087	decrease	XIAP	ENSG00000101966	16007142;16061681;12893773;
D002087	increase	ABCB1	ENSG00000085563	15711177;
D002087	increase	ALPI	ENSG00000163295	16042588;
D002087	increase	AXIN2	ENSG00000168646	16042588;

D002087	increase	BAK1	ENSG00000030110	16042588;15781658;
D002087	increase	BCL2A1	ENSG00000140379	12130515;
D002087	increase	BCL2L1	ENSG00000171552	16042588;15177505;12893773;
D002087	increase	CASP8	ENSG00000064012	16042588;15781658;12893773;
D002087	increase	CDH1	ENSG00000039068	16042588;
D002087	increase	CDKN1A	ENSG00000124762	16042588;16112107;17431121;12893773;14707268;16007142;15942645;1213051
D002087	increase	CDKN1B	ENSG00000111276	16112107;17431121;
D002087	increase	CEBPE	ENSG00000092067	12130515;
D002087	increase	CLU	ENSG00000120885	16042588;
D002087	increase	CST6	ENSG00000175315	19503093;
D002087	increase	DAPK1	ENSG00000196730	16042588;
D002087	increase	GADD45A	ENSG00000116717	16042588;
D002087	increase	GSTA1	ENSG00000243955	12896903;
D002087	increase	GSTA2	ENSG00000244067	15746163;12896903;
D002087	increase	GSTA4	ENSG00000170899	15829614;15746163;
D002087	increase	GSTM2	ENSG00000213366	12896903;15746163;
D002087	increase	GSTP1	ENSG00000084207	15746163;12896903;
D002087	increase	GSTT2	ENSG00000133433	15746163;
D002087	increase	H1F0	ENSG00000189060	16006241;
D002087	increase	HBP1	ENSG00000105856	16042588;
D002087	increase	IFIT3	ENSG00000119917	12130515;
D002087	increase	KLK3	ENSG00000142515	11059779;
D002087	increase	LGALS1	ENSG00000100097	15541394;
D002087	increase	MYC	ENSG00000136997	12414619;
D002087	increase	NR3C1	ENSG00000113580	17498915;
D002087	increase	PPARG	ENSG00000132170	16112107;
D002087	increase	PTEN	ENSG00000171862	16042588;
D002087	increase	RASSF1	ENSG00000068028	18203293;
D002087	increase	SLC5A5	ENSG00000105641	17967635;17164311;
D002087	increase	ST3GAL5	ENSG00000115525	15541394;
D002104	decrease	ADH6	ENSG00000172955	17547211;
D002104	decrease	AKR1B1	ENSG00000085662	17547211;
D002104	decrease	ALDH6A1	ENSG00000119711	17547211;
D002104	decrease	ATP2B1	ENSG00000070961	17547211;
D002104	decrease	CACYBP	ENSG00000116161	16440303;
D002104	decrease	CIDEB	ENSG00000136305	17547211;
D002104	decrease	CLGN	ENSG00000153132	17547211;
D002104	decrease	CRYAA	ENSG00000160202	19031421;
D002104	decrease	EBP	ENSG00000147155	17547211;
D002104	decrease	ESR1	ENSG00000091831	17125913;16635015;
D002104	decrease	GPER	ENSG00000164850	17547211;
D002104	decrease	GSN	ENSG00000148180	17547211;
D002104	decrease	MGAT2	ENSG00000168282	17547211;
D002104	decrease	NUCB2	ENSG00000070081	17547211;
D002104	decrease	OS9	ENSG00000135506	17547211;
D002104	decrease	PDIA2	ENSG00000185615	16440303;
D002104	decrease	PGC	ENSG00000096088	17547211;
D002104	decrease	PSPH	ENSG00000146733	17547211;
D002104	decrease	SERPINA6	ENSG00000170099	17547211;
D002104	decrease	SERPINB1	ENSG00000021355	17547211;

D002104	decrease	SERPINC1	ENSG00000117601	17547211;
D002104	decrease	SOD1	ENSG00000142168	16440303;17120531;
D002104	decrease	TNFSF10	ENSG00000121858	17547211;
D002104	increase	ABCG2	ENSG00000118777	17547211;
D002104	increase	ANXA2	ENSG00000182718	17547211;
D002104	increase	ARHGDIA	ENSG00000141522	16440303;
D002104	increase	BIRC2	ENSG00000110330	14647439;
D002104	increase	CDKN1A	ENSG00000124762	17174997;14647439;
D002104	increase	CSF2	ENSG00000164400	18082304;
D002104	increase	CYP2C9	ENSG00000138109	11551516;
D002104	increase	ETS2	ENSG00000157557	17547211;
D002104	increase	FOS	ENSG00000170345	11738272;17125913;
D002104	increase	GADD45A	ENSG00000116717	17174997;
D002104	increase	GTPBP6	ENSG00000178605	17547211;
D002104	increase	HMOX1	ENSG00000100292	16959797;17244614;12716893;17156779;17547211;14647439;
D002104	increase	HSP90B1	ENSG00000166598	16440303;
D002104	increase	HSPA1A	ENSG00000232804	16460681;17244614;16729991;
D002104	increase	HSPA4	ENSG00000170606	16959797;
D002104	increase	IFITM2	ENSG00000185201	17547211;
D002104	increase	IL10	ENSG00000136634	18082304;
D002104	increase	IL1A	ENSG00000115008	18082304;
D002104	increase	IL1B	ENSG00000125538	18082304;
D002104	increase	IL6	ENSG00000136244	18082304;
D002104	increase	ITGB3BP	ENSG00000142856	17547211;
D002104	increase	JUN	ENSG00000177606	11738272;17125913;
D002104	increase	KIF2C	ENSG00000142945	19031421;
D002104	increase	MCM2	ENSG00000073111	17547211;
D002104	increase	MT1A	ENSG00000205362	12716893;16827180;17409696;15664440;
D002104	increase	MT1E	ENSG00000169715	16827180;12908821;
D002104	increase	MT1F	ENSG00000198417	16827180;
D002104	increase	MT1G	ENSG00000125144	16827180;
D002104	increase	MT1H	ENSG00000205358	16827180;
D002104	increase	MT1X	ENSG00000187193	16827180;17409696;
D002104	increase	MT2A	ENSG00000125148	15698955;12716893;17409708;17409696;15967203;15664440;
D002104	increase	MT3	ENSG00000087250	11087412;15461142;15782327;12220637;11994011;15698955;15755312;11738272;16854967;12908821;1512
D002104	increase	MYC	ENSG00000136997	11738272;
D002104	increase	PAK3	ENSG00000077264	19031421;
D002104	increase	PBK	ENSG00000168078	19031421;
D002104	increase	PDGFA	ENSG00000197461	17125913;
D002104	increase	PPIA	ENSG00000196262	16440303;
D002104	increase	PRDX1	ENSG00000117450	16440303;
D002104	increase	PRDX6	ENSG00000117592	16440303;
D002104	increase	PTTG1	ENSG00000164611	19031421;17547211;
D002104	increase	SERPINE2	ENSG00000135919	17547211;
D002104	increase	SFRS2	ENSG00000161547	17547211;
D002104	increase	SLC46A1	ENSG00000076351	17156779;

D002104	increase	SPP1	ENSG00000118785	17547211;
D002104	increase	STMN1	ENSG00000117632	16440303;
D002104	increase	TNFAIP3	ENSG00000118503	17547211;
D002104	increase	TP53	ENSG00000141510	10531375;17174997;
D002104	increase	TPX2	ENSG00000088325	19031421;
D002104	increase	TXN	ENSG00000136810	16440303;
D002104	increase	TYMS	ENSG00000176890	17547211;
D002104	increase	VDAC1	ENSG00000213585	16440303;
D002110	decrease	ACAT2	ENSG00000120437	11793227;
D002110	decrease	ALDH18A1	ENSG00000059573	11793227;
D002110	decrease	ANG	ENSG00000214274	11793227;
D002110	decrease	ATP5J2	ENSG00000241468	11793227;
D002110	decrease	CALU	ENSG00000128595	11793227;
D002110	decrease	CDK4	ENSG00000135446	11793227;
D002110	decrease	CYC1	ENSG00000179091	11793227;
D002110	decrease	DAD1	ENSG00000129562	11793227;
D002110	decrease	GLRX	ENSG00000173221	11793227;
D002110	decrease	GLT8D1	ENSG00000016864	11793227;
D002110	decrease	GLUD1	ENSG00000148672	11793227;
D002110	decrease	GSTO1	ENSG00000148834	11793227;
D002110	decrease	LDHA	ENSG00000134333	11793227;
D002110	decrease	MGST1	ENSG00000008394	11793227;
D002110	decrease	MICB	ENSG00000231179	18395517;
D002110	decrease	PRELID1	ENSG00000169230	11793227;
D002110	decrease	PSME3	ENSG00000131467	11793227;
D002110	decrease	RPS6KA3	ENSG00000177189	11793227;
D002110	decrease	SCFD1	ENSG00000249037	11793227;
D002110	decrease	SEC13	ENSG00000157020	11793227;
D002110	decrease	SEC23A	ENSG00000100934	11793227;
D002110	decrease	SERPINA6	ENSG00000170099	11793227;
D002110	decrease	SFRS1	ENSG00000136450	11793227;
D002110	decrease	SQLE	ENSG00000104549	11793227;
D002110	decrease	STRBP	ENSG00000165209	11793227;
D002110	decrease	TFDP1	ENSG00000198176	11793227;
D002110	decrease	TM9SF1	ENSG00000100926	11793227;
D002110	decrease	TMEM97	ENSG00000109084	11793227;
D002110	decrease	UQCRC2	ENSG00000140740	11793227;
D002110	increase	ACIN1	ENSG00000100813	11793227;
D002110	increase	ACSL3	ENSG00000123983	11793227;
D002110	increase	ARL6IP1	ENSG00000170540	11793227;
D002110	increase	CDKN1A	ENSG00000124762	17977830;18347191;18223691;
D002110	increase	EIF1	ENSG00000173812	11793227;
D002110	increase	IGFBP1	ENSG00000146678	11793227;
D002110	increase	MT1G	ENSG00000125144	11793227;
D002110	increase	OAT	ENSG00000065154	11793227;
D002110	increase	RRS1	ENSG00000179041	11793227;
D002110	increase	SLC16A1	ENSG00000155380	19023563;
D002110	increase	SMOX	ENSG00000088826	11793227;
D002110	increase	TP53	ENSG00000141510	17977830;16024610;16777994;
D002110	increase	UGT1A1	ENSG00000241635	18227147;
D002112	increase	CYP24A1	ENSG00000019186	16289102;16524720;
D002117	affect	NOG	ENSG00000183691	15268896;

D002117	decrease	ABCB10	ENSG00000135776	12875902;
D002117	decrease	ADAM28	ENSG00000042980	16002434;
D002117	decrease	ADRB2	ENSG00000169252	16002434;
D002117	decrease	AFF3	ENSG00000144218	16002434;
D002117	decrease	AGAP3	ENSG00000133612	16002434;
D002117	decrease	ALG13	ENSG00000101901	16002434;
D002117	decrease	ALOX12	ENSG00000108839	16002434;
D002117	decrease	APCDD1	ENSG00000154856	16002434;
D002117	decrease	ARHGEF3	ENSG00000163947	16002434;
D002117	decrease	ARL14	ENSG00000179674	16002434;
D002117	decrease	ARL4A	ENSG00000122644	16002434;
D002117	decrease	ARSI	ENSG00000183876	16002434;
D002117	decrease	ASS1	ENSG00000130707	12875902;
D002117	decrease	ATF3	ENSG00000162772	16002434;
D002117	decrease	ATP5F1	ENSG00000116459	16847355;15951637;
D002117	decrease	ATP6V1A	ENSG00000114573	12798352;
D002117	decrease	BAG1	ENSG00000250477	12479363;
D002117	decrease	BAIAP2	ENSG00000175866	16002434;
D002117	decrease	BAZ2A	ENSG00000076108	16002434;
D002117	decrease	BCL11B	ENSG00000127152	16002434;
D002117	decrease	BCL2	ENSG00000171791	15955619;12479363;
D002117	decrease	BCL2L1	ENSG00000171552	15955619;12479363;
D002117	decrease	BIRC2	ENSG00000110330	12479363;
D002117	decrease	BIRC3	ENSG00000023445	16002434;12479363;
D002117	decrease	C1ORF125	ENSG00000162779	16002434;
D002117	decrease	C1ORF61	ENSG00000125462	16002434;
D002117	decrease	C20ORF70	ENSG00000131050	16002434;
D002117	decrease	C8ORF34	ENSG00000165084	16002434;
D002117	decrease	C9ORF169	ENSG00000197191	16002434;
D002117	decrease	CBLB	ENSG00000114423	16002434;
D002117	decrease	CBX2	ENSG00000173894	16002434;
D002117	decrease	CCL20	ENSG00000115009	16002434;
D002117	decrease	CCNA1	ENSG00000133101	12875902;
D002117	decrease	CCND1	ENSG00000110092	15955619;
D002117	decrease	CCND2	ENSG00000118971	16847355;15951637;16002434;
D002117	decrease	CD44	ENSG00000026508	16051482;
D002117	decrease	CDC25A	ENSG00000164045	16002434;
D002117	decrease	CDR2L	ENSG00000109089	16002434;
D002117	decrease	CGA	ENSG00000135346	16002434;
D002117	decrease	CGNL1	ENSG00000128849	16002434;
D002117	decrease	CLDN1	ENSG00000163347	16002434;
D002117	decrease	COL4A2	ENSG00000134871	12875902;
D002117	decrease	CPA4	ENSG00000128510	16002434;
D002117	decrease	CRLS1	ENSG00000088766	16002434;
D002117	decrease	CSE1L	ENSG00000124207	12798352;
D002117	decrease	CSF1	ENSG00000184371	16002434;
D002117	decrease	CSRNP2	ENSG00000110925	16002434;
D002117	decrease	CTNNA1	ENSG00000044115	12875902;
D002117	decrease	CXCL11	ENSG00000169248	16002434;
D002117	decrease	CXCL14	ENSG00000145824	16002434;
D002117	decrease	CXCR7	ENSG00000144476	16002434;
D002117	decrease	CYP2S1	ENSG00000167600	16002434;
D002117	decrease	CYR61	ENSG00000142871	16002434;

D002117	decrease	DAB2IP	ENSG00000136848	16002434;
D002117	decrease	DAPK1	ENSG00000196730	16002434;
D002117	decrease	DCN	ENSG00000011465	16002434;
D002117	decrease	DHRS3	ENSG00000162496	16002434;
D002117	decrease	DLK2	ENSG00000171462	16002434;
D002117	decrease	EDN1	ENSG00000078401	16002434;
D002117	decrease	EEPD1	ENSG00000122547	16002434;
D002117	decrease	EFNB2	ENSG00000125266	16002434;
D002117	decrease	EIF2S1	ENSG00000134001	16847355;
D002117	decrease	EIF2S2	ENSG00000125977	16847355;15951637;
D002117	decrease	EIF2S3	ENSG00000130741	16847355;
D002117	decrease	EIF3B	ENSG00000106263	12875902;
D002117	decrease	EIF4A1	ENSG00000161960	12798352;
D002117	decrease	EIF4EBP2	ENSG00000148730	12798352;
D002117	decrease	ELF3	ENSG00000163435	16002434;
D002117	decrease	ETS2	ENSG00000157557	16002434;
D002117	decrease	FAM57A	ENSG00000167695	16002434;
D002117	decrease	FAM65A	ENSG00000039523	16002434;
D002117	decrease	FASN	ENSG00000169710	12798352;
D002117	decrease	FBXO32	ENSG00000156804	16002434;
D002117	decrease	FDXACB1	ENSG00000086848	16002434;
D002117	decrease	FGFBP1	ENSG00000137440	16002434;
D002117	decrease	FOLH1	ENSG00000086205	18247401;
D002117	decrease	FOXQ1	ENSG00000164379	16002434;
D002117	decrease	FUT4	ENSG00000196371	16002434;
D002117	decrease	GALNTL4	ENSG00000110328	16002434;
D002117	decrease	GCNT1	ENSG00000187210	16002434;
D002117	decrease	GLS	ENSG00000115419	16002434;
D002117	decrease	GLUL	ENSG00000135821	16002434;
D002117	decrease	GPR109B	ENSG00000182782	16002434;
D002117	decrease	GPR39	ENSG00000183840	16002434;
D002117	decrease	GPRC5B	ENSG00000167191	16002434;
D002117	decrease	GREM1	ENSG00000166923	15268896;16002434;
D002117	decrease	GRHL3	ENSG00000158055	16002434;
D002117	decrease	HHIP	ENSG00000164161	16002434;
D002117	decrease	HSPB3	ENSG00000169271	16002434;
D002117	decrease	HSPD1	ENSG00000144381	12875902;
D002117	decrease	HSPE1	ENSG00000115541	12875902;
D002117	decrease	ID2	ENSG00000115738	16002434;
D002117	decrease	IER3	ENSG00000137331	16002434;
D002117	decrease	IFIT1	ENSG00000185745	16002434;
D002117	decrease	IFIT2	ENSG00000119922	16002434;
D002117	decrease	IFIT3	ENSG00000119917	16002434;
D002117	decrease	IL1RN	ENSG00000136689	16002434;
D002117	decrease	IL7R	ENSG00000168685	16002434;
D002117	decrease	INHBA	ENSG00000122641	16002434;
D002117	decrease	IPO11	ENSG00000086200	16847355;
D002117	decrease	IPO13	ENSG00000117408	16847355;
D002117	decrease	IPO5	ENSG00000065150	16847355;
D002117	decrease	IPO7	ENSG00000205339	16847355;
D002117	decrease	IPO8	ENSG00000133704	16847355;
D002117	decrease	IPO9	ENSG00000198700	16847355;
D002117	decrease	IRF1	ENSG00000125347	16002434;
D002117	decrease	IRX3	ENSG00000177508	16002434;

D002117	decrease	JAG2	ENSG00000184916	16002434;
D002117	decrease	JUND	ENSG00000130522	16002434;
D002117	decrease	KCNJ15	ENSG00000157551	16002434;
D002117	decrease	KIAA1199	ENSG00000103888	16002434;
D002117	decrease	KITLG	ENSG00000049130	16002434;
D002117	decrease	KMO	ENSG00000117009	16002434;
D002117	decrease	KPNA2	ENSG00000182481	18614847;16847355;
D002117	decrease	KPNA3	ENSG00000102753	16847355;
D002117	decrease	KPNA4	ENSG00000186432	18614847;16847355;
D002117	decrease	KPNB1	ENSG00000108424	16847355;18614847;
D002117	decrease	L3MBTL3	ENSG00000198945	16002434;
D002117	decrease	LAYN	ENSG00000204381	16002434;
D002117	decrease	LDHA	ENSG00000134333	16847355;15951637;
D002117	decrease	LFNG	ENSG00000106003	16002434;
D002117	decrease	LGALS3	ENSG00000131981	12875902;
D002117	decrease	LIMCH1	ENSG00000064042	16002434;
D002117	decrease	LPP	ENSG00000145012	12875902;
D002117	decrease	LRIG3	ENSG00000139263	16002434;
D002117	decrease	MAGI1	ENSG00000151276	16002434;
D002117	decrease	MCL1	ENSG00000143384	12479363;
D002117	decrease	MECOM	ENSG00000085276	16002434;
D002117	decrease	MITF	ENSG00000187098	16002434;
D002117	decrease	MMP2	ENSG00000087245	16002434;
D002117	decrease	MRPS12	ENSG00000128626	16002434;
D002117	decrease	MTUS1	ENSG00000129422	16002434;
D002117	decrease	MYB	ENSG00000118513	12875902;16002434;
D002117	decrease	MYBL2	ENSG00000101057	12875902;
D002117	decrease	MYC	ENSG00000136997	16051482;12414619;12875902;
D002117	decrease	MYLIP	ENSG00000007944	16002434;
D002117	decrease	NAP1L1	ENSG00000187109	12798352;
D002117	decrease	NAV1	ENSG00000134369	16002434;
D002117	decrease	NEFL	ENSG00000104725	16002434;
D002117	decrease	NFATC2	ENSG00000101096	16002434;
D002117	decrease	NFKBIZ	ENSG00000144802	16002434;
D002117	decrease	NID2	ENSG00000087303	16002434;
D002117	decrease	NME2	ENSG00000243678	16847355;15951637;
D002117	decrease	NRARP	ENSG00000198435	16002434;
D002117	decrease	NRG2	ENSG00000158458	16002434;
D002117	decrease	NRP1	ENSG00000099250	16002434;
D002117	decrease	NTRK3	ENSG00000140538	16002434;
D002117	decrease	NUMA1	ENSG00000137497	12798352;
D002117	decrease	NUP153	ENSG00000124789	16002434;
D002117	decrease	OASL	ENSG00000135114	16002434;
D002117	decrease	OLR1	ENSG00000173391	16002434;
D002117	decrease	OSTALPHA	ENSG00000163959	16002434;
D002117	decrease	P2RY10	ENSG00000078589	16002434;
D002117	decrease	PAK6	ENSG00000137843	16002434;
D002117	decrease	PALLD	ENSG00000129116	16002434;
D002117	decrease	PAX9	ENSG00000198807	16002434;
D002117	decrease	PCDH7	ENSG00000169851	16002434;
D002117	decrease	PCNA	ENSG00000132646	15951637;
D002117	decrease	PCOTH	ENSG00000205850	16002434;
D002117	decrease	PDCD5	ENSG00000105185	12875902;

D002117	decrease	PHB	ENSG00000167085	12875902;
D002117	decrease	PLAC9	ENSG00000189129	16002434;
D002117	decrease	PNKD	ENSG00000127838	16002434;
D002117	decrease	POU2AF1	ENSG00000110777	12875902;
D002117	decrease	PPFIBP1	ENSG00000110841	16002434;
D002117	decrease	PPP2R4	ENSG00000119383	16002434;
D002117	decrease	PRDX1	ENSG00000117450	12875902;
D002117	decrease	PRICKLE2	ENSG00000163637	16002434;
D002117	decrease	PSMF1	ENSG00000125818	16002434;
D002117	decrease	PTGER2	ENSG00000125384	16002434;
D002117	decrease	PTGER4	ENSG00000171522	16002434;
D002117	decrease	PTH	ENSG00000152266	15956805;15891005;
D002117	decrease	PTHLH	ENSG00000087494	16002434;16243370;
D002117	decrease	PTPN3	ENSG00000070159	16129123;
D002117	decrease	PTPRZ1	ENSG00000106278	16002434;
D002117	decrease	RGPD1	ENSG00000187627	12798352;
D002117	decrease	RNF43	ENSG00000108375	16002434;
D002117	decrease	ROR1	ENSG00000185483	16002434;
D002117	decrease	RPS6KA5	ENSG00000100784	16002434;
D002117	decrease	RRAD	ENSG00000166592	16002434;
D002117	decrease	RSAD2	ENSG00000134321	16002434;
D002117	decrease	RUNX1	ENSG00000159216	16002434;
D002117	decrease	RUNX2	ENSG00000250096	17293108;16002434;
D002117	decrease	S100A8	ENSG00000143546	16002434;
D002117	decrease	SBNO1	ENSG00000139697	16002434;
D002117	decrease	SCD	ENSG00000099194	12798352;
D002117	decrease	SCEL	ENSG00000136155	16002434;
D002117	decrease	SEMA3C	ENSG00000075223	15951637;
D002117	decrease	SERPINB13	ENSG00000197641	16002434;
D002117	decrease	SERPINB2	ENSG00000197632	16002434;
D002117	decrease	SERPINB3	ENSG00000057149	16002434;
D002117	decrease	SET	ENSG00000119335	12798352;
D002117	decrease	SEZ6L2	ENSG00000174938	16002434;
D002117	decrease	SFTPA1	ENSG00000122852	15951333;
D002117	decrease	SGK1	ENSG00000118515	16002434;
D002117	decrease	SKP2	ENSG00000145604	12954644;
D002117	decrease	SLC2A2	ENSG00000163581	16002434;
D002117	decrease	SLC2A5	ENSG00000142583	16002434;
D002117	decrease	SLC43A3	ENSG00000134802	16002434;
D002117	decrease	SLC7A1	ENSG00000139514	16002434;
D002117	decrease	SLC7A8	ENSG00000092068	16002434;
D002117	decrease	SMAD6	ENSG00000137834	16002434;
D002117	decrease	SNRPB	ENSG00000125835	12798352;
D002117	decrease	SOCS2	ENSG00000120833	16002434;
D002117	decrease	SOX9	ENSG00000125398	16002434;
D002117	decrease	SPRR1A	ENSG00000169474	16002434;
D002117	decrease	SRM	ENSG00000116649	12875902;
D002117	decrease	SRPK1	ENSG00000096063	12875902;
D002117	decrease	STOML1	ENSG00000067221	16002434;
D002117	decrease	SYNGR1	ENSG00000100321	16002434;
D002117	decrease	TCF12	ENSG00000140262	12875902;
D002117	decrease	TCF7L1	ENSG00000152284	16002434;
D002117	decrease	TFDP2	ENSG00000114126	16129123;
D002117	decrease	TGM1	ENSG00000092295	16002434;

D002117	decrease	TIMP3	ENSG00000100234	16002434;
D002117	decrease	TM4SF1	ENSG00000169908	16002434;
D002117	decrease	TMTC2	ENSG00000179104	16002434;
D002117	decrease	TNN	ENSG00000120332	16002434;
D002117	decrease	TNPO1	ENSG00000083312	16847355;
D002117	decrease	TNPO2	ENSG00000105576	18614847;16847355;
D002117	decrease	TP53I3	ENSG00000115129	12798352;
D002117	decrease	TRAM2	ENSG00000065308	16002434;
D002117	decrease	TRIM16	ENSG00000221926	16002434;
D002117	decrease	TSC22D3	ENSG00000157514	16002434;
D002117	decrease	TXNIP	ENSG00000117289	16002434;
D002117	decrease	USP46	ENSG00000109189	14996990;
D002117	decrease	VAV3	ENSG00000134215	16002434;
D002117	decrease	VIM	ENSG00000026025	16129123;
D002117	decrease	VSNL1	ENSG00000163032	16002434;
D002117	decrease	WNT7B	ENSG00000188064	16002434;
D002117	decrease	XIAP	ENSG00000101966	12479363;
D002117	decrease	XPO1	ENSG00000082898	18614847;16847355;
D002117	decrease	XPO5	ENSG00000124571	18614847;16847355;
D002117	decrease	XPO7	ENSG00000130227	18614847;16847355;
D002117	decrease	XPOT	ENSG00000184575	18614847;16847355;
D002117	decrease	XRCC5	ENSG00000079246	15951637;
D002117	decrease	XRCC6	ENSG00000196419	16847355;15951637;
D002117	decrease	ZNF362	ENSG00000160094	16002434;
D002117	decrease	ZNF488	ENSG00000165388	16002434;
D002117	decrease	ZNF674	ENSG00000251192	16002434;
D002117	decrease	ZNF697	ENSG00000143067	16002434;
D002117	increase	ABAT	ENSG00000183044	16002434;
D002117	increase	ABCA1	ENSG00000165029	16002434;
D002117	increase	ABCB1	ENSG00000085563	12387747;
D002117	increase	ABCB5	ENSG00000004846	16002434;
D002117	increase	ABCC3	ENSG00000108846	15824121;
D002117	increase	ABHD3	ENSG00000158201	16002434;
D002117	increase	ABLIM1	ENSG00000099204	16002434;
D002117	increase	ACADM	ENSG00000117054	16002434;
D002117	increase	ACIN1	ENSG00000100813	12798352;
D002117	increase	ADAM19	ENSG00000135074	16002434;
D002117	increase	ADAMTS20	ENSG00000173157	16002434;
D002117	increase	ADRA1B	ENSG00000170214	16002434;
D002117	increase	AIG1	ENSG00000146416	16002434;
D002117	increase	AKAP12	ENSG00000131016	16002434;
D002117	increase	ALMS1	ENSG00000116127	16002434;
D002117	increase	ALOX5	ENSG00000012779	16002434;16129123;
D002117	increase	ALS2	ENSG00000248657	16002434;
D002117	increase	ANKMY2	ENSG00000106524	16002434;
D002117	increase	ANKRD43	ENSG00000198944	16002434;
D002117	increase	ANO10	ENSG00000160746	16002434;
D002117	increase	AP1G1	ENSG00000166747	14996990;
D002117	increase	AREG	ENSG00000205595	11237771;16002434;14996990;
D002117	increase	AR	ENSG00000169083	18247401;10875257;
D002117	increase	ARHGAP10	ENSG00000071205	16002434;
D002117	increase	ARHGAP5	ENSG00000100852	16002434;
D002117	increase	ARHGEF16	ENSG00000130762	16002434;

D002117	increase	ARID3B	ENSG00000179361	16002434;
D002117	increase	ARL2BP	ENSG00000102931	16002434;
D002117	increase	ARRDC4	ENSG00000140450	16002434;
D002117	increase	ATF6	ENSG00000118217	16002434;
D002117	increase	ATM	ENSG00000149311	16002434;
D002117	increase	ATP8B1	ENSG00000081923	16002434;
D002117	increase	B4GALT1	ENSG00000086062	16002434;
D002117	increase	B4GALT6	ENSG00000118276	16002434;
D002117	increase	BAIAP2L2	ENSG00000128298	16002434;
D002117	increase	BARX1	ENSG00000131668	16002434;
D002117	increase	BAX	ENSG00000087088	12479363;15955619;
D002117	increase	BBS10	ENSG00000179941	16002434;
D002117	increase	BCAS1	ENSG00000064787	16002434;
D002117	increase	BCL2A1	ENSG00000140379	16847355;15951637;
D002117	increase	BDNF	ENSG00000176697	16002434;
D002117	increase	BGLAP	ENSG00000242252	17293108;12898515;16267019;
D002117	increase	BLVRA	ENSG00000106605	16002434;
D002117	increase	BMP2	ENSG00000125845	15268896;16002434;
D002117	increase	BMP4	ENSG00000125378	15268896;
D002117	increase	BMP6	ENSG00000153162	15268896;16002434;
D002117	increase	BNIP3L	ENSG00000104765	16002434;
D002117	increase	BTG1	ENSG00000133639	12875902;
D002117	increase	C11ORF54	ENSG00000182919	16002434;
D002117	increase	C13ORF31	ENSG00000179630	16002434;
D002117	increase	C14ORF149	ENSG00000126790	16002434;
D002117	increase	C16ORF45	ENSG00000166780	16002434;
D002117	increase	C16ORF62	ENSG00000103544	16002434;
D002117	increase	C20ORF196	ENSG00000171984	16002434;
D002117	increase	C22ORF29	ENSG00000215012	16002434;
D002117	increase	C5ORF54	ENSG00000221886	16002434;
D002117	increase	C6ORF118	ENSG00000112539	16002434;
D002117	increase	C9ORF72	ENSG00000147894	16002434;
D002117	increase	C9ORF85	ENSG00000155621	16002434;
D002117	increase	CA12	ENSG00000074410	14996990;
D002117	increase	CA2	ENSG00000104267	16002434;
D002117	increase	CAB39	ENSG00000135932	16002434;
D002117	increase	CALB1	ENSG00000104327	15922086;
D002117	increase	CAMP	ENSG00000164047	15985530;
D002117	increase	CAPN7	ENSG00000131375	16002434;
D002117	increase	CARHSP1	ENSG00000153048	16002434;
D002117	increase	CASK	ENSG00000147044	16002434;
D002117	increase	CASP1	ENSG00000137752	16002434;
D002117	increase	CASP4	ENSG00000196954	16002434;
D002117	increase	CCDC121	ENSG00000176714	16002434;
D002117	increase	CCDC88C	ENSG00000015133	16002434;
D002117	increase	CCDC93	ENSG00000125633	16002434;
D002117	increase	CCNC	ENSG00000112237	15890193;
D002117	increase	CCNG2	ENSG00000138764	16002434;
D002117	increase	CD14	ENSG00000170458	14729647;16002434;16766008;
D002117	increase	CD274	ENSG00000120217	16002434;
D002117	increase	CD38	ENSG00000004468	16329108;
D002117	increase	CD3G	ENSG00000160654	12875902;

D002117	increase	CD86	ENSG00000114013	16002434;
D002117	increase	CD97	ENSG00000123146	16002434;
D002117	increase	CDA	ENSG00000158825	16002434;
D002117	increase	CDH1	ENSG00000039068	16051482;
D002117	increase	CDKN1A	ENSG00000124762	15955619;16434701;16039115;
D002117	increase	CDKN1B	ENSG00000111276	12954644;16322758;16039115;
D002117	increase	CDKN2D	ENSG00000129355	16322758;
D002117	increase	CDS1	ENSG00000163624	16002434;
D002117	increase	CDYL	ENSG00000153046	16002434;
D002117	increase	CEACAM6	ENSG00000086548	14996990;
D002117	increase	CEBPB	ENSG00000172216	14729647;16002434;
D002117	increase	CHD9	ENSG00000177200	16002434;
D002117	increase	CHIC2	ENSG00000109220	16002434;
D002117	increase	CHMP4C	ENSG00000164695	16002434;
D002117	increase	CHST11	ENSG00000171310	16002434;
D002117	increase	CHST3	ENSG00000122863	16002434;
D002117	increase	CHST7	ENSG00000147119	16002434;
D002117	increase	CITED4	ENSG00000179862	16002434;
D002117	increase	CLEC2D	ENSG00000069493	16002434;
D002117	increase	CLGN	ENSG00000153132	16002434;
D002117	increase	CLIP4	ENSG00000115295	16002434;
D002117	increase	CLMN	ENSG00000165959	16002434;
D002117	increase	CMIP	ENSG00000153815	16002434;
D002117	increase	CNGB1	ENSG00000070729	16002434;
D002117	increase	CNTNAP4	ENSG00000152910	16002434;
D002117	increase	COL13A1	ENSG00000197467	16002434;
D002117	increase	COL16A1	ENSG00000084636	16002434;
D002117	increase	COL1A1	ENSG00000108821	16002434;
D002117	increase	COL22A1	ENSG00000169436	16002434;
D002117	increase	COLEC12	ENSG00000158270	16002434;
D002117	increase	CPD	ENSG00000108582	16002434;
D002117	increase	CPEB2	ENSG00000137449	16002434;
D002117	increase	CP	ENSG00000047457	14996990;
D002117	increase	CPNE8	ENSG00000139117	16002434;
D002117	increase	CREG2	ENSG00000175874	16002434;
D002117	increase	CRISPLD1	ENSG00000121005	16002434;
D002117	increase	CRYBG3	ENSG00000080200	16002434;
D002117	increase	CSMD2	ENSG00000121904	16002434;
D002117	increase	CST6	ENSG00000175315	16002434;
D002117	increase	CTSB	ENSG00000164733	16847355;15951637;
D002117	increase	CUL3	ENSG00000036257	16002434;
D002117	increase	CXADR	ENSG00000154639	16002434;
D002117	increase	CXORF57	ENSG00000147231	16002434;
D002117	increase	CYB5R2	ENSG00000166394	16002434;
D002117	increase	CYB5R4	ENSG00000065615	16002434;
D002117	increase	CYP19A1	ENSG00000137869	15862960;16002434;
D002117	increase	CYP1A1	ENSG00000140465	19070657;
D002117	increase	CYP24A1	ENSG00000019186	16289102;15955619;15867263;15919092;16039846;15538745;14996990;16061850;16002434;15890193;1599
D002117	increase	CYP26B1	ENSG00000003137	16002434;

D002117	increase	CYP27B1	ENSG00000111012	16061850;
D002117	increase	CYP3A4	ENSG00000160868	19766177;17270371;15992766;11723248;17965521;
D002117	increase	CYP3A5	ENSG00000106258	16002434;
D002117	increase	CYP3A7	ENSG00000160870	16002434;
D002117	increase	DCBLD2	ENSG00000057019	16002434;
D002117	increase	DDIT3	ENSG00000175197	16322758;
D002117	increase	DDX26B	ENSG00000165359	16002434;
D002117	increase	DEF8	ENSG00000140995	16002434;
D002117	increase	DENND1B	ENSG00000213047	16002434;
D002117	increase	DENND4C	ENSG00000137145	16002434;
D002117	increase	DGAT2	ENSG00000062282	16002434;
D002117	increase	DLGAP1	ENSG00000170579	16002434;
D002117	increase	DNAJC3	ENSG00000102580	16002434;
D002117	increase	DNER	ENSG00000187957	16002434;
D002117	increase	DOCK11	ENSG00000147251	16002434;
D002117	increase	DOCK4	ENSG00000128512	16002434;
D002117	increase	DOCK5	ENSG00000147459	16002434;
D002117	increase	DPY19L1	ENSG00000173852	16002434;
D002117	increase	DTWD1	ENSG00000104047	16002434;
D002117	increase	DTX4	ENSG00000110042	16002434;
D002117	increase	DUSP10	ENSG00000143507	16002434;
D002117	increase	DUSP5	ENSG00000138166	16002434;
D002117	increase	EAF2	ENSG00000145088	16002434;
D002117	increase	EEF1A1	ENSG00000156508	12875902;
D002117	increase	EFCAB7	ENSG00000203965	16002434;
D002117	increase	EFTUD1	ENSG00000140598	16002434;
D002117	increase	EGLN1	ENSG00000135766	16002434;
D002117	increase	EGR3	ENSG00000179388	16002434;
D002117	increase	EHBP1	ENSG00000115504	16002434;
D002117	increase	EHBP1L1	ENSG00000173442	16002434;
D002117	increase	EHD1	ENSG00000110047	16002434;
D002117	increase	EHD4	ENSG00000103966	16002434;
D002117	increase	EIF2C2	ENSG00000123908	16002434;
D002117	increase	EIF5A2	ENSG00000163577	16002434;
D002117	increase	ELF2	ENSG00000109381	16002434;
D002117	increase	ELP4	ENSG00000109911	16002434;
D002117	increase	EMB	ENSG00000170571	16002434;
D002117	increase	EMR3	ENSG00000131355	16002434;
D002117	increase	ENOSF1	ENSG00000132199	12875902;
D002117	increase	ENTPD3	ENSG00000168032	16002434;
D002117	increase	EOMES	ENSG00000163508	16002434;
D002117	increase	ERMP1	ENSG00000099219	16002434;
D002117	increase	ESR1	ENSG00000091831	16002434;
D002117	increase	ETNK2	ENSG00000143845	16002434;
D002117	increase	EVI2B	ENSG00000185862	12875902;
D002117	increase	EVI5	ENSG00000067208	16002434;
D002117	increase	F13B	ENSG00000143278	16002434;
D002117	increase	FAM105B	ENSG00000154124	16002434;
D002117	increase	FAM107B	ENSG00000065809	16002434;
D002117	increase	FAM116B	ENSG00000205593	16002434;
D002117	increase	FAM126A	ENSG00000122591	16002434;
D002117	increase	FAM129A	ENSG00000135842	16002434;
D002117	increase	FAM20C	ENSG00000177706	16002434;

D002117	increase	FAM43B	ENSG00000183114	16002434;
D002117	increase	FAM49A	ENSG00000197872	16002434;
D002117	increase	FAM83B	ENSG00000168143	16002434;
D002117	increase	FAS	ENSG00000026103	16002434;
D002117	increase	FBLIM1	ENSG00000162458	16002434;
D002117	increase	FBXO3	ENSG00000110429	16002434;
D002117	increase	FCHSD2	ENSG00000137478	16002434;
D002117	increase	FGD3	ENSG00000127084	16002434;
D002117	increase	FGF23	ENSG00000118972	15956805;16234967;15698453;15264182;15917335;
D002117	increase	FGFR2	ENSG00000066468	12761878;
D002117	increase	FHOD3	ENSG00000134775	16002434;
D002117	increase	FLRT3	ENSG00000125848	16002434;
D002117	increase	FNBP1L	ENSG00000137942	16002434;
D002117	increase	FOS	ENSG00000170345	16002434;
D002117	increase	FOSL2	ENSG00000075426	16002434;
D002117	increase	FOXK1	ENSG00000164916	16002434;
D002117	increase	FOXO1	ENSG00000150907	16002434;
D002117	increase	FUT11	ENSG00000196968	16002434;
D002117	increase	FZD1	ENSG00000157240	16002434;
D002117	increase	G0S2	ENSG00000123689	16002434;
D002117	increase	G6PD	ENSG00000160211	16002434;
D002117	increase	GAB1	ENSG00000109458	16002434;
D002117	increase	GADD45A	ENSG00000116717	16002434;
D002117	increase	GAP43	ENSG00000172020	16002434;
D002117	increase	GATA2	ENSG00000179348	16002434;
D002117	increase	GCNT3	ENSG00000140297	16002434;
D002117	increase	GDF15	ENSG00000130513	15386353;16523695;
D002117	increase	GEM	ENSG00000164949	14996990;16002434;
D002117	increase	GIMAP2	ENSG00000106560	16002434;
D002117	increase	GIMAP8	ENSG00000171115	16002434;
D002117	increase	GK5	ENSG00000175066	16002434;
D002117	increase	GPR115	ENSG00000153294	16002434;
D002117	increase	GPR50	ENSG00000102195	16002434;
D002117	increase	GPR98	ENSG00000164199	16002434;
D002117	increase	GRAMD4	ENSG00000075240	16002434;
D002117	increase	GRB2	ENSG00000177885	15951637;
D002117	increase	GRB7	ENSG00000141738	16002434;
D002117	increase	GRHL1	ENSG00000134317	16002434;
D002117	increase	GRK5	ENSG00000198873	16002434;
D002117	increase	HAL	ENSG00000084110	12798352;
D002117	increase	HAS2	ENSG00000170961	16002434;
D002117	increase	HBEGF	ENSG00000113070	16002434;
D002117	increase	HIVEP2	ENSG00000010818	16002434;
D002117	increase	HMCN1	ENSG00000143341	16002434;
D002117	increase	HOXA10	ENSG00000078399	16632680;
D002117	increase	HR	ENSG00000168453	16002434;
D002117	increase	HS3ST3A1	ENSG00000153976	16002434;
D002117	increase	HS6ST2	ENSG00000171004	16002434;
D002117	increase	HSD17B2	ENSG00000086696	16002434;
D002117	increase	HSPG2	ENSG00000142798	16002434;
D002117	increase	HTR2C	ENSG00000147246	16002434;
D002117	increase	IFLTD1	ENSG00000152936	16002434;
D002117	increase	IFNG	ENSG00000111537	15879110;

D002117	increase	IGFBP3	ENSG00000146674	15955619;16825320;
D002117	increase	IL10	ENSG00000136634	16322758;
D002117	increase	IL1R1	ENSG00000115594	16002434;
D002117	increase	IL1RL1	ENSG00000115602	16002434;
D002117	increase	IL2RA	ENSG00000134460	15879110;
D002117	increase	IL2RG	ENSG00000147168	16129123;
D002117	increase	IL4R	ENSG00000077238	16002434;
D002117	increase	IL8	ENSG00000169429	12753408;16002434;16847355;12875902;15951637;
D002117	increase	ILDR1	ENSG00000145103	16002434;
D002117	increase	INPP4B	ENSG00000109452	16002434;
D002117	increase	INSIG2	ENSG00000125629	16002434;
D002117	increase	IQCB1	ENSG00000173226	16002434;
D002117	increase	IRAK2	ENSG00000134070	16002434;
D002117	increase	IRS1	ENSG00000169047	16002434;
D002117	increase	IRX4	ENSG00000113430	16002434;
D002117	increase	ISM1	ENSG00000101230	16002434;
D002117	increase	ITGA7	ENSG00000135424	16002434;
D002117	increase	ITGAM	ENSG00000169896	14729647;
D002117	increase	ITSN2	ENSG00000198399	16002434;
D002117	increase	JUNB	ENSG00000171223	14996990;16002434;
D002117	increase	KAL1	ENSG00000011201	16002434;
D002117	increase	KCNK9	ENSG00000169427	16002434;
D002117	increase	KCNS3	ENSG00000170745	16002434;
D002117	increase	KCNT2	ENSG00000162687	16002434;
D002117	increase	KCTD12	ENSG00000178695	16002434;
D002117	increase	KIAA0776	ENSG00000014123	16002434;
D002117	increase	KIAA1024	ENSG00000169330	16129123;
D002117	increase	KIAA1671	ENSG00000197077	16002434;
D002117	increase	KIAA1797	ENSG00000188352	16002434;
D002117	increase	KIAA1804	ENSG00000143674	16002434;
D002117	increase	KIF26A	ENSG00000066735	16002434;
D002117	increase	KIF3C	ENSG00000084731	16002434;
D002117	increase	KIFAP3	ENSG00000075945	16002434;
D002117	increase	KLF11	ENSG00000172059	16002434;
D002117	increase	KLF4	ENSG00000136826	16002434;
D002117	increase	KLF8	ENSG00000102349	16002434;
D002117	increase	KLHL14	ENSG00000197705	16002434;
D002117	increase	KLHL5	ENSG00000109790	16002434;
D002117	increase	KLK13	ENSG00000167759	16002434;
D002117	increase	KLK6	ENSG00000167755	16002434;
D002117	increase	KPNA1	ENSG00000114030	16847355;18614847;
D002117	increase	KRT16	ENSG00000186832	16002434;
D002117	increase	KRT37	ENSG00000108417	16002434;
D002117	increase	KSR1	ENSG00000141068	16002434;
D002117	increase	LACTB2	ENSG00000147592	16002434;
D002117	increase	LAMB3	ENSG00000196878	16002434;
D002117	increase	LBH	ENSG00000213626	16002434;
D002117	increase	LDLRAP1	ENSG00000157978	16002434;
D002117	increase	LGALS8	ENSG00000116977	16002434;
D002117	increase	LGR4	ENSG00000205213	16002434;
D002117	increase	LHX1	ENSG00000132130	16002434;
D002117	increase	LIPG	ENSG00000101670	16002434;
D002117	increase	LMCD1	ENSG00000071282	16002434;

D002117	increase	LOX	ENSG00000113083	16002434;
D002117	increase	LPGAT1	ENSG00000123684	16002434;
D002117	increase	LRRC58	ENSG00000163428	16002434;
D002117	increase	LSP1	ENSG00000130592	12875902;
D002117	increase	LTBP2	ENSG00000119681	16002434;
D002117	increase	LYN	ENSG00000249529	12875902;
D002117	increase	LYPD5	ENSG00000159871	16002434;
D002117	increase	LYZ	ENSG00000090382	16847355;
D002117	increase	LZTS1	ENSG00000061337	16002434;
D002117	increase	MAFB	ENSG00000204103	16002434;
D002117	increase	MALL	ENSG00000144063	16002434;
D002117	increase	MAN1C1	ENSG00000117643	16002434;
D002117	increase	MAP3K8	ENSG00000107968	16002434;
D002117	increase	MAPK13	ENSG00000156711	16002434;
D002117	increase	1-Mar	ENSG00000145416	16002434;
D002117	increase	3-Mar	ENSG00000173926	16002434;
D002117	increase	MBTD1	ENSG00000011258	16002434;
D002117	increase	MDFIC	ENSG00000135272	16002434;
D002117	increase	MDM1	ENSG00000111554	16002434;
D002117	increase	METRNL	ENSG00000176845	16002434;
D002117	increase	MFI2	ENSG00000163975	16002434;
D002117	increase	MIB1	ENSG00000101752	16002434;
D002117	increase	MINPP1	ENSG00000107789	16002434;
D002117	increase	MLF1	ENSG00000178053	16002434;
D002117	increase	MMD	ENSG00000108960	16002434;
D002117	increase	MMP1	ENSG00000196611	16002434;
D002117	increase	MMP3	ENSG00000149968	16002434;
D002117	increase	MN1	ENSG00000169184	15890672;16002434;
D002117	increase	MPHOSPH6	ENSG00000135698	16002434;
D002117	increase	MRPS6	ENSG00000243927	16002434;
D002117	increase	MSX2	ENSG00000120149	16002434;
D002117	increase	MTSS1	ENSG00000170873	16002434;
D002117	increase	MUT	ENSG00000146085	16002434;
D002117	increase	MXD1	ENSG00000059728	16002434;
D002117	increase	MYCL1	ENSG00000116990	16002434;
D002117	increase	MYO1D	ENSG00000176658	16002434;
D002117	increase	NAV3	ENSG00000067798	16002434;
D002117	increase	NBPF10	ENSG00000203832	12875902;
D002117	increase	NELL2	ENSG00000184613	16002434;
D002117	increase	NET1	ENSG00000173848	16002434;
D002117	increase	NEXN	ENSG00000162614	16002434;
D002117	increase	NGF	ENSG00000134259	16002434;
D002117	increase	NHS	ENSG00000188158	16002434;
D002117	increase	NINJ1	ENSG00000131669	12875902;
D002117	increase	NLRP1	ENSG00000091592	16002434;
D002117	increase	NOD2	ENSG00000167207	16002434;
D002117	increase	NR2F1	ENSG00000175745	16002434;
D002117	increase	NRIP1	ENSG00000180530	16002434;
D002117	increase	NRP2	ENSG00000118257	16002434;
D002117	increase	NSUN6	ENSG00000241058	16002434;
D002117	increase	NUDT11	ENSG00000196368	16002434;
D002117	increase	NUDT7	ENSG00000140876	16002434;
D002117	increase	NUP133	ENSG00000069248	16002434;
D002117	increase	NUP62CL	ENSG00000198088	16002434;

D002117	increase	OMG	ENSG00000126861	12875902;
D002117	increase	ORC4L	ENSG00000115947	16002434;
D002117	increase	OSR2	ENSG00000164920	16002434;
D002117	increase	OVOS2	ENSG00000177359	16002434;
D002117	increase	OVOS	ENSG00000177359	16002434;
D002117	increase	OXTR	ENSG00000180914	16002434;
D002117	increase	P2RY2	ENSG00000175591	12875902;16002434;
D002117	increase	PADI3	ENSG00000142619	16002434;
D002117	increase	PAQR5	ENSG00000137819	16002434;
D002117	increase	PBX1	ENSG00000185630	16002434;
D002117	increase	PDCD6IP	ENSG00000170248	16002434;
D002117	increase	PDE2A	ENSG00000186642	16002434;
D002117	increase	PDE4B	ENSG00000184588	16002434;
D002117	increase	PDPN	ENSG00000162493	16002434;
D002117	increase	PDZD2	ENSG00000133401	16002434;
D002117	increase	PEX3	ENSG00000034693	16002434;
D002117	increase	PFDN5	ENSG00000123349	16847355;
D002117	increase	PHLDB2	ENSG00000144824	16002434;
D002117	increase	PIK3CA	ENSG00000121879	16002434;
D002117	increase	PINK1	ENSG00000158828	16002434;
D002117	increase	PLA2G10	ENSG00000069764	16002434;
D002117	increase	PLCXD2	ENSG00000240891	16002434;
D002117	increase	PLEKHA2	ENSG00000169499	16002434;
D002117	increase	PLEKHA7	ENSG00000166689	16002434;
D002117	increase	PLLP	ENSG00000102934	16002434;
D002117	increase	PLXNA2	ENSG00000076356	16002434;
D002117	increase	PMAIP1	ENSG00000141682	16002434;12875902;
D002117	increase	PODXL	ENSG00000128567	16002434;
D002117	increase	POFUT1	ENSG00000101346	16002434;
D002117	increase	POU3F1	ENSG00000185668	16002434;
D002117	increase	PPARD	ENSG00000112033	15890193;
D002117	increase	PPP1R1C	ENSG00000150722	16002434;
D002117	increase	PPP1R3B	ENSG00000173281	16002434;
D002117	increase	PPP2R3A	ENSG00000073711	16002434;
D002117	increase	PPP2R5A	ENSG00000066027	16002434;
D002117	increase	PPP3CA	ENSG00000138814	16002434;
D002117	increase	PPP3CB	ENSG00000107758	16002434;
D002117	increase	PRMT2	ENSG00000160310	16002434;
D002117	increase	PRNP	ENSG00000171867	16002434;
D002117	increase	PRR5L	ENSG00000135362	16002434;
D002117	increase	PSAP	ENSG00000197746	12875902;
D002117	increase	PTGS1	ENSG00000095303	16129123;
D002117	increase	PTGS2	ENSG00000073756	16002434;
D002117	increase	PTP4A1	ENSG00000112245	16002434;
D002117	increase	PTPN1	ENSG00000196396	16002434;
D002117	increase	PTPRE	ENSG00000132334	16002434;
D002117	increase	PTPRM	ENSG00000173482	16002434;
D002117	increase	RABGAP1L	ENSG00000250898	16002434;
D002117	increase	RAG1	ENSG00000166349	16002434;
D002117	increase	RAGE	ENSG00000080823	16002434;
D002117	increase	RALGPS2	ENSG00000116191	16002434;
D002117	increase	RARRES1	ENSG00000118849	14996990;
D002117	increase	RASSF3	ENSG00000153179	16002434;

D002117	increase	RB1	ENSG00000139687	14729647;12954644;16039115;
D002117	increase	RBL2	ENSG00000103479	14729647;
D002117	increase	RET	ENSG00000165731	16002434;
D002117	increase	RFFL	ENSG00000092871	16002434;
D002117	increase	RGNEF	ENSG00000214944	16002434;
D002117	increase	RHPN2	ENSG00000131941	16002434;
D002117	increase	RIPK2	ENSG00000104312	16002434;
D002117	increase	RNF128	ENSG00000133135	16002434;
D002117	increase	RNF144A	ENSG00000151692	16002434;
D002117	increase	RNF145	ENSG00000145860	16002434;
D002117	increase	RNF149	ENSG00000163162	16002434;
D002117	increase	RNF185	ENSG00000138942	16002434;
D002117	increase	RNF19A	ENSG00000034677	16002434;
D002117	increase	RNF2	ENSG00000121481	16002434;
D002117	increase	RNF39	ENSG00000237733	16002434;
D002117	increase	RNFT1	ENSG00000189050	16002434;
D002117	increase	RP2	ENSG00000102218	16002434;
D002117	increase	RPLP2	ENSG00000177600	16002434;
D002117	increase	RTN4R	ENSG00000040608	16002434;
D002117	increase	S100A3	ENSG00000188015	16002434;
D002117	increase	S100G	ENSG00000169906	16491319;
D002117	increase	S100PBP	ENSG00000116497	16002434;
D002117	increase	SCAMP1	ENSG00000085365	16002434;
D002117	increase	SCOC	ENSG00000153130	16002434;
D002117	increase	SCPEP1	ENSG00000121064	16002434;
D002117	increase	SDCBP	ENSG00000137575	12875902;
D002117	increase	SEC14L1	ENSG00000129657	16002434;
D002117	increase	SEMA3F	ENSG00000001617	16002434;
D002117	increase	SEMA4B	ENSG00000185033	16002434;
D002117	increase	SEMA6D	ENSG00000137872	16002434;
D002117	increase	8-Sep	ENSG00000164402	16002434;
D002117	increase	SERINC2	ENSG00000168528	16002434;
D002117	increase	SERPINB1	ENSG00000021355	16847355;16002434;15951637;
D002117	increase	SERPINB6	ENSG00000124570	16002434;
D002117	increase	SERPINB9	ENSG00000170542	16002434;
D002117	increase	SERPINE2	ENSG00000135919	16002434;
D002117	increase	SFRS12IP1	ENSG00000153006	16002434;
D002117	increase	SFTPB	ENSG00000168878	15951333;
D002117	increase	SFXN3	ENSG00000107819	16002434;
D002117	increase	SH3GL3	ENSG00000140600	16002434;
D002117	increase	SH3KBP1	ENSG00000147010	16002434;
D002117	increase	SH3PXD2A	ENSG00000107957	16002434;
D002117	increase	SH3PXD2B	ENSG00000174705	16002434;
D002117	increase	SH3TC1	ENSG00000125089	16002434;
D002117	increase	SHE	ENSG00000169291	16002434;
D002117	increase	SIRPA	ENSG00000198053	16002434;
D002117	increase	SLC10A2	ENSG00000125255	16002434;
D002117	increase	SLC1A1	ENSG00000106688	16002434;
D002117	increase	SLC1A6	ENSG00000105143	16002434;
D002117	increase	SLC25A27	ENSG00000153291	16002434;
D002117	increase	SLC25A4	ENSG00000151729	16002434;
D002117	increase	SLC30A1	ENSG00000170385	16002434;

D002117	increase	SLC44A5	ENSG00000137968	16002434;
D002117	increase	SLC46A1	ENSG00000076351	16002434;
D002117	increase	SLC4A7	ENSG00000033867	16002434;
D002117	increase	SLC5A3	ENSG00000198743	16002434;
D002117	increase	SLCO1A2	ENSG00000084453	16002434;
D002117	increase	SLCO1B3	ENSG00000111700	16002434;
D002117	increase	SLFN11	ENSG00000172716	16002434;
D002117	increase	SMAD7	ENSG00000101665	16002434;
D002117	increase	SNCAIP	ENSG00000064692	16002434;
D002117	increase	SNRK	ENSG00000163788	16002434;
D002117	increase	SNX18	ENSG00000178996	16002434;
D002117	increase	SNX30	ENSG00000148158	16002434;
D002117	increase	SNX9	ENSG00000130340	16002434;
D002117	increase	SOS2	ENSG00000100485	16002434;
D002117	increase	SOST	ENSG00000167941	15268896;
D002117	increase	SOX4	ENSG00000124766	16002434;
D002117	increase	SP140L	ENSG00000185404	16002434;
D002117	increase	SPAG1	ENSG00000104450	16002434;
D002117	increase	SPANXN3	ENSG00000189252	16002434;
D002117	increase	SPARCL1	ENSG00000152583	16002434;
D002117	increase	SPP1	ENSG00000118785	16267019;
D002117	increase	SPRY2	ENSG00000136158	16002434;
D002117	increase	SPTLC2	ENSG00000100596	16002434;
D002117	increase	SQLE	ENSG00000104549	16002434;
D002117	increase	SRGAP1	ENSG00000196935	16002434;
D002117	increase	SRGAP2	ENSG00000163486	16002434;
D002117	increase	SRI	ENSG00000075142	14996990;
D002117	increase	SSH1	ENSG00000084112	16002434;
D002117	increase	ST3GAL6	ENSG00000064225	16002434;
D002117	increase	ST8SIA1	ENSG00000111728	16002434;
D002117	increase	STAM2	ENSG00000115145	16002434;
D002117	increase	STAMBPL1	ENSG00000138134	16002434;
D002117	increase	STK39	ENSG00000198648	16002434;
D002117	increase	SVEP1	ENSG00000165124	16002434;
D002117	increase	SYDE1	ENSG00000105137	16002434;
D002117	increase	SYNJ2	ENSG00000078269	16002434;
D002117	increase	SYT12	ENSG00000173227	16002434;
D002117	increase	SYT1	ENSG00000067715	16002434;
D002117	increase	TACSTD2	ENSG00000184292	16002434;
D002117	increase	TAOK3	ENSG00000135090	16002434;
D002117	increase	TCEA2	ENSG00000171703	16002434;
D002117	increase	TCEAL1	ENSG00000172465	16002434;
D002117	increase	TDRKH	ENSG00000182134	16002434;
D002117	increase	TGFA	ENSG00000163235	16002434;
D002117	increase	TGFB2	ENSG00000092969	16002434;
D002117	increase	THRB	ENSG00000151090	16002434;
D002117	increase	TIMP2	ENSG00000035862	16002434;
D002117	increase	TINAG	ENSG00000137251	16002434;
D002117	increase	TINAGL1	ENSG00000142910	16002434;
D002117	increase	TMC6	ENSG00000141524	16002434;
D002117	increase	TMEM117	ENSG00000139173	16002434;
D002117	increase	TMEM156	ENSG00000121895	16002434;
D002117	increase	TMEM158	ENSG00000249992	16002434;
D002117	increase	TMEM27	ENSG00000147003	16002434;

D002117	increase	TMEM40	ENSG00000088726	16002434;
D002117	increase	TMEM57	ENSG00000204178	16002434;
D002117	increase	TMEM79	ENSG00000163472	16002434;
D002117	increase	TMOD1	ENSG00000136842	16002434;
D002117	increase	TNFAIP2	ENSG00000185215	16129123;
D002117	increase	TNFAIP8	ENSG00000145779	16002434;
D002117	increase	TNFAIP8L3	ENSG00000183578	16002434;
D002117	increase	TNFRSF19	ENSG00000127863	16002434;
D002117	increase	TNFRSF21	ENSG00000146072	16002434;
D002117	increase	TNFSF11	ENSG00000120659	17293108;
D002117	increase	TP73	ENSG00000078900	17716971;
D002117	increase	TPBG	ENSG00000146242	16002434;
D002117	increase	TPST1	ENSG00000169902	16002434;
D002117	increase	TRAF4	ENSG00000076604	16002434;
D002117	increase	TRIB1	ENSG00000173334	16002434;
D002117	increase	TRIM24	ENSG00000122779	16002434;
D002117	increase	TRIM2	ENSG00000109654	16002434;
D002117	increase	TRIM38	ENSG00000112343	16002434;
D002117	increase	TRIM56	ENSG00000169871	16002434;
D002117	increase	TRIM6	ENSG00000121236	16002434;
D002117	increase	TRPV6	ENSG00000165125	17293108;16491319;
D002117	increase	TSLP	ENSG00000251640	16002434;
D002117	increase	TUBA1A	ENSG00000167552	16002434;
D002117	increase	TXNDC11	ENSG00000153066	16002434;
D002117	increase	TYROBP	ENSG00000011600	12875902;16322758;
D002117	increase	UBIAD1	ENSG00000120942	16002434;
D002117	increase	UHRF1BP1	ENSG00000065060	16002434;
D002117	increase	ULK1	ENSG00000177169	16002434;
D002117	increase	UPRT	ENSG00000094841	16002434;
D002117	increase	USP40	ENSG00000085982	16002434;
D002117	increase	USP6NL	ENSG00000148429	16002434;
D002117	increase	UST	ENSG00000111962	16002434;
D002117	increase	VDR	ENSG00000111424	10875257;15824177;15615532;15572423;16076355;15890193;16061850;16051482;17293108;15985530;15919092;16002434;15992766;15922086;16267019;15955619;15664452;15891005;15876428;16204233;15861036;[illegible]
D002117	increase	VEGFA	ENSG00000112715	16002434;
D002117	increase	VGLL4	ENSG00000144560	16002434;
D002117	increase	WDR36	ENSG00000134987	16002434;
D002117	increase	WDR72	ENSG00000166415	16002434;
D002117	increase	WDSUB1	ENSG00000196151	16002434;
D002117	increase	WIPI1	ENSG00000070540	16002434;
D002117	increase	WNK2	ENSG00000165238	16002434;
D002117	increase	WNT5A	ENSG00000114251	16002434;
D002117	increase	WSB1	ENSG00000109046	16002434;
D002117	increase	XCL1	ENSG00000143184	16002434;
D002117	increase	XCL2	ENSG00000143185	16002434;
D002117	increase	ZBTB38	ENSG00000177311	16002434;
D002117	increase	ZC3H12C	ENSG00000149289	16002434;
D002117	increase	ZFP36	ENSG00000128016	16002434;

D002117	increase	ZNF114	ENSG00000178150	16002434;
D002117	increase	ZNF167	ENSG00000196345	16002434;
D002117	increase	ZNF185	ENSG00000147394	16002434;
D002117	increase	ZNF462	ENSG00000148143	16002434;
D002117	increase	ZNF641	ENSG00000167528	16002434;
D002117	increase	ZSWIM6	ENSG00000130449	16002434;
D002118	affect	FGF23	ENSG00000118972	16381997;15086938;
D002118	affect	MMP1	ENSG00000196611	17508023;
D002118	affect	PTGS2	ENSG00000073756	12493747;
D002118	decrease	TP53	ENSG00000141510	16043423;
D002118	increase	IL6	ENSG00000136244	11238657;
D002118	increase	IL8	ENSG00000169429	17000667;14505796;15748943;10984371;11298490;16212921;
D002118	increase	IVL	ENSG00000163207	10469331;
D002118	increase	TGM1	ENSG00000092295	10469331;
D002122	increase	PPID	ENSG00000171497	18614807;
D002122	increase	S100A8	ENSG00000143546	17214633;
D002122	increase	S100A9	ENSG00000163220	17214633;
D002166	decrease	ABCB1	ENSG00000085563	17284363;10692111;
D002166	decrease	BCL2	ENSG00000171791	19481069;10692111;
D002166	decrease	BID	ENSG00000015475	19481069;
D002166	decrease	BIRC5	ENSG00000089685	16061681;
D002166	decrease	BRCA1	ENSG00000012048	10344722;
D002166	decrease	BRCA2	ENSG00000139618	10344722;
D002166	decrease	CAB39	ENSG00000135932	17374387;
D002166	decrease	CCNB1	ENSG00000134057	16061681;
D002166	decrease	CEBPZ	ENSG00000115816	17374387;
D002166	decrease	EP300	ENSG00000100393	10344722;
D002166	decrease	LGALS1	ENSG00000100097	19483324;
D002166	decrease	MAP3K5	ENSG00000197442	17374387;
D002166	decrease	MAPT	ENSG00000186868	16930453;
D002166	decrease	NCK2	ENSG00000071051	17374387;
D002166	decrease	PRC1	ENSG00000198901	17374387;
D002166	decrease	RAD51	ENSG00000051180	10344722;
D002166	decrease	RBL1	ENSG00000080839	15585644;
D002166	decrease	THBS1	ENSG00000137801	16962673;
D002166	decrease	XIAP	ENSG00000101966	16211302;16061681;
D002166	increase	AGRN	ENSG00000188157	17374387;
D002166	increase	ANXA4	ENSG00000196975	17374387;
D002166	increase	BAX	ENSG00000087088	19481069;15665116;17555331;
D002166	increase	CASP2	ENSG00000106144	14757846;
D002166	increase	CDKN1A	ENSG00000124762	11741290;9673414;175553
D002166	increase	CEACAM1	ENSG00000079385	17374387;
D002166	increase	DPYD	ENSG00000188641	16568373;
D002166	increase	EI24	ENSG00000149547	17374387;
D002166	increase	EPM2AIP1	ENSG00000178567	17374387;
D002166	increase	FDXR	ENSG00000161513	17374387;
D002166	increase	GSTP1	ENSG00000084207	15500952;14732228;
D002166	increase	JUN	ENSG00000177606	15585644;
D002166	increase	MAP2K3	ENSG00000034152	17374387;
D002166	increase	MDM2	ENSG00000135679	17555331;
D002166	increase	PLK3	ENSG00000173846	17374387;

D002166	increase	SDC1	ENSG00000115884	17374387;
D002166	increase	TAP1	ENSG00000230705	17374387;
D002166	increase	TAX1BP3	ENSG00000213977	17374387;
D002166	increase	TNFSF9	ENSG00000125657	17374387;
D002166	increase	TP53	ENSG00000141510	19481069;17555331;12082016;9673414;11741290;106
D002166	increase	TP53I3	ENSG00000115129	17374387;
D002166	increase	TYMS	ENSG00000176890	16568373;
D002193	decrease	BCL2	ENSG00000171791	16117893;
D002193	decrease	BIRC5	ENSG00000089685	16117893;
D002193	increase	AHR	ENSG00000106546	14644660;
D002193	increase	BAX	ENSG00000087088	16117893;
D002193	increase	CYP1A1	ENSG00000140465	14644660;
D002211	decrease	ACSL3	ENSG00000123983	18533110;
D002211	decrease	APOC1	ENSG00000130208	18533110;
D002211	decrease	BCL2	ENSG00000171791	18991268;17505005;18405923;
D002211	decrease	BCL2L1	ENSG00000171552	17505005;
D002211	decrease	BIRC5	ENSG00000089685	17505005;
D002211	decrease	CCND1	ENSG00000110092	17505005;
D002211	decrease	FABP4	ENSG00000170323	18533110;
D002211	decrease	FABP5	ENSG00000164687	18533110;
D002211	decrease	FOXA1	ENSG00000129514	18533110;
D002211	decrease	IL12B	ENSG00000113302	18533110;
D002211	decrease	PCTP	ENSG00000141179	18533110;
D002211	decrease	PPARA	ENSG00000186951	18769453;
D002211	decrease	PPARD	ENSG00000112033	18769453;
D002211	decrease	PPARG	ENSG00000132170	18769453;
D002211	decrease	RXRA	ENSG00000186350	18769453;
D002211	decrease	RXRB	ENSG00000235712	18769453;
D002211	decrease	SCP2	ENSG00000251612	18533110;
D002211	decrease	TGFB1	ENSG00000105329	18533110;
D002211	decrease	TM7SF2	ENSG00000149809	18533110;
D002211	decrease	VEGFA	ENSG00000112715	17505005;
D002211	increase	ABCB1	ENSG00000085563	16674925;
D002211	increase	ACOX1	ENSG00000161533	18533110;
D002211	increase	ALB	ENSG00000163631	18991268;
D002211	increase	AR	ENSG00000169083	19059400;
D002211	increase	ATF4	ENSG00000128272	18533110;
D002211	increase	BAX	ENSG00000087088	17292493;
D002211	increase	CALCA	ENSG00000110680	18584893;16996168;
D002211	increase	CALCB	ENSG00000175868	18584893;
D002211	increase	CASP3	ENSG00000164305	17442041;17505005;18405923;18991268;
D002211	increase	CDKN2A	ENSG00000147889	18533110;
D002211	increase	COX5A	ENSG00000178741	18991268;
D002211	increase	DDIT3	ENSG00000175197	18533110;
D002211	increase	GAPDH	ENSG00000111640	18991268;
D002211	increase	GFAP	ENSG00000131095	18405923;
D002211	increase	GLUD1	ENSG00000148672	18991268;
D002211	increase	GSTP1	ENSG00000084207	15313406;
D002211	increase	HSP90B1	ENSG00000166598	18991268;
D002211	increase	IL6	ENSG00000136244	17505005;17306835;
D002211	increase	LGALS1	ENSG00000100097	18991268;

D002211	increase	MAP2	ENSG00000078018	18405923;
D002211	increase	MMP1	ENSG00000196611	17508023;
D002211	increase	NPC1	ENSG00000141458	18533110;
D002211	increase	PRDX6	ENSG00000117592	18991268;
D002211	increase	RPS6KA3	ENSG00000177189	18991268;
D002211	increase	SC4MOL	ENSG00000052802	18533110;
D002211	increase	SUMO4	ENSG00000177688	18991268;
D002211	increase	TNF	ENSG00000223952	15313406;
D002211	increase	TP53	ENSG00000141510	17292493;18991268;
D002211	increase	TRPV1	ENSG00000196689	15615864;16597917;18769453;17018028;18230619;17442041;18804451;16793902;17913835;17392452;17110039;19056576;17306835;
D002211	increase	TRPV6	ENSG00000165125	17292493;
D002217	increase	CD40	ENSG00000101017	16879495;
D002219	increase	IFNG	ENSG00000111537	19111005;
D002219	increase	IL10	ENSG00000136634	19376255;
D002219	increase	IL12A	ENSG00000168811	19376255;19111005;
D002219	increase	IL12B	ENSG00000113302	19376255;19111005;
D002219	increase	IL1B	ENSG00000125538	19376255;19111005;
D002219	increase	IL6	ENSG00000136244	19376255;19111005;
D002219	increase	IL8	ENSG00000169429	19376255;19111005;
D002219	increase	TNF	ENSG00000223952	19376255;19111005;
D002220	affect	IL6	ENSG00000136244	10700573;
D002220	affect	TNF	ENSG00000223952	10700573;
D002220	increase	CYP2B6	ENSG00000197408	14977870;
D002220	increase	CYP3A4	ENSG00000160868	15915352;15286053;12065438;9512926;12673034;12505310;17954527;16837568;10219967;
D002220	increase	CYP3A5	ENSG00000106258	12673034;
D002220	increase	NR3C1	ENSG00000113580	12673034;10219967;
D002246	increase	LPA	ENSG00000198670	12712689;
D002251	decrease	C6ORF108	ENSG00000112667	11566570;
D002251	decrease	CRYBB3	ENSG00000100053	11566570;
D002251	decrease	MAPK3	ENSG00000102882	11566570;
D002251	decrease	MUTYH	ENSG00000132781	11566570;
D002251	decrease	PFDN5	ENSG00000123349	11566570;
D002251	decrease	PMS2	ENSG00000122512	11566570;
D002251	decrease	POU2F1	ENSG00000143190	11566570;
D002251	decrease	RRAD	ENSG00000166592	11566570;
D002251	increase	ABCB1	ENSG00000085563	15120965;
D002251	increase	ACTA2	ENSG00000107796	18207697;
D002251	increase	APOA2	ENSG00000158874	11566570;
D002251	increase	COL1A1	ENSG00000108821	18207697;17030193;
D002251	increase	CRYAB	ENSG00000109846	16815347;18207697;
D002251	increase	HTR1D	ENSG00000179546	11566570;
D002251	increase	IGF2	ENSG00000167244	11566570;
D002251	increase	IL6	ENSG00000136244	11566570;
D002251	increase	MAPK12	ENSG00000188130	11566570;
D002251	increase	NR1H4	ENSG00000012504	11566570;
D002251	increase	SERPINH1	ENSG00000149257	16815347;18207697;
D002251	increase	TF	ENSG00000091513	11566570;
D002258	decrease	CIRBP	ENSG00000099622	15075239;

D002258	decrease	RBM3	ENSG00000102317	15075239;
D002259	decrease	BAX	ENSG00000087088	16039398;
D002259	decrease	BCL2L1	ENSG00000171552	16039398;
D002259	decrease	CASP8	ENSG00000064012	16039398;
D002259	decrease	CAT	ENSG00000121691	16039398;
D002259	decrease	CCNC	ENSG00000112237	16039398;
D002259	decrease	CCND1	ENSG00000110092	16039398;
D002259	decrease	CCNG1	ENSG00000113328	16039398;
D002259	decrease	DDB1	ENSG00000167986	16039398;
D002259	decrease	DNAJA1	ENSG00000086061	16039398;
D002259	decrease	EGR1	ENSG00000120738	16039398;
D002259	decrease	EPHX2	ENSG00000120915	16039398;
D002259	decrease	GADD45A	ENSG00000116717	16039398;
D002259	decrease	GSR	ENSG00000104687	16039398;
D002259	decrease	GSTM3	ENSG00000134202	16039398;
D002259	decrease	HMOX2	ENSG00000103415	16039398;
D002259	decrease	HSF1	ENSG00000185122	16039398;
D002259	decrease	HSPA1A	ENSG00000232804	16039398;
D002259	decrease	HSPA1B	ENSG00000232804	16039398;
D002259	decrease	HSPA1L	ENSG00000204390	16039398;
D002259	decrease	HSPA2	ENSG00000126803	16039398;
D002259	decrease	HSPA4	ENSG00000170606	16039398;
D002259	decrease	HSPA8	ENSG00000109971	16039398;
D002259	decrease	IL18	ENSG00000150782	16039398;
D002259	decrease	IL1A	ENSG00000115008	16039398;
D002259	decrease	MDM2	ENSG00000135679	16039398;
D002259	decrease	MT1A	ENSG00000205362	16039398;
D002259	decrease	MT1H	ENSG00000205358	16039398;
D002259	decrease	MT2A	ENSG00000125148	16039398;
D002259	decrease	NFKBIA	ENSG00000100906	16039398;
D002259	decrease	RAD23A	ENSG00000179262	16039398;
D002259	decrease	RPL13A	ENSG00000142541	16039398;
D002259	decrease	SERPINE1	ENSG00000106366	16039398;
D002259	decrease	XRCC5	ENSG00000079246	16039398;
D002259	increase	CYP1A1	ENSG00000140465	16039398;
D002259	increase	GDF15	ENSG00000130513	16039398;
D002259	increase	HMOX1	ENSG00000100292	16039398;
D002259	increase	HSPE1	ENSG00000115541	16039398;
D002259	increase	PTGS2	ENSG00000073756	16039398;
D002259	increase	SOD2	ENSG00000112096	16039398;
D002330	decrease	ACTC1	ENSG00000159251	15980968;
D002330	decrease	ACTG2	ENSG00000163017	15980968;
D002330	decrease	ACTN1	ENSG00000072110	15980968;
D002330	decrease	ADAM15	ENSG00000143537	15980968;
D002330	decrease	ADAMTS4	ENSG00000158859	15980968;
D002330	decrease	ADCYAP1	ENSG00000141433	15980968;
D002330	decrease	ADRA1B	ENSG00000170214	15980968;
D002330	decrease	AKT2	ENSG00000105221	15980968;
D002330	decrease	ALAD	ENSG00000148218	15980968;
D002330	decrease	ALOX15B	ENSG00000179593	15980968;
D002330	decrease	AMH	ENSG00000104899	15980968;
D002330	decrease	APC2	ENSG00000115266	15980968;
D002330	decrease	AQP8	ENSG00000103375	15980968;
D002330	decrease	ARPC2	ENSG00000163466	15980968;

D002330	decrease	ARTN	ENSG00000251559	15980968;
D002330	decrease	ASMTL	ENSG00000169093	15980968;
D002330	decrease	ATP1A3	ENSG00000105409	15980968;
D002330	decrease	ATP4B	ENSG00000186009	15980968;
D002330	decrease	ATP5D	ENSG00000099624	15980968;
D002330	decrease	ATP6V0C	ENSG00000185883	15980968;
D002330	decrease	ATXN2L	ENSG00000168488	15980968;
D002330	decrease	B4GALT5	ENSG00000158470	15980968;
D002330	decrease	BAIAP2	ENSG00000175866	15980968;
D002330	decrease	BAIAP3	ENSG00000007516	15980968;
D002330	decrease	BCL2	ENSG00000171791	16187019;
D002330	decrease	BCL2L1	ENSG00000171552	16187019;
D002330	decrease	BECN1	ENSG00000126581	15980968;
D002330	decrease	BICD1	ENSG00000151746	15980968;
D002330	decrease	BLK	ENSG00000136573	15980968;
D002330	decrease	BMP7	ENSG00000101144	15980968;
D002330	decrease	BRF1	ENSG00000185024	15980968;
D002330	decrease	CA6	ENSG00000131686	15980968;
D002330	decrease	CALB2	ENSG00000172137	15980968;
D002330	decrease	CD151	ENSG00000177697	15980968;
D002330	decrease	CD68	ENSG00000129226	15980968;
D002330	decrease	CD79A	ENSG00000105369	15980968;
D002330	decrease	CDK8	ENSG00000132964	15980968;
D002330	decrease	CDX4	ENSG00000131264	15980968;
D002330	decrease	CEACAM3	ENSG00000170956	15980968;
D002330	decrease	CEBPB	ENSG00000172216	15980968;
D002330	decrease	CHRM4	ENSG00000180720	15980968;
D002330	decrease	CHRNB3	ENSG00000147432	15980968;
D002330	decrease	CHRNG	ENSG00000196811	15980968;
D002330	decrease	CKM	ENSG00000104879	15980968;
D002330	decrease	COL9A1	ENSG00000112280	15980968;
D002330	decrease	COX6A2	ENSG00000156885	15980968;
D002330	decrease	CRABP1	ENSG00000166426	15980968;
D002330	decrease	CRAT	ENSG00000095321	15980968;
D002330	decrease	CRYBB1	ENSG00000100122	15980968;
D002330	decrease	CST5	ENSG00000170367	15980968;
D002330	decrease	CTF1	ENSG00000150281	15980968;
D002330	decrease	CYP19A1	ENSG00000137869	15980968;
D002330	decrease	DHX16	ENSG00000231377	15980968;
D002330	decrease	DNASE1L2	ENSG00000167968	15980968;
D002330	decrease	DVL1	ENSG00000107404	15980968;
D002330	decrease	EIF6	ENSG00000242372	15980968;
D002330	decrease	EPHX1	ENSG00000143819	15980968;
D002330	decrease	EZH2	ENSG00000106462	15980968;
D002330	decrease	FBN1	ENSG00000166147	15980968;
D002330	decrease	FKBP1A	ENSG00000088832	15980968;
D002330	decrease	FOXJ1	ENSG00000129654	15980968;
D002330	decrease	FTH1	ENSG00000167996	15980968;
D002330	decrease	FTL	ENSG00000087086	15980968;
D002330	decrease	FYN	ENSG00000010810	15980968;
D002330	decrease	GABRA2	ENSG00000151834	15980968;
D002330	decrease	GABRA6	ENSG00000145863	15980968;
D002330	decrease	GAMT	ENSG00000130005	15980968;
D002330	decrease	GAST	ENSG00000184502	15980968;

D002330	decrease	GFAP	ENSG00000131095	15980968;
D002330	decrease	GHSR	ENSG00000121853	15980968;
D002330	decrease	GOLGA2	ENSG00000167110	15980968;
D002330	decrease	GP1BB	ENSG00000203618	15980968;
D002330	decrease	GPR25	ENSG00000170128	15980968;
D002330	decrease	GPR32	ENSG00000142511	15980968;
D002330	decrease	GPR37	ENSG00000170775	15980968;
D002330	decrease	GRB7	ENSG00000141738	15980968;
D002330	decrease	GRN	ENSG00000030582	15980968;
D002330	decrease	GTF2F1	ENSG00000125651	15980968;
D002330	decrease	H2AFX	ENSG00000188486	15980968;
D002330	decrease	HADH	ENSG00000138796	15980968;
D002330	decrease	HAGH	ENSG00000063854	15980968;
D002330	decrease	HCFC1	ENSG00000172534	15980968;
D002330	decrease	HMGN2	ENSG00000198830	15980968;
D002330	decrease	HNF1A	ENSG00000135100	15980968;
D002330	decrease	HOXA4	ENSG00000197576	15980968;
D002330	decrease	ICAM5	ENSG00000105376	15980968;
D002330	decrease	IDS	ENSG00000010404	15980968;
D002330	decrease	IER3	ENSG00000137331	15980968;
D002330	decrease	IKBKG	ENSG00000073009	15980968;
D002330	decrease	INPPL1	ENSG00000165458	15980968;
D002330	decrease	INSIG1	ENSG00000186480	15980968;
D002330	decrease	INSL3	ENSG00000248099	15980968;
D002330	decrease	IRS1	ENSG00000169047	15980968;
D002330	decrease	ISLR	ENSG00000129009	15980968;
D002330	decrease	ITGA3	ENSG00000005884	15980968;
D002330	decrease	ITIH1	ENSG00000055957	15980968;
D002330	decrease	KCNH2	ENSG00000055118	15980968;
D002330	decrease	KIF5A	ENSG00000155980	15980968;
D002330	decrease	KRT17	ENSG00000128422	15980968;
D002330	decrease	KRT31	ENSG00000094796	15980968;
D002330	decrease	LAD1	ENSG00000159166	15980968;
D002330	decrease	LAMB3	ENSG00000196878	15980968;
D002330	decrease	LMX1B	ENSG00000136944	15980968;
D002330	decrease	LOXL1	ENSG00000129038	15980968;
D002330	decrease	LRCH4	ENSG00000077454	15980968;
D002330	decrease	LRMP	ENSG00000118308	15980968;
D002330	decrease	MAD1L1	ENSG00000002822	15980968;
D002330	decrease	MAP4K2	ENSG00000168067	15980968;
D002330	decrease	MAPK10	ENSG00000109339	15980968;
D002330	decrease	MAT1A	ENSG00000151224	15980968;
D002330	decrease	MDK	ENSG00000110492	15980968;
D002330	decrease	MED1	ENSG00000125686	15980968;
D002330	decrease	MGAT3	ENSG00000128268	15980968;
D002330	decrease	MSR1	ENSG00000038945	15980968;
D002330	decrease	MUC2	ENSG00000198788	15980968;
D002330	decrease	MVD	ENSG00000167508	15980968;
D002330	decrease	MYBPH	ENSG00000133055	15980968;
D002330	decrease	MYD88	ENSG00000172936	15980968;
D002330	decrease	MYOD1	ENSG00000129152	15980968;
D002330	decrease	MYOG	ENSG00000122180	15980968;
D002330	decrease	NDUFC1	ENSG00000109390	15980968;
D002330	decrease	NKX2-2	ENSG00000125820	15980968;

D002330	decrease	NPAS1	ENSG00000130751	15980968;
D002330	decrease	NPBWR2	ENSG00000125522	15980968;
D002330	decrease	NRTN	ENSG00000171119	15980968;
D002330	decrease	PAEP	ENSG00000122133	15980968;
D002330	decrease	PAX8	ENSG00000125618	15980968;
D002330	decrease	PBX2	ENSG00000236353	15980968;
D002330	decrease	PCBD1	ENSG00000166228	15980968;
D002330	decrease	PCDHGC3	ENSG00000240184	15980968;
D002330	decrease	PCOLCE	ENSG00000106333	15980968;
D002330	decrease	PCYT1A	ENSG00000161217	15980968;
D002330	decrease	PFKFB2	ENSG00000123836	15980968;
D002330	decrease	PFKM	ENSG00000152556	15980968;
D002330	decrease	PFN1	ENSG00000108518	15980968;
D002330	decrease	POLR2E	ENSG00000099817	15980968;
D002330	decrease	PPM1G	ENSG00000115241	15980968;
D002330	decrease	PRC1	ENSG00000198901	17374387;
D002330	decrease	PRL	ENSG00000172179	15980968;
D002330	decrease	PRM2	ENSG00000122304	15980968;
D002330	decrease	PTPN3	ENSG00000070159	15980968;
D002330	decrease	PZP	ENSG00000126838	15980968;
D002330	decrease	RAD23A	ENSG00000179262	15980968;
D002330	decrease	RAD51	ENSG00000051180	15980968;
D002330	decrease	RCN1	ENSG00000049449	15980968;
D002330	decrease	RGR	ENSG00000248619	15980968;
D002330	decrease	RIT2	ENSG00000152214	15980968;
D002330	decrease	5-Sep	ENSG00000184702	15980968;
D002330	decrease	SFPQ	ENSG00000116560	15980968;
D002330	decrease	SGCA	ENSG00000108823	15980968;
D002330	decrease	SHMT2	ENSG00000182199	15980968;
D002330	decrease	SHOX	ENSG00000185960	15980968;
D002330	decrease	SLC30A3	ENSG00000115194	15980968;
D002330	decrease	SLCO1A2	ENSG00000084453	15980968;
D002330	decrease	SOCS1	ENSG00000185338	17374387;
D002330	decrease	SORBS2	ENSG00000154556	15980968;
D002330	decrease	SPEG	ENSG00000072195	15980968;
D002330	decrease	SPRR2B	ENSG00000196805	15980968;
D002330	decrease	SRP14	ENSG00000140319	15980968;
D002330	decrease	SRY	ENSG00000184895	15980968;
D002330	decrease	STAM	ENSG00000136738	15980968;
D002330	decrease	STX1B	ENSG00000099365	15980968;
D002330	decrease	STXBP2	ENSG00000076944	15980968;
D002330	decrease	TAC1	ENSG00000006128	15980968;
D002330	decrease	TCF21	ENSG00000118526	15980968;
D002330	decrease	TLE3	ENSG00000140332	15980968;
D002330	decrease	TNFRSF11B	ENSG00000164761	15980968;
D002330	decrease	TRAF1	ENSG00000056558	15980968;
D002330	decrease	TSPAN4	ENSG00000214063	15980968;
D002330	decrease	TUBG1	ENSG00000131462	15980968;
D002330	decrease	UPF1	ENSG00000005007	15980968;
D002330	decrease	VGF	ENSG00000128564	15980968;
D002330	decrease	WIPF1	ENSG00000115935	15980968;
D002330	decrease	YWHAH	ENSG00000128245	15980968;
D002330	decrease	ZBTB17	ENSG00000116809	15980968;
D002330	decrease	ZYX	ENSG00000159840	15980968;

D002330	increase	ABCB1	ENSG00000085563	15239124;
D002330	increase	AVPR1A	ENSG00000166148	15980968;
D002330	increase	BCL10	ENSG00000142867	15980968;
D002330	increase	CCL2	ENSG00000108691	15980968;
D002330	increase	CD80	ENSG00000121594	15980968;
D002330	increase	CDKN1A	ENSG00000124762	15735757;
D002330	increase	CLTCL1	ENSG00000249108	15980968;
D002330	increase	CNTNAP1	ENSG00000108797	15980968;
D002330	increase	CTNNAL1	ENSG00000119326	15980968;
D002330	increase	CYTH1	ENSG00000108669	15980968;
D002330	increase	ECM2	ENSG00000106823	15980968;
D002330	increase	EEF1G	ENSG00000149016	15980968;
D002330	increase	EIF2B1	ENSG00000111361	15980968;
D002330	increase	ESRRA	ENSG00000173153	15980968;
D002330	increase	FNTA	ENSG00000168522	15980968;
D002330	increase	GCM1	ENSG00000137270	15980968;
D002330	increase	GDI1	ENSG00000203879	15980968;
D002330	increase	GDI2	ENSG00000057608	15980968;
D002330	increase	GNRHR	ENSG00000109163	15980968;
D002330	increase	GOLGA4	ENSG00000144674	15980968;
D002330	increase	GPR65	ENSG00000140030	15980968;
D002330	increase	LAIR1	ENSG00000167613	15980968;
D002330	increase	PIK3R3	ENSG00000117461	15980968;
D002330	increase	RNASE4	ENSG00000181784	15980968;
D002330	increase	SLC10A1	ENSG00000100652	15980968;
D002330	increase	SLC16A2	ENSG00000147100	15980968;
D002330	increase	SLC6A2	ENSG00000103546	15980968;
D002330	increase	SMARCD1	ENSG00000066117	15980968;
D002330	increase	SMPD2	ENSG00000135587	15980968;
D002330	increase	SNAPC1	ENSG00000023608	15980968;
D002330	increase	SNAPC3	ENSG00000164975	15980968;
D002330	increase	SNCG	ENSG00000173267	15980968;
D002330	increase	SNRPB2	ENSG00000125870	15980968;
D002330	increase	ST8SIA1	ENSG00000111728	15980968;
D002330	increase	STS	ENSG00000101846	15980968;
D002330	increase	TP53	ENSG00000141510	15735757;
D002330	increase	TUBB	ENSG00000229684	15980968;
D002330	increase	UGT8	ENSG00000174607	15980968;
D002331	increase	CPT1A	ENSG00000110090	16283381;
D002331	increase	SLC22A5	ENSG00000197375	16928358;19539806;15486076;16283381;
D002392	decrease	ABCG2	ENSG00000118777	17077187;
D002392	decrease	ARHGAP4	ENSG00000089820	15465739;
D002392	decrease	BCL2	ENSG00000171791	19557821;
D002392	decrease	BRCC3	ENSG00000185515	15465739;
D002392	decrease	CCND1	ENSG00000110092	16413414;
D002392	decrease	DEK	ENSG00000124795	15465739;
D002392	decrease	FLT3LG	ENSG00000090554	15465739;
D002392	decrease	HSPD1	ENSG00000144381	15465739;
D002392	decrease	IL2RA	ENSG00000134460	15465739;
D002392	decrease	IL32	ENSG00000008517	15465739;
D002392	decrease	IRF4	ENSG00000137265	15465739;
D002392	decrease	LYN	ENSG00000249529	15465739;
D002392	decrease	MIF	ENSG00000240972	15465739;

D002392	decrease	NFKBIA	ENSG00000100906	19557821;
D002392	decrease	NPM1	ENSG00000181163	15465739;
D002392	decrease	RAC1	ENSG00000136238	15465739;
D002392	decrease	SERPINE1	ENSG00000106366	17379280;
D002392	decrease	SF1	ENSG00000250652	15465739;
D002392	decrease	STAT1	ENSG00000115415	15465739;
D002392	decrease	TK1	ENSG00000167900	15465739;
D002392	decrease	TNFRSF8	ENSG00000120949	15465739;
D002392	decrease	TOP1	ENSG00000198900	15465739;
D002392	decrease	TOP2A	ENSG00000131747	15465739;
D002392	decrease	XCR1	ENSG00000173578	15465739;
D002392	increase	ANXA2	ENSG00000182718	15465739;
D002392	increase	ATF4	ENSG00000128272	15465739;
D002392	increase	BAT2	ENSG00000204469	15465739;
D002392	increase	BTG1	ENSG00000133639	15465739;
D002392	increase	CCL2	ENSG00000108691	17103373;
D002392	increase	CD19	ENSG00000177455	15465739;
D002392	increase	CD81	ENSG00000110651	15465739;
D002392	increase	CD9	ENSG00000010278	15465739;
D002392	increase	CEBPG	ENSG00000153879	15465739;
D002392	increase	CXCL10	ENSG00000169245	17103373;
D002392	increase	EWSR1	ENSG00000182944	15465739;
D002392	increase	FTH1	ENSG00000167996	15465739;
D002392	increase	GRN	ENSG00000030582	15465739;
D002392	increase	HCFC1	ENSG00000172534	15465739;
D002392	increase	HMOX1	ENSG00000100292	16799064;
D002392	increase	HOXD3	ENSG00000128652	15465739;
D002392	increase	ICAM1	ENSG00000090339	15465739;
D002392	increase	IL10	ENSG00000136634	15465739;
D002392	increase	IL1B	ENSG00000125538	19557821;17103373;
D002392	increase	ITGAL	ENSG00000005844	15465739;
D002392	increase	ITGB2	ENSG00000160255	17103373;
D002392	increase	MAP2K1	ENSG00000169032	15465739;
D002392	increase	NCF1	ENSG00000158517	17103373;19450372;
D002392	increase	NFE2L2	ENSG00000116044	16799064;
D002392	increase	NOTCH1	ENSG00000148400	15465739;
D002392	increase	PLAT	ENSG00000104368	11236827;
D002392	increase	PLAU	ENSG00000122861	11236827;
D002392	increase	PTGS2	ENSG00000073756	19557821;17103373;10920275;
D002392	increase	RPL6	ENSG00000089009	15465739;
D002392	increase	S100B	ENSG00000160307	17103373;
D002392	increase	SLC20A1	ENSG00000144136	15465739;
D002392	increase	STAT5B	ENSG00000173757	15465739;
D002392	increase	STAT6	ENSG00000166888	15465739;
D002392	increase	TCF7	ENSG00000081059	15465739;
D002392	increase	TNF	ENSG00000223952	19557821;17103373;
D002392	increase	ZNF593	ENSG00000142684	15465739;
D002569	decrease	HNF4A	ENSG00000101076	17992261;
D002569	decrease	SHBG	ENSG00000129214	17992261;
D002635	decrease	CORO1A	ENSG00000102879	16040207;
D002635	decrease	PON1	ENSG00000005421	16269825;
D002635	decrease	SREBF1	ENSG00000072310	16269825;

D002635	increase	ABCB11	ENSG00000073734	16423920;12525500;14684751;12519787;
D002635	increase	ABCB4	ENSG00000005471	14527955;14623915;15588777;
D002635	increase	FGF19	ENSG00000162344	16269825;
D002635	increase	NR0B2	ENSG00000131910	16037943;16269825;
D002635	increase	NR1H4	ENSG00000012504	15307955;12519787;12525500;15576845;14527955;14684751;17567710;15145977;16037943;16423920;
D002635	increase	OSTALPHA	ENSG00000163959	16423920;
D002635	increase	OSTBETA	ENSG00000186198	16423920;
D002635	increase	VIPR1	ENSG00000114812	16037943;
D002699	affect	BIRC5	ENSG00000089685	17476690;
D002699	affect	TNFRSF10A	ENSG00000104689	14614459;
D002699	decrease	BCL2	ENSG00000171791	11699414;
D002699	decrease	HIST1H4C	ENSG00000182217	16861886;15556009;
D002699	increase	ABCB1	ENSG00000085563	15239124;
D002699	increase	BAX	ENSG00000087088	11699414;
D002699	increase	BBC3	ENSG00000105327	17476690;
D002699	increase	CDKN1B	ENSG00000111276	16039525;
D002699	increase	FAS	ENSG00000026103	17476690;11675354;
D002699	increase	MDM2	ENSG00000135679	17476690;
D002699	increase	RAD51	ENSG00000051180	10473103;
D002699	increase	SFN	ENSG00000175793	18092340;
D002699	increase	TNFRSF10B	ENSG00000120889	14614459;18092340;
D002699	increase	TP53	ENSG00000141510	18092340;
D002701	increase	ATG12	ENSG00000145782	17457038;
D002706	decrease	RB1	ENSG00000139687	10048758;
D002706	increase	CYP19A1	ENSG00000137869	16996190;
D002706	increase	CYP26A1	ENSG00000095596	15589975;
D002706	increase	CYP2B6	ENSG00000197408	15548381;
D002706	increase	CYP3A4	ENSG00000160868	12417264;15548381;
D002706	increase	NR1I2	ENSG00000144852	16054614;12417264;15548381;
D002706	increase	RARB	ENSG00000077092	15589975;
D002706	increase	RARG	ENSG00000172819	15589975;
D002725	increase	ABCB1	ENSG00000085563	15120965;
D002725	increase	IL8	ENSG00000169429	11306435;
D002738	increase	CCL2	ENSG00000108691	15139008;
D002738	increase	HSPA1B	ENSG00000232804	17046822;
D002738	increase	IL8	ENSG00000169429	15139008;
D002746	decrease	SLC2A3	ENSG00000059804	15342952;
D002746	increase	C10ORF10	ENSG00000165507	15342952;
D002746	increase	FABP1	ENSG00000163586	15342952;
D002746	increase	FNDC4	ENSG00000115226	15342952;
D002746	increase	GDPD3	ENSG00000102886	15342952;
D002746	increase	HPN	ENSG00000105707	15342952;
D002746	increase	INHBE	ENSG00000139269	15342952;
D002746	increase	LSS	ENSG00000160285	15342952;
D002746	increase	NUPR1	ENSG00000176046	15342952;
D002746	increase	PHYH	ENSG00000107537	15342952;
D002746	increase	SERPINA3	ENSG00000196136	15342952;
D002746	increase	WIPI1	ENSG00000070540	15342952;
D002762	decrease	CCNE1	ENSG00000105173	12907249;

D002762	decrease	CDKN1A	ENSG00000124762	12907249;
D002762	decrease	ND4	ENSG00000198886	10491287;
D002762	decrease	VIM	ENSG00000026025	17024972;
D002762	increase	CD14	ENSG00000170458	15988048;
D002762	increase	CDA	ENSG00000158825	18537153;
D002762	increase	CDKN1B	ENSG00000111276	12907249;
D002762	increase	CDKN2D	ENSG00000129355	16582618;
D002762	increase	CYP24A1	ENSG00000019186	16207822;
D002762	increase	CYP2B6	ENSG00000197408	11991950;
D002762	increase	CYP2C9	ENSG00000138109	11991950;
D002762	increase	CYP3A4	ENSG00000160868	15005856;16207822;11991950;
D002762	increase	ITGAM	ENSG00000169896	15988048;
D002762	increase	ITGAX	ENSG00000140678	15988048;
D002762	increase	PML	ENSG00000140464	15988048;
D002762	increase	RARA	ENSG00000131759	15988048;
D002762	increase	TGFB1	ENSG00000105329	15988048;
D002762	increase	VDR	ENSG00000111424	17024972;
D002772	decrease	IL12A	ENSG00000168811	9927516;
D002772	decrease	IL12B	ENSG00000113302	9927516;
D002772	decrease	IL12RB1	ENSG00000096996	9927516;
D002772	decrease	IL12RB2	ENSG00000081985	9927516;
D002772	decrease	TNF	ENSG00000223952	9927516;
D002772	increase	BIRC3	ENSG00000023445	12837940;
D002772	increase	BIRC7	ENSG00000101197	12837940;
D002777	decrease	CYP7A1	ENSG00000167910	15145977;
D002777	increase	ABCB11	ENSG00000073734	15145977;
D002777	increase	NR0B2	ENSG00000131910	15145977;
D002784	increase	CES1	ENSG00000198848	11015575;8049197;127731
D002784	increase	FAS	ENSG00000026103	16162944;
D002784	increase	FGF7	ENSG00000140285	16162944;
D002784	increase	SCD	ENSG00000099194	16162944;
D002793	decrease	CDX2	ENSG00000165556	16990345;
D002794	affect	ALDH1A2	ENSG00000128918	17616785;
D002794	affect	ARHGAP9	ENSG00000123329	17616785;
D002794	affect	BUB1B	ENSG00000156970	17616785;
D002794	affect	CCL2	ENSG00000108691	17616785;
D002794	affect	CD163	ENSG00000177575	17616785;
D002794	affect	CD36	ENSG00000135218	17616785;
D002794	affect	CDCA8	ENSG00000134690	17616785;
D002794	affect	CHEK1	ENSG00000149554	17616785;
D002794	affect	COLEC12	ENSG00000158270	17616785;
D002794	affect	CST3	ENSG00000101439	17616785;
D002794	affect	CX3CR1	ENSG00000168329	17616785;
D002794	affect	DOCK2	ENSG00000134516	17616785;
D002794	affect	FOXA1	ENSG00000129514	17616785;
D002794	affect	GBE1	ENSG00000114480	17616785;
D002794	affect	GZMK	ENSG00000113088	17616785;
D002794	affect	HSPA5	ENSG00000044574	17616785;
D002794	affect	IL2RB	ENSG00000100385	17616785;
D002794	affect	KIF20A	ENSG00000112984	17616785;
D002794	affect	KIF23	ENSG00000137807	17616785;
D002794	affect	KLRF1	ENSG00000150045	17616785;
D002794	affect	MAL2	ENSG00000147676	17616785;

D002794	affect	1-Mar	ENSG00000145416	17616785;
D002794	affect	MGP	ENSG00000111341	17616785;
D002794	affect	MMP2	ENSG00000087245	17616785;
D002794	affect	MYOM2	ENSG00000036448	17616785;
D002794	affect	NFKBIA	ENSG00000100906	17616785;
D002794	affect	PDAP1	ENSG00000106244	17616785;
D002794	affect	PLAUR	ENSG00000011422	17616785;
D002794	affect	PRAME	ENSG00000185686	17616785;
D002794	affect	PTPRCAP	ENSG00000213402	17616785;
D002794	affect	RGS19	ENSG00000171700	17616785;
D002794	affect	RNPEPL1	ENSG00000142327	17616785;
D002794	affect	SFRP1	ENSG00000104332	17616785;
D002794	affect	SLC7A7	ENSG00000155465	17616785;
D002794	affect	SMARCA1	ENSG00000102038	17616785;
D002794	affect	TERT	ENSG00000164362	17616785;
D002794	affect	TNFAIP3	ENSG00000118503	17616785;
D002794	affect	UCK1	ENSG00000130717	17616785;
D002857	decrease	ACP2	ENSG00000134575	17547211;
D002857	decrease	ACSL1	ENSG00000151726	17547211;
D002857	decrease	AKR1B1	ENSG00000085662	17547211;
D002857	decrease	ATP2B1	ENSG00000070961	17547211;
D002857	decrease	AZGP1	ENSG00000160862	17547211;
D002857	decrease	C14ORF1	ENSG00000133935	17547211;
D002857	decrease	CALR	ENSG00000179218	17547211;
D002857	decrease	CDKN2A	ENSG00000147889	16828922;
D002857	decrease	CFB	ENSG00000243570	17547211;
D002857	decrease	CLGN	ENSG00000153132	17547211;
D002857	decrease	CLPTM1	ENSG00000104853	17547211;
D002857	decrease	CRYAA	ENSG00000160202	17547211;
D002857	decrease	CX3CL1	ENSG00000006210	17547211;
D002857	decrease	F5	ENSG00000198734	17547211;
D002857	decrease	FURIN	ENSG00000140564	17547211;
D002857	decrease	GGCX	ENSG00000115486	17547211;
D002857	decrease	GPER	ENSG00000164850	17547211;
D002857	decrease	HMGCR	ENSG00000113161	17547211;
D002857	decrease	IL17RB	ENSG00000056736	17547211;
D002857	decrease	ITIH3	ENSG00000162267	17547211;
D002857	decrease	MGAT2	ENSG00000168282	17547211;
D002857	decrease	MMP2	ENSG00000087245	16581223;
D002857	decrease	NR0B2	ENSG00000131910	17547211;
D002857	decrease	NUCB2	ENSG00000070081	17547211;
D002857	decrease	OS9	ENSG00000135506	17547211;
D002857	decrease	PDIA4	ENSG00000155660	17547211;
D002857	decrease	PGC	ENSG00000096088	17547211;
D002857	decrease	PGF	ENSG00000119630	17547211;
D002857	decrease	PNMA1	ENSG00000176903	17547211;
D002857	decrease	REPIN1	ENSG00000214022	17547211;
D002857	decrease	SERPINB1	ENSG00000021355	17547211;
D002857	decrease	SERPINC1	ENSG00000117601	17547211;
D002857	decrease	SFRS5	ENSG00000100650	17547211;
D002857	decrease	TAF7	ENSG00000178913	17547211;
D002857	decrease	TNFSF10	ENSG00000121858	17547211;
D002857	decrease	TOB1	ENSG00000141232	17547211;
D002857	increase	ABCG2	ENSG00000118777	17547211;

D002857	increase	ADM	ENSG00000148926	17547211;
D002857	increase	ANXA2	ENSG00000182718	17547211;
D002857	increase	ASNS	ENSG00000070669	17547211;
D002857	increase	CAT	ENSG00000121691	16488005;
D002857	increase	CCNB2	ENSG00000157456	17547211;
D002857	increase	CDKN1A	ENSG00000124762	17547211;
D002857	increase	CHST3	ENSG00000122863	17547211;
D002857	increase	ENPP1	ENSG00000197594	17547211;
D002857	increase	FOSL1	ENSG00000175592	17547211;
D002857	increase	HIF1A	ENSG00000100644	16386771;
D002857	increase	HIST1H4C	ENSG00000182217	17547211;
D002857	increase	HMGA1	ENSG00000137309	17547211;
D002857	increase	HMGN2	ENSG00000198830	17547211;
D002857	increase	HMOX1	ENSG00000100292	16488005;
D002857	increase	IER2	ENSG00000160888	17547211;
D002857	increase	ITGB3BP	ENSG00000142856	17547211;
D002857	increase	KLF10	ENSG00000155090	17547211;
D002857	increase	MAFF	ENSG00000185022	17547211;
D002857	increase	MCM2	ENSG00000073111	17547211;
D002857	increase	PIM1	ENSG00000137193	17547211;
D002857	increase	PPIH	ENSG00000171960	17547211;
D002857	increase	PTTG1	ENSG00000164611	17547211;
D002857	increase	RFC2	ENSG00000049541	17547211;
D002857	increase	RNASEH2A	ENSG00000104889	17547211;
D002857	increase	SFRS2	ENSG00000161547	17547211;
D002857	increase	SFRS7	ENSG00000115875	17547211;
D002857	increase	SIK1	ENSG00000142178	17547211;
D002857	increase	SNRPA	ENSG00000077312	17547211;
D002857	increase	TIMP1	ENSG00000102265	16581223;
D002857	increase	TNFAIP3	ENSG00000118503	17547211;
D002857	increase	TYMS	ENSG00000176890	17547211;
D002857	increase	UBE2C	ENSG00000175063	17547211;
D002857	increase	UGCG	ENSG00000148154	17547211;
D002857	increase	ZWINT	ENSG00000122952	17547211;
D002927	decrease	CD14	ENSG00000170458	11556524;
D002927	decrease	ITGA4	ENSG00000115232	11556524;
D002927	decrease	ITGAX	ENSG00000140678	11556524;
D002927	decrease	LRP1	ENSG00000123384	11556524;
D002927	increase	HRH2	ENSG00000113749	9681472;1912125;1223526
D002927	increase	IFNG	ENSG00000111537	10569698;
D002927	increase	IL16	ENSG00000172349	12235264;
D002927	increase	PDE4B	ENSG00000184588	11131300;
D002927	increase	TNF	ENSG00000223952	9088875;
D002939	decrease	BCL2	ENSG00000171791	12063570;
D002939	decrease	CDKN1A	ENSG00000124762	12063570;
D002939	decrease	IFNG	ENSG00000111537	16006447;
D002939	decrease	IL4	ENSG00000113520	16006447;
D002939	decrease	SLC2A1	ENSG00000117394	19022360;
D002939	increase	IL1B	ENSG00000125538	9267955;16148020;110529<br>20;12428247;
D002939	increase	IL6	ENSG00000136244	11052920;9267955;
D002939	increase	IL8	ENSG00000169429	9267955;18191106;110529
D002939	increase	MMP13	ENSG00000137745	16148020;
D002939	increase	MMP1	ENSG00000196611	16148020;12428247;

D002939	increase	MMP3	ENSG00000149968	12428247;
D002939	increase	TGFB1	ENSG00000105329	19371339;
D002939	increase	TNF	ENSG00000223952	9267955;16939481;110529
D002945	affect	CNTF	ENSG00000242689	16898872;
D002945	affect	EDN2	ENSG00000127129	16898872;
D002945	affect	GDF15	ENSG00000130513	16898872;
D002945	affect	GUK1	ENSG00000143774	16898872;
D002945	affect	IFITM1	ENSG00000185885	16898872;18202764;16217747;
D002945	affect	NOTCH4	ENSG00000204301	16898872;
D002945	affect	NTHL1	ENSG00000065057	16898872;
D002945	affect	PFDN5	ENSG00000123349	16898872;
D002945	affect	RHOC	ENSG00000155366	16898872;
D002945	affect	RPS5	ENSG00000083845	16898872;
D002945	affect	TUBA1A	ENSG00000167552	16898872;
D002945	decrease	AKR1B1	ENSG00000085662	16803524;
D002945	decrease	ARHGEF6	ENSG00000129675	16803524;
D002945	decrease	BCL2L12	ENSG00000126453	17404015;
D002945	decrease	BIRC3	ENSG00000023445	14654915;16391810;
D002945	decrease	CFLAR	ENSG00000003402	14601052;
D002945	decrease	CREBBP	ENSG00000005339	17498666;
D002945	decrease	EGFR	ENSG00000146648	17146438;15723263;18337622;15737843;
D002945	decrease	EP300	ENSG00000100393	17498666;
D002945	decrease	ERBB2	ENSG00000141736	18337622;15737843;
D002945	decrease	ERCC1	ENSG00000012061	15173087;15737843;
D002945	decrease	GCNT1	ENSG00000187210	16803524;
D002945	decrease	GSTP1	ENSG00000084207	16785472;16622263;15239142;2747627;19396019;15279901;14678959;15737843;
D002945	decrease	HIF1A	ENSG00000100644	17066447;17498666;16532342;
D002945	decrease	IGFBP3	ENSG00000146674	16061661;
D002945	decrease	KRT19	ENSG00000171345	16061661;
D002945	decrease	KRT4	ENSG00000170477	16061661;
D002945	decrease	MAD2L2	ENSG00000116670	16803524;
D002945	decrease	MGMT	ENSG00000170430	2766459;15015788;7562019;15809756;16043385;1603
D002945	decrease	MYC	ENSG00000136997	17628013;16797627;16803524;
D002945	decrease	NAIP	ENSG00000250628	11911975;14654915;
D002945	decrease	PMPCA	ENSG00000165688	16803524;
D002945	decrease	PPP3CB	ENSG00000107758	16803524;
D002945	decrease	PTK2	ENSG00000169398	16391810;
D002945	decrease	RELA	ENSG00000173039	19003803;
D002945	decrease	SERPINB4	ENSG00000206073	16803524;
D002945	decrease	SOCS1	ENSG00000185338	17374387;
D002945	decrease	TERT	ENSG00000164362	17923759;18021753;
D002945	decrease	TNFAIP3	ENSG00000118503	16803524;
D002945	decrease	TNFSF13	ENSG00000161955	18423122;
D002945	decrease	TRAM1	ENSG00000067167	16061661;
D002945	decrease	TYMP	ENSG00000025708	16568373;17638652;18728667;
D002945	decrease	UMPS	ENSG00000114491	16568373;17237621;
D002945	decrease	USP14	ENSG00000101557	16803524;

D002945	decrease	VEGFA	ENSG00000112715	17498666;
D002945	decrease	XIAP	ENSG00000101966	18071906;14601052;15981204;16391810;15863139;11911975;14654915;1614236
D002945	increase	ABCB1	ENSG00000085563	15756446;16785472;17284363;15802814;15990222;15650019;15239124;
D002945	increase	ABCC1	ENSG00000103222	15756446;15448748;15802814;17638652;18695918;
D002945	increase	ABCC2	ENSG00000023839	15756446;15985617;17982673;8797578;15688364;186
D002945	increase	AKT2	ENSG00000105221	18071906;16803524;
D002945	increase	AXL	ENSG00000167601	16061661;
D002945	increase	BAX	ENSG00000087088	16009487;17399942;14601052;15641988;14512787;17619073;16619537;
D002945	increase	BBC3	ENSG00000105327	16481741;
D002945	increase	BCL2	ENSG00000171791	16009487;15917659;14601052;15981204;17404015;14512787;17619073;1661953
D002945	increase	BCL2L1	ENSG00000171552	16020667;15917659;14601052;15981204;17399942;17619073;16619537;
D002945	increase	BIRC5	ENSG00000089685	17686279;11911975;14654915;16142363;15981204;15917659;16382892;14601052;15970709;17399942;1498
D002945	increase	C7ORF16	ENSG00000106341	16803524;
D002945	increase	CASP3	ENSG00000164305	16080519;17619073;16803524;17399942;16009487;14512787;15863139;18071906;17853063;18176112;
D002945	increase	CASP8	ENSG00000064012	17619073;16803524;16009487;18071906;
D002945	increase	CASP9	ENSG00000132906	17404015;17619073;16009487;16803524;
D002945	increase	CDC40	ENSG00000168438	16803524;
D002945	increase	CDKL5	ENSG00000008086	16803524;
D002945	increase	CDKN1A	ENSG00000124762	12807743;14601052;17355264;11099651;
D002945	increase	CDKN2A	ENSG00000147889	16797627;
D002945	increase	CFHR1	ENSG00000244414	16803524;
D002945	increase	CIDEB	ENSG00000136305	16803524;
D002945	increase	CYP3A4	ENSG00000160868	15650019;
D002945	increase	DDIT3	ENSG00000175197	17355264;
D002945	increase	DPYD	ENSG00000188641	16568373;18728667;16685392;15737843;
D002945	increase	ELMO2	ENSG00000062598	16803524;
D002945	increase	ETV4	ENSG00000175832	16061661;
D002945	increase	FANCG	ENSG00000221829	16061661;16243825;
D002945	increase	FAS	ENSG00000026103	16009487;16957513;17305640;
D002945	increase	FASLG	ENSG00000117560	16009487;16957513;
D002945	increase	FGFR3	ENSG00000068078	12066199;
D002945	increase	FOSL1	ENSG00000175592	16061661;15756446;16217747;
D002945	increase	GADD45A	ENSG00000116717	16797627;19003803;
D002945	increase	HNRNPA1	ENSG00000135486	16803524;

D002945	increase	HTRA2	ENSG00000115317	15863139;
D002945	increase	IL4R	ENSG00000077238	14678968;
D002945	increase	ITGA9	ENSG00000144668	12066199;
D002945	increase	ITGB4	ENSG00000132470	12066199;
D002945	increase	JUN	ENSG00000177606	17355264;16898872;
D002945	increase	MCM2	ENSG00000073111	16061661;
D002945	increase	MDM2	ENSG00000135679	16797627;
D002945	increase	MMP10	ENSG00000166670	12066199;
D002945	increase	MMP15	ENSG00000102996	12066199;
D002945	increase	MMP16	ENSG00000156103	12066199;
D002945	increase	MT1A	ENSG00000205362	12680227;
D002945	increase	MT2A	ENSG00000125148	12680227;16394183;
D002945	increase	MT3	ENSG00000087250	16061661;16898872;
D002945	increase	MUT	ENSG00000146085	19427250;19199343;
D002945	increase	NUDT1	ENSG00000106268	16803524;
D002945	increase	PARD6A	ENSG00000102981	16803524;
D002945	increase	PCNA	ENSG00000132646	16803524;11099651;16685392;
D002945	increase	PFKFB4	ENSG00000114268	16803524;
D002945	increase	PMAIP1	ENSG00000141682	18176112;17216584;
D002945	increase	PPIE	ENSG00000084072	16803524;
D002945	increase	PTGS2	ENSG00000073756	15846114;18695918;15517878;16322294;
D002945	increase	RPL21	ENSG00000122026	16803524;
D002945	increase	RPS4Y2	ENSG00000157828	16803524;
D002945	increase	SNAP29	ENSG00000099940	16803524;
D002945	increase	SNRPA	ENSG00000077312	16803524;
D002945	increase	STYX	ENSG00000198252	16803524;
D002945	increase	TANK	ENSG00000136560	16803524;
D002945	increase	TLR2	ENSG00000137462	16803524;
D002945	increase	TP53	ENSG00000141510	17555331;15854392;16797627;15990222;12082016;12807743;14562046;15578696;15999354;17498666;17216584;16020667;16211088;16077963;15254702;
D002945	increase	TP73	ENSG00000078900	14678968;17076661;
D002945	increase	TRAP1	ENSG00000126602	17853063;16061661;
D002945	increase	TYMS	ENSG00000176890	16568373;16685392;16622263;16424979;10482907;17638652;15737843;
D002945	increase	UGDH	ENSG00000109814	16803524;
D002955	increase	CCNE1	ENSG00000105173	15342418;
D002955	increase	CDC6	ENSG00000094804	15342418;
D002955	increase	CDK1	ENSG00000170312	15342418;
D002955	increase	CDK2	ENSG00000123374	15342418;
D002955	increase	CDKN1A	ENSG00000124762	15342418;
D002955	increase	DHFR	ENSG00000228716	16712799;
D002976	increase	IL10	ENSG00000136634	15502056;
D002976	increase	IL8	ENSG00000169429	15502056;
D002990	decrease	ABCC1	ENSG00000103222	16882163;
D002990	decrease	GCLC	ENSG00000001084	16882163;
D002990	increase	CYP3A5	ENSG00000106258	16882163;
D002994	increase	CCL2	ENSG00000108691	16600694;
D002994	increase	CYP1A1	ENSG00000140465	15521013;

D002994	increase	JUN	ENSG00000177606	16600694;
D002994	increase	RELA	ENSG00000173039	16600694;
D002995	decrease	CYP2A6	ENSG00000198077	11038160;
D002995	increase	CYP2C8	ENSG00000138115	15771232;
D002995	increase	CYP2C9	ENSG00000138109	15802389;
D002995	increase	CYP3A4	ENSG00000160868	15771232;
D002995	increase	UGT1A1	ENSG00000241635	15771232;
D002996	decrease	ITGB3	ENSG00000056345	15749485;
D002996	increase	PGR	ENSG00000082175	15749485;
D002997	decrease	AP1S1	ENSG00000106367	15342952;
D002997	decrease	SLC2A3	ENSG00000059804	15342952;
D002997	increase	C10ORF10	ENSG00000165507	15342952;
D002997	increase	FABP1	ENSG00000163586	15342952;
D002997	increase	HPN	ENSG00000105707	15342952;
D002997	increase	INHBE	ENSG00000139269	15342952;
D002997	increase	LSS	ENSG00000160285	15342952;
D002997	increase	NUPR1	ENSG00000176046	15342952;
D002997	increase	PHYH	ENSG00000107537	15342952;
D002997	increase	SERPINA3	ENSG00000196136	15342952;
D002997	increase	WIPI1	ENSG00000070540	15342952;
D003010	decrease	EZH2	ENSG00000106462	19691856;
D003010	increase	ABCA5	ENSG00000154265	19691856;
D003010	increase	ACADSB	ENSG00000196177	19691856;
D003010	increase	ACAP2	ENSG00000114331	19691856;
D003010	increase	ADRB2	ENSG00000169252	19691856;
D003010	increase	AKAP11	ENSG00000023516	19691856;
D003010	increase	AKAP9	ENSG00000127914	19691856;
D003010	increase	ANKRD12	ENSG00000101745	19691856;
D003010	increase	APC	ENSG00000134982	19691856;
D003010	increase	AR	ENSG00000169083	19691856;
D003010	increase	ARID4A	ENSG00000032219	19691856;
D003010	increase	ATG4C	ENSG00000125703	19691856;
D003010	increase	ATRX	ENSG00000085224	19691856;
D003010	increase	AZI2	ENSG00000163512	19691856;
D003010	increase	B3GNT5	ENSG00000176597	19691856;
D003010	increase	BAZ2B	ENSG00000123636	19691856;
D003010	increase	BDP1	ENSG00000145734	19691856;
D003010	increase	BRWD1	ENSG00000185658	19691856;
D003010	increase	CACNB2	ENSG00000165995	19691856;
D003010	increase	CBLN2	ENSG00000141668	19691856;
D003010	increase	CCDC112	ENSG00000164221	19691856;
D003010	increase	CCDC55	ENSG00000126653	19691856;
D003010	increase	CDH23	ENSG00000107736	19691856;
D003010	increase	CENPC1	ENSG00000145241	19691856;
D003010	increase	CEP135	ENSG00000174799	19691856;
D003010	increase	CEP290	ENSG00000198707	19691856;
D003010	increase	CEP350	ENSG00000135837	19691856;
D003010	increase	CEP70	ENSG00000114107	19691856;
D003010	increase	CFH	ENSG00000000971	19691856;
D003010	increase	CHD9	ENSG00000177200	19691856;
D003010	increase	CHIC1	ENSG00000204116	19691856;
D003010	increase	CHM	ENSG00000188419	19691856;
D003010	increase	CLGN	ENSG00000153132	19691856;
D003010	increase	CLIP1	ENSG00000130779	19691856;

D003010	increase	CNTN3	ENSG00000113805	19691856;
D003010	increase	COBLL1	ENSG00000082438	19691856;
D003010	increase	CPD	ENSG00000108582	19691856;
D003010	increase	CPNE9	ENSG00000144550	19691856;
D003010	increase	CTAGE5	ENSG00000150527	19691856;
D003010	increase	DKKL1	ENSG00000104901	19691856;
D003010	increase	DMXL1	ENSG00000172869	19691856;
D003010	increase	DNAJC10	ENSG00000077232	19691856;
D003010	increase	DST	ENSG00000151914	19691856;
D003010	increase	DYNC2H1	ENSG00000187240	19691856;
D003010	increase	DYNC2LI1	ENSG00000138036	19691856;
D003010	increase	ECHDC1	ENSG00000093144	19691856;
D003010	increase	ESCO1	ENSG00000141446	19691856;
D003010	increase	FAS	ENSG00000026103	19691856;
D003010	increase	FZD3	ENSG00000104290	19691856;
D003010	increase	GABPA	ENSG00000154727	19691856;
D003010	increase	GCC2	ENSG00000135968	19691856;
D003010	increase	GK	ENSG00000198814	19691856;
D003010	increase	GNPDA2	ENSG00000163281	19691856;
D003010	increase	GOLGA4	ENSG00000144674	19691856;
D003010	increase	GOLGB1	ENSG00000173230	19691856;
D003010	increase	HBD	ENSG00000223609	19691856;
D003010	increase	HINT3	ENSG00000111911	19691856;
D003010	increase	IBTK	ENSG00000005700	19691856;
D003010	increase	IFT80	ENSG00000068885	19691856;
D003010	increase	KLHDC1	ENSG00000197776	19691856;
D003010	increase	KLHL2	ENSG00000109466	19691856;
D003010	increase	KLK3	ENSG00000142515	19691856;
D003010	increase	KTN1	ENSG00000126777	19691856;
D003010	increase	LIG4	ENSG00000174405	19691856;
D003010	increase	LRAT	ENSG00000121207	19691856;
D003010	increase	LTB	ENSG00000236237	19691856;
D003010	increase	MAGEA3	ENSG00000221867	19691856;
D003010	increase	MANEA	ENSG00000172469	19691856;
D003010	increase	MAP9	ENSG00000164114	19691856;
D003010	increase	MBNL2	ENSG00000139793	19691856;
D003010	increase	MIPOL1	ENSG00000151338	19691856;
D003010	increase	MOBKL1A	ENSG00000173542	19691856;
D003010	increase	MON2	ENSG00000061987	19691856;
D003010	increase	MOSPD2	ENSG00000130150	19691856;
D003010	increase	MPP7	ENSG00000150054	19691856;
D003010	increase	MYO6	ENSG00000196586	19691856;
D003010	increase	NDFIP2	ENSG00000102471	19691856;
D003010	increase	NFE2L2	ENSG00000116044	19691856;
D003010	increase	NIPBL	ENSG00000164190	19691856;
D003010	increase	NPAT	ENSG00000149308	19691856;
D003010	increase	NRIP1	ENSG00000180530	19691856;
D003010	increase	NSUN3	ENSG00000178694	19691856;
D003010	increase	NT5DC1	ENSG00000178425	19691856;
D003010	increase	NUDCD1	ENSG00000120526	19691856;
D003010	increase	ODF2L	ENSG00000122417	19691856;
D003010	increase	PCM1	ENSG00000078674	19691856;
D003010	increase	PCMTD1	ENSG00000168300	19691856;
D003010	increase	PDIK1L	ENSG00000175087	19691856;

D003010	increase	PHF14	ENSG00000106443	19691856;
D003010	increase	PHF3	ENSG00000118482	19691856;
D003010	increase	PHIP	ENSG00000146247	19691856;
D003010	increase	PIK3C2A	ENSG00000011405	19691856;
D003010	increase	POLK	ENSG00000122008	19691856;
D003010	increase	PRODH2	ENSG00000250799	19691856;
D003010	increase	PTPN22	ENSG00000248536	19691856;
D003010	increase	RAD50	ENSG00000113522	19691856;
D003010	increase	RAD54B	ENSG00000197275	19691856;
D003010	increase	RAPGEF4	ENSG00000091428	19691856;
D003010	increase	RB1CC1	ENSG00000023287	19691856;
D003010	increase	REV3L	ENSG00000009413	19691856;
D003010	increase	RGPD5	ENSG00000183054	19691856;
D003010	increase	RGS5	ENSG00000143248	19691856;
D003010	increase	RICTOR	ENSG00000164327	19691856;
D003010	increase	SAMD9	ENSG00000205413	19691856;
D003010	increase	SEMA3C	ENSG00000075223	19691856;
D003010	increase	SENP6	ENSG00000112701	19691856;
D003010	increase	SENP7	ENSG00000138468	19691856;
D003010	increase	SFRS2IP	ENSG00000139218	19691856;
D003010	increase	SHOC2	ENSG00000108061	19691856;
D003010	increase	SLC4A5	ENSG00000188687	19691856;
D003010	increase	SLCO1B1	ENSG00000134538	19691856;
D003010	increase	SLITRK6	ENSG00000184564	19691856;
D003010	increase	SLK	ENSG00000065613	19691856;
D003010	increase	SMCHD1	ENSG00000101596	19691856;
D003010	increase	SOCS2	ENSG00000120833	19691856;
D003010	increase	STAG2	ENSG00000101972	19691856;
D003010	increase	STEAP2	ENSG00000157214	19691856;
D003010	increase	STXBP4	ENSG00000166263	19691856;
D003010	increase	TBC1D15	ENSG00000121749	19691856;
D003010	increase	TBC1D8B	ENSG00000133138	19691856;
D003010	increase	TCEAL7	ENSG00000182916	19691856;
D003010	increase	TEX9	ENSG00000151575	19691856;
D003010	increase	THAP2	ENSG00000173451	19691856;
D003010	increase	TMED5	ENSG00000117500	19691856;
D003010	increase	TMEM106B	ENSG00000106460	19691856;
D003010	increase	TMF1	ENSG00000144747	19691856;
D003010	increase	TRIP11	ENSG00000100815	19691856;
D003010	increase	UBE2W	ENSG00000104343	19691856;
D003010	increase	UBLCP1	ENSG00000164332	19691856;
D003010	increase	UPF3B	ENSG00000125351	19691856;
D003010	increase	VCPIP1	ENSG00000175073	19691856;
D003010	increase	VPS13A	ENSG00000197969	19691856;
D003010	increase	VPS13C	ENSG00000129003	19691856;
D003010	increase	VPS37A	ENSG00000155975	19691856;
D003010	increase	WDR17	ENSG00000150627	19691856;
D003010	increase	ZBTB10	ENSG00000205189	19691856;
D003010	increase	ZBTB1	ENSG00000126804	19691856;
D003010	increase	ZCCHC6	ENSG00000083223	19691856;
D003010	increase	ZFY	ENSG00000067646	19691856;
D003010	increase	ZFYVE16	ENSG00000039319	19691856;
D003010	increase	ZNF138	ENSG00000197008	19691856;
D003010	increase	ZNF141	ENSG00000131127	19691856;

D003010	increase	ZNF280D	ENSG00000137871	19691856;
D003010	increase	ZNF292	ENSG00000188994	19691856;
D003010	increase	ZNF449	ENSG00000173275	19691856;
D003010	increase	ZNF480	ENSG00000198464	19691856;
D003010	increase	ZNF493	ENSG00000196268	19691856;
D003010	increase	ZNF567	ENSG00000189042	19691856;
D003010	increase	ZNF605	ENSG00000196458	19691856;
D003010	increase	ZNF638	ENSG00000075292	19691856;
D003010	increase	ZNF644	ENSG00000122482	19691856;
D003010	increase	ZNF721	ENSG00000246238	19691856;
D003022	affect	NR3C1	ENSG00000113580	12673034;12695351;
D003022	affect	UGT1A1	ENSG00000241635	15849716;
D003022	increase	ABCB1	ENSG00000085563	8917702;8632764;
D003022	increase	ABCC2	ENSG00000023839	11836020;
D003022	increase	CYP2B6	ENSG00000197408	14977870;12695351;
D003022	increase	CYP3A4	ENSG00000160868	10219967;15135088;14977870;12065438;12673034;12505310;16837568;15466163;8632764;
D003022	increase	CYP3A5	ENSG00000106258	12673034;8632764;
D003022	increase	CYP3A7	ENSG00000160870	12673034;
D003022	increase	NR1I2	ENSG00000144852	14977870;15849716;16919048;15548381;12065438;12695351;
D003024	decrease	FCGR3B	ENSG00000162747	16510162;
D003024	decrease	SLC2A3	ENSG00000059804	15342952;
D003024	decrease	TAGLN	ENSG00000149591	15342952;
D003024	increase	ASAH1	ENSG00000104763	15342952;
D003024	increase	ASNS	ENSG00000070669	15342952;
D003024	increase	C10ORF10	ENSG00000165507	15342952;
D003024	increase	FABP1	ENSG00000163586	15342952;
D003024	increase	FASLG	ENSG00000117560	16510162;
D003024	increase	FNDC4	ENSG00000115226	15342952;
D003024	increase	GDPD3	ENSG00000102886	15342952;
D003024	increase	HMOX1	ENSG00000100292	18227147;
D003024	increase	HPN	ENSG00000105707	15342952;
D003024	increase	INHBE	ENSG00000139269	15342952;
D003024	increase	LSS	ENSG00000160285	15342952;
D003024	increase	NR0B2	ENSG00000131910	15342952;
D003024	increase	NUPR1	ENSG00000176046	15342952;
D003024	increase	SERPINA3	ENSG00000196136	15342952;
D003024	increase	UGT1A1	ENSG00000241635	18227147;
D003024	increase	WIPI1	ENSG00000070540	15342952;
D003033	increase	CYP1A1	ENSG00000140465	16882163;15720126;
D003033	increase	CYP1A2	ENSG00000140505	16882163;
D003033	increase	CYP1B1	ENSG00000138061	16882163;15720126;
D003033	increase	CYP2C18	ENSG00000108242	16882163;
D003033	increase	CYP2S1	ENSG00000167600	16054184;
D003033	increase	GCLC	ENSG00000001084	16882163;
D003033	increase	GSTP1	ENSG00000084207	16882163;
D003033	increase	HMOX1	ENSG00000100292	16882163;
D003033	increase	NQO1	ENSG00000181019	16882163;
D003033	increase	PTGS2	ENSG00000073756	16882163;
D003035	decrease	CAT	ENSG00000121691	16488005;

D003035	increase	EPO	ENSG00000130427	10699462;
D003035	increase	FOS	ENSG00000170345	14971665;
D003035	increase	GSTA1	ENSG00000243955	14971665;
D003035	increase	HIF1A	ENSG00000100644	16877034;16386771;
D003035	increase	HMOX1	ENSG00000100292	16488005;
D003035	increase	MT2A	ENSG00000125148	14971665;
D003035	increase	NDRG1	ENSG00000104419	16386771;
D003078	decrease	MAPT	ENSG00000186868	16930453;
D003078	decrease	TAT	ENSG00000198650	15744361;
D003078	increase	ABCB1	ENSG00000085563	16007523;15112361;15725475;15342794;
D003276	decrease	MKI67	ENSG00000148773	16019350;
D003276	decrease	PROC	ENSG00000115718	15185533;
D003276	decrease	PROS1	ENSG00000184500	15185533;
D003276	increase	PLG	ENSG00000122194	15185533;
D003277	decrease	FSHB	ENSG00000131808	11228061;
D003277	decrease	LHB	ENSG00000104826	11228061;
D003277	increase	SHBG	ENSG00000129214	16409223;
D003300	decrease	AR	ENSG00000169083	17308104;
D003300	decrease	BCL2	ENSG00000171791	16962711;
D003300	decrease	CCNA2	ENSG00000145386	15367699;
D003300	decrease	SRC	ENSG00000197122	16600465;
D003300	increase	APP	ENSG00000142192	16648635;16627626;18583042;17239395;17119284;15699049;16325427;1696271
D003300	increase	BACE1	ENSG00000186318	18583042;
D003300	increase	BAX	ENSG00000087088	16962711;
D003300	increase	CASP3	ENSG00000164305	16962711;
D003300	increase	CCS	ENSG00000173992	16627626;
D003300	increase	CP	ENSG00000047457	16842975;17242517;17032174;10949969;
D003300	increase	F3	ENSG00000117525	7600656;
D003300	increase	FOS	ENSG00000170345	17032174;
D003300	increase	HIF1A	ENSG00000100644	16973622;
D003300	increase	HMOX1	ENSG00000100292	17244614;19888908;
D003300	increase	HSPA1A	ENSG00000232804	17244614;
D003300	increase	HSPA8	ENSG00000109971	16600465;
D003300	increase	IL8	ENSG00000169429	18464055;
D003300	increase	JUN	ENSG00000177606	17032174;
D003300	increase	MMP2	ENSG00000087245	16648635;
D003300	increase	MMP3	ENSG00000149968	16648635;
D003300	increase	MT1X	ENSG00000187193	17409696;
D003300	increase	MT2A	ENSG00000125148	17409696;
D003300	increase	NOS1	ENSG00000089250	16962711;
D003300	increase	NOS2	ENSG00000007171	16962711;
D003300	increase	SOD1	ENSG00000142168	19325915;10889018;16962711;10949969;16627626;17112476;
D003345	increase	SLC1A2	ENSG00000110436	17138558;
D003374	decrease	ESR1	ENSG00000091831	17275315;
D003375	decrease	AR	ENSG00000169083	11867264;

D003375	decrease	ESR1	ENSG00000091831	16328721;11162928;16566672;11518614;9751507;15876408;10397250;15084758;11867264;16118406;17275032;12650720;
D003375	decrease	IL2	ENSG00000109471	16696175;
D003375	decrease	NFKB1	ENSG00000109320	16696175;
D003375	increase	PGR	ENSG00000082175	11867264;
D003474	affect	KDR	ENSG00000128052	17960570;
D003474	affect	MAPK14	ENSG00000112062	17151092;18357586;
D003474	affect	SELE	ENSG00000007908	17960570;
D003474	affect	TP53	ENSG00000141510	19020741;17332930;17041101;17332326;
D003474	decrease	AARS	ENSG00000090861	17198877;
D003474	decrease	ABCB1	ENSG00000085563	17121181;18414057;18439772;
D003474	decrease	ADCY9	ENSG00000162104	15713895;
D003474	decrease	AGR2	ENSG00000106541	17198877;
D003474	decrease	AKT1	ENSG00000142208	18660423;18214481;17291458;17640567;16219905;17918158;16023083;19372569;18790744;17289836;17596214;17332930;
D003474	decrease	ALDOA	ENSG00000149925	16880289;
D003474	decrease	ALDOC	ENSG00000109107	17198877;
D003474	decrease	ANKRD37	ENSG00000186352	17198877;
D003474	decrease	ANKRD50	ENSG00000151458	17999991;
D003474	decrease	ANKZF1	ENSG00000163516	17198877;
D003474	decrease	APC	ENSG00000134982	15713895;
D003474	decrease	ARHGAP30	ENSG00000186517	18421014;
D003474	decrease	ARNT	ENSG00000143437	16880289;18682687;19018768;
D003474	decrease	BIRC5	ENSG00000089685	17671737;19235267;17596214;19372569;16106398;16023083;18593936;
D003474	decrease	CASP7	ENSG00000165806	16101141;16023083;
D003474	decrease	CASP8	ENSG00000064012	17148446;17096185;14555224;16173963;
D003474	decrease	CCNB1	ENSG00000134057	18719366;19401701;17148446;
D003474	decrease	CCNE1	ENSG00000105173	17148446;18156803;
D003474	decrease	CD36	ENSG00000135218	16713233;
D003474	decrease	CD40	ENSG00000101017	16101141;
D003474	decrease	CD55	ENSG00000196352	17999991;
D003474	decrease	CDC25C	ENSG00000158402	16106398;19401701;
D003474	decrease	CDH11	ENSG00000140937	18025290;
D003474	decrease	CDH17	ENSG00000079112	17198877;
D003474	decrease	CDK1	ENSG00000170312	19401701;16106398;17148446;
D003474	decrease	CDK4	ENSG00000135446	18156803;
D003474	decrease	CDK6	ENSG00000105810	18156803;
D003474	decrease	CLCN6	ENSG00000011021	17198877;
D003474	decrease	COL4A5	ENSG00000188153	15713895;
D003474	decrease	CTGF	ENSG00000118523	17965732;
D003474	decrease	CTNNB1	ENSG00000168036	17041101;19294764;19573523;
D003474	decrease	CXCL1	ENSG00000163739	17999991;

D003474	decrease	CXCL2	ENSG00000081041	17999991;
D003474	decrease	DDIT4L	ENSG00000145358	17999991;
D003474	decrease	DHRS1	ENSG00000157379	17198877;
D003474	decrease	DVL1	ENSG00000107404	19573523;
D003474	decrease	E2F5	ENSG00000133740	15713895;
D003474	decrease	EDIL3	ENSG00000164176	18421014;
D003474	decrease	EGFR	ENSG00000146648	15129424;10851300;18214481;17918158;18719366;15486348;
D003474	decrease	EIF1	ENSG00000173812	17999991;
D003474	decrease	ELF2	ENSG00000109381	15713895;
D003474	decrease	ENO1	ENSG00000074800	16880289;
D003474	decrease	EP300	ENSG00000100393	17927689;
D003474	decrease	EPO	ENSG00000130427	16880289;
D003474	decrease	ERBB2	ENSG00000141736	17041101;17918158;18719366;
D003474	decrease	ERBB3	ENSG00000065361	17918158;15713895;
D003474	decrease	ERCC1	ENSG00000012061	17596214;
D003474	decrease	ERN1	ENSG00000178607	17999991;
D003474	decrease	ESR1	ENSG00000091831	15899841;18347134;
D003474	decrease	ETS2	ENSG00000157557	17332326;
D003474	decrease	FAM120B	ENSG00000112584	17999991;
D003474	decrease	FASLG	ENSG00000117560	16101141;
D003474	decrease	FGFR2	ENSG00000066468	15713895;
D003474	decrease	FLT1	ENSG00000102755	18593936;
D003474	decrease	FOXF2	ENSG00000137273	15713895;
D003474	decrease	FZD1	ENSG00000157240	15713895;
D003474	decrease	GPER	ENSG00000164850	17198877;
D003474	decrease	H1FX	ENSG00000184897	17198877;
D003474	decrease	HBS1L	ENSG00000112339	18421014;
D003474	decrease	HDAC1	ENSG00000116478	17927689;18161303;
D003474	decrease	HDAC2	ENSG00000196591	18421014;
D003474	decrease	HDAC3	ENSG00000171720	17927689;
D003474	decrease	HIF1A	ENSG00000100644	16880289;18682687;
D003474	decrease	IDO1	ENSG00000131203	18348204;
D003474	decrease	IFNK	ENSG00000147896	18421014;
D003474	decrease	IGF1R	ENSG00000140443	17499312;17918158;
D003474	decrease	IGFBP3	ENSG00000146674	16101141;17499312;
D003474	decrease	IGLL1	ENSG00000128322	17198877;
D003474	decrease	IKBKB	ENSG00000104365	16023083;16219905;17965732;
D003474	decrease	IL2	ENSG00000109471	19761891;
D003474	decrease	INSIG2	ENSG00000125629	18704882;
D003474	decrease	JAK2	ENSG00000096968	16959222;
D003474	decrease	JSRP1	ENSG00000167476	17198877;
D003474	decrease	KCNMB2	ENSG00000197584	17198877;
D003474	decrease	KLF10	ENSG00000155090	17999991;
D003474	decrease	KLHL24	ENSG00000114796	17198877;
D003474	decrease	KLK2	ENSG00000167751	18719366;
D003474	decrease	KRAS	ENSG00000133703	17041101;
D003474	decrease	KRTAP2-1	ENSG00000212725	17999991;
D003474	decrease	LRMP	ENSG00000118308	17198877;
D003474	decrease	MAP2K4	ENSG00000065559	15713895;

D003474	decrease	MAPK1	ENSG00000100030	18357586;11322385;19074641;18214481;18555241;18316600;
D003474	decrease	MAPK3	ENSG00000102882	18357586;11322385;19074641;18214481;18555241;18316600;
D003474	decrease	MCM7	ENSG00000166508	17198877;
D003474	decrease	MDM2	ENSG00000135679	17332326;
D003474	decrease	MFI2	ENSG00000163975	17198877;
D003474	decrease	MKI67	ENSG00000148773	19372569;18226269;
D003474	decrease	MME	ENSG00000196549	17198877;
D003474	decrease	MMP14	ENSG00000157227	18495463;
D003474	decrease	MMP2	ENSG00000087245	19189304;18226269;17996675;18495463;
D003474	decrease	MPHOSPH6	ENSG00000135698	18421014;
D003474	decrease	MVD	ENSG00000167508	17198877;
D003474	decrease	MXD3	ENSG00000213347	17198877;15713895;
D003474	decrease	NAIP	ENSG00000250628	19250217;17671737;15911101;
D003474	decrease	NANOS1	ENSG00000188613	17999991;
D003474	decrease	NFKB1	ENSG00000109320	17041101;16219905;17399992;16243823;15489888;17640567;17596214;16173963;16023083;18593936;18555241;16299251;
D003474	decrease	NFYA	ENSG00000001167	15713895;
D003474	decrease	NHLRC2	ENSG00000196865	17999991;
D003474	decrease	NKX3-1	ENSG00000167034	17303007;18719366;
D003474	decrease	NOTCH1	ENSG00000148400	17927689;18640131;
D003474	decrease	NPC2	ENSG00000119655	17198877;
D003474	decrease	NR4A2	ENSG00000153234	17999991;
D003474	decrease	OTUD7B	ENSG00000163113	18421014;
D003474	decrease	PCK1	ENSG00000124253	17198877;
D003474	decrease	PFKFB4	ENSG00000114268	17198877;
D003474	decrease	PHB	ENSG00000167085	19137819;
D003474	decrease	PMEPA1	ENSG00000124225	18719366;
D003474	decrease	POLB	ENSG00000070501	19401701;
D003474	decrease	PPARD	ENSG00000112033	19294764;
D003474	decrease	PPARG	ENSG00000132170	15713005;15486348;17965732;
D003474	decrease	PPP3CC	ENSG00000120910	17198877;
D003474	decrease	PRKDC	ENSG00000121031	17596214;
D003474	decrease	PTPN13	ENSG00000163629	15713895;
D003474	decrease	RAMP1	ENSG00000132329	17198877;
D003474	decrease	RFXAP	ENSG00000133111	17999991;
D003474	decrease	RGS7	ENSG00000182901	17999991;
D003474	decrease	RIMS2	ENSG00000176406	17999991;
D003474	decrease	RUNX2	ENSG00000250096	15129424;
D003474	decrease	SCRN2	ENSG00000141295	17198877;
D003474	decrease	SDHA	ENSG00000073578	17198877;
D003474	decrease	SFRP5	ENSG00000120057	16101141;
D003474	decrease	SFRS5	ENSG00000100650	17999991;
D003474	decrease	SFXN1	ENSG00000164466	18421014;
D003474	decrease	SLC37A4	ENSG00000137700	16777101;
D003474	decrease	SNAI2	ENSG00000019549	19573523;

D003474	decrease	SP1	ENSG00000185591	18347134;18593936;
D003474	decrease	SP3	ENSG00000172845	18593936;
D003474	decrease	SP4	ENSG00000105866	18593936;
D003474	decrease	SRC	ENSG00000197122	16959222;
D003474	decrease	STAT5A	ENSG00000126561	16959222;
D003474	decrease	STAT5B	ENSG00000173757	16959222;
D003474	decrease	STS	ENSG00000101846	17198877;
D003474	decrease	SUZ12	ENSG00000178691	18421014;
D003474	decrease	TBC1D1	ENSG00000065882	17198877;
D003474	decrease	TIMP1	ENSG00000102265	17996675;
D003474	decrease	TIMP2	ENSG00000035862	17996675;
D003474	decrease	TMPRSS2	ENSG00000184012	18719366;
D003474	decrease	TOP2A	ENSG00000131747	18348204;18414057;
D003474	decrease	TRADD	ENSG00000102871	15713895;
D003474	decrease	TREM1	ENSG00000124731	17198877;
D003474	decrease	VHL	ENSG00000134086	18682687;
D003474	decrease	WT1	ENSG00000184937	19196508;18034345;
D003474	decrease	XRCC5	ENSG00000079246	17596214;
D003474	decrease	XRCC6	ENSG00000196419	17596214;
D003474	decrease	YPEL3	ENSG00000090238	17198877;
D003474	decrease	YWHAE	ENSG00000108953	19294764;
D003474	decrease	ZFHX3	ENSG00000140836	17198877;
D003474	decrease	ZFP36L1	ENSG00000185650	15713895;
D003474	decrease	ZNF589	ENSG00000164048	18421014;
D003474	increase	A4GALT	ENSG00000128274	16819191;
D003474	increase	ABCG1	ENSG00000160179	16713233;
D003474	increase	ABCG2	ENSG00000118777	17077187;
D003474	increase	ADAM17	ENSG00000151694	17666914;
D003474	increase	ADAMTS4	ENSG00000158859	18321735;
D003474	increase	AHR	ENSG00000106546	17077187;19018768;
D003474	increase	AKAP6	ENSG00000151320	18421014;
D003474	increase	APP	ENSG00000142192	18537544;18583042;
D003474	increase	AQP3	ENSG00000165272	18214481;
D003474	increase	AR	ENSG00000169083	18676361;18719366;12497104;
D003474	increase	ATF3	ENSG00000162772	18719366;15713895;18555241;
D003474	increase	B3GALT6	ENSG00000176022	17198877;
D003474	increase	BACE1	ENSG00000186318	18583042;
D003474	increase	BAD	ENSG00000002330	19235267;
D003474	increase	BAK1	ENSG00000030110	17332930;18226269;
D003474	increase	BAX	ENSG00000087088	17332930;19235267;17671742;17096185;18226269;
D003474	increase	BBC3	ENSG00000105327	17332930;
D003474	increase	BCL2A1	ENSG00000140379	16219905;17596214;
D003474	increase	BCL2	ENSG00000171791	19250217;17671737;16243823;16219905;17640567;17596214;17671742;17332930;19235267;19372569;18226269;16023083;18593936;17096185;12497104;186401
D003474	increase	BCL2L11	ENSG00000153094	17332930;

D003474	increase	BCL2L1	ENSG00000171552	19250217;17671737;16243823;16219905;17640567;15911101;19235267;17596214;19372569;17332930;18226269;16173963;16023083;17148446;12497104;186401
D003474	increase	BCL2L2	ENSG00000129473	16101141;
D003474	increase	BCOR	ENSG00000183337	17198877;
D003474	increase	BIRC2	ENSG00000110330	17671737;16243823;16219905;17640567;17596214;16023083;
D003474	increase	BIRC3	ENSG00000023445	16243823;16219905;17596214;19372569;15911101;16101141;
D003474	increase	BIRC7	ENSG00000101197	15911101;
D003474	increase	CASP1	ENSG00000137752	16101141;
D003474	increase	CASP2	ENSG00000106144	16101141;
D003474	increase	CASP3	ENSG00000164305	17332930;17041101;17148446;19294764;19235267;17640567;17671742;17096185;17363495;19401701;16023083;16101141;18555241;
D003474	increase	CASP4	ENSG00000196954	16101141;
D003474	increase	CASP9	ENSG00000132906	16101141;17148446;17671742;14555224;16023083;18555241;
D003474	increase	CCL2	ENSG00000108691	17666914;17198877;
D003474	increase	CCND1	ENSG00000110092	17041101;16243823;16219905;15489888;17640567;19189304;15486348;19372569;16959222;17148446;19573523;18226269;16106398;16023083;18593936;18790744;18316600;18156803;186[illegible]
D003474	increase	CCPG1	ENSG00000214882	17198877;
D003474	increase	CD14	ENSG00000170458	18180316;
D003474	increase	CD44	ENSG00000026508	15713895;
D003474	increase	CDC14B	ENSG00000081377	17198877;
D003474	increase	CDH1	ENSG00000039068	19573523;
D003474	increase	CDKN1A	ENSG00000124762	17332326;18593936;17148446;18316600;18161303;15713895;18226269;1815680
D003474	increase	CDKN1B	ENSG00000111276	18593936;15713895;18226269;18156803;
D003474	increase	CDKN2A	ENSG00000147889	17148446;18156803;
D003474	increase	CFLAR	ENSG00000003402	16219905;16173963;
D003474	increase	COL2A1	ENSG00000139219	18321735;
D003474	increase	CXCL12	ENSG00000107562	18660423;
D003474	increase	CXCR4	ENSG00000121966	18660423;17198877;
D003474	increase	CYP1A1	ENSG00000140465	19018768;17012224;15841493;11454723;
D003474	increase	CYP1B1	ENSG00000138061	19018768;17637178;
D003474	increase	CYP3A4	ENSG00000160868	17270371;17965521;17210444;
D003474	increase	CYTH4	ENSG00000100055	17198877;
D003474	increase	DAXX	ENSG00000231617	16101141;
D003474	increase	DDIT3	ENSG00000175197	17171638;16613838;18719366;

D003474	increase	DNAJB9	ENSG00000128590	17999991;
D003474	increase	DUSP10	ENSG00000143507	15713895;17151092;
D003474	increase	DUSP1	ENSG00000120129	15713895;
D003474	increase	DUSP5	ENSG00000138166	17198877;
D003474	increase	DYNLL1	ENSG00000088986	17198877;
D003474	increase	EGF	ENSG00000138798	18214481;15129424;10851300;15486348;
D003474	increase	EGR1	ENSG00000120738	17999991;17198877;18316600;
D003474	increase	FDPS	ENSG00000160752	16713233;
D003474	increase	FN1	ENSG00000115414	12388107;18555241;
D003474	increase	FNTB	ENSG00000125954	17999991;
D003474	increase	FOS	ENSG00000170345	17999991;15713895;
D003474	increase	FOSL1	ENSG00000175592	17148446;
D003474	increase	FOSL2	ENSG00000075426	17148446;17999991;
D003474	increase	FUS	ENSG00000089280	17999991;17198877;
D003474	increase	GADD45A	ENSG00000116717	18719366;16101141;
D003474	increase	GCLM	ENSG00000023909	19188863;17999991;17198877;
D003474	increase	GSK3B	ENSG00000082701	18790744;19573523;
D003474	increase	GSTP1	ENSG00000084207	9463521;15999103;14555224;17449203;18414057;16101141;14742295;
D003474	increase	HIST1H2BD	ENSG00000158373	17999991;
D003474	increase	HIST1H4J	ENSG00000182217	15713895;
D003474	increase	HIST2H4B	ENSG00000182217	17198877;
D003474	increase	HIST3H2BB	ENSG00000196890	15713895;
D003474	increase	HMGCR	ENSG00000113161	16713233;
D003474	increase	HMOX1	ENSG00000100292	18357586;17464175;19188863;17999991;18719366;14742295;
D003474	increase	HOXB5	ENSG00000120075	18421014;
D003474	increase	HPRT1	ENSG00000165704	16101141;
D003474	increase	HSPA4	ENSG00000170606	11322385;
D003474	increase	ICAM1	ENSG00000090339	16243823;17666914;16219905;
D003474	increase	IFNA1	ENSG00000197919	17979888;
D003474	increase	IFNAR1	ENSG00000142166	17979888;
D003474	increase	IFNAR2	ENSG00000159110	17979888;
D003474	increase	IFNB1	ENSG00000171855	17979888;
D003474	increase	IFNG	ENSG00000111537	16959222;
D003474	increase	IL10	ENSG00000136634	17979888;
D003474	increase	IL18	ENSG00000150782	16368150;17399992;
D003474	increase	IL1B	ENSG00000125538	16819191;17291458;17273796;17151092;
D003474	increase	IL6	ENSG00000136244	19074641;18676361;18357586;17273796;17151092;17999991;18025290;
D003474	increase	IL8	ENSG00000169429	18413660;19074641;17000667;16819191;17273796;17666914;17151092;18226269;12216086;
D003474	increase	ITGB1	ENSG00000150093	17291458;
D003474	increase	IVL	ENSG00000163207	17148446;
D003474	increase	JUNB	ENSG00000171223	17148446;
D003474	increase	JUND	ENSG00000130522	17148446;

D003474	increase	JUN	ENSG00000177606	17148446;18628248;17596214;15713895;
D003474	increase	KLF6	ENSG00000067082	15713895;
D003474	increase	KLHL21	ENSG00000162413	17198877;
D003474	increase	KLK3	ENSG00000142515	18676361;18719366;12497104;
D003474	increase	KYNU	ENSG00000115919	18421014;
D003474	increase	LCP2	ENSG00000043462	17198877;
D003474	increase	LDLR	ENSG00000130164	18704882;16963807;16713233;
D003474	increase	LRRN3	ENSG00000173114	17198877;
D003474	increase	LTA	ENSG00000226979	16101141;17198877;
D003474	increase	LTB	ENSG00000236237	17198877;
D003474	increase	MAP2K3	ENSG00000034152	15713895;
D003474	increase	MAP3K1	ENSG00000095015	17148446;15713895;
D003474	increase	MAPK8	ENSG00000107643	16101141;18316600;
D003474	increase	MFNG	ENSG00000100060	17198877;
D003474	increase	MGMT	ENSG00000170430	16950796;17596214;
D003474	increase	MMP10	ENSG00000166670	15713895;
D003474	increase	MMP3	ENSG00000149968	18321735;15713895;
D003474	increase	MMP7	ENSG00000137673	17928719;
D003474	increase	MMP9	ENSG00000100985	17291458;16243823;16219905;17640567;18628248;19189304;17996675;18226269;18495463;
D003474	increase	MRPL1	ENSG00000169288	17198877;
D003474	increase	MS4A1	ENSG00000156738	17198877;18421014;
D003474	increase	MST1R	ENSG00000164078	18593918;
D003474	increase	MXD1	ENSG00000059728	15713895;
D003474	increase	MYC	ENSG00000136997	17041101;16243823;16219905;15911101;15713895;
D003474	increase	MYL12A	ENSG00000101608	17999991;
D003474	increase	NFE2L2	ENSG00000116044	17449203;
D003474	increase	NFKBIA	ENSG00000100906	18413660;16819191;17291458;17927689;16243823;15489888;17666914;16219905;16023083;17289836;16106398;12216086;17999991;1
D003474	increase	NME1	ENSG00000239672	17198877;
D003474	increase	NOS2	ENSG00000007171	17640567;
D003474	increase	NQO1	ENSG00000181019	19188863;17198877;
D003474	increase	NR1H3	ENSG00000025434	16713233;
D003474	increase	NUP98	ENSG00000110713	17198877;
D003474	increase	ODC1	ENSG00000115758	17198877;
D003474	increase	PCNA	ENSG00000132646	18226269;16101141;
D003474	increase	PER2	ENSG00000132326	17198877;
D003474	increase	PLAU	ENSG00000122861	18226269;18495463;18025290;
D003474	increase	PLAUR	ENSG00000011422	15713895;
D003474	increase	PLEKHH2	ENSG00000152527	18421014;
D003474	increase	PMAIP1	ENSG00000141682	17332930;
D003474	increase	PPIF	ENSG00000108179	17198877;
D003474	increase	PRKCD	ENSG00000163932	17171638;17148446;
D003474	increase	PRKCE	ENSG00000171132	17148446;14742295;
D003474	increase	PRKCH	ENSG00000027075	17148446;
D003474	increase	PRLR	ENSG00000113494	18421014;

D003474	increase	PTGS2	ENSG00000073756	19250217;17671737;17291458;16243823;16219905;15489888;17640567;15129424;17363495;18596194;17151092;17918158;18348204;17999991;15911101;17671742;16340194;18226269;158[illegible]
D003474	increase	PTPN6	ENSG00000111679	17198877;
D003474	increase	PTPN7	ENSG00000143851	17198877;
D003474	increase	RARA	ENSG00000131759	8940178;
D003474	increase	RASSF2	ENSG00000101265	17198877;
D003474	increase	RBM39	ENSG00000131051	17999991;
D003474	increase	RELA	ENSG00000173039	16219905;12216086;18413660;19074641;15911101;12388107;16819191;17291458;17399992;17927689;16243823;18593918;17666914;15489888;17640567;17273796;18226269;16173963;16023083;17999991;18593936;18555241;19372569;18640[illegible]
D003474	increase	REV3L	ENSG00000009413	19137819;
D003474	increase	RGS16	ENSG00000143333	17198877;
D003474	increase	RPL31	ENSG00000071082	17999991;
D003474	increase	7-Sep	ENSG00000122545	16101141;
D003474	increase	SERPINB2	ENSG00000197632	15713895;
D003474	increase	SERPINB5	ENSG00000206075	17290611;
D003474	increase	SERPINE1	ENSG00000106366	15713895;
D003474	increase	SFPQ	ENSG00000116560	17198877;
D003474	increase	SFRS1	ENSG00000136450	17999991;
D003474	increase	SFRS2	ENSG00000161547	17198877;
D003474	increase	SFRS3	ENSG00000112081	17198877;
D003474	increase	SFRS6	ENSG00000124193	17198877;
D003474	increase	SLC30A1	ENSG00000170385	17999991;
D003474	increase	SLC3A2	ENSG00000168003	17198877;
D003474	increase	SLC48A1	ENSG00000211584	17198877;
D003474	increase	SLC7A11	ENSG00000151012	17999991;
D003474	increase	SLCO1A2	ENSG00000084453	18421014;
D003474	increase	SMN2	ENSG00000172062	17962980;
D003474	increase	SPIB	ENSG00000142539	17198877;
D003474	increase	STAT1	ENSG00000115415	16959222;
D003474	increase	STAT3	ENSG00000168610	16959222;17666914;
D003474	increase	SYCP2	ENSG00000196074	18421014;
D003474	increase	TBC1D8	ENSG00000204634	17999991;
D003474	increase	TF	ENSG00000091513	15713895;
D003474	increase	TFRC	ENSG00000072274	17198877;
D003474	increase	TGFB1	ENSG00000105329	8940178;
D003474	increase	TIAF1	ENSG00000221995	18421014;
D003474	increase	TNF	ENSG00000223952	18357586;17927689;17666914;16219905;16819191;17273796;15489888;17640567;14555224;17291458;17151092;16023083;17198877;
D003474	increase	TNFRSF10A	ENSG00000104689	18226269;
D003474	increase	TNFRSF10B	ENSG00000120889	16101141;18226269;16613838;

D003474	increase	TNFRSF1A	ENSG00000067182	17666914;
D003474	increase	TNFRSF1B	ENSG00000028137	17666914;
D003474	increase	TNFSF10	ENSG00000121858	15713895;16101141;18226269;
D003474	increase	TNFSF11	ENSG00000120659	15129424;
D003474	increase	TRAF1	ENSG00000056558	16219905;19372569;16023083;
D003474	increase	TRAF2	ENSG00000127191	16101141;
D003474	increase	TRAF3	ENSG00000131323	16101141;
D003474	increase	TRAF6	ENSG00000175104	16101141;
D003474	increase	TRIB3	ENSG00000101255	18719366;
D003474	increase	TRIM16	ENSG00000221926	17999991;
D003474	increase	UBC	ENSG00000150991	16101141;
D003474	increase	UGT1A1	ENSG00000241635	16819192;14557274;
D003474	increase	UGT1A6	ENSG00000167165	16819192;
D003474	increase	UTP14A	ENSG00000156697	17198877;
D003474	increase	VASP	ENSG00000125753	17198877;
D003474	increase	VEGFA	ENSG00000112715	17960570;16368150;16243823;16219905;18596194;19294764;18390174;19372569;18593936;18226269;
D003474	increase	VIP	ENSG00000146469	19189304;
D003474	increase	XIAP	ENSG00000101966	19250217;17671737;16243823;16219905;17671742;17596214;19372569;16023083;16173963;16106398;1591
D003474	increase	ZNF287	ENSG00000141040	18421014;
D003474	increase	ZNF823	ENSG00000197933	17999991;
D003513	affect	ADI1	ENSG00000182551	17786183;
D003513	affect	BTG2	ENSG00000159388	17234770;
D003513	affect	CASP9	ENSG00000132906	16166294;
D003513	affect	CD1D	ENSG00000158473	17392484;
D003513	affect	CLMN	ENSG00000165959	16688769;
D003513	affect	EFNB2	ENSG00000125266	16688769;
D003513	affect	FARP1	ENSG00000152767	16849584;
D003513	affect	GDF15	ENSG00000130513	18801729;
D003513	affect	IFNA1	ENSG00000197919	15937643;
D003513	affect	IFNB1	ENSG00000171855	15937643;
D003513	affect	MMP13	ENSG00000137745	15138554;
D003513	affect	MN1	ENSG00000169184	15890672;
D003513	affect	NAV2	ENSG00000166833	16688769;
D003513	affect	NEDD9	ENSG00000111859	16688769;
D003513	affect	PTPRG	ENSG00000144724	16849584;
D003513	affect	RAB30	ENSG00000137502	16849584;
D003513	affect	TRIM16	ENSG00000221926	16636064;
D003513	affect	TRPV6	ENSG00000165125	16362534;
D003513	affect	TYMP	ENSG00000025708	15937643;
D003513	decrease	ABCB4	ENSG00000005471	11331069;
D003513	decrease	ABCC5	ENSG00000114770	15072547;
D003513	decrease	AHR	ENSG00000106546	15385644;
D003513	decrease	BRCA1	ENSG00000012048	10344722;
D003513	decrease	BRCA2	ENSG00000139618	10344722;
D003513	decrease	CDK2	ENSG00000123374	12800980;
D003513	decrease	CFLAR	ENSG00000003402	16077199;12663669;
D003513	decrease	CHRM1	ENSG00000168539	17624924;

D003513	decrease	ERBB2	ENSG00000141736	15228094;15072547;
D003513	decrease	ERBB3	ENSG00000065361	15228094;
D003513	decrease	FASN	ENSG00000169710	12798352;
D003513	decrease	NFKB1	ENSG00000109320	16637064;
D003513	decrease	NFKBIA	ENSG00000100906	16637064;
D003513	decrease	RELA	ENSG00000173039	16637064;
D003513	decrease	TYMS	ENSG00000176890	17172411;
D003513	decrease	UGT1A1	ENSG00000241635	15072547;
D003513	increase	ABCC3	ENSG00000108846	15072547;
D003513	increase	ADAM9	ENSG00000168615	17018608;
D003513	increase	AREG	ENSG00000205595	11237771;15228094;15072547;
D003513	increase	ATF4	ENSG00000128272	18533110;
D003513	increase	BECN1	ENSG00000126581	16882451;
D003513	increase	CASP7	ENSG00000165806	16166294;
D003513	increase	CCNA2	ENSG00000145386	15072547;
D003513	increase	CCND1	ENSG00000110092	17606477;18058799;
D003513	increase	CYP1A1	ENSG00000140465	15385644;
D003513	increase	EGFR	ENSG00000146648	15228094;
D003513	increase	EREG	ENSG00000124882	15228094;
D003513	increase	FOS	ENSG00000170345	15090535;
D003513	increase	HBEGF	ENSG00000113070	15228094;
D003513	increase	HMOX1	ENSG00000100292	18357586;
D003513	increase	ICAM1	ENSG00000090339	16087364;
D003513	increase	IER3	ENSG00000137331	16849584;
D003513	increase	IFNG	ENSG00000111537	15937643;16087364;
D003513	increase	IGFBP4	ENSG00000141753	15072547;
D003513	increase	IL8	ENSG00000169429	17606477;18308354;
D003513	increase	IRS1	ENSG00000169047	15072547;
D003513	increase	ITPR1	ENSG00000150995	17241155;
D003513	increase	KRT13	ENSG00000171401	16849584;
D003513	increase	MSR1	ENSG00000038945	9614211;
D003513	increase	MYLK	ENSG00000251351	15701621;
D003513	increase	NOV	ENSG00000136999	15072547;
D003513	increase	PMAIP1	ENSG00000141682	17216584;
D003513	increase	PTGS2	ENSG00000073756	16894348;
D003513	increase	SAT1	ENSG00000130066	16637064;
D003513	increase	SERPINB9	ENSG00000170542	10681578;
D003513	increase	SLC5A5	ENSG00000105641	17164311;
D003513	increase	SMN1	ENSG00000172062	17064354;
D003513	increase	SOCS1	ENSG00000185338	16849584;
D003513	increase	SULT1A1	ENSG00000196502	16308312;
D003513	increase	TACC1	ENSG00000147526	15072547;
D003513	increase	TFF1	ENSG00000160182	15072547;
D003513	increase	TGFA	ENSG00000163235	15228094;
D003513	increase	TJP1	ENSG00000104067	16087364;
D003513	increase	TNF	ENSG00000223952	15701621;16077199;
D003513	increase	TP53	ENSG00000141510	15016801;
D003513	increase	UGT2B15	ENSG00000196620	16690804;
D003517	increase	IL4	ENSG00000113520	12112628;
D003520	affect	BAG1	ENSG00000250477	16322899;
D003520	affect	BUB3	ENSG00000154473	16322899;
D003520	affect	CDKN1B	ENSG00000111276	16322899;
D003520	affect	CTNNBIP1	ENSG00000178585	16322899;

D003520	affect	EIF1AX	ENSG00000173674	16322899;
D003520	affect	EIF4EBP1	ENSG00000187840	16322899;
D003520	affect	GTF3C1	ENSG00000077235	16322899;
D003520	affect	ILF3	ENSG00000129351	16322899;
D003520	affect	IRS1	ENSG00000169047	16322899;
D003520	affect	MAPK14	ENSG00000112062	16322899;
D003520	affect	NAIP	ENSG00000250628	16322899;
D003520	affect	PLOD1	ENSG00000083444	16322899;
D003520	affect	PLOD3	ENSG00000106397	16322899;
D003520	affect	SRM	ENSG00000116649	16322899;
D003520	affect	ST14	ENSG00000149418	16322899;
D003520	affect	STK39	ENSG00000198648	16322899;
D003520	decrease	ACTB	ENSG00000075624	12167460;
D003520	decrease	ALOX5AP	ENSG00000132965	17403535;
D003520	decrease	ATF5	ENSG00000169136	17403535;
D003520	decrease	BIK	ENSG00000100290	17403535;
D003520	decrease	BPI	ENSG00000101425	17403535;
D003520	decrease	C13ORF27	ENSG00000151287	17403535;
D003520	decrease	CA2	ENSG00000104267	17403535;
D003520	decrease	CASP1	ENSG00000137752	17403535;
D003520	decrease	CCR8	ENSG00000179934	17403535;
D003520	decrease	COX7A2	ENSG00000112695	17403535;
D003520	decrease	CST7	ENSG00000077984	17403535;
D003520	decrease	CYP1B1	ENSG00000138061	12167460;
D003520	decrease	CYP2B6	ENSG00000197408	18496131;17502835;15248218;12498089;11389073;16183265;12872138;15769884;18212249;18633247;10919648;16322899;
D003520	decrease	EPB41L2	ENSG00000079819	17403535;
D003520	decrease	F3	ENSG00000117525	17403535;
D003520	decrease	ICAM3	ENSG00000076662	17403535;
D003520	decrease	KBTBD11	ENSG00000176595	17403535;
D003520	decrease	MDFIC	ENSG00000135272	17403535;
D003520	decrease	MGMT	ENSG00000170430	17403535;15741301;
D003520	decrease	MS4A3	ENSG00000149516	17403535;
D003520	decrease	MX2	ENSG00000183486	17403535;
D003520	decrease	P2RY2	ENSG00000175591	17403535;
D003520	decrease	PRTN3	ENSG00000196415	17403535;
D003520	decrease	RNASE2	ENSG00000169385	17403535;
D003520	decrease	RRAGA	ENSG00000155876	17403535;
D003520	decrease	S100P	ENSG00000163993	17403535;16217747;
D003520	increase	ALDH1A1	ENSG00000165092	17502835;18496131;12513786;17403535;
D003520	increase	ALDH2	ENSG00000111275	17403535;
D003520	increase	ANGPT1	ENSG00000154188	17403535;
D003520	increase	BCL2	ENSG00000171791	15940066;11723234;14503796;16322899;
D003520	increase	CASP3	ENSG00000164305	11723234;17020986;14503796;16675587;
D003520	increase	CCIN	ENSG00000185972	17475930;
D003520	increase	CTSH	ENSG00000103811	17403535;
D003520	increase	DLC1	ENSG00000164741	17403535;
D003520	increase	DNAJB6	ENSG00000105993	17475930;
D003520	increase	DZIP1	ENSG00000134874	17403535;

D003520	increase	ERG	ENSG00000157554	17403535;
D003520	increase	ESR1	ENSG00000091831	16322899;
D003520	increase	ESR2	ENSG00000140009	16322899;
D003520	increase	FKBPL	ENSG00000223666	14503796;
D003520	increase	FSCN1	ENSG00000075618	17403535;
D003520	increase	FYN	ENSG00000010810	17403535;
D003520	increase	GAS2	ENSG00000148935	17475930;
D003520	increase	GLRB	ENSG00000109738	17403535;
D003520	increase	GNG12	ENSG00000172380	17403535;
D003520	increase	HNRNPK	ENSG00000165119	17475930;
D003520	increase	LGALS1	ENSG00000100097	17403535;
D003520	increase	MAOA	ENSG00000189221	17403535;
D003520	increase	MARCKS	ENSG00000155130	17403535;
D003520	increase	PALM2-AKAP2	ENSG00000157654	17403535;
D003520	increase	PHLDA2	ENSG00000181649	17403535;
D003520	increase	PSMA5	ENSG00000143106	17475930;
D003520	increase	RAMP1	ENSG00000132329	17403535;16896004;
D003520	increase	RBPMS	ENSG00000157110	17403535;
D003520	increase	RRAS2	ENSG00000133818	17403535;
D003520	increase	RTEL1	ENSG00000026036	17475930;
D003520	increase	SPARC	ENSG00000113140	17403535;
D003520	increase	TM4SF1	ENSG00000169908	17403535;
D003520	increase	TYMP	ENSG00000025708	15150550;
D003545	decrease	CCND1	ENSG00000110092	15890017;
D003545	decrease	CDKN1B	ENSG00000111276	15890017;
D003561	decrease	C13ORF34	ENSG00000136122	17374387;
D003561	decrease	CDCA8	ENSG00000134690	17374387;
D003561	decrease	CKS2	ENSG00000123975	17374387;
D003561	decrease	DALRD3	ENSG00000178149	19194470;
D003561	decrease	DBN1	ENSG00000113758	19194470;
D003561	decrease	DUSP4	ENSG00000120875	19194470;
D003561	decrease	HIST1H4C	ENSG00000182217	19194470;
D003561	decrease	KPNA2	ENSG00000182481	17374387;
D003561	decrease	MED9	ENSG00000141026	19194470;
D003561	decrease	PARD6A	ENSG00000102981	19194470;
D003561	decrease	PDE6D	ENSG00000156973	19194470;
D003561	decrease	PRC1	ENSG00000198901	17374387;
D003561	decrease	PSRC1	ENSG00000134222	17374387;
D003561	decrease	PSTPIP2	ENSG00000152229	19194470;
D003561	decrease	RABGGTB	ENSG00000137955	19194470;
D003561	decrease	RAD23A	ENSG00000179262	19194470;
D003561	decrease	RBM34	ENSG00000188739	19194470;
D003561	decrease	RECQL5	ENSG00000108469	19194470;
D003561	decrease	SLITRK5	ENSG00000165300	19194470;
D003561	decrease	THY1	ENSG00000154096	19194470;
D003561	decrease	TK1	ENSG00000167900	12893260;
D003561	decrease	TK2	ENSG00000166548	12893260;
D003561	decrease	UBE2C	ENSG00000175063	17374387;
D003561	decrease	USP7	ENSG00000187555	19194470;
D003561	increase	ACAP1	ENSG00000072818	19194470;
D003561	increase	APOBEC3B	ENSG00000179750	19194470;
D003561	increase	ARL6IP5	ENSG00000144746	16430862;
D003561	increase	ATP10D	ENSG00000145246	19194470;

D003561	increase	BIN2	ENSG00000110934	19194470;
D003561	increase	BRCA2	ENSG00000139618	19194470;
D003561	increase	C3AR1	ENSG00000171860	19194470;
D003561	increase	CD8A	ENSG00000153563	19194470;
D003561	increase	CDKN1A	ENSG00000124762	17977830;
D003561	increase	CXCR4	ENSG00000121966	19194470;
D003561	increase	CYTH1	ENSG00000108669	19194470;
D003561	increase	DENND1A	ENSG00000119522	19194470;
D003561	increase	DHFR	ENSG00000228716	19194470;
D003561	increase	ENTPD1	ENSG00000138185	19194470;
D003561	increase	FAM111A	ENSG00000166801	19194470;
D003561	increase	FCGR2A	ENSG00000143226	19194470;
D003561	increase	FCGR2C	ENSG00000244682	19194470;
D003561	increase	GRK5	ENSG00000198873	19194470;
D003561	increase	GSTCD	ENSG00000138780	19194470;
D003561	increase	GTPBP1	ENSG00000100226	19194470;
D003561	increase	HSPA6	ENSG00000173110	19194470;
D003561	increase	LY75	ENSG00000054219	19194470;
D003561	increase	LYZ	ENSG00000090382	19194470;
D003561	increase	NDC80	ENSG00000080986	19194470;
D003561	increase	PDLIM4	ENSG00000131435	17634552;
D003561	increase	RAB5B	ENSG00000111540	19194470;
D003561	increase	RAD51	ENSG00000051180	19194470;
D003561	increase	RNASE2	ENSG00000169385	19194470;
D003561	increase	RRM2	ENSG00000171848	19194470;
D003561	increase	S100P	ENSG00000163993	19194470;
D003561	increase	SERPINA1	ENSG00000197249	19194470;
D003561	increase	SLA	ENSG00000155926	19194470;
D003561	increase	SLC43A3	ENSG00000134802	19194470;
D003561	increase	SRGN	ENSG00000122862	19194470;
D003561	increase	TFE3	ENSG00000068323	19194470;
D003561	increase	TNFSF10	ENSG00000121858	19194470;
D003561	increase	TP53	ENSG00000141510	17977830;12082016;19628630;
D003561	increase	UBL3	ENSG00000122042	19194470;
D003561	increase	VEZF1	ENSG00000136451	19194470;
D003561	increase	XRCC1	ENSG00000073050	19194470;
D003561	increase	ZNF323	ENSG00000235109	19194470;
D003562	increase	GYPA	ENSG00000170180	12181422;
D003606	increase	BIRC5	ENSG00000089685	15577328;
D003606	increase	CD38	ENSG00000004468	16078447;11583285;14669796;
D003606	increase	CD69	ENSG00000110848	17973783;14669796;
D003606	increase	IFNA1	ENSG00000197919	16078447;11583285;
D003606	increase	IFNA2	ENSG00000188379	17973783;14669796;
D003606	increase	IL8	ENSG00000169429	15123733;15026559;12939465;
D003606	increase	IRF1	ENSG00000125347	17973783;
D003606	increase	VEGFA	ENSG00000112715	15123733;12939465;15026559;
D003609	affect	CCND1	ENSG00000110092	17606477;
D003609	affect	LTC4S	ENSG00000213316	12574384;
D003609	decrease	CASP3	ENSG00000164305	16001973;
D003609	decrease	CASP7	ENSG00000165806	16001973;

D003609	decrease	CASP8	ENSG00000064012	16001973;
D003609	decrease	CDKN1A	ENSG00000124762	12807743;14601052;
D003609	decrease	CEBPB	ENSG00000172216	16001973;
D003609	decrease	CFLAR	ENSG00000003402	14601052;
D003609	decrease	FADD	ENSG00000168040	16001973;
D003609	decrease	LMNB1	ENSG00000113368	16001973;
D003609	decrease	MAP2K4	ENSG00000065559	16001973;
D003609	decrease	MAP3K7	ENSG00000135341	16001973;
D003609	decrease	MYC	ENSG00000136997	16001973;
D003609	decrease	NFKB1	ENSG00000109320	16001973;
D003609	decrease	SOS1	ENSG00000115904	16001973;
D003609	decrease	XIAP	ENSG00000101966	14601052;12430140;
D003609	increase	ADAM9	ENSG00000168615	17018608;
D003609	increase	AGT	ENSG00000135744	17214612;
D003609	increase	BCL2L1	ENSG00000171552	14601052;12430140;
D003609	increase	CD1D	ENSG00000158473	17392484;
D003609	increase	CD28	ENSG00000178562	11465111;
D003609	increase	CLMN	ENSG00000165959	16688769;
D003609	increase	CYP1A1	ENSG00000140465	11752201;
D003609	increase	CYP1B1	ENSG00000138061	11752201;
D003609	increase	CYP3A4	ENSG00000160868	15795092;
D003609	increase	DDAH2	ENSG00000225635	17977009;
D003609	increase	EFNB2	ENSG00000125266	16688769;
D003609	increase	GAPDH	ENSG00000111640	16001973;
D003609	increase	GDF15	ENSG00000130513	17257620;
D003609	increase	HMOX1	ENSG00000100292	18357586;8764571;
D003609	increase	ID1	ENSG00000125968	16494909;
D003609	increase	IER3	ENSG00000137331	16849584;
D003609	increase	IFNA1	ENSG00000197919	15937643;
D003609	increase	IFNB1	ENSG00000171855	15937643;
D003609	increase	IFNG	ENSG00000111537	15937643;16085646;
D003609	increase	IL1B	ENSG00000125538	12512699;16338976;16365456;
D003609	increase	IL8	ENSG00000169429	17606477;18308354;12039947;
D003609	increase	IRF1	ENSG00000125347	16085646;16636311;
D003609	increase	ITPR1	ENSG00000150995	17241155;
D003609	increase	MN1	ENSG00000169184	15890672;
D003609	increase	MYLK	ENSG00000251351	15701621;
D003609	increase	NAV2	ENSG00000166833	16688769;
D003609	increase	NEDD9	ENSG00000111859	16688769;
D003609	increase	PAPPA	ENSG00000182752	16338976;
D003609	increase	PLAT	ENSG00000104368	11236827;
D003609	increase	PLAU	ENSG00000122861	11236827;
D003609	increase	PTGS2	ENSG00000073756	16894348;
D003609	increase	ROS1	ENSG00000047936	16001973;
D003609	increase	SAT1	ENSG00000130066	16637064;
D003609	increase	STAT1	ENSG00000115415	16001973;
D003609	increase	SULT1A1	ENSG00000196502	16308312;
D003609	increase	TGFB1	ENSG00000105329	16365456;
D003609	increase	TNF	ENSG00000223952	16001973;15701621;16338976;
D003609	increase	TP53	ENSG00000141510	10531375;16001973;16024796;12869419;12807743;

D003609	increase	TRPV6	ENSG00000165125	16362534;
D003609	increase	TYMP	ENSG00000025708	15937643;
D003609	increase	VEGFA	ENSG00000112715	16386082;18790786;
D003613	increase	IL8	ENSG00000169429	15866594;
D003613	increase	TNF	ENSG00000223952	15866594;
D003630	decrease	CAB39	ENSG00000135932	17374387;
D003630	decrease	CEBPZ	ENSG00000115816	17374387;
D003630	decrease	MAP3K5	ENSG00000197442	17374387;
D003630	decrease	MAPT	ENSG00000186868	16930453;
D003630	decrease	NCK2	ENSG00000071051	17374387;
D003630	decrease	PRC1	ENSG00000198901	17374387;
D003630	increase	AGRN	ENSG00000188157	17374387;
D003630	increase	ANXA4	ENSG00000196975	17374387;
D003630	increase	CCNG2	ENSG00000138764	18754885;
D003630	increase	CEACAM1	ENSG00000079385	17374387;
D003630	increase	EI24	ENSG00000149547	17374387;
D003630	increase	EPM2AIP1	ENSG00000178567	17374387;
D003630	increase	FDXR	ENSG00000161513	17374387;
D003630	increase	MAP2K3	ENSG00000034152	17374387;
D003630	increase	PLK3	ENSG00000173846	17374387;
D003630	increase	SDC1	ENSG00000115884	17374387;
D003630	increase	TAP1	ENSG00000230705	17374387;
D003630	increase	TAX1BP3	ENSG00000213977	17374387;
D003630	increase	TNFSF9	ENSG00000125657	17374387;
D003630	increase	TP53I3	ENSG00000115129	17374387;
D003632	increase	ACVR1C	ENSG00000123612	19422813;
D003632	increase	ACVRL1	ENSG00000139567	19422813;
D003632	increase	ALK	ENSG00000171094	19422813;
D003632	increase	ERBB3	ENSG00000065361	19422813;
D003632	increase	KIT	ENSG00000157404	19422813;
D003632	increase	PDGFRA	ENSG00000134853	19422813;
D003632	increase	PDGFRB	ENSG00000113721	19422813;
D003633	increase	ABCB1	ENSG00000085563	15710169;
D003633	increase	ACVR1C	ENSG00000123612	19422813;
D003633	increase	ACVRL1	ENSG00000139567	19422813;
D003633	increase	ALK	ENSG00000171094	19422813;
D003633	increase	CYP19A1	ENSG00000137869	15070871;16077170;
D003633	increase	CYP1B1	ENSG00000138061	16040568;
D003633	increase	ERBB3	ENSG00000065361	19422813;
D003633	increase	IL4	ENSG00000113520	12696651;
D003633	increase	KIT	ENSG00000157404	19422813;
D003633	increase	PDGFRA	ENSG00000134853	19422813;
D003633	increase	PDGFRB	ENSG00000113721	19422813;
D003633	increase	PGR	ENSG00000082175	14579009;18262749;16859517;
D003634	decrease	GJA1	ENSG00000152661	8820588;
D003634	decrease	HSD17B1	ENSG00000108786	12858341;
D003634	decrease	IL2	ENSG00000109471	16696175;
D003634	decrease	MT2A	ENSG00000125148	12858341;
D003634	decrease	NFKB1	ENSG00000109320	16696175;
D003634	increase	ABCB1	ENSG00000085563	15710169;
D003634	increase	AHR	ENSG00000106546	17311112;
D003634	increase	CFTR	ENSG00000001626	14766015;
D003634	increase	CYP11A1	ENSG00000140459	12858341;

D003634	increase	CYP1A1	ENSG00000140465	17311112;
D003634	increase	CYP1B1	ENSG00000138061	17311112;
D003634	increase	CYP2B6	ENSG00000197408	15548381;
D003634	increase	CYP3A4	ENSG00000160868	17035600;15548381;
D003634	increase	ESR1	ENSG00000091831	12676605;15026083;16626760;15064155;11485867;16329587;
D003634	increase	GNRH1	ENSG00000147437	16329587;
D003634	increase	HSD3B1	ENSG00000203857	12858341;
D003634	increase	HSPA5	ENSG00000044574	15710169;
D003634	increase	IL4	ENSG00000113520	12696651;
D003634	increase	JUN	ENSG00000177606	14604893;
D003634	increase	PGR	ENSG00000082175	14579009;18262749;
D003634	increase	POMC	ENSG00000115138	16329587;
D003634	increase	TNF	ENSG00000223952	15603917;
D003676	decrease	CCNA2	ENSG00000145386	11468187;
D003676	decrease	CCNB1	ENSG00000134057	11468187;
D003676	decrease	CCND1	ENSG00000110092	11468187;
D003676	decrease	CCND2	ENSG00000118971	11468187;
D003676	decrease	CCND3	ENSG00000112576	11468187;
D003676	decrease	CDK2	ENSG00000123374	17631934;11468187;
D003676	increase	CCNE1	ENSG00000105173	11468187;
D003676	increase	CDC14A	ENSG00000079335	16760464;
D003676	increase	CDKN1A	ENSG00000124762	11468187;12807743;
D003676	increase	ELANE	ENSG00000197561	11919081;
D003676	increase	EPO	ENSG00000130427	10699462;
D003676	increase	GADD45A	ENSG00000116717	11468187;
D003676	increase	HIF1A	ENSG00000100644	16775626;15840558;17364964;16330433;
D003676	increase	HIF3A	ENSG00000124440	16775626;
D003676	increase	IL1B	ENSG00000125538	9851740;
D003676	increase	IL6	ENSG00000136244	9344885;
D003676	increase	IL8	ENSG00000169429	9344885;17097691;
D003676	increase	MSR1	ENSG00000038945	9614211;
D003676	increase	TFRC	ENSG00000072274	16760464;15993339;12807743;
D003676	increase	TNF	ENSG00000223952	9851740;
D003676	increase	TP53	ENSG00000141510	16357363;
D003676	increase	VEGFA	ENSG00000112715	15075239;
D003676	increase	WT1	ENSG00000184937	12738801;
D003683	increase	HMOX1	ENSG00000100292	19683516;
D003687	affect	AGR2	ENSG00000106541	17094431;
D003687	affect	DHCR24	ENSG00000116133	17094431;
D003687	affect	NTS	ENSG00000133636	17094431;
D003687	decrease	CYP17A1	ENSG00000148795	16030167;15562394;15844475;15625562;
D003687	decrease	RB1	ENSG00000139687	15752352;
D003687	increase	CDKN1A	ENSG00000124762	15752352;
D003687	increase	KLK3	ENSG00000142515	15994348;17503469;
D003687	increase	TP53	ENSG00000141510	15752352;
D003840	decrease	CYP7A1	ENSG00000167910	14684751;
D003840	increase	ABCB11	ENSG00000073734	14684751;
D003840	increase	NR1H4	ENSG00000012504	14684751;17567710;
D003891	decrease	HTR1A	ENSG00000178394	15885359;
D003891	decrease	IFNA1	ENSG00000197919	15885359;

D003891	decrease	NR3C1	ENSG00000113580	15885359;
D003902	increase	TRIM21	ENSG00000132109	9833039;
D003907	affect	HLA-DQB1	ENSG00000179344	12732289;
D003907	affect	IL10	ENSG00000136634	16357325;
D003907	affect	LYN	ENSG00000249529	12732289;
D003907	affect	NOG	ENSG00000183691	15268896;
D003907	affect	NR1I2	ENSG00000144852	16054614;12695351;12065438;
D003907	affect	NR1I3	ENSG00000143257	12695351;
D003907	affect	PRKCA	ENSG00000154229	12732289;
D003907	decrease	ACP5	ENSG00000102575	16079895;
D003907	decrease	BCL2	ENSG00000171791	15867202;
D003907	decrease	BCL2L1	ENSG00000171552	15804364;
D003907	decrease	CCR3	ENSG00000183625	15931867;
D003907	decrease	CDK2	ENSG00000123374	12800980;
D003907	decrease	CHI3L1	ENSG00000133048	16357325;
D003907	decrease	EDN1	ENSG00000078401	12206003;
D003907	decrease	ESR1	ENSG00000091831	16691199;
D003907	decrease	HLA-DMB	ENSG00000242092	16144940;
D003907	decrease	ICAM1	ENSG00000090339	17931847;11735124;
D003907	decrease	IL12RB1	ENSG00000096996	9743329;
D003907	decrease	IL12RB2	ENSG00000081985	9743329;
D003907	decrease	IL5	ENSG00000113525	14739603;11724761;16243713;16254627;10883730;15931867;12871219;1565031
D003907	decrease	IL5RA	ENSG00000091181	8049355;11724761;147396
D003907	decrease	NOS2	ENSG00000007171	12206003;
D003907	decrease	NR1D1	ENSG00000126368	17075855;
D003907	decrease	PLAU	ENSG00000122861	10467400;
D003907	decrease	PLAUR	ENSG00000011422	12732289;
D003907	decrease	PTHLH	ENSG00000087494	16278781;
D003907	decrease	SFTPA1	ENSG00000122852	16857225;
D003907	decrease	TCN2	ENSG00000185339	12732289;
D003907	decrease	TLR3	ENSG00000164342	12732289;
D003907	decrease	TNFRSF11B	ENSG00000164761	11855844;
D003907	decrease	TNFSF11	ENSG00000120659	16079895;
D003907	decrease	TP53	ENSG00000141510	19037090;
D003907	decrease	XIAP	ENSG00000101966	16144937;
D003907	increase	ABCB1	ENSG00000085563	12387747;
D003907	increase	ABCC3	ENSG00000108846	15979871;
D003907	increase	ACAN	ENSG00000157766	17502159;
D003907	increase	ADRA1B	ENSG00000170214	10229127;
D003907	increase	ADRA1D	ENSG00000171873	10229127;
D003907	increase	ALB	ENSG00000163631	16908450;14676436;
D003907	increase	BIRC3	ENSG00000023445	15804364;16144937;
D003907	increase	BMP2	ENSG00000125845	15268896;17502159;
D003907	increase	BMP4	ENSG00000125378	15268896;
D003907	increase	BMP6	ENSG00000153162	15268896;
D003907	increase	CCL26	ENSG00000006606	16045735;
D003907	increase	CCL2	ENSG00000108691	19255438;
D003907	increase	CCL5	ENSG00000161570	16908450;
D003907	increase	CCND1	ENSG00000110092	15489888;
D003907	increase	CES1	ENSG00000198848	10640517;
D003907	increase	CES2	ENSG00000172831	10640517;

D003907	increase	CHID1	ENSG00000177830	16357325;
D003907	increase	COL2A1	ENSG00000139219	17502159;
D003907	increase	CSF1R	ENSG00000182578	15679045;
D003907	increase	CSF2	ENSG00000164400	12077271;19255438;16908450;
D003907	increase	CSF3	ENSG00000108342	16144937;
D003907	increase	CXCR4	ENSG00000121966	11872090;12732289;
D003907	increase	CYBB	ENSG00000165168	9794432;
D003907	increase	CYP19A1	ENSG00000137869	15862960;16691199;15525692;
D003907	increase	CYP1A1	ENSG00000140465	10923861;16985168;15620718;
D003907	increase	CYP2A6	ENSG00000198077	17978169;10923861;
D003907	increase	CYP2B6	ENSG00000197408	12695351;15802389;15548381;
D003907	increase	CYP2C8	ENSG00000138115	15933212;
D003907	increase	CYP2C9	ENSG00000138109	16985168;15802389;
D003907	increase	CYP3A4	ENSG00000160868	12505310;8114683;16985168;12065438;18094037;10923861;9512926;12695351;17954527;16837568;15466163;10219967;10640517;155
D003907	increase	CYP3A5	ENSG00000106258	10696073;
D003907	increase	CYP4Z1	ENSG00000186160	15797250;
D003907	increase	DUSP1	ENSG00000120129	16144937;
D003907	increase	EEF1A2	ENSG00000101210	16144937;
D003907	increase	ERBB4	ENSG00000178568	16144937;
D003907	increase	EREG	ENSG00000124882	16144938;
D003907	increase	ETS2	ENSG00000157557	12732289;
D003907	increase	F2	ENSG00000180210	11733402;
D003907	increase	F2R	ENSG00000181104	11733402;
D003907	increase	F3	ENSG00000117525	11733402;
D003907	increase	FKBP5	ENSG00000096060	19255438;15679045;16144937;12732289;
D003907	increase	FPR1	ENSG00000171051	19037090;
D003907	increase	GREM1	ENSG00000166923	15268896;
D003907	increase	HLA-DPB1	ENSG00000223865	12732289;
D003907	increase	HLA-DQA1	ENSG00000196735	12732289;
D003907	increase	HLA-DRA	ENSG00000204287	12732289;
D003907	increase	HNF4A	ENSG00000101076	17978169;
D003907	increase	HPGD	ENSG00000164120	15680906;
D003907	increase	HSD11B2	ENSG00000176387	15679045;16857225;
D003907	increase	IFNG	ENSG00000111537	10883730;9743329;163573
D003907	increase	IGFBP1	ENSG00000146678	18483179;
D003907	increase	IL15	ENSG00000164136	16144937;
D003907	increase	IL1B	ENSG00000125538	16354411;16359550;15382119;
D003907	increase	IL4	ENSG00000113520	16045735;12077271;16357325;
D003907	increase	IL6	ENSG00000136244	16354411;16243974;
D003907	increase	IL8	ENSG00000169429	7522859;12077271;16354411;16364007;16908450;14676436;17235257;
D003907	increase	IRF8	ENSG00000140968	18483179;
D003907	increase	LAD1	ENSG00000159166	18483179;
D003907	increase	MAOA	ENSG00000189221	15679045;15946989;

D003907	increase	MAP2K3	ENSG00000034152	15817653;
D003907	increase	MCL1	ENSG00000143384	16144937;
D003907	increase	MGMT	ENSG00000170430	15481722;
D003907	increase	MMP12	ENSG00000110347	16359550;
D003907	increase	MT1E	ENSG00000169715	16144937;
D003907	increase	MT1G	ENSG00000125144	16144937;
D003907	increase	MYB	ENSG00000118513	16144937;
D003907	increase	MYC	ENSG00000136997	2558650;
D003907	increase	NCF1	ENSG00000158517	9794432;
D003907	increase	NFKBIA	ENSG00000100906	15489888;12732289;
D003907	increase	NR3C1	ENSG00000113580	18483179;15698552;16857225;11956172;15705660;11861974;15382119;15933212;15817653;12695351;16621434;12730620;16243974;15813899;10219967;179781
D003907	increase	OLFM4	ENSG00000102837	15679045;
D003907	increase	PER1	ENSG00000179094	15679045;
D003907	increase	PLAG1	ENSG00000181690	16144937;
D003907	increase	PRKX	ENSG00000183943	16144937;
D003907	increase	PTGS2	ENSG00000073756	16354411;19255438;15489888;15680906;
D003907	increase	RREB1	ENSG00000124782	16144937;
D003907	increase	SCEL	ENSG00000136155	16144937;
D003907	increase	SDCBP	ENSG00000137575	16144937;
D003907	increase	SFRP1	ENSG00000104332	16144937;
D003907	increase	SFTPB	ENSG00000168878	16857225;2539393;
D003907	increase	SFTPC	ENSG00000168484	2539393;
D003907	increase	SGK1	ENSG00000118515	15679045;
D003907	increase	SLC5A5	ENSG00000105641	16234306;16954431;
D003907	increase	SLC9A3	ENSG00000066230	16564041;
D003907	increase	SOCS1	ENSG00000185338	12732289;
D003907	increase	SOST	ENSG00000167941	15268896;
D003907	increase	SOX9	ENSG00000125398	17502159;
D003907	increase	TACC2	ENSG00000138162	16144939;
D003907	increase	TFF1	ENSG00000160182	15525692;
D003907	increase	TGFB1	ENSG00000105329	17502159;
D003907	increase	TGFB3	ENSG00000119699	16144937;
D003907	increase	TGFBR2	ENSG00000163513	12732289;
D003907	increase	TNF	ENSG00000223952	15489888;19255438;16243974;16769766;12721113;10883730;
D003907	increase	TSC22D3	ENSG00000157514	19255438;17081986;18483179;
D003907	increase	UGT1A1	ENSG00000241635	15557560;15560369;
D003907	increase	VCAM1	ENSG00000162692	16769766;11735124;
D003907	increase	WHSC2	ENSG00000185049	16144937;
D003958	increase	SOD2	ENSG00000112096	8760145;
D003975	affect	IL6	ENSG00000136244	10700573;
D003975	affect	TNF	ENSG00000223952	10700573;
D003976	decrease	ERCC5	ENSG00000134899	16539210;
D003976	decrease	RRM1	ENSG00000167325	16539210;
D003976	increase	ABCB1	ENSG00000085563	16221533;
D003976	increase	CALR	ENSG00000179218	16758764;

D003976	increase	CYP2C19	ENSG00000165841	15764407;11708902;16757081;
D003976	increase	CYP2D6	ENSG00000100197	16757081;
D003976	increase	CYP3A4	ENSG00000160868	15764407;11708902;16757081;
D003976	increase	CYP3A5	ENSG00000106258	16757081;
D003976	increase	TGFB3	ENSG00000119699	16758764;
D003993	decrease	SLC5A5	ENSG00000105641	16257484;
D003993	increase	AKR1C1	ENSG00000187134	18214805;
D003993	increase	CYP1B1	ENSG00000138061	16040568;
D003993	increase	INHA	ENSG00000123999	18214805;
D003994	decrease	MYC	ENSG00000136997	9187264;7798212;
D003994	increase	BDNF	ENSG00000176697	19189864;
D003994	increase	CCL3	ENSG00000006075	12538206;
D003994	increase	FOS	ENSG00000170345	9187264;7798212;
D003994	increase	IL8	ENSG00000169429	9258252;
D003994	increase	JUN	ENSG00000177606	9187264;
D003994	increase	REN	ENSG00000143839	9256163;
D003994	increase	SLC2A5	ENSG00000142583	12820898;
D004004	affect	SLC38A2	ENSG00000134294	15581851;
D004008	decrease	EPAS1	ENSG00000116016	12912967;
D004008	decrease	HIF1A	ENSG00000100644	12912967;
D004008	increase	CCND1	ENSG00000110092	15489888;15188006;
D004008	increase	EGR1	ENSG00000120738	15509713;
D004008	increase	GDF15	ENSG00000130513	15555568;
D004008	increase	HMOX1	ENSG00000100292	18227147;
D004008	increase	PTGS2	ENSG00000073756	15705740;15489888;17164136;
D004008	increase	TNF	ENSG00000223952	15489888;
D004008	increase	UGT1A1	ENSG00000241635	15334623;15593333;18227147;
D004026	decrease	GJA1	ENSG00000152661	8820588;
D004026	increase	ACVR1C	ENSG00000123612	19422813;
D004026	increase	ACVRL1	ENSG00000139567	19422813;
D004026	increase	ALK	ENSG00000171094	19422813;
D004026	increase	CYP26A1	ENSG00000095596	15589975;
D004026	increase	CYP2B6	ENSG00000197408	15548381;
D004026	increase	CYP3A4	ENSG00000160868	12417264;15548381;
D004026	increase	ERBB3	ENSG00000065361	19422813;
D004026	increase	ESR1	ENSG00000091831	14579009;10774817;16626760;9168004;9075711;15261991;15064155;11922773;
D004026	increase	ESR2	ENSG00000140009	16626760;15261991;
D004026	increase	KIT	ENSG00000157404	19422813;
D004026	increase	NR1I2	ENSG00000144852	16054614;12417264;15548381;
D004026	increase	PDGFRA	ENSG00000134853	19422813;
D004026	increase	PDGFRB	ENSG00000113721	19422813;
D004026	increase	RARB	ENSG00000077092	15589975;
D004026	increase	RARG	ENSG00000172819	15589975;
D004040	decrease	ABHD2	ENSG00000140526	17490981;
D004040	decrease	AKR1A1	ENSG00000117448	17490981;
D004040	decrease	AMIGO2	ENSG00000139211	17490981;
D004040	decrease	BNIP3	ENSG00000176171	17093179;
D004040	decrease	BTBD7	ENSG00000011114	17490981;

D004040	decrease	CA4	ENSG00000167434	17490981;
D004040	decrease	CCDC12	ENSG00000160799	17490981;
D004040	decrease	CCND2	ENSG00000118971	17490981;
D004040	decrease	CD2	ENSG00000116824	17093179;
D004040	decrease	CD34	ENSG00000174059	17490981;
D004040	decrease	CD69	ENSG00000110848	17093179;
D004040	decrease	CHST10	ENSG00000115526	17490981;
D004040	decrease	CRTAP	ENSG00000170275	17490981;
D004040	decrease	CSNK2A1	ENSG00000101266	17490981;
D004040	decrease	DDR1	ENSG00000204580	17490981;
D004040	decrease	DDX17	ENSG00000100201	17490981;
D004040	decrease	ELL2	ENSG00000118985	17490981;
D004040	decrease	ENO1	ENSG00000074800	17490981;
D004040	decrease	FAM89A	ENSG00000182118	17490981;
D004040	decrease	FDFT1	ENSG00000079459	17490981;
D004040	decrease	FXR1	ENSG00000114416	17490981;
D004040	decrease	GAS7	ENSG00000007237	17490981;
D004040	decrease	GLG1	ENSG00000090863	17490981;
D004040	decrease	GOLGA2	ENSG00000167110	17490981;
D004040	decrease	GZMA	ENSG00000145649	17093179;
D004040	decrease	H3F3B	ENSG00000163041	17490981;
D004040	decrease	IGFBP5	ENSG00000115461	17490981;
D004040	decrease	IL10	ENSG00000136634	12533503;
D004040	decrease	IL1RN	ENSG00000136689	12533503;
D004040	decrease	IL6	ENSG00000136244	12533503;
D004040	decrease	IL8	ENSG00000169429	12533503;
D004040	decrease	INSR	ENSG00000171105	17490981;
D004040	decrease	KLC1	ENSG00000126214	17490981;
D004040	decrease	KLHL23	ENSG00000213160	17490981;
D004040	decrease	KLRD1	ENSG00000134539	17093179;
D004040	decrease	KLRF1	ENSG00000150045	17093179;
D004040	decrease	LDHB	ENSG00000111716	17093179;
D004040	decrease	LEF1	ENSG00000138795	17093179;
D004040	decrease	LGALS1	ENSG00000100097	17490981;
D004040	decrease	LIPE	ENSG00000079435	17490981;
D004040	decrease	MAL	ENSG00000172005	17093179;
D004040	decrease	MOAP1	ENSG00000165943	17093179;
D004040	decrease	MRPL55	ENSG00000162910	17490981;
D004040	decrease	MRPS30	ENSG00000112996	17490981;
D004040	decrease	MTDH	ENSG00000147649	17490981;
D004040	decrease	MTMR12	ENSG00000150712	17490981;
D004040	decrease	NFKBIA	ENSG00000100906	17093179;
D004040	decrease	PAIP1	ENSG00000172239	17490981;
D004040	decrease	PDCD4	ENSG00000150593	17093179;
D004040	decrease	PDXDC1	ENSG00000179889	17490981;
D004040	decrease	PELO	ENSG00000152684	17490981;
D004040	decrease	PEX16	ENSG00000121680	17490981;
D004040	decrease	PGRMC2	ENSG00000164040	17490981;
D004040	decrease	PLXNB2	ENSG00000196576	17490981;
D004040	decrease	POLD4	ENSG00000175482	17490981;
D004040	decrease	PPIG	ENSG00000138398	17490981;
D004040	decrease	PPP1R12B	ENSG00000248526	17490981;
D004040	decrease	PRKCH	ENSG00000027075	17093179;
D004040	decrease	PRKCQ	ENSG00000065675	17093179;

D004040	decrease	RAB18	ENSG00000099246	17490981;
D004040	decrease	RAB6B	ENSG00000154917	17490981;
D004040	decrease	RASGRP1	ENSG00000172575	17093179;
D004040	decrease	SCP2	ENSG00000251612	17093179;
D004040	decrease	SDC2	ENSG00000169439	17490981;
D004040	decrease	SFRP1	ENSG00000104332	17490981;
D004040	decrease	SFRS2IP	ENSG00000139218	17490981;
D004040	decrease	SLC25A6	ENSG00000169100	17490981;
D004040	decrease	SYNCRIP	ENSG00000135316	17490981;
D004040	decrease	TCEAL1	ENSG00000172465	17490981;
D004040	decrease	TCF25	ENSG00000141002	17490981;
D004040	decrease	TCF7L2	ENSG00000148737	17490981;
D004040	decrease	TFE3	ENSG00000068323	17490981;
D004040	decrease	TMEM214	ENSG00000119777	17490981;
D004040	decrease	TNFRSF1A	ENSG00000067182	17490981;
D004040	decrease	TUSC5	ENSG00000184811	17490981;
D004040	decrease	TWIST2	ENSG00000233608	17490981;
D004040	decrease	UBA2	ENSG00000126261	17490981;
D004040	decrease	VAPB	ENSG00000124164	17490981;
D004040	decrease	WDR45L	ENSG00000141580	17490981;
D004040	decrease	WIPI2	ENSG00000248535	17490981;
D004040	decrease	YAF2	ENSG00000015153	17490981;
D004040	decrease	ZBTB4	ENSG00000174282	17490981;
D004040	decrease	ZNF791	ENSG00000173875	17490981;
D004040	increase	ADAMTS1	ENSG00000154734	17490981;
D004040	increase	AKAP8L	ENSG00000011243	17490981;
D004040	increase	APOLD1	ENSG00000178878	17490981;
D004040	increase	ARL6IP6	ENSG00000177917	17490981;
D004040	increase	ATP1B3	ENSG00000069849	17490981;
D004040	increase	BLNK	ENSG00000095585	17490981;
D004040	increase	C1ORF63	ENSG00000117616	17490981;
D004040	increase	C8ORF4	ENSG00000176907	17490981;
D004040	increase	CARD8	ENSG00000105483	17490981;
D004040	increase	CCL18	ENSG00000006074	17490981;
D004040	increase	CD14	ENSG00000170458	17093179;
D004040	increase	CD86	ENSG00000114013	17490981;
D004040	increase	CD93	ENSG00000125810	17093179;
D004040	increase	CNDP2	ENSG00000133313	17490981;
D004040	increase	CTBP2	ENSG00000175029	17490981;
D004040	increase	CYP1B1	ENSG00000138061	17490981;
D004040	increase	DAPK1	ENSG00000196730	17093179;
D004040	increase	DDIT4	ENSG00000168209	17093179;
D004040	increase	DDX5	ENSG00000108654	17490981;
D004040	increase	DHRS9	ENSG00000073737	17093179;
D004040	increase	DUSP6	ENSG00000139318	17490981;
D004040	increase	EIF4E	ENSG00000151247	17490981;
D004040	increase	EWSR1	ENSG00000182944	17490981;
D004040	increase	FABP5	ENSG00000164687	17490981;
D004040	increase	GAA	ENSG00000171298	17093179;
D004040	increase	GALNT2	ENSG00000143641	17490981;
D004040	increase	HAL	ENSG00000084110	17093179;
D004040	increase	HSPA1B	ENSG00000232804	17490981;
D004040	increase	HSPA8	ENSG00000109971	17490981;
D004040	increase	HSPE1	ENSG00000115541	17490981;

D004040	increase	IER5	ENSG00000162783	17490981;
D004040	increase	IL10RA	ENSG00000110324	17490981;
D004040	increase	IMPDH1	ENSG00000106348	17093179;
D004040	increase	KIAA0528	ENSG00000111731	17490981;
D004040	increase	KLF4	ENSG00000136826	17490981;
D004040	increase	KPNA2	ENSG00000182481	17490981;
D004040	increase	LGMN	ENSG00000100600	17490981;
D004040	increase	MAN2A1	ENSG00000112893	17490981;
D004040	increase	MBNL2	ENSG00000139793	17490981;
D004040	increase	MKNK2	ENSG00000099875	17490981;
D004040	increase	MSX1	ENSG00000163132	17490981;
D004040	increase	MYEOV2	ENSG00000172428	17490981;
D004040	increase	MYO1E	ENSG00000157483	17490981;
D004040	increase	NDFIP2	ENSG00000102471	17490981;
D004040	increase	NDNL2	ENSG00000185115	17490981;
D004040	increase	NDUFC1	ENSG00000109390	17490981;
D004040	increase	NID1	ENSG00000116962	17490981;
D004040	increase	PIK3C2B	ENSG00000133056	17490981;
D004040	increase	PLEKHB2	ENSG00000115762	17490981;
D004040	increase	PRDX6	ENSG00000117592	17490981;
D004040	increase	PSAP	ENSG00000197746	17093179;
D004040	increase	PYGL	ENSG00000100504	17093179;
D004040	increase	QTRTD1	ENSG00000151576	17490981;
D004040	increase	SAMHD1	ENSG00000101347	17490981;
D004040	increase	SGK1	ENSG00000118515	17490981;
D004040	increase	SIGLEC5	ENSG00000105501	17093179;
D004040	increase	SLC40A1	ENSG00000138449	17490981;
D004040	increase	SMOC2	ENSG00000112562	17490981;
D004040	increase	STRN	ENSG00000115808	17490981;
D004040	increase	TIA1	ENSG00000116001	17490981;
D004040	increase	TIMP3	ENSG00000100234	17490981;
D004040	increase	TMX1	ENSG00000139921	17490981;
D004040	increase	TNFSF13	ENSG00000161955	17093179;
D004040	increase	ZNF443	ENSG00000180855	17490981;
D004040	increase	ZNF573	ENSG00000189144	17490981;
D004050	decrease	CAT	ENSG00000121691	8279580;
D004050	decrease	ICAM1	ENSG00000090339	10644010;
D004050	increase	SOD1	ENSG00000142168	8279580;10644010;
D004050	increase	TP53	ENSG00000141510	10951577;
D004051	decrease	CDO1	ENSG00000129596	16223563;
D004051	decrease	MAPK1	ENSG00000100030	16455614;
D004051	decrease	MAPK3	ENSG00000102882	16455614;
D004051	decrease	MYC	ENSG00000136997	16455614;
D004051	decrease	PAPSS1	ENSG00000138801	16223563;
D004051	decrease	PAPSS2	ENSG00000198682	16223563;
D004051	decrease	PPARA	ENSG00000186951	10581215;16455614;
D004051	decrease	SUOX	ENSG00000139531	16223563;
D004051	increase	ABCB1	ENSG00000085563	17003290;
D004051	increase	CYP1A1	ENSG00000140465	16954067;
D004051	increase	CYP1B1	ENSG00000138061	16040568;
D004051	increase	NR1I2	ENSG00000144852	16054614;17003290;
D004051	increase	PPARD	ENSG00000112033	16455614;
D004054	decrease	ABCB1	ENSG00000085563	16925584;
D004054	decrease	CYP11A1	ENSG00000140459	12858341;

D004054	decrease	DLX4	ENSG00000108813	12858341;
D004054	decrease	ENO2	ENSG00000111674	15159206;
D004054	decrease	HSD17B1	ENSG00000108786	12858341;
D004054	decrease	HSD3B1	ENSG00000203857	12858341;
D004054	decrease	KLK3	ENSG00000142515	15846301;
D004054	decrease	SELENBP1	ENSG00000143416	15159206;
D004054	decrease	SLC12A2	ENSG00000064651	15159206;
D004054	decrease	TUBB4	ENSG00000104833	15924336;
D004054	decrease	WNT7A	ENSG00000154764	16534752;
D004054	increase	ADCY1	ENSG00000164742	15766595;
D004054	increase	ADORA1	ENSG00000163485	15766595;
D004054	increase	APOE	ENSG00000130203	15766595;
D004054	increase	ARHGDIA	ENSG00000141522	15159206;15766595;
D004054	increase	C6ORF108	ENSG00000112667	15766595;
D004054	increase	CAPNS1	ENSG00000126247	15766595;
D004054	increase	CDC20	ENSG00000117399	15766595;
D004054	increase	CDK10	ENSG00000185324	15766595;
D004054	increase	CDK5	ENSG00000164885	15766595;
D004054	increase	CDKN1C	ENSG00000129757	15766595;
D004054	increase	CKS1B	ENSG00000173207	15766595;
D004054	increase	CKS2	ENSG00000123975	15766595;
D004054	increase	CLU	ENSG00000120885	15766595;
D004054	increase	DAD1	ENSG00000129562	15766595;
D004054	increase	DCC	ENSG00000187323	15766595;
D004054	increase	DDIT3	ENSG00000175197	15766595;
D004054	increase	DDR1	ENSG00000204580	15766595;
D004054	increase	EGR3	ENSG00000179388	15159206;
D004054	increase	ESRRA	ENSG00000173153	11559547;
D004054	increase	ESRRB	ENSG00000119715	11559547;
D004054	increase	ESRRG	ENSG00000196482	11447273;17049190;16889744;11559547;14751226;12579585;
D004054	increase	FGR	ENSG00000000938	15766595;
D004054	increase	FLT3LG	ENSG00000090554	15766595;
D004054	increase	FOSL1	ENSG00000175592	15766595;
D004054	increase	FZD2	ENSG00000180340	15766595;
D004054	increase	HINT1	ENSG00000169567	15766595;
D004054	increase	HOXA10	ENSG00000078399	15236964;16632680;15890768;
D004054	increase	HOXA9	ENSG00000078399	16632680;
D004054	increase	IGFBP2	ENSG00000115457	15766595;
D004054	increase	IGFBP6	ENSG00000167779	15846301;
D004054	increase	ITGB7	ENSG00000139626	15766595;
D004054	increase	JAK3	ENSG00000105639	15766595;
D004054	increase	LYN	ENSG00000249529	15766595;
D004054	increase	MAOA	ENSG00000189221	10811566;
D004054	increase	MAP2K1	ENSG00000169032	15766595;
D004054	increase	MLH1	ENSG00000076242	15766595;
D004054	increase	MMP11	ENSG00000099953	15766595;
D004054	increase	MYC	ENSG00000136997	15766595;
D004054	increase	NME1	ENSG00000239672	15766595;
D004054	increase	NME2	ENSG00000243678	15766595;
D004054	increase	PDCD6	ENSG00000249915	15766595;
D004054	increase	PDZK1	ENSG00000174827	15159206;

D004054	increase	POLA1	ENSG00000101868	15766595;
D004054	increase	PRKCSH	ENSG00000130175	15766595;
D004054	increase	PTMA	ENSG00000187514	15766595;
D004054	increase	RAP1GAP	ENSG00000076864	15766595;
D004054	increase	RFC5	ENSG00000111445	15766595;
D004054	increase	RPA3	ENSG00000106399	15766595;
D004054	increase	RPS19	ENSG00000105372	15766595;
D004054	increase	RPS3A	ENSG00000145425	15766595;
D004054	increase	SERPINA3	ENSG00000196136	10811566;
D004054	increase	SKI	ENSG00000157933	15766595;
D004054	increase	SLC7A11	ENSG00000151012	15159206;
D004054	increase	STAT1	ENSG00000115415	15766595;
D004054	increase	TFF1	ENSG00000160182	11559547;10811566;15525692;
D004054	increase	TGFB3	ENSG00000119699	10811566;
D004054	increase	TNK2	ENSG00000061938	15766595;
D004054	increase	TOB1	ENSG00000141232	15766595;
D004054	increase	TRAF3	ENSG00000131323	15766595;
D004054	increase	WARS	ENSG00000140105	15159206;
D004054	increase	YWHAB	ENSG00000166913	15766595;
D004077	decrease	KCNH2	ENSG00000055118	19139152;
D004110	increase	IL12A	ENSG00000168811	15652234;
D004110	increase	IL12B	ENSG00000113302	15652234;
D004117	increase	CHRM3	ENSG00000133019	15033005;
D004121	affect	ABCB1	ENSG00000085563	12843640;
D004121	affect	CYP1A2	ENSG00000140505	12843640;
D004121	affect	CYP1B1	ENSG00000138061	12843640;
D004121	affect	CYP2A6	ENSG00000198077	12843640;
D004121	affect	CYP3A4	ENSG00000160868	12843640;
D004121	affect	CYP3A5	ENSG00000106258	12843640;
D004121	affect	CYP3A7	ENSG00000160870	12843640;
D004121	affect	GAPDH	ENSG00000111640	12843640;
D004121	affect	UGT1A6	ENSG00000167165	12843640;
D004121	affect	UGT1A9	ENSG00000241119	12843640;
D004121	decrease	DNMT1	ENSG00000130816	17065238;
D004121	decrease	EDN2	ENSG00000127129	15619356;
D004121	decrease	MYC	ENSG00000136997	7798212;
D004121	increase	BAX	ENSG00000087088	15998540;17652743;
D004121	increase	CCL2	ENSG00000108691	17529908;14576080;
D004121	increase	CD38	ENSG00000004468	15964820;
D004121	increase	CLDN6	ENSG00000184697	18661270;
D004121	increase	CLDN8	ENSG00000156284	18661270;
D004121	increase	CLDN9	ENSG00000213937	18661270;
D004121	increase	CYP1A1	ENSG00000140465	12843640;12147272;
D004121	increase	CYP27A1	ENSG00000135929	15964820;
D004121	increase	CYP2E1	ENSG00000130649	12843640;12147272;9143349;8074729;
D004121	increase	FAS	ENSG00000026103	15998540;
D004121	increase	GAD1	ENSG00000128683	17065238;
D004121	increase	GRN	ENSG00000030582	16873554;
D004121	increase	IL8	ENSG00000169429	17529908;
D004121	increase	ITGAM	ENSG00000169896	15964820;16617325;
D004121	increase	ITGAX	ENSG00000140678	15964820;
D004121	increase	ITGB2	ENSG00000160255	15964820;

D004121	increase	OCLN	ENSG00000197822	18661270;
D004121	increase	PADI4	ENSG00000159339	15964820;
D004121	increase	PDCD4	ENSG00000150593	17259349;
D004121	increase	RELN	ENSG00000189056	17065238;
D004121	increase	TGM2	ENSG00000198959	15964820;
D004121	increase	TJP1	ENSG00000104067	18661270;
D004121	increase	TNF	ENSG00000223952	14576080;
D004121	increase	TNFRSF1A	ENSG00000067182	9870925;
D004126	increase	LTF	ENSG00000012223	18063697;
D004126	increase	MPO	ENSG00000005381	18063697;
D004126	increase	NCF1	ENSG00000158517	18063697;
D004126	increase	TNFSF10	ENSG00000121858	18063697;
D004128	decrease	A2M	ENSG00000175899	17547211;
D004128	decrease	ACP2	ENSG00000134575	17547211;
D004128	decrease	ACSL1	ENSG00000151726	17547211;
D004128	decrease	ADH6	ENSG00000172955	17547211;
D004128	decrease	AKR1B1	ENSG00000085662	17547211;
D004128	decrease	ALDH18A1	ENSG00000059573	17547211;
D004128	decrease	ATP2B1	ENSG00000070961	17547211;
D004128	decrease	C14ORF1	ENSG00000133935	17547211;
D004128	decrease	CDC6	ENSG00000094804	19031421;
D004128	decrease	CETN2	ENSG00000147400	19031421;
D004128	decrease	CIDEB	ENSG00000136305	17547211;
D004128	decrease	CLPTM1	ENSG00000104853	17547211;
D004128	decrease	CRYAA	ENSG00000160202	19031421;17547211;
D004128	decrease	CX3CL1	ENSG00000006210	17547211;
D004128	decrease	DHCR7	ENSG00000172893	17547211;
D004128	decrease	EBP	ENSG00000147155	17547211;
D004128	decrease	F10	ENSG00000126218	17547211;
D004128	decrease	F5	ENSG00000198734	17547211;
D004128	decrease	FABP1	ENSG00000163586	17547211;
D004128	decrease	FDFT1	ENSG00000079459	17547211;
D004128	decrease	FDPS	ENSG00000160752	17547211;
D004128	decrease	FURIN	ENSG00000140564	17547211;
D004128	decrease	GGCX	ENSG00000115486	17547211;
D004128	decrease	GPER	ENSG00000164850	17547211;
D004128	decrease	GSN	ENSG00000148180	17547211;
D004128	decrease	HMGCR	ENSG00000113161	17547211;
D004128	decrease	HMGCS1	ENSG00000112972	17547211;
D004128	decrease	HSD17B2	ENSG00000086696	17547211;
D004128	decrease	HSD17B7	ENSG00000132196	17547211;
D004128	decrease	IL17RB	ENSG00000056736	17547211;
D004128	decrease	LIPA	ENSG00000107798	17547211;
D004128	decrease	LSS	ENSG00000160285	17547211;
D004128	decrease	MGAT2	ENSG00000168282	17547211;
D004128	decrease	MRPL49	ENSG00000149792	17547211;
D004128	decrease	MT1X	ENSG00000187193	17547211;
D004128	decrease	NDRG2	ENSG00000165795	17547211;
D004128	decrease	NFKBIA	ENSG00000100906	17547211;
D004128	decrease	NUCB2	ENSG00000070081	17547211;
D004128	decrease	PDIA4	ENSG00000155660	17547211;
D004128	decrease	PGC	ENSG00000096088	17547211;
D004128	decrease	PGF	ENSG00000119630	17547211;
D004128	decrease	PROM1	ENSG00000007062	17547211;

D004128	decrease	RAD23B	ENSG00000119318	19031421;
D004128	decrease	RELN	ENSG00000189056	17547211;
D004128	decrease	SCAND1	ENSG00000171222	17547211;
D004128	decrease	SCD	ENSG00000099194	17547211;
D004128	decrease	SERPINA5	ENSG00000188488	17547211;
D004128	decrease	SERPINA6	ENSG00000170099	17547211;
D004128	decrease	SERPINB1	ENSG00000021355	17547211;
D004128	decrease	SFRS1	ENSG00000136450	17547211;
D004128	decrease	SQLE	ENSG00000104549	17547211;
D004128	decrease	TMED2	ENSG00000086598	17547211;
D004128	decrease	TNFSF10	ENSG00000121858	17849272;
D004128	decrease	TRA2A	ENSG00000164548	17547211;
D004128	decrease	UBD	ENSG00000213886	17547211;
D004128	decrease	VTN	ENSG00000109072	17547211;
D004128	increase	ABCC3	ENSG00000108846	17547211;
D004128	increase	ABCG2	ENSG00000118777	17547211;
D004128	increase	AKAP12	ENSG00000131016	17547211;
D004128	increase	ANXA2	ENSG00000182718	17547211;
D004128	increase	AQP3	ENSG00000165272	17547211;
D004128	increase	ASNS	ENSG00000070669	17547211;
D004128	increase	BLVRB	ENSG00000090013	17547211;
D004128	increase	BUB3	ENSG00000154473	19031421;
D004128	increase	CAPN2	ENSG00000162909	17547211;
D004128	increase	CDKN1A	ENSG00000124762	17547211;
D004128	increase	CES1	ENSG00000198848	17547211;
D004128	increase	CIDEC	ENSG00000187288	17547211;
D004128	increase	CRY1	ENSG00000008405	19031421;
D004128	increase	CTSH	ENSG00000103811	17547211;
D004128	increase	DFNA5	ENSG00000105928	17547211;
D004128	increase	DUSP13	ENSG00000079393	19031421;
D004128	increase	DUSP1	ENSG00000120129	17547211;
D004128	increase	ENO2	ENSG00000111674	17547211;
D004128	increase	ENPP1	ENSG00000197594	17547211;
D004128	increase	ETS2	ENSG00000157557	17547211;
D004128	increase	FAH	ENSG00000103876	17547211;
D004128	increase	FEZ2	ENSG00000171055	17547211;
D004128	increase	FOSL1	ENSG00000175592	17547211;
D004128	increase	GADD45A	ENSG00000116717	19031421;
D004128	increase	GCLC	ENSG00000001084	17547211;
D004128	increase	GCNT3	ENSG00000140297	17547211;
D004128	increase	GLA	ENSG00000102393	17547211;
D004128	increase	GLRX	ENSG00000173221	17547211;
D004128	increase	GRB10	ENSG00000106070	17547211;
D004128	increase	HMGA1	ENSG00000137309	17547211;
D004128	increase	HPCAL1	ENSG00000115756	17547211;
D004128	increase	HPD	ENSG00000158104	17547211;
D004128	increase	ID1	ENSG00000125968	15120964;
D004128	increase	IER2	ENSG00000160888	17547211;
D004128	increase	IFITM2	ENSG00000185201	17547211;
D004128	increase	KLF6	ENSG00000067082	17547211;
D004128	increase	KPNA4	ENSG00000186432	17547211;
D004128	increase	KRT19	ENSG00000171345	17547211;
D004128	increase	LGALS1	ENSG00000100097	17547211;
D004128	increase	LGALS3	ENSG00000131981	17547211;

D004128	increase	MAFF	ENSG00000185022	17547211;
D004128	increase	MXRA7	ENSG00000182534	17547211;
D004128	increase	OGG1	ENSG00000114026	19031421;
D004128	increase	PHLDA2	ENSG00000181649	17547211;
D004128	increase	PIK3CD	ENSG00000171608	17547211;
D004128	increase	PIM1	ENSG00000137193	17547211;
D004128	increase	PTTG1	ENSG00000164611	19031421;17547211;
D004128	increase	RBPMS	ENSG00000157110	17547211;
D004128	increase	SERPINE2	ENSG00000135919	17547211;
D004128	increase	SFN	ENSG00000175793	17547211;
D004128	increase	SHC1	ENSG00000160691	19031421;
D004128	increase	SIK1	ENSG00000142178	17547211;
D004128	increase	SLC12A6	ENSG00000140199	17547211;
D004128	increase	SPP1	ENSG00000118785	17547211;
D004128	increase	SQRDL	ENSG00000137767	17547211;
D004128	increase	TIMP1	ENSG00000102265	17547211;
D004128	increase	TNFAIP8	ENSG00000145779	17547211;
D004128	increase	TNFRSF10B	ENSG00000120889	17849272;
D004128	increase	TRIB1	ENSG00000173334	17547211;
D004128	increase	UGCG	ENSG00000148154	17547211;
D004128	increase	VRK2	ENSG00000028116	17547211;
D004137	increase	ACOX2	ENSG00000168306	16314067;
D004137	increase	ADORA2B	ENSG00000170425	16314067;
D004137	increase	ANXA1	ENSG00000135046	16314067;
D004137	increase	ANXA2	ENSG00000182718	16314067;
D004137	increase	ARG2	ENSG00000081181	16314067;
D004137	increase	CCL4	ENSG00000129277	16314067;
D004137	increase	CCR7	ENSG00000126353	15737199;
D004137	increase	CD86	ENSG00000114013	19033392;15737199;
D004137	increase	CDKN1A	ENSG00000124762	16314067;
D004137	increase	CLIC4	ENSG00000169504	16314067;
D004137	increase	CTSB	ENSG00000164733	16314067;
D004137	increase	DDIT3	ENSG00000175197	17171638;
D004137	increase	DUSP1	ENSG00000120129	16314067;
D004137	increase	FTH1	ENSG00000167996	16314067;
D004137	increase	GPNMB	ENSG00000136235	16314067;
D004137	increase	HIPK2	ENSG00000064393	16314067;
D004137	increase	HMOX1	ENSG00000100292	19033392;16314067;
D004137	increase	HSPA6	ENSG00000173110	16314067;
D004137	increase	ICAM1	ENSG00000090339	15737199;
D004137	increase	IL1B	ENSG00000125538	16314067;
D004137	increase	JUN	ENSG00000177606	16314067;
D004137	increase	KEAP1	ENSG00000079999	19033392;
D004137	increase	KLF6	ENSG00000067082	16314067;
D004137	increase	MARCKS	ENSG00000155130	16314067;
D004137	increase	NFE2L2	ENSG00000116044	19033392;
D004137	increase	NINJ1	ENSG00000131669	16314067;
D004137	increase	NQO1	ENSG00000181019	19033392;16314067;
D004137	increase	PIR	ENSG00000087842	16314067;
D004137	increase	RHOC	ENSG00000155366	16314067;
D004137	increase	S100A10	ENSG00000197747	16314067;
D004137	increase	SEMA4C	ENSG00000168758	16314067;
D004137	increase	SI	ENSG00000090402	16314067;
D004137	increase	SOD2	ENSG00000112096	16314067;

D004137	increase	SPTA1	ENSG00000163554	16314067;
D004137	increase	TIMP3	ENSG00000100234	16314067;
D004144	increase	BCL2A1	ENSG00000140379	16331273;
D004144	increase	BCL2	ENSG00000171791	16331273;
D004144	increase	BCL2L1	ENSG00000171552	16331273;
D004144	increase	BIRC2	ENSG00000110330	16331273;
D004144	increase	CCND1	ENSG00000110092	16331273;
D004144	increase	CFLAR	ENSG00000003402	16331273;
D004144	increase	MMP9	ENSG00000100985	16331273;
D004144	increase	MYC	ENSG00000136997	16331273;
D004144	increase	PTGS2	ENSG00000073756	16331273;
D004144	increase	TNF	ENSG00000223952	16331273;
D004144	increase	VEGFA	ENSG00000112715	16331273;
D004147	increase	AHR	ENSG00000106546	15792794;
D004147	increase	CYP1A2	ENSG00000140505	15977188;
D004147	increase	CYP2S1	ENSG00000167600	15608128;16054184;
D004147	increase	CYP3A4	ENSG00000160868	15977188;
D004147	increase	TNF	ENSG00000223952	15792794;
D004164	decrease	BGLAP	ENSG00000242252	19597852;
D004164	decrease	COL2A1	ENSG00000139219	19597852;
D004164	decrease	COL3A1	ENSG00000168542	19597852;
D004164	decrease	COL5A3	ENSG00000080573	19597852;
D004206	decrease	NR0B2	ENSG00000131910	15342952;
D004206	increase	ASAH1	ENSG00000104763	15342952;
D004221	decrease	AR	ENSG00000169083	19789329;
D004221	decrease	CCNA2	ENSG00000145386	15367699;
D004221	decrease	MCM2	ENSG00000073111	19789329;
D004221	decrease	MCM5	ENSG00000100297	19789329;
D004221	decrease	MMP2	ENSG00000087245	18047805;
D004221	decrease	MMP9	ENSG00000100985	18047805;
D004221	increase	BAX	ENSG00000087088	17079463;
D004221	increase	CDKN1B	ENSG00000111276	17079463;
D004221	increase	MT1B	ENSG00000169688	19789329;
D004221	increase	MT1F	ENSG00000198417	19789329;
D004221	increase	MT1G	ENSG00000125144	19789329;
D004221	increase	MT1X	ENSG00000187193	19789329;
D004221	increase	MT2A	ENSG00000125148	19789329;
D004229	decrease	CCND1	ENSG00000110092	17332266;
D004229	increase	ATF3	ENSG00000162772	17332266;
D004229	increase	CCNG2	ENSG00000138764	17332266;
D004229	increase	DDIT3	ENSG00000175197	17332266;
D004229	increase	GADD45A	ENSG00000116717	17332266;
D004229	increase	HIF1A	ENSG00000100644	17658243;14695184;
D004229	increase	HSPA5	ENSG00000044574	17416481;17332266;15710169;
D004237	decrease	IFNG	ENSG00000111537	10883730;
D004237	decrease	IL5	ENSG00000113525	10883730;
D004237	decrease	TNF	ENSG00000223952	10883730;
D004237	increase	CYP3A7	ENSG00000160870	8812189;
D004280	increase	CCL3	ENSG00000006075	12818968;
D004280	increase	IL10	ENSG00000136634	15502056;
D004280	increase	IL8	ENSG00000169429	12818968;15502056;
D004280	increase	REN	ENSG00000143839	9203624;
D004281	decrease	AKT1	ENSG00000142208	17077332;

D004281	decrease	APOB	ENSG00000084674	17237316;
D004281	decrease	BCL2	ENSG00000171791	19116882;11090257;19015929;
D004281	decrease	BCL2L12	ENSG00000126453	19015929;
D004281	decrease	CASP9	ENSG00000132906	19015929;
D004281	decrease	COPS5	ENSG00000121022	17077332;
D004281	decrease	CRP	ENSG00000132693	17237316;
D004281	decrease	FAS	ENSG00000026103	19015929;
D004281	decrease	IFNA2	ENSG00000188379	17077332;
D004281	decrease	IL4	ENSG00000113520	16305726;
D004281	decrease	IL6	ENSG00000136244	16305726;16702388;
D004281	decrease	JUND	ENSG00000130522	17077332;
D004281	decrease	LEP	ENSG00000174697	15949695;
D004281	decrease	SREBF1	ENSG00000072310	15949695;
D004281	decrease	TNF	ENSG00000223952	16702388;
D004281	increase	ABCB11	ENSG00000073734	15307955;
D004281	increase	BAX	ENSG00000087088	19015929;
D004281	increase	CPT1A	ENSG00000110090	15307955;
D004281	increase	IL1B	ENSG00000125538	16305726;
D004281	increase	KNG1	ENSG00000113889	15307955;
D004281	increase	PCNA	ENSG00000132646	16870008;
D004298	decrease	BCL2	ENSG00000171791	17603292;
D004316	decrease	SLC2A3	ENSG00000059804	17567588;
D004316	decrease	TAGLN	ENSG00000149591	17567588;
D004316	increase	ASNS	ENSG00000070669	17567588;
D004316	increase	C10ORF10	ENSG00000165507	17567588;
D004316	increase	FABP1	ENSG00000163586	17567588;
D004316	increase	GDPD3	ENSG00000102886	17567588;
D004316	increase	INHBE	ENSG00000139269	17567588;
D004316	increase	LSS	ENSG00000160285	17567588;
D004316	increase	NUPR1	ENSG00000176046	17567588;
D004316	increase	SERPINA3	ENSG00000196136	17567588;
D004317	affect	ATF3	ENSG00000162772	15205334;
D004317	affect	BAG1	ENSG00000250477	16322899;
D004317	affect	BTG1	ENSG00000133639	16705698;
D004317	affect	BUB3	ENSG00000154473	16322899;
D004317	affect	CDK5R1	ENSG00000176749	15205334;
D004317	affect	CPXM1	ENSG00000088882	15205334;
D004317	affect	CRHBP	ENSG00000145708	15205334;
D004317	affect	CTNNBIP1	ENSG00000178585	16322899;
D004317	affect	CTSB	ENSG00000164733	17378599;16705698;
D004317	affect	CYP2B6	ENSG00000197408	16322899;
D004317	affect	DAXX	ENSG00000231617	12948851;
D004317	affect	DCT	ENSG00000080166	15205334;
D004317	affect	DPYSL3	ENSG00000113657	15205334;
D004317	affect	EIF1AX	ENSG00000173674	16322899;
D004317	affect	EIF4EBP1	ENSG00000187840	16322899;
D004317	affect	ELN	ENSG00000049540	15205334;
D004317	affect	EPYC	ENSG00000083782	15205334;
D004317	affect	ERBB2	ENSG00000141736	15205334;17010609;15834928;16168102;15486187;
D004317	affect	FBLN1	ENSG00000077942	15205334;
D004317	affect	FHL2	ENSG00000115641	17682292;
D004317	affect	FKBP5	ENSG00000096060	15571967;

D004317	affect	GEM	ENSG00000164949	15205334;
D004317	affect	GTF3C1	ENSG00000077235	16322899;
D004317	affect	ILF3	ENSG00000129351	16322899;
D004317	affect	IRS1	ENSG00000169047	16322899;
D004317	affect	MCM4	ENSG00000104738	15205334;
D004317	affect	MMP9	ENSG00000100985	15205334;
D004317	affect	NR4A1	ENSG00000123358	15205334;16322897;
D004317	affect	PAWR	ENSG00000177425	12948851;
D004317	affect	PLOD1	ENSG00000083444	16322899;
D004317	affect	PLOD3	ENSG00000106397	16322899;
D004317	affect	RAB21	ENSG00000080371	15205334;
D004317	affect	RAB6C	ENSG00000222014	18992151;
D004317	affect	RB1	ENSG00000139687	15205334;16537896;15141020;
D004317	affect	RGS16	ENSG00000143333	15205334;
D004317	affect	RGS1	ENSG00000090104	15205334;
D004317	affect	RRM2	ENSG00000171848	15205334;16896004;
D004317	affect	SKP2	ENSG00000145604	17893511;16217747;
D004317	affect	SPHK2	ENSG00000063176	17974990;
D004317	affect	SPON1	ENSG00000152268	15205334;
D004317	affect	SRM	ENSG00000116649	16322899;
D004317	affect	ST14	ENSG00000149418	16322899;
D004317	affect	STK39	ENSG00000198648	16322899;
D004317	affect	TCEB3	ENSG00000011007	15205334;
D004317	decrease	ADAM19	ENSG00000135074	18510171;
D004317	decrease	ADAMTS5	ENSG00000154736	16404146;
D004317	decrease	AHCY	ENSG00000101444	18510171;
D004317	decrease	AKT1	ENSG00000142208	16168102;17339365;16782806;18071906;16740780;16438844;17359293;15494689;17935137;16059641;
D004317	decrease	AZGP1	ENSG00000160862	16404146;
D004317	decrease	B4GALT1	ENSG00000086062	16404146;
D004317	decrease	BAD	ENSG00000002330	16705698;16001973;16843435;16438844;17359293;
D004317	decrease	BCL2L11	ENSG00000153094	17339365;
D004317	decrease	BID	ENSG00000015475	15289866;15870702;16705698;16868541;15812552;
D004317	decrease	BIK	ENSG00000100290	16007125;16705698;
D004317	decrease	BRCA1	ENSG00000012048	10344722;15607317;
D004317	decrease	BRCA2	ENSG00000139618	10344722;
D004317	decrease	BUB1B	ENSG00000156970	16217747;15870702;
D004317	decrease	CAPN6	ENSG00000077274	16404146;
D004317	decrease	CCND1	ENSG00000110092	16036217;
D004317	decrease	CCNE1	ENSG00000105173	16036217;
D004317	decrease	CDK1	ENSG00000170312	19074854;16036217;16537896;15205334;15870702;15141020;17320279;
D004317	decrease	CENPA	ENSG00000115163	16217747;15870702;
D004317	decrease	CFLAR	ENSG00000003402	14601052;15897917;
D004317	decrease	CHEK1	ENSG00000149554	17085670;15489221;16217747;18698031;16036217;17991895;15870702;17088865;17320279;
D004317	decrease	CLDN1	ENSG00000163347	16404146;

D004317	decrease	COL12A1	ENSG00000111799	15205334;
D004317	decrease	COL18A1	ENSG00000182871	17627616;
D004317	decrease	CREBBP	ENSG00000005339	17498666;
D004317	decrease	CRISP2	ENSG00000124490	16404146;
D004317	decrease	CXCL6	ENSG00000124875	16404146;
D004317	decrease	DGKI	ENSG00000157680	16404146;
D004317	decrease	DKK2	ENSG00000155011	16404146;
D004317	decrease	DSPP	ENSG00000152591	16404146;
D004317	decrease	EGF	ENSG00000138798	16969495;
D004317	decrease	EP300	ENSG00000100393	10344722;17498666;
D004317	decrease	ERAP1	ENSG00000164307	16404146;
D004317	decrease	FCGR3A	ENSG00000203747	17852453;
D004317	decrease	FOLR1	ENSG00000110195	16404146;15634643;
D004317	decrease	FUT4	ENSG00000196371	17852453;
D004317	decrease	GLO1	ENSG00000124767	16085563;
D004317	decrease	GSTA1	ENSG00000243955	16890185;
D004317	decrease	GULP1	ENSG00000144366	16001973;
D004317	decrease	HCRTR1	ENSG00000121764	16404146;
D004317	decrease	HK2	ENSG00000159399	16595493;
D004317	decrease	HLA-DQB1	ENSG00000179344	16404146;16322897;
D004317	decrease	ITGB4	ENSG00000132470	15205334;
D004317	decrease	JAG2	ENSG00000184916	16404146;
D004317	decrease	KCNJ13	ENSG00000115474	16404146;
D004317	decrease	KCNJ16	ENSG00000153822	16404146;
D004317	decrease	KLF6	ENSG00000067082	15205334;
D004317	decrease	LMNB1	ENSG00000113368	16001973;16217747;
D004317	decrease	LY75	ENSG00000054219	16404146;
D004317	decrease	MAD2L1	ENSG00000164109	15870702;16217747;
D004317	decrease	MAPK14	ENSG00000112062	15870702;16843435;15557793;15494689;16322899;
D004317	decrease	MAPT	ENSG00000186868	16930453;16896004;
D004317	decrease	MCL1	ENSG00000143384	17935137;
D004317	decrease	METAP2	ENSG00000111142	16404146;
D004317	decrease	MKI67	ENSG00000148773	15205334;18695136;
D004317	decrease	MLLT3	ENSG00000171843	16404146;
D004317	decrease	MME	ENSG00000196549	16997790;16217747;
D004317	decrease	MSH6	ENSG00000116062	16404146;
D004317	decrease	MYC	ENSG00000136997	18606404;16001973;11585056;18802399;15911101;
D004317	decrease	NDUFB7	ENSG00000099795	16404146;
D004317	decrease	NMBR	ENSG00000135577	16404146;
D004317	decrease	OR2F1	ENSG00000213215	16404146;
D004317	decrease	PPARD	ENSG00000112033	16404146;
D004317	decrease	PROCR	ENSG00000101000	17172434;
D004317	decrease	PTTG1	ENSG00000164611	15205334;
D004317	decrease	RAD51	ENSG00000051180	17351394;10344722;
D004317	decrease	RBL1	ENSG00000080839	16537896;
D004317	decrease	REL	ENSG00000162924	15870702;16404146;17890907;
D004317	decrease	RGS13	ENSG00000127074	16404146;
D004317	decrease	SCNN1G	ENSG00000166828	15564131;
D004317	decrease	SEC22B	ENSG00000223380	16404146;
D004317	decrease	SMAD2	ENSG00000175387	18606404;
D004317	decrease	SMAD3	ENSG00000166949	18606404;

D004317	decrease	SMAD4	ENSG00000141646	18606404;
D004317	decrease	SQLE	ENSG00000104549	15205334;
D004317	decrease	TFF1	ENSG00000160182	16579640;
D004317	decrease	THBS1	ENSG00000137801	15205334;16962673;
D004317	decrease	TNFRSF25	ENSG00000215788	16705698;
D004317	decrease	TTPA	ENSG00000137561	16404146;
D004317	decrease	TYMS	ENSG00000176890	16168113;10482907;
D004317	decrease	UGT2B4	ENSG00000156096	16404146;
D004317	decrease	UPP1	ENSG00000183696	18510171;
D004317	decrease	UQCRH	ENSG00000173660	18510171;
D004317	decrease	ZNF22	ENSG00000165512	16404146;
D004317	increase	ABCB1	ENSG00000085563	16044152;15765123;16322897;15239142;16356834;17085340;16499877;17947497;16831368;8917702;11313874;11355955;15725475;15112361;17284363;15861398;15967469;17526808;17483874;18461970;15501994;15736412;18510171;18560228;16544145;17852453;16579640;15946544;18992151;[illegible]
D004317	increase	ABCB4	ENSG00000005471	11313874;16579640;12926078;
D004317	increase	ABCC1	ENSG00000103222	15581632;17940500;11560771;10900222;15473893;12067707;12657726;12504668;15548710;15880572;18560228;17852453;15946544;17352253;16331495;
D004317	increase	ABCG2	ENSG00000118777	18382425;17273774;17938326;
D004317	increase	AIFM1	ENSG00000156709	16168113;
D004317	increase	ANGPT1	ENSG00000154188	15763944;
D004317	increase	APAF1	ENSG00000120868	15939500;16001973;
D004317	increase	ARHGEF17	ENSG00000110237	16404146;
D004317	increase	ARHGEF1	ENSG00000076928	16404146;
D004317	increase	ARL6IP5	ENSG00000144746	16430862;
D004317	increase	ATF4	ENSG00000128272	16298333;
D004317	increase	ATP6V0E1	ENSG00000113732	16579640;
D004317	increase	BAX	ENSG00000087088	15456408;17542780;15492826;16322301;16705698;15576332;14601052;16001973;17404015;15939500;17922852;17959036;17339365;16168113;15985719;
D004317	increase	BBC3	ENSG00000105327	16439685;
D004317	increase	BCL2A1	ENSG00000140379	16217747;16572199;
D004317	increase	BCL2	ENSG00000171791	17542780;16450387;16322301;17541305;16705698;15576332;14601052;15897917;16168113;18560228;16001973;17404015;16890185;17959036;11779855;15939500;15571967;15985719;163[illegible]
D004317	increase	BCL2L12	ENSG00000126453	17404015;15576332;

D004317	increase	BCL2L1	ENSG00000171552	17923112;15289866;11468182;15781649;14601052;16843435;17922852;18560228;15939500;17912235;1591
D004317	increase	BIRC2	ENSG00000110330	16217747;14666661;14601052;15359644;17521628;15571967;
D004317	increase	BIRC3	ENSG00000023445	16217747;12926068;12948851;15899819;16705698;17521628;15911101;
D004317	increase	BIRC5	ENSG00000089685	11911975;16086872;16108013;16364925;14601052;17124180;19363521;17521628;17035597;
D004317	increase	BIRC6	ENSG00000115760	17521628;
D004317	increase	BIRC7	ENSG00000101197	17521628;
D004317	increase	BIRC8	ENSG00000163098	17521628;
D004317	increase	BTG2	ENSG00000159388	16705698;
D004317	increase	C7ORF23	ENSG00000135185	15556294;18510171;
D004317	increase	CABC1	ENSG00000163050	16404146;
D004317	increase	CASP10	ENSG00000003400	16001973;
D004317	increase	CASP1	ENSG00000137752	16001973;
D004317	increase	CASP2	ENSG00000106144	15917298;17013758;16001973;14757846;
D004317	increase	CASP3	ENSG00000164305	17085670;15456408;17285121;17542780;17013758;16909308;15870702;18278454;15981920;17088416;16962673;16843435;16364925;15985719;15571967;11779855;19106633;15578696;15680309;15868924;17437844;16823846;18508926;17959036;17653088;15555623;18071906;15557793;16890185;15897917;16024631;18246814;17935137;17651020;16705698;15576332;16001973;15939500;16168113;168[illegible]
D004317	increase	CASP6	ENSG00000138794	15870702;17922852;17437844;16001973;
D004317	increase	CASP7	ENSG00000165806	15870702;16298333;19106633;17653088;16024631;16705698;16001973;1616811
D004317	increase	CASP8	ENSG00000064012	17013758;15870702;16705698;15981920;18508926;15868924;17437844;18071906;15897917;16024631;16001973;16168113;16868541;17970047;15812552;
D004317	increase	CASP9	ENSG00000132906	17285121;17339365;17013758;15870702;16705698;15981920;17404015;18508926;11779855;17437844;15868924;17935137;15555623;17653088;16311509;15557793;16024631;17651020;16364925;15939500;16001973;15576332;16168113;15812[illegible]

D004317	increase	CBR1	ENSG00000159228	18635746;18755171;
D004317	increase	CCL2	ENSG00000108691	12908082;
D004317	increase	CCNA2	ENSG00000145386	16036217;17390037;16537896;15205334;16284694;
D004317	increase	CCNB1	ENSG00000134057	16928833;17893511;16537896;15205334;15141020;17320279;
D004317	increase	CD44	ENSG00000026508	16705698;
D004317	increase	CDC25C	ENSG00000158402	19074854;17320279;
D004317	increase	CDK10	ENSG00000185324	16404146;
D004317	increase	CDKN1A	ENSG00000124762	15781256;18347191;19074854;15492826;15161699;16909308;15823547;15601469;17893511;18269916;14601052;15141020;16705698;15205334;16439685;17653088;16537896;17682292;15555623;17974990;14665630;[illegible]
D004317	increase	CDKN1B	ENSG00000111276	16705698;17893511;16036217;17935137;16322899;
D004317	increase	CDKN2A	ENSG00000147889	16705698;
D004317	increase	CEBPB	ENSG00000172216	16001973;
D004317	increase	CES2	ENSG00000172831	15205334;
D004317	increase	CHI3L1	ENSG00000133048	15205334;
D004317	increase	CLIP1	ENSG00000130779	16404146;
D004317	increase	CPT1A	ENSG00000110090	16283381;
D004317	increase	CSPG4	ENSG00000173546	16404146;
D004317	increase	CTGF	ENSG00000118523	15205334;16579640;
D004317	increase	CTSD	ENSG00000117984	18566016;
D004317	increase	CYC1	ENSG00000179091	16001973;
D004317	increase	CYCS	ENSG00000172115	16331251;16749863;16705698;16001973;16868541;
D004317	increase	CYP27B1	ENSG00000111012	17716971;
D004317	increase	DDIT3	ENSG00000175197	16298333;17912235;
D004317	increase	DEK	ENSG00000124795	16579640;
D004317	increase	DHPS	ENSG00000095059	16404146;
D004317	increase	DIABLO	ENSG00000184047	12948851;17521628;
D004317	increase	DNAJA1	ENSG00000086061	16579640;
D004317	increase	DNMT1	ENSG00000130816	17991895;17065238;17124180;
D004317	increase	DPP4	ENSG00000197635	15753397;
D004317	increase	EDN1	ENSG00000078401	17974986;
D004317	increase	EGR1	ENSG00000120738	15205334;17330857;
D004317	increase	EPHA2	ENSG00000142627	16900372;
D004317	increase	EPHX1	ENSG00000143819	15205334;
D004317	increase	ESR1	ENSG00000091831	16322899;
D004317	increase	ESR2	ENSG00000140009	16900372;16322899;
D004317	increase	F3	ENSG00000117525	18278454;18246814;18325115;
D004317	increase	F7	ENSG00000057593	18325115;
D004317	increase	FADD	ENSG00000168040	16705698;16001973;17970047;
D004317	increase	FAS	ENSG00000026103	15763944;15981920;17970047;17404015;17088865;15576332;11779855;1670569
D004317	increase	FASLG	ENSG00000117560	15763944;17088865;15981920;

D004317	increase	FASN	ENSG00000169710	17089011;
D004317	increase	FDXR	ENSG00000161513	15205334;
D004317	increase	FKBP8	ENSG00000105701	16404146;
D004317	increase	FOS	ENSG00000170345	15205334;16900372;
D004317	increase	G6PD	ENSG00000160211	16404146;
D004317	increase	GAD1	ENSG00000128683	17065238;
D004317	increase	GADD45A	ENSG00000116717	15205334;
D004317	increase	GADD45G	ENSG00000130222	16404146;
D004317	increase	GATA1	ENSG00000102145	17097070;14623254;
D004317	increase	GDF15	ENSG00000130513	16705698;15205334;
D004317	increase	GP9	ENSG00000169704	16404146;
D004317	increase	GSTP1	ENSG00000084207	14623254;16579640;15533597;15205334;15448748;16356834;18225754;16217747;15946544;10900222;
D004317	increase	HBG2	ENSG00000196565	17097070;
D004317	increase	HIF1A	ENSG00000100644	17498666;17912235;16532342;16628086;
D004317	increase	HRAS	ENSG00000174775	16001973;
D004317	increase	HSP90AA1	ENSG00000080824	17680992;
D004317	increase	HSPA4	ENSG00000170606	16311509;
D004317	increase	HSPA5	ENSG00000044574	16298333;17680992;
D004317	increase	HSPA8	ENSG00000109971	16579640;17680992;
D004317	increase	HSPA9	ENSG00000113013	17680992;
D004317	increase	HSPB1	ENSG00000106211	17680992;
D004317	increase	HSPD1	ENSG00000144381	17680992;
D004317	increase	HUWE1	ENSG00000086758	16404146;
D004317	increase	ID3	ENSG00000117318	15205334;
D004317	increase	IFNA1	ENSG00000197919	17959036;
D004317	increase	IL6	ENSG00000136244	16001973;
D004317	increase	IL8	ENSG00000169429	15899819;18510171;12908082;16217747;
D004317	increase	JUN	ENSG00000177606	15205334;15880572;15585644;18645001;
D004317	increase	KHSRP	ENSG00000088247	16404146;
D004317	increase	KLF2	ENSG00000127528	18510171;
D004317	increase	MAP2K4	ENSG00000065559	15870702;16001973;
D004317	increase	MAPK1	ENSG00000100030	17526808;18468633;16001973;17974986;15557793;
D004317	increase	MAPK8	ENSG00000107643	15870702;15880572;16890185;15917298;15494689;16868541;
D004317	increase	MDM2	ENSG00000135679	16001973;17959036;14665630;17653088;19074854;17935137;
D004317	increase	MEF2A	ENSG00000068305	16001973;
D004317	increase	MFGE8	ENSG00000140545	16404146;
D004317	increase	MRPS18A	ENSG00000096080	16404146;
D004317	increase	MT1G	ENSG00000125144	16579640;
D004317	increase	MT2A	ENSG00000125148	16579640;
D004317	increase	NAIP	ENSG00000250628	17521628;11911975;15911101;16322899;
D004317	increase	NFKB1	ENSG00000109320	15905586;15870702;15571967;15555623;16001973;17912235;18463201;1632230

D004317	increase	NFKBIA	ENSG00000100906	15870702;15899819;17935137;17542780;15571967;18463201;
D004317	increase	NOS2	ENSG00000007171	15695394;18463201;
D004317	increase	OR12D2	ENSG00000168787	16404146;
D004317	increase	P2RX2	ENSG00000187848	16404146;
D004317	increase	PAX6	ENSG00000007372	16404146;
D004317	increase	PDCD5	ENSG00000105185	16579640;
D004317	increase	PITX1	ENSG00000069011	16404146;
D004317	increase	PLAU	ENSG00000122861	15557793;12908082;16356834;16217747;
D004317	increase	PMAIP1	ENSG00000141682	17653088;
D004317	increase	POLD4	ENSG00000175482	16404146;
D004317	increase	POMT2	ENSG00000009830	16404146;
D004317	increase	PPT2	ENSG00000231618	16404146;
D004317	increase	PRKACA	ENSG00000072062	16404146;
D004317	increase	PRL	ENSG00000172179	18681966;
D004317	increase	PTEN	ENSG00000171862	11707646;17330857;17935137;16438844;17359293;15548710;
D004317	increase	PTGS2	ENSG00000073756	15846114;15205334;15501994;16127422;18498876;
D004317	increase	PTPN13	ENSG00000163629	16001973;
D004317	increase	RARB	ENSG00000077092	17608728;
D004317	increase	RBL2	ENSG00000103479	16537896;15585644;
D004317	increase	RBPMS	ENSG00000157110	16404146;
D004317	increase	RELA	ENSG00000173039	15905586;15911101;17097285;15870702;18269916;15899819;15571967;15555623;18463201;17542780;17890907;15823547;16322301;1
D004317	increase	RELN	ENSG00000189056	17065238;
D004317	increase	RHCG	ENSG00000140519	16404146;
D004317	increase	RPS27L	ENSG00000185088	18056458;
D004317	increase	SERPINE1	ENSG00000106366	16705698;15870702;16217747;
D004317	increase	SERPINH1	ENSG00000149257	17680992;
D004317	increase	SLC22A5	ENSG00000197375	16283381;
D004317	increase	SLC2A1	ENSG00000117394	16595493;
D004317	increase	SLIT3	ENSG00000184347	16404146;
D004317	increase	SMAD7	ENSG00000101665	18606404;
D004317	increase	SOD1	ENSG00000142168	16579640;
D004317	increase	SOS1	ENSG00000115904	16001973;
D004317	increase	SPARC	ENSG00000113140	15870702;
D004317	increase	SSH3	ENSG00000172830	16404146;
D004317	increase	STAT1	ENSG00000115415	16001973;17072862;
D004317	increase	STAT2	ENSG00000170581	16001973;
D004317	increase	STAT3	ENSG00000168610	16001973;17920763;
D004317	increase	TAGLN	ENSG00000149591	15870702;
D004317	increase	TERT	ENSG00000164362	16077987;15003732;18560228;18396642;
D004317	increase	TGM2	ENSG00000198959	15870702;17073438;
D004317	increase	THBD	ENSG00000178726	17172434;
D004317	increase	TIA1	ENSG00000116001	16404146;
D004317	increase	TLX3	ENSG00000164438	16404146;

D004317	increase	TNF	ENSG00000223952	15823547;16001973;15899819;12908082;17651020;
D004317	increase	TNFRSF10A	ENSG00000104689	16364925;15897917;17437844;16098063;
D004317	increase	TNFRSF10B	ENSG00000120889	16364925;16705698;17922852;15897917;16098063;
D004317	increase	TNFSF10	ENSG00000121858	16364925;19106633;
D004317	increase	TOP2A	ENSG00000131747	16026610;15205334;17351394;15765123;15239142;16217747;11470519;15753397;16969495;15486187;
D004317	increase	TP53	ENSG00000141510	15781256;15763944;17285121;16826403;15489221;19074854;17893511;19363521;17634554;17124180;18510171;15141020;16013437;16404146;15939500;17959036;16001973;17088865;14665630;12082016;15671536;16108013;11779855;15555623;15601469;17339365;10969820;16537896;17912235;17653088;16432175;17555331;19106633;16168113;15823547;17085670;15578696;17369602;15182437;17608641;18698031;18269916;17079232;17682292;16211[illegible]
D004317	increase	TP53I3	ENSG00000115129	15205334;
D004317	increase	TP73	ENSG00000078900	17716971;
D004317	increase	TRAP1	ENSG00000126602	17680992;
D004317	increase	TRIM23	ENSG00000113595	16404146;
D004317	increase	TRIM28	ENSG00000130726	17079232;
D004317	increase	TRIM34	ENSG00000239456	16404146;
D004317	increase	TUBB	ENSG00000229684	16579640;
D004317	increase	TYMP	ENSG00000025708	15150550;
D004317	increase	UGCG	ENSG00000148154	16404146;18245173;
D004317	increase	VDR	ENSG00000111424	17716971;
D004317	increase	VEGFA	ENSG00000112715	17627616;17498666;16900372;17912235;18494554;17431384;
D004317	increase	XBP1	ENSG00000100219	15205334;
D004317	increase	XIAP	ENSG00000101966	18071906;15359644;14601052;15897917;12948851;11911975;17521628;16211302;15911101;
D004317	increase	ZMAT3	ENSG00000172667	16439685;
D004317	increase	ZMYM2	ENSG00000121741	16579640;
D004318	decrease	ABCB1	ENSG00000085563	11147995;
D004318	increase	IL1B	ENSG00000125538	17267227;
D004318	increase	MMP13	ENSG00000137745	17267227;
D004318	increase	MMP3	ENSG00000149968	17267227;
D004365	decrease	BCL2	ENSG00000171791	17696246;19295228;
D004365	decrease	CCND1	ENSG00000110092	15456542;
D004365	decrease	CCND2	ENSG00000118971	15456542;
D004365	decrease	CCND3	ENSG00000112576	15456542;
D004365	decrease	CCNE1	ENSG00000105173	15456542;
D004365	decrease	IL5	ENSG00000113525	15644155;

D004365	decrease	MCL1	ENSG00000143384	15802533;
D004365	decrease	MYC	ENSG00000136997	19295228;
D004365	decrease	NOS3	ENSG00000164867	16891912;
D004365	decrease	TEP1	ENSG00000129566	19295228;
D004365	decrease	TERT	ENSG00000164362	19295228;
D004365	increase	BAX	ENSG00000087088	15456542;15802533;
D004365	increase	CASP3	ENSG00000164305	17696246;15456542;15802533;19295228;
D004365	increase	CD40	ENSG00000101017	15894585;
D004365	increase	CD80	ENSG00000121594	15894585;
D004365	increase	CD83	ENSG00000112149	15894585;
D004365	increase	CD86	ENSG00000114013	15894585;
D004365	increase	CYP11A1	ENSG00000140459	14711788;
D004365	increase	CYP19A1	ENSG00000137869	14711788;
D004365	increase	CYP3A4	ENSG00000160868	12695340;
D004365	increase	FGF2	ENSG00000138685	16313111;
D004365	increase	HSD3B2	ENSG00000203859	14711788;
D004365	increase	ICAM1	ENSG00000090339	15894585;
D004365	increase	IL10	ENSG00000136634	15894585;
D004365	increase	IL12A	ENSG00000168811	15894585;
D004365	increase	IL12B	ENSG00000113302	15894585;
D004365	increase	OLR1	ENSG00000173391	16891912;
D004365	increase	STAR	ENSG00000147465	14711788;
D004390	decrease	HYDIN	ENSG00000157423	16790487;
D004390	increase	BCR	ENSG00000186716	16790487;
D004390	increase	CLIP3	ENSG00000105270	16790487;
D004390	increase	CYP2B6	ENSG00000197408	17079358;15557345;15764407;11854147;16757081;15548381;
D004390	increase	CYP2C19	ENSG00000165841	17079358;15764407;16757081;
D004390	increase	CYP2D6	ENSG00000100197	16757081;
D004390	increase	CYP3A4	ENSG00000160868	17079358;17110060;16790556;15560889;16757081;15548381;
D004390	increase	CYP3A5	ENSG00000106258	17079358;17110060;16757081;
D004390	increase	IL6	ENSG00000136244	16790487;
D004390	increase	MAPK1	ENSG00000100030	16790487;
D004390	increase	MAPK3	ENSG00000102882	16790487;
D004390	increase	PPP2R3B	ENSG00000167393	16790487;
D004391	affect	CCL11	ENSG00000172156	17805423;
D004391	affect	IL1A	ENSG00000115008	17805423;
D004391	decrease	ACAT1	ENSG00000075239	17805423;
D004391	decrease	ACVR1B	ENSG00000135503	17805423;
D004391	decrease	ACY1	ENSG00000243989	17805423;
D004391	decrease	AGTRAP	ENSG00000177674	17805423;
D004391	decrease	ALDH2	ENSG00000111275	17805423;
D004391	decrease	AMH	ENSG00000104899	17805423;
D004391	decrease	AMHR2	ENSG00000135409	17805423;
D004391	decrease	ARF1	ENSG00000143761	17805423;
D004391	decrease	BAG1	ENSG00000250477	17805423;
D004391	decrease	BNIP3	ENSG00000176171	17805423;
D004391	decrease	CAPNS1	ENSG00000126247	17805423;
D004391	decrease	CAT	ENSG00000121691	17805423;

D004391	decrease	CCNA2	ENSG00000145386	17805423;
D004391	decrease	CCNB1	ENSG00000134057	17805423;
D004391	decrease	CCNF	ENSG00000162063	17805423;
D004391	decrease	CCNI	ENSG00000118816	17805423;
D004391	decrease	CCT2	ENSG00000166226	17805423;
D004391	decrease	CCT4	ENSG00000115484	17805423;
D004391	decrease	CD164	ENSG00000135535	17805423;
D004391	decrease	CD46	ENSG00000117335	17805423;
D004391	decrease	CD63	ENSG00000135404	17805423;
D004391	decrease	CD81	ENSG00000110651	17805423;
D004391	decrease	CDK4	ENSG00000135446	17805423;
D004391	decrease	CDKN2B	ENSG00000147883	17805423;
D004391	decrease	CDKN2C	ENSG00000123080	17805423;
D004391	decrease	COMT	ENSG00000093010	17805423;
D004391	decrease	CREB3L3	ENSG00000060566	17805423;
D004391	decrease	CRIP1	ENSG00000213145	17805423;
D004391	decrease	CXCR4	ENSG00000121966	17805423;
D004391	decrease	CYP2A7	ENSG00000198077	17805423;
D004391	decrease	DEFB1	ENSG00000164825	17805423;
D004391	decrease	EIF4A2	ENSG00000156976	17805423;
D004391	decrease	EPHX1	ENSG00000143819	17805423;
D004391	decrease	FCGR1A	ENSG00000150337	17805423;
D004391	decrease	FGF7	ENSG00000140285	17805423;
D004391	decrease	FOSL1	ENSG00000175592	17805423;
D004391	decrease	FUS	ENSG00000089280	17805423;
D004391	decrease	GDF1	ENSG00000130283	17805423;
D004391	decrease	HMGCS1	ENSG00000112972	17805423;
D004391	decrease	HSPD1	ENSG00000144381	17805423;
D004391	decrease	HSPE1	ENSG00000115541	17805423;
D004391	decrease	IFNGR1	ENSG00000027697	17805423;
D004391	decrease	IL12B	ENSG00000113302	17805423;
D004391	decrease	IRF7	ENSG00000185507	17805423;
D004391	decrease	ITGA2B	ENSG00000005961	17805423;
D004391	decrease	KRT7	ENSG00000135480	17805423;
D004391	decrease	LDHA	ENSG00000134333	17805423;
D004391	decrease	MAPK14	ENSG00000112062	17805423;
D004391	decrease	MGMT	ENSG00000170430	17805423;
D004391	decrease	MGST2	ENSG00000085871	17805423;
D004391	decrease	MRPS28	ENSG00000147586	17805423;
D004391	decrease	MT2A	ENSG00000125148	17805423;
D004391	decrease	NME4	ENSG00000103202	17805423;
D004391	decrease	NPM1	ENSG00000181163	17805423;
D004391	decrease	NTHL1	ENSG00000065057	17805423;
D004391	decrease	NTRK3	ENSG00000140538	17805423;
D004391	decrease	ODC1	ENSG00000115758	17805423;
D004391	decrease	PCNA	ENSG00000132646	17805423;
D004391	decrease	PDCD4	ENSG00000150593	17805423;
D004391	decrease	PGAM1	ENSG00000171314	17805423;
D004391	decrease	PIM1	ENSG00000137193	17805423;
D004391	decrease	PPA1	ENSG00000180817	17805423;
D004391	decrease	PSMB3	ENSG00000108294	17805423;
D004391	decrease	PSME2	ENSG00000100911	17805423;
D004391	decrease	PTDSS1	ENSG00000156471	17805423;
D004391	decrease	PTHLH	ENSG00000087494	17805423;

D004391	decrease	PTMS	ENSG00000159335	17805423;
D004391	decrease	RNASE3	ENSG00000169397	17805423;
D004391	decrease	RNASE6	ENSG00000169413	17805423;
D004391	decrease	RNH1	ENSG00000023191	17805423;
D004391	decrease	RPLP1	ENSG00000137818	17805423;
D004391	decrease	RPS15	ENSG00000115268	17805423;
D004391	decrease	SCARB1	ENSG00000073060	17805423;
D004391	decrease	SOAT2	ENSG00000167780	17805423;
D004391	decrease	TCERG1	ENSG00000113649	17805423;
D004391	decrease	TCP1	ENSG00000120438	17805423;
D004391	decrease	TP53	ENSG00000141510	17805423;
D004391	decrease	TUBA1B	ENSG00000123416	17805423;
D004391	decrease	TUBG1	ENSG00000131462	17805423;
D004391	decrease	TYMP	ENSG00000025708	17805423;
D004391	decrease	VEGFA	ENSG00000112715	17805423;
D004391	decrease	XRCC5	ENSG00000079246	17805423;
D004391	increase	ADAM17	ENSG00000151694	17805423;
D004391	increase	ADAMDEC1	ENSG00000134028	17805423;
D004391	increase	ANXA5	ENSG00000164111	17805423;
D004391	increase	BCL2A1	ENSG00000140379	17805423;
D004391	increase	BIRC3	ENSG00000023445	17805423;
D004391	increase	BMPR1A	ENSG00000107779	17805423;
D004391	increase	BTG2	ENSG00000159388	17805423;
D004391	increase	CASP1	ENSG00000137752	17805423;
D004391	increase	CCL19	ENSG00000172724	17805423;
D004391	increase	CCL1	ENSG00000108702	17805423;
D004391	increase	CCL20	ENSG00000115009	17805423;
D004391	increase	CCL24	ENSG00000106178	17805423;
D004391	increase	CCL2	ENSG00000108691	17805423;
D004391	increase	CCL3	ENSG00000006075	17805423;
D004391	increase	CCL4	ENSG00000129277	17805423;
D004391	increase	CCL5	ENSG00000161570	17805423;
D004391	increase	CCL7	ENSG00000108688	17805423;
D004391	increase	CCR5	ENSG00000160791	17805423;
D004391	increase	CD14	ENSG00000170458	17805423;
D004391	increase	CD44	ENSG00000026508	17805423;
D004391	increase	CD48	ENSG00000117091	17805423;
D004391	increase	CD55	ENSG00000196352	17805423;
D004391	increase	CD83	ENSG00000112149	17805423;
D004391	increase	CDKN1A	ENSG00000124762	17805423;
D004391	increase	CHST1	ENSG00000175264	17805423;
D004391	increase	CITED1	ENSG00000125931	17805423;
D004391	increase	CSF1	ENSG00000184371	17805423;
D004391	increase	CSF2	ENSG00000164400	17805423;
D004391	increase	CSF3	ENSG00000108342	17805423;
D004391	increase	CTGF	ENSG00000118523	15735009;
D004391	increase	CXCL10	ENSG00000169245	17805423;
D004391	increase	CXCL1	ENSG00000163739	17805423;
D004391	increase	CXCL2	ENSG00000081041	17805423;
D004391	increase	CXCL3	ENSG00000163734	17805423;
D004391	increase	CYP1A1	ENSG00000140465	15735009;
D004391	increase	CYP1B1	ENSG00000138061	17805423;15735009;
D004391	increase	CYR61	ENSG00000142871	15735009;
D004391	increase	DUSP1	ENSG00000120129	17805423;15735009;

D004391	increase	DUSP2	ENSG00000158050	17805423;
D004391	increase	DUSP5	ENSG00000138166	17805423;
D004391	increase	FCAR	ENSG00000248798	17805423;
D004391	increase	FGR	ENSG00000000938	17805423;
D004391	increase	FOS	ENSG00000170345	15735009;
D004391	increase	FTH1	ENSG00000167996	17805423;
D004391	increase	GADD45A	ENSG00000116717	17805423;
D004391	increase	GATA3	ENSG00000107485	17805423;
D004391	increase	GCLC	ENSG00000001084	17805423;
D004391	increase	GGT1	ENSG00000100031	17805423;
D004391	increase	GSS	ENSG00000100983	17805423;
D004391	increase	GSTM3	ENSG00000134202	17805423;
D004391	increase	HLA-A	ENSG00000206503	17805423;
D004391	increase	HLA-E	ENSG00000225201	17805423;
D004391	increase	HLA-F	ENSG00000204642	17805423;
D004391	increase	HSP90B1	ENSG00000166598	17805423;
D004391	increase	HSPA1A	ENSG00000232804	17805423;
D004391	increase	HSPA1B	ENSG00000232804	17805423;
D004391	increase	HSPA5	ENSG00000044574	17805423;
D004391	increase	HSPA6	ENSG00000173110	17805423;
D004391	increase	ICAM1	ENSG00000090339	17805423;
D004391	increase	IER3	ENSG00000137331	17805423;
D004391	increase	IFNG	ENSG00000111537	17805423;
D004391	increase	IFNGR2	ENSG00000159128	17805423;
D004391	increase	IGFBP3	ENSG00000146674	17805423;
D004391	increase	IL10	ENSG00000136634	17805423;
D004391	increase	IL1B	ENSG00000125538	17805423;
D004391	increase	IL23A	ENSG00000110944	17805423;
D004391	increase	IL32	ENSG00000008517	17805423;
D004391	increase	IL3	ENSG00000164399	17805423;
D004391	increase	IL6	ENSG00000136244	17805423;
D004391	increase	IL7	ENSG00000104432	17805423;
D004391	increase	IL8	ENSG00000169429	17805423;
D004391	increase	JAG1	ENSG00000101384	15735009;
D004391	increase	JUND	ENSG00000130522	17805423;
D004391	increase	JUN	ENSG00000177606	17805423;15735009;
D004391	increase	LIF	ENSG00000128342	17805423;
D004391	increase	MCL1	ENSG00000143384	15735009;
D004391	increase	MMP14	ENSG00000157227	17805423;
D004391	increase	MMP9	ENSG00000100985	17805423;
D004391	increase	MT1A	ENSG00000205362	17805423;
D004391	increase	MT1E	ENSG00000169715	17805423;
D004391	increase	MT1G	ENSG00000125144	17805423;
D004391	increase	MT1H	ENSG00000205358	17805423;
D004391	increase	MYST2	ENSG00000136504	17805423;
D004391	increase	NAMPT	ENSG00000105835	17805423;
D004391	increase	NFKB1	ENSG00000109320	17805423;
D004391	increase	NFKB2	ENSG00000077150	17805423;
D004391	increase	NFKBIA	ENSG00000100906	17805423;
D004391	increase	NQO1	ENSG00000181019	17805423;
D004391	increase	PLAU	ENSG00000122861	17805423;
D004391	increase	PLAUR	ENSG00000011422	17805423;
D004391	increase	PMAIP1	ENSG00000141682	17805423;
D004391	increase	POR	ENSG00000127948	17805423;

D004391	increase	PPARG	ENSG00000132170	15735009;
D004391	increase	PTGS2	ENSG00000073756	17805423;
D004391	increase	RASSF2	ENSG00000101265	17805423;
D004391	increase	RCN1	ENSG00000049449	17805423;
D004391	increase	RGS1	ENSG00000090104	17805423;
D004391	increase	RIPK2	ENSG00000104312	17805423;
D004391	increase	SAT1	ENSG00000130066	17805423;
D004391	increase	SERPINB2	ENSG00000197632	17805423;
D004391	increase	SLC2A1	ENSG00000117394	15735009;
D004391	increase	SOD2	ENSG00000112096	17805423;
D004391	increase	TANK	ENSG00000136560	17805423;
D004391	increase	THBS1	ENSG00000137801	17805423;
D004391	increase	TMSB4X	ENSG00000205542	17805423;
D004391	increase	TNFAIP2	ENSG00000185215	17805423;
D004391	increase	TNFAIP3	ENSG00000118503	17805423;
D004391	increase	TNFAIP6	ENSG00000123610	17805423;
D004391	increase	TNF	ENSG00000223952	17805423;
D004391	increase	TNFRSF1A	ENSG00000067182	17805423;
D004391	increase	TNFRSF1B	ENSG00000028137	17805423;
D004391	increase	TNIP1	ENSG00000145901	17805423;
D004391	increase	TRAF1	ENSG00000056558	17805423;
D004391	increase	TRAF4	ENSG00000076604	17805423;
D004391	increase	UGT2B10	ENSG00000109181	17805423;
D004391	increase	UGT2B4	ENSG00000156096	17805423;
D004394	affect	PIBF1	ENSG00000083535	15760377;
D004394	decrease	IFNG	ENSG00000111537	16045524;
D004394	decrease	TNF	ENSG00000223952	16045524;
D004394	increase	IL4	ENSG00000113520	16045524;
D004394	increase	IL6	ENSG00000136244	16045524;
D004492	increase	IL6	ENSG00000136244	11238657;
D004533	increase	BIRC3	ENSG00000023445	16154993;
D004533	increase	HMOX1	ENSG00000100292	18357586;
D004533	increase	NOS1	ENSG00000089250	16791854;
D004533	increase	TNF	ENSG00000223952	16154993;
D004610	decrease	APAF1	ENSG00000120868	12002526;
D004610	decrease	BMP6	ENSG00000153162	12002526;
D004610	decrease	BRF1	ENSG00000185024	12002526;
D004610	decrease	CALR	ENSG00000179218	12002526;
D004610	decrease	DAXX	ENSG00000231617	12002526;
D004610	decrease	DFFA	ENSG00000160049	12002526;
D004610	decrease	EGR3	ENSG00000179388	12002526;
D004610	decrease	FGFR1	ENSG00000249195	12002526;
D004610	decrease	FLNA	ENSG00000196924	12002526;
D004610	decrease	GPLD1	ENSG00000112293	12002526;
D004610	decrease	HIF1A	ENSG00000100644	12002526;
D004610	decrease	IGFBP4	ENSG00000141753	12002526;
D004610	decrease	KLK3	ENSG00000142515	12002526;
D004610	decrease	MAP1A	ENSG00000166963	12002526;
D004610	decrease	NFKB2	ENSG00000077150	12002526;
D004610	decrease	PML	ENSG00000140464	12002526;
D004610	decrease	PPARD	ENSG00000112033	12002526;
D004610	decrease	PRKG1	ENSG00000185532	12002526;
D004610	decrease	RAN	ENSG00000132341	12002526;
D004610	decrease	RELA	ENSG00000173039	12002526;

D004610	decrease	RGS5	ENSG00000143248	12002526;
D004610	decrease	SOD3	ENSG00000109610	12002526;
D004610	decrease	TAF1C	ENSG00000103168	12002526;
D004610	decrease	TFAM	ENSG00000108064	12002526;
D004610	decrease	UGP2	ENSG00000169764	12002526;
D004610	decrease	USP4	ENSG00000114316	12002526;
D004610	decrease	ZBTB16	ENSG00000109906	12002526;
D004610	increase	ACPP	ENSG00000014257	12002526;
D004610	increase	ASCL1	ENSG00000139352	12002526;
D004610	increase	ASNS	ENSG00000070669	12002526;
D004610	increase	ATF3	ENSG00000162772	12002526;
D004610	increase	ATG5	ENSG00000057663	12002526;
D004610	increase	CASP3	ENSG00000164305	12002526;
D004610	increase	E4F1	ENSG00000167967	12002526;
D004610	increase	EP300	ENSG00000100393	12002526;
D004610	increase	EPS15	ENSG00000085832	12002526;
D004610	increase	EZH1	ENSG00000108799	12002526;
D004610	increase	GSTA4	ENSG00000170899	12002526;
D004610	increase	GTF2E2	ENSG00000197265	12002526;
D004610	increase	GTF2H4	ENSG00000234370	12002526;
D004610	increase	IER2	ENSG00000160888	12002526;
D004610	increase	IGFBP2	ENSG00000115457	12002526;
D004610	increase	INPP5D	ENSG00000168918	12002526;
D004610	increase	IQGAP2	ENSG00000145703	12002526;
D004610	increase	IRF9	ENSG00000213928	12002526;
D004610	increase	LITAF	ENSG00000189067	12002526;
D004610	increase	MMD	ENSG00000108960	12002526;
D004610	increase	NNAT	ENSG00000053438	12002526;
D004610	increase	NPAS2	ENSG00000170485	12002526;
D004610	increase	PLP2	ENSG00000102007	12002526;
D004610	increase	PTN	ENSG00000105894	12002526;
D004610	increase	PTPRN2	ENSG00000155093	12002526;
D004610	increase	2-Sep	ENSG00000168385	12002526;
D004610	increase	STAT3	ENSG00000168610	12002526;
D004610	increase	TCF12	ENSG00000140262	12002526;
D004610	increase	TGFB1	ENSG00000105329	12002526;
D004610	increase	WARS	ENSG00000140105	12002526;
D004610	increase	ZMYM2	ENSG00000121741	12002526;
D004640	decrease	MAPT	ENSG00000186868	16930453;
D004642	decrease	ACTA1	ENSG00000143632	16045814;
D004642	decrease	AKT1	ENSG00000142208	16045814;17077332;
D004642	decrease	BAG5	ENSG00000166170	16045814;
D004642	decrease	BFAR	ENSG00000103429	16045814;
D004642	decrease	CANX	ENSG00000127022	16045814;
D004642	decrease	CCNB2	ENSG00000157456	16045814;
D004642	decrease	CDK4	ENSG00000135446	16045814;
D004642	decrease	CELSR3	ENSG00000008300	16045814;
D004642	decrease	COPS5	ENSG00000121022	17077332;
D004642	decrease	CTSD	ENSG00000117984	16045814;
D004642	decrease	DHFR	ENSG00000228716	16045814;
D004642	decrease	EIF2B1	ENSG00000111361	16045814;
D004642	decrease	GSTM3	ENSG00000134202	16045814;
D004642	decrease	IFNA2	ENSG00000188379	17077332;
D004642	decrease	ITPR1	ENSG00000150995	16045814;

D004642	decrease	JUND	ENSG00000130522	17077332;
D004642	decrease	KRT19	ENSG00000171345	16045814;
D004642	decrease	MACF1	ENSG00000127603	16045814;
D004642	decrease	MAPT	ENSG00000186868	16045814;
D004642	decrease	PDCL	ENSG00000136940	16045814;
D004642	decrease	PRKCD	ENSG00000163932	16045814;
D004642	decrease	PTPLAD1	ENSG00000074696	16045814;
D004642	decrease	RAF1	ENSG00000132155	16045814;
D004642	decrease	RB1	ENSG00000139687	16045814;
D004642	decrease	RHOA	ENSG00000067560	16045814;
D004642	decrease	ROCK1	ENSG00000067900	16045814;
D004642	decrease	ROCK2	ENSG00000134318	16045814;
D004642	decrease	SAE1	ENSG00000142230	16045814;
D004642	decrease	STIP1	ENSG00000168439	16045814;
D004642	decrease	TAOK3	ENSG00000135090	16045814;
D004642	decrease	TBCC	ENSG00000124659	16045814;
D004642	decrease	TUBB3	ENSG00000198211	16045814;
D004642	decrease	TXNL4B	ENSG00000140830	16045814;
D004642	decrease	VDAC1	ENSG00000213585	16045814;
D004642	increase	ATM	ENSG00000149311	16045814;
D004642	increase	CYP1A1	ENSG00000140465	17012224;14644660;
D004642	increase	CYP1B1	ENSG00000138061	14990334;
D004642	increase	DNAJB1	ENSG00000132002	16045814;
D004642	increase	E2F5	ENSG00000133740	16045814;
D004642	increase	EI24	ENSG00000149547	16045814;
D004642	increase	FAM48A	ENSG00000102710	16045814;
D004642	increase	FOXO1	ENSG00000150907	16045814;
D004642	increase	GAS2	ENSG00000148935	16045814;
D004642	increase	GSTP1	ENSG00000084207	15313406;
D004642	increase	NFKBIA	ENSG00000100906	16045814;
D004642	increase	PIK3C3	ENSG00000078142	16045814;
D004642	increase	PPM1D	ENSG00000170836	16045814;
D004642	increase	PRDX6	ENSG00000117592	16045814;
D004642	increase	RCN2	ENSG00000117906	16045814;
D004642	increase	ST8SIA1	ENSG00000111728	16045814;
D004642	increase	TNF	ENSG00000223952	15313406;
D004642	increase	TRAF2	ENSG00000127191	16045814;
D004726	affect	CYP1B1	ENSG00000138061	11358810;
D004726	decrease	ESR1	ENSG00000091831	14579009;10774817;16626760;10397250;15261991;15064155;14975756;
D004726	decrease	HSP90B1	ENSG00000166598	16884889;
D004726	decrease	HSPB1	ENSG00000106211	16884889;
D004726	increase	BCL2	ENSG00000171791	10821422;
D004726	increase	CYP1A1	ENSG00000140465	11358810;
D004726	increase	CYP26A1	ENSG00000095596	15589975;
D004726	increase	CYP2B6	ENSG00000197408	16581944;16855053;15548381;
D004726	increase	CYP3A4	ENSG00000160868	16581944;16855053;12417264;15548381;
D004726	increase	ESR2	ENSG00000140009	16626760;15261991;15064155;14975756;
D004726	increase	HSPA5	ENSG00000044574	16884889;
D004726	increase	NR1I2	ENSG00000144852	16054614;12417264;15548381;

D004726	increase	PGR	ENSG00000082175	14579009;9144411;18262749;16242299;
D004726	increase	RARB	ENSG00000077092	15589975;
D004726	increase	RARG	ENSG00000172819	15589975;
D004726	increase	TERT	ENSG00000164362	15832825;
D004726	increase	TFF1	ENSG00000160182	16242299;
D004732	increase	CYP26A1	ENSG00000095596	15589975;
D004732	increase	CYP2B6	ENSG00000197408	15548381;
D004732	increase	CYP3A4	ENSG00000160868	15548381;
D004732	increase	RARB	ENSG00000077092	15589975;
D004732	increase	RARG	ENSG00000172819	15589975;
D004786	decrease	KLRK1	ENSG00000213809	18468695;
D004786	decrease	NCR3	ENSG00000225211	18468695;
D004837	increase	CA2	ENSG00000104267	12729923;
D004837	increase	CCL3	ENSG00000006075	12538206;
D004837	increase	CTSK	ENSG00000143387	12729923;
D004837	increase	IL10	ENSG00000136634	10588509;
D004837	increase	IL1B	ENSG00000125538	10588509;
D004837	increase	IL6	ENSG00000136244	10588509;
D004837	increase	ITGAV	ENSG00000138448	12729923;
D004837	increase	SOD1	ENSG00000142168	11163531;
D004837	increase	SOD2	ENSG00000112096	11163531;
D004837	increase	TNF	ENSG00000223952	10588509;
D004917	decrease	TAGLN	ENSG00000149591	17567588;
D004958	affect	ABCA3	ENSG00000167972	14699072;
D004958	affect	ABCD3	ENSG00000117528	14699072;
D004958	affect	ABLIM1	ENSG00000099204	14699072;
D004958	affect	AGT	ENSG00000135744	14699072;17336289;
D004958	affect	AHNAK2	ENSG00000185567	14699072;
D004958	affect	AKAP1	ENSG00000121057	14699072;
D004958	affect	ALCAM	ENSG00000170017	14699072;
D004958	affect	ALDH2	ENSG00000111275	14699072;
D004958	affect	AP1G1	ENSG00000166747	14699072;
D004958	affect	ARL4C	ENSG00000188042	14699072;
D004958	affect	ARL6IP5	ENSG00000144746	14699072;
D004958	affect	BIRC3	ENSG00000023445	14699072;
D004958	affect	BMP4	ENSG00000125378	14699072;
D004958	affect	BMP5	ENSG00000112175	14699072;
D004958	affect	BTBD3	ENSG00000132640	14699072;
D004958	affect	C11ORF41	ENSG00000110427	14699072;
D004958	affect	C18ORF10	ENSG00000134779	14699072;
D004958	affect	C2CD2	ENSG00000157617	14699072;
D004958	affect	C5ORF13	ENSG00000134986	14699072;
D004958	affect	CACNB4	ENSG00000182389	14699072;
D004958	affect	CAMK2G	ENSG00000148660	14699072;
D004958	affect	CASP7	ENSG00000165806	14699072;
D004958	affect	CCL16	ENSG00000161573	14699072;
D004958	affect	CCL2	ENSG00000108691	17006379;14699072;
D004958	affect	CD83	ENSG00000112149	14699072;
D004958	affect	CDA	ENSG00000158825	17404688;
D004958	affect	CDON	ENSG00000064309	14699072;
D004958	affect	CEBPB	ENSG00000172216	17395694;
D004958	affect	CHRD	ENSG00000090539	14699072;
D004958	affect	CLASP2	ENSG00000163539	14699072;

D004958	affect	CLEC4M	ENSG00000104938	14699072;
D004958	affect	CLTB	ENSG00000175416	14699072;
D004958	affect	COL15A1	ENSG00000204291	14699072;
D004958	affect	CPE	ENSG00000109472	14699072;
D004958	affect	CRIP1	ENSG00000213145	14699072;
D004958	affect	CRKL	ENSG00000099942	14699072;
D004958	affect	CST5	ENSG00000170367	14699072;
D004958	affect	CTGF	ENSG00000118523	14699072;
D004958	affect	CTNNB1	ENSG00000168036	16053526;
D004958	affect	CXADR	ENSG00000154639	14699072;
D004958	affect	CYP27B1	ENSG00000111012	17659868;15795327;
D004958	affect	DDIT4	ENSG00000168209	14699072;
D004958	affect	DNMBP	ENSG00000107554	14699072;
D004958	affect	DYNC1I1	ENSG00000158560	14699072;
D004958	affect	ELF1	ENSG00000120690	14699072;
D004958	affect	EMP1	ENSG00000134531	14699072;
D004958	affect	ENPP2	ENSG00000136960	14699072;
D004958	affect	EPHA4	ENSG00000116106	14699072;
D004958	affect	EPHX1	ENSG00000143819	14699072;
D004958	affect	ERBB3	ENSG00000065361	14699072;16029874;
D004958	affect	EVI2A	ENSG00000126860	14699072;
D004958	affect	EXT2	ENSG00000151348	14699072;
D004958	affect	FHL2	ENSG00000115641	14699072;15666801;
D004958	affect	FLRT1	ENSG00000126500	14699072;
D004958	affect	FNBP1	ENSG00000187239	14699072;
D004958	affect	FOXO1	ENSG00000150907	14699072;
D004958	affect	FSHB	ENSG00000131808	17028438;16584521;15781295;
D004958	affect	FZD2	ENSG00000180340	14699072;
D004958	affect	FZD7	ENSG00000155760	14699072;
D004958	affect	GABBR2	ENSG00000136928	14699072;
D004958	affect	GABRA4	ENSG00000109158	16000229;
D004958	affect	GABRA5	ENSG00000186297	16000229;
D004958	affect	GABRP	ENSG00000094755	16000229;
D004958	affect	GALNT1	ENSG00000141429	14699072;
D004958	affect	GAS1	ENSG00000180447	14699072;
D004958	affect	GCH1	ENSG00000250845	14699072;
D004958	affect	GLI2	ENSG00000074047	14699072;
D004958	affect	GPER	ENSG00000164850	17088055;15090535;
D004958	affect	GPR161	ENSG00000143147	14699072;
D004958	affect	GPR176	ENSG00000166073	14699072;
D004958	affect	GPR56	ENSG00000205336	14699072;
D004958	affect	GRB10	ENSG00000106070	14699072;
D004958	affect	HAPLN1	ENSG00000145681	14699072;
D004958	affect	HAS2	ENSG00000170961	14699072;
D004958	affect	HSD17B3	ENSG00000130948	14699072;
D004958	affect	HSF2	ENSG00000025156	17395694;
D004958	affect	IGFBP6	ENSG00000167779	14699072;
D004958	affect	IL11	ENSG00000095752	14699072;
D004958	affect	IL15	ENSG00000164136	14699072;
D004958	affect	IL32	ENSG00000008517	14699072;
D004958	affect	IL7R	ENSG00000168685	14699072;
D004958	affect	JUND	ENSG00000130522	14699072;
D004958	affect	KCNN4	ENSG00000104783	14699072;

D004958	affect	KIAA0368	ENSG00000136813	14699072;
D004958	affect	KLF10	ENSG00000155090	14699072;
D004958	affect	KLRC4	ENSG00000183542	14699072;
D004958	affect	KRT15	ENSG00000171346	14699072;
D004958	affect	KRT19	ENSG00000171345	14699072;
D004958	affect	KRT75	ENSG00000170454	14699072;
D004958	affect	LAMA4	ENSG00000112769	14699072;
D004958	affect	LASS6	ENSG00000172292	14699072;
D004958	affect	LOX	ENSG00000113083	14699072;
D004958	affect	LPL	ENSG00000175445	14699072;
D004958	affect	LPO	ENSG00000167419	16584521;
D004958	affect	MAP2K5	ENSG00000137764	14699072;
D004958	affect	MDFIC	ENSG00000135272	14699072;
D004958	affect	MMP12	ENSG00000110347	14699072;
D004958	affect	MYO10	ENSG00000145555	14699072;
D004958	affect	NAB2	ENSG00000166886	14699072;
D004958	affect	NCF2	ENSG00000116701	14699072;
D004958	affect	NEFH	ENSG00000100285	14699072;
D004958	affect	NPR2	ENSG00000159899	14699072;
D004958	affect	PALLD	ENSG00000129116	14699072;
D004958	affect	PAPSS2	ENSG00000198682	14699072;
D004958	affect	PDE4B	ENSG00000184588	14699072;
D004958	affect	PDLIM3	ENSG00000154553	14699072;
D004958	affect	PITX1	ENSG00000069011	14699072;
D004958	affect	PKP2	ENSG00000057294	14699072;
D004958	affect	PLOD2	ENSG00000152952	14699072;
D004958	affect	PMAIP1	ENSG00000141682	14699072;
D004958	affect	PNP	ENSG00000198805	14699072;
D004958	affect	PPP1R12A	ENSG00000058272	14699072;
D004958	affect	PRMT7	ENSG00000132600	17404688;
D004958	affect	PTPRU	ENSG00000060656	14699072;
D004958	affect	RAB11FIP2	ENSG00000107560	14699072;
D004958	affect	RAB36	ENSG00000100228	14699072;
D004958	affect	RABGAP1L	ENSG00000250898	14699072;
D004958	affect	RAI14	ENSG00000039560	14699072;
D004958	affect	RASGRF1	ENSG00000058335	14699072;
D004958	affect	RGS13	ENSG00000127074	14699072;
D004958	affect	RHOBTB3	ENSG00000164292	14699072;
D004958	affect	RIPK2	ENSG00000104312	14699072;
D004958	affect	RLN2	ENSG00000107014	14699072;
D004958	affect	RNASE4	ENSG00000181784	14699072;
D004958	affect	RNF14	ENSG00000013561	14699072;
D004958	affect	RPS6KA2	ENSG00000071242	14699072;
D004958	affect	S100G	ENSG00000169906	16491319;
D004958	affect	SAA1	ENSG00000173432	14699072;
D004958	affect	SATB2	ENSG00000119042	14699072;
D004958	affect	SFRS2IP	ENSG00000139218	14699072;
D004958	affect	SH3BGR	ENSG00000185437	14699072;
D004958	affect	SLC27A2	ENSG00000140284	14699072;
D004958	affect	SLC35E2	ENSG00000215790	14699072;
D004958	affect	SMAD3	ENSG00000166949	14699072;
D004958	affect	SMOX	ENSG00000088826	14699072;
D004958	affect	SOSTDC1	ENSG00000171243	14699072;
D004958	affect	ST3GAL1	ENSG00000008513	14699072;

D004958	affect	STAM	ENSG00000136738	14699072;
D004958	affect	STBD1	ENSG00000118804	14699072;
D004958	affect	TANK	ENSG00000136560	14699072;
D004958	affect	TGIF1	ENSG00000177426	14699072;
D004958	affect	TH	ENSG00000180176	14699072;
D004958	affect	TIPARP	ENSG00000163659	14699072;
D004958	affect	TMOD1	ENSG00000136842	14699072;
D004958	affect	TNIP1	ENSG00000145901	14699072;
D004958	affect	TOB1	ENSG00000141232	14699072;
D004958	affect	TP53	ENSG00000141510	15870704;16053526;
D004958	affect	TRAF1	ENSG00000056558	14699072;
D004958	affect	TRPV6	ENSG00000165125	16491319;
D004958	affect	TSPAN5	ENSG00000168785	14699072;
D004958	affect	UBE2L6	ENSG00000156587	14699072;
D004958	affect	VPS8	ENSG00000156931	14699072;
D004958	affect	WFS1	ENSG00000109501	14699072;
D004958	affect	ZBTB5	ENSG00000168795	14699072;
D004958	affect	ZFP36	ENSG00000128016	14699072;
D004958	affect	ZNF185	ENSG00000147394	14699072;
D004958	affect	ZNF652	ENSG00000198740	17404688;
D004958	affect	ZNF79	ENSG00000196152	14699072;
D004958	decrease	ABCA8	ENSG00000141338	17404688;
D004958	decrease	ABCB1	ENSG00000085563	16925584;
D004958	decrease	ABCC11	ENSG00000121270	18310281;
D004958	decrease	ABCC5	ENSG00000114770	15072547;
D004958	decrease	ABCG2	ENSG00000118777	15365089;15695404;
D004958	decrease	ACPP	ENSG00000014257	12391264;17404688;
D004958	decrease	ADAM12	ENSG00000148848	17404688;
D004958	decrease	ADRA1D	ENSG00000171873	15849065;
D004958	decrease	AGPAT4	ENSG00000026652	17404688;
D004958	decrease	AGTR1	ENSG00000144891	15159206;
D004958	decrease	AKR1B10	ENSG00000198074	17404688;
D004958	decrease	ANGPT1	ENSG00000154188	16705744;
D004958	decrease	ANGPT2	ENSG00000091879	17404688;
D004958	decrease	ANGPTL2	ENSG00000136859	17404688;
D004958	decrease	APLP2	ENSG00000084234	16705744;
D004958	decrease	AQP3	ENSG00000165272	14699072;17404688;
D004958	decrease	ARAP3	ENSG00000120318	17404688;
D004958	decrease	ARHGDIA	ENSG00000141522	15757668;15159206;
D004958	decrease	ARHGEF17	ENSG00000110237	17404688;
D004958	decrease	ARNT2	ENSG00000172379	16514628;
D004958	decrease	ATP1B1	ENSG00000143153	16474171;
D004958	decrease	ATP5S	ENSG00000125375	17404688;
D004958	decrease	BASP1	ENSG00000176788	16474171;
D004958	decrease	BAX	ENSG00000087088	11896290;15870704;
D004958	decrease	BAZ2A	ENSG00000076108	16705744;
D004958	decrease	BDKRB1	ENSG00000100739	17404688;
D004958	decrease	BHLHE41	ENSG00000123095	17404688;
D004958	decrease	BNIP2	ENSG00000140299	11424952;9169520;
D004958	decrease	BTG1	ENSG00000133639	16474171;
D004958	decrease	C10ORF116	ENSG00000148671	17404688;
D004958	decrease	C10ORF72	ENSG00000165633	17404688;
D004958	decrease	C16ORF45	ENSG00000166780	17404688;
D004958	decrease	C21ORF7	ENSG00000156265	17404688;

D004958	decrease	C6ORF106	ENSG00000196821	16514628;
D004958	decrease	CA2	ENSG00000104267	14699072;17404688;
D004958	decrease	CACNA1G	ENSG00000006283	17404688;
D004958	decrease	CAMK2N1	ENSG00000162545	16474171;
D004958	decrease	CCL20	ENSG00000115009	16298037;
D004958	decrease	CCL5	ENSG00000161570	16842799;17404688;
D004958	decrease	CCNG1	ENSG00000113328	16029874;
D004958	decrease	CD58	ENSG00000116815	16705744;
D004958	decrease	CD96	ENSG00000153283	17404688;
D004958	decrease	CDH5	ENSG00000179776	17404688;
D004958	decrease	CDH6	ENSG00000113361	17404688;
D004958	decrease	CDK2AP2	ENSG00000167797	16474171;
D004958	decrease	CDKN2C	ENSG00000123080	16474171;
D004958	decrease	CDKN3	ENSG00000100526	16474171;
D004958	decrease	CEACAM5	ENSG00000105388	17404688;
D004958	decrease	CEACAM6	ENSG00000086548	17404688;
D004958	decrease	CEBPD	ENSG00000221869	14699072;16474171;
D004958	decrease	CHGA	ENSG00000100604	17404688;
D004958	decrease	CLCA4	ENSG00000016602	17404688;
D004958	decrease	CLDN4	ENSG00000189143	16474171;
D004958	decrease	CLIC2	ENSG00000155962	17404688;
D004958	decrease	CLIC3	ENSG00000169583	17404688;
D004958	decrease	CLPTM1	ENSG00000104853	16705744;
D004958	decrease	CLSTN1	ENSG00000171603	16514628;
D004958	decrease	CNIH3	ENSG00000143786	17404688;
D004958	decrease	COMT	ENSG00000093010	15288375;18192686;18497090;
D004958	decrease	CPZ	ENSG00000109625	17404688;
D004958	decrease	CRABP2	ENSG00000143320	17404688;
D004958	decrease	CSTA	ENSG00000121552	15072547;
D004958	decrease	CTBP1	ENSG00000159692	17404688;
D004958	decrease	CTSH	ENSG00000103811	16514628;
D004958	decrease	CTSW	ENSG00000172543	17404688;
D004958	decrease	CXCL14	ENSG00000145824	17404688;
D004958	decrease	CXCL3	ENSG00000163734	16298037;
D004958	decrease	CYP7B1	ENSG00000172817	16630558;
D004958	decrease	DACT1	ENSG00000165617	17404688;
D004958	decrease	DAPK1	ENSG00000196730	16202921;
D004958	decrease	DBN1	ENSG00000113758	17404688;
D004958	decrease	DCHS1	ENSG00000166341	17404688;
D004958	decrease	DDAH2	ENSG00000225635	17404688;
D004958	decrease	DENND2A	ENSG00000146966	17404688;
D004958	decrease	DRG2	ENSG00000108591	16705744;
D004958	decrease	DUSP4	ENSG00000120875	16202921;
D004958	decrease	DUSP9	ENSG00000130829	15849065;
D004958	decrease	DYRK2	ENSG00000127334	17404688;
D004958	decrease	EFNA1	ENSG00000169242	15072547;
D004958	decrease	EFNB2	ENSG00000125266	16474171;17404688;
D004958	decrease	EFS	ENSG00000100842	17404688;
D004958	decrease	EGR1	ENSG00000120738	17404688;
D004958	decrease	ELF3	ENSG00000163435	16202921;
D004958	decrease	ENC1	ENSG00000171617	14699072;17404688;
D004958	decrease	ENO2	ENSG00000111674	15159206;
D004958	decrease	ENO3	ENSG00000108515	15757668;

D004958	decrease	EPB41L4A	ENSG00000129595	17404688;
D004958	decrease	EPHB4	ENSG00000196411	16705744;
D004958	decrease	ERCC2	ENSG00000104884	16705744;
D004958	decrease	ERICH1	ENSG00000104714	15849065;
D004958	decrease	ESRRA	ENSG00000173153	15968320;
D004958	decrease	ETS2	ENSG00000157557	16474171;
D004958	decrease	F13A1	ENSG00000124491	14699072;17404688;
D004958	decrease	FANCC	ENSG00000158169	16705744;
D004958	decrease	FERMT1	ENSG00000101311	17404688;
D004958	decrease	FGF18	ENSG00000156427	17404688;
D004958	decrease	FJX1	ENSG00000179431	17404688;
D004958	decrease	FLNC	ENSG00000128591	16514628;
D004958	decrease	FLRT2	ENSG00000185070	14699072;17404688;
D004958	decrease	FSCN1	ENSG00000075618	17404688;
D004958	decrease	FST	ENSG00000134363	14699072;16298037;
D004958	decrease	GADD45A	ENSG00000116717	16474171;
D004958	decrease	GAP43	ENSG00000172020	17404688;
D004958	decrease	GBP1	ENSG00000117228	16705744;
D004958	decrease	GJA4	ENSG00000187513	17404688;
D004958	decrease	GPR17	ENSG00000144230	17404688;
D004958	decrease	GPRC5A	ENSG00000013588	14699072;16474171;
D004958	decrease	GRB14	ENSG00000115290	16202921;
D004958	decrease	GREM1	ENSG00000166923	17404688;
D004958	decrease	GSS	ENSG00000100983	16705744;
D004958	decrease	GSTT1	ENSG00000184674	17404688;
D004958	decrease	H2AFV	ENSG00000105968	17404688;
D004958	decrease	HABP2	ENSG00000148702	17404688;
D004958	decrease	HDAC1	ENSG00000116478	16029874;
D004958	decrease	HGF	ENSG00000019991	17404688;
D004958	decrease	HIST1H2AC	ENSG00000180573	16474171;
D004958	decrease	HIST2H2BE	ENSG00000184678	16474171;
D004958	decrease	HLA-DRB3	ENSG00000231679	17404688;
D004958	decrease	HM13	ENSG00000101294	16705744;
D004958	decrease	HSD11B2	ENSG00000176387	17515840;
D004958	decrease	ID3	ENSG00000117318	16474171;
D004958	decrease	IDS	ENSG00000010404	16202921;
D004958	decrease	IGFBP3	ENSG00000146674	16368782;15855258;15809018;17404688;
D004958	decrease	IGFBP5	ENSG00000115461	14699072;15159206;
D004958	decrease	IL1R1	ENSG00000115594	16202921;
D004958	decrease	IL24	ENSG00000162892	14699072;16298037;17404688;
D004958	decrease	IL2RB	ENSG00000100385	17404688;
D004958	decrease	IL33	ENSG00000137033	17404688;
D004958	decrease	IL6	ENSG00000136244	17266178;17532054;16298037;15983630;
D004958	decrease	ITGB3	ENSG00000056345	17404688;
D004958	decrease	ITGB6	ENSG00000115221	17404688;
D004958	decrease	ITM2A	ENSG00000078596	17404688;
D004958	decrease	ITM2C	ENSG00000135916	17404688;
D004958	decrease	JUP	ENSG00000173801	16474171;
D004958	decrease	KLK2	ENSG00000167751	10687981;
D004958	decrease	KLRB1	ENSG00000111796	17404688;
D004958	decrease	KRAS	ENSG00000133703	16474171;

D004958	decrease	KRT23	ENSG00000108244	17404688;
D004958	decrease	KRT8	ENSG00000170421	16705744;
D004958	decrease	L1CAM	ENSG00000198910	16474171;
D004958	decrease	LAMA3	ENSG00000053747	17404688;
D004958	decrease	LCK	ENSG00000182866	17404688;
D004958	decrease	LCN2	ENSG00000148346	15866426;
D004958	decrease	LRRC8D	ENSG00000171492	17404688;
D004958	decrease	LXN	ENSG00000079257	17404688;
D004958	decrease	LYPD1	ENSG00000150551	17404688;
D004958	decrease	MAGED4	ENSG00000154545	17404688;
D004958	decrease	MAPK1	ENSG00000100030	15930183;15292328;12441364;16901965;12948864;14710592;15358673;17088408;15090535;17689939;16840783;15185748;14975756;
D004958	decrease	MAPK8	ENSG00000107643	16029874;
D004958	decrease	MARCKS	ENSG00000155130	17404688;
D004958	decrease	MATN4	ENSG00000124159	17404688;
D004958	decrease	MED20	ENSG00000124641	16705744;
D004958	decrease	MFAP2	ENSG00000117122	17404688;
D004958	decrease	MFAP5	ENSG00000197614	17404688;
D004958	decrease	MME	ENSG00000196549	16202921;
D004958	decrease	MMP11	ENSG00000099953	17404688;
D004958	decrease	MMP14	ENSG00000157227	17404688;
D004958	decrease	MMP15	ENSG00000102996	15072547;
D004958	decrease	MMP1	ENSG00000196611	17404688;
D004958	decrease	MMP27	ENSG00000137675	17404688;
D004958	decrease	MMP3	ENSG00000149968	16009159;17404688;
D004958	decrease	MTHFD1	ENSG00000100714	16705744;
D004958	decrease	MUC4	ENSG00000145113	17404688;
D004958	decrease	MXD4	ENSG00000123933	16474171;
D004958	decrease	MYCN	ENSG00000134323	17404688;
D004958	decrease	MYH6	ENSG00000197616	16474171;
D004958	decrease	MYLK	ENSG00000251351	17404688;
D004958	decrease	NCOA3	ENSG00000124151	17962382;17872375;17395694;16474171;16739346;
D004958	decrease	NEDD9	ENSG00000111859	16474171;
D004958	decrease	NMT1	ENSG00000136448	16705744;17404688;
D004958	decrease	NOV	ENSG00000136999	15072547;
D004958	decrease	NQO1	ENSG00000181019	18497090;
D004958	decrease	NR1H2	ENSG00000131408	16029874;
D004958	decrease	NR4A1	ENSG00000123358	16514628;
D004958	decrease	NUDT6	ENSG00000170917	17404688;
D004958	decrease	ORM1	ENSG00000229314	17404688;
D004958	decrease	PA2G4	ENSG00000170515	16705744;
D004958	decrease	PBX1	ENSG00000185630	17404688;
D004958	decrease	PCDHGC3	ENSG00000240184	17404688;
D004958	decrease	PDE4C	ENSG00000105650	15849065;
D004958	decrease	PDGFA	ENSG00000197461	15866426;17404688;
D004958	decrease	PDGFB	ENSG00000100311	15866426;17404688;
D004958	decrease	PDPK1	ENSG00000140992	17404688;
D004958	decrease	PFAS	ENSG00000178921	17404688;
D004958	decrease	PIK3R2	ENSG00000105647	16705744;
D004958	decrease	PKIA	ENSG00000171033	17404688;

D004958	decrease	PLAU	ENSG00000122861	14699072;17404688;
D004958	decrease	PLEC	ENSG00000178209	14699072;16705744;
D004958	decrease	PLEK2	ENSG00000100558	17404688;
D004958	decrease	PLK2	ENSG00000145632	14699072;16202921;
D004958	decrease	PMEPA1	ENSG00000124225	16474171;
D004958	decrease	POLD4	ENSG00000175482	16474171;
D004958	decrease	POLR2J	ENSG00000005075	17404688;
D004958	decrease	PPAP2B	ENSG00000162407	16474171;
D004958	decrease	PPAP2C	ENSG00000141934	16474171;
D004958	decrease	PPFIBP2	ENSG00000166387	17404688;
D004958	decrease	PPP1R16B	ENSG00000101445	17404688;
D004958	decrease	PRAME	ENSG00000185686	17404688;
D004958	decrease	PRKCD	ENSG00000163932	15757668;
D004958	decrease	PSAP	ENSG00000197746	16202921;
D004958	decrease	PTH2R	ENSG00000144407	17404688;
D004958	decrease	PTHLH	ENSG00000087494	17676588;17404688;
D004958	decrease	PTPN13	ENSG00000163629	16202921;
D004958	decrease	PZP	ENSG00000126838	16705744;
D004958	decrease	QPCT	ENSG00000115828	17404688;
D004958	decrease	RAB15	ENSG00000139998	17404688;
D004958	decrease	RAF1	ENSG00000132155	16029874;12432273;15930183;
D004958	decrease	RANBP3	ENSG00000031823	16202921;
D004958	decrease	RARRES1	ENSG00000118849	17404688;
D004958	decrease	RASL12	ENSG00000103710	17404688;
D004958	decrease	RBBP6	ENSG00000122257	17404688;
D004958	decrease	RGS3	ENSG00000249544	17404688;
D004958	decrease	RGS4	ENSG00000117152	17404688;
D004958	decrease	RGS5	ENSG00000143248	17404688;
D004958	decrease	RPL27A	ENSG00000166441	17404688;
D004958	decrease	RPS20	ENSG00000008988	17404688;
D004958	decrease	RPS6KA5	ENSG00000100784	17404688;
D004958	decrease	RRAS	ENSG00000126458	16029874;
D004958	decrease	RTN3	ENSG00000133318	17404688;
D004958	decrease	SASH1	ENSG00000111961	17404688;
D004958	decrease	SCD	ENSG00000099194	15757668;
D004958	decrease	SELENBP1	ENSG00000143416	15159206;
D004958	decrease	SERPINB2	ENSG00000197632	16298037;17404688;
D004958	decrease	SERPINB5	ENSG00000206075	17404688;
D004958	decrease	SIGLEC15	ENSG00000197046	17404688;
D004958	decrease	SLC12A2	ENSG00000064651	15757668;15159206;
D004958	decrease	SLC14A1	ENSG00000141469	17404688;
D004958	decrease	SLC16A6	ENSG00000108932	17404688;
D004958	decrease	SLCO3A1	ENSG00000176463	17404688;
D004958	decrease	SMARCA4	ENSG00000127616	16705744;
D004958	decrease	SMG1	ENSG00000157106	16705744;
D004958	decrease	SNAI1	ENSG00000124216	15955695;
D004958	decrease	SNAI2	ENSG00000019549	15955695;
D004958	decrease	SNCAIP	ENSG00000064692	17404688;
D004958	decrease	SOX11	ENSG00000176887	17404688;
D004958	decrease	SOX2	ENSG00000181449	16474171;
D004958	decrease	SOX4	ENSG00000124766	16474171;17404688;
D004958	decrease	SOX9	ENSG00000125398	14699072;17404688;
D004958	decrease	SPON1	ENSG00000152268	17404688;

D004958	decrease	SQSTM1	ENSG00000161011	16474171;
D004958	decrease	ST7	ENSG00000004866	17404688;
D004958	decrease	STC1	ENSG00000159167	17404688;
D004958	decrease	STEAP1	ENSG00000164647	16474171;
D004958	decrease	STMN2	ENSG00000104435	17404688;
D004958	decrease	SUFU	ENSG00000107882	16202921;
D004958	decrease	SURF4	ENSG00000148248	16705744;
D004958	decrease	SYNJ2	ENSG00000078269	17404688;
D004958	decrease	TACC1	ENSG00000147526	14699072;15072547;
D004958	decrease	TBC1D19	ENSG00000109680	17404688;
D004958	decrease	TCEB2	ENSG00000103363	16514628;
D004958	decrease	TCF25	ENSG00000141002	16474171;
D004958	decrease	TCF4	ENSG00000196628	17404688;
D004958	decrease	TELO2	ENSG00000100726	16705744;
D004958	decrease	TFPI	ENSG00000003436	17029634;
D004958	decrease	TGFB2	ENSG00000092969	14699072;16029874;16474171;17404688;
D004958	decrease	TGM2	ENSG00000198959	14699072;17404688;
D004958	decrease	THY1	ENSG00000154096	14699072;17404688;
D004958	decrease	TIMP1	ENSG00000102265	16202921;
D004958	decrease	TIMP3	ENSG00000100234	16474171;
D004958	decrease	TMEM158	ENSG00000249992	17404688;
D004958	decrease	TMEM5	ENSG00000118600	17404688;
D004958	decrease	TMSB15A	ENSG00000158164	17404688;
D004958	decrease	TNFRSF11B	ENSG00000164761	14699072;16202921;17404688;
D004958	decrease	TNFRSF21	ENSG00000146072	17404688;
D004958	decrease	TNFRSF4	ENSG00000186827	17404688;
D004958	decrease	TP53I11	ENSG00000175274	15757668;
D004958	decrease	TP53INP1	ENSG00000164938	16202921;
D004958	decrease	TRIB2	ENSG00000071575	14699072;17404688;
D004958	decrease	TRIM16	ENSG00000221926	16474171;
D004958	decrease	TWIST1	ENSG00000122691	17404688;
D004958	decrease	UBD	ENSG00000213886	17404688;
D004958	decrease	UGT1A1	ENSG00000241635	15560369;15378558;15072547;17898154;15472229;15593333;15117964;15769885;16051636;
D004958	decrease	UGT1A3	ENSG00000243135	16474171;15472229;15117964;
D004958	decrease	WISP2	ENSG00000064205	14699072;17404688;
D004958	decrease	WNT7A	ENSG00000154764	16534752;
D004958	decrease	XCL1	ENSG00000143184	17404688;
D004958	decrease	XCL2	ENSG00000143185	17404688;
D004958	decrease	YPEL5	ENSG00000119801	16474171;
D004958	decrease	ZBED2	ENSG00000177494	17404688;
D004958	decrease	ZCCHC14	ENSG00000140948	17404688;
D004958	decrease	ZFP36L1	ENSG00000185650	16474171;
D004958	decrease	ZNF217	ENSG00000171940	15072547;
D004958	decrease	ZNF426	ENSG00000130818	17404688;
D004958	decrease	ZNF428	ENSG00000131116	17404688;
D004958	increase	ABAT	ENSG00000183044	14699072;17404688;
D004958	increase	ABCC3	ENSG00000108846	15072547;17404688;
D004958	increase	ACSL1	ENSG00000151726	17404688;
D004958	increase	ADAM15	ENSG00000143537	16705744;

D004958	increase	ADAMTS1	ENSG00000154734	17018655;
D004958	increase	ADAMTS8	ENSG00000134917	17404688;
D004958	increase	ADIPOR2	ENSG00000006831	17404688;
D004958	increase	ADRB1	ENSG00000043591	16202921;
D004958	increase	AFP	ENSG00000081051	14711791;18497072;
D004958	increase	AGER	ENSG00000204305	15878629;
D004958	increase	AGR2	ENSG00000106541	16474171;
D004958	increase	AHNAK	ENSG00000124942	14699072;16514628;
D004958	increase	ALPL	ENSG00000162551	12650720;17404688;
D004958	increase	ALPP	ENSG00000163283	16053526;17404688;
D004958	increase	ALPPL2	ENSG00000163286	15993745;
D004958	increase	AMY1A	ENSG00000187733	17404688;
D004958	increase	ANKRD55	ENSG00000249922	17404688;
D004958	increase	ANTXR1	ENSG00000169604	17404688;
D004958	increase	AP1S2	ENSG00000182287	16474171;
D004958	increase	APOBEC3B	ENSG00000179750	16474171;17404688;
D004958	increase	APOD	ENSG00000189058	16514628;17404688;
D004958	increase	APOM	ENSG00000226215	17404688;
D004958	increase	AREG	ENSG00000205595	12432273;16474171;16202921;15072547;16514628;16257430;
D004958	increase	AR	ENSG00000169083	17131305;15084347;14751673;12676605;15994348;14628596;12391264;11867264;15950459;15171712;9846162;11861974;11861975;15159206;15849065;
D004958	increase	ARL3	ENSG00000138175	16474171;
D004958	increase	ART3	ENSG00000156219	17404688;
D004958	increase	ASNS	ENSG00000070669	15757668;16514628;
D004958	increase	ASPM	ENSG00000066279	16474171;
D004958	increase	ASRGL1	ENSG00000162174	17404688;
D004958	increase	ASS1	ENSG00000130707	15757668;
D004958	increase	ATAD2	ENSG00000156802	16474171;17404688;
D004958	increase	ATF3	ENSG00000162772	16474171;16516039;
D004958	increase	ATP6V1A	ENSG00000114573	17404688;
D004958	increase	AURKA	ENSG00000087586	16474171;17268063;16514628;
D004958	increase	AURKB	ENSG00000178999	16474171;
D004958	increase	BCL2	ENSG00000171791	11896290;15897899;16051634;10545406;15870704;
D004958	increase	BCL2L1	ENSG00000171552	11896290;15969986;
D004958	increase	BGLAP	ENSG00000242252	17689939;
D004958	increase	BIK	ENSG00000100290	17638070;
D004958	increase	BIRC5	ENSG00000089685	16474171;16705744;
D004958	increase	BLM	ENSG00000197299	17268063;
D004958	increase	BRCA1	ENSG00000012048	12473173;11110848;16061635;16029874;16417649;17266178;
D004958	increase	BUB1B	ENSG00000156970	16474171;
D004958	increase	C10ORF10	ENSG00000165507	16298037;
D004958	increase	C4ORF31	ENSG00000173376	17404688;
D004958	increase	CA12	ENSG00000074410	14699072;16474171;15913882;
D004958	increase	CACNA1B	ENSG00000148408	17962382;
D004958	increase	CALB2	ENSG00000172137	14699072;17962382;

D004958	increase	CAPN6	ENSG00000077274	17404688;
D004958	increase	CBX1	ENSG00000108468	15757668;16514628;
D004958	increase	CCNA1	ENSG00000133101	17404688;
D004958	increase	CCNA2	ENSG00000145386	16474171;17445807;15072547;
D004958	increase	CCNB1	ENSG00000134057	17445807;
D004958	increase	CCNB2	ENSG00000157456	15897899;16474171;
D004958	increase	CCND1	ENSG00000110092	15897899;16328721;11896290;16061635;16839579;17266178;15297603;
D004958	increase	CCND3	ENSG00000112576	15897899;
D004958	increase	CCNE1	ENSG00000105173	15897899;
D004958	increase	CCNE2	ENSG00000175305	15897899;16029874;
D004958	increase	CD44	ENSG00000026508	16912569;
D004958	increase	CDC14B	ENSG00000081377	17404688;
D004958	increase	CDC20	ENSG00000117399	16474171;
D004958	increase	CDC6	ENSG00000094804	16474171;17268063;
D004958	increase	CDK1	ENSG00000170312	16474171;
D004958	increase	CDK2	ENSG00000123374	16474171;
D004958	increase	CDKN1A	ENSG00000124762	12473173;11896290;16474171;15870704;15757668;
D004958	increase	CDKN1B	ENSG00000111276	17283133;
D004958	increase	CDT1	ENSG00000167513	16474171;
D004958	increase	CECR6	ENSG00000183307	17962382;
D004958	increase	CENPA	ENSG00000115163	16474171;16514628;
D004958	increase	CENPF	ENSG00000117724	16474171;
D004958	increase	CENPJ	ENSG00000151849	16474171;
D004958	increase	CENPN	ENSG00000166451	16474171;
D004958	increase	CFTR	ENSG00000001626	15471937;17404688;
D004958	increase	CHEK1	ENSG00000149554	16474171;
D004958	increase	CHST7	ENSG00000147119	17404688;
D004958	increase	CHSY1	ENSG00000131873	16474171;
D004958	increase	CITED2	ENSG00000164442	17404688;
D004958	increase	CKB	ENSG00000166165	16621514;17404688;
D004958	increase	CKS1B	ENSG00000173207	16474171;
D004958	increase	CLCN4	ENSG00000073464	17404688;
D004958	increase	CLIC6	ENSG00000159212	16474171;
D004958	increase	CNR1	ENSG00000118432	17404688;
D004958	increase	COL12A1	ENSG00000111799	16474171;
D004958	increase	COPB2	ENSG00000184432	16514628;
D004958	increase	COX7A2L	ENSG00000115944	17266178;
D004958	increase	CRISPLD2	ENSG00000103196	17404688;
D004958	increase	CRLF1	ENSG00000006016	17404688;
D004958	increase	CRP	ENSG00000132693	12616983;
D004958	increase	CRYAB	ENSG00000109846	17404688;
D004958	increase	CSF2RA	ENSG00000198223	17404688;
D004958	increase	CSF3	ENSG00000108342	16298037;17404688;
D004958	increase	CSRP2BP	ENSG00000149474	16474171;
D004958	increase	CTPS	ENSG00000171793	16474171;
D004958	increase	CTSD	ENSG00000117984	15090535;15072547;16257430;15219790;
D004958	increase	CUX2	ENSG00000111249	17404688;
D004958	increase	CXCL10	ENSG00000169245	15557169;17404688;
D004958	increase	CXCL11	ENSG00000169248	15557169;17404688;
D004958	increase	CXCL12	ENSG00000107562	15986123;15913882;

D004958	increase	CYP11A1	ENSG00000140459	12858341;
D004958	increase	CYP19A1	ENSG00000137869	12839940;16024248;16030167;16054388;14699072;15955695;16029874;
D004958	increase	CYP1A1	ENSG00000140465	15142886;15507517;15805301;11186135;15377160;9920887;11358810;15837795;18497090;16474171;16029874;15964790;
D004958	increase	CYP1B1	ENSG00000138061	15142886;15507517;15285606;11074351;15763594;15961252;15377160;9920887;11358810;15837795;16474
D004958	increase	CYP26A1	ENSG00000095596	17404688;
D004958	increase	CYP2A6	ENSG00000198077	17646279;
D004958	increase	CYP3A4	ENSG00000160868	12505310;9047321;9714305;16790556;12417264;1565
D004958	increase	CYP4B1	ENSG00000142973	17404688;
D004958	increase	D4S234E	ENSG00000168824	17404688;
D004958	increase	DAD1	ENSG00000129562	16474171;
D004958	increase	DDX21	ENSG00000165732	16514628;
D004958	increase	DDX3Y	ENSG00000067048	16514628;
D004958	increase	DEPDC1B	ENSG00000035499	16474171;
D004958	increase	DHCR24	ENSG00000116133	15585566;
D004958	increase	DHRS2	ENSG00000100867	16474171;
D004958	increase	DHX9	ENSG00000135829	16514628;
D004958	increase	DKK1	ENSG00000107984	17404688;
D004958	increase	DLX4	ENSG00000108813	12858341;
D004958	increase	DMBT1	ENSG00000187908	15564322;17404688;
D004958	increase	DNAJA1	ENSG00000086061	16514628;
D004958	increase	DPP4	ENSG00000197635	17404688;
D004958	increase	DTL	ENSG00000143476	16474171;
D004958	increase	DTYMK	ENSG00000168393	16474171;
D004958	increase	DUSP1	ENSG00000120129	14699072;17404688;
D004958	increase	EBAG9	ENSG00000147654	17266178;
D004958	increase	EBP	ENSG00000147155	16474171;
D004958	increase	ECT2	ENSG00000114346	16474171;
D004958	increase	EGR3	ENSG00000179388	15159206;15913882;
D004958	increase	EIF2S1	ENSG00000134001	16514628;
D004958	increase	EIF2S3	ENSG00000130741	17404688;
D004958	increase	EIF5	ENSG00000100664	16514628;
D004958	increase	ELOVL5	ENSG00000012660	16514628;
D004958	increase	EMID2	ENSG00000160963	17962382;
D004958	increase	EMP2	ENSG00000213853	16474171;
D004958	increase	EPOR	ENSG00000187266	16705744;
D004958	increase	ERBB2	ENSG00000141736	16488535;15072547;16393665;

D004958	increase	ESR1	ENSG00000091831	9751507;14579009;15658860;11751013;15885261;8858116;15615701;15941852;15713566;9126867;11110848;12441364;12822488;16328721;9168004;15253041;16566672;15899841;15261991;16061840;17275032;17962382;17006379;16925584;14699072;17395694;15878629;17646279;15564322;17237823;15947004;16061635;16965913;16118406;16728493;11331656;16839579;11162928;15955695;17400923;16037132;16024245;15961563;15946813;15521089;11518614;17203231;15833885;11368622;19666091;15120416;16079615;15876408;16337670;15937998;15026083;12676605;11530281;10397250;17141713;17287592;16417649;15890768;15236964;17872375;14617632;16055089;15666801;17135255;15084758;16029874;16393665;15130599;15219790;12753421;16202921;12948864;16739346;17125913;15118252;17904202;11867264;12558164;12623674;17935960;17266178;16434996;9846162;11875334;18486176;15064155;15103026;11922773;15968319;16076840;1
D004958	increase	ESR2	ENSG00000140009	9751507;15103026;12822488;16566672;15261991;17275032;17962382;14699072;17131305;17006379;16965913;16118406;16728493;16839579;15946813;11518614;17203231;15961563;11368622;15937998;16079615;17125913;17287592;16938840;15236964;17872375;17135255;15130599;16596234;15118252;11875334;18486176;17935960;12387416;15064155;15171712;17266178;12377985;16739346;16216903;15123288;12399409;14742696;17051425;
D004958	increase	EZR	ENSG00000092820	15737688;
D004958	increase	FAIM	ENSG00000158234	17404688;
D004958	increase	FAM134B	ENSG00000154153	17404688;
D004958	increase	FANCI	ENSG00000140525	16474171;
D004958	increase	FARP1	ENSG00000152767	16849584;
D004958	increase	FBXO32	ENSG00000156804	16474171;
D004958	increase	FBXO5	ENSG00000112029	16474171;
D004958	increase	FEN1	ENSG00000168496	16474171;
D004958	increase	FGA	ENSG00000171560	17404688;

D004958	increase	FGB	ENSG00000171564	15913882;
D004958	increase	FGF7	ENSG00000140285	12697038;15291741;14999240;
D004958	increase	FGF9	ENSG00000102678	12072406;14602803;17404688;
D004958	increase	FGFR2	ENSG00000066468	14699072;14602803;18575591;
D004958	increase	FGFR3	ENSG00000068078	14602803;
D004958	increase	FGL2	ENSG00000127951	17404688;
D004958	increase	FKBP4	ENSG00000004478	14699072;16474171;
D004958	increase	FKBP5	ENSG00000096060	17404688;
D004958	increase	FOS	ENSG00000170345	15090535;11896290;16029874;17142977;15072547;
D004958	increase	FOXC1	ENSG00000054598	16029874;
D004958	increase	FYTTD1	ENSG00000122068	16514628;
D004958	increase	GAA	ENSG00000171298	16705744;
D004958	increase	GABRA3	ENSG00000011677	16000229;
D004958	increase	GABRB3	ENSG00000166206	16000229;
D004958	increase	GABRE	ENSG00000102287	16000229;
D004958	increase	GAL	ENSG00000069482	16202921;
D004958	increase	GAST	ENSG00000184502	17404688;
D004958	increase	GFRA1	ENSG00000151892	16474171;
D004958	increase	GGH	ENSG00000137563	16474171;
D004958	increase	GINS1	ENSG00000101003	16474171;
D004958	increase	GINS2	ENSG00000131153	16474171;
D004958	increase	GJA1	ENSG00000152661	15849065;
D004958	increase	GLYAT	ENSG00000149124	14699072;17404688;
D004958	increase	GMNN	ENSG00000112312	16474171;
D004958	increase	GNRH1	ENSG00000147437	17054466;10566683;
D004958	increase	GNRHR	ENSG00000109163	12574206;
D004958	increase	GOLGA5	ENSG00000066455	16514628;
D004958	increase	GPD1L	ENSG00000152642	17404688;
D004958	increase	GPR109B	ENSG00000182782	17404688;
D004958	increase	GREB1	ENSG00000196208	14699072;15986123;15849065;16474171;17404688;
D004958	increase	H2AFX	ENSG00000188486	16474171;16462019;
D004958	increase	H2AFZ	ENSG00000164032	16474171;
D004958	increase	HADH	ENSG00000138796	16474171;10600649;
D004958	increase	HAND2	ENSG00000164107	17404688;
D004958	increase	HBA1	ENSG00000206172	17404688;
D004958	increase	HBA2	ENSG00000206172	17404688;
D004958	increase	HBB	ENSG00000244734	17404688;
D004958	increase	HBD	ENSG00000223609	17404688;
D004958	increase	HBG1	ENSG00000213934	17404688;
D004958	increase	HBG2	ENSG00000196565	17404688;
D004958	increase	HIST1H1C	ENSG00000187837	17404688;
D004958	increase	HIST1H2BD	ENSG00000158373	17404688;
D004958	increase	HIST1H2BH	ENSG00000197459	17404688;
D004958	increase	HIST1H2BI	ENSG00000168242	17404688;
D004958	increase	HMGB1	ENSG00000189403	16514628;
D004958	increase	HMGB2	ENSG00000164104	16474171;
D004958	increase	HMOX1	ENSG00000100292	16298037;17404688;
D004958	increase	HNRNPA2B	ENSG00000122566	16514628;
D004958	increase	HNRNPU	ENSG00000153187	16514628;

D004958	increase	HOXA10	ENSG00000078399	15890768;15236964;16632680;
D004958	increase	HOXA11	ENSG00000005073	16632680;17404688;
D004958	increase	HRAS	ENSG00000174775	12117780;16051634;
D004958	increase	HSD17B1	ENSG00000108786	12858341;16054388;
D004958	increase	HSD17B2	ENSG00000086696	16054388;17404688;
D004958	increase	HSD3B1	ENSG00000203857	12858341;
D004958	increase	HSP90AB1	ENSG00000096384	16514628;
D004958	increase	HSP90B1	ENSG00000166598	16514628;
D004958	increase	HSPA4	ENSG00000170606	16514628;
D004958	increase	HSPA6	ENSG00000173110	16029874;17404688;
D004958	increase	HSPA8	ENSG00000109971	16514628;
D004958	increase	HSPD1	ENSG00000144381	16514628;
D004958	increase	IER3	ENSG00000137331	15913882;16849584;
D004958	increase	IFI44L	ENSG00000137959	17404688;
D004958	increase	IFRD1	ENSG00000006652	16514628;
D004958	increase	IGF1	ENSG00000017427	16368782;17872375;15855258;17484513;15762036;15809018;
D004958	increase	IGFBP1	ENSG00000146678	17484513;17404688;
D004958	increase	IGFBP4	ENSG00000141753	14699072;15757668;16474171;15072547;12399409;
D004958	increase	IL1B	ENSG00000125538	17266178;16298037;16934544;17636213;
D004958	increase	IL1RL1	ENSG00000115602	17404688;
D004958	increase	IL20RA	ENSG00000016402	17404688;
D004958	increase	IL6ST	ENSG00000134352	17404688;
D004958	increase	IL8	ENSG00000169429	15866594;14699072;12574206;16298037;
D004958	increase	ILF2	ENSG00000143621	16474171;
D004958	increase	INHBA	ENSG00000122641	17636213;
D004958	increase	IQGAP1	ENSG00000140575	16514628;
D004958	increase	IRS1	ENSG00000169047	15949379;16029874;15072547;
D004958	increase	IRS2	ENSG00000185950	15949379;17404688;
D004958	increase	IRX4	ENSG00000113430	17404688;
D004958	increase	ISG20	ENSG00000172183	14699072;15913882;
D004958	increase	ITGA2	ENSG00000164171	16912569;
D004958	increase	ITGA3	ENSG00000005884	16912569;
D004958	increase	ITGB1	ENSG00000150093	16912569;
D004958	increase	ITGBL1	ENSG00000198542	17404688;
D004958	increase	ITPK1	ENSG00000100605	16474171;
D004958	increase	JUNB	ENSG00000171223	16029874;
D004958	increase	JUN	ENSG00000177606	17395694;17088408;17266178;15930183;
D004958	increase	KBTBD11	ENSG00000176595	17404688;
D004958	increase	KCNG1	ENSG00000026559	17404688;
D004958	increase	KCNH1	ENSG00000143473	19351862;
D004958	increase	KCNJ8	ENSG00000121361	17404688;
D004958	increase	KCNMB1	ENSG00000145936	15849065;
D004958	increase	KDR	ENSG00000128052	17289903;
D004958	increase	KIAA0101	ENSG00000166803	16514628;16474171;
D004958	increase	KIF11	ENSG00000138160	16474171;
D004958	increase	KIF14	ENSG00000118193	16474171;
D004958	increase	KIF15	ENSG00000163808	16474171;

D004958	increase	KIF20A	ENSG00000112984	16474171;16514628;
D004958	increase	KIF23	ENSG00000137807	16474171;
D004958	increase	KIF4A	ENSG00000090889	16474171;
D004958	increase	KLK3	ENSG00000142515	17131305;12391264;17503469;17404077;15994348;15950459;10687981;
D004958	increase	KLRC1	ENSG00000134545	17962382;
D004958	increase	KLRC3	ENSG00000205810	17395694;17404688;
D004958	increase	KPNA2	ENSG00000182481	16474171;16514628;
D004958	increase	KPNB1	ENSG00000108424	16514628;
D004958	increase	KRT13	ENSG00000171401	16849584;
D004958	increase	KRT6A	ENSG00000205420	17404688;
D004958	increase	KRT6B	ENSG00000185479	17404688;
D004958	increase	LIPC	ENSG00000166035	15961563;
D004958	increase	LTBP1	ENSG00000049323	17404688;
D004958	increase	MAD1L1	ENSG00000002822	16474171;
D004958	increase	MADD	ENSG00000110514	16705744;
D004958	increase	MAOA	ENSG00000189221	10811566;17404688;
D004958	increase	MAOB	ENSG00000069535	17404688;
D004958	increase	MAP2K6	ENSG00000108984	17404688;
D004958	increase	MAPK3	ENSG00000102882	15930183;15615701;15292328;12441364;16901965;12948864;15358673;17088408;15090535;17689939;16840783;14975756;
D004958	increase	MASTL	ENSG00000120539	16474171;
D004958	increase	MATR3	ENSG00000015479	16514628;
D004958	increase	MCM10	ENSG00000065328	16474171;
D004958	increase	MCM2	ENSG00000073111	16474171;17268063;
D004958	increase	MCM5	ENSG00000100297	17268063;
D004958	increase	MCM6	ENSG00000076003	16474171;
D004958	increase	MCM7	ENSG00000166508	16474171;
D004958	increase	MDM2	ENSG00000135679	12168835;
D004958	increase	MED14	ENSG00000180182	16705744;
D004958	increase	MELK	ENSG00000165304	16474171;
D004958	increase	MEN1	ENSG00000133895	15090535;
D004958	increase	MGP	ENSG00000111341	16202921;16514628;
D004958	increase	MLF1	ENSG00000178053	16474171;
D004958	increase	MLF1IP	ENSG00000151725	16474171;
D004958	increase	MMP9	ENSG00000100985	12948864;17404688;
D004958	increase	MPHOSPH9	ENSG00000051825	16474171;
D004958	increase	MST4	ENSG00000134602	16514628;
D004958	increase	MT1M	ENSG00000205364	17404688;
D004958	increase	MT1X	ENSG00000187193	17404688;
D004958	increase	MT2A	ENSG00000125148	16202921;
D004958	increase	MTA3	ENSG00000057935	15955695;
D004958	increase	MYB	ENSG00000118513	16029874;16474171;
D004958	increase	MYBL1	ENSG00000185697	16202921;15913882;
D004958	increase	MYBL2	ENSG00000101057	16474171;
D004958	increase	MYC	ENSG00000136997	16839579;16474171;15072547;17266178;16051634;11896290;16155795;16093440;15833885;
D004958	increase	MYH10	ENSG00000133026	16901965;
D004958	increase	MYT1	ENSG00000196132	17268063;

D004958	increase	NARS	ENSG00000134440	16514628;
D004958	increase	NCAPD2	ENSG00000010292	16474171;
D004958	increase	NCAPG	ENSG00000109805	16474171;
D004958	increase	NCAPH	ENSG00000121152	16514628;
D004958	increase	NCL	ENSG00000115053	16514628;
D004958	increase	NCOA2	ENSG00000140396	17962382;15084758;17872375;17395694;16739346;16328721;15123288;
D004958	increase	NDC80	ENSG00000080986	16474171;
D004958	increase	NDP	ENSG00000124479	17404688;
D004958	increase	NFIB	ENSG00000147862	14699072;17404688;
D004958	increase	NFIC	ENSG00000141905	9920887;
D004958	increase	NFKBIA	ENSG00000100906	14699072;12574206;16298037;
D004958	increase	NKD1	ENSG00000140807	17962382;
D004958	increase	NKX3-1	ENSG00000167034	17131305;
D004958	increase	NOS3	ENSG00000164867	16840783;15358673;14742696;
D004958	increase	NPM1	ENSG00000181163	16514628;
D004958	increase	NPTX2	ENSG00000106236	17404688;
D004958	increase	NRIP1	ENSG00000180530	14699072;15757668;16029874;
D004958	increase	NUS1	ENSG00000153989	16514628;
D004958	increase	OLFM1	ENSG00000130558	14699072;16474171;
D004958	increase	OLFML3	ENSG00000116774	16474171;
D004958	increase	OSBPL11	ENSG00000144909	17404688;
D004958	increase	OVGP1	ENSG00000085465	15501902;
D004958	increase	P2RY14	ENSG00000174944	17404688;
D004958	increase	PAX5	ENSG00000196092	17404688;
D004958	increase	PBK	ENSG00000168078	16474171;
D004958	increase	PCK2	ENSG00000100889	15757668;
D004958	increase	PCNA	ENSG00000132646	16474171;15072547;17268063;
D004958	increase	PCP4	ENSG00000183036	16474171;
D004958	increase	PCSK6	ENSG00000140479	17404688;
D004958	increase	PDZD2	ENSG00000133401	17404688;
D004958	increase	PDZK1	ENSG00000174827	14699072;15757668;16393665;15159206;
D004958	increase	PGBD5	ENSG00000177614	17404688;
D004958	increase	PGR	ENSG00000082175	15806153;8812239;15449902;12822488;15219790;16202921;15913882;16393665;15072547;16892383;16257430;16242299;16000229;11867264;17904202;16323292;12753421;15218360;17142977;16739346;11606380;11[illegible]
D004958	increase	PID1	ENSG00000153823	17404688;
D004958	increase	PIK3CA	ENSG00000121879	16029874;
D004958	increase	PIM1	ENSG00000137193	16298037;
D004958	increase	PLAT	ENSG00000104368	15219790;
D004958	increase	PLK4	ENSG00000142731	16474171;
D004958	increase	PLOD1	ENSG00000083444	16474171;
D004958	increase	POLR2B	ENSG00000047315	16514628;
D004958	increase	POU5F1	ENSG00000235068	17404688;
D004958	increase	PPARGC1A	ENSG00000109819	17404688;

D004958	increase	PPM1D	ENSG00000170836	16514628;
D004958	increase	PPP2R2C	ENSG00000074211	16474171;
D004958	increase	PRC1	ENSG00000198901	16474171;
D004958	increase	PRDX4	ENSG00000123131	18272409;
D004958	increase	PRIM1	ENSG00000198056	16474171;
D004958	increase	PRKDC	ENSG00000121031	16514628;
D004958	increase	PRKRA	ENSG00000248314	16202921;
D004958	increase	PRKX	ENSG00000183943	17404688;
D004958	increase	PRL	ENSG00000172179	17054466;17187171;15950373;
D004958	increase	PRLR	ENSG00000113494	16202921;
D004958	increase	PRSS23	ENSG00000150687	15913882;
D004958	increase	PRUNE2	ENSG00000106772	17404688;
D004958	increase	PSAT1	ENSG00000135069	15757668;
D004958	increase	PSMD14	ENSG00000115233	16514628;
D004958	increase	PSMD6	ENSG00000163636	16514628;
D004958	increase	PSME4	ENSG00000068878	16514628;
D004958	increase	PTGER3	ENSG00000050628	14699072;16474171;
D004958	increase	PTGS1	ENSG00000095303	17404688;
D004958	increase	PTGS2	ENSG00000073756	18480072;14699072;17872375;15849065;17404688;
D004958	increase	PTMA	ENSG00000187514	9169520;
D004958	increase	PTPRG	ENSG00000144724	16849584;
D004958	increase	PTPRR	ENSG00000153233	17404688;
D004958	increase	PWP2	ENSG00000241945	16705744;
D004958	increase	RAB30	ENSG00000137502	16849584;
D004958	increase	RACGAP1	ENSG00000161800	16474171;16514628;
D004958	increase	RAD51AP1	ENSG00000111247	16474171;
D004958	increase	RAD51C	ENSG00000108384	16474171;
D004958	increase	RAD51	ENSG00000051180	16474171;
D004958	increase	RAP1A	ENSG00000116473	16514628;
D004958	increase	RBBP7	ENSG00000102054	16514628;
D004958	increase	RBBP8	ENSG00000101773	15757668;
D004958	increase	RBP4	ENSG00000138207	14699072;17456573;17404688;
D004958	increase	RGS2	ENSG00000116741	17404688;
D004958	increase	RNASEH1	ENSG00000171865	16474171;
D004958	increase	ROM1	ENSG00000149489	17962382;
D004958	increase	RPL6	ENSG00000089009	16514628;
D004958	increase	RPS6KA3	ENSG00000177189	15757668;
D004958	increase	RRM1	ENSG00000167325	16514628;
D004958	increase	RRM2	ENSG00000171848	16474171;17404688;
D004958	increase	RUNX2	ENSG00000250096	16202921;17689939;
D004958	increase	S100A2	ENSG00000196754	17404688;
D004958	increase	S100P	ENSG00000163993	15159206;
D004958	increase	SAA2	ENSG00000134339	17404688;
D004958	increase	SAC3D1	ENSG00000168061	16474171;
D004958	increase	SCGB1D1	ENSG00000168515	17404688;
D004958	increase	SCGB1D2	ENSG00000124935	17404688;
D004958	increase	SCGB2A1	ENSG00000124939	17404688;
D004958	increase	SCGB2A2	ENSG00000110484	17404688;
D004958	increase	SEC14L1	ENSG00000129657	17404688;
D004958	increase	SECTM1	ENSG00000141574	17404688;
D004958	increase	SERPINA1	ENSG00000197249	14699072;16202921;

D004958	increase	SERPINA3	ENSG00000196136	10811566;17404688;
D004958	increase	SERPINB9	ENSG00000170542	17237823;14617632;10681578;
D004958	increase	SERPINE1	ENSG00000106366	16011840;17404688;
D004958	increase	SFPQ	ENSG00000116560	16514628;
D004958	increase	SFRS1	ENSG00000136450	16514628;
D004958	increase	SFXN2	ENSG00000156398	16474171;
D004958	increase	SGK1	ENSG00000118515	16202921;
D004958	increase	SHBG	ENSG00000129214	17484513;
D004958	increase	SHMT2	ENSG00000182199	15757668;
D004958	increase	SLC15A2	ENSG00000163406	17404688;
D004958	increase	SLC1A4	ENSG00000115902	15757668;
D004958	increase	SLC26A2	ENSG00000155850	16514628;17404688;
D004958	increase	SLC2A1	ENSG00000117394	15350188;
D004958	increase	SLC39A6	ENSG00000141424	16514628;
D004958	increase	SLC39A8	ENSG00000138821	16474171;
D004958	increase	SLC47A1	ENSG00000142494	17404688;
D004958	increase	SLC4A8	ENSG00000050438	17962382;
D004958	increase	SLC6A14	ENSG00000087916	16474171;
D004958	increase	SLC7A11	ENSG00000151012	16474171;15159206;
D004958	increase	SLC7A2	ENSG00000003989	17404688;
D004958	increase	SLC7A5	ENSG00000103257	15757668;
D004958	increase	SLC7A8	ENSG00000092068	17404688;
D004958	increase	SMC2	ENSG00000136824	16474171;
D004958	increase	SMC4	ENSG00000113810	16474171;
D004958	increase	SOCS1	ENSG00000185338	16849584;17404688;
D004958	increase	SOCS2	ENSG00000120833	16029874;17272397;
D004958	increase	SOCS3	ENSG00000184557	16055089;
D004958	increase	SON	ENSG00000159140	16514628;
D004958	increase	SORBS2	ENSG00000154556	17404688;
D004958	increase	SP7	ENSG00000170374	17689939;
D004958	increase	SPARCL1	ENSG00000152583	17404688;
D004958	increase	SPATA13	ENSG00000182957	17962382;
D004958	increase	SRPX	ENSG00000101955	17404688;
D004958	increase	STAT1	ENSG00000115415	16514628;
D004958	increase	STC2	ENSG00000113739	15757668;16474171;
D004958	increase	STEAP4	ENSG00000127954	17404688;
D004958	increase	STK39	ENSG00000198648	17131305;
D004958	increase	STMN1	ENSG00000117632	16474171;16514628;
D004958	increase	TACC3	ENSG00000013810	16474171;
D004958	increase	TAF9	ENSG00000085231	16514628;
D004958	increase	TARS	ENSG00000113407	16514628;
D004958	increase	TERT	ENSG00000164362	16798746;15585566;16051634;
D004958	increase	TFF1	ENSG00000160182	15913882;14617632;17904202;16093440;16155795;18310281;14706564;10064545;15219790;15072547;16965913;16797915;16892383;16242299;16474171;15986123;15525692;15118252;16514628;16393665;12473173;10811566;15090535;12753421;11580929;11693576;9276642;17404688;12014631;

D004958	increase	TFF3	ENSG00000160180	16202921;16474171;17404688;
D004958	increase	TFPI2	ENSG00000105825	16202921;
D004958	increase	TGFA	ENSG00000163235	14699072;15120416;
D004958	increase	TGFB3	ENSG00000119699	16202921;10811566;17404688;
D004958	increase	THBD	ENSG00000178726	17404688;
D004958	increase	THBS1	ENSG00000137801	16474171;
D004958	increase	THBS2	ENSG00000186340	16514628;
D004958	increase	THSD7A	ENSG00000005108	17404688;
D004958	increase	TK1	ENSG00000167900	16474171;
D004958	increase	TLR2	ENSG00000137462	17404688;
D004958	increase	TMEM38B	ENSG00000095209	16474171;
D004958	increase	TMEM97	ENSG00000109084	16474171;
D004958	increase	TMPO	ENSG00000120802	16474171;16514628;
D004958	increase	TNF	ENSG00000223952	15866594;12574206;12547825;16030167;15866426;
D004958	increase	TNFRSF13B	ENSG00000240505	15849065;
D004958	increase	TNPO1	ENSG00000083312	16514628;
D004958	increase	TOP1	ENSG00000198900	16514628;
D004958	increase	TOP2A	ENSG00000131747	16474171;
D004958	increase	TOP2B	ENSG00000077097	16514628;
D004958	increase	TPD52L1	ENSG00000111907	15757668;
D004958	increase	TPM3	ENSG00000143549	16474171;
D004958	increase	TRAF6	ENSG00000175104	16202921;
D004958	increase	TRAK2	ENSG00000115993	16514628;
D004958	increase	TRIP13	ENSG00000071539	16474171;
D004958	increase	TSC22D3	ENSG00000157514	17404688;
D004958	increase	TUBB6	ENSG00000176014	16474171;
D004958	increase	TYMS	ENSG00000176890	16474171;
D004958	increase	UBE2C	ENSG00000175063	16474171;
D004958	increase	UBE2T	ENSG00000077152	16474171;
D004958	increase	UGT2B15	ENSG00000196620	16690804;15117964;
D004958	increase	UHRF1	ENSG00000034063	16474171;
D004958	increase	ULK1	ENSG00000177169	15757668;
D004958	increase	UNG	ENSG00000076248	14699072;16474171;16514628;
D004958	increase	USP32	ENSG00000170832	16514628;
D004958	increase	VCAM1	ENSG00000162692	12547825;
D004958	increase	VDR	ENSG00000111424	15930183;17088408;
D004958	increase	VEGFA	ENSG00000112715	17289903;18252963;17010073;16299233;
D004958	increase	WARS	ENSG00000140105	15757668;15159206;
D004958	increase	WNT10B	ENSG00000169884	12437293;
D004958	increase	WNT2	ENSG00000105989	15072547;
D004958	increase	XPO1	ENSG00000082898	16514628;
D004958	increase	XRCC5	ENSG00000079246	16514628;
D004958	increase	ZBTB16	ENSG00000109906	17404688;
D004958	increase	ZNF703	ENSG00000183779	16474171;
D004958	increase	ZWINT	ENSG00000122952	16474171;
D004963	decrease	ESR1	ENSG00000091831	15219790;
D004963	increase	CTSD	ENSG00000117984	15219790;
D004963	increase	PGR	ENSG00000082175	15219790;
D004963	increase	PLAT	ENSG00000104368	15219790;
D004963	increase	TFF1	ENSG00000160182	15219790;

D004964	decrease	AGTR1	ENSG00000144891	15159206;
D004964	decrease	ARHGDIA	ENSG00000141522	15159206;
D004964	decrease	ENO2	ENSG00000111674	15159206;
D004964	decrease	IGFBP5	ENSG00000115461	15159206;
D004964	decrease	SELENBP1	ENSG00000143416	15159206;
D004964	decrease	SLC12A2	ENSG00000064651	15159206;
D004964	increase	AFP	ENSG00000081051	18497072;
D004964	increase	AGER	ENSG00000204305	15878629;
D004964	increase	EGR3	ENSG00000179388	15159206;
D004964	increase	NOS3	ENSG00000164867	12547825;
D004964	increase	PDZK1	ENSG00000174827	15159206;
D004964	increase	SLC7A11	ENSG00000151012	15159206;
D004964	increase	TNF	ENSG00000223952	12547825;
D004964	increase	VCAM1	ENSG00000162692	12547825;
D004964	increase	WARS	ENSG00000140105	15159206;
D004966	decrease	CCL2	ENSG00000108691	16842799;
D004966	decrease	FSHB	ENSG00000131808	10843175;16236362;
D004966	decrease	IGF1	ENSG00000017427	10843175;
D004966	decrease	IGFALS	ENSG00000099769	10843175;
D004966	decrease	IGFBP3	ENSG00000146674	10843175;
D004966	decrease	LHB	ENSG00000104826	10843175;
D004966	decrease	PROS1	ENSG00000184500	15850603;
D004966	decrease	SERPINE1	ENSG00000106366	15850603;
D004966	increase	BCL2	ENSG00000171791	15779298;
D004966	increase	CRP	ENSG00000132693	17882670;15990257;
D004966	increase	F7	ENSG00000057593	15850603;
D004966	increase	GH1	ENSG00000189162	10843175;
D004967	affect	AREG	ENSG00000205595	15473135;
D004967	affect	BSN	ENSG00000164061	15473135;
D004967	affect	CDH2	ENSG00000170558	15473135;
D004967	affect	ESR1	ENSG00000091831	15664452;16051668;
D004967	affect	ESR2	ENSG00000140009	15664452;
D004967	affect	NPY1R	ENSG00000164128	15473135;
D004967	affect	RBBP8	ENSG00000101773	15473135;
D004967	affect	VDR	ENSG00000111424	15664452;
D004967	decrease	COMT	ENSG00000093010	15777990;15987428;17138778;
D004967	decrease	TNFRSF11A	ENSG00000141655	16005483;
D004967	decrease	TNFRSF11B	ENSG00000164761	16005483;
D004967	decrease	TNFSF11	ENSG00000120659	16005483;
D004967	increase	ATP7A	ENSG00000165240	17109627;
D004967	increase	BCL2	ENSG00000171791	16007339;15955616;
D004967	increase	DVL1	ENSG00000107404	18204794;
D004967	increase	ESRRA	ENSG00000173153	15821111;12960079;
D004967	increase	HRAS	ENSG00000174775	18204794;
D004967	increase	MCM2	ENSG00000073111	18204794;
D004967	increase	PGR	ENSG00000082175	15806153;
D004967	increase	TFF1	ENSG00000160182	15847785;
D004967	increase	TP53	ENSG00000141510	18204794;
D004967	increase	TP53I3	ENSG00000115129	18204794;
D004970	decrease	ABCB1	ENSG00000085563	16925584;
D004970	decrease	ARHGDIA	ENSG00000141522	15159206;
D004970	decrease	ENO2	ENSG00000111674	15159206;
D004970	decrease	PGR	ENSG00000082175	15806153;

D004970	decrease	SELENBP1	ENSG00000143416	15159206;
D004970	decrease	SLC12A2	ENSG00000064651	15159206;
D004970	increase	EGR3	ENSG00000179388	15159206;
D004970	increase	PDZK1	ENSG00000174827	15159206;
D004970	increase	S100P	ENSG00000163993	15159206;
D004970	increase	SLC7A11	ENSG00000151012	15159206;
D004970	increase	WARS	ENSG00000140105	15159206;
D004976	decrease	MAPK14	ENSG00000112062	14556662;
D004976	decrease	MAPK8	ENSG00000107643	14556662;
D004976	increase	AKR1C1	ENSG00000187134	9973208;
D004976	increase	CYP1A1	ENSG00000140465	9973208;
D004976	increase	DDIT3	ENSG00000175197	17171638;
D004976	increase	GSTP1	ENSG00000084207	15911318;9463521;9806904;15999103;14556662;12781783;10900222;2747627;11
D004976	increase	MAP2K1	ENSG00000169032	14556662;
D004976	increase	MAPK3	ENSG00000102882	14556662;
D004996	decrease	COX1	ENSG00000198804	18823003;
D004996	increase	BCL2	ENSG00000171791	15897899;
D004996	increase	CCND1	ENSG00000110092	15897899;
D004996	increase	CCNE1	ENSG00000105173	15897899;
D004996	increase	CCNE2	ENSG00000175305	15897899;
D004997	affect	ALPL	ENSG00000162551	12521655;
D004997	affect	BGLAP	ENSG00000242252	12521655;
D004997	affect	VDR	ENSG00000111424	12521655;
D004997	decrease	C4BPB	ENSG00000123843	14551147;
D004997	decrease	CD4	ENSG00000010610	10813102;
D004997	decrease	F5	ENSG00000198734	14551147;
D004997	decrease	FSHB	ENSG00000131808	12013081;11907703;10843175;17190377;16002536;16291413;15387353;1524449
D004997	decrease	GAS6	ENSG00000248501	17717130;
D004997	decrease	IGF1	ENSG00000017427	15817916;10843175;16002536;11172797;
D004997	decrease	IGFBP2	ENSG00000115457	15817916;
D004997	decrease	IGFBP3	ENSG00000146674	15817916;10843175;
D004997	decrease	IL6R	ENSG00000160712	11907703;
D004997	decrease	KLK2	ENSG00000167751	10687981;
D004997	decrease	KLK3	ENSG00000142515	10687981;
D004997	decrease	LHB	ENSG00000104826	11907703;10843175;17190377;17105841;16002536;16291413;15387353;1524449
D004997	decrease	PLAT	ENSG00000104368	12618251;15157790;15288212;
D004997	decrease	SCARB1	ENSG00000073060	17673517;
D004997	decrease	SERPINC1	ENSG00000117601	17374706;14561536;11994571;12618251;15288212;14981370;
D004997	decrease	SERPINE1	ENSG00000106366	14561536;15504374;14981370;15288212;15157790;
D004997	decrease	TFPI	ENSG00000003436	17029634;
D004997	decrease	TFRC	ENSG00000072274	16002536;
D004997	decrease	VCAM1	ENSG00000162692	17105841;15769986;17903073;
D004997	increase	AGER	ENSG00000204305	15878629;

D004997	increase	AGT	ENSG00000135744	11095476;17374706;17519148;
D004997	increase	APOB	ENSG00000084674	15769986;
D004997	increase	ATF3	ENSG00000162772	18001166;
D004997	increase	CCR1	ENSG00000163823	10770211;
D004997	increase	CCR5	ENSG00000160791	10770211;
D004997	increase	CP	ENSG00000047457	12616983;
D004997	increase	CRP	ENSG00000132693	10959719;17105841;14557435;16637303;12616983;15769986;17903073;1698222
D004997	increase	CXCR3	ENSG00000186810	10770211;
D004997	increase	ESR1	ENSG00000091831	17673517;11162928;11518614;12623674;11331656;12387416;
D004997	increase	ESR2	ENSG00000140009	11518614;10633228;16938840;12387416;
D004997	increase	F10	ENSG00000126218	14561536;11720199;
D004997	increase	F2	ENSG00000180210	16522526;15504374;15288212;
D004997	increase	F7	ENSG00000057593	12616980;14561536;12618251;15157790;15288212;
D004997	increase	F8	ENSG00000185010	11720199;12618251;
D004997	increase	FGA	ENSG00000171560	16637303;12618251;12616983;12616980;15288212;14561536;
D004997	increase	FGB	ENSG00000171564	16637303;12618251;12616983;12616980;15288212;14561536;
D004997	increase	FGG	ENSG00000171557	16637303;12618251;12616983;12616980;15288212;14561536;
D004997	increase	FN1	ENSG00000115414	15504374;
D004997	increase	GH1	ENSG00000189162	10843175;11172797;
D004997	increase	GJA1	ENSG00000152661	10633228;
D004997	increase	IGF2	ENSG00000167244	15817916;
D004997	increase	IGFALS	ENSG00000099769	10843175;15817916;
D004997	increase	IGFBP4	ENSG00000141753	15817916;
D004997	increase	LDLR	ENSG00000130164	17900441;
D004997	increase	MDM2	ENSG00000135679	12168835;
D004997	increase	PLG	ENSG00000122194	17374706;14561536;11720199;12618251;15157790;
D004997	increase	PROC	ENSG00000115718	14561536;11720199;11994571;15669648;12618251;15288212;14551147;
D004997	increase	PROS1	ENSG00000184500	17374706;11720199;11994571;16522526;12618251;15669648;14551147;1528821
D004997	increase	REN	ENSG00000143839	11095476;
D004997	increase	SCARB2	ENSG00000138760	17673517;
D004997	increase	SERPINA5	ENSG00000188488	14551147;
D004997	increase	SERPINA6	ENSG00000170099	11245553;16982228;
D004997	increase	SERPINA7	ENSG00000123561	16982228;
D004997	increase	SERPIND1	ENSG00000099937	15669648;
D004997	increase	SHBG	ENSG00000129214	11907703;17374706;10899481;14981370;12586319;17519148;11245553;16982228;10709161;

D004997	increase	SP1	ENSG00000185591	15878629;
D004997	increase	TNF	ENSG00000223952	15769986;
D004997	increase	VWF	ENSG00000110799	12616983;
D004999	decrease	BCL2	ENSG00000171791	15985719;
D004999	increase	BAX	ENSG00000087088	15985719;
D005047	affect	ATF4	ENSG00000128272	16298333;
D005047	affect	BAK1	ENSG00000030110	15215046;
D005047	affect	BCL2L1	ENSG00000171552	11468182;15917659;15215046;
D005047	affect	CASP9	ENSG00000132906	15215046;18056177;17935137;16364925;16799960;
D005047	affect	CCNA2	ENSG00000145386	17390037;
D005047	affect	DDIT3	ENSG00000175197	16298333;
D005047	affect	HSPA5	ENSG00000044574	16298333;
D005047	decrease	BCL2	ENSG00000171791	15917659;12063570;
D005047	decrease	BIRC3	ENSG00000023445	15050749;14666661;
D005047	decrease	CAB39	ENSG00000135932	17374387;
D005047	decrease	CEBPZ	ENSG00000115816	17374387;
D005047	decrease	EGF	ENSG00000138798	15228094;16969495;
D005047	decrease	EGFR	ENSG00000146648	15228094;
D005047	decrease	ERBB2	ENSG00000141736	15228094;
D005047	decrease	ERBB3	ENSG00000065361	16217747;15228094;
D005047	decrease	ERBB4	ENSG00000178568	15228094;
D005047	decrease	MAP3K5	ENSG00000197442	17374387;
D005047	decrease	MCL1	ENSG00000143384	17935137;
D005047	decrease	MMP1	ENSG00000196611	18557930;
D005047	decrease	NCK2	ENSG00000071051	17374387;
D005047	decrease	PRC1	ENSG00000198901	17374387;
D005047	decrease	TOP2A	ENSG00000131747	17575151;11676865;12569090;11470519;11531262;16969495;
D005047	decrease	UBE2C	ENSG00000175063	17374387;
D005047	increase	ABCB1	ENSG00000085563	12969965;15239124;
D005047	increase	AGRN	ENSG00000188157	17374387;
D005047	increase	ANXA4	ENSG00000196975	17374387;
D005047	increase	AREG	ENSG00000205595	15228094;
D005047	increase	BIRC2	ENSG00000110330	16217747;10815900;15050749;14970392;
D005047	increase	BRCA1	ENSG00000012048	16417649;15607317;
D005047	increase	CASP2	ENSG00000106144	14757846;
D005047	increase	CASP3	ENSG00000164305	16951922;15215046;18056177;16364925;16844113;17935137;18649362;
D005047	increase	CCL3	ENSG00000006075	10540334;
D005047	increase	CCNG2	ENSG00000138764	18754885;
D005047	increase	CD274	ENSG00000120217	17920123;
D005047	increase	CDK2	ENSG00000123374	16417649;16217747;
D005047	increase	CDKN1A	ENSG00000124762	14601052;
D005047	increase	CDKN1B	ENSG00000111276	17935137;
D005047	increase	CEACAM1	ENSG00000079385	17374387;
D005047	increase	CFLAR	ENSG00000003402	14601052;
D005047	increase	CYP27B1	ENSG00000111012	17716971;
D005047	increase	EI24	ENSG00000149547	17374387;
D005047	increase	EPM2AIP1	ENSG00000178567	17374387;
D005047	increase	EREG	ENSG00000124882	15228094;

D005047	increase	FASN	ENSG00000169710	17089011;
D005047	increase	FDXR	ENSG00000161513	17374387;
D005047	increase	HBEGF	ENSG00000113070	15228094;
D005047	increase	IL8	ENSG00000169429	10540334;
D005047	increase	MAP2K3	ENSG00000034152	17374387;
D005047	increase	PLK3	ENSG00000173846	17374387;
D005047	increase	RAD52	ENSG00000002016	16417649;
D005047	increase	RAD54L	ENSG00000085999	16417649;
D005047	increase	RPS27L	ENSG00000185088	18056458;
D005047	increase	SDC1	ENSG00000115884	17374387;
D005047	increase	TAP1	ENSG00000230705	17374387;
D005047	increase	TAX1BP3	ENSG00000213977	17374387;
D005047	increase	TGFA	ENSG00000163235	15228094;
D005047	increase	TNFRSF10B	ENSG00000120889	16364925;15964798;
D005047	increase	TNFSF9	ENSG00000125657	17374387;
D005047	increase	TP53	ENSG00000141510	15964798;12082016;18698031;
D005047	increase	TP53I3	ENSG00000115129	17374387;
D005047	increase	TP73	ENSG00000078900	17716971;
D005047	increase	VDR	ENSG00000111424	17716971;15713801;12969965;
D005047	increase	XRCC2	ENSG00000196584	16417649;
D005047	increase	XRCC3	ENSG00000126215	16417649;
D005047	increase	XRCC4	ENSG00000152422	16417649;
D005054	increase	UGDH	ENSG00000109814	16292757;
D005217	increase	IL1B	ENSG00000125538	16804125;
D005227	affect	CFTR	ENSG00000001626	14762183;
D005229	decrease	CRP	ENSG00000132693	16276071;
D005230	increase	ITGAM	ENSG00000169896	16357311;
D005231	increase	CD40	ENSG00000101017	15684433;
D005231	increase	IL12B	ENSG00000113302	15684433;
D005231	increase	PTGS2	ENSG00000073756	15684433;
D005231	increase	TNF	ENSG00000223952	15684433;17823444;
D005273	decrease	MAPT	ENSG00000186868	16930453;
D005279	increase	NGFR	ENSG00000064300	17409433;
D005280	increase	EDNRB	ENSG00000136160	16340002;
D005419	decrease	ABCC1	ENSG00000103222	15670588;15916776;15465739;
D005419	decrease	ARHGAP4	ENSG00000089820	15465739;
D005419	decrease	BCR	ENSG00000186716	15465739;
D005419	decrease	BRCC3	ENSG00000185515	15465739;
D005419	decrease	CD79A	ENSG00000105369	15465739;
D005419	decrease	CEBPG	ENSG00000153879	15465739;
D005419	decrease	FLT3LG	ENSG00000090554	15465739;
D005419	decrease	FTH1	ENSG00000167996	15465739;
D005419	decrease	GSTP1	ENSG00000084207	15465739;
D005419	decrease	HSPD1	ENSG00000144381	15465739;
D005419	decrease	IGF1	ENSG00000017427	15922007;
D005419	decrease	IRF4	ENSG00000137265	15465739;
D005419	decrease	MAP2K1	ENSG00000169032	15465739;
D005419	decrease	NPM1	ENSG00000181163	15465739;
D005419	decrease	PARP1	ENSG00000143799	15465739;
D005419	decrease	RAC1	ENSG00000136238	15465739;
D005419	decrease	RPL6	ENSG00000089009	15465739;

D005419	decrease	STAT1	ENSG00000115415	15465739;
D005419	decrease	TCF3	ENSG00000071564	15465739;
D005419	decrease	TK1	ENSG00000167900	15465739;
D005419	decrease	TOP1	ENSG00000198900	15465739;
D005419	decrease	TOP2A	ENSG00000131747	15465739;
D005419	decrease	XCL1	ENSG00000143184	15465739;
D005419	decrease	XRCC1	ENSG00000073050	15465739;
D005419	increase	BAT2	ENSG00000204469	15465739;
D005419	increase	BSG	ENSG00000172270	15465739;
D005419	increase	CD81	ENSG00000110651	15465739;
D005419	increase	CSF2RB	ENSG00000100368	15465739;
D005419	increase	CYP1A1	ENSG00000140465	15104253;
D005419	increase	ETS1	ENSG00000134954	15465739;
D005419	increase	GRN	ENSG00000030582	15465739;
D005419	increase	HCFC1	ENSG00000172534	15465739;
D005419	increase	HOXD3	ENSG00000128652	15465739;
D005419	increase	ICAM1	ENSG00000090339	15465739;
D005419	increase	IL10	ENSG00000136634	15465739;
D005419	increase	IL2RA	ENSG00000134460	15465739;
D005419	increase	IL4	ENSG00000113520	15465739;
D005419	increase	IRAK1	ENSG00000184216	15465739;
D005419	increase	IRF2	ENSG00000168310	15465739;
D005419	increase	ITGA2B	ENSG00000005961	15465739;
D005419	increase	ITGAL	ENSG00000005844	15465739;
D005419	increase	MIF	ENSG00000240972	15465739;
D005419	increase	MLF2	ENSG00000089693	15465739;
D005419	increase	NUP214	ENSG00000126883	15465739;
D005419	increase	PBX2	ENSG00000236353	15465739;
D005419	increase	POU3F2	ENSG00000184486	15465739;
D005419	increase	SELL	ENSG00000188404	15465739;
D005419	increase	SLC20A1	ENSG00000144136	15465739;
D005419	increase	STAT6	ENSG00000166888	15465739;
D005419	increase	TCF7	ENSG00000081059	15465739;
D005419	increase	TNK2	ENSG00000061938	15465739;
D005419	increase	UGT1A1	ENSG00000241635	15104253;
D005419	increase	XCR1	ENSG00000173578	15465739;
D005419	increase	ZNF593	ENSG00000142684	15465739;
D005424	decrease	SLC2A3	ENSG00000059804	15342952;
D005424	increase	ASAH1	ENSG00000104763	15342952;
D005424	increase	ASNS	ENSG00000070669	15342952;
D005424	increase	C10ORF10	ENSG00000165507	15342952;
D005424	increase	FABP1	ENSG00000163586	15342952;
D005424	increase	FNDC4	ENSG00000115226	15342952;
D005424	increase	GDPD3	ENSG00000102886	15342952;
D005424	increase	INHBE	ENSG00000139269	15342952;
D005424	increase	NUPR1	ENSG00000176046	15342952;
D005424	increase	SERPINA3	ENSG00000196136	15342952;
D005424	increase	WIPI1	ENSG00000070540	15342952;
D005438	increase	KLK3	ENSG00000142515	11956172;
D005439	increase	IL1A	ENSG00000115008	10866999;
D005439	increase	NOS2	ENSG00000007171	10866999;
D005439	increase	PPARG	ENSG00000132170	9013583;10866999;
D005439	increase	PTGS2	ENSG00000073756	10866999;
D005439	increase	TNF	ENSG00000223952	10866999;

D005467	affect	UNG	ENSG00000076248	16951200;
D005467	increase	TK1	ENSG00000167900	16951200;
D005467	increase	TP53	ENSG00000141510	19015155;
D005472	affect	AARS	ENSG00000090861	15016801;
D005472	affect	ACTL8	ENSG00000117148	15016801;
D005472	affect	ACY1	ENSG00000243989	15016801;
D005472	affect	ADAM10	ENSG00000137845	15016801;
D005472	affect	ADAMTS9	ENSG00000163638	15016801;
D005472	affect	AGR2	ENSG00000106541	15016801;
D005472	affect	AKAP12	ENSG00000131016	16584549;
D005472	affect	AKR1B10	ENSG00000198074	16584549;
D005472	affect	AKR1C1	ENSG00000187134	16217747;16584549;
D005472	affect	AKR1C3	ENSG00000196139	16217747;16584549;
D005472	affect	ALG3	ENSG00000214160	15016801;
D005472	affect	ANKRD36B	ENSG00000196912	16584549;
D005472	affect	ANP32E	ENSG00000143401	15016801;
D005472	affect	AP3S1	ENSG00000177879	15016801;
D005472	affect	ARMCX1	ENSG00000126947	15016801;
D005472	affect	ASAH1	ENSG00000104763	16217747;15016801;
D005472	affect	ATOH8	ENSG00000168874	16584549;
D005472	affect	ATP5D	ENSG00000099624	15016801;
D005472	affect	ATXN10	ENSG00000130638	15016801;
D005472	affect	BCAT1	ENSG00000060982	15016801;
D005472	affect	BCKDHA	ENSG00000248098	16584549;
D005472	affect	BDKRB2	ENSG00000168398	15016801;
D005472	affect	BNIP3	ENSG00000176171	15067352;15016801;
D005472	affect	BOP1	ENSG00000170727	15016801;
D005472	affect	C12ORF5	ENSG00000078237	15016801;
D005472	affect	C1ORF183	ENSG00000197852	15016801;
D005472	affect	C1ORF35	ENSG00000143793	16584549;
D005472	affect	C4BPB	ENSG00000123843	16584549;
D005472	affect	CA14	ENSG00000118298	15016801;
D005472	affect	CAD	ENSG00000084774	15016801;
D005472	affect	CANX	ENSG00000127022	18678097;15016801;
D005472	affect	CBS	ENSG00000160200	15016801;
D005472	affect	CCNJL	ENSG00000135083	16584549;
D005472	affect	CD38	ENSG00000004468	16584549;
D005472	affect	CD52	ENSG00000169442	16584549;15016801;
D005472	affect	CD8A	ENSG00000153563	15016801;
D005472	affect	CDC20	ENSG00000117399	15016801;
D005472	affect	CDIPT	ENSG00000103502	16584549;
D005472	affect	CDK5R1	ENSG00000176749	15205334;
D005472	affect	CDKN3	ENSG00000100526	15016801;
D005472	affect	CHRNA4	ENSG00000101204	15016801;
D005472	affect	CLTC	ENSG00000141367	18927307;15016801;
D005472	affect	COL6A3	ENSG00000163359	16217747;15016801;
D005472	affect	CPD	ENSG00000108582	16217747;15016801;
D005472	affect	CP	ENSG00000047457	15016801;
D005472	affect	CPXM1	ENSG00000088882	15205334;
D005472	affect	CRHBP	ENSG00000145708	15205334;
D005472	affect	CRYGS	ENSG00000213139	15016801;
D005472	affect	CRYL1	ENSG00000165475	15016801;
D005472	affect	CSE1L	ENSG00000124207	15016801;
D005472	affect	CSNK1E	ENSG00000213923	16584549;

D005472	affect	CST8	ENSG00000125815	16584549;
D005472	affect	CTGF	ENSG00000118523	15205334;
D005472	affect	CUTC	ENSG00000119929	16217747;15016801;
D005472	affect	CXCR4	ENSG00000121966	16584549;
D005472	affect	CXCR7	ENSG00000144476	15016801;
D005472	affect	CYC1	ENSG00000179091	15016801;
D005472	affect	CYP2F1	ENSG00000197446	16584549;
D005472	affect	CYP3A4	ENSG00000160868	16584549;
D005472	affect	DARS	ENSG00000115866	16584549;
D005472	affect	DCK	ENSG00000156136	15016801;
D005472	affect	DCLK1	ENSG00000133083	15016801;
D005472	affect	DCT	ENSG00000080166	15205334;
D005472	affect	DCXR	ENSG00000169738	15016801;
D005472	affect	DIMT1L	ENSG00000086189	15016801;
D005472	affect	DLC1	ENSG00000164741	15016801;
D005472	affect	DLK1	ENSG00000185559	15016801;
D005472	affect	DPYSL3	ENSG00000113657	15205334;
D005472	affect	DUSP14	ENSG00000161326	15016801;
D005472	affect	DUSP5	ENSG00000138166	15016801;
D005472	affect	E2F8	ENSG00000129173	16584549;
D005472	affect	EHF	ENSG00000135373	15016801;
D005472	affect	ELN	ENSG00000049540	15205334;
D005472	affect	EP400	ENSG00000183495	15016801;
D005472	affect	EPHA2	ENSG00000142627	15016801;
D005472	affect	EPYC	ENSG00000083782	15205334;
D005472	affect	ETF1	ENSG00000120705	15016801;
D005472	affect	ETS1	ENSG00000134954	15016801;
D005472	affect	FAH	ENSG00000103876	15016801;
D005472	affect	FAM102A	ENSG00000167106	16584549;
D005472	affect	FAM107B	ENSG00000065809	15016801;
D005472	affect	FAM114A1	ENSG00000197712	16584549;
D005472	affect	FAM20C	ENSG00000177706	16584549;
D005472	affect	FKBP5	ENSG00000096060	15016801;
D005472	affect	FMOD	ENSG00000122176	15016801;
D005472	affect	FOXC1	ENSG00000054598	16584549;
D005472	affect	GBAS	ENSG00000146729	15016801;
D005472	affect	GCSH	ENSG00000140905	15016801;
D005472	affect	GDF1	ENSG00000130283	15016801;
D005472	affect	GEM	ENSG00000164949	15205334;
D005472	affect	GINS2	ENSG00000131153	16584549;
D005472	affect	GP1BB	ENSG00000203618	16584549;
D005472	affect	GPR137C	ENSG00000180998	16584549;
D005472	affect	GRHL3	ENSG00000158055	16584549;
D005472	affect	GRINA	ENSG00000178719	16584549;
D005472	affect	GSPT1	ENSG00000103342	15016801;
D005472	affect	GYG2	ENSG00000056998	15016801;
D005472	affect	H2AFX	ENSG00000188486	16584549;15016801;
D005472	affect	HAND1	ENSG00000113196	15016801;
D005472	affect	HAPLN2	ENSG00000132702	16584549;
D005472	affect	HBA1	ENSG00000206172	15016801;
D005472	affect	HDGFRP3	ENSG00000166503	15016801;
D005472	affect	HERPUD1	ENSG00000051108	15016801;
D005472	affect	HHLA3	ENSG00000197568	16584549;
D005472	affect	HIST2H4A	ENSG00000182217	16584549;

D005472	affect	HM13	ENSG00000101294	16584549;
D005472	affect	HMMR	ENSG00000072571	15016801;
D005472	affect	HNRNPA2B	ENSG00000122566	15016801;
D005472	affect	HNRNPF	ENSG00000169813	15016801;
D005472	affect	HOXC11	ENSG00000123388	15331170;
D005472	affect	HPX	ENSG00000110169	15016801;
D005472	affect	HSD17B2	ENSG00000086696	16584549;
D005472	affect	HTR7	ENSG00000148680	16584549;
D005472	affect	IER5	ENSG00000162783	15016801;
D005472	affect	IFI16	ENSG00000163565	15016801;
D005472	affect	IL8	ENSG00000169429	16584549;
D005472	affect	INPP5E	ENSG00000148384	15016801;
D005472	affect	IQGAP2	ENSG00000145703	15016801;
D005472	affect	ITPR1	ENSG00000150995	15016801;
D005472	affect	KCNQ2	ENSG00000075043	15016801;
D005472	affect	KIAA0090	ENSG00000127463	16584549;
D005472	affect	KIF15	ENSG00000163808	15016801;
D005472	affect	KIF23	ENSG00000137807	15016801;
D005472	affect	KLF5	ENSG00000102554	15016801;
D005472	affect	KLK15	ENSG00000174562	16584549;
D005472	affect	KRT17	ENSG00000128422	16584549;
D005472	affect	KRT6A	ENSG00000205420	16584549;
D005472	affect	L1TD1	ENSG00000240563	15016801;
D005472	affect	LDHA	ENSG00000134333	15016801;
D005472	affect	LRBA	ENSG00000198589	15016801;
D005472	affect	LYPLA1	ENSG00000120992	15016801;
D005472	affect	MACF1	ENSG00000127603	15016801;
D005472	affect	MAP4K5	ENSG00000012983	15016801;
D005472	affect	MARS	ENSG00000166986	15016801;
D005472	affect	MCM10	ENSG00000065328	16584549;
D005472	affect	MCM4	ENSG00000104738	15205334;
D005472	affect	MCM6	ENSG00000076003	15016801;
D005472	affect	MCM7	ENSG00000166508	15548681;15016801;
D005472	affect	MDFIC	ENSG00000135272	16584549;
D005472	affect	MEF2B	ENSG00000213999	16584549;
D005472	affect	METAP2	ENSG00000111142	15016801;
D005472	affect	MFN2	ENSG00000116688	16584549;
D005472	affect	MMP1	ENSG00000196611	15016801;
D005472	affect	MMP9	ENSG00000100985	15205334;
D005472	affect	MRAS	ENSG00000158186	16584549;
D005472	affect	MYLIP	ENSG00000007944	16584549;
D005472	affect	NASP	ENSG00000132780	15016801;
D005472	affect	NBN	ENSG00000104320	15016801;
D005472	affect	NCAPG	ENSG00000109805	15016801;
D005472	affect	NEK2	ENSG00000117650	15016801;
D005472	affect	NEUROD1	ENSG00000162992	16584549;
D005472	affect	NINJ1	ENSG00000131669	15016801;
D005472	affect	NKIRAS1	ENSG00000197885	16584549;
D005472	affect	NR1D2	ENSG00000174738	15016801;
D005472	affect	NR4A1	ENSG00000123358	15205334;16557594;
D005472	affect	NUPR1	ENSG00000176046	16584549;
D005472	affect	ODC1	ENSG00000115758	15016801;
D005472	affect	OXCT1	ENSG00000083720	16217747;15016801;
D005472	affect	PAFAH1B1	ENSG00000007168	15016801;

D005472	affect	PAIP1	ENSG00000172239	15016801;
D005472	affect	PDCD1LG2	ENSG00000197646	15016801;
D005472	affect	PGLYRP1	ENSG00000008438	15016801;
D005472	affect	PHIP	ENSG00000146247	15016801;
D005472	affect	PI4KB	ENSG00000143393	15016801;
D005472	affect	PLAU	ENSG00000122861	16584549;
D005472	affect	PMAIP1	ENSG00000141682	16217747;15016801;
D005472	affect	PMF1	ENSG00000160783	15016801;
D005472	affect	PPA2	ENSG00000138777	15016801;
D005472	affect	PPP1CC	ENSG00000186298	15016801;
D005472	affect	PREPL	ENSG00000138078	15016801;
D005472	affect	PRKAG2	ENSG00000106617	15016801;
D005472	affect	PRKCI	ENSG00000163558	15016801;
D005472	affect	PRKCZ	ENSG00000067606	16584549;
D005472	affect	PRKDC	ENSG00000121031	18546291;15016801;
D005472	affect	PROCR	ENSG00000101000	15016801;
D005472	affect	PRSS2	ENSG00000204983	16584549;
D005472	affect	PUM1	ENSG00000134644	16584549;
D005472	affect	PURA	ENSG00000185129	16584549;
D005472	affect	PURG	ENSG00000172733	15016801;
D005472	affect	QSOX1	ENSG00000116260	15016801;
D005472	affect	R3HDM2	ENSG00000179912	16584549;
D005472	affect	RAB21	ENSG00000080371	15205334;
D005472	affect	RANBP1	ENSG00000099901	15016801;
D005472	affect	RAX2	ENSG00000173976	16584549;
D005472	affect	RB1	ENSG00000139687	15205334;17640669;18383818;18678097;
D005472	affect	RCBTB2	ENSG00000136161	16584549;
D005472	affect	RDX	ENSG00000137710	16584549;
D005472	affect	RECQL4	ENSG00000160957	15016801;
D005472	affect	REST	ENSG00000084093	16584549;
D005472	affect	REV1	ENSG00000135945	16584549;
D005472	affect	RFC4	ENSG00000163918	15016801;
D005472	affect	RGS16	ENSG00000143333	15205334;
D005472	affect	RGS1	ENSG00000090104	15205334;
D005472	affect	RPL10A	ENSG00000198755	15016801;
D005472	affect	RPS26	ENSG00000197728	16584549;
D005472	affect	SAMHD1	ENSG00000101347	16584549;
D005472	affect	SAP30	ENSG00000164105	15016801;
D005472	affect	SCN2B	ENSG00000149575	16584549;
D005472	affect	SCNN1B	ENSG00000168447	16584549;
D005472	affect	SEC31B	ENSG00000075826	15016801;
D005472	affect	SERPINA7	ENSG00000123561	15016801;
D005472	affect	SERPIND1	ENSG00000099937	15016801;
D005472	affect	SETD5	ENSG00000168137	16584549;
D005472	affect	SEZ6L	ENSG00000100095	15016801;
D005472	affect	SF3B3	ENSG00000189091	15016801;
D005472	affect	SFRS6	ENSG00000124193	15016801;
D005472	affect	SHMT2	ENSG00000182199	15016801;
D005472	affect	SIGLEC9	ENSG00000129450	15016801;
D005472	affect	SLC25A23	ENSG00000125648	16584549;
D005472	affect	SLC48A1	ENSG00000211584	16584549;
D005472	affect	SLCO2A1	ENSG00000174640	16584549;
D005472	affect	SP3	ENSG00000172845	15016801;

D005472	affect	SPAG5	ENSG00000076382	15016801;
D005472	affect	SPINK4	ENSG00000122711	16584549;
D005472	affect	SPON1	ENSG00000152268	15205334;
D005472	affect	SPRR1B	ENSG00000169469	16584549;
D005472	affect	SPRR3	ENSG00000163209	16584549;
D005472	affect	SRP9	ENSG00000143742	15016801;
D005472	affect	STARD7	ENSG00000084090	16584549;
D005472	affect	TAF3	ENSG00000165632	16584549;
D005472	affect	TBCD	ENSG00000141556	15016801;
D005472	affect	TCEB3	ENSG00000011007	15205334;
D005472	affect	TFF1	ENSG00000160182	15016801;
D005472	affect	TFRC	ENSG00000072274	15016801;
D005472	affect	TGFA	ENSG00000163235	15016801;
D005472	affect	TJP3	ENSG00000105289	16584549;
D005472	affect	TMEM40	ENSG00000088726	16584549;
D005472	affect	TOB1	ENSG00000141232	15016801;
D005472	affect	TPP1	ENSG00000166340	16584549;
D005472	affect	TRAP1	ENSG00000126602	15016801;
D005472	affect	TRIB3	ENSG00000101255	16584549;
D005472	affect	TROAP	ENSG00000135451	15016801;
D005472	affect	TSPO	ENSG00000100300	15016801;
D005472	affect	TSSC1	ENSG00000032389	15016801;
D005472	affect	UBE2S	ENSG00000108106	15016801;
D005472	affect	UBE2T	ENSG00000077152	16557594;
D005472	affect	UGT8	ENSG00000174607	15016801;
D005472	affect	UNC5B	ENSG00000107731	16584549;
D005472	affect	UNG	ENSG00000076248	16951200;18714155;
D005472	affect	UQCRB	ENSG00000156467	15016801;
D005472	affect	USH2A	ENSG00000042781	15016801;
D005472	affect	VANGL2	ENSG00000162738	15016801;
D005472	affect	VPREB3	ENSG00000128218	16584549;
D005472	affect	VPS35	ENSG00000069329	16584549;
D005472	affect	WARS	ENSG00000140105	15016801;
D005472	affect	WBP4	ENSG00000120688	16584549;
D005472	affect	WDR61	ENSG00000140395	15016801;
D005472	affect	WEE1	ENSG00000166483	15016801;
D005472	affect	WTAP	ENSG00000146457	15016801;
D005472	affect	YARS	ENSG00000134684	15016801;
D005472	affect	ZAK	ENSG00000091436	16584549;
D005472	affect	ZC3H3	ENSG00000014164	16584549;
D005472	affect	ZIM2	ENSG00000198300	15016801;
D005472	affect	ZMYND8	ENSG00000101040	15016801;
D005472	affect	ZNF266	ENSG00000174652	15016801;
D005472	decrease	ABCC1	ENSG00000103222	12576456;17638652;
D005472	decrease	ACTG2	ENSG00000163017	16709241;
D005472	decrease	ADCY9	ENSG00000162104	19219653;
D005472	decrease	AHNAK	ENSG00000124942	15352031;
D005472	decrease	AKT1	ENSG00000142208	18794807;17918158;
D005472	decrease	ALCAM	ENSG00000170017	15352031;
D005472	decrease	ALDH1A3	ENSG00000184254	15352031;
D005472	decrease	ALKBH8	ENSG00000137760	16510598;
D005472	decrease	AMPD2	ENSG00000116337	19219653;
D005472	decrease	ANGPT2	ENSG00000091879	17786339;
D005472	decrease	ANLN	ENSG00000011426	17982676;15016801;

D005472	decrease	ANXA1	ENSG00000135046	16217747;15352031;
D005472	decrease	ANXA4	ENSG00000196975	15109396;
D005472	decrease	AP2S1	ENSG00000042753	16391804;
D005472	decrease	AP4S1	ENSG00000100478	16510598;
D005472	decrease	ARPC3	ENSG00000111229	16709241;
D005472	decrease	ASS1	ENSG00000130707	16391804;
D005472	decrease	ATG12	ENSG00000145782	16510598;
D005472	decrease	ATP9A	ENSG00000054793	15352031;
D005472	decrease	AURKA	ENSG00000087586	16557594;
D005472	decrease	BCAR3	ENSG00000137936	15352031;
D005472	decrease	BCL2L12	ENSG00000126453	19015929;
D005472	decrease	BDH1	ENSG00000161267	16803524;15585135;
D005472	decrease	BRIX1	ENSG00000113460	16510598;
D005472	decrease	BUB1B	ENSG00000156970	16709241;16557594;
D005472	decrease	BUB1	ENSG00000169679	16709241;
D005472	decrease	BUB3	ENSG00000154473	15548681;16803524;15585135;
D005472	decrease	C13ORF34	ENSG00000136122	17374387;
D005472	decrease	CAV1	ENSG00000105974	15352031;
D005472	decrease	CAV2	ENSG00000105971	16510598;
D005472	decrease	CCNA2	ENSG00000145386	15548681;18383818;16557594;15205334;15546879;
D005472	decrease	CCNB1	ENSG00000134057	17982676;16557594;15205334;15546879;15016801;
D005472	decrease	CCNB2	ENSG00000157456	16709241;16557594;17982488;15016801;
D005472	decrease	CCNC	ENSG00000112237	16709241;
D005472	decrease	CCND1	ENSG00000110092	15352031;18930000;17982676;
D005472	decrease	CD8B	ENSG00000172116	15352031;
D005472	decrease	CDC25A	ENSG00000164045	16510598;
D005472	decrease	CDC25B	ENSG00000101224	16709241;
D005472	decrease	CDC73	ENSG00000134371	16510598;
D005472	decrease	CDCA8	ENSG00000134690	17374387;
D005472	decrease	CDH1	ENSG00000039068	15352031;
D005472	decrease	CDKN1B	ENSG00000111276	17217824;18930000;
D005472	decrease	CDKN2A	ENSG00000147889	17982676;
D005472	decrease	CDKN2C	ENSG00000123080	16557594;
D005472	decrease	CEP55	ENSG00000138180	17039268;
D005472	decrease	CKS2	ENSG00000123975	17374387;
D005472	decrease	CLDN4	ENSG00000189143	15352031;
D005472	decrease	CLEC11A	ENSG00000105472	15352031;
D005472	decrease	COL12A1	ENSG00000111799	15205334;16510598;
D005472	decrease	COL4A2	ENSG00000134871	15352031;
D005472	decrease	CPA4	ENSG00000128510	16510598;
D005472	decrease	CRYZ	ENSG00000116791	17982676;
D005472	decrease	CST7	ENSG00000077984	15352031;
D005472	decrease	CTBP2	ENSG00000175029	18927307;16391804;
D005472	decrease	CTPS	ENSG00000171793	19219653;
D005472	decrease	CTTN	ENSG00000085733	16803524;15585135;
D005472	decrease	CYTH3	ENSG00000008256	16803524;
D005472	decrease	DBF4	ENSG00000006634	16510598;
D005472	decrease	DICER1	ENSG00000100697	16510598;
D005472	decrease	DKK1	ENSG00000107984	16217747;15352031;
D005472	decrease	DUT	ENSG00000128951	19015155;16557594;

D005472	decrease	ECT2	ENSG00000114346	17982676;
D005472	decrease	EFHD2	ENSG00000142634	16510598;
D005472	decrease	EFNB2	ENSG00000125266	16217747;15352031;
D005472	decrease	EGFR	ENSG00000146648	16098254;19217205;17169374;16836928;15981280;17146438;15723263;17918158;17982676;18794807;1573
D005472	decrease	EIF2B2	ENSG00000119718	16803524;15585135;
D005472	decrease	ENTPD1	ENSG00000138185	19219653;
D005472	decrease	EPRS	ENSG00000136628	16709241;
D005472	decrease	ERBB2	ENSG00000141736	17918158;18337622;15940066;15205334;15737843;
D005472	decrease	ERBB3	ENSG00000065361	17918158;
D005472	decrease	ERCC1	ENSG00000012061	17401013;18448328;15213713;15173087;17016597;15737843;
D005472	decrease	EREG	ENSG00000124882	16510598;
D005472	decrease	ERP29	ENSG00000089248	16803524;15585135;
D005472	decrease	F12	ENSG00000131187	15352031;
D005472	decrease	F2	ENSG00000180210	16709241;
D005472	decrease	F3	ENSG00000117525	15067352;16510598;
D005472	decrease	FAM76B	ENSG00000077458	16510598;
D005472	decrease	FANCC	ENSG00000158169	16557594;
D005472	decrease	FARS2	ENSG00000145982	16101138;
D005472	decrease	FBXO5	ENSG00000112029	16557594;
D005472	decrease	FGF2	ENSG00000138685	16510598;
D005472	decrease	FGG	ENSG00000171557	16217747;17039268;
D005472	decrease	FNDC3B	ENSG00000075420	16217747;16510598;
D005472	decrease	FOLR1	ENSG00000110195	16510598;
D005472	decrease	FOXN3	ENSG00000053254	15352031;
D005472	decrease	GLI1	ENSG00000111087	18776995;
D005472	decrease	GNB2	ENSG00000172354	16709241;
D005472	decrease	GNE	ENSG00000159921	15352031;
D005472	decrease	GUCY1B3	ENSG00000061918	19219653;
D005472	decrease	HBE1	ENSG00000213931	15352031;
D005472	decrease	HDAC4	ENSG00000068024	16557594;
D005472	decrease	HIST1H4B	ENSG00000182217	17039268;15016801;
D005472	decrease	HIST1H4C	ENSG00000182217	17039268;16510598;
D005472	decrease	HK2	ENSG00000159399	16595493;18772588;
D005472	decrease	HMG20B	ENSG00000064961	16709241;
D005472	decrease	HNRNPA1	ENSG00000135486	16101138;
D005472	decrease	HS6ST2	ENSG00000171004	16510598;
D005472	decrease	HSPA1B	ENSG00000232804	15352031;
D005472	decrease	HSPA4	ENSG00000170606	15352031;
D005472	decrease	IGFBP5	ENSG00000115461	16557594;
D005472	decrease	IGFBP6	ENSG00000167779	15352031;
D005472	decrease	ITGB4	ENSG00000132470	15205334;
D005472	decrease	KCTD12	ENSG00000178695	17039268;
D005472	decrease	KIAA0101	ENSG00000166803	17039268;16391804;
D005472	decrease	KLF6	ENSG00000067082	15205334;
D005472	decrease	KLK6	ENSG00000167755	15352031;
D005472	decrease	KNG1	ENSG00000113889	16391804;
D005472	decrease	KPNA2	ENSG00000182481	17374387;15016801;
D005472	decrease	KPNA3	ENSG00000102753	16709241;
D005472	decrease	KRAS	ENSG00000133703	16803524;15585135;

D005472	decrease	KRT5	ENSG00000186081	15352031;
D005472	decrease	KRT86	ENSG00000170442	15352031;
D005472	decrease	L1CAM	ENSG00000198910	15352031;
D005472	decrease	LGALS7	ENSG00000178934	15352031;
D005472	decrease	LIPH	ENSG00000163898	16709241;
D005472	decrease	LRP8	ENSG00000157193	16101138;
D005472	decrease	MAD2L1	ENSG00000164109	16557594;17039268;
D005472	decrease	MAD2L2	ENSG00000116670	16803524;
D005472	decrease	MAGEA12	ENSG00000213401	15352031;
D005472	decrease	MAGEA2	ENSG00000184750	15352031;
D005472	decrease	MAGEA6	ENSG00000197172	15352031;
D005472	decrease	MAGEB2	ENSG00000099399	15352031;
D005472	decrease	MARCKS	ENSG00000155130	17039268;16217747;
D005472	decrease	MBNL1	ENSG00000152601	16709241;
D005472	decrease	MCM3	ENSG00000112118	16709241;15016801;
D005472	decrease	MDFI	ENSG00000112559	15352031;
D005472	decrease	MGMT	ENSG00000170430	17487405;
D005472	decrease	MKI67	ENSG00000148773	15205334;16709241;15548681;15940066;
D005472	decrease	MKX	ENSG00000150051	16510598;
D005472	decrease	MPP7	ENSG00000150054	16510598;
D005472	decrease	MSH2	ENSG00000095002	16709241;16557594;18949393;
D005472	decrease	MSX2	ENSG00000120149	15352031;
D005472	decrease	MT1X	ENSG00000187193	17039268;
D005472	decrease	MTAP	ENSG00000099810	16510598;
D005472	decrease	MYB	ENSG00000118513	16557594;15548681;
D005472	decrease	NAF1	ENSG00000145414	16510598;
D005472	decrease	NAIP	ENSG00000250628	16101138;
D005472	decrease	NBL1	ENSG00000158747	16709241;
D005472	decrease	NEK1	ENSG00000137601	16709241;
D005472	decrease	NEK4	ENSG00000114904	16709241;
D005472	decrease	NEK6	ENSG00000119408	17039268;
D005472	decrease	NEK9	ENSG00000119638	16709241;
D005472	decrease	NFIB	ENSG00000147862	16557594;
D005472	decrease	NFKB1	ENSG00000109320	15548681;16637064;
D005472	decrease	NOM1	ENSG00000146909	16510598;
D005472	decrease	NPM3	ENSG00000107833	16510598;
D005472	decrease	NTRK2	ENSG00000148053	15352031;
D005472	decrease	NTSR1	ENSG00000101188	15352031;
D005472	decrease	NUCKS1	ENSG00000069275	16803524;15585135;15016801;
D005472	decrease	PAQR8	ENSG00000170915	17039268;
D005472	decrease	PARP1	ENSG00000143799	19084572;17982676;16525653;15996812;
D005472	decrease	PCDHA5	ENSG00000204965	16803524;15585135;
D005472	decrease	PDCD4	ENSG00000150593	15109396;
D005472	decrease	PDCD6IP	ENSG00000170248	17039268;
D005472	decrease	PDIA4	ENSG00000155660	16709241;
D005472	decrease	PDXK	ENSG00000160209	16510598;
D005472	decrease	PDXP	ENSG00000241360	16709241;
D005472	decrease	PIR	ENSG00000087842	15352031;
D005472	decrease	POLA1	ENSG00000101868	19219653;
D005472	decrease	PPARG	ENSG00000132170	15352031;

D005472	decrease	PPP1R2	ENSG00000184203	17039268;16557594;
D005472	decrease	PRC1	ENSG00000198901	17374387;
D005472	decrease	PRODH2	ENSG00000250799	15352031;
D005472	decrease	PRPF18	ENSG00000165630	16101138;
D005472	decrease	PRPS2	ENSG00000101911	16510598;
D005472	decrease	PRRG1	ENSG00000130962	17039268;
D005472	decrease	PSMB10	ENSG00000205220	16510598;
D005472	decrease	PSRC1	ENSG00000134222	17374387;
D005472	decrease	PTEN	ENSG00000171862	17039268;
D005472	decrease	RAB11A	ENSG00000103769	17039268;
D005472	decrease	RAB8A	ENSG00000167461	16709241;
D005472	decrease	RAD54B	ENSG00000197275	16557594;
D005472	decrease	RAMP1	ENSG00000132329	15352031;16896004;
D005472	decrease	RARRES2	ENSG00000106538	16709241;
D005472	decrease	RBBP4	ENSG00000162521	16557594;18678097;
D005472	decrease	RBP1	ENSG00000248478	15352031;
D005472	decrease	RBPJ	ENSG00000168214	16391804;
D005472	decrease	RCC1	ENSG00000180198	16709241;
D005472	decrease	RELA	ENSG00000173039	19003803;15548681;16680372;16637064;
D005472	decrease	RGS13	ENSG00000127074	16803524;
D005472	decrease	RGS2	ENSG00000116741	16510598;
D005472	decrease	RNF4	ENSG00000063978	16709241;
D005472	decrease	RPL37	ENSG00000145592	16557594;
D005472	decrease	RPL3	ENSG00000100316	16803524;15585135;
D005472	decrease	RRBP1	ENSG00000125844	15352031;
D005472	decrease	RRP15	ENSG00000067533	16510598;
D005472	decrease	S100A3	ENSG00000188015	15352031;
D005472	decrease	S100A4	ENSG00000196154	15352031;
D005472	decrease	S100P	ENSG00000163993	15352031;18636193;
D005472	decrease	SCAMP3	ENSG00000116521	16391804;
D005472	decrease	SCG2	ENSG00000171951	15352031;
D005472	decrease	SCNN1A	ENSG00000111319	15352031;
D005472	decrease	SERF2	ENSG00000140264	16510598;
D005472	decrease	SERPINB4	ENSG00000206073	16803524;
D005472	decrease	SHH	ENSG00000164690	18776995;
D005472	decrease	SLC15A1	ENSG00000088386	16253763;
D005472	decrease	SLC16A1	ENSG00000155380	16709241;
D005472	decrease	SMN2	ENSG00000172062	16101138;
D005472	decrease	SMO	ENSG00000128602	18776995;
D005472	decrease	SNAPC1	ENSG00000023608	16803524;15585135;
D005472	decrease	SNRNP48	ENSG00000168566	16510598;
D005472	decrease	SQLE	ENSG00000104549	15205334;
D005472	decrease	SR140	ENSG00000163714	16510598;
D005472	decrease	SRP19	ENSG00000153037	16391804;
D005472	decrease	SRRM1	ENSG00000133226	16709241;
D005472	decrease	STAU1	ENSG00000124214	16709241;
D005472	decrease	SUFU	ENSG00000107882	18776995;
D005472	decrease	SUPV3L1	ENSG00000156502	17039268;
D005472	decrease	SWAP70	ENSG00000133789	16510598;
D005472	decrease	SYMPK	ENSG00000125755	16709241;
D005472	decrease	SYNCRIP	ENSG00000135316	17039268;
D005472	decrease	TAF5L	ENSG00000135801	16391804;
D005472	decrease	TAGLN	ENSG00000149591	15352031;16217747;

D005472	decrease	TERT	ENSG00000164362	17923759;16929163;16455633;17996122;15003732;18396642;15608686;1501680
D005472	decrease	TFDP1	ENSG00000198176	16510598;
D005472	decrease	THOC1	ENSG00000079134	16709241;
D005472	decrease	TK1	ENSG00000167900	16557594;15374632;15548681;
D005472	decrease	TMEM33	ENSG00000109133	16510598;
D005472	decrease	TNNI3	ENSG00000129991	15352031;
D005472	decrease	TNNT1	ENSG00000105048	15352031;
D005472	decrease	TOP1	ENSG00000198900	16709241;18509181;
D005472	decrease	TOP2A	ENSG00000131747	15205334;16557594;18465341;15016801;
D005472	decrease	TPI1	ENSG00000111669	16510598;18309519;
D005472	decrease	TSPAN8	ENSG00000127324	16391804;
D005472	decrease	TSR1	ENSG00000167721	16510598;
D005472	decrease	TTYH2	ENSG00000141540	16510598;
D005472	decrease	TUBA8	ENSG00000183785	17039268;
D005472	decrease	TUBB	ENSG00000229684	17039268;
D005472	decrease	UBE2C	ENSG00000175063	17039268;16557594;17374387;
D005472	decrease	UCK2	ENSG00000143179	19219653;15548681;
D005472	decrease	USP14	ENSG00000101557	16803524;
D005472	decrease	VAV3	ENSG00000134215	16557594;
D005472	decrease	VPS52	ENSG00000224455	16709241;
D005472	decrease	WDR4	ENSG00000160193	16510598;
D005472	decrease	YWHAE	ENSG00000108953	16709241;
D005472	decrease	ZBTB20	ENSG00000181722	16510598;
D005472	decrease	ZBTB7A	ENSG00000178951	15352031;
D005472	decrease	ZNF367	ENSG00000165244	16510598;
D005472	increase	ABCB1	ENSG00000085563	12576456;17284363;17934748;17287894;15239124;
D005472	increase	ABCC6	ENSG00000091262	15352031;
D005472	increase	ACADL	ENSG00000115361	16803524;15585135;
D005472	increase	ADSS	ENSG00000035687	16709241;16803524;15585135;
D005472	increase	AIFM1	ENSG00000156709	16168113;
D005472	increase	AKT2	ENSG00000105221	16803524;
D005472	increase	AMT	ENSG00000145020	16803524;15585135;
D005472	increase	ANGPT1	ENSG00000154188	17786339;
D005472	increase	ANKRD11	ENSG00000167522	16584549;16510598;
D005472	increase	ANKRD1	ENSG00000148677	17039268;
D005472	increase	ANXA2	ENSG00000182718	15152939;
D005472	increase	ANXA3	ENSG00000138772	17039268;
D005472	increase	APAF1	ENSG00000120868	16557594;
D005472	increase	APOBEC3C	ENSG00000244509	15352031;16510598;
D005472	increase	AREG	ENSG00000205595	15067352;16709241;
D005472	increase	ARFGAP1	ENSG00000101199	15331170;
D005472	increase	ARL4C	ENSG00000188042	16510598;
D005472	increase	ATF3	ENSG00000162772	15205334;16709241;16557594;14665630;15016801;
D005472	increase	ATP6	ENSG00000198899	16391804;
D005472	increase	AVEN	ENSG00000169857	16510598;16709241;
D005472	increase	BAK1	ENSG00000030110	18593893;16557594;15489892;

D005472	increase	BARX2	ENSG00000043039	16803524;15585135;
D005472	increase	BAT1	ENSG00000229496	16510598;
D005472	increase	BAX	ENSG00000087088	11062132;16686938;17399942;15996812;15709199;18834353;16168113;15641988;17589894;17045692;17056233;15986848;19015929;
D005472	increase	BBC3	ENSG00000105327	16481741;16557594;14665630;15489892;15016801;
D005472	increase	BCL2	ENSG00000171791	16407826;16412276;16332177;18949393;15177426;16603448;15940066;17439414;15709199;19084572;17589894;16557594;15996812;16929163;17287894;18205257;18161298;14503796;19015929;16596200;16391804;
D005472	increase	BCL2L1	ENSG00000171552	16407826;15767554;17033717;16875599;15289866;15709199;17439414;16929163;17045692;17399942;16557594;16596200;
D005472	increase	BEAN	ENSG00000166546	15352031;
D005472	increase	BIRC2	ENSG00000110330	17039268;
D005472	increase	BIRC3	ENSG00000023445	16557594;
D005472	increase	BIRC5	ENSG00000089685	15067352;16217747;18714155;17686279;15685548;16929163;16709241;15109396;17399942;16596200;
D005472	increase	BNIP3L	ENSG00000104765	15067352;15109396;16557594;16709241;17039268;
D005472	increase	BRCA1	ENSG00000012048	17982676;15607317;16101138;16557594;
D005472	increase	BTG2	ENSG00000159388	17982676;15016801;
D005472	increase	BTN3A3	ENSG00000111801	16101138;
D005472	increase	C10ORF116	ENSG00000148671	17039268;
D005472	increase	C10ORF118	ENSG00000165813	16510598;
D005472	increase	C10ORF119	ENSG00000197771	17039268;
D005472	increase	C20ORF165	ENSG00000149634	15331170;
D005472	increase	C4ORF34	ENSG00000163683	16510598;
D005472	increase	C7ORF16	ENSG00000106341	16803524;
D005472	increase	CALB2	ENSG00000172137	16510598;
D005472	increase	CASP3	ENSG00000164305	17056233;15546879;19084572;16407826;15685448;12414664;16803524;18830594;17399942;16080519;18205257;18161298;17498489;15282376;17589894;14503796;15585135;18772588;17016659;16168113;18608205;[illegible]
D005472	increase	CASP4	ENSG00000196954	16217747;15352031;
D005472	increase	CASP8	ENSG00000064012	17056233;17498489;16803524;12414664;18830594;17637740;15585135;17016659;16168113;

D005472	increase	CASP9	ENSG00000132906	17056233;16168113;16407826;12414664;15996812;16803524;18830594;17498489;15546879;17016659;1901
D005472	increase	CCND2	ENSG00000118971	15352031;15016801;
D005472	increase	CCND3	ENSG00000112576	15067352;15548681;18383818;16709241;
D005472	increase	CCNE1	ENSG00000105173	18930000;16101138;15342418;
D005472	increase	CCNG1	ENSG00000113328	16217747;16709241;17982676;16557594;15152939;
D005472	increase	CCNG2	ENSG00000138764	15067352;17039268;16510598;16557594;18754885;
D005472	increase	CD274	ENSG00000120217	17920123;
D005472	increase	CDC40	ENSG00000168438	16803524;
D005472	increase	CDC6	ENSG00000094804	16557594;17039268;16709241;15342418;
D005472	increase	CDCA5	ENSG00000146670	16709241;
D005472	increase	CDK1	ENSG00000170312	16557594;17039268;15205334;15152939;15342418;
D005472	increase	CDK2	ENSG00000123374	15548681;18383818;16557594;15342418;
D005472	increase	CDKN1A	ENSG00000124762	15067352;15161699;15331170;15942645;16686938;15375580;15546879;16168113;15489892;15625077;14665630;16434701;17355264;16557594;16584549;16709241;15205334;17039268;19098008;17699798;17982676;15016801;18834353;15342[illegible]
D005472	increase	CEBPB	ENSG00000172216	16584549;16557594;
D005472	increase	CENPQ	ENSG00000031691	15331170;
D005472	increase	CES2	ENSG00000172831	16963839;17287894;15205334;
D005472	increase	CFLAR	ENSG00000003402	16373718;15548681;16217747;17045692;15152939;
D005472	increase	CHEK1	ENSG00000149554	18698031;15016801;
D005472	increase	CHI3L1	ENSG00000133048	15205334;
D005472	increase	CIDEB	ENSG00000136305	16803524;15585135;
D005472	increase	CPE	ENSG00000109472	15352031;
D005472	increase	CRABP1	ENSG00000166426	15352031;
D005472	increase	CRIM1	ENSG00000150938	15352031;16510598;
D005472	increase	CSTF3	ENSG00000176102	15331170;
D005472	increase	CTSH	ENSG00000103811	15352031;
D005472	increase	CUL4B	ENSG00000158290	16803524;
D005472	increase	CUL5	ENSG00000166266	16557594;
D005472	increase	CYFIP2	ENSG00000055163	16217747;17982676;17039268;15016801;
D005472	increase	CYR61	ENSG00000142871	15067352;17699798;
D005472	increase	DDB2	ENSG00000134574	15331170;17982676;16584549;
D005472	increase	DDIT3	ENSG00000175197	17355264;
D005472	increase	DDIT4	ENSG00000168209	17039268;16510598;16557594;
D005472	increase	DHFR	ENSG00000228716	16557594;15109396;
D005472	increase	DHX15	ENSG00000109606	16584549;
D005472	increase	DIABLO	ENSG00000184047	18031615;16557594;

D005472	increase	DKK4	ENSG00000104371	16584549;
D005472	increase	DNER	ENSG00000187957	16510598;
D005472	increase	DPYD	ENSG00000188641	16568373;17046731;17121937;18443386;18299612;19104657;17064846;15591715;15858133;18986760;15246190;18383874;15239142;16361556;18197934;15017333;18619742;15655543;18846242;19020767;15993511;16646564;18600527;15930747;17520254;16033824;14689231;16282729;17377791;19105824;15890242;18225548;18075467;16115930;16557585;16761622;19219653;17330233;15109396;15865071;15737843;19093184;
D005472	increase	DTYMK	ENSG00000168393	15067352;15109396;15016801;
D005472	increase	DUSP6	ENSG00000139318	16510598;
D005472	increase	ECHS1	ENSG00000127884	15152939;
D005472	increase	EDN1	ENSG00000078401	19098008;
D005472	increase	EEF1A1	ENSG00000156508	17982676;
D005472	increase	EGR1	ENSG00000120738	18757417;15205334;16557594;17039268;
D005472	increase	EIF3H	ENSG00000147677	16803524;15585135;
D005472	increase	EIF4E	ENSG00000151247	16584549;15016801;
D005472	increase	EIF4H	ENSG00000106682	16803524;15585135;
D005472	increase	EIF5	ENSG00000100664	17039268;
D005472	increase	ELMO2	ENSG00000062598	16803524;
D005472	increase	EPAS1	ENSG00000116016	16557594;
D005472	increase	EPHX1	ENSG00000143819	16217747;15205334;
D005472	increase	FANCG	ENSG00000221829	15067352;15548681;15109396;
D005472	increase	FAS	ENSG00000026103	17056233;15034078;15161716;16957513;17039268;15152939;16709241;16557594;14665630;17982676;15585135;17045692;15546879;15016801;19015929;165962
D005472	increase	FBLN1	ENSG00000077942	15205334;17039268;
D005472	increase	FDXR	ENSG00000161513	17982676;16557594;15205334;14665630;15016801;
D005472	increase	FEN1	ENSG00000168496	15067352;15109396;
D005472	increase	FHL3	ENSG00000183386	17039268;
D005472	increase	FILIP1L	ENSG00000168386	15352031;
D005472	increase	FKBPL	ENSG00000223666	14503796;
D005472	increase	FOS	ENSG00000170345	15205334;17039268;
D005472	increase	FXYD3	ENSG00000089356	15152939;
D005472	increase	G0S2	ENSG00000123689	15352031;
D005472	increase	GADD45A	ENSG00000116717	17982676;16709241;15205334;19003803;19098008;15067352;15016801;
D005472	increase	GAMT	ENSG00000130005	15352031;16896004;15016801;
D005472	increase	GART	ENSG00000159131	19219653;
D005472	increase	GDF15	ENSG00000130513	15205334;15016801;

D005472	increase	GPR132	ENSG00000183484	16584549;
D005472	increase	GPR35	ENSG00000178623	15331170;
D005472	increase	GSTP1	ENSG00000084207	15205334;15213713;16622263;15737843;
D005472	increase	GSTT1	ENSG00000184674	16803524;15585135;
D005472	increase	HINT1	ENSG00000169567	16803524;15585135;
D005472	increase	HIST1H1D	ENSG00000124575	15585135;
D005472	increase	HIST1H2BK	ENSG00000197903	16101138;15352031;16217747;
D005472	increase	HLA-F	ENSG00000204642	16101138;
D005472	increase	HLA-G	ENSG00000237216	16101138;
D005472	increase	HNRNPC	ENSG00000092199	16803524;
D005472	increase	HRAS	ENSG00000174775	16557594;
D005472	increase	HSPA2	ENSG00000126803	16101138;16217747;
D005472	increase	HSPB8	ENSG00000152137	16557594;
D005472	increase	HSPE1	ENSG00000115541	15152939;
D005472	increase	HTATIP2	ENSG00000109854	16391804;
D005472	increase	HTRA1	ENSG00000166033	15352031;
D005472	increase	ID1	ENSG00000125968	16557594;
D005472	increase	ID2	ENSG00000115738	15352031;16557594;
D005472	increase	ID3	ENSG00000117318	15205334;16557594;
D005472	increase	IFI27L2	ENSG00000119632	16391804;
D005472	increase	IFI35	ENSG00000068079	16101138;
D005472	increase	IFI6	ENSG00000126709	17039268;15685448;
D005472	increase	IFIT1	ENSG00000185745	17039268;
D005472	increase	IFIT2	ENSG00000119922	16101138;
D005472	increase	IFITM1	ENSG00000185885	16101138;17039268;16510598;
D005472	increase	IFNA1	ENSG00000197919	17786339;15709199;17971768;17045692;17016659;16101138;16568373;
D005472	increase	IFNAR1	ENSG00000142166	17088986;
D005472	increase	IFNAR2	ENSG00000159110	17088986;16101138;
D005472	increase	IFNG	ENSG00000111537	15162350;16101138;17711513;16568373;15301717;
D005472	increase	IL18	ENSG00000150782	15162350;16391804;
D005472	increase	IMPA2	ENSG00000141401	16510598;
D005472	increase	IMPDH2	ENSG00000178035	19219653;
D005472	increase	INSIG1	ENSG00000186480	17039268;
D005472	increase	IRAK1	ENSG00000184216	15109396;15067352;
D005472	increase	IREB2	ENSG00000136381	16584549;
D005472	increase	IRF1	ENSG00000125347	15709199;
D005472	increase	IRF9	ENSG00000213928	16101138;
D005472	increase	ISG15	ENSG00000187608	16101138;16557594;
D005472	increase	JAG1	ENSG00000101384	16510598;
D005472	increase	JUNB	ENSG00000171223	16557594;18678097;
D005472	increase	JUN	ENSG00000177606	15205334;17355264;16557594;
D005472	increase	KHK	ENSG00000138030	15352031;
D005472	increase	KITLG	ENSG00000049130	19098008;
D005472	increase	KLF4	ENSG00000136826	17039268;16557594;
D005472	increase	KLHL24	ENSG00000114796	16510598;
D005472	increase	LMF1	ENSG00000103227	16391804;
D005472	increase	M6PR	ENSG00000003056	15585135;
D005472	increase	MAF	ENSG00000178573	16557594;

D005472	increase	MALT1	ENSG00000172175	15109396;15067352;
D005472	increase	MAMDC2	ENSG00000165072	16510598;
D005472	increase	MAML2	ENSG00000184384	16510598;
D005472	increase	MAN1A1	ENSG00000111885	16510598;
D005472	increase	MAP2	ENSG00000078018	16510598;
D005472	increase	MAPK13	ENSG00000156711	16803524;15585135;
D005472	increase	MAPKAP1	ENSG00000119487	16557594;
D005472	increase	MAST4	ENSG00000069020	16510598;
D005472	increase	MDM2	ENSG00000135679	15546879;14665630;16557594;17982676;19098008;
D005472	increase	MR1	ENSG00000153029	16101138;
D005472	increase	MT1E	ENSG00000169715	15352031;
D005472	increase	MT1M	ENSG00000205364	15352031;
D005472	increase	MVP	ENSG00000013364	15750632;
D005472	increase	MYBL2	ENSG00000101057	16557594;15548681;
D005472	increase	MYC	ENSG00000136997	16709241;16557594;16803524;15585135;18802399;17628013;
D005472	increase	MYO1E	ENSG00000157483	15331170;
D005472	increase	NBPF10	ENSG00000203832	16510598;
D005472	increase	NDUFA10	ENSG00000130414	16803524;15585135;15548681;
D005472	increase	NFIC	ENSG00000141905	16584549;
D005472	increase	NFKBIA	ENSG00000100906	16929163;16337965;16637064;
D005472	increase	NOV	ENSG00000136999	16510598;
D005472	increase	NRIP1	ENSG00000180530	16510598;16391804;
D005472	increase	NT5M	ENSG00000205309	19219653;
D005472	increase	NTN4	ENSG00000074527	17039268;
D005472	increase	OAS2	ENSG00000111335	16557594;
D005472	increase	OLFM1	ENSG00000130558	15352031;
D005472	increase	OPTN	ENSG00000123240	16101138;17039268;16217747;
D005472	increase	ORC6L	ENSG00000091651	16584549;
D005472	increase	PAICS	ENSG00000128050	19219653;
D005472	increase	PANK1	ENSG00000152782	16557594;
D005472	increase	PARP14	ENSG00000173193	17039268;
D005472	increase	PCNA	ENSG00000132646	18205257;16709241;16557594;
D005472	increase	PDAP1	ENSG00000106244	16709241;16803524;15585135;
D005472	increase	PDF	ENSG00000213380	16510598;
D005472	increase	PFN2	ENSG00000070087	15352031;15016801;
D005472	increase	PHLDA3	ENSG00000174307	16557594;
D005472	increase	PLAT	ENSG00000104368	16101138;
D005472	increase	PLEKHB1	ENSG00000021300	16510598;
D005472	increase	PLK1	ENSG00000166851	15016801;
D005472	increase	PLK2	ENSG00000145632	17982676;
D005472	increase	PLOD2	ENSG00000152952	16557594;15352031;
D005472	increase	PLRG1	ENSG00000171566	16391804;
D005472	increase	PNRC1	ENSG00000146278	16510598;
D005472	increase	PPFIBP1	ENSG00000110841	16510598;
D005472	increase	PPIE	ENSG00000084072	16803524;
D005472	increase	PPM1D	ENSG00000170836	17982676;14665630;15016801;

D005472	increase	PPP1R15A	ENSG00000087074	15331170;16709241;
D005472	increase	PPP2CB	ENSG00000104695	16709241;
D005472	increase	PPP3CA	ENSG00000138814	16803524;15585135;18927307;16391804;
D005472	increase	PROM1	ENSG00000007062	15352031;
D005472	increase	PRPS1	ENSG00000147224	16584549;
D005472	increase	PRR13	ENSG00000205352	16391804;
D005472	increase	PTCH1	ENSG00000185920	18776995;16391804;
D005472	increase	PTGR1	ENSG00000106853	15585135;
D005472	increase	PTGS2	ENSG00000073756	17918158;15205334;16340194;15846114;12414664;18695918;16322294;
D005472	increase	PTTG1	ENSG00000164611	17039268;16557594;15205334;15016801;
D005472	increase	RAD23B	ENSG00000119318	15109396;15067352;
D005472	increase	RAF1	ENSG00000132155	15152939;
D005472	increase	RBM14	ENSG00000239306	16557594;
D005472	increase	RND2	ENSG00000108830	16101138;
D005472	increase	RNF34	ENSG00000170633	16407826;16080519;16270526;16596200;
D005472	increase	RPL23	ENSG00000125691	15152939;
D005472	increase	RPRD1B	ENSG00000101413	15331170;
D005472	increase	RPS27L	ENSG00000185088	18056458;16557594;15016801;
D005472	increase	RPS28	ENSG00000233927	15152939;
D005472	increase	RRM1	ENSG00000167325	15331170;
D005472	increase	RRM2B	ENSG00000048392	16557594;15890238;15016801;
D005472	increase	RRM2	ENSG00000171848	15205334;16584549;17039268;16557594;16709241;16896004;15548681;
D005472	increase	RSRC2	ENSG00000111011	15331170;
D005472	increase	S100A10	ENSG00000197747	16557594;
D005472	increase	S100A2	ENSG00000196754	16584549;15352031;17982676;15016801;
D005472	increase	SAA1	ENSG00000173432	16101138;
D005472	increase	SAT1	ENSG00000130066	16510598;17237273;15152939;16637064;
D005472	increase	SCCPDH	ENSG00000143653	16101138;
D005472	increase	SCD	ENSG00000099194	17982676;17039268;16217747;
D005472	increase	SDCCAG8	ENSG00000054282	16510598;
D005472	increase	SEMA3A	ENSG00000075213	16510598;
D005472	increase	SERPINA5	ENSG00000188488	17039268;
D005472	increase	SERPINB2	ENSG00000197632	16709241;
D005472	increase	SERPINB5	ENSG00000206075	17982676;16331619;15016801;16391804;
D005472	increase	SERTAD1	ENSG00000197019	16584549;16557594;15016801;
D005472	increase	SESN1	ENSG00000080546	17982676;14665630;15016801;
D005472	increase	SFN	ENSG00000175793	16584549;17982676;16557594;15999354;15016801;
D005472	increase	SFRS11	ENSG00000116754	15331170;
D005472	increase	SIGLEC1	ENSG00000088827	16391804;
D005472	increase	SLC16A6	ENSG00000108932	15352031;16510598;

D005472	increase	SLC29A1	ENSG00000112759	17695509;16868479;18383843;16217747;18992248;
D005472	increase	SLC2A1	ENSG00000117394	16253763;16595493;
D005472	increase	SLC3A2	ENSG00000168003	15331170;
D005472	increase	SMARCA1	ENSG00000102038	15352031;16217747;
D005472	increase	SMCHD1	ENSG00000101596	16510598;
D005472	increase	SMOX	ENSG00000088826	17987291;
D005472	increase	SMS	ENSG00000102172	15152939;15016801;
D005472	increase	SMURF1	ENSG00000198742	16557594;
D005472	increase	SNAP29	ENSG00000099940	16803524;
D005472	increase	SNTB1	ENSG00000172164	16510598;
D005472	increase	SOCS1	ENSG00000185338	16557594;
D005472	increase	SOX4	ENSG00000124766	16510598;
D005472	increase	SPRR1A	ENSG00000169474	16584549;
D005472	increase	SPRY1	ENSG00000164056	17039268;
D005472	increase	SRP72	ENSG00000174780	16584549;
D005472	increase	STAT3	ENSG00000168610	18205257;18161298;16557594;15730734;15682485;
D005472	increase	STX5	ENSG00000162236	16391804;
D005472	increase	SYTL2	ENSG00000137501	16510598;
D005472	increase	TANK	ENSG00000136560	16803524;
D005472	increase	TAP1	ENSG00000230705	17982676;
D005472	increase	TAX1BP3	ENSG00000213977	16709241;
D005472	increase	TCF19	ENSG00000137310	15331170;
D005472	increase	TERF2	ENSG00000132604	16803524;15585135;
D005472	increase	TGFB1	ENSG00000105329	15988270;16557594;16217747;
D005472	increase	TGFBR3	ENSG00000069702	16557594;
D005472	increase	THBS1	ENSG00000137801	18757417;15205334;16217747;
D005472	increase	TK2	ENSG00000166548	19219653;
D005472	increase	TLR3	ENSG00000164342	18779317;
D005472	increase	TMEM74	ENSG00000164841	16510598;
D005472	increase	TMSB10	ENSG00000034510	15152939;
D005472	increase	TMSB4X	ENSG00000205542	15152939;16364925;
D005472	increase	TNFAIP3	ENSG00000118503	16557594;
D005472	increase	TNFRSF10A	ENSG00000104689	15585621;
D005472	increase	TNFRSF10B	ENSG00000120889	17982676;15585621;17922852;
D005472	increase	TNFRSF10C	ENSG00000173535	15585621;
D005472	increase	TNFRSF10D	ENSG00000173530	15585621;15016801;
D005472	increase	TNFRSF11B	ENSG00000164761	16557594;
D005472	increase	TNFRSF18	ENSG00000186891	16557594;
D005472	increase	TNFSF10	ENSG00000121858	16373718;19106633;15120327;
D005472	increase	TNFSF13	ENSG00000161955	18423122;
D005472	increase	TP53AIP1	ENSG00000120471	17971768;

D005472	increase	TP53	ENSG00000141510	15016801;17634554;17971768;16963839;16168113;15489892;18930000;15625077;16709241;18779317;14665630;12082016;19098008;16584549;15865071;17699798;15546879;17056233;19015155;17982676;18498133;15041737;17339891;15608686;18600534;15999354;18698031;15152939;15161716;15940066;16557594;17637740;19106633;15534911;16686938;16077963;152547
D005472	increase	TP53I3	ENSG00000115129	17982676;15205334;16709241;16557594;14665630;15016801;16217747;
D005472	increase	TP53INP1	ENSG00000164938	16510598;16557594;17039268;17982676;15016801;
D005472	increase	TRIAP1	ENSG00000170855	17982676;
D005472	increase	TRIB2	ENSG00000071575	16510598;
D005472	increase	TSC2	ENSG00000103197	16584549;
D005472	increase	TSHZ1	ENSG00000179981	16584549;
D005472	increase	TXNIP	ENSG00000117289	18930000;15016801;15548681;
D005472	increase	TYMP	ENSG00000025708	16568373;17454858;15538739;15041737;15655543;17520254;17638652;1530171
D005472	increase	TYMS	ENSG00000176890	18794807;16168113;16557585;15374632;17339891;18197934;17330233;16568373;15109396;19219653;19084572;16584549;15865071;17172411;16267625;15213713;15067352;19082493;18722050;18661526;17016601;18448328;15446554;16622263;15735113;18505590;16424979;18986760;18507058;17454858;18593893;15246190;18383874;18490900;15239142;16596248;19074750;10482907;19020767;15655543;15788669;18607850;18633250;17203168;15538739;15548681;15608557;18676755;15152939;15161716;16282729;19105824;17377791;14970324;17018589;17047489;17520254;15890242;15993511;15918040;15770397;17638652;15737843;
D005472	increase	UMPS	ENSG00000114491	16557585;19084572;17854773;19082440;16757204;16477629;19020767;17303323;19020740;15548681;1723
D005472	increase	UPP1	ENSG00000183696	15109396;15331170;15067352;
D005472	increase	UTP3	ENSG00000132467	16391804;
D005472	increase	UXT	ENSG00000126756	16803524;15585135;
D005472	increase	VAT1	ENSG00000108828	16101138;

D005472	increase	VEGFA	ENSG00000112715	17982676;17786339;17039268;18494554;
D005472	increase	XBP1	ENSG00000100219	15205334;
D005472	increase	XIAP	ENSG00000101966	16628085;
D005472	increase	ZDHHC9	ENSG00000188706	17039268;
D005472	increase	ZMAT3	ENSG00000172667	17982676;
D005472	increase	ZNF238	ENSG00000179456	16510598;
D005472	increase	ZNF408	ENSG00000175213	16391804;
D005472	increase	ZNF473	ENSG00000142528	15331170;
D005473	decrease	AP1S1	ENSG00000106367	15342952;
D005473	decrease	HTR1A	ENSG00000178394	15885359;
D005473	decrease	IFNA1	ENSG00000197919	15885359;
D005473	decrease	NR3C1	ENSG00000113580	15885359;
D005473	decrease	SLC2A3	ENSG00000059804	17567588;15342952;
D005473	decrease	TAGLN	ENSG00000149591	17567588;15342952;
D005473	increase	ASAH1	ENSG00000104763	17567588;15342952;
D005473	increase	ASNS	ENSG00000070669	17567588;15342952;
D005473	increase	C10ORF10	ENSG00000165507	17567588;15342952;
D005473	increase	FABP1	ENSG00000163586	17567588;15342952;
D005473	increase	FNDC4	ENSG00000115226	17567588;15342952;
D005473	increase	GDPD3	ENSG00000102886	17567588;
D005473	increase	HPN	ENSG00000105707	15342952;
D005473	increase	INHBE	ENSG00000139269	17567588;15342952;
D005473	increase	LSS	ENSG00000160285	17567588;15342952;
D005473	increase	NR0B2	ENSG00000131910	15342952;
D005473	increase	NUPR1	ENSG00000176046	17567588;15342952;
D005473	increase	SERPINA3	ENSG00000196136	17567588;15342952;
D005473	increase	WIPI1	ENSG00000070540	17567588;15342952;
D005475	decrease	PTGS1	ENSG00000095303	8897462;
D005475	decrease	PTGS2	ENSG00000073756	8897462;
D005480	increase	ELAVL1	ENSG00000066044	18056468;
D005480	increase	MAPK11	ENSG00000185386	18056468;
D005480	increase	MAPK14	ENSG00000112062	18056468;
D005480	increase	MAPKAPK2	ENSG00000162889	18056468;
D005480	increase	MAPKAPK3	ENSG00000114738	18056468;
D005480	increase	NGFR	ENSG00000064300	18056468;17447067;17409433;
D005485	decrease	AR	ENSG00000169083	16601080;15483189;16111029;17303007;16857237;9705896;10696776;12730620;15589981;11981028;16893599;16169144;9846162;119
D005485	decrease	FSHB	ENSG00000131808	11228061;
D005485	decrease	LHB	ENSG00000104826	11228061;
D005485	decrease	NKX3-1	ENSG00000167034	17303007;
D005485	decrease	TNFRSF11B	ENSG00000164761	17189957;
D005485	increase	GREB1	ENSG00000196208	16496412;
D005485	increase	IGF1	ENSG00000017427	16368782;
D005485	increase	IGFBP2	ENSG00000115457	16368782;
D005492	affect	ABCC1	ENSG00000103222	15657365;
D005492	affect	ABCG2	ENSG00000118777	17032904;15657365;
D005492	affect	ACTA2	ENSG00000107796	17320366;
D005492	affect	ACTC1	ENSG00000159251	17320366;
D005492	affect	AFAP1	ENSG00000196526	17320366;
D005492	affect	AK3L1	ENSG00000162433	17320366;

D005492	affect	ALDH1A1	ENSG00000165092	17868486;
D005492	affect	ANXA13	ENSG00000104537	17868486;
D005492	affect	ARHGAP29	ENSG00000137962	17320366;
D005492	affect	ARL4C	ENSG00000188042	17320366;
D005492	affect	ATF6B	ENSG00000228628	17320366;
D005492	affect	ATM	ENSG00000149311	16361273;
D005492	affect	B2M	ENSG00000166710	17868486;
D005492	affect	BAX	ENSG00000087088	16361273;
D005492	affect	BCL2	ENSG00000171791	16611376;
D005492	affect	BCL2L11	ENSG00000153094	16361273;
D005492	affect	BFAR	ENSG00000103429	16361273;
D005492	affect	BIRC3	ENSG00000023445	16361273;
D005492	affect	BNIP3	ENSG00000176171	17320366;
D005492	affect	BNIP3L	ENSG00000104765	17320366;
D005492	affect	C5ORF13	ENSG00000134986	17320366;
D005492	affect	CASP4	ENSG00000196954	16361273;
D005492	affect	CASP6	ENSG00000138794	16361273;
D005492	affect	CASP8	ENSG00000064012	16361273;
D005492	affect	CCND1	ENSG00000110092	16361273;
D005492	affect	CCNE1	ENSG00000105173	16361273;
D005492	affect	CCRL1	ENSG00000129048	17320366;
D005492	affect	CDKN1A	ENSG00000124762	16361273;
D005492	affect	CDKN1B	ENSG00000111276	16361273;
D005492	affect	CHEK1	ENSG00000149554	16361273;
D005492	affect	CIDEB	ENSG00000136305	16361273;
D005492	affect	CNTF	ENSG00000242689	16898872;
D005492	affect	CTNNB1	ENSG00000168036	16361273;
D005492	affect	CTSC	ENSG00000109861	17320366;
D005492	affect	CXCR7	ENSG00000144476	17320366;
D005492	affect	DEK	ENSG00000124795	17320366;
D005492	affect	DHFR	ENSG00000228716	17311948;
D005492	affect	DNMT3A	ENSG00000119772	17311948;
D005492	affect	DNMT3B	ENSG00000088305	17311948;
D005492	affect	DPT	ENSG00000143196	17320366;
D005492	affect	EDN2	ENSG00000127129	16898872;
D005492	affect	EPHB6	ENSG00000106123	17320366;
D005492	affect	ETS2	ENSG00000157557	16361273;
D005492	affect	FAM126A	ENSG00000122591	17320366;
D005492	affect	FAS	ENSG00000026103	16611376;
D005492	affect	FHL1	ENSG00000022267	17320366;
D005492	affect	FPGS	ENSG00000136877	17311948;
D005492	affect	FTL	ENSG00000087086	17868486;
D005492	affect	GAS6	ENSG00000248501	17320366;
D005492	affect	GDF15	ENSG00000130513	16898872;
D005492	affect	GDPD3	ENSG00000102886	17320366;
D005492	affect	GGH	ENSG00000137563	17311948;
D005492	affect	GJA1	ENSG00000152661	17320366;
D005492	affect	GUK1	ENSG00000143774	16898872;
D005492	affect	HRK	ENSG00000135116	16361273;
D005492	affect	HSPB7	ENSG00000173641	17320366;
D005492	affect	ID1	ENSG00000125968	17320366;
D005492	affect	IFITM1	ENSG00000185885	16898872;
D005492	affect	IGFBP5	ENSG00000115461	17320366;
D005492	affect	IL2	ENSG00000109471	16611376;

D005492	affect	ITGA3	ENSG00000005884	16361273;
D005492	affect	ITGA4	ENSG00000115232	17320366;
D005492	affect	ITGA5	ENSG00000161638	16361273;
D005492	affect	ITGA6	ENSG00000091409	16361273;
D005492	affect	ITGB1	ENSG00000150093	16361273;
D005492	affect	JUN	ENSG00000177606	16898872;
D005492	affect	KNTC1	ENSG00000184445	17320366;
D005492	affect	MALL	ENSG00000144063	17320366;
D005492	affect	MAP1B	ENSG00000131711	17320366;
D005492	affect	MBD2	ENSG00000134046	17311948;
D005492	affect	MCL1	ENSG00000143384	16361273;
D005492	affect	MDM2	ENSG00000135679	16361273;
D005492	affect	MGLL	ENSG00000074416	17320366;
D005492	affect	MICA	ENSG00000183214	16361273;
D005492	affect	MIF	ENSG00000240972	17320366;
D005492	affect	MME	ENSG00000196549	17320366;
D005492	affect	MMP3	ENSG00000149968	17320366;
D005492	affect	MT3	ENSG00000087250	16898872;
D005492	affect	MTHFR	ENSG00000177000	17311948;16375580;17115185;16690736;16621645;16646054;17301267;
D005492	affect	MTR	ENSG00000116984	17311948;
D005492	affect	MTRR	ENSG00000124275	17311948;
D005492	affect	MUC1	ENSG00000185499	17320366;
D005492	affect	MYO10	ENSG00000145555	17320366;
D005492	affect	NCAM1	ENSG00000149294	16361273;
D005492	affect	NDST1	ENSG00000070614	17320366;
D005492	affect	NFKB1	ENSG00000109320	16361273;
D005492	affect	NFKBIA	ENSG00000100906	16361273;16845898;
D005492	affect	NME4	ENSG00000103202	16361273;
D005492	affect	NOTCH4	ENSG00000204301	16898872;
D005492	affect	NTHL1	ENSG00000065057	16898872;
D005492	affect	NTN4	ENSG00000074527	17320366;
D005492	affect	PCOLCE2	ENSG00000163710	17320366;
D005492	affect	PDGFA	ENSG00000197461	16361273;
D005492	affect	PFDN5	ENSG00000123349	16898872;
D005492	affect	PGK1	ENSG00000102144	17320366;
D005492	affect	PGRMC2	ENSG00000164040	17320366;
D005492	affect	PIK3R1	ENSG00000145675	16361273;
D005492	affect	PLAU	ENSG00000122861	16361273;
D005492	affect	PLAUR	ENSG00000011422	16361273;
D005492	affect	PODXL	ENSG00000128567	17320366;
D005492	affect	PRKDC	ENSG00000121031	16361273;
D005492	affect	PRR16	ENSG00000184838	17320366;
D005492	affect	PTK6	ENSG00000101213	17320366;
D005492	affect	RAF1	ENSG00000132155	16361273;
D005492	affect	RASA1	ENSG00000145715	16361273;
D005492	affect	RECK	ENSG00000122707	17320366;
D005492	affect	RHOC	ENSG00000155366	16898872;
D005492	affect	RPA3	ENSG00000106399	16361273;
D005492	affect	RPS5	ENSG00000083845	16898872;
D005492	affect	S100A10	ENSG00000197747	17320366;
D005492	affect	S100A4	ENSG00000196154	16361273;
D005492	affect	SERPINB2	ENSG00000197632	16361273;

D005492	affect	SHMT1	ENSG00000176974	17311948;
D005492	affect	SLC40A1	ENSG00000138449	17868486;
D005492	affect	SLC7A7	ENSG00000155465	17868486;
D005492	affect	TAGLN	ENSG00000149591	17320366;
D005492	affect	TM4SF1	ENSG00000169908	17320366;
D005492	affect	TNFRSF10B	ENSG00000120889	16361273;
D005492	affect	TNFRSF14	ENSG00000157873	16361273;
D005492	affect	TP53	ENSG00000141510	16611376;16361273;
D005492	affect	TRAF1	ENSG00000056558	16361273;
D005492	affect	TRAF4	ENSG00000076604	16361273;
D005492	affect	TRAF6	ENSG00000175104	16361273;
D005492	affect	TRAIP	ENSG00000183763	16361273;
D005492	affect	TUBA1A	ENSG00000167552	16898872;
D005492	affect	TYMS	ENSG00000176890	17320366;17311948;
D005492	affect	USP53	ENSG00000145390	17320366;
D005492	affect	VEGFA	ENSG00000112715	16361273;
D005492	affect	VGLL3	ENSG00000206538	17320366;
D005492	affect	WISP1	ENSG00000104415	17320366;
D005492	affect	WNT5A	ENSG00000114251	17320366;
D005492	affect	ZNF219	ENSG00000165804	17320366;
D005492	decrease	AKAP12	ENSG00000131016	11723236;
D005492	decrease	CCND3	ENSG00000112576	11723236;
D005492	decrease	CDH13	ENSG00000140945	11723236;
D005492	decrease	CDH1	ENSG00000039068	17868486;
D005492	decrease	CRP	ENSG00000132693	16491109;17237316;16517955;16634837;
D005492	decrease	FOLR1	ENSG00000110195	17311948;17616776;
D005492	decrease	FOLR2	ENSG00000165457	16740727;17616776;
D005492	decrease	GPT	ENSG00000167701	16877991;
D005492	decrease	HDAC1	ENSG00000116478	18310117;
D005492	decrease	IFITM3	ENSG00000142089	11723236;
D005492	decrease	IGF1R	ENSG00000140443	16728583;
D005492	decrease	IL8	ENSG00000169429	16491109;16634837;
D005492	decrease	KRT14	ENSG00000186847	11723236;
D005492	decrease	MECP2	ENSG00000169057	18310117;
D005492	decrease	SLC19A1	ENSG00000173638	17311948;17616776;16750224;16900951;17034785;
D005492	decrease	SLC46A1	ENSG00000076351	17475902;17616776;17129779;17446347;
D005492	decrease	SOD3	ENSG00000109610	18310117;
D005492	increase	AGR2	ENSG00000106541	17868486;
D005492	increase	ANKRD1	ENSG00000148677	17868486;
D005492	increase	APOB	ENSG00000084674	17868486;17237316;
D005492	increase	BIRC2	ENSG00000110330	15211800;
D005492	increase	CCL2	ENSG00000108691	16845898;16491109;16634837;
D005492	increase	CDH3	ENSG00000062038	17868486;
D005492	increase	CLGN	ENSG00000153132	11723236;
D005492	increase	DKK1	ENSG00000107984	17320366;17868486;
D005492	increase	DNMT1	ENSG00000130816	17311948;18310117;
D005492	increase	FGA	ENSG00000171560	17868486;
D005492	increase	INSIG1	ENSG00000186480	11723236;
D005492	increase	SAT1	ENSG00000130066	17868486;
D005492	increase	SOX4	ENSG00000124766	17868486;

D005492	increase	SPINK1	ENSG00000164266	17868486;
D005492	increase	TXNIP	ENSG00000117289	11723236;
D005557	increase	CSF2	ENSG00000164400	18082304;
D005557	increase	IFNG	ENSG00000111537	18082304;
D005557	increase	IL10	ENSG00000136634	18082304;
D005557	increase	IL1A	ENSG00000115008	18082304;
D005557	increase	IL1B	ENSG00000125538	18082304;
D005557	increase	IL2	ENSG00000109471	18082304;
D005557	increase	IL6	ENSG00000136244	18082304;16860297;
D005557	increase	IL8	ENSG00000169429	16860297;
D005557	increase	TP53	ENSG00000141510	15715472;
D005576	decrease	ADAM12	ENSG00000148848	15313392;15967414;
D005576	decrease	ADAMTS1	ENSG00000154734	15313392;15967414;
D005576	decrease	APPBP2	ENSG00000062725	16751804;
D005576	decrease	ATXN3	ENSG00000066427	16751804;
D005576	decrease	EPS8	ENSG00000151491	15313392;15967414;
D005576	decrease	GAS6	ENSG00000248501	15313392;
D005576	decrease	LTBP2	ENSG00000119681	15313392;
D005576	decrease	MAF	ENSG00000178573	16751804;
D005576	decrease	MTERFD2	ENSG00000122085	16751804;
D005576	decrease	NAV1	ENSG00000134369	16751804;
D005576	decrease	NUMA1	ENSG00000137497	16751804;
D005576	decrease	PIAS1	ENSG00000033800	16751804;
D005576	decrease	SESN1	ENSG00000080546	16751804;
D005576	decrease	SLC30A7	ENSG00000162695	16751804;
D005576	decrease	SULT2A1	ENSG00000105398	17080404;
D005576	decrease	ZBTB10	ENSG00000205189	16751804;
D005576	decrease	ZFP36L2	ENSG00000152518	15967414;
D005576	increase	ABCB1	ENSG00000085563	12926078;
D005576	increase	ABCB4	ENSG00000005471	12926078;
D005576	increase	ACSL3	ENSG00000123983	16751804;
D005576	increase	AREG	ENSG00000205595	15474502;15313392;15967414;15284208;
D005576	increase	AR	ENSG00000169083	16751804;17219378;
D005576	increase	BDNF	ENSG00000176697	19189864;
D005576	increase	CAMKK2	ENSG00000110931	16751804;
D005576	increase	CDC6	ENSG00000094804	16751804;
D005576	increase	CRLS1	ENSG00000088766	16751804;
D005576	increase	CYP11A1	ENSG00000140459	14691014;17080404;
D005576	increase	CYP11B1	ENSG00000160882	12525251;17080404;
D005576	increase	CYP11B2	ENSG00000179142	17080404;
D005576	increase	CYP17A1	ENSG00000148795	15886251;17080404;15625562;
D005576	increase	CYP19A1	ENSG00000137869	14691014;16996190;16357189;17080404;
D005576	increase	DCT	ENSG00000080166	17460731;
D005576	increase	DYNLL2	ENSG00000121083	16751804;
D005576	increase	EIF2B5	ENSG00000145191	16751804;
D005576	increase	EREG	ENSG00000124882	15474502;15967414;15313392;
D005576	increase	EVI5	ENSG00000067208	16751804;
D005576	increase	FADS1	ENSG00000149485	16751804;
D005576	increase	FGF2	ENSG00000138685	10320526;
D005576	increase	FGFR1	ENSG00000249195	10320526;

D005576	increase	FGFR2	ENSG00000066468	10320526;
D005576	increase	FKBP5	ENSG00000096060	19255438;
D005576	increase	GNAI3	ENSG00000065135	16751804;
D005576	increase	HMGCR	ENSG00000113161	16751804;
D005576	increase	HSD3B2	ENSG00000203859	17080404;
D005576	increase	IFNG	ENSG00000111537	18953428;
D005576	increase	INSIG1	ENSG00000186480	16751804;
D005576	increase	ITGB5	ENSG00000082781	16751804;
D005576	increase	KLK2	ENSG00000167751	16751804;
D005576	increase	KLK3	ENSG00000142515	16751804;
D005576	increase	MAP7	ENSG00000135525	16751804;
D005576	increase	MED28	ENSG00000118579	16751804;
D005576	increase	MITF	ENSG00000187098	17460731;
D005576	increase	MMP16	ENSG00000156103	16751804;
D005576	increase	NGLY1	ENSG00000151092	16751804;
D005576	increase	NR5A2	ENSG00000116833	16357189;
D005576	increase	PAX8	ENSG00000125618	15961562;
D005576	increase	PDE4B	ENSG00000184588	15967414;
D005576	increase	PDE4D	ENSG00000113448	15967414;
D005576	increase	PLA2G2A	ENSG00000188257	18953428;
D005576	increase	PPARGC1A	ENSG00000109819	16357189;
D005576	increase	PPARGC1B	ENSG00000155846	16357189;
D005576	increase	PTGS2	ENSG00000073756	12493747;
D005576	increase	REN	ENSG00000143839	9256163;
D005576	increase	RRP12	ENSG00000052749	16751804;
D005576	increase	SELE	ENSG00000007908	8780174;
D005576	increase	SFTPC	ENSG00000168484	2539393;
D005576	increase	SLC2A5	ENSG00000142583	12820898;
D005576	increase	SLC45A2	ENSG00000164175	17460731;
D005576	increase	SLC5A5	ENSG00000105641	16439463;16954431;
D005576	increase	SPCS3	ENSG00000129128	16751804;
D005576	increase	STAR	ENSG00000147465	14691014;17080404;15967414;
D005576	increase	TGFB1	ENSG00000105329	15625562;
D005576	increase	TGS1	ENSG00000137574	16751804;
D005576	increase	TMEM50A	ENSG00000183726	16751804;
D005576	increase	TNF	ENSG00000223952	11675405;8780174;
D005576	increase	TOB1	ENSG00000141232	15313392;
D005576	increase	TYRP1	ENSG00000107165	17460731;
D005576	increase	UBE2J1	ENSG00000241519	16751804;
D005576	increase	WT1	ENSG00000184937	15961562;
D005632	decrease	ACACB	ENSG00000076555	18063403;
D005632	decrease	HNF4A	ENSG00000101076	17992261;
D005632	decrease	MYC	ENSG00000136997	2558650;
D005632	decrease	NOS3	ENSG00000164867	18508964;
D005632	decrease	PPARGC1A	ENSG00000109819	18063403;
D005632	decrease	SHBG	ENSG00000129214	17992261;
D005632	decrease	SLC2A4	ENSG00000181856	18063403;
D005632	increase	APOA4	ENSG00000110244	11983814;
D005632	increase	ICAM1	ENSG00000090339	18508964;
D005632	increase	SCD	ENSG00000099194	18063403;
D005632	increase	SLC2A5	ENSG00000142583	1634504;12820898;8554516;17710649;16960373;
D005665	increase	REN	ENSG00000143839	11095476;11046100;

D005665	increase	UGT1A1	ENSG00000241635	18227147;
D005688	decrease	NDUFB6	ENSG00000165264	19523936;
D005707	increase	GABPA	ENSG00000154727	16308312;
D005707	increase	LDLR	ENSG00000130164	12612140;
D005707	increase	MMP7	ENSG00000137673	17928719;
D005707	increase	SULT1A1	ENSG00000196502	16308312;
D005839	affect	MUT	ENSG00000146085	19427250;
D005905	increase	CYP1A1	ENSG00000140465	10799337;
D005912	increase	BIRC3	ENSG00000023445	14527959;
D005912	increase	IL8	ENSG00000169429	11527995;
D005938	decrease	BCL2	ENSG00000171791	15748212;
D005938	decrease	IL5	ENSG00000113525	15113433;15835818;
D005938	decrease	IL7R	ENSG00000168685	15748212;
D005938	increase	CDKN1A	ENSG00000124762	15034294;
D005938	increase	CDKN1B	ENSG00000111276	15034294;
D005938	increase	CDKN2A	ENSG00000147889	15034294;
D005938	increase	CDKN2B	ENSG00000147883	15034294;
D005938	increase	IL10	ENSG00000136634	16384973;
D005938	increase	MT2A	ENSG00000125148	11878821;
D005944	decrease	BCL2L1	ENSG00000171552	15972124;
D005944	increase	ACAN	ENSG00000157766	18321735;
D005944	increase	COL1A1	ENSG00000108821	18321735;
D005944	increase	COL2A1	ENSG00000139219	18321735;
D005944	increase	IL1B	ENSG00000125538	18321735;
D005947	affect	ASNS	ENSG00000070669	17409444;
D005947	affect	MYC	ENSG00000136997	2558650;
D005947	decrease	BCL2	ENSG00000171791	19557821;
D005947	decrease	FOLR1	ENSG00000110195	19616087;
D005947	decrease	HNF4A	ENSG00000101076	17992261;
D005947	decrease	HSPD1	ENSG00000144381	17855661;
D005947	decrease	NFKBIA	ENSG00000100906	19557821;
D005947	decrease	NOS1	ENSG00000089250	17855661;
D005947	decrease	NOS3	ENSG00000164867	17855661;
D005947	decrease	PPARA	ENSG00000186951	16600694;
D005947	decrease	PPARGC1A	ENSG00000109819	17855661;
D005947	decrease	SHBG	ENSG00000129214	17992261;
D005947	increase	AGER	ENSG00000204305	19557821;
D005947	increase	ARNT	ENSG00000143437	15767253;
D005947	increase	CCL2	ENSG00000108691	16600694;19158351;17655880;16143069;
D005947	increase	CD14	ENSG00000170458	18180316;
D005947	increase	ESR1	ENSG00000091831	16621514;
D005947	increase	ESR2	ENSG00000140009	16621514;
D005947	increase	FN1	ENSG00000115414	15086456;12388107;16320596;
D005947	increase	FOS	ENSG00000170345	18180316;
D005947	increase	G6PC	ENSG00000131482	15767253;
D005947	increase	GAPDH	ENSG00000111640	7826318;
D005947	increase	IGF1R	ENSG00000140443	7826318;
D005947	increase	IL1B	ENSG00000125538	19557821;
D005947	increase	INS	ENSG00000129965	16505238;17192482;19188863;18347054;16984975;11574405;
D005947	increase	INSR	ENSG00000171105	7826318;

D005947	increase	JUN	ENSG00000177606	16600694;18180316;
D005947	increase	NFKB1	ENSG00000109320	18180316;
D005947	increase	PTGS2	ENSG00000073756	19557821;
D005947	increase	RELA	ENSG00000173039	16600694;19557821;12388107;
D005947	increase	SLC2A5	ENSG00000142583	12820898;8554516;177106
D005947	increase	TGFB1	ENSG00000105329	17597607;
D005947	increase	TNF	ENSG00000223952	19557821;
D005978	affect	HMOX1	ENSG00000100292	8743975;
D005978	decrease	PROCR	ENSG00000101000	17172434;
D005978	increase	BCL2	ENSG00000171791	17640567;
D005978	increase	BCL2L1	ENSG00000171552	17640567;
D005978	increase	BIRC2	ENSG00000110330	17640567;
D005978	increase	CCND1	ENSG00000110092	17640567;
D005978	increase	DDIT3	ENSG00000175197	17171638;
D005978	increase	GCLC	ENSG00000001084	12504668;11673643;
D005978	increase	HIF1A	ENSG00000100644	16678800;17658243;
D005978	increase	IL6	ENSG00000136244	15379216;
D005978	increase	IL8	ENSG00000169429	15379216;12208513;
D005978	increase	ITGAM	ENSG00000169896	16357311;
D005978	increase	MMP9	ENSG00000100985	17015178;17640567;
D005978	increase	NOS2	ENSG00000007171	17640567;
D005978	increase	PTGS2	ENSG00000073756	17640567;
D005978	increase	TNF	ENSG00000223952	16219905;17640567;10802230;
D005990	decrease	MYC	ENSG00000136997	2558650;
D005990	increase	APOA1	ENSG00000118137	19013290;
D005996	affect	CALCA	ENSG00000110680	14659530;
D005996	decrease	EDN1	ENSG00000078401	12206003;
D005996	decrease	NOS2	ENSG00000007171	12206003;
D005996	increase	FAS	ENSG00000026103	12105852;
D005996	increase	GUCY1B3	ENSG00000061918	11742226;
D005996	increase	HMOX1	ENSG00000100292	17470570;
D005996	increase	IL6	ENSG00000136244	17470570;
D005996	increase	IL8	ENSG00000169429	17470570;
D005996	increase	MMP2	ENSG00000087245	12084592;
D005996	increase	MMP7	ENSG00000137673	12084592;
D005996	increase	MMP9	ENSG00000100985	12084592;
D006063	increase	AFP	ENSG00000081051	18497072;
D006072	decrease	CCL2	ENSG00000108691	19825521;
D006072	decrease	CXCL1	ENSG00000163739	19825521;
D006072	decrease	EPHB4	ENSG00000196411	19825521;
D006072	decrease	FGF2	ENSG00000138685	19825521;
D006072	decrease	FGFR3	ENSG00000068078	19825521;
D006072	decrease	ID1	ENSG00000125968	19825521;
D006072	decrease	LAMA5	ENSG00000130702	19825521;
D006072	decrease	MDK	ENSG00000110492	19825521;
D006072	decrease	PDGFA	ENSG00000197461	19825521;
D006072	increase	TYMP	ENSG00000025708	19825521;
D006096	decrease	KCNH2	ENSG00000055118	19139152;
D006096	decrease	MAPT	ENSG00000186868	16930453;
D006160	increase	GYPA	ENSG00000170180	17002868;
D006220	decrease	ABCB1	ENSG00000085563	15946544;
D006220	decrease	ABCC1	ENSG00000103222	15946544;

D006220	decrease	GSTP1	ENSG00000084207	15946544;
D006221	increase	CYB5A	ENSG00000166347	3689822;
D006241	increase	IL1B	ENSG00000125538	9714973;
D006241	increase	TNF	ENSG00000223952	9714973;
D006388	decrease	IL10	ENSG00000136634	17185614;
D006388	increase	IL2	ENSG00000109471	17185614;
D006388	increase	IL4	ENSG00000113520	17185614;
D006418	increase	ABCB6	ENSG00000115657	17006453;
D006418	increase	HMOX1	ENSG00000100292	17156779;
D006418	increase	SLC46A1	ENSG00000076351	17156779;
D006427	decrease	TFRC	ENSG00000072274	16760464;
D006427	increase	ARL6IP5	ENSG00000144746	16430862;
D006427	increase	BIRC2	ENSG00000110330	14647439;
D006427	increase	CDKN1A	ENSG00000124762	14647439;
D006427	increase	GSTP1	ENSG00000084207	15313425;
D006427	increase	HMOX1	ENSG00000100292	15589375;17464175;18357586;8764571;17420286;16959797;14647439;
D006533	decrease	GJA1	ENSG00000152661	8738476;
D006533	decrease	HRAS	ENSG00000174775	1771636;
D006533	decrease	MAP2K1	ENSG00000169032	9704902;15888667;
D006533	decrease	MAP3K1	ENSG00000095015	9704902;
D006533	decrease	MAPK1	ENSG00000100030	9704902;15888667;
D006533	decrease	MAPK3	ENSG00000102882	9704902;15888667;
D006533	decrease	RB1	ENSG00000139687	10048758;
D006533	decrease	TP53	ENSG00000141510	9544696;
D006534	decrease	GJA1	ENSG00000152661	8738476;
D006534	increase	PGR	ENSG00000082175	16242299;
D006534	increase	TFF1	ENSG00000160182	16242299;
D006569	increase	MMP9	ENSG00000100985	19110045;
D006569	increase	PTGS2	ENSG00000073756	19110045;
D006581	decrease	IFNG	ENSG00000111537	15319175;11266329;
D006581	decrease	IL10	ENSG00000136634	15319175;
D006581	decrease	IL5	ENSG00000113525	15319175;
D006582	decrease	CCND1	ENSG00000110092	16735606;
D006582	decrease	MYC	ENSG00000136997	16735606;
D006582	increase	CTNNB1	ENSG00000168036	16735606;
D006582	increase	SIAH1	ENSG00000196470	16735606;
D006582	increase	WNT3A	ENSG00000154342	16735606;
D006586	increase	CCL5	ENSG00000161570	11306435;
D006586	increase	CSF2	ENSG00000164400	11306435;
D006586	increase	IL8	ENSG00000169429	11306435;
D006588	decrease	CCND1	ENSG00000110092	14757120;
D006588	decrease	CCND3	ENSG00000112576	14757120;
D006588	decrease	CCNE1	ENSG00000105173	14757120;
D006588	decrease	RB1	ENSG00000139687	14757120;
D006588	increase	CCNG2	ENSG00000138764	14757120;
D006632	affect	CD14	ENSG00000170458	14499251;
D006632	affect	HRH3	ENSG00000101180	14499251;12706455;
D006632	affect	IL10	ENSG00000136634	9819373;
D006632	decrease	CD40	ENSG00000101017	11306145;
D006632	decrease	HRH4	ENSG00000134489	14499251;11181941;15843518;
D006632	decrease	PTPRC	ENSG00000081237	11306145;

D006632	increase	CSF2	ENSG00000164400	9856766;
D006632	increase	FOS	ENSG00000170345	9187264;
D006632	increase	GUSB	ENSG00000169919	11238657;
D006632	increase	HRH1	ENSG00000196639	2419744;12147327;14499251;11238657;11527950;11306145;
D006632	increase	HRH2	ENSG00000113749	9681472;12235264;15843518;9187264;14499251;11238657;10569698;9819373;11
D006632	increase	IFNG	ENSG00000111537	10569698;
D006632	increase	IL12A	ENSG00000168811	15843518;9819373;
D006632	increase	IL12B	ENSG00000113302	15843518;9819373;
D006632	increase	IL16	ENSG00000172349	12235264;
D006632	increase	IL6	ENSG00000136244	11238657;9856766;
D006632	increase	MMP1	ENSG00000196611	9856766;
D006632	increase	PDE4B	ENSG00000184588	11131300;
D006632	increase	TNF	ENSG00000223952	14499251;
D006710	affect	EDN1	ENSG00000078401	16757814;
D006710	decrease	BCL2	ENSG00000171791	16757814;
D006710	decrease	CCNA1	ENSG00000133101	17698632;
D006710	decrease	EPHB6	ENSG00000106123	17320366;
D006710	decrease	HDAC1	ENSG00000116478	18310117;
D006710	decrease	MECP2	ENSG00000169057	18310117;17698632;
D006710	decrease	NOS3	ENSG00000164867	16757814;
D006710	decrease	SOD3	ENSG00000109610	18310117;
D006710	decrease	WISP1	ENSG00000104415	17320366;
D006710	decrease	WNT5A	ENSG00000114251	17320366;
D006710	increase	BAX	ENSG00000087088	16757814;
D006710	increase	CCL2	ENSG00000108691	16845898;11356643;
D006710	increase	CCL5	ENSG00000161570	16225753;
D006710	increase	DNMT1	ENSG00000130816	17698632;18310117;
D006710	increase	MMP9	ENSG00000100985	16251475;
D006710	increase	NFKBIA	ENSG00000100906	16845898;17586618;11356643;
D006710	increase	VCAM1	ENSG00000162692	17586618;
D006812	increase	ITGA2	ENSG00000164171	19686714;
D006820	decrease	NR1D1	ENSG00000126368	17075855;
D006820	increase	HAS2	ENSG00000170961	11318944;
D006820	increase	IL1B	ENSG00000125538	11318944;
D006820	increase	IRAK3	ENSG00000090376	15728517;
D006830	decrease	ABCB5	ENSG00000004846	17183730;
D006830	decrease	ACOT2	ENSG00000119673	17183730;
D006830	decrease	ADRBK2	ENSG00000100077	17183730;
D006830	decrease	AGPAT2	ENSG00000169692	17183730;
D006830	decrease	ALOX15	ENSG00000161905	17183730;
D006830	decrease	AOAH	ENSG00000136250	17183730;
D006830	decrease	B9D1	ENSG00000108641	17183730;
D006830	decrease	CA8	ENSG00000178538	17183730;
D006830	decrease	CCDC146	ENSG00000135205	17183730;
D006830	decrease	CTCF	ENSG00000102974	17183730;
D006830	decrease	DLAT	ENSG00000150768	17183730;
D006830	decrease	ELL3	ENSG00000128886	17183730;
D006830	decrease	FOXP2	ENSG00000128573	17183730;
D006830	decrease	GPAA1	ENSG00000197858	17183730;

D006830	decrease	HBA1	ENSG00000206172	17183730;
D006830	decrease	HBB	ENSG00000244734	17183730;
D006830	decrease	HBZ	ENSG00000130656	17183730;
D006830	decrease	HEATR2	ENSG00000164818	17183730;
D006830	decrease	HEATR5B	ENSG00000008869	17183730;
D006830	decrease	IL3	ENSG00000164399	17183730;
D006830	decrease	ITGAM	ENSG00000169896	17183730;
D006830	decrease	KCNA7	ENSG00000104848	17183730;
D006830	decrease	KRT17	ENSG00000128422	17183730;
D006830	decrease	NAMPT	ENSG00000105835	17183730;
D006830	decrease	NBR1	ENSG00000188554	17183730;
D006830	decrease	NCR1	ENSG00000189430	17183730;
D006830	decrease	NET1	ENSG00000173848	17183730;
D006830	decrease	NHLRC2	ENSG00000196865	17183730;
D006830	decrease	NTSR2	ENSG00000169006	17183730;
D006830	decrease	NUDT7	ENSG00000140876	17183730;
D006830	decrease	OPRD1	ENSG00000116329	17183730;
D006830	decrease	PHC1	ENSG00000111752	17183730;
D006830	decrease	PSMD3	ENSG00000108344	17183730;
D006830	decrease	REG1A	ENSG00000115386	17183730;
D006830	decrease	RPS4Y1	ENSG00000129824	17183730;
D006830	decrease	S100A8	ENSG00000143546	17183730;
D006830	decrease	SCML1	ENSG00000047634	17183730;
D006830	decrease	SELPLG	ENSG00000110876	17183730;
D006830	decrease	SHOX2	ENSG00000168779	17183730;
D006830	decrease	SKI	ENSG00000157933	17183730;
D006830	decrease	SPRR1A	ENSG00000169474	17183730;
D006830	decrease	SPRR1B	ENSG00000169469	17183730;
D006830	decrease	STEAP2	ENSG00000157214	17183730;
D006830	decrease	TOPBP1	ENSG00000163781	17183730;
D006830	decrease	TRIM61	ENSG00000183439	17183730;
D006830	decrease	UBOX5	ENSG00000185019	17183730;
D006830	decrease	ZNF18	ENSG00000154957	17183730;
D006830	increase	ABP1	ENSG00000002726	17183730;
D006830	increase	ACAA1	ENSG00000060971	17183730;
D006830	increase	ACAD9	ENSG00000177646	17183730;
D006830	increase	ACAT2	ENSG00000120437	17183730;
D006830	increase	ACCN4	ENSG00000072182	17183730;
D006830	increase	ACIN1	ENSG00000100813	17183730;
D006830	increase	ACN9	ENSG00000196636	17183730;
D006830	increase	ADAMTS10	ENSG00000142303	17183730;
D006830	increase	ADIPOR1	ENSG00000159346	17183730;
D006830	increase	ADSL	ENSG00000239900	17183730;
D006830	increase	AES	ENSG00000104964	17183730;
D006830	increase	AFG3L2	ENSG00000141385	17183730;
D006830	increase	AGK	ENSG00000006530	17183730;
D006830	increase	AKR1A1	ENSG00000117448	17183730;
D006830	increase	AKR1C2	ENSG00000151632	17183730;
D006830	increase	AKR1C4	ENSG00000198610	17183730;
D006830	increase	AKT2	ENSG00000105221	17183730;
D006830	increase	ALDH18A1	ENSG00000059573	17183730;
D006830	increase	ALDH9A1	ENSG00000143149	17183730;
D006830	increase	ANAPC11	ENSG00000141552	17183730;
D006830	increase	ANKFN1	ENSG00000153930	17183730;

D006830	increase	ANKHD1	ENSG00000131503	17183730;
D006830	increase	ANKIB1	ENSG00000001629	17183730;
D006830	increase	ANKRD44	ENSG00000065413	17183730;
D006830	increase	ANXA2	ENSG00000182718	17183730;
D006830	increase	APCDD1L	ENSG00000198768	17183730;
D006830	increase	AQP10	ENSG00000143595	17183730;
D006830	increase	ARCN1	ENSG00000095139	17183730;
D006830	increase	ARF1	ENSG00000143761	17183730;
D006830	increase	ARHGAP23	ENSG00000225485	17183730;
D006830	increase	ARHGAP5	ENSG00000100852	17183730;
D006830	increase	ARHGEF12	ENSG00000196914	17183730;
D006830	increase	ARIH2	ENSG00000177479	17183730;
D006830	increase	ARL2	ENSG00000213465	17183730;
D006830	increase	ARL6IP5	ENSG00000144746	17183730;
D006830	increase	ART4	ENSG00000111339	17183730;
D006830	increase	ASB7	ENSG00000183475	17183730;
D006830	increase	ASPH	ENSG00000198363	17183730;
D006830	increase	ATG10	ENSG00000152348	17183730;
D006830	increase	ATP13A4	ENSG00000127249	17183730;
D006830	increase	ATP5E	ENSG00000124172	17183730;
D006830	increase	ATP5G3	ENSG00000154518	17183730;
D006830	increase	ATP5I	ENSG00000169020	17183730;
D006830	increase	ATP5J	ENSG00000154723	17183730;
D006830	increase	ATP6AP1	ENSG00000071553	17183730;
D006830	increase	ATP6V1B1	ENSG00000116039	17183730;
D006830	increase	ATP6V1C1	ENSG00000155097	17183730;
D006830	increase	ATXN2	ENSG00000204842	17183730;
D006830	increase	ATXN7L1	ENSG00000146776	17183730;
D006830	increase	AURKAIP1	ENSG00000175756	17183730;
D006830	increase	BAD	ENSG00000002330	17183730;
D006830	increase	BAG1	ENSG00000250477	17183730;
D006830	increase	BCKDK	ENSG00000103507	17183730;
D006830	increase	BOLA1	ENSG00000178096	17183730;
D006830	increase	BPGM	ENSG00000172331	17183730;
D006830	increase	BPIL1	ENSG00000078898	17183730;
D006830	increase	BRD8	ENSG00000112983	17183730;
D006830	increase	C10ORF35	ENSG00000171224	17183730;
D006830	increase	C11ORF70	ENSG00000137691	17183730;
D006830	increase	C14ORF101	ENSG00000070269	17183730;
D006830	increase	C14ORF147	ENSG00000165389	17183730;
D006830	increase	C14ORF156	ENSG00000119705	17183730;
D006830	increase	C19ORF70	ENSG00000174917	17183730;
D006830	increase	C1ORF106	ENSG00000163362	17183730;
D006830	increase	C1ORF150	ENSG00000169224	17183730;
D006830	increase	C1ORF59	ENSG00000162639	17183730;
D006830	increase	C1ORF63	ENSG00000117616	17183730;
D006830	increase	C1QTNF5	ENSG00000223953	17183730;
D006830	increase	C20ORF108	ENSG00000124098	17183730;
D006830	increase	C21ORF33	ENSG00000160221	17183730;
D006830	increase	C3ORF62	ENSG00000188315	17183730;
D006830	increase	C4A	ENSG00000244731	17183730;
D006830	increase	C5ORF13	ENSG00000134986	17183730;
D006830	increase	C5ORF45	ENSG00000161010	17183730;
D006830	increase	C6ORF125	ENSG00000137288	17183730;

D006830	increase	C6ORF132	ENSG00000188112	17183730;
D006830	increase	C7ORF28B	ENSG00000146574	17183730;
D006830	increase	C7ORF42	ENSG00000106609	17183730;
D006830	increase	C8ORF38	ENSG00000156170	17183730;
D006830	increase	C8ORF55	ENSG00000130193	17183730;
D006830	increase	C9ORF152	ENSG00000188959	17183730;
D006830	increase	C9ORF84	ENSG00000165181	17183730;
D006830	increase	CA7	ENSG00000168748	17183730;
D006830	increase	CACNG6	ENSG00000130433	17183730;
D006830	increase	CAMLG	ENSG00000164615	17183730;
D006830	increase	CANX	ENSG00000127022	17183730;
D006830	increase	CASC4	ENSG00000166734	17183730;
D006830	increase	CASD1	ENSG00000127995	17183730;
D006830	increase	CBX7	ENSG00000100307	17183730;
D006830	increase	CCDC138	ENSG00000163006	17183730;
D006830	increase	CCDC47	ENSG00000108588	17183730;
D006830	increase	CCDC56	ENSG00000183978	17183730;
D006830	increase	CCDC72	ENSG00000232112	17183730;
D006830	increase	CCDC82	ENSG00000149231	17183730;
D006830	increase	CCL22	ENSG00000102962	17183730;
D006830	increase	CCL28	ENSG00000151882	17183730;
D006830	increase	CCL4	ENSG00000129277	17183730;
D006830	increase	CCM2	ENSG00000136280	17183730;
D006830	increase	CCT2	ENSG00000166226	17183730;
D006830	increase	CCT7	ENSG00000135624	17183730;
D006830	increase	CD1C	ENSG00000158481	17183730;
D006830	increase	CD63	ENSG00000135404	17183730;
D006830	increase	CDADC1	ENSG00000102543	17183730;
D006830	increase	CDC37	ENSG00000105401	17183730;
D006830	increase	CDC42	ENSG00000070831	17183730;
D006830	increase	CDK4	ENSG00000135446	17183730;
D006830	increase	CDR2	ENSG00000140743	17183730;
D006830	increase	CEL	ENSG00000170835	17183730;
D006830	increase	CENPN	ENSG00000166451	17183730;
D006830	increase	CHCHD10	ENSG00000250479	17183730;
D006830	increase	CHCHD2	ENSG00000106153	17183730;
D006830	increase	CHD2	ENSG00000173575	17183730;
D006830	increase	CHMP5	ENSG00000086065	17183730;
D006830	increase	CHN2	ENSG00000106069	17183730;
D006830	increase	CHRNA1	ENSG00000138435	17183730;
D006830	increase	CHST13	ENSG00000180767	17183730;
D006830	increase	CIB1	ENSG00000185043	17183730;
D006830	increase	CLASP2	ENSG00000163539	17183730;
D006830	increase	CLCN5	ENSG00000171365	17183730;
D006830	increase	CLYBL	ENSG00000125246	17183730;
D006830	increase	CNNM3	ENSG00000168763	17183730;
D006830	increase	COG5	ENSG00000164597	17183730;
D006830	increase	COL1A1	ENSG00000108821	17183730;
D006830	increase	COL4A6	ENSG00000197565	17183730;
D006830	increase	COMMD1	ENSG00000173163	17183730;
D006830	increase	COMMD6	ENSG00000188243	17183730;
D006830	increase	COPE	ENSG00000105669	17183730;
D006830	increase	COPS4	ENSG00000138663	17183730;
D006830	increase	COQ4	ENSG00000167113	17183730;

D006830	increase	COX6B1	ENSG00000126267	17183730;
D006830	increase	COX7C	ENSG00000127184	17183730;
D006830	increase	CREB5	ENSG00000146592	17183730;
D006830	increase	CRYBA4	ENSG00000196431	17183730;
D006830	increase	CST4	ENSG00000101441	17183730;
D006830	increase	CTNND1	ENSG00000198561	17183730;
D006830	increase	CTSK	ENSG00000143387	17183730;
D006830	increase	CUEDC2	ENSG00000107874	17183730;
D006830	increase	CUL1	ENSG00000055130	17183730;
D006830	increase	CYB5R1	ENSG00000159348	17183730;
D006830	increase	CYB5R3	ENSG00000100243	17183730;
D006830	increase	CYFIP2	ENSG00000055163	17183730;
D006830	increase	CYP2A7	ENSG00000198077	17183730;
D006830	increase	CYP4X1	ENSG00000186377	17183730;
D006830	increase	CYP4Z1	ENSG00000186160	17183730;
D006830	increase	DACT2	ENSG00000164488	17183730;
D006830	increase	DAPK1	ENSG00000196730	15862127;
D006830	increase	DBF4B	ENSG00000161692	17183730;
D006830	increase	DBI	ENSG00000155368	17183730;
D006830	increase	DCAF8	ENSG00000132716	17183730;
D006830	increase	DCLRE1C	ENSG00000152457	17183730;
D006830	increase	DCP1A	ENSG00000162290	17183730;
D006830	increase	DDT	ENSG00000099977	17183730;
D006830	increase	DDX27	ENSG00000124228	17183730;
D006830	increase	DDX46	ENSG00000145833	17183730;
D006830	increase	DNAH14	ENSG00000185842	17183730;
D006830	increase	DNAJA3	ENSG00000103423	17183730;
D006830	increase	DNAJC3	ENSG00000102580	17183730;
D006830	increase	DNAJC8	ENSG00000126698	17183730;
D006830	increase	DNHD1	ENSG00000179532	17183730;
D006830	increase	DPY19L3	ENSG00000178904	17183730;
D006830	increase	DRD4	ENSG00000069696	17183730;
D006830	increase	DYNC1LI1	ENSG00000144635	17183730;
D006830	increase	DYNLL1	ENSG00000088986	17183730;
D006830	increase	EDDM3A	ENSG00000181562	17183730;
D006830	increase	EEF1D	ENSG00000104529	17183730;
D006830	increase	EFHB	ENSG00000163576	17183730;
D006830	increase	EFHD2	ENSG00000142634	17183730;
D006830	increase	EHMT2	ENSG00000204371	17183730;
D006830	increase	EIF1AY	ENSG00000198692	17183730;
D006830	increase	EIF4B	ENSG00000063046	17183730;
D006830	increase	EIF4G3	ENSG00000075151	17183730;
D006830	increase	ELK3	ENSG00000111145	17183730;
D006830	increase	ELL	ENSG00000105656	17183730;
D006830	increase	ELOVL6	ENSG00000170522	17183730;
D006830	increase	EMID2	ENSG00000160963	17183730;
D006830	increase	EMP3	ENSG00000142227	17183730;
D006830	increase	EPAS1	ENSG00000116016	17183730;
D006830	increase	EPC1	ENSG00000120616	17183730;
D006830	increase	EPHA8	ENSG00000070886	17183730;
D006830	increase	EPN2	ENSG00000072134	17183730;
D006830	increase	EPRS	ENSG00000136628	17183730;
D006830	increase	ERO1LB	ENSG00000086619	17183730;
D006830	increase	ESR1	ENSG00000091831	15862127;

D006830	increase	EXOC3L2	ENSG00000130201	17183730;
D006830	increase	EXOC7	ENSG00000182473	17183730;
D006830	increase	F11R	ENSG00000158769	17183730;
D006830	increase	F12	ENSG00000131187	17183730;
D006830	increase	FAM19A1	ENSG00000183662	17183730;
D006830	increase	FAM3A	ENSG00000071889	17183730;
D006830	increase	FAM46A	ENSG00000112773	17183730;
D006830	increase	FAM50A	ENSG00000071859	17183730;
D006830	increase	FBRSL1	ENSG00000112787	17183730;
D006830	increase	FBXW4	ENSG00000107829	17183730;
D006830	increase	FDPS	ENSG00000160752	17183730;
D006830	increase	FFAR3	ENSG00000185897	17183730;
D006830	increase	FHIT	ENSG00000189283	15862127;
D006830	increase	FIS1	ENSG00000214253	17183730;
D006830	increase	FLOT1	ENSG00000137312	17183730;
D006830	increase	FOXD4L1	ENSG00000184492	17183730;
D006830	increase	FOXP1	ENSG00000114861	17183730;
D006830	increase	FST	ENSG00000134363	17183730;
D006830	increase	FSTL5	ENSG00000168843	17183730;
D006830	increase	FTH1	ENSG00000167996	17183730;
D006830	increase	FTHL17	ENSG00000132446	17183730;
D006830	increase	FXYD5	ENSG00000089327	17183730;
D006830	increase	GABARAP	ENSG00000170296	17183730;
D006830	increase	GALNT7	ENSG00000109586	17183730;
D006830	increase	GALP	ENSG00000197487	17183730;
D006830	increase	GAMT	ENSG00000130005	17183730;
D006830	increase	GANAB	ENSG00000089597	17183730;
D006830	increase	GCOM1	ENSG00000137878	17183730;
D006830	increase	GFRA2	ENSG00000168546	17183730;
D006830	increase	GFRA3	ENSG00000146013	17183730;
D006830	increase	GJD4	ENSG00000177291	17183730;
D006830	increase	GLA	ENSG00000102393	17183730;
D006830	increase	GNPAT	ENSG00000116906	17183730;
D006830	increase	GOLGB1	ENSG00000173230	17183730;
D006830	increase	GP2	ENSG00000169347	17183730;
D006830	increase	GPBP1L1	ENSG00000159592	17183730;
D006830	increase	GPR125	ENSG00000152990	17183730;
D006830	increase	GPR83	ENSG00000123901	17183730;
D006830	increase	GPS2	ENSG00000132522	17183730;
D006830	increase	GRAP	ENSG00000154016	17183730;
D006830	increase	GRK4	ENSG00000125388	17183730;
D006830	increase	GRWD1	ENSG00000105447	17183730;
D006830	increase	GSN	ENSG00000148180	17183730;
D006830	increase	GSTP1	ENSG00000084207	15862127;
D006830	increase	GSTT2	ENSG00000133433	17183730;
D006830	increase	GUK1	ENSG00000143774	17183730;
D006830	increase	H3F3A	ENSG00000163041	17183730;
D006830	increase	HAAO	ENSG00000162882	17183730;
D006830	increase	HCFC1R1	ENSG00000103145	17183730;
D006830	increase	HDGFL1	ENSG00000112273	17183730;
D006830	increase	HEBP1	ENSG00000013583	17183730;
D006830	increase	HFE	ENSG00000010704	17183730;
D006830	increase	HIGD2A	ENSG00000146066	17183730;
D006830	increase	HINT1	ENSG00000169567	17183730;

D006830	increase	HINT2	ENSG00000137133	17183730;
D006830	increase	HK1	ENSG00000156515	17183730;
D006830	increase	HLA-A	ENSG00000206503	17183730;
D006830	increase	HLA-B	ENSG00000234745	17183730;
D006830	increase	HLA-DOB	ENSG00000241910	17183730;
D006830	increase	HMGCL	ENSG00000117305	17183730;
D006830	increase	HPS3	ENSG00000163755	17183730;
D006830	increase	HPSE	ENSG00000173083	17183730;
D006830	increase	HSBP1	ENSG00000230989	17183730;
D006830	increase	HSPA8	ENSG00000109971	17183730;
D006830	increase	HYI	ENSG00000178922	17183730;
D006830	increase	ICAM1	ENSG00000090339	17183730;
D006830	increase	ICMT	ENSG00000116237	17183730;
D006830	increase	IDH3G	ENSG00000067829	17183730;
D006830	increase	IFITM2	ENSG00000185201	17183730;
D006830	increase	IFRD1	ENSG00000006652	17183730;
D006830	increase	IFT57	ENSG00000114446	17183730;
D006830	increase	IGBP1	ENSG00000089289	17183730;
D006830	increase	IGF2BP2	ENSG00000073792	17183730;
D006830	increase	IGLL1	ENSG00000128322	17183730;
D006830	increase	IL1RAPL2	ENSG00000189108	17183730;
D006830	increase	INO80E	ENSG00000169592	17183730;
D006830	increase	IRF7	ENSG00000185507	17183730;
D006830	increase	IRX3	ENSG00000177508	17183730;
D006830	increase	ITSN1	ENSG00000205726	17183730;
D006830	increase	ITSN2	ENSG00000198399	17183730;
D006830	increase	JARID2	ENSG00000008083	17183730;
D006830	increase	JMJD1C	ENSG00000171988	17183730;
D006830	increase	KDELR3	ENSG00000100196	17183730;
D006830	increase	KIAA0586	ENSG00000100578	17183730;
D006830	increase	KIAA1045	ENSG00000122733	17183730;
D006830	increase	KIAA1217	ENSG00000120549	17183730;
D006830	increase	KIAA2018	ENSG00000176542	17183730;
D006830	increase	KLF6	ENSG00000067082	17183730;
D006830	increase	KLHL23	ENSG00000213160	17183730;
D006830	increase	KLHL9	ENSG00000198642	17183730;
D006830	increase	KRTAP4-7	ENSG00000240871	17183730;
D006830	increase	LASP1	ENSG00000002834	17183730;
D006830	increase	LASS5	ENSG00000139624	17183730;
D006830	increase	LEF1	ENSG00000138795	17183730;
D006830	increase	LGALS1	ENSG00000100097	17183730;
D006830	increase	LGALS2	ENSG00000100079	17183730;
D006830	increase	LGR4	ENSG00000205213	17183730;
D006830	increase	LHFPL5	ENSG00000197753	17183730;
D006830	increase	LIN37	ENSG00000188223	17183730;
D006830	increase	LPCAT4	ENSG00000176454	17183730;
D006830	increase	LPGAT1	ENSG00000123684	17183730;
D006830	increase	LPIN3	ENSG00000132793	17183730;
D006830	increase	LPP	ENSG00000145012	17183730;
D006830	increase	LRFN1	ENSG00000128011	17183730;
D006830	increase	LRP10	ENSG00000197324	17183730;
D006830	increase	LRRC40	ENSG00000066557	17183730;
D006830	increase	LRRC8E	ENSG00000171017	17183730;
D006830	increase	LSMD1	ENSG00000183011	17183730;

D006830	increase	LY86	ENSG00000112799	17183730;
D006830	increase	MAOA	ENSG00000189221	17183730;
D006830	increase	MAP2K3	ENSG00000034152	17183730;
D006830	increase	MARK3	ENSG00000075413	17183730;
D006830	increase	MAS1	ENSG00000130368	17183730;
D006830	increase	MBD6	ENSG00000166987	17183730;
D006830	increase	ME2	ENSG00000082212	17183730;
D006830	increase	MED8	ENSG00000159479	17183730;
D006830	increase	METTL3	ENSG00000165819	17183730;
D006830	increase	METTL6	ENSG00000206562	17183730;
D006830	increase	MFSD5	ENSG00000182544	17183730;
D006830	increase	MGAT1	ENSG00000131446	17183730;
D006830	increase	MGEA5	ENSG00000198408	17183730;
D006830	increase	MGMT	ENSG00000170430	15862127;
D006830	increase	MGP	ENSG00000111341	17183730;
D006830	increase	MICAL3	ENSG00000243156	17183730;
D006830	increase	MIS12	ENSG00000167842	17183730;
D006830	increase	MKS1	ENSG00000011143	17183730;
D006830	increase	MLLT10	ENSG00000078403	17183730;
D006830	increase	MMAB	ENSG00000139428	17183730;
D006830	increase	MME	ENSG00000196549	17183730;
D006830	increase	MMP10	ENSG00000166670	17183730;
D006830	increase	MMP25	ENSG00000008516	17183730;
D006830	increase	MPST	ENSG00000128309	17183730;
D006830	increase	MRPL11	ENSG00000174547	17183730;
D006830	increase	MRPL39	ENSG00000154719	17183730;
D006830	increase	MRPL45	ENSG00000174100	17183730;
D006830	increase	MRPL53	ENSG00000204822	17183730;
D006830	increase	MRPS12	ENSG00000128626	17183730;
D006830	increase	MRPS18A	ENSG00000096080	17183730;
D006830	increase	MRPS24	ENSG00000062582	17183730;
D006830	increase	MRPS5	ENSG00000144029	17183730;
D006830	increase	MRRF	ENSG00000148187	17183730;
D006830	increase	MTA3	ENSG00000057935	17183730;
D006830	increase	MTMR4	ENSG00000108389	17183730;
D006830	increase	MUC1	ENSG00000185499	17183730;
D006830	increase	MUC7	ENSG00000171195	17183730;
D006830	increase	MYBL2	ENSG00000101057	17183730;
D006830	increase	MYCBP2	ENSG00000005810	17183730;
D006830	increase	MYL6B	ENSG00000196465	17183730;
D006830	increase	MYOM1	ENSG00000101605	17183730;
D006830	increase	MYOT	ENSG00000120729	17183730;
D006830	increase	NALCN	ENSG00000102452	17183730;
D006830	increase	NAP1L1	ENSG00000187109	17183730;
D006830	increase	NBEAL1	ENSG00000144426	17183730;
D006830	increase	NCF1	ENSG00000158517	17183730;
D006830	increase	NCOA4	ENSG00000138293	17183730;
D006830	increase	NCOR1	ENSG00000141027	17183730;
D006830	increase	NDUFA13	ENSG00000186010	17183730;
D006830	increase	NDUFA1	ENSG00000125356	17183730;
D006830	increase	NDUFA3	ENSG00000170906	17183730;
D006830	increase	NDUFA5	ENSG00000128609	17183730;
D006830	increase	NDUFA6	ENSG00000184983	17183730;
D006830	increase	NDUFB10	ENSG00000140990	17183730;

D006830	increase	NDUFB1	ENSG00000183648	17183730;
D006830	increase	NDUFB2	ENSG00000090266	17183730;
D006830	increase	NDUFS5	ENSG00000168653	17183730;
D006830	increase	NEDD1	ENSG00000139350	17183730;
D006830	increase	NF1	ENSG00000249384	17183730;
D006830	increase	NFATC2	ENSG00000101096	17183730;
D006830	increase	NHP2L1	ENSG00000100138	17183730;
D006830	increase	NHS	ENSG00000188158	17183730;
D006830	increase	NMD3	ENSG00000169251	17183730;
D006830	increase	NME1	ENSG00000239672	17183730;
D006830	increase	NOD1	ENSG00000106100	17183730;
D006830	increase	NOVA1	ENSG00000248163	17183730;
D006830	increase	NPAS3	ENSG00000151322	17183730;
D006830	increase	NPDC1	ENSG00000107281	17183730;
D006830	increase	NPHP4	ENSG00000131697	17183730;
D006830	increase	NPR1	ENSG00000169418	17183730;
D006830	increase	NR1H3	ENSG00000025434	17183730;
D006830	increase	NR2C2	ENSG00000177463	17183730;
D006830	increase	NR3C1	ENSG00000113580	17183730;
D006830	increase	NRXN2	ENSG00000110076	17183730;
D006830	increase	NSDHL	ENSG00000147383	17183730;
D006830	increase	NSFL1C	ENSG00000088833	17183730;
D006830	increase	NSUN4	ENSG00000117481	17183730;
D006830	increase	NTAN1	ENSG00000157045	17183730;
D006830	increase	NUDC	ENSG00000090273	17183730;
D006830	increase	NUMB	ENSG00000133961	17183730;
D006830	increase	NUP153	ENSG00000124789	17183730;
D006830	increase	NUPR1	ENSG00000176046	17183730;
D006830	increase	NXPH3	ENSG00000182575	17183730;
D006830	increase	OAZ1	ENSG00000104904	17183730;
D006830	increase	OBSCN	ENSG00000154358	17183730;
D006830	increase	OCIAD2	ENSG00000145247	17183730;
D006830	increase	OGG1	ENSG00000114026	17183730;
D006830	increase	OGT	ENSG00000147162	17183730;
D006830	increase	OPA1	ENSG00000198836	17183730;
D006830	increase	OR9I1	ENSG00000172377	17183730;
D006830	increase	P2RX3	ENSG00000109991	17183730;
D006830	increase	PABPC1L2B	ENSG00000186288	17183730;
D006830	increase	PAFAH1B3	ENSG00000079462	17183730;
D006830	increase	PARK7	ENSG00000116288	17183730;
D006830	increase	PARP14	ENSG00000173193	17183730;
D006830	increase	PARP1	ENSG00000143799	17183730;
D006830	increase	PASD1	ENSG00000166049	17183730;
D006830	increase	PBX2	ENSG00000236353	17183730;
D006830	increase	PCBD1	ENSG00000166228	17183730;
D006830	increase	PCCB	ENSG00000114054	17183730;
D006830	increase	PCDH15	ENSG00000150275	17183730;
D006830	increase	PEBP4	ENSG00000134020	17183730;
D006830	increase	PFKFB4	ENSG00000114268	17183730;
D006830	increase	PFN1	ENSG00000108518	17183730;
D006830	increase	PGAM2	ENSG00000164708	17183730;
D006830	increase	PGM1	ENSG00000079739	17183730;
D006830	increase	PHB	ENSG00000167085	17183730;
D006830	increase	PHC2	ENSG00000134686	17183730;

D006830	increase	PIGS	ENSG00000087111	17183730;
D006830	increase	PIP	ENSG00000159763	17183730;
D006830	increase	PLCG1	ENSG00000124181	17183730;
D006830	increase	PLRG1	ENSG00000171566	17183730;
D006830	increase	PLXDC1	ENSG00000161381	17183730;
D006830	increase	PMM2	ENSG00000140650	17183730;
D006830	increase	PMVK	ENSG00000163344	17183730;
D006830	increase	POLE4	ENSG00000115350	17183730;
D006830	increase	POLR2J2	ENSG00000228049	17183730;
D006830	increase	POLR2L	ENSG00000177700	17183730;
D006830	increase	POP5	ENSG00000167272	17183730;
D006830	increase	PPIAL4A	ENSG00000198161	17183730;
D006830	increase	PPIL4	ENSG00000131013	17183730;
D006830	increase	PPP1R12A	ENSG00000058272	17183730;
D006830	increase	PPP1R9A	ENSG00000158528	17183730;
D006830	increase	PPP2R1B	ENSG00000137713	17183730;
D006830	increase	PRB3	ENSG00000197870	17183730;
D006830	increase	PRKACG	ENSG00000165059	17183730;
D006830	increase	PRKCSH	ENSG00000130175	17183730;
D006830	increase	PRKG1	ENSG00000185532	17183730;
D006830	increase	PROM2	ENSG00000155066	17183730;
D006830	increase	PRPF18	ENSG00000165630	17183730;
D006830	increase	PRR13	ENSG00000205352	17183730;
D006830	increase	PRTFDC1	ENSG00000099256	17183730;
D006830	increase	PSD3	ENSG00000156011	17183730;
D006830	increase	PSMA2	ENSG00000106588	17183730;
D006830	increase	PSMA4	ENSG00000041357	17183730;
D006830	increase	PSMB1	ENSG00000008018	17183730;
D006830	increase	PSMB2	ENSG00000126067	17183730;
D006830	increase	PSMB3	ENSG00000108294	17183730;
D006830	increase	PSMB6	ENSG00000142507	17183730;
D006830	increase	PSMB9	ENSG00000240065	17183730;
D006830	increase	PSMC6	ENSG00000100519	17183730;
D006830	increase	PSMD1	ENSG00000173692	17183730;
D006830	increase	PSMD4	ENSG00000159352	17183730;
D006830	increase	PSMD8	ENSG00000099341	17183730;
D006830	increase	PSMG3	ENSG00000157778	17183730;
D006830	increase	PTK2B	ENSG00000120899	17183730;
D006830	increase	PTMA	ENSG00000187514	17183730;
D006830	increase	PTPRJ	ENSG00000149177	17183730;
D006830	increase	PUM1	ENSG00000134644	17183730;
D006830	increase	PYCARD	ENSG00000103490	17183730;
D006830	increase	PYCRL	ENSG00000104524	17183730;
D006830	increase	RAB13	ENSG00000143545	17183730;
D006830	increase	RAB1A	ENSG00000138069	17183730;
D006830	increase	RAB30	ENSG00000137502	17183730;
D006830	increase	RAB3A	ENSG00000105649	17183730;
D006830	increase	RAB3D	ENSG00000105514	17183730;
D006830	increase	RAB6A	ENSG00000175582	17183730;
D006830	increase	RABEP2	ENSG00000177548	17183730;
D006830	increase	RABGEF1	ENSG00000154710	17183730;
D006830	increase	RABL5	ENSG00000128581	17183730;
D006830	increase	RAD21	ENSG00000164754	17183730;
D006830	increase	RALGDS	ENSG00000160271	17183730;

D006830	increase	RALGPS1	ENSG00000136828	17183730;
D006830	increase	RAP1A	ENSG00000116473	17183730;
D006830	increase	RBL1	ENSG00000080839	17183730;
D006830	increase	RBM22	ENSG00000086589	17183730;
D006830	increase	RBM4	ENSG00000173933	17183730;
D006830	increase	RCL1	ENSG00000120158	17183730;
D006830	increase	REEP4	ENSG00000168476	17183730;
D006830	increase	RER1	ENSG00000157916	17183730;
D006830	increase	RFX1	ENSG00000132005	17183730;
D006830	increase	RGS7BP	ENSG00000186479	17183730;
D006830	increase	RGS9BP	ENSG00000186326	17183730;
D006830	increase	RGS9	ENSG00000108370	17183730;
D006830	increase	RIN3	ENSG00000100599	17183730;
D006830	increase	RINL	ENSG00000187994	17183730;
D006830	increase	RIPK1	ENSG00000137275	17183730;
D006830	increase	RIPPLY1	ENSG00000147223	17183730;
D006830	increase	RMND5B	ENSG00000145916	17183730;
D006830	increase	RNF152	ENSG00000176641	17183730;
D006830	increase	RNF187	ENSG00000168159	17183730;
D006830	increase	ROBO2	ENSG00000185008	17183730;
D006830	increase	RPL10A	ENSG00000198755	17183730;
D006830	increase	RPL13	ENSG00000167526	17183730;
D006830	increase	RPL17	ENSG00000215472	17183730;
D006830	increase	RPL18A	ENSG00000105640	17183730;
D006830	increase	RPL23A	ENSG00000198242	17183730;
D006830	increase	RPL24	ENSG00000114391	17183730;
D006830	increase	RPL26	ENSG00000161970	17183730;
D006830	increase	RPL27	ENSG00000131469	17183730;
D006830	increase	RPL31	ENSG00000071082	17183730;
D006830	increase	RPL34	ENSG00000109475	17183730;
D006830	increase	RPL36	ENSG00000130255	17183730;
D006830	increase	RPL37A	ENSG00000197756	17183730;
D006830	increase	RPL39	ENSG00000198918	17183730;
D006830	increase	RPL41	ENSG00000229117	17183730;
D006830	increase	RPL7	ENSG00000147604	17183730;
D006830	increase	RPLP1	ENSG00000137818	17183730;
D006830	increase	RPP21	ENSG00000241370	17183730;
D006830	increase	RPS11	ENSG00000142534	17183730;
D006830	increase	RPS12	ENSG00000112306	17183730;
D006830	increase	RPS19BP1	ENSG00000187051	17183730;
D006830	increase	RPS27A	ENSG00000143947	17183730;
D006830	increase	RPS27	ENSG00000177954	17183730;
D006830	increase	RPS2	ENSG00000140988	17183730;
D006830	increase	RPS6KA5	ENSG00000100784	17183730;
D006830	increase	RPS9	ENSG00000170889	17183730;
D006830	increase	RSPO4	ENSG00000101282	17183730;
D006830	increase	RTN4RL1	ENSG00000185924	17183730;
D006830	increase	RWDD1	ENSG00000111832	17183730;
D006830	increase	RXRG	ENSG00000249671	17183730;
D006830	increase	S100A6	ENSG00000197956	17183730;
D006830	increase	S100PBP	ENSG00000116497	17183730;
D006830	increase	SAMD4A	ENSG00000020577	17183730;
D006830	increase	SAMM50	ENSG00000100347	17183730;
D006830	increase	SCAP	ENSG00000114650	17183730;

D006830	increase	SCARF1	ENSG00000074660	17183730;
D006830	increase	SCNN1B	ENSG00000168447	17183730;
D006830	increase	SCRN2	ENSG00000141295	17183730;
D006830	increase	SDF2L1	ENSG00000128228	17183730;
D006830	increase	SEC11C	ENSG00000166562	17183730;
D006830	increase	SEC31A	ENSG00000138674	17183730;
D006830	increase	SEMA4A	ENSG00000196189	17183730;
D006830	increase	2-Sep	ENSG00000168385	17183730;
D006830	increase	SERP2	ENSG00000151778	17183730;
D006830	increase	SERPING1	ENSG00000149131	17183730;
D006830	increase	SERTAD2	ENSG00000179833	17183730;
D006830	increase	SF1	ENSG00000250652	17183730;
D006830	increase	SF3B14	ENSG00000115128	17183730;
D006830	increase	SFRS8	ENSG00000061936	17183730;
D006830	increase	SHANK2	ENSG00000162105	17183730;
D006830	increase	SIDT2	ENSG00000149577	17183730;
D006830	increase	SIPA1L1	ENSG00000197555	17183730;
D006830	increase	SLC12A3	ENSG00000070915	17183730;
D006830	increase	SLC16A2	ENSG00000147100	17183730;
D006830	increase	SLC19A2	ENSG00000117479	17183730;
D006830	increase	SLC22A16	ENSG00000004809	17183730;
D006830	increase	SLC22A17	ENSG00000092096	17183730;
D006830	increase	SLC23A3	ENSG00000213901	17183730;
D006830	increase	SLC24A6	ENSG00000089060	17183730;
D006830	increase	SLC25A13	ENSG00000004864	17183730;
D006830	increase	SLC25A5	ENSG00000005022	17183730;
D006830	increase	SLC2A9	ENSG00000109667	17183730;
D006830	increase	SLC30A2	ENSG00000158014	17183730;
D006830	increase	SLC35C1	ENSG00000181830	17183730;
D006830	increase	SLC3A2	ENSG00000168003	17183730;
D006830	increase	SLC6A13	ENSG00000010379	17183730;
D006830	increase	SLC6A2	ENSG00000103546	17183730;
D006830	increase	SMARCC1	ENSG00000173473	17183730;
D006830	increase	SMO	ENSG00000128602	17183730;
D006830	increase	SMURF2	ENSG00000108854	17183730;
D006830	increase	SNAI3	ENSG00000185669	17183730;
D006830	increase	SNF8	ENSG00000159210	17183730;
D006830	increase	SNRNP70	ENSG00000104852	17183730;
D006830	increase	SNRPB2	ENSG00000125870	17183730;
D006830	increase	SNRPB	ENSG00000125835	17183730;
D006830	increase	SNRPD2	ENSG00000125743	17183730;
D006830	increase	SNRPG	ENSG00000143977	17183730;
D006830	increase	SOX1	ENSG00000182968	17183730;
D006830	increase	SOX9	ENSG00000125398	17183730;
D006830	increase	SPDEF	ENSG00000124664	17183730;
D006830	increase	SPINT1	ENSG00000166145	17183730;
D006830	increase	SRP72	ENSG00000174780	17183730;
D006830	increase	SRP9	ENSG00000143742	17183730;
D006830	increase	SRXN1	ENSG00000172070	17183730;
D006830	increase	SSBP4	ENSG00000130511	17183730;
D006830	increase	SSPN	ENSG00000123096	17183730;
D006830	increase	SSR1	ENSG00000124783	17183730;
D006830	increase	ST8SIA2	ENSG00000140557	17183730;
D006830	increase	STAB2	ENSG00000136011	17183730;

D006830	increase	STAG1	ENSG00000118007	17183730;
D006830	increase	STK38	ENSG00000112079	17183730;
D006830	increase	STX11	ENSG00000135604	17183730;
D006830	increase	STX5	ENSG00000162236	17183730;
D006830	increase	STYX	ENSG00000198252	17183730;
D006830	increase	SUFU	ENSG00000107882	17183730;
D006830	increase	SUMO3	ENSG00000184900	17183730;
D006830	increase	SYP	ENSG00000102003	17183730;
D006830	increase	SYT6	ENSG00000134207	17183730;
D006830	increase	TACC2	ENSG00000138162	17183730;
D006830	increase	TAF12	ENSG00000120656	17183730;
D006830	increase	TALDO1	ENSG00000177156	17183730;
D006830	increase	TAS2R5	ENSG00000127366	17183730;
D006830	increase	TBC1D1	ENSG00000065882	17183730;
D006830	increase	TBCD	ENSG00000141556	17183730;
D006830	increase	TBRG1	ENSG00000154144	17183730;
D006830	increase	TBX18	ENSG00000112837	17183730;
D006830	increase	TCEB2	ENSG00000103363	17183730;
D006830	increase	TCERG1	ENSG00000113649	17183730;
D006830	increase	TCF12	ENSG00000140262	17183730;
D006830	increase	TEC	ENSG00000135605	17183730;
D006830	increase	TFCP2	ENSG00000135457	17183730;
D006830	increase	TFDP1	ENSG00000198176	17183730;
D006830	increase	TFF2	ENSG00000160181	17183730;
D006830	increase	TGIF2	ENSG00000118707	17183730;
D006830	increase	THRAP3	ENSG00000054118	17183730;
D006830	increase	THY1	ENSG00000154096	17183730;
D006830	increase	TIMM13	ENSG00000099800	17183730;
D006830	increase	TIMM50	ENSG00000105197	17183730;
D006830	increase	TIMM8B	ENSG00000150779	17183730;
D006830	increase	TIMP2	ENSG00000035862	17183730;
D006830	increase	TIPRL	ENSG00000143155	17183730;
D006830	increase	TM7SF3	ENSG00000064115	17183730;
D006830	increase	TMCO1	ENSG00000143183	17183730;
D006830	increase	TMED3	ENSG00000166557	17183730;
D006830	increase	TMEM66	ENSG00000133872	17183730;
D006830	increase	TMEM80	ENSG00000177042	17183730;
D006830	increase	TMEM9	ENSG00000116857	17183730;
D006830	increase	TMSB10	ENSG00000034510	17183730;
D006830	increase	TNFAIP8	ENSG00000145779	17183730;
D006830	increase	TOMM20	ENSG00000173726	17183730;
D006830	increase	TOMM7	ENSG00000196683	17183730;
D006830	increase	TP53	ENSG00000141510	12082016;17183730;
D006830	increase	TPI1	ENSG00000111669	17183730;
D006830	increase	TPM2	ENSG00000198467	17183730;
D006830	increase	TPSAB1	ENSG00000172236	17183730;
D006830	increase	TPTE2	ENSG00000132958	17183730;
D006830	increase	TRAF2	ENSG00000127191	17183730;
D006830	increase	TRAPPC4	ENSG00000196655	17183730;
D006830	increase	TRAPPC6A	ENSG00000007255	17183730;
D006830	increase	TRIB2	ENSG00000071575	17183730;
D006830	increase	TRIM46	ENSG00000163462	17183730;
D006830	increase	TRIP10	ENSG00000125733	17183730;
D006830	increase	TRIP6	ENSG00000087077	17183730;

D006830	increase	TRPT1	ENSG00000149743	17183730;
D006830	increase	TSEN54	ENSG00000182173	17183730;
D006830	increase	TSPAN10	ENSG00000182612	17183730;
D006830	increase	TSPAN18	ENSG00000157570	17183730;
D006830	increase	TSPO	ENSG00000100300	17183730;
D006830	increase	TST	ENSG00000128311	17183730;
D006830	increase	TUBB6	ENSG00000176014	17183730;
D006830	increase	TYW3	ENSG00000162623	17183730;
D006830	increase	UBA52	ENSG00000221983	17183730;
D006830	increase	UBE2L6	ENSG00000156587	17183730;
D006830	increase	UGGT2	ENSG00000102595	17183730;
D006830	increase	ULBP1	ENSG00000111981	17183730;
D006830	increase	UNC45A	ENSG00000140553	17183730;
D006830	increase	UQCRB	ENSG00000156467	17183730;
D006830	increase	UQCRQ	ENSG00000164405	17183730;
D006830	increase	USP33	ENSG00000077254	17183730;
D006830	increase	USP40	ENSG00000085982	17183730;
D006830	increase	USP49	ENSG00000164663	17183730;
D006830	increase	VEGFB	ENSG00000173511	17183730;
D006830	increase	WBP2NL	ENSG00000183066	17183730;
D006830	increase	WBP4	ENSG00000120688	17183730;
D006830	increase	WDR46	ENSG00000227057	17183730;
D006830	increase	WNT1	ENSG00000125084	17183730;
D006830	increase	XYLB	ENSG00000093217	17183730;
D006830	increase	YTHDF1	ENSG00000149658	17183730;
D006830	increase	YWHAQ	ENSG00000134308	17183730;
D006830	increase	ZC3H3	ENSG00000014164	17183730;
D006830	increase	ZDHHC17	ENSG00000186908	17183730;
D006830	increase	ZDHHC7	ENSG00000153786	17183730;
D006830	increase	ZFAND2B	ENSG00000158552	17183730;
D006830	increase	ZFP62	ENSG00000196670	17183730;
D006830	increase	ZFR	ENSG00000056097	17183730;
D006830	increase	ZMPSTE24	ENSG00000084073	17183730;
D006830	increase	ZNF211	ENSG00000121417	17183730;
D006830	increase	ZNF227	ENSG00000131115	17183730;
D006830	increase	ZNF354C	ENSG00000177932	17183730;
D006830	increase	ZNF398	ENSG00000197024	17183730;
D006830	increase	ZNF439	ENSG00000171291	17183730;
D006830	increase	ZNF441	ENSG00000197044	17183730;
D006830	increase	ZNF45	ENSG00000124459	17183730;
D006830	increase	ZNF493	ENSG00000196268	17183730;
D006830	increase	ZNF519	ENSG00000175322	17183730;
D006830	increase	ZNF540	ENSG00000171817	17183730;
D006830	increase	ZNF556	ENSG00000172000	17183730;
D006830	increase	ZNF592	ENSG00000166716	17183730;
D006830	increase	ZNF621	ENSG00000172888	17183730;
D006830	increase	ZNF622	ENSG00000173545	17183730;
D006830	increase	ZNF701	ENSG00000167562	17183730;
D006830	increase	ZNF768	ENSG00000169957	17183730;
D006843	decrease	CCND1	ENSG00000110092	14757120;
D006843	decrease	CCND3	ENSG00000112576	14757120;
D006843	decrease	CCNE1	ENSG00000105173	14757120;
D006843	decrease	CYP21A2	ENSG00000231852	16650473;
D006843	decrease	HSD3B2	ENSG00000203859	16650473;

D006843	decrease	RB1	ENSG00000139687	14757120;
D006843	increase	AHR	ENSG00000106546	16650473;
D006843	increase	CCNG2	ENSG00000138764	14757120;
D006843	increase	CYP11B2	ENSG00000179142	16650473;
D006854	decrease	CD14	ENSG00000170458	17033190;
D006854	decrease	TLR4	ENSG00000136869	17033190;
D006854	increase	ABCC3	ENSG00000108846	15979871;
D006854	increase	CYP3A4	ENSG00000160868	12505310;15941677;15025748;16024008;15266218;
D006854	increase	EGF	ENSG00000138798	17805209;
D006854	increase	GSTA1	ENSG00000243955	11297599;
D006854	increase	HSD11B1	ENSG00000117594	17761898;16751407;16054388;
D006854	increase	ICAM1	ENSG00000090339	15358673;
D006854	increase	KLK3	ENSG00000142515	11956172;
D006854	increase	NR3C1	ENSG00000113580	15698552;16857225;11956172;15705660;15883974;15780662;16054388;18483179;17716631;15755863;
D006854	increase	PTGS2	ENSG00000073756	17805209;
D006854	increase	SFTPB	ENSG00000168878	2539393;
D006854	increase	SFTPC	ENSG00000168484	2539393;
D006854	increase	SLC5A5	ENSG00000105641	16439463;
D006854	increase	TGM1	ENSG00000092295	11485391;
D006854	increase	TLR2	ENSG00000137462	17033190;
D006854	increase	VCAM1	ENSG00000162692	15358673;
D006861	affect	CLDN1	ENSG00000163347	18661270;
D006861	affect	CLDN2	ENSG00000165376	18661270;
D006861	affect	CLDN7	ENSG00000181885	18661270;
D006861	affect	CLDN8	ENSG00000156284	18661270;
D006861	affect	OCLN	ENSG00000197822	18661270;
D006861	decrease	ACTB	ENSG00000075624	12419474;
D006861	decrease	ADCY3	ENSG00000138031	12414654;
D006861	decrease	ADORA1	ENSG00000163485	12419474;
D006861	decrease	AMPH	ENSG00000078053	12419474;
D006861	decrease	APC	ENSG00000134982	12419474;
D006861	decrease	ARHGDIA	ENSG00000141522	12419474;
D006861	decrease	ATF4	ENSG00000128272	12419474;
D006861	decrease	AURKB	ENSG00000178999	12414654;
D006861	decrease	BAK1	ENSG00000030110	12419474;
D006861	decrease	BCL2	ENSG00000171791	9989825;12672908;
D006861	decrease	BMP4	ENSG00000125378	12419474;
D006861	decrease	BNIP3	ENSG00000176171	12419474;
D006861	decrease	C6ORF108	ENSG00000112667	12419474;
D006861	decrease	CAPN2	ENSG00000162909	12419474;
D006861	decrease	CAPNS1	ENSG00000126247	12419474;
D006861	decrease	CASK	ENSG00000147044	12419474;
D006861	decrease	CCNA1	ENSG00000133101	15761015;
D006861	decrease	CCNA2	ENSG00000145386	12419474;12414654;
D006861	decrease	CCNT1	ENSG00000129315	12419474;
D006861	decrease	CCR1	ENSG00000163823	12419474;
D006861	decrease	CDC20	ENSG00000117399	12419474;
D006861	decrease	CDC25A	ENSG00000164045	12419474;
D006861	decrease	CDC25C	ENSG00000158402	12419474;

D006861	decrease	CDH11	ENSG00000140937	12419474;
D006861	decrease	CDH2	ENSG00000170558	12419474;
D006861	decrease	CDH3	ENSG00000062038	12419474;
D006861	decrease	CDK10	ENSG00000185324	12419474;
D006861	decrease	CDK2	ENSG00000123374	12479875;
D006861	decrease	CDK9	ENSG00000136807	12419474;
D006861	decrease	CDKN2B	ENSG00000147883	12419474;
D006861	decrease	CDKN2D	ENSG00000129355	12419474;
D006861	decrease	CENPA	ENSG00000115163	12414654;
D006861	decrease	CHEK1	ENSG00000149554	12414654;
D006861	decrease	CLDN6	ENSG00000184697	18661270;
D006861	decrease	CLDN9	ENSG00000213937	18661270;
D006861	decrease	COMT	ENSG00000093010	12419474;
D006861	decrease	CP	ENSG00000047457	19019832;
D006861	decrease	CRHR1	ENSG00000120088	12419474;
D006861	decrease	CSNK1G2	ENSG00000133275	12419474;
D006861	decrease	CTNNA1	ENSG00000044115	12419474;
D006861	decrease	CTNNB1	ENSG00000168036	12419474;
D006861	decrease	CUX1	ENSG00000160967	12419474;
D006861	decrease	CYTL1	ENSG00000170891	15761015;
D006861	decrease	DAPK1	ENSG00000196730	12419474;
D006861	decrease	DDR2	ENSG00000162733	12419474;
D006861	decrease	DYNLL1	ENSG00000088986	12419474;
D006861	decrease	ELK1	ENSG00000126767	12419474;
D006861	decrease	EPO	ENSG00000130427	7980410;
D006861	decrease	ERBB2	ENSG00000141736	12419474;
D006861	decrease	FCGR3B	ENSG00000162747	16510162;
D006861	decrease	FECH	ENSG00000066926	12419474;
D006861	decrease	FEN1	ENSG00000168496	12414654;
D006861	decrease	FGFR1	ENSG00000249195	8849686;12419474;
D006861	decrease	FMO1	ENSG00000010932	12419474;
D006861	decrease	FOXM1	ENSG00000111206	12414654;
D006861	decrease	GJA1	ENSG00000152661	15761015;
D006861	decrease	GNAS	ENSG00000087460	12419474;
D006861	decrease	GNB1	ENSG00000078369	12419474;
D006861	decrease	GNB2	ENSG00000172354	12419474;
D006861	decrease	GTF2B	ENSG00000137947	12419474;
D006861	decrease	HDAC1	ENSG00000116478	12419474;
D006861	decrease	HGFAC	ENSG00000109758	12419474;
D006861	decrease	HGF	ENSG00000019991	12419474;
D006861	decrease	HINT1	ENSG00000169567	12419474;
D006861	decrease	HIST1H4C	ENSG00000182217	12414654;
D006861	decrease	HLA-C	ENSG00000204525	12419474;
D006861	decrease	HMGA1	ENSG00000137309	12419474;
D006861	decrease	ID1	ENSG00000125968	12419474;
D006861	decrease	IL18	ENSG00000150782	12419474;
D006861	decrease	ITGA3	ENSG00000005884	12419474;
D006861	decrease	ITGA5	ENSG00000161638	12419474;
D006861	decrease	ITGA7	ENSG00000135424	12419474;
D006861	decrease	ITGAL	ENSG00000005844	12419474;
D006861	decrease	ITGAV	ENSG00000138448	12419474;
D006861	decrease	ITGB1	ENSG00000150093	12419474;
D006861	decrease	ITGB4	ENSG00000132470	12419474;
D006861	decrease	ITGB5	ENSG00000082781	12419474;

D006861	decrease	ITGB6	ENSG00000115221	12419474;
D006861	decrease	JUND	ENSG00000130522	12419474;
D006861	decrease	LBR	ENSG00000143815	12414654;
D006861	decrease	LCAT	ENSG00000213398	12419474;
D006861	decrease	MAP2K5	ENSG00000137764	12419474;
D006861	decrease	MAPK9	ENSG00000050748	12419474;15795422;
D006861	decrease	MARCKSL1	ENSG00000175130	12419474;
D006861	decrease	MCM4	ENSG00000104738	12419474;
D006861	decrease	MMP11	ENSG00000099953	12419474;
D006861	decrease	MYBL2	ENSG00000101057	12419474;
D006861	decrease	NDUFB7	ENSG00000099795	12419474;
D006861	decrease	NF2	ENSG00000186575	12419474;
D006861	decrease	NFE2L1	ENSG00000082641	12419474;
D006861	decrease	NFIX	ENSG00000008441	12419474;
D006861	decrease	NFKB1	ENSG00000109320	15905586;11961005;16230421;9973483;12419474;15710601;15090542;15322087;16007145;
D006861	decrease	NFRKB	ENSG00000170322	12419474;
D006861	decrease	NMBR	ENSG00000135577	12419474;
D006861	decrease	NR2F6	ENSG00000160113	12419474;
D006861	decrease	PAIP1	ENSG00000172239	12419474;
D006861	decrease	PDYN	ENSG00000101327	12419474;
D006861	decrease	PFDN4	ENSG00000101132	12419474;
D006861	decrease	PHB	ENSG00000167085	12419474;
D006861	decrease	PIK3R2	ENSG00000105647	12419474;
D006861	decrease	PLK1	ENSG00000166851	12414654;
D006861	decrease	POLD1	ENSG00000062822	12419474;
D006861	decrease	POLE2	ENSG00000100479	12414654;
D006861	decrease	PPP1R8	ENSG00000117751	12419474;
D006861	decrease	PSMC3	ENSG00000165916	12419474;
D006861	decrease	PTMS	ENSG00000159335	12419474;
D006861	decrease	QSOX1	ENSG00000116260	12419474;
D006861	decrease	RB1	ENSG00000139687	12419474;12621062;15242773;
D006861	decrease	RBL2	ENSG00000103479	12419474;12621062;
D006861	decrease	RNH1	ENSG00000023191	12419474;
D006861	decrease	RPA1	ENSG00000132383	12419474;
D006861	decrease	RPL13A	ENSG00000142541	12419474;
D006861	decrease	RPS9	ENSG00000170889	12419474;
D006861	decrease	SF1	ENSG00000250652	12419474;
D006861	decrease	SLC1A2	ENSG00000110436	17138558;
D006861	decrease	SLC7A5	ENSG00000103257	12419474;
D006861	decrease	SOX18	ENSG00000203883	15761015;
D006861	decrease	SPINT2	ENSG00000167642	12419474;
D006861	decrease	SPRY2	ENSG00000136158	12419474;
D006861	decrease	SRF	ENSG00000112658	12419474;
D006861	decrease	SST	ENSG00000157005	12414654;
D006861	decrease	STAB1	ENSG00000010327	15761015;
D006861	decrease	SYP	ENSG00000102003	12419474;
D006861	decrease	TANK	ENSG00000136560	12419474;
D006861	decrease	TCEA1	ENSG00000187735	12419474;
D006861	decrease	TERT	ENSG00000164362	15761015;
D006861	decrease	TJP1	ENSG00000104067	18661270;12419474;

D006861	decrease	TK1	ENSG00000167900	12414654;
D006861	decrease	TMPO	ENSG00000120802	12414654;
D006861	decrease	TPP1	ENSG00000166340	12419474;
D006861	decrease	TSC2	ENSG00000103197	12419474;
D006861	decrease	TUBA1A	ENSG00000167552	12414654;
D006861	decrease	TUBA1B	ENSG00000123416	12419474;
D006861	decrease	UBE2A	ENSG00000077721	12419474;
D006861	decrease	UBE2C	ENSG00000175063	12414654;
D006861	decrease	UNG	ENSG00000076248	12414654;
D006861	decrease	VCAM1	ENSG00000162692	12419474;
D006861	decrease	ZNF91	ENSG00000167232	12419474;
D006861	decrease	ZYX	ENSG00000159840	12419474;
D006861	increase	ACACA	ENSG00000132142	15297373;
D006861	increase	ACAT2	ENSG00000120437	18032389;
D006861	increase	ADAM9	ENSG00000168615	17342749;17018608;
D006861	increase	ADM	ENSG00000148926	18687144;
D006861	increase	AKR1C1	ENSG00000187134	9973208;
D006861	increase	AKR1C2	ENSG00000151632	12414654;
D006861	increase	ALB	ENSG00000163631	11961005;
D006861	increase	ALOX5AP	ENSG00000132965	15761015;
D006861	increase	APP	ENSG00000142192	15699049;12419474;
D006861	increase	AREG	ENSG00000205595	12414654;
D006861	increase	ARNT	ENSG00000143437	15297373;16880289;18603805;
D006861	increase	ATF3	ENSG00000162772	18687144;12414654;
D006861	increase	BAX	ENSG00000087088	12419474;17395767;12672908;
D006861	increase	BECN1	ENSG00000126581	19451193;
D006861	increase	BTG2	ENSG00000159388	18687144;12414654;
D006861	increase	CAT	ENSG00000121691	11178967;7980410;17342749;17018608;9525474;9870925;12361925;11603923;14759608;10754530;10751631;16677106;11313354;15336504;15101064;9569045;
D006861	increase	CAV1	ENSG00000105974	18385095;17108117;
D006861	increase	CCL2	ENSG00000108691	11961005;16143069;12419474;17529908;
D006861	increase	CD52	ENSG00000169442	15761015;
D006861	increase	CDKN1A	ENSG00000124762	18687144;8943236;17395767;12414654;15242773;12479875;
D006861	increase	CFLAR	ENSG00000003402	18603805;
D006861	increase	COX2	ENSG00000198712	16531283;
D006861	increase	CTAGE5	ENSG00000150527	12414654;
D006861	increase	CTGF	ENSG00000118523	15147944;
D006861	increase	CTNND1	ENSG00000198561	18603805;
D006861	increase	CYP1A1	ENSG00000140465	9973208;
D006861	increase	CYP1B1	ENSG00000138061	12414654;
D006861	increase	CYP3A4	ENSG00000160868	15056853;
D006861	increase	CYP51A1	ENSG00000001630	18032389;
D006861	increase	CYR61	ENSG00000142871	12414654;
D006861	increase	DDIT3	ENSG00000175197	12111836;12414654;
D006861	increase	DDIT4	ENSG00000168209	16008523;
D006861	increase	DDX42	ENSG00000198231	12414654;

D006861	increase	DHCR7	ENSG00000172893	18032389;
D006861	increase	DKK1	ENSG00000107984	12414654;
D006861	increase	DNMT1	ENSG00000130816	18314485;
D006861	increase	DUSP14	ENSG00000161326	18687144;
D006861	increase	DUSP1	ENSG00000120129	18687144;
D006861	increase	DUSP5	ENSG00000138166	18687144;
D006861	increase	EDN1	ENSG00000078401	18234130;12414654;11755377;
D006861	increase	EGR1	ENSG00000120738	12414654;
D006861	increase	EPAS1	ENSG00000116016	18603805;
D006861	increase	F2R	ENSG00000181104	12419474;
D006861	increase	FAS	ENSG00000026103	12414654;
D006861	increase	FASLG	ENSG00000117560	16510162;
D006861	increase	FDFT1	ENSG00000079459	18032389;
D006861	increase	FDPS	ENSG00000160752	18032389;
D006861	increase	FDXR	ENSG00000161513	12414654;
D006861	increase	FGG	ENSG00000171557	12414654;
D006861	increase	FN1	ENSG00000115414	15147944;15086456;
D006861	increase	FOS	ENSG00000170345	9525474;12419474;186871
D006861	increase	FST	ENSG00000134363	18687144;
D006861	increase	FTL	ENSG00000087086	16707465;12414654;
D006861	increase	GADD45A	ENSG00000116717	16814109;
D006861	increase	GAPDH	ENSG00000111640	12419474;11295360;
D006861	increase	GCLM	ENSG00000023909	12414654;
D006861	increase	GDF15	ENSG00000130513	18687144;12414654;
D006861	increase	GLRX	ENSG00000173221	12414654;
D006861	increase	GRIN2C	ENSG00000161509	12414654;
D006861	increase	GSTP1	ENSG00000084207	15528046;15231573;12419474;15832819;15056853;15533597;
D006861	increase	HBEGF	ENSG00000113070	12676768;
D006861	increase	HIF1A	ENSG00000100644	18603805;15297373;14695184;
D006861	increase	HIF3A	ENSG00000124440	18603805;
D006861	increase	HMGCR	ENSG00000113161	12414654;18032389;
D006861	increase	HMGCS1	ENSG00000112972	18032389;
D006861	increase	HMOX1	ENSG00000100292	18687144;10218639;12414654;
D006861	increase	HRAS	ENSG00000174775	11008122;
D006861	increase	HSPA1A	ENSG00000232804	17513788;12414654;
D006861	increase	HSPA1B	ENSG00000232804	12414654;
D006861	increase	HSPA6	ENSG00000173110	12414654;
D006861	increase	HSPB1	ENSG00000106211	11295360;
D006861	increase	IER3	ENSG00000137331	12419474;18687144;
D006861	increase	IFNB1	ENSG00000171855	18322232;
D006861	increase	IGFBP5	ENSG00000115461	12414654;
D006861	increase	IL1B	ENSG00000125538	10802230;16351573;
D006861	increase	IL8	ENSG00000169429	9707512;18687144;122078
D006861	increase	INHBB	ENSG00000163083	12414654;
D006861	increase	INSIG1	ENSG00000186480	18032389;
D006861	increase	JUN	ENSG00000177606	12419474;12414654;15795422;
D006861	increase	LDLR	ENSG00000130164	12414654;18032389;
D006861	increase	LIF	ENSG00000128342	12414654;
D006861	increase	LRP6	ENSG00000070018	12414654;

D006861	increase	MAFG	ENSG00000197063	12414654;
D006861	increase	ME1	ENSG00000065833	12414654;
D006861	increase	MMP9	ENSG00000100985	15531749;
D006861	increase	MT1G	ENSG00000125144	12414654;
D006861	increase	MT1H	ENSG00000205358	12414654;
D006861	increase	MT1X	ENSG00000187193	12414654;
D006861	increase	MT2A	ENSG00000125148	12414654;
D006861	increase	MYC	ENSG00000136997	15761015;12419474;
D006861	increase	NDUFB8	ENSG00000166136	18603805;
D006861	increase	NOS3	ENSG00000164867	18234130;
D006861	increase	NQO1	ENSG00000181019	11008122;12414654;93433
D006861	increase	NR4A1	ENSG00000123358	12414654;
D006861	increase	OGG1	ENSG00000114026	19651184;
D006861	increase	OSGIN1	ENSG00000140961	12414654;
D006861	increase	PARK7	ENSG00000116288	11811539;18377993;
D006861	increase	PDIA3	ENSG00000167004	18603805;
D006861	increase	PGGT1B	ENSG00000164219	12414654;
D006861	increase	PGK1	ENSG00000102144	18603805;
D006861	increase	PLA2R1	ENSG00000153246	12414654;
D006861	increase	PPM1D	ENSG00000170836	12414654;
D006861	increase	PPP1R15A	ENSG00000087074	18687144;
D006861	increase	PPP2CB	ENSG00000104695	12414654;
D006861	increase	PRDX1	ENSG00000117450	11295360;11469800;
D006861	increase	PRDX2	ENSG00000167815	11295360;11469800;
D006861	increase	PRDX6	ENSG00000117592	11295360;14741336;
D006861	increase	PRKAA1	ENSG00000132356	15297373;
D006861	increase	PRNP	ENSG00000171867	12419474;
D006861	increase	PSMD12	ENSG00000197170	12414654;
D006861	increase	RPS6KB1	ENSG00000108443	19451193;18603805;
D006861	increase	S100P	ENSG00000163993	12414654;
D006861	increase	SC5DL	ENSG00000109929	18032389;
D006861	increase	SDC4	ENSG00000124145	18687144;
D006861	increase	SERPINB5	ENSG00000206075	12414654;
D006861	increase	SLAMF1	ENSG00000117090	12414654;
D006861	increase	SLC7A11	ENSG00000151012	12414654;
D006861	increase	SOD2	ENSG00000112096	8760145;9569045;
D006861	increase	SP1	ENSG00000185591	17108117;
D006861	increase	SQLE	ENSG00000104549	18032389;
D006861	increase	SREBF2	ENSG00000198911	18032389;
D006861	increase	TGFB1	ENSG00000105329	15147944;
D006861	increase	TNF	ENSG00000223952	16794257;10802230;11034997;16531283;
D006861	increase	TNFRSF10B	ENSG00000120889	18687144;
D006861	increase	TNFRSF1A	ENSG00000067182	9870925;
D006861	increase	TOM1	ENSG00000100284	12414654;
D006861	increase	TP53	ENSG00000141510	10531375;12419474;15242773;9989825;17555331;15795422;8943236;
D006861	increase	VDAC1	ENSG00000213585	18802751;
D006861	increase	ZFP36	ENSG00000128016	18687144;
D006886	increase	CCL2	ENSG00000108691	15139008;
D006886	increase	IL8	ENSG00000169429	15139008;
D006918	decrease	C13ORF34	ENSG00000136122	17374387;
D006918	decrease	CDCA8	ENSG00000134690	17374387;

D006918	decrease	CKS2	ENSG00000123975	17374387;
D006918	decrease	EDN3	ENSG00000124205	15020278;
D006918	decrease	KPNA2	ENSG00000182481	17374387;
D006918	decrease	PRC1	ENSG00000198901	17374387;
D006918	decrease	PSRC1	ENSG00000134222	17374387;
D006918	decrease	UBE2C	ENSG00000175063	17374387;
D006918	increase	BAX	ENSG00000087088	16005713;
D006918	increase	CCNA2	ENSG00000145386	11468187;
D006918	increase	CCNB1	ENSG00000134057	11468187;
D006918	increase	CCND1	ENSG00000110092	11468187;
D006918	increase	CCND2	ENSG00000118971	11468187;
D006918	increase	CCND3	ENSG00000112576	11468187;
D006918	increase	CCNE1	ENSG00000105173	11468187;
D006918	increase	CCNG1	ENSG00000113328	16005713;
D006918	increase	CDKN1A	ENSG00000124762	16005713;
D006918	increase	FAS	ENSG00000026103	16005713;
D006918	increase	MECOM	ENSG00000085276	16532439;
D006918	increase	RUNX1	ENSG00000159216	16532439;
D006918	increase	TP53	ENSG00000141510	16005713;
D007052	affect	ALOX12B	ENSG00000179477	17070997;
D007052	affect	ALOXE3	ENSG00000179148	17070997;
D007052	affect	ANXA1	ENSG00000135046	17070997;
D007052	affect	ANXA2	ENSG00000182718	18351690;
D007052	affect	ANXA3	ENSG00000138772	17070997;
D007052	affect	C3	ENSG00000125730	17070997;
D007052	affect	CACNA2D3	ENSG00000157445	17070997;
D007052	affect	CDH3	ENSG00000062038	17070997;
D007052	affect	CDSN	ENSG00000204539	17070997;
D007052	affect	CEACAM1	ENSG00000079385	17070997;
D007052	affect	CLU	ENSG00000120885	17070997;
D007052	affect	COL11A1	ENSG00000060718	17070997;
D007052	affect	CX3CL1	ENSG00000006210	17070997;
D007052	affect	DDX1	ENSG00000079785	18351690;
D007052	affect	DSC1	ENSG00000134765	17070997;
D007052	affect	ENO1	ENSG00000074800	18351690;
D007052	affect	EPHB6	ENSG00000106123	17070997;
D007052	affect	FRZB	ENSG00000162998	17070997;
D007052	affect	HPGD	ENSG00000164120	17070997;
D007052	affect	ITGBL1	ENSG00000198542	17070997;
D007052	affect	LDHB	ENSG00000111716	18351690;
D007052	affect	LGALS1	ENSG00000100097	18351690;
D007052	affect	MAPRE2	ENSG00000166974	17070997;
D007052	affect	NOS1	ENSG00000089250	17070997;
D007052	affect	NQO1	ENSG00000181019	17070997;
D007052	affect	PLA2G2A	ENSG00000188257	17070997;
D007052	affect	PML	ENSG00000140464	17070997;
D007052	affect	PPIA	ENSG00000196262	18351690;
D007052	affect	PRDX6	ENSG00000117592	18351690;
D007052	affect	PTGER3	ENSG00000050628	17070997;
D007052	affect	RAN	ENSG00000132341	18351690;
D007052	affect	SOCS3	ENSG00000184557	17070997;
D007052	affect	TMPRSS4	ENSG00000137648	17070997;
D007052	decrease	ANXA9	ENSG00000143412	17070997;

D007052	decrease	APP	ENSG00000142192	11700559;12777371;14586007;15817521;
D007052	decrease	CYP19A1	ENSG00000137869	15964185;
D007052	decrease	EPAS1	ENSG00000116016	12912967;
D007052	decrease	FABP4	ENSG00000170323	17070997;
D007052	decrease	FASN	ENSG00000169710	18351690;
D007052	decrease	HIF1A	ENSG00000100644	12912967;
D007052	decrease	INSM1	ENSG00000173404	17070997;
D007052	decrease	LTBR	ENSG00000111321	17070997;
D007052	decrease	LY9	ENSG00000122224	17070997;
D007052	decrease	P2RY2	ENSG00000175591	10496880;
D007052	decrease	PYY	ENSG00000131096	17070997;
D007052	decrease	SLC2A1	ENSG00000117394	12912967;
D007052	increase	ADAMTS8	ENSG00000134917	17070997;
D007052	increase	BACE1	ENSG00000186318	14586007;
D007052	increase	BCL2L1	ENSG00000171552	18348186;
D007052	increase	CCL2	ENSG00000108691	11230753;17070997;
D007052	increase	CCND1	ENSG00000110092	15489888;
D007052	increase	CD36	ENSG00000135218	12664607;
D007052	increase	CXCL10	ENSG00000169245	19020770;
D007052	increase	EGF	ENSG00000138798	16340751;
D007052	increase	ELAVL1	ENSG00000066044	18056468;
D007052	increase	F3	ENSG00000117525	17070997;
D007052	increase	FOSB	ENSG00000125740	17070997;
D007052	increase	GCLC	ENSG00000001084	11909699;
D007052	increase	GCLM	ENSG00000023909	11909699;
D007052	increase	GDF15	ENSG00000130513	15555568;
D007052	increase	HSPA1A	ENSG00000232804	16239242;
D007052	increase	ICAM1	ENSG00000090339	16769766;17931847;
D007052	increase	IFNG	ENSG00000111537	14586007;
D007052	increase	IL10	ENSG00000136634	19020770;
D007052	increase	IL1A	ENSG00000115008	11230753;17070997;
D007052	increase	IL6	ENSG00000136244	17070997;
D007052	increase	MAL	ENSG00000172005	17070997;
D007052	increase	MAPK11	ENSG00000185386	18056468;
D007052	increase	MAPK14	ENSG00000112062	18056468;
D007052	increase	MAPKAPK2	ENSG00000162889	18056468;
D007052	increase	MAPKAPK3	ENSG00000114738	18056468;
D007052	increase	MCL1	ENSG00000143384	18348186;
D007052	increase	MMP1	ENSG00000196611	16580899;
D007052	increase	MMP3	ENSG00000149968	16580899;
D007052	increase	MMP9	ENSG00000100985	16340751;
D007052	increase	MTA2	ENSG00000149480	17070997;
D007052	increase	NGFR	ENSG00000064300	18056468;17447067;17409433;
D007052	increase	PGAM1	ENSG00000171314	18351690;
D007052	increase	PLAU	ENSG00000122861	16340751;
D007052	increase	PLEC	ENSG00000178209	18351690;
D007052	increase	PPP1CA	ENSG00000172531	18348186;
D007052	increase	PRDX2	ENSG00000167815	18351690;
D007052	increase	PTGS2	ENSG00000073756	15705740;15489888;18848576;17164136;16678543;
D007052	increase	RELA	ENSG00000173039	10328874;9890559;15489888;10602496;

D007052	increase	SAT1	ENSG00000130066	16262603;
D007052	increase	SELE	ENSG00000007908	16769766;
D007052	increase	SOD2	ENSG00000112096	17070997;
D007052	increase	THBS1	ENSG00000137801	18261746;
D007052	increase	TNC	ENSG00000041982	17070997;
D007052	increase	TNF	ENSG00000223952	14586007;10328874;9890559;15489888;10602496;16769766;11753679;
D007052	increase	TNFRSF21	ENSG00000146072	11753679;
D007052	increase	TPI1	ENSG00000111669	18351690;
D007052	increase	VCAM1	ENSG00000162692	16769766;
D007099	decrease	SLC2A3	ENSG00000059804	17567588;15342952;
D007099	decrease	TAGLN	ENSG00000149591	17567588;15342952;
D007099	increase	ASAH1	ENSG00000104763	17567588;15342952;
D007099	increase	ASNS	ENSG00000070669	17567588;15342952;
D007099	increase	C10ORF10	ENSG00000165507	17567588;15342952;
D007099	increase	DNAJC5	ENSG00000101152	11257428;
D007099	increase	FABP1	ENSG00000163586	17567588;15342952;
D007099	increase	FNDC4	ENSG00000115226	17567588;15342952;
D007099	increase	GDPD3	ENSG00000102886	17567588;15342952;
D007099	increase	HPN	ENSG00000105707	17567588;15342952;
D007099	increase	INHBE	ENSG00000139269	17567588;15342952;
D007099	increase	LSS	ENSG00000160285	17567588;15342952;
D007099	increase	NR0B2	ENSG00000131910	15342952;
D007099	increase	NUPR1	ENSG00000176046	17567588;15342952;
D007099	increase	SERPINA3	ENSG00000196136	17567588;15342952;
D007099	increase	WIPI1	ENSG00000070540	17567588;15342952;
D007213	affect	IL12A	ENSG00000168811	11024006;
D007213	affect	IL12B	ENSG00000113302	11024006;
D007213	decrease	ABCA3	ENSG00000167972	18360721;
D007213	decrease	ABCC1	ENSG00000103222	15066675;15715484;12142058;16636798;17940500;15916776;15581599;18360721;16331495;
D007213	decrease	ADAMTS1	ENSG00000154734	16984733;
D007213	decrease	ADNP	ENSG00000101126	16984733;
D007213	decrease	AEBP2	ENSG00000139154	16984733;
D007213	decrease	AKT1	ENSG00000142208	17654042;17341418;
D007213	decrease	ALS2CR4	ENSG00000155755	16984733;
D007213	decrease	AMD1	ENSG00000123505	16984733;
D007213	decrease	ANKRD13C	ENSG00000118454	16984733;
D007213	decrease	ARID1A	ENSG00000117713	16984733;
D007213	decrease	ARID5B	ENSG00000150347	16984733;
D007213	decrease	ATP8B1	ENSG00000081923	16984733;
D007213	decrease	BAD	ENSG00000002330	18678619;17409426;
D007213	decrease	BAZ1A	ENSG00000198604	16984733;
D007213	decrease	BCL2A1	ENSG00000140379	17054584;
D007213	decrease	BCL2	ENSG00000171791	11721434;10785260;18678619;
D007213	decrease	BCL2L1	ENSG00000171552	11062132;16572323;
D007213	decrease	BIRC5	ENSG00000089685	16707021;15248904;17270149;
D007213	decrease	BMP2K	ENSG00000138756	16984733;
D007213	decrease	C10ORF10	ENSG00000165507	17401462;
D007213	decrease	C12ORF35	ENSG00000174718	16984733;

D007213	decrease	CCR3	ENSG00000183625	11980903;
D007213	decrease	CDK2	ENSG00000123374	14566837;15786552;
D007213	decrease	CDK4	ENSG00000135446	15786552;
D007213	decrease	CHD1	ENSG00000153922	16984733;
D007213	decrease	CITED2	ENSG00000164442	16984733;
D007213	decrease	COL1A1	ENSG00000108821	8971655;
D007213	decrease	CPSF6	ENSG00000111605	16984733;
D007213	decrease	CREBL2	ENSG00000111269	16984733;
D007213	decrease	CSPP1	ENSG00000104218	16984733;
D007213	decrease	CTNNB1	ENSG00000168036	15963497;11756231;15188006;12813129;
D007213	decrease	CYP19A1	ENSG00000137869	15964185;
D007213	decrease	DIDO1	ENSG00000101191	16984733;
D007213	decrease	DNAJA2	ENSG00000069345	16984733;
D007213	decrease	DST	ENSG00000151914	16984733;
D007213	decrease	DUSP6	ENSG00000139318	16984733;
D007213	decrease	E2F3	ENSG00000112242	16984733;
D007213	decrease	EFR3A	ENSG00000132294	16984733;
D007213	decrease	EIF1AX	ENSG00000173674	16984733;
D007213	decrease	F3	ENSG00000117525	20200314;
D007213	decrease	FBXW2	ENSG00000119402	16984733;
D007213	decrease	FZD7	ENSG00000155760	16984733;
D007213	decrease	GBP1	ENSG00000117228	16984733;
D007213	decrease	GLS	ENSG00000115419	16984733;
D007213	decrease	GLUL	ENSG00000135821	17401462;
D007213	decrease	HACE1	ENSG00000085382	16984733;
D007213	decrease	IL1B	ENSG00000125538	8971655;12512699;15705740;10772282;10388637;
D007213	decrease	IL6ST	ENSG00000134352	16984733;
D007213	decrease	IL7	ENSG00000104432	16984733;
D007213	decrease	IL8	ENSG00000169429	16984733;
D007213	decrease	IPO5	ENSG00000065150	16984733;
D007213	decrease	ISG20L2	ENSG00000143319	16984733;
D007213	decrease	KCTD12	ENSG00000178695	16984733;
D007213	decrease	LAMC1	ENSG00000135862	15561105;
D007213	decrease	LYN	ENSG00000249529	16984733;
D007213	decrease	MAP4K4	ENSG00000071054	16984733;
D007213	decrease	MBNL1	ENSG00000152601	16984733;
D007213	decrease	MMP2	ENSG00000087245	11719597;
D007213	decrease	MMP9	ENSG00000100985	11719597;
D007213	decrease	NHSL1	ENSG00000135540	16984733;
D007213	decrease	NPM1	ENSG00000181163	10619186;
D007213	decrease	NR2F2	ENSG00000185551	16984733;
D007213	decrease	NRCAM	ENSG00000091129	16984733;
D007213	decrease	NUFIP2	ENSG00000108256	16984733;
D007213	decrease	PANK1	ENSG00000152782	16984733;
D007213	decrease	PCNA	ENSG00000132646	17054584;
D007213	decrease	PJA2	ENSG00000198961	16984733;
D007213	decrease	PKP4	ENSG00000144283	16984733;
D007213	decrease	PLK2	ENSG00000145632	16984733;
D007213	decrease	PPP4R2	ENSG00000163605	16984733;
D007213	decrease	PRKCI	ENSG00000163558	16984733;
D007213	decrease	PRPF4B	ENSG00000112739	16984733;
D007213	decrease	PTGER2	ENSG00000125384	17555711;

D007213	decrease	PTPN12	ENSG00000127947	16984733;
D007213	decrease	RAD23B	ENSG00000119318	16984733;
D007213	decrease	RAPGEF6	ENSG00000158987	16984733;
D007213	decrease	RAPH1	ENSG00000173166	16984733;
D007213	decrease	RBM15	ENSG00000162775	16984733;
D007213	decrease	REV1	ENSG00000135945	16984733;
D007213	decrease	RNF144B	ENSG00000137393	16984733;
D007213	decrease	RNF145	ENSG00000145860	16984733;
D007213	decrease	ROCK2	ENSG00000134318	16984733;
D007213	decrease	SEC63	ENSG00000025796	16984733;
D007213	decrease	SELL	ENSG00000188404	11980903;
D007213	decrease	SFRS6	ENSG00000124193	16984733;
D007213	decrease	SLC16A1	ENSG00000155380	19023563;16984733;
D007213	decrease	SOCS5	ENSG00000171150	16984733;
D007213	decrease	SON	ENSG00000159140	16984733;
D007213	decrease	SOX7	ENSG00000171056	16984733;
D007213	decrease	SPATA18	ENSG00000163071	16984733;
D007213	decrease	SPATS1	ENSG00000249481	17401462;
D007213	decrease	SR140	ENSG00000163714	16984733;
D007213	decrease	STAT1	ENSG00000115415	15631656;16572323;
D007213	decrease	STC1	ENSG00000159167	17401462;
D007213	decrease	TDG	ENSG00000139372	16984733;
D007213	decrease	TFRC	ENSG00000072274	16984733;
D007213	decrease	TGS1	ENSG00000137574	16984733;
D007213	decrease	THBD	ENSG00000178726	16984733;
D007213	decrease	TMEFF2	ENSG00000144339	16984733;
D007213	decrease	TMTC1	ENSG00000133687	16984733;
D007213	decrease	TOB1	ENSG00000141232	16984733;
D007213	decrease	TPD52L1	ENSG00000111907	16984733;
D007213	decrease	TROVE2	ENSG00000116747	16984733;
D007213	decrease	TUBGCP3	ENSG00000126216	16984733;
D007213	decrease	TXN	ENSG00000136810	17654042;
D007213	decrease	TXNIP	ENSG00000117289	16984733;
D007213	decrease	UBLCP1	ENSG00000164332	16984733;
D007213	decrease	UBR5	ENSG00000104517	16984733;
D007213	decrease	UGCG	ENSG00000148154	16984733;
D007213	decrease	UGT3A1	ENSG00000145626	16984733;
D007213	decrease	WAPAL	ENSG00000062650	16984733;
D007213	decrease	WISP1	ENSG00000104415	17054584;
D007213	decrease	YOD1	ENSG00000180667	16984733;
D007213	decrease	YTHDF2	ENSG00000198492	16984733;
D007213	decrease	YTHDF3	ENSG00000185728	16984733;
D007213	decrease	YWHAE	ENSG00000108953	18678619;17409426;
D007213	decrease	ZBTB11	ENSG00000066422	16984733;
D007213	decrease	ZBTB41	ENSG00000177888	16984733;
D007213	decrease	ZBTB44	ENSG00000196323	16984733;
D007213	decrease	ZC3H13	ENSG00000123200	16984733;
D007213	decrease	ZNF148	ENSG00000163848	16984733;
D007213	decrease	ZNF238	ENSG00000179456	16984733;
D007213	decrease	ZNF398	ENSG00000197024	16984733;
D007213	decrease	ZNHIT6	ENSG00000117174	16984733;
D007213	increase	ABCC3	ENSG00000108846	16636798;12704183;11820781;
D007213	increase	ACRBP	ENSG00000111644	16984733;

D007213	increase	ADORA3	ENSG00000121933	17568578;
D007213	increase	ALDH1L2	ENSG00000136010	17401462;
D007213	increase	AMIGO2	ENSG00000139211	16984733;
D007213	increase	ANGPTL4	ENSG00000167772	16984733;
D007213	increase	ANXA5	ENSG00000164111	18678619;
D007213	increase	APC	ENSG00000134982	15963497;
D007213	increase	APP	ENSG00000142192	11700559;16111494;14586007;
D007213	increase	ARL4C	ENSG00000188042	16984733;
D007213	increase	ARRDC3	ENSG00000113369	16984733;
D007213	increase	ASNS	ENSG00000070669	17401462;
D007213	increase	ATP5I	ENSG00000169020	16984733;
D007213	increase	BACE1	ENSG00000186318	14586007;
D007213	increase	BAX	ENSG00000087088	11062132;11005569;
D007213	increase	BIRC3	ENSG00000023445	17873517;16984733;
D007213	increase	BTG1	ENSG00000133639	16984733;
D007213	increase	C17ORF95	ENSG00000181038	16984733;
D007213	increase	C6ORF145	ENSG00000168994	16984733;
D007213	increase	C6ORF52	ENSG00000137434	16984733;
D007213	increase	CASP3	ENSG00000164305	17341418;17873517;12485869;15804060;15265368;15812552;
D007213	increase	CASP8	ENSG00000064012	15812552;15265368;
D007213	increase	CBS	ENSG00000160200	17401462;
D007213	increase	CCDC50	ENSG00000152492	16984733;
D007213	increase	CCND1	ENSG00000110092	15489888;11756231;11313997;15188006;12606626;17270149;
D007213	increase	CCNG2	ENSG00000138764	16984733;
D007213	increase	CCPG1	ENSG00000214882	17401462;
D007213	increase	CDH1	ENSG00000039068	15963497;
D007213	increase	CDKN1A	ENSG00000124762	15190207;14566837;15786552;
D007213	increase	CDKN1B	ENSG00000111276	16984733;11260862;
D007213	increase	CFLAR	ENSG00000003402	17873517;
D007213	increase	CHAC1	ENSG00000128965	17401462;
D007213	increase	CSF2	ENSG00000164400	16984733;
D007213	increase	CTH	ENSG00000116761	17401462;
D007213	increase	DDIT3	ENSG00000175197	17341418;
D007213	increase	DNAJC4	ENSG00000110011	16984733;
D007213	increase	DUSP1	ENSG00000120129	16984733;
D007213	increase	EDN1	ENSG00000078401	16133044;16984733;
D007213	increase	EGR1	ENSG00000120738	11595463;16227405;15509713;
D007213	increase	FOXQ1	ENSG00000164379	16984733;
D007213	increase	GADD45B	ENSG00000099860	16984733;
D007213	increase	GATA6	ENSG00000141448	16984733;
D007213	increase	GCLC	ENSG00000001084	11909699;11820781;
D007213	increase	GCLM	ENSG00000023909	11909699;
D007213	increase	GDF15	ENSG00000130513	15509713;17401462;16984733;18076062;15555568;15180942;
D007213	increase	HPGD	ENSG00000164120	11237475;14736730;11352223;
D007213	increase	HSPA1A	ENSG00000232804	16239242;

D007213	increase	HSPA5	ENSG00000044574	17341418;
D007213	increase	IFNG	ENSG00000111537	14586007;16407166;
D007213	increase	IL11	ENSG00000095752	16984733;
D007213	increase	IL6	ENSG00000136244	16111494;15379866;10388637;10772282;
D007213	increase	ITGAM	ENSG00000169896	11980903;
D007213	increase	JUNB	ENSG00000171223	16984733;
D007213	increase	JUN	ENSG00000177606	16984733;15804060;
D007213	increase	KEAP1	ENSG00000079999	11909699;
D007213	increase	KLF4	ENSG00000136826	16984733;
D007213	increase	LBH	ENSG00000213626	16984733;
D007213	increase	LTA	ENSG00000226979	17873517;
D007213	increase	MAP2K4	ENSG00000065559	15804060;
D007213	increase	MYC	ENSG00000136997	11756231;10403534;11260862;
D007213	increase	NEDD9	ENSG00000111859	16984733;
D007213	increase	NET1	ENSG00000173848	16984733;
D007213	increase	NGF	ENSG00000134259	16984733;
D007213	increase	NKX3-1	ENSG00000167034	16984733;
D007213	increase	NME1	ENSG00000239672	12192599;
D007213	increase	NR4A1	ENSG00000123358	10919358;
D007213	increase	NT5E	ENSG00000135318	17568578;
D007213	increase	NUAK1	ENSG00000074590	16984733;
D007213	increase	PLAU	ENSG00000122861	16984733;
D007213	increase	PMAIP1	ENSG00000141682	17401462;
D007213	increase	PMEPA1	ENSG00000124225	16984733;
D007213	increase	PPARD	ENSG00000112033	10555149;18678619;17341418;17409426;
D007213	increase	PPARG	ENSG00000132170	12485869;17602619;9013583;16407166;12065695;12606626;17341418;
D007213	increase	PRKCB	ENSG00000166501	10702201;
D007213	increase	PRKCE	ENSG00000171132	10702201;
D007213	increase	PRKCH	ENSG00000027075	10702201;
D007213	increase	PSPH	ENSG00000146733	17401462;
D007213	increase	PTGS1	ENSG00000095303	12142058;16141368;16354411;14736730;15482327;
D007213	increase	PTGS2	ENSG00000073756	12142058;16690318;15705740;15489888;10866311;10866999;15482327;
D007213	increase	RHOB	ENSG00000143878	16984733;
D007213	increase	SALL1	ENSG00000103449	16984733;
D007213	increase	SARS	ENSG00000031698	17401462;
D007213	increase	SAT1	ENSG00000130066	16262603;
D007213	increase	SC4MOL	ENSG00000052802	17401462;
D007213	increase	SCRG1	ENSG00000164106	17401462;
D007213	increase	SERPINE1	ENSG00000106366	16320596;16984733;
D007213	increase	SLC19A2	ENSG00000117479	16984733;
D007213	increase	SLC3A2	ENSG00000168003	17401462;
D007213	increase	SLC7A11	ENSG00000151012	17401462;
D007213	increase	SMAD7	ENSG00000101665	16984733;
D007213	increase	SNAI2	ENSG00000019549	16984733;
D007213	increase	SOX18	ENSG00000203883	16984733;
D007213	increase	SPANXA1	ENSG00000203926	16984733;

D007213	increase	THBS1	ENSG00000137801	16984733;18261746;16227405;
D007213	increase	TIMP1	ENSG00000102265	11719597;
D007213	increase	TMEM49	ENSG00000062716	16984733;
D007213	increase	TMSB4X	ENSG00000205542	15292456;
D007213	increase	TNF	ENSG00000223952	14586007;15489888;11753679;16407166;15379866;10772282;
D007213	increase	TNFRSF21	ENSG00000146072	11753679;
D007213	increase	TP53	ENSG00000141510	11260862;10403534;
D007213	increase	TRIB3	ENSG00000101255	17401462;
D007213	increase	TSC22D1	ENSG00000102804	16984733;
D007213	increase	TUBE1	ENSG00000074935	17401462;
D007213	increase	UCP2	ENSG00000175567	16984733;
D007213	increase	UPP1	ENSG00000183696	16984733;
D007213	increase	VEGFA	ENSG00000112715	11595463;15637740;
D007213	increase	XBP1	ENSG00000100219	17401462;
D007213	increase	XIAP	ENSG00000101966	17873517;
D007213	increase	ZC3HAV1	ENSG00000105939	16984733;
D007213	increase	ZC3HAV1L	ENSG00000146858	16984733;
D007213	increase	ZEB1	ENSG00000148516	16984733;
D007220	increase	PGR	ENSG00000082175	18262749;
D007306	increase	TRIM21	ENSG00000132109	9833039;
D007455	increase	CLDN5	ENSG00000184113	16514648;
D007455	increase	OCLN	ENSG00000197822	16514648;
D007455	increase	SLC5A5	ENSG00000105641	12602914;17332617;17967635;
D007455	increase	TF	ENSG00000091513	16772442;
D007455	increase	TFRC	ENSG00000072274	16772442;
D007455	increase	TJP1	ENSG00000104067	16514648;
D007464	decrease	APP	ENSG00000142192	16648635;
D007464	decrease	AR	ENSG00000169083	17308104;
D007464	increase	EPO	ENSG00000130427	16973622;
D007464	increase	HIF1A	ENSG00000100644	16973622;
D007464	increase	MMP2	ENSG00000087245	16648635;
D007464	increase	MMP3	ENSG00000149968	16648635;
D007464	increase	VEGFA	ENSG00000112715	16973622;
D007501	decrease	HAMP	ENSG00000105697	16460831;
D007501	decrease	IL6	ENSG00000136244	16460831;
D007501	decrease	PON1	ENSG00000005421	16052486;
D007501	increase	CALR	ENSG00000179218	11500943;
D007501	increase	CYP2A6	ENSG00000198077	11927840;
D007501	increase	EPAS1	ENSG00000116016	17973296;
D007501	increase	HIF1A	ENSG00000100644	16877034;17973296;19729006;17364964;
D007501	increase	TFRC	ENSG00000072274	16988136;17101452;16564538;
D007502	increase	VEGFA	ENSG00000112715	15075239;
D007529	affect	ALDH1A3	ENSG00000184254	16365062;
D007529	affect	ANP32A	ENSG00000140350	16365062;
D007529	affect	AOX1	ENSG00000138356	16365062;
D007529	affect	APOBEC3G	ENSG00000239713	16365062;
D007529	affect	ARHGEF1	ENSG00000076928	16365062;
D007529	affect	COL13A1	ENSG00000197467	16365062;
D007529	affect	COL1A1	ENSG00000108821	16365062;

D007529	affect	COL6A3	ENSG00000163359	16365062;
D007529	affect	DNAJB6	ENSG00000105993	16365062;
D007529	affect	DPP4	ENSG00000197635	16365062;
D007529	affect	F12	ENSG00000131187	16365062;
D007529	affect	GSTZ1	ENSG00000100577	16365062;
D007529	affect	HIST1H1C	ENSG00000187837	16365062;
D007529	affect	HIST1H2AB	ENSG00000168274	16365062;
D007529	affect	HIST1H3D	ENSG00000197153	16365062;
D007529	affect	HIST1H3H	ENSG00000197153	16365062;
D007529	affect	HIST2H2AA	ENSG00000203812	16365062;
D007529	affect	HIST2H2BE	ENSG00000184678	16365062;
D007529	affect	HPGD	ENSG00000164120	16365062;
D007529	affect	IGFBP3	ENSG00000146674	16365062;
D007529	affect	IGFBP5	ENSG00000115461	16365062;
D007529	affect	IL27RA	ENSG00000104998	16365062;
D007529	affect	IRF7	ENSG00000185507	16365062;
D007529	affect	ITGB2	ENSG00000160255	16365062;
D007529	affect	JUNB	ENSG00000171223	16365062;
D007529	affect	LDLR	ENSG00000130164	16365062;
D007529	affect	LIF	ENSG00000128342	16365062;
D007529	affect	MGST3	ENSG00000143198	16365062;
D007529	affect	NOV	ENSG00000136999	16365062;
D007529	affect	NOX5	ENSG00000137808	16365062;
D007529	affect	NPC2	ENSG00000119655	16365062;
D007529	affect	NTSR1	ENSG00000101188	16365062;
D007529	affect	PHLDA1	ENSG00000139289	16365062;
D007529	affect	PPAP2B	ENSG00000162407	16365062;
D007529	affect	PTX3	ENSG00000163661	16365062;
D007529	affect	PXN	ENSG00000089159	16365062;
D007529	affect	SPARC	ENSG00000113140	16365062;
D007529	affect	SQLE	ENSG00000104549	16365062;
D007529	affect	TFCP2	ENSG00000135457	16365062;
D007529	affect	TM7SF2	ENSG00000149809	16365062;
D007529	affect	TUFT1	ENSG00000143367	16365062;
D007529	decrease	APOBEC3B	ENSG00000179750	17374662;
D007529	decrease	ARG2	ENSG00000081181	17374662;
D007529	decrease	BCL2	ENSG00000171791	18640131;
D007529	decrease	BCL2L1	ENSG00000171552	18640131;
D007529	decrease	BTG1	ENSG00000133639	17374662;
D007529	decrease	CAMKK2	ENSG00000110931	17374662;
D007529	decrease	CCNB2	ENSG00000157456	17374662;
D007529	decrease	CCND1	ENSG00000110092	18640131;
D007529	decrease	CDKN2C	ENSG00000123080	17374662;
D007529	decrease	CEBPD	ENSG00000221869	17374662;
D007529	decrease	CLDN3	ENSG00000165215	17374662;
D007529	decrease	DHFR	ENSG00000228716	17374662;
D007529	decrease	DUSP4	ENSG00000120875	17374662;
D007529	decrease	EIF3C	ENSG00000184110	17374662;
D007529	decrease	ENDOD1	ENSG00000149218	17374662;
D007529	decrease	ENDOG	ENSG00000167136	17374662;
D007529	decrease	FN1	ENSG00000115414	16365062;
D007529	decrease	FST	ENSG00000134363	16365062;
D007529	decrease	GALNT7	ENSG00000109586	17374662;
D007529	decrease	HEBP2	ENSG00000051620	17374662;

D007529	decrease	IL8	ENSG00000169429	16365062;
D007529	decrease	INHBA	ENSG00000122641	16365062;
D007529	decrease	IQGAP2	ENSG00000145703	17374662;
D007529	decrease	KIF2C	ENSG00000142945	17374662;
D007529	decrease	KIF4A	ENSG00000090889	17374662;
D007529	decrease	KLK2	ENSG00000167751	17374662;
D007529	decrease	KLK3	ENSG00000142515	17374662;
D007529	decrease	MAF	ENSG00000178573	17374662;
D007529	decrease	MAOA	ENSG00000189221	17374662;
D007529	decrease	MMP13	ENSG00000137745	16365062;
D007529	decrease	NKX3-1	ENSG00000167034	17374662;
D007529	decrease	NOTCH1	ENSG00000148400	18640131;
D007529	decrease	PPAP2A	ENSG00000067113	17374662;
D007529	decrease	PRC1	ENSG00000198901	17374662;
D007529	decrease	RAC3	ENSG00000169750	17374662;
D007529	decrease	RACGAP1	ENSG00000161800	17374662;
D007529	decrease	SAP30	ENSG00000164105	17374662;
D007529	decrease	SLC43A1	ENSG00000149150	17374662;
D007529	decrease	TM4SF1	ENSG00000169908	16365062;17374662;
D007529	decrease	TOP2A	ENSG00000131747	17374662;
D007529	decrease	TUBB	ENSG00000229684	17374662;
D007529	decrease	UAP1	ENSG00000117143	17374662;
D007529	increase	ACSL3	ENSG00000123983	16365062;
D007529	increase	ADAM10	ENSG00000137845	17374662;
D007529	increase	ASPH	ENSG00000198363	17374662;
D007529	increase	ATP1B1	ENSG00000143153	17374662;
D007529	increase	ATP2B1	ENSG00000070961	17374662;
D007529	increase	BAMBI	ENSG00000095739	17374662;
D007529	increase	CDKN1A	ENSG00000124762	16365062;17374662;
D007529	increase	CDKN1B	ENSG00000111276	17374662;
D007529	increase	DNAJB9	ENSG00000128590	17374662;
D007529	increase	DPYSL2	ENSG00000092964	17374662;
D007529	increase	EHHADH	ENSG00000113790	17374662;
D007529	increase	FADS2	ENSG00000134824	17374662;
D007529	increase	FDFT1	ENSG00000079459	17374662;
D007529	increase	FOXO3	ENSG00000118689	17374662;
D007529	increase	FTH1	ENSG00000167996	17374662;
D007529	increase	G6PD	ENSG00000160211	17374662;
D007529	increase	GALNT10	ENSG00000164574	17374662;
D007529	increase	GCA	ENSG00000115271	17374662;
D007529	increase	GCLM	ENSG00000023909	17374662;
D007529	increase	GSTK1	ENSG00000250907	17374662;
D007529	increase	HIBCH	ENSG00000198130	17374662;
D007529	increase	HMGCS1	ENSG00000112972	17374662;
D007529	increase	IDI1	ENSG00000067064	17374662;
D007529	increase	INSIG1	ENSG00000186480	16365062;17374662;
D007529	increase	ITM2B	ENSG00000136156	17374662;
D007529	increase	JUND	ENSG00000130522	17374662;
D007529	increase	JUN	ENSG00000177606	17374662;
D007529	increase	LSS	ENSG00000160285	17374662;
D007529	increase	ME1	ENSG00000065833	17374662;
D007529	increase	NQO1	ENSG00000181019	16365062;17374662;
D007529	increase	PANK3	ENSG00000120137	17374662;
D007529	increase	PGAP1	ENSG00000197121	17374662;

D007529	increase	PGD	ENSG00000142657	17374662;
D007529	increase	PIK3C2A	ENSG00000011405	17374662;
D007529	increase	PIR	ENSG00000087842	17374662;
D007529	increase	PPP1R2	ENSG00000184203	17374662;
D007529	increase	RDX	ENSG00000137710	17374662;
D007529	increase	SCD	ENSG00000099194	16365062;17374662;
D007529	increase	SELENBP1	ENSG00000143416	17374662;
D007529	increase	SESN1	ENSG00000080546	17374662;
D007529	increase	SQSTM1	ENSG00000161011	16365062;17374662;
D007529	increase	TAX1BP1	ENSG00000106052	17374662;
D007529	increase	UGDH	ENSG00000109814	17374662;
D007530	affect	APP	ENSG00000142192	17287498;
D007530	increase	BACE1	ENSG00000186318	17287498;
D007530	increase	NCSTN	ENSG00000162736	17287498;
D007538	increase	CYP2E1	ENSG00000130649	12668988;17950035;80747
D007538	increase	PLOD2	ENSG00000152952	15997088;
D007544	increase	CDKN1A	ENSG00000124762	15190207;
D007545	decrease	CYP1A1	ENSG00000140465	15627480;
D007545	decrease	FN1	ENSG00000115414	11821714;
D007545	decrease	TGFB1	ENSG00000105329	11821714;
D007545	increase	CCL3	ENSG00000006075	12538206;
D007545	increase	IL8	ENSG00000169429	9258252;
D007545	increase	MMP9	ENSG00000100985	16574429;
D007545	increase	TNF	ENSG00000223952	8096834;8780174;
D007548	decrease	EDN1	ENSG00000078401	15719745;
D007548	increase	NOS3	ENSG00000164867	15719745;
D007550	decrease	SILV	ENSG00000185664	8043013;
D007550	decrease	TYR	ENSG00000077498	8043013;
D007550	decrease	TYRP1	ENSG00000107165	8043013;
D007631	decrease	CDH1	ENSG00000039068	11695219;
D007631	decrease	CTNNB1	ENSG00000168036	11695219;
D007631	increase	CYP2B6	ENSG00000197408	15548381;
D007631	increase	CYP3A4	ENSG00000160868	15548381;
D007654	decrease	IL4	ENSG00000113520	15284827;
D007654	decrease	IL5	ENSG00000113525	15284827;
D007654	decrease	SLC2A3	ENSG00000059804	17567588;15342952;
D007654	increase	ASNS	ENSG00000070669	15342952;
D007654	increase	C10ORF10	ENSG00000165507	15342952;
D007654	increase	FABP1	ENSG00000163586	17567588;15342952;
D007654	increase	FNDC4	ENSG00000115226	17567588;15342952;
D007654	increase	GDPD3	ENSG00000102886	15342952;
D007654	increase	HPN	ENSG00000105707	15342952;
D007654	increase	INHBE	ENSG00000139269	17567588;15342952;
D007654	increase	LSS	ENSG00000160285	17567588;15342952;
D007654	increase	NR0B2	ENSG00000131910	15342952;
D007654	increase	NUPR1	ENSG00000176046	17567588;15342952;
D007654	increase	SERPINA3	ENSG00000196136	15342952;
D007654	increase	WIPI1	ENSG00000070540	15342952;
D007660	increase	GCLC	ENSG00000001084	11909699;
D007660	increase	GCLM	ENSG00000023909	11909699;
D007660	increase	NGFR	ENSG00000064300	17409433;
D007832	decrease	MAPT	ENSG00000186868	16930453;
D007854	increase	IL8	ENSG00000169429	18464055;
D007975	decrease	HSD11B2	ENSG00000176387	18032417;

D007975	increase	BAX	ENSG00000087088	15970427;
D007975	increase	MCL1	ENSG00000143384	15970427;
D007978	increase	ALPL	ENSG00000162551	18500657;
D007978	increase	ENPP1	ENSG00000197594	18500657;
D007978	increase	PHEX	ENSG00000102174	18500657;
D007978	increase	PTGS2	ENSG00000073756	15047127;
D008012	decrease	IL6	ENSG00000136244	16378115;
D008044	increase	CYP1B1	ENSG00000138061	16040568;
D008050	increase	IFNG	ENSG00000111537	16299289;
D008050	increase	IL12A	ENSG00000168811	16299289;
D008050	increase	IL12B	ENSG00000113302	16299289;
D008050	increase	IL15	ENSG00000164136	16299289;
D008050	increase	IL18	ENSG00000150782	16299289;
D008050	increase	IL8	ENSG00000169429	16299289;
D008055	decrease	NOS3	ENSG00000164867	16891912;
D008055	increase	OLR1	ENSG00000173391	16891912;
D008063	decrease	BCL2	ENSG00000171791	16990509;
D008063	decrease	NFE2L2	ENSG00000116044	18258206;17197552;
D008063	increase	CD86	ENSG00000114013	19033392;
D008063	increase	GCLC	ENSG00000001084	16249512;17940886;
D008063	increase	GCLM	ENSG00000023909	16249512;
D008063	increase	HMOX1	ENSG00000100292	19033392;
D008063	increase	INS	ENSG00000129965	16505238;
D008063	increase	KEAP1	ENSG00000079999	19033392;
D008063	increase	NQO1	ENSG00000181019	17940886;19033392;
D008070	affect	CYBA	ENSG00000051523	18414230;
D008070	decrease	ABCB11	ENSG00000073734	15205115;
D008070	decrease	ABCC2	ENSG00000023839	15205115;
D008070	decrease	CTGF	ENSG00000118523	17965732;
D008070	decrease	LTC4S	ENSG00000213316	12574384;
D008070	decrease	SELL	ENSG00000188404	12734376;
D008070	decrease	SLC10A1	ENSG00000100652	15205115;
D008070	increase	ADORA2A	ENSG00000128271	16387843;
D008070	increase	ADRB2	ENSG00000169252	9258252;
D008070	increase	BCL2A1	ENSG00000140379	15860229;
D008070	increase	BIRC3	ENSG00000023445	16154993;15454145;
D008070	increase	CCL20	ENSG00000115009	15860229;
D008070	increase	CCL2	ENSG00000108691	17529908;16317058;15196211;15788441;
D008070	increase	CCL3	ENSG00000006075	12818968;17529908;19211157;
D008070	increase	CCL4	ENSG00000129277	17529908;19020770;15788441;19211157;
D008070	increase	CD14	ENSG00000170458	18180316;12176910;
D008070	increase	CD40	ENSG00000101017	15684433;16317058;16299289;
D008070	increase	CD80	ENSG00000121594	16299289;
D008070	increase	CD83	ENSG00000112149	17698565;16299289;
D008070	increase	CD86	ENSG00000114013	17698565;16299289;
D008070	increase	CTNNB1	ENSG00000168036	11739494;
D008070	increase	CXCL10	ENSG00000169245	19020770;17529908;15860229;
D008070	increase	CXCL1	ENSG00000163739	14997032;
D008070	increase	CXCL2	ENSG00000081041	10718115;
D008070	increase	CYBB	ENSG00000165168	17855475;

D008070	increase	DDX58	ENSG00000107201	16303604;
D008070	increase	EHD1	ENSG00000110047	15860229;
D008070	increase	FGF7	ENSG00000140285	14962112;11842940;
D008070	increase	FOS	ENSG00000170345	14572605;18180316;
D008070	increase	GADD45B	ENSG00000099860	15860229;
D008070	increase	HRH2	ENSG00000113749	10569698;
D008070	increase	ICAM1	ENSG00000090339	18414230;16317058;15358673;15860229;10328874;
D008070	increase	IFIT1	ENSG00000185745	15860229;
D008070	increase	IFIT2	ENSG00000119922	15860229;
D008070	increase	IFIT3	ENSG00000119917	15860229;
D008070	increase	IFITM1	ENSG00000185885	15860229;
D008070	increase	IFNG	ENSG00000111537	15779068;15379866;10569698;16713974;17399992;19020770;15358692;9256163;16380391;
D008070	increase	IL10	ENSG00000136634	17475839;17698565;16939481;10588509;16713974;9258252;9637507;11994432;19020770;17185614;12115622;10328874;10072544;16369138;19211157;17377406;15914193;17253143;
D008070	increase	IL12A	ENSG00000168811	17475839;15379866;15652234;11369638;9637507;12470611;15516327;19020770;16369138;16299289;10651
D008070	increase	IL12B	ENSG00000113302	17475839;15802305;15684433;15379866;15652234;11369638;9637507;11717198;15860229;12470611;15516327;19020770;15914193;10651989;16369138;16299289;19211157;18279049;1725[illegible]
D008070	increase	IL13	ENSG00000169194	19020770;
D008070	increase	IL18	ENSG00000150782	17399992;16100009;17253143;
D008070	increase	IL1A	ENSG00000115008	10866999;15860229;17929310;15779068;
D008070	increase	IL1B	ENSG00000125538	9851740;15652230;15802305;19164858;10588509;16804125;9890559;10772282;12574323;15860229;10328874;16687389;19020770;10072544;16369138;11344824;17377406;19211157;16112[illegible]
D008070	increase	IL1RN	ENSG00000136689	10072544;17377406;
D008070	increase	IL23A	ENSG00000110944	15860229;15516327;18490488;
D008070	increase	IL2	ENSG00000109471	17377406;19020770;
D008070	increase	IL4	ENSG00000113520	15698409;19020770;

D008070	increase	IL6	ENSG00000136244	16367934;15802305;15379866;17698565;18357586;16317058;10588509;17532054;16107141;11344824;15388451;15860229;11950692;19020770;17529908;16369138;9351880;10072544;19211157;16373689;17000234;17377406;12225373;17585980;16426496;16387846;15779068;[illegible]
D008070	increase	IL8	ENSG00000169429	16367934;15802305;15779068;9258252;12818968;15379866;14997032;12176910;18414230;16364007;17529908;15698409;16366002;15860229;11344824;10328874;14572605;16804125;15893496;17636245;11527995;19020770;16369138;15474070;17053167;15788441;10072544;12470611;19211157;17884996;16384605;12734376;12225373;16387846;16112155;
D008070	increase	IRF1	ENSG00000125347	11739238;
D008070	increase	ISG15	ENSG00000187608	15860229;
D008070	increase	ITGAM	ENSG00000169896	12734376;
D008070	increase	JUN	ENSG00000177606	18180316;
D008070	increase	LITAF	ENSG00000189067	17408380;
D008070	increase	MMP7	ENSG00000137673	11739494;
D008070	increase	MX1	ENSG00000157601	15860229;
D008070	increase	NFKBIA	ENSG00000100906	16815550;15652234;11527995;11739238;17053167;15671028;10072544;
D008070	increase	NOS1	ENSG00000089250	17855475;
D008070	increase	NOS2	ENSG00000007171	10866999;16235733;
D008070	increase	OASL	ENSG00000135114	15860229;
D008070	increase	PF4	ENSG00000163737	15788441;
D008070	increase	PLA2G2A	ENSG00000188257	15964894;
D008070	increase	PLAUR	ENSG00000011422	11739494;
D008070	increase	PPBP	ENSG00000163736	15316029;15788441;
D008070	increase	PTGES2	ENSG00000148334	15779068;
D008070	increase	PTGS2	ENSG00000073756	15684433;16317058;10866999;15047127;15860229;19020770;16116226;15964894;15779068;16959971;
D008070	increase	RARA	ENSG00000131759	17216566;
D008070	increase	RELA	ENSG00000173039	15905586;16815550;10328874;17399992;10531305;15358673;15047127;15893496;15652234;15389581;14572605;9973483;10640779;15964894;11739238;1581195
D008070	increase	SERPINB9	ENSG00000170542	12177049;
D008070	increase	SERPINE1	ENSG00000106366	16317058;16320596;
D008070	increase	SOD2	ENSG00000112096	15860229;
D008070	increase	TGFB1	ENSG00000105329	11344824;17253143;
D008070	increase	TLR2	ENSG00000137462	12734376;12225373;12176910;

D008070	increase	TLR3	ENSG00000164342	15191912;
D008070	increase	TLR4	ENSG00000136869	16317058;12734376;12225373;17532054;12176910;10835634;12574384;
D008070	increase	TNFAIP6	ENSG00000123610	15860229;
D008070	increase	TNF	ENSG00000223952	9851740;12805067;15802305;15779068;9721806;15684433;10328874;15379866;17092623;17698565;9088875;16939481;18357586;10588509;14499251;11994432;9637507;9890559;15388451;10772282;8096834;12406900;16804125;10640779;15860229;17929310;17408380;11344824;15893496;19020770;14962975;10072544;16973359;16369138;16154993;15914193;15358692;15474070;19211157;18279049;16373689;7769305;17585980;16387846;12574384;17253143;16112155;16318612;15893782;11699074;176787
D008070	increase	TNFRSF1A	ENSG00000067182	10072544;
D008070	increase	TNFRSF1B	ENSG00000028137	10072544;
D008070	increase	VCAM1	ENSG00000162692	16317058;15358673;
D008070	increase	VDR	ENSG00000111424	15876428;
D008070	increase	VEGFA	ENSG00000112715	16385345;
D008078	increase	MAPK1	ENSG00000100030	11414687;
D008078	increase	MAPK3	ENSG00000102882	11414687;
D008078	increase	MMP1	ENSG00000196611	11414687;
D008078	increase	TIMP1	ENSG00000102265	11414687;
D008095	decrease	ABCA1	ENSG00000165029	15883047;
D008095	decrease	SCARB1	ENSG00000073060	15883047;
D008095	increase	CYP3A4	ENSG00000160868	15708356;17965521;
D008130	decrease	SOCS1	ENSG00000185338	17374387;
D008130	increase	ABCB1	ENSG00000085563	15239124;
D008148	increase	CDKN1B	ENSG00000111276	15118409;
D008148	increase	CYP3A4	ENSG00000160868	12505310;
D008148	increase	FAS	ENSG00000026103	17088865;
D008148	increase	FASLG	ENSG00000117560	17088865;
D008148	increase	LDLR	ENSG00000130164	12524230;
D008148	increase	TP53	ENSG00000141510	17088865;
D008244	increase	LAMA3	ENSG00000053747	15541073;
D008244	increase	LAMB3	ENSG00000196878	15541073;
D008244	increase	LAMC2	ENSG00000248691	15541073;
D008294	decrease	BUB1	ENSG00000169679	16079077;
D008294	decrease	CDC20	ENSG00000117399	18214805;
D008294	decrease	CENPF	ENSG00000117724	16079077;
D008294	decrease	PLAT	ENSG00000104368	16079077;
D008294	decrease	RFC3	ENSG00000133119	16079077;
D008294	decrease	TYMS	ENSG00000176890	16079077;
D008294	increase	AKR1C1	ENSG00000187134	18214805;16079077;
D008294	increase	AKR1C2	ENSG00000151632	16079077;
D008294	increase	DVL1	ENSG00000107404	18204794;
D008294	increase	HRAS	ENSG00000174775	18204794;

D008294	increase	MCM2	ENSG00000073111	18204794;
D008294	increase	PCNA	ENSG00000132646	12762645;
D008294	increase	TP53	ENSG00000141510	12762645;18204794;19787260;
D008294	increase	TP53I3	ENSG00000115129	18204794;
D008315	increase	ABCA1	ENSG00000165029	17070507;
D008315	increase	CD36	ENSG00000135218	17070507;
D008315	increase	MT1A	ENSG00000205362	15640275;
D008315	increase	MT2A	ENSG00000125148	15640275;
D008344	decrease	BCL2L1	ENSG00000171552	15824117;
D008344	increase	BAX	ENSG00000087088	15824117;
D008345	decrease	ACO2	ENSG00000100412	16625280;
D008345	decrease	AKT1	ENSG00000142208	17043766;
D008345	decrease	FOS	ENSG00000170345	17043766;
D008345	decrease	JUN	ENSG00000177606	17043766;
D008345	decrease	MMP14	ENSG00000157227	17043766;
D008345	decrease	MMP2	ENSG00000087245	17043766;
D008345	decrease	SRC	ENSG00000197122	16600465;
D008345	increase	APP	ENSG00000142192	18583042;
D008345	increase	BACE1	ENSG00000186318	18583042;
D008345	increase	HIF1A	ENSG00000100644	16386771;
D008345	increase	HSPA8	ENSG00000109971	16600465;
D008345	increase	NDRG1	ENSG00000104419	16386771;
D008351	increase	PTGS2	ENSG00000073756	15944324;
D008456	increase	TNF	ENSG00000223952	10883730;
D008463	decrease	MAPT	ENSG00000186868	16930453;
D008463	increase	CYP1A1	ENSG00000140465	12841937;
D008463	increase	CYP1A2	ENSG00000140505	12841937;
D008464	increase	APP	ENSG00000142192	16627626;
D008464	increase	BCL2	ENSG00000171791	16835749;
D008464	increase	GATA3	ENSG00000107485	16835749;
D008464	increase	NFKB1	ENSG00000109320	16835749;
D008464	increase	PCNA	ENSG00000132646	16835749;
D008464	increase	STAT1	ENSG00000115415	16582591;16835749;
D008466	decrease	BCL2	ENSG00000171791	16738803;
D008466	decrease	ERBB2	ENSG00000141736	17932452;
D008466	decrease	LAMC2	ENSG00000248691	9504665;18095945;
D008466	increase	BAX	ENSG00000087088	16738803;
D008466	increase	GSTA2	ENSG00000244067	15778998;
D008466	increase	S100P	ENSG00000163993	15936073;
D008466	increase	TP53	ENSG00000141510	16738803;
D008468	affect	CLU	ENSG00000120885	12388792;
D008468	affect	GAL	ENSG00000069482	12388792;
D008468	affect	GAP43	ENSG00000172020	12388792;
D008468	affect	RET	ENSG00000165731	12388792;
D008468	affect	TIMP1	ENSG00000102265	12388792;
D008468	increase	CYP1A2	ENSG00000140505	15272053;
D008468	increase	NR1I3	ENSG00000143257	15272053;
D008469	increase	TNF	ENSG00000223952	10328874;
D008525	affect	FSHB	ENSG00000131808	16584521;
D008525	affect	LPO	ENSG00000167419	16584521;
D008535	affect	CLU	ENSG00000120885	12388792;
D008535	affect	GAL	ENSG00000069482	12388792;
D008535	affect	GAP43	ENSG00000172020	12388792;

D008535	affect	RET	ENSG00000165731	12388792;
D008535	affect	TIMP1	ENSG00000102265	12388792;
D008543	decrease	PLD1	ENSG00000075651	14615486;
D008543	decrease	TYR	ENSG00000077498	14615486;
D008550	decrease	CYP19A1	ENSG00000137869	16080194;
D008550	increase	CASP3	ENSG00000164305	12880480;
D008558	increase	ICAM1	ENSG00000090339	16025434;
D008558	increase	TNF	ENSG00000223952	16025434;
D008610	increase	RAP1A	ENSG00000116473	12671036;
D008610	increase	RHOA	ENSG00000067560	12671036;
D008610	increase	RHOB	ENSG00000143878	12671036;
D008623	increase	ACTA2	ENSG00000107796	17053324;
D008627	increase	ABCC1	ENSG00000103222	15585369;
D008627	increase	HMOX1	ENSG00000100292	17244614;
D008627	increase	HSPA1A	ENSG00000232804	17244614;
D008628	affect	C1ORF144	ENSG00000055070	16823088;
D008628	affect	CLK1	ENSG00000013441	16823088;
D008628	affect	POGK	ENSG00000143157	16823088;
D008628	affect	POLD2	ENSG00000106628	16823088;
D008628	affect	RNGTT	ENSG00000111880	16823088;
D008628	affect	TK2	ENSG00000166548	16823088;
D008628	decrease	A2M	ENSG00000175899	17547211;
D008628	decrease	ACP2	ENSG00000134575	17547211;
D008628	decrease	ACSL1	ENSG00000151726	17547211;
D008628	decrease	AKR1B1	ENSG00000085662	17547211;
D008628	decrease	AKT2	ENSG00000105221	16823088;
D008628	decrease	ALDH6A1	ENSG00000119711	17547211;
D008628	decrease	ARF4	ENSG00000168374	16823088;
D008628	decrease	ATP6V1A	ENSG00000114573	16823088;
D008628	decrease	AZGP1	ENSG00000160862	17547211;
D008628	decrease	BAT3	ENSG00000204463	16823088;
D008628	decrease	BUB3	ENSG00000154473	16823088;
D008628	decrease	CALM3	ENSG00000143933	16823088;
D008628	decrease	CCR2	ENSG00000121807	16823088;
D008628	decrease	CD84	ENSG00000066294	16823088;
D008628	decrease	CFB	ENSG00000243570	17547211;
D008628	decrease	CIDEB	ENSG00000136305	17547211;
D008628	decrease	CLGN	ENSG00000153132	17547211;
D008628	decrease	CLTC	ENSG00000141367	16823088;
D008628	decrease	CRYAA	ENSG00000160202	17547211;
D008628	decrease	CYB561	ENSG00000008283	16823088;
D008628	decrease	DNAH1	ENSG00000114841	16823088;
D008628	decrease	EIF3F	ENSG00000175390	16823088;
D008628	decrease	F10	ENSG00000126218	17547211;
D008628	decrease	F5	ENSG00000198734	17547211;
D008628	decrease	FCGR3A	ENSG00000203747	16823088;
D008628	decrease	FCRLA	ENSG00000132185	16823088;
D008628	decrease	FN1	ENSG00000115414	16823088;
D008628	decrease	FURIN	ENSG00000140564	17547211;
D008628	decrease	GGCX	ENSG00000115486	17547211;
D008628	decrease	GPER	ENSG00000164850	17547211;
D008628	decrease	GSN	ENSG00000148180	17547211;
D008628	decrease	HLA-DQA1	ENSG00000196735	16823088;
D008628	decrease	HLA-DQB1	ENSG00000179344	16823088;

D008628	decrease	HS1BP3	ENSG00000118960	16823088;
D008628	decrease	IFRD1	ENSG00000006652	17547211;
D008628	decrease	IKBKAP	ENSG00000070061	16823088;
D008628	decrease	IL17RB	ENSG00000056736	17547211;
D008628	decrease	IL3RA	ENSG00000185291	16823088;
D008628	decrease	ITIH3	ENSG00000162267	17547211;
D008628	decrease	IVNS1ABP	ENSG00000116679	16823088;
D008628	decrease	LANCL1	ENSG00000115365	16823088;
D008628	decrease	LEPR	ENSG00000116678	16823088;
D008628	decrease	LILRA2	ENSG00000239998	16823088;
D008628	decrease	LIPA	ENSG00000107798	17547211;
D008628	decrease	LMNA	ENSG00000160789	16823088;
D008628	decrease	MGAT2	ENSG00000168282	17547211;
D008628	decrease	MORF4L2	ENSG00000123562	16823088;
D008628	decrease	MTMR11	ENSG00000014914	16823088;
D008628	decrease	MYC	ENSG00000136997	16823088;
D008628	decrease	NDUFS2	ENSG00000158864	16823088;
D008628	decrease	OS9	ENSG00000135506	17547211;
D008628	decrease	PDIA4	ENSG00000155660	17547211;
D008628	decrease	PDS5B	ENSG00000083642	16823088;
D008628	decrease	PGC	ENSG00000096088	17547211;
D008628	decrease	PNMA1	ENSG00000176903	17547211;
D008628	decrease	PPP1R15A	ENSG00000087074	16823088;
D008628	decrease	PRDX6	ENSG00000117592	16823088;
D008628	decrease	PSPH	ENSG00000146733	17547211;
D008628	decrease	RELN	ENSG00000189056	17547211;
D008628	decrease	RNASEH1	ENSG00000171865	16823088;
D008628	decrease	RPL19	ENSG00000108298	16823088;
D008628	decrease	RPL28	ENSG00000108107	16823088;
D008628	decrease	RPS5	ENSG00000083845	16823088;
D008628	decrease	SERPINB1	ENSG00000021355	17547211;
D008628	decrease	SERPINC1	ENSG00000117601	17547211;
D008628	decrease	SHC1	ENSG00000160691	16823088;
D008628	decrease	SLIT3	ENSG00000184347	16823088;
D008628	decrease	SRF	ENSG00000112658	16823088;
D008628	decrease	SSR2	ENSG00000163479	16823088;
D008628	decrease	TMF1	ENSG00000144747	16823088;
D008628	decrease	TNFSF10	ENSG00000121858	17547211;
D008628	decrease	TSPAN7	ENSG00000156298	17547211;
D008628	decrease	TST	ENSG00000128311	17547211;
D008628	decrease	TWF2	ENSG00000247596	16823088;
D008628	decrease	UBE2N	ENSG00000177889	16823088;
D008628	decrease	VTN	ENSG00000109072	17547211;
D008628	decrease	YWHAZ	ENSG00000164924	16823088;
D008628	increase	ABCG1	ENSG00000160179	16823088;
D008628	increase	ABCG2	ENSG00000118777	17547211;
D008628	increase	ADM	ENSG00000148926	17547211;
D008628	increase	ALG3	ENSG00000214160	16823088;
D008628	increase	ANKHD1	ENSG00000131503	16823088;
D008628	increase	ANXA2	ENSG00000182718	17547211;
D008628	increase	BANK1	ENSG00000153064	16823088;
D008628	increase	BCOR	ENSG00000183337	16823088;
D008628	increase	BIRC3	ENSG00000023445	16823088;
D008628	increase	BUB1	ENSG00000169679	16823088;

D008628	increase	C13ORF18	ENSG00000102445	16823088;
D008628	increase	C4ORF34	ENSG00000163683	16823088;
D008628	increase	CA3	ENSG00000164879	16823088;
D008628	increase	CCNB2	ENSG00000157456	17547211;
D008628	increase	CD226	ENSG00000150637	16823088;
D008628	increase	CD40	ENSG00000101017	16823088;
D008628	increase	CDK5R1	ENSG00000176749	16823088;
D008628	increase	CDKN3	ENSG00000100526	17547211;
D008628	increase	CFD	ENSG00000197766	16823088;
D008628	increase	CKS2	ENSG00000123975	17547211;
D008628	increase	CLASP2	ENSG00000163539	16823088;
D008628	increase	CLPTM1	ENSG00000104853	17547211;16823088;
D008628	increase	COL4A3	ENSG00000169031	16823088;
D008628	increase	CPZ	ENSG00000109625	16823088;
D008628	increase	CSPG5	ENSG00000114646	16823088;
D008628	increase	CTBP1	ENSG00000159692	16823088;
D008628	increase	CYP2U1	ENSG00000155016	16823088;
D008628	increase	DAB1	ENSG00000173406	16823088;
D008628	increase	DOCK10	ENSG00000135905	16823088;
D008628	increase	DPYSL4	ENSG00000151640	16823088;
D008628	increase	DTNA	ENSG00000134769	16823088;
D008628	increase	EEA1	ENSG00000102189	16823088;
D008628	increase	EFNB1	ENSG00000090776	16823088;
D008628	increase	EMX2	ENSG00000170370	16823088;
D008628	increase	EN1	ENSG00000163064	16823088;
D008628	increase	ENPP1	ENSG00000197594	17547211;
D008628	increase	ESR2	ENSG00000140009	16823088;
D008628	increase	ETFB	ENSG00000105379	16823088;
D008628	increase	ETS2	ENSG00000157557	17547211;
D008628	increase	EZH1	ENSG00000108799	16823088;
D008628	increase	FABP7	ENSG00000164434	16823088;
D008628	increase	FAM125B	ENSG00000249991	16823088;
D008628	increase	FGF2	ENSG00000138685	16823088;
D008628	increase	FOSL1	ENSG00000175592	17547211;
D008628	increase	GABRA6	ENSG00000145863	16823088;
D008628	increase	GAS7	ENSG00000007237	16823088;
D008628	increase	GCLC	ENSG00000001084	17547211;
D008628	increase	GLA	ENSG00000102393	17547211;
D008628	increase	GLRA3	ENSG00000145451	16823088;
D008628	increase	GNGT1	ENSG00000127928	16823088;
D008628	increase	GNRH1	ENSG00000147437	16823088;
D008628	increase	HABP4	ENSG00000130956	16823088;
D008628	increase	HEPH	ENSG00000089472	16823088;
D008628	increase	HIST1H2AG	ENSG00000233224	16823088;
D008628	increase	HIST1H3I	ENSG00000197153	16823088;
D008628	increase	HIST1H4C	ENSG00000182217	17547211;
D008628	increase	HMGN2	ENSG00000198830	17547211;
D008628	increase	HMOX1	ENSG00000100292	17244614;17547211;
D008628	increase	HNF1A	ENSG00000135100	16823088;
D008628	increase	HNRNPH3	ENSG00000096746	16823088;
D008628	increase	HOXB6	ENSG00000108511	16823088;
D008628	increase	HSP90AA1	ENSG00000080824	16823088;
D008628	increase	HSPA1A	ENSG00000232804	17244614;
D008628	increase	HTN1	ENSG00000126550	16823088;

D008628	increase	HTR7	ENSG00000148680	16823088;
D008628	increase	IFNA8	ENSG00000120242	16823088;
D008628	increase	IMPG1	ENSG00000112706	16823088;
D008628	increase	ITGB3BP	ENSG00000142856	17547211;
D008628	increase	KCNH2	ENSG00000055118	16823088;
D008628	increase	LAMB4	ENSG00000091128	16823088;
D008628	increase	LPPR4	ENSG00000117600	16823088;
D008628	increase	LRRFIP1	ENSG00000124831	16823088;
D008628	increase	MAGEB4	ENSG00000120289	16823088;
D008628	increase	MAP2K2	ENSG00000126934	16823088;
D008628	increase	MCM2	ENSG00000073111	17547211;
D008628	increase	MED16	ENSG00000175221	16823088;
D008628	increase	MEOX2	ENSG00000106511	16823088;
D008628	increase	MFAP5	ENSG00000197614	16823088;
D008628	increase	MLL4	ENSG00000105663	16823088;
D008628	increase	MT1H	ENSG00000205358	16823088;
D008628	increase	MTF1	ENSG00000188786	16823088;
D008628	increase	MYO1B	ENSG00000128641	16823088;
D008628	increase	NAP1L1	ENSG00000187109	16823088;
D008628	increase	NAV3	ENSG00000067798	16823088;
D008628	increase	NCOA3	ENSG00000124151	16823088;
D008628	increase	NGF	ENSG00000134259	16823088;
D008628	increase	NMT2	ENSG00000152465	16823088;
D008628	increase	NUP107	ENSG00000111581	17547211;
D008628	increase	PARK7	ENSG00000116288	16823088;
D008628	increase	PDE4DIP	ENSG00000178104	16823088;
D008628	increase	PEX14	ENSG00000142655	16823088;
D008628	increase	PHOX2B	ENSG00000109132	16823088;
D008628	increase	PIK3R4	ENSG00000196455	16823088;
D008628	increase	PLGLB1	ENSG00000125551	16823088;
D008628	increase	POU2F3	ENSG00000137709	16823088;
D008628	increase	PPIA	ENSG00000196262	16823088;
D008628	increase	PPIH	ENSG00000171960	17547211;
D008628	increase	PPP1R12A	ENSG00000058272	16823088;
D008628	increase	PTTG1	ENSG00000164611	17547211;
D008628	increase	RAC1	ENSG00000136238	16823088;
D008628	increase	RACGAP1	ENSG00000161800	17547211;
D008628	increase	RBPMS	ENSG00000157110	17547211;
D008628	increase	RHOBTB2	ENSG00000008853	16823088;
D008628	increase	RNASEH2A	ENSG00000104889	16823088;17547211;
D008628	increase	RNF138	ENSG00000134758	16823088;
D008628	increase	SAA2	ENSG00000134339	16823088;
D008628	increase	SAP30	ENSG00000164105	16823088;
D008628	increase	SCG3	ENSG00000104112	16823088;
D008628	increase	SEC24C	ENSG00000176986	16823088;
D008628	increase	SENP6	ENSG00000112701	16823088;
D008628	increase	SFRS15	ENSG00000156304	16823088;
D008628	increase	SFRS2	ENSG00000161547	17547211;
D008628	increase	SFRS7	ENSG00000115875	17547211;
D008628	increase	SH3GLB2	ENSG00000148341	16823088;
D008628	increase	SH3PXD2A	ENSG00000107957	16823088;
D008628	increase	SLC9A3R2	ENSG00000065054	16823088;
D008628	increase	SMC4	ENSG00000113810	17547211;
D008628	increase	SNRPA	ENSG00000077312	17547211;

D008628	increase	SOX18	ENSG00000203883	16823088;
D008628	increase	SPP1	ENSG00000118785	17547211;
D008628	increase	SPTBN1	ENSG00000115306	16823088;
D008628	increase	TAC1	ENSG00000006128	16823088;
D008628	increase	TGFB2	ENSG00000092969	16823088;
D008628	increase	TM2D1	ENSG00000162604	16823088;
D008628	increase	TNFAIP3	ENSG00000118503	17547211;
D008628	increase	TNFAIP8	ENSG00000145779	17547211;
D008628	increase	TOX3	ENSG00000103460	16823088;
D008628	increase	TPM2	ENSG00000198467	16823088;
D008628	increase	TRIP13	ENSG00000071539	17547211;
D008628	increase	TYMS	ENSG00000176890	17547211;
D008628	increase	UBE2C	ENSG00000175063	17547211;
D008628	increase	UGCG	ENSG00000148154	17547211;
D008628	increase	UQCRB	ENSG00000156467	16823088;
D008628	increase	USP39	ENSG00000168883	16823088;
D008628	increase	USPL1	ENSG00000132952	16823088;
D008628	increase	VWA1	ENSG00000179403	16823088;
D008628	increase	YBX1	ENSG00000065978	16823088;
D008628	increase	ZBTB10	ENSG00000205189	16823088;
D008628	increase	ZBTB1	ENSG00000126804	16823088;
D008628	increase	ZNF80	ENSG00000174255	16823088;
D008628	increase	ZWINT	ENSG00000122952	17547211;
D008687	decrease	CRP	ENSG00000132693	14557435;
D008704	affect	CLU	ENSG00000120885	12388792;
D008704	affect	GAL	ENSG00000069482	12388792;
D008704	affect	GAP43	ENSG00000172020	12388792;
D008704	affect	RET	ENSG00000165731	12388792;
D008704	affect	TIMP1	ENSG00000102265	12388792;
D008714	increase	ESR2	ENSG00000140009	15064155;15130599;
D008723	increase	JUN	ENSG00000177606	15263067;
D008724	decrease	HSP90B1	ENSG00000166598	16271832;
D008724	decrease	HSPA1A	ENSG00000232804	16271832;
D008724	decrease	HSPA8	ENSG00000109971	16271832;
D008724	increase	HSPA5	ENSG00000044574	16271832;
D008727	decrease	CD4	ENSG00000010610	15476228;
D008727	decrease	CD68	ENSG00000129226	15476228;
D008727	decrease	CD8A	ENSG00000153563	15476228;
D008727	decrease	CYP3A4	ENSG00000160868	12065438;16837568;
D008727	decrease	DKK1	ENSG00000107984	17320366;
D008727	decrease	ICAM1	ENSG00000090339	15476228;
D008727	decrease	IL8	ENSG00000169429	15476228;
D008727	decrease	MMP3	ENSG00000149968	15476228;
D008727	decrease	MTR	ENSG00000116984	16598758;
D008727	decrease	PRC1	ENSG00000198901	17374387;
D008727	decrease	SELE	ENSG00000007908	15476228;
D008727	decrease	TAGLN	ENSG00000149591	17320366;
D008727	decrease	UBE2C	ENSG00000175063	17374387;
D008727	decrease	WISP1	ENSG00000104415	17320366;
D008727	increase	ABCB1	ENSG00000085563	12576456;15239124;
D008727	increase	CASP8	ENSG00000064012	17637740;
D008727	increase	CDKN1A	ENSG00000124762	17637740;
D008727	increase	MDM2	ENSG00000135679	17637740;
D008727	increase	ODC1	ENSG00000115758	16598758;

D008727	increase	TNFRSF10A	ENSG00000104689	17637740;
D008727	increase	TNFRSF10B	ENSG00000120889	17637740;
D008727	increase	TP53	ENSG00000141510	17637740;
D008727	increase	WNT5A	ENSG00000114251	17320366;
D008730	increase	CYP1A1	ENSG00000140465	9169082;
D008731	decrease	DLX4	ENSG00000108813	12858341;
D008731	decrease	HSD17B1	ENSG00000108786	12858341;
D008731	decrease	MT2A	ENSG00000125148	12858341;
D008731	decrease	SELENBP1	ENSG00000143416	15159206;
D008731	increase	CYP11A1	ENSG00000140459	12858341;
D008731	increase	CYP19A1	ENSG00000137869	16996190;
D008731	increase	CYP2B6	ENSG00000197408	9733665;9047321;1010114 7;12464242;9241656;15548
D008731	increase	CYP3A4	ENSG00000160868	9714305;15466163;155483
D008731	increase	EGR3	ENSG00000179388	15159206;
D008731	increase	ESR1	ENSG00000091831	10999957;9751507;152530 41;14579009;10774817;166 26760;15890768;10579340; 11861976;12623674;15064 155;11485867;11922773;14
D008731	increase	HOXA10	ENSG00000078399	15890768;16632680;
D008731	increase	HSD3B1	ENSG00000203857	12858341;
D008741	affect	CDKN1A	ENSG00000124762	17079232;
D008741	decrease	DNMT1	ENSG00000130816	11912126;
D008741	increase	CDX2	ENSG00000165556	16990345;
D008741	increase	JUN	ENSG00000177606	16061660;
D008741	increase	TP53	ENSG00000141510	10531375;17079232;
D008743	decrease	MKI67	ENSG00000148773	18418871;
D008743	increase	AKR1C1	ENSG00000187134	19800895;
D008743	increase	AKR1C2	ENSG00000151632	19800895;
D008743	increase	CDKN1A	ENSG00000124762	18418871;
D008743	increase	CYP1A1	ENSG00000140465	19800895;
D008743	increase	GADD45A	ENSG00000116717	18418871;
D008743	increase	MDM2	ENSG00000135679	18418871;
D008743	increase	TP53	ENSG00000141510	18418871;
D008748	decrease	CES1	ENSG00000198848	10640517;
D008748	decrease	CES2	ENSG00000172831	10640517;
D008748	decrease	CYP3A4	ENSG00000160868	10640517;
D008748	increase	AHR	ENSG00000106546	16489053;16359657;15860 653;19286049;
D008748	increase	AHRR	ENSG00000063438	12655030;14672759;
D008748	increase	AKR1A1	ENSG00000117448	9973208;
D008748	increase	AKR1C1	ENSG00000187134	9973208;
D008748	increase	AREG	ENSG00000205595	16257430;
D008748	increase	C3	ENSG00000125730	16257430;
D008748	increase	CTSD	ENSG00000117984	16489053;16257430;
D008748	increase	CYP1A1	ENSG00000140465	16489053;15620718;10799 337;9973208;14672759;162 57430;15860653;16359657; 15448115;12490585;
D008748	increase	CYP1A2	ENSG00000140505	15769886;15448115;
D008748	increase	ESR1	ENSG00000091831	16489053;16257430;
D008748	increase	PGR	ENSG00000082175	16257430;
D008748	increase	PON1	ENSG00000005421	15169886;
D008748	increase	TFF1	ENSG00000160182	16257430;

D008751	increase	MAPT	ENSG00000186868	16930453;
D008767	decrease	ANP32A	ENSG00000140350	18959786;
D008767	decrease	ASMTL	ENSG00000169093	18959786;
D008767	decrease	BTBD1	ENSG00000064726	18959786;
D008767	decrease	GNB2L1	ENSG00000204628	18959786;
D008767	decrease	VIM	ENSG00000026025	18959786;
D008767	increase	PSMC6	ENSG00000100519	18959786;
D008767	increase	WARS	ENSG00000140105	18959786;
D008769	decrease	AMBP	ENSG00000106927	12773770;
D008769	decrease	ATP1B3	ENSG00000069849	12634122;
D008769	decrease	BMP4	ENSG00000125378	12773770;
D008769	decrease	CBX5	ENSG00000094916	12634122;
D008769	decrease	CDH8	ENSG00000150394	12634122;
D008769	decrease	CDKN1A	ENSG00000124762	12634122;
D008769	decrease	CKB	ENSG00000166165	12634122;
D008769	decrease	CXCR4	ENSG00000121966	12634122;
D008769	decrease	CYB561D2	ENSG00000114395	12634122;
D008769	decrease	DEAF1	ENSG00000177030	12634122;
D008769	decrease	EIF5	ENSG00000100664	12634122;
D008769	decrease	EPHB3	ENSG00000182580	12634122;
D008769	decrease	FCGRT	ENSG00000104870	12634122;
D008769	decrease	FOSL1	ENSG00000175592	12634122;
D008769	decrease	GSS	ENSG00000100983	12634122;
D008769	decrease	HIST1H4B	ENSG00000182217	12634122;
D008769	decrease	HMGB2	ENSG00000164104	12634122;
D008769	decrease	ID1	ENSG00000125968	12634122;
D008769	decrease	ITGB4	ENSG00000132470	12634122;12773770;
D008769	decrease	KRT17	ENSG00000128422	12634122;
D008769	decrease	LIG1	ENSG00000105486	12634122;
D008769	decrease	MCM2	ENSG00000073111	12634122;
D008769	decrease	MCM4	ENSG00000104738	12634122;
D008769	decrease	MGST2	ENSG00000085871	12634122;
D008769	decrease	MLH1	ENSG00000076242	17671175;
D008769	decrease	MYLK	ENSG00000251351	12634122;
D008769	decrease	NBL1	ENSG00000158747	12634122;
D008769	decrease	PCDH7	ENSG00000169851	12634122;
D008769	decrease	PCNA	ENSG00000132646	12634122;
D008769	decrease	PTPRA	ENSG00000132670	12634122;
D008769	decrease	PTPRG	ENSG00000144724	12634122;
D008769	decrease	PTPRS	ENSG00000105426	12634122;
D008769	decrease	RAD23A	ENSG00000179262	12634122;12773770;
D008769	decrease	RARRES3	ENSG00000133321	12634122;
D008769	decrease	SFRS7	ENSG00000115875	12634122;
D008769	decrease	SMAD2	ENSG00000175387	12634122;
D008769	decrease	SPARC	ENSG00000113140	12634122;
D008769	decrease	STK39	ENSG00000198648	12634122;
D008769	decrease	TSC22D1	ENSG00000102804	12634122;
D008769	decrease	WARS	ENSG00000140105	12634122;
D008769	increase	ADAM10	ENSG00000137845	12634122;
D008769	increase	ALPL	ENSG00000162551	12634122;
D008769	increase	APC	ENSG00000134982	12634122;
D008769	increase	ARAF	ENSG00000078061	12634122;
D008769	increase	AXL	ENSG00000167601	12634122;
D008769	increase	BAG1	ENSG00000250477	12634122;

D008769	increase	BIRC2	ENSG00000110330	12634122;
D008769	increase	BRCA1	ENSG00000012048	12634122;
D008769	increase	BRCA2	ENSG00000139618	12634122;
D008769	increase	CASP4	ENSG00000196954	12634122;
D008769	increase	CCNA1	ENSG00000133101	12634122;
D008769	increase	CCNH	ENSG00000134480	12634122;
D008769	increase	CDK5	ENSG00000164885	12634122;
D008769	increase	DAB2	ENSG00000153071	12634122;12773770;
D008769	increase	DEK	ENSG00000124795	12634122;
D008769	increase	DSTN	ENSG00000249789	12634122;
D008769	increase	EIF3E	ENSG00000104408	12634122;
D008769	increase	ELK3	ENSG00000111145	12634122;
D008769	increase	EMP1	ENSG00000134531	12634122;
D008769	increase	EPCAM	ENSG00000119888	12634122;
D008769	increase	EPHA1	ENSG00000146904	12634122;
D008769	increase	ERBB2	ENSG00000141736	12634122;
D008769	increase	ERBB4	ENSG00000178568	12634122;
D008769	increase	ETS1	ENSG00000134954	12634122;
D008769	increase	FASN	ENSG00000169710	12634122;
D008769	increase	FES	ENSG00000182511	12634122;
D008769	increase	FGFR1OP	ENSG00000213066	12634122;
D008769	increase	FYN	ENSG00000010810	12634122;
D008769	increase	GDF15	ENSG00000130513	12634122;12773770;
D008769	increase	HLA-DRB1	ENSG00000196126	12634122;
D008769	increase	HMGB1	ENSG00000189403	12634122;
D008769	increase	HSP90B1	ENSG00000166598	12634122;
D008769	increase	IFITM1	ENSG00000185885	12634122;12773770;
D008769	increase	IFRD1	ENSG00000006652	12634122;
D008769	increase	KIT	ENSG00000157404	12634122;
D008769	increase	LGALS8	ENSG00000116977	12634122;
D008769	increase	MAPK4	ENSG00000141639	12634122;
D008769	increase	MAPK9	ENSG00000050748	12634122;
D008769	increase	MAX	ENSG00000125952	12634122;
D008769	increase	MCAM	ENSG00000076706	12634122;12773770;
D008769	increase	MDM2	ENSG00000135679	12634122;
D008769	increase	MECOM	ENSG00000085276	12634122;
D008769	increase	MET	ENSG00000105976	12634122;
D008769	increase	MSH2	ENSG00000095002	12634122;
D008769	increase	MYBL1	ENSG00000185697	12634122;
D008769	increase	MYL2	ENSG00000111245	12634122;
D008769	increase	MYL9	ENSG00000101335	12634122;
D008769	increase	PDGFB	ENSG00000100311	12634122;
D008769	increase	PEA15	ENSG00000162734	12634122;
D008769	increase	PGK1	ENSG00000102144	12634122;
D008769	increase	PLXNA3	ENSG00000130827	12634122;
D008769	increase	PRKD1	ENSG00000184304	12634122;
D008769	increase	PTP4A1	ENSG00000112245	12634122;
D008769	increase	RBBP5	ENSG00000117222	12634122;
D008769	increase	RBM6	ENSG00000004534	12634122;
D008769	increase	RPSA	ENSG00000168028	12773770;
D008769	increase	SERPING1	ENSG00000149131	12634122;
D008769	increase	SNRPN	ENSG00000128739	12634122;
D008769	increase	SPINK1	ENSG00000164266	12634122;
D008769	increase	SPINT2	ENSG00000167642	12634122;

D008769	increase	SQLE	ENSG00000104549	12634122;
D008769	increase	TBRG4	ENSG00000136270	12634122;
D008769	increase	TIAM1	ENSG00000156299	12634122;
D008769	increase	TNC	ENSG00000041982	12634122;
D008769	increase	TNFRSF25	ENSG00000215788	12634122;
D008769	increase	TP53	ENSG00000141510	12634122;
D008769	increase	TRAM1	ENSG00000067167	12634122;
D008769	increase	UCHL1	ENSG00000154277	12634122;
D008769	increase	VIM	ENSG00000026025	12634122;12773770;
D008769	increase	WNT2	ENSG00000105989	12634122;
D008770	decrease	CCNA2	ENSG00000145386	15870882;
D008770	decrease	CDK1	ENSG00000170312	15870882;
D008775	decrease	CYP3A4	ENSG00000160868	16837568;15964336;
D008775	increase	FCER2	ENSG00000104921	11997017;
D008775	increase	IL4	ENSG00000113520	11997017;
D008775	increase	IL4R	ENSG00000077238	11997017;
D008797	affect	NR3C1	ENSG00000113580	10219967;
D008797	increase	CYP3A4	ENSG00000160868	12505310;10219967;
D008798	affect	RAP1A	ENSG00000116473	12671036;
D008798	affect	RHOA	ENSG00000067560	12671036;
D008798	affect	RHOB	ENSG00000143878	12671036;
D008798	increase	PON1	ENSG00000005421	14500290;15690306;
D008803	increase	HTR2C	ENSG00000147246	18083778;
D008825	decrease	IL4	ENSG00000113520	15284827;
D008825	decrease	IL5	ENSG00000113525	15284827;
D008874	increase	ABCB1	ENSG00000085563	8632764;
D008911	increase	BACE1	ENSG00000186318	18583042;
D008926	decrease	AXL	ENSG00000167601	18522535;
D008926	increase	F3	ENSG00000117525	15636197;
D008926	increase	IFNG	ENSG00000111537	18953428;
D008926	increase	MAOA	ENSG00000189221	15946989;
D008926	increase	PLA2G2A	ENSG00000188257	18953428;
D008926	increase	PON1	ENSG00000005421	15690306;
D008942	affect	BIRC3	ENSG00000023445	12948851;
D008942	affect	DAXX	ENSG00000231617	12948851;
D008942	affect	PAWR	ENSG00000177425	12948851;
D008942	decrease	BIRC2	ENSG00000110330	15359644;
D008942	decrease	XIAP	ENSG00000101966	15359644;12948851;
D008942	increase	ABCB1	ENSG00000085563	15239124;
D008942	increase	ABCG2	ENSG00000118777	15695404;15875186;17032<br>904;15640379;16954373;16<br>636798;15239124;1570330
D008942	increase	CASP2	ENSG00000106144	14757846;
D008942	increase	RARB	ENSG00000077092	17608728;
D009020	decrease	BCL2	ENSG00000171791	17542780;
D009020	increase	BAX	ENSG00000087088	17542780;
D009151	affect	CD40LG	ENSG00000102245	16173061;
D009151	affect	CXCL2	ENSG00000081041	16173061;
D009151	affect	ERBB4	ENSG00000178568	16173061;
D009151	affect	FGF7	ENSG00000140285	16173061;
D009151	affect	FGF8	ENSG00000107831	16173061;
D009151	affect	FGF9	ENSG00000102678	16173061;
D009151	affect	GDF1	ENSG00000130283	16173061;
D009151	affect	IL13	ENSG00000169194	16173061;

D009151	affect	IL3	ENSG00000164399	16173061;
D009151	affect	IL7R	ENSG00000168685	16173061;
D009151	affect	NOTCH2	ENSG00000134250	16173061;
D009151	affect	PDCD10	ENSG00000114209	16173061;
D009151	affect	PDGFA	ENSG00000197461	16173061;
D009151	affect	PDGFRB	ENSG00000113721	16173061;
D009151	affect	TNFRSF10A	ENSG00000104689	16173061;
D009151	affect	TNFRSF1A	ENSG00000067182	16173061;
D009151	affect	TNFRSF1B	ENSG00000028137	16173061;
D009151	affect	VEGFB	ENSG00000173511	16173061;
D009151	affect	VEGFC	ENSG00000150630	16173061;
D009151	decrease	BCL2	ENSG00000171791	9665388;11428642;
D009151	decrease	C6ORF114	ENSG00000187461	12884408;
D009151	decrease	CETN2	ENSG00000147400	12884408;
D009151	decrease	CHST15	ENSG00000182022	12884408;
D009151	decrease	CSF3R	ENSG00000119535	16173061;
D009151	decrease	FGFR3	ENSG00000068078	16173061;
D009151	decrease	FN1	ENSG00000115414	9665388;11428642;183423
D009151	decrease	IL10RA	ENSG00000110324	16173061;
D009151	decrease	IL4R	ENSG00000077238	16173061;
D009151	decrease	IL6R	ENSG00000160712	16173061;
D009151	decrease	IL9	ENSG00000145839	16173061;
D009151	decrease	IL9R	ENSG00000124334	16173061;
D009151	decrease	ITGA6	ENSG00000091409	12880345;
D009151	decrease	ITGB4	ENSG00000132470	12880345;11428621;
D009151	decrease	KDR	ENSG00000128052	16173061;
D009151	decrease	KRT14	ENSG00000186847	12880345;18342354;14644<br>625;12884408;11428621;
D009151	decrease	LAMA5	ENSG00000130702	12880345;
D009151	decrease	LAMC2	ENSG00000248691	18095945;9504665;183003
D009151	decrease	MCL1	ENSG00000143384	12884408;
D009151	decrease	MIF	ENSG00000240972	12884408;
D009151	decrease	NBN	ENSG00000104320	12884408;
D009151	decrease	NGF	ENSG00000134259	16173061;
D009151	decrease	NTF3	ENSG00000185652	16173061;
D009151	decrease	OXSR1	ENSG00000172939	12884408;
D009151	decrease	PELO	ENSG00000152684	12884408;
D009151	decrease	POP1	ENSG00000104356	12884408;
D009151	increase	ACHE	ENSG00000087085	17229443;
D009151	increase	C3	ENSG00000125730	15953574;
D009151	increase	CALM1	ENSG00000143933	16935404;
D009151	increase	CCL11	ENSG00000172156	17620002;
D009151	increase	CCL2	ENSG00000108691	17720292;
D009151	increase	CCL3	ENSG00000006075	17720292;
D009151	increase	CCL4	ENSG00000129277	17720292;
D009151	increase	CCL5	ENSG00000161570	17620002;
D009151	increase	CCND2	ENSG00000118971	12884408;
D009151	increase	CD93	ENSG00000125810	11428648;
D009151	increase	CSF2	ENSG00000164400	17894541;
D009151	increase	CSF3	ENSG00000108342	17894541;
D009151	increase	CTGF	ENSG00000118523	16173061;
D009151	increase	EGF	ENSG00000138798	17266442;
D009151	increase	FAS	ENSG00000026103	16173061;12482751;
D009151	increase	FASLG	ENSG00000117560	16173061;12482751;

D009151	increase	GADD45A	ENSG00000116717	12884408;
D009151	increase	GNPDA1	ENSG00000113552	12884408;
D009151	increase	HBEGF	ENSG00000113070	16173061;
D009151	increase	IL10	ENSG00000136634	16173061;
D009151	increase	IL1A	ENSG00000115008	8603935;
D009151	increase	IL1B	ENSG00000125538	11428645;17524151;10025362;17266442;15251176;
D009151	increase	IL5	ENSG00000113525	17620002;
D009151	increase	IL6	ENSG00000136244	12753408;16173061;11766126;17524151;10025362;17266442;15251176;11428645;17510838;
D009151	increase	IL8	ENSG00000169429	12753408;16173061;10593592;17524151;15251176;11428645;12083423;10025362;17894541;17266442;1751
D009151	increase	KRT10	ENSG00000186395	9665388;11428642;
D009151	increase	KRT1	ENSG00000167768	12482751;9665388;114286
D009151	increase	MRPS18B	ENSG00000229861	12884408;
D009151	increase	NCKAP1	ENSG00000061676	12884408;
D009151	increase	ND3	ENSG00000198840	12884408;
D009151	increase	NOS2	ENSG00000007171	17964119;17524151;
D009151	increase	POMC	ENSG00000115138	8299707;
D009151	increase	PSMD6	ENSG00000163636	12884408;
D009151	increase	RPL27A	ENSG00000166441	12884408;
D009151	increase	RPS12	ENSG00000112306	12884408;
D009151	increase	SUMO2	ENSG00000188612	12884408;
D009151	increase	TGFB1	ENSG00000105329	17266442;15286001;
D009151	increase	TNF	ENSG00000223952	17524151;15868479;10025362;17266442;15251176;11428645;16173061;
D009151	increase	TOMM20	ENSG00000173726	12884408;
D009151	increase	TP53	ENSG00000141510	9665388;11428642;
D009173	decrease	MAPT	ENSG00000186868	16930453;
D009240	increase	IL1B	ENSG00000125538	12946449;
D009240	increase	IL6	ENSG00000136244	12946449;
D009240	increase	IL8	ENSG00000169429	12946449;17459229;
D009240	increase	ITGAM	ENSG00000169896	10215738;15774851;
D009240	increase	ITGB2	ENSG00000160255	10215738;
D009240	increase	PTGS2	ENSG00000073756	12946449;
D009240	increase	TNF	ENSG00000223952	12946449;
D009240	increase	VEGFA	ENSG00000112715	17520823;17377739;17459229;
D009268	increase	IL1B	ENSG00000125538	16148020;
D009268	increase	MMP13	ENSG00000137745	16148020;
D009281	decrease	BCL2	ENSG00000171791	17224139;
D009281	increase	TP53	ENSG00000141510	17224139;
D009288	increase	CCND1	ENSG00000110092	15489888;
D009288	increase	GCLC	ENSG00000001084	11909699;
D009288	increase	GCLM	ENSG00000023909	11909699;
D009288	increase	NGFR	ENSG00000064300	17409433;
D009288	increase	PTGS2	ENSG00000073756	15705740;15489888;17164136;
D009288	increase	TNF	ENSG00000223952	15489888;
D009496	increase	FOS	ENSG00000170345	17000667;

D009496	increase	FOSL1	ENSG00000175592	17000667;
D009496	increase	IL8	ENSG00000169429	17000667;
D009496	increase	JUNB	ENSG00000171223	17000667;
D009496	increase	NFKB1	ENSG00000109320	17000667;
D009496	increase	RELA	ENSG00000173039	17000667;
D009532	affect	STAT1	ENSG00000115415	19403854;
D009532	decrease	CHPT1	ENSG00000111666	19031421;
D009532	decrease	CLIP1	ENSG00000130779	19031421;
D009532	decrease	EHMT2	ENSG00000204371	16648469;
D009532	decrease	NEK4	ENSG00000114904	19031421;
D009532	decrease	UBE2A	ENSG00000077721	16870173;
D009532	increase	AURKA	ENSG00000087586	19031421;
D009532	increase	BIRC5	ENSG00000089685	19031421;
D009532	increase	BUB1B	ENSG00000156970	19031421;
D009532	increase	CCNB2	ENSG00000157456	19031421;
D009532	increase	CD86	ENSG00000114013	19033392;
D009532	increase	CDC25B	ENSG00000101224	19031421;
D009532	increase	CKS2	ENSG00000123975	19031421;
D009532	increase	FOS	ENSG00000170345	14971665;
D009532	increase	GSTA1	ENSG00000243955	14971665;
D009532	increase	HIF1A	ENSG00000100644	11504687;16877034;19403854;16386771;
D009532	increase	HMOX1	ENSG00000100292	19033392;
D009532	increase	KEAP1	ENSG00000079999	19033392;
D009532	increase	KIF2C	ENSG00000142945	19031421;
D009532	increase	MMP2	ENSG00000087245	16581223;
D009532	increase	MT2A	ENSG00000125148	14971665;
D009532	increase	NDRG1	ENSG00000104419	16386771;
D009532	increase	NEK2	ENSG00000117650	19031421;
D009532	increase	NQO1	ENSG00000181019	19033392;
D009532	increase	NUSAP1	ENSG00000137804	19031421;
D009532	increase	PAK3	ENSG00000077264	19031421;
D009532	increase	PBK	ENSG00000168078	19031421;
D009532	increase	PLK1	ENSG00000166851	19031421;
D009532	increase	PTTG1	ENSG00000164611	19031421;
D009532	increase	SERPINE1	ENSG00000106366	11504687;
D009532	increase	SMC4	ENSG00000113810	19031421;
D009532	increase	SPAG5	ENSG00000076382	19031421;
D009532	increase	TPX2	ENSG00000088325	19031421;
D009532	increase	UBE2E1	ENSG00000170142	16870173;
D009532	increase	VEGFA	ENSG00000112715	19403854;
D009536	affect	IL1A	ENSG00000115008	11031321;
D009536	affect	IL1R2	ENSG00000115590	11031321;
D009536	affect	IL1RN	ENSG00000136689	11031321;
D009536	decrease	IL6	ENSG00000136244	12727023;
D009536	increase	BAX	ENSG00000087088	16998810;
D009536	increase	CDKN1A	ENSG00000124762	12782109;
D009536	increase	INS	ENSG00000129965	16505238;
D009536	increase	MDM2	ENSG00000135679	16998810;
D009536	increase	TP53	ENSG00000141510	12782109;
D009538	decrease	AMHR2	ENSG00000135409	18247414;
D009538	decrease	BAD	ENSG00000002330	18247414;
D009538	decrease	BCL2A1	ENSG00000140379	18247414;
D009538	decrease	BRCA1	ENSG00000012048	18247414;

D009538	decrease	BTF3	ENSG00000145741	18247414;
D009538	decrease	BTK	ENSG00000010671	18247414;
D009538	decrease	C5AR1	ENSG00000197405	18247414;
D009538	decrease	CARTPT	ENSG00000164326	18247414;
D009538	decrease	CCL16	ENSG00000161573	18247414;
D009538	decrease	CCR6	ENSG00000112486	18247414;
D009538	decrease	CD4	ENSG00000010610	18247414;
D009538	decrease	CHRNA3	ENSG00000080644	12628473;
D009538	decrease	CHRNA5	ENSG00000169684	12628473;
D009538	decrease	CHRNA6	ENSG00000147434	12628473;
D009538	decrease	CHRNA7	ENSG00000175344	12628473;
D009538	decrease	CREB1	ENSG00000118260	18247414;
D009538	decrease	DEDD	ENSG00000158796	18247414;
D009538	decrease	DFFB	ENSG00000169598	18247414;
D009538	decrease	DNASE2	ENSG00000105612	18247414;
D009538	decrease	EDNRA	ENSG00000151617	18247414;
D009538	decrease	EPHA3	ENSG00000044524	18247414;
D009538	decrease	EPHB3	ENSG00000182580	18247414;
D009538	decrease	FLT3	ENSG00000122025	18247414;
D009538	decrease	FN1	ENSG00000115414	18247414;
D009538	decrease	HLTF	ENSG00000071794	18247414;
D009538	decrease	IFI6	ENSG00000126709	18247414;
D009538	decrease	IFNAR1	ENSG00000142166	18247414;
D009538	decrease	IL10	ENSG00000136634	18247414;
D009538	decrease	IL3RA	ENSG00000185291	18247414;
D009538	decrease	IL9	ENSG00000145839	18247414;
D009538	decrease	ITGAL	ENSG00000005844	18247414;
D009538	decrease	ITGB1	ENSG00000150093	11338295;
D009538	decrease	IVL	ENSG00000163207	16011614;
D009538	decrease	KLF11	ENSG00000172059	18247414;
D009538	decrease	LTB	ENSG00000236237	18247414;
D009538	decrease	MADD	ENSG00000110514	18247414;
D009538	decrease	MST1	ENSG00000173531	18247414;
D009538	decrease	NFKBIE	ENSG00000146232	18247414;
D009538	decrease	NFYB	ENSG00000120837	18247414;
D009538	decrease	NME3	ENSG00000103024	18247414;
D009538	decrease	OPRK1	ENSG00000082556	18247414;
D009538	decrease	POU2AF1	ENSG00000110777	18247414;
D009538	decrease	PSMB6	ENSG00000142507	18247414;
D009538	decrease	PTAFR	ENSG00000169403	18247414;
D009538	decrease	PTX3	ENSG00000163661	18247414;
D009538	decrease	RARG	ENSG00000172819	18247414;
D009538	decrease	RB1	ENSG00000139687	16011614;
D009538	decrease	RPL7A	ENSG00000148303	18247414;
D009538	decrease	RUNX1T1	ENSG00000079102	18247414;
D009538	decrease	S100A10	ENSG00000197747	18247414;
D009538	decrease	SLC19A1	ENSG00000173638	19616087;
D009538	decrease	SLC9A3R2	ENSG00000065054	18247414;
D009538	decrease	SOD1	ENSG00000142168	16627626;
D009538	decrease	SREBF1	ENSG00000072310	18247414;
D009538	decrease	STAT2	ENSG00000170581	18247414;
D009538	decrease	SUPT4H1	ENSG00000213246	18247414;
D009538	decrease	TFDP1	ENSG00000198176	18247414;
D009538	decrease	TNFRSF1A	ENSG00000067182	18247414;

D009538	decrease	TP53	ENSG00000141510	18247414;16011614;
D009538	decrease	UBE2I	ENSG00000103275	18247414;
D009538	decrease	VIM	ENSG00000026025	18247414;
D009538	increase	AATK	ENSG00000181409	18247414;
D009538	increase	APP	ENSG00000142192	16627626;
D009538	increase	CBL	ENSG00000110395	18247414;
D009538	increase	CCL5	ENSG00000161570	18247414;
D009538	increase	CCR5	ENSG00000160791	18247414;
D009538	increase	CCS	ENSG00000173992	16627626;
D009538	increase	CD59	ENSG00000085063	18247414;
D009538	increase	CDKN1A	ENSG00000124762	16011614;
D009538	increase	CSF2	ENSG00000164400	18082304;
D009538	increase	CYP1A1	ENSG00000140465	11849738;
D009538	increase	CYP1A2	ENSG00000140505	15916432;11849738;
D009538	increase	EPO	ENSG00000130427	18247414;
D009538	increase	EZR	ENSG00000092820	18247414;
D009538	increase	FASLG	ENSG00000117560	18247414;
D009538	increase	GDF1	ENSG00000130283	18247414;
D009538	increase	IL1A	ENSG00000115008	18082304;
D009538	increase	IL1B	ENSG00000125538	18082304;
D009538	increase	IL6	ENSG00000136244	18082304;
D009538	increase	IL7	ENSG00000104432	18082304;
D009538	increase	NOS2	ENSG00000007171	18247414;
D009538	increase	PLD2	ENSG00000129219	18247414;
D009538	increase	POU2F1	ENSG00000143190	18247414;
D009538	increase	PRKAR2B	ENSG00000005249	18247414;
D009538	increase	RGS1	ENSG00000090104	18247414;
D009538	increase	RPS25	ENSG00000118181	18247414;
D009538	increase	SMAD9	ENSG00000120693	18247414;
D009538	increase	SNCA	ENSG00000145335	18247414;
D009538	increase	STK25	ENSG00000115694	18247414;
D009538	increase	TSHR	ENSG00000165409	18247414;
D009538	increase	UGP2	ENSG00000169764	18247414;
D009543	affect	BCL2	ENSG00000171791	10695937;
D009543	increase	ABCB1	ENSG00000085563	8632764;
D009543	increase	CYP3A4	ENSG00000160868	15286053;15634941;15784650;16712844;15937564;15618748;8632764;17954527;16837568;
D009543	increase	FGF2	ENSG00000138685	16188231;
D009543	increase	FOS	ENSG00000170345	16188231;
D009543	increase	MYC	ENSG00000136997	10695937;
D009544	decrease	CYP19A1	ENSG00000137869	15964185;
D009569	decrease	BCL2	ENSG00000171791	15126337;
D009569	decrease	BCL2L1	ENSG00000171552	15126337;
D009569	decrease	BIRC3	ENSG00000023445	15126337;
D009569	decrease	BIRC5	ENSG00000089685	15126337;
D009569	decrease	CD86	ENSG00000114013	13678430;
D009569	decrease	EDN1	ENSG00000078401	11187978;15838368;
D009569	decrease	HIF1A	ENSG00000100644	15465035;16310922;
D009569	decrease	IL12B	ENSG00000113302	13678430;
D009569	decrease	XIAP	ENSG00000101966	15126337;
D009569	increase	ANXA5	ENSG00000164111	16024610;
D009569	increase	BBC3	ENSG00000105327	15126337;

D009569	increase	FAS	ENSG00000026103	15126337;
D009569	increase	IL8	ENSG00000169429	13678430;10619828;
D009569	increase	NOS2	ENSG00000007171	10450786;15979383;18845238;
D009569	increase	PMAIP1	ENSG00000141682	15126337;
D009569	increase	PPM1D	ENSG00000170836	15126337;
D009569	increase	TP53	ENSG00000141510	16024610;15979383;17555331;
D009569	increase	TP53I3	ENSG00000115129	15126337;
D009588	decrease	AHR	ENSG00000106546	17204746;
D009588	decrease	BRCA1	ENSG00000012048	10344722;
D009588	decrease	BRCA2	ENSG00000139618	10344722;
D009588	decrease	ERBB2	ENSG00000141736	16417271;
D009588	increase	NFE2L2	ENSG00000116044	17204746;
D009589	increase	BAX	ENSG00000087088	16041517;
D009589	increase	BCL2	ENSG00000171791	16041517;
D009589	increase	BID	ENSG00000015475	16041517;
D009589	increase	FAS	ENSG00000026103	16041517;
D009589	increase	FASLG	ENSG00000117560	16041517;
D009589	increase	JUN	ENSG00000177606	16041517;
D009589	increase	MAP3K1	ENSG00000095015	16041517;
D009589	increase	MAPK8	ENSG00000107643	16041517;
D009589	increase	MCL1	ENSG00000143384	16041517;
D009589	increase	TP53	ENSG00000141510	16041517;
D009599	decrease	BCL2	ENSG00000171791	15708893;
D009599	decrease	BIRC5	ENSG00000089685	14988404;
D009599	decrease	HIF1A	ENSG00000100644	15465035;
D009599	decrease	HSPD1	ENSG00000144381	17855661;
D009599	decrease	IFNG	ENSG00000111537	9201699;
D009599	decrease	IL4	ENSG00000113520	9201699;
D009599	decrease	MYC	ENSG00000136997	7798212;
D009599	decrease	NOS1	ENSG00000089250	17855661;
D009599	decrease	NOS3	ENSG00000164867	17855661;
D009599	decrease	PPARGC1A	ENSG00000109819	17855661;
D009599	increase	CASP3	ENSG00000164305	15708893;
D009599	increase	CASP7	ENSG00000165806	15708893;
D009599	increase	G6PD	ENSG00000160211	17018880;
D009599	increase	HMOX1	ENSG00000100292	8764571;
D009599	increase	MT1A	ENSG00000205362	17018880;
D009599	increase	MT1E	ENSG00000169715	17018880;
D009599	increase	MT1X	ENSG00000187193	17018880;
D009599	increase	MT2A	ENSG00000125148	17018880;
D009599	increase	MTF1	ENSG00000188786	17018880;
D009599	increase	SLC30A1	ENSG00000170385	17018880;
D009599	increase	TP53	ENSG00000141510	15979383;
D009602	decrease	LYNX1	ENSG00000180155	17280689;
D009602	decrease	SLURP1	ENSG00000126233	17280689;
D009637	decrease	FPR1	ENSG00000171051	17520823;17459229;
D009637	increase	CASP8	ENSG00000064012	17645780;
D009637	increase	CDH1	ENSG00000039068	18418215;
D009637	increase	CDKN2A	ENSG00000147889	18164318;
D009637	increase	FAS	ENSG00000026103	17645780;
D009637	increase	HSD11B2	ENSG00000176387	18032417;
D009637	increase	IGF1R	ENSG00000140443	18491370;

D009637	increase	IL1B	ENSG00000125538	12573452;
D009637	increase	IL8	ENSG00000169429	17459229;
D009637	increase	PTGS2	ENSG00000073756	12573452;
D009637	increase	SOD2	ENSG00000112096	8760145;
D009637	increase	TNF	ENSG00000223952	8760145;
D009637	increase	TNFRSF10B	ENSG00000120889	17645780;
D009637	increase	VEGFA	ENSG00000112715	17520823;17459229;
D009638	increase	FN1	ENSG00000115414	11821714;
D009638	increase	S100A8	ENSG00000143546	16677282;
D009638	increase	S100A9	ENSG00000163220	16677282;
D009640	affect	FSHB	ENSG00000131808	15244497;
D009640	affect	LHB	ENSG00000104826	15244497;
D009640	affect	SHBG	ENSG00000129214	10709161;
D009640	decrease	MAPT	ENSG00000186868	16930453;
D009640	decrease	SERPINC1	ENSG00000117601	14561536;
D009640	decrease	SERPINE1	ENSG00000106366	14561536;
D009640	increase	F10	ENSG00000126218	14561536;
D009640	increase	F7	ENSG00000057593	14561536;
D009640	increase	FGA	ENSG00000171560	14561536;
D009640	increase	FGB	ENSG00000171564	14561536;
D009640	increase	FGG	ENSG00000171557	14561536;
D009640	increase	PLG	ENSG00000122194	14561536;
D009640	increase	PROC	ENSG00000115718	14561536;
D009640	increase	VEGFA	ENSG00000112715	17010073;
D009641	increase	FASN	ENSG00000169710	15870863;
D009643	increase	IL1B	ENSG00000125538	16148020;
D009643	increase	MMP13	ENSG00000137745	16148020;
D009643	increase	MMP1	ENSG00000196611	16148020;
D009644	increase	KLK3	ENSG00000142515	11580929;
D009665	decrease	HIF1A	ENSG00000100644	16596621;
D009675	decrease	CDH2	ENSG00000170558	11853877;
D009675	increase	CD38	ENSG00000004468	11853877;
D009840	decrease	CCND1	ENSG00000110092	15925326;
D009840	decrease	HIF1A	ENSG00000100644	15840558;
D009840	increase	CD14	ENSG00000170458	10491287;
D009840	increase	CD38	ENSG00000004468	10491287;
D009853	affect	NR1I2	ENSG00000144852	12695340;
D009853	decrease	ABCB4	ENSG00000005471	12040753;
D009853	decrease	ADH1C	ENSG00000248144	12040753;
D009853	increase	ALDH1A3	ENSG00000184254	12040753;
D009853	increase	ALDH3A1	ENSG00000108602	12040753;
D009853	increase	CYP1A1	ENSG00000140465	11454723;12040753;17954527;15681896;12623754;11849738;15860653;1284193
D009853	increase	CYP1A2	ENSG00000140505	16035375;18332078;12040753;15681896;17954527;15802389;12623754;1284193
D009853	increase	CYP1B1	ENSG00000138061	12623754;12040753;
D009853	increase	CYP2B6	ENSG00000197408	17954527;
D009853	increase	CYP2E1	ENSG00000130649	15802389;
D009853	increase	CYP3A4	ENSG00000160868	15286053;15626586;12485723;17565714;12695340;17954527;15802389;1546616
D009921	decrease	ADRB2	ENSG00000169252	16980553;
D009942	decrease	MDM2	ENSG00000135679	17210701;

D009942	decrease	MDM4	ENSG00000198625	17210701;
D009942	increase	ANXA5	ENSG00000164111	17210701;
D009942	increase	PMAIP1	ENSG00000141682	17210701;
D009942	increase	TP53	ENSG00000141510	17210701;
D010042	decrease	KCNH2	ENSG00000055118	19139152;
D010100	affect	AKT1	ENSG00000142208	18924134;
D010100	affect	BIRC5	ENSG00000089685	16628086;
D010100	affect	HSPD1	ENSG00000144381	15583868;
D010100	affect	ITGB1	ENSG00000150093	16628086;
D010100	affect	MAPK8	ENSG00000107643	19196431;10780954;
D010100	affect	MAPK9	ENSG00000050748	19196431;
D010100	decrease	NFKBIA	ENSG00000100906	17671207;11034997;
D010100	increase	ANGPT2	ENSG00000091879	10854229;
D010100	increase	BACE1	ENSG00000186318	19196431;
D010100	increase	BIRC3	ENSG00000023445	17671207;
D010100	increase	BNIP3	ENSG00000176171	17914565;
D010100	increase	CA9	ENSG00000107159	18924134;
D010100	increase	CAT	ENSG00000121691	19196431;11306437;
D010100	increase	CCND1	ENSG00000110092	17914565;
D010100	increase	CDKN1A	ENSG00000124762	19091749;10951577;
D010100	increase	CDKN1B	ENSG00000111276	17914565;
D010100	increase	CDKN1C	ENSG00000129757	17914565;
D010100	increase	DCC	ENSG00000187323	17914565;
D010100	increase	DNMT1	ENSG00000130816	18314485;
D010100	increase	E2F3	ENSG00000112242	17914565;
D010100	increase	EGR1	ENSG00000120738	17914565;
D010100	increase	FOS	ENSG00000170345	17914565;
D010100	increase	HIF1A	ENSG00000100644	12062197;18924134;11424089;19091749;16628086;19196431;18394600;12376345;19542490;17671207;12054621;19139008;17764071;1
D010100	increase	HSF1	ENSG00000185122	11424089;
D010100	increase	HSPB1	ENSG00000106211	15583868;17914565;
D010100	increase	IL6	ENSG00000136244	11950692;
D010100	increase	JUN	ENSG00000177606	17914565;
D010100	increase	LOX	ENSG00000113083	18924134;
D010100	increase	MDK	ENSG00000110492	17914565;
D010100	increase	MOS	ENSG00000172680	17914565;
D010100	increase	MPG	ENSG00000103152	17914565;
D010100	increase	MT1X	ENSG00000187193	17914565;
D010100	increase	MT2A	ENSG00000125148	17914565;
D010100	increase	MT3	ENSG00000087250	12763630;12058024;
D010100	increase	NOL3	ENSG00000140939	17914565;
D010100	increase	PDK1	ENSG00000152256	17671207;
D010100	increase	PLCG1	ENSG00000124181	17914565;
D010100	increase	PPP1R15A	ENSG00000087074	17914565;
D010100	increase	PRKCD	ENSG00000163932	11424089;
D010100	increase	PROM1	ENSG00000007062	18985161;
D010100	increase	RRAS	ENSG00000126458	17914565;
D010100	increase	SLC2A1	ENSG00000117394	19091749;10780954;74939
D010100	increase	STC2	ENSG00000113739	18394600;
D010100	increase	TGFA	ENSG00000163235	11171960;
D010100	increase	TP53	ENSG00000141510	10951577;

D010100	increase	VCL	ENSG00000035403	17914565;
D010100	increase	VEGFA	ENSG00000112715	19091749;19542490;17914565;17671207;12563064;17764071;18575727;
D010100	increase	VEGFB	ENSG00000173511	17914565;
D010100	increase	WT1	ENSG00000184937	12738801;
D010117	increase	MMP9	ENSG00000100985	19371603;
D010126	affect	PPARA	ENSG00000186951	17355223;
D010126	affect	TFAP2A	ENSG00000137203	17355223;
D010126	decrease	CFTR	ENSG00000001626	19061877;
D010126	decrease	IL5	ENSG00000113525	10226068;
D010126	decrease	TAC1	ENSG00000006128	9310018;
D010126	increase	AHR	ENSG00000106546	19536146;
D010126	increase	APP	ENSG00000142192	15513908;
D010126	increase	BRS3	ENSG00000102239	17355223;16426703;
D010126	increase	COL1A1	ENSG00000108821	9820643;
D010126	increase	COL3A1	ENSG00000168542	9820643;
D010126	increase	CYP1A1	ENSG00000140465	19536146;
D010126	increase	CYP1A2	ENSG00000140505	19536146;
D010126	increase	CYP1B1	ENSG00000138061	19536146;
D010126	increase	HMOX1	ENSG00000100292	18524839;18274635;
D010126	increase	IL1B	ENSG00000125538	15570426;7598938;
D010126	increase	IL6	ENSG00000136244	15379216;15570426;7598938;16860297;
D010126	increase	IL8	ENSG00000169429	19059883;15379216;9820643;16819095;9297567;19162161;9124608;11781168;7598938;16860297;
D010126	increase	NQO1	ENSG00000181019	17652311;19059883;
D010126	increase	PTGS2	ENSG00000073756	9176252;15513908;
D010126	increase	SFTPA1	ENSG00000122852	15065867;14617519;11781168;
D010126	increase	SFTPA2	ENSG00000185303	15065867;14617519;11781168;
D010126	increase	TGFB1	ENSG00000105329	9820643;
D010126	increase	TNF	ENSG00000223952	16456144;14617519;15570426;11781168;7598938;17450233;19236316;15486341;
D010171	decrease	HNF4A	ENSG00000101076	17992261;
D010171	decrease	SHBG	ENSG00000129214	17992261;
D010269	decrease	BCL2L1	ENSG00000171552	15824117;
D010269	decrease	SFTPD	ENSG00000133661	17503247;
D010269	increase	APAF1	ENSG00000120868	17064354;
D010269	increase	BAX	ENSG00000087088	15824117;
D010269	increase	CASP8	ENSG00000064012	17064354;
D010269	increase	CASP9	ENSG00000132906	17064354;
D010269	increase	CTGF	ENSG00000118523	16767377;15728566;
D010269	increase	HMOX1	ENSG00000100292	17409708;15728566;16263688;
D010269	increase	ICAM1	ENSG00000090339	10644010;
D010269	increase	JUN	ENSG00000177606	16237197;
D010269	increase	LSM1	ENSG00000175324	17064354;
D010269	increase	MT2A	ENSG00000125148	14503839;
D010269	increase	NQO1	ENSG00000181019	17409708;
D010269	increase	PARK7	ENSG00000116288	11811539;11697128;14662519;

D010269	increase	PRDX1	ENSG00000117450	11697128;
D010269	increase	PRDX2	ENSG00000167815	11697128;
D010269	increase	PRDX3	ENSG00000165672	11697128;
D010269	increase	SCGB1A1	ENSG00000149021	17174135;
D010269	increase	SFTPA1	ENSG00000122852	17174135;
D010269	increase	SFTPB	ENSG00000168878	17174135;
D010269	increase	SMN1	ENSG00000172062	17064354;
D010269	increase	SNCA	ENSG00000145335	17934957;16239214;15234109;12716914;
D010269	increase	SOD1	ENSG00000142168	11590119;10644010;18686095;16738222;15824117;16677106;
D010269	increase	SOD2	ENSG00000112096	10644010;14503839;14660625;11306437;10969820;
D010269	increase	TGFB1	ENSG00000105329	15728566;
D010269	increase	TP53	ENSG00000141510	11369509;15162845;10969820;
D010269	increase	VEGFA	ENSG00000112715	15728566;
D010278	increase	DVL1	ENSG00000107404	18204794;
D010278	increase	HRAS	ENSG00000174775	18204794;
D010278	increase	MCM2	ENSG00000073111	18204794;
D010278	increase	PCNA	ENSG00000132646	12762645;
D010278	increase	TP53	ENSG00000141510	12762645;19787260;
D010278	increase	TP53I3	ENSG00000115129	18204794;
D010365	increase	IL1B	ENSG00000125538	18308354;
D010365	increase	IL8	ENSG00000169429	18308354;
D010406	increase	SLC1A2	ENSG00000110436	17138558;
D010416	affect	FOS	ENSG00000170345	12724925;
D010416	affect	TP53	ENSG00000141510	12724925;
D010416	decrease	CCR5	ENSG00000160791	18486366;
D010416	decrease	CSE1L	ENSG00000124207	11064002;
D010416	decrease	XCL2	ENSG00000143185	18486366;
D010416	increase	CCL3	ENSG00000006075	18486366;
D010416	increase	CYP1A1	ENSG00000140465	8812189;12724925;
D010416	increase	CYP2B6	ENSG00000197408	15548381;
D010416	increase	CYP3A4	ENSG00000160868	15548381;
D010416	increase	CYP3A7	ENSG00000160870	8812189;
D010416	increase	DNAJB4	ENSG00000162616	18486366;
D010416	increase	ERCC3	ENSG00000163161	18486366;
D010416	increase	ERCC4	ENSG00000175595	18486366;
D010416	increase	HSP90AA1	ENSG00000080824	18486366;
D010416	increase	IL21	ENSG00000138684	18486366;
D010416	increase	LTBR	ENSG00000111321	18486366;
D010416	increase	POR	ENSG00000127948	18486366;
D010416	increase	RAD23A	ENSG00000179262	18486366;
D010416	increase	TRADD	ENSG00000102871	18486366;
D010416	increase	UGT1A10	ENSG00000242515	18486366;
D010416	increase	XRCC4	ENSG00000152422	18486366;
D010417	increase	FTH1	ENSG00000167996	17641250;
D010417	increase	HMOX1	ENSG00000100292	17641250;
D010419	decrease	FABP1	ENSG00000163586	15342952;
D010419	decrease	LSS	ENSG00000160285	15342952;
D010419	decrease	SLC2A3	ENSG00000059804	15342952;
D010419	increase	ASNS	ENSG00000070669	15342952;
D010419	increase	C10ORF10	ENSG00000165507	15342952;

D010419	increase	FNDC4	ENSG00000115226	15342952;
D010419	increase	HPN	ENSG00000105707	15342952;
D010419	increase	INHBE	ENSG00000139269	15342952;
D010419	increase	NR0B2	ENSG00000131910	15342952;
D010419	increase	NUPR1	ENSG00000176046	15342952;
D010419	increase	SERPINA3	ENSG00000196136	15342952;
D010419	increase	WIPI1	ENSG00000070540	15342952;
D010424	increase	JUN	ENSG00000177606	15263067;
D010431	affect	CRYAB	ENSG00000109846	18207697;
D010431	affect	SERPINH1	ENSG00000149257	18207697;
D010431	increase	ACTA2	ENSG00000107796	18207697;
D010431	increase	COL1A1	ENSG00000108821	18207697;
D010431	increase	TNF	ENSG00000223952	7769305;
D010457	increase	BCL2A1	ENSG00000140379	15860229;
D010457	increase	CCL20	ENSG00000115009	15860229;15191912;
D010457	increase	CSF2	ENSG00000164400	12176910;15191912;12574323;
D010457	increase	CXCL5	ENSG00000163735	15860229;
D010457	increase	EHD1	ENSG00000110047	15860229;
D010457	increase	GADD45B	ENSG00000099860	15860229;
D010457	increase	ICAM1	ENSG00000090339	15860229;
D010457	increase	IL12B	ENSG00000113302	15860229;
D010457	increase	IL1A	ENSG00000115008	15860229;
D010457	increase	IL1B	ENSG00000125538	15860229;12574323;
D010457	increase	IL23A	ENSG00000110944	15860229;
D010457	increase	IL6	ENSG00000136244	15860229;
D010457	increase	IL8	ENSG00000169429	12176910;15893496;15860229;
D010457	increase	MMP1	ENSG00000196611	15860229;
D010457	increase	PTGS2	ENSG00000073756	15860229;
D010457	increase	SOD2	ENSG00000112096	15860229;
D010457	increase	TNFAIP6	ENSG00000123610	15860229;
D010457	increase	TNF	ENSG00000223952	15893496;15860229;
D010480	decrease	SLC2A3	ENSG00000059804	15342952;
D010480	decrease	TAGLN	ENSG00000149591	15342952;
D010480	increase	ASAH1	ENSG00000104763	15342952;
D010480	increase	C10ORF10	ENSG00000165507	15342952;
D010480	increase	FABP1	ENSG00000163586	15342952;
D010480	increase	FNDC4	ENSG00000115226	15342952;
D010480	increase	GDPD3	ENSG00000102886	15342952;
D010480	increase	HPN	ENSG00000105707	15342952;
D010480	increase	LSS	ENSG00000160285	15342952;
D010480	increase	NR0B2	ENSG00000131910	15342952;
D010480	increase	SERPINA3	ENSG00000196136	15342952;
D010480	increase	WIPI1	ENSG00000070540	15342952;
D010628	decrease	PTGS2	ENSG00000073756	14592552;
D010634	affect	CYP7A1	ENSG00000167910	15629111;
D010634	affect	IL6	ENSG00000136244	10700573;
D010634	affect	TNF	ENSG00000223952	10700573;
D010634	decrease	GSTA1	ENSG00000243955	7986210;
D010634	decrease	GSTM1	ENSG00000134184	7986210;
D010634	increase	ABCB1	ENSG00000085563	18408562;19409404;18094037;8632764;
D010634	increase	CES1	ENSG00000198848	10640517;

D010634	increase	CES2	ENSG00000172831	10640517;
D010634	increase	CYP1A1	ENSG00000140465	19409404;16354159;
D010634	increase	CYP1A2	ENSG00000140505	15272053;
D010634	increase	CYP2A6	ENSG00000198077	11038160;16857725;15629111;15448115;
D010634	increase	CYP2B6	ENSG00000197408	12695351;12870655;14977870;18332078;18094037;17954527;15802389;15629111;15548381;15448115;
D010634	increase	CYP2C19	ENSG00000165841	17304159;16789993;15629111;
D010634	increase	CYP2C9	ENSG00000138109	17304159;15629111;15802389;
D010634	increase	CYP3A4	ENSG00000160868	12695340;10219967;14977870;12065438;18094037;16837568;17954527;15802389;15629111;12505310;15833926;15466163;12673034;8632764;10640517;1554838
D010634	increase	CYP3A5	ENSG00000106258	10696073;15629111;
D010634	increase	NR1I2	ENSG00000144852	12695351;12695340;15039302;14977870;12065438;15548381;
D010634	increase	NR1I3	ENSG00000143257	15272053;12695351;
D010634	increase	NR3C1	ENSG00000113580	12695351;10219967;12673034;
D010653	increase	CCND1	ENSG00000110092	15489888;
D010653	increase	CYP3A4	ENSG00000160868	10219967;9512926;
D010653	increase	NR3C1	ENSG00000113580	10219967;
D010653	increase	PTGS2	ENSG00000073756	15489888;
D010653	increase	TNF	ENSG00000223952	10328874;15489888;
D010654	increase	CDKN1A	ENSG00000124762	10866311;18223691;16875574;
D010654	increase	IFNG	ENSG00000111537	10866311;
D010656	affect	GDF15	ENSG00000130513	19505289;
D010656	increase	CAV3	ENSG00000182533	12847114;
D010656	increase	CDKN1A	ENSG00000124762	10766810;
D010656	increase	EDN1	ENSG00000078401	12724418;
D010656	increase	EP300	ENSG00000100393	12724418;
D010656	increase	FN1	ENSG00000115414	11821714;
D010656	increase	GATA4	ENSG00000136574	12724418;
D010656	increase	MMP9	ENSG00000100985	16574429;
D010656	increase	MYH7	ENSG00000092054	12847114;
D010656	increase	TGFB1	ENSG00000105329	11821714;
D010670	decrease	FOS	ENSG00000170345	18003792;
D010670	increase	CASP9	ENSG00000132906	16447258;
D010670	increase	PPARA	ENSG00000186951	16447258;
D010670	increase	RELA	ENSG00000173039	16447258;
D010672	affect	BCL2	ENSG00000171791	11134551;10695937;
D010672	affect	IL6	ENSG00000136244	10700573;
D010672	affect	NR1I3	ENSG00000143257	15123723;15849716;
D010672	affect	TNF	ENSG00000223952	10700573;
D010672	decrease	AFP	ENSG00000081051	14741686;
D010672	decrease	BMP1	ENSG00000168487	14741686;
D010672	decrease	CAPN5	ENSG00000149260	14741686;
D010672	decrease	CCND1	ENSG00000110092	14741686;

D010672	decrease	CDKN1A	ENSG00000124762	14741686;
D010672	decrease	COL1A1	ENSG00000108821	8971655;
D010672	decrease	DAPK2	ENSG00000035664	14741686;
D010672	decrease	FRS3	ENSG00000137218	14741686;
D010672	decrease	GSTK1	ENSG00000250907	14741686;
D010672	decrease	GSTO1	ENSG00000148834	14741686;
D010672	decrease	IL1B	ENSG00000125538	8971655;9029455;
D010672	decrease	ITGA2	ENSG00000164171	15948689;
D010672	decrease	ITGB1	ENSG00000150093	15948689;
D010672	decrease	MMP2	ENSG00000087245	15948689;
D010672	decrease	MMP3	ENSG00000149968	15948689;
D010672	decrease	PLAUR	ENSG00000011422	14741686;
D010672	decrease	PLXNB2	ENSG00000196576	14741686;
D010672	decrease	STAT4	ENSG00000138378	14741686;
D010672	decrease	VEGFB	ENSG00000173511	14741686;
D010672	increase	CASP10	ENSG00000003400	14741686;
D010672	increase	COL6A1	ENSG00000142156	8224229;
D010672	increase	COL6A2	ENSG00000142173	8224229;
D010672	increase	COL6A3	ENSG00000163359	8224229;
D010672	increase	CTSB	ENSG00000164733	10914799;14741686;
D010672	increase	CTSL1	ENSG00000135047	10914799;
D010672	increase	CYP2B6	ENSG00000197408	12695351;18206661;15123723;14977870;18332078;17954527;
D010672	increase	CYP2C9	ENSG00000138109	11503012;16873909;9860067;16220110;11740344;11434505;10208645;14659971;15855726;2025243;9110362;11234294;10850388;10510154;11668218;14600250;
D010672	increase	CYP2E1	ENSG00000130649	18206661;
D010672	increase	CYP3A4	ENSG00000160868	18206661;12695340;10219967;16207822;14977870;9512926;12065438;18332078;16837568;17954527;12673034;12505310;15466163;
D010672	increase	CYP3A5	ENSG00000106258	12673034;
D010672	increase	CYP3A7	ENSG00000160870	12673034;
D010672	increase	DAP3	ENSG00000132676	14741686;
D010672	increase	EOMES	ENSG00000163508	14741686;
D010672	increase	FGFR2	ENSG00000066468	11352631;
D010672	increase	FLRT2	ENSG00000185070	14741686;
D010672	increase	GSTA1	ENSG00000243955	11134551;
D010672	increase	ITGA8	ENSG00000077943	14741686;
D010672	increase	LPIN1	ENSG00000134324	14741686;
D010672	increase	MAP1B	ENSG00000131711	14741686;
D010672	increase	MDK	ENSG00000110492	14741686;
D010672	increase	MMP1	ENSG00000196611	15948689;14741686;10914799;
D010672	increase	MYC	ENSG00000136997	10695937;
D010672	increase	NFKB1	ENSG00000109320	10700573;14741686;
D010672	increase	NR1I2	ENSG00000144852	12695351;14600250;12695340;15039302;14977870;12065438;
D010672	increase	NR3C1	ENSG00000113580	12695351;10219967;12673034;

D010672	increase	PDGFB	ENSG00000100311	9029455;8463938;
D010672	increase	PIP4K2A	ENSG00000150867	14741686;
D010672	increase	PRSS12	ENSG00000164099	14741686;
D010672	increase	RPL28	ENSG00000108107	14741686;
D010672	increase	S100A4	ENSG00000196154	14741686;
D010672	increase	SC5DL	ENSG00000109929	14741686;
D010672	increase	SP3	ENSG00000172845	14741686;
D010672	increase	TIMP1	ENSG00000102265	10914799;15948689;
D010672	increase	TP53	ENSG00000141510	11134551;
D010672	increase	UGT1A1	ENSG00000241635	15849716;10421657;
D010693	increase	CYP1A1	ENSG00000140465	17177569;
D010710	affect	AKT1	ENSG00000142208	16763222;
D010710	increase	DMP1	ENSG00000152592	20185895;
D010710	increase	FGF23	ENSG00000118972	14988389;15827108;15917335;16011465;15838626;15613425;16352682;16272628;16369891;15284207;15961556;15995295;15248822;11409890;15086938;146331
D010710	increase	MKNK1	ENSG00000079277	16763222;
D010710	increase	PHEX	ENSG00000102174	16011465;15542045;
D010712	increase	PTGS2	ENSG00000073756	15210717;
D010716	affect	MAPK1	ENSG00000100030	19013290;
D010716	affect	MAPK3	ENSG00000102882	19013290;
D010716	increase	APOA1	ENSG00000118137	19013290;
D010758	affect	CPT1A	ENSG00000110090	17715259;
D010758	affect	EDNRB	ENSG00000136160	17715259;
D010758	affect	IL11	ENSG00000095752	17715259;
D010758	affect	IL6	ENSG00000136244	17715259;
D010758	affect	NR4A1	ENSG00000123358	17715259;
D010758	affect	NR4A2	ENSG00000153234	17715259;
D010758	affect	PEX11A	ENSG00000166821	17715259;
D010758	decrease	APOE	ENSG00000130203	17715259;
D010758	decrease	CPN2	ENSG00000178772	17715259;
D010758	decrease	CTDSPL	ENSG00000144677	17715259;
D010758	decrease	CYP1B1	ENSG00000138061	17715259;
D010758	decrease	CYP2B6	ENSG00000197408	17715259;
D010758	decrease	GHRHR	ENSG00000106128	17715259;
D010758	decrease	IL1B	ENSG00000125538	17715259;
D010758	decrease	IL1F5	ENSG00000136695	17715259;
D010758	decrease	IL1RN	ENSG00000136689	17715259;
D010758	decrease	IL24	ENSG00000162892	17715259;
D010758	decrease	IL8	ENSG00000169429	17715259;
D010758	decrease	NCOR1	ENSG00000141027	17715259;
D010758	decrease	POSTN	ENSG00000133110	17715259;
D010758	decrease	RGS4	ENSG00000117152	17715259;
D010758	decrease	SERPINE1	ENSG00000106366	17715259;
D010758	decrease	THBS1	ENSG00000137801	17715259;
D010758	decrease	TNFRSF11B	ENSG00000164761	17715259;
D010758	increase	AADAC	ENSG00000114771	17715259;
D010758	increase	ALB	ENSG00000163631	17715259;
D010758	increase	ALPL	ENSG00000162551	17715259;
D010758	increase	B4GALT1	ENSG00000086062	17715259;
D010758	increase	BMP4	ENSG00000125378	17715259;

D010758	increase	CD14	ENSG00000170458	17715259;
D010758	increase	CES1	ENSG00000198848	17715259;
D010758	increase	COL15A1	ENSG00000204291	17715259;
D010758	increase	COL1A1	ENSG00000108821	17715259;
D010758	increase	CP	ENSG00000047457	17715259;
D010758	increase	CRIP1	ENSG00000213145	17715259;
D010758	increase	CST1	ENSG00000170373	17715259;
D010758	increase	CYP24A1	ENSG00000019186	17715259;
D010758	increase	DTNA	ENSG00000134769	17715259;
D010758	increase	FABP3	ENSG00000121769	17715259;
D010758	increase	FBLN2	ENSG00000163520	17715259;
D010758	increase	FGF23	ENSG00000118972	15356053;12419819;
D010758	increase	ID3	ENSG00000117318	17715259;
D010758	increase	MAP4	ENSG00000047849	17715259;
D010758	increase	MAPK13	ENSG00000156711	17715259;
D010758	increase	MCAM	ENSG00000076706	17715259;
D010758	increase	MFI2	ENSG00000163975	17715259;
D010758	increase	MGP	ENSG00000111341	17715259;
D010758	increase	MRPL33	ENSG00000243147	17715259;
D010758	increase	PTGS1	ENSG00000095303	17715259;
D010758	increase	PTGS2	ENSG00000073756	17715259;
D010758	increase	RARRES1	ENSG00000118849	17715259;
D010758	increase	SMC3	ENSG00000108055	17715259;
D010758	increase	SOD3	ENSG00000109610	17715259;
D010758	increase	SON	ENSG00000159140	17715259;
D010758	increase	SPIB	ENSG00000142539	17715259;
D010758	increase	SPP1	ENSG00000118785	17715259;
D010758	increase	TGFB3	ENSG00000119699	17715259;
D010758	increase	THBD	ENSG00000178726	17715259;
D010758	increase	TNXB	ENSG00000168477	17715259;
D010758	increase	TPR	ENSG00000047410	17715259;
D010758	increase	TREM1	ENSG00000124731	17715259;
D010758	increase	VDR	ENSG00000111424	17715259;
D010833	affect	PRKCD	ENSG00000163932	15868430;
D010833	decrease	CDKN1B	ENSG00000111276	15868430;
D010833	increase	TP53	ENSG00000141510	16357363;
D010847	decrease	GSTA1	ENSG00000243955	7986210;
D010847	decrease	GSTM1	ENSG00000134184	7986210;
D010853	increase	IL1B	ENSG00000125538	18854230;17429439;
D010853	increase	IL4	ENSG00000113520	18854230;
D010869	decrease	ADRB2	ENSG00000169252	2175014;
D010869	increase	ADRB1	ENSG00000043591	2175014;
D010882	increase	IFNG	ENSG00000111537	10414782;
D010882	increase	IL4	ENSG00000113520	10414782;
D010890	increase	CD40	ENSG00000101017	16879495;
D010894	decrease	CYP19A1	ENSG00000137869	15964185;
D010894	increase	TNF	ENSG00000223952	10328874;
D010936	affect	CRP	ENSG00000132693	17884346;
D010936	affect	GADD45A	ENSG00000116717	12878215;
D010936	affect	HSP90AA1	ENSG00000080824	12878215;
D010936	affect	HSPB1	ENSG00000106211	12878215;
D010936	affect	IL1A	ENSG00000115008	17884346;
D010936	affect	IL1B	ENSG00000125538	17884346;
D010936	affect	MDM2	ENSG00000135679	12878215;

D010936	affect	NR1I2	ENSG00000144852	12695340;15627040;17210444;
D010936	affect	ODC1	ENSG00000115758	12878215;
D010936	affect	PTCH1	ENSG00000185920	12878215;
D010936	decrease	ARHGAP4	ENSG00000089820	15465739;
D010936	decrease	BCL2L1	ENSG00000171552	17619071;
D010936	decrease	BCR	ENSG00000186716	15465739;
D010936	decrease	BRCC3	ENSG00000185515	15465739;
D010936	decrease	CASP3	ENSG00000164305	16962711;17619071;
D010936	decrease	CCNA2	ENSG00000145386	15327971;
D010936	decrease	CCR2	ENSG00000121807	12462994;
D010936	decrease	CCR3	ENSG00000183625	12462994;
D010936	decrease	CCR5	ENSG00000160791	12462994;
D010936	decrease	CD79A	ENSG00000105369	15465739;
D010936	decrease	CDK2	ENSG00000123374	15327971;
D010936	decrease	CDK6	ENSG00000105810	15930460;
D010936	decrease	CEBPG	ENSG00000153879	15465739;
D010936	decrease	DCT	ENSG00000080166	17473463;
D010936	decrease	E2F1	ENSG00000101412	15327971;
D010936	decrease	FLT3LG	ENSG00000090554	15465739;
D010936	decrease	FTH1	ENSG00000167996	15465739;
D010936	decrease	GSTP1	ENSG00000084207	15465739;
D010936	decrease	HSPD1	ENSG00000144381	15465739;
D010936	decrease	IL2	ENSG00000109471	12878215;12412711;19761891;
D010936	decrease	IL8	ENSG00000169429	16243974;
D010936	decrease	IRF4	ENSG00000137265	15465739;
D010936	decrease	MAP2K1	ENSG00000169032	15465739;
D010936	decrease	NPM1	ENSG00000181163	15465739;
D010936	decrease	PARP1	ENSG00000143799	15465739;19168119;17619071;
D010936	decrease	RAC1	ENSG00000136238	15465739;
D010936	decrease	RBL1	ENSG00000080839	15327971;
D010936	decrease	RPL6	ENSG00000089009	15465739;
D010936	decrease	STAT1	ENSG00000115415	15465739;
D010936	decrease	TCF3	ENSG00000071564	15465739;
D010936	decrease	TK1	ENSG00000167900	15465739;
D010936	decrease	TOP1	ENSG00000198900	15465739;
D010936	decrease	TOP2A	ENSG00000131747	15465739;
D010936	decrease	VEGFA	ENSG00000112715	14666653;
D010936	decrease	XCL1	ENSG00000143184	15465739;
D010936	decrease	XRCC1	ENSG00000073050	15465739;
D010936	increase	ABCB1	ENSG00000085563	15729621;18425719;15627040;18439772;
D010936	increase	ABCC1	ENSG00000103222	15465739;18351577;
D010936	increase	ABCC2	ENSG00000023839	15729621;18351577;
D010936	increase	APP	ENSG00000142192	16962711;
D010936	increase	BAT2	ENSG00000204469	15465739;
D010936	increase	BAX	ENSG00000087088	18834353;12878215;16962711;17619071;
D010936	increase	BCL2	ENSG00000171791	16962711;
D010936	increase	BIRC2	ENSG00000110330	12878215;18841906;
D010936	increase	BIRC3	ENSG00000023445	12878215;18841906;
D010936	increase	BSG	ENSG00000172270	15465739;
D010936	increase	CASP10	ENSG00000003400	18351577;

D010936	increase	CD81	ENSG00000110651	15465739;
D010936	increase	CDKN1A	ENSG00000124762	12878215;15327971;18834353;
D010936	increase	CHST3	ENSG00000122863	18351577;
D010936	increase	CSF2RB	ENSG00000100368	15465739;
D010936	increase	CYP1A1	ENSG00000140465	19794518;17177569;15560888;15629189;
D010936	increase	CYP1A2	ENSG00000140505	15025748;15629189;
D010936	increase	CYP1B1	ENSG00000138061	19794518;
D010936	increase	CYP24A1	ENSG00000019186	18351577;
D010936	increase	CYP2D6	ENSG00000100197	15025748;15930738;15974642;15900287;15992226;15629189;
D010936	increase	CYP2E1	ENSG00000130649	15900287;15974642;15629189;
D010936	increase	CYP3A4	ENSG00000160868	12695340;15627040;15900287;15992226;15974642;15025748;17210444;
D010936	increase	EPO	ENSG00000130427	18351577;
D010936	increase	ETS1	ENSG00000134954	15465739;
D010936	increase	FAS	ENSG00000026103	17619071;
D010936	increase	GCLC	ENSG00000001084	11864778;
D010936	increase	GRN	ENSG00000030582	15465739;
D010936	increase	GSTA4	ENSG00000170899	18351577;
D010936	increase	GSTT2	ENSG00000133433	18351577;
D010936	increase	HCFC1	ENSG00000172534	15465739;
D010936	increase	HMOX1	ENSG00000100292	17210444;
D010936	increase	HOXD3	ENSG00000128652	15465739;
D010936	increase	ICAM1	ENSG00000090339	19103272;15465739;
D010936	increase	IFNG	ENSG00000111537	19103272;
D010936	increase	IL10	ENSG00000136634	15465739;
D010936	increase	IL2RA	ENSG00000134460	12878215;15465739;
D010936	increase	IL4	ENSG00000113520	15465739;
D010936	increase	IL6	ENSG00000136244	16243974;
D010936	increase	IRAK1	ENSG00000184216	15465739;
D010936	increase	IRF2	ENSG00000168310	15465739;
D010936	increase	ITGA2B	ENSG00000005961	15465739;
D010936	increase	ITGAL	ENSG00000005844	15465739;
D010936	increase	LPL	ENSG00000175445	15975614;
D010936	increase	MIF	ENSG00000240972	15465739;
D010936	increase	MLF2	ENSG00000089693	15465739;
D010936	increase	MMP9	ENSG00000100985	18841906;
D010936	increase	NFKB2	ENSG00000077150	18351577;
D010936	increase	NFKBIB	ENSG00000104825	18351577;
D010936	increase	NOS1	ENSG00000089250	16962711;
D010936	increase	NOS2	ENSG00000007171	16962711;18841906;
D010936	increase	NOS3	ENSG00000164867	17278969;
D010936	increase	NR3C1	ENSG00000113580	16243974;
D010936	increase	NUP214	ENSG00000126883	15465739;
D010936	increase	PBX2	ENSG00000236353	15465739;
D010936	increase	PGR	ENSG00000082175	11368622;16076101;
D010936	increase	POU3F2	ENSG00000184486	15465739;
D010936	increase	PPARA	ENSG00000186951	15975614;16680075;
D010936	increase	PTGS2	ENSG00000073756	15997105;18841906;
D010936	increase	PTPN6	ENSG00000111679	18351577;

D010936	increase	PTPRJ	ENSG00000149177	18351577;
D010936	increase	SELL	ENSG00000188404	15465739;
D010936	increase	SLC20A1	ENSG00000144136	15465739;
D010936	increase	SOD1	ENSG00000142168	16962711;
D010936	increase	STAT6	ENSG00000166888	15465739;
D010936	increase	TCF7	ENSG00000081059	15465739;
D010936	increase	TFF1	ENSG00000160182	11368622;14706564;
D010936	increase	TGFA	ENSG00000163235	18351577;
D010936	increase	TNF	ENSG00000223952	16243974;18841906;
D010936	increase	TNK2	ENSG00000061938	15465739;
D010936	increase	TP53	ENSG00000141510	18834353;
D010936	increase	UGT2B7	ENSG00000171234	18351577;
D010936	increase	XCR1	ENSG00000173578	15465739;
D010936	increase	ZNF593	ENSG00000142684	15465739;
D010938	affect	IL1B	ENSG00000125538	16804125;
D010938	decrease	BCL2	ENSG00000171791	15961301;
D010938	increase	GADD45A	ENSG00000116717	15961301;
D010938	increase	PPARG	ENSG00000132170	15961301;
D010938	increase	PTGS2	ENSG00000073756	12672646;
D010938	increase	TP53	ENSG00000141510	15961301;
D010972	decrease	PPARA	ENSG00000186951	16386258;
D010972	increase	AGTR1	ENSG00000144891	16386258;
D010972	increase	ALOX12	ENSG00000108839	16386258;
D010972	increase	ALOX5	ENSG00000012779	16386258;
D010972	increase	CCR2	ENSG00000121807	16386258;
D010972	increase	CRP	ENSG00000132693	16386258;
D010972	increase	CX3CR1	ENSG00000168329	16386258;
D010972	increase	EGR1	ENSG00000120738	16386258;
D010972	increase	IL1A	ENSG00000115008	16386258;
D010972	increase	IL1B	ENSG00000125538	16386258;
D010972	increase	MMP13	ENSG00000137745	16386258;
D010972	increase	OLR1	ENSG00000173391	16386258;
D010972	increase	PTAFR	ENSG00000169403	16386258;
D010972	increase	PTGS1	ENSG00000095303	16386258;
D010972	increase	PTGS2	ENSG00000073756	16386258;
D010972	increase	SELE	ENSG00000007908	16386258;
D010972	increase	TIMP4	ENSG00000157150	16386258;
D010972	increase	TNF	ENSG00000223952	16386258;
D010984	increase	GSTP1	ENSG00000084207	15788669;16814760;
D011034	decrease	MAPT	ENSG00000186868	16930453;
D011070	affect	TP53	ENSG00000141510	18779317;
D011070	increase	CD83	ENSG00000112149	17698565;
D011070	increase	CD86	ENSG00000114013	17698565;
D011070	increase	EIF2AK2	ENSG00000055332	17669408;
D011070	increase	IFNB1	ENSG00000171855	17669408;18779317;
D011070	increase	IL8	ENSG00000169429	18779317;17669408;
D011070	increase	MX1	ENSG00000157601	17669408;
D011070	increase	OAS1	ENSG00000089127	17669408;
D011070	increase	TLR2	ENSG00000137462	17669408;
D011070	increase	TLR3	ENSG00000164342	17669408;18779317;
D011070	increase	TLR7	ENSG00000196664	17669408;
D011075	increase	PGR	ENSG00000082175	11693576;
D011075	increase	TFF1	ENSG00000160182	11693576;
D011078	decrease	ATF3	ENSG00000162772	16474171;

D011078	decrease	ATP1B1	ENSG00000143153	16474171;
D011078	decrease	BASP1	ENSG00000176788	16474171;
D011078	decrease	BTG1	ENSG00000133639	16474171;
D011078	decrease	CAMK2N1	ENSG00000162545	16474171;
D011078	decrease	CDK2AP2	ENSG00000167797	16474171;
D011078	decrease	CDKN1A	ENSG00000124762	16474171;
D011078	decrease	CDKN2C	ENSG00000123080	16474171;
D011078	decrease	CDKN3	ENSG00000100526	16474171;
D011078	decrease	CEBPD	ENSG00000221869	16474171;
D011078	decrease	CLDN4	ENSG00000189143	16474171;
D011078	decrease	CYP21A2	ENSG00000231852	16650473;
D011078	decrease	EFNB2	ENSG00000125266	16474171;
D011078	decrease	ETS2	ENSG00000157557	16474171;
D011078	decrease	GADD45A	ENSG00000116717	16474171;
D011078	decrease	GPRC5A	ENSG00000013588	16474171;
D011078	decrease	HIST1H2AC	ENSG00000180573	16474171;
D011078	decrease	HIST2H2BE	ENSG00000184678	16474171;
D011078	decrease	HSD3B2	ENSG00000203859	16650473;
D011078	decrease	ID3	ENSG00000117318	16474171;
D011078	decrease	JUP	ENSG00000173801	16474171;
D011078	decrease	KRAS	ENSG00000133703	16474171;
D011078	decrease	L1CAM	ENSG00000198910	16474171;
D011078	decrease	MXD4	ENSG00000123933	16474171;
D011078	decrease	MYH6	ENSG00000197616	16474171;
D011078	decrease	NCOA3	ENSG00000124151	16474171;
D011078	decrease	NEDD9	ENSG00000111859	16474171;
D011078	decrease	PMEPA1	ENSG00000124225	16474171;
D011078	decrease	POLD4	ENSG00000175482	16474171;
D011078	decrease	PPAP2B	ENSG00000162407	16474171;
D011078	decrease	PPAP2C	ENSG00000141934	16474171;
D011078	decrease	SLC7A11	ENSG00000151012	16474171;
D011078	decrease	SOX2	ENSG00000181449	16474171;
D011078	decrease	SOX4	ENSG00000124766	16474171;
D011078	decrease	SQSTM1	ENSG00000161011	16474171;
D011078	decrease	STEAP1	ENSG00000164647	16474171;
D011078	decrease	TCF25	ENSG00000141002	16474171;
D011078	decrease	TGFB2	ENSG00000092969	16474171;
D011078	decrease	TIMP3	ENSG00000100234	16474171;
D011078	decrease	TRIM16	ENSG00000221926	16474171;
D011078	decrease	UGT1A3	ENSG00000243135	16474171;
D011078	decrease	YPEL5	ENSG00000119801	16474171;
D011078	decrease	ZFP36L1	ENSG00000185650	16474171;
D011078	increase	AGR2	ENSG00000106541	16474171;
D011078	increase	AHR	ENSG00000106546	16650473;
D011078	increase	AP1S2	ENSG00000182287	16474171;
D011078	increase	APOBEC3B	ENSG00000179750	16474171;
D011078	increase	AREG	ENSG00000205595	16474171;
D011078	increase	ARL3	ENSG00000138175	16474171;
D011078	increase	ASPM	ENSG00000066279	16474171;
D011078	increase	ATAD2	ENSG00000156802	16474171;
D011078	increase	AURKA	ENSG00000087586	16474171;
D011078	increase	AURKB	ENSG00000178999	16474171;
D011078	increase	BIRC5	ENSG00000089685	16474171;
D011078	increase	BUB1B	ENSG00000156970	16474171;

D011078	increase	CA12	ENSG00000074410	16474171;
D011078	increase	CCNA2	ENSG00000145386	16474171;
D011078	increase	CCNB2	ENSG00000157456	16474171;
D011078	increase	CDC20	ENSG00000117399	16474171;
D011078	increase	CDC6	ENSG00000094804	16474171;
D011078	increase	CDK1	ENSG00000170312	16474171;
D011078	increase	CDK2	ENSG00000123374	16474171;
D011078	increase	CDT1	ENSG00000167513	16474171;
D011078	increase	CENPA	ENSG00000115163	16474171;
D011078	increase	CENPF	ENSG00000117724	16474171;
D011078	increase	CENPJ	ENSG00000151849	16474171;
D011078	increase	CENPN	ENSG00000166451	16474171;
D011078	increase	CHEK1	ENSG00000149554	16474171;
D011078	increase	CHSY1	ENSG00000131873	16474171;
D011078	increase	CKS1B	ENSG00000173207	16474171;
D011078	increase	CLIC6	ENSG00000159212	16474171;
D011078	increase	COL12A1	ENSG00000111799	16474171;
D011078	increase	CSRP2BP	ENSG00000149474	16474171;
D011078	increase	CTPS	ENSG00000171793	16474171;
D011078	increase	CYP11B2	ENSG00000179142	16650473;
D011078	increase	CYP1A1	ENSG00000140465	16474171;17112691;
D011078	increase	CYP1B1	ENSG00000138061	16474171;
D011078	increase	DAD1	ENSG00000129562	16474171;
D011078	increase	DEPDC1B	ENSG00000035499	16474171;
D011078	increase	DHRS2	ENSG00000100867	16474171;
D011078	increase	DTL	ENSG00000143476	16474171;
D011078	increase	DTYMK	ENSG00000168393	16474171;
D011078	increase	EBP	ENSG00000147155	16474171;
D011078	increase	ECT2	ENSG00000114346	16474171;
D011078	increase	EMP2	ENSG00000213853	16474171;
D011078	increase	ESR1	ENSG00000091831	12377985;
D011078	increase	ESR2	ENSG00000140009	12377985;
D011078	increase	FANCI	ENSG00000140525	16474171;
D011078	increase	FBXO32	ENSG00000156804	16474171;
D011078	increase	FBXO5	ENSG00000112029	16474171;
D011078	increase	FEN1	ENSG00000168496	16474171;
D011078	increase	FKBP4	ENSG00000004478	16474171;
D011078	increase	GFRA1	ENSG00000151892	16474171;
D011078	increase	GGH	ENSG00000137563	16474171;
D011078	increase	GINS1	ENSG00000101003	16474171;
D011078	increase	GINS2	ENSG00000131153	16474171;
D011078	increase	GMNN	ENSG00000112312	16474171;
D011078	increase	GREB1	ENSG00000196208	16474171;
D011078	increase	H2AFX	ENSG00000188486	16474171;
D011078	increase	H2AFZ	ENSG00000164032	16474171;
D011078	increase	HADH	ENSG00000138796	16474171;
D011078	increase	HMGB2	ENSG00000164104	16474171;
D011078	increase	IGFBP4	ENSG00000141753	16474171;
D011078	increase	ILF2	ENSG00000143621	16474171;
D011078	increase	ITPK1	ENSG00000100605	16474171;
D011078	increase	KIAA0101	ENSG00000166803	16474171;
D011078	increase	KIF11	ENSG00000138160	16474171;
D011078	increase	KIF14	ENSG00000118193	16474171;
D011078	increase	KIF15	ENSG00000163808	16474171;

D011078	increase	KIF20A	ENSG00000112984	16474171;
D011078	increase	KIF23	ENSG00000137807	16474171;
D011078	increase	KIF4A	ENSG00000090889	16474171;
D011078	increase	KPNA2	ENSG00000182481	16474171;
D011078	increase	MAD1L1	ENSG00000002822	16474171;
D011078	increase	MASTL	ENSG00000120539	16474171;
D011078	increase	MCM10	ENSG00000065328	16474171;
D011078	increase	MCM2	ENSG00000073111	16474171;
D011078	increase	MCM6	ENSG00000076003	16474171;
D011078	increase	MCM7	ENSG00000166508	16474171;
D011078	increase	MELK	ENSG00000165304	16474171;
D011078	increase	MLF1	ENSG00000178053	16474171;
D011078	increase	MLF1IP	ENSG00000151725	16474171;
D011078	increase	MPHOSPH9	ENSG00000051825	16474171;
D011078	increase	MYB	ENSG00000118513	16474171;
D011078	increase	MYBL2	ENSG00000101057	16474171;
D011078	increase	MYC	ENSG00000136997	16474171;
D011078	increase	NCAPD2	ENSG00000010292	16474171;
D011078	increase	NCAPG	ENSG00000109805	16474171;
D011078	increase	NDC80	ENSG00000080986	16474171;
D011078	increase	OLFM1	ENSG00000130558	16474171;
D011078	increase	OLFML3	ENSG00000116774	16474171;
D011078	increase	PBK	ENSG00000168078	16474171;
D011078	increase	PCNA	ENSG00000132646	16474171;
D011078	increase	PCP4	ENSG00000183036	16474171;
D011078	increase	PLK4	ENSG00000142731	16474171;
D011078	increase	PLOD1	ENSG00000083444	16474171;
D011078	increase	PPP2R2C	ENSG00000074211	16474171;
D011078	increase	PRC1	ENSG00000198901	16474171;
D011078	increase	PRIM1	ENSG00000198056	16474171;
D011078	increase	PTGER3	ENSG00000050628	16474171;
D011078	increase	RACGAP1	ENSG00000161800	16474171;
D011078	increase	RAD51AP1	ENSG00000111247	16474171;
D011078	increase	RAD51C	ENSG00000108384	16474171;
D011078	increase	RAD51	ENSG00000051180	16474171;
D011078	increase	RNASEH1	ENSG00000171865	16474171;
D011078	increase	RRM2	ENSG00000171848	16474171;
D011078	increase	SAC3D1	ENSG00000168061	16474171;
D011078	increase	SFXN2	ENSG00000156398	16474171;
D011078	increase	SLC39A8	ENSG00000138821	16474171;
D011078	increase	SLC6A14	ENSG00000087916	16474171;
D011078	increase	SMC2	ENSG00000136824	16474171;
D011078	increase	SMC4	ENSG00000113810	16474171;
D011078	increase	STC2	ENSG00000113739	16474171;
D011078	increase	STMN1	ENSG00000117632	16474171;
D011078	increase	TACC3	ENSG00000013810	16474171;
D011078	increase	TFF1	ENSG00000160182	16474171;
D011078	increase	TFF3	ENSG00000160180	16474171;
D011078	increase	THBS1	ENSG00000137801	16474171;
D011078	increase	TK1	ENSG00000167900	16474171;
D011078	increase	TMEM38B	ENSG00000095209	16474171;
D011078	increase	TMEM97	ENSG00000109084	16474171;
D011078	increase	TMPO	ENSG00000120802	16474171;
D011078	increase	TOP2A	ENSG00000131747	16474171;16906772;

D011078	increase	TPM3	ENSG00000143549	16474171;
D011078	increase	TRIP13	ENSG00000071539	16474171;
D011078	increase	TUBB6	ENSG00000176014	16474171;
D011078	increase	TYMS	ENSG00000176890	16474171;
D011078	increase	UBE2C	ENSG00000175063	16474171;
D011078	increase	UBE2T	ENSG00000077152	16474171;
D011078	increase	UHRF1	ENSG00000034063	16474171;
D011078	increase	UNG	ENSG00000076248	16474171;
D011078	increase	ZNF703	ENSG00000183779	16474171;
D011078	increase	ZWINT	ENSG00000122952	16474171;
D011084	decrease	CYP21A2	ENSG00000231852	16650473;
D011084	decrease	HSD3B2	ENSG00000203859	16650473;
D011084	decrease	RB1	ENSG00000139687	16002475;
D011084	increase	AHR	ENSG00000106546	16650473;
D011084	increase	AHRR	ENSG00000063438	12655030;
D011084	increase	CDKN1A	ENSG00000124762	16002475;
D011084	increase	CYP11B2	ENSG00000179142	16650473;
D011084	increase	CYP1A1	ENSG00000140465	15801491;15917149;11967624;15278913;16002475;15720126;
D011084	increase	CYP1A2	ENSG00000140505	15824912;
D011084	increase	CYP1B1	ENSG00000138061	15122594;15471096;15278913;16002475;15720126;
D011084	increase	FGF6	ENSG00000111241	16002475;
D011084	increase	FGF9	ENSG00000102678	16002475;
D011084	increase	FIGF	ENSG00000165197	16002475;
D011084	increase	FOSL1	ENSG00000175592	16002475;
D011084	increase	IL11	ENSG00000095752	16002475;
D011084	increase	IL1A	ENSG00000115008	16002475;
D011084	increase	IL6	ENSG00000136244	16002475;
D011112	increase	CD86	ENSG00000114013	15671028;
D011134	increase	CD40	ENSG00000101017	15894585;
D011134	increase	CD80	ENSG00000121594	15894585;
D011134	increase	CD83	ENSG00000112149	15894585;
D011134	increase	CD86	ENSG00000114013	15894585;
D011134	increase	ICAM1	ENSG00000090339	15894585;
D011134	increase	IL10	ENSG00000136634	15894585;
D011134	increase	IL12A	ENSG00000168811	15894585;
D011134	increase	IL12B	ENSG00000113302	15894585;
D011143	decrease	ANGPT1	ENSG00000154188	12809767;
D011188	decrease	KCNH2	ENSG00000055118	19419905;17624316;19139152;15618689;16960444;15679475;
D011188	increase	CYP11B2	ENSG00000179142	12193581;9139807;
D011192	affect	LCK	ENSG00000182866	17078813;
D011192	decrease	ACP2	ENSG00000134575	17547211;
D011192	decrease	ACSL1	ENSG00000151726	17547211;
D011192	decrease	AKR1B1	ENSG00000085662	17547211;
D011192	decrease	ATP2B1	ENSG00000070961	17547211;
D011192	decrease	AZGP1	ENSG00000160862	17547211;
D011192	decrease	C14ORF1	ENSG00000133935	17547211;
D011192	decrease	CALR	ENSG00000179218	17547211;
D011192	decrease	CFB	ENSG00000243570	17547211;
D011192	decrease	CLGN	ENSG00000153132	17547211;

D011192	decrease	CLPTM1	ENSG00000104853	17547211;
D011192	decrease	CRYAA	ENSG00000160202	17547211;
D011192	decrease	CX3CL1	ENSG00000006210	17547211;
D011192	decrease	F5	ENSG00000198734	17547211;
D011192	decrease	FURIN	ENSG00000140564	17547211;
D011192	decrease	GGCX	ENSG00000115486	17547211;
D011192	decrease	GPER	ENSG00000164850	17547211;
D011192	decrease	HMGCR	ENSG00000113161	17547211;
D011192	decrease	IL17RB	ENSG00000056736	17547211;
D011192	decrease	ITIH3	ENSG00000162267	17547211;
D011192	decrease	MGAT2	ENSG00000168282	17547211;
D011192	decrease	NR0B2	ENSG00000131910	17547211;
D011192	decrease	NUCB2	ENSG00000070081	17547211;
D011192	decrease	OS9	ENSG00000135506	17547211;
D011192	decrease	PDIA4	ENSG00000155660	17547211;
D011192	decrease	PGC	ENSG00000096088	17547211;
D011192	decrease	PGF	ENSG00000119630	17547211;
D011192	decrease	PNMA1	ENSG00000176903	17547211;
D011192	decrease	REPIN1	ENSG00000214022	17547211;
D011192	decrease	SERPINB1	ENSG00000021355	17547211;
D011192	decrease	SERPINC1	ENSG00000117601	17547211;
D011192	decrease	SFRS5	ENSG00000100650	17547211;
D011192	decrease	TAF7	ENSG00000178913	17547211;
D011192	decrease	TNFSF10	ENSG00000121858	17547211;
D011192	decrease	TOB1	ENSG00000141232	17547211;
D011192	increase	ABCG2	ENSG00000118777	17547211;
D011192	increase	ADM	ENSG00000148926	17547211;
D011192	increase	ANXA2	ENSG00000182718	17547211;
D011192	increase	ASNS	ENSG00000070669	17547211;
D011192	increase	CCNB2	ENSG00000157456	17547211;
D011192	increase	CDKN1A	ENSG00000124762	17547211;
D011192	increase	CHST3	ENSG00000122863	17547211;
D011192	increase	ENPP1	ENSG00000197594	17547211;
D011192	increase	FOSL1	ENSG00000175592	17547211;
D011192	increase	HIST1H4C	ENSG00000182217	17547211;
D011192	increase	HMGA1	ENSG00000137309	17547211;
D011192	increase	HMGN2	ENSG00000198830	17547211;
D011192	increase	HMOX1	ENSG00000100292	16775837;
D011192	increase	IER2	ENSG00000160888	17547211;
D011192	increase	IL6	ENSG00000136244	17078813;
D011192	increase	ITGB3BP	ENSG00000142856	17547211;
D011192	increase	KLF10	ENSG00000155090	17547211;
D011192	increase	MAFF	ENSG00000185022	17547211;
D011192	increase	MCM2	ENSG00000073111	17547211;
D011192	increase	PIM1	ENSG00000137193	17547211;
D011192	increase	PPIH	ENSG00000171960	17547211;
D011192	increase	PTTG1	ENSG00000164611	17547211;
D011192	increase	RFC2	ENSG00000049541	17547211;
D011192	increase	RNASEH2A	ENSG00000104889	17547211;
D011192	increase	SFRS2	ENSG00000161547	17547211;
D011192	increase	SFRS7	ENSG00000115875	17547211;
D011192	increase	SIK1	ENSG00000142178	17547211;
D011192	increase	SNRPA	ENSG00000077312	17547211;
D011192	increase	TNFAIP3	ENSG00000118503	17547211;

D011192	increase	TYMS	ENSG00000176890	17547211;
D011192	increase	UBE2C	ENSG00000175063	17547211;
D011192	increase	UGCG	ENSG00000148154	17547211;
D011192	increase	ZWINT	ENSG00000122952	17547211;
D011223	increase	IL5	ENSG00000113525	15213147;
D011224	decrease	FN1	ENSG00000115414	11821714;
D011224	increase	CYP1A1	ENSG00000140465	15627480;
D011239	decrease	IL4	ENSG00000113520	15821026;
D011239	decrease	IL5	ENSG00000113525	15821026;
D011239	increase	CYP3A4	ENSG00000160868	12673034;
D011239	increase	HPGD	ENSG00000164120	15680906;
D011241	decrease	CCL11	ENSG00000172156	15523430;
D011241	decrease	CCL13	ENSG00000181374	15523430;
D011241	decrease	IL13	ENSG00000169194	15523430;
D011241	decrease	IL5	ENSG00000113525	12722970;15523430;
D011241	decrease	TGFB1	ENSG00000105329	12722970;
D011241	increase	CDKN1A	ENSG00000124762	15034294;
D011241	increase	CDKN1B	ENSG00000111276	15034294;
D011241	increase	CDKN2A	ENSG00000147889	15034294;
D011241	increase	CDKN2B	ENSG00000147883	15034294;
D011284	increase	CYP3A4	ENSG00000160868	15650019;
D011285	increase	CYP3A4	ENSG00000160868	10219967;12505310;15541768;
D011285	increase	NR3C1	ENSG00000113580	10219967;
D011285	increase	UGT1A10	ENSG00000242515	16155002;
D011285	increase	UGT1A1	ENSG00000241635	16155002;
D011285	increase	UGT1A3	ENSG00000243135	16155002;
D011285	increase	UGT1A4	ENSG00000244474	16155002;
D011285	increase	UGT1A5	ENSG00000240224	16155002;
D011285	increase	UGT1A6	ENSG00000167165	16155002;
D011285	increase	UGT1A7	ENSG00000244122	16155002;
D011285	increase	UGT1A8	ENSG00000242366	16155002;
D011285	increase	UGT1A9	ENSG00000241119	16155002;
D011319	increase	CYP1A1	ENSG00000140465	12623754;17954527;
D011319	increase	CYP1A2	ENSG00000140505	12920490;12623754;17954527;
D011319	increase	CYP1B1	ENSG00000138061	12623754;
D011339	decrease	CYP3A4	ENSG00000160868	12065438;15915352;16837568;
D011341	increase	SELE	ENSG00000007908	16769766;
D011341	increase	TNF	ENSG00000223952	16769766;
D011341	increase	VCAM1	ENSG00000162692	16769766;
D011342	increase	HSD11B2	ENSG00000176387	15489962;
D011342	increase	WIPI1	ENSG00000070540	15342952;
D011345	decrease	CCND1	ENSG00000110092	16569247;
D011345	decrease	CRP	ENSG00000132693	16123850;
D011345	decrease	MAT2A	ENSG00000168906	16569247;
D011345	decrease	PPARA	ENSG00000186951	15299082;16569247;
D011345	decrease	SEMA6B	ENSG00000167680	16525649;
D011345	increase	ABCB4	ENSG00000005471	14685799;
D011345	increase	ABCG5	ENSG00000138075	14685799;
D011345	increase	CCL2	ENSG00000108691	16600694;
D011345	increase	CYP7A1	ENSG00000167910	14685799;
D011345	increase	JUN	ENSG00000177606	16600694;

D011345	increase	PCK2	ENSG00000100889	16569247;
D011345	increase	PON1	ENSG00000005421	16123850;
D011345	increase	RELA	ENSG00000173039	16600694;
D011345	increase	SCARB1	ENSG00000073060	16816107;
D011345	increase	SREBF2	ENSG00000198911	14685799;
D011353	increase	ABCC1	ENSG00000103222	15746575;
D011372	increase	CCND1	ENSG00000110092	15862825;
D011372	increase	CDK2	ENSG00000123374	15862825;
D011372	increase	PGR	ENSG00000082175	15845615;15862825;
D011372	increase	TP53	ENSG00000141510	15860260;
D011372	increase	VEGFA	ENSG00000112715	15860260;15845615;
D011374	affect	APOBEC3B	ENSG00000179750	17404688;
D011374	affect	CDA	ENSG00000158825	17404688;
D011374	affect	MUC1	ENSG00000185499	15971251;
D011374	affect	PRMT7	ENSG00000132600	17404688;
D011374	decrease	ABCA8	ENSG00000141338	17404688;
D011374	decrease	ABCC3	ENSG00000108846	17404688;
D011374	decrease	ABCC6	ENSG00000091262	18070364;
D011374	decrease	ABHD11	ENSG00000249672	18037150;
D011374	decrease	ACPP	ENSG00000014257	17404688;
D011374	decrease	ACTA2	ENSG00000107796	18037150;
D011374	decrease	ACTN1	ENSG00000072110	18037150;
D011374	decrease	ADAM12	ENSG00000148848	17404688;
D011374	decrease	AGAP3	ENSG00000133612	18037150;
D011374	decrease	AGPAT4	ENSG00000026652	17404688;
D011374	decrease	AKAP8	ENSG00000105127	18037150;
D011374	decrease	AKAP9	ENSG00000127914	18070364;
D011374	decrease	AKR1B10	ENSG00000198074	17404688;
D011374	decrease	ALDH3A1	ENSG00000108602	18037150;
D011374	decrease	ANGPT2	ENSG00000091879	17404688;
D011374	decrease	ANGPTL2	ENSG00000136859	17404688;
D011374	decrease	APEX1	ENSG00000100823	18037150;
D011374	decrease	APOC4	ENSG00000224916	18037150;
D011374	decrease	AQP3	ENSG00000165272	17404688;
D011374	decrease	ARAP3	ENSG00000120318	17404688;
D011374	decrease	ARHGDIA	ENSG00000141522	18070364;
D011374	decrease	ARHGEF17	ENSG00000110237	17404688;
D011374	decrease	ATP2B4	ENSG00000058668	18070364;
D011374	decrease	ATP5S	ENSG00000125375	17404688;
D011374	decrease	B4GALT2	ENSG00000117411	18037150;
D011374	decrease	BAG1	ENSG00000250477	18037150;
D011374	decrease	BAT2	ENSG00000204469	18070364;
D011374	decrease	BDKRB1	ENSG00000100739	17404688;
D011374	decrease	BDNF	ENSG00000176697	18070364;
D011374	decrease	BHLHE41	ENSG00000123095	17404688;
D011374	decrease	BID	ENSG00000015475	18037150;
D011374	decrease	C10ORF116	ENSG00000148671	17404688;
D011374	decrease	C10ORF72	ENSG00000165633	17404688;
D011374	decrease	C12ORF5	ENSG00000078237	18037150;
D011374	decrease	C16ORF45	ENSG00000166780	17404688;
D011374	decrease	C1QBP	ENSG00000108561	18037150;
D011374	decrease	C21ORF7	ENSG00000156265	17404688;
D011374	decrease	CA2	ENSG00000104267	17404688;18037150;
D011374	decrease	CACNA1G	ENSG00000006283	17404688;

D011374	decrease	CATSPERB	ENSG00000133962	18070364;
D011374	decrease	CCL5	ENSG00000161570	17404688;
D011374	decrease	CD83	ENSG00000112149	18037150;
D011374	decrease	CD96	ENSG00000153283	17404688;
D011374	decrease	CDH5	ENSG00000179776	17404688;
D011374	decrease	CDH6	ENSG00000113361	17404688;
D011374	decrease	CEACAM5	ENSG00000105388	17404688;
D011374	decrease	CEACAM6	ENSG00000086548	17404688;
D011374	decrease	CFTR	ENSG00000001626	15471937;17404688;
D011374	decrease	CHGA	ENSG00000100604	17404688;
D011374	decrease	CHRNE	ENSG00000108556	18070364;
D011374	decrease	CLCA4	ENSG00000016602	17404688;
D011374	decrease	CLDN4	ENSG00000189143	18037150;
D011374	decrease	CLIC2	ENSG00000155962	17404688;
D011374	decrease	CLIC3	ENSG00000169583	17404688;
D011374	decrease	CLIC4	ENSG00000169504	18070364;
D011374	decrease	CNIH3	ENSG00000143786	17404688;
D011374	decrease	COL11A1	ENSG00000060718	18070364;
D011374	decrease	COL16A1	ENSG00000084636	18070364;
D011374	decrease	COL1A1	ENSG00000108821	18070364;
D011374	decrease	COL1A2	ENSG00000164692	18070364;
D011374	decrease	COL4A1	ENSG00000187498	18070364;
D011374	decrease	COL5A2	ENSG00000204262	18070364;
D011374	decrease	COL6A1	ENSG00000142156	18070364;
D011374	decrease	COL8A2	ENSG00000171812	18070364;
D011374	decrease	COMT	ENSG00000093010	17138778;
D011374	decrease	COTL1	ENSG00000103187	18037150;
D011374	decrease	COX7A1	ENSG00000161281	18070364;
D011374	decrease	CPM	ENSG00000135678	17404688;18070364;
D011374	decrease	CPZ	ENSG00000109625	17404688;
D011374	decrease	CRABP2	ENSG00000143320	17404688;
D011374	decrease	CSK	ENSG00000103653	18037150;
D011374	decrease	CSPG5	ENSG00000114646	18037150;
D011374	decrease	CSTA	ENSG00000121552	18070364;
D011374	decrease	CTBP1	ENSG00000159692	17404688;
D011374	decrease	CTSW	ENSG00000172543	17404688;
D011374	decrease	CXCL14	ENSG00000145824	17404688;
D011374	decrease	CYP1B1	ENSG00000138061	18037150;
D011374	decrease	CYP27B1	ENSG00000111012	17404688;
D011374	decrease	CYP2C18	ENSG00000108242	18070364;
D011374	decrease	DACT1	ENSG00000165617	17404688;
D011374	decrease	DBN1	ENSG00000113758	17404688;
D011374	decrease	DCHS1	ENSG00000166341	17404688;
D011374	decrease	DDAH2	ENSG00000225635	17404688;
D011374	decrease	DENND2A	ENSG00000146966	17404688;
D011374	decrease	DES	ENSG00000175084	18070364;
D011374	decrease	DHX15	ENSG00000109606	18037150;
D011374	decrease	DIAPH1	ENSG00000131504	18037150;
D011374	decrease	DMBT1	ENSG00000187908	17404688;
D011374	decrease	DST	ENSG00000151914	18070364;
D011374	decrease	DUS3L	ENSG00000141994	18037150;
D011374	decrease	DYRK2	ENSG00000127334	17404688;
D011374	decrease	EFNB2	ENSG00000125266	17404688;
D011374	decrease	EFS	ENSG00000100842	17404688;

D011374	decrease	EGR1	ENSG00000120738	17404688;
D011374	decrease	EHD1	ENSG00000110047	18037150;
D011374	decrease	ELOVL1	ENSG00000066322	18037150;
D011374	decrease	ENC1	ENSG00000171617	17404688;
D011374	decrease	EPB41L4A	ENSG00000129595	17404688;
D011374	decrease	ESR1	ENSG00000091831	18037150;
D011374	decrease	F13A1	ENSG00000124491	17404688;
D011374	decrease	FAM153A	ENSG00000170074	17404688;
D011374	decrease	FAT2	ENSG00000086570	18037150;
D011374	decrease	FERMT1	ENSG00000101311	17404688;
D011374	decrease	FGF18	ENSG00000156427	18037150;17404688;
D011374	decrease	FGF9	ENSG00000102678	12072406;17404688;
D011374	decrease	FGFBP1	ENSG00000137440	18037150;
D011374	decrease	FH	ENSG00000091483	18037150;
D011374	decrease	FJX1	ENSG00000179431	17404688;
D011374	decrease	FLRT2	ENSG00000185070	17404688;
D011374	decrease	FN1	ENSG00000115414	18070364;
D011374	decrease	FRMD4A	ENSG00000151474	18070364;
D011374	decrease	FSCN1	ENSG00000075618	17404688;
D011374	decrease	GABRA5	ENSG00000186297	16000229;
D011374	decrease	GAP43	ENSG00000172020	17404688;
D011374	decrease	GEMIN6	ENSG00000152147	18037150;
D011374	decrease	GJA4	ENSG00000187513	17404688;
D011374	decrease	GLIS2	ENSG00000126603	18037150;
D011374	decrease	GMNN	ENSG00000112312	18037150;
D011374	decrease	GPR17	ENSG00000144230	17404688;
D011374	decrease	GREM1	ENSG00000166923	17404688;
D011374	decrease	GRPEL1	ENSG00000109519	18037150;
D011374	decrease	GRWD1	ENSG00000105447	18037150;
D011374	decrease	GSTT1	ENSG00000184674	17404688;
D011374	decrease	H2AFV	ENSG00000105968	17404688;
D011374	decrease	HABP2	ENSG00000148702	17404688;
D011374	decrease	HDAC1	ENSG00000116478	18037150;
D011374	decrease	HGF	ENSG00000019991	17404688;
D011374	decrease	HIPK2	ENSG00000064393	18070364;
D011374	decrease	HLA-DRB3	ENSG00000231679	17404688;
D011374	decrease	HMGA1	ENSG00000137309	17404688;
D011374	decrease	HOXA10	ENSG00000078399	18037150;
D011374	decrease	HOXA11	ENSG00000005073	17404688;
D011374	decrease	HS6ST1	ENSG00000136720	18037150;
D011374	decrease	HYAL2	ENSG00000068001	18037150;
D011374	decrease	IGF1R	ENSG00000140443	18070364;
D011374	decrease	IGFBP2	ENSG00000115457	18070364;15809018;
D011374	decrease	IGFBP3	ENSG00000146674	18070364;15809018;17404688;
D011374	decrease	IGFBP5	ENSG00000115461	18070364;
D011374	decrease	IGLL1	ENSG00000128322	18070364;
D011374	decrease	IL24	ENSG00000162892	17404688;
D011374	decrease	IL2RB	ENSG00000100385	17404688;
D011374	decrease	IL33	ENSG00000137033	17404688;
D011374	decrease	IPO4	ENSG00000196497	18037150;
D011374	decrease	ITGB1BP1	ENSG00000119185	18037150;
D011374	decrease	ITGB6	ENSG00000115221	17404688;
D011374	decrease	ITM2A	ENSG00000078596	17404688;

D011374	decrease	ITM2C	ENSG00000135916	17404688;
D011374	decrease	KCNK3	ENSG00000171303	18070364;
D011374	decrease	KLF10	ENSG00000155090	18037150;
D011374	decrease	KLRB1	ENSG00000111796	17404688;
D011374	decrease	KLRC3	ENSG00000205810	17404688;
D011374	decrease	KRT17	ENSG00000128422	18037150;
D011374	decrease	KRT23	ENSG00000108244	17404688;
D011374	decrease	L1CAM	ENSG00000198910	18070364;
D011374	decrease	LAMA3	ENSG00000053747	17404688;
D011374	decrease	LCK	ENSG00000182866	17404688;
D011374	decrease	LDHA	ENSG00000134333	18037150;
D011374	decrease	LIMA1	ENSG00000050405	18037150;
D011374	decrease	LOXL2	ENSG00000134013	18070364;
D011374	decrease	LRRC8D	ENSG00000171492	17404688;
D011374	decrease	LXN	ENSG00000079257	17404688;
D011374	decrease	LYPD1	ENSG00000150551	17404688;
D011374	decrease	MAGED4	ENSG00000154545	17404688;
D011374	decrease	MARCKS	ENSG00000155130	17404688;
D011374	decrease	MAT2A	ENSG00000168906	18037150;
D011374	decrease	MATN4	ENSG00000124159	17404688;
D011374	decrease	METAP2	ENSG00000111142	18070364;
D011374	decrease	MFAP2	ENSG00000117122	17404688;
D011374	decrease	MFAP5	ENSG00000197614	17404688;
D011374	decrease	MGP	ENSG00000111341	18070364;
D011374	decrease	MICAL2	ENSG00000133816	18070364;
D011374	decrease	MME	ENSG00000196549	18070364;
D011374	decrease	MMP11	ENSG00000099953	17404688;
D011374	decrease	MMP14	ENSG00000157227	17404688;
D011374	decrease	MMP1	ENSG00000196611	17404688;
D011374	decrease	MMP27	ENSG00000137675	17404688;
D011374	decrease	MMP2	ENSG00000087245	18070364;
D011374	decrease	MMP3	ENSG00000149968	16009159;17404688;
D011374	decrease	MMP9	ENSG00000100985	17404688;
D011374	decrease	MRPL27	ENSG00000108826	18037150;
D011374	decrease	MRPS28	ENSG00000147586	18037150;
D011374	decrease	MRTO4	ENSG00000053372	18037150;
D011374	decrease	MUC4	ENSG00000145113	17404688;
D011374	decrease	MYC	ENSG00000136997	18045962;
D011374	decrease	MYCN	ENSG00000134323	17404688;
D011374	decrease	MYH1	ENSG00000109061	18070364;
D011374	decrease	MYL9	ENSG00000101335	18070364;18037150;
D011374	decrease	MYLK	ENSG00000251351	17404688;
D011374	decrease	NANOS1	ENSG00000188613	18037150;
D011374	decrease	NCOA4	ENSG00000138293	18037150;
D011374	decrease	NEFH	ENSG00000100285	18070364;
D011374	decrease	NMB	ENSG00000197696	17404688;
D011374	decrease	NMT1	ENSG00000136448	17404688;
D011374	decrease	NOTCH2	ENSG00000134250	18070364;
D011374	decrease	NOX3	ENSG00000074771	18070364;
D011374	decrease	NUDT6	ENSG00000170917	17404688;
D011374	decrease	ODC1	ENSG00000115758	18037150;
D011374	decrease	OLFM1	ENSG00000130558	18037150;
D011374	decrease	ORM1	ENSG00000229314	16901474;17404688;
D011374	decrease	OXTR	ENSG00000180914	18070364;

D011374	decrease	PAK1IP1	ENSG00000111845	18037150;
D011374	decrease	PATZ1	ENSG00000100105	18037150;
D011374	decrease	PBX1	ENSG00000185630	17404688;
D011374	decrease	PCDHGA11	ENSG00000081853	18070364;
D011374	decrease	PCDHGA1	ENSG00000204956	18070364;
D011374	decrease	PCDHGA3	ENSG00000081853	18070364;
D011374	decrease	PCDHGC3	ENSG00000240184	17404688;
D011374	decrease	PDCL3	ENSG00000115539	18037150;
D011374	decrease	PDGFB	ENSG00000100311	17404688;
D011374	decrease	PDGFRA	ENSG00000134853	17614352;16888808;
D011374	decrease	PDLIM1	ENSG00000107438	18070364;
D011374	decrease	PDLIM7	ENSG00000196923	18070364;
D011374	decrease	PDPK1	ENSG00000140992	17404688;
D011374	decrease	PFAS	ENSG00000178921	17404688;
D011374	decrease	PKIA	ENSG00000171033	17404688;
D011374	decrease	PLAU	ENSG00000122861	17404688;18070364;
D011374	decrease	PLEC	ENSG00000178209	18070364;18037150;
D011374	decrease	PLEK2	ENSG00000100558	17404688;
D011374	decrease	POFUT1	ENSG00000101346	18037150;
D011374	decrease	POLE4	ENSG00000115350	18037150;
D011374	decrease	POLR1B	ENSG00000125630	18037150;
D011374	decrease	POLR2J	ENSG00000005075	17404688;
D011374	decrease	POLR3K	ENSG00000161980	18037150;
D011374	decrease	PPFIBP2	ENSG00000166387	17404688;
D011374	decrease	PPIF	ENSG00000108179	18037150;
D011374	decrease	PPP1R16B	ENSG00000101445	17404688;
D011374	decrease	PPP2R3C	ENSG00000092020	18037150;
D011374	decrease	PRAME	ENSG00000185686	17404688;
D011374	decrease	PSPC1	ENSG00000121390	18037150;
D011374	decrease	PTH2R	ENSG00000144407	17404688;
D011374	decrease	QPCT	ENSG00000115828	17404688;
D011374	decrease	RAB15	ENSG00000139998	17404688;
D011374	decrease	RAN	ENSG00000132341	18037150;
D011374	decrease	RARRES1	ENSG00000118849	17404688;
D011374	decrease	RARRES2	ENSG00000106538	18037150;
D011374	decrease	RASL12	ENSG00000103710	17404688;
D011374	decrease	RBBP6	ENSG00000122257	17404688;
D011374	decrease	RCC1	ENSG00000180198	17404688;18037150;
D011374	decrease	RELA	ENSG00000173039	17437195;15358673;
D011374	decrease	RGS3	ENSG00000249544	17404688;
D011374	decrease	RGS4	ENSG00000117152	17404688;
D011374	decrease	RGS5	ENSG00000143248	17404688;
D011374	decrease	ROGDI	ENSG00000067836	18037150;
D011374	decrease	RPL27A	ENSG00000166441	17404688;
D011374	decrease	RPS20	ENSG00000008988	17404688;
D011374	decrease	RPS6KA5	ENSG00000100784	17404688;
D011374	decrease	RRP9	ENSG00000114767	18037150;
D011374	decrease	RRS1	ENSG00000179041	18037150;
D011374	decrease	RTN3	ENSG00000133318	17404688;
D011374	decrease	RXRA	ENSG00000186350	18037150;
D011374	decrease	SASH1	ENSG00000111961	17404688;
D011374	decrease	SDC1	ENSG00000115884	18037150;
D011374	decrease	SEH1L	ENSG00000085415	18037150;
D011374	decrease	SERPINB2	ENSG00000197632	17404688;

D011374	decrease	SERPINB5	ENSG00000206075	17404688;
D011374	decrease	SFPQ	ENSG00000116560	18037150;
D011374	decrease	SFRS6	ENSG00000124193	18037150;
D011374	decrease	SH3BP4	ENSG00000130147	18037150;
D011374	decrease	SIGLEC15	ENSG00000197046	17404688;
D011374	decrease	SLC14A1	ENSG00000141469	17404688;
D011374	decrease	SLC16A6	ENSG00000108932	17404688;
D011374	decrease	SLC4A11	ENSG00000088836	18037150;
D011374	decrease	SLC7A5	ENSG00000103257	18037150;
D011374	decrease	SLC9A3R1	ENSG00000109062	18037150;
D011374	decrease	SLCO3A1	ENSG00000176463	17404688;
D011374	decrease	SLIT1	ENSG00000187122	18037150;
D011374	decrease	SNCAIP	ENSG00000064692	17404688;
D011374	decrease	SOX11	ENSG00000176887	17404688;
D011374	decrease	SOX4	ENSG00000124766	17404688;
D011374	decrease	SOX9	ENSG00000125398	17404688;
D011374	decrease	SPON1	ENSG00000152268	17404688;
D011374	decrease	SPON2	ENSG00000159674	18070364;
D011374	decrease	SPRR2B	ENSG00000196805	17404688;
D011374	decrease	SST	ENSG00000157005	17404688;
D011374	decrease	ST7	ENSG00000004866	17404688;
D011374	decrease	STAR	ENSG00000147465	18070364;
D011374	decrease	STC1	ENSG00000159167	17404688;
D011374	decrease	STMN2	ENSG00000104435	17404688;
D011374	decrease	SYNJ2	ENSG00000078269	17404688;
D011374	decrease	TAGLN	ENSG00000149591	18037150;
D011374	decrease	TARDBP	ENSG00000120948	18037150;
D011374	decrease	TAX1BP3	ENSG00000213977	18037150;
D011374	decrease	TBC1D19	ENSG00000109680	17404688;
D011374	decrease	TBC1D2	ENSG00000095383	18037150;
D011374	decrease	TCF4	ENSG00000196628	17404688;
D011374	decrease	TFF1	ENSG00000160182	17404688;
D011374	decrease	TGFB2	ENSG00000092969	17404688;
D011374	decrease	TGFB3	ENSG00000119699	17404688;
D011374	decrease	TGM2	ENSG00000198959	17404688;
D011374	decrease	THBS1	ENSG00000137801	18070364;18037150;
D011374	decrease	THY1	ENSG00000154096	17404688;
D011374	decrease	TMEM158	ENSG00000249992	17404688;
D011374	decrease	TMEM5	ENSG00000118600	17404688;
D011374	decrease	TMPRSS3	ENSG00000160183	17404688;
D011374	decrease	TMSB15A	ENSG00000158164	17404688;
D011374	decrease	TNFRSF11B	ENSG00000164761	17404688;
D011374	decrease	TNFRSF21	ENSG00000146072	17404688;
D011374	decrease	TNFRSF4	ENSG00000186827	17404688;
D011374	decrease	TNFSF10	ENSG00000121858	18070364;
D011374	decrease	TP53	ENSG00000141510	14529565;
D011374	decrease	TPM1	ENSG00000140416	18037150;
D011374	decrease	TRA2A	ENSG00000164548	18070364;
D011374	decrease	TRIB2	ENSG00000071575	17404688;
D011374	decrease	TRIM16	ENSG00000221926	18037150;
D011374	decrease	TTLL12	ENSG00000100304	18037150;
D011374	decrease	TUBB	ENSG00000229684	18037150;
D011374	decrease	TWIST1	ENSG00000122691	17404688;
D011374	decrease	TXNIP	ENSG00000117289	18037150;

D011374	decrease	UBD	ENSG00000213886	17404688;
D011374	decrease	UBL4A	ENSG00000102178	18037150;
D011374	decrease	UMPS	ENSG00000114491	18037150;
D011374	decrease	UNG	ENSG00000076248	18037150;
D011374	decrease	WDR3	ENSG00000065183	18037150;
D011374	decrease	WDR77	ENSG00000116455	18037150;
D011374	decrease	WISP2	ENSG00000064205	17404688;
D011374	decrease	XCL1	ENSG00000143184	17404688;
D011374	decrease	XCL2	ENSG00000143185	17404688;
D011374	decrease	ZCCHC14	ENSG00000140948	17404688;
D011374	decrease	ZFR	ENSG00000056097	18037150;
D011374	decrease	ZNF426	ENSG00000130818	17404688;
D011374	decrease	ZNF428	ENSG00000131116	17404688;
D011374	decrease	ZNF532	ENSG00000074657	18070364;
D011374	increase	ABAT	ENSG00000183044	17404688;
D011374	increase	ABCG1	ENSG00000160179	18070364;
D011374	increase	ACAT2	ENSG00000120437	18070364;
D011374	increase	ACSL1	ENSG00000151726	17404688;
D011374	increase	ADAMTS1	ENSG00000154734	17018655;
D011374	increase	ADAMTS20	ENSG00000173157	18070364;
D011374	increase	ADAMTS8	ENSG00000134917	17404688;
D011374	increase	ADIPOR2	ENSG00000006831	17404688;
D011374	increase	AFP	ENSG00000081051	18497072;
D011374	increase	AIM1L	ENSG00000176092	17404688;
D011374	increase	ALPL	ENSG00000162551	16326432;17404688;
D011374	increase	ALPP	ENSG00000163283	17404688;
D011374	increase	AMY1A	ENSG00000187733	17404688;
D011374	increase	ANKRD55	ENSG00000249922	17404688;
D011374	increase	ANTXR1	ENSG00000169604	17404688;
D011374	increase	APOD	ENSG00000189058	17404688;
D011374	increase	APOM	ENSG00000226215	17404688;
D011374	increase	AREG	ENSG00000205595	18070364;
D011374	increase	ARHGEF3	ENSG00000163947	18070364;
D011374	increase	ARL4A	ENSG00000122644	18037150;
D011374	increase	ART3	ENSG00000156219	17404688;
D011374	increase	ASNS	ENSG00000070669	18037150;
D011374	increase	ASPM	ENSG00000066279	18070364;
D011374	increase	ASRGL1	ENSG00000162174	17404688;
D011374	increase	ATAD2	ENSG00000156802	17404688;
D011374	increase	ATF3	ENSG00000162772	15674352;
D011374	increase	ATF4	ENSG00000128272	18037150;
D011374	increase	ATG2B	ENSG00000066739	18070364;
D011374	increase	ATP1A1	ENSG00000163399	18037150;
D011374	increase	ATP6V1A	ENSG00000114573	17404688;
D011374	increase	AURKA	ENSG00000087586	16888808;
D011374	increase	BAG3	ENSG00000151929	18037150;
D011374	increase	BBS10	ENSG00000179941	18070364;
D011374	increase	BBS7	ENSG00000138686	18070364;
D011374	increase	BCL2	ENSG00000171791	15779298;
D011374	increase	BET1	ENSG00000105829	18037150;
D011374	increase	BIN1	ENSG00000136717	18037150;
D011374	increase	C10ORF10	ENSG00000165507	16123073;18070364;
D011374	increase	C14ORF104	ENSG00000165506	18070364;
D011374	increase	C14ORF109	ENSG00000153485	18070364;

D011374	increase	C18ORF1	ENSG00000168675	17404688;
D011374	increase	C2ORF67	ENSG00000144445	18070364;
D011374	increase	C4ORF31	ENSG00000173376	17404688;
D011374	increase	C5ORF4	ENSG00000170271	18070364;
D011374	increase	CAMP	ENSG00000164047	17701593;
D011374	increase	CAPN6	ENSG00000077274	17404688;
D011374	increase	CAV1	ENSG00000105974	15674352;
D011374	increase	CCNA1	ENSG00000133101	17404688;
D011374	increase	CCNB1	ENSG00000134057	18037150;18070364;
D011374	increase	CCNB2	ENSG00000157456	18070364;
D011374	increase	CCND1	ENSG00000110092	18070364;16888808;
D011374	increase	CCRL1	ENSG00000129048	18070364;
D011374	increase	CDC14B	ENSG00000081377	17404688;
D011374	increase	CDC20	ENSG00000117399	18070364;
D011374	increase	CDC42EP3	ENSG00000163171	17404688;
D011374	increase	CDC6	ENSG00000094804	18070364;
D011374	increase	CDK1	ENSG00000170312	18070364;
D011374	increase	CDK8	ENSG00000132964	16888808;
D011374	increase	CDK9	ENSG00000136807	16888808;
D011374	increase	CDKN1A	ENSG00000124762	18045962;
D011374	increase	CDKN2AIP	ENSG00000168564	18070364;
D011374	increase	CDKN2C	ENSG00000123080	18070364;
D011374	increase	CEBPD	ENSG00000221869	18037150;
D011374	increase	CEBPG	ENSG00000153879	18037150;
D011374	increase	CENPF	ENSG00000117724	18070364;
D011374	increase	CENPN	ENSG00000166451	18070364;
D011374	increase	CENPQ	ENSG00000031691	18070364;
D011374	increase	CEP55	ENSG00000138180	18070364;
D011374	increase	CEP57	ENSG00000166037	18070364;
D011374	increase	CEP76	ENSG00000101624	18070364;
D011374	increase	CHAC1	ENSG00000128965	18037150;
D011374	increase	CHORDC1	ENSG00000110172	18037150;
D011374	increase	CHPT1	ENSG00000111666	17404688;
D011374	increase	CHST7	ENSG00000147119	17404688;
D011374	increase	CITED2	ENSG00000164442	17404688;
D011374	increase	CKB	ENSG00000166165	17404688;
D011374	increase	CLCN4	ENSG00000073464	17404688;
D011374	increase	CLDN5	ENSG00000184113	17404688;
D011374	increase	CNR1	ENSG00000118432	17404688;
D011374	increase	CRISPLD2	ENSG00000103196	17404688;
D011374	increase	CRLF1	ENSG00000006016	17404688;
D011374	increase	CRYAB	ENSG00000109846	17404688;
D011374	increase	CSF2RA	ENSG00000198223	17404688;
D011374	increase	CSF3	ENSG00000108342	17404688;
D011374	increase	CSRNP2	ENSG00000110925	18037150;
D011374	increase	CTSE	ENSG00000196188	17404688;
D011374	increase	CUX2	ENSG00000111249	18070364;17404688;
D011374	increase	CXCL10	ENSG00000169245	15557169;17404688;
D011374	increase	CXCL11	ENSG00000169248	17404688;15557169;
D011374	increase	CXCL5	ENSG00000163735	18070364;
D011374	increase	CYB5B	ENSG00000103018	18070364;
D011374	increase	CYP26A1	ENSG00000095596	17404688;
D011374	increase	CYP4B1	ENSG00000142973	17404688;
D011374	increase	CYP4Z1	ENSG00000186160	15797250;

D011374	increase	CYP51A1	ENSG00000001630	18070364;
D011374	increase	D4S234E	ENSG00000168824	17404688;
D011374	increase	DDIT3	ENSG00000175197	18037150;
D011374	increase	DDIT4	ENSG00000168209	18037150;
D011374	increase	DEFA1	ENSG00000240247	17701593;
D011374	increase	DEFA3	ENSG00000239839	17701593;
D011374	increase	DHCR7	ENSG00000172893	18070364;
D011374	increase	DKK1	ENSG00000107984	16449346;17404688;
D011374	increase	DLC1	ENSG00000164741	15674352;
D011374	increase	DLX4	ENSG00000108813	18070364;
D011374	increase	DNAJB1	ENSG00000132002	18037150;
D011374	increase	DNAJB9	ENSG00000128590	18070364;18037150;
D011374	increase	DPP4	ENSG00000197635	17404688;
D011374	increase	DUSP1	ENSG00000120129	17404688;
D011374	increase	DUSP22	ENSG00000112679	18037150;
D011374	increase	DUSP5	ENSG00000138166	18037150;
D011374	increase	DUSP6	ENSG00000139318	18037150;
D011374	increase	EBP	ENSG00000147155	18070364;
D011374	increase	EFNA1	ENSG00000169242	18037150;
D011374	increase	EGF	ENSG00000138798	16175315;
D011374	increase	EGFR	ENSG00000146648	16175315;
D011374	increase	EIF2S3	ENSG00000130741	17404688;
D011374	increase	EIF5	ENSG00000100664	18037150;
D011374	increase	ELOVL6	ENSG00000170522	18070364;
D011374	increase	ENPEP	ENSG00000138792	17404688;
D011374	increase	ERBB2IP	ENSG00000112851	18037150;
D011374	increase	ESR2	ENSG00000140009	17683929;
D011374	increase	ESRRA	ENSG00000173153	15968320;
D011374	increase	ETV1	ENSG00000006468	18070364;
D011374	increase	EVI2A	ENSG00000126860	18070364;
D011374	increase	EZH2	ENSG00000106462	18070364;
D011374	increase	F3	ENSG00000117525	15562024;
D011374	increase	FAAH	ENSG00000117480	16154199;
D011374	increase	FADS1	ENSG00000149485	18070364;
D011374	increase	FADS2	ENSG00000134824	18070364;
D011374	increase	FAIM	ENSG00000158234	17404688;
D011374	increase	FAM105A	ENSG00000145569	18070364;
D011374	increase	FAM107A	ENSG00000168309	17404688;
D011374	increase	FAM134B	ENSG00000154153	17404688;
D011374	increase	FANCI	ENSG00000140525	18070364;
D011374	increase	FBLN1	ENSG00000077942	15774544;
D011374	increase	FBLN2	ENSG00000163520	17404688;
D011374	increase	FBXO11	ENSG00000250152	18037150;
D011374	increase	FDFT1	ENSG00000079459	18070364;
D011374	increase	FDPS	ENSG00000160752	18070364;
D011374	increase	FGA	ENSG00000171560	17404688;
D011374	increase	FGL2	ENSG00000127951	17404688;
D011374	increase	FKBP5	ENSG00000096060	17404688;18037150;
D011374	increase	FNDC3A	ENSG00000102531	18037150;
D011374	increase	FOS	ENSG00000170345	16175315;
D011374	increase	GABRA2	ENSG00000151834	16000229;
D011374	increase	GABRG3	ENSG00000182256	16000229;
D011374	increase	GADD45A	ENSG00000116717	18037150;
D011374	increase	GALK2	ENSG00000156958	18070364;

D011374	increase	GARS	ENSG00000106105	18037150;
D011374	increase	GAST	ENSG00000184502	17404688;
D011374	increase	GDF15	ENSG00000130513	18037150;
D011374	increase	GFPT2	ENSG00000131459	18037150;
D011374	increase	GK	ENSG00000198814	18070364;
D011374	increase	GLYAT	ENSG00000149124	17404688;
D011374	increase	GNRH1	ENSG00000147437	10566683;
D011374	increase	GOT1	ENSG00000120053	18037150;
D011374	increase	GPD1L	ENSG00000152642	17404688;
D011374	increase	GPR109B	ENSG00000182782	17404688;
D011374	increase	GPR126	ENSG00000112414	18070364;
D011374	increase	GREB1	ENSG00000196208	17404688;
D011374	increase	GSX1	ENSG00000169840	18070364;
D011374	increase	GTF2IRD1	ENSG00000006704	18037150;
D011374	increase	HAND2	ENSG00000164107	17404688;18070364;
D011374	increase	HBA1	ENSG00000206172	17404688;
D011374	increase	HBA2	ENSG00000206172	17404688;
D011374	increase	HBB	ENSG00000244734	17404688;18070364;
D011374	increase	HBD	ENSG00000223609	18070364;17404688;
D011374	increase	HBG1	ENSG00000213934	17404688;
D011374	increase	HBG2	ENSG00000196565	17404688;
D011374	increase	HBP1	ENSG00000105856	18070364;
D011374	increase	HBS1L	ENSG00000112339	18070364;
D011374	increase	HERPUD1	ENSG00000051108	18037150;
D011374	increase	HIST1H1C	ENSG00000187837	17404688;
D011374	increase	HIST1H2BD	ENSG00000158373	17404688;
D011374	increase	HIST1H2BH	ENSG00000197459	17404688;
D011374	increase	HIST1H2BI	ENSG00000168242	17404688;
D011374	increase	HIST4H4	ENSG00000182217	15857753;16888808;
D011374	increase	HJURP	ENSG00000123485	18070364;
D011374	increase	HLA-G	ENSG00000237216	16210391;
D011374	increase	HMGB2	ENSG00000164104	18070364;
D011374	increase	HMGCR	ENSG00000113161	18070364;
D011374	increase	HMGCS1	ENSG00000112972	18070364;
D011374	increase	HMGN3	ENSG00000118418	18070364;
D011374	increase	HMMR	ENSG00000072571	18070364;
D011374	increase	HMOX1	ENSG00000100292	18070364;17404688;
D011374	increase	HSD11B2	ENSG00000176387	18032417;18070364;15857753;
D011374	increase	HSD17B11	ENSG00000198189	18070364;
D011374	increase	HSD17B2	ENSG00000086696	17404688;
D011374	increase	HSPA1B	ENSG00000232804	18037150;
D011374	increase	HSPA6	ENSG00000173110	17404688;
D011374	increase	HYOU1	ENSG00000149428	18037150;
D011374	increase	ICAM1	ENSG00000090339	15358673;
D011374	increase	ID1	ENSG00000125968	18045962;18037150;
D011374	increase	ID2	ENSG00000115738	18037150;
D011374	increase	ID3	ENSG00000117318	18037150;
D011374	increase	IDH1	ENSG00000138413	18070364;
D011374	increase	IDI1	ENSG00000067064	18070364;
D011374	increase	IER5L	ENSG00000188483	18037150;
D011374	increase	IFI44L	ENSG00000137959	17404688;
D011374	increase	IFNAR2	ENSG00000159110	18070364;
D011374	increase	IFRD1	ENSG00000006652	18037150;

D011374	increase	IFT74	ENSG00000096872	18070364;
D011374	increase	IGFBP1	ENSG00000146678	15501902;17484513;17404688;
D011374	increase	IL15	ENSG00000164136	17404688;
D011374	increase	IL1B	ENSG00000125538	17437195;17636213;
D011374	increase	IL1RL1	ENSG00000115602	17404688;
D011374	increase	IL20RA	ENSG00000016402	17404688;
D011374	increase	IL6ST	ENSG00000134352	17404688;
D011374	increase	IL8	ENSG00000169429	15780010;15866594;
D011374	increase	INHBA	ENSG00000122641	18070364;17636213;
D011374	increase	INSIG1	ENSG00000186480	18070364;
D011374	increase	IRAK1	ENSG00000184216	17701593;
D011374	increase	IRAK2	ENSG00000134070	17701593;
D011374	increase	IRAK3	ENSG00000090376	17404688;
D011374	increase	IRAK4	ENSG00000198001	17701593;
D011374	increase	IRS2	ENSG00000185950	15949379;17404688;
D011374	increase	IRX4	ENSG00000113430	17404688;
D011374	increase	ITGA7	ENSG00000135424	18037150;
D011374	increase	ITGB3	ENSG00000056345	17404688;16403803;
D011374	increase	ITGBL1	ENSG00000198542	17404688;
D011374	increase	JDP2	ENSG00000140044	18037150;
D011374	increase	JUND	ENSG00000130522	18037150;
D011374	increase	KBTBD11	ENSG00000176595	17404688;
D011374	increase	KCNG1	ENSG00000026559	17404688;
D011374	increase	KCNJ16	ENSG00000153822	18070364;
D011374	increase	KCNJ8	ENSG00000121361	17404688;
D011374	increase	KDSR	ENSG00000119537	18070364;
D011374	increase	KIAA0101	ENSG00000166803	18070364;
D011374	increase	KIAA0895	ENSG00000164542	18070364;
D011374	increase	KIAA1009	ENSG00000135315	18070364;
D011374	increase	KIAA1609	ENSG00000140950	17404688;
D011374	increase	KIF20A	ENSG00000112984	18070364;
D011374	increase	KIF4A	ENSG00000090889	18070364;
D011374	increase	KIR2DS1	ENSG00000125498	17404688;
D011374	increase	KLF5	ENSG00000102554	18070364;18037150;
D011374	increase	KRT6A	ENSG00000205420	17404688;
D011374	increase	KRT6B	ENSG00000185479	17404688;
D011374	increase	LARP4	ENSG00000161813	18070364;
D011374	increase	LDLR	ENSG00000130164	18070364;18037150;
D011374	increase	LGALS8	ENSG00000116977	18070364;
D011374	increase	LIPG	ENSG00000101670	18070364;
D011374	increase	LMCD1	ENSG00000071282	17404688;
D011374	increase	LMO4	ENSG00000143013	18037150;
D011374	increase	LPIN1	ENSG00000134324	18070364;
D011374	increase	LRRC1	ENSG00000137269	17404688;
D011374	increase	LSS	ENSG00000160285	18070364;
D011374	increase	LTBP1	ENSG00000049323	17404688;
D011374	increase	MAF	ENSG00000178573	17404688;
D011374	increase	MAOA	ENSG00000189221	17404688;
D011374	increase	MAOB	ENSG00000069535	17404688;
D011374	increase	MAP2	ENSG00000078018	18037150;
D011374	increase	MAP2K6	ENSG00000108984	17404688;
D011374	increase	MAP3K8	ENSG00000107968	18070364;
D011374	increase	MAP7	ENSG00000135525	18070364;

D011374	increase	MARS	ENSG00000166986	18037150;
D011374	increase	MATK	ENSG00000007264	17492661;
D011374	increase	MDM1	ENSG00000111554	18070364;
D011374	increase	METTL7A	ENSG00000185432	18070364;
D011374	increase	MKI67	ENSG00000148773	16888808;
D011374	increase	MKNK2	ENSG00000099875	18037150;
D011374	increase	MLF1IP	ENSG00000151725	18070364;
D011374	increase	MON2	ENSG00000061987	18070364;
D011374	increase	MPZL2	ENSG00000149573	17404688;
D011374	increase	MRPS6	ENSG00000243927	18037150;
D011374	increase	MSLN	ENSG00000102854	17404688;
D011374	increase	MT1A	ENSG00000205362	18037150;
D011374	increase	MT1B	ENSG00000169688	18037150;
D011374	increase	MT1E	ENSG00000169715	18037150;
D011374	increase	MT1G	ENSG00000125144	18037150;
D011374	increase	MT1H	ENSG00000205358	18037150;
D011374	increase	MT1M	ENSG00000205364	17404688;18070364;
D011374	increase	MT1X	ENSG00000187193	18037150;17404688;
D011374	increase	MT2A	ENSG00000125148	18037150;
D011374	increase	MTA1	ENSG00000182979	16888808;
D011374	increase	MTF2	ENSG00000143033	18070364;
D011374	increase	MTHFD2	ENSG00000065911	18037150;
D011374	increase	MUC5B	ENSG00000117983	18070364;
D011374	increase	MVK	ENSG00000110921	18070364;
D011374	increase	MYLIP	ENSG00000007944	18037150;
D011374	increase	MYO16	ENSG00000041515	17404688;
D011374	increase	NCAPG	ENSG00000109805	18070364;
D011374	increase	NDC80	ENSG00000080986	18070364;
D011374	increase	NDP	ENSG00000124479	17404688;
D011374	increase	NFIB	ENSG00000147862	17404688;
D011374	increase	NFKBIA	ENSG00000100906	17437195;18037150;
D011374	increase	NKG7	ENSG00000105374	18070364;
D011374	increase	NLGN4X	ENSG00000146938	17404688;
D011374	increase	NME2	ENSG00000243678	15674352;
D011374	increase	NOS3	ENSG00000164867	15358673;
D011374	increase	NPTX2	ENSG00000106236	17404688;
D011374	increase	NPY1R	ENSG00000164128	18070364;
D011374	increase	NPY	ENSG00000122585	18070364;
D011374	increase	NR1I2	ENSG00000144852	16054614;15650019;18098064;
D011374	increase	NR4A1	ENSG00000123358	18037150;
D011374	increase	NSDHL	ENSG00000147383	18070364;
D011374	increase	NUP160	ENSG00000030066	18070364;
D011374	increase	NUSAP1	ENSG00000137804	18070364;
D011374	increase	OGT	ENSG00000147162	18070364;18037150;
D011374	increase	OPN3	ENSG00000054277	18037150;
D011374	increase	ORC4L	ENSG00000115947	18070364;
D011374	increase	OSBPL11	ENSG00000144909	17404688;
D011374	increase	OVGP1	ENSG00000085465	15501902;
D011374	increase	P2RY14	ENSG00000174944	17404688;
D011374	increase	PAX5	ENSG00000196092	17404688;
D011374	increase	PBK	ENSG00000168078	18070364;
D011374	increase	PCSK6	ENSG00000140479	17404688;18070364;
D011374	increase	PDAP1	ENSG00000106244	16888808;

D011374	increase	PDGFA	ENSG00000197461	16888808;17404688;
D011374	increase	PDS5A	ENSG00000121892	18070364;
D011374	increase	PDZD2	ENSG00000133401	17404688;
D011374	increase	PEG10	ENSG00000242265	18070364;
D011374	increase	PER1	ENSG00000179094	18037150;
D011374	increase	PGBD5	ENSG00000177614	17404688;
D011374	increase	PGM3	ENSG00000013375	18037150;
D011374	increase	PGR	ENSG00000082175	9144411;14579009;18262749;9126867;8812239;16326432;1544902;15189034;158
D011374	increase	PHLDA2	ENSG00000181649	18037150;
D011374	increase	PIBF1	ENSG00000083535	17701593;
D011374	increase	PID1	ENSG00000153823	17404688;
D011374	increase	PLA2G4A	ENSG00000116711	18070364;
D011374	increase	POU5F1	ENSG00000235068	17404688;
D011374	increase	PPARGC1A	ENSG00000109819	17404688;
D011374	increase	PPL	ENSG00000118898	18070364;
D011374	increase	PPP1R15A	ENSG00000087074	18037150;
D011374	increase	PRC1	ENSG00000198901	18070364;
D011374	increase	PRDM12	ENSG00000130711	18070364;
D011374	increase	PRKAG2	ENSG00000106617	17404688;
D011374	increase	PRKX	ENSG00000183943	17404688;
D011374	increase	PRL	ENSG00000172179	17187171;16403803;
D011374	increase	PRNP	ENSG00000171867	18037150;
D011374	increase	PRPF40A	ENSG00000196504	18037150;
D011374	increase	PRUNE2	ENSG00000106772	17404688;
D011374	increase	PSAT1	ENSG00000135069	18037150;
D011374	increase	PTGS1	ENSG00000095303	17404688;
D011374	increase	PTGS2	ENSG00000073756	17404688;
D011374	increase	PTHLH	ENSG00000087494	18070364;17404688;
D011374	increase	PTPRR	ENSG00000153233	17404688;
D011374	increase	RB1CC1	ENSG00000023287	18037150;
D011374	increase	RBP4	ENSG00000138207	17404688;
D011374	increase	RGS2	ENSG00000116741	17404688;18070364;
D011374	increase	RHOU	ENSG00000116574	18037150;
D011374	increase	RIF1	ENSG00000080345	18070364;
D011374	increase	RIMBP2	ENSG00000060709	17404688;
D011374	increase	RORB	ENSG00000198963	17404688;
D011374	increase	RRM2	ENSG00000171848	18070364;17404688;
D011374	increase	S100A2	ENSG00000196754	17404688;
D011374	increase	SAA2	ENSG00000134339	17404688;
D011374	increase	SAMD9	ENSG00000205413	18070364;
D011374	increase	SARS	ENSG00000031698	18037150;
D011374	increase	SC4MOL	ENSG00000052802	18070364;
D011374	increase	SCD	ENSG00000099194	18070364;
D011374	increase	SCGB1D1	ENSG00000168515	17404688;
D011374	increase	SCGB1D2	ENSG00000124935	17404688;
D011374	increase	SCGB2A1	ENSG00000124939	17404688;
D011374	increase	SCGB2A2	ENSG00000110484	17404688;
D011374	increase	SDF2L1	ENSG00000128228	18037150;
D011374	increase	SEC14L1	ENSG00000129657	18070364;18037150;17404688;
D011374	increase	SECTM1	ENSG00000141574	17404688;
D011374	increase	SERPINA3	ENSG00000196136	17404688;

D011374	increase	SERPINE1	ENSG00000106366	17404688;
D011374	increase	SESN2	ENSG00000130766	18037150;
D011374	increase	SGK1	ENSG00000118515	18070364;
D011374	increase	SGK3	ENSG00000104205	18070364;
D011374	increase	SHCBP1	ENSG00000171241	18070364;
D011374	increase	SHMT1	ENSG00000176974	18070364;
D011374	increase	SIPA1L1	ENSG00000197555	18037150;
D011374	increase	SLC15A2	ENSG00000163406	17404688;
D011374	increase	SLC26A2	ENSG00000155850	17404688;
D011374	increase	SLC2A1	ENSG00000117394	15350188;
D011374	increase	SLC38A2	ENSG00000134294	18037150;
D011374	increase	SLC47A1	ENSG00000142494	17404688;
D011374	increase	SLC7A11	ENSG00000151012	18037150;
D011374	increase	SLC7A2	ENSG00000003989	17404688;
D011374	increase	SLC7A8	ENSG00000092068	17404688;
D011374	increase	SMC2	ENSG00000136824	18070364;
D011374	increase	SMPDL3A	ENSG00000172594	18070364;
D011374	increase	SOCS1	ENSG00000185338	17404688;
D011374	increase	SORBS2	ENSG00000154556	17404688;
D011374	increase	SPARCL1	ENSG00000152583	17404688;
D011374	increase	SPRY4	ENSG00000187678	18037150;
D011374	increase	SQSTM1	ENSG00000161011	18037150;
D011374	increase	SRPX	ENSG00000101955	17404688;
D011374	increase	SRY	ENSG00000184895	18037150;
D011374	increase	STAT5A	ENSG00000126561	16175315;
D011374	increase	STAT5B	ENSG00000173757	16175315;
D011374	increase	STC2	ENSG00000113739	18037150;
D011374	increase	STEAP4	ENSG00000127954	17404688;
D011374	increase	STX3	ENSG00000166900	18037150;
D011374	increase	SUMO1	ENSG00000116030	18070364;
D011374	increase	SYNPO2L	ENSG00000166317	18070364;
D011374	increase	SYVN1	ENSG00000162298	18037150;
D011374	increase	TACC2	ENSG00000138162	18037150;
D011374	increase	TBC1D1	ENSG00000065882	18037150;
D011374	increase	TBC1D25	ENSG00000068354	18070364;
D011374	increase	TBCCD1	ENSG00000113838	18070364;
D011374	increase	TDO2	ENSG00000151790	18070364;
D011374	increase	TFF3	ENSG00000160180	17404688;
D011374	increase	TGFB1	ENSG00000105329	16403803;
D011374	increase	TGIF1	ENSG00000177426	18037150;
D011374	increase	THBD	ENSG00000178726	17404688;18070364;
D011374	increase	THSD7A	ENSG00000005108	17404688;
D011374	increase	TK1	ENSG00000167900	18070364;
D011374	increase	TLL2	ENSG00000095587	18070364;
D011374	increase	TLR2	ENSG00000137462	17404688;
D011374	increase	TLR4	ENSG00000136869	17701593;
D011374	increase	TLR5	ENSG00000187554	17701593;
D011374	increase	TLR7	ENSG00000196664	17701593;
D011374	increase	TMEM47	ENSG00000147027	18037150;
D011374	increase	TMEM97	ENSG00000109084	18070364;
D011374	increase	TNFAIP6	ENSG00000123610	18070364;
D011374	increase	TNF	ENSG00000223952	15866594;16891052;16318612;
D011374	increase	TOP2A	ENSG00000131747	18070364;

D011374	increase	TPBG	ENSG00000146242	18037150;
D011374	increase	TRIB3	ENSG00000101255	18037150;
D011374	increase	TRPC4	ENSG00000133107	18070364;
D011374	increase	TSC22D3	ENSG00000157514	17404688;
D011374	increase	TTC30A	ENSG00000197557	18070364;
D011374	increase	TTF2	ENSG00000116830	18070364;
D011374	increase	TYMS	ENSG00000176890	18070364;
D011374	increase	UBE2H	ENSG00000186591	18037150;
D011374	increase	UGT1A1	ENSG00000241635	18098064;
D011374	increase	USO1	ENSG00000138768	18037150;
D011374	increase	VCAM1	ENSG00000162692	15358673;
D011374	increase	VEGFA	ENSG00000112715	16299233;16888808;17010073;
D011374	increase	WDHD1	ENSG00000198554	18070364;
D011374	increase	WT1	ENSG00000184937	14662165;
D011374	increase	XBP1	ENSG00000100219	18037150;
D011374	increase	XRCC4	ENSG00000152422	18070364;
D011374	increase	YTHDC2	ENSG00000047188	18070364;
D011374	increase	ZBED2	ENSG00000177494	17404688;18070364;
D011374	increase	ZBTB16	ENSG00000109906	17404688;
D011374	increase	ZCCHC10	ENSG00000155329	18070364;
D011374	increase	ZFP36L1	ENSG00000185650	18037150;
D011374	increase	ZNF268	ENSG00000090612	18070364;
D011374	increase	ZNF652	ENSG00000198740	17404688;
D011397	increase	ACSL1	ENSG00000151726	15103026;
D011397	increase	AP1G1	ENSG00000166747	15103026;
D011397	increase	C12ORF5	ENSG00000078237	15103026;
D011397	increase	DLG5	ENSG00000151208	15103026;
D011397	increase	GLUL	ENSG00000135821	15103026;
D011397	increase	GPSM2	ENSG00000121957	15103026;
D011397	increase	MT1B	ENSG00000169688	15103026;
D011397	increase	MT1X	ENSG00000187193	15103026;
D011397	increase	MT2A	ENSG00000125148	15103026;
D011397	increase	MYO10	ENSG00000145555	15103026;
D011397	increase	QSOX1	ENSG00000116260	15103026;
D011397	increase	RBBP7	ENSG00000102054	15103026;
D011397	increase	S100P	ENSG00000163993	15103026;
D011397	increase	SP1	ENSG00000185591	16807381;
D011397	increase	SP3	ENSG00000172845	16807381;
D011411	decrease	TNF	ENSG00000223952	17585980;17355946;
D011429	increase	TGM2	ENSG00000198959	18295389;
D011433	increase	CCL3	ENSG00000006075	12538206;
D011433	increase	CYP1A1	ENSG00000140465	15627480;
D011433	increase	FN1	ENSG00000115414	11821714;
D011433	increase	IL1B	ENSG00000125538	11854442;
D011433	increase	MMP9	ENSG00000100985	16574429;
D011433	increase	PTGS2	ENSG00000073756	11854442;15210717;
D011691	increase	CYP1A1	ENSG00000140465	15385644;
D011691	increase	LDLR	ENSG00000130164	12524230;
D011719	decrease	GSTA1	ENSG00000243955	7986210;
D011719	decrease	GSTM1	ENSG00000134184	7986210;
D011720	increase	IL2	ENSG00000109471	11592964;
D011736	affect	SHMT1	ENSG00000176974	17482557;

D011794	affect	CTNNB1	ENSG00000168036	15670774;19814765;19440933;
D011794	affect	CYCS	ENSG00000172115	16059641;
D011794	affect	EIF3A	ENSG00000107581	15221776;
D011794	affect	HSPD1	ENSG00000144381	15583868;
D011794	affect	IL10	ENSG00000136634	17717114;18184041;
D011794	affect	MAPK13	ENSG00000156711	15221776;
D011794	affect	MRPS22	ENSG00000175110	15221776;
D011794	affect	NR1I2	ENSG00000144852	12695340;17210444;19034627;
D011794	affect	PPIL4	ENSG00000131013	15221776;
D011794	affect	RELA	ENSG00000173039	19557821;15113938;16794257;17588137;18958421;18852136;
D011794	affect	RPSA	ENSG00000168028	15221776;
D011794	affect	TCF4	ENSG00000196628	15670774;
D011794	decrease	ABCA1	ENSG00000165029	17639512;
D011794	decrease	ABCC4	ENSG00000125257	16156793;16004972;18624906;
D011794	decrease	ACE2	ENSG00000130234	17639512;
D011794	decrease	ACPP	ENSG00000014257	12391264;
D011794	decrease	ADAM15	ENSG00000143537	15309432;
D011794	decrease	ADRM1	ENSG00000130706	15309432;
D011794	decrease	ALPI	ENSG00000163295	17639512;
D011794	decrease	AMPD2	ENSG00000116337	15309432;
D011794	decrease	ANPEP	ENSG00000166825	17639512;
D011794	decrease	AOC3	ENSG00000131471	15309432;
D011794	decrease	APAF1	ENSG00000120868	15309432;
D011794	decrease	APOB	ENSG00000084674	17475462;12612140;17639512;
D011794	decrease	APOM	ENSG00000226215	17639512;
D011794	decrease	AQP3	ENSG00000165272	17639512;
D011794	decrease	ARVCF	ENSG00000099889	15309432;
D011794	decrease	ATP6V0B	ENSG00000117410	15309432;
D011794	decrease	ATP6V1C1	ENSG00000155097	15309432;
D011794	decrease	BCL2	ENSG00000171791	15975156;19557821;12672908;16280456;18787224;16049707;15741050;16059641;18852136;16619537;
D011794	decrease	BCL2L14	ENSG00000121380	17639512;
D011794	decrease	BCL2L1	ENSG00000171552	18787224;16049707;16619537;
D011794	decrease	BCR	ENSG00000186716	14715546;
D011794	decrease	CAMK1	ENSG00000134072	15309432;
D011794	decrease	CASP1	ENSG00000137752	15309432;19729006;
D011794	decrease	CAT	ENSG00000121691	15782287;10656621;16126241;15221776;
D011794	decrease	CCL5	ENSG00000161570	16797527;
D011794	decrease	CCNB1	ENSG00000134057	19009557;18331776;19397994;10601582;16049707;
D011794	decrease	CCND3	ENSG00000112576	14715546;
D011794	decrease	CCNE2	ENSG00000175305	14715546;
D011794	decrease	CCNF	ENSG00000162063	14715546;
D011794	decrease	CD74	ENSG00000019582	18818744;
D011794	decrease	CDC16	ENSG00000130177	14715546;
D011794	decrease	CDC37	ENSG00000105401	15309432;

D011794	decrease	CDC7	ENSG00000097046	14715546;
D011794	decrease	CDK1	ENSG00000170312	10601582;14715546;19009557;
D011794	decrease	CEACAM5	ENSG00000105388	15309432;
D011794	decrease	CFB	ENSG00000243570	17639512;
D011794	decrease	CFLAR	ENSG00000003402	18980244;
D011794	decrease	CITED1	ENSG00000125931	15309432;
D011794	decrease	CLDN10	ENSG00000134873	15309432;
D011794	decrease	COL3A1	ENSG00000168542	17086741;
D011794	decrease	CSF2	ENSG00000164400	18346843;15180920;10718847;
D011794	decrease	CTSO	ENSG00000151792	17639512;
D011794	decrease	DNASE1	ENSG00000213918	17639512;
D011794	decrease	DPP4	ENSG00000197635	17639512;
D011794	decrease	DRG2	ENSG00000108591	15309432;
D011794	decrease	E2F2	ENSG00000007968	14715546;
D011794	decrease	E2F3	ENSG00000112242	14715546;
D011794	decrease	EGF	ENSG00000138798	15135307;16619521;12469199;
D011794	decrease	EGR1	ENSG00000120738	15309432;
D011794	decrease	EPHX1	ENSG00000143819	17639512;
D011794	decrease	EPHX2	ENSG00000120915	17639512;
D011794	decrease	ERBB3	ENSG00000065361	15741050;
D011794	decrease	ESR1	ENSG00000091831	16118406;15185748;14706564;15182386;9751507;15937998;11502892;
D011794	decrease	F3	ENSG00000117525	12871381;
D011794	decrease	FABP6	ENSG00000170231	17639512;
D011794	decrease	FASN	ENSG00000169710	19729006;
D011794	decrease	FBP1	ENSG00000165140	18331776;
D011794	decrease	FGL2	ENSG00000127951	15309432;
D011794	decrease	FMO1	ENSG00000010932	17639512;
D011794	decrease	FMO5	ENSG00000131781	17639512;
D011794	decrease	FN1	ENSG00000115414	15309432;
D011794	decrease	G0S2	ENSG00000123689	17639512;
D011794	decrease	GALNT1	ENSG00000141429	15309432;
D011794	decrease	GDF15	ENSG00000130513	15309432;
D011794	decrease	GLS	ENSG00000115419	17639512;
D011794	decrease	GLUD2	ENSG00000182890	15221776;
D011794	decrease	GPER	ENSG00000164850	15090535;15309432;
D011794	decrease	GRB14	ENSG00000115290	15309432;
D011794	decrease	GRPEL1	ENSG00000109519	15221776;
D011794	decrease	GSK3A	ENSG00000105723	16968065;
D011794	decrease	GSK3B	ENSG00000082701	16968061;16968065;
D011794	decrease	GTF2H1	ENSG00000110768	17639512;
D011794	decrease	H1FX	ENSG00000184897	15309432;
D011794	decrease	HBG2	ENSG00000196565	15221776;
D011794	decrease	HDC	ENSG00000140287	17191106;17191019;
D011794	decrease	HEPH	ENSG00000089472	17639512;
D011794	decrease	HIST1H2AC	ENSG00000180573	15309432;
D011794	decrease	HMGCS1	ENSG00000112972	19729006;
D011794	decrease	HNRPDL	ENSG00000152795	15309432;
D011794	decrease	HRAS	ENSG00000174775	10652438;
D011794	decrease	HSPA1B	ENSG00000232804	15221776;
D011794	decrease	IDI1	ENSG00000067064	19729006;

D011794	decrease	IFNGR2	ENSG00000159128	15309432;
D011794	decrease	IL1R1	ENSG00000115594	15309432;
D011794	decrease	IL2	ENSG00000109471	19761891;
D011794	decrease	IL4R	ENSG00000077238	15309432;
D011794	decrease	KEAP1	ENSG00000079999	17462537;
D011794	decrease	KIAA1751	ENSG00000142609	15221776;
D011794	decrease	KIF15	ENSG00000163808	15221776;
D011794	decrease	KIF20B	ENSG00000138182	15309432;
D011794	decrease	KLK2	ENSG00000167751	11238180;
D011794	decrease	KRAS	ENSG00000133703	19424582;10652438;
D011794	decrease	LEF1	ENSG00000138795	17148581;
D011794	decrease	LGALS3BP	ENSG00000108679	15309432;
D011794	decrease	LMNA	ENSG00000160789	15221776;
D011794	decrease	LMNB1	ENSG00000113368	15221776;
D011794	decrease	MAD2L1	ENSG00000164109	15309432;
D011794	decrease	MAP2K4	ENSG00000065559	15309432;
D011794	decrease	MAP3K12	ENSG00000139625	15309432;
D011794	decrease	MAP4K1	ENSG00000104814	15309432;
D011794	decrease	MDM2	ENSG00000135679	17973296;
D011794	decrease	MEP1A	ENSG00000112818	17639512;
D011794	decrease	MLL	ENSG00000118058	15309432;17468513;
D011794	decrease	MRPL13	ENSG00000172172	15221776;
D011794	decrease	MST1	ENSG00000173531	17639512;
D011794	decrease	MYB	ENSG00000118513	14715546;
D011794	decrease	MYD88	ENSG00000172936	19729006;
D011794	decrease	MYO1A	ENSG00000166866	17639512;
D011794	decrease	NCOA1	ENSG00000084676	15309432;
D011794	decrease	NDUFA4	ENSG00000189043	15309432;
D011794	decrease	NDUFS1	ENSG00000023228	15221776;
D011794	decrease	NFKBIA	ENSG00000100906	19557821;18982426;17588137;17184768;18852136;
D011794	decrease	NIN	ENSG00000100503	15221776;
D011794	decrease	NKX3-1	ENSG00000167034	11238180;
D011794	decrease	NME4	ENSG00000103202	15309432;15221776;
D011794	decrease	NOS2	ENSG00000007171	12144868;17184768;
D011794	decrease	NOS3	ENSG00000164867	16891912;15740983;
D011794	decrease	NRAS	ENSG00000213281	10652438;
D011794	decrease	NT5E	ENSG00000135318	17643826;
D011794	decrease	ODC1	ENSG00000115758	11238180;
D011794	decrease	P4HB	ENSG00000185624	15221776;
D011794	decrease	PA2G4	ENSG00000170515	15221776;
D011794	decrease	PAX8	ENSG00000125618	15309432;
D011794	decrease	PCNA	ENSG00000132646	14715546;
D011794	decrease	PDK2	ENSG00000005882	15309432;
D011794	decrease	PKM2	ENSG00000067225	15221776;
D011794	decrease	PKP2	ENSG00000057294	15309432;
D011794	decrease	POLA1	ENSG00000101868	15309432;
D011794	decrease	POLD2	ENSG00000106628	15309432;
D011794	decrease	PPP1R1A	ENSG00000135447	15309432;
D011794	decrease	PPP2R4	ENSG00000119383	15309432;
D011794	decrease	PSCA	ENSG00000167653	18331776;
D011794	decrease	RALA	ENSG00000006451	15309432;
D011794	decrease	RARB	ENSG00000077092	15309432;
D011794	decrease	RBM3	ENSG00000102317	15309432;

D011794	decrease	RBM4	ENSG00000173933	15221776;
D011794	decrease	RBMS1	ENSG00000153250	15309432;
D011794	decrease	RBMS2	ENSG00000076067	15309432;
D011794	decrease	RELB	ENSG00000104856	15309432;
D011794	decrease	RGN	ENSG00000130988	15309432;
D011794	decrease	RGS7	ENSG00000182901	15309432;
D011794	decrease	RQCD1	ENSG00000144580	15309432;
D011794	decrease	RSAD2	ENSG00000134321	19729006;
D011794	decrease	S100G	ENSG00000169906	17639512;
D011794	decrease	S100P	ENSG00000163993	15309432;
D011794	decrease	SAMD9	ENSG00000205413	19729006;
D011794	decrease	SART1	ENSG00000175467	15309432;
D011794	decrease	SDHC	ENSG00000143252	15309432;15221776;
D011794	decrease	SEMA3C	ENSG00000075223	19729006;
D011794	decrease	SERPINE1	ENSG00000106366	17379280;
D011794	decrease	SFRP1	ENSG00000104332	15309432;
D011794	decrease	SIAH1	ENSG00000196470	15309432;
D011794	decrease	SIAH2	ENSG00000181788	15309432;
D011794	decrease	SI	ENSG00000090402	17639512;
D011794	decrease	SMAD5	ENSG00000113658	15309432;
D011794	decrease	STAT1	ENSG00000115415	16077199;15309432;
D011794	decrease	STAT2	ENSG00000170581	16797527;
D011794	decrease	STMN1	ENSG00000117632	15221776;
D011794	decrease	SULT2A1	ENSG00000105398	17639512;
D011794	decrease	TCEB2	ENSG00000103363	15309432;
D011794	decrease	TCF7	ENSG00000081059	17148581;
D011794	decrease	TCF7L2	ENSG00000148737	19440933;
D011794	decrease	TF	ENSG00000091513	16507316;17639512;
D011794	decrease	TFF2	ENSG00000160181	17639512;
D011794	decrease	THBS1	ENSG00000137801	19729006;
D011794	decrease	TMPRSS6	ENSG00000187045	17639512;
D011794	decrease	TNNC1	ENSG00000114854	19729006;
D011794	decrease	TSPAN3	ENSG00000140391	15309432;
D011794	decrease	TYMS	ENSG00000176890	16573383;
D011794	decrease	UGT2B15	ENSG00000196620	17639512;
D011794	decrease	VRK2	ENSG00000028116	15309432;
D011794	decrease	WWTR1	ENSG00000018408	17639512;
D011794	decrease	XPNPEP2	ENSG00000122121	17639512;
D011794	decrease	ZFP161	ENSG00000198081	15309432;
D011794	decrease	ZW10	ENSG00000086827	15309432;
D011794	increase	ABCB1	ENSG00000085563	15112361;15969930;15266218;16059641;18283591;18624906;
D011794	increase	ABCC1	ENSG00000103222	16156793;16004972;15041478;15670588;11836020;
D011794	increase	ABCG2	ENSG00000118777	17077187;18624906;
D011794	increase	ACADVL	ENSG00000072778	15221776;
D011794	increase	ADAM12	ENSG00000148848	15309432;
D011794	increase	AGER	ENSG00000204305	19557821;
D011794	increase	AHR	ENSG00000106546	17077187;19286049;19299563;14644660;15169886;19794518;19034627;
D011794	increase	AK3L1	ENSG00000162433	19729006;

D011794	increase	AKT1	ENSG00000142208	16059641;19464278;16619521;19244381;16794257;15741050;17973296;15829497;14715546;
D011794	increase	ALDOA	ENSG00000149925	17292933;
D011794	increase	ANXA1	ENSG00000135046	15221776;17639512;
D011794	increase	ANXA2	ENSG00000182718	15221776;
D011794	increase	ANXA5	ENSG00000164111	18377872;
D011794	increase	APC	ENSG00000134982	14715546;
D011794	increase	AR	ENSG00000169083	15661808;11238180;16239064;12391264;
D011794	increase	ARF3	ENSG00000134287	17292933;
D011794	increase	ARHGDIB	ENSG00000111348	17639512;
D011794	increase	ATF3	ENSG00000162772	17639512;
D011794	increase	AURKB	ENSG00000178999	17639512;
D011794	increase	BAD	ENSG00000002330	14767529;
D011794	increase	BAK1	ENSG00000030110	16280456;
D011794	increase	BAX	ENSG00000087088	15975156;17652743;12672908;16280456;17876056;16059641;16049707;1661953
D011794	increase	BIRC3	ENSG00000023445	17639512;
D011794	increase	BIRC5	ENSG00000089685	18377872;19440933;14988404;
D011794	increase	BMP1	ENSG00000168487	15309432;
D011794	increase	BMP2	ENSG00000125845	16996034;
D011794	increase	BNIP3	ENSG00000176171	19729006;
D011794	increase	BPTF	ENSG00000171634	19729006;
D011794	increase	BRCA1	ENSG00000012048	14715546;
D011794	increase	BRCA2	ENSG00000139618	15309432;
D011794	increase	BRDT	ENSG00000137948	15309432;
D011794	increase	BUB1B	ENSG00000156970	15309432;
D011794	increase	BUB1	ENSG00000169679	17639512;
D011794	increase	CASP3	ENSG00000164305	15975156;15583868;19464278;18385095;15795422;12672908;17929310;16280456;18377872;17876056;18563345;18787224;19397994;15741050;18331776;14767529;16049707;18980244;
D011794	increase	CASP7	ENSG00000165806	18787224;14767529;
D011794	increase	CAV1	ENSG00000105974	17876056;18385095;
D011794	increase	CCL2	ENSG00000108691	17103373;18489909;16794257;17880982;17191019;
D011794	increase	CCND1	ENSG00000110092	17148581;12589822;18787224;19440933;15309432;18323654;12469199;1471554
D011794	increase	CCND2	ENSG00000118971	14715546;17639512;
D011794	increase	CCNE1	ENSG00000105173	12589822;12934110;
D011794	increase	CD40	ENSG00000101017	16601352;
D011794	increase	CDC6	ENSG00000094804	15309432;17639512;
D011794	increase	CDH11	ENSG00000140937	15309432;
D011794	increase	CDH2	ENSG00000170558	15309432;
D011794	increase	CDK2	ENSG00000123374	12589822;11322924;14715546;
D011794	increase	CDK4	ENSG00000135446	12589822;15309432;14715546;
D011794	increase	CDK8	ENSG00000132964	14715546;

D011794	increase	CDKN1A	ENSG00000124762	16059641;11790449;16280456;12934110;11322924;14715546;19397994;18323654;19009557;16049707;1258
D011794	increase	CDKN1B	ENSG00000111276	11322924;15829497;
D011794	increase	CDKN1C	ENSG00000129757	14715546;
D011794	increase	CDKN2C	ENSG00000123080	18331776;
D011794	increase	CEBPB	ENSG00000172216	11779194;
D011794	increase	CEBPD	ENSG00000221869	11779194;
D011794	increase	CFL1	ENSG00000172757	17292933;
D011794	increase	CIT	ENSG00000122966	17639512;
D011794	increase	CLDN4	ENSG00000189143	18492835;
D011794	increase	COL1A1	ENSG00000108821	19814765;
D011794	increase	CREBBP	ENSG00000005339	14715546;
D011794	increase	CRP	ENSG00000132693	18184041;17388968;17184768;
D011794	increase	CTNNA1	ENSG00000044115	15309432;
D011794	increase	CTSD	ENSG00000117984	15090535;
D011794	increase	CUL4B	ENSG00000158290	14715546;
D011794	increase	CXCL10	ENSG00000169245	17103373;
D011794	increase	CYP19A1	ENSG00000137869	16023337;16965912;15319488;
D011794	increase	CYP1A1	ENSG00000140465	15760569;19766177;15618000;14633740;17177569;16271822;18456490;19794518;17639512;
D011794	increase	CYP1A2	ENSG00000140505	16544949;10620351;12949046;15916432;19034627;
D011794	increase	CYP1B1	ENSG00000138061	16271822;17637178;19794518;
D011794	increase	CYP3A4	ENSG00000160868	12695340;15266218;19766177;16442130;11502731;
D011794	increase	DARS	ENSG00000115866	19729006;
D011794	increase	DDIT3	ENSG00000175197	19428936;
D011794	increase	DDIT4	ENSG00000168209	19729006;
D011794	increase	DEFA1	ENSG00000240247	19814765;
D011794	increase	DENR	ENSG00000139726	19729006;
D011794	increase	DLGAP5	ENSG00000126787	17639512;
D011794	increase	EDN1	ENSG00000078401	11755377;18234130;
D011794	increase	EDNRB	ENSG00000136160	15309432;
D011794	increase	EFEMP1	ENSG00000115380	15309432;
D011794	increase	EGLN3	ENSG00000129521	17639512;
D011794	increase	EIF4A1	ENSG00000161960	17292933;
D011794	increase	EMP1	ENSG00000134531	17639512;
D011794	increase	EMP3	ENSG00000142227	17639512;
D011794	increase	ENO1	ENSG00000074800	17292933;
D011794	increase	EPAS1	ENSG00000116016	17973296;
D011794	increase	EPO	ENSG00000130427	19729006;
D011794	increase	ERBB2	ENSG00000141736	15741050;14715546;
D011794	increase	ERP29	ENSG00000089248	17292933;
D011794	increase	ESR2	ENSG00000140009	16118406;14706564;15182386;11502892;9751507;15937998;18937077;16596234;18955141;
D011794	increase	F2	ENSG00000180210	14628445;
D011794	increase	FBLN2	ENSG00000163520	15309432;

D011794	increase	FCGR2B	ENSG00000072694	15309432;
D011794	increase	FGF7	ENSG00000140285	15309432;
D011794	increase	FH	ENSG00000091483	17292933;
D011794	increase	FOS	ENSG00000170345	15090535;18982426;
D011794	increase	FPR2	ENSG00000171049	15309432;
D011794	increase	FZD2	ENSG00000180340	17639512;
D011794	increase	GABPB2	ENSG00000143458	15309432;
D011794	increase	GADD45A	ENSG00000116717	16211300;
D011794	increase	GADD45B	ENSG00000099860	18331776;
D011794	increase	GATA1	ENSG00000102145	10656287;
D011794	increase	GBE1	ENSG00000114480	19729006;
D011794	increase	GCLC	ENSG00000001084	11864778;
D011794	increase	GCLM	ENSG00000023909	19576171;
D011794	increase	GIPC1	ENSG00000123159	15309432;
D011794	increase	GLO1	ENSG00000124767	17292933;
D011794	increase	GNA15	ENSG00000060558	15309432;
D011794	increase	GNB2L1	ENSG00000204628	15221776;17292933;
D011794	increase	GPI	ENSG00000105220	19729006;
D011794	increase	GSTA1	ENSG00000243955	12756216;17639512;
D011794	increase	GYS1	ENSG00000104812	15309432;
D011794	increase	HEBP2	ENSG00000051620	17292933;
D011794	increase	HIF1A	ENSG00000100644	17364964;17973296;19729006;11424089;12054621;
D011794	increase	HK2	ENSG00000159399	15661808;
D011794	increase	HMBS	ENSG00000149397	10656287;
D011794	increase	HMOX1	ENSG00000100292	19576171;17210444;16799064;
D011794	increase	HNRNPA2B	ENSG00000122566	17292933;
D011794	increase	HNRNPH3	ENSG00000096746	17292933;
D011794	increase	HOOK2	ENSG00000095066	15221776;
D011794	increase	HSF1	ENSG00000185122	11424089;
D011794	increase	HSF4	ENSG00000102878	15309432;
D011794	increase	HSP90B1	ENSG00000166598	15221776;
D011794	increase	HSPA1A	ENSG00000232804	17639512;
D011794	increase	HSPA5	ENSG00000044574	19428936;15221776;
D011794	increase	HSPB1	ENSG00000106211	15583868;17639512;
D011794	increase	HSPE1	ENSG00000115541	17292933;
D011794	increase	ICAM1	ENSG00000090339	18982426;15113938;18489909;15322261;14628445;17880982;
D011794	increase	IER3	ENSG00000137331	17639512;
D011794	increase	IFNG	ENSG00000111537	12431781;
D011794	increase	IL1A	ENSG00000115008	17929310;
D011794	increase	IL1B	ENSG00000125538	19557821;12871381;18982426;19367675;15180920;17103373;17388968;18958421;17588137;15309432;
D011794	increase	IL4	ENSG00000113520	12789233;12431781;
D011794	increase	IL5	ENSG00000113525	12789233;16797527;
D011794	increase	IL6	ENSG00000136244	18184041;16426496;17388968;18958421;17588137;11893605;17191106;1679752
D011794	increase	IL8	ENSG00000169429	17717114;18184041;15180920;17884996;16794257;18958421;17588137;1530943

D011794	increase	IMMT	ENSG00000132305	15221776;
D011794	increase	INS	ENSG00000129965	16505238;
D011794	increase	IQCF1	ENSG00000173389	15221776;
D011794	increase	ITGA6	ENSG00000091409	15309432;
D011794	increase	ITGB2	ENSG00000160255	17103373;
D011794	increase	JUN	ENSG00000177606	15795422;18982426;18628248;10620351;
D011794	increase	KIF23	ENSG00000137807	17639512;
D011794	increase	KLC4	ENSG00000137171	19729006;
D011794	increase	KLK3	ENSG00000142515	11238180;15661808;12391264;
D011794	increase	KRT1	ENSG00000167768	17292933;
D011794	increase	KRT20	ENSG00000171431	15309432;
D011794	increase	LGALS1	ENSG00000100097	17639512;
D011794	increase	MAFF	ENSG00000185022	17639512;
D011794	increase	MALT1	ENSG00000172175	17639512;
D011794	increase	MAPK1	ENSG00000100030	18377872;19464278;11414687;19557821;19367675;17103373;12589822;18628248;16996034;10718847;18419748;18346843;17379280;1
D011794	increase	MAPK3	ENSG00000102882	15090535;18377872;19464278;11414687;19557821;19367675;17103373;12589822;18628248;16996034;10718847;18419748;16059641;17379280;18346843;
D011794	increase	MCM4	ENSG00000104738	17639512;
D011794	increase	MEN1	ENSG00000133895	15090535;
D011794	increase	MIF	ENSG00000240972	19729006;
D011794	increase	MITF	ENSG00000187098	15309432;
D011794	increase	MKI67	ENSG00000148773	17639512;
D011794	increase	MMP1	ENSG00000196611	11414687;12724357;
D011794	increase	MMP7	ENSG00000137673	15725655;17639512;
D011794	increase	MMP9	ENSG00000100985	15135307;12589822;19804834;18628248;
D011794	increase	MSH2	ENSG00000095002	14715546;
D011794	increase	MT2A	ENSG00000125148	10656621;
D011794	increase	MTMR15	ENSG00000198690	19729006;
D011794	increase	MUC2	ENSG00000198788	15309432;
D011794	increase	MYBL2	ENSG00000101057	17639512;
D011794	increase	MYC	ENSG00000136997	15670774;16948901;15309432;
D011794	increase	MYO5A	ENSG00000197535	15221776;
D011794	increase	NCF1	ENSG00000158517	17103373;19450372;
D011794	increase	NDRG1	ENSG00000104419	19729006;
D011794	increase	NEDD8	ENSG00000129559	17292933;
D011794	increase	NFE2	ENSG00000123405	10656287;
D011794	increase	NFE2L2	ENSG00000116044	19576171;17462537;16799064;
D011794	increase	NQO1	ENSG00000181019	11275421;
D011794	increase	OGG1	ENSG00000114026	19651184;
D011794	increase	OLR1	ENSG00000173391	16891912;17639512;
D011794	increase	OMG	ENSG00000126861	15309432;
D011794	increase	P2RY2	ENSG00000175591	15309432;
D011794	increase	PAFAH1B1	ENSG00000007168	15221776;

D011794	increase	PARP1	ENSG00000143799	16280456;18377872;17876056;17884996;18787224;15741050;16059641;
D011794	increase	PBRM1	ENSG00000163939	15221776;
D011794	increase	PDK4	ENSG00000004799	15309432;
D011794	increase	PEBP1	ENSG00000089220	17292933;
D011794	increase	PFKFB3	ENSG00000170525	19729006;
D011794	increase	PFKP	ENSG00000067057	19729006;
D011794	increase	PGAM1	ENSG00000171314	17292933;
D011794	increase	PGK1	ENSG00000102144	19729006;
D011794	increase	PHLDA1	ENSG00000139289	17639512;
D011794	increase	PHLDA2	ENSG00000181649	17639512;
D011794	increase	PI4KA	ENSG00000249793	15309432;
D011794	increase	PITX2	ENSG00000164093	15309432;
D011794	increase	PLA2G16	ENSG00000176485	17639512;
D011794	increase	PLAT	ENSG00000104368	18419748;11236827;
D011794	increase	PLAU	ENSG00000122861	11236827;12724357;
D011794	increase	PLK1	ENSG00000166851	17639512;
D011794	increase	PON1	ENSG00000005421	15169886;
D011794	increase	PPIA	ENSG00000196262	17292933;
D011794	increase	PRDX1	ENSG00000117450	17292933;
D011794	increase	PRDX2	ENSG00000167815	17292933;
D011794	increase	PRDX4	ENSG00000123131	15221776;
D011794	increase	PRKCQ	ENSG00000065675	15309432;
D011794	increase	PTEN	ENSG00000171862	14715546;15829497;
D011794	increase	PTGFRN	ENSG00000134247	19729006;
D011794	increase	PTGS2	ENSG00000073756	12144868;19557821;12672646;17103373;10783318;19804834;14988445;15225597;15155531;17184768;1092
D011794	increase	RAB3B	ENSG00000169213	17639512;
D011794	increase	RAD54B	ENSG00000197275	17639512;
D011794	increase	RB1	ENSG00000139687	14767529;19009557;16049707;14715546;
D011794	increase	RGS1	ENSG00000090104	15309432;
D011794	increase	RIT2	ENSG00000152214	15309432;
D011794	increase	ROCK1	ENSG00000067900	15221776;
D011794	increase	RPA3	ENSG00000106399	16797527;
D011794	increase	RUNX2	ENSG00000250096	16996034;
D011794	increase	RXRG	ENSG00000249671	15309432;
D011794	increase	S100B	ENSG00000160307	17103373;
D011794	increase	SELE	ENSG00000007908	15113938;
D011794	increase	SELP	ENSG00000174175	14628445;
D011794	increase	SERPINB3	ENSG00000057149	15309432;
D011794	increase	SERPINB9	ENSG00000170542	15309432;
D011794	increase	SFN	ENSG00000175793	18331776;
D011794	increase	SH3BP2	ENSG00000087266	15309432;
D011794	increase	SOD1	ENSG00000142168	15782287;10656621;18686095;
D011794	increase	SOD2	ENSG00000112096	18852136;
D011794	increase	SP1	ENSG00000185591	18419748;18377872;15661808;18980244;18852136;
D011794	increase	SPP1	ENSG00000118785	16996034;
D011794	increase	STC1	ENSG00000159167	19729006;
D011794	increase	STK17B	ENSG00000081320	17639512;

D011794	increase	SUB1	ENSG00000113387	15309432;
D011794	increase	TAF9	ENSG00000085231	15309432;
D011794	increase	TFF1	ENSG00000160182	15090535;14706564;
D011794	increase	TFRC	ENSG00000072274	17364964;
D011794	increase	TGFA	ENSG00000163235	10783318;
D011794	increase	TGFB1	ENSG00000105329	17086741;17639512;
D011794	increase	TGFBR2	ENSG00000163513	14715546;
D011794	increase	THBS2	ENSG00000186340	15309432;
D011794	increase	TIMP1	ENSG00000102265	11414687;11506819;
D011794	increase	TIMP2	ENSG00000035862	11506819;15309432;
D011794	increase	TM4SF1	ENSG00000169908	17639512;
D011794	increase	TMEM45A	ENSG00000181458	19729006;
D011794	increase	TNF	ENSG00000223952	19557821;17929310;17103373;15113938;16794257;12589822;12871381;18489909;15322261;14628445;18958421;17588137;17880982;
D011794	increase	TNFRSF10B	ENSG00000120889	17876056;18980244;
D011794	increase	TNFRSF11B	ENSG00000164761	15309432;
D011794	increase	TNFRSF1B	ENSG00000028137	15309432;
D011794	increase	TNFSF10	ENSG00000121858	17876056;18377872;17639512;18980244;
D011794	increase	TP53BP2	ENSG00000143514	15309432;
D011794	increase	TP53	ENSG00000141510	18323654;11522280;15795422;19424582;16948901;16720314;14715546;1797329
D011794	increase	TP53I3	ENSG00000115129	18331776;19397994;
D011794	increase	TP63	ENSG00000073282	18331776;19397994;
D011794	increase	TP73	ENSG00000078900	18331776;19397994;
D011794	increase	TPI1	ENSG00000111669	17292933;
D011794	increase	TRIB3	ENSG00000101255	17639512;
D011794	increase	TSC1	ENSG00000165699	14715546;
D011794	increase	TSC2	ENSG00000103197	14715546;
D011794	increase	TSFM	ENSG00000123297	15309432;15221776;
D011794	increase	TSG101	ENSG00000074319	15309432;
D011794	increase	TUFM	ENSG00000178952	17292933;
D011794	increase	UGT1A10	ENSG00000242515	14557274;17639512;
D011794	increase	UGT1A1	ENSG00000241635	12756216;11442279;14557274;
D011794	increase	UGT1A6	ENSG00000167165	18094037;
D011794	increase	UGT1A9	ENSG00000241119	14557274;17639512;
D011794	increase	VCAM1	ENSG00000162692	15113938;18489909;17880982;
D011794	increase	VDAC1	ENSG00000213585	17292933;
D011794	increase	VEGFA	ENSG00000112715	19005980;19729006;12469199;17973296;
D011794	increase	VHL	ENSG00000134086	17973296;14715546;
D011794	increase	VIPR1	ENSG00000114812	15309432;
D011794	increase	WEE1	ENSG00000166483	16280456;
D011794	increase	WSB1	ENSG00000109046	19729006;
D011794	increase	ZNF165	ENSG00000197279	17639512;
D011794	increase	ZNF624	ENSG00000197566	15221776;
D011802	decrease	SLC2A3	ENSG00000059804	17567588;15342952;
D011802	decrease	TAGLN	ENSG00000149591	17567588;
D011802	increase	ASNS	ENSG00000070669	15342952;

D011802	increase	C10ORF10	ENSG00000165507	17567588;
D011802	increase	FABP1	ENSG00000163586	17567588;
D011802	increase	INHBE	ENSG00000139269	15342952;
D011802	increase	NUPR1	ENSG00000176046	17567588;
D011804	increase	CCNB1	ENSG00000134057	15826604;
D011899	affect	GUSB	ENSG00000169919	11238657;
D011899	affect	IL6	ENSG00000136244	11238657;
D011899	decrease	IL12A	ENSG00000168811	9819373;
D011899	decrease	IL12B	ENSG00000113302	9819373;
D011899	increase	IFNG	ENSG00000111537	10569698;
D011929	increase	ABCB1	ENSG00000085563	18385176;
D012110	increase	ABCB1	ENSG00000085563	10617675;8632764;
D012110	increase	CYP1A1	ENSG00000140465	15627480;
D012110	increase	CYP3A4	ENSG00000160868	16837568;8632764;
D012110	increase	CYP3A5	ENSG00000106258	8632764;
D012172	decrease	BTC	ENSG00000174808	16470170;
D012172	increase	AREG	ENSG00000205595	16470170;
D012172	increase	CYP26A1	ENSG00000095596	17526768;
D012172	increase	HBEGF	ENSG00000113070	16470170;
D012176	decrease	MMP1	ENSG00000196611	16055107;
D012176	decrease	TERT	ENSG00000164362	16482212;
D012176	increase	CDKN2D	ENSG00000129355	16582618;
D012176	increase	CYP17A1	ENSG00000148795	15914525;
D012176	increase	ELN	ENSG00000049540	16055107;
D012176	increase	FBN1	ENSG00000166147	16055107;
D012176	increase	MUC1	ENSG00000185499	11287445;
D012176	increase	PLAT	ENSG00000104368	7556191;
D012176	increase	STAR	ENSG00000147465	15914525;
D012254	increase	SLC6A4	ENSG00000108576	16476009;
D012256	decrease	CRP	ENSG00000132693	16517955;
D012293	decrease	ABCA1	ENSG00000165029	15883047;
D012293	decrease	CYP7A1	ENSG00000167910	15629111;
D012293	decrease	PPARA	ENSG00000186951	16292757;
D012293	decrease	SCARB1	ENSG00000073060	15883047;
D012293	increase	ABCB1	ENSG00000085563	15964336;17191263;18094037;15710169;14722322;17003290;8632764;
D012293	increase	ABCC1	ENSG00000103222	11836020;17191263;
D012293	increase	ABCC2	ENSG00000023839	15652233;12206135;11836020;
D012293	increase	ABCG1	ENSG00000160179	12040753;
D012293	increase	ADH1C	ENSG00000248144	12040753;
D012293	increase	CES1	ENSG00000198848	10640517;
D012293	increase	CES2	ENSG00000172831	10640517;
D012293	increase	CYP1A1	ENSG00000140465	10923861;19409404;12040753;
D012293	increase	CYP1A2	ENSG00000140505	10923861;15977188;17590308;
D012293	increase	CYP24A1	ENSG00000019186	15630458;
D012293	increase	CYP2A6	ENSG00000198077	16857725;15629111;14709624;
D012293	increase	CYP2B6	ENSG00000197408	12695351;14977870;18332078;17954527;15802389;12040753;15629111;18094037;15548381;14709624;

D012293	increase	CYP2C19	ENSG00000165841	16985168;15629111;17954527;14709624;
D012293	increase	CYP2C8	ENSG00000138115	15771232;15933212;17954527;12040753;
D012293	increase	CYP2C9	ENSG00000138109	14600250;16985168;14722322;15919766;15802389;17954527;15629111;17590308;14709624;
D012293	increase	CYP2E1	ENSG00000130649	18071298;
D012293	increase	CYP3A4	ENSG00000160868	16146350;12695340;10219967;15964336;16985168;12417264;16632523;18332078;12065438;14977870;9512926;17270371;15771232;14636322;14722322;18094037;15554232;12642470;12673034;15855724;15802389;15769886;12040753;16565514;15977188;15629111;16837568;17954527;15681896;15833926;15710169;15075359;15466163;12505310;14600250;17590308;15548381;8632764;10640517;14709[illegible]
D012293	increase	CYP3A5	ENSG00000106258	12673034;15629111;15681896;12040753;
D012293	increase	CYP3A7	ENSG00000160870	12673034;
D012293	increase	NR0B2	ENSG00000131910	15670600;
D012293	increase	NR1I2	ENSG00000144852	12695351;15670600;14600250;12695340;15705662;14977870;15864135;15933212;15849716;15883047;15630458;15833926;16857725;12417264;16632523;18692084;15802384;15885729;14636322;16565514;15548381;12065438;19249324;18098[illegible]
D012293	increase	NR3C1	ENSG00000113580	12695351;10219967;12673034;
D012293	increase	RXRA	ENSG00000186350	16632523;16292757;
D012293	increase	SLCO1B1	ENSG00000134538	15670600;15652233;
D012293	increase	UGDH	ENSG00000109814	16292757;
D012293	increase	UGT1A1	ENSG00000241635	15849716;15104253;15771232;18098064;
D012293	increase	UGT1A6	ENSG00000167165	18094037;
D012294	decrease	GSTM1	ENSG00000134184	7986210;
D012294	increase	GSTA1	ENSG00000243955	7986210;
D012402	affect	HAX1	ENSG00000143575	19763263;
D012402	decrease	ACAD9	ENSG00000177646	16565515;
D012402	decrease	ARL2	ENSG00000213465	17651460;
D012402	decrease	CDH1	ENSG00000039068	17596522;
D012402	decrease	CP	ENSG00000047457	19019832;
D012402	decrease	FAS	ENSG00000026103	8902626;
D012402	decrease	FN1	ENSG00000115414	15086456;17651460;
D012402	decrease	HSPA9	ENSG00000113013	16565515;
D012402	decrease	MAPT	ENSG00000186868	16930453;
D012402	decrease	TNFRSF11B	ENSG00000164761	17651460;
D012402	increase	ACTA2	ENSG00000107796	17596522;
D012402	increase	ANPEP	ENSG00000166825	8833408;

D012402	increase	APAF1	ENSG00000120868	17064354;
D012402	increase	ATF4	ENSG00000128272	19233273;
D012402	increase	ATM	ENSG00000149311	18191903;
D012402	increase	ATP5E	ENSG00000124172	18191903;
D012402	increase	ATP5G3	ENSG00000154518	18191903;
D012402	increase	ATP5I	ENSG00000169020	18191903;
D012402	increase	ATP5J	ENSG00000154723	18191903;
D012402	increase	ATP5L	ENSG00000167283	18191903;
D012402	increase	ATP5O	ENSG00000241837	18191903;
D012402	increase	ATR	ENSG00000175054	18191903;
D012402	increase	ATXN3	ENSG00000066427	18191903;
D012402	increase	BACE1	ENSG00000186318	19196431;
D012402	increase	BARD1	ENSG00000138376	18191903;
D012402	increase	BCL2	ENSG00000171791	12730627;16815102;
D012402	increase	BECN1	ENSG00000126581	18032788;
D012402	increase	BIRC3	ENSG00000023445	17671207;
D012402	increase	BRCA1	ENSG00000012048	18191903;
D012402	increase	BRCA2	ENSG00000139618	18191903;
D012402	increase	CAT	ENSG00000121691	15904944;
D012402	increase	CCND1	ENSG00000110092	15897899;
D012402	increase	CCNH	ENSG00000134480	18191903;
D012402	increase	CD14	ENSG00000170458	10491287;
D012402	increase	CD38	ENSG00000004468	10491287;8833408;
D012402	increase	CDKN1A	ENSG00000124762	19091749;
D012402	increase	CHEK1	ENSG00000149554	18191903;
D012402	increase	CNOT7	ENSG00000198791	18191903;
D012402	increase	COX4I1	ENSG00000131143	18191903;
D012402	increase	COX5A	ENSG00000178741	18191903;
D012402	increase	COX5B	ENSG00000135940	18191903;
D012402	increase	COX7A2	ENSG00000112695	18191903;
D012402	increase	COX7B	ENSG00000131174	18191903;
D012402	increase	DDIT3	ENSG00000175197	12111836;19233273;
D012402	increase	EIF2S1	ENSG00000134001	19233273;
D012402	increase	EXO1	ENSG00000174371	18191903;
D012402	increase	FANCA	ENSG00000187741	18191903;
D012402	increase	FANCC	ENSG00000158169	18191903;
D012402	increase	FANCD2	ENSG00000144554	18191903;
D012402	increase	FANCE	ENSG00000112039	18191903;
D012402	increase	FANCF	ENSG00000183161	18191903;
D012402	increase	FANCG	ENSG00000221829	18191903;
D012402	increase	FTH1	ENSG00000167996	18325346;
D012402	increase	HIF1A	ENSG00000100644	12376345;19542490;19091749;12062197;
D012402	increase	HTRA2	ENSG00000115317	19763263;
D012402	increase	HUS1	ENSG00000136273	18191903;
D012402	increase	ICAM1	ENSG00000090339	12952965;
D012402	increase	IDH3A	ENSG00000166411	19763263;
D012402	increase	IL6	ENSG00000136244	11950692;
D012402	increase	IL8	ENSG00000169429	12208513;
D012402	increase	MRE11A	ENSG00000020922	18191903;
D012402	increase	MRPS9	ENSG00000135972	18191903;
D012402	increase	MSR1	ENSG00000038945	9614211;
D012402	increase	MT2A	ENSG00000125148	16402917;
D012402	increase	MUS81	ENSG00000172732	18191903;

D012402	increase	NDUFA10	ENSG00000130414	18191903;
D012402	increase	NDUFA3	ENSG00000170906	18191903;
D012402	increase	NDUFA4	ENSG00000189043	18191903;
D012402	increase	NDUFA6	ENSG00000184983	18191903;
D012402	increase	NDUFA9	ENSG00000139180	18191903;
D012402	increase	NDUFB1	ENSG00000183648	18191903;
D012402	increase	NDUFB2	ENSG00000090266	18191903;
D012402	increase	NDUFB3	ENSG00000119013	18191903;
D012402	increase	NDUFB4	ENSG00000065518	18191903;
D012402	increase	NDUFB5	ENSG00000136521	18191903;
D012402	increase	NDUFB6	ENSG00000165264	18191903;
D012402	increase	NDUFB7	ENSG00000099795	18191903;
D012402	increase	NDUFB8	ENSG00000166136	18191903;
D012402	increase	NDUFS4	ENSG00000164258	18191903;
D012402	increase	NDUFS6	ENSG00000145494	18191903;
D012402	increase	NDUFS8	ENSG00000110717	18191903;
D012402	increase	NUDT1	ENSG00000106268	18191903;
D012402	increase	PARK7	ENSG00000116288	18377993;18331584;
D012402	increase	PDHB	ENSG00000168291	19763263;
D012402	increase	PDK1	ENSG00000152256	17671207;
D012402	increase	POLA1	ENSG00000101868	18191903;
D012402	increase	POLE2	ENSG00000100479	18191903;
D012402	increase	PPID	ENSG00000171497	18614807;
D012402	increase	PRKAA1	ENSG00000132356	17651460;
D012402	increase	PROM1	ENSG00000007062	18985161;
D012402	increase	RAD17	ENSG00000152942	18191903;
D012402	increase	RAD18	ENSG00000070950	18191903;
D012402	increase	RAD1	ENSG00000113456	18191903;
D012402	increase	RAD50	ENSG00000113522	18191903;
D012402	increase	RAD51AP1	ENSG00000111247	18191903;
D012402	increase	RAD51	ENSG00000051180	18191903;
D012402	increase	RAD54B	ENSG00000197275	18191903;
D012402	increase	RAD9A	ENSG00000172613	18191903;
D012402	increase	RFC4	ENSG00000163918	18191903;
D012402	increase	RFC5	ENSG00000111445	18191903;
D012402	increase	SLC2A1	ENSG00000117394	19091749;10780954;
D012402	increase	SLC6A12	ENSG00000111181	16303854;
D012402	increase	SMN1	ENSG00000172062	17064354;
D012402	increase	SNAI1	ENSG00000124216	17596522;
D012402	increase	SNCA	ENSG00000145335	16239214;19114014;15114628;17131421;17041725;18514418;12151787;
D012402	increase	SOD2	ENSG00000112096	15904944;18032788;
D012402	increase	SREBF1	ENSG00000072310	17651460;
D012402	increase	TK2	ENSG00000166548	16565515;
D012402	increase	TP53	ENSG00000141510	18191903;
D012402	increase	UBE2N	ENSG00000177889	18191903;
D012402	increase	UQCRB	ENSG00000156467	18191903;
D012402	increase	UQCRC1	ENSG00000010256	18191903;
D012402	increase	VDAC1	ENSG00000213585	18802751;
D012402	increase	VEGFA	ENSG00000112715	19091749;19542490;17671207;
D012413	decrease	KCNH2	ENSG00000055118	19139152;
D012428	decrease	MDM2	ENSG00000135679	17210701;

D012428	decrease	MDM4	ENSG00000198625	17210701;
D012428	increase	ANXA5	ENSG00000164111	17210701;
D012428	increase	PMAIP1	ENSG00000141682	17210701;
D012428	increase	TP53	ENSG00000141510	17210701;
D012430	affect	MMP1	ENSG00000196611	17508023;
D012431	affect	BAX	ENSG00000087088	15975156;
D012431	decrease	BCL2	ENSG00000171791	15975156;19557821;
D012431	decrease	NFKBIA	ENSG00000100906	19557821;
D012431	increase	AGER	ENSG00000204305	19557821;
D012431	increase	IL1B	ENSG00000125538	19557821;18958421;
D012431	increase	IL6	ENSG00000136244	18958421;
D012431	increase	IL8	ENSG00000169429	18958421;
D012431	increase	PTGS2	ENSG00000073756	19557821;
D012431	increase	TNF	ENSG00000223952	19557821;18958421;
D012435	decrease	COL1A1	ENSG00000108821	15983038;
D012435	decrease	COL1A2	ENSG00000164692	15983038;
D012436	decrease	COL1A1	ENSG00000108821	15983038;
D012436	decrease	COL1A2	ENSG00000164692	15983038;
D012459	decrease	MMP7	ENSG00000137673	15725655;
D012460	decrease	IL1B	ENSG00000125538	15955209;
D012460	decrease	IL8	ENSG00000169429	15955209;
D012460	decrease	RELA	ENSG00000173039	15955209;
D012631	increase	JUN	ENSG00000177606	15263067;
D012642	increase	CASP3	ENSG00000164305	12880480;
D012642	increase	PARK2	ENSG00000185345	12880480;
D012642	increase	SNCA	ENSG00000145335	15114628;12880480;
D012643	decrease	AASS	ENSG00000008311	19244175;
D012643	decrease	ACP1	ENSG00000143727	19244175;
D012643	decrease	ADAMTS5	ENSG00000154736	19244175;
D012643	decrease	ADD3	ENSG00000148700	19244175;
D012643	decrease	ALCAM	ENSG00000170017	19244175;
D012643	decrease	AMPD3	ENSG00000133805	19244175;
D012643	decrease	AMY2A	ENSG00000243480	19244175;
D012643	decrease	ANGPT1	ENSG00000154188	19244175;
D012643	decrease	ANK3	ENSG00000151150	19244175;
D012643	decrease	ANKRA2	ENSG00000164331	19244175;
D012643	decrease	ANP32B	ENSG00000136938	19244175;
D012643	decrease	APC	ENSG00000134982	19244175;
D012643	decrease	APPBP2	ENSG00000062725	19244175;
D012643	decrease	APP	ENSG00000142192	19244175;
D012643	decrease	AR	ENSG00000169083	14729601;16648561;
D012643	decrease	ARG2	ENSG00000081181	19244175;
D012643	decrease	ARIH1	ENSG00000166233	19244175;
D012643	decrease	ARL5A	ENSG00000162980	19244175;
D012643	decrease	ASXL1	ENSG00000171456	19244175;
D012643	decrease	ASXL2	ENSG00000143970	19244175;
D012643	decrease	ATG12	ENSG00000145782	19244175;
D012643	decrease	ATP10D	ENSG00000145246	19244175;
D012643	decrease	ATP2C1	ENSG00000017260	19244175;
D012643	decrease	ATP5H	ENSG00000167863	19244175;
D012643	decrease	ATP5I	ENSG00000169020	19244175;
D012643	decrease	ATP5J	ENSG00000154723	19244175;
D012643	decrease	ATP6V1C1	ENSG00000155097	19244175;
D012643	decrease	ATRX	ENSG00000085224	19244175;

D012643	decrease	ATXN1	ENSG00000124788	19244175;
D012643	decrease	ATXN7	ENSG00000163635	19244175;
D012643	decrease	AVL9	ENSG00000105778	19244175;
D012643	decrease	AXL	ENSG00000167601	19244175;
D012643	decrease	BARD1	ENSG00000138376	19244175;
D012643	decrease	BAT2L2	ENSG00000117523	19244175;
D012643	decrease	BBX	ENSG00000114439	19244175;
D012643	decrease	BCL11A	ENSG00000119866	19244175;
D012643	decrease	BCL2	ENSG00000171791	17523932;
D012643	decrease	BDH2	ENSG00000164039	19244175;
D012643	decrease	BICD1	ENSG00000151746	19244175;
D012643	decrease	C11ORF10	ENSG00000134825	19244175;
D012643	decrease	C11ORF30	ENSG00000158636	19244175;
D012643	decrease	C11ORF48	ENSG00000162194	19244175;
D012643	decrease	C12ORF41	ENSG00000139620	19244175;
D012643	decrease	C14ORF138	ENSG00000100483	19244175;
D012643	decrease	C16ORF61	ENSG00000103121	19244175;
D012643	decrease	C1ORF107	ENSG00000117597	19244175;
D012643	decrease	C1ORF50	ENSG00000164008	19244175;
D012643	decrease	C1ORF54	ENSG00000118292	19244175;
D012643	decrease	C1ORF63	ENSG00000117616	19244175;
D012643	decrease	C20ORF27	ENSG00000101220	19244175;
D012643	decrease	C3ORF63	ENSG00000163946	19244175;
D012643	decrease	C5ORF30	ENSG00000181751	19244175;
D012643	decrease	C5ORF4	ENSG00000170271	19244175;
D012643	decrease	C7ORF23	ENSG00000135185	19244175;
D012643	decrease	C7ORF25	ENSG00000136197	19244175;
D012643	decrease	CALD1	ENSG00000122786	19244175;
D012643	decrease	CALR	ENSG00000179218	19244175;
D012643	decrease	CAP2	ENSG00000112186	19244175;
D012643	decrease	CAPN7	ENSG00000131375	19244175;
D012643	decrease	CASP8	ENSG00000064012	19244175;12432279;17970047;
D012643	decrease	CBLB	ENSG00000114423	19244175;
D012643	decrease	CBX5	ENSG00000094916	19244175;
D012643	decrease	CCDC53	ENSG00000120860	19244175;
D012643	decrease	CCDC72	ENSG00000232112	19244175;
D012643	decrease	CCNB1	ENSG00000134057	11925459;
D012643	decrease	CCNC	ENSG00000112237	11925459;
D012643	decrease	CCND2	ENSG00000118971	11925459;
D012643	decrease	CDC14B	ENSG00000081377	19244175;
D012643	decrease	CDC16	ENSG00000130177	19244175;
D012643	decrease	CDC23	ENSG00000094880	19244175;
D012643	decrease	CDC42BPA	ENSG00000143776	19244175;
D012643	decrease	CDC73	ENSG00000134371	19244175;
D012643	decrease	CDH11	ENSG00000140937	19244175;
D012643	decrease	CDKN1A	ENSG00000124762	18583539;
D012643	decrease	CDKN1C	ENSG00000129757	19244175;
D012643	decrease	CDS1	ENSG00000163624	19244175;
D012643	decrease	CEP290	ENSG00000198707	19244175;
D012643	decrease	CEP57	ENSG00000166037	19244175;
D012643	decrease	CFH	ENSG00000000971	19244175;
D012643	decrease	CHD1	ENSG00000153922	19244175;
D012643	decrease	CHD9	ENSG00000177200	19244175;

D012643	decrease	CHORDC1	ENSG00000110172	19244175;
D012643	decrease	CKAP2	ENSG00000136108	19244175;
D012643	decrease	CKS2	ENSG00000123975	19244175;
D012643	decrease	CLCC1	ENSG00000121940	19244175;
D012643	decrease	CNIH4	ENSG00000143771	19244175;
D012643	decrease	CNIH	ENSG00000100528	19244175;
D012643	decrease	CNOT8	ENSG00000155508	19244175;
D012643	decrease	CNPY2	ENSG00000144785	19244175;
D012643	decrease	COL14A1	ENSG00000187955	19244175;
D012643	decrease	COL4A3	ENSG00000169031	19244175;
D012643	decrease	COMMD3	ENSG00000148444	19244175;
D012643	decrease	COX6A1	ENSG00000111775	19244175;
D012643	decrease	COX7A1	ENSG00000161281	19244175;
D012643	decrease	COX7A2	ENSG00000112695	19244175;
D012643	decrease	COX7B	ENSG00000131174	19244175;
D012643	decrease	COX7C	ENSG00000127184	19244175;
D012643	decrease	CPA3	ENSG00000163751	19244175;
D012643	decrease	CPD	ENSG00000108582	19244175;
D012643	decrease	CRBN	ENSG00000113851	19244175;
D012643	decrease	CREB1	ENSG00000118260	19244175;
D012643	decrease	CREBBP	ENSG00000005339	19244175;
D012643	decrease	CSNK1A1	ENSG00000113712	19244175;
D012643	decrease	CSNK2A1	ENSG00000101266	19244175;
D012643	decrease	CSTA	ENSG00000121552	19244175;
D012643	decrease	CTBP2	ENSG00000175029	19244175;
D012643	decrease	CTBS	ENSG00000117151	19244175;
D012643	decrease	CTSO	ENSG00000151792	19244175;
D012643	decrease	CUL3	ENSG00000036257	19244175;
D012643	decrease	CUL4A	ENSG00000139842	19244175;
D012643	decrease	CXCL9	ENSG00000138755	18997278;
D012643	decrease	CXORF40A	ENSG00000197620	19244175;
D012643	decrease	CYORF15B	ENSG00000131002	19244175;
D012643	decrease	CYP3A4	ENSG00000160868	19244175;
D012643	decrease	CYP3A5	ENSG00000106258	19244175;
D012643	decrease	DAAM1	ENSG00000100592	19244175;
D012643	decrease	DACT1	ENSG00000165617	19244175;
D012643	decrease	DDX18	ENSG00000088205	19244175;
D012643	decrease	DDX24	ENSG00000089737	19244175;
D012643	decrease	DDX3Y	ENSG00000067048	19244175;
D012643	decrease	DERA	ENSG00000023697	19244175;
D012643	decrease	DFNA5	ENSG00000105928	19244175;
D012643	decrease	DICER1	ENSG00000100697	19244175;
D012643	decrease	DIP2C	ENSG00000151240	19244175;
D012643	decrease	DLG1	ENSG00000075711	19244175;
D012643	decrease	DNAJA3	ENSG00000103423	19244175;
D012643	decrease	DNAJB4	ENSG00000162616	19244175;
D012643	decrease	DNAJC7	ENSG00000168259	19244175;
D012643	decrease	DNAJC9	ENSG00000213551	19244175;
D012643	decrease	DPYD	ENSG00000188641	19244175;
D012643	decrease	DR1	ENSG00000117505	19244175;
D012643	decrease	DSC2	ENSG00000134755	19244175;
D012643	decrease	DSE	ENSG00000111817	19244175;
D012643	decrease	DYM	ENSG00000141627	19244175;
D012643	decrease	DYNLRB1	ENSG00000125971	19244175;

D012643	decrease	ECM2	ENSG00000106823	19244175;
D012643	decrease	EFHC1	ENSG00000096093	19244175;
D012643	decrease	EGFR	ENSG00000146648	17390030;
D012643	decrease	EHBP1	ENSG00000115504	19244175;
D012643	decrease	EIF2AK2	ENSG00000055332	19244175;
D012643	decrease	EIF5A	ENSG00000132507	19244175;
D012643	decrease	EPB41L4B	ENSG00000095203	19244175;
D012643	decrease	EVI5	ENSG00000067208	19244175;
D012643	decrease	EXOSC8	ENSG00000120699	19244175;
D012643	decrease	EXT2	ENSG00000151348	19244175;
D012643	decrease	EZR	ENSG00000092820	19244175;
D012643	decrease	FAM105A	ENSG00000145569	19244175;
D012643	decrease	FAM111A	ENSG00000166801	19244175;
D012643	decrease	FAM115A	ENSG00000198420	19244175;
D012643	decrease	FAM127B	ENSG00000203950	19244175;
D012643	decrease	FAM129A	ENSG00000135842	19244175;
D012643	decrease	FAM158A	ENSG00000100908	19244175;
D012643	decrease	FAM164A	ENSG00000104427	19244175;
D012643	decrease	FAM190B	ENSG00000107771	19244175;
D012643	decrease	FAM21C	ENSG00000172661	19244175;
D012643	decrease	FAT4	ENSG00000196159	19244175;
D012643	decrease	FBXL5	ENSG00000118564	19244175;
D012643	decrease	FBXO11	ENSG00000250152	19244175;
D012643	decrease	FECH	ENSG00000066926	19244175;
D012643	decrease	FERMT1	ENSG00000101311	19244175;
D012643	decrease	FHOD3	ENSG00000134775	19244175;
D012643	decrease	FIG4	ENSG00000112367	19244175;
D012643	decrease	FKBP3	ENSG00000100442	19244175;
D012643	decrease	FLRT3	ENSG00000125848	19244175;
D012643	decrease	FOLH1	ENSG00000086205	19244175;
D012643	decrease	FOXF2	ENSG00000137273	19244175;
D012643	decrease	FXR1	ENSG00000114416	19244175;
D012643	decrease	FZD7	ENSG00000155760	19244175;
D012643	decrease	GALNT1	ENSG00000141429	19244175;
D012643	decrease	GBP1	ENSG00000117228	19244175;
D012643	decrease	GFAP	ENSG00000131095	18583539;
D012643	decrease	GGPS1	ENSG00000152904	19244175;
D012643	decrease	GLMN	ENSG00000174842	19244175;
D012643	decrease	GMNN	ENSG00000112312	19244175;
D012643	decrease	GNAS	ENSG00000087460	19244175;
D012643	decrease	GNS	ENSG00000135677	19244175;
D012643	decrease	GOSR1	ENSG00000108587	19244175;
D012643	decrease	GPR137B	ENSG00000077585	19244175;
D012643	decrease	GPRIN2	ENSG00000204175	19244175;
D012643	decrease	GRIA2	ENSG00000120251	19244175;
D012643	decrease	GRINL1A	ENSG00000137878	19244175;
D012643	decrease	GTPBP8	ENSG00000163607	19244175;
D012643	decrease	GULP1	ENSG00000144366	19244175;
D012643	decrease	H3F3A	ENSG00000163041	19244175;
D012643	decrease	HADHA	ENSG00000084754	19244175;
D012643	decrease	HBA2	ENSG00000206172	19244175;
D012643	decrease	HEPH	ENSG00000089472	19244175;
D012643	decrease	HIPK2	ENSG00000064393	19244175;
D012643	decrease	HIST1H4F	ENSG00000182217	19244175;

D012643	decrease	HLF	ENSG00000108924	19244175;
D012643	decrease	HMGB1	ENSG00000189403	19244175;
D012643	decrease	HNMT	ENSG00000150540	19244175;
D012643	decrease	HOMER1	ENSG00000152413	19244175;
D012643	decrease	HOOK1	ENSG00000134709	19244175;
D012643	decrease	HS3ST3A1	ENSG00000153976	19244175;
D012643	decrease	HSP90AA1	ENSG00000080824	19244175;
D012643	decrease	HSPA9	ENSG00000113013	19244175;
D012643	decrease	HSPB2	ENSG00000170276	18997278;
D012643	decrease	HSPBAP1	ENSG00000169087	19244175;
D012643	decrease	ID2	ENSG00000115738	19244175;
D012643	decrease	IFNA10	ENSG00000186803	19244175;
D012643	decrease	IFT74	ENSG00000096872	19244175;
D012643	decrease	IFT81	ENSG00000122970	19244175;
D012643	decrease	IGF2R	ENSG00000197081	19244175;
D012643	decrease	IKBKAP	ENSG00000070061	19244175;
D012643	decrease	IL6ST	ENSG00000134352	19244175;
D012643	decrease	INTS6	ENSG00000249168	19244175;
D012643	decrease	IQCB1	ENSG00000173226	19244175;
D012643	decrease	IQCK	ENSG00000174628	19244175;
D012643	decrease	ITGAE	ENSG00000083457	19244175;
D012643	decrease	KIAA0528	ENSG00000111731	19244175;
D012643	decrease	KIAA0831	ENSG00000126775	19244175;
D012643	decrease	KIAA0892	ENSG00000129933	19244175;
D012643	decrease	KIAA1012	ENSG00000153339	19244175;
D012643	decrease	KIF5C	ENSG00000168280	19244175;
D012643	decrease	KLF9	ENSG00000119138	19244175;
D012643	decrease	KLHDC10	ENSG00000128607	19244175;
D012643	decrease	KLHL20	ENSG00000076321	19244175;
D012643	decrease	KLK3	ENSG00000142515	14729601;
D012643	decrease	KPNA1	ENSG00000114030	19244175;
D012643	decrease	KTN1	ENSG00000126777	19244175;
D012643	decrease	LIMCH1	ENSG00000064042	19244175;
D012643	decrease	LIPT1	ENSG00000144182	19244175;
D012643	decrease	LPIN1	ENSG00000134324	19244175;
D012643	decrease	LRRFIP1	ENSG00000124831	19244175;
D012643	decrease	LUC7L2	ENSG00000146963	19244175;
D012643	decrease	LUC7L3	ENSG00000108848	19244175;
D012643	decrease	LYPLA1	ENSG00000120992	19244175;
D012643	decrease	MACF1	ENSG00000127603	19244175;
D012643	decrease	MAGI2	ENSG00000187391	19244175;
D012643	decrease	MALT1	ENSG00000172175	19244175;
D012643	decrease	MAP3K5	ENSG00000197442	19244175;
D012643	decrease	MAP3K7	ENSG00000135341	19244175;
D012643	decrease	MAP3K8	ENSG00000107968	19244175;
D012643	decrease	MAPK1	ENSG00000100030	18583539;
D012643	decrease	MAPK3	ENSG00000102882	18583539;
D012643	decrease	MAPK9	ENSG00000050748	18583539;
D012643	decrease	MBD1	ENSG00000141644	19244175;
D012643	decrease	MBD4	ENSG00000129071	19244175;
D012643	decrease	MBNL1	ENSG00000152601	19244175;
D012643	decrease	MCCC1	ENSG00000078070	19244175;
D012643	decrease	MDM1	ENSG00000111554	19244175;
D012643	decrease	ME1	ENSG00000065833	19244175;

D012643	decrease	MED13L	ENSG00000123066	19244175;
D012643	decrease	METAP2	ENSG00000111142	19244175;
D012643	decrease	MIOS	ENSG00000164654	19244175;
D012643	decrease	MIS12	ENSG00000167842	19244175;
D012643	decrease	MMP14	ENSG00000157227	17390030;
D012643	decrease	MMP15	ENSG00000102996	17390030;
D012643	decrease	MMP16	ENSG00000156103	17390030;
D012643	decrease	MMP9	ENSG00000100985	17390030;
D012643	decrease	MON2	ENSG00000061987	19244175;
D012643	decrease	MRPL44	ENSG00000135900	19244175;
D012643	decrease	MRPS33	ENSG00000090263	19244175;
D012643	decrease	MSH5	ENSG00000227314	19244175;
D012643	decrease	MTF2	ENSG00000143033	19244175;
D012643	decrease	MTMR15	ENSG00000198690	19244175;
D012643	decrease	MTMR9	ENSG00000104643	19244175;
D012643	decrease	MUT	ENSG00000146085	19244175;
D012643	decrease	MYO6	ENSG00000196586	19244175;
D012643	decrease	N4BP2L2	ENSG00000244754	19244175;
D012643	decrease	NACA	ENSG00000196531	19244175;
D012643	decrease	NAV2	ENSG00000166833	19244175;
D012643	decrease	NCOA2	ENSG00000140396	19244175;
D012643	decrease	NDUFA1	ENSG00000125356	19244175;
D012643	decrease	NDUFA4	ENSG00000189043	19244175;
D012643	decrease	NDUFA7	ENSG00000167774	19244175;
D012643	decrease	NDUFB1	ENSG00000183648	19244175;
D012643	decrease	NDUFB2	ENSG00000090266	19244175;
D012643	decrease	NDUFS4	ENSG00000164258	19244175;
D012643	decrease	NEK1	ENSG00000137601	19244175;
D012643	decrease	NEK2	ENSG00000117650	19244175;
D012643	decrease	NFAT5	ENSG00000102908	19244175;
D012643	decrease	NFE2L2	ENSG00000116044	19244175;
D012643	decrease	NFS1	ENSG00000244005	19244175;
D012643	decrease	NID2	ENSG00000087303	19244175;
D012643	decrease	NIPBL	ENSG00000164190	19244175;
D012643	decrease	NKTR	ENSG00000114857	19244175;
D012643	decrease	NOC3L	ENSG00000173145	19244175;
D012643	decrease	NOL3	ENSG00000140939	19244175;
D012643	decrease	NOL8	ENSG00000198000	19244175;
D012643	decrease	NOLC1	ENSG00000166197	19244175;
D012643	decrease	NR2C1	ENSG00000120798	19244175;
D012643	decrease	NR3C1	ENSG00000113580	19244175;
D012643	decrease	NSMAF	ENSG00000035681	19244175;
D012643	decrease	OLA1	ENSG00000138430	19244175;
D012643	decrease	OPA1	ENSG00000198836	19244175;
D012643	decrease	OSBPL1A	ENSG00000141447	19244175;
D012643	decrease	PA2G4	ENSG00000170515	19244175;
D012643	decrease	PAGE4	ENSG00000101951	19244175;
D012643	decrease	PALLD	ENSG00000129116	19244175;
D012643	decrease	PAM	ENSG00000145730	19244175;
D012643	decrease	PAOX	ENSG00000148832	19244175;
D012643	decrease	PCM1	ENSG00000078674	19244175;
D012643	decrease	PCNA	ENSG00000132646	11925459;
D012643	decrease	PCP4	ENSG00000183036	19244175;
D012643	decrease	PCSK5	ENSG00000099139	19244175;

D012643	decrease	PCYOX1L	ENSG00000145882	19244175;
D012643	decrease	PDE8A	ENSG00000073417	19244175;
D012643	decrease	PDPK1	ENSG00000140992	19244175;
D012643	decrease	PDSS2	ENSG00000164494	19244175;
D012643	decrease	PER3	ENSG00000049246	19244175;
D012643	decrease	PEX1	ENSG00000127980	19244175;
D012643	decrease	PEX3	ENSG00000034693	19244175;
D012643	decrease	PGAP1	ENSG00000197121	19244175;
D012643	decrease	PGM3	ENSG00000013375	19244175;
D012643	decrease	PHACTR2	ENSG00000112419	19244175;
D012643	decrease	PHF3	ENSG00000118482	19244175;
D012643	decrease	PHLDA1	ENSG00000139289	19244175;
D012643	decrease	PIAS4	ENSG00000105229	19244175;
D012643	decrease	PIGG	ENSG00000174227	19244175;
D012643	decrease	PIGK	ENSG00000142892	19244175;
D012643	decrease	PIGP	ENSG00000185808	19244175;
D012643	decrease	PKN2	ENSG00000065243	19244175;
D012643	decrease	PLA2G12A	ENSG00000123739	19244175;
D012643	decrease	PLCB4	ENSG00000101333	19244175;
D012643	decrease	PLEKHA5	ENSG00000052126	19244175;
D012643	decrease	PLOD2	ENSG00000152952	19244175;
D012643	decrease	PMPCB	ENSG00000105819	19244175;
D012643	decrease	PMS1	ENSG00000064933	19244175;
D012643	decrease	POLR2K	ENSG00000147669	19244175;
D012643	decrease	PON2	ENSG00000105854	19244175;
D012643	decrease	POSTN	ENSG00000133110	19244175;
D012643	decrease	PPAP2B	ENSG00000162407	19244175;
D012643	decrease	PPAT	ENSG00000128059	19244175;
D012643	decrease	PPFIBP1	ENSG00000110841	19244175;
D012643	decrease	PPIG	ENSG00000138398	19244175;
D012643	decrease	PPP1R9A	ENSG00000158528	19244175;
D012643	decrease	PPP2R5E	ENSG00000154001	19244175;
D012643	decrease	PPPDE1	ENSG00000121644	19244175;
D012643	decrease	PRKCB	ENSG00000166501	19244175;
D012643	decrease	PRKCI	ENSG00000163558	19244175;
D012643	decrease	PRMT3	ENSG00000185238	19244175;
D012643	decrease	PRPF40A	ENSG00000196504	19244175;
D012643	decrease	PRPF4B	ENSG00000112739	19244175;
D012643	decrease	PSIP1	ENSG00000164985	19244175;
D012643	decrease	PTPRO	ENSG00000151490	19244175;
D012643	decrease	PWP1	ENSG00000136045	19244175;
D012643	decrease	PYCRL	ENSG00000104524	19244175;
D012643	decrease	R3HDM1	ENSG00000048991	19244175;
D012643	decrease	RAB14	ENSG00000119396	19244175;
D012643	decrease	RABGAP1	ENSG00000011454	19244175;
D012643	decrease	RAD23B	ENSG00000119318	19244175;
D012643	decrease	RALBP1	ENSG00000017797	19244175;
D012643	decrease	RALGPS1	ENSG00000136828	19244175;
D012643	decrease	RANBP2	ENSG00000153201	19244175;
D012643	decrease	RANBP9	ENSG00000010017	19244175;
D012643	decrease	RAPGEF6	ENSG00000158987	19244175;
D012643	decrease	RBBP6	ENSG00000122257	19244175;
D012643	decrease	RBM25	ENSG00000119707	19244175;
D012643	decrease	RBM4	ENSG00000173933	19244175;

D012643	decrease	RCHY1	ENSG00000163743	19244175;
D012643	decrease	RCOR3	ENSG00000117625	19244175;
D012643	decrease	RDH11	ENSG00000072042	19244175;
D012643	decrease	RECK	ENSG00000122707	19244175;
D012643	decrease	RECQL	ENSG00000004700	19244175;
D012643	decrease	RGPD8	ENSG00000169629	19244175;
D012643	decrease	RHOBTB1	ENSG00000072422	19244175;
D012643	decrease	RHOBTB3	ENSG00000164292	19244175;
D012643	decrease	RNASEH1	ENSG00000171865	19244175;
D012643	decrease	RND3	ENSG00000115963	19244175;
D012643	decrease	RNF111	ENSG00000157450	19244175;
D012643	decrease	RPL10A	ENSG00000198755	19244175;
D012643	decrease	RPL14	ENSG00000188846	19244175;
D012643	decrease	RPL23	ENSG00000125691	19244175;
D012643	decrease	RPL27	ENSG00000131469	19244175;
D012643	decrease	RPL31	ENSG00000071082	19244175;
D012643	decrease	RPL37A	ENSG00000197756	19244175;
D012643	decrease	RPL38	ENSG00000172809	19244175;
D012643	decrease	RPL39	ENSG00000198918	19244175;
D012643	decrease	RPL7	ENSG00000147604	19244175;
D012643	decrease	RPS20	ENSG00000008988	19244175;
D012643	decrease	RPS21	ENSG00000171858	19244175;
D012643	decrease	RPS25	ENSG00000118181	19244175;
D012643	decrease	RPS27	ENSG00000177954	19244175;
D012643	decrease	RPS27L	ENSG00000185088	19244175;
D012643	decrease	RPS29	ENSG00000213741	19244175;
D012643	decrease	RPS7	ENSG00000171863	19244175;
D012643	decrease	RRBP1	ENSG00000125844	19244175;
D012643	decrease	RSRC2	ENSG00000111011	19244175;
D012643	decrease	RUFY3	ENSG00000018189	19244175;
D012643	decrease	RUNX1T1	ENSG00000079102	19244175;
D012643	decrease	RYBP	ENSG00000163602	19244175;
D012643	decrease	SACS	ENSG00000151835	19244175;
D012643	decrease	SAMM50	ENSG00000100347	19244175;
D012643	decrease	SART3	ENSG00000075856	19244175;
D012643	decrease	SATB1	ENSG00000182568	19244175;
D012643	decrease	SCAMP1	ENSG00000085365	19244175;
D012643	decrease	SCAPER	ENSG00000140386	19244175;
D012643	decrease	SCML1	ENSG00000047634	19244175;
D012643	decrease	SCML2	ENSG00000102098	19244175;
D012643	decrease	SCRG1	ENSG00000164106	19244175;
D012643	decrease	SDCCAG8	ENSG00000054282	19244175;
D012643	decrease	SEC16A	ENSG00000148396	19244175;
D012643	decrease	SEC22B	ENSG00000223380	19244175;
D012643	decrease	SEC24B	ENSG00000138802	19244175;
D012643	decrease	SEC31A	ENSG00000138674	19244175;
D012643	decrease	SECISBP2L	ENSG00000138593	19244175;
D012643	decrease	SERBP1	ENSG00000142864	19244175;
D012643	decrease	SERTAD2	ENSG00000179833	19244175;
D012643	decrease	SETBP1	ENSG00000152217	19244175;
D012643	decrease	SETD6	ENSG00000103037	19244175;
D012643	decrease	SET	ENSG00000119335	19244175;
D012643	decrease	SETMAR	ENSG00000170364	19244175;
D012643	decrease	SF1	ENSG00000250652	19244175;

D012643	decrease	SF3B1	ENSG00000115524	19244175;
D012643	decrease	SFRS11	ENSG00000116754	19244175;
D012643	decrease	SFRS15	ENSG00000156304	19244175;
D012643	decrease	SFRS18	ENSG00000132424	19244175;
D012643	decrease	SFRS2IP	ENSG00000139218	19244175;
D012643	decrease	SFRS7	ENSG00000115875	19244175;
D012643	decrease	SHANK2	ENSG00000162105	19244175;
D012643	decrease	SHFM1	ENSG00000127922	19244175;
D012643	decrease	SIAH1	ENSG00000196470	19244175;
D012643	decrease	SIRT1	ENSG00000096717	19244175;
D012643	decrease	SLC11A2	ENSG00000110911	19244175;
D012643	decrease	SLC12A2	ENSG00000064651	19244175;
D012643	decrease	SLC19A1	ENSG00000173638	19244175;
D012643	decrease	SLC19A2	ENSG00000117479	19244175;
D012643	decrease	SLC1A1	ENSG00000106688	19244175;
D012643	decrease	SLC25A12	ENSG00000115840	19244175;
D012643	decrease	SLC25A16	ENSG00000122912	19244175;
D012643	decrease	SLC25A17	ENSG00000100372	19244175;
D012643	decrease	SLC25A36	ENSG00000114120	19244175;
D012643	decrease	SLC35D1	ENSG00000116704	19244175;
D012643	decrease	SLC4A4	ENSG00000080493	19244175;
D012643	decrease	SLC4A7	ENSG00000033867	19244175;
D012643	decrease	SLC6A2	ENSG00000103546	19244175;
D012643	decrease	SLIT2	ENSG00000145147	19244175;
D012643	decrease	SMARCA5	ENSG00000153147	19244175;
D012643	decrease	SMARCC1	ENSG00000173473	19244175;
D012643	decrease	SMCHD1	ENSG00000101596	19244175;
D012643	decrease	SMURF2	ENSG00000108854	19244175;
D012643	decrease	SNAP25	ENSG00000132639	19244175;
D012643	decrease	SNRNP27	ENSG00000124380	19244175;
D012643	decrease	SOBP	ENSG00000112320	19244175;
D012643	decrease	SON	ENSG00000159140	19244175;
D012643	decrease	SOS1	ENSG00000115904	19244175;
D012643	decrease	SOX4	ENSG00000124766	19244175;
D012643	decrease	SOX9	ENSG00000125398	19244175;
D012643	decrease	SP100	ENSG00000067066	19244175;
D012643	decrease	SPCS2	ENSG00000118363	19244175;
D012643	decrease	SPEN	ENSG00000065526	19244175;
D012643	decrease	SRPK1	ENSG00000096063	19244175;
D012643	decrease	SRRM2	ENSG00000167978	19244175;
D012643	decrease	SS18L2	ENSG00000008324	19244175;
D012643	decrease	SSX2IP	ENSG00000117155	19244175;
D012643	decrease	STAG2	ENSG00000101972	19244175;
D012643	decrease	STK38L	ENSG00000211455	19244175;
D012643	decrease	SULF1	ENSG00000137573	19244175;
D012643	decrease	SUPT7L	ENSG00000119760	19244175;
D012643	decrease	SUZ12	ENSG00000178691	19244175;
D012643	decrease	SYNCRIP	ENSG00000135316	19244175;
D012643	decrease	TAF2	ENSG00000064313	19244175;
D012643	decrease	TAF5L	ENSG00000135801	19244175;
D012643	decrease	TARP	ENSG00000211689	19244175;
D012643	decrease	TAS2R14	ENSG00000212127	19244175;
D012643	decrease	TBC1D30	ENSG00000111490	19244175;
D012643	decrease	TBC1D8B	ENSG00000133138	19244175;

D012643	decrease	TBL1X	ENSG00000101849	19244175;
D012643	decrease	TCEB3	ENSG00000011007	19244175;
D012643	decrease	TCERG1	ENSG00000113649	19244175;
D012643	decrease	TCF12	ENSG00000140262	19244175;
D012643	decrease	TCF7L2	ENSG00000148737	19244175;
D012643	decrease	TCTN1	ENSG00000204852	19244175;
D012643	decrease	TERF1	ENSG00000147601	19244175;
D012643	decrease	TERT	ENSG00000164362	17948765;
D012643	decrease	TFB2M	ENSG00000162851	19244175;
D012643	decrease	TGFBR3	ENSG00000069702	19244175;
D012643	decrease	TGOLN2	ENSG00000152291	19244175;
D012643	decrease	THBD	ENSG00000178726	19244175;
D012643	decrease	THOC2	ENSG00000125676	19244175;
D012643	decrease	THSD7A	ENSG00000005108	19244175;
D012643	decrease	THUMPD1	ENSG00000066654	19244175;
D012643	decrease	TIA1	ENSG00000116001	19244175;
D012643	decrease	TIMM8A	ENSG00000126953	19244175;
D012643	decrease	TIMM8B	ENSG00000150779	19244175;
D012643	decrease	TIMM9	ENSG00000100575	19244175;
D012643	decrease	TIMP1	ENSG00000102265	17390030;
D012643	decrease	TIMP2	ENSG00000035862	17390030;
D012643	decrease	TIMP4	ENSG00000157150	17390030;
D012643	decrease	TM2D1	ENSG00000162604	19244175;
D012643	decrease	TMC5	ENSG00000103534	19244175;
D012643	decrease	TMEM168	ENSG00000146802	19244175;
D012643	decrease	TMF1	ENSG00000144747	19244175;
D012643	decrease	TMSB15A	ENSG00000158164	19244175;
D012643	decrease	TMSB15B	ENSG00000158164	19244175;
D012643	decrease	TNFSF10	ENSG00000121858	19244175;
D012643	decrease	TNPO2	ENSG00000105576	19244175;
D012643	decrease	TOB2	ENSG00000183864	19244175;
D012643	decrease	TOMM7	ENSG00000196683	19244175;
D012643	decrease	TOP1	ENSG00000198900	19244175;
D012643	decrease	TPR	ENSG00000047410	19244175;
D012643	decrease	TRIM24	ENSG00000122779	19244175;
D012643	decrease	TRIM27	ENSG00000234495	19244175;
D012643	decrease	TRIM36	ENSG00000152503	19244175;
D012643	decrease	TRIM38	ENSG00000112343	19244175;
D012643	decrease	TRIM52	ENSG00000183718	19244175;
D012643	decrease	TRIT1	ENSG00000043514	19244175;
D012643	decrease	TRMT11	ENSG00000066651	19244175;
D012643	decrease	TRMT61B	ENSG00000171103	19244175;
D012643	decrease	TROVE2	ENSG00000116747	19244175;
D012643	decrease	TRPC4	ENSG00000133107	19244175;
D012643	decrease	TTC17	ENSG00000052841	19244175;
D012643	decrease	TTC31	ENSG00000115282	19244175;
D012643	decrease	TTC37	ENSG00000198677	19244175;
D012643	decrease	TTC3	ENSG00000182670	19244175;
D012643	decrease	TULP3	ENSG00000078246	19244175;
D012643	decrease	UBA52	ENSG00000221983	19244175;
D012643	decrease	UBAP2	ENSG00000137073	19244175;
D012643	decrease	UBE2V1	ENSG00000244687	19244175;
D012643	decrease	UBL3	ENSG00000122042	19244175;
D012643	decrease	UGCG	ENSG00000148154	19244175;

D012643	decrease	UHRF1BP1L	ENSG00000111647	19244175;
D012643	decrease	UPF3A	ENSG00000169062	19244175;
D012643	decrease	UPF3B	ENSG00000125351	19244175;
D012643	decrease	UQCR10	ENSG00000184076	19244175;
D012643	decrease	UQCRB	ENSG00000156467	19244175;
D012643	decrease	UQCRH	ENSG00000173660	19244175;
D012643	decrease	UQCRQ	ENSG00000164405	19244175;
D012643	decrease	USP33	ENSG00000077254	19244175;
D012643	decrease	USP3	ENSG00000140455	19244175;
D012643	decrease	USP47	ENSG00000170242	19244175;
D012643	decrease	USP4	ENSG00000114316	19244175;
D012643	decrease	USP9X	ENSG00000124486	19244175;
D012643	decrease	USPL1	ENSG00000132952	19244175;
D012643	decrease	UTP11L	ENSG00000183520	19244175;
D012643	decrease	WDR19	ENSG00000157796	19244175;
D012643	decrease	WDR5B	ENSG00000196981	19244175;
D012643	decrease	WSB1	ENSG00000109046	19244175;
D012643	decrease	WWP1	ENSG00000123124	19244175;
D012643	decrease	XPO6	ENSG00000169180	19244175;
D012643	decrease	YLPM1	ENSG00000119596	19244175;
D012643	decrease	YWHAE	ENSG00000108953	19244175;
D012643	decrease	ZBTB11	ENSG00000066422	19244175;
D012643	decrease	ZBTB44	ENSG00000196323	19244175;
D012643	decrease	ZC3H15	ENSG00000065548	19244175;
D012643	decrease	ZC3HAV1	ENSG00000105939	19244175;
D012643	decrease	ZCCHC24	ENSG00000165424	19244175;
D012643	decrease	ZCCHC2	ENSG00000141664	19244175;
D012643	decrease	ZCCHC6	ENSG00000083223	19244175;
D012643	decrease	ZCCHC8	ENSG00000033030	19244175;
D012643	decrease	ZEB1	ENSG00000148516	19244175;
D012643	decrease	ZKSCAN1	ENSG00000106261	19244175;
D012643	decrease	ZMYM2	ENSG00000121741	19244175;
D012643	decrease	ZMYM4	ENSG00000146463	19244175;
D012643	decrease	ZNF12	ENSG00000164631	19244175;
D012643	decrease	ZNF131	ENSG00000172262	19244175;
D012643	decrease	ZNF148	ENSG00000163848	19244175;
D012643	decrease	ZNF180	ENSG00000167384	19244175;
D012643	decrease	ZNF195	ENSG00000005801	19244175;
D012643	decrease	ZNF211	ENSG00000121417	19244175;
D012643	decrease	ZNF264	ENSG00000083844	19244175;
D012643	decrease	ZNF26	ENSG00000198393	19244175;
D012643	decrease	ZNF274	ENSG00000171606	19244175;
D012643	decrease	ZNF337	ENSG00000130684	19244175;
D012643	decrease	ZNF492	ENSG00000229676	19244175;
D012643	decrease	ZNF552	ENSG00000178935	19244175;
D012643	decrease	ZNF573	ENSG00000189144	19244175;
D012643	decrease	ZNF586	ENSG00000083828	19244175;
D012643	decrease	ZNF692	ENSG00000171163	19244175;
D012643	decrease	ZNF839	ENSG00000022976	19244175;
D012643	decrease	ZNF85	ENSG00000105750	19244175;
D012643	decrease	ZNF91	ENSG00000167232	17293063;
D012643	decrease	ZWINT	ENSG00000122952	19244175;
D012643	increase	A2M	ENSG00000175899	18997278;
D012643	increase	AAMP	ENSG00000127837	19244175;

D012643	increase	AATF	ENSG00000108270	19244175;
D012643	increase	ABCA7	ENSG00000064687	19244175;
D012643	increase	ABCA9	ENSG00000154258	18583539;
D012643	increase	ABCF3	ENSG00000161204	19244175;
D012643	increase	ABHD2	ENSG00000140526	19244175;
D012643	increase	ABR	ENSG00000159842	19244175;
D012643	increase	ACAA2	ENSG00000167315	19244175;
D012643	increase	ACADS	ENSG00000122971	19244175;
D012643	increase	ACIN1	ENSG00000100813	19244175;
D012643	increase	ACO2	ENSG00000100412	19244175;
D012643	increase	ACOT2	ENSG00000119673	19244175;
D012643	increase	ACTB	ENSG00000075624	19244175;
D012643	increase	ACTG1	ENSG00000184009	19244175;
D012643	increase	ACTN1	ENSG00000072110	19244175;
D012643	increase	ACTN4	ENSG00000130402	19244175;
D012643	increase	ACTR1A	ENSG00000138107	19244175;
D012643	increase	ACTR1B	ENSG00000115073	19244175;
D012643	increase	ACY1	ENSG00000243989	19244175;
D012643	increase	ADCY2	ENSG00000078295	19244175;
D012643	increase	ADCY6	ENSG00000174233	19244175;
D012643	increase	ADD1	ENSG00000087274	19244175;
D012643	increase	ADH1B	ENSG00000196616	19244175;18583539;
D012643	increase	ADRA2C	ENSG00000184160	19244175;
D012643	increase	ADRM1	ENSG00000130706	19244175;
D012643	increase	AGPAT2	ENSG00000169692	19244175;
D012643	increase	AGRN	ENSG00000188157	19244175;
D012643	increase	AIM1L	ENSG00000176092	19244175;
D012643	increase	AIP	ENSG00000110711	19244175;
D012643	increase	AK1	ENSG00000106992	19244175;
D012643	increase	AK2	ENSG00000004455	19244175;
D012643	increase	AK3	ENSG00000147853	19244175;
D012643	increase	AKAP13	ENSG00000170776	19244175;
D012643	increase	AKT1	ENSG00000142208	17339365;19244175;18583539;
D012643	increase	ALAS1	ENSG00000023330	19244175;
D012643	increase	ALDH4A1	ENSG00000159423	19244175;
D012643	increase	ALDOA	ENSG00000149925	19244175;
D012643	increase	ALG3	ENSG00000214160	19244175;
D012643	increase	ALG9	ENSG00000086848	19244175;
D012643	increase	ALS2CL	ENSG00000178038	19244175;
D012643	increase	ANAPC2	ENSG00000176248	19244175;
D012643	increase	ANKRD30B	ENSG00000180777	18583539;
D012643	increase	ANPEP	ENSG00000166825	19244175;
D012643	increase	AP1B1	ENSG00000100280	19244175;
D012643	increase	AP1M2	ENSG00000129354	19244175;
D012643	increase	AP2A2	ENSG00000183020	19244175;
D012643	increase	AP2M1	ENSG00000161203	19244175;
D012643	increase	AP2S1	ENSG00000042753	19244175;
D012643	increase	APEH	ENSG00000164062	19244175;
D012643	increase	APLP2	ENSG00000084234	19244175;
D012643	increase	APOD	ENSG00000189058	18583539;
D012643	increase	APOE	ENSG00000130203	19244175;
D012643	increase	APOL1	ENSG00000100342	19244175;
D012643	increase	APOL3	ENSG00000128284	19244175;

D012643	increase	AQP3	ENSG00000165272	19244175;
D012643	increase	ARAF	ENSG00000078061	19244175;
D012643	increase	ARF5	ENSG00000004059	19244175;
D012643	increase	ARFGAP2	ENSG00000149182	19244175;
D012643	increase	ARFIP2	ENSG00000132254	19244175;
D012643	increase	ARFRP1	ENSG00000101246	19244175;
D012643	increase	ARHGDIA	ENSG00000141522	19244175;
D012643	increase	ARL6IP4	ENSG00000182196	19244175;
D012643	increase	ARPC1A	ENSG00000241685	19244175;
D012643	increase	ARPC1B	ENSG00000130429	19244175;
D012643	increase	ARPC4	ENSG00000241553	19244175;
D012643	increase	ASAP3	ENSG00000088280	19244175;
D012643	increase	ASB1	ENSG00000065802	19244175;
D012643	increase	ASCC2	ENSG00000100325	19244175;
D012643	increase	ASL	ENSG00000126522	19244175;
D012643	increase	ASMTL	ENSG00000169093	19244175;
D012643	increase	ASS1	ENSG00000130707	19244175;
D012643	increase	ATF4	ENSG00000128272	19244175;
D012643	increase	ATF5	ENSG00000169136	19244175;
D012643	increase	ATG9A	ENSG00000198925	19244175;
D012643	increase	ATMIN	ENSG00000166454	19244175;
D012643	increase	ATP2B4	ENSG00000058668	19244175;
D012643	increase	ATP6AP1	ENSG00000071553	19244175;
D012643	increase	ATP6V0D1	ENSG00000159720	19244175;
D012643	increase	B3GAT3	ENSG00000149541	19244175;
D012643	increase	BAG1	ENSG00000250477	19244175;
D012643	increase	BAI1	ENSG00000181790	19244175;
D012643	increase	BAP1	ENSG00000163930	19244175;
D012643	increase	BAT1	ENSG00000229496	19244175;
D012643	increase	BAT3	ENSG00000204463	19244175;
D012643	increase	BATF	ENSG00000156127	19244175;
D012643	increase	BAX	ENSG00000087088	16307838;
D012643	increase	BAZ1B	ENSG00000009954	19244175;
D012643	increase	BCL2L11	ENSG00000153094	17339365;
D012643	increase	BCL2L1	ENSG00000171552	19244175;
D012643	increase	BCL3	ENSG00000069399	19244175;
D012643	increase	BCL7B	ENSG00000106635	19244175;
D012643	increase	BCLAF1	ENSG00000029363	19244175;
D012643	increase	BIN1	ENSG00000136717	19244175;
D012643	increase	BLVRB	ENSG00000090013	19244175;
D012643	increase	BRD2	ENSG00000204256	19244175;
D012643	increase	BRF2	ENSG00000104221	19244175;
D012643	increase	BRMS1	ENSG00000174744	19244175;
D012643	increase	BSCL2	ENSG00000168000	19244175;
D012643	increase	BST2	ENSG00000130303	19244175;
D012643	increase	BYSL	ENSG00000112578	19244175;
D012643	increase	C11ORF51	ENSG00000110200	19244175;
D012643	increase	C11ORF68	ENSG00000175573	19244175;
D012643	increase	C16ORF42	ENSG00000007520	19244175;
D012643	increase	C18ORF1	ENSG00000168675	19244175;
D012643	increase	C19ORF21	ENSG00000099812	19244175;
D012643	increase	C1QB	ENSG00000173369	19244175;
D012643	increase	C2ORF42	ENSG00000115998	19244175;
D012643	increase	C6ORF106	ENSG00000196821	19244175;

D012643	increase	C7ORF26	ENSG00000146576	19244175;
D012643	increase	C7ORF43	ENSG00000146826	19244175;
D012643	increase	CABIN1	ENSG00000099991	19244175;
D012643	increase	CALCOCO1	ENSG00000012822	19244175;
D012643	increase	CALHM2	ENSG00000138172	19244175;
D012643	increase	CALM3	ENSG00000143933	19244175;
D012643	increase	CALU	ENSG00000128595	19244175;
D012643	increase	CAMK2B	ENSG00000058404	19244175;
D012643	increase	CAPG	ENSG00000042493	19244175;
D012643	increase	CAPN1	ENSG00000014216	19244175;
D012643	increase	CAPNS1	ENSG00000126247	19244175;
D012643	increase	CAPZB	ENSG00000077549	19244175;
D012643	increase	CBX7	ENSG00000100307	19244175;
D012643	increase	CCDC101	ENSG00000176476	19244175;
D012643	increase	CCDC64	ENSG00000135127	19244175;
D012643	increase	CCHCR1	ENSG00000204536	19244175;
D012643	increase	CCL2	ENSG00000108691	16143069;
D012643	increase	CCL3L1	ENSG00000205021	17293063;
D012643	increase	CCND1	ENSG00000110092	19244175;
D012643	increase	CCRL1	ENSG00000129048	18583539;
D012643	increase	CD2BP2	ENSG00000169217	19244175;
D012643	increase	CD44	ENSG00000026508	18196609;
D012643	increase	CD74	ENSG00000019582	19244175;
D012643	increase	CD99	ENSG00000002586	19244175;
D012643	increase	CDC42EP4	ENSG00000179604	19244175;
D012643	increase	CDH3	ENSG00000062038	19244175;
D012643	increase	CDK10	ENSG00000185324	19244175;
D012643	increase	CDK1	ENSG00000170312	11925459;18583539;
D012643	increase	CDK2AP2	ENSG00000167797	19244175;
D012643	increase	CDK2	ENSG00000123374	11925459;18583539;
D012643	increase	CDK4	ENSG00000135446	11925459;19244175;
D012643	increase	CEL	ENSG00000170835	19244175;
D012643	increase	CEP350	ENSG00000135837	19244175;
D012643	increase	CES1	ENSG00000198848	19244175;
D012643	increase	CFD	ENSG00000197766	18583539;
D012643	increase	CHD3	ENSG00000170004	19244175;
D012643	increase	CHD4	ENSG00000111642	19244175;
D012643	increase	CHKA	ENSG00000110721	19244175;
D012643	increase	CHMP1A	ENSG00000131165	19244175;
D012643	increase	CHMP2A	ENSG00000130724	19244175;
D012643	increase	CHN2	ENSG00000106069	19244175;
D012643	increase	CHPF	ENSG00000123989	19244175;
D012643	increase	CHRNA4	ENSG00000101204	19244175;
D012643	increase	CHRNE	ENSG00000108556	19244175;
D012643	increase	CHST3	ENSG00000122863	19244175;
D012643	increase	CHST6	ENSG00000183196	19244175;
D012643	increase	CIZ1	ENSG00000148337	19244175;
D012643	increase	CKB	ENSG00000166165	19244175;
D012643	increase	CLASP1	ENSG00000074054	18583539;
D012643	increase	CLCN3	ENSG00000109572	19244175;
D012643	increase	CLDN4	ENSG00000189143	19244175;
D012643	increase	CLDN5	ENSG00000184113	16514648;
D012643	increase	CLEC3B	ENSG00000163815	18583539;
D012643	increase	CLN3	ENSG00000188603	19244175;

D012643	increase	CLPTM1	ENSG00000104853	19244175;
D012643	increase	CLTB	ENSG00000175416	19244175;
D012643	increase	CLU	ENSG00000120885	19244175;
D012643	increase	CMTM6	ENSG00000091317	18583539;
D012643	increase	CNN2	ENSG00000064666	19244175;
D012643	increase	CNN3	ENSG00000117519	19244175;
D012643	increase	CNTNAP3	ENSG00000106714	18583539;
D012643	increase	COG4	ENSG00000103051	19244175;
D012643	increase	COG7	ENSG00000168434	19244175;
D012643	increase	COG8	ENSG00000213380	19244175;
D012643	increase	COL18A1	ENSG00000182871	19244175;
D012643	increase	COL4A3BP	ENSG00000113163	19244175;
D012643	increase	COL6A2	ENSG00000142173	19244175;
D012643	increase	COMMD4	ENSG00000140365	19244175;
D012643	increase	COPE	ENSG00000105669	19244175;
D012643	increase	COPG	ENSG00000181789	19244175;
D012643	increase	COPS7A	ENSG00000111652	19244175;
D012643	increase	CPSF1	ENSG00000071894	19244175;
D012643	increase	CRAT	ENSG00000095321	19244175;
D012643	increase	CREB3	ENSG00000107175	19244175;
D012643	increase	CRELD1	ENSG00000163703	19244175;
D012643	increase	CSDA	ENSG00000060138	19244175;
D012643	increase	CSF1	ENSG00000184371	19244175;
D012643	increase	CSK	ENSG00000103653	19244175;
D012643	increase	CSNK1E	ENSG00000213923	19244175;
D012643	increase	CSNK2B	ENSG00000224398	19244175;
D012643	increase	CSPG4	ENSG00000173546	19244175;
D012643	increase	CSPP1	ENSG00000104218	19244175;
D012643	increase	CST3	ENSG00000101439	19244175;
D012643	increase	CTBP1	ENSG00000159692	19244175;
D012643	increase	CTNNA1	ENSG00000044115	19244175;
D012643	increase	CTNNBL1	ENSG00000132792	19244175;
D012643	increase	CTSB	ENSG00000164733	19244175;
D012643	increase	CUEDC2	ENSG00000107874	19244175;
D012643	increase	CUL7	ENSG00000044090	19244175;
D012643	increase	CUTC	ENSG00000119929	19244175;
D012643	increase	CXCL1	ENSG00000163739	17293063;
D012643	increase	CXCL2	ENSG00000081041	17293063;
D012643	increase	CXXC1	ENSG00000154832	19244175;
D012643	increase	CYB561D2	ENSG00000114395	19244175;
D012643	increase	DAPK3	ENSG00000167657	19244175;
D012643	increase	DCTN1	ENSG00000204843	19244175;
D012643	increase	DCXR	ENSG00000169738	19244175;
D012643	increase	DDAH2	ENSG00000225635	19244175;
D012643	increase	DDOST	ENSG00000244038	19244175;
D012643	increase	DDX17	ENSG00000100201	19244175;
D012643	increase	DDX41	ENSG00000183258	19244175;
D012643	increase	DDX49	ENSG00000105671	19244175;
D012643	increase	DEDD	ENSG00000158796	19244175;
D012643	increase	DES	ENSG00000175084	19244175;
D012643	increase	DGCR14	ENSG00000100056	19244175;
D012643	increase	DGCR6	ENSG00000183628	19244175;
D012643	increase	DGCR8	ENSG00000128191	19244175;
D012643	increase	DHCR24	ENSG00000116133	19244175;

D012643	increase	DHCR7	ENSG00000172893	19244175;
D012643	increase	DHPS	ENSG00000095059	19244175;
D012643	increase	DHRS4	ENSG00000157326	19244175;
D012643	increase	DHRS7B	ENSG00000109016	19244175;
D012643	increase	DHX30	ENSG00000132153	19244175;
D012643	increase	DMTF1	ENSG00000135164	19244175;
D012643	increase	DMWD	ENSG00000185800	19244175;
D012643	increase	DNPEP	ENSG00000123992	19244175;
D012643	increase	DOHH	ENSG00000129932	19244175;
D012643	increase	DOLPP1	ENSG00000167130	19244175;
D012643	increase	DOM3Z	ENSG00000224313	19244175;
D012643	increase	DRG2	ENSG00000108591	19244175;
D012643	increase	DULLARD	ENSG00000175826	19244175;
D012643	increase	DUOX1	ENSG00000137857	19244175;
D012643	increase	DUSP8	ENSG00000184545	19244175;
D012643	increase	E2F1	ENSG00000101412	19244175;
D012643	increase	E2F4	ENSG00000205250	19244175;
D012643	increase	E2F6	ENSG00000169016	19244175;
D012643	increase	E4F1	ENSG00000167967	19244175;
D012643	increase	ECSIT	ENSG00000130159	19244175;
D012643	increase	EDF1	ENSG00000107223	19244175;
D012643	increase	EDNRA	ENSG00000151617	19244175;
D012643	increase	EEF1A2	ENSG00000101210	19244175;
D012643	increase	EEF1D	ENSG00000104529	19244175;
D012643	increase	EFEMP2	ENSG00000172638	19244175;
D012643	increase	EFHD2	ENSG00000142634	19244175;
D012643	increase	EHD2	ENSG00000024422	19244175;
D012643	increase	EIF1AY	ENSG00000198692	18583539;
D012643	increase	EIF3B	ENSG00000106263	19244175;
D012643	increase	EIF4G1	ENSG00000114867	19244175;
D012643	increase	EIF6	ENSG00000242372	19244175;
D012643	increase	EMILIN1	ENSG00000138080	19244175;
D012643	increase	EML2	ENSG00000125746	19244175;
D012643	increase	EMP2	ENSG00000213853	19244175;
D012643	increase	EMP3	ENSG00000142227	19244175;
D012643	increase	ENDOG	ENSG00000167136	19244175;
D012643	increase	ENG	ENSG00000106991	19244175;
D012643	increase	ENO2	ENSG00000111674	19244175;
D012643	increase	EPHB6	ENSG00000106123	19244175;
D012643	increase	EPHX2	ENSG00000120915	19244175;
D012643	increase	EPN2	ENSG00000072134	19244175;
D012643	increase	ERBB2	ENSG00000141736	19244175;
D012643	increase	ERCC1	ENSG00000012061	19244175;
D012643	increase	ERGIC3	ENSG00000125991	19244175;
D012643	increase	ESRP2	ENSG00000103067	19244175;
D012643	increase	EVC	ENSG00000072840	19244175;
D012643	increase	EWSR1	ENSG00000182944	19244175;
D012643	increase	EXOSC4	ENSG00000178896	19244175;
D012643	increase	EXT1	ENSG00000182197	19244175;
D012643	increase	EYA2	ENSG00000064655	19244175;
D012643	increase	EYA3	ENSG00000158161	18583539;
D012643	increase	F11R	ENSG00000158769	19244175;
D012643	increase	F2R	ENSG00000181104	19244175;
D012643	increase	FAAH	ENSG00000117480	19244175;

D012643	increase	FABP3	ENSG00000121769	18583539;
D012643	increase	FADD	ENSG00000168040	17970047;
D012643	increase	FADS3	ENSG00000221968	19244175;
D012643	increase	FAM107A	ENSG00000168309	19244175;
D012643	increase	FAM108A1	ENSG00000129968	19244175;
D012643	increase	FAM134A	ENSG00000144567	19244175;
D012643	increase	FAM149A	ENSG00000109794	19244175;
D012643	increase	FAM32A	ENSG00000105058	19244175;
D012643	increase	FAM38A	ENSG00000103335	19244175;
D012643	increase	FAM3A	ENSG00000071889	19244175;
D012643	increase	FAM50A	ENSG00000071859	19244175;
D012643	increase	FAM53B	ENSG00000189319	19244175;
D012643	increase	FARP1	ENSG00000152767	19244175;
D012643	increase	FARSA	ENSG00000179115	19244175;
D012643	increase	FASN	ENSG00000169710	19244175;
D012643	increase	FBLN1	ENSG00000077942	19244175;
D012643	increase	FBLN5	ENSG00000140092	19244175;
D012643	increase	FBXL8	ENSG00000135722	19244175;
D012643	increase	FBXO17	ENSG00000104835	19244175;
D012643	increase	FBXO28	ENSG00000143756	19244175;
D012643	increase	FBXO2	ENSG00000116661	19244175;
D012643	increase	FCGRT	ENSG00000104870	19244175;
D012643	increase	FFAR2	ENSG00000126262	17293063;
D012643	increase	FGFR1	ENSG00000249195	18583539;
D012643	increase	FIS1	ENSG00000214253	19244175;
D012643	increase	FKBP1A	ENSG00000088832	19244175;
D012643	increase	FLII	ENSG00000177731	19244175;
D012643	increase	FLNA	ENSG00000196924	19244175;
D012643	increase	FLOT1	ENSG00000137312	19244175;
D012643	increase	FLOT2	ENSG00000132589	19244175;
D012643	increase	FMO5	ENSG00000131781	19244175;
D012643	increase	FRAT1	ENSG00000165879	19244175;
D012643	increase	FTL	ENSG00000087086	19244175;
D012643	increase	FUS	ENSG00000089280	19244175;
D012643	increase	FZR1	ENSG00000105325	19244175;
D012643	increase	G0S2	ENSG00000123689	17293063;
D012643	increase	GADD45A	ENSG00000116717	19244175;
D012643	increase	GANAB	ENSG00000089597	19244175;
D012643	increase	GAPDH	ENSG00000111640	19244175;
D012643	increase	GAS8	ENSG00000141013	19244175;
D012643	increase	GATAD2A	ENSG00000167491	19244175;
D012643	increase	GCAT	ENSG00000100116	19244175;
D012643	increase	GGT1	ENSG00000100031	19244175;
D012643	increase	GIPC1	ENSG00000123159	19244175;
D012643	increase	GJB1	ENSG00000169562	19244175;
D012643	increase	GLG1	ENSG00000090863	19244175;
D012643	increase	GLI2	ENSG00000074047	19244175;
D012643	increase	GLTSCR1	ENSG00000063169	19244175;
D012643	increase	GMPPA	ENSG00000144591	19244175;
D012643	increase	GMPR2	ENSG00000100938	19244175;
D012643	increase	GNAI2	ENSG00000114353	19244175;
D012643	increase	GNB2	ENSG00000172354	19244175;
D012643	increase	GNL1	ENSG00000228581	19244175;
D012643	increase	GNRH2	ENSG00000125787	19244175;

D012643	increase	GOLGA3	ENSG00000090615	19244175;
D012643	increase	GOLGA8A	ENSG00000175265	19244175;
D012643	increase	GORASP1	ENSG00000114745	19244175;
D012643	increase	GPI	ENSG00000105220	19244175;
D012643	increase	GPKOW	ENSG00000068394	19244175;
D012643	increase	GPR107	ENSG00000148358	19244175;
D012643	increase	GPR137	ENSG00000173264	19244175;
D012643	increase	GRINA	ENSG00000178719	19244175;
D012643	increase	GRK6	ENSG00000198055	19244175;
D012643	increase	GRN	ENSG00000030582	19244175;
D012643	increase	GSK3A	ENSG00000105723	19244175;
D012643	increase	GSK3B	ENSG00000082701	17339365;19244175;
D012643	increase	GSN	ENSG00000148180	19244175;
D012643	increase	GSTP1	ENSG00000084207	19244175;
D012643	increase	GTF2F1	ENSG00000125651	19244175;
D012643	increase	GTF2H2	ENSG00000145736	19244175;
D012643	increase	GTF3C1	ENSG00000077235	19244175;
D012643	increase	H2AFX	ENSG00000188486	19244175;
D012643	increase	H2AFY2	ENSG00000099284	19244175;
D012643	increase	H6PD	ENSG00000049239	19244175;
D012643	increase	HAGH	ENSG00000063854	19244175;
D012643	increase	HARS	ENSG00000170445	19244175;
D012643	increase	HAUS3	ENSG00000214367	19244175;
D012643	increase	HAUS4	ENSG00000092036	19244175;
D012643	increase	HDGF	ENSG00000143321	19244175;
D012643	increase	HDLBP	ENSG00000115677	19244175;
D012643	increase	HES1	ENSG00000114315	19244175;
D012643	increase	HGS	ENSG00000185359	19244175;
D012643	increase	HHIP	ENSG00000164161	18997278;
D012643	increase	HIP1R	ENSG00000130787	19244175;
D012643	increase	HIRIP3	ENSG00000149929	19244175;
D012643	increase	HLA-B	ENSG00000234745	19244175;
D012643	increase	HLA-C	ENSG00000204525	19244175;
D012643	increase	HLA-DPA1	ENSG00000168384	19244175;
D012643	increase	HLA-DQB1	ENSG00000179344	19244175;
D012643	increase	HLA-DRA	ENSG00000204287	19244175;
D012643	increase	HLA-DRB3	ENSG00000231679	19244175;
D012643	increase	HLA-E	ENSG00000225201	19244175;
D012643	increase	HLA-F	ENSG00000204642	19244175;
D012643	increase	HLA-G	ENSG00000237216	19244175;
D012643	increase	HMBS	ENSG00000149397	19244175;
D012643	increase	HMGXB3	ENSG00000113716	19244175;
D012643	increase	HMX1	ENSG00000215612	19244175;
D012643	increase	HPCAL1	ENSG00000115756	19244175;
D012643	increase	HPN	ENSG00000105707	19244175;
D012643	increase	HRAS	ENSG00000174775	19244175;
D012643	increase	HSD17B8	ENSG00000232357	19244175;
D012643	increase	HSPA1A	ENSG00000232804	19244175;
D012643	increase	HSPA4	ENSG00000170606	19244175;
D012643	increase	HSPB1	ENSG00000106211	19244175;
D012643	increase	HTR4	ENSG00000164270	19244175;
D012643	increase	IARS	ENSG00000196305	19244175;
D012643	increase	ICA1	ENSG00000003147	19244175;
D012643	increase	ID1	ENSG00000125968	19244175;

D012643	increase	IDH3B	ENSG00000101365	19244175;
D012643	increase	IDH3G	ENSG00000067829	19244175;
D012643	increase	IDUA	ENSG00000127415	19244175;
D012643	increase	IER3	ENSG00000137331	17293063;
D012643	increase	IFI35	ENSG00000068079	19244175;
D012643	increase	IFI6	ENSG00000126709	19244175;
D012643	increase	IFITM1	ENSG00000185885	19244175;
D012643	increase	IFITM2	ENSG00000185201	19244175;
D012643	increase	IFITM3	ENSG00000142089	19244175;
D012643	increase	IFRD2	ENSG00000214706	19244175;
D012643	increase	IGFBP2	ENSG00000115457	19244175;
D012643	increase	IGFBP3	ENSG00000146674	18997278;15318950;
D012643	increase	IGFBP4	ENSG00000141753	19244175;
D012643	increase	IGFBP6	ENSG00000167779	19244175;
D012643	increase	IK	ENSG00000113141	19244175;
D012643	increase	IL17RC	ENSG00000163702	19244175;
D012643	increase	IL1B	ENSG00000125538	10802230;18196609;17293063;
D012643	increase	IL4R	ENSG00000077238	19244175;
D012643	increase	IL8	ENSG00000169429	17293063;
D012643	increase	ILF3	ENSG00000129351	19244175;
D012643	increase	ILK	ENSG00000166333	19244175;
D012643	increase	INTS1	ENSG00000164880	19244175;
D012643	increase	INTS3	ENSG00000143624	19244175;
D012643	increase	IRF3	ENSG00000126456	19244175;
D012643	increase	ISYNA1	ENSG00000105655	19244175;
D012643	increase	ITGA3	ENSG00000005884	19244175;
D012643	increase	ITM2C	ENSG00000135916	19244175;
D012643	increase	KCNJ15	ENSG00000157551	17293063;
D012643	increase	KCTD17	ENSG00000100379	19244175;
D012643	increase	KDM5D	ENSG00000012817	18583539;
D012643	increase	KDM6B	ENSG00000132510	19244175;
D012643	increase	KEAP1	ENSG00000079999	19244175;
D012643	increase	KIAA0100	ENSG00000007202	19244175;
D012643	increase	KIAA0494	ENSG00000159658	19244175;
D012643	increase	KIAA0652	ENSG00000175224	19244175;
D012643	increase	KIF1C	ENSG00000129250	19244175;
D012643	increase	KL	ENSG00000133116	19244175;
D012643	increase	KLF12	ENSG00000118922	17293063;
D012643	increase	KLHL24	ENSG00000114796	19244175;
D012643	increase	KLHL9	ENSG00000198642	19244175;
D012643	increase	KPNA6	ENSG00000025800	19244175;
D012643	increase	KPNB1	ENSG00000108424	19244175;
D012643	increase	KRT17	ENSG00000128422	19244175;
D012643	increase	KRT18	ENSG00000111057	19244175;
D012643	increase	KRT19	ENSG00000171345	19244175;
D012643	increase	KRT7	ENSG00000135480	19244175;
D012643	increase	KRT8	ENSG00000170421	19244175;
D012643	increase	LAMB3	ENSG00000196878	19244175;
D012643	increase	LAMP1	ENSG00000185896	19244175;
D012643	increase	LARP4B	ENSG00000107929	19244175;
D012643	increase	LIME1	ENSG00000203896	19244175;
D012643	increase	LITAF	ENSG00000189067	19244175;
D012643	increase	LMAN2	ENSG00000169223	19244175;

D012643	increase	LMNA	ENSG00000160789	19244175;
D012643	increase	LRRC41	ENSG00000132128	19244175;
D012643	increase	LRRC47	ENSG00000130764	19244175;
D012643	increase	LSP1	ENSG00000130592	19244175;
D012643	increase	LSR	ENSG00000105699	19244175;
D012643	increase	LTBP3	ENSG00000168056	19244175;
D012643	increase	LTBP4	ENSG00000090006	19244175;
D012643	increase	LTBR	ENSG00000111321	19244175;
D012643	increase	LY6E	ENSG00000160932	19244175;
D012643	increase	LYPLA2	ENSG00000011009	19244175;
D012643	increase	MAGED2	ENSG00000102316	19244175;
D012643	increase	MALL	ENSG00000144063	19244175;
D012643	increase	MAN1B1	ENSG00000177239	19244175;
D012643	increase	MAN2B1	ENSG00000104774	19244175;
D012643	increase	MAP2K2	ENSG00000126934	19244175;
D012643	increase	MAP2K3	ENSG00000034152	19244175;
D012643	increase	MAP4	ENSG00000047849	19244175;
D012643	increase	MAP7D1	ENSG00000116871	19244175;
D012643	increase	MAPK11	ENSG00000185386	19244175;
D012643	increase	2-Mar	ENSG00000099785	19244175;
D012643	increase	6-Mar	ENSG00000145495	19244175;18583539;
D012643	increase	MARCKS	ENSG00000155130	19244175;
D012643	increase	MAX	ENSG00000125952	19244175;
D012643	increase	MAZ	ENSG00000103495	19244175;
D012643	increase	MBD3	ENSG00000071655	19244175;
D012643	increase	MCF2L	ENSG00000126217	19244175;
D012643	increase	MCM5	ENSG00000100297	19244175;
D012643	increase	MCM7	ENSG00000166508	19244175;
D012643	increase	MCRS1	ENSG00000187778	19244175;
D012643	increase	MDK	ENSG00000110492	19244175;
D012643	increase	MED15	ENSG00000099917	19244175;
D012643	increase	MED24	ENSG00000008838	19244175;
D012643	increase	MFAP2	ENSG00000117122	19244175;
D012643	increase	MFGE8	ENSG00000140545	19244175;
D012643	increase	MFSD10	ENSG00000109736	19244175;
D012643	increase	MGAT1	ENSG00000131446	19244175;
D012643	increase	MGP	ENSG00000111341	18583539;
D012643	increase	MIIP	ENSG00000116691	19244175;
D012643	increase	MINK1	ENSG00000141503	19244175;
D012643	increase	MLEC	ENSG00000110917	19244175;
D012643	increase	MLF2	ENSG00000089693	19244175;
D012643	increase	MLL4	ENSG00000105663	19244175;
D012643	increase	MMP23B	ENSG00000189409	18583539;
D012643	increase	MMP24	ENSG00000125966	17390030;19244175;
D012643	increase	MMP2	ENSG00000087245	17390030;19244175;
D012643	increase	MON1B	ENSG00000103111	19244175;
D012643	increase	MPST	ENSG00000128309	19244175;
D012643	increase	MPV17	ENSG00000115204	19244175;
D012643	increase	MRPL12	ENSG00000183093	19244175;
D012643	increase	MRPL2	ENSG00000112651	19244175;
D012643	increase	MRPL34	ENSG00000130312	19244175;
D012643	increase	MRPL49	ENSG00000149792	19244175;
D012643	increase	MRPL4	ENSG00000105364	19244175;
D012643	increase	MRPL9	ENSG00000143436	19244175;

D012643	increase	MSN	ENSG00000147065	19244175;
D012643	increase	MTCH2	ENSG00000109919	19244175;
D012643	increase	MTF1	ENSG00000188786	19244175;
D012643	increase	MUC4	ENSG00000145113	19244175;
D012643	increase	MVK	ENSG00000110921	19244175;
D012643	increase	MVP	ENSG00000013364	19244175;
D012643	increase	MX1	ENSG00000157601	19244175;
D012643	increase	MYC	ENSG00000136997	11925459;17948765;18583539;
D012643	increase	MYH9	ENSG00000100345	19244175;
D012643	increase	MYL6	ENSG00000092841	19244175;
D012643	increase	MZF1	ENSG00000099326	19244175;
D012643	increase	NAGK	ENSG00000124357	19244175;
D012643	increase	NAGLU	ENSG00000108784	19244175;
D012643	increase	NANOG	ENSG00000111704	18583539;
D012643	increase	NAPA	ENSG00000105402	19244175;
D012643	increase	NASP	ENSG00000132780	19244175;
D012643	increase	NCDN	ENSG00000020129	19244175;
D012643	increase	NDUFS3	ENSG00000213619	19244175;
D012643	increase	NDUFV1	ENSG00000167792	19244175;
D012643	increase	NEDD8	ENSG00000129559	19244175;
D012643	increase	NES	ENSG00000132688	18583539;
D012643	increase	NF2	ENSG00000186575	19244175;
D012643	increase	NFIC	ENSG00000141905	19244175;
D012643	increase	NFKBIB	ENSG00000104825	19244175;
D012643	increase	NHP2L1	ENSG00000100138	19244175;
D012643	increase	NINJ1	ENSG00000131669	19244175;
D012643	increase	NLGN4Y	ENSG00000165246	18583539;
D012643	increase	NLRP3	ENSG00000162711	17293063;
D012643	increase	NOMO3	ENSG00000103226	19244175;
D012643	increase	NOSIP	ENSG00000142546	19244175;
D012643	increase	NOTCH3	ENSG00000074181	19244175;
D012643	increase	NPEPL1	ENSG00000215440	19244175;
D012643	increase	NSMCE2	ENSG00000156831	18583539;
D012643	increase	NT5DC2	ENSG00000168268	19244175;
D012643	increase	NUCB1	ENSG00000104805	19244175;
D012643	increase	NUDC	ENSG00000090273	19244175;
D012643	increase	NUP93	ENSG00000102900	19244175;
D012643	increase	NUTF2	ENSG00000102898	19244175;
D012643	increase	OAZ1	ENSG00000104904	19244175;
D012643	increase	OAZ2	ENSG00000180304	19244175;
D012643	increase	OBSL1	ENSG00000124006	19244175;
D012643	increase	OCLN	ENSG00000197822	16514648;
D012643	increase	OGFR	ENSG00000060491	19244175;
D012643	increase	OPTN	ENSG00000123240	19244175;
D012643	increase	OS9	ENSG00000135506	19244175;
D012643	increase	OSBP2	ENSG00000184792	19244175;
D012643	increase	OSGEP	ENSG00000092094	19244175;
D012643	increase	OVGP1	ENSG00000085465	19244175;
D012643	increase	PAFAH1B1	ENSG00000007168	19244175;
D012643	increase	PAFAH1B3	ENSG00000079462	19244175;
D012643	increase	PAK4	ENSG00000130669	19244175;
D012643	increase	PALM2	ENSG00000243444	19244175;
D012643	increase	PANK4	ENSG00000157881	19244175;

D012643	increase	PCBP1	ENSG00000169564	19244175;
D012643	increase	PCDHGC3	ENSG00000240184	19244175;
D012643	increase	PC	ENSG00000173599	19244175;
D012643	increase	PCOLCE	ENSG00000106333	19244175;
D012643	increase	PCSK7	ENSG00000160613	19244175;
D012643	increase	PDGFA	ENSG00000197461	19244175;
D012643	increase	PEA15	ENSG00000162734	19244175;
D012643	increase	PEF1	ENSG00000162517	19244175;
D012643	increase	PES1	ENSG00000100029	19244175;
D012643	increase	PEX14	ENSG00000142655	19244175;
D012643	increase	PEX7	ENSG00000112357	19244175;
D012643	increase	PFKL	ENSG00000141959	19244175;
D012643	increase	PFN1	ENSG00000108518	19244175;
D012643	increase	PGAP2	ENSG00000249611	19244175;
D012643	increase	PIH1D1	ENSG00000104872	19244175;
D012643	increase	PIK3CD	ENSG00000171608	19244175;
D012643	increase	PITPNA	ENSG00000174238	19244175;
D012643	increase	PITPNM1	ENSG00000110697	19244175;
D012643	increase	PITX1	ENSG00000069011	19244175;
D012643	increase	PJA1	ENSG00000181191	19244175;
D012643	increase	PKD1	ENSG00000008710	19244175;
D012643	increase	PKM2	ENSG00000067225	19244175;
D012643	increase	PLA2G16	ENSG00000176485	19244175;
D012643	increase	PLA2G2A	ENSG00000188257	18583539;
D012643	increase	PLEC	ENSG00000178209	19244175;
D012643	increase	PLEKHJ1	ENSG00000104886	19244175;
D012643	increase	PLEKHO1	ENSG00000023902	19244175;
D012643	increase	PLLP	ENSG00000102934	19244175;
D012643	increase	PLVAP	ENSG00000130300	19244175;
D012643	increase	PLXNB2	ENSG00000196576	19244175;
D012643	increase	PML	ENSG00000140464	19244175;
D012643	increase	PNKP	ENSG00000039650	19244175;
D012643	increase	PNPLA6	ENSG00000032444	19244175;
D012643	increase	POLA2	ENSG00000014138	19244175;
D012643	increase	POLDIP3	ENSG00000100227	19244175;
D012643	increase	POLL	ENSG00000166169	19244175;
D012643	increase	POLR2A	ENSG00000181222	19244175;
D012643	increase	POLR2C	ENSG00000102978	19244175;
D012643	increase	POR	ENSG00000127948	19244175;
D012643	increase	POU5F1	ENSG00000235068	18583539;
D012643	increase	PPIB	ENSG00000166794	19244175;
D012643	increase	PPIL2	ENSG00000100023	19244175;
D012643	increase	PPL	ENSG00000118898	18583539;
D012643	increase	PPM1J	ENSG00000155367	19244175;
D012643	increase	PPME1	ENSG00000214517	19244175;
D012643	increase	PPP1CA	ENSG00000172531	19244175;
D012643	increase	PPP1R10	ENSG00000235291	19244175;
D012643	increase	PPP1R14B	ENSG00000173457	19244175;
D012643	increase	PPP2R1A	ENSG00000105568	19244175;
D012643	increase	PPP2R4	ENSG00000119383	19244175;
D012643	increase	PPP2R5D	ENSG00000112640	19244175;
D012643	increase	PPP4C	ENSG00000149923	19244175;
D012643	increase	PRAF2	ENSG00000243279	19244175;
D012643	increase	PRDM2	ENSG00000116731	19244175;

D012643	increase	PRKAG1	ENSG00000181929	19244175;
D012643	increase	PRKD2	ENSG00000105287	19244175;
D012643	increase	PRMT1	ENSG00000126457	19244175;
D012643	increase	PRMT2	ENSG00000160310	19244175;
D012643	increase	PRMT5	ENSG00000100462	19244175;
D012643	increase	PRPF6	ENSG00000101161	19244175;
D012643	increase	PRPF8	ENSG00000174231	19244175;
D012643	increase	PRSS8	ENSG00000052344	19244175;
D012643	increase	PRUNE	ENSG00000143363	19244175;
D012643	increase	PRX	ENSG00000105227	19244175;
D012643	increase	PSAP	ENSG00000197746	19244175;
D012643	increase	PSMB10	ENSG00000205220	19244175;
D012643	increase	PSMB4	ENSG00000159377	19244175;
D012643	increase	PSMC1	ENSG00000100764	19244175;
D012643	increase	PSMC4	ENSG00000013275	19244175;
D012643	increase	PSMD4	ENSG00000159352	19244175;
D012643	increase	PSMD6	ENSG00000163636	19244175;
D012643	increase	PSMD8	ENSG00000099341	19244175;
D012643	increase	PSME1	ENSG00000092010	19244175;
D012643	increase	PTGDS	ENSG00000107317	19244175;
D012643	increase	PTGES	ENSG00000148344	19244175;
D012643	increase	PTMS	ENSG00000159335	19244175;
D012643	increase	PTOV1	ENSG00000104960	19244175;
D012643	increase	PTPN18	ENSG00000072135	19244175;
D012643	increase	PTPN6	ENSG00000111679	19244175;
D012643	increase	PTRF	ENSG00000177469	19244175;
D012643	increase	PTX3	ENSG00000163661	17293063;
D012643	increase	PVRL2	ENSG00000130202	19244175;
D012643	increase	PYCARD	ENSG00000103490	19244175;
D012643	increase	PYGB	ENSG00000100994	19244175;
D012643	increase	QTRT1	ENSG00000213339	19244175;
D012643	increase	RAB11B	ENSG00000185236	19244175;
D012643	increase	RAB25	ENSG00000132698	19244175;
D012643	increase	RABAC1	ENSG00000105404	19244175;
D012643	increase	RAD23A	ENSG00000179262	19244175;
D012643	increase	RALY	ENSG00000125970	19244175;
D012643	increase	RANBP3	ENSG00000031823	19244175;
D012643	increase	RANGAP1	ENSG00000100401	19244175;
D012643	increase	RASSF1	ENSG00000068028	19244175;
D012643	increase	RASSF7	ENSG00000099849	19244175;
D012643	increase	RBBP4	ENSG00000162521	19244175;
D012643	increase	RBCK1	ENSG00000125826	19244175;
D012643	increase	RBM10	ENSG00000182872	19244175;
D012643	increase	RBM14	ENSG00000239306	19244175;
D012643	increase	RBM42	ENSG00000126254	19244175;
D012643	increase	RBM5	ENSG00000003756	19244175;
D012643	increase	RBMS1	ENSG00000153250	19244175;
D012643	increase	RBPMS	ENSG00000157110	19244175;
D012643	increase	RECQL5	ENSG00000108469	19244175;
D012643	increase	RELA	ENSG00000173039	19244175;
D012643	increase	RERG	ENSG00000134533	18583539;
D012643	increase	RFTN1	ENSG00000131378	19244175;
D012643	increase	RGS13	ENSG00000127074	18583539;
D012643	increase	RHBDD3	ENSG00000100263	19244175;

D012643	increase	RHOG	ENSG00000177105	19244175;
D012643	increase	RHOU	ENSG00000116574	18583539;
D012643	increase	RING1	ENSG00000231115	19244175;
D012643	increase	RND1	ENSG00000172602	19244175;
D012643	increase	RNF220	ENSG00000187147	19244175;
D012643	increase	RNF40	ENSG00000103549	19244175;
D012643	increase	RNH1	ENSG00000023191	19244175;
D012643	increase	RNPEP	ENSG00000176393	19244175;
D012643	increase	RNPS1	ENSG00000205937	19244175;
D012643	increase	RPRD2	ENSG00000163125	19244175;
D012643	increase	RPS4Y1	ENSG00000129824	18583539;
D012643	increase	RPS6KA1	ENSG00000117676	19244175;
D012643	increase	RPS6KA2	ENSG00000071242	19244175;
D012643	increase	RRP1	ENSG00000160214	19244175;
D012643	increase	RRP7A	ENSG00000189306	19244175;
D012643	increase	RSAD1	ENSG00000136444	19244175;
D012643	increase	RUNX3	ENSG00000020633	18583539;
D012643	increase	RUSC2	ENSG00000198853	19244175;
D012643	increase	RUVBL1	ENSG00000175792	19244175;
D012643	increase	RUVBL2	ENSG00000183207	19244175;
D012643	increase	RXRA	ENSG00000186350	15318950;
D012643	increase	S100A11	ENSG00000163191	19244175;
D012643	increase	S100A6	ENSG00000197956	19244175;
D012643	increase	SAFB2	ENSG00000130254	19244175;
D012643	increase	SAFB	ENSG00000160633	19244175;
D012643	increase	SAPS2	ENSG00000100239	19244175;
D012643	increase	SARDH	ENSG00000123453	19244175;
D012643	increase	SART1	ENSG00000175467	19244175;
D012643	increase	SCAMP3	ENSG00000116521	19244175;
D012643	increase	SCARA5	ENSG00000168079	18583539;
D012643	increase	SCN9A	ENSG00000169432	18583539;
D012643	increase	SCO2	ENSG00000130489	19244175;
D012643	increase	SDC3	ENSG00000162512	19244175;
D012643	increase	SDK1	ENSG00000146555	18583539;
D012643	increase	SEC13	ENSG00000157020	19244175;
D012643	increase	SEC23B	ENSG00000101310	19244175;
D012643	increase	SELENBP1	ENSG00000143416	16380993;
D012643	increase	7-Sep	ENSG00000122545	18583539;
D012643	increase	SERPINF1	ENSG00000132386	19244175;
D012643	increase	SF3A2	ENSG00000104897	19244175;
D012643	increase	SF3B4	ENSG00000143368	19244175;
D012643	increase	SFRS1	ENSG00000136450	19244175;
D012643	increase	SFRS9	ENSG00000111786	19244175;
D012643	increase	SGSH	ENSG00000181523	19244175;
D012643	increase	SGSM3	ENSG00000100359	19244175;
D012643	increase	SHB	ENSG00000107338	19244175;
D012643	increase	SIGIRR	ENSG00000185187	19244175;
D012643	increase	SIN3B	ENSG00000127511	19244175;
D012643	increase	SIPA1L3	ENSG00000105738	19244175;
D012643	increase	SIRPA	ENSG00000198053	19244175;
D012643	increase	SIRT5	ENSG00000124523	18583539;
D012643	increase	SKI	ENSG00000157933	19244175;
D012643	increase	SLC22A18	ENSG00000110628	19244175;
D012643	increase	SLC25A1	ENSG00000100075	19244175;

D012643	increase	SLC25A22	ENSG00000177542	19244175;
D012643	increase	SLC25A37	ENSG00000147454	19244175;
D012643	increase	SLC25A6	ENSG00000169100	19244175;
D012643	increase	SLC29A1	ENSG00000112759	19244175;
D012643	increase	SLC39A1	ENSG00000143570	19244175;
D012643	increase	SLC39A7	ENSG00000224399	19244175;
D012643	increase	SLC3A2	ENSG00000168003	19244175;
D012643	increase	SLC44A4	ENSG00000204385	19244175;
D012643	increase	SLC6A8	ENSG00000130821	19244175;
D012643	increase	SLC7A5	ENSG00000103257	19244175;
D012643	increase	SLC7A8	ENSG00000092068	19244175;
D012643	increase	SMARCA4	ENSG00000127616	19244175;
D012643	increase	SMARCD2	ENSG00000108604	19244175;
D012643	increase	SMARCD3	ENSG00000082014	19244175;
D012643	increase	SMG5	ENSG00000198952	19244175;
D012643	increase	SMG7	ENSG00000116698	19244175;
D012643	increase	SMOX	ENSG00000088826	19244175;
D012643	increase	SMTN	ENSG00000183963	19244175;
D012643	increase	SMURF1	ENSG00000198742	19244175;
D012643	increase	SNRNP35	ENSG00000184209	19244175;
D012643	increase	SNRPB	ENSG00000125835	19244175;
D012643	increase	SNX17	ENSG00000115234	19244175;
D012643	increase	SNX1	ENSG00000028528	19244175;
D012643	increase	SOD2	ENSG00000112096	19074884;19528373;17293063;
D012643	increase	SORBS3	ENSG00000120896	19244175;
D012643	increase	SOX2	ENSG00000181449	18583539;
D012643	increase	SPG7	ENSG00000197912	19244175;
D012643	increase	SPINT1	ENSG00000166145	19244175;
D012643	increase	SPSB3	ENSG00000162032	19244175;
D012643	increase	SPTAN1	ENSG00000197694	19244175;
D012643	increase	SREBF2	ENSG00000198911	19244175;
D012643	increase	SSNA1	ENSG00000176101	19244175;
D012643	increase	SSRP1	ENSG00000149136	19244175;
D012643	increase	ST14	ENSG00000149418	19244175;
D012643	increase	ST5	ENSG00000166444	19244175;
D012643	increase	STAB1	ENSG00000010327	19244175;
D012643	increase	STARD5	ENSG00000172345	19244175;
D012643	increase	STBD1	ENSG00000118804	19244175;
D012643	increase	STEAP4	ENSG00000127954	18583539;
D012643	increase	STK11	ENSG00000118046	19244175;
D012643	increase	STK24	ENSG00000102572	19244175;
D012643	increase	STOML2	ENSG00000165283	19244175;
D012643	increase	STUB1	ENSG00000103266	19244175;
D012643	increase	STYXL1	ENSG00000127952	19244175;
D012643	increase	SUMO2	ENSG00000188612	19244175;
D012643	increase	SUPT5H	ENSG00000196235	19244175;
D012643	increase	SYNJ2	ENSG00000078269	19244175;
D012643	increase	SYNPO	ENSG00000171992	19244175;
D012643	increase	TAF10	ENSG00000166337	19244175;
D012643	increase	TAGLN2	ENSG00000158710	19244175;
D012643	increase	TALDO1	ENSG00000177156	19244175;
D012643	increase	TAP1	ENSG00000230705	19244175;
D012643	increase	TAPBP	ENSG00000206208	19244175;

D012643	increase	TARBP2	ENSG00000139546	19244175;
D012643	increase	TAX1BP3	ENSG00000213977	19244175;
D012643	increase	TBC1D9B	ENSG00000197226	19244175;
D012643	increase	TBL3	ENSG00000183751	19244175;
D012643	increase	TCEA2	ENSG00000171703	19244175;
D012643	increase	TEAD3	ENSG00000007866	19244175;
D012643	increase	TENC1	ENSG00000111077	19244175;
D012643	increase	TEP1	ENSG00000129566	18583539;
D012643	increase	TFEB	ENSG00000112561	19244175;
D012643	increase	THAP11	ENSG00000168286	19244175;
D012643	increase	THAP7	ENSG00000184436	19244175;
D012643	increase	THYN1	ENSG00000151500	19244175;
D012643	increase	TIAF1	ENSG00000221995	19244175;
D012643	increase	TIMP3	ENSG00000100234	17390030;19244175;
D012643	increase	TJP1	ENSG00000104067	16514648;
D012643	increase	TKT	ENSG00000163931	19244175;
D012643	increase	TLE2	ENSG00000065717	19244175;
D012643	increase	TLN1	ENSG00000137076	19244175;
D012643	increase	TM9SF1	ENSG00000100926	19244175;
D012643	increase	TM9SF4	ENSG00000101337	19244175;
D012643	increase	TMEM222	ENSG00000186501	19244175;
D012643	increase	TMEM53	ENSG00000126106	19244175;
D012643	increase	TMEM63A	ENSG00000196187	19244175;
D012643	increase	TMPRSS2	ENSG00000184012	19244175;
D012643	increase	TMPRSS6	ENSG00000187045	19244175;
D012643	increase	TNFAIP2	ENSG00000185215	19244175;
D012643	increase	TNFAIP6	ENSG00000123610	17293063;
D012643	increase	TNF	ENSG00000223952	10802230;18196609;17293063;
D012643	increase	TNFRSF10B	ENSG00000120889	12432279;
D012643	increase	TNFRSF10C	ENSG00000173535	17293063;
D012643	increase	TNFRSF12A	ENSG00000006327	19244175;
D012643	increase	TNK1	ENSG00000174292	19244175;
D012643	increase	TOR3A	ENSG00000186283	19244175;
D012643	increase	TOX4	ENSG00000092203	19244175;
D012643	increase	TP53	ENSG00000141510	17523932;18583539;19244175;
D012643	increase	TPI1	ENSG00000111669	19244175;
D012643	increase	TPM2	ENSG00000198467	19244175;
D012643	increase	TPP1	ENSG00000166340	19244175;
D012643	increase	TRA2A	ENSG00000164548	19244175;17293063;
D012643	increase	TRAF4	ENSG00000076604	19244175;
D012643	increase	TRAFD1	ENSG00000135148	19244175;
D012643	increase	TREX1	ENSG00000213689	19244175;
D012643	increase	TRIB2	ENSG00000071575	19244175;
D012643	increase	TRIM28	ENSG00000130726	19244175;
D012643	increase	TRIM29	ENSG00000137699	19244175;
D012643	increase	TRIP6	ENSG00000087077	19244175;
D012643	increase	TRO	ENSG00000067445	19244175;
D012643	increase	TRRAP	ENSG00000196367	19244175;
D012643	increase	TSC2	ENSG00000103197	19244175;
D012643	increase	TSPAN14	ENSG00000108219	19244175;
D012643	increase	TSSC4	ENSG00000184281	19244175;
D012643	increase	TSTA3	ENSG00000104522	19244175;

D012643	increase	TUBB4	ENSG00000104833	19244175;
D012643	increase	TUBB	ENSG00000229684	19244175;
D012643	increase	TYMP	ENSG00000025708	19244175;
D012643	increase	U2AF1	ENSG00000160201	19244175;
D012643	increase	UBA1	ENSG00000130985	19244175;
D012643	increase	UBAP2L	ENSG00000143569	19244175;
D012643	increase	UBE2L3	ENSG00000185651	19244175;
D012643	increase	UBE2Z	ENSG00000159202	19244175;
D012643	increase	UBE4B	ENSG00000130939	19244175;
D012643	increase	UBN1	ENSG00000118900	19244175;
D012643	increase	UBXN1	ENSG00000162191	19244175;
D012643	increase	UFD1L	ENSG00000070010	19244175;
D012643	increase	UNC5B	ENSG00000107731	19244175;
D012643	increase	UQCRC1	ENSG00000010256	19244175;
D012643	increase	USF2	ENSG00000105698	19244175;
D012643	increase	USP9Y	ENSG00000114374	18583539;
D012643	increase	VAMP8	ENSG00000118640	19244175;
D012643	increase	VARS2	ENSG00000137411	19244175;
D012643	increase	VAT1	ENSG00000108828	19244175;
D012643	increase	VGF	ENSG00000128564	19244175;
D012643	increase	VILL	ENSG00000136059	19244175;
D012643	increase	VIM	ENSG00000026025	19244175;
D012643	increase	VIPR1	ENSG00000114812	19244175;
D012643	increase	VKORC1	ENSG00000167397	19244175;
D012643	increase	VPS28	ENSG00000160948	19244175;
D012643	increase	VPS39	ENSG00000166887	19244175;
D012643	increase	VPS4A	ENSG00000132612	19244175;
D012643	increase	VRK3	ENSG00000105053	18583539;
D012643	increase	WASF2	ENSG00000158195	19244175;
D012643	increase	WBP2	ENSG00000132471	19244175;
D012643	increase	WDR1	ENSG00000071127	19244175;
D012643	increase	WDR45	ENSG00000196998	19244175;
D012643	increase	WDR6	ENSG00000178252	19244175;
D012643	increase	WFDC2	ENSG00000101443	19244175;
D012643	increase	WHSC2	ENSG00000185049	19244175;
D012643	increase	WNT5B	ENSG00000111186	19244175;
D012643	increase	WRAP53	ENSG00000141499	19244175;
D012643	increase	YIPF3	ENSG00000137207	19244175;
D012643	increase	YWHAZ	ENSG00000164924	19244175;
D012643	increase	ZCWPW1	ENSG00000078487	19244175;
D012643	increase	ZER1	ENSG00000160445	19244175;
D012643	increase	ZFP42	ENSG00000179059	18583539;
D012643	increase	ZNF212	ENSG00000170260	19244175;
D012643	increase	ZNF219	ENSG00000165804	19244175;
D012643	increase	ZNF324	ENSG00000083812	19244175;
D012643	increase	ZNF358	ENSG00000198816	19244175;
D012643	increase	ZNF500	ENSG00000103199	19244175;
D012643	increase	ZNF571	ENSG00000180479	18583539;
D012643	increase	ZNF580	ENSG00000213015	19244175;
D012643	increase	ZNF768	ENSG00000169957	19244175;
D012643	increase	ZYX	ENSG00000159840	19244175;
D012643	increase	ZZEF1	ENSG00000074755	19244175;
D012645	affect	ACD	ENSG00000102977	17222674;
D012645	affect	ACO2	ENSG00000100412	17222674;

D012645	affect	ATF2	ENSG00000115966	17222674;
D012645	affect	ATP6V0A1	ENSG00000033627	17222674;
D012645	affect	CBX6	ENSG00000183741	17222674;
D012645	affect	CFL2	ENSG00000165410	18444160;
D012645	affect	CLSTN3	ENSG00000139182	17222674;
D012645	affect	CNNM3	ENSG00000168763	17222674;
D012645	affect	COL6A1	ENSG00000142156	17222674;
D012645	affect	CRMP1	ENSG00000072832	17222674;
D012645	affect	CYHR1	ENSG00000187954	17222674;
D012645	affect	DST	ENSG00000151914	17222674;
D012645	affect	DUSP3	ENSG00000108861	17222674;
D012645	affect	ECE2	ENSG00000145194	17222674;
D012645	affect	ECSIT	ENSG00000130159	17222674;
D012645	affect	EHD1	ENSG00000110047	17222674;
D012645	affect	FAM102A	ENSG00000167106	17222674;
D012645	affect	GRK6	ENSG00000198055	17222674;
D012645	affect	HPN	ENSG00000105707	17222674;
D012645	affect	HSPE1	ENSG00000115541	18444160;
D012645	affect	IKBKB	ENSG00000104365	17222674;
D012645	affect	ITM2C	ENSG00000135916	17222674;
D012645	affect	KLHDC3	ENSG00000124702	17222674;
D012645	affect	L3MBTL4	ENSG00000154655	17222674;
D012645	affect	LASP1	ENSG00000002834	17222674;
D012645	affect	LTBR	ENSG00000111321	17222674;
D012645	affect	MAP3K11	ENSG00000173327	17222674;
D012645	affect	MRPL54	ENSG00000183617	17222674;
D012645	affect	NARF	ENSG00000141562	17222674;
D012645	affect	NDUFV1	ENSG00000167792	17222674;
D012645	affect	NME6	ENSG00000172113	18444160;
D012645	affect	PARVB	ENSG00000188677	17222674;
D012645	affect	PCK2	ENSG00000100889	17222674;
D012645	affect	PCNXL3	ENSG00000197136	17222674;
D012645	affect	PIGU	ENSG00000101464	17222674;
D012645	affect	PLG	ENSG00000122194	17222674;
D012645	affect	PMVK	ENSG00000163344	17222674;
D012645	affect	POLL	ENSG00000166169	17222674;
D012645	affect	PPM1B	ENSG00000138032	17222674;
D012645	affect	PRKCZ	ENSG00000067606	17222674;
D012645	affect	RAP2A	ENSG00000125249	17222674;
D012645	affect	RPUSD1	ENSG00000007376	17222674;
D012645	affect	SCARB1	ENSG00000073060	17222674;
D012645	affect	SEMA5A	ENSG00000112902	17222674;
D012645	affect	SHC1	ENSG00000160691	17222674;
D012645	affect	SLC25A1	ENSG00000100075	17222674;
D012645	affect	SRRT	ENSG00000087087	17222674;
D012645	affect	SSBP1	ENSG00000106028	18444160;
D012645	affect	STX17	ENSG00000136874	17222674;
D012645	affect	TAOK2	ENSG00000149930	17222674;
D012645	affect	TBX1	ENSG00000184058	17222674;
D012645	affect	TEAD3	ENSG00000007866	17222674;
D012645	affect	TREX1	ENSG00000213689	17222674;
D012645	affect	TSC22D4	ENSG00000166925	17222674;
D012645	affect	UBE2S	ENSG00000108106	17222674;
D012645	affect	VPS4A	ENSG00000132612	17222674;

D012645	affect	WWP2	ENSG00000198373	17222674;
D012645	decrease	KLK3	ENSG00000142515	17205524;15141018;
D012645	increase	AR	ENSG00000169083	17205524;16239064;
D012645	increase	EGFR	ENSG00000146648	17205524;
D012645	increase	GSTP1	ENSG00000084207	17465221;18676679;
D012645	increase	IL8	ENSG00000169429	14572605;
D012645	increase	TP53	ENSG00000141510	17205524;17100737;
D012701	affect	IL6	ENSG00000136244	15802305;
D012701	affect	IL8	ENSG00000169429	15802305;
D012721	affect	AHR	ENSG00000106546	9169082;9853009;9858662;<br>17553063;
D012721	increase	CYP1A1	ENSG00000140465	9169082;11226373;
D012721	increase	DDIT3	ENSG00000175197	11226373;
D012721	increase	FOS	ENSG00000170345	11226373;
D012721	increase	FSHB	ENSG00000131808	16413094;
D012721	increase	HSPA5	ENSG00000044574	11226373;
D012721	increase	STAR	ENSG00000147465	16413094;
D012822	increase	MINA	ENSG00000170854	15897898;
D012834	increase	HMOX1	ENSG00000100292	17244614;
D012834	increase	HSPA1A	ENSG00000232804	16460681;17244614;
D012835	increase	HMOX1	ENSG00000100292	17244614;
D012835	increase	HSPA1A	ENSG00000232804	16460681;17244614;
D012838	increase	ABCG2	ENSG00000118777	17077187;
D012838	increase	AHR	ENSG00000106546	17077187;16076518;
D012838	increase	MGMT	ENSG00000170430	16950796;
D012839	decrease	IFNG	ENSG00000111537	10883730;
D012839	decrease	TNF	ENSG00000223952	10883730;
D012839	increase	CYP19A1	ENSG00000137869	11675267;10746939;
D012839	increase	NR5A1	ENSG00000136931	17520059;
D012906	affect	NOS3	ENSG00000164867	15178637;
D012906	decrease	ALPL	ENSG00000162551	18035141;
D012906	decrease	HDAC2	ENSG00000196591	18421014;
D012906	decrease	PTGIS	ENSG00000124212	17255567;
D012906	decrease	PTGS1	ENSG00000095303	17255567;
D012906	decrease	PTH	ENSG00000152266	18035141;
D012906	increase	AHR	ENSG00000106546	17012224;
D012906	increase	CYP1A1	ENSG00000140465	17012224;
D012906	increase	PLA2G4A	ENSG00000116711	17255567;
D012906	increase	PTGS2	ENSG00000073756	17255567;
D012964	decrease	KCNH2	ENSG00000055118	19139152;
D012965	increase	SLC6A12	ENSG00000111181	16303854;
D012967	increase	IL1B	ENSG00000125538	9856837;
D012967	increase	TNF	ENSG00000223952	9856837;
D012969	increase	LPO	ENSG00000167419	9504181;
D012977	increase	IL1B	ENSG00000125538	16581224;
D012977	increase	IL8	ENSG00000169429	16581224;
D012977	increase	TNF	ENSG00000223952	16581224;
D012980	increase	ICAM1	ENSG00000090339	10328874;
D012980	increase	IL10	ENSG00000136634	10328874;
D012980	increase	IL1B	ENSG00000125538	10328874;
D012980	increase	IL8	ENSG00000169429	10328874;
D012980	increase	RELA	ENSG00000173039	10328874;
D012980	increase	SCARB1	ENSG00000073060	16816107;
D012980	increase	TNF	ENSG00000223952	10328874;

D013004	increase	SSTR1	ENSG00000139874	18575731;
D013004	increase	SSTR2	ENSG00000180616	18575731;
D013004	increase	SSTR3	ENSG00000183473	18575731;
D013004	increase	SSTR4	ENSG00000132671	18575731;
D013004	increase	SSTR5	ENSG00000162009	18575731;
D013015	decrease	ASNS	ENSG00000070669	15342952;
D013015	decrease	LSS	ENSG00000160285	15342952;
D013015	decrease	NR0B2	ENSG00000131910	15342952;
D013015	increase	PHYH	ENSG00000107537	17567588;
D013015	increase	SLC2A3	ENSG00000059804	15342952;
D013110	increase	CYP17A1	ENSG00000148795	15666826;
D013148	increase	CYP3A4	ENSG00000160868	12505310;
D013185	increase	PON1	ENSG00000005421	14500290;
D013196	affect	AGR2	ENSG00000106541	17094431;
D013196	affect	CYP27B1	ENSG00000111012	17659868;
D013196	affect	DHCR24	ENSG00000116133	17094431;
D013196	affect	NTS	ENSG00000133636	17094431;
D013196	decrease	ACPP	ENSG00000014257	12391264;
D013196	decrease	AGPS	ENSG00000018510	16631469;
D013196	decrease	ATP1B3	ENSG00000069849	16631469;
D013196	decrease	CBS	ENSG00000160200	17854288;
D013196	decrease	CCND1	ENSG00000110092	15322241;
D013196	decrease	CDKN1A	ENSG00000124762	15322241;
D013196	decrease	CHRM1	ENSG00000168539	17624924;
D013196	decrease	CYP7B1	ENSG00000172817	16630558;
D013196	decrease	FBXL16	ENSG00000127585	16631469;
D013196	decrease	FBXO28	ENSG00000143756	16631469;
D013196	decrease	KIAA1217	ENSG00000120549	17624924;
D013196	decrease	PTHLH	ENSG00000087494	17676588;
D013196	decrease	SMAD4	ENSG00000141646	16597033;
D013196	decrease	TMPRSS2	ENSG00000184012	12711008;
D013196	decrease	TMSB4X	ENSG00000205542	17916567;
D013196	decrease	TNFRSF11B	ENSG00000164761	17189957;
D013196	decrease	WBSCR28	ENSG00000175877	17624924;
D013196	increase	ACYP2	ENSG00000170634	16631469;
D013196	increase	ADAM9	ENSG00000168615	17342749;
D013196	increase	ADAMTS1	ENSG00000154734	17018655;
D013196	increase	ADCYAP1R	ENSG00000078549	17254854;
D013196	increase	ALDH1A3	ENSG00000184254	17526768;

D013196	increase	AR	ENSG00000169083	14579009;11861976;15483189;15840436;12676605;16107550;11861974;12377982;15336702;16169144;14532843;17342749;17526768;15466214;10402478;10696776;16125883;17980950;14628596;15064155;9182867;14751673;11956172;10875257;16111029;15994348;9705896;12730620;11985887;16857237;11222873;10910998;16266977;17804755;17218635;16893599;18487222;16648561;16020486;16951154;12391264;12711008;12368053;15171712;10188193;11861975;16621434;9846162;16877366;18922931;18324645;11241555;1749999
D013196	increase	ATF3	ENSG00000162772	16516039;
D013196	increase	C20ORF194	ENSG00000088854	17254854;
D013196	increase	CCL2	ENSG00000108691	16317058;
D013196	increase	CCNA2	ENSG00000145386	15647840;
D013196	increase	CD40	ENSG00000101017	16317058;
D013196	increase	CD83	ENSG00000112149	17254854;
D013196	increase	CDK2	ENSG00000123374	15647840;
D013196	increase	CHPF	ENSG00000123989	16631469;
D013196	increase	CREB1	ENSG00000118260	15466214;
D013196	increase	CYP24A1	ENSG00000019186	16524720;15538745;
D013196	increase	CYP26A1	ENSG00000095596	17526768;
D013196	increase	E2F1	ENSG00000101412	15647840;
D013196	increase	EIF1AY	ENSG00000198692	16631469;
D013196	increase	EMP2	ENSG00000213853	16631469;
D013196	increase	FOLH1	ENSG00000086205	12711008;
D013196	increase	FOS	ENSG00000170345	15466214;
D013196	increase	FRAT1	ENSG00000165879	16631469;
D013196	increase	GDF15	ENSG00000130513	16523695;
D013196	increase	GREB1	ENSG00000196208	16496412;
D013196	increase	HOXD11	ENSG00000128713	17254854;
D013196	increase	ICAM1	ENSG00000090339	16317058;
D013196	increase	ID1	ENSG00000125968	16541417;
D013196	increase	ID2	ENSG00000115738	16541417;
D013196	increase	ID3	ENSG00000117318	16541417;
D013196	increase	ID4	ENSG00000172201	16541417;
D013196	increase	IGF1	ENSG00000017427	16368782;
D013196	increase	IGF1R	ENSG00000140443	18491370;
D013196	increase	IGFBP2	ENSG00000115457	16368782;
D013196	increase	IGFBP3	ENSG00000146674	16368782;16825320;
D013196	increase	IL6	ENSG00000136244	14532843;16317058;
D013196	increase	INTS4	ENSG00000149262	17254854;
D013196	increase	KLK3	ENSG00000142515	15336702;15950459;16648561;18487222;16951154;12711008;12391264;17624924;17503469;16140617;17131305;17075824;11580929;18922931;15994348;120322

D013196	increase	KLK7	ENSG00000169035	17254854;
D013196	increase	LRRC28	ENSG00000168904	16631469;
D013196	increase	MAK	ENSG00000111837	16951154;
D013196	increase	MAPK6	ENSG00000069956	16631469;
D013196	increase	ME1	ENSG00000065833	16631469;
D013196	increase	MEF2A	ENSG00000068305	16631469;
D013196	increase	MYBL2	ENSG00000101057	17254854;
D013196	increase	MYOD1	ENSG00000129152	17440010;
D013196	increase	MYOG	ENSG00000122180	17440010;
D013196	increase	NCOA1	ENSG00000084676	16648561;16877366;17804755;
D013196	increase	NCOA2	ENSG00000140396	16648561;15336702;16877366;
D013196	increase	NCOA3	ENSG00000124151	16877366;
D013196	increase	NKX3-1	ENSG00000167034	12711008;17131305;
D013196	increase	NUDT11	ENSG00000196368	16631469;
D013196	increase	PHB	ENSG00000167085	18487222;
D013196	increase	PHOSPHO2	ENSG00000144362	16631469;
D013196	increase	PIGF	ENSG00000151665	16631469;
D013196	increase	PIP	ENSG00000159763	10402478;
D013196	increase	PMEPA1	ENSG00000124225	12711008;
D013196	increase	PTGS2	ENSG00000073756	16317058;
D013196	increase	PTS	ENSG00000150787	16631469;
D013196	increase	SCGB2A1	ENSG00000124939	16020486;
D013196	increase	SELENBP1	ENSG00000143416	16631469;
D013196	increase	SERPINE1	ENSG00000106366	16317058;
D013196	increase	SMAD3	ENSG00000166949	16597033;
D013196	increase	SNAI1	ENSG00000124216	16541421;
D013196	increase	SNAI2	ENSG00000019549	16541421;
D013196	increase	SPA17	ENSG00000064199	16631469;
D013196	increase	SREBF1	ENSG00000072310	16541421;
D013196	increase	SREBF2	ENSG00000198911	16541421;
D013196	increase	STK39	ENSG00000198648	17131305;
D013196	increase	TLR4	ENSG00000136869	16317058;
D013196	increase	TNF	ENSG00000223952	16317058;11241555;
D013196	increase	TRAP1	ENSG00000126602	16631469;
D013196	increase	TRIOBP	ENSG00000100106	17254854;
D013196	increase	UFM1	ENSG00000120686	16631469;
D013196	increase	UGT2B15	ENSG00000196620	16690804;
D013196	increase	VCAM1	ENSG00000162692	16317058;
D013196	increase	ZBTB26	ENSG00000171448	16631469;
D013256	increase	CYP3A4	ENSG00000160868	15955870;
D013324	increase	SPP1	ENSG00000118785	16886220;
D013395	increase	INS	ENSG00000129965	1805284;
D013420	decrease	PHYH	ENSG00000107537	15342952;
D013431	increase	IL8	ENSG00000169429	18464055;
D013442	affect	NR3C1	ENSG00000113580	10219967;
D013442	increase	CYP2B6	ENSG00000197408	14977870;
D013442	increase	CYP3A4	ENSG00000160868	10219967;12065438;9512926;16837568;17954527;
D013449	increase	CCNB1	ENSG00000134057	15826604;
D013449	increase	NOTCH1	ENSG00000148400	19147571;
D013467	affect	AKT1	ENSG00000142208	18924134;
D013467	affect	CLU	ENSG00000120885	12388792;

D013467	affect	CSK	ENSG00000103653	18644992;
D013467	affect	GAL	ENSG00000069482	12388792;
D013467	affect	GAP43	ENSG00000172020	12388792;
D013467	affect	RET	ENSG00000165731	12388792;
D013467	affect	TIMP1	ENSG00000102265	12388792;
D013467	decrease	AKT2	ENSG00000105221	11906190;
D013467	decrease	ATP2A1	ENSG00000196296	11906190;
D013467	decrease	AURKA	ENSG00000087586	11906190;
D013467	decrease	BCL2	ENSG00000171791	14610217;15793875;
D013467	decrease	BCL2L1	ENSG00000171552	11062132;
D013467	decrease	BIRC5	ENSG00000089685	16707021;14610217;11906190;18156316;
D013467	decrease	BRCA1	ENSG00000012048	16273229;
D013467	decrease	BRD4	ENSG00000141867	11906190;
D013467	decrease	BZW2	ENSG00000136261	11093808;
D013467	decrease	C21ORF33	ENSG00000160221	11906190;
D013467	decrease	CA9	ENSG00000107159	18924134;
D013467	decrease	CALM3	ENSG00000143933	11906190;
D013467	decrease	CCNA2	ENSG00000145386	16488075;
D013467	decrease	CCNB1	ENSG00000134057	11906190;
D013467	decrease	CCNE1	ENSG00000105173	16488075;
D013467	decrease	CCNJ	ENSG00000107443	11906190;
D013467	decrease	CDK1	ENSG00000170312	16273229;
D013467	decrease	CDK3	ENSG00000250506	11906190;
D013467	decrease	CDK4	ENSG00000135446	18291362;17292881;
D013467	decrease	CDK6	ENSG00000105810	16488075;
D013467	decrease	CPNE1	ENSG00000214078	11906190;
D013467	decrease	CSNK2A1	ENSG00000101266	11906190;
D013467	decrease	CTNNB1	ENSG00000168036	18291362;11751639;
D013467	decrease	DCN	ENSG00000011465	11507085;
D013467	decrease	E2F1	ENSG00000101412	16488075;
D013467	decrease	EHMT2	ENSG00000204371	11906190;
D013467	decrease	ERCC5	ENSG00000134899	11906190;
D013467	decrease	HIF1A	ENSG00000100644	18924134;
D013467	decrease	HLCS	ENSG00000159267	11906190;
D013467	decrease	HMMR	ENSG00000072571	11906190;
D013467	decrease	ICAM1	ENSG00000090339	18059344;
D013467	decrease	ITGB4	ENSG00000132470	12628509;
D013467	decrease	LOX	ENSG00000113083	18924134;
D013467	decrease	NAP1L1	ENSG00000187109	11507085;
D013467	decrease	NAT1	ENSG00000171428	11828994;
D013467	decrease	NDST2	ENSG00000166507	11906190;
D013467	decrease	NFKBIA	ENSG00000100906	15489888;11591705;18059344;
D013467	decrease	ORC4L	ENSG00000115947	11906190;
D013467	decrease	PCDH17	ENSG00000118946	11906190;
D013467	decrease	PLAT	ENSG00000104368	12628509;
D013467	decrease	RB1	ENSG00000139687	16488075;
D013467	decrease	RNASE1	ENSG00000129538	11906190;
D013467	decrease	RNF26	ENSG00000173456	11906190;
D013467	decrease	SUMO3	ENSG00000184900	11906190;
D013467	decrease	TIMP3	ENSG00000100234	12628509;
D013467	decrease	TM7SF3	ENSG00000064115	11093808;
D013467	decrease	TMED9	ENSG00000184840	11906190;

D013467	decrease	TNS3	ENSG00000136205	11906190;
D013467	decrease	TRIT1	ENSG00000043514	11906190;
D013467	decrease	TSNAX	ENSG00000116918	11906190;
D013467	decrease	UNG	ENSG00000076248	11906190;
D013467	increase	ABCC1	ENSG00000103222	11820781;
D013467	increase	ABCC3	ENSG00000108846	11820781;
D013467	increase	ACP2	ENSG00000134575	11906190;
D013467	increase	AIFM2	ENSG00000042286	11906190;
D013467	increase	ALOX15	ENSG00000161905	10904086;11406566;
D013467	increase	ANKRD11	ENSG00000167522	11906190;
D013467	increase	APP	ENSG00000142192	15190207;
D013467	increase	ARGLU1	ENSG00000134884	11906190;
D013467	increase	ARRDC3	ENSG00000113369	11906190;
D013467	increase	ASNS	ENSG00000070669	11906190;
D013467	increase	ATF3	ENSG00000162772	11906190;
D013467	increase	ATF4	ENSG00000128272	11906190;
D013467	increase	BAX	ENSG00000087088	11062132;
D013467	increase	BBC3	ENSG00000105327	11906190;
D013467	increase	BCAP29	ENSG00000075790	11906190;
D013467	increase	C1ORF52	ENSG00000162642	11906190;
D013467	increase	CASP3	ENSG00000164305	15713900;
D013467	increase	CASP9	ENSG00000132906	15713900;
D013467	increase	CCL5	ENSG00000161570	16159934;
D013467	increase	CCND1	ENSG00000110092	18291362;15489888;11593435;17292881;
D013467	increase	CDC42EP1	ENSG00000128283	11906190;
D013467	increase	CDKN1A	ENSG00000124762	17292881;18644992;15190207;14566837;11507085;18291362;12628509;
D013467	increase	CNNM4	ENSG00000158158	11906190;
D013467	increase	CSNK2A2	ENSG00000070770	11906190;
D013467	increase	CTSB	ENSG00000164733	15190207;
D013467	increase	CYP1A1	ENSG00000140465	17985343;
D013467	increase	CYP1A2	ENSG00000140505	17985343;
D013467	increase	CYP1B1	ENSG00000138061	17985343;
D013467	increase	DDIT3	ENSG00000175197	12628509;
D013467	increase	DGCR14	ENSG00000100056	11906190;
D013467	increase	DPYS	ENSG00000147647	11906190;
D013467	increase	ETS1	ENSG00000134954	11906190;
D013467	increase	FBXO42	ENSG00000037637	11906190;
D013467	increase	FHL2	ENSG00000115641	11906190;
D013467	increase	FTH1	ENSG00000167996	11093808;
D013467	increase	GADD45A	ENSG00000116717	11906190;
D013467	increase	GBA	ENSG00000177628	15190207;
D013467	increase	GCLC	ENSG00000001084	11820781;15946948;
D013467	increase	GCNT1	ENSG00000187210	11093808;
D013467	increase	GDF15	ENSG00000130513	11906190;15180942;
D013467	increase	GRB10	ENSG00000106070	11906190;
D013467	increase	GRN	ENSG00000030582	15190207;
D013467	increase	GTF2B	ENSG00000137947	11906190;
D013467	increase	HERPUD1	ENSG00000051108	11093808;
D013467	increase	HIST2H4B	ENSG00000182217	11906190;
D013467	increase	IL1B	ENSG00000125538	11318944;16159934;10328874;12628509;

D013467	increase	IL24	ENSG00000162892	15713900;
D013467	increase	IL32	ENSG00000008517	15190207;
D013467	increase	INPP5E	ENSG00000148384	11906190;
D013467	increase	ITGA2	ENSG00000164171	11906190;
D013467	increase	ITGB3	ENSG00000056345	15190207;
D013467	increase	JUND	ENSG00000130522	11906190;
D013467	increase	KIAA1324L	ENSG00000164659	11906190;
D013467	increase	KIF14	ENSG00000118193	11906190;
D013467	increase	KLF5	ENSG00000102554	11906190;
D013467	increase	LAD1	ENSG00000159166	11507085;
D013467	increase	LARP4	ENSG00000161813	11906190;
D013467	increase	LDB1	ENSG00000198728	11906190;
D013467	increase	LFNG	ENSG00000106003	11906190;
D013467	increase	LGALS3BP	ENSG00000108679	15190207;
D013467	increase	LGALS3	ENSG00000131981	15190207;
D013467	increase	MAP4K3	ENSG00000011566	11906190;
D013467	increase	MAPK8	ENSG00000107643	17292881;15713900;
D013467	increase	MED15	ENSG00000099917	11906190;
D013467	increase	MIIP	ENSG00000116691	11906190;
D013467	increase	MTHFD2	ENSG00000065911	11906190;
D013467	increase	MYC	ENSG00000136997	18291362;17292881;12414619;
D013467	increase	NDRG1	ENSG00000104419	16273229;
D013467	increase	NEU1	ENSG00000227129	11906190;
D013467	increase	NFE2L1	ENSG00000082641	11906190;
D013467	increase	NFIL3	ENSG00000165030	11906190;
D013467	increase	NPC1	ENSG00000141458	11906190;
D013467	increase	NQO1	ENSG00000181019	17985343;11093808;
D013467	increase	OSTF1	ENSG00000134996	11906190;
D013467	increase	OTUD1	ENSG00000165312	11906190;
D013467	increase	PCK2	ENSG00000100889	11906190;
D013467	increase	PDLIM7	ENSG00000196923	11906190;
D013467	increase	PKD1	ENSG00000008710	11906190;
D013467	increase	PLEKHF1	ENSG00000166289	11906190;
D013467	increase	PLXNA3	ENSG00000130827	11906190;
D013467	increase	PML	ENSG00000140464	16273229;
D013467	increase	PRKCDBP	ENSG00000170955	11906190;
D013467	increase	PTGS2	ENSG00000073756	11820781;15793875;15489888;
D013467	increase	RELA	ENSG00000173039	18059344;10328874;15489888;16159934;16273229;
D013467	increase	SAA1	ENSG00000173432	15190207;
D013467	increase	SAT1	ENSG00000130066	16262603;14506281;
D013467	increase	SERPINB1	ENSG00000021355	11507085;
D013467	increase	SHC1	ENSG00000160691	15190207;
D013467	increase	SIRT1	ENSG00000096717	11906190;
D013467	increase	SLC16A6	ENSG00000108932	11906190;
D013467	increase	SLC30A1	ENSG00000170385	11906190;
D013467	increase	SLC38A2	ENSG00000134294	11906190;
D013467	increase	SLC3A2	ENSG00000168003	11906190;
D013467	increase	SLC7A1	ENSG00000139514	11906190;
D013467	increase	SNX21	ENSG00000124104	11906190;
D013467	increase	SNX25	ENSG00000109762	11906190;
D013467	increase	SOD2	ENSG00000112096	15190207;

D013467	increase	SQSTM1	ENSG00000161011	11906190;
D013467	increase	STC2	ENSG00000113739	11906190;
D013467	increase	STXBP2	ENSG00000076944	11906190;
D013467	increase	TBC1D10B	ENSG00000169221	11906190;
D013467	increase	TBK1	ENSG00000183735	11093808;
D013467	increase	TBX3	ENSG00000135111	11906190;
D013467	increase	TGM2	ENSG00000198959	15190207;
D013467	increase	TMEM127	ENSG00000135956	11906190;
D013467	increase	TMEM39A	ENSG00000176142	11906190;
D013467	increase	TNF	ENSG00000223952	10328874;15489888;16159934;
D013467	increase	TP53	ENSG00000141510	15713900;
D013467	increase	TRIM2	ENSG00000109654	11906190;
D013467	increase	UGGT2	ENSG00000102595	11906190;
D013467	increase	UGT1A1	ENSG00000241635	17985343;15843492;
D013467	increase	WDR3	ENSG00000065183	11906190;
D013467	increase	XBP1	ENSG00000100219	11906190;
D013467	increase	ZNF165	ENSG00000197279	11093808;
D013467	increase	ZNF354A	ENSG00000169131	11906190;
D013481	decrease	AR	ENSG00000169083	16928829;
D013496	increase	GCLC	ENSG00000001084	11909699;
D013496	increase	GCLM	ENSG00000023909	11909699;
D013498	increase	FGF2	ENSG00000138685	8394679;
D013498	increase	FGF5	ENSG00000138675	8394679;
D013498	increase	IL10	ENSG00000136634	15914193;
D013498	increase	IL12B	ENSG00000113302	15914193;
D013498	increase	TNF	ENSG00000223952	15914193;
D013498	increase	VDR	ENSG00000111424	15930183;
D013605	decrease	ACAN	ENSG00000157766	18196609;16624732;
D013605	decrease	CD44	ENSG00000026508	18196609;
D013605	decrease	HAS2	ENSG00000170961	18196609;
D013605	increase	ADAMTS5	ENSG00000154736	18196609;
D013605	increase	BAX	ENSG00000087088	16307838;19541794;
D013605	increase	IL1B	ENSG00000125538	18196609;
D013605	increase	TNF	ENSG00000223952	18196609;
D013629	affect	ABLIM3	ENSG00000173210	14699072;
D013629	affect	ACSL1	ENSG00000151726	14699072;
D013629	affect	ADAM9	ENSG00000168615	14699072;
D013629	affect	AGT	ENSG00000135744	14699072;
D013629	affect	ALCAM	ENSG00000170017	14699072;
D013629	affect	ANGPTL2	ENSG00000136859	14699072;
D013629	affect	AP1G1	ENSG00000166747	14699072;
D013629	affect	ARHGAP29	ENSG00000137962	14699072;
D013629	affect	ARID5B	ENSG00000150347	14699072;
D013629	affect	ATP9A	ENSG00000054793	14699072;
D013629	affect	AZIN1	ENSG00000155096	14699072;
D013629	affect	BACH1	ENSG00000156273	14699072;
D013629	affect	BAZ1A	ENSG00000198604	14699072;
D013629	affect	BDNF	ENSG00000176697	14699072;
D013629	affect	BMP2K	ENSG00000138756	14699072;
D013629	affect	BMP5	ENSG00000112175	14699072;
D013629	affect	BMP7	ENSG00000101144	14699072;
D013629	affect	BTF3	ENSG00000145741	14699072;
D013629	affect	C18ORF1	ENSG00000168675	14699072;

D013629	affect	C2CD2	ENSG00000157617	14699072;
D013629	affect	CA12	ENSG00000074410	14699072;
D013629	affect	CAP2	ENSG00000112186	14699072;
D013629	affect	CAPN2	ENSG00000162909	14699072;
D013629	affect	CASP7	ENSG00000165806	14699072;
D013629	affect	CAV1	ENSG00000105974	14699072;
D013629	affect	CCDC86	ENSG00000110104	14699072;
D013629	affect	CD44	ENSG00000026508	14699072;
D013629	affect	CDH11	ENSG00000140937	14699072;
D013629	affect	CDH2	ENSG00000170558	14699072;
D013629	affect	CDK1	ENSG00000170312	14699072;
D013629	affect	CEBPB	ENSG00000172216	14699072;
D013629	affect	CHRD	ENSG00000090539	14699072;
D013629	affect	CLTB	ENSG00000175416	14699072;
D013629	affect	COL11A1	ENSG00000060718	14699072;
D013629	affect	COL3A1	ENSG00000168542	14699072;
D013629	affect	COL6A3	ENSG00000163359	14699072;
D013629	affect	CPE	ENSG00000109472	14699072;
D013629	affect	CSTB	ENSG00000160213	14699072;
D013629	affect	CXCR7	ENSG00000144476	14699072;
D013629	affect	CYP26A1	ENSG00000095596	14699072;
D013629	affect	DAXX	ENSG00000231617	14699072;
D013629	affect	DDIT4	ENSG00000168209	14699072;
D013629	affect	DDX21	ENSG00000165732	14699072;
D013629	affect	DNMBP	ENSG00000107554	14699072;
D013629	affect	DRD5	ENSG00000169676	14699072;
D013629	affect	DUSP1	ENSG00000120129	14699072;
D013629	affect	EDIL3	ENSG00000164176	14699072;
D013629	affect	EFEMP1	ENSG00000115380	14699072;
D013629	affect	EIF2C2	ENSG00000123908	14699072;
D013629	affect	EMP1	ENSG00000134531	14699072;
D013629	affect	ENC1	ENSG00000171617	14699072;
D013629	affect	EPB41L2	ENSG00000079819	14699072;
D013629	affect	EPHX1	ENSG00000143819	14699072;
D013629	affect	F13A1	ENSG00000124491	14699072;
D013629	affect	F2R	ENSG00000181104	14699072;
D013629	affect	F3	ENSG00000117525	14699072;
D013629	affect	FAP	ENSG00000078098	14699072;
D013629	affect	FHL2	ENSG00000115641	14699072;15666801;
D013629	affect	FLNB	ENSG00000136068	14699072;
D013629	affect	FLNC	ENSG00000128591	14699072;
D013629	affect	FLOT1	ENSG00000137312	14699072;
D013629	affect	FLRT1	ENSG00000126500	14699072;
D013629	affect	FMNL1	ENSG00000184922	14699072;
D013629	affect	FN1	ENSG00000115414	14699072;
D013629	affect	FOXC1	ENSG00000054598	14699072;
D013629	affect	FOXO1	ENSG00000150907	14699072;
D013629	affect	FZD6	ENSG00000164930	14699072;
D013629	affect	G0S2	ENSG00000123689	14699072;
D013629	affect	G6PD	ENSG00000160211	14699072;
D013629	affect	GALT	ENSG00000213930	14699072;
D013629	affect	GAS1	ENSG00000180447	14699072;
D013629	affect	GATA2	ENSG00000179348	14699072;
D013629	affect	GJA1	ENSG00000152661	14699072;

D013629	affect	GLRB	ENSG00000109738	14699072;
D013629	affect	GLUD2	ENSG00000182890	14699072;
D013629	affect	GLYAT	ENSG00000149124	14699072;
D013629	affect	GPC1	ENSG00000063660	14699072;
D013629	affect	GPR56	ENSG00000205336	14699072;
D013629	affect	GRK5	ENSG00000198873	14699072;
D013629	affect	H1F0	ENSG00000189060	14699072;
D013629	affect	HAPLN1	ENSG00000145681	14699072;
D013629	affect	HEG1	ENSG00000173706	14699072;
D013629	affect	HRK	ENSG00000135116	14699072;
D013629	affect	HTR2A	ENSG00000102468	14699072;
D013629	affect	HTR3A	ENSG00000166736	14699072;
D013629	affect	ID1	ENSG00000125968	14699072;
D013629	affect	IDH2	ENSG00000182054	14699072;
D013629	affect	IER3	ENSG00000137331	14699072;
D013629	affect	IFIT2	ENSG00000119922	14699072;
D013629	affect	IGFBP4	ENSG00000141753	14699072;
D013629	affect	IGFBP5	ENSG00000115461	14699072;
D013629	affect	IL4R	ENSG00000077238	14699072;
D013629	affect	IRS1	ENSG00000169047	14699072;
D013629	affect	ISG20	ENSG00000172183	14699072;
D013629	affect	ITM2B	ENSG00000136156	14699072;
D013629	affect	ITPR3	ENSG00000096433	14699072;
D013629	affect	KCNJ8	ENSG00000121361	14699072;
D013629	affect	KCNN4	ENSG00000104783	14699072;
D013629	affect	KIAA1279	ENSG00000198954	14699072;
D013629	affect	KIF5B	ENSG00000170759	14699072;
D013629	affect	KLKB1	ENSG00000164344	14699072;
D013629	affect	KLRC4	ENSG00000183542	14699072;
D013629	affect	LAMA5	ENSG00000130702	14699072;
D013629	affect	LAMC1	ENSG00000135862	14699072;
D013629	affect	LASS6	ENSG00000172292	14699072;
D013629	affect	LCN2	ENSG00000148346	14699072;
D013629	affect	LGALS3	ENSG00000131981	14699072;
D013629	affect	LGR5	ENSG00000139292	14699072;
D013629	affect	LMNB2	ENSG00000176619	14699072;
D013629	affect	LPL	ENSG00000175445	14699072;
D013629	affect	LRIG1	ENSG00000144749	14699072;
D013629	affect	LRRFIP1	ENSG00000124831	14699072;
D013629	affect	LTBP1	ENSG00000049323	14699072;
D013629	affect	LTBP2	ENSG00000119681	14699072;
D013629	affect	MAF	ENSG00000178573	14699072;
D013629	affect	MAFF	ENSG00000185022	14699072;
D013629	affect	MAGEA8	ENSG00000156009	14699072;
D013629	affect	MAN2B2	ENSG00000013288	14699072;
D013629	affect	MAPK9	ENSG00000050748	14699072;
D013629	affect	MARCKSL1	ENSG00000175130	14699072;
D013629	affect	MDFIC	ENSG00000135272	14699072;
D013629	affect	MMP9	ENSG00000100985	14699072;
D013629	affect	MORF4L2	ENSG00000123562	14699072;
D013629	affect	MRPL33	ENSG00000243147	14699072;
D013629	affect	NAB1	ENSG00000138386	14699072;
D013629	affect	NCF2	ENSG00000116701	14699072;
D013629	affect	NCOA4	ENSG00000138293	14699072;

D013629	affect	NEFH	ENSG00000100285	14699072;
D013629	affect	NEK7	ENSG00000151414	14699072;
D013629	affect	NFIL3	ENSG00000165030	14699072;
D013629	affect	NNMT	ENSG00000166741	14699072;
D013629	affect	NPR2	ENSG00000159899	14699072;
D013629	affect	NR1D2	ENSG00000174738	14699072;
D013629	affect	OPTN	ENSG00000123240	14699072;
D013629	affect	OSMR	ENSG00000145623	14699072;
D013629	affect	PALLD	ENSG00000129116	14699072;
D013629	affect	PCBP1	ENSG00000169564	14699072;
D013629	affect	PDE4B	ENSG00000184588	14699072;
D013629	affect	PF4V1	ENSG00000109272	14699072;
D013629	affect	PLEC	ENSG00000178209	14699072;
D013629	affect	PLSCR1	ENSG00000188313	14699072;
D013629	affect	PNP	ENSG00000198805	14699072;
D013629	affect	PODXL	ENSG00000128567	14699072;
D013629	affect	PPP1R12B	ENSG00000248526	14699072;
D013629	affect	PPP1R2	ENSG00000184203	14699072;
D013629	affect	PRDX3	ENSG00000165672	14699072;
D013629	affect	PROS1	ENSG00000184500	15850603;
D013629	affect	PRPF40A	ENSG00000196504	14699072;
D013629	affect	PTHLH	ENSG00000087494	14699072;
D013629	affect	PTN	ENSG00000105894	14699072;
D013629	affect	PTPN4	ENSG00000088179	14699072;
D013629	affect	PTPRE	ENSG00000132334	14699072;
D013629	affect	PTPRH	ENSG00000080031	14699072;
D013629	affect	QKI	ENSG00000112531	14699072;
D013629	affect	RAB31	ENSG00000168461	14699072;
D013629	affect	RAB5A	ENSG00000144566	14699072;
D013629	affect	RASA1	ENSG00000145715	14699072;
D013629	affect	RASGRF1	ENSG00000058335	14699072;
D013629	affect	RBPMS	ENSG00000157110	14699072;
D013629	affect	RCOR1	ENSG00000089902	14699072;
D013629	affect	RFK	ENSG00000250683	14699072;
D013629	affect	RFTN1	ENSG00000131378	14699072;
D013629	affect	RGS10	ENSG00000148908	14699072;
D013629	affect	RGS13	ENSG00000127074	14699072;
D013629	affect	RGS4	ENSG00000117152	14699072;
D013629	affect	RHOBTB3	ENSG00000164292	14699072;
D013629	affect	RHOQ	ENSG00000119729	14699072;
D013629	affect	RYR1	ENSG00000196218	14699072;
D013629	affect	SALL1	ENSG00000103449	14699072;
D013629	affect	SDCBP	ENSG00000137575	14699072;
D013629	affect	SEMA3C	ENSG00000075223	14699072;
D013629	affect	SFRS2IP	ENSG00000139218	14699072;
D013629	affect	SHB	ENSG00000107338	14699072;
D013629	affect	SMAD7	ENSG00000101665	14699072;
D013629	affect	SNCA	ENSG00000145335	14699072;
D013629	affect	SOSTDC1	ENSG00000171243	14699072;
D013629	affect	SOX9	ENSG00000125398	14699072;
D013629	affect	SPP1	ENSG00000118785	14699072;
D013629	affect	STARD8	ENSG00000130052	14699072;
D013629	affect	STK24	ENSG00000102572	14699072;
D013629	affect	TACC1	ENSG00000147526	14699072;

D013629	affect	TGFA	ENSG00000163235	14699072;
D013629	affect	TGFB2	ENSG00000092969	14699072;
D013629	affect	TGM2	ENSG00000198959	14699072;
D013629	affect	THBS1	ENSG00000137801	14699072;
D013629	affect	TM4SF1	ENSG00000169908	14699072;
D013629	affect	TMEM158	ENSG00000249992	14699072;
D013629	affect	TMOD1	ENSG00000136842	14699072;
D013629	affect	TOB1	ENSG00000141232	14699072;
D013629	affect	TP53I11	ENSG00000175274	14699072;
D013629	affect	TPM1	ENSG00000140416	14699072;
D013629	affect	TPST2	ENSG00000128294	14699072;
D013629	affect	TRIB2	ENSG00000071575	14699072;
D013629	affect	TTC35	ENSG00000104412	14699072;
D013629	affect	UBR5	ENSG00000104517	14699072;
D013629	affect	UPF1	ENSG00000005007	14699072;
D013629	affect	WDR1	ENSG00000071127	14699072;
D013629	affect	ZFP36	ENSG00000128016	14699072;
D013629	affect	ZMIZ1	ENSG00000108175	14699072;
D013629	decrease	ABCB1	ENSG00000085563	16925584;
D013629	decrease	ABCG2	ENSG00000118777	15695404;
D013629	decrease	AIPL1	ENSG00000129221	15849065;
D013629	decrease	AP1S1	ENSG00000106367	17567588;
D013629	decrease	APC2	ENSG00000115266	15590111;
D013629	decrease	CALR	ENSG00000179218	16202921;
D013629	decrease	CCL20	ENSG00000115009	16298037;
D013629	decrease	CD6	ENSG00000013725	15590111;
D013629	decrease	CRYM	ENSG00000103316	15590111;
D013629	decrease	CSF3	ENSG00000108342	16298037;
D013629	decrease	CTRL	ENSG00000141086	15590111;
D013629	decrease	CXCL3	ENSG00000163734	16298037;
D013629	decrease	CYP27B1	ENSG00000111012	15590111;
D013629	decrease	DHX38	ENSG00000140829	15590111;
D013629	decrease	DKK4	ENSG00000104371	15590111;
D013629	decrease	E2F1	ENSG00000101412	15590111;
D013629	decrease	FST	ENSG00000134363	14699072;16298037;
D013629	decrease	FUS	ENSG00000089280	16202921;
D013629	decrease	GAL	ENSG00000069482	15590111;
D013629	decrease	GLUD1	ENSG00000148672	17098229;
D013629	decrease	GYPE	ENSG00000197465	15590111;
D013629	decrease	HCRTR2	ENSG00000137252	15590111;
D013629	decrease	HMBS	ENSG00000149397	15849065;
D013629	decrease	HMOX1	ENSG00000100292	16298037;
D013629	decrease	HNRNPAB	ENSG00000197451	17098229;
D013629	decrease	HNRNPH3	ENSG00000096746	17098229;
D013629	decrease	HOMER2	ENSG00000103942	14699072;15590111;
D013629	decrease	HTR7	ENSG00000148680	15590111;
D013629	decrease	IFI6	ENSG00000126709	16202921;
D013629	decrease	IGF1	ENSG00000017427	15809018;16847815;
D013629	decrease	IL1B	ENSG00000125538	15180920;16298037;
D013629	decrease	IL24	ENSG00000162892	16298037;
D013629	decrease	IL4	ENSG00000113520	15590111;
D013629	decrease	IL6	ENSG00000136244	16298037;
D013629	decrease	IL8	ENSG00000169429	16298037;
D013629	decrease	IVL	ENSG00000163207	14699072;15590111;

D013629	decrease	KCNA5	ENSG00000130037	15590111;
D013629	decrease	KRT86	ENSG00000170442	15590111;
D013629	decrease	MAPK11	ENSG00000185386	15590111;
D013629	decrease	MAT1A	ENSG00000151224	15590111;
D013629	decrease	MC2R	ENSG00000185231	15590111;
D013629	decrease	MKI67	ENSG00000148773	18855134;15767642;16002280;
D013629	decrease	MLANA	ENSG00000120215	15590111;
D013629	decrease	MTHFR	ENSG00000177000	15849065;
D013629	decrease	NANS	ENSG00000095380	17098229;
D013629	decrease	NFIX	ENSG00000008441	15849065;
D013629	decrease	NFKBIA	ENSG00000100906	14699072;15489888;16298037;
D013629	decrease	NOVA1	ENSG00000248163	15590111;
D013629	decrease	PGC	ENSG00000096088	15590111;
D013629	decrease	POU6F1	ENSG00000184271	15590111;
D013629	decrease	PPIA	ENSG00000196262	17098229;
D013629	decrease	PPP2CA	ENSG00000113575	15590111;
D013629	decrease	PROC	ENSG00000115718	15850603;
D013629	decrease	PRPS1L1	ENSG00000229937	15590111;
D013629	decrease	PTK2B	ENSG00000120899	15590111;
D013629	decrease	RGS9	ENSG00000108370	15590111;
D013629	decrease	RND2	ENSG00000108830	15590111;
D013629	decrease	SERPINB2	ENSG00000197632	16298037;
D013629	decrease	SERPINE1	ENSG00000106366	15850603;
D013629	decrease	SF3A2	ENSG00000104897	15590111;
D013629	decrease	SLC2A3	ENSG00000059804	14699072;17567588;15342952;
D013629	decrease	STX18	ENSG00000168818	15590111;
D013629	decrease	TAGLN	ENSG00000149591	17567588;15342952;
D013629	decrease	TFF3	ENSG00000160180	16202921;
D013629	decrease	TFPI	ENSG00000003436	17029634;
D013629	decrease	UPK3A	ENSG00000100373	15590111;
D013629	decrease	WAS	ENSG00000015285	15590111;
D013629	decrease	WNT10B	ENSG00000169884	15590111;
D013629	decrease	ZBTB22	ENSG00000237056	15590111;
D013629	increase	ABCC11	ENSG00000121270	18310281;
D013629	increase	APBA3	ENSG00000011132	15590111;
D013629	increase	APEX2	ENSG00000169188	15590111;
D013629	increase	AREG	ENSG00000205595	16202921;
D013629	increase	ASAH1	ENSG00000104763	17567588;15342952;
D013629	increase	ASNS	ENSG00000070669	17567588;
D013629	increase	ATP6V0A1	ENSG00000033627	15590111;
D013629	increase	BARD1	ENSG00000138376	15590111;
D013629	increase	BCL2	ENSG00000171791	16051634;
D013629	increase	BIK	ENSG00000100290	17638070;
D013629	increase	BRPF1	ENSG00000156983	15590111;
D013629	increase	C10ORF10	ENSG00000165507	16298037;15342952;
D013629	increase	CCND1	ENSG00000110092	14699072;15489888;16202921;18855134;
D013629	increase	CDKN1A	ENSG00000124762	12866041;
D013629	increase	CECR6	ENSG00000183307	17962382;
D013629	increase	COL11A2	ENSG00000235708	15590111;
D013629	increase	CSPG4	ENSG00000173546	15590111;

D013629	increase	DHFR	ENSG00000228716	15590111;
D013629	increase	DMBT1	ENSG00000187908	15564322;
D013629	increase	DST	ENSG00000151914	16202921;
D013629	increase	EEF1A1	ENSG00000156508	17098229;
D013629	increase	ESR1	ENSG00000091831	14699072;17395694;16051668;11162928;15521089;15842231;11518614;12765246;15026083;15950373;15666801;16328721;15840436;12676605;15713566;12377985;14975756;17513867;15135306;15103026;17962382;16010412;19351862;18855[illegible]
D013629	increase	ESR2	ENSG00000140009	14699072;17638070;16010412;15821116;11518614;15950373;12377985;15135306;16202921;16739346;
D013629	increase	F8	ENSG00000185010	15850603;
D013629	increase	F9	ENSG00000101981	15850603;
D013629	increase	FABP1	ENSG00000163586	17567588;15342952;
D013629	increase	FICD	ENSG00000198855	15590111;
D013629	increase	FNDC4	ENSG00000115226	15342952;
D013629	increase	FOXN3	ENSG00000053254	15849065;
D013629	increase	GDPD3	ENSG00000102886	17567588;15342952;
D013629	increase	GPSM2	ENSG00000121957	15590111;
D013629	increase	HIP1	ENSG00000127946	15590111;
D013629	increase	HPN	ENSG00000105707	15342952;
D013629	increase	IGFBP1	ENSG00000146678	16847815;
D013629	increase	IGFBP3	ENSG00000146674	16847815;
D013629	increase	INHBE	ENSG00000139269	17567588;
D013629	increase	KCNH1	ENSG00000143473	19351862;
D013629	increase	KIF23	ENSG00000137807	15590111;
D013629	increase	KLRC1	ENSG00000134545	17962382;
D013629	increase	KLRC3	ENSG00000205810	14699072;17395694;
D013629	increase	LEF1	ENSG00000138795	15590111;
D013629	increase	LSS	ENSG00000160285	15590111;17567588;15342952;
D013629	increase	MAP1A	ENSG00000166963	15590111;
D013629	increase	MAP2K4	ENSG00000065559	15590111;
D013629	increase	MBP	ENSG00000197971	14645110;
D013629	increase	MCM6	ENSG00000076003	17098229;
D013629	increase	MTMR2	ENSG00000087053	15849065;
D013629	increase	MYCBP	ENSG00000214114	15590111;
D013629	increase	MYC	ENSG00000136997	14699072;16051634;
D013629	increase	MYH10	ENSG00000133026	16901965;
D013629	increase	MYO5A	ENSG00000197535	17098229;
D013629	increase	NCOA2	ENSG00000140396	17395694;16739346;
D013629	increase	NKD1	ENSG00000140807	17962382;
D013629	increase	NR0B2	ENSG00000131910	15342952;
D013629	increase	NUPR1	ENSG00000176046	17567588;
D013629	increase	PARG	ENSG00000227345	15590111;
D013629	increase	PGR	ENSG00000082175	15832264;16000581;16002280;16739346;18855134;
D013629	increase	PIM1	ENSG00000137193	16298037;
D013629	increase	POLR2J	ENSG00000005075	15849065;

D013629	increase	PRPF4B	ENSG00000112739	15590111;
D013629	increase	PSMA1	ENSG00000129084	17098229;
D013629	increase	PTGS2	ENSG00000073756	16127422;15489888;
D013629	increase	RPS15	ENSG00000115268	15590111;
D013629	increase	RRP9	ENSG00000114767	15590111;
D013629	increase	RUNX1	ENSG00000159216	15590111;
D013629	increase	RUNX1T1	ENSG00000079102	16202921;
D013629	increase	SERPINA3	ENSG00000196136	15342952;
D013629	increase	SERPINB9	ENSG00000170542	10681578;
D013629	increase	SFPQ	ENSG00000116560	17098229;
D013629	increase	SLC4A8	ENSG00000050438	17962382;
D013629	increase	SPATA13	ENSG00000182957	17962382;
D013629	increase	SPATA2	ENSG00000158480	15590111;
D013629	increase	STAB1	ENSG00000010327	15849065;
D013629	increase	SYNGR4	ENSG00000105467	15590111;
D013629	increase	TAF4	ENSG00000130699	15590111;
D013629	increase	TERT	ENSG00000164362	16051634;18021753;
D013629	increase	TFDP2	ENSG00000114126	15590111;
D013629	increase	TFF1	ENSG00000160182	18310281;
D013629	increase	TFF2	ENSG00000160181	15590111;
D013629	increase	TIE1	ENSG00000066056	15590111;
D013629	increase	TNF	ENSG00000223952	15489888;
D013629	increase	TNFRSF11B	ENSG00000164761	17420779;
D013629	increase	TNR	ENSG00000116147	15590111;
D013629	increase	VWF	ENSG00000110799	15850603;
D013629	increase	WIPI1	ENSG00000070540	15342952;
D013629	increase	XRCC3	ENSG00000126215	15590111;
D013654	decrease	ADORA2A	ENSG00000128271	16579726;
D013654	decrease	BID	ENSG00000015475	16579726;
D013654	decrease	BMPR2	ENSG00000204217	16579726;
D013654	decrease	CHN1	ENSG00000128656	16579726;
D013654	decrease	CXCR3	ENSG00000186810	16579726;
D013654	decrease	DDAH1	ENSG00000250352	16579726;
D013654	decrease	FGFR1	ENSG00000249195	16579726;
D013654	decrease	GRM7	ENSG00000196277	16579726;
D013654	decrease	GUCY1A3	ENSG00000164116	16579726;
D013654	decrease	HMGB1	ENSG00000189403	16579726;
D013654	decrease	IGFBP3	ENSG00000146674	16579726;
D013654	decrease	LYN	ENSG00000249529	16579726;
D013654	decrease	MS4A6A	ENSG00000110077	16579726;
D013654	decrease	OR51E2	ENSG00000167332	16579726;
D013654	decrease	PAG1	ENSG00000076641	16579726;
D013654	decrease	PAK4	ENSG00000130669	16579726;
D013654	decrease	PPP2R5E	ENSG00000154001	16579726;
D013654	decrease	PRKAR2B	ENSG00000005249	16579726;
D013654	decrease	PSEN2	ENSG00000143801	16579726;
D013654	decrease	PTPRK	ENSG00000152894	16579726;
D013654	decrease	RAB5A	ENSG00000144566	16579726;
D013654	decrease	RAPGEF2	ENSG00000109756	16579726;
D013654	decrease	SRP54	ENSG00000100883	16579726;
D013654	decrease	STAT3	ENSG00000168610	16579726;
D013654	decrease	TACR1	ENSG00000115353	16579726;
D013654	decrease	TP53I3	ENSG00000115129	16579726;
D013654	decrease	TYK2	ENSG00000105397	16579726;

D013654	decrease	VCP	ENSG00000165280	16579726;
D013654	increase	ABL2	ENSG00000143322	16579726;
D013654	increase	ADAP2	ENSG00000184060	16579726;
D013654	increase	ADRA1B	ENSG00000170214	16579726;
D013654	increase	CAP2	ENSG00000112186	16579726;
D013654	increase	CGA	ENSG00000135346	16579726;
D013654	increase	COPS2	ENSG00000166200	16579726;
D013654	increase	DGKZ	ENSG00000149091	16579726;
D013654	increase	EI24	ENSG00000149547	16579726;
D013654	increase	KRIT1	ENSG00000001631	16579726;
D013654	increase	LANCL1	ENSG00000115365	16579726;
D013654	increase	POLB	ENSG00000070501	16579726;
D013654	increase	POLD1	ENSG00000062822	16579726;
D013654	increase	POLM	ENSG00000122678	16579726;
D013654	increase	PPP1R16B	ENSG00000101445	16579726;
D013654	increase	RAD51C	ENSG00000108384	16579726;
D013654	increase	RAD9A	ENSG00000172613	16579726;
D013654	increase	RASGRP1	ENSG00000172575	16579726;
D013654	increase	RPS6KA2	ENSG00000071242	16579726;
D013654	increase	SCG5	ENSG00000166922	16579726;
D013654	increase	SMC3	ENSG00000108055	16579726;
D013654	increase	SNAPC5	ENSG00000174446	16579726;
D013654	increase	SPRY2	ENSG00000136158	16579726;
D013654	increase	TEK	ENSG00000120156	16579726;
D013654	increase	XRCC4	ENSG00000152422	16579726;
D013713	decrease	MYC	ENSG00000136997	11585056;
D013713	decrease	ODC1	ENSG00000115758	11585056;
D013726	affect	MT2A	ENSG00000125148	9973176;
D013726	decrease	CRH	ENSG00000147571	17127497;
D013726	increase	ADRA1B	ENSG00000170214	10229127;
D013726	increase	ADRA1D	ENSG00000171873	10229127;
D013726	increase	ADRB2	ENSG00000169252	16395068;11429395;16153394;9258252;11675405;9277405;12920204;17292646;16648137;16024912;10516654;10952688;11753577;12826277;1280742;16980553;1
D013726	increase	IL10	ENSG00000136634	9258252;12417450;
D013726	increase	IL1B	ENSG00000125538	9258252;
D013726	increase	IL8	ENSG00000169429	9258252;16384605;
D013726	increase	MAPK1	ENSG00000100030	11675405;12826277;16980553;
D013726	increase	MAPK3	ENSG00000102882	11675405;12826277;16980553;
D013726	increase	NR3C1	ENSG00000113580	9973176;
D013726	increase	REN	ENSG00000143839	9256163;9203624;
D013726	increase	SFTPB	ENSG00000168878	2539393;
D013726	increase	TNF	ENSG00000223952	11675405;9256163;
D013739	affect	INSR	ENSG00000171105	17162844;
D013739	decrease	ADIPOQ	ENSG00000181092	16793964;
D013739	decrease	BAX	ENSG00000087088	18030663;
D013739	decrease	CASP3	ENSG00000164305	18030663;
D013739	decrease	CASP9	ENSG00000132906	18030663;
D013739	decrease	CYP17A1	ENSG00000148795	15625562;
D013739	decrease	HOXA10	ENSG00000078399	16632680;

[truncated: 710,829 more chars]
