# Supplementary material for: Preferential regulation of miRNA targets by environmental chemicals in the human genome
Source: BMC Genomics. 2011 May 18;12:244. doi: 10.1186/1471-2164-12-244 (PMC3118786; doi:10.1186/1471-2164-12-244)
Supplement: Additional file 2 — Table S2: The list of miRNA targets predicted by PicTar, TargetScan5.1, both programs of PicTar and TargetScan5.1 (intersections), and by PITA. [file 1471-2164-12-244-S2.PDF]

PicTar	TargetScan	Intersections	PITA
ENSG00000109819	ENSG00000109819	ENSG00000109819	ENSG00000109819
ENSG00000152778	ENSG00000152778	ENSG00000152778	ENSG00000152778
ENSG00000069011	ENSG00000182575	ENSG00000069011	ENSG00000161057
ENSG00000197969	ENSG00000138443	ENSG00000182575	ENSG00000197969
ENSG00000182575	ENSG00000188906	ENSG00000160013	ENSG00000196409
ENSG00000160013	ENSG00000124151	ENSG00000124151	ENSG00000182575
ENSG00000135750	ENSG00000127329	ENSG00000135750	ENSG00000138443
ENSG00000124151	ENSG00000095637	ENSG00000127329	ENSG00000051596
ENSG00000127329	ENSG00000189241	ENSG00000095637	ENSG00000188906
ENSG00000095637	ENSG00000109103	ENSG00000109103	ENSG00000124151
ENSG00000163449	ENSG00000100796	ENSG00000006283	ENSG00000127329
ENSG00000109103	ENSG00000135541	ENSG00000100796	ENSG00000095637
ENSG00000136925	ENSG00000105701	ENSG00000105701	ENSG00000189241
ENSG00000006283	ENSG00000143376	ENSG00000120925	ENSG00000182511
ENSG00000100796	ENSG00000048052	ENSG00000143376	ENSG00000109103
ENSG00000105701	ENSG00000163602	ENSG00000204962	ENSG00000100450
ENSG00000120925	ENSG00000062282	ENSG00000048052	ENSG00000135541
ENSG00000143376	ENSG00000196155	ENSG00000163602	ENSG00000249948
ENSG00000204962	ENSG00000142871	ENSG00000141367	ENSG00000105701
ENSG00000163602	ENSG00000157216	ENSG00000142871	ENSG00000143376
ENSG00000048052	ENSG00000006468	ENSG00000157216	ENSG00000163602
ENSG00000141367	ENSG00000175130	ENSG00000182450	ENSG00000048052
ENSG00000142871	ENSG00000142539	ENSG00000006468	ENSG00000062282
ENSG00000157216	ENSG00000165240	ENSG00000142539	ENSG00000196155
ENSG00000182450	ENSG00000114654	ENSG00000172020	ENSG00000142871
ENSG00000006468	ENSG00000172020	ENSG00000186432	ENSG00000157216
ENSG00000142539	ENSG00000115486	ENSG00000143507	ENSG00000006468
ENSG00000172020	ENSG00000196458	ENSG00000122786	ENSG00000175130
ENSG00000186432	ENSG00000029639	ENSG00000166405	ENSG00000182368
ENSG00000124562	ENSG00000132604	ENSG00000132604	ENSG00000165240
ENSG00000143507	ENSG00000139218	ENSG00000139218	ENSG00000142539
ENSG00000136542	ENSG00000111218	ENSG00000111218	ENSG00000114654
ENSG00000122786	ENSG00000162676	ENSG00000107954	ENSG00000172020
ENSG00000166405	ENSG00000131724	ENSG00000131437	ENSG00000115486
ENSG00000139218	ENSG00000183117	ENSG00000177370	ENSG00000163870
ENSG00000132604	ENSG00000168564	ENSG00000162676	ENSG00000196458
ENSG00000107954	ENSG00000005884	ENSG00000131724	ENSG00000029639
ENSG00000133135	ENSG00000128595	ENSG00000183117	ENSG00000139218
ENSG00000111218	ENSG00000086102	ENSG00000184408	ENSG00000132604
ENSG00000131437	ENSG00000136531	ENSG00000168564	ENSG00000178372
ENSG00000177370	ENSG00000140455	ENSG00000005884	ENSG00000117477
ENSG00000142185	ENSG00000175220	ENSG00000128595	ENSG00000133135
ENSG00000131724	ENSG00000186479	ENSG00000136531	ENSG00000111218
ENSG00000162676	ENSG00000168283	ENSG00000140455	ENSG00000142185
ENSG00000122390	ENSG00000120029	ENSG00000175220	ENSG00000131724
ENSG00000183117	ENSG00000034510	ENSG00000168283	ENSG00000162676
ENSG00000184408	ENSG00000136161	ENSG00000159267	ENSG00000183117
ENSG00000168564	ENSG00000141956	ENSG00000101126	ENSG00000164794
ENSG00000101194	ENSG00000153922	ENSG00000117394	ENSG00000149483
ENSG00000005884	ENSG00000170561	ENSG00000124333	ENSG00000112812
ENSG00000128595	ENSG00000120332	ENSG00000085741	ENSG00000005884
ENSG00000152939	ENSG00000145103	ENSG00000120029	ENSG00000128595
ENSG00000136531	ENSG00000102805	ENSG00000034510	ENSG00000086102
ENSG00000140455	ENSG00000090539	ENSG00000141956	ENSG00000140455
ENSG00000175220	ENSG00000081052	ENSG00000183242	ENSG00000183617
ENSG00000159267	ENSG00000119314	ENSG00000008197	ENSG00000136159
ENSG00000168283	ENSG00000134757	ENSG00000170561	ENSG00000175220
ENSG00000101126	ENSG00000100379	ENSG00000169967	ENSG00000112981
ENSG00000117394	ENSG00000196652	ENSG00000064490	ENSG00000136161

ENSG00000124333	ENSG00000008283	ENSG00000141298	ENSG00000034510
ENSG00000085741	ENSG00000108468	ENSG00000144119	ENSG00000120029
ENSG00000120029	ENSG00000164114	ENSG00000165566	ENSG00000141956
ENSG00000034510	ENSG00000050628	ENSG00000168259	ENSG00000187608
ENSG00000141956	ENSG00000158109	ENSG00000099864	ENSG00000153922
ENSG00000183242	ENSG00000054523	ENSG00000046647	ENSG00000170561
ENSG00000008197	ENSG00000151136	ENSG00000090539	ENSG00000120332
ENSG00000152932	ENSG00000136044	ENSG00000049245	ENSG00000100652
ENSG00000170561	ENSG00000163898	ENSG00000081052	ENSG00000075673
ENSG00000169967	ENSG00000102057	ENSG00000119314	ENSG00000167536
ENSG00000174684	ENSG00000155846	ENSG00000164077	ENSG00000145103
ENSG00000064490	ENSG00000179776	ENSG00000100379	ENSG00000085265
ENSG00000141298	ENSG00000155760	ENSG00000165527	ENSG00000102805
ENSG00000144119	ENSG00000134516	ENSG00000108468	ENSG00000090539
ENSG00000165566	ENSG00000125266	ENSG00000050628	ENSG00000081052
ENSG00000168259	ENSG00000168274	ENSG00000134250	ENSG00000166211
ENSG00000099864	ENSG00000110721	ENSG00000054523	ENSG00000134757
ENSG00000046647	ENSG00000107404	ENSG00000151136	ENSG00000119314
ENSG00000090539	ENSG00000178826	ENSG00000166111	ENSG00000100379
ENSG00000049245	ENSG00000177098	ENSG00000136044	ENSG00000183625
ENSG00000081052	ENSG00000105829	ENSG00000135148	ENSG00000196652
ENSG00000119314	ENSG00000080709	ENSG00000102057	ENSG00000100568
ENSG00000164077	ENSG00000165985	ENSG00000155846	ENSG00000008283
ENSG00000100379	ENSG00000139211	ENSG00000153560	ENSG00000241127
ENSG00000100568	ENSG00000196220	ENSG00000179776	ENSG00000108468
ENSG00000165527	ENSG00000131238	ENSG00000106070	ENSG00000050628
ENSG00000031544	ENSG00000088451	ENSG00000155760	ENSG00000185615
ENSG00000108468	ENSG00000081019	ENSG00000125266	ENSG00000054523
ENSG00000050628	ENSG00000188816	ENSG00000123607	ENSG00000151136
ENSG00000182310	ENSG00000115904	ENSG00000167191	ENSG00000163898
ENSG00000134250	ENSG00000115963	ENSG00000177098	ENSG00000189132
ENSG00000054523	ENSG00000160199	ENSG00000105829	ENSG00000102057
ENSG00000151136	ENSG00000178567	ENSG00000080709	ENSG00000130714
ENSG00000136044	ENSG00000131153	ENSG00000106541	ENSG00000155846
ENSG00000166111	ENSG00000101146	ENSG00000196220	ENSG00000241563
ENSG00000135148	ENSG00000106993	ENSG00000158186	ENSG00000179776
ENSG00000102057	ENSG00000005073	ENSG00000081019	ENSG00000186628
ENSG00000155846	ENSG00000164330	ENSG00000140564	ENSG00000155760
ENSG00000153560	ENSG00000058453	ENSG00000003249	ENSG00000134516
ENSG00000130349	ENSG00000100056	ENSG00000123636	ENSG00000125266
ENSG00000161011	ENSG00000105053	ENSG00000115963	ENSG00000168274
ENSG00000141968	ENSG00000121671	ENSG00000160199	ENSG00000065154
ENSG00000106070	ENSG00000154822	ENSG00000154642	ENSG00000110721
ENSG00000179776	ENSG00000131686	ENSG00000154556	ENSG00000184220
ENSG00000155760	ENSG00000183840	ENSG00000101146	ENSG00000132669
ENSG00000125266	ENSG00000136026	ENSG00000106993	ENSG00000107404
ENSG00000135697	ENSG00000134873	ENSG00000005073	ENSG00000178826
ENSG00000088888	ENSG00000145439	ENSG00000104332	ENSG00000105829
ENSG00000184220	ENSG00000134215	ENSG00000164330	ENSG00000177098
ENSG00000132669	ENSG00000078114	ENSG00000182149	ENSG00000080709
ENSG00000123607	ENSG00000169291	ENSG00000122335	ENSG00000244537
ENSG00000167191	ENSG00000177045	ENSG00000121671	ENSG00000140829
ENSG00000148334	ENSG00000196363	ENSG00000154822	ENSG00000160111
ENSG00000105829	ENSG00000181852	ENSG00000136026	ENSG00000165985
ENSG00000177098	ENSG00000116863	ENSG00000080815	ENSG00000169026
ENSG00000080709	ENSG00000180592	ENSG00000196712	ENSG00000139211
ENSG00000177164	ENSG00000117533	ENSG00000116922	ENSG00000131238
ENSG00000106541	ENSG00000181938	ENSG00000138758	ENSG00000196220
ENSG00000196220	ENSG00000096433	ENSG00000198729	ENSG00000125755
ENSG00000125755	ENSG00000082258	ENSG00000134215	ENSG00000077463
ENSG00000158186	ENSG00000104327	ENSG00000101323	ENSG00000138109
ENSG00000165512	ENSG00000142279	ENSG00000078114	ENSG00000081019
ENSG00000081019	ENSG00000157388	ENSG00000184611	ENSG00000165512
ENSG00000140564	ENSG00000186577	ENSG00000175348	ENSG00000156049

ENSG00000003249	ENSG00000008710	ENSG00000136352	ENSG00000188816
ENSG00000180849	ENSG00000082556	ENSG00000168924	ENSG00000176046
ENSG00000043462	ENSG00000156050	ENSG00000181852	ENSG00000043462
ENSG00000123636	ENSG00000154222	ENSG00000135269	ENSG00000115963
ENSG00000115963	ENSG00000213341	ENSG00000106571	ENSG00000155792
ENSG00000160199	ENSG00000188419	ENSG00000180592	ENSG00000160199
ENSG00000154642	ENSG00000142784	ENSG00000117533	ENSG00000178567
ENSG00000154556	ENSG00000184156	ENSG00000096433	ENSG00000141378
ENSG00000101146	ENSG00000150403	ENSG00000082258	ENSG00000121898
ENSG00000106993	ENSG00000128881	ENSG00000165338	ENSG00000099337
ENSG00000005073	ENSG00000051341	ENSG00000104327	ENSG00000090376
ENSG00000196559	ENSG00000142235	ENSG00000107581	ENSG00000101146
ENSG00000104332	ENSG00000143217	ENSG00000157388	ENSG00000106993
ENSG00000164330	ENSG00000106714	ENSG00000008710	ENSG00000005073
ENSG00000172954	ENSG00000168676	ENSG00000125505	ENSG00000120242
ENSG00000182149	ENSG00000162636	ENSG00000120129	ENSG00000174130
ENSG00000122335	ENSG00000115159	ENSG00000100296	ENSG00000058453
ENSG00000125449	ENSG00000156675	ENSG00000142784	ENSG00000105053
ENSG00000121671	ENSG00000196176	ENSG00000184156	ENSG00000100056
ENSG00000154822	ENSG00000044115	ENSG00000159335	ENSG00000160285
ENSG00000136026	ENSG00000169946	ENSG00000128881	ENSG00000121671
ENSG00000080815	ENSG00000118245	ENSG00000154864	ENSG00000154822
ENSG00000196712	ENSG00000173545	ENSG00000051341	ENSG00000131686
ENSG00000116922	ENSG00000186714	ENSG00000143217	ENSG00000100577
ENSG00000138758	ENSG00000143921	ENSG00000106714	ENSG00000183840
ENSG00000198729	ENSG00000144791	ENSG00000115159	ENSG00000136026
ENSG00000134215	ENSG00000089041	ENSG00000156675	ENSG00000133665
ENSG00000101323	ENSG00000110987	ENSG00000196176	ENSG00000134873
ENSG00000078114	ENSG00000155189	ENSG00000164742	ENSG00000145439
ENSG00000136352	ENSG00000080573	ENSG00000044115	ENSG00000134215
ENSG00000175348	ENSG00000151079	ENSG00000109158	ENSG00000078114
ENSG00000184611	ENSG00000145362	ENSG00000169946	ENSG00000169291
ENSG00000168924	ENSG00000198211	ENSG00000177508	ENSG00000177045
ENSG00000181852	ENSG00000143457	ENSG00000118245	ENSG00000196363
ENSG00000135269	ENSG00000174332	ENSG00000173545	ENSG00000181852
ENSG00000106571	ENSG00000132612	ENSG00000143921	ENSG00000165905
ENSG00000205707	ENSG00000185269	ENSG00000174953	ENSG00000132139
ENSG00000117533	ENSG00000100557	ENSG00000089041	ENSG00000205707
ENSG00000180592	ENSG00000176142	ENSG00000110987	ENSG00000073009
ENSG00000096433	ENSG00000180979	ENSG00000155189	ENSG00000117533
ENSG00000082258	ENSG00000164402	ENSG00000080573	ENSG00000082258
ENSG00000116062	ENSG00000198892	ENSG00000151079	ENSG00000096433
ENSG00000165338	ENSG00000170214	ENSG00000145362	ENSG00000163283
ENSG00000107581	ENSG00000145868	ENSG00000106025	ENSG00000104327
ENSG00000104327	ENSG00000125633	ENSG00000198211	ENSG00000076344
ENSG00000157388	ENSG00000145012	ENSG00000143457	ENSG00000142279
ENSG00000111666	ENSG00000182359	ENSG00000119725	ENSG00000157388
ENSG00000008710	ENSG00000157306	ENSG00000152601	ENSG00000148300
ENSG00000125505	ENSG00000178585	ENSG00000158805	ENSG00000008710
ENSG00000006114	ENSG00000196169	ENSG00000155926	ENSG00000197943
ENSG00000100296	ENSG00000145022	ENSG00000174332	ENSG00000082556
ENSG00000120129	ENSG00000179841	ENSG00000132612	ENSG00000154222
ENSG00000184156	ENSG00000204160	ENSG00000185269	ENSG00000213341
ENSG00000142784	ENSG00000115112	ENSG00000185149	ENSG00000188419
ENSG00000110619	ENSG00000115310	ENSG00000176142	ENSG00000184156
ENSG00000159335	ENSG00000119669	ENSG00000180979	ENSG00000142784
ENSG00000128881	ENSG00000117862	ENSG00000135740	ENSG00000150403
ENSG00000154864	ENSG00000131408	ENSG00000198901	ENSG00000128881
ENSG00000051341	ENSG00000119919	ENSG00000125633	ENSG00000051341
ENSG00000126785	ENSG00000105851	ENSG00000145012	ENSG00000142235
ENSG00000143217	ENSG00000186063	ENSG00000025039	ENSG00000164291
ENSG00000106714	ENSG00000136908	ENSG00000178585	ENSG00000126785
ENSG00000115159	ENSG00000158296	ENSG00000016082	ENSG00000143217
ENSG00000118508	ENSG00000179087	ENSG00000118513	ENSG00000168676

ENSG00000139323	ENSG00000010319	ENSG00000007312	ENSG00000106714
ENSG00000156675	ENSG00000074356	ENSG00000123600	ENSG00000115159
ENSG00000196176	ENSG00000145861	ENSG00000185070	ENSG00000162636
ENSG00000078725	ENSG00000007384	ENSG00000102230	ENSG00000156675
ENSG00000109158	ENSG00000165861	ENSG00000163466	ENSG00000196176
ENSG00000044115	ENSG00000242515	ENSG00000142347	ENSG00000044115
ENSG00000164742	ENSG00000159200	ENSG00000064999	ENSG00000146109
ENSG00000169946	ENSG00000074803	ENSG00000204160	ENSG00000169946
ENSG00000177508	ENSG00000149657	ENSG00000172216	ENSG00000118245
ENSG00000118245	ENSG00000144026	ENSG00000115310	ENSG00000173545
ENSG00000173545	ENSG00000175518	ENSG00000100711	ENSG00000135966
ENSG00000155287	ENSG00000137501	ENSG00000180138	ENSG00000138944
ENSG00000143921	ENSG00000116459	ENSG00000119669	ENSG00000186714
ENSG00000174953	ENSG00000006715	ENSG00000117862	ENSG00000130700
ENSG00000089041	ENSG00000167619	ENSG00000123989	ENSG00000100196
ENSG00000110987	ENSG00000167264	ENSG00000167778	ENSG00000169006
ENSG00000155189	ENSG00000081985	ENSG00000136908	ENSG00000187766
ENSG00000080573	ENSG00000137842	ENSG00000181291	ENSG00000114480
ENSG00000151079	ENSG00000069345	ENSG00000158296	ENSG00000089041
ENSG00000145362	ENSG00000148229	ENSG00000143466	ENSG00000144791
ENSG00000198211	ENSG00000115484	ENSG00000010319	ENSG00000110987
ENSG00000106025	ENSG00000188811	ENSG00000186834	ENSG00000155189
ENSG00000125695	ENSG00000129152	ENSG00000158458	ENSG00000130751
ENSG00000143457	ENSG00000175066	ENSG00000142920	ENSG00000080573
ENSG00000119725	ENSG00000103184	ENSG00000141448	ENSG00000145362
ENSG00000152601	ENSG00000143545	ENSG00000074356	ENSG00000151079
ENSG00000158805	ENSG00000221978	ENSG00000165861	ENSG00000198211
ENSG00000117595	ENSG00000104880	ENSG00000108100	ENSG00000183060
ENSG00000155926	ENSG00000175906	ENSG00000159200	ENSG00000135898
ENSG00000174332	ENSG00000149328	ENSG00000149657	ENSG00000143457
ENSG00000132612	ENSG00000204291	ENSG00000179455	ENSG00000130270
ENSG00000185269	ENSG00000163823	ENSG00000146414	ENSG00000117595
ENSG00000185149	ENSG00000170989	ENSG00000108753	ENSG00000174332
ENSG00000163481	ENSG00000154710	ENSG00000115738	ENSG00000132612
ENSG00000176142	ENSG00000132470	ENSG00000175518	ENSG00000100557
ENSG00000136247	ENSG00000161955	ENSG00000144228	ENSG00000185269
ENSG00000073614	ENSG00000162739	ENSG00000071626	ENSG00000163481
ENSG00000180979	ENSG00000065054	ENSG00000211455	ENSG00000214226
ENSG00000135740	ENSG00000164733	ENSG00000123353	ENSG00000176142
ENSG00000198901	ENSG00000212864	ENSG00000048405	ENSG00000141759
ENSG00000125633	ENSG00000168228	ENSG00000139190	ENSG00000180979
ENSG00000206456	ENSG00000198939	ENSG00000167264	ENSG00000164402
ENSG00000108671	ENSG00000124164	ENSG00000069345	ENSG00000170214
ENSG00000145012	ENSG00000105514	ENSG00000115419	ENSG00000116785
ENSG00000025039	ENSG00000135677	ENSG00000129152	ENSG00000145012
ENSG00000178585	ENSG00000145386	ENSG00000185344	ENSG00000206305
ENSG00000016082	ENSG00000204308	ENSG00000106397	ENSG00000157306
ENSG00000118513	ENSG00000134186	ENSG00000007168	ENSG00000182359
ENSG00000077312	ENSG00000166435	ENSG00000170191	ENSG00000178585
ENSG00000007312	ENSG00000167094	ENSG00000104880	ENSG00000162931
ENSG00000123600	ENSG00000198887	ENSG00000175906	ENSG00000164483
ENSG00000102230	ENSG00000164600	ENSG00000099365	ENSG00000077312
ENSG00000185070	ENSG00000124406	ENSG00000136122	ENSG00000168671
ENSG00000163466	ENSG00000168148	ENSG00000204291	ENSG00000196169
ENSG00000142347	ENSG00000166974	ENSG00000115295	ENSG00000145022
ENSG00000064999	ENSG00000069493	ENSG00000170989	ENSG00000179841
ENSG00000172216	ENSG00000140650	ENSG00000154710	ENSG00000204160
ENSG00000204160	ENSG00000123146	ENSG00000126768	ENSG00000115112
ENSG00000115310	ENSG00000130856	ENSG00000132470	ENSG00000115310
ENSG00000100711	ENSG00000184787	ENSG00000161955	ENSG00000186191
ENSG00000105639	ENSG00000132475	ENSG00000162739	ENSG00000105639
ENSG00000117862	ENSG00000162704	ENSG00000103653	ENSG00000117862
ENSG00000170876	ENSG00000180694	ENSG00000065054	ENSG00000170876
ENSG00000180138	ENSG00000175305	ENSG00000164733	ENSG00000119669

ENSG00000119669	ENSG00000177301	ENSG00000138592	ENSG00000131408
ENSG00000123989	ENSG00000173826	ENSG00000070614	ENSG00000119919
ENSG00000167778	ENSG00000115474	ENSG00000164604	ENSG00000173480
ENSG00000136908	ENSG00000171396	ENSG00000198939	ENSG00000105851
ENSG00000181291	ENSG00000204764	ENSG00000124164	ENSG00000136908
ENSG00000158296	ENSG00000196374	ENSG00000105514	ENSG00000171840
ENSG00000143466	ENSG00000107566	ENSG00000135677	ENSG00000158296
ENSG00000186834	ENSG00000162873	ENSG00000145386	ENSG00000179087
ENSG00000158458	ENSG00000132406	ENSG00000204308	ENSG00000010319
ENSG00000010319	ENSG00000184792	ENSG00000172350	ENSG00000008086
ENSG00000142920	ENSG00000078053	ENSG00000134186	ENSG00000101181
ENSG00000074356	ENSG00000167994	ENSG00000137273	ENSG00000241484
ENSG00000141448	ENSG00000186787	ENSG00000169057	ENSG00000074356
ENSG00000143207	ENSG00000170873	ENSG00000162599	ENSG00000007384
ENSG00000165861	ENSG00000151746	ENSG00000164600	ENSG00000145861
ENSG00000108100	ENSG00000150540	ENSG00000139998	ENSG00000165861
ENSG00000159200	ENSG00000064042	ENSG00000166974	ENSG00000184384
ENSG00000102595	ENSG00000167693	ENSG00000069493	ENSG00000074803
ENSG00000149657	ENSG00000146426	ENSG00000130856	ENSG00000149657
ENSG00000179455	ENSG00000128683	ENSG00000102218	ENSG00000114378
ENSG00000146414	ENSG00000013297	ENSG00000132475	ENSG00000112499
ENSG00000206254	ENSG00000182896	ENSG00000162704	ENSG00000144026
ENSG00000140534	ENSG00000128573	ENSG00000175305	ENSG00000129559
ENSG00000108753	ENSG00000141084	ENSG00000177301	ENSG00000073146
ENSG00000129559	ENSG00000168646	ENSG00000115474	ENSG00000175518
ENSG00000115738	ENSG00000106278	ENSG00000088812	ENSG00000137501
ENSG00000211449	ENSG00000115282	ENSG00000204764	ENSG00000116459
ENSG00000175518	ENSG00000171680	ENSG00000107566	ENSG00000162894
ENSG00000148702	ENSG00000197406	ENSG00000162873	ENSG00000126233
ENSG00000144228	ENSG00000113391	ENSG00000132406	ENSG00000204604
ENSG00000071626	ENSG00000104442	ENSG00000184792	ENSG00000041353
ENSG00000211455	ENSG00000101680	ENSG00000189056	ENSG00000006715
ENSG00000123353	ENSG00000253304	ENSG00000078053	ENSG00000196152
ENSG00000048405	ENSG00000106610	ENSG00000066583	ENSG00000143799
ENSG00000143799	ENSG00000137692	ENSG00000170873	ENSG00000137699
ENSG00000137699	ENSG00000076321	ENSG00000172201	ENSG00000167619
ENSG00000139190	ENSG00000117505	ENSG00000150540	ENSG00000167264
ENSG00000167264	ENSG00000151718	ENSG00000035403	ENSG00000081985
ENSG00000078808	ENSG00000186594	ENSG00000146426	ENSG00000137842
ENSG00000069345	ENSG00000108771	ENSG00000128683	ENSG00000069345
ENSG00000101773	ENSG00000186153	ENSG00000063438	ENSG00000101773
ENSG00000105835	ENSG00000105402	ENSG00000013297	ENSG00000105835
ENSG00000129152	ENSG00000137449	ENSG00000173221	ENSG00000115484
ENSG00000115419	ENSG00000133065	ENSG00000073350	ENSG00000148229
ENSG00000109618	ENSG00000110841	ENSG00000163935	ENSG00000129152
ENSG00000185344	ENSG00000153721	ENSG00000128573	ENSG00000103184
ENSG00000106397	ENSG00000197471	ENSG00000159792	ENSG00000205867
ENSG00000147256	ENSG00000198380	ENSG00000147162	ENSG00000143545
ENSG00000007168	ENSG00000152904	ENSG00000141084	ENSG00000092470
ENSG00000170191	ENSG00000115762	ENSG00000185650	ENSG00000221978
ENSG00000101084	ENSG00000122966	ENSG00000168646	ENSG00000250722
ENSG00000166143	ENSG00000177728	ENSG00000106278	ENSG00000112299
ENSG00000119688	ENSG00000159110	ENSG00000143458	ENSG00000125885
ENSG00000104880	ENSG00000129595	ENSG00000171680	ENSG00000100814
ENSG00000175906	ENSG00000126882	ENSG00000197406	ENSG00000166143
ENSG00000155622	ENSG00000204231	ENSG00000113391	ENSG00000119688
ENSG00000091592	ENSG00000151876	ENSG00000104442	ENSG00000104880
ENSG00000099365	ENSG00000168918	ENSG00000155858	ENSG00000175906
ENSG00000136122	ENSG00000112319	ENSG00000137692	ENSG00000183134
ENSG00000204291	ENSG00000125355	ENSG00000076321	ENSG00000204291
ENSG00000115295	ENSG00000152558	ENSG00000130635	ENSG00000163823
ENSG00000170989	ENSG00000063176	ENSG00000136295	ENSG00000188257
ENSG00000132470	ENSG00000134897	ENSG00000104722	ENSG00000108381
ENSG00000154710	ENSG00000180305	ENSG00000136279	ENSG00000154710

ENSG00000126768	ENSG00000020181	ENSG00000148358	ENSG00000132470
ENSG00000161955	ENSG00000152763	ENSG00000172375	ENSG00000161955
ENSG00000103653	ENSG00000116957	ENSG00000140403	ENSG00000110680
ENSG00000162739	ENSG00000113838	ENSG00000100987	ENSG00000182118
ENSG00000065054	ENSG00000110427	ENSG00000117505	ENSG00000162739
ENSG00000164733	ENSG00000170340	ENSG00000151718	ENSG00000139168
ENSG00000138592	ENSG00000131381	ENSG00000129292	ENSG00000065054
ENSG00000070614	ENSG00000148426	ENSG00000110514	ENSG00000164733
ENSG00000164604	ENSG00000135624	ENSG00000186594	ENSG00000105642
ENSG00000187815	ENSG00000178966	ENSG00000137449	ENSG00000168228
ENSG00000198939	ENSG00000146830	ENSG00000197471	ENSG00000198939
ENSG00000124164	ENSG00000137054	ENSG00000115762	ENSG00000124164
ENSG00000105514	ENSG00000172765	ENSG00000177732	ENSG00000105514
ENSG00000145386	ENSG00000115944	ENSG00000122966	ENSG00000151332
ENSG00000151332	ENSG00000144712	ENSG00000177728	ENSG00000135677
ENSG00000135677	ENSG00000070961	ENSG00000129595	ENSG00000134186
ENSG00000204308	ENSG00000155886	ENSG00000126882	ENSG00000166435
ENSG00000172350	ENSG00000138668	ENSG00000091656	ENSG00000167094
ENSG00000134186	ENSG00000115266	ENSG00000204231	ENSG00000187003
ENSG00000163781	ENSG00000164896	ENSG00000112319	ENSG00000198887
ENSG00000137273	ENSG00000069667	ENSG00000130699	ENSG00000164600
ENSG00000151116	ENSG00000113558	ENSG00000125355	ENSG00000175164
ENSG00000169057	ENSG00000158022	ENSG00000108406	ENSG00000124406
ENSG00000162599	ENSG00000004864	ENSG00000113263	ENSG00000168148
ENSG00000164600	ENSG00000168411	ENSG00000063176	ENSG00000166974
ENSG00000088320	ENSG00000151846	ENSG00000149923	ENSG00000069493
ENSG00000166974	ENSG00000139613	ENSG00000118242	ENSG00000140650
ENSG00000139998	ENSG00000145191	ENSG00000145819	ENSG00000123146
ENSG00000069493	ENSG00000146005	ENSG00000143093	ENSG00000130856
ENSG00000187876	ENSG00000167182	ENSG00000163067	ENSG00000184787
ENSG00000102218	ENSG00000100425	ENSG00000134897	ENSG00000132475
ENSG00000130856	ENSG00000073150	ENSG00000020181	ENSG00000162704
ENSG00000132475	ENSG00000011426	ENSG00000124466	ENSG00000173302
ENSG00000162704	ENSG00000157657	ENSG00000112592	ENSG00000180694
ENSG00000173302	ENSG00000095794	ENSG00000211448	ENSG00000177301
ENSG00000115808	ENSG00000198797	ENSG00000113838	ENSG00000175305
ENSG00000177301	ENSG00000092421	ENSG00000116754	ENSG00000096006
ENSG00000175305	ENSG00000113580	ENSG00000170340	ENSG00000115474
ENSG00000115474	ENSG00000169122	ENSG00000148426	ENSG00000180316
ENSG00000088812	ENSG00000104853	ENSG00000087338	ENSG00000171396
ENSG00000177408	ENSG00000111652	ENSG00000105695	ENSG00000204764
ENSG00000204764	ENSG00000134595	ENSG00000170242	ENSG00000196374
ENSG00000107566	ENSG00000149600	ENSG00000132382	ENSG00000107566
ENSG00000145919	ENSG00000095321	ENSG00000136381	ENSG00000114859
ENSG00000162873	ENSG00000128512	ENSG00000146830	ENSG00000162873
ENSG00000132406	ENSG00000081923	ENSG00000172765	ENSG00000132406
ENSG00000184792	ENSG00000174151	ENSG00000105967	ENSG00000184792
ENSG00000189056	ENSG00000064726	ENSG00000070961	ENSG00000078053
ENSG00000204955	ENSG00000165417	ENSG00000155886	ENSG00000167994
ENSG00000139767	ENSG00000166928	ENSG00000115266	ENSG00000170873
ENSG00000078053	ENSG00000183783	ENSG00000164896	ENSG00000151746
ENSG00000066583	ENSG00000170921	ENSG00000157240	ENSG00000065613
ENSG00000172201	ENSG00000146826	ENSG00000069667	ENSG00000150540
ENSG00000170873	ENSG00000164985	ENSG00000113558	ENSG00000104320
ENSG00000150540	ENSG00000134308	ENSG00000158022	ENSG00000147138
ENSG00000065613	ENSG00000076685	ENSG00000140382	ENSG00000167656
ENSG00000196975	ENSG00000139915	ENSG00000119537	ENSG00000167693
ENSG00000168329	ENSG00000112183	ENSG00000168411	ENSG00000128683
ENSG00000035403	ENSG00000137857	ENSG00000108604	ENSG00000146426
ENSG00000146426	ENSG00000074657	ENSG00000151846	ENSG00000013297
ENSG00000128683	ENSG00000168528	ENSG00000105948	ENSG00000116030
ENSG00000063438	ENSG00000081059	ENSG00000139613	ENSG00000182896
ENSG00000110700	ENSG00000143365	ENSG00000170374	ENSG00000128573
ENSG00000013297	ENSG00000011566	ENSG00000145191	ENSG00000187187

ENSG00000116030	ENSG00000014216	ENSG00000146005	ENSG00000162066
ENSG00000073350	ENSG00000143882	ENSG00000167182	ENSG00000141084
ENSG00000173221	ENSG00000093134	ENSG00000100425	ENSG00000178665
ENSG00000163935	ENSG00000140943	ENSG00000073150	ENSG00000106278
ENSG00000004487	ENSG00000111785	ENSG00000011426	ENSG00000014914
ENSG00000128573	ENSG00000139874	ENSG00000175893	ENSG00000168646
ENSG00000198205	ENSG00000152049	ENSG00000095794	ENSG00000141371
ENSG00000159792	ENSG00000066032	ENSG00000198797	ENSG00000115282
ENSG00000141084	ENSG00000082898	ENSG00000092421	ENSG00000144045
ENSG00000147162	ENSG00000145741	ENSG00000164736	ENSG00000185527
ENSG00000185650	ENSG00000156113	ENSG00000113580	ENSG00000171680
ENSG00000106278	ENSG00000138413	ENSG00000169122	ENSG00000197406
ENSG00000168646	ENSG00000080493	ENSG00000100505	ENSG00000104442
ENSG00000143458	ENSG00000079689	ENSG00000104853	ENSG00000101680
ENSG00000171680	ENSG00000154188	ENSG00000112305	ENSG00000146223
ENSG00000197406	ENSG00000166689	ENSG00000111652	ENSG00000184280
ENSG00000113391	ENSG00000231925	ENSG00000115207	ENSG00000073754
ENSG00000104442	ENSG00000165671	ENSG00000144674	ENSG00000076321
ENSG00000155858	ENSG00000197951	ENSG00000142949	ENSG00000128713
ENSG00000124191	ENSG00000253950	ENSG00000159592	ENSG00000152078
ENSG00000137692	ENSG00000131378	ENSG00000082438	ENSG00000117505
ENSG00000076321	ENSG00000119714	ENSG00000120088	ENSG00000151718
ENSG00000130635	ENSG00000133740	ENSG00000174151	ENSG00000170175
ENSG00000189419	ENSG00000198912	ENSG00000065883	ENSG00000108771
ENSG00000066135	ENSG00000196453	ENSG00000064726	ENSG00000080823
ENSG00000136295	ENSG00000116106	ENSG00000162461	ENSG00000105402
ENSG00000136279	ENSG00000171310	ENSG00000165417	ENSG00000186153
ENSG00000104722	ENSG00000092096	ENSG00000183783	ENSG00000069849
ENSG00000148358	ENSG00000154217	ENSG00000146826	ENSG00000137449
ENSG00000172375	ENSG00000165655	ENSG00000143379	ENSG00000110841
ENSG00000140403	ENSG00000136100	ENSG00000164985	ENSG00000133065
ENSG00000100987	ENSG00000163104	ENSG00000134308	ENSG00000141505
ENSG00000117505	ENSG00000048392	ENSG00000164649	ENSG00000132446
ENSG00000151718	ENSG00000125875	ENSG00000145244	ENSG00000153721
ENSG00000129292	ENSG00000177951	ENSG00000196132	ENSG00000197471
ENSG00000110514	ENSG00000106355	ENSG00000109787	ENSG00000135476
ENSG00000186594	ENSG00000125844	ENSG00000148925	ENSG00000141569
ENSG00000080823	ENSG00000116525	ENSG00000139915	ENSG00000198380
ENSG00000117091	ENSG00000168490	ENSG00000113384	ENSG00000197912
ENSG00000069849	ENSG00000135090	ENSG00000186354	ENSG00000011143
ENSG00000137449	ENSG00000093167	ENSG00000112183	ENSG00000152904
ENSG00000091490	ENSG00000167766	ENSG00000137857	ENSG00000115762
ENSG00000197471	ENSG00000196284	ENSG00000074657	ENSG00000164035
ENSG00000115762	ENSG00000091831	ENSG00000181541	ENSG00000249709
ENSG00000177732	ENSG00000186310	ENSG00000136261	ENSG00000239672
ENSG00000166869	ENSG00000163644	ENSG00000179922	ENSG00000122966
ENSG00000122966	ENSG00000181781	ENSG00000168528	ENSG00000244165
ENSG00000177728	ENSG00000164932	ENSG00000081059	ENSG00000177728
ENSG00000129595	ENSG00000109047	ENSG00000143365	ENSG00000148942
ENSG00000204231	ENSG00000131370	ENSG00000143970	ENSG00000129595
ENSG00000126882	ENSG00000198722	ENSG00000011566	ENSG00000159110
ENSG00000091656	ENSG00000146802	ENSG00000143882	ENSG00000126882
ENSG00000138379	ENSG00000176022	ENSG00000049323	ENSG00000151876
ENSG00000164764	ENSG00000075945	ENSG00000176641	ENSG00000168918
ENSG00000206052	ENSG00000158352	ENSG00000093134	ENSG00000112319
ENSG00000130699	ENSG00000150764	ENSG00000140943	ENSG00000206052
ENSG00000125355	ENSG00000175866	ENSG00000184232	ENSG00000125355
ENSG00000112319	ENSG00000138434	ENSG00000111785	ENSG00000152558
ENSG00000108406	ENSG00000127241	ENSG00000139874	ENSG00000188986
ENSG00000113263	ENSG00000124356	ENSG00000066032	ENSG00000063176
ENSG00000063176	ENSG00000050438	ENSG00000082898	ENSG00000197181
ENSG00000149923	ENSG00000118946	ENSG00000145741	ENSG00000136098
ENSG00000118242	ENSG00000108557	ENSG00000156113	ENSG00000134897
ENSG00000145819	ENSG00000198597	ENSG00000138413	ENSG00000020181

ENSG00000163067	ENSG00000137266	ENSG00000080493	ENSG00000180305
ENSG00000143093	ENSG00000062194	ENSG00000154188	ENSG00000240403
ENSG00000134897	ENSG00000179195	ENSG00000166689	ENSG00000152763
ENSG00000020181	ENSG00000172531	ENSG00000165671	ENSG00000040933
ENSG00000148481	ENSG00000164168	ENSG00000184613	ENSG00000116957
ENSG00000124466	ENSG00000071054	ENSG00000131378	ENSG00000113838
ENSG00000040933	ENSG00000165891	ENSG00000133740	ENSG00000110427
ENSG00000211448	ENSG00000117266	ENSG00000196453	ENSG00000112237
ENSG00000112592	ENSG00000146247	ENSG00000116106	ENSG00000132703
ENSG00000113838	ENSG00000070367	ENSG00000174238	ENSG00000131381
ENSG00000116754	ENSG00000151952	ENSG00000119979	ENSG00000162526
ENSG00000170340	ENSG00000092931	ENSG00000171310	ENSG00000148426
ENSG00000112237	ENSG00000146731	ENSG00000092096	ENSG00000137054
ENSG00000162526	ENSG00000112312	ENSG00000154217	ENSG00000166664
ENSG00000148426	ENSG00000121653	ENSG00000165655	ENSG00000115944
ENSG00000105695	ENSG00000108839	ENSG00000163104	ENSG00000178966
ENSG00000087338	ENSG00000101265	ENSG00000078369	ENSG00000172765
ENSG00000170242	ENSG00000103855	ENSG00000177951	ENSG00000116212
ENSG00000132382	ENSG00000148468	ENSG00000124788	ENSG00000132026
ENSG00000136381	ENSG00000211452	ENSG00000165072	ENSG00000144712
ENSG00000146830	ENSG00000157350	ENSG00000138138	ENSG00000172568
ENSG00000172765	ENSG00000198837	ENSG00000106355	ENSG00000070961
ENSG00000105967	ENSG00000187147	ENSG00000143632	ENSG00000189057
ENSG00000116212	ENSG00000102678	ENSG00000105401	ENSG00000138802
ENSG00000070961	ENSG00000176986	ENSG00000125844	ENSG00000115266
ENSG00000155886	ENSG00000182379	ENSG00000172059	ENSG00000158022
ENSG00000166862	ENSG00000197329	ENSG00000120992	ENSG00000143536
ENSG00000138802	ENSG00000160963	ENSG00000168490	ENSG00000004864
ENSG00000115266	ENSG00000198756	ENSG00000135090	ENSG00000168411
ENSG00000157240	ENSG00000144824	ENSG00000093167	ENSG00000151846
ENSG00000164896	ENSG00000155629	ENSG00000196284	ENSG00000174080
ENSG00000069509	ENSG00000197312	ENSG00000117713	ENSG00000139613
ENSG00000069667	ENSG00000126107	ENSG00000091831	ENSG00000206075
ENSG00000158022	ENSG00000179295	ENSG00000186310	ENSG00000131165
ENSG00000140382	ENSG00000172575	ENSG00000130985	ENSG00000146005
ENSG00000113558	ENSG00000059915	ENSG00000152520	ENSG00000167182
ENSG00000119537	ENSG00000180543	ENSG00000164932	ENSG00000100425
ENSG00000168411	ENSG00000198498	ENSG00000109047	ENSG00000011426
ENSG00000108604	ENSG00000168077	ENSG00000101445	ENSG00000073150
ENSG00000105948	ENSG00000106665	ENSG00000131370	ENSG00000157657
ENSG00000151846	ENSG00000135213	ENSG00000138670	ENSG00000078814
ENSG00000139613	ENSG00000083099	ENSG00000106852	ENSG00000047188
ENSG00000181396	ENSG00000152266	ENSG00000150625	ENSG00000095794
ENSG00000145191	ENSG00000165355	ENSG00000198722	ENSG00000198797
ENSG00000206075	ENSG00000165152	ENSG00000146802	ENSG00000092421
ENSG00000170374	ENSG00000111319	ENSG00000137522	ENSG00000187266
ENSG00000206327	ENSG00000180611	ENSG00000178184	ENSG00000113580
ENSG00000131165	ENSG00000186566	ENSG00000179152	ENSG00000104853
ENSG00000146005	ENSG00000129472	ENSG00000068383	ENSG00000111652
ENSG00000167182	ENSG00000164124	ENSG00000150764	ENSG00000158864
ENSG00000100425	ENSG00000091140	ENSG00000114648	ENSG00000168268
ENSG00000133612	ENSG00000126353	ENSG00000120162	ENSG00000134595
ENSG00000011426	ENSG00000166444	ENSG00000175866	ENSG00000166710
ENSG00000073150	ENSG00000138073	ENSG00000138434	ENSG00000051825
ENSG00000175893	ENSG00000069122	ENSG00000127241	ENSG00000149600
ENSG00000162543	ENSG00000126464	ENSG00000124491	ENSG00000095321
ENSG00000047188	ENSG00000115053	ENSG00000124356	ENSG00000078142
ENSG00000198797	ENSG00000076555	ENSG00000115365	ENSG00000128512
ENSG00000095794	ENSG00000166598	ENSG00000146376	ENSG00000148604
ENSG00000092421	ENSG00000123472	ENSG00000108557	ENSG00000081923
ENSG00000156831	ENSG00000116670	ENSG00000137266	ENSG00000174151
ENSG00000164736	ENSG00000161526	ENSG00000062194	ENSG00000064726
ENSG00000113580	ENSG00000165209	ENSG00000125944	ENSG00000165417
ENSG00000169122	ENSG00000187531	ENSG00000172531	ENSG00000170921

ENSG00000104853	ENSG00000241685	ENSG00000071054	ENSG00000164985
ENSG00000100505	ENSG00000061337	ENSG00000165891	ENSG00000134308
ENSG00000112305	ENSG00000132872	ENSG00000079277	ENSG00000048740
ENSG00000111652	ENSG00000101255	ENSG00000117266	ENSG00000166797
ENSG00000115207	ENSG00000168772	ENSG00000126016	ENSG00000170439
ENSG00000171243	ENSG00000187172	ENSG00000146247	ENSG00000175809
ENSG00000144674	ENSG00000130734	ENSG00000070367	ENSG00000076685
ENSG00000188649	ENSG00000171735	ENSG00000118473	ENSG00000047579
ENSG00000142949	ENSG00000111011	ENSG00000092931	ENSG00000112183
ENSG00000051825	ENSG00000167468	ENSG00000205423	ENSG00000137857
ENSG00000120088	ENSG00000071242	ENSG00000153310	ENSG00000074657
ENSG00000159592	ENSG00000198624	ENSG00000175707	ENSG00000165140
ENSG00000082438	ENSG00000112715	ENSG00000121653	ENSG00000186297
ENSG00000065883	ENSG00000078618	ENSG00000101265	ENSG00000143365
ENSG00000174151	ENSG00000172292	ENSG00000187147	ENSG00000168528
ENSG00000064726	ENSG00000170044	ENSG00000104728	ENSG00000081059
ENSG00000165417	ENSG00000153395	ENSG00000102678	ENSG00000011566
ENSG00000162461	ENSG00000170289	ENSG00000117013	ENSG00000014216
ENSG00000183783	ENSG00000145040	ENSG00000176986	ENSG00000143882
ENSG00000143379	ENSG00000122584	ENSG00000197329	ENSG00000063761
ENSG00000146826	ENSG00000183960	ENSG00000198756	ENSG00000093134
ENSG00000164985	ENSG00000090269	ENSG00000144824	ENSG00000140943
ENSG00000103540	ENSG00000107262	ENSG00000137507	ENSG00000111785
ENSG00000048740	ENSG00000158985	ENSG00000122025	ENSG00000139874
ENSG00000134308	ENSG00000015676	ENSG00000155629	ENSG00000152049
ENSG00000166797	ENSG00000164296	ENSG00000074219	ENSG00000082898
ENSG00000164649	ENSG00000154342	ENSG00000114999	ENSG00000066032
ENSG00000145244	ENSG00000128271	ENSG00000126107	ENSG00000156113
ENSG00000196132	ENSG00000156860	ENSG00000172575	ENSG00000138413
ENSG00000109787	ENSG00000120314	ENSG00000059915	ENSG00000080493
ENSG00000047579	ENSG00000173705	ENSG00000180543	ENSG00000079689
ENSG00000148925	ENSG00000086666	ENSG00000105281	ENSG00000154188
ENSG00000139915	ENSG00000109466	ENSG00000113083	ENSG00000166689
ENSG00000186354	ENSG00000185272	ENSG00000106665	ENSG00000165671
ENSG00000113384	ENSG00000143294	ENSG00000144357	ENSG00000197951
ENSG00000137857	ENSG00000107864	ENSG00000103429	ENSG00000188801
ENSG00000112183	ENSG00000176731	ENSG00000137073	ENSG00000169344
ENSG00000181541	ENSG00000180011	ENSG00000180801	ENSG00000119714
ENSG00000074657	ENSG00000164438	ENSG00000152266	ENSG00000133740
ENSG00000136261	ENSG00000116793	ENSG00000165355	ENSG00000198912
ENSG00000164066	ENSG00000163882	ENSG00000165349	ENSG00000196453
ENSG00000179922	ENSG00000170085	ENSG00000111319	ENSG00000116106
ENSG00000081059	ENSG00000081026	ENSG00000179449	ENSG00000092096
ENSG00000186297	ENSG00000150477	ENSG00000085832	ENSG00000154217
ENSG00000168528	ENSG00000101000	ENSG00000198846	ENSG00000165655
ENSG00000143365	ENSG00000143315	ENSG00000180611	ENSG00000136100
ENSG00000118181	ENSG00000179915	ENSG00000186566	ENSG00000172594
ENSG00000143970	ENSG00000241119	ENSG00000105223	ENSG00000163104
ENSG00000011566	ENSG00000129353	ENSG00000129472	ENSG00000048392
ENSG00000143882	ENSG00000203782	ENSG00000126353	ENSG00000121716
ENSG00000049323	ENSG00000135776	ENSG00000102125	ENSG00000070601
ENSG00000093134	ENSG00000125744	ENSG00000166444	ENSG00000125875
ENSG00000176641	ENSG00000198221	ENSG00000069122	ENSG00000177951
ENSG00000140943	ENSG00000196917	ENSG00000072832	ENSG00000106355
ENSG00000139874	ENSG00000076356	ENSG00000105656	ENSG00000125844
ENSG00000184232	ENSG00000117519	ENSG00000170515	ENSG00000148795
ENSG00000111785	ENSG00000204120	ENSG00000162366	ENSG00000134460
ENSG00000145741	ENSG00000140511	ENSG00000166598	ENSG00000101439
ENSG00000082898	ENSG00000166049	ENSG00000123472	ENSG00000107165
ENSG00000156113	ENSG00000168936	ENSG00000161526	ENSG00000116525
ENSG00000066032	ENSG00000160888	ENSG00000165209	ENSG00000168490
ENSG00000138413	ENSG00000180901	ENSG00000164283	ENSG00000135090
ENSG00000080493	ENSG00000143333	ENSG00000204590	ENSG00000242612
ENSG00000154188	ENSG00000204176	ENSG00000187531	ENSG00000198049

ENSG00000166689	ENSG00000138386	ENSG00000061337	ENSG00000167766
ENSG00000170396	ENSG00000143179	ENSG00000180891	ENSG00000196284
ENSG00000165671	ENSG00000010610	ENSG00000132872	ENSG00000091831
ENSG00000184613	ENSG00000138207	ENSG00000169105	ENSG00000116151
ENSG00000131378	ENSG00000174243	ENSG00000137872	ENSG00000163644
ENSG00000188801	ENSG00000072401	ENSG00000130734	ENSG00000137274
ENSG00000169344	ENSG00000171862	ENSG00000132463	ENSG00000164932
ENSG00000133740	ENSG00000101542	ENSG00000171735	ENSG00000131370
ENSG00000196453	ENSG00000127804	ENSG00000178177	ENSG00000149300
ENSG00000164830	ENSG00000151881	ENSG00000196876	ENSG00000198722
ENSG00000116106	ENSG00000136630	ENSG00000111011	ENSG00000176022
ENSG00000174238	ENSG00000121578	ENSG00000204673	ENSG00000117906
ENSG00000181631	ENSG00000165494	ENSG00000071242	ENSG00000075945
ENSG00000138593	ENSG00000205089	ENSG00000172292	ENSG00000158352
ENSG00000119979	ENSG00000118496	ENSG00000164011	ENSG00000145781
ENSG00000111305	ENSG00000110148	ENSG00000170289	ENSG00000150764
ENSG00000171310	ENSG00000132522	ENSG00000145040	ENSG00000071203
ENSG00000092096	ENSG00000150527	ENSG00000108797	ENSG00000160055
ENSG00000154217	ENSG00000035681	ENSG00000174059	ENSG00000138434
ENSG00000165655	ENSG00000184863	ENSG00000184602	ENSG00000175866
ENSG00000163104	ENSG00000130997	ENSG00000122584	ENSG00000138483
ENSG00000174373	ENSG00000168743	ENSG00000183960	ENSG00000127241
ENSG00000070601	ENSG00000132383	ENSG00000080822	ENSG00000124356
ENSG00000177951	ENSG00000129003	ENSG00000015676	ENSG00000050438
ENSG00000078369	ENSG00000165661	ENSG00000141542	ENSG00000136883
ENSG00000124788	ENSG00000165219	ENSG00000145808	ENSG00000118946
ENSG00000138138	ENSG00000091844	ENSG00000103710	ENSG00000108557
ENSG00000165072	ENSG00000153064	ENSG00000154342	ENSG00000123136
ENSG00000106355	ENSG00000139546	ENSG00000128271	ENSG00000213934
ENSG00000143632	ENSG00000119866	ENSG00000156860	ENSG00000198597
ENSG00000105401	ENSG00000183386	ENSG00000086666	ENSG00000009724
ENSG00000125844	ENSG00000121775	ENSG00000134001	ENSG00000150753
ENSG00000171724	ENSG00000121068	ENSG00000109466	ENSG00000168884
ENSG00000107165	ENSG00000186603	ENSG00000143294	ENSG00000179195
ENSG00000172059	ENSG00000173065	ENSG00000107864	ENSG00000172531
ENSG00000120992	ENSG00000123560	ENSG00000112339	ENSG00000170502
ENSG00000168490	ENSG00000170482	ENSG00000180011	ENSG00000007908
ENSG00000135090	ENSG00000101464	ENSG00000164438	ENSG00000071054
ENSG00000168389	ENSG00000007944	ENSG00000107968	ENSG00000166321
ENSG00000093167	ENSG00000134686	ENSG00000085511	ENSG00000165891
ENSG00000206495	ENSG00000077092	ENSG00000170085	ENSG00000124588
ENSG00000196284	ENSG00000171067	ENSG00000075391	ENSG00000076258
ENSG00000117713	ENSG00000182255	ENSG00000123572	ENSG00000117266
ENSG00000091831	ENSG00000242441	ENSG00000109079	ENSG00000139714
ENSG00000130985	ENSG00000167578	ENSG00000179915	ENSG00000070367
ENSG00000186310	ENSG00000240694	ENSG00000175470	ENSG00000242247
ENSG00000152520	ENSG00000135951	ENSG00000129353	ENSG00000006128
ENSG00000164932	ENSG00000167004	ENSG00000138347	ENSG00000151952
ENSG00000109047	ENSG00000174899	ENSG00000153162	ENSG00000137880
ENSG00000101445	ENSG00000134817	ENSG00000125744	ENSG00000146731
ENSG00000131370	ENSG00000101974	ENSG00000139116	ENSG00000108839
ENSG00000150625	ENSG00000140548	ENSG00000116874	ENSG00000112312
ENSG00000106852	ENSG00000084073	ENSG00000115825	ENSG00000121653
ENSG00000138670	ENSG00000066455	ENSG00000076356	ENSG00000136305
ENSG00000198722	ENSG00000171055	ENSG00000173812	ENSG00000211452
ENSG00000146802	ENSG00000198563	ENSG00000117519	ENSG00000101265
ENSG00000137522	ENSG00000141013	ENSG00000204120	ENSG00000103855
ENSG00000117906	ENSG00000243725	ENSG00000168936	ENSG00000106086
ENSG00000178184	ENSG00000118217	ENSG00000160888	ENSG00000157350
ENSG00000179152	ENSG00000160633	ENSG00000143333	ENSG00000198837
ENSG00000197079	ENSG00000164924	ENSG00000175395	ENSG00000102678
ENSG00000068383	ENSG00000198740	ENSG00000138639	ENSG00000176986
ENSG00000150764	ENSG00000135625	ENSG00000138386	ENSG00000241320
ENSG00000114648	ENSG00000138757	ENSG00000177519	ENSG00000182379

ENSG00000175866	ENSG00000047932	ENSG00000143179	ENSG00000169302
ENSG00000138434	ENSG00000140326	ENSG00000121481	ENSG00000055950
ENSG00000120162	ENSG00000142867	ENSG00000010610	ENSG00000197329
ENSG00000162191	ENSG00000187288	ENSG00000144452	ENSG00000160963
ENSG00000102900	ENSG00000011304	ENSG00000072401	ENSG00000198756
ENSG00000124491	ENSG00000147679	ENSG00000171862	ENSG00000144824
ENSG00000127241	ENSG00000154975	ENSG00000101542	ENSG00000155629
ENSG00000124356	ENSG00000156471	ENSG00000164258	ENSG00000197312
ENSG00000110917	ENSG00000135698	ENSG00000125834	ENSG00000179295
ENSG00000115365	ENSG00000105991	ENSG00000176871	ENSG00000126107
ENSG00000146376	ENSG00000187720	ENSG00000127804	ENSG00000172575
ENSG00000172995	ENSG00000198650	ENSG00000136630	ENSG00000059915
ENSG00000108557	ENSG00000102054	ENSG00000167325	ENSG00000168077
ENSG00000137266	ENSG00000197930	ENSG00000165494	ENSG00000086288
ENSG00000150753	ENSG00000072736	ENSG00000151704	ENSG00000139631
ENSG00000062194	ENSG00000112096	ENSG00000118496	ENSG00000162441
ENSG00000125944	ENSG00000129194	ENSG00000110148	ENSG00000188056
ENSG00000172531	ENSG00000143469	ENSG00000132522	ENSG00000085514
ENSG00000071054	ENSG00000134121	ENSG00000150527	ENSG00000083099
ENSG00000134480	ENSG00000119596	ENSG00000116337	ENSG00000196923
ENSG00000166321	ENSG00000121871	ENSG00000147724	ENSG00000152266
ENSG00000165891	ENSG00000148680	ENSG00000035681	ENSG00000185559
ENSG00000126016	ENSG00000141642	ENSG00000092068	ENSG00000165355
ENSG00000079277	ENSG00000214575	ENSG00000111321	ENSG00000176681
ENSG00000117266	ENSG00000173262	ENSG00000162551	ENSG00000185359
ENSG00000146247	ENSG00000163697	ENSG00000165661	ENSG00000165152
ENSG00000070367	ENSG00000149554	ENSG00000106089	ENSG00000111319
ENSG00000146007	ENSG00000147140	ENSG00000180008	ENSG00000204947
ENSG00000118473	ENSG00000151239	ENSG00000137944	ENSG00000167759
ENSG00000006128	ENSG00000161243	ENSG00000165219	ENSG00000114854
ENSG00000092931	ENSG00000167202	ENSG00000139546	ENSG00000180611
ENSG00000205423	ENSG00000179057	ENSG00000119866	ENSG00000168952
ENSG00000153310	ENSG00000147475	ENSG00000183386	ENSG00000172732
ENSG00000175707	ENSG00000080819	ENSG00000121775	ENSG00000129472
ENSG00000121653	ENSG00000182318	ENSG00000072315	ENSG00000091140
ENSG00000101265	ENSG00000145194	ENSG00000121068	ENSG00000164124
ENSG00000106086	ENSG00000204531	ENSG00000153012	ENSG00000140675
ENSG00000102678	ENSG00000106636	ENSG00000104863	ENSG00000126353
ENSG00000104728	ENSG00000164619	ENSG00000023909	ENSG00000084110
ENSG00000187147	ENSG00000135317	ENSG00000198925	ENSG00000166444
ENSG00000117013	ENSG00000118507	ENSG00000173065	ENSG00000138073
ENSG00000176986	ENSG00000147488	ENSG00000123560	ENSG00000135845
ENSG00000055950	ENSG00000143127	ENSG00000109919	ENSG00000069122
ENSG00000197329	ENSG00000130749	ENSG00000170522	ENSG00000125319
ENSG00000198756	ENSG00000005810	ENSG00000105568	ENSG00000115053
ENSG00000122025	ENSG00000204963	ENSG00000101464	ENSG00000147206
ENSG00000137507	ENSG00000163812	ENSG00000007944	ENSG00000076555
ENSG00000144824	ENSG00000122783	ENSG00000134686	ENSG00000126464
ENSG00000155629	ENSG00000100095	ENSG00000182985	ENSG00000166598
ENSG00000074219	ENSG00000108518	ENSG00000077092	ENSG00000123472
ENSG00000126107	ENSG00000143033	ENSG00000171067	ENSG00000116670
ENSG00000114999	ENSG00000160014	ENSG00000182255	ENSG00000161526
ENSG00000172575	ENSG00000123360	ENSG00000123066	ENSG00000185168
ENSG00000059915	ENSG00000222040	ENSG00000171056	ENSG00000165209
ENSG00000180543	ENSG00000132793	ENSG00000167004	ENSG00000110900
ENSG00000113083	ENSG00000076248	ENSG00000101974	ENSG00000061337
ENSG00000105281	ENSG00000131503	ENSG00000140548	ENSG00000096088
ENSG00000106665	ENSG00000184481	ENSG00000170854	ENSG00000127743
ENSG00000103429	ENSG00000241994	ENSG00000084073	ENSG00000132872
ENSG00000144357	ENSG00000107874	ENSG00000066455	ENSG00000101255
ENSG00000137073	ENSG00000179097	ENSG00000123411	ENSG00000168772
ENSG00000180801	ENSG00000100836	ENSG00000154764	ENSG00000171735
ENSG00000152266	ENSG00000138029	ENSG00000145687	ENSG00000197168
ENSG00000165355	ENSG00000135454	ENSG00000160633	ENSG00000167468

ENSG00000165349	ENSG00000134453	ENSG00000167971	ENSG00000103546
ENSG00000111319	ENSG00000004455	ENSG00000127124	ENSG00000071242
ENSG00000108272	ENSG00000077782	ENSG00000164924	ENSG00000198624
ENSG00000085832	ENSG00000173947	ENSG00000135625	ENSG00000172292
ENSG00000179449	ENSG00000119760	ENSG00000138757	ENSG00000179817
ENSG00000198846	ENSG00000073670	ENSG00000198142	ENSG00000175634
ENSG00000180611	ENSG00000213420	ENSG00000182195	ENSG00000170044
ENSG00000186566	ENSG00000125462	ENSG00000185519	ENSG00000138115
ENSG00000105223	ENSG00000120756	ENSG00000011304	ENSG00000105376
ENSG00000129472	ENSG00000166847	ENSG00000154975	ENSG00000170289
ENSG00000167701	ENSG00000182591	ENSG00000198824	ENSG00000145040
ENSG00000126353	ENSG00000129480	ENSG00000156471	ENSG00000110955
ENSG00000102125	ENSG00000048028	ENSG00000100060	ENSG00000122584
ENSG00000166444	ENSG00000138036	ENSG00000105991	ENSG00000130383
ENSG00000072832	ENSG00000164076	ENSG00000187720	ENSG00000204572
ENSG00000069122	ENSG00000160883	ENSG00000153201	ENSG00000183960
ENSG00000105656	ENSG00000182944	ENSG00000105647	ENSG00000090269
ENSG00000170515	ENSG00000106415	ENSG00000166478	ENSG00000107262
ENSG00000162366	ENSG00000168079	ENSG00000102054	ENSG00000105205
ENSG00000123472	ENSG00000073060	ENSG00000072736	ENSG00000158985
ENSG00000166598	ENSG00000111266	ENSG00000134121	ENSG00000089060
ENSG00000161526	ENSG00000185950	ENSG00000121871	ENSG00000015676
ENSG00000165209	ENSG00000196591	ENSG00000138675	ENSG00000136895
ENSG00000204590	ENSG00000170091	ENSG00000170961	ENSG00000121410
ENSG00000164283	ENSG00000099953	ENSG00000148680	ENSG00000164296
ENSG00000187531	ENSG00000129071	ENSG00000173262	ENSG00000125746
ENSG00000061337	ENSG00000198668	ENSG00000163697	ENSG00000128271
ENSG00000180891	ENSG00000145495	ENSG00000149554	ENSG00000154342
ENSG00000132872	ENSG00000156983	ENSG00000102858	ENSG00000156860
ENSG00000169105	ENSG00000047578	ENSG00000197386	ENSG00000173705
ENSG00000137872	ENSG00000067167	ENSG00000151617	ENSG00000120314
ENSG00000130734	ENSG00000214046	ENSG00000114423	ENSG00000196611
ENSG00000132463	ENSG00000125285	ENSG00000095587	ENSG00000198715
ENSG00000171735	ENSG00000170681	ENSG00000110066	ENSG00000164087
ENSG00000196876	ENSG00000121989	ENSG00000167202	ENSG00000137124
ENSG00000178177	ENSG00000172053	ENSG00000179456	ENSG00000180346
ENSG00000111011	ENSG00000163132	ENSG00000179057	ENSG00000180245
ENSG00000204673	ENSG00000101888	ENSG00000147475	ENSG00000109466
ENSG00000071242	ENSG00000146463	ENSG00000145194	ENSG00000152219
ENSG00000172292	ENSG00000108759	ENSG00000169184	ENSG00000185272
ENSG00000164011	ENSG00000166090	ENSG00000164619	ENSG00000143294
ENSG00000170289	ENSG00000070371	ENSG00000124198	ENSG00000006607
ENSG00000145040	ENSG00000163840	ENSG00000118507	ENSG00000178307
ENSG00000174059	ENSG00000006047	ENSG00000134769	ENSG00000088782
ENSG00000108797	ENSG00000132819	ENSG00000147488	ENSG00000107864
ENSG00000111669	ENSG00000072195	ENSG00000159714	ENSG00000205777
ENSG00000184602	ENSG00000132591	ENSG00000122591	ENSG00000176731
ENSG00000122584	ENSG00000158079	ENSG00000116128	ENSG00000154451
ENSG00000181894	ENSG00000168813	ENSG00000143127	ENSG00000180011
ENSG00000183960	ENSG00000135374	ENSG00000005810	ENSG00000164438
ENSG00000113811	ENSG00000177239	ENSG00000143375	ENSG00000163882
ENSG00000080822	ENSG00000184012	ENSG00000167977	ENSG00000170085
ENSG00000170456	ENSG00000170017	ENSG00000035862	ENSG00000081026
ENSG00000015676	ENSG00000053372	ENSG00000204963	ENSG00000150477
ENSG00000141542	ENSG00000077063	ENSG00000163812	ENSG00000101000
ENSG00000155085	ENSG00000099364	ENSG00000101311	ENSG00000143315
ENSG00000136895	ENSG00000099250	ENSG00000108518	ENSG00000163497
ENSG00000145808	ENSG00000134443	ENSG00000081853	ENSG00000179915
ENSG00000125746	ENSG00000141526	ENSG00000143033	ENSG00000099284
ENSG00000103710	ENSG00000107147	ENSG00000160014	ENSG00000129353
ENSG00000128271	ENSG00000155090	ENSG00000123360	ENSG00000104823
ENSG00000154342	ENSG00000145990	ENSG00000196353	ENSG00000135776
ENSG00000156860	ENSG00000101298	ENSG00000119396	ENSG00000143226
ENSG00000186812	ENSG00000111046	ENSG00000102931	ENSG00000125744

ENSG00000086666	ENSG00000121057	ENSG00000162300	ENSG00000185507
ENSG00000134001	ENSG00000131368	ENSG00000131503	ENSG00000165810
ENSG00000108848	ENSG00000110756	ENSG00000184481	ENSG00000184492
ENSG00000109466	ENSG00000013573	ENSG00000107874	ENSG00000177383
ENSG00000143294	ENSG00000158578	ENSG00000120705	ENSG00000196917
ENSG00000203961	ENSG00000188322	ENSG00000137825	ENSG00000102030
ENSG00000178307	ENSG00000163703	ENSG00000125347	ENSG00000076356
ENSG00000107864	ENSG00000165194	ENSG00000100836	ENSG00000117154
ENSG00000112339	ENSG00000114520	ENSG00000138029	ENSG00000117519
ENSG00000180011	ENSG00000124226	ENSG00000119125	ENSG00000162482
ENSG00000164438	ENSG00000120963	ENSG00000128594	ENSG00000172955
ENSG00000107968	ENSG00000182400	ENSG00000125977	ENSG00000120262
ENSG00000185555	ENSG00000175928	ENSG00000004455	ENSG00000166049
ENSG00000085511	ENSG00000184677	ENSG00000077782	ENSG00000140511
ENSG00000170085	ENSG00000129116	ENSG00000073670	ENSG00000168936
ENSG00000173193	ENSG00000076928	ENSG00000141668	ENSG00000160888
ENSG00000075391	ENSG00000139197	ENSG00000156463	ENSG00000115524
ENSG00000143771	ENSG00000185651	ENSG00000078304	ENSG00000143333
ENSG00000123572	ENSG00000103496	ENSG00000120756	ENSG00000180901
ENSG00000175470	ENSG00000183814	ENSG00000152779	ENSG00000185361
ENSG00000179915	ENSG00000005238	ENSG00000173868	ENSG00000169919
ENSG00000109079	ENSG00000099814	ENSG00000114354	ENSG00000168765
ENSG00000129353	ENSG00000092871	ENSG00000197081	ENSG00000204176
ENSG00000138347	ENSG00000154134	ENSG00000182591	ENSG00000138386
ENSG00000143226	ENSG00000164603	ENSG00000048028	ENSG00000118113
ENSG00000153066	ENSG00000100075	ENSG00000133424	ENSG00000143179
ENSG00000153162	ENSG00000175544	ENSG00000068615	ENSG00000007541
ENSG00000125744	ENSG00000171791	ENSG00000164076	ENSG00000112273
ENSG00000165792	ENSG00000121101	ENSG00000160883	ENSG00000184857
ENSG00000139116	ENSG00000129696	ENSG00000122877	ENSG00000010610
ENSG00000116874	ENSG00000198576	ENSG00000168481	ENSG00000174243
ENSG00000115825	ENSG00000184983	ENSG00000182944	ENSG00000072401
ENSG00000076356	ENSG00000196526	ENSG00000168079	ENSG00000138207
ENSG00000173812	ENSG00000130522	ENSG00000073060	ENSG00000171862
ENSG00000117519	ENSG00000169851	ENSG00000111266	ENSG00000101542
ENSG00000204120	ENSG00000111605	ENSG00000027075	ENSG00000127804
ENSG00000168936	ENSG00000037280	ENSG00000185950	ENSG00000121578
ENSG00000169609	ENSG00000177688	ENSG00000091640	ENSG00000167992
ENSG00000115524	ENSG00000173918	ENSG00000196591	ENSG00000165494
ENSG00000178057	ENSG00000156398	ENSG00000170091	ENSG00000136522
ENSG00000160888	ENSG00000179361	ENSG00000099953	ENSG00000011600
ENSG00000143333	ENSG00000044524	ENSG00000144959	ENSG00000118496
ENSG00000175395	ENSG00000114302	ENSG00000111737	ENSG00000129317
ENSG00000138639	ENSG00000197142	ENSG00000169564	ENSG00000110148
ENSG00000075234	ENSG00000197965	ENSG00000145495	ENSG00000132522
ENSG00000138386	ENSG00000198879	ENSG00000156983	ENSG00000168065
ENSG00000177519	ENSG00000062725	ENSG00000067167	ENSG00000150527
ENSG00000143179	ENSG00000239306	ENSG00000125954	ENSG00000035681
ENSG00000115239	ENSG00000065809	ENSG00000125285	ENSG00000125485
ENSG00000121481	ENSG00000117697	ENSG00000121989	ENSG00000049541
ENSG00000067066	ENSG00000072163	ENSG00000172053	ENSG00000184863
ENSG00000010610	ENSG00000196628	ENSG00000101888	ENSG00000130997
ENSG00000144452	ENSG00000163064	ENSG00000121005	ENSG00000168743
ENSG00000072401	ENSG00000172733	ENSG00000097021	ENSG00000132383
ENSG00000171862	ENSG00000143702	ENSG00000146463	ENSG00000105298
ENSG00000101542	ENSG00000189221	ENSG00000139220	ENSG00000129003
ENSG00000164258	ENSG00000160785	ENSG00000166090	ENSG00000180209
ENSG00000176871	ENSG00000183943	ENSG00000102287	ENSG00000165661
ENSG00000125834	ENSG00000196482	ENSG00000188549	ENSG00000118271
ENSG00000127804	ENSG00000170144	ENSG00000006047	ENSG00000166896
ENSG00000136630	ENSG00000198728	ENSG00000196914	ENSG00000165219
ENSG00000167325	ENSG00000214113	ENSG00000012048	ENSG00000139546
ENSG00000162630	ENSG00000100625	ENSG00000164815	ENSG00000119866
ENSG00000171100	ENSG00000196236	ENSG00000132819	ENSG00000183386

ENSG00000165494	ENSG00000185946	ENSG00000134758	ENSG00000121775
ENSG00000151704	ENSG00000115616	ENSG00000089225	ENSG00000121068
ENSG00000132716	ENSG00000139173	ENSG00000165197	ENSG00000123560
ENSG00000118496	ENSG00000160209	ENSG00000135374	ENSG00000173065
ENSG00000108523	ENSG00000133101	ENSG00000177239	ENSG00000170482
ENSG00000110148	ENSG00000173153	ENSG00000100811	ENSG00000139572
ENSG00000132522	ENSG00000168958	ENSG00000170017	ENSG00000186446
ENSG00000150527	ENSG00000104723	ENSG00000134684	ENSG00000042753
ENSG00000116337	ENSG00000114120	ENSG00000161021	ENSG00000198336
ENSG00000147724	ENSG00000137474	ENSG00000171940	ENSG00000134686
ENSG00000035681	ENSG00000164010	ENSG00000151702	ENSG00000007944
ENSG00000147647	ENSG00000111667	ENSG00000138448	ENSG00000164972
ENSG00000168614	ENSG00000177697	ENSG00000077063	ENSG00000077092
ENSG00000115254	ENSG00000137656	ENSG00000099364	ENSG00000171067
ENSG00000092068	ENSG00000072415	ENSG00000099250	ENSG00000182255
ENSG00000111321	ENSG00000106617	ENSG00000183020	ENSG00000132744
ENSG00000197043	ENSG00000162804	ENSG00000108788	ENSG00000240694
ENSG00000162551	ENSG00000162616	ENSG00000136156	ENSG00000167578
ENSG00000197822	ENSG00000253313	ENSG00000151240	ENSG00000158571
ENSG00000165661	ENSG00000170381	ENSG00000171044	ENSG00000135951
ENSG00000106089	ENSG00000204681	ENSG00000141526	ENSG00000178828
ENSG00000140632	ENSG00000174807	ENSG00000108932	ENSG00000167004
ENSG00000118271	ENSG00000124789	ENSG00000135298	ENSG00000174899
ENSG00000180008	ENSG00000182107	ENSG00000155090	ENSG00000131143
ENSG00000165219	ENSG00000105556	ENSG00000145990	ENSG00000101974
ENSG00000137944	ENSG00000104205	ENSG00000101298	ENSG00000140548
ENSG00000111445	ENSG00000175274	ENSG00000104419	ENSG00000084073
ENSG00000139546	ENSG00000116096	ENSG00000111046	ENSG00000197530
ENSG00000101276	ENSG00000006634	ENSG00000167528	ENSG00000141013
ENSG00000183386	ENSG00000105419	ENSG00000121057	ENSG00000243725
ENSG00000119866	ENSG00000145332	ENSG00000131368	ENSG00000182583
ENSG00000121775	ENSG00000198478	ENSG00000110756	ENSG00000160633
ENSG00000072315	ENSG00000155304	ENSG00000198363	ENSG00000118217
ENSG00000078328	ENSG00000145708	ENSG00000095397	ENSG00000128185
ENSG00000121068	ENSG00000179454	ENSG00000163703	ENSG00000184056
ENSG00000153012	ENSG00000143450	ENSG00000171502	ENSG00000178821
ENSG00000023909	ENSG00000145536	ENSG00000114520	ENSG00000164924
ENSG00000104863	ENSG00000123454	ENSG00000124226	ENSG00000198740
ENSG00000198925	ENSG00000145349	ENSG00000120963	ENSG00000135625
ENSG00000173065	ENSG00000165023	ENSG00000132405	ENSG00000138757
ENSG00000123560	ENSG00000177889	ENSG00000166224	ENSG00000047932
ENSG00000109919	ENSG00000137573	ENSG00000175928	ENSG00000140326
ENSG00000139549	ENSG00000130962	ENSG00000184677	ENSG00000142867
ENSG00000170522	ENSG00000130054	ENSG00000102038	ENSG00000187288
ENSG00000105568	ENSG00000165071	ENSG00000129116	ENSG00000011304
ENSG00000042753	ENSG00000185567	ENSG00000139197	ENSG00000154975
ENSG00000101464	ENSG00000179163	ENSG00000185651	ENSG00000156471
ENSG00000007944	ENSG00000179010	ENSG00000103496	ENSG00000146085
ENSG00000182985	ENSG00000123091	ENSG00000183814	ENSG00000162458
ENSG00000134686	ENSG00000087299	ENSG00000171621	ENSG00000135698
ENSG00000164972	ENSG00000198643	ENSG00000005238	ENSG00000105991
ENSG00000077092	ENSG00000091527	ENSG00000079432	ENSG00000187720
ENSG00000171067	ENSG00000184811	ENSG00000092871	ENSG00000198650
ENSG00000168452	ENSG00000140470	ENSG00000084693	ENSG00000197930
ENSG00000182255	ENSG00000160803	ENSG00000135960	ENSG00000072736
ENSG00000173581	ENSG00000150760	ENSG00000164603	ENSG00000112096
ENSG00000123066	ENSG00000234545	ENSG00000100075	ENSG00000129194
ENSG00000137496	ENSG00000101444	ENSG00000165476	ENSG00000105392
ENSG00000100575	ENSG00000204271	ENSG00000147533	ENSG00000143469
ENSG00000178828	ENSG00000179564	ENSG00000171791	ENSG00000134121
ENSG00000171056	ENSG00000132801	ENSG00000121101	ENSG00000121542
ENSG00000167004	ENSG00000125386	ENSG00000105854	ENSG00000119596
ENSG00000198300	ENSG00000153551	ENSG00000104435	ENSG00000121871
ENSG00000101974	ENSG00000134917	ENSG00000100221	ENSG00000175792

ENSG00000140548	ENSG00000076043	ENSG00000156639	ENSG00000169696
ENSG00000170854	ENSG00000141579	ENSG00000171604	ENSG00000148680
ENSG00000084073	ENSG00000050030	ENSG00000198576	ENSG00000171291
ENSG00000066455	ENSG00000124786	ENSG00000141433	ENSG00000141642
ENSG00000154764	ENSG00000138311	ENSG00000146425	ENSG00000214575
ENSG00000123411	ENSG00000163380	ENSG00000196526	ENSG00000173262
ENSG00000145687	ENSG00000175197	ENSG00000130522	ENSG00000198417
ENSG00000160633	ENSG00000175602	ENSG00000169851	ENSG00000163697
ENSG00000013725	ENSG00000244005	ENSG00000111605	ENSG00000006282
ENSG00000167971	ENSG00000116852	ENSG00000174780	ENSG00000197496
ENSG00000132517	ENSG00000105429	ENSG00000156398	ENSG00000135318
ENSG00000127124	ENSG00000093072	ENSG00000161405	ENSG00000149554
ENSG00000164924	ENSG00000151532	ENSG00000148935	ENSG00000181036
ENSG00000135625	ENSG00000005955	ENSG00000165802	ENSG00000147140
ENSG00000127528	ENSG00000129682	ENSG00000117090	ENSG00000179673
ENSG00000138757	ENSG00000056097	ENSG00000179361	ENSG00000172724
ENSG00000198142	ENSG00000115306	ENSG00000044524	ENSG00000174407
ENSG00000099797	ENSG00000038210	ENSG00000095951	ENSG00000163736
ENSG00000182195	ENSG00000063244	ENSG00000019505	ENSG00000161243
ENSG00000185519	ENSG00000164849	ENSG00000124216	ENSG00000138050
ENSG00000011304	ENSG00000116285	ENSG00000114302	ENSG00000132010
ENSG00000154975	ENSG00000146433	ENSG00000198746	ENSG00000167202
ENSG00000198824	ENSG00000113273	ENSG00000197965	ENSG00000179057
ENSG00000100060	ENSG00000186231	ENSG00000062725	ENSG00000147475
ENSG00000156471	ENSG00000100012	ENSG00000067560	ENSG00000080819
ENSG00000114126	ENSG00000111276	ENSG00000070886	ENSG00000135114
ENSG00000105991	ENSG00000198909	ENSG00000196632	ENSG00000145194
ENSG00000187720	ENSG00000100604	ENSG00000196628	ENSG00000106636
ENSG00000153201	ENSG00000105323	ENSG00000103194	ENSG00000164619
ENSG00000105647	ENSG00000124177	ENSG00000043591	ENSG00000118507
ENSG00000166478	ENSG00000099956	ENSG00000163064	ENSG00000147488
ENSG00000102054	ENSG00000235718	ENSG00000172733	ENSG00000185100
ENSG00000168005	ENSG00000166987	ENSG00000185591	ENSG00000187808
ENSG00000072736	ENSG00000106688	ENSG00000143702	ENSG00000150681
ENSG00000134121	ENSG00000203879	ENSG00000160785	ENSG00000124733
ENSG00000138675	ENSG00000109220	ENSG00000196482	ENSG00000143127
ENSG00000121871	ENSG00000117174	ENSG00000198728	ENSG00000005810
ENSG00000170961	ENSG00000165102	ENSG00000142453	ENSG00000125375
ENSG00000148680	ENSG00000178149	ENSG00000107104	ENSG00000229183
ENSG00000082146	ENSG00000141279	ENSG00000148297	ENSG00000086548
ENSG00000173262	ENSG00000189298	ENSG00000185946	ENSG00000163812
ENSG00000197496	ENSG00000167695	ENSG00000139173	ENSG00000162399
ENSG00000163697	ENSG00000134245	ENSG00000183873	ENSG00000011295
ENSG00000135318	ENSG00000124201	ENSG00000173153	ENSG00000085563
ENSG00000149554	ENSG00000136205	ENSG00000136848	ENSG00000182156
ENSG00000179673	ENSG00000177058	ENSG00000165006	ENSG00000100095
ENSG00000198457	ENSG00000166960	ENSG00000104723	ENSG00000161091
ENSG00000197386	ENSG00000197557	ENSG00000175779	ENSG00000112029
ENSG00000102858	ENSG00000182902	ENSG00000105700	ENSG00000108518
ENSG00000151617	ENSG00000167081	ENSG00000155380	ENSG00000172156
ENSG00000114423	ENSG00000183578	ENSG00000135924	ENSG00000123360
ENSG00000095587	ENSG00000213983	ENSG00000111667	ENSG00000160014
ENSG00000167202	ENSG00000146006	ENSG00000177697	ENSG00000222040
ENSG00000110066	ENSG00000085831	ENSG00000072415	ENSG00000204577
ENSG00000179057	ENSG00000143401	ENSG00000055070	ENSG00000076248
ENSG00000147475	ENSG00000186318	ENSG00000151090	ENSG00000132793
ENSG00000179456	ENSG00000112855	ENSG00000158301	ENSG00000127362
ENSG00000197929	ENSG00000153531	ENSG00000106617	ENSG00000104714
ENSG00000145194	ENSG00000166716	ENSG00000164117	ENSG00000131503
ENSG00000169184	ENSG00000127946	ENSG00000138092	ENSG00000154611
ENSG00000138663	ENSG00000163026	ENSG00000162616	ENSG00000213619
ENSG00000164619	ENSG00000183160	ENSG00000112033	ENSG00000241994
ENSG00000124198	ENSG00000242108	ENSG00000124789	ENSG00000179097
ENSG00000118507	ENSG00000083844	ENSG00000165478	ENSG00000205846

ENSG00000134769	ENSG00000125841	ENSG00000170616	ENSG00000163141
ENSG00000147488	ENSG00000147601	ENSG00000105556	ENSG00000100836
ENSG00000147571	ENSG00000136052	ENSG00000104205	ENSG00000174599
ENSG00000159714	ENSG00000120087	ENSG00000078098	ENSG00000138029
ENSG00000124733	ENSG00000139719	ENSG00000006634	ENSG00000112280
ENSG00000143127	ENSG00000120833	ENSG00000156976	ENSG00000135454
ENSG00000122591	ENSG00000144583	ENSG00000147813	ENSG00000185803
ENSG00000116128	ENSG00000114805	ENSG00000198478	ENSG00000134453
ENSG00000005810	ENSG00000174628	ENSG00000155304	ENSG00000004455
ENSG00000125375	ENSG00000117500	ENSG00000148730	ENSG00000077782
ENSG00000143375	ENSG00000156920	ENSG00000161654	ENSG00000165688
ENSG00000167977	ENSG00000167173	ENSG00000179454	ENSG00000080166
ENSG00000035862	ENSG00000178691	ENSG00000163126	ENSG00000119760
ENSG00000163812	ENSG00000158402	ENSG00000143450	ENSG00000173947
ENSG00000204963	ENSG00000163352	ENSG00000118564	ENSG00000073670
ENSG00000181110	ENSG00000005194	ENSG00000131459	ENSG00000125462
ENSG00000171530	ENSG00000204439	ENSG00000145349	ENSG00000213420
ENSG00000177735	ENSG00000154127	ENSG00000165023	ENSG00000120756
ENSG00000011295	ENSG00000137672	ENSG00000172349	ENSG00000138964
ENSG00000101311	ENSG00000162688	ENSG00000096746	ENSG00000105136
ENSG00000108518	ENSG00000183691	ENSG00000177889	ENSG00000166847
ENSG00000123360	ENSG00000095139	ENSG00000137573	ENSG00000182591
ENSG00000081853	ENSG00000075891	ENSG00000204228	ENSG00000151466
ENSG00000143033	ENSG00000175262	ENSG00000130962	ENSG00000129480
ENSG00000160014	ENSG00000169710	ENSG00000177551	ENSG00000048028
ENSG00000196353	ENSG00000179965	ENSG00000135482	ENSG00000138036
ENSG00000102931	ENSG00000184743	ENSG00000128191	ENSG00000188229
ENSG00000119396	ENSG00000162980	ENSG00000130054	ENSG00000159674
ENSG00000162300	ENSG00000152484	ENSG00000151348	ENSG00000187461
ENSG00000131503	ENSG00000146122	ENSG00000198324	ENSG00000160883
ENSG00000184481	ENSG00000183682	ENSG00000179010	ENSG00000164076
ENSG00000107874	ENSG00000118729	ENSG00000177685	ENSG00000127399
ENSG00000120705	ENSG00000132823	ENSG00000131653	ENSG00000106415
ENSG00000158220	ENSG00000138175	ENSG00000123091	ENSG00000073060
ENSG00000137825	ENSG00000079482	ENSG00000125447	ENSG00000168079
ENSG00000125347	ENSG00000173482	ENSG00000105887	ENSG00000111266
ENSG00000100836	ENSG00000117682	ENSG00000158850	ENSG00000185950
ENSG00000138029	ENSG00000169045	ENSG00000136874	ENSG00000196591
ENSG00000112280	ENSG00000108405	ENSG00000091527	ENSG00000185177
ENSG00000174599	ENSG00000157045	ENSG00000153714	ENSG00000099953
ENSG00000119125	ENSG00000119950	ENSG00000176454	ENSG00000129071
ENSG00000128594	ENSG00000100994	ENSG00000107140	ENSG00000198668
ENSG00000185803	ENSG00000065882	ENSG00000135678	ENSG00000145495
ENSG00000125977	ENSG00000244486	ENSG00000150760	ENSG00000156983
ENSG00000004455	ENSG00000198131	ENSG00000149658	ENSG00000067167
ENSG00000077782	ENSG00000105173	ENSG00000142611	ENSG00000047578
ENSG00000073670	ENSG00000189308	ENSG00000054611	ENSG00000214046
ENSG00000156463	ENSG00000186480	ENSG00000148798	ENSG00000100395
ENSG00000141668	ENSG00000170180	ENSG00000101444	ENSG00000125285
ENSG00000120756	ENSG00000134198	ENSG00000179564	ENSG00000121989
ENSG00000078304	ENSG00000136720	ENSG00000132801	ENSG00000243708
ENSG00000152779	ENSG00000130638	ENSG00000163032	ENSG00000163132
ENSG00000173868	ENSG00000144671	ENSG00000125386	ENSG00000101888
ENSG00000154114	ENSG00000125398	ENSG00000140859	ENSG00000228083
ENSG00000114354	ENSG00000241360	ENSG00000076043	ENSG00000108759
ENSG00000197081	ENSG00000114757	ENSG00000139636	ENSG00000146463
ENSG00000151466	ENSG00000110768	ENSG00000124786	ENSG00000070371
ENSG00000182591	ENSG00000066027	ENSG00000117620	ENSG00000107014
ENSG00000048028	ENSG00000183317	ENSG00000152455	ENSG00000163840
ENSG00000133424	ENSG00000163083	ENSG00000146535	ENSG00000181562
ENSG00000068615	ENSG00000118432	ENSG00000183508	ENSG00000006047
ENSG00000160883	ENSG00000094804	ENSG00000111653	ENSG00000072195
ENSG00000122877	ENSG00000132254	ENSG00000163347	ENSG00000038945
ENSG00000164076	ENSG00000168615	ENSG00000138650	ENSG00000132591

ENSG00000168481	ENSG00000133193	ENSG00000151532	ENSG00000205358
ENSG00000153923	ENSG00000109101	ENSG00000005955	ENSG00000135374
ENSG00000182944	ENSG00000174516	ENSG00000070269	ENSG00000158079
ENSG00000117600	ENSG00000196422	ENSG00000175497	ENSG00000168813
ENSG00000168079	ENSG00000177300	ENSG00000140519	ENSG00000177239
ENSG00000167685	ENSG00000196935	ENSG00000129682	ENSG00000184012
ENSG00000073060	ENSG00000163359	ENSG00000138162	ENSG00000170017
ENSG00000027075	ENSG00000089154	ENSG00000056097	ENSG00000077063
ENSG00000111266	ENSG00000103202	ENSG00000155876	ENSG00000099364
ENSG00000185950	ENSG00000121060	ENSG00000115306	ENSG00000099250
ENSG00000091640	ENSG00000249158	ENSG00000109089	ENSG00000134443
ENSG00000172775	ENSG00000124610	ENSG00000038210	ENSG00000108691
ENSG00000196591	ENSG00000196711	ENSG00000120805	ENSG00000141526
ENSG00000170091	ENSG00000173452	ENSG00000164849	ENSG00000107147
ENSG00000099953	ENSG00000186472	ENSG00000116285	ENSG00000170619
ENSG00000144959	ENSG00000197448	ENSG00000182504	ENSG00000155090
ENSG00000111737	ENSG00000095370	ENSG00000135519	ENSG00000145990
ENSG00000106049	ENSG00000197601	ENSG00000186231	ENSG00000101298
ENSG00000167720	ENSG00000139291	ENSG00000111276	ENSG00000144891
ENSG00000169564	ENSG00000090104	ENSG00000049759	ENSG00000111046
ENSG00000164329	ENSG00000075624	ENSG00000198909	ENSG00000104671
ENSG00000145495	ENSG00000034152	ENSG00000105323	ENSG00000121057
ENSG00000176909	ENSG00000101670	ENSG00000124177	ENSG00000110756
ENSG00000156983	ENSG00000174640	ENSG00000174502	ENSG00000131368
ENSG00000067167	ENSG00000152784	ENSG00000149503	ENSG00000126259
ENSG00000125954	ENSG00000157450	ENSG00000170027	ENSG00000013573
ENSG00000125285	ENSG00000102878	ENSG00000177575	ENSG00000158578
ENSG00000121989	ENSG00000135365	ENSG00000198858	ENSG00000188322
ENSG00000108669	ENSG00000164916	ENSG00000099956	ENSG00000163703
ENSG00000123595	ENSG00000167716	ENSG00000145780	ENSG00000165194
ENSG00000172053	ENSG00000182871	ENSG00000137267	ENSG00000114520
ENSG00000101888	ENSG00000136732	ENSG00000166987	ENSG00000120963
ENSG00000121005	ENSG00000167323	ENSG00000171150	ENSG00000159527
ENSG00000146463	ENSG00000226979	ENSG00000106688	ENSG00000182400
ENSG00000097021	ENSG00000079557	ENSG00000169016	ENSG00000175928
ENSG00000139220	ENSG00000135070	ENSG00000203879	ENSG00000203618
ENSG00000166090	ENSG00000113648	ENSG00000171443	ENSG00000184677
ENSG00000102287	ENSG00000152402	ENSG00000186648	ENSG00000139197
ENSG00000188549	ENSG00000215386	ENSG00000109220	ENSG00000129116
ENSG00000006047	ENSG00000241973	ENSG00000058262	ENSG00000185651
ENSG00000196914	ENSG00000240583	ENSG00000173898	ENSG00000183814
ENSG00000012048	ENSG00000181409	ENSG00000072274	ENSG00000005238
ENSG00000164815	ENSG00000159082	ENSG00000108175	ENSG00000185627
ENSG00000132819	ENSG00000106330	ENSG00000155324	ENSG00000099814
ENSG00000140367	ENSG00000137393	ENSG00000070366	ENSG00000092871
ENSG00000089225	ENSG00000149480	ENSG00000162231	ENSG00000155269
ENSG00000134758	ENSG00000166592	ENSG00000116698	ENSG00000136824
ENSG00000196275	ENSG00000133466	ENSG00000178149	ENSG00000100122
ENSG00000165197	ENSG00000101417	ENSG00000133401	ENSG00000071894
ENSG00000165521	ENSG00000129535	ENSG00000110958	ENSG00000165506
ENSG00000038945	ENSG00000167549	ENSG00000144560	ENSG00000100075
ENSG00000135374	ENSG00000184160	ENSG00000141279	ENSG00000180210
ENSG00000170017	ENSG00000164543	ENSG00000147526	ENSG00000175544
ENSG00000100811	ENSG00000174672	ENSG00000134508	ENSG00000171791
ENSG00000177239	ENSG00000182747	ENSG00000167695	ENSG00000121101
ENSG00000134684	ENSG00000158792	ENSG00000134245	ENSG00000129696
ENSG00000151702	ENSG00000113262	ENSG00000124201	ENSG00000184983
ENSG00000161021	ENSG00000168758	ENSG00000172530	ENSG00000198576
ENSG00000171940	ENSG00000198420	ENSG00000138622	ENSG00000146918
ENSG00000138448	ENSG00000108587	ENSG00000136205	ENSG00000196526
ENSG00000113048	ENSG00000131941	ENSG00000118515	ENSG00000130522
ENSG00000077063	ENSG00000113328	ENSG00000177058	ENSG00000111605
ENSG00000099364	ENSG00000165807	ENSG00000166960	ENSG00000169851
ENSG00000099250	ENSG00000181830	ENSG00000131188	ENSG00000037280

ENSG00000183020	ENSG00000136425	ENSG00000182902	ENSG00000177688
ENSG00000108788	ENSG00000149016	ENSG00000048540	ENSG00000178597
ENSG00000136156	ENSG00000170941	ENSG00000167081	ENSG00000156398
ENSG00000151240	ENSG00000149972	ENSG00000183578	ENSG00000103355
ENSG00000108691	ENSG00000155099	ENSG00000114853	ENSG00000173918
ENSG00000171044	ENSG00000144597	ENSG00000107372	ENSG00000179361
ENSG00000141526	ENSG00000167601	ENSG00000084628	ENSG00000044524
ENSG00000108932	ENSG00000170677	ENSG00000186318	ENSG00000205517
ENSG00000173702	ENSG00000167123	ENSG00000144749	ENSG00000114302
ENSG00000155090	ENSG00000149269	ENSG00000185909	ENSG00000083812
ENSG00000135298	ENSG00000162073	ENSG00000129219	ENSG00000064692
ENSG00000145990	ENSG00000155052	ENSG00000073803	ENSG00000197142
ENSG00000101298	ENSG00000185875	ENSG00000070061	ENSG00000151151
ENSG00000104419	ENSG00000116819	ENSG00000163026	ENSG00000198879
ENSG00000111046	ENSG00000146216	ENSG00000206053	ENSG00000197965
ENSG00000167528	ENSG00000125170	ENSG00000074416	ENSG00000062725
ENSG00000121057	ENSG00000047644	ENSG00000001626	ENSG00000151790
ENSG00000131368	ENSG00000174697	ENSG00000125798	ENSG00000239306
ENSG00000110756	ENSG00000159184	ENSG00000136052	ENSG00000065809
ENSG00000100829	ENSG00000178607	ENSG00000164692	ENSG00000170369
ENSG00000198363	ENSG00000115183	ENSG00000101384	ENSG00000072163
ENSG00000095397	ENSG00000180667	ENSG00000100934	ENSG00000143727
ENSG00000165501	ENSG00000196588	ENSG00000120087	ENSG00000196628
ENSG00000163703	ENSG00000116741	ENSG00000120833	ENSG00000163064
ENSG00000171502	ENSG00000213699	ENSG00000173926	ENSG00000172733
ENSG00000114520	ENSG00000067955	ENSG00000076770	ENSG00000131591
ENSG00000124226	ENSG00000135617	ENSG00000151893	ENSG00000073737
ENSG00000120963	ENSG00000152578	ENSG00000174628	ENSG00000189221
ENSG00000197995	ENSG00000015133	ENSG00000117500	ENSG00000143702
ENSG00000132405	ENSG00000167085	ENSG00000167173	ENSG00000106560
ENSG00000166224	ENSG00000131979	ENSG00000137693	ENSG00000129988
ENSG00000184677	ENSG00000108256	ENSG00000204439	ENSG00000183943
ENSG00000175928	ENSG00000158008	ENSG00000154127	ENSG00000196482
ENSG00000102038	ENSG00000137285	ENSG00000137672	ENSG00000172215
ENSG00000129116	ENSG00000147324	ENSG00000183691	ENSG00000198728
ENSG00000139197	ENSG00000166734	ENSG00000095139	ENSG00000173041
ENSG00000206247	ENSG00000109705	ENSG00000075891	ENSG00000099849
ENSG00000185651	ENSG00000163281	ENSG00000168066	ENSG00000100625
ENSG00000103496	ENSG00000150907	ENSG00000169710	ENSG00000115616
ENSG00000171621	ENSG00000114107	ENSG00000136068	ENSG00000205670
ENSG00000183814	ENSG00000173786	ENSG00000163683	ENSG00000139173
ENSG00000079432	ENSG00000101452	ENSG00000119812	ENSG00000160209
ENSG00000005238	ENSG00000162367	ENSG00000143797	ENSG00000133101
ENSG00000092871	ENSG00000051009	ENSG00000184743	ENSG00000173153
ENSG00000084693	ENSG00000110079	ENSG00000026025	ENSG00000152454
ENSG00000134107	ENSG00000132464	ENSG00000162980	ENSG00000185686
ENSG00000163377	ENSG00000105939	ENSG00000173020	ENSG00000104723
ENSG00000162711	ENSG00000137871	ENSG00000152484	ENSG00000128218
ENSG00000135960	ENSG00000134970	ENSG00000029993	ENSG00000114120
ENSG00000165476	ENSG00000100314	ENSG00000173166	ENSG00000164010
ENSG00000164603	ENSG00000175746	ENSG00000158156	ENSG00000111667
ENSG00000100075	ENSG00000175414	ENSG00000118729	ENSG00000177697
ENSG00000147533	ENSG00000166507	ENSG00000132823	ENSG00000163362
ENSG00000204914	ENSG00000100413	ENSG00000138175	ENSG00000123500
ENSG00000164484	ENSG00000034713	ENSG00000173482	ENSG00000103249
ENSG00000171791	ENSG00000152465	ENSG00000117682	ENSG00000072415
ENSG00000121101	ENSG00000141384	ENSG00000169045	ENSG00000105339
ENSG00000105854	ENSG00000149577	ENSG00000184564	ENSG00000106617
ENSG00000170540	ENSG00000167654	ENSG00000148848	ENSG00000162804
ENSG00000104435	ENSG00000157470	ENSG00000108405	ENSG00000104848
ENSG00000155330	ENSG00000063241	ENSG00000119950	ENSG00000162616
ENSG00000100221	ENSG00000111371	ENSG00000182010	ENSG00000170381
ENSG00000171604	ENSG00000167112	ENSG00000065882	ENSG00000124102
ENSG00000156639	ENSG00000143157	ENSG00000143420	ENSG00000137804

ENSG00000198576	ENSG00000179008	ENSG00000198739	ENSG00000174807
ENSG00000141433	ENSG00000205531	ENSG00000171522	ENSG00000071909
ENSG00000146425	ENSG00000138083	ENSG00000189308	ENSG00000124789
ENSG00000147257	ENSG00000168872	ENSG00000105173	ENSG00000182107
ENSG00000196526	ENSG00000216490	ENSG00000116991	ENSG00000175287
ENSG00000130522	ENSG00000131737	ENSG00000186480	ENSG00000104205
ENSG00000169851	ENSG00000108684	ENSG00000186298	ENSG00000175274
ENSG00000111605	ENSG00000169689	ENSG00000137947	ENSG00000116096
ENSG00000174780	ENSG00000163166	ENSG00000143384	ENSG00000104892
ENSG00000206376	ENSG00000134996	ENSG00000118707	ENSG00000135778
ENSG00000156398	ENSG00000111241	ENSG00000134198	ENSG00000105419
ENSG00000161405	ENSG00000159214	ENSG00000130638	ENSG00000006634
ENSG00000148935	ENSG00000196782	ENSG00000151150	ENSG00000145332
ENSG00000165802	ENSG00000135968	ENSG00000116473	ENSG00000140030
ENSG00000117090	ENSG00000187049	ENSG00000125398	ENSG00000080298
ENSG00000179943	ENSG00000114904	ENSG00000114757	ENSG00000182141
ENSG00000179361	ENSG00000020633	ENSG00000110768	ENSG00000198478
ENSG00000095951	ENSG00000104133	ENSG00000066027	ENSG00000221983
ENSG00000044524	ENSG00000038295	ENSG00000100335	ENSG00000108679
ENSG00000019505	ENSG00000172867	ENSG00000105245	ENSG00000145708
ENSG00000114302	ENSG00000165914	ENSG00000163083	ENSG00000142661
ENSG00000124216	ENSG00000106991	ENSG00000168214	ENSG00000130475
ENSG00000198746	ENSG00000139842	ENSG00000088179	ENSG00000109390
ENSG00000083812	ENSG00000181192	ENSG00000196730	ENSG00000143450
ENSG00000064692	ENSG00000225190	ENSG00000118432	ENSG00000145536
ENSG00000197965	ENSG00000184113	ENSG00000094804	ENSG00000221878
ENSG00000067560	ENSG00000092020	ENSG00000132254	ENSG00000123454
ENSG00000062725	ENSG00000101017	ENSG00000168615	ENSG00000145349
ENSG00000070886	ENSG00000111344	ENSG00000133193	ENSG00000165023
ENSG00000184922	ENSG00000128965	ENSG00000082458	ENSG00000177889
ENSG00000196632	ENSG00000183775	ENSG00000109101	ENSG00000130962
ENSG00000196628	ENSG00000166272	ENSG00000137809	ENSG00000137573
ENSG00000103194	ENSG00000123728	ENSG00000139318	ENSG00000165071
ENSG00000043591	ENSG00000152217	ENSG00000174516	ENSG00000185567
ENSG00000163064	ENSG00000177150	ENSG00000163630	ENSG00000179163
ENSG00000172733	ENSG00000164400	ENSG00000161642	ENSG00000116171
ENSG00000162885	ENSG00000155034	ENSG00000156011	ENSG00000179010
ENSG00000143702	ENSG00000187145	ENSG00000196422	ENSG00000123091
ENSG00000185591	ENSG00000100280	ENSG00000196935	ENSG00000099721
ENSG00000106560	ENSG00000158715	ENSG00000163359	ENSG00000087299
ENSG00000160785	ENSG00000174567	ENSG00000179284	ENSG00000175325
ENSG00000196482	ENSG00000101115	ENSG00000165660	ENSG00000091527
ENSG00000198728	ENSG00000141510	ENSG00000007047	ENSG00000160961
ENSG00000142453	ENSG00000175482	ENSG00000141540	ENSG00000010626
ENSG00000167487	ENSG00000100345	ENSG00000173452	ENSG00000141655
ENSG00000107104	ENSG00000159713	ENSG00000145715	ENSG00000184811
ENSG00000148297	ENSG00000092531	ENSG00000095370	ENSG00000181617
ENSG00000185946	ENSG00000180440	ENSG00000176406	ENSG00000120256
ENSG00000140395	ENSG00000183643	ENSG00000197601	ENSG00000140470
ENSG00000139173	ENSG00000004779	ENSG00000090104	ENSG00000150760
ENSG00000183873	ENSG00000109756	ENSG00000075624	ENSG00000160803
ENSG00000173153	ENSG00000137090	ENSG00000034152	ENSG00000167377
ENSG00000033011	ENSG00000172061	ENSG00000174640	ENSG00000101444
ENSG00000136848	ENSG00000105856	ENSG00000143248	ENSG00000204271
ENSG00000165006	ENSG00000063015	ENSG00000154358	ENSG00000182687
ENSG00000104723	ENSG00000157693	ENSG00000188827	ENSG00000179564
ENSG00000175779	ENSG00000198890	ENSG00000130147	ENSG00000132801
ENSG00000113643	ENSG00000170962	ENSG00000152784	ENSG00000174788
ENSG00000167183	ENSG00000096070	ENSG00000157450	ENSG00000174343
ENSG00000155659	ENSG00000069431	ENSG00000135365	ENSG00000125386
ENSG00000105700	ENSG00000184216	ENSG00000182718	ENSG00000153551
ENSG00000155380	ENSG00000120875	ENSG00000167716	ENSG00000134917
ENSG00000135924	ENSG00000167110	ENSG00000182871	ENSG00000108700
ENSG00000111667	ENSG00000119321	ENSG00000117625	ENSG00000076043

ENSG00000177697	ENSG00000139200	ENSG00000171316	ENSG00000141579
ENSG00000163362	ENSG00000135870	ENSG00000167323	ENSG00000050030
ENSG00000123500	ENSG00000165646	ENSG00000081791	ENSG00000154227
ENSG00000055070	ENSG00000145863	ENSG00000142459	ENSG00000138311
ENSG00000072415	ENSG00000164398	ENSG00000077157	ENSG00000163380
ENSG00000158301	ENSG00000144847	ENSG00000135070	ENSG00000175197
ENSG00000106617	ENSG00000172340	ENSG00000113648	ENSG00000244005
ENSG00000151090	ENSG00000214128	ENSG00000137055	ENSG00000116852
ENSG00000164117	ENSG00000128059	ENSG00000163661	ENSG00000105429
ENSG00000138092	ENSG00000168487	ENSG00000155506	ENSG00000093072
ENSG00000162616	ENSG00000156858	ENSG00000152402	ENSG00000151532
ENSG00000142186	ENSG00000101310	ENSG00000121753	ENSG00000164081
ENSG00000112033	ENSG00000130066	ENSG00000173068	ENSG00000129682
ENSG00000071909	ENSG00000103978	ENSG00000165795	ENSG00000189169
ENSG00000124789	ENSG00000188636	ENSG00000107295	ENSG00000056097
ENSG00000163806	ENSG00000214655	ENSG00000159082	ENSG00000115306
ENSG00000196141	ENSG00000175556	ENSG00000107863	ENSG00000153563
ENSG00000105556	ENSG00000185518	ENSG00000106330	ENSG00000038210
ENSG00000104205	ENSG00000086506	ENSG00000137393	ENSG00000063244
ENSG00000170616	ENSG00000120802	ENSG00000166592	ENSG00000104140
ENSG00000165478	ENSG00000185262	ENSG00000133466	ENSG00000164849
ENSG00000197179	ENSG00000066629	ENSG00000101417	ENSG00000116285
ENSG00000078098	ENSG00000110871	ENSG00000100439	ENSG00000146433
ENSG00000006634	ENSG00000182866	ENSG00000129535	ENSG00000113273
ENSG00000196166	ENSG00000040633	ENSG00000167549	ENSG00000186231
ENSG00000156976	ENSG00000061938	ENSG00000169228	ENSG00000147168
ENSG00000147813	ENSG00000115073	ENSG00000036530	ENSG00000100012
ENSG00000080298	ENSG00000147570	ENSG00000184160	ENSG00000068654
ENSG00000155304	ENSG00000167978	ENSG00000164543	ENSG00000197172
ENSG00000183473	ENSG00000106123	ENSG00000174672	ENSG00000111276
ENSG00000198478	ENSG00000204856	ENSG00000102753	ENSG00000154856
ENSG00000148730	ENSG00000168356	ENSG00000121774	ENSG00000177599
ENSG00000179454	ENSG00000100354	ENSG00000067606	ENSG00000100604
ENSG00000130475	ENSG00000189050	ENSG00000097033	ENSG00000198909
ENSG00000161654	ENSG00000168118	ENSG00000168758	ENSG00000184903
ENSG00000163126	ENSG00000187097	ENSG00000198420	ENSG00000124177
ENSG00000074660	ENSG00000071189	ENSG00000108587	ENSG00000105383
ENSG00000143450	ENSG00000175567	ENSG00000113328	ENSG00000235718
ENSG00000118564	ENSG00000141905	ENSG00000182533	ENSG00000171819
ENSG00000206156	ENSG00000162896	ENSG00000170633	ENSG00000106688
ENSG00000113194	ENSG00000147255	ENSG00000181830	ENSG00000166987
ENSG00000131459	ENSG00000104725	ENSG00000136425	ENSG00000188766
ENSG00000145349	ENSG00000213057	ENSG00000149016	ENSG00000203879
ENSG00000165023	ENSG00000108950	ENSG00000170941	ENSG00000128311
ENSG00000196218	ENSG00000197380	ENSG00000155099	ENSG00000183569
ENSG00000172349	ENSG00000134982	ENSG00000165084	ENSG00000147588
ENSG00000096746	ENSG00000125850	ENSG00000144597	ENSG00000198471
ENSG00000177889	ENSG00000122705	ENSG00000172260	ENSG00000165102
ENSG00000166855	ENSG00000112655	ENSG00000119408	ENSG00000093009
ENSG00000204228	ENSG00000198252	ENSG00000167601	ENSG00000178149
ENSG00000137573	ENSG00000100592	ENSG00000170677	ENSG00000100138
ENSG00000130962	ENSG00000184402	ENSG00000167123	ENSG00000186792
ENSG00000177551	ENSG00000174903	ENSG00000149269	ENSG00000141279
ENSG00000135482	ENSG00000128656	ENSG00000125170	ENSG00000101365
ENSG00000128191	ENSG00000146904	ENSG00000043143	ENSG00000125207
ENSG00000151348	ENSG00000213071	ENSG00000129451	ENSG00000167695
ENSG00000130054	ENSG00000166813	ENSG00000196932	ENSG00000134245
ENSG00000198324	ENSG00000088367	ENSG00000165775	ENSG00000124201
ENSG00000179010	ENSG00000126858	ENSG00000146151	ENSG00000136205
ENSG00000123091	ENSG00000085721	ENSG00000174697	ENSG00000180116
ENSG00000177685	ENSG00000144724	ENSG00000122958	ENSG00000091704
ENSG00000131653	ENSG00000076604	ENSG00000196588	ENSG00000143190
ENSG00000125447	ENSG00000198937	ENSG00000116741	ENSG00000166960
ENSG00000158850	ENSG00000181449	ENSG00000067955	ENSG00000100448

ENSG00000105887	ENSG00000102967	ENSG00000135617	ENSG00000145604
ENSG00000136874	ENSG00000179119	ENSG00000177352	ENSG00000197557
ENSG00000196873	ENSG00000101150	ENSG00000154124	ENSG00000182902
ENSG00000170714	ENSG00000173614	ENSG00000167085	ENSG00000167081
ENSG00000091527	ENSG00000109956	ENSG00000131979	ENSG00000213983
ENSG00000115339	ENSG00000140287	ENSG00000108256	ENSG00000183578
ENSG00000153714	ENSG00000182578	ENSG00000088543	ENSG00000146006
ENSG00000176454	ENSG00000128872	ENSG00000158008	ENSG00000143401
ENSG00000120256	ENSG00000154330	ENSG00000137285	ENSG00000186318
ENSG00000107140	ENSG00000071051	ENSG00000188120	ENSG00000102898
ENSG00000135678	ENSG00000101955	ENSG00000166734	ENSG00000153531
ENSG00000150760	ENSG00000158125	ENSG00000135537	ENSG00000166716
ENSG00000149658	ENSG00000183726	ENSG00000163281	ENSG00000127946
ENSG00000142611	ENSG00000185000	ENSG00000150907	ENSG00000119673
ENSG00000054611	ENSG00000170776	ENSG00000166823	ENSG00000087077
ENSG00000169957	ENSG00000160185	ENSG00000173786	ENSG00000187730
ENSG00000101444	ENSG00000117152	ENSG00000101452	ENSG00000157570
ENSG00000148798	ENSG00000004897	ENSG00000107223	ENSG00000240344
ENSG00000179564	ENSG00000025800	ENSG00000162367	ENSG00000183160
ENSG00000132801	ENSG00000125895	ENSG00000049239	ENSG00000242108
ENSG00000163032	ENSG00000134153	ENSG00000051009	ENSG00000156414
ENSG00000125386	ENSG00000169330	ENSG00000021826	ENSG00000105388
ENSG00000140859	ENSG00000113532	ENSG00000099715	ENSG00000093144
ENSG00000076043	ENSG00000214517	ENSG00000132694	ENSG00000108270
ENSG00000139636	ENSG00000074370	ENSG00000182173	ENSG00000083844
ENSG00000124786	ENSG00000151789	ENSG00000104067	ENSG00000171124
ENSG00000117620	ENSG00000131558	ENSG00000133627	ENSG00000172037
ENSG00000152455	ENSG00000205937	ENSG00000137871	ENSG00000162998
ENSG00000146535	ENSG00000167552	ENSG00000181744	ENSG00000092850
ENSG00000183508	ENSG00000105409	ENSG00000115318	ENSG00000136052
ENSG00000174562	ENSG00000184992	ENSG00000134970	ENSG00000120087
ENSG00000103066	ENSG00000146242	ENSG00000149582	ENSG00000139719
ENSG00000111653	ENSG00000021762	ENSG00000100314	ENSG00000160211
ENSG00000102362	ENSG00000073536	ENSG00000100523	ENSG00000173673
ENSG00000157764	ENSG00000181804	ENSG00000166507	ENSG00000120833
ENSG00000163347	ENSG00000165246	ENSG00000034713	ENSG00000215305
ENSG00000179750	ENSG00000221946	ENSG00000152465	ENSG00000144583
ENSG00000138650	ENSG00000196199	ENSG00000185668	ENSG00000162542
ENSG00000120509	ENSG00000182836	ENSG00000130427	ENSG00000114805
ENSG00000151532	ENSG00000166831	ENSG00000162775	ENSG00000117500
ENSG00000115641	ENSG00000198799	ENSG00000109111	ENSG00000156920
ENSG00000070269	ENSG00000122299	ENSG00000076554	ENSG00000158402
ENSG00000175497	ENSG00000125871	ENSG00000143387	ENSG00000167173
ENSG00000005955	ENSG00000070950	ENSG00000157470	ENSG00000178691
ENSG00000129682	ENSG00000104164	ENSG00000063241	ENSG00000154065
ENSG00000140519	ENSG00000127688	ENSG00000143157	ENSG00000163352
ENSG00000056097	ENSG00000075415	ENSG00000205531	ENSG00000005194
ENSG00000138162	ENSG00000144043	ENSG00000138083	ENSG00000012779
ENSG00000115306	ENSG00000052126	ENSG00000115594	ENSG00000137672
ENSG00000155876	ENSG00000101493	ENSG00000168872	ENSG00000162688
ENSG00000197632	ENSG00000114353	ENSG00000108684	ENSG00000157191
ENSG00000153563	ENSG00000131236	ENSG00000122566	ENSG00000095139
ENSG00000109089	ENSG00000146859	ENSG00000162627	ENSG00000183691
ENSG00000120805	ENSG00000187391	ENSG00000114904	ENSG00000075891
ENSG00000038210	ENSG00000113161	ENSG00000020633	ENSG00000188305
ENSG00000164849	ENSG00000129159	ENSG00000038295	ENSG00000148377
ENSG00000104140	ENSG00000187325	ENSG00000172867	ENSG00000175262
ENSG00000110925	ENSG00000117479	ENSG00000139842	ENSG00000169710
ENSG00000116285	ENSG00000184203	ENSG00000006530	ENSG00000182405
ENSG00000182504	ENSG00000101188	ENSG00000170035	ENSG00000167964
ENSG00000132376	ENSG00000122068	ENSG00000186081	ENSG00000139626
ENSG00000032514	ENSG00000159322	ENSG00000172731	ENSG00000135596
ENSG00000135519	ENSG00000168899	ENSG00000128965	ENSG00000179965
ENSG00000186231	ENSG00000128510	ENSG00000125520	ENSG00000188878

ENSG00000154856	ENSG00000176887	ENSG00000122884	ENSG00000162980
ENSG00000111276	ENSG00000175048	ENSG00000123728	ENSG00000139357
ENSG00000049759	ENSG00000169410	ENSG00000056487	ENSG00000152484
ENSG00000198909	ENSG00000099785	ENSG00000176749	ENSG00000172179
ENSG00000185238	ENSG00000091129	ENSG00000009830	ENSG00000164761
ENSG00000105323	ENSG00000132005	ENSG00000152217	ENSG00000126067
ENSG00000184903	ENSG00000198680	ENSG00000177150	ENSG00000146122
ENSG00000124177	ENSG00000128254	ENSG00000017621	ENSG00000147119
ENSG00000003756	ENSG00000203791	ENSG00000174371	ENSG00000183682
ENSG00000174502	ENSG00000153443	ENSG00000164400	ENSG00000106400
ENSG00000149503	ENSG00000090054	ENSG00000157168	ENSG00000118729
ENSG00000177575	ENSG00000127418	ENSG00000180318	ENSG00000119917
ENSG00000170027	ENSG00000004838	ENSG00000042429	ENSG00000132823
ENSG00000092067	ENSG00000164796	ENSG00000100280	ENSG00000142227
ENSG00000198858	ENSG00000183615	ENSG00000158715	ENSG00000173482
ENSG00000099956	ENSG00000146674	ENSG00000100345	ENSG00000162975
ENSG00000171819	ENSG00000138356	ENSG00000159713	ENSG00000138175
ENSG00000137267	ENSG00000121879	ENSG00000180776	ENSG00000079482
ENSG00000145780	ENSG00000141753	ENSG00000092531	ENSG00000120860
ENSG00000106688	ENSG00000100216	ENSG00000054116	ENSG00000117682
ENSG00000171150	ENSG00000065970	ENSG00000164620	ENSG00000138193
ENSG00000166987	ENSG00000101144	ENSG00000109099	ENSG00000108405
ENSG00000171453	ENSG00000104826	ENSG00000169594	ENSG00000119950
ENSG00000169016	ENSG00000167193	ENSG00000137090	ENSG00000100994
ENSG00000203879	ENSG00000185414	ENSG00000149294	ENSG00000143633
ENSG00000171443	ENSG00000103932	ENSG00000140992	ENSG00000065882
ENSG00000115504	ENSG00000136574	ENSG00000175376	ENSG00000205221
ENSG00000186648	ENSG00000170011	ENSG00000172061	ENSG00000198131
ENSG00000131269	ENSG00000163291	ENSG00000105856	ENSG00000244486
ENSG00000109220	ENSG00000177464	ENSG00000063015	ENSG00000105173
ENSG00000136603	ENSG00000179603	ENSG00000170962	ENSG00000186480
ENSG00000147588	ENSG00000187033	ENSG00000198719	ENSG00000170180
ENSG00000058262	ENSG00000147400	ENSG00000006042	ENSG00000134198
ENSG00000173898	ENSG00000197467	ENSG00000184216	ENSG00000136720
ENSG00000072274	ENSG00000168000	ENSG00000196323	ENSG00000130638
ENSG00000166133	ENSG00000166920	ENSG00000120875	ENSG00000197766
ENSG00000108175	ENSG00000079337	ENSG00000079739	ENSG00000144671
ENSG00000155324	ENSG00000169255	ENSG00000168959	ENSG00000125398
ENSG00000070366	ENSG00000198881	ENSG00000118518	ENSG00000241360
ENSG00000162231	ENSG00000164494	ENSG00000185033	ENSG00000110768
ENSG00000178149	ENSG00000125753	ENSG00000128228	ENSG00000114757
ENSG00000116698	ENSG00000173950	ENSG00000183955	ENSG00000066027
ENSG00000133401	ENSG00000167588	ENSG00000145335	ENSG00000183317
ENSG00000110958	ENSG00000114279	ENSG00000139200	ENSG00000163083
ENSG00000147526	ENSG00000204442	ENSG00000165646	ENSG00000107020
ENSG00000141279	ENSG00000157851	ENSG00000182108	ENSG00000100156
ENSG00000144560	ENSG00000008277	ENSG00000164398	ENSG00000188373
ENSG00000134508	ENSG00000138792	ENSG00000111540	ENSG00000112619
ENSG00000134245	ENSG00000081014	ENSG00000125821	ENSG00000118432
ENSG00000167695	ENSG00000108510	ENSG00000172915	ENSG00000094804
ENSG00000172530	ENSG00000163428	ENSG00000157978	ENSG00000132254
ENSG00000138622	ENSG00000112294	ENSG00000172340	ENSG00000168615
ENSG00000124201	ENSG00000153944	ENSG00000128059	ENSG00000133193
ENSG00000136205	ENSG00000130304	ENSG00000174483	ENSG00000109101
ENSG00000118515	ENSG00000116833	ENSG00000158555	ENSG00000174516
ENSG00000177058	ENSG00000178449	ENSG00000168487	ENSG00000196422
ENSG00000166960	ENSG00000218336	ENSG00000130066	ENSG00000117791
ENSG00000196345	ENSG00000173821	ENSG00000103978	ENSG00000196935
ENSG00000145604	ENSG00000134871	ENSG00000188636	ENSG00000163359
ENSG00000131188	ENSG00000132305	ENSG00000114030	ENSG00000089154
ENSG00000048540	ENSG00000161649	ENSG00000033050	ENSG00000103202
ENSG00000182902	ENSG00000244122	ENSG00000066629	ENSG00000121060
ENSG00000167081	ENSG00000033867	ENSG00000171540	ENSG00000124610
ENSG00000183578	ENSG00000184368	ENSG00000168438	ENSG00000160593

ENSG00000107372	ENSG00000244617	ENSG00000124783	ENSG00000153802
ENSG00000114853	ENSG00000089685	ENSG00000040633	ENSG00000121552
ENSG00000084628	ENSG00000023572	ENSG00000114850	ENSG00000186472
ENSG00000186318	ENSG00000175137	ENSG00000164270	ENSG00000182352
ENSG00000092820	ENSG00000196230	ENSG00000061938	ENSG00000197448
ENSG00000102898	ENSG00000119685	ENSG00000115073	ENSG00000095370
ENSG00000144749	ENSG00000164181	ENSG00000147570	ENSG00000150244
ENSG00000185909	ENSG00000163638	ENSG00000167978	ENSG00000075624
ENSG00000129219	ENSG00000147364	ENSG00000106123	ENSG00000139291
ENSG00000179950	ENSG00000150459	ENSG00000168356	ENSG00000090104
ENSG00000073803	ENSG00000213204	ENSG00000189050	ENSG00000034152
ENSG00000070061	ENSG00000148926	ENSG00000168118	ENSG00000168026
ENSG00000163026	ENSG00000163378	ENSG00000163600	ENSG00000160049
ENSG00000206053	ENSG00000187164	ENSG00000187097	ENSG00000174640
ENSG00000001626	ENSG00000114125	ENSG00000071189	ENSG00000101670
ENSG00000074416	ENSG00000115902	ENSG00000109381	ENSG00000152784
ENSG00000157570	ENSG00000104408	ENSG00000112062	ENSG00000157450
ENSG00000206207	ENSG00000089195	ENSG00000147255	ENSG00000164122
ENSG00000156414	ENSG00000048707	ENSG00000185860	ENSG00000102878
ENSG00000125648	ENSG00000114416	ENSG00000111186	ENSG00000135365
ENSG00000106537	ENSG00000135604	ENSG00000196072	ENSG00000164916
ENSG00000125798	ENSG00000119471	ENSG00000123374	ENSG00000167716
ENSG00000162520	ENSG00000182606	ENSG00000197380	ENSG00000182871
ENSG00000162998	ENSG00000198792	ENSG00000126091	ENSG00000100784
ENSG00000136052	ENSG00000167565	ENSG00000134982	ENSG00000136732
ENSG00000164692	ENSG00000134056	ENSG00000125850	ENSG00000167323
ENSG00000101384	ENSG00000186075	ENSG00000122705	ENSG00000079557
ENSG00000168917	ENSG00000121361	ENSG00000100592	ENSG00000113648
ENSG00000120087	ENSG00000132635	ENSG00000101911	ENSG00000152402
ENSG00000100934	ENSG00000174206	ENSG00000179632	ENSG00000215386
ENSG00000120833	ENSG00000214021	ENSG00000167604	ENSG00000241973
ENSG00000173926	ENSG00000108924	ENSG00000162545	ENSG00000117475
ENSG00000076770	ENSG00000114439	ENSG00000184402	ENSG00000240583
ENSG00000151893	ENSG00000136868	ENSG00000174903	ENSG00000113248
ENSG00000174628	ENSG00000171282	ENSG00000138744	ENSG00000181409
ENSG00000117500	ENSG00000166783	ENSG00000128656	ENSG00000012124
ENSG00000081913	ENSG00000107651	ENSG00000173681	ENSG00000159082
ENSG00000167173	ENSG00000172977	ENSG00000146904	ENSG00000106330
ENSG00000204375	ENSG00000022355	ENSG00000141150	ENSG00000149480
ENSG00000154065	ENSG00000156466	ENSG00000165462	ENSG00000186115
ENSG00000137693	ENSG00000055130	ENSG00000173917	ENSG00000166592
ENSG00000204439	ENSG00000119383	ENSG00000088367	ENSG00000133466
ENSG00000172409	ENSG00000135932	ENSG00000126858	ENSG00000162390
ENSG00000154127	ENSG00000148396	ENSG00000111450	ENSG00000075399
ENSG00000135749	ENSG00000160539	ENSG00000131788	ENSG00000167363
ENSG00000137672	ENSG00000162290	ENSG00000144724	ENSG00000101417
ENSG00000157191	ENSG00000109670	ENSG00000163719	ENSG00000129535
ENSG00000157927	ENSG00000119280	ENSG00000076604	ENSG00000151470
ENSG00000183691	ENSG00000163879	ENSG00000181449	ENSG00000167549
ENSG00000095139	ENSG00000126950	ENSG00000179119	ENSG00000184160
ENSG00000075891	ENSG00000144642	ENSG00000101150	ENSG00000164543
ENSG00000181195	ENSG00000160271	ENSG00000154654	ENSG00000146276
ENSG00000168066	ENSG00000014824	ENSG00000149599	ENSG00000174672
ENSG00000089094	ENSG00000107623	ENSG00000140287	ENSG00000135502
ENSG00000169710	ENSG00000145623	ENSG00000182621	ENSG00000186818
ENSG00000136068	ENSG00000124217	ENSG00000182578	ENSG00000182747
ENSG00000163683	ENSG00000161800	ENSG00000128872	ENSG00000132514
ENSG00000160218	ENSG00000115840	ENSG00000166575	ENSG00000173269
ENSG00000135596	ENSG00000244405	ENSG00000101955	ENSG00000113262
ENSG00000149548	ENSG00000165588	ENSG00000113916	ENSG00000165272
ENSG00000119812	ENSG00000163444	ENSG00000170776	ENSG00000168758
ENSG00000143797	ENSG00000187566	ENSG00000169118	ENSG00000108587
ENSG00000026025	ENSG00000151117	ENSG00000117152	ENSG00000131941
ENSG00000184743	ENSG00000122641	ENSG00000025800	ENSG00000113328

ENSG00000196437	ENSG00000103326	ENSG00000128652	ENSG00000136425
ENSG00000162980	ENSG00000164116	ENSG00000125895	ENSG00000181830
ENSG00000173020	ENSG00000187098	ENSG00000134153	ENSG00000121031
ENSG00000152484	ENSG00000137414	ENSG00000113532	ENSG00000170941
ENSG00000029993	ENSG00000130340	ENSG00000173846	ENSG00000149972
ENSG00000173166	ENSG00000158104	ENSG00000120063	ENSG00000155099
ENSG00000158156	ENSG00000115461	ENSG00000131558	ENSG00000170889
ENSG00000118729	ENSG00000140463	ENSG00000156599	ENSG00000144597
ENSG00000132823	ENSG00000169247	ENSG00000162992	ENSG00000167601
ENSG00000138175	ENSG00000127603	ENSG00000167552	ENSG00000170677
ENSG00000173482	ENSG00000100319	ENSG00000105409	ENSG00000161513
ENSG00000169045	ENSG00000204688	ENSG00000132326	ENSG00000149269
ENSG00000117682	ENSG00000214063	ENSG00000125166	ENSG00000162073
ENSG00000138193	ENSG00000213760	ENSG00000146242	ENSG00000154265
ENSG00000184564	ENSG00000221866	ENSG00000118971	ENSG00000221829
ENSG00000108405	ENSG00000166669	ENSG00000021762	ENSG00000185875
ENSG00000148848	ENSG00000182979	ENSG00000175115	ENSG00000116711
ENSG00000119950	ENSG00000063322	ENSG00000181804	ENSG00000155052
ENSG00000182010	ENSG00000135720	ENSG00000135686	ENSG00000116819
ENSG00000065882	ENSG00000180432	ENSG00000068323	ENSG00000125170
ENSG00000198739	ENSG00000188215	ENSG00000182836	ENSG00000146216
ENSG00000143420	ENSG00000050820	ENSG00000023516	ENSG00000137074
ENSG00000171522	ENSG00000070778	ENSG00000146360	ENSG00000047644
ENSG00000105173	ENSG00000185338	ENSG00000075785	ENSG00000174697
ENSG00000189308	ENSG00000011105	ENSG00000198799	ENSG00000159184
ENSG00000116991	ENSG00000114737	ENSG00000122299	ENSG00000178607
ENSG00000186480	ENSG00000112164	ENSG00000153233	ENSG00000115183
ENSG00000186298	ENSG00000114503	ENSG00000158163	ENSG00000180667
ENSG00000137947	ENSG00000156218	ENSG00000184307	ENSG00000150722
ENSG00000134198	ENSG00000174106	ENSG00000027869	ENSG00000116741
ENSG00000143384	ENSG00000189067	ENSG00000070950	ENSG00000196588
ENSG00000118707	ENSG00000179886	ENSG00000081087	ENSG00000213699
ENSG00000130638	ENSG00000101460	ENSG00000104164	ENSG00000067955
ENSG00000151150	ENSG00000126773	ENSG00000123612	ENSG00000136856
ENSG00000116473	ENSG00000128709	ENSG00000166886	ENSG00000135617
ENSG00000125398	ENSG00000129355	ENSG00000127688	ENSG00000137288
ENSG00000110768	ENSG00000183307	ENSG00000133104	ENSG00000152578
ENSG00000114757	ENSG00000074590	ENSG00000075415	ENSG00000015133
ENSG00000066027	ENSG00000165782	ENSG00000144043	ENSG00000167085
ENSG00000163083	ENSG00000115839	ENSG00000143126	ENSG00000181977
ENSG00000105245	ENSG00000160050	ENSG00000052126	ENSG00000131979
ENSG00000100335	ENSG00000134317	ENSG00000101350	ENSG00000108256
ENSG00000168214	ENSG00000007062	ENSG00000148516	ENSG00000158008
ENSG00000088179	ENSG00000120738	ENSG00000110076	ENSG00000137285
ENSG00000149212	ENSG00000148835	ENSG00000114353	ENSG00000147324
ENSG00000196730	ENSG00000119614	ENSG00000131236	ENSG00000166734
ENSG00000118432	ENSG00000156687	ENSG00000091972	ENSG00000109705
ENSG00000173418	ENSG00000197283	ENSG00000146859	ENSG00000163281
ENSG00000094804	ENSG00000105825	ENSG00000187391	ENSG00000140105
ENSG00000132254	ENSG00000155966	ENSG00000113161	ENSG00000101452
ENSG00000168615	ENSG00000121749	ENSG00000170382	ENSG00000173786
ENSG00000133193	ENSG00000131013	ENSG00000145901	ENSG00000162367
ENSG00000109101	ENSG00000084112	ENSG00000116871	ENSG00000172935
ENSG00000082458	ENSG00000145020	ENSG00000187325	ENSG00000110079
ENSG00000137809	ENSG00000186564	ENSG00000117479	ENSG00000151065
ENSG00000174516	ENSG00000152620	ENSG00000184203	ENSG00000132464
ENSG00000139318	ENSG00000113361	ENSG00000106459	ENSG00000105939
ENSG00000136933	ENSG00000107485	ENSG00000167767	ENSG00000130177
ENSG00000196422	ENSG00000133142	ENSG00000187123	ENSG00000134970
ENSG00000163630	ENSG00000182870	ENSG00000122068	ENSG00000167863
ENSG00000161642	ENSG00000163545	ENSG00000159322	ENSG00000100314
ENSG00000156011	ENSG00000116977	ENSG00000152242	ENSG00000166507
ENSG00000155319	ENSG00000119121	ENSG00000128510	ENSG00000100413
ENSG00000196935	ENSG00000151778	ENSG00000099785	ENSG00000175414

ENSG00000163359	ENSG00000184575	ENSG00000091129	ENSG00000034713
ENSG00000179284	ENSG00000198626	ENSG00000169410	ENSG00000152465
ENSG00000165660	ENSG00000113448	ENSG00000132005	ENSG00000141384
ENSG00000007047	ENSG00000197324	ENSG00000074266	ENSG00000101199
ENSG00000141540	ENSG00000169223	ENSG00000139508	ENSG00000149577
ENSG00000173452	ENSG00000172782	ENSG00000203791	ENSG00000167654
ENSG00000145715	ENSG00000163938	ENSG00000155011	ENSG00000157470
ENSG00000176406	ENSG00000168807	ENSG00000127452	ENSG00000063241
ENSG00000095370	ENSG00000130037	ENSG00000137221	ENSG00000111371
ENSG00000197601	ENSG00000116005	ENSG00000135124	ENSG00000167112
ENSG00000075624	ENSG00000214706	ENSG00000090054	ENSG00000117054
ENSG00000090104	ENSG00000101544	ENSG00000004838	ENSG00000137634
ENSG00000034152	ENSG00000131779	ENSG00000164796	ENSG00000156313
ENSG00000068745	ENSG00000117676	ENSG00000043355	ENSG00000143157
ENSG00000174640	ENSG00000152503	ENSG00000108588	ENSG00000205531
ENSG00000143248	ENSG00000170345	ENSG00000142961	ENSG00000138083
ENSG00000188827	ENSG00000187714	ENSG00000146674	ENSG00000151725
ENSG00000152784	ENSG00000161940	ENSG00000130204	ENSG00000216490
ENSG00000130147	ENSG00000160097	ENSG00000078269	ENSG00000168872
ENSG00000031003	ENSG00000204619	ENSG00000065970	ENSG00000108684
ENSG00000154358	ENSG00000152804	ENSG00000137193	ENSG00000096654
ENSG00000157450	ENSG00000139880	ENSG00000167193	ENSG00000169689
ENSG00000099992	ENSG00000163743	ENSG00000101438	ENSG00000134996
ENSG00000121579	ENSG00000112624	ENSG00000154162	ENSG00000111241
ENSG00000157870	ENSG00000108395	ENSG00000136574	ENSG00000159214
ENSG00000135365	ENSG00000139324	ENSG00000170011	ENSG00000196782
ENSG00000182718	ENSG00000197779	ENSG00000177464	ENSG00000135968
ENSG00000167716	ENSG00000172757	ENSG00000179603	ENSG00000114904
ENSG00000182871	ENSG00000139055	ENSG00000147400	ENSG00000020633
ENSG00000117625	ENSG00000198830	ENSG00000085491	ENSG00000038295
ENSG00000171316	ENSG00000128294	ENSG00000197467	ENSG00000186827
ENSG00000100784	ENSG00000164163	ENSG00000168000	ENSG00000165912
ENSG00000167323	ENSG00000115526	ENSG00000171160	ENSG00000127952
ENSG00000081791	ENSG00000148120	ENSG00000100027	ENSG00000165914
ENSG00000135070	ENSG00000187889	ENSG00000118402	ENSG00000139842
ENSG00000113648	ENSG00000070476	ENSG00000156515	ENSG00000106991
ENSG00000142459	ENSG00000120586	ENSG00000164494	ENSG00000180479
ENSG00000077157	ENSG00000139263	ENSG00000114279	ENSG00000181192
ENSG00000137055	ENSG00000108551	ENSG00000157851	ENSG00000163295
ENSG00000138756	ENSG00000102098	ENSG00000184058	ENSG00000167791
ENSG00000163661	ENSG00000124212	ENSG00000008277	ENSG00000225190
ENSG00000155506	ENSG00000156531	ENSG00000138792	ENSG00000173253
ENSG00000152402	ENSG00000119906	ENSG00000081014	ENSG00000137225
ENSG00000017373	ENSG00000117523	ENSG00000100151	ENSG00000014123
ENSG00000121753	ENSG00000165996	ENSG00000066427	ENSG00000184113
ENSG00000165795	ENSG00000182473	ENSG00000133112	ENSG00000101017
ENSG00000107295	ENSG00000115935	ENSG00000108510	ENSG00000183775
ENSG00000173068	ENSG00000175183	ENSG00000112294	ENSG00000128965
ENSG00000159082	ENSG00000133657	ENSG00000153944	ENSG00000132436
ENSG00000106330	ENSG00000154917	ENSG00000039319	ENSG00000100865
ENSG00000107863	ENSG00000108984	ENSG00000105971	ENSG00000166272
ENSG00000134490	ENSG00000125149	ENSG00000116833	ENSG00000106009
ENSG00000137393	ENSG00000149218	ENSG00000166292	ENSG00000123728
ENSG00000166592	ENSG00000132879	ENSG00000134871	ENSG00000152217
ENSG00000133466	ENSG00000173114	ENSG00000196189	ENSG00000177150
ENSG00000075399	ENSG00000073282	ENSG00000132305	ENSG00000189171
ENSG00000101417	ENSG00000167461	ENSG00000138069	ENSG00000155034
ENSG00000185602	ENSG00000111799	ENSG00000135111	ENSG00000078900
ENSG00000129535	ENSG00000169756	ENSG00000136280	ENSG00000132840
ENSG00000100439	ENSG00000104756	ENSG00000090520	ENSG00000120693
ENSG00000167549	ENSG00000061936	ENSG00000033867	ENSG00000187145
ENSG00000169228	ENSG00000140320	ENSG00000184368	ENSG00000100280
ENSG00000036530	ENSG00000163110	ENSG00000162695	ENSG00000112578
ENSG00000184160	ENSG00000182752	ENSG00000121075	ENSG00000158715

ENSG00000164543	ENSG00000168748	ENSG00000166326	ENSG00000174567
ENSG00000135502	ENSG00000105464	ENSG00000165434	ENSG00000101115
ENSG00000166681	ENSG00000137747	ENSG00000070423	ENSG00000141510
ENSG00000174672	ENSG00000144476	ENSG00000164107	ENSG00000175482
ENSG00000102753	ENSG00000079387	ENSG00000182934	ENSG00000100345
ENSG00000121774	ENSG00000115956	ENSG00000175137	ENSG00000156735
ENSG00000067606	ENSG00000156508	ENSG00000119685	ENSG00000130702
ENSG00000106246	ENSG00000169554	ENSG00000163638	ENSG00000092531
ENSG00000143479	ENSG00000058063	ENSG00000021574	ENSG00000107960
ENSG00000097033	ENSG00000180155	ENSG00000083168	ENSG00000183643
ENSG00000198420	ENSG00000104517	ENSG00000126767	ENSG00000170866
ENSG00000108587	ENSG00000204392	ENSG00000135837	ENSG00000109756
ENSG00000168758	ENSG00000168763	ENSG00000165125	ENSG00000137090
ENSG00000113328	ENSG00000155666	ENSG00000073598	ENSG00000162929
ENSG00000083857	ENSG00000181885	ENSG00000148926	ENSG00000172061
ENSG00000170633	ENSG00000155850	ENSG00000163378	ENSG00000105856
ENSG00000136425	ENSG00000154920	ENSG00000187164	ENSG00000063015
ENSG00000182533	ENSG00000100979	ENSG00000114125	ENSG00000198890
ENSG00000181830	ENSG00000110367	ENSG00000115902	ENSG00000157693
ENSG00000178467	ENSG00000073921	ENSG00000114416	ENSG00000170962
ENSG00000170941	ENSG00000002746	ENSG00000182606	ENSG00000096070
ENSG00000149016	ENSG00000137491	ENSG00000198792	ENSG00000167220
ENSG00000184650	ENSG00000110713	ENSG00000167565	ENSG00000069431
ENSG00000155099	ENSG00000111752	ENSG00000100311	ENSG00000185823
ENSG00000131023	ENSG00000204138	ENSG00000004399	ENSG00000184216
ENSG00000068781	ENSG00000183856	ENSG00000109083	ENSG00000121022
ENSG00000165084	ENSG00000122779	ENSG00000149930	ENSG00000120875
ENSG00000144597	ENSG00000164307	ENSG00000108924	ENSG00000167110
ENSG00000172260	ENSG00000165794	ENSG00000114439	ENSG00000104129
ENSG00000119408	ENSG00000131626	ENSG00000136868	ENSG00000119321
ENSG00000167601	ENSG00000156374	ENSG00000204634	ENSG00000139200
ENSG00000170677	ENSG00000100644	ENSG00000166783	ENSG00000152591
ENSG00000167123	ENSG00000115942	ENSG00000107651	ENSG00000165646
ENSG00000149269	ENSG00000134287	ENSG00000102078	ENSG00000145863
ENSG00000149043	ENSG00000160606	ENSG00000022355	ENSG00000135870
ENSG00000198718	ENSG00000172667	ENSG00000099904	ENSG00000183684
ENSG00000125170	ENSG00000008516	ENSG00000139926	ENSG00000164398
ENSG00000043143	ENSG00000104938	ENSG00000147676	ENSG00000164707
ENSG00000129451	ENSG00000182676	ENSG00000156466	ENSG00000167721
ENSG00000137074	ENSG00000132024	ENSG00000055130	ENSG00000081760
ENSG00000196932	ENSG00000148483	ENSG00000119383	ENSG00000144847
ENSG00000146151	ENSG00000008083	ENSG00000135932	ENSG00000143942
ENSG00000165775	ENSG00000143641	ENSG00000160539	ENSG00000172340
ENSG00000174697	ENSG00000169129	ENSG00000162290	ENSG00000128059
ENSG00000020577	ENSG00000162706	ENSG00000109670	ENSG00000204495
ENSG00000122958	ENSG00000183735	ENSG00000119280	ENSG00000168487
ENSG00000183648	ENSG00000069974	ENSG00000172660	ENSG00000101310
ENSG00000116741	ENSG00000198374	ENSG00000144642	ENSG00000103978
ENSG00000196588	ENSG00000185633	ENSG00000160271	ENSG00000188636
ENSG00000067955	ENSG00000166398	ENSG00000014824	ENSG00000175556
ENSG00000180828	ENSG00000153814	ENSG00000107623	ENSG00000126803
ENSG00000198246	ENSG00000091073	ENSG00000145623	ENSG00000171295
ENSG00000135617	ENSG00000170500	ENSG00000115840	ENSG00000185518
ENSG00000177352	ENSG00000123352	ENSG00000124140	ENSG00000120802
ENSG00000154124	ENSG00000115896	ENSG00000085998	ENSG00000066629
ENSG00000167085	ENSG00000145555	ENSG00000107984	ENSG00000185262
ENSG00000131979	ENSG00000182087	ENSG00000165588	ENSG00000144736
ENSG00000158008	ENSG00000171208	ENSG00000163444	ENSG00000182866
ENSG00000088543	ENSG00000186642	ENSG00000151117	ENSG00000105640
ENSG00000108256	ENSG00000179314	ENSG00000122641	ENSG00000040633
ENSG00000137285	ENSG00000143363	ENSG00000103326	ENSG00000061938
ENSG00000188120	ENSG00000110315	ENSG00000101290	ENSG00000115073
ENSG00000166734	ENSG00000144868	ENSG00000175040	ENSG00000100373
ENSG00000183340	ENSG00000198961	ENSG00000187098	ENSG00000147570

ENSG00000135537	ENSG00000163686	ENSG00000106789	ENSG00000167978
ENSG00000163281	ENSG00000097096	ENSG00000137414	ENSG00000109475
ENSG00000103723	ENSG00000017797	ENSG00000115461	ENSG00000154040
ENSG00000150907	ENSG00000147113	ENSG00000147854	ENSG00000106123
ENSG00000166823	ENSG00000115317	ENSG00000102144	ENSG00000168356
ENSG00000101452	ENSG00000100522	ENSG00000134046	ENSG00000109991
ENSG00000107223	ENSG00000219438	ENSG00000127603	ENSG00000100354
ENSG00000173786	ENSG00000240021	ENSG00000197381	ENSG00000167291
ENSG00000162367	ENSG00000164574	ENSG00000108829	ENSG00000106809
ENSG00000049239	ENSG00000179542	ENSG00000108001	ENSG00000168118
ENSG00000051009	ENSG00000173334	ENSG00000166669	ENSG00000198001
ENSG00000021826	ENSG00000071564	ENSG00000137075	ENSG00000171201
ENSG00000099715	ENSG00000179933	ENSG00000147471	ENSG00000187097
ENSG00000132694	ENSG00000126653	ENSG00000065361	ENSG00000071189
ENSG00000133627	ENSG00000204569	ENSG00000171570	ENSG00000175567
ENSG00000182173	ENSG00000128283	ENSG00000135720	ENSG00000168152
ENSG00000104067	ENSG00000016864	ENSG00000184867	ENSG00000141905
ENSG00000137871	ENSG00000177485	ENSG00000151657	ENSG00000162664
ENSG00000115128	ENSG00000176974	ENSG00000184009	ENSG00000162896
ENSG00000008294	ENSG00000173588	ENSG00000166170	ENSG00000147255
ENSG00000181744	ENSG00000128604	ENSG00000188215	ENSG00000126778
ENSG00000115318	ENSG00000118412	ENSG00000104635	ENSG00000104725
ENSG00000149582	ENSG00000136881	ENSG00000070778	ENSG00000182986
ENSG00000134970	ENSG00000088833	ENSG00000162368	ENSG00000161055
ENSG00000100314	ENSG00000163684	ENSG00000185338	ENSG00000169126
ENSG00000197885	ENSG00000123124	ENSG00000179387	ENSG00000134982
ENSG00000100523	ENSG00000180259	ENSG00000011105	ENSG00000125850
ENSG00000166507	ENSG00000148204	ENSG00000112164	ENSG00000122705
ENSG00000034713	ENSG00000001629	ENSG00000079156	ENSG00000112655
ENSG00000185668	ENSG00000140941	ENSG00000114503	ENSG00000198252
ENSG00000130427	ENSG00000112394	ENSG00000162959	ENSG00000213085
ENSG00000152465	ENSG00000167165	ENSG00000156218	ENSG00000100592
ENSG00000162775	ENSG00000160691	ENSG00000174106	ENSG00000184923
ENSG00000109111	ENSG00000215421	ENSG00000189067	ENSG00000184402
ENSG00000143387	ENSG00000183798	ENSG00000101460	ENSG00000162032
ENSG00000076554	ENSG00000146469	ENSG00000072849	ENSG00000174903
ENSG00000063241	ENSG00000064651	ENSG00000172432	ENSG00000128656
ENSG00000157470	ENSG00000196396	ENSG00000183307	ENSG00000156411
ENSG00000156313	ENSG00000196850	ENSG00000074590	ENSG00000146904
ENSG00000143157	ENSG00000157554	ENSG00000165782	ENSG00000126858
ENSG00000205531	ENSG00000198814	ENSG00000160050	ENSG00000113494
ENSG00000138083	ENSG00000198265	ENSG00000134317	ENSG00000142330
ENSG00000115594	ENSG00000163041	ENSG00000135164	ENSG00000166813
ENSG00000168872	ENSG00000174307	ENSG00000120738	ENSG00000088367
ENSG00000108684	ENSG00000127445	ENSG00000148835	ENSG00000144724
ENSG00000185339	ENSG00000187653	ENSG00000156687	ENSG00000085721
ENSG00000122566	ENSG00000119844	ENSG00000197283	ENSG00000076604
ENSG00000162627	ENSG00000180354	ENSG00000105825	ENSG00000137601
ENSG00000111300	ENSG00000010704	ENSG00000129007	ENSG00000198937
ENSG00000091436	ENSG00000141985	ENSG00000155966	ENSG00000181449
ENSG00000020633	ENSG00000197006	ENSG00000121749	ENSG00000102967
ENSG00000114904	ENSG00000011478	ENSG00000084112	ENSG00000120094
ENSG00000038295	ENSG00000188984	ENSG00000197746	ENSG00000172270
ENSG00000172867	ENSG00000143669	ENSG00000140391	ENSG00000179119
ENSG00000168944	ENSG00000148908	ENSG00000145020	ENSG00000212128
ENSG00000139842	ENSG00000163512	ENSG00000085224	ENSG00000110717
ENSG00000196531	ENSG00000171444	ENSG00000152620	ENSG00000101150
ENSG00000173120	ENSG00000184194	ENSG00000101407	ENSG00000173614
ENSG00000129514	ENSG00000142627	ENSG00000107485	ENSG00000109956
ENSG00000173253	ENSG00000112964	ENSG00000163545	ENSG00000140287
ENSG00000006530	ENSG00000099804	ENSG00000108821	ENSG00000141434
ENSG00000170035	ENSG00000001617	ENSG00000138032	ENSG00000182578
ENSG00000186081	ENSG00000139436	ENSG00000167703	ENSG00000128872
ENSG00000182054	ENSG00000125691	ENSG00000119121	ENSG00000143595

ENSG00000172731	ENSG00000124693	ENSG00000198626	ENSG00000154330
ENSG00000125520	ENSG00000133477	ENSG00000184254	ENSG00000071051
ENSG00000128965	ENSG00000067221	ENSG00000113448	ENSG00000158125
ENSG00000122884	ENSG00000185122	ENSG00000197324	ENSG00000136861
ENSG00000123728	ENSG00000100028	ENSG00000072110	ENSG00000176953
ENSG00000056487	ENSG00000124772	ENSG00000101868	ENSG00000183726
ENSG00000176749	ENSG00000171004	ENSG00000130037	ENSG00000160185
ENSG00000009830	ENSG00000166037	ENSG00000133275	ENSG00000170776
ENSG00000174371	ENSG00000114098	ENSG00000134371	ENSG00000004897
ENSG00000017621	ENSG00000049246	ENSG00000116005	ENSG00000117152
ENSG00000177150	ENSG00000165175	ENSG00000101544	ENSG00000025800
ENSG00000152217	ENSG00000023228	ENSG00000117676	ENSG00000165609
ENSG00000164400	ENSG00000068024	ENSG00000152503	ENSG00000125895
ENSG00000141048	ENSG00000108654	ENSG00000170345	ENSG00000141873
ENSG00000157168	ENSG00000164414	ENSG00000187714	ENSG00000134153
ENSG00000188352	ENSG00000087470	ENSG00000160097	ENSG00000147592
ENSG00000180318	ENSG00000110422	ENSG00000172803	ENSG00000168634
ENSG00000108773	ENSG00000105928	ENSG00000176928	ENSG00000113532
ENSG00000042429	ENSG00000143845	ENSG00000204619	ENSG00000169330
ENSG00000100280	ENSG00000100030	ENSG00000105576	ENSG00000167065
ENSG00000158715	ENSG00000064393	ENSG00000064300	ENSG00000214517
ENSG00000179146	ENSG00000141376	ENSG00000152804	ENSG00000074370
ENSG00000100345	ENSG00000137177	ENSG00000139880	ENSG00000153896
ENSG00000159713	ENSG00000136653	ENSG00000152683	ENSG00000158683
ENSG00000180776	ENSG00000241322	ENSG00000108395	ENSG00000131558
ENSG00000092531	ENSG00000137216	ENSG00000197779	ENSG00000205937
ENSG00000156735	ENSG00000154146	ENSG00000172757	ENSG00000105409
ENSG00000054116	ENSG00000105519	ENSG00000001084	ENSG00000146242
ENSG00000198876	ENSG00000159588	ENSG00000198830	ENSG00000021762
ENSG00000164620	ENSG00000112242	ENSG00000164163	ENSG00000073536
ENSG00000169594	ENSG00000150054	ENSG00000174282	ENSG00000181804
ENSG00000109099	ENSG00000120341	ENSG00000115526	ENSG00000185594
ENSG00000140992	ENSG00000113300	ENSG00000187889	ENSG00000165246
ENSG00000149294	ENSG00000153147	ENSG00000115993	ENSG00000221946
ENSG00000137090	ENSG00000164111	ENSG00000131446	ENSG00000182836
ENSG00000175376	ENSG00000238227	ENSG00000120586	ENSG00000117010
ENSG00000140521	ENSG00000179335	ENSG00000139263	ENSG00000166831
ENSG00000172061	ENSG00000136754	ENSG00000108551	ENSG00000198799
ENSG00000105856	ENSG00000140450	ENSG00000124212	ENSG00000167741
ENSG00000081320	ENSG00000067840	ENSG00000156531	ENSG00000122299
ENSG00000063015	ENSG00000116783	ENSG00000166200	ENSG00000160959
ENSG00000170962	ENSG00000143442	ENSG00000159256	ENSG00000125871
ENSG00000167220	ENSG00000164327	ENSG00000119906	ENSG00000070950
ENSG00000198719	ENSG00000181904	ENSG00000115935	ENSG00000104164
ENSG00000006042	ENSG00000160113	ENSG00000154917	ENSG00000167920
ENSG00000184216	ENSG00000101246	ENSG00000135052	ENSG00000075415
ENSG00000196323	ENSG00000204956	ENSG00000108984	ENSG00000127688
ENSG00000079739	ENSG00000135248	ENSG00000109654	ENSG00000143320
ENSG00000120875	ENSG00000121380	ENSG00000125149	ENSG00000144043
ENSG00000198833	ENSG00000128346	ENSG00000127152	ENSG00000112983
ENSG00000168959	ENSG00000182827	ENSG00000173114	ENSG00000052126
ENSG00000118518	ENSG00000132912	ENSG00000073282	ENSG00000101493
ENSG00000185033	ENSG00000084070	ENSG00000080503	ENSG00000198538
ENSG00000128228	ENSG00000099994	ENSG00000167461	ENSG00000114353
ENSG00000183955	ENSG00000175591	ENSG00000157110	ENSG00000131236
ENSG00000145335	ENSG00000175416	ENSG00000132718	ENSG00000139579
ENSG00000139200	ENSG00000149357	ENSG00000124767	ENSG00000146859
ENSG00000165646	ENSG00000130032	ENSG00000111799	ENSG00000007264
ENSG00000167721	ENSG00000166887	ENSG00000104756	ENSG00000187391
ENSG00000182108	ENSG00000105613	ENSG00000061936	ENSG00000113161
ENSG00000164398	ENSG00000116539	ENSG00000140320	ENSG00000148803
ENSG00000111540	ENSG00000152154	ENSG00000197651	ENSG00000166377
ENSG00000125821	ENSG00000130311	ENSG00000156486	ENSG00000113205
ENSG00000204227	ENSG00000158417	ENSG00000182752	ENSG00000162604

ENSG00000172915	ENSG00000057019	ENSG00000168748	ENSG00000117479
ENSG00000172340	ENSG00000197977	ENSG00000157557	ENSG00000184203
ENSG00000157978	ENSG00000188612	ENSG00000137747	ENSG00000184363
ENSG00000128059	ENSG00000117362	ENSG00000144476	ENSG00000122068
ENSG00000174483	ENSG00000025423	ENSG00000079387	ENSG00000101188
ENSG00000134463	ENSG00000011347	ENSG00000123388	ENSG00000172500
ENSG00000158555	ENSG00000138185	ENSG00000156508	ENSG00000159322
ENSG00000168487	ENSG00000173621	ENSG00000169635	ENSG00000171817
ENSG00000130066	ENSG00000139624	ENSG00000169554	ENSG00000158669
ENSG00000103978	ENSG00000082293	ENSG00000104517	ENSG00000168899
ENSG00000188636	ENSG00000096717	ENSG00000151612	ENSG00000128510
ENSG00000126803	ENSG00000161813	ENSG00000168763	ENSG00000176887
ENSG00000172031	ENSG00000004939	ENSG00000135443	ENSG00000175048
ENSG00000114030	ENSG00000100359	ENSG00000164056	ENSG00000145384
ENSG00000033050	ENSG00000112782	ENSG00000136859	ENSG00000111704
ENSG00000066629	ENSG00000090060	ENSG00000155850	ENSG00000099785
ENSG00000171540	ENSG00000155087	ENSG00000023287	ENSG00000169410
ENSG00000168438	ENSG00000151208	ENSG00000073921	ENSG00000091129
ENSG00000124783	ENSG00000105737	ENSG00000002746	ENSG00000132005
ENSG00000133059	ENSG00000162623	ENSG00000110713	ENSG00000198680
ENSG00000040633	ENSG00000146278	ENSG00000111752	ENSG00000128254
ENSG00000099991	ENSG00000119414	ENSG00000183856	ENSG00000185013
ENSG00000114850	ENSG00000160967	ENSG00000148704	ENSG00000196793
ENSG00000164270	ENSG00000181143	ENSG00000147649	ENSG00000153443
ENSG00000061938	ENSG00000130707	ENSG00000165280	ENSG00000090054
ENSG00000115073	ENSG00000180914	ENSG00000131626	ENSG00000127418
ENSG00000147570	ENSG00000175354	ENSG00000174574	ENSG00000004838
ENSG00000167978	ENSG00000123933	ENSG00000156374	ENSG00000164796
ENSG00000106123	ENSG00000151849	ENSG00000100644	ENSG00000146674
ENSG00000168356	ENSG00000164190	ENSG00000134287	ENSG00000121879
ENSG00000189050	ENSG00000132622	ENSG00000087053	ENSG00000141753
ENSG00000106809	ENSG00000183044	ENSG00000172667	ENSG00000151414
ENSG00000168118	ENSG00000184675	ENSG00000008516	ENSG00000100216
ENSG00000163600	ENSG00000120068	ENSG00000183741	ENSG00000065970
ENSG00000187097	ENSG00000153234	ENSG00000135338	ENSG00000101144
ENSG00000071189	ENSG00000082482	ENSG00000115806	ENSG00000135185
ENSG00000136518	ENSG00000176273	ENSG00000132024	ENSG00000131650
ENSG00000109381	ENSG00000163701	ENSG00000153253	ENSG00000104826
ENSG00000162664	ENSG00000122042	ENSG00000008083	ENSG00000167193
ENSG00000112062	ENSG00000183638	ENSG00000162687	ENSG00000177842
ENSG00000147255	ENSG00000173805	ENSG00000169129	ENSG00000103932
ENSG00000126778	ENSG00000157782	ENSG00000143641	ENSG00000185414
ENSG00000185860	ENSG00000123892	ENSG00000162706	ENSG00000186222
ENSG00000111186	ENSG00000075651	ENSG00000183735	ENSG00000136574
ENSG00000196072	ENSG00000127995	ENSG00000069974	ENSG00000163291
ENSG00000123374	ENSG00000100744	ENSG00000011454	ENSG00000170011
ENSG00000126091	ENSG00000135341	ENSG00000166398	ENSG00000177464
ENSG00000197380	ENSG00000102531	ENSG00000153814	ENSG00000179603
ENSG00000134982	ENSG00000134207	ENSG00000091073	ENSG00000187033
ENSG00000125850	ENSG00000013016	ENSG00000123352	ENSG00000188536
ENSG00000122705	ENSG00000163092	ENSG00000115896	ENSG00000147400
ENSG00000177752	ENSG00000074527	ENSG00000112972	ENSG00000107618
ENSG00000101911	ENSG00000103264	ENSG00000145555	ENSG00000124490
ENSG00000100592	ENSG00000164713	ENSG00000182087	ENSG00000197467
ENSG00000179632	ENSG00000196517	ENSG00000011021	ENSG00000168000
ENSG00000167604	ENSG00000171914	ENSG00000198947	ENSG00000138002
ENSG00000162545	ENSG00000120727	ENSG00000171208	ENSG00000079337
ENSG00000184402	ENSG00000020129	ENSG00000186642	ENSG00000185437
ENSG00000138744	ENSG00000174370	ENSG00000143363	ENSG00000108242
ENSG00000185742	ENSG00000134258	ENSG00000110315	ENSG00000198881
ENSG00000149654	ENSG00000204839	ENSG00000160741	ENSG00000164494
ENSG00000174903	ENSG00000077097	ENSG00000156642	ENSG00000125753
ENSG00000128656	ENSG00000155097	ENSG00000144868	ENSG00000140263
ENSG00000182667	ENSG00000180182	ENSG00000198961	ENSG00000173950

ENSG00000173681	ENSG00000139537	ENSG00000127947	ENSG00000167588
ENSG00000146904	ENSG00000179388	ENSG00000163686	ENSG00000114279
ENSG00000165462	ENSG00000157349	ENSG00000186260	ENSG00000157851
ENSG00000141150	ENSG00000137575	ENSG00000017797	ENSG00000008277
ENSG00000204379	ENSG00000109016	ENSG00000177283	ENSG00000138792
ENSG00000173917	ENSG00000102104	ENSG00000100522	ENSG00000081014
ENSG00000142330	ENSG00000109171	ENSG00000164574	ENSG00000006788
ENSG00000126858	ENSG00000132932	ENSG00000179542	ENSG00000100473
ENSG00000175334	ENSG00000137801	ENSG00000163297	ENSG00000163428
ENSG00000113494	ENSG00000198574	ENSG00000173334	ENSG00000167286
ENSG00000088367	ENSG00000118267	ENSG00000151014	ENSG00000112294
ENSG00000111450	ENSG00000173281	ENSG00000180219	ENSG00000153944
ENSG00000131788	ENSG00000170748	ENSG00000168906	ENSG00000170222
ENSG00000144724	ENSG00000197140	ENSG00000119042	ENSG00000161664
ENSG00000123610	ENSG00000133731	ENSG00000068366	ENSG00000130304
ENSG00000163719	ENSG00000174136	ENSG00000087303	ENSG00000116833
ENSG00000076604	ENSG00000182240	ENSG00000204569	ENSG00000218336
ENSG00000137601	ENSG00000149177	ENSG00000100503	ENSG00000198056
ENSG00000181449	ENSG00000173264	ENSG00000085978	ENSG00000162753
ENSG00000184208	ENSG00000075388	ENSG00000016864	ENSG00000134871
ENSG00000179119	ENSG00000083444	ENSG00000177485	ENSG00000163576
ENSG00000101150	ENSG00000159216	ENSG00000176974	ENSG00000187801
ENSG00000154654	ENSG00000099282	ENSG00000144677	ENSG00000241468
ENSG00000140287	ENSG00000110200	ENSG00000074696	ENSG00000144451
ENSG00000149599	ENSG00000174225	ENSG00000006025	ENSG00000161649
ENSG00000182578	ENSG00000117245	ENSG00000018236	ENSG00000130762
ENSG00000182621	ENSG00000111530	ENSG00000151514	ENSG00000171643
ENSG00000128872	ENSG00000169213	ENSG00000101400	ENSG00000033867
ENSG00000166575	ENSG00000124749	ENSG00000173674	ENSG00000167236
ENSG00000182542	ENSG00000149596	ENSG00000139625	ENSG00000100883
ENSG00000101955	ENSG00000143553	ENSG00000170004	ENSG00000089685
ENSG00000113916	ENSG00000107938	ENSG00000151834	ENSG00000141519
ENSG00000170776	ENSG00000171408	ENSG00000140941	ENSG00000175137
ENSG00000169118	ENSG00000124939	ENSG00000060749	ENSG00000135847
ENSG00000117152	ENSG00000075035	ENSG00000112394	ENSG00000144161
ENSG00000025800	ENSG00000140543	ENSG00000153187	ENSG00000164181
ENSG00000128652	ENSG00000138061	ENSG00000169718	ENSG00000119685
ENSG00000125895	ENSG00000182931	ENSG00000167165	ENSG00000147364
ENSG00000134153	ENSG00000171475	ENSG00000160691	ENSG00000163638
ENSG00000173846	ENSG00000222028	ENSG00000100991	ENSG00000150459
ENSG00000113532	ENSG00000198700	ENSG00000183798	ENSG00000167972
ENSG00000120063	ENSG00000164970	ENSG00000064651	ENSG00000174586
ENSG00000162666	ENSG00000198807	ENSG00000146469	ENSG00000213204
ENSG00000131558	ENSG00000169359	ENSG00000126603	ENSG00000105486
ENSG00000156599	ENSG00000189377	ENSG00000178403	ENSG00000128165
ENSG00000117868	ENSG00000120137	ENSG00000196396	ENSG00000204071
ENSG00000162992	ENSG00000109065	ENSG00000196850	ENSG00000248098
ENSG00000167552	ENSG00000049130	ENSG00000157554	ENSG00000148926
ENSG00000125166	ENSG00000099194	ENSG00000111674	ENSG00000187164
ENSG00000105409	ENSG00000168071	ENSG00000198814	ENSG00000163378
ENSG00000132326	ENSG00000183527	ENSG00000163041	ENSG00000177225
ENSG00000118971	ENSG00000151835	ENSG00000174307	ENSG00000114125
ENSG00000146242	ENSG00000146072	ENSG00000111670	ENSG00000118596
ENSG00000021762	ENSG00000170606	ENSG00000115041	ENSG00000015285
ENSG00000175115	ENSG00000165124	ENSG00000079215	ENSG00000115902
ENSG00000181804	ENSG00000160613	ENSG00000116141	ENSG00000048707
ENSG00000135686	ENSG00000158445	ENSG00000127445	ENSG00000114416
ENSG00000068323	ENSG00000166086	ENSG00000102189	ENSG00000135604
ENSG00000182836	ENSG00000176595	ENSG00000104765	ENSG00000119471
ENSG00000092841	ENSG00000203950	ENSG00000007174	ENSG00000182606
ENSG00000023516	ENSG00000179134	ENSG00000101180	ENSG00000183963
ENSG00000075785	ENSG00000114127	ENSG00000119844	ENSG00000174776
ENSG00000146360	ENSG00000168806	ENSG00000067113	ENSG00000127463
ENSG00000198799	ENSG00000175087	ENSG00000180354	ENSG00000167565

ENSG00000122299	ENSG00000157617	ENSG00000197006	ENSG00000188610
ENSG00000153233	ENSG00000198795	ENSG00000141985	ENSG00000186075
ENSG00000164038	ENSG00000169155	ENSG00000152795	ENSG00000121361
ENSG00000160959	ENSG00000121742	ENSG00000100528	ENSG00000124596
ENSG00000158163	ENSG00000104341	ENSG00000163512	ENSG00000177030
ENSG00000184307	ENSG00000130675	ENSG00000110497	ENSG00000137821
ENSG00000027869	ENSG00000100722	ENSG00000142627	ENSG00000175766
ENSG00000070950	ENSG00000189212	ENSG00000112964	ENSG00000239756
ENSG00000171462	ENSG00000105325	ENSG00000188763	ENSG00000171793
ENSG00000081087	ENSG00000060762	ENSG00000168067	ENSG00000214021
ENSG00000123612	ENSG00000140577	ENSG00000120071	ENSG00000108924
ENSG00000104164	ENSG00000157613	ENSG00000099804	ENSG00000136868
ENSG00000172725	ENSG00000173991	ENSG00000001617	ENSG00000171282
ENSG00000166886	ENSG00000185963	ENSG00000147439	ENSG00000146733
ENSG00000133104	ENSG00000160654	ENSG00000139436	ENSG00000107651
ENSG00000127688	ENSG00000065491	ENSG00000198910	ENSG00000022355
ENSG00000171766	ENSG00000145794	ENSG00000006576	ENSG00000184374
ENSG00000075415	ENSG00000053438	ENSG00000170537	ENSG00000156466
ENSG00000205830	ENSG00000197576	ENSG00000170162	ENSG00000055130
ENSG00000144043	ENSG00000143155	ENSG00000133477	ENSG00000100033
ENSG00000143126	ENSG00000133895	ENSG00000067221	ENSG00000090932
ENSG00000052126	ENSG00000144785	ENSG00000136383	ENSG00000169551
ENSG00000101350	ENSG00000152413	ENSG00000162735	ENSG00000188818
ENSG00000148516	ENSG00000165983	ENSG00000170271	ENSG00000119383
ENSG00000053524	ENSG00000082701	ENSG00000124772	ENSG00000187244
ENSG00000110076	ENSG00000000457	ENSG00000171004	ENSG00000135932
ENSG00000114353	ENSG00000139496	ENSG00000166037	ENSG00000160539
ENSG00000185115	ENSG00000087258	ENSG00000114098	ENSG00000109670
ENSG00000131236	ENSG00000074317	ENSG00000096060	ENSG00000162290
ENSG00000091972	ENSG00000186523	ENSG00000049246	ENSG00000119280
ENSG00000146859	ENSG00000119446	ENSG00000144445	ENSG00000101161
ENSG00000187391	ENSG00000155975	ENSG00000175582	ENSG00000163879
ENSG00000113161	ENSG00000174007	ENSG00000131061	ENSG00000126950
ENSG00000170382	ENSG00000129347	ENSG00000165175	ENSG00000144642
ENSG00000188933	ENSG00000172940	ENSG00000068024	ENSG00000160271
ENSG00000145901	ENSG00000109184	ENSG00000108654	ENSG00000014824
ENSG00000162604	ENSG00000157593	ENSG00000125952	ENSG00000107623
ENSG00000116871	ENSG00000077809	ENSG00000164414	ENSG00000145623
ENSG00000187325	ENSG00000115514	ENSG00000087470	ENSG00000163993
ENSG00000065457	ENSG00000035664	ENSG00000143845	ENSG00000124217
ENSG00000184203	ENSG00000131018	ENSG00000100030	ENSG00000161800
ENSG00000106459	ENSG00000155893	ENSG00000064393	ENSG00000115840
ENSG00000117479	ENSG00000102359	ENSG00000141376	ENSG00000149452
ENSG00000184363	ENSG00000196476	ENSG00000085433	ENSG00000125900
ENSG00000168890	ENSG00000179362	ENSG00000136653	ENSG00000067248
ENSG00000167767	ENSG00000092964	ENSG00000144063	ENSG00000244405
ENSG00000111196	ENSG00000091664	ENSG00000154146	ENSG00000165588
ENSG00000122068	ENSG00000181218	ENSG00000089280	ENSG00000221859
ENSG00000187123	ENSG00000181656	ENSG00000112242	ENSG00000187566
ENSG00000159322	ENSG00000100330	ENSG00000120341	ENSG00000171631
ENSG00000152242	ENSG00000159346	ENSG00000113300	ENSG00000151117
ENSG00000128510	ENSG00000165359	ENSG00000164111	ENSG00000110799
ENSG00000091129	ENSG00000146083	ENSG00000153147	ENSG00000122641
ENSG00000120318	ENSG00000135272	ENSG00000102921	ENSG00000103326
ENSG00000099785	ENSG00000167702	ENSG00000179335	ENSG00000164116
ENSG00000169410	ENSG00000141401	ENSG00000140450	ENSG00000197915
ENSG00000132005	ENSG00000134748	ENSG00000184162	ENSG00000187098
ENSG00000074266	ENSG00000165283	ENSG00000170265	ENSG00000137414
ENSG00000139508	ENSG00000109790	ENSG00000180329	ENSG00000130340
ENSG00000203791	ENSG00000165288	ENSG00000078399	ENSG00000092377
ENSG00000155011	ENSG00000162437	ENSG00000067840	ENSG00000158104
ENSG00000120437	ENSG00000125430	ENSG00000143442	ENSG00000115461
ENSG00000127452	ENSG00000132792	ENSG00000163909	ENSG00000198754
ENSG00000137221	ENSG00000123908	ENSG00000181904	ENSG00000169247

ENSG00000135124	ENSG00000204248	ENSG00000160113	ENSG00000196466
ENSG00000090054	ENSG00000111199	ENSG00000204956	ENSG00000127603
ENSG00000004838	ENSG00000106638	ENSG00000126698	ENSG00000100319
ENSG00000164796	ENSG00000133687	ENSG00000182827	ENSG00000214063
ENSG00000043355	ENSG00000167770	ENSG00000132912	ENSG00000221866
ENSG00000108588	ENSG00000141026	ENSG00000084070	ENSG00000166669
ENSG00000142961	ENSG00000104221	ENSG00000077147	ENSG00000182979
ENSG00000146674	ENSG00000130725	ENSG00000111875	ENSG00000013392
ENSG00000130204	ENSG00000122257	ENSG00000170579	ENSG00000135720
ENSG00000078269	ENSG00000115380	ENSG00000175591	ENSG00000058091
ENSG00000065970	ENSG00000198642	ENSG00000141682	ENSG00000180432
ENSG00000137193	ENSG00000119801	ENSG00000149357	ENSG00000178229
ENSG00000135185	ENSG00000132128	ENSG00000198435	ENSG00000173530
ENSG00000131650	ENSG00000003987	ENSG00000152592	ENSG00000188215
ENSG00000167193	ENSG00000144579	ENSG00000130032	ENSG00000050820
ENSG00000101438	ENSG00000175318	ENSG00000097007	ENSG00000070778
ENSG00000154162	ENSG00000187772	ENSG00000183337	ENSG00000157399
ENSG00000136574	ENSG00000117395	ENSG00000166887	ENSG00000185338
ENSG00000170011	ENSG00000134333	ENSG00000032444	ENSG00000011105
ENSG00000177464	ENSG00000112282	ENSG00000048140	ENSG00000114737
ENSG00000179603	ENSG00000175093	ENSG00000136051	ENSG00000112164
ENSG00000147400	ENSG00000204394	ENSG00000143162	ENSG00000164729
ENSG00000197467	ENSG00000172828	ENSG00000173726	ENSG00000121417
ENSG00000085491	ENSG00000137076	ENSG00000204371	ENSG00000156218
ENSG00000168000	ENSG00000171094	ENSG00000152154	ENSG00000174106
ENSG00000171160	ENSG00000040531	ENSG00000181061	ENSG00000189067
ENSG00000100027	ENSG00000178776	ENSG00000158417	ENSG00000179886
ENSG00000119640	ENSG00000109084	ENSG00000057019	ENSG00000187513
ENSG00000118402	ENSG00000248485	ENSG00000197977	ENSG00000101460
ENSG00000156515	ENSG00000139505	ENSG00000117362	ENSG00000128050
ENSG00000176842	ENSG00000179051	ENSG00000168538	ENSG00000128709
ENSG00000164494	ENSG00000176907	ENSG00000004948	ENSG00000126773
ENSG00000140263	ENSG00000101977	ENSG00000011347	ENSG00000129355
ENSG00000114279	ENSG00000149948	ENSG00000173621	ENSG00000074590
ENSG00000157851	ENSG00000134109	ENSG00000139624	ENSG00000183307
ENSG00000008277	ENSG00000103522	ENSG00000082293	ENSG00000162063
ENSG00000138792	ENSG00000155363	ENSG00000096717	ENSG00000165782
ENSG00000184058	ENSG00000173218	ENSG00000145860	ENSG00000115839
ENSG00000081014	ENSG00000205929	ENSG00000004939	ENSG00000160050
ENSG00000100151	ENSG00000185432	ENSG00000112782	ENSG00000134317
ENSG00000066427	ENSG00000131508	ENSG00000036672	ENSG00000106511
ENSG00000133112	ENSG00000125084	ENSG00000090060	ENSG00000166510
ENSG00000100473	ENSG00000140557	ENSG00000147408	ENSG00000105248
ENSG00000108510	ENSG00000142599	ENSG00000136636	ENSG00000129028
ENSG00000013561	ENSG00000198060	ENSG00000136842	ENSG00000007062
ENSG00000008405	ENSG00000177469	ENSG00000155087	ENSG00000120738
ENSG00000112294	ENSG00000113578	ENSG00000151208	ENSG00000206562
ENSG00000039319	ENSG00000116729	ENSG00000123444	ENSG00000148835
ENSG00000153944	ENSG00000169047	ENSG00000105737	ENSG00000178201
ENSG00000105971	ENSG00000104808	ENSG00000050405	ENSG00000105825
ENSG00000170222	ENSG00000066294	ENSG00000065000	ENSG00000156687
ENSG00000116833	ENSG00000186335	ENSG00000146278	ENSG00000155966
ENSG00000166292	ENSG00000188338	ENSG00000174576	ENSG00000121749
ENSG00000134871	ENSG00000074211	ENSG00000188706	ENSG00000131013
ENSG00000196189	ENSG00000163814	ENSG00000180964	ENSG00000130772
ENSG00000132305	ENSG00000163625	ENSG00000101333	ENSG00000204178
ENSG00000138069	ENSG00000146809	ENSG00000119414	ENSG00000084112
ENSG00000135111	ENSG00000134075	ENSG00000100258	ENSG00000145020
ENSG00000136280	ENSG00000146267	ENSG00000182704	ENSG00000186564
ENSG00000090520	ENSG00000198088	ENSG00000180914	ENSG00000152620
ENSG00000033867	ENSG00000079308	ENSG00000175354	ENSG00000113361
ENSG00000184368	ENSG00000107816	ENSG00000011275	ENSG00000166922
ENSG00000166326	ENSG00000052850	ENSG00000123933	ENSG00000107485
ENSG00000121075	ENSG00000112414	ENSG00000143418	ENSG00000182870

ENSG00000162695	ENSG00000174705	ENSG00000130703	ENSG00000197162
ENSG00000165434	ENSG00000138604	ENSG00000164190	ENSG00000163545
ENSG00000137313	ENSG00000196937	ENSG00000130827	ENSG00000116977
ENSG00000167934	ENSG00000112146	ENSG00000183044	ENSG00000184500
ENSG00000070423	ENSG00000173226	ENSG00000155657	ENSG00000160953
ENSG00000164107	ENSG00000196542	ENSG00000067191	ENSG00000119121
ENSG00000182934	ENSG00000122375	ENSG00000197959	ENSG00000184575
ENSG00000175137	ENSG00000183475	ENSG00000184675	ENSG00000198626
ENSG00000119685	ENSG00000102760	ENSG00000120068	ENSG00000113448
ENSG00000163638	ENSG00000120903	ENSG00000153234	ENSG00000140522
ENSG00000073331	ENSG00000030110	ENSG00000082482	ENSG00000197324
ENSG00000021574	ENSG00000048828	ENSG00000176273	ENSG00000169223
ENSG00000106351	ENSG00000146143	ENSG00000140350	ENSG00000172782
ENSG00000167972	ENSG00000181751	ENSG00000125851	ENSG00000105808
ENSG00000126767	ENSG00000119707	ENSG00000163701	ENSG00000161204
ENSG00000083168	ENSG00000112218	ENSG00000143630	ENSG00000168807
ENSG00000135837	ENSG00000135916	ENSG00000122042	ENSG00000130037
ENSG00000148926	ENSG00000174514	ENSG00000006062	ENSG00000104825
ENSG00000073598	ENSG00000136238	ENSG00000173805	ENSG00000116005
ENSG00000165125	ENSG00000135144	ENSG00000137992	ENSG00000214706
ENSG00000187164	ENSG00000099860	ENSG00000157782	ENSG00000198298
ENSG00000163378	ENSG00000132388	ENSG00000123892	ENSG00000131779
ENSG00000118596	ENSG00000124766	ENSG00000075651	ENSG00000117676
ENSG00000114125	ENSG00000176438	ENSG00000083799	ENSG00000170345
ENSG00000115902	ENSG00000166025	ENSG00000127995	ENSG00000152503
ENSG00000114416	ENSG00000167861	ENSG00000100744	ENSG00000048545
ENSG00000182606	ENSG00000105255	ENSG00000135341	ENSG00000016391
ENSG00000183963	ENSG00000187122	ENSG00000102531	ENSG00000187714
ENSG00000174226	ENSG00000243156	ENSG00000134207	ENSG00000161940
ENSG00000159352	ENSG00000149262	ENSG00000103160	ENSG00000160097
ENSG00000198792	ENSG00000090857	ENSG00000013016	ENSG00000166682
ENSG00000167565	ENSG00000100023	ENSG00000131386	ENSG00000139880
ENSG00000100311	ENSG00000148672	ENSG00000162105	ENSG00000163743
ENSG00000004399	ENSG00000171224	ENSG00000188428	ENSG00000108395
ENSG00000109083	ENSG00000136931	ENSG00000074527	ENSG00000138755
ENSG00000149930	ENSG00000104388	ENSG00000138615	ENSG00000112624
ENSG00000108924	ENSG00000153989	ENSG00000103264	ENSG00000139324
ENSG00000114439	ENSG00000151224	ENSG00000164713	ENSG00000197779
ENSG00000204386	ENSG00000169439	ENSG00000171914	ENSG00000120279
ENSG00000136868	ENSG00000105270	ENSG00000120727	ENSG00000172757
ENSG00000204634	ENSG00000089486	ENSG00000020129	ENSG00000198830
ENSG00000146733	ENSG00000140265	ENSG00000174370	ENSG00000128294
ENSG00000107651	ENSG00000096093	ENSG00000138495	ENSG00000164163
ENSG00000166783	ENSG00000001460	ENSG00000105855	ENSG00000115526
ENSG00000102078	ENSG00000253873	ENSG00000077097	ENSG00000165732
ENSG00000022355	ENSG00000196505	ENSG00000119820	ENSG00000187889
ENSG00000099904	ENSG00000186470	ENSG00000105866	ENSG00000148120
ENSG00000139926	ENSG00000122644	ENSG00000187189	ENSG00000070476
ENSG00000156466	ENSG00000162552	ENSG00000068971	ENSG00000139263
ENSG00000147676	ENSG00000142798	ENSG00000180182	ENSG00000108551
ENSG00000055130	ENSG00000106477	ENSG00000157349	ENSG00000102098
ENSG00000119383	ENSG00000136938	ENSG00000137575	ENSG00000124212
ENSG00000187244	ENSG00000088356	ENSG00000039068	ENSG00000107959
ENSG00000197208	ENSG00000146676	ENSG00000179388	ENSG00000156531
ENSG00000135932	ENSG00000125835	ENSG00000140479	ENSG00000165996
ENSG00000160539	ENSG00000171864	ENSG00000114346	ENSG00000117523
ENSG00000109670	ENSG00000111860	ENSG00000153487	ENSG00000186448
ENSG00000162290	ENSG00000196787	ENSG00000166257	ENSG00000182473
ENSG00000196785	ENSG00000087510	ENSG00000102104	ENSG00000175183
ENSG00000119280	ENSG00000121904	ENSG00000132932	ENSG00000115935
ENSG00000176055	ENSG00000142039	ENSG00000137801	ENSG00000108984
ENSG00000172660	ENSG00000102893	ENSG00000175161	ENSG00000198189
ENSG00000168781	ENSG00000072364	ENSG00000198369	ENSG00000133657
ENSG00000160271	ENSG00000171105	ENSG00000137817	ENSG00000154917

ENSG00000173714	ENSG00000176542	ENSG00000170748	ENSG00000161265
ENSG00000144642	ENSG00000118257	ENSG00000171303	ENSG00000128309
ENSG00000014824	ENSG00000131094	ENSG00000067715	ENSG00000149218
ENSG00000107623	ENSG00000106006	ENSG00000113649	ENSG00000132879
ENSG00000033030	ENSG00000106013	ENSG00000182240	ENSG00000230522
ENSG00000145623	ENSG00000143252	ENSG00000107560	ENSG00000197903
ENSG00000136003	ENSG00000005812	ENSG00000178741	ENSG00000173114
ENSG00000165059	ENSG00000126010	ENSG00000149177	ENSG00000133103
ENSG00000163002	ENSG00000131069	ENSG00000168461	ENSG00000167461
ENSG00000115840	ENSG00000148053	ENSG00000173264	ENSG00000111799
ENSG00000111057	ENSG00000005700	ENSG00000120694	ENSG00000061936
ENSG00000124140	ENSG00000213445	ENSG00000060138	ENSG00000104756
ENSG00000085998	ENSG00000111269	ENSG00000166006	ENSG00000081665
ENSG00000107984	ENSG00000111696	ENSG00000083444	ENSG00000140320
ENSG00000187912	ENSG00000104177	ENSG00000159216	ENSG00000163110
ENSG00000165588	ENSG00000133083	ENSG00000129675	ENSG00000182752
ENSG00000163444	ENSG00000172985	ENSG00000099282	ENSG00000168748
ENSG00000151117	ENSG00000123119	ENSG00000107745	ENSG00000137747
ENSG00000122641	ENSG00000185928	ENSG00000110200	ENSG00000105464
ENSG00000175040	ENSG00000205302	ENSG00000162004	ENSG00000079387
ENSG00000103326	ENSG00000103018	ENSG00000058866	ENSG00000115956
ENSG00000101290	ENSG00000181965	ENSG00000162298	ENSG00000156508
ENSG00000106789	ENSG00000143398	ENSG00000111530	ENSG00000058063
ENSG00000187098	ENSG00000172869	ENSG00000175564	ENSG00000167800
ENSG00000137414	ENSG00000138095	ENSG00000012983	ENSG00000180155
ENSG00000115461	ENSG00000113583	ENSG00000124749	ENSG00000133063
ENSG00000147854	ENSG00000132549	ENSG00000149596	ENSG00000168763
ENSG00000102144	ENSG00000198758	ENSG00000153774	ENSG00000129932
ENSG00000134046	ENSG00000123552	ENSG00000107938	ENSG00000137392
ENSG00000127603	ENSG00000180573	ENSG00000171408	ENSG00000155666
ENSG00000197381	ENSG00000145029	ENSG00000008735	ENSG00000181885
ENSG00000108829	ENSG00000141127	ENSG00000140543	ENSG00000155850
ENSG00000115657	ENSG00000132313	ENSG00000163596	ENSG00000138621
ENSG00000108001	ENSG00000154727	ENSG00000160087	ENSG00000154920
ENSG00000137075	ENSG00000184840	ENSG00000138061	ENSG00000167311
ENSG00000147471	ENSG00000062650	ENSG00000171475	ENSG00000100979
ENSG00000166669	ENSG00000167670	ENSG00000158055	ENSG00000213512
ENSG00000065361	ENSG00000129657	ENSG00000155744	ENSG00000110367
ENSG00000058091	ENSG00000112419	ENSG00000164970	ENSG00000073921
ENSG00000171570	ENSG00000116663	ENSG00000169359	ENSG00000002746
ENSG00000135720	ENSG00000153898	ENSG00000116205	ENSG00000137491
ENSG00000151657	ENSG00000132466	ENSG00000158321	ENSG00000111752
ENSG00000184867	ENSG00000123104	ENSG00000172007	ENSG00000110713
ENSG00000184009	ENSG00000107551	ENSG00000049130	ENSG00000204138
ENSG00000166501	ENSG00000168246	ENSG00000149115	ENSG00000183856
ENSG00000166170	ENSG00000103415	ENSG00000159251	ENSG00000165794
ENSG00000188215	ENSG00000101935	ENSG00000099194	ENSG00000177398
ENSG00000104635	ENSG00000090924	ENSG00000141258	ENSG00000095110
ENSG00000070778	ENSG00000112658	ENSG00000115109	ENSG00000131626
ENSG00000162368	ENSG00000069956	ENSG00000132182	ENSG00000156374
ENSG00000185338	ENSG00000101972	ENSG00000151835	ENSG00000152556
ENSG00000179387	ENSG00000196405	ENSG00000146072	ENSG00000100644
ENSG00000011105	ENSG00000105204	ENSG00000170606	ENSG00000115942
ENSG00000112164	ENSG00000102181	ENSG00000160613	ENSG00000104901
ENSG00000156795	ENSG00000136167	ENSG00000158445	ENSG00000134287
ENSG00000079156	ENSG00000138081	ENSG00000146555	ENSG00000196071
ENSG00000114503	ENSG00000184083	ENSG00000114127	ENSG00000130921
ENSG00000164729	ENSG00000104131	ENSG00000175087	ENSG00000186207
ENSG00000162959	ENSG00000173599	ENSG00000170113	ENSG00000008516
ENSG00000156218	ENSG00000116329	ENSG00000089123	ENSG00000182676
ENSG00000174106	ENSG00000110344	ENSG00000157617	ENSG00000104938
ENSG00000189067	ENSG00000168484	ENSG00000198795	ENSG00000132024
ENSG00000101460	ENSG00000050426	ENSG00000169155	ENSG00000146618
ENSG00000072849	ENSG00000165617	ENSG00000163577	ENSG00000100387

ENSG00000172432	ENSG00000215788	ENSG00000172348	ENSG00000008083
ENSG00000184434	ENSG00000117069	ENSG00000130675	ENSG00000186031
ENSG00000183307	ENSG00000150457	ENSG00000100722	ENSG00000143641
ENSG00000074590	ENSG00000187678	ENSG00000060762	ENSG00000069974
ENSG00000162063	ENSG00000221988	ENSG00000157613	ENSG00000198374
ENSG00000165782	ENSG00000134146	ENSG00000173991	ENSG00000166398
ENSG00000160050	ENSG00000163734	ENSG00000185963	ENSG00000153814
ENSG00000134317	ENSG00000104472	ENSG00000065491	ENSG00000091073
ENSG00000106511	ENSG00000157227	ENSG00000164292	ENSG00000160282
ENSG00000135164	ENSG00000116035	ENSG00000145794	ENSG00000188155
ENSG00000129028	ENSG00000066405	ENSG00000053438	ENSG00000123352
ENSG00000120738	ENSG00000171033	ENSG00000095303	ENSG00000170500
ENSG00000150687	ENSG00000118733	ENSG00000197576	ENSG00000115896
ENSG00000148835	ENSG00000179476	ENSG00000143155	ENSG00000182087
ENSG00000105825	ENSG00000152223	ENSG00000120093	ENSG00000145555
ENSG00000156687	ENSG00000108375	ENSG00000147852	ENSG00000171208
ENSG00000197283	ENSG00000112308	ENSG00000133895	ENSG00000186642
ENSG00000129007	ENSG00000213676	ENSG00000166402	ENSG00000132664
ENSG00000155966	ENSG00000101224	ENSG00000177427	ENSG00000143363
ENSG00000121749	ENSG00000139517	ENSG00000196642	ENSG00000110315
ENSG00000084112	ENSG00000044446	ENSG00000144785	ENSG00000135451
ENSG00000197746	ENSG00000141570	ENSG00000074047	ENSG00000144868
ENSG00000126214	ENSG00000113070	ENSG00000165983	ENSG00000198961
ENSG00000140391	ENSG00000133247	ENSG00000082701	ENSG00000143184
ENSG00000145020	ENSG00000153071	ENSG00000081842	ENSG00000163686
ENSG00000152620	ENSG00000156284	ENSG00000000457	ENSG00000097096
ENSG00000085224	ENSG00000010818	ENSG00000198315	ENSG00000017797
ENSG00000101407	ENSG00000198911	ENSG00000140199	ENSG00000147113
ENSG00000107485	ENSG00000186766	ENSG00000087258	ENSG00000115317
ENSG00000163545	ENSG00000136213	ENSG00000162374	ENSG00000198734
ENSG00000108821	ENSG00000154229	ENSG00000074317	ENSG00000219438
ENSG00000138032	ENSG00000137831	ENSG00000132854	ENSG00000006756
ENSG00000167703	ENSG00000186439	ENSG00000161544	ENSG00000100522
ENSG00000119121	ENSG00000122547	ENSG00000091157	ENSG00000240021
ENSG00000198626	ENSG00000161835	ENSG00000143324	ENSG00000164574
ENSG00000163492	ENSG00000243284	ENSG00000103257	ENSG00000179542
ENSG00000113448	ENSG00000253797	ENSG00000155975	ENSG00000173334
ENSG00000184254	ENSG00000197122	ENSG00000036549	ENSG00000179751
ENSG00000197324	ENSG00000174348	ENSG00000065534	ENSG00000071564
ENSG00000161204	ENSG00000162631	ENSG00000197646	ENSG00000179933
ENSG00000072110	ENSG00000167842	ENSG00000036257	ENSG00000126653
ENSG00000101868	ENSG00000070814	ENSG00000109184	ENSG00000163060
ENSG00000130037	ENSG00000197629	ENSG00000183166	ENSG00000128283
ENSG00000133275	ENSG00000170638	ENSG00000103275	ENSG00000203724
ENSG00000134371	ENSG00000128915	ENSG00000157593	ENSG00000016864
ENSG00000116005	ENSG00000130558	ENSG00000077809	ENSG00000177485
ENSG00000101544	ENSG00000253731	ENSG00000116157	ENSG00000188000
ENSG00000117676	ENSG00000147419	ENSG00000108511	ENSG00000176974
ENSG00000170345	ENSG00000179151	ENSG00000035664	ENSG00000128604
ENSG00000152503	ENSG00000043514	ENSG00000131018	ENSG00000131944
ENSG00000187714	ENSG00000117586	ENSG00000092964	ENSG00000168594
ENSG00000160097	ENSG00000101367	ENSG00000179362	ENSG00000118412
ENSG00000172803	ENSG00000165555	ENSG00000091664	ENSG00000177202
ENSG00000176928	ENSG00000133997	ENSG00000115307	ENSG00000136881
ENSG00000204619	ENSG00000124074	ENSG00000167580	ENSG00000167483
ENSG00000064300	ENSG00000239779	ENSG00000181656	ENSG00000103168
ENSG00000105576	ENSG00000061273	ENSG00000100330	ENSG00000170509
ENSG00000170279	ENSG00000015171	ENSG00000159346	ENSG00000163684
ENSG00000152804	ENSG00000134247	ENSG00000175931	ENSG00000088833
ENSG00000112474	ENSG00000143772	ENSG00000165359	ENSG00000131914
ENSG00000139880	ENSG00000103353	ENSG00000197757	ENSG00000094841
ENSG00000152683	ENSG00000103479	ENSG00000146083	ENSG00000180259
ENSG00000108395	ENSG00000186265	ENSG00000135272	ENSG00000148204
ENSG00000197779	ENSG00000101343	ENSG00000167702	ENSG00000099821

ENSG00000001084	ENSG00000116194	ENSG00000147894	ENSG00000103811
ENSG00000172757	ENSG00000196083	ENSG00000134748	ENSG00000001629
ENSG00000198830	ENSG00000115919	ENSG00000186532	ENSG00000140941
ENSG00000164163	ENSG00000144460	ENSG00000109790	ENSG00000047457
ENSG00000115526	ENSG00000166484	ENSG00000139219	ENSG00000160223
ENSG00000174282	ENSG00000196866	ENSG00000112079	ENSG00000188603
ENSG00000187889	ENSG00000077327	ENSG00000011114	ENSG00000099399
ENSG00000115993	ENSG00000178301	ENSG00000167395	ENSG00000160691
ENSG00000120586	ENSG00000169035	ENSG00000165288	ENSG00000215421
ENSG00000131446	ENSG00000133392	ENSG00000162437	ENSG00000171421
ENSG00000108551	ENSG00000136144	ENSG00000125430	ENSG00000146469
ENSG00000139263	ENSG00000188133	ENSG00000123908	ENSG00000183798
ENSG00000124212	ENSG00000153006	ENSG00000204248	ENSG00000064651
ENSG00000156531	ENSG00000214014	ENSG00000173391	ENSG00000148488
ENSG00000166200	ENSG00000111331	ENSG00000111199	ENSG00000185664
ENSG00000159256	ENSG00000168300	ENSG00000112902	ENSG00000197563
ENSG00000152942	ENSG00000005339	ENSG00000124479	ENSG00000196396
ENSG00000119906	ENSG00000120656	ENSG00000174804	ENSG00000090487
ENSG00000115935	ENSG00000182890	ENSG00000167770	ENSG00000196850
ENSG00000108984	ENSG00000124593	ENSG00000198836	ENSG00000157554
ENSG00000198189	ENSG00000118454	ENSG00000161960	ENSG00000198814
ENSG00000135052	ENSG00000107872	ENSG00000122257	ENSG00000163041
ENSG00000154917	ENSG00000037749	ENSG00000198642	ENSG00000198265
ENSG00000151883	ENSG00000214140	ENSG00000133703	ENSG00000174307
ENSG00000109654	ENSG00000100483	ENSG00000119801	ENSG00000186891
ENSG00000125149	ENSG00000105447	ENSG00000178904	ENSG00000127445
ENSG00000127152	ENSG00000127337	ENSG00000132128	ENSG00000114248
ENSG00000173114	ENSG00000166863	ENSG00000137962	ENSG00000164068
ENSG00000080503	ENSG00000004139	ENSG00000110881	ENSG00000137760
ENSG00000073282	ENSG00000163932	ENSG00000178252	ENSG00000156170
ENSG00000167461	ENSG00000135045	ENSG00000204618	ENSG00000170855
ENSG00000184937	ENSG00000087586	ENSG00000144579	ENSG00000180354
ENSG00000177494	ENSG00000215012	ENSG00000187772	ENSG00000010704
ENSG00000164647	ENSG00000132821	ENSG00000135446	ENSG00000197006
ENSG00000157110	ENSG00000170571	ENSG00000163482	ENSG00000141985
ENSG00000147160	ENSG00000166135	ENSG00000143952	ENSG00000011478
ENSG00000132718	ENSG00000072803	ENSG00000103966	ENSG00000188984
ENSG00000124767	ENSG00000142208	ENSG00000175093	ENSG00000148908
ENSG00000111799	ENSG00000187957	ENSG00000204394	ENSG00000171847
ENSG00000061936	ENSG00000100401	ENSG00000108389	ENSG00000143669
ENSG00000104756	ENSG00000011201	ENSG00000137076	ENSG00000146453
ENSG00000140320	ENSG00000114062	ENSG00000040531	ENSG00000163512
ENSG00000197651	ENSG00000133961	ENSG00000171094	ENSG00000171444
ENSG00000156486	ENSG00000177548	ENSG00000065978	ENSG00000142627
ENSG00000130158	ENSG00000146872	ENSG00000151135	ENSG00000184194
ENSG00000182752	ENSG00000127948	ENSG00000109084	ENSG00000116198
ENSG00000168748	ENSG00000164197	ENSG00000152700	ENSG00000112964
ENSG00000157557	ENSG00000106686	ENSG00000113369	ENSG00000150627
ENSG00000137747	ENSG00000170270	ENSG00000139505	ENSG00000099804
ENSG00000170315	ENSG00000177733	ENSG00000196821	ENSG00000001617
ENSG00000144476	ENSG00000184634	ENSG00000101193	ENSG00000139436
ENSG00000079387	ENSG00000198785	ENSG00000176907	ENSG00000125691
ENSG00000156508	ENSG00000184261	ENSG00000123178	ENSG00000082996
ENSG00000123388	ENSG00000149922	ENSG00000120265	ENSG00000244414
ENSG00000169635	ENSG00000175229	ENSG00000184838	ENSG00000135426
ENSG00000169554	ENSG00000167491	ENSG00000101977	ENSG00000110887
ENSG00000104517	ENSG00000175193	ENSG00000090975	ENSG00000124693
ENSG00000168763	ENSG00000164651	ENSG00000135999	ENSG00000060642
ENSG00000151612	ENSG00000106367	ENSG00000134109	ENSG00000108474
ENSG00000135443	ENSG00000149380	ENSG00000155363	ENSG00000138400
ENSG00000197053	ENSG00000182636	ENSG00000173218	ENSG00000133477
ENSG00000143674	ENSG00000187017	ENSG00000185432	ENSG00000067221
ENSG00000164056	ENSG00000133812	ENSG00000065485	ENSG00000185122
ENSG00000104865	ENSG00000141867	ENSG00000131508	ENSG00000100028

ENSG00000136859	ENSG00000124208	ENSG00000125084	ENSG00000178150
ENSG00000136244	ENSG00000177963	ENSG00000178568	ENSG00000050344
ENSG00000140987	ENSG00000197991	ENSG00000079819	ENSG00000177830
ENSG00000155850	ENSG00000144619	ENSG00000142599	ENSG00000115593
ENSG00000023287	ENSG00000205213	ENSG00000157103	ENSG00000145545
ENSG00000073921	ENSG00000122482	ENSG00000010278	ENSG00000171004
ENSG00000002746	ENSG00000168556	ENSG00000165731	ENSG00000124772
ENSG00000111752	ENSG00000137713	ENSG00000177469	ENSG00000166037
ENSG00000110713	ENSG00000168522	ENSG00000113578	ENSG00000114098
ENSG00000183856	ENSG00000155366	ENSG00000116729	ENSG00000049246
ENSG00000104549	ENSG00000166997	ENSG00000169047	ENSG00000117593
ENSG00000148704	ENSG00000144331	ENSG00000075213	ENSG00000162843
ENSG00000147649	ENSG00000112701	ENSG00000030419	ENSG00000165175
ENSG00000165280	ENSG00000198455	ENSG00000034063	ENSG00000023228
ENSG00000131626	ENSG00000164164	ENSG00000141034	ENSG00000108654
ENSG00000174574	ENSG00000152760	ENSG00000188338	ENSG00000068024
ENSG00000156374	ENSG00000198753	ENSG00000183114	ENSG00000163519
ENSG00000074706	ENSG00000083307	ENSG00000074211	ENSG00000175489
ENSG00000152556	ENSG00000178860	ENSG00000179604	ENSG00000067992
ENSG00000100644	ENSG00000054277	ENSG00000163814	ENSG00000183273
ENSG00000134287	ENSG00000122180	ENSG00000163625	ENSG00000164414
ENSG00000087053	ENSG00000142677	ENSG00000110025	ENSG00000110422
ENSG00000172667	ENSG00000203883	ENSG00000146809	ENSG00000105928
ENSG00000008516	ENSG00000184545	ENSG00000146267	ENSG00000143845
ENSG00000135338	ENSG00000008853	ENSG00000144909	ENSG00000115289
ENSG00000183741	ENSG00000100968	ENSG00000165490	ENSG00000100030
ENSG00000115806	ENSG00000198945	ENSG00000005889	ENSG00000064393
ENSG00000132024	ENSG00000152767	ENSG00000143995	ENSG00000141376
ENSG00000100387	ENSG00000159374	ENSG00000117009	ENSG00000102854
ENSG00000153253	ENSG00000132003	ENSG00000169813	ENSG00000137177
ENSG00000008083	ENSG00000151468	ENSG00000196715	ENSG00000136653
ENSG00000162687	ENSG00000113971	ENSG00000079308	ENSG00000213689
ENSG00000169129	ENSG00000177200	ENSG00000107816	ENSG00000137216
ENSG00000143641	ENSG00000162407	ENSG00000052850	ENSG00000154146
ENSG00000162706	ENSG00000187231	ENSG00000064652	ENSG00000088727
ENSG00000183735	ENSG00000113739	ENSG00000112414	ENSG00000163975
ENSG00000069974	ENSG00000164385	ENSG00000135775	ENSG00000183347
ENSG00000011454	ENSG00000167635	ENSG00000138604	ENSG00000105519
ENSG00000166398	ENSG00000147535	ENSG00000196937	ENSG00000159588
ENSG00000153814	ENSG00000130958	ENSG00000173207	ENSG00000112110
ENSG00000091073	ENSG00000184381	ENSG00000171723	ENSG00000189023
ENSG00000188155	ENSG00000131196	ENSG00000183475	ENSG00000180881
ENSG00000123352	ENSG00000196700	ENSG00000102760	ENSG00000112242
ENSG00000115896	ENSG00000119630	ENSG00000153291	ENSG00000164111
ENSG00000112972	ENSG00000185920	ENSG00000155508	ENSG00000150054
ENSG00000118939	ENSG00000136104	ENSG00000048828	ENSG00000113300
ENSG00000182087	ENSG00000005001	ENSG00000181751	ENSG00000102313
ENSG00000011021	ENSG00000036828	ENSG00000112218	ENSG00000238227
ENSG00000145555	ENSG00000100403	ENSG00000102119	ENSG00000087191
ENSG00000144655	ENSG00000164082	ENSG00000135916	ENSG00000136754
ENSG00000198947	ENSG00000150672	ENSG00000105137	ENSG00000179335
ENSG00000186642	ENSG00000170581	ENSG00000046653	ENSG00000140450
ENSG00000171208	ENSG00000171435	ENSG00000136238	ENSG00000067840
ENSG00000110315	ENSG00000197933	ENSG00000135144	ENSG00000116783
ENSG00000143363	ENSG00000122515	ENSG00000124766	ENSG00000176293
ENSG00000160741	ENSG00000197724	ENSG00000132388	ENSG00000143442
ENSG00000156642	ENSG00000138623	ENSG00000145242	ENSG00000160113
ENSG00000144868	ENSG00000204103	ENSG00000106462	ENSG00000101246
ENSG00000198961	ENSG00000009307	ENSG00000105255	ENSG00000164327
ENSG00000089351	ENSG00000187823	ENSG00000168209	ENSG00000121380
ENSG00000127947	ENSG00000124608	ENSG00000187122	ENSG00000128346
ENSG00000163686	ENSG00000165672	ENSG00000090857	ENSG00000182827
ENSG00000186260	ENSG00000196208	ENSG00000198960	ENSG00000132912
ENSG00000017797	ENSG00000073417	ENSG00000126583	ENSG00000134640

ENSG00000177283	ENSG00000167130	ENSG00000148672	ENSG00000164221
ENSG00000006756	ENSG00000168264	ENSG00000136931	ENSG00000112249
ENSG00000100522	ENSG00000182150	ENSG00000153956	ENSG00000125245
ENSG00000164574	ENSG00000143322	ENSG00000104388	ENSG00000099994
ENSG00000179542	ENSG00000185652	ENSG00000206527	ENSG00000143819
ENSG00000151014	ENSG00000148143	ENSG00000153989	ENSG00000175591
ENSG00000173334	ENSG00000101892	ENSG00000124440	ENSG00000175416
ENSG00000163297	ENSG00000134899	ENSG00000116273	ENSG00000149357
ENSG00000180219	ENSG00000240184	ENSG00000151224	ENSG00000083814
ENSG00000124207	ENSG00000164220	ENSG00000169439	ENSG00000110921
ENSG00000168906	ENSG00000115556	ENSG00000105270	ENSG00000177971
ENSG00000175520	ENSG00000125510	ENSG00000089486	ENSG00000130032
ENSG00000068366	ENSG00000197128	ENSG00000160058	ENSG00000166887
ENSG00000119042	ENSG00000140285	ENSG00000169429	ENSG00000178078
ENSG00000204569	ENSG00000124429	ENSG00000170486	ENSG00000176826
ENSG00000087303	ENSG00000156172	ENSG00000196505	ENSG00000112486
ENSG00000138660	ENSG00000128052	ENSG00000122644	ENSG00000116539
ENSG00000100503	ENSG00000042445	ENSG00000162552	ENSG00000095319
ENSG00000083097	ENSG00000135472	ENSG00000142798	ENSG00000130311
ENSG00000016864	ENSG00000152092	ENSG00000146574	ENSG00000158417
ENSG00000156381	ENSG00000126777	ENSG00000106477	ENSG00000117650
ENSG00000085978	ENSG00000155465	ENSG00000136938	ENSG00000057019
ENSG00000177485	ENSG00000173915	ENSG00000088356	ENSG00000197977
ENSG00000176974	ENSG00000204842	ENSG00000146676	ENSG00000124780
ENSG00000144677	ENSG00000102882	ENSG00000105810	ENSG00000188612
ENSG00000074696	ENSG00000104957	ENSG00000139793	ENSG00000130203
ENSG00000175198	ENSG00000103126	ENSG00000087510	ENSG00000114054
ENSG00000018236	ENSG00000004776	ENSG00000121904	ENSG00000117362
ENSG00000006025	ENSG00000143158	ENSG00000185164	ENSG00000025423
ENSG00000151514	ENSG00000126070	ENSG00000142039	ENSG00000071655
ENSG00000101400	ENSG00000181924	ENSG00000166963	ENSG00000162594
ENSG00000173674	ENSG00000183283	ENSG00000072364	ENSG00000138185
ENSG00000139625	ENSG00000157152	ENSG00000171105	ENSG00000011347
ENSG00000131914	ENSG00000166509	ENSG00000118257	ENSG00000173621
ENSG00000094841	ENSG00000132356	ENSG00000131094	ENSG00000139624
ENSG00000170004	ENSG00000169862	ENSG00000106006	ENSG00000166145
ENSG00000151834	ENSG00000126457	ENSG00000112378	ENSG00000082293
ENSG00000140941	ENSG00000084636	ENSG00000136448	ENSG00000096717
ENSG00000060749	ENSG00000183423	ENSG00000134504	ENSG00000157326
ENSG00000112394	ENSG00000158863	ENSG00000174437	ENSG00000161813
ENSG00000153187	ENSG00000187595	ENSG00000143252	ENSG00000004939
ENSG00000028310	ENSG00000003393	ENSG00000005812	ENSG00000127054
ENSG00000128524	ENSG00000144426	ENSG00000135823	ENSG00000112782
ENSG00000169718	ENSG00000127564	ENSG00000148053	ENSG00000152404
ENSG00000167165	ENSG00000171450	ENSG00000107625	ENSG00000090060
ENSG00000160691	ENSG00000172578	ENSG00000163904	ENSG00000151208
ENSG00000100991	ENSG00000006459	ENSG00000111269	ENSG00000155087
ENSG00000165416	ENSG00000148719	ENSG00000181090	ENSG00000105737
ENSG00000183798	ENSG00000117859	ENSG00000111696	ENSG00000175879
ENSG00000146469	ENSG00000204267	ENSG00000104177	ENSG00000146278
ENSG00000064651	ENSG00000173852	ENSG00000086289	ENSG00000105171
ENSG00000126603	ENSG00000008441	ENSG00000067798	ENSG00000119414
ENSG00000178403	ENSG00000186314	ENSG00000121848	ENSG00000130707
ENSG00000196396	ENSG00000112679	ENSG00000133083	ENSG00000173272
ENSG00000196850	ENSG00000007237	ENSG00000198794	ENSG00000180914
ENSG00000157554	ENSG00000113758	ENSG00000123119	ENSG00000096395
ENSG00000176092	ENSG00000165188	ENSG00000205302	ENSG00000142082
ENSG00000111674	ENSG00000006712	ENSG00000140280	ENSG00000175354
ENSG00000198814	ENSG00000196268	ENSG00000181965	ENSG00000170807
ENSG00000163041	ENSG00000177807	ENSG00000167178	ENSG00000123933
ENSG00000144635	ENSG00000110693	ENSG00000143398	ENSG00000151849
ENSG00000174307	ENSG00000163793	ENSG00000172869	ENSG00000164190
ENSG00000111670	ENSG00000102996	ENSG00000125812	ENSG00000163584
ENSG00000115041	ENSG00000149679	ENSG00000113583	ENSG00000132622

ENSG00000079215	ENSG00000104967	ENSG00000054803	ENSG00000139053
ENSG00000196421	ENSG00000140854	ENSG00000107672	ENSG00000154252
ENSG00000116141	ENSG00000125775	ENSG00000128591	ENSG00000183044
ENSG00000127445	ENSG00000136807	ENSG00000198492	ENSG00000172046
ENSG00000102189	ENSG00000184371	ENSG00000124225	ENSG00000082482
ENSG00000104765	ENSG00000125676	ENSG00000145029	ENSG00000153234
ENSG00000133808	ENSG00000100678	ENSG00000154727	ENSG00000120068
ENSG00000007174	ENSG00000100206	ENSG00000184840	ENSG00000184675
ENSG00000102710	ENSG00000131323	ENSG00000062650	ENSG00000176273
ENSG00000103067	ENSG00000168418	ENSG00000121741	ENSG00000163701
ENSG00000164068	ENSG00000122592	ENSG00000166266	ENSG00000122042
ENSG00000165973	ENSG00000124535	ENSG00000136011	ENSG00000183638
ENSG00000137760	ENSG00000169245	ENSG00000167670	ENSG00000122194
ENSG00000067113	ENSG00000143858	ENSG00000101945	ENSG00000173805
ENSG00000101180	ENSG00000057657	ENSG00000152952	ENSG00000157782
ENSG00000119844	ENSG00000181619	ENSG00000104885	ENSG00000175455
ENSG00000170855	ENSG00000174021	ENSG00000108947	ENSG00000170909
ENSG00000180354	ENSG00000077254	ENSG00000197457	ENSG00000123892
ENSG00000141985	ENSG00000006652	ENSG00000132466	ENSG00000075651
ENSG00000197006	ENSG00000064547	ENSG00000169744	ENSG00000182224
ENSG00000152795	ENSG00000125814	ENSG00000123104	ENSG00000170310
ENSG00000136040	ENSG00000163431	ENSG00000107551	ENSG00000135341
ENSG00000100528	ENSG00000198963	ENSG00000168246	ENSG00000100744
ENSG00000163512	ENSG00000070413	ENSG00000132507	ENSG00000127995
ENSG00000172366	ENSG00000189060	ENSG00000134318	ENSG00000132740
ENSG00000110497	ENSG00000156959	ENSG00000101935	ENSG00000138674
ENSG00000142627	ENSG00000120709	ENSG00000103415	ENSG00000102531
ENSG00000057935	ENSG00000138107	ENSG00000076864	ENSG00000243989
ENSG00000188763	ENSG00000198952	ENSG00000112658	ENSG00000134207
ENSG00000112964	ENSG00000115084	ENSG00000090061	ENSG00000013016
ENSG00000055208	ENSG00000102934	ENSG00000101972	ENSG00000186940
ENSG00000168067	ENSG00000179546	ENSG00000069966	ENSG00000145945
ENSG00000120071	ENSG00000138111	ENSG00000105204	ENSG00000074527
ENSG00000099804	ENSG00000101019	ENSG00000136167	ENSG00000162572
ENSG00000001617	ENSG00000176358	ENSG00000130733	ENSG00000249311
ENSG00000139436	ENSG00000055955	ENSG00000131791	ENSG00000103264
ENSG00000147439	ENSG00000157368	ENSG00000102908	ENSG00000196517
ENSG00000198910	ENSG00000183454	ENSG00000137807	ENSG00000171914
ENSG00000082996	ENSG00000086589	ENSG00000144278	ENSG00000196754
ENSG00000006576	ENSG00000172638	ENSG00000138081	ENSG00000020129
ENSG00000170537	ENSG00000128590	ENSG00000184083	ENSG00000174370
ENSG00000170162	ENSG00000149527	ENSG00000090447	ENSG00000151498
ENSG00000133477	ENSG00000160460	ENSG00000104131	ENSG00000134258
ENSG00000065665	ENSG00000110711	ENSG00000084234	ENSG00000204839
ENSG00000067221	ENSG00000122707	ENSG00000102755	ENSG00000077097
ENSG00000136383	ENSG00000165168	ENSG00000121058	ENSG00000105246
ENSG00000162735	ENSG00000143622	ENSG00000152284	ENSG00000155097
ENSG00000170271	ENSG00000163681	ENSG00000173599	ENSG00000180182
ENSG00000050344	ENSG00000165156	ENSG00000163029	ENSG00000187754
ENSG00000124772	ENSG00000079385	ENSG00000121964	ENSG00000100554
ENSG00000171004	ENSG00000105538	ENSG00000164548	ENSG00000157349
ENSG00000166037	ENSG00000153558	ENSG00000197111	ENSG00000137575
ENSG00000096060	ENSG00000213171	ENSG00000174469	ENSG00000179388
ENSG00000114098	ENSG00000127838	ENSG00000110344	ENSG00000130234
ENSG00000049246	ENSG00000198964	ENSG00000107263	ENSG00000197134
ENSG00000144445	ENSG00000051180	ENSG00000197321	ENSG00000109016
ENSG00000175582	ENSG00000085733	ENSG00000083937	ENSG00000181350
ENSG00000165175	ENSG00000173210	ENSG00000050426	ENSG00000141627
ENSG00000131061	ENSG00000128731	ENSG00000165617	ENSG00000102104
ENSG00000108654	ENSG00000010017	ENSG00000117069	ENSG00000132932
ENSG00000068024	ENSG00000106443	ENSG00000150457	ENSG00000109171
ENSG00000125952	ENSG00000019186	ENSG00000131051	ENSG00000137801
ENSG00000164414	ENSG00000179528	ENSG00000187678	ENSG00000148175
ENSG00000087470	ENSG00000107859	ENSG00000103423	ENSG00000198574

ENSG00000143845	ENSG00000177994	ENSG00000123159	ENSG00000140986
ENSG00000100030	ENSG00000116521	ENSG00000171587	ENSG00000118267
ENSG00000064393	ENSG00000160051	ENSG00000169306	ENSG00000123213
ENSG00000177769	ENSG00000186111	ENSG00000157227	ENSG00000145526
ENSG00000141376	ENSG00000165699	ENSG00000162928	ENSG00000186407
ENSG00000085433	ENSG00000213614	ENSG00000054267	ENSG00000173281
ENSG00000136653	ENSG00000120253	ENSG00000171033	ENSG00000170748
ENSG00000143620	ENSG00000140807	ENSG00000108375	ENSG00000150201
ENSG00000144063	ENSG00000076641	ENSG00000112308	ENSG00000174136
ENSG00000130687	ENSG00000107679	ENSG00000101224	ENSG00000154678
ENSG00000089280	ENSG00000070159	ENSG00000058272	ENSG00000206172
ENSG00000154146	ENSG00000165443	ENSG00000136888	ENSG00000178084
ENSG00000204410	ENSG00000165821	ENSG00000006432	ENSG00000182240
ENSG00000112242	ENSG00000154493	ENSG00000113070	ENSG00000106404
ENSG00000120341	ENSG00000103222	ENSG00000135862	ENSG00000184157
ENSG00000164111	ENSG00000135423	ENSG00000163637	ENSG00000149177
ENSG00000153147	ENSG00000108813	ENSG00000135414	ENSG00000075290
ENSG00000113300	ENSG00000089692	ENSG00000133247	ENSG00000173264
ENSG00000102921	ENSG00000125740	ENSG00000158158	ENSG00000075388
ENSG00000179335	ENSG00000122122	ENSG00000153071	ENSG00000082929
ENSG00000140450	ENSG00000119403	ENSG00000010818	ENSG00000162618
ENSG00000184162	ENSG00000187398	ENSG00000119938	ENSG00000083444
ENSG00000170265	ENSG00000197594	ENSG00000148187	ENSG00000163564
ENSG00000180329	ENSG00000102096	ENSG00000154229	ENSG00000159216
ENSG00000067840	ENSG00000132911	ENSG00000069275	ENSG00000099282
ENSG00000078399	ENSG00000150967	ENSG00000186439	ENSG00000174225
ENSG00000163909	ENSG00000113712	ENSG00000172572	ENSG00000140993
ENSG00000143442	ENSG00000120885	ENSG00000159423	ENSG00000110200
ENSG00000160113	ENSG00000090581	ENSG00000161835	ENSG00000171298
ENSG00000181904	ENSG00000162613	ENSG00000058085	ENSG00000117245
ENSG00000126698	ENSG00000166233	ENSG00000116199	ENSG00000111530
ENSG00000169562	ENSG00000064218	ENSG00000170689	ENSG00000178234
ENSG00000204956	ENSG00000162522	ENSG00000183773	ENSG00000169213
ENSG00000160801	ENSG00000183496	ENSG00000165995	ENSG00000175352
ENSG00000128845	ENSG00000140497	ENSG00000128564	ENSG00000124749
ENSG00000182827	ENSG00000170430	ENSG00000078401	ENSG00000149596
ENSG00000132912	ENSG00000117385	ENSG00000130558	ENSG00000121764
ENSG00000084070	ENSG00000167106	ENSG00000147419	ENSG00000107938
ENSG00000072864	ENSG00000088808	ENSG00000179151	ENSG00000092929
ENSG00000112249	ENSG00000136634	ENSG00000077585	ENSG00000171408
ENSG00000077147	ENSG00000164754	ENSG00000043514	ENSG00000124939
ENSG00000140525	ENSG00000186522	ENSG00000066382	ENSG00000124578
ENSG00000111875	ENSG00000117036	ENSG00000183780	ENSG00000151650
ENSG00000170579	ENSG00000185722	ENSG00000117586	ENSG00000163608
ENSG00000154328	ENSG00000185989	ENSG00000177380	ENSG00000138061
ENSG00000204376	ENSG00000151694	ENSG00000101367	ENSG00000182931
ENSG00000175591	ENSG00000126261	ENSG00000106615	ENSG00000131100
ENSG00000149357	ENSG00000177511	ENSG00000070759	ENSG00000071082
ENSG00000141682	ENSG00000116133	ENSG00000134152	ENSG00000198700
ENSG00000198435	ENSG00000173064	ENSG00000133997	ENSG00000164970
ENSG00000152592	ENSG00000173208	ENSG00000166825	ENSG00000198807
ENSG00000097007	ENSG00000103042	ENSG00000108387	ENSG00000205176
ENSG00000130032	ENSG00000139266	ENSG00000197587	ENSG00000120137
ENSG00000183337	ENSG00000203811	ENSG00000115415	ENSG00000049130
ENSG00000166887	ENSG00000112130	ENSG00000110911	ENSG00000109065
ENSG00000141325	ENSG00000164463	ENSG00000108556	ENSG00000112799
ENSG00000032444	ENSG00000144802	ENSG00000163349	ENSG00000099194
ENSG00000048140	ENSG00000112039	ENSG00000181481	ENSG00000198342
ENSG00000136051	ENSG00000138795	ENSG00000139154	ENSG00000142973
ENSG00000197056	ENSG00000183862	ENSG00000061273	ENSG00000085415
ENSG00000120333	ENSG00000110060	ENSG00000015171	ENSG00000170606
ENSG00000176635	ENSG00000125966	ENSG00000039560	ENSG00000146072
ENSG00000143162	ENSG00000168769	ENSG00000124942	ENSG00000182326
ENSG00000075043	ENSG00000160791	ENSG00000197562	ENSG00000188976

ENSG00000204371	ENSG00000150281	ENSG00000011258	ENSG00000165124
ENSG00000173726	ENSG00000186442	ENSG00000102572	ENSG00000160613
ENSG00000137731	ENSG00000135638	ENSG00000134247	ENSG00000158445
ENSG00000152154	ENSG00000118418	ENSG00000143772	ENSG00000166086
ENSG00000181061	ENSG00000122126	ENSG00000125354	ENSG00000176595
ENSG00000158417	ENSG00000137309	ENSG00000103353	ENSG00000203950
ENSG00000117650	ENSG00000064655	ENSG00000103479	ENSG00000179134
ENSG00000197977	ENSG00000162511	ENSG00000117016	ENSG00000114127
ENSG00000057019	ENSG00000185697	ENSG00000116194	ENSG00000168806
ENSG00000168216	ENSG00000067704	ENSG00000156384	ENSG00000175087
ENSG00000117362	ENSG00000103647	ENSG00000163848	ENSG00000198795
ENSG00000168538	ENSG00000106772	ENSG00000166484	ENSG00000121742
ENSG00000004948	ENSG00000163320	ENSG00000198373	ENSG00000104341
ENSG00000011347	ENSG00000111912	ENSG00000178301	ENSG00000244274
ENSG00000173621	ENSG00000123268	ENSG00000204217	ENSG00000189212
ENSG00000139624	ENSG00000001561	ENSG00000102978	ENSG00000105325
ENSG00000082293	ENSG00000073584	ENSG00000136144	ENSG00000115297
ENSG00000206318	ENSG00000180398	ENSG00000137203	ENSG00000060762
ENSG00000096717	ENSG00000124721	ENSG00000188133	ENSG00000140577
ENSG00000136143	ENSG00000101474	ENSG00000092439	ENSG00000173991
ENSG00000004939	ENSG00000112379	ENSG00000138293	ENSG00000157613
ENSG00000145860	ENSG00000164684	ENSG00000153006	ENSG00000185963
ENSG00000112782	ENSG00000187715	ENSG00000127220	ENSG00000160654
ENSG00000036672	ENSG00000163946	ENSG00000072609	ENSG00000180855
ENSG00000136636	ENSG00000100321	ENSG00000111331	ENSG00000065491
ENSG00000147408	ENSG00000168546	ENSG00000125414	ENSG00000145794
ENSG00000136842	ENSG00000118058	ENSG00000005339	ENSG00000149136
ENSG00000090060	ENSG00000164920	ENSG00000150471	ENSG00000148288
ENSG00000155087	ENSG00000091009	ENSG00000010322	ENSG00000197576
ENSG00000151208	ENSG00000112245	ENSG00000120656	ENSG00000053438
ENSG00000105737	ENSG00000147202	ENSG00000154783	ENSG00000143155
ENSG00000123444	ENSG00000139438	ENSG00000182890	ENSG00000038002
ENSG00000050405	ENSG00000011523	ENSG00000124593	ENSG00000133895
ENSG00000065000	ENSG00000077274	ENSG00000118454	ENSG00000184471
ENSG00000175879	ENSG00000166923	ENSG00000037749	ENSG00000104763
ENSG00000146278	ENSG00000198522	ENSG00000176105	ENSG00000116221
ENSG00000180964	ENSG00000076826	ENSG00000116514	ENSG00000125538
ENSG00000188706	ENSG00000101752	ENSG00000169641	ENSG00000184313
ENSG00000174576	ENSG00000079841	ENSG00000100483	ENSG00000152413
ENSG00000119414	ENSG00000197879	ENSG00000127337	ENSG00000165983
ENSG00000101333	ENSG00000135469	ENSG00000165675	ENSG00000000457
ENSG00000142178	ENSG00000109436	ENSG00000163932	ENSG00000139496
ENSG00000100258	ENSG00000172819	ENSG00000135045	ENSG00000186523
ENSG00000166347	ENSG00000042286	ENSG00000132821	ENSG00000087258
ENSG00000182704	ENSG00000140153	ENSG00000180530	ENSG00000074317
ENSG00000180914	ENSG00000095485	ENSG00000170571	ENSG00000119446
ENSG00000175354	ENSG00000084731	ENSG00000166135	ENSG00000174007
ENSG00000011275	ENSG00000254108	ENSG00000072803	ENSG00000115290
ENSG00000123933	ENSG00000100226	ENSG00000110786	ENSG00000118197
ENSG00000130703	ENSG00000167550	ENSG00000180304	ENSG00000109184
ENSG00000143418	ENSG00000137259	ENSG00000134369	ENSG00000172940
ENSG00000164190	ENSG00000198301	ENSG00000187957	ENSG00000157593
ENSG00000130827	ENSG00000072571	ENSG00000123700	ENSG00000077809
ENSG00000183044	ENSG00000106366	ENSG00000114062	ENSG00000115514
ENSG00000155657	ENSG00000137343	ENSG00000127914	ENSG00000215790
ENSG00000067191	ENSG00000151012	ENSG00000133961	ENSG00000035664
ENSG00000197959	ENSG00000183137	ENSG00000178425	ENSG00000131018
ENSG00000184675	ENSG00000139620	ENSG00000197694	ENSG00000155893
ENSG00000120068	ENSG00000102385	ENSG00000107829	ENSG00000188868
ENSG00000153234	ENSG00000118705	ENSG00000146872	ENSG00000196476
ENSG00000082482	ENSG00000185015	ENSG00000145824	ENSG00000166126
ENSG00000010219	ENSG00000124831	ENSG00000185728	ENSG00000179362
ENSG00000140350	ENSG00000168887	ENSG00000177733	ENSG00000092964
ENSG00000125851	ENSG00000136813	ENSG00000082641	ENSG00000091664

ENSG00000176273	ENSG00000113368	ENSG00000198785	ENSG00000181218
ENSG00000163701	ENSG00000130830	ENSG00000175229	ENSG00000181656
ENSG00000143630	ENSG00000126461	ENSG00000129521	ENSG00000159346
ENSG00000122042	ENSG00000101337	ENSG00000164651	ENSG00000100330
ENSG00000006062	ENSG00000214013	ENSG00000106367	ENSG00000172070
ENSG00000173805	ENSG00000129946	ENSG00000182636	ENSG00000165359
ENSG00000137992	ENSG00000130669	ENSG00000187017	ENSG00000146083
ENSG00000157782	ENSG00000184979	ENSG00000133812	ENSG00000141401
ENSG00000075651	ENSG00000134765	ENSG00000006007	ENSG00000167702
ENSG00000123892	ENSG00000185619	ENSG00000141867	ENSG00000135272
ENSG00000115998	ENSG00000111707	ENSG00000197256	ENSG00000134748
ENSG00000182224	ENSG00000166595	ENSG00000069869	ENSG00000105993
ENSG00000083799	ENSG00000133884	ENSG00000124208	ENSG00000234906
ENSG00000178209	ENSG00000168256	ENSG00000151715	ENSG00000109790
ENSG00000138674	ENSG00000138449	ENSG00000166068	ENSG00000080618
ENSG00000100744	ENSG00000151491	ENSG00000174718	ENSG00000134014
ENSG00000135341	ENSG00000125686	ENSG00000168542	ENSG00000026950
ENSG00000127995	ENSG00000135378	ENSG00000177614	ENSG00000175806
ENSG00000102531	ENSG00000131095	ENSG00000197991	ENSG00000165288
ENSG00000103160	ENSG00000198518	ENSG00000072042	ENSG00000162437
ENSG00000134207	ENSG00000069329	ENSG00000144619	ENSG00000125430
ENSG00000013016	ENSG00000130592	ENSG00000205213	ENSG00000132792
ENSG00000131386	ENSG00000122176	ENSG00000131773	ENSG00000123908
ENSG00000162105	ENSG00000154370	ENSG00000122482	ENSG00000064703
ENSG00000188428	ENSG00000136002	ENSG00000167371	ENSG00000205810
ENSG00000138615	ENSG00000171126	ENSG00000139722	ENSG00000111199
ENSG00000074527	ENSG00000189182	ENSG00000168556	ENSG00000106638
ENSG00000181368	ENSG00000089220	ENSG00000115649	ENSG00000133687
ENSG00000206398	ENSG00000142303	ENSG00000104093	ENSG00000198523
ENSG00000162641	ENSG00000131669	ENSG00000197343	ENSG00000166199
ENSG00000103264	ENSG00000168904	ENSG00000137713	ENSG00000167770
ENSG00000164713	ENSG00000138246	ENSG00000168522	ENSG00000141026
ENSG00000171914	ENSG00000092529	ENSG00000155366	ENSG00000104221
ENSG00000120727	ENSG00000134533	ENSG00000087245	ENSG00000166946
ENSG00000020129	ENSG00000198720	ENSG00000133318	ENSG00000130725
ENSG00000174370	ENSG00000091164	ENSG00000144331	ENSG00000130943
ENSG00000138495	ENSG00000113805	ENSG00000112701	ENSG00000170954
ENSG00000105855	ENSG00000178573	ENSG00000198815	ENSG00000122257
ENSG00000077097	ENSG00000174744	ENSG00000198455	ENSG00000115380
ENSG00000119820	ENSG00000205155	ENSG00000114857	ENSG00000187583
ENSG00000105866	ENSG00000174652	ENSG00000132424	ENSG00000198642
ENSG00000068971	ENSG00000138760	ENSG00000101216	ENSG00000119801
ENSG00000187189	ENSG00000133131	ENSG00000164164	ENSG00000100297
ENSG00000180182	ENSG00000148123	ENSG00000101782	ENSG00000132128
ENSG00000039068	ENSG00000103549	ENSG00000108352	ENSG00000003987
ENSG00000137575	ENSG00000185009	ENSG00000169714	ENSG00000158077
ENSG00000157349	ENSG00000163162	ENSG00000152760	ENSG00000135372
ENSG00000179388	ENSG00000205269	ENSG00000083307	ENSG00000187772
ENSG00000206115	ENSG00000159842	ENSG00000122180	ENSG00000175318
ENSG00000140479	ENSG00000167723	ENSG00000164093	ENSG00000144579
ENSG00000114346	ENSG00000121486	ENSG00000184545	ENSG00000204007
ENSG00000130234	ENSG00000111790	ENSG00000111961	ENSG00000163961
ENSG00000153487	ENSG00000148343	ENSG00000100968	ENSG00000042980
ENSG00000183461	ENSG00000102935	ENSG00000156925	ENSG00000134333
ENSG00000181350	ENSG00000144061	ENSG00000152767	ENSG00000175093
ENSG00000166257	ENSG00000180447	ENSG00000204965	ENSG00000126460
ENSG00000102104	ENSG00000197114	ENSG00000151468	ENSG00000172828
ENSG00000132932	ENSG00000166197	ENSG00000000003	ENSG00000137076
ENSG00000137801	ENSG00000177674	ENSG00000113971	ENSG00000171094
ENSG00000148482	ENSG00000221914	ENSG00000177200	ENSG00000166788
ENSG00000175161	ENSG00000138614	ENSG00000162407	ENSG00000040531
ENSG00000198369	ENSG00000117899	ENSG00000187231	ENSG00000149131
ENSG00000179399	ENSG00000164318	ENSG00000077044	ENSG00000133773
ENSG00000137817	ENSG00000172296	ENSG00000167635	ENSG00000186094

ENSG00000131981	ENSG00000144366	ENSG00000167941	ENSG00000154639
ENSG00000170748	ENSG00000136451	ENSG00000130958	ENSG00000109084
ENSG00000171303	ENSG00000106483	ENSG00000160145	ENSG00000248485
ENSG00000122711	ENSG00000125246	ENSG00000119630	ENSG00000155561
ENSG00000067715	ENSG00000166002	ENSG00000196700	ENSG00000139505
ENSG00000169592	ENSG00000127511	ENSG00000185920	ENSG00000179051
ENSG00000113649	ENSG00000197565	ENSG00000082805	ENSG00000106211
ENSG00000112234	ENSG00000112139	ENSG00000165322	ENSG00000177047
ENSG00000182240	ENSG00000106348	ENSG00000158856	ENSG00000176907
ENSG00000178741	ENSG00000075856	ENSG00000005001	ENSG00000196387
ENSG00000107560	ENSG00000109738	ENSG00000036828	ENSG00000101977
ENSG00000184157	ENSG00000134058	ENSG00000100403	ENSG00000149948
ENSG00000149177	ENSG00000205056	ENSG00000168348	ENSG00000134109
ENSG00000106404	ENSG00000116260	ENSG00000150672	ENSG00000100150
ENSG00000168461	ENSG00000183172	ENSG00000155111	ENSG00000103522
ENSG00000204919	ENSG00000155592	ENSG00000157077	ENSG00000155363
ENSG00000120694	ENSG00000122861	ENSG00000119953	ENSG00000156931
ENSG00000144362	ENSG00000101236	ENSG00000197724	ENSG00000205929
ENSG00000060138	ENSG00000186020	ENSG00000179600	ENSG00000173218
ENSG00000173264	ENSG00000072422	ENSG00000198010	ENSG00000184029
ENSG00000166006	ENSG00000108840	ENSG00000204103	ENSG00000131508
ENSG00000082929	ENSG00000144857	ENSG00000009307	ENSG00000185432
ENSG00000162733	ENSG00000100916	ENSG00000116667	ENSG00000125084
ENSG00000083444	ENSG00000150893	ENSG00000187823	ENSG00000136450
ENSG00000129675	ENSG00000136770	ENSG00000170819	ENSG00000142599
ENSG00000159216	ENSG00000185610	ENSG00000170802	ENSG00000140557
ENSG00000099282	ENSG00000173598	ENSG00000167130	ENSG00000197816
ENSG00000107745	ENSG00000010244	ENSG00000168264	ENSG00000101003
ENSG00000162004	ENSG00000108061	ENSG00000183255	ENSG00000196683
ENSG00000197619	ENSG00000151461	ENSG00000185652	ENSG00000198060
ENSG00000110200	ENSG00000168495	ENSG00000143390	ENSG00000113578
ENSG00000129084	ENSG00000135632	ENSG00000182968	ENSG00000177469
ENSG00000058866	ENSG00000101280	ENSG00000101892	ENSG00000169047
ENSG00000111530	ENSG00000198399	ENSG00000148143	ENSG00000116729
ENSG00000162298	ENSG00000099622	ENSG00000133026	ENSG00000172116
ENSG00000175564	ENSG00000116604	ENSG00000178695	ENSG00000066294
ENSG00000012983	ENSG00000171617	ENSG00000010810	ENSG00000186335
ENSG00000152670	ENSG00000101846	ENSG00000164220	ENSG00000188338
ENSG00000175352	ENSG00000221870	ENSG00000163660	ENSG00000185252
ENSG00000153774	ENSG00000055609	ENSG00000115556	ENSG00000169905
ENSG00000149596	ENSG00000126088	ENSG00000110436	ENSG00000074211
ENSG00000124749	ENSG00000173230	ENSG00000165556	ENSG00000143627
ENSG00000107938	ENSG00000116032	ENSG00000153885	ENSG00000147223
ENSG00000171408	ENSG00000168135	ENSG00000143344	ENSG00000163814
ENSG00000122694	ENSG00000110046	ENSG00000141522	ENSG00000163625
ENSG00000008735	ENSG00000168282	ENSG00000162769	ENSG00000085274
ENSG00000140543	ENSG00000170473	ENSG00000168795	ENSG00000146809
ENSG00000163596	ENSG00000100304	ENSG00000164458	ENSG00000134075
ENSG00000174695	ENSG00000112759	ENSG00000160570	ENSG00000174945
ENSG00000160087	ENSG00000114573	ENSG00000140285	ENSG00000079308
ENSG00000138061	ENSG00000147883	ENSG00000128052	ENSG00000122687
ENSG00000158055	ENSG00000161573	ENSG00000124126	ENSG00000107816
ENSG00000171475	ENSG00000198648	ENSG00000152092	ENSG00000163218
ENSG00000131100	ENSG00000156299	ENSG00000135472	ENSG00000167508
ENSG00000038219	ENSG00000119772	ENSG00000126777	ENSG00000091947
ENSG00000155744	ENSG00000090238	ENSG00000108219	ENSG00000175077
ENSG00000010030	ENSG00000183155	ENSG00000166747	ENSG00000052850
ENSG00000164970	ENSG00000176058	ENSG00000132485	ENSG00000112414
ENSG00000169359	ENSG00000070831	ENSG00000204842	ENSG00000118434
ENSG00000116205	ENSG00000182446	ENSG00000143437	ENSG00000205038
ENSG00000158321	ENSG00000104643	ENSG00000103126	ENSG00000167523
ENSG00000172007	ENSG00000139364	ENSG00000138771	ENSG00000174705
ENSG00000151665	ENSG00000136828	ENSG00000205339	ENSG00000196937
ENSG00000049130	ENSG00000213066	ENSG00000135547	ENSG00000138604

ENSG00000149115	ENSG00000171388	ENSG00000004776	ENSG00000173226
ENSG00000159251	ENSG00000035928	ENSG00000127554	ENSG00000196542
ENSG00000099194	ENSG00000133606	ENSG00000197535	ENSG00000112146
ENSG00000141258	ENSG00000162139	ENSG00000126070	ENSG00000122375
ENSG00000115109	ENSG00000161847	ENSG00000144668	ENSG00000183475
ENSG00000130876	ENSG00000142166	ENSG00000157152	ENSG00000030110
ENSG00000177143	ENSG00000108094	ENSG00000183283	ENSG00000120903
ENSG00000085415	ENSG00000164104	ENSG00000181924	ENSG00000088926
ENSG00000132182	ENSG00000028839	ENSG00000164631	ENSG00000197901
ENSG00000151835	ENSG00000109572	ENSG00000173548	ENSG00000176920
ENSG00000170606	ENSG00000152270	ENSG00000132356	ENSG00000213096
ENSG00000146072	ENSG00000118526	ENSG00000130201	ENSG00000119707
ENSG00000160613	ENSG00000071967	ENSG00000169862	ENSG00000135916
ENSG00000158445	ENSG00000175374	ENSG00000138430	ENSG00000139278
ENSG00000116117	ENSG00000134780	ENSG00000084636	ENSG00000174514
ENSG00000146555	ENSG00000102221	ENSG00000163393	ENSG00000136238
ENSG00000114127	ENSG00000164406	ENSG00000158863	ENSG00000008438
ENSG00000089123	ENSG00000161267	ENSG00000003393	ENSG00000164816
ENSG00000175087	ENSG00000144746	ENSG00000171450	ENSG00000135144
ENSG00000170113	ENSG00000113231	ENSG00000148719	ENSG00000099860
ENSG00000157617	ENSG00000105819	ENSG00000117859	ENSG00000124766
ENSG00000198795	ENSG00000141738	ENSG00000204267	ENSG00000132388
ENSG00000169155	ENSG00000095739	ENSG00000186591	ENSG00000176438
ENSG00000163577	ENSG00000165119	ENSG00000173757	ENSG00000197620
ENSG00000172348	ENSG00000064787	ENSG00000132471	ENSG00000166025
ENSG00000130675	ENSG00000147059	ENSG00000119242	ENSG00000167861
ENSG00000100722	ENSG00000137269	ENSG00000198561	ENSG00000105255
ENSG00000154415	ENSG00000111962	ENSG00000007237	ENSG00000136546
ENSG00000115297	ENSG00000064961	ENSG00000113758	ENSG00000243156
ENSG00000060762	ENSG00000167548	ENSG00000165188	ENSG00000149262
ENSG00000173991	ENSG00000166908	ENSG00000006712	ENSG00000243489
ENSG00000157613	ENSG00000177885	ENSG00000177807	ENSG00000100023
ENSG00000170775	ENSG00000162430	ENSG00000153130	ENSG00000148672
ENSG00000185963	ENSG00000126216	ENSG00000110693	ENSG00000171224
ENSG00000130589	ENSG00000186417	ENSG00000129245	ENSG00000136931
ENSG00000065491	ENSG00000113441	ENSG00000101665	ENSG00000148296
ENSG00000164292	ENSG00000183655	ENSG00000122545	ENSG00000165475
ENSG00000145794	ENSG00000172789	ENSG00000179348	ENSG00000151224
ENSG00000149136	ENSG00000154734	ENSG00000183770	ENSG00000169439
ENSG00000095303	ENSG00000071073	ENSG00000149679	ENSG00000089486
ENSG00000197576	ENSG00000153827	ENSG00000140854	ENSG00000145217
ENSG00000177791	ENSG00000161202	ENSG00000109689	ENSG00000096093
ENSG00000053438	ENSG00000245848	ENSG00000168092	ENSG00000139370
ENSG00000143155	ENSG00000167615	ENSG00000104960	ENSG00000001460
ENSG00000120093	ENSG00000176444	ENSG00000163995	ENSG00000196505
ENSG00000133895	ENSG00000100243	ENSG00000054118	ENSG00000186470
ENSG00000147852	ENSG00000107562	ENSG00000136807	ENSG00000122644
ENSG00000166402	ENSG00000138735	ENSG00000184371	ENSG00000142798
ENSG00000177427	ENSG00000137460	ENSG00000063245	ENSG00000162552
ENSG00000118816	ENSG00000181104	ENSG00000134294	ENSG00000125484
ENSG00000196642	ENSG00000060982	ENSG00000134954	ENSG00000106477
ENSG00000144785	ENSG00000122643	ENSG00000131323	ENSG00000136938
ENSG00000074047	ENSG00000151502	ENSG00000074695	ENSG00000088356
ENSG00000165983	ENSG00000162104	ENSG00000124535	ENSG00000146676
ENSG00000082701	ENSG00000114742	ENSG00000057657	ENSG00000155367
ENSG00000081842	ENSG00000179270	ENSG00000049192	ENSG00000125835
ENSG00000198315	ENSG00000100442	ENSG00000174021	ENSG00000171864
ENSG00000000457	ENSG00000105085	ENSG00000064547	ENSG00000111860
ENSG00000140199	ENSG00000107854	ENSG00000077254	ENSG00000143412
ENSG00000074317	ENSG00000227372	ENSG00000163431	ENSG00000149968
ENSG00000162374	ENSG00000162761	ENSG00000125814	ENSG00000196787
ENSG00000087258	ENSG00000159788	ENSG00000070413	ENSG00000178719
ENSG00000161544	ENSG00000172845	ENSG00000189060	ENSG00000163157
ENSG00000091157	ENSG00000168672	ENSG00000198963	ENSG00000196659

ENSG00000132854	ENSG00000248383	ENSG00000138107	ENSG00000087510
ENSG00000143324	ENSG00000103569	ENSG00000120709	ENSG00000179909
ENSG00000103257	ENSG00000116649	ENSG00000198952	ENSG00000179855
ENSG00000148541	ENSG00000064989	ENSG00000115216	ENSG00000121904
ENSG00000155975	ENSG00000180370	ENSG00000170624	ENSG00000180785
ENSG00000036549	ENSG00000052344	ENSG00000113522	ENSG00000006704
ENSG00000178722	ENSG00000144218	ENSG00000115084	ENSG00000102893
ENSG00000065534	ENSG00000105711	ENSG00000115520	ENSG00000072364
ENSG00000197646	ENSG00000184557	ENSG00000114166	ENSG00000171105
ENSG00000036257	ENSG00000166454	ENSG00000198915	ENSG00000176542
ENSG00000183166	ENSG00000118804	ENSG00000085382	ENSG00000118257
ENSG00000109184	ENSG00000125637	ENSG00000138111	ENSG00000183597
ENSG00000103275	ENSG00000100346	ENSG00000119138	ENSG00000131094
ENSG00000157593	ENSG00000162384	ENSG00000055955	ENSG00000106006
ENSG00000077809	ENSG00000154174	ENSG00000101019	ENSG00000111247
ENSG00000152042	ENSG00000155511	ENSG00000157368	ENSG00000101811
ENSG00000116157	ENSG00000083290	ENSG00000137745	ENSG00000166736
ENSG00000108511	ENSG00000203759	ENSG00000167332	ENSG00000178502
ENSG00000035664	ENSG00000082153	ENSG00000139597	ENSG00000167083
ENSG00000131018	ENSG00000133805	ENSG00000086589	ENSG00000101443
ENSG00000179362	ENSG00000113140	ENSG00000063046	ENSG00000143252
ENSG00000092964	ENSG00000143569	ENSG00000168779	ENSG00000005812
ENSG00000091664	ENSG00000164031	ENSG00000143870	ENSG00000083842
ENSG00000167580	ENSG00000111850	ENSG00000129250	ENSG00000126010
ENSG00000115307	ENSG00000138764	ENSG00000174996	ENSG00000164120
ENSG00000181656	ENSG00000101198	ENSG00000128590	ENSG00000143125
ENSG00000159346	ENSG00000171206	ENSG00000171533	ENSG00000105696
ENSG00000100330	ENSG00000096092	ENSG00000112530	ENSG00000131069
ENSG00000175931	ENSG00000254147	ENSG00000177694	ENSG00000148053
ENSG00000172070	ENSG00000075618	ENSG00000122707	ENSG00000170373
ENSG00000172062	ENSG00000143167	ENSG00000163681	ENSG00000111269
ENSG00000197757	ENSG00000010318	ENSG00000101294	ENSG00000163738
ENSG00000165359	ENSG00000110107	ENSG00000165156	ENSG00000111696
ENSG00000146083	ENSG00000148154	ENSG00000187416	ENSG00000104177
ENSG00000167702	ENSG00000119969	ENSG00000153558	ENSG00000188996
ENSG00000135272	ENSG00000156603	ENSG00000184486	ENSG00000168040
ENSG00000147894	ENSG00000117215	ENSG00000127838	ENSG00000126368
ENSG00000134748	ENSG00000175898	ENSG00000198964	ENSG00000099725
ENSG00000105993	ENSG00000187210	ENSG00000162927	ENSG00000204941
ENSG00000109790	ENSG00000171219	ENSG00000085733	ENSG00000185928
ENSG00000184014	ENSG00000186047	ENSG00000128731	ENSG00000130755
ENSG00000139219	ENSG00000181072	ENSG00000173210	ENSG00000205302
ENSG00000186532	ENSG00000175105	ENSG00000005249	ENSG00000103018
ENSG00000100288	ENSG00000124214	ENSG00000010017	ENSG00000181965
ENSG00000112079	ENSG00000141699	ENSG00000069702	ENSG00000143398
ENSG00000011114	ENSG00000173542	ENSG00000068028	ENSG00000172869
ENSG00000149451	ENSG00000156026	ENSG00000104313	ENSG00000113583
ENSG00000167395	ENSG00000004478	ENSG00000169139	ENSG00000138095
ENSG00000175806	ENSG00000137843	ENSG00000107859	ENSG00000140025
ENSG00000165288	ENSG00000030582	ENSG00000116521	ENSG00000110660
ENSG00000162437	ENSG00000130988	ENSG00000171017	ENSG00000132549
ENSG00000125430	ENSG00000152977	ENSG00000205250	ENSG00000123552
ENSG00000123908	ENSG00000197860	ENSG00000165699	ENSG00000198758
ENSG00000204248	ENSG00000108018	ENSG00000181555	ENSG00000180573
ENSG00000173391	ENSG00000144152	ENSG00000140807	ENSG00000091536
ENSG00000157312	ENSG00000099985	ENSG00000144815	ENSG00000178980
ENSG00000112902	ENSG00000168938	ENSG00000076641	ENSG00000100365
ENSG00000111199	ENSG00000144935	ENSG00000178562	ENSG00000145029
ENSG00000124479	ENSG00000115234	ENSG00000107679	ENSG00000132313
ENSG00000174804	ENSG00000178537	ENSG00000070159	ENSG00000100097
ENSG00000186635	ENSG00000145016	ENSG00000153094	ENSG00000154727
ENSG00000101162	ENSG00000159167	ENSG00000108506	ENSG00000154263
ENSG00000198836	ENSG00000161653	ENSG00000165443	ENSG00000062650
ENSG00000167770	ENSG00000135299	ENSG00000024422	ENSG00000177084

ENSG00000005156	ENSG00000129534	ENSG00000154493	ENSG00000129657
ENSG00000161960	ENSG00000137815	ENSG00000103222	ENSG00000167670
ENSG00000164944	ENSG00000090615	ENSG00000129968	ENSG00000112419
ENSG00000126970	ENSG00000079332	ENSG00000135423	ENSG00000116663
ENSG00000122257	ENSG00000173404	ENSG00000006118	ENSG00000153898
ENSG00000204964	ENSG00000137478	ENSG00000125740	ENSG00000132466
ENSG00000198642	ENSG00000155096	ENSG00000134852	ENSG00000123104
ENSG00000119801	ENSG00000164885	ENSG00000183092	ENSG00000142619
ENSG00000133703	ENSG00000132256	ENSG00000137502	ENSG00000171401
ENSG00000178904	ENSG00000108379	ENSG00000129757	ENSG00000107551
ENSG00000137962	ENSG00000106609	ENSG00000102096	ENSG00000128944
ENSG00000132128	ENSG00000165376	ENSG00000143867	ENSG00000103415
ENSG00000110881	ENSG00000108852	ENSG00000150967	ENSG00000101935
ENSG00000178252	ENSG00000054793	ENSG00000113712	ENSG00000077943
ENSG00000204618	ENSG00000164741	ENSG00000136267	ENSG00000126895
ENSG00000144579	ENSG00000101098	ENSG00000005102	ENSG00000090924
ENSG00000198889	ENSG00000186187	ENSG00000170325	ENSG00000167531
ENSG00000187772	ENSG00000229117	ENSG00000120885	ENSG00000112658
ENSG00000135446	ENSG00000138867	ENSG00000162613	ENSG00000101972
ENSG00000163482	ENSG00000163558	ENSG00000197892	ENSG00000069956
ENSG00000204007	ENSG00000166016	ENSG00000183779	ENSG00000196405
ENSG00000143952	ENSG00000150093	ENSG00000044459	ENSG00000105204
ENSG00000103966	ENSG00000175084	ENSG00000099203	ENSG00000106266
ENSG00000204394	ENSG00000187191	ENSG00000139112	ENSG00000102181
ENSG00000175093	ENSG00000114982	ENSG00000166233	ENSG00000136167
ENSG00000108389	ENSG00000168610	ENSG00000064218	ENSG00000197747
ENSG00000185082	ENSG00000139433	ENSG00000183496	ENSG00000138081
ENSG00000137076	ENSG00000147044	ENSG00000140497	ENSG00000184083
ENSG00000171094	ENSG00000163412	ENSG00000119318	ENSG00000101096
ENSG00000065978	ENSG00000168439	ENSG00000167106	ENSG00000108666
ENSG00000040531	ENSG00000242366	ENSG00000117616	ENSG00000107643
ENSG00000053747	ENSG00000133027	ENSG00000117385	ENSG00000089327
ENSG00000151135	ENSG00000176049	ENSG00000136634	ENSG00000196420
ENSG00000109084	ENSG00000133639	ENSG00000087274	ENSG00000173599
ENSG00000152700	ENSG00000132109	ENSG00000079102	ENSG00000116329
ENSG00000090861	ENSG00000149591	ENSG00000088808	ENSG00000196911
ENSG00000113369	ENSG00000150455	ENSG00000164754	ENSG00000105127
ENSG00000139505	ENSG00000123505	ENSG00000168710	ENSG00000110344
ENSG00000196821	ENSG00000179300	ENSG00000117036	ENSG00000050426
ENSG00000101193	ENSG00000155130	ENSG00000185722	ENSG00000165617
ENSG00000139921	ENSG00000048544	ENSG00000123576	ENSG00000215788
ENSG00000176907	ENSG00000111725	ENSG00000151694	ENSG00000196616
ENSG00000123178	ENSG00000177542	ENSG00000034677	ENSG00000172322
ENSG00000120265	ENSG00000110906	ENSG00000177105	ENSG00000150457
ENSG00000101977	ENSG00000071205	ENSG00000126261	ENSG00000117069
ENSG00000184838	ENSG00000176597	ENSG00000116133	ENSG00000187678
ENSG00000156076	ENSG00000254221	ENSG00000177511	ENSG00000163734
ENSG00000156127	ENSG00000183918	ENSG00000166793	ENSG00000134146
ENSG00000090975	ENSG00000213079	ENSG00000120549	ENSG00000126953
ENSG00000135999	ENSG00000099331	ENSG00000173208	ENSG00000165805
ENSG00000179630	ENSG00000138071	ENSG00000092098	ENSG00000011590
ENSG00000134109	ENSG00000128655	ENSG00000153767	ENSG00000170959
ENSG00000149532	ENSG00000105216	ENSG00000103042	ENSG00000181444
ENSG00000155363	ENSG00000175311	ENSG00000139266	ENSG00000104472
ENSG00000156931	ENSG00000184588	ENSG00000138031	ENSG00000157227
ENSG00000173218	ENSG00000104415	ENSG00000175387	ENSG00000116035
ENSG00000185432	ENSG00000122012	ENSG00000166275	ENSG00000143228
ENSG00000065485	ENSG00000077514	ENSG00000116678	ENSG00000130770
ENSG00000131508	ENSG00000143443	ENSG00000164463	ENSG00000066405
ENSG00000148384	ENSG00000160293	ENSG00000144802	ENSG00000172236
ENSG00000178568	ENSG00000158941	ENSG00000138795	ENSG00000124678
ENSG00000125084	ENSG00000181666	ENSG00000176087	ENSG00000171033
ENSG00000079819	ENSG00000088876	ENSG00000121104	ENSG00000118733
ENSG00000196116	ENSG00000125818	ENSG00000149927	ENSG00000179476

ENSG00000136450	ENSG00000133110	ENSG00000172939	ENSG00000152223
ENSG00000164309	ENSG00000169762	ENSG00000182674	ENSG00000108375
ENSG00000142599	ENSG00000178053	ENSG00000184182	ENSG00000112308
ENSG00000157103	ENSG00000126746	ENSG00000118418	ENSG00000188785
ENSG00000198359	ENSG00000111711	ENSG00000003147	ENSG00000101224
ENSG00000010278	ENSG00000158636	ENSG00000122126	ENSG00000139517
ENSG00000167103	ENSG00000143153	ENSG00000064655	ENSG00000127083
ENSG00000177469	ENSG00000143499	ENSG00000103647	ENSG00000138772
ENSG00000113578	ENSG00000118007	ENSG00000163320	ENSG00000044446
ENSG00000165731	ENSG00000114948	ENSG00000136643	ENSG00000141570
ENSG00000116729	ENSG00000117122	ENSG00000111912	ENSG00000089012
ENSG00000169047	ENSG00000124785	ENSG00000106069	ENSG00000134297
ENSG00000034063	ENSG00000176204	ENSG00000157916	ENSG00000113070
ENSG00000075213	ENSG00000149150	ENSG00000001561	ENSG00000138463
ENSG00000030419	ENSG00000151338	ENSG00000073584	ENSG00000182768
ENSG00000141034	ENSG00000086570	ENSG00000180398	ENSG00000133247
ENSG00000188338	ENSG00000204991	ENSG00000176532	ENSG00000153071
ENSG00000183114	ENSG00000179165	ENSG00000205581	ENSG00000100092
ENSG00000184324	ENSG00000071794	ENSG00000023902	ENSG00000171130
ENSG00000074211	ENSG00000092201	ENSG00000112379	ENSG00000197785
ENSG00000113810	ENSG00000054356	ENSG00000187715	ENSG00000156284
ENSG00000179604	ENSG00000122507	ENSG00000140750	ENSG00000112941
ENSG00000110777	ENSG00000130816	ENSG00000125817	ENSG00000198911
ENSG00000163814	ENSG00000107738	ENSG00000100321	ENSG00000198816
ENSG00000163625	ENSG00000156395	ENSG00000112245	ENSG00000139410
ENSG00000110025	ENSG00000154781	ENSG00000147202	ENSG00000110074
ENSG00000085274	ENSG00000105176	ENSG00000125869	ENSG00000120800
ENSG00000146809	ENSG00000163914	ENSG00000139438	ENSG00000136213
ENSG00000146267	ENSG00000122756	ENSG00000198522	ENSG00000137831
ENSG00000144909	ENSG00000171121	ENSG00000166923	ENSG00000154229
ENSG00000165490	ENSG00000152822	ENSG00000077274	ENSG00000187630
ENSG00000005889	ENSG00000196547	ENSG00000197879	ENSG00000186439
ENSG00000117009	ENSG00000168702	ENSG00000185049	ENSG00000161835
ENSG00000143995	ENSG00000147454	ENSG00000005893	ENSG00000243284
ENSG00000169813	ENSG00000205726	ENSG00000092847	ENSG00000124237
ENSG00000196715	ENSG00000132205	ENSG00000159479	ENSG00000197122
ENSG00000079308	ENSG00000179761	ENSG00000118640	ENSG00000108702
ENSG00000100014	ENSG00000132824	ENSG00000118407	ENSG00000115085
ENSG00000107816	ENSG00000134138	ENSG00000172819	ENSG00000122432
ENSG00000100897	ENSG00000153790	ENSG00000178235	ENSG00000174348
ENSG00000052850	ENSG00000169218	ENSG00000140153	ENSG00000162631
ENSG00000112414	ENSG00000196872	ENSG00000084731	ENSG00000167842
ENSG00000064652	ENSG00000070731	ENSG00000108946	ENSG00000109911
ENSG00000135775	ENSG00000143971	ENSG00000043039	ENSG00000070814
ENSG00000104824	ENSG00000135334	ENSG00000100226	ENSG00000198483
ENSG00000196937	ENSG00000112893	ENSG00000167550	ENSG00000197629
ENSG00000138604	ENSG00000160445	ENSG00000183900	ENSG00000095777
ENSG00000171490	ENSG00000114656	ENSG00000106366	ENSG00000128915
ENSG00000173207	ENSG00000184226	ENSG00000198301	ENSG00000143436
ENSG00000171723	ENSG00000127334	ENSG00000111837	ENSG00000130558
ENSG00000102760	ENSG00000089916	ENSG00000137343	ENSG00000147419
ENSG00000183475	ENSG00000053254	ENSG00000184304	ENSG00000124827
ENSG00000026103	ENSG00000134253	ENSG00000151012	ENSG00000043514
ENSG00000153291	ENSG00000181418	ENSG00000139620	ENSG00000117586
ENSG00000155508	ENSG00000035499	ENSG00000118705	ENSG00000188191
ENSG00000048828	ENSG00000062598	ENSG00000008282	ENSG00000213931
ENSG00000181751	ENSG00000162337	ENSG00000124831	ENSG00000085662
ENSG00000176980	ENSG00000146670	ENSG00000100325	ENSG00000101367
ENSG00000176927	ENSG00000104859	ENSG00000089818	ENSG00000239900
ENSG00000104228	ENSG00000113013	ENSG00000113368	ENSG00000187688
ENSG00000112218	ENSG00000068831	ENSG00000137727	ENSG00000178460
ENSG00000135916	ENSG00000087074	ENSG00000162188	ENSG00000133997
ENSG00000102119	ENSG00000179241	ENSG00000119004	ENSG00000213160
ENSG00000105137	ENSG00000239389	ENSG00000206561	ENSG00000132481

ENSG00000046653	ENSG00000120837	ENSG00000126461	ENSG00000239779
ENSG00000136238	ENSG00000170260	ENSG00000101337	ENSG00000130413
ENSG00000135144	ENSG00000170836	ENSG00000130669	ENSG00000015171
ENSG00000086598	ENSG00000164542	ENSG00000145833	ENSG00000180999
ENSG00000132388	ENSG00000172264	ENSG00000134765	ENSG00000143772
ENSG00000124766	ENSG00000197818	ENSG00000173653	ENSG00000134247
ENSG00000145242	ENSG00000171560	ENSG00000185619	ENSG00000089847
ENSG00000106462	ENSG00000124422	ENSG00000205542	ENSG00000159189
ENSG00000187122	ENSG00000112977	ENSG00000081189	ENSG00000103479
ENSG00000168209	ENSG00000064115	ENSG00000111707	ENSG00000186265
ENSG00000105255	ENSG00000082684	ENSG00000185551	ENSG00000213246
ENSG00000169241	ENSG00000124782	ENSG00000156273	ENSG00000101343
ENSG00000090857	ENSG00000070182	ENSG00000133884	ENSG00000116194
ENSG00000047365	ENSG00000137709	ENSG00000111897	ENSG00000185973
ENSG00000198960	ENSG00000008056	ENSG00000168256	ENSG00000185104
ENSG00000126583	ENSG00000102452	ENSG00000101040	ENSG00000115919
ENSG00000148672	ENSG00000165323	ENSG00000151491	ENSG00000196083
ENSG00000136931	ENSG00000179583	ENSG00000125686	ENSG00000166743
ENSG00000104388	ENSG00000100601	ENSG00000138449	ENSG00000144460
ENSG00000153956	ENSG00000139645	ENSG00000182512	ENSG00000160446
ENSG00000153989	ENSG00000172175	ENSG00000197442	ENSG00000125457
ENSG00000206527	ENSG00000140848	ENSG00000170006	ENSG00000160255
ENSG00000124440	ENSG00000102781	ENSG00000185090	ENSG00000135315
ENSG00000116273	ENSG00000169884	ENSG00000163430	ENSG00000166484
ENSG00000151224	ENSG00000188783	ENSG00000147130	ENSG00000196866
ENSG00000169439	ENSG00000144285	ENSG00000122176	ENSG00000077327
ENSG00000105270	ENSG00000164659	ENSG00000136002	ENSG00000169035
ENSG00000089486	ENSG00000143847	ENSG00000163145	ENSG00000187054
ENSG00000145217	ENSG00000000005	ENSG00000126458	ENSG00000133392
ENSG00000160058	ENSG00000170801	ENSG00000171126	ENSG00000136144
ENSG00000169429	ENSG00000152193	ENSG00000134444	ENSG00000107447
ENSG00000170486	ENSG00000130803	ENSG00000155970	ENSG00000111331
ENSG00000196505	ENSG00000253293	ENSG00000138246	ENSG00000168300
ENSG00000161217	ENSG00000166349	ENSG00000092529	ENSG00000005339
ENSG00000122644	ENSG00000076984	ENSG00000134533	ENSG00000120656
ENSG00000162552	ENSG00000102069	ENSG00000198720	ENSG00000130021
ENSG00000142798	ENSG00000169252	ENSG00000131507	ENSG00000182890
ENSG00000206466	ENSG00000136478	ENSG00000091164	ENSG00000151806
ENSG00000146574	ENSG00000105227	ENSG00000165934	ENSG00000118454
ENSG00000136527	ENSG00000153879	ENSG00000128045	ENSG00000107872
ENSG00000165899	ENSG00000112699	ENSG00000104343	ENSG00000037749
ENSG00000106477	ENSG00000135525	ENSG00000166128	ENSG00000175104
ENSG00000136938	ENSG00000054967	ENSG00000050748	ENSG00000134910
ENSG00000088356	ENSG00000166794	ENSG00000198586	ENSG00000214140
ENSG00000123975	ENSG00000183908	ENSG00000137834	ENSG00000100483
ENSG00000115275	ENSG00000112640	ENSG00000138760	ENSG00000105447
ENSG00000146676	ENSG00000141349	ENSG00000153048	ENSG00000166863
ENSG00000105810	ENSG00000156671	ENSG00000133131	ENSG00000127337
ENSG00000155367	ENSG00000154096	ENSG00000148123	ENSG00000182809
ENSG00000160404	ENSG00000130202	ENSG00000148842	ENSG00000213402
ENSG00000139793	ENSG00000131242	ENSG00000164411	ENSG00000182180
ENSG00000087510	ENSG00000153575	ENSG00000103549	ENSG00000004139
ENSG00000180561	ENSG00000145723	ENSG00000163795	ENSG00000099749
ENSG00000121904	ENSG00000177106	ENSG00000185009	ENSG00000163932
ENSG00000185164	ENSG00000078140	ENSG00000062716	ENSG00000143578
ENSG00000148985	ENSG00000179833	ENSG00000162576	ENSG00000138378
ENSG00000142039	ENSG00000181649	ENSG00000081277	ENSG00000135045
ENSG00000166963	ENSG00000117280	ENSG00000159842	ENSG00000087586
ENSG00000072364	ENSG00000104375	ENSG00000139946	ENSG00000215012
ENSG00000171105	ENSG00000143079	ENSG00000167723	ENSG00000166135
ENSG00000121577	ENSG00000167772	ENSG00000121486	ENSG00000072803
ENSG00000118257	ENSG00000000938	ENSG00000122254	ENSG00000186866
ENSG00000166226	ENSG00000166913	ENSG00000148343	ENSG00000142208
ENSG00000131094	ENSG00000178033	ENSG00000102935	ENSG00000086717

ENSG00000106006	ENSG00000174915	ENSG00000180447	ENSG00000100401
ENSG00000112378	ENSG00000122367	ENSG00000158882	ENSG00000011201
ENSG00000136448	ENSG00000172081	ENSG00000197114	ENSG00000114062
ENSG00000174437	ENSG00000154059	ENSG00000105880	ENSG00000175206
ENSG00000134504	ENSG00000100241	ENSG00000170759	ENSG00000145293
ENSG00000143252	ENSG00000128710	ENSG00000104783	ENSG00000133961
ENSG00000005812	ENSG00000033122	ENSG00000179820	ENSG00000173013
ENSG00000164120	ENSG00000032219	ENSG00000188786	ENSG00000177548
ENSG00000135823	ENSG00000134072	ENSG00000164318	ENSG00000146872
ENSG00000115966	ENSG00000100100	ENSG00000171786	ENSG00000159228
ENSG00000148053	ENSG00000170577	ENSG00000144366	ENSG00000134352
ENSG00000107625	ENSG00000103266	ENSG00000136451	ENSG00000175894
ENSG00000182885	ENSG00000167904	ENSG00000125246	ENSG00000127948
ENSG00000111269	ENSG00000100124	ENSG00000166002	ENSG00000150361
ENSG00000163904	ENSG00000214717	ENSG00000171596	ENSG00000137648
ENSG00000111696	ENSG00000135744	ENSG00000131467	ENSG00000106686
ENSG00000181090	ENSG00000133872	ENSG00000143156	ENSG00000021461
ENSG00000104177	ENSG00000125848	ENSG00000100931	ENSG00000170270
ENSG00000121848	ENSG00000166260	ENSG00000127511	ENSG00000184634
ENSG00000086289	ENSG00000178217	ENSG00000158966	ENSG00000162585
ENSG00000067798	ENSG00000176531	ENSG00000084676	ENSG00000198785
ENSG00000133083	ENSG00000118160	ENSG00000112139	ENSG00000149922
ENSG00000126368	ENSG00000112851	ENSG00000106348	ENSG00000196335
ENSG00000198794	ENSG00000205420	ENSG00000120159	ENSG00000175229
ENSG00000123119	ENSG00000105220	ENSG00000130559	ENSG00000175193
ENSG00000205302	ENSG00000198951	ENSG00000169783	ENSG00000143476
ENSG00000140280	ENSG00000093000	ENSG00000114867	ENSG00000167491
ENSG00000181965	ENSG00000168477	ENSG00000197982	ENSG00000106367
ENSG00000143398	ENSG00000096063	ENSG00000156097	ENSG00000164651
ENSG00000167178	ENSG00000173451	ENSG00000075856	ENSG00000182636
ENSG00000172869	ENSG00000171471	ENSG00000141480	ENSG00000149380
ENSG00000113583	ENSG00000173456	ENSG00000172071	ENSG00000133812
ENSG00000125812	ENSG00000112531	ENSG00000127585	ENSG00000141867
ENSG00000054803	ENSG00000172780	ENSG00000109738	ENSG00000187017
ENSG00000178980	ENSG00000176244	ENSG00000107331	ENSG00000143631
ENSG00000107672	ENSG00000171951	ENSG00000122861	ENSG00000081248
ENSG00000128591	ENSG00000112335	ENSG00000120685	ENSG00000104972
ENSG00000198492	ENSG00000254122	ENSG00000103769	ENSG00000172465
ENSG00000124225	ENSG00000012963	ENSG00000101236	ENSG00000177963
ENSG00000145029	ENSG00000116254	ENSG00000164023	ENSG00000179921
ENSG00000100365	ENSG00000186340	ENSG00000072422	ENSG00000176194
ENSG00000100097	ENSG00000115756	ENSG00000108840	ENSG00000166396
ENSG00000154727	ENSG00000128578	ENSG00000120659	ENSG00000205213
ENSG00000188707	ENSG00000068308	ENSG00000144857	ENSG00000144619
ENSG00000184840	ENSG00000188580	ENSG00000100916	ENSG00000168427
ENSG00000062650	ENSG00000125656	ENSG00000150893	ENSG00000059588
ENSG00000166266	ENSG00000183087	ENSG00000185610	ENSG00000115648
ENSG00000121741	ENSG00000181588	ENSG00000165300	ENSG00000122482
ENSG00000101945	ENSG00000083635	ENSG00000136770	ENSG00000203705
ENSG00000136011	ENSG00000166340	ENSG00000010244	ENSG00000137713
ENSG00000167670	ENSG00000080845	ENSG00000173598	ENSG00000168522
ENSG00000152952	ENSG00000106004	ENSG00000108061	ENSG00000155366
ENSG00000104885	ENSG00000146373	ENSG00000075413	ENSG00000178226
ENSG00000119523	ENSG00000158480	ENSG00000141404	ENSG00000185947
ENSG00000108947	ENSG00000163534	ENSG00000166317	ENSG00000155833
ENSG00000197457	ENSG00000103534	ENSG00000151461	ENSG00000188931
ENSG00000132466	ENSG00000168778	ENSG00000118260	ENSG00000174944
ENSG00000123104	ENSG00000083312	ENSG00000168495	ENSG00000112701
ENSG00000169744	ENSG00000108828	ENSG00000135632	ENSG00000198455
ENSG00000107551	ENSG00000179918	ENSG00000198399	ENSG00000144589
ENSG00000051108	ENSG00000185345	ENSG00000171492	ENSG00000057663
ENSG00000168246	ENSG00000118900	ENSG00000137218	ENSG00000164164
ENSG00000132507	ENSG00000143434	ENSG00000116604	ENSG00000152760
ENSG00000144136	ENSG00000132286	ENSG00000171617	ENSG00000142937

ENSG00000134318	ENSG00000128645	ENSG00000037965	ENSG00000166548
ENSG00000103415	ENSG00000106683	ENSG00000055609	ENSG00000179091
ENSG00000101935	ENSG00000002834	ENSG00000153574	ENSG00000198753
ENSG00000076864	ENSG00000186501	ENSG00000168135	ENSG00000241399
ENSG00000103200	ENSG00000106460	ENSG00000168282	ENSG00000083307
ENSG00000112658	ENSG00000119139	ENSG00000170473	ENSG00000172123
ENSG00000090061	ENSG00000158457	ENSG00000172380	ENSG00000160298
ENSG00000101972	ENSG00000158796	ENSG00000106144	ENSG00000167755
ENSG00000069966	ENSG00000197226	ENSG00000099957	ENSG00000178860
ENSG00000105204	ENSG00000129422	ENSG00000117222	ENSG00000122180
ENSG00000136167	ENSG00000164338	ENSG00000155313	ENSG00000054277
ENSG00000129009	ENSG00000111261	ENSG00000165029	ENSG00000147485
ENSG00000130733	ENSG00000147576	ENSG00000198648	ENSG00000203883
ENSG00000131791	ENSG00000146842	ENSG00000119772	ENSG00000142677
ENSG00000102908	ENSG00000085185	ENSG00000090238	ENSG00000184545
ENSG00000137807	ENSG00000114541	ENSG00000183155	ENSG00000100968
ENSG00000159199	ENSG00000173838	ENSG00000070831	ENSG00000008853
ENSG00000128708	ENSG00000185504	ENSG00000182446	ENSG00000205593
ENSG00000144278	ENSG00000134853	ENSG00000140157	ENSG00000171469
ENSG00000184083	ENSG00000187689	ENSG00000171388	ENSG00000198945
ENSG00000138081	ENSG00000040731	ENSG00000133606	ENSG00000176978
ENSG00000090447	ENSG00000033327	ENSG00000135956	ENSG00000152767
ENSG00000104131	ENSG00000137070	ENSG00000183833	ENSG00000132003
ENSG00000135953	ENSG00000170627	ENSG00000141458	ENSG00000167850
ENSG00000102755	ENSG00000178764	ENSG00000161847	ENSG00000151468
ENSG00000084234	ENSG00000092208	ENSG00000108094	ENSG00000113971
ENSG00000107643	ENSG00000130313	ENSG00000186716	ENSG00000177200
ENSG00000121058	ENSG00000152894	ENSG00000158887	ENSG00000162407
ENSG00000152284	ENSG00000182580	ENSG00000158545	ENSG00000187231
ENSG00000173599	ENSG00000086730	ENSG00000165915	ENSG00000174529
ENSG00000163029	ENSG00000132964	ENSG00000028839	ENSG00000113739
ENSG00000164548	ENSG00000117472	ENSG00000109572	ENSG00000164385
ENSG00000121964	ENSG00000117000	ENSG00000152270	ENSG00000167635
ENSG00000197111	ENSG00000244038	ENSG00000118526	ENSG00000147535
ENSG00000174469	ENSG00000176563	ENSG00000134780	ENSG00000184381
ENSG00000134049	ENSG00000005020	ENSG00000102221	ENSG00000131196
ENSG00000110344	ENSG00000023041	ENSG00000140538	ENSG00000137171
ENSG00000197321	ENSG00000104365	ENSG00000140262	ENSG00000119630
ENSG00000107263	ENSG00000159873	ENSG00000132341	ENSG00000198930
ENSG00000083937	ENSG00000172728	ENSG00000113231	ENSG00000185920
ENSG00000050426	ENSG00000093183	ENSG00000162946	ENSG00000005001
ENSG00000165617	ENSG00000139428	ENSG00000141738	ENSG00000089639
ENSG00000117069	ENSG00000157680	ENSG00000095739	ENSG00000102837
ENSG00000150457	ENSG00000164715	ENSG00000165119	ENSG00000152433
ENSG00000131051	ENSG00000070610	ENSG00000064787	ENSG00000188243
ENSG00000187678	ENSG00000159658	ENSG00000157869	ENSG00000036828
ENSG00000128989	ENSG00000183072	ENSG00000147059	ENSG00000164082
ENSG00000103423	ENSG00000120686	ENSG00000188687	ENSG00000100403
ENSG00000123159	ENSG00000109586	ENSG00000019991	ENSG00000150672
ENSG00000171587	ENSG00000215262	ENSG00000171877	ENSG00000188677
ENSG00000126953	ENSG00000118960	ENSG00000137269	ENSG00000187260
ENSG00000157227	ENSG00000133105	ENSG00000170049	ENSG00000170581
ENSG00000169306	ENSG00000125945	ENSG00000106392	ENSG00000161944
ENSG00000115020	ENSG00000106633	ENSG00000111962	ENSG00000011422
ENSG00000162928	ENSG00000077279	ENSG00000166908	ENSG00000171435
ENSG00000054267	ENSG00000152936	ENSG00000177885	ENSG00000174292
ENSG00000171033	ENSG00000074319	ENSG00000162430	ENSG00000068903
ENSG00000174498	ENSG00000143061	ENSG00000047849	ENSG00000198917
ENSG00000108375	ENSG00000147689	ENSG00000126216	ENSG00000197724
ENSG00000112308	ENSG00000118689	ENSG00000099875	ENSG00000138623
ENSG00000101224	ENSG00000137959	ENSG00000186417	ENSG00000204103
ENSG00000138772	ENSG00000188994	ENSG00000169758	ENSG00000009307
ENSG00000136888	ENSG00000162734	ENSG00000113441	ENSG00000163607
ENSG00000058272	ENSG00000118503	ENSG00000132004	ENSG00000187823

ENSG00000113070	ENSG00000120896	ENSG00000154734	ENSG00000124608
ENSG00000006432	ENSG00000111145	ENSG00000172789	ENSG00000165672
ENSG00000135862	ENSG00000141380	ENSG00000183655	ENSG00000115325
ENSG00000163637	ENSG00000100726	ENSG00000071073	ENSG00000196208
ENSG00000135414	ENSG00000108349	ENSG00000103888	ENSG00000073417
ENSG00000138463	ENSG00000057294	ENSG00000161202	ENSG00000167130
ENSG00000133247	ENSG00000197971	ENSG00000134222	ENSG00000185231
ENSG00000158158	ENSG00000146197	ENSG00000167615	ENSG00000168264
ENSG00000153071	ENSG00000182541	ENSG00000176444	ENSG00000108576
ENSG00000100092	ENSG00000110888	ENSG00000100243	ENSG00000093217
ENSG00000112941	ENSG00000108231	ENSG00000143013	ENSG00000130764
ENSG00000010818	ENSG00000137198	ENSG00000107562	ENSG00000182150
ENSG00000119938	ENSG00000065150	ENSG00000138735	ENSG00000102886
ENSG00000087206	ENSG00000132953	ENSG00000137460	ENSG00000143322
ENSG00000148187	ENSG00000099995	ENSG00000060982	ENSG00000185652
ENSG00000102241	ENSG00000066248	ENSG00000122643	ENSG00000240184
ENSG00000154229	ENSG00000151062	ENSG00000204256	ENSG00000101892
ENSG00000187630	ENSG00000134884	ENSG00000162104	ENSG00000164220
ENSG00000122674	ENSG00000161647	ENSG00000100442	ENSG00000123609
ENSG00000069275	ENSG00000182324	ENSG00000114742	ENSG00000115556
ENSG00000186439	ENSG00000160305	ENSG00000105767	ENSG00000143278
ENSG00000159423	ENSG00000157064	ENSG00000107854	ENSG00000125510
ENSG00000172572	ENSG00000110243	ENSG00000162761	ENSG00000115685
ENSG00000161835	ENSG00000180233	ENSG00000172845	ENSG00000110811
ENSG00000058085	ENSG00000128917	ENSG00000168672	ENSG00000186205
ENSG00000108702	ENSG00000108344	ENSG00000116649	ENSG00000140285
ENSG00000122432	ENSG00000111716	ENSG00000064989	ENSG00000124429
ENSG00000116199	ENSG00000065675	ENSG00000103569	ENSG00000128052
ENSG00000170689	ENSG00000127080	ENSG00000180370	ENSG00000156172
ENSG00000183773	ENSG00000080546	ENSG00000167114	ENSG00000135472
ENSG00000165995	ENSG00000122692	ENSG00000184557	ENSG00000126777
ENSG00000128564	ENSG00000168310	ENSG00000118804	ENSG00000155465
ENSG00000078401	ENSG00000064195	ENSG00000114784	ENSG00000149305
ENSG00000143436	ENSG00000113575	ENSG00000100346	ENSG00000173915
ENSG00000130558	ENSG00000174016	ENSG00000125637	ENSG00000141293
ENSG00000147419	ENSG00000145391	ENSG00000155511	ENSG00000204842
ENSG00000124827	ENSG00000159202	ENSG00000154174	ENSG00000133119
ENSG00000042317	ENSG00000075884	ENSG00000149397	ENSG00000102882
ENSG00000179151	ENSG00000164048	ENSG00000176697	ENSG00000131080
ENSG00000077585	ENSG00000100417	ENSG00000083290	ENSG00000163528
ENSG00000043514	ENSG00000155252	ENSG00000133805	ENSG00000090989
ENSG00000066382	ENSG00000077522	ENSG00000187672	ENSG00000103126
ENSG00000177380	ENSG00000130528	ENSG00000136717	ENSG00000143811
ENSG00000117586	ENSG00000153250	ENSG00000172379	ENSG00000004776
ENSG00000183780	ENSG00000133256	ENSG00000113140	ENSG00000126070
ENSG00000101367	ENSG00000138101	ENSG00000163606	ENSG00000181924
ENSG00000106615	ENSG00000112116	ENSG00000149295	ENSG00000183283
ENSG00000070759	ENSG00000215455	ENSG00000138741	ENSG00000157152
ENSG00000133997	ENSG00000141431	ENSG00000182199	ENSG00000166509
ENSG00000134152	ENSG00000116774	ENSG00000170185	ENSG00000132356
ENSG00000157985	ENSG00000025796	ENSG00000164031	ENSG00000169862
ENSG00000166825	ENSG00000157734	ENSG00000138764	ENSG00000100142
ENSG00000108387	ENSG00000057757	ENSG00000171206	ENSG00000021776
ENSG00000197587	ENSG00000170791	ENSG00000168994	ENSG00000084636
ENSG00000115415	ENSG00000211445	ENSG00000109339	ENSG00000136943
ENSG00000180771	ENSG00000101082	ENSG00000100393	ENSG00000144035
ENSG00000110911	ENSG00000131759	ENSG00000166833	ENSG00000127564
ENSG00000108556	ENSG00000162694	ENSG00000110107	ENSG00000003393
ENSG00000163349	ENSG00000196470	ENSG00000107862	ENSG00000144426
ENSG00000181481	ENSG00000171928	ENSG00000119969	ENSG00000171450
ENSG00000139154	ENSG00000112837	ENSG00000118655	ENSG00000148719
ENSG00000130413	ENSG00000056661	ENSG00000156603	ENSG00000140990
ENSG00000067182	ENSG00000184205	ENSG00000122557	ENSG00000172578
ENSG00000061273	ENSG00000152527	ENSG00000196313	ENSG00000117859

ENSG00000015171	ENSG00000119782	ENSG00000187210	ENSG00000173852
ENSG00000120533	ENSG00000126351	ENSG00000186047	ENSG00000149735
ENSG00000124942	ENSG00000167711	ENSG00000124214	ENSG00000186895
ENSG00000039560	ENSG00000092203	ENSG00000166888	ENSG00000008441
ENSG00000011258	ENSG00000162639	ENSG00000141699	ENSG00000112679
ENSG00000139641	ENSG00000151692	ENSG00000156026	ENSG00000111801
ENSG00000102572	ENSG00000176619	ENSG00000158161	ENSG00000007237
ENSG00000197562	ENSG00000074201	ENSG00000004478	ENSG00000113758
ENSG00000143772	ENSG00000112695	ENSG00000154845	ENSG00000197993
ENSG00000134247	ENSG00000167987	ENSG00000137843	ENSG00000006712
ENSG00000125354	ENSG00000135838	ENSG00000152977	ENSG00000165188
ENSG00000103353	ENSG00000170365	ENSG00000108018	ENSG00000196268
ENSG00000103479	ENSG00000082497	ENSG00000197860	ENSG00000177807
ENSG00000117016	ENSG00000163939	ENSG00000136870	ENSG00000110693
ENSG00000116194	ENSG00000130939	ENSG00000168938	ENSG00000171195
ENSG00000185104	ENSG00000163930	ENSG00000144935	ENSG00000163793
ENSG00000156384	ENSG00000134030	ENSG00000107105	ENSG00000102996
ENSG00000125457	ENSG00000146592	ENSG00000099985	ENSG00000213996
ENSG00000163848	ENSG00000084733	ENSG00000115234	ENSG00000205111
ENSG00000182490	ENSG00000196188	ENSG00000178537	ENSG00000128274
ENSG00000166484	ENSG00000047597	ENSG00000159167	ENSG00000198829
ENSG00000198373	ENSG00000204580	ENSG00000172354	ENSG00000149679
ENSG00000178301	ENSG00000125780	ENSG00000102003	ENSG00000140854
ENSG00000204217	ENSG00000163389	ENSG00000014164	ENSG00000104967
ENSG00000102978	ENSG00000101222	ENSG00000110880	ENSG00000125775
ENSG00000136144	ENSG00000172986	ENSG00000137815	ENSG00000136807
ENSG00000137203	ENSG00000020256	ENSG00000090615	ENSG00000184164
ENSG00000138293	ENSG00000178685	ENSG00000185905	ENSG00000142765
ENSG00000092439	ENSG00000178075	ENSG00000164054	ENSG00000184371
ENSG00000188133	ENSG00000183878	ENSG00000079332	ENSG00000105982
ENSG00000153006	ENSG00000132563	ENSG00000182132	ENSG00000100678
ENSG00000127220	ENSG00000181274	ENSG00000163788	ENSG00000100206
ENSG00000072609	ENSG00000170915	ENSG00000173404	ENSG00000131323
ENSG00000125414	ENSG00000144681	ENSG00000137478	ENSG00000168418
ENSG00000111331	ENSG00000138834	ENSG00000124181	ENSG00000145491
ENSG00000005339	ENSG00000117751	ENSG00000186660	ENSG00000122592
ENSG00000010322	ENSG00000151552	ENSG00000164885	ENSG00000117834
ENSG00000120656	ENSG00000140092	ENSG00000155096	ENSG00000125999
ENSG00000150471	ENSG00000163867	ENSG00000141141	ENSG00000169245
ENSG00000154783	ENSG00000144730	ENSG00000135473	ENSG00000124535
ENSG00000130021	ENSG00000070770	ENSG00000108379	ENSG00000186509
ENSG00000087460	ENSG00000130711	ENSG00000165244	ENSG00000057657
ENSG00000182890	ENSG00000107742	ENSG00000106609	ENSG00000143858
ENSG00000124593	ENSG00000136146	ENSG00000108852	ENSG00000181619
ENSG00000118454	ENSG00000105722	ENSG00000165376	ENSG00000174021
ENSG00000122741	ENSG00000213859	ENSG00000148572	ENSG00000077254
ENSG00000100650	ENSG00000170248	ENSG00000138166	ENSG00000206450
ENSG00000037749	ENSG00000143393	ENSG00000099246	ENSG00000125814
ENSG00000175104	ENSG00000066777	ENSG00000164741	ENSG00000163431
ENSG00000176105	ENSG00000125351	ENSG00000101098	ENSG00000198963
ENSG00000116514	ENSG00000136758	ENSG00000186187	ENSG00000189060
ENSG00000134910	ENSG00000157014	ENSG00000114019	ENSG00000070413
ENSG00000100483	ENSG00000168397	ENSG00000139651	ENSG00000120709
ENSG00000169641	ENSG00000158747	ENSG00000003056	ENSG00000138107
ENSG00000127337	ENSG00000105974	ENSG00000153214	ENSG00000123080
ENSG00000165675	ENSG00000101825	ENSG00000138867	ENSG00000112186
ENSG00000163932	ENSG00000121210	ENSG00000163558	ENSG00000198952
ENSG00000135045	ENSG00000101327	ENSG00000173327	ENSG00000184895
ENSG00000138378	ENSG00000133789	ENSG00000158019	ENSG00000189283
ENSG00000077264	ENSG00000126581	ENSG00000183715	ENSG00000076662
ENSG00000132821	ENSG00000136404	ENSG00000150093	ENSG00000166441
ENSG00000180530	ENSG00000163605	ENSG00000175084	ENSG00000115084
ENSG00000170571	ENSG00000144290	ENSG00000111252	ENSG00000179546
ENSG00000166135	ENSG00000080371	ENSG00000187191	ENSG00000173145

ENSG00000072803	ENSG00000179059	ENSG00000168610	ENSG00000055955
ENSG00000153815	ENSG00000169085	ENSG00000069399	ENSG00000183454
ENSG00000154080	ENSG00000141837	ENSG00000139433	ENSG00000070031
ENSG00000180304	ENSG00000118985	ENSG00000169490	ENSG00000172638
ENSG00000110786	ENSG00000125878	ENSG00000170745	ENSG00000086589
ENSG00000134369	ENSG00000168993	ENSG00000108179	ENSG00000026036
ENSG00000187957	ENSG00000154447	ENSG00000168439	ENSG00000096872
ENSG00000114062	ENSG00000124523	ENSG00000174306	ENSG00000128590
ENSG00000123700	ENSG00000009335	ENSG00000157106	ENSG00000149527
ENSG00000138801	ENSG00000133937	ENSG00000187961	ENSG00000221864
ENSG00000127914	ENSG00000106100	ENSG00000116731	ENSG00000160460
ENSG00000133961	ENSG00000177463	ENSG00000133639	ENSG00000213809
ENSG00000197694	ENSG00000206560	ENSG00000176049	ENSG00000170890
ENSG00000178425	ENSG00000124574	ENSG00000149591	ENSG00000110711
ENSG00000107829	ENSG00000145920	ENSG00000173376	ENSG00000122707
ENSG00000146872	ENSG00000198160	ENSG00000153786	ENSG00000165168
ENSG00000134352	ENSG00000103319	ENSG00000123505	ENSG00000143622
ENSG00000150361	ENSG00000099204	ENSG00000179300	ENSG00000163681
ENSG00000130283	ENSG00000149260	ENSG00000141179	ENSG00000173369
ENSG00000145824	ENSG00000144485	ENSG00000155130	ENSG00000118307
ENSG00000115263	ENSG00000165185	ENSG00000111725	ENSG00000165156
ENSG00000185728	ENSG00000002587	ENSG00000177542	ENSG00000079385
ENSG00000177733	ENSG00000196581	ENSG00000132535	ENSG00000105668
ENSG00000082641	ENSG00000141140	ENSG00000136193	ENSG00000153558
ENSG00000162585	ENSG00000171703	ENSG00000165449	ENSG00000213171
ENSG00000198785	ENSG00000136986	ENSG00000110906	ENSG00000127838
ENSG00000175229	ENSG00000166401	ENSG00000130429	ENSG00000051180
ENSG00000129521	ENSG00000175344	ENSG00000174839	ENSG00000114021
ENSG00000106367	ENSG00000100629	ENSG00000176597	ENSG00000085733
ENSG00000164651	ENSG00000123364	ENSG00000157404	ENSG00000113318
ENSG00000182636	ENSG00000164530	ENSG00000183918	ENSG00000128731
ENSG00000006007	ENSG00000183864	ENSG00000151322	ENSG00000173210
ENSG00000187017	ENSG00000166483	ENSG00000099331	ENSG00000010017
ENSG00000141867	ENSG00000078674	ENSG00000128655	ENSG00000106443
ENSG00000133812	ENSG00000168172	ENSG00000105327	ENSG00000197362
ENSG00000197256	ENSG00000188158	ENSG00000105216	ENSG00000019186
ENSG00000069869	ENSG00000095539	ENSG00000104415	ENSG00000107859
ENSG00000124208	ENSG00000154429	ENSG00000143443	ENSG00000179528
ENSG00000151715	ENSG00000077458	ENSG00000158941	ENSG00000116521
ENSG00000081248	ENSG00000129473	ENSG00000160932	ENSG00000177683
ENSG00000166068	ENSG00000166889	ENSG00000071246	ENSG00000134363
ENSG00000174718	ENSG00000187866	ENSG00000125818	ENSG00000165699
ENSG00000172465	ENSG00000134955	ENSG00000135486	ENSG00000186111
ENSG00000168542	ENSG00000198838	ENSG00000169762	ENSG00000213614
ENSG00000177614	ENSG00000167196	ENSG00000057608	ENSG00000120253
ENSG00000119787	ENSG00000176753	ENSG00000178053	ENSG00000112761
ENSG00000072042	ENSG00000120251	ENSG00000126746	ENSG00000164465
ENSG00000090266	ENSG00000150782	ENSG00000100697	ENSG00000140807
ENSG00000197991	ENSG00000182168	ENSG00000111711	ENSG00000076641
ENSG00000144619	ENSG00000156453	ENSG00000163520	ENSG00000165799
ENSG00000205213	ENSG00000185274	ENSG00000158636	ENSG00000070159
ENSG00000168427	ENSG00000185112	ENSG00000112320	ENSG00000107679
ENSG00000059588	ENSG00000170925	ENSG00000143153	ENSG00000165443
ENSG00000131773	ENSG00000116580	ENSG00000077684	ENSG00000187116
ENSG00000151690	ENSG00000165887	ENSG00000185985	ENSG00000165821
ENSG00000122482	ENSG00000090889	ENSG00000118007	ENSG00000132581
ENSG00000168556	ENSG00000163950	ENSG00000158258	ENSG00000103222
ENSG00000167371	ENSG00000123342	ENSG00000114948	ENSG00000154493
ENSG00000139722	ENSG00000197702	ENSG00000117122	ENSG00000128609
ENSG00000128607	ENSG00000197147	ENSG00000124205	ENSG00000135423
ENSG00000104093	ENSG00000170558	ENSG00000124785	ENSG00000108813
ENSG00000115649	ENSG00000186106	ENSG00000176204	ENSG00000132698
ENSG00000197343	ENSG00000130540	ENSG00000184508	ENSG00000171446
ENSG00000137713	ENSG00000138316	ENSG00000151338	ENSG00000089692

ENSG00000168522	ENSG00000078967	ENSG00000086570	ENSG00000006659
ENSG00000155366	ENSG00000169871	ENSG00000115677	ENSG00000119986
ENSG00000087245	ENSG00000198198	ENSG00000153904	ENSG00000125740
ENSG00000144331	ENSG00000119729	ENSG00000143643	ENSG00000119403
ENSG00000133318	ENSG00000108819	ENSG00000071794	ENSG00000163017
ENSG00000112701	ENSG00000138942	ENSG00000092201	ENSG00000187398
ENSG00000198815	ENSG00000129625	ENSG00000120519	ENSG00000144820
ENSG00000198455	ENSG00000135913	ENSG00000141622	ENSG00000197594
ENSG00000144589	ENSG00000121766	ENSG00000100084	ENSG00000102096
ENSG00000114857	ENSG00000159140	ENSG00000162642	ENSG00000132911
ENSG00000132424	ENSG00000112425	ENSG00000130816	ENSG00000150967
ENSG00000057663	ENSG00000113621	ENSG00000156395	ENSG00000168061
ENSG00000101216	ENSG00000170390	ENSG00000105176	ENSG00000113712
ENSG00000188290	ENSG00000145423	ENSG00000163914	ENSG00000120885
ENSG00000101782	ENSG00000160789	ENSG00000122756	ENSG00000119326
ENSG00000164164	ENSG00000222014	ENSG00000152822	ENSG00000090581
ENSG00000108352	ENSG00000137312	ENSG00000196547	ENSG00000118849
ENSG00000169714	ENSG00000117758	ENSG00000168702	ENSG00000162613
ENSG00000152760	ENSG00000111361	ENSG00000147454	ENSG00000161277
ENSG00000139644	ENSG00000131748	ENSG00000205726	ENSG00000185420
ENSG00000083307	ENSG00000150086	ENSG00000132205	ENSG00000129810
ENSG00000152684	ENSG00000070808	ENSG00000198898	ENSG00000100603
ENSG00000122180	ENSG00000003989	ENSG00000134138	ENSG00000162522
ENSG00000186684	ENSG00000127616	ENSG00000132824	ENSG00000145113
ENSG00000184545	ENSG00000143341	ENSG00000082397	ENSG00000125734
ENSG00000111961	ENSG00000163251	ENSG00000151474	ENSG00000196839
ENSG00000164093	ENSG00000181039	ENSG00000161533	ENSG00000140497
ENSG00000100968	ENSG00000169826	ENSG00000076053	ENSG00000117707
ENSG00000156925	ENSG00000124171	ENSG00000186174	ENSG00000213054
ENSG00000126561	ENSG00000135407	ENSG00000078747	ENSG00000170430
ENSG00000152767	ENSG00000145907	ENSG00000196872	ENSG00000117385
ENSG00000204965	ENSG00000139132	ENSG00000112893	ENSG00000167106
ENSG00000151468	ENSG00000244474	ENSG00000135334	ENSG00000136634
ENSG00000000003	ENSG00000162951	ENSG00000160445	ENSG00000088808
ENSG00000162407	ENSG00000171302	ENSG00000184226	ENSG00000164754
ENSG00000177200	ENSG00000180929	ENSG00000127334	ENSG00000186522
ENSG00000113971	ENSG00000198355	ENSG00000089916	ENSG00000117036
ENSG00000187231	ENSG00000130766	ENSG00000053254	ENSG00000185989
ENSG00000077044	ENSG00000148090	ENSG00000128641	ENSG00000185722
ENSG00000065600	ENSG00000161958	ENSG00000105983	ENSG00000151694
ENSG00000148824	ENSG00000105698	ENSG00000151640	ENSG00000126261
ENSG00000167635	ENSG00000133069	ENSG00000035499	ENSG00000173612
ENSG00000167941	ENSG00000166415	ENSG00000062598	ENSG00000116133
ENSG00000130958	ENSG00000130338	ENSG00000162337	ENSG00000177511
ENSG00000137171	ENSG00000161714	ENSG00000146670	ENSG00000173064
ENSG00000119630	ENSG00000101412	ENSG00000112511	ENSG00000173208
ENSG00000196700	ENSG00000147145	ENSG00000104859	ENSG00000068400
ENSG00000160145	ENSG00000172534	ENSG00000113013	ENSG00000140600
ENSG00000185920	ENSG00000142864	ENSG00000141198	ENSG00000139266
ENSG00000082805	ENSG00000108309	ENSG00000166900	ENSG00000112130
ENSG00000107077	ENSG00000131374	ENSG00000068831	ENSG00000144802
ENSG00000165322	ENSG00000160917	ENSG00000152430	ENSG00000112039
ENSG00000005001	ENSG00000103056	ENSG00000179241	ENSG00000184945
ENSG00000158856	ENSG00000204033	ENSG00000120837	ENSG00000138795
ENSG00000102837	ENSG00000227802	ENSG00000170836	ENSG00000158815
ENSG00000140416	ENSG00000165383	ENSG00000170260	ENSG00000183258
ENSG00000036828	ENSG00000104081	ENSG00000172818	ENSG00000183862
ENSG00000038358	ENSG00000047621	ENSG00000113196	ENSG00000110060
ENSG00000100403	ENSG00000135636	ENSG00000118922	ENSG00000125966
ENSG00000105894	ENSG00000197106	ENSG00000198821	ENSG00000153292
ENSG00000198637	ENSG00000223501	ENSG00000132670	ENSG00000150281
ENSG00000168348	ENSG00000125816	ENSG00000197818	ENSG00000167874
ENSG00000155111	ENSG00000115414	ENSG00000088832	ENSG00000110324
ENSG00000150672	ENSG00000128298	ENSG00000104812	ENSG00000160124

ENSG00000157077	ENSG00000112297	ENSG00000148943	ENSG00000186442
ENSG00000119953	ENSG00000214827	ENSG00000171560	ENSG00000123999
ENSG00000011422	ENSG00000146063	ENSG00000112977	ENSG00000187045
ENSG00000197724	ENSG00000067057	ENSG00000124782	ENSG00000243710
ENSG00000179600	ENSG00000166925	ENSG00000137709	ENSG00000135638
ENSG00000198010	ENSG00000105726	ENSG00000185386	ENSG00000151005
ENSG00000204103	ENSG00000075275	ENSG00000179583	ENSG00000176473
ENSG00000009307	ENSG00000107831	ENSG00000144339	ENSG00000122126
ENSG00000132153	ENSG00000171843	ENSG00000169884	ENSG00000137309
ENSG00000116667	ENSG00000142623	ENSG00000145681	ENSG00000064655
ENSG00000149179	ENSG00000035141	ENSG00000188783	ENSG00000081181
ENSG00000138294	ENSG00000168137	ENSG00000164659	ENSG00000162747
ENSG00000187823	ENSG00000162745	ENSG00000011638	ENSG00000140297
ENSG00000172671	ENSG00000101624	ENSG00000143933	ENSG00000118162
ENSG00000170819	ENSG00000168291	ENSG00000152193	ENSG00000185697
ENSG00000115325	ENSG00000213064	ENSG00000171885	ENSG00000162511
ENSG00000198641	ENSG00000169181	ENSG00000171132	ENSG00000067704
ENSG00000170802	ENSG00000135392	ENSG00000166349	ENSG00000103647
ENSG00000167130	ENSG00000128849	ENSG00000184916	ENSG00000168530
ENSG00000166718	ENSG00000097046	ENSG00000163113	ENSG00000163320
ENSG00000168264	ENSG00000092199	ENSG00000015532	ENSG00000172315
ENSG00000108576	ENSG00000179862	ENSG00000169252	ENSG00000111912
ENSG00000183255	ENSG00000003137	ENSG00000136478	ENSG00000123268
ENSG00000185652	ENSG00000118873	ENSG00000116132	ENSG00000001561
ENSG00000143390	ENSG00000071462	ENSG00000186908	ENSG00000073584
ENSG00000148143	ENSG00000151726	ENSG00000105227	ENSG00000180398
ENSG00000101892	ENSG00000146910	ENSG00000189120	ENSG00000104529
ENSG00000182968	ENSG00000103426	ENSG00000109458	ENSG00000162692
ENSG00000133026	ENSG00000187824	ENSG00000153879	ENSG00000071539
ENSG00000164220	ENSG00000084090	ENSG00000135525	ENSG00000124721
ENSG00000010810	ENSG00000176623	ENSG00000152256	ENSG00000101474
ENSG00000178695	ENSG00000196576	ENSG00000131475	ENSG00000047230
ENSG00000163660	ENSG00000146950	ENSG00000166794	ENSG00000172893
ENSG00000115556	ENSG00000131089	ENSG00000171988	ENSG00000112379
ENSG00000110436	ENSG00000171314	ENSG00000183908	ENSG00000086504
ENSG00000165556	ENSG00000206190	ENSG00000169375	ENSG00000164684
ENSG00000166024	ENSG00000162772	ENSG00000112640	ENSG00000187715
ENSG00000153885	ENSG00000140090	ENSG00000165637	ENSG00000111052
ENSG00000115685	ENSG00000143590	ENSG00000125733	ENSG00000100321
ENSG00000143344	ENSG00000185483	ENSG00000164418	ENSG00000163946
ENSG00000162769	ENSG00000197136	ENSG00000163376	ENSG00000168546
ENSG00000168795	ENSG00000123689	ENSG00000156671	ENSG00000118058
ENSG00000141522	ENSG00000163516	ENSG00000111642	ENSG00000164920
ENSG00000164458	ENSG00000110171	ENSG00000197948	ENSG00000067836
ENSG00000160570	ENSG00000143851	ENSG00000088247	ENSG00000172006
ENSG00000140285	ENSG00000116761	ENSG00000102024	ENSG00000112245
ENSG00000124126	ENSG00000086991	ENSG00000131242	ENSG00000147202
ENSG00000128052	ENSG00000087365	ENSG00000049247	ENSG00000123870
ENSG00000013455	ENSG00000145703	ENSG00000145723	ENSG00000011523
ENSG00000135472	ENSG00000164099	ENSG00000177106	ENSG00000077274
ENSG00000152092	ENSG00000156535	ENSG00000081041	ENSG00000139438
ENSG00000126777	ENSG00000170485	ENSG00000078140	ENSG00000166923
ENSG00000166747	ENSG00000182795	ENSG00000197622	ENSG00000101752
ENSG00000108219	ENSG00000109452	ENSG00000197451	ENSG00000079841
ENSG00000132485	ENSG00000158470	ENSG00000197183	ENSG00000223572
ENSG00000204842	ENSG00000185442	ENSG00000078403	ENSG00000197879
ENSG00000197445	ENSG00000152954	ENSG00000104375	ENSG00000162129
ENSG00000143437	ENSG00000158813	ENSG00000167772	ENSG00000135469
ENSG00000103126	ENSG00000018625	ENSG00000143079	ENSG00000109436
ENSG00000138771	ENSG00000106588	ENSG00000130779	ENSG00000196660
ENSG00000135547	ENSG00000119508	ENSG00000166913	ENSG00000122986
ENSG00000205339	ENSG00000113552	ENSG00000135655	ENSG00000171428
ENSG00000127554	ENSG00000135842	ENSG00000111142	ENSG00000172819
ENSG00000004776	ENSG00000099968	ENSG00000154640	ENSG00000095485

ENSG00000197535	ENSG00000155816	ENSG00000122367	ENSG00000140153
ENSG00000204117	ENSG00000115541	ENSG00000152969	ENSG00000084731
ENSG00000126070	ENSG00000049769	ENSG00000150630	ENSG00000159339
ENSG00000181924	ENSG00000139117	ENSG00000154059	ENSG00000019485
ENSG00000123384	ENSG00000071537	ENSG00000088538	ENSG00000100226
ENSG00000183283	ENSG00000180198	ENSG00000100241	ENSG00000198301
ENSG00000144668	ENSG00000188042	ENSG00000128710	ENSG00000072571
ENSG00000157152	ENSG00000179774	ENSG00000033122	ENSG00000106366
ENSG00000164631	ENSG00000154945	ENSG00000032219	ENSG00000100564
ENSG00000141551	ENSG00000150593	ENSG00000134072	ENSG00000213523
ENSG00000132356	ENSG00000147650	ENSG00000100100	ENSG00000161905
ENSG00000173548	ENSG00000137571	ENSG00000117308	ENSG00000183137
ENSG00000169862	ENSG00000179593	ENSG00000170577	ENSG00000120324
ENSG00000130201	ENSG00000101448	ENSG00000162670	ENSG00000090339
ENSG00000138430	ENSG00000187475	ENSG00000182831	ENSG00000151012
ENSG00000163393	ENSG00000197635	ENSG00000167904	ENSG00000139620
ENSG00000084636	ENSG00000134323	ENSG00000134283	ENSG00000102385
ENSG00000158863	ENSG00000151025	ENSG00000100124	ENSG00000185015
ENSG00000113100	ENSG00000102316	ENSG00000148660	ENSG00000124831
ENSG00000003393	ENSG00000153207	ENSG00000113721	ENSG00000221972
ENSG00000171450	ENSG00000197217	ENSG00000079805	ENSG00000136813
ENSG00000148719	ENSG00000107341	ENSG00000185634	ENSG00000113368
ENSG00000186591	ENSG00000101282	ENSG00000066117	ENSG00000130830
ENSG00000117859	ENSG00000168385	ENSG00000125848	ENSG00000101337
ENSG00000204267	ENSG00000050327	ENSG00000164040	ENSG00000214013
ENSG00000173757	ENSG00000169221	ENSG00000163947	ENSG00000129946
ENSG00000132471	ENSG00000101138	ENSG00000178217	ENSG00000134802
ENSG00000119242	ENSG00000102445	ENSG00000115884	ENSG00000142733
ENSG00000198561	ENSG00000138698	ENSG00000112851	ENSG00000130669
ENSG00000007237	ENSG00000157353	ENSG00000106771	ENSG00000134765
ENSG00000113758	ENSG00000107554	ENSG00000130829	ENSG00000171159
ENSG00000131233	ENSG00000184825	ENSG00000105220	ENSG00000178988
ENSG00000165188	ENSG00000117408	ENSG00000117643	ENSG00000129673
ENSG00000006712	ENSG00000158987	ENSG00000164506	ENSG00000185619
ENSG00000177807	ENSG00000117410	ENSG00000091039	ENSG00000183668
ENSG00000153130	ENSG00000089050	ENSG00000102606	ENSG00000111707
ENSG00000110693	ENSG00000131966	ENSG00000100320	ENSG00000189180
ENSG00000171195	ENSG00000118482	ENSG00000150776	ENSG00000060558
ENSG00000129245	ENSG00000183748	ENSG00000148400	ENSG00000166595
ENSG00000101665	ENSG00000159147	ENSG00000211450	ENSG00000107821
ENSG00000122545	ENSG00000085449	ENSG00000168477	ENSG00000133884
ENSG00000110696	ENSG00000078081	ENSG00000096063	ENSG00000159685
ENSG00000183770	ENSG00000135926	ENSG00000120708	ENSG00000168256
ENSG00000179348	ENSG00000157890	ENSG00000143924	ENSG00000167261
ENSG00000149679	ENSG00000198663	ENSG00000068796	ENSG00000151491
ENSG00000140854	ENSG00000145246	ENSG00000072135	ENSG00000138449
ENSG00000109689	ENSG00000108799	ENSG00000173456	ENSG00000125686
ENSG00000168092	ENSG00000156140	ENSG00000089693	ENSG00000171236
ENSG00000104960	ENSG00000156504	ENSG00000112531	ENSG00000135378
ENSG00000181007	ENSG00000164070	ENSG00000172780	ENSG00000198518
ENSG00000196930	ENSG00000156427	ENSG00000171951	ENSG00000131095
ENSG00000163995	ENSG00000136816	ENSG00000028277	ENSG00000069329
ENSG00000054118	ENSG00000163071	ENSG00000149474	ENSG00000130592
ENSG00000183574	ENSG00000120539	ENSG00000111481	ENSG00000171798
ENSG00000136807	ENSG00000175215	ENSG00000139289	ENSG00000122176
ENSG00000184371	ENSG00000096264	ENSG00000012963	ENSG00000136002
ENSG00000063245	ENSG00000198382	ENSG00000165495	ENSG00000171126
ENSG00000134294	ENSG00000203813	ENSG00000141644	ENSG00000089220
ENSG00000134954	ENSG00000144355	ENSG00000186340	ENSG00000142303
ENSG00000131323	ENSG00000170448	ENSG00000115756	ENSG00000232125
ENSG00000173040	ENSG00000197037	ENSG00000150768	ENSG00000131669
ENSG00000179772	ENSG00000038382	ENSG00000107882	ENSG00000196172
ENSG00000074695	ENSG00000130695	ENSG00000068308	ENSG00000138246
ENSG00000124535	ENSG00000009780	ENSG00000165806	ENSG00000168904

ENSG00000105066	ENSG00000122565	ENSG00000084463	ENSG00000092529
ENSG00000057657	ENSG00000160867	ENSG00000181588	ENSG00000134533
ENSG00000174021	ENSG00000161677	ENSG00000198408	ENSG00000091164
ENSG00000049192	ENSG00000125967	ENSG00000164778	ENSG00000101052
ENSG00000064547	ENSG00000237441	ENSG00000166340	ENSG00000198720
ENSG00000077254	ENSG00000166452	ENSG00000089558	ENSG00000197548
ENSG00000102466	ENSG00000073910	ENSG00000108443	ENSG00000113805
ENSG00000125814	ENSG00000168096	ENSG00000110975	ENSG00000178573
ENSG00000163431	ENSG00000074621	ENSG00000080845	ENSG00000174744
ENSG00000070413	ENSG00000186517	ENSG00000106004	ENSG00000205155
ENSG00000189060	ENSG00000101938	ENSG00000104290	ENSG00000106952
ENSG00000198963	ENSG00000030066	ENSG00000133818	ENSG00000174652
ENSG00000120709	ENSG00000182389	ENSG00000185670	ENSG00000138760
ENSG00000138107	ENSG00000089057	ENSG00000146373	ENSG00000197253
ENSG00000198952	ENSG00000113742	ENSG00000158480	ENSG00000133131
ENSG00000115216	ENSG00000125845	ENSG00000108582	ENSG00000128606
ENSG00000170624	ENSG00000004975	ENSG00000100234	ENSG00000156345
ENSG00000113522	ENSG00000155229	ENSG00000173511	ENSG00000196734
ENSG00000115084	ENSG00000152128	ENSG00000083312	ENSG00000147434
ENSG00000115520	ENSG00000124875	ENSG00000166446	ENSG00000117448
ENSG00000114166	ENSG00000185332	ENSG00000108828	ENSG00000103549
ENSG00000163285	ENSG00000129270	ENSG00000162650	ENSG00000089335
ENSG00000198915	ENSG00000160325	ENSG00000165997	ENSG00000185009
ENSG00000085382	ENSG00000168502	ENSG00000179918	ENSG00000163162
ENSG00000110583	ENSG00000133454	ENSG00000118900	ENSG00000172345
ENSG00000055955	ENSG00000153982	ENSG00000110172	ENSG00000159842
ENSG00000119138	ENSG00000136099	ENSG00000143434	ENSG00000167723
ENSG00000101019	ENSG00000110944	ENSG00000132286	ENSG00000121486
ENSG00000138111	ENSG00000182500	ENSG00000128645	ENSG00000144061
ENSG00000157368	ENSG00000182175	ENSG00000106683	ENSG00000148343
ENSG00000137745	ENSG00000163382	ENSG00000002834	ENSG00000102935
ENSG00000167332	ENSG00000133704	ENSG00000106336	ENSG00000145506
ENSG00000139597	ENSG00000145743	ENSG00000187239	ENSG00000180447
ENSG00000064763	ENSG00000001167	ENSG00000128918	ENSG00000197114
ENSG00000086589	ENSG00000134108	ENSG00000072858	ENSG00000177674
ENSG00000063046	ENSG00000185883	ENSG00000106460	ENSG00000221914
ENSG00000159086	ENSG00000198920	ENSG00000158457	ENSG00000138614
ENSG00000114988	ENSG00000113119	ENSG00000158796	ENSG00000117899
ENSG00000168779	ENSG00000110429	ENSG00000160679	ENSG00000197465
ENSG00000129250	ENSG00000089902	ENSG00000129422	ENSG00000144366
ENSG00000143870	ENSG00000196449	ENSG00000197226	ENSG00000106483
ENSG00000174996	ENSG00000177981	ENSG00000133030	ENSG00000125246
ENSG00000128590	ENSG00000092148	ENSG00000177888	ENSG00000133661
ENSG00000171533	ENSG00000214882	ENSG00000167680	ENSG00000127511
ENSG00000166503	ENSG00000166828	ENSG00000072201	ENSG00000197565
ENSG00000112530	ENSG00000143367	ENSG00000198053	ENSG00000103051
ENSG00000177694	ENSG00000163624	ENSG00000204713	ENSG00000112139
ENSG00000122707	ENSG00000174939	ENSG00000049449	ENSG00000106348
ENSG00000163681	ENSG00000189403	ENSG00000085185	ENSG00000138685
ENSG00000101294	ENSG00000188315	ENSG00000143850	ENSG00000161939
ENSG00000165156	ENSG00000172137	ENSG00000185504	ENSG00000104687
ENSG00000139679	ENSG00000170075	ENSG00000157827	ENSG00000168333
ENSG00000187416	ENSG00000143614	ENSG00000166526	ENSG00000075856
ENSG00000153558	ENSG00000184560	ENSG00000134853	ENSG00000105048
ENSG00000127838	ENSG00000108292	ENSG00000139746	ENSG00000205056
ENSG00000184486	ENSG00000182601	ENSG00000104497	ENSG00000249670
ENSG00000198964	ENSG00000205916	ENSG00000092445	ENSG00000155592
ENSG00000085733	ENSG00000130956	ENSG00000121966	ENSG00000122861
ENSG00000162927	ENSG00000111859	ENSG00000040731	ENSG00000102048
ENSG00000173210	ENSG00000235568	ENSG00000033327	ENSG00000101236
ENSG00000128731	ENSG00000157483	ENSG00000135108	ENSG00000186020
ENSG00000154553	ENSG00000166448	ENSG00000089597	ENSG00000072422
ENSG00000005249	ENSG00000099381	ENSG00000178764	ENSG00000110077
ENSG00000010017	ENSG00000132361	ENSG00000164976	ENSG00000178623

ENSG00000069702	ENSG00000109920	ENSG00000182580	ENSG00000108840
ENSG00000068028	ENSG00000116396	ENSG00000113658	ENSG00000144857
ENSG00000104313	ENSG00000106236	ENSG00000117000	ENSG00000100916
ENSG00000169139	ENSG00000107021	ENSG00000176563	ENSG00000150893
ENSG00000107859	ENSG00000118363	ENSG00000005020	ENSG00000136770
ENSG00000116521	ENSG00000181929	ENSG00000095203	ENSG00000185610
ENSG00000205250	ENSG00000065802	ENSG00000023041	ENSG00000010244
ENSG00000171017	ENSG00000166450	ENSG00000104365	ENSG00000181240
ENSG00000134363	ENSG00000174083	ENSG00000159873	ENSG00000173598
ENSG00000147842	ENSG00000174099	ENSG00000172728	ENSG00000108061
ENSG00000165699	ENSG00000007866	ENSG00000116983	ENSG00000151461
ENSG00000181555	ENSG00000176788	ENSG00000157680	ENSG00000168495
ENSG00000140807	ENSG00000169926	ENSG00000164715	ENSG00000100285
ENSG00000144815	ENSG00000106799	ENSG00000070610	ENSG00000130810
ENSG00000076641	ENSG00000185022	ENSG00000005379	ENSG00000135632
ENSG00000178562	ENSG00000140945	ENSG00000120686	ENSG00000198399
ENSG00000070159	ENSG00000176018	ENSG00000205189	ENSG00000101280
ENSG00000153094	ENSG00000083520	ENSG00000109586	ENSG00000099622
ENSG00000107679	ENSG00000236279	ENSG00000133105	ENSG00000170233
ENSG00000108506	ENSG00000169908	ENSG00000117560	ENSG00000116604
ENSG00000206265	ENSG00000154319	ENSG00000204967	ENSG00000171617
ENSG00000165443	ENSG00000123643	ENSG00000139350	ENSG00000101846
ENSG00000024422	ENSG00000127314	ENSG00000100068	ENSG00000221870
ENSG00000154493	ENSG00000158092	ENSG00000183023	ENSG00000055609
ENSG00000103222	ENSG00000113722	ENSG00000125945	ENSG00000126088
ENSG00000129968	ENSG00000165406	ENSG00000145730	ENSG00000147081
ENSG00000135423	ENSG00000187634	ENSG00000196535	ENSG00000173230
ENSG00000171446	ENSG00000104969	ENSG00000176973	ENSG00000116032
ENSG00000006118	ENSG00000126106	ENSG00000077279	ENSG00000144659
ENSG00000134852	ENSG00000137497	ENSG00000074319	ENSG00000230366
ENSG00000125740	ENSG00000149308	ENSG00000012171	ENSG00000168135
ENSG00000005302	ENSG00000110108	ENSG00000147689	ENSG00000102225
ENSG00000183092	ENSG00000158201	ENSG00000118689	ENSG00000164818
ENSG00000141342	ENSG00000149792	ENSG00000174595	ENSG00000186910
ENSG00000021488	ENSG00000158769	ENSG00000162734	ENSG00000170473
ENSG00000137502	ENSG00000103707	ENSG00000146729	ENSG00000100304
ENSG00000129757	ENSG00000088854	ENSG00000120896	ENSG00000136487
ENSG00000168824	ENSG00000160179	ENSG00000111145	ENSG00000112759
ENSG00000104064	ENSG00000183434	ENSG00000118503	ENSG00000114573
ENSG00000102096	ENSG00000204262	ENSG00000145864	ENSG00000147883
ENSG00000143867	ENSG00000103502	ENSG00000158270	ENSG00000161573
ENSG00000178531	ENSG00000072182	ENSG00000141380	ENSG00000156299
ENSG00000150967	ENSG00000179104	ENSG00000108349	ENSG00000198648
ENSG00000136267	ENSG00000122359	ENSG00000197971	ENSG00000119772
ENSG00000113712	ENSG00000123191	ENSG00000011028	ENSG00000120440
ENSG00000005102	ENSG00000102974	ENSG00000146197	ENSG00000090238
ENSG00000170325	ENSG00000182040	ENSG00000182541	ENSG00000171962
ENSG00000120885	ENSG00000088038	ENSG00000110888	ENSG00000078902
ENSG00000197892	ENSG00000170266	ENSG00000108231	ENSG00000130377
ENSG00000162613	ENSG00000130287	ENSG00000174611	ENSG00000183155
ENSG00000183779	ENSG00000036565	ENSG00000161671	ENSG00000176058
ENSG00000044459	ENSG00000176490	ENSG00000066248	ENSG00000070831
ENSG00000099203	ENSG00000143624	ENSG00000132953	ENSG00000104643
ENSG00000166233	ENSG00000072134	ENSG00000152495	ENSG00000182446
ENSG00000139112	ENSG00000176225	ENSG00000182324	ENSG00000139364
ENSG00000064218	ENSG00000115946	ENSG00000157064	ENSG00000136828
ENSG00000204916	ENSG00000067082	ENSG00000160305	ENSG00000171388
ENSG00000183496	ENSG00000157445	ENSG00000110243	ENSG00000035928
ENSG00000140497	ENSG00000120868	ENSG00000159784	ENSG00000133606
ENSG00000119318	ENSG00000008513	ENSG00000162813	ENSG00000141556
ENSG00000117385	ENSG00000089116	ENSG00000180233	ENSG00000162139
ENSG00000117616	ENSG00000147465	ENSG00000128917	ENSG00000128833
ENSG00000167106	ENSG00000116478	ENSG00000181222	ENSG00000116455
ENSG00000088808	ENSG00000138160	ENSG00000108344	ENSG00000108094

ENSG00000087274	ENSG00000163472	ENSG00000090686	ENSG00000142166
ENSG00000136634	ENSG00000108578	ENSG00000103740	ENSG00000161847
ENSG00000079102	ENSG00000142655	ENSG00000111716	ENSG00000169704
ENSG00000164754	ENSG00000167768	ENSG00000137764	ENSG00000164104
ENSG00000137345	ENSG00000151892	ENSG00000198440	ENSG00000151475
ENSG00000168710	ENSG00000072657	ENSG00000080546	ENSG00000121454
ENSG00000117036	ENSG00000147459	ENSG00000168310	ENSG00000028839
ENSG00000185722	ENSG00000106034	ENSG00000064195	ENSG00000109572
ENSG00000151694	ENSG00000135097	ENSG00000133226	ENSG00000152270
ENSG00000123576	ENSG00000145375	ENSG00000153707	ENSG00000100083
ENSG00000034677	ENSG00000110675	ENSG00000113575	ENSG00000118526
ENSG00000177105	ENSG00000105583	ENSG00000109133	ENSG00000071967
ENSG00000126261	ENSG00000157654	ENSG00000145391	ENSG00000006747
ENSG00000177511	ENSG00000158865	ENSG00000159202	ENSG00000102221
ENSG00000116133	ENSG00000062485	ENSG00000100417	ENSG00000108306
ENSG00000169136	ENSG00000104814	ENSG00000188613	ENSG00000125337
ENSG00000173208	ENSG00000114268	ENSG00000153250	ENSG00000161267
ENSG00000166793	ENSG00000128585	ENSG00000134575	ENSG00000144746
ENSG00000120549	ENSG00000010361	ENSG00000138101	ENSG00000113231
ENSG00000092098	ENSG00000012232	ENSG00000186073	ENSG00000197487
ENSG00000134901	ENSG00000135517	ENSG00000025796	ENSG00000160200
ENSG00000150961	ENSG00000100003	ENSG00000116774	ENSG00000105819
ENSG00000187251	ENSG00000157150	ENSG00000179562	ENSG00000095739
ENSG00000153767	ENSG00000168916	ENSG00000170791	ENSG00000134313
ENSG00000103042	ENSG00000115507	ENSG00000135903	ENSG00000064787
ENSG00000068400	ENSG00000081237	ENSG00000143537	ENSG00000181374
ENSG00000140600	ENSG00000185787	ENSG00000211445	ENSG00000135902
ENSG00000139266	ENSG00000064309	ENSG00000132780	ENSG00000042088
ENSG00000138031	ENSG00000160710	ENSG00000131759	ENSG00000137269
ENSG00000175387	ENSG00000138823	ENSG00000115468	ENSG00000111962
ENSG00000166275	ENSG00000175826	ENSG00000196470	ENSG00000167548
ENSG00000116678	ENSG00000176102	ENSG00000162694	ENSG00000064961
ENSG00000144802	ENSG00000166961	ENSG00000040341	ENSG00000182372
ENSG00000164463	ENSG00000106701	ENSG00000112144	ENSG00000094755
ENSG00000184945	ENSG00000056345	ENSG00000116685	ENSG00000177885
ENSG00000138795	ENSG00000158859	ENSG00000116584	ENSG00000162430
ENSG00000176087	ENSG00000103150	ENSG00000056661	ENSG00000126216
ENSG00000121104	ENSG00000188725	ENSG00000184205	ENSG00000113441
ENSG00000149927	ENSG00000172262	ENSG00000152527	ENSG00000186417
ENSG00000172939	ENSG00000164100	ENSG00000074181	ENSG00000240230
ENSG00000167874	ENSG00000107186	ENSG00000143140	ENSG00000154734
ENSG00000182674	ENSG00000164691	ENSG00000159388	ENSG00000144792
ENSG00000184182	ENSG00000198431	ENSG00000108774	ENSG00000183655
ENSG00000118418	ENSG00000183579	ENSG00000140406	ENSG00000172789
ENSG00000003147	ENSG00000113520	ENSG00000126351	ENSG00000071073
ENSG00000122126	ENSG00000103196	ENSG00000065029	ENSG00000153827
ENSG00000187975	ENSG00000130508	ENSG00000131771	ENSG00000149716
ENSG00000064655	ENSG00000196767	ENSG00000070214	ENSG00000161202
ENSG00000081181	ENSG00000173692	ENSG00000106829	ENSG00000115204
ENSG00000103647	ENSG00000121274	ENSG00000151692	ENSG00000245848
ENSG00000136643	ENSG00000072682	ENSG00000074201	ENSG00000167615
ENSG00000163320	ENSG00000187840	ENSG00000103994	ENSG00000176444
ENSG00000157916	ENSG00000213977	ENSG00000154620	ENSG00000100243
ENSG00000111912	ENSG00000114450	ENSG00000118200	ENSG00000138735
ENSG00000106069	ENSG00000090971	ENSG00000170365	ENSG00000107562
ENSG00000001561	ENSG00000007314	ENSG00000082497	ENSG00000197872
ENSG00000073584	ENSG00000114554	ENSG00000174840	ENSG00000206506
ENSG00000137078	ENSG00000160202	ENSG00000141664	ENSG00000166821
ENSG00000180398	ENSG00000137040	ENSG00000130939	ENSG00000181104
ENSG00000176532	ENSG00000004660	ENSG00000164305	ENSG00000060982
ENSG00000185275	ENSG00000178996	ENSG00000163930	ENSG00000206288
ENSG00000186458	ENSG00000196104	ENSG00000187091	ENSG00000122643
ENSG00000047230	ENSG00000164442	ENSG00000134030	ENSG00000151502
ENSG00000023902	ENSG00000120889	ENSG00000065989	ENSG00000112984

ENSG00000205581	ENSG00000162782	ENSG00000197375	ENSG00000162104
ENSG00000112379	ENSG00000179041	ENSG00000146592	ENSG00000100442
ENSG00000187715	ENSG00000155984	ENSG00000171365	ENSG00000114742
ENSG00000140750	ENSG00000103404	ENSG00000084733	ENSG00000107854
ENSG00000100321	ENSG00000165389	ENSG00000026508	ENSG00000227372
ENSG00000125817	ENSG00000140443	ENSG00000174943	ENSG00000159788
ENSG00000196435	ENSG00000124209	ENSG00000163877	ENSG00000162761
ENSG00000112245	ENSG00000204977	ENSG00000125492	ENSG00000135722
ENSG00000125869	ENSG00000169992	ENSG00000161203	ENSG00000172845
ENSG00000147202	ENSG00000132874	ENSG00000204580	ENSG00000168672
ENSG00000198522	ENSG00000137845	ENSG00000125780	ENSG00000248383
ENSG00000077274	ENSG00000168591	ENSG00000047597	ENSG00000064989
ENSG00000139438	ENSG00000108433	ENSG00000163389	ENSG00000116649
ENSG00000166923	ENSG00000100106	ENSG00000101222	ENSG00000103569
ENSG00000133597	ENSG00000101166	ENSG00000178685	ENSG00000163563
ENSG00000075702	ENSG00000116918	ENSG00000157800	ENSG00000180370
ENSG00000197879	ENSG00000211456	ENSG00000204843	ENSG00000073969
ENSG00000185049	ENSG00000122435	ENSG00000183878	ENSG00000005486
ENSG00000005893	ENSG00000086189	ENSG00000119661	ENSG00000159069
ENSG00000092847	ENSG00000145632	ENSG00000170915	ENSG00000105705
ENSG00000122986	ENSG00000103254	ENSG00000144681	ENSG00000052344
ENSG00000159479	ENSG00000185418	ENSG00000003400	ENSG00000144218
ENSG00000118407	ENSG00000011332	ENSG00000138834	ENSG00000184557
ENSG00000118640	ENSG00000154736	ENSG00000117751	ENSG00000105711
ENSG00000172819	ENSG00000105438	ENSG00000149182	ENSG00000105610
ENSG00000092969	ENSG00000075539	ENSG00000159461	ENSG00000229453
ENSG00000178235	ENSG00000225697	ENSG00000061676	ENSG00000145996
ENSG00000084731	ENSG00000171307	ENSG00000140092	ENSG00000143486
ENSG00000140153	ENSG00000100596	ENSG00000103671	ENSG00000034239
ENSG00000185002	ENSG00000140873	ENSG00000186918	ENSG00000100346
ENSG00000108946	ENSG00000179023	ENSG00000163867	ENSG00000125637
ENSG00000043039	ENSG00000147457	ENSG00000144730	ENSG00000162384
ENSG00000100226	ENSG00000099889	ENSG00000142875	ENSG00000004700
ENSG00000167550	ENSG00000180772	ENSG00000070770	ENSG00000155511
ENSG00000183900	ENSG00000130584	ENSG00000130711	ENSG00000154174
ENSG00000198301	ENSG00000072952	ENSG00000162624	ENSG00000083290
ENSG00000106366	ENSG00000178104	ENSG00000068137	ENSG00000203759
ENSG00000111837	ENSG00000136014	ENSG00000107742	ENSG00000082153
ENSG00000171097	ENSG00000100348	ENSG00000176390	ENSG00000133805
ENSG00000137343	ENSG00000149761	ENSG00000065618	ENSG00000135355
ENSG00000184304	ENSG00000172404	ENSG00000105722	ENSG00000113140
ENSG00000151012	ENSG00000101057	ENSG00000179292	ENSG00000080224
ENSG00000139620	ENSG00000114744	ENSG00000143393	ENSG00000143569
ENSG00000167986	ENSG00000139629	ENSG00000066777	ENSG00000214097
ENSG00000118705	ENSG00000113657	ENSG00000043093	ENSG00000164031
ENSG00000008282	ENSG00000007545	ENSG00000115652	ENSG00000111850
ENSG00000124831	ENSG00000163485	ENSG00000143549	ENSG00000138764
ENSG00000100325	ENSG00000022840	ENSG00000166147	ENSG00000096092
ENSG00000089818	ENSG00000176783	ENSG00000168397	ENSG00000171206
ENSG00000113368	ENSG00000174721	ENSG00000119681	ENSG00000168028
ENSG00000137727	ENSG00000170647	ENSG00000158747	ENSG00000162910
ENSG00000162188	ENSG00000154359	ENSG00000105974	ENSG00000153498
ENSG00000119004	ENSG00000161558	ENSG00000101825	ENSG00000075618
ENSG00000126461	ENSG00000135535	ENSG00000121210	ENSG00000179913
ENSG00000206561	ENSG00000135506	ENSG00000116266	ENSG00000135801
ENSG00000101337	ENSG00000144524	ENSG00000126581	ENSG00000143167
ENSG00000124496	ENSG00000172493	ENSG00000184144	ENSG00000010318
ENSG00000145833	ENSG00000148735	ENSG00000146776	ENSG00000110107
ENSG00000130669	ENSG00000171813	ENSG00000101331	ENSG00000100519
ENSG00000134765	ENSG00000162909	ENSG00000163605	ENSG00000156603
ENSG00000178988	ENSG00000177764	ENSG00000144290	ENSG00000173762
ENSG00000099256	ENSG00000177000	ENSG00000080371	ENSG00000117215
ENSG00000102010	ENSG00000113327	ENSG00000130150	ENSG00000175581
ENSG00000173653	ENSG00000141429	ENSG00000171246	ENSG00000187210

ENSG00000185619	ENSG00000204539	ENSG00000169085	ENSG00000171219
ENSG00000205542	ENSG00000152944	ENSG00000127837	ENSG00000186047
ENSG00000133858	ENSG00000138468	ENSG00000162923	ENSG00000175105
ENSG00000111707	ENSG00000130294	ENSG00000168724	ENSG00000205441
ENSG00000081189	ENSG00000168395	ENSG00000116857	ENSG00000124214
ENSG00000185551	ENSG00000134762	ENSG00000196639	ENSG00000156802
ENSG00000138018	ENSG00000154380	ENSG00000130940	ENSG00000173542
ENSG00000111897	ENSG00000115252	ENSG00000118985	ENSG00000143194
ENSG00000133884	ENSG00000133116	ENSG00000168993	ENSG00000171431
ENSG00000156273	ENSG00000145949	ENSG00000154447	ENSG00000004478
ENSG00000101040	ENSG00000134531	ENSG00000117569	ENSG00000137843
ENSG00000168256	ENSG00000165525	ENSG00000178538	ENSG00000030582
ENSG00000151491	ENSG00000198366	ENSG00000122085	ENSG00000130988
ENSG00000125686	ENSG00000169071	ENSG00000164032	ENSG00000121933
ENSG00000138449	ENSG00000233034	ENSG00000133937	ENSG00000152977
ENSG00000182512	ENSG00000065243	ENSG00000177463	ENSG00000197860
ENSG00000197442	ENSG00000182093	ENSG00000198855	ENSG00000108018
ENSG00000170006	ENSG00000070882	ENSG00000206560	ENSG00000126861
ENSG00000163430	ENSG00000172992	ENSG00000144567	ENSG00000197555
ENSG00000185090	ENSG00000116001	ENSG00000124574	ENSG00000240251
ENSG00000147130	ENSG00000171204	ENSG00000198160	ENSG00000168938
ENSG00000171798	ENSG00000103356	ENSG00000065308	ENSG00000144935
ENSG00000122176	ENSG00000106948	ENSG00000103319	ENSG00000099985
ENSG00000136002	ENSG00000124228	ENSG00000114349	ENSG00000114026
ENSG00000163145	ENSG00000138617	ENSG00000099204	ENSG00000115234
ENSG00000126458	ENSG00000173540	ENSG00000149260	ENSG00000178537
ENSG00000171126	ENSG00000143494	ENSG00000131873	ENSG00000159167
ENSG00000104413	ENSG00000025434	ENSG00000002587	ENSG00000145016
ENSG00000134444	ENSG00000090661	ENSG00000196581	ENSG00000161653
ENSG00000155970	ENSG00000169955	ENSG00000141140	ENSG00000135299
ENSG00000118096	ENSG00000100605	ENSG00000131711	ENSG00000011243
ENSG00000138246	ENSG00000187555	ENSG00000136986	ENSG00000129534
ENSG00000134533	ENSG00000165424	ENSG00000123364	ENSG00000137815
ENSG00000092529	ENSG00000162892	ENSG00000103089	ENSG00000090615
ENSG00000165934	ENSG00000141736	ENSG00000164530	ENSG00000186103
ENSG00000091164	ENSG00000141503	ENSG00000124194	ENSG00000079332
ENSG00000131507	ENSG00000227500	ENSG00000120616	ENSG00000173404
ENSG00000198720	ENSG00000106078	ENSG00000166483	ENSG00000173585
ENSG00000197548	ENSG00000125730	ENSG00000130590	ENSG00000137478
ENSG00000128045	ENSG00000081377	ENSG00000168172	ENSG00000155096
ENSG00000166128	ENSG00000174950	ENSG00000196498	ENSG00000108379
ENSG00000104343	ENSG00000248871	ENSG00000188158	ENSG00000116771
ENSG00000050748	ENSG00000106692	ENSG00000175832	ENSG00000132256
ENSG00000198586	ENSG00000011405	ENSG00000095539	ENSG00000161281
ENSG00000137834	ENSG00000154118	ENSG00000154429	ENSG00000180644
ENSG00000138760	ENSG00000100065	ENSG00000077458	ENSG00000108852
ENSG00000153048	ENSG00000163874	ENSG00000129473	ENSG00000165376
ENSG00000133131	ENSG00000148737	ENSG00000166889	ENSG00000054793
ENSG00000148123	ENSG00000197798	ENSG00000166165	ENSG00000178950
ENSG00000128606	ENSG00000139832	ENSG00000187866	ENSG00000173566
ENSG00000148842	ENSG00000158467	ENSG00000124795	ENSG00000164741
ENSG00000164411	ENSG00000187764	ENSG00000138835	ENSG00000186451
ENSG00000103549	ENSG00000198900	ENSG00000134955	ENSG00000101098
ENSG00000117448	ENSG00000108107	ENSG00000198838	ENSG00000186187
ENSG00000163795	ENSG00000015479	ENSG00000177103	ENSG00000229117
ENSG00000185009	ENSG00000168447	ENSG00000167196	ENSG00000138867
ENSG00000062716	ENSG00000133056	ENSG00000101746	ENSG00000163558
ENSG00000162576	ENSG00000071127	ENSG00000120251	ENSG00000166016
ENSG00000081277	ENSG00000168175	ENSG00000182168	ENSG00000150093
ENSG00000159842	ENSG00000011007	ENSG00000091879	ENSG00000145241
ENSG00000139946	ENSG00000167996	ENSG00000112559	ENSG00000175084
ENSG00000167723	ENSG00000124743	ENSG00000156453	ENSG00000141040
ENSG00000121486	ENSG00000126003	ENSG00000163053	ENSG00000168610
ENSG00000122254	ENSG00000064419	ENSG00000185112	ENSG00000139433

ENSG00000102935	ENSG00000198894	ENSG00000123342	ENSG00000147044
ENSG00000148343	ENSG00000198929	ENSG00000163950	ENSG00000139344
ENSG00000180447	ENSG00000114455	ENSG00000129204	ENSG00000168439
ENSG00000158882	ENSG00000167257	ENSG00000196233	ENSG00000163412
ENSG00000197114	ENSG00000005882	ENSG00000170558	ENSG00000116191
ENSG00000105880	ENSG00000041982	ENSG00000175505	ENSG00000075218
ENSG00000170759	ENSG00000050767	ENSG00000196776	ENSG00000133027
ENSG00000104783	ENSG00000242689	ENSG00000130540	ENSG00000092330
ENSG00000188786	ENSG00000121039	ENSG00000176148	ENSG00000133639
ENSG00000179820	ENSG00000243646	ENSG00000076716	ENSG00000176049
ENSG00000163866	ENSG00000068697	ENSG00000185305	ENSG00000132109
ENSG00000164318	ENSG00000185340	ENSG00000160392	ENSG00000149591
ENSG00000171786	ENSG00000169855	ENSG00000156298	ENSG00000141552
ENSG00000144366	ENSG00000152661	ENSG00000066136	ENSG00000150455
ENSG00000136451	ENSG00000148248	ENSG00000205426	ENSG00000123505
ENSG00000174428	ENSG00000056998	ENSG00000138942	ENSG00000179300
ENSG00000125246	ENSG00000176340	ENSG00000121766	ENSG00000155130
ENSG00000166002	ENSG00000182611	ENSG00000135913	ENSG00000111725
ENSG00000157212	ENSG00000169914	ENSG00000159140	ENSG00000048544
ENSG00000171596	ENSG00000156103	ENSG00000078699	ENSG00000149273
ENSG00000131467	ENSG00000187664	ENSG00000008323	ENSG00000177542
ENSG00000143156	ENSG00000196277	ENSG00000149091	ENSG00000198040
ENSG00000100931	ENSG00000114739	ENSG00000118523	ENSG00000110906
ENSG00000127511	ENSG00000142634	ENSG00000112425	ENSG00000071205
ENSG00000158966	ENSG00000031691	ENSG00000160216	ENSG00000179403
ENSG00000084676	ENSG00000243943	ENSG00000115844	ENSG00000167333
ENSG00000106348	ENSG00000145365	ENSG00000198146	ENSG00000176597
ENSG00000112139	ENSG00000116406	ENSG00000145423	ENSG00000183918
ENSG00000120159	ENSG00000116688	ENSG00000125618	ENSG00000109846
ENSG00000138685	ENSG00000119878	ENSG00000160789	ENSG00000008324
ENSG00000130559	ENSG00000163069	ENSG00000071575	ENSG00000102145
ENSG00000169783	ENSG00000013523	ENSG00000179912	ENSG00000213079
ENSG00000114867	ENSG00000173575	ENSG00000064601	ENSG00000099331
ENSG00000161939	ENSG00000107897	ENSG00000029534	ENSG00000138071
ENSG00000197982	ENSG00000122863	ENSG00000117758	ENSG00000128655
ENSG00000111087	ENSG00000112195	ENSG00000126217	ENSG00000173638
ENSG00000156097	ENSG00000163872	ENSG00000114270	ENSG00000105216
ENSG00000075856	ENSG00000172590	ENSG00000114770	ENSG00000132357
ENSG00000141480	ENSG00000160994	ENSG00000150086	ENSG00000104415
ENSG00000172071	ENSG00000112773	ENSG00000127616	ENSG00000175311
ENSG00000174749	ENSG00000157214	ENSG00000070808	ENSG00000184588
ENSG00000181210	ENSG00000102081	ENSG00000166181	ENSG00000077514
ENSG00000127585	ENSG00000239704	ENSG00000143341	ENSG00000143443
ENSG00000107331	ENSG00000243364	ENSG00000160714	ENSG00000160293
ENSG00000109738	ENSG00000076513	ENSG00000169826	ENSG00000158941
ENSG00000176754	ENSG00000090372	ENSG00000086062	ENSG00000181666
ENSG00000111879	ENSG00000165548	ENSG00000145907	ENSG00000004534
ENSG00000122861	ENSG00000100201	ENSG00000139132	ENSG00000088876
ENSG00000120685	ENSG00000136152	ENSG00000196557	ENSG00000125818
ENSG00000103769	ENSG00000088448	ENSG00000198216	ENSG00000100949
ENSG00000101236	ENSG00000160685	ENSG00000182732	ENSG00000133110
ENSG00000164023	ENSG00000100228	ENSG00000162951	ENSG00000169762
ENSG00000072422	ENSG00000053108	ENSG00000148090	ENSG00000178053
ENSG00000108840	ENSG00000053918	ENSG00000161958	ENSG00000126746
ENSG00000186702	ENSG00000115350	ENSG00000105698	ENSG00000133019
ENSG00000120659	ENSG00000112739	ENSG00000168081	ENSG00000111711
ENSG00000189030	ENSG00000206418	ENSG00000124222	ENSG00000169418
ENSG00000144857	ENSG00000151623	ENSG00000143801	ENSG00000158636
ENSG00000100916	ENSG00000189184	ENSG00000130338	ENSG00000198692
ENSG00000150893	ENSG00000196177	ENSG00000186416	ENSG00000143153
ENSG00000185610	ENSG00000168843	ENSG00000186469	ENSG00000136628
ENSG00000165300	ENSG00000007923	ENSG00000177169	ENSG00000071553
ENSG00000136770	ENSG00000114686	ENSG00000101412	ENSG00000163286
ENSG00000010244	ENSG00000110801	ENSG00000147145	ENSG00000143499

ENSG00000173598	ENSG00000169193	ENSG00000134532	ENSG00000118007
ENSG00000108061	ENSG00000124098	ENSG00000142864	ENSG00000101251
ENSG00000075413	ENSG00000137135	ENSG00000156052	ENSG00000114948
ENSG00000141404	ENSG00000153485	ENSG00000172346	ENSG00000105472
ENSG00000151461	ENSG00000134874	ENSG00000139083	ENSG00000164331
ENSG00000166317	ENSG00000134321	ENSG00000108309	ENSG00000117122
ENSG00000118260	ENSG00000253953	ENSG00000160917	ENSG00000124785
ENSG00000168495	ENSG00000130713	ENSG00000121281	ENSG00000106803
ENSG00000123095	ENSG00000133265	ENSG00000103056	ENSG00000177595
ENSG00000100285	ENSG00000008118	ENSG00000006451	ENSG00000176204
ENSG00000135632	ENSG00000112561	ENSG00000155545	ENSG00000149150
ENSG00000171492	ENSG00000197697	ENSG00000111328	ENSG00000151338
ENSG00000198399	ENSG00000087916	ENSG00000104081	ENSG00000099769
ENSG00000137218	ENSG00000204186	ENSG00000102158	ENSG00000086570
ENSG00000162755	ENSG00000178802	ENSG00000047621	ENSG00000204991
ENSG00000116604	ENSG00000143319	ENSG00000105552	ENSG00000145214
ENSG00000171617	ENSG00000081803	ENSG00000115414	ENSG00000117480
ENSG00000037965	ENSG00000101191	ENSG00000146063	ENSG00000139618
ENSG00000164576	ENSG00000100433	ENSG00000166925	ENSG00000071794
ENSG00000115137	ENSG00000113761	ENSG00000106682	ENSG00000179165
ENSG00000055609	ENSG00000120688	ENSG00000075275	ENSG00000092201
ENSG00000153574	ENSG00000133316	ENSG00000171843	ENSG00000054356
ENSG00000184470	ENSG00000059728	ENSG00000035141	ENSG00000160870
ENSG00000168135	ENSG00000134755	ENSG00000168291	ENSG00000186334
ENSG00000102225	ENSG00000145354	ENSG00000135392	ENSG00000130816
ENSG00000168282	ENSG00000131016	ENSG00000128849	ENSG00000107738
ENSG00000170473	ENSG00000150051	ENSG00000072310	ENSG00000156395
ENSG00000162869	ENSG00000168497	ENSG00000012660	ENSG00000154781
ENSG00000172380	ENSG00000162344	ENSG00000152642	ENSG00000105176
ENSG00000140015	ENSG00000168280	ENSG00000173698	ENSG00000148671
ENSG00000185246	ENSG00000109501	ENSG00000092199	ENSG00000122756
ENSG00000099957	ENSG00000145358	ENSG00000179862	ENSG00000163914
ENSG00000106144	ENSG00000169933	ENSG00000118873	ENSG00000171121
ENSG00000117222	ENSG00000178163	ENSG00000003137	ENSG00000137860
ENSG00000155313	ENSG00000112796	ENSG00000155755	ENSG00000152822
ENSG00000165029	ENSG00000177034	ENSG00000148700	ENSG00000169903
ENSG00000198648	ENSG00000136928	ENSG00000151726	ENSG00000196547
ENSG00000119772	ENSG00000164326	ENSG00000109113	ENSG00000168702
ENSG00000090238	ENSG00000138814	ENSG00000103426	ENSG00000147454
ENSG00000183155	ENSG00000197614	ENSG00000136490	ENSG00000242866
ENSG00000070831	ENSG00000075240	ENSG00000146950	ENSG00000205726
ENSG00000182446	ENSG00000198327	ENSG00000100941	ENSG00000132205
ENSG00000140157	ENSG00000102034	ENSG00000180488	ENSG00000179761
ENSG00000171388	ENSG00000186575	ENSG00000184005	ENSG00000100266
ENSG00000133606	ENSG00000132849	ENSG00000171314	ENSG00000132824
ENSG00000135956	ENSG00000148948	ENSG00000206190	ENSG00000134138
ENSG00000183833	ENSG00000180616	ENSG00000162772	ENSG00000108599
ENSG00000116455	ENSG00000162378	ENSG00000148110	ENSG00000169218
ENSG00000141458	ENSG00000149798	ENSG00000004799	ENSG00000188501
ENSG00000161847	ENSG00000179218	ENSG00000100461	ENSG00000070731
ENSG00000108094	ENSG00000110048	ENSG00000140090	ENSG00000112893
ENSG00000186716	ENSG00000169230	ENSG00000143590	ENSG00000197857
ENSG00000158887	ENSG00000166407	ENSG00000185483	ENSG00000184226
ENSG00000158545	ENSG00000130165	ENSG00000110848	ENSG00000114656
ENSG00000188263	ENSG00000116984	ENSG00000183576	ENSG00000143149
ENSG00000165915	ENSG00000150401	ENSG00000123689	ENSG00000127334
ENSG00000151475	ENSG00000101189	ENSG00000163516	ENSG00000089916
ENSG00000121454	ENSG00000099308	ENSG00000104695	ENSG00000137868
ENSG00000111684	ENSG00000147437	ENSG00000163618	ENSG00000131943
ENSG00000028839	ENSG00000140259	ENSG00000110171	ENSG00000053254
ENSG00000109572	ENSG00000196368	ENSG00000157540	ENSG00000120952
ENSG00000152270	ENSG00000181826	ENSG00000135912	ENSG00000134253
ENSG00000100083	ENSG00000127863	ENSG00000125257	ENSG00000181418
ENSG00000118526	ENSG00000147251	ENSG00000114209	ENSG00000146966

ENSG00000178558	ENSG00000133216	ENSG00000086991	ENSG00000035499
ENSG00000134780	ENSG00000173825	ENSG00000156990	ENSG00000162337
ENSG00000102221	ENSG00000169239	ENSG00000145703	ENSG00000062598
ENSG00000162961	ENSG00000100302	ENSG00000170485	ENSG00000146670
ENSG00000197494	ENSG00000196981	ENSG00000182795	ENSG00000184881
ENSG00000140538	ENSG00000105821	ENSG00000158470	ENSG00000104859
ENSG00000108306	ENSG00000172831	ENSG00000185442	ENSG00000113013
ENSG00000140262	ENSG00000145451	ENSG00000060069	ENSG00000215825
ENSG00000132341	ENSG00000101152	ENSG00000152954	ENSG00000059377
ENSG00000162946	ENSG00000138326	ENSG00000158813	ENSG00000068831
ENSG00000113231	ENSG00000130396	ENSG00000106588	ENSG00000087074
ENSG00000106105	ENSG00000111817	ENSG00000148339	ENSG00000150990
ENSG00000141738	ENSG00000189319	ENSG00000018625	ENSG00000105968
ENSG00000095739	ENSG00000131738	ENSG00000112081	ENSG00000179241
ENSG00000165119	ENSG00000136160	ENSG00000119508	ENSG00000120837
ENSG00000064787	ENSG00000132640	ENSG00000099968	ENSG00000170836
ENSG00000147059	ENSG00000176165	ENSG00000115541	ENSG00000170260
ENSG00000157869	ENSG00000163581	ENSG00000139117	ENSG00000164542
ENSG00000188687	ENSG00000109132	ENSG00000164185	ENSG00000198099
ENSG00000019991	ENSG00000188761	ENSG00000071537	ENSG00000197818
ENSG00000137269	ENSG00000162889	ENSG00000095752	ENSG00000148814
ENSG00000171877	ENSG00000172840	ENSG00000180198	ENSG00000170324
ENSG00000170049	ENSG00000148331	ENSG00000188042	ENSG00000104356
ENSG00000106392	ENSG00000075151	ENSG00000172057	ENSG00000186860
ENSG00000111962	ENSG00000142686	ENSG00000179774	ENSG00000047410
ENSG00000118579	ENSG00000112697	ENSG00000154945	ENSG00000203730
ENSG00000101473	ENSG00000165804	ENSG00000147650	ENSG00000171560
ENSG00000166908	ENSG00000105221	ENSG00000150593	ENSG00000100884
ENSG00000177885	ENSG00000177051	ENSG00000102468	ENSG00000124422
ENSG00000162430	ENSG00000205808	ENSG00000137571	ENSG00000112977
ENSG00000126216	ENSG00000132561	ENSG00000178951	ENSG00000146926
ENSG00000047849	ENSG00000101882	ENSG00000180875	ENSG00000064115
ENSG00000186417	ENSG00000101745	ENSG00000181690	ENSG00000104918
ENSG00000173662	ENSG00000117289	ENSG00000197635	ENSG00000184544
ENSG00000099875	ENSG00000075142	ENSG00000136153	ENSG00000082684
ENSG00000113441	ENSG00000148219	ENSG00000134323	ENSG00000124782
ENSG00000169758	ENSG00000206579	ENSG00000197217	ENSG00000151655
ENSG00000132004	ENSG00000100307	ENSG00000188760	ENSG00000070182
ENSG00000183655	ENSG00000130822	ENSG00000168385	ENSG00000186925
ENSG00000172789	ENSG00000028203	ENSG00000107341	ENSG00000164199
ENSG00000154734	ENSG00000179262	ENSG00000169221	ENSG00000008056
ENSG00000145107	ENSG00000170653	ENSG00000157502	ENSG00000137709
ENSG00000071073	ENSG00000204463	ENSG00000068078	ENSG00000120327
ENSG00000103888	ENSG00000102786	ENSG00000102445	ENSG00000179583
ENSG00000121644	ENSG00000090097	ENSG00000177303	ENSG00000100601
ENSG00000161202	ENSG00000146648	ENSG00000138698	ENSG00000165323
ENSG00000134222	ENSG00000054965	ENSG00000110237	ENSG00000172175
ENSG00000167615	ENSG00000165259	ENSG00000135116	ENSG00000139645
ENSG00000173914	ENSG00000106785	ENSG00000157353	ENSG00000102781
ENSG00000176444	ENSG00000176769	ENSG00000117408	ENSG00000140848
ENSG00000100243	ENSG00000113851	ENSG00000116497	ENSG00000169884
ENSG00000163931	ENSG00000145416	ENSG00000158987	ENSG00000188783
ENSG00000143013	ENSG00000113456	ENSG00000152291	ENSG00000145975
ENSG00000138735	ENSG00000102984	ENSG00000175592	ENSG00000144285
ENSG00000107562	ENSG00000119946	ENSG00000146540	ENSG00000164659
ENSG00000197872	ENSG00000162607	ENSG00000115145	ENSG00000126890
ENSG00000137460	ENSG00000163479	ENSG00000118482	ENSG00000143847
ENSG00000060982	ENSG00000125820	ENSG00000120053	ENSG00000000005
ENSG00000166189	ENSG00000102897	ENSG00000055917	ENSG00000171557
ENSG00000122643	ENSG00000158825	ENSG00000166912	ENSG00000170801
ENSG00000204256	ENSG00000125868	ENSG00000078081	ENSG00000166596
ENSG00000112984	ENSG00000183808	ENSG00000085449	ENSG00000166173
ENSG00000162104	ENSG00000031823	ENSG00000135926	ENSG00000130803
ENSG00000100442	ENSG00000177272	ENSG00000157890	ENSG00000178498

ENSG00000114742	ENSG00000171532	ENSG00000145246	ENSG00000198092
ENSG00000107854	ENSG00000124762	ENSG00000108799	ENSG00000166349
ENSG00000105767	ENSG00000137878	ENSG00000156140	ENSG00000076382
ENSG00000162761	ENSG00000063601	ENSG00000188177	ENSG00000076984
ENSG00000172845	ENSG00000156162	ENSG00000156427	ENSG00000204920
ENSG00000110195	ENSG00000141750	ENSG00000164070	ENSG00000197891
ENSG00000168672	ENSG00000196781	ENSG00000136816	ENSG00000144962
ENSG00000187741	ENSG00000169760	ENSG00000120539	ENSG00000151353
ENSG00000103569	ENSG00000188176	ENSG00000175215	ENSG00000109519
ENSG00000064989	ENSG00000136999	ENSG00000204335	ENSG00000169252
ENSG00000116649	ENSG00000171574	ENSG00000073792	ENSG00000136478
ENSG00000180370	ENSG00000100239	ENSG00000177606	ENSG00000105227
ENSG00000073969	ENSG00000151229	ENSG00000147862	ENSG00000153879
ENSG00000167114	ENSG00000149926	ENSG00000033170	ENSG00000112699
ENSG00000184557	ENSG00000109320	ENSG00000198382	ENSG00000135525
ENSG00000105370	ENSG00000173930	ENSG00000181315	ENSG00000130305
ENSG00000132646	ENSG00000167775	ENSG00000144355	ENSG00000147183
ENSG00000164941	ENSG00000204310	ENSG00000170448	ENSG00000166794
ENSG00000114784	ENSG00000135503	ENSG00000109762	ENSG00000183908
ENSG00000118804	ENSG00000143178	ENSG00000108960	ENSG00000112640
ENSG00000143486	ENSG00000198018	ENSG00000132434	ENSG00000159596
ENSG00000100346	ENSG00000147416	ENSG00000197037	ENSG00000150048
ENSG00000125637	ENSG00000185053	ENSG00000038382	ENSG00000141349
ENSG00000157335	ENSG00000204694	ENSG00000130695	ENSG00000156671
ENSG00000127990	ENSG00000011376	ENSG00000009780	ENSG00000154096
ENSG00000155511	ENSG00000145725	ENSG00000171109	ENSG00000130202
ENSG00000154174	ENSG00000241839	ENSG00000122565	ENSG00000131242
ENSG00000149397	ENSG00000198863	ENSG00000162236	ENSG00000153575
ENSG00000176697	ENSG00000089356	ENSG00000157954	ENSG00000172250
ENSG00000083290	ENSG00000103174	ENSG00000114796	ENSG00000177106
ENSG00000187672	ENSG00000150394	ENSG00000125967	ENSG00000117984
ENSG00000133805	ENSG00000221823	ENSG00000113597	ENSG00000188938
ENSG00000136717	ENSG00000136854	ENSG00000166452	ENSG00000181649
ENSG00000172379	ENSG00000182909	ENSG00000117461	ENSG00000179833
ENSG00000113140	ENSG00000180628	ENSG00000073910	ENSG00000117280
ENSG00000205571	ENSG00000033178	ENSG00000168096	ENSG00000099385
ENSG00000136738	ENSG00000007341	ENSG00000136689	ENSG00000167772
ENSG00000149295	ENSG00000080854	ENSG00000188486	ENSG00000000938
ENSG00000138741	ENSG00000188021	ENSG00000073756	ENSG00000143079
ENSG00000182199	ENSG00000158006	ENSG00000152229	ENSG00000015413
ENSG00000163606	ENSG00000163249	ENSG00000182389	ENSG00000166913
ENSG00000170185	ENSG00000128203	ENSG00000179837	ENSG00000128335
ENSG00000164031	ENSG00000170881	ENSG00000106546	ENSG00000174446
ENSG00000138764	ENSG00000101306	ENSG00000160294	ENSG00000174915
ENSG00000171206	ENSG00000172738	ENSG00000176463	ENSG00000164008
ENSG00000168994	ENSG00000029363	ENSG00000113742	ENSG00000122367
ENSG00000109339	ENSG00000154001	ENSG00000175874	ENSG00000125861
ENSG00000173959	ENSG00000130544	ENSG00000135945	ENSG00000172081
ENSG00000100393	ENSG00000151067	ENSG00000004975	ENSG00000143473
ENSG00000135801	ENSG00000186493	ENSG00000029364	ENSG00000129437
ENSG00000110107	ENSG00000086544	ENSG00000155229	ENSG00000154059
ENSG00000166833	ENSG00000024526	ENSG00000152128	ENSG00000100241
ENSG00000169094	ENSG00000144040	ENSG00000104219	ENSG00000154262
ENSG00000100519	ENSG00000155754	ENSG00000164930	ENSG00000128710
ENSG00000119969	ENSG00000160999	ENSG00000160325	ENSG00000033122
ENSG00000107862	ENSG00000189334	ENSG00000004059	ENSG00000032219
ENSG00000118655	ENSG00000166206	ENSG00000152749	ENSG00000134072
ENSG00000156603	ENSG00000085365	ENSG00000009413	ENSG00000166136
ENSG00000122557	ENSG00000091127	ENSG00000168502	ENSG00000198598
ENSG00000196313	ENSG00000131795	ENSG00000133454	ENSG00000062822
ENSG00000187210	ENSG00000038427	ENSG00000100412	ENSG00000103266
ENSG00000083622	ENSG00000143258	ENSG00000153982	ENSG00000170577
ENSG00000186047	ENSG00000139514	ENSG00000149925	ENSG00000167904
ENSG00000124214	ENSG00000198105	ENSG00000115421	ENSG00000196748

ENSG00000166888	ENSG00000164151	ENSG00000136099	ENSG00000135744
ENSG00000141699	ENSG00000107819	ENSG00000137770	ENSG00000104921
ENSG00000156802	ENSG00000111554	ENSG00000110944	ENSG00000137077
ENSG00000156026	ENSG00000242616	ENSG00000136457	ENSG00000156234
ENSG00000158161	ENSG00000243232	ENSG00000183723	ENSG00000125848
ENSG00000154582	ENSG00000212747	ENSG00000182500	ENSG00000166260
ENSG00000004478	ENSG00000102007	ENSG00000172466	ENSG00000176531
ENSG00000143452	ENSG00000103494	ENSG00000182175	ENSG00000178217
ENSG00000154845	ENSG00000163833	ENSG00000103489	ENSG00000118160
ENSG00000137843	ENSG00000130254	ENSG00000166341	ENSG00000112851
ENSG00000152977	ENSG00000137166	ENSG00000133704	ENSG00000205420
ENSG00000197860	ENSG00000108830	ENSG00000145743	ENSG00000170584
ENSG00000108018	ENSG00000140279	ENSG00000057252	ENSG00000105220
ENSG00000133983	ENSG00000169282	ENSG00000001167	ENSG00000198951
ENSG00000126861	ENSG00000136378	ENSG00000124615	ENSG00000112137
ENSG00000197555	ENSG00000130244	ENSG00000134108	ENSG00000093000
ENSG00000136870	ENSG00000121900	ENSG00000185883	ENSG00000137185
ENSG00000099985	ENSG00000116668	ENSG00000147246	ENSG00000110075
ENSG00000168938	ENSG00000203784	ENSG00000110429	ENSG00000104731
ENSG00000107105	ENSG00000243414	ENSG00000121083	ENSG00000096063
ENSG00000144935	ENSG00000112333	ENSG00000198218	ENSG00000173451
ENSG00000115234	ENSG00000004866	ENSG00000059378	ENSG00000160336
ENSG00000178537	ENSG00000133794	ENSG00000005007	ENSG00000173456
ENSG00000159167	ENSG00000012822	ENSG00000124493	ENSG00000172780
ENSG00000172354	ENSG00000169604	ENSG00000152214	ENSG00000112531
ENSG00000102003	ENSG00000204970	ENSG00000132825	ENSG00000176244
ENSG00000014164	ENSG00000134851	ENSG00000157766	ENSG00000171951
ENSG00000105520	ENSG00000102804	ENSG00000196449	ENSG00000112335
ENSG00000205268	ENSG00000173077	ENSG00000092148	ENSG00000163634
ENSG00000110880	ENSG00000112232	ENSG00000117114	ENSG00000131931
ENSG00000137815	ENSG00000111049	ENSG00000138780	ENSG00000229809
ENSG00000090615	ENSG00000196428	ENSG00000163624	ENSG00000116254
ENSG00000185905	ENSG00000137409	ENSG00000083642	ENSG00000221838
ENSG00000079332	ENSG00000165685	ENSG00000189403	ENSG00000116745
ENSG00000164054	ENSG00000146700	ENSG00000172137	ENSG00000186340
ENSG00000182132	ENSG00000185624	ENSG00000170075	ENSG00000162728
ENSG00000163788	ENSG00000104522	ENSG00000143614	ENSG00000100299
ENSG00000204917	ENSG00000173933	ENSG00000184560	ENSG00000109736
ENSG00000173404	ENSG00000165120	ENSG00000182601	ENSG00000100336
ENSG00000137478	ENSG00000135605	ENSG00000205916	ENSG00000115756
ENSG00000124181	ENSG00000006210	ENSG00000137710	ENSG00000170956
ENSG00000164885	ENSG00000163348	ENSG00000130956	ENSG00000185298
ENSG00000186660	ENSG00000174255	ENSG00000111859	ENSG00000104537
ENSG00000155096	ENSG00000138030	ENSG00000164252	ENSG00000068308
ENSG00000141141	ENSG00000110934	ENSG00000157483	ENSG00000128578
ENSG00000137311	ENSG00000114735	ENSG00000179242	ENSG00000163754
ENSG00000135473	ENSG00000124641	ENSG00000174871	ENSG00000183087
ENSG00000108379	ENSG00000115364	ENSG00000147382	ENSG00000083635
ENSG00000165244	ENSG00000137941	ENSG00000116396	ENSG00000111325
ENSG00000128654	ENSG00000109189	ENSG00000109920	ENSG00000166340
ENSG00000106609	ENSG00000109046	ENSG00000106236	ENSG00000213029
ENSG00000165376	ENSG00000169032	ENSG00000154153	ENSG00000128802
ENSG00000108852	ENSG00000100034	ENSG00000107021	ENSG00000181873
ENSG00000148572	ENSG00000137265	ENSG00000181788	ENSG00000080845
ENSG00000099246	ENSG00000196182	ENSG00000171552	ENSG00000106004
ENSG00000138166	ENSG00000183207	ENSG00000157224	ENSG00000164253
ENSG00000166582	ENSG00000115758	ENSG00000007866	ENSG00000197888
ENSG00000164741	ENSG00000110435	ENSG00000176788	ENSG00000136929
ENSG00000186451	ENSG00000163288	ENSG00000169926	ENSG00000144481
ENSG00000101098	ENSG00000001631	ENSG00000106799	ENSG00000158480
ENSG00000186187	ENSG00000136802	ENSG00000111885	ENSG00000189164
ENSG00000114019	ENSG00000243279	ENSG00000185022	ENSG00000163534
ENSG00000139651	ENSG00000136940	ENSG00000141580	ENSG00000103534
ENSG00000131778	ENSG00000182197	ENSG00000140945	ENSG00000083312

ENSG00000003056	ENSG00000117632	ENSG00000132142	ENSG00000108828
ENSG00000153214	ENSG00000138641	ENSG00000083520	ENSG00000139187
ENSG00000163558	ENSG00000131746	ENSG00000112406	ENSG00000179918
ENSG00000138867	ENSG00000198822	ENSG00000154319	ENSG00000185345
ENSG00000173327	ENSG00000135250	ENSG00000186868	ENSG00000118900
ENSG00000158019	ENSG00000146828	ENSG00000138136	ENSG00000143434
ENSG00000183715	ENSG00000122729	ENSG00000123643	ENSG00000132286
ENSG00000150093	ENSG00000152332	ENSG00000127314	ENSG00000005436
ENSG00000175084	ENSG00000166342	ENSG00000158092	ENSG00000128645
ENSG00000138315	ENSG00000160310	ENSG00000165406	ENSG00000002834
ENSG00000111252	ENSG00000128596	ENSG00000141232	ENSG00000106683
ENSG00000187191	ENSG00000129244	ENSG00000152192	ENSG00000118894
ENSG00000185751	ENSG00000198812	ENSG00000135597	ENSG00000181552
ENSG00000141040	ENSG00000121297	ENSG00000187498	ENSG00000106460
ENSG00000168610	ENSG00000080644	ENSG00000137497	ENSG00000119139
ENSG00000069399	ENSG00000101210	ENSG00000102780	ENSG00000158796
ENSG00000139433	ENSG00000185924	ENSG00000110108	ENSG00000158457
ENSG00000169490	ENSG00000204406	ENSG00000158201	ENSG00000129422
ENSG00000170745	ENSG00000136231	ENSG00000183580	ENSG00000111261
ENSG00000108179	ENSG00000169764	ENSG00000160179	ENSG00000164338
ENSG00000168439	ENSG00000049319	ENSG00000128016	ENSG00000123106
ENSG00000174306	ENSG00000135018	ENSG00000204262	ENSG00000090006
ENSG00000157106	ENSG00000146038	ENSG00000103502	ENSG00000147576
ENSG00000116191	ENSG00000175782	ENSG00000170425	ENSG00000085185
ENSG00000130787	ENSG00000164879	ENSG00000072182	ENSG00000114541
ENSG00000116731	ENSG00000163462	ENSG00000179104	ENSG00000185504
ENSG00000187961	ENSG00000086712	ENSG00000122359	ENSG00000181634
ENSG00000176049	ENSG00000164938	ENSG00000102974	ENSG00000134853
ENSG00000133639	ENSG00000169860	ENSG00000187735	ENSG00000083544
ENSG00000149591	ENSG00000181856	ENSG00000088038	ENSG00000040731
ENSG00000173376	ENSG00000183722	ENSG00000166261	ENSG00000165511
ENSG00000153786	ENSG00000066739	ENSG00000130287	ENSG00000033327
ENSG00000123505	ENSG00000180861	ENSG00000072134	ENSG00000137070
ENSG00000179300	ENSG00000157193	ENSG00000170525	ENSG00000178764
ENSG00000141179	ENSG00000070018	ENSG00000151327	ENSG00000130313
ENSG00000155130	ENSG00000172469	ENSG00000091542	ENSG00000152894
ENSG00000111725	ENSG00000148200	ENSG00000115946	ENSG00000182580
ENSG00000177542	ENSG00000104904	ENSG00000067082	ENSG00000104899
ENSG00000136193	ENSG00000066044	ENSG00000117020	ENSG00000117472
ENSG00000132535	ENSG00000087495	ENSG00000169031	ENSG00000086730
ENSG00000165449	ENSG00000145192	ENSG00000120868	ENSG00000117000
ENSG00000130429	ENSG00000177666	ENSG00000157445	ENSG00000244038
ENSG00000110906	ENSG00000109881	ENSG00000116560	ENSG00000183091
ENSG00000144283	ENSG00000132031	ENSG00000142192	ENSG00000023041
ENSG00000174839	ENSG00000153823	ENSG00000168476	ENSG00000104365
ENSG00000176597	ENSG00000181789	ENSG00000147606	ENSG00000172728
ENSG00000167333	ENSG00000188487	ENSG00000187838	ENSG00000170906
ENSG00000157404	ENSG00000108424	ENSG00000108578	ENSG00000071991
ENSG00000183918	ENSG00000184939	ENSG00000158615	ENSG00000157680
ENSG00000023191	ENSG00000139826	ENSG00000167768	ENSG00000139428
ENSG00000151322	ENSG00000198081	ENSG00000187257	ENSG00000164715
ENSG00000102145	ENSG00000126562	ENSG00000106689	ENSG00000070610
ENSG00000147164	ENSG00000100647	ENSG00000072657	ENSG00000159658
ENSG00000091428	ENSG00000196549	ENSG00000147459	ENSG00000183072
ENSG00000099331	ENSG00000114251	ENSG00000196090	ENSG00000120686
ENSG00000128655	ENSG00000182481	ENSG00000106034	ENSG00000167216
ENSG00000105327	ENSG00000159692	ENSG00000147231	ENSG00000109586
ENSG00000105216	ENSG00000107736	ENSG00000133874	ENSG00000118960
ENSG00000104415	ENSG00000139725	ENSG00000135097	ENSG00000133105
ENSG00000132357	ENSG00000141639	ENSG00000131002	ENSG00000125945
ENSG00000143443	ENSG00000143878	ENSG00000143776	ENSG00000106633
ENSG00000158941	ENSG00000198183	ENSG00000185896	ENSG00000077279
ENSG00000071246	ENSG00000133636	ENSG00000146374	ENSG00000074319
ENSG00000160932	ENSG00000160190	ENSG00000129654	ENSG00000196091

ENSG00000178662	ENSG00000181467	ENSG00000110675	ENSG00000143061
ENSG00000004534	ENSG00000198162	ENSG00000157654	ENSG00000198883
ENSG00000125818	ENSG00000076351	ENSG00000158865	ENSG00000244752
ENSG00000135486	ENSG00000095564	ENSG00000062485	ENSG00000118689
ENSG00000153807	ENSG00000185760	ENSG00000143603	ENSG00000147689
ENSG00000169762	ENSG00000176658	ENSG00000154645	ENSG00000137959
ENSG00000057608	ENSG00000116017	ENSG00000177479	ENSG00000188994
ENSG00000178053	ENSG00000089177	ENSG00000114268	ENSG00000162734
ENSG00000126746	ENSG00000130222	ENSG00000128585	ENSG00000101916
ENSG00000100697	ENSG00000100664	ENSG00000010361	ENSG00000118503
ENSG00000111711	ENSG00000105877	ENSG00000115363	ENSG00000120896
ENSG00000163520	ENSG00000171551	ENSG00000012232	ENSG00000158169
ENSG00000158636	ENSG00000124920	ENSG00000100105	ENSG00000141380
ENSG00000149418	ENSG00000067177	ENSG00000175984	ENSG00000100726
ENSG00000112320	ENSG00000125629	ENSG00000100003	ENSG00000108349
ENSG00000143153	ENSG00000109107	ENSG00000155827	ENSG00000057294
ENSG00000185985	ENSG00000099917	ENSG00000115507	ENSG00000117143
ENSG00000077684	ENSG00000181982	ENSG00000185787	ENSG00000185247
ENSG00000118007	ENSG00000135048	ENSG00000160710	ENSG00000077150
ENSG00000158258	ENSG00000159363	ENSG00000064309	ENSG00000197971
ENSG00000114948	ENSG00000162971	ENSG00000138823	ENSG00000182541
ENSG00000164331	ENSG00000053702	ENSG00000076067	ENSG00000146197
ENSG00000117122	ENSG00000168661	ENSG00000175826	ENSG00000162413
ENSG00000124205	ENSG00000124193	ENSG00000176102	ENSG00000108231
ENSG00000124785	ENSG00000011009	ENSG00000155729	ENSG00000120647
ENSG00000176204	ENSG00000166471	ENSG00000106701	ENSG00000171161
ENSG00000106803	ENSG00000144118	ENSG00000056345	ENSG00000137198
ENSG00000158023	ENSG00000101346	ENSG00000144893	ENSG00000066248
ENSG00000149573	ENSG00000213672	ENSG00000116016	ENSG00000099995
ENSG00000099139	ENSG00000100078	ENSG00000175029	ENSG00000132953
ENSG00000156171	ENSG00000026652	ENSG00000182247	ENSG00000151062
ENSG00000151338	ENSG00000134698	ENSG00000158859	ENSG00000170291
ENSG00000198793	ENSG00000159263	ENSG00000120913	ENSG00000161647
ENSG00000184508	ENSG00000164251	ENSG00000156304	ENSG00000182324
ENSG00000086570	ENSG00000171873	ENSG00000164100	ENSG00000160305
ENSG00000183675	ENSG00000120742	ENSG00000116903	ENSG00000157064
ENSG00000153904	ENSG00000160007	ENSG00000173402	ENSG00000129562
ENSG00000115677	ENSG00000169398	ENSG00000198353	ENSG00000119650
ENSG00000119686	ENSG00000060656	ENSG00000029725	ENSG00000180233
ENSG00000117480	ENSG00000108176	ENSG00000136709	ENSG00000128917
ENSG00000145214	ENSG00000136830	ENSG00000146477	ENSG00000165730
ENSG00000143643	ENSG00000059804	ENSG00000114923	ENSG00000142156
ENSG00000071794	ENSG00000185658	ENSG00000164691	ENSG00000108344
ENSG00000092201	ENSG00000057704	ENSG00000137824	ENSG00000196497
ENSG00000120519	ENSG00000188483	ENSG00000183688	ENSG00000203837
ENSG00000141622	ENSG00000161048	ENSG00000038274	ENSG00000196689
ENSG00000100084	ENSG00000002919	ENSG00000072682	ENSG00000143994
ENSG00000162642	ENSG00000156030	ENSG00000187840	ENSG00000065675
ENSG00000130816	ENSG00000146457	ENSG00000111880	ENSG00000127080
ENSG00000156395	ENSG00000117155	ENSG00000075461	ENSG00000156500
ENSG00000105176	ENSG00000145734	ENSG00000114554	ENSG00000080546
ENSG00000122756	ENSG00000086200	ENSG00000004660	ENSG00000122692
ENSG00000163914	ENSG00000197409	ENSG00000054983	ENSG00000168310
ENSG00000073734	ENSG00000181704	ENSG00000104886	ENSG00000166750
ENSG00000152822	ENSG00000163013	ENSG00000107518	ENSG00000064195
ENSG00000204218	ENSG00000116717	ENSG00000198356	ENSG00000113575
ENSG00000196547	ENSG00000115525	ENSG00000095787	ENSG00000094631
ENSG00000168702	ENSG00000189079	ENSG00000198689	ENSG00000198573
ENSG00000147454	ENSG00000175175	ENSG00000196104	ENSG00000174016
ENSG00000205726	ENSG00000103021	ENSG00000164442	ENSG00000145391
ENSG00000132205	ENSG00000163235	ENSG00000185352	ENSG00000159202
ENSG00000100266	ENSG00000127325	ENSG00000144645	ENSG00000164048
ENSG00000198898	ENSG00000003096	ENSG00000165389	ENSG00000100417
ENSG00000132824	ENSG00000067208	ENSG00000140443	ENSG00000077522

ENSG00000134138	ENSG00000178974	ENSG00000204977	ENSG00000130528
ENSG00000108599	ENSG00000072501	ENSG00000124209	ENSG00000204561
ENSG00000082397	ENSG00000118762	ENSG00000169992	ENSG00000153250
ENSG00000151474	ENSG00000107443	ENSG00000182552	ENSG00000188293
ENSG00000161533	ENSG00000177465	ENSG00000137845	ENSG00000133256
ENSG00000076053	ENSG00000156017	ENSG00000136536	ENSG00000138101
ENSG00000186174	ENSG00000196358	ENSG00000168591	ENSG00000198788
ENSG00000078747	ENSG00000155980	ENSG00000108433	ENSG00000166902
ENSG00000196872	ENSG00000136826	ENSG00000100106	ENSG00000112116
ENSG00000112893	ENSG00000105976	ENSG00000101166	ENSG00000240563
ENSG00000135334	ENSG00000065135	ENSG00000158050	ENSG00000215455
ENSG00000160445	ENSG00000082213	ENSG00000211456	ENSG00000142230
ENSG00000184226	ENSG00000108688	ENSG00000140396	ENSG00000141431
ENSG00000143149	ENSG00000131389	ENSG00000107362	ENSG00000071859
ENSG00000127334	ENSG00000170871	ENSG00000131067	ENSG00000025796
ENSG00000164386	ENSG00000124120	ENSG00000122435	ENSG00000116774
ENSG00000089916	ENSG00000187939	ENSG00000145632	ENSG00000007933
ENSG00000128641	ENSG00000124302	ENSG00000163468	ENSG00000157734
ENSG00000105983	ENSG00000134070	ENSG00000011332	ENSG00000132570
ENSG00000053254	ENSG00000144381	ENSG00000143756	ENSG00000057757
ENSG00000151640	ENSG00000173276	ENSG00000172795	ENSG00000170791
ENSG00000035499	ENSG00000054654	ENSG00000154736	ENSG00000011083
ENSG00000080839	ENSG00000180287	ENSG00000105438	ENSG00000211445
ENSG00000162337	ENSG00000119541	ENSG00000102290	ENSG00000100749
ENSG00000062598	ENSG00000162989	ENSG00000171307	ENSG00000164363
ENSG00000146670	ENSG00000213023	ENSG00000196338	ENSG00000101082
ENSG00000112511	ENSG00000197713	ENSG00000100596	ENSG00000131759
ENSG00000104859	ENSG00000160233	ENSG00000140873	ENSG00000126752
ENSG00000167634	ENSG00000143842	ENSG00000147457	ENSG00000140950
ENSG00000113013	ENSG00000119927	ENSG00000138829	ENSG00000196470
ENSG00000170276	ENSG00000157895	ENSG00000119231	ENSG00000177731
ENSG00000141198	ENSG00000118193	ENSG00000099889	ENSG00000162694
ENSG00000166900	ENSG00000183763	ENSG00000099942	ENSG00000125826
ENSG00000068831	ENSG00000109606	ENSG00000110274	ENSG00000171928
ENSG00000152430	ENSG00000164889	ENSG00000158406	ENSG00000112837
ENSG00000171189	ENSG00000170498	ENSG00000180772	ENSG00000056661
ENSG00000179241	ENSG00000007402	ENSG00000130584	ENSG00000073712
ENSG00000105968	ENSG00000173273	ENSG00000140575	ENSG00000184205
ENSG00000120837	ENSG00000133195	ENSG00000072952	ENSG00000162723
ENSG00000170260	ENSG00000103512	ENSG00000177432	ENSG00000154269
ENSG00000170836	ENSG00000172613	ENSG00000165886	ENSG00000152527
ENSG00000172818	ENSG00000173171	ENSG00000178104	ENSG00000119782
ENSG00000113196	ENSG00000139910	ENSG00000136014	ENSG00000106012
ENSG00000178694	ENSG00000113645	ENSG00000163214	ENSG00000140740
ENSG00000146166	ENSG00000196562	ENSG00000143815	ENSG00000100324
ENSG00000118922	ENSG00000213658	ENSG00000113282	ENSG00000126351
ENSG00000198821	ENSG00000073464	ENSG00000100348	ENSG00000183844
ENSG00000132670	ENSG00000205560	ENSG00000088305	ENSG00000149201
ENSG00000177627	ENSG00000188582	ENSG00000172404	ENSG00000164638
ENSG00000197818	ENSG00000143515	ENSG00000101057	ENSG00000167711
ENSG00000148943	ENSG00000143761	ENSG00000114744	ENSG00000165553
ENSG00000104812	ENSG00000070404	ENSG00000131462	ENSG00000164265
ENSG00000088832	ENSG00000108312	ENSG00000113657	ENSG00000122679
ENSG00000047410	ENSG00000006125	ENSG00000135074	ENSG00000151692
ENSG00000171560	ENSG00000109062	ENSG00000007545	ENSG00000176619
ENSG00000100884	ENSG00000128266	ENSG00000176783	ENSG00000205097
ENSG00000112977	ENSG00000241852	ENSG00000170647	ENSG00000074201
ENSG00000124782	ENSG00000164161	ENSG00000103035	ENSG00000188033
ENSG00000181798	ENSG00000105369	ENSG00000154359	ENSG00000143575
ENSG00000137709	ENSG00000104154	ENSG00000135535	ENSG00000131808
ENSG00000185386	ENSG00000185621	ENSG00000140983	ENSG00000167987
ENSG00000144339	ENSG00000015592	ENSG00000176407	ENSG00000135838
ENSG00000179583	ENSG00000075711	ENSG00000172493	ENSG00000196967
ENSG00000169884	ENSG00000204952	ENSG00000144524	ENSG00000123329

ENSG00000188783	ENSG00000116747	ENSG00000157020	ENSG00000102076
ENSG00000145681	ENSG00000173801	ENSG00000006194	ENSG00000170365
ENSG00000147050	ENSG00000178202	ENSG00000171813	ENSG00000142173
ENSG00000164659	ENSG00000134243	ENSG00000162909	ENSG00000082497
ENSG00000167968	ENSG00000128714	ENSG00000177764	ENSG00000099910
ENSG00000011638	ENSG00000134825	ENSG00000185585	ENSG00000102890
ENSG00000143933	ENSG00000066422	ENSG00000177000	ENSG00000163930
ENSG00000152193	ENSG00000126903	ENSG00000113327	ENSG00000130939
ENSG00000171885	ENSG00000088881	ENSG00000186998	ENSG00000155959
ENSG00000171132	ENSG00000067064	ENSG00000141429	ENSG00000134900
ENSG00000178498	ENSG00000205683	ENSG00000152944	ENSG00000134030
ENSG00000198092	ENSG00000156575	ENSG00000147010	ENSG00000146592
ENSG00000166349	ENSG00000025772	ENSG00000130294	ENSG00000104805
ENSG00000184916	ENSG00000128567	ENSG00000138468	ENSG00000084733
ENSG00000163113	ENSG00000127561	ENSG00000164219	ENSG00000147383
ENSG00000015532	ENSG00000197177	ENSG00000168395	ENSG00000163825
ENSG00000147507	ENSG00000146576	ENSG00000134762	ENSG00000196188
ENSG00000120733	ENSG00000115355	ENSG00000154380	ENSG00000125780
ENSG00000169252	ENSG00000196331	ENSG00000133116	ENSG00000134389
ENSG00000136478	ENSG00000169131	ENSG00000177917	ENSG00000047597
ENSG00000116132	ENSG00000089682	ENSG00000160551	ENSG00000020256
ENSG00000105227	ENSG00000205629	ENSG00000134531	ENSG00000178685
ENSG00000186908	ENSG00000174132	ENSG00000135363	ENSG00000183878
ENSG00000189120	ENSG00000172794	ENSG00000070882	ENSG00000178075
ENSG00000109458	ENSG00000117305	ENSG00000116001	ENSG00000132563
ENSG00000153879	ENSG00000069998	ENSG00000106948	ENSG00000181274
ENSG00000135525	ENSG00000108854	ENSG00000173540	ENSG00000144681
ENSG00000152256	ENSG00000112290	ENSG00000138617	ENSG00000170915
ENSG00000166794	ENSG00000164379	ENSG00000143494	ENSG00000154719
ENSG00000131475	ENSG00000134202	ENSG00000025434	ENSG00000198466
ENSG00000171988	ENSG00000149575	ENSG00000187555	ENSG00000138834
ENSG00000183908	ENSG00000009765	ENSG00000100605	ENSG00000117751
ENSG00000140598	ENSG00000181163	ENSG00000165424	ENSG00000151552
ENSG00000169375	ENSG00000160469	ENSG00000120458	ENSG00000151164
ENSG00000112640	ENSG00000048649	ENSG00000141503	ENSG00000153832
ENSG00000165637	ENSG00000141469	ENSG00000106078	ENSG00000136110
ENSG00000150048	ENSG00000078018	ENSG00000124571	ENSG00000140092
ENSG00000125733	ENSG00000064313	ENSG00000081377	ENSG00000163867
ENSG00000164418	ENSG00000089723	ENSG00000171456	ENSG00000144730
ENSG00000163376	ENSG00000089250	ENSG00000174950	ENSG00000122386
ENSG00000111642	ENSG00000161956	ENSG00000120254	ENSG00000146707
ENSG00000156671	ENSG00000188778	ENSG00000109943	ENSG00000070770
ENSG00000100141	ENSG00000178761	ENSG00000106692	ENSG00000130711
ENSG00000088247	ENSG00000178732	ENSG00000132334	ENSG00000126432
ENSG00000197948	ENSG00000198589	ENSG00000134986	ENSG00000005448
ENSG00000102024	ENSG00000144644	ENSG00000099940	ENSG00000136146
ENSG00000131242	ENSG00000143786	ENSG00000008300	ENSG00000107742
ENSG00000049247	ENSG00000138821	ENSG00000162981	ENSG00000169876
ENSG00000145723	ENSG00000183785	ENSG00000100065	ENSG00000105607
ENSG00000136485	ENSG00000165832	ENSG00000154118	ENSG00000213859
ENSG00000177106	ENSG00000125249	ENSG00000130224	ENSG00000105722
ENSG00000081041	ENSG00000103043	ENSG00000163874	ENSG00000170248
ENSG00000078140	ENSG00000172943	ENSG00000111783	ENSG00000101441
ENSG00000197622	ENSG00000114861	ENSG00000148737	ENSG00000066777
ENSG00000197451	ENSG00000114316	ENSG00000197798	ENSG00000180176
ENSG00000197183	ENSG00000138594	ENSG00000102100	ENSG00000125351
ENSG00000127955	ENSG00000213853	ENSG00000158467	ENSG00000023608
ENSG00000078403	ENSG00000116213	ENSG00000142669	ENSG00000091181
ENSG00000104375	ENSG00000129173	ENSG00000187764	ENSG00000136758
ENSG00000143079	ENSG00000174963	ENSG00000198900	ENSG00000157014
ENSG00000167772	ENSG00000139737	ENSG00000184845	ENSG00000105088
ENSG00000130779	ENSG00000119684	ENSG00000108107	ENSG00000158747
ENSG00000166913	ENSG00000128245	ENSG00000015479	ENSG00000185885
ENSG00000135655	ENSG00000179094	ENSG00000133056	ENSG00000105974

ENSG00000111142	ENSG00000185046	ENSG00000071127	ENSG00000159261
ENSG00000154640	ENSG00000170903	ENSG00000167996	ENSG00000101825
ENSG00000178836	ENSG00000079435	ENSG00000169180	ENSG00000167757
ENSG00000122367	ENSG00000056558	ENSG00000126003	ENSG00000183395
ENSG00000088930	ENSG00000131148	ENSG00000064419	ENSG00000161326
ENSG00000147955	ENSG00000102302	ENSG00000196586	ENSG00000221963
ENSG00000150630	ENSG00000119514	ENSG00000198894	ENSG00000132906
ENSG00000154059	ENSG00000204003	ENSG00000063169	ENSG00000101327
ENSG00000152969	ENSG00000167930	ENSG00000170743	ENSG00000133789
ENSG00000088538	ENSG00000109163	ENSG00000070756	ENSG00000115386
ENSG00000153037	ENSG00000122420	ENSG00000169895	ENSG00000126581
ENSG00000100241	ENSG00000177570	ENSG00000005882	ENSG00000160075
ENSG00000128710	ENSG00000120049	ENSG00000164171	ENSG00000136404
ENSG00000136997	ENSG00000253910	ENSG00000167257	ENSG00000176014
ENSG00000033122	ENSG00000137766	ENSG00000150995	ENSG00000205744
ENSG00000134072	ENSG00000119720	ENSG00000101079	ENSG00000144290
ENSG00000032219	ENSG00000151292	ENSG00000063978	ENSG00000080371
ENSG00000100100	ENSG00000013588	ENSG00000164045	ENSG00000103121
ENSG00000117308	ENSG00000176422	ENSG00000050767	ENSG00000179059
ENSG00000106524	ENSG00000129990	ENSG00000182901	ENSG00000169085
ENSG00000170577	ENSG00000244754	ENSG00000121039	ENSG00000163785
ENSG00000162670	ENSG00000105738	ENSG00000141756	ENSG00000141837
ENSG00000182831	ENSG00000170927	ENSG00000141446	ENSG00000118985
ENSG00000167904	ENSG00000197170	ENSG00000125434	ENSG00000125878
ENSG00000134283	ENSG00000129993	ENSG00000130758	ENSG00000168993
ENSG00000122483	ENSG00000123338	ENSG00000164061	ENSG00000154447
ENSG00000163689	ENSG00000149311	ENSG00000150594	ENSG00000164175
ENSG00000100124	ENSG00000087095	ENSG00000185340	ENSG00000124523
ENSG00000148660	ENSG00000054219	ENSG00000068697	ENSG00000180921
ENSG00000198752	ENSG00000152409	ENSG00000169855	ENSG00000009335
ENSG00000113721	ENSG00000126453	ENSG00000152661	ENSG00000188629
ENSG00000137077	ENSG00000137726	ENSG00000197712	ENSG00000163518
ENSG00000143787	ENSG00000185129	ENSG00000148248	ENSG00000161681
ENSG00000079805	ENSG00000149639	ENSG00000056998	ENSG00000177463
ENSG00000185634	ENSG00000149557	ENSG00000172113	ENSG00000206560
ENSG00000066117	ENSG00000124145	ENSG00000156103	ENSG00000181264
ENSG00000156234	ENSG00000112210	ENSG00000139372	ENSG00000104147
ENSG00000125848	ENSG00000142149	ENSG00000196361	ENSG00000124574
ENSG00000163947	ENSG00000168612	ENSG00000169220	ENSG00000115523
ENSG00000164040	ENSG00000033800	ENSG00000187664	ENSG00000163114
ENSG00000178217	ENSG00000028528	ENSG00000196277	ENSG00000039537
ENSG00000115884	ENSG00000163818	ENSG00000142634	ENSG00000145920
ENSG00000132170	ENSG00000113594	ENSG00000031691	ENSG00000198160
ENSG00000112851	ENSG00000102543	ENSG00000145365	ENSG00000103319
ENSG00000106771	ENSG00000128463	ENSG00000116406	ENSG00000058729
ENSG00000130829	ENSG00000108342	ENSG00000198873	ENSG00000099204
ENSG00000105220	ENSG00000180660	ENSG00000163069	ENSG00000149260
ENSG00000117643	ENSG00000124104	ENSG00000185052	ENSG00000144485
ENSG00000099783	ENSG00000104043	ENSG00000013523	ENSG00000002587
ENSG00000128389	ENSG00000182534	ENSG00000107897	ENSG00000196581
ENSG00000164506	ENSG00000171368	ENSG00000149485	ENSG00000166220
ENSG00000091039	ENSG00000109180	ENSG00000114626	ENSG00000171703
ENSG00000204655	ENSG00000142700	ENSG00000163872	ENSG00000241600
ENSG00000102606	ENSG00000142856	ENSG00000070444	ENSG00000139574
ENSG00000100320	ENSG00000164112	ENSG00000018189	ENSG00000198083
ENSG00000112137	ENSG00000186197	ENSG00000103034	ENSG00000187272
ENSG00000150776	ENSG00000134940	ENSG00000172590	ENSG00000136986
ENSG00000148400	ENSG00000133107	ENSG00000112773	ENSG00000091732
ENSG00000211450	ENSG00000253928	ENSG00000102081	ENSG00000166401
ENSG00000110075	ENSG00000163171	ENSG00000153046	ENSG00000175344
ENSG00000168477	ENSG00000177565	ENSG00000178074	ENSG00000100629
ENSG00000096063	ENSG00000196961	ENSG00000140299	ENSG00000123364
ENSG00000120708	ENSG00000149541	ENSG00000076201	ENSG00000111537
ENSG00000088899	ENSG00000204084	ENSG00000169682	ENSG00000185689

ENSG00000135643	ENSG00000156875	ENSG00000147100	ENSG00000164530
ENSG00000143924	ENSG00000132321	ENSG00000076513	ENSG00000183864
ENSG00000068796	ENSG00000136878	ENSG00000090372	ENSG00000166483
ENSG00000167754	ENSG00000101986	ENSG00000146021	ENSG00000078674
ENSG00000072135	ENSG00000184640	ENSG00000136152	ENSG00000135390
ENSG00000105443	ENSG00000174165	ENSG00000160685	ENSG00000168172
ENSG00000173456	ENSG00000127249	ENSG00000088448	ENSG00000066697
ENSG00000089693	ENSG00000188153	ENSG00000197771	ENSG00000165046
ENSG00000112531	ENSG00000006327	ENSG00000066468	ENSG00000188158
ENSG00000172780	ENSG00000213626	ENSG00000009694	ENSG00000095539
ENSG00000151287	ENSG00000087111	ENSG00000170604	ENSG00000077458
ENSG00000171951	ENSG00000143006	ENSG00000115350	ENSG00000129473
ENSG00000149474	ENSG00000101220	ENSG00000168488	ENSG00000154429
ENSG00000028277	ENSG00000178772	ENSG00000151623	ENSG00000105755
ENSG00000111481	ENSG00000007376	ENSG00000185811	ENSG00000002745
ENSG00000139289	ENSG00000166123	ENSG00000180957	ENSG00000188641
ENSG00000138709	ENSG00000163154	ENSG00000162433	ENSG00000198838
ENSG00000131931	ENSG00000133935	ENSG00000189184	ENSG00000134955
ENSG00000165495	ENSG00000086619	ENSG00000185818	ENSG00000167196
ENSG00000012963	ENSG00000008196	ENSG00000168843	ENSG00000117360
ENSG00000141644	ENSG00000047056	ENSG00000114686	ENSG00000185966
ENSG00000186340	ENSG00000088387	ENSG00000124882	ENSG00000150782
ENSG00000115756	ENSG00000106635	ENSG00000178726	ENSG00000120251
ENSG00000106031	ENSG00000120948	ENSG00000100167	ENSG00000168818
ENSG00000174607	ENSG00000105997	ENSG00000147144	ENSG00000182168
ENSG00000181492	ENSG00000090905	ENSG00000169193	ENSG00000157884
ENSG00000150768	ENSG00000160410	ENSG00000173486	ENSG00000156453
ENSG00000151729	ENSG00000167306	ENSG00000124098	ENSG00000185274
ENSG00000107882	ENSG00000143768	ENSG00000137135	ENSG00000185112
ENSG00000068308	ENSG00000064225	ENSG00000116990	ENSG00000116580
ENSG00000165806	ENSG00000124701	ENSG00000134874	ENSG00000163950
ENSG00000163754	ENSG00000197153	ENSG00000116750	ENSG00000090889
ENSG00000084463	ENSG00000125503	ENSG00000120949	ENSG00000123342
ENSG00000181588	ENSG00000166619	ENSG00000204564	ENSG00000197702
ENSG00000198408	ENSG00000113387	ENSG00000112561	ENSG00000121064
ENSG00000115241	ENSG00000132692	ENSG00000087916	ENSG00000197147
ENSG00000164778	ENSG00000131398	ENSG00000189159	ENSG00000170558
ENSG00000166340	ENSG00000107290	ENSG00000178802	ENSG00000149651
ENSG00000089558	ENSG00000130227	ENSG00000113761	ENSG00000178522
ENSG00000108443	ENSG00000170370	ENSG00000100433	ENSG00000186106
ENSG00000110975	ENSG00000062038	ENSG00000120688	ENSG00000117724
ENSG00000080845	ENSG00000158435	ENSG00000198477	ENSG00000130540
ENSG00000076650	ENSG00000101843	ENSG00000134755	ENSG00000139985
ENSG00000106004	ENSG00000088826	ENSG00000149547	ENSG00000078967
ENSG00000166323	ENSG00000132639	ENSG00000090470	ENSG00000138316
ENSG00000104290	ENSG00000063587	ENSG00000171119	ENSG00000169871
ENSG00000133818	ENSG00000133138	ENSG00000131016	ENSG00000198198
ENSG00000185670	ENSG00000170043	ENSG00000103044	ENSG00000105198
ENSG00000158480	ENSG00000078804	ENSG00000175426	ENSG00000197711
ENSG00000146373	ENSG00000145721	ENSG00000150051	ENSG00000132676
ENSG00000108582	ENSG00000198768	ENSG00000162344	ENSG00000119729
ENSG00000100234	ENSG00000187736	ENSG00000103449	ENSG00000108819
ENSG00000173511	ENSG00000074054	ENSG00000155961	ENSG00000138942
ENSG00000083312	ENSG00000149257	ENSG00000151914	ENSG00000129625
ENSG00000166446	ENSG00000131504	ENSG00000131149	ENSG00000183888
ENSG00000108828	ENSG00000069011	ENSG00000116641	ENSG00000135913
ENSG00000124253	ENSG00000160013	ENSG00000112796	ENSG00000121766
ENSG00000162650	ENSG00000135750	ENSG00000136928	ENSG00000159140
ENSG00000179148	ENSG00000177096	ENSG00000171169	ENSG00000025770
ENSG00000179918	ENSG00000101849	ENSG00000065911	ENSG00000112425
ENSG00000165997	ENSG00000128602	ENSG00000138814	ENSG00000170390
ENSG00000137161	ENSG00000006283	ENSG00000197614	ENSG00000110852
ENSG00000118900	ENSG00000135919	ENSG00000158246	ENSG00000198169
ENSG00000110172	ENSG00000106780	ENSG00000115107	ENSG00000145423

ENSG00000168701	ENSG00000120925	ENSG00000102034	ENSG00000226492
ENSG00000132286	ENSG00000204962	ENSG00000119699	ENSG00000182223
ENSG00000143434	ENSG00000141367	ENSG00000111432	ENSG00000182256
ENSG00000111907	ENSG00000134452	ENSG00000186575	ENSG00000222014
ENSG00000128645	ENSG00000182450	ENSG00000165410	ENSG00000160789
ENSG00000002834	ENSG00000213213	ENSG00000110851	ENSG00000058804
ENSG00000106683	ENSG00000176845	ENSG00000148948	ENSG00000111361
ENSG00000187239	ENSG00000186432	ENSG00000116489	ENSG00000117758
ENSG00000106336	ENSG00000143507	ENSG00000085063	ENSG00000131748
ENSG00000128918	ENSG00000122786	ENSG00000113108	ENSG00000150086
ENSG00000106460	ENSG00000166405	ENSG00000149798	ENSG00000127616
ENSG00000072858	ENSG00000107954	ENSG00000110048	ENSG00000003989
ENSG00000158457	ENSG00000167244	ENSG00000104490	ENSG00000070808
ENSG00000160679	ENSG00000213901	ENSG00000120075	ENSG00000119041
ENSG00000158796	ENSG00000131437	ENSG00000166407	ENSG00000108591
ENSG00000043822	ENSG00000177370	ENSG00000130165	ENSG00000143341
ENSG00000197226	ENSG00000154274	ENSG00000101189	ENSG00000131042
ENSG00000129422	ENSG00000070495	ENSG00000163817	ENSG00000150275
ENSG00000133030	ENSG00000158710	ENSG00000147548	ENSG00000163251
ENSG00000177888	ENSG00000103005	ENSG00000196368	ENSG00000203667
ENSG00000167680	ENSG00000184408	ENSG00000140044	ENSG00000147003
ENSG00000072201	ENSG00000058673	ENSG00000127863	ENSG00000135407
ENSG00000198053	ENSG00000133134	ENSG00000147251	ENSG00000124171
ENSG00000204713	ENSG00000091128	ENSG00000133216	ENSG00000139132
ENSG00000049449	ENSG00000159267	ENSG00000119283	ENSG00000100294
ENSG00000085185	ENSG00000251380	ENSG00000148840	ENSG00000106565
ENSG00000143850	ENSG00000101126	ENSG00000123358	ENSG00000162951
ENSG00000185504	ENSG00000124333	ENSG00000100302	ENSG00000171302
ENSG00000157827	ENSG00000117394	ENSG00000119689	ENSG00000180929
ENSG00000134853	ENSG00000016402	ENSG00000137094	ENSG00000198355
ENSG00000166526	ENSG00000085741	ENSG00000146834	ENSG00000148090
ENSG00000139746	ENSG00000077080	ENSG00000120149	ENSG00000161958
ENSG00000104497	ENSG00000183242	ENSG00000144455	ENSG00000130766
ENSG00000092445	ENSG00000008197	ENSG00000138131	ENSG00000163040
ENSG00000121966	ENSG00000163131	ENSG00000101152	ENSG00000171711
ENSG00000040731	ENSG00000169967	ENSG00000106484	ENSG00000105698
ENSG00000170166	ENSG00000149256	ENSG00000122691	ENSG00000204488
ENSG00000033327	ENSG00000173436	ENSG00000168785	ENSG00000133069
ENSG00000135108	ENSG00000064490	ENSG00000163531	ENSG00000166415
ENSG00000178764	ENSG00000109971	ENSG00000111817	ENSG00000153303
ENSG00000089597	ENSG00000141298	ENSG00000101928	ENSG00000162643
ENSG00000134343	ENSG00000144119	ENSG00000163694	ENSG00000205758
ENSG00000164976	ENSG00000165566	ENSG00000189319	ENSG00000130338
ENSG00000182580	ENSG00000182872	ENSG00000136160	ENSG00000054179
ENSG00000117000	ENSG00000143032	ENSG00000135316	ENSG00000161714
ENSG00000113658	ENSG00000129566	ENSG00000168575	ENSG00000173641
ENSG00000176563	ENSG00000168259	ENSG00000132640	ENSG00000156873
ENSG00000095203	ENSG00000099864	ENSG00000176165	ENSG00000101412
ENSG00000005020	ENSG00000046647	ENSG00000025293	ENSG00000162062
ENSG00000123415	ENSG00000185088	ENSG00000168286	ENSG00000134020
ENSG00000023041	ENSG00000049245	ENSG00000109132	ENSG00000172534
ENSG00000104365	ENSG00000213015	ENSG00000119402	ENSG00000142864
ENSG00000112763	ENSG00000164077	ENSG00000162889	ENSG00000141562
ENSG00000159873	ENSG00000165527	ENSG00000165030	ENSG00000204370
ENSG00000172728	ENSG00000126500	ENSG00000072071	ENSG00000047936
ENSG00000116983	ENSG00000243709	ENSG00000172840	ENSG00000131374
ENSG00000157680	ENSG00000134250	ENSG00000148331	ENSG00000160917
ENSG00000164715	ENSG00000101842	ENSG00000116679	ENSG00000103056
ENSG00000070610	ENSG00000166111	ENSG00000075151	ENSG00000132688
ENSG00000005379	ENSG00000196664	ENSG00000185630	ENSG00000186832
ENSG00000085276	ENSG00000135148	ENSG00000165804	ENSG00000124508
ENSG00000120686	ENSG00000001630	ENSG00000112697	ENSG00000154814
ENSG00000109586	ENSG00000121067	ENSG00000132561	ENSG00000081818
ENSG00000205189	ENSG00000107338	ENSG00000101745	ENSG00000104081

ENSG00000069424	ENSG00000153560	ENSG00000134375	ENSG00000165383
ENSG00000102226	ENSG00000115425	ENSG00000117289	ENSG00000135636
ENSG00000117560	ENSG00000011260	ENSG00000169925	ENSG00000047621
ENSG00000204967	ENSG00000106070	ENSG00000075142	ENSG00000197106
ENSG00000133105	ENSG00000145390	ENSG00000148219	ENSG00000115414
ENSG00000197085	ENSG00000123607	ENSG00000136935	ENSG00000125816
ENSG00000139350	ENSG00000167191	ENSG00000100307	ENSG00000214827
ENSG00000100068	ENSG00000139182	ENSG00000179262	ENSG00000112297
ENSG00000145730	ENSG00000087086	ENSG00000163884	ENSG00000128298
ENSG00000125945	ENSG00000170296	ENSG00000028203	ENSG00000067057
ENSG00000183023	ENSG00000164663	ENSG00000110092	ENSG00000146063
ENSG00000196535	ENSG00000106541	ENSG00000102786	ENSG00000105726
ENSG00000176973	ENSG00000158186	ENSG00000204463	ENSG00000166925
ENSG00000077279	ENSG00000215712	ENSG00000120899	ENSG00000075275
ENSG00000135040	ENSG00000184007	ENSG00000146648	ENSG00000107831
ENSG00000074319	ENSG00000140564	ENSG00000104331	ENSG00000171843
ENSG00000196091	ENSG00000003249	ENSG00000124313	ENSG00000184348
ENSG00000012171	ENSG00000123636	ENSG00000198844	ENSG00000142623
ENSG00000147689	ENSG00000154642	ENSG00000176769	ENSG00000168137
ENSG00000118689	ENSG00000163704	ENSG00000137404	ENSG00000162745
ENSG00000205437	ENSG00000122824	ENSG00000080802	ENSG00000177700
ENSG00000174595	ENSG00000154556	ENSG00000145416	ENSG00000168291
ENSG00000162734	ENSG00000104332	ENSG00000102984	ENSG00000169181
ENSG00000146729	ENSG00000213533	ENSG00000163346	ENSG00000213064
ENSG00000101916	ENSG00000182149	ENSG00000115665	ENSG00000135392
ENSG00000101004	ENSG00000049540	ENSG00000162607	ENSG00000128849
ENSG00000120896	ENSG00000122335	ENSG00000119946	ENSG00000097046
ENSG00000118503	ENSG00000185127	ENSG00000175073	ENSG00000179862
ENSG00000111145	ENSG00000223380	ENSG00000163479	ENSG00000164129
ENSG00000204487	ENSG00000196712	ENSG00000125820	ENSG00000118873
ENSG00000145864	ENSG00000080815	ENSG00000102897	ENSG00000003137
ENSG00000158270	ENSG00000116922	ENSG00000144354	ENSG00000151726
ENSG00000141380	ENSG00000138758	ENSG00000147655	ENSG00000188064
ENSG00000108349	ENSG00000198729	ENSG00000138759	ENSG00000143751
ENSG00000197971	ENSG00000204175	ENSG00000186350	ENSG00000146910
ENSG00000011028	ENSG00000101323	ENSG00000031823	ENSG00000103426
ENSG00000146197	ENSG00000136352	ENSG00000181472	ENSG00000173908
ENSG00000164180	ENSG00000175348	ENSG00000177272	ENSG00000084090
ENSG00000182541	ENSG00000184611	ENSG00000171532	ENSG00000153294
ENSG00000108231	ENSG00000168924	ENSG00000124762	ENSG00000196576
ENSG00000110888	ENSG00000106571	ENSG00000137878	ENSG00000176623
ENSG00000174611	ENSG00000135269	ENSG00000101109	ENSG00000183709
ENSG00000161671	ENSG00000165338	ENSG00000156162	ENSG00000178021
ENSG00000066248	ENSG00000179083	ENSG00000141750	ENSG00000131089
ENSG00000132953	ENSG00000107581	ENSG00000164983	ENSG00000113790
ENSG00000152495	ENSG00000176381	ENSG00000041988	ENSG00000083750
ENSG00000182324	ENSG00000124486	ENSG00000196781	ENSG00000242110
ENSG00000135324	ENSG00000125505	ENSG00000169760	ENSG00000171314
ENSG00000160305	ENSG00000100296	ENSG00000136999	ENSG00000066557
ENSG00000157064	ENSG00000120129	ENSG00000188176	ENSG00000206190
ENSG00000110243	ENSG00000144711	ENSG00000096968	ENSG00000162772
ENSG00000159784	ENSG00000159335	ENSG00000100239	ENSG00000143590
ENSG00000162813	ENSG00000203727	ENSG00000151229	ENSG00000140090
ENSG00000005022	ENSG00000085872	ENSG00000149926	ENSG00000185483
ENSG00000186838	ENSG00000154864	ENSG00000109320	ENSG00000182645
ENSG00000180233	ENSG00000013503	ENSG00000173930	ENSG00000197136
ENSG00000128917	ENSG00000100147	ENSG00000204310	ENSG00000172543
ENSG00000181222	ENSG00000176274	ENSG00000167775	ENSG00000186468
ENSG00000108344	ENSG00000198046	ENSG00000100478	ENSG00000123689
ENSG00000090686	ENSG00000109158	ENSG00000135503	ENSG00000169857
ENSG00000103740	ENSG00000164742	ENSG00000183049	ENSG00000114956
ENSG00000111716	ENSG00000102962	ENSG00000169856	ENSG00000143851
ENSG00000137764	ENSG00000177508	ENSG00000105662	ENSG00000110171
ENSG00000198440	ENSG00000174953	ENSG00000147416	ENSG00000175445

ENSG00000080546	ENSG00000165282	ENSG00000198018	ENSG00000145777
ENSG00000168310	ENSG00000106025	ENSG00000185053	ENSG00000116761
ENSG00000166750	ENSG00000161381	ENSG00000185532	ENSG00000181191
ENSG00000064195	ENSG00000152601	ENSG00000075223	ENSG00000165583
ENSG00000133226	ENSG00000119725	ENSG00000129351	ENSG00000083457
ENSG00000113575	ENSG00000158805	ENSG00000174111	ENSG00000086991
ENSG00000153707	ENSG00000155926	ENSG00000124702	ENSG00000087365
ENSG00000109133	ENSG00000185149	ENSG00000125968	ENSG00000164099
ENSG00000145391	ENSG00000135740	ENSG00000198959	ENSG00000145703
ENSG00000159202	ENSG00000198901	ENSG00000145725	ENSG00000170485
ENSG00000100417	ENSG00000182263	ENSG00000183853	ENSG00000156535
ENSG00000023318	ENSG00000104369	ENSG00000125675	ENSG00000182795
ENSG00000152910	ENSG00000025039	ENSG00000089356	ENSG00000243477
ENSG00000188613	ENSG00000016082	ENSG00000175573	ENSG00000109452
ENSG00000153250	ENSG00000118513	ENSG00000103174	ENSG00000158470
ENSG00000134575	ENSG00000007312	ENSG00000136854	ENSG00000189042
ENSG00000138101	ENSG00000123600	ENSG00000180628	ENSG00000152954
ENSG00000126878	ENSG00000102230	ENSG00000033178	ENSG00000158813
ENSG00000130513	ENSG00000185070	ENSG00000007341	ENSG00000167617
ENSG00000204889	ENSG00000105135	ENSG00000189043	ENSG00000108528
ENSG00000204918	ENSG00000163466	ENSG00000142188	ENSG00000106588
ENSG00000186073	ENSG00000187175	ENSG00000188021	ENSG00000018625
ENSG00000116774	ENSG00000142347	ENSG00000066855	ENSG00000113552
ENSG00000025796	ENSG00000099954	ENSG00000167889	ENSG00000119508
ENSG00000179562	ENSG00000116147	ENSG00000163249	ENSG00000099968
ENSG00000164451	ENSG00000064999	ENSG00000081154	ENSG00000155816
ENSG00000170791	ENSG00000172216	ENSG00000114315	ENSG00000115541
ENSG00000143537	ENSG00000100711	ENSG00000113430	ENSG00000146233
ENSG00000135903	ENSG00000180138	ENSG00000170881	ENSG00000049769
ENSG00000211445	ENSG00000107807	ENSG00000101306	ENSG00000139117
ENSG00000132780	ENSG00000123989	ENSG00000029363	ENSG00000071537
ENSG00000104427	ENSG00000167778	ENSG00000136158	ENSG00000180198
ENSG00000131759	ENSG00000181291	ENSG00000154001	ENSG00000188042
ENSG00000115468	ENSG00000143466	ENSG00000151067	ENSG00000154945
ENSG00000196470	ENSG00000158458	ENSG00000186493	ENSG00000179774
ENSG00000162694	ENSG00000186834	ENSG00000086544	ENSG00000147650
ENSG00000203685	ENSG00000141448	ENSG00000184986	ENSG00000150593
ENSG00000112144	ENSG00000142920	ENSG00000144040	ENSG00000137571
ENSG00000040341	ENSG00000186687	ENSG00000115008	ENSG00000117335
ENSG00000196892	ENSG00000108100	ENSG00000181773	ENSG00000172716
ENSG00000151572	ENSG00000175662	ENSG00000176624	ENSG00000179593
ENSG00000116685	ENSG00000149932	ENSG00000166206	ENSG00000112706
ENSG00000056661	ENSG00000179455	ENSG00000085365	ENSG00000101448
ENSG00000116584	ENSG00000146414	ENSG00000176884	ENSG00000134376
ENSG00000073712	ENSG00000128268	ENSG00000091127	ENSG00000187475
ENSG00000184205	ENSG00000188807	ENSG00000038427	ENSG00000144115
ENSG00000152527	ENSG00000108753	ENSG00000070087	ENSG00000134323
ENSG00000074181	ENSG00000115738	ENSG00000138336	ENSG00000197635
ENSG00000143140	ENSG00000130741	ENSG00000118495	ENSG00000049089
ENSG00000159388	ENSG00000144228	ENSG00000034053	ENSG00000151025
ENSG00000108774	ENSG00000205927	ENSG00000143258	ENSG00000101203
ENSG00000140406	ENSG00000071626	ENSG00000139514	ENSG00000102316
ENSG00000189015	ENSG00000211455	ENSG00000072121	ENSG00000197217
ENSG00000126351	ENSG00000088827	ENSG00000198105	ENSG00000153207
ENSG00000065029	ENSG00000123353	ENSG00000111554	ENSG00000235109
ENSG00000131771	ENSG00000048405	ENSG00000167074	ENSG00000101282
ENSG00000107771	ENSG00000163811	ENSG00000102007	ENSG00000107341
ENSG00000165553	ENSG00000156269	ENSG00000008952	ENSG00000168385
ENSG00000070214	ENSG00000155256	ENSG00000137166	ENSG00000050327
ENSG00000008256	ENSG00000101076	ENSG00000140279	ENSG00000169221
ENSG00000106829	ENSG00000174137	ENSG00000169282	ENSG00000147124
ENSG00000151692	ENSG00000166411	ENSG00000168874	ENSG00000101138
ENSG00000074201	ENSG00000139190	ENSG00000136378	ENSG00000102445
ENSG00000103994	ENSG00000115419	ENSG00000121900	ENSG00000095383

ENSG00000154620	ENSG00000185344	ENSG00000116668	ENSG00000119401
ENSG00000170365	ENSG00000106397	ENSG00000112333	ENSG00000138698
ENSG00000118200	ENSG00000007168	ENSG00000167535	ENSG00000164867
ENSG00000142173	ENSG00000170191	ENSG00000127022	ENSG00000196705
ENSG00000181085	ENSG00000244462	ENSG00000186815	ENSG00000157353
ENSG00000082497	ENSG00000125255	ENSG00000168539	ENSG00000206292
ENSG00000174840	ENSG00000099365	ENSG00000133794	ENSG00000197050
ENSG00000141664	ENSG00000136122	ENSG00000198231	ENSG00000125046
ENSG00000187091	ENSG00000115295	ENSG00000012822	ENSG00000107554
ENSG00000164305	ENSG00000214872	ENSG00000095066	ENSG00000162194
ENSG00000130939	ENSG00000126768	ENSG00000169604	ENSG00000117408
ENSG00000163930	ENSG00000103653	ENSG00000075340	ENSG00000001497
ENSG00000155959	ENSG00000089289	ENSG00000112038	ENSG00000173535
ENSG00000134030	ENSG00000138592	ENSG00000204970	ENSG00000158987
ENSG00000134900	ENSG00000070614	ENSG00000165659	ENSG00000102981
ENSG00000065989	ENSG00000164604	ENSG00000102804	ENSG00000117410
ENSG00000197375	ENSG00000213047	ENSG00000104881	ENSG00000089050
ENSG00000146592	ENSG00000185278	ENSG00000134851	ENSG00000131966
ENSG00000084733	ENSG00000172350	ENSG00000124507	ENSG00000118482
ENSG00000171365	ENSG00000108439	ENSG00000145685	ENSG00000158525
ENSG00000026508	ENSG00000164609	ENSG00000112232	ENSG00000085449
ENSG00000125492	ENSG00000137273	ENSG00000111049	ENSG00000078081
ENSG00000174943	ENSG00000169057	ENSG00000198055	ENSG00000135926
ENSG00000163877	ENSG00000162599	ENSG00000137409	ENSG00000105655
ENSG00000161203	ENSG00000119411	ENSG00000196428	ENSG00000157890
ENSG00000125780	ENSG00000184678	ENSG00000167118	ENSG00000163491
ENSG00000047597	ENSG00000120784	ENSG00000168140	ENSG00000196344
ENSG00000204580	ENSG00000139998	ENSG00000126062	ENSG00000145246
ENSG00000163389	ENSG00000102218	ENSG00000175264	ENSG00000198663
ENSG00000101222	ENSG00000130299	ENSG00000135622	ENSG00000112664
ENSG00000178685	ENSG00000169896	ENSG00000165685	ENSG00000108799
ENSG00000183878	ENSG00000066336	ENSG00000146700	ENSG00000066185
ENSG00000157800	ENSG00000168453	ENSG00000185624	ENSG00000156140
ENSG00000204843	ENSG00000088812	ENSG00000117525	ENSG00000134538
ENSG00000119661	ENSG00000165195	ENSG00000091409	ENSG00000164070
ENSG00000144681	ENSG00000189056	ENSG00000092051	ENSG00000156427
ENSG00000170915	ENSG00000172201	ENSG00000153113	ENSG00000136816
ENSG00000050130	ENSG00000066583	ENSG00000006210	ENSG00000163071
ENSG00000003400	ENSG00000164440	ENSG00000067646	ENSG00000120539
ENSG00000138834	ENSG00000035403	ENSG00000185774	ENSG00000175215
ENSG00000117751	ENSG00000063438	ENSG00000163348	ENSG00000203813
ENSG00000185133	ENSG00000055813	ENSG00000186951	ENSG00000198382
ENSG00000151164	ENSG00000073350	ENSG00000134324	ENSG00000144355
ENSG00000159461	ENSG00000173221	ENSG00000137941	ENSG00000170448
ENSG00000149182	ENSG00000163935	ENSG00000109189	ENSG00000135346
ENSG00000061676	ENSG00000185825	ENSG00000157087	ENSG00000197037
ENSG00000140092	ENSG00000159792	ENSG00000109046	ENSG00000147654
ENSG00000136110	ENSG00000147162	ENSG00000169032	ENSG00000038382
ENSG00000103671	ENSG00000185650	ENSG00000137265	ENSG00000130695
ENSG00000186918	ENSG00000084764	ENSG00000100034	ENSG00000009780
ENSG00000163867	ENSG00000143458	ENSG00000159164	ENSG00000135002
ENSG00000197696	ENSG00000121769	ENSG00000060718	ENSG00000122565
ENSG00000144730	ENSG00000155858	ENSG00000074410	ENSG00000213516
ENSG00000142875	ENSG00000061987	ENSG00000181495	ENSG00000160867
ENSG00000204182	ENSG00000130635	ENSG00000196182	ENSG00000161677
ENSG00000162881	ENSG00000179889	ENSG00000153339	ENSG00000173627
ENSG00000161547	ENSG00000136295	ENSG00000110435	ENSG00000166452
ENSG00000070770	ENSG00000136279	ENSG00000106723	ENSG00000105321
ENSG00000130711	ENSG00000104722	ENSG00000001631	ENSG00000163159
ENSG00000126432	ENSG00000010404	ENSG00000136940	ENSG00000073910
ENSG00000162624	ENSG00000172375	ENSG00000136802	ENSG00000074621
ENSG00000068137	ENSG00000148358	ENSG00000182197	ENSG00000198400
ENSG00000107242	ENSG00000100987	ENSG00000117632	ENSG00000133067
ENSG00000107742	ENSG00000140403	ENSG00000073711	ENSG00000186517

ENSG00000176390	ENSG00000129292	ENSG00000115194	ENSG00000030066
ENSG00000065618	ENSG00000110514	ENSG00000107282	ENSG00000182389
ENSG00000105722	ENSG00000162148	ENSG00000198822	ENSG00000101938
ENSG00000179292	ENSG00000177732	ENSG00000176971	ENSG00000204174
ENSG00000143393	ENSG00000149428	ENSG00000083896	ENSG00000089057
ENSG00000066777	ENSG00000160408	ENSG00000125970	ENSG00000152611
ENSG00000023608	ENSG00000160683	ENSG00000131375	ENSG00000104889
ENSG00000043093	ENSG00000091656	ENSG00000049618	ENSG00000188171
ENSG00000115652	ENSG00000130699	ENSG00000152332	ENSG00000113742
ENSG00000166147	ENSG00000113263	ENSG00000166342	ENSG00000125845
ENSG00000143549	ENSG00000108406	ENSG00000075826	ENSG00000004975
ENSG00000168397	ENSG00000249992	ENSG00000197584	ENSG00000155229
ENSG00000119681	ENSG00000149923	ENSG00000141646	ENSG00000124875
ENSG00000184524	ENSG00000105707	ENSG00000128596	ENSG00000171759
ENSG00000158747	ENSG00000145819	ENSG00000165572	ENSG00000152128
ENSG00000105974	ENSG00000118242	ENSG00000129244	ENSG00000185332
ENSG00000101825	ENSG00000163067	ENSG00000121297	ENSG00000129270
ENSG00000161326	ENSG00000143093	ENSG00000010803	ENSG00000072041
ENSG00000121210	ENSG00000175595	ENSG00000107779	ENSG00000160325
ENSG00000160844	ENSG00000173546	ENSG00000186283	ENSG00000168502
ENSG00000116266	ENSG00000124466	ENSG00000136231	ENSG00000133454
ENSG00000126581	ENSG00000105643	ENSG00000204406	ENSG00000136099
ENSG00000179796	ENSG00000211448	ENSG00000169764	ENSG00000160323
ENSG00000113302	ENSG00000112592	ENSG00000135018	ENSG00000137700
ENSG00000090534	ENSG00000116754	ENSG00000104888	ENSG00000110944
ENSG00000146776	ENSG00000157796	ENSG00000146038	ENSG00000182175
ENSG00000184144	ENSG00000125124	ENSG00000166924	ENSG00000163382
ENSG00000176014	ENSG00000203852	ENSG00000160094	ENSG00000133028
ENSG00000101331	ENSG00000172461	ENSG00000140798	ENSG00000133704
ENSG00000163605	ENSG00000087338	ENSG00000123983	ENSG00000145743
ENSG00000144290	ENSG00000170242	ENSG00000164879	ENSG00000001167
ENSG00000080371	ENSG00000105695	ENSG00000172663	ENSG00000134108
ENSG00000130150	ENSG00000084754	ENSG00000163462	ENSG00000178690
ENSG00000112742	ENSG00000132382	ENSG00000086712	ENSG00000185883
ENSG00000171246	ENSG00000136381	ENSG00000164938	ENSG00000198920
ENSG00000148459	ENSG00000138035	ENSG00000181856	ENSG00000126790
ENSG00000169085	ENSG00000105967	ENSG00000126821	ENSG00000110429
ENSG00000127837	ENSG00000139668	ENSG00000103064	ENSG00000158517
ENSG00000168724	ENSG00000157240	ENSG00000183722	ENSG00000089902
ENSG00000162923	ENSG00000163406	ENSG00000066739	ENSG00000165633
ENSG00000163785	ENSG00000119537	ENSG00000138041	ENSG00000140379
ENSG00000125247	ENSG00000140382	ENSG00000157193	ENSG00000196449
ENSG00000116857	ENSG00000108604	ENSG00000142002	ENSG00000092148
ENSG00000196639	ENSG00000105948	ENSG00000070018	ENSG00000177981
ENSG00000130940	ENSG00000170374	ENSG00000081479	ENSG00000214882
ENSG00000114331	ENSG00000116095	ENSG00000165879	ENSG00000061918
ENSG00000118985	ENSG00000184260	ENSG00000148200	ENSG00000166828
ENSG00000168993	ENSG00000143409	ENSG00000019549	ENSG00000171467
ENSG00000117569	ENSG00000100997	ENSG00000066044	ENSG00000143367
ENSG00000154447	ENSG00000175893	ENSG00000087495	ENSG00000174939
ENSG00000178538	ENSG00000164736	ENSG00000163659	ENSG00000164304
ENSG00000173467	ENSG00000112305	ENSG00000132031	ENSG00000189403
ENSG00000122085	ENSG00000100505	ENSG00000196411	ENSG00000171241
ENSG00000164032	ENSG00000115207	ENSG00000170832	ENSG00000188315
ENSG00000133937	ENSG00000144674	ENSG00000119703	ENSG00000164089
ENSG00000177463	ENSG00000142949	ENSG00000153823	ENSG00000160695
ENSG00000161681	ENSG00000120088	ENSG00000181789	ENSG00000172137
ENSG00000198855	ENSG00000082438	ENSG00000108424	ENSG00000119636
ENSG00000206560	ENSG00000159592	ENSG00000174938	ENSG00000170075
ENSG00000144567	ENSG00000164808	ENSG00000167258	ENSG00000143614
ENSG00000124574	ENSG00000065883	ENSG00000180818	ENSG00000243130
ENSG00000115523	ENSG00000239264	ENSG00000108953	ENSG00000214160
ENSG00000135766	ENSG00000162461	ENSG00000139970	ENSG00000184560
ENSG00000198160	ENSG00000143379	ENSG00000168314	ENSG00000108292

ENSG00000103319	ENSG00000164649	ENSG00000172239	ENSG00000182601
ENSG00000065308	ENSG00000196132	ENSG00000139826	ENSG00000173163
ENSG00000149260	ENSG00000115694	ENSG00000180228	ENSG00000138297
ENSG00000142513	ENSG00000145244	ENSG00000171720	ENSG00000130956
ENSG00000099204	ENSG00000109787	ENSG00000044574	ENSG00000111859
ENSG00000114349	ENSG00000147224	ENSG00000114251	ENSG00000116703
ENSG00000131873	ENSG00000148925	ENSG00000196549	ENSG00000157483
ENSG00000002587	ENSG00000007202	ENSG00000182481	ENSG00000235568
ENSG00000196581	ENSG00000088756	ENSG00000157514	ENSG00000166448
ENSG00000141140	ENSG00000186354	ENSG00000188987	ENSG00000099381
ENSG00000131711	ENSG00000113384	ENSG00000107736	ENSG00000132361
ENSG00000136986	ENSG00000197429	ENSG00000143878	ENSG00000127423
ENSG00000178550	ENSG00000181541	ENSG00000141639	ENSG00000160396
ENSG00000091732	ENSG00000136261	ENSG00000164303	ENSG00000109920
ENSG00000125772	ENSG00000179922	ENSG00000067141	ENSG00000116396
ENSG00000206437	ENSG00000143970	ENSG00000151276	ENSG00000171155
ENSG00000123364	ENSG00000058668	ENSG00000198162	ENSG00000204648
ENSG00000111537	ENSG00000049323	ENSG00000095564	ENSG00000140939
ENSG00000185689	ENSG00000176641	ENSG00000116017	ENSG00000106236
ENSG00000166979	ENSG00000184232	ENSG00000176658	ENSG00000074964
ENSG00000103089	ENSG00000066056	ENSG00000185760	ENSG00000166800
ENSG00000164530	ENSG00000109861	ENSG00000073849	ENSG00000171566
ENSG00000124194	ENSG00000142892	ENSG00000089177	ENSG00000107021
ENSG00000120616	ENSG00000087263	ENSG00000100813	ENSG00000111640
ENSG00000117614	ENSG00000111224	ENSG00000100664	ENSG00000181929
ENSG00000166483	ENSG00000184613	ENSG00000125107	ENSG00000130347
ENSG00000130590	ENSG00000105426	ENSG00000136535	ENSG00000065802
ENSG00000135390	ENSG00000174238	ENSG00000165379	ENSG00000158604
ENSG00000168172	ENSG00000123240	ENSG00000124920	ENSG00000126705
ENSG00000180758	ENSG00000157765	ENSG00000125629	ENSG00000166450
ENSG00000066697	ENSG00000119979	ENSG00000119899	ENSG00000174099
ENSG00000196498	ENSG00000019995	ENSG00000099917	ENSG00000007866
ENSG00000006638	ENSG00000139163	ENSG00000135048	ENSG00000176788
ENSG00000175832	ENSG00000124788	ENSG00000048991	ENSG00000180638
ENSG00000095539	ENSG00000078369	ENSG00000116251	ENSG00000165309
ENSG00000188158	ENSG00000165072	ENSG00000159363	ENSG00000169926
ENSG00000154429	ENSG00000138138	ENSG00000124193	ENSG00000106799
ENSG00000077458	ENSG00000143632	ENSG00000011009	ENSG00000116791
ENSG00000129473	ENSG00000242265	ENSG00000161791	ENSG00000185022
ENSG00000166889	ENSG00000105401	ENSG00000164088	ENSG00000239886
ENSG00000166165	ENSG00000138587	ENSG00000144118	ENSG00000140945
ENSG00000187866	ENSG00000172059	ENSG00000162702	ENSG00000176018
ENSG00000124795	ENSG00000134057	ENSG00000101346	ENSG00000169908
ENSG00000138835	ENSG00000120992	ENSG00000135333	ENSG00000010438
ENSG00000188641	ENSG00000182782	ENSG00000100078	ENSG00000102271
ENSG00000177103	ENSG00000123307	ENSG00000026652	ENSG00000123643
ENSG00000134955	ENSG00000117713	ENSG00000159263	ENSG00000127314
ENSG00000198838	ENSG00000168298	ENSG00000100207	ENSG00000184985
ENSG00000167196	ENSG00000130985	ENSG00000134698	ENSG00000126775
ENSG00000170312	ENSG00000152520	ENSG00000169398	ENSG00000158092
ENSG00000143195	ENSG00000166780	ENSG00000060656	ENSG00000129226
ENSG00000101746	ENSG00000198570	ENSG00000120742	ENSG00000136696
ENSG00000156110	ENSG00000136827	ENSG00000136830	ENSG00000165406
ENSG00000154978	ENSG00000101445	ENSG00000059804	ENSG00000113722
ENSG00000120251	ENSG00000138670	ENSG00000185658	ENSG00000101447
ENSG00000182168	ENSG00000150625	ENSG00000057704	ENSG00000187634
ENSG00000091879	ENSG00000106852	ENSG00000188483	ENSG00000104969
ENSG00000112559	ENSG00000253767	ENSG00000187079	ENSG00000137497
ENSG00000156453	ENSG00000137522	ENSG00000161048	ENSG00000126106
ENSG00000163053	ENSG00000178184	ENSG00000019144	ENSG00000171448
ENSG00000185112	ENSG00000179152	ENSG00000156030	ENSG00000149308
ENSG00000123342	ENSG00000068383	ENSG00000141568	ENSG00000133742
ENSG00000163950	ENSG00000251664	ENSG00000075420	ENSG00000110108
ENSG00000167475	ENSG00000131482	ENSG00000184489	ENSG00000158201

ENSG00000129204	ENSG00000108423	ENSG00000146457	ENSG00000149792
ENSG00000196233	ENSG00000114648	ENSG00000181220	ENSG00000158769
ENSG00000170558	ENSG00000105219	ENSG00000117155	ENSG00000169692
ENSG00000178522	ENSG00000120162	ENSG00000139687	ENSG00000103707
ENSG00000175505	ENSG00000124491	ENSG00000086200	ENSG00000160179
ENSG00000095574	ENSG00000163513	ENSG00000181704	ENSG00000088854
ENSG00000130540	ENSG00000168734	ENSG00000086475	ENSG00000107281
ENSG00000196776	ENSG00000115365	ENSG00000116717	ENSG00000007216
ENSG00000136048	ENSG00000198948	ENSG00000115525	ENSG00000111843
ENSG00000205765	ENSG00000146376	ENSG00000167614	ENSG00000183434
ENSG00000176148	ENSG00000204086	ENSG00000104447	ENSG00000204262
ENSG00000185305	ENSG00000125944	ENSG00000155849	ENSG00000103502
ENSG00000076716	ENSG00000196504	ENSG00000175175	ENSG00000137207
ENSG00000066136	ENSG00000126016	ENSG00000161638	ENSG00000072182
ENSG00000156298	ENSG00000079277	ENSG00000163235	ENSG00000122359
ENSG00000160392	ENSG00000118473	ENSG00000108370	ENSG00000179104
ENSG00000166529	ENSG00000101558	ENSG00000122008	ENSG00000123191
ENSG00000205426	ENSG00000205423	ENSG00000189266	ENSG00000102974
ENSG00000138942	ENSG00000153310	ENSG00000171812	ENSG00000140505
ENSG00000121766	ENSG00000175707	ENSG00000150938	ENSG00000111906
ENSG00000135913	ENSG00000013619	ENSG00000067208	ENSG00000182040
ENSG00000159140	ENSG00000104728	ENSG00000174799	ENSG00000128928
ENSG00000078699	ENSG00000117013	ENSG00000109332	ENSG00000088038
ENSG00000008323	ENSG00000221949	ENSG00000118762	ENSG00000170266
ENSG00000172927	ENSG00000122025	ENSG00000072501	ENSG00000198203
ENSG00000112425	ENSG00000137507	ENSG00000107443	ENSG00000127125
ENSG00000118523	ENSG00000111249	ENSG00000111731	ENSG00000105708
ENSG00000149091	ENSG00000074219	ENSG00000101557	ENSG00000036565
ENSG00000164105	ENSG00000114999	ENSG00000165458	ENSG00000176490
ENSG00000110852	ENSG00000113083	ENSG00000196358	ENSG00000143624
ENSG00000198146	ENSG00000105281	ENSG00000168939	ENSG00000072134
ENSG00000115844	ENSG00000186652	ENSG00000182489	ENSG00000204983
ENSG00000160216	ENSG00000166263	ENSG00000177468	ENSG00000176383
ENSG00000145423	ENSG00000144357	ENSG00000152782	ENSG00000099812
ENSG00000144535	ENSG00000103429	ENSG00000171223	ENSG00000176225
ENSG00000125618	ENSG00000137073	ENSG00000158286	ENSG00000067082
ENSG00000160789	ENSG00000130309	ENSG00000155980	ENSG00000164744
ENSG00000071575	ENSG00000180801	ENSG00000121892	ENSG00000157445
ENSG00000179912	ENSG00000068305	ENSG00000136826	ENSG00000120868
ENSG00000058804	ENSG00000100364	ENSG00000105976	ENSG00000168374
ENSG00000064601	ENSG00000181031	ENSG00000065135	ENSG00000089116
ENSG00000029534	ENSG00000165349	ENSG00000132155	ENSG00000107317
ENSG00000114270	ENSG00000085832	ENSG00000136021	ENSG00000185730
ENSG00000117758	ENSG00000179449	ENSG00000108688	ENSG00000147465
ENSG00000126217	ENSG00000198846	ENSG00000131389	ENSG00000172717
ENSG00000114770	ENSG00000076706	ENSG00000196371	ENSG00000116478
ENSG00000104980	ENSG00000105223	ENSG00000116237	ENSG00000138160
ENSG00000150086	ENSG00000241878	ENSG00000101695	ENSG00000106327
ENSG00000127616	ENSG00000149231	ENSG00000124302	ENSG00000108578
ENSG00000070808	ENSG00000168243	ENSG00000007392	ENSG00000142655
ENSG00000166181	ENSG00000102125	ENSG00000144381	ENSG00000167768
ENSG00000130241	ENSG00000134259	ENSG00000173276	ENSG00000138075
ENSG00000143341	ENSG00000072832	ENSG00000147027	ENSG00000151892
ENSG00000120594	ENSG00000170515	ENSG00000074755	ENSG00000179934
ENSG00000160714	ENSG00000105656	ENSG00000054654	ENSG00000072657
ENSG00000151445	ENSG00000162366	ENSG00000180287	ENSG00000171345
ENSG00000169826	ENSG00000204590	ENSG00000119541	ENSG00000147459
ENSG00000086062	ENSG00000164283	ENSG00000108262	ENSG00000115687
ENSG00000147003	ENSG00000180891	ENSG00000162989	ENSG00000109610
ENSG00000145907	ENSG00000136631	ENSG00000115226	ENSG00000135097
ENSG00000139132	ENSG00000169105	ENSG00000094975	ENSG00000145375
ENSG00000100294	ENSG00000137872	ENSG00000158711	ENSG00000110675
ENSG00000196557	ENSG00000132463	ENSG00000197713	ENSG00000105583
ENSG00000129055	ENSG00000164953	ENSG00000106077	ENSG00000062485

ENSG00000198216	ENSG00000178177	ENSG00000143842	ENSG00000104814
ENSG00000182732	ENSG00000196876	ENSG00000140937	ENSG00000114268
ENSG00000162951	ENSG00000175097	ENSG00000119927	ENSG00000177455
ENSG00000141452	ENSG00000204673	ENSG00000107249	ENSG00000128585
ENSG00000148090	ENSG00000166860	ENSG00000006831	ENSG00000120942
ENSG00000161958	ENSG00000225830	ENSG00000078295	ENSG00000106819
ENSG00000163040	ENSG00000156587	ENSG00000163599	ENSG00000012232
ENSG00000105698	ENSG00000164011	ENSG00000109606	ENSG00000135517
ENSG00000152076	ENSG00000072210	ENSG00000136240	ENSG00000156475
ENSG00000168081	ENSG00000174059	ENSG00000007402	ENSG00000100003
ENSG00000143801	ENSG00000108797	ENSG00000133195	ENSG00000157150
ENSG00000124222	ENSG00000184602	ENSG00000122912	ENSG00000081237
ENSG00000100181	ENSG00000197024	ENSG00000151247	ENSG00000168916
ENSG00000138382	ENSG00000113734	ENSG00000103512	ENSG00000115507
ENSG00000130338	ENSG00000173214	ENSG00000173171	ENSG00000185787
ENSG00000100593	ENSG00000080822	ENSG00000139910	ENSG00000167562
ENSG00000186416	ENSG00000127951	ENSG00000113645	ENSG00000124444
ENSG00000205758	ENSG00000141542	ENSG00000119048	ENSG00000160710
ENSG00000173641	ENSG00000082196	ENSG00000188582	ENSG00000064309
ENSG00000186469	ENSG00000121236	ENSG00000021645	ENSG00000138823
ENSG00000156873	ENSG00000198908	ENSG00000049656	ENSG00000102524
ENSG00000177169	ENSG00000145808	ENSG00000143761	ENSG00000175826
ENSG00000101412	ENSG00000103710	ENSG00000181915	ENSG00000176102
ENSG00000147145	ENSG00000103495	ENSG00000109685	ENSG00000185669
ENSG00000134532	ENSG00000091986	ENSG00000109062	ENSG00000056345
ENSG00000142864	ENSG00000140691	ENSG00000006125	ENSG00000106701
ENSG00000156052	ENSG00000134001	ENSG00000108312	ENSG00000140319
ENSG00000172346	ENSG00000054938	ENSG00000165238	ENSG00000180846
ENSG00000139083	ENSG00000204969	ENSG00000128266	ENSG00000158859
ENSG00000108309	ENSG00000116161	ENSG00000139146	ENSG00000103150
ENSG00000160917	ENSG00000112339	ENSG00000135387	ENSG00000137673
ENSG00000121281	ENSG00000116574	ENSG00000164161	ENSG00000071243
ENSG00000103056	ENSG00000166228	ENSG00000137101	ENSG00000115596
ENSG00000006451	ENSG00000107968	ENSG00000133121	ENSG00000172262
ENSG00000155545	ENSG00000185477	ENSG00000170419	ENSG00000164100
ENSG00000111328	ENSG00000085511	ENSG00000164949	ENSG00000127527
ENSG00000104081	ENSG00000167397	ENSG00000104154	ENSG00000107186
ENSG00000102158	ENSG00000018510	ENSG00000015592	ENSG00000164691
ENSG00000047621	ENSG00000075391	ENSG00000116747	ENSG00000125363
ENSG00000105552	ENSG00000143862	ENSG00000173801	ENSG00000064607
ENSG00000115414	ENSG00000123572	ENSG00000119227	ENSG00000116350
ENSG00000146063	ENSG00000175470	ENSG00000134243	ENSG00000198431
ENSG00000166925	ENSG00000109079	ENSG00000128714	ENSG00000183579
ENSG00000106682	ENSG00000138347	ENSG00000079335	ENSG00000150750
ENSG00000075275	ENSG00000153162	ENSG00000134825	ENSG00000130508
ENSG00000171843	ENSG00000164627	ENSG00000107187	ENSG00000103196
ENSG00000111775	ENSG00000139116	ENSG00000168036	ENSG00000173692
ENSG00000035141	ENSG00000115825	ENSG00000066422	ENSG00000196767
ENSG00000177700	ENSG00000116874	ENSG00000126903	ENSG00000166816
ENSG00000168291	ENSG00000158717	ENSG00000066827	ENSG00000121274
ENSG00000164897	ENSG00000173812	ENSG00000205683	ENSG00000152213
ENSG00000172602	ENSG00000129187	ENSG00000112238	ENSG00000139540
ENSG00000135392	ENSG00000175395	ENSG00000164823	ENSG00000187840
ENSG00000072310	ENSG00000143570	ENSG00000127561	ENSG00000184436
ENSG00000128849	ENSG00000138639	ENSG00000128567	ENSG00000159455
ENSG00000012660	ENSG00000177519	ENSG00000162783	ENSG00000213977
ENSG00000152642	ENSG00000119922	ENSG00000115355	ENSG00000114450
ENSG00000173698	ENSG00000121481	ENSG00000103187	ENSG00000213930
ENSG00000092199	ENSG00000137776	ENSG00000169131	ENSG00000007314
ENSG00000169446	ENSG00000171954	ENSG00000198026	ENSG00000196406
ENSG00000179862	ENSG00000144452	ENSG00000163820	ENSG00000142515
ENSG00000118873	ENSG00000104381	ENSG00000172794	ENSG00000114554
ENSG00000003137	ENSG00000164258	ENSG00000160712	ENSG00000160202
ENSG00000102901	ENSG00000176871	ENSG00000069998	ENSG00000137040

ENSG00000155755	ENSG00000125834	ENSG00000170142	ENSG00000004660
ENSG00000148700	ENSG00000187109	ENSG00000108854	ENSG00000134184
ENSG00000151726	ENSG00000130226	ENSG00000112290	ENSG00000164841
ENSG00000156973	ENSG00000167325	ENSG00000164379	ENSG00000072694
ENSG00000109113	ENSG00000095015	ENSG00000149575	ENSG00000137364
ENSG00000103426	ENSG00000172399	ENSG00000168334	ENSG00000140488
ENSG00000073905	ENSG00000114529	ENSG00000119638	ENSG00000165496
ENSG00000146950	ENSG00000151704	ENSG00000117411	ENSG00000196104
ENSG00000136490	ENSG00000116337	ENSG00000198732	ENSG00000198178
ENSG00000100941	ENSG00000147724	ENSG00000135723	ENSG00000164442
ENSG00000075292	ENSG00000163735	ENSG00000147421	ENSG00000120889
ENSG00000113790	ENSG00000092068	ENSG00000181163	ENSG00000179041
ENSG00000180488	ENSG00000111321	ENSG00000160469	ENSG00000103404
ENSG00000171314	ENSG00000159459	ENSG00000129038	ENSG00000165389
ENSG00000184005	ENSG00000171777	ENSG00000065559	ENSG00000135972
ENSG00000206190	ENSG00000022567	ENSG00000048649	ENSG00000140443
ENSG00000162772	ENSG00000162551	ENSG00000141469	ENSG00000124209
ENSG00000148110	ENSG00000159650	ENSG00000123144	ENSG00000169992
ENSG00000004799	ENSG00000144852	ENSG00000078018	ENSG00000137845
ENSG00000100461	ENSG00000106089	ENSG00000153914	ENSG00000132874
ENSG00000010295	ENSG00000254245	ENSG00000077454	ENSG00000204844
ENSG00000140090	ENSG00000180008	ENSG00000076108	ENSG00000108433
ENSG00000143590	ENSG00000137944	ENSG00000089723	ENSG00000100106
ENSG00000185483	ENSG00000188488	ENSG00000166949	ENSG00000116918
ENSG00000110848	ENSG00000154914	ENSG00000185019	ENSG00000085871
ENSG00000123689	ENSG00000196743	ENSG00000161956	ENSG00000197701
ENSG00000183576	ENSG00000072315	ENSG00000188778	ENSG00000211456
ENSG00000163516	ENSG00000015153	ENSG00000178761	ENSG00000114446
ENSG00000104695	ENSG00000153012	ENSG00000182568	ENSG00000122435
ENSG00000163618	ENSG00000023909	ENSG00000135222	ENSG00000134779
ENSG00000110171	ENSG00000104863	ENSG00000100246	ENSG00000183309
ENSG00000175445	ENSG00000198925	ENSG00000198589	ENSG00000185418
ENSG00000135912	ENSG00000088992	ENSG00000165233	ENSG00000011332
ENSG00000157540	ENSG00000178718	ENSG00000132842	ENSG00000154736
ENSG00000125257	ENSG00000138696	ENSG00000103342	ENSG00000105438
ENSG00000114209	ENSG00000109919	ENSG00000138821	ENSG00000075539
ENSG00000086991	ENSG00000204923	ENSG00000165832	ENSG00000225697
ENSG00000141720	ENSG00000154832	ENSG00000170779	ENSG00000171307
ENSG00000145703	ENSG00000170522	ENSG00000107719	ENSG00000109686
ENSG00000156990	ENSG00000105568	ENSG00000145309	ENSG00000100596
ENSG00000170485	ENSG00000182985	ENSG00000125249	ENSG00000179023
ENSG00000182795	ENSG00000123066	ENSG00000086300	ENSG00000140873
ENSG00000158470	ENSG00000172830	ENSG00000117640	ENSG00000148346
ENSG00000185442	ENSG00000171056	ENSG00000172943	ENSG00000147457
ENSG00000060069	ENSG00000169750	ENSG00000114861	ENSG00000099889
ENSG00000101844	ENSG00000170854	ENSG00000169436	ENSG00000134193
ENSG00000166482	ENSG00000122870	ENSG00000181790	ENSG00000180772
ENSG00000121413	ENSG00000154764	ENSG00000138594	ENSG00000198954
ENSG00000152954	ENSG00000123411	ENSG00000129173	ENSG00000072952
ENSG00000158813	ENSG00000131899	ENSG00000174963	ENSG00000182004
ENSG00000196167	ENSG00000145687	ENSG00000139737	ENSG00000154079
ENSG00000059758	ENSG00000197417	ENSG00000128245	ENSG00000178104
ENSG00000167157	ENSG00000167971	ENSG00000179094	ENSG00000116690
ENSG00000025156	ENSG00000009950	ENSG00000100284	ENSG00000136014
ENSG00000148339	ENSG00000113812	ENSG00000154175	ENSG00000144029
ENSG00000018625	ENSG00000127124	ENSG00000185046	ENSG00000100348
ENSG00000106588	ENSG00000218891	ENSG00000017260	ENSG00000101057
ENSG00000107521	ENSG00000198142	ENSG00000198791	ENSG00000172404
ENSG00000119508	ENSG00000204682	ENSG00000102302	ENSG00000068976
ENSG00000112081	ENSG00000101997	ENSG00000173473	ENSG00000086061
ENSG00000099968	ENSG00000145476	ENSG00000167930	ENSG00000137100
ENSG00000109270	ENSG00000182195	ENSG00000196792	ENSG00000114744
ENSG00000115541	ENSG00000185519	ENSG00000122420	ENSG00000178921
ENSG00000146233	ENSG00000121897	ENSG00000166579	ENSG00000113657

ENSG00000139117	ENSG00000175550	ENSG00000089006	ENSG00000139629
ENSG00000164185	ENSG00000100060	ENSG00000157933	ENSG00000163485
ENSG00000095752	ENSG00000198824	ENSG00000120049	ENSG00000007545
ENSG00000071537	ENSG00000165724	ENSG00000183662	ENSG00000176783
ENSG00000180198	ENSG00000153201	ENSG00000115561	ENSG00000170647
ENSG00000188042	ENSG00000136869	ENSG00000151292	ENSG00000174721
ENSG00000172057	ENSG00000105647	ENSG00000013588	ENSG00000154359
ENSG00000179774	ENSG00000038532	ENSG00000176422	ENSG00000196812
ENSG00000154945	ENSG00000182771	ENSG00000008130	ENSG00000135535
ENSG00000150593	ENSG00000166478	ENSG00000105186	ENSG00000135506
ENSG00000147650	ENSG00000166289	ENSG00000153993	ENSG00000166948
ENSG00000102468	ENSG00000101134	ENSG00000197170	ENSG00000172493
ENSG00000137571	ENSG00000139131	ENSG00000198612	ENSG00000144524
ENSG00000180875	ENSG00000138675	ENSG00000100227	ENSG00000196227
ENSG00000178951	ENSG00000170961	ENSG00000129993	ENSG00000173610
ENSG00000181690	ENSG00000253485	ENSG00000184185	ENSG00000140368
ENSG00000144115	ENSG00000139547	ENSG00000110047	ENSG00000148735
ENSG00000136153	ENSG00000164512	ENSG00000154237	ENSG00000183048
ENSG00000197635	ENSG00000197386	ENSG00000140451	ENSG00000162909
ENSG00000134323	ENSG00000102858	ENSG00000149311	ENSG00000076003
ENSG00000197217	ENSG00000114779	ENSG00000164695	ENSG00000177764
ENSG00000188760	ENSG00000151617	ENSG00000087095	ENSG00000177000
ENSG00000168385	ENSG00000114423	ENSG00000126453	ENSG00000113327
ENSG00000107341	ENSG00000095587	ENSG00000115170	ENSG00000161921
ENSG00000171612	ENSG00000110066	ENSG00000185129	ENSG00000160180
ENSG00000169221	ENSG00000179456	ENSG00000137726	ENSG00000141429
ENSG00000068078	ENSG00000155962	ENSG00000149639	ENSG00000138468
ENSG00000157502	ENSG00000158773	ENSG00000151615	ENSG00000130294
ENSG00000102445	ENSG00000169184	ENSG00000166167	ENSG00000115252
ENSG00000177303	ENSG00000203772	ENSG00000124145	ENSG00000134762
ENSG00000138698	ENSG00000134769	ENSG00000142149	ENSG00000168395
ENSG00000119401	ENSG00000124198	ENSG00000112210	ENSG00000154380
ENSG00000110237	ENSG00000159714	ENSG00000028528	ENSG00000133116
ENSG00000135116	ENSG00000116128	ENSG00000033800	ENSG00000136783
ENSG00000157353	ENSG00000122591	ENSG00000198944	ENSG00000103507
ENSG00000117408	ENSG00000143375	ENSG00000174233	ENSG00000111732
ENSG00000116497	ENSG00000035862	ENSG00000113594	ENSG00000113303
ENSG00000158987	ENSG00000167977	ENSG00000128463	ENSG00000100614
ENSG00000152291	ENSG00000179111	ENSG00000108342	ENSG00000134531
ENSG00000175592	ENSG00000104218	ENSG00000180660	ENSG00000169071
ENSG00000115145	ENSG00000111077	ENSG00000177426	ENSG00000142046
ENSG00000146540	ENSG00000137812	ENSG00000124104	ENSG00000162763
ENSG00000118482	ENSG00000145934	ENSG00000110931	ENSG00000065243
ENSG00000120053	ENSG00000101311	ENSG00000181722	ENSG00000070882
ENSG00000055917	ENSG00000140474	ENSG00000111816	ENSG00000172992
ENSG00000166912	ENSG00000081853	ENSG00000176994	ENSG00000171204
ENSG00000078081	ENSG00000196353	ENSG00000134644	ENSG00000116001
ENSG00000085449	ENSG00000100219	ENSG00000101266	ENSG00000103356
ENSG00000135926	ENSG00000102931	ENSG00000142856	ENSG00000106948
ENSG00000157890	ENSG00000119396	ENSG00000132589	ENSG00000138617
ENSG00000145246	ENSG00000161180	ENSG00000187486	ENSG00000173540
ENSG00000108799	ENSG00000180190	ENSG00000171451	ENSG00000090661
ENSG00000164134	ENSG00000162300	ENSG00000169242	ENSG00000187555
ENSG00000156140	ENSG00000172458	ENSG00000163171	ENSG00000100605
ENSG00000188177	ENSG00000120705	ENSG00000151320	ENSG00000128519
ENSG00000156427	ENSG00000205838	ENSG00000149541	ENSG00000162892
ENSG00000164070	ENSG00000129515	ENSG00000196961	ENSG00000141736
ENSG00000136816	ENSG00000137825	ENSG00000114383	ENSG00000170820
ENSG00000177646	ENSG00000125347	ENSG00000204084	ENSG00000141503
ENSG00000120539	ENSG00000123096	ENSG00000115540	ENSG00000106078
ENSG00000175215	ENSG00000119125	ENSG00000156875	ENSG00000227500
ENSG00000204335	ENSG00000151413	ENSG00000151967	ENSG00000204503
ENSG00000150045	ENSG00000128594	ENSG00000163873	ENSG00000143196
ENSG00000183530	ENSG00000196644	ENSG00000136878	ENSG00000163507

ENSG00000073792	ENSG00000125977	ENSG00000101986	ENSG00000066933
ENSG00000147862	ENSG00000186352	ENSG00000198121	ENSG00000081377
ENSG00000177606	ENSG00000141668	ENSG00000107758	ENSG00000135069
ENSG00000033170	ENSG00000156463	ENSG00000135127	ENSG00000174950
ENSG00000198382	ENSG00000078304	ENSG00000162302	ENSG00000136872
ENSG00000144355	ENSG00000152779	ENSG00000120690	ENSG00000011405
ENSG00000181315	ENSG00000173868	ENSG00000188153	ENSG00000162419
ENSG00000170448	ENSG00000114354	ENSG00000006327	ENSG00000091262
ENSG00000109762	ENSG00000197081	ENSG00000139890	ENSG00000196372
ENSG00000132434	ENSG00000112599	ENSG00000087111	ENSG00000100065
ENSG00000108960	ENSG00000133424	ENSG00000164626	ENSG00000154118
ENSG00000197037	ENSG00000068615	ENSG00000198286	ENSG00000163874
ENSG00000038382	ENSG00000122877	ENSG00000171634	ENSG00000242498
ENSG00000147654	ENSG00000163486	ENSG00000010270	ENSG00000148737
ENSG00000130695	ENSG00000143653	ENSG00000139209	ENSG00000197798
ENSG00000009780	ENSG00000078246	ENSG00000166123	ENSG00000157601
ENSG00000171109	ENSG00000166169	ENSG00000151490	ENSG00000139832
ENSG00000165943	ENSG00000168481	ENSG00000104936	ENSG00000187764
ENSG00000122565	ENSG00000027075	ENSG00000163154	ENSG00000198900
ENSG00000157954	ENSG00000091640	ENSG00000182742	ENSG00000108107
ENSG00000162236	ENSG00000188026	ENSG00000138279	ENSG00000100341
ENSG00000114796	ENSG00000144959	ENSG00000008196	ENSG00000133056
ENSG00000113597	ENSG00000111737	ENSG00000086619	ENSG00000071127
ENSG00000125967	ENSG00000169564	ENSG00000047056	ENSG00000011007
ENSG00000166452	ENSG00000152990	ENSG00000088387	ENSG00000133243
ENSG00000189311	ENSG00000125954	ENSG00000106635	ENSG00000156804
ENSG00000117461	ENSG00000185010	ENSG00000100376	ENSG00000167996
ENSG00000073910	ENSG00000121005	ENSG00000182158	ENSG00000124743
ENSG00000168096	ENSG00000188001	ENSG00000204576	ENSG00000173406
ENSG00000136689	ENSG00000097021	ENSG00000184047	ENSG00000126003
ENSG00000188486	ENSG00000139220	ENSG00000120948	ENSG00000064419
ENSG00000073756	ENSG00000169174	ENSG00000105997	ENSG00000136867
ENSG00000152229	ENSG00000167460	ENSG00000168675	ENSG00000179142
ENSG00000182389	ENSG00000010539	ENSG00000171603	ENSG00000007129
ENSG00000179837	ENSG00000102287	ENSG00000065357	ENSG00000198894
ENSG00000106546	ENSG00000188549	ENSG00000163635	ENSG00000154768
ENSG00000160294	ENSG00000196914	ENSG00000090905	ENSG00000121931
ENSG00000176463	ENSG00000012048	ENSG00000160410	ENSG00000114455
ENSG00000173744	ENSG00000164815	ENSG00000153561	ENSG00000198929
ENSG00000165801	ENSG00000111181	ENSG00000179431	ENSG00000100351
ENSG00000113742	ENSG00000089225	ENSG00000124701	ENSG00000186166
ENSG00000175874	ENSG00000134758	ENSG00000205944	ENSG00000136950
ENSG00000135945	ENSG00000165197	ENSG00000065526	ENSG00000005882
ENSG00000004975	ENSG00000106263	ENSG00000166619	ENSG00000183403
ENSG00000029364	ENSG00000055163	ENSG00000125503	ENSG00000041982
ENSG00000155229	ENSG00000184517	ENSG00000173848	ENSG00000169372
ENSG00000198862	ENSG00000162620	ENSG00000179981	ENSG00000239474
ENSG00000152128	ENSG00000100811	ENSG00000113387	ENSG00000242689
ENSG00000105663	ENSG00000134684	ENSG00000156521	ENSG00000050767
ENSG00000072041	ENSG00000108187	ENSG00000170370	ENSG00000121039
ENSG00000104219	ENSG00000151702	ENSG00000130227	ENSG00000113555
ENSG00000164930	ENSG00000171940	ENSG00000177879	ENSG00000159915
ENSG00000160325	ENSG00000161021	ENSG00000158435	ENSG00000188921
ENSG00000004059	ENSG00000138448	ENSG00000005483	ENSG00000243646
ENSG00000206484	ENSG00000183020	ENSG00000112182	ENSG00000068697
ENSG00000152749	ENSG00000108788	ENSG00000088826	ENSG00000146909
ENSG00000009413	ENSG00000136156	ENSG00000179674	ENSG00000185340
ENSG00000168502	ENSG00000151240	ENSG00000128285	ENSG00000169855
ENSG00000100412	ENSG00000171044	ENSG00000169194	ENSG00000142102
ENSG00000133454	ENSG00000108932	ENSG00000112562	ENSG00000152661
ENSG00000153982	ENSG00000143520	ENSG00000132639	ENSG00000172331
ENSG00000149925	ENSG00000135298	ENSG00000129691	ENSG00000176340
ENSG00000115421	ENSG00000104419	ENSG00000050165	ENSG00000148248
ENSG00000136099	ENSG00000167528	ENSG00000146963	ENSG00000056998

ENSG00000171878	ENSG00000198363	ENSG00000159399	ENSG00000169914
ENSG00000137770	ENSG00000095397	ENSG00000170852	ENSG00000156103
ENSG00000160323	ENSG00000171502	ENSG00000078804	ENSG00000089876
ENSG00000110944	ENSG00000132405	ENSG00000170043	ENSG00000100823
ENSG00000136457	ENSG00000166224	ENSG00000131725	ENSG00000198934
ENSG00000183723	ENSG00000102038	ENSG00000187736	ENSG00000187664
ENSG00000149187	ENSG00000180263	ENSG00000074054	ENSG00000196277
ENSG00000182500	ENSG00000171621	ENSG00000131504	ENSG00000114739
ENSG00000172466	ENSG00000079432		ENSG00000142634
ENSG00000182175	ENSG00000084693		ENSG00000243943
ENSG00000103489	ENSG00000159445		ENSG00000198870
ENSG00000166341	ENSG00000113600		ENSG00000145365
ENSG00000133704	ENSG00000198176		ENSG00000119878
ENSG00000145743	ENSG00000135960		ENSG00000116688
ENSG00000057252	ENSG00000165476		ENSG00000163069
ENSG00000001167	ENSG00000156150		ENSG00000099624
ENSG00000124615	ENSG00000151148		ENSG00000121858
ENSG00000134108	ENSG00000147533		ENSG00000013523
ENSG00000047648	ENSG00000110442		ENSG00000107897
ENSG00000087494	ENSG00000105854		ENSG00000173575
ENSG00000137936	ENSG00000104435		ENSG00000122863
ENSG00000185883	ENSG00000169918		ENSG00000089127
ENSG00000147246	ENSG00000100221		ENSG00000163872
ENSG00000110429	ENSG00000171604		ENSG00000112195
ENSG00000165633	ENSG00000156639		ENSG00000172590
ENSG00000198218	ENSG00000132773		ENSG00000112773
ENSG00000121083	ENSG00000141433		ENSG00000102081
ENSG00000059378	ENSG00000146425		ENSG00000157214
ENSG00000005007	ENSG00000121905		ENSG00000144908
ENSG00000143512	ENSG00000174780		ENSG00000107593
ENSG00000124493	ENSG00000152268		ENSG00000078898
ENSG00000115953	ENSG00000148935		ENSG00000243364
ENSG00000132825	ENSG00000161405		ENSG00000239704
ENSG00000152214	ENSG00000165802		ENSG00000188690
ENSG00000157766	ENSG00000117090		ENSG00000184148
ENSG00000196449	ENSG00000095951		ENSG00000090372
ENSG00000092148	ENSG00000019505		ENSG00000165548
ENSG00000117114	ENSG00000230989		ENSG00000127588
ENSG00000171467	ENSG00000204252		ENSG00000140961
ENSG00000138780	ENSG00000124216		ENSG00000023839
ENSG00000173027	ENSG00000198746		ENSG00000100201
ENSG00000163624	ENSG00000100385		ENSG00000136152
ENSG00000083642	ENSG00000067560		ENSG00000088448
ENSG00000167674	ENSG00000070886		ENSG00000160685
ENSG00000160584	ENSG00000103111		ENSG00000087995
ENSG00000164304	ENSG00000118322		ENSG00000100228
ENSG00000189403	ENSG00000171992		ENSG00000053918
ENSG00000137200	ENSG00000196632		ENSG00000166557
ENSG00000160695	ENSG00000155016		ENSG00000053108
ENSG00000172137	ENSG00000103194		ENSG00000115350
ENSG00000170075	ENSG00000043591		ENSG00000112739
ENSG00000119636	ENSG00000137494		ENSG00000206418
ENSG00000143614	ENSG00000185591		ENSG00000118785
ENSG00000184560	ENSG00000176387		ENSG00000027001
ENSG00000185404	ENSG00000142453		ENSG00000156413
ENSG00000182601	ENSG00000107104		ENSG00000159899
ENSG00000205916	ENSG00000161013		ENSG00000151623
ENSG00000161692	ENSG00000148297		ENSG00000089775
ENSG00000137710	ENSG00000227345		ENSG00000189184
ENSG00000130956	ENSG00000137500		ENSG00000206308
ENSG00000111859	ENSG00000110400		ENSG00000120697
ENSG00000164252	ENSG00000138769		ENSG00000196177
ENSG00000174738	ENSG00000183873		ENSG00000188425
ENSG00000157483	ENSG00000136848		ENSG00000140481

ENSG00000162972	ENSG00000165006	ENSG00000168843
ENSG00000132704	ENSG00000175779	ENSG00000114686
ENSG00000179242	ENSG00000155380	ENSG00000007923
ENSG00000174871	ENSG00000110042	ENSG00000125903
ENSG00000116396	ENSG00000105700	ENSG00000110801
ENSG00000109920	ENSG00000135924	ENSG00000186272
ENSG00000147382	ENSG00000197245	ENSG00000110723
ENSG00000171155	ENSG00000055070	ENSG00000124098
ENSG00000106236	ENSG00000158301	ENSG00000137135
ENSG00000074964	ENSG00000151090	ENSG00000198488
ENSG00000154153	ENSG00000101335	ENSG00000134874
ENSG00000107021	ENSG00000164117	ENSG00000134321
ENSG00000137869	ENSG00000138092	ENSG00000166426
ENSG00000111640	ENSG00000186074	ENSG00000130713
ENSG00000181788	ENSG00000112033	ENSG00000133265
ENSG00000171552	ENSG00000136197	ENSG00000131097
ENSG00000157224	ENSG00000165478	ENSG00000008118
ENSG00000007866	ENSG00000170616	ENSG00000163636
ENSG00000176788	ENSG00000165868	ENSG00000140905
ENSG00000147642	ENSG00000078098	ENSG00000112561
ENSG00000162852	ENSG00000186150	ENSG00000197697
ENSG00000169926	ENSG00000112041	ENSG00000087916
ENSG00000106799	ENSG00000186184	ENSG00000204186
ENSG00000185022	ENSG00000156976	ENSG00000198551
ENSG00000111885	ENSG00000147813	ENSG00000177854
ENSG00000141580	ENSG00000166159	ENSG00000136840
ENSG00000140945	ENSG00000173320	ENSG00000178802
ENSG00000132142	ENSG00000148730	ENSG00000152672
ENSG00000083520	ENSG00000161654	ENSG00000081803
ENSG00000112406	ENSG00000110375	ENSG00000143319
ENSG00000186868	ENSG00000150347	ENSG00000101191
ENSG00000154319	ENSG00000163126	ENSG00000100433
ENSG00000102271	ENSG00000174748	ENSG00000113761
ENSG00000138136	ENSG00000118564	ENSG00000178199
ENSG00000123643	ENSG00000083123	ENSG00000120688
ENSG00000126775	ENSG00000163902	ENSG00000059728
ENSG00000127314	ENSG00000131459	ENSG00000134755
ENSG00000184985	ENSG00000172349	ENSG00000240505
ENSG00000158092	ENSG00000096746	ENSG00000065548
ENSG00000165406	ENSG00000240428	ENSG00000131016
ENSG00000101447	ENSG00000204228	ENSG00000168497
ENSG00000141232	ENSG00000177551	ENSG00000169733
ENSG00000152192	ENSG00000135482	ENSG00000162344
ENSG00000187498	ENSG00000179397	ENSG00000189367
ENSG00000135597	ENSG00000151348	ENSG00000110031
ENSG00000137497	ENSG00000128191	ENSG00000103642
ENSG00000171448	ENSG00000198324	ENSG00000088836
ENSG00000102780	ENSG00000177685	ENSG00000168280
ENSG00000204356	ENSG00000131653	ENSG00000109501
ENSG00000140688	ENSG00000125447	ENSG00000145358
ENSG00000204344	ENSG00000105887	ENSG00000105676
ENSG00000110108	ENSG00000158850	ENSG00000157542
ENSG00000158201	ENSG00000136874	ENSG00000169933
ENSG00000183580	ENSG00000153714	ENSG00000162882
ENSG00000160179	ENSG00000176454	ENSG00000108515
ENSG00000164332	ENSG00000172159	ENSG00000177425
ENSG00000128016	ENSG00000135678	ENSG00000112796
ENSG00000204262	ENSG00000107140	ENSG00000177034
ENSG00000103502	ENSG00000132970	ENSG00000070010
ENSG00000170425	ENSG00000149658	ENSG00000136928
ENSG00000072182	ENSG00000137819	ENSG00000138814
ENSG00000120526	ENSG00000142611	ENSG00000197614
ENSG00000122359	ENSG00000054611	ENSG00000167780
ENSG00000179104	ENSG00000148798	ENSG00000102034

ENSG00000102974	ENSG00000175727	ENSG00000159409
ENSG00000187735	ENSG00000163032	ENSG00000172554
ENSG00000088038	ENSG00000168090	ENSG00000186575
ENSG00000166261	ENSG00000140859	ENSG00000159079
ENSG00000130287	ENSG00000146066	ENSG00000141030
ENSG00000127125	ENSG00000188800	ENSG00000090621
ENSG00000072134	ENSG00000177613	ENSG00000180537
ENSG00000091542	ENSG00000139636	ENSG00000132849
ENSG00000151327	ENSG00000117620	ENSG00000180616
ENSG00000170525	ENSG00000146535	ENSG00000110651
ENSG00000101421	ENSG00000152455	ENSG00000197021
ENSG00000127989	ENSG00000183508	ENSG00000162378
ENSG00000115946	ENSG00000176208	ENSG00000149798
ENSG00000067082	ENSG00000111653	ENSG00000179218
ENSG00000188529	ENSG00000163347	ENSG00000110048
ENSG00000117020	ENSG00000138650	ENSG00000154721
ENSG00000169031	ENSG00000175497	ENSG00000166407
ENSG00000120868	ENSG00000070269	ENSG00000089505
ENSG00000157445	ENSG00000145779	ENSG00000198429
ENSG00000168374	ENSG00000140519	ENSG00000183098
ENSG00000116560	ENSG00000138162	ENSG00000144566
ENSG00000142192	ENSG00000155876	ENSG00000116984
ENSG00000168476	ENSG00000109089	ENSG00000142409
ENSG00000181458	ENSG00000120805	ENSG00000130165
ENSG00000106327	ENSG00000175182	ENSG00000145626
ENSG00000147606	ENSG00000182504	ENSG00000150401
ENSG00000187838	ENSG00000135519	ENSG00000102383
ENSG00000108578	ENSG00000100146	ENSG00000101189
ENSG00000158615	ENSG00000049759	ENSG00000099308
ENSG00000167768	ENSG00000174502	ENSG00000169435
ENSG00000187257	ENSG00000149503	ENSG00000117528
ENSG00000106689	ENSG00000179088	ENSG00000196368
ENSG00000072657	ENSG00000177575	ENSG00000181826
ENSG00000147459	ENSG00000170027	ENSG00000127863
ENSG00000172273	ENSG00000198858	ENSG00000133216
ENSG00000196090	ENSG00000137267	ENSG00000173825
ENSG00000147231	ENSG00000145780	ENSG00000142632
ENSG00000106034	ENSG00000171150	ENSG00000169239
ENSG00000133874	ENSG00000100842	ENSG00000059122
ENSG00000135097	ENSG00000169016	ENSG00000100302
ENSG00000131002	ENSG00000095261	ENSG00000213937
ENSG00000143776	ENSG00000171443	ENSG00000186431
ENSG00000185896	ENSG00000186648	ENSG00000196981
ENSG00000129654	ENSG00000099901	ENSG00000184697
ENSG00000110675	ENSG00000171848	ENSG00000172831
ENSG00000146374	ENSG00000058262	ENSG00000145451
ENSG00000158865	ENSG00000173898	ENSG00000101152
ENSG00000157654	ENSG00000072274	ENSG00000173110
ENSG00000143603	ENSG00000116218	ENSG00000154889
ENSG00000062485	ENSG00000108175	ENSG00000130396
ENSG00000188803	ENSG00000155324	ENSG00000111817
ENSG00000177479	ENSG00000162231	ENSG00000133943
ENSG00000154645	ENSG00000070366	ENSG00000189319
ENSG00000114268	ENSG00000116698	ENSG00000122574
ENSG00000128585	ENSG00000133401	ENSG00000157823
ENSG00000010361	ENSG00000110958	ENSG00000136160
ENSG00000115363	ENSG00000147526	ENSG00000132640
ENSG00000106819	ENSG00000144560	ENSG00000170615
ENSG00000012232	ENSG00000134508	ENSG00000163581
ENSG00000100105	ENSG00000233224	ENSG00000109132
ENSG00000175984	ENSG00000138622	ENSG00000177943
ENSG00000156475	ENSG00000172530	ENSG00000162889
ENSG00000155827	ENSG00000118515	ENSG00000188566
ENSG00000100003	ENSG00000144827	ENSG00000176945

ENSG00000204613	ENSG00000204469	ENSG00000165716
ENSG00000115507	ENSG00000115760	ENSG00000177710
ENSG00000185787	ENSG00000116701	ENSG00000172840
ENSG00000064309	ENSG00000131188	ENSG00000205784
ENSG00000160710	ENSG00000048540	ENSG00000148331
ENSG00000158089	ENSG00000174951	ENSG00000075151
ENSG00000138823	ENSG00000084774	ENSG00000165804
ENSG00000076067	ENSG00000114853	ENSG00000112697
ENSG00000175826	ENSG00000107372	ENSG00000176236
ENSG00000176102	ENSG00000084628	ENSG00000177051
ENSG00000155729	ENSG00000144749	ENSG00000105221
ENSG00000106701	ENSG00000185909	ENSG00000197905
ENSG00000056345	ENSG00000129219	ENSG00000132561
ENSG00000144893	ENSG00000073803	ENSG00000205808
ENSG00000116016	ENSG00000102387	ENSG00000165471
ENSG00000175029	ENSG00000070061	ENSG00000101882
ENSG00000158859	ENSG00000001626	ENSG00000101745
ENSG00000182247	ENSG00000074416	ENSG00000103995
ENSG00000071243	ENSG00000206053	ENSG00000117289
ENSG00000109061	ENSG00000081051	ENSG00000075142
ENSG00000120913	ENSG00000129277	ENSG00000148219
ENSG00000156304	ENSG00000101638	ENSG00000206579
ENSG00000006740	ENSG00000144840	ENSG00000228716
ENSG00000164100	ENSG00000125798	ENSG00000104660
ENSG00000116903	ENSG00000168234	ENSG00000130822
ENSG00000173402	ENSG00000243056	ENSG00000100307
ENSG00000198353	ENSG00000187778	ENSG00000028203
ENSG00000136709	ENSG00000164692	ENSG00000179262
ENSG00000130052	ENSG00000101384	ENSG00000197893
ENSG00000029725	ENSG00000100934	ENSG00000106631
ENSG00000146477	ENSG00000178965	ENSG00000113946
ENSG00000114923	ENSG00000173926	ENSG00000170653
ENSG00000164691	ENSG00000197430	ENSG00000102786
ENSG00000145014	ENSG00000117115	ENSG00000090097
ENSG00000174579	ENSG00000076770	ENSG00000146648
ENSG00000198161	ENSG00000151893	ENSG00000115828
ENSG00000137824	ENSG00000132702	ENSG00000139797
ENSG00000116350	ENSG00000142449	ENSG00000106785
ENSG00000183688	ENSG00000067048	ENSG00000105251
ENSG00000038274	ENSG00000115009	ENSG00000176393
ENSG00000139540	ENSG00000079112	ENSG00000171953
ENSG00000072682	ENSG00000137693	ENSG00000100867
ENSG00000187840	ENSG00000187068	ENSG00000176769
ENSG00000111880	ENSG00000039600	ENSG00000164404
ENSG00000075461	ENSG00000168066	ENSG00000145879
ENSG00000179036	ENSG00000136068	ENSG00000113851
ENSG00000170385	ENSG00000163683	ENSG00000177873
ENSG00000114554	ENSG00000196950	ENSG00000145416
ENSG00000004660	ENSG00000100852	ENSG00000113456
ENSG00000104886	ENSG00000136108	ENSG00000162607
ENSG00000054983	ENSG00000100426	ENSG00000132386
ENSG00000165113	ENSG00000119812	ENSG00000119946
ENSG00000107518	ENSG00000184271	ENSG00000163479
ENSG00000198356	ENSG00000143797	ENSG00000125820
ENSG00000153317	ENSG00000026025	ENSG00000158825
ENSG00000177744	ENSG00000173020	ENSG00000135077
ENSG00000095787	ENSG00000078237	ENSG00000142748
ENSG00000140488	ENSG00000029993	ENSG00000125868
ENSG00000196104	ENSG00000149636	ENSG00000183808
ENSG00000198689	ENSG00000173166	ENSG00000113212
ENSG00000164442	ENSG00000147123	ENSG00000031823
ENSG00000134025	ENSG00000158156	ENSG00000181991
ENSG00000185352	ENSG00000163435	ENSG00000035720
ENSG00000144645	ENSG00000148848	ENSG00000141385

ENSG00000165389	ENSG00000184564	ENSG00000169715
ENSG00000140443	ENSG00000006757	ENSG00000160207
ENSG00000141741	ENSG00000182010	ENSG00000146147
ENSG00000204977	ENSG00000143420	ENSG00000177272
ENSG00000124209	ENSG00000198739	ENSG00000171532
ENSG00000169992	ENSG00000162736	ENSG00000124762
ENSG00000137845	ENSG00000171522	ENSG00000156162
ENSG00000182552	ENSG00000104964	ENSG00000144591
ENSG00000168591	ENSG00000108861	ENSG00000196781
ENSG00000136536	ENSG00000116991	ENSG00000141750
ENSG00000108433	ENSG00000126883	ENSG00000134248
ENSG00000100106	ENSG00000137947	ENSG00000169760
ENSG00000101166	ENSG00000186298	ENSG00000136999
ENSG00000158050	ENSG00000167208	ENSG00000140876
ENSG00000211456	ENSG00000067900	ENSG00000171574
ENSG00000176095	ENSG00000118707	ENSG00000100239
ENSG00000140396	ENSG00000143384	ENSG00000151229
ENSG00000107362	ENSG00000151150	ENSG00000149926
ENSG00000131067	ENSG00000116473	ENSG00000104267
ENSG00000122435	ENSG00000180353	ENSG00000109320
ENSG00000145632	ENSG00000105245	ENSG00000167775
ENSG00000163468	ENSG00000100335	ENSG00000142794
ENSG00000143756	ENSG00000088179	ENSG00000173930
ENSG00000011332	ENSG00000168214	ENSG00000135503
ENSG00000172795	ENSG00000005469	ENSG00000065621
ENSG00000154736	ENSG00000196730	ENSG00000143178
ENSG00000105438	ENSG00000135709	ENSG00000180767
ENSG00000184274	ENSG00000196226	ENSG00000198018
ENSG00000102290	ENSG00000150076	ENSG00000147416
ENSG00000132768	ENSG00000137809	ENSG00000185053
ENSG00000171307	ENSG00000082458	ENSG00000198553
ENSG00000196338	ENSG00000139318	ENSG00000011376
ENSG00000105372	ENSG00000163630	ENSG00000136688
ENSG00000100596	ENSG00000156011	ENSG00000145725
ENSG00000140873	ENSG00000161642	ENSG00000198464
ENSG00000147457	ENSG00000112149	ENSG00000178093
ENSG00000138829	ENSG00000179284	ENSG00000146094
ENSG00000099889	ENSG00000165660	ENSG00000198863
ENSG00000099942	ENSG00000155158	ENSG00000089356
ENSG00000119231	ENSG00000007047	ENSG00000159640
ENSG00000110274	ENSG00000141540	ENSG00000103174
ENSG00000180772	ENSG00000145715	ENSG00000150394
ENSG00000161849	ENSG00000130332	ENSG00000221823
ENSG00000158406	ENSG00000130164	ENSG00000100890
ENSG00000130584	ENSG00000176406	ENSG00000120563
ENSG00000140575	ENSG00000180596	ENSG00000136854
ENSG00000203956	ENSG00000124818	ENSG00000206452
ENSG00000072952	ENSG00000187510	ENSG00000182909
ENSG00000177432	ENSG00000143248	ENSG00000151611
ENSG00000165886	ENSG00000106290	ENSG00000206455
ENSG00000178104	ENSG00000130147	ENSG00000180628
ENSG00000136014	ENSG00000188827	ENSG00000007341
ENSG00000163214	ENSG00000154358	ENSG00000152467
ENSG00000143815	ENSG00000082512	ENSG00000080854
ENSG00000182220	ENSG00000182718	ENSG00000158006
ENSG00000113282	ENSG00000128342	ENSG00000188021
ENSG00000100348	ENSG00000171316	ENSG00000148290
ENSG00000088305	ENSG00000117625	ENSG00000148057
ENSG00000101057	ENSG00000117153	ENSG00000170881
ENSG00000172404	ENSG00000131969	ENSG00000184445
ENSG00000086061	ENSG00000081791	ENSG00000142675
ENSG00000137100	ENSG00000142459	ENSG00000101306
ENSG00000114744	ENSG00000077157	ENSG00000154001
ENSG00000131462	ENSG00000137055	ENSG00000029363

ENSG00000113657	ENSG00000163661	ENSG00000130544
ENSG00000095596	ENSG00000155506	ENSG00000151067
ENSG00000135074	ENSG00000121753	ENSG00000100632
ENSG00000007545	ENSG00000107295	ENSG00000151365
ENSG00000176783	ENSG00000165795	ENSG00000183801
ENSG00000103035	ENSG00000173068	ENSG00000112877
ENSG00000170647	ENSG00000221968	ENSG00000143147
ENSG00000154359	ENSG00000107863	ENSG00000086544
ENSG00000007001	ENSG00000102471	ENSG00000153044
ENSG00000135535	ENSG00000138796	ENSG00000143171
ENSG00000140983	ENSG00000111846	ENSG00000024526
ENSG00000176407	ENSG00000100439	ENSG00000144040
ENSG00000196227	ENSG00000169228	ENSG00000113396
ENSG00000172493	ENSG00000036530	ENSG00000160999
ENSG00000144524	ENSG00000102753	ENSG00000146476
ENSG00000109680	ENSG00000121774	ENSG00000189334
ENSG00000157020	ENSG00000087448	ENSG00000166206
ENSG00000006194	ENSG00000166897	ENSG00000133460
ENSG00000162909	ENSG00000067606	ENSG00000085365
ENSG00000171813	ENSG00000097033	ENSG00000214253
ENSG00000177764	ENSG00000170633	ENSG00000091127
ENSG00000177000	ENSG00000182533	ENSG00000131795
ENSG00000185585	ENSG00000099625	ENSG00000180549
ENSG00000113327	ENSG00000119865	ENSG00000110011
ENSG00000167279	ENSG00000157379	ENSG00000124249
ENSG00000186998	ENSG00000163421	ENSG00000143258
ENSG00000141429	ENSG00000165084	ENSG00000139514
ENSG00000152944	ENSG00000172260	ENSG00000146757
ENSG00000147010	ENSG00000119408	ENSG00000100290
ENSG00000138468	ENSG00000129451	ENSG00000198105
ENSG00000130294	ENSG00000043143	ENSG00000108826
ENSG00000148411	ENSG00000196932	ENSG00000164151
ENSG00000164219	ENSG00000146151	ENSG00000123575
ENSG00000154380	ENSG00000165775	ENSG00000242616
ENSG00000134762	ENSG00000109929	ENSG00000111554
ENSG00000168395	ENSG00000122958	ENSG00000107819
ENSG00000133116	ENSG00000002330	ENSG00000089472
ENSG00000111732	ENSG00000204946	ENSG00000212747
ENSG00000103507	ENSG00000106244	ENSG00000153093
ENSG00000177917	ENSG00000179869	ENSG00000102007
ENSG00000100614	ENSG00000124813	ENSG00000130254
ENSG00000206489	ENSG00000172403	ENSG00000163833
ENSG00000160551	ENSG00000177352	ENSG00000137166
ENSG00000134531	ENSG00000154124	ENSG00000108830
ENSG00000114790	ENSG00000088543	ENSG00000095970
ENSG00000142046	ENSG00000103226	ENSG00000140279
ENSG00000135363	ENSG00000188120	ENSG00000138039
ENSG00000100815	ENSG00000135537	ENSG00000169282
ENSG00000070882	ENSG00000205445	ENSG00000197837
ENSG00000136750	ENSG00000166823	ENSG00000163617
ENSG00000166971	ENSG00000107223	ENSG00000136378
ENSG00000116001	ENSG00000049239	ENSG00000116668
ENSG00000170145	ENSG00000021826	ENSG00000121900
ENSG00000105648	ENSG00000099715	ENSG00000130244
ENSG00000106948	ENSG00000132694	ENSG00000189162
ENSG00000138617	ENSG00000182173	ENSG00000203784
ENSG00000173540	ENSG00000133627	ENSG00000243414
ENSG00000143494	ENSG00000104067	ENSG00000112333
ENSG00000025434	ENSG00000167851	ENSG00000141577
ENSG00000206329	ENSG00000111877	ENSG00000004866
ENSG00000187555	ENSG00000181744	ENSG00000158874
ENSG00000100605	ENSG00000115318	ENSG00000075131
ENSG00000165424	ENSG00000129235	ENSG00000012822
ENSG00000141503	ENSG00000106299	ENSG00000174374

ENSG00000120458	ENSG00000149582	ENSG00000169604
ENSG00000106078	ENSG00000120278	ENSG00000178952
ENSG00000134909	ENSG00000100523	ENSG00000102804
ENSG00000124571	ENSG00000130427	ENSG00000179526
ENSG00000066933	ENSG00000185668	ENSG00000150551
ENSG00000171456	ENSG00000109111	ENSG00000173077
ENSG00000081377	ENSG00000133706	ENSG00000112232
ENSG00000120254	ENSG00000162775	ENSG00000120910
ENSG00000109943	ENSG00000076554	ENSG00000137409
ENSG00000174950	ENSG00000143387	ENSG00000196428
ENSG00000072133	ENSG00000114738	ENSG00000105427
ENSG00000106692	ENSG00000176076	ENSG00000068878
ENSG00000132334	ENSG00000170348	ENSG00000198841
ENSG00000162419	ENSG00000115594	ENSG00000165685
ENSG00000134986	ENSG00000122566	ENSG00000146700
ENSG00000099940	ENSG00000162434	ENSG00000083067
ENSG00000008300	ENSG00000162627	ENSG00000104522
ENSG00000162981	ENSG00000180483	ENSG00000163728
ENSG00000100065	ENSG00000125810	ENSG00000163808
ENSG00000130224	ENSG00000166432	ENSG00000130653
ENSG00000154118	ENSG00000110090	ENSG00000173933
ENSG00000163874	ENSG00000130382	ENSG00000135605
ENSG00000111783	ENSG00000170035	ENSG00000006210
ENSG00000197798	ENSG00000006530	ENSG00000174255
ENSG00000148737	ENSG00000139445	ENSG00000163348
ENSG00000102100	ENSG00000186081	ENSG00000130167
ENSG00000136715	ENSG00000172731	ENSG00000143502
ENSG00000096467	ENSG00000221986	ENSG00000138030
ENSG00000158467	ENSG00000125520	ENSG00000182903
ENSG00000120820	ENSG00000122884	ENSG00000110934
ENSG00000142669	ENSG00000176749	ENSG00000114735
ENSG00000187764	ENSG00000056487	ENSG00000128739
ENSG00000184845	ENSG00000009830	ENSG00000140107
ENSG00000198900	ENSG00000017621	ENSG00000142945
ENSG00000108107	ENSG00000174371	ENSG00000115364
ENSG00000116044	ENSG00000100626	ENSG00000109189
ENSG00000015479	ENSG00000157168	ENSG00000109046
ENSG00000133056	ENSG00000180318	ENSG00000150656
ENSG00000071127	ENSG00000153107	ENSG00000169032
ENSG00000133243	ENSG00000132541	ENSG00000137265
ENSG00000156804	ENSG00000042429	ENSG00000100034
ENSG00000167996	ENSG00000177082	ENSG00000158122
ENSG00000169180	ENSG00000180776	ENSG00000104998
ENSG00000126003	ENSG00000171227	ENSG00000070985
ENSG00000136867	ENSG00000117748	ENSG00000196182
ENSG00000064419	ENSG00000054116	ENSG00000183207
ENSG00000196586	ENSG00000164620	ENSG00000198276
ENSG00000198894	ENSG00000169594	ENSG00000110435
ENSG00000063169	ENSG00000109099	ENSG00000164050
ENSG00000170743	ENSG00000119778	ENSG00000163288
ENSG00000070756	ENSG00000149294	ENSG00000001631
ENSG00000169895	ENSG00000140992	ENSG00000136940
ENSG00000167257	ENSG00000175376	ENSG00000243279
ENSG00000005882	ENSG00000163933	ENSG00000136802
ENSG00000136950	ENSG00000072786	ENSG00000120907
ENSG00000164171	ENSG00000198719	ENSG00000117632
ENSG00000150995	ENSG00000006042	ENSG00000138641
ENSG00000101079	ENSG00000243667	ENSG00000131746
ENSG00000063978	ENSG00000079739	ENSG00000196976
ENSG00000141449	ENSG00000196323	ENSG00000111727
ENSG00000164045	ENSG00000110104	ENSG00000198822
ENSG00000182901	ENSG00000168959	ENSG00000135250
ENSG00000050767	ENSG00000118518	ENSG00000184451
ENSG00000141756	ENSG00000159625	ENSG00000196646

ENSG00000121039	ENSG00000196532	ENSG00000100368
ENSG00000141446	ENSG00000185033	ENSG00000146828
ENSG00000125434	ENSG00000128228	ENSG00000110245
ENSG00000130758	ENSG00000184454	ENSG00000122729
ENSG00000164061	ENSG00000107937	ENSG00000152332
ENSG00000185340	ENSG00000183955	ENSG00000166342
ENSG00000150594	ENSG00000183431	ENSG00000153930
ENSG00000068697	ENSG00000145335	ENSG00000160310
ENSG00000169855	ENSG00000213347	ENSG00000129244
ENSG00000152661	ENSG00000130402	ENSG00000198812
ENSG00000197712	ENSG00000182108	ENSG00000136271
ENSG00000116815	ENSG00000111540	ENSG00000121297
ENSG00000056998	ENSG00000125821	ENSG00000080644
ENSG00000148248	ENSG00000172915	ENSG00000176679
ENSG00000172113	ENSG00000157978	ENSG00000197016
ENSG00000156103	ENSG00000174483	ENSG00000101210
ENSG00000139372	ENSG00000214753	ENSG00000113504
ENSG00000196361	ENSG00000158555	ENSG00000129646
ENSG00000169220	ENSG00000196376	ENSG00000185924
ENSG00000187664	ENSG00000114030	ENSG00000089101
ENSG00000196277	ENSG00000176853	ENSG00000136231
ENSG00000031691	ENSG00000087903	ENSG00000154646
ENSG00000142634	ENSG00000033050	ENSG00000196843
ENSG00000145365	ENSG00000171540	ENSG00000169764
ENSG00000116406	ENSG00000124783	ENSG00000049319
ENSG00000087502	ENSG00000168438	ENSG00000198331
ENSG00000198873	ENSG00000164270	ENSG00000129465
ENSG00000163069	ENSG00000114850	ENSG00000135018
ENSG00000185052	ENSG00000151923	ENSG00000146038
ENSG00000013523	ENSG00000142528	ENSG00000160213
ENSG00000149485	ENSG00000101321	ENSG00000016602
ENSG00000107897	ENSG00000165409	ENSG00000142252
ENSG00000114626	ENSG00000153789	ENSG00000130598
ENSG00000070444	ENSG00000140830	ENSG00000171199
ENSG00000163872	ENSG00000162174	ENSG00000175782
ENSG00000103034	ENSG00000163600	ENSG00000186790
ENSG00000018189	ENSG00000109381	ENSG00000164879
ENSG00000172590	ENSG00000140995	ENSG00000116039
ENSG00000112773	ENSG00000123836	ENSG00000166762
ENSG00000102081	ENSG00000112062	ENSG00000086712
ENSG00000078898	ENSG00000185860	ENSG00000163462
ENSG00000153046	ENSG00000129636	ENSG00000169860
ENSG00000140299	ENSG00000111186	ENSG00000164938
ENSG00000178074	ENSG00000196072	ENSG00000181856
ENSG00000076201	ENSG00000134278	ENSG00000183722
ENSG00000147100	ENSG00000113240	ENSG00000180861
ENSG00000169682	ENSG00000123374	ENSG00000157193
ENSG00000076513	ENSG00000126091	ENSG00000070018
ENSG00000090372	ENSG00000094796	ENSG00000171483
ENSG00000140961	ENSG00000101911	ENSG00000130717
ENSG00000105366	ENSG00000179632	ENSG00000175224
ENSG00000107130	ENSG00000162545	ENSG00000172469
ENSG00000146021	ENSG00000167604	ENSG00000104904
ENSG00000088448	ENSG00000138744	ENSG00000148200
ENSG00000136152	ENSG00000181577	ENSG00000066044
ENSG00000160685	ENSG00000119682	ENSG00000102924
ENSG00000009694	ENSG00000173681	ENSG00000087495
ENSG00000197771	ENSG00000157500	ENSG00000177666
ENSG00000066468	ENSG00000105723	ENSG00000109881
ENSG00000115350	ENSG00000164749	ENSG00000132031
ENSG00000170604	ENSG00000131171	ENSG00000165457
ENSG00000168488	ENSG00000165462	ENSG00000187922
ENSG00000189290	ENSG00000141150	ENSG00000153823
ENSG00000101413	ENSG00000173917	ENSG00000113749

ENSG00000151623	ENSG00000090659	ENSG00000160439
ENSG00000185811	ENSG00000111450	ENSG00000120329
ENSG00000180957	ENSG00000131788	ENSG00000188487
ENSG00000162433	ENSG00000163719	ENSG00000181789
ENSG00000189184	ENSG00000213654	ENSG00000178287
ENSG00000197967	ENSG00000042781	ENSG00000108424
ENSG00000185818	ENSG00000152785	ENSG00000023445
ENSG00000179709	ENSG00000065609	ENSG00000184939
ENSG00000168843	ENSG00000154654	ENSG00000139826
ENSG00000114686	ENSG00000149599	ENSG00000184224
ENSG00000178726	ENSG00000122203	ENSG00000213218
ENSG00000124882	ENSG00000182621	ENSG00000134597
ENSG00000100167	ENSG00000166575	ENSG00000185069
ENSG00000067829	ENSG00000113916	ENSG00000198081
ENSG00000167807	ENSG00000166313	ENSG00000130948
ENSG00000147144	ENSG00000132623	ENSG00000126562
ENSG00000173486	ENSG00000169118	ENSG00000100647
ENSG00000169193	ENSG00000128652	ENSG00000196549
ENSG00000163864	ENSG00000135404	ENSG00000114251
ENSG00000186272	ENSG00000142319	ENSG00000159692
ENSG00000157625	ENSG00000173846	ENSG00000204979
ENSG00000137135	ENSG00000120063	ENSG00000139725
ENSG00000124098	ENSG00000156599	ENSG00000107736
ENSG00000174456	ENSG00000162992	ENSG00000143878
ENSG00000116990	ENSG00000125166	ENSG00000141639
ENSG00000166295	ENSG00000132326	ENSG00000198183
ENSG00000134874	ENSG00000118971	ENSG00000148677
ENSG00000116750	ENSG00000181035	ENSG00000133636
ENSG00000120949	ENSG00000175115	ENSG00000160190
ENSG00000204564	ENSG00000105559	ENSG00000181467
ENSG00000112561	ENSG00000135686	ENSG00000159840
ENSG00000105996	ENSG00000068323	ENSG00000198162
ENSG00000087916	ENSG00000087152	ENSG00000095564
ENSG00000156876	ENSG00000023516	ENSG00000185760
ENSG00000189159	ENSG00000075785	ENSG00000116017
ENSG00000187446	ENSG00000146360	ENSG00000176658
ENSG00000177854	ENSG00000137802	ENSG00000063127
ENSG00000178802	ENSG00000216937	ENSG00000105877
ENSG00000113761	ENSG00000241978	ENSG00000115705
ENSG00000100433	ENSG00000023892	ENSG00000100664
ENSG00000182463	ENSG00000153233	ENSG00000154997
ENSG00000120688	ENSG00000158163	ENSG00000171551
ENSG00000114978	ENSG00000184307	ENSG00000196074
ENSG00000134755	ENSG00000111110	ENSG00000067177
ENSG00000198477	ENSG00000027869	ENSG00000124920
ENSG00000078668	ENSG00000081087	ENSG00000125629
ENSG00000149547	ENSG00000123612	ENSG00000180083
ENSG00000090470	ENSG00000179639	ENSG00000109107
ENSG00000171119	ENSG00000101363	ENSG00000135048
ENSG00000065548	ENSG00000013306	ENSG00000159363
ENSG00000131016	ENSG00000166886	ENSG00000053702
ENSG00000103044	ENSG00000113595	ENSG00000124193
ENSG00000175426	ENSG00000133104	ENSG00000214320
ENSG00000116883	ENSG00000143126	ENSG00000011009
ENSG00000111664	ENSG00000101350	ENSG00000125122
ENSG00000204382	ENSG00000148516	ENSG00000166471
ENSG00000177932	ENSG00000110244	ENSG00000144118
ENSG00000150051	ENSG00000162949	ENSG00000101346
ENSG00000162344	ENSG00000110076	ENSG00000213672
ENSG00000103449	ENSG00000248541	ENSG00000100526
ENSG00000110031	ENSG00000091972	ENSG00000145782
ENSG00000155961	ENSG00000175166	ENSG00000101958
ENSG00000103642	ENSG00000170382	ENSG00000100078
ENSG00000133958	ENSG00000145901	ENSG00000026652

ENSG00000151914	ENSG00000116871	ENSG00000134698
ENSG00000188368	ENSG00000143067	ENSG00000159263
ENSG00000131149	ENSG00000106459	ENSG00000001036
ENSG00000157837	ENSG00000187676	ENSG00000186862
ENSG00000116641	ENSG00000167767	ENSG00000164251
ENSG00000177425	ENSG00000187123	ENSG00000090273
ENSG00000112796	ENSG00000186364	ENSG00000171873
ENSG00000136928	ENSG00000183513	ENSG00000120742
ENSG00000065911	ENSG00000175793	ENSG00000160007
ENSG00000138814	ENSG00000142065	ENSG00000169398
ENSG00000171169	ENSG00000206384	ENSG00000060656
ENSG00000197614	ENSG00000152242	ENSG00000026751
ENSG00000158246	ENSG00000129749	ENSG00000059804
ENSG00000115107	ENSG00000187742	ENSG00000188624
ENSG00000102034	ENSG00000217128	ENSG00000185658
ENSG00000159409	ENSG00000167230	ENSG00000057704
ENSG00000119699	ENSG00000141424	ENSG00000188483
ENSG00000111432	ENSG00000074266	ENSG00000156030
ENSG00000186575	ENSG00000139508	ENSG00000002919
ENSG00000197463	ENSG00000155011	ENSG00000146457
ENSG00000165410	ENSG00000135373	ENSG00000117155
ENSG00000180537	ENSG00000131043	ENSG00000188643
ENSG00000141030	ENSG00000127452	ENSG00000162645
ENSG00000148948	ENSG00000135124	ENSG00000188076
ENSG00000110851	ENSG00000137221	ENSG00000197409
ENSG00000116489	ENSG00000043355	ENSG00000086200
ENSG00000085063	ENSG00000177108	ENSG00000181704
ENSG00000113108	ENSG00000108588	ENSG00000163013
ENSG00000149798	ENSG00000142961	ENSG00000116717
ENSG00000110048	ENSG00000130204	ENSG00000115525
ENSG00000104490	ENSG00000078269	ENSG00000171115
ENSG00000120075	ENSG00000137193	ENSG00000189079
ENSG00000166407	ENSG00000056277	ENSG00000175175
ENSG00000183098	ENSG00000101438	ENSG00000163235
ENSG00000144566	ENSG00000154162	ENSG00000174600
ENSG00000130165	ENSG00000126247	ENSG00000172653
ENSG00000131355	ENSG00000154122	ENSG00000113141
ENSG00000163817	ENSG00000085491	ENSG00000118263
ENSG00000102383	ENSG00000198087	ENSG00000003096
ENSG00000125976	ENSG00000171160	ENSG00000067208
ENSG00000101189	ENSG00000100027	ENSG00000106624
ENSG00000147548	ENSG00000185800	ENSG00000140836
ENSG00000196368	ENSG00000118402	ENSG00000178974
ENSG00000140044	ENSG00000163626	ENSG00000169474
ENSG00000131831	ENSG00000156515	ENSG00000151792
ENSG00000127863	ENSG00000162456	ENSG00000118762
ENSG00000148840	ENSG00000213186	ENSG00000107443
ENSG00000119283	ENSG00000033627	ENSG00000164610
ENSG00000147251	ENSG00000184058	ENSG00000094916
ENSG00000133216	ENSG00000100151	ENSG00000177465
ENSG00000123358	ENSG00000066427	ENSG00000156017
ENSG00000100302	ENSG00000133112	ENSG00000177302
ENSG00000205837	ENSG00000172209	ENSG00000196358
ENSG00000008226	ENSG00000039319	ENSG00000155980
ENSG00000084674	ENSG00000105971	ENSG00000136826
ENSG00000106554	ENSG00000180834	ENSG00000126838
ENSG00000146834	ENSG00000151773	ENSG00000105976
ENSG00000100281	ENSG00000121743	ENSG00000065135
ENSG00000119689	ENSG00000166292	ENSG00000102109
ENSG00000137094	ENSG00000170417	ENSG00000158806
ENSG00000120149	ENSG00000196189	ENSG00000105609
ENSG00000163751	ENSG00000138069	ENSG00000164211
ENSG00000170634	ENSG00000174482	ENSG00000165025
ENSG00000114993	ENSG00000135111	ENSG00000221995

ENSG00000144455	ENSG00000136280	ENSG00000131389
ENSG00000158793	ENSG00000134717	ENSG00000170871
ENSG00000096401	ENSG00000114638	ENSG00000120457
ENSG00000101152	ENSG00000090520	ENSG00000144214
ENSG00000138131	ENSG00000135100	ENSG00000124302
ENSG00000106484	ENSG00000166326	ENSG00000134070
ENSG00000154889	ENSG00000121075	ENSG00000165533
ENSG00000134602	ENSG00000162695	ENSG00000173276
ENSG00000174015	ENSG00000165434	ENSG00000162592
ENSG00000122691	ENSG00000060339	ENSG00000178928
ENSG00000168785	ENSG00000198554	ENSG00000134987
ENSG00000163531	ENSG00000167005	ENSG00000088325
ENSG00000111817	ENSG00000070423	ENSG00000054654
ENSG00000101928	ENSG00000164107	ENSG00000165507
ENSG00000163694	ENSG00000182934	ENSG00000173267
ENSG00000189319	ENSG00000157107	ENSG00000119541
ENSG00000135316	ENSG00000213588	ENSG00000180287
ENSG00000136160	ENSG00000142657	ENSG00000125454
ENSG00000168575	ENSG00000143028	ENSG00000205795
ENSG00000132640	ENSG00000121864	ENSG00000120160
ENSG00000134815	ENSG00000055732	ENSG00000143198
ENSG00000176165	ENSG00000021574	ENSG00000109944
ENSG00000025293	ENSG00000103248	ENSG00000162989
ENSG00000168286	ENSG00000126767	ENSG00000213023
ENSG00000109132	ENSG00000083168	ENSG00000141447
ENSG00000119402	ENSG00000253846	ENSG00000035115
ENSG00000162889	ENSG00000135837	ENSG00000197713
ENSG00000165030	ENSG00000073598	ENSG00000160233
ENSG00000172840	ENSG00000165125	ENSG00000143842
ENSG00000072071	ENSG00000152102	ENSG00000119927
ENSG00000148331	ENSG00000100311	ENSG00000118193
ENSG00000116679	ENSG00000004399	ENSG00000157895
ENSG00000075151	ENSG00000109083	ENSG00000164889
ENSG00000187094	ENSG00000149930	ENSG00000170498
ENSG00000112697	ENSG00000204634	ENSG00000175324
ENSG00000185630	ENSG00000249915	ENSG00000007402
ENSG00000165804	ENSG00000109265	ENSG00000156697
ENSG00000197905	ENSG00000102078	ENSG00000173273
ENSG00000132561	ENSG00000134440	ENSG00000133195
ENSG00000143382	ENSG00000136877	ENSG00000172613
ENSG00000171681	ENSG00000129493	ENSG00000103512
ENSG00000101745	ENSG00000139926	ENSG00000173171
ENSG00000151176	ENSG00000099904	ENSG00000139910
ENSG00000134375	ENSG00000147676	ENSG00000113645
ENSG00000117289	ENSG00000152428	ENSG00000196562
ENSG00000169925	ENSG00000115977	ENSG00000213658
ENSG00000075142	ENSG00000137275	ENSG00000073464
ENSG00000148219	ENSG00000172660	ENSG00000205560
ENSG00000111644	ENSG00000107164	ENSG00000188582
ENSG00000136935	ENSG00000124140	ENSG00000143515
ENSG00000104660	ENSG00000085998	ENSG00000143761
ENSG00000100307	ENSG00000107984	ENSG00000166527
ENSG00000163884	ENSG00000130881	ENSG00000070404
ENSG00000145431	ENSG00000136319	ENSG00000179021
ENSG00000179262	ENSG00000126804	ENSG00000108312
ENSG00000028203	ENSG00000101290	ENSG00000109062
ENSG00000204463	ENSG00000175040	ENSG00000006125
ENSG00000102786	ENSG00000106789	ENSG00000118855
ENSG00000110092	ENSG00000175868	ENSG00000128266
ENSG00000120899	ENSG00000112874	ENSG00000125445
ENSG00000104331	ENSG00000147854	ENSG00000120156
ENSG00000146648	ENSG00000102144	ENSG00000241852
ENSG00000124313	ENSG00000134046	ENSG00000164161
ENSG00000106304	ENSG00000197381	ENSG00000105369

ENSG00000198844	ENSG00000145087	ENSG00000107099
ENSG00000176769	ENSG00000108829	ENSG00000168582
ENSG00000137404	ENSG00000159377	ENSG00000104154
ENSG00000080802	ENSG00000108001	ENSG00000239732
ENSG00000081177	ENSG00000147471	ENSG00000015592
ENSG00000145416	ENSG00000137075	ENSG00000185621
ENSG00000163346	ENSG00000065361	ENSG00000075711
ENSG00000115665	ENSG00000101457	ENSG00000204952
ENSG00000102984	ENSG00000171570	ENSG00000116747
ENSG00000162607	ENSG00000107731	ENSG00000173801
ENSG00000119946	ENSG00000022267	ENSG00000166046
ENSG00000175073	ENSG00000151657	ENSG00000182707
ENSG00000163479	ENSG00000184867	ENSG00000188269
ENSG00000125820	ENSG00000184009	ENSG00000178202
ENSG00000102897	ENSG00000166170	ENSG00000134243
ENSG00000144354	ENSG00000165757	ENSG00000128714
ENSG00000147655	ENSG00000104635	ENSG00000066422
ENSG00000138759	ENSG00000108961	ENSG00000163629
ENSG00000186350	ENSG00000162368	ENSG00000198681
ENSG00000096474	ENSG00000165186	ENSG00000126903
ENSG00000031823	ENSG00000179387	ENSG00000088881
ENSG00000181472	ENSG00000079156	ENSG00000067064
ENSG00000141385	ENSG00000162959	ENSG00000205683
ENSG00000157429	ENSG00000119547	ENSG00000156575
ENSG00000177181	ENSG00000072849	ENSG00000130656
ENSG00000177272	ENSG00000148180	ENSG00000154143
ENSG00000171532	ENSG00000172432	ENSG00000025772
ENSG00000124762	ENSG00000080824	ENSG00000127561
ENSG00000101109	ENSG00000054598	ENSG00000128567
ENSG00000137878	ENSG00000137168	ENSG00000168056
ENSG00000156162	ENSG00000135164	ENSG00000167612
ENSG00000206391	ENSG00000118369	ENSG00000133641
ENSG00000055118	ENSG00000171700	ENSG00000197177
ENSG00000196781	ENSG00000182791	ENSG00000146576
ENSG00000041988	ENSG00000132465	ENSG00000196329
ENSG00000141750	ENSG00000129007	ENSG00000169131
ENSG00000164983	ENSG00000197746	ENSG00000163219
ENSG00000134248	ENSG00000166265	ENSG00000205629
ENSG00000169760	ENSG00000140391	ENSG00000092621
ENSG00000136999	ENSG00000085224	ENSG00000172794
ENSG00000188176	ENSG00000101407	ENSG00000117305
ENSG00000100239	ENSG00000149289	ENSG00000139174
ENSG00000096968	ENSG00000108821	ENSG00000204571
ENSG00000151229	ENSG00000138032	ENSG00000108854
ENSG00000149926	ENSG00000128791	ENSG00000112290
ENSG00000109320	ENSG00000167703	ENSG00000137210
ENSG00000104267	ENSG00000165813	ENSG00000164379
ENSG00000204310	ENSG00000184254	ENSG00000134202
ENSG00000173930	ENSG00000007372	ENSG00000149575
ENSG00000167775	ENSG00000111206	ENSG00000239305
ENSG00000142794	ENSG00000072110	ENSG00000112208
ENSG00000100478	ENSG00000082515	ENSG00000179331
ENSG00000161914	ENSG00000101868	ENSG00000182362
ENSG00000135503	ENSG00000133275	ENSG00000122140
ENSG00000131620	ENSG00000126952	ENSG00000181163
ENSG00000183049	ENSG00000134371	ENSG00000160469
ENSG00000169856	ENSG00000100139	ENSG00000197937
ENSG00000198018	ENSG00000197520	ENSG00000141469
ENSG00000147416	ENSG00000070785	ENSG00000078018
ENSG00000105662	ENSG00000172803	ENSG00000122490
ENSG00000185053	ENSG00000176928	ENSG00000064313
ENSG00000112115	ENSG00000253958	ENSG00000092853
ENSG00000075223	ENSG00000212916	ENSG00000089723
ENSG00000185532	ENSG00000064300	ENSG00000089250

ENSG00000129351	ENSG00000105576	ENSG00000100558
ENSG00000174111	ENSG00000113211	ENSG00000116882
ENSG00000198553	ENSG00000177311	ENSG00000161956
ENSG00000125968	ENSG00000136237	ENSG00000188778
ENSG00000124702	ENSG00000152683	ENSG00000178761
ENSG00000099260	ENSG00000162496	ENSG00000178732
ENSG00000198959	ENSG00000165917	ENSG00000198589
ENSG00000151693	ENSG00000180787	ENSG00000106028
ENSG00000183853	ENSG00000001084	ENSG00000143786
ENSG00000145725	ENSG00000143891	ENSG00000165643
ENSG00000206427	ENSG00000174282	ENSG00000144644
ENSG00000178093	ENSG00000115993	ENSG00000022277
ENSG00000125675	ENSG00000131446	ENSG00000138821
ENSG00000089356	ENSG00000166200	ENSG00000183785
ENSG00000159640	ENSG00000159256	ENSG00000036448
ENSG00000175573	ENSG00000171823	ENSG00000165832
ENSG00000103174	ENSG00000166523	ENSG00000099937
ENSG00000165061	ENSG00000135052	ENSG00000111886
ENSG00000136854	ENSG00000122733	ENSG00000184619
ENSG00000180628	ENSG00000109654	ENSG00000125249
ENSG00000033178	ENSG00000100485	ENSG00000103043
ENSG00000007341	ENSG00000127152	ENSG00000172943
ENSG00000189043	ENSG00000163328	ENSG00000114861
ENSG00000142188	ENSG00000140323	ENSG00000166105
ENSG00000066855	ENSG00000080503	ENSG00000197272
ENSG00000188021	ENSG00000120057	ENSG00000103024
ENSG00000167889	ENSG00000073067	ENSG00000114316
ENSG00000163249	ENSG00000160753	ENSG00000073050
ENSG00000183291	ENSG00000157110	ENSG00000244094
ENSG00000081154	ENSG00000132718	ENSG00000138594
ENSG00000113430	ENSG00000124767	ENSG00000064666
ENSG00000114315	ENSG00000065413	ENSG00000213853
ENSG00000170881	ENSG00000197651	ENSG00000186226
ENSG00000101306	ENSG00000156486	ENSG00000185324
ENSG00000029363	ENSG00000221899	ENSG00000129173
ENSG00000154001	ENSG00000072062	ENSG00000119915
ENSG00000136158	ENSG00000157557	ENSG00000174963
ENSG00000151067	ENSG00000123388	ENSG00000151575
ENSG00000186493	ENSG00000169635	ENSG00000119684
ENSG00000143147	ENSG00000185219	ENSG00000128245
ENSG00000086544	ENSG00000151612	ENSG00000179094
ENSG00000184986	ENSG00000135443	ENSG00000170367
ENSG00000144040	ENSG00000164056	ENSG00000079435
ENSG00000100767	ENSG00000136859	ENSG00000170903
ENSG00000115008	ENSG00000138411	ENSG00000185046
ENSG00000181773	ENSG00000157992	ENSG00000056558
ENSG00000176624	ENSG00000108733	ENSG00000130487
ENSG00000166206	ENSG00000153820	ENSG00000131148
ENSG00000146476	ENSG00000023287	ENSG00000102302
ENSG00000085365	ENSG00000094963	ENSG00000119514
ENSG00000091127	ENSG00000116675	ENSG00000166091
ENSG00000176884	ENSG00000173905	ENSG00000204003
ENSG00000038427	ENSG00000148704	ENSG00000109163
ENSG00000166530	ENSG00000147649	ENSG00000234616
ENSG00000070087	ENSG00000165280	ENSG00000122420
ENSG00000138336	ENSG00000183161	ENSG00000114115
ENSG00000118495	ENSG00000174574	ENSG00000100888
ENSG00000034053	ENSG00000180998	ENSG00000177570
ENSG00000143258	ENSG00000181817	ENSG00000243024
ENSG00000139514	ENSG00000073861	ENSG00000078596
ENSG00000072121	ENSG00000087053	ENSG00000120049
ENSG00000198105	ENSG00000198000	ENSG00000179168
ENSG00000111554	ENSG00000135338	ENSG00000137766
ENSG00000102007	ENSG00000183741	ENSG00000151292

ENSG00000167074	ENSG00000115806	ENSG00000013588
ENSG00000008952	ENSG00000101577	ENSG00000129990
ENSG00000137166	ENSG00000153253	ENSG00000176422
ENSG00000140279	ENSG00000162687	ENSG00000214102
ENSG00000169282	ENSG00000011454	ENSG00000105738
ENSG00000168874	ENSG00000138669	ENSG00000174547
ENSG00000136378	ENSG00000137204	ENSG00000170927
ENSG00000121900	ENSG00000240224	ENSG00000197170
ENSG00000116668	ENSG00000112972	ENSG00000123338
ENSG00000112333	ENSG00000133393	ENSG00000129993
ENSG00000167535	ENSG00000011021	ENSG00000125843
ENSG00000150867	ENSG00000198947	ENSG00000174808
ENSG00000127022	ENSG00000005844	ENSG00000149311
ENSG00000186815	ENSG00000160741	ENSG00000054219
ENSG00000168539	ENSG00000100101	ENSG00000087095
ENSG00000133794	ENSG00000156642	ENSG00000152409
ENSG00000198231	ENSG00000182095	ENSG00000126453
ENSG00000012822	ENSG00000127947	ENSG00000158477
ENSG00000197093	ENSG00000106302	ENSG00000185129
ENSG00000095066	ENSG00000165325	ENSG00000137726
ENSG00000169604	ENSG00000186260	ENSG00000152457
ENSG00000075340	ENSG00000183287	ENSG00000149639
ENSG00000204970	ENSG00000094880	ENSG00000149557
ENSG00000112038	ENSG00000177283	ENSG00000124145
ENSG00000134851	ENSG00000109814	ENSG00000137955
ENSG00000124507	ENSG00000134489	ENSG00000112210
ENSG00000102804	ENSG00000196296	ENSG00000142149
ENSG00000165659	ENSG00000151014	ENSG00000157017
ENSG00000104881	ENSG00000163297	ENSG00000122224
ENSG00000145685	ENSG00000180219	ENSG00000033800
ENSG00000087085	ENSG00000168309	ENSG00000028528
ENSG00000113296	ENSG00000113389	ENSG00000168612
ENSG00000112232	ENSG00000166428	ENSG00000142552
ENSG00000111049	ENSG00000168906	ENSG00000077549
ENSG00000122585	ENSG00000068366	ENSG00000125384
ENSG00000198055	ENSG00000119042	ENSG00000241644
ENSG00000137409	ENSG00000087303	ENSG00000163818
ENSG00000196428	ENSG00000070190	ENSG00000250423
ENSG00000167118	ENSG00000137714	ENSG00000113594
ENSG00000068878	ENSG00000100503	ENSG00000144554
ENSG00000126062	ENSG00000085978	ENSG00000128463
ENSG00000160767	ENSG00000123684	ENSG00000102543
ENSG00000168140	ENSG00000079150	ENSG00000108342
ENSG00000175264	ENSG00000144677	ENSG00000180660
ENSG00000135622	ENSG00000074696	ENSG00000203909
ENSG00000165685	ENSG00000006025	ENSG00000124104
ENSG00000146700	ENSG00000018236	ENSG00000104043
ENSG00000185624	ENSG00000151514	ENSG00000182534
ENSG00000115548	ENSG00000101400	ENSG00000171368
ENSG00000171425	ENSG00000173674	ENSG00000109180
ENSG00000163728	ENSG00000158423	ENSG00000168961
ENSG00000117525	ENSG00000139625	ENSG00000100253
ENSG00000091409	ENSG00000112200	ENSG00000142700
ENSG00000092051	ENSG00000170004	ENSG00000142856
ENSG00000153113	ENSG00000151834	ENSG00000164112
ENSG00000182253	ENSG00000060749	ENSG00000137513
ENSG00000006210	ENSG00000196507	ENSG00000186197
ENSG00000067646	ENSG00000198919	ENSG00000134940
ENSG00000185774	ENSG00000153187	ENSG00000099958
ENSG00000106121	ENSG00000197121	ENSG00000133107
ENSG00000163348	ENSG00000146090	ENSG00000163171
ENSG00000186951	ENSG00000169718	ENSG00000177565
ENSG00000138439	ENSG00000159450	ENSG00000149050
ENSG00000128739	ENSG00000100991	ENSG00000149541

ENSG00000103742	ENSG00000166033	ENSG00000196961
ENSG00000137941	ENSG00000126603	ENSG00000187726
ENSG00000157087	ENSG00000178403	ENSG00000197265
ENSG00000109189	ENSG00000111674	ENSG00000163798
ENSG00000134324	ENSG00000120675	ENSG00000180424
ENSG00000109046	ENSG00000111670	ENSG00000204084
ENSG00000167447	ENSG00000215568	ENSG00000174989
ENSG00000169032	ENSG00000115041	ENSG00000050555
ENSG00000205003	ENSG00000079215	ENSG00000156875
ENSG00000059145	ENSG00000116141	ENSG00000186510
ENSG00000100034	ENSG00000102870	ENSG00000172766
ENSG00000137265	ENSG00000102189	ENSG00000203835
ENSG00000177182	ENSG00000104765	ENSG00000136878
ENSG00000159164	ENSG00000007174	ENSG00000101986
ENSG00000074410	ENSG00000067113	ENSG00000152492
ENSG00000060718	ENSG00000101180	ENSG00000122497
ENSG00000181495	ENSG00000152795	ENSG00000184640
ENSG00000196182	ENSG00000180626	ENSG00000169189
ENSG00000173200	ENSG00000089159	ENSG00000174165
ENSG00000100982	ENSG00000100528	ENSG00000146833
ENSG00000153339	ENSG00000186638	ENSG00000127249
ENSG00000110435	ENSG00000131116	ENSG00000188153
ENSG00000106723	ENSG00000197705	ENSG00000182518
ENSG00000164050	ENSG00000110497	ENSG00000006327
ENSG00000001631	ENSG00000188763	ENSG00000213626
ENSG00000136940	ENSG00000168067	ENSG00000087111
ENSG00000136802	ENSG00000120071	ENSG00000143006
ENSG00000182197	ENSG00000172382	ENSG00000125630
ENSG00000155903	ENSG00000147439	ENSG00000169683
ENSG00000117632	ENSG00000198910	ENSG00000172817
ENSG00000073711	ENSG00000172936	ENSG00000164975
ENSG00000107282	ENSG00000006576	ENSG00000188959
ENSG00000115194	ENSG00000170537	ENSG00000101220
ENSG00000198822	ENSG00000132763	ENSG00000178772
ENSG00000176971	ENSG00000170162	ENSG00000007376
ENSG00000083896	ENSG00000136383	ENSG00000166123
ENSG00000125970	ENSG00000162735	ENSG00000136897
ENSG00000131375	ENSG00000141068	ENSG00000163154
ENSG00000049618	ENSG00000133048	ENSG00000185220
ENSG00000169018	ENSG00000142606	ENSG00000213759
ENSG00000152332	ENSG00000170271	ENSG00000159337
ENSG00000075826	ENSG00000096060	ENSG00000133935
ENSG00000166342	ENSG00000149313	ENSG00000086619
ENSG00000197584	ENSG00000144445	ENSG00000008196
ENSG00000196456	ENSG00000175582	ENSG00000047056
ENSG00000141646	ENSG00000131061	ENSG00000141337
ENSG00000178445	ENSG00000125952	ENSG00000050730
ENSG00000128596	ENSG00000148826	ENSG00000088387
ENSG00000165572	ENSG00000120280	ENSG00000196684
ENSG00000129244	ENSG00000138606	ENSG00000213918
ENSG00000121297	ENSG00000155368	ENSG00000106635
ENSG00000010803	ENSG00000182185	ENSG00000183103
ENSG00000107779	ENSG00000085433	ENSG00000120948
ENSG00000186283	ENSG00000144063	ENSG00000105997
ENSG00000152382	ENSG00000089280	ENSG00000198039
ENSG00000204406	ENSG00000135549	ENSG00000127957
ENSG00000136231	ENSG00000156564	ENSG00000090905
ENSG00000186261	ENSG00000102921	ENSG00000167306
ENSG00000169764	ENSG00000184162	ENSG00000112159
ENSG00000135018	ENSG00000170265	ENSG00000241690
ENSG00000104888	ENSG00000180329	ENSG00000160410
ENSG00000146038	ENSG00000078399	ENSG00000143742
ENSG00000166924	ENSG00000176108	ENSG00000136811
ENSG00000104951	ENSG00000047634	ENSG00000105352

ENSG00000160094	ENSG00000163909	ENSG00000066230
ENSG00000140798	ENSG00000106080	ENSG00000160471
ENSG00000164879	ENSG00000126698	ENSG00000143768
ENSG00000123983	ENSG00000092108	ENSG00000064225
ENSG00000164902	ENSG00000215440	ENSG00000124701
ENSG00000172663	ENSG00000137310	ENSG00000129195
ENSG00000163462	ENSG00000165060	ENSG00000125503
ENSG00000086712	ENSG00000077147	ENSG00000166619
ENSG00000164938	ENSG00000111875	ENSG00000113387
ENSG00000181856	ENSG00000170579	ENSG00000120699
ENSG00000103064	ENSG00000153283	ENSG00000222046
ENSG00000126821	ENSG00000168229	ENSG00000155957
ENSG00000183722	ENSG00000189369	ENSG00000132692
ENSG00000169902	ENSG00000197846	ENSG00000131398
ENSG00000155636	ENSG00000141682	ENSG00000107290
ENSG00000066739	ENSG00000140332	ENSG00000130227
ENSG00000138041	ENSG00000115232	ENSG00000170370
ENSG00000157193	ENSG00000198435	ENSG00000062038
ENSG00000142002	ENSG00000152592	ENSG00000158435
ENSG00000070018	ENSG00000097007	ENSG00000101843
ENSG00000175224	ENSG00000183337	ENSG00000182983
ENSG00000081479	ENSG00000032444	ENSG00000088826
ENSG00000165879	ENSG00000048140	ENSG00000132639
ENSG00000109471	ENSG00000132329	ENSG00000063587
ENSG00000148200	ENSG00000136051	ENSG00000154545
ENSG00000019549	ENSG00000065833	ENSG00000078804
ENSG00000066044	ENSG00000143162	ENSG00000170043
ENSG00000185023	ENSG00000204371	ENSG00000145721
ENSG00000087495	ENSG00000173726	ENSG00000074054
ENSG00000163659	ENSG00000133488	ENSG00000149257
ENSG00000132031	ENSG00000181061	ENSG00000131504
ENSG00000196411	ENSG00000174796	ENSG00000069011
ENSG00000170832	ENSG00000160716	ENSG00000160013
ENSG00000153823	ENSG00000147443	ENSG00000012504
ENSG00000119703	ENSG00000136937	ENSG00000135750
ENSG00000181789	ENSG00000059573	ENSG00000167394
ENSG00000183486	ENSG00000004948	ENSG00000167380
ENSG00000108424	ENSG00000168538	ENSG00000101849
ENSG00000111231	ENSG00000104983	ENSG00000177096
ENSG00000174938	ENSG00000006075	ENSG00000128602
ENSG00000167258	ENSG00000145860	ENSG00000240891
ENSG00000180818	ENSG00000036672	ENSG00000106780
ENSG00000139970	ENSG00000136842	ENSG00000120925
ENSG00000108953	ENSG00000136636	ENSG00000120328
ENSG00000183784	ENSG00000147408	ENSG00000169087
ENSG00000168314	ENSG00000123444	ENSG00000159403
ENSG00000172239	ENSG00000050405	ENSG00000141367
ENSG00000072518	ENSG00000065000	ENSG00000134452
ENSG00000139826	ENSG00000174576	ENSG00000182450
ENSG00000180228	ENSG00000101333	ENSG00000167633
ENSG00000171720	ENSG00000180964	ENSG00000213213
ENSG00000112493	ENSG00000188706	ENSG00000176845
ENSG00000165650	ENSG00000111596	ENSG00000186432
ENSG00000163207	ENSG00000100258	ENSG00000143507
ENSG00000044574	ENSG00000148341	ENSG00000124562
ENSG00000122550	ENSG00000182704	ENSG00000223609
ENSG00000114251	ENSG00000167785	ENSG00000120669
ENSG00000196549	ENSG00000164039	ENSG00000122786
ENSG00000157514	ENSG00000179520	ENSG00000156009
ENSG00000182481	ENSG00000011275	ENSG00000166405
ENSG00000188987	ENSG00000113638	ENSG00000107954
ENSG00000107736	ENSG00000130703	ENSG00000167244
ENSG00000143878	ENSG00000143418	ENSG00000131015
ENSG00000141639	ENSG00000130827	ENSG00000213901

ENSG00000164303	ENSG00000152254	ENSG00000166947
ENSG00000159840	ENSG00000155657	ENSG00000131437
ENSG00000067141	ENSG00000067191	ENSG00000177370
ENSG00000198162	ENSG00000197959	ENSG00000154274
ENSG00000151276	ENSG00000010671	ENSG00000158710
ENSG00000134255	ENSG00000125851	ENSG00000103005
ENSG00000095564	ENSG00000140350	ENSG00000184408
ENSG00000116017	ENSG00000143630	ENSG00000058673
ENSG00000176658	ENSG00000006062	ENSG00000129128
ENSG00000185760	ENSG00000137992	ENSG00000183196
ENSG00000073849	ENSG00000108639	ENSG00000167232
ENSG00000089177	ENSG00000153179	ENSG00000091128
ENSG00000101463	ENSG00000139352	ENSG00000159267
ENSG00000100813	ENSG00000083799	ENSG00000251380
ENSG00000100664	ENSG00000103160	ENSG00000101126
ENSG00000125107	ENSG00000131386	ENSG00000016402
ENSG00000136535	ENSG00000167522	ENSG00000114650
ENSG00000165379	ENSG00000125965	ENSG00000117394
ENSG00000124920	ENSG00000162105	ENSG00000085741
ENSG00000125629	ENSG00000188428	ENSG00000077080
ENSG00000163399	ENSG00000138615	ENSG00000183242
ENSG00000119899	ENSG00000196235	ENSG00000008197
ENSG00000099917	ENSG00000146112	ENSG00000205362
ENSG00000135048	ENSG00000092758	ENSG00000163131
ENSG00000048991	ENSG00000180332	ENSG00000169967
ENSG00000116251	ENSG00000088682	ENSG00000149256
ENSG00000159363	ENSG00000130477	ENSG00000173436
ENSG00000124193	ENSG00000090020	ENSG00000064490
ENSG00000011009	ENSG00000105855	ENSG00000109971
ENSG00000164088	ENSG00000138495	ENSG00000132429
ENSG00000161791	ENSG00000174123	ENSG00000141298
ENSG00000144118	ENSG00000119820	ENSG00000171872
ENSG00000162702	ENSG00000174564	ENSG00000144119
ENSG00000101346	ENSG00000176903	ENSG00000143032
ENSG00000135333	ENSG00000168032	ENSG00000165702
ENSG00000165966	ENSG00000105866	ENSG00000168259
ENSG00000101958	ENSG00000188542	ENSG00000099864
ENSG00000169607	ENSG00000068971	ENSG00000188770
ENSG00000100078	ENSG00000187189	ENSG00000213015
ENSG00000026652	ENSG00000112167	ENSG00000049245
ENSG00000159263	ENSG00000039068	ENSG00000183624
ENSG00000134698	ENSG00000140479	ENSG00000109743
ENSG00000100207	ENSG00000114346	ENSG00000164077
ENSG00000090273	ENSG00000153487	ENSG00000126500
ENSG00000120742	ENSG00000166257	ENSG00000071282
ENSG00000060656	ENSG00000082175	ENSG00000165527
ENSG00000169398	ENSG00000175161	ENSG00000031544
ENSG00000136830	ENSG00000198369	ENSG00000142089
ENSG00000088882	ENSG00000137817	ENSG00000250834
ENSG00000059804	ENSG00000171303	ENSG00000243709
ENSG00000185658	ENSG00000067715	ENSG00000206150
ENSG00000057704	ENSG00000113649	ENSG00000134250
ENSG00000188483	ENSG00000135452	ENSG00000185176
ENSG00000187079	ENSG00000178741	ENSG00000101842
ENSG00000161048	ENSG00000107560	ENSG00000166111
ENSG00000019144	ENSG00000168461	ENSG00000196664
ENSG00000156030	ENSG00000060138	ENSG00000116824
ENSG00000075420	ENSG00000120694	ENSG00000135148
ENSG00000141568	ENSG00000166006	ENSG00000184709
ENSG00000184489	ENSG00000129675	ENSG00000166959
ENSG00000146457	ENSG00000107745	ENSG00000001630
ENSG00000181220	ENSG00000162004	ENSG00000159579
ENSG00000117155	ENSG00000058866	ENSG00000121067
ENSG00000164654	ENSG00000162298	ENSG00000153560

ENSG00000139687	ENSG00000152766	ENSG00000107338
ENSG00000052723	ENSG00000175564	ENSG00000115425
ENSG00000086200	ENSG00000134775	ENSG00000221957
ENSG00000181704	ENSG00000012983	ENSG00000161011
ENSG00000086475	ENSG00000153774	ENSG00000106070
ENSG00000116717	ENSG00000243789	ENSG00000135697
ENSG00000115525	ENSG00000008735	ENSG00000145390
ENSG00000167614	ENSG00000141441	ENSG00000128253
ENSG00000104447	ENSG00000163596	ENSG00000164109
ENSG00000155849	ENSG00000160087	ENSG00000167191
ENSG00000175175	ENSG00000158055	ENSG00000139182
ENSG00000161638	ENSG00000155744	ENSG00000089169
ENSG00000163235	ENSG00000116205	ENSG00000148334
ENSG00000164176	ENSG00000158321	ENSG00000087086
ENSG00000108370	ENSG00000175646	ENSG00000179477
ENSG00000122008	ENSG00000172007	ENSG00000053371
ENSG00000189266	ENSG00000149115	ENSG00000170296
ENSG00000171812	ENSG00000159251	ENSG00000164663
ENSG00000150938	ENSG00000141258	ENSG00000106541
ENSG00000118263	ENSG00000115109	ENSG00000106113
ENSG00000205624	ENSG00000144320	ENSG00000134809
ENSG00000067208	ENSG00000187605	ENSG00000158186
ENSG00000140836	ENSG00000100372	ENSG00000184007
ENSG00000174799	ENSG00000170234	ENSG00000122378
ENSG00000109332	ENSG00000140264	ENSG00000140564
ENSG00000114933	ENSG00000132182	ENSG00000003249
ENSG00000118762	ENSG00000162738	ENSG00000123636
ENSG00000072501	ENSG00000146555	ENSG00000154642
ENSG00000107443	ENSG00000089123	ENSG00000163704
ENSG00000111731	ENSG00000170113	ENSG00000122824
ENSG00000101557	ENSG00000172348	ENSG00000154556
ENSG00000165458	ENSG00000163577	ENSG00000196559
ENSG00000196358	ENSG00000204070	ENSG00000104332
ENSG00000168939	ENSG00000186629	ENSG00000127084
ENSG00000182489	ENSG00000183778	ENSG00000164597
ENSG00000177468	ENSG00000164292	ENSG00000182149
ENSG00000152782	ENSG00000095303	ENSG00000213533
ENSG00000162891	ENSG00000123243	ENSG00000049540
ENSG00000171223	ENSG00000120093	ENSG00000122335
ENSG00000158286	ENSG00000147852	ENSG00000005381
ENSG00000140612	ENSG00000166402	ENSG00000185127
ENSG00000155980	ENSG00000175938	ENSG00000125449
ENSG00000121892	ENSG00000177427	ENSG00000107798
ENSG00000136826	ENSG00000196642	ENSG00000223380
ENSG00000105976	ENSG00000074047	ENSG00000196712
ENSG00000065135	ENSG00000170703	ENSG00000080815
ENSG00000132155	ENSG00000081842	ENSG00000124570
ENSG00000136021	ENSG00000198315	ENSG00000168630
ENSG00000108688	ENSG00000140199	ENSG00000067445
ENSG00000131389	ENSG00000162374	ENSG00000116922
ENSG00000163875	ENSG00000164933	ENSG00000138758
ENSG00000196371	ENSG00000068724	ENSG00000198729
ENSG00000148634	ENSG00000132854	ENSG00000101323
ENSG00000116237	ENSG00000091157	ENSG00000188313
ENSG00000124302	ENSG00000161544	ENSG00000175348
ENSG00000101695	ENSG00000143324	ENSG00000184611
ENSG00000007392	ENSG00000103257	ENSG00000137877
ENSG00000145888	ENSG00000187800	ENSG00000168924
ENSG00000142327	ENSG00000121207	ENSG00000135269
ENSG00000144381	ENSG00000036549	ENSG00000106571
ENSG00000173276	ENSG00000065534	ENSG00000156261
ENSG00000147027	ENSG00000121895	ENSG00000182308
ENSG00000167642	ENSG00000186130	ENSG00000165338
ENSG00000178928	ENSG00000010072	ENSG00000236613

ENSG00000074755	ENSG00000197646	ENSG00000168509
ENSG00000054654	ENSG00000036257	ENSG00000196419
ENSG00000180287	ENSG00000183166	ENSG00000204611
ENSG00000119541	ENSG00000103275	ENSG00000124486
ENSG00000108262	ENSG00000116157	ENSG00000120129
ENSG00000162989	ENSG00000179222	ENSG00000100296
ENSG00000115226	ENSG00000108511	ENSG00000119285
ENSG00000094975	ENSG00000152377	ENSG00000110619
ENSG00000141447	ENSG00000135094	ENSG00000144711
ENSG00000158711	ENSG00000142556	ENSG00000159335
ENSG00000197713	ENSG00000214022	ENSG00000096996
ENSG00000106077	ENSG00000148158	ENSG00000154864
ENSG00000143842	ENSG00000115307	ENSG00000085872
ENSG00000140937	ENSG00000167580	ENSG00000013503
ENSG00000119927	ENSG00000175931	ENSG00000139323
ENSG00000107249	ENSG00000240065	ENSG00000176274
ENSG00000006831	ENSG00000197757	ENSG00000196963
ENSG00000139351	ENSG00000064102	ENSG00000111639
ENSG00000078295	ENSG00000147894	ENSG00000198046
ENSG00000160183	ENSG00000167566	ENSG00000164742
ENSG00000163599	ENSG00000134326	ENSG00000109158
ENSG00000109606	ENSG00000139219	ENSG00000196511
ENSG00000136240	ENSG00000186532	ENSG00000177508
ENSG00000175324	ENSG00000138722	ENSG00000102962
ENSG00000116209	ENSG00000183570	ENSG00000155287
ENSG00000007402	ENSG00000011114	ENSG00000175745
ENSG00000162222	ENSG00000112079	ENSG00000180096
ENSG00000133195	ENSG00000131828	ENSG00000174953
ENSG00000122912	ENSG00000167395	ENSG00000125148
ENSG00000103512	ENSG00000173391	ENSG00000196966
ENSG00000151247	ENSG00000112902	ENSG00000165282
ENSG00000173171	ENSG00000124479	ENSG00000122965
ENSG00000188730	ENSG00000174804	ENSG00000106025
ENSG00000139910	ENSG00000168830	ENSG00000186193
ENSG00000113645	ENSG00000143340	ENSG00000130724
ENSG00000183619	ENSG00000198836	ENSG00000167900
ENSG00000119048	ENSG00000131876	ENSG00000161381
ENSG00000188582	ENSG00000161960	ENSG00000119725
ENSG00000021645	ENSG00000185436	ENSG00000152601
ENSG00000049656	ENSG00000133703	ENSG00000158805
ENSG00000143761	ENSG00000178904	ENSG00000155926
ENSG00000179021	ENSG00000075568	ENSG00000185149
ENSG00000155640	ENSG00000040608	ENSG00000136247
ENSG00000181915	ENSG00000137962	ENSG00000135740
ENSG00000109062	ENSG00000110881	ENSG00000182263
ENSG00000108312	ENSG00000178252	ENSG00000198901
ENSG00000006125	ENSG00000204618	ENSG00000105374
ENSG00000109685	ENSG00000198517	ENSG00000025039
ENSG00000113966	ENSG00000135446	ENSG00000145337
ENSG00000165238	ENSG00000156650	ENSG00000104369
ENSG00000118639	ENSG00000163482	ENSG00000016082
ENSG00000128266	ENSG00000125378	ENSG00000118513
ENSG00000139146	ENSG00000143952	ENSG00000183837
ENSG00000135387	ENSG00000103966	ENSG00000132437
ENSG00000164161	ENSG00000005961	ENSG00000007312
ENSG00000137101	ENSG00000108389	ENSG00000134461
ENSG00000133121	ENSG00000011451	ENSG00000184330
ENSG00000164949	ENSG00000167799	ENSG00000102230
ENSG00000104154	ENSG00000065978	ENSG00000185070
ENSG00000170419	ENSG00000151135	ENSG00000105135
ENSG00000182022	ENSG00000110448	ENSG00000163466
ENSG00000015592	ENSG00000152700	ENSG00000187175
ENSG00000116747	ENSG00000113369	ENSG00000142347
ENSG00000173801	ENSG00000196821	ENSG00000124449

ENSG00000144224	ENSG00000106346	ENSG00000099954
ENSG00000108264	ENSG00000101193	ENSG00000116147
ENSG00000119227	ENSG00000066926	ENSG00000160193
ENSG00000134243	ENSG00000179813	ENSG00000124103
ENSG00000128714	ENSG00000123178	ENSG00000064999
ENSG00000134825	ENSG00000248857	ENSG00000136436
ENSG00000107187	ENSG00000120265	ENSG00000172216
ENSG00000079335	ENSG00000105516	ENSG00000104894
ENSG00000066422	ENSG00000184838	ENSG00000100711
ENSG00000184771	ENSG00000166562	ENSG00000180138
ENSG00000168036	ENSG00000135999	ENSG00000107807
ENSG00000141698	ENSG00000090975	ENSG00000123989
ENSG00000163629	ENSG00000155066	ENSG00000167778
ENSG00000126903	ENSG00000119487	ENSG00000181291
ENSG00000066827	ENSG00000170454	ENSG00000082126
ENSG00000205683	ENSG00000143373	ENSG00000143466
ENSG00000157873	ENSG00000065485	ENSG00000186834
ENSG00000112238	ENSG00000178568	ENSG00000158458
ENSG00000164823	ENSG00000079819	ENSG00000142920
ENSG00000127561	ENSG00000157103	ENSG00000141448
ENSG00000128567	ENSG00000156194	ENSG00000196326
ENSG00000162783	ENSG00000112541	ENSG00000085552
ENSG00000124802	ENSG00000176915	ENSG00000175662
ENSG00000115355	ENSG00000010278	ENSG00000166157
ENSG00000103187	ENSG00000141485	ENSG00000213762
ENSG00000169131	ENSG00000165731	ENSG00000077380
ENSG00000198026	ENSG00000041515	ENSG00000130768
ENSG00000161896	ENSG00000182952	ENSG00000110203
ENSG00000172794	ENSG00000030419	ENSG00000179455
ENSG00000160712	ENSG00000034063	ENSG00000103343
ENSG00000163820	ENSG00000075213	ENSG00000146414
ENSG00000069998	ENSG00000185085	ENSG00000128268
ENSG00000139174	ENSG00000141034	ENSG00000174004
ENSG00000170142	ENSG00000183114	ENSG00000140832
ENSG00000169231	ENSG00000179604	ENSG00000140534
ENSG00000140465	ENSG00000110025	ENSG00000108753
ENSG00000108854	ENSG00000144909	ENSG00000115738
ENSG00000112290	ENSG00000143321	ENSG00000148702
ENSG00000164379	ENSG00000165490	ENSG00000130741
ENSG00000149575	ENSG00000143954	ENSG00000205927
ENSG00000168334	ENSG00000005889	ENSG00000071626
ENSG00000117411	ENSG00000143995	ENSG00000122971
ENSG00000119638	ENSG00000119335	ENSG00000211455
ENSG00000128908	ENSG00000117009	ENSG00000088827
ENSG00000112208	ENSG00000112293	ENSG00000123353
ENSG00000135723	ENSG00000169813	ENSG00000163811
ENSG00000198732	ENSG00000196715	ENSG00000156269
ENSG00000147421	ENSG00000113312	ENSG00000129538
ENSG00000181163	ENSG00000119718	ENSG00000155256
ENSG00000160469	ENSG00000132603	ENSG00000145331
ENSG00000119698	ENSG00000164756	ENSG00000101076
ENSG00000129038	ENSG00000064652	ENSG00000174137
ENSG00000065559	ENSG00000135775	ENSG00000139190
ENSG00000048649	ENSG00000173207	ENSG00000166411
ENSG00000141469	ENSG00000171723	ENSG00000078808
ENSG00000123144	ENSG00000163501	ENSG00000103510
ENSG00000153914	ENSG00000153291	ENSG00000121406
ENSG00000078018	ENSG00000155508	ENSG00000196757
ENSG00000077454	ENSG00000102119	ENSG00000115419
ENSG00000117139	ENSG00000128923	ENSG00000100565
ENSG00000076108	ENSG00000101856	ENSG00000041880
ENSG00000166949	ENSG00000105137	ENSG00000185344
ENSG00000185019	ENSG00000204130	ENSG00000130175
ENSG00000089723	ENSG00000046653	ENSG00000106397

ENSG00000100558	ENSG00000102172	ENSG00000007168
ENSG00000161956	ENSG00000166848	ENSG00000170191
ENSG00000188778	ENSG00000107201	ENSG00000244462
ENSG00000100242	ENSG00000145242	ENSG00000101084
ENSG00000178761	ENSG00000114745	ENSG00000125255
ENSG00000135222	ENSG00000106462	ENSG00000105364
ENSG00000100246	ENSG00000168209	ENSG00000173992
ENSG00000182568	ENSG00000240849	ENSG00000134760
ENSG00000198589	ENSG00000198960	ENSG00000214872
ENSG00000165233	ENSG00000165138	ENSG00000132199
ENSG00000132842	ENSG00000126583	ENSG00000126768
ENSG00000113269	ENSG00000153956	ENSG00000060491
ENSG00000138821	ENSG00000206527	ENSG00000103653
ENSG00000103342	ENSG00000124440	ENSG00000089289
ENSG00000165832	ENSG00000116273	ENSG00000138592
ENSG00000170779	ENSG00000138398	ENSG00000070614
ENSG00000107719	ENSG00000160058	ENSG00000164604
ENSG00000055044	ENSG00000047346	ENSG00000187815
ENSG00000145309	ENSG00000124067	ENSG00000213047
ENSG00000125249	ENSG00000169429	ENSG00000172350
ENSG00000197214	ENSG00000164466	ENSG00000108439
ENSG00000086300	ENSG00000170486	ENSG00000164609
ENSG00000117640	ENSG00000105357	ENSG00000163781
ENSG00000172943	ENSG00000146574	ENSG00000137273
ENSG00000114861	ENSG00000113763	ENSG00000088256
ENSG00000169436	ENSG00000105810	ENSG00000169057
ENSG00000115459	ENSG00000174775	ENSG00000162599
ENSG00000181790	ENSG00000111913	ENSG00000119411
ENSG00000138594	ENSG00000139793	ENSG00000163209
ENSG00000185324	ENSG00000082269	ENSG00000184678
ENSG00000129173	ENSG00000127954	ENSG00000167513
ENSG00000119915	ENSG00000169891	ENSG00000120784
ENSG00000168386	ENSG00000185164	ENSG00000088320
ENSG00000174963	ENSG00000119457	ENSG00000170477
ENSG00000139737	ENSG00000166963	ENSG00000139998
ENSG00000128245	ENSG00000176533	ENSG00000130299
ENSG00000179094	ENSG00000131470	ENSG00000102218
ENSG00000100284	ENSG00000129933	ENSG00000144648
ENSG00000154175	ENSG00000112378	ENSG00000169896
ENSG00000017260	ENSG00000136448	ENSG00000066336
ENSG00000185046	ENSG00000134504	ENSG00000115808
ENSG00000198791	ENSG00000174437	ENSG00000088812
ENSG00000135930	ENSG00000197045	ENSG00000168453
ENSG00000102302	ENSG00000107815	ENSG00000087250
ENSG00000173473	ENSG00000188227	ENSG00000189056
ENSG00000138678	ENSG00000147394	ENSG00000165195
ENSG00000140307	ENSG00000135823	ENSG00000126251
ENSG00000077721	ENSG00000107625	ENSG00000066583
ENSG00000102683	ENSG00000163904	ENSG00000160882
ENSG00000111786	ENSG00000243978	ENSG00000172201
ENSG00000167930	ENSG00000181090	ENSG00000166118
ENSG00000196792	ENSG00000121848	ENSG00000196975
ENSG00000205673	ENSG00000067798	ENSG00000164440
ENSG00000122420	ENSG00000086289	ENSG00000168329
ENSG00000198513	ENSG00000128340	ENSG00000035403
ENSG00000166579	ENSG00000143621	ENSG00000055332
ENSG00000128322	ENSG00000198794	ENSG00000063438
ENSG00000162701	ENSG00000187969	ENSG00000055813
ENSG00000089006	ENSG00000140280	ENSG00000173221
ENSG00000157933	ENSG00000167178	ENSG00000073350
ENSG00000120049	ENSG00000125812	ENSG00000163935
ENSG00000183662	ENSG00000155660	ENSG00000163885
ENSG00000115561	ENSG00000147509	ENSG00000111335
ENSG00000206407	ENSG00000242732	ENSG00000185825

ENSG00000151292	ENSG00000163714	ENSG00000213585
ENSG00000013588	ENSG00000160307	ENSG00000159792
ENSG00000176422	ENSG00000173706	ENSG00000185650
ENSG00000008130	ENSG00000163508	ENSG00000084764
ENSG00000105186	ENSG00000054803	ENSG00000147162
ENSG00000153993	ENSG00000128591	ENSG00000143458
ENSG00000205309	ENSG00000107672	ENSG00000121769
ENSG00000197170	ENSG00000198492	ENSG00000197405
ENSG00000198612	ENSG00000124225	ENSG00000155858
ENSG00000129993	ENSG00000135083	ENSG00000231924
ENSG00000100227	ENSG00000198242	ENSG00000170949
ENSG00000184185	ENSG00000100036	ENSG00000187848
ENSG00000110047	ENSG00000121741	ENSG00000139988
ENSG00000154237	ENSG00000166266	ENSG00000169814
ENSG00000149311	ENSG00000137413	ENSG00000250312
ENSG00000140451	ENSG00000100292	ENSG00000105605
ENSG00000164695	ENSG00000101945	ENSG00000061987
ENSG00000087095	ENSG00000136011	ENSG00000130635
ENSG00000126453	ENSG00000198742	ENSG00000131495
ENSG00000115170	ENSG00000152952	ENSG00000136295
ENSG00000204420	ENSG00000007952	ENSG00000104722
ENSG00000185129	ENSG00000104885	ENSG00000136279
ENSG00000137726	ENSG00000157510	ENSG00000010404
ENSG00000166167	ENSG00000108947	ENSG00000145740
ENSG00000151615	ENSG00000197457	ENSG00000148358
ENSG00000149639	ENSG00000169744	ENSG00000100987
ENSG00000124145	ENSG00000126814	ENSG00000140403
ENSG00000142149	ENSG00000205336	ENSG00000129292
ENSG00000112210	ENSG00000132507	ENSG00000204001
ENSG00000124357	ENSG00000134318	ENSG00000110514
ENSG00000033800	ENSG00000076864	ENSG00000117091
ENSG00000028528	ENSG00000090061	ENSG00000162148
ENSG00000174233	ENSG00000187942	ENSG00000177732
ENSG00000198944	ENSG00000114374	ENSG00000128284
ENSG00000113594	ENSG00000069966	ENSG00000149428
ENSG00000128463	ENSG00000179029	ENSG00000166589
ENSG00000108342	ENSG00000168242	ENSG00000160408
ENSG00000180660	ENSG00000130733	ENSG00000091656
ENSG00000177426	ENSG00000131791	ENSG00000168004
ENSG00000110931	ENSG00000129354	ENSG00000138379
ENSG00000124104	ENSG00000102908	ENSG00000130699
ENSG00000181827	ENSG00000137807	ENSG00000182393
ENSG00000171385	ENSG00000090447	ENSG00000173409
ENSG00000135362	ENSG00000144278	ENSG00000113263
ENSG00000181722	ENSG00000084234	ENSG00000108406
ENSG00000111816	ENSG00000102755	ENSG00000249992
ENSG00000134644	ENSG00000121058	ENSG00000149923
ENSG00000176994	ENSG00000167747	ENSG00000034971
ENSG00000101266	ENSG00000159648	ENSG00000105707
ENSG00000115091	ENSG00000152284	ENSG00000118242
ENSG00000197579	ENSG00000163029	ENSG00000145819
ENSG00000142856	ENSG00000197111	ENSG00000154438
ENSG00000140623	ENSG00000164548	ENSG00000163067
ENSG00000187486	ENSG00000121964	ENSG00000143093
ENSG00000132589	ENSG00000174469	ENSG00000124466
ENSG00000126945	ENSG00000137261	ENSG00000173546
ENSG00000099958	ENSG00000197321	ENSG00000213401
ENSG00000171451	ENSG00000107263	ENSG00000105643
ENSG00000169242	ENSG00000083937	ENSG00000083845
ENSG00000163171	ENSG00000009954	ENSG00000112592
ENSG00000151320	ENSG00000185666	ENSG00000211448
ENSG00000149541	ENSG00000168491	ENSG00000116754
ENSG00000196961	ENSG00000131051	ENSG00000125124
ENSG00000197265	ENSG00000119421	ENSG00000157796

ENSG00000114383	ENSG00000103423	ENSG00000172461
ENSG00000204084	ENSG00000123159	ENSG00000106153
ENSG00000024862	ENSG00000171587	ENSG00000170242
ENSG00000115540	ENSG00000169306	ENSG00000105695
ENSG00000156875	ENSG00000159733	ENSG00000087338
ENSG00000151967	ENSG00000181754	ENSG00000146192
ENSG00000136878	ENSG00000218823	ENSG00000084754
ENSG00000163873	ENSG00000168398	ENSG00000021355
ENSG00000198121	ENSG00000135093	ENSG00000132382
ENSG00000101986	ENSG00000162928	ENSG00000136381
ENSG00000107758	ENSG00000054267	ENSG00000138035
ENSG00000122497	ENSG00000169299	ENSG00000105967
ENSG00000135127	ENSG00000204301	ENSG00000166862
ENSG00000162302	ENSG00000058272	ENSG00000139668
ENSG00000120690	ENSG00000136888	ENSG00000157240
ENSG00000188153	ENSG00000135862	ENSG00000163406
ENSG00000006327	ENSG00000006432	ENSG00000140382
ENSG00000166337	ENSG00000163637	ENSG00000108604
ENSG00000139890	ENSG00000117318	ENSG00000105948
ENSG00000087111	ENSG00000135414	ENSG00000116095
ENSG00000164626	ENSG00000068793	ENSG00000170374
ENSG00000198286	ENSG00000158158	ENSG00000204219
ENSG00000171634	ENSG00000167113	ENSG00000163762
ENSG00000010270	ENSG00000119938	ENSG00000143409
ENSG00000139209	ENSG00000173559	ENSG00000169688
ENSG00000166123	ENSG00000148187	ENSG00000100997
ENSG00000151490	ENSG00000165689	ENSG00000175893
ENSG00000104936	ENSG00000069275	ENSG00000164736
ENSG00000158560	ENSG00000121388	ENSG00000100505
ENSG00000160877	ENSG00000172572	ENSG00000112305
ENSG00000163154	ENSG00000159423	ENSG00000115207
ENSG00000182742	ENSG00000058085	ENSG00000144674
ENSG00000138279	ENSG00000115592	ENSG00000171243
ENSG00000008196	ENSG00000186897	ENSG00000142949
ENSG00000086619	ENSG00000116199	ENSG00000082438
ENSG00000047056	ENSG00000170689	ENSG00000159592
ENSG00000198646	ENSG00000183773	ENSG00000120088
ENSG00000088387	ENSG00000165995	ENSG00000065883
ENSG00000106635	ENSG00000127129	ENSG00000239264
ENSG00000100376	ENSG00000128564	ENSG00000164808
ENSG00000184047	ENSG00000114013	ENSG00000162461
ENSG00000204576	ENSG00000078401	ENSG00000143379
ENSG00000182158	ENSG00000103241	ENSG00000134419
ENSG00000120948	ENSG00000162402	ENSG00000125962
ENSG00000105997	ENSG00000077585	ENSG00000164649
ENSG00000168675	ENSG00000066382	ENSG00000115694
ENSG00000065357	ENSG00000183780	ENSG00000196132
ENSG00000171603	ENSG00000112118	ENSG00000145244
ENSG00000127957	ENSG00000177380	ENSG00000109787
ENSG00000163635	ENSG00000120158	ENSG00000147224
ENSG00000090905	ENSG00000106615	ENSG00000197345
ENSG00000160410	ENSG00000070759	ENSG00000148925
ENSG00000153561	ENSG00000134152	ENSG00000007202
ENSG00000136811	ENSG00000103091	ENSG00000088756
ENSG00000179431	ENSG00000166825	ENSG00000125870
ENSG00000124701	ENSG00000007516	ENSG00000113384
ENSG00000205944	ENSG00000108387	ENSG00000186354
ENSG00000065526	ENSG00000197587	ENSG00000197429
ENSG00000166619	ENSG00000115415	ENSG00000065717
ENSG00000125503	ENSG00000110911	ENSG00000198346
ENSG00000113387	ENSG00000108556	ENSG00000181541
ENSG00000179981	ENSG00000163349	ENSG00000136261
ENSG00000173848	ENSG00000181481	ENSG00000143970
ENSG00000156521	ENSG00000139154	ENSG00000049323

ENSG00000130227	ENSG00000124942	ENSG00000058668
ENSG00000170370	ENSG00000039560	ENSG00000176641
ENSG00000177879	ENSG00000102572	ENSG00000169727
ENSG00000158435	ENSG00000011258	ENSG00000123143
ENSG00000005483	ENSG00000197562	ENSG00000184232
ENSG00000112182	ENSG00000165131	ENSG00000116721
ENSG00000123685	ENSG00000184343	ENSG00000066056
ENSG00000204961	ENSG00000125354	ENSG00000163554
ENSG00000088826	ENSG00000136944	ENSG00000109861
ENSG00000151917	ENSG00000117016	ENSG00000142892
ENSG00000179674	ENSG00000116031	ENSG00000104213
ENSG00000128285	ENSG00000196890	ENSG00000087263
ENSG00000169194	ENSG00000156384	ENSG00000135929
ENSG00000112562	ENSG00000110484	ENSG00000111224
ENSG00000132639	ENSG00000152137	ENSG00000203859
ENSG00000129691	ENSG00000163848	ENSG00000184613
ENSG00000146963	ENSG00000187855	ENSG00000105426
ENSG00000050165	ENSG00000198373	ENSG00000164830
ENSG00000159399	ENSG00000204217	ENSG00000169740
ENSG00000170852	ENSG00000137145	ENSG00000174238
ENSG00000170043	ENSG00000178645	ENSG00000123240
ENSG00000078804	ENSG00000180251	ENSG00000157765
ENSG00000131725	ENSG00000102978	ENSG00000181631
ENSG00000187736	ENSG00000137203	ENSG00000119979
ENSG00000074054	ENSG00000138293	ENSG00000111305
ENSG00000204968	ENSG00000092439	ENSG00000019995
ENSG00000131504	ENSG00000127220	ENSG00000139163
	ENSG00000177189	ENSG00000196805
	ENSG00000072609	ENSG00000005421
	ENSG00000125414	ENSG00000078369
	ENSG00000010322	ENSG00000124788
	ENSG00000150471	ENSG00000086967
	ENSG00000154783	ENSG00000138138
	ENSG00000154478	ENSG00000165072
	ENSG00000113360	ENSG00000169379
	ENSG00000176105	ENSG00000143632
	ENSG00000116514	ENSG00000242265
	ENSG00000158195	ENSG00000105401
	ENSG00000138074	ENSG00000112936
	ENSG00000196459	ENSG00000172059
	ENSG00000169641	ENSG00000134057
	ENSG00000164024	ENSG00000135521
	ENSG00000165675	ENSG00000120992
	ENSG00000168404	ENSG00000115761
	ENSG00000180530	ENSG00000182782
	ENSG00000110786	ENSG00000123307
	ENSG00000180304	ENSG00000117713
	ENSG00000134369	ENSG00000168298
	ENSG00000123700	ENSG00000152520
	ENSG00000127914	ENSG00000166780
	ENSG00000088726	ENSG00000136827
	ENSG00000178425	ENSG00000125779
	ENSG00000197694	ENSG00000101445
	ENSG00000107829	ENSG00000171606
	ENSG00000213741	ENSG00000138670
	ENSG00000213578	ENSG00000106852
	ENSG00000145824	ENSG00000150625
	ENSG00000162415	ENSG00000137522
	ENSG00000185728	ENSG00000149564
	ENSG00000128891	ENSG00000178184
	ENSG00000082641	ENSG00000023734
	ENSG00000203710	ENSG00000179152
	ENSG00000186732	ENSG00000068383
	ENSG00000129521	ENSG00000131482

ENSG00000006007
ENSG00000197256
ENSG00000069869
ENSG00000151715
ENSG00000166068
ENSG00000174718
ENSG00000168542
ENSG00000177614
ENSG00000184277
ENSG00000072042
ENSG00000168267
ENSG00000112473
ENSG00000142910
ENSG00000131773
ENSG00000167371
ENSG00000139722
ENSG00000184574
ENSG00000163539
ENSG00000104093
ENSG00000115649
ENSG00000159723
ENSG00000197343
ENSG00000087245
ENSG00000133318
ENSG00000182035
ENSG00000198815
ENSG00000114857
ENSG00000132424
ENSG00000136541
ENSG00000101216
ENSG00000101782
ENSG00000108352
ENSG00000169714
ENSG00000103942
ENSG00000164093
ENSG00000111961
ENSG00000157423
ENSG00000177125
ENSG00000145284
ENSG00000163081
ENSG00000156925
ENSG00000089091
ENSG00000178188
ENSG00000124575
ENSG00000204965
ENSG00000000003
ENSG00000171867
ENSG00000213265
ENSG00000077044
ENSG00000196305
ENSG00000122304
ENSG00000087302
ENSG00000167941
ENSG00000096384
ENSG00000234186
ENSG00000160145
ENSG00000082805
ENSG00000213190
ENSG00000165322
ENSG00000158856
ENSG00000123064
ENSG00000116138
ENSG00000141576
ENSG00000221890
ENSG00000114648
ENSG00000120162
ENSG00000071994
ENSG00000124491
ENSG00000163513
ENSG00000168734
ENSG00000090382
ENSG00000115365
ENSG00000198948
ENSG00000146376
ENSG00000204086
ENSG00000127318
ENSG00000196504
ENSG00000164434
ENSG00000126016
ENSG00000079277
ENSG00000146007
ENSG00000118473
ENSG00000101558
ENSG00000178935
ENSG00000153310
ENSG00000175707
ENSG00000188958
ENSG00000104728
ENSG00000221949
ENSG00000198932
ENSG00000137507
ENSG00000122025
ENSG00000074219
ENSG00000114999
ENSG00000146215
ENSG00000095906
ENSG00000134061
ENSG00000105281
ENSG00000147174
ENSG00000186652
ENSG00000110013
ENSG00000104833
ENSG00000103429
ENSG00000166263
ENSG00000137073
ENSG00000130309
ENSG00000180801
ENSG00000068305
ENSG00000120915
ENSG00000120662
ENSG00000100364
ENSG00000181031
ENSG00000173261
ENSG00000085832
ENSG00000170054
ENSG00000198846
ENSG00000076706
ENSG00000105223
ENSG00000100802
ENSG00000241878
ENSG00000204193
ENSG00000149231
ENSG00000168243
ENSG00000167701
ENSG00000147905
ENSG00000102125
ENSG00000164488
ENSG00000072832

ENSG00000155111	ENSG00000170515
ENSG00000168348	ENSG00000105656
ENSG00000157077	ENSG00000162366
ENSG00000119953	ENSG00000167600
ENSG00000179600	ENSG00000164283
ENSG00000198010	ENSG00000180891
ENSG00000116667	ENSG00000196475
ENSG00000170819	ENSG00000167910
ENSG00000160219	ENSG00000143753
ENSG00000170802	ENSG00000183032
ENSG00000170323	ENSG00000110328
ENSG00000185250	ENSG00000197653
ENSG00000155100	ENSG00000140104
ENSG00000183255	ENSG00000137872
ENSG00000198089	ENSG00000112053
ENSG00000143390	ENSG00000132463
ENSG00000182968	ENSG00000165923
ENSG00000133026	ENSG00000196876
ENSG00000010810	ENSG00000178177
ENSG00000178695	ENSG00000175097
ENSG00000124134	ENSG00000204673
ENSG00000163660	ENSG00000166860
ENSG00000110436	ENSG00000156587
ENSG00000109066	ENSG00000177628
ENSG00000165556	ENSG00000127507
ENSG00000153885	ENSG00000164011
ENSG00000143344	ENSG00000155438
ENSG00000141522	ENSG00000072210
ENSG00000162769	ENSG00000090674
ENSG00000168795	ENSG00000184432
ENSG00000164458	ENSG00000174059
ENSG00000160570	ENSG00000108797
ENSG00000148773	ENSG00000111669
ENSG00000124126	ENSG00000169976
ENSG00000111676	ENSG00000197024
ENSG00000140386	ENSG00000184602
ENSG00000134882	ENSG00000173214
ENSG00000147912	ENSG00000181894
ENSG00000118702	ENSG00000151360
ENSG00000198558	ENSG00000196408
ENSG00000166747	ENSG00000168515
ENSG00000108219	ENSG00000114491
ENSG00000132485	ENSG00000080822
ENSG00000213240	ENSG00000127951
ENSG00000152359	ENSG00000173141
ENSG00000170464	ENSG00000082196
ENSG00000143437	ENSG00000141542
ENSG00000134590	ENSG00000121236
ENSG00000138771	ENSG00000134291
ENSG00000205339	ENSG00000198908
ENSG00000135547	ENSG00000145808
ENSG00000127554	ENSG00000072756
ENSG00000197535	ENSG00000178700
ENSG00000178878	ENSG00000103710
ENSG00000198783	ENSG00000176956
ENSG00000144668	ENSG00000122735
ENSG00000164631	ENSG00000167840
ENSG00000146049	ENSG00000103495
ENSG00000173548	ENSG00000088386
ENSG00000130201	ENSG00000186812
ENSG00000138430	ENSG00000186009
ENSG00000163393	ENSG00000140691
ENSG00000143337	ENSG00000176435
ENSG00000144369	ENSG00000134001

ENSG00000130720	ENSG00000213145
ENSG00000186591	ENSG00000177602
ENSG00000106341	ENSG00000158828
ENSG00000173757	ENSG00000107789
ENSG00000132471	ENSG00000104760
ENSG00000198561	ENSG00000116161
ENSG00000119242	ENSG00000023171
ENSG00000153130	ENSG00000116574
ENSG00000129245	ENSG00000112339
ENSG00000101665	ENSG00000169251
ENSG00000198743	ENSG00000136235
ENSG00000122545	ENSG00000166228
ENSG00000183770	ENSG00000107968
ENSG00000179348	ENSG00000085511
ENSG00000109689	ENSG00000167397
ENSG00000189337	ENSG00000173193
ENSG00000168092	ENSG00000160856
ENSG00000091651	ENSG00000018510
ENSG00000196533	ENSG00000075391
ENSG00000104960	ENSG00000117450
ENSG00000054118	ENSG00000143862
ENSG00000163995	ENSG00000133980
ENSG00000063245	ENSG00000143771
ENSG00000134294	ENSG00000167981
ENSG00000134954	ENSG00000123572
ENSG00000204352	ENSG00000187514
ENSG00000017427	ENSG00000109079
ENSG00000160208	ENSG00000175470
ENSG00000074695	ENSG00000138347
ENSG00000049192	ENSG00000153162
ENSG00000215301	ENSG00000139116
ENSG00000184897	ENSG00000171102
ENSG00000214114	ENSG00000164627
ENSG00000115216	ENSG00000115825
ENSG00000172752	ENSG00000116874
ENSG00000170624	ENSG00000151948
ENSG00000113522	ENSG00000158717
ENSG00000114166	ENSG00000173812
ENSG00000115520	ENSG00000171757
ENSG00000198604	ENSG00000129187
ENSG00000198915	ENSG00000069188
ENSG00000085382	ENSG00000197852
ENSG00000112992	ENSG00000180089
ENSG00000119138	ENSG00000174791
ENSG00000137745	ENSG00000169609
ENSG00000167332	ENSG00000120055
ENSG00000105649	ENSG00000070729
ENSG00000139597	ENSG00000018408
ENSG00000138080	ENSG00000175395
ENSG00000063046	ENSG00000143570
ENSG00000106003	ENSG00000138639
ENSG00000168779	ENSG00000177519
ENSG00000139567	ENSG00000183640
ENSG00000143870	ENSG00000240857
ENSG00000129250	ENSG00000115239
ENSG00000174996	ENSG00000121481
ENSG00000126822	ENSG00000119922
ENSG00000138496	ENSG00000067066
ENSG00000221837	ENSG00000144452
ENSG00000171533	ENSG00000171954
ENSG00000112530	ENSG00000104381
ENSG00000177694	ENSG00000122145
ENSG00000174928	ENSG00000164258
ENSG00000101294	ENSG00000125834

ENSG00000122862	ENSG00000176871
ENSG00000187416	ENSG00000174442
ENSG00000184486	ENSG00000187109
ENSG00000162927	ENSG00000130226
ENSG00000159217	ENSG00000162630
ENSG00000005249	ENSG00000241794
ENSG00000069702	ENSG00000171100
ENSG00000146701	ENSG00000095015
ENSG00000068028	ENSG00000172399
ENSG00000169139	ENSG00000180806
ENSG00000104313	ENSG00000114529
ENSG00000116299	ENSG00000151704
ENSG00000205250	ENSG00000005243
ENSG00000154518	ENSG00000172269
ENSG00000171017	ENSG00000085788
ENSG00000110321	ENSG00000174100
ENSG00000140694	ENSG00000160401
ENSG00000181555	ENSG00000140660
ENSG00000144815	ENSG00000135441
ENSG00000178562	ENSG00000116337
ENSG00000153094	ENSG00000163735
ENSG00000074603	ENSG00000213024
ENSG00000108506	ENSG00000125037
ENSG00000024422	ENSG00000147647
ENSG00000132518	ENSG00000109906
ENSG00000129968	ENSG00000131477
ENSG00000080345	ENSG00000092068
ENSG00000090530	ENSG00000111321
ENSG00000124243	ENSG00000164978
ENSG00000006118	ENSG00000159459
ENSG00000134852	ENSG00000197043
ENSG00000189091	ENSG00000171777
ENSG00000183092	ENSG00000022567
ENSG00000137502	ENSG00000164172
ENSG00000129757	ENSG00000146013
ENSG00000184702	ENSG00000162551
ENSG00000143297	ENSG00000165474
ENSG00000143867	ENSG00000130414
ENSG00000087266	ENSG00000159650
ENSG00000131471	ENSG00000144852
ENSG00000136267	ENSG00000182816
ENSG00000005102	ENSG00000106089
ENSG00000170325	ENSG00000162976
ENSG00000197892	ENSG00000117281
ENSG00000183779	ENSG00000180008
ENSG00000044459	ENSG00000160949
ENSG00000099203	ENSG00000188488
ENSG00000139112	ENSG00000111445
ENSG00000177374	ENSG00000196743
ENSG00000196544	ENSG00000154914
ENSG00000119318	ENSG00000101276
ENSG00000117616	ENSG00000132915
ENSG00000120696	ENSG00000102349
ENSG00000166348	ENSG00000072315
ENSG00000079102	ENSG00000153012
ENSG00000087274	ENSG00000015153
ENSG00000128829	ENSG00000023909
ENSG00000168710	ENSG00000104863
ENSG00000079691	ENSG00000198925
ENSG00000167088	ENSG00000088992
ENSG00000148337	ENSG00000178718
ENSG00000123576	ENSG00000138696
ENSG00000170899	ENSG00000109919
ENSG00000034677	ENSG00000154832

ENSG00000177105	ENSG00000170522
ENSG00000166793	ENSG00000139549
ENSG00000120549	ENSG00000105568
ENSG00000092098	ENSG00000184221
ENSG00000153767	ENSG00000111701
ENSG00000138031	ENSG00000204873
ENSG00000102053	ENSG00000182985
ENSG00000175387	ENSG00000172922
ENSG00000116678	ENSG00000144231
ENSG00000166275	ENSG00000172830
ENSG00000242372	ENSG00000137496
ENSG00000176087	ENSG00000196865
ENSG00000121104	ENSG00000125901
ENSG00000149927	ENSG00000171056
ENSG00000108963	ENSG00000169750
ENSG00000172939	ENSG00000198028
ENSG00000101230	ENSG00000166451
ENSG00000124784	ENSG00000171794
ENSG00000182674	ENSG00000170854
ENSG00000137558	ENSG00000122870
ENSG00000184182	ENSG00000123411
ENSG00000003147	ENSG00000154764
ENSG00000107957	ENSG00000131899
ENSG00000112276	ENSG00000145687
ENSG00000181585	ENSG00000095981
ENSG00000136643	ENSG00000167971
ENSG00000106069	ENSG00000013725
ENSG00000157916	ENSG00000009950
ENSG00000117298	ENSG00000132517
ENSG00000178031	ENSG00000127124
ENSG00000196747	ENSG00000113812
ENSG00000153936	ENSG00000127528
ENSG00000180878	ENSG00000218891
ENSG00000176532	ENSG00000073008
ENSG00000163739	ENSG00000204682
ENSG00000205581	ENSG00000101997
ENSG00000023902	ENSG00000145476
ENSG00000110844	ENSG00000182195
ENSG00000140750	ENSG00000159131
ENSG00000125817	ENSG00000185519
ENSG00000162999	ENSG00000121897
ENSG00000070718	ENSG00000100060
ENSG00000112576	ENSG00000165724
ENSG00000125869	ENSG00000155761
ENSG00000173567	ENSG00000161860
ENSG00000188997	ENSG00000153201
ENSG00000185049	ENSG00000136869
ENSG00000168505	ENSG00000105647
ENSG00000005893	ENSG00000182771
ENSG00000128011	ENSG00000166478
ENSG00000143514	ENSG00000127586
ENSG00000124459	ENSG00000166289
ENSG00000092847	ENSG00000149084
ENSG00000159479	ENSG00000101134
ENSG00000118640	ENSG00000135900
ENSG00000118407	ENSG00000139131
ENSG00000178235	ENSG00000159763
ENSG00000108946	ENSG00000138675
ENSG00000043039	ENSG00000170961
ENSG00000125657	ENSG00000198521
ENSG00000183900	ENSG00000180448
ENSG00000011485	ENSG00000170100
ENSG00000111837	ENSG00000102858
ENSG00000176678	ENSG00000114779

ENSG00000184304	ENSG00000151617
ENSG00000162517	ENSG00000128394
ENSG00000130176	ENSG00000114423
ENSG00000008282	ENSG00000095587
ENSG00000165949	ENSG00000169738
ENSG00000100325	ENSG00000203747
ENSG00000089818	ENSG00000182858
ENSG00000137727	ENSG00000100578
ENSG00000162188	ENSG00000110066
ENSG00000119004	ENSG00000179456
ENSG00000172115	ENSG00000214107
ENSG00000206561	ENSG00000100024
ENSG00000213694	ENSG00000155962
ENSG00000198682	ENSG00000162040
ENSG00000167384	ENSG00000158773
ENSG00000021852	ENSG00000167554
ENSG00000105329	ENSG00000104231
ENSG00000145833	ENSG00000157911
ENSG00000186675	ENSG00000169184
ENSG00000184900	ENSG00000226650
ENSG00000122223	ENSG00000203772
ENSG00000173653	ENSG00000188095
ENSG00000205542	ENSG00000134769
ENSG00000081189	ENSG00000124198
ENSG00000154144	ENSG00000147571
ENSG00000185551	ENSG00000159714
ENSG00000156273	ENSG00000116128
ENSG00000197157	ENSG00000143375
ENSG00000111897	ENSG00000179111
ENSG00000101040	ENSG00000035862
ENSG00000151553	ENSG00000167977
ENSG00000167207	ENSG00000104218
ENSG00000182512	ENSG00000137812
ENSG00000197442	ENSG00000111077
ENSG00000170006	ENSG00000165164
ENSG00000054690	ENSG00000130701
ENSG00000185090	ENSG00000145934
ENSG00000163430	ENSG00000140474
ENSG00000138180	ENSG00000134470
ENSG00000147130	ENSG00000196353
ENSG00000154655	ENSG00000081721
ENSG00000198736	ENSG00000100219
ENSG00000163145	ENSG00000119396
ENSG00000126458	ENSG00000102931
ENSG00000136111	ENSG00000170236
ENSG00000213551	ENSG00000162300
ENSG00000250120	ENSG00000172458
ENSG00000068885	ENSG00000187483
ENSG00000134444	ENSG00000101182
ENSG00000155970	ENSG00000120705
ENSG00000182287	ENSG00000093010
ENSG00000165934	ENSG00000129515
ENSG00000131507	ENSG00000137825
ENSG00000162746	ENSG00000125347
ENSG00000128045	ENSG00000127533
ENSG00000104343	ENSG00000123096
ENSG00000166128	ENSG00000091138
ENSG00000125912	ENSG00000151413
ENSG00000050748	ENSG00000119125
ENSG00000198586	ENSG00000119929
ENSG00000137834	ENSG00000128594
ENSG00000196187	ENSG00000168883
ENSG00000153048	ENSG00000125977
ENSG00000164929	ENSG00000186352

ENSG00000170290	ENSG00000175315
ENSG00000148842	ENSG00000156463
ENSG00000179071	ENSG00000141668
ENSG00000164411	ENSG00000078304
ENSG00000163795	ENSG00000152779
ENSG00000175267	ENSG00000178381
ENSG00000062716	ENSG00000173868
ENSG00000162576	ENSG00000108846
ENSG00000081277	ENSG00000114354
ENSG00000139946	ENSG00000112599
ENSG00000122254	ENSG00000197081
ENSG00000151812	ENSG00000101213
ENSG00000158882	ENSG00000133424
ENSG00000105880	ENSG00000101901
ENSG00000181333	ENSG00000179178
ENSG00000170759	ENSG00000068615
ENSG00000104783	ENSG00000143653
ENSG00000188786	ENSG00000122877
ENSG00000179820	ENSG00000163486
ENSG00000221818	ENSG00000078246
ENSG00000189292	ENSG00000166169
ENSG00000171786	ENSG00000168481
ENSG00000158714	ENSG00000133561
ENSG00000171596	ENSG00000127415
ENSG00000131467	ENSG00000241598
ENSG00000143156	ENSG00000167685
ENSG00000196092	ENSG00000091640
ENSG00000100931	ENSG00000144959
ENSG00000158966	ENSG00000111737
ENSG00000105953	ENSG00000106049
ENSG00000165055	ENSG00000169469
ENSG00000084676	ENSG00000167720
ENSG00000187792	ENSG00000196653
ENSG00000120159	ENSG00000123901
ENSG00000139734	ENSG00000125954
ENSG00000130559	ENSG00000185010
ENSG00000114867	ENSG00000123595
ENSG00000171320	ENSG00000121005
ENSG00000169783	ENSG00000196154
ENSG00000197982	ENSG00000097021
ENSG00000156097	ENSG00000010539
ENSG00000141480	ENSG00000167460
ENSG00000172071	ENSG00000169174
ENSG00000127585	ENSG00000251247
ENSG00000107331	ENSG00000102287
ENSG00000181513	ENSG00000188549
ENSG00000067225	ENSG00000196914
ENSG00000101349	ENSG00000012048
ENSG00000102178	ENSG00000111181
ENSG00000103769	ENSG00000164815
ENSG00000138028	ENSG00000132164
ENSG00000128563	ENSG00000140367
ENSG00000120685	ENSG00000168275
ENSG00000164023	ENSG00000134758
ENSG00000120659	ENSG00000089225
ENSG00000118972	ENSG00000196275
ENSG00000165300	ENSG00000165521
ENSG00000163815	ENSG00000165197
ENSG00000116127	ENSG00000197568
ENSG00000103269	ENSG00000206461
ENSG00000075413	ENSG00000120451
ENSG00000141404	ENSG00000055163
ENSG00000166317	ENSG00000184517
ENSG00000118260	ENSG00000172014

ENSG00000171492	ENSG00000100811
ENSG00000154305	ENSG00000134684
ENSG00000137218	ENSG00000172748
ENSG00000037965	ENSG00000171940
ENSG00000148153	ENSG00000151702
ENSG00000153574	ENSG00000161021
ENSG00000131910	ENSG00000138448
ENSG00000011198	ENSG00000113048
ENSG00000113356	ENSG00000183020
ENSG00000172380	ENSG00000108788
ENSG00000099957	ENSG00000161980
ENSG00000106144	ENSG00000136156
ENSG00000117222	ENSG00000176160
ENSG00000155313	ENSG00000151240
ENSG00000165029	ENSG00000171044
ENSG00000138152	ENSG00000108932
ENSG00000144895	ENSG00000135298
ENSG00000103365	ENSG00000173702
ENSG00000204435	ENSG00000143520
ENSG00000140157	ENSG00000104419
ENSG00000213923	ENSG00000189401
ENSG00000135956	ENSG00000189058
ENSG00000183833	ENSG00000167528
ENSG00000141458	ENSG00000187682
ENSG00000186716	ENSG00000203943
ENSG00000197921	ENSG00000198363
ENSG00000158887	ENSG00000165501
ENSG00000103657	ENSG00000095397
ENSG00000158545	ENSG00000171502
ENSG00000165915	ENSG00000132405
ENSG00000213465	ENSG00000166224
ENSG00000064995	ENSG00000141506
ENSG00000116962	ENSG00000102038
ENSG00000086758	ENSG00000143869
ENSG00000175329	ENSG00000105146
ENSG00000140538	ENSG00000133878
ENSG00000140262	ENSG00000180263
ENSG00000132341	ENSG00000171621
ENSG00000204599	ENSG00000079432
ENSG00000162946	ENSG00000167657
ENSG00000135447	ENSG00000144771
ENSG00000157869	ENSG00000206073
ENSG00000123395	ENSG00000163377
ENSG00000019991	ENSG00000162711
ENSG00000188687	ENSG00000159445
ENSG00000171877	ENSG00000198176
ENSG00000170049	ENSG00000113600
ENSG00000188312	ENSG00000135960
ENSG00000106392	ENSG00000165476
ENSG00000154930	ENSG00000196811
ENSG00000206538	ENSG00000095002
ENSG00000126001	ENSG00000151148
ENSG00000047849	ENSG00000156150
ENSG00000099875	ENSG00000147533
ENSG00000169758	ENSG00000110442
ENSG00000132004	ENSG00000143119
ENSG00000129255	ENSG00000100221
ENSG00000166333	ENSG00000156639
ENSG00000103888	ENSG00000171604
ENSG00000143554	ENSG00000132773
ENSG00000134222	ENSG00000141433
ENSG00000143013	ENSG00000062096
ENSG00000185933	ENSG00000146425
ENSG00000164118	ENSG00000147257

ENSG00000022976	ENSG00000131849
ENSG00000143368	ENSG00000121905
ENSG00000204256	ENSG00000174780
ENSG00000105767	ENSG00000152268
ENSG00000079931	ENSG00000197841
ENSG00000110395	ENSG00000148935
ENSG00000198168	ENSG00000165802
ENSG00000188157	ENSG00000117090
ENSG00000055483	ENSG00000159166
ENSG00000108091	ENSG00000019505
ENSG00000167114	ENSG00000230989
ENSG00000162144	ENSG00000124216
ENSG00000167645	ENSG00000181027
ENSG00000196814	ENSG00000100385
ENSG00000114784	ENSG00000198851
ENSG00000176697	ENSG00000067560
ENSG00000149397	ENSG00000070886
ENSG00000144468	ENSG00000101200
ENSG00000187672	ENSG00000172000
ENSG00000166225	ENSG00000118322
ENSG00000172379	ENSG00000103111
ENSG00000136717	ENSG00000176732
ENSG00000183826	ENSG00000171992
ENSG00000107175	ENSG00000196632
ENSG00000182199	ENSG00000184922
ENSG00000138741	ENSG00000155016
ENSG00000149295	ENSG00000103194
ENSG00000163606	ENSG00000224201
ENSG00000170185	ENSG00000156206
ENSG00000132680	ENSG00000043591
ENSG00000168994	ENSG00000079393
ENSG00000109339	ENSG00000167543
ENSG00000047249	ENSG00000163216
ENSG00000100393	ENSG00000162885
ENSG00000139800	ENSG00000137494
ENSG00000116830	ENSG00000185591
ENSG00000166833	ENSG00000135063
ENSG00000175356	ENSG00000176387
ENSG00000145147	ENSG00000037637
ENSG00000107862	ENSG00000142453
ENSG00000118655	ENSG00000167487
ENSG00000122557	ENSG00000161013
ENSG00000196313	ENSG00000227345
ENSG00000141582	ENSG00000135211
ENSG00000182973	ENSG00000110400
ENSG00000166888	ENSG00000149476
ENSG00000066735	ENSG00000169676
ENSG00000158161	ENSG00000180992
ENSG00000154845	ENSG00000085719
ENSG00000135828	ENSG00000138769
ENSG00000136870	ENSG00000182566
ENSG00000107105	ENSG00000132661
ENSG00000172354	ENSG00000183873
ENSG00000102003	ENSG00000119965
ENSG00000014164	ENSG00000033011
ENSG00000110880	ENSG00000136848
ENSG00000243444	ENSG00000165006
ENSG00000167470	ENSG00000160182
ENSG00000185905	ENSG00000169297
ENSG00000164054	ENSG00000155659
ENSG00000182132	ENSG00000155380
ENSG00000163788	ENSG00000110042
ENSG00000176170	ENSG00000135924
ENSG00000124181	ENSG00000197245

ENSG00000186660	ENSG00000213139
ENSG00000141141	ENSG00000055070
ENSG00000095627	ENSG00000158301
ENSG00000135473	ENSG00000151090
ENSG00000100731	ENSG00000164117
ENSG00000101442	ENSG00000101335
ENSG00000184990	ENSG00000132274
ENSG00000165244	ENSG00000132952
ENSG00000140545	ENSG00000142186
ENSG00000148572	ENSG00000126856
ENSG00000138166	ENSG00000186074
ENSG00000111615	ENSG00000112033
ENSG00000146938	ENSG00000085465
ENSG00000099246	ENSG00000117151
ENSG00000129474	ENSG00000136197
ENSG00000148408	ENSG00000165478
ENSG00000114019	ENSG00000165868
ENSG00000139651	ENSG00000078098
ENSG00000003056	ENSG00000198015
ENSG00000153214	ENSG00000112041
ENSG00000173327	ENSG00000186150
ENSG00000158019	ENSG00000156976
ENSG00000183715	ENSG00000166159
ENSG00000111252	ENSG00000147813
ENSG00000184270	ENSG00000173320
ENSG00000069399	ENSG00000069535
ENSG00000178971	ENSG00000188888
ENSG00000169490	ENSG00000148730
ENSG00000166508	ENSG00000161654
ENSG00000170745	ENSG00000110375
ENSG00000108179	ENSG00000150347
ENSG00000174306	ENSG00000163126
ENSG00000157106	ENSG00000074660
ENSG00000187961	ENSG00000118564
ENSG00000116731	ENSG00000138738
ENSG00000162512	ENSG00000174748
ENSG00000173376	ENSG00000083123
ENSG00000138303	ENSG00000163902
ENSG00000153786	ENSG00000131459
ENSG00000141179	ENSG00000196218
ENSG00000104856	ENSG00000172349
ENSG00000104613	ENSG00000144134
ENSG00000132535	ENSG00000165621
ENSG00000136193	ENSG00000131480
ENSG00000165449	ENSG00000177551
ENSG00000130429	ENSG00000198390
ENSG00000174839	ENSG00000135482
ENSG00000135424	ENSG00000179397
ENSG00000157404	ENSG00000128191
ENSG00000151322	ENSG00000151348
ENSG00000173338	ENSG00000161610
ENSG00000175155	ENSG00000198324
ENSG00000128422	ENSG00000131653
ENSG00000105327	ENSG00000125447
ENSG00000104903	ENSG00000158850
ENSG00000160932	ENSG00000105887
ENSG00000170777	ENSG00000136874
ENSG00000071246	ENSG00000143543
ENSG00000135486	ENSG00000187607
ENSG00000057608	ENSG00000115339
ENSG00000120217	ENSG00000153714
ENSG00000100697	ENSG00000172159
ENSG00000163520	ENSG00000135678
ENSG00000105202	ENSG00000107140

ENSG00000112320	ENSG00000197483
ENSG00000101871	ENSG00000175121
ENSG00000077684	ENSG00000132970
ENSG00000185985	ENSG00000105379
ENSG00000004961	ENSG00000149658
ENSG00000138459	ENSG00000073111
ENSG00000158258	ENSG00000104368
ENSG00000124205	ENSG00000142611
ENSG00000184886	ENSG00000137819
ENSG00000184508	ENSG00000054611
ENSG00000196367	ENSG00000169957
ENSG00000115677	ENSG00000148798
ENSG00000153904	ENSG00000143493
ENSG00000143643	ENSG00000175727
ENSG00000198826	ENSG00000104412
ENSG00000211460	ENSG00000168597
ENSG00000120519	ENSG00000177335
ENSG00000172497	ENSG00000064270
ENSG00000141622	ENSG00000163032
ENSG00000100084	ENSG00000168090
ENSG00000162642	ENSG00000140859
ENSG00000104870	ENSG00000146066
ENSG00000105879	ENSG00000163530
ENSG00000130119	ENSG00000177613
ENSG00000198898	ENSG00000106128
ENSG00000160161	ENSG00000139636
ENSG00000082397	ENSG00000133115
ENSG00000151474	ENSG00000117620
ENSG00000161533	ENSG00000146535
ENSG00000076053	ENSG00000152455
ENSG00000186174	ENSG00000183508
ENSG00000078747	ENSG00000140398
ENSG00000100422	ENSG00000174562
ENSG00000128641	ENSG00000133800
ENSG00000105983	ENSG00000111653
ENSG00000151640	ENSG00000102362
ENSG00000072958	ENSG00000179750
ENSG00000112511	ENSG00000163347
ENSG00000166439	ENSG00000059691
ENSG00000167595	ENSG00000138650
ENSG00000141198	ENSG00000120509
ENSG00000166900	ENSG00000070269
ENSG00000197808	ENSG00000175497
ENSG00000152430	ENSG00000140519
ENSG00000163673	ENSG00000145779
ENSG00000172818	ENSG00000197714
ENSG00000113196	ENSG00000155876
ENSG00000215252	ENSG00000197632
ENSG00000118922	ENSG00000109089
ENSG00000132670	ENSG00000120805
ENSG00000198821	ENSG00000135519
ENSG00000104812	ENSG00000198077
ENSG00000088832	ENSG00000178922
ENSG00000148943	ENSG00000100146
ENSG00000143318	ENSG00000049759
ENSG00000241553	ENSG00000185238
ENSG00000156256	ENSG00000003756
ENSG00000205352	ENSG00000174502
ENSG00000119392	ENSG00000149503
ENSG00000185386	ENSG00000179088
ENSG00000144339	ENSG00000177575
ENSG00000162493	ENSG00000170027
ENSG00000145681	ENSG00000198858
ENSG00000139899	ENSG00000137267

ENSG00000011638	ENSG00000145780
ENSG00000143933	ENSG00000171150
ENSG00000171885	ENSG00000100842
ENSG00000133111	ENSG00000169016
ENSG00000171132	ENSG00000095261
ENSG00000147036	ENSG00000171443
ENSG00000124635	ENSG00000131269
ENSG00000184916	ENSG00000115504
ENSG00000163113	ENSG00000183971
ENSG00000167107	ENSG00000156510
ENSG00000015532	ENSG00000099901
ENSG00000116132	ENSG00000102312
ENSG00000165678	ENSG00000188389
ENSG00000186908	ENSG00000149124
ENSG00000189120	ENSG00000068079
ENSG00000109458	ENSG00000171848
ENSG00000152256	ENSG00000058262
ENSG00000171988	ENSG00000173898
ENSG00000131475	ENSG00000072274
ENSG00000169375	ENSG00000124587
ENSG00000165637	ENSG00000116218
ENSG00000125733	ENSG00000155324
ENSG00000139437	ENSG00000178130
ENSG00000154928	ENSG00000070366
ENSG00000243135	ENSG00000162231
ENSG00000164418	ENSG00000116698
ENSG00000163376	ENSG00000110958
ENSG00000111642	ENSG00000185513
ENSG00000088247	ENSG00000133401
ENSG00000197948	ENSG00000144560
ENSG00000102024	ENSG00000147526
ENSG00000049247	ENSG00000134508
ENSG00000203812	ENSG00000172530
ENSG00000152518	ENSG00000138622
ENSG00000081041	ENSG00000117594
ENSG00000197622	ENSG00000144827
ENSG00000197451	ENSG00000188833
ENSG00000197183	ENSG00000115760
ENSG00000078403	ENSG00000152705
ENSG00000173209	ENSG00000188452
ENSG00000130779	ENSG00000205809
ENSG00000196998	ENSG00000196345
ENSG00000135655	ENSG00000184999
ENSG00000173894	ENSG00000131188
ENSG00000167658	ENSG00000048540
ENSG00000011465	ENSG00000066923
ENSG00000111142	ENSG00000084774
ENSG00000154640	ENSG00000174951
ENSG00000073169	ENSG00000107372
ENSG00000132749	ENSG00000092820
ENSG00000187323	ENSG00000144749
ENSG00000173124	ENSG00000185909
ENSG00000161904	ENSG00000129219
ENSG00000152969	ENSG00000073803
ENSG00000150630	ENSG00000102387
ENSG00000160602	ENSG00000070061
ENSG00000088538	ENSG00000074416
ENSG00000112246	ENSG00000001626
ENSG00000006377	ENSG00000081051
ENSG00000119522	ENSG00000129277
ENSG00000170088	ENSG00000101638
ENSG00000215021	ENSG00000164946
ENSG00000148429	ENSG00000113068
ENSG00000117308	ENSG00000125648

ENSG00000162670	ENSG00000106537
ENSG00000169083	ENSG00000109917
ENSG00000182831	ENSG00000144840
ENSG00000134283	ENSG00000125798
ENSG00000148660	ENSG00000164587
ENSG00000113721	ENSG00000015520
ENSG00000136819	ENSG00000242252
ENSG00000079805	ENSG00000187778
ENSG00000085377	ENSG00000164692
ENSG00000185634	ENSG00000101384
ENSG00000066117	ENSG00000100934
ENSG00000140931	ENSG00000168917
ENSG00000164040	ENSG00000178965
ENSG00000163947	ENSG00000173926
ENSG00000115884	ENSG00000117115
ENSG00000106771	ENSG00000152689
ENSG00000130829	ENSG00000076770
ENSG00000117643	ENSG00000151893
ENSG00000007968	ENSG00000090013
ENSG00000136521	ENSG00000142449
ENSG00000091039	ENSG00000067048
ENSG00000164506	ENSG00000140374
ENSG00000102606	ENSG00000079112
ENSG00000100320	ENSG00000184984
ENSG00000150776	ENSG00000145826
ENSG00000148400	ENSG00000137693
ENSG00000211450	ENSG00000114113
ENSG00000213865	ENSG00000135749
ENSG00000186487	ENSG00000039600
ENSG00000120708	ENSG00000156222
ENSG00000143924	ENSG00000168066
ENSG00000068796	ENSG00000072954
ENSG00000156853	ENSG00000069812
ENSG00000100380	ENSG00000136068
ENSG00000072135	ENSG00000165714
ENSG00000089693	ENSG00000196950
ENSG00000028277	ENSG00000100852
ENSG00000149474	ENSG00000136108
ENSG00000156253	ENSG00000064201
ENSG00000111481	ENSG00000145041
ENSG00000139289	ENSG00000149548
ENSG00000165495	ENSG00000100426
ENSG00000101654	ENSG00000119812
ENSG00000154027	ENSG00000184271
ENSG00000141644	ENSG00000147889
ENSG00000150768	ENSG00000078237
ENSG00000107882	ENSG00000142224
ENSG00000135821	ENSG00000173020
ENSG00000165806	ENSG00000196437
ENSG00000135631	ENSG00000072840
ENSG00000084463	ENSG00000130775
ENSG00000184988	ENSG00000184502
ENSG00000198408	ENSG00000029993
ENSG00000164778	ENSG00000167346
ENSG00000089558	ENSG00000173166
ENSG00000108443	ENSG00000147123
ENSG00000110975	ENSG00000169413
ENSG00000104290	ENSG00000158156
ENSG00000162341	ENSG00000183747
ENSG00000132394	ENSG00000163435
ENSG00000133818	ENSG00000184564
ENSG00000185670	ENSG00000148848
ENSG00000253159	ENSG00000171564
ENSG00000099219	ENSG00000167625

ENSG00000108582	ENSG00000006757
ENSG00000173511	ENSG00000161640
ENSG00000100234	ENSG00000162736
ENSG00000148484	ENSG00000198739
ENSG00000115361	ENSG00000143420
ENSG00000166446	ENSG00000104964
ENSG00000162650	ENSG00000171522
ENSG00000046651	ENSG00000108861
ENSG00000158528	ENSG00000116991
ENSG00000165997	ENSG00000126883
ENSG00000182472	ENSG00000137947
ENSG00000162777	ENSG00000160226
ENSG00000241058	ENSG00000189190
ENSG00000110172	ENSG00000186298
ENSG00000091482	ENSG00000143384
ENSG00000187239	ENSG00000118707
ENSG00000106336	ENSG00000067900
ENSG00000128918	ENSG00000141977
ENSG00000072858	ENSG00000151150
ENSG00000160679	ENSG00000116473
ENSG00000133030	ENSG00000125498
ENSG00000177888	ENSG00000133574
ENSG00000175785	ENSG00000083454
ENSG00000186530	ENSG00000151376
ENSG00000167680	ENSG00000180353
ENSG00000072201	ENSG00000105245
ENSG00000198053	ENSG00000088179
ENSG00000204713	ENSG00000149212
ENSG00000049449	ENSG00000005469
ENSG00000143850	ENSG00000135709
ENSG00000100362	ENSG00000196730
ENSG00000140948	ENSG00000173418
ENSG00000157827	ENSG00000161270
ENSG00000240764	ENSG00000167740
ENSG00000139746	ENSG00000137809
ENSG00000166526	ENSG00000082458
ENSG00000104497	ENSG00000139318
ENSG00000092445	ENSG00000136933
ENSG00000122121	ENSG00000156011
ENSG00000121966	ENSG00000163630
ENSG00000135108	ENSG00000112149
ENSG00000116786	ENSG00000122218
ENSG00000089597	ENSG00000179284
ENSG00000164976	ENSG00000007047
ENSG00000162614	ENSG00000141540
ENSG00000175463	ENSG00000125743
ENSG00000214078	ENSG00000185940
ENSG00000113658	ENSG00000145715
ENSG00000169499	ENSG00000130332
ENSG00000149499	ENSG00000182919
ENSG00000185008	ENSG00000183978
ENSG00000095203	ENSG00000130164
ENSG00000196262	ENSG00000176406
ENSG00000075975	ENSG00000180596
ENSG00000116983	ENSG00000124818
ENSG00000169432	ENSG00000106290
ENSG00000173457	ENSG00000143248
ENSG00000005379	ENSG00000228314
ENSG00000164144	ENSG00000188827
ENSG00000028137	ENSG00000130147
ENSG00000205189	ENSG00000154358
ENSG00000087157	ENSG00000197641
ENSG00000004848	ENSG00000082512
ENSG00000117560	ENSG00000157870

ENSG00000127328	ENSG00000182718
ENSG00000204967	ENSG00000128342
ENSG00000164615	ENSG00000198796
ENSG00000139350	ENSG00000171316
ENSG00000100068	ENSG00000117625
ENSG00000183023	ENSG00000117153
ENSG00000145730	ENSG00000178363
ENSG00000196150	ENSG00000109854
ENSG00000196535	ENSG00000081791
ENSG00000176973	ENSG00000077157
ENSG00000134013	ENSG00000142459
ENSG00000137486	ENSG00000137055
ENSG00000183379	ENSG00000175063
ENSG00000012171	ENSG00000163661
ENSG00000150712	ENSG00000080031
ENSG00000115464	ENSG00000183145
ENSG00000174595	ENSG00000155506
ENSG00000146729	ENSG00000090612
ENSG00000013293	ENSG00000121753
ENSG00000184357	ENSG00000165795
ENSG00000159921	ENSG00000163424
ENSG00000145864	ENSG00000173068
ENSG00000131845	ENSG00000107295
ENSG00000100410	ENSG00000107863
ENSG00000158270	ENSG00000134490
ENSG00000205981	ENSG00000102471
ENSG00000167797	ENSG00000130055
ENSG00000011028	ENSG00000111846
ENSG00000172661	ENSG00000100439
ENSG00000243335	ENSG00000250995
ENSG00000174611	ENSG00000169228
ENSG00000161671	ENSG00000131055
ENSG00000158786	ENSG00000036530
ENSG00000152495	ENSG00000101639
ENSG00000159784	ENSG00000075073
ENSG00000162813	ENSG00000166681
ENSG00000181222	ENSG00000102753
ENSG00000090686	ENSG00000087448
ENSG00000100350	ENSG00000121774
ENSG00000198393	ENSG00000067606
ENSG00000103740	ENSG00000106246
ENSG00000137764	ENSG00000097033
ENSG00000198440	ENSG00000151882
ENSG00000231852	ENSG00000170633
ENSG00000133226	ENSG00000182533
ENSG00000153707	ENSG00000099625
ENSG00000109133	ENSG00000157379
ENSG00000135457	ENSG00000184650
ENSG00000188613	ENSG00000079459
ENSG00000169314	ENSG00000163421
ENSG00000134575	ENSG00000165084
ENSG00000156958	ENSG00000172260
ENSG00000132002	ENSG00000119408
ENSG00000133739	ENSG00000129596
ENSG00000101190	ENSG00000115155
ENSG00000187990	ENSG00000149043
ENSG00000186073	ENSG00000043143
ENSG00000101751	ENSG00000129451
ENSG00000179562	ENSG00000196932
ENSG00000184788	ENSG00000165775
ENSG00000125898	ENSG00000146151
ENSG00000186017	ENSG00000092295
ENSG00000135903	ENSG00000171209
ENSG00000143537	ENSG00000148688

ENSG00000132780	ENSG00000109929
ENSG00000115468	ENSG00000122958
ENSG00000147065	ENSG00000179058
ENSG00000040341	ENSG00000002330
ENSG00000112144	ENSG00000186567
ENSG00000105011	ENSG00000176248
ENSG00000116685	ENSG00000106244
ENSG00000116584	ENSG00000204946
ENSG00000198108	ENSG00000179869
ENSG00000170445	ENSG00000124813
ENSG00000074181	ENSG00000198246
ENSG00000143140	ENSG00000172403
ENSG00000173349	ENSG00000154124
ENSG00000241697	ENSG00000088543
ENSG00000159388	ENSG00000103226
ENSG00000108774	ENSG00000008311
ENSG00000140406	ENSG00000249481
ENSG00000065029	ENSG00000183340
ENSG00000131771	ENSG00000135537
ENSG00000179364	ENSG00000205445
ENSG00000070214	ENSG00000103723
ENSG00000106829	ENSG00000169248
ENSG00000140464	ENSG00000156219
ENSG00000103994	ENSG00000162654
ENSG00000154620	ENSG00000166823
ENSG00000118200	ENSG00000107223
ENSG00000253537	ENSG00000221955
ENSG00000084444	ENSG00000049239
ENSG00000174840	ENSG00000144837
ENSG00000141664	ENSG00000182544
ENSG00000187091	ENSG00000150787
ENSG00000164305	ENSG00000021826
ENSG00000065989	ENSG00000099715
ENSG00000173889	ENSG00000181523
ENSG00000197375	ENSG00000132694
ENSG00000104112	ENSG00000104067
ENSG00000171365	ENSG00000133627
ENSG00000026508	ENSG00000182173
ENSG00000174943	ENSG00000167851
ENSG00000125492	ENSG00000187173
ENSG00000163877	ENSG00000131068
ENSG00000161203	ENSG00000115318
ENSG00000031698	ENSG00000181744
ENSG00000204843	ENSG00000106299
ENSG00000157800	ENSG00000198625
ENSG00000122484	ENSG00000149582
ENSG00000119661	ENSG00000197885
ENSG00000082781	ENSG00000120278
ENSG00000003400	ENSG00000100523
ENSG00000108064	ENSG00000148399
ENSG00000168255	ENSG00000185668
ENSG00000075407	ENSG00000130427
ENSG00000159461	ENSG00000133706
ENSG00000149182	ENSG00000109111
ENSG00000061676	ENSG00000135973
ENSG00000103671	ENSG00000186395
ENSG00000186918	ENSG00000127928
ENSG00000165629	ENSG00000076554
ENSG00000142875	ENSG00000143387
ENSG00000131668	ENSG00000114738
ENSG00000162624	ENSG00000176076
ENSG00000080189	ENSG00000002822
ENSG00000068137	ENSG00000170348
ENSG00000151503	ENSG00000104883

ENSG00000176390	ENSG00000115594
ENSG00000157219	ENSG00000013288
ENSG00000137033	ENSG00000185339
ENSG00000065618	ENSG00000123473
ENSG00000179292	ENSG00000176125
ENSG00000160796	ENSG00000122566
ENSG00000104946	ENSG00000162434
ENSG00000043093	ENSG00000180483
ENSG00000115652	ENSG00000166927
ENSG00000143549	ENSG00000143355
ENSG00000166147	ENSG00000204828
ENSG00000135925	ENSG00000086232
ENSG00000119681	ENSG00000140678
ENSG00000167186	ENSG00000125810
ENSG00000116266	ENSG00000166432
ENSG00000167419	ENSG00000129514
ENSG00000123562	ENSG00000110090
ENSG00000204381	ENSG00000130382
ENSG00000112078	ENSG00000224586
ENSG00000184144	ENSG00000170035
ENSG00000146776	ENSG00000142273
ENSG00000188386	ENSG00000167749
ENSG00000101331	ENSG00000173372
ENSG00000075426	ENSG00000182054
ENSG00000101966	ENSG00000186081
ENSG00000130150	ENSG00000139445
ENSG00000171246	ENSG00000172731
ENSG00000127837	ENSG00000013375
ENSG00000162923	ENSG00000133863
ENSG00000168724	ENSG00000221986
ENSG00000116857	ENSG00000125520
ENSG00000156711	ENSG00000122884
ENSG00000196639	ENSG00000056487
ENSG00000066084	ENSG00000176749
ENSG00000115255	ENSG00000009830
ENSG00000130940	ENSG00000248144
ENSG00000117569	ENSG00000174371
ENSG00000178538	ENSG00000017621
ENSG00000122085	ENSG00000100626
ENSG00000164032	ENSG00000157168
ENSG00000198855	ENSG00000109445
ENSG00000144567	ENSG00000180318
ENSG00000102796	ENSG00000153107
ENSG00000065308	ENSG00000137996
ENSG00000114349	ENSG00000176746
ENSG00000131873	ENSG00000184227
ENSG00000179299	ENSG00000223802
ENSG00000074800	ENSG00000118965
ENSG00000176428	ENSG00000042429
ENSG00000099822	ENSG00000205642
ENSG00000131711	ENSG00000102103
ENSG00000189410	ENSG00000134201
ENSG00000151458	ENSG00000177082
ENSG00000041357	ENSG00000137574
ENSG00000136141	ENSG00000181867
ENSG00000154803	ENSG00000117748
ENSG00000107796	ENSG00000171227
ENSG00000156467	ENSG00000054116
ENSG00000103089	ENSG00000169594
ENSG00000169442	ENSG00000109099
ENSG00000129911	ENSG00000119778
ENSG00000124194	ENSG00000172888
ENSG00000146858	ENSG00000140992
ENSG00000196924	ENSG00000149294

ENSG00000120616	ENSG00000140521
ENSG00000130590	ENSG00000163933
ENSG00000159023	ENSG00000081320
ENSG00000196498	ENSG00000160862
ENSG00000164051	ENSG00000072786
ENSG00000175832	ENSG00000006116
ENSG00000158106	ENSG00000198719
ENSG00000166165	ENSG00000006042
ENSG00000124795	ENSG00000079739
ENSG00000138835	ENSG00000136273
ENSG00000177103	ENSG00000110104
ENSG00000101746	ENSG00000134339
ENSG00000158292	ENSG00000168959
ENSG00000091879	ENSG00000118518
ENSG00000112559	ENSG00000159625
ENSG00000177556	ENSG00000215048
ENSG00000163053	ENSG00000185033
ENSG00000186288	ENSG00000007306
ENSG00000129204	ENSG00000184454
ENSG00000196233	ENSG00000183955
ENSG00000175505	ENSG00000134627
ENSG00000111262	ENSG00000183431
ENSG00000196776	ENSG00000150991
ENSG00000138613	ENSG00000213347
ENSG00000134690	ENSG00000145335
ENSG00000176148	ENSG00000130402
ENSG00000076716	ENSG00000119900
ENSG00000185305	ENSG00000182108
ENSG00000160392	ENSG00000111540
ENSG00000156298	ENSG00000125821
ENSG00000066136	ENSG00000172915
ENSG00000176371	ENSG00000198542
ENSG00000171608	ENSG00000185043
ENSG00000108106	ENSG00000169994
ENSG00000205426	ENSG00000157978
ENSG00000144747	ENSG00000147133
ENSG00000078699	ENSG00000134463
ENSG00000008323	ENSG00000174483
ENSG00000197063	ENSG00000139192
ENSG00000118523	ENSG00000158555
ENSG00000149091	ENSG00000196376
ENSG00000198946	ENSG00000196565
ENSG00000115844	ENSG00000167641
ENSG00000160216	ENSG00000114030
ENSG00000198146	ENSG00000176853
ENSG00000125618	ENSG00000134028
ENSG00000071575	ENSG00000182810
ENSG00000179912	ENSG00000033050
ENSG00000064601	ENSG00000087903
ENSG00000109805	ENSG00000171540
ENSG00000029534	ENSG00000119723
ENSG00000126217	ENSG00000124783
ENSG00000114270	ENSG00000168438
ENSG00000114770	ENSG00000099991
ENSG00000127359	ENSG00000111275
ENSG00000166181	ENSG00000183751
ENSG00000104321	ENSG00000164270
ENSG00000196878	ENSG00000114850
ENSG00000160714	ENSG00000151923
ENSG00000086062	ENSG00000163956
ENSG00000124160	ENSG00000142528
ENSG00000196407	ENSG00000101321
ENSG00000196557	ENSG00000165409
ENSG00000182732	ENSG00000123453

ENSG00000198216	ENSG00000153789
ENSG00000197323	ENSG00000156136
ENSG00000049167	ENSG00000081386
ENSG00000169509	ENSG00000140830
ENSG00000168081	ENSG00000163600
ENSG00000124222	ENSG00000162174
ENSG00000143801	ENSG00000198585
ENSG00000175854	ENSG00000109381
ENSG00000090565	ENSG00000123836
ENSG00000186416	ENSG00000112062
ENSG00000162241	ENSG00000167515
ENSG00000186469	ENSG00000002726
ENSG00000177169	ENSG00000129636
ENSG00000134532	ENSG00000111186
ENSG00000156052	ENSG00000196072
ENSG00000172346	ENSG00000113240
ENSG00000139083	ENSG00000134278
ENSG00000164007	ENSG00000123374
ENSG00000121281	ENSG00000174827
ENSG00000129048	ENSG00000126091
ENSG00000006451	ENSG00000198034
ENSG00000221869	ENSG00000119535
ENSG00000155545	ENSG00000105929
ENSG00000111328	ENSG00000101911
ENSG00000102158	ENSG00000179632
ENSG00000105552	ENSG00000037897
ENSG00000159495	ENSG00000188321
ENSG00000106682	ENSG00000162545
ENSG00000068650	ENSG00000149654
ENSG00000150316	ENSG00000182667
ENSG00000168876	ENSG00000119682
ENSG00000072310	ENSG00000173681
ENSG00000012660	ENSG00000157500
ENSG00000152642	ENSG00000105723
ENSG00000173698	ENSG00000164749
ENSG00000186007	ENSG00000131171
ENSG00000155755	ENSG00000141150
ENSG00000148700	ENSG00000165462
ENSG00000172197	ENSG00000162924
ENSG00000109113	ENSG00000143612
ENSG00000213281	ENSG00000173917
ENSG00000136490	ENSG00000090659
ENSG00000100941	ENSG00000229972
ENSG00000180488	ENSG00000175334
ENSG00000130545	ENSG00000131788
ENSG00000184005	ENSG00000117592
ENSG00000233822	ENSG00000042781
ENSG00000148110	ENSG00000141437
ENSG00000004799	ENSG00000065609
ENSG00000100461	ENSG00000138135
ENSG00000157259	ENSG00000152785
ENSG00000181045	ENSG00000154654
ENSG00000110848	ENSG00000149599
ENSG00000183576	ENSG00000122203
ENSG00000104695	ENSG00000182621
ENSG00000163618	ENSG00000166575
ENSG00000135912	ENSG00000132330
ENSG00000157540	ENSG00000146416
ENSG00000125257	ENSG00000196636
ENSG00000185739	ENSG00000129221
ENSG00000178171	ENSG00000197497
ENSG00000114209	ENSG00000113916
ENSG00000112214	ENSG00000132623
ENSG00000155329	ENSG00000105612

ENSG00000156990	ENSG00000166313
ENSG00000137504	ENSG00000165215
ENSG00000170153	ENSG00000169118
ENSG00000160563	ENSG00000171487
ENSG00000167034	ENSG00000128652
ENSG00000060069	ENSG00000118094
ENSG00000206026	ENSG00000184599
ENSG00000065320	ENSG00000135404
ENSG00000068001	ENSG00000173846
ENSG00000091317	ENSG00000142319
ENSG00000148339	ENSG00000120063
ENSG00000112081	ENSG00000069248
ENSG00000215271	ENSG00000182551
ENSG00000136379	ENSG00000156599
ENSG00000164185	ENSG00000162992
ENSG00000095752	ENSG00000132326
ENSG00000100077	ENSG00000125166
ENSG00000113088	ENSG00000118971
ENSG00000103061	ENSG00000148843
ENSG00000172057	ENSG00000181035
ENSG00000102468	ENSG00000197769
ENSG00000178951	ENSG00000186889
ENSG00000180875	ENSG00000175115
ENSG00000181690	ENSG00000148690
ENSG00000136153	ENSG00000068323
ENSG00000102763	ENSG00000100116
ENSG00000186212	ENSG00000106823
ENSG00000188760	ENSG00000023516
ENSG00000165271	ENSG00000087152
ENSG00000157502	ENSG00000092841
ENSG00000068078	ENSG00000146360
ENSG00000151576	ENSG00000168301
ENSG00000177303	ENSG00000137802
ENSG00000134744	ENSG00000144191
ENSG00000110237	ENSG00000162571
ENSG00000135116	ENSG00000241978
ENSG00000088986	ENSG00000111203
ENSG00000127870	ENSG00000023892
ENSG00000116497	ENSG00000153233
ENSG00000220205	ENSG00000158163
ENSG00000131473	ENSG00000184307
ENSG00000197894	ENSG00000111110
ENSG00000152291	ENSG00000027869
ENSG00000175592	ENSG00000081087
ENSG00000115145	ENSG00000131379
ENSG00000146540	ENSG00000179639
ENSG00000120053	ENSG00000123612
ENSG00000055917	ENSG00000172725
ENSG00000166912	ENSG00000101363
ENSG00000188130	ENSG00000113595
ENSG00000176946	ENSG00000166886
ENSG00000130821	ENSG00000133104
ENSG00000188177	ENSG00000171766
ENSG00000111012	ENSG00000247746
ENSG00000174231	ENSG00000196388
ENSG00000175548	ENSG00000130706
ENSG00000204335	ENSG00000143126
ENSG00000073792	ENSG00000101350
ENSG00000147862	ENSG00000148516
ENSG00000177606	ENSG00000213215
ENSG00000033170	ENSG00000053524
ENSG00000181315	ENSG00000089063
ENSG00000163755	ENSG00000110244
ENSG00000109762	ENSG00000145908

ENSG00000108960	ENSG00000162949
ENSG00000132434	ENSG00000110076
ENSG00000184828	ENSG00000185115
ENSG00000105549	ENSG00000107890
ENSG00000147180	ENSG00000141560
ENSG00000171109	ENSG00000091972
ENSG00000175606	ENSG00000150636
ENSG00000162236	ENSG00000182584
ENSG00000157954	ENSG00000070019
ENSG00000114796	ENSG00000149196
ENSG00000113597	ENSG00000143067
ENSG00000066654	ENSG00000065457
ENSG00000117461	ENSG00000106459
ENSG00000163155	ENSG00000170955
ENSG00000136689	ENSG00000187676
ENSG00000188486	ENSG00000167767
ENSG00000073756	ENSG00000186364
ENSG00000154511	ENSG00000135046
ENSG00000152229	ENSG00000168237
ENSG00000108509	ENSG00000100665
ENSG00000179837	ENSG00000175793
ENSG00000106546	ENSG00000129749
ENSG00000081800	ENSG00000152242
ENSG00000160294	ENSG00000206384
ENSG00000176463	ENSG00000171865
ENSG00000118046	ENSG00000217128
ENSG00000165275	ENSG00000124469
ENSG00000105229	ENSG00000124370
ENSG00000175874	ENSG00000167230
ENSG00000135945	ENSG00000113569
ENSG00000018280	ENSG00000141424
ENSG00000029364	ENSG00000074266
ENSG00000158483	ENSG00000139508
ENSG00000104219	ENSG00000155011
ENSG00000164588	ENSG00000115568
ENSG00000164930	ENSG00000131043
ENSG00000004059	ENSG00000164256
ENSG00000152749	ENSG00000135373
ENSG00000009413	ENSG00000127452
ENSG00000162817	ENSG00000135124
ENSG00000100412	ENSG00000137221
ENSG00000149925	ENSG00000130312
ENSG00000039523	ENSG00000167895
ENSG00000115421	ENSG00000135679
ENSG00000163888	ENSG00000043355
ENSG00000137770	ENSG00000108588
ENSG00000136457	ENSG00000177108
ENSG00000183723	ENSG00000142961
ENSG00000172466	ENSG00000221926
ENSG00000145569	ENSG00000130204
ENSG00000103489	ENSG00000078269
ENSG00000166341	ENSG00000135917
ENSG00000057252	ENSG00000187048
ENSG00000124615	ENSG00000137193
ENSG00000130517	ENSG00000185761
ENSG00000139726	ENSG00000105397
ENSG00000167653	ENSG00000087088
ENSG00000147246	ENSG00000101438
ENSG00000181652	ENSG00000154162
ENSG00000112685	ENSG00000147381
ENSG00000157184	ENSG00000163611
ENSG00000198218	ENSG00000126247
ENSG00000121083	ENSG00000154122
ENSG00000168269	ENSG00000085491

ENSG00000059378	ENSG00000122376
ENSG00000005007	ENSG00000198087
ENSG00000124493	ENSG00000178882
ENSG00000132825	ENSG00000171174
ENSG00000152214	ENSG00000100027
ENSG00000157766	ENSG00000119640
ENSG00000213625	ENSG00000183695
ENSG00000161179	ENSG00000185800
ENSG00000117114	ENSG00000118402
ENSG00000179588	ENSG00000156515
ENSG00000138780	ENSG00000149488
ENSG00000155093	ENSG00000213186
ENSG00000141219	ENSG00000033627
ENSG00000083642	ENSG00000184058
ENSG00000171357	ENSG00000100151
ENSG00000137710	ENSG00000066427
ENSG00000109771	ENSG00000133112
ENSG00000162409	ENSG00000186583
ENSG00000164252	ENSG00000013561
ENSG00000143977	ENSG00000039319
ENSG00000179242	ENSG00000180834
ENSG00000174871	ENSG00000105971
ENSG00000115875	ENSG00000204815
ENSG00000147382	ENSG00000151773
ENSG00000213928	ENSG00000121743
ENSG00000154153	ENSG00000166292
ENSG00000136297	ENSG00000116176
ENSG00000117597	ENSG00000196189
ENSG00000181788	ENSG00000138069
ENSG00000171552	ENSG00000167862
ENSG00000157224	ENSG00000135111
ENSG00000111863	ENSG00000112175
ENSG00000134086	ENSG00000068394
ENSG00000141314	ENSG00000134717
ENSG00000136699	ENSG00000114638
ENSG00000099800	ENSG00000056736
ENSG00000152348	ENSG00000143303
ENSG00000105849	ENSG00000142684
ENSG00000111885	ENSG00000121075
ENSG00000141580	ENSG00000162695
ENSG00000005108	ENSG00000166326
ENSG00000132142	ENSG00000182853
ENSG00000162490	ENSG00000165434
ENSG00000197061	ENSG00000128040
ENSG00000112406	ENSG00000167005
ENSG00000186868	ENSG00000198554
ENSG00000102743	ENSG00000164107
ENSG00000164323	ENSG00000070423
ENSG00000138136	ENSG00000157107
ENSG00000176410	ENSG00000182934
ENSG00000141232	ENSG00000142657
ENSG00000152192	ENSG00000159398
ENSG00000187498	ENSG00000196968
ENSG00000135597	ENSG00000122787
ENSG00000113073	ENSG00000143028
ENSG00000102780	ENSG00000121864
ENSG00000067533	ENSG00000073331
ENSG00000183580	ENSG00000021574
ENSG00000117481	ENSG00000103248
ENSG00000175699	ENSG00000118898
ENSG00000128016	ENSG00000137509
ENSG00000180357	ENSG00000083168
ENSG00000185499	ENSG00000126767
ENSG00000170425	ENSG00000186912

ENSG00000153132	ENSG00000135837
ENSG00000122778	ENSG00000143185
ENSG00000204645	ENSG00000073598
ENSG00000092607	ENSG00000100311
ENSG00000065060	ENSG00000125971
ENSG00000187735	ENSG00000056291
ENSG00000166261	ENSG00000004399
ENSG00000205060	ENSG00000105507
ENSG00000170275	ENSG00000132965
ENSG00000170525	ENSG00000109083
ENSG00000151327	ENSG00000224510
ENSG00000091542	ENSG00000149930
ENSG00000239388	ENSG00000173258
ENSG00000198825	ENSG00000125089
ENSG00000117020	ENSG00000204634
ENSG00000169031	ENSG00000144713
ENSG00000189007	ENSG00000134440
ENSG00000136875	ENSG00000102078
ENSG00000197019	ENSG00000136286
ENSG00000114698	ENSG00000136877
ENSG00000116560	ENSG00000130812
ENSG00000142192	ENSG00000099904
ENSG00000188647	ENSG00000139926
ENSG00000204624	ENSG00000147676
ENSG00000168476	ENSG00000152428
ENSG00000213463	ENSG00000129282
ENSG00000187838	ENSG00000197208
ENSG00000147606	ENSG00000115977
ENSG00000158615	ENSG00000099795
ENSG00000187257	ENSG00000137275
ENSG00000106689	ENSG00000197084
ENSG00000196090	ENSG00000172660
ENSG00000186326	ENSG00000168781
ENSG00000147231	ENSG00000173714
ENSG00000133874	ENSG00000089737
ENSG00000131002	ENSG00000033030
ENSG00000185896	ENSG00000117228
ENSG00000143776	ENSG00000164961
ENSG00000129654	ENSG00000107164
ENSG00000146374	ENSG00000162438
ENSG00000143603	ENSG00000159176
ENSG00000177479	ENSG00000165059
ENSG00000154645	ENSG00000124140
ENSG00000198182	ENSG00000083720
ENSG00000142541	ENSG00000085998
ENSG00000184117	ENSG00000107984
ENSG00000115363	ENSG00000130881
ENSG00000100105	ENSG00000136319
ENSG00000175984	ENSG00000126804
ENSG00000134042	ENSG00000101290
ENSG00000155827	ENSG00000175040
ENSG00000111728	ENSG00000060140
ENSG00000124529	ENSG00000171649
ENSG00000184672	ENSG00000100804
ENSG00000189143	ENSG00000106789
ENSG00000152207	ENSG00000079101
ENSG00000076067	ENSG00000080511
ENSG00000114646	ENSG00000108255
ENSG00000155729	ENSG00000174606
ENSG00000110492	ENSG00000112874
ENSG00000116016	ENSG00000147854
ENSG00000144893	ENSG00000146409
ENSG00000175029	ENSG00000013583
ENSG00000182247	ENSG00000102144

ENSG00000175538	ENSG00000134046
ENSG00000120913	ENSG00000163803
ENSG00000156304	ENSG00000197381
ENSG00000116903	ENSG00000213949
ENSG00000173402	ENSG00000145087
ENSG00000198353	ENSG00000108829
ENSG00000136709	ENSG00000108001
ENSG00000029725	ENSG00000137075
ENSG00000146477	ENSG00000147471
ENSG00000114923	ENSG00000065361
ENSG00000183558	ENSG00000248099
ENSG00000137824	ENSG00000101457
ENSG00000183688	ENSG00000171570
ENSG00000234127	ENSG00000131781
ENSG00000038274	ENSG00000107731
ENSG00000176153	ENSG00000022267
ENSG00000099949	ENSG00000184867
ENSG00000160191	ENSG00000151657
ENSG00000111880	ENSG00000184009
ENSG00000075461	ENSG00000244509
ENSG00000054983	ENSG00000165757
ENSG00000104886	ENSG00000166170
ENSG00000107518	ENSG00000104635
ENSG00000198356	ENSG00000159387
ENSG00000095787	ENSG00000011677
ENSG00000198689	ENSG00000179387
ENSG00000183831	ENSG00000165186
ENSG00000185352	ENSG00000079156
ENSG00000144645	ENSG00000185347
ENSG00000127481	ENSG00000182050
ENSG00000182552	ENSG00000119547
ENSG00000183762	ENSG00000167434
ENSG00000136536	ENSG00000148180
ENSG00000215717	ENSG00000172432
ENSG00000158050	ENSG00000184434
ENSG00000140396	ENSG00000080824
ENSG00000107362	ENSG00000100889
ENSG00000131067	ENSG00000179715
ENSG00000144229	ENSG00000054598
ENSG00000163468	ENSG00000135164
ENSG00000106261	ENSG00000137168
ENSG00000143756	ENSG00000118369
ENSG00000172795	ENSG00000171700
ENSG00000102290	ENSG00000182791
ENSG00000196338	ENSG00000132465
ENSG00000108039	ENSG00000171772
ENSG00000112727	ENSG00000129007
ENSG00000138829	ENSG00000089234
ENSG00000119231	ENSG00000197746
ENSG00000099942	ENSG00000166265
ENSG00000110274	ENSG00000126214
ENSG00000158406	ENSG00000140391
ENSG00000140575	ENSG00000085224
ENSG00000140553	ENSG00000101407
ENSG00000177432	ENSG00000149289
ENSG00000165886	ENSG00000019582
ENSG00000163214	ENSG00000108821
ENSG00000143815	ENSG00000060566
ENSG00000113282	ENSG00000126870
ENSG00000175216	ENSG00000138032
ENSG00000088305	ENSG00000128791
ENSG00000131462	ENSG00000167703
ENSG00000171403	ENSG00000237289
ENSG00000135074	ENSG00000165813

ENSG00000103035
ENSG00000140983
ENSG00000176407
ENSG00000104299
ENSG00000157020
ENSG00000006194
ENSG00000185585
ENSG00000158014
ENSG00000185960
ENSG00000186998
ENSG00000111271
ENSG00000147010
ENSG00000164219
ENSG00000158042
ENSG00000196724
ENSG00000002016
ENSG00000177917
ENSG00000160551
ENSG00000135363
ENSG00000187837
ENSG00000183484
ENSG00000120458
ENSG00000011132
ENSG00000124571
ENSG00000171456
ENSG00000120254
ENSG00000109943
ENSG00000162591
ENSG00000132334
ENSG00000134986
ENSG00000109832
ENSG00000099940
ENSG00000008300
ENSG00000162981
ENSG00000130224
ENSG00000111783
ENSG00000102100
ENSG00000142669
ENSG00000184845
ENSG00000048471
ENSG00000169180
ENSG00000166881
ENSG00000196586
ENSG00000063169
ENSG00000060237
ENSG00000170743
ENSG00000173275
ENSG00000070756
ENSG00000196704
ENSG00000169895
ENSG00000164171
ENSG00000159173
ENSG00000150995
ENSG00000003436
ENSG00000101079
ENSG00000215397
ENSG00000063978
ENSG00000247596
ENSG00000164045
ENSG00000182901
ENSG00000141756
ENSG00000133985
ENSG00000141446
ENSG00000125434
ENSG00000184254
ENSG00000007372
ENSG00000111206
ENSG00000185917
ENSG00000160973
ENSG00000023843
ENSG00000072110
ENSG00000177947
ENSG00000090512
ENSG00000148606
ENSG00000126952
ENSG00000133275
ENSG00000174175
ENSG00000134371
ENSG00000070785
ENSG00000204936
ENSG00000177992
ENSG00000176928
ENSG00000215127
ENSG00000212916
ENSG00000160948
ENSG00000113211
ENSG00000151651
ENSG00000064300
ENSG00000105576
ENSG00000177311
ENSG00000180879
ENSG00000136237
ENSG00000142583
ENSG00000178789
ENSG00000146350
ENSG00000152683
ENSG00000162496
ENSG00000165917
ENSG00000180787
ENSG00000001084
ENSG00000143891
ENSG00000174282
ENSG00000177025
ENSG00000115993
ENSG00000106976
ENSG00000131446
ENSG00000159871
ENSG00000186654
ENSG00000112337
ENSG00000166200
ENSG00000159256
ENSG00000158716
ENSG00000204363
ENSG00000171823
ENSG00000179580
ENSG00000115970
ENSG00000166523
ENSG00000122733
ENSG00000109654
ENSG00000100485
ENSG00000135905
ENSG00000127152
ENSG00000100416
ENSG00000101425
ENSG00000163328
ENSG00000140323
ENSG00000080503
ENSG00000035687

ENSG00000130758	ENSG00000184937
ENSG00000164061	ENSG00000143314
ENSG00000068354	ENSG00000177494
ENSG00000150594	ENSG00000120057
ENSG00000197712	ENSG00000073067
ENSG00000172113	ENSG00000157110
ENSG00000139372	ENSG00000160753
ENSG00000196361	ENSG00000132718
ENSG00000169220	ENSG00000124767
ENSG00000089163	ENSG00000065413
ENSG00000198873	ENSG00000138777
ENSG00000134438	ENSG00000130158
ENSG00000185052	ENSG00000156486
ENSG00000149485	ENSG00000197651
ENSG00000114626	ENSG00000072062
ENSG00000070444	ENSG00000157557
ENSG00000018189	ENSG00000164342
ENSG00000103034	ENSG00000123388
ENSG00000149534	ENSG00000169635
ENSG00000213639	ENSG00000166535
ENSG00000099290	ENSG00000185219
ENSG00000153046	ENSG00000161542
ENSG00000178074	ENSG00000135443
ENSG00000140299	ENSG00000164056
ENSG00000076201	ENSG00000136859
ENSG00000147100	ENSG00000138411
ENSG00000169682	ENSG00000140987
ENSG00000160991	ENSG00000175003
ENSG00000123992	ENSG00000108733
ENSG00000151033	ENSG00000128805
ENSG00000146021	ENSG00000099817
ENSG00000170734	ENSG00000023287
ENSG00000126524	ENSG00000094963
ENSG00000150977	ENSG00000162714
ENSG00000066468	ENSG00000116675
ENSG00000009694	ENSG00000173905
ENSG00000197771	ENSG00000148704
ENSG00000168907	ENSG00000147649
ENSG00000170604	ENSG00000183077
ENSG00000168488	ENSG00000165280
ENSG00000052795	ENSG00000183161
ENSG00000185811	ENSG00000140968
ENSG00000180957	ENSG00000180998
ENSG00000162433	ENSG00000181817
ENSG00000185818	ENSG00000213297
ENSG00000099998	ENSG00000073861
ENSG00000178726	ENSG00000087053
ENSG00000124882	ENSG00000198482
ENSG00000100167	ENSG00000198000
ENSG00000197444	ENSG00000183741
ENSG00000084652	ENSG00000115806
ENSG00000147144	ENSG00000101577
ENSG00000173486	ENSG00000153253
ENSG00000237190	ENSG00000162687
ENSG00000116990	ENSG00000011454
ENSG00000090432	ENSG00000138669
ENSG00000213390	ENSG00000137204
ENSG00000116750	ENSG00000112972
ENSG00000120949	ENSG00000133393
ENSG00000167037	ENSG00000011021
ENSG00000204564	ENSG00000132837
ENSG00000127483	ENSG00000198947
ENSG00000147573	ENSG00000244607
ENSG00000189159	ENSG00000143919

ENSG00000137642	ENSG00000005844
ENSG00000106992	ENSG00000160741
ENSG00000108239	ENSG00000177663
ENSG00000198477	ENSG00000127947
ENSG00000090470	ENSG00000046774
ENSG00000149547	ENSG00000106302
ENSG00000171119	ENSG00000186260
ENSG00000205208	ENSG00000165325
ENSG00000103044	ENSG00000183287
ENSG00000175426	ENSG00000094880
ENSG00000197776	ENSG00000177283
ENSG00000170412	ENSG00000109814
ENSG00000204388	ENSG00000134489
ENSG00000159307	ENSG00000196296
ENSG00000184584	ENSG00000151014
ENSG00000103449	ENSG00000163297
ENSG00000155961	ENSG00000105750
ENSG00000175895	ENSG00000168309
ENSG00000005513	ENSG00000104312
ENSG00000151914	ENSG00000113389
ENSG00000148218	ENSG00000180219
ENSG00000131149	ENSG00000105697
ENSG00000116641	ENSG00000124207
ENSG00000204065	ENSG00000166428
ENSG00000197859	ENSG00000099866
ENSG00000171169	ENSG00000168906
ENSG00000065911	ENSG00000119042
ENSG00000158246	ENSG00000068366
ENSG00000115107	ENSG00000175520
ENSG00000119699	ENSG00000087303
ENSG00000111432	ENSG00000070190
ENSG00000092978	ENSG00000241837
ENSG00000197273	ENSG00000137714
ENSG00000146047	ENSG00000108602
ENSG00000165410	ENSG00000100503
ENSG00000169519	ENSG00000083097
ENSG00000110851	ENSG00000085978
ENSG00000116489	ENSG00000123684
ENSG00000183354	ENSG00000079150
ENSG00000085063	ENSG00000144677
ENSG00000146285	ENSG00000074696
ENSG00000167881	ENSG00000018236
ENSG00000164591	ENSG00000006025
ENSG00000113108	ENSG00000175198
ENSG00000060709	ENSG00000153246
ENSG00000138308	ENSG00000151514
ENSG00000130726	ENSG00000165695
ENSG00000104490	ENSG00000101400
ENSG00000120075	ENSG00000173674
ENSG00000198691	ENSG00000158423
ENSG00000020922	ENSG00000139625
ENSG00000163817	ENSG00000112200
ENSG00000129667	ENSG00000170004
ENSG00000147548	ENSG00000221947
ENSG00000159556	ENSG00000196507
ENSG00000175265	ENSG00000060749
ENSG00000140044	ENSG00000198919
ENSG00000119283	ENSG00000197121
ENSG00000148840	ENSG00000140459
ENSG00000123358	ENSG00000028310
ENSG00000172932	ENSG00000146090
ENSG00000119689	ENSG00000169718
ENSG00000137094	ENSG00000128524
ENSG00000146834	ENSG00000159450

ENSG00000120149	ENSG00000213494
ENSG00000144455	ENSG00000165416
ENSG00000072778	ENSG00000100991
ENSG00000144741	ENSG00000166033
ENSG00000138131	ENSG00000126603
ENSG00000108021	ENSG00000178403
ENSG00000106331	ENSG00000196109
ENSG00000106484	ENSG00000176092
ENSG00000168785	ENSG00000163239
ENSG00000122691	ENSG00000111674
ENSG00000163531	ENSG00000120675
ENSG00000101928	ENSG00000144635
ENSG00000163694	ENSG00000111670
ENSG00000131747	ENSG00000185480
ENSG00000135119	ENSG00000115041
ENSG00000130529	ENSG00000215568
ENSG00000135316	ENSG00000079215
ENSG00000168575	ENSG00000116141
ENSG00000125459	ENSG00000133808
ENSG00000025293	ENSG00000102189
ENSG00000168286	ENSG00000102870
ENSG00000134077	ENSG00000104765
ENSG00000119402	ENSG00000213199
ENSG00000143107	ENSG00000174236
ENSG00000090316	ENSG00000007174
ENSG00000163827	ENSG00000102710
ENSG00000169258	ENSG00000165973
ENSG00000165030	ENSG00000160447
ENSG00000072071	ENSG00000157119
ENSG00000242028	ENSG00000067113
ENSG00000007350	ENSG00000101180
ENSG00000116679	ENSG00000152795
ENSG00000186143	ENSG00000180626
ENSG00000185630	ENSG00000089159
ENSG00000172901	ENSG00000157181
ENSG00000134375	ENSG00000100528
ENSG00000115457	ENSG00000197705
ENSG00000169925	ENSG00000119655
ENSG00000146587	ENSG00000203857
ENSG00000144233	ENSG00000188763
ENSG00000136935	ENSG00000166148
ENSG00000253305	ENSG00000055208
ENSG00000163884	ENSG00000168067
ENSG00000110092	ENSG00000177238
ENSG00000120899	ENSG00000236515
ENSG00000104331	ENSG00000120071
ENSG00000124313	ENSG00000172382
ENSG00000198844	ENSG00000147439
ENSG00000137404	ENSG00000172164
ENSG00000080802	ENSG00000198910
ENSG00000103351	ENSG00000223496
ENSG00000118276	ENSG00000172936
ENSG00000164855	ENSG00000006576
ENSG00000163346	ENSG00000170537
ENSG00000115665	ENSG00000112077
ENSG00000118420	ENSG00000132763
ENSG00000175073	ENSG00000170162
ENSG00000144354	ENSG00000131142
ENSG00000147655	ENSG00000077942
ENSG00000138759	ENSG00000160299
ENSG00000186350	ENSG00000136383
ENSG00000181472	ENSG00000162735
ENSG00000203814	ENSG00000141068
ENSG00000164683	ENSG00000133048

ENSG00000144834	ENSG00000113525
ENSG00000134709	ENSG00000142606
ENSG00000101109	ENSG00000170271
ENSG00000164983	ENSG00000170860
ENSG00000166845	ENSG00000096060
ENSG00000041988	ENSG00000149313
ENSG00000063660	ENSG00000214216
ENSG00000136444	ENSG00000120832
ENSG00000096968	ENSG00000175582
ENSG00000085644	ENSG00000131061
ENSG00000100478	ENSG00000133020
ENSG00000103047	ENSG00000125952
ENSG00000162521	ENSG00000148826
ENSG00000164284	ENSG00000152034
ENSG00000134569	ENSG00000070526
ENSG00000183049	ENSG00000120280
ENSG00000169856	ENSG00000138606
ENSG00000105662	ENSG00000121903
ENSG00000140526	ENSG00000154102
ENSG00000185532	ENSG00000155368
ENSG00000075223	ENSG00000182185
ENSG00000129351	ENSG00000085433
ENSG00000198673	ENSG00000197355
ENSG00000174111	ENSG00000144063
ENSG00000125968	ENSG00000089280
ENSG00000124702	ENSG00000135549
ENSG00000188554	ENSG00000160256
ENSG00000198959	ENSG00000130193
ENSG00000183853	ENSG00000156564
ENSG00000125675	ENSG00000125458
ENSG00000175573	ENSG00000102921
ENSG00000142188	ENSG00000170265
ENSG00000189043	ENSG00000180329
ENSG00000184349	ENSG00000239382
ENSG00000166803	ENSG00000176108
ENSG00000066855	ENSG00000078399
ENSG00000167889	ENSG00000047634
ENSG00000182572	ENSG00000163909
ENSG00000081154	ENSG00000106080
ENSG00000114315	ENSG00000126698
ENSG00000113430	ENSG00000169562
ENSG00000009844	ENSG00000128845
ENSG00000104432	ENSG00000215440
ENSG00000140795	ENSG00000105989
ENSG00000181322	ENSG00000072864
ENSG00000136327	ENSG00000165060
ENSG00000136158	ENSG00000088205
ENSG00000032389	ENSG00000111875
ENSG00000184986	ENSG00000170579
ENSG00000115008	ENSG00000153283
ENSG00000111254	ENSG00000189068
ENSG00000181773	ENSG00000154328
ENSG00000176624	ENSG00000168229
ENSG00000039987	ENSG00000189369
ENSG00000104142	ENSG00000104818
ENSG00000165169	ENSG00000141682
ENSG00000176884	ENSG00000115232
ENSG00000198771	ENSG00000140332
ENSG00000070087	ENSG00000152592
ENSG00000034053	ENSG00000065328
ENSG00000118495	ENSG00000097007
ENSG00000138336	ENSG00000183337
ENSG00000122642	ENSG00000141325
ENSG00000072121	ENSG00000032444

ENSG00000058404	ENSG00000174500
ENSG00000158526	ENSG00000138798
ENSG00000167074	ENSG00000048140
ENSG00000008952	ENSG00000182670
ENSG00000168874	ENSG00000136051
ENSG00000188015	ENSG00000150628
ENSG00000167105	ENSG00000197056
ENSG00000167535	ENSG00000065833
ENSG00000116985	ENSG00000120333
ENSG00000127022	ENSG00000079785
ENSG00000067842	ENSG00000176635
ENSG00000164142	ENSG00000166311
ENSG00000186815	ENSG00000075043
ENSG00000168539	ENSG00000143162
ENSG00000198231	ENSG00000173726
ENSG00000095066	ENSG00000137731
ENSG00000075340	ENSG00000133488
ENSG00000112038	ENSG00000181061
ENSG00000165704	ENSG00000174796
ENSG00000152953	ENSG00000160716
ENSG00000165659	ENSG00000119973
ENSG00000104881	ENSG00000147443
ENSG00000124507	ENSG00000136937
ENSG00000130307	ENSG00000243772
ENSG00000145685	ENSG00000160284
ENSG00000049768	ENSG00000059573
ENSG00000140743	ENSG00000004948
ENSG00000184347	ENSG00000120289
ENSG00000198055	ENSG00000168995
ENSG00000167118	ENSG00000006075
ENSG00000184571	ENSG00000172336
ENSG00000113615	ENSG00000036672
ENSG00000126062	ENSG00000041802
ENSG00000168140	ENSG00000164430
ENSG00000175264	ENSG00000077800
ENSG00000135622	ENSG00000136636
ENSG00000182508	ENSG00000136842
ENSG00000144021	ENSG00000123444
ENSG00000117525	ENSG00000050405
ENSG00000091409	ENSG00000065000
ENSG00000092051	ENSG00000105492
ENSG00000153113	ENSG00000174576
ENSG00000138100	ENSG00000180964
ENSG00000067646	ENSG00000188706
ENSG00000185774	ENSG00000101333
ENSG00000186951	ENSG00000111596
ENSG00000111424	ENSG00000148341
ENSG00000128886	ENSG00000167785
ENSG00000120334	ENSG00000243509
ENSG00000147316	ENSG00000164039
ENSG00000167984	ENSG00000179520
ENSG00000134324	ENSG00000113638
ENSG00000157087	ENSG00000130703
ENSG00000120215	ENSG00000143418
ENSG00000135218	ENSG00000130827
ENSG00000159164	ENSG00000164821
ENSG00000060718	ENSG00000152254
ENSG00000074410	ENSG00000155657
ENSG00000182963	ENSG00000214026
ENSG00000164091	ENSG00000067191
ENSG00000181495	ENSG00000197959
ENSG00000168394	ENSG00000186026
ENSG00000153339	ENSG00000010671
ENSG00000170549	ENSG00000010219

ENSG00000106723	ENSG00000125851
ENSG00000115947	ENSG00000143630
ENSG00000073711	ENSG00000122136
ENSG00000115194	ENSG00000006062
ENSG00000107282	ENSG00000137992
ENSG00000107614	ENSG00000108639
ENSG00000136695	ENSG00000146243
ENSG00000103550	ENSG00000153179
ENSG00000106328	ENSG00000139352
ENSG00000176971	ENSG00000083799
ENSG00000083896	ENSG00000164694
ENSG00000187650	ENSG00000103160
ENSG00000125970	ENSG00000131386
ENSG00000162510	ENSG00000167522
ENSG00000131375	ENSG00000125965
ENSG00000049618	ENSG00000162105
ENSG00000075826	ENSG00000139269
ENSG00000197584	ENSG00000188428
ENSG00000141646	ENSG00000161574
ENSG00000145817	ENSG00000198208
ENSG00000145675	ENSG00000196235
ENSG00000165572	ENSG00000092758
ENSG00000221994	ENSG00000180332
ENSG00000010803	ENSG00000088682
ENSG00000140853	ENSG00000130477
ENSG00000107779	ENSG00000090020
ENSG00000186283	ENSG00000101158
ENSG00000157093	ENSG00000105855
ENSG00000102245	ENSG00000169385
ENSG00000182957	ENSG00000119820
ENSG00000104888	ENSG00000176903
ENSG00000166924	ENSG00000105866
ENSG00000160094	ENSG00000168032
ENSG00000140798	ENSG00000132498
ENSG00000123983	ENSG00000056586
ENSG00000172663	ENSG00000172243
ENSG00000177354	ENSG00000187189
ENSG00000103064	ENSG00000068971
ENSG00000198933	ENSG00000138231
ENSG00000126821	ENSG00000112167
ENSG00000174740	ENSG00000039068
ENSG00000138041	ENSG00000140479
ENSG00000142002	ENSG00000114346
ENSG00000164244	ENSG00000153487
ENSG00000165879	ENSG00000116752
ENSG00000081479	ENSG00000082175
ENSG00000019549	ENSG00000166257
ENSG00000242419	ENSG00000074771
ENSG00000101574	ENSG00000198369
ENSG00000163659	ENSG00000006071
ENSG00000196411	ENSG00000137817
ENSG00000170832	ENSG00000179399
ENSG00000119703	ENSG00000171303
ENSG00000125813	ENSG00000166473
ENSG00000142197	ENSG00000067715
ENSG00000174938	ENSG00000113649
ENSG00000167258	ENSG00000135452
ENSG00000180818	ENSG00000099810
ENSG00000108953	ENSG00000107560
ENSG00000139970	ENSG00000178852
ENSG00000168314	ENSG00000168461
ENSG00000172239	ENSG00000085840
ENSG00000180228	ENSG00000187180
ENSG00000125991	ENSG00000060138

ENSG00000171466	ENSG00000197748
ENSG00000183718	ENSG00000120694
ENSG00000171720	ENSG00000243696
ENSG00000147099	ENSG00000166006
ENSG00000044574	ENSG00000162733
ENSG00000157514	ENSG00000137841
ENSG00000188987	ENSG00000129675
ENSG00000080603	ENSG00000107745
ENSG00000164303	ENSG00000153208
ENSG00000067141	ENSG00000058866
ENSG00000151276	ENSG00000162298
ENSG00000253148	ENSG00000152766
ENSG00000099377	ENSG00000144821
ENSG00000073849	ENSG00000175564
ENSG00000116704	ENSG00000153976
ENSG00000100813	ENSG00000134775
ENSG00000173442	ENSG00000012983
ENSG00000004142	ENSG00000152670
ENSG00000189325	ENSG00000119715
ENSG00000203880	ENSG00000153774
ENSG00000125107	ENSG00000185736
ENSG00000136535	ENSG00000008735
ENSG00000214193	ENSG00000124935
ENSG00000165379	ENSG00000141441
ENSG00000119899	ENSG00000163596
ENSG00000102401	ENSG00000160087
ENSG00000118514	ENSG00000169393
ENSG00000048991	ENSG00000112343
ENSG00000116251	ENSG00000158055
ENSG00000138380	ENSG00000105641
ENSG00000096696	ENSG00000155744
ENSG00000161791	ENSG00000010030
ENSG00000164088	ENSG00000116205
ENSG00000165669	ENSG00000158321
ENSG00000102317	ENSG00000175646
ENSG00000162702	ENSG00000188910
ENSG00000135333	ENSG00000151665
ENSG00000180509	ENSG00000172007
ENSG00000100207	ENSG00000187021
ENSG00000251322	ENSG00000149115
ENSG00000121594	ENSG00000159251
ENSG00000090776	ENSG00000205364
ENSG00000187079	ENSG00000144320
ENSG00000019144	ENSG00000115109
ENSG00000250510	ENSG00000140718
ENSG00000141568	ENSG00000100372
ENSG00000075420	ENSG00000177143
ENSG00000184489	ENSG00000174156
ENSG00000181220	ENSG00000132182
ENSG00000139687	ENSG00000140264
ENSG00000180900	ENSG00000162738
ENSG00000169067	ENSG00000242173
ENSG00000086475	ENSG00000146555
ENSG00000167614	ENSG00000181638
ENSG00000104447	ENSG00000170113
ENSG00000155849	ENSG00000163577
ENSG00000161638	ENSG00000147614
ENSG00000108370	ENSG00000204070
ENSG00000144843	ENSG00000171262
ENSG00000122008	ENSG00000124256
ENSG00000189266	ENSG00000070501
ENSG00000171812	ENSG00000154415
ENSG00000150938	ENSG00000154743
ENSG00000215454	ENSG00000170775

ENSG00000174799	ENSG00000183778
ENSG00000109332	ENSG00000164292
ENSG00000175970	ENSG00000177791
ENSG00000168297	ENSG00000095303
ENSG00000111731	ENSG00000123243
ENSG00000101557	ENSG00000120093
ENSG00000143222	ENSG00000213337
ENSG00000165458	ENSG00000166402
ENSG00000100599	ENSG00000177427
ENSG00000168939	ENSG00000198759
ENSG00000182489	ENSG00000121440
ENSG00000177468	ENSG00000196642
ENSG00000152782	ENSG00000074047
ENSG00000171223	ENSG00000250571
ENSG00000158286	ENSG00000107902
ENSG00000121892	ENSG00000170703
ENSG00000239697	ENSG00000198315
ENSG00000132155	ENSG00000140199
ENSG00000136021	ENSG00000162374
ENSG00000134594	ENSG00000164933
ENSG00000152056	ENSG00000166704
ENSG00000079313	ENSG00000102547
ENSG00000196371	ENSG00000068724
ENSG00000162849	ENSG00000115163
ENSG00000116237	ENSG00000091157
ENSG00000101695	ENSG00000161544
ENSG00000007392	ENSG00000077420
ENSG00000163964	ENSG00000143324
ENSG00000095209	ENSG00000103257
ENSG00000147027	ENSG00000036549
ENSG00000204052	ENSG00000126456
ENSG00000074755	ENSG00000121207
ENSG00000108262	ENSG00000187800
ENSG00000115226	ENSG00000065534
ENSG00000134551	ENSG00000186130
ENSG00000174197	ENSG00000010072
ENSG00000159210	ENSG00000197646
ENSG00000094975	ENSG00000036257
ENSG00000158711	ENSG00000183166
ENSG00000106077	ENSG00000103275
ENSG00000166569	ENSG00000116157
ENSG00000140937	ENSG00000182492
ENSG00000107249	ENSG00000179222
ENSG00000006831	ENSG00000108511
ENSG00000158290	ENSG00000152377
ENSG00000078295	ENSG00000170465
ENSG00000163599	ENSG00000135094
ENSG00000136240	ENSG00000142556
ENSG00000244687	ENSG00000148158
ENSG00000122912	ENSG00000183495
ENSG00000153902	ENSG00000214022
ENSG00000151247	ENSG00000128951
ENSG00000119048	ENSG00000102043
ENSG00000167995	ENSG00000115307
ENSG00000203778	ENSG00000167580
ENSG00000021645	ENSG00000171595
ENSG00000049656	ENSG00000175931
ENSG00000181915	ENSG00000197757
ENSG00000109685	ENSG00000064102
ENSG00000165238	ENSG00000140682
ENSG00000181026	ENSG00000147894
ENSG00000187140	ENSG00000127252
ENSG00000139146	ENSG00000167566
ENSG00000135387	ENSG00000074071

ENSG00000137101	ENSG00000145075
ENSG00000160201	ENSG00000139219
ENSG00000137942	ENSG00000186532
ENSG00000133121	ENSG00000183570
ENSG00000170419	ENSG00000100288
ENSG00000164949	ENSG00000138722
ENSG00000119227	ENSG00000133710
ENSG00000079335	ENSG00000112079
ENSG00000107187	ENSG00000011114
ENSG00000168036	ENSG00000131828
ENSG00000124203	ENSG00000149451
ENSG00000066827	ENSG00000167395
ENSG00000112238	ENSG00000178913
ENSG00000164823	ENSG00000173391
ENSG00000162783	ENSG00000112902
ENSG00000101132	ENSG00000124479
ENSG00000103187	ENSG00000174804
ENSG00000198026	ENSG00000168679
ENSG00000078061	ENSG00000197415
ENSG00000160712	ENSG00000101162
ENSG00000163820	ENSG00000150337
ENSG00000146386	ENSG00000130561
ENSG00000170142	ENSG00000136930
ENSG00000075790	ENSG00000198836
ENSG00000152818	ENSG00000131876
ENSG00000015475	ENSG00000138442
ENSG00000168334	ENSG00000198454
ENSG00000117411	ENSG00000005156
ENSG00000119638	ENSG00000161960
ENSG00000198732	ENSG00000168101
ENSG00000182013	ENSG00000185436
ENSG00000135723	ENSG00000166839
ENSG00000166188	ENSG00000203896
ENSG00000027847	ENSG00000014257
ENSG00000136504	ENSG00000133703
ENSG00000147421	ENSG00000075568
ENSG00000159212	ENSG00000178904
ENSG00000129038	ENSG00000040608
ENSG00000065559	ENSG00000137962
ENSG00000205279	ENSG00000125651
ENSG00000170523	ENSG00000110881
ENSG00000123144	ENSG00000178252
ENSG00000153914	ENSG00000162383
ENSG00000178919	ENSG00000198517
ENSG00000077454	ENSG00000165091
ENSG00000169504	ENSG00000135446
ENSG00000162069	ENSG00000027697
ENSG00000076108	ENSG00000156650
ENSG00000132478	ENSG00000163482
ENSG00000204304	ENSG00000143952
ENSG00000166949	ENSG00000125378
ENSG00000109118	ENSG00000170631
ENSG00000185019	ENSG00000103966
ENSG00000197070	ENSG00000173212
ENSG00000127526	ENSG00000108389
ENSG00000128242	ENSG00000005961
ENSG00000138767	ENSG00000011451
ENSG00000184292	ENSG00000167799
ENSG00000143546	ENSG00000065978
ENSG00000116544	ENSG00000053747
ENSG00000198704	ENSG00000151135
ENSG00000100246	ENSG00000110448
ENSG00000182568	ENSG00000005801
ENSG00000135222	ENSG00000152700

ENSG00000132842	ENSG00000090861
ENSG00000204116	ENSG00000113369
ENSG00000165233	ENSG00000110318
ENSG00000103342	ENSG00000106346
ENSG00000114395	ENSG00000196821
ENSG00000215193	ENSG00000101193
ENSG00000170779	ENSG00000066926
ENSG00000107719	ENSG00000123178
ENSG00000145309	ENSG00000189280
ENSG00000169062	ENSG00000120265
ENSG00000169570	ENSG00000105516
ENSG00000086300	ENSG00000156076
ENSG00000117640	ENSG00000135999
ENSG00000092345	ENSG00000090975
ENSG00000169436	ENSG00000155066
ENSG00000166387	ENSG00000198569
ENSG00000181790	ENSG00000119487
ENSG00000049283	ENSG00000170454
ENSG00000132294	ENSG00000081692
ENSG00000186767	ENSG00000143373
ENSG00000100284	ENSG00000203780
ENSG00000154175	ENSG00000148384
ENSG00000184388	ENSG00000178568
ENSG00000017260	ENSG00000079819
ENSG00000197461	ENSG00000157103
ENSG00000198791	ENSG00000112541
ENSG00000146232	ENSG00000156194
ENSG00000124374	ENSG00000167618
ENSG00000214435	ENSG00000158473
ENSG00000173473	ENSG00000167103
ENSG00000196792	ENSG00000141485
ENSG00000112769	ENSG00000165731
ENSG00000166579	ENSG00000182952
ENSG00000138688	ENSG00000044012
ENSG00000089006	ENSG00000185085
ENSG00000185515	ENSG00000030419
ENSG00000164508	ENSG00000075213
ENSG00000157933	ENSG00000034063
ENSG00000141161	ENSG00000141034
ENSG00000091137	ENSG00000159917
ENSG00000183662	ENSG00000183114
ENSG00000119397	ENSG00000110777
ENSG00000115561	ENSG00000179604
ENSG00000154767	ENSG00000110025
ENSG00000124092	ENSG00000100567
ENSG00000008130	ENSG00000151304
ENSG00000105186	ENSG00000144909
ENSG00000153993	ENSG00000143321
ENSG00000198612	ENSG00000143954
ENSG00000184185	ENSG00000005889
ENSG00000100227	ENSG00000117009
ENSG00000110047	ENSG00000143995
ENSG00000154237	ENSG00000119335
ENSG00000140451	ENSG00000112293
ENSG00000164695	ENSG00000196715
ENSG00000115170	ENSG00000113312
ENSG00000151615	ENSG00000119718
ENSG00000166167	ENSG00000132603
ENSG00000137331	ENSG00000119013
ENSG00000232810	ENSG00000077238
ENSG00000137492	ENSG00000164756
ENSG00000170967	ENSG00000064652
ENSG00000162377	ENSG00000135775
ENSG00000006015	ENSG00000155621

ENSG00000146263	ENSG00000160321
ENSG00000198944	ENSG00000171490
ENSG00000174233	ENSG00000171723
ENSG00000062524	ENSG00000026103
ENSG00000198853	ENSG00000153291
ENSG00000177426	ENSG00000163501
ENSG00000181392	ENSG00000087008
ENSG00000006695	ENSG00000130433
ENSG00000110931	ENSG00000013810
ENSG00000181722	ENSG00000036473
ENSG00000111816	ENSG00000160224
ENSG00000176994	ENSG00000155508
ENSG00000134644	ENSG00000223526
ENSG00000183520	ENSG00000108622
ENSG00000101266	ENSG00000104228
ENSG00000132589	ENSG00000102119
ENSG00000187486	ENSG00000128923
ENSG00000111665	ENSG00000101856
ENSG00000171451	ENSG00000105137
ENSG00000169242	ENSG00000037042
ENSG00000151320	ENSG00000204130
ENSG00000204899	ENSG00000089053
ENSG00000040487	ENSG00000046653
ENSG00000077713	ENSG00000082074
ENSG00000142168	ENSG00000113719
ENSG00000114383	ENSG00000179941
ENSG00000115540	ENSG00000086598
ENSG00000151967	ENSG00000102172
ENSG00000163873	ENSG00000166848
ENSG00000146411	ENSG00000107201
ENSG00000198121	ENSG00000145242
ENSG00000107758	ENSG00000114745
ENSG00000103460	ENSG00000106462
ENSG00000135127	ENSG00000168209
ENSG00000162302	ENSG00000169241
ENSG00000120690	ENSG00000130303
ENSG00000066322	ENSG00000132781
ENSG00000139890	ENSG00000198960
ENSG00000121940	ENSG00000168930
ENSG00000164626	ENSG00000165138
ENSG00000198286	ENSG00000115661
ENSG00000171634	ENSG00000126583
ENSG00000182346	ENSG00000131732
ENSG00000163590	ENSG00000131037
ENSG00000162065	ENSG00000153956
ENSG00000010270	ENSG00000206527
ENSG00000139209	ENSG00000124440
ENSG00000151490	ENSG00000116273
ENSG00000104936	ENSG00000139180
ENSG00000182742	ENSG00000121310
ENSG00000138279	ENSG00000160058
ENSG00000174013	ENSG00000168827
ENSG00000100376	ENSG00000047346
ENSG00000182158	ENSG00000124067
ENSG00000204576	ENSG00000169429
ENSG00000184047	ENSG00000164466
ENSG00000222047	ENSG00000197728
ENSG00000177853	ENSG00000161217
ENSG00000168675	ENSG00000100029
ENSG00000171603	ENSG00000105357
ENSG00000065357	ENSG00000165392
ENSG00000165526	ENSG00000146574
ENSG00000163635	ENSG00000172023
ENSG00000153561	ENSG00000113763

ENSG00000079246	ENSG00000165899
ENSG00000179431	ENSG00000169515
ENSG00000205944	ENSG00000123975
ENSG00000065526	ENSG00000171960
ENSG00000179981	ENSG00000105810
ENSG00000173848	ENSG00000174775
ENSG00000156521	ENSG00000170613
ENSG00000146250	ENSG00000139793
ENSG00000177879	ENSG00000160404
ENSG00000005483	ENSG00000165269
ENSG00000112182	ENSG00000082269
ENSG00000128285	ENSG00000127954
ENSG00000179674	ENSG00000169891
ENSG00000169194	ENSG00000125735
ENSG00000241635	ENSG00000185164
ENSG00000112562	ENSG00000160678
ENSG00000052802	ENSG00000080572
ENSG00000129691	ENSG00000166963
ENSG00000243244	ENSG00000176533
ENSG00000050165	ENSG00000058335
ENSG00000146963	ENSG00000131470
ENSG00000175203	ENSG00000166226
ENSG00000159399	ENSG00000138385
ENSG00000182923	ENSG00000112378
ENSG00000170852	ENSG00000129933
ENSG00000131725	ENSG00000136448
ENSG00000119509	ENSG00000134504
	ENSG00000174460
	ENSG00000174437
	ENSG00000197045
	ENSG00000110063
	ENSG00000135899
	ENSG00000188227
	ENSG00000147394
	ENSG00000077348
	ENSG00000135823
	ENSG00000108786
	ENSG00000182885
	ENSG00000163904
	ENSG00000243978
	ENSG00000181090
	ENSG00000067798
	ENSG00000086289
	ENSG00000078795
	ENSG00000196415
	ENSG00000128340
	ENSG00000143621
	ENSG00000113319
	ENSG00000198794
	ENSG00000187969
	ENSG00000123965
	ENSG00000140280
	ENSG00000167178
	ENSG00000164399
	ENSG00000186847
	ENSG00000147509
	ENSG00000162444
	ENSG00000242732
	ENSG00000126215
	ENSG00000112818
	ENSG00000163714
	ENSG00000173706
	ENSG00000160307
	ENSG00000163508

ENSG00000054803
ENSG00000167383
ENSG00000128591
ENSG00000198492
ENSG00000133313
ENSG00000135083
ENSG00000187621
ENSG00000198242
ENSG00000128581
ENSG00000100036
ENSG00000188707
ENSG00000166266
ENSG00000143110
ENSG00000100292
ENSG00000100031
ENSG00000101945
ENSG00000136011
ENSG00000198742
ENSG00000007952
ENSG00000152952
ENSG00000100342
ENSG00000113905
ENSG00000120211
ENSG00000119523
ENSG00000106258
ENSG00000108947
ENSG00000115850
ENSG00000130595
ENSG00000197457
ENSG00000169744
ENSG00000126814
ENSG00000205336
ENSG00000105664
ENSG00000132507
ENSG00000144136
ENSG00000134318
ENSG00000102021
ENSG00000106018
ENSG00000090061
ENSG00000187942
ENSG00000114374
ENSG00000069966
ENSG00000129009
ENSG00000179029
ENSG00000130733
ENSG00000131791
ENSG00000129354
ENSG00000105063
ENSG00000102908
ENSG00000119328
ENSG00000128708
ENSG00000144278
ENSG00000090447
ENSG00000164125
ENSG00000084234
ENSG00000102755
ENSG00000121058
ENSG00000167771
ENSG00000159648
ENSG00000133835
ENSG00000152284
ENSG00000183733
ENSG00000083817
ENSG00000163029

ENSG00000105371
ENSG00000117877
ENSG00000164548
ENSG00000197111
ENSG00000121964
ENSG00000134049
ENSG00000174469
ENSG00000137261
ENSG00000197321
ENSG00000107263
ENSG00000083937
ENSG00000009954
ENSG00000136960
ENSG00000185666
ENSG00000183346
ENSG00000168491
ENSG00000138629
ENSG00000103423
ENSG00000140093
ENSG00000123159
ENSG00000171587
ENSG00000184465
ENSG00000169306
ENSG00000159733
ENSG00000218823
ENSG00000135093
ENSG00000168398
ENSG00000162928
ENSG00000054267
ENSG00000169299
ENSG00000206302
ENSG00000077498
ENSG00000058272
ENSG00000204519
ENSG00000136888
ENSG00000006432
ENSG00000135862
ENSG00000163637
ENSG00000135414
ENSG00000068793
ENSG00000008382
ENSG00000158158
ENSG00000167113
ENSG00000188404
ENSG00000119938
ENSG00000095713
ENSG00000160349
ENSG00000173559
ENSG00000148187
ENSG00000108469
ENSG00000165689
ENSG00000122674
ENSG00000069275
ENSG00000118777
ENSG00000121388
ENSG00000159423
ENSG00000172572
ENSG00000058085
ENSG00000132746
ENSG00000115592
ENSG00000186897
ENSG00000116199
ENSG00000170689
ENSG00000165995

ENSG00000165816
ENSG00000127129
ENSG00000123094
ENSG00000128564
ENSG00000114013
ENSG00000078401
ENSG00000103241
ENSG00000105404
ENSG00000162402
ENSG00000077585
ENSG00000066382
ENSG00000177380
ENSG00000183780
ENSG00000120158
ENSG00000106615
ENSG00000105499
ENSG00000198258
ENSG00000070759
ENSG00000103091
ENSG00000134152
ENSG00000198798
ENSG00000206181
ENSG00000166825
ENSG00000007516
ENSG00000108387
ENSG00000197587
ENSG00000115415
ENSG00000180771
ENSG00000110911
ENSG00000105483
ENSG00000108556
ENSG00000163349
ENSG00000181481
ENSG00000139154
ENSG00000039560
ENSG00000124942
ENSG00000197562
ENSG00000011258
ENSG00000102572
ENSG00000165131
ENSG00000241595
ENSG00000125354
ENSG00000188694
ENSG00000175189
ENSG00000117016
ENSG00000116031
ENSG00000121570
ENSG00000156384
ENSG00000110484
ENSG00000152137
ENSG00000188060
ENSG00000163848
ENSG00000187855
ENSG00000108849
ENSG00000181784
ENSG00000213471
ENSG00000198373
ENSG00000197299
ENSG00000204217
ENSG00000137145
ENSG00000178645
ENSG00000102978
ENSG00000180251
ENSG00000137203

ENSG00000138293
ENSG00000092439
ENSG00000127220
ENSG00000072609
ENSG00000177189
ENSG00000010322
ENSG00000150471
ENSG00000154783
ENSG00000160194
ENSG00000087460
ENSG00000167315
ENSG00000154478
ENSG00000139405
ENSG00000113360
ENSG00000176105
ENSG00000223953
ENSG00000158195
ENSG00000138074
ENSG00000196459
ENSG00000169641
ENSG00000164024
ENSG00000198685
ENSG00000156509
ENSG00000168404
ENSG00000077264
ENSG00000180530
ENSG00000143183
ENSG00000180304
ENSG00000110786
ENSG00000134369
ENSG00000185608
ENSG00000123700
ENSG00000138801
ENSG00000127914
ENSG00000203663
ENSG00000088726
ENSG00000197694
ENSG00000178425
ENSG00000213741
ENSG00000213578
ENSG00000162415
ENSG00000145824
ENSG00000173335
ENSG00000115263
ENSG00000185728
ENSG00000082641
ENSG00000111981
ENSG00000203710
ENSG00000186732
ENSG00000129521
ENSG00000069869
ENSG00000151715
ENSG00000166068
ENSG00000172678
ENSG00000174718
ENSG00000168542
ENSG00000243566
ENSG00000177614
ENSG00000184277
ENSG00000008517
ENSG00000197479
ENSG00000072042
ENSG00000113492
ENSG00000142910

ENSG00000069206
ENSG00000124157
ENSG00000131773
ENSG00000111412
ENSG00000167371
ENSG00000139722
ENSG00000115649
ENSG00000163539
ENSG00000104093
ENSG00000159723
ENSG00000197343
ENSG00000154258
ENSG00000087245
ENSG00000133318
ENSG00000197119
ENSG00000198815
ENSG00000114857
ENSG00000101216
ENSG00000101782
ENSG00000108352
ENSG00000169714
ENSG00000185024
ENSG00000103942
ENSG00000152684
ENSG00000146678
ENSG00000164093
ENSG00000111961
ENSG00000157423
ENSG00000005981
ENSG00000145284
ENSG00000177125
ENSG00000134007
ENSG00000077616
ENSG00000089091
ENSG00000156925
ENSG00000126561
ENSG00000178795
ENSG00000124575
ENSG00000163810
ENSG00000108244
ENSG00000000003
ENSG00000171867
ENSG00000213265
ENSG00000077044
ENSG00000133619
ENSG00000196305
ENSG00000232112
ENSG00000148824
ENSG00000122304
ENSG00000167941
ENSG00000096384
ENSG00000160145
ENSG00000163958
ENSG00000213190
ENSG00000165322
ENSG00000158856
ENSG00000123064
ENSG00000116138
ENSG00000141576
ENSG00000140416
ENSG00000170848
ENSG00000168348
ENSG00000221890
ENSG00000155111

ENSG00000157077
ENSG00000105699
ENSG00000119953
ENSG00000139835
ENSG00000179600
ENSG00000198010
ENSG00000100600
ENSG00000109323
ENSG00000116667
ENSG00000082212
ENSG00000148600
ENSG00000149179
ENSG00000172671
ENSG00000133250
ENSG00000137270
ENSG00000170819
ENSG00000160219
ENSG00000167014
ENSG00000170802
ENSG00000168703
ENSG00000157131
ENSG00000155100
ENSG00000183255
ENSG00000143390
ENSG00000182968
ENSG00000104450
ENSG00000129170
ENSG00000133026
ENSG00000010810
ENSG00000178695
ENSG00000124134
ENSG00000110436
ENSG00000109066
ENSG00000198271
ENSG00000165556
ENSG00000153885
ENSG00000143344
ENSG00000141522
ENSG00000168795
ENSG00000092200
ENSG00000164458
ENSG00000160570
ENSG00000148773
ENSG00000205869
ENSG00000124126
ENSG00000111676
ENSG00000188522
ENSG00000147912
ENSG00000118702
ENSG00000198558
ENSG00000166747
ENSG00000108219
ENSG00000213240
ENSG00000170464
ENSG00000143437
ENSG00000178028
ENSG00000134590
ENSG00000138771
ENSG00000205339
ENSG00000135547
ENSG00000127554
ENSG00000131864
ENSG00000163517
ENSG00000197535

ENSG00000178878
ENSG00000144668
ENSG00000164631
ENSG00000138375
ENSG00000141551
ENSG00000244482
ENSG00000146049
ENSG00000163393
ENSG00000143337
ENSG00000130720
ENSG00000107611
ENSG00000186591
ENSG00000106341
ENSG00000173757
ENSG00000132471
ENSG00000213366
ENSG00000181019
ENSG00000143498
ENSG00000119242
ENSG00000198561
ENSG00000070540
ENSG00000117691
ENSG00000153130
ENSG00000117322
ENSG00000129245
ENSG00000198743
ENSG00000101665
ENSG00000122545
ENSG00000110696
ENSG00000183770
ENSG00000179348
ENSG00000006074
ENSG00000109689
ENSG00000196136
ENSG00000091651
ENSG00000104960
ENSG00000196533
ENSG00000163995
ENSG00000054118
ENSG00000063245
ENSG00000163098
ENSG00000187955
ENSG00000134294
ENSG00000204352
ENSG00000134954
ENSG00000017427
ENSG00000173040
ENSG00000075089
ENSG00000074695
ENSG00000141574
ENSG00000134830
ENSG00000055211
ENSG00000104237
ENSG00000153822
ENSG00000049192
ENSG00000215301
ENSG00000184897
ENSG00000214114
ENSG00000115216
ENSG00000134824
ENSG00000166192
ENSG00000157211
ENSG00000170624
ENSG00000113522

ENSG00000160781
ENSG00000114544
ENSG00000244067
ENSG00000124557
ENSG00000167705
ENSG00000115520
ENSG00000163285
ENSG00000198604
ENSG00000198915
ENSG00000085382
ENSG00000162729
ENSG00000112992
ENSG00000109758
ENSG00000119138
ENSG00000158481
ENSG00000137745
ENSG00000167332
ENSG00000105649
ENSG00000196357
ENSG00000138080
ENSG00000106333
ENSG00000063046
ENSG00000187871
ENSG00000151364
ENSG00000114988
ENSG00000131127
ENSG00000159086
ENSG00000106003
ENSG00000139567
ENSG00000168779
ENSG00000129250
ENSG00000143870
ENSG00000174996
ENSG00000163631
ENSG00000126822
ENSG00000221837
ENSG00000198125
ENSG00000171533
ENSG00000112530
ENSG00000239402
ENSG00000175600
ENSG00000177694
ENSG00000100360
ENSG00000174928
ENSG00000101294
ENSG00000187416
ENSG00000184486
ENSG00000159217
ENSG00000005249
ENSG00000170608
ENSG00000136492
ENSG00000101307
ENSG00000214530
ENSG00000069702
ENSG00000146701
ENSG00000068028
ENSG00000169139
ENSG00000104313
ENSG00000116299
ENSG00000229859
ENSG00000168878
ENSG00000160688
ENSG00000171017
ENSG00000154518

ENSG00000205250
ENSG00000110321
ENSG00000140694
ENSG00000181555
ENSG00000144815
ENSG00000178562
ENSG00000078070
ENSG00000153094
ENSG00000074603
ENSG00000108506
ENSG00000162396
ENSG00000024422
ENSG00000129968
ENSG00000090530
ENSG00000197745
ENSG00000006118
ENSG00000124243
ENSG00000134852
ENSG00000161509
ENSG00000189091
ENSG00000137502
ENSG00000106268
ENSG00000184702
ENSG00000143297
ENSG00000137960
ENSG00000087266
ENSG00000143867
ENSG00000178531
ENSG00000131471
ENSG00000136267
ENSG00000196867
ENSG00000005102
ENSG00000170325
ENSG00000167302
ENSG00000197892
ENSG00000173464
ENSG00000183779
ENSG00000099203
ENSG00000139112
ENSG00000147853
ENSG00000197996
ENSG00000160838
ENSG00000177374
ENSG00000196544
ENSG00000206460
ENSG00000119318
ENSG00000117616
ENSG00000120696
ENSG00000079102
ENSG00000087274
ENSG00000166348
ENSG00000128829
ENSG00000168710
ENSG00000117983
ENSG00000136270
ENSG00000167088
ENSG00000197102
ENSG00000123576
ENSG00000170899
ENSG00000006744
ENSG00000177105
ENSG00000169136
ENSG00000120549
ENSG00000166793

ENSG00000092098
ENSG00000134901
ENSG00000118997
ENSG00000150961
ENSG00000153767
ENSG00000165828
ENSG00000103485
ENSG00000138031
ENSG00000102053
ENSG00000175387
ENSG00000166275
ENSG00000116678
ENSG00000172482
ENSG00000101405
ENSG00000176087
ENSG00000149927
ENSG00000111245
ENSG00000126549
ENSG00000108963
ENSG00000172939
ENSG00000101230
ENSG00000124784
ENSG00000182674
ENSG00000137558
ENSG00000184182
ENSG00000003147
ENSG00000107957
ENSG00000160345
ENSG00000159958
ENSG00000176171
ENSG00000104976
ENSG00000112276
ENSG00000165388
ENSG00000181585
ENSG00000136643
ENSG00000106069
ENSG00000157916
ENSG00000178031
ENSG00000152022
ENSG00000137078
ENSG00000169188
ENSG00000196747
ENSG00000153936
ENSG00000114670
ENSG00000180878
ENSG00000163191
ENSG00000196584
ENSG00000166866
ENSG00000163739
ENSG00000134249
ENSG00000205581
ENSG00000101546
ENSG00000140750
ENSG00000110844
ENSG00000125817
ENSG00000162999
ENSG00000070718
ENSG00000149054
ENSG00000112576
ENSG00000125869
ENSG00000173567
ENSG00000108878
ENSG00000133597
ENSG00000075702

ENSG00000122678
ENSG00000185049
ENSG00000168505
ENSG00000160886
ENSG00000128011
ENSG00000143514
ENSG00000143001
ENSG00000103490
ENSG00000092847
ENSG00000159479
ENSG00000118407
ENSG00000118640
ENSG00000178235
ENSG00000159708
ENSG00000108946
ENSG00000043039
ENSG00000125657
ENSG00000183900
ENSG00000061794
ENSG00000128534
ENSG00000011485
ENSG00000126264
ENSG00000111837
ENSG00000171097
ENSG00000139973
ENSG00000184304
ENSG00000176678
ENSG00000169684
ENSG00000162517
ENSG00000130176
ENSG00000167986
ENSG00000008282
ENSG00000163682
ENSG00000165949
ENSG00000100325
ENSG00000089818
ENSG00000070748
ENSG00000137727
ENSG00000148082
ENSG00000162188
ENSG00000119004
ENSG00000172115
ENSG00000206561
ENSG00000198682
ENSG00000167384
ENSG00000021852
ENSG00000105329
ENSG00000124496
ENSG00000145833
ENSG00000186675
ENSG00000122223
ENSG00000184900
ENSG00000102010
ENSG00000099256
ENSG00000173653
ENSG00000205542
ENSG00000081189
ENSG00000154144
ENSG00000131183
ENSG00000103260
ENSG00000185551
ENSG00000111897
ENSG00000156273
ENSG00000197157

ENSG00000182512
ENSG00000197442
ENSG00000188822
ENSG00000197020
ENSG00000054690
ENSG00000130520
ENSG00000185090
ENSG00000138180
ENSG00000163430
ENSG00000147130
ENSG00000154655
ENSG00000198736
ENSG00000197863
ENSG00000163145
ENSG00000126458
ENSG00000136111
ENSG00000213551
ENSG00000147402
ENSG00000180185
ENSG00000134444
ENSG00000068885
ENSG00000155970
ENSG00000118096
ENSG00000155307
ENSG00000182287
ENSG00000131507
ENSG00000165934
ENSG00000102970
ENSG00000148513
ENSG00000128045
ENSG00000166128
ENSG00000104343
ENSG00000125912
ENSG00000050748
ENSG00000198586
ENSG00000137834
ENSG00000196187
ENSG00000131059
ENSG00000164929
ENSG00000153048
ENSG00000116726
ENSG00000170290
ENSG00000148842
ENSG00000165684
ENSG00000164411
ENSG00000105072
ENSG00000062716
ENSG00000175267
ENSG00000135773
ENSG00000197150
ENSG00000162576
ENSG00000081277
ENSG00000154016
ENSG00000139946
ENSG00000150637
ENSG00000122254
ENSG00000100721
ENSG00000126522
ENSG00000180938
ENSG00000158882
ENSG00000181333
ENSG00000177042
ENSG00000143774
ENSG00000170759

ENSG00000104783
ENSG00000115286
ENSG00000185245
ENSG00000188786
ENSG00000179820
ENSG00000163866
ENSG00000182325
ENSG00000221818
ENSG00000171786
ENSG00000139194
ENSG00000158714
ENSG00000196946
ENSG00000174428
ENSG00000133106
ENSG00000171596
ENSG00000131467
ENSG00000196092
ENSG00000124275
ENSG00000100931
ENSG00000158966
ENSG00000170421
ENSG00000165055
ENSG00000105953
ENSG00000084676
ENSG00000187792
ENSG00000120159
ENSG00000169972
ENSG00000130559
ENSG00000139734
ENSG00000049883
ENSG00000171320
ENSG00000156097
ENSG00000141480
ENSG00000172071
ENSG00000181513
ENSG00000127585
ENSG00000107331
ENSG00000177504
ENSG00000176754
ENSG00000067225
ENSG00000101349
ENSG00000075336
ENSG00000102178
ENSG00000120685
ENSG00000128563
ENSG00000103769
ENSG00000138028
ENSG00000166685
ENSG00000164023
ENSG00000101353
ENSG00000120659
ENSG00000189030
ENSG00000118972
ENSG00000165300
ENSG00000116127
ENSG00000129744
ENSG00000103269
ENSG00000168928
ENSG00000075413
ENSG00000166317
ENSG00000141404
ENSG00000118260
ENSG00000009790
ENSG00000171492

ENSG00000154305
ENSG00000137218
ENSG00000037965
ENSG00000164576
ENSG00000221826
ENSG00000148153
ENSG00000153574
ENSG00000203737
ENSG00000131910
ENSG00000114631
ENSG00000184470
ENSG00000011198
ENSG00000113356
ENSG00000173156
ENSG00000164967
ENSG00000172380
ENSG00000185246
ENSG00000140015
ENSG00000106144
ENSG00000155313
ENSG00000117222
ENSG00000104044
ENSG00000165029
ENSG00000105717
ENSG00000120798
ENSG00000158552
ENSG00000144895
ENSG00000103365
ENSG00000140157
ENSG00000135480
ENSG00000091583
ENSG00000179409
ENSG00000084092
ENSG00000213923
ENSG00000134398
ENSG00000135956
ENSG00000183833
ENSG00000141458
ENSG00000154277
ENSG00000197921
ENSG00000228049
ENSG00000186716
ENSG00000168038
ENSG00000158887
ENSG00000103657
ENSG00000165915
ENSG00000167613
ENSG00000150783
ENSG00000213465
ENSG00000085117
ENSG00000114204
ENSG00000172987
ENSG00000064995
ENSG00000108883
ENSG00000116962
ENSG00000086758
ENSG00000109511
ENSG00000140538
ENSG00000185222
ENSG00000140262
ENSG00000132341
ENSG00000111332
ENSG00000162946
ENSG00000106105

ENSG00000162595
ENSG00000135447
ENSG00000139193
ENSG00000162598
ENSG00000110080
ENSG00000157869
ENSG00000123395
ENSG00000188687
ENSG00000019991
ENSG00000171877
ENSG00000170049
ENSG00000160951
ENSG00000167792
ENSG00000106392
ENSG00000149021
ENSG00000118579
ENSG00000154930
ENSG00000206538
ENSG00000126001
ENSG00000047849
ENSG00000169758
ENSG00000173662
ENSG00000099875
ENSG00000145107
ENSG00000129255
ENSG00000103888
ENSG00000166333
ENSG00000174946
ENSG00000147166
ENSG00000134222
ENSG00000173914
ENSG00000184674
ENSG00000163931
ENSG00000143013
ENSG00000175676
ENSG00000204748
ENSG00000101204
ENSG00000129103
ENSG00000164118
ENSG00000143368
ENSG00000170891
ENSG00000187166
ENSG00000079931
ENSG00000198467
ENSG00000057593
ENSG00000158901
ENSG00000110395
ENSG00000110195
ENSG00000103037
ENSG00000184908
ENSG00000187741
ENSG00000055483
ENSG00000188157
ENSG00000124568
ENSG00000108091
ENSG00000167114
ENSG00000167645
ENSG00000162144
ENSG00000148225
ENSG00000171503
ENSG00000160972
ENSG00000114784
ENSG00000127990
ENSG00000090554

ENSG00000149397
ENSG00000176697
ENSG00000007038
ENSG00000107018
ENSG00000144468
ENSG00000185873
ENSG00000180113
ENSG00000136717
ENSG00000166225
ENSG00000172379
ENSG00000183826
ENSG00000163093
ENSG00000107175
ENSG00000136738
ENSG00000163606
ENSG00000138741
ENSG00000149295
ENSG00000182199
ENSG00000170185
ENSG00000132680
ENSG00000196954
ENSG00000168994
ENSG00000047249
ENSG00000109339
ENSG00000100393
ENSG00000139800
ENSG00000166833
ENSG00000116830
ENSG00000175356
ENSG00000107862
ENSG00000118655
ENSG00000183628
ENSG00000196313
ENSG00000198931
ENSG00000140009
ENSG00000141582
ENSG00000123200
ENSG00000182973
ENSG00000166888
ENSG00000146054
ENSG00000066735
ENSG00000158161
ENSG00000154845
ENSG00000104835
ENSG00000154133
ENSG00000018869
ENSG00000136697
ENSG00000108590
ENSG00000122970
ENSG00000156265
ENSG00000213999
ENSG00000135828
ENSG00000182307
ENSG00000213892
ENSG00000136870
ENSG00000106536
ENSG00000107105
ENSG00000010327
ENSG00000137752
ENSG00000101342
ENSG00000014164
ENSG00000102003
ENSG00000166153
ENSG00000205268

ENSG00000110880
ENSG00000165028
ENSG00000243444
ENSG00000167470
ENSG00000182132
ENSG00000163788
ENSG00000176170
ENSG00000164828
ENSG00000130950
ENSG00000124181
ENSG00000108384
ENSG00000186660
ENSG00000166851
ENSG00000141141
ENSG00000095627
ENSG00000141965
ENSG00000103313
ENSG00000100731
ENSG00000101442
ENSG00000100714
ENSG00000162086
ENSG00000165244
ENSG00000140545
ENSG00000148572
ENSG00000138166
ENSG00000099246
ENSG00000129474
ENSG00000111615
ENSG00000146938
ENSG00000079263
ENSG00000125931
ENSG00000148408
ENSG00000114019
ENSG00000131778
ENSG00000139651
ENSG00000003056
ENSG00000153214
ENSG00000173327
ENSG00000158019
ENSG00000138315
ENSG00000185201
ENSG00000184270
ENSG00000104953
ENSG00000069399
ENSG00000178971
ENSG00000169490
ENSG00000160808
ENSG00000108179
ENSG00000174306
ENSG00000157106
ENSG00000131050
ENSG00000130787
ENSG00000187961
ENSG00000116731
ENSG00000162512
ENSG00000151748
ENSG00000186665
ENSG00000138303
ENSG00000225855
ENSG00000153786
ENSG00000141179
ENSG00000168062
ENSG00000124116
ENSG00000132535

ENSG00000136193
ENSG00000165449
ENSG00000135424
ENSG00000125611
ENSG00000157404
ENSG00000023191
ENSG00000139144
ENSG00000151322
ENSG00000143793
ENSG00000173338
ENSG00000111802
ENSG00000203797
ENSG00000175155
ENSG00000160352
ENSG00000128422
ENSG00000105327
ENSG00000104903
ENSG00000071246
ENSG00000160932
ENSG00000170777
ENSG00000057608
ENSG00000120217
ENSG00000100697
ENSG00000163520
ENSG00000158869
ENSG00000149418
ENSG00000101871
ENSG00000185985
ENSG00000077684
ENSG00000198730
ENSG00000004961
ENSG00000138459
ENSG00000158258
ENSG00000120438
ENSG00000137098
ENSG00000124205
ENSG00000157653
ENSG00000184886
ENSG00000165115
ENSG00000158955
ENSG00000138435
ENSG00000056972
ENSG00000186038
ENSG00000184508
ENSG00000196367
ENSG00000153904
ENSG00000115677
ENSG00000102243
ENSG00000143643
ENSG00000198826
ENSG00000211460
ENSG00000197753
ENSG00000120519
ENSG00000141622
ENSG00000100084
ENSG00000150676
ENSG00000162642
ENSG00000105879
ENSG00000104870
ENSG00000101440
ENSG00000130119
ENSG00000073734
ENSG00000124391
ENSG00000198898

ENSG00000082397
ENSG00000160161
ENSG00000151474
ENSG00000076053
ENSG00000161533
ENSG00000078747
ENSG00000186174
ENSG00000100422
ENSG00000100938
ENSG00000128641
ENSG00000105983
ENSG00000151640
ENSG00000080839
ENSG00000072958
ENSG00000109208
ENSG00000166439
ENSG00000152430
ENSG00000105771
ENSG00000163673
ENSG00000058799
ENSG00000172818
ENSG00000101981
ENSG00000113196
ENSG00000215252
ENSG00000118922
ENSG00000132670
ENSG00000198821
ENSG00000177627
ENSG00000104812
ENSG00000088832
ENSG00000148943
ENSG00000064205
ENSG00000187051
ENSG00000143318
ENSG00000187223
ENSG00000241553
ENSG00000156256
ENSG00000205352
ENSG00000185386
ENSG00000144339
ENSG00000162493
ENSG00000145681
ENSG00000139899
ENSG00000114473
ENSG00000143933
ENSG00000138134
ENSG00000128408
ENSG00000133111
ENSG00000171885
ENSG00000112667
ENSG00000171132
ENSG00000130511
ENSG00000147036
ENSG00000124635
ENSG00000184916
ENSG00000165874
ENSG00000163113
ENSG00000239839
ENSG00000015532
ENSG00000147507
ENSG00000116132
ENSG00000165678
ENSG00000186908
ENSG00000109458

ENSG00000189120
ENSG00000108465
ENSG00000152256
ENSG00000171988
ENSG00000131475
ENSG00000169375
ENSG00000168350
ENSG00000165390
ENSG00000165637
ENSG00000125733
ENSG00000139437
ENSG00000168903
ENSG00000154928
ENSG00000164418
ENSG00000163376
ENSG00000111642
ENSG00000221857
ENSG00000088247
ENSG00000197948
ENSG00000102024
ENSG00000049247
ENSG00000152518
ENSG00000143416
ENSG00000170727
ENSG00000197622
ENSG00000197183
ENSG00000127955
ENSG00000078403
ENSG00000159063
ENSG00000173209
ENSG00000196998
ENSG00000135655
ENSG00000173894
ENSG00000214265
ENSG00000167658
ENSG00000111142
ENSG00000011465
ENSG00000132749
ENSG00000187323
ENSG00000086159
ENSG00000173124
ENSG00000161904
ENSG00000088538
ENSG00000160602
ENSG00000112246
ENSG00000006377
ENSG00000119522
ENSG00000088053
ENSG00000215021
ENSG00000148429
ENSG00000117308
ENSG00000178386
ENSG00000106524
ENSG00000169083
ENSG00000162670
ENSG00000104879
ENSG00000134283
ENSG00000148660
ENSG00000113721
ENSG00000136250
ENSG00000185721
ENSG00000136819
ENSG00000079805
ENSG00000128626

ENSG00000085377
ENSG00000185634
ENSG00000066117
ENSG00000102309
ENSG00000111348
ENSG00000105509
ENSG00000140931
ENSG00000163947
ENSG00000169981
ENSG00000164040
ENSG00000187908
ENSG00000115884
ENSG00000132170
ENSG00000106771
ENSG00000130829
ENSG00000187535
ENSG00000117643
ENSG00000183621
ENSG00000007968
ENSG00000164506
ENSG00000136521
ENSG00000161634
ENSG00000091039
ENSG00000102606
ENSG00000100320
ENSG00000150776
ENSG00000157551
ENSG00000148400
ENSG00000101850
ENSG00000213865
ENSG00000186487
ENSG00000135643
ENSG00000120708
ENSG00000143924
ENSG00000068796
ENSG00000156853
ENSG00000100380
ENSG00000167754
ENSG00000072135
ENSG00000089693
ENSG00000163344
ENSG00000103335
ENSG00000163794
ENSG00000149474
ENSG00000028277
ENSG00000184925
ENSG00000111481
ENSG00000117262
ENSG00000139289
ENSG00000165495
ENSG00000154027
ENSG00000101654
ENSG00000141644
ENSG00000106031
ENSG00000150768
ENSG00000151729
ENSG00000204527
ENSG00000107882
ENSG00000135821
ENSG00000165806
ENSG00000135631
ENSG00000084463
ENSG00000198408
ENSG00000115241

ENSG00000241186
ENSG00000089558
ENSG00000164778
ENSG00000108443
ENSG00000139287
ENSG00000110975
ENSG00000180884
ENSG00000134539
ENSG00000104290
ENSG00000111713
ENSG00000132394
ENSG00000171311
ENSG00000133818
ENSG00000185670
ENSG00000108582
ENSG00000173511
ENSG00000100234
ENSG00000148484
ENSG00000115361
ENSG00000197361
ENSG00000166446
ENSG00000124253
ENSG00000162650
ENSG00000046651
ENSG00000158528
ENSG00000179148
ENSG00000182472
ENSG00000162777
ENSG00000165997
ENSG00000169224
ENSG00000110172
ENSG00000117971
ENSG00000091482
ENSG00000111907
ENSG00000221843
ENSG00000187239
ENSG00000106336
ENSG00000128918
ENSG00000072858
ENSG00000163515
ENSG00000177888
ENSG00000175785
ENSG00000180697
ENSG00000156689
ENSG00000167680
ENSG00000186530
ENSG00000072201
ENSG00000198053
ENSG00000049449
ENSG00000170484
ENSG00000136573
ENSG00000172005
ENSG00000143850
ENSG00000100170
ENSG00000229676
ENSG00000100362
ENSG00000140948
ENSG00000157827
ENSG00000178999
ENSG00000156738
ENSG00000129450
ENSG00000166526
ENSG00000104497
ENSG00000160799

ENSG00000092445
ENSG00000122121
ENSG00000121966
ENSG00000170166
ENSG00000135108
ENSG00000100429
ENSG00000116786
ENSG00000089597
ENSG00000164976
ENSG00000162614
ENSG00000181135
ENSG00000144550
ENSG00000214078
ENSG00000113658
ENSG00000169499
ENSG00000149499
ENSG00000154473
ENSG00000095203
ENSG00000185008
ENSG00000123415
ENSG00000196262
ENSG00000112763
ENSG00000176912
ENSG00000148344
ENSG00000075975
ENSG00000116983
ENSG00000168925
ENSG00000138646
ENSG00000064012
ENSG00000169432
ENSG00000173457
ENSG00000170122
ENSG00000147896
ENSG00000164144
ENSG00000005379
ENSG00000028137
ENSG00000205189
ENSG00000004848
ENSG00000087157
ENSG00000180205
ENSG00000102226
ENSG00000069424
ENSG00000136449
ENSG00000117560
ENSG00000127328
ENSG00000198610
ENSG00000164615
ENSG00000139350
ENSG00000100068
ENSG00000124155
ENSG00000183023
ENSG00000069020
ENSG00000196150
ENSG00000196535
ENSG00000176973
ENSG00000172232
ENSG00000163633
ENSG00000134013
ENSG00000183379
ENSG00000137486
ENSG00000012171
ENSG00000161298
ENSG00000150712
ENSG00000115464

ENSG00000174595
ENSG00000124664
ENSG00000146729
ENSG00000166573
ENSG00000184357
ENSG00000159921
ENSG00000131845
ENSG00000145864
ENSG00000100410
ENSG00000158270
ENSG00000205981
ENSG00000167797
ENSG00000132846
ENSG00000077235
ENSG00000011028
ENSG00000105058
ENSG00000172661
ENSG00000162897
ENSG00000243335
ENSG00000174611
ENSG00000158786
ENSG00000126860
ENSG00000115604
ENSG00000176720
ENSG00000162813
ENSG00000181222
ENSG00000090686
ENSG00000198393
ENSG00000100350
ENSG00000103740
ENSG00000137764
ENSG00000198440
ENSG00000133226
ENSG00000153707
ENSG00000109133
ENSG00000152910
ENSG00000141934
ENSG00000162365
ENSG00000100453
ENSG00000188613
ENSG00000183439
ENSG00000183036
ENSG00000169314
ENSG00000134575
ENSG00000156958
ENSG00000133739
ENSG00000132002
ENSG00000166394
ENSG00000214050
ENSG00000101190
ENSG00000130513
ENSG00000186073
ENSG00000101751
ENSG00000179562
ENSG00000184788
ENSG00000186017
ENSG00000143537
ENSG00000135903
ENSG00000164935
ENSG00000132780
ENSG00000149929
ENSG00000115468
ENSG00000203685
ENSG00000040341

ENSG00000112144
ENSG00000147065
ENSG00000105011
ENSG00000116685
ENSG00000116584
ENSG00000148356
ENSG00000196242
ENSG00000074181
ENSG00000170445
ENSG00000173349
ENSG00000143140
ENSG00000159388
ENSG00000140406
ENSG00000108774
ENSG00000065029
ENSG00000179115
ENSG00000131771
ENSG00000070214
ENSG00000179364
ENSG00000106829
ENSG00000140464
ENSG00000103994
ENSG00000142698
ENSG00000117148
ENSG00000169964
ENSG00000105497
ENSG00000154620
ENSG00000136514
ENSG00000118200
ENSG00000084444
ENSG00000181085
ENSG00000142959
ENSG00000141664
ENSG00000164305
ENSG00000241233
ENSG00000065989
ENSG00000173889
ENSG00000197375
ENSG00000104112
ENSG00000171365
ENSG00000026508
ENSG00000163877
ENSG00000174943
ENSG00000125492
ENSG00000161203
ENSG00000181284
ENSG00000181778
ENSG00000243452
ENSG00000031698
ENSG00000212938
ENSG00000187242
ENSG00000095464
ENSG00000157800
ENSG00000112365
ENSG00000204843
ENSG00000099960
ENSG00000082781
ENSG00000003400
ENSG00000107438
ENSG00000108064
ENSG00000168255
ENSG00000075407
ENSG00000128699
ENSG00000061676

ENSG00000103671
ENSG00000186918
ENSG00000162385
ENSG00000197696
ENSG00000128513
ENSG00000139515
ENSG00000142875
ENSG00000100347
ENSG00000131668
ENSG00000162881
ENSG00000161547
ENSG00000080189
ENSG00000162624
ENSG00000080561
ENSG00000107242
ENSG00000110057
ENSG00000176390
ENSG00000157219
ENSG00000065618
ENSG00000197472
ENSG00000160796
ENSG00000132681
ENSG00000043093
ENSG00000115652
ENSG00000143549
ENSG00000166147
ENSG00000135925
ENSG00000119681
ENSG00000184524
ENSG00000187556
ENSG00000148450
ENSG00000167186
ENSG00000197008
ENSG00000197961
ENSG00000166140
ENSG00000086696
ENSG00000160844
ENSG00000116266
ENSG00000167419
ENSG00000141524
ENSG00000179796
ENSG00000123562
ENSG00000204381
ENSG00000113302
ENSG00000090534
ENSG00000184144
ENSG00000188386
ENSG00000101331
ENSG00000075426
ENSG00000112742
ENSG00000171246
ENSG00000130150
ENSG00000163161
ENSG00000127837
ENSG00000162923
ENSG00000103852
ENSG00000125247
ENSG00000116857
ENSG00000197249
ENSG00000156711
ENSG00000196639
ENSG00000066084
ENSG00000115255
ENSG00000130940

ENSG00000058600
ENSG00000139178
ENSG00000117569
ENSG00000178538
ENSG00000213231
ENSG00000172689
ENSG00000122085
ENSG00000164032
ENSG00000135766
ENSG00000065308
ENSG00000012061
ENSG00000114349
ENSG00000131873
ENSG00000074800
ENSG00000176428
ENSG00000175054
ENSG00000166965
ENSG00000205649
ENSG00000099822
ENSG00000131711
ENSG00000127884
ENSG00000151458
ENSG00000189410
ENSG00000041357
ENSG00000118557
ENSG00000136141
ENSG00000154803
ENSG00000107796
ENSG00000136243
ENSG00000169021
ENSG00000156467
ENSG00000166979
ENSG00000103089
ENSG00000129911
ENSG00000169442
ENSG00000124194
ENSG00000196924
ENSG00000117614
ENSG00000130590
ENSG00000159023
ENSG00000196498
ENSG00000006638
ENSG00000175832
ENSG00000158106
ENSG00000166165
ENSG00000124795
ENSG00000138835
ENSG00000177103
ENSG00000105428
ENSG00000161570
ENSG00000172183
ENSG00000100225
ENSG00000156110
ENSG00000101746
ENSG00000147082
ENSG00000091879
ENSG00000111358
ENSG00000177556
ENSG00000112559
ENSG00000163053
ENSG00000171729
ENSG00000108784
ENSG00000103199
ENSG00000129204

ENSG00000196233
ENSG00000175505
ENSG00000125731
ENSG00000196776
ENSG00000111262
ENSG00000103197
ENSG00000186160
ENSG00000176155
ENSG00000111700
ENSG00000134690
ENSG00000176148
ENSG00000076716
ENSG00000185305
ENSG00000156298
ENSG00000066136
ENSG00000171217
ENSG00000176371
ENSG00000171608
ENSG00000159516
ENSG00000172890
ENSG00000187372
ENSG00000144747
ENSG00000008323
ENSG00000078699
ENSG00000197063
ENSG00000172927
ENSG00000118523
ENSG00000149091
ENSG00000122133
ENSG00000160216
ENSG00000115844
ENSG00000144535
ENSG00000125618
ENSG00000139531
ENSG00000071575
ENSG00000176029
ENSG00000179912
ENSG00000064601
ENSG00000029534
ENSG00000126217
ENSG00000114270
ENSG00000127359
ENSG00000114770
ENSG00000168685
ENSG00000166181
ENSG00000174325
ENSG00000104321
ENSG00000120594
ENSG00000196878
ENSG00000179930
ENSG00000160714
ENSG00000086062
ENSG00000124160
ENSG00000196407
ENSG00000196557
ENSG00000129055
ENSG00000198216
ENSG00000182732
ENSG00000100099
ENSG00000185862
ENSG00000197323
ENSG00000250361
ENSG00000124659
ENSG00000049167

ENSG00000152076
ENSG00000117118
ENSG00000124839
ENSG00000168081
ENSG00000076924
ENSG00000143801
ENSG00000124222
ENSG00000175854
ENSG00000090565
ENSG00000115866
ENSG00000186416
ENSG00000013275
ENSG00000167555
ENSG00000186469
ENSG00000162241
ENSG00000126709
ENSG00000177169
ENSG00000106052
ENSG00000182612
ENSG00000134532
ENSG00000156052
ENSG00000172346
ENSG00000139083
ENSG00000121281
ENSG00000164007
ENSG00000129048
ENSG00000006451
ENSG00000104738
ENSG00000221869
ENSG00000155545
ENSG00000111328
ENSG00000105552
ENSG00000159495
ENSG00000119559
ENSG00000068650
ENSG00000111775
ENSG00000165487
ENSG00000168876
ENSG00000172602
ENSG00000187456
ENSG00000012660
ENSG00000072310
ENSG00000179826
ENSG00000152642
ENSG00000173698
ENSG00000151366
ENSG00000162398
ENSG00000102901
ENSG00000155755
ENSG00000214324
ENSG00000148700
ENSG00000156973
ENSG00000109113
ENSG00000141200
ENSG00000107949
ENSG00000213281
ENSG00000064932
ENSG00000169717
ENSG00000186090
ENSG00000136490
ENSG00000178602
ENSG00000100941
ENSG00000170458
ENSG00000180488

ENSG00000130545
ENSG00000184005
ENSG00000147869
ENSG00000131115
ENSG00000233822
ENSG00000148110
ENSG00000100461
ENSG00000004799
ENSG00000157259
ENSG00000181045
ENSG00000110848
ENSG00000105287
ENSG00000183576
ENSG00000104695
ENSG00000163618
ENSG00000135912
ENSG00000157540
ENSG00000125257
ENSG00000185739
ENSG00000178171
ENSG00000155329
ENSG00000156990
ENSG00000141720
ENSG00000180066
ENSG00000170153
ENSG00000074935
ENSG00000156482
ENSG00000116661
ENSG00000179085
ENSG00000010256
ENSG00000167034
ENSG00000060069
ENSG00000065320
ENSG00000133321
ENSG00000165863
ENSG00000101844
ENSG00000166482
ENSG00000133816
ENSG00000203785
ENSG00000091317
ENSG00000077232
ENSG00000025156
ENSG00000148339
ENSG00000163012
ENSG00000107521
ENSG00000112081
ENSG00000136379
ENSG00000095752
ENSG00000100077
ENSG00000188379
ENSG00000113088
ENSG00000103061
ENSG00000172057
ENSG00000102468
ENSG00000178951
ENSG00000180875
ENSG00000084072
ENSG00000181690
ENSG00000182749
ENSG00000136153
ENSG00000102763
ENSG00000203985
ENSG00000113889
ENSG00000165271

ENSG00000160214
ENSG00000157502
ENSG00000068078
ENSG00000151576
ENSG00000177303
ENSG00000134744
ENSG00000110237
ENSG00000135116
ENSG00000175711
ENSG00000233276
ENSG00000099869
ENSG00000158764
ENSG00000088986
ENSG00000127870
ENSG00000116497
ENSG00000220205
ENSG00000177294
ENSG00000114812
ENSG00000131473
ENSG00000197894
ENSG00000152291
ENSG00000079974
ENSG00000175592
ENSG00000115145
ENSG00000120053
ENSG00000166912
ENSG00000055917
ENSG00000188130
ENSG00000169059
ENSG00000136560
ENSG00000164134
ENSG00000130821
ENSG00000188177
ENSG00000111012
ENSG00000177646
ENSG00000175548
ENSG00000142687
ENSG00000150045
ENSG00000204335
ENSG00000073792
ENSG00000147862
ENSG00000177606
ENSG00000184956
ENSG00000033170
ENSG00000163755
ENSG00000181315
ENSG00000109762
ENSG00000198590
ENSG00000132434
ENSG00000108960
ENSG00000197353
ENSG00000184828
ENSG00000105549
ENSG00000171109
ENSG00000175606
ENSG00000157954
ENSG00000162236
ENSG00000114796
ENSG00000118004
ENSG00000066654
ENSG00000117461
ENSG00000127472
ENSG00000163155
ENSG00000137757

ENSG00000136689
ENSG00000149806
ENSG00000188486
ENSG00000073756
ENSG00000154511
ENSG00000152229
ENSG00000108509
ENSG00000179837
ENSG00000081800
ENSG00000165970
ENSG00000125788
ENSG00000106546
ENSG00000176463
ENSG00000118046
ENSG00000185479
ENSG00000165275
ENSG00000105229
ENSG00000175874
ENSG00000029364
ENSG00000018280
ENSG00000005075
ENSG00000124299
ENSG00000158483
ENSG00000104219
ENSG00000164588
ENSG00000143889
ENSG00000214087
ENSG00000164930
ENSG00000004059
ENSG00000009413
ENSG00000152749
ENSG00000162817
ENSG00000100412
ENSG00000149925
ENSG00000039523
ENSG00000115421
ENSG00000163888
ENSG00000137770
ENSG00000136457
ENSG00000183723
ENSG00000172466
ENSG00000109472
ENSG00000185379
ENSG00000145569
ENSG00000103489
ENSG00000183690
ENSG00000166341
ENSG00000169397
ENSG00000135702
ENSG00000057252
ENSG00000124615
ENSG00000047648
ENSG00000087494
ENSG00000130517
ENSG00000005100
ENSG00000139726
ENSG00000089820
ENSG00000167653
ENSG00000054148
ENSG00000147246
ENSG00000181652
ENSG00000112685
ENSG00000198218
ENSG00000059378

ENSG00000168269
ENSG00000005007
ENSG00000124493
ENSG00000152214
ENSG00000132825
ENSG00000157766
ENSG00000213625
ENSG00000117114
ENSG00000179588
ENSG00000138780
ENSG00000155093
ENSG00000141219
ENSG00000163202
ENSG00000186474
ENSG00000171357
ENSG00000184933
ENSG00000100427
ENSG00000051620
ENSG00000065615
ENSG00000071889
ENSG00000137710
ENSG00000161692
ENSG00000183150
ENSG00000109771
ENSG00000162409
ENSG00000205413
ENSG00000164252
ENSG00000174738
ENSG00000163535
ENSG00000132704
ENSG00000179242
ENSG00000104611
ENSG00000174871
ENSG00000174669
ENSG00000115875
ENSG00000110876
ENSG00000158623
ENSG00000136297
ENSG00000178038
ENSG00000117597
ENSG00000137869
ENSG00000139971
ENSG00000181788
ENSG00000171552
ENSG00000157224
ENSG00000111863
ENSG00000121351
ENSG00000197838
ENSG00000042493
ENSG00000134086
ENSG00000141314
ENSG00000099800
ENSG00000152348
ENSG00000137440
ENSG00000105849
ENSG00000141580
ENSG00000111885
ENSG00000167969
ENSG00000206013
ENSG00000117601
ENSG00000005108
ENSG00000132142
ENSG00000162490
ENSG00000197061

ENSG00000188152
ENSG00000112406
ENSG00000164323
ENSG00000186868
ENSG00000138136
ENSG00000087237
ENSG00000138642
ENSG00000167967
ENSG00000141232
ENSG00000171045
ENSG00000152192
ENSG00000168803
ENSG00000135597
ENSG00000187498
ENSG00000113073
ENSG00000103375
ENSG00000067533
ENSG00000140688
ENSG00000163705
ENSG00000183580
ENSG00000158488
ENSG00000168517
ENSG00000117481
ENSG00000126602
ENSG00000164332
ENSG00000083828
ENSG00000175699
ENSG00000180357
ENSG00000128016
ENSG00000153132
ENSG00000126749
ENSG00000165105
ENSG00000120526
ENSG00000122778
ENSG00000010932
ENSG00000163807
ENSG00000092607
ENSG00000187735
ENSG00000102554
ENSG00000166261
ENSG00000205060
ENSG00000119431
ENSG00000170275
ENSG00000149742
ENSG00000101421
ENSG00000239388
ENSG00000091542
ENSG00000170525
ENSG00000127989
ENSG00000067365
ENSG00000198825
ENSG00000183421
ENSG00000157303
ENSG00000213965
ENSG00000130684
ENSG00000117020
ENSG00000169031
ENSG00000197019
ENSG00000136875
ENSG00000114698
ENSG00000134242
ENSG00000116560
ENSG00000188647
ENSG00000142192

ENSG00000168476
ENSG00000204624
ENSG00000106868
ENSG00000147606
ENSG00000187838
ENSG00000213463
ENSG00000141456
ENSG00000197928
ENSG00000136147
ENSG00000158615
ENSG00000187257
ENSG00000106689
ENSG00000159224
ENSG00000152926
ENSG00000114200
ENSG00000196090
ENSG00000147231
ENSG00000186326
ENSG00000133874
ENSG00000131002
ENSG00000185896
ENSG00000143776
ENSG00000146374
ENSG00000129654
ENSG00000143603
ENSG00000154645
ENSG00000177479
ENSG00000198182
ENSG00000121390
ENSG00000142541
ENSG00000022556
ENSG00000115353
ENSG00000184117
ENSG00000084453
ENSG00000122543
ENSG00000111679
ENSG00000175984
ENSG00000134042
ENSG00000186377
ENSG00000155827
ENSG00000111728
ENSG00000189143
ENSG00000152207
ENSG00000244025
ENSG00000158089
ENSG00000168434
ENSG00000076067
ENSG00000114646
ENSG00000180730
ENSG00000023697
ENSG00000197586
ENSG00000083838
ENSG00000134240
ENSG00000155729
ENSG00000110492
ENSG00000144893
ENSG00000116016
ENSG00000175029
ENSG00000182247
ENSG00000103599
ENSG00000175538
ENSG00000040275
ENSG00000120913
ENSG00000156304

ENSG00000173402
ENSG00000198353
ENSG00000116903
ENSG00000136709
ENSG00000146477
ENSG00000029725
ENSG00000130052
ENSG00000114923
ENSG00000106538
ENSG00000203668
ENSG00000145014
ENSG00000170893
ENSG00000183688
ENSG00000176399
ENSG00000178096
ENSG00000102001
ENSG00000127377
ENSG00000176153
ENSG00000054392
ENSG00000099949
ENSG00000171346
ENSG00000182898
ENSG00000111880
ENSG00000075461
ENSG00000104886
ENSG00000054983
ENSG00000105197
ENSG00000107518
ENSG00000165113
ENSG00000213022
ENSG00000198356
ENSG00000095787
ENSG00000198689
ENSG00000104897
ENSG00000130844
ENSG00000137875
ENSG00000183831
ENSG00000185352
ENSG00000144645
ENSG00000109576
ENSG00000169900
ENSG00000141741
ENSG00000143556
ENSG00000145692
ENSG00000182552
ENSG00000183762
ENSG00000158050
ENSG00000186496
ENSG00000169403
ENSG00000140396
ENSG00000144229
ENSG00000143756
ENSG00000183828
ENSG00000106261
ENSG00000172795
ENSG00000102290
ENSG00000132768
ENSG00000196338
ENSG00000198842
ENSG00000101074
ENSG00000108039
ENSG00000112727
ENSG00000138829
ENSG00000119231

ENSG00000137038
ENSG00000099942
ENSG00000110274
ENSG00000177689
ENSG00000158406
ENSG00000140575
ENSG00000115392
ENSG00000165886
ENSG00000177432
ENSG00000169598
ENSG00000163214
ENSG00000244682
ENSG00000182220
ENSG00000143815
ENSG00000088305
ENSG00000175216
ENSG00000131462
ENSG00000095596
ENSG00000171403
ENSG00000135074
ENSG00000103035
ENSG00000007001
ENSG00000140983
ENSG00000176407
ENSG00000109680
ENSG00000183647
ENSG00000243927
ENSG00000185189
ENSG00000140835
ENSG00000006194
ENSG00000173660
ENSG00000179407
ENSG00000092010
ENSG00000173227
ENSG00000158014
ENSG00000185585
ENSG00000203942
ENSG00000130640
ENSG00000186998
ENSG00000131697
ENSG00000147010
ENSG00000006555
ENSG00000111271
ENSG00000139133
ENSG00000158042
ENSG00000196724
ENSG00000178773
ENSG00000002016
ENSG00000177917
ENSG00000160551
ENSG00000135363
ENSG00000136750
ENSG00000197408
ENSG00000126947
ENSG00000159348
ENSG00000167136
ENSG00000120458
ENSG00000183484
ENSG00000124571
ENSG00000011132
ENSG00000123977
ENSG00000171456
ENSG00000109943
ENSG00000072133

ENSG00000162591
ENSG00000132334
ENSG00000174405
ENSG00000141338
ENSG00000134986
ENSG00000099940
ENSG00000108278
ENSG00000111536
ENSG00000008300
ENSG00000023330
ENSG00000162981
ENSG00000130224
ENSG00000130208
ENSG00000111783
ENSG00000102100
ENSG00000118194
ENSG00000136715
ENSG00000158373
ENSG00000113249
ENSG00000142669
ENSG00000184845
ENSG00000137080
ENSG00000116044
ENSG00000182134
ENSG00000169180
ENSG00000168955
ENSG00000147684
ENSG00000196586
ENSG00000100271
ENSG00000063169
ENSG00000060237
ENSG00000170743
ENSG00000173275
ENSG00000070756
ENSG00000169895
ENSG00000164171
ENSG00000159173
ENSG00000003436
ENSG00000150995
ENSG00000213903
ENSG00000137975
ENSG00000101079
ENSG00000214897
ENSG00000064687
ENSG00000215397
ENSG00000063978
ENSG00000164045
ENSG00000182901
ENSG00000141756
ENSG00000141446
ENSG00000133985
ENSG00000130758
ENSG00000125434
ENSG00000164061
ENSG00000180425
ENSG00000068354
ENSG00000131435
ENSG00000150594
ENSG00000172113
ENSG00000139372
ENSG00000196361
ENSG00000169220
ENSG00000198873
ENSG00000087502

ENSG00000134438
ENSG00000146039
ENSG00000157881
ENSG00000185052
ENSG00000239713
ENSG00000149485
ENSG00000070444
ENSG00000103034
ENSG00000018189
ENSG00000149534
ENSG00000213639
ENSG00000198093
ENSG00000153046
ENSG00000140299
ENSG00000076201
ENSG00000147100
ENSG00000123992
ENSG00000105366
ENSG00000107130
ENSG00000146021
ENSG00000151033
ENSG00000126524
ENSG00000170734
ENSG00000122952
ENSG00000066468
ENSG00000197771
ENSG00000009694
ENSG00000122188
ENSG00000168907
ENSG00000170604
ENSG00000168488
ENSG00000165819
ENSG00000132693
ENSG00000180957
ENSG00000185811
ENSG00000162433
ENSG00000076242
ENSG00000178726
ENSG00000124882
ENSG00000100167
ENSG00000067829
ENSG00000197444
ENSG00000187790
ENSG00000141012
ENSG00000214274
ENSG00000084652
ENSG00000163864
ENSG00000128973
ENSG00000116990
ENSG00000159618
ENSG00000166295
ENSG00000160868
ENSG00000213390
ENSG00000116750
ENSG00000121680
ENSG00000120949
ENSG00000085982
ENSG00000103145
ENSG00000147573
ENSG00000127483
ENSG00000105996
ENSG00000026559
ENSG00000105398
ENSG00000189159

ENSG00000156876
ENSG00000126243
ENSG00000186810
ENSG00000182463
ENSG00000106992
ENSG00000108239
ENSG00000078668
ENSG00000090470
ENSG00000149547
ENSG00000165682
ENSG00000171119
ENSG00000103044
ENSG00000249315
ENSG00000175426
ENSG00000197776
ENSG00000095059
ENSG00000111664
ENSG00000170412
ENSG00000177932
ENSG00000188493
ENSG00000166840
ENSG00000197992
ENSG00000159307
ENSG00000103449
ENSG00000144230
ENSG00000152315
ENSG00000155961
ENSG00000140057
ENSG00000175895
ENSG00000005513
ENSG00000151914
ENSG00000131149
ENSG00000148218
ENSG00000116641
ENSG00000183671
ENSG00000118961
ENSG00000068438
ENSG00000204065
ENSG00000197859
ENSG00000065911
ENSG00000157538
ENSG00000158246
ENSG00000115107
ENSG00000111432
ENSG00000119699
ENSG00000092978
ENSG00000197273
ENSG00000109072
ENSG00000146047
ENSG00000165410
ENSG00000012223
ENSG00000145936
ENSG00000083223
ENSG00000169519
ENSG00000110851
ENSG00000183354
ENSG00000116489
ENSG00000085063
ENSG00000146285
ENSG00000243543
ENSG00000164591
ENSG00000167881
ENSG00000102265
ENSG00000113108

ENSG00000187758
ENSG00000138308
ENSG00000060709
ENSG00000175106
ENSG00000130726
ENSG00000104490
ENSG00000120075
ENSG00000198691
ENSG00000020922
ENSG00000131355
ENSG00000163817
ENSG00000129667
ENSG00000147548
ENSG00000159556
ENSG00000175265
ENSG00000203963
ENSG00000164674
ENSG00000140044
ENSG00000131831
ENSG00000119283
ENSG00000148840
ENSG00000123358
ENSG00000008226
ENSG00000106554
ENSG00000084674
ENSG00000189299
ENSG00000119689
ENSG00000137094
ENSG00000196263
ENSG00000129991
ENSG00000120149
ENSG00000235631
ENSG00000163751
ENSG00000160783
ENSG00000144455
ENSG00000114993
ENSG00000072778
ENSG00000162722
ENSG00000096401
ENSG00000144741
ENSG00000158793
ENSG00000138131
ENSG00000108021
ENSG00000106331
ENSG00000165584
ENSG00000111237
ENSG00000106484
ENSG00000105732
ENSG00000134602
ENSG00000168785
ENSG00000137675
ENSG00000122691
ENSG00000163531
ENSG00000124602
ENSG00000101928
ENSG00000131747
ENSG00000130529
ENSG00000168575
ENSG00000179133
ENSG00000151929
ENSG00000134815
ENSG00000125459
ENSG00000025293
ENSG00000168286

ENSG00000134077
ENSG00000119402
ENSG00000143107
ENSG00000163827
ENSG00000197446
ENSG00000185163
ENSG00000174527
ENSG00000072071
ENSG00000007350
ENSG00000116679
ENSG00000163220
ENSG00000187094
ENSG00000185630
ENSG00000186143
ENSG00000171681
ENSG00000143382
ENSG00000103528
ENSG00000133317
ENSG00000134375
ENSG00000115457
ENSG00000049249
ENSG00000169925
ENSG00000146587
ENSG00000111644
ENSG00000136935
ENSG00000057149
ENSG00000145431
ENSG00000163884
ENSG00000126267
ENSG00000163072
ENSG00000110092
ENSG00000120899
ENSG00000104331
ENSG00000124313
ENSG00000106304
ENSG00000123569
ENSG00000166455
ENSG00000198844
ENSG00000067596
ENSG00000103351
ENSG00000080802
ENSG00000118276
ENSG00000105669
ENSG00000163346
ENSG00000115665
ENSG00000006534
ENSG00000175073
ENSG00000144354
ENSG00000147655
ENSG00000138759
ENSG00000186350
ENSG00000181472
ENSG00000143748
ENSG00000203814
ENSG00000161551
ENSG00000157429
ENSG00000172889
ENSG00000164683
ENSG00000134709
ENSG00000144834
ENSG00000180104
ENSG00000101109
ENSG00000117407
ENSG00000055118

ENSG00000166845
ENSG00000164983
ENSG00000041988
ENSG00000063660
ENSG00000214562
ENSG00000136444
ENSG00000096968
ENSG00000085644
ENSG00000153933
ENSG00000116183
ENSG00000196391
ENSG00000100478
ENSG00000161914
ENSG00000141316
ENSG00000184786
ENSG00000103047
ENSG00000162521
ENSG00000164284
ENSG00000134569
ENSG00000183049
ENSG00000161973
ENSG00000164128
ENSG00000169856
ENSG00000105662
ENSG00000140526
ENSG00000109193
ENSG00000185532
ENSG00000075223
ENSG00000129351
ENSG00000176101
ENSG00000174111
ENSG00000198673
ENSG00000078177
ENSG00000125968
ENSG00000124702
ENSG00000099260
ENSG00000188554
ENSG00000198959
ENSG00000125846
ENSG00000183853
ENSG00000145592
ENSG00000125675
ENSG00000175573
ENSG00000165061
ENSG00000188282
ENSG00000124812
ENSG00000142188
ENSG00000185215
ENSG00000184349
ENSG00000189043
ENSG00000243137
ENSG00000166803
ENSG00000066855
ENSG00000167889
ENSG00000182572
ENSG00000081154
ENSG00000113430
ENSG00000114315
ENSG00000181322
ENSG00000034533
ENSG00000136327
ENSG00000013563
ENSG00000136158
ENSG00000125304

ENSG00000171497
ENSG00000166262
ENSG00000065183
ENSG00000034693
ENSG00000032389
ENSG00000118308
ENSG00000130957
ENSG00000126934
ENSG00000128383
ENSG00000204414
ENSG00000130300
ENSG00000100767
ENSG00000111254
ENSG00000115008
ENSG00000181773
ENSG00000103245
ENSG00000039987
ENSG00000132958
ENSG00000104142
ENSG00000165169
ENSG00000176884
ENSG00000173578
ENSG00000198771
ENSG00000070087
ENSG00000034053
ENSG00000122642
ENSG00000072121
ENSG00000058404
ENSG00000159111
ENSG00000176020
ENSG00000167074
ENSG00000143839
ENSG00000164167
ENSG00000187010
ENSG00000168874
ENSG00000188015
ENSG00000167105
ENSG00000142512
ENSG00000165171
ENSG00000167535
ENSG00000116985
ENSG00000185482
ENSG00000127022
ENSG00000067842
ENSG00000186815
ENSG00000125741
ENSG00000168539
ENSG00000187569
ENSG00000198231
ENSG00000103363
ENSG00000197093
ENSG00000095066
ENSG00000130935
ENSG00000180340
ENSG00000075340
ENSG00000165704
ENSG00000112038
ENSG00000152953
ENSG00000124507
ENSG00000165659
ENSG00000104881
ENSG00000130307
ENSG00000177868
ENSG00000049768

ENSG00000145685
ENSG00000087085
ENSG00000140743
ENSG00000184347
ENSG00000198055
ENSG00000169347
ENSG00000161328
ENSG00000197363
ENSG00000184571
ENSG00000113615
ENSG00000140284
ENSG00000126062
ENSG00000175264
ENSG00000135622
ENSG00000182508
ENSG00000171425
ENSG00000021300
ENSG00000091409
ENSG00000092051
ENSG00000153113
ENSG00000138100
ENSG00000147274
ENSG00000067646
ENSG00000185774
ENSG00000180347
ENSG00000186951
ENSG00000167815
ENSG00000111424
ENSG00000188937
ENSG00000128886
ENSG00000147316
ENSG00000163900
ENSG00000157087
ENSG00000134324
ENSG00000167447
ENSG00000120215
ENSG00000212710
ENSG00000135218
ENSG00000159164
ENSG00000060718
ENSG00000074410
ENSG00000182963
ENSG00000181495
ENSG00000173200
ENSG00000168060
ENSG00000153339
ENSG00000170549
ENSG00000106723
ENSG00000115947
ENSG00000073711
ENSG00000107282
ENSG00000115194
ENSG00000136695
ENSG00000111341
ENSG00000231260
ENSG00000106328
ENSG00000083896
ENSG00000187650
ENSG00000125970
ENSG00000162510
ENSG00000049618
ENSG00000131375
ENSG00000147155
ENSG00000196456

ENSG00000141294
ENSG00000197584
ENSG00000149089
ENSG00000117543
ENSG00000141646
ENSG00000145817
ENSG00000178445
ENSG00000156574
ENSG00000145675
ENSG00000165572
ENSG00000221994
ENSG00000110218
ENSG00000010803
ENSG00000006611
ENSG00000105675
ENSG00000107779
ENSG00000138078
ENSG00000196418
ENSG00000186283
ENSG00000164362
ENSG00000135406
ENSG00000102245
ENSG00000104888
ENSG00000182957
ENSG00000140798
ENSG00000182916
ENSG00000181092
ENSG00000123983
ENSG00000172663
ENSG00000177354
ENSG00000103064
ENSG00000198933
ENSG00000171135
ENSG00000126821
ENSG00000169902
ENSG00000174740
ENSG00000138041
ENSG00000142002
ENSG00000125879
ENSG00000073578
ENSG00000081479
ENSG00000165879
ENSG00000134365
ENSG00000019549
ENSG00000101574
ENSG00000130818
ENSG00000163659
ENSG00000181610
ENSG00000196411
ENSG00000170832
ENSG00000147121
ENSG00000183486
ENSG00000125813
ENSG00000142197
ENSG00000167258
ENSG00000180818
ENSG00000174938
ENSG00000108953
ENSG00000139970
ENSG00000154957
ENSG00000183784
ENSG00000172239
ENSG00000168314
ENSG00000072518

ENSG00000180228
ENSG00000125991
ENSG00000197558
ENSG00000171466
ENSG00000183718
ENSG00000165650
ENSG00000123737
ENSG00000171720
ENSG00000131096
ENSG00000147099
ENSG00000163207
ENSG00000044574
ENSG00000122550
ENSG00000157514
ENSG00000160326
ENSG00000242885
ENSG00000080603
ENSG00000188987
ENSG00000221932
ENSG00000164303
ENSG00000131409
ENSG00000004468
ENSG00000067141
ENSG00000151276
ENSG00000180745
ENSG00000099377
ENSG00000160072
ENSG00000073849
ENSG00000100813
ENSG00000004142
ENSG00000203880
ENSG00000125107
ENSG00000136535
ENSG00000214193
ENSG00000166801
ENSG00000165379
ENSG00000163399
ENSG00000227460
ENSG00000119899
ENSG00000102401
ENSG00000118514
ENSG00000048991
ENSG00000174837
ENSG00000116251
ENSG00000215915
ENSG00000178927
ENSG00000197919
ENSG00000197566
ENSG00000138380
ENSG00000096696
ENSG00000161791
ENSG00000164088
ENSG00000165669
ENSG00000102317
ENSG00000162702
ENSG00000135333
ENSG00000165966
ENSG00000180509
ENSG00000122859
ENSG00000100207
ENSG00000144306
ENSG00000242019
ENSG00000184178
ENSG00000157005

ENSG00000130348
ENSG00000148950
ENSG00000074966
ENSG00000121594
ENSG00000204104
ENSG00000090776
ENSG00000187079
ENSG00000019144
ENSG00000250510
ENSG00000075420
ENSG00000141568
ENSG00000184489
ENSG00000154760
ENSG00000171611
ENSG00000133246
ENSG00000112494
ENSG00000139687
ENSG00000180900
ENSG00000147403
ENSG00000086475
ENSG00000104447
ENSG00000167614
ENSG00000155849
ENSG00000144848
ENSG00000123815
ENSG00000161638
ENSG00000164176
ENSG00000108370
ENSG00000144843
ENSG00000189266
ENSG00000010310
ENSG00000171812
ENSG00000161920
ENSG00000150938
ENSG00000215454
ENSG00000174799
ENSG00000109332
ENSG00000166343
ENSG00000228258
ENSG00000115138
ENSG00000150667
ENSG00000168297
ENSG00000111731
ENSG00000184210
ENSG00000187554
ENSG00000101557
ENSG00000143222
ENSG00000128694
ENSG00000165458
ENSG00000138794
ENSG00000100599
ENSG00000182489
ENSG00000177468
ENSG00000152782
ENSG00000162891
ENSG00000171223
ENSG00000153015
ENSG00000158286
ENSG00000121892
ENSG00000119711
ENSG00000149516
ENSG00000239697
ENSG00000132155
ENSG00000136021

ENSG00000129965
ENSG00000079313
ENSG00000152056
ENSG00000101940
ENSG00000172771
ENSG00000196371
ENSG00000148634
ENSG00000162849
ENSG00000136866
ENSG00000168757
ENSG00000187862
ENSG00000116237
ENSG00000167608
ENSG00000170542
ENSG00000149809
ENSG00000101695
ENSG00000130783
ENSG00000142327
ENSG00000188739
ENSG00000145888
ENSG00000007392
ENSG00000163964
ENSG00000095209
ENSG00000141959
ENSG00000147027
ENSG00000204052
ENSG00000074755
ENSG00000131730
ENSG00000108262
ENSG00000159720
ENSG00000133055
ENSG00000104524
ENSG00000168496
ENSG00000115226
ENSG00000116353
ENSG00000159210
ENSG00000134551
ENSG00000147127
ENSG00000094975
ENSG00000174197
ENSG00000163357
ENSG00000158711
ENSG00000147596
ENSG00000106077
ENSG00000166569
ENSG00000144048
ENSG00000140937
ENSG00000175294
ENSG00000107249
ENSG00000006831
ENSG00000158290
ENSG00000078295
ENSG00000160183
ENSG00000101347
ENSG00000163599
ENSG00000134545
ENSG00000136240
ENSG00000244687
ENSG00000116209
ENSG00000152583
ENSG00000162222
ENSG00000122912
ENSG00000153902
ENSG00000151247

ENSG00000188730
ENSG00000119048
ENSG00000021645
ENSG00000049656
ENSG00000235739
ENSG00000155640
ENSG00000109685
ENSG00000165238
ENSG00000105523
ENSG00000123496
ENSG00000139146
ENSG00000137101
ENSG00000160201
ENSG00000137942
ENSG00000133121
ENSG00000164949
ENSG00000105550
ENSG00000169777
ENSG00000159055
ENSG00000112031
ENSG00000168003
ENSG00000119227
ENSG00000079335
ENSG00000107187
ENSG00000168036
ENSG00000141698
ENSG00000144488
ENSG00000169567
ENSG00000184302
ENSG00000123739
ENSG00000157873
ENSG00000141497
ENSG00000147378
ENSG00000112238
ENSG00000142484
ENSG00000239789
ENSG00000183230
ENSG00000162783
ENSG00000198502
ENSG00000101132
ENSG00000103187
ENSG00000014919
ENSG00000078061
ENSG00000158315
ENSG00000163820
ENSG00000160712
ENSG00000146386
ENSG00000170142
ENSG00000169231
ENSG00000075790
ENSG00000140465
ENSG00000152818
ENSG00000015475
ENSG00000124343
ENSG00000117411
ENSG00000119638
ENSG00000198732
ENSG00000166188
ENSG00000205439
ENSG00000027847
ENSG00000159212
ENSG00000136504
ENSG00000147421
ENSG00000138813

ENSG00000129038
ENSG00000065559
ENSG00000089248
ENSG00000101197
ENSG00000205279
ENSG00000186010
ENSG00000153914
ENSG00000178919
ENSG00000077454
ENSG00000169504
ENSG00000162069
ENSG00000076108
ENSG00000177409
ENSG00000185019
ENSG00000166949
ENSG00000109118
ENSG00000127526
ENSG00000103253
ENSG00000128242
ENSG00000138767
ENSG00000184292
ENSG00000108798
ENSG00000100242
ENSG00000116544
ENSG00000198704
ENSG00000180773
ENSG00000182568
ENSG00000100246
ENSG00000104499
ENSG00000221867
ENSG00000165233
ENSG00000204116
ENSG00000132842
ENSG00000171792
ENSG00000206505
ENSG00000148362
ENSG00000103342
ENSG00000141086
ENSG00000215193
ENSG00000170779
ENSG00000107719
ENSG00000114902
ENSG00000165118
ENSG00000101605
ENSG00000105650
ENSG00000169062
ENSG00000169570
ENSG00000086300
ENSG00000092345
ENSG00000117640
ENSG00000198185
ENSG00000169436
ENSG00000162267
ENSG00000181790
ENSG00000166387
ENSG00000049283
ENSG00000148308
ENSG00000163499
ENSG00000172367
ENSG00000161572
ENSG00000100284
ENSG00000154175
ENSG00000244411
ENSG00000017260

ENSG00000198791
ENSG00000197461
ENSG00000135930
ENSG00000146232
ENSG00000124374
ENSG00000214435
ENSG00000173473
ENSG00000140307
ENSG00000102683
ENSG00000077721
ENSG00000196792
ENSG00000164485
ENSG00000112769
ENSG00000138688
ENSG00000166579
ENSG00000144867
ENSG00000089006
ENSG00000128322
ENSG00000157933
ENSG00000175787
ENSG00000141161
ENSG00000091137
ENSG00000136488
ENSG00000119397
ENSG00000183662
ENSG00000115561
ENSG00000154767
ENSG00000105290
ENSG00000204793
ENSG00000124092
ENSG00000008130
ENSG00000105186
ENSG00000205309
ENSG00000153993
ENSG00000198612
ENSG00000184185
ENSG00000100227
ENSG00000110047
ENSG00000154237
ENSG00000167880
ENSG00000164695
ENSG00000115170
ENSG00000175467
ENSG00000166167
ENSG00000151615
ENSG00000137492
ENSG00000170967
ENSG00000162377
ENSG00000124357
ENSG00000006015
ENSG00000146263
ENSG00000174233
ENSG00000198944
ENSG00000062524
ENSG00000170468
ENSG00000198853
ENSG00000169550
ENSG00000177054
ENSG00000110931
ENSG00000006695
ENSG00000171385
ENSG00000167751
ENSG00000181722
ENSG00000111816

ENSG00000134644
ENSG00000176994
ENSG00000183520
ENSG00000115602
ENSG00000101266
ENSG00000197579
ENSG00000115091
ENSG00000140623
ENSG00000132589
ENSG00000117400
ENSG00000187486
ENSG00000111665
ENSG00000103148
ENSG00000235103
ENSG00000169242
ENSG00000151320
ENSG00000040487
ENSG00000142168
ENSG00000114383
ENSG00000141076
ENSG00000115540
ENSG00000164405
ENSG00000185838
ENSG00000151967
ENSG00000163873
ENSG00000146411
ENSG00000107758
ENSG00000160993
ENSG00000135127
ENSG00000162757
ENSG00000162302
ENSG00000066322
ENSG00000120690
ENSG00000064199
ENSG00000037474
ENSG00000166337
ENSG00000164776
ENSG00000139890
ENSG00000121940
ENSG00000180815
ENSG00000063180
ENSG00000100926
ENSG00000164626
ENSG00000198286
ENSG00000003402
ENSG00000171634
ENSG00000103175
ENSG00000182346
ENSG00000112651
ENSG00000064835
ENSG00000173421
ENSG00000000971
ENSG00000163590
ENSG00000010270
ENSG00000139209
ENSG00000151490
ENSG00000104936
ENSG00000158560
ENSG00000182742
ENSG00000138279
ENSG00000163687
ENSG00000172016
ENSG00000111602
ENSG00000151418

ENSG00000174013
ENSG00000172318
ENSG00000100376
ENSG00000184047
ENSG00000182158
ENSG00000222047
ENSG00000168675
ENSG00000065357
ENSG00000171603
ENSG00000165526
ENSG00000163635
ENSG00000105865
ENSG00000079246
ENSG00000179431
ENSG00000153002
ENSG00000205944
ENSG00000065526
ENSG00000173848
ENSG00000179981
ENSG00000051128
ENSG00000156521
ENSG00000146250
ENSG00000177879
ENSG00000112182
ENSG00000005483
ENSG00000204961
ENSG00000137251
ENSG00000128285
ENSG00000112562
ENSG00000169194
ENSG00000196156
ENSG00000052802
ENSG00000243244
ENSG00000129691
ENSG00000146963
ENSG00000050165
ENSG00000175203
ENSG00000164078
ENSG00000159399
ENSG00000182923
ENSG00000178026
ENSG00000170852
ENSG00000239961
ENSG00000131725
ENSG00000119509
