# Supplementary figures and images for: Preferential regulation of miRNA targets by environmental chemicals in the human genome
Source: BMC Genomics. 2011 May 18;12:244. doi: 10.1186/1471-2164-12-244 (PMC3118786; doi:10.1186/1471-2164-12-244)

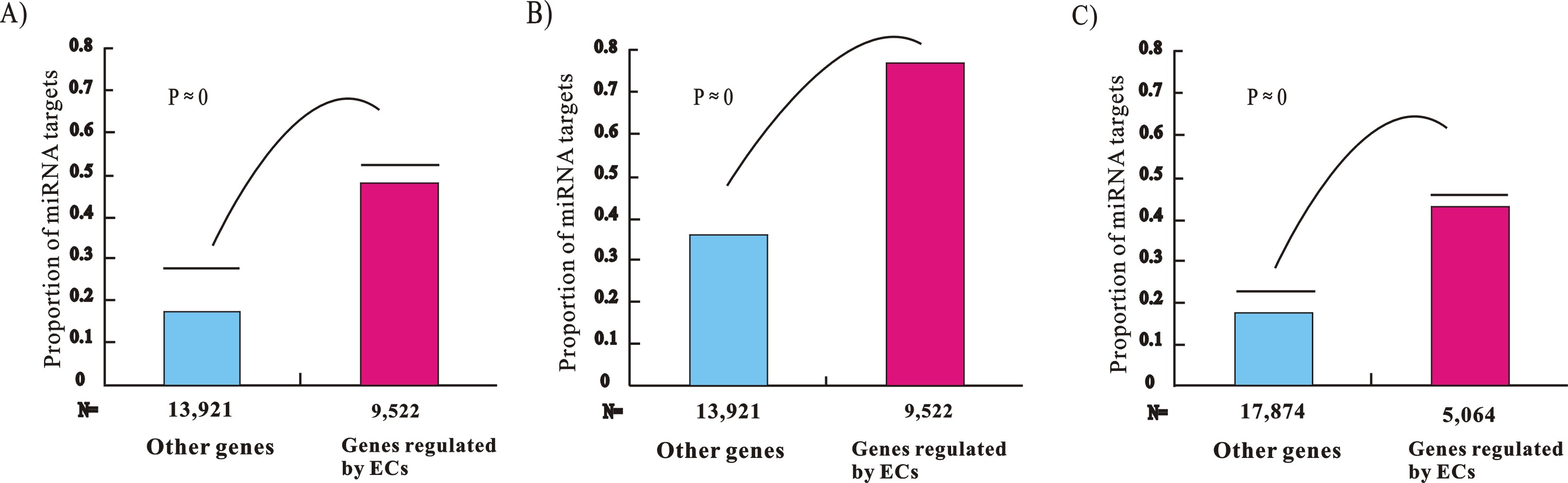

Supplement: Additional file 3 — Figure S1: miRNA targets are enriched among EC-genes in Mus musculus and Rattus norvegicus. This figure shows the proportion of miRNA targets predicted (A) by Mus musculus TargetScan5.1, (B) by Mus musculus PITA and (C) by Rattus norvegicus TargetScan5.1. As there are no miRNA targets from the PicTar prediction for both species and PITA prediction for Rattus norvegicus, this figure shows the proportion of miRNA targets predicted by TargetScan5.1 and the Rattus norvegicus PITA. The horizontal lines above histogram bars represent the proportion of miRNA targets using genes with human orthologs as background. [file 1471-2164-12-244-S3.JPEG]

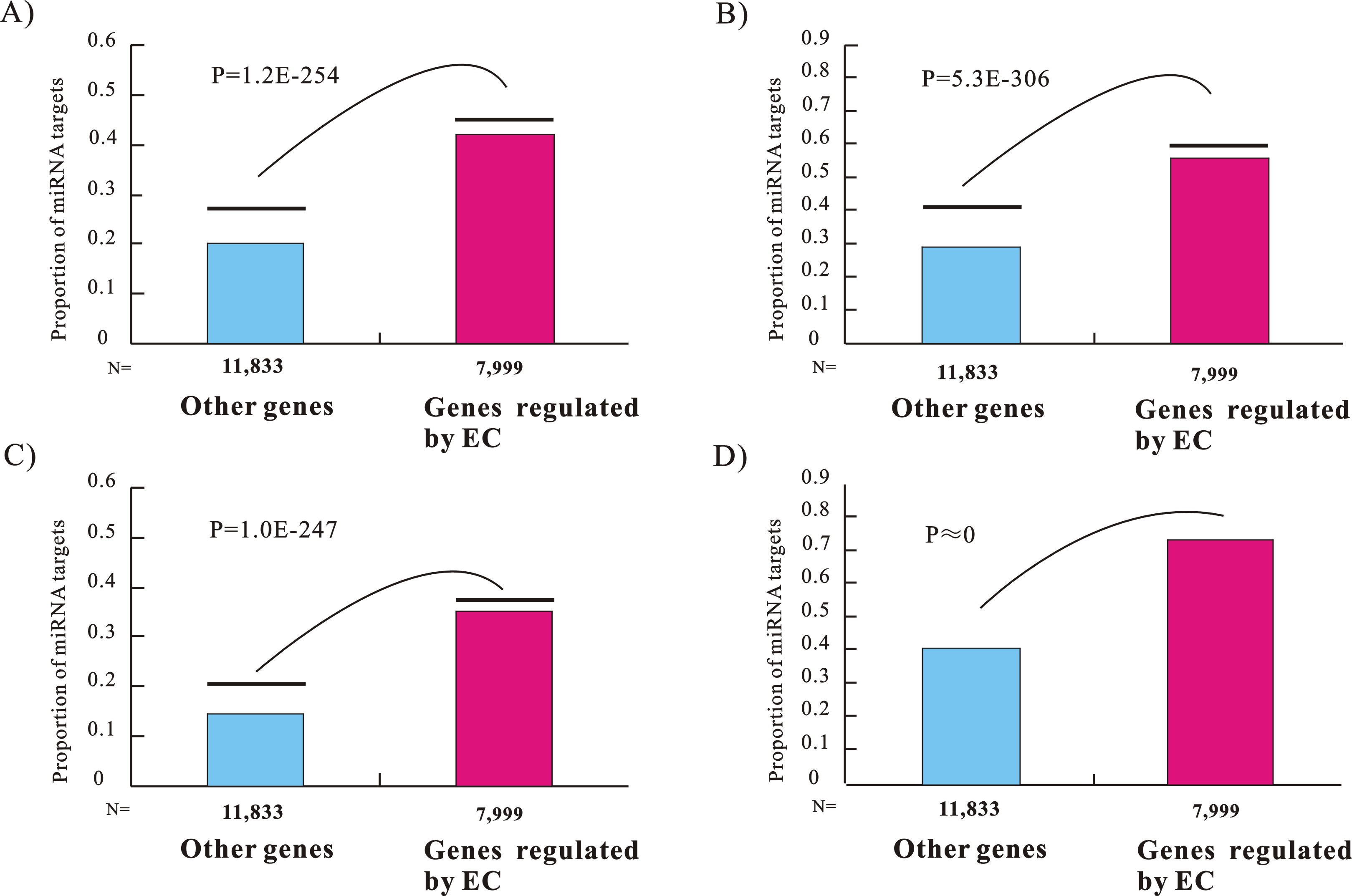

Supplement: Additional file 5 — Figure S2: miRNA targets are enriched among human EC-genes after filtering out the cancer-related genes in simulations. This figure shows the proportion of miRNA targets predicted by PicTar, TargetScan5.1, both programs of PicTar and TargetScan5.1 (intersections), and PITA. The horizontal lines above the histogram bars represent the proportion of miRNA targets using genes with mouse orthologs as background. [file 1471-2164-12-244-S5.JPEG]

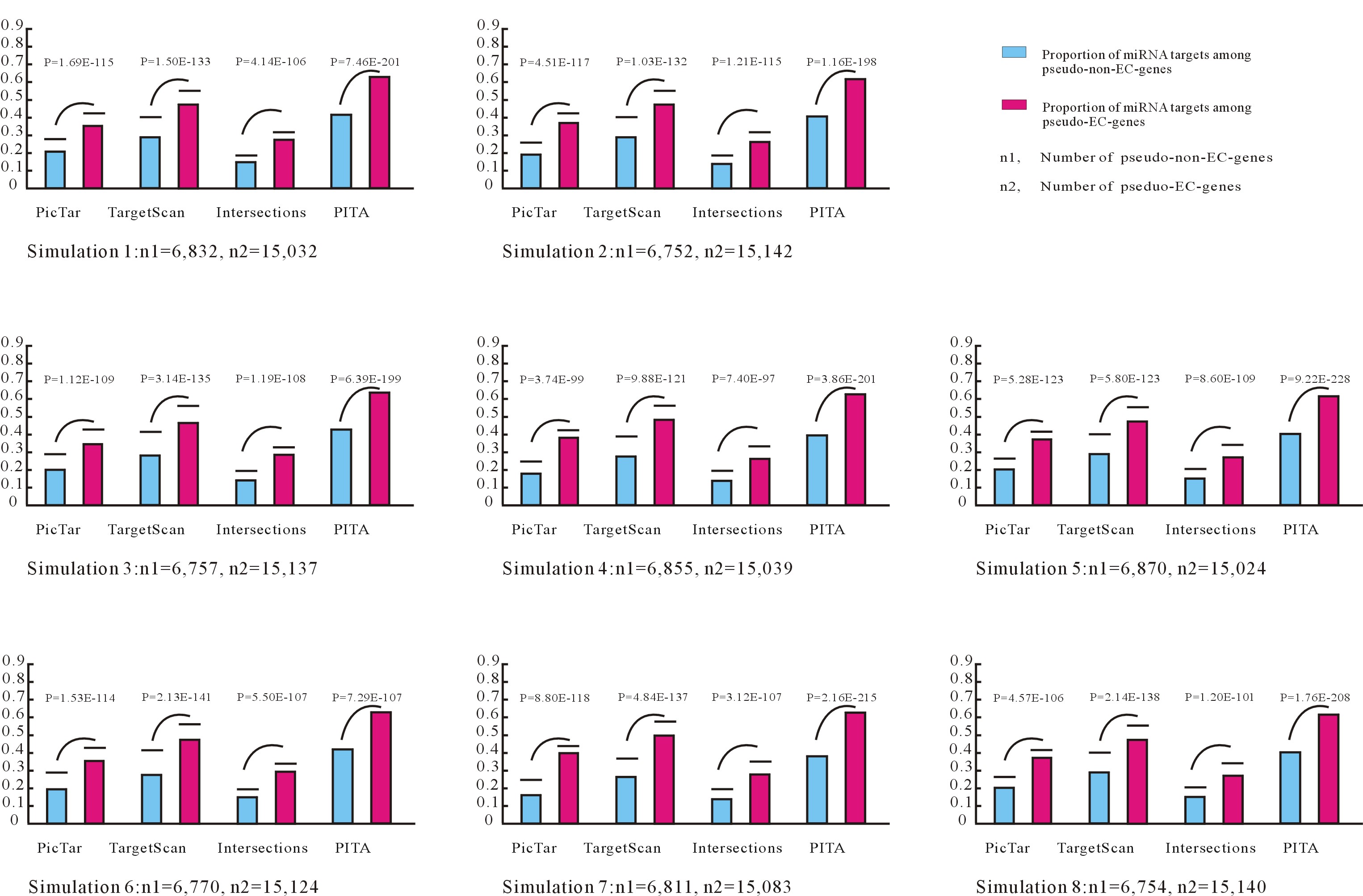

Supplement: Additional file 6 — Figure S3: miRNA targets are enriched among human EC-genes after controlling for potential false-negatives of non-EC-genes. This figure shows the proportion of miRNA targets predicted by PicTar, TargetScan5.1, both programs of PicTar and TargetScan5.1 (intersections), and PITA. The horizontal lines above the histogram bars represent the proportion of miRNA targets using genes with mouse orthologs as background. [file 1471-2164-12-244-S6.JPEG]

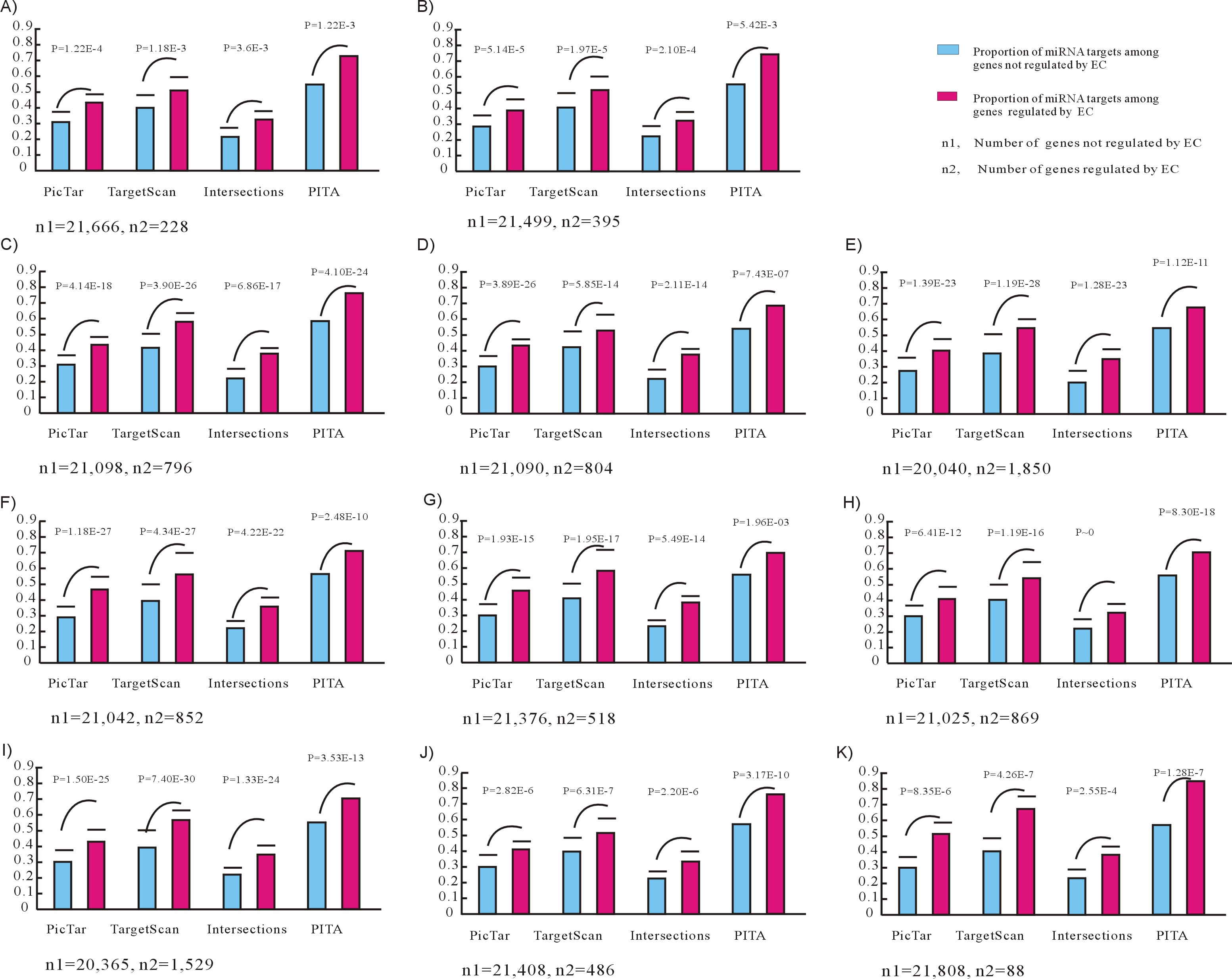

Supplement: Additional file 7 — Figure S4: miRNA targets are enriched among human EC-genes in 11 raw-data-based microarray datasets. This figure shows the proportion of miRNA targets between differentially vs. non-differentially regulated genes where (A) A549 cells treated with asbestos, (B) Beas-2B cells treated with asbestos, (C) Dendritic cells treated with AM580, (D) Dendritic cells treated with rosiglitazone, (E) Epithelia cells treated with selenium, (F) Epithelia cells treated with Vitamin E, (G) Stromal cells treated with selenium, (H) HCT116 cells treated with fluorouracil, (I) HCT116 cells treated with SN38, (J) HepG2 cells treated with NMN and (K) HepG2 cells treated with phenol. The horizontal lines above the histogram bars represent the proportion of miRNA targets using genes with mouse orthologs as background. [file 1471-2164-12-244-S7.JPEG]

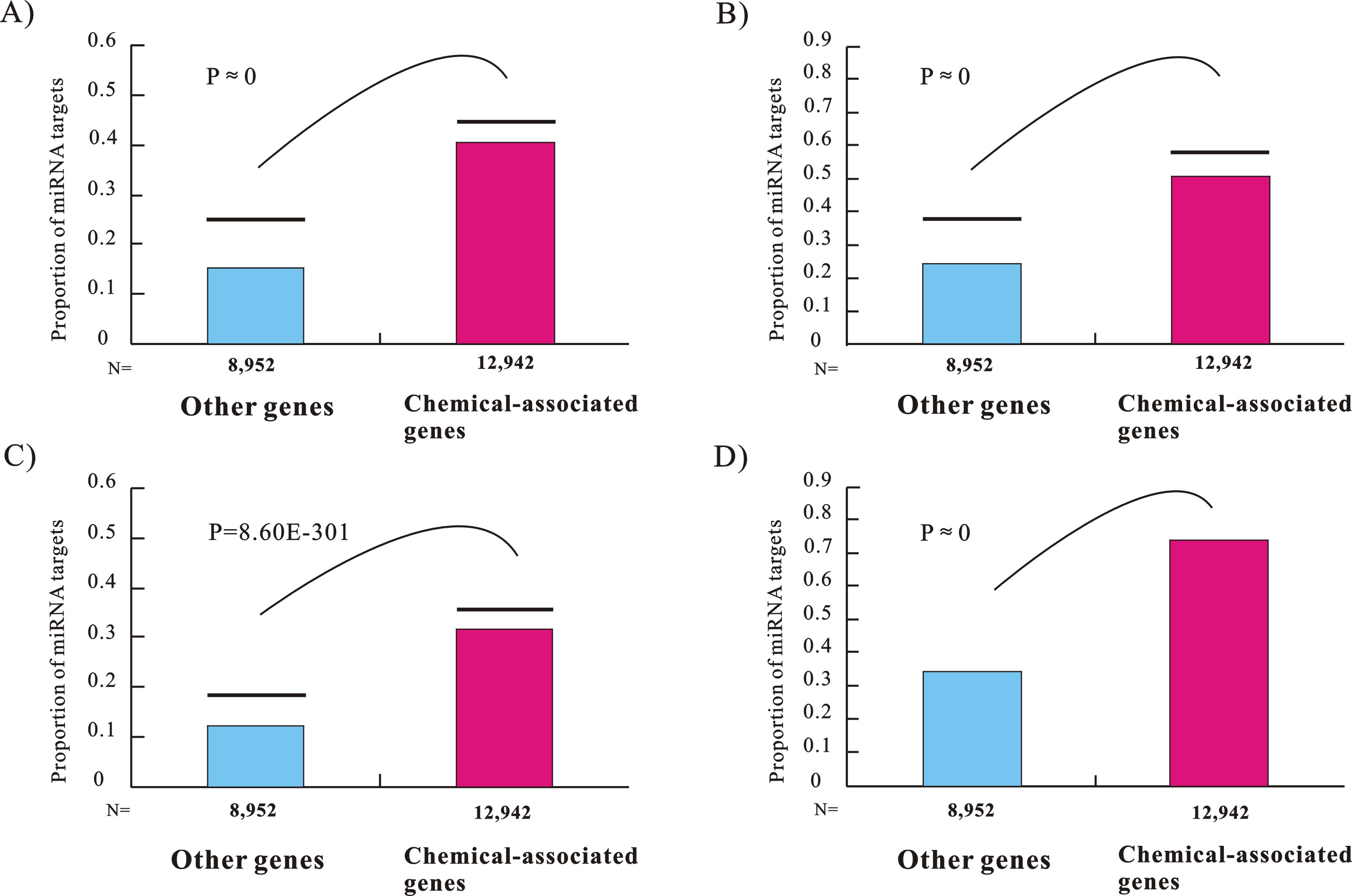

Supplement: Additional file 10 — Figure S5: miRNA targets are enriched among human EC-associated genes by STITCH2.0. This figure shows the proportion of miRNA targets predicted (A) by PicTar, (B) by TargetScan5.1, (C) by both programs of PicTar and TargetScan5.1 (intersections), and (D) by PITA. The horizontal lines above the histogram bars represent the proportion of miRNA targets using genes with mouse orthologs as background. [file 1471-2164-12-244-S10.JPEG]
