# Supplementary material for: Preferential regulation of miRNA targets by environmental chemicals in the human genome
Source: BMC Genomics. 2011 May 18;12:244. doi: 10.1186/1471-2164-12-244 (PMC3118786; doi:10.1186/1471-2164-12-244)
Supplement: Additional file 4 — Table S3: The 4,126 papers investigating the expression profiles of human genes in response to ECs. [file 1471-2164-12-244-S4.PDF]

| PubMed Id | Number of regulatory relationships among ECs and Genes | miRNA related or not | Title |
|---|---|---|---|
| 16325577 | 3 | Yes | A minicircuitry comprised of microRNA-223 and transcription factors NFI-A and C/EBPalpha regulates human granulopoiesis. |
| 17283129 | 1 | Yes | Differential patterns of microRNA expression in neuroblastoma are correlated with prognosis, differentiation, and apoptosis. |
| 17621267 | 24 | Yes | Epigenetic regulation of microRNA-370 by interleukin-6 in malignant human cholangiocytes. |
| 18347134 | 2 | Yes | Curcumin (diferuloylmethane) alters the expression profiles of microRNAs in human pancreatic cancer cells. |
| 19116145 | 4 | Yes | Epigenetic therapy upregulates the tumor suppressor microRNA-126 and its host gene EGFL7 in human cancer cells. |
| 10025362 | 4 | No | Response of normal human keratinocytes to sulfur mustard (HD): cytokine release using a non-enzymatic detachment procedure. |
| 10048758 | 3 | No | Effect of the chlorinated hydrocarbons heptachlor, chlordane, and toxaphene on retinoblastoma tumor suppressor in human lymphocytes. |
| 10064545 | 7 | No | Estrogenic potential of certain pyrethroid compounds in the MCF-7 human breast carcinoma cell line. |
| 10072544 | 56 | No | Selective regulation of cytokine induction by adenoviral gene transfer of IkappaBalpha into human macrophages: lipopolysaccharide-induced, but not |
| 10076546 | 5 | No | Differential modulation of pro- and anti-inflammatory cytokine receptors by N-(4-trifluoromethylphenyl)-2-cyano-3-hydroxy-crotonic acid amide (A77 1726), the |
| 10202824 | 3 | No | Persistent alterations in T-cell repertoire, cytokine and chemokine receptor gene expression after 1 year of highly active antiretroviral therapy. |
| 10215738 | 7 | No | Plaunotol prevents indomethacin-induced gastric mucosal injury in rats by inhibiting neutrophil activation. |
| 10218491 | 28 | No | Stimulation of peripheral cannabinoid receptor CB2 induces MCP-1 and IL-8 gene expression in human promyelocytic cell line HL60. |
| 10218639 | 1 | No | Cyclooxygenase dependent release of heme from microsomal hemeproteins correlates with induction of heme oxygenase 1 transcription in human fibroblasts. |
| 10219967 | 31 | No | these inducers act through post-transcriptional mechanisms. 6. These results highlight differences in the molecular mechanisms of induction of CYP3A4 by the |
| 10224351 | 8 | No | Regulation of IL-5 and IL-5 receptor expression in the bone marrow of allergic asthmatics. |

| | | | |
|---|---|---|---|
| 10226068 | 1 | No | Interleukin-5 production by human airway epithelial cells. |
| 10229127 | 8 | No | Neuroendocrine mediators up-regulate alpha1b- and alpha1d-adrenergic receptor subtypes in human monocytes. |
| 10231603 | 1 | No | Molecular mechanisms of the antiproliferative effect of vitamin K3 on Jurkat cells. |
| 10320526 | 4 | No | Parathyroid hormone regulates the expression of fibroblast growth factor-2 mRNA and fibroblast growth factor receptor mRNA in osteoblastic cells. |
| 10328874 | 51 | No | Non-steroidal anti-inflammatory drugs inhibit the expression of cytokines and induce HSP70 in human monocytes. |
| 10338361 | 1 | No | Inhibitory effects of nitric oxide on the expression and activity of aromatase in human granulosa cells. |
| 10344722 | 24 | No | Coordinate alterations in the expression of BRCA1, BRCA2, p300, and Rad51 in response to genotoxic and other stresses in human prostate cancer cells. |
| 10370019 | 1 | No | Lung hypoplasia caused by nitrofen is mediated by down-regulation of thyroid transcription factor TTF-1. |
| 10377187 | 2 | No | Lipoxin (LX)A4 and aspirin-triggered 15-epi-LXA4 inhibit tumor necrosis factor 1alpha-initiated neutrophil responses and trafficking: regulators of a |
| 10402478 | 11 | No | Regulation of GCDFP-15 expression in human mammary cancer cells. |
| 10403534 | 3 | No | Differential apoptosis by indomethacin in gastric epithelial cells through the constitutive expression of wild-type p53 and/or up-regulation of c-myc. |
| 10414782 | 4 | No | Pyrethroids and piperonyl-butoxide affect human T-lymphocytes in vitro. |
| 10421657 | 2 | No | Expression and inducibility of the human bilirubin UDP-glucuronosyltransferase UGT1A1 in liver and cultured primary hepatocytes: evidence for both genetic and |
| 10428506 | 6 | No | Modulation of histidine decarboxylase activity and cytokine synthesis in human leukemic cell lines: relationship with basophilic and/or megakaryocytic |
| 10432307 | 3 | No | Thioredoxin reductase is the major selenoprotein expressed in human umbilical-vein endothelial cells and is regulated by protein kinase C. |
| 10467400 | 2 | No | Overexpression of urokinase-type plasminogen activator in pancreatic adenocarcinoma is regulated by constitutively activated RelA. |
| 10469331 | 6 | No | raise intracellular Ca2+ and stimulate differentiation in normal human foreskin keratinocytes. In the presence of 0.03 mM Ca2+, NPS R-467 increased the |
| 10473103 | 1 | No | Chlorambucil induction of HsRad51 in B-cell chronic lymphocytic leukemia. |

| | | | |
|---|---|---|---|
| 10485483 | 2 | No | Increased cyclooxygenase-2 expression in human pancreatic carcinomas and cell lines: growth inhibition by nonsteroidal anti-inflammatory drugs. |
| 10491287 | 7 | No | Inhibition of mitochondrial function in HL60 cells is associated with an increased apoptosis and expression of CD14. |
| 10496880 | 6 | No | Pseudomonas aeruginosa quorum-sensing signal molecule N-(3-oxododecanoyl)-L-homoserine lactone inhibits expression of P2Y receptors in |
| 10498757 | 1 | No | Retinoic acid induces Gpx2 gene expression in MCF-7 human breast cancer cells. |
| 10531375 | 15 | No | Cadmium induces conformational modifications of wild-type p53 and suppresses p53 response to DNA damage in cultured cells. |
| 10540334 | 12 | No | Expression and release of chemokines associated with apoptotic cell death in human promonocytic U937 cells and peripheral blood mononuclear cells. |
| 10545406 | 9 | No | Effects of environmental estrogens on tumor necrosis factor alpha-mediated apoptosis in MCF-7 cells. |
| 10564678 | 2 | No | DT-Diaphorase expression and tumor cell sensitivity to 17-allylamino, 17-demethoxygeldanamycin, an inhibitor of heat shock protein 90. |
| 10566683 | 2 | No | Sex steroids and odorants modulate gonadotropin-releasing hormone secretion in primary cultures of human olfactory cells. |
| 10567916 | 1 | No | Effects of retinoic acid on N-glycosylation and mRNA stability of the liver/bone/kidney alkaline phosphatase in neuronal cells. |
| 10569698 | 27 | No | Histamine and histamine-receptor antagonists modify gene expression and biosynthesis of interferon gamma in peripheral human blood mononuclear cells and |
| 10571726 | 2 | No | Altered gene expression in melanocytes exposed to 4-tertiary butyl phenol (4-TBP): upregulation of the A2b adenosine receptor 1. |
| 10588509 | 8 | No | Attenuation of catecholamine-induced immunosuppression in whole blood from patients with sepsis. |
| 10593592 | 2 | No | Sulfur mustard upregulates the expression of interleukin-8 in cultured human keratinocytes. |
| 10599054 | 1 | No | Effects of time and cholinesterase inhibitor treatment on multiple cerebrospinal fluid parameters in Alzheimer's disease. |
| 10601582 | 2 | No | Effects of genistein and structurally related phytoestrogens on cell cycle kinetics and apoptosis in MDA-MB-468 human breast cancer cells. |
| 10619186 | 1 | No | Decrease in nucleophosmin/B23 mRNA and telomerase activity during indomethacin-induced apoptosis of gastric KATO-III cancer cells. |
| 10619828 | 1 | No | Nitric oxide synthase inhibitors attenuate ozone-induced airway inflammation in guinea pigs. Possible role of interleukin-8. |

| | | | |
|---|---|---|---|
| 10633228 | 9 | No | Increased expression of estrogen receptor beta in human uterine smooth muscle at term. |
| 10637131 | 2 | No | Lung mucin production is stimulated by the air pollutant residual oil fly ash. |
| 10640517 | 12 | No | Dexamethasone differentially regulates expression of carboxylesterase genes in humans and rats. |
| 10640773 | 16 | No | Oxidative stress causes mucin synthesis via transactivation of epidermal growth factor receptor: role of neutrophils. |
| 10640779 | 10 | No | NF-kappa B modulates TNF-alpha production by alveolar macrophages in asymptomatic HIV-seropositive individuals. |
| 10644010 | 11 | No | SODs are involved in the regulation of ICAM-1 expression in human melanoma and endothelial cells. |
| 10651068 | 1 | No | Inhibition of beta-globin gene expression by 3'-azido-3'-deoxythymidine in human erythroid progenitor cells. |
| 10651989 | 2 | No | Human CD4+ T lymphocytes with remarkable regulatory functions on dendritic cells and nickel-specific Th1 immune responses. |
| 10652438 | 6 | No | Quercetin inhibits p21-RAS expression in human colon cancer cell lines and in primary colorectal tumors. |
| 10656287 | 42 | No | Oxidative stress involvement in chemically induced differentiation of K562 cells. |
| 10656621 | 5 | No | Expression of antioxidant proteins in human intestinal Caco-2 cells treated with dietary flavonoids. |
| 10681578 | 10 | No | Proteinase inhibitor 9, an inhibitor of granzyme B-mediated apoptosis, is a primary estrogen-inducible gene in human liver cells. |
| 10687981 | 10 | No | Dramatic suppression of plasma and urinary prostate specific antigen and human glandular kallikrein by antiandrogens in male-to-female transsexuals. |
| 10691972 | 10 | No | Characterization of the 5'-flanking region of the human multidrug resistance protein 2 (MRP2) gene and its regulation in comparison withthe multidrug |
| 10692111 | 2 | No | p53-dependent apoptosis in melanoma cells after treatment with camptothecin. |
| 10695937 | 4 | No | Immunolocalizaiton of c-Myc and bcl-2 proto-oncogene products in gingival hyperplasia induced by nifedipine and phenytoin. |
| 10696073 | 20 | No | Induction and regulation of xenobiotic-metabolizing cytochrome P450s in the human A549 lung adenocarcinoma cell line. |
| 10699462 | 2 | No | Erythropoietin induction in Hep3B cells is not affected by inhibition of heme biosynthesis. |

| | | | |
|---|---|---|---|
| 10700573 | 22 | No | Sodium valproate inhibits production of TNF-alpha and IL-6 and activation of NF-kappaB. |
| 10702201 | 4 | No | Overexpression of protein kinase C-beta1 isoenzyme suppresses indomethacin-induced apoptosis in gastric epithelial cells. |
| 10709161 | 3 | No | A double-blind, placebo-controlled evaluation of the effect of oral doses of rizatriptan 10 mg on oral contraceptive pharmacokinetics in healthy female |
| 10712238 | 17 | No | ERK signaling mediates the induction of inflammatory cytokines by bufalin in human monocytic cells. |
| 10718115 | 7 | No | Expression and regulation of macrophage inflammatory protein-2 gene by vanadium in mouse macrophages. |
| 10718847 | 3 | No | Effects of luteolin, quercetin and baicalein on immunoglobulin E-mediated mediator release from human cultured mast cells. |
| 10720412 | 1 | No | NADH oxidase activation is involved in arsenite-induced oxidative DNA damage in human vascular smooth muscle cells. |
| 10739673 | 28 | No | A role for serine proteases in mediating phorbol ester-induced differentiation of HL-60 cells. |
| 10746939 | 3 | No | 2-Chloro-s-triazine herbicides induce aromatase (CYP19) activity in H295R human adrenocortical carcinoma cells: a novel mechanism for estrogenicity? |
| 10763856 | 4 | No | beta-Adrenoceptor stimulation up-regulates phosphodiesterase 4 activity and reduces prostaglandin E2-inhibitory effects in human neutrophils. |
| 10766810 | 2 | No | Alpha(1) adrenergic agonist induction of p21(waf1/cip1) mRNA stability in transfected HepG2 cells correlates with the increased binding of an AU-rich |
| 10769631 | 7 | No | Correlation of chemopreventive efficacy data from the human epidermal cell assay with in vivo data. |
| 10770211 | 6 | No | In vivo effects of sex steroids on lymphocyte responsiveness and immunoglobulin levels in humans. |
| 10771139 | 8 | No | Activation of the hsp70 promoter by environmental inorganic and organic chemicals: relationships with cytotoxicity and lipophilicity. |
| 10772776 | 1 | No | Effect of the serine protease inhibitor N-tosyl-l-phenylalanine-chloromethyl ketone (TPCK) on MCF-7 mammary tumour cells growth and differentiation. |
| 10779383 | 1 | No | Role for early growth response-1 protein in alpha(1)-adrenergic stimulation of fibroblast growth factor-2 promoter activity in cardiac myocytes. |
| 10780527 | 1 | No | Bisphosphonates induce apoptosis in human breast cancer cell lines. |
| 10780954 | 1 | No | GLUT-1 reduces hypoxia-induced apoptosis and JNK pathway activation. |

| | | | |
|---|---|---|---|
| 10783318 | 16 | No | Suppression of cyclooxygenase-2 promoter-dependent transcriptional activity in colon cancer cells by chemopreventive agents with a resorcin-type structure. |
| 10785260 | 2 | No | Indomethacin induces apoptosis and inhibits proliferation in chronic myeloid leukemia cells. |
| 10799337 | 6 | No | Diflubenzuron, a benzoyl-urea insecticide, is a potent inhibitor of TCDD-induced CYP1A1 expression in HepG2 cells. |
| 10806356 | 2 | No | Structural organization of the human gastrointestinal glutathione peroxidase (GPX2) promoter and 3'-nontranscribed region: transcriptional response to |
| 10807402 | 3 | No | The effect of NSAIDs and a COX-2 specific inhibitor on Helicobacter pylori-induced PGE2 and HGF in human gastric fibroblasts. |
| 10811566 | 12 | No | Assaying estrogenicity by quantitating the expression levels of endogenous estrogen-regulated genes. |
| 10813102 | 5 | No | A comparative study of the effects of two oral contraceptives containing dienogest or desogestrel on the human immune system. |
| 10821422 | 1 | No | Evidence for the induction of apoptosis by endosulfan in a human T-cell leukemic line. |
| 10821843 | 16 | No | Genomic and functional changes induced by the activation of the peripheral cannabinoid receptor CB2 in the promyelocytic cells HL-60. Possible involvement |
| 10822086 | 2 | No | Effects of trovafloxacin on the IL-1-dependent activation of E-selectin in human endothelial cells in vitro. |
| 10822281 | 14 | No | Susceptibility to drug-induced apoptosis correlates with differential modulation of Bad, Bcl-2 and Bcl-xL protein levels. |
| 10833474 | 4 | No | Par-4, a proapoptotic gene, is regulated by NSAIDs in human colon carcinoma cells. |
| 10836612 | 8 | No | Alterations of extracellular matrix induced by tobacco smoke extract. |
| 10843175 | 18 | No | Estrogens exert route- and dose-dependent effects on insulin-like growth factor (IGF)-binding protein-3 and the acid-labile subunit of the IGF ternary complex. |
| 10843967 | 4 | No | Neutrophil elastase induces MUC5AC messenger RNA expression by an oxidant-dependent mechanism. |
| 10851300 | 1 | No | Therapeutic potential of curcumin in human prostate cancer. II. Curcumin inhibits tyrosine kinase activity of epidermal growth factor receptor and depletes the |
| 10854229 | 1 | No | Hypoxia-inducible angiopoietin-2 expression is mimicked by iodonium compounds and occurs in the rat brain and skin in response to systemic hypoxia and tissue |
| 10856522 | 12 | No | Glycation amplifies lipoprotein(a)-induced alterations in the generation of fibrinolytic regulators from human vascular endothelial cells. |

| | | | |
|---|---|---|---|
| 10866311 | 4 | No | Regrowth of 5-fluorouracil-treated human colon cancer cells is prevented by the combination of interferon gamma, indomethacin, and phenylbutyrate. |
| 10866818 | 4 | No | Mechanisms of induction of human tissue inhibitor of metalloproteinases-1 (TIMP-1) gene expression by all-trans retinoic acid in combination with basic |
| 10866999 | 29 | No | Two opposing effects of non-steroidal anti-inflammatory drugs on the expression of the inducible cyclooxygenase. Mediation through different signaling pathways. |
| 10874123 | 1 | No | Multiple levels of regulation of selenoprotein biosynthesis revealed from the analysis of human glioma cell lines. |
| 10875257 | 1 | No | 1alpha,25-dihydroxyvitamin D3 inhibits prostate cancer cell growth by androgen-dependent and androgen-independent mechanisms. |
| 10877050 | 1 | No | Theophylline, pentostatin (Nipent), and chlorambucil: a dose-escalation study targeting intrinsic biologic resistance mechanisms in patients with relapsed |
| 10883730 | 16 | No | Effects of selected herbicides on cytokine production in vitro. |
| 10890505 | 5 | No | Hydroquinone inhibits PMA-induced activation of NFkappaB in primary human CD19+ B lymphocytes. |
| 10898742 | 3 | No | Iron-ascorbate alters the efficiency of Caco-2 cells to assemble and secrete lipoproteins. |
| 10899481 | 2 | No | Evidence that treatment with monophasic oral contraceptive formulations containing ethinylestradiol plus gestodene reduces bone resorption in young |
| 10904086 | 2 | No | 15-LOX-1: a novel molecular target of nonsteroidal anti-inflammatory drug-induced apoptosis in colorectal cancer cells. |
| 10905503 | 3 | No | DNA damage and activation of c-ras in human embryo lung cells exposed to chrysotile and cigarette smoking solution. |
| 10910034 | 1 | No | Exisulind induction of apoptosis involves guanosine 3',5'-cyclic monophosphate phosphodiesterase inhibition, protein kinase G activation, and attenuated |
| 10910098 | 7 | No | p53-mediated differentiation of the erythroleukemia cell line K562. |
| 10914799 | 6 | No | Phenytoin and cyclosporin A suppress the expression of MMP-1, TIMP-1, and cathepsin L, but not cathepsin B in cultured gingival fibroblasts. |
| 10919358 | 4 | No | Transcriptional induction of Nur77 by indomethacin that results in apoptosis of colon cancer cells. |
| 10920275 | 4 | No | Suppression by flavonoids of cyclooxygenase-2 promoter-dependent transcriptional activity in colon cancer cells: structure-activity relationship. |
| 10928463 | 6 | No | A randomized cross-over study on the effects of levonorgestrel- and desogestrel-containing oral contraceptives on the anticoagulant pathways. |

| | | | |
|---|---|---|---|
| 10950785 | 12 | No | Expression of the chemokine receptors CXCR1 and CXCR2 on granulocytes in human endotoxemia and tuberculosis: involvement of the p38 mitogen-activated protein |
| 10950852 | 2 | No | Induction of UDP-glucuronosyltransferase UGT1A1 by the flavonoid chrysin in the human hepatoma cell line hep G2. |
| 10951577 | 14 | No | Redox regulation of p53 during hypoxia. |
| 10952721 | 6 | No | Residual type 1 immunity in patients genetically deficient for interleukin 12 receptor beta1 (IL-12Rbeta1): evidence for an IL-12Rbeta1-independent pathway of |
| 10959719 | 1 | No | Oral ethinyl estradiol, but not transdermal 17beta-estradiol, increases plasma C-reactive protein levels in men. |
| 10969820 | 2 | No | Deregulated manganese superoxide dismutase expression and resistance to oxidative injury in p53-deficient cells. |
| 11005569 | 1 | No | Indomethacin-induced apoptosis in esophageal adenocarcinoma cells involves upregulation of Bax and translocation of mitochondrial cytochrome C independent |
| 11008122 | 10 | No | Profiles of antioxidant/electrophile response element (ARE/EpRE) nuclear protein binding and c-Ha-ras transactivation in vascular smooth muscle cells treated with |
| 11024006 | 10 | No | Anti-inflammatory effects of sodium butyrate on human monocytes: potent inhibition of IL-12 and up-regulation of IL-10 production. |
| 11031321 | 21 | No | Il-1-related cytokine responses of nonimmune skin cells subjected to CEES exposure with and without potential vesicant antagonists. |
| 11034997 | 1 | No | Hyperoxia inhibits oxidant-induced apoptosis in lung epithelial cells. |
| 11038160 | 4 | No | CYP2A5/CYP2A6 expression in mouse and human hepatocytes treated with various in vivo inducers. |
| 11041225 | 4 | No | The effect of progestins on vascular endothelial growth factor, oestrogen receptor and progesterone receptor immunoreactivity and endothelial cell density |
| 11046123 | 8 | No | Inhibition of endothelial cell activation by nitric oxide donors. |
| 11052920 | 6 | No | Effect of ciprofloxacin on the activation of the transcription factors nuclear factor kappaB, activator protein-1 and nuclear factor-interleukin-6, and |
| 11056131 | 2 | No | Effect of the Yuzpe regimen of emergency contraception on markers of endometrial receptivity. |
| 11059779 | 3 | No | Ligand-independent activation of the androgen receptor by the differentiation agent butyrate in human prostate cancer cells. |
| 11062132 | 5 | No | Role of BAX in the apoptotic response to anticancer agents. |

| | | | |
|---|---|---|---|
| 11064002 | 3 | No | Different cell death mechanisms and gene expression in human cells induced by pentachlorophenol and its major metabolite, tetrachlorohydroquinone. |
| 11080718 | 1 | No | Down-regulation of IL-12 by topical corticosteroids in chronic atopic dermatitis. |
| 11090257 | 1 | No | Docosahexaenoic acid is a potent inducer of apoptosis in HT-29 colon cancer cells. |
| 11093808 | 8 | No | Growth-suppressive effect of non-steroidal anti-inflammatory drugs on 11 colon-cancer cell lines and fluorescence differential display of genes whose |
| 11095261 | 1 | No | Chemosensitisation of malignant melanoma by BCL2 antisense therapy. |
| 11095476 | 4 | No | Influence of the M235T polymorphism of human angiotensinogen (AGT) on plasma AGT and renin concentrations after ethinylestradiol administration. |
| 11099651 | 2 | No | Toxicogenomics-based discrimination of toxic mechanism in HepG2 human hepatoma cells. |
| 11108730 | 2 | No | Modulation of hepatic lipoprotein synthesis and secretion by taxifolin, a plant flavonoid. |
| 11110672 | 1 | No | Functional expression of CCR1, CCR3, CCR4, and CXCR4 chemokine receptors on human platelets. |
| 11110848 | 3 | No | Indole-3-carbinol is a negative regulator of estrogen receptor-alpha signaling in human tumor cells. |
| 11127411 | 1 | No | Individual variability in the zinc inducibility of metallothionein-IIA mRNA in human lymphocytes. |
| 11131300 | 3 | No | Histamine up-regulates phosphodiesterase 4 activity and reduces prostaglandin E2-inhibitory effects in human neutrophils. |
| 11134551 | 2 | No | Effects of phenytoin on glutathione status and oxidative stress biomarker gene mRNA levels in cultured precision human liver slices. |
| 11134558 | 32 | No | Application of cDNA microarray to the study of arsenic-induced liver diseases in the population of Guizhou, China. |
| 11134660 | 1 | No | Indirubin inhibits inflammatory reactions in delayed-type hypersensitivity. |
| 11135700 | 3 | No | [An experimental study on arsenic trioxide-selectively induced human hepatocarcinoma cell lines apoptosis and its related genes] |
| 11139446 | 8 | No | Human renal mesangial cells are a target for the anti-inflammatory action of 9-cis retinoic acid. |
| 11145710 | 8 | No | Actin filaments are involved in the regulation of trafficking of two closely related chemokine receptors, CXCR1 and CXCR2. |

| | | | |
|---|---|---|---|
| 11147995 | 1 | No | Functional and histochemical analysis of MDR3 P-glycoprotein in a tetracycline-controlled gene expression system. |
| 11154737 | 1 | No | NRH:quinone oxidoreductase2 (NQO2). |
| 11159045 | 8 | No | Vanadium stimulates human bronchial epithelial cells to produce heparin-binding epidermal growth factor-like growth factor: a mitogen for lung fibroblasts. |
| 11162468 | 3 | No | Maf genes are involved in multiple stress response in human. |
| 11163531 | 4 | No | Epinephrine upregulates superoxide dismutase in human coronary artery endothelial cells. |
| 11168427 | 7 | No | Metallothionein isogene transcription in red blood cell precursors from human cord blood. |
| 11171960 | 2 | No | Role of transforming growth factor-alpha in von Hippel--Lindau (VHL)(-/-) clear cell renal carcinoma cell proliferation: a possible mechanism coupling VHL tumor |
| 11172797 | 6 | No | Effects of two oral contraceptives on plasma levels of insulin-like growth factor I (IGF-I) and growth hormone (hGH). |
| 11181447 | 2 | No | Changes in DNA 8-hydroxyguanine levels, 8-hydroxyguanine repair activity, and hOGG1 and hMTH1 mRNA expression in human lung alveolar epithelial cells induced |
| 11198217 | 5 | No | Chrysotile-induced cell transformation and transcriptional changes of c-myc oncogene in human embryo lung cells. |
| 11226373 | 6 | No | Induction of cytochrome P450 1A1 gene expression, oxidative stress, and genotoxicity by carbaryl and thiabendazole in transfected human HepG2 and |
| 11227219 | 15 | No | Suicidal differential housekeeping gene activity in apoptosis induced by DCNP. |
| 11228061 | 10 | No | Hormonal and clinical effects of GnRH agonist alone, or in combination with a combined oral contraceptive or flutamide in women with severe hirsutism. |
| 11230554 | 4 | No | Effects of styrene-7,8-oxide over p53, p21, bcl-2 and bax expression in human lymphocyte cultures. |
| 11236827 | 24 | No | Polyphyenolics increase t-PA and u-PA gene transcription in cultured human endothelial cells. |
| 11237475 | 4 | No | Indomethacin increases 15-PGDH mRNA expression in HL60 cells differentiated by PMA. |
| 11237771 | 5 | No | Amphiregulin is a vitamin D3 target gene in squamous cell and breast carcinoma. |
| 11238180 | 9 | No | Quercetin inhibits the expression and function of the androgen receptor in LNCaP prostate cancer cells. |

| | | | |
|---|---|---|---|
| 11238657 | 36 | No | Histamine induces exocytosis and IL-6 production from human lung macrophages through interaction with H1 receptors. |
| 11245553 | 4 | No | A 1-year pharmacokinetic investigation of a novel oral contraceptive containing drospirenone in healthy female volunteers. |
| 11250057 | 7 | No | Effects of crocidolite asbestos on human bronchoepithelial-dependent fibroblast stimulation in coculture: the role of IL-6 and GM-CSF. |
| 11257428 | 4 | No | Induction of cysteine string protein after chronic antidepressant treatment in rat frontal cortex. |
| 11260862 | 3 | No | Effect of the nonsteroidal anti-inflammatory drug indomethacin on proliferation and apoptosis of colon carcinoma cells. |
| 11264000 | 2 | No | Reactive oxygen species-related induction of multidrug resistance-associated protein 2 expression in primary hepatocytes exposed to sulforaphane. |
| 11266329 | 1 | No | Associations of blood levels of PCB, HCHS, and HCB with numbers of lymphocyte subpopulations, in vitro lymphocyte response, plasma cytokine levels, and |
| 11275421 | 2 | No | Induction of human NAD(P)H:quinone oxidoreductase (NQO1) gene expression by the flavonol quercetin. |
| 11287445 | 1 | No | Effects of retinoids on cancerous phenotype and apoptosis in organotypic cultures of ovarian carcinoma. |
| 11288978 | 1 | No | Amiodarone stimulates interleukin-6 production in cultured human thyrocytes, exerting cytotoxic effects on thyroid follicles in suspension culture. |
| 11295360 | 15 | No | Variants of peroxiredoxins expression in response to hydroperoxide stress. |
| 11297035 | 4 | No | Platelet CD40 ligand (CD40L)--subcellular localization, regulation of expression, and inhibition by clopidogrel. |
| 11297599 | 2 | No | Overexpression of glutathione-S-transferase A1 in benign adrenocortical adenomas from patients with Cushing's syndrome. |
| 11304534 | 2 | No | Characterization of a novel airway epithelial cell-specific short chain alcohol dehydrogenase/reductase gene whose expression is up-regulated by retinoids and is |
| 11306145 | 25 | No | Inhibition of effects of endogenously synthesized histamine disturbs in vitro human dendritic cell differentiation. |
| 11306435 | 37 | No | Benzene-extracted components are important for the major activity of diesel exhaust particles: effect on interleukin-8 gene expression in human bronchial |
| 11306982 | 2 | No | Regulation of IL-5 receptor on eosinophil progenitors in allergic inflammation: role of retinoic acid. |
| 11307925 | 2 | No | Aberrant expression of cyclin D1 in pulmonary proliferative lesions induced by high doses of urethane in transgenic mice carrying the human prototype c-H-ras |

| | | | |
|---|---|---|---|
| 11313354 | 4 | No | Human alveolar macrophages and granulocyte-macrophage colony-stimulating factor-induced monocyte-derived macrophages are resistant to H2O2 via their high |
| 11313997 | 4 | No | The nonsteroidal anti-inflammatory drugs aspirin and indomethacin attenuate beta-catenin/TCF-4 signaling. |
| 11318944 | 2 | No | Regulation of renal proximal tubular epithelial cell hyaluronan generation: implications for diabetic nephropathy. |
| 11322385 | 8 | No | Alternative activation of extracellular signal-regulated protein kinases in curcumin and arsenite-induced HSP70 gene expression in human colorectal carcinoma |
| 11322924 | 6 | No | Effects of structurally related flavonoids on cell cycle progression of human melanoma cells: regulation of cyclin-dependent kinases CDK2 and CDK1. |
| 11331069 | 7 | No | Protein kinase C-mediated down-regulation of MDR3 mRNA expression in Chang liver cells. |
| 11338295 | 1 | No | Effect of nicotine on fibroblast beta 1 integrin expression and distribution in vitro. |
| 11342237 | 2 | No | SeO(2) induces apoptosis with down-regulation of Bcl-2 and up-regulation of P53 expression in both immortal human hepatic cell line and hepatoma cell line. |
| 11343241 | 22 | No | Intracellular signaling pathways involved in acetaldehyde-induced collagen and fibronectin gene expression in human hepatic stellate cells. |
| 11344824 | 16 | No | Cytokine response and oxidative stress produced by ethanol, acetaldehyde and endotoxin treatment in HepG2 cells. |
| 11346484 | 2 | No | Protective effects of coffee diterpenes against aflatoxin B1-induced genotoxicity: mechanisms in rat and human cells. |
| 11352223 | 1 | No | Indomethacin, a cox inhibitor, enhances 15-PGDH and decreases human tumoral C cells proliferation. |
| 11352631 | 4 | No | Up-regulation of keratinocyte growth factor and receptor: a possible mechanism of action of phenytoin in wound healing. |
| 11356643 | 6 | No | Homocysteine induces monocyte chemoattractant protein-1 expression by activating NF-kappaB in THP-1 macrophages. |
| 11358810 | 10 | No | Differential regulation of cytochrome P450 1A1 and 1B1 by a combination of dioxin and pesticides in the breast tumor cell line MCF-7. |
| 11360202 | 6 | No | Repression of GADD153/CHOP by NF-kappaB: a possible cellular defense against endoplasmic reticulum stress-induced cell death. |
| 11368622 | 6 | No | Evaluation of estrogenic activity of plant extracts for the potential treatment of menopausal symptoms. |
| 11369509 | 2 | No | Increased susceptibility of copper-deficient neuroblastoma cells to oxidative stress-mediated apoptosis. |

| | | | |
|---|---|---|---|
| 11369638 | 1 | No | Prostaglandin E(2) is a selective inducer of interleukin-12 p40 (IL-12p40) production and an inhibitor of bioactive IL-12p70 heterodimer. |
| 11372388 | 6 | No | [Zafirlukast inhibition of leukotriene C4 induced endothelin-1 expression in human airway structural cell] |
| 11378265 | 1 | No | UCN-01 induces cytotoxicity toward human CLL cells through a p53-independent mechanism. |
| 11401523 | 1 | No | CaT1 expression correlates with tumor grade in prostate cancer. |
| 11403239 | 3 | No | Interleukin-6 expression by osteoblast-like MG63 cells challenged with four acrylic bone cements. |
| 11406566 | 6 | No | Nonsteroidal anti-inflammatory drugs induce apoptosis in esophageal cancer cells by restoring 15-lipoxygenase-1 expression. |
| 11408547 | 2 | No | Arsenic induces expression of the multidrug resistance-associated protein 2 (MRP2) gene in primary rat and human hepatocytes. |
| 11409192 | 9 | No | Transcriptional activation of stress genes and cytotoxicity in human liver carcinoma cells (HepG2) exposed to 2,4,6-trinitrotoluene, 2,4-dinitrotoluene, and |
| 11409902 | 4 | No | Cloning of the human cholesteryl ester hydrolase promoter: identification of functional peroxisomal proliferator-activated receptor responsive elements. |
| 11410519 | 10 | No | Interleukin-6 induces androgen responsiveness in prostate cancer cells through up-regulation of androgen receptor expression. |
| 11414687 | 27 | No | Quercetin inhibits matrix metalloproteinase-1 expression in human vascular endothelial cells through extracellular signal-regulated kinase. |
| 11424089 | 22 | No | Role of protein kinase Cdelta in transmitting hypoxia signal to HSF and HIF-1. |
| 11424952 | 1 | No | Estrogens, apoptosis and cells of neural origin. |
| 11425850 | 5 | No | The human type 2 iodothyronine deiodinase is a selenoprotein highly expressed in a mesothelioma cell line. |
| 11428619 | 4 | No | Effects of CEES on inflammatory mediators, heat shock protein 70A, histology and ultrastructure in two skin models. |
| 11428621 | 2 | No | Effects of sulfur mustard on the basal cell adhesion complex. |
| 11428642 | 5 | No | Calmodulin, poly(ADP-ribose)polymerase and p53 are targets for modulating the effects of sulfur mustard. |
| 11428645 | 4 | No | Response of normal human keratinocytes to sulfur mustard: cytokine release. |

| | | | |
|---|---|---|---|
| 11428648 | 1 | No | Exposure of human epidermal keratinocyte cell cultures to sulfur mustard promotes binding of complement C1q: implications for toxicity and medical countermeasures. |
| 11437639 | 2 | No | Mercuric ion attenuates nuclear factor-kappaB activation and DNA binding in normal rat kidney epithelial cells: implications for mercury-induced |
| 11438525 | 1 | No | Silencer activity of NFATc2 in the interleukin-12 receptor beta 2 proximal promoter in human T helper cells. |
| 11441113 | 2 | No | Functional expression of IL-12 receptor by human eosinophils: IL-12 promotes eosinophil apoptosis. |
| 11441983 | 1 | No | Singlet oxygen inhibits agonist-induced P-selectin expression and formation of platelet aggregates. |
| 11442279 | 2 | No | Induction of UDP-glucuronosyltransferase UGT1A1 by the flavonoid chrysin in Caco-2 cells--potential role in carcinogen bioinactivation. |
| 11442481 | 1 | No | Protein S levels are lower in women receiving desogestrel-containing combined oral contraceptives (COCs) than in women receiving levonorgestrel-containing COCs |
| 11448926 | 8 | No | Destabilization of steroid receptors by heat shock protein 90-binding drugs: a ligand-independent approach to hormonal therapy of breast cancer. |
| 11454723 | 14 | No | The use of a high-volume screening procedure to assess the effects of dietary flavonoids on human cyp1a1 expression. |
| 11465111 | 5 | No | Immunobiology of CD28 expression on human neutrophils. I. CD28 regulates neutrophil migration by modulating CXCR-1 expression. |
| 11468187 | 24 | No | The potential of iron chelators of the pyridoxal isonicotinoyl hydrazone class as effective antiproliferative agents, IV: The mechanisms involved in inhibiting |
| 11472980 | 4 | No | Geldanamycin inhibits NF-kappaB activation and interleukin-8 gene expression in cultured human respiratory epithelium. |
| 11478917 | 3 | No | Discovery of a novel compound: insight into mechanisms for acrylamide-induced axonopathy and colchicine-induced apoptotic neuronal cell death. |
| 11485391 | 28 | No | Keratinocyte differentiation marker suppression by arsenic: mediation by AP1 response elements and antagonism by tetradecanoylphorbol acetate. |
| 11489473 | 3 | No | Effects of Matrine on proliferation and differentiation in K-562 cells. |
| 11490238 | 1 | No | Safety and efficacy of exisulind for treatment of recurrent prostate cancer after radical prostatectomy. |
| 11494140 | 9 | No | Detection of differentially expressed genes in human colon carcinoma cells treated with a selective COX-2 inhibitor. |
| 11500943 | 2 | No | Iron-induced oxidative stress up-regulates calreticulin levels in intestinal epithelial (Caco-2) cells. |

| | | | |
|---|---|---|---|
| 11502892 | 4 | No | Estrogen receptor alpha mediates the proliferative but not the cytotoxic dose-dependent effects of two major phytoestrogens on human breast cancer cells. |
| 11504687 | 9 | No | Nickel requires hypoxia-inducible factor-1 alpha, not redox signaling, to induce plasminogen activator inhibitor-1. |
| 11506819 | 2 | No | Dietary supplementation with the anti-tumour promoter quercetin: its effects on matrix metalloproteinase gene regulation. |
| 11507085 | 5 | No | p21(WAF1/cip1) is an important determinant of intestinal cell response to sulindac in vitro and in vivo. |
| 11507245 | 6 | No | ATM status confers sensitivity to arsenic cytotoxic effects. |
| 11514583 | 1 | No | Regulation of the hypoxia-inducible factor 1alpha by the inflammatory mediators nitric oxide and tumor necrosis factor-alpha in contrast to desferroxamine and |
| 11522280 | 2 | No | Do wine polyphenols modulate p53 gene expression in human cancer cell lines? |
| 11527995 | 8 | No | Regulation of interleukin-8 gene expression after phagocytosis of zymosan by human monocytic cells. |
| 11531949 | 2 | No | Down-regulation of CXCR1 and CXCR2 expression on human neutrophils upon activation of whole blood by S. aureus is mediated by TNF-alpha. |
| 11545736 | 7 | No | Transcription factor MIZ-1 is regulated via microtubule association. |
| 11551516 | 1 | No | Relationships between non-occupational cadmium exposure and expression of nine cytochrome P450 forms in human liver and kidney cortex samples. |
| 11556524 | 12 | No | Increased histidine decarboxylase expression during in vitro monocyte maturation; a possible role of endogenously synthesised histamine in monocyte/macrophage |
| 11559547 | 6 | No | Transcriptional regulation of the estrogen-inducible pS2 breast cancer marker gene by the ERR family of orphan nuclear receptors. |
| 11559570 | 2 | No | Sulindac sulfide-induced apoptosis involves death receptor 5 and the caspase 8-dependent pathway in human colon and prostate cancer cells. |
| 11564622 | 1 | No | Green fluorescent protein (GFP) as a marker of aryl hydrocarbon receptor (AhR) function in developing zebrafish (Danio rerio). |
| 11566570 | 23 | No | The use of genomics technology to investigate gene expression changes in cultured human liver cells. |
| 11580929 | 12 | No | Effects of natural products and nutraceuticals on steroid hormone-regulated gene expression. |
| 11583285 | 2 | No | Therapeutic implications of the kinetics of immunomodulation during single or combined treatment of melanoma patients with dacarbazine and interferon-alpha. |

| | | | |
|---|---|---|---|
| 11585056 | 5 | No | Suppression of c-myc expression and c-Myc function in response to sustained DNA damage in MCF-7 breast tumor cells. |
| 11589617 | 1 | No | Studies of apoptosis of malignant lymphoma cells induced by arsenic trioxide. |
| 11592964 | 5 | No | Potent inhibition of NFAT activation and T cell cytokine production by novel low molecular weight pyrazole compounds. |
| 11593435 | 2 | No | Dysregulation of integrin-linked kinase (ILK) signaling in colonic polyposis. |
| 11595463 | 8 | No | NSAIDs inhibit the activation of egr-1 gene in microvascular endothelial cells. A key to inhibition of angiogenesis? |
| 11602670 | 4 | No | Cyclic GMP-dependent protein kinase activation and induction by exisulind and CP461 in colon tumor cells. |
| 11603923 | 3 | No | Reactive oxygen species mediate cyclic strain-induced endothelin-1 gene expression via Ras/Raf/extracellular signal-regulated kinase pathway in |
| 11606380 | 6 | No | Estrogenic and antiestrogenic properties of resveratrol in mammary tumor models. |
| 11673643 | 4 | No | Induction of glutathione synthesis in human keratinocytes by Ginkgo biloba extract (EGb761). |
| 11675267 | 4 | No | Effects of chloro-s-triazine herbicides and metabolites on aromatase activity in various human cell lines and on vitellogenin production in male carp hepatocytes. |
| 11675354 | 2 | No | Caspase 8 activation independent of Fas (CD95/APO-1) signaling may mediate killing of B-chronic lymphocytic leukemia cells by cytotoxic drugs or gamma |
| 11675405 | 47 | No | Activation of beta(2)-adrenoceptor prevents shiga toxin 2-induced TNF-alpha gene transcription. |
| 11678611 | 24 | No | Atrazine potentiation of arsenic trioxide-induced cytotoxicity and gene expression in human liver carcinoma cells (HepG2). |
| 11679177 | 1 | No | Decrease of cyclin D1 in the human lung adenocarcinoma cell line A-427 by 7-hydroxycoumarin. |
| 11689710 | 3 | No | Transcriptional induction of MKP-1 in response to stress is associated with histone H3 phosphorylation-acetylation. |
| 11693492 | 5 | No | Acute exposure to arsenite induces metallothionein isoform-specific gene expression in human proximal tubule cells. |
| 11693576 | 8 | No | Estrogen-like properties of brominated analogs of bisphenol A in the MCF-7 human breast cancer cell line. |
| 11695219 | 2 | No | Kepone (chlordecone) disrupts adherens junctions in human breast epithelial cells cultured on matrigel. |

| | | | |
|---|---|---|---|
| 11697128 | 4 | No | Oxidized forms of peroxiredoxins and DJ-1 on two-dimensional gels increased in response to sublethal levels of paraquat. |
| 11698162 | 3 | No | Mechanisms of isocyanate sensitisation. An in vitro approach. |
| 11698246 | 44 | No | Combined modulation of the mesangial machinery for monocyte recruitment by inhibition of NF-kappaB. |
| 11699414 | 2 | No | Bcl-2 antisense oligonucleotides enhance the cytotoxicity of chlorambucil in B-cell chronic lymphocytic leukaemia cells. |
| 11710721 | 2 | No | Oxidative stress in scleroderma: maintenance of scleroderma fibroblast phenotype by the constitutive up-regulation of reactive oxygen species generation through |
| 11714746 | 7 | No | Arsenic inhibition of telomerase transcription leads to genetic instability. |
| 11717198 | 1 | No | CD16+ and CD16- human blood monocyte subsets differentiate in vitro to dendritic cells with different abilities to stimulate CD4+ T cells. |
| 11719597 | 6 | No | Matrix metalloproteinase (MMP)-2 and MMP-9 and their inhibitor, TIMP-1, in human term decidua and fetal membranes: the effect of prostaglandin F(2alpha) and |
| 11720199 | 4 | No | Effects of two oral contraceptives, containing 30 or 20 microg of ethinyl estradiol in combination with gestodene, on blood coagulation and fibrinolysis in |
| 11721434 | 1 | No | [Study of apoptosis induced by indomethacin in chronic myeloid leukemia] |
| 11723236 | 8 | No | Impact of extracellular folate levels on global gene expression. |
| 11723248 | 3 | No | Transcriptional control of intestinal cytochrome P-4503A by 1alpha,25-dihydroxy vitamin D3. |
| 11724761 | 2 | No | allergen exposure or spontaneous worsening of asthma. We investigated the expression of IL-5 and IL-5Ralpha receptor in circulating CD34(+) progenitor |
| 11733402 | 6 | No | Sex steroids used in hormonal treatment increase vascular procoagulant activity by inducing thrombin receptor (PAR-1) expression: role of the glucocorticoid |
| 11735124 | 6 | No | NF-kappaB activation for constitutive expression of VCAM-1 and ICAM-1 on B lymphocytes and plasma cells. |
| 11738272 | 5 | No | Transient induction of metallothionein isoform 3 (MT-3), c-fos, c-jun and c-myc in human proximal tubule cells exposed to cadmium. |
| 11739238 | 7 | No | (IRF)-1 in human umbilical vein endothelial cells (HUVECs). 2. LPS stimulated a time- and concentration-dependent increase in IRF-1 protein expression, an effect |
| 11739494 | 11 | No | Regulation and possible function of beta-catenin in human monocytes. |

| | | | |
|---|---|---|---|
| 11741290 | 3 | No | Camptothecin and Zeocin can increase p53 levels during all cell cycle stages. |
| 11742226 | 1 | No | Glyceryl trinitrate treatment up-regulates soluble guanylyl cyclase in rat dura mater. |
| 11752201 | 7 | No | Aryl hydrocarbon receptor mediates sensitivity of MCF-7 breast cancer cells to antitumor agent 2-(4-amino-3-methylphenyl) benzothiazole. |
| 11753679 | 11 | No | Tumor necrosis factor-alpha induces the expression of DR6, a member of the TNF receptor family, through activation of NF-kappaB. |
| 11755377 | 8 | No | Induction of endothelin-1 expression by oxidative stress in vascular smooth muscle cells. |
| 11756231 | 7 | No | Indomethacin induces differential expression of beta-catenin, gamma-catenin and T-cell factor target genes in human colorectal cancer cells. |
| 11762555 | 1 | No | Pharmacodynamics and pharmacokinetics of omapatrilat in heart failure. |
| 11766126 | 2 | No | The role of interleukin-6 (IL-6) in human sulfur mustard (HD) toxicology. |
| 11774253 | 7 | No | Mitotic arrest induced by XK469, a novel antitumor agent, is correlated with the inhibition of cyclin B1 ubiquitination. |
| 11775218 | 7 | No | Overexpression of Bcl-2 partly inhibits apoptosis of human cervical cancer SiHa cells induced by arsenic trioxide. |
| 11776067 | 1 | No | Effect of selenium on human myocardial glutathione peroxidase gene expression. |
| 11779194 | 8 | No | Proteasome inhibitors activate the transcription factors C/EBP-beta and delta in human intestinal epithelial cells. |
| 11779855 | 10 | No | Doxorubicin induces apoptosis and CD95 gene expression in human primary endothelial cells through a p53-dependent mechanism. |
| 11780464 | 1 | No | Apoptosis inducing effects of arsenic trioxide on human bladder cancer cell line BIU-87. |
| 11781168 | 10 | No | The effect of ozone exposure on the ability of human surfactant protein a variants to stimulate cytokine production. |
| 11790449 | 7 | No | Effect of flavonoids on cell cycle progression in prostate cancer cells. |
| 11793227 | 115 | No | Gene expression changes associated with cytotoxicity identified using cDNA arrays. |
| 11800023 | 3 | No | Stereospecific antitumor activity of radicicol oxime derivatives. |

| | | | |
|---|---|---|---|
| 11811539 | 2 | No | DJ-1 is an indicator for endogenous reactive oxygen species elicited by endotoxin. |
| 11813266 | 3 | No | Sodium arsenite suppresses human papillomavirus-16 E6 gene and enhances apoptosis in E6-transfected human lymphoblastoid cells. |
| 11820781 | 15 | No | Induction of multidrug resistance proteins MRP1 and MRP3 and gamma-glutamylcysteine synthetase gene expression by nonsteroidal |
| 11821714 | 8 | No | The regulation of human vascular smooth muscle extracellular matrix protein production by alpha- and beta-adrenoceptor stimulation. |
| 11825526 | 2 | No | [Effects of inorganic arsenicals on the methylation of p16 gene CpG islands and the expression of p16 gene in BEP2D cells] |
| 11828994 | 1 | No | Sulindac inhibited gene expression and activity of arylamine N-acetyltransferase and DNA-2-aminofluorene adduct formation in T24 human bladder tumor cells. |
| 11830524 | 31 | No | Identification of molecular targets associated with selenium-induced growth inhibition in human breast cells using cDNA microarrays. |
| 11835400 | 4 | No | Arsenite stimulates cyclooxygenase-2 expression through activating IkappaB kinase and nuclear factor kappaB in primary and ECV304 endothelial cells. |
| 11836020 | 12 | No | Influence of redox-active compounds and PXR-activators on human MRP1 and MRP2 gene expression. |
| 11842940 | 2 | No | Keratinocyte growth factor (KGF)-1 and -2 protein and gene expression in human gingival fibroblasts. |
| 11845989 | 7 | No | Androgen sensitivity related proteins in hormone-sensitive and hormone-insensitive prostate cancer cell lines treated by androgen antagonist |
| 11849738 | 8 | No | Induction of CYP1A1 and CYP1A2 expressions by prototypic and atypical inducers in the human lung. |
| 11853877 | 3 | No | Novobiocin is a novel inducer of CD38 on cells of the myelomonocytic lineage. |
| 11854442 | 11 | No | Regulation of cyclooxygenase-2 expression by phospholipase D in human amnion-derived WISH cells. |
| 11855844 | 14 | No | Bisphosphonates pamidronate and zoledronic acid stimulate osteoprotegerin production by primary human osteoblasts. |
| 11859000 | 2 | No | The synthesis and selective IL-2 inhibitory activity of bis piperazine-phenol Mannich adducts. |
| 11861401 | 10 | No | GATA-6 transcriptional regulation of 15-lipoxygenase-1 during NSAID-induced apoptosis in colorectal cancer cells. |
| 11861792 | 2 | No | Leukotriene B4 production in human mononuclear phagocytes is modulated by interleukin-4-induced 15-lipoxygenase. |

| | | | |
|---|---|---|---|
| 11864778 | 4 | No | Flavonoids increase the intracellular glutathione level by transactivation of the gamma-glutamylcysteine synthetase catalytical subunit promoter. |
| 11867264 | 14 | No | Effects of the environmental estrogens bisphenol A, o,p'-DDT, p-tert-octylphenol and coumestrol on apoptosis induction, cell proliferation and the expression of |
| 11872090 | 2 | No | CXCR4 expression on monocytes is up-regulated by dexamethasone and is modulated by autologous CD3+ T cells. |
| 11875189 | 10 | No | Transcriptional and physiological responses of HepG2 cells exposed to diethyl maleate: time course analysis. |
| 11875746 | 1 | No | Anti-angiogenic effects of the thienopyridine SR 25989 in vitro and in vivo in a murine pulmonary metastasis model. |
| 11877046 | 1 | No | [p15 gene expression in acute lymphoblastic leukemia cell line Molt4 induced by arsenic trioxide] |
| 11877450 | 6 | No | Kaurane diterpene, kamebakaurin, inhibits NF-kappa B by directly targeting the DNA-binding activity of p50 and blocks the expression of antiapoptotic NF-kappa B |
| 11878821 | 1 | No | Metallothionein protects retinal pigment epithelial cells against apoptosis and oxidative stress. |
| 11882518 | 1 | No | Shedding of TNF-alpha receptors, blood pressure, and insulin sensitivity in type 2 diabetes mellitus. |
| 11884474 | 1 | No | Pathogenesis of murine experimental allergic rhinitis: a study of local and systemic consequences of IL-5 deficiency. |
| 11888521 | 21 | No | Inhibition of TNF-alpha induced ICAM-1, VCAM-1 and E-selectin expression by selenium. |
| 11893605 | 13 | No | Proteasome inhibitors induce heat shock response and increase IL-6 expression in human intestinal epithelial cells. |
| 11896290 | 13 | No | Acetaminophen-induced proliferation of estrogen-responsive breast cancer cells is associated with increases in c-myc RNA expression and NF-kappaB activity. |
| 11906190 | 216 | No | Expression profile analysis of colon cancer cells in response to sulindac or aspirin. |
| 11907703 | 4 | No | Oral contraceptives moderately effect bone resorption markers and serum-soluble interleukin-6 receptor concentrations. |
| 11909699 | 19 | No | Redox-sensitive interaction between KIAA0132 and Nrf2 mediates indomethacin-induced expression of gamma-glutamylcysteine synthetase. |
| 11911975 | 4 | No | Resistance to diverse apoptotic triggers in multidrug resistant HL60 cells and its possible relationship to the expression of P-glycoprotein, Fas and of the |
| 11912124 | 2 | No | Requirement of BAX for TRAIL/Apo2L-induced apoptosis of colorectal cancers: synergism with sulindac-mediated inhibition of Bcl-x(L). |

| | | | |
|---|---|---|---|
| 11912126 | 2 | No | Selective depletion of human DNA-methyltransferase DNMT1 proteins by sulfonate-derived methylating agents. |
| 11918509 | 4 | No | Severe interaction between ritonavir and acenocoumarol. |
| 11919081 | 6 | No | Neutrophil elastase induces MUC5AC gene expression in airway epithelium via a pathway involving reactive oxygen species. |
| 11922773 | 17 | No | Assessment of interactions of diverse ternary mixtures in an estrogen receptor-alpha reporter assay. |
| 11925459 | 8 | No | Selenite and selenomethionine promote HL-60 cell cycle progression. |
| 11925476 | 5 | No | Diallyl disulfide (DADS) induces the antitumorigenic NSAID-activated gene (NAG-1) by a p53-dependent mechanism in human colorectal HCT 116 cells. |
| 11927840 | 1 | No | Variation in coumarin 7-hydroxylase activity associated with genetic polymorphism of cytochrome P450 2A6 and the body status of iron stores in adult Thai males and |
| 11948457 | 8 | No | PPARgamma-mediated antineoplastic effect of NSAID sulindac on human oral squamous carcinoma cells. |
| 11950692 | 12 | No | Role of mitochondrial oxidant generation in endothelial cell responses to hypoxia. |
| 11956172 | 2 | No | A glucocorticoid-responsive mutant androgen receptor exhibits unique ligand specificity: therapeutic implications for androgen-independent prostate cancer. |
| 11960367 | 8 | No | Induction of human MDR1 gene expression by 2-acetylaminofluorene is mediated by effectors of the phosphoinositide 3-kinase pathway that activate NF-kappaB |
| 11961005 | 8 | No | Protein overload-induced NF-kappaB activation in proximal tubular cells requires H(2)O(2) through a PKC-dependent pathway. |
| 11967624 | 1 | No | Susceptibility factors and DNA adducts in peripheral blood mononuclear cells of aluminium smelter workers exposed to polycyclic aromatic hydrocarbons. |
| 11978551 | 5 | No | Acetaldehyde activates Jun/AP-1 expression and DNA binding activity in human oral keratinocytes. |
| 11980903 | 11 | No | Indomethacin causes prostaglandin D(2)-like and eotaxin-like selective responses in eosinophils and basophils. |
| 11983814 | 1 | No | Fructose intake increases hyperlipidemia and modifies apolipoprotein expression in apolipoprotein AI-CIII-AIV transgenic mice. |
| 11991950 | 3 | No | Expression of CYP3A4, CYP2B6, and CYP2C9 is regulated by the vitamin D receptor pathway in primary human hepatocytes. |
| 11994432 | 8 | No | Evidence for a dual mechanism for IL-10 suppression of TNF-alpha production that does not involve inhibition of p38 mitogen-activated protein kinase or NF-kappa B |

| | | | |
|---|---|---|---|
| 11994442 | 1 | No | Patterns of chemokine receptor expression on peripheral blood gamma delta T lymphocytes: strong expression of CCR5 is a selective feature of V delta 2/V |
| 11996209 | 3 | No | Effect of two oral contraceptives containing ethinylestradiol and levonorgestrel on serum and urinary surrogate markers of endothelial function. |
| 11996908 | 10 | No | Bone cell mitogenic action of fluoroaluminate and aluminum fluoride but not that of sodium fluoride involves upregulation of the insulin-like growth factor |
| 11997017 | 8 | No | Corticosteroid inhibits IL-4 signaling through down-regulation of IL-4 receptor and STAT6 activity. |
| 12002526 | 140 | No | Interactive gene expression pattern in prostate cancer cells exposed to phenolic antioxidants. |
| 12007958 | 6 | No | Activation of the nuclear transcription factor kappaB (NFkappaB) and differential gene expression in U87 glioma cells after exposure to the cytoprotector |
| 12013081 | 1 | No | Successful pregnancy in a 42-year-old woman with imminent ovarian failure following ovulation induction with ethinyl estradiol without gonadotropins and in |
| 12014631 | 3 | No | Functional repression of estrogen receptor a by arsenic trioxide in human breast cancer cells. |
| 12015078 | 1 | No | [Study of the effects of quercetin on PML gene and protein expression and localization in leukemia cells] |
| 12015082 | 3 | No | [Study on gene expression changes of arsenic sulfide treated NB4 cells] |
| 12016129 | 33 | No | IL-17 stimulates inflammatory responses via NF-kappaB and MAP kinase pathways in human colonic myofibroblasts. |
| 12016162 | 107 | No | Changes in gene expression profiles of human fibroblasts in response to sodium arsenite treatment. |
| 12032296 | 11 | No | Vitamin E succinate inhibits the function of androgen receptor and the expression of prostate-specific antigen in prostate cancer cells. |
| 12039947 | 10 | No | Phospholipase D activation by sphingosine 1-phosphate regulates interleukin-8 secretion in human bronchial epithelial cells. |
| 12040753 | 14 | No | Evaluation of gene induction of drug-metabolizing enzymes and transporters in primary culture of human hepatocytes using high-sensitivity real-time reverse |
| 12044887 | 8 | No | Flavonoids differentially regulate IFN gamma-induced ICAM-1 expression in human keratinocytes: molecular mechanisms of action. |
| 12045164 | 6 | No | Prevention of inflammation-induced endothelial dysfunction: a novel vasculo-protective action of aspirin. |
| 12047756 | 1 | No | Antibody to Cryptococcus neoformans capsular glucuronoxylomannan promotes expression of interleukin-12Rbeta2 subunit on human T cells in vitro through |

| | | | |
|---|---|---|---|
| 12054621 | 2 | No | The dietary flavonoid quercetin modulates HIF-1 alpha activity in endothelial cells. |
| 12062197 | 5 | No | Inhibitors of mitochondrial complex I attenuate the accumulation of hypoxia-inducible factor-1 during hypoxia in Hep3B cells. |
| 12063570 | 3 | No | Ciprofloxacin inhibits cell growth and synergises the effect of etoposide in hormone resistant prostate cancer cells. |
| 12066199 | 6 | No | Up-regulated gene expression of angiogenesis factors in post-chemotherapeutic lung cancer tissues determined by cDNA macroarray. |
| 12067895 | 4 | No | Reversal of thrombin-induced deactivation of CD39/ATPDase in endothelial cells by HMG-CoA reductase inhibition: effects on Rho-GTPase and adenosine nucleotide |
| 12072406 | 2 | No | Fibroblast growth factor-9 is an endometrial stromal growth factor. |
| 12082016 | 10 | No | High-throughput measurement of the Tp53 response to anticancer drugs and random compounds using a stably integrated Tp53-responsive luciferase reporter. |
| 12083423 | 3 | No | Suppression of sulfur mustard-increased IL-8 in human keratinocyte cell cultures by serine protease inhibitors: implications for toxicity and medical |
| 12084592 | 3 | No | Nitroglycerin upregulates matrix metalloproteinase expression by human macrophages. |
| 12084617 | 2 | No | Acrolein causes transcriptional induction of phase II genes by activation of Nrf2 in human lung type II epithelial (A549) cells. |
| 12084619 | 2 | No | Amiodarone induces apoptosis in L-132 human lung epithelial cell line. |
| 12085989 | 2 | No | Induction of hepatic inducible nitric oxide synthase by cholesterol in vivo and in vitro. |
| 12086014 | 2 | No | Enhancement by cyclosporin A of taxol-induced apoptosis of human urinary bladder cancer cells. |
| 12105852 | 1 | No | Influence of the nitric oxide donor glyceryl trinitrate on apoptotic pathways in human colon cancer cells. |
| 12111696 | 35 | No | Mechanisms of the growth inhibitory effects of the isoflavonoid biochanin A on LNCaP cells and xenografts. |
| 12111836 | 1 | No | Specific up-regulation of GADD153/CHOP in 1-methyl-4-phenyl-pyridinium-treated SH-SY5Y cells. |
| 12112628 | 3 | No | The influence of maternal exposure to volatile organic compounds on the cytokine secretion profile of neonatal T cells. |
| 12115622 | 2 | No | Increased IL-10 production during spontaneous apoptosis of monocytes. |

| | | | |
|---|---|---|---|
| 12126518 | 1 | No | [Apoptosis of drug-resistant human ovarian carcinoma cell line 3AO/cDDP induced by arsenic trioxide and its mechanism] |
| 12126965 | 6 | No | Arsenite-induced reactive oxygen species and the repression of alpha-tocopherol in the MGC-803 cells. |
| 12127262 | 2 | No | Induction and inhibition of aromatase (CYP19) activity by various classes of pesticides in H295R human adrenocortical carcinoma cells. |
| 12130515 | 42 | No | Targeted removal of PML-RARalpha protein is required prior to inhibition of histone deacetylase for overcoming all-trans retinoic acid differentiation |
| 12144868 | 6 | No | Downregulation of COX-2 and iNOS by amentoflavone and quercetin in A549 human lung adenocarcinoma cell line. |
| 12147272 | 9 | No | CYP1A1 induction by pyridine and its metabolites in HepG2 cells. |
| 12151360 | 3 | No | Sulforaphane and its glutathione conjugate but not sulforaphane nitrile induce UDP-glucuronosyl transferase (UGT1A1) and glutathione transferase (GSTA1) in |
| 12160929 | 6 | No | Possible involvement of glutathione and p53 in trichloroethylene- and perchloroethylene-induced lipid peroxidation and apoptosis in human lung cancer |
| 12167460 | 2 | No | Effect of cyclophosphamide on gene expression of cytochromes p450 and beta-actin in the HL-60 cell line. |
| 12168835 | 4 | No | Detection of MDM2 alterations in cultured human hepatocytes treated with 17beta-estradiol or 17alpha-ethinylestradiol. |
| 12176910 | 3 | No | Role of toll-like receptor 2 (TLR2) in neutrophil activation: GM-CSF enhances TLR2 expression and TLR2-mediated interleukin 8 responses in neutrophils. |
| 12177049 | 7 | No | Modulators of inflammation use nuclear factor-kappa B and activator protein-1 sites to induce the caspase-1 and granzyme B inhibitor, proteinase inhibitor 9. |
| 12181422 | 8 | No | A77 1726 induces differentiation of human myeloid leukemia K562 cells by depletion of intracellular CTP pools. |
| 12183065 | 5 | No | Volatile organic compounds cytotoxicity and expression of HSP72, HSP90 and GRP78 stress proteins in cultured human cells. |
| 12189556 | 2 | No | Retinoids cause apoptosis in pancreatic cancer cells via activation of RAR-gamma and altered expression of Bcl-2/Bax. |
| 12192599 | 1 | No | Exposure of human breast cancer cells to the anti-inflammatory agent indomethacin alters choline phospholipid metabolites and Nm23 expression. |
| 12193581 | 5 | No | Calmodulin-dependent kinase I regulates adrenal cell expression of aldosterone synthase. |
| 12203115 | 8 | No | Apo2L/TRAIL differentially modulates the apoptotic effects of sulindac and a COX-2 selective non-steroidal anti-inflammatory agent in Bax-deficient cells. |

| | | | |
|---|---|---|---|
| 12206003 | 4 | No | [Effects of nitroglycerin and dexamethasone on nitric oxide and endothelin derived from alveolar macrophages in patients with mild and middle asthma] |
| 12206135 | 1 | No | The drug efflux pump MRP2: regulation of expression in physiopathological situations and by endogenous and exogenous compounds. |
| 12207893 | 5 | No | The antioxidant cocktail effective microorganism X (EM-X) inhibits oxidant-induced interleukin-8 release and the peroxidation of phospholipids in |
| 12208513 | 44 | No | Proteasome inhibitors stimulate activator protein-1 pathway via reactive oxygen species production. |
| 12208739 | 2 | No | Cyclooxygenase-2 overexpression inhibits death receptor 5 expression and confers resistance to tumor necrosis factor-related apoptosis-inducing ligand-induced |
| 12208782 | 17 | No | apoptosis and promotes cell survival, was identified to be chiefly down-regulated in a dose-dependent manner following CEES treatment. Functional analysis showed |
| 12216086 | 4 | No | Curcumin inhibits interleukin 8 production and enhances interleukin 8 receptor expression on the cell surface:impact on human pancreatic carcinoma cell growth |
| 12220541 | 1 | No | Multiple basic-leucine zipper proteins regulate induction of the mouse heme oxygenase-1 gene by arsenite. |
| 12224597 | 6 | No | Baicalein inhibits DMBA-DNA adduct formation by modulating CYP1A1 and CYP1B1 activities. |
| 12235264 | 22 | No | Histamine h(4) and h(2) receptors control histamine-induced interleukin-16 release from human CD8(+) T cells. |
| 12239597 | 2 | No | Influence of allergen-specific immunotherapy on IL-4-dependent IL-12 production by monocytes. |
| 12241537 | 4 | No | Arsenite inhibits interleukin-6 production in human intestinal epithelial cells by down-regulating nuclear factor-kappaB activity. |
| 12359242 | 2 | No | Acute arsenite-induced 8-hydroxyguanine is associated with inhibition of repair activity in cultured human cells. |
| 12376534 | 6 | No | Involvement of proteasome in the dynamic assembly of the androgen receptor transcription complex. |
| 12377979 | 65 | No | Coordination of altered DNA repair and damage pathways in arsenite-exposed keratinocytes. |
| 12377985 | 10 | No | In vitro antiestrogenic effects of aryl methyl sulfone metabolites of polychlorinated biphenyls and 2,2-bis(4-chlorophenyl)-1,1-dichloroethene on |
| 12387747 | 5 | No | Influence of different chemicals on MDR-1 P-glycoprotein expression and activity in the HK-2 proximal tubular cell line. |
| 12388107 | 23 | No | High glucose-induced, endothelin-dependent fibronectin synthesis is mediated via NF-kappa B and AP-1. |

| | | | |
|---|---|---|---|
| 12388546 | 1 | No | Arsenic trioxide promotes histone H3 phosphoacetylation at the chromatin of CASPASE-10 in acute promyelocytic leukemia cells. |
| 12388792 | 25 | No | Discovery of molecular mechanisms of neuroprotection using cell-based bioassays and oligonucleotide arrays. |
| 12391264 | 14 | No | The mutant androgen receptor T877A mediates the proliferative but not the cytotoxic dose-dependent effects of genistein and quercetin on human LNCaP |
| 12391285 | 2 | No | Peroxisome proliferator-activated receptor-gamma is a target of nonsteroidal anti-inflammatory drugs mediating cyclooxygenase-independent inhibition of lung |
| 12392815 | 4 | No | Pro-apoptotic actions of exisulind and CP461 in SW480 colon tumor cells involve beta-catenin and cyclin D1 down-regulation. |
| 12393170 | 4 | No | Polycyclic aromatic hydrocarbons induce IL-8 expression through nuclear factor kappaB activation in A549 cell line. |
| 12393423 | 4 | No | Expression of the inhibitor of apoptosis (IAP) family members in human neutrophils: up-regulation of cIAP2 by granulocyte colony-stimulating factor and |
| 12396874 | 3 | No | Estrogenicity of organophosphorus and pyrethroid pesticides. |
| 12399409 | 8 | No | Characterization of the biological roles of the estrogen receptors, ERalpha and ERbeta, in estrogen target tissues in vivo through the use of an |
| 12401803 | 4 | No | The link module from human TSG-6 inhibits neutrophil migration in a hyaluronan- and inter-alpha -inhibitor-independent manner. |
| 12404063 | 1 | No | Fibroblast growth factor 8 expression in breast carcinoma: associations with androgen receptor and prostate-specific antigen expressions. |
| 12406900 | 1 | No | Extracellular ubiquitin inhibits the TNF-alpha response to endotoxin in peripheral blood mononuclear cells and regulates endotoxin hyporesponsiveness in |
| 12412711 | 1 | No | In vitro immunomodulatory effects of herbal products. |
| 12414619 | 4 | No | Novel detection and differential utilization of a c-myc transcriptional block in colon cancer chemoprevention. |
| 12414654 | 210 | No | Gene expression after treatment with hydrogen peroxide, menadione, or t-butyl hydroperoxide in breast cancer cells. |
| 12417264 | 9 | No | PXR-dependent induction of human CYP3A4 gene expression by organochlorine pesticides. |
| 12417450 | 1 | No | Oral terbutaline differentially affects cytokine (IL-10, IL-12, TNF, IFNg) release in multiple sclerosis patients and controls. |
| 12419474 | 439 | No | Microarray analysis of H2O2-, HNE-, or tBH-treated ARPE-19 cells. |

| | | | |
|---|---|---|---|
| 12421474 | 2 | No | Apoptotic effect of As2S2 on K562 cells and its mechanism. |
| 12423647 | 1 | No | In vitro effects of monophthalates on cytokine expression in the monocytic cell line THP-1 and in peripheral blood mononuclear cells from allergic and |
| 12426128 | 2 | No | Sodium arsenite-induced stress-related gene expression in normal human epidermal, HaCaT, and HEL30 keratinocytes. |
| 12428247 | 6 | No | Ciprofloxacin enhances the stimulation of matrix metalloproteinase 3 expression by interleukin-1beta in human tendon-derived cells. A potential mechanism of |
| 12429649 | 1 | No | Zinc inhibits nuclear factor-kappa B activation and sensitizes prostate cancer cells to cytotoxic agents. |
| 12430140 | 2 | No | Synergy is achieved by complementation with Apo2L/TRAIL and actinomycin D in Apo2L/TRAIL-mediated apoptosis of prostate cancer cells: role of XIAP in |
| 12431781 | 4 | No | The flavonoid, quercetin, differentially regulates Th-1 (IFNgamma) and Th-2 (IL4) cytokine gene expression by normal peripheral blood mononuclear cells. |
| 12432273 | 1 | No | The effects of beta-estradiol on Raf activity, cell cycle progression and growth factor synthesis in the MCF-7 breast cancer cell line. |
| 12432279 | 1 | No | Death receptor 5 regulation during selenium-mediated apoptosis in human prostate cancer cells. |
| 12437293 | 5 | No | Effects of pyrethroid insecticides and estrogen on WNT10B proto-oncogene expression. |
| 12446695 | 9 | No | Increased protein stability as a mechanism that enhances Nrf2-mediated transcriptional activation of the antioxidant response element. Degradation of |
| 12452020 | 2 | No | [Primary study of arsenic trioxide inhibits abdomino-metastatic tumor formation of human ovarian carcinoma in nude mice and its mechanisms] |
| 12460908 | 4 | No | A mycobacterial iron chelator, desferri-exochelin, induces hypoxia-inducible factors 1 and 2, NIP3, and vascular endothelial growth factor in cancer cell |
| 12462994 | 8 | No | Grape seed extract proanthocyanidins downregulate HIV-1 entry coreceptors, CCR2b, CCR3 and CCR5 gene expression by normal peripheral blood mononuclear cells. |
| 12466137 | 4 | No | Constitutive nuclear factor-kappaB activity is crucial for human retinoblastoma cell viability. |
| 12469199 | 3 | No | Quercetin regulates growth of Ishikawa cells through the suppression of EGF and cyclin D1. |
| 12470298 | 12 | No | Reactive oxygen species mediate the down-regulation of mitochondrial transcripts and proteins by tumour necrosis factor-alpha in L929 cells. |
| 12470611 | 10 | No | Trichostatin A, a histone deacetylase inhibitor, down-regulates interleukin-12 transcription in SV-40-transformed lung epithelial cells. |

| | | | |
|---|---|---|---|
| 12473173 | 30 | No | Inhibition of E2-induced expression of BRCA1 by persistent organochlorines. |
| 12478894 | 4 | No | [Impact of arsenic trioxide on proliferation and metastasis of drug-resistant human ovarian carcinoma cell line] |
| 12479363 | 7 | No | Apoptosis induction by 1alpha,25-dihydroxyvitamin D3 in prostate cancer. |
| 12479875 | 3 | No | Down-regulation and decreased activity of cyclin-dependent kinase 2 in H2O2-induced premature senescence. |
| 12482751 | 2 | No | Expression of dominant-negative Fas-associated death domain blocks human keratinocyte apoptosis and vesication induced by sulfur mustard. |
| 12482858 | 23 | No | Evidence for a role of p38 kinase in hypoxia-inducible factor 1-independent induction of vascular endothelial growth factor expression by sodium arsenite. |
| 12490120 | 2 | No | [Arsenic trioxide induced apoptosis and expression of p53 and bcl-2 genes in human small cell lung cancer cells] |
| 12490308 | 2 | No | The combined treatment of aspirin and radiation induces apoptosis by the regulation of bcl-2 and caspase-3 in human cervical cancer cell. |
| 12490585 | 4 | No | Blockage of multidrug resistance-associated proteins potentiates the inhibitory effects of arsenic trioxide on CYP1A1 induction by polycyclic aromatic |
| 12492458 | 1 | No | Treatment with atorvastatin alters the ratio of interleukin-12/interleukin-10 gene expression [corrected]. |
| 12493747 | 24 | No | Treatment of human T cells with bisperoxovanadium phosphotyrosyl phosphatase inhibitors leads to activation of cyclooxygenase-2 gene. |
| 12496413 | 1 | No | Activated STAT4 and a functional role for IL-12 in human Peyer's patches. |
| 12497104 | 4 | No | Therapeutic potential of curcumin in human prostate cancer-I. curcumin induces apoptosis in both androgen-dependent and androgen-independent prostate cancer |
| 12504091 | 6 | No | Vitamin E succinate inhibits human prostate cancer cell growth via modulating cell cycle regulatory machinery. |
| 12505310 | 19 | No | Receptor-dependent regulation of the CYP3A4 gene. |
| 12513809 | 3 | No | [Study on the role of PML-RARalpha and RARalpha fusion proteins in NB4 cell apoptosis induced by arsenic trisulfide] |
| 12517777 | 90 | No | Delineation of the molecular basis for selenium-induced growth arrest in human prostate cancer cells by oligonucleotide array. |
| 12517783 | 28 | No | Flavopiridol down-regulates antiapoptotic proteins and sensitizes human breast cancer cells to epothilone B-induced apoptosis. |

| | | | |
|---|---|---|---|
| 12519787 | 6 | No | Retinoid X receptor (RXR) agonist-induced antagonism of farnesoid X receptor (FXR) activity due to absence of coactivator recruitment and decreased DNA |
| 12521655 | 8 | No | Vitamin D receptor gene polymorphism and bone metabolism during low-dose oral contraceptive use in young women. |
| 12524230 | 11 | No | Synergistic activation of human LDL receptor expression by SCAP ligand and cytokine oncostatin M. |
| 12525500 | 7 | No | Guggulsterone is a farnesoid X receptor antagonist in coactivator association assays but acts to enhance transcription of bile salt export pump. |
| 12533503 | 5 | No | Carbohydrate ingestion influences skeletal muscle cytokine mRNA and plasma cytokine levels after a 3-h run. |
| 12534559 | 2 | No | Metalloproteinase-9 is increased after toluene diisocyanate exposure in the induced sputum from patients with toluene diisocyanate-induced asthma. |
| 12536650 | 2 | No | [Relationship between sex hormone levels and blood calcitonin gene-related peptide/endothelin-1 in postmenopausal women with coronary heart disease] |
| 12538206 | 11 | No | Adrenaline inhibits lipopolysaccharide-induced macrophage inflammatory protein-1 alpha in human monocytes: the role of beta-adrenergic receptors. |
| 12542540 | 8 | No | Influence of 5-aminolevulinic acid and red light on collagen metabolism of human dermal fibroblasts. |
| 12547825 | 18 | No | 17-epiestriol, an estrogen metabolite, is more potent than estradiol in inhibiting vascular cell adhesion molecule 1 (VCAM-1) mRNA expression. |
| 12547826 | 14 | No | Tumor promoter arsenite stimulates histone H3 phosphoacetylation of proto-oncogenes c-fos and c-jun chromatin in human diploid fibroblasts. |
| 12556443 | 2 | No | The C-terminal fragment of presenilin 2 triggers p53-mediated staurosporine-induced apoptosis, a function independent of the |
| 12556972 | 1 | No | Effect of a novel somatostatin analogue combined with cytotoxic drugs on human tumour xenografts and metastasis of B16 melanoma. |
| 12560223 | 7 | No | Arsenic/interferon specifically reverses 2 distinct gene networks critical for the survival of HTLV-1-infected leukemic cells. |
| 12563064 | 1 | No | Vascular endothelial growth factor in the cerebrospinal fluid of infants who died of sudden infant death syndrome: evidence for antecedent hypoxia. |
| 12566991 | 2 | No | Genomic structure, gene expression, and promoter analysis of human multidrug resistance-associated protein 7. |
| 12569548 | 3 | No | Decreased DNA repair gene expression among individuals exposed to arsenic in United States drinking water. |
| 12569576 | 60 | No | Differential expression of genes induced by resveratrol in LNCaP cells: P53-mediated molecular targets. |

| | | | |
|---|---|---|---|
| 12572858 | 1 | No | Regulation of the glyoxalase pathway in human brain microvascular endothelium: effects of troglitazone and tertiary butylhydroperoxide. |
| 12573452 | 16 | No | Inhibition by eicosapentaenoic acid of IL-1beta-induced PGHS-2 expression in human microvascular endothelial cells: involvement of lipoxygenase-derived |
| 12574206 | 14 | No | Tumor necrosis factor-alpha-induced interleukin-8 (IL-8) expression in endometriotic stromal cells, probably through nuclear factor-kappa B activation: |
| 12574323 | 4 | No | Cutting edge: distinct Toll-like receptor 2 activators selectively induce different classes of mediator production from human mast cells. |
| 12574384 | 12 | No | Lipopolysaccharide down-regulates the leukotriene C4 synthase gene in the monocyte-like cell line, THP-1. |
| 12586319 | 4 | No | Serum distribution of the major metabolites of norgestimate in relation to its pharmacological properties. |
| 12587719 | 3 | No | Mechanistic pathways of antioxidant cytoprotection by a novel IH636 grape seed proanthocyanidin extract. |
| 12589822 | 12 | No | Quercetin exerts multiple inhibitory effects on vascular smooth muscle cells: role of ERK1/2, cell-cycle regulation, and matrix metalloproteinase-9. |
| 12606626 | 1 | No | Activation of peroxisome proliferator-activated receptor gamma does not explain the antiproliferative activity of the nonsteroidal anti-inflammatory drug |
| 12612140 | 3 | No | Red wine polyphenolics increase LDL receptor expression and activity and suppress the secretion of ApoB100 from human HepG2 cells. |
| 12612910 | 2 | No | Acetaldehyde impairs mitochondrial glutathione transport in HepG2 cells through endoplasmic reticulum stress. |
| 12616980 | 15 | No | Genetic polymorphisms modify the response of factor VII to oral contraceptive use: an example of gene-environment interaction. |
| 12616983 | 19 | No | Pro-inflammatory effects of oestrogens during use of oral contraceptives and hormone replacement treatment. |
| 12618251 | 70 | No | The effects of seven monophasic oral contraceptive regimens on hemostatic variables: conclusions from a large randomized multicenter study. |
| 12621035 | 3 | No | Stimulation of beta 2-adrenergic receptor increases cystic fibrosis transmembrane conductance regulator expression in human airway epithelial cells through a |
| 12623754 | 14 | No | line, HepG2. Besides the positive control beta -naphthoflavone, the H(+)/K(+)-ATPase inhibitors omeprazole, lansoprazole, pantoprazole and |
| 12623786 | 1 | No | 15-deoxy-delta 12,14-prostaglandin J2 and laminar fluid shear stress stabilize c-IAP1 in vascular endothelial cells. |
| 12628473 | 4 | No | Nicotine-induced Ca2+ signaling and down-regulation of nicotinic acetylcholine receptor subunit expression in the CEM human leukemic T-cell line. |

| | | | |
|---|---|---|---|
| 12628509 | 16 | No | New TNF-alpha releasing inhibitors as cancer preventive agents from traditional herbal medicine and combination cancer prevention study with EGCG and sulindac or |
| 12632063 | 1 | No | Genome-scale analysis of resveratrol-induced gene expression profile in human ovarian cancer cells using a cDNA microarray. |
| 12634122 | 461 | No | Characterization of gene expression changes associated with MNNG, arsenic, or metal mixture treatment in human keratinocytes: application of cDNA microarray |
| 12642470 | 10 | No | Comparative effects of thiazolidinediones on in vitro P450 enzyme induction and inhibition. |
| 12644474 | 3 | No | Par-4 transcriptionally regulates Bcl-2 through a WT1-binding site on the bcl-2 promoter. |
| 12655030 | 3 | No | Expression of aryl hydrocarbon receptor repressor in normal human tissues and inducibility by polycyclic aromatic hydrocarbons in human tumor-derived cell |
| 12660819 | 1 | No | Immediate-early gene induction by the stresses anisomycin and arsenite in human osteosarcoma cells involves MAPK cascade signaling to Elk-1, CREB and SRF. |
| 12663510 | 16 | No | Synergy between sulforaphane and selenium in the induction of thioredoxin reductase 1 requires both transcriptional and translational modulation. |
| 12663669 | 3 | No | Inhibition of phosphatidylinositol 3-kinase sensitizes vascular endothelial cells to cytokine-initiated cathepsin-dependent apoptosis. |
| 12664607 | 2 | No | Regulation of CD36 expression in human melanoma cells. |
| 12670519 | 1 | No | Molecular epidemiology of plasma oncoproteins in vinyl chloride monomer workers in Taiwan. |
| 12671036 | 62 | No | Monoterpene regulation of Ras and Ras-related protein expression. |
| 12672646 | 6 | No | Protection by quercetin against cooking oil fumes-induced DNA damage in human lung adenocarcinoma CL-3 cells: role of COX-2. |
| 12672908 | 10 | No | Polyphenolic flavonoids differ in their antiapoptotic efficacy in hydrogen peroxide-treated human vascular endothelial cells. |
| 12673034 | 17 | No | Induction of CYP3As in HepG2 cells by several drugs. Association between induction of CYP3A4 and expression of glucocorticoid receptor. |
| 12676607 | 5 | No | Phosphorylation of p53 protein in A549 human pulmonary epithelial cells exposed to asbestos fibers. |
| 12676768 | 12 | No | Vanadium-induced HB-EGF expression in human lung fibroblasts is oxidant dependent and requires MAP kinases. |
| 12676771 | 28 | No | Serine protease inhibitors modulate chemotactic cytokine production by human lung fibroblasts in vitro. |

| | | | |
|---|---|---|---|
| 12676792 | 4 | No | Inhibition of NF-kappaB essentially contributes to arsenic-induced apoptosis. |
| 12679007 | 3 | No | [Arsenic trioxide inhibits P-glycoprotein expression in multidrug-resistant human leukemia K562/ADM cell line that overexpresses mdr-1 gene and enhances their |
| 12679051 | 27 | No | Low-level arsenite induced gene expression in HEK293 cells. |
| 12679171 | 2 | No | Effect of chromium on apolipoprotein A-I expression in HepG2 cells. |
| 12680227 | 2 | No | Expression pattern of cisplatin-induced metallothionein isoforms in squamous cell carcinoma. |
| 12694875 | 2 | No | Antitumor effect of the cinnamaldehyde derivative CB403 through the arrest of cell cycle progression in the G2/M phase. |
| 12695340 | 19 | No | Regulation of CYP3A4 expression in human hepatocytes by pharmaceuticals and natural products. |
| 12695351 | 76 | No | Glucocorticoid receptor enhancement of pregnane X receptor-mediated CYP2B6 regulation in primary human hepatocytes. |
| 12696651 | 2 | No | Associations of dichlorodiphenyltrichloroethane (DDT) 4.4 and dichlorodiphenyldichloroethylene (DDE) 4.4 blood levels with plasma IL-4. |
| 12703962 | 5 | No | Low concentrations of arsenic induce vascular endothelial growth factor and nitric oxide release and stimulate angiogenesis in vitro. |
| 12705905 | 9 | No | Guggulsterone antagonizes farnesoid X receptor induction of bile salt export pump but activates pregnane X receptor to inhibit cholesterol 7alpha-hydroxylase gene. |
| 12711008 | 28 | No | Characterisation of gene expression patterns in 22RV1 cells for determination of environmental androgenic/antiandrogenic compounds. |
| 12712689 | 1 | No | [Effects of long-term exposure to lower concentration of carbon disulfide on cardiovascular system of workers in a viscose rayon factory] |
| 12716893 | 16 | No | Chromium(VI) down-regulates heavy metal-induced metallothionein gene transcription by modifying transactivation potential of the key transcription |
| 12720008 | 6 | No | Ethanol and acetone stimulate the proliferation of HaCaT keratinocytes: the possible role of alcohol in exacerbating psoriasis. |
| 12721113 | 5 | No | phospholipase A(2) with anti-inflammatory properties. In this work, we have studied the mechanism of action of this compound in the inflammatory response |
| 12722970 | 2 | No | Cytokine abnormalities in a patient with eosinophilic fasciitis. |
| 12724357 | 6 | No | PTHrP [67-86] regulates the expression of stress proteins in breast cancer cells inducing modifications in urokinase-plasminogen activator and MMP-1 expression. |

| | | | |
|---|---|---|---|
| 12724418 | 3 | No | Cardiac p300 is involved in myocyte growth with decompensated heart failure. |
| 12724925 | 3 | No | CYP1a1, HSP70, P53, and c-fos expression in human liver carcinoma cells (HepG2) exposed to pentachlorophenol. |
| 12727023 | 2 | No | Downregulation of NF-kappaB activation in human keratinocytes by melanogenic inhibitors. |
| 12727804 | 108 | No | Global alteration of gene expression in human keratinocytes by inorganic arsenic. |
| 12729831 | 3 | No | Tumor necrosis factor-alpha production-regulating activity of phthalimide derivatives in genetically modified murine melanoma cells B78H1. |
| 12729923 | 3 | No | Effects of beta-adrenergic agonists on bone-resorbing activity in human osteoclast-like cells. |
| 12730239 | 12 | No | Increased AMP:ATP ratio and AMP-activated protein kinase activity during cellular senescence linked to reduced HuR function. |
| 12732289 | 17 | No | Glucocorticoid regulation of human eosinophil gene expression. |
| 12734198 | 60 | No | Gene modulation by the cyclooxygenase inhibitor, sulindac sulfide, in human colorectal carcinoma cells: possible link to apoptosis. |
| 12734376 | 2 | No | Selective roles for Toll-like receptor (TLR)2 and TLR4 in the regulation of neutrophil activation and life span. |
| 12738801 | 3 | No | Oxygen-regulated expression of the Wilms' tumor suppressor Wt1 involves hypoxia-inducible factor-1 (HIF-1). |
| 12749819 | 8 | No | Patterns of gene expressions induced by arsenic trioxide in cultured human fibroblasts. |
| 12749906 | 25 | No | Hydroquinone and catechol interfere with T cell cycle entry and progression through the G1 phase. |
| 12753421 | 12 | No | Effects of the environmental oestrogens bisphenol A, tetrachlorobisphenol A, tetrabromobisphenol A, 4-hydroxybiphenyl and 4,4'-dihydroxybiphenyl on oestrogen |
| 12756216 | 4 | No | Absorption/metabolism of sulforaphane and quercetin, and regulation of phase II enzymes, in human jejunum in vivo. |
| 12760830 | 274 | No | Genomic and proteomic profiling of responses to toxic metals in human lung cells. |
| 12761878 | 1 | No | Vitamin D enhances mitogenesis mediated by keratinocyte growth factor receptor in keratinocytes. |
| 12762645 | 12 | No | Organophosphorous pesticides in breast cancer progression. |

| | | | |
|---|---|---|---|
| 12763630 | 1 | No | Expression of metallothionein-III induced by hypoxia attenuates hypoxia-induced cell death in vitro. |
| 12766334 | 1 | No | Retinoids and cancer: antitumoral effects of ATRA, 9-cis RA and the new retinoid IIF on the HL-60 leukemic cell line. |
| 12766906 | 7 | No | Identification of distinct and common gene expression changes after oxidative stress and gamma and ultraviolet radiation. |
| 12773770 | 32 | No | Gene expression patterns as potential molecular biomarkers for malignant transformation in human keratinocytes treated with MNNG, arsenic, or a metal |
| 12775586 | 2 | No | Role of the cytosolic chaperones Hsp70 and Hsp90 in maturation of the cardiac potassium channel HERG. |
| 12776498 | 3 | No | [Investigation on NB4 cell responses to realgar by cDNA microarray] |
| 12782109 | 9 | No | The effect of tertiary butylhydroperoxide and nicotinamide on human cortical neurons. |
| 12783709 | 2 | No | [Effect of arsenic trioxide on the expression of cyclins gene in HL60 cells] |
| 12788061 | 13 | No | Triphenyltin enhances the neutrophilic differentiation of promyelocytic HL-60 cells. |
| 12789233 | 38 | No | Fisetin, a flavonol, inhibits TH2-type cytokine production by activated human basophils. |
| 12798352 | 17 | No | Inhibition of fatty acid synthase expression by 1alpha,25-dihydroxyvitamin D3 in prostate cancer cells. |
| 12799645 | 1 | No | Zoledronic acid induces antiproliferative and apoptotic effects in human pancreatic cancer cells in vitro. |
| 12800980 | 4 | No | CDK2 is involved in the S-phase lengthening induced by glucocorticoids in normal human lymphocytes. |
| 12804770 | 4 | No | Fibroblast growth factor-10 upregulates Na,K-ATPase via the MAPK pathway. |
| 1280742 | 1 | No | Terbutaline-induced downregulation of beta 2-adrenoceptors without Gi-protein alterations in human lymphocytes. |
| 12807730 | 1 | No | CpG methylation-dependent repression of the human O6-methylguanine-DNA methyltransferase gene linked to chromatin structure alteration. |
| 12807743 | 17 | No | Potent iron chelators increase the mRNA levels of the universal cyclin-dependent kinase inhibitor p21(CIP1/WAF1), but paradoxically inhibit its translation: a |
| 12807757 | 1 | No | Modulation of benzo[a]pyrene-induced p53 DNA activity by acrolein. |

| | | | |
|---|---|---|---|
| 12809767 | 1 | No | Modulation of angiogenic functions in human macrophages by biomaterials. |
| 12818968 | 12 | No | Dobutamine modulates lipopolysaccharide-induced macrophage inflammatory protein-1alpha and interleukin-8 production in human monocytes. |
| 12820898 | 10 | No | Fructose modulates GLUT5 mRNA stability in differentiated Caco-2 cells: role of cAMP-signalling pathway and PABP (polyadenylated-binding protein)-interacting |
| 12821135 | 1 | No | Dephosphorylation of p53 during cell death by N-alpha-tosyl-L-phenylalanyl chloromethyl ketone. |
| 12822488 | 6 | No | [Studies on estrogenic activities of food additives with human breast cancer MCF-7 cells and mechanism of estrogenicity by BHA and OPP] |
| 12824081 | 2 | No | Effect of beta1- and beta2-adrenergic stimulation on energy expenditure, substrate oxidation, and UCP3 expression in humans. |
| 12832416 | 37 | No | Disruption of the c-JUN-JNK complex by a cell-permeable peptide containing the c-JUN delta domain induces apoptosis and affects a distinct set of |
| 12837940 | 5 | No | Inhibition of apoptosis in normal and transformed intestinal epithelial cells by cAMP through induction of inhibitor of apoptosis protein (IAP)-2. |
| 12839939 | 10 | No | Microarray analysis and RNA silencing link fra-1 to cd44 and c-met expression in mesothelioma. |
| 12841937 | 4 | No | The effects of mebendazole on P4501A activity in rat hepatocytes and HepG2 cells. Comparison with tiabendazole and omeprazole. |
| 12843640 | 12 | No | Effects of dimethyl sulfoxide on the gene induction of cytochrome P450 isoforms, UGT-dependent glucuronosyl transferase isoforms, and ABCB1 in primary culture of |
| 12844480 | 16 | No | Elucidation of molecular targets of mammary cancer chemoprevention in the rat by organoselenium compounds using cDNA microarray. |
| 12845720 | 1 | No | Preliminary study of the in vitro growth inhibition of human bladder cancer cell line BIU-87 by arsenic trioxide. |
| 12847114 | 2 | No | Adenovirus-mediated overexpression of caveolin-3 inhibits rat cardiomyocyte hypertrophy. |
| 12847216 | 5 | No | Cutting edge: sanglifehrin A, a novel cyclophilin-binding immunosuppressant blocks bioactive IL-12 production by human dendritic cells. |
| 12852829 | 19 | No | Gene expression profile changes in NB4 cells induced by arsenic trioxide. |
| 12858341 | 19 | No | Estrogenic endocrine disruptive components interfere with calcium handling and differentiation of human trophoblast cells. |
| 12864811 | 4 | No | Phenotypic switching of Cryptococcus neoformans can influence the outcome of the human immune response. |

| | | | |
|---|---|---|---|
| 12866041 | 2 | No | Additive effects of tamoxifen and the farnesyl transferase inhibitor FTI-277 on inhibition of MCF-7 breast cancer cell-cycle progression. |
| 12867288 | 1 | No | Selenium regulates transcription factor NF-kappaB activation during the acute phase reaction. |
| 12869419 | 4 | No | The effect of potent iron chelators on the regulation of p53: examination of the expression, localization and DNA-binding activity of p53 and the transactivation |
| 12870655 | 2 | No | Transcript profiling of cytochrome P450 genes in HL-60 human leukemic cells: upregulation of CYP1B1 by all-trans-retinoic acid. |
| 12871219 | 1 | No | Evidence for post-transcriptional regulation of interleukin-5 by dexamethasone. |
| 12871381 | 2 | No | Resveratrol and quercetin down-regulate tissue factor expression by human stimulated vascular cells. |
| 12872138 | 1 | No | Sustained P450 expression and prodrug activation in bolus cyclophosphamide-treated cultured tumor cells. Impact of prodrug schedule on P450 |
| 12875902 | 74 | No | Comparison of the gene expression profiles of monocytic versus granulocytic lineages of HL-60 leukemia cell differentiation by DNA microarray analysis. |
| 12878215 | 12 | No | Anti-HIV activity of olive leaf extract (OLE) and modulation of host cell gene expression by HIV-1 infection and OLE treatment. |
| 12880345 | 6 | No | Multiphoton microscopy: an optical approach to understanding and resolving sulfur mustard lesions. |
| 12880480 | 16 | No | Metallothionein attenuates 3-morpholinosydnonimine (SIN-1)-induced oxidative stress in dopaminergic neurons. |
| 12883691 | 1 | No | P53-induced gene 11 (PIG11) involved in arsenic trioxide-induced apoptosis in human gastric cancer MGC-803 cells. |
| 12884408 | 24 | No | Effects of sulfur mustard on transcription in human epidermal keratinocytes: analysis by mRNA differential display. |
| 12888115 | 1 | No | Blockage of transcription as a trigger for p53 accumulation by 2-acetylaminofluorene DNA-adducts. |
| 12890387 | 40 | No | Gene expression profile changes in NB4 cells induced by realgar. |
| 12893085 | 3 | No | Inhibition of p53, p21 and Bax by pifithrin-alpha does not affect UV induced apoptotic response in CS-B cells. |
| 12893260 | 2 | No | Arabinosylcytosine downregulates thymidine kinase and induces cross-resistance to zidovudine in T-lymphoid cells. |
| 12893773 | 41 | No | The proteasome inhibitor bortezomib interacts synergistically with histone deacetylase inhibitors to induce apoptosis in Bcr/Abl+ cells sensitive and |

| | | | |
|---|---|---|---|
| 12896903 | 5 | No | Expression of glutathione S-transferases (GSTs) in human colon cells and inducibility of GSTM2 by butyrate. |
| 12896980 | 2 | No | Selenite-induced survival of HuH7 hepatoma cells involves activation of focal adhesion kinase-phosphatidylinositol 3-kinase-Akt pathway and Rac1. |
| 12898515 | 11 | No | 9-cis retinoic acid accelerates calcitriol-induced osteocalcin production and promotes degradation of both vitamin D receptor and retinoid X receptor in human |
| 12899209 | 3 | No | Molecular pathology of skin carcinogenesis due to arsenicalism from coal-burning. |
| 12903497 | 1 | No | [Arsenic trioxide induced human esophageal cancer Ec109 cell apoptosis with downregulation of c-myc gene expression] |
| 12903512 | 2 | No | [Arsenic trioxide induces human tumor cell apoptosis and G2 + M arrest whereas causes HPV16 DNA immortalized cervical epithelial cells G1 block] |
| 12907249 | 19 | No | Inhibition of MG-63 cell cycle progression by synthetic vitamin D3 analogs mediated by p27, Cdk2, cyclin E, and the retinoblastoma protein. |
| 12907618 | 5 | No | The biological and biochemical effects of CP-654577, a selective erbB2 kinase inhibitor, on human breast cancer cells. |
| 12908082 | 4 | No | Induction of TNF-alpha, uPA, IL-8 and MCP-1 by doxorubicin in human lung carcinoma cells. |
| 12910240 | 2 | No | Down-regulation of RPE65 protein expression and promoter activity by retinoic acid. |
| 12912967 | 7 | No | Ibuprofen-mediated reduction of hypoxia-inducible factors HIF-1alpha and HIF-2alpha in prostate cancer cells. |
| 12914969 | 1 | No | Wilms' tumor suppressor (WT1) is a mediator of neuronal degeneration associated with the pathogenesis of Alzheimer's disease. |
| 12926078 | 8 | No | Reduction of MDR3 mRNA levels by forskolin in Chang liver cells. |
| 12928151 | 7 | No | Gene expression of inflammatory molecules in circulating lymphocytes from arsenic-exposed human subjects. |
| 12930308 | 1 | No | Nickel-induced keratinocyte proliferation and up-modulation of the keratinocyte growth factor receptor expression. |
| 12932289 | 2 | No | Vgamma9/Vdelta2 T lymphocytes in Italian patients with Beh莽et's disease: evidence for expansion, and tumour necrosis factor receptor II and interleukin-12 receptor |
| 12934110 | 2 | No | Quercetin elevates p27(Kip1) and arrests both primary and HPV16 E6/E7 transformed human keratinocytes in G1. |
| 12934402 | 4 | No | [Renal protective effect of nilestriol in aged women with hypertension] |

| | | | |
|---|---|---|---|
| 12938025 | 1 | No | Inhibition of energy metabolism down-regulates the Alzheimer related presenilin 2 gene. |
| 12939465 | 4 | No | Dacarbazine causes transcriptional up-regulation of interleukin 8 and vascular endothelial growth factor in melanoma cells: a possible escape mechanism from |
| 12946449 | 52 | No | Suppressive effect of selective cyclooxygenase-2 inhibitor on cytokine release in human neutrophils. |
| 12948851 | 29 | No | Synergistic effects of chemotherapeutic drugs in lymphoma cells are associated with down-regulation of inhibitor of apoptosis proteins (IAPs), |
| 12948864 | 9 | No | Long-term exposure to beta-hexachlorocyclohexane (beta-HCH) promotes transformation and invasiveness of MCF-7 human breast cancer cells. |
| 12949046 | 4 | No | Sulforaphane and quercetin modulate PhIP-DNA adduct formation in human HepG2 cells and hepatocytes. |
| 12949356 | 4 | No | Thioredoxin reductase in human hepatoma cells is transcriptionally regulated by sulforaphane and other electrophiles via an antioxidant response element. |
| 12952965 | 11 | No | Mitochondrial cytochrome c release mediates ceramide-induced activator protein 2 activation and gene expression in keratinocytes. |
| 12954644 | 1 | No | Vitamin D inhibits G1 to S progression in LNCaP prostate cancer cells through p27Kip1 stabilization and Cdk2 mislocalization to the cytoplasm. |
| 12958071 | 349 | No | ST1926, a novel and orally active retinoid-related molecule inducing apoptosis in myeloid leukemia cells: modulation of intracellular calcium homeostasis. |
| 12960079 | 1 | No | Estrogen stimulates estrogen-related receptor alpha gene expression through conserved hormone response elements. |
| 12963989 | 2 | No | A novel ginseng saponin metabolite induces apoptosis and down-regulates fibroblast growth factor receptor 3 in myeloma cells. |
| 12969788 | 7 | No | Apoptosis and modulation of cell cycle control by synthetic derivatives of ursodeoxycholic acid and chenodeoxycholic acid in human prostate cancer cells. |
| 12970779 | 9 | No | A new selective AKT pharmacological inhibitor reduces resistance to chemotherapeutic drugs, TRAIL, all-trans-retinoic acid, and ionizing radiation of |
| 12974195 | 2 | No | Localization of inducible nitric oxide synthase and endothelial constitutive nitric oxide synthase in airway mucosa of toluene diisocyanate-induced asthma. |
| 13678430 | 6 | No | Regulatory role of nitric oxide on monocyte-derived dendritic cell functions. |
| 14499251 | 16 | No | Histamine downregulates CD14 expression via H2 receptors on human monocytes. |
| 14500290 | 10 | No | Simvastatin modulates expression of the PON1 gene and increases serum paraoxonase: a role for sterol regulatory element-binding protein-2. |

| | | | |
|---|---|---|---|
| 14503796 | 9 | No | The differential effects of cyclophosphamide, epirubicin and 5-fluorouracil on apoptotic marker (CPP-32), pro-apoptotic protein (p21(WAF-1)) and anti-apoptotic |
| 14503839 | 2 | No | Differential induction of Mn-containing superoxide dismutase by paraquat in peripheral lymphocytes of normal subjects and gastric cancer patients. |
| 14504107 | 1 | No | Annexin II mediates plasminogen-dependent matrix invasion by human monocytes: enhanced expression by macrophages. |
| 14506281 | 6 | No | Cyclooxygenase-independent induction of apoptosis by sulindac sulfone is mediated by polyamines in colon cancer. |
| 14508237 | 1 | No | A novel capsaicin derivative VOA induced relaxation in rat mesenteric and aortic arteries: involvement of CGRP, NO, cGMP, and endothelium-dependent activities. |
| 14512787 | 2 | No | Induction of apoptosis and/or necrosis following exposure to antitumour agents in a melanoma cell line, probably through modulation of Bcl-2 family proteins. |
| 14516785 | 4 | No | Expression of minichromosome maintenance proteins in vascular smooth muscle cells is ERK/MAPK dependent. |
| 14518702 | 5 | No | Mitochondrial proliferation, DNA depletion and adipocyte differentiation in subcutaneous adipose tissue of HIV-positive HAART recipients. |
| 14523996 | 2 | No | Sodium arsenite downregulates transcriptional activity of AP-1 and CRE binding proteins in IL-1beta-treated Caco-2 cells by increasing the expression of the |
| 14527955 | 6 | No | Farnesoid X receptor activates transcription of the phospholipid pump MDR3. |
| 14527959 | 10 | No | Induction of cIAP-2 in human colon cancer cells through PKC delta/NF-kappa B. |
| 14529565 | 1 | No | Overexpression of wild-type p53 gene renders MCF-7 breast cancer cells more sensitive to the antiproliferative effect of progesterone. |
| 14532843 | 13 | No | Androgen dependent regulation of bacillus Calmette-Guerin induced interleukin-6 expression in human transitional carcinoma cell lines. |
| 14551147 | 15 | No | Effect of second- and third-generation oral contraceptives on the protein C system in the absence or presence of the factor VLeiden mutation: a randomized |
| 14555224 | 4 | No | Induction of apoptosis by curcumin: mediation by glutathione S-transferase P1-1 inhibition. |
| 14555285 | 5 | No | Bisphenol A significantly enhances the neutrophilic differentiation of promyelocytic HL-60 cells. |
| 14555400 | 6 | No | Metallothionein isoform 1 and 2 gene expression in a human urothelial cell line (UROtsa) exposed to CdCl2 and NaAsO2. |
| 14555707 | 6 | No | Sulindac metabolites induce caspase- and proteasome-dependent degradation of beta-catenin protein in human colon cancer cells. |

| | | | |
|---|---|---|---|
| 14556662 | 5 | No | Characterization of cell death induced by ethacrynic acid in a human colon cancer cell line DLD-1 and suppression by N-acetyl-L-cysteine. |
| 14557435 | 3 | No | Metformin reduces serum C-reactive protein levels in women with polycystic ovary syndrome. |
| 14561536 | 24 | No | Comparative study of the effects of two once-a-month injectable contraceptives (Cyclofem and Mesigyna) and one oral contraceptive (Ortho-Novum 1/35) on |
| 14562121 | 2 | No | Increased expression of fibroblast growth factor receptor 3 in CD34+ BCR-ABL+ cells from patients with chronic myeloid leukemia. |
| 14572605 | 21 | No | Selenium-containing compounds attenuate peroxynitrite-mediated NF-kappaB and AP-1 activation and interleukin-8 gene and protein expression in human leukocytes. |
| 14572618 | 12 | No | Effects of TNFalpha on expression of ICAM-1 in human airway epithelial cells in vitro: oxidant-mediated pathways and transcription factors. |
| 14576064 | 1 | No | Geldanamycin and herbimycin A induce apoptotic killing of B chronic lymphocytic leukemia cells and augment the cells' sensitivity to cytotoxic drugs. |
| 14576080 | 15 | No | Superoxide, H2O2, and iron are required for TNF-alpha-induced MCP-1 gene expression in endothelial cells: role of Rac1 and NADPH oxidase. |
| 14576182 | 4 | No | Valproate potentiates androgen biosynthesis in human ovarian theca cells. |
| 14577614 | 1 | No | Effects of 2-amino-4,6-dinitrotoluene on p53 tumor suppressor gene expression. |
| 14581372 | 8 | No | Exisulind and related compounds inhibit expression and function of the androgen receptor in human prostate cancer cells. |
| 14583596 | 87 | No | Gene expression profile analysis of 4-phenylbutyrate treatment of IB3-1 bronchial epithelial cell line demonstrates a major influence on heat-shock proteins. |
| 14586007 | 23 | No | Nonsteroidal anti-inflammatory drugs and peroxisome proliferator-activated receptor-gamma agonists modulate immunostimulated processing of amyloid precursor |
| 14592552 | 1 | No | Effects of phenacetin and its metabolite p-phenetidine on COX-1 and COX-2 activities and expression in vitro. |
| 14592851 | 1 | No | Decreased macrophage paraoxonase 2 expression in patients with hypercholesterolemia is the result of their increased cellular cholesterol |
| 14595851 | 8 | No | Naphthalene exposure: effects on gene expression and proliferation in human cord blood cells. |
| 14600250 | 10 | No | Induction of human CYP2C9 by rifampicin, hyperforin, and phenobarbital is mediated by the pregnane X receptor. |
| 14600836 | 2 | No | Human osteoclasts express different CXC chemokines depending on cell culture substrate: molecular and immunocytochemical evidence of high levels of CXCL10 and |

| | | | |
|---|---|---|---|
| 14601052 | 8 | No | Selective and nonselective toxicity of TRAIL/Apo2L combined with chemotherapy in human bone tumour cells vs. normal human cells. |
| 14602803 | 4 | No | Expression and mitogenic effect of fibroblast growth factor-9 in human endometriotic implant is regulated by aberrant production of estrogen. |
| 14604893 | 1 | No | Mechanism of AP-1-mediated gene expression by select organochlorines through the p38 MAPK pathway. |
| 14610217 | 2 | No | The chemopreventive agent sulindac attenuates expression of the antiapoptotic protein survivin in colorectal carcinoma cells. |
| 14612938 | 5 | No | Induction of Bax and activation of caspases during beta-sitosterol-mediated apoptosis in human colon cancer cells. |
| 14614459 | 4 | No | Role of the TRAIL/APO2-L death receptors in chlorambucil- and fludarabine-induced apoptosis in chronic lymphocytic leukemia. |
| 14615486 | 2 | No | Involvement of phospholipase D1 in melanogenesis of mouse B16 melanoma cells. |
| 14617519 | 28 | No | Human SP-A genetic variants and bleomycin-induced cytokine production by THP-1 cells: effect of ozone-induced SP-A oxidation. |
| 14617632 | 4 | No | Interplay between estrogen response element sequence and ligands controls in vivo binding of estrogen receptor to regulated genes. |
| 14617803 | 73 | No | Diverse effects of methylseleninic acid on the transcriptional program of human prostate cancer cells. |
| 14619337 | 1 | No | Montelukast inhibits interleukin-5 mRNA expression and cysteinyl leukotriene production in ragweed and mite-stimulated peripheral blood mononuclear cells from |
| 14623254 | 6 | No | Expression of glutathione S-transferase P1-1 in differentiating K562: role of GATA-1. |
| 14623915 | 2 | No | Hepatoprotection by the farnesoid X receptor agonist GW4064 in rat models of intra- and extrahepatic cholestasis. |
| 14628445 | 4 | No | [Effects of quercetin on platelet-endothelial cell adhesion and their expression of adhesion molecules] |
| 14633654 | 14 | No | Gene modulation by Cox-1 and Cox-2 specific inhibitors in human colorectal carcinoma cancer cells. |
| 14633667 | 6 | No | Tea polyphenol (-)-epigallocatechin-3-gallate inhibits DNA methyltransferase and reactivates methylation-silenced genes in cancer cell lines. |
| 14633726 | 2 | No | Arsenic trioxide inhibits translation of mRNA of bcr-abl, resulting in attenuation of Bcr-Abl levels and apoptosis of human leukemia cells. |
| 14636322 | 4 | No | Dose-dependent induction of cytochrome P450 (CYP) 3A4 and activation of pregnane X receptor by topiramate. |

| | | | |
|---|---|---|---|
| 14642128 | 1 | No | Arsenic trioxide inhibits p-glycoprotein expression in multidrug-resistant human leukemia cells that overexpress the MDR1 gene. |
| 14642406 | 22 | No | Regulation of selenoprotein GPx4 expression and activity in human endothelial cells by fatty acids, cytokines and antioxidants. |
| 14644359 | 1 | No | Heat shock proteins hsp32 and hsp70 as biomarkers of an early response? In vitro induction of heat shock proteins after exposure of cell culture to carcinogenic |
| 14644660 | 6 | No | Flavonoids as aryl hydrocarbon receptor agonists/antagonists: effects of structure and cell context. |
| 14644929 | 3 | No | Natural porcine surfactant augments airway inflammation after allergen challenge in patients with asthma. |
| 14645110 | 3 | No | Tamoxifen inhibits cell proliferation via mitogen-activated protein kinase cascades in human ovarian cancer cell lines in a manner not dependent on the |
| 14646600 | 4 | No | Real-time PCR analysis of the apoptosis related genes in ATRA treated APL t(15;17) patients. |
| 14647418 | 3 | No | Induction of apoptosis in human leukemia cells by the tyrosine kinase inhibitor adaphostin proceeds through a RAF-1/MEK/ERK- and AKT-dependent process. |
| 14647439 | 22 | No | Upregulation of heme oxygenase-1 and p21 confers resistance to apoptosis in human gastric cancer cells. |
| 14649730 | 3 | No | cDNA microarray analysis of changes in gene expression associated with MPP+ toxicity in SH-SY5Y cells. |
| 14653808 | 16 | No | The expression of retinoblastoma and Sp1 is increased by low concentrations of cyclin-dependent kinase inhibitors. |
| 14654083 | 2 | No | Increased cyclooxygenase-2 (COX-2): a potential role in the pathogenesis of lymphoma. |
| 14656992 | 7 | No | Hair growth induction by the Tellurium immunomodulator AS101: association with delayed terminal differentiation of follicular keratinocytes and ras-dependent |
| 14659530 | 1 | No | NO-induced migraine attack: strong increase in plasma calcitonin gene-related peptide (CGRP) concentration and negative correlation with platelet serotonin |
| 14659972 | 3 | No | 2', 3'-Dideoxycytidine represses thymidine kinases 1 and 2 expression in T-lymphoid cells. |
| 14660655 | 2 | No | 2'-benzoyloxycinnamaldehyde induces apoptosis in human carcinoma via reactive oxygen species. |
| 14662165 | 3 | No | Progesterone up-regulates WT1 mRNA and protein, and alters the relative expression of WT1 transcripts in cultured endometrial stromal cells. |
| 14662519 | 1 | No | The expression of DJ-1 (PARK7) in normal human CNS and idiopathic Parkinson's disease. |

| | | | |
|---|---|---|---|
| 14665630 | 19 | No | Regulation of p53 stability and function in HCT116 colon cancer cells. |
| 14666653 | 1 | No | Antitumor activity of herbal supplements in human prostate cancer xenografts implanted in immunodeficient mice. |
| 14666661 | 3 | No | c-IAP1 is overexpressed in HL-60 cells selected for doxorubicin resistance: effects on etoposide-induced apoptosis. |
| 14668052 | 34 | No | Toxicogenomics of non-viral vectors for gene therapy: a microarray study of lipofectin- and oligofectamine-induced gene expression changes in human |
| 14668793 | 9 | No | Arsenic enhances the activation of Stat1 by interferon gamma leading to synergistic expression of IRF-1. |
| 14669796 | 4 | No | IL-2-mediated augmentation of NK-cell activity and activation antigen expression on NK- and T-cell subsets in patients with metastatic melanoma treated with |
| 14672615 | 5 | No | 4-Hydroxynonenal as a selective pro-fibrogenic stimulus for activated human hepatic stellate cells. |
| 14672759 | 2 | No | Characteristic expression of aryl hydrocarbon receptor repressor gene in human tissues: organ-specific distribution and variable induction patterns in |
| 14673504 | 1 | No | [Effects of sodium selenite on telomerase activity and telomere length] |
| 14673790 | 5 | No | Monitoring protein expression by proteomics: human plasma exposed to benzene. |
| 14676436 | 9 | No | Exposure to toluene diisocyanate (TDI) induces IL-8 production from bronchial epithelial cells: effect of pro-inflammatory cytokines. |
| 14678968 | 3 | No | Identification of the interleukin 4 receptor alpha gene as a direct target for p73. |
| 14682389 | 6 | No | Arsenic trioxide-induced transcriptional activation of stress genes and expression of related proteins in human liver carcinoma cells (HepG2). |
| 14684751 | 11 | No | The farnesoid X receptor controls gene expression in a ligand- and promoter-selective fashion. |
| 14685799 | 12 | No | Fibrates modify the expression of key factors involved in bile-acid synthesis and biliary-lipid secretion in gallstone patients. |
| 14687023 | 4 | No | Long-acting beta2-adrenergic formoterol and salmeterol induce the apoptosis of B-chronic lymphocytic leukaemia cells. |
| 14687228 | 4 | No | Effects of eight antibacterial agents on cell survival and expression of epithelial-cell- or cell-adhesion-related genes in human gingival epithelial |
| 14688026 | 6 | No | Low dose induction of micronuclei by lindane. |

| | | | |
|---|---|---|---|
| 14691014 | 5 | No | A benzimidazole fungicide, benomyl, and its metabolite, carbendazim, induce aromatase activity in a human ovarian granulose-like tumor cell line (KGN). |
| 14695184 | 8 | No | Phorbol ester stimulates the nonhypoxic induction of a novel hypoxia-inducible factor 1alpha isoform: implications for tumor promotion. |
| 14699072 | 663 | No | Estradiol and selective estrogen receptor modulators differentially regulate target genes with estrogen receptors alpha and beta. |
| 14703492 | 12 | No | [Construction of subtracted cDNA library in human Jurkat T cell line induced by arsenic trioxide in vitro] |
| 14704367 | 2 | No | Expression and synthesis of fibroblast growth factor-9 in human gammadelta T-lymphocytes. Response to isopentenyl pyrophosphate and TGF-beta1/IL-15. |
| 14706564 | 5 | No | Estrogenic activities of Ginkgo biloba extracts. |
| 14707267 | 3 | No | Induction of CYP1A1 in tumor cells by the antitumor agent 2-[4-amino-3-methylphenyl]-5-fluoro-benzothiazole: a potential surrogate marker |
| 14707268 | 2 | No | Simultaneous activation of the intrinsic and extrinsic pathways by histone deacetylase (HDAC) inhibitors and tumor necrosis factor-related |
| 14709624 | 7 | No | The olivacine derivative s 16020 (9-hydroxy-5,6-dimethyl-N-[2-(dimethylamino)ethyl)-6H-pyrido(4,3-B)-carbazole-1-c |
| 14710233 | 5 | No | Sulindac targets nuclear beta-catenin accumulation and Wnt signalling in adenomas of patients with familial adenomatous polyposis and in human colorectal cancer |
| 14711788 | 5 | No | Upregulation of steroidogenic enzymes and ovarian 17beta-estradiol in human granulosa-lutein cells by Cordyceps sinensis mycelium. |
| 14715546 | 32 | No | Inhibition of prostate cancer cell colony formation by the flavonoid quercetin correlates with modulation of specific regulatory genes. |
| 14716737 | 1 | No | Analysis of the inflammatory network in benign prostate hyperplasia and prostate cancer. |
| 14722322 | 5 | No | Induction of drug metabolism enzymes and MDR1 using a novel human hepatocyte cell line. |
| 14726529 | 7 | No | PI3K/Akt is required for heat shock proteins to protect hypoxia-inducible factor 1alpha from pVHL-independent degradation. |
| 14729601 | 4 | No | Prostate specific antigen expression is down-regulated by selenium through disruption of androgen receptor signaling. |
| 14729647 | 42 | No | Retinoblastoma protein and CCAAT/enhancer-binding protein beta are required for 1,25-dihydroxyvitamin D3-induced monocytic differentiation of HL60 cells. |
| 14736730 | 2 | No | Tumor growth inhibition by indomethacin in a mouse model of human medullary thyroid cancer: implication of cyclooxygenases and 15-hydroxyprostaglandin |

| | | | |
|---|---|---|---|
| 14738865 | 2 | No | RXRalpha mRNA expression is associated with cell proliferation and cell cycle regulation in Hep3B cell. |
| 14739078 | 1 | No | Expression of phosphoenolpyruvate carboxykinase gene in human adipose tissue: induction by rosiglitazone and genetic analyses of the adipocyte-specific region |
| 14739603 | 2 | No | Differential control of eosinophil survival by glucocorticoids. |
| 14741686 | 31 | No | Role of phenytoin in wound healing: microarray analysis of early transcriptional responses in human dermal fibroblasts. |
| 14742295 | 2 | No | Curcumin-induced apoptosis in scleroderma lung fibroblasts: role of protein kinase cepsilon. |
| 14742696 | 13 | No | Augmented endothelial nitric oxide synthase (eNOS) protein expression in human pregnant myometrium: possible involvement of eNOS promoter activation by estrogen |
| 14749477 | 11 | No | Resveratrol modifies the expression of apoptotic regulatory proteins and sensitizes non-Hodgkin's lymphoma and multiple myeloma cell lines to |
| 14751568 | 2 | No | The nitrogen-containing bisphosphonate, zoledronic acid, increases mineralisation of human bone-derived cells in vitro. |
| 14752856 | 4 | No | Decreasing the number of MOPP courses reduces gonadal damage in survivors of childhood Hodgkin disease. |
| 14753746 | 6 | No | The nitrogen-containing bisphosphonate, zoledronic acid, influences RANKL expression in human osteoblast-like cells by activating TNF-alpha converting |
| 14757120 | 20 | No | Differentiation-inducing factor-1-induced growth arrest of K562 leukemia cells involves the reduction of ERK1/2 activity. |
| 14757314 | 26 | No | Inhibitory effect of tellimagrandin I on chemically induced differentiation of human leukemia K562 cells. |
| 14757846 | 6 | No | Topoisomerase I and II inhibitors control caspase-2 pre-messenger RNA splicing in human cells. |
| 14758092 | 4 | No | Growth inhibitory activity of extracts and purified components of black cohosh on human breast cancer cells. |
| 14762183 | 1 | No | Association of cystic fibrosis with abnormalities in fatty acid metabolism. |
| 14762343 | 3 | No | Potentiation of paclitaxel cytotoxicity in lung and esophageal cancer cells by pharmacologic inhibition of the phosphoinositide 3-kinase/protein kinase B |
| 14766015 | 9 | No | Concentration-dependent effects of endogenous S-nitrosoglutathione on gene regulation by specificity proteins Sp3 and Sp1. |
| 14767529 | 4 | No | Quercetin-induced growth inhibition and cell death in nasopharyngeal carcinoma cells are associated with increase in Bad and hypophosphorylated retinoblastoma |

| | | | |
|---|---|---|---|
| 14767865 | 1 | No | An omega-3 polyunsaturated fatty acid concentrate increases plasma high-density lipoprotein 2 cholesterol and paraoxonase levels in patients with familial |
| 14769150 | 2 | No | The effect of IFN-gamma and TNF-alpha on the eosinophilic differentiation and NADPH oxidase activation of human HL-60 clone 15 cells. |
| 14769215 | 19 | No | Effects of chebulinic acid on differentiation of human leukemia K562 cells. |
| 14871245 | 4 | No | Preclinical studies of fibroblast growth factor receptor 3 as a therapeutic target in multiple myeloma. |
| 14962509 | 2 | No | Polychlorinated biphenyl 126 stimulates basal and inducible aldosterone biosynthesis of human adrenocortical H295R cells. |
| 14962975 | 2 | No | High levels of catalase and glutathione peroxidase activity dampen H2O2 signaling in human alveolar macrophages. |
| 14970392 | 2 | No | Translation of cellular inhibitor of apoptosis protein 1 (c-IAP1) mRNA is IRES mediated and regulated during cell stress. |
| 14971644 | 2 | No | Arsenite induces HIF-1alpha and VEGF through PI3K, Akt and reactive oxygen species in DU145 human prostate carcinoma cells. |
| 14971657 | 5 | No | Lead-induced cytotoxicity and transcriptional activation of stress genes in human liver carcinoma (HepG2) cells. |
| 14971665 | 18 | No | Effect of the militarily-relevant heavy metals, depleted uranium and heavy metal tungsten-alloy on gene expression in human liver carcinoma cells (HepG2). |
| 14976336 | 1 | No | Human CD34+ hematopoietic progenitor cells are sensitive targets for toxicity induced by 1,4-benzoquinone. |
| 14977855 | 6 | No | RWJ-241947 (MCC-555), a unique peroxisome proliferator-activated receptor-gamma ligand with antitumor activity against human prostate cancer in vitro and in |
| 14977870 | 8 | No | Regulation of CYP2B6 in primary human hepatocytes by prototypical inducers. |
| 14979067 | 1 | No | [Anti-p53 antibodies as markers of carcinogenesis in exposures to vinyl chloride] |
| 14981370 | 9 | No | Sex hormone--binding globulin--a surrogate marker for the prothrombotic effects of combined oral contraceptives. |
| 14981915 | 3 | No | Cytotoxicity and apoptosis induction by butylated hydroxyanisole (BHA) and butylated hydroxytoluene (BHT). |
| 14988404 | 15 | No | Down-regulation of survivin in nitric oxide-induced cell growth inhibition and apoptosis of the human lung carcinoma cells. |
| 14988445 | 2 | No | Quercetin metabolites downregulate cyclooxygenase-2 transcription in human lymphocytes ex vivo but not in vivo. |

| | | | |
|---|---|---|---|
| 14989901 | 1 | No | [Molecular genetic mechanism of sodium arsenite on growth and development] |
| 14990334 | 3 | No | Evaluation of a real-time polymerase chain reaction method for the quantification of CYP1B1 gene expression in MCF-7 human breast carcinoma cells. |
| 14994387 | 4 | No | Expression of interleukin 12 receptor (IL-12R) and IL-18R on CD4+ T cells from patients with rheumatoid arthritis. |
| 14996990 | 13 | No | DNA microarray analysis of vitamin D-induced gene expression in a human colon carcinoma cell line. |
| 14997032 | 10 | No | P38 mitogen activated protein kinase is involved in the downregulation of granulocyte CXC chemokine receptors 1 and 2 during human endotoxemia. |
| 14998682 | 1 | No | Diepoxybutane induces caspase and p53-mediated apoptosis in human lymphoblasts. |
| 14999240 | 2 | No | Relationship between upregulated oestrogen receptors and expression of growth factors in cultured, human, prostatic stromal cells exposed to estradiol or |
| 15003732 | 2 | No | Effect of antineoplastic agents on the expression of human telomerase reverse transcriptase beta plus transcript in MCF-7 cells. |
| 15015580 | 1 | No | PCR and flow cytometric analysis of paclitaxel-inhibited arylamine N-acetyltransferase activity and gene expression in human osteogenic sarcoma |
| 15016631 | 1 | No | Asymmetric dimethylarginine upregulates LOX-1 in activated macrophages: role in foam cell formation. |
| 15016801 | 430 | No | p53-regulated transcriptional program associated with genotoxic stress-induced apoptosis. |
| 15020278 | 1 | No | Circulating endothelin-3 levels in patients with sickle cell disease during hydroxyurea treatment. |
| 15026559 | 2 | No | Correspondence re: DC Lev et al., Dacarbazine causes transcriptional up-regulation of interleukin 8 and vascular endothelial growth factor in melanoma |
| 15027113 | 2 | No | Inhibition of MUC1 expression by indole-3-carbinol. |
| 15031205 | 6 | No | Treatment with arsenic trioxide (ATO) and MEK1 inhibitor activates the p73-p53AIP1 apoptotic pathway in leukemia cells. |
| 15033005 | 1 | No | [Expression of M3 receptor gene in peripheral blood lymphocytes of workers exposed to dimethoate] |
| 15034078 | 1 | No | Treatment of melanoma with 5-fluorouracil or dacarbazine in vitro sensitizes cells to antigen-specific CTL lysis through perforin/granzyme- and Fas-mediated |
| 15034294 | 8 | No | Glucocorticoids induce G1 arrest of lymphoblastic cells through retinoblastoma protein Rb1 dephosphorylation in childhood acute lymphoblastic leukemia in vivo. |

| | | | |
|---|---|---|---|
| 15036117 | 2 | No | Proteomic analysis of plasma proteins of workers exposed to benzene. |
| 15037631 | 1 | No | p38 MAPK activation selectively induces cell death in K-ras-mutated human colon cancer cells through regulation of vitamin D receptor. |
| 15047127 | 6 | No | Levamisole modulates prostaglandin E2 production and cyclooxygenase II gene expression in human colonic cancer cells. |
| 15049342 | 4 | No | Cytoprotection following endoplasmic reticulum stress protein induction in continuous cell lines. |
| 15050405 | 2 | No | A comparative study of the effect of oxidative stress on the cytoskeleton in human cortical neurons. |
| 15050414 | 18 | No | 4-Hydroxy estradiol but not 2-hydroxy estradiol induces expression of hypoxia-inducible factor 1alpha and vascular endothelial growth factor A through |
| 15051476 | 2 | No | Retinoic acid and dexamethasone regulate the expression of PEDF in retinal and endothelial cells. |
| 15056853 | 4 | No | Oxidative stress induces GSTP1 and CYP3A4 expression in the human erythroleukemia cell line, K562. |
| 15057142 | 2 | No | Down-regulation of thymidylate synthase expression and its steady-state mRNA by oxaliplatin in colon cancer cells. |
| 15059897 | 23 | No | Identification of retinoid-modulated proteins in squamous carcinoma cells using high-throughput immunoblotting. |
| 15059916 | 12 | No | The histone deacetylase inhibitor MS-275 interacts synergistically with fludarabine to induce apoptosis in human leukemia cells. |
| 15061398 | 8 | No | Antihistamines added to an antileukotriene in treating seasonal allergic rhinitis: histamine and leukotriene antagonism. |
| 15065019 | 8 | No | Phosphatidylinositol 3-kinase inhibition down-regulates survivin and facilitates TRAIL-mediated apoptosis in neuroblastomas. |
| 15070760 | 26 | No | Role of NADPH oxidase in arsenic-induced reactive oxygen species formation and cytotoxicity in myeloid leukemia cells. |
| 15072547 | 102 | No | Estrogen regulation in human breast cancer cells of new downstream gene targets involved in estrogen metabolism, cell proliferation and cell transformation. |
| 15072578 | 1 | No | Regulation of parathyroid hormone-related protein gene expression by epidermal growth factor-family ligands in primary human keratinocytes. |
| 15075239 | 8 | No | Oxygen-regulated expression of the RNA-binding proteins RBM3 and CIRP by a HIF-1-independent mechanism. |
| 15075359 | 3 | No | Guggulsterone activates multiple nuclear receptors and induces CYP3A gene expression through the pregnane X receptor. |

| | | | |
|---|---|---|---|
| 15078984 | 1 | No | Flavopiridol enhances human tumor cell radiosensitivity and prolongs expression of gammaH2AX foci. |
| 15081272 | 5 | No | Effect of nickel and iron co-exposure on human lung cells. |
| 15083329 | 9 | No | Vitamin C rescues in part the effects of nitrofen on cultured human pneumocytes. |
| 15086456 | 6 | No | Reactive oxygen species amplify protein kinase C signaling in high glucose-induced fibronectin expression by human peritoneal mesothelial cells. |
| 15086938 | 2 | No | FGF-23 in patients with end-stage renal disease on hemodialysis. |
| 15089887 | 1 | No | Enhanced expression of interleukin-8 and activation of nuclear factor kappa-B in endoscopy-negative gastroesophageal reflux disease. |
| 15090468 | 15 | No | Interactions between sulforaphane and apigenin in the induction of UGT1A1 and GSTA1 in CaCo-2 cells. |
| 15090535 | 60 | No | The G protein-coupled receptor GPR30 mediates c-fos up-regulation by 17beta-estradiol and phytoestrogens in breast cancer cells. |
| 15090542 | 22 | No | Protein farnesyltransferase inhibitor (SCH 66336) abolishes NF-kappaB activation induced by various carcinogens and inflammatory stimuli leading to suppression of |
| 15093275 | 2 | No | Aryl-hydrocarbon receptor-dependent pathway and toxic effects of TCDD in humans: a population-based study in Seveso, Italy. |
| 15100154 | 2 | No | Imatinib mesylate inhibits T-cell proliferation in vitro and delayed-type hypersensitivity in vivo. |
| 15100173 | 4 | No | Induction and inhibition of cytochromes P450 by the St. John's wort constituent hyperforin in human hepatocyte cultures. |
| 15101064 | 1 | No | Profiling catalase gene expression in Drosophila melanogaster during development and aging. |
| 15103026 | 43 | No | Short-chain fatty acids enhance nuclear receptor activity through mitogen-activated protein kinase activation and histone deacetylase inhibition. |
| 15104253 | 6 | No | The induction of human UDP-glucuronosyltransferase 1A1 mediated through a distal enhancer module by flavonoids and xenobiotics. |
| 15109396 | 14 | No | Comparison of gene expression in HCT116 treatment derivatives generated by two different 5-fluorouracil exposure protocols. |
| 15113938 | 36 | No | Flavones mitigate tumor necrosis factor-alpha-induced adhesion molecule upregulation in cultured human endothelial cells: role of nuclear factor-kappa B. |
| 15114628 | 12 | No | 1-Benzyl-1,2,3,4-tetrahydroisoquinoline, a Parkinsonism-inducing endogenous toxin, increases alpha-synuclein expression and causes nuclear damage in human |

| | | | |
|---|---|---|---|
| 15115777 | 12 | No | 2-Arachidonoylglycerol, an endogenous cannabinoid receptor ligand, induces accelerated production of chemokines in HL-60 cells. |
| 15117409 | 1 | No | [HCO3-]-regulated expression and activity of soluble adenylyl cyclase in corneal endothelial and Calu-3 cells. |
| 15118252 | 6 | No | Assessing estrogenic activity of pyrethroid insecticides using in vitro combination assays. |
| 15118409 | 1 | No | Maintenance of G1 checkpoint controls in telomerase-immortalized endothelial cells. |
| 15120327 | 1 | No | Overcoming acquired resistance to TRAIL by chemotherapeutic agents and calpain inhibitor I through distinct mechanisms. |
| 15120416 | 2 | No | Estrogenic effects of resveratrol in breast cancer cells expressing mutant and wild-type estrogen receptors: role of AF-1 and AF-2. |
| 15120964 | 5 | No | Comparison of basal gene expression profiles and effects of hepatocarcinogens on gene expression in cultured primary human hepatocytes and HepG2 cells. |
| 15120965 | 4 | No | Applications of microarrays with toxicologically relevant genes (tox genes) for the evaluation of chemical toxicants in Sprague Dawley rats in vivo and human |
| 15123723 | 3 | No | Human constitutive androstane receptor mediates induction of CYP2B6 gene expression by phenytoin. |
| 15123733 | 4 | No | Exposure of melanoma cells to dacarbazine results in enhanced tumor growth and metastasis in vivo. |
| 15126337 | 11 | No | Apoptotic signaling pathways induced by nitric oxide in human lymphoblastoid cells expressing wild-type or mutant p53. |
| 15129424 | 3 | No | Therapeutic potential of curcumin in prostate cancer--V: Interference with the osteomimetic properties of hormone refractory C4-2B prostate cancer cells. |
| 15130599 | 14 | No | Effects of the pesticides prochloraz and methiocarb on human estrogen receptor alpha and beta mRNA levels analyzed by on-line RT-PCR. |
| 15132777 | 2 | No | The expressions of p21 and pRB may be good indicators for the sensitivity of esophageal squamous cell cancers to CPT-11: Cell proliferation activity |
| 15135645 | 48 | No | Equine estrogen metabolite 4-hydroxyequilenin induces anchorage-independent growth of human mammary epithelial MCF-10A cells: differential gene expression. |
| 15138554 | 6 | No | Regulation of matrix metalloproteinase 13 expression by androgen in prostate cancer. |
| 15139008 | 12 | No | Reactive oxygen species mediate chloroquine-induced expression of chemokines by human astroglial cells. |
| 15141018 | 7 | No | Methyl selenium metabolites decrease prostate-specific antigen expression by inducing protein degradation and suppressing androgen-stimulated transcription. |

| | | | |
|---|---|---|---|
| 15141020 | 1 | No | Topoisomerase poisons differentially activate DNA damage checkpoints through ataxia-telangiectasia mutated-dependent and -independent mechanisms. |
| 15142168 | 4 | No | Growth inhibition and cell cycle arrest effects of epigallocatechin gallate in the NBT-II bladder tumour cell line. |
| 15142886 | 4 | No | Resveratrol inhibits TCDD-induced expression of CYP1A1 and CYP1B1 and catechol estrogen-mediated oxidative DNA damage in cultured human mammary epithelial |
| 15144595 | 1 | No | [Effects of selective cyclooxygenase-2 inhibitor NS-398 on 5-fluorouracil chemotherapy and progression of colon cells: an experimental study] |
| 15144885 | 1 | No | alpha-Tocopheryl succinate inhibits proliferation of mesothelioma cells by selective down-regulation of fibroblast growth factor receptors. |
| 15145977 | 6 | No | Identification of intermediates in the bile acid synthetic pathway as ligands for the farnesoid X receptor. |
| 15147944 | 6 | No | Expression of connective tissue growth factor, a biomarker in senescence of human diploid fibroblasts, is up-regulated by a transforming growth |
| 15150550 | 6 | No | Modulation of thymidine phosphorylase by neoadjuvant chemotherapy in primary breast cancer. |
| 15152939 | 40 | No | 5-Fluorouracil: identification of novel downstream mediators of tumour response. |
| 15155531 | 3 | No | Synthetic and naturally occurring COX-2 inhibitors suppress proliferation in a human oesophageal adenocarcinoma cell line (OE33) by inducing apoptosis and cell |
| 15155752 | 31 | No | The Wilms tumor suppressor-1 target gene podocalyxin is transcriptionally repressed by p53. |
| 15157790 | 12 | No | The contraceptive vaginal ring (NuvaRing) and hemostasis: a comparative study. |
| 15157999 | 1 | No | 2,3,7,8-Tetrachlorodibenzo-p-dioxin inhibits cell proliferation through arylhydrocarbon receptor-mediated G1 arrest in SK-N-SH human neuronal cells. |
| 15159206 | 60 | No | Using a customized DNA microarray for expression profiling of the estrogen-responsive genes to evaluate estrogen activity among natural estrogens |
| 15161716 | 16 | No | The roles of thymidylate synthase and p53 in regulating Fas-mediated apoptosis in response to antimetabolites. |
| 15161907 | 4 | No | beta-D-Glucoside suppresses tumor necrosis factor-induced activation of nuclear transcription factor kappaB but potentiates apoptosis. |
| 15162144 | 4 | No | Resveratrol inhibits benzo[a]pyrene-DNA adduct formation in human bronchial epithelial cells. |
| 15162350 | 2 | No | Constitutive expression of interleukin-18 in head and neck squamous carcinoma cells. |

| | | | |
|---|---|---|---|
| 15162845 | 1 | No | The involvement of p53 in paraquat-induced apoptosis in human lung epithelial-like cells. |
| 15169886 | 9 | No | Dietary polyphenols increase paraoxonase 1 gene expression by an aryl hydrocarbon receptor-dependent mechanism. |
| 15169887 | 2 | No | Epstein-Barr virus latent membrane protein 1 induces synthesis of hypoxia-inducible factor 1 alpha. |
| 15173892 | 1 | No | The IL-12Rbeta2 gene functions as a tumor suppressor in human B cell malignancies. |
| 15174162 | 1 | No | A novel bis-aziridinylnaphthoquinone with anti-solid tumor activity in which induced apoptosis is associated with altered expression of Bcl-2 protein. |
| 15177505 | 7 | No | Sodium butyrate induces apoptosis in human hepatoma cells by a mitochondria/caspase pathway, associated with degradation of beta-catenin, pRb |
| 15178637 | 3 | No | Atorvastatin restores endothelial function in normocholesterolemic smokers independent of changes in low-density lipoprotein. |
| 15179185 | 8 | No | Deacetylase inhibition in malignant melanomas: impact on cell cycle regulation and survival. |
| 15180942 | 9 | No | Non-steroidal anti-inflammatory drug activated gene (NAG-1) expression is closely related to death receptor-4 and -5 induction, which may explain sulindac sulfide |
| 15180955 | 3 | No | Local cyclin-dependent kinase inhibition by flavopiridol inhibits coronary artery smooth muscle cell proliferation and migration: Implications for the |
| 15182386 | 2 | No | Effect of dietary flavonols on oestrogen receptor transactivation and cell death induction. |
| 15182955 | 4 | No | Differential regulation of CYP1A1 and CYP1B1 expression in resveratrol-treated human medulloblastoma cells. |
| 15183442 | 5 | No | Comparison of the response of primary human blood monocytes and the U937 human monocytic cell line to two different sizes of alumina ceramic particles. |
| 15185533 | 3 | No | [Effect of low-dose oral contraceptive regulon-Gedeon Richter on some hemostatic variables] |
| 15188006 | 6 | No | Effect of nonsteroidal anti-inflammatory drugs on beta-catenin protein levels and catenin-related transcription in human colorectal cancer cells. |
| 15189689 | 5 | No | [Effects of vitamin A on the differentiation, maturation and functions of dendritic cells from cord blood] |
| 15190207 | 25 | No | Induction of transcription by p21Waf1/Cip1/Sdi1: role of NFkappaB and effect of non-steroidal anti-inflammatory drugs. |
| 15191659 | 2 | No | [Re-expression of p16 gene in myeloma cell line U266 by arsenic trioxide] |

| | | | |
|---|---|---|---|
| 15191912 | 5 | No | Activation of airway epithelial cells by toll-like receptor agonists. |
| 15194426 | 9 | No | Retinoic acid regulates cell cycle progression and cell differentiation in human monocytic THP-1 cells. |
| 15196572 | 4 | No | Candida albicans and Saccharomyces cerevisiae induce interleukin-8 production from intestinal epithelial-like Caco-2 cells in the presence of butyric acid. |
| 15202011 | 32 | No | Synthetic bile acid derivatives inhibit cell proliferation and induce apoptosis in HT-29 human colon cancer cells. |
| 15204521 | 7 | No | Thermal enhancement of oxaliplatin-induced inhibition of cell proliferation and cell cycle progression in human carcinoma cell lines. |
| 15205115 | 4 | No | LPS-induced downregulation of MRP2 and BSEP in human liver is due to a |
| 15205334 | 102 | No | Cell-type-specific responses to chemotherapeutics in breast cancer. |
| 15210717 | 22 | No | Phospholipase D isozymes mediate epigallocatechin gallate-induced cyclooxygenase-2 expression in astrocyte cells. |
| 15211800 | 2 | No | [Homocysteine-induced apoptosis of endothelial cell and its antagonism by folic acid--the roles of caspase3, c-IAP1 and c-IAP2] |
| 15212811 | 11 | No | Response of human cord blood cells to styrene exposure: evaluation of its effects on apoptosis and gene expression by genomic technology. |
| 15213147 | 1 | No | Chemotherapy for schistosomiasis in Ugandan fishermen: treatment can cause a rapid increase in interleukin-5 levels in plasma but decreased levels of |
| 15213310 | 4 | No | 9-beta-D-arabinofuranosyl-2-fluoroadenine inhibits expression of vascular endothelial growth factor through hypoxia-inducible factor-1 in human ovarian |
| 15215046 | 4 | No | CD44 promotes resistance to apoptosis in human colon cancer cells. |
| 15215653 | 2 | No | Dracorhodin perchlorate induces apoptosis via activation of caspases and generation of reactive oxygen species. |
| 15218360 | 4 | No | Estrogen responsiveness of IBEP-2, a new human cell line derived from breast carcinoma. |
| 15219790 | 10 | No | From ligand structure to biological activity: modified estratrienes and their estrogenic and antiestrogenic effects in MCF-7 cells. |
| 15219929 | 8 | No | Clusters of biotin-responsive genes in human peripheral blood mononuclear cells. |
| 15221776 | 44 | No | Protein expression profiling identifies molecular targets of quercetin as a major dietary flavonoid in human colon cancer cells. |

| | | | |
|---|---|---|---|
| 15225597 | 4 | No | Effect of flavonoids and vitamin E on cyclooxygenase-2 (COX-2) transcription. |
| 15225615 | 1 | No | A P53 target gene, PIG11, contributes to chemosensitivity of cells to arsenic trioxide. |
| 15225641 | 8 | No | Vitamin E activates CRABP-II gene expression in cultured human fibroblasts, role of protein kinase C. |
| 15226459 | 58 | No | Investigation of lymphocyte gene expression for use as biomarkers for zinc status in humans. |
| 15228094 | 17 | No | The DNA damaging agent VP16 induces the expression of a subset of ligands from the EGF system in bladder cancer cells, whereas none of the four EGF receptors |
| 15231455 | 2 | No | Mechanism of sulforaphane-induced cell cycle arrest and apoptosis in human colon cancer cells. |
| 15236964 | 2 | No | A HOXA10 estrogen response element (ERE) is differentially regulated by 17 beta-estradiol and diethylstilbestrol (DES). |
| 15239124 | 14 | No | Enhanced in vitro invasiveness and drug resistance with altered gene expression patterns in a human lung carcinoma cell line after pulse selection with |
| 15242773 | 1 | No | Inhibition of phosphatidylinostol 3-kinase uncouples H2O2-induced senescent phenotype and cell cycle arrest in normal human diploid fibroblasts. |
| 15244497 | 6 | No | concentration on cycle day 2 of the third cycle was lower than that of the second cycle. CONCLUSIONS: Oral tazarotene up to 6 mg once daily does not affect the |
| 15246556 | 3 | No | Inhibition of cell cycle progression on HepG2 cells by hypsiziprenol A9, isolated from Hypsizigus marmoreus. |
| 15247267 | 2 | No | Metalloproteinase-dependent transforming growth factor-alpha release mediates neurotensin-stimulated MAP kinase activation in human colonic epithelial cells. |
| 15247282 | 3 | No | Proteasome-dependent degradation of cyclin D1 in 1-methyl-4-phenylpyridinium ion (MPP+)-induced cell cycle arrest. |
| 15248904 | 1 | No | [Indomethacin induces apoptosis through inhibition of survivin regulated by beta-catenin/TCF4 in human colorectal cancer cells] |
| 15250830 | 8 | No | Regulation of keratinocyte growth factor and scatter factor in cyclosporin-induced gingival overgrowth. |
| 15251176 | 12 | No | An inhibitor of p38 MAP kinase downregulates cytokine release induced by sulfur mustard exposure in human epidermal keratinocytes. |
| 15254749 | 10 | No | The PPARgamma ligands PGJ2 and rosiglitazone show a differential ability to inhibit proliferation and to induce apoptosis and differentiation of human |
| 15256057 | 33 | No | Regulation of gene expression and inhibition of experimental prostate cancer bone metastasis by dietary genistein. |

| | | | |
|---|---|---|---|
| 15257099 | 9 | No | Genistein-induced changes in gene expression in Panc 1 cells at physiological concentrations of genistein. |
| 15258199 | 1 | No | Bezafibrate stimulates canalicular localization of NBD-labeled PC in HepG2 cells by PPARalpha-mediated redistribution of ABCB4. |
| 15261991 | 18 | No | Effect of in vitro estrogenic pesticides on human oestrogen receptor alpha and beta mRNA levels. |
| 15263067 | 17 | No | Inhibition of activator protein 1 by barbiturates is mediated by differential effects on mitogen-activated protein kinases and the small G proteins ras and |
| 15265368 | 2 | No | Upregulation and activation of caspase-3 or caspase-8 and elevation of intracellular free calcium mediated apoptosis of indomethacin-induced K562 cells. |
| 15266218 | 4 | No | In vitro interaction of the HIV protease inhibitor ritonavir with herbal constituents: changes in P-gp and CYP3A4 activity. |
| 15268896 | 54 | No | Unique regulation of SOST, the sclerosteosis gene, by BMPs and steroid hormones in human osteoblasts. |
| 15269186 | 12 | No | Tocotrienols activate the steroid and xenobiotic receptor, SXR, and selectively regulate expression of its target genes. |
| 15272053 | 18 | No | Meclizine is an agonist ligand for mouse constitutive androstane receptor (CAR) and an inverse agonist for human CAR. |
| 15276080 | 3 | No | Participation of cyclin D1 deregulation in TNP-470-mediated cytostatic effect: involvement of senescence. |
| 15278913 | 2 | No | Effect of artificial mixtures of environmental polycyclic aromatic hydrocarbons present in coal tar, urban dust, and diesel exhaust particulates on MCF-7 cells |
| 15284208 | 4 | No | The epidermal growth factor-like growth factor amphiregulin is strongly induced by the adenosine 3',5'-monophosphate pathway in various cell types. |
| 15284827 | 10 | No | [Antimycotics suppress interleukin-4 and interleukin-5 production in anti-CD3 plus anti-CD28-stimulated T cells from patients with atopic dermatitis] |
| 15286001 | 1 | No | Fibrogenic cytokine levels in bronchoalveolar lavage aspirates 15 years after exposure to sulfur mustard. |
| 15286717 | 5 | No | Inhibitory effects of cyclosporin A on calcium mobilization-dependent interleukin-8 expression and invasive potential of human glioblastoma U251MG |
| 15288212 | 42 | No | Effect of four oral contraceptives on hemostatic parameters. |
| 15288519 | 3 | No | The soy isoflavone, genistein, protects human cortical neuronal cells from oxidative stress. |
| 15291358 | 2 | No | Nuclear retinoid receptors are involved in N-(4-hydroxyphenyl) retinamide (Fenretinide)-induced gene expression and growth inhibition in HL-60 acute |

| | | | |
|---|---|---|---|
| 15291740 | 1 | No | Thyroid hormone regulation of prohormone convertase 1 (PC1): regional expression in rat brain and in vitro characterization of negative thyroid hormone response |
| 15291876 | 3 | No | Caffeic acid phenethyl ester-induced PC-3 cell apoptosis is caspase-dependent and mediated through the loss of inhibitors of apoptosis proteins. |
| 15292067 | 15 | No | Immunomodulatory derivative of thalidomide (IMiD CC-4047) induces a shift in lineage commitment by suppressing erythropoiesis and promoting myelopoiesis. |
| 15292203 | 2 | No | Heat shock induces preferential translation of ERGIC-53 and affects its recycling pathway. |
| 15292456 | 3 | No | Selective nonsteroidal anti-inflammatory drugs induce thymosin beta-4 and alter actin cytoskeletal organization in human colorectal cancer cells. |
| 15292961 | 5 | No | The zinc finger transcription factor Egr-1 is upregulated in arsenite-treated human keratinocytes. |
| 15297370 | 2 | No | Tobacco smoke induces CYP1B1 in the aerodigestive tract. |
| 15297373 | 7 | No | AMP-activated protein kinase activity is required for vanadate-induced hypoxia-inducible factor 1alpha expression in DU145 cells. |
| 15297405 | 4 | No | Phase I trial of the cyclin-dependent kinase inhibitor flavopiridol in combination with docetaxel in patients with metastatic breast cancer. |
| 15297603 | 1 | No | Mitogenic activity of estrogens in human breast cancer cells does not rely on direct induction of mitogen-activated protein kinase/extracellularly regulated |
| 15301717 | 4 | No | [Enhancement effect of interferon gamma on the sensitivity of RT4 bladder cancer cells to 5'-deoxy-5-fluorouridine,and 5-fluorouracil through up-regulation of |
| 15302583 | 2 | No | TIS21/BTG2/PC3 is expressed through PKC-delta pathway and inhibits binding of cyclin B1-Cdc2 and its activity, independent of p53 expression. |
| 15304546 | 4 | No | Molecular dissection of the butyrate action revealed the involvement of mitogen-activated protein kinase in cystic fibrosis transmembrane conductance |
| 15307955 | 17 | No | Polyunsaturated fatty acids are FXR ligands and differentially regulate expression of FXR targets. |
| 15309432 | 123 | No | Integrated assessment by multiple gene expression analysis of quercetin bioactivity on anticancer-related mechanisms in colon cancer cells in vitro. |
| 15309715 | 3 | No | Effect of lamivudine treatment on plasma levels of transforming growth factor beta1, tissue inhibitor of metalloproteinases-1 and metalloproteinase-1 in |
| 15313166 | 3 | No | Inhibition of the MEK-1/p42 MAP kinase reduces aryl hydrocarbon receptor-DNA interactions. |
| 15313392 | 8 | No | Drug development for ovarian hyper-stimulation and anti-cancer treatment: blocking of gonadotropin signaling for epiregulin and amphiregulin biosynthesis. |

| | | | |
|---|---|---|---|
| 15313406 | 17 | No | Effect of chemopreventive agents on glutathione S-transferase P1-1 gene expression mechanisms via activating protein 1 and nuclear factor kappaB |
| 15313425 | 1 | No | Increased glutathione S-transferase P1-1 expression by mRNA stabilization in hemin-induced differentiation of K562 cells. |
| 15316029 | 4 | No | Induction and antimicrobial activity of platelet basic protein derivatives in human monocytes. |
| 15316568 | 2 | No | Analysis of resveratrol as a lung cancer chemopreventive agent in A/J mice exposed to benzo[a]pyrene. |
| 15317450 | 1 | No | Determinants of retinoid X receptor transcriptional antagonism. |
| 15318540 | 4 | No | [Effect of the monophase oral contraceptive combination with 20 ug ethinyl estradiol/150 ug desogestrel on haemostasis] |
| 15318936 | 16 | No | A retinoid X receptor (RXR)-selective retinoid reveals that RXR-alpha is potentially a therapeutic target in breast cancer cell lines, and that it |
| 15318950 | 2 | No | Cross-species global and subset gene expression profiling identifies genes involved in prostate cancer response to selenium. |
| 15319175 | 12 | No | Limited effect of selected organic pollutants on cytokine production by peripheral blood leukocytes. |
| 15319488 | 2 | No | Induction and inhibition of aromatase (CYP19) activity by natural and synthetic flavonoid compounds in H295R human adrenocortical carcinoma cells. |
| 15319539 | 1 | No | Interaction of the PAS B domain with HSP90 accelerates hypoxia-inducible factor-1alpha stabilization. |
| 15322087 | 24 | No | Guggulsterone inhibits NF-kappaB and IkappaBalpha kinase activation, suppresses expression of anti-apoptotic gene products, and enhances apoptosis. |
| 15322241 | 4 | No | 2,3,7,8-Tetrachlorodibenzo-p-dioxin blocks androgen-dependent cell proliferation of LNCaP cells through modulation of pRB phosphorylation. |
| 15322261 | 36 | No | Flavonoids inhibit tumor necrosis factor-alpha-induced up-regulation of intercellular adhesion molecule-1 (ICAM-1) in respiratory epithelial cells |
| 15324309 | 11 | No | Zoledronic acid up-regulates bone sialoprotein expression in osteoblastic cells through Rho GTPase inhibition. |
| 15327971 | 5 | No | Unique natural antioxidants (NAOs) and derived purified components inhibit cell cycle progression by downregulation of ppRb and E2F in human PC3 prostate cancer |
| 15329907 | 1 | No | Sensitivity to imatinib therapy may be predicted by testing Wilms tumor gene expression and colony growth after a short in vitro incubation. |
| 15331170 | 19 | No | Identification of novel genes associated with the response to 5-FU treatment in gastric cancer cell lines using a cDNA microarray. |

| | | | |
|---|---|---|---|
| 15331199 | 1 | No | Vascular endothelial growth factor in diabetes induced early retinal abnormalities. |
| 15331595 | 12 | No | The functional consequences of cross-talk between the vitamin D receptor and ERK signaling pathways are cell-specific. |
| 15333513 | 4 | No | Effects of avasimibe on cytochrome P450 2C9 expression in vitro and in vivo. |
| 15333708 | 2 | No | Biotin supplementation increases expression of the cytochrome P450 1B1 gene in Jurkat cells, increasing the occurrence of single-stranded DNA breaks. |
| 15334063 | 4 | No | Luteolin sensitizes tumor necrosis factor-alpha-induced apoptosis in human tumor cells. |
| 15334457 | 2 | No | Induction of RANKL expression and osteoclast maturation by the binding of fibroblast growth factor 2 to heparan sulfate proteoglycan on rheumatoid synovial |
| 15336504 | 978 | No | Comparison of characteristics of peroxide-conditioned immortal human lens-epithelial cell lines with their murine counterparts. |
| 15336539 | 1 | No | Drug uptake and pharmacological modulation of drug sensitivity in leukemia by AQP9. |
| 15336702 | 28 | No | Partial agonist/antagonist properties of androstenedione and 4-androsten-3beta,17beta-diol. |
| 15339911 | 12 | No | Critical role of endogenous Akt/IAPs and MEK1/ERK pathways in counteracting endoplasmic reticulum stress-induced cell death. |
| 15342418 | 18 | No | P21Cip1 is a critical mediator of the cytotoxic action of thymidylate synthase inhibitors in colorectal carcinoma cells. |
| 15342794 | 2 | No | Analysis of ATP-binding cassette transporter expression in drug-selected cell lines by a microarray dedicated to multidrug resistance. |
| 15342952 | 245 | No | A toxicogenomic approach to drug-induced phospholipidosis: analysis of its induction mechanism and establishment of a novel in vitro screening system. |
| 15345368 | 11 | No | Gene interaction network suggests dioxin induces a significant linkage between aryl hydrocarbon receptor and retinoic acid receptor beta. |
| 15345676 | 8 | No | Peroxisome proliferator-activated receptor gamma-dependent activation of p21 in Panc-28 pancreatic cancer cells involves Sp1 and Sp4 proteins. |
| 15350188 | 4 | No | Differential regulation of glucose transporter expression by estrogen and progesterone in Ishikawa endometrial cancer cells. |
| 15352031 | 77 | No | Dissecting progressive stages of 5-fluorouracil resistance in vitro using RNA expression profiling. |
| 15355884 | 5 | No | Fluorinated 2-(4-amino-3-methylphenyl)benzothiazoles induce CYP1A1 expression, become metabolized, and bind to macromolecules in sensitive human cancer cells. |

| | | | |
|---|---|---|---|
| 15356053 | 1 | No | FGF-23 is elevated by chronic hyperphosphatemia. |
| 15356918 | 4 | No | Stimulation of endothelial IL-8 (eIL-8) production and apoptosis by phenolic metabolites of benzene in HL-60 cells and human bone marrow endothelial cells. |
| 15358673 | 46 | No | Differential signal transduction of progesterone and medroxyprogesterone acetate in human endothelial cells. |
| 15358692 | 6 | No | Inhaled IFN-gamma for persistent nontuberculous mycobacterial pulmonary disease due to functional IFN-gamma deficiency. |
| 15359644 | 6 | No | Upon drug-induced apoptosis in lymphoma cells X-linked inhibitor of apoptosis (XIAP) translocates from the cytosol to the nucleus. |
| 15364798 | 1 | No | Possible association of heart failure status with synthetic balance between aldosterone and dehydroepiandrosterone in human heart. |
| 15367699 | 2 | No | Disulfiram inhibits activating transcription factor/cyclic AMP-responsive element binding protein and human melanoma growth in a metal-dependent manner in vitro, |
| 15368248 | 1 | No | Bisphenol A from dental polycarbonate crown upregulates the expression of hTERT. |
| 15369734 | 2 | No | Methylene chloride fraction of Scutellaria barbata induces apoptosis in human U937 leukemia cells via the mitochondrial signaling pathway. |
| 15371228 | 18 | No | Asbestos induces tissue factor in Beas-2B cells via PI3 kinase-PKC-mediated signaling. |
| 15371785 | 1 | No | Inhibition of mitogenic signaling and induction of apoptosis in human bladder smooth muscle cells treated with doxazosin. |
| 15374632 | 6 | No | Geraniol, a component of plant essential oils, modulates DNA synthesis and potentiates 5-fluorouracil efficacy on human colon tumor xenografts. |
| 15375546 | 12 | No | Breast cancer progression in MCF10A series of cell lines is associated with alterations in retinoic acid and retinoid X receptors and with differential |
| 15377668 | 2 | No | Cooperation between fibroblast growth factor receptor-4 and ErbB2 in regulation of cyclin D1 translation. |
| 15377855 | 1 | No | The experimental antitumor agents Phortress and doxorubicin are equiactive against human-derived breast carcinoma xenograft models. |
| 15378274 | 1 | No | Reversal of multidrug resistance of cancer through inhibition of P-glycoprotein by 5-bromotetrandrine. |
| 15378487 | 6 | No | The androgen receptor co-activator CBP is up-regulated following androgen withdrawal and is highly expressed in advanced prostate cancer. |
| 15378649 | 27 | No | Using DNA microarray analyses to elucidate the effects of genistein in androgen-responsive prostate cancer cells: identification of novel targets. |

| | | | |
|---|---|---|---|
| 15379216 | 8 | No | Effect of ozone exposure on intracellular glutathione redox state in cultured human airway epithelial cells. |
| 15380616 | 9 | No | Possible involvement of protein kinase C activation in differentiation of human umbilical vein endothelium-derived cell into smooth muscle-like cell. |
| 15382040 | 2 | No | Arsenic trioxide sensitizes CD95/Fas-induced apoptosis through ROS-mediated upregulation of CD95/Fas by NF-kappaB activation. |
| 15382119 | 42 | No | Interleukin 1beta inhibits CAR-induced expression of hepatic genes involved in drug and bilirubin clearance. |
| 15385644 | 29 | No | Superinduction of CYP1A1 in MCF10A cultures by cycloheximide, anisomycin, and puromycin: a process independent of effects on protein translation and unrelated |
| 15385934 | 1 | No | Potent antileukemic interactions between flavopiridol and TRAIL/Apo2L involve flavopiridol-mediated XIAP downregulation. |
| 15386353 | 2 | No | Calcitriol-induced prostate-derived factor: autocrine control of prostate cancer cell growth. |
| 15387353 | 6 | No | Pharmacodynamics and pharmacokinetics of oral contraceptives co-administered with alosetron (Lotronex). |
| 15388451 | 7 | No | Human immunodeficiency virus type 1 protease inhibitors block toll-like receptor 2 (TLR2)- and TLR4-Induced NF-kappaB activation. |
| 15447660 | 3 | No | 1-trichloromethyl-1,2,3,4-tetrahydro-beta-carboline-induced apoptosis in the human neuroblastoma cell line SK-N-SH. |
| 15448115 | 20 | No | Liver grafts preserved in Celsior solution as source of hepatocytes for drug metabolism studies: comparison with surgical liver biopsies. |
| 15450938 | 1 | No | Differentiation-inducing quinolines as experimental breast cancer agents in the MCF-7 human breast cancer cell model. |
| 15451026 | 8 | No | Signalling pathways in the induction of proteasome expression by proteolysis-inducing factor in murine myotubes. |
| 15452088 | 29 | No | Survival of retinal pigment epithelium after exposure to prolonged oxidative injury: a detailed gene expression and cellular analysis. |
| 15454121 | 3 | No | Para-Bromophenacyl bromide alleviates airway hyperresponsiveness and modulates cytokines, IgE and eosinophil levels in ovalbumin-sensitized and -challenged |
| 15454145 | 2 | No | Endotoxin reduces CD95-induced neutrophil apoptosis by cIAP-2-mediated caspase-3 degradation. |
| 15456083 | 3 | No | ATP binding cassette multidrug transporters limit the anti-HIV activity of zidovudine and indinavir in infected human macrophages. |
| 15456542 | 5 | No | Je-chun-jun induced apoptosis of human cervical carcinoma HeLa cells. |

| | | | |
|---|---|---|---|
| 15458977 | 7 | No | Induction of the paraoxonase-1 gene expression by resveratrol. |
| 15464216 | 2 | No | The differentiation potential of human foetal neuronal progenitor cells in vitro. |
| 15465035 | 6 | No | The inhibitory effect of sodium nitroprusside on HIF-1 activation is not dependent on nitric oxide-soluble guanylyl cyclase pathway. |
| 15465739 | 213 | No | Epicatechin and a cocoa polyphenolic extract modulate gene expression in human Caco-2 cells. |
| 15466163 | 10 | No | High volume bioassays to assess CYP3A4-mediated drug interactions: induction and inhibition in a single cell line. |
| 15466214 | 13 | No | Changes in androgen receptor nongenotropic signaling correlate with transition of LNCaP cells to androgen independence. |
| 15470086 | 2 | No | Retinoic acid down-regulates aldehyde dehydrogenase and increases cytotoxicity of 4-hydroperoxycyclophosphamide and acetaldehyde. |
| 15471096 | 2 | No | Occupational exposure to polycyclic aromatic hydrocarbons suppresses constitutive expression of CYP1B1 on the transcript level in human leukocytes. |
| 15471937 | 4 | No | Expression of cystic fibrosis transmembrane conductance regulator in human endometrium. |
| 15473135 | 5 | No | Expressional regulation of neuronal and cancer-related genes by estrogen in adult female rats. |
| 15474070 | 2 | No | Lipopolysaccharide-promoted proliferation of endometriotic stromal cells via induction of tumor necrosis factor alpha and interleukin-8 expression. |
| 15474452 | 2 | No | SU5416 inhibited VEGF and HIF-1alpha expression through the PI3K/AKT/p70S6K1 signaling pathway. |
| 15474502 | 2 | No | EGF-like factor epiregulin and amphiregulin expression is regulated by gonadotropins/cAMP in human ovarian follicular cells. |
| 15475434 | 1 | No | Leptin interferes with the effects of the antiestrogen ICI 182,780 in MCF-7 breast cancer cells. |
| 15475462 | 23 | No | Synergistic effects of acyclic retinoid and OSI-461 on growth inhibition and gene expression in human hepatoma cells. |
| 15476228 | 7 | No | Reduction of synovial sublining layer inflammation and proinflammatory cytokine expression in psoriatic arthritis treated with methotrexate. |
| 15478392 | 4 | No | Increased levels of IgG to cytokeratin 19 in sera of patients with toluene diisocyanate-induced asthma. |
| 15481722 | 4 | No | Induction of the DNA repair gene O6-methylguanine-DNA methyltransferase by dexamethasone in glioblastomas. |

| | | | |
|---|---|---|---|
| 15482327 | 4 | No | Nimesulide and indomethacin induce apoptosis in head and neck cancer cells. |
| 15485790 | 2 | No | [Apoptosis and regulation of expressions of apoptosis-related gene Bcl-2 and p53 induced by selenium dioxide in three leukemia cell lines] |
| 15486348 | 3 | No | Activation of PPAR{gamma} by curcumin inhibits Moser cell growth and mediates |
| 15486966 | 2 | No | Ferulic acid inhibits endothelial cell proliferation through NO down-regulating ERK1/2 pathway. |
| 15486991 | 3 | No | Induction of AP-1 activity by androgen activation of the androgen receptor in LNCaP human prostate carcinoma cells. |
| 15486995 | 1 | No | Apigenin drives the production of reactive oxygen species and initiates a mitochondrial mediated cell death pathway in prostate epithelial cells. |
| 15489888 | 48 | No | Nonsteroidal anti-inflammatory agents differ in their ability to suppress NF-kappaB activation, inhibition of expression of cyclooxygenase-2 and cyclin D1, |
| 15489892 | 15 | No | Inhibition of p53-mediated transcriptional responses by mithramycin A. |
| 15489962 | 2 | No | Epigenetic regulation of 11 beta-hydroxysteroid dehydrogenase type 2 expression. |
| 15498116 | 1 | No | [CML cell line K562 cell apoptosis induced by mangiferin.] |
| 15498119 | 2 | No | [Study on the relationship between the inhibitors of apoptosis proteins and the apoptosis of myelodysplastic syndrome cell line cells induced by aclacinomycin in |
| 15498850 | 4 | No | 15-Deoxy-delta 12,14-prostaglandin J2 induces apoptosis in human malignant B cells: an effect associated with inhibition of NF-kappa B activity and |
| 15500952 | 1 | No | GSTP1 affects chemoresistance against camptothecin in human lung adenocarcinoma cells. |
| 15501902 | 5 | No | Progesterone inhibits insulin-like growth factor binding protein-1 (IGFBP-1) production by explants of the Fallopian tube. |
| 15501958 | 3 | No | Induction of thyroid cancer cell apoptosis by a novel nuclear factor kappaB inhibitor, dehydroxymethylepoxyquinomicin. |
| 15501990 | 1 | No | Aberrant methylation of DPYD promoter, DPYD expression, and cellular sensitivity to 5-fluorouracil in cancer cells. |
| 15501994 | 5 | No | Impact of the cyclooxygenase system on doxorubicin-induced functional multidrug resistance 1 overexpression and doxorubicin sensitivity in acute myeloid leukemic |
| 15502056 | 10 | No | Dobutamine inhibits phorbol-myristate-acetate-induced activation of nuclear factor-kappaB in human T lymphocytes in vitro. |

| | | | |
|---|---|---|---|
| 15504374 | 4 | No | Clinical and metabolic aspects of the continuous use of a contraceptive association of ethinyl estradiol (30 microg) and gestodene (75 microg). |
| 15507505 | 13 | No | Thyroid hormone responsive genes in cultured human fibroblasts. |
| 15509713 | 11 | No | Cyclooxygenase inhibitors induce the expression of the tumor suppressor gene EGR-1, which results in the up-regulation of NAG-1, an antitumorigenic protein. |
| 15513908 | 4 | No | Brain inflammation and Alzheimer's-like pathology in individuals exposed to severe air pollution. |
| 15516327 | 6 | No | Expression and regulation of interleukin-23 subunits in human peripheral blood mononuclear cells and hematopoietic cell lines in response to various inducers. |
| 15517594 | 1 | No | Differentiation and drug resistance relationships in leukemia cells. |
| 15519653 | 6 | No | Peripheral benzodiazepine receptor ligands induce apoptosis and cell cycle arrest in human hepatocellular carcinoma cells and enhance chemosensitivity to |
| 15520213 | 6 | No | Casein kinase I attenuates tumor necrosis factor-related apoptosis-inducing ligand-induced apoptosis by regulating the recruitment of fas-associated death |
| 15520497 | 4 | No | Preparation of cardiovascular disease-related genes microarray and its application in exploring ligustrazine-induced changes in endothelial gene |
| 15521013 | 5 | No | Evidence for a new human CYP1A1 regulation pathway involving PPAR-alpha and 2 PPRE sites. |
| 15521073 | 4 | No | Transcriptional regulation of thioredoxin reductase 1 expression by cadmium in vascular endothelial cells: role of NF-E2-related factor-2. |
| 15523430 | 4 | No | Alterations in eotaxin, monocyte chemoattractant protein-4, interleukin-5, and interleukin-13 after systemic steroid treatment for nasal polyps. |
| 15525692 | 15 | No | Co-culture of primary human mammary fibroblasts and MCF-7 cells as an in vitro breast cancer model. |
| 15526378 | 5 | No | Involvement of extracellular signal-regulated kinase/mitogen-activated protein kinase pathway in multidrug resistance induced by HBx in hepatoma cell line. |
| 15528975 | 32 | No | Investigation of HMN-176 anticancer activity in human tumor specimens in vitro and the effects of HMN-176 on differential gene expression. |
| 15531295 | 6 | No | Piperine is a potent inhibitor of nuclear factor-kappaB (NF-kappaB), c-Fos, CREB, ATF-2 and proinflammatory cytokine gene expression in B16F-10 melanoma cells. |
| 15531749 | 19 | No | Metalloproteinases mediate mucin 5AC expression by epidermal growth factor receptor activation. |
| 15533597 | 4 | No | Expression of glutathione S-transferase P1-1 in leukemic cells is regulated by inducible AP-1 binding. |

| | | | |
|---|---|---|---|
| 15538745 | 3 | No | 5alpha-dihydrotestosterone inhibits 1alpha,25-dihydroxyvitamin D3-induced expression of CYP24 in human prostate cancer cells. |
| 15541073 | 9 | No | Increase of laminin 5 synthesis in human keratinocytes by acute wound fluid, inflammatory cytokines and growth factors, and lysophospholipids. |
| 15541394 | 4 | No | Butyrate regulation of glycosylation-related gene expression: evidence for galectin-1 upregulation in human intestinal epithelial goblet cells. |
| 15541416 | 12 | No | Proliferation-stimulating effects of icaritin and desmethylicaritin in MCF-7 cells. |
| 15541768 | 1 | No | Gene regulation for the senescence marker protein DHEA-sulfotransferase by the xenobiotic-activated nuclear pregnane X receptor (PXR). |
| 15542045 | 3 | No | Bone as a source of FGF23: regulation by phosphate? |
| 15542103 | 1 | No | Intra- and intercellular variations in the repair efficiency of O6-methylguanine, and their contribution to kinetic complexity. |
| 15542774 | 6 | No | Inhibitory effect of epidermal growth factor on resveratrol-induced apoptosis in prostate cancer cells is mediated by protein kinase C-alpha. |
| 15543232 | 3 | No | Efficacy and mechanism of action of the proteasome inhibitor PS-341 in T-cell lymphomas and HTLV-I associated adult T-cell leukemia/lymphoma. |
| 15543656 | 3 | No | HMBA induces cell death and potentiates doxorubicin toxicity in malignant mesothelioma cells. |
| 15546879 | 54 | No | Combination of 5-fluorouracil and N1,N11-diethylnorspermine markedly activates spermidine/spermine N1-acetyltransferase expression, depletes polyamines, and |
| 15547111 | 49 | No | 5-Aza-cytidine is a potent inhibitor of DNA methyltransferase 3a and induces apoptosis in HCT-116 colon cancer cells via Gadd45- and p53-dependent mechanisms. |
| 15547672 | 2 | No | Arg-Gly-Asp (RGD) peptide ameliorates carbon tetrachloride-induced liver fibrosis via inhibition of collagen production and acceleration of collagenase activity. |
| 15547680 | 3 | No | Induction of apoptosis by dideoxypetrosynol A, a polyacetylene from the sponge Petrosia sp., in human skin melanoma cells. |
| 15547720 | 6 | No | Equiguard suppresses androgen-dependent LNCaP prostate cancer cell proliferation by targeting cell cycle control via down regulation of the retinoblastoma protein |
| 15547725 | 2 | No | Extracellular signal-regulated kinase induces cyclin D1 and Cdk-2 expression and phosphorylation of retinoblastoma in hepatocellular carcinoma. |
| 15548381 | 36 | No | A PXR reporter gene assay in a stable cell culture system: CYP3A4 and CYP2B6 induction by pesticides. |
| 15553829 | 2 | No | [Study on effect of arsenic trioxide on adhesion and invasion of human hepatocarcinoma cells in vitro] |

| | | | |
|---|---|---|---|
| 15554232 | 2 | No | Rifampicin, a keystone inducer of drug metabolism: from Herbert Remmer's pioneering ideas to modern concepts. |
| 15555568 | 5 | No | Cyclooxygenase inhibitors induce apoptosis in oral cavity cancer cells by increased expression of nonsteroidal anti-inflammatory drug-activated gene. |
| 15555623 | 8 | No | Nuclear factor-kappa B and apoptosis inducing factor activation by doxorubicin analog WP744 in SH-SY5Y neuroblastoma cells. |
| 15556009 | 2 | No | Arresting cancer proliferation by small-molecule gene regulation. |
| 15556294 | 2 | No | MM-TRAG (MGC4175), a novel intracellular mitochondrial protein, is associated with the taxol- and doxorubicin-resistant phenotype in human cancer cell lines. |
| 15557169 | 9 | No | Recruitment of uterine NK cells: induction of CXC chemokine ligands 10 and 11 in human endometrium by estradiol and progesterone. |
| 15557560 | 2 | No | Transcriptional regulation of human UGT1A1 gene expression: activated glucocorticoid receptor enhances constitutive androstane receptor/pregnane X |
| 15557793 | 10 | No | Involvement of ERK1/2 and p38 MAP kinase in doxorubicin-induced uPA expression in human RC-K8 lymphoma and NCI-H69 small cell lung carcinoma cells. |
| 15560369 | 1 | No | Stimulation of transcriptional expression of human UDP-glucuronosyltransferase 1A1 by dexamethasone. |
| 15561105 | 3 | No | The cyclooxygenase inhibitor indomethacin modulates gene expression and represses the extracellular matrix protein laminin gamma1 in human glioblastoma cells. |
| 15561972 | 2 | No | Biotin uptake by human proximal tubular epithelial cells: cellular and molecular aspects. |
| 15562024 | 4 | No | Progesterone increases tissue factor gene expression, procoagulant activity, and invasion in the breast cancer cell line ZR-75-1. |
| 15564131 | 2 | No | Dual therapeutic utility of proteasome modulating agents for pharmaco-gene therapy of the cystic fibrosis airway. |
| 15564322 | 5 | No | The putative tumor suppressor deleted in malignant brain tumors 1 is an estrogen-regulated gene in rodent and primate endometrial epithelium. |
| 15566391 | 15 | No | effect of vitamin E on the apoptotic signalling mechanism induced by cinnamaldehyde in human hepatoma PLC/PRF/5 cells. 2. Using the XTT assay, |
| 15566942 | 42 | No | Induction of gene expression of xenobiotic metabolism enzymes and ABC-transport proteins by PAH and a reconstituted PAH mixture in human Caco-2 cells. |
| 15569413 | 3 | No | Interleukin-12 was not involved in promotion of T helper cell differentiation induced by theophylline. |
| 15569515 | 1 | No | [Study on the transcriptional modulation of cytochrome P450 3A4 expression by zearalenone.] |

| | | | |
|---|---|---|---|
| 15570426 | 3 | No | Ameliorative effect of ozone on cytokine production in mice injected with human rheumatoid arthritis synovial fibroblast cells. |
| 15571967 | 13 | No | Rapamycin inhibits doxorubicin-induced NF-kappaB/Rel nuclear activity and enhances the apoptosis of melanoma cells. |
| 15572027 | 3 | No | G1 cell cycle arrest due to the inhibition of erbB family receptor tyrosine kinases does not require the retinoblastoma protein. |
| 15576159 | 5 | No | Prevention of the epoxy resin-based root canal sealers-induced cyclooxygenase-2 expression and cytotoxicity of human osteoblastic cells by various antioxidants. |
| 15576332 | 10 | No | mRNA expression analysis of a variety of apoptosis-related genes, including the novel gene of the BCL2-family, BCL2L12, in HL-60 leukemia cells after treatment |
| 15576448 | 3 | No | Mechanisms of arsenite-mediated decreases in benzo[k]fluoranthene-induced human cytochrome P4501A1 levels in HepG2 cells. |
| 15577328 | 1 | No | Serum bcl-2 and survivin levels in melanoma. |
| 15579764 | 2 | No | Raloxifene modulates interleukin-6 and tumor necrosis factor-alpha synthesis in vivo: results from a pilot clinical study. |
| 15581851 | 1 | No | The synthesis of SNAT2 transporters is required for the hypertonic stimulation of system A transport activity. |
| 15582268 | 9 | No | Resveratrol-induced cell growth inhibition and apoptosis is associated with modulation of phosphoglycerate mutase B in human prostate cancer cells: |
| 15582351 | 1 | No | Analysis of oxidative stress in SK-N-MC neurons exposed to styrene-7,8-oxide. |
| 15583814 | 3 | No | Resveratrol inhibits cell cycle progression in U937 cells. |
| 15583868 | 10 | No | Pharmacological attenuation of apoptosis in reoxygenated endothelial cells. |
| 15585135 | 34 | No | Proteomic analysis of antiproliferative effects by treatment of 5-fluorouracil in cervical cancer cells. |
| 15585362 | 60 | No | Effect of single wall carbon nanotubes on human HEK293 cells. |
| 15585369 | 4 | No | Mercury induces multidrug resistance-associated protein gene through p38 mitogen-activated protein kinase. |
| 15585566 | 6 | No | Estrogen and selective estrogen receptor modulators exert neuroprotective effects and stimulate the expression of selective Alzheimer's disease indicator-1, a |
| 15585599 | 2 | No | Regulation of regional expression in rat brain PC2 by thyroid hormone/characterization of novel negative thyroid hormone response elements in |

| | | | |
|---|---|---|---|
| 15585621 | 4 | No | Partial contribution of tumor necrosis factor-related apoptosis-inducing ligand (TRAIL)/TRAIL receptor pathway to antitumor effects of |
| 15585644 | 3 | No | pRb2/p130 decreases sensitivity to apoptosis induced by camptothecin and doxorubicin but not by taxol. |
| 15585952 | 2 | No | Down regulation of hepatic PPARalpha function by AhR ligand. |
| 15587394 | 2 | No | Arsenic trioxide inhibits proliferation in K562 cells by changing cell cycle and survivin expression. |
| 15588777 | 4 | No | Multidrug resistance3 is in situ detected in the liver of patients with primary biliary cirrhosis, and induced in human hepatoma cells by bezafibrate. |
| 15589975 | 28 | No | Activation of retinoic acid receptor-dependent transcription by organochlorine pesticides. |
| 15590111 | 67 | No | Gene expression changes induced by estrogen and selective estrogen receptor modulators in primary-cultured human endometrial cells: signals that distinguish |
| 15592527 | 4 | No | Arsenic trioxide induces autophagic cell death in malignant glioma cells by upregulation of mitochondrial cell death protein BNIP3. |
| 15596250 | 2 | No | CYP1A1 and CYP1B1 genotypes, haplotypes, and TCDD-induced gene expression in subjects from Seveso, Italy. |
| 15596260 | 6 | No | Hydroxychalcones exhibit differential effects on XRE transactivation. |
| 15601469 | 4 | No | Role of the p53/p21 system in the response of human colon carcinoma cells to Doxorubicin. |
| 15601553 | 1 | No | [nhibitory effect of adenosine analogues on invasion of human ovarian cancer cell line HO-8910PM] |
| 15603917 | 4 | No | Xenobiotic-induced TNF-alpha expression and apoptosis through the p38 MAPK signaling pathway. |
| 15606011 | 6 | No | [Effect of ginsenoside Rg1 on expression of p21, cyclin E and CDK2 in the process of cell senescence] |
| 15607317 | 4 | No | Development and applications of a real-time quantitative RT-PCR method (QRT-PCR) for BRCA1 mRNA. |
| 15607904 | 7 | No | 4-Hydroxynonenal modulation of p53 family gene expression in the SK-N-BE neuroblastoma cell line. |
| 15608128 | 1 | No | Human cytochrome p450 2s1: lack of activity in the metabolic activation of several cigarette smoke carcinogens and in the metabolism of nicotine. |
| 15608132 | 1 | No | tert-Butylhydroquinone is a novel aryl hydrocarbon receptor ligand. |

| | | | |
|---|---|---|---|
| 15611089 | 5 | No | Hypoxia-inducible factor-1-dependent overexpression of myeloid cell factor-1 protects hypoxic cells against tert-butyl hydroperoxide-induced apoptosis. |
| 15613425 | 1 | No | Fibroblast growth factor-23 relationship to dietary phosphate and renal phosphate handling in healthy young men. |
| 15614042 | 6 | No | Effect of serum and antioxidants on the immunogenicity of protein kinase C-activated chronic lymphocytic leukemia cells. |
| 15617745 | 1 | No | In vitro effect of cyclosporin A, mitomycin C and prednisolone on cell kinetics in cultured human umbilical vein endothelial cells. |
| 15618017 | 8 | No | The possible role of heat shock factor-1 in the negative regulation of heme oxygenase-1. |
| 15618473 | 18 | No | Circulating endothelial progenitor cells in multiple myeloma: implications and significance. |
| 15618651 | 2 | No | Selective suppressions of human CYP3A forms, CYP3A5 and CYP3A7, by troglitazone in HepG2 cells. |
| 15619356 | 1 | No | Endothelin-2 down-regulation occurs in parallel with the anti-proliferative effect of dimethylsulfoxide in BeWO human choriocarcinoma cell line. |
| 15620718 | 4 | No | The effect of synthetic glucocorticoid, dexamethasone on CYP1A1 inducibility in adult rat and human hepatocytes. |
| 15622447 | 10 | No | Hyperforin, the active component of St. John's wort, induces IL-8 expression in human intestinal epithelial cells via a MAPK-dependent, NF-kappaB-independent |
| 15622746 | 1 | No | [Preliminary study on the arsenic trioxide-induced NB4 cell apoptosis and its molecular mechanisms] |
| 15623590 | 5 | No | Down-regulation of intratumoral aromatase messenger RNA levels by docetaxel in human breast cancers. |
| 15623601 | 1 | No | Characterization of gastrin-induced proangiogenic effects in vivo in orthotopic U373 experimental human glioblastomas and in vitro in human umbilical vein |
| 15625562 | 6 | No | Inhibition of CYP17 expression by adrenal androgens and transforming growth factor beta in adrenocortical cells. |
| 15627480 | 13 | No | Role of adrenoceptor-linked signaling pathways in the regulation of CYP1A1 gene expression. |
| 15629111 | 14 | No | Species-specific mechanisms for cholesterol 7alpha-hydroxylase (CYP7A1) regulation by drugs and bile acids. |
| 15629189 | 11 | No | Cytochrome P450 expression and activities in human tongue cells and their modulation by green tea extract. |
| 15630080 | 6 | No | Arsenite inhibition of CYP1A1 induction by 2,3,7,8-tetrachlorodibenzo-p-dioxin is independent of cell cycle arrest. |

| | | | |
|---|---|---|---|
| 15630458 | 2 | No | Possible involvement of pregnane X receptor-enhanced CYP24 expression in drug-induced osteomalacia. |
| 15631656 | 1 | No | [Proliferation-inhibiting effect of indomethacin on chronic myeliod leukemia cells is related to the supression of STAT signal transduction pathway] |
| 15631803 | 8 | No | [Activation of HIF-1 by bFGF in breast cancer: role of PI-3K and MEK1/ERK pathways] |
| 15634505 | 1 | No | [Relationship of hypoxia-inducible factor-1alpha expression and vascular endothelial growth factor in SKOV-3 ovarian cancer model] |
| 15634547 | 2 | No | [The effect of inhaled glucocorticosteroid on protein kinase C alpha expression and interleukin-5 production in induced sputum inflammatory cells of asthma |
| 15634644 | 6 | No | The small-molecule Bcl-2 inhibitor HA14-1 interacts synergistically with flavopiridol to induce mitochondrial injury and apoptosis in human myeloma cells |
| 15634944 | 1 | No | Efflux of depsipeptide FK228 (FR901228, NSC-630176) is mediated by P-glycoprotein and multidrug resistance-associated protein 1. |
| 15635043 | 3 | No | Regulation of human hepatic hydroxysteroid sulfotransferase gene expression by the peroxisome proliferator-activated receptor alpha transcription factor. |
| 15635149 | 3 | No | A mechanistic basis for the role of cycle arrest in the genetic toxicology of the dietary carcinogen 2-amino-1-methyl-6-phenylimidazo[4,5-b]pyridine (PhIP). |
| 15636197 | 6 | No | Asbestos induces tissue factor in Beas-2B human lung bronchial epithelial cells in vitro. |
| 15637740 | 1 | No | Indomethacin suppresses growth of colon cancer via inhibition of angiogenesis in vivo. |
| 15638997 | 77 | No | Microarray analysis of bicalutamide action on telomerase activity, p53 pathway and viability of prostate carcinoma cell lines. |
| 15640275 | 2 | No | Exercise-induced metallothionein expression in human skeletal muscle fibres. |
| 15641079 | 5 | No | Inhibition of interleukin-1beta-induced cyclooxygenase 2 expression in human synovial fibroblasts by 15-deoxy-Delta12,14-prostaglandin J2 through a histone |
| 15641988 | 6 | No | Enhanced Bax in oral SCC in relation to antitumor effects of chemotherapy. |
| 15644155 | 1 | No | [Therapeutic effect of Xincang Decoction on chronic airway inflammation in children with bronchial asthma in remission stage] |
| 15647279 | 2 | No | Trichostatin A induces transforming growth factor beta type II receptor promoter activity and acetylation of Sp1 by recruitment of PCAF/p300 to a Sp1.NF-Y |
| 15647840 | 4 | No | Bone morphogenetic protein-2 induces hypophosphorylation of Rb protein and repression of E2F in androgen-treated LNCaP human prostate cancer cells. |

| | | | |
|---|---|---|---|
| 15649653 | 1 | No | Modulation of Cyp3a11 mRNA expression by alpha-tocopherol but not gamma-tocotrienol in mice. |
| 15650019 | 18 | No | The pregnane X receptor regulates gene expression in a ligand- and promoter-selective fashion. |
| 15650315 | 1 | No | Pranlukast, a leukotriene receptor antagonist, inhibits interleukin-5 production via a mechanism distinct from leukotriene receptor antagonism. |
| 15650393 | 9 | No | Inhibition of NFkappaB activation and IL-8 expression in human bronchial epithelial cells by acrolein. |
| 15652230 | 6 | No | Inhibition of interleukin-8 (CXCL8/IL-8) responses by repertaxin, a new inhibitor of the chemokine receptors CXCR1 and CXCR2. |
| 15652234 | 14 | No | Inhibition of interleukin-12 expression in diltiazem-treated dendritic cells through the reduction of nuclear factor-kappa B transcriptional activity. |
| 15652504 | 7 | No | Induction of thioredoxin reductase as an adaptive response to acrolein in human umbilical vein endothelial cells. |
| 15654950 | 4 | No | Interleukin-6-type cytokines upregulate expression of multidrug resistance-associated proteins in NHEK and dermal fibroblasts. |
| 15655410 | 1 | No | The antitumor drug candidate 2-(4-amino-3-methylphenyl)-5-fluorobenzothiazole induces NF-kappaB activity in drug-sensitive MCF-7 cells. |
| 15657354 | 3 | No | Epigenetic regulation of O6-methylguanine-DNA methyltransferase gene expression by histone acetylation and methyl-CpG binding proteins. |
| 15657356 | 4 | No | Epigallocatechin-3-gallate induces apoptosis in estrogen receptor-negative human breast carcinoma cells via modulation in protein expression of p53 and Bax and |
| 15657365 | 2 | No | Cytoplasmic confinement of breast cancer resistance protein (BCRP/ABCG2) as a novel mechanism of adaptation to short-term folate deprivation. |
| 15658607 | 5 | No | Inhibitory effect of inflammatory cytokines production from activated mast cells by Gamisopoonghwanghyul-tang. |
| 15659568 | 1 | No | Cytochrome P450 1A1 and 1B1 in human blood lymphocytes are not suitable as biomarkers of exposure to dioxin-like compounds: polymorphisms and |
| 15659783 | 2 | No | Analysis of the effects of different alcohols on MCF-7 human breast cancer cells. |
| 15661808 | 10 | No | Involvement of transcription factor Sp1 in quercetin-mediated inhibitory effect on the androgen receptor in human prostate cancer cells. |
| 15661813 | 6 | No | 5,7-Dimethoxyflavone downregulates CYP1A1 expression and benzo[a]pyrene-induced DNA binding in Hep G2 cells. |
| 15663903 | 5 | No | Triptolide suppresses CD80 and CD86 expressions and IL-12 production in THP-1 cells. |

| | | | |
|---|---|---|---|
| 15664440 | 2 | No | Increased levels of metallothionein in placenta of smokers. |
| 15664452 | 7 | No | Regulation of the human vitamin D3 receptor promoter in breast cancer cells is mediated through Sp1 sites. |
| 15665116 | 6 | No | Pharmacologic inhibitors of PI3K/Akt potentiate the apoptotic action of the antileukemic drug arsenic trioxide via glutathione depletion and increased |
| 15665275 | 8 | No | The dual ErbB1/ErbB2 inhibitor, lapatinib (GW572016), cooperates with tamoxifen to inhibit both cell proliferation- and estrogen-dependent gene expression in |
| 15666826 | 2 | No | ACTH regulates steroidogenic gene expression and cortisol biosynthesis in the human adrenal cortex via sphingolipid metabolism. |
| 15666830 | 7 | No | On the control of the hCYP11B2 gene expressing cytochrome P450 aldosterone synthase. |
| 15668717 | 5 | No | A methoxy derivative of resveratrol analogue selectively induced activation of the mitochondrial apoptotic pathway in transformed fibroblasts. |
| 15669648 | 6 | No | [The effect of two low dose oral contraceptive with gestodene on the systems of natural inhibitors of coagulation] |
| 15670600 | 2 | No | Identification of pregnane X receptor binding sites in the regulatory regions of genes involved in bile acid homeostasis. |
| 15670636 | 2 | No | Resveratrol interference with the cell cycle protects human neuroblastoma SH-SY5Y cell from paclitaxel-induced apoptosis. |
| 15670751 | 5 | No | Indole-3-carbinol and 3,3'-diindolylmethane induce expression of NAG-1 in a p53-independent manner. |
| 15670774 | 4 | No | Quercetin, a potent inhibitor against beta-catenin/Tcf signaling in SW480 colon cancer cells. |
| 15670817 | 8 | No | Differentiative pathway activated by 3-aminobenzamide, an inhibitor of PARP, in human osteosarcoma MG-63 cells. |
| 15670848 | 2 | No | Effects of Se-depletion on glutathione peroxidase and selenoprotein W gene expression in the colon. |
| 15671028 | 1 | No | Direct effects of polymyxin B on human dendritic cells maturation. The role of IkappaB-alpha/NF-kappaB and ERK1/2 pathways and adhesion. |
| 15671536 | 1 | No | Visualization of endogenous p53-mediated transcription in vivo using sodium iodide symporter. |
| 15672453 | 488 | No | Transcriptomic and proteomic responses of human renal HEK293 cells to uranium toxicity. |
| 15672752 | 11 | No | and sulforaphane (SFN) protect animals against chemically induced tumours. Putative chemoprotective mechanisms include modulated expression of hepatic |

| | | | |
|---|---|---|---|
| 15673847 | 2 | No | Effects of 2,2',4,4'-tetrachlorobiphenyl on granulocytic HL-60 cell function and expression of cyclooxygenase-2. |
| 15674352 | 12 | No | Identification of ATF-3, caveolin-1, DLC-1, and NM23-H2 as putative antitumorigenic, progesterone-regulated genes for ovarian cancer cells by gene |
| 15677771 | 2 | No | Keratinocyte growth factor expression by fibroblasts in pulmonary fibrosis: poor response to interleukin-1beta. |
| 15678371 | 1 | No | Ispaghula (Plantago ovata) seed husk polysaccharides promote proliferation of human epithelial cells (skin keratinocytes and fibroblasts) via enhanced growth |
| 15679045 | 13 | No | SGK1, a potential regulator of c-fms related breast cancer aggressiveness. |
| 15679716 | 2 | No | Effect of inhaled corticosteroid on an immunoreactive thymus and activation-regulated chemokine expression in the bronchial biopsies from |
| 15679717 | 1 | No | Biochemical interaction between effects of beclomethasone dipropionate and salbutamol or formoterol in sputum cells from mild to moderate asthmatics. |
| 15680906 | 12 | No | 15-Hydroxyprostaglandin dehydrogenase can be induced by dexamethasone and other glucocorticoids at the therapeutic level in A549 human lung adenocarcinoma cells. |
| 15681896 | 12 | No | Effects of NO-1886 (Ibrolipim), a lipoprotein lipase-promoting agent, on gene induction of cytochrome P450s, carboxylesterases, and sulfotransferases in |
| 15684433 | 24 | No | Polyunsaturated fatty acids block dendritic cell activation and function independently of NF-kappaB activation. |
| 15684474 | 4 | No | Dracorhodin perchlorate induces A375-S2 cell apoptosis via accumulation of p53 and activation of caspases. |
| 15687330 | 8 | No | Thalidomide inhibits tumor necrosis factor-alpha-induced interleukin-8 expression in endometriotic stromal cells, possibly through suppression of nuclear |
| 15688415 | 1 | No | Resveratrol-induced apoptosis in MCF-7 human breast cancer cells involves a caspase-independent mechanism with downregulation of Bcl-2 and NF-kappaB. |
| 15688416 | 1 | No | Effect of resveratrol on the development of spontaneous mammary tumors in HER-2/neu transgenic mice. |
| 15690306 | 11 | No | Effect of pitavastatin on transactivation of human serum paraoxonase 1 gene. |
| 15691296 | 1 | No | Ursodeoxycholic acid reduces increased circulating endothelin 2 in primary biliary cirrhosis. |
| 15695372 | 5 | No | Regulation of hypoxia-inducible factor (HIF)-1 activity and expression of HIF hydroxylases in response to insulin-like growth factor I. |
| 15695394 | 2 | No | Nitric oxide reverts the resistance to doxorubicin in human colon cancer cells by inhibiting the drug efflux. |

| | | | |
|---|---|---|---|
| 15695403 | 2 | No | Retinoic acid inhibits the proliferative response induced by CD40 activation and interleukin-4 in mantle cell lymphoma. |
| 15695404 | 5 | No | Estrogen-mediated post transcriptional down-regulation of breast cancer resistance protein/ABCG2. |
| 15695405 | 7 | No | Identification of a novel small-molecule inhibitor of the hypoxia-inducible factor 1 pathway. |
| 15698409 | 8 | No | Differential modulation of interleukin 8 by interleukin 4 and interleukin 10 in HepG2 cells treated with acetaldehyde. |
| 15698582 | 18 | No | Role of cell signaling in B[a]P-induced apoptosis: characterization of unspecific effects of cell signaling inhibitors and apoptotic effects of B[a]P metabolites. |
| 15699255 | 1 | No | Retinoids and pulmonary hypertension. |
| 15701621 | 6 | No | Mechanism of TNF-{alpha} modulation of Caco-2 intestinal epithelial tight junction barrier: role of myosin light-chain kinase protein expression. |
| 15703175 | 12 | No | Na+/Ca2+ exchanger activity modulates connective tissue growth factor mRNA expression in transforming growth factor beta1- and Des-Arg10-kallidin-stimulated |
| 15703266 | 4 | No | PCB126 induces differential changes in androgen, cortisol, and aldosterone biosynthesis in human adrenocortical H295R cells. |
| 15703815 | 3 | No | Promoter hypermethylation and inactivation of O(6)-methylguanine-DNA methyltransferase in esophageal squamous cell carcinomas and its reactivation in |
| 15705601 | 1 | No | Epigallocatechin-3-gallate induces mitochondrial membrane depolarization and caspase-dependent apoptosis in pancreatic cancer cells. |
| 15705907 | 8 | No | Activator protein accelerates dihydropyrimidine dehydrogenase gene transcription in cancer cells. |
| 15707588 | 2 | No | Proteasome inhibitors induce peroxisome proliferator-activated receptor transactivation through RXR accumulation and a protein kinase C-dependent |
| 15708893 | 3 | No | Effect of nitric oxide on mitochondrial respiratory activity of human articular chondrocytes. |
| 15709199 | 8 | No | Combination of IFN-alpha and 5-fluorouracil induces apoptosis through IFN-alpha/beta receptor in human hepatocellular carcinoma cells. |
| 15710169 | 7 | No | Differential effects of the organochlorine pesticide DDT and its metabolite p,p'-DDE on p-glycoprotein activity and expression. |
| 15710601 | 28 | No | Evodiamine abolishes constitutive and inducible NF-kappaB activation by inhibiting IkappaBalpha kinase activation, thereby suppressing |
| 15711177 | 2 | No | Effect of sodium butyrate on doxorubicin resistance and expression of multidrug resistance genes in thyroid carcinoma cells. |

| | | | |
|---|---|---|---|
| 15713371 | 8 | No | Effects of suberoylanilide hydroxamic acid and trichostatin A on induction of cytochrome P450 enzymes and benzo[a]pyrene DNA adduct formation in human cells. |
| 15713895 | 38 | No | Gene expression profiling identifies activating transcription factor 3 as a novel contributor to the proapoptotic effect of curcumin. |
| 15713900 | 7 | No | Sulindac enhances adenoviral vector expressing mda-7/IL-24-mediated apoptosis in human lung cancer. |
| 15715472 | 2 | No | The role of intracellularly released formaldehyde and butyric acid in the anticancer activity of acyloxyalkyl esters. |
| 15719745 | 6 | No | [Effect of compound salvia injection on nitrate ester tolerance] |
| 15720126 | 4 | No | Effect of a standardized complex mixture derived from coal tar on the metabolic activation of carcinogenic polycyclic aromatic hydrocarbons in human cells in |
| 15721411 | 1 | No | Effects of prolonged tamoxifen treatment on receptor expression and apoptosis of ovarian cancer cells. |
| 15723263 | 18 | No | Gefitinib ("Iressa", ZD1839) inhibits SN38-triggered EGF signals and IL-8 production in gastric cancer cells. |
| 15724410 | 1 | No | [Effects of Epimedium pubescens icariine on proliferation and differentiation of human osteoblasts] |
| 15724842 | 1 | No | 3-amino thioacridone inhibits DNA synthesis and induce DNA damage in T-cell acute lymphoblastic leukemia (T-ALL) in a p16-dependent manner. |
| 15725085 | 66 | No | Changes in gene expression profiles of multiple myeloma cells induced by arsenic trioxide (ATO): possible mechanisms to explain ATO resistance in vivo. |
| 15725655 | 5 | No | Nobiletin, a citrus flavonoid, down-regulates matrix metalloproteinase-7 (matrilysin) expression in HT-29 human colorectal cancer cells. |
| 15725661 | 4 | No | Vaticanol C-induced cell death is associated with inhibition of pro-survival signaling in HL60 human leukemia cell line. |
| 15728517 | 1 | No | Tumor cells deactivate human monocytes by up-regulating IL-1 receptor associated kinase-M expression via CD44 and TLR4. |
| 15728566 | 10 | No | Vascular endothelial growth factor reduced and connective tissue growth factor induced by triamcinolone in ARPE19 cells under oxidative stress. |
| 15729293 | 1 | No | A dopamine D4 receptor antagonist attenuates ischemia-induced neuronal cell damage via upregulation of neuronal apoptosis inhibitory protein. |
| 15729575 | 35 | No | Thiazolidinediones inhibit proliferation of microvascular and macrovascular cells by a PPARgamma-independent mechanism. |
| 15729620 | 4 | No | Isoliquiritigenin inhibits cell proliferation and induces apoptosis in human hepatoma cells. |

| | | | |
|---|---|---|---|
| 15733867 | 3 | No | Histone deacetylase inhibitor BL1521 induces a G1-phase arrest in neuroblastoma cells through altered expression of cell cycle proteins. |
| 15734734 | 1 | No | Homocysteine down-regulates cellular glutathione peroxidase (GPx1) by decreasing translation. |
| 15735009 | 46 | No | Altered gene expression patterns in MCF-7 cells induced by the urban dust particulate complex mixture standard reference material 1649a. |
| 15735044 | 3 | No | Induction of apoptosis in primary meningioma cultures by fenretinide. |
| 15735708 | 2 | No | The cancer chemopreventive agent resveratrol induces tensin, a cell-matrix adhesion protein with signaling and antitumor activities. |
| 15735757 | 6 | No | Inactivated MGMT by O6-benzylguanine is associated with prolonged G2/M arrest in cancer cells treated with BCNU. |
| 15736412 | 1 | No | Quantification of the expression of multidrug resistance-related genes in human tumour cell lines grown with free doxorubicin or doxorubicin encapsulated in |
| 15737199 | 8 | No | Redox imbalance induced by contact sensitizers triggers the maturation of dendritic cells. |
| 15737688 | 1 | No | Estradiol-induced ezrin overexpression in ovarian cancer: a new signaling domain for estrogen. |
| 15737843 | 12 | No | Downregulation of TS, DPD, ERCC1, GST-Pi, EGFR, and HER2 gene expression after neoadjuvant three-modality treatment in patients with esophageal cancer. |
| 15740016 | 13 | No | Sulforaphane, erucin, and iberin up-regulate thioredoxin reductase 1 expression in human MCF-7 cells. |
| 15740068 | 3 | No | Induction of apoptosis by the Anthocyanidins through regulation of Bcl-2 gene and activation of c-Jun N-terminal kinase cascade in hepatoma cells. |
| 15740983 | 8 | No | A blend of polyphenolic compounds explains the stimulatory effect of red wine on human endothelial NO synthase. |
| 15741050 | 3 | No | Quercetin decreases the expression of ErbB2 and ErbB3 proteins in HT-29 human colon cancer cells. |
| 15741166 | 16 | No | Small interference RNA-mediated gene silencing of human biliverdin reductase, but not that of heme oxygenase-1, attenuates arsenite-mediated induction of the |
| 15741222 | 12 | No | Thalidomide-induced antiangiogenic action is mediated by ceramide through depletion of VEGF receptors, and is antagonized by sphingosine-1-phosphate. |
| 15743794 | 1 | No | Functional erythropoietin autocrine loop in melanoma. |
| 15744361 | 3 | No | Microtubule disarray in primary cultures of human hepatocytes inhibits transcriptional activity of the glucocorticoid receptor via activation of c-jun |

| | | | |
|---|---|---|---|
| 15746163 | 5 | No | Butyrate may enhance toxicological defence in primary, adenoma and tumor human colon cells by favourably modulating expression of glutathione S-transferases |
| 15746575 | 1 | No | High cytotoxic sensitivity of the human small cell lung doxorubicin-resistant carcinoma (GLC4/ADR) cell line to prodigiosin through apoptosis activation. |
| 15748212 | 2 | No | Increased peripheral blood T-cell apoptosis and decreased Bcl-2 in chronic obstructive pulmonary disease. |
| 15748509 | 1 | No | [Expression of protein p53 in workers occupationally exposed to benzidine and bladder cancer patients.] |
| 15748703 | 6 | No | Mitochondrial-dependent, reactive oxygen species-independent apoptosis by myricetin: roles of protein kinase C, cytochrome c, and caspase cascade. |
| 15749485 | 2 | No | Differential expression of endometrial integrins and progesterone receptor during the window of implantation in normo-ovulatory women treated with clomiphene |
| 15749674 | 2 | No | Changes in expression of WT1 isoforms during induced differentiation of the NB4 cell line. |
| 15750632 | 2 | No | YB-1 facilitates basal and 5-fluorouracil-inducible expression of the human major vault protein (MVP) gene. |
| 15752352 | 3 | No | Dehydroepiandrosterone inhibits the proliferation of human umbilical vein endothelial cells by enhancing the expression of p53 and p21, restricting the |
| 15753397 | 4 | No | Regulation of p38 phosphorylation and topoisomerase IIalpha expression in the B-cell lymphoma line Jiyoye by CD26/dipeptidyl peptidase IV is associated with |
| 15753651 | 4 | No | Cdk9 regulates neural differentiation and its expression correlates with the differentiation grade of neuroblastoma and PNET tumors. |
| 15753895 | 4 | No | Glucocorticoid-induced surface expression of annexin 1 blocks beta2-integrin adhesion of human eosinophils to intercellular adhesion molecule 1 surrogate |
| 15754000 | 2 | No | Retinoid receptor mRNA expression profiles in human bladder cancer specimens. |
| 15754008 | 8 | No | Genistein suppresses the invasive potential of human breast cancer cells through transcriptional regulation of metalloproteinases and their tissue inhibitors. |
| 15754350 | 4 | No | Androgen receptor signaling and vitamin D receptor action in prostate cancer cells. |
| 15756023 | 4 | No | Growth inhibition of multiple myeloma cells by a novel IkappaB kinase inhibitor. |
| 15757668 | 59 | No | Expression profiling of the estrogen responsive genes in response to phytoestrogens using a customized DNA microarray. |
| 15760377 | 1 | No | The impact of dydrogesterone supplementation on hormonal profile and progesterone-induced blocking factor concentrations in women with threatened |

| | | | |
|---|---|---|---|
| 15761015 | 242 | No | Arsenic suppresses gene expression in promyelocytic leukemia cells partly through Sp1 oxidation. |
| 15761118 | 3 | No | Antimalarial artemisinin drugs induce cytochrome P450 and MDR1 expression by activation of xenosensors pregnane X receptor and constitutive androstane |
| 15762036 | 3 | No | Effects of 17beta-estradiol, tamoxifen and raloxifene on the protein and mRNA expression of interleukin-6, transforming growth factor-beta1 and insulin-like |
| 15762874 | 7 | No | 4-(Methylnitrosamino)-1-(3-pyridyl)-1-butanone, a component of tobacco smoke, modulates mediator release from human bronchial and alveolar epithelial cells. |
| 15763656 | 5 | No | Resveratrol-mediated sensitisation to TRAIL-induced apoptosis depends on death receptor and mitochondrial signalling. |
| 15763944 | 13 | No | Angiopoietin-1 inhibits doxorubicin-induced human umbilical vein endothelial cell death by modulating fas expression and via the PI3K/Akt pathway. |
| 15764585 | 6 | No | Characterization of activating signal cointegrator-2 as a novel transcriptional coactivator of the xenobiotic nuclear receptor constitutive androstane receptor. |
| 15764647 | 3 | No | Ablation of either p21 or Bax prevents p53-dependent apoptosis induced by green tea polyphenol epigallocatechin-3-gallate. |
| 15766417 | 2 | No | [The inhibitory effect of deoxynivalenol on TAP-1 expression in human peripheral blood mononuclear cells in vitro] |
| 15766595 | 50 | No | Analysis of gene expression induced by diethylstilbestrol (DES) in human primitive Mullerian duct cells using microarray. |
| 15767253 | 3 | No | Intestinal glucose-dependent expression of glucose-6-phosphatase: involvement of the aryl receptor nuclear translocator transcription factor. |
| 15767336 | 57 | No | Resveratrol-induced gene expression profiles in human prostate cancer cells. |
| 15767558 | 6 | No | The conventional nonsteroidal anti-inflammatory drug sulindac sulfide arrests ovarian cancer cell growth via the expression of NAG-1/MIC-1/GDF-15. |
| 15767642 | 4 | No | Biomarker changes during neoadjuvant anastrozole, tamoxifen, or the combination: influence of hormonal status and HER-2 in breast cancer--a study from the IMPACT |
| 15769886 | 4 | No | In vivo induction of human cytochrome P450 enzymes expressed in chimeric mice with humanized liver. |
| 15769986 | 8 | No | Oral contraceptives improve endothelial function in amenorrheic athletes. |
| 15770523 | 6 | No | Sequence dependent potentiation of gemcitabine by flavopiridol in human breast cancer cells. |
| 15770638 | 20 | No | Analysis of telomerase activity and RNA expression in a patient with acute promyelocytic leukemia treated with all-trans retinoic acid. |

| | | | |
|---|---|---|---|
| 15771232 | 16 | No | Comparative effects of fibrates on drug metabolizing enzymes in human hepatocytes. |
| 15774544 | 10 | No | Progesterone induces the fibulin-1 expression in human endometrial stromal cells. |
| 15774851 | 1 | No | Neonatal eosinophils possess efficient Eotaxin/IL-5- and N-formyl-methionyl-leucyl-phenylalanine-induced transmigration in vitro. |
| 15777837 | 1 | No | The distinct erythropoietin functions that promote cell survival and proliferation are affected by aluminum exposure through mechanisms involving |
| 15777968 | 1 | No | Identification of intelectin overexpression in malignant pleural mesothelioma by serial analysis of gene expression (SAGE). |
| 15778998 | 1 | No | Overexpression of GSTA2 protects against cell cycle arrest and apoptosis induced by the DNA inter-strand crosslinking nitrogen mustard, mechlorethamine. |
| 15779068 | 78 | No | Effects of resveratrol, piceatannol, tri-acetoxystilbene, and genistein on the inflammatory response of human peripheral blood leukocytes. |
| 15779298 | 2 | No | [Protein bcl-2 expression in the secretory endometrium after oral estrogen-progesterone replacement] |
| 15781256 | 10 | No | Two glucosylceramide synthase inhibitors attenuate doxorubicin-induced p21Cip1/Waf1 upregulation in HepG2 cells, irrespective of their differential |
| 15781649 | 10 | No | Predominant Bcl-XL knockdown disables antiapoptotic mechanisms: tumor necrosis factor-related apoptosis-inducing ligand-based triple chemotherapy overcomes |
| 15781658 | 6 | No | Coadministration of histone deacetylase inhibitors and perifosine synergistically induces apoptosis in human leukemia cells through Akt and ERK1/2 inactivation and |
| 15782132 | 7 | No | Rapamycin induces apoptosis of JN-DSRCT-1 cells by increasing the Bax : Bcl-xL ratio through concurrent mechanisms dependent and independent of its mTOR |
| 15782287 | 2 | No | BACKGROUND: Dietary polyphenols like quercetin and rutin are considered beneficial because of their potential protective role in the pathogenesis of |
| 15784690 | 115 | No | Zidovudine induces S-phase arrest and cell cycle gene expression changes in human cells. |
| 15786421 | 3 | No | Chemosensitization by STI571 targeting the platelet-derived growth factor/platelet-derived growth factor receptor-signaling pathway in the tumor |
| 15786552 | 3 | No | Effect of indomethacin on cell cycle proteins in colon cancer cell lines. |
| 15788148 | 2 | No | Etiopathogenesis of atopic dermatitis--an overview. |
| 15788408 | 12 | No | The oxidative stressor arsenite activates vascular endothelial growth factor mRNA transcription by an ATF4-dependent mechanism. |

| | | | |
|---|---|---|---|
| 15788441 | 6 | No | Regulated expression of platelet factor 4 in human monocytes--role of PARs as a quantitatively important monocyte activation pathway. |
| 15788722 | 2 | No | Post-transcriptional regulation of metallothionein isoform 1 and 2 expression in the human breast and the MCF-10A cell line. |
| 15790403 | 15 | No | Casodex treatment induces hypoxia-related gene expression in the LNCaP prostate cancer progression model. |
| 15790450 | 1 | No | Sensitization of cervical cancer cell lines to low-dose radiation by retinoic acid does not require functional p53. |
| 15792794 | 4 | No | ERK-dependent induction of TNFalpha expression by the environmental contaminant benzo(a)pyrene in primary human macrophages. |
| 15793875 | 8 | No | Apoptosis of human gastric cancer SGC-7901 cells induced by mitomycin combined with sulindac. |
| 15794184 | 1 | No | Valproate-induced eosinophilia in children with epilepsy: role of interleukin-5. |
| 15795092 | 4 | No | Transcriptional induction of CYP3A4 by o,p'-DDT in HepG2 cells. |
| 15795327 | 4 | No | 25-hydroxyvitamin D3-1alpha-hydroxylase is expressed in human vascular smooth muscle cells and is upregulated by parathyroid hormone and estrogenic compounds. |
| 15796204 | 2 | No | N-acetyltransferase is involved in baicalein-induced N-acetylation of 2-aminofluorene and DNA-2-aminofluorene adduct formation in human leukemia HL-60 |
| 15797241 | 1 | No | Repression of 5-aminolevulinate synthase gene by the potent tumor promoter, TPA, involves multiple signal transduction pathways. |
| 15797250 | 7 | No | Conditional regulation of the human CYP4X1 and CYP4Z1 genes. |
| 15797623 | 4 | No | Effects of 3-methylcholanthrene on the transcriptional activity and mRNA accumulation of the oncogene hWAPL. |
| 15799245 | 1 | No | JWA--a novel environmental-responsive gene, involved in estrogen receptor-associated signal pathway in MCF-7 and MDA-MB-231 breast carcinoma |
| 15802305 | 2 | No | 5-Hydroxytryptamine modulates cytokine and chemokine production in LPS-primed human monocytes via stimulation of different 5-HTR subtypes. |
| 15802384 | 4 | No | Identification of HMG-CoA reductase inhibitors as activators for human, mouse and rat constitutive androstane receptor. |
| 15802389 | 23 | No | Expression and induction potential of cytochromes P450 in human cryopreserved hepatocytes. |
| 15802533 | 4 | No | The natural product honokiol induces caspase-dependent apoptosis in B-cell chronic lymphocytic leukemia (B-CLL) cells. |

| | | | |
|---|---|---|---|
| 15803460 | 4 | No | PLAC1 expression increases during trophoblast differentiation: evidence for regulatory interactions with the fibroblast growth factor-7 (FGF-7) axis. |
| 15804060 | 1 | No | [Indomethacin-induced HL-60 leukemic cell apoptosis and the activation of C-jun NH2- terminal kinase signal transduction pathway] |
| 15804364 | 1 | No | Dexamethasone inhibits the HSV-tk/ ganciclovir bystander effect in malignant glioma cells. |
| 15804745 | 1 | No | CXCR2 Expression on neutrophils is upregulated during the relapsing phase of ocular Behcet disease. |
| 15806153 | 15 | No | Estrogen-induced loss of progesterone receptor expression in normal and malignant ovarian surface epithelial cells. |
| 15808406 | 11 | No | Transcriptional signatures of environmentally relevant exposures in normal human mammary epithelial cells: benzo[a]pyrene. |
| 15808407 | 2 | No | Induction of CYP1A1 and CYP1B1 and formation of carcinogen-DNA adducts in normal human mammary epithelial cells treated with benzo[a]pyrene. |
| 15809756 | 1 | No | Cisplatin represses transcriptional activity from the minimal promoter of the O6-methylguanine methyltransferase gene and increases sensitivity of human |
| 15811958 | 22 | No | Indole-3-carbinol suppresses NF-kappaB and IkappaBalpha kinase activation, causing inhibition of expression of NF-kappaB-regulated antiapoptotic and |
| 15813022 | 1 | No | [Mechanism of dracorhodin perchlorate-induced Hela cell apoptosis] |
| 15816529 | 2 | No | Wogonin inhibits N-acetyltransferase activity and gene expression in human leukemia HL-60 cells. |
| 15816534 | 2 | No | Hydroquinone-induced apoptosis in HL-60 cells. |
| 15816839 | 7 | No | 4-Tertiary butyl phenol exposure sensitizes human melanocytes to dendritic cell-mediated killing: relevance to vitiligo. |
| 15817521 | 2 | No | Acute treatment with the PPARgamma agonist pioglitazone and ibuprofen reduces glial inflammation and Abeta1-42 levels in APPV717I transgenic mice. |
| 15817653 | 2 | No | p38 Mitogen-activated protein kinase (MAPK) is a key mediator in glucocorticoid-induced apoptosis of lymphoid cells: correlation between p38 MAPK |
| 15817916 | 8 | No | Ethinylestradiol and testosterone have divergent effects on circulating IGF system components in adolescents with constitutional tall stature. |
| 15821026 | 4 | No | JTP-27536 [(+)-1,3-dihydroxy-2-hydroxymethylpropyl-2-ammonium 2-[(R)-3-cyclo-hexyl-1-phenylpropyl]-1,3-dioxo-2,3-dihydro-1H-isoindole-5-carboxy |
| 15821111 | 1 | No | Estrogen-related receptor-gamma and peroxisome proliferator-activated receptor-gamma coactivator-1alpha regulate estrogen-related receptor-alpha gene |

| | | | |
|---|---|---|---|
| 15823547 | 2 | No | TNF-alpha promotes Doxorubicin-induced cell apoptosis and anti-cancer effect through downregulation of p21 in p53-deficient tumor cells. |
| 15823556 | 6 | No | Selenoprotein W as molecular target of methylmercury in human neuronal cells is down-regulated by GSH depletion. |
| 15824117 | 4 | No | Overexpression of superoxide dismutase or glutathione peroxidase protects against the paraquat + maneb-induced Parkinson disease phenotype. |
| 15824121 | 1 | No | Vitamin D receptor-dependent regulation of colon multidrug resistance-associated protein 3 gene expression by bile acids. |
| 15824912 | 1 | No | Influence of cigarette smoking on melatonin levels in man. |
| 15826604 | 3 | No | G2 arrest and apoptosis by 2-amino-N-quinoline-8-yl-benzenesulfonamide (QBS), a novel cytotoxic compound. |
| 15827326 | 1 | No | Pentameric procyanidin from Theobroma cacao selectively inhibits growth of human breast cancer cells. |
| 15829497 | 10 | No | Phytoestrogen exposure elevates PTEN levels. |
| 15829614 | 2 | No | Genotoxicity of 4-hydroxy-2-nonenal in human colon tumor cells is associated with cellular levels of glutathione and the modulation of glutathione S-transferase A4 |
| 15831526 | 1 | No | Regulation of cytochrome b5 gene transcription by Sp3, GATA-6, and steroidogenic factor 1 in human adrenal NCI-H295A cells. |
| 15832819 | 1 | No | Protection of LLC-PK1 cells against hydrogen peroxide-induced cell death by modulation of ceramide level. |
| 15832825 | 1 | No | TERT mRNA expression is up-regulated in MCF-7 cells and a mouse mammary organ culture (MMOC) system by endosulfan treatment. |
| 15833885 | 6 | No | Selenium disrupts estrogen signaling by altering estrogen receptor expression and ligand binding in human breast cancer cells. |
| 15833893 | 2 | No | Chemoresistance to depsipeptide FK228 [(E)-(1S,4S,10S,21R)-7-[(Z)-ethylidene]-4,21-diisopropyl-2-oxa-12,13-dithia-5,8,2 |
| 15833926 | 16 | No | Arsenite decreases CYP3A4 and RXRalpha in primary human hepatocytes. |
| 15835818 | 1 | No | [Effect of intranasal glucosteroid on the gene expression of interleukin-5 in nasal polyps] |
| 15837074 | 2 | No | Benzo[a]pyrene, but not 2,3,7,8-TCDD, induces G2/M cell cycle arrest, p21CIP1 and p53 phosphorylation in human choriocarcinoma JEG-3 cells: a distinct signaling |
| 15837528 | 1 | No | 1,25-dihydroxyvitamin D inhibits human ANP gene promoter activity. |

| | | | |
|---|---|---|---|
| 15837795 | 6 | No | ER alpha-AHR-ARNT protein-protein interactions mediate estradiol-dependent transrepression of dioxin-inducible gene transcription. |
| 15838368 | 1 | No | Nitric oxide inhalation modulates endothelin-1 plasma concentration gradients following left ventricular assist device implantation. |
| 15838626 | 1 | No | The role of fibroblast growth factor 23 for hypophosphatemia and abnormal regulation of vitamin D metabolism in patients with McCune-Albright syndrome. |
| 15840558 | 2 | No | Oligomycin inhibits HIF-1alpha expression in hypoxic tumor cells. |
| 15841493 | 3 | No | Antioxidant and antigenotoxic effects of plant cell wall hydroxycinnamic acids in cultured HT-29 cells. |
| 15843042 | 1 | No | Prostaglandin D(2) and J(2) induce apoptosis in human leukemia cells via activation of the caspase 3 cascade and production of reactive oxygen species. |
| 15843497 | 2 | No | The p53 inhibitor pifithrin-alpha is a potent agonist of the aryl hydrocarbon receptor. |
| 15843518 | 26 | No | Histamine H4 receptor stimulation suppresses IL-12p70 production and mediates chemotaxis in human monocyte-derived dendritic cells. |
| 15843754 | 1 | No | [Frequent epigenetic inactivation of XAF1 by promotor hypermethylation in human colon cancers] |
| 15845615 | 8 | No | Proliferation of endothelial and tumor epithelial cells by progestin-induced vascular endothelial growth factor from human breast cancer cells: paracrine and |
| 15845643 | 8 | No | Type I and type II interferons delay human neutrophil apoptosis via activation of STAT3 and up-regulation of cellular inhibitor of apoptosis 2. |
| 15846114 | 2 | No | Rapid up-regulation of cyclooxygenase-2 by 5-fluorouracil in human solid tumors. |
| 15846301 | 3 | No | Insulin-like growth factor binding protein-6 inhibits prostate cancer cell proliferation: implication for anticancer effect of diethylstilbestrol in hormone |
| 15847785 | 1 | No | [Cytosolic pS2 levels and cellular proliferation in ER-positive and PgR-positive infiltrating ductal carcinomas of the breast] |
| 15849065 | 28 | No | Identification of transcriptional biomarkers induced by SERMS in human endometrial cells using multivariate analysis of DNA microarrays. |
| 15849716 | 7 | No | Modulation of UDP-glucuronosyltransferase 1A1 in primary human hepatocytes by prototypical inducers. |
| 15849726 | 5 | No | Antitumor effects of histone deacetylase inhibitor on Ewing's family tumors. |
| 15849732 | 64 | No | Potential anticancer activity of tanshinone IIA against human breast cancer. |

| | | | |
|---|---|---|---|
| 15850603 | 13 | No | Short-term effects of estrogen, tamoxifen and raloxifene on hemostasis: a randomized-controlled study and review of the literature. |
| 15855258 | 3 | No | Comparison of the metabolic effects of raloxifene and oral estrogen in postmenopausal and growth hormone-deficient women. |
| 15855670 | 1 | No | Hormonal regulation of circulating C-reactive protein in men. |
| 15855724 | 2 | No | Induction of human CYP1A2 and CYP3A4 in primary culture of hepatocytes from chimeric mice with humanized liver. |
| 15857753 | 7 | No | Inhibition of the 26S proteasome blocks progesterone receptor-dependent transcription through failed recruitment of RNA polymerase II. |
| 15858615 | 5 | No | Antimyeloma activity of two novel N-substituted and tetraflourinated thalidomide analogs. |
| 15860229 | 40 | No | Gene-inducing program of human dendritic cells in response to BCG cell-wall skeleton (CWS), which reflects adjuvancy required for tumor immunotherapy. |
| 15860260 | 10 | No | p53-dependent inhibition of progestin-induced VEGF expression in human breast cancer cells. |
| 15860653 | 5 | No | Functional analysis of six human aryl hydrocarbon receptor variants in a Japanese population. |
| 15862127 | 5 | No | A phase I study of hydralazine to demethylate and reactivate the expression of tumor suppressor genes. |
| 15862825 | 2 | No | Integration of progesterone receptor mediated rapid signaling and nuclear actions in breast cancer cell models: role of mitogen-activated protein kinases and cell |
| 15862954 | 4 | No | Heat-induced degradation of overexpressed glucocorticoid receptor Separate protective roles of hsp90 and hsp70. |
| 15863139 | 2 | No | Regulation of HtrA2/Omi by X-linked inhibitor of apoptosis protein in chemoresistance in human ovarian cancer cells. |
| 15863507 | 2 | No | Selective induction of the tumor marker glutathione S-transferase P1 by proteasome inhibitors. |
| 15863893 | 4 | No | Beta-D-xylopyranosyl-(1-->3)-beta-D-glucuronopyranosyl echinocystic acid isolated from the roots of Codonopsis lanceolata induces caspase-dependent apoptosis in |
| 15865071 | 3 | No | Expression of dihydropyrimidine dehydrogenase, thymidylate synthase, p53 and p21 in metastatic liver tumor from colorectal cancer after 5-fluorouracil-based |
| 15866426 | 5 | No | Estradiol and medroxyprogesterone acetate regulated genes in T47D breast cancer cells. |
| 15866594 | 34 | No | Progesterone and progestational compounds attenuate tumor necrosis factor alpha-induced interleukin-8 production via nuclear factor kappa B inactivation in |

| | | | |
|---|---|---|---|
| 15867097 | 2 | No | Sustained expansion of NKT cells and antigen-specific T cells after injection of alpha-galactosyl-ceramide loaded mature dendritic cells in cancer patients. |
| 15867202 | 9 | No | Phase II study of G3139, a Bcl-2 antisense oligonucleotide, in combination with dexamethasone and thalidomide in relapsed multiple myeloma patients. |
| 15867382 | 16 | No | Motexafin gadolinium disrupts zinc metabolism in human cancer cell lines. |
| 15868430 | 2 | No | Inositol hexaphosphate (IP6) blocks proliferation of human breast cancer cells through a PKCdelta-dependent increase in p27Kip1 and decrease in retinoblastoma |
| 15868441 | 5 | No | Characterization of a human breast cancer cell line, MCF-7/RU58R-1, resistant to the pure antiestrogen RU 58,668. |
| 15868479 | 3 | No | TNF-alpha expression patterns as potential molecular biomarker for human skin cells exposed to vesicant chemical warfare agents: sulfur mustard (HD) and |
| 15870702 | 9 | No | Two distinct modes of cell death induced by doxorubicin: apoptosis and cell death through mitotic catastrophe accompanied by senescence-like phenotype. |
| 15870704 | 9 | No | Opposite effects of estrogen receptors alpha and beta on MCF-7 sensitivity to the cytotoxic action of TNF and p53 activity. |
| 15870863 | 1 | No | The estrogenic activity of synthetic progestins used in oral contraceptives enhances fatty acid synthase-dependent breast cancer cell proliferation and |
| 15870882 | 2 | No | Deficient MGMT and proficient hMLH1 expression renders gallbladder carcinoma cells sensitive to alkylating agents through G2-M cell cycle arrest. |
| 15876410 | 2 | No | Anti-growth factor activities of benzothiophenes in human breast cancer cells. |
| 15876428 | 4 | No | Lipopolysaccharide negatively modulates vitamin D action by down-regulating expression of vitamin D-induced VDR in human monocytic THP-1 cells. |
| 15878629 | 11 | No | Differential effect of estrogen receptor alpha and beta agonists on the receptor for advanced glycation end product expression in human microvascular endothelial |
| 15878651 | 22 | No | Altered gene expression in human hepatoma HepG2 cells exposed to low-level 2,4-dichlorophenoxyacetic acid and potassium nitrate. |
| 15878867 | 2 | No | Alpha-tocopheryl succinate inhibits malignant mesothelioma by disrupting the fibroblast growth factor autocrine loop: mechanism and the role of oxidative |
| 15879110 | 15 | No | Transcriptional profiling of gamma delta T cells identifies a role for vitamin D in the immunoregulation of the V gamma 9V delta 2 response to |
| 15880572 | 1 | No | Doxorubicin induces expression of multidrug resistance-associated protein 1 in human small cell lung cancer cell lines by the c-jun N-terminal kinase pathway. |
| 15882436 | 1 | No | Inhibitory effects of anti-oxidants on apoptosis of a human polyclonal T-cell line, MT-2, induced by an asbestos, chrysotile-A. |

| | | | |
|---|---|---|---|
| 15883045 | 1 | No | Alpha-tocopheryl succinate induces cytostasis and apoptosis in osteosarcoma cells: the role of E2F1. |
| 15883047 | 8 | No | Pregnane X receptor-agonists down-regulate hepatic ATP-binding cassette transporter A1 and scavenger receptor class B type I. |
| 15885359 | 20 | No | Interferon-alpha-induced modulation of glucocorticoid and serotonin receptors as a mechanism of depression. |
| 15885616 | 1 | No | Gas-inducible product gene expression in bioreactors. |
| 15886251 | 1 | No | Resistin stimulation of 17alpha-hydroxylase activity in ovarian theca cells in vitro: relevance to polycystic ovary syndrome. |
| 15887248 | 4 | No | Androgen receptor regulates Cdc6 in synchronized LNCaP cells progressing from G1 to S phase. |
| 15890017 | 2 | No | Differential susceptibility of nonmalignant human breast epithelial cells and breast cancer cells to thiol antioxidant-induced G(1)-delay. |
| 15890193 | 3 | No | The human peroxisome proliferator-activated receptor delta gene is a primary target of 1alpha,25-dihydroxyvitamin D3 and its nuclear receptor. |
| 15890477 | 9 | No | Induction of phase-1 metabolizing enzymes by oltipraz, flavone and indole-3-carbinol enhance the formation and transport of benzo[a]pyrene sulfate |
| 15890672 | 5 | No | The 1,25(OH)2D3-regulated transcription factor MN1 stimulates vitamin D receptor-mediated transcription and inhibits osteoblastic cell proliferation. |
| 15890768 | 4 | No | Methoxychlor disrupts uterine Hoxa10 gene expression. |
| 15891005 | 1 | No | Parathyroid glands in calcium regulation and human disease. |
| 15892717 | 5 | No | Dynamic regulation of the human dopachrome tautomerase promoter by MITF, ER-alpha and chromatin remodelers during proliferation and senescence of human |
| 15893423 | 6 | No | CYP1A1 and CYP1B1 expressions in medulloblastoma cells are AhR-independent and have no direct link with resveratrol-induced differentiation and apoptosis. |
| 15893496 | 10 | No | Microbial compounds induce the expression of pro-inflammatory cytokines, chemokines and human beta-defensin-2 in vaginal epithelial cells. |
| 15894134 | 1 | No | Effect of bee venom on aromatase expression and activity in leukaemic FLG 29.1 and primary osteoblastic cells. |
| 15894585 | 80 | No | Polysaccharide purified from Ganoderma lucidum induced activation and maturation of human monocyte-derived dendritic cells by the NF-kappaB and p38 |
| 15896333 | 18 | No | Role of PI3K/Akt and MEK/ERK signaling pathways in sulforaphane- and erucin-induced phase II enzymes and MRP2 transcription, G2/M arrest and cell |

| | | | |
|---|---|---|---|
| 15897233 | 9 | No | The anti-invasive activity of cyclooxygenase inhibitors is regulated by the transcription factor ATF3 (activating transcription factor 3). |
| 15897249 | 9 | No | Irinotecan pharmacokinetic and pharmacogenomic alterations induced by methylselenocysteine in human head and neck xenograft tumors. |
| 15897583 | 2 | No | Dietary flaxseed alters tumor biological markers in postmenopausal breast cancer. |
| 15897598 | 14 | No | Novel histone deacetylase inhibitors in the treatment of thyroid cancer. |
| 15897898 | 1 | No | The Human mineral dust-induced gene, mdig, is a cell growth regulating gene associated with lung cancer. |
| 15897899 | 18 | No | Estrogen-induced G1/S transition of G0-arrested estrogen-dependent breast cancer cells is regulated by mitochondrial oxidant signaling. |
| 15897917 | 3 | No | Subtoxic concentration of doxorubicin enhances TRAIL-induced apoptosis in human prostate cancer cell line LNCaP. |
| 15899475 | 10 | No | Human epidermal cell protein responses to arsenite treatment in culture. |
| 15899819 | 18 | No | Nuclear factor-kappaB induced by doxorubicin is deficient in phosphorylation and acetylation and represses nuclear factor-kappaB-dependent transcription in cancer |
| 15899923 | 5 | No | Induction and activation of the aryl hydrocarbon receptor by IL-4 in B cells. |
| 15904944 | 11 | No | Effect of fraxetin on antioxidant defense and stress proteins in human neuroblastoma cell model of rotenone neurotoxicity. Comparative study with |
| 15905203 | 16 | No | Preferential induction of CYP1B1 by benzo[a]pyrene in human oral epithelial cells: impact on DNA adduct formation and prevention by polyphenols. |
| 15905586 | 42 | No | Identification of a novel blocker of I kappa B alpha kinase that enhances cellular apoptosis and inhibits cellular invasion through suppression of NF-kappa |
| 15908479 | 17 | No | Peroxisome proliferator-activated receptor-gamma ligands suppress fibronectin gene expression in human lung carcinoma cells: involvement of both CRE and Sp1. |
| 15909811 | 1 | No | [Comparison of letrozole and exemestane used in non-adjuvant therapy of endometrial carcinoma] |
| 15911101 | 21 | No | Antitumor effects of curcumin, alone or in combination with cisplatin or doxorubicin, on human hepatic cancer cells. Analysis of their possible |
| 15911693 | 5 | No | The methyl transferase PRMT1 functions as co-activator of farnesoid X receptor (FXR)/9-cis retinoid X receptor and regulates transcription of FXR responsive |
| 15913882 | 10 | No | Gene regulation in an MCF-7 cell line that naturally expresses an estrogen receptor unable to directly bind DNA. |

| | | | |
|---|---|---|---|
| 15914193 | 6 | No | Biological inactivation and impaired detection of IL-10 by suramin. |
| 15914525 | 2 | No | Retinoids and retinol differentially regulate steroid biosynthesis in ovarian theca cells isolated from normal cycling women and women with polycystic ovary |
| 15916722 | 5 | No | Differential regulation of survivin by p53 contributes to cell cycle dependent apoptosis. |
| 15916743 | 8 | No | Peroxisome proliferator-activated receptor gamma ligands induce cell cycle arrest and apoptosis in human renal carcinoma cell lines. |
| 15917307 | 10 | No | Identification of BCRP as transporter of benzo[a]pyrene conjugates metabolically formed in Caco-2 cells and its induction by Ah-receptor agonists. |
| 15919092 | 1 | No | Spatio-temporal activation of chromatin on the human CYP24 gene promoter in the presence of 1alpha,25-Dihydroxyvitamin D3. |
| 15919668 | 2 | No | Inhibition of platelet-derived growth factor-induced cell growth signaling by a short interfering RNA for EWS-Fli1 via down-regulation of phospholipase D2 in |
| 15919766 | 1 | No | The nuclear receptors constitutive androstane receptor and pregnane X receptor cross-talk with hepatic nuclear factor 4alpha to synergistically activate the |
| 15920492 | 1 | No | Long-term thalidomide therapy resulted in lack of mdr1 gene expression in a patient with primary resistant multiple myeloma. |
| 15922007 | 2 | No | Regulation of IGF-I production and proliferation of human leiomyomal smooth muscle cells by Scutellaria barbata D. Don in vitro: isolation of flavonoids of |
| 15922086 | 12 | No | Calbindin-D28k (CaBP28k) identification and regulation by 1,25-dihydroxyvitamin D3 in human choriocarcinoma cell line JEG-3. |
| 15922381 | 2 | No | Upregulation of estrogen receptor expression in the uterus of ovariectomized B6C3F1 mice and Ishikawa cells treated with bromoethane. |
| 15922390 | 3 | No | Bee venom induces apoptosis through caspase-3 activation in synovial fibroblasts of patients with rheumatoid arthritis. |
| 15924336 | 4 | No | Estrogen effects on tubulin expression and taxane mediated cytotoxicity in prostate cancer cells. |
| 15925326 | 1 | No | Regulation of the cell cycle in response to inhibition of mitochondrial generated energy. |
| 15927959 | 3 | No | Functional characterization of JMJD2A, a histone deacetylase- and retinoblastoma-binding protein. |
| 15929907 | 56 | No | Discovery of novel biomarkers by microarray analysis of peripheral blood mononuclear cell gene expression in benzene-exposed workers. |
| 15930183 | 15 | No | Regulation of vitamin D receptor expression via estrogen-induced activation of the ERK 1/2 signaling pathway in colon and breast cancer cells. |

| | | | |
|---|---|---|---|
| 15930297 | 5 | No | Topotecan blocks hypoxia-inducible factor-1alpha and vascular endothelial growth factor expression induced by insulin-like growth factor-I in neuroblastoma cells. |
| 15930308 | 5 | No | Pharmacologic inhibition of RAF-->MEK-->ERK signaling elicits pancreatic cancer cell cycle arrest through induced expression of p27Kip1. |
| 15930313 | 11 | No | Apoptosis induction by a novel retinoid-related molecule requires nuclear factor-kappaB activation. |
| 15930460 | 1 | No | Mechanisms of action and antiproliferative properties of Brassica oleracea juice in human breast cancer cell lines. |
| 15931867 | 2 | No | [Inhibitory effects of Galectin-3 on the inflammatory cytokines and chemokines in guinea pig asthma models] |
| 15933212 | 3 | No | Human CYP2C8 is transcriptionally regulated by the nuclear receptors constitutive androstane receptor, pregnane X receptor, glucocorticoid receptor, and hepatic |
| 15936073 | 2 | No | S100P is selectively upregulated in tumor cell lines challenged with DNA cross-linking agents. |
| 15937643 | 18 | No | Interferons upregulate thymidine phosphorylase expression via JAK-STAT-dependent transcriptional activation and mRNA stabilization in human glioblastoma cells. |
| 15939500 | 22 | No | Pluronic block copolymers alter apoptotic signal transduction of doxorubicin in drug-resistant cancer cells. |
| 15939924 | 9 | No | Thalidomide downregulates angiogenic genes in bone marrow endothelial cells of patients with active multiple myeloma. |
| 15941851 | 32 | No | Trialkyltin compounds bind retinoid X receptor to alter human placental endocrine functions. |
| 15942663 | 2 | No | GSK-3beta reactivation with LY294002 sensitizes hepatoma cells to chemotherapy-induced apoptosis. |
| 15942716 | 1 | No | Upregulation of BNIP3 by 5-aza-2'-deoxycytidine sensitizes pancreatic cancer cells to hypoxia-mediated cell death. |
| 15944324 | 2 | No | Mannose-containing molecular patterns are strong inducers of cyclooxygenase-2 expression and prostaglandin E2 production in human macrophages. |
| 15945276 | 2 | No | Saturated free fatty acid, palmitic acid, induces apoptosis in fetal hepatocytes in culture. |
| 15946544 | 3 | No | [Reversal effect of haloperidol on doxorubicin resistance and chloride channel inhibition in erythroleukemic cell K562/Dox] |
| 15946813 | 2 | No | Royal jelly has estrogenic effects in vitro and in vivo. |
| 15946948 | 2 | No | Delayed mechanism for induction of gamma-glutamylcysteine synthetase heavy subunit mRNA stability by oxidative stress involving p38 mitogen-activated |

| | | | |
|---|---|---|---|
| 15946989 | 6 | No | Monoamine oxidase-A is a major target gene for glucocorticoids in human skeletal muscle cells. |
| 15947024 | 3 | No | The role of protein kinase C in regulation of TCDD-mediated CYP1A1 gene expression. |
| 15947208 | 4 | No | Apigenin inhibits expression of vascular endothelial growth factor and angiogenesis in human lung cancer cells: implication of chemoprevention of lung |
| 15947480 | 6 | No | Leukotriene D4 induces production of transforming growth factor-beta1 by eosinophils. |
| 15948689 | 6 | No | Impaired degradation of matrix collagen in human gingival fibroblasts by the antiepileptic drug phenytoin. |
| 15948978 | 5 | No | Tacrolimus decreases the expression of eotaxin, CCR3, RANTES and interleukin-5 in atopic dermatitis. |
| 15949261 | 6 | No | [Detection of gene expression alteration of myeloma cells treated with arsenic trioxide] |
| 15949266 | 2 | No | [Effects of dexamethasone on arsenic trioxide induced apoptosis, NF-kappaB activation and gene expression in lymphoma cell line] |
| 15949379 | 8 | No | [Effects of estradiol and progesterone on the expression of insulin receptor substrate in human osteoblasts] |
| 15949478 | 5 | No | PKC delta-induced activation of MAPK pathway is required for bFGF-stimulated proliferation of coronary smooth muscle cells. |
| 15949688 | 2 | No | The effect of beta-carotene and its derivatives on cytotoxicity, differentiation, proliferative potential and apoptosis on the three human acute leukemia cell |
| 15949695 | 11 | No | Fatty acids and expression of adipokines. |
| 15949805 | 2 | No | Grifolin, a potential antitumor natural product from the mushroom Albatrellus confluens, inhibits tumor cell growth by inducing apoptosis in vitro. |
| 15950373 | 2 | No | Differential effects of estrogen receptor antagonists on pituitary lactotroph proliferation and prolactin release. |
| 15950459 | 2 | No | Divergent biological effects of estradiol and diethylstilbestrol in the prostate cancer cell line MOP. |
| 15951333 | 4 | No | Regulation of human pulmonary surfactant protein gene expression by 1alpha,25-dihydroxyvitamin D3. |
| 15951637 | 14 | No | 1,25-Dihydroxyvitamin D3 suppresses gene expression of eukaryotic translation initiation factor 2 in human promyelocytic leukemia HL-60 cells. |
| 15952644 | 6 | No | [Study of IL-8 overexpression in A549 cells induced by crocidolite fibers] |

| | | | |
|---|---|---|---|
| 15953574 | 2 | No | Long-term hematological and immunological complications of sulfur mustard poisoning in Iranian veterans. |
| 15953582 | 2 | No | 2,3,7,8-tetrachlorodibenzo-p-dioxin induces CYP1B1 expression in human luteinized granulosa cells. |
| 15955085 | 2 | No | Towards dissecting the pathogenesis of retinoid-induced hair loss: all-trans retinoic acid induces premature hair follicle regression (catagen) by |
| 15955209 | 3 | No | Sulfasalazine inhibits activation of nuclear factor-kappaB in patients with ulcerative colitis. |
| 15955616 | 1 | No | The effects of hormone therapy, estrogen therapy and tibolone on apoptosis and cyclin D1 expression in postmenopausal vaginal epithelium. |
| 15955619 | 22 | No | Genistein potentiates the growth inhibitory effects of 1,25-dihydroxyvitamin D3 in DU145 human prostate cancer cells: role of the direct inhibition of CYP24 |
| 15955695 | 3 | No | Positive and negative transcriptional regulation of aromatase expression in human breast cancer tissue. |
| 15955870 | 2 | No | CYP3A5 mRNA degradation by nonsense-mediated mRNA decay. |
| 15956029 | 1 | No | Chemiluminescence quantitative immunohistochemical determination of MRP2 in liver biopsies. |
| 15956246 | 296 | No | In vitro and in vivo irinotecan-induced changes in expression profiles of cell cycle and apoptosis-associated genes in acute myeloid leukemia cells. |
| 15956251 | 4 | No | Plasma vascular endothelial growth factor and interleukin-8 as biomarkers of antitumor efficacy of a prototypical erbB family tyrosine kinase inhibitor. |
| 15956805 | 2 | No | Intravenous calcitriol therapy increases serum concentrations of fibroblast growth factor-23 in dialysis patients with secondary hyperparathyroidism. |
| 15958590 | 1 | No | Calcium/calmodulin-dependent kinase I and calcium/calmodulin-dependent kinase kinase participate in the control of cell cycle progression in MCF-7 human breast |
| 15958644 | 7 | No | Activation of p53-dependent apoptosis by acute ablation of glycogen synthase kinase-3beta in colorectal cancer cells. |
| 15958646 | 4 | No | Systemic treatment with tetra-O-methyl nordihydroguaiaretic acid suppresses the growth of human xenograft tumors. |
| 15958647 | 29 | No | The transforming growth factor-beta family members bone morphogenetic protein-2 and macrophage inhibitory cytokine-1 as mediators of the antiangiogenic activity |
| 15959780 | 16 | No | Hyperacetylation enhances the growth-inhibitory effect of all-trans retinoic acid by the restoration of retinoic acid receptor beta expression in head and neck |
| 15960896 | 2 | No | Regulating expressions of cyclin D1, pRb, and anti-cancer effects of deguelin on human Burkitt's lymphoma Daudi cells in vitro. |

| | | | |
|---|---|---|---|
| 15961160 | 14 | No | Nanomolar aluminum induces pro-inflammatory and pro-apoptotic gene expression in human brain cells in primary culture. |
| 15961274 | 10 | No | As2O3-induced c-Src/EGFR/ERK signaling is via Sp1 binding sites to stimulate p21WAF1/CIP1 expression in human epidermoid carcinoma A431 cells. |
| 15961301 | 15 | No | Bitter gourd seed fatty acid rich in 9c,11t,13t-conjugated linolenic acid induces apoptosis and up-regulates the GADD45, p53 and PPARgamma in human colon cancer |
| 15961562 | 3 | No | Expression, regulation, and function of paired-box gene 8 in the human placenta and placental cancer cell lines. |
| 15961563 | 2 | No | Bazedoxifene acetate: a selective estrogen receptor modulator with improved selectivity. |
| 15961672 | 4 | No | Selective effects of the anticancer drug Yondelis (ET-743) on cell-cycle promoters. |
| 15963385 | 2 | No | Monitoring of hematological, inflammatory and oxidative reactions to acute oral iron exposure in human volunteers: preliminary screening for selection of |
| 15963497 | 4 | No | Effect of indomethacin on E-cadherin and beta-catenin expression in HT-29 colon cancer cells. |
| 15963507 | 6 | No | The p53 pathway is synergized by p38 MAPK signaling to mediate 11,11'-dideoxyverticillin-induced G2/M arrest. |
| 15963989 | 23 | No | Comparison of replicative senescence and stress-induced premature senescence combining differential display and low-density DNA arrays. |
| 15964118 | 3 | No | Effects of anthocyanidin on the inhibition of proliferation and induction of apoptosis in human gastric adenocarcinoma cells. |
| 15964185 | 10 | No | Translational studies on aromatase, cyclooxygenases, and enzyme inhibitors in breast cancer. |
| 15964220 | 4 | No | Effects of coumarin and 7OH-coumarin on bcl-2 and Bax expression in two human lung cancer cell lines in vitro. |
| 15964311 | 3 | No | Cinnamaldehyde-induced apoptosis in human PLC/PRF/5 cells through activation of the proapoptotic Bcl-2 family proteins and MAPK pathway. |
| 15964557 | 4 | No | TCR pathway involves ICBP90 gene down-regulation via E2F binding sites. |
| 15964790 | 1 | No | Aryl hydrocarbon receptor-mediated transcription: ligand-dependent recruitment of estrogen receptor alpha to 2,3,7,8-tetrachlorodibenzo-p-dioxin-responsive |
| 15964798 | 4 | No | Transcription factor NF-kappaB differentially regulates death receptor 5 expression involving histone deacetylase 1. |
| 15964800 | 5 | No | Blockade of histone deacetylase inhibitor-induced RelA/p65 acetylation and NF-kappaB activation potentiates apoptosis in leukemia cells through a process |

| | | | |
|---|---|---|---|
| 15964820 | 38 | No | Arginine methylation provides epigenetic transcription memory for retinoid-induced differentiation in myeloid cells. |
| 15964894 | 3 | No | Impact of CFTR DeltaF508 mutation on prostaglandin E2 production and type IIA phospholipase A2 expression by pulmonary epithelial cells. |
| 15965100 | 6 | No | Effects of gonadotrophin-releasing hormone agonists on apoptosis of granulosa cells. |
| 15965230 | 4 | No | Protein phosphatase 2A regulates estrogen receptor alpha (ER) expression through modulation of ER mRNA stability. |
| 15967203 | 1 | No | Metallothionein gene expression in peripheral lymphocytes and renal dysfunction in a population environmentally exposed to cadmium. |
| 15967204 | 7 | No | Molecular markers of trichloroethylene-induced toxicity in human kidney cells. |
| 15967414 | 22 | No | Differential expression of genes coding for EGF-like factors and ADAMTS1 following gonadotropin stimulation in normal and transformed human granulosa |
| 15968319 | 4 | No | [Regulation of genistein on the levels of ERalpha, ERbeta mRNA in uterine endometrial cancer cells] |
| 15968320 | 4 | No | [Regulation of orphan receptor ERR alpha by estrogen and progesterone in endometrial carcinoma cell line] |
| 15968715 | 2 | No | Anticancer effect and apoptosis induction of gambogic acid in human gastric cancer line BGC-823. |
| 15969986 | 2 | No | Induction of thyroid papillary carcinoma cell proliferation by estrogen is associated with an altered expression of Bcl-xL. |
| 15970291 | 2 | No | Effects of raloxifene, hormone therapy, and soy isoflavone on serum high-sensitive C-reactive protein in postmenopausal women. |
| 15970427 | 12 | No | The anti-apoptotic effect of leukotriene B4 in neutrophils: a role for phosphatidylinositol 3-kinase, extracellular signal-regulated kinase and Mcl-1. |
| 15970518 | 4 | No | Docetaxel induces p53-dependent apoptosis and synergizes with farnesyl transferase inhibitor r115777 in human epithelial cancer cells. |
| 15970668 | 4 | No | Chronic imatinib mesylate exposure leads to reduced intracellular drug accumulation by induction of the ABCG2 (BCRP) and ABCB1 (MDR1) drug transport |
| 15970678 | 2 | No | High concentrations of retinoids induce differentiation and late apoptosis in pancreatic cancer cells in vitro. |
| 15970709 | 2 | No | Changes in survivin messenger RNA level during chemotherapy treatment in ovarian cancer cells. |
| 15971251 | 1 | No | The expression pattern of MUC1 glycoforms and other biomarkers of endometrial receptivity in fertile and infertile women. |

| | | | |
|---|---|---|---|
| 15972124 | 1 | No | [Role of Bcl-xL in the cathepsin D-associated apoptosis of K562 cells] |
| 15972126 | 2 | No | [Effect of realgar on expression of survivin in leukemia cell lines and its significance] |
| 15972136 | 1 | No | [Study of triptolide-induced apoptosis in MUTZ-1 cells and its allied mechanism] |
| 15972296 | 1 | No | Genomic structure and functional characterization of the human ADAM10 promoter. |
| 15972445 | 7 | No | Transcription inhibition by flavopiridol: mechanism of chronic lymphocytic leukemia cell death. |
| 15972578 | 1 | No | Overexpression of the Wnt5b gene in leiomyoma cells: implications for a role of the Wnt signaling pathway in the uterine benign tumor. |
| 15972851 | 1 | No | Methylation silencing of the Apaf-1 gene in acute leukemia. |
| 15972972 | 4 | No | 13-cis retinoic acid inhibits development and progression of chronic allograft nephropathy. |
| 15975156 | 8 | No | Differential inhibition of oxidized LDL-induced apoptosis in human endothelial cells treated with different flavonoids. |
| 15975614 | 6 | No | Salacia oblonga root improves postprandial hyperlipidemia and hepatic steatosis in Zucker diabetic fatty rats: activation of PPAR-alpha. |
| 15976015 | 5 | No | Inhibitory effects of 12-O-tetradecanoylphorbol-13-acetate alone or in combination with all-trans retinoic acid on the growth of cultured human pancreas |
| 15977188 | 24 | No | Effect of silybin and its glycosides on the expression of cytochromes P450 1A2 and 3A4 in primary cultures of human hepatocytes. |
| 15978632 | 22 | No | Arsenic trioxide induces Hsp70 expression via reactive oxygen species and JNK pathway in MDA231 cells. |
| 15978937 | 11 | No | Signal transduction of phorbol 12-myristate 13-acetate (PMA)-induced growth inhibition of human monocytic leukemia THP-1 cells is reactive oxygen dependent. |
| 15978972 | 1 | No | Comparison of effects of the rise in serum testosterone by raloxifene and oral testosterone on serum insulin-like growth factor-1 and insulin-like growth factor |
| 15979257 | 1 | No | Vinclozolin, a widely used fungizide, enhanced BaP-induced micronucleus formation in human derived hepatoma cells by increasing CYP1A1 expression. |
| 15979383 | 2 | No | Nitric oxide induces oral squamous cell carcinoma cells apoptosis with p53 accumulation. |
| 15979871 | 3 | No | Differential regulation of the human MRP2 and MRP3 gene expression by glucocorticoids. |

| | | | |
|---|---|---|---|
| 15979894 | 6 | No | Arsenic trioxide overcomes apoptosis inhibition in K562/ADM cells by regulating vital components in apoptotic pathway. |
| 15980968 | 215 | No | Gene expression profile induced by BCNU in human glioma cell lines with differential MGMT expression. |
| 15981204 | 2 | No | Cisplatin activates Akt in small cell lung cancer cells and attenuates apoptosis  by survivin upregulation. |
| 15981920 | 2 | No | HPMA copolymer-bound doxorubicin induces apoptosis in human ovarian carcinoma cells by a Fas-independent pathway. |
| 15982314 | 426 | No | Retinoic acid and its 4-oxo metabolites are functionally active in human skin cells in vitro. |
| 15982688 | 4 | No | 2,3,7,8-tetrachlorodibenzo-p-dioxin (TCDD) induces matrix metalloproteinase (MMP) expression and invasion in A2058 melanoma cells. |
| 15982805 | 3 | No | A thioredoxin reductase inhibitor induces growth inhibition and apoptosis in five cultured human carcinoma cell lines. |
| 15982930 | 5 | No | Thalidomide inhibits growth of tumors through COX-2 degradation independent of antiangiogenesis. |
| 15983038 | 6 | No | S-adenosylmethionine blocks collagen I production by preventing transforming growth factor-beta induction of the COL1A2 promoter. |
| 15985530 | 3 | No | Human cathelicidin antimicrobial peptide (CAMP) gene is a direct target of the vitamin D receptor and is strongly up-regulated in myeloid cells by |
| 15985719 | 4 | No | Induction of caspase 3 activity, bcl-2 bax and p65 gene expression modulation in human acute promyelocytic leukemia HL-60 cells by doxorubicin with amifostine. |
| 15986123 | 3 | No | GREB 1 is a critical regulator of hormone dependent breast cancer growth. |
| 15986355 | 2 | No | NF-kappaB protects Beh莽et's disease T cells against CD95-induced apoptosis up-regulating antiapoptotic proteins. |
| 15986848 | 3 | No | Apoptosis induced by aspirin and 5-fluorouracil in human colonic adenocarcinoma cells. |
| 15988048 | 15 | No | Differentiation response of acute promyelocytic leukemia cells and PML/RARa leukemogenic activity studies by real-time RT-PCR. |
| 15990222 | 4 | No | Induction of p53 and drug resistance following treatment with cisplatin or paclitaxel in ovarian cancer cell lines. |
| 15990257 | 5 | No | Effects of estrogen, raloxifene, and hormone replacement therapy on serum C-reactive protein and homocysteine levels. |
| 15990778 | 1 | No | Expression of functional cysteinyl leukotriene receptors by human basophils. |

| | | | |
|---|---|---|---|
| 15992766 | 2 | No | Promoter-, cell-, and ligand-specific transactivation responses of the VDRB1 isoform. |
| 15993080 | 2 | No | 8-Azapurines as new inhibitors of cyclin-dependent kinases. |
| 15993333 | 2 | No | Activation of c-Jun-N-terminal kinase is required for apoptosis triggered by glutathione disulfide in neuroblastoma cells. |
| 15993339 | 1 | No | Desferal inhibits breast tumor growth and does not interfere with the tumoricidal activity of doxorubicin. |
| 15993743 | 2 | No | Sanguinarine activates polycyclic aromatic hydrocarbon associated metabolic pathways in human oral keratinocytes and tissues. |
| 15993745 | 3 | No | Estrogenic and genotoxic potential of equol and two hydroxylated metabolites of Daidzein in cultured human Ishikawa cells. |
| 15993841 | 5 | No | Rebamipide inhibits gastric cancer growth by targeting survivin and Aurora-B. |
| 15993843 | 2 | No | Inhibition of melanoma cell proliferation by resveratrol is correlated with upregulation of quinone reductase 2 and p53. |
| 15993848 | 2 | No | 17-Allylamino-17-demethoxygeldanamycin overcomes TRAIL resistance in colon cancer cell lines. |
| 15994153 | 2 | No | Effects of 9-cis-retinoic acid on the insulin-like growth factor axis in former smokers. |
| 15994225 | 12 | No | The ErbB3-binding protein Ebp1 suppresses androgen receptor-mediated gene transcription and tumorigenesis of prostate cancer cells. |
| 15994348 | 3 | No | Direct agonist/antagonist functions of dehydroepiandrosterone. |
| 15995977 | 1 | No | Xanthohumol induces apoptosis in cultured 40-16 human colon cancer cells by activation of the death receptor- and mitochondrial pathway. |
| 15996700 | 3 | No | Arsenic, mode of action at biologically plausible low doses: what are the implications for low dose cancer risk? |
| 15996779 | 1 | No | Fraxetin prevents rotenone-induced apoptosis by induction of endogenous glutathione in human neuroblastoma cells. |
| 15996812 | 4 | No | DNA damaging drugs-induced down-regulation of Bcl-2 is essential for induction of apoptosis in high-risk HPV-positive HEp-2 and KB cells. |
| 15996861 | 3 | No | Expression of 15-lipoxygenase-1 in human nasal epithelium: its implication in mucociliary differentiation. |
| 15997088 | 3 | No | Identification of differentially expressed genes in hepatic HepG2 cells treated with acetaminophen using suppression subtractive hybridization. |

| | | | |
|---|---|---|---|
| 15997105 | 3 | No | Anti-inflammatory activities of aqueous extract from Radix Ophiopogon japonicus and its two constituents. |
| 15998540 | 1 | No | Dimethyl sulfoxide potentiates death receptor-mediated apoptosis in the human myeloid leukemia U937 cell line through enhancement of mitochondrial membrane |
| 16000229 | 10 | No | Gonadal steroids regulate GABAA receptor subunit mRNA expression in NT2-N neurons. |
| 16001973 | 55 | No | Response rate of fibrosarcoma cells to cytotoxic drugs on the expression level correlates to the therapeutic response rate of fibrosarcomas and is mediated by |
| 16002045 | 3 | No | Regulation of ionizing radiation-induced apoptosis by a manganese porphyrin complex. |
| 16002280 | 10 | No | Aromatase inhibitors: cellular and molecular effects. |
| 16002434 | 686 | No | Large-scale in silico and microarray-based identification of direct 1,25-dihydroxyvitamin D3 target genes. |
| 16002475 | 15 | No | Induction of fibroblast growth factor-9 and interleukin-1alpha gene expression by motorcycle exhaust particulate extracts and benzo(a)pyrene in human lung |
| 16002536 | 10 | No | Influence of exogenous oestrogen or (anti-) androgen administration on soluble transferrin receptor in human plasma. |
| 16002567 | 1 | No | Intestinal and hepatic expression of BNIP3 in necrotizing enterocolitis: |
| 16003486 | 3 | No | Antiproliferative activity of olomoucine II, a novel 2,6,9-trisubstituted purine cyclin-dependent kinase inhibitor. |
| 16003560 | 1 | No | A pilot study on the safety of combining chrysin, a non-absorbable inducer of UGT1A1, and irinotecan (CPT-11) to treat metastatic colorectal cancer. |
| 16003723 | 2 | No | Targeted chemotherapy with cytotoxic bombesin analogue AN-215 inhibits growth of experimental human prostate cancers. |
| 16004971 | 5 | No | Combination of cyclooxygenase-2 inhibitors and oxaliplatin increases the growth inhibition and death in human colon cancer cells. |
| 16005427 | 1 | No | Epigallocatechin gallate inhibits HIF-1alpha degradation in prostate cancer cells. |
| 16005483 | 6 | No | Changes in RANKL/OPG/RANK gene expression in peripheral mononuclear cells following treatment with estrogen or raloxifene. |
| 16005588 | 4 | No | Potential for interaction of kava and St. John's wort with drugs. |
| 16005713 | 5 | No | Hydroxyurea (HU)-induced apoptosis in the mouse fetal lung. |

| | | | |
|---|---|---|---|
| 16006241 | 3 | No | Characterisation of human histone H1x. |
| 16006447 | 5 | No | Differential effects of three antibiotics on T helper cell cytokine expression. |
| 16006969 | 1 | No | Enhanced transgene expression in urothelial cancer gene therapy with histone deacetylase inhibitor. |
| 16007142 | 5 | No | Sodium butyrate sensitizes human glioma cells to TRAIL-mediated apoptosis through inhibition of Cdc2 and the subsequent downregulation of survivin and XIAP. |
| 16007145 | 14 | No | Zerumbone abolishes NF-kappaB and IkappaBalpha kinase activation leading to suppression of antiapoptotic and metastatic gene expression, upregulation of |
| 16007148 | 1 | No | The role of Mcl-1 downregulation in the proapoptotic activity of the multikinase inhibitor BAY 43-9006. |
| 16007152 | 2 | No | Differential responses of Mcl-1 in photosensitized epithelial vs lymphoid-derived human cancer cells. |
| 16007204 | 27 | No | Synergistic induction of the MUC4 mucin gene by interferon-gamma and retinoic acid in human pancreatic tumour cells involves a reprogramming of signalling |
| 16007219 | 15 | No | Maspin sensitizes prostate cancer cells to doxazosin-induced apoptosis. |
| 16007339 | 1 | No | Effect of osteoblast-targeted expression of bcl-2 in bone: differential response in male and female mice. |
| 16008107 | 1 | No | Induction of drug metabolizing enzymes by vitamin E. |
| 16008523 | 6 | No | Arsenite induces a cell stress-response gene, RTP801, through reactive oxygen species and transcription factors Elk-1 and CCAAT/enhancer-binding protein. |
| 16008847 | 1 | No | [Hypermethylation of CpG island of p16 gene and arsenic trioxide induced p16 gene demethylation in multiple myeloma.] |
| 16009009 | 6 | No | [A study on the cell differentiation induced by tanshinone IIA and its molecular mechanism in retinoic acid: resistant acute promyelocytic leukemia] |
| 16009159 | 4 | No | Reduced expression of progesterone receptor-B in the endometrium of women with endometriosis and in cocultures of endometrial cells exposed to |
| 16009171 | 3 | No | Antiproliferative and proapoptotic effects of raloxifene on uterine leiomyomas in postmenopausal women. |
| 16009178 | 3 | No | Effect of mifepristone on proliferation and apoptosis of Ishikawa endometrial adenocarcinoma cells. |
| 16009488 | 10 | No | SK-7041, a new histone deacetylase inhibitor, induces G2-M cell cycle arrest and apoptosis in pancreatic cancer cell lines. |

| | | | |
|---|---|---|---|
| 16010410 | 1 | No | INSL3 in the benign hyperplastic and neoplastic human prostate gland. |
| 16010412 | 1 | No | Potential of endogenous estrogen receptor beta to influence the selective ER modulator ERbeta complex. |
| 16010430 | 4 | No | Induction of apoptosis by trichostatin A, a histone deacetylase inhibitor, is associated with inhibition of cyclooxygenase-2 activity in human non-small cell |
| 16010437 | 4 | No | Arsenic trioxide mediates intrinsic and extrinsic pathways of apoptosis and cell cycle arrest in acute megakaryocytic leukemia. |
| 16010555 | 13 | No | Sensitivity of human cord blood cells to tetrachloroethylene: cellular and molecular endpoints. |
| 16011614 | 4 | No | Effects of nicotine on proliferation, cell cycle, and differentiation in immortalized and malignant oral keratinocytes. |
| 16011840 | 6 | No | Tibolone and its metabolites enhance tissue factor and PAI-1 expression in human endometrial stromal cells: Evidence of progestogenic effects. |
| 16012519 | 26 | No | The retinoid anticancer signal: mechanisms of target gene regulation. |
| 16012733 | 5 | No | Induction of apoptosis by Chan Su, a traditional Chinese medicine, in human bladder carcinoma T24 cells. |
| 16012738 | 2 | No | Molecular mechanism of ursolic acid induced apoptosis in poorly differentiated endometrial cancer HEC108 cells. |
| 16012748 | 1 | No | Effect of estradiol and raloxifene on MUC1 expression and adhesive properties of Ishikawa cells. |
| 16012788 | 2 | No | Enhanced P53 and BAX gene expression and apoptosis in A549 cells by cis-Pt(II) complex of 3-aminoflavone in comparison with cis-DDP. |
| 16012789 | 2 | No | A study of cytotoxic synergy of UCN-01 and flavopiridol in syngeneic pair of cell lines. |
| 16013437 | 1 | No | Targeted disruption of p53 attenuates doxorubicin-induced cardiac toxicity in mice. |
| 16014322 | 1 | No | Regulation of the expression of the oncogene EVI1 through the use of alternative mRNA 5'-ends. |
| 16014403 | 8 | No | Corticotropin-releasing hormone (CRH) and urocortin act through type 1 CRH receptors to stimulate dehydroepiandrosterone sulfate production in human fetal |
| 16014563 | 6 | No | MDM2 antagonists induce p53-dependent apoptosis in AML: implications for leukemia therapy. |
| 16015594 | 1 | No | Complete androgen ablation suppresses prostate stem cell antigen (PSCA) mRNA expression in human prostate carcinoma. |

| | | | |
|---|---|---|---|
| 16019205 | 12 | No | Regulation of osteoblastic phenotype and gene expression by hop-derived phytoestrogens. |
| 16019350 | 3 | No | Effect of menstrual cycle and hormonal treatment on ki-67 and bcl-2 expression and adenomyosis. |
| 16019488 | 1 | No | Apoptotic effect of oridonin on NB4 cells and its mechanism. |
| 16019513 | 2 | No | Assessment of the cellular response to the induced expression of defensin sense and antisense cDNA in acute promyelocytic leukemia cell lines. |
| 16019514 | 1 | No | Cyclo-oxygenase 2 inhibitor, nabumetone, inhibits proliferation in chronic myeloid leukemia cell lines. |
| 16019559 | 3 | No | Upregulated hoxC4 induces CD14 expression during the differentiation of acute promyelocytic leukemia cells. |
| 16019850 | 1 | No | A hydrogen peroxide-generating agent, 6-formylpterin, enhances heat-induced apoptosis. |
| 16020486 | 3 | No | Secretoglobin 2A1 is under selective androgen control mediated by a peculiar binding site for Sp family transcription factors. |
| 16020662 | 18 | No | Androgen receptor signaling intensity is a key factor in determining the sensitivity of prostate cancer cells to selenium inhibition of growth and |
| 16021636 | 11 | No | 3,5,3'-triiodothyronine (T3) is a survival factor for pancreatic beta-cells undergoing apoptosis. |
| 16022909 | 2 | No | Reduction in BCL-2 levels by 26S proteasome inhibition with bortezomib is associated with induction of apoptosis in small cell lung cancer. |
| 16023083 | 11 | No | Curcumin (diferuloylmethane) inhibits constitutive NF-kappaB activation, induces G1/S arrest, suppresses proliferation, and induces apoptosis in mantle cell |
| 16023288 | 2 | No | Anti-proliferative effect of apigenin and its apoptotic induction in human Hep G2 cells. |
| 16023994 | 7 | No | Regulation of adiponectin receptor 1 in human hepatocytes by agonists of nuclear receptors. |
| 16024066 | 8 | No | Epigenetic-mediated upregulation of progesterone receptor B gene in endometrial cancer cell lines. |
| 16024248 | 1 | No | Aromatase in endometriosis and uterine leiomyomata. |
| 16024531 | 4 | No | Oxaliplatin, a potent inhibitor of survivin, enhances paclitaxel-induced apoptosis and mitotic catastrophe in colon cancer cells. |
| 16024610 | 2 | No | Nitric oxide-induced apoptosis in lymphoblastoid and fibroblast cells dependent on the phosphorylation and activation of p53. |

| | | | |
|---|---|---|---|
| 16024613 | 2 | No | Epidermal growth factor-induced signaling in breast cancer cells results in selective target gene activation by orphan nuclear receptor estrogen-related |
| 16024631 | 5 | No | Differential regulation of noxa in normal melanocytes and melanoma cells by proteasome inhibition: therapeutic implications. |
| 16024633 | 5 | No | Susceptibility of cholangiocarcinoma cells to parthenolide-induced apoptosis. |
| 16024638 | 8 | No | Activation of nuclear factor-kappaB contributes to induction of death receptors and apoptosis by the synthetic retinoid CD437 in DU145 human prostate cancer |
| 16024653 | 16 | No | Retinoid targeting of different D-type cyclins through distinct chemopreventive mechanisms. |
| 16024918 | 3 | No | The human ABCG1 gene: identification of LXR response elements that modulate expression in macrophages and liver. |
| 16025287 | 2 | No | TP53 promoter methylation in human gliomas. |
| 16025434 | 2 | No | The nitrogen mustard melphalan activates mitogen-activated phosphorylated kinases (MAPK), nuclear factor-kappaB and inflammatory response in lung epithelial cells. |
| 16025520 | 11 | No | Early response of alpha2(I) collagen to acetaldehyde in human hepatic stellate cells is TGF-beta independent. |
| 16026610 | 4 | No | Cancer cell adaptation to chemotherapy. |
| 16026872 | 2 | No | Hypoxia down-regulates DNA double strand break repair gene expression in prostate cancer cells. |
| 16027227 | 11 | No | The repression of E2F-1 is critical for the activity of Minerval against cancer. |
| 16027529 | 6 | No | The anti-proliferative inhibition of ellipticine in human breast mda-mb-231 cancer cells is through cell cycle arrest and apoptosis induction. |
| 16027726 | 4 | No | Inhibition of casein kinase I delta alters mitotic spindle formation and induces apoptosis in trophoblast cells. |
| 16029572 | 3 | No | [An experimental study on the mechanism of therapy for hemangioma with glucocorticoid] |
| 16029599 | 2 | No | [Primary research on arsenic trioxide inhibiting human breast cancer cells growth and its mechanisms] |
| 16029656 | 2 | No | [Effects of all-trans retinoic acid on expression of connexin genes and gap junction communication in hepatocellular carcinoma cell lines] |
| 16029874 | 35 | No | Gene expression profiling reveals novel regulation by bisphenol-A in estrogen receptor-alpha-positive human cells. |

| | | | |
|---|---|---|---|
| 16033089 | 12 | No | Stimulation of endometrial glandular cells with genistein and daidzein and their effects on ERalpha- and ERbeta-mRNA and protein expresion. |
| 16033772 | 8 | No | Effect of sulforaphane on metallothionein expression and induction of apoptosis in human hepatoma HepG2 cells. |
| 16033851 | 8 | No | Dual inhibition of mTOR and estrogen receptor signaling in vitro induces cell death in models of breast cancer. |
| 16036217 | 5 | No | Cdc2 and Cdk2 play critical roles in low dose doxorubicin-induced cell death through mitotic catastrophe but not in high dose doxorubicin-induced apoptosis. |
| 16037384 | 2 | No | Resveratrol regulates insulin-like growth factor-II in breast cancer cells. |
| 16037943 | 6 | No | VPAC1 expression is regulated by FXR agonists in the human gallbladder epithelium. |
| 16038477 | 2 | No | Reverse transcriptase inhibitors alter uncoupling protein-1 and mitochondrial biogenesis in brown adipocytes. |
| 16038730 | 1 | No | Ionic currents in multidrug resistant K562 human leukemic cells. |
| 16039115 | 4 | No | Inhibitory effect of 22-oxa-1,25-dihydroxyvitamin D3, maxacalcitol, on the proliferation of pancreatic cancer cell lines. |
| 16039398 | 97 | No | Gene expression changes in human small airway epithelial cells exposed to Delta9-tetrahydrocannabinol. |
| 16039525 | 7 | No | Disruption of gene expression and induction of apoptosis in prostate cancer cells by a DNA-damaging agent tethered to an androgen receptor ligand. |
| 16039551 | 2 | No | Chemically modified tetracyclines induce apoptosis in cultured mast cells. |
| 16039846 | 1 | No | Vitamin D and skin cancer: a problem in gene regulation. |
| 16039940 | 1 | No | Molecular events associated with arsenic-induced malignant transformation of human prostatic epithelial cells: aberrant genomic DNA methylation and K-ras |
| 16040207 | 3 | No | Downregulation of TACO gene transcription restricts mycobacterial entry/survival within human macrophages. |
| 16040568 | 8 | No | An in vivo bioassay for detecting antiandrogens using humanized transgenic mice coexpressing the tetracycline-controlled transactivator and human CYP1B1 gene. |
| 16041517 | 32 | No | Gaseous nitrogen oxide repressed benzo[a]pyrene-induced human lung fibroblast cell apoptosis via inhibiting JNK1 signals. |
| 16042588 | 23 | No | The importance of colonic butyrate transport to the regulation of genes associated with colonic tissue homoeostasis. |

| | | | |
|---|---|---|---|
| 16043219 | 3 | No | 5-Aza-2'-deoxycytidine (decitabine) can relieve p21WAF1 repression in human acute myeloid leukemia by a mechanism involving release of histone deacetylase 1 |
| 16043385 | 2 | No | Role of O6-methylguanine-DNA methyltransferase and effect of O6-benzylguanine on the anti-tumor activity of cis-diaminedichloroplatinum(II) in oral cancer cell |
| 16043423 | 1 | No | Differentiating human keratinocytes are deficient in p53 but retain global nucleotide excision repair following ultraviolet radiation. |
| 16043647 | 4 | No | Upregulation of nitric oxide production in vascular endothelial cells by all-trans retinoic acid through the phosphoinositide 3-kinase/Akt pathway. |
| 16045524 | 13 | No | Modulation of cytokine production by dydrogesterone in lymphocytes from women with recurrent miscarriage. |
| 16045735 | 6 | No | Interleukin-4 and interleukin-13 enhance CCL26 production in a human keratinocyte cell line, HaCaT cells. |
| 16045814 | 48 | No | Gene expression alteration during redox-dependent enhancement of arsenic cytotoxicity by emodin in HeLa cells. |
| 16046213 | 1 | No | Activation of the aryl hydrocarbon receptor by berberine in HepG2 and H4IIE cells: Biphasic effect on CYP1A1. |
| 16046235 | 7 | No | Etodolac induces apoptosis and inhibits cell adhesion to bone marrow stromal cells in human myeloma cells. |
| 16046582 | 1 | No | Comparative assessment in young and elderly men of the gonadotropin response to aromatase inhibition. |
| 16046716 | 3 | No | Grape polyphenols exert a cardioprotective effect in pre- and postmenopausal women by lowering plasma lipids and reducing oxidative stress. |
| 16047355 | 2 | No | Targeted therapy with a cytotoxic somatostatin analog, AN-238, inhibits growth of human experimental endometrial carcinomas expressing multidrug resistance protein |
| 16049271 | 4 | No | Comparison of TCDD and PCB CYP1A induction sensitivities in fresh hepatocytes from human donors, sprague-dawley rats, and rhesus monkeys and HepG2 cells. |
| 16049707 | 7 | No | Quercetin-induced growth inhibition and cell death in prostatic carcinoma cells (PC-3) are associated with increase in p21 and hypophosphorylated retinoblastoma |
| 16050810 | 4 | No | Regulation of ERK1 gene expression by coactivator proteins. |
| 16051184 | 2 | No | Resveratrol inhibits polyphosphoinositide metabolism in activated platelets. |
| 16051188 | 3 | No | Role of hTERT in apoptosis of cervical cancer induced by histone deacetylase inhibitor. |
| 16051281 | 7 | No | Molecular mechanisms of the 2,3,7,8-tetrachlorodibenzo-p-dioxin-induced inverted U-shaped dose responsiveness in anchorage independent growth and cell |

| | | | |
|---|---|---|---|
| 16051289 | 24 | No | Enhancement of esculetin on Taxol-induced apoptosis in human hepatoma HepG2 cells. |
| 16051428 | 5 | No | Inhibition of breast cancer cell growth and induction of cell death by 1,1-bis(3'-indolyl)methane (DIM) and 5,5'-dibromoDIM. |
| 16051478 | 2 | No | Experimental therapy of human endometrial cancers with a targeted cytotoxic bombesin analog AN-215: low induction of multidrug resistance proteins. |
| 16051482 | 3 | No | Chemoprevention of chemically-induced mammary and colon carcinogenesis by 1alpha-hydroxyvitamin D5. |
| 16051634 | 16 | No | Inhibition of estradiol-induced mammary proliferation by dibenzoylmethane through the E2-ER-ERE-dependent pathway. |
| 16051641 | 3 | No | The neurotrophin receptor TrkB cooperates with c-Met in enhancing neuroblastoma invasiveness. |
| 16052481 | 2 | No | Garcinol modulates tyrosine phosphorylation of FAK and subsequently induces apoptosis through down-regulation of Src, ERK, and Akt survival signaling in |
| 16052486 | 5 | No | Iron-ascorbic acid-induced oxidant stress and its quenching by paraoxonase 1 in HDL and the liver: comparison between humans and rats. |
| 16052530 | 26 | No | ZD6474 inhibits tumor growth and intraperitoneal dissemination in a highly metastatic orthotopic gastric cancer model. |
| 16053526 | 4 | No | P53 and beta-catenin activity during estrogen treatment of osteoblasts. |
| 16054126 | 16 | No | Effects of antioxidants and caspase-3 inhibitor on the phenylethyl isothiocyanate-induced apoptotic signaling pathways in human PLC/PRF/5 cells. |
| 16054129 | 143 | No | Retinoic acid-induced downmodulation of telomerase activity in human cancer cells. |
| 16054184 | 2 | No | CYP2S1: a short review. |
| 16054331 | 3 | No | Expressions of galectin-3, glutathione S-transferase A2 and peroxiredoxin-1 by nonylphenol-incubated Caco-2 cells and reduction in transepithelial electrical |
| 16054388 | 2 | No | Steroid signalling in the ovarian surface epithelium. |
| 16055089 | 6 | No | Estrogen receptor-alpha regulates SOCS-3 expression in human breast cancer cells. |
| 16055107 | 6 | No | The effects of a novel synthetic retinoid, seletinoid G, on the expression of extracellular matrix proteins in aged human skin in vivo. |
| 16055262 | 3 | No | Induction of apoptosis by puerarin in colon cancer HT-29 cells. |

| | | | |
|---|---|---|---|
| 16055726 | 3 | No | Glycogen synthase kinase 3-dependent phosphorylation of Mdm2 regulates p53 abundance. |
| 16059641 | 4 | No | Flavonoid quercetin, but not apigenin or luteolin, induced apoptosis in human myeloid leukemia cells and their resistant variants. |
| 16061222 | 1 | No | The cyclopentenone 15-deoxy-delta(12,14)-prostaglandin J2 inhibits G1/S transition and retinoblastoma protein phosphorylation in immortalized lymphocytes |
| 16061352 | 2 | No | Diazene JK-279 induces apoptosis-like cell death in human cervical carcinoma cells. |
| 16061374 | 1 | No | Altered thioredoxin subcellular localization and redox status in MCF-7 cells following 1,25-dihydroxyvitamin D3 treatment. |
| 16061648 | 4 | No | Mechanism of cell cycle regulation by FIP200 in human breast cancer cells. |
| 16061660 | 4 | No | Inhibition of c-Jun-N-terminal-kinase sensitizes tumor cells to CD95-induced apoptosis and induces G2/M cell cycle arrest. |
| 16061661 | 11 | No | Altered ErbB receptor signaling and gene expression in cisplatin-resistant ovarian cancer. |
| 16061671 | 2 | No | Molecular basis for G2 arrest induced by 2'-C-cyano-2'-deoxy-1-beta-D-arabino-pentofuranosylcytosine and consequences of |
| 16061681 | 6 | No | It's about time: scheduling alters effect of histone deacetylase inhibitors on camptothecin-treated cells. |
| 16061850 | 2 | No | Vitamin D(3) metabolism in human glioblastoma multiforme: functionality of CYP27B1 splice variants, metabolism of calcidiol, and effect of calcitriol. |
| 16061869 | 2 | No | A phase II trial with pharmacodynamic endpoints of the proteasome inhibitor bortezomib in patients with metastatic colorectal cancer. |
| 16061882 | 4 | No | Hsp90 inhibitor 17-allylamino-17-demethoxygeldanamycin prevents synovial sarcoma proliferation via apoptosis in in vitro models. |
| 16076101 | 5 | No | Comparison of the in vitro estrogenic activities of compounds from hops (Humulus lupulus) and red clover (Trifolium pratense). |
| 16076378 | 2 | No | Sevelamer hydrochloride and calcium bicarbonate reduce serum fibroblast growth factor 23 levels in dialysis patients. |
| 16076840 | 6 | No | Glycogen synthase kinase-3 interacts with and phosphorylates estrogen receptor alpha and is involved in the regulation of receptor activity. |
| 16076985 | 1 | No | Troglitazone-induced changes in adiponectin do not affect endothelial function in diabetes. |
| 16077170 | 1 | No | DDE-induced changes in aromatase activity in endometrial stromal cells in culture. |

| | | | |
|---|---|---|---|
| 16077199 | 1 | No | Position of STAT-1 alpha in cycloheximide-dependent apoptosis triggered by TNF-alpha in human colorectal COLO 205 cancer cell line; role of polyphenolic |
| 16077929 | 1 | No | Increased level of the p67phox subunit of NADPH oxidase by 4HPR in head and neck squamous carcinoma cells. |
| 16077934 | 3 | No | Transient activation of EGFR/AKT cell survival pathway and expression of survivin contribute to reduced sensitivity of human melanoma cells to betulinic acid. |
| 16077967 | 6 | No | Modulation of bcl-2 family proteins in MAPK independent apoptosis induced by a cdc25 phosphatase inhibitor Cpd 5 in renal cancer cells. |
| 16077987 | 1 | No | Doxorubicin modulates telomerase activity in Ewing's sarcoma in vitro and in vivo. |
| 16078447 | 2 | No | [Correlation between functional capability and phenotypic characteristics of peripheral blood lymphocytes in patients with malignant melanoma] |
| 16079077 | 7 | No | Differential gene expression in normal human mammary epithelial cells treated with malathion monitored by DNA microarrays. |
| 16079301 | 30 | No | Transcriptional regulation of activating transcription factor 3 involves the early growth response-1 gene. |
| 16079895 | 4 | No | The combination of intermediate doses of thalidomide with dexamethasone is an effective treatment for patients with refractory/relapsed multiple myeloma and |
| 16080190 | 7 | No | Induction of tubulin by docetaxel is associated with p53 status in human non small cell lung cancer cell lines. |
| 16080194 | 1 | No | Melatonin inhibits the growth of DMBA-induced mammary tumors by decreasing the local biosynthesis of estrogens through the modulation of aromatase activity. |
| 16082211 | 15 | No | Molecular targets and anticancer potential of indole-3-carbinol and its derivatives. |
| 16084059 | 4 | No | Effect of resveratrol on matrix metalloproteinase-2 (MMP-2) and Secreted Protein Acidic and Rich in Cysteine (SPARC) on human cultured glioblastoma cells. |
| 16084531 | 92 | No | Molecular mechanisms of action of angiopreventive anti-oxidants on endothelial cells: microarray gene expression analyses. |
| 16085347 | 26 | No | Caffeic acid phenethyl ester (CAPE) prevents transformation of human cells by arsenite (As) and suppresses growth of As-transformed cells. |
| 16085563 | 2 | No | Troglitazone overcomes doxorubicin-resistance in resistant K562 leukemia cells. |
| 16085646 | 28 | No | Physiological and receptor-selective retinoids modulate interferon gamma signaling by increasing the expression, nuclear localization, and functional |
| 16086235 | 2 | No | Benzo-[a]-pyrene increases invasion in MDA-MB-231 breast cancer cells via increased COX-II expression and prostaglandin E2 (PGE2) output. |

| | | | |
|---|---|---|---|
| 16086872 | 2 | No | [Effect of inhibiting survivin expression with antisense oligodeoxynucleotides on sensitivity of hepatocellular carcinoma cell lines HepG2 and HepG2/ADM to |
| 16087364 | 6 | No | Effect of cytokines on ICAM-1 and ZO-1 expression on human airway epithelial cells. |
| 16088125 | 2 | No | Induction of apoptosis by the adenosine derivative IB-MECA in parental or multidrug-resistant HL-60 leukemia cells: possible relationship to the effects on |
| 16091005 | 1 | No | Effect of resveratrol on angiogenesis and platelet/fibrin-accelerated tumor growth in the chick chorioallantoic membrane model. |
| 16091123 | 16 | No | Signal pathway of cytokines produced by reactive oxygen species generated from phorbol myristate acetate-stimulated HMC-1 cells. |
| 16091736 | 5 | No | Both PPARgamma and PPARdelta influence sulindac sulfide-mediated p21WAF1/CIP1 upregulation in a human prostate epithelial cell line. |
| 16091745 | 2 | No | BBC3 mediates fenretinide-induced cell death in neuroblastoma. |
| 16091755 | 5 | No | Combining lapatinib (GW572016), a small molecule inhibitor of ErbB1 and ErbB2 tyrosine kinases, with therapeutic anti-ErbB2 antibodies enhances apoptosis of |
| 16093440 | 18 | No | Selenium disrupts estrogen receptor (alpha) signaling and potentiates tamoxifen antagonism in endometrial cancer cells and tamoxifen-resistant breast cancer |
| 16096431 | 1 | No | Vitamin K analog (compound 5) induces apoptosis in human hepatocellular carcinoma independent of the caspase pathway. |
| 16099050 | 1 | No | Prognostic significance of low serum levels of Clara cell phospholipid-binding protein in occupational aluminium neurotoxicity. |
| 16100009 | 6 | No | Endotoxin up-regulates interleukin-18: potential role for gram-negative colonization in sarcoidosis. |
| 16101138 | 30 | No | 5-Fluorouracil up-regulates interferon pathway gene expression in esophageal cancer cells. |
| 16101141 | 26 | No | Expression profiles of apoptotic genes induced by curcumin in human breast cancer and mammary epithelial cell lines. |
| 16104877 | 4 | No | Etodolac inhibits EBER expression and induces Bcl-2-regulated apoptosis in Burkitt's lymphoma cells. |
| 16105982 | 4 | No | NF-kappaB and FLIP in arsenic trioxide (ATO)-induced apoptosis in myelodysplastic syndromes (MDSs). |
| 16106398 | 5 | No | Curcumin (diferuloylmethane) inhibits constitutive active NF-kappaB, leading to suppression of cell growth of human T-cell leukemia virus type I-infected T-cell |
| 16107141 | 4 | No | Discovery of substituted maleimides as liver X receptor agonists and determination of a ligand-bound crystal structure. |

| | | | |
|---|---|---|---|
| 16108013 | 3 | No | Small interfering RNA targeting survivin sensitizes lung cancer cell with mutant p53 to adriamycin. |
| 16109544 | 12 | No | [Receptor-related mechanism of proliferation inhibilion and apoptosis induetion of human tongue squamous cell line Tca8113 by retinoids] |
| 16109552 | 6 | No | [Effects of all-trans-retinoic acid, acitretin and tazarotene on apoptosis and Bax/Bcl-2 expressions of human melanoma cells A375 and the significance] |
| 16111494 | 2 | No | Microglial responses to amyloid beta peptide opsonization and indomethacin treatment. |
| 16112107 | 5 | No | Activation of PPARgamma is not involved in butyrate-induced epithelial cell differentiation. |
| 16112155 | 2 | No | Molecular mechanisms underlying mancozeb-induced inhibition of TNF-alpha production. |
| 16112689 | 4 | No | 2- and 4-Aminobiphenyls induce oxidative DNA damage in human hepatoma (Hep G2) cells via different mechanisms. |
| 16114553 | 4 | No | [Effect of nylestriol and levonorgestrel on the expression of Opg/OPGL in human osteosarcoma MG-63 cell lines] |
| 16115123 | 4 | No | Indirubin, a Chinese anti-leukaemia drug, promotes neutrophilic differentiation of human myelocytic leukaemia HL-60 cells. |
| 16116186 | 25 | No | G(i)-protein-dependent inhibition of IL-12 production is mediated by activation of the phosphatidylinositol 3-kinase-protein 3 kinase B/Akt pathway and JNK. |
| 16116226 | 1 | No | Specific inhibition of MyD88-independent signaling pathways of TLR3 and TLR4 by resveratrol: molecular targets are TBK1 and RIP1 in TRIF complex. |
| 16116958 | 1 | No | Role of activator protein-1 in the transcription of interleukin-5 gene regulated by protein kinase C signal in asthmatic human T lymphocytes. |
| 16117893 | 3 | No | [Apoptosis induced by cantharidin in human pulmonary carcinoma cells A549 and its molecular mechanisms] |
| 16118212 | 1 | No | Unsaturated fatty acids phosphorylate and destabilize ABCA1 through a phospholipase D2 pathway. |
| 16118546 | 1 | No | The effects of bivalirudin compared with those of unfractionated heparin plus eptifibatide on inflammation and thrombin generation and activity during coronary |
| 16123073 | 3 | No | A novel protein Depp, which is induced by progesterone in human endometrial stromal cells activates Elk-1 transcription factor. |
| 16123214 | 2 | No | Multitarget inhibition of drug-resistant multiple myeloma cell lines by dimethyl-celecoxib (DMC), a non-COX-2 inhibitory analog of celecoxib. |
| 16123850 | 2 | No | The effect of fenofibrate on serum paraoxonase activity and inflammatory markers in patients with combined hyperlipidemia. |

| | | | |
|---|---|---|---|
| 16124896 | 8 | No | [The role of reactive oxygen species in N-[4-hydroxyphenyl] retinamide induced apoptosis in bladder cancer cell lineT24] |
| 16127424 | 1 | No | F2-isoprostanes stimulate collagen synthesis in activated hepatic stellate cells: a link with liver fibrosis? |
| 16129045 | 1 | No | [Study on the mechanisms of telomerase regulations during apoptosis of the human MDS-RAEB cell line MUTZ-1 cells induced by arsenic trioxide] |
| 16129123 | 74 | No | Immediate up-regulation of the calcium-binding protein S100P and its involvement in the cytokinin-induced differentiation of human myeloid leukemia cells. |
| 16129430 | 3 | No | Enhanced expression of heat shock proteins in gradually dying cells and their release from necrotically dead cells. |
| 16131326 | 1 | No | Regulation of type 1 iodothyronine deiodinase in health and disease. |
| 16134060 | 5 | No | Chemically modified tetracycline (CMT)-3 inhibits histamine release and cytokine production in mast cells: possible involvement of protein kinase C. |
| 16135400 | 5 | No | Apoptotic events induced by naturally occurring retinoids ATRA and 13-cis retinoic acid on human hepatoma cell lines Hep3B and HepG2. |
| 16136269 | 4 | No | Conversion of Fas-resistant to Fas-sensitive MCF-7 breast cancer cells by the synergistic interaction of interferon-gamma and all-trans retinoic acid. |
| 16139439 | 12 | No | Antiandrogen-like actions of an antioxidant on survivin, Bcl-2 and PSA in human prostate cancer cells. |
| 16140617 | 1 | No | Acquisition of androgen independence by human prostate epithelial cells during arsenic-induced malignant transformation. |
| 16140939 | 4 | No | Accumulation of p53 and reductions in XIAP abundance promote the apoptosis of prostate cancer cells. |
| 16140950 | 2 | No | Protein kinase C inhibition and x-linked inhibitor of apoptosis protein degradation contribute to the sensitization effect of luteolin on tumor necrosis |
| 16140954 | 15 | No | Benzodithiophenes induce differentiation and apoptosis in human leukemia cells. |
| 16140955 | 186 | No | Benzodithiophenes potentiate differentiation of acute promyelocytic leukemia cells by lowering the threshold for ligand-mediated corepressor/coactivator |
| 16142401 | 1 | No | All-trans retinoic acid regulates CXCL16/SR-PSOX expression. |
| 16142410 | 21 | No | Liver X receptor and retinoic X receptor agonists modulate the expression of genes involved in lipid metabolism in human endothelial cells. |
| 16142594 | 2 | No | Immediate effects of fluvastain on circulating soluble endothelial protein C and free tissue factor pathway inhibitor in acute coronary syndromes. |

| | | | |
|---|---|---|---|
| 16143069 | 15 | No | [Effect of selenium on expression of P38 mitogen-activated protein kinase and monocyte chemoattractant protein-1 in diabetic endothelial cells] |
| 16144937 | 22 | No | Glucocorticoids inhibit cell death in ovarian cancer and up-regulate caspase inhibitor cIAP2. |
| 16144938 | 1 | No | Down-regulation of signal transducer and activator of transcription 3 expression using vector-based small interfering RNAs suppresses growth of human prostate |
| 16144939 | 1 | No | Identification of novel human CTL epitopes and their agonist epitopes of mesothelin. |
| 16144940 | 1 | No | Chromosomal radiosensitivity in two cell lineages derived from clinically radiosensitive cancer patients. |
| 16144943 | 11 | No | Activity of suberoylanilide hydroxamic Acid against human breast cancer cells with amplification of her-2. |
| 16148020 | 16 | No | Contrasting effects of fluoroquinolone antibiotics on the expression of the collagenases, matrix metalloproteinases (MMP)-1 and -13, in human tendon-derived |
| 16149052 | 13 | No | Genistein-induced neuronal differentiation is associated with activation of extracellular signal-regulated kinases and upregulation of p21 and N-cadherin. |
| 16150056 | 6 | No | Increased processing of APLP2 and APP with concomitant formation of APP intracellular domains in BDNF and retinoic acid-differentiated human |
| 16153628 | 2 | No | Effect of nandrolone decanonate on paraoxonase activity in hemodialysis patients. |
| 16154193 | 5 | No | Regulation of neurokinin-1 receptor messenger RNA expression in synovial fibroblasts of patients with rheumatoid arthritis. |
| 16154199 | 2 | No | Further insights into the regulation of human FAAH by progesterone and leptin implications for endogenous levels of anandamide and apoptosis of immune and |
| 16154495 | 15 | No | Resveratrol suppresses interferon-gamma-induced biochemical pathways in human peripheral blood mononuclear cells in vitro. |
| 16154993 | 25 | No | Distinct role of calmodulin and calmodulin-dependent protein kinase-II in lipopolysaccharide and tumor necrosis factor-alpha-mediated suppression of |
| 16155002 | 24 | No | Tissue-specific, inducible, and hormonal control of the human UDP-glucuronosyltransferase-1 (UGT1) locus. |
| 16155795 | 8 | No | Attenuation of estrogen receptor alpha (ERalpha) signaling by selenium in breast cancer cells via downregulation of ERalpha gene expression. |
| 16157052 | 3 | No | Mechanism of retinoid receptors in inhibiting proliferation and inducing apoptosis of human melanoma cell line A375. |
| 16158052 | 11 | No | Retinoic acid receptors and tissue-transglutaminase mediate short-term effect of retinoic acid on migration and invasion of neuroblastoma SH-SY5Y cells. |

| | | | |
|---|---|---|---|
| 16158929 | 1 | No | Intracellular glutathione levels determine cell sensitivity to apoptosis induced by the antineoplasic agent N-(4-hydroxyphenyl) retinamide. |
| 16159934 | 4 | No | Sulindac suppresses nuclear factor-kappaB activation and RANTES gene and protein expression in endometrial stromal cells from women with endometriosis. |
| 16162944 | 22 | No | KGF induces lipogenic genes through a PI3K and JNK/SREBP-1 pathway in H292 cells. |
| 16163705 | 26 | No | Ircinin-1 induces cell cycle arrest and apoptosis in SK-MEL-2 human melanoma cells. |
| 16166294 | 11 | No | Suppression of mammary carcinoma growth by retinoic acid: proapoptotic genes are targets for retinoic acid receptor and cellular retinoic acid-binding protein II |
| 16166323 | 8 | No | Activation of protein kinase G up-regulates expression of 15-lipoxygenase-1 in human colon cancer cells. |
| 16166596 | 2 | No | IGF-1 receptor tyrosine kinase inhibition by the cyclolignan PPP induces G2/M-phase accumulation and apoptosis in multiple myeloma cells. |
| 16168113 | 26 | No | Addition of 5-fluorouracil to doxorubicin-paclitaxel sequence increases caspase-dependent apoptosis in breast cancer cell lines. |
| 16168501 | 8 | No | Retinoic acid represses a cassette of candidate pluripotency chromosome 12p genes during induced loss of human embryonal carcinoma tumorigenicity. |
| 16170020 | 7 | No | Tissue transglutaminase 2 inhibition promotes cell death and chemosensitivity in glioblastomas. |
| 16170024 | 4 | No | Potentiation of paclitaxel-induced apoptosis by the novel cyclin-dependent kinase inhibitor NU6140: a possible role for survivin down-regulation. |
| 16173056 | 7 | No | Gene and protein expressions in human cord blood cells after exposure to acrylonitrile. |
| 16173061 | 79 | No | Modulation of sulfur mustard induced cell death in human epidermal keratinocytes using IL-10 and TNF-alpha. |
| 16173963 | 6 | No | Effects of nuclear factor-kappaB inhibitors and its implication on natural killer T-cell lymphoma cells. |
| 16174765 | 4 | No | Efficient intervention of growth and infiltration of primary adult T-cell leukemia cells by an HIV protease inhibitor, ritonavir. |
| 16174796 | 2 | No | Arsenic trioxide induces apoptosis of human monocytes during macrophagic differentiation through nuclear factor-kappaB-related survival pathway |
| 16175315 | 7 | No | Progesterone pre-treatment potentiates EGF pathway signaling in the breast cancer cell line ZR-75. |
| 16176868 | 6 | No | Retinoids interfere with the AP1 signalling pathway in human breast cancer cells. |

| | | | |
|---|---|---|---|
| 16177187 | 7 | No | Carotenoid transport is decreased and expression of the lipid transporters SR-BI, NPC1L1, and ABCA1 is downregulated in Caco-2 cells treated with ezetimibe. |
| 16179969 | 10 | No | JNK1-dependent antimitotic activity of thiazolidin compounds in human non-small-cell lung and colon cancer cells. |
| 16183391 | 10 | No | Additive estrogenic effects of mixtures of frequently used UV filters on pS2-gene transcription in MCF-7 cells. |
| 16184548 | 101 | No | Id-1 gene downregulation by sulindac sulfide and its upregulation during tumor development in gastric cancer. |
| 16185265 | 11 | No | Matrix metalloproteinases of epithelial origin in facial sebum of patients with acne and their regulation by isotretinoin. |
| 16186334 | 6 | No | beta-Carotene conversion into vitamin A in human retinal pigment epithelial cells. |
| 16187019 | 6 | No | Retinamide-induced apoptosis in glioblastomas is associated with down-regulation of Bcl-xL and Bcl-2 proteins. |
| 16188231 | 5 | No | The fibroblast growth factor receptors, FGFR-1 and FGFR-2, mediate two independent signalling pathways in human retinal pigment epithelial cells. |
| 16189296 | 3 | No | The histone deacetylase inhibitor LAQ824 induces human leukemia cell death through a process involving XIAP down-regulation, oxidative injury, and the acid |
| 16192631 | 4 | No | Hepatocyte growth factor receptor signaling mediates the anti-fibrotic action of 9-cis-retinoic acid in glomerular mesangial cells. |
| 16194896 | 6 | No | Regulation of a highly specific retinoic acid-4-hydroxylase (CYP26A1) enzyme and all-trans-retinoic acid metabolism in human intestinal, liver, endothelial, and |
| 16196285 | 6 | No | Effects of ATRA, acitretin and tazarotene on growth and apoptosis of Tca8113 cells. |
| 16197459 | 1 | No | All-trans retinoic acid-induced downregulation of annexin II expression in myeloid leukaemia cell lines is not confined to acute promyelocytic leukaemia. |
| 16201850 | 6 | No | Fucoidan extracted from Cladosiphon okamuranus Tokida induces apoptosis of human T-cell leukemia virus type 1-infected T-cell lines and primary adult T-cell |
| 16202731 | 2 | No | The effects of 12 weeks of HMR 3339, a novel selective estrogen receptor modulator, on markers of coagulation and fibrinolysis: a randomized, |
| 16202921 | 71 | No | Analysis of estrogen agonism and antagonism of tamoxifen, raloxifene, and ICI182780 in endometrial cancer cells: a putative role for the epidermal growth |
| 16204068 | 12 | No | Effect of p53 status and STAT1 on chemotherapy-induced, Fas-mediated apoptosis in colorectal cancer. |
| 16204082 | 8 | No | Endoplasmic reticulum stress signal mediators are targets of selenium action. |

| | | | |
|---|---|---|---|
| 16205645 | 17 | No | Enhanced selenium effect on growth arrest by BiP/GRP78 knockdown in p53-null human prostate cancer cells. |
| 16206244 | 1 | No | Interaction of PP2A catalytic subunit with Rb2/p130 is required for all-trans retinoic acid suppression of ovarian carcinoma cell growth. |
| 16206674 | 4 | No | [The impact of arsenic trioxide or all-trans retinoic acid treatment on coagulopathy in acute promyelocytic leukemia] |
| 16207822 | 2 | No | Intestinal and hepatic CYP3A4 catalyze hydroxylation of 1alpha,25-dihydroxyvitamin D(3): implications for drug-induced osteomalacia. |
| 16209266 | 3 | No | [Research on resveratrol's effects on suppressing growth and inducing apoptosis of GBC cells] |
| 16210391 | 6 | No | Progesterone enhances HLA-G gene expression in JEG-3 choriocarcinoma cells and human cytotrophoblasts in vitro. |
| 16211219 | 4 | No | Enhancement of the caspase-independent apoptotic sensitivity of pancreatic cancer cells by DHMEQ, an NF-kappaB inhibitor. |
| 16211241 | 3 | No | Targeted inhibition of transient activation of the EGFR-mediated cell survival pathway enhances paclitaxel-induced ovarian cancer cell death. |
| 16211300 | 2 | No | Quercetin induces gadd45 expression through a p53-independent pathway. |
| 16213582 | 1 | No | The Hsp90 inhibitor 17-allylamide-17-demethoxygeldanamycin induces apoptosis and differentiation of Kasumi-1 harboring the Asn822Lys KIT mutation and |
| 16215670 | 3 | No | Bcl-2 attenuates anticancer agents-induced apoptosis by sustained activation of Akt/protein kinase B in U937 cells. |
| 16216300 | 35 | No | Effects of PCBs and MeSO2-PCBs on adrenocortical steroidogenesis in H295R human adrenocortical carcinoma cells. |
| 16219905 | 28 | No | Curcumin (diferuloylmethane) down-regulates expression of cell proliferation and antiapoptotic and metastatic gene products through suppression of IkappaBalpha |
| 16221533 | 1 | No | Effect of organophosphate pesticide diazinon on expression and activity of intestinal P-glycoprotein. |
| 16222118 | 2 | No | Calcitonin induces apoptosis resistance in prostate cancer cell lines against cytotoxic drugs via the Akt/survivin pathway. |
| 16223563 | 12 | No | The effect of plasticisers on "sulphate supply" enzymes. |
| 16223781 | 17 | No | Up-regulation of MDR1 and induction of doxorubicin resistance by histone deacetylase inhibitor depsipeptide (FK228) and ATRA in acute promyelocytic |
| 16224106 | 4 | No | Neutrophil sphingosine 1-phosphate and lysophosphatidic acid receptors in pneumonia. |

| | | | |
|---|---|---|---|
| 16225753 | 1 | No | Chemokine RANTES is upregulated in monocytes from patients with hyperhomocysteinemia. |
| 16225760 | 1 | No | Effect of protein kinase C alpha, caspase-3, and survivin on apoptosis of oral cancer cells induced by staurosporine. |
| 16226872 | 6 | No | C/EBPalpha inactivation in FAK-overexpressed HL-60 cells impairs cell differentiation. |
| 16227395 | 4 | No | Resveratrol inhibits hypoxia-induced accumulation of hypoxia-inducible factor-1alpha and VEGF expression in human tongue squamous cell carcinoma and |
| 16227405 | 9 | No | Suppression of tumor cell invasion by cyclooxygenase inhibitors is mediated by thrombospondin-1 via the early growth response gene Egr-1. |
| 16227409 | 5 | No | Role of p21waf1/cip1 in effects of oxaliplatin in colorectal cancer cells. |
| 16230394 | 3 | No | Roscovitine is an effective inducer of apoptosis of Ewing's sarcoma family tumor cells in vitro and in vivo. |
| 16230421 | 22 | No | N-(4-hydroxyphenyl)retinamide inhibits invasion, suppresses osteoclastogenesis, and potentiates apoptosis through down-regulation of I(kappa)B(alpha) kinase and |
| 16234306 | 6 | No | Dexamethasone stimulation of retinoic Acid-induced sodium iodide symporter expression and cytotoxicity of 131-I in breast cancer cells. |
| 16234967 | 1 | No | Fibroblast growth factor-23 is regulated by 1alpha,25-dihydroxyvitamin D. |
| 16235733 | 1 | No | The effect of ethanol and nitric oxide on the N-nitrosodimethylamine formation in HepG2 cells overexpressing CYP2E1. |
| 16236362 | 2 | No | Effect of estrogen/progesterone hormone replacement therapy on natural killer cell cytotoxicity and immunoregulatory cytokine release by peripheral blood |
| 16237197 | 3 | No | Potentiation of apoptosis by heat stress plus pesticide exposure in stress resistant human B-lymphoma cells and its attenuation through interaction with |
| 16239064 | 1 | No | Selenium- or quercetin-induced retardation of DNA synthesis in primary prostate cells occurs in the presence of a concomitant reduction in androgen-receptor |
| 16239242 | 4 | No | Induction of HSP70 expression and recruitment of HSC70 and HSP70 in the nucleus reduce aggregation of a polyalanine expansion mutant of PABPN1 in HeLa cells. |
| 16239567 | 2 | No | Effects of Anaplasma phagocytophilum on host cell ferritin mRNA and protein levels. |
| 16241859 | 18 | No | Immunomodulatory drugs (IMiDs) increase the production of IL-2 from stimulated T cells by increasing PKC-theta activation and enhancing the DNA-binding activity |
| 16242299 | 24 | No | Serum free BG-1 cell proliferation assay: a sensitive method for determining organochlorine pesticide estrogen receptor activation at the nanomolar range. |

| | | | |
|---|---|---|---|
| 16242776 | 18 | No | Transforming growth factor-beta1 inhibits all-trans retinoic acid-induced apoptosis. |
| 16243370 | 7 | No | Prostate cancer cell type-specific involvement of the VDR and RXR in regulation of the human PTHrP gene via a negative VDRE. |
| 16243713 | 1 | No | The conserved lymphokine element 0 is a powerful activator and target for corticosteroid inhibition in human interleukin-5 transcription. |
| 16243823 | 24 | No | Curcumin suppresses the paclitaxel-induced nuclear factor-kappaB pathway in breast cancer cells and inhibits lung metastasis of human breast cancer in nude |
| 16243974 | 19 | No | A fully dissociated compound of plant origin for inflammatory gene repression. |
| 16247790 | 4 | No | Notable effects of angiotensin II receptor blocker, valsartan, on acute cardiotoxic changes after standard chemotherapy with cyclophosphamide, |
| 16249480 | 141 | No | Effect of retinoic acid on gene expression in human conjunctival epithelium: secretory phospholipase A2 mediates retinoic acid induction of MUC16. |
| 16249512 | 2 | No | (R)-alpha-lipoic acid protects retinal pigment epithelial cells from oxidative damage. |
| 16251475 | 7 | No | Regulation of homocysteine-induced MMP-9 by ERK1/2 pathway. |
| 16253226 | 6 | No | Vasodilatory and anti-inflammatory effects of the 1,2,3,4,6-penta-O-galloyl-beta-D-glucose (PGG) via a nitric oxide-cGMP pathway. |
| 16253763 | 2 | No | Regulation of human peptide transporter 1 (PEPT1) in gastric cancer cells by anticancer drugs. |
| 16254145 | 1 | No | Regulation of survivin expression through Bcr-Abl/MAPK cascade: targeting survivin overcomes imatinib resistance and increases imatinib sensitivity in |
| 16254627 | 1 | No | Dexamethasone suppresses human interleukin-5 gene promoter. |
| 16257430 | 9 | No | Aryl hydrocarbon receptor-independent activation of estrogen receptor-dependent transcription by 3-methylcholanthrene. |
| 16257484 | 4 | No | The promoter of the human sodium/iodide symporter responds to certain phthalate plasticisers. |
| 16258175 | 15 | No | Potent inhibition of carcinogen-bioactivating cytochrome P450 1B1 by the p53 inhibitor pifithrin alpha. |
| 16260419 | 4 | No | Interferon-alpha-induced expression of phospholipid scramblase 1 through STAT1 requires the sequential activation of protein kinase Cdelta and JNK. |
| 16262603 | 6 | No | Induction of spermidine/spermine N1-acetyltransferase (SSAT) by aspirin in Caco-2 colon cancer cells. |

| | | | |
|---|---|---|---|
| 16263688 | 1 | No | The bipyridyl herbicide paraquat produces oxidative stress-mediated toxicity in human neuroblastoma SH-SY5Y cells: relevance to the dopaminergic pathogenesis. |
| 16267019 | 32 | No | Resveratrol inhibits myeloma cell growth, prevents osteoclast formation, and promotes osteoblast differentiation. |
| 16269825 | 17 | No | A role for FXR and human FGF-19 in the repression of paraoxonase-1 gene expression by bile acids. |
| 16270382 | 1 | No | Inhibitory effects of N-(4-hydrophenyl) retinamide on liver cancer and malignant melanoma cells. |
| 16270723 | 1 | No | The effect of fexofenadine on expression of intercellular adhesion molecule 1 and induction of apoptosis on peripheral eosinophils. |
| 16271822 | 2 | No | Inhibition of human cytochrome CYP 1 enzymes by flavonoids of St. John's wort. |
| 16271832 | 16 | No | Relationship between toxicity of selected insecticides and expression of stress proteins (HSP, GRP) in cultured human cells: effects of commercial formulations |
| 16272461 | 2 | No | Oxidant generation promotes iron sequestration in BEAS-2B cells exposed to asbestos. |
| 16273229 | 5 | No | Differential gene expression of sulindac-treated human breast epithelial cells. |
| 16273314 | 4 | No | Receptor-selective retinoids inhibit the growth of normal and malignant breast cells by inducing G1 cell cycle blockade. |
| 16275619 | 2 | No | All-trans-retinoic acid accelerates the differentiation of human B lymphocytes maturing into plasma cells. |
| 16275999 | 8 | No | Potentiation of the lethality of the histone deacetylase inhibitor LAQ824 by the cyclin-dependent kinase inhibitor roscovitine in human leukemia cells. |
| 16276071 | 1 | No | Effect of a Mediterranean meal on postprandial carotenoids, paraoxonase activity and C-reactive protein levels. |
| 16277677 | 3 | No | Raloxifene reduces urokinase-type plasminogen activator-dependent proliferation of synoviocytes from patients with rheumatoid arthritis. |
| 16278781 | 2 | No | Bone microenvironment-related growth factors, zoledronic acid and dexamethasone differentially modulate PTHrP expression in PC-3 prostate cancer cells. |
| 16279945 | 5 | No | Shedding of the amyloid precursor protein-like protein APLP2 by disintegrin-metalloproteinases. |
| 16280456 | 8 | No | Inhibition of lung cancer cell growth by quercetin glucuronides via G2/M arrest and induction of apoptosis. |
| 16281067 | 2 | No | Overcoming MDR-associated chemoresistance in HL-60 acute myeloid leukemia cells by targeting sphingosine kinase-1. |

| | | | |
|---|---|---|---|
| 16283381 | 4 | No | Effects of doxorubicin-containing chemotherapy and a combination with L-carnitine on oxidative metabolism in patients with non-Hodgkin lymphoma. |
| 16283521 | 2 | No | Sodium arsenite-induced inhibition of eukaryotic translation initiation factor 4E (eIF4E) results in cytotoxicity and cell death. |
| 16283617 | 1 | No | Differentiation of rhabdomyosarcoma cell lines using retinoic acid. |
| 16284694 | 1 | No | The effect of doxorubicin on the expression of cyclin A in K-562 leukemia cell line. |
| 16285558 | 1 | No | [Effects of arsenic trioxideon the cell apoptosis and hTERT mRNA of human tongue cancer cells] |
| 16286479 | 1 | No | Fibroblast growth factor-2 is a downstream mediator of phosphatidylinositol 3-kinase-Akt signaling in 14,15-epoxyeicosatrienoic acid-induced angiogenesis. |
| 16286641 | 2 | No | Decreased levels of CXC-chemokines in serum of benzene-exposed workers identified by array-based proteomics. |
| 16287099 | 2 | No | Proteasome inhibitor MG132 sensitizes HPV-positive human cervical cancer cells to rhTRAIL-induced apoptosis. |
| 16287870 | 1 | No | Impaired retinoic acid (RA) signal leads to RARbeta2 epigenetic silencing and RA resistance. |
| 16288038 | 7 | No | Suppression of urokinase receptor expression by thalidomide is associated with inhibition of nuclear factor kappaB activation and subsequently suppressed |
| 16288207 | 4 | No | Regulation of p53 and suppression of apoptosis by the soluble guanylyl cyclase/cGMP pathway in human ovarian cancer cells. |
| 16288218 | 1 | No | Increased expression of proapoptotic BMCC1, a novel gene with the BNIP2 and Cdc42GAP homology (BCH) domain, is associated with favorable prognosis in human |
| 16289102 | 18 | No | Retinoic acid via RARalpha inhibits the expression of 24-hydroxylase in human prostate stromal cells. |
| 16289960 | 8 | No | Upregulation of neuropilin-1 by basic fibroblast growth factor enhances vascular smooth muscle cell migration in response to VEGF. |
| 16291413 | 10 | No | Pharmacokinetic interaction of solifenacin with an oral contraceptive containing ethinyl estradiol and levonorgestrel in healthy women: a double-blind, |
| 16292516 | 4 | No | All-trans-retinoic acid suppresses interferon-gamma and tumor necrosis factor-alpha; a possible therapeutic agent for rheumatoid arthritis. |
| 16292757 | 12 | No | Effects of xenobiotics and peroxisome proliferator-activated receptor-alpha on the human UDPglucose dehydrogenase gene expression. |
| 16298037 | 45 | No | ERE-independent ERalpha target genes differentially expressed in human breast tumors. |

| | | | |
|---|---|---|---|
| 16298333 | 17 | No | Doxorubicin prevents endoplasmic reticulum stress-induced apoptosis. |
| 16299233 | 1 | No | Positive correlation between estradiol and vascular endothelial growth factor but not fibroblast growth factor-2 in normal human breast tissue in vivo. |
| 16299251 | 2 | No | Inhibition of nuclear translocation of nuclear factor-kappaB despite lack of functional IkappaBalpha protein overcomes multiple defects in apoptosis signaling |
| 16299289 | 96 | No | Synergistic effect of Nod1 and Nod2 agonists with toll-like receptor agonists on human dendritic cells to generate interleukin-12 and T helper type 1 cells. |
| 16299307 | 3 | No | Th2-associated local reactions to the acellular diphtheria-tetanus-pertussis vaccine in 4- to 6-year-old children. |
| 16302272 | 2 | No | The androgen receptor pathway is by-passed in prostate cancer cells generated after prolonged treatment with bicalutamide. |
| 16303604 | 7 | No | Effect of peroxisome proliferator-activated receptor-gamma ligands on the expression of retinoic acid-inducible gene-I in endothelial cells stimulated with |
| 16303787 | 16 | No | Maturation of human monocyte-derived dendritic cells (MoDCs) in the presence of prostaglandin E2 optimizes CD4 and CD8 T cell-mediated responses to protein |
| 16303854 | 5 | No | Mitochondrial reactive oxygen species contribute to high NaCl-induced activation of the transcription factor TonEBP/OREBP. |
| 16305726 | 14 | No | Omega-3 fatty acids inhibit an increase of proinflammatory cytokines in patients with active Crohn's disease compared with omega-6 fatty acids. |
| 16307838 | 3 | No | T-2 toxin induces apoptosis, and selenium partly blocks, T-2 toxin induced apoptosis in chondrocytes through modulation of the Bax/Bcl-2 ratio. |
| 16308212 | 28 | No | Polypropylenimine dendrimer-induced gene expression changes: the effect of complexation with DNA, dendrimer generation and cell type. |
| 16308312 | 13 | No | Involvement of p38 MAPK and Nrf2 in phenolic acid-induced P-form phenol sulfotransferase expression in human hepatoma HepG2 cells. |
| 16309724 | 104 | No | Glial inflammation and neurodegeneration induced by candoxin, a novel neurotoxin from Bungarus candidus venom: global gene expression analysis using microarray. |
| 16310922 | 1 | No | Nitric oxide inhibits HIF-1alpha protein accumulation under hypoxic conditions: implication of 2-oxoglutarate and iron. |
| 16311067 | 5 | No | Oral submucous fibrosis: review on aetiology and pathogenesis. |
| 16311509 | 6 | No | Pharmacological induction of Hsp70 protects apoptosis-prone cells from doxorubicin: comparison with caspase-inhibitor- and cycle-arrest-mediated |
| 16313111 | 1 | No | [Effect of arnebia root oil in promoting the tissue recovery of surface of wound and basic fibroblast growth factor (bFGF) mRNA expression] |

| | | | |
|---|---|---|---|
| 16314022 | 25 | No | Chemokine regulation in response to beryllium exposure in human peripheral blood mononuclear and dendritic cells. |
| 16314067 | 100 | No | MIP-1beta, a novel biomarker for in vitro sensitization test using human monocytic cell line. |
| 16314733 | 2 | No | Effect of inhibition of aloe-emodin on N-acetyltransferase activity and gene expression in human malignant melanoma cells (A375.S2). |
| 16317058 | 90 | No | Dihydrotestosterone decreases tumor necrosis factor-alpha and lipopolysaccharide-induced inflammatory response in human endothelial cells. |
| 16317118 | 18 | No | Resveratrol inhibits TNF-alpha-induced proliferation and matrix metalloproteinase expression in human vascular smooth muscle cells. |
| 16318612 | 4 | No | Hydroxyprogesterone caproate and progesterone increase tumor necrosis factor-alpha production in lipopolysaccharide stimulated whole blood from |
| 16322068 | 7 | No | All-trans retinoic acid inhibits vascular endothelial growth factor expression in a cell model of neutrophil activation. |
| 16322294 | 2 | No | High cyclooxygenase-2 expression following neoadjuvant radiochemotherapy is associated with minor histopathologic response and poor prognosis in esophageal |
| 16322342 | 10 | No | Zoledronic acid cooperates with a cyclooxygenase-2 inhibitor and gefitinib in inhibiting breast and prostate cancer. |
| 16322690 | 2 | No | Downregulation of XIAP and induction of apoptosis by the synthetic cyclin-dependent kinase inhibitor GW8510 in non-small cell lung cancer cells. |
| 16322758 | 10 | No | Convergence of vitamin D and retinoic acid signalling at a common hormone response element. |
| 16322899 | 40 | No | Gene expression patterns for doxorubicin (Adriamycin) and cyclophosphamide (cytoxan) (AC) response and resistance. |
| 16323292 | 9 | No | Estrogenic effects of two derivatives of icariin on human breast cancer MCF-7 cells. |
| 16326432 | 5 | No | Pyrethroid insecticides, fenvalerate and permethrin, inhibit progesterone-induced alkaline phosphatase activity in T47D human breast cancer cells. |
| 16328441 | 10 | No | Mitogen-activated protein kinases mediate arsenic-induced down-regulation of survivin in human lung adenocarcinoma cells. |
| 16329108 | 11 | No | Retinoic acid-induced CD38 expression in HL-60 myeloblastic leukemia cells regulates cell differentiation or viability depending on expression levels. |
| 16329587 | 6 | No | [Effects of residues of organochlorine pesticides on reproductive endocrine in human] |
| 16330358 | 209 | No | Discrimination of vanadium from zinc using gene profiling in human bronchial epithelial cells. |

| | | | |
|---|---|---|---|
| 16330433 | 7 | No | Hypoxia-simulating agents and selective stimulation of arsenic trioxide-induced growth arrest and cell differentiation in acute promyelocytic leukemic cells. |
| 16330548 | 2 | No | Neuron restrictive silencer factor NRSF/REST is a transcriptional repressor of neuropilin-1 and diminishes the ability of semaphorin 3A to inhibit keratinocyte |
| 16331273 | 25 | No | Diosgenin inhibits osteoclastogenesis, invasion, and proliferation through the downregulation of Akt, I kappa B kinase activation and NF-kappa B-regulated gene |
| 16331495 | 2 | No | Indomethacin overcomes doxorubicin resistance with inhibiting multi-drug resistance protein 1 (MRP1). |
| 16332659 | 6 | No | Soy-isoflavone-enriched foods and inflammatory biomarkers of cardiovascular disease risk in postmenopausal women: interactions with genotype and equol |
| 16338976 | 12 | No | Cytokine stimulation of pregnancy-associated plasma protein A expression in human coronary artery smooth muscle cells: inhibition by resveratrol. |
| 16340002 | 1 | No | beta2-Adrenoceptor agonist modulates endothelin-1 receptors in human isolated bronchi. |
| 16340194 | 4 | No | Synergistic inhibitory effects of curcumin and 5-fluorouracil on the growth of the human colon cancer cell line HT-29. |
| 16340750 | 15 | No | Investigation of the mechanisms by which EB1089 abrogates apoptosis induced by 9-cis retinoic acid in pancreatic cancer cells. |
| 16340751 | 8 | No | Epidermal growth factor promotes invasiveness of pancreatic cancer cells through NF-kappaB-mediated proteinase productions. |
| 16341940 | 21 | No | Impact of venlafaxine on gene expression profile in lymphocytes of the elderly with major depression--evolution of antidepressants and the role of the |
| 16343431 | 4 | No | Luteolin, a flavonoid, inhibits AP-1 activation by basophils. |
| 16344495 | 4 | No | Immunological consequences of thalidomide treatment in Sj枚gren's syndrome. |
| 16351512 | 4 | No | Anti-angiogenesis efficacy of the garlic ingredient alliin and antioxidants: role of nitric oxide and p53. |
| 16351573 | 2 | No | Phosphorylation of Ser158 regulates inflammatory redox-dependent hepatocyte nuclear factor-4alpha transcriptional activity. |
| 16352669 | 1 | No | Effect of medical castration on CD4+ CD25+ T cells, CD8+ T cell IFN-gamma expression, and NK cells: a physiological role for testosterone and/or its |
| 16352735 | 1 | No | Anti-inflammatory effects of moxifloxacin on IL-8, IL-1beta and TNF-alpha secretion and NFkappaB and MAP-kinase activation in human monocytes stimulated |
| 16352814 | 8 | No | ATRA resolves the differentiation block in t(15;17) acute myeloid leukemia by restoring PU.1 expression. |

| | | | |
|---|---|---|---|
| 16354159 | 1 | No | Cytochrome P450 expression-induction profile and chemically mediated alterations of the WIF-B9 cell line. |
| 16354411 | 24 | No | Inhibition by licochalcone A, a novel flavonoid isolated from liquorice root, of IL-1beta-induced PGE2 production in human skin fibroblasts. |
| 16356831 | 4 | No | 2-Methoxyestradiol and paclitaxel have similar effects on the cell cycle and induction of apoptosis in prostate cancer cells. |
| 16357062 | 9 | No | Heregulin-alpha and heregulin-beta expression is linked to a COX-2-PGE2 pathway |
| 16357189 | 21 | No | Coactivation of liver receptor homologue-1 by peroxisome proliferator-activated receptor gamma coactivator-1alpha on aromatase promoter II and its inhibition by |
| 16357311 | 7 | No | Elevated concentrations of nonesterified fatty acids increase monocyte expression of CD11b and adhesion to endothelial cells. |
| 16357325 | 12 | No | Novel stabilin-1 interacting chitinase-like protein (SI-CLP) is up-regulated in alternatively activated macrophages and secreted via lysosomal pathway. |
| 16357363 | 12 | No | P53 mediates amosite asbestos-induced alveolar epithelial cell mitochondria-regulated apoptosis. |
| 16359550 | 10 | No | Induction and regulation of matrix metalloproteinase-12 in human airway smooth muscle cells. |
| 16359657 | 1 | No | Antiproliferative and antiangiogenic effects of 3-methylcholanthrene, an aryl-hydrocarbon receptor agonist, in human umbilical vascular endothelial cells. |
| 16360360 | 3 | No | The effects of atorvastatin (10 mg) on systemic inflammation in heart failure. |
| 16361081 | 4 | No | TGF-beta regulates differentially the proliferation of fetal and adult human skin fibroblasts via the activation of PKA and the autocrine action of FGF-2. |
| 16361273 | 48 | No | Effects of folate deficiency on gene expression in the apoptosis and cancer pathways in colon cancer cells. |
| 16362534 | 8 | No | 1,25-Dihydroxyvitamin D and 25-hydroxyvitamin D--mediated regulation of TRPV6 (a putative epithelial calcium channel) mRNA expression in Caco-2 cells. |
| 16363279 | 3 | No | The response of skin perfusion and of rheological and immunological variables to intravenous prostanoid administration in Raynaud's phenomenon secondary to |
| 16364007 | 4 | No | Alterations in syncytiotrophoblast cytokine expression following treatment with lipopolysaccharide. |
| 16364386 | 10 | No | p38 Mitogen-activated protein kinase mediates IL-8 induction by the ribotoxin deoxynivalenol in human monocytes. |
| 16365062 | 54 | No | Soy isoflavones alter expression of genes associated with cancer progression, including interleukin-8, in androgen-independent PC-3 human prostate cancer |

| | | | |
|---|---|---|---|
| 16365456 | 7 | No | NF-kappaB and activator protein 1 response elements and the role of histone modifications in IL-1beta-induced TGF-beta1 gene transcription. |
| 16367923 | 68 | No | Chemical genomic screening for methylation-silenced genes in gastric cancer cell lines using 5-aza-2'-deoxycytidine treatment and oligonucleotide microarray. |
| 16368150 | 4 | No | Interleukin-18 induces the production of vascular endothelial growth factor (VEGF) in rheumatoid arthritis synovial fibroblasts via AP-1-dependent pathways. |
| 16368197 | 1 | No | Imiquimod: a potential weapon against Dupuytren contracture. |
| 16368305 | 1 | No | Effect of atorvastatin on circulating proinflammatory T-lymphocyte subsets and soluble CD40 ligand in patients with stable coronary artery disease--a |
| 16368782 | 17 | No | DHT and testosterone, but not DHEA or E2, differentially modulate IGF-I, IGFBP-2, and IGFBP-3 in human prostatic stromal cells. |
| 16369138 | 7 | No | Mild hypothermia promotes pro-inflammatory cytokine production in monocytes. |
| 16369916 | 2 | No | Time-dependent resveratrol-mediated mRNA and protein expression associated with cell cycle in WR-21 cells containing mutated human c-Ha-Ras. |
| 16369997 | 8 | No | Apple flavonoids inhibit growth of HT29 human colon cancer cells and modulate expression of genes involved in the biotransformation of xenobiotics. |
| 16371897 | 2 | No | Human anulus fibrosis and nucleus pulposus cells of the intervertebral disc: effect of degeneration and culture system on cell phenotype. |
| 16373414 | 9 | No | Prostacyclin receptor up-regulates the expression of angiogenic genes in human endometrium via cross talk with epidermal growth factor Receptor and the |
| 16373662 | 1 | No | Thalidomide derivative CC-4047 inhibits osteoclast formation by down-regulation of PU.1. |
| 16373669 | 14 | No | Zn2+-induced IL-8 expression involves AP-1, JNK, and ERK activities in human airway epithelial cells. |
| 16373703 | 4 | No | Sensitization of DNA damage-induced apoptosis by the proteasome inhibitor PS-341 is p53 dependent and involves target proteins 14-3-3sigma and survivin. |
| 16373718 | 3 | No | Chemotherapy and TRAIL-mediated colon cancer cell death: the roles of p53, TRAIL receptors, and c-FLIP. |
| 16374459 | 2 | No | Regulation of skin microvasculature angiogenesis, cell migration, and permeability by a specific inhibitor of PKCalpha. |
| 16375908 | 6 | No | Simvastatin induces heat shock factor 1 in vascular endothelial cells. |
| 16376386 | 6 | No | Scoparone inhibits PMA-induced IL-8 and MCP-1 production through suppression of NF-kappaB activation in U937 cells. |

| | | | |
|---|---|---|---|
| 16377638 | 4 | No | Suberoylanilide hydroxamic acid potentiates apoptosis, inhibits invasion, and abolishes osteoclastogenesis by suppressing nuclear factor-kappaB activation. |
| 16378115 | 1 | No | [Influence of lidocaine on systemic inflammation in perioperative patients undergoing cardiopulmonary bypass] |
| 16380391 | 2 | No | Cytokine-induced metabolic effects in human adipocytes are independent of endogenous nitric oxide. |
| 16380446 | 4 | No | NF-kappaB is essential for the progression of KSHV- and EBV-infected lymphomas in vivo. |
| 16380993 | 4 | No | Selenium binding protein 1 in ovarian cancer. |
| 16381670 | 1 | No | Benzo[A]pyrene-induced oral carcinogenesis and chemoprevention: studies in bioengineered human tissue. |
| 16381673 | 1 | No | The role of pregnane X receptor in 2-acetylaminofluorene-mediated induction of drug transport and -metabolizing enzymes in mice. |
| 16381997 | 1 | No | Regulation of plasma fibroblast growth factor 23 by calcium in primary hyperparathyroidism. |
| 16382148 | 4 | No | Tissue transglutaminase serves as an inhibitor of apoptosis by cross-linking caspase 3 in thapsigargin-treated cells. |
| 16384973 | 1 | No | Multiplex cytokine detection versus ELISA for aqueous humor: IL-5, IL-10, and IFNgamma profiles in uveitis. |
| 16385345 | 2 | No | Interaction between genetic control of vascular endothelial growth factor production and retinoid responsiveness in psoriasis. |
| 16386082 | 6 | No | Retinoic acid induces VEGF gene expression in human retinal pigment epithelial cells (ARPE-19). |
| 16386258 | 32 | No | Oxidized phospholipid: POVPC binds to platelet-activating-factor receptor on human macrophages. Implications in atherosclerosis. |
| 16386771 | 19 | No | Effects of 12 metal ions on iron regulatory protein 1 (IRP-1) and hypoxia-inducible factor-1 alpha (HIF-1alpha) and HIF-regulated genes. |
| 16387843 | 2 | No | Differential expression of adenosine receptors in human neutrophils: up-regulation by specific Th1 cytokines and lipopolysaccharide. |
| 16387846 | 14 | No | Differential effects of statins on relevant functions of human monocyte-derived dendritic cells. |
| 16388738 | 4 | No | [The effect on the differentiation and maturation of dendritic cells by lupane acetate of cortex periplocae] |
| 16391493 | 8 | No | Effect of berberine on interleukin 8 and monocyte chemotactic protein 1 expression in a human retinal pigment epithelial cell line. |

| | | | |
|---|---|---|---|
| 16391804 | 54 | No | in vitro synergistic antitumor activity of a combination of 5-fluorouracil and irinotecan in human colon cancer. |
| 16391810 | 4 | No | Down-regulation of FAK and IAPs by laminin during cisplatin-induced apoptosis in testicular germ cell tumors. |
| 16391822 | 2 | No | The selective cyclooxygenase-1 inhibitor SC-560 suppresses cell proliferation and induces apoptosis in human hepatocellular carcinoma cells. |
| 16391850 | 3 | No | Antitumor effects of IDN5109 on head and neck squamous cell carcinoma. |
| 16392133 | 2 | No | Chemokine gene activation in human bone marrow-derived osteoblasts following exposure to particulate wear debris. |
| 16393665 | 20 | No | Estrogen-like properties of fluorotelomer alcohols as revealed by mcf-7 breast cancer cell proliferation. |
| 16393696 | 5 | No | Insulin-like growth factor II mediates resveratrol stimulatory effect on cathepsin D in breast cancer cells. |
| 16396990 | 19 | No | Mechanistic study of polychlorinated biphenyl 126-induced CYP11B1 and CYP11B2 up-regulation. |
| 16399772 | 6 | No | Molecular targets of the chemopreventive agent 1,4-phenylenebis (methylene)-selenocyanate in human non-small cell lung cancer. |
| 16402917 | 3 | No | Metallothionein isoform 2A expression is inducible and protects against ROS-mediated cell death in rotenone-treated HeLa cells. |
| 16403803 | 3 | No | Progesterone-dependent release of transforming growth factor-beta1 from epithelial cells enhances the endometrial decidualization by turning on the Smad |
| 16404146 | 65 | No | Gamma-irradiation and doxorubicin treatment of normal human cells cause cell cycle arrest via different pathways. |
| 16406510 | 2 | No | Responsiveness to phytoestrogens in primary human osteoblasts is modulated differentially by a "less-calcemic" analog of 1,25 dihydroxyvitamin D(3): JK |
| 16407166 | 3 | No | Nonsteroidal anti-inflammatory drugs repress beta-secretase gene promoter activity by the activation of PPARgamma. |
| 16407826 | 16 | No | Overexpression of hRFI inhibits 5-fluorouracil-induced apoptosis in colorectal cancer cells via activation of NF-kappaB and upregulation of BCL-2 and BCL-XL. |
| 16409223 | 1 | No | Impact of oral contraceptives on sex hormone-binding globulin and androgen levels: a retrospective study in women with sexual dysfunction. |
| 16411021 | 1 | No | Down regulation of N-acetylglucosaminyltransferase V facilitates all-transretinoic acid to induce apoptosis of human hepatocarcinoma cells. |
| 16411658 | 2 | No | Competing roles of aldo-keto reductase 1A1 and cytochrome P4501B1 in benzo[a]pyrene-7,8-diol activation in human bronchoalveolar H358 cells: role of |

| | | | |
|---|---|---|---|
| 16413094 | 3 | No | Carbaryl inhibits basal and FSH-induced progesterone biosynthesis of primary human granulosa-lutein cells. |
| 16413414 | 1 | No | The reaction of flavanols with nitrous acid protects against N-nitrosamine formation and leads to the formation of nitroso derivatives which inhibit cancer |
| 16413505 | 6 | No | Epidermal growth factor receptor inhibitor (PD168393) potentiates cytotoxic effects of paclitaxel against androgen-independent prostate cancer cells. |
| 16413536 | 1 | No | mtDNA controls expression of the Death Associated Protein 3. |
| 16417216 | 10 | No | Involvement of PPARgamma in oxidative stress-mediated prostaglandin E(2) production in SZ95 human sebaceous gland cells. |
| 16417271 | 2 | No | PNA-nitrogen mustard conjugates are effective suppressors of HER-2/neu and biological tools for recognition of PNA/DNA interactions. |
| 16417524 | 2 | No | Akt phosphorylates and suppresses the transactivation of retinoic acid receptor alpha. |
| 16417649 | 18 | No | New role for nuclear hormone receptors and coactivators in regulation of BRCA1-mediated DNA repair in breast cancer cell lines. |
| 16421274 | 2 | No | Nucleolin links to arsenic-induced stabilization of GADD45alpha mRNA. |
| 16423920 | 9 | No | Upregulation of a basolateral FXR-dependent bile acid efflux transporter |
| 16424399 | 3 | No | Selenium supplementation restores the antioxidative capacity and prevents cell damage in bone marrow stromal cells in vitro. |
| 16426233 | 8 | No | Role of Nrf2 in the regulation of the Mrp2 (ABCC2) gene. |
| 16426496 | 4 | No | The inhibitory effect of quercetin on IL-6 production by LPS-stimulated neutrophils. |
| 16426703 | 1 | No | Wound repair and proliferation of bronchial epithelial cells enhanced by bombesin receptor subtype 3 activation. |
| 16427187 | 2 | No | Expression of Lea in gastric cancer cell lines depends on FUT3 expression regulated by promoter methylation. |
| 16430862 | 10 | No | JWA, a novel signaling molecule, involved in the induction of differentiation of human myeloid leukemia cells. |
| 16432175 | 8 | No | Benzodiazepinedione inhibitors of the Hdm2:p53 complex suppress human tumor cell proliferation in vitro and sensitize tumors to doxorubicin in vivo. |
| 16432534 | 1 | No | Effects of geldanamycin on HIF-1alpha mediated angiogenesis and invasion in prostate cancer cells. |

| | | | |
|---|---|---|---|
| 16434701 | 2 | No | Regulation of the human p21(waf1/cip1) gene promoter via multiple binding sites for p53 and the vitamin D3 receptor. |
| 16434996 | 18 | No | BRCA1 and BRCA2 as molecular targets for phytochemicals indole-3-carbinol and genistein in breast and prostate cancer cells. |
| 16438844 | 6 | No | [Sensitivity of PTEN gene-transfected endometrial carcinoma cell line to doxorubicin-induced apoptosis] |
| 16438929 | 1 | No | Validation of real-time reverse-transcription-polymerase chain reaction for quantification of capecitabine-metabolizing enzymes. |
| 16439135 | 1 | No | Chlamydocin analogs bearing carbonyl group as possible ligand toward zinc atom in histone deacetylases. |
| 16439309 | 6 | No | Retinoic acid induces expression of SLP-76: expression with c-FMS enhances ERK activation and retinoic acid-induced differentiation/G0 arrest of HL-60 cells. |
| 16439463 | 10 | No | Hydrocortisone and purinergic signaling stimulate sodium/iodide symporter (NIS)-mediated iodide transport in breast cancer cells. |
| 16439677 | 11 | No | Functional integrity of the p53-mediated apoptotic pathway induced by the nongenotoxic agent nutlin-3 in B-cell chronic lymphocytic leukemia (B-CLL). |
| 16439685 | 22 | No | MDM2 antagonists activate p53 and synergize with genotoxic drugs in B-cell chronic lymphocytic leukemia cells. |
| 16440303 | 20 | No | Effects of cadmium on structure and enzymatic activity of Cu,Zn-SOD and oxidative status in neural cells. |
| 16442130 | 7 | No | MDR- and CYP3A4-mediated drug-herbal interactions. |
| 16443240 | 1 | No | Transport and toxic mechanism for aluminum citrate in human neuroblastoma SH-SY5Y cells. |
| 16443354 | 68 | No | Retinoic acid and ascorbic acid act synergistically in inhibiting human breast cancer cell proliferation. |
| 16447258 | 3 | No | Human glioblastoma ADF cells express tyrosinase, L-tyrosine hydroxylase and melanosomes and are sensitive to L-tyrosine and phenylthiourea. |
| 16449346 | 6 | No | Dickkopf-1, an inhibitor of Wnt signaling, is regulated by progesterone in human endometrial stromal cells. |
| 16449370 | 3 | No | Analgesic action of acetaminophen in symptomatic osteoarthritis of the knee. |
| 16449964 | 5 | No | Co-resistance to retinoic acid and TRAIL by insertion mutagenesis into RAM. |
| 16454849 | 3 | No | Thalidomide in the treatment of chronic hepatitis C unresponsive to alfa-interferon and ribavirin. |

| | | | |
|---|---|---|---|
| 16455614 | 5 | No | Effects of di(2-ethylhexyl) phthalate, a widely used peroxisome proliferator and plasticizer, on cell growth in the human keratinocyte cell line NCTC 2544. |
| 16455633 | 2 | No | Visualization of telomerase reverse transcriptase (hTERT) promoter activity using a trimodality fusion reporter construct. |
| 16457948 | 4 | No | Induction of thymidine phosphorylase expression by AZT contributes to enhancement of 5'-DFUR cytotoxicity. |
| 16460831 | 2 | No | Hepcidin generated by hepatoma cells inhibits iron export from co-cultured THP1 monocytes. |
| 16461808 | 3 | No | Effect of all-trans retinoic acid on tissue dynamics of choriocarcinoma cell lines: an organotypic model. |
| 16462019 | 2 | No | DNA damage caused by bisphenol A and estradiol through estrogenic activity. |
| 16462769 | 1 | No | Heme oxygenase-1 protects tumor cells against photodynamic therapy-mediated cytotoxicity. |
| 16465378 | 3 | No | Insulin-like growth factor-I promotes migration in human androgen-independent prostate cancer cells via the alphavbeta3 integrin and PI3-K/Akt signaling. |
| 16467109 | 2 | No | Histone deacetylase inhibitor-mediated radiosensitization of human cancer cells: class differences and the potential influence of p53. |
| 16467208 | 6 | No | MEK1 inhibition sensitizes primary acute myelogenous leukemia to arsenic trioxide-induced apoptosis. |
| 16467367 | 4 | No | Retinoic acid and oncostatin M combine to promote cartilage degradation via matrix metalloproteinase-13 expression in bovine but not human chondrocytes. |
| 16468075 | 9 | No | JWA, a novel signaling molecule, involved in all-trans retinoic acid induced differentiation of HL-60 cells. |
| 16470170 | 9 | No | Retinoid-induced epidermal hyperplasia is mediated by epidermal growth factor receptor activation via specific induction of its ligands heparin-binding EGF and |
| 16472761 | 13 | No | EGFR signaling pathway negatively regulates PSA expression and secretion via the PI3K-Akt pathway in LNCaP prostate cancer cells. |
| 16472982 | 14 | No | Zinc at pharmacologic concentrations affects cytokine expression and induces apoptosis of human peripheral blood mononuclear cells. |
| 16473406 | 8 | No | ATRA can enhance apoptosis that is induced by Flt3 tyrosine kinase inhibition in Flt3-ITD positive cells. |
| 16473924 | 7 | No | Retinoic acid can induce markers of endocrine transdifferentiation in pancreatic ductal adenocarcinoma: preliminary observations from an in vitro cell line model. |
| 16474171 | 692 | No | Convergent transcriptional profiles induced by endogenous estrogen and distinct xenoestrogens in breast cancer cells. |

| | | | |
|---|---|---|---|
| 16474264 | 2 | No | Plasma mutant-p53 protein and anti-p53 antibody as a marker: an experience in vinyl chloride workers in Italy. |
| 16475674 | 2 | No | Effects of zoledronic acid on proteinase plasma levels in patients with bone metastases. |
| 16475708 | 5 | No | Bone-related growth factors and zoledronic acid regulate the PTHrP/PTH.1 receptor bioregulation systems in MG-63 human osteosarcoma cells. |
| 16476009 | 6 | No | Serotonin transporter mRNA expression is decreased by lamivudine and ribavirin and increased by interferon in immune cells. |
| 16480277 | 3 | No | Novel sulfonanilide analogues suppress aromatase expression and activity in breast cancer cells independent of COX-2 inhibition. |
| 16480812 | 11 | No | The aryl hydrocarbon receptor activates the retinoic acid receptoralpha through SMRT antagonism. |
| 16481741 | 6 | No | Administration of PUMA adenovirus increases the sensitivity of esophageal cancer cells to anticancer drugs. |
| 16482212 | 3 | No | Telomerase targeting by retinoids in cells from patients with myeloid leukemias of various subtypes, not only APL. |
| 16484594 | 6 | No | Synthetic retinoid Am80 reduces scavenger receptor expression and atherosclerosis in mice by inhibiting IL-6. |
| 16487037 | 3 | No | Induction of endogenous Nrf2/small maf heterodimers by arsenic-mediated stress in placental choriocarcinoma cells. |
| 16488005 | 6 | No | Effect of cobalt and chromium ions on human MG-63 osteoblasts in vitro: morphology, cytotoxicity, and oxidative stress. |
| 16488075 | 5 | No | Sulindac induces specific degradation of the HPV oncoprotein E7 and causes growth arrest and apoptosis in cervical carcinoma cells. |
| 16488535 | 18 | No | Bifunctional effect of resveratrol on the expression of ErbB2 in human breast cancer cell. |
| 16489027 | 6 | No | Role of protein kinase CK2 in the regulation of tumor necrosis factor-related apoptosis inducing ligand-induced apoptosis in prostate cancer cells. |
| 16489032 | 1 | No | SKI-606 decreases growth and motility of colorectal cancer cells by preventing pp60(c-Src)-dependent tyrosine phosphorylation of beta-catenin and its nuclear |
| 16489053 | 18 | No | 3-Methylcholanthrene and other aryl hydrocarbon receptor agonists directly activate estrogen receptor alpha. |
| 16490592 | 6 | No | Resveratrol downregulates the constitutional activation of nuclear factor-kappaB in multiple myeloma cells, leading to suppression of proliferation and invasion, |
| 16490937 | 17 | No | Effects of nitrofen and vitamins A, C and E on maturation of cultured human H441 pneumocytes. |

| | | | |
|---|---|---|---|
| 16491109 | 3 | No | Effect of short-term folic acid supplementation on insulin sensitivity and inflammatory markers in overweight subjects. |
| 16491319 | 10 | No | Effect of 17beta-oestradiol on transepithelial calcium transport in human intestinal-like Caco-2 cells and its interactions with |
| 16494909 | 7 | No | Expression of the helix-loop-helix protein inhibitor of DNA binding-1 (ID-1) is activated by all-trans retinoic acid in normal human keratinocytes. |
| 16496412 | 11 | No | GREB1 is a novel androgen-regulated gene required for prostate cancer growth. |
| 16497706 | 16 | No | Induction of apoptosis and cell cycle arrest by a chalcone panduratin A isolated from Kaempferia pandurata in androgen-independent human prostate cancer cells PC3 |
| 16497877 | 48 | No | Raloxifene-induced myeloma cell apoptosis: a study of nuclear factor-kappaB inhibition and gene expression signature. |
| 16500647 | 79 | No | Cannabinoid derivatives induce cell death in pancreatic MIA PaCa-2 cells via a receptor-independent mechanism. |
| 16504308 | 3 | No | Evidence that hypoxia-inducible factor-1 (HIF-1) mediates transcriptional activation of interleukin-1beta (IL-1beta) in astrocyte cultures. |
| 16505103 | 11 | No | Berberine, a natural product, induces G1-phase cell cycle arrest and caspase-3-dependent apoptosis in human prostate carcinoma cells. |
| 16505238 | 18 | No | MafA expression and insulin promoter activity are induced by nicotinamide and related compounds in INS-1 pancreatic beta-cells. |
| 16507397 | 6 | No | Study of COX-2, Ki67, and p53 expression to predict effectiveness of 5-flurouracil, epirubicin and cyclophosphamide with celecoxib treatment in breast |
| 16507463 | 66 | No | Distinct gene expression profiles in immortalized human urothelial cells exposed to inorganic arsenite and its methylated trivalent metabolites. |
| 16510162 | 11 | No | Increased FasL expression correlates with apoptotic changes in granulocytes cultured with oxidized clozapine. |
| 16510598 | 125 | No | Pharmacogenomic identification of novel determinants of response to chemotherapy in colon cancer. |
| 16513093 | 8 | No | Organotin compounds enhance 17beta-hydroxysteroid dehydrogenase type I activity in human choriocarcinoma JAr cells: potential promotion of 17beta-estradiol |
| 16513826 | 2 | No | Thiazolidinediones and rexinoids induce peroxisome proliferator-activated receptor-coactivator (PGC)-1alpha gene transcription: an autoregulatory loop |
| 16514628 | 320 | No | Comparative gene expression profiling reveals partially overlapping but distinct genomic actions of different antiestrogens in human breast cancer cells. |
| 16514648 | 29 | No | Synergistic regulation of endothelial tight junctions by antioxidant (Se) and polyunsaturated lipid (GLA) via Claudin-5 modulation. |

| | | | |
|---|---|---|---|
| 16516039 | 4 | No | The expression of transcription factor activating transcription factor 3 in the human prostate and its regulation by androgen in prostate cancer. |
| 16517099 | 1 | No | Identification and characterization of the human retinoid X receptor alpha gene promoter. |
| 16517207 | 6 | No | Increased expression of cyclin A1 protein is associated with all-trans retinoic acid-induced apoptosis. |
| 16517955 | 12 | No | Antioxidant supplementation with or without B-group vitamins after acute ischemic stroke: a randomized controlled trial. |
| 16522526 | 2 | No | Effect of a combined oral contraceptive containing 20 microg ethinyl estradiol and 75 microg gestodene on hemostatic parameters. |
| 16523695 | 6 | No | [Transcriptional regulation of placental transforming growth factor-beta by calcitriol in prostate cancer cells is androgen-independent] |
| 16524720 | 3 | No | Androgen enhances the antiproliferative activity of vitamin D3 by suppressing 24-hydroxylase expression in LNCaP cells. |
| 16525649 | 12 | No | Effects of PPAR and RXR ligands in semaphorin 6B gene expression of human MCF-7 breast cancer cells. |
| 16525653 | 2 | No | Effects of progesterone and anti-progestin (mifepristone) treatment on proliferation and apoptosis of the human ovarian cancer cell line, OVCAR-3. |
| 16529693 | 1 | No | [Changes of gene expression profile of multiple myeloma cell line RPMI 8226 treated by arsenic trioxide] |
| 16530516 | 3 | No | All-trans retinoic acid induces XAF1 expression through an interferon regulatory factor-1 element in colon cancer. |
| 16530937 | 14 | No | Cytochrome P450 1B1, a novel chemopreventive target for benzo[a]pyrene-initiated human esophageal cancer. |
| 16531263 | 5 | No | Stimulation of erythropoiesis by thalidomide in multiple myeloma patients: its influence on FasL, TRAIL and their receptors on erythroblasts. |
| 16531283 | 8 | No | A2E selectively induces cox-2 in ARPE-19 and human neural cells. |
| 16531609 | 1 | No | Mechanisms of the copper-dependent turnover of the copper chaperone for superoxide dismutase. |
| 16532439 | 2 | No | t(3;21)(q26;q22) in myeloid leukemia: an aggressive syndrome of blast transformation associated with hydroxyurea or antimetabolite therapy. |
| 16534752 | 6 | No | Estrogens modulate the gene expression of Wnt-7a in cultured endometrial adenocarcinoma cells. |
| 1653643 | 2 | No | Effects of chronic amiodarone treatment on human myocardial beta adrenoceptor density and adenylate cyclase response. |

| | | | |
|---|---|---|---|
| 16536755 | 1 | No | Raloxifene, an oestrogen-receptor-beta-targeted therapy, inhibits androgen-independent prostate cancer growth: results from preclinical studies and |
| 16537896 | 4 | No | Primary and compensatory roles for RB family members at cell cycle gene promoters that are deacetylated and downregulated in doxorubicin-induced senescence of |
| 16539210 | 2 | No | Altered gene expression in human cells treated with the insecticide diazinon: correlation with decreased DNA excision repair capacity. |
| 16540676 | 7 | No | 4-oxo-fenretinide, a recently identified fenretinide metabolite, induces marked G2-M cell cycle arrest and apoptosis in fenretinide-sensitive and |
| 16541309 | 2 | No | Stimulatory effect of genistein and apigenin on the growth of breast cancer cells correlates with their ability to activate ER alpha. |
| 16541416 | 1 | No | KLK31P is a novel androgen regulated and transcribed pseudogene of kallikreins that is expressed at lower levels in prostate cancer cells than in normal |
| 16541417 | 4 | No | Non-redundant inhibitor of differentiation (Id) gene expression and function in human prostate epithelial cells. |
| 16541421 | 4 | No | Androgen regulation of prostasin gene expression is mediated by sterol-regulatory element-binding proteins and SLUG. |
| 16544145 | 6 | No | Reversal of multidrug resistance by two nordihydroguaiaretic acid derivatives, M4N and maltose-M3N, and their use in combination with doxorubicin or paclitaxel. |
| 16546976 | 6 | No | Resveratrol inhibits Src and Stat3 signaling and induces the apoptosis of malignant cells containing activated Stat3 protein. |
| 16546985 | 5 | No | NIK-333 inhibits growth of human T-cell leukemia virus type I-infected T-cell lines and adult T-cell leukemia cells in association with blockade of nuclear |
| 16557585 | 16 | No | Synergistic effects of docetaxel and S-1 by modulating the expression of metabolic enzymes of 5-fluorouracil in human gastric cancer cell lines. |
| 16557594 | 100 | No | Transcriptional profiling of MCF7 breast cancer cells in response to 5-Fluorouracil: relationship with cell cycle changes and apoptosis, and |
| 16562669 | 2 | No | [Effect of realgar on the gene expression profile of multiple myeloma cell line RPMI 8226] |
| 16563497 | 2 | No | The effect of particulate material on the regulation of chemokine receptor expression in leukocytes. |
| 16563610 | 2 | No | Hypoxia regulation of expression and angiogenic effects of vasoactive intestinal peptide (VIP) and VIP receptors in LNCaP prostate cancer cells. |
| 16564041 | 1 | No | Dexamethasone increases fluid absorption via Na+/H+ exchanger (NHE) 3 activation in normal human middle ear epithelial cells. |
| 16565514 | 6 | No | Identification of new human pregnane X receptor ligands among pesticides using a stable reporter cell system. |

| | | | |
|---|---|---|---|
| 16565515 | 3 | No | Proteomic identification of a stress protein, mortalin/mthsp70/GRP75: relevance to Parkinson disease. |
| 16565789 | 8 | No | Lycopene inhibition of IGF-induced cancer cell growth depends on the level of cyclin D1. |
| 16566921 | 2 | No | Regulation of bcl-2 expression by Ubc9. |
| 16568373 | 19 | No | Influence of chemotherapeutic agents and cytokines on the expression of 5-fluorouracil-associated enzymes in human colon cancer cell lines. |
| 16569247 | 4 | No | In vitro and in vivo effects of the PPAR-alpha agonists fenofibrate and retinoic acid in endometrial cancer. |
| 16569592 | 3 | No | Epigenetic heterochromatin markers distinguish terminally differentiated leukocytes from incompletely differentiated leukemia cells in human blood. |
| 16570282 | 51 | No | 4-HPR modulates gene expression in ovarian cells. |
| 16572199 | 5 | No | Upregulation of Bfl-1/A1 in leukemia cells undergoing differentiation by all-trans retinoic acid treatment attenuates chemotherapeutic agent-induced |
| 16572323 | 3 | No | Antiproliferative effect of indomethacin on CML cells is related to the suppression of STATs/Bcl-XL signal pathway. |
| 16573383 | 1 | No | Quercetin induces necrosis and apoptosis in SCC-9 oral cancer cells. |
| 16573735 | 6 | No | Peroxisome proliferator-activated receptor gamma ligands stimulate myeloid differentiation and lipogenensis in human leukemia NB4 cells. |
| 16574066 | 3 | No | Transcriptional up-regulation of restin by all-trans retinoic acid through STAT1 in cancer cell differentiation process. |
| 16574167 | 4 | No | CyclinB2 and BIRC5 genes as surrogate biomarkers for neurite outgrowth in SH-SY5Y subclonal cells. |
| 16574429 | 5 | No | Adrenoceptor blockade alters plasma gelatinase activity in patients with heart failure and MMP-9 promoter activity in a human cell line (ECV304). |
| 16575387 | 5 | No | 13-cis Retinoic acid induces apoptosis and cell cycle arrest in human SEB-1 sebocytes. |
| 16579640 | 37 | No | Alteration of genomic responses to doxorubicin and prevention of MDR in breast cancer cells by a polymer excipient: pluronic P85. |
| 16579726 | 53 | No | Taurine-responsive genes related to signal transduction as identified by cDNA microarray analyses of HepG2 cells. |
| 16579968 | 6 | No | The zinc chelator, N,N,N',N'-tetrakis (2-pyridylmethyl) ethylenediamine, increases the level of nonfunctional HIF-1alpha protein in normoxic cells. |

| | | | |
|---|---|---|---|
| 16580899 | 7 | No | Rofecoxib regulates the expression of genes related to the matrix metalloproteinase pathway in humans: implication for the adverse effects of |
| 16581223 | 3 | No | Matrix metalloproteinases and their inhibitors as biomarkers for metal toxicity in vitro. |
| 16581224 | 3 | No | Sodium nitrite-induced cytotoxicity in cultured human gastric epithelial cells. |
| 16581535 | 1 | No | Occupational toluene exposure induces cytochrome P450 2E1 mRNA expression in peripheral lymphocytes. |
| 16581781 | 6 | No | GA-binding protein and p300 are essential components of a retinoic acid-induced enhanceosome in myeloid cells. |
| 16581943 | 8 | No | Regulation of CYP1A1 gene expression by the antioxidant tert-butylhydroquinone. |
| 16582591 | 10 | No | Nicotinic receptors mediate tumorigenic action of tobacco-derived nitrosamines on immortalized oral epithelial cells. |
| 16582618 | 2 | No | p19INK4D and cell death. |
| 16584521 | 6 | No | Effects of oestradiol and oestroprogestin on erythrocyte antioxidative enzyme system activity in postmenopausal women. |
| 16584549 | 106 | No | Multi-level gene expression profiles affected by thymidylate synthase and 5-fluorouracil in colon cancer. |
| 16584924 | 1 | No | Detection of cell cycle- and differentiation stage-dependent human telomerase reverse transcriptase expression in single living cancer cells. |
| 16595493 | 5 | No | Stunning and its effect on 3H-FDG uptake and key gene expression in breast cancer cells undergoing chemotherapy. |
| 16595896 | 310 | No | Toxicogenomics of kojic acid on gene expression profiling of a375 human malignant melanoma cells. |
| 16596200 | 8 | No | hRFI overexpressed in HCT116 cells modulates Bcl-2 family proteins when treated with 5-fluorouracil. |
| 16596234 | 5 | No | Modulation of estrogen receptor-beta isoforms by phytoestrogens in breast cancer cells. |
| 16596235 | 2 | No | Acyclic retinoid, a novel synthetic retinoid, induces growth inhibition, apoptosis, and changes in mRNA expression of cell cycle- and |
| 16596621 | 1 | No | Evaluation of the HER2/neu-derived peptide GP2 for use in a peptide-based breast cancer vaccine trial. |
| 16597033 | 2 | No | [Effect of dihydrotestosterone on the transcriptions and expressions of Smad3 and Smad4 in LNCaP cell line] |

| | | | |
|---|---|---|---|
| 16598420 | 3 | No | Inhibition of matrix degrading enzymes and invasion in human glioblastoma (U87MG) cells by isoflavones. |
| 16598758 | 11 | No | Methotrexate induced differentiation in colon cancer cells is primarily due to purine deprivation. |
| 16600465 | 8 | No | Copper(II) and manganese(III) complexes of N'-[(2-hydroxy phenyl) carbonothioyl] pyridine-2-carbohydrazide: novel therapeutic agents for cancer. |
| 16600567 | 2 | No | Effects of sodium arsenite on catalase activity, gene and protein expression in HaCaT cells. |
| 16600694 | 36 | No | Aspirin and PPAR-alpha activators inhibit monocyte chemoattractant protein-1 expression induced by high glucose concentration in human endothelial cells. |
| 16601352 | 19 | No | Luteolin, a flavonoid, inhibits CD40 ligand expression by activated human basophils. |
| 16603237 | 2 | No | JHDM2A, a JmjC-containing H3K9 demethylase, facilitates transcription activation by androgen receptor. |
| 16608438 | 2 | No | All-trans retinoic acid down-regulates human albumin gene expression through the induction of C/EBPbeta-LIP. |
| 16610002 | 1 | No | Up-regulation of NAD(P)H quinone oxidoreductase 1 during human liver injury. |
| 16610086 | 6 | No | [Acitretin induces apoptosis and changes of relative signaling pathway in epidermoid carcinoma cell line A431] |
| 16611376 | 4 | No | In vitro folate deficiency induces apoptosis by a p53, Fas (Apo-1, CD95) independent, bcl-2 related mechanism in phytohaemagglutinin-stimulated human |
| 16611624 | 8 | No | c-Src is the primary signaling mediator of polychlorinated biphenyl-induced interleukin-8 expression in a human microvascular endothelial cell line. |
| 16611627 | 1 | No | The red wine polyphenol resveratrol displays bilevel inhibition on aromatase in breast cancer cells. |
| 16611738 | 18 | No | Adenosine downregulates DPPIV on HT-29 colon cancer cells by stimulating protein tyrosine phosphatase(s) and reducing ERK1/2 activity via a novel pathway. |
| 16613838 | 5 | No | Curcumin sensitizes tumor necrosis factor-related apoptosis-inducing ligand (TRAIL)-mediated apoptosis through CHOP-independent DR5 upregulation. |
| 16613989 | 1 | No | Down-regulation of the tumor suppressor gene retinoic acid receptor beta2 through the phosphoinositide 3-kinase/Akt signaling pathway. |
| 16614140 | 4 | No | Resveratrol suppresses tumor necrosis factor-alpha-induced fractalkine expression in endothelial cells. |
| 16614850 | 2 | No | RNAi-mediated knockdown of aldehyde dehydrogenase class-1A1 and class-3A1 is specific and reveals that each contributes equally to the resistance against |

| | | | |
|---|---|---|---|
| 16616026 | 1 | No | Effects of colesevelam hydrochloride on low-density lipoprotein cholesterol and high-sensitivity C-reactive protein when added to statins in patients with |
| 16616857 | 1 | No | Thalidomide alters c-MYB and PIM-1 signaling in K-562 cells. |
| 16617325 | 9 | No | The role of the nuclear Akt activation and Akt inhibitors in all-trans-retinoic acid-differentiated HL-60 cells. |
| 16618747 | 2 | No | Retinoic acid induces p27Kip1 nuclear accumulation by modulating its phosphorylation. |
| 16619537 | 6 | No | Role of mitochondria in quercetin-enhanced chemotherapeutic response in human non-small cell lung carcinoma H-520 cells. |
| 16620711 | 3 | No | [Effects of p53 inhibitor-alpha on the proliferation and apoptosis in large intestinal epithelial cells damaged by hyperthermic chemotherapy] |
| 16621245 | 1 | No | Differential effects of bisphosphonates on breast cancer cell lines. |
| 16621454 | 2 | No | ATP2C1 is specifically localized in the basal layer of normal epidermis and its depletion triggers keratinocyte differentiation. |
| 16621514 | 4 | No | Responsiveness to estradiol-17beta and to phytoestrogens in primary human osteoblasts is modulated differentially by high glucose concentration. |
| 16621909 | 3 | No | Use of organ culture to study the human fetal testis development: effect of retinoic acid. |
| 16622835 | 15 | No | Comparative proteomic analysis of hypoxia-treated and untreated human leukemic U937 cells. |
| 16624241 | 7 | No | Strategies for comparing gene expression profiles from different microarray platforms: application to a case-control experiment. |
| 16624386 | 8 | No | Antiproliferative and apoptotic effects of zinc-citrate compound (CIZAR(R)) on human epithelial ovarian cancer cell line, OVCAR-3. |
| 16624393 | 8 | No | Arsenic trioxide (As(2)O(3)) inhibits peritoneal invasion of ovarian carcinoma cells in vitro and in vivo. |
| 16624485 | 2 | No | Methylation status of the Ep-CAM promoter region in human breast cancer cell lines and breast cancer tissue. |
| 16624732 | 2 | No | [Protective effect of selenium against T-2 toxin-induced inhibition of chondrocyte aggrecan and collagen II synthesis] |
| 16625280 | 4 | No | Manganese antagonizes iron blocking mitochondrial aconitase expression in human prostate carcinoma cells. |
| 16627626 | 18 | No | Nicotine attenuates beta-amyloid-induced neurotoxicity by regulating metal homeostasis. |

| | | | |
|---|---|---|---|
| 16628085 | 2 | No | XIAP is related to the chemoresistance and inhibited its expression by RNA interference sensitize pancreatic carcinoma cells to chemotherapeutics. |
| 16628086 | 8 | No | Effect of antisense hypoxia-inducible factor 1alpha on progression, metastasis, and chemosensitivity of pancreatic cancer. |
| 16630558 | 3 | No | Regulation of steroid hydroxylase CYP7B1 by androgens and estrogens in prostate cancer LNCaP cells. |
| 16631469 | 38 | No | Differentially expressed genes in the prostate cancer cell line LNCaP after exposure to androgen and anti-androgen. |
| 16632112 | 4 | No | Involvement of selenoprotein P in protection of human astrocytes from oxidative damage. |
| 16632523 | 24 | No | Retinoids activate the RXR/SXR-mediated pathway and induce the endogenous CYP3A4 activity in Huh7 human hepatoma cells. |
| 16632680 | 13 | No | Endocrine regulation of HOX genes. |
| 16636064 | 5 | No | The estrogen-responsive B box protein is a novel regulator of the retinoid signal. |
| 16636310 | 4 | No | Nuclear accumulations of p53 and Mdm2 are accompanied by reductions in c-Abl and p300 in zinc-depleted human hepatoblastoma cells. |
| 16636311 | 23 | No | Retinoic acid exerts dual regulatory actions on the expression and nuclear localization of interferon regulatory factor-1. |
| 16636651 | 10 | No | The role of metallothionein IIa in defending lens epithelial cells against cadmium and TBHP induced oxidative stress. |
| 16637064 | 20 | No | Inactivation of IkappaB contributes to transcriptional activation of spermidine/spermine N(1)-acetyltransferase. |
| 16637073 | 3 | No | Conditional expression of PTEN alters the androgen responsiveness of prostate cancer cells. |
| 16638120 | 2 | No | Characterization of hARD2, a processed hARD1 gene duplicate, encoding a human protein N-alpha-acetyltransferase. |
| 16638750 | 5 | No | Mechanisms regulating tumor angiogenesis by 12-lipoxygenase in prostate cancer cells. |
| 16641320 | 2 | No | Inhibition of beta-defensin gene expression in airway epithelial cells by low doses of residual oil fly ash is mediated by vanadium. |
| 16646664 | 8 | No | Transcription Factor IIA tau is associated with undifferentiated cells and its gene expression is repressed in primary neurons at the chromatin level in vivo. |
| 16647867 | 5 | No | Retinoic acid-induced protein ISGylation is dependent on interferon signal transduction. |

| | | | |
|---|---|---|---|
| 16648469 | 1 | No | Nickel ions increase histone H3 lysine 9 dimethylation and induce transgene silencing. |
| 16648635 | 12 | No | Degradation of the Alzheimer disease amyloid beta-peptide by metal-dependent up-regulation of metalloprotease activity. |
| 16649251 | 1 | No | Soluble nickel inhibits HIF-prolyl-hydroxylases creating persistent hypoxic signaling in A549 cells. |
| 16650406 | 2 | No | Inhibition of MHC class II gene expression in uveal melanoma cells is due to methylation of the CIITA gene or an upstream activator. |
| 16650473 | 32 | No | Alteration of steroidogenesis in H295R cells by organic sediment contaminants and relationships to other endocrine disrupting effects. |
| 16650747 | 4 | No | Conjugated linoleic acid enhances transepithelial calcium transport in human |
| 16652382 | 1 | No | Protein tyrosine phosphatase PTP1B is involved in neuroendocrine differentiation of prostate cancer. |
| 16671099 | 51 | No | Regulation of lipocalin-2 gene by the cancer chemopreventive retinoid 4-HPR. |
| 16674925 | 2 | No | Effects of capsaicin on P-gp function and expression in Caco-2 cells. |
| 16677282 | 4 | No | Norepinephrine stimulates calprotectin expression in human monocytic cells. |
| 16678543 | 2 | No | Genetically mediated interindividual variation in analgesic responses to cyclooxygenase inhibitory drugs. |
| 16678800 | 23 | No | Critical roles of AMP-activated protein kinase in the carcinogenic metal-induced expression of VEGF and HIF-1 proteins in DU145 prostate carcinoma. |
| 16680075 | 3 | No | Paullinia pinnata extracts rich in polyphenols promote vascular relaxation via endothelium-dependent mechanisms. |
| 16681930 | 8 | No | [Effects of norcantharidin on angiogenesis of human gallbladder carcinoma and its anti-angiogenic mechanisms] |
| 16682453 | 15 | No | Hypoxia-inducible factor-1-dependent and -independent regulation of insulin-like growth factor-1-stimulated vascular endothelial growth factor secretion. |
| 16685379 | 23 | No | Identification of genes linked to gefitinib treatment in prostate cancer cell lines with or without resistance to androgen: a clue to application of gefitinib |
| 16685529 | 5 | No | Thalidomide and celecoxib as potential modulators of irinotecan's activity in cancer patients. |
| 16687389 | 3 | No | Toll-like receptor priming sensitizes macrophages to proinflammatory cytokine gene induction by deoxynivalenol and other toxicants. |

| | | | |
|---|---|---|---|
| 16687628 | 3 | No | Transcriptional regulation of nephrin gene by peroxisome proliferator-activated receptor-gamma agonist: molecular mechanism of the antiproteinuric effect of |
| 16688769 | 20 | No | Role of all-trans retinoic acid in neurite outgrowth and axonal elongation. |
| 16690736 | 2 | No | Predictors of vitamin B6 and folate concentrations in older persons: the InCHIANTI study. |
| 16690804 | 18 | No | Estrogen regulation of the glucuronidation enzyme UGT2B15 in estrogen receptor-positive breast cancer cells. |
| 16691199 | 2 | No | The modulation of aromatase and estrogen receptor alpha in cultured human dermal papilla cells by dexamethasone: a novel mechanism for selective action of |
| 16691315 | 2 | No | Garlic compound, diallyl disulfide induces cell cycle arrest in prostate cancer cell line PC-3. |
| 16696175 | 9 | No | Coumestrol, bisphenol-A, DDT, and TCDD modulation of interleukin-2 expression in activated CD+4 Jurkat T cells. |
| 16699180 | 2 | No | The adenomatous polyposis coli tumor suppressor gene regulates expression of cyclooxygenase-2 by a mechanism that involves retinoic acid. |
| 16701883 | 1 | No | Aberrant DNA demethylation in promoter region and aberrant expression of mRNA of PAX4 gene in hematologic malignancies. |
| 16702388 | 12 | No | A phase II study with antioxidants, both in the diet and supplemented, pharmaconutritional support, progestagen, and anti-cyclooxygenase-2 showing |
| 16704987 | 123 | No | Arachidonic acid-induced gene expression in colon cancer cells. |
| 16705456 | 54 | No | Distinct effects of different concentrations of sodium selenite on apoptosis, cell cycle, and gene expression profile in acute promyeloytic leukemia-derived |
| 16705669 | 12 | No | Modulation of different stress pathways after styrene and styrene-7,8-oxide exposure in HepG2 cell line and normal human hepatocytes. |
| 16705698 | 24 | No | Doxorubicin induces cell senescence preferentially over apoptosis in the FU-SY-1 synovial sarcoma cell line. |
| 16705744 | 149 | No | Changes in gene expressions elicited by physiological concentrations of genistein on human endometrial cancer cells. |
| 16705839 | 6 | No | Cytotoxicity and expression of c-fos, HSP70, and GADD45/153 proteins in human liver carcinoma (HepG2) cells exposed to dinitrotoluenes. |
| 16707021 | 2 | No | Downregulation of survivin expression and concomitant induction of apoptosis by celecoxib and its non-cyclooxygenase-2-inhibitory analog, dimethyl-celecoxib |
| 16707465 | 6 | No | c-Jun NH(2)-terminal kinase signaling axis regulates diallyl trisulfide-induced generation of reactive oxygen species and cell cycle arrest in human prostate |

| | | | |
|---|---|---|---|
| 16709241 | 65 | No | Cellular response to 5-fluorouracil (5-FU) in 5-FU-resistant colon cancer cell lines during treatment and recovery. |
| 16712799 | 1 | No | Leucovorin-induced resistance against FDH growth suppressor effects occurs through DHFR up-regulation. |
| 16712844 | 12 | No | CYP4A11 is repressed by retinoic acid in human liver cells. |
| 16712875 | 6 | No | Differential regulation of CC chemokine receptors by 9-cis retinoic acid in the human mast cell line, HMC-1. |
| 16713074 | 2 | No | Arsenic inhibits induction of cytochrome P450 1A1 by 2,3,7,8-tetrachlorodibenzo-p-dioxin in human hepatoma cells. |
| 16713233 | 6 | No | Curcumin induces changes in expression of genes involved in cholesterol homeostasis. |
| 16713974 | 6 | No | IFN-gamma suppresses IL-10 production and synergizes with TLR2 by regulating GSK3 and CREB/AP-1 proteins. |
| 16719502 | 7 | No | Water-soluble genistin glycoside isoflavones up-regulate antioxidant metallothionein expression and scavenge free radicals. |
| 16720286 | 7 | No | Comparing the effect of ATRA, 4-HPR, and CD437 in bladder cancer cells. |
| 16720314 | 1 | No | Growth inhibitory effects of quercetin on bladder cancer cell. |
| 16728583 | 4 | No | Folic acid and its metabolites modulate IGF-I receptor gene expression in colon cancer cells in a p53-dependent manner. |
| 16729991 | 5 | No | Phenotypic anchoring of arsenic and cadmium toxicity in three hepatic-related cell systems reveals compound- and cell-specific selective up-regulation of |
| 16730335 | 1 | No | Effects of short- and long-term risperidone treatment on prolactin levels in children with autism. |
| 16730657 | 2 | No | Unliganded estrogen receptor-alpha activates transcription of the mammary gland Na+/I- symporter gene. |
| 16730733 | 13 | No | Unsaturated fatty acids suppress the expression of the ATP-binding cassette transporter G1 (ABCG1) and ABCA1 genes via an LXR/RXR responsive element. |
| 16730829 | 1 | No | Specific activation of the human HSP70 promoter by copper sulfate in mosaic transgenic zebrafish. |
| 16731751 | 1 | No | Exisulind and guanylyl cyclase C induce distinct antineoplastic signaling mechanisms in human colon cancer cells. |
| 16731767 | 5 | No | Resveratrol-caused apoptosis of human prostate carcinoma LNCaP cells is mediated via modulation of phosphatidylinositol 3'-kinase/Akt pathway and Bcl-2 family |

| | | | |
|---|---|---|---|
| 16735606 | 9 | No | Hexachlorophene inhibits Wnt/beta-catenin pathway by promoting Siah-mediated beta-catenin degradation. |
| 16737972 | 8 | No | Alcohol metabolism-mediated oxidative stress down-regulates hepcidin transcription and leads to increased duodenal iron transporter expression. |
| 16738222 | 8 | No | Negative regulation of superoxide dismutase-1 promoter by thyroid hormone. |
| 16738803 | 3 | No | Inhibition of caspase-dependent mitochondrial permeability transition protects airway epithelial cells against mustard-induced apoptosis. |
| 16739346 | 9 | No | Effects of the antiestrogens tamoxifen and raloxifene on the estrogen receptor transactivation machinery. |
| 16740359 | 1 | No | Retinoic acid attenuates promyelocytic leukemia protein-induced cell death in breast cancer cells by activation of the ubiquitin-proteasome pathway. |
| 16740653 | 3 | No | Mullerian inhibiting substance regulates androgen-induced gene expression and growth in prostate cancer cells through a nuclear factor-kappaB-dependent |
| 16740727 | 14 | No | Synergistic induction of folate receptor beta by all-trans retinoic acid and histone deacetylase inhibitors in acute myelogenous leukemia cells: mechanism and |
| 16740773 | 8 | No | Sulindac sulfide and exisulind inhibit expression of the estrogen and progesterone receptors in human breast cancer cells. |
| 16749867 | 4 | No | HPMA copolymer-bound doxorubicin induces apoptosis in ovarian carcinoma cells by the disruption of mitochondrial function. |
| 16750269 | 1 | No | Plasticity of Ly49g expression is due to epigenetics. |
| 16751804 | 94 | No | Identification of genes targeted by the androgen and PKA signaling pathways in prostate cancer cells. |
| 16752155 | 4 | No | Retinoic acid elicits cytostatic, cytotoxic and immunomodulatory effects on uveal melanoma cells. |
| 16757081 | 9 | No | Diazinon, chlorpyrifos and parathion are metabolised by multiple cytochromes P450 in human liver. |
| 16757381 | 2 | No | Retinoic acids and trichostatin A (TSA), a histone deacetylase inhibitor, induce human pyruvate dehydrogenase kinase 4 (PDK4) gene expression. |
| 16757516 | 37 | No | Enhancement of tumor invasion depends on transdifferentiation of skin fibroblasts mediated by reactive oxygen species. |
| 16757814 | 15 | No | Phytoestrogen alpha-zearalanol antagonizes homocysteine-induced imbalance of nitric oxide/endothelin-1 and apoptosis in human umbilical vein endothelial |
| 16758764 | 2 | No | Alteration of gene expression in human cells treated with the agricultural chemical diazinon: possible interaction in fetal development. |

| | | | |
|---|---|---|---|
| 16759640 | 4 | No | Regulation of proliferation and gene expression in cultured human aortic smooth muscle cells by resveratrol and standardized grape extracts. |
| 16759981 | 1 | No | Chronic arsenic exposure and oxidative stress: OGG1 expression and arsenic exposure, nail selenium, and skin hyperkeratosis in Inner Mongolia. |
| 16760464 | 4 | No | Iron regulation and the cell cycle: identification of an iron-responsive element in the 3'-untranslated region of human cell division cycle 14A mRNA by a refined |
| 16760641 | 1 | No | 9-cis retinoic acid induces insulin-like growth factor binding protein-3 through DR-8 retinoic acid responsive elements. |
| 16761963 | 9 | No | Resveratrol inhibits proliferation of human epidermoid carcinoma A431 cells by modulating MEK1 and AP-1 signalling pathways. |
| 16763222 | 3 | No | Elevated inorganic phosphate stimulates Akt-ERK1/2-Mnk1 signaling in human lung cells. |
| 16763620 | 2 | No | Regulation of myofibroblast transdifferentiation by DNA methylation and MeCP2: implications for wound healing and fibrogenesis. |
| 16763719 | 2 | No | Effects of 9-cis retinoic acid on human homeobox gene NKX3.1 expression in prostate cancer cell line LNCaP. |
| 16764927 | 2 | No | beta2 Integrins are characteristically absent in acute promyelocytic leukemia and rapidly upregulated in vivo upon differentiation with all-trans retinoic acid. |
| 16765349 | 1 | No | Increased p21 expression and complex formation with cyclin E/CDK2 in retinoid-induced pre-B lymphoma cell apoptosis. |
| 16766008 | 25 | No | Enhancement of caffeic acid phenethyl ester on all-trans retinoic acid-induced differentiation in human leukemia HL-60 cells. |
| 16767377 | 2 | No | Induction of connective tissue growth factor in retinal pigment epithelium cells by oxidative stress. |
| 16769766 | 34 | No | Tripterine inhibits the expression of adhesion molecules in activated endothelial cells. |
| 16772442 | 4 | No | Salt dual-fortified with iodine and micronized ground ferric pyrophosphate affects iron status but not hemoglobin in children in Cote d'Ivoire. |
| 16775175 | 1 | No | Effects of raloxifene on circulating prolactin and estradiol levels in premenopausal women at high risk for developing breast cancer. |
| 16775626 | 6 | No | Hypoxia upregulates hypoxia inducible factor (HIF)-3alpha expression in lung epithelial cells: characterization and comparison with HIF-1alpha. |
| 16775837 | 6 | No | Chromium (VI) inhibits heme oxygenase-1 expression in vivo and in arsenic-exposed human airway epithelial cells. |
| 16777101 | 1 | No | Silencing of the human microsomal glucose-6-phosphate translocase induces glioma cell death: potential new anticancer target for curcumin. |

| | | | |
|---|---|---|---|
| 16777994 | 2 | No | Silibinin activates p53-caspase 2 pathway and causes caspase-mediated cleavage of Cip1/p21 in apoptosis induction in bladder transitional-cell papilloma RT4 cells: |
| 16778083 | 5 | No | Interplay between epidermal growth factor receptor and Janus kinase 3 regulates polychlorinated biphenyl-induced matrix metalloproteinase-3 expression and |
| 16778211 | 2 | No | Apoptosis induction by retinoids in eosinophilic leukemia cells: implication of retinoic acid receptor-alpha signaling in all-trans-retinoic acid |
| 16778962 | 2 | No | [Mechanism of tetra-arsenic tetra-sulfide in inducing apoptosis of acute promyelocytic leukemia cells] |
| 16780807 | 4 | No | Ras inhibition results in growth arrest and death of androgen-dependent and androgen-independent prostate cancer cells. |
| 16780908 | 252 | No | Gene expression signatures in CD34+-progenitor-derived dendritic cells exposed to the chemical contact allergen nickel sulfate. |
| 16782888 | 2 | No | Btg2 enhances retinoic acid-induced differentiation by modulating histone H4 methylation and acetylation. |
| 16783407 | 2 | No | Adenosine A(2A) receptors are expressed on rat and human hepatic stellate cell lines and adenosine A(2A) receptor occupancy promotes collagen production by |
| 16787719 | 1 | No | Raloxifene therapy interacts with serum osteoprotegerin in postmenopausal women. |
| 16790487 | 16 | No | The common insecticides cyfluthrin and chlorpyrifos alter the expression of a subset of genes with diverse functions in primary human astrocytes. |
| 16791854 | 4 | No | Involvement of the nitric oxide/protein kinase G pathway in polychlorinated biphenyl-induced cell death in SH-SY 5Y neuroblastoma cells. |
| 16793012 | 2 | No | Molecular and metabolic retinoid pathways in human amniotic membranes. |
| 16793964 | 1 | No | Serum adiponectin and leptin levels in relation to the metabolic syndrome, androgenic profile and somatotropic axis in healthy non-diabetic elderly men. |
| 16794257 | 12 | No | Quercetin blocks airway epithelial cell chemokine expression. |
| 16797527 | 28 | No | Anti-oxidant sensitivity of donor age-related gene expression in cultured fibroblasts. |
| 16797627 | 14 | No | Azidothymidine and cisplatin increase p14ARF expression in OVCAR-3 ovarian cancer cell line. |
| 16797915 | 4 | No | Flow cytometric cell cycle analysis allows for rapid screening of estrogenicity in MCF-7 breast cancer cells. |
| 16798617 | 5 | No | Hypoxia and ionizing radiation: changes in adhesive properties and cell adhesion molecule expression in MG-63 three-dimensional tumor spheroids. |

| | | | |
|---|---|---|---|
| 16798746 | 2 | No | Raloxifene increases proliferation and up-regulates telomerase activity in human umbilical vein endothelial cells. |
| 16799064 | 14 | No | Flavonoids protect human retinal pigment epithelial cells from oxidative-stress-induced death. |
| 16799634 | 2 | No | 5-Aza-2'-deoxycytidine-mediated reductions in G9A histone methyltransferase and histone H3 K9 di-methylation levels are linked to tumor suppressor gene |
| 16800735 | 3 | No | The human kallikrein 10 promoter contains a functional retinoid response element. |
| 16803524 | 84 | No | Analysis of the in vitro synergistic effect of 5-fluorouracil and cisplatin on cervical carcinoma cells. |
| 16803582 | 2 | No | Neisseria gonorrhoeae delays the onset of apoptosis in polymorphonuclear leukocytes. |
| 16804125 | 18 | No | Olive oil-based lipid emulsion's neutral effects on neutrophil functions and leukocyte-endothelial cell interactions. |
| 16804330 | 2 | No | Expression of IL-1beta, IL-1 receptor type I and IL-1 receptor antagonist in human aortic smooth muscle cells: effects of all-trans-retinoic acid. |
| 16806455 | 12 | No | Ni(II) activates the Nrf2 signaling pathway in human monocytic cells. |
| 16806904 | 5 | No | Dutasteride affects progesterone metabolizing enzyme activity/expression in human breast cell lines resulting in suppression of cell proliferation and detachment. |
| 16807381 | 5 | No | SP1 and SP3 mediate progesterone-dependent induction of the 17beta hydroxysteroid dehydrogenase type 2 gene in human endometrium. |
| 16809336 | 3 | No | Cyclooxygenase-2 induction by arsenite is through a nuclear factor of activated T-cell-dependent pathway and plays an antiapoptotic role in Beas-2B cells. |
| 16814109 | 6 | No | As(III) transcriptionally activates the gadd45a gene via the formation of H2O2. |
| 16814256 | 6 | No | Adiponectin protects human neuroblastoma SH-SY5Y cells against acetaldehyde-induced cytotoxicity. |
| 16814760 | 4 | No | Acridine Orange based platinum(II) complexes inducing cytotoxicity and cell cycle perturbation in spite of GSTP1 up-regulation. |
| 16815102 | 2 | No | Mitochondrial disease activates transcripts of the unfolded protein response and cell cycle and inhibits vesicular secretion and oligodendrocyte-specific |
| 16815347 | 2 | No | Human liver slices as an in vitro model to study toxicity-induced hepatic stellate cell activation in a multicellular milieu. |
| 16815550 | 1 | No | 1-BP inhibits NF-kappaB activity and Bcl-xL expression in astrocytes in vitro and reduces Bcl-xL expression in the brains of rats in vivo. |

| | | | |
|---|---|---|---|
| 16816107 | 9 | No | Aspirin regulates expression and function of scavenger receptor-BI in macrophages: studies in primary human macrophages and in mice. |
| 16817226 | 3 | No | Transcriptional regulation of the homeobox gene NKX3.1 by all-trans retinoic acid in prostate cancer cells. |
| 16818652 | 3 | No | Role of the p38 mitogen-activated protein kinase pathway in the generation of arsenic trioxide-dependent cellular responses. |
| 16819095 | 3 | No | Ozone enhances diesel exhaust particles (DEP)-induced interleukin-8 (IL-8) gene expression in human airway epithelial cells through activation of nuclear |
| 16819191 | 7 | No | Curcumin decreases binding of Shiga-like toxin-1B on human intestinal epithelial cell line HT29 stimulated with TNF-alpha and IL-1beta: suppression of p38, JNK |
| 16819192 | 4 | No | Turmeric and curcumin modulate the conjugation of 1-naphthol in Caco-2 cells. |
| 16823088 | 162 | No | Microarray analysis of mercury-induced changes in gene expression in human liver carcinoma (HepG2) cells: importance in immune responses. |
| 16823509 | 54 | No | Differential effects of omega-3 and omega-6 Fatty acids on gene expression in breast cancer cells. |
| 16825320 | 5 | No | Growth inhibitory concentrations of androgens up-regulate insulin-like growth factor binding protein-3 expression via an androgen response element in LNCaP |
| 16826196 | 22 | No | Coupling mitochondrial respiratory chain to cell death: an essential role of mitochondrial complex I in the interferon-beta and retinoic acid-induced cancer |
| 16826403 | 1 | No | Mode of interaction between butyroyloxymethyl-diethyl phosphate (AN-7) and doxorubicin in MCF-7 and resistant MCF-7/Dx cell lines. |
| 16827160 | 5 | No | Comparative effects of hypoxia on normal and immortalized human diploid fibroblasts. |
| 16827180 | 6 | No | Metallothionein 1 isoform gene expression induced by cadmium in human peripheral blood lymphocytes. |
| 16828922 | 1 | No | The reduced expression and aberrant methylation of p16(INK4a) in chromate workers with lung cancer. |
| 16829687 | 1 | No | Potent protective effect of isoimperatorin against aflatoxin B1-inducible cytotoxicity in H4IIE cells: bifunctional effects on glutathione S-transferase |
| 16832676 | 3 | No | PML-RARalpha and AML1-ETO translocations are rarely associated with methylation of the RARbeta2 promoter. |
| 16835338 | 49 | No | Gene expression profiles in peripheral lymphocytes by arsenic exposure and skin lesion status in a Bangladeshi population. |
| 16835749 | 34 | No | The nicotinic receptor antagonists abolish pathobiologic effects of tobacco-derived nitrosamines on BEP2D cells. |

| | | | |
|---|---|---|---|
| 16837568 | 24 | No | Use of immortalized human hepatocytes to predict the magnitude of clinical drug-drug interactions caused by CYP3A4 induction. |
| 16839579 | 2 | No | Nuclear type II [3H]estradiol binding site ligands: inhibition of ER-positive and ER-negative cell proliferation and c-Myc and cyclin D1 gene expression. |
| 16842799 | 4 | No | Circulating chemoattractants RANTES, negatively related to endogenous androgens, and MCP-1 are differentially suppressed by hormone therapy and raloxifene. |
| 16843435 | 3 | No | p38 MAPK downregulates phosphorylation of Bad in doxorubicin-induced endothelial apoptosis. |
| 16844113 | 1 | No | Hypoxia protects HepG2 cells against etoposide-induced apoptosis via a HIF-1-independent pathway. |
| 16845256 | 2 | No | Greater inhibitory effects of bivalirudin compared with unfractionated heparin plus eptifibitide on thrombin-induced platelet activation. |
| 16845898 | 5 | No | Folic acid inhibits homocysteine-induced superoxide anion production and nuclear factor kappa B activation in macrophages. |
| 16847355 | 31 | No | DNA microarray analysis of changes in gene expression induced by 1,25-dihydroxyvitamin D3 in human promyelocytic leukemia HL-60 cells. |
| 16847815 | 8 | No | Influence of bicalutamide with or without tamoxifen or anastrozole on insulin-like growth factor 1 and binding proteins in prostate cancer patients. |
| 16848764 | 6 | No | EGFR-mediated expression of aquaporin-3 is involved in human skin fibroblast migration. |
| 16849523 | 3 | No | An autocrine loop involving ret and glial cell-derived neurotrophic factor mediates retinoic acid-induced neuroblastoma cell differentiation. |
| 16849584 | 102 | No | Gene expression preferentially regulated by tamoxifen in breast cancer cells and correlations with clinical outcome. |
| 16849586 | 3 | No | Peroxisome proliferator-activated receptor gamma as a molecular target of resveratrol-induced modulation of polyamine metabolism. |
| 16854221 | 3 | No | HEX expression and localization in normal mammary gland and breast carcinoma. |
| 16854967 | 2 | No | Enhanced expression of metallothionein isoform 3 protein in tumor heterotransplants derived from As+3- and Cd+2-transformed human urothelial cells. |
| 16854989 | 6 | No | Retinoic acid regulates the expression of photoreceptor transcription factor NRL. |
| 16856976 | 2 | No | Clopidogrel increases expression of chemokines in peripheral blood mononuclear cells in patients with coronary artery disease: results of a double-blind |
| 16857225 | 6 | No | Reduction of glucocorticoid receptor ligand binding by the 11-beta hydroxysteroid dehydrogenase type 2 inhibitor, Thiram. |

| | | | |
|---|---|---|---|
| 16857237 | 8 | No | Antiandrogenic activity of pyrethroid pesticides and their metabolite in reporter gene assay. |
| 16857725 | 3 | No | Induction of human CYP2A6 is mediated by the pregnane X receptor with peroxisome proliferator-activated receptor-gamma coactivator 1alpha. |
| 16859120 | 2 | No | [CoCl2-induced chemotherapy resistance in SW480 cells and its mechanism] |
| 16859517 | 1 | No | Estrogenic microenvironment generated by organochlorine residues in adipose mammary tissue modulates biomarker expression in ERalpha-positive breast |
| 16860297 | 6 | No | Atmospheric photochemical transformations enhance 1,3-butadiene-induced inflammatory responses in human epithelial cells: The role of ozone and other |
| 16861886 | 1 | No | A two-hit mechanism for pre-mitotic arrest of cancer cell proliferation by a polyamide-alkylator conjugate. |
| 16864444 | 1 | No | Dracorhodin perchlorate induces apoptosis in HL-60 cells. |
| 16865256 | 5 | No | Histone deacetylase inhibitor, trichostatin A, increases the chemosensitivity of anticancer drugs in gastric cancer cell lines. |
| 16865672 | 38 | No | Molecular signatures of soy-derived phytochemicals in androgen-responsive prostate cancer cells: a comparison study using DNA microarray. |
| 16865689 | 4 | No | Identification of novel genes associated with astrocytoma progression using suppression subtractive hybridization and real-time reverse |
| 16867262 | 6 | No | Role of oxidative stress in the apoptosis of hepatocellular carcinoma induced by combination of arsenic trioxide and ascorbic acid. |
| 16868479 | 2 | No | Schedule-dependent therapeutic effects of gemcitabine combined with uracil-tegafur in a human pancreatic cancer xenograft model. |
| 16868541 | 4 | No | Phellinus linteus sensitises apoptosis induced by doxorubicin in prostate cancer. |
| 16870006 | 14 | No | Soya isoflavones suppress phorbol 12-myristate 13-acetate-induced COX-2 expression in MCF-7 cells. |
| 16870008 | 2 | No | The effect of docosahexaenoic acid and folic acid supplementation on placental apoptosis and proliferation. |
| 16870173 | 4 | No | Ni(II) affects ubiquitination of core histones H2B and H2A. |
| 16870260 | 125 | No | Cultured lymphocytes from autistic children and non-autistic siblings up-regulate heat shock protein RNA in response to thimerosal challenge. |
| 16872382 | 5 | No | Effect of all-trans-retinoic acid on the differentiation, maturation and functions of dendritic cells derived from cord blood monocytes. |

| | | | |
|---|---|---|---|
| 16872482 | 6 | No | Macrophage migration inhibitory factor: a mediator of matrix metalloproteinase-2 production in rheumatoid arthritis. |
| 16873375 | 3 | No | Androgen induction of prostate cancer cell invasion is mediated by ezrin. |
| 16873554 | 3 | No | Regulation of progranulin expression in myeloid cells. |
| 16874460 | 1 | No | CK2 phosphorylation of SAG at Thr10 regulates SAG stability, but not its E3 ligase activity. |
| 16875574 | 1 | No | [Phenol butyrate inhibits cell cycle of leukemia cell lines through up-regulation of p21WAF1/CIP1 gene] |
| 16877366 | 9 | No | Androgen receptor remains critical for cell-cycle progression in androgen-independent CWR22 prostate cancer cells. |
| 16877991 | 1 | No | Alanine amino transferase concentrations are linked to folate intakes and methylenetetrahydrofolate reductase polymorphism in obese adolescent girls. |
| 16879495 | 9 | No | Muscarinic cholinoceptor activation modulates DNA synthesis and CD40 expression in fibroblast cells. |
| 16880289 | 5 | No | Curcumin inhibits hypoxia-inducible factor-1 by degrading aryl hydrocarbon receptor nuclear translocator: a mechanism of tumor growth inhibition. |
| 16882163 | 19 | No | Regulation of cutaneous drug-metabolizing enzymes and cytoprotective gene expression by topical drugs in human skin in vivo. |
| 16882451 | 4 | No | Arsenic trioxide induces not only apoptosis but also autophagic cell death in leukemia cell lines via up-regulation of Beclin-1. |
| 16882708 | 1 | No | Results of a randomized study of 3 schedules of low-dose decitabine in higher-risk myelodysplastic syndrome and chronic myelomonocytic leukemia. |
| 16884364 | 1 | No | Arsenic trioxide exposure to ovarian carcinoma cells leads to decreased level of topoisomerase II and cytotoxicity. |
| 16884889 | 9 | No | Effect of selected insecticides on growth rate and stress protein expression in cultured human A549 and SH-SY5Y cells. |
| 16886220 | 1 | No | Osteoprecursor cell response to strontium-containing hydroxyapatite ceramics. |
| 16886892 | 54 | No | Gene signature of breast cancer cell lines treated with lycopene. |
| 16887905 | 12 | No | Epigenetic regulation of human trophoblastic cell migration and invasion. |
| 16888099 | 2 | No | Diminished proteasomal degradation results in accumulation of Gfi1 protein in monocytes. |

| | | | |
|---|---|---|---|
| 16888198 | 3 | No | Retinoic acid-induced human secretin gene expression in neuronal cells is mediated by cyclin-dependent kinase 1. |
| 16888681 | 3 | No | Dual androgen-response elements mediate androgen regulation of MMP-2 expression in prostate cancer cells. |
| 16888808 | 12 | No | Elucidating progesterone effects in breast cancer: cross talk with PDGF signaling pathway in smooth muscle cell. |
| 16890185 | 3 | No | Glutathione S-transferases as antioxidant enzymes: small cell lung cancer (H69) cells transfected with hGSTA1 resist doxorubicin-induced apoptosis. |
| 16891912 | 12 | No | Flavonoids from seabuckthorn protect endothelial cells (EA.hy926) from oxidized low-density lipoprotein induced injuries via regulation of LOX-1 and eNOS |
| 16892383 | 18 | No | Prenylflavonoids as nonsteroidal phytoestrogens and related structure-activity relationships. |
| 16894348 | 17 | No | Kinase-dependent, retinoic acid receptor-independent up-regulation of cyclooxygenase-2 by all-trans retinoic acid in human mesangial cells. |
| 16894629 | 37 | No | Morphological and molecular course of mitochondrial pathology in cultured human cells exposed long-term to Zidovudine. |
| 16895544 | 11 | No | tension remains unclear. 2. The present study shows that rats on a diet rich in either saturated or unsaturated fat had higher blood pressure compared with |
| 16898872 | 26 | No | Intrinsic cisplatin resistance in lung and ovarian cancer cells propagating in medium acutely depleted of folate. |
| 16900372 | 4 | No | Additive antitumor effect of concurrent treatment of 4-hydroxy tamoxifen with 5-fluorouracil but not with doxorubicin in estrogen receptor-positive breast |
| 16901463 | 6 | No | Resveratrol regulates the expression of LXR-alpha in human macrophages. |
| 16901965 | 5 | No | Estrogen regulates epithelial cell deformability by modulation of cortical actomyosin through phosphorylation of nonmuscle myosin heavy-chain II-B |
| 16903866 | 1 | No | Suppression of microtubule dynamics by benomyl decreases tension across kinetochore pairs and induces apoptosis in cancer cells. |
| 16904648 | 1 | No | Arsenic trioxide induces gallbladder carcinoma cell apoptosis via downregulation of Bcl-2. |
| 16906525 | 3 | No | Aluminum toxicity elicits a dysfunctional TCA cycle and succinate accumulation in hepatocytes. |
| 16908450 | 47 | No | Toluene diisocyanate (TDI) induces production of inflammatory cytokines and chemokines by bronchial epithelial cells via the epidermal growth factor receptor |
| 16909596 | 12 | No | [Mechanism of receptor for retinoids inducing apoptosis of human melanoma cell line A375] |

| | | | |
|---|---|---|---|
| 16912569 | 4 | No | Influence of phytoestrogens on the proliferation and expression of adhesion receptors in human mammary epithelial cells in vitro. |
| 16912660 | 3 | No | Effect of long-term treatment with raloxifene on mammary density in postmenopausal women. |
| 16914093 | 1 | No | Arsenic enhances the apoptosis induced by interferon gamma: key role of IRF-1. |
| 16914095 | 2 | No | Comparative effects of interferon-gamma and all- trans retinoic acid on secreted and surface-associated matrix metalloproteinase-9 expression of human monocytes. |
| 16914592 | 1 | No | Cetrorelix suppression test to assess the source of androgen overproduction in postmenopausal hirsutism. |
| 16918137 | 7 | No | Gene expression profile of multiple myeloma cell line treated by realgar. |
| 16918696 | 14 | No | Inhibition of monocytic differentiation by phosphorylation-deficient Stat1 is associated with impaired expression of Stat2, ICSBP/IRF8 and C/EBPepsilon. |
| 16920672 | 1 | No | Mechanism of fiber carcinogenesis: from reactive radical species to silencing of the beta igH3 gene. |
| 16920920 | 4 | No | All-trans retinoic acid stimulates IL-2-mediated proliferation of human T lymphocytes: early induction of cyclin D3. |
| 16921485 | 1 | No | The expression of metastasis suppressor MIM/MTSS1 is regulated by DNA methylation. |
| 16921510 | 23 | No | Restoring chemotherapy and hormone therapy sensitivity by parthenolide in a xenograft hormone refractory prostate cancer model. |
| 16922813 | 2 | No | binding to a nuclear receptor that interacts with retinoic acid-response elements (RARE) and then activates the mitogen-activated protein kinase signal pathway. |
| 16925584 | 8 | No | Estrogen-mediated post transcriptional down-regulation of P-glycoprotein in MDR1-transduced human breast cancer cells. |
| 16928304 | 1 | No | [Inhibited proliferation of B-lymphoma Raji cells and down-regulated expression of VEGF by arsenic trioxide] |
| 16928326 | 3 | No | [Hydroquinone inhibits NF-kappaB expression in human bone marrow stromal cells in vitro] |
| 16928824 | 5 | No | Resveratrol-induced cyclooxygenase-2 facilitates p53-dependent apoptosis in human breast cancer cells. |
| 16928829 | 35 | No | Inhibition of androgen receptor signaling by selenite and methylseleninic acid in prostate cancer cells: two distinct mechanisms of action. |
| 16929163 | 19 | No | Antagonistic interactions between gemcitabine and 5-fluorouracil in the human pancreatic carcinoma cell line Capan-2. |

| | | | |
|---|---|---|---|
| 16930294 | 1 | No | Involvement of the multidrug resistance P-glycoprotein in acetaminophen-induced toxicity in hepatoma-derived HepG2 and Hep3B cells. |
| 16930453 | 28 | No | Pharmacologic reductions of total tau levels; implications for the role of microtubule dynamics in regulating tau expression. |
| 16930873 | 6 | No | Toxic effect of zinc on NF-kappaB, IL-2, IL-2 receptor alpha, and TNF-alpha in HUT-78 (Th(0)) cells. |
| 16932349 | 1 | No | Jak3- and JNK-dependent vascular endothelial growth factor expression in cutaneous T-cell lymphoma. |
| 16934159 | 101 | No | Transcriptome profiling of human hepatocytes treated with Aroclor 1254 reveals transcription factor regulatory networks and clusters of regulated genes. |
| 16935404 | 1 | No | Calmodulin mediates sulfur mustard toxicity in human keratinocytes. |
| 16935849 | 1 | No | All trans-retinoic acid induces apoptosis via p38 and caspase pathways in |
| 16936753 | 1 | No | Rb2/p130 and protein phosphatase 2A: key mediators of ovarian carcinoma cell growth suppression by all-trans retinoic acid. |
| 16939895 | 7 | No | [Progesterone-modulated proteins in human endometrial cancer cell line Ishikawa] |
| 16940060 | 3 | No | In vitro cytotoxicity and mitochondrial toxicity of tenofovir alone and in combination with other antiretrovirals in human renal proximal tubule cells. |
| 16946128 | 6 | No | p53 mediates particulate matter-induced alveolar epithelial cell mitochondria-regulated apoptosis. |
| 16948474 | 2 | No | Induction of quinone reductase NQO1 by resveratrol in human K562 cells involves the antioxidant response element ARE and is accompanied by nuclear translocation |
| 16948901 | 2 | No | [Quercetin inhibits growth and induces apoptosis of human gastric carcinoma cells] |
| 16949795 | 4 | No | Androgen dependent regulation of protein kinase A subunits in prostate cancer cells. |
| 16950796 | 18 | No | Increased expression of the MGMT repair protein mediated by cysteine prodrugs and chemopreventative natural products in human lymphocytes and tumor cell lines. |
| 16951154 | 1 | No | Male germ cell-associated kinase, a male-specific kinase regulated by androgen, is a coactivator of androgen receptor in prostate cancer cells. |
| 16951191 | 182 | No | Agonist and antagonist of retinoic acid receptors cause similar changes in gene expression and induce senescence-like growth arrest in MCF-7 breast carcinoma |
| 16951200 | 4 | No | Fluorodeoxyuridine modulates cellular expression of the DNA base excision repair enzyme uracil-DNA glycosylase. |

| | | | |
|---|---|---|---|
| 16954067 | 1 | No | Modes of action and species-specific effects of di-(2-ethylhexyl)phthalate in the liver. |
| 16954206 | 1 | No | Germ cell nuclear factor is a repressor of CRIPTO-1 and CRIPTO-3. |
| 16954373 | 9 | No | Aberrant promoter methylation of the ABCG2 gene in renal carcinoma. |
| 16954431 | 17 | No | Enhancement of sodium/iodide symporter expression in thyroid and breast cancer. |
| 16954435 | 4 | No | Combination therapy for treating breast cancer using antiestrogen, ERA-923, and the mammalian target of rapamycin inhibitor, temsirolimus. |
| 16955220 | 6 | No | Growth inhibition and induction of apoptosis and differentiation of tanshinone IIA in human glioma cells. |
| 16957513 | 14 | No | Influence of antitumor drugs on the expression of Fas system in SW480 colon cancer cells. |
| 16959222 | 10 | No | Curcumin regulates signal transducer and activator of transcription (STAT) expression in K562 cells. |
| 16959797 | 33 | No | The antioxidant role of a reagent, 2',7'-dichlorodihydrofluorescin diacetate, detecting reactive-oxygen species and blocking the induction of heme oxygenase-1 |
| 16959971 | 9 | No | Characterization of cellular retinoid-binding proteins in human myometrium during pregnancy. |
| 16960172 | 2 | No | Vitamin A supplementation in children with poor vitamin A and iron status increases erythropoietin and hemoglobin concentrations without changing total |
| 16960373 | 1 | No | Retinoic acid improves a hybridoma culture in a fructose-based medium by up-regulation of fructose incorporation via retinoid nuclear receptors. |
| 16960866 | 3 | No | NF-kB inhibition as a strategy to enhance etoposide-induced apoptosis in K562 cell line. |
| 16962673 | 2 | No | The C-terminal CD47/IAP-binding domain of thrombospondin-1 prevents camptothecin- and doxorubicin-induced apoptosis in human thyroid carcinoma cells. |
| 16962711 | 37 | No | Fermented papaya preparation attenuates beta-amyloid precursor protein: beta-amyloid-mediated copper neurotoxicity in beta-amyloid precursor protein and |
| 16963248 | 60 | No | Dietary isoflavones differentially induce gene expression changes in lymphocytes from postmenopausal women who form equol as compared with those who do not. |
| 16963807 | 2 | No | Regulation of LDL receptor expression by the effect of curcumin on sterol regulatory element pathway. |
| 16963839 | 6 | No | Transcriptional activation of the carboxylesterase 2 gene by the p53 pathway. |

| | | | |
|---|---|---|---|
| 16964283 | 3 | No | RalA regulates vascular endothelial growth factor-C (VEGF-C) synthesis in prostate cancer cells during androgen ablation. |
| 16965912 | 8 | No | Phytoestrogens and their low dose combinations inhibit mRNA expression and activity of aromatase in human granulosa-luteal cells. |
| 16965913 | 3 | No | Isoflavone metabolites and their in vitro dual functions: they can act as an estrogenic agonist or antagonist depending on the estrogen concentration. |
| 16966095 | 35 | No | Exit from arsenite-induced mitotic arrest is p53 dependent. |
| 16966277 | 14 | No | Down-regulation of wt1 expression in leukemia cell lines as part of apoptotic effect in arsenic treatment using two compounds. |
| 16966607 | 3 | No | Crocidolite asbestos and SV40 are cocarcinogens in human mesothelial cells and in causing mesothelioma in hamsters. |
| 16968061 | 3 | No | Modulation of key elements of the Wnt pathway by apple polyphenols. |
| 16968065 | 5 | No | Impact of quercetin and EGCG on key elements of the Wnt pathway in human colon carcinoma cells. |
| 16968895 | 6 | No | Relationship of expression of aquaglyceroporin 9 with arsenic uptake and sensitivity in leukemia cells. |
| 16969495 | 6 | No | Epidermal growth factor induction of resistance to topoisomerase II toxins in human squamous carcinoma A431 cells. |
| 16972258 | 23 | No | N-(4-hydroxyphenyl)retinamide induces apoptosis in human retinal pigment epithelial cells: retinoic acid receptors regulate apoptosis, reactive oxygen |
| 16973179 | 5 | No | Identification of avarol derivatives as potential antipsoriatic drugs using an in vitro model for keratinocyte growth and differentiation. |
| 16973256 | 6 | No | A comparative study of the effect of raloxifene and gosereline on uterine leiomyoma volume changes and estrogen receptor, progesterone receptor, bcl-2 and |
| 16973359 | 3 | No | Inhibition of Tpl2 kinase and TNFalpha production with quinoline-3-carbonitriles for the treatment of rheumatoid arthritis. |
| 16973558 | 1 | No | Delayed biosynthesis of varicella-zoster virus glycoprotein C: upregulation by hexamethylene bisacetamide and retinoic acid treatment of infected cells. |
| 16973622 | 7 | No | Clioquinol, a Cu(II)/Zn(II) chelator, inhibits both ubiquitination and asparagine hydroxylation of hypoxia-inducible factor-1alpha, leading to expression of |
| 16973825 | 10 | No | Resveratrol attenuates TNF-alpha-induced activation of coronary arterial endothelial cells: role of NF-kappaB inhibition. |
| 16979140 | 2 | No | Inhibition of phosphatidylinositol 3-kinase-mediated glucose metabolism coincides with resveratrol-induced cell cycle arrest in human diffuse large B-cell |

| | | | |
|---|---|---|---|
| 16979867 | 1 | No | Aluminum toxicity triggers the nuclear translocation of HIF-1alpha and promotes anaerobiosis in hepatocytes. |
| 16979875 | 250 | No | Evidence for reprogramming global gene expression during zinc deficiency in the HUT-78 cell line. |
| 16980553 | 4 | No | Pleiotropic beta-agonist-promoted receptor conformations and signals independent of intrinsic activity. |
| 16982228 | 12 | No | Effects of transdermal and oral contraceptives on estrogen-sensitive hepatic proteins. |
| 16982755 | 3 | No | Epigenetic silencing of occludin promotes tumorigenic and metastatic properties of cancer cells via modulations of unique sets of apoptosis-associated genes. |
| 16982809 | 207 | No | PPARgamma controls CD1d expression by turning on retinoic acid synthesis in developing human dendritic cells. |
| 16984259 | 3 | No | MMP-21 is expressed by macrophages and fibroblasts in vivo and in culture. |
| 16984733 | 148 | No | Mechanisms of indomethacin-induced alterations in the choline phospholipid metabolism of breast cancer cells. |
| 16985168 | 59 | No | Differentiation-specific factors modulate epidermal CYP1-4 gene expression in human skin in response to retinoic acid and classic aryl hydrocarbon receptor |
| 16987005 | 2 | No | NAD(P)H oxidase activity of Nox4 in chondrocytes is both inducible and involved in collagenase expression. |
| 16987298 | 12 | No | The natural compound n-butylidenephthalide derived from Angelica sinensis inhibits malignant brain tumor growth in vitro and in vivo. |
| 16988133 | 1 | No | Vitamin A supplementation reduces the monocyte chemoattractant protein-1 intestinal immune response of Mexican children. |
| 16988136 | 1 | No | Milk folate secretion is not impaired during iron deficiency in humans. |
| 16990345 | 9 | No | Regulation of Cdx2 expression by promoter methylation, and effects of Cdx2 transfection on morphology and gene expression of human esophageal epithelial |
| 16990509 | 1 | No | Reactive oxygen species mediate caspase activation and apoptosis induced by lipoic acid in human lung epithelial cancer cells through Bcl-2 down-regulation. |
| 16996034 | 3 | No | Quercetin, a flavonoid, inhibits proliferation and increases osteogenic differentiation in human adipose stromal cells. |
| 16996129 | 81 | No | Inhibitors of the mevalonate pathway as potential therapeutic agents in multiple myeloma. |
| 16996168 | 2 | No | Stimulated release of calcitonin gene-related peptide from the human right atrium in patients with and without diabetes mellitus. |

| | | | |
|---|---|---|---|
| 16996190 | 6 | No | Modulation of aromatase activity and mRNA by various selected pesticides in the human choriocarcinoma JEG-3 cell line. |
| 16996685 | 8 | No | Combination of adenoviral vector-mediated neurotrophin-3 gene transfer and retinoic acid promotes adult bone marrow cells to differentiate into neuronal |
| 16997790 | 2 | No | PMA and doxorubicin decrease viability, MTT activity and expression of CD10 marker on NALM-1 leukemic cells. |
| 16998810 | 4 | No | SIRT1 interacts with p73 and suppresses p73-dependent transcriptional activity. |
| 17000667 | 38 | No | Curcumin inhibits neurotensin-mediated interleukin-8 production and migration of HCT116 human colon cancer cells. |
| 17000900 | 2 | No | Inhibitors of DNA methylation and histone deacetylation independently relieve AML1/ETO-mediated lysozyme repression. |
| 17002868 | 1 | No | GTP induces S-phase cell-cycle arrest and inhibits DNA synthesis in K562 cells but not in normal human peripheral lymphocytes. |
| 17003102 | 3 | No | Aryl hydrocarbon receptor activation impairs extracellular matrix remodeling during zebra fish fin regeneration. |
| 17003290 | 4 | No | The endocrine disrupting chemical, diethylhexyl phthalate, activates MDR1 gene expression in human colon cancer LS174T cells. |
| 17003459 | 3 | No | Sustained versus transient ERK1/2 signaling underlies the anti- and proapoptotic effects of oxidative stress in human RPE cells. |
| 17005224 | 2 | No | Induction of cyclin D1 by submicromolar concentrations of arsenite in human epidermal keratinocytes. |
| 17005281 | 7 | No | Mapping of the RXRalpha binding elements involved in retinoic acid induced transcriptional activation of the human SOX3 gene. |
| 17006453 | 2 | No | Identification of a mammalian mitochondrial porphyrin transporter. |
| 17007014 | 2 | No | Resveratrol engages selective apoptotic signals in gastric adenocarcinoma cells. |
| 17010073 | 13 | No | Effects of 17beta-estradiol, progesterone, synthetic progestins, tibolone, and raloxifene on vascular endothelial growth factor and Thrombospondin-1 messenger |
| 17010196 | 74 | No | Analysis of the prostate cancer cell line LNCaP transcriptome using a sequencing-by-synthesis approach. |
| 17010675 | 188 | No | Gene expression signature-based chemical genomic prediction identifies a novel class of HSP90 pathway modulators. |
| 17012224 | 28 | No | Identification of kaempferol as an inhibitor of cigarette smoke-induced activation of the aryl hydrocarbon receptor and cell transformation. |

| | | | |
|---|---|---|---|
| 17013896 | 1 | No | Inactivation of RASSF2A by promoter methylation correlates with lymph node metastasis in nasopharyngeal carcinoma. |
| 17015749 | 10 | No | Deguelin, an Akt inhibitor, suppresses IkappaBalpha kinase activation leading to suppression of NF-kappaB-regulated gene expression, potentiation of apoptosis, |
| 17016597 | 1 | No | The optimal schedule for 5-fluorouracil radiosensitization in colon cancer cell lines. |
| 17016690 | 1 | No | Hypermethylation of Cyclin D2 is associated with loss of mRNA expression and tumor development in prostate cancer. |
| 17017122 | 5 | No | PKCdelta alternatively spliced isoforms modulate cellular apoptosis in retinoic acid-induced differentiation of human NT2 cells and mouse embryonic stem cells. |
| 17018608 | 9 | No | Oxidative stress induces ADAM9 protein expression in human prostate cancer cells. |
| 17018655 | 32 | No | Regulation of A Disintegrin And Metalloproteinase with ThromboSpondin repeats-1 expression in human endometrial stromal cells by gonadal steroids involves |
| 17018880 | 41 | No | Cytotoxicity of nitric oxide is alleviated by zinc-mediated expression of antioxidant genes. |
| 17019711 | 10 | No | Epigenetic modulation of endogenous tumor suppressor expression in lung cancer xenografts suppresses tumorigenicity. |
| 17023940 | 1 | No | Intestinal absorption of vitamins. |
| 17024233 | 3 | No | Enhancement of adenoviral MDA-7-mediated cell killing in human lung cancer cells by geldanamycin and its 17-allyl- amino-17-demethoxy analogue. |
| 17024972 | 8 | No | Cholecalciferol (vitamin D3) and the retinoid N-(4-hydroxyphenyl)retinamide (4-HPR) are synergistic for chemoprevention of prostate cancer. |
| 17029595 | 6 | No | Microtubules are required for NF-kappaB nuclear translocation in neuroblastoma IMR-32 cells: modulation by zinc. |
| 17029634 | 12 | No | Estrogens, selective estrogen receptor modulators, and a selective estrogen receptor down-regulator inhibit endothelial production of tissue factor pathway |
| 17030193 | 33 | No | Acetaldehyde inhibits PPARgamma via H2O2-mediated c-Abl activation in human hepatic stellate cells. |
| 17030235 | 6 | No | Fungal zymosan induces leukotriene production by human mast cells through a dectin-1-dependent mechanism. |
| 17031849 | 1 | No | Apoptosis induced by all-trans retinoic acid in N-acetylglucosaminyltransferase V repressed human hepatocarcinoma cells is mediated through endoplasmic reticulum |
| 17032174 | 20 | No | Regulation of ceruloplasmin in human hepatic cells by redox active copper: identification of a novel AP-1 site in the ceruloplasmin gene. |

| | | | |
|---|---|---|---|
| 17033190 | 8 | No | Differential regulation of Toll-like receptor and CD14 pathways by retinoids and corticosteroids in human sebocytes. |
| 17033922 | 1 | No | Characterization of molecular and structural determinants of selective estrogen receptor downregulators. |
| 17034590 | 11 | No | Hypoxia induces expression of connective tissue growth factor in scleroderma skin fibroblasts. |
| 17034753 | 282 | No | Beta-carotene and apocarotenals promote retinoid signaling in BEAS-2B human bronchioepithelial cells. |
| 17034788 | 15 | No | CYP2E1 induced by ethanol causes oxidative stress, proteasome inhibition and cytokeratin aggresome (Mallory body-like) formation. |
| 17035597 | 8 | No | Down-regulation of inhibitor of apoptosis proteins by deguelin selectively induces apoptosis in breast cancer cells. |
| 17035600 | 3 | No | Pregnane X receptor-dependent induction of the CYP3A4 gene by o,p'-1,1,1,-trichloro-2,2-bis (p-chlorophenyl)ethane. |
| 17036391 | 2 | No | Polaprezinc protects human colon cells from oxidative injury induced by hydrogen peroxide: relevant to cytoprotective heat shock proteins. |
| 17039268 | 196 | No | Gene expression profiling of breast cancer cells in response to gemcitabine: NF-kappaB pathway activation as a potential mechanism of resistance. |
| 17041101 | 14 | No | Synthesis and biological analysis of new curcumin analogues bearing an enhanced potential for the medicinal treatment of cancer. |
| 17041912 | 2 | No | The effect of zoledronic acid incorporated in a poly(D,L-lactide) implant coating on osteoblasts in vitro. |
| 17042913 | 3 | No | selective serotonin re-uptake inhibitors (SSRI) inhibit 5-HT internalization. Therefore, the aim of the present study was to investigate the protective effect |
| 17043665 | 1 | No | DNA methylation-dependent silencing of CST6 in human breast cancer cell lines. |
| 17043766 | 5 | No | Signaling pathways mediating manganese-induced toxicity in human glioblastoma cells (u87). |
| 17044645 | 2 | No | Alterations of FHIT gene and P16 gene in nickel transformed human bronchial epithelial cells. |
| 17044934 | 1 | No | Resveratrol interferes with AKT activity and triggers apoptosis in human uterine cancer cells. |
| 17045167 | 36 | No | Effect of all-trans retinoic acid on sodium/iodide symporter expression, radioiodine uptake and gene expression profiles in a human anaplastic thyroid |
| 17045692 | 13 | No | Role of the Fas/FasL pathway in combination therapy with interferon-alpha and fluorouracil against hepatocellular carcinoma in vitro. |

| | | | |
|---|---|---|---|
| 17045927 | 20 | No | Mitochondrial reactive oxygen species and nitric oxide-mediated cancer cell apoptosis in 2-butylamino-2-demethoxyhypocrellin B photodynamic treatment. |
| 17045963 | 4 | No | Expression of the human ABCC6 gene is induced by retinoids through the retinoid X receptor. |
| 17046822 | 5 | No | CpG-B oligodeoxynucleotide promotes cell survival via up-regulation of Hsp70 to increase Bcl-xL and to decrease apoptosis-inducing factor translocation. |
| 17049120 | 6 | No | Resveratrol as a novel agent for treatment of multiple myeloma with matrix metalloproteinase inhibitory activity. |
| 17049495 | 22 | No | Elevated gadd153/chop expression during resveratrol-induced apoptosis in human colon cancer cells. |
| 17049657 | 2 | No | Promoter hypermethylation-mediated down-regulation of LATS1 and LATS2 in human astrocytoma. |
| 17050345 | 7 | No | mRNA induction and cytokine release of inflammatory mediators during in vitro exposure of human nasal respiratory epithelia to acetaldehyde. |
| 17050787 | 8 | No | Mechanisms involved in resveratrol-induced apoptosis and cell cycle arrest in prostate cancer-derived cell lines. |
| 17051425 | 6 | No | Lycopene and other carotenoids inhibit estrogenic activity of 17beta-estradiol and genistein in cancer cells. |
| 17051635 | 6 | No | Profilin 1 obtained by proteomic analysis in all-trans retinoic acid-treated hepatocarcinoma cell lines is involved in inhibition of cell proliferation and |
| 17052796 | 3 | No | Effects of selenium supplementation on expression of glutathione peroxidase isoforms in cultured human lung adenocarcinoma cell lines. |
| 17052855 | 2 | No | Resveratrol exerts its antiproliferative effect on HepG2 hepatocellular carcinoma cells, by inducing cell cycle arrest, and NOS activation. |
| 17053167 | 1 | No | Ozonized low density lipoprotein (ozLDL) inhibits NF-kappaB and IRAK-1-associated signaling. |
| 17053324 | 3 | No | Regulation of alpha-smooth muscle actin protein expression in adipose-derived stem cells. |
| 17054584 | 6 | No | Effect of indomethacin on Bfl-1, WISP-1 and proliferating cell nuclear antigen in colon cancer cell line HCT116 cells. |
| 17055343 | 11 | No | Resveratrol inhibits macrophage expression of EMMPRIN by activating PPARgamma. |
| 17056112 | 1 | No | Reversal of p15/INK4b hypermethylation in AML1/ETO-positive and -negative myeloid leukemia cell lines. |
| 17056233 | 3 | No | Inhibition of Fas expression by RNAi modulates 5-fluorouracil-induced apoptosis in HCT116 cells expressing wild-type p53. |

| | | | |
|---|---|---|---|
| 17056473 | 1 | No | Plasma-transforming growth factor-alpha expression in residents of an arseniasis area in Taiwan. |
| 17060630 | 1 | No | Complement activation by photooxidation products of A2E, a lipofuscin constituent of the retinal pigment epithelium. |
| 17064354 | 13 | No | Mitochondrial damage modulates alternative splicing in neuronal cells: implications for neurodegeneration. |
| 17064661 | 44 | No | 5-Aza-2'-deoxycytidine and depsipeptide synergistically induce expression of BIK (BCL2-interacting killer). |
| 17065238 | 11 | No | DNA methyltransferase inhibitors coordinately induce expression of the human reelin and glutamic acid decarboxylase 67 genes. |
| 17065600 | 11 | No | Phospholipase D1 as a key enzyme for decidualization in human endometrial stromal cells. |
| 17068819 | 1 | No | Discovery of deregulation of zinc homeostasis and its associated genes in esophageal squamous cell carcinoma using cDNA microarray. |
| 17069765 | 7 | No | Differentiation of human embryonic stem cells into smooth muscle cells in adherent monolayer culture. |
| 17069899 | 3 | No | Transcriptional regulation of the human Sia-alpha2,3-Gal-beta1,4-GlcNAc-R:alpha2,8-sialyltransferase (hST8Sia III) by |
| 17070308 | 1 | No | Antitumor effects of aminobisphosphonates on renal cell carcinoma cell lines. |
| 17070507 | 30 | No | Oxidative stress influences cholesterol efflux in THP-1 macrophages: role of ATP-binding cassette A1 and nuclear factors. |
| 17070997 | 134 | No | Rofecoxib modulates multiple gene expression pathways in a clinical model of acute inflammatory pain. |
| 17075824 | 2 | No | Estrogen signaling and disruption of androgen metabolism in acquired androgen-independence during cadmium carcinogenesis in human prostate epithelial |
| 17075855 | 2 | No | Functional characterization of an orphan nuclear receptor, Rev-ErbAalpha, in chondrocytes and its potential role in osteoarthritis. |
| 17076661 | 1 | No | TAp73 isoforms antagonize Notch signalling in SH-SY5Y neuroblastomas and in primary neurones. |
| 17077187 | 31 | No | Phytochemicals induce breast cancer resistance protein in Caco-2 cells and enhance the transport of benzo[a]pyrene-3-sulfate. |
| 17077328 | 9 | No | MEK blockade converts AML differentiating response to retinoids into extensive apoptosis. |
| 17077332 | 18 | No | Emodin and DHA potently increase arsenic trioxide interferon-alpha-induced cell death of HTLV-I-transformed cells by generation of reactive oxygen species and |

| | | | |
|---|---|---|---|
| 17078813 | 6 | No | Cr(VI)-stimulated STAT3 tyrosine phosphorylation and nuclear translocation in human airway epithelial cells requires Lck. |
| 17079232 | 6 | No | Doxorubicin down-regulates Kruppel-associated box domain-associated protein 1 sumoylation that relieves its transcription repression on p21WAF1/CIP1 in breast |
| 17079450 | 6 | No | A repressive epigenetic domino effect confers susceptibility to breast epithelial cell transformation: implications for predicting breast cancer risk. |
| 17079463 | 2 | No | Disulfiram, a clinically used anti-alcoholism drug and copper-binding agent, induces apoptotic cell death in breast cancer cultures and xenografts via |
| 17080404 | 29 | No | Steroidogenic gene expression in H295R cells and the human adrenal gland: adrenotoxic effects of lindane in vitro. |
| 17081986 | 3 | No | Role of SUMO-interacting motif in Daxx SUMO modification, subnuclear localization, and repression of sumoylated transcription factors. |
| 17082639 | 17 | No | A novel role of hypoxia-inducible factor in cobalt chloride- and hypoxia-mediated expression of IL-8 chemokine in human endothelial cells. |
| 17085092 | 39 | No | Neurotrophin-directed differentiation of human adult marrow stromal cells to dopaminergic-like neurons. |
| 17085670 | 1 | No | Schedule-dependent synergy between the heat shock protein 90 inhibitor 17-(dimethylaminoethylamino)-17-demethoxygeldanamycin and doxorubicin restores |
| 17086741 | 3 | No | Influence of quercetin and x-ray on collagen synthesis of cultured human keloid-derived fibroblasts. |
| 17088408 | 17 | No | Phytoestrogens regulate transcription and translation of vitamin D receptor in colon cancer cells. |
| 17088865 | 9 | No | Lovastatin protects human endothelial cells from the genotoxic and cytotoxic effects of the anticancer drugs doxorubicin and etoposide. |
| 17088986 | 4 | No | The up-regulation of type I interferon receptor gene plays a key role in hepatocellular carcinoma cells in the synergistic antiproliferative effect by |
| 17088997 | 5 | No | Resveratrol induces apoptosis in transformed follicular lymphoma OCI-LY8 cells: evidence for a novel mechanism involving inhibition of BCL6 signaling. |
| 17089011 | 3 | No | DNA topoisomerase IIalpha (TOP2A) inhibitors up-regulate fatty acid synthase gene expression in SK-Br3 breast cancer cells: in vitro evidence for a 'functional |
| 17092368 | 13 | No | Conjugated linoleic acid, unlike other unsaturated fatty acids, strongly induces glutathione synthesis without any lipoperoxidation. |
| 17093138 | 1 | No | Xenoestrogen exposure imprints expression of genes (Hoxa10) required for normal uterine development. |
| 17093179 | 58 | No | High-protein and high-carbohydrate breakfasts differentially change the transcriptome of human blood cells. |

| | | | |
|---|---|---|---|
| 17094431 | 6 | No | Comparative effects of DHEA and DHT on gene expression in human LNCaP prostate cancer cells. |
| 17096185 | 2 | No | Curcumin-induced apoptosis in human leukemia cell HL-60 is associated with inhibition of telomerase activity. |
| 17096884 | 8 | No | [Effect of cyclosporine A, raloxifene and their combination on the reversion of multidrug resistance of K562/A02 line] |
| 17097070 | 4 | No | Accumulation of gamma-globin mRNA and induction of irreversible erythroid differentiation after treatment of CML cell line K562 with new doxorubicin |
| 17097691 | 7 | No | Involvement of protein kinase Cdelta in iron chelator-induced IL-8 production in human intestinal epithelial cells. |
| 17098216 | 2 | No | Effect of chromium(VI) on the status of plasma lipid peroxidation and erythrocyte antioxidant enzymes in chromium plating workers. |
| 17098229 | 56 | No | Synergistic effects of retinoic acid and tamoxifen on human breast cancer cells: proteomic characterization. |
| 17103032 | 283 | No | Toxicogenomics of A375 human malignant melanoma cells treated with arbutin. |
| 17103373 | 36 | No | Effects of flavonoids on the expression of the pro-inflammatory response in human monocytes induced by ligation of the receptor for AGEs. |
| 17103461 | 1 | No | RIZ1 is epigenetically inactivated by promoter hypermethylation in thyroid carcinoma. |
| 17105841 | 24 | No | Effects of simvastatin and oral contraceptive agent on polycystic ovary syndrome: prospective, randomized, crossover trial. |
| 17107666 | 4 | No | All-trans retinoic acid negatively regulates cytotoxic activities of nature killer cell line 92. |
| 17107852 | 2 | No | Molecular epidemiologic evidence for diabetogenic effects of dioxin exposure in U.S. Air force veterans of the Vietnam war. |
| 17108117 | 4 | No | Oxidative stress induces premature senescence by stimulating caveolin-1 gene transcription through p38 mitogen-activated protein kinase/Sp1-mediated |
| 17109623 | 4 | No | Derivation of Motor Neurons from three Clonal Human Embryonic Stem Cell Lines. |
| 17109627 | 2 | No | Hormonal regulation of the Menkes and Wilson copper-transporting ATPases in human placental Jeg-3 cells. |
| 17110918 | 3 | No | Fenretinide-induced neuronal differentiation of ARPE-19 human retinal pigment epithelial cells is associated with the differential expression of Hsp70, 14-3-3, |
| 17112805 | 2 | No | Impact of P-glycoprotein on clopidogrel absorption. |

| | | | |
|---|---|---|---|
| 17116725 | 2 | No | Resveratrol induces cell death in colorectal cancer cells by a novel pathway involving lysosomal cathepsin D. |
| 17118196 | 38 | No | Direct and indirect effects of retinoic acid on human Th2 cytokine and chemokine expression by human T lymphocytes. |
| 17119257 | 4 | No | Progress of epidemiological and molecular epidemiological studies on benzene in China. |
| 17119383 | 2 | No | Impaired intestinal iron absorption in Crohn's disease correlates with disease activity and markers of inflammation. |
| 17120531 | 2 | No | Plasma lipid peroxidation and erythrocyte antioxidant enzymes status in workers exposed to cadmium. |
| 17121181 | 1 | No | Inhibitory effect of curcumin on MDR1 gene expression in patient leukemic cells. |
| 17121930 | 1 | No | Gallium-induced cell death in lymphoma: role of transferrin receptor cycling, involvement of Bax and the mitochondria, and effects of proteasome inhibition. |
| 17124180 | 2 | No | Molecular mechanisms of transactivation and doxorubicin-mediated repression of survivin gene in cancer cells. |
| 17125913 | 21 | No | Cadmium induces mitogenic signaling in breast cancer cell by an ERalpha-dependent mechanism. |
| 17127497 | 1 | No | Terbutaline inhibits corticotropin-releasing hormone (CRH) expression in human trophoblast cells. |
| 17131305 | 36 | No | 17beta-Estradiol differentially regulates androgen-responsive genes through estrogen receptor-beta- and extracellular-signal regulated kinase-dependent |
| 17131421 | 2 | No | RNA interference-mediated knockdown of alpha-synuclein protects human dopaminergic neuroblastoma cells from MPP(+) toxicity and reduces dopamine |
| 17133271 | 5 | No | Methylation of tumour suppressor genes APAF-1 and DAPK-1 and in vitro effects of demethylating agents in bladder and kidney cancer. |
| 17134824 | 3 | No | Establishment and characterization of cell lines from three human thyroid carcinomas: responses to all-trans-retinoic acid and mutations in the BRAF gene. |
| 17138558 | 9 | No | Translational control of glial glutamate transporter EAAT2 expression. |
| 17138778 | 4 | No | Regulation of catechol-O-methyltransferase expression in human myometrial cells. |
| 17138841 | 10 | No | Pioglitazone inhibits androgen production in NCI-H295R cells by regulating gene expression of CYP17 and HSD3B2. |
| 17138961 | 44 | No | Constitutive gene expression predisposes morphogen-mediated cell fate responses of NT2/D1 and 27X-1 human embryonal carcinoma cells. |

| | | | |
|---|---|---|---|
| 17142977 | 8 | No | Intestinal bacteria activate estrogenic effect of main constituents puerarin and daidzin of Pueraria thunbergiana. |
| 17143537 | 4 | No | Gonadotropin-releasing hormone agonists reduce the migratory and the invasive behavior of androgen-independent prostate cancer cells by interfering with the |
| 17145863 | 66 | No | Epigenetic silencing of novel tumor suppressors in malignant melanoma. |
| 17145897 | 10 | No | Effects of novel retinoic acid metabolism blocking agent (VN/14-1) on letrozole-insensitive breast cancer cells. |
| 17146438 | 2 | No | Role of epidermal growth factor receptor degradation in gemcitabine-mediated cytotoxicity. |
| 17147502 | 1 | No | Short communication: expression of human endogenous retrovirus-R gene links to differentiation of squamous cells. |
| 17148446 | 43 | No | Curcumin suppresses AP1 transcription factor-dependent differentiation and activates apoptosis in human epidermal keratinocytes. |
| 17148459 | 8 | No | Differential regulation of clusterin and its isoforms by androgens in prostate cells. |
| 17148581 | 5 | No | Epigenetic regulation of Wnt-signaling pathway in acute lymphoblastic leukemia. |
| 17150329 | 4 | No | Selenium and sulforaphane modify the expression of selenoenzymes in the human endothelial cell line EAhy926 and protect cells from oxidative damage. |
| 17151092 | 12 | No | Chemopreventive anti-inflammatory activities of curcumin and other phytochemicals mediated by MAP kinase phosphatase-5 in prostate cells. |
| 17152098 | 109 | No | Evaluation of an in vitro model of androgen ablation and identification of the androgen responsive proteome in LNCaP cells. |
| 17154495 | 3 | No | Identification of small molecule agonists of the orphan nuclear receptors liver receptor homolog-1 and steroidogenic factor-1. |
| 17155983 | 3 | No | S-allylcysteine, a water-soluble garlic derivative, suppresses the growth of a human androgen-independent prostate cancer xenograft, CWR22R, under in vivo |
| 17156779 | 7 | No | Haem carrier protein 1 (HCP1): Expression and functional studies in cultured cells. |
| 17157164 | 4 | No | The HSP90 inhibitor 17-AAG synergizes with doxorubicin and U0126 in anaplastic large cell lymphoma irrespective of ALK expression. |
| 17157857 | 8 | No | The new oral immunomodulating drug DiNAC induces brachial artery vasodilatation at rest and during hyperemia in hypercholesterolemic subjects, likely by a nitric |
| 17159502 | 5 | No | Anti-proliferative activity of fenretinide in human hepatoma cells in vitro and in vivo. |

| | | | |
|---|---|---|---|
| 17162254 | 2 | No | Correction of interleukin-2 gene expression by in vitro zinc addition to mononuclear cells from zinc-deficient human subjects: a specific test for zinc |
| 17162844 | 1 | No | [Testosterone's role in regulating expression of genes of several proliferation factors] |
| 17164311 | 3 | No | Protein synthesis inhibitors, in synergy with 5-azacytidine, restore sodium/iodide symporter gene expression in human thyroid adenoma cell line, |
| 17164350 | 8 | No | Resveratrol inhibits proliferation, induces apoptosis, and overcomes chemoresistance through down-regulation of STAT3 and nuclear |
| 17166419 | 9 | No | [Effects of vitamin C on apoptosis and proliferation inhibition of human peripheral blood mononuclear cells induced by deoxynivalenol in vitro] |
| 17166736 | 6 | No | Mycotoxins nivalenol and deoxynivalenol differentially modulate cytokine mRNA expression in Jurkat T cells. |
| 17169568 | 5 | No | Glutathione depletion in antioxidant defense of differentiated NT2-LHON cybrids. |
| 17170093 | 6 | No | Corticotropin-releasing hormone inhibits progesterone production in cultured human placental trophoblasts. |
| 17170094 | 9 | No | All-trans retinoic acid induces in vitro angiogenesis via retinoic acid receptor: possible involvement of paracrine effects of endogenous vascular endothelial |
| 17171638 | 30 | No | Curcumin-induced GADD153 upregulation: modulation by glutathione. |
| 17171646 | 2 | No | Proliferation of human breast cancer cells and anti-cancer action of doxorubicin and vinblastine are independent of PKC-alpha. |
| 17172411 | 11 | No | Histone deacetylase inhibitor enhances 5-fluorouracil cytotoxicity by down-regulating thymidylate synthase in human cancer cells. |
| 17172416 | 3 | No | Terbinafine inhibits endothelial cell migration through suppression of the Rho-mediated pathway. |
| 17172434 | 6 | No | Effects of the chemotherapeutic agent doxorubicin on the protein C anticoagulant pathway. |
| 17174135 | 3 | No | Pneumoproteins as markers of paraquat lung injury: a clinical case. |
| 17174366 | 13 | No | Thyroid hormone is a MAPK-dependent growth factor for thyroid cancer cells and is anti-apoptotic. |
| 17174997 | 4 | No | p53-Dependent but ATM-independent inhibition of DNA synthesis and G2 arrest in cadmium-treated human fibroblasts. |
| 17175104 | 6 | No | Acetaminophen selectively suppresses peripheral prostaglandin E2 release and increases COX-2 gene expression in a clinical model of acute inflammation. |

| | | | |
|---|---|---|---|
| 17177569 | 24 | No | Cytochrome P450 1A1 expression and activity in Caco-2 cells: modulation by apple juice extract and certain apple polyphenols. |
| 17178900 | 73 | No | Identification of biomarkers modulated by the rexinoid LGD1069 (bexarotene) in human breast cells using oligonucleotide arrays. |
| 17179735 | 3 | No | Biphasic pattern of exercise-induced proteinuria in sedentary and trained men. |
| 17181942 | 4 | No | [Differential proteomic expression in human liver cells stimulated by hydroquinone] |
| 17183730 | 1510 | No | A proof-of-principle study of epigenetic therapy added to neoadjuvant doxorubicin cyclophosphamide for locally advanced breast cancer. |
| 17184768 | 12 | No | The anti-inflammatory flavones quercetin and kaempferol cause inhibition of inducible nitric oxide synthase, cyclooxygenase-2 and reactive C-protein, and |
| 17185360 | 6 | No | Noncoding RNA synthesis and loss of Polycomb group repression accompanies the colinear activation of the human HOXA cluster. |
| 17185465 | 24 | No | Chromatin-modifying agents permit human hematopoietic stem cells to undergo multiple cell divisions while retaining their repopulating potential. |
| 17185614 | 17 | No | Tetradecylselenoacetic acid, a PPAR ligand with antioxidant, antiinflammatory, and hypolipidemic properties. |
| 17187171 | 4 | No | Analysis on the promoter region of human decidual prolactin gene in the progesterone-induced decidualization and cAMP-induced decidualization of human |
| 17189669 | 6 | No | Trichostatin A and 5-aza-2'-deoxycytidine switch S1P from an inhibitor to a stimulator of motility through epigenetic regulation of S1P receptors. |
| 17189957 | 3 | No | Androgen decreases osteoprotegerin expression in prostate cancer cells. |
| 17190377 | 8 | No | Influence of avosentan (SPP3OI) on the pharmacokinetics of a second generation oral contraceptive containing ethinylestradiol and levonorgestrel in healthy |
| 17191019 | 1 | No | Inhibitory effect of quercetin on tryptase and MCP-1 chemokine release, and histidine decarboxylase mRNA transcription by human mast cell-1 cell line. |
| 17191021 | 14 | No | Resveratrol, a polyphenolic phytostilbene, inhibits endothelial monocyte chemotactic protein-1 synthesis and secretion. |
| 17191106 | 3 | No | Inhibitory effect of quercetin on tryptase and interleukin-6 release, and histidine decarboxylase mRNA transcription by human mast cell-1 cell line. |
| 17191263 | 4 | No | Venlafaxine induces P-glycoprotein in human Caco-2 cells. |
| 17194187 | 13 | No | A genome-wide screen for promoter methylation in lung cancer identifies novel methylation markers for multiple malignancies. |

| | | | |
|---|---|---|---|
| 17195088 | 1 | No | Methylation and gene silencing of the Ras-related GTPase gene in lung and breast cancers. |
| 17195906 | 4 | No | Effect of combined therapy with low-dose 5-aza-2'-deoxycytidine and irinotecan on colon cancer cell line HCT-15. |
| 17196394 | 12 | No | Human oligodendrocytes derived from embryonic stem cells: Effect of noggin on phenotypic differentiation in vitro and on myelination in vivo. |
| 17196940 | 4 | No | The acetaminophen-derived bioactive N-acylphenolamine AM404 inhibits NFAT by targeting nuclear regulatory events. |
| 17197552 | 3 | No | Acrolein, a toxicant in cigarette smoke, causes oxidative damage and mitochondrial dysfunction in RPE cells: protection by (R)-alpha-lipoic acid. |
| 17198877 | 76 | No | Gene-expression profiling during curcumin-induced apoptosis reveals downregulation of CXCR4. |
| 17199430 | 2 | No | Thyroid hormone regulates endogenous amyloid-beta precursor protein gene expression and processing in both in vitro and in vivo models. |
| 17200013 | 2 | No | [Expression of S100A2 in secondarily radioresistant pancreatic cancer cells and significance thereof] |
| 17200146 | 2 | No | Expression and function of cystine/glutamate transporter in neutrophils. |
| 17200365 | 1 | No | Inhibition of breast cancer cell growth in vitro and in vivo: effect of restoration of Wwox expression. |
| 17200665 | 4 | No | Stimulation of apical and basolateral VEGF-A and VEGF-C secretion by oxidative stress in polarized retinal pigment epithelial cells. |
| 17202136 | 1 | No | A histidine-rich cluster mediates the ubiquitination and degradation of the human zinc transporter, hZIP4, and protects against zinc cytotoxicity. |
| 17202838 | 6 | No | p53 overexpression represses androgen-mediated induction of NKX3.1 in a prostate cancer cell line. |
| 17204703 | 1 | No | Androgen-dependent expression of the gastrin-releasing peptide receptor in human prostate tumor xenografts. |
| 17204746 | 6 | No | Bifunctional alkylating agent-induced p53 and nonclassical nuclear factor kappaB responses and cell death are altered by caffeic acid phenethyl ester: a potential |
| 17205524 | 11 | No | Differential effects of naturally occurring and synthetic organoselenium compounds on biomarkers in androgen responsive and androgen independent human |
| 17210444 | 9 | No | Modulation of pregnane X receptor- and electrophile responsive element-mediated gene expression by dietary polyphenolic compounds. |
| 17210701 | 17 | No | An organometallic protein kinase inhibitor pharmacologically activates p53 and induces apoptosis in human melanoma cells. |

| | | | |
|---|---|---|---|
| 17210717 | 4 | No | Inhibition of histone deacetylation does not block resilencing of p16 after 5-aza-2'-deoxycytidine treatment. |
| 17214612 | 8 | No | Amiodarone induces angiotensinogen gene expression in lung alveolar epithelial cells through activation protein-1. |
| 17214633 | 13 | No | Regulation of calprotectin expression by interleukin-1alpha and transforming growth factor-beta in human gingival keratinocytes. |
| 17216566 | 5 | No | The expression of retinoic acid receptors in lymph nodes of young children and the effect of all-trans-retinoic acid on the B cells from lymph nodes. |
| 17216584 | 21 | No | Role of Noxa in p53-independent fenretinide-induced apoptosis of neuroectodermal tumours. |
| 17218384 | 158 | No | Pharmacogenomic analysis of acute promyelocytic leukemia cells highlights CYP26 cytochrome metabolism in differential all-trans retinoic acid sensitivity. |
| 17218635 | 13 | No | Suberoylanilide hydroxamic acid (vorinostat) represses androgen receptor expression and acts synergistically with an androgen receptor antagonist to |
| 17219422 | 10 | No | Retinol decreases beta-catenin protein levels in retinoic acid-resistant colon cancer cell lines. |
| 17220163 | 3 | No | Concentration-dependent effects of a selective estrogen receptor modulator raloxifene on proliferation and apoptosis in human uterine leiomyoma cells |
| 17220478 | 1 | No | Proteomics analysis of the interactome of N-myc downstream regulated gene 1 and its interactions with the androgen response program in prostate cancer cells. |
| 17222674 | 55 | No | Selenomethionine induced transcriptional programs in human prostate cancer cells. |
| 17224139 | 2 | No | Disparity in the induction of glutathione depletion, ROS formation, poly(ADP-ribose) polymerase-1 activation, and apoptosis by quinonoid derivatives |
| 17225872 | 5 | No | Retinoic acid, GABA-ergic, and TGF-beta signaling systems are involved in human cleft palate fibroblast phenotype. |
| 17225922 | 1 | No | Solubility, uptake and biocompatibility of lutein and zeaxanthin delivered to cultured human retinal pigment epithelial cells in tween40 micelles. |
| 17229153 | 5 | No | All-trans-retinoic acid inhibits collapsin response mediator protein-2 transcriptional activity during SH-SY5Y neuroblastoma cell differentiation. |
| 17229443 | 1 | No | Apoptosis in sulfur mustard treated A549 cell cultures. |
| 17229644 | 53 | No | Induction of class II major histocompatibility complex expression in human multiple myeloma cells by retinoid. |
| 17230511 | 6 | No | The use of histone deacetylase inhibitor FK228 and DNA hypomethylation agent 5-azacytidine in human bladder cancer therapy. |

| | | | |
|---|---|---|---|
| 17233834 | 1 | No | Downregulation of RECK by promoter methylation correlates with lymph node metastasis in non-small cell lung cancer. |
| 17234736 | 1 | No | The C/EBPdelta tumor suppressor is silenced by hypermethylation in acute myeloid leukemia. |
| 17234770 | 7 | No | Suppression of mammary carcinoma cell growth by retinoic acid: the cell cycle control gene Btg2 is a direct target for retinoic acid receptor signaling. |
| 17237267 | 12 | No | Antitumor effect of the histone deacetylase inhibitor LAQ824 in combination with 13-cis-retinoic acid in human malignant melanoma. |
| 17237273 | 10 | No | The role of spermidine/spermine N1-acetyltransferase in determining response to chemotherapeutic agents in colorectal cancer cells. |
| 17237316 | 12 | No | Intake of fish oil, oleic acid, folic acid, and vitamins B-6 and E for 1 year decreases plasma C-reactive protein and reduces coronary heart disease risk |
| 17237823 | 11 | No | Interplay between the levels of estrogen and estrogen receptor controls the level of the granzyme inhibitor, proteinase inhibitor 9 and susceptibility to immune |
| 17241155 | 18 | No | Alcohol-induced blood-brain barrier dysfunction is mediated via inositol 1,4,5-triphosphate receptor (IP3R)-gated intracellular calcium release. |
| 17241391 | 2 | No | Adhesion to the extracellular matrix is positively regulated by retinoic acid in HepG2 cells. |
| 17244614 | 20 | No | Cadmium-responsive element of the human heme oxygenase-1 gene mediates heat shock factor 1-dependent transcriptional activation. |
| 17244623 | 6 | No | Epstein-Barr virus lytic infection induces retinoic acid-responsive genes through induction of a retinol-metabolizing enzyme, DHRS9. |
| 17253143 | 14 | No | Retinoic acid enhances the production of IL-10 while reducing the synthesis of IL-12 and TNF-alpha from LPS-stimulated monocytes/macrophages. |
| 17254854 | 36 | No | Effects of aromatase inhibitors on human osteoblast and osteoblast-like cells: a possible androgenic bone protective effects induced by exemestane. |
| 17255567 | 9 | No | Prostacyclin prevents pulmonary endothelial cell apoptosis induced by cigarette smoke. |
| 17257079 | 1 | No | Uptake of anti-anemic substance ferric-sorbitol-citrate by normal and malignant cells and its effects on expression of transferrin receptor 1 and ferritin. |
| 17257620 | 20 | No | Resveratrol inhibits pancreatic cancer cell proliferation through transcriptional induction of macrophage inhibitory cytokine-1. |
| 17258074 | 16 | No | An approach to elucidate potential mechanism of renal toxicity of arsenic trioxide. |
| 17259349 | 18 | No | Programmed cell death-4 tumor suppressor protein contributes to retinoic acid-induced terminal granulocytic differentiation of human myeloid leukemia |

| | | | |
|---|---|---|---|
| 17260099 | 2 | No | Downregulation of TSLC1 and DAL-1 expression occurs frequently in breast cancer. |
| 17261084 | 11 | No | Resveratrol modulates mRNA transcripts of genes related to redox metabolism and cell proliferation in non-small-cell lung carcinoma cells. |
| 17261132 | 2 | No | Effects of all-trans-retinoic on human gastric cancer cells BGC-823. |
| 17266178 | 18 | No | Phytoestrogens activate estrogen receptor beta1 and estrogenic responses in human breast and bone cancer cell lines. |
| 17266442 | 6 | No | Levels of cytokine in bronchoalveolar lavage (BAL) fluid in patients with pulmonary fibrosis due to sulfur mustard gas inhalation. |
| 17267090 | 1 | No | Intestinal transport and metabolism of acrylamide. |
| 17267227 | 12 | No | Synthesis and in vitro evaluation of targeted tetracycline derivatives: effects on inhibition of matrix metalloproteinases. |
| 17267411 | 2 | No | Dual role of DNA methylation inside and outside of CTCF-binding regions in the transcriptional regulation of the telomerase hTERT gene. |
| 17268063 | 18 | No | Modulation of the expression of bloom helicase by estrogenic agents. |
| 17270149 | 12 | No | Celecoxib inhibits the expression of survivin via the suppression of promoter activity in human colon cancer cells. |
| 17270371 | 8 | No | Possible inhibitory mechanism of Curcuma drugs on CYP3A4 in 1alpha,25 dihydroxyvitamin D3 treated Caco-2 cells. |
| 17272397 | 1 | No | Regulation of growth hormone signaling by selective estrogen receptor modulators occurs through suppression of protein tyrosine phosphatases. |
| 17273765 | 7 | No | The low-toxicity 9-cis UAB30 novel retinoid down-regulates the DNA methyltransferases and has anti-telomerase activity in human breast cancer cells. |
| 17273769 | 7 | No | Fenretinide up-regulates DR5/TRAIL-R2 expression via the induction of the transcription factor CHOP and combined treatment with fenretinide and TRAIL |
| 17273796 | 9 | No | Curcumin attenuates the expression of IL-1beta, IL-6, and TNF-alpha as well as cyclin E in TNF-alpha-treated HaCaT cells; NF-kappaB and MAPKs as potential |
| 17275315 | 1 | No | Synthesis, structure, and estrogenic activity of 4-amino-3-(2-methylbenzyl)coumarins on human breast carcinoma cells. |
| 17278969 | 2 | No | Activation of endothelial nitric oxide synthase by red wine polyphenols: impact of grape cultivars, growing area and the vinification process. |
| 17280689 | 2 | No | SLURP-1 and -2 in normal, immortalized and malignant oral keratinocytes. |

| | | | |
|---|---|---|---|
| 17283133 | 6 | No | Transforming growth factor-beta, estrogen, and progesterone converge on the regulation of p27Kip1 in the normal and malignant endometrium. |
| 17284363 | 2 | No | Gene amplification and expression in lung cancer cells with acquired paclitaxel resistance. |
| 17285121 | 3 | No | Caspase-dependent and -independent suppression of apoptosis by monoHER in Doxorubicin treated cells. |
| 17287390 | 6 | No | Resveratrol in human hepatoma HepG2 cells: metabolism and inducibility of detoxifying enzymes. |
| 17287498 | 5 | No | The inhalation anesthetic isoflurane induces a vicious cycle of apoptosis and amyloid beta-protein accumulation. |
| 17287894 | 3 | No | Schedule-dependent cytotoxicity of 5-fluorouracil and irinotecan in a colon cancer cell line. |
| 17289836 | 6 | No | Curcumin [1,7-bis(4-hydroxy-3-methoxyphenyl)-1-6-heptadine-3,5-dione; C21H20O6] sensitizes human prostate cancer cells to tumor necrosis factor-related |
| 17289903 | 2 | No | Flaxseed and its lignans inhibit estradiol-induced growth, angiogenesis, and secretion of vascular endothelial growth factor in human breast cancer xenografts |
| 17290406 | 14 | No | MAGE-A9 mRNA and protein expression in bladder cancer. |
| 17290611 | 1 | No | [Up-regulates the expression of maspin gene in prostate cancer cell line LNCaP] |
| 17291458 | 11 | No | Suppression of NF-kappaB activation by curcumin leads to inhibition of expression of cyclo-oxygenase-2 and matrix metalloproteinase-9 in human articular |
| 17292493 | 9 | No | TRPV6 mediates capsaicin-induced apoptosis in gastric cancer cells--Mechanisms behind a possible new "hot" cancer treatment. |
| 17292881 | 7 | No | JNK1 is required for sulindac-mediated inhibition of cell proliferation and induction of apoptosis in vitro and in vivo. |
| 17292933 | 43 | No | Differential protein expression of peroxiredoxin I and II by benzo(a)pyrene and quercetin treatment in 22Rv1 and PrEC prostate cell lines. |
| 17293063 | 18 | No | Changes in gene expression profiles in response to selenium supplementation among individuals with arsenic-induced pre-malignant skin lesions. |
| 17293108 | 3 | No | 1,25-Dihydroxyvitamin D3/VDR-mediated induction of FGF23 as well as transcriptional control of other bone anabolic and catabolic genes that |
| 17295091 | 8 | No | Heme oxygenase-1 and interleukin-11 are overexpressed in stress-induced premature senescence of human WI-38 fibroblasts induced by tert-butylhydroperoxide and |
| 17297452 | 46 | No | Gene expression and copy number profiling suggests the importance of allelic imbalance in 19p in asbestos-associated lung cancer. |

| | | | |
|---|---|---|---|
| 17303007 | 6 | No | Curcumin downregulates homeobox gene NKX3.1 in prostate cancer cell LNCaP. |
| 17303924 | 1 | No | The short-term effect of mustard gas on the serum immunoglobulin levels. |
| 17305640 | 1 | No | Effects of cisplatin, alpha-interferon, and 13-cis retinoic acid on the expression of Fas (CD95), intercellular adhesion molecule-1 (ICAM-1), and |
| 17306607 | 2 | No | Downregulation of connexin 43 in nasopharyngeal carcinoma cells is related to promoter methylation. |
| 17306764 | 2 | No | Retinoic acid activates human inducible nitric oxide synthase gene through binding of RARalpha/RXRalpha heterodimer to a novel retinoic acid response |
| 17306835 | 10 | No | Capsaicin induces the production of IL-6 in human upper respiratory epithelial cells. |
| 17307735 | 1 | No | Integration of the activation of the human hyaluronan synthase 2 gene promoter by common cofactors of the transcription factors retinoic acid receptor and nuclear |
| 17308104 | 2 | No | Clioquinol, a therapeutic agent for Alzheimer's disease, has proteasome-inhibitory, androgen receptor-suppressing, apoptosis-inducing, and |
| 17310985 | 1 | No | Id2 gene-targeted crosstalk between Wnt and retinoid signaling regulates proliferation in human keratinocytes. |
| 17311112 | 10 | No | The effect of indole-3-carbinol on the expression of CYP1A1, CYP1B1 and AhR genes and proliferation of MCF-7 cells. |
| 17311948 | 14 | No | Folate deficiency induces cell-specific changes in the steady-state transcript levels of genes involved in folate metabolism and 1-carbon transfer reactions in |
| 17318229 | 6 | No | Nucleophosmin acts as a novel AP2alpha-binding transcriptional corepressor during cell differentiation. |
| 17320279 | 3 | No | PKC inhibitor Go6976 induces mitosis and enhances doxorubicin-paclitaxel cytotoxicity in urinary bladder carcinoma cells. |
| 17320366 | 67 | No | Folate deficiency in normal human fibroblasts leads to altered expression of genes primarily linked to cell signaling, the cytoskeleton and extracellular |
| 17325745 | 3 | No | Androgen induces adaptation to oxidative stress in prostate cancer: implications for treatment with radiation therapy. |
| 17327447 | 4 | No | The small polyphenolic molecule kaempferol increases cellular energy expenditure and thyroid hormone activation. |
| 17329077 | 1 | No | Effects of trichlorfon on progesterone production in cultured human granulosa-lutein cells. |
| 17330099 | 76 | No | Discovery of epigenetically silenced genes in acute myeloid leukemias. |

| | | | |
|---|---|---|---|
| 17330233 | 2 | No | Thymidylate synthase and dihydropyrimidine dehydrogenase mRNA expression after administration of 5-fluorouracil to patients with colorectal cancer. |
| 17330857 | 9 | No | Histone deacetylase inhibitor trichostatin a potentiates doxorubicin-induced apoptosis by up-regulating PTEN expression. |
| 17331233 | 254 | No | Gene expression profiles in asbestos-exposed epithelial and mesothelial lung cell lines. |
| 17332266 | 32 | No | Transient receptor potential vanilloid 1 agonists cause endoplasmic reticulum stress and cell death in human lung cells. |
| 17332326 | 8 | No | Curcumin, a dietary component, has anticancer, chemosensitization, and radiosensitization effects by down-regulating the MDM2 oncogene through the |
| 17332617 | 1 | No | Enhanced expression of adenovirus-mediated sodium iodide symporter gene in MCF-7 breast cancer cells with retinoic acid treatment. |
| 17332930 | 10 | No | Involvement of Bcl-2 family members, phosphatidylinositol 3'-kinase/AKT and mitochondrial p53 in curcumin (diferulolylmethane)-induced apoptosis in prostate |
| 17334394 | 1 | No | SGNE1/7B2 is epigenetically altered and transcriptionally downregulated in human medulloblastomas. |
| 17337101 | 18 | No | Lycopene differentially induces quiescence and apoptosis in androgen-responsive and -independent prostate cancer cell lines. |
| 17339365 | 5 | No | Selenium sensitizes MCF-7 breast cancer cells to doxorubicin-induced apoptosis through modulation of phospho-Akt and its downstream substrates. |
| 17341418 | 22 | No | Indomethacin induces apoptosis in 786-O renal cell carcinoma cells by activating mitogen-activated protein kinases and AKT. |
| 17341627 | 5 | No | Retinoic acid and histone deacetylase inhibitor BML-210 inhibit proliferation of human cervical cancer HeLa cells. |
| 17342749 | 7 | No | Reactive oxygen species mediate androgen receptor- and serum starvation-elicited downstream signaling of ADAM9 expression in human prostate cancer cells. |
| 17344919 | 2 | No | Aberrant DNA methylation of the Src kinase Hck, but not of Lyn, in Philadelphia chromosome negative acute lymphocytic leukemia. |
| 17346688 | 5 | No | Sodium selenite inhibits interleukin-6-mediated androgen receptor activation in prostate cancer cells via upregulation of c-Jun. |
| 17349212 | 8 | No | Down-regulation of CD44 contributes to the differentiation of HL-60 cells induced by ATRA or HMBA. |
| 17351394 | 2 | No | Differential regulation of DNA repair protein Rad51 in human tumour cell lines exposed to doxorubicin. |
| 17352252 | 3 | No | (2E)-N,N-dibutyl-3-(4-hydroxy-3-methoxyphenyl)acrylamide induces apoptosis and cell cycle arrest in HL-60 cells. |

| | | | |
|---|---|---|---|
| 17353921 | 23 | No | Targeting homeostatic mechanisms of endoplasmic reticulum stress to increase susceptibility of cancer cells to fenretinide-induced apoptosis: the role of |
| 17355223 | 5 | No | PPARalpha and AP-2alpha regulate bombesin receptor subtype 3 expression in ozone-stressed bronchial epithelial cells. |
| 17355264 | 6 | No | Predictive value of GADD153, p21 and c-Jun for chemotherapy response in gastric cancer. |
| 17355822 | 1 | No | Icariine stimulates proliferation and differentiation of human osteoblasts by increasing production of bone morphogenetic protein 2. |
| 17355946 | 1 | No | 3,4-dichloropropionaniline suppresses normal macrophage function. |
| 17360047 | 4 | No | Arvanil and anandamide up-regulate CD36 expression in human peripheral blood mononuclear cells. |
| 17360324 | 10 | No | A genomic screen for activators of the antioxidant response element. |
| 17361223 | 3 | No | Lentiviral PU.1 overexpression restores differentiation in myeloid leukemic blasts. |
| 17363495 | 3 | No | Curcumin potentiates the apoptotic effects of chemotherapeutic agents and cytokines through down-regulation of nuclear factor-kappaB and nuclear |
| 17363696 | 6 | No | Acrolein induces cyclooxygenase-2 and prostaglandin production in human umbilical vein endothelial cells: roles of p38 MAP kinase. |
| 17364588 | 1 | No | Treatment with beta2-adrenoceptor agonist in vivo induces human clock gene, Per1, mRNA expression in peripheral blood. |
| 17364964 | 14 | No | The flavonoid quercetin induces hypoxia-inducible factor-1alpha (HIF-1alpha) and inhibits cell proliferation by depleting intracellular iron. |
| 17365036 | 1 | No | Change of heme oxygenase-1 expression in lung injury induced by chrysotile asbestos in vivo and in vitro. |
| 17366569 | 2 | No | Activation of transcription factor IL-6 (NF-IL-6) and nuclear factor-kappaB (NF-kappaB) by lipid ozonation products is crucial to interleukin-8 gene |
| 17374387 | 95 | No | Characterization of DNA reactive and non-DNA reactive anticancer drugs by gene expression profiling. |
| 17374662 | 81 | No | Soy isoflavones exert differential effects on androgen responsive genes in LNCaP human prostate cancer cells. |
| 17374706 | 4 | No | Effects of oral and transvaginal ethinyl estradiol on hemostatic factors and hepatic proteins in a randomized, crossover study. |
| 17377739 | 4 | No | A novel lipoxygenase inhibitor Nordy attenuates malignant human glioma cell responses to chemotactic and growth stimulating factors. |

| | | | |
|---|---|---|---|
| 17379067 | 1 | No | RTP801 is a novel retinoic acid-responsive gene associated with myeloid differentiation. |
| 17379280 | 10 | No | Polyphenols downregulate PAI-1 gene expression in cultured human coronary artery endothelial cells: molecular contributor to cardiovascular protection. |
| 17384146 | 2 | No | Epigenetic regulation of human alpha1d-adrenergic receptor gene expression: a role for DNA methylation in Sp1-dependent regulation. |
| 17384664 | 1 | No | The tumor suppressor Wnt inhibitory factor 1 is frequently methylated in nasopharyngeal and esophageal carcinomas. |
| 17387344 | 13 | No | Novel retinoic acid metabolism blocking agents have potent inhibitory activities on human breast cancer cells and tumour growth. |
| 17388968 | 24 | No | Effect of wine phenolics on cytokine-induced C-reactive protein expression. |
| 17389721 | 9 | No | Stress chaperones, mortalin, and pex19p mediate 5-aza-2' deoxycytidine-induced senescence of cancer cells by DNA methylation-independent pathway. |
| 17390030 | 11 | No | Inhibition of invasion and induction of apoptosis by selenium in human malignant brain tumour cells in vitro. |
| 17390037 | 2 | No | Expression of cyclin A in human leukemia cell line HL-60 following treatment with doxorubicin and etoposide: the potential involvement of cyclin A in apoptosis. |
| 17392484 | 10 | No | Retinoic acid regulates CD1d gene expression at the transcriptional level in human and rodent monocytic cells. |
| 17395010 | 2 | No | Antioxidants tiron and N-acetyl-L-cysteine differentially mediate apoptosis in melanoma cells via a reactive oxygen species-independent NF-kappaB pathway. |
| 17395694 | 19 | No | Multiple transcription factor elements collaborate with estrogen receptor alpha to activate an inducible estrogen response element in the NKG2E gene. |
| 17395767 | 7 | No | Beryllium induces premature senescence in human fibroblasts. |
| 17397030 | 1 | No | Frequent inactivation of SPARC by promoter hypermethylation in colon cancers. |
| 17399942 | 5 | No | Characterization of cell death events induced by anti-neoplastic drugs cisplatin, paclitaxel and 5-fluorouracil on human hepatoma cell lines: Possible mechanisms |
| 17399992 | 10 | No | UVB-induced IL-18 production in human keratinocyte cell line NCTC 2544 through NF-kappaB activation. |
| 17401462 | 21 | No | Anti-inflammatory agent indomethacin reduces invasion and alters metabolism in a human breast cancer cell line. |
| 17402216 | 1 | No | Serum Clara cell protein as an indicator of pulmonary impairment in occupational exposure at aluminum foundry. |

| | | | |
|---|---|---|---|
| 17403535 | 46 | No | Comparative gene expression analysis of a chronic myelogenous leukemia cell line resistant to cyclophosphamide using oligonucleotide arrays and response to |
| 17404015 | 10 | No | Breast cancer cells response to the antineoplastic agents cisplatin, carboplatin, and doxorubicin at the mRNA expression levels of distinct apoptosis-related |
| 17404069 | 4 | No | Resveratrol inhibits IL-1 beta-induced stimulation of caspase-3 and cleavage of PARP in human articular chondrocytes in vitro. |
| 17404077 | 7 | No | Beta-2-microglobulin is an androgen-regulated secreted protein elevated in serum of patients with advanced prostate cancer. |
| 17404688 | 766 | No | Progesterone regulation of implantation-related genes: new insights into the role of oestrogen. |
| 17408380 | 10 | No | Upregulation of LITAF mRNA expression upon exposure to TiAlV and polyethylene wear particles in THP-1 macrophages. |
| 17409426 | 18 | No | Nonsteroidal anti-inflammatory drugs induce colorectal cancer cell apoptosis by suppressing 14-3-3epsilon. |
| 17409433 | 6 | No | The aryl propionic acid R-flurbiprofen selectively induces p75NTR-dependent decreased survival of prostate tumor cells. |
| 17409444 | 1 | No | Enhanced expression of asparagine synthetase under glucose-deprived conditions protects pancreatic cancer cells from apoptosis induced by glucose deprivation |
| 17409696 | 11 | No | [Heavy metal responses of the human metallothionein isoform genes] |
| 17409708 | 10 | No | [Visualization and evaluation of the promoter activities of genes for stress-inducible proteins in response to environmental pollutants] |
| 17410615 | 7 | No | Combined treatment with Ad-hTRAIL and DTIC or SAHA is associated with increased mitochondrial-mediated apoptosis in human melanoma cell lines. |
| 17415525 | 9 | No | The anti-cancer compound Nordy inhibits CXCR4-mediated production of IL-8 and VEGF by malignant human glioma cells. |
| 17415710 | 1 | No | Epigenetic silencing of the candidate tumor suppressor gene PROX1 in sporadic breast cancer. |
| 17416481 | 5 | No | Effect of endoplasmic reticulum stress preconditioning on cytotoxicity of clinically relevant nephrotoxins in renal cell lines. |
| 17417771 | 24 | No | Histone deacetylase inhibitor, suberoylanilide hydroxamic acid (Vorinostat, SAHA) profoundly inhibits the growth of human pancreatic cancer cells. |
| 17418620 | 3 | No | The upregulation of metallothionein-1 expression in areca quid chewing-associated oral squamous cell carcinomas. |
| 17419000 | 4 | No | Mechanism of tert-butylhydroperoxide induced cytotoxicity in U-937 macrophages by alteration of mitochondrial function and generation of ROS. |

| | | | |
|---|---|---|---|
| 17419801 | 1 | No | DNA methylation regulates tissue-specific expression of Shank3. |
| 17420286 | 8 | No | Identification of heat shock protein 32 (Hsp32) as a novel survival factor and therapeutic target in neoplastic mast cells. |
| 17420779 | 3 | No | Differential effects of selective oestrogen receptor modulators (SERMs) tamoxifen, ospemifene and raloxifene on human osteoclasts in vitro. |
| 17426705 | 4 | No | In vivo inhibition of angiogenesis by sulphamoylated derivatives of 2-methoxyoestradiol. |
| 17431108 | 20 | No | The RET oncogene is a critical component of transcriptional programs associated with retinoic acid-induced differentiation in neuroblastoma. |
| 17431121 | 40 | No | Combined effects of retinoic acid and histone deacetylase inhibitors on human neuroblastoma SH-SY5Y cells. |
| 17431384 | 2 | No | Combination of cyclooxygenase-2 inhibitor and doxorubicin increases the growth inhibition and apoptosis in human hepatocellular carcinoma cells. |
| 17431504 | 2 | No | CaMKII regulates retinoic acid receptor transcriptional activity and the differentiation of myeloid leukemia cells. |
| 17435603 | 1 | No | Retinoic acid induces functional c-Ret tyrosine kinase in human neuroblastoma. |
| 17437195 | 1 | No | Impact of progesterone on cytokine-stimulated nuclear factor-kappaB signaling in HeLa cells. |
| 17437844 | 1 | No | Enhancement of death receptor 4 mediated apoptosis and cytotoxicity in renal cell carcinoma cells by subtoxic concentrations of doxorubicin. |
| 17438526 | 1 | No | Frequent methylation-associated silencing of a candidate tumor-suppressor, CRABP1, in esophageal squamous-cell carcinoma. |
| 17439724 | 2 | No | Nitrofen suppresses cell proliferation and promotes mitochondria-mediated apoptosis in type II pneumocytes. |
| 17440010 | 505 | No | Transcriptional profiling of testosterone-regulated genes in the skeletal muscle of human immunodeficiency virus-infected men experiencing weight loss. |
| 17442928 | 1 | No | Down-regulation of IL-7Ralpha expression in human T cells via DNA methylation. |
| 17443689 | 1 | No | Epigenetic inactivation of the metastasis suppressor RECK enhances invasion of human colon cancer cells. |
| 17445807 | 16 | No | Raloxifene increases proliferation of human endothelial cells in association with increased gene expression of cyclins A and B1. |
| 17447067 | 11 | No | A comparison of the effectiveness of selected non-steroidal anti-inflammatory drugs and their derivatives against cancer cells in vitro. |

| | | | |
|---|---|---|---|
| 17449203 | 3 | No | Curcumin activates human glutathione S-transferase P1 expression through antioxidant response element. |
| 17449538 | 12 | No | Inhibition of progesterone production in human luteinized granulosa cells treated with LXR agonists. |
| 17450233 | 2 | No | Parental smoking modifies the relation between genetic variation in tumor necrosis factor-alpha (TNF) and childhood asthma. |
| 17450800 | 7 | No | Analysis of stress responsive genes induced by single-walled carbon nanotubes in BJ Foreskin cells. |
| 17452250 | 1 | No | High-resolution analysis of 3p deletion in neuroblastoma and differential methylation of the SEMA3B tumor suppressor gene. |
| 17452981 | 1 | No | Hypermethylation of the nel-like 1 gene is a common and early event and is associated with poor prognosis in early-stage esophageal adenocarcinoma. |
| 17453147 | 3 | No | All-trans-retinoic acid induces cell growth arrest in a human medulloblastoma cell line. |
| 17454143 | 2 | No | [6]-Gingerol prevents UVB-induced ROS production and COX-2 expression in vitro and in vivo. |
| 17456367 | 16 | No | [Upstream signaling pathway of neutrophil elastase-induced mucous hypersecretion] |
| 17456573 | 2 | No | Raised serum, adipocyte, and adipose tissue retinol-binding protein 4 in overweight women with polycystic ovary syndrome: effects of gonadal and adrenal |
| 17457038 | 1 | No | Mitochondrial DNA deletions and chloramphenicol treatment stimulate the autophagic transcript ATG12. |
| 17459161 | 60 | No | Genomic analysis of human lung fibroblasts exposed to vanadium pentoxide to identify candidate genes for occupational bronchitis. |
| 17459229 | 7 | No | [Nordy inhibits cell proliferation and angiogenic factor production of malignant human glioma cells mediated by formylpeptide receptor] |
| 17460065 | 1 | No | Neuregulin1 (NRG1) signaling through Fyn modulates NMDA receptor phosphorylation: differential synaptic function in NRG1+/- knock-outs compared with wild-type |
| 17460731 | 9 | No | Post-transcriptional regulation of melanin biosynthetic enzymes by cAMP and resveratrol in human melanocytes. |
| 17461445 | 7 | No | Rosiglitazone enhances fluorouracil-induced apoptosis of HT-29 cells by activating peroxisome proliferator-activated receptor gamma. |
| 17462537 | 3 | No | Action of Nrf2 and Keap1 in ARE-mediated NQO1 expression by quercetin. |
| 17464175 | 6 | No | Curcumin reduces cold storage-induced damage in human cardiac myoblasts. |

| | | | |
|---|---|---|---|
| 17464989 | 2 | No | Tissue factor pathway inhibitor-2 as a frequently silenced tumor suppressor gene in hepatocellular carcinoma. |
| 17468514 | 1 | No | Carcinogen-induced histone alteration in normal human mammary epithelial cells. |
| 17470570 | 4 | No | Nitric oxide production is a proximal signaling event controlling exercise-induced mRNA expression in human skeletal muscle. |
| 17471463 | 4 | No | Transcriptional silencing of the TMS1/ASC tumour suppressor gene by an epigenetic mechanism in hepatocellular carcinoma cells. |
| 17473185 | 2 | No | Overexpression of human papillomavirus type 16 oncoproteins enhances hypoxia-inducible factor 1 alpha protein accumulation and vascular endothelial |
| 17473450 | 1 | No | Preventive effect of trimidox on oxidative stress in U937 cell line. |
| 17473463 | 4 | No | New constituent from Podocarpus macrophyllus var. macrophyllus shows anti-tyrosinase effect and regulates tyrosinase-related proteins and mRNA in |
| 17474084 | 3 | No | GADD45A is a mediator of CD437 induced apoptosis in ovarian carcinoma cells. |
| 17475324 | 2 | No | RXRalpha regulates the pregnancy-specific glycoprotein 5 gene transcription through a functional retinoic acid responsive element. |
| 17475839 | 50 | No | 9-cis-Retinoic acid (9cRA), a retinoid X receptor (RXR) ligand, exerts immunosuppressive effects on dendritic cells by RXR-dependent activation: |
| 17475930 | 7 | No | Chronic cyclophosphamide exposure alters the profile of rat sperm nuclear matrix proteins. |
| 17476690 | 4 | No | Differential gene expression induction by TRAIL in B chronic lymphocytic leukemia (B-CLL) cells showing high versus low levels of Zap-70. |
| 17482557 | 1 | No | Effect of vitamin B6 availability on serine hydroxymethyltransferase in MCF-7 cells. |
| 17482571 | 3 | No | Tryptanthrin inhibits MDR1 and reverses doxorubicin resistance in breast cancer cells. |
| 17483544 | 2 | No | Squalene synthase, a determinant of Raft-associated cholesterol and modulator of cancer cell proliferation. |
| 17484513 | 5 | No | Oral 17beta-estradiol and sequential progesterone in menopause: effects on insulin-like growth factors and their binding proteins. |
| 17485853 | 3 | No | Short-term hyperhomocysteinemia-induced oxidative stress activates retinal glial cells and increases vascular endothelial growth factor expression in rat retina. |
| 17486130 | 27 | No | Molecular targeting of retinoic acid metabolism in neuroblastoma: the role of the CYP26 inhibitor R116010 in vitro and in vivo. |

| | | | |
|---|---|---|---|
| 17486135 | 3 | No | Non-genomic action of resveratrol on androgen and oestrogen receptors in prostate cancer: modulation of the phosphoinositide 3-kinase pathway. |
| 17487405 | 2 | No | Depletion of O6-methylguanine-DNA methyltransferase by O6-benzylguanine enhances 5-FU cytotoxicity in colon and oral cancer cell lines. |
| 17490514 | 2 | No | [Effect of deguelin on expression of nup98 in K562 cells] |
| 17490981 | 123 | No | Dietary carbohydrate modification induces alterations in gene expression in abdominal subcutaneous adipose tissue in persons with the metabolic syndrome: the |
| 17492661 | 7 | No | Progesterone increases csk homologous kinase in HMC-1560 human mast cells and reduces cell proliferation. |
| 17498489 | 2 | No | [Effects of Chinese herbal recipe Weichang'an in inducing apoptosis and related gene expression in human gastric cancer grafted onto nude mice] |
| 17498666 | 9 | No | Cisplatin and doxorubicin repress Vascular Endothelial Growth Factor expression and differentially down-regulate Hypoxia-inducible Factor I activity in human |
| 17498915 | 12 | No | Trichostatin A, sodium butyrate, and 5-aza-2'-deoxycytidine alter the expression of glucocorticoid receptor alpha and beta isoforms in Hut-78 T- and Raji |
| 17499312 | 1 | No | The potentiation of curcumin on insulin-like growth factor-1 action in MCF-7 human breast carcinoma cells. |
| 17499557 | 2 | No | Icariin enhances endothelial nitric-oxide synthase expression on human endothelial cells in vitro. |
| 17499741 | 2 | No | Resveratrol inhibits expression and binding activity of the monocyte chemotactic protein-1 receptor, CCR2, on THP-1 monocytes. |
| 17499812 | 4 | No | resulted in down-regulation of phospho-extracellular signal-regulated protein kinase (Erk1/2) and phospho-p38 MAPK expressions. We conclude that TF induces |
| 17499975 | 8 | No | Immunomodulatory effect of selective serotonin reuptake inhibitors (SSRIs) on human T lymphocyte function and gene expression. |
| 17499997 | 1 | No | The anti-androgen effect of ganoderol B isolated from the fruiting body of Ganoderma lucidum. |
| 17502159 | 12 | No | Chondrogenic potential of human synovial mesenchymal stem cells in alginate. |
| 17502362 | 6 | No | Epigenetic regulation of leptin affects MMP-13 expression in osteoarthritic chondrocytes: possible molecular target for osteoarthritis therapeutic |
| 17503247 | 1 | No | Plasma surfactant D in patients following acute paraquat intoxication. |
| 17503469 | 28 | No | Androgen receptor or estrogen receptor-beta blockade alters DHEA-, DHT-, and E(2)-induced proliferation and PSA production in human prostate cancer cells. |

| | | | |
|---|---|---|---|
| 17505005 | 5 | No | Capsaicin is a novel blocker of constitutive and interleukin-6-inducible STAT3 activation. |
| 17505938 | 3 | No | Antiandrogenic activity of norgestimate in a human androgen-dependent stable-transfected cell line. |
| 17507084 | 2 | No | Histamine downregulates monocyte CCL2 production through the histamine H4 receptor. |
| 17507666 | 3 | No | Differential expression and oxidation of MKP-1 modulates TNF-alpha gene expression. |
| 17508023 | 9 | No | Transient receptor potential vanilloid-1 mediates heat-shock-induced matrix metalloproteinase-1 expression in human epidermal keratinocytes. |
| 17508937 | 1 | No | Small-scale immunopurification of cytochrome c oxidase for a high-throughput multiplexing analysis of enzyme activity and amount. |
| 17510436 | 49 | No | Intraprostatic androgens and androgen-regulated gene expression persist after testosterone suppression: therapeutic implications for castration-resistant |
| 17510524 | 4 | No | 2-hydroxycinnamaldehyde inhibits SW620 colon cancer cell growth through AP-1 inactivation. |
| 17512462 | 2 | No | Selenium deficiency alters epithelial cell morphology and responses to influenza. |
| 17513609 | 3 | No | Mitochondria-mediated apoptosis by diallyl trisulfide in human prostate cancer cells is associated with generation of reactive oxygen species and regulated by |
| 17513867 | 8 | No | Forkhead proteins are critical for bone morphogenetic protein-2 regulation and anti-tumor activity of resveratrol. |
| 17515571 | 5 | No | Gonadal function in males after chemotherapy for early-stage Hodgkin's lymphoma treated in four subsequent trials by the European Organisation for Research and |
| 17515840 | 1 | No | 11-Beta hydroxysteroid dehydrogenase type 2 in human adult and fetal lung and its regulation by sex steroids. |
| 17516867 | 3 | No | Molecular mechanism of anti-prostate cancer activity of Scutellaria baicalensis extract. |
| 17517105 | 8 | No | Effect of tecastemizole on pulmonary and cutaneous allergic inflammatory responses. |
| 17519148 | 8 | No | Comparison of the impact of vaginal and oral administration of combined hormonal contraceptives on hepatic proteins sensitive to estrogen. |
| 17520059 | 6 | No | Atrazine-induced aromatase expression is SF-1 dependent: implications for endocrine disruption in wildlife and reproductive cancers in humans. |
| 17520823 | 3 | No | [Effect of nordy on FPR function of malignant human glioma cell line U87] |

| | | | |
|---|---|---|---|
| 17521618 | 4 | No | De novo ceramide biosynthesis is associated with resveratrol-induced inhibition of ornithine decarboxylase activity. |
| 17521628 | 9 | No | Rapid induction of IAP family proteins and Smac/DIABLO expression after proapoptotic stimulation with doxorubicin in RPMI 8226 multiple myeloma cells. |
| 17523932 | 2 | No | Lack of effect of oral selenite on p53 associated gene expression during TL01 therapy of psoriasis patients. |
| 17524151 | 27 | No | Inhibition of sulfur mustard-induced cytotoxicity and inflammation by the macrolide antibiotic roxithromycin in human respiratory epithelial cells. |
| 17526768 | 13 | No | Androgen regulation of aldehyde dehydrogenase 1A3 (ALDH1A3) in the androgen-responsive human prostate cancer cell line LNCaP. |
| 17529908 | 18 | No | Mechanisms of oxidant regulation of monocyte chemotactic protein 1 production in human whole blood and isolated mononuclear cells. |
| 17531458 | 1 | No | Acute and chronic effects of some dietary bioactive compounds on folic acid uptake and on the expression of folic acid transporters by the human trophoblast |
| 17533736 | 6 | No | [Expression of ER alpha in chemically induced MDA-MB-435 cells and its responsiveness to endocrine] |
| 17534123 | 2 | No | Resveratrol-induced apoptosis is associated with activation of p53 and inhibition of protein translation in T47D human breast cancer cells. |
| 17536041 | 2 | No | Genetic polymorphisms in the human selenoprotein P gene determine the response of selenoprotein markers to selenium supplementation in a gender-specific manner |
| 17541156 | 18 | No | The chemopreventive effect of taxifolin is exerted through ARE-dependent gene regulation. |
| 17541959 | 6 | No | The role of androgen in determining differentiation and regulation of androgen receptor expression in the human prostatic epithelium transient amplifying |
| 17542780 | 10 | No | Morphine inhibits doxorubicin-induced reactive oxygen species generation and nuclear factor kappaB transcriptional activation in neuroblastoma SH-SY5Y cells. |
| 17545523 | 1 | No | Cytokeratin-18 is a useful serum biomarker for early determination of response of breast carcinomas to chemotherapy. |
| 17545540 | 1 | No | Functional up-regulation of human leukocyte antigen class I antigens expression by 5-aza-2'-deoxycytidine in cutaneous melanoma: immunotherapeutic implications. |
| 17547211 | 815 | No | Classification of heavy-metal toxicity by human DNA microarray analysis. |
| 17554206 | 1 | No | Resveratrol-induced growth inhibition in MDA-MB-231 breast cancer cells is associated with mitogen-activated protein kinase signaling and protein |
| 17555331 | 11 | No | Human p53 is inhibited by glutathionylation of cysteines present in the proximal DNA-binding domain during oxidative stress. |

| | | | |
|---|---|---|---|
| 17555711 | 2 | No | Indomethacin decreases EP2 prostanoid receptor expression in colon cancer cells. |
| 17557751 | 1 | No | Brassinolide, a plant sterol from pollen of Brassica napus L., induces apoptosis in human prostate cancer PC-3 cells. |
| 17558435 | 4 | No | Virodhamine and CP55,940 modulate cAMP production and IL-8 release in human bronchial epithelial cells. |
| 17562444 | 6 | No | The in vitro anti-tumour activity of zoledronic acid and docetaxel at clinically achievable concentrations in prostate cancer. |
| 17566705 | 2 | No | Survivin is a downstream target and effector of sulindac-sensitive oncogenic Stat3 signalling in head and neck cancer. |
| 17567588 | 111 | No | In vitro detection of drug-induced phospholipidosis using gene expression and fluorescent phospholipid based methodologies. |
| 17568578 | 3 | No | Indomethacin stimulates activity and expression of ecto-5'-nucleotidase/CD73 in glioma cell lines. |
| 17571247 | 1 | No | Potential advantages of DNA methyltransferase 1 (DNMT1)-targeted inhibition for cancer therapy. |
| 17572062 | 116 | No | Identification of human cell responses to benzene and benzene metabolites. |
| 17582780 | 1 | No | Raloxifene inhibits matrix metalloproteinases expression and activity in macrophages and smooth muscle cells. |
| 17584828 | 198 | No | Mifepristone induced progesterone withdrawal reveals novel regulatory pathways in human endometrium. |
| 17586618 | 10 | No | Homocysteine induces VCAM-1 gene expression through NF-kappaB and NAD(P)H oxidase activation: protective role of Mediterranean diet polyphenolic antioxidants. |
| 17588137 | 20 | No | Quercetin inhibits expression of inflammatory cytokines through attenuation of NF-kappaB and p38 MAPK in HMC-1 human mast cell line. |
| 17589894 | 6 | No | Effect of targeted magnetic nanoparticles containing 5-FU on expression of bcl-2, bax and caspase 3 in nude mice with transplanted human liver cancer. |
| 17590308 | 4 | No | Molecular and functional characterization of drug-metabolizing enzymes and transporter expression in the novel spontaneously immortalized human hepatocyte |
| 17596214 | 12 | No | Curcumin suppresses growth and chemoresistance of human glioblastoma cells via AP-1 and NFkappaB transcription factors. |
| 17596522 | 37 | No | Aldosterone induces epithelial-mesenchymal transition via ROS of mitochondrial origin. |
| 17597607 | 12 | No | Effect of protocatechualdehyde on receptor for advanced glycation end products and TGF-beta1 expression in human lens epithelial cells cultured under diabetic |

| | | | |
|---|---|---|---|
| 17599054 | 3 | No | Therapeutic potential of CDK inhibitor NU2058 in androgen-independent prostate cancer. |
| 17599376 | 9 | No | Up-regulation of early growth response gene 1 (EGR-1) via ERK1/2 signals attenuates sulindac sulfide-mediated cytotoxicity in the human intestinal |
| 17602619 | 6 | No | Desmethyl derivatives of indomethacin and sulindac as probes for cyclooxygenase-dependent biology. |
| 17603292 | 3 | No | Resveratrol protects SH-SY5Y neuroblastoma cells from apoptosis induced by dopamine. |
| 17606477 | 4 | No | Interleukin-8 signaling promotes translational regulation of cyclin D in androgen-independent prostate cancer cells. |
| 17608728 | 5 | No | The DNA-binding epidermal growth factor-receptor inhibitor PD153035 and other DNA-intercalating cytotoxic drugs reactivate the expression of the retinoic acid |
| 17611699 | 1 | No | DPD is a molecular determinant of capecitabine efficacy in colorectal cancer. |
| 17612628 | 1 | No | Suppression of DPYD expression in RKO cells via DNA methylation in the regulatory region of the DPYD promoter: a potentially important epigenetic mechanism |
| 17614352 | 6 | No | Progesterone sensitizes breast cancer MCF7 cells to imatinib inhibitory effects. |
| 17616700 | 3 | No | Delivery of 5-aza-2'-deoxycytidine to cells using oligodeoxynucleotides. |
| 17616702 | 2 | No | Effects of chromatin-modifying agents on CD34+ cells from patients with idiopathic myelofibrosis. |
| 17616776 | 5 | No | Effect of folate oversupplementation on folate uptake by human intestinal and renal epithelial cells. |
| 17616785 | 40 | No | Lymphocyte gene expression in subjects fed a low-choline diet differs between those who develop organ dysfunction and those who do not. |
| 17619071 | 3 | No | Induction of apoptosis by Uncaria tomentosa through reactive oxygen species production, cytochrome c release, and caspases activation in human leukemia |
| 17619073 | 12 | No | Solamargine induces apoptosis and sensitizes breast cancer cells to cisplatin. |
| 17620002 | 3 | No | Relationship between eosinophilia and levels of chemokines (CCL5 and CCL11) and IL-5 in bronchoalveolar lavage fluid of patients with mustard gas-induced |
| 17624924 | 10 | No | Androgen receptor-mediated repression of novel target genes. |
| 17625110 | 1 | No | Promoter methylation regulates estrogen receptor 2 in human endometrium and endometriosis. |

| | | | |
|---|---|---|---|
| 17626249 | 1 | No | Androgen-dependent regulation of medium and long chain fatty acids uptake in prostate cancer. |
| 17627616 | 3 | No | Gene transfer of endostatin enhances the efficacy of doxorubicin to suppress human hepatocellular carcinomas in mice. |
| 17627974 | 7 | No | Induction of hepatobiliary efflux transporters in acetaminophen-induced acute liver failure cases. |
| 17634039 | 1 | No | [The effect of mangiferin on telomerase activity and apoptosis in leukemic K562 cells] |
| 17634552 | 2 | No | Effect of cytarabine and decitabine in combination in human leukemic cell lines. |
| 17634554 | 2 | No | Regulation of p53 expression in response to 5-fluorouracil in human cancer RKO cells. |
| 17636213 | 5 | No | Interleukin 1beta and progesterone stimulate activin a expression and secretion from cultured human endometrial stromal cells. |
| 17636245 | 9 | No | Transcriptional regulation of deoxynivalenol-induced IL-8 expression in human monocytes. |
| 17636412 | 11 | No | Effects of dutasteride on the expression of genes related to androgen metabolism and related pathway in human prostate cancer cell lines. |
| 17636462 | 10 | No | Molecular mechanisms of resveratrol (3,4,5-trihydroxy-trans-stilbene) and its interaction with TNF-related apoptosis inducing ligand (TRAIL) in |
| 17637178 | 5 | No | Novel methoxylated flavone inhibitors of cytochrome P450 1B1 in SCC-9 human oral cancer cells. |
| 17637740 | 16 | No | Cytotoxic drug-induced, p53-mediated upregulation of caspase-8 in tumor cells. |
| 17638070 | 3 | No | Estrogen receptor beta increases the efficacy of antiestrogens by effects on apoptosis and cell cycling in breast cancer cells. |
| 17638652 | 6 | No | Comparison of pretherapeutic and posttherapeutic expression levels of chemotherapy-associated genes in adenocarcinomas of the esophagus treated by |
| 17638900 | 11 | No | Inhibition of nuclear factor-kappaB activity by temozolomide involves O6-methylguanine induced inhibition of p65 DNA binding. |
| 17639512 | 162 | No | Pathway and single gene analyses of inhibited Caco-2 differentiation by ascorbate-stabilized quercetin suggest enhancement of cellular processes |
| 17640567 | 42 | No | Role of pro-oxidants and antioxidants in the anti-inflammatory and apoptotic effects of curcumin (diferuloylmethane). |
| 17640750 | 35 | No | Up-regulation of cyclooxygenase-2 by cobalt chloride-induced hypoxia is mediated by phospholipase D isozymes in human astroglioma cells. |

| | | | |
|---|---|---|---|
| 17641250 | 2 | No | Pentaerythrityl tetranitrate and nitroglycerin, but not isosorbide mononitrate, prevent endothelial dysfunction induced by ischemia and reperfusion. |
| 17643826 | 1 | No | Ecto-5'-nucleotidase/CD73 inhibition by quercetin in the human U138MG glioma cell line. |
| 17645780 | 5 | No | Lipoxygenase inhibitors induce death receptor 5/TRAIL-R2 expression and sensitize malignant tumor cells to TRAIL-induced apoptosis. |
| 17645796 | 1 | No | Evaluation of insulin-like growth factor-I in postmenopausal women with breast cancer treated with raloxifene. |
| 17646279 | 7 | No | Human CYP2A6 is induced by estrogen via estrogen receptor. |
| 17651460 | 6 | No | Mitochondrial DNA deletions induce the adenosine monophosphate-activated protein kinase energy stress pathway and result in decreased secretion of some proteins. |
| 17651959 | 4 | No | Resveratrol inhibits heregulin-beta1-mediated matrix metalloproteinase-9 expression and cell invasion in human breast cancer cells. |
| 17652743 | 4 | No | Responses of human lens epithelial cells to quercetin and DMSO. |
| 17653088 | 5 | No | Stress via p53 pathway causes apoptosis by mitochondrial Noxa upregulation in doxorubicin-treated neuroblastoma cells. |
| 17653094 | 1 | No | Reversal of methylation silencing of Apo2L/TRAIL receptor 1 (DR4) expression overcomes resistance of SK-MEL-3 and SK-MEL-28 melanoma cells to interferons |
| 17654042 | 3 | No | Thioredoxin-1 attenuates indomethacin-induced gastric mucosal injury in mice. |
| 17655880 | 8 | No | D-Psicose inhibits the expression of MCP-1 induced by high-glucose stimulation in HUVECs. |
| 17658243 | 9 | No | Accumulation of hypoxia-inducible factor-1alpha through a novel electrophilic, thiol antioxidant-sensitive mechanism. |
| 17658692 | 10 | No | Identification of palmitate-regulated genes in HepG2 cells by applying microarray analysis. |
| 17659868 | 5 | No | 25 hydroxy-vitamin D(3)-1alpha hydroxylase expression and activity in cultured human osteoblasts and their modulation by parathyroid hormone, estrogenic |
| 17666914 | 32 | No | Curcumin attenuates inflammatory responses of TNF-alpha-stimulated human endothelial cells. |
| 17668557 | 88 | No | Some HIV antiretrovirals increase oxidative stress and alter chemokine, cytokine or adiponectin production in human adipocytes and macrophages. |
| 17669387 | 8 | No | Role of NAD(P)H:quinone oxidoreductase 1 on tumor necrosis factor-alpha-induced migration of human vascular smooth muscle cells. |

| | | | |
|---|---|---|---|
| 17669408 | 7 | No | Antiviral responses of human Fallopian tube epithelial cells to toll-like receptor 3 agonist poly(I:C). |
| 17669516 | 1 | No | Transcriptional and epigenetic regulation of the integrin collagen receptor locus ITGA1-PELO-ITGA2. |
| 17671175 | 1 | No | Exposure to the tobacco smoke constituent 4-aminobiphenyl induces chromosomal instability in human cancer cells. |
| 17671207 | 16 | No | Dual role of mitochondrial reactive oxygen species in hypoxia signaling: activation of nuclear factor-{kappa}B via c-SRC and oxidant-dependent cell death. |
| 17671737 | 13 | No | The antitumor activities of curcumin and of its isoxazole analogue are not affected by multiple gene expression changes in an MDR model of the MCF-7 breast |
| 17671742 | 8 | No | Curcumin induces apoptosis and inhibits prostaglandin E(2) production in synovial fibroblasts of patients with rheumatoid arthritis. |
| 17673517 | 4 | No | Regulation of alternative splicing of liver scavenger receptor class B gene by estrogen and the involved regulatory splicing factors. |
| 17674193 | 2 | No | Mechanisms of acquired resistance to 2-(4-Amino-3-methylphenyl)benzothiazole in breast cancer cell lines. |
| 17676588 | 3 | No | Parathyroid hormone-related protein varies with sex and androgen status in nonsmall cell lung cancer. |
| 17679169 | 6 | No | The comparative effects of gene modulators on thyroid-specific genes and radioiodine uptake. |
| 17679729 | 1 | No | Phase I study of decitabine alone or in combination with valproic acid in acute myeloid leukemia. |
| 17680992 | 8 | No | Inhibition of heat shock proteins (HSP) expression by quercetin and differential doxorubicin sensitization in neuroblastoma and Ewing's sarcoma cell lines. |
| 17682292 | 6 | No | FHL2 regulates cell cycle-dependent and doxorubicin-induced p21Cip1/Waf1 expression in breast cancer cells. |
| 17683926 | 3 | No | [6]-Gingerol inhibits metastasis of MDA-MB-231 human breast cancer cells. |
| 17683929 | 2 | No | Regulation of estrogen receptor (ER) levels in MCF-7 cells by progesterone metabolites. |
| 17683969 | 1 | No | Effects of folate cycle disruption by the green tea polyphenol epigallocatechin-3-gallate. |
| 17689939 | 33 | No | Resveratrol enhances proliferation and osteoblastic differentiation in human mesenchymal stem cells via ER-dependent ERK1/2 activation. |
| 17690521 | 1 | No | Detection of mutant p53 protein in workers occupationally exposed to benzidine. |

| | | | |
|---|---|---|---|
| 17690560 | 1 | No | The synergistic effect of 5-aza-2'-deoxycytidine and 5-fluorouracil on drug-resistant tumors. |
| 17690738 | 6 | No | Glucan and resveratrol complex--possible synergistic effects on immune system. |
| 17696246 | 6 | No | Effects of AZT and RNA-protein complex (FA-2-b-beta) extracted from Liang Jin mushroom on apoptosis of gastric cancer cells. |
| 17698565 | 26 | No | Expression and function of mixed lineage kinases in dendritic cells. |
| 17698632 | 6 | No | Homocysteine inhibits endothelial cell growth via DNA hypomethylation of the cyclin A gene. |
| 17699798 | 8 | No | Regulation of genes of the circadian clock in human colon cancer: reduced period-1 and dihydropyrimidine dehydrogenase transcription correlates in |
| 17701593 | 23 | No | Expression and modulation of progesterone induced blocking factor (PIBF) and innate immune factors in human leukemia cell lines by progesterone and |
| 17707346 | 8 | No | Modulation of early growth response gene 1 and interleukin-8 expression by ribotoxin deoxynivalenol (vomitoxin) via ERK1/2 in human epithelial intestine 407 |
| 17709599 | 16 | No | CHOP transcription factor mediates IL-8 signaling in cystic fibrosis bronchial epithelial cells. |
| 17715259 | 123 | No | Elevated phosphorus modulates vitamin D receptor-mediated gene expression in human vascular smooth muscle cells. |
| 1771636 | 1 | No | The effect of the insecticide heptachlor on ras proto-oncogene expression in human myeloblastic leukemia (ML-1) cells. |
| 17716971 | 14 | No | Differential regulation of vitamin D receptor (VDR) by the p53 Family: p73-dependent induction of VDR upon DNA damage. |
| 17717114 | 2 | No | Quercetin's influence on exercise-induced changes in plasma cytokines and muscle and leukocyte cytokine mRNA. |
| 17717130 | 2 | No | Development of a novel immunoassay for the assessment of plasma Gas6 concentrations and their variation with hormonal status. |
| 17720292 | 3 | No | Elevated levels of MCP-1, MIP-alpha and MIP-1 beta in the bronchoalveolar lavage (BAL) fluid of patients with mustard gas-induced pulmonary fibrosis. |
| 17726078 | 2 | No | Anti-Mullerian hormone is a sensitive serum marker for gonadal function in women treated for Hodgkin's lymphoma during childhood. |
| 17762189 | 3 | No | Aluminum-induced mitochondrial dysfunction leads to lipid accumulation in human hepatocytes: a link to obesity. |
| 17762391 | 59 | No | Establishment and gene analysis of an oxaliplatin-resistant colon cancer cell line THC8307/L-OHP. |

| | | | |
|---|---|---|---|
| 17762496 | 1 | No | High expressions of neuronatin isoforms in favorable neuroblastoma. |
| 17764071 | 9 | No | Identification of novel antiangiogenic anticancer activities of deguelin targeting hypoxia-inducible factor-1 alpha. |
| 17766065 | 1 | No | Pharmacological concentration of resveratrol suppresses aromatase in JEG-3 cells. |
| 17785578 | 25 | No | Decitabine up-regulates S100A2 expression and synergizes with IFN-gamma to kill uveal melanoma cells. |
| 17786183 | 4 | No | Expression and function of the human androgen-responsive gene ADI1 in prostate cancer. |
| 17786339 | 9 | No | Combination therapy of interferon-alpha and 5-fluorouracil inhibits tumor angiogenesis in human hepatocellular carcinoma cells by regulating vascular |
| 17804521 | 4 | No | The effect of resveratrol on a cell model of human aging. |
| 17804729 | 1 | No | Identifying the estrogen receptor coactivator PELP1 in autophagosomes. |
| 17804756 | 4 | No | Inhibition of prostate cancer growth by muscadine grape skin extract and resveratrol through distinct mechanisms. |
| 17805209 | 2 | No | Hydrocortisone and indomethacin negatively modulate EGF-R signaling in human fetal intestine. |
| 17805423 | 207 | No | Differential response of Mono Mac 6, BEAS-2B, and Jurkat cells to indoor dust. |
| 17822822 | 104 | No | Expression profile of human cells in culture exposed to glycidamide, a reactive metabolite of the heat-induced food carcinogen acrylamide. |
| 17823083 | 2 | No | Influence of the selective oestrogen receptor modulator (raloxifene hydrochloride) on IL-6, TNF-alpha, TGF-beta1 and bone turnover markers in the |
| 17823924 | 1 | No | Regulation of global gene expression in the bone marrow microenvironment by androgen: androgen ablation increases insulin-like growth factor binding |
| 17823925 | 1 | No | Resveratrol sensitizes androgen independent prostate cancer cells to death-receptor mediated apoptosis through multiple mechanisms. |
| 17827067 | 4 | No | Enhancement of Th1 type cytokine production and primary T cell activation by PBI-1393. |
| 17847021 | 6 | No | Methylseleninic acid inhibits microvascular endothelial G1 cell cycle progression and decreases tumor microvessel density. |
| 17849272 | 2 | No | The Effect of N-nitrosodimethylamine on TRAIL and DR5 expression in human neutrophils--preliminary study. |

| | | | |
|---|---|---|---|
| 17851650 | 43 | No | Detection of early signals of hepatotoxicity by gene expression profiling studies with cultures of metabolically competent human hepatocytes. |
| 17852453 | 4 | No | Upregulation of multi drug resistance genes in doxorubicin resistant human acute myelogeneous leukemia cells and reversal of the resistance. |
| 17853063 | 2 | No | Tumor necrosis factor-associated protein 1 (TRAP-1) protects cells from oxidative stress and apoptosis. |
| 17854288 | 1 | No | Testosterone regulation of homocysteine metabolism modulates redox status in human prostate cancer cells. |
| 17855475 | 21 | No | Neuroprotective effects of glyceryl nonivamide against microglia-like cells and 6-hydroxydopamine-induced neurotoxicity in SH-SY5Y human dopaminergic |
| 17855661 | 12 | No | Mitochondria are an essential mediator of nitric oxide/cyclic guanosine 3',5'-monophosphate blocking of glucose depletion induced cytotoxicity in human |
| 17868486 | 17 | No | High folic acid increases cell turnover and lowers differentiation and iron content in human HT29 colon cancer cells. |
| 17868649 | 4 | No | Resveratrol-induced apoptosis in human T-cell acute lymphoblastic leukaemia MOLT-4 cells. |
| 17872375 | 8 | No | Mechanism of the divergent effects of estrogen on the cell proliferation of human umbilical endothelial versus aortic smooth muscle cells. |
| 17873517 | 6 | No | Indomethacin enhances the cytotoxicity of recombinant human lymphotoxin alpha on tumor cells by suppressing NFkappaB signaling. |
| 17875398 | 5 | No | Depletion of WRN enhances DNA damage in HeLa cells exposed to the benzene metabolite, hydroquinone. |
| 17876054 | 2 | No | Selenium inhibition of survivin expression by preventing Sp1 binding to its promoter. |
| 17879943 | 1 | No | Resveratrol induces catalytic bioscavenger paraoxonase 1 expression and protects against chemical warfare nerve agent toxicity in human cell lines. |
| 17880982 | 36 | No | Comparative effects of quercetin and its predominant human metabolites on adhesion molecule expression in activated human vascular endothelial cells. |
| 17882670 | 9 | No | Effects of hormone treatment on hemostasis variables. |
| 17884346 | 7 | No | Interleukin-1 genotype-selective inhibition of inflammatory mediators by a botanical: a nutrigenetics proof of concept. |
| 17891453 | 44 | No | Trichostatin A and 5 Aza-2' deoxycytidine decrease estrogen receptor mRNA stability in ER positive MCF7 cells through modulation of HuR. |
| 17893511 | 6 | No | Differential effects of doxorubicin treatment on cell cycle arrest and Skp2 expression in breast cancer cells. |

| | | | |
|---|---|---|---|
| 17894541 | 3 | No | Increased granulocyte-colony stimulating factor (G-CSF) and granulocyte-macrophage colony stimulating factor (GM-CSF) levels in BAL fluid |
| 17900441 | 7 | No | Study of low-density lipoprotein receptor regulation by oral (steroid) contraceptives: desogestrel, levonorgestrel and ethinyl estradiol in JEG-3 cell |
| 17903073 | 4 | No | Combined continuous ethinyl estradiol/norethindrone acetate does not improve forearm blood flow in postmenopausal women at risk for cardiovascular events: a |
| 17904202 | 10 | No | Investigation of the estrogenic activities of pesticides from Pal-dang reservoir by in vitro assay. |
| 17904787 | 4 | No | Effects of capecitabine and vinorelbine on cell proliferation, metabolism and COX2 and p16 expression in breast cancer cell lines and solid tumour tissues. |
| 17908484 | 49 | No | Gene induction and apoptosis in human hepatocellular carci-noma cells SMMC-7721 exposed to 5-aza-2'-deoxycytidine. |
| 17912235 | 18 | No | RNA aptamer-targeted inhibition of NF-kappa B suppresses non-small cell lung cancer resistance to doxorubicin. |
| 17914565 | 23 | No | Metallothionein is up-regulated under hypoxia and promotes the survival of human prostate cancer cells. |
| 17914584 | 2 | No | Resveratrol induces pro-apoptotic endoplasmic reticulum stress in human colon cancer cells. |
| 17916567 | 4 | No | Downregulation of thymosin beta4 expression by androgen in prostate cancer LNCaP cells. |
| 17916643 | 7 | No | Biological role, protein expression, subcellular localization, and oxidative |
| 17918158 | 26 | No | Curcumin enhances the effects of 5-fluorouracil and oxaliplatin in mediating growth inhibition of colon cancer cells by modulating EGFR and IGF-1R. |
| 17918750 | 2 | No | Rap2 regulates androgen sensitivity in human prostate cancer cells. |
| 17919812 | 10 | No | Hypoxia enhances LPA-induced HIF-1alpha and VEGF expression: their inhibition by resveratrol. |
| 17920123 | 3 | No | Chemopreventive agents induce programmed death-1-ligand 1 (PD-L1) surface expression in breast cancer cells and promote PD-L1-mediated T cell apoptosis. |
| 17920763 | 1 | No | Inhibition of Stat3 increases doxorubicin sensitivity in a human metastatic breast cancer cell line. |
| 17922852 | 4 | No | Low concentrations of doxorubicin sensitizes human solid cancer cells to tumor necrosis factor-related apoptosis-inducing ligand (TRAIL)-receptor (R) 2-mediated |
| 17923031 | 10 | No | [The role of secreted Wnt-antagonist genes hypermethylation in early detection of colorectal tumor] |

| | | | |
|---|---|---|---|
| 17923759 | 3 | No | Change of the expression of human telomerase reverse transcriptase mRNA and human telomerase RNA after cisplatin and 5-fluorouracil exposure in head and neck |
| 17927689 | 15 | No | Curcumin, both histone deacetylase and p300/CBP-specific inhibitor, represses the activity of nuclear factor kappa B and Notch 1 in Raji cells. |
| 17928716 | 10 | No | Cytokine responses of intestinal epithelial-like Caco-2 cells to non-pathogenic and opportunistic pathogenic yeasts in the presence of butyric acid. |
| 17928719 | 19 | No | Modifying effects of dietary factors on (-)-epigallocatechin-3-gallate-induced pro-matrix metalloproteinase-7 production in HT-29 human colorectal cancer cells. |
| 17929310 | 10 | No | Resveratrol and quercetin, two natural polyphenols, reduce apoptotic neuronal cell death induced by neuroinflammation. |
| 17931847 | 7 | No | Upregulation of ICAM-1 expression in bronchial epithelial cells by airway secretions in bronchiectasis. |
| 17932452 | 1 | No | Down-regulation of HER-2 expression in human breast cancer cell HBC-4 and ZR75-1 by nitrogen-mustard-N-oxide. |
| 17934066 | 12 | No | The role of acetaldehyde in mediating effects of alcohol on expression of endogenous opioid system genes in a neuroblastoma cell line. |
| 17934748 | 1 | No | Mitomycin C induces multidrug resistance in glaucoma surgery. |
| 17934957 | 1 | No | The bipyridyl herbicide paraquat induces proteasome dysfunction in human neuroblastoma SH-SY5Y cells. |
| 17935137 | 12 | No | Phosphoinositide 3-kinase/Akt pathway plays an important role in chemoresistance of gastric cancer cells against etoposide and doxorubicin induced cell death. |
| 17935668 | 6 | No | Antimyeloma effects of resveratrol through inhibition of angiogenesis. |
| 17938187 | 13 | No | Resveratrol modulates phagocytosis of bacteria through an NF-kappaB-dependent gene program. |
| 17940511 | 7 | No | Genetic and epigenetic changes in the common 1p36 deletion in neuroblastoma tumours. |
| 17940886 | 2 | No | Potent upregulation of glutathione and NAD(P)H:quinone oxidoreductase 1 by alpha-lipoic acid in human neuroblastoma SH-SY5Y cells: protection against |
| 17948765 | 2 | No | Inhibitory effects of selenium on telomerase activity and hTERT expression in cadmium-transformed 16HBE cells. |
| 17949227 | 2 | No | Modulation of HLA-G expression. |
| 17949413 | 9 | No | Hydroxytyrosol protects retinal pigment epithelial cells from acrolein-induced oxidative stress and mitochondrial dysfunction. |

| | | | |
|---|---|---|---|
| 17951470 | 139 | No | Conjugated linoleic acid alters global gene expression in human intestinal-like Caco-2 cells in an isomer-specific manner. |
| 17954527 | 22 | No | HepaRG cells as an in vitro model for evaluation of cytochrome P450 induction in humans. |
| 17956190 | 2 | No | Resveratrol induces the heat-shock response and protects human cells from severe heat stress. |
| 17957036 | 1 | No | Does impaired mitochondrial function affect insulin signaling and action in cultured human skeletal muscle cells? |
| 17959036 | 15 | No | Interferon-alpha enhances sensitivity of human osteosarcoma U2OS cells to doxorubicin by p53-dependent apoptosis. |
| 17959153 | 12 | No | JNK inhibitor SP600125 is a partial agonist of human aryl hydrocarbon receptor and induces CYP1A1 and CYP1A2 genes in primary human hepatocytes. |
| 17959154 | 5 | No | Regulation of inflammation signalling by resveratrol in human chondrocytes in vitro. |
| 17959518 | 1 | No | [Geldanamycin inhibits proliferation and motility of human HER2/neu-overexpressing breast cancer cell line SKBr3] |
| 17960570 | 6 | No | Opposing effects of curcuminoids on serum stimulated and unstimulated angiogenic response. |
| 17962382 | 21 | No | Differential regulation of native estrogen receptor-regulatory elements by estradiol, tamoxifen, and raloxifene. |
| 17962980 | 6 | No | Induction of full-length survival motor neuron by polyphenol botanical compounds. |
| 17964119 | 2 | No | Sulfur mustard downregulates iNOS expression to inhibit wound healing in a human keratinocyte model. |
| 17965521 | 10 | No | Rapid and drastic induction of CYP3A4 mRNA expression via vitamin D receptor in human intestinal LS180 cells. |
| 17965732 | 9 | No | Curcumin inhibits connective tissue growth factor gene expression in activated hepatic stellate cells in vitro by blocking NF-kappaB and ERK signalling. |
| 17965791 | 4 | No | Gonadal function and fertility in male survivors treated for Hodgkin's disease in Iran. |
| 17967635 | 7 | No | Increased iodine uptake in thyroid carcinoma after treatment with sodium butyrate and decitabine (5-Aza-dC). |
| 17970047 | 1 | No | Doxorubicin and selenium cooperatively induce fas signaling in the absence of Fas/Fas ligand interaction. |
| 17971768 | 4 | No | Combination therapy with PEG-IFN-alpha and 5-FU inhibits HepG2 tumour cell growth in nude mice by apoptosis of p53. |

| | | | |
|---|---|---|---|
| 17973296 | 25 | No | Flavonoids-induced accumulation of hypoxia-inducible factor (HIF)-1alpha/2alpha is mediated through chelation of iron. |
| 17973783 | 9 | No | CD69 on CD56+ NK cells and response to chemoimmunotherapy in metastatic melanoma. |
| 17974986 | 5 | No | The endothelin receptor blocker bosentan inhibits doxorubicin-induced cardiomyopathy. |
| 17974990 | 3 | No | Involvement of sphingosine kinase 2 in p53-independent induction of p21 by the chemotherapeutic drug doxorubicin. |
| 17975199 | 11 | No | Gremlin-mediated decrease in bone morphogenetic protein signaling promotes pulmonary fibrosis. |
| 17977009 | 4 | No | Nebivolol reduces asymmetric dimethylarginine in endothelial cells by increasing dimethylarginine dimethylaminohydrolase 2 (DDAH2) expression and activity. |
| 17977830 | 15 | No | An ATM- and Rad3-related (ATR) signaling pathway and a phosphorylation-acetylation cascade are involved in activation of |
| 17978169 | 9 | No | Dexamethasone-mediated up-regulation of human CYP2A6 involves the glucocorticoid receptor and increased binding of hepatic nuclear factor 4 alpha to the proximal |
| 17979888 | 12 | No | Curcumin modulation of IFN-beta and IL-12 signalling and cytokine induction in human T cells. |
| 17981817 | 2 | No | Inhibin B is superior to FSH as a serum marker for spermatogenesis in men treated for Hodgkin's lymphoma with chemotherapy during childhood. |
| 17982488 | 1 | No | Wild-type p53 and p73 negatively regulate expression of proliferation related genes. |
| 17982676 | 104 | No | Apoptosis, cell cycle progression and gene expression in TP53-depleted HCT116 colon cancer cells in response to short-term 5-fluorouracil treatment. |
| 17985343 | 12 | No | Sulindac and its metabolites induce carcinogen metabolizing enzymes in human colon cancer cells. |
| 17986852 | 6 | No | Regulation of the CLDN3 gene in ovarian cancer cells. |
| 17987291 | 4 | No | Polyamine catabolism in colorectal cancer cells following treatment with oxaliplatin, 5-fluorouracil and N1, N11 diethylnorspermine. |
| 17988526 | 4 | No | [Interferon alpha enhances the sensitivity of SMMC-7721 hepatocellular carcinoma cells to 5'-deoxy-5-fluorouridine related to up-regulation of thymidine |
| 17991895 | 12 | No | DNA methylation inhibitor 5-Aza-2'-deoxycytidine induces reversible genome-wide DNA damage that is distinctly influenced by DNA methyltransferases 1 and 3B. |
| 17992120 | 8 | No | Melanoma vasculogenic mimicry is strongly related to reactive oxygen species level. |

| | | | |
|---|---|---|---|
| 17992261 | 18 | No | Monosaccharide-induced lipogenesis regulates the human hepatic sex hormone-binding globulin gene. |
| 17993253 | 6 | No | Release of MCP-1 and IL-8 from lung epithelial cells exposed to volatile organic compounds. |
| 17996122 | 2 | No | Coupled down-regulation of mTOR and telomerase activity during fluorouracil-induced apoptosis of hepatocarcinoma cells. |
| 17996675 | 4 | No | Anti-inflammatory effect of curcumin involves downregulation of MMP-9 in blood mononuclear cells. |
| 17999991 | 49 | No | Curcumin downregulates the inflammatory cytokines CXCL1 and -2 in breast cancer cells via NFkappaB. |
| 18001166 | 2 | No | Estradiol upregulates activating transcription factor 3, a candidate gene in the etiology of hypospadias. |
| 18001288 | 2 | No | Restoration of impaired phosphorylation of cyclic AMP response element-binding protein (CREB) by EGb 761 and its constituents in Abeta-expressing neuroblastoma |
| 18003597 | 2 | No | PPARalpha and PPARbeta are differentially affected by ethanol and the ethanol metabolite acetaldehyde in the MCF-7 breast cancer cell line. |
| 18003792 | 2 | No | Role of CCK1 and Y2 receptors in activation of hindbrain neurons induced by intragastric administration of bitter taste receptor ligands. |
| 18006205 | 1 | No | Ribotoxic mycotoxin deoxynivalenol induces G2/M cell cycle arrest via p21Cip/WAF1 mRNA stabilization in human epithelial cells. |
| 18006877 | 7 | No | Acrolein-activated matrix metalloproteinase 9 contributes to persistent mucin production. |
| 18020914 | 5 | No | Amiodarone reversibly decreases sodium-iodide symporter mRNA expression at therapeutic concentrations and induces antioxidant responses at |
| 18021753 | 2 | No | Epigenetic silencing of telomerase and a non-alkylating agent as a novel therapeutic approach for glioma. |
| 18025290 | 28 | No | Extended treatment with physiologic concentrations of dietary phytochemicals results in altered gene expression, reduced growth, and apoptosis of cancer |
| 18030663 | 27 | No | In vitro and in vivo modulation of testosterone mediated alterations in apoptosis related proteins by [6]-gingerol. |
| 18032389 | 24 | No | Quercetin 3-glucoside protects neuroblastoma (SH-SY5Y) cells in vitro against oxidative damage by inducing sterol regulatory element-binding protein-2-mediated |
| 18032417 | 19 | No | Effect of the interaction between lipoxygenase pathway and progesterone on the regulation of hydroxysteroid 11-Beta dehydrogenase 2 in cultured human term |
| 18032788 | 10 | No | Mitochondrial electron-transport-chain inhibitors of complexes I and II induce autophagic cell death mediated by reactive oxygen species. |

| | | | |
|---|---|---|---|
| 18033804 | 1 | No | CpG methylation plays a vital role in determining tissue- and cell-specific expression of the human cell-death-inducing DFF45-like effector A gene through |
| 18034345 | 6 | No | Effect of pure curcumin, demethoxycurcumin, and bisdemethoxycurcumin on WT1 gene expression in leukemic cell lines. |
| 18035141 | 2 | No | Maternal smoking and the vitamin D-parathyroid hormone system during the perinatal period. |
| 18036774 | 3 | No | Induction of oxidative stress in human Chang liver cells by octachlorostyrene, the persistent and bioaccumulative toxicant. |
| 18037150 | 204 | No | Gene expression in endometrial cancer cells (Ishikawa) after short time high dose exposure to progesterone. |
| 18039032 | 11 | No | Activation of inflammation/NF-kappaB signaling in infants born to arsenic-exposed mothers. |
| 18045574 | 10 | No | Alpha anomer of 5-aza-2'-deoxycytidine down-regulates hTERT mRNA expression in human leukemia HL-60 cells. |
| 18045962 | 3 | No | Growth inhibition of human salivary gland tumor cells by introduction of progesterone (Pg) receptor and Pg treatment. |
| 18047684 | 6 | No | Epigenetic regulation of RhoB loss of expression in lung cancer. |
| 18047805 | 2 | No | Disulfiram suppresses invasive ability of osteosarcoma cells via the inhibition of MMP-2 and MMP-9 expression. |
| 18048804 | 13 | No | Acrolein induces heme oxygenase-1 through PKC-delta and PI3K in human bronchial epithelial cells. |
| 1805284 | 3 | No | Immunoreactive beta-endorphin increases after an aspartame chocolate drink in healthy human subjects. |
| 18055817 | 32 | No | MMP-14 and TIMP-2 overexpression protects against hydroquinone-induced oxidant injury in RPE: implications for extracellular matrix turnover. |
| 18056458 | 3 | No | Ribosomal protein S27-like, a p53-inducible modulator of cell fate in response to genotoxic stress. |
| 18056468 | 30 | No | The p38 MAPK pathway mediates aryl propionic acid induced messenger rna stability of p75 NTR in prostate cancer cells. |
| 18058799 | 11 | No | Multiple mechanisms are involved in 6-gingerol-induced cell growth arrest and apoptosis in human colorectal cancer cells. |
| 18059344 | 5 | No | The proapoptotic effects of sulindac, sulindac sulfone and indomethacin are mediated by nucleolar translocation of the RelA(p65) subunit of NF-kappaB. |
| 18063403 | 4 | No | Effects of four-week high-fructose diet on gene expression in skeletal muscle of healthy men. |

| | | | |
|---|---|---|---|
| 18063697 | 5 | No | TNF-related apoptosis-inducing ligand (TRAIL) is expressed throughout myeloid development, resulting in a broad distribution among neutrophil granules. |
| 18070364 | 213 | No | Coordinate up-regulation of TMEM97 and cholesterol biosynthesis genes in normal ovarian surface epithelial cells treated with progesterone: implications for |
| 18071298 | 1 | No | Rifampicin exacerbates isoniazid-induced toxicity in human but not in rat hepatocytes in tissue-like cultures. |
| 18071906 | 4 | No | Akt and XIAP regulate the sensitivity of human uterine cancer cells to cisplatin, doxorubicin and taxol. |
| 18076062 | 1 | No | Cyclooxygenase inhibitors induce apoptosis in sinonasal cancer cells by increased expression of nonsteroidal anti-inflammatory drug-activated gene. |
| 18080171 | 1 | No | Risperidone versus haloperidol in children and adolescents with AD : a randomized, controlled, double-blind trial. |
| 18082045 | 1 | No | [5-azacytidine enhances anti-tumor efficacy of doxorubicin to neuroblastoma cell lines] |
| 18082304 | 23 | No | An in vitro approach to assess the toxicity of inhaled tobacco smoke components: nicotine, cadmium, formaldehyde and urethane. |
| 18083778 | 7 | No | Inverse agonist and neutral antagonist actions of antidepressants at recombinant and native 5-hydroxytryptamine2C receptors: differential modulation of cell |
| 18089816 | 2 | No | A novel bioluminescent mouse model and effective therapy for adult T-cell leukemia/lymphoma. |
| 18089846 | 9 | No | The effects of cyclooxygenase-2 expression in prostate cancer cells: modulation of response to cytotoxic agents. |
| 18092340 | 10 | No | Differential effects of chemotherapeutic drugs versus the MDM-2 antagonist nutlin-3 on cell cycle progression and induction of apoptosis in SKW6.4 |
| 18092350 | 13 | No | DNA demethylation and histone deacetylation inhibition co-operate to re-express estrogen receptor beta and induce apoptosis in prostate cancer cell-lines. |
| 18094037 | 11 | No | Induction of metabolism and transport in human intestine: validation of precision-cut slices as a tool to study induction of drug metabolism in human |
| 18098064 | 4 | No | Regulation of UDP-glucuronosyltransferase (UGT) 1A1 by progesterone and its impact on labetalol elimination. |
| 18156316 | 4 | No | Combined effects of sulindac and suberoylanilide hydroxamic acid on apoptosis induction in human lung cancer cells. |
| 18156803 | 5 | No | Linkage of curcumin-induced cell cycle arrest and apoptosis by cyclin-dependent kinase inhibitor p21(/WAF1/CIP1). |
| 18161298 | 7 | No | [Difference of gene expression profile in human gastric cancer grafted onto nude mice treated with WCA] |

| | | | |
|---|---|---|---|
| 18161303 | 3 | No | [Inhibition of curcumin on histone deacetylase and expression promotion of P21 (WAF1/CIP1) in HepG2 cells] |
| 18161920 | 1 | No | Inhibitory effect of schisandrin B on gastric cancer cells in vitro. |
| 18164318 | 2 | No | Reactivation of methylation-silenced tumor suppressor gene p16INK4a by nordihydroguaiaretic acid and its implication in G1 cell cycle arrest. |
| 18165598 | 2 | No | The differential effects of bupivacaine and lidocaine on prostaglandin E2 release, cyclooxygenase gene expression and pain in a clinical pain model. |
| 18174752 | 5 | No | A role for DNA methylation in regulating the growth suppressor PMEPA1 gene in prostate cancer. |
| 18175754 | 1142 | No | Supplementation of healthy volunteers with nutritionally relevant amounts of selenium increases the expression of lymphocyte protein biosynthesis genes. |
| 18176112 | 1 | No | Inhibition of apoptosis facilitates necrosis induced by cisplatin in gastric cancer cells. |
| 18180316 | 23 | No | High glucose enhances lipopolysaccharide-stimulated CD14 expression in U937 mononuclear cells by increasing nuclear factor kappaB and AP-1 activities. |
| 18182997 | 11 | No | Pharmacological targeting of NF-kappaB potentiates the effect of the topoisomerase inhibitor CPT-11 on colon cancer cells. |
| 18184656 | 1 | No | Phosphorylation of CtBP1 by cAMP-dependent protein kinase modulates induction of CYP17 by stimulating partnering of CtBP1 and 2. |
| 18191903 | 64 | No | Sequential and concerted gene expression changes in a chronic in vitro model of parkinsonism. |
| 18192686 | 8 | No | Soy isoflavones decrease the catechol-O-methyltransferase-mediated inactivation of 4-hydroxyestradiol in cultured MCF-7 cells. |
| 18196609 | 15 | No | Promotion of the articular cartilage proteoglycan degradation by T-2 toxin and selenium protective effect. |
| 18197826 | 6 | No | Gene profiling of normal human bronchial epithelial cells in response to asbestos and benzo(a)pyrene diol epoxide (BPDE). |
| 18197934 | 2 | No | Relationship between expression of 5-fluorouracil metabolic enzymes and 5-fluorouracil sensitivity in esophageal carcinoma cell lines. |
| 18201443 | 4 | No | The cigarette smoke component acrolein inhibits expression of the innate immune components IL-8 and human beta-defensin 2 by sinonasal epithelial cells. |
| 18203293 | 6 | No | Regulation of demethylation and re-expression of RASSF1A gene in gastric cancer cell lines by combined treatment of 5-Aza-CdR and NaB. |
| 18204794 | 20 | No | Cancer genes induced by malathion and parathion in the presence of estrogen in breast cells. |

| | | | |
|---|---|---|---|
| 18205257 | 8 | No | Effects of Wei Chang An on expression of multiple genes in human gastric cancer grafted onto nude mice. |
| 18206652 | 4 | No | Differential epigenetic regulation of Aiolos expression in human tumoral cell lines and primary cells. |
| 18206661 | 16 | No | Roles of nitric oxide in inflammatory downregulation of human cytochromes P450. |
| 18207697 | 12 | No | Liver slices as a model to study fibrogenesis and test the effects of anti-fibrotic drugs on fibrogenic cells in human liver. |
| 18214481 | 11 | No | Curcumin attenuates EGF-induced AQP3 up-regulation and cell migration in human ovarian cancer cells. |
| 18214805 | 8 | No | Application of oligonucleotide microarray technology to toxic occupational exposures. |
| 18218830 | 2 | No | Thiopental protects human T lymphocytes from apoptosis in vitro via the expression of heat shock protein 70. |
| 18223236 | 1 | No | STX140 is efficacious in vitro and in vivo in taxane-resistant breast carcinoma cells. |
| 18223691 | 29 | No | p21(WAF1/CIP1) induction by 5-azacytosine nucleosides requires DNA damage. |
| 18225533 | 3 | No | A sequential treatment of depsipeptide followed by 5-azacytidine enhances Gadd45beta expression in hepatocellular carcinoma cells. |
| 18225567 | 3 | No | In vitro evaluation of zoledronic acid resistance developed in MCF-7 cells. |
| 18226269 | 47 | No | Curcumin sensitizes TRAIL-resistant xenografts: molecular mechanisms of apoptosis, metastasis and angiogenesis. |
| 18227147 | 36 | No | Markers of electrophilic stress caused by chemically reactive metabolites in human hepatocytes. |
| 18232056 | 11 | No | Unique proteomic features induced by a potential antiglioma agent, Nordy (dl-nordihydroguaiaretic acid), in glioma cells. |
| 18234130 | 9 | No | Effects of dietary polyphenols on gene expression in human vascular endothelial cells. |
| 18240144 | 9 | No | The DNA demethylating agent 5-aza-2'-deoxycytidine activates NY-ESO-1 antigenicity in orthotopic human glioma. |
| 18245173 | 6 | No | A role for ceramide in driving cancer cell resistance to doxorubicin. |
| 18247401 | 3 | No | 1alpha,25-Dihydroxyvitamin D3 down-regulates expression of prostate specific membrane antigen in prostate cancer cells. |

| | | | |
|---|---|---|---|
| 18247414 | 241 | No | Effects of tobacco compounds on gene expression in fetal lung fibroblasts. |
| 18250044 | 1 | No | [Toluene diisocyanate increases vascular endothelial growth factor expression in human bronchial epithelial cells] |
| 18258206 | 5 | No | Lipoamide protects retinal pigment epithelial cells from oxidative stress and mitochondrial dysfunction. |
| 18261746 | 17 | No | Growth compensatory role of sulindac sulfide-induced thrombospondin-1 linked with ERK1/2 and RhoA GTPase signaling pathways. |
| 18262749 | 19 | No | Screening of some anti-progestin endocrine disruptors using a recombinant yeast based in vitro bioassay. |
| 18264100 | 4 | No | Thromboxane synthase mutations in an increased bone density disorder (Ghosal syndrome). |
| 18264126 | 2 | No | 5-Aza-2'-deoxycytidine suppresses human renal carcinoma cell growth in a xenograft model via up-regulation of the connexin 32 gene. |
| 18264725 | 6 | No | ER alpha negative breast cancer cells restore response to endocrine therapy by combination treatment with both HDAC inhibitor and DNMT inhibitor. |
| 18269916 | 7 | No | Induction of p21 by p65 in p53 null cells treated with Doxorubicin. |
| 18272409 | 4 | No | Involvement of peroxiredoxin IV in the 16alpha-hydroxyestrone-induced proliferation of human MCF-7 breast cancer cells. |
| 18274635 | 2 | No | Ozonation of human blood induces a remarkable upregulation of heme oxygenase-1 and heat stress protein-70. |
| 18283591 | 2 | No | The synergistic reversal effect of multidrug resistance by quercetin and hyperthermia in doxorubicin-resistant human myelogenous leukemia cells. |
| 18291362 | 11 | No | Sulindac suppresses beta-catenin expression in human cancer cells. |
| 18291368 | 1 | No | Epigenetic silencing of maspin expression occurs early in the conversion of keratocytes to fibroblasts. |
| 18292947 | 2 | No | Polysaccharopeptide enhances the anticancer activity of doxorubicin and etoposide on human breast cancer cells ZR-75-30. |
| 18295389 | 7 | No | NF-kappaB-activated tissue transglutaminase is involved in ethanol-induced hepatic injury and the possible role of propolis in preventing fibrogenesis. |
| 18304466 | 1 | No | Effect of all-trans retinoic acid on drug sensitivity and expression of survivin in LoVo cells. |
| 18308354 | 18 | No | Both direct and indirect effects account for the pro-inflammatory activity of enteropathogenic mycotoxins on the human intestinal epithelium: stimulation of |

| | | | |
|---|---|---|---|
| 18310046 | 2 | No | Longitudinal effects of thymidine analogues on mtDNA, mtRNA and multidrug resistance (MDR-1) induction in cultured cells. |
| 18310117 | 37 | No | Homocysteine-induced extracellular superoxide dismutase and its epigenetic mechanisms in monocytes. |
| 18310281 | 11 | No | ABCC11 expression is regulated by estrogen in MCF7 cells, correlated with estrogen receptor alpha expression in postmenopausal breast tumors and |
| 18313098 | 13 | No | Effects of fifteen PBDE metabolites, DE71, DE79 and TBBPA on steroidogenesis in the H295R cell line. |
| 18314483 | 8 | No | Epigenetic down-regulation and suppressive role of DCBLD2 in gastric cancer cell proliferation and invasion. |
| 18314485 | 2 | No | DNMT1 as a molecular target in a multimodality-resistant phenotype in tumor cells. |
| 18314491 | 10 | No | Kruppel-like factor 4 is a novel mediator of selenium in growth inhibition. |
| 18314537 | 9 | No | Asbestos-induced MKP-3 expression augments TNF-alpha gene expression in human monocytes. |
| 18316174 | 4 | No | Influences of aflatoxin B1 on reactive oxygen species generation and chemotaxis of human polymorphonuclear leukocytes. |
| 18316600 | 17 | No | p21 Waf1/Cip1 expression by curcumin in U-87MG human glioma cells: role of early growth response-1 expression. |
| 18320071 | 7 | No | Pharmacological inhibition of DNA methylation induces proinvasive and prometastatic genes in vitro and in vivo. |
| 18321735 | 21 | No | Comparison between chondroprotective effects of glucosamine, curcumin, and diacerein in IL-1beta-stimulated C-28/I2 chondrocytes. |
| 18322232 | 11 | No | STAT-1 signaling in human lung fibroblasts is induced by vanadium pentoxide through an IFN-beta autocrine loop. |
| 18323654 | 3 | No | Stabilization of p53 is involved in quercetin-induced cell cycle arrest and apoptosis in HepG2 cells. |
| 18324645 | 10 | No | Chronic azacitidine treatment results in differentiating effects, sensitizes against bicalutamide in androgen-independent prostate cancer cells. |
| 18325115 | 11 | No | Tissue factor/FVIIa activates Bcl-2 and prevents doxorubicin-induced apoptosis in neuroblastoma cells. |
| 18325346 | 2 | No | Role and regulation of ferritin H in rotenone-mediated mitochondrial oxidative stress. |
| 18325701 | 9 | No | Suppressive effects of Houttuynia cordata Thunb (Saururaceae) extract on Th2 immune response. |

| | | | |
|---|---|---|---|
| 18331776 | 58 | No | Flavones and flavonols exert cytotoxic effects on a human oesophageal adenocarcinoma cell line (OE33) by causing G2/M arrest and inducing apoptosis. |
| 18332078 | 14 | No | Comparison of immortalized Fa2N-4 cells and human hepatocytes as in vitro models for cytochrome P450 induction. |
| 18336853 | 6 | No | Nitrophenols isolated from diesel exhaust particles regulate steroidogenic gene expression and steroid synthesis in the human H295R adrenocortical cell line. |
| 18337055 | 2 | No | Inhibition of DNA methyltransferase activity upregulates Fyn tyrosine kinase expression in Hut-78 T-lymphoma cells. |
| 18347146 | 3 | No | Synergistic antitumor effect of S-1 and the epidermal growth factor receptor inhibitor gefitinib in non-small cell lung cancer cell lines: role of |
| 18347191 | 7 | No | Ataxia telangiectasia mutated and p21CIP1 modulate cell survival of drug-induced senescent tumor cells: implications for chemotherapy. |
| 18348186 | 46 | No | Protein phosphatase 1 activation and alternative splicing of Bcl-X and Mcl-1 by EGCG + ibuprofen. |
| 18348204 | 6 | No | Curcumin downregulates H19 gene transcription in tumor cells. |
| 18351577 | 28 | No | Apple polyphenols modulate expression of selected genes related to toxicological defence and stress response in human colon adenoma cells. |
| 18351690 | 18 | No | Protein profile in neuroblastoma cells incubated with S- and R-enantiomers of ibuprofen by iTRAQ-coupled 2-D LC-MS/MS analysis: possible action of induced |
| 18357371 | 1 | No | Expression of rTSbeta as a 5-fluorouracil resistance marker in patients with primary breast cancer. |
| 18357586 | 24 | No | Heme oxygenase-1 mediates the anti-inflammatory effect of Curcumin within LPS-stimulated human monocytes. |
| 18360721 | 2 | No | Alleviation of the drug-resistant phenotype in idarubicin and cytosine arabinoside double-resistant acute myeloid leukemia cells by indomethacin. |
| 18377727 | 1 | No | The novel gene LRP15 is regulated by DNA methylation and confers increased efficiency of DNA repair of ultraviolet-induced DNA damage. |
| 18377872 | 8 | No | Quercetin augments TRAIL-induced apoptotic death: involvement of the ERK signal transduction pathway. |
| 18377993 | 6 | No | Oxidative insults induce DJ-1 upregulation and redistribution: implications for neuroprotection. |
| 18382425 | 2 | No | Single-step doxorubicin-selected cancer cells overexpress the ABCG2 drug transporter through epigenetic changes. |
| 18383843 | 4 | No | Pretreatment with S-1, an oral derivative of 5-fluorouracil, enhances gemcitabine effects in pancreatic cancer xenografts. |

| | | | |
|---|---|---|---|
| 18384088 | 11 | No | 6-Shogaol induces apoptosis in human colorectal carcinoma cells via ROS production, caspase activation, and GADD 153 expression. |
| 18385095 | 6 | No | The protective effect of quercetin against oxidative stress in the human RPE in vitro. |
| 18385176 | 6 | No | Asbestos induces doxorubicin resistance in MM98 mesothelioma cells via HIF-1alpha. |
| 18390174 | 1 | No | [Curcumin inhibits the expression of vascular endothelial growth factor and androgen-independent prostate cancer cell line PC-3 in vitro] |
| 18394438 | 2 | No | Effect of cytochrome p450 polymorphisms on platelet reactivity after treatment with clopidogrel in acute coronary syndrome. |
| 18394600 | 5 | No | Epigenetic and HIF-1 regulation of stanniocalcin-2 expression in human cancer cells. |
| 18395517 | 3 | No | Induction of MHC class I-related chain B (MICB) by 5-aza-2'-deoxycytidine. |
| 18398839 | 2 | No | Persistent downregulation of the pancarcinoma-associated epithelial cell adhesion molecule via active intranuclear methylation. |
| 18400727 | 5 | No | Functional effects of a common single-nucleotide polymorphism (GPX4c718t) in the glutathione peroxidase 4 gene: interaction with sex. |
| 18404433 | 23 | No | Residual platelet ADP reactivity after clopidogrel treatment is dependent on activation of both the unblocked P2Y(1) and the P2Y (12) receptor and is |
| 18404682 | 4 | No | DNA methylation and histone modifications cause silencing of Wnt antagonist gene in human renal cell carcinoma cell lines. |
| 18405881 | 4 | No | Treatment of lung cancer cells with cytotoxic levels of sodium selenite: effects on the thioredoxin system. |
| 18405923 | 3 | No | Capsaicin induces apoptosis and terminal differentiation in human glioma A172 cells. |
| 18410530 | 1 | No | Progressive silencing of p14ARF in oesophageal adenocarcinoma. |
| 18413660 | 12 | No | Inhibition of Nod2 signaling and target gene expression by curcumin. |
| 18414057 | 11 | No | Synergistic effects of the purine analog sulfinosine and curcumin on the multidrug resistant human non-small cell lung carcinoma cell line (NCI-H460/R). |
| 18414230 | 6 | No | Nicotinamide adenine dinucleotide phosphate (reduced form) oxidase is important for LPS-induced endothelial cell activation. |
| 18414638 | 203 | No | Drinking-water arsenic exposure modulates gene expression in human lymphocytes from a U.S. population. |

| | | | |
|---|---|---|---|
| 18418215 | 2 | No | Nordihydroguaiaretic acid restores expression of silenced E-cadherin gene in human breast cancer cell lines and xenografts. |
| 18418871 | 16 | No | Effects of model organophosphorous pesticides on DNA damage and proliferation of HepG2 cells. |
| 18419197 | 6 | No | Estrogenic activity of lambda-cyhalothrin in the MCF-7 human breast carcinoma cell line. |
| 18419748 | 3 | No | Quercetin induced tissue-type plasminogen activator expression is mediated through Sp1 and p38 mitogen-activated protein kinase in human endothelial cells. |
| 18421014 | 25 | No | Curcumin restores corticosteroid function in monocytes exposed to oxidants by maintaining HDAC2. |
| 18422750 | 28 | No | Interaction of Ganoderma triterpenes with doxorubicin and proteomic characterization of the possible molecular targets of Ganoderma triterpenes. |
| 18423122 | 2 | No | [Expression of APRIL in colorectal carcinoma tissues and effects of chemotherapeutic agents on APRIL expression in colorectal carcinoma SW480 cells] |
| 18423386 | 15 | No | Potential role of phospholipase D2 in increasing interleukin-2 production by T-lymphocytes through activation of mitogen-activated protein kinases ERK1/ERK2. |
| 18424558 | 6 | No | Small-molecule RETRA suppresses mutant p53-bearing cancer cells through a p73-dependent salvage pathway. |
| 18425332 | 1 | No | Reduced T-cadherin expression and promoter methylation are associated with the development and progression of hepatocellular carcinoma. |
| 18425338 | 3 | No | CDK inhibitor enhances the sensitivity to 5-fluorouracil in colorectal cancer cells. |
| 18431742 | 17 | No | Genistein mediated histone acetylation and demethylation activates tumor suppressor genes in prostate cancer cells. |
| 18439772 | 4 | No | Curcuma drugs and curcumin regulate the expression and function of P-gp in Caco-2 cells in completely opposite ways. |
| 18440631 | 2 | No | Oct4 is epigenetically regulated by methylation in normal placenta and gestational trophoblastic disease. |
| 18443271 | 7 | No | The effect of azacitidine on interleukin-6 signaling and nuclear factor-kappaB activation and its in vitro and in vivo activity against multiple myeloma. |
| 18444160 | 8 | No | Effects of naturally occurring and synthetic organoselenium compounds on protein profiling in androgen responsive and androgen independent human prostate cancer |
| 18448484 | 9 | No | Epigenetic remodeling during arsenical-induced malignant transformation. |
| 18454446 | 6 | No | Hormonal regulation of beta2-adrenergic receptor level in prostate cancer. |

| | | | |
|---|---|---|---|
| 18454857 | 2 | No | Importance of dose-schedule of 5-aza-2'-deoxycytidine for epigenetic therapy of cancer. |
| 18456490 | 2 | No | Preferential induction of cytochrome P450 1A1 over cytochrome P450 1B1 in human breast epithelial cells following exposure to quercetin. |
| 18457675 | 19 | No | A p38-p65 transcription complex induced by endothelin-1 mediates signal transduction in cancer cells. |
| 18458674 | 1 | No | SPARC promoter hypermethylation in colorectal cancers can be reversed by 5-Aza-2'deoxycytidine to increase SPARC expression and improve therapy response. |
| 18460780 | 3 | No | [Analysis of in vitro anti-leukemia effect of 5-aza-2'-deoxycitydine] |
| 18461179 | 38 | No | The herbicide atrazine activates endocrine gene networks via non-steroidal NR5A nuclear receptors in fish and mammalian cells. |
| 18463201 | 4 | No | Activation of nuclear factor-kappa B pathway by simvastatin and RhoA silencing increases doxorubicin cytotoxicity in human colon cancer HT29 cells. |
| 18464055 | 5 | No | Source apportionment of particulate matter in the U.S. and associations with lung inflammatory markers. |
| 18468680 | 2 | No | Semenogelin I expression in myeloma cells can be upregulated pharmacologically. |
| 18468695 | 2 | No | Expression of NK cells activation receptors after occupational exposure to toxics: a preliminary study. |
| 18472964 | 2 | No | Decitabine-induced demethylation of 5' CpG island in GADD45A leads to apoptosis in osteosarcoma cells. |
| 18480072 | 19 | No | 17-Beta estradiol and hydroxyestradiols interact via the NF-kappa B pathway to elevate cyclooxygenase 2 expression and prostaglandin E2 secretion in human |
| 18482162 | 3 | No | Alcohol induces DNA damage and the Fanconi anemia D2 protein implicating FANCD2 in the DNA damage response pathways in brain. |
| 18483179 | 8 | No | Glucocorticoid receptor phosphorylation differentially affects target gene expression. |
| 18485432 | 20 | No | Hypo-responsiveness of interleukin-8 production in human embryonic epithelial intestine 407 cells independent of NF-kappaB pathway: new lessons from endotoxin |
| 18486366 | 24 | No | Modulation of signal transduction pathways in lymphocytes due to sub-lethal toxicity of chlorinated phenol. |
| 18487222 | 7 | No | Prohibitin and the SWI/SNF ATPase subunit BRG1 are required for effective androgen antagonist-mediated transcriptional repression of androgen |
| 18488220 | 3 | No | Systemic immune effects of adjuvant chemotherapy with 5-fluorouracil, epirubicin and cyclophosphamide and/or radiotherapy in breast cancer: a longitudinal study. |

| | | | |
|---|---|---|---|
| 18489909 | 30 | No | Physiologically relevant metabolites of quercetin have no effect on adhesion molecule or chemokine expression in human vascular smooth muscle cells. |
| 18490488 | 135 | No | Differential regulation of interleukin 12 and interleukin 23 production in human dendritic cells. |
| 18491370 | 6 | No | Inhibitory effects of nordihydroguaiaretic acid (NDGA) on the IGF-1 receptor and androgen dependent growth of LAPC-4 prostate cancer cells. |
| 18492835 | 2 | No | Quercetin enhances epithelial barrier function and increases claudin-4 expression in Caco-2 cells. |
| 18495333 | 2 | No | Anticancer effect of tetrandrine on primary cancer cells isolated from ascites and pleural fluids. |
| 18495463 | 5 | No | Curcumin, demethoxycurcumin and bisdemethoxycurcumin differentially inhibit cancer cell invasion through the down-regulation of MMPs and uPA. |
| 18497072 | 4 | No | Hormones of pregnancy, alpha-feto protein, and reduction of breast cancer risk. |
| 18497090 | 15 | No | Phytoestrogens modulate the expression of 17alpha-estradiol metabolizing enzymes in cultured MCF-7 cells. |
| 18497984 | 9 | No | Epigenetic modification is involved in aberrant expression of class III beta-tubulin, TUBB3, in ovarian cancer cells. |
| 18498645 | 1 | No | Epigenetic changes within the promoter region of the HLA-G gene in ovarian tumors. |
| 18500657 | 11 | No | The role of tissue-nonspecific alkaline phosphatase in the phosphate-induced activation of alkaline phosphatase and mineralization in SaOS-2 human |
| 18506536 | 6 | No | Effects of combined administration of DPD-inhibitory oral fluoropyrimidine, S-1, plus paclitaxel on gene expressions of fluoropyrimidine metabolism-related |
| 18508827 | 32 | No | Haem oxygenase-1 plays a central role in NNK-mediated lung carcinogenesis. |
| 18508964 | 7 | No | Fructose induces the inflammatory molecule ICAM-1 in endothelial cells. |
| 18510171 | 10 | No | Multiple pathways are involved in drug resistance to doxorubicin in an osteosarcoma cell line. |
| 18514395 | 39 | No | Se-methylselenocysteine alters collagen gene and protein expression in human prostate cells. |
| 18514500 | 5 | No | In vitro models for the assessment of inflammatory and immuno-modulatory effects of the volatile organic compound chlorobenzene. |
| 18516295 | 8 | No | Cyclic pifithrin-alpha sensitizes wild type p53 tumor cells to antimicrotubule agent-induced apoptosis. |

| | | | |
|---|---|---|---|
| 18522535 | 2 | No | The human receptor tyrosine kinase Axl gene--promoter characterization and regulation of constitutive expression by Sp1, Sp3 and CpG methylation. |
| 18533110 | 19 | No | Induction of the endoplasmic reticulum stress protein GADD153/CHOP by capsaicin in prostate PC-3 cells: a microarray study. |
| 18537153 | 1 | No | Dihydropyrimidine dehydrogenases and cytidine-deaminase gene polymorphisms as outcome predictors in resected gastric cancer patients treated with |
| 18538015 | 2 | No | Involvement of TSC genes and differential expression of other members of the mTOR signaling pathway in oral squamous cell carcinoma. |
| 18538736 | 30 | No | DACT3 is an epigenetic regulator of Wnt/beta-catenin signaling in colorectal cancer and is a therapeutic target of histone modifications. |
| 18539414 | 4 | No | Upregulation of I-CAM1 in response to beryllium exposure in small airway epithelial cells. |
| 18541696 | 7 | No | Down-regulation of the carcinogen-metabolizing enzyme cytochrome P450 1a1 by vanadium. |
| 18544619 | 14 | No | Deoxyribonucleic acid methyltransferase 3B promotes epigenetic silencing through histone 3 chromatin modifications in pituitary cells. |
| 18548127 | 214 | No | Microarray Data Mining for Potential Selenium Targets in Chemoprevention of Prostate Cancer. |
| 18552516 | 3 | No | The mechanism of hyperoside protection of ECV-304 cells against tert-butyl hydroperoxide-induced injury. |
| 18553387 | 2 | No | Genetic and epigenetic inactivation of T-cadherin in human hepatocellular carcinoma cells. |
| 18555005 | 1 | No | Hypermethylation of the Keap1 gene in human lung cancer cell lines and lung cancer tissues. |
| 18555241 | 8 | No | Curcumin, a nutritional supplement with antineoplastic activity, enhances leiomyoma cell apoptosis and decreases fibronectin expression. |
| 18557828 | 1 | No | 5-Azacytidine-treated human mesenchymal stem/progenitor cells derived from umbilical cord, cord blood and bone marrow do not generate cardiomyocytes in |
| 18557930 | 3 | No | Basal and UV-induced MMP-1 expression are inhibited by p53 in human dermal fibroblasts. |
| 18559491 | 4 | No | Frequently methylated tumor suppressor genes in head and neck squamous cell carcinoma. |
| 18559593 | 2 | No | ssDNA-binding protein 2 is frequently hypermethylated and suppresses cell growth in human prostate cancer. |
| 18559595 | 5 | No | Down-regulation of hedgehog-interacting protein through genetic and epigenetic alterations in human hepatocellular carcinoma. |

| | | | |
|---|---|---|---|
| 18560228 | 5 | No | Effect of doxorubicin on telomerase activity and apoptotic gene expression in doxorubicin-resistant and -sensitive MCF-7 cells: an experimental study. |
| 18566016 | 1 | No | Cathepsin D protects human neuroblastoma cells from doxorubicin-induced cell death. |
| 18567002 | 4 | No | Prostate-derived Ets transcription factor as a favorable prognostic marker in ovarian cancer patients. |
| 18567616 | 16 | No | Epigenetic inheritance of DNA methylation limits activation-induced expression of FOXP3 in conventional human CD25-CD4+ T cells. |
| 18570159 | 5 | No | (PG) and thiabendazole (TB), on cytochrome P450 (CYP) forms in cultured human hepatocytes. 2. Treatment of human hepatocytes for 72 h with 2-200 microM TB |
| 18571826 | 2 | No | Impact of para-phenylenediamine on cyclooxygenases expression and prostaglandin formation in human immortalized keratinocytes (HaCaT). |
| 18572023 | 10 | No | Arsenic-induced malignant transformation of human keratinocytes: involvement of Nrf2. |
| 18573879 | 2 | No | Selection against PUMA gene expression in Myc-driven B-cell lymphomagenesis. |
| 18575591 | 1 | No | Silencing of keratinocyte growth factor receptor restores 5-fluorouracil and tamoxifen efficacy on responsive cancer cells. |
| 18575727 | 4 | No | Deguelin inhibits human hepatocellular carcinoma by antiangiogenesis and apoptosis. |
| 18575731 | 7 | No | Incubation with somatostatin, 5-aza decitabine and trichostatin up-regulates somatostatin receptor expression in prostate cancer cells. |
| 18577749 | 1 | No | CHD5, a tumor suppressor gene deleted from 1p36.31 in neuroblastomas. |
| 18583042 | 24 | No | Exposure to metal ions regulates mRNA levels of APP and BACE1 in PC12 cells: blockage by curcumin. |
| 18583539 | 75 | No | IFATS collection: Selenium induces improvement of stem cell behaviors in human adipose-tissue stromal cells via SAPK/JNK and stemness acting signals. |
| 18584348 | 6 | No | Histone H3 (lys-9) deacetylation is associated with transcriptional silencing of E-cadherin in colorectal cancer cell lines. |
| 18584893 | 6 | No | Transient receptor potential vanilloid 1-mediated expression and secretion of endothelial cell-derived calcitonin gene-related peptide. |
| 18586549 | 2 | No | Proteasomal inhibition reduces parkin mRNA in PC12 and SH-SY5Y cells. |
| 18593368 | 1 | No | Enhancement radiosensitization of breast cancer cells by deguelin. |

| | | | |
|---|---|---|---|
| 18593918 | 6 | No | Curcumin blocks RON tyrosine kinase-mediated invasion of breast carcinoma cells. |
| 18593936 | 12 | No | Curcumin decreases specificity protein expression in bladder cancer cells. |
| 18593937 | 6 | No | Activation of protein kinase G Increases the expression of p21CIP1, p27KIP1, and histidine triad protein 1 through Sp1. |
| 18596194 | 20 | No | Curcumin inhibits VEGF-mediated angiogenesis in human intestinal microvascular endothelial cells through COX-2 and MAPK inhibition. |
| 18597608 | 4 | No | Thrombin activates Ras-CREB/ATF-1 signaling and stimulates c-fos, c-jun, and c-myc expression in human gingival fibroblasts. |
| 18599499 | 16 | No | Double-stranded RNA-activated protein kinase mediates induction of interleukin-8 expression by deoxynivalenol, Shiga toxin 1, and ricin in monocytes. |
| 18600534 | 2 | No | Functional inactivity and mutations of p53 differentially affect sensitivity to 5-fluorouracil and antifolate inhibitors of thymidylate synthase (TS) by altering |
| 18601766 | 2 | No | Zoledronic acid effects interleukin-6 expression in hormone-independent prostate cancer cell lines. |
| 18602074 | 2 | No | Bisacurone inhibits adhesion of inflammatory monocytes or cancer cells to endothelial cells through down-regulation of VCAM-1 expression. |
| 18603805 | 26 | No | PGK1 induction by a hydrogen peroxide treatment is suppressed by antioxidants in human colon carcinoma cells. |
| 18606404 | 4 | No | Doxorubicin inhibits TGF-beta signaling in human lung carcinoma A549 cells. |
| 18607998 | 3 | No | Additive effects of oral fluoropyrimidine derivative S-1 and radiation on human hypopharyngeal cancer xenografts. |
| 18608210 | 8 | No | 5-Aza-2'-deoxycytidine restores proapoptotic function of p53 in cancer cells resistant to p53-induced apoptosis. |
| 18614807 | 4 | No | Mitochondrial defects and dysfunction in calcium regulation in glaucomatous trabecular meshwork cells. |
| 18614847 | 18 | No | Differentiation-associated alteration in gene expression of importins and exportins in human leukemia HL-60 cells. |
| 18622747 | 6 | No | Mammalian target of rapamycin pathway inhibition enhances the effects of 5-aza-dC on suppressing cell proliferation in human gastric cancer cell lines. |
| 18624906 | 18 | No | Activation of beta-catenin signalling by GSK-3 inhibition increases p-glycoprotein expression in brain endothelial cells. |
| 18628248 | 25 | No | Quercetin inhibition of tumor invasion via suppressing PKC delta/ERK/AP-1-dependent matrix metalloproteinase-9 activation in breast |

| | | | |
|---|---|---|---|
| 18630517 | 12 | No | Changes of gene expression of thymidine phosphorylase, thymidylate synthase, dihydropyrimidine dehydrogenase after the administration of |
| 18636160 | 6 | No | Downmodulation of dimethyl transferase activity enhances tumor necrosis factor-related apoptosis-inducing ligand-induced apoptosis in prostate cancer |
| 18636205 | 3 | No | Analysis of the methylation status of genes up-regulated by the demethylating agent, 5-aza-2'-deoxycytidine, in esophageal squamous cell carcinoma. |
| 18640131 | 10 | No | Synergistic effects of multiple natural products in pancreatic cancer cells. |
| 18644992 | 3 | No | Antiproliferative effect of sulindac in colonic neoplasia prevention: role of COOH-terminal Src kinase. |
| 18645022 | 47 | No | Differential antiproliferative mechanisms of novel derivative of benzimidazo[1,2-alpha]quinoline in colon cancer cells depending on their p53 |
| 18645721 | 5 | No | Chlorobenzene induces the NF-kappa B and p38 MAP kinase pathways in lung epithelial cells. |
| 18649362 | 6 | No | Role of myofibroblasts in innate chemoresistance of pancreatic carcinoma--epigenetic downregulation of caspases. |
| 18660423 | 2 | No | Curcumin modulates SDF-1alpha/CXCR4-induced migration of human retinal endothelial cells (HRECs). |
| 18661270 | 27 | No | Prevention of murine experimental corneal trauma by epigenetic events regulating claudin 6 and claudin 9. |
| 18664505 | 15 | No | DNA hypermethylation regulates the expression of members of the Mu-class glutathione S-transferases and glutathione peroxidases in Barrett's |
| 18665581 | 6 | No | Sulindac derivatives that activate the peroxisome proliferator-activated receptor gamma but lack cyclooxygenase inhibition. |
| 18666234 | 2 | No | Epigenetic identification of ubiquitin carboxyl-terminal hydrolase L1 as a functional tumor suppressor and biomarker for hepatocellular carcinoma and other |
| 18673531 | 4 | No | Epigenetic mechanisms involved in differential MDR1 mRNA expression between gastric and colon cancer cell lines and rationales for clinical chemotherapy. |
| 18676361 | 17 | No | Curcumin blocks the activation of androgen and interlukin-6 on prostate-specific antigen expression in human prostatic carcinoma cells. |
| 18676679 | 18 | No | Selenite reactivates silenced genes by modifying DNA methylation and histones in prostate cancer cells. |
| 18678619 | 16 | No | Nonsteroidal anti-inflammatory drugs induced endothelial apoptosis by perturbing peroxisome proliferator-activated receptor-delta transcriptional pathway. |
| 18679414 | 4 | No | Promoter hypomethylation regulates CD133 expression in human gliomas. |

| | | | |
|---|---|---|---|
| 18681966 | 2 | No | Prolactin receptor antagonism reduces the clonogenic capacity of breast cancer cells and potentiates doxorubicin and paclitaxel cytotoxicity. |
| 18682687 | 14 | No | EF24, a novel curcumin analog, disrupts the microtubule cytoskeleton and inhibits HIF-1. |
| 18686095 | 6 | No | A high-throughput reporter gene assay to prove the ability of natural compounds to modulate glutathione peroxidase, superoxide dismutase and catalase gene |
| 18687144 | 379 | No | Novel functional view of the crocidolite asbestos-treated A549 human lung epithelial transcriptome reveals an intricate network of pathways with opposing |
| 18688853 | 1 | No | Promoter methylation of the bone morphogenetic protein-6 gene in association with adult T-cell leukemia. |
| 18690847 | 4 | No | Celecoxib upregulates multidrug resistance proteins in colon cancer: lack of synergy with standard chemotherapy. |
| 18695136 | 4 | No | Antitumor effects of doxorubicin followed by zoledronic acid in a mouse model of breast cancer. |
| 18695918 | 4 | No | In vitro susceptibility to anticancer agents of the human KB carcinoma cell line transfected with COX-2 cDNA. |
| 18701604 | 1 | No | Potential link between estrogen receptor-alpha gene hypomethylation and uterine fibroid formation. |
| 18701766 | 1 | No | Bivalirudin decreases NO bioavailability by vascular immobilization of myeloperoxidase. |
| 18703563 | 5 | No | Potential relationship between hepatobiliary osteopontin and peroxisome proliferator-activated receptor alpha expression following ethanol-associated |
| 18704418 | 1 | No | Complete cytogenetic remission after decitabine treatment in a patient with secondary AML harbouring high p15INK4b gene methylation and high global DNA |
| 18704882 | 4 | No | Curcumin up-regulates LDL receptor expression via the sterol regulatory element pathway in HepG2 cells. |
| 18706393 | 12 | No | Epigenetic mechanisms and Sp1 regulate mitochondrial citrate carrier gene expression. |
| 18709470 | 1 | No | Mesothelioma epidemiology, carcinogenesis, and pathogenesis. |
| 18715546 | 6 | No | Flavonoid glycosides isolated from Salicornia herbacea inhibit matrix metalloproteinase in HT1080 cells. |
| 18719366 | 19 | No | Androgen responsive and refractory prostate cancer cells exhibit distinct curcumin regulated transcriptome. |
| 18720364 | 40 | No | Combination of 5-azacytidine and thalidomide for the treatment of myelodysplastic syndromes and acute myeloid leukemia. |

| | | | |
|---|---|---|---|
| 18724895 | 5 | No | [mRNA expression levels of p53 and DNA damage and repair genes in peripheral blood lymphocytes of benzene-exposed workers] |
| 18728404 | 1 | No | The JNK signaling pathway is involved in sodium-selenite-induced apoptosis mediated by reactive oxygen in HepG2 cells. |
| 18729198 | 1 | No | Promoter hypermethylation of CDH13 is a common, early event in human esophageal adenocarcinogenesis and correlates with clinical risk factors. |
| 18754885 | 5 | No | Cotylenin A, a new differentiation inducer, and rapamycin cooperatively inhibit growth of cancer cells through induction of cyclin G2. |
| 18755171 | 2 | No | Reduction of doxorubicin and oracin and induction of carbonyl reductase in human breast carcinoma MCF-7 cells. |
| 18757417 | 13 | No | Molecular basis for the induction of an angiogenesis inhibitor, thrombospondin-1, by 5-fluorouracil. |
| 18757430 | 2 | No | LRRC3B, encoding a leucine-rich repeat-containing protein, is a putative tumor suppressor gene in gastric cancer. |
| 18760546 | 2 | No | Selenite treatment inhibits LAPC-4 tumor growth and prostate-specific antigen secretion in a xenograft model of human prostate cancer. |
| 18769453 | 5 | No | Transient receptor potential vanilloid-1 signaling as a regulator of human sebocyte biology. |
| 18776995 | 6 | No | Down-regulation of Sonic hedgehog signaling pathway activity is involved in 5-fluorouracil-induced apoptosis and motility inhibition in Hep3B cells. |
| 18778717 | 3 | No | The effects of 1,4-benzoquinone on c-Myb and topoisomerase II in K-562 cells. |
| 18779317 | 13 | No | p53 regulates Toll-like receptor 3 expression and function in human epithelial cell lines. |
| 18787224 | 4 | No | Constitutive activation of the Wnt canonical pathway in mantle cell lymphoma. |
| 18790744 | 3 | No | Curcumin inhibits Akt/mammalian target of rapamycin signaling through protein phosphatase-dependent mechanism. |
| 18790786 | 14 | No | Effect of chemotherapeutic stress on induction of vascular endothelial growth factor family members and receptors in human colorectal cancer cells. |
| 18791715 | 8 | No | Treatment of ovarian cancer cell lines with 5-aza-2'-deoxycytidine upregulates the expression of cancer-testis antigens and class I major histocompatibility |
| 18794807 | 4 | No | Inhibition of Src tyrosine kinase reverts chemoresistance toward 5-fluorouracil in human pancreatic carcinoma cells: an involvement of epidermal growth factor |
| 18801729 | 19 | No | Nonsteroidal anti-inflammatory drug-activated gene (NAG-1/GDF15) expression is increased by the histone deacetylase inhibitor trichostatin A. |

| | | | |
|---|---|---|---|
| 18802751 | 9 | No | Protective effects of asiatic acid on rotenone- or H2O2-induced injury in SH-SY5Y cells. |
| 18803327 | 16 | No | Identification of candidate tumor suppressor genes inactivated by promoter methylation in melanoma. |
| 18813790 | 55 | No | Histone deacetylase inhibitor scriptaid induces cell cycle arrest and epigenetic change in colon cancer cells. |
| 18815942 | 1 | No | Epigenetic silencing of LRRC3B in colorectal cancer. |
| 18817749 | 1 | No | PCFT/SLC46A1 promoter methylation and restoration of gene expression in human leukemia cells. |
| 18818525 | 8 | No | Dihydrocapsaicin (DHC), a saturated structural analog of capsaicin, induces autophagy in human cancer cells in a catalase-regulated manner. |
| 18818744 | 4 | No | Suppressive Effects of Selected Food Phytochemicals on CD74 Expression in NCI-N87 Gastric Carcinoma Cells. |
| 18819746 | 6 | No | Aberrant methylation of candidate tumor suppressor genes in neuroblastoma. |
| 18822394 | 1 | No | Role of TRP channels and NCX in mediating hypoxia-induced [Ca(2+)](i) elevation in PC12 cells. |
| 18823003 | 2 | No | Assessment of mitochondrial toxicity by analysis of mitochondrial protein expression in mononuclear cells. |
| 18823661 | 2 | No | Demethylating agent 5-aza-2'-deoxycytidine activates HLA-G expression in human leukemia cell lines. |
| 18829568 | 1 | No | Abnormal DNA methylation of CD133 in colorectal and glioblastoma tumors. |
| 18829986 | 5 | No | Methylation of CIITA promoter IV causes loss of HLA-II inducibility by IFN-gamma in promyelocytic cells. |
| 18830594 | 6 | No | Zebularine suppresses the apoptotic potential of 5-fluorouracil via cAMP/PKA/CREB pathway against human oral squamous cell carcinoma cells. |
| 18834353 | 6 | No | Effect of Bupleuri Radix extracts on the toxicity of 5-fluorouracil in HepG2 hepatoma cells and normal human lymphocytes. |
| 18841906 | 20 | No | Phenolic constituents of Amorpha fruticosa that inhibit NF-kappaB activation and related gene expression. |
| 18845238 | 13 | No | Mechanism of alcohol-induced oxidative stress and neuronal injury. |
| 18845559 | 15 | No | Induction of intestinalization in human esophageal keratinocytes is a multistep process. |

| | | | |
|---|---|---|---|
| 18847459 | 85 | No | The molecular basis of genistein-induced mitotic arrest and exit of self-renewal in embryonal carcinoma and primary cancer cell lines. |
| 18848576 | 13 | No | Selective COX-2 inhibitors modulate cellular senescence in human dermal fibroblasts in a catalytic activity-independent manner. |
| 18851785 | 27 | No | [Impact of epigallocatechin gallate on gene expression profiles of human hepatocellular carcinoma cell lines BEL7404/ADM and BEL7402/5-FU] |
| 18852136 | 12 | No | Natural polyphenols facilitate elimination of HT-29 colorectal cancer xenografts by chemoradiotherapy: a Bcl-2- and superoxide dismutase 2-dependent mechanism. |
| 18852534 | 2 | No | Retinoic acid receptor beta2 re-expression and growth inhibition in thyroid carcinoma cell lines after 5-aza-2'-deoxycytidine treatment. |
| 18854230 | 6 | No | Effect of orally administered KF66490, a phosphodiesterase 4 inhibitor, on dermatitis in mouse models. |
| 18855134 | 13 | No | Combination of methylselenocysteine with tamoxifen inhibits MCF-7 breast cancer xenografts in nude mice through elevated apoptosis and reduced angiogenesis. |
| 18922617 | 3 | No | Epigenetic silencing of the interferon regulatory factor ICSBP/IRF8 in human multiple myeloma. |
| 18922931 | 10 | No | A novel dietary flavonoid fisetin inhibits androgen receptor signaling and tumor growth in athymic nude mice. |
| 18924134 | 15 | No | NO-sulindac inhibits the hypoxia response of PC-3 prostate cancer cells via the Akt signalling pathway. |
| 18927307 | 263 | No | Clinical determinants of response to irinotecan-based therapy derived from cell line models. |
| 18927491 | 7 | No | Induction of autophagic cell death by a novel molecule is increased by hypoxia. |
| 18930000 | 22 | No | Rare sugar D-allose enhances anti-tumor effect of 5-fluorouracil on the human hepatocellular carcinoma cell line HuH-7. |
| 18938139 | 4 | No | Aberrant methylation of human L- and M-fructose 1,6-bisphosphatase genes in cancer. |
| 18945643 | 10 | No | DNA methylation inhibition increases T cell KIR expression through effects on both promoter methylation and transcription factors. |
| 18949370 | 1 | No | PAX4 has the potential to function as a tumor suppressor in human melanoma. |
| 18949431 | 1 | No | The aberrant promoter methylation of BMP3b and BMP6 in malignant pleural mesotheliomas. |
| 18953428 | 27 | No | Involvement of epigenetic mechanisms in the regulation of secreted phospholipase A2 expressions in Jurkat leukemia cells. |

| | | | |
|---|---|---|---|
| 18958421 | 44 | No | Flavonoids inhibit histamine release and expression of proinflammatory cytokines in mast cells. |
| 18959786 | 7 | No | Mercury immune toxicity in harbour seals: links to in vitro toxicity. |
| 18972406 | 2 | No | Selenoprotein P expression is controlled through interaction of the coactivator PGC-1alpha with FoxO1a and hepatocyte nuclear factor 4alpha transcription |
| 18974268 | 4 | No | Epigenetic regulation of E-cadherin controls endometrial receptivity. |
| 18974383 | 6 | No | Amiodarone inhibits arterial thrombus formation and tissue factor translation. |
| 18978557 | 3 | No | AZGP1 autoantibody predicts survival and histone deacetylase inhibitors increase expression in lung adenocarcinoma. |
| 18980195 | 1 | No | Cocultures of osteoblasts and osteoclasts are influenced by local application of zoledronic acid incorporated in a poly(D,L-lactide) implant coating. |
| 18980244 | 9 | No | Quercetin sensitizes human hepatoma cells to TRAIL-induced apoptosis via Sp1-mediated DR5 up-regulation and proteasome-mediated c-FLIPS down-regulation. |
| 18981723 | 2 | No | Reactivation of IGFBP7 by DNA demethylation inhibits human colon cancer cell growth in vitro. |
| 18982426 | 14 | No | Quercetin inhibits IL-1 beta-induced ICAM-1 expression in pulmonary epithelial cell line A549 through the MAPK pathways. |
| 18985161 | 3 | No | CD133 is a marker of bioenergetic stress in human glioma. |
| 18989703 | 1 | No | Differentiation of human adipose-derived stem cells towards cardiomyocytes is facilitated by laminin. |
| 18991268 | 12 | No | A comparative proteomic analysis for capsaicin-induced apoptosis between human hepatocarcinoma (HepG2) and human neuroblastoma (SK-N-SH) cells. |
| 18992151 | 5 | No | Methylation of WTH3, a possible drug resistant gene, inhibits p53 regulated expression. |
| 18997278 | 5 | No | Selenium is critical for cancer-signaling gene expression but not cell proliferation in human colon Caco-2 cells. |
| 19003803 | 4 | No | Adenoviral-mediated gene transfer of Gadd45a results in suppression by inducing apoptosis and cell cycle arrest in pancreatic cancer cell. |
| 19005980 | 6 | No | Inhibition of cell growth and VEGF expression in ovarian cancer cells by flavonoids. |
| 19009557 | 4 | No | Effects of low dose quercetin: cancer cell-specific inhibition of cell cycle progression. |

| | | | |
|---|---|---|---|
| 19010910 | 6 | No | p53-inducible ribonucleotide reductase (p53R2/RRM2B) is a DNA hypomethylation-independent decitabine gene target that correlates with clinical |
| 19013290 | 21 | No | Phosphatidylinositol acts through mitogen-activated protein kinase to stimulate hepatic apolipoprotein A-I secretion. |
| 19015155 | 9 | No | Regulation of human dUTPase gene expression and p53-mediated transcriptional repression in response to oxaliplatin-induced DNA damage. |
| 19015929 | 13 | No | The effect of combination treatment with docosahexaenoic acid and 5-fluorouracil on the mRNA expression of apoptosis-related genes, including the novel gene |
| 19018768 | 12 | No | Curcumin attenuates cytochrome P450 induction in response to 2,3,7,8-tetrachlorodibenzo-p-dioxin by ROS-dependently degrading AhR and ARNT. |
| 19019832 | 6 | No | Reactive oxygen species regulate ceruloplasmin by a novel mRNA decay mechanism involving its 3'-untranslated region: implications in neurodegenerative diseases. |
| 19020770 | 19 | No | Essential nutrients suppress inflammation by modulating key inflammatory gene expression. |
| 19022360 | 2 | No | Disturbance of cellular glucose transport by two prevalently used fluoroquinolone antibiotics ciprofloxacin and levofloxacin involves glucose transporter type 1. |
| 19023563 | 3 | No | Modulation of butyrate transport in Caco-2 cells. |
| 19026728 | 1 | No | Transcriptional activity of the novel identified human yy2 promoter is modified by DNA methylation. |
| 19027488 | 1 | No | Aberrant methylation of ADAMTS1 in non-small cell lung cancer. |
| 19027835 | 10 | No | Silencing of the CKII alpha and CKII alpha' genes during cellular senescence is mediated by DNA methylation. |
| 19030781 | 6 | No | Synergistic induction of NY-ESO-1 antigen expression by a novel histone deacetylase inhibitor, valproic acid, with 5-aza-2'-deoxycytidine in glioma |
| 19031421 | 138 | No | Comparison of gene expression profiles in HepG2 cells exposed to arsenic, cadmium, nickel, and three model carcinogens for investigating the mechanisms of |
| 19032668 | 2 | No | Expression and promoter methylation status of mismatch repair gene hMLH1 and hMSH2 in epithelial ovarian cancer. |
| 19033020 | 2 | No | Selenium supplementation improves antioxidant capacity in vitro and in vivo in patients with coronary artery disease The SElenium Therapy in Coronary Artery |
| 19033392 | 44 | No | HMOX1 and NQO1 genes are upregulated in response to contact sensitizers in dendritic cells and THP-1 cell line: role of the Keap1/Nrf2 pathway. |
| 19034627 | 19 | No | Bioactive terpenoids and flavonoids from Ginkgo biloba extract induce the expression of hepatic drug-metabolizing enzymes through pregnane X receptor, |

| | | | |
|---|---|---|---|
| 19036117 | 39 | No | Effect of zoledronic acid on oral fibroblasts and epithelial cells: a potential mechanism of bisphosphonate-associated osteonecrosis. |
| 19036374 | 4 | No | Acetaldehyde stimulates monocyte adhesion in a P-selectin- and TNFalpha-dependent manner. |
| 19037090 | 6 | No | Regulation of the leucocyte chemoattractant receptor FPR in glioblastoma cells by cell differentiation. |
| 19037991 | 6 | No | DNA methyltransferase inhibitor 5-aza-CdR enhances the radiosensitivity of gastric cancer cells. |
| 19040731 | 5 | No | Endothelin receptor B antagonists decrease glioma cell viability independently of their cognate receptor. |
| 19059400 | 11 | No | Capsaicin, a component of red peppers, induces expression of androgen receptor via PI3K and MAPK pathways in prostate LNCaP cells. |
| 19059883 | 5 | No | NAD(P)H quinone oxidoreductase 1 is essential for ozone-induced oxidative stress in mice and humans. |
| 19061877 | 12 | No | Ozone stress down-regulates the expression of cystic fibrosis transmembrane conductance regulator in human bronchial epithelial cells. |
| 19070657 | 3 | No | CYP1A1 induction and CYP3A4 inhibition by the fungicide imazalil in the human intestinal Caco-2 cells-comparison with other conazole pesticides. |
| 19074641 | 24 | No | Effect of curcumin on acidic pH-induced expression of IL-6 and IL-8 in human |
| 19074854 | 7 | No | Overexpression of the dual specificity phosphatase, Cdc25C, confers sensitivity on tumor cells to doxorubicin-induced cell death. |
| 19077051 | 2 | No | Combination of DNA methylation inhibitor 5-azacytidine and arsenic trioxide has synergistic activity in myeloma. |
| 19080631 | 1 | No | [Histone H3 lysine 9 methylation is associated with the expression of hMLH1 and DNA methylation in gastric cancer cells] |
| 19084572 | 16 | No | Synergistic effect of 5-fluorouracil with gambogic acid on BGC-823 human gastric carcinoma. |
| 19087683 | 1 | No | [5-aza-2'-deoxycytidine induces changes of histone H3-lysine 9 methylation in bladder tumor cells] |
| 19090986 | 1 | No | Immune defects in Alzheimer's disease: new medications development. |
| 19091749 | 20 | No | Methylalpinumisoflavone inhibits hypoxia-inducible factor-1 (HIF-1) activation by simultaneously targeting multiple pathways. |
| 19096014 | 2 | No | Hypomethylating drugs convert HA-1-negative solid tumors into targets for stem cell-based immunotherapy. |

| | | | |
|---|---|---|---|
| 19098008 | 23 | No | The essential role of p53 in hyperpigmentation of the skin via regulation of paracrine melanogenic cytokine receptor signaling. |
| 19100240 | 1 | No | Epigenetic inactivation of SLIT2 in human hepatocellular carcinomas. |
| 19101521 | 4 | No | Epithelial cell survival by activating transcription factor 3 (ATF3) in response to chemical ribosome-inactivating stress. |
| 19103272 | 8 | No | Effect of Pinus massoniana bark extract on IFN-gamma-induced ICAM-1 expression in HaCaT human keratinocytes. |
| 19103299 | 2 | No | Docetaxel/zoledronic acid combination triggers apoptosis synergistically through downregulating antiapoptotic Bcl-2 protein level in hormone-refractory prostate |
| 19106633 | 12 | No | TNFSF10 (TRAIL), a p53 target gene that mediates p53-dependent cell death. |
| 19108891 | 1 | No | Epigenetic control of differential expression of specific ERG isoforms in acute T-lymphoblastic leukemia. |
| 19110045 | 9 | No | Hesperidin inhibited acetaldehyde-induced matrix metalloproteinase-9 gene expression in human hepatocellular carcinoma cells. |
| 19111005 | 14 | No | Isocyanates induces DNA damage, apoptosis, oxidative stress, and inflammation in cultured human lymphocytes. |
| 19111594 | 1 | No | DNA damage, signalling and repair after exposure of cells to the sulphur mustard analogue 2-chloroethyl ethyl sulphide. |
| 19114014 | 1 | No | Chronic, low-dose rotenone reproduces Lewy neurites found in early stages of Parkinson's disease, reduces mitochondrial movement and slowly kills |
| 19116882 | 1 | No | Growth inhibition and induction of apoptosis of colon cancer cell lines by applying marine phospholipid. |
| 19117987 | 13 | No | Inhibition of DNA methyltransferase activates tumor necrosis factor alpha-induced monocytic differentiation in acute myeloid leukemia cells. |
| 19127263 | 4 | No | Promoter CpG methylation in cancer cells contributes to the regulation of MUC4. |
| 19137819 | 2 | No | [Changes of nuclear matrix proteins during apoptosis of human osteosarcoma MG-63 cells induced by curcumin] |
| 19139008 | 36 | No | A novel derivative of the natural agent deguelin for cancer chemoprevention and therapy. |
| 19139132 | 2 | No | Human concentrative nucleoside transporter 1-mediated uptake of 5-azacytidine enhances DNA demethylation. |
| 19139152 | 9 | No | Intracellular potassium stabilizes human ether-膝-go-go-related gene channels for export from endoplasmic reticulum. |

| | | | |
|---|---|---|---|
| 19147557 | 2 | No | Down-regulation of Rap1GAP via promoter hypermethylation promotes melanoma cell proliferation, survival, and migration. |
| 19147571 | 8 | No | gamma-Secretase inhibitors abrogate oxaliplatin-induced activation of the Notch-1 signaling pathway in colon cancer cells resulting in enhanced chemosensitivity. |
| 19148494 | 16 | No | The anti-leukemic effect of a novel histone deacetylase inhibitor MCT-1 and 5-aza-cytidine involves augmentation of Nur77 and inhibition of MMP-9 expression. |
| 19151715 | 27 | No | The human colon cancer methylome shows similar hypo- and hypermethylation at conserved tissue-specific CpG island shores. |
| 19152342 | 8 | No | Nevirapine restores androgen signaling in hormone-refractory human prostate carcinoma cells both in vitro and in vivo. |
| 19155497 | 1 | No | Methylation status of CpG islands flanking a cAMP response element motif on the protein phosphatase 2Ac alpha promoter determines CREB binding and activity. |
| 19162161 | 6 | No | Nitroxide radical TEMPO reduces ozone-induced chemokine IL-8 production in lung epithelial cells. |
| 19164858 | 2 | No | Acetaminophen-induced hepatotoxicity in mice is dependent on Tlr9 and the Nalp3 inflammasome. |
| 1916698 | 1 | No | A new insight into the histogenesis of 'mesodermomas'--malignant mesotheliomas. |
| 19168569 | 5 | No | Arsenic activates EGFR pathway signaling in the lung. |
| 19172108 | 2 | No | Study of 5-Aza-CdR on transcription regulation of RASSF1A gene in the BIU87 cell line. |
| 19177197 | 3 | No | Aberrant promoter methylation of SPARC in ovarian cancer. |
| 19181744 | 1 | No | Cap-independent translation of human SP-A 5'-UTR variants: a double-loop structure and cis-element contribution. |
| 19188863 | 18 | No | Induction of antioxidant enzymes by curcumin and its analogues in human islets: implications in transplantation. |
| 19189304 | 2 | No | Vasoactive intestinal peptide behaves as a pro-metastatic factor in human prostate cancer cells. |
| 19189864 | 10 | No | Effects of 1-bromopropane, a substitute for chlorofluorocarbons, on BDNF expression. |
| 19190346 | 6 | No | Methylation-mediated repression of GADD45alpha in prostate cancer and its role as a potential therapeutic target. |
| 19194470 | 253 | No | The DNA methyltransferase inhibitors azacitidine, decitabine and zebularine exert differential effects on cancer gene expression in acute myeloid leukemia cells. |

| | | | |
|---|---|---|---|
| 19196431 | 20 | No | The up-regulation of BACE1 mediated by hypoxia and ischemic injury: role of oxidative stress and HIF1alpha. |
| 19196508 | 2 | No | Wilms' tumour gene 1 (WT1) as a target in curcumin treatment of pancreatic cancer cells. |
| 19199343 | 6 | No | Gene induction for the treatment of methylmalonic aciduria. |
| 19208741 | 1 | No | Inactivation of the MAL gene in breast cancer is a common event that predicts benefit from adjuvant chemotherapy. |
| 19208748 | 3 | No | EFEMP1 expression promotes in vivo tumor growth in human pancreatic adenocarcinoma. |
| 19214542 | 2 | No | Stem-cell-like glioma cells are resistant to TRAIL/Apo2L and exhibit down-regulation of caspase-8 by promoter methylation. |
| 19215824 | 5 | No | A systematic assessment of radiation dose enhancement by 5-Aza-2'-deoxycytidine and histone deacetylase inhibitors in head-and-neck squamous cell carcinoma. |
| 19219653 | 15 | No | Expression profiling of nucleotide metabolism-related genes in human breast cancer cells after treatment with 5-fluorouracil. |
| 19221000 | 9 | No | BTG3 tumor suppressor gene promoter demethylation, histone modification and cell cycle arrest by genistein in renal cancer. |
| 19223499 | 2 | No | Unique methylation pattern of oncostatin m receptor gene in cancers of colorectum and other digestive organs. |
| 19225199 | 4 | No | Short-term amiodarone treatment attenuates the production of monocyte cytokines and chemokines by C-reactive protein and improves cardiac function in patients |
| 19233273 | 5 | No | Inhibition of mitochondrial function induces an integrated stress response in oligodendroglia. |
| 19234609 | 19 | No | Integrative analysis of epigenetic modulation in melanoma cell response to decitabine: clinical implications. |
| 19235267 | 10 | No | Curcumin induces apoptosis through the mitochondria-mediated apoptotic pathway in HT-29 cells. |
| 19235587 | 2 | No | Androgen receptor CpG island methylation status in human leukemia cancer cells. |
| 19240160 | 2 | No | Interleukin-6 increases prostate cancer cells resistance to bicalutamide via TIF2. |
| 19244175 | 3504 | No | Selenium and vitamin E: cell type- and intervention-specific tissue effects in prostate cancer. |
| 19249324 | 9 | No | Pyrethroid insecticides: isoform-dependent hydrolysis, induction of cytochrome P450 3A4 and evidence on the involvement of the pregnane X receptor. |

| | | | |
|---|---|---|---|
| 19250217 | 10 | No | Curcumin as a possible lead compound against hormone-independent, multidrug-resistant breast cancer. |
| 19250671 | 1 | No | Aberrant expression of TSC2 gene in the newly diagnosed acute leukemia. |
| 19255438 | 18 | No | Discovery of selective glucocorticoid receptor modulators by multiplexed reporter screening. |
| 19259094 | 6 | No | Combined inhibition of DNA methylation and histone acetylation enhances gene re-expression and drug sensitivity in vivo. |
| 19276161 | 1 | No | In vivo molecular mediators of cancer growth suppression and apoptosis by selenium in mammary and prostate models: lack of involvement of gadd genes. |
| 19276365 | 1 | No | Epigenetic regulation of c-ROS receptor tyrosine kinase expression in malignant gliomas. |
| 19277044 | 2 | No | Epigenetic silencing of EphA1 expression in colorectal cancer is correlated with poor survival. |
| 19281909 | 1 | No | and function are unclear. OBJECTIVE: To investigate expression, regulation, and function of the H(4)R on human CD4(+) T cells. METHODS: Histamine receptor 4 |
| 19283074 | 2 | No | Discovery of novel hypermethylated genes in prostate cancer using genomic CpG island microarrays. |
| 19283680 | 4 | No | Inflammatory response to isocyanates and onset of genomic instability in cultured human lung fibroblasts. |
| 19289100 | 2 | No | Differential expression of stromal cell-derived factor 1 in human brain microvascular endothelial cells and pericytes involves histone modifications. |
| 19293009 | 1 | No | Phenol peels as a novel therapeutic approach for actinic keratosis and Bowen disease: prospective pilot trial with assessment of clinical, histologic, and |
| 19294695 | 50 | No | Synergistic effect of trichostatin A and 5-aza-2'-deoxycytidine on growth inhibition of pancreatic endocrine tumour cell lines: a proteomic study. |
| 19294764 | 6 | No | Curcumin suppresses PPARdelta expression and related genes in HT-29 cells. |
| 19295137 | 4 | No | In utero exposure to methyl isocyanate in the Bhopal gas disaster: evidence of persisting hyperactivation of immune system two decades later. |
| 19295228 | 4 | No | Phyllanthus urinaria increases apoptosis and reduces telomerase activity in human nasopharyngeal carcinoma cells. |
| 19318562 | 16 | No | Genome-wide impact of androgen receptor trapped clone-27 loss on androgen-regulated transcription in prostate cancer cells. |
| 19329758 | 2 | No | Promoter CpG island hypermethylation- and H3K9me3 and H3K27me3-mediated epigenetic silencing targets the deleted in colon cancer (DCC) gene in colorectal |

| | | | |
|---|---|---|---|
| 19330836 | 3 | No | Lysyl oxidase-like 2 promotes migration in noninvasive breast cancer cells but not in normal breast epithelial cells. |
| 19331143 | 4 | No | Epigenetic modifications of the Estrogen receptor beta gene in epithelial ovarian cancer cells. |
| 19336726 | 6 | No | Liposomal encapsulation of deguelin: evidence for enhanced antitumor activity in tobacco carcinogen-induced and oncogenic K-ras-induced lung tumorigenesis. |
| 19351862 | 18 | No | Estrogens and human papilloma virus oncogenes regulate human ether-脿-go-go-1 potassium channel expression. |
| 19358275 | 2 | No | The antiretroviral nucleoside analogue Abacavir reduces cell growth and promotes differentiation of human medulloblastoma cells. |
| 19359544 | 1 | No | Development of a second-generation antiandrogen for treatment of advanced prostate cancer. |
| 19360359 | 3 | No | Se-methylselenocysteine sensitized TRAIL-mediated apoptosis via down-regulation of Bcl-2 expression. |
| 19360757 | 7 | No | Potential of deoxynivalenol to induce transcription factors in human hepatoma cells. |
| 19363521 | 148 | No | DNA methylation inhibits p53-mediated survivin repression. |
| 19365708 | 8 | No | Involvement of SRC-3 in deguelin-induced apoptosis in Jurkat cells. |
| 19367675 | 4 | No | Kaempferol and quercetin, essential ingredients in Ginkgo biloba extract, inhibit interleukin-1beta-induced MUC5AC gene expression in human airway epithelial |
| 19371339 | 1 | No | Ciprofloxacin decreases survival in HT-29 cells via the induction of TGF-beta1 secretion and enhances the anti-proliferative effect of 5-fluorouracil. |
| 19371603 | 11 | No | Acrolein activates matrix metalloproteinases by increasing reactive oxygen species in macrophages. |
| 19372550 | 4 | No | Growth inhibition of human cancer cells by 5-aza-2'-deoxycytidine does not correlate with its effects on INK4a/ARF expression or initial promoter |
| 19372569 | 13 | No | Curcumin circumvents chemoresistance in vitro and potentiates the effect of thalidomide and bortezomib against human multiple myeloma in nude mice model. |
| 19376255 | 7 | No | Induction of genomic instability in cultured human colon epithelial cells following exposure to isocyanates. |
| 19376845 | 9 | No | Resveratrol inhibits dioxin-induced expression of human CYP1A1 and CYP1B1 by inhibiting recruitment of the aryl hydrocarbon receptor complex and RNA |
| 19387464 | 7 | No | Wnt signaling pathway is epigenetically regulated by methylation of Wnt antagonists in acute myeloid leukemia. |

| | | | |
|---|---|---|---|
| 19387567 | 1 | No | Selenoprotein W modulates control of cell cycle entry. |
| 19394699 | 8 | No | Demethylating treatment suppresses natural killer cell cytolytic activity. |
| 19396019 | 4 | No | GSTP1 determines cis-platinum cytotoxicity in gastric adenocarcinoma MGC803 cells: regulation by promoter methylation and extracellular regulated kinase |
| 19397994 | 53 | No | Cytotoxicity of flavones and flavonols to a human esophageal squamous cell carcinoma cell line (KYSE-510) by induction of G2/M arrest and apoptosis. |
| 19400555 | 2 | No | Induction of gamma delta T cells using zoledronate plus interleukin-2 in patients with metastatic cancer. |
| 19401350 | 19 | No | Treatment of chronic lymphocytic leukemia with a hypomethylating agent induces expression of NXF2, an immunogenic cancer testis antigen. |
| 19401701 | 3 | No | Activation of ATM/Chk1 by curcumin causes cell cycle arrest and apoptosis in human pancreatic cancer cells. |
| 19403302 | 2 | No | Epigenetic down-regulation of BIM expression is associated with reduced optimal responses to imatinib treatment in chronic myeloid leukaemia. |
| 19403854 | 24 | No | Signal transducer and activator of transcription 1 (STAT1) is essential for chromium silencing of gene induction in human airway epithelial cells. |
| 19409404 | 8 | No | Toxicogenomic effects common to triazole antifungals and conserved between rats and humans. |
| 19417133 | 8 | No | A new class of quinoline-based DNA hypomethylating agents reactivates tumor suppressor genes by blocking DNA methyltransferase 1 activity and inducing its |
| 19422044 | 3 | No | Blockade of MEK signaling potentiates 5-Aza-2'-deoxycytidine-induced apoptosis and upregulation of p21(waf1) in acute myelogenous leukemia cells. |
| 19422813 | 35 | No | Differential effects exerted on human mammary epithelial cells by environmentally relevant organochlorine pesticides either individually or in combination. |
| 19424577 | 2 | No | CpG hypermethylation of collagen type I alpha 2 contributes to proliferation and migration activity of human bladder cancer. |
| 19426720 | 2 | No | Andrographolide could inhibit human colorectal carcinoma Lovo cells migration and invasion via down-regulation of MMP-7 expression. |
| 19427250 | 12 | No | Stop codon read-through of a methylmalonic aciduria mutation. |
| 19428936 | 34 | No | Protective effect of quercetin on ER stress caused by calcium dynamics dysregulation in intestinal epithelial cells. |
| 19438726 | 11 | No | Overexpression of human histone methylase MLL1 upon exposure to a food contaminant mycotoxin, deoxynivalenol. |

| | | | |
|---|---|---|---|
| 19440673 | 3 | No | Additive effects of 5-aza-2'-deoxycytidine and irradiation on clonogenic survival of human medulloblastoma cell lines. |
| 19440933 | 5 | No | Quercetin inhibit human SW480 colon cancer growth in association with inhibition of cyclin D1 and survivin expression through Wnt/beta-catenin signaling pathway. |
| 19442645 | 2 | No | Quantification of regional DNA methylation by liquid chromatography/tandem mass spectrometry. |
| 19445022 | 1 | No | T-lymphocyte maturation-associated protein gene as a candidate metastasis suppressor for head and neck squamous cell carcinomas. |
| 19446661 | 6 | No | Defective gammadelta T-cell function and granzyme B gene polymorphism in a cohort of newly diagnosed breast cancer patients. |
| 19450372 | 14 | No | Effects of red grape juice polyphenols in NADPH oxidase subunit expression in human neutrophils and mononuclear blood cells. |
| 19450571 | 6 | No | Panaxydol inhibits the proliferation and induces the differentiation of human hepatocarcinoma cell line HepG2. |
| 19451193 | 1 | No | Oxidative stress induces parallel autophagy and mitochondria dysfunction in human glioma U251 cells. |
| 19451745 | 5 | No | Effect of c-MYC and E2F1 gene silencing and of 5-azacytidine treatment on telomerase activity in pancreatic cancer-derived cell lines. |
| 19458056 | 23 | No | Interaction of TFAP2C with the estrogen receptor-alpha promoter is controlled by chromatin structure. |
| 19464277 | 2 | No | Estrogen-related receptor alpha (ERRalpha) inverse agonist XCT-790 induces cell death in chemotherapeutic resistant cancer cells. |
| 19470736 | 2 | No | Decitabine effect on tumor global DNA methylation and other parameters in a phase I trial in refractory solid tumors and lymphomas. |
| 19481069 | 16 | No | Combined application of camptothecin and the guanylate cyclase activator YC-1: Impact on cell death and apoptosis-related proteins in ovarian carcinoma cell |
| 19481070 | 10 | No | [6]-Gingerol induces reactive oxygen species regulated mitochondrial cell death pathway in human epidermoid carcinoma A431 cells. |
| 19483324 | 2 | No | Camptothecin-induced cell proliferation inhibition and apoptosis enhanced by DNA methyltransferase inhibitor, 5-aza-2'-deoxycytidine. |
| 19497413 | 5 | No | The selenium analog of the chemopreventive compound S,S'-(1,4-phenylenebis[1,2-ethanediyl])bisisothiourea is a remarkable inducer of |
| 19503093 | 4 | No | Frequent loss of cystatin E/M expression implicated in the progression of prostate cancer. |
| 19505289 | 1 | No | The haplotype of the growth-differentiation factor 15 gene is associated with left ventricular hypertrophy in human essential hypertension. |

| | | | |
|---|---|---|---|
| 19506552 | 1 | No | Epigenetic silencing of TTF-1/NKX2-1 through DNA hypermethylation and histone H3 modulation in thyroid carcinomas. |
| 19513903 | 4 | No | Mitochondrial oxidative stress elicits chromosomal instability after exposure to isocyanates in human kidney epithelial cells. |
| 19519261 | 1 | No | Aripiprazole in pervasive developmental disorder not otherwise specified and Asperger's disorder: a 14-week, prospective, open-label study. |
| 19523936 | 13 | No | N-acetylcysteine, coenzyme Q10 and superoxide dismutase mimetic prevent mitochondrial cell dysfunction and cell death induced by d-galactosamine in |
| 19524134 | 14 | No | Re-differentiation of thyroid carcinoma cell lines treated with 5-Aza-2'-deoxycytidine and retinoic acid. |
| 19530242 | 9 | No | Cyr61 downmodulation potentiates the anticancer effects of zoledronic acid in androgen-independent prostate cancer cells. |
| 19531572 | 2 | No | Distinct roles for histone methyltransferases G9a and GLP in cancer germ-line antigen gene regulation in human cancer cells and murine embryonic stem cells. |
| 19536146 | 19 | No | Aryl hydrocarbon receptor is an ozone sensor in human skin. |
| 19542490 | 2 | No | Hypoxia induces B-type natriuretic peptide release in cell lines derived from human cardiomyocytes. |
| 19549553 | 6 | No | The mycotoxin deoxynivalenol inhibits the cell surface expression of activation markers in human macrophages. |
| 19549761 | 1 | No | Resveratrol alters proliferative responses and apoptosis in human activated B lymphocytes in vitro. |
| 19551406 | 3 | No | DNA methylation regulates constitutive expression of Stat6 regulatory genes SOCS-1 and SHP-1 in colon cancer cells. |
| 19557821 | 95 | No | Naturally occurring flavonoids attenuate high glucose-induced expression of proinflammatory cytokines in human monocytic THP-1 cells. |
| 19559774 | 186 | No | Alteration of transcriptional profile in human bronchial epithelial cells induced by cigarette smoke condensate. |
| 19573523 | 6 | No | Potent growth suppressive activity of curcumin in human breast cancer cells: Modulation of Wnt/beta-catenin signaling. |
| 19576171 | 3 | No | Essential role of Nrf2 in keratinocyte protection from UVA by quercetin. |
| 19577553 | 21 | No | Sodium arsenite-induced DAPK promoter hypermethylation and autophagy via ERK1/2 phosphorylation in human uroepithelial cells. |
| 19584167 | 4 | No | 15-hydroxyprostaglandin dehydrogenase is down-regulated in gastric cancer. |

| | | | |
|---|---|---|---|
| 19591856 | 9 | No | HuR/ELAVL1 RNA binding protein modulates interleukin-8 induction by muco-active ribotoxin deoxynivalenol. |
| 19596284 | 15 | No | Acrolein induces a cellular stress response and triggers mitochondrial apoptosis in A549 cells. |
| 19597852 | 8 | No | Expression profile and synthesis of different collagen types I, II, III, and V of human gingival fibroblasts, osteoblasts, and SaOS-2 cells after bisphosphonate |
| 19616087 | 6 | No | Folic acid uptake by the human syncytiotrophoblast: interference by pharmacotherapy, drugs of abuse and pathological conditions. |
| 19616521 | 4 | No | Suillin from the mushroom Suillus placidus as potent apoptosis inducer in human hepatoma HepG2 cells. |
| 19625696 | 8 | No | Methylselenol, a selenium metabolite, induces cell cycle arrest in G1 phase and apoptosis via the extracellular-regulated kinase 1/2 pathway and other cancer |
| 19631781 | 4 | No | Trichlorfon induces apoptosis in SH-SY5Y neuroblastoma cells via the endoplasmic reticulum? |
| 19631782 | 2 | No | SAHA treatment overcomes the anti-apoptotic effects of Bcl-2 and is associated with the formation of mature PML nuclear bodies in human leukemic U937 cells. |
| 19632212 | 2 | No | A proteomic investigation into the human cervical cancer cell line HeLa treated with dicitratoytterbium (III) complex. |
| 19651184 | 4 | No | Quercetin inhibits hydrogen peroxide-induced DNA damage and enhances DNA repair in Caco-2 cells. |
| 19667106 | 3 | No | Nevirapine increases high-density lipoprotein cholesterol concentration by stimulation of apolipoprotein A-I production. |
| 19682441 | 8 | No | Osthole, a potential antidiabetic agent, alleviates hyperglycemia in db/db mice. |
| 19682730 | 198 | No | Identification of selective inhibitors of cancer stem cells by high-throughput screening. |
| 19683516 | 23 | No | Generation of oxidant response to copper and iron nanoparticles and salts: Stimulation by ascorbate. |
| 19686714 | 1 | No | In vitro enhancement effect of humic acid on the progression of lung cancer cells. |
| 19691856 | 162 | No | Anti-oncogenic and pro-differentiation effects of clorgyline, a monoamine oxidase A inhibitor, on high grade prostate cancer cells. |
| 19729006 | 35 | No | Hypoxia-inducible factor-1 (HIF-1) pathway activation by quercetin in human lens epithelial cells. |
| 19733639 | 6 | No | A biphasic effect of the fungicide prochloraz on aldosterone, but not cortisol, secretion in human adrenal H295R cells--underlying mechanisms. |

| | | | |
|---|---|---|---|
| 19759027 | 2 | No | Identification of the GATA factor TRPS1 as a repressor of the osteocalcin promoter. |
| 19761891 | 4 | No | Select phytochemicals suppress human T-lymphocytes and mouse splenocytes suggesting their use in autoimmunity and transplantation. |
| 19763263 | 20 | No | Novel mitochondrial substrates of omi indicate a new regulatory role in neurodegenerative disorders. |
| 19774075 | 547 | No | Identification by automated screening of a small molecule that selectively eliminates neural stem cells derived from hESCs but not dopamine neurons. |
| 19787260 | 4 | No | Organophosphorous pesticides and estrogen induce transformation of breast cells affecting p53 and c-Ha-ras genes. |
| 19789329 | 23 | No | High-throughput cell-based screening of 4910 known drugs and drug-like small molecules identifies disulfiram as an inhibitor of prostate cancer cell growth. |
| 19794518 | 10 | No | Modulation of CYP1B1 and CYP1A1 gene expression and activation of aryl hydrocarbon receptor by Ginkgo biloba extract in MCF-10A human mammary epithelial |
| 19800895 | 17 | No | Organophosphorus pesticides enhance the genotoxicity of benzo(a)pyrene by modulating its metabolism. |
| 19804834 | 21 | No | Contribution of reactive oxygen species to migration/invasion of human glioblastoma cells U87 via ERK-dependent COX-2/PGE(2) activation. |
| 19805378 | 2 | No | Reduction of phosphoinositide-phospholipase C beta1 methylation predicts the responsiveness to azacitidine in high-risk MDS. |
| 19814765 | 4 | No | Alpha-defensins increase lung fibroblast proliferation and collagen synthesis via the beta-catenin signaling pathway. |
| 19819230 | 4 | No | Mevalonate pathway intermediates downregulate zoledronic acid-induced isopentenyl pyrophosphate and ATP analog formation in human breast cancer cells. |
| 19825521 | 26 | No | Combined gossypol and zoledronic acid treatment results in synergistic induction of cell death and regulates angiogenic molecules in ovarian cancer cells. |
| 19846905 | 4 | No | STX140 and STX641 cause apoptosis via the intrinsic mitochondrial pathway and down-regulate survivin and XIAP expression in ovarian and prostate cancer cells. |
| 19888908 | 1 | No | Regulation of heme synthesis and proteasomal activity by copper: possible implications for Wilson's disease. |
| 19891054 | 3 | No | Changes in biomarkers of cardiovascular risk after a switch to abacavir in HIV-1-infected individuals receiving combination antiretroviral therapy. |
| 19915386 | 1 | No | Zoledronic acid decreases mRNA six-transmembrane epithelial antigen of prostate protein expression in prostate cancer cells. |
| 19915711 | 7 | No | Change in mRNA Expression after Atenolol, a Beta-adrenergic Receptor Antagonist and Association with Pharmacological Response. |

| | | | |
|---|---|---|---|
| 19929230 | 2 | No | The hemostatic balance in HIV-infected patients with and without antiretroviral therapy: partial restoration with antiretroviral therapy. |
| 19944065 | 20 | No | (-)-Epigallocatechin-3-gallate decreases thrombin/paclitaxel-induced endothelial tissue factor expression via the inhibition of c-Jun terminal NH2 kinase |
| 20032539 | 2 | No | Adipocyte differentiation, mitochondrial gene expression and fat distribution: differences between zidovudine and tenofovir after 6 months. |
| 2015207 | 1 | No | Biological effect monitoring of occupational exposure to 1,3-dichloropropene: effects on liver and renal function and on glutathione conjugation. |
| 20181660 | 3 | No | Hematopoietic cell kinase associates with the 40S ribosomal subunit and mediates the ribotoxic stress response to deoxynivalenol in mononuclear phagocytes. |
| 20185895 | 3 | No | Inorganic phosphate stimulates DMP1 expression in human periodontal ligament fibroblasts embedded in three-dimensional collagen gels. |
| 20196486 | 2 | No | [The discovery of an antiparkinsonian drug, zonisamide] |
| 20200314 | 4 | No | Platelet tissue factor synthesis in type 2 diabetic patients is resistant to inhibition by insulin. |
| 20349282 | 36 | No | Regulation of apoptosis-related molecules by synergistic combination of all-trans retinoic acid and zoledronic acid in hormone-refractory prostate cancer cell |
| 20370541 | 220 | No | Expression profiles of adult T-cell leukemia-lymphoma and associations with clinical responses to zidovudine and interferon alpha. |
| 2175014 | 2 | No | Effects of chronic pindolol treatment on human myocardial beta 1- and beta 2-adrenoceptor function. |
| 2539393 | 6 | No | Regulation of messenger RNAs for the hydrophobic surfactant proteins in human lung. |
| 2558650 | 8 | No | c-myc gene expression in human cells is controlled by glucose. |
| 3689822 | 3 | No | Modulation of the reductive metabolism of halothane by microsomal cytochrome b5 in rat liver. |
| 3782137 | 1 | No | Complementary DNA and protein sequences of ethanol-inducible rat and human cytochrome P-450s. Transcriptional and post-transcriptional regulation of the rat |
| 7493931 | 2 | No | Hypoxia and mitochondrial inhibitors regulate expression of glucose transporter-1 via distinct Cis-acting sequences. |
| 7556191 | 9 | No | Involvement of retinoic acid receptor alpha in the stimulation of tissue-type plasminogen-activator gene expression in human endothelial cells. |
| 7576814 | 1 | No | Activation of alpha-human tumour necrosis factor (TNF-alpha) by human monocytes (THP-1) exposed to 2-chloroethyl ethyl sulphide (H-MG). |

| | | | |
|---|---|---|---|
| 7579340 | 4 | No | Changing the differentiation program of hematopoietic cells: retinoic acid-induced shift of eosinophil-committed cells to neutrophils. |
| 7598938 | 4 | No | Ozone stimulates synthesis of inflammatory cytokines by alveolar macrophages in vitro. |
| 7600656 | 3 | No | Copper-induced tissue factor expression in human monocytic THP-1 cells and its inhibition by antioxidants. |
| 7606800 | 8 | No | Down-regulation of interleukin-2 receptor gene activation and protein expression by dideoxynucleoside analogs. |
| 7614465 | 1 | No | Pulmonary expression of glutathione S-transferase M3 in lung cancer patients: association with GSTM1 polymorphism, smoking, and asbestos exposure. |
| 7680553 | 3 | No | Modulation of fibroblast growth factor receptor expression and signalling during retinoic acid-induced differentiation of Tera-2 teratocarcinoma cells. |
| 7769305 | 3 | No | High-dose pentoxifylline in patients with AIDS: inhibition of tumor necrosis factor production. National Institute of Allergy and Infectious Diseases AIDS |
| 7798212 | 4 | No | Isolation and characterization of HL-60 cells resistant to nitroprusside-induced differentiation. |
| 7826318 | 3 | No | Glucose-induced stimulation of human insulin-receptor mRNA and tyrosine kinase activity in cultured cells. |
| 7849699 | 1 | No | Deficiency of the human mitochondrial transcription factor h-mtTFA in infantile mitochondrial myopathy is associated with mtDNA depletion. |
| 7934638 | 2 | No | Acetaldehyde regulates the gene expression of matrix-metalloproteinase-1 and -2 in human fat-storing cells. |
| 7961698 | 1 | No | Insulin and dietary fructose induce stearoyl-CoA desaturase 1 gene expression of diabetic mice. |
| 7963561 | 3 | No | Signal transduction for nuclear factor-kappa B activation. Proposed location of antioxidant-inhibitable step. |
| 7980410 | 7 | No | Role of hydrogen peroxide in hypoxia-induced erythropoietin production. |
| 7986210 | 10 | No | The influence of picolines on glutathione transferase activity and subunit composition in human liver derived Hep G2 cells. |
| 8043013 | 3 | No | Reduction of DNA synthesis, pigment synthesis, pigmentation gene mRNA and resistance to UVB in human melanoma cells treated with analogues of a histamine |
| 8049197 | 1 | No | Purification, cloning, and expression of a human enzyme with acyl coenzyme A: cholesterol acyltransferase activity, which is identical to liver |
| 8049355 | 1 | No | Interleukin-5 receptor alpha chain mRNA is down-regulated by transforming growth factor beta 1. |

| | | | |
|---|---|---|---|
| 8074729 | 6 | No | Ethanol increases content and activity of human cytochrome P4502E1 in a transduced HepG2 cell line. |
| 8096834 | 3 | No | Molecular pharmacology of the beta-adrenergic receptor on THP-1 cells. |
| 8135774 | 8 | No | Regulation of undulin synthesis and gene expression in human fat-storing cells by acetaldehyde and transforming growth factor-beta 1: comparison with fibronectin. |
| 8224229 | 3 | No | Increased expression of type VI collagen genes in drug-induced gingival enlargement. |
| 8279580 | 2 | No | Effects of inhibition of catalase and superoxide dismutase activity on antioxidant enzyme mRNA levels. |
| 8299707 | 1 | No | Time course of changes in free thyroid indices, rT3, TSH, cortisol and ACTH following exposure to sulfur mustard. |
| 8394679 | 2 | No | Increased expression of fibroblast growth factors (FGFs) and their receptor by protamine and suramin on Kaposi's sarcoma-derived cells. |
| 8463938 | 1 | No | Phenytoin increases gene expression for platelet-derived growth factor B chain in macrophages and monocytes. |
| 8554516 | 5 | No | Sugar-dependent expression of the fructose transporter GLUT5 in Caco-2 cells. |
| 8599995 | 2 | No | 3'-Azido-3'-deoxythymidine inhibits erythroid-specific transcription factors in human erythroid K562 leukemia cells. |
| 8603935 | 1 | No | Appearance of interleukin 1 alpha relates DNA interstrand cross-links and cytotoxicity in cultured human keratinocytes exposed to bis-(2-chloroethyl) |
| 8621535 | 14 | No | Generation of 8-epiprostaglandin F2alpha by human monocytes. Discriminate production by reactive oxygen species and prostaglandin endoperoxide synthase-2. |
| 8629860 | 2 | No | Tumor necrosis factor alpha gene expression in human monocytic THP-1 cells exposed to beryllium. |
| 8632764 | 14 | No | Modulators and substrates of P-glycoprotein and cytochrome P4503A coordinately up-regulate these proteins in human colon carcinoma cells. |
| 8652659 | 3 | No | Transcriptional regulation of the human NAD(P)H:quinone oxidoreductase (NQO1) gene by monofunctional inducers. |
| 8708960 | 1 | No | Cyclosporine A upregulates platelet-derived growth factor B chain in hyperplastic human gingiva. |
| 8719445 | 3 | No | Mechanism of the antiproliferative action of leflunomide. A77 1726, the active metabolite of leflunomide, does not block T-cell receptor-mediated signal |
| 8738476 | 2 | No | Inhibition of gap junctional intercellular communication in heptachlor- and heptachlor epoxide-treated normal human breast epithelial cells. |

| | | | |
|---|---|---|---|
| 8743975 | 4 | No | Lipid metabolite involvement in the activation of the human heme oxygenase-1 gene. |
| 8760145 | 9 | No | Redox regulation of manganese superoxide dismutase. |
| 8764571 | 7 | No | Expression of heme oxygenase isozyme mRNAs in the human brain and induction of heme oxygenase-1 by nitric oxide donors. |
| 8780174 | 20 | No | Action of cAMP on expression and release of adhesion molecules in human endothelial cells. |
| 8798623 | 1 | No | Ethanol cytotoxicity to a transfected HepG2 cell line expressing human cytochrome P4502E1. |
| 8812189 | 5 | No | Hierarchical cluster analysis of environmental pollutants through P450 induction in cultured hepatic cells. |
| 8820588 | 9 | No | Inhibition of gap junctional intercellular communication in normal human breast epithelial cells after treatment with pesticides, PCBs, and PBBs, alone or in |
| 8833408 | 2 | No | Rotenone, a mitochondrial NADH dehydrogenase inhibitor, induces cell surface expression of CD13 and CD38 and apoptosis in HL-60 cells. |
| 8897462 | 2 | No | Mitochondrial involvement in schizophrenia and other functional psychoses. |
| 8902626 | 2 | No | Expression of the apoptosis-mediator Fas is enhanced by dysfunctional mitochondria. |
| 8940178 | 4 | No | Transcriptional regulation by transforming growth factor beta of the expression of retinoic acid and retinoid X receptor genes in osteoblastic cells is mediated |
| 8941356 | 1 | No | Further study of the mechanism underlying the cellular resistance to AZT. |
| 8943236 | 10 | No | Induction of p21 mediated by reactive oxygen species formed during the metabolism of aziridinylbenzoquinones by HCT116 cells. |
| 8971655 | 10 | No | Interleukin-1 beta and phenytoin reduce alpha 1 (I) procollagen mRNA expression in human gingival fibroblasts. |
| 9007151 | 4 | No | Esterase inhibitors diminish the modulation of gene expression by butyric acid derivative, pivaloyloxymethyl butyrate (AN-9). |
| 9012323 | 1 | No | Immunomodulatory effects of occupational exposure to mancozeb. |
| 9029048 | 9 | No | Human NAD(P)H:quinone oxidoreductase induction in human hepatoma cells after exposure to industrial acrylates, phenolics, and metals. |
| 9029455 | 1 | No | Phenytoin and cyclosporine A specifically regulate macrophage phenotype and expression of platelet-derived growth factor and interleukin-1 in vitro and in |

| | | | |
|---|---|---|---|
| 9070347 | 6 | No | The organic component of diesel exhaust particles and phenanthrene, a major polyaromatic hydrocarbon constituent, enhances IgE production by IgE-secreting |
| 9088875 | 5 | No | Efficacy of a selective histamine H2 receptor agonist, dimaprit, in experimental models of endotoxin shock and hepatitis in mice. |
| 9124608 | 2 | No | Ozone-induced IL-8 expression and transcription factor binding in respiratory epithelial cells. |
| 9139807 | 2 | No | Angiotensin II and potassium regulate human CYP11B2 transcription through common cis-elements. |
| 9144411 | 18 | No | Several synthetic chemicals inhibit progesterone receptor-mediated transactivation in yeast. |
| 9148857 | 1 | No | Induction of the multidrug-transporter P-glycoprotein by 3'-azido-3'-deoxythymidine (AZT) treatment in tumor cell lines. |
| 9169082 | 7 | No | Carbaryl induces CYP1A1 gene expression in HepG2 and HaCaT cells but is not a ligand of the human hepatic Ah receptor. |
| 9169520 | 2 | No | Identification of estrogen-responsive genes in neuroblastoma SK-ER3 cells. |
| 9187264 | 23 | No | Histamine modulates the expression of c-fos through cyclic AMP production via the H2 receptor in the human promonocytic cell line U937. |
| 9201699 | 2 | No | Analysis of the in vitro effect of exogenous nitric oxide on human lymphocytes. |
| 9203624 | 2 | No | Beta-adrenoceptor activation-induced placental prorenin secretion is mediated by increased renin messenger RNA and protein synthesis. |
| 9256163 | 2 | No | Beta-adrenergic regulation of renin expression in differentiated U-937 monocytic cells. |
| 9258252 | 25 | No | Beta 2-adrenergic activation enhances interleukin-8 production by human monocytes. |
| 9262057 | 1 | No | Factors associated with the therapeutic efficacy of retinoic acids on malignant lymphomas. |
| 9267955 | 6 | No | Effect of ciprofloxacin on the accumulation of interleukin-6, interleukin-8, and nitrite from a human endothelial cell model of sepsis. |
| 9276643 | 7 | No | Effect of toxaphene on estrogen receptor functions in human breast cancer cells. |
| 9297567 | 2 | No | Respiratory epithelial cells display polarity in their release of the chemokine IL-8 after exposure to ozone. |
| 9310018 | 1 | No | Effects of ozone on epithelium and sensory nerves in the bronchial mucosa of healthy humans. |

| | | | |
|---|---|---|---|
| 9343371 | 7 | No | The role of NAD(P)H:quinone oxidoreductase in quinone-mediated p21 induction in human colon carcinoma cells. |
| 9344885 | 18 | No | Cytokine production by human airway epithelial cells after exposure to an air pollution particle is metal-dependent. |
| 9351880 | 6 | No | An in vitro and in vivo study of cytokines in the acute-phase response associated with bisphosphonates. |
| 9447704 | 4 | No | Angiogenesis inhibitor SR 25989 upregulates thrombospondin-1 expression in human vascular endothelial cells and foreskin fibroblasts. |
| 9504181 | 1 | No | [Effects of local treatment with sodium fluoride mouthrinse on peroxidase and hypothiocyanite saliva levels in adolescent]/. |
| 9504665 | 2 | No | Histopathologic and immunohistochemical features in human skin after exposure to nitrogen and sulfur mustard. |
| 9516142 | 11 | No | Evidence for the involvement of both retinoic acid receptor- and retinoic X receptor-dependent signaling pathways in the induction of tissue transglutaminase |
| 9525474 | 4 | No | Increase of reactive oxygen species (ROS) in endothelial cells by shear flow and involvement of ROS in shear-induced c-fos expression. |
| 9544696 | 1 | No | The effect of heptachlor, a chlorinated hydrocarbon insecticide, on p53 tumor suppressor in human lymphocytes. |
| 9586815 | 3 | No | Anticancer quinones induce pRb-preventable G2/M cell cycle arrest and apoptosis. |
| 9614211 | 28 | No | Reactive oxygen species regulate macrophage scavenger receptor type I, but not type II, in the human monocytic cell line THP-1. |
| 9628260 | 18 | No | Anthralin stimulates keratinocyte-derived proinflammatory cytokines via generation of reactive oxygen species. |
| 9637507 | 9 | No | Molecular mechanisms of the induction of IL-12 and its inhibition by IL-10. |
| 9665388 | 5 | No | Sulfur mustard induces markers of terminal differentiation and apoptosis in keratinocytes via a Ca2+-calmodulin and caspase-dependent pathway. |
| 9673414 | 2 | No | Role of wild-type p53 in the enhancement of camptothecin cytotoxicity against human prostate tumor cells. |
| 9681472 | 2 | No | Constitutive activity and structural instability of the wild-type human H2 receptor. |
| 9704902 | 4 | No | Heptachlor and the mitogen-activated protein kinase module in human lymphocytes. |
| 9707512 | 23 | No | Toxic metals stimulate inflammatory cytokines in hepatocytes through oxidative stress mechanisms. |

| | | | |
|---|---|---|---|
| 9712728 | 7 | No | Effects of triiodothyronine and amiodarone on the promoter of the human LDL receptor gene. |
| 9714973 | 2 | No | Epidermal cytokine mRNA expression induced by hapten differs from that induced by primary irritant in human skin organ culture system. |
| 9716179 | 4 | No | Increased cdc2 and cdk2 kinase activity by retinoid X receptor gamma-mediated transcriptional down-regulation of the cyclin-dependent kinase inhibitor |
| 9721806 | 12 | No | Butylated hydroxytoluene and N-acetylcysteine attenuates tumor necrosis factor-alpha (TNF-alpha) secretion and TNF-alpha mRNA expression in alveolar |
| 9743329 | 6 | No | Prostaglandin E2 and dexamethasone inhibit IL-12 receptor expression and IL-12 responsiveness. |
| 9756745 | 5 | No | Restoration of immune response by a cationic amphiphilic drug (AY 9944) in vitro: a new approach To chemotherapy against human immunodeficiency virus type 1. |
| 9794432 | 6 | No | Dexamethasone but not indomethacin inhibits human phagocyte nicotinamide adenine dinucleotide phosphate oxidase activity by down-regulating expression of genes |
| 9819373 | 18 | No | Histamine inhibits the production of interleukin-12 through interaction with H2 receptors. |
| 9820643 | 3 | No | Interactions between human bronchoepithelial cells and lung fibroblasts after ozone exposure in vitro. |
| 9833039 | 6 | No | Estrogenic xenobiotics increase expression of SS-A/Ro autoantigens in cultured human epidermal cells. |
| 9834970 | 2 | No | NADH: ubiquinone oxidoreductase inhibitors block induction of ornithine decarboxylase activity in MCF-7 human breast cancer cells. |
| 9850737 | 1 | No | Azidothymidine resistance of H9 human T-cell lymphoma cells is associated with decreased sensitivity to antitumor agents and inhibition of apoptosis. |
| 9851254 | 1 | No | Transcriptional activity of quinone methides derived from the tumor promoter butylated hydroxytoluene in HepG2 cells. |
| 9851740 | 15 | No | Differential regulation of human alveolar macrophage-derived interleukin-1beta and tumor necrosis factor-alpha by iron. |
| 9856766 | 3 | No | Human corneal epithelial cell functional responses to inflammatory agents and their antagonists. |
| 9856837 | 5 | No | Epidermal cytokines IL-1beta, TNF-alpha, and IL-12 in patients with atopic dermatitis: response to application of house dust mite antigens. |
| 9860237 | 2 | No | In vitro effects of the pyrethroid S-bioallethrin on lymphocytes and basophils from atopic and nonatopic subjects. |
| 9870925 | 20 | No | Hydrogen peroxide enhances shedding of type I soluble tumor necrosis factor receptor from pulmonary epithelial cells. |

| | | | |
|---|---|---|---|
| 9920887 | 10 | No | Estrogen receptor reduces CYP1A1 induction in cultured human endometrial cells. |
| 9927516 | 7 | No | Cholera toxin suppresses interleukin (IL)-12 production and IL-12 receptor beta1 and beta2 chain expression. |
| 9973176 | 7 | No | Interaction between glucocorticoids and beta2-agonists: alpha and beta glucocorticoid-receptor mRNA expression in human bronchial epithelial cells. |
| 9973208 | 19 | No | Isoform-specific induction of a human aldo-keto reductase by polycyclic aromatic hydrocarbons (PAHs), electrophiles, and oxidative stress: implications for the |
| 9973483 | 3 | No | Immunosuppressive leflunomide metabolite (A77 1726) blocks TNF-dependent nuclear factor-kappa B activation and gene expression. |
| 9989825 | 1 | No | Hydrogen peroxide-induced apoptosis is CD95-independent, requires the release of mitochondria-derived reactive oxygen species and the activation of NF-kappaB. |
