# Supplementary material for: Preferential regulation of miRNA targets by environmental chemicals in the human genome
Source: BMC Genomics. 2011 May 18;12:244. doi: 10.1186/1471-2164-12-244 (PMC3118786; doi:10.1186/1471-2164-12-244)
Supplement: Additional file 8 — Table S4: The 1,842 pairs of significantly concurrent EC-miRNA based on the TargetScan5.1. [file 1471-2164-12-244-S8.PDF]

Concurrent miRNA-EC pair	Number of genes regulated by both of EC and miRNA (overlapped number)	The mean of overlapped numbers in random simulations	The standard deviation of overlapped numbers in random simulations	P-value	Q-value
hsa-miR-371-5p_D011285	12	1.08	0.271293199	0	0
hsa-miR-371-5p_C016766	5	1.016949153	0.1290809	5.3973E-207	1.24E-203
hsa-miR-1260_D011285	12	1.25	0.433012702	1.3469E-134	2.06E-131
hsa-miR-203_C076852	3	1.007246377	0.084816666	6.391E-120	7.34E-117
hsa-miR-1271_C040115	5	1.042253521	0.201166998	1.76821E-84	1.62E-81
hsa-miR-221_C402665	3	1.013888889	0.117029858	9.79827E-63	7.50E-60
hsa-miR-1297_D000431	7	1.134831461	0.372993094	2.17118E-54	1.42E-51
hsa-miR-302a_D009538	6	1.128787879	0.334965015	1.4223E-46	8.16E-44
hsa-miR-302a_D000111	12	1.381974249	0.744087689	3.2497E-45	1.66E-42
hsa-miR-302a_C007262	22	2.326388889	1.447362837	2.08791E-41	9.59E-39
hsa-miR-302a_C093642	18	1.803680982	1.212708249	6.09205E-40	2.54E-37
hsa-miR-592_C007350	6	1.230769231	0.421325044	1.42067E-28	5.44E-26
hsa-miR-632_C086511	3	1.034482759	0.182465608	1.38961E-25	4.91E-23
hsa-miR-1228_D005472	298	10.23387097	28.24935308	4.14023E-25	1.36E-22
hsa-miR-576-3p_D017382	3	1.037037037	0.188852575	7.32037E-24	2.24E-21
hsa-miR-143_D000452	3	1.039370079	0.194473843	1.74177E-22	4.80E-20
hsa-miR-302a_D008070	33	2.973214286	3.063202752	1.77677E-22	4.80E-20
hsa-miR-936_C090046	4	2.02020202	0.199989797	1.04586E-21	2.67E-19
hsa-miR-612_C070515	4	1.985507246	0.208010146	8.2473E-21	1.99E-18
hsa-miR-509-3-5p_D015741	20	2.832946636	1.830377947	1.72588E-20	3.96E-18
hsa-miR-520f_C498475	3	1.043478261	0.20393112	2.01377E-20	4.40E-18
hsa-miR-220c_D001564	18	2.120481928	1.791193923	1.91116E-18	3.99E-16
hsa-miR-7_C516024	2	1.0125	0.11110243	2.5151E-17	5.02E-15
hsa-miR-190_D004958	40	3.848214286	4.277661706	2.88464E-17	5.52E-15
hsa-miR-1228_D004317	49	4.484918794	5.375818499	9.57167E-17	1.76E-14
hsa-miR-576-3p_D005472	294	10.83935743	35.41892571	1.48922E-16	2.63E-14
hsa-miR-576-3p_D013755	26	2.768392371	2.814575411	2.27744E-16	3.84E-14
hsa-miR-632_D004958	47	6.180894309	4.989603094	2.34447E-16	3.84E-14
hsa-miR-1284_D006533	2	1.013333333	0.11469767	2.96797E-16	4.70E-14
hsa-miR-130b_D013467	17	1.897058824	1.828207945	3.30853E-16	5.06E-14
hsa-miR-940_C494622	2	1.01369863	0.116236731	7.96096E-16	1.18E-13
hsa-miR-1237_C482884	2	1.013888889	0.117029858	1.30364E-15	1.87E-13
hsa-miR-190_D013629	15	1.848580442	1.626604388	1.56537E-15	2.18E-13
hsa-miR-520f_C007262	25	3.322946176	2.710374641	1.89703E-15	2.56E-13
hsa-miR-561_D005472	288	17.418	35.46969518	2.59567E-15	3.40E-13
hsa-let-7d_D005472	286	17.524	35.32825249	3.25171E-15	4.11E-13
hsa-miR-383_C093973	20	2.775641026	2.16491167	3.31119E-15	4.11E-13
hsa-miR-503_C063261	2	1.014285714	0.118666055	3.49481E-15	4.22E-13
hsa-let-7i_D013467	18	1.712328767	2.053926757	4.29525E-15	5.06E-13
hsa-miR-576-3p_C074153	5	1.193548387	0.469684509	4.64555E-15	5.28E-13
hsa-miR-576-3p_D000111	11	1.562753036	1.181465034	4.71623E-15	5.28E-13
hsa-miR-190_D020849	13	1.880116959	1.403925986	6.77279E-15	7.40E-13
hsa-miR-654-5p_C066851	4	2	0.246182982	7.54978E-15	8.06E-13
hsa-miR-503_C404397	2	1.014705882	0.120372835	9.36519E-15	9.77E-13
hsa-miR-558_D013467	22	2.537688442	2.516745263	1.6383E-14	1.67E-12

hsa-miR-151-3p_C016837	2	1.015151515	0.12215542	2.50857E-14	2.50E-12
hsa-miR-335_D003633	3	1.063291139	0.243485874	2.99267E-14	2.92E-12
hsa-miR-539_C023035	6	2.92	0.391918359	3.95012E-14	3.78E-12
hsa-miR-576-3p_C014347	73	7.543434343	8.808721133	4.62977E-14	4.34E-12
hsa-miR-649_D011374	29	5.850505051	3.059324998	4.80831E-14	4.41E-12
hsa-miR-1247_D014212	17	1.962616822	1.980867523	6.17007E-14	5.55E-12
hsa-miR-7_D034261	4	2.032258065	0.251943538	8.98722E-14	7.93E-12
hsa-miR-196a_D000079	22	2.290598291	2.628118267	9.30194E-14	8.06E-12
hsa-miR-410_D000432	2	1.015873016	0.124984252	1.09882E-13	9.17E-12
hsa-miR-147_C056516	2	1.015873016	0.124984252	1.09882E-13	9.17E-12
hsa-miR-1231_D004958	49	6.373469388	5.805109484	1.34527E-13	1.10E-11
hsa-miR-499-5p_D008769	7	1.509615385	0.729355076	2.70997E-13	2.18E-11
hsa-miR-1827_C006680	2	1.016393443	0.126983061	2.93988E-13	2.33E-11
hsa-miR-302a_D001554	6	1.330612245	0.620139577	3.14315E-13	2.45E-11
hsa-miR-503_D006588	5	1.815789474	0.420229458	3.23419E-13	2.47E-11
hsa-miR-143_D019308	2	1.016666667	0.128019096	4.80782E-13	3.62E-11
hsa-miR-1228_C014347	67	8.536144578	8.216919732	4.93549E-13	3.65E-11
hsa-miR-302a_C072553	8	1.56122449	0.869744585	5.76347E-13	4.20E-11
hsa-miR-22_C014347	110	20.752	12.73257617	6.71608E-13	4.82E-11
hsa-miR-623_D001388	2	1.016949153	0.1290809	7.86155E-13	5.55E-11
hsa-miR-520f_C063261	7	1.131578947	0.800363491	1.05573E-12	7.34E-11
hsa-miR-143_D002392	11	1.73853211	1.278072535	1.23532E-12	8.46E-11
hsa-miR-1226_C477330	2	1.017241379	0.130169559	1.28531E-12	8.55E-11
hsa-miR-1271_D014750	2	1.017241379	0.130169559	1.28531E-12	8.55E-11
hsa-miR-184_C034613	2	1.01754386	0.131286224	2.10109E-12	1.38E-10
hsa-miR-564_C011890	3	1.073394495	0.260782943	2.15232E-12	1.39E-10
hsa-miR-370_D013467	22	3.140877598	2.67673514	2.47803E-12	1.58E-10
hsa-miR-512-5p_C025462	15	2.252830189	1.820284425	4.91932E-12	3.09E-10
hsa-miR-493_D001554	5	1.275	0.519013487	5.01627E-12	3.11E-10
hsa-miR-432_C089730	9	1.660194175	1.038608087	5.49156E-12	3.35E-10
hsa-miR-641_C494622	2	1.018181818	0.133608531	5.61209E-12	3.35E-10
hsa-miR-643_C086511	2	1.018181818	0.133608531	5.61209E-12	3.35E-10
hsa-miR-576-3p_D010938	5	1.103448276	0.547396823	7.23801E-12	4.26E-10
hsa-miR-576-5p_D009151	19	1.838095238	2.491090701	7.90932E-12	4.60E-10
hsa-miR-181c_C017557	2	1.018518519	0.13481685	9.16985E-12	5.26E-10
hsa-miR-590-5p_D005472	24	3.289655172	3.049591436	1.26413E-11	7.16E-10
hsa-miR-576-3p_C105934	9	2.2	0.979795897	1.41422E-11	7.91791E-10
hsa-miR-526b_C432165	19	2.123966942	2.481569245	1.45772E-11	8.06312E-10
hsa-miR-640_C423222	2	1.018867925	0.136058539	1.49805E-11	8.19E-10
hsa-miR-497_D004054	4	1.131578947	0.408530913	1.92573E-11	1.04012E-09
hsa-miR-1255a_C006632	11	1.825825826	1.342197895	2.12824E-11	1.13613E-09
hsa-miR-632_D020849	15	2.635955056	1.821855283	2.18423E-11	1.15262E-09
hsa-miR-30d_C008493	8	1.5	0.946129637	2.37685E-11	1.24E-09
hsa-miR-576-3p_C115354	2	1.019230769	0.137335162	2.44692E-11	1.26E-09
hsa-miR-1231_D013629	17	2.528255528	2.147751845	2.57063E-11	1.31E-09
hsa-miR-632_D013629	17	2.554216867	2.145620432	2.6686E-11	1.35E-09
hsa-miR-520f_D001599	5	1.306122449	0.534678299	3.22593E-11	1.61E-09
hsa-miR-371-5p_C041517	3	1.083333333	0.276385399	5.20699E-11	2.54312E-09
hsa-miR-936_C001478	3	1.083333333	0.276385399	5.20699E-11	2.54312E-09
hsa-miR-1228_D017273	2	1.02	0.14	6.5248E-11	3.1532E-09
hsa-miR-371-5p_D013749	12	1.74025974	1.543616354	6.59943E-11	3.15604E-09
hsa-miR-1826_C501280	2	1.020408163	0.141391903	1.06517E-10	4.99E-09
hsa-miR-1261_C065250	2	1.020408163	0.141391903	1.06517E-10	4.99E-09
hsa-miR-22_D004317	62	9.606425703	8.353492817	1.37011E-10	6.35371E-09

hsa-miR-224_D002259	2	1.020833333	0.142826138	1.73853E-10	7.98159E-09
hsa-miR-22_C037219	7	1.546938776	0.8347313	2.5841E-10	1.17E-08
hsa-miR-101_C507164	2	1.021276596	0.144304893	2.83698E-10	1.28E-08
hsa-miR-758_D013755	21	2.714714715	2.892414174	2.89272E-10	1.29E-08
hsa-miR-576-3p_C080955	13	1.915662651	1.73694663	3.29685E-10	1.46E-08
hsa-miR-22_D005472	295	25.386	46.20568151	3.48933E-10	1.52567E-08
hsa-miR-509-3-5p_D013196	25	3.236363636	3.492660545	4.22922E-10	1.83173E-08
hsa-miR-1180_D014635	3	1.091666667	0.288554828	4.39797E-10	1.87156E-08
hsa-miR-632_C070081	13	1.817460317	1.765636688	4.40271E-10	1.87156E-08
hsa-miR-199a-5p_C010953	2	1.02173913	0.14583052	4.62845E-10	1.91434E-08
hsa-miR-1231_C086511	2	1.02173913	0.14583052	4.62845E-10	1.91434E-08
hsa-miR-181a_C017461	2	1.02173913	0.14583052	4.62845E-10	1.91434E-08
hsa-miR-1260_D013749	13	1.918367347	1.752394685	4.71753E-10	1.93377E-08
hsa-miR-1827_D010936	13	2.651960784	1.634927017	4.87892E-10	1.98222E-08
hsa-miR-660_D013629	15	2.326190476	2.021498681	5.75279E-10	2.31676E-08
hsa-miR-558_C063509	3	1.060606061	0.295357404	5.86256E-10	2.34044E-08
hsa-miR-365_D019287	6	1.422857143	0.7119734	5.94065E-10	2.35117E-08
hsa-miR-1279_D002699	3	1.045454545	0.298065387	6.15555E-10	2.4154E-08
hsa-miR-1228_D001374	13	2.141025641	1.734942736	7.16003E-10	2.78574E-08
hsa-miR-572_D005492	3	1.094339623	0.29230063	8.04299E-10	3.10297E-08
hsa-miR-371-5p_D020111	8	1.888607595	0.966657734	8.6347E-10	3.30349E-08
hsa-miR-660_D004958	36	5.671487603	5.017071379	9.23197E-10	3.50281E-08
hsa-miR-7_C446520	2	1.02247191	0.148212426	9.64096E-10	3.628E-08
hsa-miR-619_C013690	3	1.095238095	0.293543524	9.77678E-10	3.6492E-08
hsa-miR-151-3p_D009569	2	1.022727273	0.149032694	1.23111E-09	4.52162E-08
hsa-miR-632_D004726	2	1.022727273	0.149032694	1.23111E-09	4.52162E-08
hsa-miR-646_D004996	3	1.096446701	0.29520287	1.26391E-09	4.60525E-08
hsa-miR-1231_D020849	14	2.588807786	1.855817784	1.32534E-09	4.78389E-08
hsa-miR-199a-5p_D011794	43	9.360160966	5.640206909	1.33378E-09	4.78389E-08
hsa-miR-576-3p_C017947	26	4.619148936	3.54029319	1.35475E-09	4.82144E-08
hsa-miR-302a_D005978	5	1.177777778	0.606955568	1.60835E-09	5.67995E-08
hsa-miR-495_C419708	5	1.358381503	0.577919064	1.64824E-09	5.77639E-08
hsa-miR-579_C432165	23	2.677419355	3.388901209	1.82757E-09	6.35634E-08
hsa-miR-558_D013755	31	4.147540984	4.523088031	1.95935E-09	6.76344E-08
hsa-miR-1243_D004966	2	1.023255814	0.1507149	2.0071E-09	6.82563E-08
hsa-miR-140-5p_C423915	2	1.023255814	0.1507149	2.0071E-09	6.82563E-08
hsa-miR-432_C118258	4	1.965517241	0.319779948	2.02603E-09	6.83934E-08
hsa-miR-1244_C093973	14	2.455958549	1.909718325	2.42786E-09	8.13599E-08
hsa-miR-520f_C423917	7	1.179104478	0.945142698	2.44895E-09	8.1472E-08
hsa-miR-520f_C093642	19	3.175710594	2.644311177	2.52473E-09	8.33887E-08
hsa-miR-7_C501280	2	1.023529412	0.151577632	2.5625E-09	8.40317E-08
hsa-miR-1180_D006830	3	1.1	0.3	2.59282E-09	8.4423E-08
hsa-miR-298_D013196	27	3.880952381	3.918225916	2.80501E-09	9.06887E-08
hsa-miR-558_C007610	3	1.076923077	0.30493268	3.02112E-09	9.69927E-08
hsa-miR-569_D009840	2	1.023809524	0.152455339	3.27138E-09	1.04298E-07
hsa-miR-576-3p_D003474	31	4.475446429	4.548285075	3.61396E-09	1.14426E-07
hsa-miR-432_D005947	22	3.189393939	3.198324788	3.8432E-09	1.2085E-07
hsa-miR-1228_D015215	8	1.816091954	1.019301116	3.98329E-09	1.24403E-07
hsa-miR-302a_D014108	4	1.266666667	0.442216639	4.56314E-09	1.4155E-07
hsa-miR-503_D004996	3	1.103448276	0.304543478	4.96415E-09	1.52956E-07
hsa-miR-512-3p_D000677	2	1.024390244	0.154257447	5.33058E-09	1.63151E-07
hsa-miR-130a_D013196	25	3.551204819	3.709370062	5.90504E-09	1.79537E-07
hsa-miR-143_D000111	10	1.888283379	1.363617982	6.0547E-09	1.82489E-07
hsa-miR-600_C432165	20	2.605555556	2.991649902	6.08165E-09	1.82489E-07

hsa-miR-20a_D002857	3	1.06122449	0.313516153	6.31842E-09	1.88363E-07
hsa-miR-632_C002979	5	1.483050847	0.578323862	6.42995E-09	1.90451E-07
hsa-miR-140-3p_D010672	7	1.533018868	0.913134938	7.19105E-09	2.11629E-07
hsa-miR-302a_D006861	12	2.456359102	1.620847877	7.29205E-09	2.13234E-07
hsa-miR-132_D013467	18	2.374251497	2.69409343	7.33936E-09	2.1326E-07
hsa-miR-181d_D004958	37	5.858627859	5.507203126	8.25376E-09	2.38321E-07
hsa-miR-151-3p_D013853	2	1.025	0.15612495	8.68351E-09	2.47615E-07
hsa-miR-144_D017962	2	1.025	0.15612495	8.68351E-09	2.47615E-07
hsa-miR-628-3p_D008628	4	1.212290503	0.460343331	9.43332E-09	2.67336E-07
hsa-miR-561_C014347	71	15.238	10.10471949	9.63009E-09	2.71238E-07
hsa-miR-520f_D008070	33	4.230769231	5.102650935	9.78166E-09	2.73827E-07
hsa-miR-143_C113580	10	2.09122807	1.347679058	9.84198E-09	2.73846E-07
hsa-miR-548c-3p_D005472	299	37.694	50.2155789	1.04734E-08	2.89659E-07
hsa-miR-302a_C093973	13	2.582417582	1.797837126	1.13541E-08	3.12136E-07
hsa-miR-298_C021751	5	1.381578947	0.606263475	1.21083E-08	3.30888E-07
hsa-miR-1258_D013755	17	2.062761506	2.613177563	1.22649E-08	3.33184E-07
hsa-miR-143_D000079	18	2.459143969	2.726024608	1.28228E-08	3.46291E-07
hsa-miR-383_C017947	18	3.626326964	2.525552449	1.46205E-08	3.92531E-07
hsa-miR-610_C103505	3	1.110169492	0.313100902	1.56396E-08	4.1745E-07
hsa-miR-302a_D003300	16	2.833333333	2.317805475	1.69243E-08	4.4913E-07
hsa-let-7d_C014347	71	14.708	10.41089506	1.71758E-08	4.53185E-07
hsa-let-7i_C014347	26	3.144356955	4.103343611	1.78151E-08	4.67366E-07
hsa-miR-22_D002110	7	1.5	0.944564362	1.83369E-08	4.78322E-07
hsa-miR-151-3p_C097240	5	1.166666667	0.651615818	1.87068E-08	4.85214E-07
hsa-miR-1244_C085911	9	1.957446809	1.21965197	1.88193E-08	4.8539E-07
hsa-miR-133a_C004984	7	1.816625917	0.891753778	2.06137E-08	5.28701E-07
hsa-miR-575_C004541	4	1.16	0.48	2.07973E-08	5.30447E-07
hsa-miR-1259_D014212	31	6.130879346	4.503491879	2.11619E-08	5.3577E-07
hsa-miR-1271_D005978	5	1.233576642	0.642335766	2.12394E-08	5.3577E-07
hsa-miR-561_D019256	9	1.964864865	1.226446463	2.32951E-08	5.84414E-07
hsa-miR-1205_D019328	15	1.515151515	2.401025187	2.3502E-08	5.864E-07
hsa-let-7i_C008493	10	1.777777778	1.445512426	2.60134E-08	6.45369E-07
hsa-miR-520f_D001728	3	1.970149254	0.170175437	2.61465E-08	6.45369E-07
hsa-miR-1227_C063002	4	1.237623762	0.469853882	2.64968E-08	6.50518E-07
hsa-miR-383_D003561	3	1.113636364	0.317369092	2.67869E-08	6.54142E-07
hsa-miR-503_C400082	12	2.901785714	1.61075157	2.92318E-08	7.1007E-07
hsa-miR-23a_D002117	46	19.892	4.798368056	3.09917E-08	7.48857E-07
hsa-miR-126_D006861	7	1.418181818	0.975908138	3.21921E-08	7.7379E-07
hsa-miR-219-1-3p_C475919	8	1.470149254	1.150537178	3.51127E-08	8.39596E-07
hsa-miR-220c_D013749	14	2.163498099	2.130682589	3.72411E-08	8.85875E-07
hsa-miR-1265_C025299	3	1.11627907	0.320559273	3.9497E-08	9.31587E-07
hsa-miR-576-3p_D004317	35	4.137254902	5.755711938	3.95686E-08	9.31587E-07
hsa-miR-320a_D003907	27	5.026315789	4.051121842	4.0242E-08	9.42607E-07
hsa-miR-106b_C017947	11	1.767326733	1.653176295	4.07145E-08	9.48834E-07
hsa-miR-660_C012589	18	3.279461279	2.681652786	4.25805E-08	9.87309E-07
hsa-let-7c_C025462	9	1.673076923	1.304152449	4.28186E-08	9.8784E-07
hsa-miR-335_D011374	27	6.748995984	3.749062667	4.9111E-08	1.12734E-06
hsa-miR-576-3p_C045651	15	2.305882353	2.312914447	4.96375E-08	1.13376E-06
hsa-miR-520f_C490728	5	1.51369863	0.610819138	5.50932E-08	1.25214E-06
hsa-miR-99a_C011890	3	1.103448276	0.326404402	5.70179E-08	1.2895E-06
hsa-miR-569_D009288	2	1.027777778	0.16433555	6.09572E-08	1.36514E-06
hsa-miR-384_C423915	2	1.027777778	0.16433555	6.09572E-08	1.36514E-06
hsa-miR-302a_D000119	7	3	0.707106781	6.34912E-08	1.40989E-06
hsa-miR-516a-3p_D002392	9	1.579487179	1.338805339	6.35695E-08	1.40989E-06

hsa-miR-98_D013196	10	2.052238806	1.441998842	7.00546E-08	1.54625E-06
hsa-miR-576-3p_D014212	38	8.677484787	5.623357562	8.84415E-08	1.94275E-06
hsa-miR-653_D011374	25	5.973790323	3.5945353	9.15143E-08	1.99363E-06
hsa-miR-220c_D004051	3	1.089552239	0.333741489	9.16261E-08	1.99363E-06
hsa-miR-520f_C072553	11	2.330935252	1.593098827	9.30319E-08	2.01467E-06
hsa-miR-202_D005472	320	45.636	57.95625509	9.36285E-08	2.01807E-06
hsa-miR-199a-3p_C089730	10	2.263285024	1.417882626	9.63819E-08	2.06771E-06
hsa-miR-1307_D005472	13	1.608108108	2.116968559	9.70796E-08	2.07299E-06
hsa-miR-20a_D011794	17	2.772108844	2.66607006	9.78845E-08	2.0805E-06
hsa-let-7b_C501280	2	1.028571429	0.166598626	9.91362E-08	2.08777E-06
hsa-miR-570_D010416	2	1.028571429	0.166598626	9.91362E-08	2.08777E-06
hsa-miR-744_D000111	4	1.183333333	0.499722145	1.00802E-07	2.11316E-06
hsa-miR-330-3p_D004121	4	1.19047619	0.499432785	1.07327E-07	2.23972E-06
hsa-miR-1228_D002166	7	1.473282443	1.006302614	1.1177E-07	2.32188E-06
hsa-miR-922_D011192	5	1.403389831	0.645707102	1.13194E-07	2.34087E-06
hsa-miR-302a_D003676	5	1.454545455	0.637984948	1.22995E-07	2.53215E-06
hsa-miR-1827_C477819	5	1.240963855	0.678239419	1.25687E-07	2.57602E-06
hsa-miR-145_C423222	4	1.228070175	0.496215285	1.34636E-07	2.74717E-06
hsa-miR-1205_D002104	8	1.689873418	1.163266346	1.39847E-07	2.84087E-06
hsa-miR-151-3p_C018021	17	2.164179104	2.826299806	1.46686E-07	2.96668E-06
hsa-miR-495_C095105	7	1.740740741	0.965807764	1.50302E-07	3.02648E-06
hsa-miR-618_C025160	2	1.029411765	0.168957725	1.61166E-07	3.23105E-06
hsa-let-7i_D014212	21	3.578817734	3.351706364	1.61869E-07	3.23105E-06
hsa-miR-29a_D000241	10	2.28125	1.44123157	1.63574E-07	3.25095E-06
hsa-miR-28-3p_D019833	14	3	2.082210582	1.66778E-07	3.30034E-06
hsa-miR-503_C085911	15	3.18251928	2.248130404	1.77399E-07	3.49545E-06
hsa-miR-199a-5p_C030110	12	2.634259259	1.76790638	1.81637E-07	3.56366E-06
hsa-miR-632_C012589	17	3.414860681	2.606498588	1.93197E-07	3.77433E-06
hsa-miR-146b-3p_D004958	44	8.432	7.097420376	1.97856E-07	3.84897E-06
hsa-miR-655_D008345	3	1.111940299	0.33813465	2.00237E-07	3.87885E-06
hsa-miR-182_D002193	2	1.029850746	0.170175437	2.0546E-07	3.94672E-06
hsa-miR-7_C480030	2	1.029850746	0.170175437	2.0546E-07	3.94672E-06
hsa-miR-302e_C030110	8	1.60311284	1.199087704	2.19806E-07	4.20193E-06
hsa-miR-190_C070081	12	1.777777778	1.949992086	2.20576E-07	4.20193E-06
hsa-miR-519b-3p_D013467	17	2.101449275	2.884968176	2.23707E-07	4.24396E-06
hsa-miR-1294_C030371	3	1.12987013	0.336160496	2.25849E-07	4.26697E-06
hsa-miR-619_D020058	3	1.130434783	0.336781161	2.40884E-07	4.51387E-06
hsa-let-7i_C496197	3	1.130434783	0.336781161	2.40884E-07	4.51387E-06
hsa-miR-454_D000431	7	1.414634146	1.04707746	2.52459E-07	4.71154E-06
hsa-miR-613_D017292	8	4.02173913	0.736889185	2.53798E-07	4.71736E-06
hsa-miR-130b_D002939	2	1.03030303	0.171419826	2.61901E-07	4.80955E-06
hsa-miR-371-3p_D001194	2	1.03030303	0.171419826	2.61901E-07	4.80955E-06
hsa-miR-518d-5p_C017557	2	1.03030303	0.171419826	2.61901E-07	4.80955E-06
hsa-miR-133a_D019207	7	1.855421687	0.963584299	2.67455E-07	4.89198E-06
hsa-miR-371-5p_D010672	6	1.473404255	0.846981566	2.9565E-07	5.38623E-06
hsa-miR-520f_C482884	3	1.132596685	0.339138326	3.06712E-07	5.56567E-06
hsa-miR-633_D009599	8	1.94047619	1.147991937	3.09739E-07	5.59847E-06
hsa-miR-222_C013038	3	1.113043478	0.343010138	3.11967E-07	5.61663E-06
hsa-miR-146b-3p_D014284	5	1.443478261	0.661898512	3.24168E-07	5.8135E-06
hsa-miR-181d_D013629	14	2.477386935	2.242405655	3.28541E-07	5.869E-06
hsa-miR-1225-5p_D004610	2	1.030769231	0.172691879	3.33811E-07	5.88885E-06
hsa-miR-328_D016729	2	1.030769231	0.172691879	3.33811E-07	5.88885E-06
hsa-miR-130a_D002211	7	1.408888889	1.059102812	3.34437E-07	5.88885E-06
hsa-miR-302a_D015032	8	1.833887043	1.172348003	3.34783E-07	5.88885E-06

hsa-miR-519d_C070515	4	1.912087912	0.382253344	3.46764E-07	6.07631E-06
hsa-miR-503_C107676	14	3.534059946	2.034661666	3.5232E-07	6.15019E-06
hsa-miR-520f_D005978	6	1.382978723	0.87039222	3.55712E-07	6.18589E-06
hsa-miR-337-3p_D013629	16	3.065909091	2.544641713	3.84729E-07	6.66477E-06
hsa-miR-143_D003474	38	8.187372709	6.074346195	3.86153E-07	6.66477E-06
hsa-miR-193b_C451735	2	1.03125	0.173992636	4.25417E-07	7.28092E-06
hsa-miR-144_D003033	2	1.03125	0.173992636	4.25417E-07	7.28092E-06
hsa-miR-510_D002117	12	3.141649049	1.723797809	4.2661E-07	7.28092E-06
hsa-miR-302a_D013755	19	2.979885057	3.197007938	4.40185E-07	7.48478E-06
hsa-miR-558_C006552	5	1.302325581	0.699996137	4.97214E-07	8.42328E-06
hsa-miR-151-3p_D002857	3	1.1	0.351188458	5.00508E-07	8.44791E-06
hsa-miR-1_D014284	7	1.832	0.993869207	5.39749E-07	9.07688E-06
hsa-miR-632_D005492	7	1.838095238	0.993219644	5.47839E-07	9.1793E-06
hsa-miR-23b_D017313	6	1.537037037	0.854464592	5.56221E-07	9.28586E-06
hsa-miR-583_C048460	5	1.140350877	0.736006209	5.78735E-07	9.62671E-06
hsa-miR-132_C459604	4	1.291187739	0.509938513	5.8343E-07	9.66977E-06
hsa-miR-520f_C054649	9	1.296296296	1.510820597	5.96855E-07	9.8567E-06
hsa-miR-1237_D001599	2	1.032	0.176	6.11918E-07	1.00692E-05
hsa-miR-643_D004958	36	6.316532258	6.179653209	6.30583E-07	1.03393E-05
hsa-miR-199a-5p_C009687	10	2.228571429	1.528504671	6.35753E-07	1.0387E-05
hsa-miR-770-5p_C055494	4	1.191489362	0.531489191	6.48629E-07	1.0551E-05
hsa-miR-520f_C477941	3	1.123188406	0.350008251	6.5039E-07	1.0551E-05
hsa-miR-646_C047948	5	1.458083832	0.676626835	6.61224E-07	1.0689E-05
hsa-miR-605_D020111	7	1.866834171	0.994889215	6.6451E-07	1.07044E-05
hsa-miR-106b_C030110	6	1.225806452	0.923166969	6.73082E-07	1.07857E-05
hsa-miR-649_D004726	4	1.134328358	0.54349676	6.74254E-07	1.07857E-05
hsa-miR-199a-5p_C014036	10	5.388888889	0.890623308	6.76842E-07	1.07895E-05
hsa-miR-576-3p_C451735	2	1.032258065	0.176684696	6.90707E-07	1.09346E-05
hsa-miR-34c-5p_C422648	2	1.032258065	0.176684696	6.90707E-07	1.09346E-05
hsa-miR-132_C108123	3	1.140625	0.347634304	7.04079E-07	0.000011108
hsa-miR-1244_D017638	16	4.643145161	2.278863563	7.0819E-07	1.11272E-05
hsa-miR-1253_C095105	4	1.239631336	0.523807271	7.10146E-07	1.11272E-05
hsa-miR-320a_D011374	54	21.054	6.941979833	7.38719E-07	1.15356E-05
hsa-miR-224_C006780	7	1.88372093	0.99581481	7.42573E-07	1.15565E-05
hsa-miR-144_C080955	15	2.364341085	2.55311934	7.50084E-07	1.1615E-05
hsa-miR-383_D011794	22	4.232941176	3.643507949	7.51397E-07	1.1615E-05
hsa-miR-936_C054852	4	1.962962963	0.38311409	7.56147E-07	1.16492E-05
hsa-miR-22_C066851	6	2.170212766	0.738576807	7.83189E-07	1.20255E-05
hsa-miR-302a_D001599	2	1.032608696	0.177610159	8.11722E-07	1.24221E-05
hsa-miR-20a_C018021	17	2.444444444	2.970638207	8.21797E-07	1.25074E-05
hsa-miR-520f_D003676	7	1.759689922	1.025074728	8.22749E-07	1.25074E-05
hsa-miR-143_D002945	11	2.751677852	1.646252773	8.5745E-07	1.29883E-05
hsa-let-7b_D047630	4	1.307692308	0.514100966	8.60038E-07	1.29883E-05
hsa-miR-558_D000111	11	1.914754098	1.821352053	8.65874E-07	1.30335E-05
hsa-miR-371-5p_C022921	3	1.142857143	0.349927106	8.72205E-07	1.30433E-05
hsa-miR-302a_D007464	3	1.142857143	0.349927106	8.72205E-07	1.30433E-05
hsa-miR-365_C031477	2	1.032786885	0.178078369	8.79945E-07	1.31163E-05
hsa-miR-646_C400082	14	3.814009662	2.05637852	9.1185E-07	1.35274E-05
hsa-miR-524-5p_C050414	9	2.513978495	1.28528407	9.16363E-07	1.35274E-05
hsa-miR-524-5p_C106014	9	2.513978495	1.28528407	9.16363E-07	1.35274E-05
hsa-miR-944_D004391	9	2.274231678	1.340573538	1.01808E-06	1.49808E-05
hsa-miR-383_C412373	5	1.4	0.7	1.02917E-06	1.50956E-05
hsa-miR-22_C120275	9	1.6875	1.465149116	1.0616E-06	1.55217E-05
hsa-miR-520f_D013726	8	3.074380165	0.9720498	1.09025E-06	1.589E-05

hsa-miR-199a-5p_D010100	32	4.971659919	5.75294384	1.11676E-06	1.61317E-05
hsa-miR-576-3p_C066515	2	1.033333333	0.179505494	1.12089E-06	1.61317E-05
hsa-miR-139-3p_C029938	2	1.033333333	0.179505494	1.12089E-06	1.61317E-05
hsa-miR-520f_C477330	2	1.033333333	0.179505494	1.12089E-06	1.61317E-05
hsa-miR-140-3p_D012293	9	2.2	1.36381817	1.16903E-06	1.67719E-05
hsa-miR-1259_D011374	18	4.50931677	2.788882125	1.18667E-06	1.6972E-05
hsa-miR-520f_D001554	9	2.448430493	1.314215056	1.21827E-06	1.73698E-05
hsa-miR-216b_D013755	19	3.13681592	3.314176967	1.27539E-06	1.81279E-05
hsa-miR-503_C047948	4	1.288888889	0.525873758	0.000001284	1.81829E-05
hsa-miR-147b_D005576	4	1.185185185	0.546845299	1.28718E-06	1.81829E-05
hsa-miR-612_D013755	18	2.901492537	3.151758218	1.31541E-06	1.85247E-05
hsa-miR-22_D019256	10	2.379227053	1.54440032	1.33311E-06	1.87165E-05
hsa-miR-944_D005047	9	1.935275081	1.428677055	1.36834E-06	1.9105E-05
hsa-miR-221_C088658	8	1.900826446	1.225790072	1.3691E-06	1.9105E-05
hsa-miR-892b_C016601	7	1.692028986	1.061210793	1.38837E-06	1.93152E-05
hsa-miR-151-3p_D010100	21	3.012048193	3.797570485	1.41065E-06	1.94972E-05
hsa-miR-381_D004958	46	10.72	7.695817046	1.41617E-06	1.94972E-05
hsa-miR-1261_D005047	8	1.557377049	1.2997426	0.000001418	1.94972E-05
hsa-miR-520f_D009496	20	1.872340426	3.829432608	1.41844E-06	1.94972E-05
hsa-miR-140-5p_C013598	2	1.033898305	0.180967428	1.42762E-06	1.95648E-05
hsa-miR-199a-3p_C057693	9	1.731343284	1.475323621	1.44905E-06	1.97793E-05
hsa-miR-558_C045651	17	2.861736334	2.955758814	1.45189E-06	1.97793E-05
hsa-miR-1293_D000079	12	1.9	2.081932863	1.48573E-06	2.01804E-05
hsa-miR-337-3p_D004958	37	7.303212851	6.439588724	1.49286E-06	2.02175E-05
hsa-miR-143_C095591	3	1.115942029	0.36260858	1.51074E-06	2.03994E-05
hsa-miR-503_D002794	3	1.149350649	0.356433771	1.56688E-06	2.10954E-05
hsa-miR-520a-5p_D004128	4	1.283185841	0.531636754	1.60139E-06	2.1497E-05
hsa-miR-770-5p_D007213	11	2.702031603	1.701591455	1.6071E-06	2.15108E-05
hsa-miR-300_D019287	6	1.653846154	0.867163414	1.61515E-06	2.15455E-05
hsa-miR-660_D020849	12	2.466183575	1.967751218	1.62015E-06	2.15455E-05
hsa-miR-648_C065180	3	1.131782946	0.360444277	1.62377E-06	2.15455E-05
hsa-miR-1260_C041517	3	1.109090909	0.365450023	1.67709E-06	2.21888E-05
hsa-miR-1827_C080955	13	2.176744186	2.250874477	1.68974E-06	2.22919E-05
hsa-miR-22_D001374	14	3.045454545	2.282554511	1.74174E-06	2.29121E-05
hsa-miR-1827_D016627	4	1.836734694	0.421219744	1.77425E-06	2.32731E-05
hsa-miR-130a_D015741	13	3.004524887	2.077672161	1.8093E-06	2.338E-05
hsa-miR-497_C016104	2	1.034482759	0.182465608	1.81805E-06	2.338E-05
hsa-let-7e_D004391	2	1.034482759	0.182465608	1.81805E-06	2.338E-05
hsa-miR-148b_C106014	2	1.034482759	0.182465608	1.81805E-06	2.338E-05
hsa-miR-767-5p_D004164	2	1.034482759	0.182465608	1.81805E-06	2.338E-05
hsa-miR-148b_C050414	2	1.034482759	0.182465608	1.81805E-06	2.338E-05
hsa-miR-155_C467566	2	1.034482759	0.182465608	1.81805E-06	2.338E-05
hsa-miR-520f_D000111	14	2.946428571	2.309101888	1.8262E-06	2.34192E-05
hsa-miR-569_D005978	5	1.277310924	0.743875495	1.9546E-06	2.4996E-05
hsa-miR-17_D009538	7	1.721212121	1.070756622	1.9659E-06	2.50533E-05
hsa-miR-22_C423222	3	1.110429448	0.36748415	1.96999E-06	2.50533E-05
hsa-miR-18a_D003907	16	2.831460674	2.783993663	1.98538E-06	2.51792E-05
hsa-miR-770-5p_D011794	20	4.263888889	3.361656292	2.0709E-06	2.61915E-05
hsa-let-7b_D003474	23	3.747395833	4.154094803	2.08065E-06	2.62425E-05
hsa-miR-520f_C057416	2	1.034883721	0.183485277	2.13584E-06	2.68648E-05
hsa-miR-643_D013629	14	2.548926014	2.415022108	2.16727E-06	2.71856E-05
hsa-miR-656_D013629	28	8.112	4.308997099	2.19161E-06	2.7416E-05
hsa-miR-519b-3p_C008493	9	1.941176471	1.457589611	2.21091E-06	2.75823E-05
hsa-miR-22_C121565	9	2.13836478	1.416337679	2.25526E-06	2.80594E-05

hsa-miR-604_C047426	2	1.035087719	0.184001552	2.31494E-06	2.86556E-05
hsa-miR-576-3p_D011794	22	4.648648649	3.744422034	2.31567E-06	2.86556E-05
hsa-miR-132_D005047	8	1.581005587	1.32356261	2.35381E-06	2.90493E-05
hsa-miR-302a_D019256	5	1.396624473	0.725419418	2.41221E-06	2.96902E-05
hsa-miR-874_D013629	18	4.175732218	2.960421806	2.47989E-06	3.04416E-05
hsa-miR-656_C439584	2	1.035294118	0.184522202	2.50902E-06	3.07171E-05
hsa-miR-143_D011794	33	8.192307692	5.474403221	2.53168E-06	3.09121E-05
hsa-miR-143_C045651	18	3.305851064	3.163886618	2.6113E-06	3.17997E-05
hsa-miR-143_D013755	26	4.714285714	4.674413019	2.68192E-06	3.25733E-05
hsa-miR-202_D016729	4	1.307958478	0.537814672	2.68909E-06	3.25742E-05
hsa-miR-144_C443641	3	1.13986014	0.366449581	2.76489E-06	3.34042E-05
hsa-miR-22_D002166	8	1.879699248	1.268695345	2.78058E-06	3.35056E-05
hsa-miR-548m_D014212	45	13.642	7.040584919	2.79074E-06	3.354E-05
hsa-miR-1260_C016766	5	1.173076923	0.777598716	2.82277E-06	3.38364E-05
hsa-miR-145_C030371	3	1.103174603	0.374364331	2.83748E-06	3.39241E-05
hsa-miR-660_D003907	15	2.614942529	2.657889091	2.89353E-06	3.45044E-05
hsa-miR-614_C004648	2	1.035714286	0.185576872	2.94721E-06	3.48728E-05
hsa-miR-101_D012176	2	1.035714286	0.185576872	2.94721E-06	3.48728E-05
hsa-miR-203_C471843	2	1.035714286	0.185576872	2.94721E-06	3.48728E-05
hsa-miR-23a_D011374	47	19.682	6.109736165	2.97655E-06	3.51294E-05
hsa-miR-556-3p_D011374	33	12.158	4.601851367	3.04646E-06	3.58623E-05
hsa-let-7i_D004317	22	2.868020305	4.235796528	3.49923E-06	4.10869E-05
hsa-miR-571_C099555	4	1.293577982	0.546930589	3.51401E-06	4.11552E-05
hsa-miR-516a-3p_D011794	31	7.480808081	5.265394491	3.52364E-06	4.11629E-05
hsa-miR-632_C008493	11	2.615740741	1.783549418	3.55662E-06	4.14428E-05
hsa-miR-133a_C475919	15	3.474576271	2.494780532	3.70998E-06	4.30367E-05
hsa-miR-22_D001728	4	1.942307692	0.412005486	3.71216E-06	4.30367E-05
hsa-miR-143_D005947	19	3.480349345	3.411489431	3.7497E-06	4.33624E-05
hsa-miR-548b-3p_D013629	18	3.731914894	3.123766574	3.7663E-06	4.34449E-05
hsa-miR-383_C030110	7	1.614785992	1.127760449	3.95649E-06	4.55244E-05
hsa-miR-302a_D010126	12	1.902439024	2.184254944	4.1777E-06	4.79496E-05
hsa-miR-1231_C070081	14	2.231343284	2.566762811	4.23222E-06	4.84542E-05
hsa-miR-98_D015735	5	1.38647343	0.745622088	4.24898E-06	4.8525E-05
hsa-miR-519b-3p_D003010	4	1.274390244	0.555748446	4.29507E-06	4.89297E-05
hsa-miR-302a_D013726	5	2.657142857	0.474664221	4.30837E-06	4.89545E-05
hsa-miR-20a_C085911	7	1.787878788	1.094270914	4.31857E-06	4.89545E-05
hsa-miR-133a_C041819	4	1.333333333	0.543500648	4.34749E-06	4.91398E-05
hsa-miR-199a-5p_C085911	12	2.792763158	1.986752242	4.35633E-06	4.91398E-05
hsa-miR-503_D006843	5	1.956521739	0.624030439	4.3707E-06	4.91811E-05
hsa-miR-22_D009536	4	1.848484848	0.434930306	4.45356E-06	4.99909E-05
hsa-miR-199a-5p_D009532	21	3.659340659	3.872188183	4.55103E-06	5.07405E-05
hsa-miR-26a_D011374	36	12.538	5.324336203	4.55126E-06	5.07405E-05
hsa-miR-1231_C002979	4	1.462264151	0.517149814	4.55349E-06	5.07405E-05
hsa-miR-509-3-5p_C053541	9	2.079617834	1.477010186	4.6185E-06	5.13403E-05
hsa-miR-617_C086566	4	1.152941176	0.584221601	4.75766E-06	5.26954E-05
hsa-miR-1228_D009589	2	1.037037037	0.188852575	4.77484E-06	5.26954E-05
hsa-miR-199a-5p_C116890	2	1.037037037	0.188852575	4.77484E-06	5.26954E-05
hsa-miR-143_C019248	3	1.163636364	0.369945272	4.81109E-06	5.29681E-05
hsa-miR-576-3p_D002392	5	1.369747899	0.754432796	4.95893E-06	5.44652E-05
hsa-miR-17_D014212	64	24.908	9.177120245	4.98899E-06	5.46646E-05
hsa-miR-597_D007213	9	2.257425743	1.442999251	5.01885E-06	5.48608E-05
hsa-miR-573_C010327	5	1.51910828	0.723546974	5.19615E-06	5.6664E-05
hsa-miR-22_D047310	5	1.370535714	0.756653628	5.31804E-06	5.78557E-05
hsa-miR-1324_C118258	4	1.779661017	0.453528412	5.49221E-06	5.95964E-05

hsa-miR-520f_C113580	11	2.811557789	1.777064115	0.000005504	5.95964E-05
hsa-miR-152_D007213	6	1.5	0.949571163	5.58087E-06	6.02865E-05
hsa-miR-1244_D017239	9	2.171428571	1.470064092	5.60054E-06	6.02876E-05
hsa-miR-503_C477330	2	1.0375	0.189983552	5.60723E-06	6.02876E-05
hsa-miR-106b_D003474	11	1.844155844	2.003665051	5.81946E-06	6.24232E-05
hsa-miR-548d-3p_C066075	9	2.103274559	1.489480953	5.92015E-06	6.33553E-05
hsa-miR-632_C496751	3	1.166666667	0.372677996	5.95132E-06	6.35407E-05
hsa-miR-1260_C061133	3	1.149253731	0.376699387	6.07272E-06	6.44245E-05
hsa-miR-1243_D016729	2	1.037735849	0.190556697	6.07619E-06	6.44245E-05
hsa-miR-324-3p_C108606	2	1.037735849	0.190556697	6.07619E-06	6.44245E-05
hsa-miR-1205_D006427	7	1.512820513	1.173899809	6.12037E-06	6.47434E-05
hsa-miR-605_D008628	7	2.002439024	1.064960277	6.1893E-06	6.5322E-05
hsa-miR-216b_D013804	3	1.08	0.391918359	6.25433E-06	6.5857E-05
hsa-miR-576-3p_D019833	17	4.347648262	2.826582766	6.29224E-06	6.61045E-05
hsa-miR-22_D002104	8	1.914691943	1.31787646	7.09826E-06	7.44021E-05
hsa-miR-219-5p_C063002	5	1.530944625	0.731906675	7.21422E-06	7.54453E-05
hsa-miR-449a_C015854	2	1.038461538	0.192307692	7.73094E-06	8.03003E-05
hsa-miR-22_D003687	2	1.038461538	0.192307692	7.73094E-06	8.03003E-05
hsa-miR-1263_C042577	2	1.038461538	0.192307692	7.73094E-06	8.03003E-05
hsa-miR-199a-5p_D004229	4	1.41025641	0.541505438	7.9556E-06	8.24473E-05
hsa-miR-151-3p_C044387	5	1.173913043	0.81572448	8.16822E-06	8.44601E-05
hsa-miR-649_D004970	4	1.127659574	0.605542531	8.56944E-06	8.84097E-05
hsa-miR-510_C025462	11	2.188571429	1.963636776	8.61789E-06	8.87102E-05
hsa-miR-1271_C516024	2	1.038834951	0.193201444	8.71978E-06	8.95582E-05
hsa-miR-1827_C045651	21	4.424507659	3.832272698	9.01844E-06	9.24189E-05
hsa-let-7c_D004391	2	1.038961039	0.193502135	9.07665E-06	0.000092602
hsa-miR-432_C086511	2	1.038961039	0.193502135	9.07665E-06	0.000092602
hsa-miR-558_D014212	40	12.562	6.536830731	9.11425E-06	9.27794E-05
hsa-miR-548b-3p_D020849	16	3.800424628	2.776623649	9.23644E-06	9.38153E-05
hsa-miR-576-5p_D013726	6	2.7875	0.683625446	9.35234E-06	9.47828E-05
hsa-miR-325_D011374	11	2.668246445	1.859747283	9.40014E-06	9.50574E-05
hsa-miR-604_D047630	4	1.352112676	0.559138965	9.62854E-06	9.7153E-05
hsa-miR-1284_D004054	4	1.31092437	0.56876542	9.81842E-06	9.88517E-05
hsa-miR-646_D004144	6	1.884169884	0.888635014	9.86146E-06	9.90678E-05
hsa-miR-22_D003609	8	2.200564972	1.274211717	9.94099E-06	9.96487E-05
hsa-miR-26b_C024746	4	1.297619048	0.572791034	1.02209E-05	0.000102231
hsa-miR-22_D013467	19	4.023404255	3.470465832	1.03891E-05	0.000103688
hsa-miR-183_D003907	16	3.201591512	2.940018877	1.04135E-05	0.000103706
hsa-miR-362-3p_C496197	3	1.162011173	0.38332328	1.05911E-05	0.000105246
hsa-miR-143_C080955	12	2.047619048	2.259773432	1.08378E-05	0.000107465
hsa-let-7i_D006861	7	1.744897959	1.154505146	1.09502E-05	0.000108346
hsa-miR-664_C432165	19	2.995708155	3.735340852	1.10232E-05	0.000108833
hsa-miR-660_C070081	11	2.099099099	2.011027952	1.10562E-05	0.000108925
hsa-miR-503_C095104	3	1.135294118	0.390235489	1.12567E-05	0.000110663
hsa-miR-181d_D020849	11	2.456359102	1.928579054	1.13278E-05	0.000111124
hsa-miR-1276_D007529	8	2.284634761	1.264548856	0.000011566	0.000113219
hsa-miR-548c-3p_D019256	12	3.338297872	1.95981471	1.16704E-05	0.000113998
hsa-miR-561_D004317	33	6.879591837	6.308707283	1.19806E-05	0.000116779
hsa-let-7b_C506002	4	2.034482759	0.413793103	1.21546E-05	0.000118224
hsa-miR-874_D004958	42	11.2811245	7.510720928	1.23812E-05	0.00011978
hsa-miR-1826_C104586	5	2.0125	0.642139977	0.000012392	0.00011978
hsa-miR-320a_D012643	63	35.552	6.663879951	1.23928E-05	0.00011978
hsa-miR-1225-3p_D005632	2	1.04	0.195959179	1.25087E-05	0.000120455
hsa-miR-302a_D010936	6	1.497512438	0.988110303	1.25151E-05	0.000120455

hsa-miR-548c-3p_C017947	44	18.39	6.216582663	1.32459E-05	0.000127222
hsa-miR-646_D006588	5	1.881118881	0.674091053	1.32944E-05	0.000127333
hsa-miR-7_C016517	6	1.932692308	0.890812325	0.000013313	0.000127333
hsa-miR-190_C016601	4	1.234782609	0.594619861	1.35085E-05	0.000128935
hsa-miR-144_C006552	5	1.475728155	0.767604522	1.37525E-05	0.000130944
hsa-miR-22_D003907	19	3.949880668	3.545795686	1.37761E-05	0.000130944
hsa-miR-221_C475919	7	1.602870813	1.202147754	0.000013934	0.000132172
hsa-miR-27a_D001564	19	4.953441296	3.298855509	1.39806E-05	0.00013234
hsa-miR-381_D018021	4	1.471910112	0.542360377	1.40787E-05	0.000132995
hsa-miR-181a_D019284	14	2.82010582	2.594136243	1.42485E-05	0.000134322
hsa-miR-220c_D011084	3	1.103448276	0.402134613	0.000014676	0.000138069
hsa-miR-365_C056507	7	1.945205479	1.126157377	1.49438E-05	0.000140301
hsa-miR-891b_D011374	27	8.994	4.310216236	1.50257E-05	0.000140782
hsa-miR-1205_D002083	3	1.098765432	0.403837709	1.51966E-05	0.000142093
hsa-miR-539_D013749	10	2.192073171	1.783135248	1.53546E-05	0.000143278
hsa-miR-22_D002330	9	2.754756871	1.409385482	0.000015421	0.000143606
hsa-miR-623_D003907	16	3.320954907	2.982781914	1.59475E-05	0.000148208
hsa-miR-143_C416282	4	2.086956522	0.40786224	1.63364E-05	0.000151516
hsa-miR-503_C015329	13	3.166666667	2.283191399	1.63884E-05	0.000151692
hsa-miR-643_D020849	12	2.72234763	2.147416295	1.64359E-05	0.000151825
hsa-miR-668_C025462	11	2.403389831	1.983830066	1.68209E-05	0.00015507
hsa-miR-1227_C496197	4	1.325490196	0.580683552	1.70039E-05	0.000156443
hsa-miR-219-1-3p_D000079	8	1.573333333	1.46217494	1.74163E-05	0.000159664
hsa-miR-340_D009151	21	3.860465116	4.127664287	1.74236E-05	0.000159664
hsa-miR-576-3p_D019256	6	1.547826087	0.993399769	1.74638E-05	0.000159714
hsa-miR-24_C065180	5	1.534328358	0.763642536	1.76009E-05	0.000160567
hsa-miR-1228_D004365	7	1.673684211	1.200369288	0.000017627	0.000160567
hsa-miR-429_C024746	4	1.318584071	0.583936697	1.80198E-05	0.00016382
hsa-miR-590-5p_D004317	18	2.805970149	3.641546358	1.81679E-05	0.00016484
hsa-miR-1231_C006632	11	2.666666667	1.930539854	1.85846E-05	0.000168288
hsa-miR-130a_C496197	5	1.565079365	0.758896176	1.87125E-05	0.000169112
hsa-miR-744_D000079	5	1.428571429	0.791107035	1.89286E-05	0.000170729
hsa-miR-583_D002392	8	1.642857143	1.45277122	1.90862E-05	0.000171813
hsa-miR-181a_C480030	2	1.041666667	0.199826313	2.02242E-05	0.000180641
hsa-miR-507_C108606	2	1.041666667	0.199826313	2.02242E-05	0.000180641
hsa-miR-545_C096064	2	1.041666667	0.199826313	2.02242E-05	0.000180641
hsa-miR-659_C015854	2	1.041666667	0.199826313	2.02242E-05	0.000180641
hsa-miR-298_D002211	7	1.589473684	1.229703175	2.02955E-05	0.000180926
hsa-miR-383_D004391	4	1.31441048	0.58869423	2.05068E-05	0.000182455
hsa-miR-199a-3p_D017638	21	8.348	3.024383574	2.08996E-05	0.00018559
hsa-miR-516a-3p_C109238	4	1.356557377	0.57963919	0.000020965	0.000185811
hsa-miR-20a_C044387	5	1.19047619	0.85183542	2.12623E-05	0.000188083
hsa-miR-532-3p_D002330	6	1.75136612	0.955819559	2.13846E-05	0.000188801
hsa-miR-340_C016104	5	1.521172638	0.775463249	2.19374E-05	0.00019331
hsa-miR-504_C063002	4	1.338983051	0.585420883	2.22272E-05	0.000195182
hsa-miR-548n_D000966	4	1.374045802	0.577383911	2.22774E-05	0.000195182
hsa-miR-548n_D000965	4	1.374045802	0.577383911	2.22774E-05	0.000195182
hsa-miR-573_D004997	8	2.053030303	1.366894256	2.26387E-05	0.00019797
hsa-miR-151-3p_C085911	7	1.990196078	1.141851764	2.30834E-05	0.000201475
hsa-miR-1237_D015735	9	2.444954128	1.517239541	2.32628E-05	0.000202656
hsa-miR-561_D017239	10	2.710365854	1.697814912	2.33359E-05	0.000202907
hsa-miR-892a_D004958	43	11.132	8.15907936	2.37998E-05	0.00020655
hsa-miR-641_C104586	6	2.189944134	0.857246141	2.38953E-05	0.000206987
hsa-miR-576-3p_D017313	9	2.141818182	1.594842929	2.41391E-05	0.000208706

hsa-miR-1228_D001205	4	1.327956989	0.591128971	2.46807E-05	0.000212987
hsa-miR-513a-5p_D009151	20	3.78125	3.979643841	2.47995E-05	0.000213548
hsa-miR-1200_C090046	4	2.09039548	0.415475171	2.48387E-05	0.000213548
hsa-miR-151-3p_D005978	3	1.121212121	0.408810229	2.52958E-05	0.000217071
hsa-miR-219-1-3p_C089730	5	1.375	0.81620658	0.000025462	0.00021809
hsa-miR-574-3p_C029938	2	1.042553191	0.20184751	2.57083E-05	0.000219752
hsa-miR-632_C088658	7	1.784037559	1.198719441	2.57518E-05	0.000219752
hsa-miR-576-3p_C018021	14	2.389312977	2.797415708	2.59075E-05	0.00022067
hsa-miR-506_D024505	4	1.289156627	0.601806927	2.60261E-05	0.00022127
hsa-miR-520f_C060506	5	2.127659574	0.63971474	2.61306E-05	0.000221748
hsa-miR-202_C015329	16	4.795454545	2.696320572	2.63276E-05	0.00022295
hsa-miR-641_D034261	4	2.090909091	0.41659779	2.63694E-05	0.00022295
hsa-miR-30b_D003907	15	3.303191489	2.826280622	2.69413E-05	0.00022695
hsa-miR-144_D009288	2	1.042735043	0.202259138	2.69714E-05	0.00022695
hsa-miR-130b_C492909	5	1.195121951	0.861635206	2.69908E-05	0.00022695
hsa-miR-302f_D001280	9	1.8	1.691153453	2.73365E-05	0.000229437
hsa-miR-17_C475919	11	2.780045351	1.946952033	2.76004E-05	0.000231229
hsa-miR-576-3p_C007845	6	2.088607595	0.888603989	2.78567E-05	0.000232759
hsa-miR-1228_D006861	11	2.660714286	1.97811689	2.78844E-05	0.000232759
hsa-miR-143_D017239	10	2.666666667	1.726947258	2.80559E-05	0.000233765
hsa-miR-219-2-3p_C006632	10	2.403800475	1.79293541	2.81554E-05	0.000234169
hsa-miR-1260_D020111	7	2.044444444	1.143095213	2.89572E-05	0.000240402
hsa-miR-569_D007213	16	4.835390947	2.703005215	2.91346E-05	0.000241439
hsa-miR-556-3p_D003474	33	8.392354125	6.294018582	3.03909E-05	0.000251396
hsa-miR-1183_C057823	4	1.928571429	0.457366017	3.06535E-05	0.000253112
hsa-miR-181d_C070081	12	2.260869565	2.348309129	3.12759E-05	0.000257788
hsa-miR-15b_C050414	3	1.134715026	0.410341759	3.16806E-05	0.000260189
hsa-miR-15b_C106014	3	1.134715026	0.410341759	3.16806E-05	0.000260189
hsa-miR-208a_D008769	3	1.170103093	0.402227035	3.17766E-05	0.000260511
hsa-miR-940_D034261	4	2.092592593	0.420252064	3.19277E-05	0.000261284
hsa-miR-558_C018021	17	2.855421687	3.499165236	3.22677E-05	0.000263596
hsa-miR-143_D003632	2	1.043478261	0.20393112	3.26729E-05	0.00026596
hsa-miR-196a_C498475	2	1.043478261	0.20393112	3.26729E-05	0.00026596
hsa-miR-199a-5p_C018021	20	3.174107143	4.218480717	3.32008E-05	0.000269779
hsa-miR-558_D003609	6	1.645833333	1.006014897	3.39268E-05	0.000275191
hsa-miR-576-3p_C012655	6	1.333333333	1.083791112	3.46687E-05	0.000280713
hsa-miR-320a_C004541	5	1.574923547	0.781894455	3.47537E-05	0.000280905
hsa-miR-1201_C099555	4	1.369747899	0.592183628	3.50416E-05	0.000282735
hsa-miR-200b_C057823	4	2.055555556	0.431048105	3.52932E-05	0.000284265
hsa-miR-20a_D010100	21	3.727272727	4.35870934	3.56045E-05	0.00028627
hsa-miR-1228_C027576	7	1.964194373	1.178992072	3.69658E-05	0.000296426
hsa-miR-125b_C061133	4	1.323529412	0.6049107	3.69968E-05	0.000296426
hsa-miR-576-3p_C477819	4	1.173076923	0.642146456	3.84507E-05	0.000307539
hsa-miR-376c_C029938	6	1.832904884	0.967517186	3.86473E-05	0.000308574
hsa-miR-520a-3p_D003474	14	2.529147982	2.835995834	3.94186E-05	0.000314185
hsa-miR-202_C014347	98	39.472	16.33919264	3.99417E-05	0.000317711
hsa-miR-18a_C029938	8	2.417040359	1.32112068	3.99993E-05	0.000317711
hsa-miR-632_C051890	13	4.039215686	2.182967398	4.00822E-05	0.000317819
hsa-miR-144_C004532	2	1.044444444	0.206080411	4.15153E-05	0.000328615
hsa-miR-506_D013196	26	6.055201699	5.143908804	4.21734E-05	0.00033325
hsa-miR-381_D013629	16	3.783898305	3.046934284	4.23156E-05	0.000333799
hsa-miR-1207-5p_C053079	4	2.095238095	0.42591771	4.25245E-05	0.000334871
hsa-miR-146b-3p_D013629	14	3.183035714	2.679698424	4.31058E-05	0.000338868
hsa-miR-582-3p_C005460	6	1.755043228	0.993028544	0.000043241	0.00033935

hsa-miR-641_D019284	13	3	2.467624764	4.39046E-05	0.00034342
hsa-miR-520g_D016912	5	1.42364532	0.829168087	4.39093E-05	0.00034342
hsa-miR-582-3p_D014501	6	1.754285714	0.99551648	4.49996E-05	0.000351349
hsa-miR-224_D010758	4	1.316176471	0.614795589	4.72152E-05	0.000368022
hsa-miR-921_C002202	3	1.203947368	0.402930316	0.000047991	0.000372937
hsa-miR-198_D019259	4	1.35625	0.605669825	4.80082E-05	0.000372937
hsa-miR-1299_C002669	4	1.171875	0.6508333	4.86603E-05	0.000377364
hsa-miR-1228_C090937	4	1.6875	0.526634361	4.92533E-05	0.000381239
hsa-miR-576-3p_D003609	6	1.640243902	1.02936871	0.000049326	0.000381239
hsa-miR-520a-3p_C400082	5	1.88372093	0.722054637	4.98465E-05	0.000384614
hsa-miR-558_C492909	5	1.211111111	0.888124671	5.01552E-05	0.000386347
hsa-miR-561_C017947	27	8.859719439	4.709641405	0.000050858	0.000391104
hsa-miR-646_C506002	8	2.747572816	1.257097236	5.13678E-05	0.000394364
hsa-miR-1307_C006632	7	1.425	1.339542832	5.16078E-05	0.000395545
hsa-let-7e_D009151	19	2.1875	4.347682572	5.19392E-05	0.000397421
hsa-miR-1182_C009277	2	1.045454545	0.208298895	5.27389E-05	0.000400205
hsa-miR-297_C065180	2	1.045454545	0.208298895	5.27389E-05	0.000400205
hsa-miR-1179_D012721	2	1.045454545	0.208298895	5.27389E-05	0.000400205
hsa-miR-365_D004996	2	1.045454545	0.208298895	5.27389E-05	0.000400205
hsa-miR-132_D014112	2	1.045454545	0.208298895	5.27389E-05	0.000400205
hsa-miR-633_D002857	4	1.235772358	0.638612582	5.33631E-05	0.000404274
hsa-miR-370_C014347	47	14.46	8.875381682	5.41813E-05	0.000409796
hsa-miR-219-2-3p_D015032	6	1.628458498	1.038916848	5.49078E-05	0.000414608
hsa-miR-20a_C017947	9	2.121037464	1.683036595	5.58816E-05	0.000421268
hsa-miR-558_C080955	12	2.486486486	2.378071233	5.61606E-05	0.000422678
hsa-miR-1228_D015032	7	1.985163205	1.204499251	5.70241E-05	0.000428474
hsa-miR-506_C086566	6	1.6625	1.033123299	5.74297E-05	0.000430402
hsa-miR-509-5p_D001205	3	1.208	0.405876829	5.74882E-05	0.000430402
hsa-miR-219-5p_D004128	5	1.549689441	0.810844527	5.75619E-05	0.000430402
hsa-miR-569_D011794	27	7.609756098	5.108392817	5.80777E-05	0.000433552
hsa-miR-520a-3p_C001277	3	1.208333333	0.406116431	5.83295E-05	0.000434725
hsa-miR-98_D011374	11	2.752969121	2.048293878	5.87945E-05	0.000437147
hsa-miR-383_C059514	15	3.58490566	2.898008179	5.88449E-05	0.000437147
hsa-miR-575_D009637	4	1.836363636	0.49593388	5.92096E-05	0.000439146
hsa-miR-526b_D009637	4	1.796875	0.505584053	5.94029E-05	0.000439411
hsa-miR-622_C047426	2	1.045977011	0.209435255	5.94367E-05	0.000439411
hsa-miR-646_C107676	14	4.444685466	2.399312754	5.98087E-05	0.00044145
hsa-miR-584_C008493	10	2.324786325	1.901397266	6.07546E-05	0.000447712
hsa-miR-221_D020111	5	1.580327869	0.806290773	6.14256E-05	0.000451825
hsa-let-7b_D002087	8	2.068965517	1.446304076	6.15096E-05	0.000451825
hsa-miR-548b-3p_D004958	37	9.611222445	7.445806496	0.000061778	0.000452173
hsa-miR-365_C017185	2	1.046153846	0.20981818	6.18525E-05	0.000452173
hsa-miR-301b_D001151	2	1.046153846	0.20981818	6.18525E-05	0.000452173
hsa-miR-151-3p_D011794	15	3.245762712	3.00170321	6.22046E-05	0.000454024
hsa-miR-936_D014212	42	15.666	7.154470211	6.37379E-05	0.000464226
hsa-miR-140-3p_C496197	5	1.589665653	0.805763553	6.38045E-05	0.000464226
hsa-miR-514_C066075	7	1.591666667	1.316534804	6.56089E-05	0.000476599
hsa-miR-1280_C103303	7	1.829787234	1.255950076	6.63925E-05	0.000481529
hsa-miR-1200_D002706	2	1.046511628	0.210590352	6.69812E-05	0.000483507
hsa-miR-340_C008461	2	1.046511628	0.210590352	6.69812E-05	0.000483507
hsa-miR-934_D015081	2	1.046511628	0.210590352	6.69812E-05	0.000483507
hsa-miR-519b-3p_D004317	21	2.979166667	4.774452776	6.73657E-05	0.00048552
hsa-miR-1279_C024352	3	1.178082192	0.416852714	6.80538E-05	0.00048971
hsa-miR-558_D017382	3	1.212121212	0.408810229	6.85724E-05	0.00049267

hsa-miR-744_D018038	6	1.752265861	1.021943236	0.000069166	0.000495822
hsa-miR-576-3p_D001564	6	1.618257261	1.056259092	6.92272E-05	0.000495822
hsa-miR-219-2-3p_D005472	18	4.477178423	3.515894249	6.95929E-05	0.000497609
hsa-miR-655_D013755	22	5.262054507	4.421275793	6.96934E-05	0.000497609
hsa-miR-595_C056507	6	1.730650155	1.028339254	7.01296E-05	0.000499391
hsa-miR-140-5p_C030110	10	2.612009238	1.84304294	7.01605E-05	0.000499391
hsa-miR-22_C482205	9	3.126315789	1.445443696	7.16499E-05	0.000508728
hsa-miR-324-5p_D001194	3	1.175572519	0.41866593	0.000071694	0.000508728
hsa-miR-1237_D013196	14	2.932692308	2.844014485	7.22194E-05	0.000511666
hsa-miR-922_D002857	5	1.508143322	0.832424428	7.23704E-05	0.000511945
hsa-miR-506_D017373	8	1.554285714	1.598632068	7.36141E-05	0.000519942
hsa-miR-1205_D017313	11	2.853233831	2.052992474	7.39812E-05	0.000521732
hsa-miR-376b_D018038	4	1.234693878	0.651544796	7.50561E-05	0.000528501
hsa-miR-1291_D003907	14	2.851393189	2.876652506	7.59447E-05	0.000533939
hsa-miR-520a-3p_D019833	8	1.929530201	1.503374431	7.64439E-05	0.000535798
hsa-miR-143_D004317	31	6.616182573	6.681241744	7.65161E-05	0.000535798
hsa-miR-100_D003907	6	1.416666667	1.114924013	7.65593E-05	0.000535798
hsa-miR-143_C085746	6	2.043478261	0.954543409	7.76992E-05	0.000542948
hsa-miR-632_C001277	3	1.215384615	0.411088899	7.84568E-05	0.000547409
hsa-miR-605_D017638	16	5.938	2.58421284	7.88011E-05	0.000548977
hsa-miR-548d-3p_C059514	31	10.344	5.604432531	7.99172E-05	0.000555909
hsa-miR-509-3-5p_D017373	7	1.438356164	1.374785676	8.10763E-05	0.000563118
hsa-miR-885-3p_D005492	8	2.464285714	1.368405647	8.14831E-05	0.000565089
hsa-miR-132_D004317	26	4.682808717	5.809620464	8.18761E-05	0.000566617
hsa-let-7d_D004317	33	7.530737705	7.043125199	8.19503E-05	0.000566617
hsa-miR-199a-5p_D007213	17	5.98989899	2.857896908	8.35576E-05	0.000576862
hsa-miR-1207-5p_D007052	16	3.395705521	3.304558103	8.36927E-05	0.000576927
hsa-miR-1_D009288	2	1.047619048	0.212958855	8.50491E-05	0.000582777
hsa-miR-576-3p_C417521	2	1.047619048	0.212958855	8.50491E-05	0.000582777
hsa-miR-518d-5p_C451735	2	1.047619048	0.212958855	8.50491E-05	0.000582777
hsa-miR-576-3p_C014036	6	5.047619048	0.212958855	8.50491E-05	0.000582777
hsa-miR-106b_D015735	3	1.166666667	0.424918293	8.51776E-05	0.000582787
hsa-miR-548c-3p_C014347	80	31.918	14.14621066	8.74229E-05	0.00059726
hsa-miR-1258_C034613	3	1.171428571	0.42442438	8.75865E-05	0.000597488
hsa-miR-130b_C120275	6	1.5	1.103026141	8.79349E-05	0.000597658
hsa-miR-381_C012589	17	4.263027295	3.354238497	8.79432E-05	0.000597658
hsa-miR-885-3p_D004958	41	11.958	8.16946975	8.80019E-05	0.000597658
hsa-miR-519a_C105686	3	1.191256831	0.420161372	8.98107E-05	0.000609041
hsa-miR-504_C048460	5	1.236363636	0.913805954	0.000090479	0.000611938
hsa-miR-188-5p_D017638	14	5.078947368	2.294122508	9.05045E-05	0.000611938
hsa-miR-520f_D000001	11	3.753623188	1.83686719	9.06618E-05	0.000612101
hsa-miR-1257_C048460	5	1.243243243	0.91253746	0.000091284	0.000614402
hsa-miR-1321_C008261	3	1.17989418	0.423412678	0.000091538	0.000614402
hsa-miR-1321_C023600	3	1.17989418	0.423412678	0.000091538	0.000614402
hsa-miR-1321_C017133	3	1.17989418	0.423412678	0.000091538	0.000614402
hsa-miR-375_D013749	9	1.913934426	1.798442658	9.43876E-05	0.000632604
hsa-miR-503_D020123	7	1.926530612	1.259539195	9.49441E-05	0.000635406
hsa-miR-142-3p_D003907	13	3.098159509	2.57695003	0.000096322	0.000643689
hsa-miR-558_C025462	13	3.265508685	2.531484412	9.69636E-05	0.000646698
hsa-miR-302a_C472791	4	1.401433692	0.619397981	0.000097054	0.000646698
hsa-miR-370_C016837	3	1.17816092	0.425326186	9.72947E-05	0.000647362
hsa-miR-202_C066515	2	1.048275862	0.214348555	9.74735E-05	0.000647613
hsa-let-7d_D003561	4	1.326732673	0.638824894	9.83952E-05	0.000652792
hsa-miR-1243_C086511	2	1.048387097	0.214582818	9.97132E-05	0.000660582

hsa-miR-483-3p_D013749	9	1.791946309	1.840174954	0.000100996	0.00066804
hsa-miR-1179_D004128	4	1.366255144	0.629858212	0.00010113	0.00066804
hsa-miR-338-5p_C018021	17	3.166666667	3.707418171	0.000102012	0.000672898
hsa-miR-337-3p_D020849	12	3.127192982	2.302088491	0.000103045	0.000678737
hsa-miR-330-3p_D002794	3	1.192982456	0.423239933	0.000103765	0.0006825
hsa-miR-143_C105934	7	2.365384615	1.152402944	0.000106453	0.000699178
hsa-miR-569_C018021	17	2.80104712	3.826361983	0.00010665	0.000699472
hsa-miR-7_D005047	8	1.917602996	1.541179161	0.000107367	0.000700082
hsa-miR-383_D006861	8	2.213513514	1.461332632	0.000107499	0.000700082
hsa-miR-875-3p_D009288	2	1.048780488	0.215408802	0.000107963	0.000700082
hsa-miR-1228_C060327	2	1.048780488	0.215408802	0.000107963	0.000700082
hsa-miR-130b_C479128	2	1.048780488	0.215408802	0.000107963	0.000700082
hsa-miR-567_C022838	2	1.048780488	0.215408802	0.000107963	0.000700082
hsa-miR-558_C029728	2	1.048780488	0.215408802	0.000107963	0.000700082
hsa-miR-302e_C061133	2	1.048780488	0.215408802	0.000107963	0.000700082
hsa-miR-519b-3p_D014212	15	3.713942308	2.993967948	0.000109403	0.000708419
hsa-miR-324-3p_C086566	5	1.337078652	0.898735944	0.00010972	0.000709471
hsa-miR-383_D019833	14	3.987525988	2.633513876	0.000110042	0.000710399
hsa-miR-526b_C031278	5	1.395833333	0.883637919	0.000110173	0.000710399
hsa-miR-511_D005947	15	3.327433628	3.111333772	0.000112636	0.000725262
hsa-miR-516a-3p_C017185	2	1.049019608	0.215908976	0.000113236	0.000728104
hsa-miR-1244_D001194	3	1.183333333	0.427849922	0.000113425	0.0007283
hsa-miR-27a_D004391	13	4.770791075	2.138746857	0.000113754	0.000728914
hsa-miR-1231_C496751	3	1.225	0.417582327	0.000113954	0.000728914
hsa-miR-299-5p_D004958	39	12.63	7.523636089	0.000113997	0.000728914
hsa-miR-410_C031927	17	3.558333333	3.628121262	0.000114994	0.000733406
hsa-miR-646_D006843	5	1.982248521	0.73358464	0.000115019	0.000733406
hsa-miR-633_D007501	6	1.875	1.023169096	0.000115215	0.000733637
hsa-miR-641_C004984	4	1.403773585	0.625846525	0.000116845	0.000742985
hsa-miR-450b-3p_C010327	3	1.20610687	0.422958631	0.000117068	0.000743374
hsa-miR-574-5p_D001241	5	1.453781513	0.872048676	0.000117345	0.000744103
hsa-miR-199a-3p_D011794	27	8.153225806	5.238342441	0.000117719	0.000745445
hsa-miR-1826_C085911	7	1.887804878	1.288636095	0.00011838	0.000748599
hsa-miR-206_C034613	3	1.193548387	0.426490165	0.000118924	0.000751004
hsa-miR-520f_C107135	4	2.10619469	0.448455202	0.000119325	0.000751986
hsa-miR-140-5p_D003474	32	8.886178862	6.545280994	0.000119407	0.000751986
hsa-miR-576-3p_D002945	8	2.180412371	1.483040842	0.000121904	0.000766659
hsa-miR-143_C056507	8	2.339491917	1.440982703	0.000123441	0.000775264
hsa-miR-1251_D005492	5	1.604255319	0.835885935	0.000124473	0.000780677
hsa-miR-633_C018021	17	2.814569536	3.874528766	0.000126463	0.000792076
hsa-miR-23a_C086566	5	1.485815603	0.867954436	0.000126691	0.000792423
hsa-miR-573_C053541	6	1.713043478	1.073516913	0.00012801	0.000799584
hsa-miR-573_C063002	3	1.170588235	0.43421966	0.000128466	0.00080127
hsa-miR-29a_C016527	3	1.196202532	0.427812522	0.000128629	0.00080127
hsa-miR-1207-5p_C045651	17	3.789976134	3.594452733	0.000129535	0.00080582
hsa-miR-548c-3p_D004317	49	15.272	9.970657752	0.000131041	0.000813786
hsa-miR-497_C022838	3	1.160377358	0.437333094	0.00013117	0.000813786
hsa-miR-194_C009277	3	1.178082192	0.432972072	0.00013167	0.000815785
hsa-miR-122_D004958	35	8.366733467	7.706006789	0.000131897	0.000816091
hsa-miR-1228_D002211	5	1.299363057	0.920156233	0.000133293	0.000823618
hsa-miR-1827_C095591	3	1.137254902	0.443674843	0.000133721	0.000825152
hsa-miR-1228_C059514	16	4.247826087	3.177963467	0.000134647	0.000829751
hsa-miR-492_D011374	13	3.495555556	2.529159329	0.000135315	0.00083275
hsa-miR-920_C009131	2	1.05	0.217944947	0.000137014	0.000840951

hsa-miR-302a_C031721	2	1.05	0.217944947	0.000137014	0.000840951
hsa-miR-33a_D019800	5	1.613772455	0.839119596	0.000138315	0.000847803
hsa-miR-575_D014212	27	7.860040568	5.405155453	0.000139725	0.000855303
hsa-miR-370_C120275	6	1.393939394	1.166174631	0.000140149	0.000856756
hsa-miR-27a_D009151	19	4.008421053	4.152352869	0.000141949	0.000866606
hsa-miR-130b_C012589	14	3.707936508	2.767802497	0.000143296	0.000873668
hsa-miR-1827_C025299	5	1.625514403	0.838678685	0.000145173	0.000883938
hsa-miR-643_C070081	11	2.090566038	2.372663828	0.000145827	0.000886744
hsa-miR-558_D004317	27	5.406113537	6.188886871	0.000146463	0.000887813
hsa-miR-885-5p_D003010	5	1.637426901	0.836032358	0.000146522	0.000887813
hsa-miR-653_D004726	4	1.197368421	0.688624427	0.000146583	0.000887813
hsa-miR-503_D024505	4	1.294573643	0.66359487	0.00014782	0.000892446
hsa-miR-205_D005047	8	1.823275862	1.60246888	0.000147883	0.000892446
hsa-miR-520f_D010126	11	2.091603053	2.374966072	0.000147931	0.000892446
hsa-miR-576-3p_C059514	16	4.041860465	3.264056013	0.000148812	0.000896583
hsa-miR-606_D003010	5	1.636103152	0.837490282	0.000149489	0.000898424
hsa-miR-520f_D002087	12	3.264339152	2.327318006	0.000149509	0.000898424
hsa-miR-143_D010938	5	1.36	0.91126286	0.000150153	0.000901114
hsa-miR-539_D008748	6	2.666666667	0.829993307	0.000151168	0.000906021
hsa-miR-641_D019207	4	1.437037037	0.627840596	0.00015292	0.000915327
hsa-miR-199a-3p_D005947	16	3.582568807	3.408713714	0.000153709	0.000918852
hsa-miR-1228_D003609	5	1.49537037	0.876639655	0.000154012	0.000919466
hsa-miR-1228_C496197	4	1.387351779	0.641267044	0.000154677	0.000922237
hsa-miR-302e_C017947	14	3.668103448	2.79889446	0.000156656	0.000932825
hsa-miR-558_C004999	2	1.050847458	0.219686125	0.00016058	0.000954198
hsa-miR-574-3p_C103303	6	1.555555556	1.133115447	0.000160661	0.000954198
hsa-miR-1294_D013749	10	1.896694215	2.159370657	0.000161687	0.00095905
hsa-miR-205_C084904	3	1.235294118	0.424182503	0.000164074	0.000971953
hsa-miR-1825_C023844	2	1.051020408	0.220039374	0.000165757	0.000979396
hsa-miR-494_C487689	2	1.051020408	0.220039374	0.000165757	0.000979396
hsa-miR-29b_D014212	29	9.114228457	5.722453224	0.00016637	0.000981754
hsa-miR-145_D019821	3	1.193277311	0.43535576	0.000166826	0.000983181
hsa-miR-199a-3p_C034613	5	1.651515152	0.840137511	0.000168697	0.00099232
hsa-miR-148a_D013629	27	9.076	5.116856848	0.000168809	0.00099232
hsa-miR-122_D013629	14	3.2	2.958961734	0.00017256	0.001013073
hsa-miR-576-3p_D013467	9	2.062686567	1.836352308	0.000172954	0.001013961
hsa-miR-20a_D009532	11	2.391304348	2.317028277	0.000173153	0.001013961
hsa-miR-320a_C023888	3	1.191616766	0.436838557	0.0001735	0.001014699
hsa-miR-432_C482884	2	1.051282051	0.220572443	0.000173834	0.001015359
hsa-miR-494_C115461	3	1.18556701	0.438598915	0.000174834	0.001019902
hsa-miR-1276_D001194	4	1.404669261	0.641964388	0.00017551	0.001022461
hsa-miR-1246_C071834	3	1.17218543	0.44217388	0.000175718	0.001022461
hsa-miR-138_D004610	5	1.670623145	0.837615609	0.000176645	0.001026553
hsa-miR-576-3p_D016718	4	1.369863014	0.651801493	0.00017826	0.001034629
hsa-miR-1179_D000966	3	1.205128205	0.434386522	0.000180153	0.001042979
hsa-miR-1179_D000965	3	1.205128205	0.434386522	0.000180153	0.001042979
hsa-miR-935_D020122	19	8.734	2.811270887	0.000180435	0.001043296
hsa-miR-548e_D001194	4	1.400673401	0.644374427	0.000181267	0.001046788
hsa-miR-22_C492909	6	1.370860927	1.193891151	0.000181711	0.001048034
hsa-miR-924_C432165	19	3.085106383	4.528067491	0.000183029	0.001054311
hsa-miR-450b-3p_D005047	7	1.530612245	1.430028412	0.000185829	0.001068504
hsa-miR-432_C006632	12	3.564210526	2.279792963	0.000186156	0.001068504
hsa-miR-656_D004958	58	23.41	10.61686865	0.000186191	0.001068504
hsa-miR-1237_D015741	9	2.487864078	1.725690604	0.000186935	0.001071434

hsa-miR-576-3p_C403304	2	1.051724138	0.221469527	0.000188176	0.001075861
hsa-miR-649_C023888	2	1.051724138	0.221469527	0.000188176	0.001075861
hsa-miR-1228_D011070	5	2.611111111	0.590563656	0.000189016	0.001079319
hsa-miR-28-5p_C029938	5	1.553264605	0.873285062	0.000189276	0.001079461
hsa-miR-558_C477819	4	1.234042553	0.690752472	0.000190465	0.001084894
hsa-miR-770-5p_D001241	5	1.485943775	0.892181126	0.000191307	0.00108834
hsa-miR-20a_C097240	5	1.4	0.916515139	0.000194312	0.0011031
hsa-miR-122_D020849	13	3.33037694	2.65109852	0.000194382	0.0011031
hsa-let-7d_D000079	14	2.716723549	3.134901	0.000195721	0.001108305
hsa-miR-548c-3p_D010416	2	1.051948052	0.221922175	0.000195782	0.001108305
hsa-miR-1205_D019256	7	2.00872093	1.304034256	0.000201537	0.001139478
hsa-miR-503_C497101	4	1.509615385	0.620099128	0.000202328	0.001142543
hsa-miR-660_C016601	4	1.370860927	0.657266001	0.000203524	0.001147885
hsa-miR-548g_C010327	7	2.31403118	1.220392433	0.000205545	0.001157861
hsa-miR-370_D002110	4	1.299435028	0.677023827	0.000206655	0.001161467
hsa-miR-637_D005047	7	1.657608696	1.405403678	0.000206691	0.001161467
hsa-miR-221_D019833	13	4.128834356	2.42974219	0.000209269	0.001174516
hsa-miR-649_C070081	13	2.674267101	2.864716681	0.000210201	0.001178306
hsa-miR-569_D010100	22	4.271111111	5.158148773	0.000210459	0.001178314
hsa-miR-410_D002699	3	1.136363636	0.456812528	0.000212358	0.001187498
hsa-miR-190_C012589	11	2.838862559	2.224935514	0.000214779	0.001199575
hsa-miR-556-3p_C059514	22	7.096774194	4.277509365	0.000215672	0.001203099
hsa-miR-1226_C497103	2	1.052631579	0.223296878	0.000220484	0.001224892
hsa-miR-567_D019800	2	1.052631579	0.223296878	0.000220484	0.001224892
hsa-miR-130b_C403900	2	1.052631579	0.223296878	0.000220484	0.001224892
hsa-miR-143_D008070	18	3.588571429	4.132365039	0.000220646	0.001224892
hsa-miR-509-3-5p_C063002	4	1.406593407	0.651479387	0.000221786	0.001227224
hsa-miR-221_D002330	4	1.387543253	0.656601177	0.000221849	0.001227224
hsa-miR-17_D015474	8	2.46	1.469149414	0.000221868	0.001227224
hsa-miR-558_C105934	7	2.297619048	1.232301002	0.000222963	0.001231797
hsa-miR-633_D010100	21	4.039215686	4.943976798	0.000224508	0.001238842
hsa-miR-339-5p_D014810	19	7.932	3.106666381	0.000225145	0.001240865
hsa-miR-199a-3p_C093973	12	3.237113402	2.413193386	0.000226482	0.001246646
hsa-miR-9_C031463	4	1.980769231	0.499630041	0.000226737	0.001246646
hsa-miR-1259_D019833	12	3.446351931	2.351676782	0.000227382	0.001248697
hsa-miR-181a_D002794	3	1.197452229	0.443487775	0.000232677	0.001276249
hsa-miR-558_C085746	5	1.641025641	0.861935196	0.000233174	0.001277448
hsa-miR-199a-5p_C017947	25	9.118236473	4.618084181	0.000233482	0.001277611
hsa-miR-1827_D013755	26	7.240890688	5.541586249	0.000233847	0.001278085
hsa-miR-148a_D001151	12	4.442424242	2.063687536	0.000236591	0.001291545
hsa-let-7i_D019833	8	2.105571848	1.57900393	0.000237964	0.001296062
hsa-miR-1294_D003513	5	1.6875	0.850398191	0.000237983	0.001296062
hsa-miR-370_C492909	5	1.224299065	0.979303693	0.000241064	0.001310782
hsa-let-7e_D013755	9	1.96875	1.911876941	0.000241257	0.001310782
hsa-miR-548i_D006861	7	1.919354839	1.347619013	0.000242607	0.001316559
hsa-miR-519b-3p_C006632	8	1.959375	1.624492108	0.000244171	0.001323482
hsa-miR-25_D007052	10	2.226277372	2.13415978	0.0002458	0.00133074
hsa-miR-22_C012589	26	8.277777778	5.23511018	0.000247444	0.001337483
hsa-miR-548m_C045651	13	2.692957746	2.899019939	0.000247628	0.001337483
hsa-miR-548c-3p_C009687	10	2.847682119	1.952238804	0.000248746	0.001341942
hsa-miR-455-3p_C533894	5	1.66568915	0.859462703	0.000250296	0.001345959
hsa-miR-455-3p_C062198	5	1.66568915	0.859462703	0.000250296	0.001345959
hsa-miR-146b-3p_D020849	12	3.242888403	2.431575381	0.00025037	0.001345959
hsa-miR-143_C084656	4	1.385135135	0.662954573	0.000251899	0.001352595

hsa-miR-224_D011078	5	1.662790698	0.860779327	0.000252414	0.001353776
hsa-miR-576-3p_D007213	10	3.03271028	1.900284076	0.00025294	0.001355015
hsa-miR-1200_D010672	8	2.232375979	1.550135628	0.000253748	0.001357759
hsa-miR-638_D006861	5	1.41509434	0.930286267	0.000255675	0.001366468
hsa-miR-151-3p_C009687	6	1.862745098	1.085006693	0.000255971	0.001366468
hsa-miR-576-3p_C026105	2	1.053571429	0.225170004	0.000258304	0.001374129
hsa-miR-298_D003375	2	1.053571429	0.225170004	0.000258304	0.001374129
hsa-miR-576-3p_C049639	2	1.053571429	0.225170004	0.000258304	0.001374129
hsa-miR-576-3p_C030110	8	1.908783784	1.646594727	0.000258662	0.001374441
hsa-let-7c_C055162	3	1.25	0.433012702	0.00026162	0.001388552
hsa-miR-146b-3p_C070081	11	2.294303797	2.427186308	0.000264403	0.001401702
hsa-miR-144_D015118	6	1.4375	1.208461611	0.000265117	0.001403866
hsa-miR-1291_D016729	2	1.053763441	0.22555029	0.000266609	0.00141014
hsa-miR-143_C088658	9	2.592896175	1.743325703	0.000266976	0.001410457
hsa-miR-573_C015329	7	1.801932367	1.391623018	0.000267745	0.001412893
hsa-miR-199a-5p_D018038	24	11.466	3.615085614	0.000270679	0.001426736
hsa-miR-513a-3p_C075773	5	1.678378378	0.861687576	0.000274718	0.001446365
hsa-let-7i_D020122	8	2.651315789	1.438474468	0.000275917	0.001451014
hsa-miR-191_D005576	4	1.311827957	0.687499672	0.000277811	0.001459302
hsa-miR-632_C016340	6	2.56	0.897997773	0.000289125	0.001516115
hsa-miR-509-3-5p_D002211	6	1.526570048	1.190955985	0.000289287	0.001516115
hsa-miR-519b-3p_C014347	24	3.669154229	6.18757331	0.000291762	0.001527342
hsa-miR-518d-5p_C106014	3	1.205607477	0.448013638	0.000292544	0.001527952
hsa-miR-518d-5p_C050414	3	1.205607477	0.448013638	0.000292544	0.001527952
hsa-miR-192_C004648	8	2.67887931	1.439253441	0.000298278	0.00155613
hsa-miR-132_C093973	10	2.830985915	1.988128491	0.000301293	0.001569919
hsa-miR-503_D003609	7	2.183060109	1.293885339	0.000301605	0.001569919
hsa-miR-569_C017947	21	7.125	4.083367621	0.000303895	0.001579652
hsa-miR-633_C097240	5	1.326315789	0.967276211	0.000304163	0.001579652
hsa-miR-1827_C116926	7	2.301801802	1.260593453	0.000304851	0.001581436
hsa-miR-503_C479128	3	1.191082803	0.45334929	0.000307091	0.001591258
hsa-miR-576-3p_D003513	5	1.587878788	0.894345054	0.000308059	0.001594474
hsa-miR-299-5p_C469298	3	1.255813953	0.436317745	0.000309792	0.001601639
hsa-miR-558_D003474	26	6.691511387	5.882745799	0.000310459	0.001603282
hsa-miR-365_C446520	2	1.054794521	0.227578736	0.000314766	0.001623697
hsa-miR-221_C027576	5	1.717579251	0.859466302	0.000315799	0.001627198
hsa-miR-558_C120275	6	1.371428571	1.24408806	0.000316487	0.001628915
hsa-miR-370_C121565	6	1.932773109	1.082566919	0.000317232	0.001628974
hsa-let-7d_D019256	7	2.040214477	1.340638061	0.000317353	0.001628974
hsa-let-7i_D002110	4	1.243243243	0.713021944	0.000317563	0.001628974
hsa-miR-143_D015735	10	3.176972281	1.893942369	0.000320127	0.001640294
hsa-miR-199a-5p_C032881	7	3.428571429	0.942207568	0.000321242	0.001644172
hsa-miR-193a-5p_D000079	12	2.316831683	2.771605293	0.000321875	0.001645577
hsa-miR-519a_C044387	6	1.440559441	1.226869218	0.000325898	0.001664291
hsa-miR-520f_D000119	7	3.044444444	1.053155165	0.000327456	0.001670389
hsa-miR-28-3p_D011794	14	3.414168937	3.06471806	0.000334028	0.001702023
hsa-miR-1228_C017947	17	5.099173554	3.483502936	0.000334571	0.0017029
hsa-miR-599_C062198	4	1.436293436	0.662564462	0.000337714	0.001715094
hsa-miR-599_C533894	4	1.436293436	0.662564462	0.000337714	0.001715094
hsa-miR-1826_C111237	8	2.339130435	1.556958889	0.000345203	0.00175119
hsa-miR-200b_D007099	3	1.21875	0.449609205	0.000346583	0.00175625
hsa-miR-17_D003300	15	3.53125	3.357060734	0.00034721	0.001757487
hsa-miR-203_C066075	8	2.312807882	1.565698666	0.00034765	0.001757525
hsa-miR-380_C065180	3	1.195979899	0.455876118	0.000347983	0.001757525

hsa-miR-583_D004958	40	12.576	8.795238712	0.00035116	0.001770518
hsa-miR-219-1-3p_D005947	15	2.904761905	3.562210326	0.000351327	0.001770518
hsa-miR-1825_C030110	10	2.991091314	1.96628847	0.000353351	0.001776096
hsa-miR-601_C007262	7	2.321100917	1.269838782	0.000354035	0.001776096
hsa-miR-10b_D024483	2	1.055555556	0.229061424	0.000354368	0.001776096
hsa-let-7b_C403304	2	1.055555556	0.229061424	0.000354368	0.001776096
hsa-miR-299-3p_C013038	2	1.055555556	0.229061424	0.000354368	0.001776096
hsa-miR-520a-3p_C111237	7	2.032258065	1.355606541	0.000356992	0.001787094
hsa-miR-1258_D016718	3	1.214285714	0.451753951	0.000357341	0.001787094
hsa-miR-548c-3p_D047310	5	1.613013699	0.89761966	0.000359852	0.001797694
hsa-miR-1274b_C008261	3	1.17037037	0.464131858	0.000362959	0.001807315
hsa-miR-1274b_C023600	3	1.17037037	0.464131858	0.000362959	0.001807315
hsa-miR-1274b_C017133	3	1.17037037	0.464131858	0.000362959	0.001807315
hsa-miR-144_D020122	43	26.916	4.891313116	0.000366033	0.001820647
hsa-miR-298_C008493	20	7.098393574	3.843885959	0.00037144	0.001845542
hsa-miR-520d-5p_D005047	10	2.503778338	2.126505591	0.000375703	0.001864705
hsa-miR-26a_D004040	7	2.37360179	1.260844237	0.00037727	0.001869433
hsa-miR-200b_C010327	11	4.123732252	1.936859471	0.00037747	0.001869433
hsa-miR-885-3p_D013629	16	4.405349794	3.425018726	0.000378192	0.001870991
hsa-miR-1274b_D013654	3	1.21978022	0.452154739	0.00037977	0.001876485
hsa-miR-1259_D013196	11	2.339712919	2.491017723	0.00038012	0.001876485
hsa-miR-22_D002087	11	3.162318841	2.234713057	0.000380727	0.001877463
hsa-miR-1307_D004317	15	2.32	3.781216735	0.000381389	0.001878536
hsa-miR-144_D013755	27	8.218181818	5.83033453	0.000381763	0.001878536
hsa-miR-143_D010758	4	1.397660819	0.679521092	0.000383664	0.001885869
hsa-miR-196a_C015329	8	2.065359477	1.653568937	0.000384989	0.001890358
hsa-miR-325_C063002	2	1.034090909	0.235915222	0.000387294	0.001899644
hsa-miR-202_C074153	4	1.429078014	0.671300105	0.000388215	0.001902129
hsa-miR-494_D014635	15	6.54	2.43318721	0.000388728	0.001902612
hsa-miR-34c-3p_C075773	3	1.207650273	0.456341412	0.000390671	0.001910086
hsa-miR-7_D000082	4	1.462295082	0.662376233	0.000391269	0.001910974
hsa-miR-298_D017373	7	1.54368932	1.51225864	0.000393052	0.001917643
hsa-miR-576-3p_D015118	5	1.28	1.00079968	0.000398467	0.001941336
hsa-miR-561_C030110	9	2.541760722	1.817880483	0.000398754	0.001941336
hsa-miR-519b-3p_C004648	5	1.704485488	0.879107209	0.000403034	0.001960094
hsa-miR-224_D019800	4	1.46779661	0.662414863	0.000404226	0.001962679
hsa-miR-668_C008493	10	2.861297539	2.029646296	0.000404766	0.001962679
hsa-miR-22_D005473	3	1.265625	0.441665438	0.000404848	0.001962679
hsa-miR-632_C006632	10	2.775555556	2.058225293	0.000409343	0.001982377
hsa-miR-940_C055494	6	1.723214286	1.166571759	0.000412528	0.001994256
hsa-miR-632_D015032	6	1.795918367	1.145465924	0.00041389	0.001994256
hsa-miR-1231_C016340	6	2.592592593	0.913246519	0.000414385	0.001994256
hsa-miR-1826_C487689	2	1.056603774	0.231083938	0.000414973	0.001994256
hsa-let-7b_C049639	2	1.056603774	0.231083938	0.000414973	0.001994256
hsa-miR-425_D002259	2	1.056603774	0.231083938	0.000414973	0.001994256
hsa-miR-944_D015084	2	1.056603774	0.231083938	0.000414973	0.001994256
hsa-miR-199a-3p_D016718	5	1.728323699	0.874266708	0.000415271	0.001994256
hsa-miR-1260_C022921	3	1.266666667	0.442216639	0.00041601	0.001995718
hsa-miR-503_C023617	3	1.211640212	0.457358366	0.000417403	0.00200031
hsa-miR-22_C070515	4	2.050847458	0.501649105	0.000419017	0.002005951
hsa-miR-653_C070081	12	2.586440678	2.755300447	0.000422723	0.002021585
hsa-miR-520f_D014108	4	1.403225806	0.682965012	0.000423899	0.002025099
hsa-miR-143_D017313	9	2.705128205	1.778136264	0.000426098	0.002031458
hsa-miR-143_C007610	3	1.123893805	0.482118176	0.000426115	0.002031458

hsa-miR-875-3p_D013755	22	5.804979253	5.00780885	0.000426813	0.002032675
hsa-miR-181a_D047630	5	1.694852941	0.886319211	0.000430207	0.002046715
hsa-let-7f_D013739	7	2.25	1.311805372	0.000432455	0.002053445
hsa-miR-34c-5p_C006632	9	2.45049505	1.858889467	0.000432516	0.002053445
hsa-miR-199a-5p_D015032	10	3.099118943	1.968104506	0.000433644	0.002056673
hsa-miR-627_D014635	7	2.413333333	1.264137827	0.000436992	0.002068413
hsa-miR-1244_D019833	14	4.604887984	2.758193965	0.000437339	0.002068413
hsa-miR-130a_D017373	7	1.486842105	1.543180423	0.000437471	0.002068413
hsa-miR-519a_C018021	17	3.712041885	4.036588098	0.000438316	0.002070276
hsa-miR-1300_D016912	4	1.417322835	0.680908898	0.000440333	0.002075919
hsa-miR-558_D002110	4	1.260869565	0.725361957	0.000440415	0.002075919
hsa-miR-379_C008493	9	2.252659574	1.924780549	0.000444724	0.002093679
hsa-let-7i_C120275	6	1.419354839	1.26425277	0.000445095	0.002093679
hsa-miR-494_D006830	15	6.578	2.45192088	0.000446114	0.002095857
hsa-miR-302a_D014051	6	1.444444444	1.257078722	0.000446471	0.002095857
hsa-miR-205_D014212	40	15.646	7.926202369	0.000448533	0.002103386
hsa-miR-665_C423222	2	1.057142857	0.232115383	0.000449031	0.002103573
hsa-let-7b_C400082	6	2.032967033	1.083853012	0.000453869	0.00212407
hsa-miR-616_D011078	4	1.412451362	0.684117073	0.000456337	0.002133028
hsa-miR-503_C059514	29	11.404	5.529989512	0.00045672	0.002133028
hsa-miR-299-5p_D019259	4	1.463157895	0.669807927	0.000457177	0.002133028
hsa-miR-221_D004958	27	7.541082164	6.186789827	0.000458472	0.00213475
hsa-miR-302a_D001564	5	1.555555556	0.931280812	0.000458476	0.00213475
hsa-miR-520f_C417207	5	1.275862069	1.013581963	0.000460854	0.002143648
hsa-miR-1255a_D004317	16	2.633858268	4.086153313	0.00046358	0.002154146
hsa-miR-601_C472791	3	1.193939394	0.465760632	0.000465233	0.002159641
hsa-miR-181c_C025462	10	2.563432836	2.149211624	0.000466471	0.0021632
hsa-miR-504_C034028	4	1.447811448	0.675300351	0.000467533	0.002165937
hsa-miR-302a_D009151	9	1.603550296	2.137905116	0.000469694	0.002173755
hsa-miR-646_D017255	5	1.809859155	0.859356938	0.000472375	0.002183961
hsa-miR-1270_C103303	7	1.905063291	1.42663619	0.000475413	0.002195796
hsa-miR-381_D020849	13	3.885416667	2.692078626	0.000480468	0.002216913
hsa-miR-1228_C067311	6	1.470588235	1.257961955	0.000485382	0.002236295
hsa-miR-576-3p_C007610	2	1.057692308	0.233160686	0.000485866	0.002236295
hsa-miR-617_D002117	12	4.061099796	2.317104998	0.000486277	0.002236295
hsa-miR-1827_C105934	7	2.450331126	1.264328431	0.000486617	0.002236295
hsa-miR-627_D006830	7	2.422566372	1.272835507	0.000487271	0.002237061
hsa-miR-1293_D007529	4	1.423469388	0.684567646	0.000489161	0.002243495
hsa-miR-579_C085911	10	2.74702381	2.10264645	0.000494733	0.002264951
hsa-miR-509-3-5p_C008493	15	4.855371901	3.036239802	0.000494826	0.002264951
hsa-miR-632_D016572	6	1.925373134	1.123871677	0.000496493	0.002270318
hsa-miR-212_D013755	12	2.471223022	2.836852664	0.00049904	0.002279694
hsa-miR-206_D016718	3	1.225609756	0.45966875	0.000504437	0.002302058
hsa-miR-365_C422648	2	1.057971014	0.23368863	0.000505394	0.002304135
hsa-miR-576-3p_C002055	6	2.9375	0.826797285	0.000506117	0.002305142
hsa-miR-615-5p_C011890	5	1.756476684	0.880280231	0.000510693	0.002323678
hsa-miR-132_D002087	8	2.367924528	1.600857504	0.000511477	0.002324941
hsa-miR-558_D015118	5	1.293103448	1.017241379	0.000512788	0.002328595
hsa-miR-558_D047311	5	1.6328125	0.917461629	0.000516902	0.002344958
hsa-miR-1231_D016572	6	1.8	1.166190379	0.000521931	0.002363343
hsa-miR-325_D013196	10	2.344	2.241799277	0.000522065	0.002363343
hsa-miR-519a_C097240	6	1.622568093	1.219793159	0.000522499	0.002363343
hsa-miR-330-3p_D002392	8	2.130434783	1.678023447	0.000523894	0.00236732
hsa-miR-655_C484327	2	1.058252427	0.234220157	0.000525701	0.00237315

hsa-miR-184_D017638	5	1.491582492	0.960900525	0.000528959	0.002385512
hsa-miR-199a-3p_D006861	12	3.838574423	2.408305959	0.000531362	0.002393997
hsa-miR-513c_D019207	3	1.247311828	0.455690755	0.000536893	0.002416545
hsa-miR-154_D003010	4	1.457142857	0.680036013	0.000539697	0.00242534
hsa-miR-568_C109238	3	1.213592233	0.46531095	0.000540238	0.00242534
hsa-miR-181d_D003010	4	1.459558824	0.679411367	0.000540432	0.00242534
hsa-miR-30d_D019284	8	2.2	1.661324773	0.000541722	0.002428756
hsa-miR-936_C095105	4	1.45112782	0.682390374	0.000546112	0.002446049
hsa-miR-1243_C056507	5	1.53877551	0.949503915	0.000546913	0.002447249
hsa-let-7i_D002087	6	1.932432432	1.131025379	0.00054816	0.002450441
hsa-miR-196a_C088658	8	2.292358804	1.634625771	0.000549569	0.002453164
hsa-miR-184_D003474	12	2.333333333	2.90960684	0.000549838	0.002453164
hsa-miR-132_D017239	8	2.428571429	1.593508787	0.00055473	0.002472588
hsa-miR-20a_C009687	6	1.892857143	1.144530026	0.000557199	0.002481184
hsa-miR-519a_C022838	5	1.725761773	0.896052308	0.000561276	0.002496917
hsa-miR-548p_D020122	39	23.746	4.828818075	0.000562552	0.002500171
hsa-miR-1826_D002038	2	1.058823529	0.235294118	0.000568778	0.002515665
hsa-miR-1826_D008464	2	1.058823529	0.235294118	0.000568778	0.002515665
hsa-miR-379_D019308	2	1.058823529	0.235294118	0.000568778	0.002515665
hsa-miR-148a_C430898	2	1.058823529	0.235294118	0.000568778	0.002515665
hsa-miR-22_C060327	2	1.058823529	0.235294118	0.000568778	0.002515665
hsa-miR-7_C077990	9	3.122807018	1.693505055	0.000571249	0.002523284
hsa-miR-1300_D004997	9	2.356223176	1.936140886	0.0005716	0.002523284
hsa-miR-558_C059514	18	5.408247423	3.913278218	0.000575594	0.002538475
hsa-miR-520f_C109799	4	2.129032258	0.4913402	0.000576624	0.002540577
hsa-miR-582-5p_D010936	8	2.574162679	1.55458956	0.000580908	0.002556998
hsa-miR-212_C025462	9	2.396551724	1.927027863	0.000583635	0.002566541
hsa-miR-199a-3p_D003513	6	1.838827839	1.165606674	0.00058465	0.002566997
hsa-miR-410_D004958	61	25.55	12.53919854	0.000584857	0.002566997
hsa-miR-130a_C053541	8	2.247093023	1.658309847	0.000585995	0.002569535
hsa-miR-935_D014212	30	10.54233871	6.366037483	0.000586729	0.002570299
hsa-miR-298_D015741	12	3.93153527	2.403636881	0.000593255	0.00259641
hsa-miR-221_C053541	6	1.639130435	1.22837437	0.000595394	0.002603289
hsa-miR-22_C514580	3	1.215189873	0.468525456	0.000601182	0.002624498
hsa-miR-30b_C056507	8	2.621681416	1.544550456	0.000601388	0.002624498
hsa-miR-1307_D002117	3	1.173913043	0.480233088	0.000602175	0.002625437
hsa-miR-936_D013605	3	1.205882353	0.471506457	0.000607344	0.002644159
hsa-miR-219-1-3p_C051890	8	2.639705882	1.540337343	0.000607621	0.002644159
hsa-miR-1200_D012293	9	2.586206897	1.877060614	0.000619644	0.002693926
hsa-miR-1237_C095104	2	1.05952381	0.236602463	0.000625121	0.002715166
hsa-miR-1304_C088658	6	1.76371308	1.195575409	0.000626685	0.002719386
hsa-miR-181a_C034028	7	2.436241611	1.296987463	0.000630032	0.002731329
hsa-miR-548m_C006780	6	2	1.124135931	0.000631974	0.002737163
hsa-miR-1285_D000079	12	2.348148148	2.94723604	0.000634779	0.00274672
hsa-miR-140-5p_C107676	8	2.886861314	1.469262565	0.000636741	0.002752616
hsa-miR-149_C472511	3	1.199052133	0.475164279	0.000637795	0.002754578
hsa-miR-576-3p_C023617	2	1.059701493	0.236932953	0.000640052	0.002756547
hsa-miR-649_D003633	2	1.059701493	0.236932953	0.000640052	0.002756547
hsa-miR-302a_D006918	2	1.059701493	0.236932953	0.000640052	0.002756547
hsa-miR-202_D015735	18	8.386	2.93955847	0.000645506	0.00277743
hsa-miR-579_C031278	5	1.427480916	0.996409848	0.000647199	0.002782107
hsa-miR-151-3p_C017947	10	2.839416058	2.128134705	0.000652392	0.002801807
hsa-miR-558_C408604	3	1.285714286	0.451753951	0.000659307	0.002827786
hsa-miR-576-3p_D009240	6	4.157894737	0.488085184	0.000659673	0.002827786

hsa-miR-381_C025463	5	2.038095238	0.815607393	0.000669399	0.002859486
hsa-miR-940_C477361	7	2.723809524	1.214948465	0.000670424	0.002859486
hsa-miR-1205_D000111	7	2.046575342	1.425989893	0.000670819	0.002859486
hsa-miR-484_D001280	9	2.092783505	2.051313254	0.000671167	0.002859486
hsa-miR-524-5p_C423222	3	1.224299065	0.469806308	0.000671255	0.002859486
hsa-miR-130a_C106014	4	1.465408805	0.689360968	0.000671428	0.002859486
hsa-miR-130a_C050414	4	1.465408805	0.689360968	0.000671428	0.002859486
hsa-miR-548d-3p_D014212	53	24.822	9.847147607	0.00067532	0.002872491
hsa-miR-1200_D000082	6	2.007874016	1.128230419	0.000675733	0.002872491
hsa-miR-548i_C011890	4	1.43083004	0.700196262	0.00067953	0.00288596
hsa-miR-381_C004984	3	1.225433526	0.469953217	0.000680223	0.002886062
hsa-miR-610_C004648	7	2.458695652	1.299009001	0.00068133	0.002886062
hsa-miR-100_D011374	4	1.408602151	0.706902362	0.00068144	0.002886062
hsa-let-7i_C006632	7	1.906896552	1.472139198	0.000682134	0.002886338
hsa-miR-320a_D020122	40	25.264	4.751242364	0.000684371	0.002893137
hsa-miR-1224-3p_C095104	2	1.060240964	0.237932743	0.000687003	0.002901592
hsa-miR-124_C103505	8	2.846982759	1.492896814	0.000691436	0.002915508
hsa-miR-659_C017557	3	1.223300971	0.471148954	0.000691568	0.002915508
hsa-miR-514_D001205	4	1.493506494	0.682940737	0.000694315	0.002921377
hsa-miR-132_D024483	3	1.227272727	0.470185474	0.000694869	0.002921377
hsa-miR-892b_C112765	3	1.227272727	0.470185474	0.000694869	0.002921377
hsa-miR-646_C404397	2	1.060344828	0.238124609	0.000696327	0.002924828
hsa-miR-143_C457499	4	1.338461538	0.729107422	0.000699085	0.002933729
hsa-miR-143_C477819	4	1.257425743	0.753256499	0.000700391	0.002935186
hsa-miR-944_C093642	10	2.674698795	2.19725148	0.000700711	0.002935186
hsa-miR-7_D002945	9	2.703592814	1.861729034	0.000703475	0.002944078
hsa-miR-1_D010269	5	1.666666667	0.930949336	0.000704766	0.002944443
hsa-miR-219-1-3p_C099555	3	1.211764706	0.475125188	0.000704845	0.002944443
hsa-miR-26a_D003633	3	1.206349206	0.476719283	0.000705735	0.002945481
hsa-miR-149_C066851	4	2.079365079	0.513363861	0.000709653	0.002959143
hsa-miR-1237_D011374	21	8.106212425	4.112489102	0.000711636	0.002964719
hsa-let-7i_C012589	10	2.588888889	2.230567663	0.000716758	0.002983351
hsa-miR-888_C006780	5	1.735795455	0.911462638	0.000718013	0.0029841
hsa-miR-199a-3p_C025462	13	3.792056075	2.834406839	0.000719053	0.0029841
hsa-miR-520f_C103436	2	1.060606061	0.238606299	0.000720188	0.0029841
hsa-miR-130b_C514580	2	1.060606061	0.238606299	0.000720188	0.0029841
hsa-miR-520f_C074279	2	1.060606061	0.238606299	0.000720188	0.0029841
hsa-miR-548b-3p_D008769	4	1.434482759	0.702839493	0.000725712	0.003004278
hsa-miR-302a_D012645	3	1.173228346	0.487553562	0.000731887	0.003026725
hsa-miR-181a_D004137	4	1.452054795	0.698159928	0.000732453	0.003026725
hsa-miR-130b_D002110	4	1.323529412	0.736469649	0.000734207	0.003031245
hsa-miR-558_D010936	6	1.862542955	1.181910792	0.000736686	0.003034688
hsa-miR-1251_D011078	3	1.228070175	0.472056984	0.000736816	0.003034688
hsa-miR-202_D011374	55	32.016	7.905424973	0.000737024	0.003034688
hsa-miR-561_D002211	5	1.47037037	0.994918502	0.000741542	0.003050555
hsa-miR-505_C007262	7	2.275862069	1.367014157	0.000744446	0.00305976
hsa-miR-632_D004317	17	3.595174263	4.320247025	0.000749647	0.00307838
hsa-miR-198_D008628	6	2.085918854	1.11499534	0.000754657	0.003093015
hsa-miR-646_D003474	49	22.28	9.414754378	0.000755132	0.003093015
hsa-miR-1207-5p_C077990	10	3.379888268	1.980586784	0.000755232	0.003093015
hsa-miR-199a-5p_D020123	6	1.846153846	1.189912518	0.000757147	0.003098094
hsa-miR-221_D006861	8	2.309782609	1.678415388	0.000758912	0.003102551
hsa-miR-576-3p_D017239	7	2.189054726	1.397965687	0.000765073	0.003124956
hsa-miR-144_C105934	7	2.5	1.299830927	0.000766362	0.003127438

hsa-miR-299-3p_D005480	9	2.384615385	1.982169035	0.000767473	0.003129191
hsa-miR-668_D019284	9	2.25	2.028810348	0.000776204	0.003159581
hsa-miR-140-3p_D003022	4	1.214285714	0.77261813	0.000776303	0.003159581
hsa-miR-548c-3p_D004642	4	1.482428115	0.692620515	0.000778817	0.003167005
hsa-miR-429_D019833	11	3.372631579	2.321997421	0.000779839	0.003168355
hsa-miR-133a_C053079	4	2.134831461	0.501481205	0.00078847	0.003200589
hsa-miR-802_C051890	18	7.36437247	3.361009149	0.000794386	0.003221755
hsa-miR-874_C070081	11	2.774647887	2.528692715	0.000795117	0.003221873
hsa-miR-1231_C012589	13	3.623333333	2.923717193	0.000797081	0.0032223
hsa-miR-513b_C006780	8	2.745140389	1.545512955	0.000797085	0.0032223
hsa-miR-1324_D003520	4	1.448905109	0.703963419	0.000797328	0.0032223
hsa-miR-130b_D004317	22	4.160326087	6.004423361	0.000804637	0.003248978
hsa-miR-503_D003474	37	13.31262525	8.286361596	0.000809301	0.003264939
hsa-let-7b_D001599	2	1.061538462	0.240315375	0.000810276	0.003265663
hsa-miR-646_C497101	4	1.560240964	0.671707007	0.000810903	0.003265663
hsa-miR-556-3p_C030110	8	2.445238095	1.645875419	0.000814949	0.003277821
hsa-miR-221_C006632	10	3.192477876	2.057930067	0.00081535	0.003277821
hsa-miR-571_C472791	3	1.210810811	0.480868462	0.000817916	0.003284836
hsa-miR-520f_D013755	25	7.543259557	5.872097497	0.000818526	0.003284836
hsa-miR-576-3p_D002211	5	1.441558442	1.012820499	0.00082209	0.003295068
hsa-miR-371-5p_D000079	13	2.777777778	3.228629312	0.000822511	0.003295068
hsa-miR-22_D010656	3	1.25	0.469929073	0.000826982	0.003310091
hsa-miR-22_C066515	2	1.061728395	0.240661589	0.000829593	0.003312027
hsa-miR-885-3p_C065250	2	1.061728395	0.240661589	0.000829593	0.003312027
hsa-miR-133a_C002202	6	2.074879227	1.127905528	0.00082963	0.003312027
hsa-miR-503_D005492	12	4.747474747	2.210824767	0.000830886	0.00331416
hsa-miR-425_D008769	3	1.244019139	0.47196537	0.000833885	0.003323234
hsa-miR-22_C439285	6	2.267175573	1.068811336	0.000838189	0.00333749
hsa-let-7i_C029938	4	1.349282297	0.736935308	0.000839504	0.003339829
hsa-miR-1271_D003474	31	10.3507014	7.127753099	0.000842346	0.003348234
hsa-miR-1274a_C475919	8	2.305970149	1.696767245	0.000843124	0.003348428
hsa-miR-1827_D013749	10	2.61618799	2.259057565	0.000845526	0.003355065
hsa-miR-576-3p_C472777	12	2	3.16227766	0.000850037	0.003370052
hsa-miR-518a-5p_C016527	2	1.061946903	0.241059088	0.00085223	0.003375831
hsa-miR-512-3p_C012589	22	7.023109244	4.968545134	0.000854331	0.003381236
hsa-miR-365_D006861	10	2.939814815	2.152966937	0.000856523	0.003386991
hsa-miR-22_C017947	27	11.534	5.155661354	0.0008601	0.003398209
hsa-miR-638_D005047	7	1.71875	1.565933727	0.00086343	0.003408433
hsa-miR-151-3p_D014284	4	1.5	0.693375245	0.000865023	0.003411787
hsa-miR-219-1-3p_D015741	6	1.684410646	1.255869995	0.000866573	0.003413534
hsa-miR-558_D002945	8	2.362637363	1.682814341	0.000866953	0.003413534
hsa-miR-633_C085911	7	2.176211454	1.418803153	0.000868788	0.003417828
hsa-miR-194_C006780	6	2.05787037	1.138095836	0.000869815	0.003418939
hsa-miR-17_C034534	8	3.602040816	1.283647513	0.000877995	0.003447861
hsa-miR-597_C008493	9	2.638954869	1.924415659	0.000879167	0.003447861
hsa-miR-583_C012589	16	4.635294118	3.660430893	0.000879426	0.003447861
hsa-miR-495_C066075	8	2.346341463	1.691311738	0.00088362	0.003458495
hsa-miR-518a-5p_D000452	3	1.216216216	0.482213466	0.000883645	0.003458495
hsa-miR-409-3p_D004610	3	1.242105263	0.474969018	0.00089079	0.00348349
hsa-miR-892a_D013629	14	3.792207792	3.256047304	0.000899523	0.00350967
hsa-miR-335_D004726	4	1.246575342	0.772486147	0.000899964	0.00350967
hsa-miR-202_D004317	47	17.744	10.72354717	0.000900174	0.00350967
hsa-miR-320a_C012589	26	9.574	5.550182339	0.000900811	0.00350967
hsa-miR-376c_C006780	4	1.491749175	0.698251879	0.000901307	0.00350967

hsa-miR-524-3p_D014810	10	3.526205451	1.967728331	0.000904783	0.003520219
hsa-miR-582-5p_D009538	6	1.81626506	1.219559678	0.000910339	0.003522626
hsa-miR-1183_C017185	2	1.0625	0.242061459	0.00091154	0.003522626
hsa-miR-10b_C050229	2	1.0625	0.242061459	0.00091154	0.003522626
hsa-miR-1271_C446520	2	1.0625	0.242061459	0.00091154	0.003522626
hsa-miR-483-3p_D013849	2	1.0625	0.242061459	0.00091154	0.003522626
hsa-miR-15b_C002055	4	3.0625	0.242061459	0.00091154	0.003522626
hsa-miR-326_D005632	2	1.0625	0.242061459	0.00091154	0.003522626
hsa-miR-548d-3p_D019810	2	1.0625	0.242061459	0.00091154	0.003522626
hsa-miR-597_C459604	3	1.214285714	0.484046912	0.000913504	0.003527247
hsa-miR-574-3p_D017638	3	1.222222222	0.481766298	0.000914482	0.003528056
hsa-miR-657_C005460	7	2.51011236	1.318157119	0.000915333	0.003528374
hsa-miR-7_C025299	4	1.426136364	0.719215848	0.00091836	0.003535251
hsa-miR-492_D013196	10	2.45890411	2.332400469	0.000918657	0.003535251
hsa-miR-130a_C075773	4	1.427244582	0.719133138	0.000922185	0.003545855
hsa-miR-595_C006632	11	3.543897216	2.306238679	0.000929608	0.003571406
hsa-miR-346_C496197	3	1.213483146	0.485101868	0.000933167	0.003582082
hsa-miR-370_C024746	6	2.09223301	1.134728856	0.000934803	0.003585364
hsa-miR-132_D019833	15	5.218623482	3.119219728	0.000936585	0.0035892
hsa-miR-1826_C001277	3	1.252252252	0.473980052	0.00093894	0.003595224
hsa-miR-10a_C084656	3	1.205607477	0.487954298	0.000946291	0.003620352
hsa-miR-1243_D014810	17	7.244	3.11519887	0.000949917	0.003631198
hsa-miR-552_D001554	4	1.46350365	0.710029907	0.000951531	0.003634342
hsa-miR-569_C085911	9	2.563492063	1.96596449	0.000954465	0.003642518
hsa-miR-1243_C107676	6	2.251908397	1.086654024	0.000958111	0.003652163
hsa-miR-22_C477361	7	2.693693694	1.264859732	0.000959157	0.003652163
hsa-miR-513a-5p_D005480	9	2.368715084	2.032782726	0.000959379	0.003652163
hsa-miR-1207-5p_D034261	4	2.138888889	0.508416812	0.000965867	0.003673816
hsa-miR-548d-3p_D024483	4	1.492401216	0.702213182	0.000966898	0.003674693
hsa-miR-135a_D014212	63	33.098	11.1324928	0.00097202	0.003691103
hsa-miR-23a_C113580	9	2.778067885	1.89828545	0.00097662	0.003705506
hsa-miR-323-3p_C059514	23	9.032064128	4.676613666	0.000985966	0.003737878
hsa-miR-143_C025462	12	3.638248848	2.63624378	0.000989226	0.003747142
hsa-miR-183_D004642	3	1.197916667	0.492015237	0.000990577	0.003747459
hsa-miR-944_D012402	6	1.979228487	1.176998395	0.000990942	0.003747459
hsa-miR-646_D002794	3	1.256521739	0.474869398	0.000993633	0.003753354
hsa-miR-548i_D020122	9	3.528688525	1.650632244	0.000994136	0.003753354
hsa-miR-503_C038864	4	1.6484375	0.65657544	0.000995767	0.003756423
hsa-miR-548a-5p_C029938	3	1.212765957	0.488281262	0.00100697	0.003795566
hsa-miR-1178_D001104	6	2.060606061	1.153108945	0.001010777	0.00380679
hsa-miR-634_D020122	24	12.932	3.614882571	0.001016625	0.003824183
hsa-miR-613_C017947	17	5.725409836	3.69070746	0.001017061	0.003824183
hsa-miR-1206_C007262	9	2.425	2.026542622	0.001019471	0.003830107
hsa-miR-576-3p_D008070	15	3.090909091	3.927020194	0.001022854	0.003839675
hsa-miR-940_C002669	4	1.3	0.764852927	0.001026463	0.003850075
hsa-miR-184_D020122	5	1.80952381	0.917619583	0.00103082	0.003863261
hsa-miR-548c-3p_C412373	5	1.718592965	0.946406072	0.001033613	0.003870569
hsa-miR-503_D015474	8	2.608374384	1.631533117	0.001039752	0.003885663
hsa-miR-143_D016685	4	1.560344828	0.685927425	0.001041492	0.003885663
hsa-miR-885-3p_C004742	4	1.441295547	0.722397004	0.001042104	0.003885663
hsa-miR-140-3p_C050414	4	1.506578947	0.702408549	0.001042332	0.003885663
hsa-miR-140-3p_C106014	4	1.506578947	0.702408549	0.001042332	0.003885663
hsa-miR-1248_C479799	2	1.063636364	0.244104029	0.001042722	0.003885663
hsa-miR-20a_D006861	6	1.796208531	1.243441499	0.001057731	0.003931404

hsa-miR-520f_C067713	6	1.333333333	1.394433378	0.001057945	0.003931404
hsa-miR-548o_D012643	25	12.57	4.137765097	0.00105817	0.003931404
hsa-miR-1825_C065180	4	1.412969283	0.732079922	0.001058422	0.003931404
hsa-miR-132_D014212	27	9.702	6.014083139	0.001060042	0.003932496
hsa-miR-132_C016517	5	1.930232558	0.882087434	0.001060429	0.003932496
hsa-miR-323-3p_D002117	27	14.766	4.067338688	0.001064182	0.003943228
hsa-miR-576-3p_D006918	2	1.063829787	0.244449474	0.001066338	0.003944849
hsa-miR-181c_D004642	2	1.063829787	0.244449474	0.001066338	0.003944849
hsa-miR-1258_C059514	9	2.533536585	1.999718805	0.001069773	0.00395437
hsa-miR-548c-3p_D002104	7	1.986531987	1.510843107	0.001073055	0.003963311
hsa-miR-298_C053541	9	2.657701711	1.959291224	0.001080074	0.003986029
hsa-miR-1271_C059514	23	8.837349398	4.809592428	0.001086184	0.004005358
hsa-miR-512-3p_C103303	11	3.209638554	2.462617593	0.001087511	0.004007033
hsa-miR-1827_D002945	11	3.555555556	2.342933593	0.001093592	0.004026208
hsa-miR-548b-3p_C012589	15	4.457894737	3.453574165	0.001094685	0.004027002
hsa-let-7i_D002330	3	1.2265625	0.487577618	0.00109676	0.004031405
hsa-miR-132_C014347	30	8.512048193	7.747058998	0.001099462	0.004037926
hsa-miR-132_C065250	2	1.064102564	0.244935554	0.001100293	0.004037926
hsa-miR-650_D012645	4	1.399193548	0.739114113	0.001105267	0.00405294
hsa-miR-582-5p_D003300	15	3.808743169	3.697810614	0.001106676	0.004054868
hsa-miR-590-5p_C051890	8	2.556650246	1.659572208	0.001108658	0.004058891
hsa-miR-646_C030110	14	5.531062124	2.709838283	0.0011144	0.004074052
hsa-miR-503_C017947	29	13.158	5.479510562	0.001114574	0.004074052
hsa-miR-144_D000111	10	3.081023454	2.16530163	0.001117404	0.004079286
hsa-miR-512-3p_D004958	50	20.072	11.40415784	0.001117783	0.004079286
hsa-miR-7_C002979	5	1.903345725	0.895023392	0.001121214	0.004088557
hsa-miR-183_C496197	5	1.797843666	0.928492206	0.001122998	0.004090432
hsa-miR-556-3p_D013755	18	4.968421053	4.397254243	0.00112351	0.004090432
hsa-miR-605_C027576	6	2.154394299	1.135650295	0.001136823	0.004135622
hsa-miR-558_C074153	3	1.20212766	0.49636524	0.00113831	0.004137752
hsa-miR-299-5p_C070081	11	2.753246753	2.638160734	0.001142204	0.004145996
hsa-miR-770-5p_C008493	10	3.110864745	2.160139173	0.001142384	0.004145996
hsa-miR-1228_D011794	18	5.372844828	4.256966361	0.001151413	0.004162185
hsa-miR-22_D008769	6	2.176610979	1.129904623	0.001152092	0.004162185
hsa-miR-1200_C054852	4	2.041237113	0.545223415	0.001152615	0.004162185
hsa-miR-1224-3p_C484027	2	1.064516129	0.2456701	0.001153248	0.004162185
hsa-miR-296-5p_D019438	2	1.064516129	0.2456701	0.001153248	0.004162185
hsa-miR-613_C430898	2	1.064516129	0.2456701	0.001153248	0.004162185
hsa-miR-520f_C070515	4	2.045454545	0.543979487	0.001153334	0.004162185
hsa-miR-885-3p_C070081	11	2.811989101	2.62041204	0.001154421	0.004162185
hsa-miR-1278_C045651	8	2.005464481	1.853949462	0.001155004	0.004162185
hsa-miR-657_D014501	7	2.541387025	1.338622903	0.001162114	0.004184196
hsa-miR-1243_C088658	6	1.850828729	1.237026321	0.001162935	0.004184196
hsa-miR-140-3p_D010634	5	3.142857143	0.515078754	0.001164454	0.004186381
hsa-miR-133a_D007052	17	5.025345622	4.016303525	0.001166199	0.004189374
hsa-miR-1228_D019256	5	1.577946768	1.002187894	0.001169931	0.004199494
hsa-let-7g_D000082	3	1.237762238	0.487006583	0.001175261	0.004214953
hsa-miR-148a_D004958	59	26.774	12.55017625	0.001176276	0.004214953
hsa-miR-548d-3p_D003513	7	2.292011019	1.422997682	0.001176992	0.004214953
hsa-miR-374b_D014810	9	3.184615385	1.796512071	0.00117792	0.004214989
hsa-miR-27a_D017638	41	25.516	5.380868331	0.001180182	0.004219794
hsa-miR-656_D020849	20	8.47	3.861230374	0.001196559	0.004275021
hsa-miR-640_D020111	4	1.476351351	0.72090985	0.001207804	0.00431168
hsa-miR-492_D015735	5	1.6	0.998180162	0.001208698	0.00431168

hsa-miR-365_D015215	6	2.012084592	1.189012718	0.001210616	0.004315169
hsa-miR-1827_D015118	5	1.35106383	1.078882862	0.001213422	0.00432111
hsa-miR-330-3p_C103303	14	4.559498956	3.090173759	0.001214165	0.00432111
hsa-miR-138_C018021	17	3.680851064	4.554351281	0.001217034	0.004326064
hsa-miR-1265_D014212	16	4.224669604	3.962679338	0.001217523	0.004326064
hsa-miR-885-3p_D020849	14	4.603271984	3.075410347	0.001218384	0.004326064
hsa-miR-219-2-3p_D004317	15	3.193452381	3.975529912	0.001219924	0.004328185
hsa-miR-135a_C040937	4	2.134615385	0.519942532	0.001230166	0.004361152
hsa-miR-361-3p_D012645	3	1.255474453	0.483738439	0.001236372	0.004379771
hsa-miR-576-5p_D008070	17	3.77540107	4.529503311	0.001241107	0.004393155
hsa-miR-875-3p_D007213	17	7.320564516	3.187687452	0.001245246	0.004403023
hsa-miR-936_C095104	2	1.065217391	0.246909059	0.00124718	0.004403023
hsa-miR-1324_D004966	2	1.065217391	0.246909059	0.00124718	0.004403023
hsa-miR-412_C058317	3	1.230769231	0.491545885	0.001247904	0.004403023
hsa-miR-432_D011794	16	4.855932203	3.736048451	0.00124869	0.004403023
hsa-miR-519a_D009599	7	2.262589928	1.441710284	0.001251324	0.004408924
hsa-miR-450b-3p_D006861	6	1.643902439	1.315952549	0.001265497	0.004455442
hsa-miR-944_C095105	4	1.525316456	0.708889014	0.001270701	0.004470336
hsa-miR-520f_D019833	28	14.098	4.809614953	0.001272292	0.004472506
hsa-miR-576-3p_D002330	4	1.481228669	0.722969695	0.001276781	0.004482143
hsa-miR-299-5p_C012589	15	4.587699317	3.470793722	0.001276986	0.004482143
hsa-miR-22_D000111	9	2.59478673	2.017523076	0.001280561	0.004491257
hsa-miR-151-3p_C072553	4	1.516129032	0.712603937	0.001287692	0.00451282
hsa-miR-297_D020111	3	1.221052632	0.495855398	0.001290115	0.004517863
hsa-miR-646_C056507	13	5.620481928	2.364934211	0.001296474	0.00453489
hsa-miR-577_C095512	2	1.06557377	0.247535555	0.001296953	0.00453489
hsa-miR-224_C410733	3	1.234177215	0.492574244	0.001311565	0.004582492
hsa-miR-1228_C093642	8	2.258064516	1.792476666	0.001315851	0.004593971
hsa-miR-548b-3p_C070081	11	2.567975831	2.750018151	0.001318519	0.004599788
hsa-miR-613_C093973	9	2.838983051	1.939641497	0.00132532	0.004620003
hsa-miR-944_C065179	2	1.065789474	0.247913733	0.001327757	0.004624987
hsa-miR-641_D007213	15	6.038306452	2.947207596	0.001329639	0.004628031
hsa-miR-299-5p_D004365	5	1.705882353	0.97319487	0.001332939	0.004636002
hsa-miR-302a_D014107	3	1.222222222	0.496903995	0.001333991	0.004636149
hsa-miR-1265_D005419	3	1.321428571	0.467024887	0.001337932	0.004646328
hsa-miR-1324_D020123	5	1.671052632	0.985261893	0.001344081	0.004661054
hsa-miR-302f_D001241	5	1.688212928	0.97970559	0.001344203	0.004661054
hsa-miR-200b_D002945	11	3.71624714	2.340966691	0.001346833	0.004666649
hsa-miR-1207-5p_D019319	4	2.010204082	0.562336561	0.001355515	0.00469319
hsa-miR-371-5p_D047310	3	1.236220472	0.49336851	0.001356756	0.004693946
hsa-miR-1294_D010126	8	1.805970149	1.956533685	0.00135858	0.004696717
hsa-miR-548o_D008769	4	1.454887218	0.735661552	0.0013652	0.00471408
hsa-miR-1308_D015032	5	1.505208333	1.040819968	0.001365656	0.00471408
hsa-miR-1259_D015735	5	1.695402299	0.979257054	0.001371392	0.004727048
hsa-miR-548c-3p_D002211	7	2.09273183	1.513157765	0.001371472	0.004727048
hsa-miR-646_C049639	3	1.255172414	0.48819598	0.00137576	0.00473827
hsa-miR-338-5p_D010100	21	5.084415584	5.675707289	0.001378462	0.004744017
hsa-miR-375_D019256	5	1.671328671	0.987872078	0.001382827	0.004753956
hsa-miR-365_D000431	3	1.221374046	0.49870177	0.001383421	0.004753956
hsa-miR-520a-3p_D007213	5	1.621848739	1.004122566	0.001384732	0.004754902
hsa-miR-143_D013467	12	3.113953488	2.934722474	0.001388348	0.004763756
hsa-miR-1259_D015474	4	1.458598726	0.735717041	0.001390366	0.004765537
hsa-miR-425_C113580	6	1.858064516	1.256922559	0.001391877	0.004765537
hsa-miR-600_C031278	5	1.5	1.044465936	0.001391981	0.004765537

hsa-miR-29a_D006861	24	11.288	4.40330058	0.001403955	0.004802949
hsa-miR-122_C031278	5	1.372881356	1.087654997	0.001411039	0.004823589
hsa-miR-299-5p_D013629	14	4.495867769	3.172385934	0.00141431	0.004831174
hsa-miR-520f_C093973	14	4.363636364	3.223037483	0.001417541	0.00483861
hsa-miR-1826_D003474	20	5.514541387	5.121271052	0.001426522	0.00486296
hsa-miR-944_C093973	12	3.741477273	2.712279752	0.001426793	0.00486296
hsa-miR-27a_D013749	14	3.987315011	3.370034564	0.001433613	0.00488258
hsa-miR-7_D017239	9	2.720588235	1.998864297	0.001435852	0.00488658
hsa-miR-503_D013196	19	5.283482143	4.820204354	0.00144368	0.004909581
hsa-miR-409-5p_D020148	6	2.051282051	1.197191542	0.00144685	0.00491672
hsa-miR-130b_C108123	2	1.066666667	0.249443826	0.001458398	0.004922142
hsa-miR-644_C056516	2	1.066666667	0.249443826	0.001458398	0.004922142
hsa-miR-548f_C002202	2	1.066666667	0.249443826	0.001458398	0.004922142
hsa-let-7i_D011192	2	1.066666667	0.249443826	0.001458398	0.004922142
hsa-miR-181a_D012721	2	1.066666667	0.249443826	0.001458398	0.004922142
hsa-miR-133b_D013467	2	1.066666667	0.249443826	0.001458398	0.004922142
hsa-miR-744_C063509	2	1.066666667	0.249443826	0.001458398	0.004922142
hsa-miR-184_D016718	2	1.066666667	0.249443826	0.001458398	0.004922142
hsa-miR-638_D008769	2	1.066666667	0.249443826	0.001458398	0.004922142
hsa-miR-583_D013629	15	4.469008264	3.575783774	0.001459167	0.004922142
hsa-miR-34c-5p_D004317	15	3.208588957	4.072131815	0.001480377	0.004990023
hsa-miR-335_C070081	11	2.736070381	2.727209661	0.001483716	0.004997608
hsa-miR-130b_C008493	9	2.708144796	2.010915169	0.001484934	0.004998044
hsa-miR-888_D011078	4	1.496815287	0.728178985	0.001488091	0.004999878
hsa-miR-27a_C059514	41	20.72	7.59642021	0.001488171	0.004999878
hsa-miR-1264_D001151	8	3.059322034	1.537896279	0.001488746	0.004999878
hsa-miR-576-3p_C093642	8	2.077777778	1.881062216	0.001493257	0.005011362
hsa-miR-1271_D017313	10	3.298642534	2.160201089	0.001502033	0.005037132
hsa-miR-1308_C006632	7	1.978787879	1.567522186	0.001505041	0.005043535
hsa-miR-509-3-5p_D018038	17	7.84	3.067637527	0.001506483	0.005044685
hsa-miR-20a_C006632	7	1.982578397	1.567208499	0.001513935	0.005065944
hsa-miR-590-5p_C006632	7	2.099722992	1.52759472	0.001521975	0.005088221
hsa-miR-16_D005419	4	1.554404145	0.71141236	0.001522809	0.005088221
hsa-miR-1207-5p_D017965	4	1.264150943	0.804490778	0.001527923	0.005101596
hsa-miR-130b_D002087	6	1.910179641	1.251711869	0.001531754	0.00511067
hsa-miR-891b_C070081	12	3.017811705	3.007571158	0.001534211	0.005115151
hsa-miR-576-3p_D001241	5	1.615384615	1.016803416	0.001540475	0.005131107
hsa-miR-610_C116926	3	1.220930233	0.503503142	0.001541232	0.005131107
hsa-miR-143_C097613	6	1.798165138	1.290614081	0.001543344	0.005134415
hsa-miR-22_D017239	10	3.227621483	2.193189696	0.001546288	0.005140484
hsa-miR-1271_D012645	4	1.493506494	0.73189625	0.00154774	0.005141552
hsa-miR-22_D003561	4	1.4375	0.749846798	0.001548849	0.005141552
hsa-miR-425_D006861	7	2.115384615	1.52556407	0.001553571	0.0051535
hsa-miR-646_D001599	4	1.537604457	0.718328181	0.001559263	0.005168647
hsa-miR-26a_C012589	19	6.376068376	4.434666265	0.001564652	0.005182769
hsa-miR-7_D007052	12	3.25	2.928547232	0.001569485	0.005195029
hsa-miR-582-5p_C475919	9	2.713953488	2.021866671	0.001571072	0.005196536
hsa-miR-144_C496197	7	2.613839286	1.356362484	0.00157678	0.00520844
hsa-miR-520f_C023635	2	1.06741573	0.250740602	0.00157694	0.00520844
hsa-let-7d_C074153	3	1.254545455	0.494264626	0.001580984	0.005218043
hsa-miR-656_D001241	8	2.768539326	1.649759636	0.00158475	0.005224466
hsa-miR-503_D012402	7	2.350828729	1.447463729	0.001585206	0.005224466
hsa-miR-548c-3p_D008345	3	1.240566038	0.498730143	0.001586806	0.005225987
hsa-miR-1827_D003513	7	2.335135135	1.454204755	0.001598746	0.005261536

hsa-miR-553_C006780	2	1.067567568	0.251002373	0.001601771	0.005267715
hsa-miR-1243_D015032	6	1.687022901	1.334131902	0.001608093	0.005284721
hsa-miR-503_C506002	6	2.52195122	1.052641786	0.001614343	0.005300223
hsa-miR-936_D002117	22	11.054	3.786698298	0.001615119	0.005300223
hsa-let-7i_D001374	6	1.64556962	1.350461475	0.001632295	0.005352628
hsa-miR-1228_C088658	6	1.958333333	1.243734296	0.00163342	0.005352628
hsa-miR-22_D010972	2	1.06779661	0.251396559	0.001639744	0.005369518
hsa-miR-569_C072553	6	2	1.23049758	0.001645124	0.005383296
hsa-miR-891b_D004726	4	1.273584906	0.807196626	0.00164674	0.005384746
hsa-miR-548l_C037219	4	1.43373494	0.755585962	0.001650938	0.005394631
hsa-miR-633_C044387	6	1.555555556	1.383322178	0.001653738	0.005399937
hsa-miR-142-5p_C112765	4	1.486772487	0.739000223	0.001662612	0.005420479
hsa-miR-379_C024746	3	1.260416667	0.494654586	0.001663458	0.005420479
hsa-miR-184_D007052	8	1.875	1.977213949	0.001663571	0.005420479
hsa-miR-558_D005947	14	3.370588235	3.677957346	0.001665822	0.005423964
hsa-miR-1231_D004317	18	3.515850144	5.239690804	0.001668356	0.005428365
hsa-miR-340_C009277	4	1.498452012	0.735540125	0.001669756	0.005429072
hsa-miR-199a-3p_C021751	3	1.218446602	0.508041657	0.001677845	0.005449566
hsa-miR-1258_D002945	6	1.939393939	1.253827931	0.001679434	0.005449566
hsa-miR-338-3p_D037742	3	1.229268293	0.504730743	0.00167962	0.005449566
hsa-miR-629_C088658	6	1.808510638	1.298744039	0.00168128	0.005451099
hsa-miR-936_C070515	4	2.072	0.55390974	0.001685216	0.005460005
hsa-miR-758_D001554	3	1.232067511	0.504144372	0.001690278	0.005472543
hsa-miR-143_C002979	5	1.842323651	0.951103964	0.001695046	0.005484113
hsa-miR-182_D005947	21	5.848837209	5.533654837	0.001698318	0.005486432
hsa-let-7f_C475919	6	1.609375	1.368741676	0.001698919	0.005486432
hsa-miR-130b_D047310	3	1.234567901	0.503606866	0.001699348	0.005486432
hsa-miR-655_D009589	2	1.068181818	0.252057648	0.001705027	0.005498412
hsa-miR-1307_D015032	4	1.269230769	0.811347043	0.001705454	0.005498412
hsa-miR-151-3p_D009532	11	2.838709677	2.736807349	0.001708678	0.005504941
hsa-miR-548c-3p_D006632	8	2.796296296	1.654171266	0.001711663	0.005507642
hsa-miR-646_C015329	13	4.838174274	2.737998176	0.001713553	0.005507642
hsa-miR-155_D014810	32	18.828	4.717034662	0.001713944	0.005507642
hsa-miR-101_D004040	9	3.641975309	1.70915214	0.001714315	0.005507642
hsa-miR-202_C002979	8	2.877358491	1.626449021	0.001720252	0.005522851
hsa-miR-224_C084656	3	1.243697479	0.501533557	0.001728708	0.00554612
hsa-miR-365_D003474	21	5.938947368	5.511562864	0.001730378	0.005547602
hsa-miR-432_D005492	6	2.075208914	1.211561482	0.001733068	0.005552348
hsa-miR-1231_D003520	3	1.245901639	0.50100678	0.001734879	0.005554274
hsa-miR-1_D013467	12	3.409502262	2.907658825	0.001745632	0.005584806
hsa-miR-1308_D015081	2	1.068493151	0.252590259	0.001759102	0.005623981
hsa-miR-193b_C475919	6	1.677824268	1.35095266	0.001768398	0.005649767
hsa-miR-760_C024746	6	2.078571429	1.213424064	0.001774311	0.005664716
hsa-miR-576-3p_C006552	3	1.272727273	0.49376275	0.001778682	0.005672037
hsa-miR-1827_D010938	4	1.274725275	0.812889759	0.001779075	0.005672037
hsa-miR-365_C403304	2	1.068627451	0.252819548	0.001782794	0.00567995
hsa-miR-548p_D004958	58	28.382	12.29943397	0.001785758	0.00568267
hsa-miR-494_C057693	7	1.852216749	1.642367783	0.001787021	0.00568267
hsa-miR-140-5p_D013792	6	2.023696682	1.233193205	0.001787548	0.00568267
hsa-miR-410_D013629	23	9.046	5.072660446	0.001788599	0.00568267
hsa-miR-33a_D017638	18	8.58	3.238456422	0.001791729	0.00568513
hsa-miR-133a_C031927	15	4.236842105	3.768353057	0.00179185	0.00568513
hsa-miR-1827_D000082	5	1.784741144	0.976557026	0.001808408	0.005733702
hsa-miR-650_C063002	4	1.535384615	0.729263226	0.001810678	0.005736938

hsa-miR-802_D020122	20	10.714	3.191896615	0.001815492	0.005748223
hsa-miR-323-3p_C113580	7	2.318318318	1.480867226	0.001819968	0.005757354
hsa-miR-132_C111237	8	2.544303797	1.755984115	0.001820884	0.005757354
hsa-miR-520f_D003474	35	14.16633267	8.11974441	0.00182731	0.005773696
hsa-miR-513a-5p_D016718	9	3.454356846	1.789489745	0.00183118	0.005781945
hsa-miR-590-5p_D014635	5	1.866161616	0.950686309	0.001833589	0.005785572
hsa-miR-337-5p_D018038	4	1.391489362	0.777292022	0.001839952	0.005797427
hsa-miR-143_C063509	2	1.068965517	0.253395491	0.001843416	0.005797427
hsa-miR-365_C115354	2	1.068965517	0.253395491	0.001843416	0.005797427
hsa-miR-554_D000082	2	1.068965517	0.253395491	0.001843416	0.005797427
hsa-miR-202_C055162	5	2.178571429	0.847415037	0.00184366	0.005797427
hsa-miR-770-5p_D014212	21	7.318548387	4.983373435	0.001847891	0.005806754
hsa-miR-1291_C027576	6	2.186480186	1.181719328	0.001849245	0.005807034
hsa-miR-133a_C045651	18	5.692144374	4.414885296	0.001855021	0.00581481
hsa-miR-500_D001374	7	2.081180812	1.567914004	0.001855474	0.00581481
hsa-miR-1323_D013804	3	1.153846154	0.53293871	0.001855521	0.00581481
hsa-miR-525-5p_D005576	6	1.81938326	1.310067278	0.001872042	0.005862582
hsa-miR-130b_C014347	30	6.642561983	9.34233712	0.001875421	0.00586916
hsa-miR-151-3p_D014638	5	1.733333333	0.997775303	0.001880747	0.005878843
hsa-miR-548k_D001151	7	2.665217391	1.364490252	0.001881076	0.005878843
hsa-miR-199a-5p_C006632	17	7.048289738	3.471308397	0.001886794	0.005892702
hsa-miR-543_C030110	10	3.16985138	2.268781901	0.001892775	0.005907362
hsa-miR-558_C025463	5	2.085271318	0.880723872	0.001895598	0.005910022
hsa-miR-509-3p_D019833	10	3.22147651	2.250067772	0.001896515	0.005910022
hsa-miR-503_C111237	11	3.537735849	2.507251853	0.001897489	0.005910022
hsa-miR-892b_C025299	3	1.25203252	0.503540246	0.001915066	0.005960724
hsa-miR-223_D001194	3	1.235955056	0.508853567	0.001925897	0.005990375
hsa-miR-646_C085911	13	4.557986871	2.888410914	0.001928934	0.005995759
hsa-miR-500_D008345	2	1.069444444	0.254208406	0.001931733	0.006000397
hsa-miR-204_D004958	39	15.524	9.454598035	0.001934038	0.006003495
hsa-miR-145_D004997	9	2.942424242	1.989294119	0.001944017	0.006027825
hsa-miR-146b-3p_D019259	3	1.295081967	0.490709165	0.001944502	0.006027825
hsa-miR-17_C501280	2	1.069565217	0.254412849	0.00195446	0.006052989
hsa-miR-125b_C086566	5	1.647482014	1.030912911	0.001955516	0.006052989
hsa-miR-23a_C070081	17	5.20083682	4.23936515	0.001956575	0.006052989
hsa-miR-199a-3p_C412373	4	1.489583333	0.749927659	0.001961219	0.006061201
hsa-miR-613_D003474	19	5.776371308	4.835761508	0.00196187	0.006061201
hsa-miR-656_C004742	5	1.813513514	0.975555881	0.001972126	0.00608879
hsa-miR-1245_C050414	3	1.251396648	0.505208353	0.001977456	0.006097045
hsa-miR-1245_C106014	3	1.251396648	0.505208353	0.001977456	0.006097045
hsa-miR-524-3p_D012643	9	3.350318471	1.844943426	0.001989085	0.006120086
hsa-miR-668_D007213	8	2.624708625	1.745175226	0.001990715	0.006120086
hsa-miR-544_C475919	7	2.05915493	1.588477654	0.001991046	0.006120086
hsa-miR-1244_D002784	3	1.346153846	0.475742957	0.001992142	0.006120086
hsa-let-7i_D004137	2	1.069767442	0.254754678	0.001992927	0.006120086
hsa-miR-1304_C422648	2	1.069767442	0.254754678	0.001992927	0.006120086
hsa-miR-520d-5p_D009588	4	2.090909091	0.556702214	0.002002791	0.006143619
hsa-miR-216b_D001241	5	1.613207547	1.045241581	0.002003947	0.006143619
hsa-miR-646_C059514	37	19.198	6.859358279	0.002004605	0.006143619
hsa-miR-646_D001241	12	4.838383838	2.410863757	0.002007153	0.006143905
hsa-miR-632_D003520	3	1.283687943	0.495744174	0.002008666	0.006143905
hsa-miR-613_C004648	11	4.708249497	2.084095265	0.002008713	0.006143905
hsa-miR-365_D005576	7	2.080701754	1.584069664	0.00202752	0.006196751
hsa-miR-548p_D013853	3	1.266129032	0.501752911	0.002029491	0.006196751

hsa-miR-411_D018038	8	2.908898305	1.646183714	0.00203004	0.006196751
hsa-miR-365_C049639	2	1.07	0.255147016	0.002037809	0.006216333
hsa-miR-511_D015741	10	3.395744681	2.206562499	0.002051021	0.006252482
hsa-miR-339-5p_D008769	3	1.226130653	0.515216306	0.002064725	0.006290081
hsa-miR-576-3p_D015054	7	2.086956522	1.585615872	0.002069833	0.006301461
hsa-miR-1255a_D003474	12	2.819727891	3.210021287	0.002081493	0.00633276
hsa-miR-623_C066075	6	1.777777778	1.340983345	0.002092936	0.006363357
hsa-miR-590-5p_D006830	5	1.871859296	0.962180345	0.002101303	0.006384568
hsa-miR-575_C053541	6	1.73046875	1.358426169	0.002102802	0.006384897
hsa-miR-1259_C014347	17	5.224948875	4.27654841	0.002106574	0.006389289
hsa-miR-221_D020122	14	6.594	2.51975475	0.002107032	0.006389289
hsa-miR-513c_C410733	3	1.223529412	0.51737961	0.002123398	0.006431809
hsa-miR-376c_D004040	4	1.462046205	0.764979198	0.002123856	0.006431809
hsa-miR-335_C006780	5	1.782089552	0.99415086	0.002129966	0.00644606
hsa-miR-944_C029938	10	4.17107943	1.927203332	0.00213571	0.006459186
hsa-miR-892b_C475919	6	1.772727273	1.346099354	0.00213954	0.006465539
hsa-miR-338-3p_D013849	3	1.266990291	0.503943353	0.002140627	0.006465539
hsa-miR-577_D004128	4	1.565789474	0.731536349	0.002149405	0.006487783
hsa-miR-875-3p_C025160	2	1.070588235	0.256135777	0.00215444	0.006498708
hsa-miR-376c_C459604	3	1.257142857	0.507628875	0.002166138	0.006529704
hsa-miR-302a_C020972	5	1.871794872	0.965547199	0.002171982	0.006543025
hsa-miR-301a_D017638	11	4.577777778	2.154045082	0.002174689	0.00654593
hsa-miR-522_C056507	6	2.07591623	1.241514053	0.002175798	0.00654593
hsa-miR-181d_C012589	11	3.261589404	2.660935476	0.002184657	0.006568278
hsa-miR-1305_D016572	6	2.060810811	1.247843282	0.002191778	0.006585375
hsa-miR-576-3p_D005947	12	3.034482759	3.148428821	0.002197793	0.006599129
hsa-miR-659_D018038	30	18.124	4.349784362	0.00220683	0.006618903
hsa-miR-432_D000079	11	2.418181818	2.997115234	0.002207262	0.006618903
hsa-miR-1251_C006780	3	1.265306122	0.506105991	0.002216307	0.006641688
hsa-miR-647_C022838	3	1.269005848	0.505095272	0.002224293	0.006661271
hsa-miR-24_D019287	5	1.824207493	0.984427242	0.002227489	0.006666494
hsa-miR-576-3p_C067311	6	1.618181818	1.407653226	0.002229917	0.006669413
hsa-miR-583_C004742	4	1.504	0.754972847	0.002236273	0.006684069
hsa-miR-561_D006861	12	4.304703476	2.652929539	0.002239345	0.006688896
hsa-miR-577_D000079	12	2.8	3.251736936	0.002241801	0.006691878
hsa-miR-151-3p_D007501	4	1.55	0.739932429	0.00224404	0.006694209
hsa-miR-323-3p_C088658	9	2.88317757	2.047427654	0.002246697	0.006697783
hsa-miR-365_C002979	5	1.836956522	0.981094929	0.002249789	0.00670004
hsa-miR-199a-3p_C101044	4	1.404761905	0.788595008	0.002250373	0.00670004
hsa-miR-144_D017382	3	1.226190476	0.519871561	0.002275199	0.006769565
hsa-miR-622_D013849	3	1.272727273	0.505138881	0.002283216	0.006789019
hsa-miR-199a-3p_D013755	17	4.750542299	4.534828432	0.002290571	0.00680244
hsa-miR-1228_C030110	6	1.820433437	1.33954516	0.002290693	0.00680244
hsa-miR-632_C014347	20	5.596707819	5.478907316	0.002298951	0.00682255
hsa-miR-641_D004391	6	2.168674699	1.216545347	0.002301598	0.006825993
hsa-miR-936_C053541	8	2.408963585	1.856392783	0.002304327	0.006829674
hsa-miR-616_C006780	4	1.506451613	0.756663083	0.002310919	0.006844793
hsa-miR-646_D002087	13	4.508810573	2.98139043	0.002317975	0.006861266
hsa-miR-197_C103303	7	2.111913357	1.598205995	0.002322802	0.006862655
hsa-let-7a_C029728	2	1.048192771	0.264512029	0.002327091	0.006862655
hsa-miR-576-3p_C095591	2	1.071428571	0.257539377	0.002328908	0.006862655
hsa-miR-936_D006533	2	1.071428571	0.257539377	0.002328908	0.006862655
hsa-miR-1183_C054919	2	1.071428571	0.257539377	0.002328908	0.006862655
hsa-miR-199a-5p_D019772	2	1.071428571	0.257539377	0.002328908	0.006862655

hsa-miR-212_D014107	2	1.071428571	0.257539377	0.002328908	0.006862655
hsa-miR-26a_D004726	4	1.277372263	0.834737836	0.002340242	0.00689163
hsa-miR-199a-5p_D014638	7	2.196969697	1.56882215	0.002344195	0.006898846
hsa-miR-512-3p_D014212	40	18.872	8.663002713	0.002353007	0.006920343
hsa-miR-10a_C004541	3	1.24260355	0.516320932	0.002356766	0.006926961
hsa-miR-223_D017638	12	5.046370968	2.383551442	0.002374507	0.00697464
hsa-miR-592_D014801	4	1.450331126	0.777913168	0.002383828	0.00698956
hsa-miR-509-3-5p_D005576	7	2.149152542	1.589624503	0.002385205	0.00698956
hsa-miR-516a-3p_C006632	14	5.397959184	3.038325922	0.002386258	0.00698956
hsa-miR-9_D043371	4	1.520710059	0.754385658	0.002387199	0.00698956
hsa-miR-9_D015525	4	1.520710059	0.754385658	0.002387199	0.00698956
hsa-miR-141_C109238	4	1.519637462	0.754888112	0.002391628	0.006998065
hsa-miR-126_D020122	5	1.8525	0.982722621	0.002404072	0.007029997
hsa-miR-629_D004317	16	3.498498498	4.686332197	0.00242537	0.007085001
hsa-miR-148a_D037742	4	1.53164557	0.751966924	0.002426321	0.007085001
hsa-miR-143_D000082	4	1.474264706	0.771314417	0.002427883	0.007085001
hsa-miR-646_C403304	3	1.271186441	0.508587558	0.002429055	0.007085001
hsa-miR-1205_C022838	3	1.26	0.512249939	0.002431977	0.00708902
hsa-miR-600_C015001	5	1.897435897	0.968606501	0.002436885	0.007098819
hsa-miR-503_D006918	3	1.23902439	0.519323222	0.002447159	0.007124228
hsa-miR-569_C059514	17	6.172839506	3.974062377	0.002453943	0.00713945
hsa-miR-1260_D012293	6	2.125	1.241448018	0.002462334	0.007159326
hsa-miR-135a_C057693	8	2.221518987	1.944233335	0.002477429	0.007198656
hsa-miR-520f_C067431	4	2.031496063	0.587762444	0.002488923	0.007224556
hsa-miR-1228_C006632	11	3.751046025	2.514965448	0.002490919	0.007224556
hsa-miR-643_C016601	4	1.376404494	0.806507587	0.002491159	0.007224556
hsa-miR-532-3p_C459604	4	1.505084746	0.763005593	0.002492637	0.007224556
hsa-miR-381_D014801	5	1.728643216	1.030555136	0.002510361	0.007271336
hsa-miR-503_D004391	7	2.649237473	1.416158873	0.002513251	0.007273236
hsa-miR-204_D020849	15	5.715151515	3.342060202	0.002517041	0.007273236
hsa-miR-181c_D000966	2	1.072289157	0.258966087	0.002517354	0.007273236
hsa-miR-181c_D000965	2	1.072289157	0.258966087	0.002517354	0.007273236
hsa-miR-1243_C015329	6	1.710059172	1.394819026	0.00252519	0.007289879
hsa-miR-339-5p_C034613	3	1.276470588	0.508781025	0.00252629	0.007289879
hsa-miR-7_C058305	4	1.4	0.8	0.00253631	0.007309604
hsa-miR-519b-3p_D013749	4	1.4	0.8	0.00253631	0.007309604
hsa-miR-569_C097240	5	1.495575221	1.11406053	0.002542897	0.00732399
hsa-miR-1307_C059514	7	1.766666667	1.745152015	0.002548802	0.007336395
hsa-miR-520f_C111237	11	3.785202864	2.510440375	0.002556551	0.007354089
hsa-miR-335_D004970	4	1.295081967	0.836547595	0.002559352	0.007355486
hsa-miR-144_D016718	7	2.606126915	1.435028225	0.002560241	0.007355486
hsa-miR-202_C067311	6	1.691919192	1.403831465	0.002562129	0.007356307
hsa-miR-302a_C419708	3	1.263157895	0.513888369	0.002567744	0.00736782
hsa-let-7b_D012643	14	6.53507014	2.611879855	0.002571452	0.007373851
hsa-miR-145_D013629	24	9.984	5.410706423	0.002573586	0.007374756
hsa-miR-519b-3p_D002087	6	1.844444444	1.349165265	0.00257498	0.007374756
hsa-miR-570_D002117	26	13.96	4.531489821	0.002580647	0.0073834
hsa-miR-199a-5p_D020122	26	15.968	3.667557225	0.002581215	0.0073834
hsa-miR-512-5p_C034613	3	1.259259259	0.515866232	0.002604981	0.007446742
hsa-miR-143_C007845	6	2.25	1.204736025	0.002606631	0.007446822
hsa-miR-98_D001104	3	1.266094421	0.513768146	0.00261142	0.007455864
hsa-miR-143_C115354	2	1.072727273	0.259688306	0.00261716	0.007461837
hsa-miR-410_D010457	2	1.072727273	0.259688306	0.00261716	0.007461837
hsa-miR-497_C063002	3	1.247368421	0.51998828	0.002618388	0.007461837

hsa-miR-646_C001277	5	1.86440678	0.987909288	0.002622013	0.007467532
hsa-miR-202_C007845	7	2.652631579	1.422390533	0.002626739	0.007476354
hsa-miR-199a-5p_C093642	10	2.613707165	2.589277969	0.002634369	0.007493425
hsa-miR-202_C030371	3	1.230769231	0.525752691	0.002637052	0.007496412
hsa-miR-376a_D005492	5	1.818770227	1.00460289	0.002638935	0.007497123
hsa-miR-128_D000450	13	6.794117647	2.125190812	0.002641398	0.00749948
hsa-miR-512-3p_C070081	14	4.182448037	3.591942036	0.002650876	0.00751969
hsa-miR-323-3p_D004967	4	1.515021459	0.764551048	0.002651792	0.00751969
hsa-miR-148a_D020849	22	9.97995992	4.544987681	0.002658134	0.00753302
hsa-miR-633_C072553	5	1.783333333	1.01803187	0.002662113	0.007537525
hsa-miR-570_C113580	7	2.306306306	1.552636774	0.002663007	0.007537525
hsa-miR-330-3p_C027576	11	4.947580645	2.068697725	0.002669775	0.007549133
hsa-miR-1207-5p_C059041	4	1.582417582	0.742226145	0.002670397	0.007549133
hsa-miR-659_C004999	2	1.072992701	0.260124521	0.002678914	0.00756855
hsa-miR-503_D007213	19	8.83	3.748746457	0.002684417	0.007579433
hsa-miR-196a_C078903	3	1.25	0.5204165	0.002686116	0.007579569
hsa-miR-337-3p_C070081	10	2.59245283	2.606222529	0.002695841	0.007602338
hsa-miR-130b_C014026	2	1.073170732	0.260416543	0.002720882	0.007663976
hsa-miR-597_C025462	8	2.282868526	1.944361892	0.002721037	0.007663976
hsa-miR-1293_C045651	8	2.066225166	2.028501793	0.002726775	0.007675429
hsa-miR-576-3p_D014810	21	10.746	3.795455704	0.002733413	0.007688297
hsa-miR-144_C045651	15	4.768907563	3.785914805	0.002734696	0.007688297
hsa-miR-220c_D004391	5	1.772727273	1.02499748	0.002738447	0.007694131
hsa-miR-338-5p_C051890	25	13.052	4.540627269	0.002755884	0.007738387
hsa-miR-1228_D002945	7	2.267241379	1.574898785	0.002771129	0.007776438
hsa-miR-142-3p_C475919	7	2.062874251	1.65214399	0.002778	0.007790958
hsa-miR-206_D007052	9	2.243055556	2.357748832	0.002785907	0.007808363
hsa-miR-579_C475919	8	2.420918367	1.897600841	0.002790489	0.007816434
hsa-miR-18a_D020122	18	9.556	3.044809354	0.002800888	0.007840779
hsa-miR-202_D008727	6	1.885245902	1.348121875	0.002806967	0.007853008
hsa-miR-663_D018038	6	2.15920398	1.249687469	0.002837755	0.007934308
hsa-miR-515-5p_C017557	2	1.073684211	0.261256287	0.002844411	0.007948077
hsa-miR-936_D005419	4	1.612021858	0.737198774	0.002849984	0.007956976
hsa-miR-588_D020111	5	1.822335025	1.012063595	0.002851062	0.007956976
hsa-miR-548m_D007052	10	2.630769231	2.612509732	0.002858213	0.007972087
hsa-miR-758_C496197	3	1.269035533	0.517418017	0.002863108	0.007977527
hsa-miR-548c-3p_C093973	15	5.391304348	3.546386615	0.002864446	0.007977527
hsa-miR-569_C034613	4	1.548387097	0.759401703	0.002866025	0.007977527
hsa-miR-130b_D014212	20	6.905349794	5.090870973	0.002867114	0.007977527
hsa-miR-135a_D019800	6	2.309255079	1.196903505	0.002871554	0.007982982
hsa-miR-101_C025462	17	6.201232033	4.063227003	0.002872552	0.007982982
hsa-miR-519a_D002857	5	1.793650794	1.023409544	0.002880059	0.007999002
hsa-miR-299-5p_D020849	13	4.817073171	2.949967819	0.002885654	0.00800623
hsa-miR-302a_D015054	7	2.058823529	1.661699634	0.002886149	0.00800623
hsa-miR-142-3p_D017313	8	2.58490566	1.842940542	0.002888333	0.00800745
hsa-miR-513a-5p_C103303	17	7.117171717	3.670027121	0.002894754	0.008017642
hsa-miR-760_D005480	9	2.479452055	2.276199603	0.002895502	0.008017642
hsa-miR-632_D019833	10	3.485106383	2.274463109	0.002900195	0.008025796
hsa-miR-1243_D013739	6	2.227272727	1.228394549	0.002905975	0.008036947
hsa-miR-548n_C056516	3	1.265957447	0.519318947	0.002913509	0.008052705
hsa-miR-143_D015232	5	1.666666667	1.0701865	0.002916072	0.008052705
hsa-miR-1276_C013038	3	1.276995305	0.515748588	0.002916935	0.008052705
hsa-let-7i_C492909	5	1.380952381	1.174174096	0.002939536	0.008108517
hsa-miR-661_C065382	2	1.074074074	0.2618914	0.002940684	0.008108517

hsa-miR-766_C004541	3	1.270408163	0.518378395	0.002943522	0.00811147
hsa-miR-548a-3p_D002330	4	1.484496124	0.783645784	0.002946333	0.00811211
hsa-miR-561_D017632	3	1.375	0.484122918	0.002947331	0.00811211
hsa-miR-503_D014212	51	27.314	10.4703106	0.002949055	0.00811211
hsa-let-7d_D001564	8	2.167539267	2.011275472	0.002960597	0.008138983
hsa-miR-432_C107773	3	1.301204819	0.508599299	0.002964264	0.008139827
hsa-miR-613_C016104	2	1.056074766	0.267622824	0.002964983	0.008139827
hsa-miR-558_D011794	19	6.567901235	4.817931881	0.002966223	0.008139827
hsa-miR-583_C024746	5	1.81300813	1.020252321	0.002974051	0.008156433
hsa-miR-503_C056507	9	3.493801653	1.885668768	0.002978035	0.00815706
hsa-miR-1206_D019800	3	1.254716981	0.524158279	0.002978353	0.00815706
hsa-miR-563_D012643	9	3.415778252	1.916110155	0.00297961	0.00815706
hsa-miR-570_C051890	21	10.632	3.900842986	0.002990505	0.008182007
hsa-miR-130b_C118739	4	1.451612903	0.796715422	0.003005671	0.008218604
hsa-miR-148a_D019256	7	2.425581395	1.531922796	0.003016224	0.008241913
hsa-miR-200b_C034192	2	1.074380165	0.26238856	0.003017786	0.008241913
hsa-miR-576-3p_C025462	9	2.663492063	2.217055217	0.003029336	0.008260796
hsa-miR-576-3p_C106014	3	1.281105991	0.516334719	0.003030098	0.008260796
hsa-miR-576-3p_C050414	3	1.281105991	0.516334719	0.003030098	0.008260796
hsa-miR-519b-3p_C012589	10	2.989847716	2.488531762	0.003032619	0.008262762
hsa-miR-1197_D000638	7	2.767590618	1.404885913	0.003037011	0.008264967
hsa-miR-143_D006861	11	3.95329087	2.503913031	0.003037029	0.008264967
hsa-miR-588_C015329	8	2.327272727	1.956383913	0.003046167	0.008284925
hsa-miR-503_D013792	7	2.419254658	1.536714877	0.003053781	0.008300716
hsa-miR-132_D002110	4	1.347826087	0.834510825	0.00306335	0.008321799
hsa-miR-27a_D001104	17	9.098	2.860139157	0.00306917	0.008332679
hsa-miR-204_D013629	16	5.526209677	3.964982629	0.003072101	0.008335558
hsa-miR-320a_C070081	17	5.620408163	4.3695131	0.003073862	0.008335558
hsa-miR-802_C066075	6	1.747081712	1.414984169	0.003079582	0.008344797
hsa-miR-155_C108373	2	1.074626866	0.262788311	0.003080904	0.008344797
hsa-miR-302a_D012402	4	1.532374101	0.770892767	0.003082774	0.008344938
hsa-miR-377_C093642	10	2.720125786	2.606288473	0.003095403	0.008374187
hsa-miR-302a_D000001	11	4.52173913	2.281676828	0.003105651	0.008396963
hsa-miR-497_D019256	4	1.433497537	0.805926987	0.003107903	0.008398106
hsa-miR-199a-5p_C113580	7	2.208333333	1.62063846	0.003111472	0.008402805
hsa-miR-221_D003513	4	1.476470588	0.791149136	0.00311425	0.008405363
hsa-miR-1205_D000171	7	2.726315789	1.424907053	0.003117069	0.008408028
hsa-miR-219-5p_D004958	43	19.018	10.79118511	0.003128721	0.008434503
hsa-miR-1827_D003474	32	13.566	7.802669031	0.00313801	0.00845458
hsa-miR-506_D015741	15	6.370741483	3.178211401	0.003147258	0.008474523
hsa-miR-181c_C016104	2	1.050632911	0.270897906	0.003170852	0.008533049
hsa-miR-576-3p_C047948	2	1.075	0.263391344	0.003178039	0.008537377
hsa-miR-1179_D002038	2	1.075	0.263391344	0.003178039	0.008537377
hsa-miR-1179_D008464	2	1.075	0.263391344	0.003178039	0.008537377
hsa-miR-888_D014810	25	13.988	4.229876594	0.003183036	0.0085458
hsa-miR-646_C023617	3	1.279863481	0.519422082	0.003191181	0.008556619
hsa-miR-940_D013755	21	6.602851324	5.811631712	0.003191371	0.008556619
hsa-miR-200b_D019800	6	2.312785388	1.21082296	0.003192657	0.008556619
hsa-miR-498_D000079	13	3.151515152	3.715008154	0.003198035	0.008566032
hsa-miR-489_D016718	4	1.501992032	0.785194177	0.003222069	0.008618804
hsa-miR-432_C103303	7	2.316176471	1.586719729	0.003223017	0.008618804
hsa-miR-146b-3p_C012589	12	3.722689076	3.040135915	0.003223369	0.008618804
hsa-miR-512-3p_D020849	18	7.324649299	4.090020803	0.003234789	0.008644305
hsa-miR-561_D002166	5	1.673469388	1.081151221	0.003245646	0.008667867

hsa-miR-509-5p_D015081	2	1.075268817	0.263824605	0.00324927	0.008667867
hsa-miR-885-3p_C086511	2	1.075268817	0.263824605	0.00324927	0.008667867
hsa-miR-203_D004958	56	27.646	13.50291391	0.003258415	0.008686936
hsa-miR-1283_D020122	33	21.766	4.348706014	0.003261627	0.008686936
hsa-miR-576-3p_D015232	4	1.388888889	0.825892708	0.003262095	0.008686936
hsa-miR-519b-3p_C492909	6	1.64	1.466424222	0.003273877	0.008713258
hsa-miR-671-5p_D037742	3	1.275229358	0.522372141	0.003278007	0.008719195
hsa-miR-106b_D014212	9	2.393501805	2.351824166	0.003280898	0.008721831
hsa-miR-19a_D003561	5	1.888888889	1.004656428	0.003285181	0.008723638
hsa-miR-558_C457499	4	1.435897436	0.810029179	0.003285378	0.008723638
hsa-miR-548e_C065180	3	1.273858921	0.523019129	0.003289814	0.008730368
hsa-miR-653_C023888	2	1.075471698	0.264150943	0.003303727	0.008762225
hsa-miR-182_C056068	2	1.062068966	0.268345882	0.003306774	0.008762607
hsa-miR-520a-3p_C490728	3	1.384615385	0.486504255	0.003309597	0.008762607
hsa-miR-1293_D005944	3	1.384615385	0.486504255	0.003309597	0.008762607
hsa-miR-132_C492909	5	1.384615385	1.190030911	0.003319901	0.008782705
hsa-miR-1271_D001151	9	3.648033126	1.852850544	0.003321321	0.008782705
hsa-miR-181a_C104586	5	2.245192308	0.878414197	0.003322927	0.008782705
hsa-miR-22_D015474	7	2.530456853	1.513169362	0.00336105	0.008878355
hsa-miR-622_D008769	4	1.477966102	0.797467984	0.003367811	0.008891099
hsa-miR-874_D020849	12	4.354037267	2.79398186	0.003376725	0.008909508
hsa-miR-1827_D000111	9	2.77753304	2.20558504	0.003380924	0.00891183
hsa-miR-655_C039961	2	1.075757576	0.264609836	0.003381487	0.00891183
hsa-miR-140-5p_D016912	4	1.5	0.790569415	0.003400147	0.008955866
hsa-miR-145_D020849	22	10.448	4.529822955	0.003408759	0.008971423
hsa-miR-646_D004317	42	18.12	10.97969034	0.003413099	0.008971423
hsa-miR-27a_C089730	12	5.420841683	2.354215614	0.003413147	0.008971423
hsa-miR-520f_D005492	11	4.824242424	2.189833761	0.00341522	0.008971423
hsa-miR-484_D012643	26	15.11	4.226807306	0.003415824	0.008971423
hsa-miR-1228_C121565	6	1.974025974	1.348149535	0.003424998	0.008978935
hsa-miR-1297_D018038	10	4.18647541	2.044600415	0.003425614	0.008978935
hsa-miR-16_C005460	8	3.288659794	1.610268358	0.003428875	0.008978935
hsa-miR-219-5p_D013629	18	6.819277108	4.363442264	0.003430491	0.008978935
hsa-miR-1244_D011794	14	4.719376392	3.507116985	0.003430709	0.008978935
hsa-miR-338-5p_C097240	5	1.527472527	1.142011521	0.003431723	0.008978935
hsa-miR-1228_C065250	2	1.075949367	0.264917083	0.00343433	0.008978935
hsa-miR-15a_C031721	2	1.075949367	0.264917083	0.00343433	0.008978935
hsa-miR-302a_D003474	14	4.04	3.811536667	0.003443843	0.008998681
hsa-miR-410_C012589	22	8.681632653	5.379920092	0.003462256	0.009039843
hsa-miR-548c-3p_C030110	12	4.750507099	2.63780918	0.003463534	0.009039843
hsa-miR-608_D002330	4	1.529616725	0.781979659	0.003471755	0.009053144
hsa-miR-1183_D003632	2	1.076086957	0.265137194	0.003472574	0.009053144
hsa-miR-96_C024746	9	3.788934426	1.809163971	0.003482255	0.00907323
hsa-miR-512-5p_C007262	9	2.482758621	2.33593649	0.003484691	0.009074428
hsa-miR-650_C105686	2	1.076190476	0.265302634	0.003501533	0.009107954
hsa-miR-202_D010416	2	1.076190476	0.265302634	0.003501533	0.009107954
hsa-miR-122_D006861	7	2.372972973	1.582445704	0.003507975	0.009119543
hsa-miR-202_C053541	13	5.208924949	2.875891512	0.003535466	0.009185809
hsa-miR-140-3p_C034613	4	1.519480519	0.787242262	0.003539407	0.009187406
hsa-miR-558_C012655	6	1.6	1.496662955	0.003540083	0.009187406
hsa-miR-346_D004391	3	1.258883249	0.532196647	0.003553685	0.00921065
hsa-miR-1244_D002117	14	6.340681363	2.822073091	0.003554601	0.00921065
hsa-miR-132_D006861	8	2.743142145	1.832474882	0.003555058	0.00921065
hsa-miR-520f_D015032	11	4.232032854	2.448139841	0.003568562	0.009240422

hsa-miR-203_C006632	21	10.146	4.244135248	0.003571916	0.009243566
hsa-miR-1231_D018021	3	1.391304348	0.488042268	0.003573803	0.009243566
hsa-miR-578_C071834	3	1.276923077	0.52645965	0.003576167	0.009244472
hsa-miR-203_D020849	22	9.968	4.7978095	0.003582637	0.009253093
hsa-miR-145_C002979	6	2.237681159	1.256300233	0.003583533	0.009253093
hsa-miR-1207-5p_D007455	4	1.407407407	0.828173325	0.00358712	0.009253793
hsa-miR-936_C089730	6	2.096774194	1.309656639	0.003588843	0.009253793
hsa-miR-646_C031477	2	1.076502732	0.265800798	0.003589851	0.009253793
hsa-miR-202_D002166	7	2.426630435	1.567345982	0.003605076	0.009287825
hsa-miR-143_D014212	34	16.178	7.72672738	0.003611307	0.00929866
hsa-miR-601_C099555	3	1.222797927	0.545446003	0.003621383	0.00931116
hsa-miR-150_D002330	4	1.498281787	0.796791549	0.003621889	0.00931116
hsa-miR-769-5p_D014635	7	2.828025478	1.413515339	0.003622246	0.00931116
hsa-miR-449a_C017947	10	3.056179775	2.52875397	0.003636356	0.009337675
hsa-let-7i_D047310	3	1.192307692	0.556032011	0.003636629	0.009337675
hsa-miR-576-3p_C459604	3	1.268085106	0.530397837	0.00363937	0.00933949
hsa-let-7b_D011374	10	3.783613445	2.229817127	0.003672434	0.009419075
hsa-miR-199a-5p_C093973	11	3.674418605	2.694952229	0.0036802	0.009433723
hsa-miR-1300_C442659	2	1.076923077	0.266469355	0.003711042	0.009459963
hsa-miR-559_D013654	2	1.076923077	0.266469355	0.003711042	0.009459963
hsa-miR-648_C007997	2	1.076923077	0.266469355	0.003711042	0.009459963
hsa-miR-526b_D019377	3	2.076923077	0.266469355	0.003711042	0.009459963
hsa-miR-367_D000965	2	1.076923077	0.266469355	0.003711042	0.009459963
hsa-miR-21_C065180	2	1.076923077	0.266469355	0.003711042	0.009459963
hsa-miR-1294_D000841	2	1.076923077	0.266469355	0.003711042	0.009459963
hsa-miR-890_C095512	2	1.076923077	0.266469355	0.003711042	0.009459963
hsa-miR-1228_C052498	2	1.076923077	0.266469355	0.003711042	0.009459963
hsa-miR-367_D000966	2	1.076923077	0.266469355	0.003711042	0.009459963
hsa-miR-137_C011890	18	10.066	2.961358472	0.003721687	0.009475623
hsa-miR-101_D005947	17	4.829652997	4.899237455	0.003722085	0.009475623
hsa-miR-302a_D003609	4	1.535714286	0.785984802	0.003723377	0.009475623
hsa-miR-558_C014347	32	10.94567404	9.587207817	0.003732177	0.009492756
hsa-miR-24_D001280	9	2.347058824	2.418705937	0.003753274	0.009540164
hsa-miR-641_D004610	4	1.52	0.792326838	0.003754972	0.009540164
hsa-miR-556-3p_C017947	20	8.49	4.592809598	0.003758719	0.009540273
hsa-miR-143_C059514	18	6.951318458	4.375116722	0.003759171	0.009540273
hsa-miR-1200_D008345	3	1.301020408	0.521182897	0.003770284	0.00956319
hsa-miR-1228_D017313	7	2.236111111	1.651960582	0.003776372	0.009573343
hsa-let-7e_D008070	17	4	5.318431563	0.003782044	0.00957718
hsa-miR-138_D010100	22	5.99044586	6.845187844	0.003782058	0.00957718
hsa-miR-22_D005492	10	4.190283401	2.073601846	0.003798569	0.009609029
hsa-miR-628-5p_D014635	10	4.523232323	1.938177228	0.003798821	0.009609029
hsa-miR-381_C070081	10	2.637681159	2.724930604	0.003805364	0.009620279
hsa-miR-370_D004317	23	7.109278351	6.793448671	0.003808009	0.009621667
hsa-miR-664_D008628	7	2.798283262	1.434581055	0.00381436	0.009632413
hsa-miR-892a_C070081	11	2.853372434	3.066548597	0.003817029	0.009633854
hsa-miR-548b-3p_C024746	4	1.586092715	0.770442355	0.003824049	0.009646269
hsa-miR-19a_C022838	5	1.868493151	1.0306105	0.003828349	0.009651812
hsa-miR-656_C025462	15	5.200426439	3.812547994	0.003846546	0.009692367
hsa-miR-590-5p_D017313	5	1.671052632	1.104871484	0.003857758	0.009715286
hsa-miR-22_C074153	3	1.229050279	0.547257962	0.003879351	0.00976431
hsa-miR-553_D005576	4	1.333333333	0.862531943	0.003886384	0.009772909
hsa-miR-383_C006632	9	3.00678733	2.1562178	0.003887025	0.009772909
hsa-miR-520d-5p_D008769	7	2.871035941	1.410571078	0.003898905	0.009797413

hsa-miR-23a_C056516	3	1.235294118	0.54550697	0.003905158	0.009807757
hsa-miR-27a_C068073	8	3.241735537	1.658136053	0.003918414	0.00983567
hsa-miR-1207-5p_D000082	4	1.562913907	0.781204359	0.003933854	0.00986903
hsa-miR-548c-3p_D019833	33	19.316	5.705448624	0.003940123	0.009879358
hsa-miR-302e_D003609	4	1.513888889	0.799184577	0.003952751	0.009900853
hsa-miR-211_C051890	7	2.529100529	1.546612525	0.003953009	0.009900853
hsa-miR-1827_D005419	5	1.874100719	1.032900375	0.003963406	0.009915397
hsa-miR-19a_D008769	8	3.298568507	1.638249975	0.003964359	0.009915397
hsa-miR-379_C093642	6	1.888888889	1.406807489	0.003965295	0.009915397
hsa-miR-624_C025462	9	2.717877095	2.283353401	0.003968653	0.009918392
hsa-miR-641_D001151	8	3.258403361	1.654644842	0.003972193	0.009921838
hsa-miR-769-5p_D006830	7	2.855319149	1.420339862	0.003976067	0.009926114
hsa-miR-1243_D004317	15	3.454285714	4.664384379	0.003996125	0.009970692
hsa-miR-216b_D014810	23	12.586	4.125845853	0.00399896	0.009970692
hsa-miR-1271_C085911	8	2.703264095	1.879395074	0.004000439	0.009970692
